# Population Structure of the Invasive Asian Tiger Mosquito, *Aedes albopictus*, in the Americas

**DOI:** 10.3390/insects17080755

**Published:** 2026-07-23

**Authors:** Margaret K. Corley, Luciano Veiga Cosme, Peter A. Armbruster, Patrick F. Reilly, Ademir Jesus Martins, Kim Medley, Catalina Alfonso-Parra, Douglas G. Barron, Andrea Gloria-Soria, María Victoria Micieli, Tyler Pohlenz, John Soghigian, Thomas N. Verna, Xiaoming Wang, Katie M. Westby, Guiyun Yan, Adalgisa Caccone

**Affiliations:** 1 Department of Ecology and Evolutionary Biology, Yale University, New Haven, CT 06511, USA; 2 Department of Entomology, University of California, Riverside, CA 92521, USA; 3 Department of Biology, Georgetown University, Washington, DC 20057, USA; paa9@georgetown.edu; 4 Department of Anthropology, Yale University, New Haven, CT 06511, USA; patrick.f.reilly@yale.edu; 5 Laboratório de Biologia, Controle e Vigilância de Insetos Vetores, Instituto Oswaldo Cruz (IOC), Fundação Oswaldo Cruz (FIOCRUZ), Rio de Janeiro 21040-900, RJ, Brazil; 6 Tyson Research Center, Washington University in Saint Louis, St. Louis, MO 63130, USA; 7 Max Planck Tandem Group in Mosquito Reproductive Biology, Universidad de Antioquia, Medellín 050010, Antioquia, Colombia; 8 Instituto Colombiano de Medicina Tropical, Universidad CES, Sabaneta 055450, Antioquia, Colombia; 9 Department of Biological and Earth Sciences, Arkansas Tech University, Russellville, AR 72801, USA; douglasgbarron@gmail.com; 10 Department of Entomology, The Connecticut Agricultural Experiment Station, New Haven, CT 06511, USA; andrea.gloria-soria@ct.gov; 11 Centro de Estudios Parasitológicos y de Vectores (CEPAVE-CCT-La Plata-CONICET-UNLP), Universidad Nacional de La Plata (CONICET-UNLP), La Plata 1900, Argentina; 12 Department of Entomology, Minnie Belle Heep Center, Texas A & M University, College Station, TX 77843-2475, USA; pohlenztyler@icloud.com; 13 Faculty of Veterinary Medicine, University of Calgary, Calgary, AB T2N 1N4, Canada; 14 Burlington County Mosquito Control, Division of Public Health, Lumberton, NJ 08060, USA; 15 Department of Population Health and Disease Prevention, University of California, Irvine, CA 92697, USA

**Keywords:** *Aedes albopictus*, mosquito, invasive species, population structure, population genomics, SNP chip, disease vector

## Abstract

The Asian tiger mosquito (*Aedes albopictus*) is an invasive species that has become widespread throughout the Americas. It is a vector for several arboviruses, making it an increasing threat to human health as its range expands. To characterize population genetic structure and gain insight into the origin and spread of this invasive mosquito, we genotyped 959 individuals using ~20k SNPs. We included 50 localities across the Americas, and previously genotyped mosquitoes from 28 localities in Asia and 14 in Europe. Our analyses revealed that populations in North America were distinct from populations in South America, and this relationship was consistently supported across analyses. Most North American mosquitoes shared a common origin with those from Japan and Southern Europe (e.g., Italy, Spain), with the exception of individuals from California, which clustered with more southern areas of East Asia (China and Vietnam), as well as Greece. Brazilian and Argentinian populations were distinct from all other sampled locations, including those in the native range, but were most closely related to mosquitoes from more tropical locations in South and Southeast Asia. By revealing the likely sources of invasions in the Americas, our findings have important implications for understanding invasion dynamics of this species.

## Simple Summary

The Asian tiger mosquito (*Aedes albopictus*) is an invasive species that has become widespread throughout the Americas. It can transmit viruses, such as dengue, chikungunya, and Zika, and has the potential to spread epidemics of these diseases into new areas of the world, making it a substantial concern to global public health. In this study we gain insights into the origin and spread of this mosquito by describing the genetic structure across populations from the invasive range throughout the Americas (50 locations), Europe (14 locations), and the native range (28 locations). Our analyses revealed that invasive populations in the Americas are the results of numerous introduction events from multiple distinct regions of the native range. Populations in South America were genetically distinct from populations in more temperate regions, such as North America and Europe. We also found evidence that after the initial events that introduced them into the Americas, invasive mosquito populations mixed with one another, likely facilitated by human transport. By revealing the likely sources of invasions in the Americas, our findings contribute to a better understanding of the dynamics of biological invasions, and our results have implications for the management of this important globally distributed disease-carrying mosquito.

## 1. Introduction

The Asian tiger mosquito, *Aedes albopictus* (Skuse 1894), is a highly invasive species native to Southeast Asia. Through human-mediated dispersal, it has spread to all continents except Antarctica, mostly within the last 50 years [1,2]. *Ae. albopictus* is a competent vector for at least 26 arboviruses, including dengue, chikungunya, and Zika [3,4], and it is also an opportunistic daytime feeder and an aggressive human biter throughout its invasive range [5,6]. Its expansion thus has the potential to spread epidemics of vector-borne diseases into new areas of the world, making it a substantial public health concern [7].

*Ae. albopictus* spread widely to countries outside of Asia in the 1980s and 1990s mainly through maritime sea transport [8]. Specifically, it is believed that invasive populations were introduced to the Americas and Europe through shipments of tires and bamboo [9,10]. Subsequently, both trade and passive human-mediated transport via ground vehicles are thought to have contributed to their dispersal to new areas on both continents and continue to facilitate their on-going spread [8,11,12].

In North America the first widespread presence of *Ae. albopictus* was identified in Harris County, Texas in 1985 [13,14]. By 1986 the species was found in numerous states throughout the Southeast and Midwest regions of the United States (US) [15]. Within a decade it had spread across much of the eastern US [16,17] and northern states of Mexico [18]. As early as 2000 it had expanded its range as far north as New England (Massachusetts and Connecticut), and was present in areas on the west coast (California) by at least 2001 [19,20]. Currently, it has been found in more than 30 states in the US, at least 22 states in Mexico [21], and has been consistently reported in southwestern Ontario, Canada since 2016 [22,23].

Outside of continental North America, *Ae. albopictus* reached the Caribbean by at least 1993, when it was identified in the Dominican Republic [24]. Since then, it has spread to Cuba [25], The Cayman Islands [9], Bermuda [26], Trinidad and Tobago [27], Haiti [28], and Jamaica [29]. Most recently, it was detected in Saint Barthélemy (French West Indies) for the first time in 2025 and it is expected to continue expanding its range in the Caribbean [30].

*Ae. albopictus* was first confirmed in South America in 1986 in southeastern Brazil [31]. Over the next decade it spread throughout Brazil, and by the mid-1990s it had reached the far western state of Amazonas. Soon after, it was recorded in Colombia [32,33], and by the early 2000s it had been spread to nearly all Brazilian states, northern Argentina, and Uruguay [34,35,36,37]. It was subsequently confirmed in Venezuela, Bolivia, and Ecuador as well [38,39,40,41]. In Central America it was introduced to Guatemala by the mid-1990s [42], and was confirmed in most Central American countries by 2010 [43,44].

The ecological plasticity of *Ae. albopictus* has likely facilitated its successful establishment of new populations across a wide range of climates throughout the Americas [4]. As its range has expanded, there is evidence that *Ae. albopictus* has undergone local adaptation [45,46,47], and temperature has been shown to influence characteristics such as longevity and the developmental speed of populations [48]. In temperate areas of its invasive range it has developed the ability to withstand colder temperatures and overwinter by undergoing diapause [49,50,51,52]. This has allowed invasive populations to become firmly established, with no year-to-year population turnover, even at the northern extent of its invasive range [53]. Since the vector competence of *Ae. albopictus* persists in individuals throughout the invasive range [54,55,56], this ability to overwinter suggests diseases once restricted to tropical regions may become endemic in more temperate areas. In parallel, tropical America experienced an unprecedented surge in dengue cases in 2024, followed more recently by a marked increase in chikungunya transmission [57,58]. Although *Aedes aegypti* is widely recognized as the primary arbovirus vector in tropical America, the rapid spread of vector-competent *Ae. albopictus* may be contributing to the observed increase in arbovirus transmission in tropical regions [59]. There is also substantial evidence that *Ae. albopictus* in various locations throughout the invasive range have developed mutations conferring resistance to various pesticides [60,61,62,63,64,65]. Combined, these characteristics make it a priority for surveillance and pest control management.

Determining the invasion routes for *Ae. albopictus* populations and understanding how mosquitoes across the invasive range are related to one another can inform management strategies [4] and shed light on the underlying evolutionary and ecological processes involved in the spread of invasive species in general [66]. While historical and observational data are useful for reconstructing aspects of invasive species’ spread [55], they are often incomplete and can be misleading. Invasion histories may be complicated: some locations may have experienced multiple independent invasions or founding events that are still ongoing, and sources of invasions may originate from other previously invaded areas, rather than directly from the native range. For example, some invasive populations of *Ae. albopictus* in the Americas have been linked to the establishment of invasive populations in Europe [67,68] and remote Pacific Islands [69].

Population genetic studies can reveal nuances and complexities of invasion histories and distinguish between possible scenarios that direct observation cannot. In fact, population genetic data have been essential in shaping our understanding of the invasion histories of various invasive *Ae. albopictus* populations in the Americas. Research using a variety of genetic markers (allozymes, microsatellites, mtDNA, and genome-wide single nucleotide polymorphisms (SNPs)) has consistently found that mosquitoes from multiple locations in the central and eastern United States are most genetically similar to those from temperate East Asia, specifically Japan [47,53,68,70,71,72,73]. These US populations of Japanese origin also share genetic similarity with invasive populations in Europe, likely as the result of invasive mosquitoes in the US being introduced to Italy and other areas around the Mediterranean [68,74,75]. In contrast, populations from the west coast of the US have been linked to more southern parts of the native range (south China, Taiwan, Singapore), indicating separate introductions for eastern and western populations in North American [20].

The origin of *Ae. albopictus* in South America and the Caribbean is less straightforward. Studies using mitochondrial DNA (mtDNA) found that Brazil harbored private haplotypes not shared with the US or Europe, indicating at least one separate introduction of *Ae. albopictus* into Brazil [76,77,78]. Studies using SNP data also found that Brazilian populations were distinct from those in the US [71,73]. Limited evidence points to a potential tropical southeast Asian origin for certain Brazilian populations [77,79], but the exact source(s) of *Ae. albopictus* populations in most of South America and the Caribbean remains unresolved.

Overall, studies including mosquitoes from various areas of North and South America indicate a complex colonization history with multiple independent invasions [71,80], but more comprehensive sampling is necessary to elucidate the full story of the origins and current population genetic structure in the Americas. While previous studies have provided valuable insights into potential origins of specific populations, many relied on a limited number of microsatellite or mitochondrial markers, which may lack the resolution to distinguish closely related source populations and may not allow for the detection of fine-scale admixture. To date, there have been no widespread population genetic analyses encompassing multiple populations from the full invasive range of *Ae. albopictus* in the Americas using genome-wide genetic markers. As a result, it remains unclear how many source populations contributed to the American invasion, and whether genetic structure within the invasive range primarily reflects independent introductions from the native range or secondary spread and admixture among invasive regions.

We hypothesized that there have been multiple independent introductions of *Ae. albopictus* populations into the Americas, derived from genetically distinct source populations in the native range. If this first hypothesis is correct, we predict that there will be distinct native range clusters that are represented differentially across American regions. We also hypothesized that genetic structure within the Americas has been shaped by secondary, human-mediated spread among regions. If this second hypothesis is correct, we predict that there will be genetic similarity and admixture among regions within the invasive range, independent of geographic distance.

To test these hypotheses and address outstanding questions regarding the origin and spread of *Ae. albopictus* in the Americas, we characterized the genetic structure across North America, South America, the Caribbean, its native range, and other invasive populations in Europe, using single nucleotide polymorphisms (SNPs). Our first objective was to investigate the ancestry of *Ae. albopictus* populations in the Americas to determine which populations in the native range are the most likely sources of invasion(s). Our second objective was to characterize the regional genetic structure of populations throughout their invasive range in the Americas.

To achieve these objectives we used the Aealbo SNP chip [81], a species-specific genotyping tool that targets 175,296 SNPs and has previously been shown to successfully infer population structure across the native range and Europe [82]. This SNP chip provides an extensive set of informative genetic markers across the entire genome, allowing us to detect fine-scale structure that may not be evident when using less sensitive genetic markers. Specifically, we characterized genetic structure for 959 wild-caught mosquitoes from 92 locations (30 in North America, 17 in South America, 3 in the Caribbean, 28 in the native range, and 14 in Europe). We used these data to assess patterns and levels of genomic differentiation and diversity across the Americas and between regions of the Americas and the native range (Asia) and Europe.

## 2. Materials and Methods

### 2.1. Sample Collection and Extraction

*Ae. albopictus* mosquitoes were collected from the wild between 1995 and 2021 and were shipped by collaborators (Supplemental File S5) to Yale University, following all established shipping and permit requirements. The DNeasy Blood and Tissue kit (Qiagen, Valencia, CA, USA) was used to extract DNA from *Ae. albopictus* adults following a modified version of the manufacturer’s protocol for the purification of total DNA from insects, as described in previous publications [81,82].

**Figure 1:**
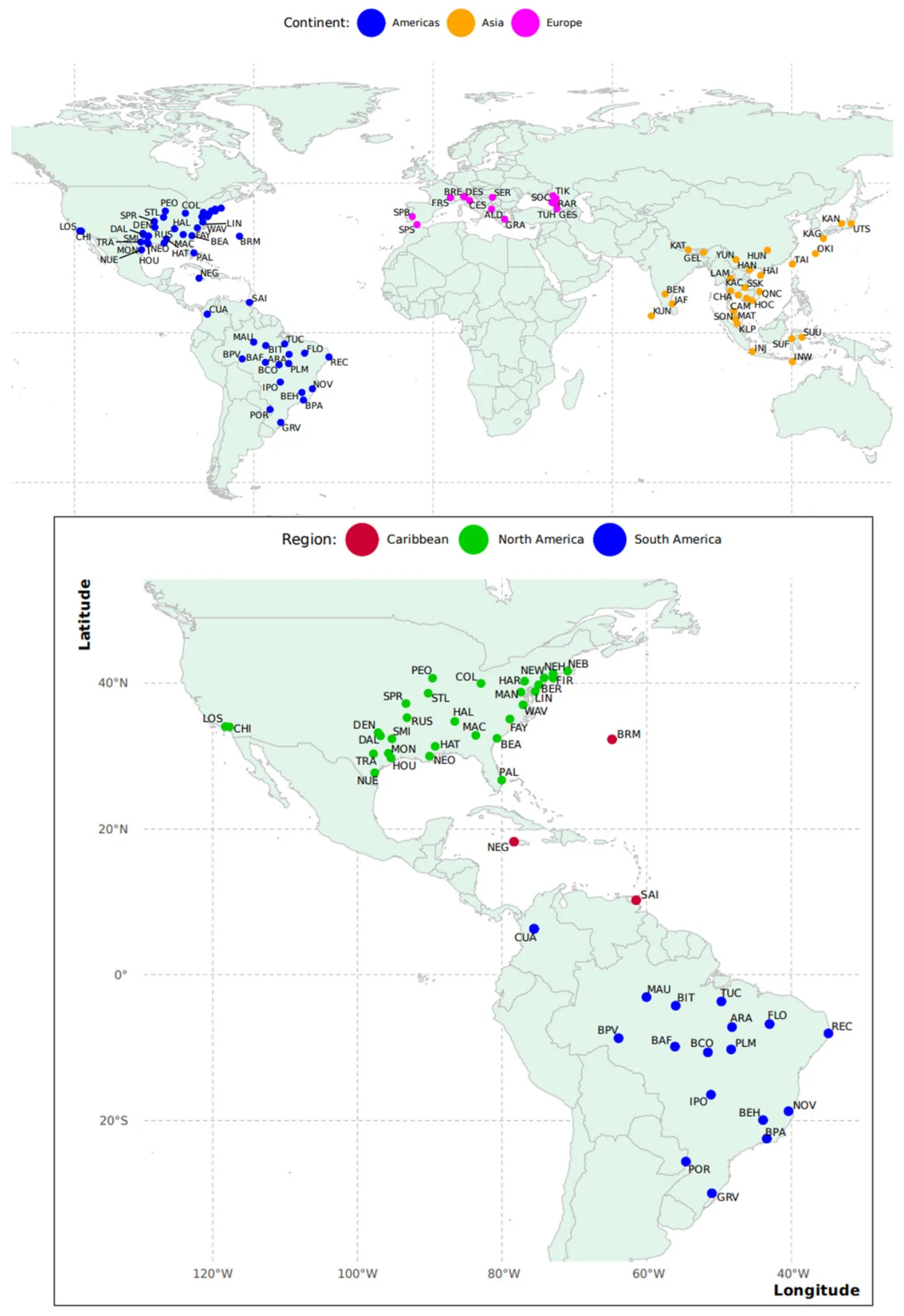
Maps of sampling locations for all *Aedes albopictus* mosquitoes genotyped. The top panel shows the 92 locations, by region, for all samples in the Global dataset. Below is a zoomed in view showing the 50 sampling sites in the American dataset. Each locality is identified by a unique three-letter abbreviation identifying the location. Details on the location, year of collection, and the number of samples per locality are reported in Table S2.

We extracted DNA from 573 mosquitoes from 50 localities in the Americas (Figure 1): 357 individuals from North America (30 localities), 184 individuals from South America (17 localities), and 32 individuals from the Caribbean (3 localities) (Table S1). To gain insights into the origins of invasive populations in the Americas, we included an additional 266 individuals from 28 localities in Asia and 169 individuals from 14 localities in Europe, all of which had been previously extracted for analyses [81,82]. The 14 European locations were selected because they had previously been found to be somewhat genetically differentiated from one another [82], and including them allowed us to explore links between invasive populations in Europe and the Americas. In total, we utilized DNA from 1008 mosquitoes across 92 localities for genotyping (Figure 1).

### 2.2. SNP Chip Genotyping

All samples were genotyped with the Aealbo microarray (Affymetrix, Santa Clara, CA, USA) in the Functional Genomics Core at the University of North Carolina at Chapel Hill. The Aeablo microarray was designed using 819 whole genome sequences (WGS) representing both native and invasive populations worldwide, and it has probes that target SNPs in coding and non-coding regions across all three chromosomes [81]. We used Axiom Analysis Suite Software v.5.1.1 to obtain genotype calls, running the “Best Practices Workflow” and the off-target variants (OTV) caller algorithm to identify miscalled clusters. The thresholds and other criteria used for quality control during data processing in Axiom Analysis Suite have been described previously [81] and are detailed in Supplemental Files S3 and S4.

We performed two distinct genotype calls: one for all mosquitoes from Asia, Europe and the Americas combined; and one for only the mosquitoes from American locations. We refer to these as the “Global” and “American” datasets, respectively. The genotype data for both datasets were exported in VCF format and used for subsequent quality control filtering. The VCF files and the raw data used to obtain these genotype calls are available at Zenodo (https://doi.org/10.5281/zenodo.20646598).

### 2.3. Statistical Analysis

#### 2.3.1. Quality Control

We used Plink v 1.9 or v 2.0 [83] and BCFtools v1.16 [84] to perform additional quality control measures on the genotyped data by filtering both variants and individuals in each dataset (Global and American). We removed variants that did not uniquely map to autosomes or failed segregation tests based on laboratory crosses [81]. Following previously validated studies [81,82] we then filtered variants, removing loci with more than 10% missingness, loci with a minor allele frequency smaller than 1%, and loci that failed Hardy–Weinberg tests (with a threshold of 0.000001 for each population). We then filtered individual mosquitoes, removing those with more than 20% missing loci, those whose expected heterozygosity values deviated more than ±4 standard deviations from the mean of all samples, and those related with a KING kinship coefficient of >0.354.

To account for linkage disequilibrium (LD) we calculated the half distance of maximum r^2^ (LD half-life). We then created an SNP dataset with LD pruning using the Plink command --indep-pairwise 5 1 0.01 (5 kb window, step 1 SNP, r^2^ < 0.01), and removed SNPs with a minor allele frequency (MAF) of ≤1%. We retained SNPs with MAF > 1% to preserve relatively rare alleles that may allow us to better distinguish between sampling locations. This SNP Set (filtered for LD half-life r^2^ < 0.01 and MAF > 0.01) was used for all downstream analyses described below, unless otherwise noted. In cases where we conducted additional filtering, we specify the criteria used in the text and in the Supplementary RMarkdown Files (S6–S10).

#### 2.3.2. Population Structure and Differentiation

We utilized several complementary methods to characterize the population structure of mosquitoes in our Global (*N* = 933) and American (*N* = 545) datasets. First, to visualize major patterns of variation and assess the relationship between mosquitoes collected in the Americas and those collected in the native range and Europe, we used principal component analysis (PCA), as implemented in the R package LEA [85]. We also performed Discriminant Analysis of Principal Components (DAPC), using the cross-validation function in the R package adegenet v. 2.1.11 to choose the number of principal components (PCs) to retain [86,87]. For both PCA and DAPC, we carried out analyses on both the Global and American datasets independently. Step-by-step procedures for these analyses are presented in Supplemental Files S8 and S9.

To further evaluate population structure, we used Bayesian inference-based clustering algorithms. To ensure our findings were robust and to check for consistency across methodologies, we carried out these analyses in two different algorithms: fastStructure [88] and LEA [85]. To identify the best number of clusters (K) for each run, we explored K ranging from 2 to 30 and selected the K that maximized marginal likelihood as calculated with the chooseK.py function in fastStructure, and the K for which the cross-entropy criterion was minimized in LEA. The specific parameters used to run each algorithm on the Global and American datasets are listed in Table S5, and step-by-step procedures for these analyses are presented in the Supplemental Files S8 and S9.

To quantify genetic differentiation among sampling locations we estimated the pairwise genetic differentiation (FST) using the R package StAMPP [89]. This method estimates FST values for each locus using the Weir & Cockerham estimator [90], using 100 bootstraps to estimate *p*-values and confidence intervals. We selected this method because it is robust for small sample sizes and unequal sampling numbers. For all FST analyses we used only locations with at least four individuals. For use in subsequent analyses (i.e., Local PCA) we also created a neighbor-joining tree based on pairwise FST estimated using the Hudson method [91,92]. We visualized the tree in FigTree v1.4.4 [93] and assigned populations to one of five major clades (Figure S17). We also examined patterns of isolation by distance (IBD) among American populations. We first used the R package adegenet [86] to calculate genetic distances based on gene frequencies, and then used a Mantel test to assess the correlation between genetic and geographic distances using the mantel.randtest function in the R package ade4 [94]. Step-by-step procedures for these analyses are presented in the Supplemental Files S8 and S9.

To further examine the evolutionary relationships among mosquitos from the native and invasive ranges, we created trees for both the Global and American datasets using two methods. First, we used IQ-TREE v2.4.0 [95] to generate trees for the Global and American datasets, using SNPs in intergenic regions of the genome. Since our goal was to assess demographic history and gain insights into the origins of invasive populations, we used intergenic SNPs (14,419 variants for the Global dataset, 12,772 for the American dataset) for these analyses to minimize the influence of selection on our results. To find the best tree for each dataset, we used the ModelFinder option [96] with 1000 bootstrap replicates and the UFBoot option -bnni to reduce the risk of overestimating branch supports. Trees were then visualized and edited in FigTree v1.4.4 [93]. After obtaining the best trees for each dataset, we repeated the analysis using only a subset of 204 individuals (with ~2 mosquitoes per locality) from the Global dataset, to more easily visualize the relationships among populations in the tree (Figure S13B).

To evaluate possible historical gene flow events amongst the ancestors of these populations, we inferred admixture graphs using TreeMix v1.13 [97]. We chose this method because it allows for both population splits and gene flow. To streamline analyses and constrain the space of possible admixture graphs, we used a subset of populations and grouped them by clusters within each region. Clusters and representative populations were identified based on our previous analyses (e.g., PCA and fastStructure), and Sulawesi, Indonesia was always used as the outgroup. We selected Sulawesi as an outgroup because our other analyses (e.g., PCA, DAPC, and fastStructure) indicated that Indonesian populations were genetically distinct from all other sampled regions and showed no evidence of contributing to invasive populations. Next, we created separate admixture graphs to examine the relationships between the native range and (1) populations in North America and Europe, and (2) populations in South America. In all analyses we tested a range of 0–5 migration edges (*m*) and identified the best *m* using the Evanno method as implemented with the R package *OptM* [98]. Confidence in the final admixture graph was evaluated with 1000 bootstrap replicates using a python wrapper (modified from https://github.com/mgharvey/misc_python/blob/master/bin/TreeMix/treemix_tree_with_bootstraps.py; accessed on 23 October 2025) and the *sumtrees* function of DendroPy v. 4.3.0 [99]. Step-by-step procedures for these analyses are presented in the Supplemental Files S8 and S9.

#### 2.3.3. Local PCA and Enrichment Analysis

Since the SNP chip contains variants distributed across the *Ae. albopictus* genome, we also examined how population structure differed along the genome. Patterns of genetic structure can vary depending on which portion of the genome is being analyzed. For example, in invasive species, migrants to new habitats may experience strong selection against alleles associated with genes that were locally adapted to the native environment. To describe variation in structural patterns across the genome, we used Local PCA as implemented in the R package lostruct [100]. Specifically, we conducted a multidimensional scaling (MSDS) analysis and identified outlier windows using the “run_lostruct.R” script from the lostruct package with windows set to 50 SNPs. This method does not specifically identify outlier loci, but instead describes larger-scale variation shared by multiple parts of the genome. We then ran a KEGG enrichment analysis using an over-representation test (ORA) as implemented in the R package clusterProfiler [101]. After Benjamini–Hochberg (BH) correction [102], we used an adjusted *p*-value cutoff of 0.05 to identify enriched pathways in the 5% most extreme outlier windows in each of the three sets of corners identified by our local PCA (3–4 windows in each corner). These windows represent regions of the genome with the most divergent patterns of genetic variation, which may be attributable to linked selection or to structural variants like chromosomal inversions. Step-by-step procedures for these analyses are presented in the Supplemental File S10.

## 3. Results

### 3.1. Genotyping and Quality Control

Of the 1008 samples in the Global dataset subjected to genotyping, 959 mosquitoes passed the quality control criteria of the Axiom Analysis Suite Software’s (v.5.1.1) “Best Practices Workflow” and 110,392 variants were recommended after running the Off Target Variant (OTV) caller. Of the 573 samples in the American dataset that underwent genotyping, 555 passed all quality control criteria, and 112,855 variants were recommended by the OTV caller. Additional details of the quality control methods implemented in Axiom Analysis Suite are outlined in the Supplemental Files S3 and S4.

The exported VCF files were subjected to additional quality control measures (see Supplemental Files S6 and S7). No individuals were missing >20% of variants. In the Global dataset we removed eight individuals with heterozygosity more than 4 SD from the mean and 18 individuals with high relatedness (>0.354), leaving 933 individuals in the final Global dataset. In the American dataset, no samples had heterozygosity > 4 SD from the mean, but we removed ten individuals with high relatedness (>0.354), leaving 545 individuals in this dataset for subsequent analyses (Table S3).

Removing the SNPs that failed the segregation test, variants missing in more than 10% of samples, and variants with a minor allele frequency <1% resulted in a SNP set of 96,268 variants for the Global dataset and 95,921 variants for the American dataset. For both datasets, all variants passed the Hardy–Weinberg Equilibrium test. After LD pruning (removing SNPs with a half distance of maximum r^2^ < 0.01), 20,825 SNPs remained in our Global dataset and 18,964 in our American dataset. Additional details of the SNP distributions in each dataset are shown in Tables S3 and S4.

### 3.2. Genetic Ancestry

#### 3.2.1. Global Dataset

**Figure 2:**
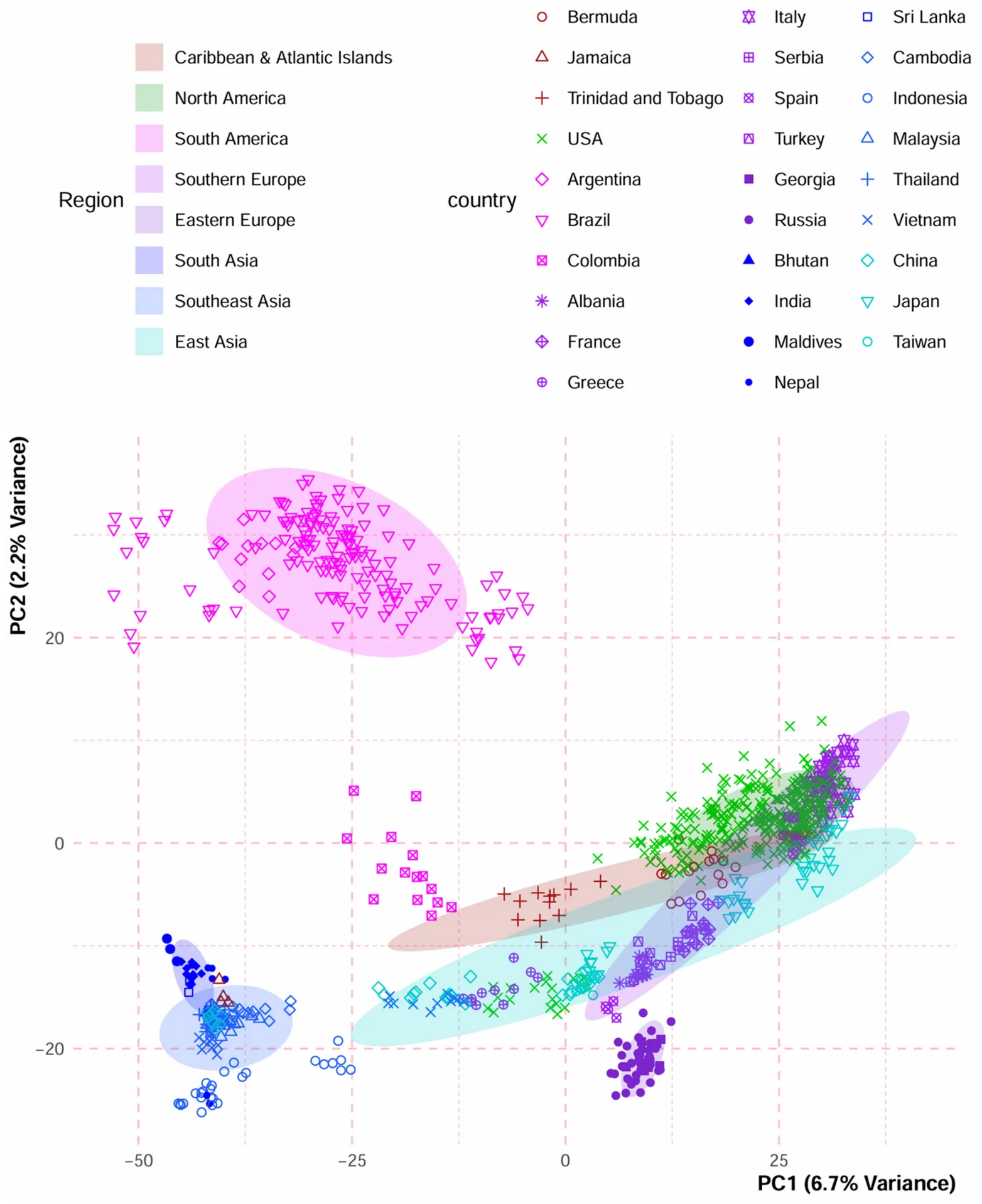
Scatterplots of principal component analysis (PCA) for 933 *Ae. albopictus* mosquitos from 92 sampling sites in the Global dataset. The *x*-axis is PC 1 and the *y*-axis is PC 2. Each symbol represents a mosquito, and the color and shape of the symbol indicates the location where they were sampled. The ellipses mark the area covering 80% of the samples. Additional plots (Figures SA-SE), and details of PCA and Discriminant Analysis of Principal components (DAPC) analyses are shown in Supplementary Files.

**Figure 3:**
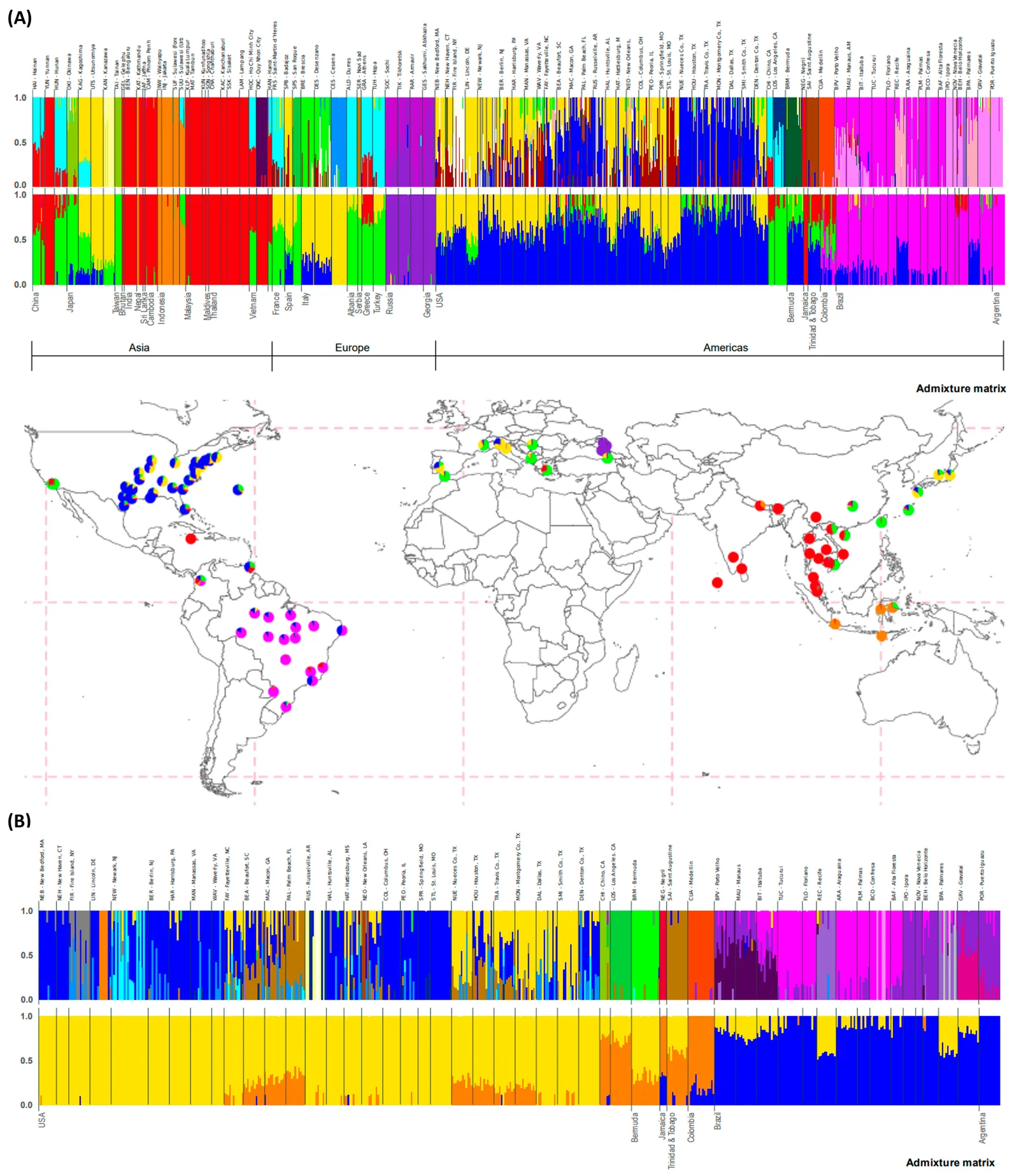
Populations structure of 933 *Ae. albopictus* mosquitos from 92 sampling sites in the Global dataset (**A**), and 545 mosquitos from 50 sampling sites in the American dataset (**B**). Plots were created from the Q matrices obtained from the fastStructure clustering algorithm run with simple prior. Each vertical bar represents one mosquito, with the height indicating the proportion of admixture from each ancestral genetic group for that individual. In (**A**) the bar plots show the Q matrices for K = 27 (**top panel**) and K = 7 (**bottom panel**), and the colored pies on the map reflect the proportion of the seven clusters found at each sampling location. In (**B**), the bar plots show the Q matrices representing K = 23 (**top panel**) and K = 3 (**bottom panel**).

The PCA of the Global dataset indicated substantial overlap of many samples from the Americas with those from the native range and Europe (Figure 2 and Figure S1). Mosquitoes from South and Southeast Asia clustered together, along with those from Jamaica, whereas mosquitoes from East Asia (Japan, Taiwan, and eastern China) were spread along PC 1, and overlapped with the European and North American samples. Most samples from the United States (US), excluding California, clustered tightly together, grouping with individuals from Japan, Italy, Bermuda, and Spain. Samples from California instead grouped with individuals from parts of East Asia (China, Taiwan, and Okinawa) and more eastern parts of southern Europe, especially Greece. However, several locations did not cluster with any populations in the native range. Samples from Eastern Europe (Russia and Georgia) and Colombia were somewhat differentiated from all other locations, and samples from Brazil and Argentina were the most differentiated from other locations, forming a diffuse cluster that was completely distinct from all other samples (Figure 2 and Figure S1). A DAPC of the Global dataset confirmed seven clusters, similar to the pattern identified by the PCA (Figures S3 and S4; Table S6). Since seven clusters were distinguished by these analyses, we also plotted the ancestry matrices for K = 7 when visualizing the results of our Bayesian clustering algorithms (Figure 3).

#### 3.2.2. American Dataset

When American locations were analyzed on their own, the cluster composition was similar to those identified when they were analyzed as part of the Global dataset. The PCA of the American dataset showed that all mosquitoes from Brazil and Argentina formed a distinct cluster, separated from other populations along PC1. Mosquitoes from Colombia, Jamaica, California, and Trinidad and Tobago formed their own diffuse cluster, and all other populations from the United States (except those from California) grouped together in a third cluster, which overlapped somewhat with samples from Bermuda (Figures S2 and S5). DAPC also identified three main clusters in the American dataset: one with all samples from Brazil and Argentina, a second with samples from Colombia, the Western US (California), Jamaica, and Trinidad and Tobago, and a third containing all samples from the Eastern and Central US and Bermuda (Figures S5 and S6; Table S6B).

As with the Global dataset, the Bayesian clustering algorithms identified finer substructure in the American dataset compared to the PCA or DAPC. For the complete American dataset, the number of ancestral clusters identified as best ranged from 20 to 23 (Figure 3B and Figure S8). Figure 3B shows a typical clustering result for K = 23 (the best K identified by maximum marginal likelihood most frequently over the 100 fastStructure runs). While most sampling locations, particularly in North America, showed some admixture, there was again clear evidence of population structure. Since three major clusters were distinguished by our PCA and DAPC analyses, we also plotted the ancestry matrices for the three clusters to visualize how different American populations grouped with one another. The populations in the K = 3 admixture plot roughly grouped into the same three clusters identified by the DAPC (Figure S5). The admixture plots generated by both the fastStructure and LEA algorithms were similar (Figure 3B and Figure S8).

#### 3.2.3. Genetic Differentiation

We estimated genetic differentiation (FST) for all localities with at least four individuals (N = 89). When locations in the Global dataset were examined together, mean FST ranged from 0.13 in the Americas to 0.16 in Europe. At the regional level, mean FST was lowest in Western Europe (0.11) and North America (0.12) and highest for Eastern Europe (0.17). The highest mean FST for a location was similar in each continent: 0.24 in the native range (for Kunfunadhoo, Maldives and Wainyapu, Indonesia), 0.24 in Europe (for Novi Sad, Serbia), and 0.23 in the Americas (for Negril, Jamaica) (Figure S10, Table S7). When American locations were examined individually, the mean FST by region was 0.10 for North America, 0.13 for South America, and 0.16 for the Caribbean (Table S7). The mean FST ranged from 0.07 in Beaufort and Houston (USA) to 0.23 in Negril (Jamaica) (Figure S10, Table S7).

**Figure 4:**
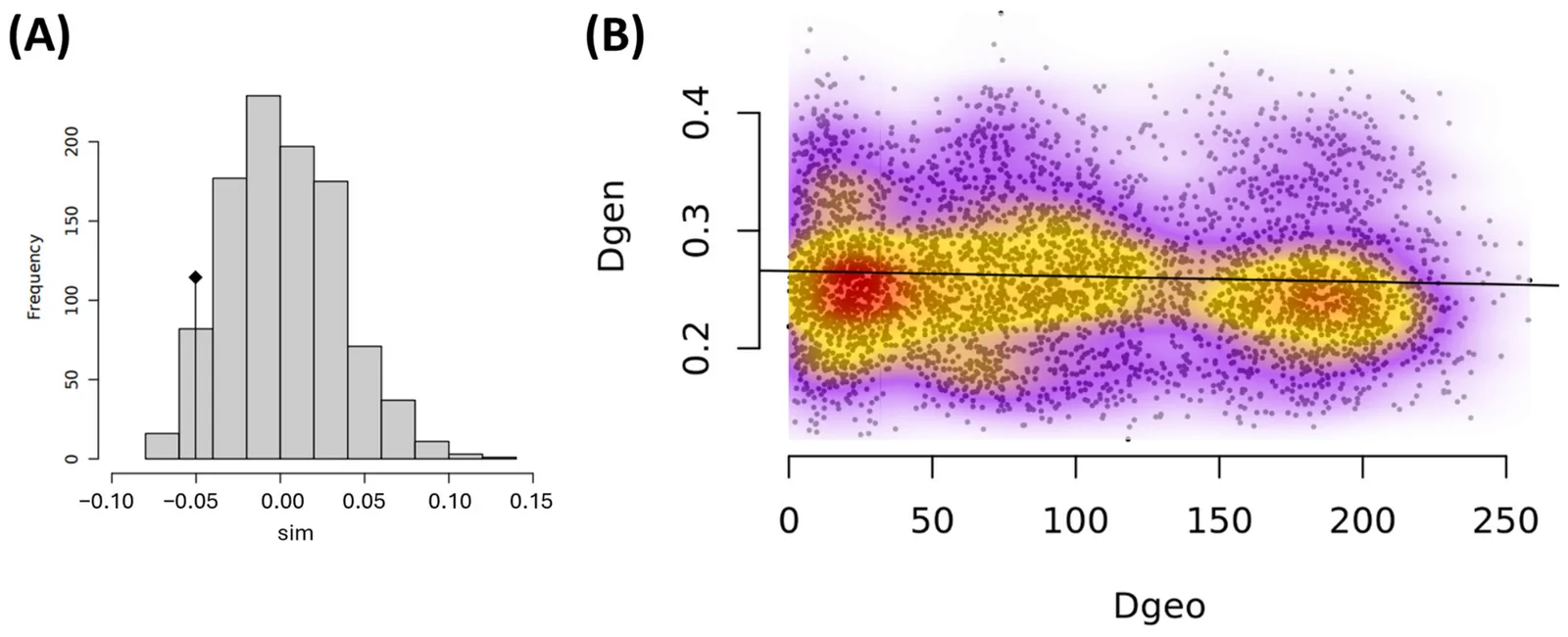
Plots of the Mantel test for isolation-by-distance (IBD) using the 89 Global populations with at least four mosquitoes. (**A**) The histogram shows the distribution of correlation coefficients between genetic (Dgen) and geographic (Dgeo) distances obtained from 999 random permutations. The arrow indicates the observed correlation coefficient (−0.05) derived from the data, which suggests no significant deviation from random expectations (*p* < 0.94). (**B**) The scatterplot shows geographic distance (Dgeo) against genetic distance (Dgen). The line from the linear regression model fit to the data had an R^2^ = 0.00. The density of overlapping points is represented by color, with warmer shades indicating more overlap.

When we plotted FST values against geographical distance for all American locations and fit a linear regression, there was no strong correlation (R^2^ for FST vs. distance = 0.0), indicating that variation in FST could not be attributed to geographic distance (Figure S11). There was also no strong evidence for isolation by distance (IBD) in the Americas when allele frequencies were used to estimate Angular (Euclidean) distance using the dist.genpop function in the R package adegenet [86]. When we fit a linear regression model for all 50 locations, the coefficient of determination was 0, indicating that variation in genetic distance cannot be explained by geographic distance amongst American populations (Figure 4). Mantel test observations were very small and not statistically significant (0.07, *p*-value = 0.12), indicating that we cannot reject the null hypothesis that geographic and genetic distance are uncorrelated. There was also no strong evidence for IBD when we looked at subsets of populations within the same country (i.e., Brazil and USA) (Figure S12). Step-by-step procedures for the analyses are detailed in Supplementary File S8.

#### 3.2.4. Evolutionary Trees and Admixture Graphs

**Figure 5:**
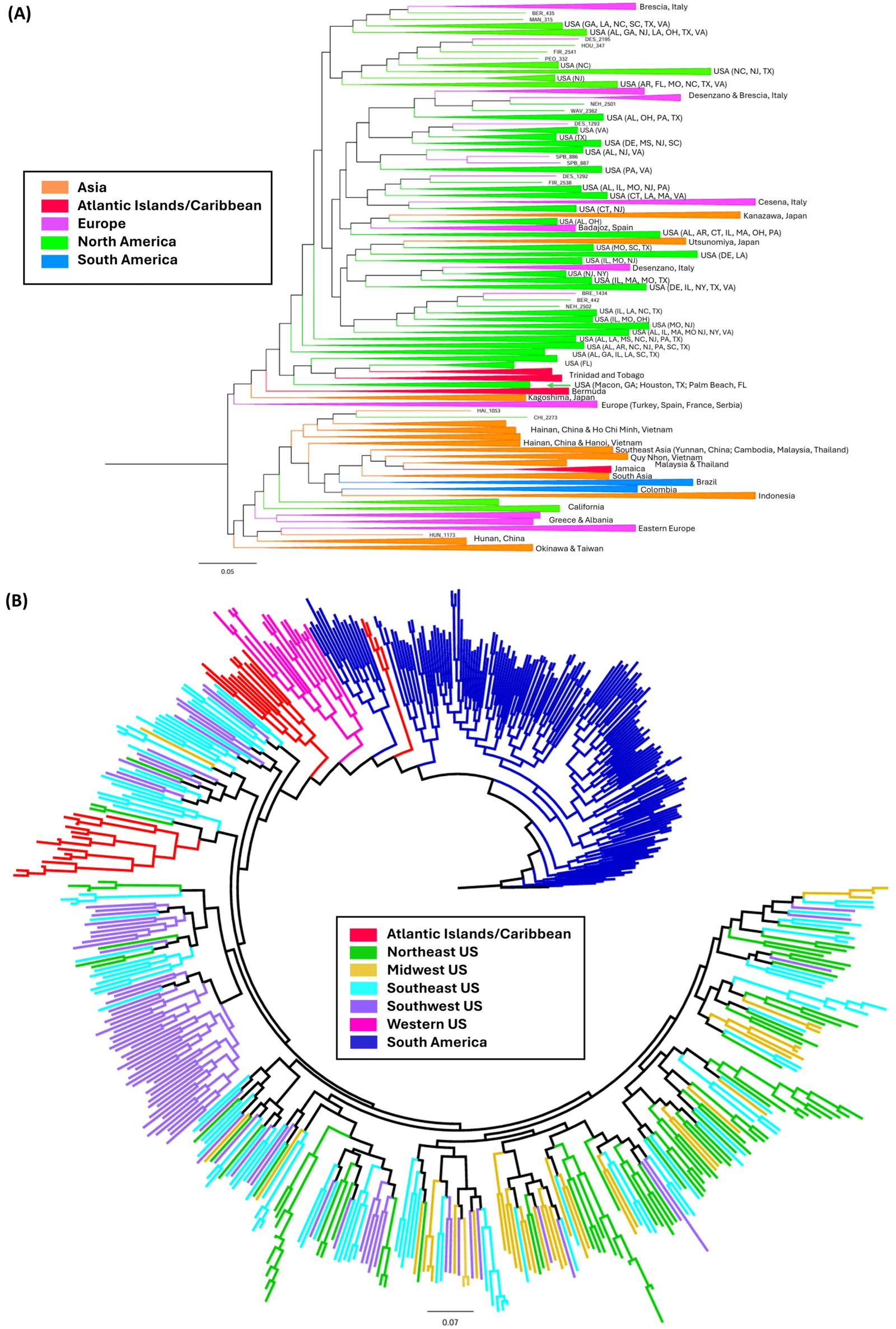
(**A**) Tree created with IQ-TREE for all individuals in the Global dataset, colored by region (orange = Asia, red = Atlantic Islands/Caribbean, purple = Europe, green = North America, blue = South America). Clades with multiple mosquitoes from the same location have been collapsed into a single branch for visualization. The full tree showing the placement of each individual mosquito in the Global dataset is shown in Figure S13. (**B**) Tree created with IQ-TREE for all individuals in the American dataset, with labels colored by subregion (red = Atlantic Islands/Caribbean; green = Northeast, US; gold = Midwest, US; cyan = Southeast, US; purple = Southwest, US; magenta = Western, US; blue = South America).

The relationships among populations identified with IQ-TREE were generally consistent with the structure identified by other methods (PCA, DAPC, and Bayesian clustering algorithms). In the Global dataset, the populations were divided into two major groups. The first contained all mosquitos from all locations in South and Southeast Asia, South America, and the Western United States (Chino and Los Angeles, CA, USA), and a subset of populations from East Asia (China, Taiwan, and Okinawa, Japan), the Caribbean (Jamaica), and Europe (all individuals from Greece, Albania, Georgia, and Russia, and one sample from France). All Brazilian samples clustered into a distinct clade, most closely associated with the branch containing mosquitoes from South and Southeast Asia and Jamaica. The Colombian samples all grouped together and were most closely associated with mosquitoes from Indonesia. The Western US (Californian) samples grouped with samples from East Asia (especially Taiwan and Hunan, China), the population from Ho Chi Minh, Vietnam, and the samples from Greece, Albania, and far Eastern Europe (Figure 5A and Figure S13).

The second major group contained all individuals from the three northernmost Japanese populations, as well as Bermuda, Trinidad and Tobago, all North American populations outside of California, and the majority of European populations (Turkey, Serbia, and all of Western and Southern Europe, except Albania and Greece). Within this second major group, mosquitos from Turkey, Spain, Serbia, and most of France clustered together, and were most closely associated with Kagoshima, Japan. The Bermuda samples clustered between these populations and the cluster that contained Trinidad and Tobago and the rest of North America. In many locations within the United States, mosquitoes from a single location were distributed across multiple subclades. Interspersed among the United States samples were mosquitos from the three Italian locations and the two remaining Japanese locations (Kanazawa and Utsunomiya, Figure 5A and Figure S13). The tree of the American dataset was consistent with the placement of American populations within the Global dataset’s tree (Figure 5B and Figure S14).

The admixture graphs generated in TreeMix using both the subset of populations from North America and Europe and the subset from South America confirmed the general clustering patterns observed in the PCA, fastStructure, and IQ-TREE analyses. For the subset comprising North America and Europe the optimal number of migration edges (*m*) identified was four, indicating multiple admixture events both between the native and invasive ranges and among populations within the invasive range (Figure S15). In the graphs of South America and the native range subset, the optimal number of migration edges (*m*) was one, between Japan and Eastern Brazil (Figure S16).

### 3.3. Local PCA

**Figure 6:**
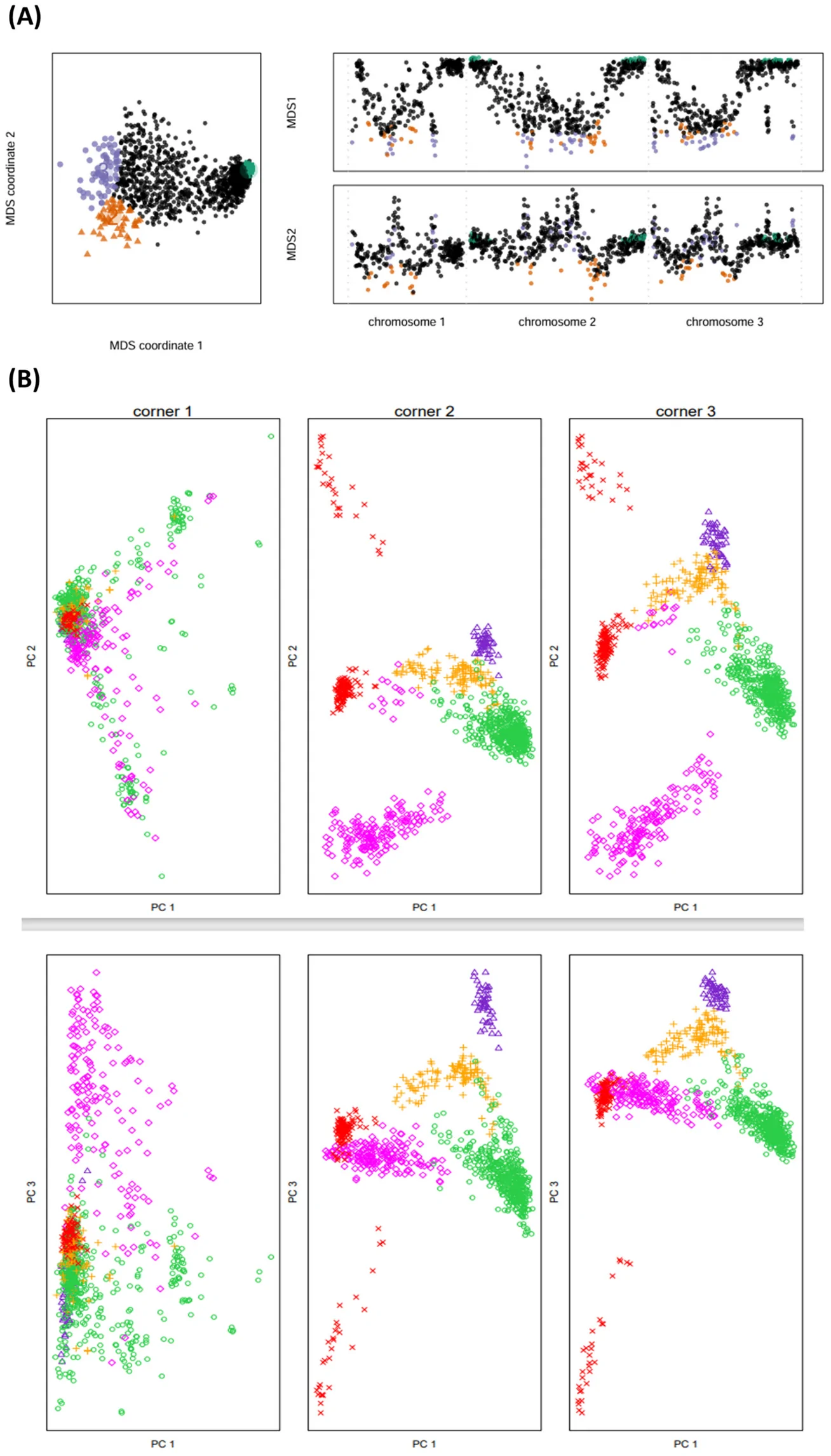
Results of local PCA run using the R package lostruct for *Aedes albopictus*. (**A**) (**Left**): Multidimensional scaling coordinates (MDS 1 on *x*-axis, MDS 2 on *y*-axis) for 50 SNP windows, with the outlier points in the three set of extreme genomic windows (“corners”) highlighted in green, orange, and purple. (**Right**): MDS1 (**top**) and MDS2 (**bottom**) values for windows across the genome for each of the three chromosomes. (**B**) Principal component plots for each of the three “corners” colored in panel (**A**): the first column shows the combined PCA plots for windows in corner 1 (green points in panel (**A**)), the second is for corner 2 (orange points in panel (**A**)); and the third is for corner 3 (purple points in panel (**A**)). In all PCA plots, each point represents a sample, and the colors and shapes of the point represent its population group/clade in the neighbor-joining tree shown in Figure S17.

To complement our genome-wide analyses, we used local PCA to investigate how population structure differed along the genome. The local PCA approach of lostruct [100] performs PCAs on a set of non-overlapping genomic windows (one PCA per window), and then clusters genomic windows based on a multidimensional scaling (MDS) analysis of the dissimilarity between these local PCAs. For our dataset, the majority of variation between local PCAs was be explained by the first MDS coordinate, which broadly differentiates the beginning and end of chromosome 2 and the latter half of chromosome 3 (“corner 1”, green points in Figure 6A and Figure S18) from the rest of the genome (“corner 2”, orange points, and “corner 3”, purple points, in Figure 6A and Figure S18).

Principal component analyses from these three corners are shown in Figure 6B. For corners 2 and 3, the local PCAs generally resembled the global PCA (Figure 2), with PC1 separating populations from tropical regions (i.e., South and Southeast Asia, South America) from more temperate regions (Europe and North America). In contrast, for corner 1, PC3 separated points from South America from other regions, especially the most temperate regions, North America and Europe. Plotting PC3 from corner 1 against latitude confirmed a statistically significant correlation (*R* = −0.76, *p* = 2.03 × 10^−176^) when all regions were included, but this trend was driven primarily by the South American region and a significant correlation between PC3 and latitude in the Americas was no longer observed when South America was excluded (Figure S19).

### 3.4. Enrichment Analysis

We ran a KEGG Over-Representation Analysis (ORA) on the top 5% of outliers in each corner identified by our local PCA. This identified a total of eight significant pathways (six in corner 1, two in corner 2, and none in corner 3). For corner 1, the significant pathways identified were related to apoptosis (two), sphingolipid metabolism, mitophagy, glycerophospholipid metabolism, and efferocytosis, and for corner 2 the pathways involved biosynthesis of amino acids and ECM-receptor interaction (Table S8).

## 4. Discussion

Population genetic analyses of *Ae. albopictus* in the Americas revealed both clear regional differentiation and widespread admixture, suggesting an invasion history shaped by multiple introductions and ongoing gene flow. Thus, both of our hypotheses were supported. Our multiple analytic approaches (PCA, Bayesian clustering, and phylogenetic inference) consistently indicated invasive populations from North America showed genetic affinity with populations from both the native range and Europe, but were distinct from invasive South American populations. Overall, our findings highlight the importance of repeated introductions and subsequent admixture in shaping the genetic structure of populations of *Ae. albopictus* across their invasive range.

### 4.1. Global Population Structure and the Origins of Invasions

North American populations in our study all showed genetic affinity with East Asia and parts of Europe, but fell into two broad groups: one genetically similar to Japan, and the other genetically similar to more southeastern parts of the native range. Specifically, our findings across multiple analyses indicate that *Ae. albopictus* across the eastern half of the United States, from Texas to New England, are most genetically similar to populations sampled in Japan and parts of southwestern Europe (Italy, Spain) (Figure 2 and Figure 3). On the other hand, our two populations from the western United States were genetically similar to eastern China, Taiwan, Okinawa and more eastern areas of southern Europe (Greece, Albania and Turkey) (Figure 2 and Figure 3). This general pattern was consistent with a previous study which found that mtDNA haplotypes from California populations were distinct from those collected in the eastern United States [20].

Our findings that the central and eastern United States share genetic ancestry with Japan and parts of Europe reinforce the results of studies based on SNPs from ddRAD that also found evidence of genetic linkage between these regions [47,73]. This pattern is also consistent with older studies that used other types of genetic markers (e.g., mtDNA haplotypes, microsatellites, allozymes) and found that populations from Texas [70], the Northeast [53], Midwest [72], and Southeast [74,77] regions of the United States all shared haplotypes with *Ae. albopictus* in Japan and/or Europe. It is thought that North American populations served as a source for the invasion of *Ae. albopictus* into Europe, specifically the introduction of populations to northern Italy around 1990 [103,104]. Our results are consistent with this proposed scenario, in which mosquitoes likely introduced into the United States from Japan [47,53,68,70,71,72,73] may have served as a source for further invasions into Europe and other areas of the central and eastern United States.

In contrast to North America, mosquitoes from parts of the Caribbean and South America typically showed ancestry distinct from the invasive populations in North America and Europe. Jamaica clearly clustered with South and Southeast Asia, and while Colombia and Trinidad and Tobago both showed evidence of substantial admixture, components of their genetic ancestry were shared with South and Southeast Asian populations as well (Figure 2 and Figure 3). This pattern was also observed in our phylogenetic analyses, which indicated two major global lineages that generally separated tropical populations in South and Southeast Asia and South America from more temperate ones in Japan, Europe, and most of North America. There were, however, a few notable exceptions to this pattern, such as California, which was placed with populations from South and Southeast Asia rather than with the other populations from the US (Figure 5). This finding further supports a separate introduction of *Ae. albopictus* to the west coast of North America, unrelated to the introduction(s) from Japan that originated the invasive populations in the eastern half of the continent [47,53,68,70,71,72,73].

One of our most striking findings across all analyses was that Brazilian and Argentinian populations consistently formed their own distinct cluster. This could be due to either an independent introduction history from population(s) in the native range that were not included in our dataset, or processes such as founder effects, genetic drift, and/or local adaptation that have occurred post-introduction. Our TreeMix analysis of South American populations, which identified fewer migration edges compared to North America and Europe, further supports a scenario of reduced gene flow between Brazil and Argentina and other areas following their initial colonization (Figures S15 and S16).

Our finding that *Ae. albopictus* from South America are genetically distinct from other populations sampled in either the invasive or native ranges is consistent with the results of several previous studies. Analyses of SNPs obtained from ddRAD found that mosquitoes from Brazil formed their own cluster, distinct from those in the US, Europe, and parts of East Asia [73]. Likewise, earlier analyzes that utilized mtDNA found unique haplotypes in Brazil [76,77,78], and high genetic differentiation between Brazilian and North American samples [105]. Brazilian populations were also found to differ phenotypically from North American *Ae. albopictus*, in that they lacked the ability to undergo photoperiodic diapause [51]. Within Brazil, our analyses indicated admixture in most populations, and a degree of substructure (Figure 3B). This is consistent with previous studies that identified high genetic diversity among Brazilian populations, pointing to multiple introduction events within South America [80,105].

Although mosquitoes from South America were differentiated from all other invasive populations sampled in our study, our maximum likelihood tree shows that they were most closely related to the populations in our dataset from South and Southeast Asia (Figure 5). This is consistent with some analyses using mtDNA haplotypes which found that populations from southeastern Brazil clustered most closely with populations from Thailand and Bhutan [106], and a study that found that populations of mosquitoes in both the north and south of Brazil shared a voltage-gated sodium channel haplotype with Malaysian *Ae. albopictus* [80].

We observed substantial admixture across many populations, particularly in eastern and central North America, the Caribbean, and parts of Europe (Figure 3). This admixture suggests that these introduced populations have experienced repeated introductions and gene flow. In addition to the results of our Bayesian clustering analyses, our phylogenetic and admixture graph analyses, which identified multiple migration edges linking populations across continents, further support this interpretation (Figure 5 and Figure S15). Overall, it seems likely that different regions of the Americas experienced independent introduction events from distinct sources, supporting our first hypothesis.

### 4.2. Regional Population Structure Within the Americas

When we analyzed American populations independently, we identified three main clusters: (1) the eastern and central United States along with Bermuda, (2) a group including the Caribbean, Colombia, and the western United States (California), and (3) Brazil and Argentina (Figure 3). The high levels of admixture within eastern North American populations indicate ongoing connectivity among regions, supporting our second hypothesis.

We found no strong evidence for isolation by distance within the Americas (Figure 4). This, along with the absence of strong geographic structuring within the United States, aside from the differentiation of California, is consistent with a scenario in which *Ae. albopictus* has undergone a recent rapid expansion from a common source and/or spread across each continent by long-distance, human-mediated dispersal. Dispersal through long-distance jumps can rapidly connect distant populations, contribute to admixture, and obscure geographic patterns of differentiation, and has been observed in other invasive insect species [107,108,109]. This finding has important implications for both evolutionary dynamics and management strategies. It implies that local populations may not be evolving in isolation and that adaptive alleles may spread across large geographic areas. It also highlights the importance of monitoring transportation networks, as they may play a more significant role than geographic proximity in shaping range expansion and population structure of *Ae. albopictus* in the Americas.

### 4.3. Variation in Structure Along the Genome and Candidates for Selection

Our local PCA analyses suggest that population structure is not uniform across the genome. While most genomic regions recapitulated the general pattern identified by the global PCA, a subset of windows (“corner 1”) exhibited a distinct pattern, with South American populations clustering separately from others (Figure 6). While the strong correlation between this signal and latitude was driven largely by South American mosquitoes, it is nonetheless consistent with the hypothesis that region-specific processes may be influencing these genomic segments. The heterogeneity in structure we observed along the genome could be explained by selection, reduced recombination, or structural variants that maintain locally adapted allele combinations. Our findings highlight the importance of examining fine-scale genomic variation, as examining genome-wide summaries alone may obscure these patterns.

Our KEGG Over-Representation Analysis (ORA) identified enrichment of several pathways involved in processes such as the cellular stress responses and metabolism, which may be linked to environmental tolerance and development. Specifically, in our corner 1 outlier windows, which represented regions of the genome where structure was most dissimilar to that which we identified using genome-wide SNPs, our ORA identified significant pathways related to apoptosis (two), sphingolipid metabolism, mitophagy, glycerophospholipid metabolism, and efferocytosis. Of particular interest are the pathways related to sphingolipids and glycerophospholipid metabolism, which may be involved in adaptations to cold tolerance, and the pathway related to mitophagy, which could be involved in adaptations enabling metabolic suppression during diapause.

Diapause is an alternative developmental trajectory, in which development is arrested and metabolism is suppressed in anticipation of unfavorable seasonal conditions [110]. It is thought to be key factor that has allowed many insects, including *Ae. albopictus*, to adapt to temperate climates and expand their ranges to higher latitudes [49,111,112,113]. It has also been shown that strong selection and rapid evolution of diapause in mosquitoes can be caused by differences in winter conditions [50]. While diapause is a complex program that may involve the expression of multiple traits through a variety of mechanisms [114], there is evidence from other insect species that increased mitophagy is an important enabler of metabolic suppression during diapause [115,116]. Similarly, KEGG analyses in other insects have identified glycerophospholipid and sphingolipid metabolism as potentially important for stress resistance and maintaining energy reserves during diapause [117]. Glycerophospholipids can also act as cryoprotectants to improve cold tolerance in diapausing insects [117,118].

Overall, the fact that our ORA for the extreme regions of the genome, which strongly separated tropical and temperate invasive populations of *Ae. albopictus*, were enriched in pathways potentially involved in diapause and cold tolerance suggests that there may have been selection for these traits which has allowed mosquitoes to adapt to novel environments and successfully establish invasive populations. This is consistent with previous findings that eggs of *Ae. albopictus* in temperate populations are more cold-tolerant than those from more tropical locations [119,120,121]. While further analyses and functional validation are needed to confirm this possibility, our identification of these candidate pathways adds to a foundation for future studies aimed at identifying traits relevant to the invasion success of *Ae. albopictus*.

### 4.4. Limitations and Future Directions

Though our dataset contained populations from more than 90 locations, some regions remain underrepresented, particularly within the native range, which may have limited our ability to precisely identify source populations. Uneven and relatively small sample sizes for some locations, and known limitations of methods for estimating network structure from *f*-statistics, may have also influenced our population structure results to an extent [122,123]. Additionally, given that the genetic makeup of invasive populations may change over time, the temporal heterogeneity of our samples may have influenced our inferences. While our analyses identified candidate regions and pathways that may be under selection, additional analyses are needed to determine the biological significance of the genomic regions identified by our local PCA. Selection scans using whole genome sequencing data and functional validation would be particularly beneficial for identifying regions of genome that may be undergoing local adaptation in invasive populations.

Future work that incorporates denser geographic sampling, particularly in the native range, Caribbean, Mexico, and Central America, would be especially useful, as it would help refine our understanding of how *Ae. albopictus* has invaded these regions, and potentially elucidate the origins of the genetically distinct populations we identified in Brazil and Argentina. Additionally, having temporal data to track changes in population structure over time would shed light on the ongoing spread of *Ae. albopictus* as it continues to experience human-mediate gene flow and expand its range in the Americas. Integrating genomic data with ecological and environmental information would also further enhance our understanding of the processes driving invasion and local adaptation. This could be informative for understanding the substructure we observed in some regions, particularly invasive Brazilian populations (Figure 3B), which occupy a wide range of biomes, from Amazonian forest to urban environments.

## 5. Conclusions

Our results demonstrate that invasive *Ae. albopictus* mosquito populations in the Americas have arisen through numerous introduction events from multiple regions of the native range, followed by extensive admixture and gene flow. Our findings are broadly consistent with previous studies, but our use of genome-wide data and local PCA approaches provide greater resolution. The pronounced differentiation of South American populations, the distinctiveness of California and Jamaica from other populations in the same region, and the absence of isolation by distance all point to a complex invasion history shaped by repeated introductions and ongoing long-distance dispersals facilitated by humans. The heterogeneous patterns of differentiation across the genome and the identification of candidate pathways suggest that local adaptation may play an important role in the continued spread and persistence of these populations, and can provide hypotheses for future functional validation studies. Together, our findings provide new insights into the dynamics of biological invasions and have implications for the management of an important globally distributed disease vector.

## Data Availability

This research involved international partnerships with scientists across many countries. All collaborators providing genetic samples were invited to be co-authors, and the data and results have been made available in public databases. The raw data files used in the analyses are available on Zenodo (Americas: https://doi.org/10.5281/zenodo.20646598, native range: https://zenodo.org/records/10048029 (accessed on 18 June 2026), Europe: https://doi.org/10.5281/zenodo.13760661). The codes describing the step-by-step of all analyses are also available at https://doi.org/10.5281/zenodo.20646598. An overview of the analyses contained in each RMarkdown file is provided in Table S1.

## Supplementary Materials

The following supporting information can be downloaded at: https://www.mdpi.com/article/10.3390/insects17080755/s1, An overview of supplementary files is provided in Table S1.
