# Supplementary material for: Population Structure of the Invasive Asian Tiger Mosquito, Aedes albopictus, in the Americas
Source: Insects. 2026 Jul 23;17(8):755. doi: 10.3390/insects17080755 (PMC13512968; doi:10.3390/insects17080755)
Supplement: Supplementary file 1 [file insects-17-00755-s001.zip › RMarkdowns/S10 - Local PCA and enrichment analyses.html]

Local PCA using lostruct & KEGG Enrichment Analysis using clusterProfiler


# Local PCA using lostruct & KEGG Enrichment Analysis using clusterProfiler

#### Margaret Corley

#### 2026-02-04

# A. Local PCA

# 1. Install and load packages

Information on Local PCA (lostruct package): https://github.com/petrelharp/local\_pca https://academic.oup.com/genetics/article/211/1/289/5931130

```
library(devtools)
```

```
## Loading required package: usethis
```

```
library(data.table)
#devtools::install_github("petrelharp/local_pca/lostruct") #install lostruct
library(lostruct)
library(here)
```

```
## here() starts at /gpfs/gibbs/pi/caccone/mkc54/albo
```

```
#install_github("petrelharp/templater")
library(templater)
```

We will use the template available on GitHub for the lostruct
analysis. I downloaded the zip file from GitHub, then copied it to the R
project directory. We can use the example files in the template to make
sure we have our input files formatted correctly.

We are using the SNP set with MAF 1% & LD 10% pruning (r2\_0.1\_b)
We will use all the populations first, then we can split them according
to their genetic distance (based on N-J tree).

```
cd /gpfs/gibbs/pi/caccone/mkc54/albo
ls local_pca-master/templated
```

```
## README.md
## bcf_and_index.sh
## data
## joined_data
## lostruct_results
## run_lostruct.R
## summarize_run.Rmd
```

Check the bcf\_and\_index.sh

```
#chmod +x local_pca-master/templated/bcf_and_index.sh #changer permissions on script
./local_pca-master/templated/bcf_and_index.sh
```

Okay. We can create a vcf for each chromosome now.

# 2. Create files for each chromosome

We’re using the files after all QC (filtering the LD 10% and MAF
1%)

## 2.1 Create tped files for each chromosome

```
cd /gpfs/gibbs/pi/caccone/mkc54/albo/
module load PLINK/1.9b_6.21-x86_64

# For chromosome 1
plink \
--allow-extra-chr \
--keep-allele-order \
--bfile manuscript1/output/snps_sets/r2_0.1_b \
--chr 1 \
--recode transpose \
--out manuscript1/output/local_pca/chrom1 \
--silent;
grep "people\|variants" manuscript1/output/local_pca/chrom1.log
```

14332 out of 61574 variants loaded from .bim file. 933 people (93
males, 61 females, 779 ambiguous) loaded from .fam. 14332 variants and
933 people pass filters and QC.

```
# For chromosome 2
plink \
--allow-extra-chr \
--keep-allele-order \
--bfile manuscript1/output/snps_sets/r2_0.1_b \
--chr 2 \
--recode transpose \
--output-missing-genotype 0 \
--silent \
--out manuscript1/output/local_pca/chrom2;
grep "people\|variants" manuscript1/output/local_pca/chrom2.log
```

25373 out of 61574 variants loaded from .bim file. 933 people (93
males, 61 females, 779 ambiguous) loaded from .fam. 25373 variants and
933 people pass filters and QC.

```
# For chromosome 3
plink \
--allow-extra-chr \
--keep-allele-order \
--bfile manuscript1/output/snps_sets/r2_0.1_b \
--chr 3 \
--recode transpose \
--output-missing-genotype 0 \
--silent \
--out manuscript1/output/local_pca/chrom3;
grep "people\|variants" manuscript1/output/local_pca/chrom3.log
```

21869 out of 61574 variants loaded from .bim file. 933 people (93
males, 61 females, 779 ambiguous) loaded from .fam. 21869 variants and
933 people pass filters and QC.

Compress them

```
# For chromosome 1
gzip -c manuscript1/output/local_pca/chrom1.tped > manuscript1/output/local_pca/chrom1.tped.gz

# For chromosome 2
gzip -c manuscript1/output/local_pca/chrom2.tped > manuscript1/output/local_pca/chrom2.tped.gz

# For chromosome 3
gzip -c manuscript1/output/local_pca/chrom3.tped > manuscript1/output/local_pca/chrom3.tped.gz
```

Check the files

```
list.files(path = "/gpfs/gibbs/pi/caccone/mkc54/albo/manuscript1/output/local_pca/")
```

## 2.2 Create vcf files for each chromosome

```
cd /gpfs/gibbs/pi/caccone/mkc54/albo

# For chromosome 1, converting to VCF format
plink \
--allow-extra-chr \
--keep-allele-order \
--tfile manuscript1/output/local_pca/chrom1 \
--recode vcf \
--out manuscript1/output/local_pca/chrom1 \
--silent;

# For chromosome 2, converting to VCF format
plink \
--allow-extra-chr \
--keep-allele-order \
--tfile manuscript1/output/local_pca/chrom2 \
--recode vcf \
--out manuscript1/output/local_pca/chrom2 \
--silent;

# For chromosome 3, converting to VCF format
plink \
--allow-extra-chr \
--keep-allele-order \
--tfile manuscript1/output/local_pca/chrom3 \
--recode vcf \
--out manuscript1/output/local_pca/chrom3 \
--silent;

# all chromosomes
plink \
--allow-extra-chr \
--keep-allele-order \
--bfile manuscript1/output/snps_sets/r2_0.1_b \
--recode vcf \
--out manuscript1/output/local_pca/all_chrs \
--silent;
```

Load bcftools

```
module load BCFtools/1.21-GCC-12.2.0
```

Use the script they gave us to convert to bcf and index it

```
./local_pca-master/templated/bcf_and_index.sh manuscript1/output/local_pca/chrom1.vcf manuscript1/output/local_pca/chrom2.vcf manuscript1/output/local_pca/chrom3.vcf manuscript1/output/local_pca/all_chrs.vcf
```

Converting manuscript1/output/local\_pca/chrom1.vcf to
manuscript1/output/local\_pca/chrom1.bcf (and indexing). Converting
manuscript1/output/local\_pca/chrom2.vcf to
manuscript1/output/local\_pca/chrom2.bcf (and indexing). Converting
manuscript1/output/local\_pca/chrom3.vcf to
manuscript1/output/local\_pca/chrom3.bcf (and indexing). Converting
manuscript1/output/local\_pca/all\_chrs.vcf to
manuscript1/output/local\_pca/all\_chrs.bcf (and indexing).

Check the example data

```
ls /gpfs/gibbs/pi/caccone/mkc54/albo/local_pca-master/templated/data
```

```
## sample_info.tsv
```

They also have a sample\_info.tsv file, with population names in the
“data” directory

Now we have to create a “sample\_info.tsv” file for our data. We need
to get the population information based on our neighbor-joining tree
first.

## 2.3 Create sample\_info file

Using new NJ-trees created with Hudson’s Fst We will cluster by the
NJ tree: Open the NJ tree we created. Then select the clade, hit ctrl C
and then ctrl V in the tree\_string between the curly brackets. Do this
for each clade in the tree and create a tsv file for each.

Cluster 1: STL-MAN - USA, Japan, Europe (green)

```
import pandas as pd
import os
import re  # Import the regex module

# Create the directory if it doesn't exist
output_dir = 'manuscript1/output/local_pca'
os.makedirs(output_dir, exist_ok=True)

# Tree string provided by the user
tree_string = """
(((STL:0.02027605921,WAV:0.01654665847):0.0006691027535,BER:0.01787895303):0.0009787291906,((((((KAN:0.05730248568,LIN:0.02375582915):0.003206260512,(UTS:0.02611568351,SPR:0.02655496265):0.004746127175):0.002362946117,SPB:0.01758671584):0.001377670772,((BRE:0.03951895281,DES:0.02973274957):0.005537559583,CES:0.06801341828):0.005988969664):0.001366573382,SER:0.121140836):0.001095982671,FRS:0.02189222148):0.005322066292,KAG:0.03797241891):0.004359406809,BRM:0.05420383263):0.002835025919):0.0008812922605,((HAL:0.008088857207,COL:0.01555197128):0.0006433426898,PEO:0.01002437864):0.002684888583):0.0007119770991,(((NEH:0.009078229121,NEB:0.01565743674):0.002962480039,FIR:0.04099330256):0.00051150751,((((((DEN:0.009145436159,NEW:0.02091024743):0.0005337312006,HAT:0.01707014259):0.001459816864,(((((TRA:0.006987650047,BEA:0.007860368684):0.001129344919,SAI:0.03308377804):0.004070113589,PAL:0.02381658766):0.002875175997,MAC:0.0144877373):0.0008738602746,HOU:0.005850604941):0.002683830883):0.002394193128,FAY:0.01036197637):0.0007847556679,(((NUE:0.009191605252,MON:0.004249458821):0.003035619099,SMI:0.01054598726):0.001932327627,DAL:0.006040811836):0.005902332822):0.0005925741119,NEO:0.01964720886):0.001628206779):0.001397308334,RUS:0.04286069001):0.000942123184,HAR:0.02264539474):0.001075995907,MAN:0.0108553762):0.0007084902383):0.000333465455);
"""

# Extract population names using regex (before each ":")
populations = re.findall(r'([A-Z]+):', tree_string)

# Create a DataFrame with one column
df_populations = pd.DataFrame(populations, columns=["Population"])

# Save the DataFrame to a TSV file with one column
df_populations.to_csv(f'{output_dir}/clade_1_STL-MAN_green.tsv', sep='\t', index=False, header=False)
```

Cluster 2: Eastern Europe - SOC-TIK (purple)

```
import pandas as pd
import os
import re  # Import the regex module

# Create the directory if it doesn't exist
output_dir = 'manuscript1/output/local_pca'
os.makedirs(output_dir, exist_ok=True)

# Tree string provided by the user
tree_string = """
tree tree_1 = [&R] (((SOC:0.004409216633,GES:0.008773911394):0.004098662725,RAR:0.01266153153):0.00766894537,TIK:0.02647323205):0.03862187738):0.002047598679,
"""

# Extract population names using regex (before each ":")
populations = re.findall(r'([A-Z]+):', tree_string)

# Create a DataFrame with one column
df_populations = pd.DataFrame(populations, columns=["Population"])

# Save the DataFrame to a TSV file with one column
df_populations.to_csv(f'{output_dir}/clade_2_SOC_TIK_purple.tsv', sep='\t', index=False, header=False)
```

Cluster 3: HAI-SPS (orange)

```
import pandas as pd
import os
import re  # Import the regex module

# Create the directory if it doesn't exist
output_dir = 'manuscript1/output/local_pca'
os.makedirs(output_dir, exist_ok=True)

# Tree string provided by the user
tree_string ="""
tree tree_1 = [&R] (((((((((((((HAI:0.0153553169,HOC:0.01973058195):0.0006568269264,HAN:0.01979846137):0.006375908939):0.003111311371,CHI:0.03580228465):0.004577226844,GRA:0.02070793239):0.00239092865,LOS:0.04073920687):0.0008308021212,HUN:0.01316245579):0.002441884503,(OKI:0.04146199965,TAI:0.08545584167):0.00546606298):0.002087522716,((TUH:0.02355035574,SPS:0.05737880171):0.001385928768,ALD:0.04752471786):0.002476919485):0.0007971402015,
"""

# Extract population names using regex (before each ":")
populations = re.findall(r'([A-Z]+):', tree_string)

# Create a DataFrame with one column
df_populations = pd.DataFrame(populations, columns=["Population"])

# Save the DataFrame to a TSV file with one column
df_populations.to_csv(f'{output_dir}/clade_3_HAI-SPS_orange.tsv', sep='\t', index=False, header=False)
```

Cluster 4: Asia 1 - SSK-KAT (red)

```
import pandas as pd
import os
import re  # Import the regex module

# Create the directory if it doesn't exist
output_dir = 'manuscript1/output/local_pca'
os.makedirs(output_dir, exist_ok=True)

# Tree string provided by the user
tree_string = """
tree tree_1 = [&R] ((((((((((((SSK:0.00561958891,CHA:0.004163813741):0.0006483530416,YUN:0.004833555297):0.0002948873398,LAM:0.00744133066):0.0005186073855,KAC:0.005791858786):0.0007502673395,BEN:0.01695339946):0.001583440573,MAT:0.01561699499):0.002292825007,CAM:0.003888670202):0.002596614229,QNC:0.06087137731):0.003584805305,KLP:0.04077762541):0.001228637193,NEG:0.1070018398):0.001082113955,((((INW:0.05461642978,INJ:0.012689059):0.0103781405,SUF:0.02650153503):0.02210700187,SUU:0.008414202169):0.01930025251,KAT:0.01148861292):0.02301938234):0.01226600054,
"""

# Extract population names using regex (before each ":")
populations = re.findall(r'([A-Z]+):', tree_string)

# Create a DataFrame with one column
df_populations = pd.DataFrame(populations, columns=["Population"])

# Save the DataFrame to a TSV file with one column
df_populations.to_csv(f'{output_dir}/clade_4_SSK-KAT_red.tsv', sep='\t', index=False, header=False)
```

Clade 5: South America = REC-MED (magenta)

```
import pandas as pd
import os
import re  # Import the regex module

# Create the directory if it doesn't exist
output_dir = 'manuscript1/output/local_pca'
os.makedirs(output_dir, exist_ok=True)

# Tree string provided by the user
tree_string = """
tree tree_1 = [&R] (((((((REC:0.0107061996,BPA:0.01708733394):0.01276584045,(((((((FLO:0.01023835546,PLM:0.009776625179):0.002127349737,BAF:0.005152329207):0.002358324577,(TUC:0.007082839017,ARA:0.003995829435):0.00237903607):0.0008409067814,BCO:0.014645935):0.01050882309,BIT:0.02268067145):0.001655226313,((((IPO:0.0218734182,NOV:0.01218874585):0.01574630824,POR:0.00917905715):0.006094342874,BEH:0.01678421638):0.01201513205,GRV:0.0195139899):0.008600460076):0.001905355706,(MAU:0.0118163193,BPV:0.02171092625):0.005042065554):0.01503801787):0.01352654459,(CUA:0.007031355639,MED:0.004434725905):0.0479736998):0.008563050821,
"""

# Extract population names using regex (before each ":")
populations = re.findall(r'([A-Z]+):', tree_string)

# Create a DataFrame with one column
df_populations = pd.DataFrame(populations, columns=["Population"])

# Save the DataFrame to a TSV file with one column
df_populations.to_csv(f'{output_dir}/clade_5_REC_MED_magenta.tsv', sep='\t', index=False, header=False)
```

Now we can create a sample\_info.tsv using the tsv files we created
for each clade. We first need to create a file with all the names of our
samples. Then we need to create short names for our “populations” that
we selected based on the NJ tree. First, let’s create a file with all
the samples in our dataset

```
bcftools query -l manuscript1/output/local_pca/chrom1.bcf > manuscript1/output/local_pca/samples.txt;
wc -l manuscript1/output/local_pca/samples.txt;
head manuscript1/output/local_pca/samples.txt
```

Create files with new names

```
cd /gpfs/gibbs/pi/caccone/mkc54/albo
cp manuscript1/output/local_pca/clade_1_STL-MAN_green.tsv manuscript1/output/local_pca/clade1.txt;
cp manuscript1/output/local_pca/clade_2_SOC_TIK_purple.tsv manuscript1/output/local_pca/clade2.txt;
cp manuscript1/output/local_pca/clade_3_HAI-SPS_orange.tsv manuscript1/output/local_pca/clade3.txt;
cp manuscript1/output/local_pca/clade_4_SSK-KAT_red.tsv manuscript1/output/local_pca/clade4.txt;
cp manuscript1/output/local_pca/clade_5_REC_MED_magenta.tsv manuscript1/output/local_pca/clade5.txt;
```

Let’s check the files we have (you should have only the samples.txt
and the new file names we created above)

```
ls /gpfs/gibbs/pi/caccone/mkc54/albo/manuscript1/output/local_pca/*.txt
```

The samples\_info.tsv file is a tab-delimited file with two columns.
The first one is already created in the first column of the samples.txt,
and it is the individual id. We now have to create a second column. We
can create python code to do the following. Split the string from the
first column by “*” since the samples names are like ”OKI\_1001”.
Then, we will use the first string before the ”*” to store it in a
dictionary, named “population”. Next, we will create new dictionaries
with each of the text files. The name of the dictionary will be the file
name without the extension “.txt”.

Now, to create the value for the second column of our
sample\_info.tsv, we will have to match the strings in the dictionary
“populations” with the strings of each dictionary we created from the
text files that we categorize as tree\_topology. For example, the string
in the text file samples.txt was OKI\_1001. Then we split it by “\_“, and
stored the first string in the dictionary”population”. Then, after
create the other dictionaries from the text files, we search for the
string “OKI” in each dictionary we created from the text files. Next, we
enter the name of the dictionary we found a match as the value in the
second column of our samples\_info.tsv. So, the output for the example
could be be “OKI BR3”, if the match was found in the dictionary BR3 that
we created from the text file.

```
import os
import pandas as pd

# Load the sample file
samples_df = pd.read_csv('/gpfs/gibbs/pi/caccone/mkc54/albo/manuscript1/output/local_pca/samples.txt', sep='\t', header=None, names=['ID'])

# Create a dictionary to hold population data
populations = {sample_id: sample_id.split('_')[0] for sample_id in samples_df['ID']}

# Directory containing the population files (tree topology group)
directory = '/gpfs/gibbs/pi/caccone/mkc54/albo/manuscript1/output/local_pca'
tree_topology_files = [
    'clade1.txt', 'clade2.txt', 'clade3.txt', 'clade4.txt', 'clade5.txt'
]

# Create dictionaries for each file in tree topology
tree_topology = {}
for filename in tree_topology_files:
    filepath = os.path.join(directory, filename)
    dict_name = filename.replace('.txt', '')  # Dictionary name without extension
    with open(filepath, 'r') as file:
        tree_topology[dict_name] = set(line.strip() for line in file)

# Create the second column by matching population prefixes with tree topology dictionaries
samples_df['Source'] = samples_df['ID'].apply(lambda x: next(
    (key for key, ids in tree_topology.items() if populations[x] in ids), 'Unknown'
))

# Save the result to samples_info.tsv
output_path = os.path.join(directory, 'sample_info.tsv')
samples_df.to_csv(output_path, sep='\t', index=False, header=False)
```

Let’s check the output

```
head /gpfs/gibbs/pi/caccone/mkc54/albo/manuscript1/output/local_pca/sample_info.tsv
```

```
## OKI_1001 clade3
## OKI_1002 clade3
## OKI_1003 clade3
## OKI_1004 clade3
## OKI_1005 clade3
## OKI_1006 clade3
## OKI_1007 clade3
## OKI_1008 clade3
## OKI_1009 clade3
## OKI_1011 clade3
```

We need to add a header to the file

```
cd /gpfs/gibbs/pi/caccone/mkc54/albo
# First, add the header to a new temporary file, then append the original file
printf "ID\tpopulation\n" | cat - manuscript1/output/local_pca/sample_info.tsv > manuscript1/output/local_pca/sample_info_with_header.tsv;
```

Move it to the right directory

```
mv manuscript1/output/local_pca/sample_info_with_header.tsv manuscript1/output/local_pca/templated/data/sample_info.tsv;
#head manuscript1/output/local_pca/templated/data/sample_info.tsv
```

Check it

```
head /gpfs/gibbs/pi/caccone/mkc54/albo/manuscript1/output/local_pca/templated/data/sample_info.tsv
```

```
## ID   population
## OKI_1001 clade3
## OKI_1002 clade3
## OKI_1003 clade3
## OKI_1004 clade3
## OKI_1005 clade3
## OKI_1006 clade3
## OKI_1007 clade3
## OKI_1008 clade3
## OKI_1009 clade3
```

# 3. Run lostruct on data

On the terminal

```
salloc -p scavenge --cpus-per-task=5 -t 2:00:00 --mem-per-cpu=10000  #need to request resources to install lostruct
module load R/4.4.1-foss-2022b
R
library(optparse)
library(lostruct) 
library(devtools)
library(templater)
library(colorspace)
library(RColorBrewer)
library(codetools)
q()
```

Run for 100 SNP windows

```
cd /gpfs/gibbs/pi/caccone/mkc54/albo/manuscript1/output/local_pca/templated;
./run_lostruct.R -i data -t snp -s 100 -I data/sample_info.tsv -j 0001
```

Run for 50 SNP windows

```
cd /gpfs/gibbs/pi/caccone/mkc54/albo/manuscript1/output/local_pca/templated;
./run_lostruct.R -i data -t snp -s 50 -I data/sample_info.tsv -j 0001
```

Finding PCs for chromsome chr1 in file
/gpfs/gibbs/pi/caccone/mkc54/albo/manuscript1/output/local\_pca/templated/data/chr1.bcf
and writing out to
lostruct\_results/type\_snp\_size\_50\_weights\_none\_jobid\_0001/chr1.pca.csv
and lostruct\_r
esults/type\_snp\_size\_50\_weights\_none\_jobid\_0001/chr1.regions.csv Finding
PCs for chromsome chr2 in file
/gpfs/gibbs/pi/caccone/mkc54/albo/manuscript1/output/local\_pca/templated/data/chr2.bcf
and writing out to
lostruct\_results/type\_snp\_size\_50\_weights\_none\_jobid\_0001/chr2.pca.csv
and lostruct\_r
esults/type\_snp\_size\_50\_weights\_none\_jobid\_0001/chr2.regions.csv Finding
PCs for chromsome chr3 in file
/gpfs/gibbs/pi/caccone/mkc54/albo/manuscript1/output/local\_pca/templated/data/chr3.bcf
and writing out to
lostruct\_results/type\_snp\_size\_50\_weights\_none\_jobid\_0001/chr3.pca.csv
and lostruct\_r
esults/type\_snp\_size\_50\_weights\_none\_jobid\_0001/chr3.regions.csv Warning
messages: 1: In any(chrom.wins) : coercing argument of type ‘double’ to
logical 2: In vcf\_windower\_snp(file = file, sites = sites, size = size,
samples = samples) : Trimming from chromosome ends: 1: 32 SNPs. 3: In
any(chrom.wins) : coercing argument of type ‘double’ to logical 4: In
vcf\_windower\_snp(file = file, sites = sites, size = size, samples =
samples) : Trimming from chromosome ends: 2: 23 SNPs. 5: In
any(chrom.wins) : coercing argument of type ‘double’ to logical 6: In
vcf\_windower\_snp(file = file, sites = sites, size = size, samples =
samples) : Trimming from chromosome ends: 3: 19 SNPs. Done finding PCs,
computing distances. user system elapsed 133.047 0.214 133.711 Done
computing distances, running MDS and writing results to
lostruct\_results/type\_snp\_size\_50\_weights\_none\_jobid\_0001/mds\_coords.csv
All done![1] “2026-02-17 09:53:55 EST”

Now we can check the results it generated

```
cd /gpfs/gibbs/pi/caccone/mkc54/albo
ls manuscript1/output/local_pca/templated/lostruct_results/type_snp_size_50_weights_none_jobid_0001/
```

```
## cache
## chr1.pca.csv
## chr1.regions.csv
## chr1_outlier_positions.pdf
## chr1_outlier_windows_colored.pdf
## chr2.pca.csv
## chr2.regions.csv
## chr2_outlier_positions.pdf
## chr2_outlier_windows_colored.pdf
## chr3.pca.csv
## chr3.regions.csv
## chr3_outlier_positions.pdf
## chr3_outlier_windows_colored.pdf
## config.json
## figure
## mds_coords.csv
## run_summary.html
## run_summary.md
```

cache chr1.pca.csv chr1.regions.csv chr2.pca.csv chr2.regions.csv
chr3.pca.csv chr3.regions.csv config.json figure mds\_coords.csv
run\_summary.html run\_summary.md

It works. Now we can summarize the output using the templater
library

```
library(templater)
```

On the terminal run (make sure you have pandoc installed).

```
module load Pandoc/3.1.2
module load BCFtools/1.21-GCC-12.2.0
cd /gpfs/gibbs/pi/caccone/mkc54/albo/manuscript1/output/local_pca/templated;
Rscript -e 'templater::render_template("summarize_run.Rmd",output="lostruct_results/type_snp_size_50_weights_none_jobid_0001/run_summary.html",change.rootdir=TRUE)'
```

# 4. Customized plots

```
system("which bcftools")
```

Can get the code from the “summarize\_run.Rmd” file

## 4.1 For 100 SNP windows

Here are the number of windows per chromsome, and the computed MDS
coordinates, colored by chromosome:

```
table(regions$chrom)
```

```
## 
##   1   2   3 
## 143 253 218
```

```
pairs( mds.coords[,mds.cols], pch=20, col=adjustcolor(chrom.cols,0.75) )
```

```
if (do.pdfs) { pdf_copy() }
```

```
pdf("/gpfs/gibbs/pi/caccone/mkc54/albo/manuscript1/output/local_pca/output/final_100kb/MDS_chrom_scatter.pdf", width=6, height=6)
table(regions$chrom)
pairs( mds.coords[,mds.cols], pch=20, col=adjustcolor(chrom.cols,0.75) )
dev.off()
```

Here are the extreme windows in the MDS plot:

```
mds.corners <- corners( mds.coords[,mds.cols[1:2]], prop=.05 )
# set up colors and pchs for corners
corner.cols <- brewer.pal(3,"Dark2")
corner.pch <- c(15,17,19)
ccols <- rep("black",nrow(mds.coords))
cpch <- rep(20,nrow(mds.coords))
for (k in 1:ncol(mds.corners)) {
    ccols[ mds.corners[,k] ] <- corner.cols[k]
    cpch[ mds.corners[,k] ] <- corner.pch[k]
}
# centroids of the corners in MDS space
corner.mds <- do.call(rbind, lapply(1:ncol(mds.corners), 
                function (ii){
                    colMeans(mds.coords[mds.corners[,ii],-(1:2)])
                } ) )
```

```
spacing <- 1
opar <- par(mar=c(4,4,2,1)+.1,mgp=c(2.5,0.8,0))
layout(matrix(c(rep(1,length(mds.cols)),1+seq_along(mds.cols)),ncol=2),
        widths=c(1,2), heights=layout_heights(length(mds.cols),dl=spacing,ncol=2))
plot( mds.coords[,mds.cols[1:2]], pch=cpch, 
     col=adjustcolor(ccols,0.75),  asp=1,
     xaxt='n', yaxt='n',
     xlab="MDS coordinate 1", ylab="MDS coordinate 2" )
points( corner.mds, pch=20, cex=5,
        col=adjustcolor(corner.cols,0.25))
text( corner.mds, labels=seq_len(nrow(corner.mds)), 
        col=corner.cols, cex=2, lwd=2 )
opar2 <- par(mar=c(par("mar"),spacing/2)[c(5,2,3,4)])
for (k in mds.cols) {
    lastone <- (k==mds.cols[length(mds.cols)])
    if (lastone) { par(mar=c(par("mar"),opar2$mar[1])[c(5,2,3,4)]) }
    chrom.plot( mds.coords[,k], pch=20, 
            xlab=if (lastone) { "Position (Mb)"} else { "" }, # main=paste("MDS coordinate",match(k,mds.cols)),
            chrom.labels=lastone,
            ylab=colnames(mds.coords)[k],
            col=adjustcolor(ccols,0.75) )
    # do this for all but first
    par(mar=c(par("mar"),spacing/2)[c(1,2,5,4)])
}
```

```
par(opar)
```

Now, we’ll look at PCA plots from the extracted corners. (this is
done without a ton of memory by accumulating the covariance matrix in
`running_cov`):

```
corner.npc <- 4
corner.regions <- vector(mode='list', length=ncol(mds.corners))
for (k in seq_along(corner.regions)) {
    corner.regions[[k]] <- regions[ mds.corners[,k],]
}
corner.covmats <- vector(mode='list', length=ncol(mds.corners))
for (k in seq_along(corner.covmats)) {
    reg <- regions[ mds.corners[,k], ]
    qfun <- multi_vcf_query_fn( chrom.list=chroms, file=bcf.files, regions=reg )
    corner.covmats[[k]] <- running_cov(qfun,1:nrow(reg), normalize.rows=TRUE)
}
corner.pca <- vector(mode='list', length=ncol(mds.corners))
for (k in seq_along(corner.pca)) {
    corner.pca[[k]] <- cov_pca(covmat=corner.covmats[[k]], k=corner.npc, w=opt$weights)
}
```

Color scheme

```
pop.names <- levels(samps$population)
pop.colors <- c("#2BCE48", "purple3", "orange", "red", "magenta")
pop.pch <- seq_len(nlevels(samps$population))
plot( rep(1,length(pop.names)), seq_along(pop.names), 
       cex=4, pch=pop.pch, col=pop.colors, xlim=c(0,length(pop.names)),
       xlab='', ylab='', xaxt='n', yaxt='n' )
text( rep(1,length(pop.names)), seq_along(pop.names), labels=pop.names, pos=4 )
```

```
for (i in 1:(corner.npc-1)) {
  for (j in (i+1):corner.npc) {

    layout(t(1:3))  # 3 panels horizontally
    for (k in 1:ncol(mds.corners)) {
      vectors <- matrix(corner.pca[[k]][-(1:(1+corner.npc))], ncol = corner.npc)[, c(i, j)]
      colnames(vectors) <- paste("PC", c(i, j))
      par(mgp = c(0.7, 0.7, 0), mar = c(2,2,2,0)+0.1)
      plot(vectors, 
           pch = pop.pch[samps$population], 
           col = pop.colors[samps$population],
           xaxt = 'n', yaxt = 'n')
      if (i == 1 && j == 2) {
        mtext(paste("corner", k), side = 3)
      }
    }

  }
}
```

```
top_regions_MSD1 <- head(regions[order(mds.coords$MDS1, decreasing = TRUE), ], 10)
#write.csv(top_regions_MSD1, file = "/gpfs/gibbs/pi/caccone/mkc54/albo/manuscript1/output/local_pca/output/final_100kb/top_regions_mds1.csv", row.names = FALSE)
```

```
top_regions_MSD2 <- head(regions[order(mds.coords$MDS2, decreasing = TRUE), ], 10)
#write.csv(top_regions_MSD2, file = "/gpfs/gibbs/pi/caccone/mkc54/albo/manuscript1/output/local_pca/output/final_100kb/top_regions_mds2.csv", row.names = FALSE)
```

### 4.1.1 Look at correlation between latitude and PCs

Save the order of samps

```
# Save the original order
original_ids <- samps$id
```

Extract pop names from samps

```
samps$pop_prefix <- sub("_.*", "", samps$id)
```

Import file with latitude

```
sampling_loc <- readRDS(here("scripts", "RMarkdowns", "output", "sampling_loc_all_pops_Oct2025.rds"))
head(sampling_loc)
```

```
##      Pop_City Country Latitude Longitude Continent Abbreviation  Year    Region
## 1      Hainan   China  19.2000  109.6000      Asia          HAI  2014 East Asia
## 2      Yunnan   China  24.4753  101.3431      Asia          YUN  2014 East Asia
## 3       Hunan   China  27.6253  111.8569      Asia          HUN  2001 East Asia
## 4     Okinawa   Japan  26.5013  127.9454      Asia          OKI 2018? East Asia
## 5 Tanegashima   Japan  30.4564  130.9784      Asia          TAN  2008 East Asia
## 6   Kagoshima   Japan  31.5602  130.5581      Asia          KAG  2018 East Asia
##   Subregion order order2 orderold AM_order state FINAL_order Clade
## 1     China    55     47       47       NA                 1     3
## 2     China    56     48       48       NA                 2     4
## 3     China    57     49       49       NA                 3     3
## 4  Southern    54     46       46       NA                 4     3
## 5  Southern    NA     NA       NA       NA                 5    NA
## 6  Southern    53     45       45       NA                 6     1
```

Merge

```
samps <- merge(samps, sampling_loc, by.x = "pop_prefix", by.y = "Abbreviation", all.x = TRUE)
samps <- samps[match(original_ids, samps$id), ]
```

Correlate PC3 with latitude

```
length(corner.pca)
```

```
## [1] 3
```

```
str(corner.pca[[1]], max.level = 1)
```

```
##  num [1:3737] 1055.34 12.94 11.8 9.53 7.8 ...
```

Need to rebuild the matrix for corner 1

```
vectors <- matrix(corner.pca[[1]][-(1:(1 + corner.npc))], ncol = corner.npc)
```

Extract PC3 for corner 1

```
pc3_scores <- vectors[, 3]
```

Test correlation

```
cor_test <- cor.test(pc3_scores, samps$Latitude, method = "pearson")
print(cor_test)
```

```
## 
##  Pearson's product-moment correlation
## 
## data:  pc3_scores and samps$Latitude
## t = -35.681, df = 931, p-value < 2.2e-16
## alternative hypothesis: true correlation is not equal to 0
## 95 percent confidence interval:
##  -0.7858542 -0.7315055
## sample estimates:
##        cor 
## -0.7600053
```

Plot

```
plot(samps$Latitude, pc3_scores,
     pch = 21, bg = adjustcolor(pop.colors[samps$Abbreviation], 0.7),
     xlab = "Latitude",
     ylab = "PC3 score",
     main = paste("PC3 vs Latitude\nr =", round(cor_test$estimate, 3),
                  ", p =", signif(cor_test$p.value, 3)))
```

```
## Warning in matrix(offset, nrow = 4L, ncol = ncol(x)): non-empty data for
## zero-extent matrix
```

```
abline(lm(pc3_scores ~ samps$Latitude), col = "red", lwd = 2)
```

Color by clades

```
# Define your custom colors
pop.colors <- c("#2BCE48", "purple3", "orange", "red", "magenta")

# Make sure the order of colors matches the order of unique regions
region.levels <- unique(samps$Clade)
region.colors <- setNames(pop.colors[seq_along(region.levels)], region.levels)

# Plot PC3 vs Latitude colored by region
plot(samps$Latitude, pc3_scores,
     pch = 21,
     bg = adjustcolor(region.colors[samps$Clade], 0.7),
     xlab = "Latitude",
     ylab = "PC3 score",
     main = paste("PC3 vs Latitude\nr =", round(cor_test$estimate, 3),
                  ", p =", signif(cor_test$p.value, 3)))

# Add regression line across all points
abline(lm(pc3_scores ~ samps$Latitude), col = "red", lwd = 2)

# Add a legend for regions
legend("topright",
       legend = names(region.colors),
       pt.bg = region.colors,
       pch = 21,
       title = "Clade")
```

By Regions

```
# Define your custom colors
pop.colors <- c("red", "purple3", "cyan", "blue", "purple3", "magenta", "orange", "#2BCE48", "purple3")

# Make sure the order of colors matches the order of unique regions
region.levels <- unique(samps$Region)
region.colors <- setNames(pop.colors[seq_along(region.levels)], region.levels)

# Plot PC3 vs Latitude colored by region
plot(samps$Latitude, pc3_scores,
     pch = 21,
     bg = adjustcolor(region.colors[samps$Region], 0.7),
     xlab = "Latitude",
     ylab = "PC3 score",
     main = paste("PC3 vs Latitude\nr =", round(cor_test$estimate, 3),
                  ", p =", signif(cor_test$p.value, 3)))

# Add regression line across all points
abline(lm(pc3_scores ~ samps$Latitude), col = "red", lwd = 2)
```

Regions in Americas only

```
keep_regions <- c("South America", "Caribbean", "North America")
samps_sub <- subset(samps, Region %in% keep_regions)
pc3_scores_sub <- pc3_scores[samps$Region %in% keep_regions]

# Define new custom colors
pop.colors <- c("magenta", "orange", "#2BCE48")

region.levels <- keep_regions
region.colors <- setNames(pop.colors, region.levels)

# Calculate correlation for the subset
cor_test_sub <- cor.test(samps_sub$Latitude, pc3_scores_sub)

plot(samps_sub$Latitude, pc3_scores_sub,
     pch = 21,
     bg = adjustcolor(region.colors[samps_sub$Region], 0.7),
     xlab = "Latitude",
     ylab = "PC3 score",
     main = paste("PC3 vs Latitude\nr =", round(cor_test_sub$estimate, 3),
                  ", p =", signif(cor_test_sub$p.value, 3)))
abline(lm(pc3_scores_sub ~ samps_sub$Latitude), col = "red", lwd = 2)

legend("topright",
       legend = names(region.colors),
       pt.bg = region.colors,
       pch = 21,
       title = "Region")
```

Exclude regions

Just North American and Caribbean

```
keep_regions <- c("Caribbean", "North America")
samps_sub <- subset(samps, Region %in% keep_regions)
pc3_scores_sub <- pc3_scores[samps$Region %in% keep_regions]

pop.colors <- c("orange", "#2BCE48")
region.levels <- keep_regions
region.colors <- setNames(pop.colors, region.levels)
```

Re-calcualte correlation for subset

```
cor_test_sub <- cor.test(samps_sub$Latitude, pc3_scores_sub)
```

```
plot(samps_sub$Latitude, pc3_scores_sub,
     pch = 21,
     bg = adjustcolor(region.colors[samps_sub$Region], 0.7),
     xlab = "Latitude",
     ylab = "PC3 score",
     main = paste(
       "PC3 vs Latitude (subset)\n",
       "r =", round(cor_test_sub$estimate, 3),
       ", p =", signif(cor_test_sub$p.value, 3)
     ))
abline(lm(pc3_scores_sub ~ samps_sub$Latitude), col = "red", lwd = 2)

legend("topright",
       legend = names(region.colors),
       pt.bg = region.colors,
       pch = 21,
       title = "Region")
```

Only Asia

```
keep_regions <- c("East Asia", "Southeast Asia", "South Asia")
samps_sub2 <- subset(samps, Region %in% keep_regions)
pc3_scores_sub2 <- pc3_scores[samps$Region %in% keep_regions]

pop.colors <- c("red", "blue", "cyan")
region.levels <- keep_regions
region.colors <- setNames(pop.colors, region.levels)
```

Re-calcualte correlation for subset

```
cor_test_sub2 <- cor.test(samps_sub2$Latitude, pc3_scores_sub2)
```

```
plot(samps_sub2$Latitude, pc3_scores_sub2,
     pch = 21,
     bg = adjustcolor(region.colors[samps_sub2$Region], 0.7),
     xlab = "Latitude",
     ylab = "PC3 score",
     main = paste(
       "PC3 vs Latitude (subset)\n",
       "r =", round(cor_test_sub2$estimate, 3),
       ", p =", signif(cor_test_sub2$p.value, 3)
     ))
abline(lm(pc3_scores_sub2 ~ samps_sub2$Latitude), col = "red", lwd = 2)

legend("topright",
       legend = names(region.colors),
       pt.bg = region.colors,
       pch = 21,
       title = "Region")
```

Just South America

```
keep_regions <- c("South America")
samps_sub <- subset(samps, Region %in% keep_regions)
pc3_scores_sub <- pc3_scores[samps$Region %in% keep_regions]

pop.colors <- c("magenta")
region.levels <- keep_regions
region.colors <- setNames(pop.colors, region.levels)
```

Re-calcualte correlation for subset

```
cor_test_sub <- cor.test(samps_sub$Latitude, pc3_scores_sub)
```

```
plot(samps_sub$Latitude, pc3_scores_sub,
     pch = 21,
     bg = adjustcolor(region.colors[samps_sub$Region], 0.7),
     xlab = "Latitude",
     ylab = "PC3 score",
     main = paste(
       "PC3 vs Latitude (subset)\n",
       "r =", round(cor_test_sub$estimate, 3),
       ", p =", signif(cor_test_sub$p.value, 3)
     ))
abline(lm(pc3_scores_sub ~ samps_sub$Latitude), col = "red", lwd = 2)

legend("topright",
       legend = names(region.colors),
       pt.bg = region.colors,
       pch = 21,
       title = "Region")
```

## 4.2 Repeat for 50 SNP windows

Here are the number of windows per chromsome, and the computed MDS
coordinates, colored by chromosome:

```
table(regions$chrom)
```

```
## 
##   1   2   3 
## 286 507 437
```

```
pairs( mds.coords[,mds.cols], pch=20, col=adjustcolor(chrom.cols,0.75) )
```

```
if (do.pdfs) { pdf_copy() }
```

1 2 3 286 507 437

```
pdf("/gpfs/gibbs/pi/caccone/mkc54/albo/manuscript1/output/local_pca/output/MDS_chrom_scatter_50.pdf", width=6, height=6)
table(regions$chrom)
```

```
## 
##   1   2   3 
## 286 507 437
```

```
pairs( mds.coords[,mds.cols], pch=20, col=adjustcolor(chrom.cols,0.75) )
dev.off()
```

```
## png 
##   2
```

Here are the extreme windows in the MDS plot:

```
mds.corners <- corners( mds.coords[,mds.cols[1:2]], prop=.05 )
# set up colors and pchs for corners
corner.cols <- brewer.pal(3,"Dark2")
corner.pch <- c(15,17,19)
ccols <- rep("black",nrow(mds.coords))
cpch <- rep(20,nrow(mds.coords))
for (k in 1:ncol(mds.corners)) {
    ccols[ mds.corners[,k] ] <- corner.cols[k]
    cpch[ mds.corners[,k] ] <- corner.pch[k]
}
# centroids of the corners in MDS space
corner.mds <- do.call(rbind, lapply(1:ncol(mds.corners), 
                function (ii){
                    colMeans(mds.coords[mds.corners[,ii],-(1:2)])
                } ) )
```

```
# plot corners and MDS along the chromosome
spacing <- 1
opar <- par(mar=c(4,4,2,1)+.1,mgp=c(2.5,0.8,0))
layout(matrix(c(rep(1,length(mds.cols)),1+seq_along(mds.cols)),ncol=2),
        widths=c(1,2), heights=layout_heights(length(mds.cols),dl=spacing,ncol=2))
plot( mds.coords[,mds.cols[1:2]], pch=cpch, 
     col=adjustcolor(ccols,0.75),  asp=1,
     xaxt='n', yaxt='n',
     xlab="MDS coordinate 1", ylab="MDS coordinate 2" )
points( corner.mds, pch=20, cex=5,
        col=adjustcolor(corner.cols,0.25))
text( corner.mds, labels=seq_len(nrow(corner.mds)), 
        col=corner.cols, cex=2, lwd=2 )
opar2 <- par(mar=c(par("mar"),spacing/2)[c(5,2,3,4)])
for (k in mds.cols) {
    lastone <- (k==mds.cols[length(mds.cols)])
    if (lastone) { par(mar=c(par("mar"),opar2$mar[1])[c(5,2,3,4)]) }
    chrom.plot( mds.coords[,k], pch=20, 
            xlab=if (lastone) { "Position (Mb)"} else { "" }, # main=paste("MDS coordinate",match(k,mds.cols)),
            chrom.labels=lastone,
            ylab=colnames(mds.coords)[k],
            col=adjustcolor(ccols,0.75) )
    # do this for all but first
    par(mar=c(par("mar"),spacing/2)[c(1,2,5,4)])
}
```

```
par(opar)
```

Now, we’ll look at PCA plots from the extracted corners. (this is
done without a ton of memory by accumulating the covariance matrix in
`running_cov`):

```
corner.npc <- 4
corner.regions <- vector(mode='list', length=ncol(mds.corners))
for (k in seq_along(corner.regions)) {
    corner.regions[[k]] <- regions[ mds.corners[,k],]
}
corner.covmats <- vector(mode='list', length=ncol(mds.corners))
for (k in seq_along(corner.covmats)) {
    reg <- regions[ mds.corners[,k], ]
    qfun <- multi_vcf_query_fn( chrom.list=chroms, file=bcf.files, regions=reg )
    corner.covmats[[k]] <- running_cov(qfun,1:nrow(reg), normalize.rows=TRUE)
}
corner.pca <- vector(mode='list', length=ncol(mds.corners))
for (k in seq_along(corner.pca)) {
    corner.pca[[k]] <- cov_pca(covmat=corner.covmats[[k]], k=corner.npc, w=opt$weights)
}
```

Create color scheme (to match tree)

```
pop.names <- levels(samps$population)
pop.colors <- c("#2BCE48", "purple3", "orange", "red", "magenta")
pop.pch <- seq_len(nlevels(samps$population))
plot( rep(1,length(pop.names)), seq_along(pop.names), 
       cex=4, pch=pop.pch, col=pop.colors, xlim=c(0,length(pop.names)),
       xlab='', ylab='', xaxt='n', yaxt='n' )
text( rep(1,length(pop.names)), seq_along(pop.names), labels=pop.names, pos=4 )
```

```
for (i in 1:(corner.npc-1)) {
  for (j in (i+1):corner.npc) {

    layout(t(1:3))  # 3 panels horizontally
    for (k in 1:ncol(mds.corners)) {
      vectors <- matrix(corner.pca[[k]][-(1:(1+corner.npc))], ncol = corner.npc)[, c(i, j)]
      colnames(vectors) <- paste("PC", c(i, j))
      par(mgp = c(0.7, 0.7, 0), mar = c(2,2,2,0)+0.1)
      plot(vectors, 
           pch = pop.pch[samps$population], 
           col = pop.colors[samps$population],
           xaxt = 'n', yaxt = 'n')
      if (i == 1 && j == 2) {
        mtext(paste("corner", k), side = 3)
      }
    }

  }
}
```

```
pdf("/gpfs/gibbs/pi/caccone/mkc54/albo/manuscript1/output/local_pca/output/all_corner_plots_colors_50.pdf", width = 8, height = 6)

for (i in 1:(corner.npc-1)) {
  for (j in (i+1):corner.npc) {

    layout(t(1:3))  # 3 panels horizontally
    for (k in 1:ncol(mds.corners)) {
      vectors <- matrix(corner.pca[[k]][-(1:(1+corner.npc))], ncol = corner.npc)[, c(i, j)]
      colnames(vectors) <- paste("PC", c(i, j))
      par(mgp = c(0.7, 0.7, 0), mar = c(2,2,2,0)+0.1)
      plot(vectors, 
           pch = pop.pch[samps$population], 
           col = pop.colors[samps$population],
           xaxt = 'n', yaxt = 'n')
      if (i == 1 && j == 2) {
        mtext(paste("corner", k), side = 3)
      }
    }

  }
}
dev.off()
```

```
# Assign colors/shapes by population
library(RColorBrewer)
pops <- levels(samps$population)
pop.cols <- setNames(brewer.pal(length(pops), "Set1"), pops)
pop.pchs <- setNames(rep(21:25, length.out=length(pops)), pops)

ccols <- pop.cols[as.character(samps$population)]
cpch <- pop.pchs[as.character(samps$population)]

# Plot layout
spacing <- 1
opar <- par(mar = c(4,4,2,1) + .1, mgp = c(2.5,0.8,0))
layout(matrix(c(rep(1,length(mds.cols)),1+seq_along(mds.cols)),ncol=2),
       widths = c(1,2), heights = layout_heights(length(mds.cols), dl = spacing, ncol = 2))

# Main MDS plot
plot(mds.coords[, mds.cols[1:2]],
     pch = cpch,
     bg = adjustcolor(ccols, 0.75),
     col = "black",
     asp = 1,
     xaxt = 'n', yaxt = 'n',
     xlab = "MDS coordinate 1", ylab = "MDS coordinate 2"
)
points(corner.mds, pch=21, cex=3, bg="white", col="red", lwd=2)
text(corner.mds, labels=seq_len(nrow(corner.mds)), col="red", cex=2, lwd=2)

# Add legend
legend("topright", legend = pops, pt.bg = pop.cols, pch = 21, title = "Population", bty = "n")
```

```
colnames(mds.coords)
```

```
## [1] "chrom"  "window" "MDS1"   "MDS2"
```

```
#"chrom"  "window" "MDS1"   "MDS2"
```

```
head(regions[order(mds.coords[["MDS1"]], decreasing = TRUE), ], 10)
```

```
##     chrom     start       end       pos
## 336     2  28619713  30629866 426064768
## 298     2   8121141   8628614 404814856
## 764     2 565730543 566887307 962748903
## 311     2  12608134  13292460 409390275
## 323     2  17361635  19200812 414721202
## 770     2 571353426 571891730 968062556
## 780     2 576885784 577794352 973780046
## 305     2  10131691  10457122 406734384
## 750     2 552237455 553207758 949162584
## 783     2 578654225 579012557 975273369
```

```
head(regions[order(mds.coords[["MDS2"]], decreasing = TRUE), ], 10)
```

```
##     chrom     start       end        pos
## 578     2 343563725 344056244  740249962
## 586     2 354735720 355074062  751344869
## 580     2 345552510 346371680  742402073
## 561     2 328436918 328694024  725005449
## 99      1 142987141 144017882  143502512
## 579     2 344056503 345541153  741238806
## 940     3 183505620 184494417 1190650140
## 975     3 222007053 223966802 1229637050
## 439     2 181238257 181892947  578005580
## 538     2 292636370 293427888  689472107
```

```
top_regions_MSD1 <- head(regions[order(mds.coords$MDS1, decreasing = TRUE), ], 10)
#write.csv(top_regions_MSD1, file = "/gpfs/gibbs/pi/caccone/mkc54/albo/manuscript1/output/local_pca/output/top_regions_mds1_50.csv", row.names = FALSE)
```

```
top_regions_MSD2 <- head(regions[order(mds.coords$MDS2, decreasing = TRUE), ], 10)
#write.csv(top_regions_MSD2, file = "/gpfs/gibbs/pi/caccone/mkc54/albo/manuscript1/output/local_pca/output/top_regions_mds2_50.csv", row.names = FALSE)
```

# 5. Outliers for each corner

```
center <- colMeans(mds.coords[, c("MDS1","MDS2")])

mds.coords$dist <- sqrt(
  (mds.coords$MDS1 - center[1])^2 +
  (mds.coords$MDS2 - center[2])^2
)
```

Add corner labels

```
mds.coords$corner <- NA

for (k in 1:ncol(mds.corners)) {
  mds.coords$corner[mds.corners[, k]] <- k
}
```

## 5.1 Corner 1

Subset corner 1

```
mds.coords$corner <- as.numeric(as.character(mds.coords$corner))
corner1_windows <- mds.coords[
  !is.na(mds.coords$corner) & mds.coords$corner == 1,
]
```

Get top 5% in corner 1

```
cutoff <- quantile(corner1_windows$dist, 0.95)

top5_corner1 <- corner1_windows[corner1_windows$dist >= cutoff, ]
```

```
regions$chrom <- paste0("chr", regions$chrom)

regions$window <- ave(regions$start, regions$chrom, FUN = seq_along)
```

```
top_5_corner1_full <- merge(
  top5_corner1,
  regions[, c("chrom","window","start","end","pos")],
  by = c("chrom","window"),
  all.x = TRUE
)
```

Order and save

```
top_5_corner1_full <- top_5_corner1_full[
  order(top_5_corner1_full$chrom,
        top_5_corner1_full$window),
]

final_extreme1 <- top_5_corner1_full[, c(
  "chrom",
  "window",
  "start",
  "end",
  "pos",
  "MDS1",
  "MDS2",
  "dist"
)]
```

Remove ‘chr’ from chromosome name

```
# Remove "chr" prefix from chromosome names
final_extreme1$chrom <- sub("^chr", "", final_extreme1$chrom)
```

```
write.csv(
  final_extreme1,
  "/gpfs/gibbs/pi/caccone/mkc54/albo/manuscript1/output/local_pca/output/corner1_top_5_percent_extreme_windows_50_final.csv",
  row.names = FALSE
)
```

```
library(GenomicRanges)
library(rtracklayer)
library(dplyr)
```

```
# Top 5% extreme windows (chromosome-level coordinates)
corner1_top5 <- read.csv("/gpfs/gibbs/pi/caccone/mkc54/albo/manuscript1/output/local_pca/output/corner1_top_5_percent_extreme_windows_50_final.csv")
```

Import scaffold sizes

Note: it is very important to import the scaffold names as character
(not numeric), because the scaffolds are named 1.1, 1.10, 1.100, 1.101
etc. Importing them as numeric collapses 1.1, 1.10, 1.100 into the same
scaffold name (1.100)

```
scaffolds <- read.table(
  "/gpfs/gibbs/pi/caccone/mkc54/albo/manuscript1/output/local_pca/scaffold_sizes.txt",
  header = FALSE,
  colClasses = c("character", "numeric")
)

colnames(scaffolds) <- c("scaffold", "length")

head(scaffolds)
```

```
##   scaffold   length
## 1      1.1   351198
## 2     1.10 11939576
## 3    1.100  3389100
## 4    1.101   470438
## 5    1.102  2525157
## 6    1.103   150026
```

Double check it

```
any(duplicated(scaffolds$scaffold))
```

```
## [1] FALSE
```

The scaffold size data frame looks good. Now we need to create
cumulative chromosome coordinates (resetting from 1 for each chromosome
to match the window coordinates)

```
scaffolds <- scaffolds %>%
  mutate(chrom = sub("\\..*$", "", scaffold)) %>%
  group_by(chrom) %>%
  mutate(
    cum_start = cumsum(lag(length, default = 0)) + 1,
    cum_end   = cumsum(length)
  ) %>%
  ungroup()
```

Make sure chroms are in same format for corner1\_top5 and
scaffolds

```
corner1_top5$chrom <- as.character(corner1_top5$chrom)
scaffolds$chrom <- as.character(scaffolds$chrom)
```

Check how many windows per chromosome

```
table(corner1_top5$chrom)
```

```
## 
## 2 
## 4
```

Only 4 windows, all on chrm 2 (which is what we expect)

Map windows to scaffolds

```
map_window <- function(chr, win_start, win_end) {

  scaf_chr <- scaffolds %>% filter(chrom == chr)

  overlaps <- scaf_chr %>%
    filter(cum_end >= win_start & cum_start <= win_end) %>%
    mutate(
      scaffold_start = pmax(win_start, cum_start) - cum_start + 1,
      scaffold_end   = pmin(win_end, cum_end) - cum_start + 1,
      window_chr     = chr,
      window_start   = win_start,
      window_end     = win_end
    )

  if (nrow(overlaps) == 0) return(NULL)
  overlaps
}

mapped_windows_corner1 <- do.call(rbind,
  lapply(1:nrow(corner1_top5), function(i)
    map_window(corner1_top5$chrom[i], corner1_top5$start[i], corner1_top5$end[i])
  )
)
```

Check it

```
table(mapped_windows_corner1$window_chr)
```

```
## 
##  2 
## 10
```

Some windows span multiple scaffolds, so this is correct.

Double check for the window on chr 2

```
mapped_windows_corner1 %>%
  filter(window_chr == "2") %>%
  select(scaffold, scaffold_start, scaffold_end)
```

```
## # A tibble: 10 × 3
##    scaffold scaffold_start scaffold_end
##    <chr>             <dbl>        <dbl>
##  1 2.103            410278       917751
##  2 2.103           4897271      5581597
##  3 2.107           8061470      8620481
##  4 2.108                 1       391310
##  5 2.109                 1       371853
##  6 2.11                  1       501773
##  7 2.110                 1        62790
##  8 2.111                 1       112530
##  9 2.112                 1        10886
## 10 2.97            7151071      8059639
```

Import the GFF3 and remove the chr prefix

```
genes <- import("AALBF3_annotation.gff3")

unique(seqnames(genes)) #Check scaffold names
```

```
##   [1] chr3.9   chr3.7   chr3.11  chr3.12  chr3.16  chr3.133 chr1.72  chr3.165
##   [9] chr3.19  chr3.20  chr3.126 chr3.27  chr3.62  chr3.28  chr3.127 chr3.29 
##  [17] chr3.30  chr3.35  chr3.32  chr3.33  chr3.39  chr3.40  chr3.97  chr3.45 
##  [25] chr3.46  chr3.41  chr1.67  chr2.176 chr3.187 chr3.49  chr3.1   chr2.125
##  [33] chr2.119 chr2.126 chr2.127 chr2.131 chr2.130 chr2.107 chr2.115 chr2.96 
##  [41] chr2.106 chr2.52  chr2.100 chr2.98  chr2.99  chr2.97  chr1.165 chr1.113
##  [49] chr3.143 chr1.27  chr1.8   chr3.152 chr2.158 chr3.129 chr1.156 chr3.108
##  [57] chr2.5   chr3.43  chr3.192 chr3.134 chr3.75  chr3.91  chr1.108 chr2.128
##  [65] chr2.180 chr2.186 chr1.69  chr1.74  chr1.73  chr1.36  chr1.35  chr1.164
##  [73] chr1.85  chr2.170 chr1.46  chr1.145 chr2.85  chr1.87  chr3.115 chr2.200
##  [81] chr2.32  chr2.28  chr1.96  chr1.105 chr3.102 chr2.138 chr2.82  chr2.150
##  [89] chr3.186 chr3.137 chr1.29  chr2.62  chr3.6   chr3.15  chr3.72  chr3.74 
##  [97] chr3.21  chr2.188 chr3.63  chr3.150 chr2.204 chr3.76  chr1.135 chr1.138
## [105] chr1.82  chr2.154 chr2.195 chr2.205 chr3.111 chr2.105 chr2.80  chr3.181
## [113] chr2.101 chr1.26  chr1.140 chr3.2   chr1.139 chr1.95  chr1.106 chr3.156
## [121] chr2.198 chr2.6   chr2.9   chr2.78  chr2.197 chr2.193 chr2.196 chr1.170
## [129] chr3.191 chr3.189 chr2.202 chr3.14  chr2.33  chr3.89  chr1.81  chr3.95 
## [137] chr2.185 chr2.159 chr1.124 chr2.190 chr2.177 chr1.131 chr1.23  chr3.142
## [145] chr2.20  chr3.139 chr3.138 chr1.98  chr3.136 chr3.121 chr3.122 chr3.146
## [153] chr3.119 chr1.112 chr3.22  chr2.203 chr1.136 chr2.189 chr2.137 chr2.135
## [161] chr1.5   chr3.34  chr3.47  chr2.206 chr1.19  chr1.15  chr1.57  chr1.11 
## [169] chr2.155 chr2.69  chr2.83  chr2.92  chr2.76  chr2.72  chr2.77  chr2.70 
## [177] chr2.75  chr2.79  chr2.73  chr2.68  chr2.71  chr1.144 chr2.8   chr2.87 
## [185] chr3.147 chr3.94  chr1.133 chr1.128 chr1.1   chr1.41  chr1.90  chr1.91 
## [193] chr1.93  chr1.92  chr3.135 chr3.26  chr3.10  chr2.121 chr2.113 chr2.151
## [201] chr1.2   chr1.142 chr2.40  chr2.42  chr1.51  chr1.58  chr1.17  chr2.123
## [209] chr1.134 chr1.148 chr1.45  chr1.149 chr1.147 chr1.152 chr1.154 chr2.161
## [217] chr3.132 chr3.79  chr2.171 chr1.162 chr1.59  chr2.54  chr3.188 chr1.62 
## [225] chr3.106 chr1.77  chr1.75  chr1.30  chr2.1   chr3.92  chr3.68  chr3.70 
## [233] chr3.66  chr3.65  chr3.61  chr3.85  chr3.69  chr3.8   chr2.157 chr2.201
## [241] chr2.31  chr2.24  chr1.43  chr3.100 chr3.52  chr2.181 chr2.194 chr1.150
## [249] chr2.118 chr2.30  chr2.122 chr3.174 chr3.163 chr2.91  chr2.60  chr2.162
## [257] chr2.64  chr2.153 chr2.103 chr1.168 chr3.64  chr3.73  chr2.46  chr2.38 
## [265] chr2.36  chr2.27  chr2.17  chr2.19  chr2.143 chr2.22  chr2.182 chr2.160
## [273] chr3.51  chr3.173 chr3.54  chr3.197 chr3.58  chr3.56  chr3.50  chr2.58 
## [281] chr3.93  chr3.128 chr3.67  chr2.108 chr2.65  chr2.179 chr1.117 chr3.23 
## [289] chr1.71  chr2.112 chr2.102 chr1.28  chr1.16  chr1.102 chr3.80  chr1.21 
## [297] chr2.11  chr1.31  chr1.32  chr3.184 chr3.81  chr3.144 chr3.77  chr2.39 
## [305] chr1.151 chr3.151 chr2.140 chr1.34  chr2.129 chr1.48  chr2.178 chr1.20 
## [313] chr3.31  chr1.114 chr3.101 chr3.44  chr2.15  chr1.158 chr1.88  chr2.174
## [321] chr1.64  chr1.76  chr1.86  chr2.139 chr2.144 chr1.78  chr2.90  chr2.16 
## [329] chr3.194 chr3.131 chr3.53  chr1.80  chr2.51  chr2.156 chr1.60  chr3.118
## [337] chr3.190 chr3.145 chr3.157 chr3.155 chr3.159 chr3.166 chr3.160 chr3.154
## [345] chr3.168 chr3.169 chr3.170 chr3.176 chr3.178 chr3.180 chr2.48  chr2.29 
## [353] chr2.47  chr3.107 chr2.192 chr3.110 chr3.167 chr2.114 chr3.5   chr1.22 
## [361] chr1.120 chr3.177 chr3.105 chr1.129 chr2.63  chr2.104 chr3.88  chr3.59 
## [369] chr3.78  chr3.38  chr3.161 chr1.50  chr1.37  chr2.164 chr3.36  chr1.89 
## [377] chr3.193 chr1.104 chr1.56  chr3.148 chr1.166 chr2.148 chr3.82  chr3.18 
## [385] chr2.167 chr1.99  chr1.63  chr2.141 chr1.160 chr2.67  chr2.184 chr2.57 
## [393] chr1.119 chr2.56  chr2.49  chr1.42  chr2.89  chr2.199 chr2.95  chr2.111
## [401] chr2.136 chr3.17  chr2.88  chr2.14  chr3.24  chr3.112 chr3.25  chr3.182
## [409] chr1.68  chr1.61  chr1.54  chr1.55  chr2.147 chr2.145 chr3.130 chr1.84 
## [417] chr1.97  chr1.155 chr1.44  chr3.198 chr1.12  chr2.50  chr2.37  chr1.24 
## [425] chr3.3   chr2.61  chr3.162 chr3.48  chr3.96  chr1.9   chr1.53  chr2.66 
## [433] chr1.14  chr2.10  chr1.33  chr2.175 chr3.83  chr2.172 chr2.168 chr2.4  
## [441] chr2.163 chr2.165 chr2.166 chr1.7   chr2.59  chr1.49  chr3.196 chr1.167
## [449] chr1.109 chr2.152 chr1.13  chr3.179 chr3.86  chr2.26  chr2.13  chr1.39 
## [457] chr3.175 chr3.87  chr1.65  chr1.66  chr3.90  chr1.3   chr1.153 chr2.169
## [465] chr3.71  chr2.132 chr1.38  chr1.52  chr1.25  chr1.10  chr2.18  chr3.104
## [473] chr3.37  chr2.74  chr2.12  chr2.120 chr3.124 chr3.125 chr1.83  chr2.55 
## [481] chr2.41  chr2.94  chr1.94  chr2.109 chr2.7   chr1.110 chr3.164 chr3.57 
## [489] chr2.116 chr3.113 chr1.111 chr1.101 chr2.23  chr1.103 chr3.114 chr3.116
## [497] chr3.117 chr3.109 chr2.134 chr2.86  chr2.93  chr1.70  chr1.107 chr2.44 
## [505] chr3.13  chr1.6   chr2.25  chr3.99  chr2.84  chr3.149 chr1.143 chr1.40 
## [513] chr3.84  chr1.169 chr2.43  chr3.140 chr1.127 chr3.171 chr3.172 chr2.133
## [521] chr2.146 chr2.142 chr2.124 chr1.157 chr2.21  chr1.18  chr3.103 chr2.173
## [529] chr2.81  chr2.110 chr2.45  chr1.47  chr3.158 chr3.183 chr1.125 chr1.141
## [537] chr1.137 chr3.123 chr1.132 chr1.161 chr1.126 chr1.123 chr1.121 chr1.115
## [545] chr1.116 chr2.2   chr2.149 chr1.159 chr1.130 chr3.141 chr2.187 chr3.185
## [553] chr1.4   chr1.163 chr3.153 chr3.195 chr3.42  chr3.55  chr3.120 chr2.117
## [561] chr3.98  chr3.60  chr2.53  chr1.79  chr2.35 
## 565 Levels: chr1.1 chr1.10 chr1.101 chr1.102 chr1.103 chr1.104 ... chr3.99
```

Remove “chr” prefix to match chrom names in scaffold\_sizes

```
seqlevels(genes) <- gsub("^chr", "", seqlevels(genes))
seqnames(genes)  <- gsub("^chr", "", seqnames(genes))
```

Build GRanges

```
windows_gr <- GRanges(
  seqnames = mapped_windows_corner1$scaffold,
  ranges   = IRanges(
    start = mapped_windows_corner1$scaffold_start,
    end   = mapped_windows_corner1$scaffold_end
  )
)

genes_gr <- genes   # already cleaned
```

Find overlaps

```
overlaps <- findOverlaps(windows_gr, genes_gr)

length(overlaps)
```

```
## [1] 581
```

Export genes in 5% extreme windows of corner 1

```
corner1_genes_in_top5 <- data.frame(
  scaffold   = seqnames(genes_gr)[subjectHits(overlaps)],
  gene_id    = mcols(genes_gr)$ID[subjectHits(overlaps)],
  gene_start = start(genes_gr)[subjectHits(overlaps)],
  gene_end   = end(genes_gr)[subjectHits(overlaps)],
  chr = mapped_windows_corner1$window_chr[queryHits(overlaps)]
)

corner1_genes_in_top5 <- distinct(corner1_genes_in_top5)

#write.csv(
#  corner1_genes_in_top5,
#  "/gpfs/gibbs/pi/caccone/mkc54/albo/manuscript1/output/local_pca/output/corner1_genes_in_top5_percent_windows_scaffold_mapped.csv",
#  row.names = FALSE
#)
```

## 5.2 Corner 2

Subset corner 2

```
mds.coords$corner <- as.numeric(as.character(mds.coords$corner))
corner2_windows <- mds.coords[
  !is.na(mds.coords$corner) & mds.coords$corner == 2,
]
```

Get top 5% in corner 2

```
cutoff <- quantile(corner2_windows$dist, 0.95)

top5_corner2 <- corner2_windows[corner2_windows$dist >= cutoff, ]
```

```
top_5_corner2_full <- merge(
  top5_corner2,
  regions[, c("chrom","window","start","end","pos")],
  by = c("chrom","window"),
  all.x = TRUE
)
```

Order and save

```
top_5_corner2_full <- top_5_corner2_full[
  order(top_5_corner2_full$chrom,
        top_5_corner2_full$window),
]

final_extreme2 <- top_5_corner2_full[, c(
  "chrom",
  "window",
  "start",
  "end",
  "pos",
  "MDS1",
  "MDS2",
  "dist"
)]
```

Remove ‘chr’ from chromosome name

```
# Remove "chr" prefix from chromosome names
final_extreme2$chrom <- sub("^chr", "", final_extreme2$chrom)
```

```
write.csv(
  final_extreme2,
  "/gpfs/gibbs/pi/caccone/mkc54/albo/manuscript1/output/local_pca/output/corner2_top_5_percent_extreme_windows_50_final.csv",
  row.names = FALSE
)
```

```
library(GenomicRanges)
library(rtracklayer)
library(dplyr)
```

```
# Top 5% extreme windows (chromosome-level coordinates)
corner2_top5 <- read.csv("/gpfs/gibbs/pi/caccone/mkc54/albo/manuscript1/output/local_pca/output/corner2_top_5_percent_extreme_windows_50_final.csv")
```

Import scaffold sizes

Note: it is very important to import the scaffold names as character
(not numeric), because the scaffolds are named 1.1, 1.10, 1.100, 1.101
etc. Importing them as numeric collapses 1.1, 1.10, 1.100 into the same
scaffold name (1.100)

```
scaffolds <- read.table(
  "/gpfs/gibbs/pi/caccone/mkc54/albo/manuscript1/output/local_pca/scaffold_sizes.txt",
  header = FALSE,
  colClasses = c("character", "numeric")
)

colnames(scaffolds) <- c("scaffold", "length")

head(scaffolds)
```

```
##   scaffold   length
## 1      1.1   351198
## 2     1.10 11939576
## 3    1.100  3389100
## 4    1.101   470438
## 5    1.102  2525157
## 6    1.103   150026
```

Double check it

```
any(duplicated(scaffolds$scaffold))
```

```
## [1] FALSE
```

The scaffold size data frame looks good. Now we need to create
cumulative chromosome coordinates (resetting from 1 for each chromosome
to match the window coordinates)

```
scaffolds <- scaffolds %>%
  mutate(chrom = sub("\\..*$", "", scaffold)) %>%
  group_by(chrom) %>%
  mutate(
    cum_start = cumsum(lag(length, default = 0)) + 1,
    cum_end   = cumsum(length)
  ) %>%
  ungroup()
```

Make sure chroms are in same format for corner1\_top5 and
scaffolds

```
corner2_top5$chrom <- as.character(corner2_top5$chrom)
scaffolds$chrom <- as.character(scaffolds$chrom)
```

Check how many windows per chromosome

```
table(corner2_top5$chrom)
```

```
## 
## 1 2 
## 1 2
```

Only 3 windows, 1 on chrom 1 and 2 on chrm 2

Map windows to scaffolds

```
map_window <- function(chr, win_start, win_end) {

  scaf_chr <- scaffolds %>% filter(chrom == chr)

  overlaps <- scaf_chr %>%
    filter(cum_end >= win_start & cum_start <= win_end) %>%
    mutate(
      scaffold_start = pmax(win_start, cum_start) - cum_start + 1,
      scaffold_end   = pmin(win_end, cum_end) - cum_start + 1,
      window_chr     = chr,
      window_start   = win_start,
      window_end     = win_end
    )

  if (nrow(overlaps) == 0) return(NULL)
  overlaps
}

mapped_windows_corner2 <- do.call(rbind,
  lapply(1:nrow(corner2_top5), function(i)
    map_window(corner2_top5$chrom[i], corner2_top5$start[i], corner2_top5$end[i])
  )
)
```

Check it

```
table(mapped_windows_corner2$window_chr)
```

```
## 
## 1 2 
## 1 4
```

Some windows span multiple scaffolds, so this is correct.

Double check for the window on chr 1

```
mapped_windows_corner2 %>%
  filter(window_chr == "1") %>%
  select(scaffold, scaffold_start, scaffold_end)
```

```
## # A tibble: 1 × 3
##   scaffold scaffold_start scaffold_end
##   <chr>             <dbl>        <dbl>
## 1 1.147           2378798      4398604
```

Import the GFF3 and remove the chr prefix

```
genes <- import("AALBF3_annotation.gff3")

unique(seqnames(genes)) #Check scaffold names
```

```
##   [1] chr3.9   chr3.7   chr3.11  chr3.12  chr3.16  chr3.133 chr1.72  chr3.165
##   [9] chr3.19  chr3.20  chr3.126 chr3.27  chr3.62  chr3.28  chr3.127 chr3.29 
##  [17] chr3.30  chr3.35  chr3.32  chr3.33  chr3.39  chr3.40  chr3.97  chr3.45 
##  [25] chr3.46  chr3.41  chr1.67  chr2.176 chr3.187 chr3.49  chr3.1   chr2.125
##  [33] chr2.119 chr2.126 chr2.127 chr2.131 chr2.130 chr2.107 chr2.115 chr2.96 
##  [41] chr2.106 chr2.52  chr2.100 chr2.98  chr2.99  chr2.97  chr1.165 chr1.113
##  [49] chr3.143 chr1.27  chr1.8   chr3.152 chr2.158 chr3.129 chr1.156 chr3.108
##  [57] chr2.5   chr3.43  chr3.192 chr3.134 chr3.75  chr3.91  chr1.108 chr2.128
##  [65] chr2.180 chr2.186 chr1.69  chr1.74  chr1.73  chr1.36  chr1.35  chr1.164
##  [73] chr1.85  chr2.170 chr1.46  chr1.145 chr2.85  chr1.87  chr3.115 chr2.200
##  [81] chr2.32  chr2.28  chr1.96  chr1.105 chr3.102 chr2.138 chr2.82  chr2.150
##  [89] chr3.186 chr3.137 chr1.29  chr2.62  chr3.6   chr3.15  chr3.72  chr3.74 
##  [97] chr3.21  chr2.188 chr3.63  chr3.150 chr2.204 chr3.76  chr1.135 chr1.138
## [105] chr1.82  chr2.154 chr2.195 chr2.205 chr3.111 chr2.105 chr2.80  chr3.181
## [113] chr2.101 chr1.26  chr1.140 chr3.2   chr1.139 chr1.95  chr1.106 chr3.156
## [121] chr2.198 chr2.6   chr2.9   chr2.78  chr2.197 chr2.193 chr2.196 chr1.170
## [129] chr3.191 chr3.189 chr2.202 chr3.14  chr2.33  chr3.89  chr1.81  chr3.95 
## [137] chr2.185 chr2.159 chr1.124 chr2.190 chr2.177 chr1.131 chr1.23  chr3.142
## [145] chr2.20  chr3.139 chr3.138 chr1.98  chr3.136 chr3.121 chr3.122 chr3.146
## [153] chr3.119 chr1.112 chr3.22  chr2.203 chr1.136 chr2.189 chr2.137 chr2.135
## [161] chr1.5   chr3.34  chr3.47  chr2.206 chr1.19  chr1.15  chr1.57  chr1.11 
## [169] chr2.155 chr2.69  chr2.83  chr2.92  chr2.76  chr2.72  chr2.77  chr2.70 
## [177] chr2.75  chr2.79  chr2.73  chr2.68  chr2.71  chr1.144 chr2.8   chr2.87 
## [185] chr3.147 chr3.94  chr1.133 chr1.128 chr1.1   chr1.41  chr1.90  chr1.91 
## [193] chr1.93  chr1.92  chr3.135 chr3.26  chr3.10  chr2.121 chr2.113 chr2.151
## [201] chr1.2   chr1.142 chr2.40  chr2.42  chr1.51  chr1.58  chr1.17  chr2.123
## [209] chr1.134 chr1.148 chr1.45  chr1.149 chr1.147 chr1.152 chr1.154 chr2.161
## [217] chr3.132 chr3.79  chr2.171 chr1.162 chr1.59  chr2.54  chr3.188 chr1.62 
## [225] chr3.106 chr1.77  chr1.75  chr1.30  chr2.1   chr3.92  chr3.68  chr3.70 
## [233] chr3.66  chr3.65  chr3.61  chr3.85  chr3.69  chr3.8   chr2.157 chr2.201
## [241] chr2.31  chr2.24  chr1.43  chr3.100 chr3.52  chr2.181 chr2.194 chr1.150
## [249] chr2.118 chr2.30  chr2.122 chr3.174 chr3.163 chr2.91  chr2.60  chr2.162
## [257] chr2.64  chr2.153 chr2.103 chr1.168 chr3.64  chr3.73  chr2.46  chr2.38 
## [265] chr2.36  chr2.27  chr2.17  chr2.19  chr2.143 chr2.22  chr2.182 chr2.160
## [273] chr3.51  chr3.173 chr3.54  chr3.197 chr3.58  chr3.56  chr3.50  chr2.58 
## [281] chr3.93  chr3.128 chr3.67  chr2.108 chr2.65  chr2.179 chr1.117 chr3.23 
## [289] chr1.71  chr2.112 chr2.102 chr1.28  chr1.16  chr1.102 chr3.80  chr1.21 
## [297] chr2.11  chr1.31  chr1.32  chr3.184 chr3.81  chr3.144 chr3.77  chr2.39 
## [305] chr1.151 chr3.151 chr2.140 chr1.34  chr2.129 chr1.48  chr2.178 chr1.20 
## [313] chr3.31  chr1.114 chr3.101 chr3.44  chr2.15  chr1.158 chr1.88  chr2.174
## [321] chr1.64  chr1.76  chr1.86  chr2.139 chr2.144 chr1.78  chr2.90  chr2.16 
## [329] chr3.194 chr3.131 chr3.53  chr1.80  chr2.51  chr2.156 chr1.60  chr3.118
## [337] chr3.190 chr3.145 chr3.157 chr3.155 chr3.159 chr3.166 chr3.160 chr3.154
## [345] chr3.168 chr3.169 chr3.170 chr3.176 chr3.178 chr3.180 chr2.48  chr2.29 
## [353] chr2.47  chr3.107 chr2.192 chr3.110 chr3.167 chr2.114 chr3.5   chr1.22 
## [361] chr1.120 chr3.177 chr3.105 chr1.129 chr2.63  chr2.104 chr3.88  chr3.59 
## [369] chr3.78  chr3.38  chr3.161 chr1.50  chr1.37  chr2.164 chr3.36  chr1.89 
## [377] chr3.193 chr1.104 chr1.56  chr3.148 chr1.166 chr2.148 chr3.82  chr3.18 
## [385] chr2.167 chr1.99  chr1.63  chr2.141 chr1.160 chr2.67  chr2.184 chr2.57 
## [393] chr1.119 chr2.56  chr2.49  chr1.42  chr2.89  chr2.199 chr2.95  chr2.111
## [401] chr2.136 chr3.17  chr2.88  chr2.14  chr3.24  chr3.112 chr3.25  chr3.182
## [409] chr1.68  chr1.61  chr1.54  chr1.55  chr2.147 chr2.145 chr3.130 chr1.84 
## [417] chr1.97  chr1.155 chr1.44  chr3.198 chr1.12  chr2.50  chr2.37  chr1.24 
## [425] chr3.3   chr2.61  chr3.162 chr3.48  chr3.96  chr1.9   chr1.53  chr2.66 
## [433] chr1.14  chr2.10  chr1.33  chr2.175 chr3.83  chr2.172 chr2.168 chr2.4  
## [441] chr2.163 chr2.165 chr2.166 chr1.7   chr2.59  chr1.49  chr3.196 chr1.167
## [449] chr1.109 chr2.152 chr1.13  chr3.179 chr3.86  chr2.26  chr2.13  chr1.39 
## [457] chr3.175 chr3.87  chr1.65  chr1.66  chr3.90  chr1.3   chr1.153 chr2.169
## [465] chr3.71  chr2.132 chr1.38  chr1.52  chr1.25  chr1.10  chr2.18  chr3.104
## [473] chr3.37  chr2.74  chr2.12  chr2.120 chr3.124 chr3.125 chr1.83  chr2.55 
## [481] chr2.41  chr2.94  chr1.94  chr2.109 chr2.7   chr1.110 chr3.164 chr3.57 
## [489] chr2.116 chr3.113 chr1.111 chr1.101 chr2.23  chr1.103 chr3.114 chr3.116
## [497] chr3.117 chr3.109 chr2.134 chr2.86  chr2.93  chr1.70  chr1.107 chr2.44 
## [505] chr3.13  chr1.6   chr2.25  chr3.99  chr2.84  chr3.149 chr1.143 chr1.40 
## [513] chr3.84  chr1.169 chr2.43  chr3.140 chr1.127 chr3.171 chr3.172 chr2.133
## [521] chr2.146 chr2.142 chr2.124 chr1.157 chr2.21  chr1.18  chr3.103 chr2.173
## [529] chr2.81  chr2.110 chr2.45  chr1.47  chr3.158 chr3.183 chr1.125 chr1.141
## [537] chr1.137 chr3.123 chr1.132 chr1.161 chr1.126 chr1.123 chr1.121 chr1.115
## [545] chr1.116 chr2.2   chr2.149 chr1.159 chr1.130 chr3.141 chr2.187 chr3.185
## [553] chr1.4   chr1.163 chr3.153 chr3.195 chr3.42  chr3.55  chr3.120 chr2.117
## [561] chr3.98  chr3.60  chr2.53  chr1.79  chr2.35 
## 565 Levels: chr1.1 chr1.10 chr1.101 chr1.102 chr1.103 chr1.104 ... chr3.99
```

Remove “chr” prefix to match chrom names in scaffold\_sizes

```
seqlevels(genes) <- gsub("^chr", "", seqlevels(genes))
seqnames(genes)  <- gsub("^chr", "", seqnames(genes))
```

Build GRanges

```
windows_gr <- GRanges(
  seqnames = mapped_windows_corner2$scaffold,
  ranges   = IRanges(
    start = mapped_windows_corner2$scaffold_start,
    end   = mapped_windows_corner2$scaffold_end
  )
)

genes_gr <- genes   # already cleaned
```

Find overlaps

```
overlaps <- findOverlaps(windows_gr, genes_gr)

length(overlaps)
```

```
## [1] 1693
```

1693

Export genes in 5% extreme windows of corner 2

```
corner2_genes_in_top5 <- data.frame(
  scaffold   = seqnames(genes_gr)[subjectHits(overlaps)],
  gene_id    = mcols(genes_gr)$ID[subjectHits(overlaps)],
  gene_start = start(genes_gr)[subjectHits(overlaps)],
  gene_end   = end(genes_gr)[subjectHits(overlaps)],
  chr = mapped_windows_corner2$window_chr[queryHits(overlaps)]
)

corner2_genes_in_top5 <- distinct(corner2_genes_in_top5)
```

```
write.csv(
  corner2_genes_in_top5,
  "/gpfs/gibbs/pi/caccone/mkc54/albo/manuscript1/output/local_pca/output/corner2_genes_in_top5_percent_windows_scaffold_mapped.csv",
  row.names = FALSE
)
```

## 5.3 Corner 3

Subset corner 3

```
mds.coords$corner <- as.numeric(as.character(mds.coords$corner))
corner3_windows <- mds.coords[
  !is.na(mds.coords$corner) & mds.coords$corner == 3,
]
```

Get top 5% in corner 3

```
cutoff <- quantile(corner3_windows$dist, 0.95)

top5_corner3 <- corner3_windows[corner3_windows$dist >= cutoff, ]
```

```
regions$chrom <- paste0("chr", regions$chrom)

regions$window <- ave(regions$start, regions$chrom, FUN = seq_along)
```

```
top_5_corner3_full <- merge(
  top5_corner3,
  regions[, c("chrom","window","start","end","pos")],
  by = c("chrom","window"),
  all.x = TRUE
)
```

Order and save

```
top_5_corner3_full <- top_5_corner3_full[
  order(top_5_corner3_full$chrom,
        top_5_corner3_full$window),
]

final_extreme3 <- top_5_corner3_full[, c(
  "chrom",
  "window",
  "start",
  "end",
  "pos",
  "MDS1",
  "MDS2",
  "dist"
)]
```

Remove ‘chr’ from chromosome name

```
# Remove "chr" prefix from chromosome names
final_extreme3$chrom <- sub("^chr", "", final_extreme3$chrom)
```

```
write.csv(
  final_extreme3,
  "/gpfs/gibbs/pi/caccone/mkc54/albo/manuscript1/output/local_pca/output/corner3_top_5_percent_extreme_windows_50_final.csv",
  row.names = FALSE
)
```

```
library(GenomicRanges)
library(rtracklayer)
library(dplyr)
```

```
# Top 5% extreme windows (chromosome-level coordinates)
corner3_top5 <- read.csv("/gpfs/gibbs/pi/caccone/mkc54/albo/manuscript1/output/local_pca/output/corner3_top_5_percent_extreme_windows_50_final.csv")
```

Import scaffold sizes Note: it is very important to import the
scaffold names as character (not numeric), because the scaffolds are
named 1.1, 1.10, 1.100, 1.101 etc. Importing them as numeric collapses
1.1, 1.10, 1.100 into the same scaffold name (1.100)

```
scaffolds <- read.table(
  "/gpfs/gibbs/pi/caccone/mkc54/albo/manuscript1/output/local_pca/scaffold_sizes.txt",
  header = FALSE,
  colClasses = c("character", "numeric")
)

colnames(scaffolds) <- c("scaffold", "length")

head(scaffolds)
```

```
##   scaffold   length
## 1      1.1   351198
## 2     1.10 11939576
## 3    1.100  3389100
## 4    1.101   470438
## 5    1.102  2525157
## 6    1.103   150026
```

Double check it

```
any(duplicated(scaffolds$scaffold))
```

```
## [1] FALSE
```

The scaffold size data frame looks good. Now we need to create
cumulative chromosome coordinates (resetting from 1 for each chromosome
to match the window coordinates)

```
scaffolds <- scaffolds %>%
  mutate(chrom = sub("\\..*$", "", scaffold)) %>%
  group_by(chrom) %>%
  mutate(
    cum_start = cumsum(lag(length, default = 0)) + 1,
    cum_end   = cumsum(length)
  ) %>%
  ungroup()
```

Make sure chroms are in same format for corner1\_top5 and
scaffolds

```
corner3_top5$chrom <- as.character(corner3_top5$chrom)
scaffolds$chrom <- as.character(scaffolds$chrom)
```

Check how many windows per chromosome

```
table(corner3_top5$chrom)
```

```
## 
## 1 2 
## 1 3
```

Only 4 windows, 1 on chrom 1 and 3 on chrm 2

Map windows to scaffolds

```
map_window <- function(chr, win_start, win_end) {

  scaf_chr <- scaffolds %>% filter(chrom == chr)

  overlaps <- scaf_chr %>%
    filter(cum_end >= win_start & cum_start <= win_end) %>%
    mutate(
      scaffold_start = pmax(win_start, cum_start) - cum_start + 1,
      scaffold_end   = pmin(win_end, cum_end) - cum_start + 1,
      window_chr     = chr,
      window_start   = win_start,
      window_end     = win_end
    )

  if (nrow(overlaps) == 0) return(NULL)
  overlaps
}

mapped_windows_corner3 <- do.call(rbind,
  lapply(1:nrow(corner3_top5), function(i)
    map_window(corner3_top5$chrom[i], corner3_top5$start[i], corner3_top5$end[i])
  )
)
```

Check it

```
table(mapped_windows_corner3$window_chr)
```

```
## 
## 1 2 
## 1 5
```

Some windows span multiple scaffolds, so this is correct.

Double check for the window on chr 1

```
mapped_windows_corner3 %>%
  filter(window_chr == "1") %>%
  select(scaffold, scaffold_start, scaffold_end)
```

```
## # A tibble: 1 × 3
##   scaffold scaffold_start scaffold_end
##   <chr>             <dbl>        <dbl>
## 1 1.7            13008599     14561828
```

Import the GFF3 and remove the chr prefix

```
genes <- import("AALBF3_annotation.gff3")

unique(seqnames(genes)) #Check scaffold names
```

```
##   [1] chr3.9   chr3.7   chr3.11  chr3.12  chr3.16  chr3.133 chr1.72  chr3.165
##   [9] chr3.19  chr3.20  chr3.126 chr3.27  chr3.62  chr3.28  chr3.127 chr3.29 
##  [17] chr3.30  chr3.35  chr3.32  chr3.33  chr3.39  chr3.40  chr3.97  chr3.45 
##  [25] chr3.46  chr3.41  chr1.67  chr2.176 chr3.187 chr3.49  chr3.1   chr2.125
##  [33] chr2.119 chr2.126 chr2.127 chr2.131 chr2.130 chr2.107 chr2.115 chr2.96 
##  [41] chr2.106 chr2.52  chr2.100 chr2.98  chr2.99  chr2.97  chr1.165 chr1.113
##  [49] chr3.143 chr1.27  chr1.8   chr3.152 chr2.158 chr3.129 chr1.156 chr3.108
##  [57] chr2.5   chr3.43  chr3.192 chr3.134 chr3.75  chr3.91  chr1.108 chr2.128
##  [65] chr2.180 chr2.186 chr1.69  chr1.74  chr1.73  chr1.36  chr1.35  chr1.164
##  [73] chr1.85  chr2.170 chr1.46  chr1.145 chr2.85  chr1.87  chr3.115 chr2.200
##  [81] chr2.32  chr2.28  chr1.96  chr1.105 chr3.102 chr2.138 chr2.82  chr2.150
##  [89] chr3.186 chr3.137 chr1.29  chr2.62  chr3.6   chr3.15  chr3.72  chr3.74 
##  [97] chr3.21  chr2.188 chr3.63  chr3.150 chr2.204 chr3.76  chr1.135 chr1.138
## [105] chr1.82  chr2.154 chr2.195 chr2.205 chr3.111 chr2.105 chr2.80  chr3.181
## [113] chr2.101 chr1.26  chr1.140 chr3.2   chr1.139 chr1.95  chr1.106 chr3.156
## [121] chr2.198 chr2.6   chr2.9   chr2.78  chr2.197 chr2.193 chr2.196 chr1.170
## [129] chr3.191 chr3.189 chr2.202 chr3.14  chr2.33  chr3.89  chr1.81  chr3.95 
## [137] chr2.185 chr2.159 chr1.124 chr2.190 chr2.177 chr1.131 chr1.23  chr3.142
## [145] chr2.20  chr3.139 chr3.138 chr1.98  chr3.136 chr3.121 chr3.122 chr3.146
## [153] chr3.119 chr1.112 chr3.22  chr2.203 chr1.136 chr2.189 chr2.137 chr2.135
## [161] chr1.5   chr3.34  chr3.47  chr2.206 chr1.19  chr1.15  chr1.57  chr1.11 
## [169] chr2.155 chr2.69  chr2.83  chr2.92  chr2.76  chr2.72  chr2.77  chr2.70 
## [177] chr2.75  chr2.79  chr2.73  chr2.68  chr2.71  chr1.144 chr2.8   chr2.87 
## [185] chr3.147 chr3.94  chr1.133 chr1.128 chr1.1   chr1.41  chr1.90  chr1.91 
## [193] chr1.93  chr1.92  chr3.135 chr3.26  chr3.10  chr2.121 chr2.113 chr2.151
## [201] chr1.2   chr1.142 chr2.40  chr2.42  chr1.51  chr1.58  chr1.17  chr2.123
## [209] chr1.134 chr1.148 chr1.45  chr1.149 chr1.147 chr1.152 chr1.154 chr2.161
## [217] chr3.132 chr3.79  chr2.171 chr1.162 chr1.59  chr2.54  chr3.188 chr1.62 
## [225] chr3.106 chr1.77  chr1.75  chr1.30  chr2.1   chr3.92  chr3.68  chr3.70 
## [233] chr3.66  chr3.65  chr3.61  chr3.85  chr3.69  chr3.8   chr2.157 chr2.201
## [241] chr2.31  chr2.24  chr1.43  chr3.100 chr3.52  chr2.181 chr2.194 chr1.150
## [249] chr2.118 chr2.30  chr2.122 chr3.174 chr3.163 chr2.91  chr2.60  chr2.162
## [257] chr2.64  chr2.153 chr2.103 chr1.168 chr3.64  chr3.73  chr2.46  chr2.38 
## [265] chr2.36  chr2.27  chr2.17  chr2.19  chr2.143 chr2.22  chr2.182 chr2.160
## [273] chr3.51  chr3.173 chr3.54  chr3.197 chr3.58  chr3.56  chr3.50  chr2.58 
## [281] chr3.93  chr3.128 chr3.67  chr2.108 chr2.65  chr2.179 chr1.117 chr3.23 
## [289] chr1.71  chr2.112 chr2.102 chr1.28  chr1.16  chr1.102 chr3.80  chr1.21 
## [297] chr2.11  chr1.31  chr1.32  chr3.184 chr3.81  chr3.144 chr3.77  chr2.39 
## [305] chr1.151 chr3.151 chr2.140 chr1.34  chr2.129 chr1.48  chr2.178 chr1.20 
## [313] chr3.31  chr1.114 chr3.101 chr3.44  chr2.15  chr1.158 chr1.88  chr2.174
## [321] chr1.64  chr1.76  chr1.86  chr2.139 chr2.144 chr1.78  chr2.90  chr2.16 
## [329] chr3.194 chr3.131 chr3.53  chr1.80  chr2.51  chr2.156 chr1.60  chr3.118
## [337] chr3.190 chr3.145 chr3.157 chr3.155 chr3.159 chr3.166 chr3.160 chr3.154
## [345] chr3.168 chr3.169 chr3.170 chr3.176 chr3.178 chr3.180 chr2.48  chr2.29 
## [353] chr2.47  chr3.107 chr2.192 chr3.110 chr3.167 chr2.114 chr3.5   chr1.22 
## [361] chr1.120 chr3.177 chr3.105 chr1.129 chr2.63  chr2.104 chr3.88  chr3.59 
## [369] chr3.78  chr3.38  chr3.161 chr1.50  chr1.37  chr2.164 chr3.36  chr1.89 
## [377] chr3.193 chr1.104 chr1.56  chr3.148 chr1.166 chr2.148 chr3.82  chr3.18 
## [385] chr2.167 chr1.99  chr1.63  chr2.141 chr1.160 chr2.67  chr2.184 chr2.57 
## [393] chr1.119 chr2.56  chr2.49  chr1.42  chr2.89  chr2.199 chr2.95  chr2.111
## [401] chr2.136 chr3.17  chr2.88  chr2.14  chr3.24  chr3.112 chr3.25  chr3.182
## [409] chr1.68  chr1.61  chr1.54  chr1.55  chr2.147 chr2.145 chr3.130 chr1.84 
## [417] chr1.97  chr1.155 chr1.44  chr3.198 chr1.12  chr2.50  chr2.37  chr1.24 
## [425] chr3.3   chr2.61  chr3.162 chr3.48  chr3.96  chr1.9   chr1.53  chr2.66 
## [433] chr1.14  chr2.10  chr1.33  chr2.175 chr3.83  chr2.172 chr2.168 chr2.4  
## [441] chr2.163 chr2.165 chr2.166 chr1.7   chr2.59  chr1.49  chr3.196 chr1.167
## [449] chr1.109 chr2.152 chr1.13  chr3.179 chr3.86  chr2.26  chr2.13  chr1.39 
## [457] chr3.175 chr3.87  chr1.65  chr1.66  chr3.90  chr1.3   chr1.153 chr2.169
## [465] chr3.71  chr2.132 chr1.38  chr1.52  chr1.25  chr1.10  chr2.18  chr3.104
## [473] chr3.37  chr2.74  chr2.12  chr2.120 chr3.124 chr3.125 chr1.83  chr2.55 
## [481] chr2.41  chr2.94  chr1.94  chr2.109 chr2.7   chr1.110 chr3.164 chr3.57 
## [489] chr2.116 chr3.113 chr1.111 chr1.101 chr2.23  chr1.103 chr3.114 chr3.116
## [497] chr3.117 chr3.109 chr2.134 chr2.86  chr2.93  chr1.70  chr1.107 chr2.44 
## [505] chr3.13  chr1.6   chr2.25  chr3.99  chr2.84  chr3.149 chr1.143 chr1.40 
## [513] chr3.84  chr1.169 chr2.43  chr3.140 chr1.127 chr3.171 chr3.172 chr2.133
## [521] chr2.146 chr2.142 chr2.124 chr1.157 chr2.21  chr1.18  chr3.103 chr2.173
## [529] chr2.81  chr2.110 chr2.45  chr1.47  chr3.158 chr3.183 chr1.125 chr1.141
## [537] chr1.137 chr3.123 chr1.132 chr1.161 chr1.126 chr1.123 chr1.121 chr1.115
## [545] chr1.116 chr2.2   chr2.149 chr1.159 chr1.130 chr3.141 chr2.187 chr3.185
## [553] chr1.4   chr1.163 chr3.153 chr3.195 chr3.42  chr3.55  chr3.120 chr2.117
## [561] chr3.98  chr3.60  chr2.53  chr1.79  chr2.35 
## 565 Levels: chr1.1 chr1.10 chr1.101 chr1.102 chr1.103 chr1.104 ... chr3.99
```

Remove “chr” prefix to match chrom names in scaffold\_sizes

```
seqlevels(genes) <- gsub("^chr", "", seqlevels(genes))
seqnames(genes)  <- gsub("^chr", "", seqnames(genes))
```

Build GRanges

```
windows_gr <- GRanges(
  seqnames = mapped_windows_corner3$scaffold,
  ranges   = IRanges(
    start = mapped_windows_corner3$scaffold_start,
    end   = mapped_windows_corner3$scaffold_end
  )
)

genes_gr <- genes   # already cleaned
```

Find overlaps

```
overlaps <- findOverlaps(windows_gr, genes_gr)

length(overlaps)
```

```
## [1] 1652
```

Export genes in 5% extreme windows of corner 3

```
corner3_genes_in_top5 <- data.frame(
  scaffold   = seqnames(genes_gr)[subjectHits(overlaps)],
  gene_id    = mcols(genes_gr)$ID[subjectHits(overlaps)],
  gene_start = start(genes_gr)[subjectHits(overlaps)],
  gene_end   = end(genes_gr)[subjectHits(overlaps)],
  chr = mapped_windows_corner3$window_chr[queryHits(overlaps)]
)

corner3_genes_in_top5 <- distinct(corner3_genes_in_top5)
```

```
write.csv(
  corner3_genes_in_top5,
  "/gpfs/gibbs/pi/caccone/mkc54/albo/manuscript1/output/local_pca/output/corner3_genes_in_top5_percent_windows_scaffold_mapped.csv",
  row.names = FALSE
)
```

# B. Enrichment (KEGG-ORA)

Based on script from P. Armbruster

Install packages

```
#Install packages
#rm -rf ~/R/x86_64-pc-linux-gnu-library/4.2/00LOCK-*

#Need older version of ggplot2 for ggtree to install correctly
#remove.packages("ggplot2")
#install.packages("remotes")
#remotes::install_version("ggplot2", version = "3.4.4", repos = "https://cran.rstudio.com/")
#remotes::install_github("YuLab-SMU/ggtree", dependencies = TRUE) # install the development version of ggtree from GitHub
#BiocManager::install("clusterProfiler", dependencies = TRUE)
```

```
# Load required libraries
library(clusterProfiler)
library(enrichplot)
library(ggplot2)
```

The SNP chip has at least one SNP in each gene model, so for the
background list we will use all the gene models. I am using the
“universal\_LOC\_list.txt” provided by Peter Armbruster.

Lostruct clusters genomic windows based on a multidimensional scaling
(MDS) analysis of the dissimilarity between these local PCAs and
identifies extreme “corners” by selecting the top 5% of windows with the
most extreme MDS coordinates. So we will start by using the genes in the
top 5% outlier windows in each corner. We will use the results from the
localpca analysis performed with 50 SNP windows.

# 1. Corner 1 - Top 5% outliers for 50 SNP windows

## 1.1 Load data

```
library(dplyr)

outliers_1 <- read.csv("manuscript1/output/enrichment/corner1_genes_in_top5_percent_windows_scaffold_mapped.csv")
head(outliers_1)
```

```
##   scaffold               gene_id gene_start gene_end chr
## 1    2.103     gene-LOC115266684     582297   584009   2
## 2    2.103    rna-XM_029873207.1     582297   584009   2
## 3    2.103 exon-XM_029873207.1-1     583793   584009   2
## 4    2.103 exon-XM_029873207.1-2     582676   582789   2
## 5    2.103 exon-XM_029873207.1-3     582297   582620   2
## 6    2.103    cds-XP_029729067.1     583793   584009   2
```

```
# Should have: corner, chrom, gene_id, gene_start, gene_end
```

```
# Extract LOC IDs; everything else becomes NA
outliers_1 <- outliers_1 %>%
  mutate(core_gene_id = ifelse(grepl("LOC\\d+", gene_id),
                               sub(".*?(LOC\\d+).*", "\\1", gene_id),
                               NA))

# Check
head(outliers_1[, c("gene_id", "core_gene_id")])
```

```
##                 gene_id core_gene_id
## 1     gene-LOC115266684 LOC115266684
## 2    rna-XM_029873207.1         <NA>
## 3 exon-XM_029873207.1-1         <NA>
## 4 exon-XM_029873207.1-2         <NA>
## 5 exon-XM_029873207.1-3         <NA>
## 6    cds-XP_029729067.1         <NA>
```

Now the core\_gene\_id column should match the format of our
“universal\_gene\_file”

Export it

```
core_ids_unique <- outliers_1 %>%
  pull(core_gene_id) %>%
  na.omit() %>%
  unique()
```

```
write.table(core_ids_unique,
            file = "/gpfs/gibbs/pi/caccone/mkc54/albo/manuscript1/output/enrichment/corner1_outlier_5_genes_coreIDs_50.txt",
            quote = FALSE,
            row.names = FALSE,
            col.names = FALSE)
```

```
cat("Loading gene lists...\n")
```

```
## Loading gene lists...
```

```
# Specify input files
input_gene_file <- "/gpfs/gibbs/pi/caccone/mkc54/albo/manuscript1/output/enrichment/corner1_outlier_5_genes_coreIDs_50.txt"  # Change this to your gene list of interest
universe_gene_file <- "/gpfs/gibbs/pi/caccone/mkc54/albo/manuscript1/output/enrichment/universal_LOC_list.txt"  # Change this to your background/universe list

# Load input gene list
inputgenelist <- read.table(
  input_gene_file,
  header = FALSE,
  stringsAsFactors = FALSE,
  col.names = "gene_id"
)

# Load universe gene list
universegenelist <- read.table(
  universe_gene_file,
  header = FALSE,
  stringsAsFactors = FALSE,
  col.names = "gene_id"
)
```

Convert LOC IDs to NCBI gene IDs by removing “LOC” prefix

```
inputgenelist$ncbi_geneid <- gsub("^LOC", "", inputgenelist$gene_id)
universegenelist$ncbi_geneid <- gsub("^LOC", "", universegenelist$gene_id)

cat("Input genes loaded:", nrow(inputgenelist), "\n")
```

```
## Input genes loaded: 46
```

```
cat("Universe genes loaded:", nrow(universegenelist), "\n")
```

```
## Universe genes loaded: 21553
```

Input genes loaded: 46  
Universe genes loaded: 21553

Now our gene list of interest is ~0.2% of the total

## 1.2 KEGG Over-Representation Analysis (ORA)

### 1.2.1 Using BH correction and p<0.05 threshold

```
options(rstudio.notebook.plot.cache = FALSE)

cat("Running KEGG Over-Representation Analysis (ORA)...\n")
```

```
## Running KEGG Over-Representation Analysis (ORA)...
```

```
# Perform KEGG ORA
kegg_ora <- enrichKEGG(
  gene = inputgenelist$ncbi_geneid,
  universe = universegenelist$ncbi_geneid,
  organism = "aalb",
  pvalueCutoff = 0.05,
  pAdjustMethod = "BH",
  keyType = "ncbi-geneid"
)
```

```
## Reading KEGG annotation online: "https://rest.kegg.jp/link/aalb/pathway"...
```

```
## Reading KEGG annotation online: "https://rest.kegg.jp/list/pathway/aalb"...
```

```
## Reading KEGG annotation online: "https://rest.kegg.jp/conv/ncbi-geneid/aalb"...
```

```
# View results
print(head(kegg_ora))
```

```
##                  ID
## aalb04214 aalb04214
## aalb04215 aalb04215
## aalb00600 aalb00600
## aalb04137 aalb04137
## aalb00564 aalb00564
## aalb04148 aalb04148
##                                                                        Description
## aalb04214                Apoptosis - fly - Aedes albopictus (Asian tiger mosquito)
## aalb04215   Apoptosis - multiple species - Aedes albopictus (Asian tiger mosquito)
## aalb00600        Sphingolipid metabolism - Aedes albopictus (Asian tiger mosquito)
## aalb04137             Mitophagy - animal - Aedes albopictus (Asian tiger mosquito)
## aalb00564 Glycerophospholipid metabolism - Aedes albopictus (Asian tiger mosquito)
## aalb04148                  Efferocytosis - Aedes albopictus (Asian tiger mosquito)
##           GeneRatio BgRatio       pvalue     p.adjust       qvalue
## aalb04214      4/11 56/3977 1.081909e-05 0.0001731054 0.0001138851
## aalb04215      2/11 28/3977 2.528285e-03 0.0198316953 0.0130471680
## aalb00600      2/11 34/3977 3.718443e-03 0.0198316953 0.0130471680
## aalb04137      2/11 59/3977 1.092069e-02 0.0384480304 0.0252947568
## aalb00564      2/11 62/3977 1.201501e-02 0.0384480304 0.0252947568
## aalb04148      2/11 74/3977 1.685340e-02 0.0449424033 0.0295673706
##                                            geneID Count
## aalb04214 109408179/115257864/109428244/115271029     4
## aalb04215                     109428244/115271029     2
## aalb00600                     115266598/109430628     2
## aalb04137                     109408179/109427928     2
## aalb00564                     115266598/109430628     2
## aalb04148                     109428244/115271029     2
```

```
# Save ORA results to file
if (!is.null(kegg_ora) && nrow(kegg_ora@result) > 0) {
  write.table(
    as.data.frame(kegg_ora),
    file = "/gpfs/gibbs/pi/caccone/mkc54/albo/manuscript1/output/enrichment/corner1/kegg_ora_results.tsv",
    sep = "\t",
    row.names = FALSE,
    quote = FALSE
  )
  cat("ORA results saved to kegg_ora_results_corner1.tsv\n")

  # Visualizations for ORA
  # Dot plot
  pdf("/gpfs/gibbs/pi/caccone/mkc54/albo/manuscript1/output/enrichment/corner1/kegg_ora_dotplot.pdf", width = 10, height = 8)
  print(dotplot(kegg_ora, showCategory = 20, title = "KEGG ORA - Dot Plot"))
  dev.off()

  # Bar plot
  pdf("/gpfs/gibbs/pi/caccone/mkc54/albo/manuscript1/output/enrichment/corner1/kegg_ora_barplot.pdf", width = 10, height = 8)
  print(barplot(kegg_ora, showCategory = 20, title = "KEGG ORA - Bar Plot"))
  dev.off()

# Enrichment map (if multiple pathways are enriched)
  # Need at least 2 significant pathways for enrichment map
  significant_pathways <- sum(kegg_ora@result$p.adjust < 0.05)
  if (significant_pathways >= 2) {
    tryCatch({
      kegg_ora_sim <- pairwise_termsim(kegg_ora)
      pdf("/gpfs/gibbs/pi/caccone/mkc54/albo/manuscript1/output/enrichment/corner1/kegg_ora_emaplot.pdf", width = 12, height = 10)
      # Suppress warnings about deprecated arguments
      suppressWarnings(print(emapplot(kegg_ora_sim, showCategory = 20)))
      dev.off()
      cat("Enrichment map saved\n")
    }, error = function(e) {
      cat("Note: Could not create enrichment map -", conditionMessage(e), "\n")
    })
  } else {
    cat("Note: Need at least 2 significant pathways for enrichment map (found:", significant_pathways, ")\n")
  }

  cat("ORA visualizations saved\n")
} else {
  cat("No significant pathways found in ORA\n")
}
```

```
## ORA results saved to kegg_ora_results_corner1.tsv
```

```
## Warning: `aes_string()` was deprecated in ggplot2 3.0.0.
## ℹ Please use tidy evaluation idioms with `aes()`.
## ℹ See also `vignette("ggplot2-in-packages")` for more information.
## ℹ The deprecated feature was likely used in the enrichplot package.
##   Please report the issue at
##   <https://github.com/GuangchuangYu/enrichplot/issues>.
## This warning is displayed once per session.
## Call `lifecycle::last_lifecycle_warnings()` to see where this warning was
## generated.
```

```
## Warning in fortify(object, showCategory = showCategory, by = x, ...): Arguments in `...` must be used.
## ✖ Problematic argument:
## • by = x
## ℹ Did you misspell an argument name?
```

```
## Enrichment map saved
## ORA visualizations saved
```

6 pathways are significant for corner 1

# 2. Corner 2 - Top 5% outliers for 50 SNP windows

## 2.1 Load data

```
library(dplyr)

outliers_2 <- read.csv("manuscript1/output/enrichment/corner2_genes_in_top5_percent_windows_scaffold_mapped.csv")
head(outliers_2)
```

```
##   scaffold               gene_id gene_start gene_end chr
## 1    1.147     gene-LOC109406400    4395029  4402025   1
## 2    1.147    rna-XM_029867665.1    4395029  4402025   1
## 3    1.147 exon-XM_029867665.1-1    4395029  4395281   1
## 4    1.147 exon-XM_029867665.1-2    4395373  4396195   1
## 5    1.147 exon-XM_029867665.1-3    4396198  4397372   1
## 6    1.147 exon-XM_029867665.1-4    4397374  4397476   1
```

```
# Should have: corner, chrom, gene_id, gene_start, gene_end
```

```
# Extract LOC IDs; everything else becomes NA
outliers_2 <- outliers_2 %>%
  mutate(core_gene_id = ifelse(grepl("LOC\\d+", gene_id),
                               sub(".*?(LOC\\d+).*", "\\1", gene_id),
                               NA))

# Check
head(outliers_2[, c("gene_id", "core_gene_id")])
```

```
##                 gene_id core_gene_id
## 1     gene-LOC109406400 LOC109406400
## 2    rna-XM_029867665.1         <NA>
## 3 exon-XM_029867665.1-1         <NA>
## 4 exon-XM_029867665.1-2         <NA>
## 5 exon-XM_029867665.1-3         <NA>
## 6 exon-XM_029867665.1-4         <NA>
```

Now the core\_gene\_id column should match the format of our
“universal\_gene\_file”

Export it

```
core_ids_unique <- outliers_2 %>%
  pull(core_gene_id) %>%
  na.omit() %>%
  unique()
```

```
write.table(core_ids_unique,
            file = "/gpfs/gibbs/pi/caccone/mkc54/albo/manuscript1/output/enrichment/corner2_outlier_5_genes_coreIDs_50.txt",
            quote = FALSE,
            row.names = FALSE,
            col.names = FALSE)
```

```
cat("Loading gene lists...\n")
```

```
## Loading gene lists...
```

```
# Specify input files
input_gene_file <- "/gpfs/gibbs/pi/caccone/mkc54/albo/manuscript1/output/enrichment/corner2_outlier_5_genes_coreIDs_50.txt"  # Change this to your gene list of interest
universe_gene_file <- "/gpfs/gibbs/pi/caccone/mkc54/albo/manuscript1/output/enrichment/universal_LOC_list.txt"  # Change this to your background/universe list

# Load input gene list
inputgenelist <- read.table(
  input_gene_file,
  header = FALSE,
  stringsAsFactors = FALSE,
  col.names = "gene_id"
)

# Load universe gene list
universegenelist <- read.table(
  universe_gene_file,
  header = FALSE,
  stringsAsFactors = FALSE,
  col.names = "gene_id"
)
```

Convert LOC IDs to NCBI gene IDs by removing “LOC” prefix

```
inputgenelist$ncbi_geneid <- gsub("^LOC", "", inputgenelist$gene_id)
universegenelist$ncbi_geneid <- gsub("^LOC", "", universegenelist$gene_id)

cat("Input genes loaded:", nrow(inputgenelist), "\n")
```

```
## Input genes loaded: 106
```

```
cat("Universe genes loaded:", nrow(universegenelist), "\n")
```

```
## Universe genes loaded: 21553
```

Input genes loaded: 106  
Universe genes loaded: 21553

Now our gene list of interest is ~0.5% of the total

## 2.2. KEGG Over-Representation Analysis (ORA)

### 2.2.1 Using BH correction and p<0.05 threshold

```
options(rstudio.notebook.plot.cache = FALSE)

cat("Running KEGG Over-Representation Analysis (ORA)...\n")
```

```
## Running KEGG Over-Representation Analysis (ORA)...
```

```
# Perform KEGG ORA
kegg_ora <- enrichKEGG(
  gene = inputgenelist$ncbi_geneid,
  universe = universegenelist$ncbi_geneid,
  organism = "aalb",
  pvalueCutoff = 0.05,
  pAdjustMethod = "BH",
  keyType = "ncbi-geneid"
)

# View results
print(head(kegg_ora))
```

```
##                  ID
## aalb01230 aalb01230
## aalb04512 aalb04512
##                                                                     Description
## aalb01230 Biosynthesis of amino acids - Aedes albopictus (Asian tiger mosquito)
## aalb04512    ECM-receptor interaction - Aedes albopictus (Asian tiger mosquito)
##           GeneRatio BgRatio      pvalue   p.adjust     qvalue
## aalb01230      3/18 57/3977 0.001955593 0.03412762 0.02927125
## aalb04512      2/18 17/3977 0.002527972 0.03412762 0.02927125
##                                  geneID Count
## aalb01230 109423532/109423534/109410517     3
## aalb04512           109426653/115265272     2
```

```
# Save ORA results to file
if (!is.null(kegg_ora) && nrow(kegg_ora@result) > 0) {
  write.table(
    as.data.frame(kegg_ora),
    file = "/gpfs/gibbs/pi/caccone/mkc54/albo/manuscript1/output/enrichment/corner2/kegg_ora_results.tsv",
    sep = "\t",
    row.names = FALSE,
    quote = FALSE
  )
  cat("ORA results saved to kegg_ora_results_corner2.tsv\n")

  # Visualizations for ORA
  # Dot plot
  pdf("/gpfs/gibbs/pi/caccone/mkc54/albo/manuscript1/output/enrichment/corner2/kegg_ora_dotplot.pdf", width = 10, height = 8)
  print(dotplot(kegg_ora, showCategory = 20, title = "KEGG ORA - Dot Plot"))
  dev.off()

  # Bar plot
  pdf("/gpfs/gibbs/pi/caccone/mkc54/albo/manuscript1/output/enrichment/corner2/kegg_ora_barplot.pdf", width = 10, height = 8)
  print(barplot(kegg_ora, showCategory = 20, title = "KEGG ORA - Bar Plot"))
  dev.off()

# Enrichment map (if multiple pathways are enriched)
  # Need at least 2 significant pathways for enrichment map
  significant_pathways <- sum(kegg_ora@result$p.adjust < 0.05)
  if (significant_pathways >= 2) {
    tryCatch({
      kegg_ora_sim <- pairwise_termsim(kegg_ora)
      pdf("/gpfs/gibbs/pi/caccone/mkc54/albo/manuscript1/output/enrichment/corner2/kegg_ora_emaplot.pdf", width = 12, height = 10)
      # Suppress warnings about deprecated arguments
      suppressWarnings(print(emapplot(kegg_ora_sim, showCategory = 20)))
      dev.off()
      cat("Enrichment map saved\n")
    }, error = function(e) {
      cat("Note: Could not create enrichment map -", conditionMessage(e), "\n")
    })
  } else {
    cat("Note: Need at least 2 significant pathways for enrichment map (found:", significant_pathways, ")\n")
  }

  cat("ORA visualizations saved\n")
} else {
  cat("No significant pathways found in ORA\n")
}
```

```
## ORA results saved to kegg_ora_results_corner2.tsv
```

```
## Warning in fortify(object, showCategory = showCategory, by = x, ...): Arguments in `...` must be used.
## ✖ Problematic argument:
## • by = x
## ℹ Did you misspell an argument name?
```

```
## Note: Could not create enrichment map - Viewport has zero dimension(s) 
## ORA visualizations saved
```

2 significant pathways - aalb01230 & aalb04512

# 3. Corner 3 - Top 5% outliers for 50 SNP windows

## 3.1 Load data

```
library(dplyr)

outliers_3 <- read.csv("manuscript1/output/enrichment/corner3_genes_in_top5_percent_windows_scaffold_mapped.csv")
head(outliers_3)
```

```
##   scaffold               gene_id gene_start gene_end chr
## 1      1.7     gene-LOC109408932   14561597 14571818   1
## 2      1.7    rna-XM_019682329.2   14561597 14571818   1
## 3      1.7 exon-XM_019682329.2-3   14561597 14562457   1
## 4      1.7    cds-XP_019537874.2   14561750 14562457   1
## 5      1.7     gene-LOC109408933   14535181 14561699   1
## 6      1.7    rna-XM_019682330.2   14535181 14561699   1
```

```
# Should have: corner, chrom, gene_id, gene_start, gene_end
```

```
# Extract LOC IDs; everything else becomes NA
outliers_3 <- outliers_3 %>%
  mutate(core_gene_id = ifelse(grepl("LOC\\d+", gene_id),
                               sub(".*?(LOC\\d+).*", "\\1", gene_id),
                               NA))

# Check
head(outliers_3[, c("gene_id", "core_gene_id")])
```

```
##                 gene_id core_gene_id
## 1     gene-LOC109408932 LOC109408932
## 2    rna-XM_019682329.2         <NA>
## 3 exon-XM_019682329.2-3         <NA>
## 4    cds-XP_019537874.2         <NA>
## 5     gene-LOC109408933 LOC109408933
## 6    rna-XM_019682330.2         <NA>
```

Now the core\_gene\_id column should match the format of our
“universal\_gene\_file”

Export it

```
core_ids_unique <- outliers_3 %>%
  pull(core_gene_id) %>%
  na.omit() %>%
  unique()
```

```
write.table(core_ids_unique,
            file = "/gpfs/gibbs/pi/caccone/mkc54/albo/manuscript1/output/enrichment/corner3_outlier_5_genes_coreIDs_50.txt",
            quote = FALSE,
            row.names = FALSE,
            col.names = FALSE)
```

```
cat("Loading gene lists...\n")
```

```
## Loading gene lists...
```

```
# Specify input files
input_gene_file <- "/gpfs/gibbs/pi/caccone/mkc54/albo/manuscript1/output/enrichment/corner3_outlier_5_genes_coreIDs_50.txt"  # Change this to your gene list of interest
universe_gene_file <- "/gpfs/gibbs/pi/caccone/mkc54/albo/manuscript1/output/enrichment/universal_LOC_list.txt"  # Change this to your background/universe list

# Load input gene list
inputgenelist <- read.table(
  input_gene_file,
  header = FALSE,
  stringsAsFactors = FALSE,
  col.names = "gene_id"
)

# Load universe gene list
universegenelist <- read.table(
  universe_gene_file,
  header = FALSE,
  stringsAsFactors = FALSE,
  col.names = "gene_id"
)
```

Convert LOC IDs to NCBI gene IDs by removing “LOC” prefix

```
inputgenelist$ncbi_geneid <- gsub("^LOC", "", inputgenelist$gene_id)
universegenelist$ncbi_geneid <- gsub("^LOC", "", universegenelist$gene_id)

cat("Input genes loaded:", nrow(inputgenelist), "\n")
```

```
## Input genes loaded: 97
```

```
cat("Universe genes loaded:", nrow(universegenelist), "\n")
```

```
## Universe genes loaded: 21553
```

Input genes loaded: 97  
Universe genes loaded: 21553

Now our gene list of interest is ~0.45% of the total

## 3.2. KEGG Over-Representation Analysis (ORA)

### 3.2.1 Using BH correction and p<0.05 threshold

```
options(rstudio.notebook.plot.cache = FALSE)

cat("Running KEGG Over-Representation Analysis (ORA)...\n")
```

```
## Running KEGG Over-Representation Analysis (ORA)...
```

```
# Perform KEGG ORA
kegg_ora <- enrichKEGG(
  gene = inputgenelist$ncbi_geneid,
  universe = universegenelist$ncbi_geneid,
  organism = "aalb",
  pvalueCutoff = 0.05,
  pAdjustMethod = "BH",
  keyType = "ncbi-geneid"
)

# View results
print(head(kegg_ora))
```

```
## [1] ID          Description GeneRatio   BgRatio     pvalue      p.adjust   
## [7] qvalue      geneID      Count      
## <0 rows> (or 0-length row.names)
```

```
# Save ORA results to file
if (!is.null(kegg_ora) && nrow(kegg_ora@result) > 0) {
  write.table(
    as.data.frame(kegg_ora),
    file = "/gpfs/gibbs/pi/caccone/mkc54/albo/manuscript1/output/enrichment/corner3/kegg_ora_results.tsv",
    sep = "\t",
    row.names = FALSE,
    quote = FALSE
  )
  cat("ORA results saved to kegg_ora_results_corner3.tsv\n")

  # Visualizations for ORA
  # Dot plot
  pdf("/gpfs/gibbs/pi/caccone/mkc54/albo/manuscript1/output/enrichment/corner3/kegg_ora_dotplot.pdf", width = 10, height = 8)
  print(dotplot(kegg_ora, showCategory = 20, title = "KEGG ORA - Dot Plot"))
  dev.off()

  # Bar plot
  pdf("/gpfs/gibbs/pi/caccone/mkc54/albo/manuscript1/output/enrichment/corner3/kegg_ora_barplot.pdf", width = 10, height = 8)
  print(barplot(kegg_ora, showCategory = 20, title = "KEGG ORA - Bar Plot"))
  dev.off()

# Enrichment map (if multiple pathways are enriched)
  # Need at least 2 significant pathways for enrichment map
  significant_pathways <- sum(kegg_ora@result$p.adjust < 0.05)
  if (significant_pathways >= 2) {
    tryCatch({
      kegg_ora_sim <- pairwise_termsim(kegg_ora)
      pdf("/gpfs/gibbs/pi/caccone/mkc54/albo/manuscript1/output/enrichment/corner3/kegg_ora_emaplot.pdf", width = 12, height = 10)
      # Suppress warnings about deprecated arguments
      suppressWarnings(print(emapplot(kegg_ora_sim, showCategory = 20)))
      dev.off()
      cat("Enrichment map saved\n")
    }, error = function(e) {
      cat("Note: Could not create enrichment map -", conditionMessage(e), "\n")
    })
  } else {
    cat("Note: Need at least 2 significant pathways for enrichment map (found:", significant_pathways, ")\n")
  }

  cat("ORA visualizations saved\n")
} else {
  cat("No significant pathways found in ORA\n")
}
```

```
## ORA results saved to kegg_ora_results_corner3.tsv
```

```
## Warning in fortify(object, showCategory = showCategory, by = x, ...): Arguments in `...` must be used.
## ✖ Problematic argument:
## • by = x
## ℹ Did you misspell an argument name?
```

```
## Note: Need at least 2 significant pathways for enrichment map (found: 0 )
## ORA visualizations saved
```

0 found

END
