# Supplementary material for: Population Structure of the Invasive Asian Tiger Mosquito, Aedes albopictus, in the Americas
Source: Insects. 2026 Jul 23;17(8):755. doi: 10.3390/insects17080755 (PMC13512968; doi:10.3390/insects17080755)
Supplement: Supplementary file 1 [file insects-17-00755-s001.zip › RMarkdowns/S6 - QC for American dataset.html]

QC for Americas dataset


# QC for Americas dataset

#### Margaret Corley

#### 2026-05-03

- Note: This file creates two different sets of SNPs - 1 with Minor
  Allele Frequency of 10% (MAF 0.1) and another 1% (MAF 0.01) (code
  starting in section 9). As stated in the text of the Methods section,
  the SNP set with 1% MAF pruning is used for all analyses, unless
  otherwise specified.

# 1. Load R libraries and software

```
library(tidyverse)
```

```
## Warning: Your system is mis-configured: '/etc/localtime' is not a symlink
```

```
library(here)
library(dplyr)
library(ggplot2)
library(colorout)
library(extrafont)
library(reticulate)
library(scales)
library(stringr)
library(grid)
library(flextable)
library(devtools)
library(readr)
library(purrr)
library(ggtext)
library(ggvenn)
library(data.table)
library(RColorBrewer)
library(ggrepel)
```

### 1.2 Get software for quality control

Check Plink version

```
module spider plink
#Versions on the cluster:
        #LINK/1.9b_6.21-x86_64
        #LINK/2_avx2_20221024
module load PLINK/2_avx2_20221024

plink2 --version
#PLINK v2.00a3.7LM AVX2 Intel (24 Oct 2022)
```

Check BCFtools version

```
module spider BCFtools
#BCFtools/1.16-GCCcore-10.2.0

module load BCFtools/1.16-GCCcore-10.2.0
```

### 1.3 Check if the data is in the correct location

```
cd /gpfs/gibbs/pi/caccone/mkc54/albo

ls data/americas/* # we use to truncate the name of the file showing all files
```

```
## data/americas/albo_manuscript1_19Aug2024.vcf
## data/americas/albo_manuscript1_lowadmix_fs_23Aug2024_newprior.vcf
## data/americas/all_americas_1Apr2024_newprior.vcf
## data/americas/americas_low_admix_fs_10Sep2024.vcf
## data/americas/asia_americas_all_28Mar2024_newpriors.vcf
## data/americas/lowadmix_asia_euro_americas_fs_15Sep2024.vcf
## data/americas/native_euro_lowadmix_man1_16Sep2024.vcf
## data/americas/native_europe_man1_12Sep2024.vcf
## 
## data/americas/north_america:
## albo_usa_local_adapt_2Oct2024.vcf
## albo_usa_lowadmix_fs_1Jul2024_newprior.vcf
## asia_north_america_1Apr2024_newprior.vcf
## north_america_28Mar2024_newprior.vcf
## north_america_carib_28Mar2024_newprior.vcf
## 
## data/americas/south_america:
## albo_south_america_lowadmix_fs_1Jul2024_newprior.vcf
## asia_south_america_carib_1Apr2024_newprior.vcf
## south_america_28Mar2024_newprior.vcf
## south_america_carib_28Mar2024_newprior.vcf
```

Check how many sample names we have in our vcf

```
# make sure you have all the .CEL samples in your family file
bcftools query -l /gpfs/gibbs/pi/caccone/mkc54/albo/data/americas/all_americas_1Apr2024_newprior.vcf | wc -l
#555
```

555

# 2. The data

## 2.1 Use Plink2 to convert to bed format

```
cd /gpfs/gibbs/pi/caccone/mkc54/albo

plink2 \
--allow-extra-chr \
--vcf data/americas/all_americas_1Apr2024_newprior.vcf \
--const-fid \
--make-bed \
--exclude americas/albopictus_SNPs_fail_segregation.txt \
--fa /gpfs/gibbs/project/caccone/mkc54/albo/genome/albo.fasta.gz \
--ref-from-fa 'force' `# sets REF alleles when it can be done unambiguously, we use force to change the alleles` \
--out americas/output/file1 \
--silent;
# --keep-allele-order \ if you use Plink 1.9
grep "variants" americas/output/file1.log; # to get the number of variants from the log file.
```

–vcf: 112855 variants scanned. 112855 variants loaded from
americas/output/file1-temporary.pvar.zst. –exclude: 111397 variants
remaining. 111397 variants remaining after main filters. –ref-from-fa
force: 0 variants changed, 111397 validated.

Check the fam file

```
head americas/output/file1.fam
```

```
## MAU  153 0   0   0   -9
## MAU  154 0   0   0   -9
## MAU  155 0   0   0   -9
## MAU  156 0   0   0   -9
## MAU  158 0   0   0   -9
## MAU  16  0   0   0   -9
## TUC  174 0   0   0   -9
## REC  175 0   0   0   -9
## REC  176 0   0   0   -9
## REC  177 0   0   0   -9
```

```
head -n 5 americas/sample_ped_info_ALLPOPS_1Apr2024.txt
```

```
## Sample Filename  Family_ID   Individual_ID   Father_ID   Mother_ID   Sex Affection Status
## 1_LabCross_KF2.CEL   LAB 1   0   0   2   -9
## 2_LabCross_MF81.CEL  LAB 2   0   0   2   -9
## 3_LabCross_MF71.CEL  LAB 3   0   0   2   -9
## 4_LabCross_KF42.CEL  LAB 4   0   0   2   -9
```

Import the fam file we use with Axiom Suite

```
# the order of the rows in this file does not matter
samples <-
  read.delim(
    file   = here("americas", "sample_ped_info_ALLPOPS_1Apr2024.txt"
    ),
    header = TRUE
  )
head(samples)
```

```
##       Sample.Filename Family_ID Individual_ID Father_ID Mother_ID Sex
## 1  1_LabCross_KF2.CEL       LAB             1         0         0   2
## 2 2_LabCross_MF81.CEL       LAB             2         0         0   2
## 3 3_LabCross_MF71.CEL       LAB             3         0         0   2
## 4 4_LabCross_KF42.CEL       LAB             4         0         0   2
## 5 5_LabCross_KF57.CEL       LAB             5         0         0   2
## 6 6_LabCross_MF32.CEL       LAB             6         0         0   2
##   Affection.Status
## 1               -9
## 2               -9
## 3               -9
## 4               -9
## 5               -9
## 6               -9
```

Import .fam file we created once we created the bed file using
Plink2

```
#The fam file is the same for both data sets with the default or new priors
fam1 <-
  read.delim(
    file   = here("americas/output/file1.fam"),
    header = FALSE,
    
  )
head(fam1)
```

```
##    V1  V2 V3 V4 V5 V6
## 1 MAU 153  0  0  0 -9
## 2 MAU 154  0  0  0 -9
## 3 MAU 155  0  0  0 -9
## 4 MAU 156  0  0  0 -9
## 5 MAU 158  0  0  0 -9
## 6 MAU  16  0  0  0 -9
```

Merge the tibbles

```
# Extract the number part from the columns
fam1_temp <- fam1 |>
  mutate(num_id = as.numeric(str_extract(V2, "^\\d+")))

samples_temp <- samples |>
  mutate(num_id = as.numeric(str_extract(Sample.Filename, "^\\d+")))

# Perform the left join using the num_id columns and keep the order of fam1
df <- fam1_temp |>
  dplyr::left_join(samples_temp, by = "num_id") |>
  dplyr::select(-num_id) |>
  dplyr::select(8:13)

# check the data frame
head(df)
```

```
##   Family_ID Individual_ID Father_ID Mother_ID Sex Affection.Status
## 1       MAU           153         0         0   0               -9
## 2       MAU           154         0         0   0               -9
## 3       MAU           155         0         0   0               -9
## 4       MAU           156         0         0   0               -9
## 5       MAU           158         0         0   0               -9
## 6       MAU            16         0         0   0               -9
```

Check how many samples we have in our file

```
nrow(df)
```

```
## [1] 555
```

```
#555
```

Before saving the new fam file, change the original file to a
different name, to compare the order later. If you want to repeat the
steps above after you save the new file1.fam, you will need to import
the vcf again

```
# Save and override the .fam file for dp
write.table(
  df,
  file      = here("americas/output/file1.fam"),
  sep       = "\t",
  row.names = FALSE,
  col.names = FALSE,
  quote     = FALSE
)
```

Check the new .fam file to see if has the order and the sample
attributes we want.

```
# you can open the file on a text editor and double check the sample order and information.
head -n 5 americas/output/file1.fam
```

```
## MAU  153 0   0   0   -9
## MAU  154 0   0   0   -9
## MAU  155 0   0   0   -9
## MAU  156 0   0   0   -9
## MAU  158 0   0   0   -9
```

## 2.3 Remove duplicates

We had 4 samples genotyped 3 times each. We can check for the
triplicates in our dataset

```
# Check triplicates
grep "a\|b\|c" americas/output/file1.fam
```

There are no duplicates, so we can skip to next step.

Create a new bed removing the SNPs that failed the segretation test.
We can also make sure the reference alleles match the reference
genome

```
# I created a fam file with the information about each sample, but first we import the data and create a bed file setting the family id constant
plink2 \
--allow-extra-chr \
--bfile americas/output/file1 \
--make-bed \
--fa /gpfs/gibbs/project/caccone/mkc54/albo/genome/albo.fasta.gz \
--ref-from-fa 'force' `# sets REF alleles when it can be done unambiguously, we use force to change the alleles` \
--exclude europe/output/files/albopictus_SNPs_fail_segregation.txt \
--out americas/output/file1b \
--silent;
# --keep-allele-order \ if you use Plink 1.9
grep "samples\|variants" americas/output/file1b.log # to get the number of variants from the log file.
```

555 samples (33 females, 32 males, 490 ambiguous; 555 founders)
loaded from 111397 variants loaded from americas/output/file1.bim.
–exclude: 111397 variants remaining. 111397 variants remaining after
main filters. –ref-from-fa force: 0 variants changed, 111397
validated.

Check the headings of the the files we will work on.

```
head -n 5 americas/output/file1b.fam
```

```
## MAU  153 0   0   0   -9
## MAU  154 0   0   0   -9
## MAU  155 0   0   0   -9
## MAU  156 0   0   0   -9
## MAU  158 0   0   0   -9
```

Check how many samples are in the .fam file

```
wc -l americas/output/file1b.fam
#555
```

```
## 555 americas/output/file1b.fam
```

## 2.4 Check the number of samples and localities

This part is very important. Make sure there are no NAs or something
that is not what you would expect. For example, the number of mosquitoes
per population.

```
awk '{print $1}' americas/output/file1b.fam | sort | uniq -c | awk '{print $2, $1}'
```

```
## ARA 12
## BAF 7
## BCO 12
## BEA 12
## BEH 9
## BER 12
## BIT 12
## BPA 12
## BPV 12
## BRM 16
## CHI 7
## COL 10
## CUA 8
## DAL 12
## DEN 12
## FAY 11
## FIR 12
## FLO 8
## GRV 12
## HAL 10
## HAR 12
## HAT 11
## HOU 12
## IPO 8
## LIN 12
## LOS 14
## MAC 12
## MAN 12
## MAU 12
## MED 7
## MON 12
## NEB 11
## NEG 4
## NEH 7
## NEO 12
## NEW 22
## NOV 4
## NUE 12
## PAL 11
## PEO 10
## PLM 7
## POR 12
## REC 12
## RUS 12
## SAI 12
## SMI 12
## SPR 8
## STL 12
## TRA 12
## TUC 14
## WAV 7
```

# 3. Quality control steps

## 3.1 Missingness

```
plink2 \
--allow-extra-chr \
--bfile americas/output/file1b \
--geno 0.1                `# we set genotype missiningness to 10% with this option` \
--make-bed \
--out americas/output/file2 \
--silent \
--missing;                 # --missing produces sample-based and variant-based missing data reports. If run with --within/--family, the variant-based report is stratified by cluster.
grep "variants" americas/output/file2.log
```

111397 variants loaded from americas/output/file1b.bim. –geno: 9408
variants removed due to missing genotype data. 101989 variants remaining
after main filters.

Make plot

```
#   ____________________________________________________________________________
#   import individual missingness                                           ####
indmiss <-                                              # name of the data frame we are creating
  read.delim(                                           # use function read table
    file   = here(
      "americas/output/file2.smiss"
    ),                                                  # we use library here to import file2.imiss from data/QC
    header = TRUE                                       # we do have headers in our file
  )
#   ____________________________________________________________________________
#   import SNP missingness                                                  ####
snpmiss <-
  read.delim(
    file   = here(
      "americas/output/file2.vmiss"
    ),
    header = TRUE
  )
#
```

Plot individual missingness

```
# load plotting theme
source(
  here("scripts", "RMarkdowns", "my_theme2.R"
  )
)

ggplot( # Start a ggplot object with the data and aesthetic mappings
  indmiss,
  aes(
    x = F_MISS
  )
) +
  geom_histogram( # Add a histogram layer
    color            = "black",
    fill             = "lightgray",
    bins             = 6
  ) +
  geom_text(
    # Add text labels for bin counts
    stat             = "bin",
    aes(
    label = after_stat(count)
  ),
    vjust            = -0.5,
    color            = "black",
    size             = 3,
    bins             = 6
  ) +
  geom_vline(
    # Add a vertical line at the mean of F_MISS
    aes(
    xintercept = mean(F_MISS)),
    color            = "red",
    linetype         = "dotted",
    linewidth        = .5
  ) +
  geom_text(
    # Add a text label for the mean of F_MISS
    aes(
      x = mean(F_MISS),
      y = 75,
      label = paste0(
        "Mean \n",
        scales::percent(mean(F_MISS),
          accuracy = 0.01
        )
      )
    ),
    size = 3,
    color = "red",
    hjust = -.1
  ) +
  labs( # Add axis labels
    x                = "Individual Missingness (%)",
    y                = "Frequency (n)"
  ) +
  my_theme() +
  scale_x_continuous( # Scale the x-axis to display percentages
    labels           = scales::percent,
    n.breaks         = 6
  )
```

```
## Warning in geom_text(aes(x = mean(F_MISS), y = 75, label = paste0("Mean \n", : All aesthetics have length 1, but the data has 555 rows.
## ℹ Please consider using `annotate()` or provide this layer with data containing
##   a single row.
```

Save Plot

```
#
# save the plot
ggsave(
  here("scripts", "RMarkdowns", "output", "americas", "americas_all", "figures" , "individual_missingness10_americas_all.pdf"
  ),
  width              = 7,
  height             = 5,
  units              = "in"
)
```

```
# Define a function to customize the theme
my_theme <- function() {
  theme_minimal(base_size = 12, base_family = "") +
    theme(
      panel.grid.major = element_line(
        linetype = "dashed",
        linewidth = 0.2,
        color = "gray"
      ),
      panel.grid.minor = element_line(
        linetype = "dashed",
        linewidth = 0.2,
        color = "gray"
      ),
      # Customize the x-axis label
      axis.title.x = element_text(
        angle          = 0,
        hjust          = 1,
        face           = "bold"
      ),
      # Customize the y-axis label
      axis.title.y = element_text(
        angle          = 90,
        hjust          = 1,
        face           = "bold"
      )
    )
}
# we can save the function to source it later
dump(                                                    # check ?dump for more information
  "my_theme",                                            # the object we want to save
  here("scripts", "RMarkdowns", 
   "my_theme2.R")                # use here to save it our function as .R 
)
```

Plot variant missingness

```
# This plot takes a while to compute
# This code creates a histogram from the snpmiss data set using the F_MISS column.
# ggplot builds a histogram of individual missingness data
ggplot(
  snpmiss,
  aes(
    x = F_MISS
  )
) +
  geom_histogram(
    color = "black",
    fill = "lightgray",
    bins = 6
  ) +
  stat_bin(
    geom = "text",
    aes(
      label = format(
        after_stat(count),
        big.mark = ",",
        scientific = FALSE
      )
    ),
    vjust = -0.5,
    color = "black",
    size = 2,
    bins = 6
  ) +
  geom_vline(
    aes(
      xintercept = mean(F_MISS)
    ),
    color = "red",
    linetype = "dotted",
    linewidth = 0.5
  ) +
  geom_text(
    aes(
      x = mean(F_MISS),
      y = 16000,
      label = paste0(
        "Mean \n",
        scales::percent(mean(F_MISS),
          accuracy = 0.01
        )
      )
    ),
    size = 3,
    color = "red",
    # hjust = 1.5,
    vjust = -.2
  ) +
  labs(
    x = "Variant Missingness (%)",
    y = "Frequency (n)"
  ) +
  # theme_minimal(
  #   base_size = 12,
  #   base_family = "Roboto Condensed"
  # ) +
  scale_x_continuous(
    labels = scales::percent,
    n.breaks = 6
  ) +
  scale_y_continuous(
    labels = scales::label_comma(),
    n.breaks = 5
  ) +
  my_theme()
```

```
## Warning in geom_text(aes(x = mean(F_MISS), y = 16000, label = paste0("Mean \n", : All aesthetics have length 1, but the data has 111397 rows.
## ℹ Please consider using `annotate()` or provide this layer with data containing
##   a single row.
```

Save plot

```
# save the plot
ggsave(
  here("scripts", "RMarkdowns", "output", "americas", "americas_all",  "figures" , "SNPs_missingness10_americas_all.pdf"
  ),
  width  = 7,
  height = 5,
  units  = "in"
)
```

Remove individuals missing more than 20% of SNPs. You can use the
threshold you want, change the flag –mind

```
plink2 \
--allow-extra-chr \
--bfile americas/output/file2 \
--mind 0.2               `# here we set the individual missingness threshold of 20%`\
--make-bed \
--out americas/output/file3 \
--silent;
grep "samples\|variants" americas/output/file3.log
```

555 samples (33 females, 32 males, 490 ambiguous; 555 founders)
loaded from 101989 variants loaded from americas/output/file2.bim. 0
samples removed due to missing genotype data (–mind). 555 samples (33
females, 32 males, 490 ambiguous; 555 founders) remaining after

## 3.2 Minor allele frequency

First, estimate the allele frequency with Plink

```
plink2 \
--allow-extra-chr \
--bfile americas/output/file3 \
--freq \
--out americas/output/MAF_check \
--silent
```

Now apply the MAF filter

```
# We will use MAF of 10%
plink2 \
--allow-extra-chr \
--bfile americas/output/file3 \
--maf 0.1 \
--make-bed \
--out americas/output/file4 \
--silent;
grep "variants" americas/output/file4.log
```

101989 variants loaded from americas/output/file3.bim. 17930 variants
removed due to allele frequency threshold(s) 84059 variants remaining
after main filters.

Then plot with ggplot

```
#   Import MAF data                                                         ####
maf_freq <-
  read.delim(
    here(
      "americas/output/MAF_check.afreq"
    ),
    header = TRUE
  )
```

Make MAF plot

```
#   make the plot                                                           ####
ggplot(
  maf_freq,
  aes(ALT_FREQS)
) +
  geom_histogram(
    colour = "black",
    fill = "lightgray",
    bins = 40
  ) +
  labs(
    x = "Minor Allele Frequency (MAF)",
    y = "Frequency (n)",
    caption = "<span style='color:red;'><i>Red</i></span> <span style='color:black;'><i>line at</i></span><span style='color:red;'><i> MAF 10%</i></span><span style='color:black;'><i> threshold</i></span>."
  ) +
  geom_text(
    aes(
      x = .1,
      y = 8000,
      label = paste0("17,930 SNPs")
    ),
    size = 3,
    color = "red",
    vjust = -.2
  ) +
  geom_vline(xintercept = 0.1, color = "red") +
  my_theme() +
  theme(plot.caption = element_markdown()) +
  scale_y_continuous(label = scales::number_format(big.mark = ",")) +
  scale_x_continuous(breaks = c(0, 0.1, 0.2, 0.4, 0.6, 0.8, 1))
```

```
## Warning in geom_text(aes(x = 0.1, y = 8000, label = paste0("17,930 SNPs")), : All aesthetics have length 1, but the data has 101989 rows.
## ℹ Please consider using `annotate()` or provide this layer with data containing
##   a single row.
```

```
# save the plot
ggsave(
  here("scripts", "RMarkdowns", "output", "americas", "americas_all",  "figures" , "MAF_10_freq_plot_americas_all.pdf"
  ),
  width  = 7,
  height = 5,
  units  = "in"
)
```

We removed 17,930 variants due to the MAF filter. Next we will
excludes markers which deviate from Hardy–Weinberg equilibrium (HWE). It
is a common indicator of genotyping error, but may also indicate
evolutionary selection. We have to do it for each population
individually. We cannot do it for all populations at once. Therefore,
the first step is create a new bed file with Plink keeping only one
population. I like to create a new directory and name it “hardy”, and
copy the “file4” there.

```
mkdir -p americas/output/files/hardy;
cp americas/output/file4.* americas/output/files/hardy/
```

## 3.3 HWE test

Now we can run the HWE test. However, we will need to apply the SNP
missingness again for each population. If we do not, the HWE will vary
widely. With the bash script below, we will create a new file for each
population, run the HWE test with HWE p value <1e‐6 (HWE p value
<1e‐6). Then, we ask Plink to generate a list of SNPs that passed the
test for each population.

```
for fam in $(awk '{print $1}' americas/output/files/hardy/file4.fam | sort | uniq); 
do 
echo $fam | \
plink2 \
--allow-extra-chr \
--silent \
--keep-allele-order \
--bfile americas/output/files/hardy/file4 \
--keep-fam /dev/stdin \
--make-bed \
--out americas/output/files/hardy/$fam \
--hwe 0.000001 \
--geno 0.1 \
--write-snplist; \
done
```

Next, we use “cat” and “awk” to concatenate the SNP list from all
populations, and remove duplicates. Once we have a list of SNPs that
passed the test for each population, we can use Plink to create a new
bed file keeping only the SNPs that passed the test in each population.
First, lets get the list of SNPs, and count how many passed:

```
cd /gpfs/gibbs/pi/caccone/mkc54/albo
cat americas/output/files/hardy/*.snplist | awk '!a[$0]++' > americas/output/files/passed_hwe.txt;
wc -l americas/output/files/passed_hwe.txt
```

All variants passed HWE test. If some failed, next time we could
remove the variants that did not pass HWE test, using the –extract flag,
extracting only those that passed HWE.

## 3.4 Linkage analysis

We can analyse the LD patterns for each populations using different
approaches. If we want to look at general patterns we can get an
estimate for each population, for example, the half of the distance of
the r2 max. We can use Plink2 to estimates some statistics for us. One
important consideration to is how good is our genome assembly. Does it
matter if we use the 574 scaffolds or assign each scaffold to a
chromosome or not. So, if you want to know if it matters, you would need
to create a new chromosomal scale and perform the calculations. Since,
we want to get only an general approximate estimate of the linkage
levels, we can look at the largest scaffolds. Since chromosome 1 has the
M locus, we will get the largest scaffold of chromosome 2 and 3. We
create a new chromosomal scale when we want to plot the data. For now,
we can import a file that LC created with the size of each scaffold
(data/genome/scaffold\_sizes.txt). The code below imports the file and
get the 3 largest scaffolds from each chromosome.

```
#   import the file with the scaffold sizes                                 ####
scaffold_bps <-
  read_delim(
    here("scripts", "RMarkdowns", "scaffold_sizes.txt"
    ),
    col_names      = FALSE,
    show_col_types = FALSE,
    col_types      = "cn"
  )
#
# set column names
colnames(
  scaffold_bps
) <- c(
  "Scaffold", "Size"
)
#   ____________________________________________________________________________
#   create new column with the chromosome number                            ####
sizes <-
  scaffold_bps |>
  mutate(
    Chromosome = case_when( # we use mutate to create a new column called Chromosome
      startsWith(
        Scaffold, "1"
      ) ~ "1",              # use startsWith to get Scaffold rows starting with 1 and output 1 on Chromosome column
      startsWith(
        Scaffold, "2"
      ) ~ "2",
      startsWith(
        Scaffold, "3"
      ) ~ "3"
    )
  )
#
# group by Chromosome and get row with highest Size value
df_max <-
  sizes |>
  group_by(
    Chromosome
  ) |>
  slice_max(
    Size
  )
#
# print result
df_max |>
  mutate(
    Size_Mb = Size/1e6
  )
```

```
## # A tibble: 3 × 4
## # Groups:   Chromosome [3]
##   Scaffold     Size Chromosome Size_Mb
##   <chr>       <dbl> <chr>        <dbl>
## 1 1.85     28902815 1             28.9
## 2 2.17     43762705 2             43.8
## 3 3.75     25123842 3             25.1
```

The largest scaffolds of chromosome 2 is 43Mb, and chromosome 3 is
25Mb. We can also take a look at the size distribution of the scaffolds
using ggplot2.

```
# create histogram using ggplot2
library(scales)
ggplot(
  sizes,
  aes(
    x = Size
  )
) +
  geom_histogram(
    binwidth = 1e6,
    aes(fill = factor(
      Chromosome
    )),
    color = "lightgray"
  ) +
  labs(
    title = "Scaffold sizes per chromosome",
    x = "Scaffold Size (Mb)",
    y = "Count (n)"
  ) +
  # xlab("Scaffold Size (Mb)") +
  # ylab("Count (n)") +
  my_theme() +
  scale_x_continuous(
    labels = comma_format(
      scale = 1e-6
    )
  ) +
  facet_wrap(
    ~Chromosome,
    ncol = 3,
    scales = "free_x"
  ) +
  scale_fill_manual(
    values = c(
      "pink", "green", "orange"
    )
  ) +
  guides(fill = "none")
```

Now, we can create a list of SNPs from these two large scaffolds. We
hope they will give us an average LD statistic that represents the
entire genome. We can import the .bim file and get the list of variants
from scaffolds 2.17 (43Mb) and 3.75 (25Mb)

Import the .bim file with the SNPs

```
#   import the bim file with the SNP data                                   ####
snps <-
  read_delim(                    # to learn about the options use here, run ?read_delim on the console.
    here(
      "americas/output/file4.bim" #output/populations/file4.bim
    ),                           # use library here to load it
    col_names      = FALSE,      # we don't have header in the input file
    show_col_types = FALSE,      # suppress message from read_delim
    col_types      = "ccidcc"    # set the class of each column
  )
#
# set column names
colnames(
  snps
) <-                             # to add a header in our tibble
  c(
    "Scaffold", "SNP", "Cm", "Position", "Allele1", "Allele2"
  )
#
# check the tibble
head(snps)
```

```
## # A tibble: 6 × 6
##   Scaffold SNP             Cm Position Allele1 Allele2
##   <chr>    <chr>        <int>    <dbl> <chr>   <chr>  
## 1 1.1      AX-583035163     0   315386 A       G      
## 2 1.1      AX-583033356     0   315674 C       T      
## 3 1.1      AX-583033370     0   330057 G       A      
## 4 1.1      AX-583035194     0   330265 A       G      
## 5 1.1      AX-583033387     0   331288 C       T      
## 6 1.10     AX-583035257     0    91677 T       C
```

Write a function to import the bim files

```
#   function to import bim files                                            ####
#
import_bim <- function(file) {
  # import as a tibble and set columns as integers
  bim <-
    read_delim(
      file,
      col_names      = FALSE,
      show_col_types = FALSE,
      col_types      = "ccidcc"
    )
  # rename the columns by index
  bim <- bim |>
    rename(
      Scaffold = 1,
      SNP      = 2,
      Cm       = 3,
      Position = 4,
      Allele1  = 5,
      Allele2  = 6
    )
  return(bim)
}
# we can save the function to source it later
dump(                                                     # check ?dump for more information
  "import_bim",                                           # the object we want to save
  here("scripts", "RMarkdowns", "analyses", "import_bim.R")       # use here to save it our function as .R 
)
```

Now create the list of SNPs we want to use for the general linkage
analysis

```
snps_4ld <-
  snps |>
  filter( # scaffolds 2.17 (43Mb) and 3.75 (25Mb)
    Scaffold == "2.17" | Scaffold == "3.75"
  ) |> 
  select(
    "Scaffold",
    "SNP"
  )
nrow(snps_4ld)
```

```
## [1] 5762
```

We have 5762 SNPs to estimate LD that are located in the two largest
scaffolds of chromosome 2 and 3.

```
#   save calculation to load later                                          ####
write.table(
  snps_4ld,
  file      = here(
    "americas/output/files/ld/snps_4ld.txt"
  ),
  sep       = "\t",
  row.names = FALSE,
  col.names = FALSE,
  quote     = FALSE
)
```

Now we have to select the populations for which we want to estimate
the LD statistic. Let only estimate LD for populations that we have 12
or more individuals.

```
awk '{print $1}' americas/output/file4.fam | sort | uniq -c | awk '{print $2, $1}' | awk '$2 >= 12 {print}'
```

```
## ARA 12
## BCO 12
## BEA 12
## BER 12
## BIT 12
## BPA 12
## BPV 12
## BRM 16
## DAL 12
## DEN 12
## FIR 12
## GRV 12
## HAR 12
## HOU 12
## LIN 12
## LOS 14
## MAC 12
## MAN 12
## MAU 12
## MON 12
## NEO 12
## NEW 22
## NUE 12
## POR 12
## REC 12
## RUS 12
## SAI 12
## SMI 12
## STL 12
## TRA 12
## TUC 14
```

Now we can create a list of populations that have 12 or more
individuals. We can use the command above but only print the list of
families. We can create a new directory for the LD analysis.

Create list of populations

```
awk '{print $1}' americas/output/file4.fam | sort | uniq -c | awk '{print $2, $1}' | awk '$2 >= 12 {print}' | awk '{print $1}' > americas/output/files/ld/pops_4ld.txt
```

### 3.4.1 Half distance r^2 max

Now use Plink1.9 to estimate LD and then bin the data

```
#load plink 1.9
module load PLINK/1.9b_6.21-x86_64
plink --version
#PLINK v1.90b6.21 64-bit (19 Oct 2020)
```

```
for fam in $(awk '{print $1}' americas/output/files/ld/pops_4ld.txt | sort | uniq); 
do 
echo $fam | \
plink \
--allow-extra-chr \
--keep-allele-order \
--extract americas/output/files/ld/snps_4ld.txt \
--bfile americas/output/file4 \
--keep-fam /dev/stdin \
--maf 0.1 \
--r2 \
--out americas/output/files/ld/$fam \
--geno 0.2 \
--write-snplist \
--silent
done;
#
rm americas/output/files/ld/*.nosex
```

Once we subset the bed files per population, we set a MAF and
genotype missingness of 10% as before but now we have smaller sample
size and not all populations will have all the variants.

```
import os

# Replace with the directory containing the .snplist files
directory = "americas/output/files/ld"

# Get a list of all .snplist files in the directory
file_list = [filename for filename in os.listdir(directory) if filename.endswith(".snplist")]

# Create a set to store the unique variants
unique_variants = set()

# Loop over each file and add the variants to the set
for filename in file_list:
    with open(os.path.join(directory, filename), "r") as file:
        for line in file:
            unique_variants.add(line.strip())

# Create a dictionary to store the number of files each variant appears in
variant_counts = {variant: 0 for variant in unique_variants}

# Loop over each file again and count the variants
for filename in file_list:
    with open(os.path.join(directory, filename), "r") as file:
        for line in file:
            variant = line.strip()
            variant_counts[variant] += 1

# Calculate the threshold for the number of files a variant must appear in
threshold = int(len(file_list) * 0.50)

# Get the list of unique variants that appear in at least 80% of files
all_variants = [variant for variant, count in variant_counts.items() if count >= threshold]

# Save the list of variants to a new file
with open("americas/output/files/ld/all_unique_variants_50pct.txt", "w") as file:
    file.write("\n".join(all_variants))
```

```
## 67859
```

```
#67859
```

Check how many SNPs

```
cat americas/output/files/ld/all_unique_variants_50pct.txt | sort | awk '!seen[gensub(/^[[:space:]]+|[[:space:]]+$/, "", "g")]++' | wc -l
#5220
```

```
## 5220
```

Now we can estimate LD again using the variants that are present in
at least 50% of the populations.

Now we can use Plink1.9 to estimate LD

```
for fam in $(awk '{print $1}' americas/output/files/ld/pops_4ld.txt | sort | uniq); 
do 
echo $fam | \
plink \
--allow-extra-chr \
--keep-allele-order \
--extract americas/output/files/ld/all_unique_variants_50pct.txt \
--bfile americas/output/file4 \
--keep-fam /dev/stdin \
--maf 0.1 \
--r2 \
--out americas/output/files/ld/$fam \
--geno 0.2 \
--write-snplist \
--silent
done;
#
rm americas/output/files/ld/*.nosex
```

Check one of the files

```
head /gpfs/gibbs/pi/caccone/mkc54/albo/americas/output/files/ld/MAU.ld
```

```
##  CHR_A BP_A SNP_A CHR_B BP_B SNP_B R2 
##  2.17 56405 AX-584670672 2.17 70977 AX-584670684 1 
##  2.17 56405 AX-584670672 2.17 71211 AX-585179355 1 
##  2.17 56405 AX-584670672 2.17 71656 AX-585179364 1 
##  2.17 56405 AX-584670672 2.17 71887 AX-584670706 1 
##  2.17 56405 AX-584670672 2.17 87654 AX-584670822 1 
##  2.17 56405 AX-584670672 2.17 114613 AX-584670867 1 
##  2.17 56405 AX-584670672 2.17 115267 AX-585179548 0.897436 
##  2.17 56405 AX-584670672 2.17 146817 AX-584670926 1 
##  2.17 70977 AX-584670684 2.17 71211 AX-585179355 1
```

Clean the LD files from Plink

```
ld_files <- list.files(path = "americas/output/files/ld", pattern = "\\.ld$", full.names = TRUE)

for (file in ld_files) {
  system(paste("awk -i inplace '{gsub(/[[:blank:]]+/, \" \")}1'", file))
}
```

Check one of the files

```
head americas/output/files/ld/MAU.ld
```

```
##  CHR_A BP_A SNP_A CHR_B BP_B SNP_B R2 
##  2.17 56405 AX-584670672 2.17 70977 AX-584670684 1 
##  2.17 56405 AX-584670672 2.17 71211 AX-585179355 1 
##  2.17 56405 AX-584670672 2.17 71656 AX-585179364 1 
##  2.17 56405 AX-584670672 2.17 71887 AX-584670706 1 
##  2.17 56405 AX-584670672 2.17 87654 AX-584670822 1 
##  2.17 56405 AX-584670672 2.17 114613 AX-584670867 1 
##  2.17 56405 AX-584670672 2.17 115267 AX-585179548 0.897436 
##  2.17 56405 AX-584670672 2.17 146817 AX-584670926 1 
##  2.17 70977 AX-584670684 2.17 71211 AX-585179355 1
```

Function to import LD data

```
import_ld_files <- function(directory) {
  ld_files <-
    list.files(
      path = directory,
      pattern = "\\.ld$",
      full.names = TRUE
    )

  ld_tibbles <- list()

  for (file in ld_files) {
    ld_name <- gsub(".ld", "", basename(file))
    ld_data <- read_delim(
      file,
      col_names = TRUE,
      delim = " ",
      show_col_types = FALSE
    ) %>%
      select(c("CHR_A", "BP_A", "SNP_A", "CHR_B", "BP_B", "SNP_B", "R2"))
    ld_tibbles[[ld_name]] <- ld_data
  }

  return(ld_tibbles)
}

# we can save the function to source it later
dump(                                                     # check ?dump for more information
  "import_ld_files",                                           # the object we want to save
  here("scripts", "RMarkdowns", "analyses", "import_ld_files.R")       # use here to save it our function as .R 
)
```

Import the LD data

```
my_ld_tibbles <- import_ld_files("americas/output/files/ld/")


head(my_ld_tibbles$MAU)
```

```
## # A tibble: 6 × 7
##   CHR_A  BP_A SNP_A        CHR_B   BP_B SNP_B           R2
##   <dbl> <dbl> <chr>        <dbl>  <dbl> <chr>        <dbl>
## 1  2.17 56405 AX-584670672  2.17  70977 AX-584670684     1
## 2  2.17 56405 AX-584670672  2.17  71211 AX-585179355     1
## 3  2.17 56405 AX-584670672  2.17  71656 AX-585179364     1
## 4  2.17 56405 AX-584670672  2.17  71887 AX-584670706     1
## 5  2.17 56405 AX-584670672  2.17  87654 AX-584670822     1
## 6  2.17 56405 AX-584670672  2.17 114613 AX-584670867     1
```

Estimate half distance of maximum R2 value.

Since we have several populations, it is better to write a
function

```
get_half_pop <- function(ld_tibble) {
  # Split the tibble into sub-tibbles by scaffold
  ld_split <- split(ld_tibble, ld_tibble[, 1])
  # Remove rows with missing data
  ld_no_nan <- lapply(ld_split, function(x) {
    x[complete.cases(x), ]
  })
  # Calculate the distance between SNPs
  ld_dist <-
    lapply(ld_no_nan, function(x) {
      cbind(x, x[, 5] - x[, 2])
    })
  # Sort the sub-tibbles by distance
  ld_dist_order <- lapply(ld_dist, function(x) {
    x[order(x[, 8]), ]
  })
  # Combine the sub-tibbles into one tibble
  ld_proc <- do.call(rbind, ld_dist_order)
  # Sort the tibble by distance
  ld_sorted <- ld_proc[order(ld_proc[, 8]), ]
  # Bin the data by distance
  bin.1000 <-
    cut(ld_sorted[, 8], (c(0:1000) * 1000), include.lowest = TRUE)
  # Calculate the mean R2 for each bin
  ld_bin_1000_sorted <-
    tapply(ld_sorted[, 7], bin.1000, function(x) {
      mean(x)
    })
  # Fit a LOESS curve to the binned data
  lo.1000.ld <- loess(ld_bin_1000_sorted ~ c(1:1000))
  # Find the value of the binned R2 that corresponds to half the maximum R2 value
  d.pop <- max(ld_bin_1000_sorted, na.rm = TRUE)
  half.pop <- which(ld_bin_1000_sorted <= (d.pop / 2))[1]
  return(half.pop)
}
# we can save the function to source it later
dump(
  "get_half_pop",
  here("scripts", "RMarkdowns", "analyses", "get_half_pop.R")
)
```

Test the function, it should return the same value as we got
above

```
# for one population, you can change the population name to see other populations
half_pal<- get_half_pop(my_ld_tibbles$MAU)
half_pal
```

```
## (2e+03,3e+03] 
##             3
```

Estimates for all populations

```
# Create an empty data.frame to store the results
half_pops_df <-
  data.frame(
    row.names = names(my_ld_tibbles),
    half_distance = numeric(length(my_ld_tibbles)),
    stringsAsFactors = FALSE
  )

# Calculate the hald distance of max r^2 value for each tibble and store in the data.frame
for (tib_name in names(my_ld_tibbles)) {
  tib_half <- get_half_pop(my_ld_tibbles[[tib_name]])
  half_pops_df[tib_name, "half_distance"] <- tib_half
}

# Print the resulting data.frame
half_pops_df
```

```
##     half_distance
## ARA             2
## BCO             2
## BEA             3
## BER             1
## BIT             4
## BPA             3
## BPV             2
## BRM             1
## DAL            36
## DEN             8
## FIR             2
## GRV             1
## HAR            13
## HOU            55
## LIN             3
## LOS             1
## MAC             1
## MAN             1
## MAU             3
## MON             2
## NEO             4
## NEW             1
## NUE             5
## POR             1
## REC             1
## RUS             3
## SAI             1
## SMI             6
## STL             2
## TRA             4
## TUC             2
```

The half distance of the maximum r^2 for all populations vary from 1
to 55kb\* when we bin the data. We do see larger blocks but most of the
ld blocks are small. You can use the entire genome to estimate LD
instead of the larger scaffolds, but the result will be almost the same.
I have done it, but I included only the larger scaffolds to make it
easier for anyone to repeat the analysis. Once we start using the entire
genome, we need to set a LD window, otherwise we will have billions of
estimates since we would have to estimate r^2 for all pairs of SNPs on
each chromosome. Let’s say we would have 500 SNPs on chromosome 1, we
would have 2^500, which is 3.273391e+150 pairwise estimates.

Make a plot for all populations

```
# Calculate half distances for each population
populations <- names(my_ld_tibbles)
half_distances <- sapply(populations, function(population) {
  ld_tibble <- my_ld_tibbles[[population]]
  half_pop <- get_half_pop(ld_tibble)
  return(half_pop)
})

# Create a tibble to store the half distances
half_distance_tibble <- data.frame(
  population = populations,
  half_distance = half_distances
)

# set the order of populations, you can change it on Excel then use it as a vector name pop_order
pop_order <-
  c(
    unique(
      half_distance_tibble$population
    )
  )

# set the order by continent (Africa, Americas, Asia, Europe) - check Excel spreadsheet named Pops_ld_order.xlxs or make your own. For example, range or continent.
half_distance_tibble$population <-
  factor(half_distance_tibble$population, levels = pop_order)

# Plot the half distances for each population
ggplot(half_distance_tibble, aes(x = population, y = half_distance)) +
  geom_bar(
    stat = "identity",
    fill = "lightgray",
    alpha = 0.8
  ) +
  geom_text(
    aes(label = round(half_distance, 2)),
    vjust = -0.5,
    size = 4,
    color = "red"
  ) +
  labs(x = "Population", y = expression(bold("Half Distance of Max " ~ r^
    2 ~ " (kb)"))) +
  my_theme() +
  my_theme() +
  theme(
    axis.text.x = element_text(
      angle = 90,
      hjust = 1,
      size = 14
    ),
    axis.text.y = element_text(size = 14),
    axis.title.x = element_text(size = 16, face = "bold"),
    axis.title.y = element_text(size = 16, face = "bold")
  )
```

```
# save the plot
ggsave(
  here("scripts", "RMarkdowns", "output", "americas", "americas_all", "figures", "linkage_half_distance_LONG_scaffolds_americas_all.pdf"
  ),
  width  = 12,
  height = 6,
  units  = "in"
)
```

We can also plot the distribution of LD blocks across all scaffolds.
Later we will create a new chromosomal scale, but for now lets estimate
LD blocks using the genome as it is.

### 3.4.2 Estimate LD blocks using scaffolds

```
mkdir -p americas/output/files/ld/blocks
```

We can use Plink1.9 to estimate LD blocks for the populations with
more than 12 individuals. We will use the entire genome for this part
instead of the larger scaffolds only. We will set max distance of LD
blocks of 200kb. We found out that the average half distance of r^2 max
is relatively small

```
for fam in $(awk '{print $1}' americas/output/files/ld/pops_4ld.txt | sort | uniq); 
do 
echo $fam | \
plink \
--allow-extra-chr \
--keep-allele-order \
--bfile americas/output/file4 \
--keep-fam /dev/stdin \
--maf 0.1 \
--blocks no-pheno-req \
--blocks-max-kb 200 \
--out americas/output/files/ld/blocks/$fam \
--geno 0.1 \
--silent
done;
#
rm americas/output/files/ld/blocks/*.nosex
```

Now get some data from our .log files

```
echo "Population,n,nVariants,geno,maf,passQC" > americas/output/files/ld/blocks/table_ld_stats.csv
for file in americas/output/files/ld/blocks/*.log
do
  variants=$(grep -oE '([0-9]+) variants loaded from \.bim file' $file | grep -oE '[0-9]+')
  geno=$(grep -oE '([0-9]+) variants removed due to missing genotype data \(--geno\)' $file | grep -oE '[0-9]+')
  maf=$(grep -oE '([0-9]+) variants removed due to minor allele threshold\(s\)' $file | grep -oE '[0-9]+')
  pass=$(grep -oE '([0-9]+) variants and [0-9]+ people pass filters and QC\.' $file | grep -oE '[0-9]+' | head -1)
  n=$(grep -oE '([0-9]+) variants and [0-9]+ people pass filters and QC\.' $file | grep -oE '[0-9]+' | tail -1)
  filename=$(basename $file .log)
  echo "$filename,$n,$variants,$geno,$maf,$pass" >> americas/output/files/ld/blocks/table_ld_stats.csv
done;
head -n 5 americas/output/files/ld/blocks/table_ld_stats.csv
```

```
## Population,n,nVariants,geno,maf,passQC
## ARA,12,84059,5823,23548,54688
## BCO,12,84059,8545,24396,51118
## BEA,12,84059,3651,13281,67127
## BER,12,84059,4249,16940,62870
```

Check it out

```
# Load data from the table_ld_stats.csv file
ld_blocks <- read.csv(
  here(
    "americas/output/files/ld/blocks/table_ld_stats.csv"
    ),
  header = TRUE,
  sep = ","
)

# Create the flextable
ft <- flextable(ld_blocks)

# Apply zebra theme
ft <- theme_zebra(ft)

# Add a caption to the table
ft <- flextable::add_header_lines(ft, "Table 1: Summary of quality control for all American populations data.")

ft
```

| Table 1: Summary of quality control for all American populations data. | | | | | |
| --- | --- | --- | --- | --- | --- |
| Population | n | nVariants | geno | maf | passQC |
| ARA | 12 | 84,059 | 5,823 | 23,548 | 54,688 |
| BCO | 12 | 84,059 | 8,545 | 24,396 | 51,118 |
| BEA | 12 | 84,059 | 3,651 | 13,281 | 67,127 |
| BER | 12 | 84,059 | 4,249 | 16,940 | 62,870 |
| BIT | 12 | 84,059 | 5,938 | 24,578 | 53,543 |
| BPA | 12 | 84,059 | 7,465 | 22,044 | 54,550 |
| BPV | 12 | 84,059 | 9,692 | 23,829 | 50,538 |
| BRM | 16 | 84,059 | 6,435 | 20,927 | 56,697 |
| DAL | 12 | 84,059 | 3,486 | 15,241 | 65,332 |
| DEN | 12 | 84,059 | 5,514 | 14,887 | 63,658 |
| FIR | 12 | 84,059 | 4,359 | 21,798 | 57,902 |
| GRV | 12 | 84,059 | 5,260 | 24,712 | 54,087 |
| HAR | 12 | 84,059 | 4,338 | 17,565 | 62,156 |
| HOU | 12 | 84,059 | 3,994 | 13,158 | 66,907 |
| LIN | 12 | 84,059 | 4,870 | 18,778 | 60,411 |
| LOS | 14 | 84,059 | 6,268 | 21,805 | 55,986 |
| MAC | 12 | 84,059 | 6,390 | 15,140 | 62,529 |
| MAN | 12 | 84,059 | 6,358 | 14,811 | 62,890 |
| MAU | 12 | 84,059 | 8,936 | 20,893 | 54,230 |
| MON | 12 | 84,059 | 5,428 | 14,228 | 64,403 |
| NEO | 12 | 84,059 | 3,724 | 16,621 | 63,714 |
| NEW | 22 | 84,059 | 4,296 | 15,837 | 63,926 |
| NUE | 12 | 84,059 | 3,992 | 15,120 | 64,947 |
| POR | 12 | 84,059 | 6,932 | 27,711 | 49,416 |
| REC | 12 | 84,059 | 5,808 | 20,614 | 57,637 |
| RUS | 12 | 84,059 | 4,946 | 19,707 | 59,406 |
| SAI | 12 | 84,059 | 5,172 | 19,336 | 59,551 |
| SMI | 12 | 84,059 | 4,296 | 16,320 | 63,443 |
| STL | 12 | 84,059 | 3,984 | 18,459 | 61,616 |
| TRA | 12 | 84,059 | 4,182 | 13,536 | 66,341 |
| TUC | 14 | 84,059 | 7,158 | 21,282 | 55,619 |

```
# Save it to a Word document
officer::read_docx() |>
  body_add_flextable(ft) |>
  print(target = here::here("scripts", "RMarkdowns", "output", "americas", "americas_all", "summary_maf_geno_filter_population_americas_all.docx"))
```

```
cd americas/output/files/ld/blocks
wc -l *.blocks | \
  awk '{population = gensub(/\.blocks/, "", "g", $2); print population "\t" $1}' | \
  sed 's#/##' | \
  sed '$d' > populations_block_counts.csv;
head -n 30 populations_block_counts.csv
```

```
## ARA  90
## BCO  125
## BEA  73
## BER  126
## BIT  151
## BPA  146
## BPV  91
## BRM  589
## DAL  106
## DEN  96
## FIR  186
## GRV  181
## HAR  140
## HOU  69
## LIN  152
## LOS  216
## MAC  98
## MAN  76
## MAU  107
## MON  71
## NEO  97
## NEW  847
## NUE  76
## POR  104
## REC  147
## RUS  205
## SAI  82
## SMI  108
## STL  163
## TRA  85
```

Now add the number of blocks to the table we made

```
# Load data from the table_ld_stats.csv file
ld_blocks <- read.csv(
    "americas/output/files/ld/blocks/table_ld_stats.csv"
    ,
  header = TRUE,
  sep = ","
)

# Load the population counts data from the CSV file
pop_counts <-
  read.delim(
      "americas/output/files/ld/blocks/populations_block_counts.csv"
    ,
    header = F,
    sep = "\t"
  ) |>
  rename(
    Population       = 1,
    nBlocks           = 2
  )

# Merge the population counts with the table data
ld_blocks <- merge(ld_blocks, pop_counts, by = "Population")

# Create the flextable
ft <- flextable(ld_blocks)

# Apply zebra theme
ft <- theme_zebra(ft)

# Add a caption to the table
ft <- flextable::add_header_lines(ft, "Table 2: Number of linkage blocks detected with Plink for populations with at least 12 individuals.")

ft
```

| Table 2: Number of linkage blocks detected with Plink for populations with at least 12 individuals. | | | | | | |
| --- | --- | --- | --- | --- | --- | --- |
| Population | n | nVariants | geno | maf | passQC | nBlocks |
| ARA | 12 | 84,059 | 5,823 | 23,548 | 54,688 | 90 |
| BCO | 12 | 84,059 | 8,545 | 24,396 | 51,118 | 125 |
| BEA | 12 | 84,059 | 3,651 | 13,281 | 67,127 | 73 |
| BER | 12 | 84,059 | 4,249 | 16,940 | 62,870 | 126 |
| BIT | 12 | 84,059 | 5,938 | 24,578 | 53,543 | 151 |
| BPA | 12 | 84,059 | 7,465 | 22,044 | 54,550 | 146 |
| BPV | 12 | 84,059 | 9,692 | 23,829 | 50,538 | 91 |
| BRM | 16 | 84,059 | 6,435 | 20,927 | 56,697 | 589 |
| DAL | 12 | 84,059 | 3,486 | 15,241 | 65,332 | 106 |
| DEN | 12 | 84,059 | 5,514 | 14,887 | 63,658 | 96 |
| FIR | 12 | 84,059 | 4,359 | 21,798 | 57,902 | 186 |
| GRV | 12 | 84,059 | 5,260 | 24,712 | 54,087 | 181 |
| HAR | 12 | 84,059 | 4,338 | 17,565 | 62,156 | 140 |
| HOU | 12 | 84,059 | 3,994 | 13,158 | 66,907 | 69 |
| LIN | 12 | 84,059 | 4,870 | 18,778 | 60,411 | 152 |
| LOS | 14 | 84,059 | 6,268 | 21,805 | 55,986 | 216 |
| MAC | 12 | 84,059 | 6,390 | 15,140 | 62,529 | 98 |
| MAN | 12 | 84,059 | 6,358 | 14,811 | 62,890 | 76 |
| MAU | 12 | 84,059 | 8,936 | 20,893 | 54,230 | 107 |
| MON | 12 | 84,059 | 5,428 | 14,228 | 64,403 | 71 |
| NEO | 12 | 84,059 | 3,724 | 16,621 | 63,714 | 97 |
| NEW | 22 | 84,059 | 4,296 | 15,837 | 63,926 | 847 |
| NUE | 12 | 84,059 | 3,992 | 15,120 | 64,947 | 76 |
| POR | 12 | 84,059 | 6,932 | 27,711 | 49,416 | 104 |
| REC | 12 | 84,059 | 5,808 | 20,614 | 57,637 | 147 |
| RUS | 12 | 84,059 | 4,946 | 19,707 | 59,406 | 205 |
| SAI | 12 | 84,059 | 5,172 | 19,336 | 59,551 | 82 |
| SMI | 12 | 84,059 | 4,296 | 16,320 | 63,443 | 108 |
| STL | 12 | 84,059 | 3,984 | 18,459 | 61,616 | 163 |
| TRA | 12 | 84,059 | 4,182 | 13,536 | 66,341 | 85 |
| TUC | 14 | 84,059 | 7,158 | 21,282 | 55,619 | 135 |

```
# Save it to a Word document
officer::read_docx() |>
  body_add_flextable(ft) |>
  print(target = here::here("scripts", "RMarkdowns", "output", "americas", "americas_all", "summary_ld_blocks_americas_all.docx"))
```

Get the size of each block from the .block.det files

```
get_kb_column <- function(dir_path) {
  # obtain the list of files with extension .blocks.det
  file_names <- list.files(path = dir_path, pattern = "\\.blocks\\.det$", full.names = TRUE)
  
  # create an empty list to hold the data frames
  block_list <- list()
  
  # loop through the files and read the data into the list
  for (file in file_names) {
    df <- read.table(file, header = TRUE, check.names = FALSE, stringsAsFactors = FALSE)
    # select only the KB column and add it to the block_list with the file name
    block_list[[file]] <- df %>% select(KB) %>% add_column(file = file, .before = 1)
  }
  
  # combine the data frames in the block_list into a single data frame
  blocks <- bind_rows(block_list)
  
  # clean up the file name column
  blocks$file <- str_remove(blocks$file, "^.*\\/ld\\/blocks\\/")
  
  return(blocks)
}

# example usage: replace dir_path with your directory path
dir_path <- here("americas/output/files/ld/blocks")

blocks<- 
  get_kb_column(dir_path) |>
  mutate(file = str_remove(file, ".blocks.det")) |>
  as_tibble() |>
  rename(
    Population = 1
  )
```

Create density plot of the size of the LD blocks Plink found

```
# to check how many colors we need
# n_distinct(blocks$Population) 
#48

# define the color palette with 38 color blind colors
library(RColorBrewer)
n <- 50
qual_col_pals = brewer.pal.info[brewer.pal.info$category == 'qual',]
col_vector = unlist(mapply(brewer.pal, qual_col_pals$maxcolors, rownames(qual_col_pals)))
pie(rep(1,n), col=sample(col_vector, n))
```

```
col_vector
```

```
##  [1] "#7FC97F" "#BEAED4" "#FDC086" "#FFFF99" "#386CB0" "#F0027F" "#BF5B17"
##  [8] "#666666" "#1B9E77" "#D95F02" "#7570B3" "#E7298A" "#66A61E" "#E6AB02"
## [15] "#A6761D" "#666666" "#A6CEE3" "#1F78B4" "#B2DF8A" "#33A02C" "#FB9A99"
## [22] "#E31A1C" "#FDBF6F" "#FF7F00" "#CAB2D6" "#6A3D9A" "#FFFF99" "#B15928"
## [29] "#FBB4AE" "#B3CDE3" "#CCEBC5" "#DECBE4" "#FED9A6" "#FFFFCC" "#E5D8BD"
## [36] "#FDDAEC" "#F2F2F2" "#B3E2CD" "#FDCDAC" "#CBD5E8" "#F4CAE4" "#E6F5C9"
## [43] "#FFF2AE" "#F1E2CC" "#CCCCCC" "#E41A1C" "#377EB8" "#4DAF4A" "#984EA3"
## [50] "#FF7F00" "#FFFF33" "#A65628" "#F781BF" "#999999" "#66C2A5" "#FC8D62"
## [57] "#8DA0CB" "#E78AC3" "#A6D854" "#FFD92F" "#E5C494" "#B3B3B3" "#8DD3C7"
## [64] "#FFFFB3" "#BEBADA" "#FB8072" "#80B1D3" "#FDB462" "#B3DE69" "#FCCDE5"
## [71] "#D9D9D9" "#BC80BD" "#CCEBC5" "#FFED6F"
```

```
#colors <- 
#  c("#52ef99", "#146c45", "#75d5e1", "#FB8072", "#2c4a5e", "#FFED7F", "#6a8fe0", "#FDCDAC", "#8c61cd", "#f365e7", "#871550","#f6c8de", "#a113b2", "#BF5B17", "#1F78B4", "#cf749b", "#FFFF33", "#FF7F00","#2524f9", "#cddb9b","#799d10", "#CCCCCC", "#B3E2CD", "#a7e831", "#984EA3", "#754819", "#fda547", "#a41415", "#fd5917", "#fd4e8b", "#ead624", "#6A3D9A","#FDC086", "#21a708", "#332288", "#51f310", "#9d8d88", "#66C2A5")


#colors <- 
#  c("#52ef99", "#146c45", "#75d5e1", "#2c4a5e", "#6a8fe0", "#8c61cd", "#f365e7", "#871550", "#f6c8de", "#a113b2", "#cf749b", "#2524f9",  "#cddb9b", "#799d10", "#a7e831", "#754819", "#fda547", "#a41415", "#fd5917", "#fd4e8b", "#ead624", "#21a708", "#332288", "#51f310", "#9d8d88")


# make plot using the sample y scale for all populations
ggplot(blocks, aes(x = KB)) +
  stat_density(
    aes(y = after_stat(count), fill = factor(Population)),
    linewidth = .5,
    alpha = .4,
    position = "identity"
  ) +
  scale_fill_manual(values = col_vector) +
  scale_x_continuous(name = "Block length (kb)") +
  scale_y_continuous(name = "Count") +
  theme(
    plot.title = element_text(hjust = 0.5, size = 18, face = "bold"),
    axis.text = element_text(size = 12),
    axis.title = element_text(size = 16, face = "bold"),
    strip.text = element_text(size = 14, face = "bold"),
    plot.background = element_rect(fill = "white"),
    panel.background = element_rect(fill = 'white', colour = 'black')
  ) +
  guides(fill = "none") +
  facet_wrap( ~ Population, ncol = 3) + my_theme()
```

```
# save the plot as a PDF using ggsave
ggsave(
  here("scripts",
    "RMarkdowns",
    "output",
    "americas",
    "americas_all",
    "figures",
    "block_density_y_scale_fixed_americas_all.pdf"
  ),
  width = 10,
  height = 10,
  units = "in"
)
```

Make plot allowing the y axis scale free

```
ggplot(blocks, aes(x = KB)) +
  stat_density(
    aes(y = after_stat(count), fill = factor(Population)),
    linewidth = .5,
    alpha = .4,
    position = "identity"
  ) +
  scale_fill_manual(values = col_vector) +
  scale_x_continuous(name = "Block length (kb)") +
  scale_y_continuous(name = "Count") +
  theme(
    plot.title = element_text(hjust = 0.5, size = 18, face = "bold"),
    axis.text = element_text(size = 12),
    axis.title = element_text(size = 16, face = "bold"),
    strip.text = element_text(size = 14, face = "bold"),
    plot.background = element_rect(fill = "white"),
    panel.background = element_rect(fill = 'white', colour = 'black')
  ) +
  guides(fill = "none") +
  facet_wrap( ~ Population, ncol = 3, scales = "free_y") +
  my_theme()
```

```
# save the plot as a PDF using ggsave
ggsave(
  here("scripts",
    "RMarkdowns",
    "output",
    "americas",
    "americas_all",
    "figures",
    "block_density_free_scale_americas_all.pdf"
  ),
  width = 20,
  height = 18,
  units = "in"
)
```

## 3.5 LD pruning

Since we do not have to remove any SNPs due to deviation from HWE, we
can proceed with heterozygosity estimates. The first step is to “prune”
our data set. We will check the pairwise linkage estimates for all SNPs.
We can work with file4. We will use “`indep-pairwise`” to
check if there are SNPs above a certain linkage disequilibrium (LD)
threshold. Check Plink documentation for more details https://www.cog-genomics.org/plink/1.9/ld I used
“`--indep-pairwise 5 1 0.1`” , which indicates according to
the documentation:
`--indep-pairphase <window size>['kb'] <step size (variant ct)> <r^2 threshold>`
We will check in a window of 5kb if there is any pair of SNPs with r2
estimates above 0.1, then we will move our window 1 SNP and check again
for SNPs above the threshold. We will repeat this procedure until we
check the entire genome.

Switch back to plink2

```
module load PLINK/2_avx2_20221024
#PLINK/1.9b_6.21-x86_64 => PLINK/2_avx2_20221024
plink2 --version
```

```
plink2 \
--allow-extra-chr \
--bfile americas/output/file4 \
--extract americas/output/files/passed_hwe.txt \
--indep-pairwise 5 1 0.1 \
--out americas/output/indepSNP \
--silent;
grep 'pairwise\|variants\|samples' americas/output/indepSNP.log
```

–indep-pairwise 5 1 0.1 555 samples (33 females, 32 males, 490
ambiguous; 555 founders) loaded from 84059 variants loaded from
americas/output/file4.bim. –extract: 84059 variants remaining. 84059
variants remaining after main filters. –indep-pairwise (28 compute
threads): 40102/84059 variants removed.

Remember, the SNPs are not removed from our data set. Plink created 3
files when we ran the code above. One is the “indepSNP.log” file, and
the other two are: “indepSNP.prune.in” -> list of SNPs with squared
correlation smaller than our r2 threshold of 0.1. “indepSNP.prune.out”
-> list of SNPs with squared correlation greater than our r2
threshold of 0.1. For our heterozygosity estimates, we want to use the
set of SNPs that are below our r2 threshold of 0.1. We consider that
they are randomly associated. We can use Plink to estimate the
heterozygosity using the “indepSNP.prune.in” file.

## 3.6 Heterozygosity

```
plink2 \
--allow-extra-chr \
--bfile americas/output/file4 \
--extract americas/output/indepSNP.prune.in \
--het \
--out americas/output/R_check \
--silent;
grep 'variants' americas/output/R_check.log
```

84059 variants loaded from americas/output/file4.bim. –extract: 43957
variants remaining. 43957 variants remaining after main filters.

```
#   find individuals with high heterozygosity                              ####
# import the data from Plink
het <- read.delim(
  here(
    "americas/output/R_check.het"
  ),
  head = TRUE
)
#
# check head of the file
colnames(het)
```

```
## [1] "X.FID"  "IID"    "O.HOM." "E.HOM." "OBS_CT" "F"
```

Estimate heterozygosity

```
# create a column named HET_RATE and calculate the heterozygosity rate
het$HET_RATE <- (het$"OBS_CT" - het$"O.HOM") / het$"OBS_CT"
#
# use subset function to get values deviating from 4sd of the mean heterozygosity rate.
het_fail <-
  subset(
    het, (het$HET_RATE < mean(
      het$HET_RATE
    ) - 4 * sd(
      het$HET_RATE
    )) |
      (het$HET_RATE > mean(
        het$HET_RATE
      ) + 4 * sd(
        het$HET_RATE
      ))
  )
#
# get the list of individuals that failed our threshold of 4sd from the mean.
het_fail$HET_DST <-
  (het_fail$HET_RATE - mean(
    het$HET_RATE
  )) / sd(
    het$HET_RATE
  )
```

Save the files to use with Plink

```
#   save the data to use with Plink2                                        ####
#
write.table(
  het_fail,
  here(
    "americas/output/fail-het-qc.txt"
  ),
  row.names = FALSE
)
```

Make plot

```
#   make a heterozygosity plot                                              ####
#
ggplot(
  het,
  aes(
    HET_RATE
  )
) +
  geom_histogram(
    colour           = "black",
    fill             = "lightgray",
    bins             = 40
  ) +
  labs(
    x                = "Heterozygosity Rate",
    y                = "Number of Individuals"
  ) +
  geom_vline(
    aes(
      xintercept     = mean(
        HET_RATE
      )
    ),
    col              = "red",
    linewidth        = 1.5
  ) +
  geom_vline(
    aes(
      xintercept     = mean(
        HET_RATE
      ) + 4 * sd(
        HET_RATE
      )
    ),
    col              = "#BFB9B9",
    linewidth        = 1
  ) +
  geom_vline(
    aes(
      xintercept     = mean(
        HET_RATE
      ) - 4 * sd(
        HET_RATE
      )
    ),
    col              = "#BFB9B9",
    linewidth        = 1
  ) + 
  my_theme() +
  scale_y_continuous(
  )
```

```
#   save the heterozygosity plot                                            ####
ggsave(
  here("scripts", "RMarkdowns", "output", "americas", "americas_all", "figures", "Heterozygosity_americas_all.pdf"
  ),
  width  = 5,
  height = 4,
  units  = "in"
)
```

The red line in the plot above indicates the mean, and the gray lines
indicate 4 standard deviation from the mean. We can see that some
mosquitoes do have excess heterozygous sites. We will remove them.

We can get their ID from the file “fail-het-qc.txt”. We can use the
bash script below to parse the file to use with Plink

```
sed 's/"// g' americas/output/fail-het-qc.txt | awk '{print$1, $2}'> americas/output/het_fail_ind.txt;
echo 'How many mosquitoes we need to remove from our data set:';
cat americas/output/het_fail_ind.txt | tail -n +2 | wc -l;
echo 'Which mosquitoes we have to remove:';
tail -n +2 americas/output/het_fail_ind.txt
```

```
## How many mosquitoes we need to remove from our data set:
## 1
## Which mosquitoes we have to remove:
## LOS 355
```

Heterozygosity is outside the SD threshold in 1 individual (LOS 355).
We will remove it using Plink

```
plink2 \
--allow-extra-chr \
--bfile americas/output/file4 \
--remove americas/output/het_fail_ind.txt \
--make-bed \
--out americas/output/file5 \
--silent;
grep 'variants\|samples' americas/output/file5.log
```

555 samples (33 females, 32 males, 490 ambiguous; 555 founders)
loaded from 84059 variants loaded from americas/output/file4.bim.
–remove: 554 samples remaining. 554 samples (33 females, 32 males, 489
ambiguous; 554 founders) remaining after

## 3.7 Relatedness

Check for cryptic relatedness. Check Plink2 documentation https://www.cog-genomics.org/plink/2.0/distance You can
download King directly https://www.kingrelatedness.com/manual.shtml or check
their manuscript https://www.ncbi.nlm.populations.gov/pmc/articles/PMC3025716/pdf/btq559.pdf
From Plink2 documentation: “Note that KING kinship coefficients are
scaled such that duplicate samples have kinship 0.5, not 1. First-degree
relations (parent-child, full siblings) correspond to ~0.25,
second-degree relations correspond to ~0.125, etc. It is conventional to
use a cutoff of ~0.354 (the geometric mean of 0.5 and 0.25) to screen
for monozygotic twins and duplicate samples, ~0.177 to add first-degree
relations, etc.” There two options. One is to run only –make-king and
another one is to use –make-king-table We will use the threshold of
0.354 and create a table.

```
# Plink2 will create a file with extension .king
plink2 \
--allow-extra-chr \
--bfile americas/output/file5 \
--extract americas/output/indepSNP.prune.in \
--make-king-table rel-check \
--king-table-filter 0.354 \
--out americas/output/file5 \
--silent;
grep 'variants\|samples' americas/output/file5.log
```

554 samples (33 females, 32 males, 489 ambiguous; 554 founders)
loaded from 84059 variants loaded from americas/output/file5.bim.
–extract: 43957 variants remaining. 43957 variants remaining after main
filters.

Check the individuals that did not pass filtering

```
head -n 20 americas/output/file5.kin0
```

```
## #FID1    IID1    FID2    IID2    NSNP    HETHET  IBS0    KINSHIP
## BPA  2460    BPA 2452    40810   0.223401    0.0115413   0.365469
## CHI  2274    CHI 2269    42024   0.227584    0.0141824   0.358898
## HAT  2395    HAT 2393    42339   0.284253    0.00481825  0.437943
## IPO  2590    IPO 2585    42152   0.213916    0.00794743  0.397197
## LOS  366 LOS 365 42400   0.202193    0   0.498661
## LOS  367 LOS 365 42350   0.202267    0   0.498428
## LOS  367 LOS 366 42331   0.201743    0   0.497695
## NEB  2549    NEB 2546    40880   0.289188    0.00335127  0.402043
## NEW  423 NEW 413 42561   0.248091    0.0121238   0.365219
## NEW  423 NEW 420 42558   0.249918    0.0151088   0.355654
## REC  179 REC 175 42331   0.254164    0.00845716  0.395623
## SPR  465 SPR 460 42393   0.256339    0.0090581   0.383346
```

We want to remove one of the individuals of the pairs

```
plink2 \
--allow-extra-chr \
--bfile americas/output/file5 \
--extract americas/output/indepSNP.prune.in \
--make-king triangle bin \
--out americas/output/file6 \
--silent;
grep 'variants\|samples' americas/output/file6.log
```

554 samples (33 females, 32 males, 489 ambiguous; 554 founders)
loaded from 84059 variants loaded from americas/output/file5.bim.
–extract: 43957 variants remaining. 43957 variants remaining after main
filters. –make-king pass 1/1: Scanning for rare variants… done. 0
variants handled by initial scan (43957 remaining). –make-king: 43957
variants processed.

Now we can use Plink2 to remove one of the mosquitoes from the pair
with high kinship. It will remove samples with high relatedness (2460,
2274, 2395, 366, 367, 2590, 2549, 423, 179, 465). Plink2 always tries to
maximize the number of samples passing the filters.

```
plink2 \
--allow-extra-chr \
--bfile americas/output/file5 \
--king-cutoff americas/output/file6 0.354 \
--make-bed \
--out americas/output/file7 \
--silent;
grep 'samples\|variants\|remaining' americas/output/file7.log
```

554 samples (33 females, 32 males, 489 ambiguous; 554 founders)
loaded from 84059 variants loaded from americas/output/file5.bim.
americas/output/file7.king.cutoff.out.id , and 544 remaining sample IDs
written

# 4. Create chromosomal scale

Import the .bim file with the SNPs to create a new chromosomal
scale.

```
#   ____________________________________________________________________________
#   import the bim file with the SNP data                                   ####
snps <-
  read_delim(                    # to learn about the options use here, run ?read_delim on the console.
    here(
      "americas/output/file7.bim"
    ),                           # use library here to load it
    col_names      = FALSE,      # we don't have header in the input file
    show_col_types = FALSE,      # suppress message from read_delim
    col_types      = "ccidcc"    # set the class of each column
  )
#
# set column names
colnames(
  snps
) <-                             # to add a header in our tibble
  c(
    "Scaffold", "SNP", "Cm", "Position", "Allele1", "Allele2"
  )
#
# check the tibble
head(snps)
```

```
## # A tibble: 6 × 6
##   Scaffold SNP             Cm Position Allele1 Allele2
##   <chr>    <chr>        <int>    <dbl> <chr>   <chr>  
## 1 1        AX-583035163     0   315386 A       G      
## 2 1        AX-583033356     0   315674 C       T      
## 3 1        AX-583033370     0   330057 G       A      
## 4 1        AX-583035194     0   330265 A       G      
## 5 1        AX-583033387     0   331288 C       T      
## 6 1        AX-583035257     0   442875 T       C
```

Separate the tibbles into each chromosome

```
#   separate the SNP data per chromosome                                    
# chr1
chr1_snps <-
  snps |>
  filter(
    str_detect(
      Scaffold, "^1."
    )
  ) |> # here we get only Scaffold rows starting with 1
  as_tibble() # save as tibble
#
# chr2
chr2_snps <-
  snps |>
  filter(
    str_detect(
      Scaffold, "^2."
    )
  ) |>
  as_tibble()
#
# chr3
chr3_snps <-
  snps |>
  filter(
    str_detect(
      Scaffold, "^3."
    )
  ) |>
  as_tibble()
```

Import the file with sizes of each scaffold.

```
#   import the file with the scaffold sizes                                 ####
sizes <-
  read_delim(
    here(
      "/gpfs/gibbs/project/caccone/mkc54/albo/genome/scaffold_sizes.txt"
    ),
    col_names      = FALSE,
    show_col_types = FALSE,
    col_types      = "cd"
  )
#
# set column names
colnames(
  sizes
) <- c(
  "Scaffold", "Size"
)
#   ____________________________________________________________________________
#   create new column with the chromosome number                            ####
sizes <- 
  sizes |>
  mutate(
    Chromosome = case_when( # we use mutate to create a new column called Chromosome
      startsWith(
        Scaffold, "1"
      ) ~ "1", # use startsWith to get Scaffold rows starting with 1 and output 1 on Chromosome column
      startsWith(
        Scaffold, "2"
      ) ~ "2",
      startsWith(
        Scaffold, "3"
      ) ~ "3"
    )
  ) |>
  arrange(
    Scaffold
  )                   # to sort the order of the scaffolds, fixing the problem we have with scaffold 1.86
# check it
head(sizes)
```

```
## # A tibble: 6 × 3
##   Scaffold     Size Chromosome
##   <chr>       <dbl> <chr>     
## 1 1.1        351198 1         
## 2 1.10     11939576 1         
## 3 1.100     3389100 1         
## 4 1.101      470438 1         
## 5 1.102     2525157 1         
## 6 1.103      150026 1
```

Create new scale. Get the scaffolds for each chromosome.

```
#   separate the scaffold sizes tibble per chromosome                       ####
# chr1
chr1_scaffolds <- 
  sizes |>
  filter(
    str_detect(
      Scaffold, "^1" # we use library stringr to get scaffolds starting with 1 (chromosome 1)
    )
  ) |> 
  as_tibble()
#
# chr2
chr2_scaffolds <-
  sizes |>
  filter(
    str_detect(
      Scaffold, "^2" # we use library stringr to get scaffolds starting with 2 (chromosome 2)
    )
  ) |> 
  as_tibble()
#
# # chr3
chr3_scaffolds <-
  sizes |>
  filter(
    str_detect(
      Scaffold, "^3" # we use library stringr to get scaffolds starting with 3 (chromosome 3)
    )
  ) |>
  as_tibble()
```

Create a scale for each chromosome.

```
#   create a new scale for each chromosome                                  ####
# chr1
chr1_scaffolds$overall_size_before_bp <-
  0                                                                        # we create a new column with zeros
for (i in 2:nrow(
  chr1_scaffolds
)
) {                                                                        # loop to start on second line
  chr1_scaffolds$overall_size_before_bp[i] <-                              # set position on the scale
    chr1_scaffolds$overall_size_before_bp[i - 1] + chr1_scaffolds$Size[i - # add the scaffold size and the location to get position on new scale
      1]
}
#
# chr2
chr2_scaffolds$overall_size_before_bp <- 0
for (i in 2:nrow(
  chr2_scaffolds
)
) {
  chr2_scaffolds$overall_size_before_bp[i] <-
    chr2_scaffolds$overall_size_before_bp[i - 1] + chr2_scaffolds$Size[i -
      1]
}
#
# chr3
chr3_scaffolds$overall_size_before_bp <- 0
for (i in 2:nrow(
  chr3_scaffolds
)
) {
  chr3_scaffolds$overall_size_before_bp[i] <-
    chr3_scaffolds$overall_size_before_bp[i - 1] + chr3_scaffolds$Size[i -
      1]
}
```

Merge the data frames scaffolds and SNPs.

```
#   merge the data sets using the tidyverse function left_join              ####
# chr1
chr1_scale <-
  chr1_snps |>          # create data frame for each chromosome, get chr1_snps
  left_join(            # use lef_join function to merge it with chr1_scaffolds
    chr1_scaffolds,
    by = "Scaffold"
  ) |>                  # set column to use for merging (Scaffold in this case)
  na.omit() |>          # remove NAs, we don't have SNPs in every scaffold
  mutate(
    midPos_fullseq = as.numeric(
      Position
    ) +                 # make new columns numeric
      as.numeric(
        overall_size_before_bp
      )
  )
#
# chr2
chr2_scale <-
  chr2_snps |>
  left_join(
    chr2_scaffolds,
    by = "Scaffold"
  ) |>
  na.omit() |>
  mutate(
    midPos_fullseq = as.numeric(
      Position
    ) +
      as.numeric(
        overall_size_before_bp
      )
  )
#
# chr3
chr3_scale <-
  chr3_snps |>
  left_join(
    chr3_scaffolds,
    by = "Scaffold"
  ) |>
  na.omit() |>
  mutate(
    midPos_fullseq = as.numeric(
      Position
    ) +
      as.numeric(
        overall_size_before_bp
      )
  )
```

Merge all chromosome scales.

```
#   merge the data sets, and select only the columns we need                ####
chroms <- rbind(
  chr1_scale, chr2_scale, chr3_scale
) |>
  dplyr::select(
    Chromosome, SNP, Cm, midPos_fullseq, Allele1, Allele2
  )
# check it
head(chroms)
```

```
## # A tibble: 0 × 6
## # ℹ 6 variables: Chromosome <chr>, SNP <chr>, Cm <int>, midPos_fullseq <dbl>,
## #   Allele1 <chr>, Allele2 <chr>
```

Save the new .bim file

```
#   ____________________________________________________________________________
#   save the new bim file with a new name, I added "B"                      ####
write.table(
  chroms,
  file      = here(
    "americas/output/file7B.bim"
  ),
  sep       = "\t",
  row.names = FALSE,
  col.names = FALSE,
  quote     = FALSE
)
```

Rename the .bim files

```
# change the name of the first .bim file, for example, append _backup.bim, and then replace the original file
mv americas/output/file7.bim americas/output/file7_backup.bim;
# than change the new bim we create to the original name (do it only once, otherwise it will mess up)
mv americas/output/file7B.bim americas/output/file7.bim
```

Create a new bed file with Plink2 to see if it works. For example, to
see if the variants are in the right order. Plink2 will give us a
warning.

```
cd /gpfs/gibbs/pi/caccone/mkc54/albo

plink2 \
--bfile americas/output/file7 \
--make-bed \
--out americas/output/test01;
# then we remove the files 
rm americas/output/test01.*
```

PLINK v2.00a3.7LM AVX2 Intel (24 Oct 2022)
www.cog-genomics.org/plink/2.0/ (C) 2005-2022 Shaun Purcell, Christopher
Chang GNU General Public License v3 Logging to
americas/output/test01.log. Options in effect: –bfile
americas/output/file7 –make-bed –out americas/output/test01 Start time:
Wed Apr 3 15:09:34 2024 257321 MiB RAM detected; reserving 128660 MiB
for main workspace. Using up to 32 threads (change this with –threads).
544 samples (33 females, 31 males, 480 ambiguous; 544 founders) loaded
from americas/output/file7.fam. 84059 variants loaded from
americas/output/file7.bim. Note: No phenotype data present. Writing
americas/output/test01.fam … done. Writing americas/output/test01.bim …
done. Writing americas/output/test01.bed … done. End time: Wed Apr 3
15:09:34 2024 [mkc54@login2.mccleary albo]$ # then we remove the
files [mkc54@login2.mccleary albo]$ rm
americas/output/test01.\*

## 4.1 Estimate LD blocks using chromosomal scale

```
mkdir -p americas/output/files/ld/blocks_chr
```

```
module load PLINK/1.9b_6.21-x86_64
```

We can use Plink1.9 to estimate LD blocks for the populations with
more than 12 individuals. We will use the entire genome for this part
instead of the larger scaffolds only. We will set max distance of LD
blocks of 500kb. We found out that the average half distance of r^2 max
is small, from 1 to 5kb

```
for fam in $(awk '{print $1}' americas/output/files/ld/pops_4ld.txt | sort | uniq); 
do 
echo $fam | \
plink \
--allow-extra-chr \
--keep-allele-order \
--bfile americas/output/file7 \
--keep-fam /dev/stdin \
--maf 0.1 \
--blocks no-pheno-req \
--blocks-max-kb 200 \
--out americas/output/files/ld/blocks_chr/$fam \
--geno 0.1 \
--silent
done;
#
rm americas/output/files/ld/blocks_chr/*.nosex
```

Get some data from our .log files

```
echo "Population,n,nVariants,geno,maf,passQC" > americas/output/files/ld/blocks_chr/table_ld_stats.csv
for file in americas/output/files/ld/blocks_chr/*.log
do
  variants=$(grep -oE '([0-9]+) variants loaded from \.bim file' $file | grep -oE '[0-9]+')
  geno=$(grep -oE '([0-9]+) variants removed due to missing genotype data \(--geno\)' $file | grep -oE '[0-9]+')
  maf=$(grep -oE '([0-9]+) variants removed due to minor allele threshold\(s\)' $file | grep -oE '[0-9]+')
  pass=$(grep -oE '([0-9]+) variants and [0-9]+ people pass filters and QC\.' $file | grep -oE '[0-9]+' | head -1)
  n=$(grep -oE '([0-9]+) variants and [0-9]+ people pass filters and QC\.' $file | grep -oE '[0-9]+' | tail -1)
  filename=$(basename $file .log)
  echo "$filename,$n,$variants,$geno,$maf,$pass" >> americas/output/files/ld/blocks_chr/table_ld_stats.csv
done;
head -n 5 americas/output/files/ld/blocks_chr/table_ld_stats.csv
```

Check it out

```
# Load data from the table_ld_stats.csv file
ld_blocks <- read.csv(
  here(
    "americas/output/files/ld/blocks_chr/table_ld_stats.csv"
    ),
  header = TRUE,
  sep = ","
)

# Create the flextable
ft <- flextable(ld_blocks)

# Apply zebra theme
ft <- theme_zebra(ft)

# Add a caption to the table
ft <- flextable::add_header_lines(ft, "Table 1: Summary of quality control for all American samples dataset.")

ft
```

| Table 1: Summary of quality control for all American samples dataset. | | | | | |
| --- | --- | --- | --- | --- | --- |
| Population | n | nVariants | geno | maf | passQC |
| ARA | 12 | 84,059 | 5,823 | 23,548 | 54,688 |
| BCO | 12 | 84,059 | 8,545 | 24,396 | 51,118 |
| BEA | 12 | 84,059 | 3,651 | 13,281 | 67,127 |
| BER | 12 | 84,059 | 4,249 | 16,940 | 62,870 |
| BIT | 12 | 84,059 | 5,938 | 24,578 | 53,543 |
| BPA | 11 | 84,059 | 6,809 | 21,983 | 55,267 |
| BPV | 12 | 84,059 | 9,692 | 23,829 | 50,538 |
| BRM | 16 | 84,059 | 6,435 | 20,927 | 56,697 |
| DAL | 12 | 84,059 | 3,486 | 15,241 | 65,332 |
| DEN | 12 | 84,059 | 5,514 | 14,887 | 63,658 |
| FIR | 12 | 84,059 | 4,359 | 21,798 | 57,902 |
| GRV | 12 | 84,059 | 5,260 | 24,712 | 54,087 |
| HAR | 12 | 84,059 | 4,338 | 17,565 | 62,156 |
| HOU | 12 | 84,059 | 3,994 | 13,158 | 66,907 |
| LIN | 12 | 84,059 | 4,870 | 18,778 | 60,411 |
| LOS | 11 | 84,059 | 4,565 | 23,010 | 56,484 |
| MAC | 12 | 84,059 | 6,390 | 15,140 | 62,529 |
| MAN | 12 | 84,059 | 6,358 | 14,811 | 62,890 |
| MAU | 12 | 84,059 | 8,936 | 20,893 | 54,230 |
| MON | 12 | 84,059 | 5,428 | 14,228 | 64,403 |
| NEO | 12 | 84,059 | 3,724 | 16,621 | 63,714 |
| NEW | 21 | 84,059 | 3,950 | 16,049 | 64,060 |
| NUE | 12 | 84,059 | 3,992 | 15,120 | 64,947 |
| POR | 12 | 84,059 | 6,932 | 27,711 | 49,416 |
| REC | 11 | 84,059 | 5,140 | 21,385 | 57,534 |
| RUS | 12 | 84,059 | 4,946 | 19,707 | 59,406 |
| SAI | 12 | 84,059 | 5,172 | 19,336 | 59,551 |
| SMI | 12 | 84,059 | 4,296 | 16,320 | 63,443 |
| STL | 12 | 84,059 | 3,984 | 18,459 | 61,616 |
| TRA | 12 | 84,059 | 4,182 | 13,536 | 66,341 |
| TUC | 14 | 84,059 | 7,158 | 21,282 | 55,619 |

```
# Save it to a Word document
officer::read_docx() |>
  body_add_flextable(ft) |>
  print(target = here::here("americas/output/summary_blocks_chr.docx"))
```

Now count how many blocks we found in each population

```
cd americas/output/files/ld/blocks_chr
wc -l *.blocks | \
  awk '{population = gensub(/\.blocks_chr/, "", "g", $2); print population "\t" $1}' | \
  sed 's#americas/output/files/ld/blocks_chr/##' | \
  sed 's/.blocks//' | \
  sed '$d' > populations_block_counts.csv;
head -n 79 populations_block_counts.csv
```

```
## ARA  90
## BCO  125
## BEA  73
## BER  126
## BIT  151
## BPA  107
## BPV  91
## BRM  589
## DAL  106
## DEN  96
## FIR  186
## GRV  181
## HAR  140
## HOU  69
## LIN  152
## LOS  78
## MAC  98
## MAN  76
## MAU  107
## MON  71
## NEO  97
## NEW  753
## NUE  76
## POR  104
## REC  106
## RUS  205
## SAI  82
## SMI  108
## STL  163
## TRA  85
## TUC  135
```

Add the number of blocks to the table we made

```
# Load data from the table_ld_stats.csv file
ld_blocks <- read.csv(
  here(
    "americas/output/files/ld/blocks_chr/table_ld_stats.csv"
    ),
  header = TRUE,
  sep = ","
)

# Load the population counts data from the CSV file
pop_counts <-
  read.delim(
    here(
      "americas/output/files/ld/blocks_chr/populations_block_counts.csv"
    ),
    header = F,
    sep = "\t"
  ) |>
  rename(
    Population       = 1,
    nBlocks          = 2
  )

# Merge the population counts with the table data
ld_blocks <- merge(ld_blocks, pop_counts, by = "Population")

# Create the flextable
ft <- flextable(ld_blocks)

# Apply zebra theme
ft <- theme_zebra(ft)

# Add a caption to the table
ft <- flextable::add_header_lines(ft, "Table 2: Number of linkage blocks detected with Plink for populations with at least 10 individuals.")

ft
```

| Table 2: Number of linkage blocks detected with Plink for populations with at least 10 individuals. | | | | | | |
| --- | --- | --- | --- | --- | --- | --- |
| Population | n | nVariants | geno | maf | passQC | nBlocks |
| ARA | 12 | 84,059 | 5,823 | 23,548 | 54,688 | 90 |
| BCO | 12 | 84,059 | 8,545 | 24,396 | 51,118 | 125 |
| BEA | 12 | 84,059 | 3,651 | 13,281 | 67,127 | 73 |
| BER | 12 | 84,059 | 4,249 | 16,940 | 62,870 | 126 |
| BIT | 12 | 84,059 | 5,938 | 24,578 | 53,543 | 151 |
| BPA | 11 | 84,059 | 6,809 | 21,983 | 55,267 | 107 |
| BPV | 12 | 84,059 | 9,692 | 23,829 | 50,538 | 91 |
| BRM | 16 | 84,059 | 6,435 | 20,927 | 56,697 | 589 |
| DAL | 12 | 84,059 | 3,486 | 15,241 | 65,332 | 106 |
| DEN | 12 | 84,059 | 5,514 | 14,887 | 63,658 | 96 |
| FIR | 12 | 84,059 | 4,359 | 21,798 | 57,902 | 186 |
| GRV | 12 | 84,059 | 5,260 | 24,712 | 54,087 | 181 |
| HAR | 12 | 84,059 | 4,338 | 17,565 | 62,156 | 140 |
| HOU | 12 | 84,059 | 3,994 | 13,158 | 66,907 | 69 |
| LIN | 12 | 84,059 | 4,870 | 18,778 | 60,411 | 152 |
| LOS | 11 | 84,059 | 4,565 | 23,010 | 56,484 | 78 |
| MAC | 12 | 84,059 | 6,390 | 15,140 | 62,529 | 98 |
| MAN | 12 | 84,059 | 6,358 | 14,811 | 62,890 | 76 |
| MAU | 12 | 84,059 | 8,936 | 20,893 | 54,230 | 107 |
| MON | 12 | 84,059 | 5,428 | 14,228 | 64,403 | 71 |
| NEO | 12 | 84,059 | 3,724 | 16,621 | 63,714 | 97 |
| NEW | 21 | 84,059 | 3,950 | 16,049 | 64,060 | 753 |
| NUE | 12 | 84,059 | 3,992 | 15,120 | 64,947 | 76 |
| POR | 12 | 84,059 | 6,932 | 27,711 | 49,416 | 104 |
| REC | 11 | 84,059 | 5,140 | 21,385 | 57,534 | 106 |
| RUS | 12 | 84,059 | 4,946 | 19,707 | 59,406 | 205 |
| SAI | 12 | 84,059 | 5,172 | 19,336 | 59,551 | 82 |
| SMI | 12 | 84,059 | 4,296 | 16,320 | 63,443 | 108 |
| STL | 12 | 84,059 | 3,984 | 18,459 | 61,616 | 163 |
| TRA | 12 | 84,059 | 4,182 | 13,536 | 66,341 | 85 |
| TUC | 14 | 84,059 | 7,158 | 21,282 | 55,619 | 135 |

```
# Save it to a Word document
officer::read_docx() |>
  body_add_flextable(ft) |>
  print(target = here::here("americas/output/summary_ld_blocks_chr.docx"))
```

Get the size of each block from the .block.det files

```
get_kb_column <- function(dir_path) {
  # obtain the list of files with extension .blocks.det
  file_names <- list.files(path = dir_path, pattern = "\\.blocks\\.det$", full.names = TRUE)
  
  # create an empty list to hold the data frames
  block_list <- list()
  
  # loop through the files and read the data into the list
  for (file in file_names) {
    df <- read.table(file, header = TRUE, check.names = FALSE, stringsAsFactors = FALSE)
    # select only the KB column and add it to the block_list with the file name
    block_list[[file]] <- df %>% dplyr::select(KB) %>% add_column(file = file, .before = 1)
  }
  
  # combine the data frames in the block_list into a single data frame
  blocks <- bind_rows(block_list)
  
  # clean up the file name column
  blocks$file <- str_remove(blocks$file, "^.*\\/ld\\/blocks\\/")
  
  return(blocks)
}

# example usage: replace dir_path with your directory path
dir_path <- here("/gpfs/gibbs/pi/caccone/mkc54/albo/americas/output/files/ld/blocks_chr")

blocks<- 
  get_kb_column(dir_path) |>
  mutate(file = str_remove(file, "/gpfs/gibbs/pi/caccone/mkc54/albo/americas/output/files/ld/blocks_chr/")) |>
  mutate(file = str_remove(file, ".blocks.det")) |>

  as_tibble() |>
  rename(
    Population = 1
  )
```

Create density plot of the size of the LD blocks Plink found

```
# to check how many colors we need
# n_distinct(blocks$Population) #24

source(
  here("scripts", "RMarkdowns",
    "my_theme2.R"
  )
)

n <- 50
qual_col_pals = brewer.pal.info[brewer.pal.info$category == 'qual',]
col_vector = unlist(mapply(brewer.pal, qual_col_pals$maxcolors, rownames(qual_col_pals)))
pie(rep(1,n), col=sample(col_vector, n))
```

```
col_vector
```

```
##  [1] "#7FC97F" "#BEAED4" "#FDC086" "#FFFF99" "#386CB0" "#F0027F" "#BF5B17"
##  [8] "#666666" "#1B9E77" "#D95F02" "#7570B3" "#E7298A" "#66A61E" "#E6AB02"
## [15] "#A6761D" "#666666" "#A6CEE3" "#1F78B4" "#B2DF8A" "#33A02C" "#FB9A99"
## [22] "#E31A1C" "#FDBF6F" "#FF7F00" "#CAB2D6" "#6A3D9A" "#FFFF99" "#B15928"
## [29] "#FBB4AE" "#B3CDE3" "#CCEBC5" "#DECBE4" "#FED9A6" "#FFFFCC" "#E5D8BD"
## [36] "#FDDAEC" "#F2F2F2" "#B3E2CD" "#FDCDAC" "#CBD5E8" "#F4CAE4" "#E6F5C9"
## [43] "#FFF2AE" "#F1E2CC" "#CCCCCC" "#E41A1C" "#377EB8" "#4DAF4A" "#984EA3"
## [50] "#FF7F00" "#FFFF33" "#A65628" "#F781BF" "#999999" "#66C2A5" "#FC8D62"
## [57] "#8DA0CB" "#E78AC3" "#A6D854" "#FFD92F" "#E5C494" "#B3B3B3" "#8DD3C7"
## [64] "#FFFFB3" "#BEBADA" "#FB8072" "#80B1D3" "#FDB462" "#B3DE69" "#FCCDE5"
## [71] "#D9D9D9" "#BC80BD" "#CCEBC5" "#FFED6F"
```

```
# make plot using the sample y scale for all populations
ggplot(blocks, aes(x = KB)) +
  stat_density(
    aes(y = after_stat(count), fill = factor(Population)),
    linewidth = .5,
    alpha = .4,
    position = "identity"
  ) +
  scale_fill_manual(values = col_vector) +
  scale_x_continuous(name = "Block length (kb)") +
  scale_y_continuous(name = "Count") +
  theme(
    plot.title = element_text(hjust = 0.5, size = 18, face = "bold"),
    axis.text = element_text(size = 12),
    axis.title = element_text(size = 16, face = "bold"),
    strip.text = element_text(size = 14, face = "bold"),
    plot.background = element_rect(fill = "white"),
    panel.background = element_rect(fill = 'white', colour = 'black')
  ) +
  guides(fill = "none") +
  facet_wrap( ~ Population, ncol = 3) + my_theme()
```

```
# save the plot as a PDF using ggsave
ggsave(
  here("/gpfs/gibbs/pi/caccone/mkc54/albo/scripts/RMarkdowns/output/americas/americas_all/figures/block_density_y_scale_fixed_chr_americas_all.pdf"
  ),
  width = 10,
  height = 10,
  units = "in"
)
```

Make plot allowing the y axis scale free

```
ggplot(blocks, aes(x = KB)) +
  stat_density(
    aes(y = after_stat(count), fill = factor(Population)),
    linewidth = .5,
    alpha = .4,
    position = "identity"
  ) +
  scale_fill_manual(values = col_vector) +
  scale_x_continuous(name = "Block length (kb)") +
  scale_y_continuous(name = "Count") +
  theme(
    plot.title = element_text(hjust = 0.5, size = 18, face = "bold"),
    axis.text = element_text(size = 12),
    axis.title = element_text(size = 16, face = "bold"),
    strip.text = element_text(size = 14, face = "bold"),
    plot.background = element_rect(fill = "white"),
    panel.background = element_rect(fill = 'white', colour = 'black')
  ) +
  guides(fill = "none") +
  facet_wrap( ~ Population, ncol = 3, scales = "free_y") +
  my_theme()
```

```
# save the plot as a PDF using ggsave
ggsave(
  here("scripts/RMarkdowns/output/americas/americas_all/figures/block_density_free_scale_chr_americas_all.pdf"
  ),
  width = 20,
  height = 18,
  units = "in"
)
```

# 5. Plot SNP density

After quality control with approximately 85k SNPs

```
# load the function that we saved earlier
source(
  here("scripts", "RMarkdowns",
    "analyses", "import_bim.R"
  ),
  local = knitr::knit_global()
)
# import the file
snp_den_qc <- import_bim(
  here(
    "americas/output/file7.bim"
  )
)
```

Make plot of the SNP density

```
#   ____________________________________________________________________________
#   plot SNP density after QC                                               ####
snp_den_qc |>
  rename(
    Chromosome = 1
  ) |>
  mutate(
    Position           = as.numeric(
      Position
    )
  ) |>
  ggplot(
    aes(
      x                = Position
    ),
    label              = sprintf(
      "%0.2f",
      round(
        a,
        digits         = 0
      )
    )
  ) +
  geom_histogram(
    aes(
      y                = after_stat(
        count
      )
    ),
    binwidth           = 1e6
  ) +
  facet_wrap(
    vars(
      Chromosome
    ),
    scales             = "free_x"
  ) +
  labs(
    title              = "SNP Density after QC",
    x                  = expression(
      "Position in the genome (Mb)"
    ),
    y                  = expression(
      "Number of SNPs"
    )
  ) +
  scale_x_continuous(
    labels             = function(x) {
      format(
        x / 1e6,
        big.mark       = ",", 
        scientific     = FALSE
      )
    }
  ) +
  geom_density(
  aes(
    y = 1e6 * after_stat(count)
  ),
  color = "red",
  linewidth = .75,
  alpha = .4,
  fill = "pink"
  ) +
    theme(
    panel.grid.major   = element_line(
      linetype         = "dashed",
      linewidth        = 0.2
    ),
    panel.grid.minor   = element_line(
      linetype         = "dashed",
      linewidth        = 0.2
    ),
    panel.spacing      = unit(0.5, "lines"),
    strip.text         = element_text(
      face             = "bold", hjust = .5
    ),
    strip.background.x = element_rect(
      color            = "gray"
    )
  )
```

```
## Warning in fortify(data, ...): Arguments in `...` must be used.
## ✖ Problematic argument:
## • label = sprintf("%0.2f", round(a, digits = 0))
## ℹ Did you misspell an argument name?
```

```
#   save the density plot                                            ####
ggsave(
  here("scripts/RMarkdowns/output/americas/americas_all/figures/snp_density_after_qc_americas_all.pdf"
  ),
  width  = 10,
  height = 6,
  units  = "in"
)
```

SNPs per chromosome

```
# we can use dplyr "count" to get the number of SNPs for each chromosome
# lets get the data we need
snps_per_chrm <- 
  snp_den_qc |>
  count(
    Scaffold) |>
  rename(
    Chromosome = 1,
    "SNPs (N) " = 2
  )

# Create the flextable
ft <- flextable::flextable(snps_per_chrm)

# Apply zebra theme
ft <- flextable::theme_zebra(ft)

# Add a caption to the table
ft <- flextable::add_header_lines(ft, "SNPs per chromosome after quality control")
ft
```

| SNPs per chromosome after quality control | |
| --- | --- |
| Chromosome | SNPs (N) |
| 1 | 18,633 |
| 2 | 35,103 |
| 3 | 30,323 |

Get the mean number of SNPs per chromosome for the entire genome

```
# we first use dplyr cut_width to get the number of SNPs per 1Mb window
albo_den <- 
  snp_den_qc |>
  dplyr::select(
    Scaffold, Position
  ) |>
  group_by(
    Scaffold,
    windows               = cut_width(
      Position,
      width               = 1e6,
      boundary            = 0
    )
  ) |>
  summarise(
    n                     = n(),
    .groups               = "keep"
  ) |>
  group_by(
    Scaffold
  ) |>
  summarise(
    mean                  = mean(n),
    n                     = n(),
    .groups               = "keep"
  ) |>
  rename(
    Chromosome            = 1,
    "SNPs per 1Mb window" = 2,
    "Number of windows"   = 3
  )
#
# check the results
snp_table <-
  flextable(
    albo_den
  )
snp_table <- colformat_double(
  x        = snp_table,
  big.mark = ",",
  digits   = 2,
  na_str   = "N/A"
)
snp_table
```

| Chromosome | SNPs per 1Mb window | Number of windows |
| --- | --- | --- |
| 1 | 50.77 | 367 |
| 2 | 60.63 | 579 |
| 3 | 62.01 | 489 |

Merge objects

```
# we can merge the two data sets we created above into one table
after_qc <-
  snps_per_chrm |>
  left_join(
    albo_den,
    by = "Chromosome"
  )
snp_table2 <- flextable(
  after_qc)
snp_table2 <- colformat_double(
  x        = snp_table2,
  big.mark = ",",
  digits   = 2, 
  na_str   = "N/A"
  )
snp_table2
```

| Chromosome | SNPs (N) | SNPs per 1Mb window | Number of windows |
| --- | --- | --- | --- |
| 1 | 18,633 | 50.77 | 367 |
| 2 | 35,103 | 60.63 | 579 |
| 3 | 30,323 | 62.01 | 489 |

```
# Save it to a Word document
officer::read_docx() |>
  body_add_flextable(snp_table2) |>
  print(target = here::here("americas/output/snps_per_chromosome_table_americas.docx"))
```

# 6. LD pruning using the chromosomal scale

```
# we set a window of variants of 5 and move the window 1 variant per time, removing 1 of the variants with lowest MAF from a pair above the threshold of r^2 > 0.1
# the mean distance is 203kb across the tested populations. Try --indep-pairwise 200kb 1 0.1
plink2 \
--allow-extra-chr \
--bfile americas/output/file7 \
--indep-pairwise 5 1 0.1 \
--out americas/output/indepSNP_chr \
--silent;
grep 'pairwise\|variants\|samples' americas/output/indepSNP_chr.log
```

–indep-pairwise 5 1 0.1 544 samples (33 females, 31 males, 480
ambiguous; 544 founders) loaded from 84059 variants loaded from
americas/output/file7.bim. –indep-pairwise (3 compute threads):
40257/84059 variants removed.

Do the scaffold again

First need to import same SNP list Import the .bim file with the
SNPs

```
#   import the bim file with the SNP data                                   ####
snps <-
  read_delim(                    # to learn about the options use here, run ?read_delim on the console.
    here(
      "americas/output/file7_backup.bim" #output/populations/file4.bim
    ),                           # use library here to load it
    col_names      = FALSE,      # we don't have header in the input file
    show_col_types = FALSE,      # suppress message from read_delim
    col_types      = "ccidcc"    # set the class of each column
  )
#
# set column names
colnames(
  snps
) <-                             # to add a header in our tibble
  c(
    "Scaffold", "SNP", "Cm", "Position", "Allele1", "Allele2"
  )
#
# check the tibble
head(snps)
```

```
## # A tibble: 6 × 6
##   Scaffold SNP             Cm Position Allele1 Allele2
##   <chr>    <chr>        <int>    <dbl> <chr>   <chr>  
## 1 1.1      AX-583035163     0   315386 A       G      
## 2 1.1      AX-583033356     0   315674 C       T      
## 3 1.1      AX-583033370     0   330057 G       A      
## 4 1.1      AX-583035194     0   330265 A       G      
## 5 1.1      AX-583033387     0   331288 C       T      
## 6 1.10     AX-583035257     0    91677 T       C
```

```
write.table(
  snps,
  file      = here(
    "americas/output/snps_all_scaffolds_4ld.txt"
  ),
  sep       = "\t",
  row.names = FALSE,
  col.names = FALSE,
  quote     = FALSE
)

nrow(snps)
#84059
```

```
plink2 \
--allow-extra-chr \
--bfile americas/output/file5 \
--king-cutoff americas/output/file6 0.354 \
--make-bed \
--out americas/output/file11a \
--silent;
grep 'samples\|variants\|remaining' americas/output/file11a.log
```

554 samples (33 females, 32 males, 489 ambiguous; 554 founders)
loaded from 84059 variants loaded from americas/output/file5.bim.
americas/output/file11a.king.cutoff.out.id , and 544 remaining sample
IDs

```
# we set a window of variants of 5 and move the window 1 variant each time, removing 1 of the variants with lowest MAF from a pair above the threshold of r^2 > 0.1
# the mean distance is 203kb across the tested populations. We used --indep-pairwise 5 1 0.1 before. We can use the same values from the mean half distance max r2
plink2 \
--allow-extra-chr \
--bfile americas/output/file11a \
--indep-pairwise 5 1 0.1 \
--out americas/output/indepSNP_scaffolds \
--silent;
grep 'pairwise\|variants\|samples' americas/output/indepSNP_scaffolds.log
```

–indep-pairwise 5 1 0.1 544 samples (33 females, 31 males, 480
ambiguous; 544 founders) loaded from 84059 variants loaded from
americas/output/file11a.bim. –indep-pairwise (28 compute threads):
40229/84059 variants removed.

Now compare the two sets of SNPs using scaffolds or chromosomal
scale

Create Venn diagram of SNPs removed due to LD

```
# Read in the two files as vectors
prunout_chr <-
  read_delim(
    here(
      "americas/output/indepSNP_chr.prune.out"
    ),
    delim = " ",
    col_names = FALSE,
    show_col_types = FALSE
  )

prunout_scaffolds <-
  read_delim(
    here(
      "americas/output/indepSNP_scaffolds.prune.out"
    ),
    delim = " ",
    col_names = FALSE,
    show_col_types = FALSE
  )

# Convert the list to vector
prunout_scaffolds <- unlist(prunout_scaffolds)
prunout_chr <- unlist(prunout_chr)


# Calculate shared values
prunout <- intersect(prunout_chr, prunout_scaffolds)

# Create Venn diagram
venn_data1 <- list(
  "Chromosomal" = prunout_chr,
  "Scaffolds" = prunout_scaffolds
)

# create plot
venn_plot1 <- ggvenn(venn_data1, fill_color = c("steelblue", "darkorange"), show_percentage = TRUE)
```

```
## Warning: Using `size` aesthetic for lines was deprecated in ggplot2 3.4.0.
## ℹ Please use `linewidth` instead.
## ℹ The deprecated feature was likely used in the ggvenn package.
##   Please report the issue to the authors.
## This warning is displayed once every 8 hours.
## Call `lifecycle::last_lifecycle_warnings()` to see where this warning was
## generated.
```

```
# Add a title
venn_plot1 <- venn_plot1 +
  ggtitle("Comparison of genomic scales for linked SNPs") +
  theme(plot.title = element_text(hjust = .5))

# Display the Venn diagram
print(venn_plot1)
```

```
# Save Venn diagram to PDF
output_path <- here("scripts/RMarkdowns/output/americas/americas_all/figures/SNPs_linked_comparison_americas_all.pdf")
ggsave(output_path, venn_plot1, height = 5, width = 5, dpi = 300)
```

# 7. Pruning r2 for LD-pruned datasets

We already have a dataset made using the chromosomal scale that
pruned at 0.1 (made in step 9).

If needed, can create it again using the code below

```
plink2 \
--allow-extra-chr \
--bfile americas/output/file7 \
--indep-pairwise 5 1 0.1 \
--out americas/output/indepSNP_chr \
--silent;
grep 'pairwise\|variants\|samples' americas/output/indepSNP_chr.log
```

–indep-pairwise 5 1 0.1 544 samples (33 females, 31 males, 480
ambiguous; 544 founders) loaded from 84059 variants loaded from
americas/output/file7.bim. –indep-pairwise (3 compute threads):
40257/84059 variants removed.

*The indepSNP\_chr.prune.in file produced here = those SNPs <
our 0.1 threshold, that we want to use*

Make pruned dataset for 0.01. Can use file7

```
plink2 \
--allow-extra-chr \
--bfile americas/output/file7 \
--indep-pairwise 5 1 0.01 \
--out americas/output/indepSNP_chr_r01 \
--silent;
grep 'pairwise\|variants\|samples' americas/output/indepSNP_chr_r01.log
```

–indep-pairwise 5 1 0.01 544 samples (33 females, 31 males, 480
ambiguous; 544 founders) loaded from 84059 variants loaded from
americas/output/file7.bim. –indep-pairwise (3 compute threads):
68095/84059 variants removed.

Use the SNP set the LD pruning we just did and extract them from
file7

```
plink2 \
--keep-allele-order \
--bfile americas/output/file7 \
--make-bed \
--export vcf \
--out americas/output/snps_sets/r2_0.1 \
--extract americas/output/indepSNP_chr.prune.in \
--silent
grep "variants\|samples" americas/output/snps_sets/r2_0.1.log
```

544 samples (33 females, 31 males, 480 ambiguous; 544 founders)
loaded from 84059 variants loaded from americas/output/file7.bim.
–extract: 43802 variants remaining. 43802 variants remaining after main
filters.

Repeat for 0.01 dataset

```
plink2 \
--keep-allele-order \
--bfile americas/output/file7 \
--make-bed \
--export vcf \
--out americas/output/snps_sets/r2_0.01 \
--extract americas/output/indepSNP_chr_r01.prune.in \
--silent
grep "variants\|samples" americas/output/snps_sets/r2_0.01.log
```

544 samples (33 females, 31 males, 480 ambiguous; 544 founders)
loaded from 84059 variants loaded from americas/output/file7.bim.
–extract: 15964 variants remaining. 15964 variants remaining after main
filters.

These snp set can now be used for subsequent pop gen analyses.

# 8. Run PCA with LD-pruned dataset

```
plink2 \
--allow-extra-chr \
--bfile americas/output/snps_sets/r2_0.1 \
--pca allele-wts \
--freq \
--out americas/output/pca_pops_1 \
--silent;
grep 'samples\|variants' americas/output/pca_pops_1.log
```

544 samples (33 females, 31 males, 480 ambiguous; 544 founders)
loaded from 43802 variants loaded from
americas/output/snps\_sets/r2\_0.1.bim.

Check the files

```
head -n 2 americas/output/pca_pops_1.eigenvec
```

```
## #FID IID PC1 PC2 PC3 PC4 PC5 PC6 PC7 PC8 PC9 PC10
## MAU  153 -0.0501094  0.0191435   -0.0232742  -0.0141601  -0.00750308 0.0276132   0.0354234   -0.0142406  -0.00617247 0.0216437
```

```
head -n 10 americas/output/pca_pops_1.eigenval
```

```
## 38.7946
## 7.99382
## 6.79599
## 6.41753
## 5.82164
## 5.42499
## 5.28792
## 4.9997
## 4.68428
## 4.55087
```

Import PCA data

```
# import the data from Plink
pca <- read.delim(
  here(
    "americas/output/pca_pops_1.eigenvec"
  ),
  head = TRUE
)
 
# check head of the file
head(pca)
```

```
##   X.FID IID        PC1        PC2         PC3         PC4         PC5
## 1   MAU 153 -0.0501094 0.01914350 -0.02327420 -0.01416010 -0.00750308
## 2   MAU 154 -0.0481246 0.01401430 -0.02503260  0.00706932 -0.03027340
## 3   MAU 155 -0.0428834 0.01002240  0.01301580  0.00317885  0.01438070
## 4   MAU 156 -0.0480644 0.00761829 -0.03285610  0.01557050 -0.04476120
## 5   MAU 158 -0.0494621 0.01623050 -0.00186854  0.03216720 -0.00862696
## 6   MAU  16 -0.0527168 0.01282840 -0.03610950  0.00914322 -0.02108160
##           PC6       PC7         PC8          PC9        PC10
## 1  0.02761320 0.0354234 -0.01424060 -0.006172470  0.02164370
## 2 -0.02028980 0.0469852 -0.00730572 -0.015434300 -0.01655050
## 3 -0.01232650 0.0235913 -0.01726910 -0.019289400  0.02933210
## 4 -0.04091380 0.0285620 -0.01474340  0.000802796 -0.01474800
## 5 -0.00191078 0.0555682 -0.00551643 -0.013135100 -0.02209600
## 6 -0.01947270 0.0219928 -0.00635999  0.000134328 -0.00361387
```

Import sample attributes

```
# import sample attributes
samples2 <- read.delim(
  here("scripts", "RMarkdowns", "population_metadata_all_1Apr2024.txt"
  ),
  head = TRUE
)
#
# check head of the file
head(samples2)
```

```
##              geo    range continent         region             country pop
## 1      Caribbean invasive Caribbean      Caribbean             Bermuda BRM
## 2      Caribbean invasive Caribbean      Caribbean             Jamaica JAM
## 3      Caribbean invasive Caribbean      Caribbean Trinidad and Tobago SAI
## 4 Central Africa invasive    Africa Central Africa               Gabon GAB
## 5    East Africa invasive    Africa    East Africa          Madagascar DGV
## 6    East Africa invasive    Africa    East Africa          Madagascar ANT
##          pop_city               state   Subregion
## 1         Bermuda             Bermuda   Caribbean
## 2          Negirl             Jamaica   Caribbean
## 3 Saint Augustine Trinidad and Tobago   Caribbean
## 4     Franceville                <NA>      Africa
## 5     Diego ville                <NA> East Africa
## 6    Antananarivo                <NA> East Africa
```

Check number of samples per population

```
pops_pca <- 
  pca |>
  group_by(X.FID) |>
  summarize(count_distinct = n_distinct(IID))

# check it
head(pops_pca)
```

```
## # A tibble: 6 × 2
##   X.FID count_distinct
##   <chr>          <int>
## 1 ARA               12
## 2 BAF                7
## 3 BCO               12
## 4 BEA               12
## 5 BEH                9
## 6 BER               12
```

Merge the data

```
df4 <-
  merge(
    pca,
    samples2,
    by.x = "X.FID",
    by.y = "pop",
    all.x = T,
    all.y = F
  ) 
head(df4)
```

```
##   X.FID  IID        PC1       PC2        PC3          PC4         PC5
## 1   ARA 2581 -0.0727905 0.0368180  0.0129636 -0.013651000  0.03939610
## 2   ARA 2582 -0.0603525 0.0417421  0.0129047 -0.000794396  0.01242390
## 3   ARA 2571 -0.0620801 0.0266160  0.0429298 -0.039753100 -0.02222500
## 4   ARA 2572 -0.0682431 0.0238515 -0.0108020  0.002857400 -0.00555243
## 5   ARA 2573 -0.0640657 0.0229639  0.0395067 -0.025809000 -0.01363930
## 6   ARA 2574 -0.0597342 0.0444424  0.0623465 -0.014877000  0.03306560
##           PC6         PC7         PC8         PC9        PC10           geo
## 1  0.01532100  0.01750270  0.03320530  0.06338280 -0.00447642 South America
## 2 -0.00742101  0.02425410 -0.00264248 -0.00345076  0.00824394 South America
## 3 -0.01162230 -0.00136024  0.01629550  0.04409920 -0.01088620 South America
## 4 -0.01862630 -0.02265190  0.00240911  0.01560300  0.01128210 South America
## 5 -0.01127180 -0.01005320  0.03071840  0.05214210 -0.02082330 South America
## 6  0.00838980 -0.01617310  0.04856780  0.07293420 -0.01283380 South America
##      range     continent        region country  pop_city      state
## 1 invasive South America South America  Brazil Araguaína TO, Brazil
## 2 invasive South America South America  Brazil Araguaína TO, Brazil
## 3 invasive South America South America  Brazil Araguaína TO, Brazil
## 4 invasive South America South America  Brazil Araguaína TO, Brazil
## 5 invasive South America South America  Brazil Araguaína TO, Brazil
## 6 invasive South America South America  Brazil Araguaína TO, Brazil
##       Subregion
## 1 South America
## 2 South America
## 3 South America
## 4 South America
## 5 South America
## 6 South America
```

Get some data for the PCA plot

```
# How many populations
length(unique(df4$X.FID))
```

```
## [1] 51
```

```
# How many countries
length(unique(df4$country))
```

```
## [1] 7
```

```
# How many subregions
length(unique(df4$Subregion))
```

```
## [1] 7
```

```
# How many states
length(unique(df4$state))
```

```
## [1] 36
```

Check the states

```
unique(df4$state)
```

```
##  [1] "TO, Brazil"          "MT, Brazil"          "SC"                 
##  [4] "MG, Brazil"          "NJ"                  "PA, Brazil"         
##  [7] "RO, Brazil"          "Bermuda"             "CA"                 
## [10] "OH"                  "Colombia"            "TX"                 
## [13] "NC"                  "NY"                  "PI, Brazil"         
## [16] "RS, Brazil"          "AL"                  "PA"                 
## [19] "MS"                  "GO, Brazil"          "DE"                 
## [22] "GA"                  "VA"                  "AM, Brazil"         
## [25] "MA"                  "Jamaica"             "CT"                 
## [28] "LA"                  "ES, Brazil"          "FL"                 
## [31] "IL"                  "Argentina"           "PE, Brazil"         
## [34] "AR"                  "Trinidad and Tobago" "MO"
```

Check the countries

```
unique(df4$country)
```

```
## [1] "Brazil"              "USA"                 "Bermuda"            
## [4] "Colombia"            "Jamaica"             "Argentina"          
## [7] "Trinidad and Tobago"
```

Make plot1

```
# source the plotting function
source(here("scripts", "RMarkdowns", "my_theme2.R"))

# Shapes
N = 100
M = 1000
good.shapes = c(1:25, 33:127)

# Colors
palette1 <- brewer.pal(12, "Paired")
palette2 <- brewer.pal(11, "Set3")
palette23 <- c(palette1, palette2)
colors2 <- 
  c("#52ef99", "#146c45", "#75d5e1", "#FB8072", "#2c4a5e", "#6a8fe0", "#8c61cd", "#f365e7", "#871550", "#a113b2", "#BF5B17", "#1F78B4", "#cf749b",  "#FF7F00","#2524f9", "#799d10", "#B3E2CD",  "#984EA3", "#754819", "#fda547", "#a41415", "#fd5917", "#fd4e8b", "#ead624", "#6A3D9A", "#21a708", "#332288", "#51f310", "#9d8d88", "#66C2A5", "#E41A1C", "#BC80BD", "#E7297A", "darkgray", "orange2", "magenta", "gold4", "purple", "#a7e831")


# Compute the count for each country
state_count <- df4 |>
  group_by(state) |>
  summarize(count = n())

# Merge the count back to the main data
df4 <- df4 |>
  left_join(state_count, by = "state")

# Create a custom label for the legend
df4$state_label <-
  paste(df4$state, " (", df4$count, ")", sep = "")

# Define the color and shape manually
#palette23 <- c(palette1, palette2)
colors <- setNames(colors2, unique(df4$state_label))
shapes <-
  setNames(good.shapes[c(1:25, 28:31, 55:57, 1:4)], unique(df4$state_label))


# Compute the center of ellipses for each continent
ellipse_centers <- df4 |>
  group_by(country) |>
  summarise(PC1_center = mean(PC1), PC2_center = mean(PC2))

# # Calculate the number of samples per region
country_count <- df4 |>
  group_by(country) |>
  summarise(count = n())
country_labels <-
  setNames(
    paste(country_count$country, " (", country_count$count, ")", sep = ""),
    country_count$country
  )

# Define the colors you want for each continent
#continent_colors <-
  #c("yellow", "pink", "#6a8fe0", "gold3", "#FF6F90", "lightpink3", "lightblue","blue") # Adjust these colors to your preference

country_colors <-
  c("pink", "#fdc547", "#ffffaa", "#baffc9", "#9bf6ff", "#a0c4ff", "#bdb2ff")# Adjust these colors to your preference

  
# Create the plot
ggplot(df4, aes(PC1, PC2)) +
  geom_point(aes(shape = state_label, color = state_label)) +
  stat_ellipse(
    aes(fill = country, group = country),
    geom = "polygon",
    alpha = 0.25,
    level = 0.8,
    segments = 40,
    color = "darkgray"
  ) +
  stat_ellipse(
    aes(group = country),
    geom = "path",
    level = 0.8,
    segments = 40,
    color = "darkgray"
  ) + # Line around the ellipse
  geom_text_repel(
    data = ellipse_centers,
    aes(x = PC1_center, y = PC2_center, label = country),
    color = "black"
  ) + # Add labels using ggrepel
  xlab("PC1 (38.8% Variance)") + 
  ylab("PC2 (8.0% Variance)") +
  labs(caption = "Principal Component Analysis with 43,802 SNPs \n of mosquitoes from 51 localities across the Americas.") + 
  guides(
    color = guide_legend(
      title = "State",
      order = 2,
      ncol = 2
    ),
    shape = guide_legend(
      title = "State",
      order = 2,
      ncol = 2
    ),
    fill = guide_legend(
      title = "Region",
      order = 1,
      ncol = 1
    )
  ) +
  scale_fill_manual(values = country_colors, labels = country_labels) +
  scale_color_manual(values = colors) +
  scale_shape_manual(values = shapes) +
  guides(fill=guide_legend(ncol=2)) +
  my_theme() +
  theme(
    plot.caption = element_text(face = "italic"),
    legend.position = "right",
    legend.justification = "top",
    legend.box.just = "center",
    legend.box.background = element_blank(),
    legend.text=element_text(size=6),
    plot.margin = margin(5.5, 30, 5.5, 5.5, "points"),
    legend.margin = margin(5, 5, 5, 40) # move the legend a bit up
  )
```

```
#   save the pca plot                                                       ####
ggsave(
  here("scripts/RMarkdowns/output/americas/americas_all/figures/PCA_americas_all_r1_plink_bystate_PC1&2.pdf"
  ),
  width  = 10,
  height = 7,
  units  = "in"
)
```

Make plot2

```
# source the plotting function
source(here("scripts", "RMarkdowns", "my_theme2.R"))

# Shapes
N = 100
M = 1000
good.shapes = c(1:25, 33:127)

# Colors
palette1 <- brewer.pal(12, "Paired")
palette2 <- brewer.pal(11, "Set3")
palette23 <- c(palette1, palette2)
colors2 <- 
  c("#52ef99", "#146c45", "#75d5e1", "#FB8072", "#2c4a5e", "#6a8fe0", "#8c61cd", "#f365e7", "#871550", "#a113b2", "#BF5B17", "#1F78B4", "#cf749b",  "#FF7F00","#2524f9", "#799d10", "#B3E2CD",  "#984EA3", "#754819", "#fda547", "#a41415", "#fd5917", "#fd4e8b", "#ead624", "#6A3D9A", "#21a708", "#332288", "#51f310", "#9d8d88", "#66C2A5", "#E41A1C", "#BC80BD", "#E7297A", "darkgray", "orange2", "magenta", "gold4", "purple", "#a7e831")


# Compute the count for each country
state_count <- df4 |>
  group_by(state) |>
  summarize(count = n())

# Merge the count back to the main data
df4 <- df4 |>
  left_join(state_count, by = "state")

# Create a custom label for the legend
df4$state_label <-
  paste(df4$state, " (", df4$count, ")", sep = "")

# Define the color and shape manually
#palette23 <- c(palette1, palette2)
colors <- setNames(colors2, unique(df4$state_label))
shapes <-
  setNames(good.shapes[c(1:25, 28:31, 55:57, 1:4)], unique(df4$state_label))


# Compute the center of ellipses for each continent
ellipse_centers <- df4 |>
  group_by(country) |>
  summarise(PC1_center = mean(PC1), PC3_center = mean(PC3))

# # Calculate the number of samples per region
country_count <- df4 |>
  group_by(country) |>
  summarise(count = n())
country_labels <-
  setNames(
    paste(country_count$country, " (", country_count$count, ")", sep = ""),
    country_count$country
  )

# Define the colors you want for each continent
#continent_colors <-
  #c("yellow", "pink", "#6a8fe0", "gold3", "#FF6F90", "lightpink3", "lightblue","blue") # Adjust these colors to your preference


country_colors <-
  c("pink", "#fdc547", "#ffffaa", "#baffc9", "#9bf6ff", "#a0c4ff", "#bdb2ff")# Adjust these colors to your preference

  
# Create the plot
ggplot(df4, aes(PC1, PC3)) +
  geom_point(aes(shape = state_label, color = state_label)) +
  stat_ellipse(
    aes(fill = country, group = country),
    geom = "polygon",
    alpha = 0.25,
    level = 0.8,
    segments = 40,
    color = "darkgray"
  ) +
  stat_ellipse(
    aes(group = country),
    geom = "path",
    level = 0.8,
    segments = 40,
    color = "darkgray"
  ) + # Line around the ellipse
  geom_text_repel(
    data = ellipse_centers,
    aes(x = PC1_center, y = PC3_center, label = country),
    color = "black"
  ) + # Add labels using ggrepel
  xlab("PC1 (38.8% Variance)") + 
  ylab("PC3 (6.8% Variance)") +
  labs(caption = "Principal Component Analysis with 43,802 SNPs \n of mosquitoes from 51 localities across the Americas.") + 
  guides(
    color = guide_legend(
      title = "State",
      order = 2,
      ncol = 2
    ),
    shape = guide_legend(
      title = "State",
      order = 2,
      ncol = 2
    ),
    fill = guide_legend(
      title = "Region",
      order = 1,
      ncol = 1
    )
  ) +
  scale_fill_manual(values = country_colors, labels = country_labels) +
  scale_color_manual(values = colors) +
  scale_shape_manual(values = shapes) +
  guides(fill=guide_legend(ncol=2)) +
  my_theme() +
  theme(
    plot.caption = element_text(face = "italic"),
    legend.position = "right",
    legend.justification = "top",
    legend.box.just = "center",
    legend.box.background = element_blank(),
    legend.text=element_text(size=6),
    plot.margin = margin(5.5, 30, 5.5, 5.5, "points"),
    legend.margin = margin(5, 5, 5, 40) # move the legend a bit up
  )
```

```
#   save the pca plot                                                       ####
ggsave(
  here("scripts/RMarkdowns/output/americas/americas_all/figures/PCA_americas_all_r1_plink_PCS1&3.pdf"
  ),
  width  = 10,
  height = 7,
  units  = "in"
)
```

# 9. Make a new snp set with Minor Allele Frequency of 1% (MAF 0.01)

## 9.1 Minor allele frequency

First, estimate the allele frequency with Plink

```
cd /gpfs/gibbs/pi/caccone/mkc54/albo
plink2 \
--allow-extra-chr \
--bfile americas/output/file3 \
--freq \
--out americas/output/MAF_check \
--silent
```

Now apply the MAF filter for 0.01

```
# We will use MAF of 1%
plink2 \
--allow-extra-chr \
--bfile americas/output/file3 \
--maf 0.01 \
--make-bed \
--out americas/output/file4b \
--silent;
grep "variants" americas/output/file4b.log
```

101989 variants loaded from americas/output/file3.bim. 6068 variants
removed due to allele frequency threshold(s) 95921 variants remaining
after main filters.

Plot it with ggplot

```
#   Import MAF data                                                         ####
maf_freq <-
  read.delim(
    here(
      "americas/output/MAF_check.afreq"
    ),
    header = TRUE
  )
```

Make MAF plot

```
#   make the plot                                                           ####
ggplot(
  maf_freq,
  aes(ALT_FREQS)
) +
  geom_histogram(
    colour = "black",
    fill = "lightgray",
    bins = 40
  ) +
  labs(
    x = "Minor Allele Frequency (MAF)",
    y = "Frequency (n)",
    caption = "<span style='color:red;'><i>Red</i></span> <span style='color:black;'><i>line at</i></span><span style='color:red;'><i> MAF 1%</i></span><span style='color:black;'><i> threshold</i></span>."
  ) +
  geom_text(
    aes(
      x = .01,
      y = 8000,
      label = paste0("6,068 SNPs")
    ),
    size = 3,
    color = "red",
    vjust = -.2
  ) +
  geom_vline(xintercept = 0.01, color = "red") +
  my_theme() +
  theme(plot.caption = element_markdown()) +
  scale_y_continuous(label = scales::number_format(big.mark = ",")) +
  scale_x_continuous(breaks = c(0, 0.1, 0.2, 0.4, 0.6, 0.8, 1.0))
```

```
## Warning in geom_text(aes(x = 0.01, y = 8000, label = paste0("6,068 SNPs")), : All aesthetics have length 1, but the data has 101989 rows.
## ℹ Please consider using `annotate()` or provide this layer with data containing
##   a single row.
```

```
# save the plot
ggsave(
  here("scripts", "RMarkdowns",
    "output", "americas", "americas_all", "figures", "MAF_0.01_freq_plot_all_americas.pdf"
  ),
  width  = 7,
  height = 5,
  units  = "in"
)
```

We removed 6,068 variants due to the MAF filter.

## 9.2 HWE test

Now we can run the HWE test. However, we will need to apply the SNP
missingness again for each population. If we do not, the HWE will vary
widely. With the bash script below, we will create a new file for each
population, run the HWE test with HWE p value <1e‐6 (HWE p value
<1e‐6). Then, we ask Plink to generate a list of SNPs that passed the
test for each population.

```
mkdir -p americas/output/files/hardyb;
cp americas/output/file4b.* americas/output/files/hardyb/
```

```
for fam in $(awk '{print $1}' americas/output/files/hardyb/file4b.fam | sort | uniq); 
do 
echo $fam | \
plink2 \
--allow-extra-chr \
--silent \
--keep-allele-order \
--bfile americas/output/files/hardyb/file4b \
--keep-fam /dev/stdin \
--make-bed \
--out americas/output/files/hardyb/$fam \
--hwe 0.000001 \
--geno 0.1 \
--write-snplist; \
done
```

Next, we use “cat” and “awk” to concatenate the SNP list from all
populations, and remove duplicates. Once we have a list of SNPs that
passed the test for each population, we can use Plink to create a new
bed file keeping only the SNPs that passed the test in each population.
First, lets get the list of SNPs, and count how many passed:

```
cd /gpfs/gibbs/pi/caccone/mkc54/albo
cat americas/output/files/hardyb/*.snplist | awk '!a[$0]++' > americas/output/files/passed_hwe_b.txt;
wc -l americas/output/files/passed_hwe_b.txt
```

95921 americas/output/files/passed\_hwe\_b.txt

All 95921 variants passed HWE test. If any failed, we could remove
the variants that did not pass HWE test, using the –extract flag,
extracting only those that passed HWE.

## 9.3 LD pruning

Since we do not have to remove any SNPs due to deviation from HWE, we
can proceed with heterozygosity estimates. The first step is to “prune”
our data set. We will check the pairwise linkage estimates for all SNPs.
We can work with file4. We will use “`indep-pairwise`” to
check if there are SNPs above a certain linkage disequilibrium (LD)
threshold. Check Plink documentation for more details https://www.cog-genomics.org/plink/1.9/ld I used
“`--indep-pairwise 5 1 0.1`” , which indicates according to
the documentation:
`--indep-pairphase <window size>['kb'] <step size (variant ct)> <r^2 threshold>`
We will check in a window of 5kb if there is any pair of SNPs with r2
estimates above 0.1, then we will move our window 1 SNP and check again
for SNPs above the threshold. We will repeat this procedure until we
check the entire genome.

Switch back to plink2

```
module load PLINK/2_avx2_20221024
#PLINK/1.9b_6.21-x86_64 => PLINK/2_avx2_20221024
plink2 --version
```

```
plink2 \
--allow-extra-chr \
--bfile americas/output/file4b \
--extract americas/output/files/passed_hwe_b.txt \
--indep-pairwise 5 1 0.1 \
--out americas/output/indepSNPb \
--silent;
grep 'pairwise\|variants\|samples' americas/output/indepSNPb.log
```

–indep-pairwise 5 1 0.1 555 samples (33 females, 32 males, 490
ambiguous; 555 founders) loaded from 95921 variants loaded from
americas/output/file4b.bim. –extract: 95921 variants remaining. 95921
variants remaining after main filters. –indep-pairwise (27 compute
threads): 42133/95921 variants removed.

Remember, the SNPs are not removed from our data set. Plink created 3
files when we ran the code above. One is the “indepSNP.log” file, and
the other two are: “indepSNP.prune.in” -> list of SNPs with squared
correlation smaller than our r2 threshold of 0.1. “indepSNP.prune.out”
-> list of SNPs with squared correlation greater than our r2
threshold of 0.1. For our heterozygosity estimates, we want to use the
set of SNPs that are below our r2 threshold of 0.1. We consider that
they are randomly associated. We can use Plink to estimate the
heterozygosity using the “indepSNP.prune.in” file.

## 9.4 Heterozygosity

```
plink2 \
--allow-extra-chr \
--bfile americas/output/file4b \
--extract americas/output/indepSNPb.prune.in \
--het \
--out americas/output/R_checkb \
--silent;
grep 'variants' americas/output/R_checkb.log
```

95921 variants loaded from americas/output/file4b.bim. –extract:
53788 variants remaining. 53788 variants remaining after main
filters.

```
#   find individuals with high heterozygosity                              ####
# import the data from Plink
het <- read.delim(
  here(
    "americas/output/R_checkb.het"
  ),
  head = TRUE
)
#
# check head of the file
colnames(het)
```

```
## [1] "X.FID"  "IID"    "O.HOM." "E.HOM." "OBS_CT" "F"
```

Estimate heterozygosity

```
# create a column named HET_RATE and calculate the heterozygosity rate
het$HET_RATE <- (het$"OBS_CT" - het$"O.HOM") / het$"OBS_CT"
#
# use subset function to get values deviating from 4sd of the mean heterozygosity rate.
het_fail <-
  subset(
    het, (het$HET_RATE < mean(
      het$HET_RATE
    ) - 4 * sd(
      het$HET_RATE
    )) |
      (het$HET_RATE > mean(
        het$HET_RATE
      ) + 4 * sd(
        het$HET_RATE
      ))
  )
#
# get the list of individuals that failed our threshold of 4sd from the mean.
het_fail$HET_DST <-
  (het_fail$HET_RATE - mean(
    het$HET_RATE
  )) / sd(
    het$HET_RATE
  )
```

Save the files to use with Plink

```
#   save the data to use with Plink2                                        ####
#
write.table(
  het_fail,
  here(
    "americas/output/fail-het-qc_b.txt"
  ),
  row.names = FALSE
)
```

Make plot

```
#   make a heterozygosity plot                                              ####
#
ggplot(
  het,
  aes(
    HET_RATE
  )
) +
  geom_histogram(
    colour           = "black",
    fill             = "lightgray",
    bins             = 40
  ) +
  labs(
    x                = "Heterozygosity Rate",
    y                = "Number of Individuals"
  ) +
  geom_vline(
    aes(
      xintercept     = mean(
        HET_RATE
      )
    ),
    col              = "red",
    linewidth        = 1.5
  ) +
  geom_vline(
    aes(
      xintercept     = mean(
        HET_RATE
      ) + 4 * sd(
        HET_RATE
      )
    ),
    col              = "#BFB9B9",
    linewidth        = 1
  ) +
  geom_vline(
    aes(
      xintercept     = mean(
        HET_RATE
      ) - 4 * sd(
        HET_RATE
      )
    ),
    col              = "#BFB9B9",
    linewidth        = 1
  ) + 
  my_theme() +
  scale_y_continuous(
  )
```

```
#   save the heterozygosity plot                                            ####
ggsave(
  here("scripts", "RMarkdowns",
    "output", "americas", "americas_all", "figures", "Heterozygosity_all_MAF_0.01.pdf"
  ),
  width  = 5,
  height = 4,
  units  = "in"
)
```

The red line in the plot above indicates the mean, and the gray lines
indicate 4 standard deviation from the mean. We can see that some
mosquitoes do have excess heterozygous sites. We will remove them. We
can get their ID from the file “fail-het-qc.txt”. We can use the bash
script below to parse the file to use with Plink

```
sed 's/"// g' americas/output/fail-het-qc_b.txt | awk '{print$1, $2}'> americas/output/het_fail_ind_b.txt;
echo 'How many mosquitoes we need to remove from our data set:';
cat americas/output/het_fail_ind_b.txt | tail -n +2 | wc -l;
echo 'Which mosquitoes we have to remove:';
tail -n +2 americas/output/het_fail_ind_b.txt
```

```
## How many mosquitoes we need to remove from our data set:
## 0
## Which mosquitoes we have to remove:
```

None need to be removed

Heterozygosity is outside the SD threshold in 0 individuals; so we
don’t really need to remove any. Let’s create file 5 though, to be
consistent with the file names for other datasets

```
plink2 \
--allow-extra-chr \
--bfile americas/output/file4b \
--remove americas/output/het_fail_ind_b.txt \
--make-bed \
--out americas/output/file5b \
--silent;
grep 'variants\|samples' americas/output/file5b.log
```

555 samples (33 females, 32 males, 490 ambiguous; 555 founders)
loaded from 95921 variants loaded from americas/output/file4b.bim.
–remove: 555 samples remaining. 555 samples (33 females, 32 males, 490
ambiguous; 555 founders) remaining after

## 9.5 Relatedness

Check for cryptic relatedness. Check Plink2 documentation https://www.cog-genomics.org/plink/2.0/distance You can
download King directly https://www.kingrelatedness.com/manual.shtml or check
their manuscript https://www.ncbi.nlm.populations.gov/pmc/articles/PMC3025716/pdf/btq559.pdf
From Plink2 documentation: “Note that KING kinship coefficients are
scaled such that duplicate samples have kinship 0.5, not 1. First-degree
relations (parent-child, full siblings) correspond to ~0.25,
second-degree relations correspond to ~0.125, etc. It is conventional to
use a cutoff of ~0.354 (the geometric mean of 0.5 and 0.25) to screen
for monozygotic twins and duplicate samples, ~0.177 to add first-degree
relations, etc.” There two options. One is to run only –make-king and
another one is to use –make-king-table We will use the threshold of
0.354 and create a table.

```
# Plink2 will create a file with extension .king
plink2 \
--allow-extra-chr \
--bfile americas/output/file5b \
--extract americas/output/indepSNPb.prune.in \
--make-king-table rel-check \
--king-table-filter 0.354 \
--out americas/output/file5b \
--silent;
grep 'variants\|samples' americas/output/file5b.log
```

555 samples (33 females, 32 males, 490 ambiguous; 555 founders)
loaded from 95921 variants loaded from americas/output/file5b.bim.
–extract: 53788 variants remaining. 53788 variants remaining after main
filters.

Check the individuals that did not pass our filtering

```
head -n 20 americas/output/file5b.kin0
```

```
## #FID1    IID1    FID2    IID2    NSNP    HETHET  IBS0    KINSHIP
## BPA  2460    BPA 2452    50155   0.196232    0.0101884   0.364882
## CHI  2274    CHI 2269    51431   0.205596    0.0129105   0.35565
## HAT  2395    HAT 2393    51880   0.252776    0.00422128  0.437638
## IPO  2590    IPO 2585    51632   0.190483    0.00704989  0.397711
## LOS  366 LOS 365 51915   0.185862    0   0.498527
## LOS  367 LOS 365 51847   0.185777    0   0.49824
## LOS  367 LOS 366 51833   0.185287    0   0.497485
## NEB  2549    NEB 2546    50274   0.255997    0.00288419  0.402883
## NEW  423 NEW 413 52176   0.220312    0.0107521   0.365234
## NEW  423 NEW 420 52172   0.221326    0.0131296   0.35638
## REC  179 REC 175 51895   0.22401 0.0072454   0.397193
## SPR  465 SPR 460 51975   0.227532    0.00794613  0.383078
```

We want to remove one of the individuals of the pairs.

```
plink2 \
--allow-extra-chr \
--bfile americas/output/file5b \
--extract americas/output/indepSNPb.prune.in \
--make-king triangle bin \
--out americas/output/file6b \
--silent;
grep 'variants\|samples' americas/output/file6b.log
```

555 samples (33 females, 32 males, 490 ambiguous; 555 founders)
loaded from 95921 variants loaded from americas/output/file5b.bim.
–extract: 53788 variants remaining. 53788 variants remaining after main
filters. –make-king pass 1/1: Scanning for rare variants… done. 241
variants handled by initial scan (53547 remaining). –make-king: 53788
variants processed.

Now we can use Plink2 to remove one of the mosquitoes from the pair
with high kinship. We had 12. Plink2 always tries to maximize the number
of samples passing the filters.

```
plink2 \
--allow-extra-chr \
--bfile americas/output/file5b \
--king-cutoff americas/output/file6b 0.354 \
--make-bed \
--out americas/output/file7b \
--silent;
grep 'samples\|variants\|remaining' americas/output/file7b.log
```

555 samples (33 females, 32 males, 490 ambiguous; 555 founders)
loaded from 95921 variants loaded from americas/output/file5b.bim.
americas/output/file7b.king.cutoff.out.id , and 545 remaining sample
IDs

It removed 10 individuals

## 9.6 Create chromosomal scale for MAF 0.01 files

Import the .bim file with the SNPs to create a new chromosomal
scale.

```
#   ____________________________________________________________________________
#   import the bim file with the SNP data                                   ####
snps <-
  read_delim(                    # to learn about the options use here, run ?read_delim on the console.
    here(
      "americas/output/file7b.bim"
    ),                           # use library here to load it
    col_names      = FALSE,      # we don't have header in the input file
    show_col_types = FALSE,      # suppress message from read_delim
    col_types      = "ccidcc"    # set the class of each column
  )
#
# set column names
colnames(
  snps
) <-                             # to add a header in our tibble
  c(
    "Scaffold", "SNP", "Cm", "Position", "Allele1", "Allele2"
  )
#
# check the tibble
head(snps)
```

```
## # A tibble: 6 × 6
##   Scaffold SNP             Cm Position Allele1 Allele2
##   <chr>    <chr>        <int>    <dbl> <chr>   <chr>  
## 1 1        AX-583033342     0   315059 C       G      
## 2 1        AX-583035163     0   315386 A       G      
## 3 1        AX-583033356     0   315674 C       T      
## 4 1        AX-583033370     0   330057 G       A      
## 5 1        AX-583035194     0   330265 A       G      
## 6 1        AX-583033387     0   331288 C       T
```

Separate the tibbles into each chromosome

```
#   separate the SNP data per chromosome                                    
# chr1
chr1_snps <-
  snps |>
  filter(
    str_detect(
      Scaffold, "^1."
    )
  ) |> # here we get only Scaffold rows starting with 1
  as_tibble() # save as tibble
#
# chr2
chr2_snps <-
  snps |>
  filter(
    str_detect(
      Scaffold, "^2."
    )
  ) |>
  as_tibble()
#
# chr3
chr3_snps <-
  snps |>
  filter(
    str_detect(
      Scaffold, "^3."
    )
  ) |>
  as_tibble()
```

Import the file with sizes of each scaffold.

```
#   import the file with the scaffold sizes                                 ####
sizes <-
  read_delim(
    here(
      "/gpfs/gibbs/project/caccone/mkc54/albo/genome/scaffold_sizes.txt"
    ),
    col_names      = FALSE,
    show_col_types = FALSE,
    col_types      = "cd"
  )
#
# set column names
colnames(
  sizes
) <- c(
  "Scaffold", "Size"
)
#   ____________________________________________________________________________
#   create new column with the chromosome number                            ####
sizes <- 
  sizes |>
  mutate(
    Chromosome = case_when( # we use mutate to create a new column called Chromosome
      startsWith(
        Scaffold, "1"
      ) ~ "1", # use startsWith to get Scaffold rows starting with 1 and output 1 on Chromosome column
      startsWith(
        Scaffold, "2"
      ) ~ "2",
      startsWith(
        Scaffold, "3"
      ) ~ "3"
    )
  ) |>
  arrange(
    Scaffold
  )                   # to sort the order of the scaffolds, fixing the problem we have with scaffold 1.86
# check it
head(sizes)
```

```
## # A tibble: 6 × 3
##   Scaffold     Size Chromosome
##   <chr>       <dbl> <chr>     
## 1 1.1        351198 1         
## 2 1.10     11939576 1         
## 3 1.100     3389100 1         
## 4 1.101      470438 1         
## 5 1.102     2525157 1         
## 6 1.103      150026 1
```

Create new scale. Get the scaffolds for each chromosome.

```
#   separate the scaffold sizes tibble per chromosome                       ####
# chr1
chr1_scaffolds <- 
  sizes |>
  filter(
    str_detect(
      Scaffold, "^1" # we use library stringr to get scaffolds starting with 1 (chromosome 1)
    )
  ) |> 
  as_tibble()
#
# chr2
chr2_scaffolds <-
  sizes |>
  filter(
    str_detect(
      Scaffold, "^2" # we use library stringr to get scaffolds starting with 2 (chromosome 2)
    )
  ) |> 
  as_tibble()
#
# # chr3
chr3_scaffolds <-
  sizes |>
  filter(
    str_detect(
      Scaffold, "^3" # we use library stringr to get scaffolds starting with 3 (chromosome 3)
    )
  ) |>
  as_tibble()
```

Create a scale for each chromosome.

```
#   create a new scale for each chromosome                                  ####
# chr1
chr1_scaffolds$overall_size_before_bp <-
  0                                                                        # we create a new column with zeros
for (i in 2:nrow(
  chr1_scaffolds
)
) {                                                                        # loop to start on second line
  chr1_scaffolds$overall_size_before_bp[i] <-                              # set position on the scale
    chr1_scaffolds$overall_size_before_bp[i - 1] + chr1_scaffolds$Size[i - # add the scaffold size and the location to get position on new scale
      1]
}
#
# chr2
chr2_scaffolds$overall_size_before_bp <- 0
for (i in 2:nrow(
  chr2_scaffolds
)
) {
  chr2_scaffolds$overall_size_before_bp[i] <-
    chr2_scaffolds$overall_size_before_bp[i - 1] + chr2_scaffolds$Size[i -
      1]
}
#
# chr3
chr3_scaffolds$overall_size_before_bp <- 0
for (i in 2:nrow(
  chr3_scaffolds
)
) {
  chr3_scaffolds$overall_size_before_bp[i] <-
    chr3_scaffolds$overall_size_before_bp[i - 1] + chr3_scaffolds$Size[i -
      1]
}
```

Merge the data frames scaffolds and SNPs.

```
#   merge the data sets using the tidyverse function left_join              ####
# chr1
chr1_scale <-
  chr1_snps |>          # create data frame for each chromosome, get chr1_snps
  left_join(            # use lef_join function to merge it with chr1_scaffolds
    chr1_scaffolds,
    by = "Scaffold"
  ) |>                  # set column to use for merging (Scaffold in this case)
  na.omit() |>          # remove NAs, we don't have SNPs in every scaffold
  mutate(
    midPos_fullseq = as.numeric(
      Position
    ) +                 # make new columns numeric
      as.numeric(
        overall_size_before_bp
      )
  )
#
# chr2
chr2_scale <-
  chr2_snps |>
  left_join(
    chr2_scaffolds,
    by = "Scaffold"
  ) |>
  na.omit() |>
  mutate(
    midPos_fullseq = as.numeric(
      Position
    ) +
      as.numeric(
        overall_size_before_bp
      )
  )
#
# chr3
chr3_scale <-
  chr3_snps |>
  left_join(
    chr3_scaffolds,
    by = "Scaffold"
  ) |>
  na.omit() |>
  mutate(
    midPos_fullseq = as.numeric(
      Position
    ) +
      as.numeric(
        overall_size_before_bp
      )
  )
```

Merge all chromosome scales.

```
#   merge the data sets, and select only the columns we need                ####
chroms <- rbind(
  chr1_scale, chr2_scale, chr3_scale
) |>
  dplyr::select(
    Chromosome, SNP, Cm, midPos_fullseq, Allele1, Allele2
  )
# check it
head(chroms)
```

```
## # A tibble: 0 × 6
## # ℹ 6 variables: Chromosome <chr>, SNP <chr>, Cm <int>, midPos_fullseq <dbl>,
## #   Allele1 <chr>, Allele2 <chr>
```

Save the new .bim file

```
#   ____________________________________________________________________________
#   save the new bim file with a new name, I added "B"                      ####
write.table(
  chroms,
  file      = here(
    "americas/output/file7C.bim"
  ),
  sep       = "\t",
  row.names = FALSE,
  col.names = FALSE,
  quote     = FALSE
)
```

Rename the .bim files

```
cd /gpfs/gibbs/pi/caccone/mkc54/albo
# change the name of the first .bim file, for example, append _backup.bim, and then replace the original file
mv americas/output/file7b.bim americas/output/file7b_backup.bim;
# than change the new bim we create to the original name (do it only once, otherwise it will mess up)
mv americas/output/file7C.bim americas/output/file7b.bim
```

Create a new bed file with Plink2 to see if it works. For example, to
see if the variants are in the right order. Plink2 will give us a
warning.

```
cd /gpfs/gibbs/pi/caccone/mkc54/albo

plink2 \
--bfile americas/output/file7b \
--make-bed \
--out americas/output/test01;
# then we remove the files 
rm americas/output/test01.*
```

PLINK v2.00a3.7LM AVX2 Intel (24 Oct 2022)
www.cog-genomics.org/plink/2.0/ (C) 2005-2022 Shaun Purcell, Christopher
Chang GNU General Public License v3 Logging to
americas/output/test01.log. Options in effect: –bfile
americas/output/file7b –make-bed –out americas/output/test01 Start time:
Fri Jun 21 10:34:22 2024 257321 MiB RAM detected; reserving 128660 MiB
for main workspace. Using up to 32 threads (change this with –threads).
545 samples (33 females, 31 males, 481 ambiguous; 545 founders) loaded
from americas/output/file7b.fam. 95921 variants loaded from
americas/output/file7b.bim. Note: No phenotype data present. Writing
americas/output/test01.fam … done. Writing americas/output/test01.bim …
done. Writing americas/output/test01.bed … done.

### 9.6.1 Estimate LD blocks using chromosomal scale

```
mkdir -p americas/output/files/ld/blocks_chr_b
```

```
module load PLINK/1.9b_6.21-x86_64
```

We can use Plink1.9 to estimate LD blocks for the populations with at
least 10 individuals. We will use the entire genome for this part
instead of the larger scaffolds only. We will set max distance of LD
blocks of 200kb. We found out that the average half distance of r^2 max
is small, from 1 to 5kb

```
for fam in $(awk '{print $1}' americas/output/files/ld/pops_4ld.txt | sort | uniq); 
do 
echo $fam | \
plink \
--allow-extra-chr \
--keep-allele-order \
--bfile americas/output/file7b \
--keep-fam /dev/stdin \
--maf 0.01 \
--blocks no-pheno-req \
--blocks-max-kb 200 \
--out americas/output/files/ld/blocks_chr_b/$fam \
--geno 0.1 \
--silent
done;
#
rm americas/output/files/ld/blocks_chr_b/*.nosex
```

Now we can get some data from our .log files

```
echo "Population,n,nVariants,geno,maf,passQC" > americas/output/files/ld/blocks_chr_b/table_ld_statsb.csv
for file in americas/output/files/ld/blocks_chr_b/*.log
do
  variants=$(grep -oE '([0-9]+) variants loaded from \.bim file' $file | grep -oE '[0-9]+')
  geno=$(grep -oE '([0-9]+) variants removed due to missing genotype data \(--geno\)' $file | grep -oE '[0-9]+')
  maf=$(grep -oE '([0-9]+) variants removed due to minor allele threshold\(s\)' $file | grep -oE '[0-9]+')
  pass=$(grep -oE '([0-9]+) variants and [0-9]+ people pass filters and QC\.' $file | grep -oE '[0-9]+' | head -1)
  n=$(grep -oE '([0-9]+) variants and [0-9]+ people pass filters and QC\.' $file | grep -oE '[0-9]+' | tail -1)
  filename=$(basename $file .log)
  echo "$filename,$n,$variants,$geno,$maf,$pass" >> americas/output/files/ld/blocks_chr_b/table_ld_statsb.csv
done;
head -n 5 americas/output/files/ld/blocks_chr_b/table_ld_statsb.csv
```

```
## Population,n,nVariants,geno,maf,passQC
## ARA,12,95921,6469,15665,73787
## BCO,12,95921,9430,17449,69042
## BEA,12,95921,4133,6434,85354
## BER,12,95921,4718,10081,81122
```

Population,n,nVariants,geno,maf,passQC ARA,12,95921,6469,15665,73787
BCO,12,95921,9430,17449,69042 BEA,12,95921,4133,6434,85354
BER,12,95921,4718,10081,81122

We can check it

```
# Load data from the table_ld_stats.csv file
ld_blocks <- read.csv(
  here(
    "americas/output/files/ld/blocks_chr_b/table_ld_statsb.csv"
    ),
  header = TRUE,
  sep = ","
)

# Create the flextable
ft <- flextable(ld_blocks)

# Apply zebra theme
ft <- theme_zebra(ft)

# Add a caption to the table
ft <- flextable::add_header_lines(ft, "Table 1: Summary of quality control for all populations (MAF 1%).")

ft
```

| Table 1: Summary of quality control for all populations (MAF 1%). | | | | | |
| --- | --- | --- | --- | --- | --- |
| Population | n | nVariants | geno | maf | passQC |
| ARA | 12 | 95,921 | 6,469 | 15,665 | 73,787 |
| BCO | 12 | 95,921 | 9,430 | 17,449 | 69,042 |
| BEA | 12 | 95,921 | 4,133 | 6,434 | 85,354 |
| BER | 12 | 95,921 | 4,718 | 10,081 | 81,122 |
| BIT | 12 | 95,921 | 6,608 | 16,946 | 72,367 |
| BPA | 11 | 95,921 | 7,463 | 17,311 | 71,147 |
| BPV | 12 | 95,921 | 10,645 | 16,579 | 68,697 |
| BRM | 16 | 95,921 | 7,223 | 14,785 | 73,913 |
| DAL | 12 | 95,921 | 3,870 | 8,400 | 83,651 |
| DEN | 12 | 95,921 | 6,165 | 8,786 | 80,970 |
| FIR | 12 | 95,921 | 4,874 | 15,125 | 75,922 |
| GRV | 12 | 95,921 | 5,819 | 17,982 | 72,120 |
| HAR | 12 | 95,921 | 4,914 | 10,898 | 80,109 |
| HOU | 12 | 95,921 | 4,461 | 6,404 | 85,056 |
| LIN | 12 | 95,921 | 5,471 | 12,761 | 77,689 |
| LOS | 12 | 95,921 | 6,524 | 14,649 | 74,748 |
| MAC | 12 | 95,921 | 7,002 | 7,976 | 80,943 |
| MAN | 12 | 95,921 | 6,965 | 8,950 | 80,006 |
| MAU | 12 | 95,921 | 9,795 | 13,592 | 72,534 |
| MON | 12 | 95,921 | 6,113 | 6,631 | 83,177 |
| NEO | 12 | 95,921 | 4,203 | 9,580 | 82,138 |
| NEW | 21 | 95,921 | 4,351 | 7,553 | 84,017 |
| NUE | 12 | 95,921 | 4,518 | 7,612 | 83,791 |
| POR | 12 | 95,921 | 7,640 | 19,711 | 68,570 |
| REC | 11 | 95,921 | 5,715 | 14,981 | 75,225 |
| RUS | 12 | 95,921 | 5,527 | 16,938 | 73,456 |
| SAI | 12 | 95,921 | 5,855 | 10,102 | 79,964 |
| SMI | 12 | 95,921 | 4,750 | 8,492 | 82,679 |
| STL | 12 | 95,921 | 4,474 | 11,646 | 79,801 |
| TRA | 12 | 95,921 | 4,717 | 7,213 | 83,991 |
| TUC | 14 | 95,921 | 7,871 | 14,831 | 73,219 |

```
# Save it to a Word document
officer::read_docx() |>
  body_add_flextable(ft) |>
  print(target = here::here("americas/output/summary_blocks_chr_b.docx"))
```

Now we can count how many blocks we found in each population

```
cd americas/output/files/ld/blocks_chr_b
wc -l *.blocks | \
  awk '{population = gensub(/\.blocks_chr/, "", "g", $2); print population "\t" $1}' | \
  sed 's#all_pops/output/files/ld/blocks_chr_b/##' | \
  sed 's/.blocks//' | \
  sed '$d' > populations_block_counts_b.csv;
head -n 79 populations_block_counts_b.csv
```

```
## ARA  82
## BCO  109
## BEA  70
## BER  113
## BIT  132
## BPA  97
## BPV  80
## BRM  535
## DAL  94
## DEN  91
## FIR  167
## GRV  162
## HAR  120
## HOU  64
## LIN  143
## LOS  123
## MAC  90
## MAN  67
## MAU  100
## MON  63
## NEO  92
## NEW  714
## NUE  65
## POR  95
## REC  99
## RUS  195
## SAI  78
## SMI  91
## STL  155
## TRA  77
## TUC  118
```

Now we can add the number of blocks to the table we made

```
# Load data from the table_ld_stats.csv file
ld_blocks <- read.csv(
  here(
    "americas/output/files/ld/blocks_chr_b/table_ld_statsb.csv"
    ),
  header = TRUE,
  sep = ","
)

# Load the population counts data from the CSV file
pop_counts <-
  read.delim(
    here(
      "americas/output/files/ld/blocks_chr_b/populations_block_counts_b.csv"
    ),
    header = F,
    sep = "\t"
  ) |>
  rename(
    Population       = 1,
    nBlocks          = 2
  )

# Merge the population counts with the table data
ld_blocks <- merge(ld_blocks, pop_counts, by = "Population")

# Create the flextable
ft <- flextable(ld_blocks)

# Apply zebra theme
ft <- theme_zebra(ft)

# Add a caption to the table
ft <- flextable::add_header_lines(ft, "Table 2: Number of linkage blocks detected with Plink for populations with at least 10 individuals.")

ft
```

| Table 2: Number of linkage blocks detected with Plink for populations with at least 10 individuals. | | | | | | |
| --- | --- | --- | --- | --- | --- | --- |
| Population | n | nVariants | geno | maf | passQC | nBlocks |
| ARA | 12 | 95,921 | 6,469 | 15,665 | 73,787 | 82 |
| BCO | 12 | 95,921 | 9,430 | 17,449 | 69,042 | 109 |
| BEA | 12 | 95,921 | 4,133 | 6,434 | 85,354 | 70 |
| BER | 12 | 95,921 | 4,718 | 10,081 | 81,122 | 113 |
| BIT | 12 | 95,921 | 6,608 | 16,946 | 72,367 | 132 |
| BPA | 11 | 95,921 | 7,463 | 17,311 | 71,147 | 97 |
| BPV | 12 | 95,921 | 10,645 | 16,579 | 68,697 | 80 |
| BRM | 16 | 95,921 | 7,223 | 14,785 | 73,913 | 535 |
| DAL | 12 | 95,921 | 3,870 | 8,400 | 83,651 | 94 |
| DEN | 12 | 95,921 | 6,165 | 8,786 | 80,970 | 91 |
| FIR | 12 | 95,921 | 4,874 | 15,125 | 75,922 | 167 |
| GRV | 12 | 95,921 | 5,819 | 17,982 | 72,120 | 162 |
| HAR | 12 | 95,921 | 4,914 | 10,898 | 80,109 | 120 |
| HOU | 12 | 95,921 | 4,461 | 6,404 | 85,056 | 64 |
| LIN | 12 | 95,921 | 5,471 | 12,761 | 77,689 | 143 |
| LOS | 12 | 95,921 | 6,524 | 14,649 | 74,748 | 123 |
| MAC | 12 | 95,921 | 7,002 | 7,976 | 80,943 | 90 |
| MAN | 12 | 95,921 | 6,965 | 8,950 | 80,006 | 67 |
| MAU | 12 | 95,921 | 9,795 | 13,592 | 72,534 | 100 |
| MON | 12 | 95,921 | 6,113 | 6,631 | 83,177 | 63 |
| NEO | 12 | 95,921 | 4,203 | 9,580 | 82,138 | 92 |
| NEW | 21 | 95,921 | 4,351 | 7,553 | 84,017 | 714 |
| NUE | 12 | 95,921 | 4,518 | 7,612 | 83,791 | 65 |
| POR | 12 | 95,921 | 7,640 | 19,711 | 68,570 | 95 |
| REC | 11 | 95,921 | 5,715 | 14,981 | 75,225 | 99 |
| RUS | 12 | 95,921 | 5,527 | 16,938 | 73,456 | 195 |
| SAI | 12 | 95,921 | 5,855 | 10,102 | 79,964 | 78 |
| SMI | 12 | 95,921 | 4,750 | 8,492 | 82,679 | 91 |
| STL | 12 | 95,921 | 4,474 | 11,646 | 79,801 | 155 |
| TRA | 12 | 95,921 | 4,717 | 7,213 | 83,991 | 77 |
| TUC | 14 | 95,921 | 7,871 | 14,831 | 73,219 | 118 |

```
# Save it to a Word document
officer::read_docx() |>
  body_add_flextable(ft) |>
  print(target = here::here("americas/output/summary_blocks_chr_b.docx"))
```

Get the size of each block from the .block.det files

```
get_kb_column <- function(dir_path) {
  # obtain the list of files with extension .blocks.det
  file_names <- list.files(path = dir_path, pattern = "\\.blocks\\.det$", full.names = TRUE)
  
  # create an empty list to hold the data frames
  block_list <- list()
  
  # loop through the files and read the data into the list
  for (file in file_names) {
    df <- read.table(file, header = TRUE, check.names = FALSE, stringsAsFactors = FALSE)
    # select only the KB column and add it to the block_list with the file name
    block_list[[file]] <- df %>% dplyr::select(KB) %>% add_column(file = file, .before = 1)
  }
  
  # combine the data frames in the block_list into a single data frame
  blocks <- bind_rows(block_list)
  
  # clean up the file name column
  blocks$file <- str_remove(blocks$file, "^.*\\/ld\\/blocks\\/")
  
  return(blocks)
}

# example usage: replace dir_path with your directory path
dir_path <- here("americas/output/files/ld/blocks_chr_b")

blocks<- 
  get_kb_column(dir_path) |>
  mutate(file = str_remove(file, "americas/output/files/ld/blocks_chr_b/")) |>
  mutate(file = str_remove(file, ".blocks.det")) |>

  as_tibble() |>
  rename(
    Population = 1
  )
```

## 9.7 Plot SNP density

After quality control with approximately 100k SNPs

```
# load the function that we saved earlier
source(
  here("scripts", "RMarkdowns",
    "analyses", "import_bim.R"
  ),
  local = knitr::knit_global()
)
# import the file
snp_den_qc <- import_bim(
  here(
    "americas/output/file7b.bim"
  )
)
```

Make plot of the SNP density

```
#   ____________________________________________________________________________
#   plot SNP density after QC                                               ####
snp_den_qc |>
  rename(
    Chromosome = 1
  ) |>
  mutate(
    Position           = as.numeric(
      Position
    )
  ) |>
  ggplot(
    aes(
      x                = Position
    ),
    label              = sprintf(
      "%0.2f",
      round(
        a,
        digits         = 0
      )
    )
  ) +
  geom_histogram(
    aes(
      y                = after_stat(
        count
      )
    ),
    binwidth           = 1e6
  ) +
  facet_wrap(
    vars(
      Chromosome
    ),
    scales             = "free_x"
  ) +
  labs(
    title              = "SNP Density after QC",
    x                  = expression(
      "Position in the genome (Mb)"
    ),
    y                  = expression(
      "Number of SNPs"
    )
  ) +
  scale_x_continuous(
    labels             = function(x) {
      format(
        x / 1e6,
        big.mark       = ",", 
        scientific     = FALSE
      )
    }
  ) +
  geom_density(
  aes(
    y = 1e6 * after_stat(count)
  ),
  color = "red",
  linewidth = .75,
  alpha = .4,
  fill = "pink"
  ) +
    theme(
    panel.grid.major   = element_line(
      linetype         = "dashed",
      linewidth        = 0.2
    ),
    panel.grid.minor   = element_line(
      linetype         = "dashed",
      linewidth        = 0.2
    ),
    panel.spacing      = unit(0.5, "lines"),
    strip.text         = element_text(
      face             = "bold", hjust = .5
    ),
    strip.background.x = element_rect(
      color            = "gray"
    )
  )
```

```
## Warning in fortify(data, ...): Arguments in `...` must be used.
## ✖ Problematic argument:
## • label = sprintf("%0.2f", round(a, digits = 0))
## ℹ Did you misspell an argument name?
```

```
#   save the density plot                                            ####
ggsave(
  here("scripts/RMarkdowns/output/americas/americas_all/figures/snp_density_after_qc_all_b.pdf"
  ),
  width  = 10,
  height = 6,
  units  = "in"
)
```

SNPs per chromosome

```
# we can use dplyr "count" to get the number of SNPs for each chromosome
# lets get the data we need
snps_per_chrm <- 
  snp_den_qc |>
  count(
    Scaffold) |>
  rename(
    Chromosome = 1,
    "SNPs (N) " = 2
  )

# Create the flextable
ft <- flextable::flextable(snps_per_chrm)

# Apply zebra theme
ft <- flextable::theme_zebra(ft)

# Add a caption to the table
ft <- flextable::add_header_lines(ft, "SNPs per chromosome after quality control")
ft
```

| SNPs per chromosome after quality control | |
| --- | --- |
| Chromosome | SNPs (N) |
| 1 | 21,283 |
| 2 | 40,171 |
| 3 | 34,467 |

We can get the mean number of SNPs per chromosome for the entire
genome

```
# we first use dplyr cut_width to get the number of SNPs per 1Mb window
albo_den <- 
  snp_den_qc |>
  dplyr::select(
    Scaffold, Position
  ) |>
  group_by(
    Scaffold,
    windows               = cut_width(
      Position,
      width               = 1e6,
      boundary            = 0
    )
  ) |>
  summarise(
    n                     = n(),
    .groups               = "keep"
  ) |>
  group_by(
    Scaffold
  ) |>
  summarise(
    mean                  = mean(n),
    n                     = n(),
    .groups               = "keep"
  ) |>
  rename(
    Chromosome            = 1,
    "SNPs per 1Mb window" = 2,
    "Number of windows"   = 3
  )
#
# check the results
snp_table <-
  flextable(
    albo_den
  )
snp_table <- colformat_double(
  x        = snp_table,
  big.mark = ",",
  digits   = 2,
  na_str   = "N/A"
)
snp_table
```

| Chromosome | SNPs per 1Mb window | Number of windows |
| --- | --- | --- |
| 1 | 57.83 | 368 |
| 2 | 69.14 | 581 |
| 3 | 70.48 | 489 |

Merge objects

```
# we can merge the two data sets we created above into one table
after_qc <-
  snps_per_chrm |>
  left_join(
    albo_den,
    by = "Chromosome"
  )
snp_table2 <- flextable(
  after_qc)
snp_table2 <- colformat_double(
  x        = snp_table2,
  big.mark = ",",
  digits   = 2, 
  na_str   = "N/A"
  )
snp_table2
```

| Chromosome | SNPs (N) | SNPs per 1Mb window | Number of windows |
| --- | --- | --- | --- |
| 1 | 21,283 | 57.83 | 368 |
| 2 | 40,171 | 69.14 | 581 |
| 3 | 34,467 | 70.48 | 489 |

```
# Save it to a Word document
officer::read_docx() |>
  body_add_flextable(snp_table2) |>
  print(target = here::here("americas/output/snps_per_chromosome_table_all_pops_MAF01.docx"))
```

## 9.8 LD pruning using the chromosomal scale for MAF 0.01

```
# we set a window of variants of 5 and move the window 1 variant per time, removing 1 of the variants with lowest MAF from a pair above the threshold of r^2 > 0.1
plink2 \
--allow-extra-chr \
--bfile americas/output/file7b \
--indep-pairwise 5 1 0.1 \
--out americas/output/indepSNP_chrb \
--silent;
grep 'pairwise\|variants\|samples' americas/output/indepSNP_chrb.log
```

–indep-pairwise 5 1 0.1 545 samples (33 females, 31 males, 481
ambiguous; 545 founders) loaded from 95921 variants loaded from
americas/output/file7b.bim. –indep-pairwise (3 compute threads):
42283/95921 variants removed.

Do the scaffold again

First need to import same SNP list Import the .bim file with the
SNPs

```
#   import the bim file with the SNP data                                   ####
snps <-
  read_delim(                    # to learn about the options use here, run ?read_delim on the console.
    here(
      "americas/output/file7b_backup.bim" #output/populations/file4.bim
    ),                           # use library here to load it
    col_names      = FALSE,      # we don't have header in the input file
    show_col_types = FALSE,      # suppress message from read_delim
    col_types      = "ccidcc"    # set the class of each column
  )
#
# set column names
colnames(
  snps
) <-                             # to add a header in our tibble
  c(
    "Scaffold", "SNP", "Cm", "Position", "Allele1", "Allele2"
  )
#
# check the tibble
head(snps)
```

```
## # A tibble: 6 × 6
##   Scaffold SNP             Cm Position Allele1 Allele2
##   <chr>    <chr>        <int>    <dbl> <chr>   <chr>  
## 1 1.1      AX-583033342     0   315059 C       G      
## 2 1.1      AX-583035163     0   315386 A       G      
## 3 1.1      AX-583033356     0   315674 C       T      
## 4 1.1      AX-583033370     0   330057 G       A      
## 5 1.1      AX-583035194     0   330265 A       G      
## 6 1.1      AX-583033387     0   331288 C       T
```

```
write.table(
  snps,
  file      = here(
    "americas/output/snps_all_scaffolds_4ld_b.txt"
  ),
  sep       = "\t",
  row.names = FALSE,
  col.names = FALSE,
  quote     = FALSE
)

nrow(snps)
#97472
```

```
plink2 \
--allow-extra-chr \
--bfile americas/output/file5b \
--king-cutoff americas/output/file6b 0.354 \
--make-bed \
--out americas/output/file11b \
--silent;
grep 'samples\|variants\|remaining' americas/output/file11b.log
```

555 samples (33 females, 32 males, 490 ambiguous; 555 founders)
loaded from 95921 variants loaded from americas/output/file5b.bim.
americas/output/file11b.king.cutoff.out.id , and 545 remaining sample
IDs

```
# we set a window of variants of 5 and move the window 1 variant each time, removing 1 of the variants with lowest MAF from a pair above the threshold of r^2 > 0.1
plink2 \
--allow-extra-chr \
--bfile americas/output/file11b \
--indep-pairwise 5 1 0.1 \
--out americas/output/indepSNP_scaffoldsb \
--silent;
grep 'pairwise\|variants\|samples' americas/output/indepSNP_scaffoldsb.log
```

–indep-pairwise 5 1 0.1 545 samples (33 females, 31 males, 481
ambiguous; 545 founders) loaded from 95921 variants loaded from
americas/output/file11b.bim. –indep-pairwise (27 compute threads):
42257/95921 variants removed.

Now we can compare the two sets of SNPs using scaffolds or
chromosomal scale

Create Venn diagram of SNPs removed due to LD

```
# Read in the two files as vectors
prunout_chr <-
  read_delim(
    here(
      "americas/output/indepSNP_chrb.prune.out"
    ),
    delim = " ",
    col_names = FALSE,
    show_col_types = FALSE
  )

prunout_scaffolds <-
  read_delim(
    here(
      "americas/output/indepSNP_scaffoldsb.prune.out"
    ),
    delim = " ",
    col_names = FALSE,
    show_col_types = FALSE
  )

# Convert the list to vector
prunout_scaffolds <- unlist(prunout_scaffolds)
prunout_chr <- unlist(prunout_chr)


# Calculate shared values
prunout <- intersect(prunout_chr, prunout_scaffolds)

# Create Venn diagram
venn_data1 <- list(
  "Chromosomal" = prunout_chr,
  "Scaffolds" = prunout_scaffolds
)

# create plot
venn_plot1 <- ggvenn(venn_data1, fill_color = c("steelblue", "darkorange"), show_percentage = TRUE)

# Add a title
venn_plot1 <- venn_plot1 +
  ggtitle("Comparison of genomic scales for linked SNPs MAF 0.01") +
  theme(plot.title = element_text(hjust = .5))

# Display the Venn diagram
print(venn_plot1)
```

```
# Save Venn diagram to PDF
output_path <- here("scripts/RMarkdowns/output/americas/americas_all/figures/SNPs_linked_comparison_all_b.pdf")
ggsave(output_path, venn_plot1, height = 5, width = 5, dpi = 300)
```

Now compare MAF 0.01 to MAF 0.1 pruning Create Venn diagram of SNPs
removed due to LD

```
# Read in the two files as vectors
prunout_chr_0.01 <-
  read_delim(
    here(
      "americas/output/indepSNP_chrb.prune.out"
    ),
    delim = " ",
    col_names = FALSE,
    show_col_types = FALSE
  )

prunout_chr_0.1 <-
  read_delim(
    here(
      "americas/output/indepSNP_chr.prune.out"
    ),
    delim = " ",
    col_names = FALSE,
    show_col_types = FALSE
  )

# Convert the list to vector
prunout_chr_0.01 <- unlist(prunout_chr_0.01)
prunout_chr_0.1 <- unlist(prunout_chr_0.1)


# Calculate shared values
prunout <- intersect(prunout_chr_0.01, prunout_chr_0.1)

# Create Venn diagram
venn_data1 <- list(
  "MAF 1%" = prunout_chr_0.01,
  "MAF 10%" = prunout_chr_0.1
)

# create plot
venn_plot1 <- ggvenn(venn_data1, fill_color = c("steelblue", "darkorange"), show_percentage = TRUE)

# Add a title
venn_plot1 <- venn_plot1 +
  ggtitle("Comparison of SNPs sets pruned for MAF 1% and MAF 10%") +
  theme(plot.title = element_text(hjust = .5))

# Display the Venn diagram
print(venn_plot1)
```

```
# Save Venn diagram to PDF
output_path <- here("scripts/RMarkdowns/output/americas/americas_all/figures/SNPs_comparison_all_MAF01.pdf")
ggsave(output_path, venn_plot1, height = 5, width = 5, dpi = 300)
```

## 9.9 Pruning r2 for LD-pruned datasets with MAF 1%

If needed, can create it again using the code below

```
plink2 \
--allow-extra-chr \
--bfile americas/output/file7b \
--indep-pairwise 5 1 0.1 \
--out americas/output/indepSNP_chrb \
--silent;
grep 'pairwise\|variants\|samples' americas/output/indepSNP_chrb.log
```

–indep-pairwise 5 1 0.1 545 samples (33 females, 31 males, 481
ambiguous; 545 founders) loaded from 95921 variants loaded from
americas/output/file7b.bim. –indep-pairwise (3 compute threads):
42283/95921 variants removed.

*The indepSNP\_chrb.prune.in file produced here = those SNPs <
our LD 0.1 threshold, that we want to use*

Now we need to make pruned dataset for 0.01. Can use file7b

```
plink2 \
--allow-extra-chr \
--bfile americas/output/file7b \
--indep-pairwise 5 1 0.01 \
--out americas/output/indepSNP_chrb_r01 \
--silent;
grep 'pairwise\|variants\|samples' americas/output/indepSNP_chrb_r01.log
```

–indep-pairwise 5 1 0.01 545 samples (33 females, 31 males, 481
ambiguous; 545 founders) loaded from 95921 variants loaded from
americas/output/file7b.bim. –indep-pairwise (3 compute threads):
76957/95921 variants removed.

We can use the SNP set the LD pruning we just did and extract them
from file7

```
plink2 \
--keep-allele-order \
--bfile americas/output/file7b \
--make-bed \
--export vcf \
--out americas/output/snps_sets/r2_0.1_b \
--extract americas/output/indepSNP_chrb.prune.in \
--silent
grep "variants\|samples" americas/output/snps_sets/r2_0.1_b.log
```

545 samples (33 females, 31 males, 481 ambiguous; 545 founders)
loaded from 95921 variants loaded from americas/output/file7b.bim.
–extract: 53638 variants remaining. 53638 variants remaining after main
filters.

Repeat for 0.01 dataset

```
plink2 \
--keep-allele-order \
--bfile americas/output/file7b \
--make-bed \
--export vcf \
--out americas/output/snps_sets/r2_0.01_b \
--extract americas/output/indepSNP_chrb_r01.prune.in \
--silent
grep "variants\|samples" americas/output/snps_sets/r2_0.01_b.log
```

545 samples (33 females, 31 males, 481 ambiguous; 545 founders)
loaded from 95921 variants loaded from americas/output/file7b.bim.
–extract: 18964 variants remaining. 18964 variants remaining after main
filters.

These snp set can now be used for subsequent pop gen analyses.

Create Venn diagram of SNPs removed due to LD

```
# Read in the two files as vectors
prunout_chr_0.01 <-
  read_delim(
    here(
      "americas/output/indepSNP_chrb_r01.prune.in"
    ),
    delim = " ",
    col_names = FALSE,
    show_col_types = FALSE
  )

prunout_chr_0.1 <-
  read_delim(
    here(
      "americas/output/indepSNP_chr_r01.prune.in"
    ),
    delim = " ",
    col_names = FALSE,
    show_col_types = FALSE
  )

# Convert the list to vector
prunout_chr_0.01 <- unlist(prunout_chr_0.01)
prunout_chr_0.1 <- unlist(prunout_chr_0.1)


# Calculate shared values
prunout <- intersect(prunout_chr_0.01, prunout_chr_0.1)

# Create Venn diagram
venn_data1 <- list(
  "MAF 1%" = prunout_chr_0.01,
  "MAF 10%" = prunout_chr_0.1
)

# create plot
venn_plot1 <- ggvenn(venn_data1, fill_color = c("steelblue", "darkorange"), show_percentage = TRUE)

# Add a title
venn_plot1 <- venn_plot1 +
  ggtitle("Comparison of LD 0.01 SNPs sets for MAF 1% and MAF 10%") +
  theme(plot.title = element_text(hjust = .5))

# Display the Venn diagram
print(venn_plot1)
```

```
# Save Venn diagram to PDF
output_path <- here("scripts/RMarkdowns/output/americas/americas_all/figures/SNPs_comparison_all_pops_MAFs_r2_0.01.pdf")
ggsave(output_path, venn_plot1, height = 6, width = 5, dpi = 300)
```
