# Supplementary material for: Population Structure of the Invasive Asian Tiger Mosquito, Aedes albopictus, in the Americas
Source: Insects. 2026 Jul 23;17(8):755. doi: 10.3390/insects17080755 (PMC13512968; doi:10.3390/insects17080755)
Supplement: Supplementary file 1 [file insects-17-00755-s001.zip › RMarkdowns/S7 - QC for Global dataset.html]

QC for Global dataset


# QC for Global dataset

#### Margaret Corley

#### 2026-05-05

- Note: This file creates two different sets of SNPs - 1 with Minor
  Allele Frequency of 10% (MAF 0.1) and another 1% (MAF 0.01) (code
  starting in section 9). As stated in the text of the Methods section,
  the SNP set with 1% MAF pruning is used for all analyses, unless
  otherwise specified.

# 1. Load R libraries and software

```
library(tidyverse)
```

```
## Warning in system("timedatectl", intern = TRUE): running command 'timedatectl'
## had status 1
```

```
## Warning: Your system is mis-configured: '/etc/localtime' is not a symlink
```

```
library(here)
library(dplyr)
library(ggplot2)
library(colorout)
library(extrafont)
library(reticulate)
#library(scales)
library(stringr)
library(grid)
library(flextable)
library(devtools)
library(readr)
library(purrr)
library(ggtext)
library(ggvenn)
library(data.table)
library(RColorBrewer)
library(ggrepel)
```

### 1.2 Get software for quality control

Check your Plink version.

```
module spider plink
#Versions on the cluster:
        #LINK/1.9b_6.21-x86_64
        #LINK/2_avx2_20221024
module load PLINK/2_avx2_20221024

plink2 --version
#PLINK v2.00a3.7LM AVX2 Intel (24 Oct 2022)
```

Check your BCFtools version.

```
module spider BCFtools

module load BCFtools/1.16-GCCcore-10.2.0
```

### 1.3 Check if the data is in the correct location

```
cd /gpfs/gibbs/pi/caccone/mkc54/albo

ls data/americas/* # we use * to truncate the name of the file showing all files
```

Check how many sample names we have in our vcf

```
# make sure you have all the .CEL samples in your family file
bcftools query -l /gpfs/gibbs/pi/caccone/mkc54/albo/data/americas/albo_manuscript1_19Aug2024.vcf | wc -l
#959
```

# 2. The data

## 2.1 Use Plink2 to convert to bed format

```
cd /gpfs/gibbs/pi/caccone/mkc54/albo

plink2 \
--allow-extra-chr \
--vcf data/americas/albo_manuscript1_19Aug2024.vcf \
--const-fid \
--make-bed \
--exclude europe/output/files/albopictus_SNPs_fail_segregation.txt \
--fa /gpfs/gibbs/project/caccone/mkc54/albo/genome/albo.fasta.gz \
--ref-from-fa 'force' `# sets REF alleles when it can be done unambiguously, we use force to change the alleles` \
--out manuscript1/output/file1 \
--silent;
# --keep-allele-order \ if you use Plink 1.9
grep "variants" manuscript1/output/file1.log; # to get the number of variants from the log file.
```

–vcf: 110392 variants scanned. 110392 variants loaded from
manuscript1/output/file1-temporary.pvar.zst. –exclude: 108992 variants
remaining. 108992 variants remaining after main filters. –ref-from-fa
force: 0 variants changed, 108992 validated.

Check the fam file

```
head manuscript1/output/file1.fam
```

```
## OKI  1001    0   0   2   -9
## OKI  1002    0   0   2   -9
## OKI  1003    0   0   2   -9
## OKI  1004    0   0   2   -9
## OKI  1005    0   0   2   -9
## OKI  1006    0   0   1   -9
## OKI  1007    0   0   1   -9
## OKI  1008    0   0   1   -9
## OKI  1009    0   0   1   -9
## OKI  1010    0   0   1   -9
```

```
head -n 5 scripts/RMarkdowns/sample_ped_info_ALLPOPS_1Apr2024.txt
```

```
## Sample Filename  Family_ID   Individual_ID   Father_ID   Mother_ID   Sex Affection Status
## 1_LabCross_KF2.CEL   LAB 1   0   0   2   -9
## 2_LabCross_MF81.CEL  LAB 2   0   0   2   -9
## 3_LabCross_MF71.CEL  LAB 3   0   0   2   -9
## 4_LabCross_KF42.CEL  LAB 4   0   0   2   -9
```

Import the fam file we use with Axiom Suite

```
# the order of the rows in this file does not matter
samples <-
  read.delim(
    file   = here("scripts", "RMarkdowns", "sample_ped_info_ALLPOPS_1Apr2024.txt"
    ),
    header = TRUE
  )
head(samples)
```

```
##       Sample.Filename Family_ID Individual_ID Father_ID Mother_ID Sex
## 1  1_LabCross_KF2.CEL       LAB             1         0         0   2
## 2 2_LabCross_MF81.CEL       LAB             2         0         0   2
## 3 3_LabCross_MF71.CEL       LAB             3         0         0   2
## 4 4_LabCross_KF42.CEL       LAB             4         0         0   2
## 5 5_LabCross_KF57.CEL       LAB             5         0         0   2
## 6 6_LabCross_MF32.CEL       LAB             6         0         0   2
##   Affection.Status
## 1               -9
## 2               -9
## 3               -9
## 4               -9
## 5               -9
## 6               -9
```

Import .fam file we created when we created the bed file using
Plink2

```
#The fam file is the same for both data sets with the default or new priors
fam1 <-
  read.delim(
    file   = here("manuscript1/output/file1.fam"),
    header = FALSE,
    
  )
head(fam1)
```

```
##    V1   V2 V3 V4 V5 V6
## 1 OKI 1001  0  0  2 -9
## 2 OKI 1002  0  0  2 -9
## 3 OKI 1003  0  0  2 -9
## 4 OKI 1004  0  0  2 -9
## 5 OKI 1005  0  0  2 -9
## 6 OKI 1006  0  0  1 -9
```

Merge the tibbles

```
# Extract the number part from the columns
fam1_temp <- fam1 |>
  mutate(num_id = as.numeric(str_extract(V2, "^\\d+")))

samples_temp <- samples |>
  mutate(num_id = as.numeric(str_extract(Sample.Filename, "^\\d+")))

# Perform the left join using the num_id columns and keep the order of fam1
df <- fam1_temp |>
  dplyr::left_join(samples_temp, by = "num_id") |>
  dplyr::select(-num_id) |>
  dplyr::select(8:13)

# check the data frame
head(df)
```

```
##   Family_ID Individual_ID Father_ID Mother_ID Sex Affection.Status
## 1       OKI          1001         0         0   2               -9
## 2       OKI          1002         0         0   2               -9
## 3       OKI          1003         0         0   2               -9
## 4       OKI          1004         0         0   2               -9
## 5       OKI          1005         0         0   2               -9
## 6       OKI          1006         0         0   1               -9
```

We can check how many samples we have in our file

```
nrow(df)
```

```
## [1] 959
```

```
#959
```

Before saving the new fam file, you can change the original file to a
different name, to compare the order later. If you want to repeat the
steps above after you saving the new file1.fam, you will need to import
the vcf again.

```
# Save and override the .fam file for dp
write.table(
  df,
  file      = here("manuscript1/output/file1.fam"),
  sep       = "\t",
  row.names = FALSE,
  col.names = FALSE,
  quote     = FALSE
)
```

Check the new .fam file to see if has the order and the sample
attributes we want.

```
# you can open the file on a text editor and double check the sample order and information.
head -n 5 manuscript1/output/file1.fam
```

```
## OKI  1001    0   0   2   -9
## OKI  1002    0   0   2   -9
## OKI  1003    0   0   2   -9
## OKI  1004    0   0   2   -9
## OKI  1005    0   0   2   -9
```

## 2.3 Check for duplicates

We had 5 samples genotyped 3 times each. I removed all but one of the
duplicates for each, but We can double check for them in our dataset

```
# Check triplicates
grep "a\|b\|c" manuscript1/output/file1.fam
```

There are no duplicates remaining

Create a new bed removing the SNPs that failed the segretation test.
We can also make sure the reference alleles match the reference
genome

```
# I created a fam file with the information about each sample, but first we import the data and create a bed file setting the family id constant
plink2 \
--allow-extra-chr \
--bfile manuscript1/output/file1 \
--make-bed \
--fa /gpfs/gibbs/project/caccone/mkc54/albo/genome/albo.fasta.gz \
--ref-from-fa 'force' `# sets REF alleles when it can be done unambiguously, we use force to change the alleles` \
--exclude europe/output/files/albopictus_SNPs_fail_segregation.txt \
--out manuscript1/output/file1b \
--silent;
# --keep-allele-order \ if you use Plink 1.9
grep "samples\|variants" manuscript1/output/file1b.log # to get the number of variants from the log file.
```

959 samples (62 females, 101 males, 796 ambiguous; 959 founders)
loaded from 108992 variants loaded from manuscript1/output/file1.bim.
–exclude: 108992 variants remaining. 108992 variants remaining after
main filters. –ref-from-fa force: 0 variants changed, 108992
validated.

Check the headings of the the files we will work on.

```
head -n 5 manuscript1/output/file1b.fam
```

```
## OKI  1001    0   0   2   -9
## OKI  1002    0   0   2   -9
## OKI  1003    0   0   2   -9
## OKI  1004    0   0   2   -9
## OKI  1005    0   0   2   -9
```

Check how many samples are in the .fam file

```
wc -l manuscript1/output/file1b.fam
#959
```

```
## 959 manuscript1/output/file1b.fam
```

## 2.4 Checking the number of samples and localities

This part is very important. Make sure there are no NAs or something
that is not what you would expect. For example, the number of mosquitoes
per population.

```
awk '{print $1}' manuscript1/output/file1b.fam | sort | uniq -c | awk '{print $2, $1}'
```

```
## ALD 10
## ARA 12
## BAF 7
## BCO 12
## BEA 12
## BEH 9
## BEN 14
## BER 12
## BIT 12
## BPA 12
## BPV 12
## BRE 13
## BRM 16
## CAM 12
## CES 15
## CHA 12
## CHI 7
## COL 11
## CUA 8
## DAL 12
## DEN 12
## DES 17
## FAY 12
## FIR 12
## FLO 8
## FRS 12
## GEL 2
## GES 12
## GRA 12
## GRV 12
## HAI 12
## HAL 10
## HAN 4
## HAR 12
## HAT 11
## HOC 7
## HOU 12
## HUN 12
## INJ 11
## INW 4
## IPO 8
## JAF 2
## KAC 6
## KAG 14
## KAN 12
## KAT 10
## KLP 4
## KUN 4
## LAM 9
## LIN 12
## LOS 14
## MAC 12
## MAN 12
## MAT 12
## MAU 12
## MED 7
## MON 12
## NEB 11
## NEG 4
## NEH 7
## NEO 12
## NEW 22
## NOV 4
## NUE 12
## OKI 12
## PAL 11
## PEO 10
## PLM 7
## POR 12
## QNC 12
## RAR 12
## REC 12
## RUS 12
## SAI 12
## SER 4
## SMI 12
## SOC 12
## SON 3
## SPB 8
## SPR 8
## SPS 9
## SSK 12
## STL 12
## SUF 6
## SUU 6
## TAI 7
## TIK 12
## TRA 12
## TUC 14
## TUH 12
## UTS 12
## WAV 7
## YUN 9
```

# 3. Quality control steps

## 3.1 Missingness

```
plink2 \
--allow-extra-chr \
--bfile manuscript1/output/file1b \
--geno 0.1                `# we set genotype missiningness to 10% with this option` \
--make-bed \
--out manuscript1/output/file2 \
--silent \
--missing;                 # --missing produces sample-based and variant-based missing data reports. If run with --within/--family, the variant-based report is stratified by cluster.
grep "variants" manuscript1/output/file2.log
```

108992 variants loaded from manuscript1/output/file1b.bim. –geno:
9303 variants removed due to missing genotype data. 99689 variants
remaining after main filters.

Make plot

```
#   ____________________________________________________________________________
#   import individual missingness                                           ####
indmiss <-                                              # name of the data frame we are creating
  read.delim(                                           # use function read table
    file   = here(
      "manuscript1/output/file2.smiss"
    ),                                                  # we use library here to import file2.imiss from data/QC
    header = TRUE                                       # we do have headers in our file
  )
#   ____________________________________________________________________________
#   import SNP missingness                                                  ####
snpmiss <-
  read.delim(
    file   = here(
      "manuscript1/output/file2.vmiss"
    ),
    header = TRUE
  )
#
```

Plot individual missingness

```
# load plotting theme
source(
  here("scripts", "RMarkdowns", "my_theme2.R"
  )
)

ggplot( # Start a ggplot object with the data and aesthetic mappings
  indmiss,
  aes(
    x = F_MISS
  )
) +
  geom_histogram( # Add a histogram layer
    color            = "black",
    fill             = "lightgray",
    bins             = 6
  ) +
  geom_text(
    # Add text labels for bin counts
    stat             = "bin",
    aes(
    label = after_stat(count)
  ),
    vjust            = -0.5,
    color            = "black",
    size             = 3,
    bins             = 6
  ) +
  geom_vline(
    # Add a vertical line at the mean of F_MISS
    aes(
    xintercept = mean(F_MISS)),
    color            = "red",
    linetype         = "dotted",
    linewidth        = .5
  ) +
  geom_text(
    # Add a text label for the mean of F_MISS
    aes(
      x = mean(F_MISS),
      y = 75,
      label = paste0(
        "Mean \n",
        scales::percent(mean(F_MISS),
          accuracy = 0.01
        )
      )
    ),
    size = 3,
    color = "red",
    hjust = -.1
  ) +
  labs( # Add axis labels
    x                = "Individual Missingness (%)",
    y                = "Frequency (n)"
  ) +
  my_theme() +
  scale_x_continuous( # Scale the x-axis to display percentages
    labels           = scales::percent,
    n.breaks         = 6
  )
```

```
## Warning in geom_text(aes(x = mean(F_MISS), y = 75, label = paste0("Mean \n", : All aesthetics have length 1, but the data has 959 rows.
## ℹ Please consider using `annotate()` or provide this layer with data containing
##   a single row.
```

```
#
# save the plot
ggsave(
  here("scripts", "RMarkdowns", "output", "americas", "manuscript1", "figures", "individual_missingness10_man1.pdf"
  ),
  width              = 7,
  height             = 5,
  units              = "in"
)
```

```
# Define a function to customize the theme
my_theme <- function() {
  theme_minimal(base_size = 12, base_family = "") +
    theme(
      panel.grid.major = element_line(
        linetype = "dashed",
        linewidth = 0.2,
        color = "gray"
      ),
      panel.grid.minor = element_line(
        linetype = "dashed",
        linewidth = 0.2,
        color = "gray"
      ),
      # Customize the x-axis label
      axis.title.x = element_text(
        angle          = 0,
        hjust          = 1,
        face           = "bold"
      ),
      # Customize the y-axis label
      axis.title.y = element_text(
        angle          = 90,
        hjust          = 1,
        face           = "bold"
      )
    )
}
# we can save the function to source it later
dump(                                                    # check ?dump for more information
  "my_theme",                                            # the object we want to save
  here("scripts", "RMarkdowns", 
   "my_theme2.R")                # use here to save it our function as .R 
)
```

Plot variant missingness

```
# This code creates a histogram from the snpmiss data set using the F_MISS column.
# ggplot builds a histogram of individual missingness data
ggplot(
  snpmiss,
  aes(
    x = F_MISS
  )
) +
  geom_histogram(
    color = "black",
    fill = "lightgray",
    bins = 6
  ) +
  stat_bin(
    geom = "text",
    aes(
      label = format(
        after_stat(count),
        big.mark = ",",
        scientific = FALSE
      )
    ),
    vjust = -0.5,
    color = "black",
    size = 2,
    bins = 6
  ) +
  geom_vline(
    aes(
      xintercept = mean(F_MISS)
    ),
    color = "red",
    linetype = "dotted",
    linewidth = 0.5
  ) +
  geom_text(
    aes(
      x = mean(F_MISS),
      y = 16000,
      label = paste0(
        "Mean \n",
        scales::percent(mean(F_MISS),
          accuracy = 0.01
        )
      )
    ),
    size = 3,
    color = "red",
    # hjust = 1.5,
    vjust = -.2
  ) +
  labs(
    x = "Variant Missingness (%)",
    y = "Frequency (n)"
  ) +
  # theme_minimal(
  #   base_size = 12,
  #   base_family = "Roboto Condensed"
  # ) +
  scale_x_continuous(
    labels = scales::percent,
    n.breaks = 6
  ) +
  scale_y_continuous(
    labels = scales::label_comma(),
    n.breaks = 5
  ) +
  my_theme()
```

```
## Warning in geom_text(aes(x = mean(F_MISS), y = 16000, label = paste0("Mean \n", : All aesthetics have length 1, but the data has 108992 rows.
## ℹ Please consider using `annotate()` or provide this layer with data containing
##   a single row.
```

```
# save the plot
ggsave(
  here("scripts", "RMarkdowns", "output", "americas", "manuscript1", "figures", "SNPs_missingness10_man1.pdf"
  ),
  width  = 7,
  height = 5,
  units  = "in"
)
```

Remove individuals missing more than 20% of SNPs. You can use the
threshold you want, change the flag –mind

```
plink2 \
--allow-extra-chr \
--bfile manuscript1/output/file2 \
--mind 0.2               `# here we set the individual missingness threshold of 20%`\
--make-bed \
--out manuscript1/output/file3 \
--silent;
grep "samples\|variants" manuscript1/output/file3.log
```

959 samples (62 females, 101 males, 796 ambiguous; 959 founders)
loaded from 99689 variants loaded from manuscript1/output/file2.bim. 0
samples removed due to missing genotype data (–mind). 959 samples (62
females, 101 males, 796 ambiguous; 959 founders) remaining

## 3.2 Minor allele frequency

First, we estimate the allele frequency with Plink.

```
plink2 \
--allow-extra-chr \
--bfile manuscript1/output/file3 \
--freq \
--out manuscript1/output/MAF_check \
--silent
```

Now we apply the MAF filter.

```
# We will use MAF of 10%
plink2 \
--allow-extra-chr \
--bfile manuscript1/output/file3 \
--maf 0.1 \
--make-bed \
--out manuscript1/output/file4 \
--silent;
grep "variants" manuscript1/output/file4.log
```

99689 variants loaded from manuscript1/output/file3.bim. 14228
variants removed due to allele frequency threshold(s) 85461 variants
remaining after main filters.

Then we plot it with ggplot.

```
#   Import MAF data                                                         ####
maf_freq <-
  read.delim(
    here(
      "manuscript1/output/MAF_check.afreq"
    ),
    header = TRUE
  )
```

Make MAF plot

```
#   make the plot                                                           ####
ggplot(
  maf_freq,
  aes(ALT_FREQS)
) +
  geom_histogram(
    colour = "black",
    fill = "lightgray",
    bins = 40
  ) +
  labs(
    x = "Minor Allele Frequency (MAF)",
    y = "Frequency (n)",
    caption = "<span style='color:red;'><i>Red</i></span> <span style='color:black;'><i>line at</i></span><span style='color:red;'><i> MAF 10%</i></span><span style='color:black;'><i> threshold</i></span>."
  ) +
  geom_text(
    aes(
      x = .1,
      y = 8000,
      label = paste0("14,228 SNPs")
    ),
    size = 3,
    color = "red",
    vjust = -.2
  ) +
  geom_vline(xintercept = 0.1, color = "red") +
  my_theme() +
  theme(plot.caption = element_markdown()) +
  scale_y_continuous(label = scales::number_format(big.mark = ",")) +
  scale_x_continuous(breaks = c(0, 0.1, 0.2, 0.4, 0.6, 0.8, 1))
```

```
## Warning in geom_text(aes(x = 0.1, y = 8000, label = paste0("14,228 SNPs")), : All aesthetics have length 1, but the data has 99689 rows.
## ℹ Please consider using `annotate()` or provide this layer with data containing
##   a single row.
```

```
# save the plot
ggsave(
  here("scripts", "RMarkdowns", "output", "americas",  "manuscript1", "figures", "MAF_10_freq_plot_man1.pdf"
  ),
  width  = 7,
  height = 5,
  units  = "in"
)
```

We removed 14,228 variants due to the 10% MAF filter.

Next we will excludes markers which deviate from Hardy–Weinberg
equilibrium (HWE). It is a common indicator of genotyping error, but may
also indicate evolutionary selection. We have to do it for each
population individually. We cannot do it for all populations at once.
Therefore, the first step is create a new bed file with Plink keeping
only one population. We will create a new directory and name it “hardy”,
and copy the “file4” there.

```
mkdir -p manuscript1/output/files/hardy;
cp manuscript1/output/file4.* manuscript1/output/files/hardy/
```

## 3.3 HWE test

Run the HWE test. However, we will need to apply the SNP missingness
again for each population. If we do not, the HWE will vary widely. The
bash script below creates a new file for each population; run the HWE
test with HWE p value <1e‐6 (HWE p value <1e‐6). Then, we use
Plink to generate a list of SNPs that passed the test for each
population.

```
for fam in $(awk '{print $1}' manuscript1/output/files/hardy/file4.fam | sort | uniq); 
do 
echo $fam | \
plink2 \
--allow-extra-chr \
--silent \
--keep-allele-order \
--bfile manuscript1/output/files/hardy/file4 \
--keep-fam /dev/stdin \
--make-bed \
--out manuscript1/output/files/hardy/$fam \
--hwe 0.000001 \
--geno 0.1 \
--write-snplist; \
done
```

Use “cat” and “awk” to concatenate the SNP list from all populations,
and remove duplicates. Once we have a list of SNPs that passed the test
for each population, we can use Plink to create a new bed file keeping
only the SNPs that passed the test in each population. First, lets get
the list of SNPs, and count how many passed:

```
cd /gpfs/gibbs/pi/caccone/mkc54/albo
cat manuscript1/output/files/hardy/*.snplist | awk '!a[$0]++' > manuscript1/output/files/passed_hwe.txt;
wc -l manuscript1/output/files/passed_hwe.txt
```

85,461 passed, which means all variants passed HWE test. If some
failed, next time we could remove the variants that did not pass HWE
test, using the –extract flag, extracting only those that passed
HWE.

## 3.4 Linkage Estimates

We can analyze the LD patterns for each populations using different
approaches. If we want to look at general patterns we can get an
estimate for each population, for example, the half of the distance of
the r2 max. We can use Plink2 to estimates some statistics for us. One
important consideration to is how good is our genome assembly. Does it
matter if we use the 574 scaffolds or assign each scaffold to a
chromosome or not. So, if you want to know if it matters, you would need
to create a new chromosomal scale and perform the calculations. Since,
we want to get only an general approximate estimate of the linkage
levels, we can look at the largest scaffolds. Since chromosome 1 has the
M locus, we will get the largest scaffold of chromosome 2 and 3. We
create a new chromosomal scale when we want to plot the data. For now,
we can import a file that LC created with the size of each scaffold
(data/genome/scaffold\_sizes.txt). The code below imports the file and
get the 3 largest scaffolds from each chromosome.

```
#   import the file with the scaffold sizes                                 ####
scaffold_bps <-
  read_delim(
    here("scripts", "RMarkdowns", "scaffold_sizes.txt"
    ),
    col_names      = FALSE,
    show_col_types = FALSE,
    col_types      = "cn"
  )
#
# set column names
colnames(
  scaffold_bps
) <- c(
  "Scaffold", "Size"
)
#   ____________________________________________________________________________
#   create new column with the chromosome number                            ####
sizes <-
  scaffold_bps |>
  mutate(
    Chromosome = case_when( # we use mutate to create a new column called Chromosome
      startsWith(
        Scaffold, "1"
      ) ~ "1",              # use startsWith to get Scaffold rows starting with 1 and output 1 on Chromosome column
      startsWith(
        Scaffold, "2"
      ) ~ "2",
      startsWith(
        Scaffold, "3"
      ) ~ "3"
    )
  )
#
# group by Chromosome and get row with highest Size value
df_max <-
  sizes |>
  group_by(
    Chromosome
  ) |>
  slice_max(
    Size
  )
#
# print result
df_max |>
  mutate(
    Size_Mb = Size/1e6
  )
```

```
## # A tibble: 3 × 4
## # Groups:   Chromosome [3]
##   Scaffold     Size Chromosome Size_Mb
##   <chr>       <dbl> <chr>        <dbl>
## 1 1.85     28902815 1             28.9
## 2 2.17     43762705 2             43.8
## 3 3.75     25123842 3             25.1
```

The largest scaffolds of chromosome 2 is 43Mb, and chromosome 3 is
25Mb. We can also take a look at the size distribution of the scaffolds
using ggplot2.

```
# create histogram using ggplot2
library(scales)
```

```
## 
## Attaching package: 'scales'
```

```
## The following object is masked from 'package:purrr':
## 
##     discard
```

```
## The following object is masked from 'package:readr':
## 
##     col_factor
```

```
ggplot(
  sizes,
  aes(
    x = Size
  )
) +
  geom_histogram(
    binwidth = 1e6,
    aes(fill = factor(
      Chromosome
    )),
    color = "lightgray"
  ) +
  labs(
    title = "Scaffold sizes per chromosome",
    x = "Scaffold Size (Mb)",
    y = "Count (n)"
  ) +
  # xlab("Scaffold Size (Mb)") +
  # ylab("Count (n)") +
  my_theme() +
  scale_x_continuous(
    labels = comma_format(
      scale = 1e-6
    )
  ) +
  facet_wrap(
    ~Chromosome,
    ncol = 3,
    scales = "free_x"
  ) +
  scale_fill_manual(
    values = c(
      "pink", "green", "orange"
    )
  ) +
  guides(fill = "none")
```

Now, we can create a list of SNPs from these two large scaffolds. We
hope they will give us an average LD statistic that represents the
entire genome. We can import the .bim file and get the list of variants
from scaffolds 2.17 (43Mb) and 3.75 (25Mb)

Import the .bim file with the SNPs

```
#   import the bim file with the SNP data                                   ####
snps <-
  read_delim(                    # to learn about the options use here, run ?read_delim on the console.
    here(
      "manuscript1/output/file4.bim" 
    ),                           # use library here to load it
    col_names      = FALSE,      # we don't have header in the input file
    show_col_types = FALSE,      # suppress message from read_delim
    col_types      = "ccidcc"    # set the class of each column
  )
#
# set column names
colnames(
  snps
) <-                             # to add a header in our tibble
  c(
    "Scaffold", "SNP", "Cm", "Position", "Allele1", "Allele2"
  )
#
# check the tibble
head(snps)
```

```
## # A tibble: 6 × 6
##   Scaffold SNP             Cm Position Allele1 Allele2
##   <chr>    <chr>        <int>    <dbl> <chr>   <chr>  
## 1 1.1      AX-583035163     0   315386 A       G      
## 2 1.1      AX-583033356     0   315674 C       T      
## 3 1.1      AX-583033370     0   330057 G       A      
## 4 1.1      AX-583035194     0   330265 A       G      
## 5 1.1      AX-583033387     0   331288 C       T      
## 6 1.10     AX-583035257     0    91677 T       C
```

Write a function to import the bim files.

```
#   function to import bim files                                            ####
#
import_bim <- function(file) {
  # import as a tibble and set columns as integers
  bim <-
    read_delim(
      file,
      col_names      = FALSE,
      show_col_types = FALSE,
      col_types      = "ccidcc"
    )
  # rename the columns by index
  bim <- bim |>
    rename(
      Scaffold = 1,
      SNP      = 2,
      Cm       = 3,
      Position = 4,
      Allele1  = 5,
      Allele2  = 6
    )
  return(bim)
}
# we can save the function to source it later
dump(                                                     # check ?dump for more information
  "import_bim",                                           # the object we want to save
  here("scripts", "RMarkdowns", "analyses", "import_bim.R")       # use here to save it our function as .R 
)
```

Create the list of SNPs we want to use for the general linkage
analysis.

```
snps_4ld <-
  snps |>
  filter( # scaffolds 2.17 (43Mb) and 3.75 (25Mb)
    Scaffold == "2.17" | Scaffold == "3.75"
  ) |> 
  select(
    "Scaffold",
    "SNP"
  )
nrow(snps_4ld)
```

```
## [1] 5838
```

```
#5838
```

We have 5,838 SNPs to estimate LD that are located in the two largest
scaffolds of chromosome 2 and 3.

```
#   save calculation to load later                                          ####
write.table(
  snps_4ld,
  file      = here(
    "manuscript1/output/files/ld/snps_4ld.txt"
  ),
  sep       = "\t",
  row.names = FALSE,
  col.names = FALSE,
  quote     = FALSE
)
```

Select the populations for which we want to estimate the LD
statistic. Let only estimate LD for populations that we have 12 or more
individuals.

```
awk '{print $1}' manuscript1/output/file4.fam | sort | uniq -c | awk '{print $2, $1}' | awk '$2 >= 12 {print}'
```

```
## ARA 12
## BCO 12
## BEA 12
## BEN 14
## BER 12
## BIT 12
## BPA 12
## BPV 12
## BRE 13
## BRM 16
## CAM 12
## CES 15
## CHA 12
## DAL 12
## DEN 12
## DES 17
## FAY 12
## FIR 12
## FRS 12
## GES 12
## GRA 12
## GRV 12
## HAI 12
## HAR 12
## HOU 12
## HUN 12
## KAG 14
## KAN 12
## LIN 12
## LOS 14
## MAC 12
## MAN 12
## MAT 12
## MAU 12
## MON 12
## NEO 12
## NEW 22
## NUE 12
## OKI 12
## POR 12
## QNC 12
## RAR 12
## REC 12
## RUS 12
## SAI 12
## SMI 12
## SOC 12
## SSK 12
## STL 12
## TIK 12
## TRA 12
## TUC 14
## TUH 12
## UTS 12
```

Now we can create a list of populations that have 12 or more
individuals. We can use the command above but only print the list of
families. We can create a new directory for the LD analysis.

Create list of populations

```
awk '{print $1}' manuscript1/output/file4.fam | sort | uniq -c | awk '{print $2, $1}' | awk '$2 >= 12 {print}' | awk '{print $1}' > manuscript1/output/files/ld/pops_4ld.txt
```

### 3.4.1 Half distance r^2 max

Use Plink1.9 to estimate LD and then bin the data

```
#load plink 1.9
module load PLINK/1.9b_6.21-x86_64
plink --version
#PLINK v1.90b6.21 64-bit (19 Oct 2020)
```

```
for fam in $(awk '{print $1}' manuscript1/output/files/ld/pops_4ld.txt | sort | uniq); 
do 
echo $fam | \
plink \
--allow-extra-chr \
--keep-allele-order \
--extract manuscript1/output/files/ld/snps_4ld.txt \
--bfile all_pops/output/file4 \
--keep-fam /dev/stdin \
--maf 0.1 \
--r2 \
--out manuscript1/output/files/ld/$fam \
--geno 0.2 \
--write-snplist \
--silent
done;
#
rm manuscript1/output/files/ld/*.nosex
```

Once we subset the bed files per population, we set a MAF and
genotype missingness of 10% as before but now we have smaller sample
size and not all populations will have all the variants.

```
import os

# Replace with the directory containing the .snplist files
directory = "manuscript1/output/files/ld"

# Get a list of all .snplist files in the directory
file_list = [filename for filename in os.listdir(directory) if filename.endswith(".snplist")]

# Create a set to store the unique variants
unique_variants = set()

# Loop over each file and add the variants to the set
for filename in file_list:
    with open(os.path.join(directory, filename), "r") as file:
        for line in file:
            unique_variants.add(line.strip())

# Create a dictionary to store the number of files each variant appears in
variant_counts = {variant: 0 for variant in unique_variants}

# Loop over each file again and count the variants
for filename in file_list:
    with open(os.path.join(directory, filename), "r") as file:
        for line in file:
            variant = line.strip()
            variant_counts[variant] += 1

# Calculate the threshold for the number of files a variant must appear in
threshold = int(len(file_list) * 0.50)

# Get the list of unique variants that appear in at least 80% of files
all_variants = [variant for variant, count in variant_counts.items() if count >= threshold]

# Save the list of variants to a new file
with open("manuscript1/output/files/ld/all_unique_variants_50pct.txt", "w") as file:
    file.write("\n".join(all_variants))
    
#63,153
```

```
## 63153
```

Check how many SNPs

```
cat manuscript1/output/files/ld/all_unique_variants_50pct.txt | sort | awk '!seen[gensub(/^[[:space:]]+|[[:space:]]+$/, "", "g")]++' | wc -l
#4,858
```

```
## 4858
```

Now we can estimate LD again using the variants that are present in
at least 50% of the populations. Use Plink1.9 to estimate LD

```
for fam in $(awk '{print $1}' manuscript1/output/files/ld/pops_4ld.txt | sort | uniq); 
do 
echo $fam | \
plink \
--allow-extra-chr \
--keep-allele-order \
--extract manuscript1/output/files/ld/all_unique_variants_50pct.txt \
--bfile all_pops/output/file4 \
--keep-fam /dev/stdin \
--maf 0.1 \
--r2 \
--out manuscript1/output/files/ld/$fam \
--geno 0.2 \
--write-snplist \
--silent
done;
#
rm manuscript1/output/files/ld/*.nosex
```

Check one of the files

```
head /gpfs/gibbs/pi/caccone/mkc54/albo/manuscript1/output/files/ld/MAU.ld
```

```
##  CHR_A BP_A SNP_A CHR_B BP_B SNP_B R2 
##  2.17 56405 AX-584670672 2.17 70977 AX-584670684 1 
##  2.17 56405 AX-584670672 2.17 71211 AX-585179355 1 
##  2.17 56405 AX-584670672 2.17 71656 AX-585179364 1 
##  2.17 56405 AX-584670672 2.17 87654 AX-584670822 1 
##  2.17 56405 AX-584670672 2.17 114613 AX-584670867 1 
##  2.17 56405 AX-584670672 2.17 115267 AX-585179548 0.897436 
##  2.17 56405 AX-584670672 2.17 146817 AX-584670926 1 
##  2.17 56405 AX-584670672 2.17 161344 AX-584670951 1 
##  2.17 70977 AX-584670684 2.17 71211 AX-585179355 1
```

Clean the LD files from Plink

```
ld_files <- list.files(path = "manuscript1/output/files/ld", pattern = "\\.ld$", full.names = TRUE)

for (file in ld_files) {
  system(paste("awk -i inplace '{gsub(/[[:blank:]]+/, \" \")}1'", file))
}
```

Check one of the files

```
head manuscript1/output/files/ld/MAU.ld
```

```
##  CHR_A BP_A SNP_A CHR_B BP_B SNP_B R2 
##  2.17 56405 AX-584670672 2.17 70977 AX-584670684 1 
##  2.17 56405 AX-584670672 2.17 71211 AX-585179355 1 
##  2.17 56405 AX-584670672 2.17 71656 AX-585179364 1 
##  2.17 56405 AX-584670672 2.17 87654 AX-584670822 1 
##  2.17 56405 AX-584670672 2.17 114613 AX-584670867 1 
##  2.17 56405 AX-584670672 2.17 115267 AX-585179548 0.897436 
##  2.17 56405 AX-584670672 2.17 146817 AX-584670926 1 
##  2.17 56405 AX-584670672 2.17 161344 AX-584670951 1 
##  2.17 70977 AX-584670684 2.17 71211 AX-585179355 1
```

Function to import LD data

```
import_ld_files <- function(directory) {
  ld_files <-
    list.files(
      path = directory,
      pattern = "\\.ld$",
      full.names = TRUE
    )

  ld_tibbles <- list()

  for (file in ld_files) {
    ld_name <- gsub(".ld", "", basename(file))
    ld_data <- read_delim(
      file,
      col_names = TRUE,
      delim = " ",
      show_col_types = FALSE
    ) %>%
      select(c("CHR_A", "BP_A", "SNP_A", "CHR_B", "BP_B", "SNP_B", "R2"))
    ld_tibbles[[ld_name]] <- ld_data
  }

  return(ld_tibbles)
}

# we can save the function to source it later
dump(                                                     # check ?dump for more information
  "import_ld_files",                                           # the object we want to save
  here("scripts", "RMarkdowns", "analyses", "import_ld_files.R")       # use here to save it our function as .R 
)
```

Import the LD data

```
my_ld_tibbles <- import_ld_files("manuscript1/output/files/ld/")


head(my_ld_tibbles$MAU)
```

```
## # A tibble: 6 × 7
##   CHR_A  BP_A SNP_A        CHR_B   BP_B SNP_B           R2
##   <dbl> <dbl> <chr>        <dbl>  <dbl> <chr>        <dbl>
## 1  2.17 56405 AX-584670672  2.17  70977 AX-584670684 1    
## 2  2.17 56405 AX-584670672  2.17  71211 AX-585179355 1    
## 3  2.17 56405 AX-584670672  2.17  71656 AX-585179364 1    
## 4  2.17 56405 AX-584670672  2.17  87654 AX-584670822 1    
## 5  2.17 56405 AX-584670672  2.17 114613 AX-584670867 1    
## 6  2.17 56405 AX-584670672  2.17 115267 AX-585179548 0.897
```

Estimate half distance of maximum R2 value.

Since we have several populations, it is better to write a
function

```
get_half_pop <- function(ld_tibble) {
  # Split the tibble into sub-tibbles by scaffold
  ld_split <- split(ld_tibble, ld_tibble[, 1])
  # Remove rows with missing data
  ld_no_nan <- lapply(ld_split, function(x) {
    x[complete.cases(x), ]
  })
  # Calculate the distance between SNPs
  ld_dist <-
    lapply(ld_no_nan, function(x) {
      cbind(x, x[, 5] - x[, 2])
    })
  # Sort the sub-tibbles by distance
  ld_dist_order <- lapply(ld_dist, function(x) {
    x[order(x[, 8]), ]
  })
  # Combine the sub-tibbles into one tibble
  ld_proc <- do.call(rbind, ld_dist_order)
  # Sort the tibble by distance
  ld_sorted <- ld_proc[order(ld_proc[, 8]), ]
  # Bin the data by distance
  bin.1000 <-
    cut(ld_sorted[, 8], (c(0:1000) * 1000), include.lowest = TRUE)
  # Calculate the mean R2 for each bin
  ld_bin_1000_sorted <-
    tapply(ld_sorted[, 7], bin.1000, function(x) {
      mean(x)
    })
  # Fit a LOESS curve to the binned data
  lo.1000.ld <- loess(ld_bin_1000_sorted ~ c(1:1000))
  # Find the value of the binned R2 that corresponds to half the maximum R2 value
  d.pop <- max(ld_bin_1000_sorted, na.rm = TRUE)
  half.pop <- which(ld_bin_1000_sorted <= (d.pop / 2))[1]
  return(half.pop)
}
# we can save the function to source it later
dump(
  "get_half_pop",
  here("scripts", "RMarkdowns", "analyses", "get_half_pop.R")
)
```

Test the function, it should return the same value as we got
above

```
# for one population, you can change the population name to see other populations
half_pal<- get_half_pop(my_ld_tibbles$MAU)
half_pal
```

```
## (6.1e+04,6.2e+04] 
##                62
```

Estimates for all populations

```
# Create an empty data.frame to store the results
half_pops_df <-
  data.frame(
    row.names = names(my_ld_tibbles),
    half_distance = numeric(length(my_ld_tibbles)),
    stringsAsFactors = FALSE
  )

# Calculate the hald distance of max r^2 value for each tibble and store in the data.frame
for (tib_name in names(my_ld_tibbles)) {
  tib_half <- get_half_pop(my_ld_tibbles[[tib_name]])
  half_pops_df[tib_name, "half_distance"] <- tib_half
}

# Print the resulting data.frame
half_pops_df
```

```
##     half_distance
## ARA             2
## BCO             2
## BEA             1
## BEN             1
## BER             2
## BIT             4
## BPA             2
## BPV             2
## BRE             2
## BRM             1
## CAM             1
## CES             4
## CHA             1
## DAL             3
## DEN             3
## DES             9
## FAY             1
## FIR             2
## FRS             1
## GES             2
## GRA             3
## GRV             2
## HAI             1
## HAR             1
## HOU            50
## HUN             1
## KAG             2
## KAN             5
## LIN             4
## LOS             1
## MAC             3
## MAN             1
## MAT             1
## MAU            62
## MON             1
## NEO             4
## NEW             2
## NUE             3
## OKI             1
## POR             1
## QNC             1
## RAR             2
## REC             1
## RUS             3
## SAI             1
## SMI             1
## SOC             2
## SSK             1
## STL             4
## TIK             3
## TRA             2
## TUC             2
## TUH             1
## UTS            34
```

The half distance of the maximum r^2 for all populations vary from 1
to 9kb\* when we bin the data (except for HOU, MAU, & UTS which are
outliers). We do see larger blocks but most of the ld blocks are small.
You can use the entire genome to estimate LD instead of the larger
scaffolds, but the result will be almost the same. I have done it, but I
included only the larger scaffolds to make it easier for anyone to
repeat the analysis. Once we start using the entire genome, we need to
set a LD window, otherwise we will have billions of estimates since we
would have to estimate r^2 for all pairs of SNPs on each chromosome.
Let’s say we would have 500 SNPs on chromosome 1, we would have 2^500,
which is 3.273391e+150 pairwise estimates.

Make a plot for all populations

```
# Calculate half distances for each population
populations <- names(my_ld_tibbles)
half_distances <- sapply(populations, function(population) {
  ld_tibble <- my_ld_tibbles[[population]]
  half_pop <- get_half_pop(ld_tibble)
  return(half_pop)
})

# Create a tibble to store the half distances
half_distance_tibble <- data.frame(
  population = populations,
  half_distance = half_distances
)

# set the order of populations, you can change it on Excel then use it as a vector name pop_order
pop_order <-
  c(
    unique(
      half_distance_tibble$population
    )
  )

# set the order by continent (Africa, Americas, Asia, Europe) - check Excel spreadsheet named Pops_ld_order.xlxs or make your own. For example, range or continent.
half_distance_tibble$population <-
  factor(half_distance_tibble$population, levels = pop_order)

# Plot the half distances for each population
ggplot(half_distance_tibble, aes(x = population, y = half_distance)) +
  geom_bar(
    stat = "identity",
    fill = "lightgray",
    alpha = 0.8
  ) +
  geom_text(
    aes(label = round(half_distance, 2)),
    vjust = -0.5,
    size = 4,
    color = "red"
  ) +
  labs(x = "Population", y = expression(bold("Half Distance of Max " ~ r^
    2 ~ " (kb)"))) +
  my_theme() +
  my_theme() +
  theme(
    axis.text.x = element_text(
      angle = 90,
      hjust = 1,
      size = 14
    ),
    axis.text.y = element_text(size = 14),
    axis.title.x = element_text(size = 16, face = "bold"),
    axis.title.y = element_text(size = 16, face = "bold")
  )
```

```
# save the plot
ggsave(
  here("scripts", "RMarkdowns", "output", "americas", "manuscript1", "figures", "linkage_half_distance_LONG_scaffolds_man1.pdf"
  ),
  width  = 12,
  height = 6,
  units  = "in"
)
```

We can also plot the distribution of LD blocks across all scaffolds.
Later we will create a new chromosomal scale, but for now lets estimate
LD blocks using the genome as it is.

### 3.4.2 Estimate LD blocks using scaffolds

```
mkdir -p manuscript1/output/files/ld/blocks
```

Use Plink1.9 to estimate LD blocks for the populations with more than
12 individuals. We will use the entire genome for this part instead of
the larger scaffolds only. We will set max distance of LD blocks of
200kb. We found out that the average half distance of r^2 max is
relatively small

```
for fam in $(awk '{print $1}' manuscript1/output/files/ld/pops_4ld.txt | sort | uniq); 
do 
echo $fam | \
plink \
--allow-extra-chr \
--keep-allele-order \
--bfile manuscript1/output/file4 \
--keep-fam /dev/stdin \
--maf 0.1 \
--blocks no-pheno-req \
--blocks-max-kb 200 \
--out manuscript1/output/files/ld/blocks/$fam \
--geno 0.1 \
--silent
done;
#
rm manuscript1/output/files/ld/blocks/*.nosex
```

Now we can get some data from our .log files

```
echo "Population,n,nVariants,geno,maf,passQC" > manuscript1/output/files/ld/blocks/table_ld_stats.csv
for file in manuscript1/output/files/ld/blocks/*.log
do
  variants=$(grep -oE '([0-9]+) variants loaded from \.bim file' $file | grep -oE '[0-9]+')
  geno=$(grep -oE '([0-9]+) variants removed due to missing genotype data \(--geno\)' $file | grep -oE '[0-9]+')
  maf=$(grep -oE '([0-9]+) variants removed due to minor allele threshold\(s\)' $file | grep -oE '[0-9]+')
  pass=$(grep -oE '([0-9]+) variants and [0-9]+ people pass filters and QC\.' $file | grep -oE '[0-9]+' | head -1)
  n=$(grep -oE '([0-9]+) variants and [0-9]+ people pass filters and QC\.' $file | grep -oE '[0-9]+' | tail -1)
  filename=$(basename $file .log)
  echo "$filename,$n,$variants,$geno,$maf,$pass" >> manuscript1/output/files/ld/blocks/table_ld_stats.csv
done;
head -n 5 manuscript1/output/files/ld/blocks/table_ld_stats.csv
```

```
## Population,n,nVariants,geno,maf,passQC
## ARA,12,85461,6429,25218,53814
## BCO,12,85461,9704,25825,49932
## BEA,12,85461,3477,14783,67201
## BEN,14,85461,7041,28804,49616
```

We can check the it out

```
# Load data from the table_ld_stats.csv file
ld_blocks <- read.csv(
  here(
    "manuscript1/output/files/ld/blocks/table_ld_stats.csv"
    ),
  header = TRUE,
  sep = ","
)

# Create the flextable
ft <- flextable(ld_blocks)

# Apply zebra theme
ft <- theme_zebra(ft)

# Add a caption to the table
ft <- flextable::add_header_lines(ft, "Table 1: Summary of quality control for all populations.")

ft
```

| Table 1: Summary of quality control for all populations. | | | | | |
| --- | --- | --- | --- | --- | --- |
| Population | n | nVariants | geno | maf | passQC |
| ARA | 12 | 85,461 | 6,429 | 25,218 | 53,814 |
| BCO | 12 | 85,461 | 9,704 | 25,825 | 49,932 |
| BEA | 12 | 85,461 | 3,477 | 14,783 | 67,201 |
| BEN | 14 | 85,461 | 7,041 | 28,804 | 49,616 |
| BER | 12 | 85,461 | 4,209 | 18,464 | 62,788 |
| BIT | 12 | 85,461 | 7,086 | 26,101 | 52,274 |
| BPA | 12 | 85,461 | 8,639 | 23,611 | 53,211 |
| BPV | 12 | 85,461 | 10,732 | 25,218 | 49,511 |
| BRE | 13 | 85,461 | 9,293 | 22,756 | 53,412 |
| BRM | 16 | 85,461 | 6,863 | 22,214 | 56,384 |
| CAM | 12 | 85,461 | 5,493 | 26,900 | 53,068 |
| CES | 15 | 85,461 | 16,335 | 23,434 | 45,692 |
| CHA | 12 | 85,461 | 4,558 | 29,511 | 51,392 |
| DAL | 12 | 85,461 | 3,602 | 17,076 | 64,783 |
| DEN | 12 | 85,461 | 6,833 | 16,419 | 62,209 |
| DES | 17 | 85,461 | 16,769 | 19,582 | 49,110 |
| FAY | 12 | 85,461 | 14,783 | 14,545 | 56,133 |
| FIR | 12 | 85,461 | 4,649 | 23,337 | 57,475 |
| FRS | 12 | 85,461 | 4,091 | 19,572 | 61,798 |
| GES | 12 | 85,461 | 4,586 | 26,478 | 54,397 |
| GRA | 12 | 85,461 | 5,571 | 20,194 | 59,696 |
| GRV | 12 | 85,461 | 5,137 | 26,328 | 53,996 |
| HAI | 12 | 85,461 | 4,427 | 20,621 | 60,413 |
| HAR | 12 | 85,461 | 4,490 | 19,079 | 61,892 |
| HOU | 12 | 85,461 | 3,824 | 14,629 | 67,008 |
| HUN | 12 | 85,461 | 4,222 | 18,256 | 62,983 |
| KAG | 14 | 85,461 | 5,420 | 21,533 | 58,508 |
| KAN | 12 | 85,461 | 4,963 | 27,404 | 53,094 |
| LIN | 12 | 85,461 | 4,918 | 20,177 | 60,366 |
| LOS | 14 | 85,461 | 5,868 | 22,650 | 56,943 |
| MAC | 12 | 85,461 | 6,335 | 16,658 | 62,468 |
| MAN | 12 | 85,461 | 6,247 | 16,448 | 62,766 |
| MAT | 12 | 85,461 | 4,887 | 30,076 | 50,498 |
| MAU | 12 | 85,461 | 9,420 | 22,455 | 53,586 |
| MON | 12 | 85,461 | 6,644 | 15,754 | 63,063 |
| NEO | 12 | 85,461 | 3,883 | 18,203 | 63,375 |
| NEW | 22 | 85,461 | 4,216 | 17,590 | 63,655 |
| NUE | 12 | 85,461 | 4,386 | 16,713 | 64,362 |
| OKI | 12 | 85,461 | 5,915 | 25,782 | 53,764 |
| POR | 12 | 85,461 | 6,839 | 29,230 | 49,392 |
| QNC | 12 | 85,461 | 6,492 | 35,688 | 43,281 |
| RAR | 12 | 85,461 | 4,582 | 27,465 | 53,414 |
| REC | 12 | 85,461 | 6,444 | 22,253 | 56,764 |
| RUS | 12 | 85,461 | 5,031 | 21,090 | 59,340 |
| SAI | 12 | 85,461 | 4,673 | 20,439 | 60,349 |
| SMI | 12 | 85,461 | 5,061 | 18,074 | 62,326 |
| SOC | 12 | 85,461 | 4,594 | 25,985 | 54,882 |
| SSK | 12 | 85,461 | 4,769 | 29,646 | 51,046 |
| STL | 12 | 85,461 | 4,662 | 20,118 | 60,681 |
| TIK | 12 | 85,461 | 4,599 | 28,317 | 52,545 |
| TRA | 12 | 85,461 | 5,004 | 15,125 | 65,332 |
| TUC | 14 | 85,461 | 7,453 | 23,040 | 54,968 |
| TUH | 12 | 85,461 | 4,999 | 20,524 | 59,938 |
| UTS | 12 | 85,461 | 3,892 | 21,897 | 59,672 |

```
# Save it to a Word document
officer::read_docx() |>
  body_add_flextable(ft) |>
  print(target = here::here("scripts", "RMarkdowns", "output", "americas", "manuscript1", "summary_maf_geno_filter_population_manuscript1.docx"))
```

```
cd manuscript1/output/files/ld/blocks
wc -l *.blocks | \
  awk '{population = gensub(/\.blocks/, "", "g", $2); print population "\t" $1}' | \
  sed 's#/##' | \
  sed '$d' > populations_block_counts.csv;
head -n 30 populations_block_counts.csv
```

```
## ARA  85
## BCO  115
## BEA  75
## BEN  18
## BER  115
## BIT  142
## BPA  132
## BPV  84
## BRE  306
## BRM  559
## CAM  12
## CES  448
## CHA  9
## DAL  101
## DEN  96
## DES  546
## FAY  62
## FIR  182
## FRS  62
## GES  174
## GRA  34
## GRV  176
## HAI  58
## HAR  138
## HOU  69
## HUN  48
## KAG  193
## KAN  308
## LIN  158
## LOS  203
```

Add the number of blocks to the table we made

```
# Load data from the table_ld_stats.csv file
ld_blocks <- read.csv(
    "manuscript1/output/files/ld/blocks/table_ld_stats.csv"
    ,
  header = TRUE,
  sep = ","
)

# Load the population counts data from the CSV file
pop_counts <-
  read.delim(
      "manuscript1/output/files/ld/blocks/populations_block_counts.csv"
    ,
    header = F,
    sep = "\t"
  ) |>
  rename(
    Population       = 1,
    nBlocks           = 2
  )

# Merge the population counts with the table data
ld_blocks <- merge(ld_blocks, pop_counts, by = "Population")

# Create the flextable
ft <- flextable(ld_blocks)

# Apply zebra theme
ft <- theme_zebra(ft)

# Add a caption to the table
ft <- flextable::add_header_lines(ft, "Table 2: Number of linkage blocks detected with Plink for populations with at least 12 individuals.")

ft
```

| Table 2: Number of linkage blocks detected with Plink for populations with at least 12 individuals. | | | | | | |
| --- | --- | --- | --- | --- | --- | --- |
| Population | n | nVariants | geno | maf | passQC | nBlocks |
| ARA | 12 | 85,461 | 6,429 | 25,218 | 53,814 | 85 |
| BCO | 12 | 85,461 | 9,704 | 25,825 | 49,932 | 115 |
| BEA | 12 | 85,461 | 3,477 | 14,783 | 67,201 | 75 |
| BEN | 14 | 85,461 | 7,041 | 28,804 | 49,616 | 18 |
| BER | 12 | 85,461 | 4,209 | 18,464 | 62,788 | 115 |
| BIT | 12 | 85,461 | 7,086 | 26,101 | 52,274 | 142 |
| BPA | 12 | 85,461 | 8,639 | 23,611 | 53,211 | 132 |
| BPV | 12 | 85,461 | 10,732 | 25,218 | 49,511 | 84 |
| BRE | 13 | 85,461 | 9,293 | 22,756 | 53,412 | 306 |
| BRM | 16 | 85,461 | 6,863 | 22,214 | 56,384 | 559 |
| CAM | 12 | 85,461 | 5,493 | 26,900 | 53,068 | 12 |
| CES | 15 | 85,461 | 16,335 | 23,434 | 45,692 | 448 |
| CHA | 12 | 85,461 | 4,558 | 29,511 | 51,392 | 9 |
| DAL | 12 | 85,461 | 3,602 | 17,076 | 64,783 | 101 |
| DEN | 12 | 85,461 | 6,833 | 16,419 | 62,209 | 96 |
| DES | 17 | 85,461 | 16,769 | 19,582 | 49,110 | 546 |
| FAY | 12 | 85,461 | 14,783 | 14,545 | 56,133 | 62 |
| FIR | 12 | 85,461 | 4,649 | 23,337 | 57,475 | 182 |
| FRS | 12 | 85,461 | 4,091 | 19,572 | 61,798 | 62 |
| GES | 12 | 85,461 | 4,586 | 26,478 | 54,397 | 174 |
| GRA | 12 | 85,461 | 5,571 | 20,194 | 59,696 | 34 |
| GRV | 12 | 85,461 | 5,137 | 26,328 | 53,996 | 176 |
| HAI | 12 | 85,461 | 4,427 | 20,621 | 60,413 | 58 |
| HAR | 12 | 85,461 | 4,490 | 19,079 | 61,892 | 138 |
| HOU | 12 | 85,461 | 3,824 | 14,629 | 67,008 | 69 |
| HUN | 12 | 85,461 | 4,222 | 18,256 | 62,983 | 48 |
| KAG | 14 | 85,461 | 5,420 | 21,533 | 58,508 | 193 |
| KAN | 12 | 85,461 | 4,963 | 27,404 | 53,094 | 308 |
| LIN | 12 | 85,461 | 4,918 | 20,177 | 60,366 | 158 |
| LOS | 14 | 85,461 | 5,868 | 22,650 | 56,943 | 203 |
| MAC | 12 | 85,461 | 6,335 | 16,658 | 62,468 | 104 |
| MAN | 12 | 85,461 | 6,247 | 16,448 | 62,766 | 74 |
| MAT | 12 | 85,461 | 4,887 | 30,076 | 50,498 | 16 |
| MAU | 12 | 85,461 | 9,420 | 22,455 | 53,586 | 102 |
| MON | 12 | 85,461 | 6,644 | 15,754 | 63,063 | 68 |
| NEO | 12 | 85,461 | 3,883 | 18,203 | 63,375 | 94 |
| NEW | 22 | 85,461 | 4,216 | 17,590 | 63,655 | 834 |
| NUE | 12 | 85,461 | 4,386 | 16,713 | 64,362 | 78 |
| OKI | 12 | 85,461 | 5,915 | 25,782 | 53,764 | 137 |
| POR | 12 | 85,461 | 6,839 | 29,230 | 49,392 | 96 |
| QNC | 12 | 85,461 | 6,492 | 35,688 | 43,281 | 35 |
| RAR | 12 | 85,461 | 4,582 | 27,465 | 53,414 | 155 |
| REC | 12 | 85,461 | 6,444 | 22,253 | 56,764 | 136 |
| RUS | 12 | 85,461 | 5,031 | 21,090 | 59,340 | 194 |
| SAI | 12 | 85,461 | 4,673 | 20,439 | 60,349 | 77 |
| SMI | 12 | 85,461 | 5,061 | 18,074 | 62,326 | 107 |
| SOC | 12 | 85,461 | 4,594 | 25,985 | 54,882 | 126 |
| SSK | 12 | 85,461 | 4,769 | 29,646 | 51,046 | 7 |
| STL | 12 | 85,461 | 4,662 | 20,118 | 60,681 | 156 |
| TIK | 12 | 85,461 | 4,599 | 28,317 | 52,545 | 194 |
| TRA | 12 | 85,461 | 5,004 | 15,125 | 65,332 | 89 |
| TUC | 14 | 85,461 | 7,453 | 23,040 | 54,968 | 124 |
| TUH | 12 | 85,461 | 4,999 | 20,524 | 59,938 | 112 |
| UTS | 12 | 85,461 | 3,892 | 21,897 | 59,672 | 147 |

```
# Save it to a Word document
officer::read_docx() |>
  body_add_flextable(ft) |>
  print(target = here::here("scripts", "RMarkdowns", "output", "americas", "manuscript1", "summary_ld_blocks_manuscript1.docx"))
```

Get the size of each block from the .block.det files

```
get_kb_column <- function(dir_path) {
  # obtain the list of files with extension .blocks.det
  file_names <- list.files(path = dir_path, pattern = "\\.blocks\\.det$", full.names = TRUE)
  
  # create an empty list to hold the data frames
  block_list <- list()
  
  # loop through the files and read the data into the list
  for (file in file_names) {
    df <- read.table(file, header = TRUE, check.names = FALSE, stringsAsFactors = FALSE)
    # select only the KB column and add it to the block_list with the file name
    block_list[[file]] <- df %>% select(KB) %>% add_column(file = file, .before = 1)
  }
  
  # combine the data frames in the block_list into a single data frame
  blocks <- bind_rows(block_list)
  
  # clean up the file name column
  blocks$file <- str_remove(blocks$file, "^.*\\/ld\\/blocks\\/")
  
  return(blocks)
}

# example usage: replace dir_path with your directory path
dir_path <- here("manuscript1/output/files/ld/blocks")

blocks<- 
  get_kb_column(dir_path) |>
  mutate(file = str_remove(file, ".blocks.det")) |>
  as_tibble() |>
  rename(
    Population = 1
  )
```

Create density plot of the size of the LD blocks Plink found

```
# to check how many colors we need
# n_distinct(blocks$Population) 
#89

# define the color palette with 89 color blind colors
library(RColorBrewer)
#n <- 89
#qual_col_pals = brewer.pal.info[brewer.pal.info$category == 'qual',]
#col_vector = unlist(mapply(brewer.pal, qual_col_pals$maxcolors, rownames(qual_col_pals)))
#pie(rep(1,n), col=sample(col_vector, n))
#col_vector

col_vector <- read.delim(
  file = here("scripts/RMarkdowns/colors89.txt"), header = FALSE)
head(col_vector)
```

```
##        V1
## 1 #FB000D
## 2 #0DFF00
## 3 #2A0DFC
## 4 #FFD0D0
## 5 #FE0DDE
## 6 #16D1FE
```

```
col_vector <- as.vector(col_vector$V1)

# make plot using the sample y scale for all populations
ggplot(blocks, aes(x = KB)) +
  stat_density(
    aes(y = after_stat(count), fill = factor(Population)),
    linewidth = .5,
    alpha = .4,
    position = "identity"
  ) +
  scale_fill_manual(values = col_vector) +
  scale_x_continuous(name = "Block length (kb)") +
  scale_y_continuous(name = "Count") +
  theme(
    plot.title = element_text(hjust = 0.5, size = 18, face = "bold"),
    axis.text = element_text(size = 12),
    axis.title = element_text(size = 16, face = "bold"),
    strip.text = element_text(size = 14, face = "bold"),
    plot.background = element_rect(fill = "white"),
    panel.background = element_rect(fill = 'white', colour = 'black')
  ) +
  guides(fill = "none") +
  facet_wrap( ~ Population, ncol = 3) + my_theme()
```

```
# save the plot as a PDF using ggsave
ggsave(
  here("scripts",
    "RMarkdowns",
    "output",
    "americas",
    "manuscript1",
    "figures",
    "block_density_y_scale_fixed_man1.pdf"
  ),
  width = 10,
  height = 20,
  units = "in"
)
```

Make plot allowing the y axis scale free

```
ggplot(blocks, aes(x = KB)) +
  stat_density(
    aes(y = after_stat(count), fill = factor(Population)),
    linewidth = .5,
    alpha = .4,
    position = "identity"
  ) +
  scale_fill_manual(values = col_vector) +
  scale_x_continuous(name = "Block length (kb)") +
  scale_y_continuous(name = "Count") +
  theme(
    plot.title = element_text(hjust = 0.5, size = 18, face = "bold"),
    axis.text = element_text(size = 12),
    axis.title = element_text(size = 16, face = "bold"),
    strip.text = element_text(size = 14, face = "bold"),
    plot.background = element_rect(fill = "white"),
    panel.background = element_rect(fill = 'white', colour = 'black')
  ) +
  guides(fill = "none") +
  facet_wrap( ~ Population, ncol = 3, scales = "free_y") +
  my_theme()
```

```
# save the plot as a PDF using ggsave
ggsave(
  here("scripts",
    "RMarkdowns",
    "output",
    "americas",
    "manuscript1",
    "figures",
    "block_density_free_scale_man1.pdf"
  ),
  width = 20,
  height = 18,
  units = "in"
)
```

## 3.5 LD pruning

Since we do not have to remove any SNPs due to deviation from HWE, we
can proceed with heterozygosity estimates. The first step is to “prune”
our data set. We will check the pairwise linkage estimates for all SNPs.
We can work with file4. We will use “`indep-pairwise`” to
check if there are SNPs above a certain linkage disequilibrium (LD)
threshold. Check Plink documentation for more details https://www.cog-genomics.org/plink/1.9/ld I used
“`--indep-pairwise 5 1 0.1`” , which indicates according to
the documentation:
`--indep-pairphase <window size>['kb'] <step size (variant ct)> <r^2 threshold>`
We will check in a window of 5kb if there is any pair of SNPs with r2
estimates above 0.1, then we will move our window 1 SNP and check again
for SNPs above the threshold. We will repeat this procedure until we
check the entire genome.

Need to switch back to plink2

```
module load PLINK/2_avx2_20221024
#PLINK/1.9b_6.21-x86_64 => PLINK/2_avx2_20221024
plink2 --version
```

```
plink2 \
--allow-extra-chr \
--bfile manuscript1/output/file4 \
--extract manuscript1/output/files/passed_hwe.txt \
--indep-pairwise 5 1 0.1 \
--out manuscript1/output/indepSNP \
--silent;
grep 'pairwise\|variants\|samples' manuscript1/output/indepSNP.log
```

–indep-pairwise 5 1 0.1 959 samples (62 females, 101 males, 796
ambiguous; 959 founders) loaded from 85461 variants loaded from
manuscript1/output/file4.bim. –extract: 85461 variants remaining. 85461
variants remaining after main filters. –indep-pairwise (28 compute
threads): 32369/85461 variants removed.

Remember, the SNPs are not removed from our data set. Plink created 3
files when we ran the code above. One is the “indepSNP.log” file, and
the other two are: “indepSNP.prune.in” -> list of SNPs with squared
correlation smaller than our r2 threshold of 0.1. “indepSNP.prune.out”
-> list of SNPs with squared correlation greater than our r2
threshold of 0.1. For our heterozygosity estimates, we want to use the
set of SNPs that are below our r2 threshold of 0.1. We consider that
they are randomly associated. We can use Plink to estimate the
heterozygosity using the “indepSNP.prune.in” file.

## 3.6 Heterozygosity

```
plink2 \
--allow-extra-chr \
--bfile manuscript1/output/file4 \
--extract manuscript1/output/indepSNP.prune.in \
--het \
--out manuscript1/output/R_check \
--silent;
grep 'variants' manuscript1/output/R_check.log
```

85461 variants loaded from manuscript1/output/file4.bim. –extract:
53092 variants remaining. 53092 variants remaining after main
filters.

```
#   find individuals with high heterozygosity                              ####
# import the data from Plink
het <- read.delim(
  here(
    "manuscript1/output/R_check.het"
  ),
  head = TRUE
)
#
# check head of the file
colnames(het)
```

```
## [1] "X.FID"  "IID"    "O.HOM." "E.HOM." "OBS_CT" "F"
```

Estimate heterozygosity

```
# create a column named HET_RATE and calculate the heterozygosity rate
het$HET_RATE <- (het$"OBS_CT" - het$"O.HOM") / het$"OBS_CT"
#
# use subset function to get values deviating from 4sd of the mean heterozygosity rate.
het_fail <-
  subset(
    het, (het$HET_RATE < mean(
      het$HET_RATE
    ) - 4 * sd(
      het$HET_RATE
    )) |
      (het$HET_RATE > mean(
        het$HET_RATE
      ) + 4 * sd(
        het$HET_RATE
      ))
  )
#
# get the list of individuals that failed our threshold of 4sd from the mean.
het_fail$HET_DST <-
  (het_fail$HET_RATE - mean(
    het$HET_RATE
  )) / sd(
    het$HET_RATE
  )
```

Save the files to use with Plink

```
#   save the data to use with Plink2                                        ####
#
write.table(
  het_fail,
  here(
    "manuscript1/output/fail-het-qc.txt"
  ),
  row.names = FALSE
)
```

Make plot

```
#   make a heterozygosity plot                                              ####
#
ggplot(
  het,
  aes(
    HET_RATE
  )
) +
  geom_histogram(
    colour           = "black",
    fill             = "lightgray",
    bins             = 40
  ) +
  labs(
    x                = "Heterozygosity Rate",
    y                = "Number of Individuals"
  ) +
  geom_vline(
    aes(
      xintercept     = mean(
        HET_RATE
      )
    ),
    col              = "red",
    linewidth        = 1.5
  ) +
  geom_vline(
    aes(
      xintercept     = mean(
        HET_RATE
      ) + 4 * sd(
        HET_RATE
      )
    ),
    col              = "#BFB9B9",
    linewidth        = 1
  ) +
  geom_vline(
    aes(
      xintercept     = mean(
        HET_RATE
      ) - 4 * sd(
        HET_RATE
      )
    ),
    col              = "#BFB9B9",
    linewidth        = 1
  ) + 
  my_theme() +
  scale_y_continuous(
  )
```

```
#   save the heterozygosity plot                                            ####
ggsave(
  here("scripts", "RMarkdowns", "output", "americas", "manuscript1", "figures", "Heterozygosity_man1.pdf"
  ),
  width  = 5,
  height = 4,
  units  = "in"
)
```

The red line in the plot above indicates the mean, and the gray lines
indicate 4 standard deviation from the mean. We can see that some
mosquitoes do have excess heterozygous sites. We will remove them. We
can get their ID from the file “fail-het-qc.txt”. We can use the bash
script below to parse the file to use with Plink

```
sed 's/"// g' manuscript1/output/fail-het-qc.txt | awk '{print$1, $2}'> manuscript1/output/het_fail_ind.txt;
echo 'How many mosquitoes we need to remove from our data set:';
cat manuscript1/output/het_fail_ind.txt | tail -n +2 | wc -l;
echo 'Which mosquitoes we have to remove:';
tail -n +2 manuscript1/output/het_fail_ind.txt
```

```
## How many mosquitoes we need to remove from our data set:
## 8
## Which mosquitoes we have to remove:
## QNC 1248
## COL 471
## KAT 601
## KAT 605
## KAT 606
## KAT 608
## KUN 682
## GRA 734
```

Heterozygosity is outside the SD threshold in 8 individuals. QNC 1248
COL 471 KAT 601 KAT 605 KAT 606 KAT 608 KUN 682 GRA 734 We will remove
them using Plink:

```
plink2 \
--allow-extra-chr \
--bfile manuscript1/output/file4 \
--remove manuscript1/output/het_fail_ind.txt \
--make-bed \
--out manuscript1/output/file5 \
--silent;
grep 'variants\|samples' manuscript1/output/file5.log
```

959 samples (62 females, 101 males, 796 ambiguous; 959 founders)
loaded from 85461 variants loaded from manuscript1/output/file4.bim.
–remove: 951 samples remaining. 951 samples (61 females, 96 males, 794
ambiguous; 951 founders) remaining after

## 3.7 Relatedness

Check for cryptic relatedness. Check Plink2 documentation https://www.cog-genomics.org/plink/2.0/distance You can
download King directly https://www.kingrelatedness.com/manual.shtml or check
their manuscript https://www.ncbi.nlm.populations.gov/pmc/articles/PMC3025716/pdf/btq559.pdf
From Plink2 documentation: “Note that KING kinship coefficients are
scaled such that duplicate samples have kinship 0.5, not 1. First-degree
relations (parent-child, full siblings) correspond to ~0.25,
second-degree relations correspond to ~0.125, etc. It is conventional to
use a cutoff of ~0.354 (the geometric mean of 0.5 and 0.25) to screen
for monozygotic twins and duplicate samples, ~0.177 to add first-degree
relations, etc.” There two options. One is to run only –make-king and
another one is to use –make-king-table We will use the threshold of
0.354 and create a table.

```
# Plink2 will create a file with extension .king
plink2 \
--allow-extra-chr \
--bfile manuscript1/output/file5 \
--extract manuscript1/output/indepSNP.prune.in \
--make-king-table rel-check \
--king-table-filter 0.354 \
--out manuscript1/output/file5 \
--silent;
grep 'variants\|samples' manuscript1/output/file5.log
```

951 samples (61 females, 96 males, 794 ambiguous; 951 founders)
loaded from 85461 variants loaded from manuscript1/output/file5.bim.
–extract: 53092 variants remaining. 53092 variants remaining after main
filters.

Check the individuals that did not pass our filtering.

```
head -n 30 manuscript1/output/file5.kin0
```

```
## #FID1    IID1    FID2    IID2    NSNP    HETHET  IBS0    KINSHIP
## BEN  259 BEN 258 50787   0.24589 0   0.493896
## BEN  260 BEN 258 51190   0.249111    0   0.497112
## BEN  260 BEN 259 50839   0.246681    0   0.493795
## BPA  2460    BPA 2452    49052   0.21414 0.011498    0.362447
## CHI  2274    CHI 2269    50896   0.22155 0.013223    0.358999
## DES  1445    DES 1292    51292   0.262887    0.00382126  0.453077
## HAT  2395    HAT 2393    50843   0.267195    0.00430738  0.436993
## IPO  2590    IPO 2585    50733   0.206   0.0075099   0.396444
## KAG  228 KAG 227 51782   0.27552 0   0.498881
## KAG  229 KAG 227 51687   0.27533 0   0.49865
## KAG  229 KAG 228 51713   0.275521    0   0.49846
## KAN  1327    KAN 1325    51492   0.237862    0.00792356  0.400495
## LOS  366 LOS 365 51334   0.198835    0   0.498802
## LOS  367 LOS 365 51260   0.198615    0   0.498042
## LOS  367 LOS 366 51245   0.198107    0   0.497352
## NEB  2549    NEB 2546    49299   0.274427    0.00290067  0.401603
## NEW  423 NEW 413 51536   0.237601    0.0111767   0.366153
## NEW  423 NEW 420 51525   0.239088    0.0139544   0.356606
## OKI  1010    OKI 1006    50619   0.225607    0.0127225   0.359407
## REC  179 REC 175 50950   0.243317    0.00798822  0.396319
## SPR  465 SPR 460 51299   0.244683    0.00799236  0.384376
## SPS  915 SPS 914 51250   0.216878    0.0102634   0.365102
```

We want to remove one of the individuals of the pairs.

```
plink2 \
--allow-extra-chr \
--bfile manuscript1/output/file5 \
--extract manuscript1/output/indepSNP.prune.in \
--make-king triangle bin \
--out manuscript1/output/file6 \
--silent;
grep 'variants\|samples' manuscript1/output/file6.log
```

951 samples (61 females, 96 males, 794 ambiguous; 951 founders)
loaded from 85461 variants loaded from manuscript1/output/file5.bim.
–extract: 53092 variants remaining. 53092 variants remaining after main
filters. –make-king pass 1/1: Scanning for rare variants… done. 0
variants handled by initial scan (53092 remaining). –make-king: 53092
variants processed.

Now use Plink2 to remove one of the mosquitoes from the pair with
high kinship. It will remove samples with high relatedness (2460, 2274,
2395, 366, 367, 2590, 2549, 423, 179, 465). Plink2 always tries to
maximize the number of samples passing the filters.

```
plink2 \
--allow-extra-chr \
--bfile manuscript1/output/file5 \
--king-cutoff manuscript1/output/file6 0.354 \
--make-bed \
--out manuscript1/output/file7 \
--silent;
grep 'samples\|variants\|remaining' manuscript1/output/file7.log
```

951 samples (61 females, 96 males, 794 ambiguous; 951 founders)
loaded from 85461 variants loaded from manuscript1/output/file5.bim.
manuscript1/output/file7.king.cutoff.out.id , and 933 remaining sample
IDs

# 4. Create chromosomal scale

Import the .bim file with the SNPs to create a new chromosomal
scale.

```
#   ____________________________________________________________________________
#   import the bim file with the SNP data                                   ####
snps <-
  read_delim(                    # to learn about the options use here, run ?read_delim on the console.
    here(
      "manuscript1/output/file7.bim"
    ),                           # use library here to load it
    col_names      = FALSE,      # we don't have header in the input file
    show_col_types = FALSE,      # suppress message from read_delim
    col_types      = "ccidcc"    # set the class of each column
  )
#
# set column names
colnames(
  snps
) <-                             # to add a header in our tibble
  c(
    "Scaffold", "SNP", "Cm", "Position", "Allele1", "Allele2"
  )
#
# check the tibble
head(snps)
```

```
## # A tibble: 6 × 6
##   Scaffold SNP             Cm Position Allele1 Allele2
##   <chr>    <chr>        <int>    <dbl> <chr>   <chr>  
## 1 1        AX-583035163     0   315386 A       G      
## 2 1        AX-583033356     0   315674 C       T      
## 3 1        AX-583033370     0   330057 G       A      
## 4 1        AX-583035194     0   330265 A       G      
## 5 1        AX-583033387     0   331288 C       T      
## 6 1        AX-583035257     0   442875 T       C
```

Separate the tibbles into each chromosome

```
#   separate the SNP data per chromosome                                    
# chr1
chr1_snps <-
  snps |>
  filter(
    str_detect(
      Scaffold, "^1."
    )
  ) |> # here we get only Scaffold rows starting with 1
  as_tibble() # save as tibble
#
# chr2
chr2_snps <-
  snps |>
  filter(
    str_detect(
      Scaffold, "^2."
    )
  ) |>
  as_tibble()
#
# chr3
chr3_snps <-
  snps |>
  filter(
    str_detect(
      Scaffold, "^3."
    )
  ) |>
  as_tibble()
```

Import the file with sizes of each scaffold.

```
#   import the file with the scaffold sizes                                 ####
sizes <-
  read_delim(
    here(
      "/gpfs/gibbs/project/caccone/mkc54/albo/genome/scaffold_sizes.txt"
    ),
    col_names      = FALSE,
    show_col_types = FALSE,
    col_types      = "cd"
  )
#
# set column names
colnames(
  sizes
) <- c(
  "Scaffold", "Size"
)
#   ____________________________________________________________________________
#   create new column with the chromosome number                            ####
sizes <- 
  sizes |>
  mutate(
    Chromosome = case_when( # we use mutate to create a new column called Chromosome
      startsWith(
        Scaffold, "1"
      ) ~ "1", # use startsWith to get Scaffold rows starting with 1 and output 1 on Chromosome column
      startsWith(
        Scaffold, "2"
      ) ~ "2",
      startsWith(
        Scaffold, "3"
      ) ~ "3"
    )
  ) |>
  arrange(
    Scaffold
  )                   # to sort the order of the scaffolds, fixing the problem we have with scaffold 1.86
# check it
head(sizes)
```

```
## # A tibble: 6 × 3
##   Scaffold     Size Chromosome
##   <chr>       <dbl> <chr>     
## 1 1.1        351198 1         
## 2 1.10     11939576 1         
## 3 1.100     3389100 1         
## 4 1.101      470438 1         
## 5 1.102     2525157 1         
## 6 1.103      150026 1
```

Create new scale. Get the scaffolds for each chromosome.

```
#   separate the scaffold sizes tibble per chromosome                       ####
# chr1
chr1_scaffolds <- 
  sizes |>
  filter(
    str_detect(
      Scaffold, "^1" # we use library stringr to get scaffolds starting with 1 (chromosome 1)
    )
  ) |> 
  as_tibble()
#
# chr2
chr2_scaffolds <-
  sizes |>
  filter(
    str_detect(
      Scaffold, "^2" # we use library stringr to get scaffolds starting with 2 (chromosome 2)
    )
  ) |> 
  as_tibble()
#
# # chr3
chr3_scaffolds <-
  sizes |>
  filter(
    str_detect(
      Scaffold, "^3" # we use library stringr to get scaffolds starting with 3 (chromosome 3)
    )
  ) |>
  as_tibble()
```

Create a scale for each chromosome.

```
#   create a new scale for each chromosome                                  ####
# chr1
chr1_scaffolds$overall_size_before_bp <-
  0                                                                        # we create a new column with zeros
for (i in 2:nrow(
  chr1_scaffolds
)
) {                                                                        # loop to start on second line
  chr1_scaffolds$overall_size_before_bp[i] <-                              # set position on the scale
    chr1_scaffolds$overall_size_before_bp[i - 1] + chr1_scaffolds$Size[i - # add the scaffold size and the location to get position on new scale
      1]
}
#
# chr2
chr2_scaffolds$overall_size_before_bp <- 0
for (i in 2:nrow(
  chr2_scaffolds
)
) {
  chr2_scaffolds$overall_size_before_bp[i] <-
    chr2_scaffolds$overall_size_before_bp[i - 1] + chr2_scaffolds$Size[i -
      1]
}
#
# chr3
chr3_scaffolds$overall_size_before_bp <- 0
for (i in 2:nrow(
  chr3_scaffolds
)
) {
  chr3_scaffolds$overall_size_before_bp[i] <-
    chr3_scaffolds$overall_size_before_bp[i - 1] + chr3_scaffolds$Size[i -
      1]
}
```

Merge the data frames scaffolds and SNPs.

```
#   merge the data sets using the tidyverse function left_join              ####
# chr1
chr1_scale <-
  chr1_snps |>          # create data frame for each chromosome, get chr1_snps
  left_join(            # use lef_join function to merge it with chr1_scaffolds
    chr1_scaffolds,
    by = "Scaffold"
  ) |>                  # set column to use for merging (Scaffold in this case)
  na.omit() |>          # remove NAs, we don't have SNPs in every scaffold
  mutate(
    midPos_fullseq = as.numeric(
      Position
    ) +                 # make new columns numeric
      as.numeric(
        overall_size_before_bp
      )
  )
#
# chr2
chr2_scale <-
  chr2_snps |>
  left_join(
    chr2_scaffolds,
    by = "Scaffold"
  ) |>
  na.omit() |>
  mutate(
    midPos_fullseq = as.numeric(
      Position
    ) +
      as.numeric(
        overall_size_before_bp
      )
  )
#
# chr3
chr3_scale <-
  chr3_snps |>
  left_join(
    chr3_scaffolds,
    by = "Scaffold"
  ) |>
  na.omit() |>
  mutate(
    midPos_fullseq = as.numeric(
      Position
    ) +
      as.numeric(
        overall_size_before_bp
      )
  )
```

Merge all chromosome scales.

```
#   merge the data sets, and select only the columns we need                ####
chroms <- rbind(
  chr1_scale, chr2_scale, chr3_scale
) |>
  dplyr::select(
    Chromosome, SNP, Cm, midPos_fullseq, Allele1, Allele2
  )
# check it
head(chroms)
```

```
## # A tibble: 0 × 6
## # ℹ 6 variables: Chromosome <chr>, SNP <chr>, Cm <int>, midPos_fullseq <dbl>,
## #   Allele1 <chr>, Allele2 <chr>
```

Save the new .bim file

```
#   ____________________________________________________________________________
#   save the new bim file with a new name, I added "B"                      ####
write.table(
  chroms,
  file      = here(
    "manuscript1/output/file7B.bim"
  ),
  sep       = "\t",
  row.names = FALSE,
  col.names = FALSE,
  quote     = FALSE
)
```

Rename the .bim files

```
# change the name of the first .bim file, for example, append _backup.bim, and then replace the original file
mv manuscript1/output/file7.bim manuscript1/output/file7_backup.bim;
# than change the new bim we create to the original name (do it only once, otherwise it will mess up)
mv manuscript1/output/file7B.bim manuscript1/output/file7.bim
```

Create a new bed file with Plink2 to see if it works. For example, to
see if the variants are in the right order. Plink2 will give us a
warning.

```
plink2 \
--bfile manuscript1/output/file7 \
--make-bed \
--out manuscript1/output/test01;
# then we remove the files 
rm manuscript1/output/test01.*
```

PLINK v2.00a3.7LM AVX2 Intel (24 Oct 2022)
www.cog-genomics.org/plink/2.0/ (C) 2005-2022 Shaun Purcell, Christopher
Chang GNU General Public License v3 Logging to
manuscript1/output/test01.log. Options in effect: –bfile
manuscript1/output/file7 –make-bed –out manuscript1/output/test01 Start
time: Tue Aug 20 15:31:46 2024 257321 MiB RAM detected; reserving 128660
MiB for main workspace. Using up to 32 threads (change this with
–threads). 933 samples (61 females, 93 males, 779 ambiguous; 933
founders) loaded from manuscript1/output/file7.fam. 85461 variants
loaded from manuscript1/output/file7.bim. Note: No phenotype data
present. Writing manuscript1/output/test01.fam … done. Writing
manuscript1/output/test01.bim … done. Writing
manuscript1/output/test01.bed … done. End time: Tue Aug 20 15:31:46
2024

## 4.1 Estimate LD blocks using chromosomal scale

```
mkdir -p manuscript1/output/files/ld/blocks_chr
```

```
module load PLINK/1.9b_6.21-x86_64
```

Use Plink1.9 to estimate LD blocks for the populations with more than
12 individuals. We will use the entire genome for this part instead of
the larger scaffolds only. We will set max distance of LD blocks of
500kb. We found out that the average half distance of r^2 max is small,
from 1 to 5kb

```
for fam in $(awk '{print $1}' manuscript1/output/files/ld/pops_4ld.txt | sort | uniq); 
do 
echo $fam | \
plink \
--allow-extra-chr \
--keep-allele-order \
--bfile manuscript1/output/file7 \
--keep-fam /dev/stdin \
--maf 0.1 \
--blocks no-pheno-req \
--blocks-max-kb 200 \
--out manuscript1/output/files/ld/blocks_chr/$fam \
--geno 0.1 \
--silent
done;
#
rm manuscript1/output/files/ld/blocks_chr/*.nosex
```

Get some data from our .log files

```
echo "Population,n,nVariants,geno,maf,passQC" > manuscript1/output/files/ld/blocks_chr/table_ld_stats.csv
for file in manuscript1/output/files/ld/blocks_chr/*.log
do
  variants=$(grep -oE '([0-9]+) variants loaded from \.bim file' $file | grep -oE '[0-9]+')
  geno=$(grep -oE '([0-9]+) variants removed due to missing genotype data \(--geno\)' $file | grep -oE '[0-9]+')
  maf=$(grep -oE '([0-9]+) variants removed due to minor allele threshold\(s\)' $file | grep -oE '[0-9]+')
  pass=$(grep -oE '([0-9]+) variants and [0-9]+ people pass filters and QC\.' $file | grep -oE '[0-9]+' | head -1)
  n=$(grep -oE '([0-9]+) variants and [0-9]+ people pass filters and QC\.' $file | grep -oE '[0-9]+' | tail -1)
  filename=$(basename $file .log)
  echo "$filename,$n,$variants,$geno,$maf,$pass" >> manuscript1/output/files/ld/blocks_chr/table_ld_stats.csv
done;
head -n 5 manuscript1/output/files/ld/blocks_chr/table_ld_stats.csv
```

Check it

```
# Load data from the table_ld_stats.csv file
ld_blocks <- read.csv(
  here(
    "manuscript1/output/files/ld/blocks_chr/table_ld_stats.csv"
    ),
  header = TRUE,
  sep = ","
)

# Create the flextable
ft <- flextable(ld_blocks)

# Apply zebra theme
ft <- theme_zebra(ft)

# Add a caption to the table
ft <- flextable::add_header_lines(ft, "Table 1: Summary of quality control for all populations dataset.")

ft
```

| Table 1: Summary of quality control for all populations dataset. | | | | | |
| --- | --- | --- | --- | --- | --- |
| Population | n | nVariants | geno | maf | passQC |
| ARA | 12 | 85,461 | 6,429 | 25,218 | 53,814 |
| BCO | 12 | 85,461 | 9,704 | 25,825 | 49,932 |
| BEA | 12 | 85,461 | 3,477 | 14,783 | 67,201 |
| BEN | 12 | 85,461 | 5,921 | 31,214 | 48,326 |
| BER | 12 | 85,461 | 4,209 | 18,464 | 62,788 |
| BIT | 12 | 85,461 | 7,086 | 26,101 | 52,274 |
| BPA | 11 | 85,461 | 7,935 | 23,594 | 53,932 |
| BPV | 12 | 85,461 | 10,732 | 25,218 | 49,511 |
| BRE | 13 | 85,461 | 9,293 | 22,756 | 53,412 |
| BRM | 16 | 85,461 | 6,863 | 22,214 | 56,384 |
| CAM | 12 | 85,461 | 5,493 | 26,900 | 53,068 |
| CES | 15 | 85,461 | 16,335 | 23,434 | 45,692 |
| CHA | 12 | 85,461 | 4,558 | 29,511 | 51,392 |
| DAL | 12 | 85,461 | 3,602 | 17,076 | 64,783 |
| DEN | 12 | 85,461 | 6,833 | 16,419 | 62,209 |
| DES | 16 | 85,461 | 16,348 | 19,501 | 49,612 |
| FAY | 12 | 85,461 | 14,783 | 14,545 | 56,133 |
| FIR | 12 | 85,461 | 4,649 | 23,337 | 57,475 |
| FRS | 12 | 85,461 | 4,091 | 19,572 | 61,798 |
| GES | 12 | 85,461 | 4,586 | 26,478 | 54,397 |
| GRA | 11 | 85,461 | 4,532 | 21,294 | 59,635 |
| GRV | 12 | 85,461 | 5,137 | 26,328 | 53,996 |
| HAI | 12 | 85,461 | 4,427 | 20,621 | 60,413 |
| HAR | 12 | 85,461 | 4,490 | 19,079 | 61,892 |
| HOU | 12 | 85,461 | 3,824 | 14,629 | 67,008 |
| HUN | 12 | 85,461 | 4,222 | 18,256 | 62,983 |
| KAG | 12 | 85,461 | 4,624 | 22,331 | 58,506 |
| KAN | 11 | 85,461 | 4,405 | 28,843 | 52,213 |
| LIN | 12 | 85,461 | 4,918 | 20,177 | 60,366 |
| LOS | 12 | 85,461 | 5,284 | 23,527 | 56,650 |
| MAC | 12 | 85,461 | 6,335 | 16,658 | 62,468 |
| MAN | 12 | 85,461 | 6,247 | 16,448 | 62,766 |
| MAT | 12 | 85,461 | 4,887 | 30,076 | 50,498 |
| MAU | 12 | 85,461 | 9,420 | 22,455 | 53,586 |
| MON | 12 | 85,461 | 6,644 | 15,754 | 63,063 |
| NEO | 12 | 85,461 | 3,883 | 18,203 | 63,375 |
| NEW | 21 | 85,461 | 3,824 | 17,811 | 63,826 |
| NUE | 12 | 85,461 | 4,386 | 16,713 | 64,362 |
| OKI | 11 | 85,461 | 5,277 | 26,235 | 53,949 |
| POR | 12 | 85,461 | 6,839 | 29,230 | 49,392 |
| QNC | 11 | 85,461 | 5,469 | 36,726 | 43,266 |
| RAR | 12 | 85,461 | 4,582 | 27,465 | 53,414 |
| REC | 11 | 85,461 | 5,725 | 23,044 | 56,692 |
| RUS | 12 | 85,461 | 5,031 | 21,090 | 59,340 |
| SAI | 12 | 85,461 | 4,673 | 20,439 | 60,349 |
| SMI | 12 | 85,461 | 5,061 | 18,074 | 62,326 |
| SOC | 12 | 85,461 | 4,594 | 25,985 | 54,882 |
| SSK | 12 | 85,461 | 4,769 | 29,646 | 51,046 |
| STL | 12 | 85,461 | 4,662 | 20,118 | 60,681 |
| TIK | 12 | 85,461 | 4,599 | 28,317 | 52,545 |
| TRA | 12 | 85,461 | 5,004 | 15,125 | 65,332 |
| TUC | 14 | 85,461 | 7,453 | 23,040 | 54,968 |
| TUH | 12 | 85,461 | 4,999 | 20,524 | 59,938 |
| UTS | 12 | 85,461 | 3,892 | 21,897 | 59,672 |

```
# Save it to a Word document
officer::read_docx() |>
  body_add_flextable(ft) |>
  print(target = here::here("manuscript1/output/summary_blocks_chr.docx"))
```

Count how many blocks we found in each population

```
cd manuscript1/output/files/ld/blocks_chr
wc -l *.blocks | \
  awk '{population = gensub(/\.blocks_chr/, "", "g", $2); print population "\t" $1}' | \
  sed 's#manuscript1/output/files/ld/blocks_chr/##' | \
  sed 's/.blocks//' | \
  sed '$d' > populations_block_counts.csv;
head -n 79 populations_block_counts.csv
```

```
## ARA  85
## BCO  115
## BEA  75
## BEN  10
## BER  115
## BIT  142
## BPA  89
## BPV  84
## BRE  306
## BRM  559
## CAM  12
## CES  448
## CHA  9
## DAL  101
## DEN  96
## DES  430
## FAY  62
## FIR  182
## FRS  62
## GES  174
## GRA  30
## GRV  176
## HAI  58
## HAR  138
## HOU  69
## HUN  48
## KAG  115
## KAN  228
## LIN  158
## LOS  124
## MAC  104
## MAN  74
## MAT  16
## MAU  102
## MON  68
## NEO  94
## NEW  746
## NUE  78
## OKI  99
## POR  96
## QNC  25
## RAR  155
## REC  98
## RUS  194
## SAI  77
## SMI  107
## SOC  126
## SSK  7
## STL  156
## TIK  194
## TRA  89
## TUC  125
## TUH  112
## UTS  147
```

Add the number of blocks to the table we made

```
# Load data from the table_ld_stats.csv file
ld_blocks <- read.csv(
  here(
    "manuscript1/output/files/ld/blocks_chr/table_ld_stats.csv"
    ),
  header = TRUE,
  sep = ","
)

# Load the population counts data from the CSV file
pop_counts <-
  read.delim(
    here(
      "manuscript1/output/files/ld/blocks_chr/populations_block_counts.csv"
    ),
    header = F,
    sep = "\t"
  ) |>
  rename(
    Population       = 1,
    nBlocks          = 2
  )

# Merge the population counts with the table data
ld_blocks <- merge(ld_blocks, pop_counts, by = "Population")

# Create the flextable
ft <- flextable(ld_blocks)

# Apply zebra theme
ft <- theme_zebra(ft)

# Add a caption to the table
ft <- flextable::add_header_lines(ft, "Table 2: Number of linkage blocks detected with Plink for populations with at least 10 individuals.")

ft
```

| Table 2: Number of linkage blocks detected with Plink for populations with at least 10 individuals. | | | | | | |
| --- | --- | --- | --- | --- | --- | --- |
| Population | n | nVariants | geno | maf | passQC | nBlocks |
| ARA | 12 | 85,461 | 6,429 | 25,218 | 53,814 | 85 |
| BCO | 12 | 85,461 | 9,704 | 25,825 | 49,932 | 115 |
| BEA | 12 | 85,461 | 3,477 | 14,783 | 67,201 | 75 |
| BEN | 12 | 85,461 | 5,921 | 31,214 | 48,326 | 10 |
| BER | 12 | 85,461 | 4,209 | 18,464 | 62,788 | 115 |
| BIT | 12 | 85,461 | 7,086 | 26,101 | 52,274 | 142 |
| BPA | 11 | 85,461 | 7,935 | 23,594 | 53,932 | 89 |
| BPV | 12 | 85,461 | 10,732 | 25,218 | 49,511 | 84 |
| BRE | 13 | 85,461 | 9,293 | 22,756 | 53,412 | 306 |
| BRM | 16 | 85,461 | 6,863 | 22,214 | 56,384 | 559 |
| CAM | 12 | 85,461 | 5,493 | 26,900 | 53,068 | 12 |
| CES | 15 | 85,461 | 16,335 | 23,434 | 45,692 | 448 |
| CHA | 12 | 85,461 | 4,558 | 29,511 | 51,392 | 9 |
| DAL | 12 | 85,461 | 3,602 | 17,076 | 64,783 | 101 |
| DEN | 12 | 85,461 | 6,833 | 16,419 | 62,209 | 96 |
| DES | 16 | 85,461 | 16,348 | 19,501 | 49,612 | 430 |
| FAY | 12 | 85,461 | 14,783 | 14,545 | 56,133 | 62 |
| FIR | 12 | 85,461 | 4,649 | 23,337 | 57,475 | 182 |
| FRS | 12 | 85,461 | 4,091 | 19,572 | 61,798 | 62 |
| GES | 12 | 85,461 | 4,586 | 26,478 | 54,397 | 174 |
| GRA | 11 | 85,461 | 4,532 | 21,294 | 59,635 | 30 |
| GRV | 12 | 85,461 | 5,137 | 26,328 | 53,996 | 176 |
| HAI | 12 | 85,461 | 4,427 | 20,621 | 60,413 | 58 |
| HAR | 12 | 85,461 | 4,490 | 19,079 | 61,892 | 138 |
| HOU | 12 | 85,461 | 3,824 | 14,629 | 67,008 | 69 |
| HUN | 12 | 85,461 | 4,222 | 18,256 | 62,983 | 48 |
| KAG | 12 | 85,461 | 4,624 | 22,331 | 58,506 | 115 |
| KAN | 11 | 85,461 | 4,405 | 28,843 | 52,213 | 228 |
| LIN | 12 | 85,461 | 4,918 | 20,177 | 60,366 | 158 |
| LOS | 12 | 85,461 | 5,284 | 23,527 | 56,650 | 124 |
| MAC | 12 | 85,461 | 6,335 | 16,658 | 62,468 | 104 |
| MAN | 12 | 85,461 | 6,247 | 16,448 | 62,766 | 74 |
| MAT | 12 | 85,461 | 4,887 | 30,076 | 50,498 | 16 |
| MAU | 12 | 85,461 | 9,420 | 22,455 | 53,586 | 102 |
| MON | 12 | 85,461 | 6,644 | 15,754 | 63,063 | 68 |
| NEO | 12 | 85,461 | 3,883 | 18,203 | 63,375 | 94 |
| NEW | 21 | 85,461 | 3,824 | 17,811 | 63,826 | 746 |
| NUE | 12 | 85,461 | 4,386 | 16,713 | 64,362 | 78 |
| OKI | 11 | 85,461 | 5,277 | 26,235 | 53,949 | 99 |
| POR | 12 | 85,461 | 6,839 | 29,230 | 49,392 | 96 |
| QNC | 11 | 85,461 | 5,469 | 36,726 | 43,266 | 25 |
| RAR | 12 | 85,461 | 4,582 | 27,465 | 53,414 | 155 |
| REC | 11 | 85,461 | 5,725 | 23,044 | 56,692 | 98 |
| RUS | 12 | 85,461 | 5,031 | 21,090 | 59,340 | 194 |
| SAI | 12 | 85,461 | 4,673 | 20,439 | 60,349 | 77 |
| SMI | 12 | 85,461 | 5,061 | 18,074 | 62,326 | 107 |
| SOC | 12 | 85,461 | 4,594 | 25,985 | 54,882 | 126 |
| SSK | 12 | 85,461 | 4,769 | 29,646 | 51,046 | 7 |
| STL | 12 | 85,461 | 4,662 | 20,118 | 60,681 | 156 |
| TIK | 12 | 85,461 | 4,599 | 28,317 | 52,545 | 194 |
| TRA | 12 | 85,461 | 5,004 | 15,125 | 65,332 | 89 |
| TUC | 14 | 85,461 | 7,453 | 23,040 | 54,968 | 125 |
| TUH | 12 | 85,461 | 4,999 | 20,524 | 59,938 | 112 |
| UTS | 12 | 85,461 | 3,892 | 21,897 | 59,672 | 147 |

```
# Save it to a Word document
officer::read_docx() |>
  body_add_flextable(ft) |>
  print(target = here::here("manuscript1/output/summary_ld_blocks_chr.docx"))
```

Get the size of each block from the .block.det files

```
get_kb_column <- function(dir_path) {
  # obtain the list of files with extension .blocks.det
  file_names <- list.files(path = dir_path, pattern = "\\.blocks\\.det$", full.names = TRUE)
  
  # create an empty list to hold the data frames
  block_list <- list()
  
  # loop through the files and read the data into the list
  for (file in file_names) {
    df <- read.table(file, header = TRUE, check.names = FALSE, stringsAsFactors = FALSE)
    # select only the KB column and add it to the block_list with the file name
    block_list[[file]] <- df %>% dplyr::select(KB) %>% add_column(file = file, .before = 1)
  }
  
  # combine the data frames in the block_list into a single data frame
  blocks <- bind_rows(block_list)
  
  # clean up the file name column
  blocks$file <- str_remove(blocks$file, "^.*\\/ld\\/blocks\\/")
  
  return(blocks)
}

# example usage: replace dir_path with your directory path
dir_path <- here("/gpfs/gibbs/pi/caccone/mkc54/albo/manuscript1/output/files/ld/blocks_chr")

blocks<- 
  get_kb_column(dir_path) |>
  mutate(file = str_remove(file, "/gpfs/gibbs/pi/caccone/mkc54/albo/manuscript1/output/files/ld/blocks_chr/")) |>
  mutate(file = str_remove(file, ".blocks.det")) |>

  as_tibble() |>
  rename(
    Population = 1
  )
```

Create density plot of the size of the LD blocks Plink found

```
source(
  here("scripts", "RMarkdowns",
    "my_theme2.R"
  )
)

col_vector <- read.delim(
  file = here("scripts/RMarkdowns/colors89.txt"), header = FALSE)
head(col_vector)
```

```
##        V1
## 1 #FB000D
## 2 #0DFF00
## 3 #2A0DFC
## 4 #FFD0D0
## 5 #FE0DDE
## 6 #16D1FE
```

```
col_vector <- as.vector(col_vector$V1)

# make plot using the sample y scale for all populations
ggplot(blocks, aes(x = KB)) +
  stat_density(
    aes(y = after_stat(count), fill = factor(Population)),
    linewidth = .5,
    alpha = .4,
    position = "identity"
  ) +
  scale_fill_manual(values = col_vector) +
  scale_x_continuous(name = "Block length (kb)") +
  scale_y_continuous(name = "Count") +
  theme(
    plot.title = element_text(hjust = 0.5, size = 18, face = "bold"),
    axis.text = element_text(size = 12),
    axis.title = element_text(size = 16, face = "bold"),
    strip.text = element_text(size = 14, face = "bold"),
    plot.background = element_rect(fill = "white"),
    panel.background = element_rect(fill = 'white', colour = 'black')
  ) +
  guides(fill = "none") +
  facet_wrap( ~ Population, ncol = 3) + my_theme()
```

```
# save the plot as a PDF using ggsave
ggsave(
  here("scripts/RMarkdowns/output/americas/manuscript1/figures/block_density_y_scale_fixed_chr_man1.pdf"
  ),
  width = 20,
  height = 18,
  units = "in"
)
```

Make plot allowing the y axis scale free

```
ggplot(blocks, aes(x = KB)) +
  stat_density(
    aes(y = after_stat(count), fill = factor(Population)),
    linewidth = .5,
    alpha = .4,
    position = "identity"
  ) +
  scale_fill_manual(values = col_vector) +
  scale_x_continuous(name = "Block length (kb)") +
  scale_y_continuous(name = "Count") +
  theme(
    plot.title = element_text(hjust = 0.5, size = 18, face = "bold"),
    axis.text = element_text(size = 12),
    axis.title = element_text(size = 16, face = "bold"),
    strip.text = element_text(size = 14, face = "bold"),
    plot.background = element_rect(fill = "white"),
    panel.background = element_rect(fill = 'white', colour = 'black')
  ) +
  guides(fill = "none") +
  facet_wrap( ~ Population, ncol = 3, scales = "free_y") +
  my_theme()
```

```
# save the plot as a PDF using ggsave
ggsave(
  here("scripts/RMarkdowns/output/americas/manuscript1/figures/block_density_free_scale_chr_man1.pdf"
  ),
  width = 20,
  height = 18,
  units = "in"
)
```

# 5. Plot SNP density

After quality control with approximately 85k SNPs

```
# load the function that we saved earlier
source(
  here("scripts", "RMarkdowns",
    "analyses", "import_bim.R"
  ),
  local = knitr::knit_global()
)
# import the file
snp_den_qc <- import_bim(
  here(
    "manuscript1/output/file7.bim"
  )
)
```

Make plot of the SNP density

```
#   ____________________________________________________________________________
#   plot SNP density after QC                                               ####
snp_den_qc |>
  rename(
    Chromosome = 1
  ) |>
  mutate(
    Position           = as.numeric(
      Position
    )
  ) |>
  ggplot(
    aes(
      x                = Position
    ),
    label              = sprintf(
      "%0.2f",
      round(
        a,
        digits         = 0
      )
    )
  ) +
  geom_histogram(
    aes(
      y                = after_stat(
        count
      )
    ),
    binwidth           = 1e6
  ) +
  facet_wrap(
    vars(
      Chromosome
    ),
    scales             = "free_x"
  ) +
  labs(
    title              = "SNP Density after QC",
    x                  = expression(
      "Position in the genome (Mb)"
    ),
    y                  = expression(
      "Number of SNPs"
    )
  ) +
  scale_x_continuous(
    labels             = function(x) {
      format(
        x / 1e6,
        big.mark       = ",", 
        scientific     = FALSE
      )
    }
  ) +
  geom_density(
  aes(
    y = 1e6 * after_stat(count)
  ),
  color = "red",
  linewidth = .75,
  alpha = .4,
  fill = "pink"
  ) +
    theme(
    panel.grid.major   = element_line(
      linetype         = "dashed",
      linewidth        = 0.2
    ),
    panel.grid.minor   = element_line(
      linetype         = "dashed",
      linewidth        = 0.2
    ),
    panel.spacing      = unit(0.5, "lines"),
    strip.text         = element_text(
      face             = "bold", hjust = .5
    ),
    strip.background.x = element_rect(
      color            = "gray"
    )
  )
```

```
#   save the density plot                                            ####
ggsave(
  here("scripts/RMarkdowns/output/americas/manuscript1/figures/snp_density_after_qc_man1.pdf"
  ),
  width  = 10,
  height = 6,
  units  = "in"
)
```

SNPs per chromosome

```
# we can use dplyr "count" to get the number of SNPs for each chromosome
# lets get the data we need
snps_per_chrm <- 
  snp_den_qc |>
  count(
    Scaffold) |>
  rename(
    Chromosome = 1,
    "SNPs (N) " = 2
  )

# Create the flextable
ft <- flextable::flextable(snps_per_chrm)

# Apply zebra theme
ft <- flextable::theme_zebra(ft)

# Add a caption to the table
ft <- flextable::add_header_lines(ft, "SNPs per chromosome after quality control")
ft
```

| SNPs per chromosome after quality control | |
| --- | --- |
| Chromosome | SNPs (N) |
| 1 | 18,973 |
| 2 | 35,799 |
| 3 | 30,689 |

Get the mean number of SNPs per chromosome for the entire genome

```
# we first use dplyr cut_width to get the number of SNPs per 1Mb window
albo_den <- 
  snp_den_qc |>
  dplyr::select(
    Scaffold, Position
  ) |>
  group_by(
    Scaffold,
    windows               = cut_width(
      Position,
      width               = 1e6,
      boundary            = 0
    )
  ) |>
  summarise(
    n                     = n(),
    .groups               = "keep"
  ) |>
  group_by(
    Scaffold
  ) |>
  summarise(
    mean                  = mean(n),
    n                     = n(),
    .groups               = "keep"
  ) |>
  rename(
    Chromosome            = 1,
    "SNPs per 1Mb window" = 2,
    "Number of windows"   = 3
  )
#
# check the results
snp_table <-
  flextable(
    albo_den
  )
snp_table <- colformat_double(
  x        = snp_table,
  big.mark = ",",
  digits   = 2,
  na_str   = "N/A"
)
snp_table
```

Merge objects

```
# we can merge the two data sets we created above into one table
after_qc <-
  snps_per_chrm |>
  left_join(
    albo_den,
    by = "Chromosome"
  )
snp_table2 <- flextable(
  after_qc)
snp_table2 <- colformat_double(
  x        = snp_table2,
  big.mark = ",",
  digits   = 2, 
  na_str   = "N/A"
  )
snp_table2
```

```
# Save it to a Word document
officer::read_docx() |>
  body_add_flextable(snp_table2) |>
  print(target = here::here("manuscript1/output/snps_per_chromosome_table_manuscript1.docx"))
```

# 6. LD pruning using the chromosomal scale

```
# we set a window of variants of 5 and move the window 1 variant per time, removing 1 of the variants with lowest MAF from a pair above the threshold of r^2 > 0.1
plink2 \
--allow-extra-chr \
--bfile manuscript1/output/file7 \
--indep-pairwise 5 1 0.1 \
--out manuscript1/output/indepSNP_chr \
--silent;
grep 'pairwise\|variants\|samples' manuscript1/output/indepSNP_chr.log
```

–indep-pairwise 5 1 0.1 933 samples (61 females, 93 males, 779
ambiguous; 933 founders) loaded from 85461 variants loaded from
manuscript1/output/file7.bim. –indep-pairwise (3 compute threads):
32492/85461 variants removed.

Do the scaffold again

First need to import same SNP list Import the .bim file with the
SNPs

```
#   import the bim file with the SNP data                                   ####
snps <-
  read_delim(                    # to learn about the options use here, run ?read_delim on the console.
    here(
      "manuscript1/output/file7_backup.bim" #output/populations/file4.bim
    ),                           # use library here to load it
    col_names      = FALSE,      # we don't have header in the input file
    show_col_types = FALSE,      # suppress message from read_delim
    col_types      = "ccidcc"    # set the class of each column
  )
#
# set column names
colnames(
  snps
) <-                             # to add a header in our tibble
  c(
    "Scaffold", "SNP", "Cm", "Position", "Allele1", "Allele2"
  )
#
# check the tibble
head(snps)
```

```
## # A tibble: 6 × 6
##   Scaffold SNP             Cm Position Allele1 Allele2
##   <chr>    <chr>        <int>    <dbl> <chr>   <chr>  
## 1 1.1      AX-583035163     0   315386 A       G      
## 2 1.1      AX-583033356     0   315674 C       T      
## 3 1.1      AX-583033370     0   330057 G       A      
## 4 1.1      AX-583035194     0   330265 A       G      
## 5 1.1      AX-583033387     0   331288 C       T      
## 6 1.10     AX-583035257     0    91677 T       C
```

```
write.table(
  snps,
  file      = here(
    "manuscript1/output/snps_all_scaffolds_4ld.txt"
  ),
  sep       = "\t",
  row.names = FALSE,
  col.names = FALSE,
  quote     = FALSE
)

nrow(snps)
#85,461
```

```
plink2 \
--allow-extra-chr \
--bfile manuscript1/output/file5 \
--king-cutoff manuscript1/output/file6 0.354 \
--make-bed \
--out manuscript1/output/file11a \
--silent;
grep 'samples\|variants\|remaining' manuscript1/output/file11a.log
```

951 samples (61 females, 96 males, 794 ambiguous; 951 founders)
loaded from 85461 variants loaded from manuscript1/output/file5.bim.
manuscript1/output/file11a.king.cutoff.out.id , and 933 remaining sample
IDs

```
# we set a window of variants of 5 and move the window 1 variant each time, removing 1 of the variants with lowest MAF from a pair above the threshold of r^2 > 0.1
plink2 \
--allow-extra-chr \
--bfile manuscript1/output/file11a \
--indep-pairwise 5 1 0.1 \
--out manuscript1/output/indepSNP_scaffolds \
--silent;
grep 'pairwise\|variants\|samples' manuscript1/output/indepSNP_scaffolds.log
```

–indep-pairwise 5 1 0.1 933 samples (61 females, 93 males, 779
ambiguous; 933 founders) loaded from 85461 variants loaded from
manuscript1/output/file11a.bim. –indep-pairwise (28 compute threads):
32477/85461 variants removed.

Now compare the two sets of SNPs using scaffolds or chromosomal
scale

Create Venn diagram of SNPs removed due to LD

```
# Read in the two files as vectors
prunout_chr <-
  read_delim(
    here(
      "manuscript1/output/indepSNP_chr.prune.out"
    ),
    delim = " ",
    col_names = FALSE,
    show_col_types = FALSE
  )

prunout_scaffolds <-
  read_delim(
    here(
      "manuscript1/output/indepSNP_scaffolds.prune.out"
    ),
    delim = " ",
    col_names = FALSE,
    show_col_types = FALSE
  )

# Convert the list to vector
prunout_scaffolds <- unlist(prunout_scaffolds)
prunout_chr <- unlist(prunout_chr)


# Calculate shared values
prunout <- intersect(prunout_chr, prunout_scaffolds)

# Create Venn diagram
venn_data1 <- list(
  "Chromosomal" = prunout_chr,
  "Scaffolds" = prunout_scaffolds
)

# create plot
venn_plot1 <- ggvenn(venn_data1, fill_color = c("steelblue", "darkorange"), show_percentage = TRUE)

# Add a title
venn_plot1 <- venn_plot1 +
  ggtitle("Comparison of genomic scales for linked SNPs") +
  theme(plot.title = element_text(hjust = .5))

# Display the Venn diagram
print(venn_plot1)
```

```
# Save Venn diagram to PDF
output_path <- here("scripts/RMarkdowns/output/americas/manuscript1/figures/SNPs_linked_comparison_man1.pdf")
ggsave(output_path, venn_plot1, height = 5, width = 5, dpi = 300)
```

# 7. Pruning r2 for LD-pruned datasets

We already have a dataset made using the chromosomal scale that
pruned at 0.1 (made in step 9). If needed, can create it again using the
code below

```
plink2 \
--allow-extra-chr \
--bfile manuscript1/output/file7 \
--indep-pairwise 5 1 0.1 \
--out manuscript1/output/indepSNP_chr \
--silent;
grep 'pairwise\|variants\|samples' manuscript1/output/indepSNP_chr.log
```

–indep-pairwise 5 1 0.1 933 samples (61 females, 93 males, 779
ambiguous; 933 founders) loaded from 85461 variants loaded from
manuscript1/output/file7.bim. –indep-pairwise (3 compute threads):
32492/85461 variants removed.

Now we need to make pruned dataset for 0.01. Can use file7

```
plink2 \
--allow-extra-chr \
--bfile manuscript1/output/file7 \
--indep-pairwise 5 1 0.01 \
--out manuscript1/output/indepSNP_chr_r01 \
--silent;
grep 'pairwise\|variants\|samples' manuscript1/output/indepSNP_chr_r01.log
```

–indep-pairwise 5 1 0.01 933 samples (61 females, 93 males, 779
ambiguous; 933 founders) loaded from 85461 variants loaded from
manuscript1/output/file7.bim. –indep-pairwise (3 compute threads):
67382/85461 variants removed.

We can use the SNP set the LD pruning we just did and extract them
from file7

```
plink2 \
--keep-allele-order \
--bfile manuscript1/output/file7 \
--make-bed \
--export vcf \
--out manuscript1/output/snps_sets/r2_0.1 \
--extract manuscript1/output/indepSNP_chr.prune.in \
--silent
grep "variants\|samples" manuscript1/output/snps_sets/r2_0.1.log
```

933 samples (61 females, 93 males, 779 ambiguous; 933 founders)
loaded from 85461 variants loaded from manuscript1/output/file7.bim.
–extract: 52969 variants remaining. 52969 variants remaining after main
filters.

Repeat for 0.01 dataset

```
plink2 \
--keep-allele-order \
--bfile manuscript1/output/file7 \
--make-bed \
--export vcf \
--out manuscript1/output/snps_sets/r2_0.01 \
--extract manuscript1/output/indepSNP_chr_r01.prune.in \
--silent
grep "variants\|samples" manuscript1/output/snps_sets/r2_0.01.log
```

933 samples (61 females, 93 males, 779 ambiguous; 933 founders)
loaded from 85461 variants loaded from manuscript1/output/file7.bim.
–extract: 18079 variants remaining. 18079 variants remaining after main
filters.

These snp set can now be used for subsequent pop gen analyses.

# 8. Run PCA with LD-pruned dataset

```
plink2 \
--allow-extra-chr \
--bfile manuscript1/output/snps_sets/r2_0.01 \
--pca allele-wts \
--freq \
--out manuscript1/output/pca_pops_01 \
--silent;
grep 'samples\|variants' manuscript1/output/pca_pops_01.log
```

933 samples (61 females, 93 males, 779 ambiguous; 933 founders)
loaded from 18079 variants loaded from
manuscript1/output/snps\_sets/r2\_0.01.bim.

Check the files

```
head -n 2 manuscript1/output/pca_pops_01.eigenvec
```

```
## #FID IID PC1 PC2 PC3 PC4 PC5 PC6 PC7 PC8 PC9 PC10
## OKI  1001    0.00349156  -0.0268474  0.0200066   -0.0330706  0.0373854   0.0423607   -0.00898562 -0.00193913 -0.0140887  -0.0273157
```

```
head -n 10 manuscript1/output/pca_pops_01.eigenval
```

```
## 75.5724
## 27.09
## 16.2228
## 9.39242
## 9.04022
## 8.4542
## 7.36081
## 6.64457
## 6.43132
## 6.10407
```

Import PCA data

```
# import the data from Plink
pca <- read.delim(
  here(
    "manuscript1/output/pca_pops_01.eigenvec"
  ),
  head = TRUE
)
 
# check head of the file
head(pca)
```

```
##   X.FID  IID        PC1        PC2       PC3        PC4       PC5       PC6
## 1   OKI 1001 0.00349156 -0.0268474 0.0200066 -0.0330706 0.0373854 0.0423607
## 2   OKI 1002 0.00289618 -0.0294323 0.0162885 -0.0278071 0.0331991 0.0419769
## 3   OKI 1003 0.00351340 -0.0280753 0.0193459 -0.0302897 0.0382998 0.0496153
## 4   OKI 1004 0.00632866 -0.0213497 0.0138200 -0.0606556 0.0301422 0.0494885
## 5   OKI 1005 0.00592163 -0.0224315 0.0146760 -0.0653543 0.0323706 0.0476036
## 6   OKI 1006 0.00393091 -0.0229728 0.0177528 -0.0328802 0.0312586 0.0415294
##           PC7         PC8         PC9       PC10
## 1 -0.00898562 -0.00193913 -0.01408870 -0.0273157
## 2 -0.01012320 -0.00102705 -0.01503750 -0.0169791
## 3 -0.01948780  0.00277972 -0.02438790 -0.0373294
## 4  0.01624510  0.01816430 -0.00022433 -0.0750729
## 5  0.01103320  0.01512080 -0.00312499 -0.0769569
## 6 -0.02239840 -0.01205220 -0.02864100 -0.0468231
```

Import sample attributes

```
# import sample attributes
samples2 <- read.delim(
  here("scripts", "RMarkdowns", "population_metadata_all_1Apr2024.txt"
  ),
  head = TRUE
)
#
# check head of the file
head(samples2)
```

```
##              geo    range continent         region             country pop
## 1      Caribbean invasive Caribbean      Caribbean             Bermuda BRM
## 2      Caribbean invasive Caribbean      Caribbean             Jamaica JAM
## 3      Caribbean invasive Caribbean      Caribbean Trinidad and Tobago SAI
## 4 Central Africa invasive    Africa Central Africa               Gabon GAB
## 5    East Africa invasive    Africa    East Africa          Madagascar DGV
## 6    East Africa invasive    Africa    East Africa          Madagascar ANT
##          pop_city               state   Subregion
## 1         Bermuda             Bermuda   Caribbean
## 2          Negirl             Jamaica   Caribbean
## 3 Saint Augustine Trinidad and Tobago   Caribbean
## 4     Franceville                <NA>      Africa
## 5     Diego ville                <NA> East Africa
## 6    Antananarivo                <NA> East Africa
```

Check number of samples per population

```
pops_pca <- 
  pca |>
  group_by(X.FID) |>
  summarize(count_distinct = n_distinct(IID))

# check it
head(pops_pca)
```

```
## # A tibble: 6 × 2
##   X.FID count_distinct
##   <chr>          <int>
## 1 ALD               10
## 2 ARA               12
## 3 BAF                7
## 4 BCO               12
## 5 BEA               12
## 6 BEH                9
```

Merge the data

```
df4 <-
  merge(
    pca,
    samples2,
    by.x = "X.FID",
    by.y = "pop",
    all.x = T,
    all.y = F
  ) 
head(df4)
```

```
##   X.FID IID       PC1        PC2       PC3         PC4       PC5       PC6
## 1   ALD 802 0.0123187 -0.0241695 0.0228197  0.00366512 0.0633966 0.0596583
## 2   ALD 803 0.0102396 -0.0271658 0.0279268  0.00260196 0.0704725 0.0730473
## 3   ALD 804 0.0108586 -0.0271392 0.0302391 -0.00523062 0.0535795 0.0526822
## 4   ALD 806 0.0107240 -0.0262354 0.0259617 -0.00615988 0.0681342 0.0584090
## 5   ALD 807 0.0123584 -0.0269610 0.0254099 -0.00780090 0.0743480 0.0641394
## 6   ALD 808 0.0105688 -0.0229107 0.0184793 -0.00342716 0.0562444 0.0463262
##          PC7         PC8         PC9       PC10             geo    range
## 1 -0.0227893 -0.02497720  0.00273265 0.01659930 Southern Europe invasive
## 2 -0.0167810 -0.01197000 -0.00771944 0.02414200 Southern Europe invasive
## 3 -0.0260679 -0.02166750 -0.01458460 0.01995300 Southern Europe invasive
## 4 -0.0193865 -0.00938591 -0.01715120 0.01338780 Southern Europe invasive
## 5 -0.0219957 -0.01002570 -0.01525270 0.00985423 Southern Europe invasive
## 6 -0.0193876 -0.01314210 -0.00364543 0.01483740 Southern Europe invasive
##   continent          region country pop_city state   Subregion
## 1    Europe Southern Europe Albania   Durres  <NA> East Europe
## 2    Europe Southern Europe Albania   Durres  <NA> East Europe
## 3    Europe Southern Europe Albania   Durres  <NA> East Europe
## 4    Europe Southern Europe Albania   Durres  <NA> East Europe
## 5    Europe Southern Europe Albania   Durres  <NA> East Europe
## 6    Europe Southern Europe Albania   Durres  <NA> East Europe
```

Get some data for the PCA plot

```
# How many populations
length(unique(df4$X.FID)) #93
```

```
## [1] 93
```

```
# How many countries
length(unique(df4$country)) #29
```

```
## [1] 29
```

```
# How many regions
length(unique(df4$region)) #9
```

```
## [1] 9
```

```
# How many Continents
length(unique(df4$continent)) #5
```

```
## [1] 5
```

Check the countries

```
unique(df4$country)
```

```
##  [1] "Albania"             "Brazil"              "USA"                
##  [4] "India"               "Italy"               "Bermuda"            
##  [7] "Cambodia"            "Thailand"            "Colombia"           
## [10] "France"              "Bhutan"              "Georgia"            
## [13] "Greece"              "China"               "Vietnam"            
## [16] "Indonesia"           "Sri Lanka"           "Japan"              
## [19] "Nepal"               "Malaysia"            "Maldives"           
## [22] "Jamaica"             "Argentina"           "Russia"             
## [25] "Trinidad and Tobago" "Serbia"              "Spain"              
## [28] "Taiwan"              "Turkey"
```

Check the regions

```
unique(df4$region)
```

```
## [1] "Southern Europe" "South America"   "North America"   "South Asia"     
## [5] "Caribbean"       "Southeast Asia"  "Western Europe"  "Eastern Europe" 
## [9] "East Asia"
```

Make plot1

```
# source the plotting function
source(here("scripts", "RMarkdowns", "my_theme2.R"))

# Shapes
N = 100
M = 1000
good.shapes = c(1:25, 33:127)


# Compute the count for each country
country_count <- df4 |>
  group_by(country) |>
  summarize(count = n())

# Merge the count back to the main data
df4 <- df4 |>
  left_join(country_count, by = "country")

# Create a custom label for the legend
df4$country_label <-
  paste(df4$country, " (", df4$count, ")", sep = "")

# Define the color and shape manually
#palette23 <- c(palette1, palette2)
colors <- setNames(col_vector, unique(df4$country_label))
shapes <-
  setNames(good.shapes[c(1:25, 28:31, 55:57, 1:13)], unique(df4$country_label))


# Compute the center of ellipses for each continent
ellipse_centers <- df4 |>
  group_by(region) |>
  summarise(PC1_center = mean(PC1), PC2_center = mean(PC2))

# # Calculate the number of samples per region
continent_count <- df4 |>
  group_by(region) |>
  summarise(count = n())
continent_labels <-
  setNames(
    paste(continent_count$region, " (", continent_count$count, ")", sep = ""),
    continent_count$region
  )

continent_colors <-
  c("pink","#ffffaa","#bdb2ff","#9bf6ff","#baffc9","#ffffaa","#ffffaa","#bdb2ff","#bdb2ff")
  #Adjust these colors to your preference

#country_colors <-
#c("#bdb2ff","#baffc9","pink","#ffffaa","#baffc9","#ffffaa","#ffffaa","#baffc9","#bdb2ff","#bdb2ff","#bdb2ff","#ffffaa", "#ffffaa","#bdb2ff","pink","#ffffaa","#ffffaa","#ffffaa","#ffffaa","#bdb2ff","#bdb2ff","#bdb2ff","#ffffaa","#ffffaa","#ffffaa","pink","#ffffaa","#9bf6ff","#ffffaa")   #Adjust these colors to your preference

# Create the plot
ggplot(df4, aes(PC1, PC2)) +
  geom_point(aes(shape = country_label, color = country_label)) +
  stat_ellipse(
    aes(fill = region, group = region),
    geom = "polygon",
    alpha = 0.2,
    level = 0.8,
    segments = 40,
    color = "darkgray"
  ) +
  stat_ellipse(
    aes(group = region),
    geom = "path",
    level = 0.8,
    segments = 40,
    color = "darkgray"
  ) + # Line around the ellipse
  geom_text_repel(
    data = ellipse_centers,
    aes(x = PC1_center, y = PC2_center, label = region),
    color = "black"
  ) + # Add labels using ggrepel
  xlab("PC1 (75.57% Variance)") + 
  ylab("PC2 (27.09% Variance)") +
  labs(caption = "Principal Component Analysis with 18,079 SNPs \n of mosquitoes from 93 localities across 29 countries in Asia, the Americas, and Europe.") + 
guides(
    color = guide_legend(
      title = "Country",
      order = 2,
      ncol = 2
    ),
    shape = guide_legend(
      title = "Country",
      order = 2,
      ncol = 2
    ),
    fill = guide_legend(
      title = "Continent",
      order = 1,
      ncol = 1
    )
  ) +
  scale_fill_manual(values = continent_colors, labels = continent_labels) +
  scale_color_manual(values = colors) +
  scale_shape_manual(values = shapes) +
  guides(fill=guide_legend(ncol=2)) +
  my_theme() +
  theme(
    plot.caption = element_text(face = "italic"),
    legend.position = "right",
    legend.justification = "top",
    legend.box.just = "center",
    legend.box.background = element_blank(),
    legend.text=element_text(size=6),
    plot.margin = margin(5.5, 30, 5.5, 5.5, "points"),
    legend.margin = margin(5, 5, 5, 40) # move the legend a bit up
  )
```

```
#   save the pca plot                                                       ####
ggsave(
  here("scripts", "RMarkdowns",
    "output", "americas", "manuscript1", "figures", "PCA_manuscript1_r01_plink_PC1_2.pdf"
  ),
  width  = 10,
  height = 10,
  units  = "in"
)
```

Make plot 1 & 3

```
# source the plotting function
source(here("scripts", "RMarkdowns", "my_theme2.R"))

# Shapes
N = 100
M = 1000
good.shapes = c(1:25, 33:127)


# Compute the count for each country
country_count <- df4 |>
  group_by(country) |>
  summarize(count = n())

# Merge the count back to the main data
df4 <- df4 |>
  left_join(country_count, by = "country")

# Create a custom label for the legend
df4$country_label <-
  paste(df4$country, " (", df4$count, ")", sep = "")

# Define the color and shape manually
#palette23 <- c(palette1, palette2)
colors <- setNames(col_vector, unique(df4$country_label))
shapes <-
  setNames(good.shapes[c(1:25, 28:31, 55:57, 1:13)], unique(df4$country_label))


# Compute the center of ellipses for each continent
ellipse_centers <- df4 |>
  group_by(region) |>
  summarise(PC1_center = mean(PC1), PC3_center = mean(PC3))

# # Calculate the number of samples per region
continent_count <- df4 |>
  group_by(region) |>
  summarise(count = n())
continent_labels <-
  setNames(
    paste(continent_count$region, " (", continent_count$count, ")", sep = ""),
    continent_count$region
  )

continent_colors <-
  c("pink","#ffffaa","#bdb2ff","#9bf6ff","#baffc9","#ffffaa","#ffffaa","#bdb2ff","#bdb2ff")
  #Adjust these colors to your preference

#country_colors <-
#c("#bdb2ff","#baffc9","pink","#ffffaa","#baffc9","#ffffaa","#ffffaa","#baffc9","#bdb2ff","#bdb2ff","#bdb2ff","#ffffaa", "#ffffaa","#bdb2ff","pink","#ffffaa","#ffffaa","#ffffaa","#ffffaa","#bdb2ff","#bdb2ff","#bdb2ff","#ffffaa","#ffffaa","#ffffaa","pink","#ffffaa","#9bf6ff","#ffffaa")   #Adjust these colors to your preference

# Create the plot
ggplot(df4, aes(PC1, PC3)) +
  geom_point(aes(shape = country_label, color = country_label)) +
  stat_ellipse(
    aes(fill = region, group = region),
    geom = "polygon",
    alpha = 0.2,
    level = 0.8,
    segments = 40,
    color = "darkgray"
  ) +
  stat_ellipse(
    aes(group = region),
    geom = "path",
    level = 0.8,
    segments = 40,
    color = "darkgray"
  ) + # Line around the ellipse
  geom_text_repel(
    data = ellipse_centers,
    aes(x = PC1_center, y = PC3_center, label = region),
    color = "black"
  ) + # Add labels using ggrepel
  xlab("PC1 (75.57% Variance)") + 
  ylab("PC3 (16.22% Variance)") +
  labs(caption = "Principal Component Analysis with 18,079 SNPs \n of mosquitoes from 93 localities across 29 countries in Asia, the Americas, and Europe.") + 
guides(
    color = guide_legend(
      title = "Country",
      order = 2,
      ncol = 2
    ),
    shape = guide_legend(
      title = "Country",
      order = 2,
      ncol = 2
    ),
    fill = guide_legend(
      title = "Continent",
      order = 1,
      ncol = 1
    )
  ) +
  scale_fill_manual(values = continent_colors, labels = continent_labels) +
  scale_color_manual(values = colors) +
  scale_shape_manual(values = shapes) +
  guides(fill=guide_legend(ncol=2)) +
  my_theme() +
  theme(
    plot.caption = element_text(face = "italic"),
    legend.position = "right",
    legend.justification = "top",
    legend.box.just = "center",
    legend.box.background = element_blank(),
    legend.text=element_text(size=6),
    plot.margin = margin(5.5, 30, 5.5, 5.5, "points"),
    legend.margin = margin(5, 5, 5, 40) # move the legend a bit up
  )
```

```
#   save the pca plot                                                       ####
ggsave(
  here("scripts", "RMarkdowns",
    "output", "americas", "manuscript1", "figures", "PCA_manuscript1_r01_plink_PC1_3.pdf"
  ),
  width  = 10,
  height = 10,
  units  = "in"
)
```

Make plot 1 & 4

```
# source the plotting function
source(here("scripts", "RMarkdowns", "my_theme2.R"))

# Shapes
N = 100
M = 1000
good.shapes = c(1:25, 33:127)


# Compute the count for each country
country_count <- df4 |>
  group_by(country) |>
  summarize(count = n())

# Merge the count back to the main data
df4 <- df4 |>
  left_join(country_count, by = "country")

# Create a custom label for the legend
df4$country_label <-
  paste(df4$country, " (", df4$count, ")", sep = "")

# Define the color and shape manually
#palette23 <- c(palette1, palette2)
colors <- setNames(col_vector, unique(df4$country_label))
shapes <-
  setNames(good.shapes[c(1:25, 28:31, 55:57, 1:13)], unique(df4$country_label))


# Compute the center of ellipses for each continent
ellipse_centers <- df4 |>
  group_by(region) |>
  summarise(PC1_center = mean(PC1), PC4_center = mean(PC4))

# # Calculate the number of samples per region
continent_count <- df4 |>
  group_by(region) |>
  summarise(count = n())
continent_labels <-
  setNames(
    paste(continent_count$region, " (", continent_count$count, ")", sep = ""),
    continent_count$region
  )

continent_colors <-
  c("pink","#ffffaa","#bdb2ff","#9bf6ff","#baffc9","#ffffaa","#ffffaa","#bdb2ff","#bdb2ff")
  #Adjust these colors to your preference

#country_colors <-
#c("#bdb2ff","#baffc9","pink","#ffffaa","#baffc9","#ffffaa","#ffffaa","#baffc9","#bdb2ff","#bdb2ff","#bdb2ff","#ffffaa", "#ffffaa","#bdb2ff","pink","#ffffaa","#ffffaa","#ffffaa","#ffffaa","#bdb2ff","#bdb2ff","#bdb2ff","#ffffaa","#ffffaa","#ffffaa","pink","#ffffaa","#9bf6ff","#ffffaa")   #Adjust these colors to your preference

# Create the plot
ggplot(df4, aes(PC1, PC4)) +
  geom_point(aes(shape = country_label, color = country_label)) +
  stat_ellipse(
    aes(fill = region, group = region),
    geom = "polygon",
    alpha = 0.2,
    level = 0.8,
    segments = 40,
    color = "darkgray"
  ) +
  stat_ellipse(
    aes(group = region),
    geom = "path",
    level = 0.8,
    segments = 40,
    color = "darkgray"
  ) + # Line around the ellipse
  geom_text_repel(
    data = ellipse_centers,
    aes(x = PC1_center, y = PC4_center, label = region),
    color = "black"
  ) + # Add labels using ggrepel
  xlab("PC1 (75.57% Variance)") + 
  ylab("PC4 (9.39% Variance)") +
  labs(caption = "Principal Component Analysis with 18,079 SNPs \n of mosquitoes from 93 localities across 29 countries in Asia, the Americas, and Europe.") + 
guides(
    color = guide_legend(
      title = "Country",
      order = 2,
      ncol = 2
    ),
    shape = guide_legend(
      title = "Country",
      order = 2,
      ncol = 2
    ),
    fill = guide_legend(
      title = "Continent",
      order = 1,
      ncol = 1
    )
  ) +
  scale_fill_manual(values = continent_colors, labels = continent_labels) +
  scale_color_manual(values = colors) +
  scale_shape_manual(values = shapes) +
  guides(fill=guide_legend(ncol=2)) +
  my_theme() +
  theme(
    plot.caption = element_text(face = "italic"),
    legend.position = "right",
    legend.justification = "top",
    legend.box.just = "center",
    legend.box.background = element_blank(),
    legend.text=element_text(size=6),
    plot.margin = margin(5.5, 30, 5.5, 5.5, "points"),
    legend.margin = margin(5, 5, 5, 40) # move the legend a bit up
  )
```

```
#   save the pca plot                                                       ####
ggsave(
  here("scripts", "RMarkdowns",
    "output", "americas", "manuscript1", "figures", "PCA_manuscript1_r01_plink_PC1_4.pdf"
  ),
  width  = 10,
  height = 10,
  units  = "in"
)
```

# 9. Make a new snp set with Minor Allele Frequency of 1% (MAF 0.01)

## 9.1 Minor allele frequency

First, estimate the allele frequency with Plink.

```
cd /gpfs/gibbs/pi/caccone/mkc54/albo
plink2 \
--allow-extra-chr \
--bfile manuscript1/output/file3 \
--freq \
--out manuscript1/output/MAF_check \
--silent
```

Now apply the MAF filter for 0.01

```
# We will use MAF of 1%
plink2 \
--allow-extra-chr \
--bfile manuscript1/output/file3 \
--maf 0.01 \
--make-bed \
--out manuscript1/output/file4b \
--silent;
grep "variants" manuscript1/output/file4b.log
```

99689 variants loaded from manuscript1/output/file3.bim. 3421
variants removed due to allele frequency threshold(s) 96268 variants
remaining after main filters.

Plot it with ggplot.

```
#   Import MAF data                                                         ####
maf_freq <-
  read.delim(
    here(
      "manuscript1/output/MAF_check.afreq"
    ),
    header = TRUE
  )
```

Make MAF plot

```
#   make the plot                                                           ####
ggplot(
  maf_freq,
  aes(ALT_FREQS)
) +
  geom_histogram(
    colour = "black",
    fill = "lightgray",
    bins = 40
  ) +
  labs(
    x = "Minor Allele Frequency (MAF)",
    y = "Frequency (n)",
    caption = "<span style='color:red;'><i>Red</i></span> <span style='color:black;'><i>line at</i></span><span style='color:red;'><i> MAF 1%</i></span><span style='color:black;'><i> threshold</i></span>."
  ) +
  geom_text(
    aes(
      x = .01,
      y = 8000,
      label = paste0("3,421 SNPs")
    ),
    size = 3,
    color = "red",
    vjust = -.2
  ) +
  geom_vline(xintercept = 0.01, color = "red") +
  my_theme() +
  theme(plot.caption = element_markdown()) +
  scale_y_continuous(label = scales::number_format(big.mark = ",")) +
  scale_x_continuous(breaks = c(0, 0.1, 0.2, 0.4, 0.6, 0.8, 1.0))
```

```
## Warning in geom_text(aes(x = 0.01, y = 8000, label = paste0("3,421 SNPs")), : All aesthetics have length 1, but the data has 99689 rows.
## ℹ Please consider using `annotate()` or provide this layer with data containing
##   a single row.
```

```
# save the plot
ggsave(
  here("scripts", "RMarkdowns",
    "output", "americas", "manuscript1", "figures", "MAF_0.01_freq_plot_manuscript1.pdf"
  ),
  width  = 7,
  height = 5,
  units  = "in"
)
```

We removed 3,421 variants due to the MAF filter.

## 9.2 HWE test

Now we can run the HWE test. However, we will need to apply the SNP
missingness again for each population. If we do not, the HWE will vary
widely. With the bash script below, we will create a new file for each
population, run the HWE test with HWE p value <1e‐6 (HWE p value
<1e‐6). Then, we ask Plink to generate a list of SNPs that passed the
test for each population.

```
mkdir -p manuscript1/output/files/hardyb;
cp manuscript1/output/file4b.* manuscript1/output/files/hardyb/
```

```
for fam in $(awk '{print $1}' manuscript1/output/files/hardyb/file4b.fam | sort | uniq); 
do 
echo $fam | \
plink2 \
--allow-extra-chr \
--silent \
--keep-allele-order \
--bfile manuscript1/output/files/hardyb/file4b \
--keep-fam /dev/stdin \
--make-bed \
--out manuscript1/output/files/hardyb/$fam \
--hwe 0.000001 \
--geno 0.1 \
--write-snplist; \
done
```

Next, use “cat” and “awk” to concatenate the SNP list from all
populations, and remove duplicates. Once we have a list of SNPs that
passed the test for each population, we can use Plink to create a new
bed file keeping only the SNPs that passed the test in each population.
First, lets get the list of SNPs, and count how many passed:

```
cd /gpfs/gibbs/pi/caccone/mkc54/albo
cat manuscript1/output/files/hardyb/*.snplist | awk '!a[$0]++' > manuscript1/output/files/passed_hwe_b.txt;
wc -l manuscript1/output/files/passed_hwe_b.txt
```

All 96,268 variants passed HWE test. If any failed, we could remove
the variants that did not pass HWE test, using the –extract flag,
extracting only those that passed HWE.

## 9.3 LD pruning

Since we do not have to remove any SNPs due to deviation from HWE, we
can proceed with heterozygosity estimates. The first step is to “prune”
our data set. We will check the pairwise linkage estimates for all SNPs.
We can work with file4. We will use “`indep-pairwise`” to
check if there are SNPs above a certain linkage disequilibrium (LD)
threshold. Check Plink documentation for more details https://www.cog-genomics.org/plink/1.9/ld I used
“`--indep-pairwise 5 1 0.1`” , which indicates according to
the documentation:
`--indep-pairphase <window size>['kb'] <step size (variant ct)> <r^2 threshold>`
We will check in a window of 5kb if there is any pair of SNPs with r2
estimates above 0.1, then we will move our window 1 SNP and check again
for SNPs above the threshold. We will repeat this procedure until we
check the entire genome.

Need to switch back to plink2

```
module load PLINK/2_avx2_20221024
#PLINK/1.9b_6.21-x86_64 => PLINK/2_avx2_20221024
plink2 --version
```

```
plink2 \
--allow-extra-chr \
--bfile manuscript1/output/file4b \
--extract manuscript1/output/files/passed_hwe_b.txt \
--indep-pairwise 5 1 0.1 \
--out manuscript1/output/indepSNPb \
--silent;
grep 'pairwise\|variants\|samples' manuscript1/output/indepSNPb.log
```

–indep-pairwise 5 1 0.1 959 samples (62 females, 101 males, 796
ambiguous; 959 founders) loaded from 96268 variants loaded from
manuscript1/output/file4b.bim. –extract: 96268 variants remaining. 96268
variants remaining after main filters. –indep-pairwise (27 compute
threads): 34628/96268 variants removed.

Remember, the SNPs are not removed from our data set. Plink created 3
files when we ran the code above. One is the “indepSNP.log” file, and
the other two are: “indepSNP.prune.in” -> list of SNPs with squared
correlation smaller than our r2 threshold of 0.1. “indepSNP.prune.out”
-> list of SNPs with squared correlation greater than our r2
threshold of 0.1. For our heterozygosity estimates, we want to use the
set of SNPs that are below our r2 threshold of 0.1. We consider that
they are randomly associated. We can use Plink to estimate the
heterozygosity using the “indepSNP.prune.in” file.

## 9.4 Heterozygosity

```
plink2 \
--allow-extra-chr \
--bfile manuscript1/output/file4b \
--extract manuscript1/output/indepSNPb.prune.in \
--het \
--out manuscript1/output/R_checkb \
--silent;
grep 'variants' manuscript1/output/R_checkb.log
```

96268 variants loaded from manuscript1/output/file4b.bim. –extract:
61640 variants remaining. 61640 variants remaining after main
filters.

```
#   find individuals with high heterozygosity                              ####
# import the data from Plink
het <- read.delim(
  here(
    "manuscript1/output/R_checkb.het"
  ),
  head = TRUE
)
#
# check head of the file
colnames(het)
```

```
## [1] "X.FID"  "IID"    "O.HOM." "E.HOM." "OBS_CT" "F"
```

Estimate heterozygosity

```
# create a column named HET_RATE and calculate the heterozygosity rate
het$HET_RATE <- (het$"OBS_CT" - het$"O.HOM") / het$"OBS_CT"
#
# use subset function to get values deviating from 4sd of the mean heterozygosity rate.
het_fail <-
  subset(
    het, (het$HET_RATE < mean(
      het$HET_RATE
    ) - 4 * sd(
      het$HET_RATE
    )) |
      (het$HET_RATE > mean(
        het$HET_RATE
      ) + 4 * sd(
        het$HET_RATE
      ))
  )
#
# get the list of individuals that failed our threshold of 4sd from the mean.
het_fail$HET_DST <-
  (het_fail$HET_RATE - mean(
    het$HET_RATE
  )) / sd(
    het$HET_RATE
  )
```

Save the files to use with Plink

```
#   save the data to use with Plink2                                        ####
#
write.table(
  het_fail,
  here(
    "manuscript1/output/fail-het-qc_b.txt"
  ),
  row.names = FALSE
)
```

Make plot

```
#   make a heterozygosity plot                                              ####
#
ggplot(
  het,
  aes(
    HET_RATE
  )
) +
  geom_histogram(
    colour           = "black",
    fill             = "lightgray",
    bins             = 40
  ) +
  labs(
    x                = "Heterozygosity Rate",
    y                = "Number of Individuals"
  ) +
  geom_vline(
    aes(
      xintercept     = mean(
        HET_RATE
      )
    ),
    col              = "red",
    linewidth        = 1.5
  ) +
  geom_vline(
    aes(
      xintercept     = mean(
        HET_RATE
      ) + 4 * sd(
        HET_RATE
      )
    ),
    col              = "#BFB9B9",
    linewidth        = 1
  ) +
  geom_vline(
    aes(
      xintercept     = mean(
        HET_RATE
      ) - 4 * sd(
        HET_RATE
      )
    ),
    col              = "#BFB9B9",
    linewidth        = 1
  ) + 
  my_theme() +
  scale_y_continuous(
  )
```

```
#   save the heterozygosity plot                                            ####
ggsave(
  here("scripts", "RMarkdowns",
    "output", "americas", "manuscript1", "figures", "Heterozygosity_man1_MAF_0.01.pdf"
  ),
  width  = 5,
  height = 4,
  units  = "in"
)
```

The red line in the plot above indicates the mean, and the gray lines
indicate 4 standard deviation from the mean. We can see that some
mosquitoes do have excess heterozygous sites. We will remove them. We
can get their ID from the file “fail-het-qc.txt”. We can use the bash
script below to parse the file to use with Plink

```
sed 's/"// g' manuscript1/output/fail-het-qc_b.txt | awk '{print$1, $2}'> manuscript1/output/het_fail_ind_b.txt;
echo 'How many mosquitoes we need to remove from our data set:';
cat manuscript1/output/het_fail_ind_b.txt | tail -n +2 | wc -l;
echo 'Which mosquitoes we have to remove:';
tail -n +2 manuscript1/output/het_fail_ind_b.txt
```

```
## How many mosquitoes we need to remove from our data set:
## 8
## Which mosquitoes we have to remove:
## QNC 1248
## COL 471
## KAT 601
## KAT 605
## KAT 606
## KAT 608
## KUN 682
## GRA 734
```

Heterozygosity is outside the SD threshold in 4 KAT individuals (601,
605, 606, 608), 1 QNC (1248), 1 GRA (734), 1 KUN (682), and 1 COL (471).
We will remove these 8 individuals.

Remove these mosquitoes from our data set using Plink:

```
plink2 \
--allow-extra-chr \
--bfile manuscript1/output/file4b \
--remove manuscript1/output/het_fail_ind_b.txt \
--make-bed \
--out manuscript1/output/file5b \
--silent;
grep 'variants\|samples' manuscript1/output/file5b.log
```

959 samples (62 females, 101 males, 796 ambiguous; 959 founders)
loaded from 96268 variants loaded from manuscript1/output/file4b.bim.
–remove: 951 samples remaining. 951 samples (61 females, 96 males, 794
ambiguous; 951 founders) remaining after

## 9.5 Relatedness

Check for cryptic relatedness. Check Plink2 documentation https://www.cog-genomics.org/plink/2.0/distance You can
download King directly https://www.kingrelatedness.com/manual.shtml or check
their manuscript https://www.ncbi.nlm.populations.gov/pmc/articles/PMC3025716/pdf/btq559.pdf
From Plink2 documentation: “Note that KING kinship coefficients are
scaled such that duplicate samples have kinship 0.5, not 1. First-degree
relations (parent-child, full siblings) correspond to ~0.25,
second-degree relations correspond to ~0.125, etc. It is conventional to
use a cutoff of ~0.354 (the geometric mean of 0.5 and 0.25) to screen
for monozygotic twins and duplicate samples, ~0.177 to add first-degree
relations, etc.” There two options. One is to run only –make-king and
another one is to use –make-king-table We will use the threshold of
0.354 and create a table.

```
# Plink2 will create a file with extension .king
plink2 \
--allow-extra-chr \
--bfile manuscript1/output/file5b \
--extract manuscript1/output/indepSNPb.prune.in \
--make-king-table rel-check \
--king-table-filter 0.354 \
--out manuscript1/output/file5b \
--silent;
grep 'variants\|samples' manuscript1/output/file5b.log
```

951 samples (61 females, 96 males, 794 ambiguous; 951 founders)
loaded from 96268 variants loaded from manuscript1/output/file5b.bim.
–extract: 61640 variants remaining. 61640 variants remaining after main
filters.

Check the individuals that did not pass our filtering.

```
head -n 20 manuscript1/output/file5b.kin0
```

```
## #FID1    IID1    FID2    IID2    NSNP    HETHET  IBS0    KINSHIP
## BEN  259 BEN 258 58962   0.227808    0   0.493593
## BEN  260 BEN 258 59431   0.231007    0   0.496883
## BEN  260 BEN 259 59022   0.228644    0   0.493684
## BPA  2460    BPA 2452    57160   0.193055    0.0103044   0.362755
## CHI  2274    CHI 2269    59122   0.202497    0.0120936   0.357325
## DES  1445    DES 1292    59635   0.237612    0.00353819  0.452319
## HAT  2395    HAT 2393    59109   0.242653    0.00407721  0.435765
## IPO  2590    IPO 2585    58971   0.187397    0.006783    0.396773
## KAG  228 KAG 227 60171   0.250486    0   0.498742
## KAG  229 KAG 227 60071   0.250287    0   0.498607
## KAG  229 KAG 228 60094   0.250458    0   0.498327
## KAN  1327    KAN 1325    59838   0.215749    0.00715265  0.399697
## LOS  366 LOS 365 59647   0.183781    0   0.498567
## LOS  367 LOS 365 59571   0.183579    0   0.497791
## LOS  367 LOS 366 59543   0.183061    0   0.497078
## NEB  2549    NEB 2546    57485   0.247525    0.00257458  0.402187
## NEW  423 NEW 413 59879   0.215869    0.0101204   0.366618
## NEW  423 NEW 420 59880   0.21655 0.0127088   0.355892
## OKI  1010    OKI 1006    58881   0.206094    0.0118035   0.357799
```

We want to remove one of the individuals of the pairs.

```
plink2 \
--allow-extra-chr \
--bfile manuscript1/output/file5b \
--extract manuscript1/output/indepSNPb.prune.in \
--make-king triangle bin \
--out manuscript1/output/file6b \
--silent;
grep 'variants\|samples' manuscript1/output/file6b.log
```

951 samples (61 females, 96 males, 794 ambiguous; 951 founders)
loaded from 96268 variants loaded from manuscript1/output/file5b.bim.
–extract: 61640 variants remaining. 61640 variants remaining after main
filters. –make-king pass 1/1: Scanning for rare variants… done. 175
variants handled by initial scan (61465 remaining). –make-king: 61640
variants processed.

Use Plink2 to remove one of the mosquitoes from the pair with high
kinship. It will remove samples since we had 2 samples in some
populations with high relatedness, and we could remove 1 and keep the
other two. Plink2 always tries to maximize the number of samples passing
the filters.

```
plink2 \
--allow-extra-chr \
--bfile manuscript1/output/file5b \
--king-cutoff manuscript1/output/file6b 0.354 \
--make-bed \
--out manuscript1/output/file7b \
--silent;
grep 'samples\|variants\|remaining' manuscript1/output/file7b.log
```

951 samples (61 females, 96 males, 794 ambiguous; 951 founders)
loaded from 96268 variants loaded from manuscript1/output/file5b.bim.
manuscript1/output/file7b.king.cutoff.out.id , and 933 remaining sample
IDs

## 9.6 Create chromosomal scale for MAF 0.01 files

Import the .bim file with the SNPs to create a new chromosomal
scale.

```
#   ____________________________________________________________________________
#   import the bim file with the SNP data                                   ####
snps <-
  read_delim(                    # to learn about the options use here, run ?read_delim on the console.
    here(
      "manuscript1/output/file7b.bim"
    ),                           # use library here to load it
    col_names      = FALSE,      # we don't have header in the input file
    show_col_types = FALSE,      # suppress message from read_delim
    col_types      = "ccidcc"    # set the class of each column
  )
#
# set column names
colnames(
  snps
) <-                             # to add a header in our tibble
  c(
    "Scaffold", "SNP", "Cm", "Position", "Allele1", "Allele2"
  )
#
# check the tibble
head(snps)
```

```
## # A tibble: 6 × 6
##   Scaffold SNP             Cm Position Allele1 Allele2
##   <chr>    <chr>        <int>    <dbl> <chr>   <chr>  
## 1 1        AX-583033342     0   315059 C       G      
## 2 1        AX-583035163     0   315386 A       G      
## 3 1        AX-583033356     0   315674 C       T      
## 4 1        AX-583033370     0   330057 G       A      
## 5 1        AX-583035194     0   330265 A       G      
## 6 1        AX-583033387     0   331288 C       T
```

Separate the tibbles into each chromosome

```
#   separate the SNP data per chromosome                                    
# chr1
chr1_snps <-
  snps |>
  filter(
    str_detect(
      Scaffold, "^1."
    )
  ) |> # here we get only Scaffold rows starting with 1
  as_tibble() # save as tibble
#
# chr2
chr2_snps <-
  snps |>
  filter(
    str_detect(
      Scaffold, "^2."
    )
  ) |>
  as_tibble()
#
# chr3
chr3_snps <-
  snps |>
  filter(
    str_detect(
      Scaffold, "^3."
    )
  ) |>
  as_tibble()
```

Import the file with sizes of each scaffold.

```
#   import the file with the scaffold sizes                                 ####
sizes <-
  read_delim(
    here(
      "/gpfs/gibbs/project/caccone/mkc54/albo/genome/scaffold_sizes.txt"
    ),
    col_names      = FALSE,
    show_col_types = FALSE,
    col_types      = "cd"
  )
#
# set column names
colnames(
  sizes
) <- c(
  "Scaffold", "Size"
)
#   ____________________________________________________________________________
#   create new column with the chromosome number                            ####
sizes <- 
  sizes |>
  mutate(
    Chromosome = case_when( # we use mutate to create a new column called Chromosome
      startsWith(
        Scaffold, "1"
      ) ~ "1", # use startsWith to get Scaffold rows starting with 1 and output 1 on Chromosome column
      startsWith(
        Scaffold, "2"
      ) ~ "2",
      startsWith(
        Scaffold, "3"
      ) ~ "3"
    )
  ) |>
  arrange(
    Scaffold
  )                   # to sort the order of the scaffolds, fixing the problem we have with scaffold 1.86
# check it
head(sizes)
```

```
## # A tibble: 6 × 3
##   Scaffold     Size Chromosome
##   <chr>       <dbl> <chr>     
## 1 1.1        351198 1         
## 2 1.10     11939576 1         
## 3 1.100     3389100 1         
## 4 1.101      470438 1         
## 5 1.102     2525157 1         
## 6 1.103      150026 1
```

Create new scale. Get the scaffolds for each chromosome.

```
#   separate the scaffold sizes tibble per chromosome                       ####
# chr1
chr1_scaffolds <- 
  sizes |>
  filter(
    str_detect(
      Scaffold, "^1" # we use library stringr to get scaffolds starting with 1 (chromosome 1)
    )
  ) |> 
  as_tibble()
#
# chr2
chr2_scaffolds <-
  sizes |>
  filter(
    str_detect(
      Scaffold, "^2" # we use library stringr to get scaffolds starting with 2 (chromosome 2)
    )
  ) |> 
  as_tibble()
#
# # chr3
chr3_scaffolds <-
  sizes |>
  filter(
    str_detect(
      Scaffold, "^3" # we use library stringr to get scaffolds starting with 3 (chromosome 3)
    )
  ) |>
  as_tibble()
```

Create a scale for each chromosome.

```
#   create a new scale for each chromosome                                  ####
# chr1
chr1_scaffolds$overall_size_before_bp <-
  0                                                                        # we create a new column with zeros
for (i in 2:nrow(
  chr1_scaffolds
)
) {                                                                        # loop to start on second line
  chr1_scaffolds$overall_size_before_bp[i] <-                              # set position on the scale
    chr1_scaffolds$overall_size_before_bp[i - 1] + chr1_scaffolds$Size[i - # add the scaffold size and the location to get position on new scale
      1]
}
#
# chr2
chr2_scaffolds$overall_size_before_bp <- 0
for (i in 2:nrow(
  chr2_scaffolds
)
) {
  chr2_scaffolds$overall_size_before_bp[i] <-
    chr2_scaffolds$overall_size_before_bp[i - 1] + chr2_scaffolds$Size[i -
      1]
}
#
# chr3
chr3_scaffolds$overall_size_before_bp <- 0
for (i in 2:nrow(
  chr3_scaffolds
)
) {
  chr3_scaffolds$overall_size_before_bp[i] <-
    chr3_scaffolds$overall_size_before_bp[i - 1] + chr3_scaffolds$Size[i -
      1]
}
```

Merge the data frames scaffolds and SNPs.

```
#   merge the data sets using the tidyverse function left_join              ####
# chr1
chr1_scale <-
  chr1_snps |>          # create data frame for each chromosome, get chr1_snps
  left_join(            # use lef_join function to merge it with chr1_scaffolds
    chr1_scaffolds,
    by = "Scaffold"
  ) |>                  # set column to use for merging (Scaffold in this case)
  na.omit() |>          # remove NAs, we don't have SNPs in every scaffold
  mutate(
    midPos_fullseq = as.numeric(
      Position
    ) +                 # make new columns numeric
      as.numeric(
        overall_size_before_bp
      )
  )
#
# chr2
chr2_scale <-
  chr2_snps |>
  left_join(
    chr2_scaffolds,
    by = "Scaffold"
  ) |>
  na.omit() |>
  mutate(
    midPos_fullseq = as.numeric(
      Position
    ) +
      as.numeric(
        overall_size_before_bp
      )
  )
#
# chr3
chr3_scale <-
  chr3_snps |>
  left_join(
    chr3_scaffolds,
    by = "Scaffold"
  ) |>
  na.omit() |>
  mutate(
    midPos_fullseq = as.numeric(
      Position
    ) +
      as.numeric(
        overall_size_before_bp
      )
  )
```

Merge all chromosome scales.

```
#   merge the data sets, and select only the columns we need                ####
chroms <- rbind(
  chr1_scale, chr2_scale, chr3_scale
) |>
  dplyr::select(
    Chromosome, SNP, Cm, midPos_fullseq, Allele1, Allele2
  )
# check it
head(chroms)
```

```
## # A tibble: 0 × 6
## # ℹ 6 variables: Chromosome <chr>, SNP <chr>, Cm <int>, midPos_fullseq <dbl>,
## #   Allele1 <chr>, Allele2 <chr>
```

Save the new .bim file

```
#   ____________________________________________________________________________
#   save the new bim file with a new name, I added "B"                      ####
write.table(
  chroms,
  file      = here(
    "manuscript1/output/file7C.bim"
  ),
  sep       = "\t",
  row.names = FALSE,
  col.names = FALSE,
  quote     = FALSE
)
```

Rename the .bim files

```
# change the name of the first .bim file, for example, append _backup.bim, and then replace the original file
mv manuscript1/output/file7b.bim manuscript1/output/file7b_backup.bim;
# than change the new bim we create to the original name (do it only once, otherwise it will mess up)
mv manuscript1/output/file7C.bim manuscript1/output/file7b.bim
```

Create a new bed file with Plink2 to see if it works. For example, to
see if the variants are in the right order. Plink2 will give us a
warning.

```
cd /gpfs/gibbs/pi/caccone/mkc54/albo

plink2 \
--bfile manuscript1/output/file7b \
--make-bed \
--out manuscript1/output/test01;
# then we remove the files 
rm manuscript1/output/test01.*
```

PLINK v2.00a3.7LM AVX2 Intel (24 Oct 2022)
www.cog-genomics.org/plink/2.0/ (C) 2005-2022 Shaun Purcell, Christopher
Chang GNU General Public License v3 Logging to
manuscript1/output/test01.log. Options in effect: –bfile
manuscript1/output/file7b –make-bed –out manuscript1/output/test01 Start
time: Wed Aug 21 09:53:01 2024 257321 MiB RAM detected; reserving 128660
MiB for main workspace. Using up to 32 threads (change this with
–threads). 933 samples (61 females, 93 males, 779 ambiguous; 933
founders) loaded from manuscript1/output/file7b.fam. 96268 variants
loaded from manuscript1/output/file7b.bim. Note: No phenotype data
present. Writing manuscript1/output/test01.fam … done. Writing
manuscript1/output/test01.bim … done. Writing
manuscript1/output/test01.bed … done. End time: Wed Aug 21 09:53:01
2024

### 9.6.1 Estimate LD blocks using chromosomal scale

```
mkdir -p manuscript1/output/files/ld/blocks_chr_b
```

```
module load PLINK/1.9b_6.21-x86_64
```

Use Plink1.9 to estimate LD blocks for the populations with more than
12 individuals. We will use the entire genome for this part instead of
the larger scaffolds only. We will set max distance of LD blocks of
500kb. We found out that the average half distance of r^2 max is small,
from 1 to 5kb

```
for fam in $(awk '{print $1}' manuscript1/output/files/ld/pops_4ld.txt | sort | uniq); 
do 
echo $fam | \
plink \
--allow-extra-chr \
--keep-allele-order \
--bfile manuscript1/output/file7b \
--keep-fam /dev/stdin \
--maf 0.01 \
--blocks no-pheno-req \
--blocks-max-kb 200 \
--out manuscript1/output/files/ld/blocks_chr_b/$fam \
--geno 0.1 \
--silent
done;
#
rm manuscript1/output/files/ld/blocks_chr_b/*.nosex
```

Get some data from our .log files

```
echo "Population,n,nVariants,geno,maf,passQC" > manuscript1/output/files/ld/blocks_chr_b/table_ld_statsb.csv
for file in manuscript1/output/files/ld/blocks_chr_b/*.log
do
  variants=$(grep -oE '([0-9]+) variants loaded from \.bim file' $file | grep -oE '[0-9]+')
  geno=$(grep -oE '([0-9]+) variants removed due to missing genotype data \(--geno\)' $file | grep -oE '[0-9]+')
  maf=$(grep -oE '([0-9]+) variants removed due to minor allele threshold\(s\)' $file | grep -oE '[0-9]+')
  pass=$(grep -oE '([0-9]+) variants and [0-9]+ people pass filters and QC\.' $file | grep -oE '[0-9]+' | head -1)
  n=$(grep -oE '([0-9]+) variants and [0-9]+ people pass filters and QC\.' $file | grep -oE '[0-9]+' | tail -1)
  filename=$(basename $file .log)
  echo "$filename,$n,$variants,$geno,$maf,$pass" >> manuscript1/output/files/ld/blocks_chr_b/table_ld_statsb.csv
done;
head -n 5 manuscript1/output/files/ld/blocks_chr_b/table_ld_statsb.csv
```

```
## Population,n,nVariants,geno,maf,passQC
## ARA,12,96268,6982,17263,72023
## BCO,12,96268,10481,18826,66961
## BEA,12,96268,3844,8087,84337
## BEN,12,96268,6640,19170,70458
```

Check it

```
# Load data from the table_ld_stats.csv file
ld_blocks <- read.csv(
  here(
    "manuscript1/output/files/ld/blocks_chr_b/table_ld_statsb.csv"
    ),
  header = TRUE,
  sep = ","
)

# Create the flextable
ft <- flextable(ld_blocks)

# Apply zebra theme
ft <- theme_zebra(ft)

# Add a caption to the table
ft <- flextable::add_header_lines(ft, "Table 1: Summary of quality control for manuscript1 populations (MAF 1%).")

ft
```

| Table 1: Summary of quality control for manuscript1 populations (MAF 1%). | | | | | |
| --- | --- | --- | --- | --- | --- |
| Population | n | nVariants | geno | maf | passQC |
| ARA | 12 | 96,268 | 6,982 | 17,263 | 72,023 |
| BCO | 12 | 96,268 | 10,481 | 18,826 | 66,961 |
| BEA | 12 | 96,268 | 3,844 | 8,087 | 84,337 |
| BEN | 12 | 96,268 | 6,640 | 19,170 | 70,458 |
| BER | 12 | 96,268 | 4,608 | 11,708 | 79,952 |
| BIT | 12 | 96,268 | 7,708 | 18,445 | 70,115 |
| BPA | 11 | 96,268 | 8,576 | 18,779 | 68,913 |
| BPV | 12 | 96,268 | 11,666 | 17,884 | 66,718 |
| BRE | 13 | 96,268 | 9,999 | 19,824 | 66,445 |
| BRM | 16 | 96,268 | 7,570 | 16,277 | 72,421 |
| CAM | 12 | 96,268 | 6,081 | 13,376 | 76,811 |
| CES | 15 | 96,268 | 17,721 | 20,549 | 57,998 |
| CHA | 12 | 96,268 | 5,095 | 16,628 | 74,545 |
| DAL | 12 | 96,268 | 3,949 | 10,055 | 82,264 |
| DEN | 12 | 96,268 | 7,456 | 10,465 | 78,347 |
| DES | 16 | 96,268 | 17,748 | 14,122 | 64,398 |
| FAY | 12 | 96,268 | 15,872 | 8,589 | 71,807 |
| FIR | 12 | 96,268 | 5,123 | 16,735 | 74,410 |
| FRS | 12 | 96,268 | 4,508 | 11,621 | 80,139 |
| GES | 12 | 96,268 | 5,029 | 21,734 | 69,505 |
| GRA | 11 | 96,268 | 4,982 | 11,370 | 79,916 |
| GRV | 12 | 96,268 | 5,561 | 19,521 | 71,186 |
| HAI | 12 | 96,268 | 4,884 | 10,671 | 80,713 |
| HAR | 12 | 96,268 | 4,938 | 12,565 | 78,765 |
| HOU | 12 | 96,268 | 4,208 | 8,039 | 84,021 |
| HUN | 12 | 96,268 | 4,650 | 9,626 | 81,992 |
| KAG | 12 | 96,268 | 5,116 | 16,028 | 75,124 |
| KAN | 11 | 96,268 | 4,842 | 22,845 | 68,581 |
| LIN | 12 | 96,268 | 5,436 | 14,360 | 76,472 |
| LOS | 12 | 96,268 | 5,890 | 16,184 | 74,194 |
| MAC | 12 | 96,268 | 6,867 | 9,509 | 79,892 |
| MAN | 12 | 96,268 | 6,737 | 10,597 | 78,934 |
| MAT | 12 | 96,268 | 5,499 | 18,213 | 72,556 |
| MAU | 12 | 96,268 | 10,172 | 15,166 | 70,930 |
| MON | 12 | 96,268 | 7,299 | 8,388 | 80,581 |
| NEO | 12 | 96,268 | 4,327 | 11,148 | 80,793 |
| NEW | 21 | 96,268 | 4,160 | 9,224 | 82,884 |
| NUE | 12 | 96,268 | 4,866 | 9,285 | 82,117 |
| OKI | 11 | 96,268 | 5,792 | 19,032 | 71,444 |
| POR | 12 | 96,268 | 7,437 | 21,137 | 67,694 |
| QNC | 11 | 96,268 | 6,061 | 33,435 | 56,772 |
| RAR | 12 | 96,268 | 5,060 | 21,835 | 69,373 |
| REC | 11 | 96,268 | 6,266 | 16,524 | 73,478 |
| RUS | 12 | 96,268 | 5,563 | 18,455 | 72,250 |
| SAI | 12 | 96,268 | 5,176 | 11,292 | 79,800 |
| SMI | 12 | 96,268 | 5,537 | 10,213 | 80,518 |
| SOC | 12 | 96,268 | 5,030 | 19,244 | 71,994 |
| SSK | 12 | 96,268 | 5,350 | 16,907 | 74,011 |
| STL | 12 | 96,268 | 5,094 | 13,312 | 77,862 |
| TIK | 12 | 96,268 | 5,039 | 22,775 | 68,454 |
| TRA | 12 | 96,268 | 5,492 | 8,897 | 81,879 |
| TUC | 14 | 96,268 | 8,095 | 16,358 | 71,815 |
| TUH | 12 | 96,268 | 5,527 | 12,945 | 77,796 |
| UTS | 12 | 96,268 | 4,292 | 15,811 | 76,165 |

```
# Save it to a Word document
officer::read_docx() |>
  body_add_flextable(ft) |>
  print(target = here::here("manuscript1/output/summary_blocks_chr_b.docx"))
```

Count how many blocks we found in each population

```
cd manuscript1/output/files/ld/blocks_chr_b
wc -l *.blocks | \
  awk '{population = gensub(/\.blocks_chr/, "", "g", $2); print population "\t" $1}' | \
  sed 's#manuscript1/output/files/ld/blocks_chr_b/##' | \
  sed 's/.blocks//' | \
  sed '$d' > populations_block_counts_b.csv;
head -n 79 populations_block_counts_b.csv
```

```
## ARA  75
## BCO  106
## BEA  68
## BEN  9
## BER  104
## BIT  124
## BPA  80
## BPV  75
## BRE  279
## BRM  517
## CAM  12
## CES  422
## CHA  9
## DAL  94
## DEN  90
## DES  385
## FAY  61
## FIR  167
## FRS  53
## GES  162
## GRA  30
## GRV  164
## HAI  51
## HAR  120
## HOU  63
## HUN  43
## KAG  109
## KAN  207
## LIN  146
## LOS  117
## MAC  93
## MAN  66
## MAT  15
## MAU  95
## MON  61
## NEO  86
## NEW  699
## NUE  71
## OKI  96
## POR  92
## QNC  21
## RAR  148
## REC  90
## RUS  187
## SAI  73
## SMI  90
## SOC  117
## SSK  6
## STL  148
## TIK  184
## TRA  81
## TUC  110
## TUH  106
## UTS  141
```

Add the number of blocks to the table we made

```
# Load data from the table_ld_stats.csv file
ld_blocks <- read.csv(
  here(
    "manuscript1/output/files/ld/blocks_chr_b/table_ld_statsb.csv"
    ),
  header = TRUE,
  sep = ","
)

# Load the population counts data from the CSV file
pop_counts <-
  read.delim(
    here(
      "manuscript1/output/files/ld/blocks_chr_b/populations_block_counts_b.csv"
    ),
    header = F,
    sep = "\t"
  ) |>
  rename(
    Population       = 1,
    nBlocks          = 2
  )

# Merge the population counts with the table data
ld_blocks <- merge(ld_blocks, pop_counts, by = "Population")

# Create the flextable
ft <- flextable(ld_blocks)

# Apply zebra theme
ft <- theme_zebra(ft)

# Add a caption to the table
ft <- flextable::add_header_lines(ft, "Table 2: Number of linkage blocks detected with Plink for populations with at least 10 individuals.")

ft
```

| Table 2: Number of linkage blocks detected with Plink for populations with at least 10 individuals. | | | | | | |
| --- | --- | --- | --- | --- | --- | --- |
| Population | n | nVariants | geno | maf | passQC | nBlocks |
| ARA | 12 | 96,268 | 6,982 | 17,263 | 72,023 | 75 |
| BCO | 12 | 96,268 | 10,481 | 18,826 | 66,961 | 106 |
| BEA | 12 | 96,268 | 3,844 | 8,087 | 84,337 | 68 |
| BEN | 12 | 96,268 | 6,640 | 19,170 | 70,458 | 9 |
| BER | 12 | 96,268 | 4,608 | 11,708 | 79,952 | 104 |
| BIT | 12 | 96,268 | 7,708 | 18,445 | 70,115 | 124 |
| BPA | 11 | 96,268 | 8,576 | 18,779 | 68,913 | 80 |
| BPV | 12 | 96,268 | 11,666 | 17,884 | 66,718 | 75 |
| BRE | 13 | 96,268 | 9,999 | 19,824 | 66,445 | 279 |
| BRM | 16 | 96,268 | 7,570 | 16,277 | 72,421 | 517 |
| CAM | 12 | 96,268 | 6,081 | 13,376 | 76,811 | 12 |
| CES | 15 | 96,268 | 17,721 | 20,549 | 57,998 | 422 |
| CHA | 12 | 96,268 | 5,095 | 16,628 | 74,545 | 9 |
| DAL | 12 | 96,268 | 3,949 | 10,055 | 82,264 | 94 |
| DEN | 12 | 96,268 | 7,456 | 10,465 | 78,347 | 90 |
| DES | 16 | 96,268 | 17,748 | 14,122 | 64,398 | 385 |
| FAY | 12 | 96,268 | 15,872 | 8,589 | 71,807 | 61 |
| FIR | 12 | 96,268 | 5,123 | 16,735 | 74,410 | 167 |
| FRS | 12 | 96,268 | 4,508 | 11,621 | 80,139 | 53 |
| GES | 12 | 96,268 | 5,029 | 21,734 | 69,505 | 162 |
| GRA | 11 | 96,268 | 4,982 | 11,370 | 79,916 | 30 |
| GRV | 12 | 96,268 | 5,561 | 19,521 | 71,186 | 164 |
| HAI | 12 | 96,268 | 4,884 | 10,671 | 80,713 | 51 |
| HAR | 12 | 96,268 | 4,938 | 12,565 | 78,765 | 120 |
| HOU | 12 | 96,268 | 4,208 | 8,039 | 84,021 | 63 |
| HUN | 12 | 96,268 | 4,650 | 9,626 | 81,992 | 43 |
| KAG | 12 | 96,268 | 5,116 | 16,028 | 75,124 | 109 |
| KAN | 11 | 96,268 | 4,842 | 22,845 | 68,581 | 207 |
| LIN | 12 | 96,268 | 5,436 | 14,360 | 76,472 | 146 |
| LOS | 12 | 96,268 | 5,890 | 16,184 | 74,194 | 117 |
| MAC | 12 | 96,268 | 6,867 | 9,509 | 79,892 | 93 |
| MAN | 12 | 96,268 | 6,737 | 10,597 | 78,934 | 66 |
| MAT | 12 | 96,268 | 5,499 | 18,213 | 72,556 | 15 |
| MAU | 12 | 96,268 | 10,172 | 15,166 | 70,930 | 95 |
| MON | 12 | 96,268 | 7,299 | 8,388 | 80,581 | 61 |
| NEO | 12 | 96,268 | 4,327 | 11,148 | 80,793 | 86 |
| NEW | 21 | 96,268 | 4,160 | 9,224 | 82,884 | 699 |
| NUE | 12 | 96,268 | 4,866 | 9,285 | 82,117 | 71 |
| OKI | 11 | 96,268 | 5,792 | 19,032 | 71,444 | 96 |
| POR | 12 | 96,268 | 7,437 | 21,137 | 67,694 | 92 |
| QNC | 11 | 96,268 | 6,061 | 33,435 | 56,772 | 21 |
| RAR | 12 | 96,268 | 5,060 | 21,835 | 69,373 | 148 |
| REC | 11 | 96,268 | 6,266 | 16,524 | 73,478 | 90 |
| RUS | 12 | 96,268 | 5,563 | 18,455 | 72,250 | 187 |
| SAI | 12 | 96,268 | 5,176 | 11,292 | 79,800 | 73 |
| SMI | 12 | 96,268 | 5,537 | 10,213 | 80,518 | 90 |
| SOC | 12 | 96,268 | 5,030 | 19,244 | 71,994 | 117 |
| SSK | 12 | 96,268 | 5,350 | 16,907 | 74,011 | 6 |
| STL | 12 | 96,268 | 5,094 | 13,312 | 77,862 | 148 |
| TIK | 12 | 96,268 | 5,039 | 22,775 | 68,454 | 184 |
| TRA | 12 | 96,268 | 5,492 | 8,897 | 81,879 | 81 |
| TUC | 14 | 96,268 | 8,095 | 16,358 | 71,815 | 110 |
| TUH | 12 | 96,268 | 5,527 | 12,945 | 77,796 | 106 |
| UTS | 12 | 96,268 | 4,292 | 15,811 | 76,165 | 141 |

```
# Save it to a Word document
officer::read_docx() |>
  body_add_flextable(ft) |>
  print(target = here::here("manuscript1/output/summary_blocks_chr_b.docx"))
```

Get the size of each block from the .block.det files

```
get_kb_column <- function(dir_path) {
  # obtain the list of files with extension .blocks.det
  file_names <- list.files(path = dir_path, pattern = "\\.blocks\\.det$", full.names = TRUE)
  
  # create an empty list to hold the data frames
  block_list <- list()
  
  # loop through the files and read the data into the list
  for (file in file_names) {
    df <- read.table(file, header = TRUE, check.names = FALSE, stringsAsFactors = FALSE)
    # select only the KB column and add it to the block_list with the file name
    block_list[[file]] <- df %>% dplyr::select(KB) %>% add_column(file = file, .before = 1)
  }
  
  # combine the data frames in the block_list into a single data frame
  blocks <- bind_rows(block_list)
  
  # clean up the file name column
  blocks$file <- str_remove(blocks$file, "^.*\\/ld\\/blocks\\/")
  
  return(blocks)
}

# example usage: replace dir_path with your directory path
dir_path <- here("manuscript1/output/files/ld/blocks_chr_b")

blocks<- 
  get_kb_column(dir_path) |>
  mutate(file = str_remove(file, "manuscript1/output/files/ld/blocks_chr_b/")) |>
  mutate(file = str_remove(file, ".blocks.det")) |>

  as_tibble() |>
  rename(
    Population = 1
  )
```

## 9.7 Plot SNP density

```
# load the function that we saved earlier
source(
  here("scripts", "RMarkdowns",
    "analyses", "import_bim.R"
  ),
  local = knitr::knit_global()
)
# import the file
snp_den_qc <- import_bim(
  here(
    "manuscript1/output/file7b.bim"
  )
)
```

Make plot of the SNP density

```
#   ____________________________________________________________________________
#   plot SNP density after QC                                               ####
snp_den_qc |>
  rename(
    Chromosome = 1
  ) |>
  mutate(
    Position           = as.numeric(
      Position
    )
  ) |>
  ggplot(
    aes(
      x                = Position
    ),
    label              = sprintf(
      "%0.2f",
      round(
        a,
        digits         = 0
      )
    )
  ) +
  geom_histogram(
    aes(
      y                = after_stat(
        count
      )
    ),
    binwidth           = 1e6
  ) +
  facet_wrap(
    vars(
      Chromosome
    ),
    scales             = "free_x"
  ) +
  labs(
    title              = "SNP Density after QC",
    x                  = expression(
      "Position in the genome (Mb)"
    ),
    y                  = expression(
      "Number of SNPs"
    )
  ) +
  scale_x_continuous(
    labels             = function(x) {
      format(
        x / 1e6,
        big.mark       = ",", 
        scientific     = FALSE
      )
    }
  ) +
  geom_density(
  aes(
    y = 1e6 * after_stat(count)
  ),
  color = "red",
  linewidth = .75,
  alpha = .4,
  fill = "pink"
  ) +
    theme(
    panel.grid.major   = element_line(
      linetype         = "dashed",
      linewidth        = 0.2
    ),
    panel.grid.minor   = element_line(
      linetype         = "dashed",
      linewidth        = 0.2
    ),
    panel.spacing      = unit(0.5, "lines"),
    strip.text         = element_text(
      face             = "bold", hjust = .5
    ),
    strip.background.x = element_rect(
      color            = "gray"
    )
  )
```

```
## Warning in fortify(data, ...): Arguments in `...` must be used.
## ✖ Problematic argument:
## • label = sprintf("%0.2f", round(a, digits = 0))
## ℹ Did you misspell an argument name?
```

```
#   save the density plot                                            ####
ggsave(
  here("scripts/RMarkdowns/output/americas/manuscript1/figures/snp_density_after_qc_manuscript1_b.pdf"
  ),
  width  = 10,
  height = 6,
  units  = "in"
)
```

SNPs per chromosome

```
# we can use dplyr "count" to get the number of SNPs for each chromosome
# lets get the data we need
snps_per_chrm <- 
  snp_den_qc |>
  count(
    Scaffold) |>
  rename(
    Chromosome = 1,
    "SNPs (N) " = 2
  )

# Create the flextable
ft <- flextable::flextable(snps_per_chrm)

# Apply zebra theme
ft <- flextable::theme_zebra(ft)

# Add a caption to the table
ft <- flextable::add_header_lines(ft, "SNPs per chromosome after quality control")
ft
```

| SNPs per chromosome after quality control | |
| --- | --- |
| Chromosome | SNPs (N) |
| 1 | 21,337 |
| 2 | 40,408 |
| 3 | 34,523 |

Get the mean number of SNPs per chromosome for the entire genome

```
# we first use dplyr cut_width to get the number of SNPs per 1Mb window
albo_den <- 
  snp_den_qc |>
  dplyr::select(
    Scaffold, Position
  ) |>
  group_by(
    Scaffold,
    windows               = cut_width(
      Position,
      width               = 1e6,
      boundary            = 0
    )
  ) |>
  summarise(
    n                     = n(),
    .groups               = "keep"
  ) |>
  group_by(
    Scaffold
  ) |>
  summarise(
    mean                  = mean(n),
    n                     = n(),
    .groups               = "keep"
  ) |>
  rename(
    Chromosome            = 1,
    "SNPs per 1Mb window" = 2,
    "Number of windows"   = 3
  )
#
# check the results
snp_table <-
  flextable(
    albo_den
  )
snp_table <- colformat_double(
  x        = snp_table,
  big.mark = ",",
  digits   = 2,
  na_str   = "N/A"
)
snp_table
```

| Chromosome | SNPs per 1Mb window | Number of windows |
| --- | --- | --- |
| 1 | 57.98 | 368 |
| 2 | 69.67 | 580 |
| 3 | 70.60 | 489 |

Merge objects

```
# we can merge the two data sets we created above into one table
after_qc <-
  snps_per_chrm |>
  left_join(
    albo_den,
    by = "Chromosome"
  )
snp_table2 <- flextable(
  after_qc)
snp_table2 <- colformat_double(
  x        = snp_table2,
  big.mark = ",",
  digits   = 2, 
  na_str   = "N/A"
  )
snp_table2
```

| Chromosome | SNPs (N) | SNPs per 1Mb window | Number of windows |
| --- | --- | --- | --- |
| 1 | 21,337 | 57.98 | 368 |
| 2 | 40,408 | 69.67 | 580 |
| 3 | 34,523 | 70.60 | 489 |

```
# Save it to a Word document
officer::read_docx() |>
  body_add_flextable(snp_table2) |>
  print(target = here::here("manuscript1/output/snps_per_chromosome_table_manuscript1_MAF01.docx"))
```

## 9.8 LD pruning using the chromosomal scale for MAF 0.01

```
# we set a window of variants of 5 and move the window 1 variant per time, removing 1 of the variants with lowest MAF from a pair above the threshold of r^2 > 0.1
plink2 \
--allow-extra-chr \
--bfile manuscript1/output/file7b \
--indep-pairwise 5 1 0.1 \
--out manuscript1/output/indepSNP_chrb \
--silent;
grep 'pairwise\|variants\|samples' manuscript1/output/indepSNP_chrb.log
```

–indep-pairwise 5 1 0.1 933 samples (61 females, 93 males, 779
ambiguous; 933 founders) loaded from 96268 variants loaded from
manuscript1/output/file7b.bim. –indep-pairwise (3 compute threads):
34694/96268 variants removed.

Do the scaffold again

First need to import same SNP list Import the .bim file with the
SNPs

```
#   import the bim file with the SNP data                                   ####
snps <-
  read_delim(                    # to learn about the options use here, run ?read_delim on the console.
    here(
      "manuscript1/output/file7b_backup.bim" #output/populations/file4.bim
    ),                           # use library here to load it
    col_names      = FALSE,      # we don't have header in the input file
    show_col_types = FALSE,      # suppress message from read_delim
    col_types      = "ccidcc"    # set the class of each column
  )
#
# set column names
colnames(
  snps
) <-                             # to add a header in our tibble
  c(
    "Scaffold", "SNP", "Cm", "Position", "Allele1", "Allele2"
  )
#
# check the tibble
head(snps)
```

```
## # A tibble: 6 × 6
##   Scaffold SNP             Cm Position Allele1 Allele2
##   <chr>    <chr>        <int>    <dbl> <chr>   <chr>  
## 1 1.1      AX-583033342     0   315059 C       G      
## 2 1.1      AX-583035163     0   315386 A       G      
## 3 1.1      AX-583033356     0   315674 C       T      
## 4 1.1      AX-583033370     0   330057 G       A      
## 5 1.1      AX-583035194     0   330265 A       G      
## 6 1.1      AX-583033387     0   331288 C       T
```

```
write.table(
  snps,
  file      = here(
    "manuscript1/output/snps_all_scaffolds_4ld_b.txt"
  ),
  sep       = "\t",
  row.names = FALSE,
  col.names = FALSE,
  quote     = FALSE
)

nrow(snps)
#96,268
```

```
plink2 \
--allow-extra-chr \
--bfile manuscript1/output/file5b \
--king-cutoff manuscript1/output/file6b 0.354 \
--make-bed \
--out manuscript1/output/file11b \
--silent;
grep 'samples\|variants\|remaining' manuscript1/output/file11b.log
```

951 samples (61 females, 96 males, 794 ambiguous; 951 founders)
loaded from 96268 variants loaded from manuscript1/output/file5b.bim.
manuscript1/output/file11b.king.cutoff.out.id , and 933 remaining sample
IDs

```
# we set a window of variants of 5 and move the window 1 variant each time, removing 1 of the variants with lowest MAF from a pair above the threshold of r^2 > 0.1
plink2 \
--allow-extra-chr \
--bfile manuscript1/output/file11b \
--indep-pairwise 5 1 0.1 \
--out manuscript1/output/indepSNP_scaffoldsb \
--silent;
grep 'pairwise\|variants\|samples' manuscript1/output/indepSNP_scaffoldsb.log
```

–indep-pairwise 5 1 0.1 933 samples (61 females, 93 males, 779
ambiguous; 933 founders) loaded from 96268 variants loaded from
manuscript1/output/file11b.bim. –indep-pairwise (27 compute threads):
34675/96268 variants removed.

Compare the two sets of SNPs using scaffolds or chromosomal scale

Create Venn diagram of SNPs removed due to LD

```
# Read in the two files as vectors
prunout_chr <-
  read_delim(
    here(
      "manuscript1/output/indepSNP_chrb.prune.out"
    ),
    delim = " ",
    col_names = FALSE,
    show_col_types = FALSE
  )

prunout_scaffolds <-
  read_delim(
    here(
      "manuscript1/output/indepSNP_scaffoldsb.prune.out"
    ),
    delim = " ",
    col_names = FALSE,
    show_col_types = FALSE
  )

# Convert the list to vector
prunout_scaffolds <- unlist(prunout_scaffolds)
prunout_chr <- unlist(prunout_chr)


# Calculate shared values
prunout <- intersect(prunout_chr, prunout_scaffolds)

# Create Venn diagram
venn_data1 <- list(
  "Chromosomal" = prunout_chr,
  "Scaffolds" = prunout_scaffolds
)

# create plot
venn_plot1 <- ggvenn(venn_data1, fill_color = c("steelblue", "darkorange"), show_percentage = TRUE)

# Add a title
venn_plot1 <- venn_plot1 +
  ggtitle("Comparison of genomic scales for linked SNPs MAF 0.01") +
  theme(plot.title = element_text(hjust = .5))

# Display the Venn diagram
print(venn_plot1)
```

```
# Save Venn diagram to PDF
output_path <- here("scripts/RMarkdowns/output/americas/manuscript1/figures/SNPs_linked_comparison_man1_b.pdf")
ggsave(output_path, venn_plot1, height = 5, width = 5, dpi = 300)
```

Now compare MAF 0.01 to MAF 0.1 pruning

Create Venn diagram of SNPs removed due to LD

```
# Read in the two files as vectors
prunout_chr_0.01 <-
  read_delim(
    here(
      "manuscript1/output/indepSNP_chrb.prune.out"
    ),
    delim = " ",
    col_names = FALSE,
    show_col_types = FALSE
  )

prunout_chr_0.1 <-
  read_delim(
    here(
      "manuscript1/output/indepSNP_chr.prune.out"
    ),
    delim = " ",
    col_names = FALSE,
    show_col_types = FALSE
  )

# Convert the list to vector
prunout_chr_0.01 <- unlist(prunout_chr_0.01)
prunout_chr_0.1 <- unlist(prunout_chr_0.1)


# Calculate shared values
prunout <- intersect(prunout_chr_0.01, prunout_chr_0.1)

# Create Venn diagram
venn_data1 <- list(
  "MAF 1%" = prunout_chr_0.01,
  "MAF 10%" = prunout_chr_0.1
)

# create plot
venn_plot1 <- ggvenn(venn_data1, fill_color = c("steelblue", "darkorange"), show_percentage = TRUE)

# Add a title
venn_plot1 <- venn_plot1 +
  ggtitle("Comparison of SNPs sets pruned for MAF 1% and MAF 10%") +
  theme(plot.title = element_text(hjust = .5))

# Display the Venn diagram
print(venn_plot1)
```

```
# Save Venn diagram to PDF
output_path <- here("scripts/RMarkdowns/output/americas/manuscript1/figures/SNPs_comparison_man1_MAF01.pdf")
ggsave(output_path, venn_plot1, height = 5, width = 5, dpi = 300)
```

## 9.9 Pruning r2 for LD-pruned datasets with MAF 1%

If needed, can create it again using the code below

```
plink2 \
--allow-extra-chr \
--bfile manuscript1/output/file7b \
--indep-pairwise 5 1 0.1 \
--out manuscript1/output/indepSNP_chrb \
--silent;
grep 'pairwise\|variants\|samples' manuscript1/output/indepSNP_chrb.log
```

–indep-pairwise 5 1 0.1 933 samples (61 females, 93 males, 779
ambiguous; 933 founders) loaded from 96268 variants loaded from
manuscript1/output/file7b.bim. –indep-pairwise (3 compute threads):
34694/96268 variants removed.

*The indepSNP\_chrb.prune.in file produced here = those SNPs <
our LD 0.1 threshold, that we want to use*

Make a pruned dataset for 0.01. Can use file7b

```
plink2 \
--allow-extra-chr \
--bfile manuscript1/output/file7b \
--indep-pairwise 5 1 0.01 \
--out manuscript1/output/indepSNP_chrb_r01 \
--silent;
grep 'pairwise\|variants\|samples' manuscript1/output/indepSNP_chrb_r01.log
```

–indep-pairwise 5 1 0.01 933 samples (61 females, 93 males, 779
ambiguous; 933 founders) loaded from 96268 variants loaded from
manuscript1/output/file7b.bim. –indep-pairwise (3 compute threads):
75443/96268 variants removed.

Use the SNP set with the LD pruning we just did and extract them from
file7

```
plink2 \
--keep-allele-order \
--bfile manuscript1/output/file7b \
--make-bed \
--export vcf \
--out manuscript1/output/snps_sets/r2_0.1_b \
--extract manuscript1/output/indepSNP_chrb.prune.in \
--silent
grep "variants\|samples" manuscript1/output/snps_sets/r2_0.1_b.log
```

933 samples (61 females, 93 males, 779 ambiguous; 933 founders)
loaded from 96268 variants loaded from manuscript1/output/file7b.bim.
–extract: 61574 variants remaining. 61574 variants remaining after main
filters.

Repeat for 0.01 dataset

```
plink2 \
--keep-allele-order \
--bfile manuscript1/output/file7b \
--make-bed \
--export vcf \
--out manuscript1/output/snps_sets/r2_0.01_b \
--extract manuscript1/output/indepSNP_chrb_r01.prune.in \
--silent
grep "variants\|samples" manuscript1/output/snps_sets/r2_0.01_b.log
```

933 samples (61 females, 93 males, 779 ambiguous; 933 founders)
loaded from 96268 variants loaded from manuscript1/output/file7b.bim.
–extract: 20825 variants remaining. 20825 variants remaining after main
filters.

These snp set can now be used for subsequent pop gen analyses.

Create Venn diagram of SNPs removed due to LD

```
# Read in the two files as vectors
prunout_chr_0.01 <-
  read_delim(
    here(
      "manuscript1/output/indepSNP_chrb_r01.prune.in"
    ),
    delim = " ",
    col_names = FALSE,
    show_col_types = FALSE
  )

prunout_chr_0.1 <-
  read_delim(
    here(
      "manuscript1/output/indepSNP_chr_r01.prune.in"
    ),
    delim = " ",
    col_names = FALSE,
    show_col_types = FALSE
  )

# Convert the list to vector
prunout_chr_0.01 <- unlist(prunout_chr_0.01)
prunout_chr_0.1 <- unlist(prunout_chr_0.1)


# Calculate shared values
prunout <- intersect(prunout_chr_0.01, prunout_chr_0.1)

# Create Venn diagram
venn_data1 <- list(
  "MAF 1%" = prunout_chr_0.01,
  "MAF 10%" = prunout_chr_0.1
)

# create plot
venn_plot1 <- ggvenn(venn_data1, fill_color = c("steelblue", "darkorange"), show_percentage = TRUE)

# Add a title
venn_plot1 <- venn_plot1 +
  ggtitle("Comparison of LD 0.01 SNPs sets for MAF 1% and MAF 10%") +
  theme(plot.title = element_text(hjust = .5))

# Display the Venn diagram
print(venn_plot1)
```

```
# Save Venn diagram to PDF
output_path <- here("scripts/RMarkdowns/output/americas/manuscript1/figures/SNPs_comparison_manuscript1_MAFs_r2_0.01.pdf")
ggsave(output_path, venn_plot1, height = 6, width = 5, dpi = 300)
```

## 9.10 Run PCA with LD-pruned dataset for MAF 0.01

```
plink2 \
--allow-extra-chr \
--bfile manuscript1/output/snps_sets/r2_0.01_b \
--pca allele-wts \
--freq \
--out manuscript1/output/pca_pops_01_b \
--silent;
grep 'samples\|variants' manuscript1/output/pca_pops_01_b.log
```

933 samples (61 females, 93 males, 779 ambiguous; 933 founders)
loaded from 20825 variants loaded from
manuscript1/output/snps\_sets/r2\_0.01\_b.bim.

Check the files

```
head -n 2 manuscript1/output/pca_pops_01_b.eigenvec
```

```
## #FID IID PC1 PC2 PC3 PC4 PC5 PC6 PC7 PC8 PC9 PC10
## OKI  1001    0.00372 -0.0256312  0.0249291   -0.0133598  -0.0150662  -0.0598764  -0.00730545 -0.00368111 0.0176557   -0.0213382
```

```
head -n 10 manuscript1/output/pca_pops_01_b.eigenval
```

```
## 74.9741
## 27.2383
## 16.5536
## 11.8164
## 9.00932
## 8.61948
## 7.07637
## 6.41528
## 6.17938
## 5.88318
```

Import PCA data

```
# import the data from Plink
pca <- read.delim(
  here(
    "manuscript1/output/pca_pops_01_b.eigenvec"
  ),
  head = TRUE
)
 
# check head of the file
head(pca)
```

```
##   X.FID  IID        PC1        PC2       PC3        PC4        PC5        PC6
## 1   OKI 1001 0.00372000 -0.0256312 0.0249291 -0.0133598 -0.0150662 -0.0598764
## 2   OKI 1002 0.00277544 -0.0278244 0.0233629 -0.0127780 -0.0130537 -0.0562631
## 3   OKI 1003 0.00354341 -0.0270703 0.0229562 -0.0105642 -0.0150406 -0.0662643
## 4   OKI 1004 0.00623197 -0.0205134 0.0181432 -0.0160257 -0.0426669 -0.0657675
## 5   OKI 1005 0.00590760 -0.0213982 0.0196279 -0.0195062 -0.0455870 -0.0657807
## 6   OKI 1006 0.00379198 -0.0235685 0.0211274 -0.0106995 -0.0194568 -0.0586173
##           PC7         PC8        PC9       PC10
## 1 -0.00730545 -0.00368111 0.01765570 -0.0213382
## 2 -0.00786066 -0.00557507 0.01233320 -0.0180832
## 3 -0.01864640 -0.01005520 0.02064830 -0.0415701
## 4  0.01541570 -0.02590500 0.00325770 -0.0489583
## 5  0.01043700 -0.02213060 0.00606282 -0.0542690
## 6 -0.02154170  0.00109869 0.02857090 -0.0517220
```

Import sample attributes

```
# import sample attributes
samples2 <- read.delim(
  here("scripts", "RMarkdowns", "population_metadata_all_1Apr2024.txt"
  ),
  head = TRUE
)
#
# check head of the file
head(samples2)
```

```
##              geo    range continent         region             country pop
## 1      Caribbean invasive Caribbean      Caribbean             Bermuda BRM
## 2      Caribbean invasive Caribbean      Caribbean             Jamaica JAM
## 3      Caribbean invasive Caribbean      Caribbean Trinidad and Tobago SAI
## 4 Central Africa invasive    Africa Central Africa               Gabon GAB
## 5    East Africa invasive    Africa    East Africa          Madagascar DGV
## 6    East Africa invasive    Africa    East Africa          Madagascar ANT
##          pop_city               state   Subregion
## 1         Bermuda             Bermuda   Caribbean
## 2          Negirl             Jamaica   Caribbean
## 3 Saint Augustine Trinidad and Tobago   Caribbean
## 4     Franceville                <NA>      Africa
## 5     Diego ville                <NA> East Africa
## 6    Antananarivo                <NA> East Africa
```

Check number of samples per population

```
pops_pca <- 
  pca |>
  group_by(X.FID) |>
  summarize(count_distinct = n_distinct(IID))

# check it
head(pops_pca)
```

```
## # A tibble: 6 × 2
##   X.FID count_distinct
##   <chr>          <int>
## 1 ALD               10
## 2 ARA               12
## 3 BAF                7
## 4 BCO               12
## 5 BEA               12
## 6 BEH                9
```

Merge the data

```
df4 <-
  merge(
    pca,
    samples2,
    by.x = "X.FID",
    by.y = "pop",
    all.x = T,
    all.y = F
  ) 
head(df4)
```

```
##   X.FID IID        PC1        PC2       PC3         PC4       PC5        PC6
## 1   ALD 802 0.01219580 -0.0234713 0.0272435 -0.00618730 0.0301107 -0.0816297
## 2   ALD 803 0.01052130 -0.0258215 0.0294054 -0.00506896 0.0325099 -0.0957621
## 3   ALD 804 0.01086530 -0.0253772 0.0338470 -0.00726068 0.0194989 -0.0739442
## 4   ALD 806 0.00991576 -0.0252500 0.0297633 -0.00922047 0.0236407 -0.0862424
## 5   ALD 807 0.01165220 -0.0253242 0.0298490 -0.01142570 0.0247250 -0.0951329
## 6   ALD 808 0.01014360 -0.0217679 0.0216053 -0.00996684 0.0231941 -0.0705673
##          PC7        PC8       PC9      PC10             geo    range continent
## 1 -0.0190815 0.03009150 0.0114123 0.0238378 Southern Europe invasive    Europe
## 2 -0.0127487 0.01524100 0.0136302 0.0328984 Southern Europe invasive    Europe
## 3 -0.0196301 0.02346740 0.0241072 0.0250425 Southern Europe invasive    Europe
## 4 -0.0150293 0.00853551 0.0273907 0.0237191 Southern Europe invasive    Europe
## 5 -0.0174092 0.00970653 0.0233102 0.0189202 Southern Europe invasive    Europe
## 6 -0.0155357 0.01977960 0.0111917 0.0219364 Southern Europe invasive    Europe
##            region country pop_city state   Subregion
## 1 Southern Europe Albania   Durres  <NA> East Europe
## 2 Southern Europe Albania   Durres  <NA> East Europe
## 3 Southern Europe Albania   Durres  <NA> East Europe
## 4 Southern Europe Albania   Durres  <NA> East Europe
## 5 Southern Europe Albania   Durres  <NA> East Europe
## 6 Southern Europe Albania   Durres  <NA> East Europe
```

Get some data for the PCA plot

```
# How many populations
length(unique(df4$X.FID)) #93
```

```
## [1] 93
```

```
# How many countries
length(unique(df4$country)) #29
```

```
## [1] 29
```

```
# How many regions
length(unique(df4$region)) #9
```

```
## [1] 9
```

```
# How many Continents
length(unique(df4$continent)) #5
```

```
## [1] 5
```

Check the countries

```
unique(df4$country)
```

```
##  [1] "Albania"             "Brazil"              "USA"                
##  [4] "India"               "Italy"               "Bermuda"            
##  [7] "Cambodia"            "Thailand"            "Colombia"           
## [10] "France"              "Bhutan"              "Georgia"            
## [13] "Greece"              "China"               "Vietnam"            
## [16] "Indonesia"           "Sri Lanka"           "Japan"              
## [19] "Nepal"               "Malaysia"            "Maldives"           
## [22] "Jamaica"             "Argentina"           "Russia"             
## [25] "Trinidad and Tobago" "Serbia"              "Spain"              
## [28] "Taiwan"              "Turkey"
```

Check the regions

```
unique(df4$region)
```

```
## [1] "Southern Europe" "South America"   "North America"   "South Asia"     
## [5] "Caribbean"       "Southeast Asia"  "Western Europe"  "Eastern Europe" 
## [9] "East Asia"
```

Make plot1

```
# source the plotting function
source(here("scripts", "RMarkdowns", "my_theme2.R"))

# Shapes
N = 100
M = 1000
good.shapes = c(1:25, 33:127)


# Compute the count for each country
country_count <- df4 |>
  group_by(country) |>
  summarize(count = n())

# Merge the count back to the main data
df4 <- df4 |>
  left_join(country_count, by = "country")

# Create a custom label for the legend
df4$country_label <-
  paste(df4$country, " (", df4$count, ")", sep = "")

# Define the color and shape manually
#palette23 <- c(palette1, palette2)
colors <- setNames(col_vector, unique(df4$country_label))
shapes <-
  setNames(good.shapes[c(1:25, 28:31, 55:57, 1:13)], unique(df4$country_label))


# Compute the center of ellipses for each continent
ellipse_centers <- df4 |>
  group_by(region) |>
  summarise(PC1_center = mean(PC1), PC2_center = mean(PC2))

# # Calculate the number of samples per region
continent_count <- df4 |>
  group_by(region) |>
  summarise(count = n())
continent_labels <-
  setNames(
    paste(continent_count$region, " (", continent_count$count, ")", sep = ""),
    continent_count$region
  )

continent_colors <-
  c("pink","#ffffaa","#bdb2ff","#9bf6ff","#baffc9","#ffffaa","#ffffaa","#bdb2ff","#bdb2ff")
  #Adjust these colors to your preference

#country_colors <-
#c("#bdb2ff","#baffc9","pink","#ffffaa","#baffc9","#ffffaa","#ffffaa","#baffc9","#bdb2ff","#bdb2ff","#bdb2ff","#ffffaa", "#ffffaa","#bdb2ff","pink","#ffffaa","#ffffaa","#ffffaa","#ffffaa","#bdb2ff","#bdb2ff","#bdb2ff","#ffffaa","#ffffaa","#ffffaa","pink","#ffffaa","#9bf6ff","#ffffaa")   #Adjust these colors to your preference

# Create the plot
ggplot(df4, aes(PC1, PC2)) +
  geom_point(aes(shape = country_label, color = country_label)) +
  stat_ellipse(
    aes(fill = region, group = region),
    geom = "polygon",
    alpha = 0.2,
    level = 0.8,
    segments = 40,
    color = "darkgray"
  ) +
  stat_ellipse(
    aes(group = region),
    geom = "path",
    level = 0.8,
    segments = 40,
    color = "darkgray"
  ) + # Line around the ellipse
  geom_text_repel(
    data = ellipse_centers,
    aes(x = PC1_center, y = PC2_center, label = region),
    color = "black"
  ) + # Add labels using ggrepel
  xlab("PC1 (74.97% Variance)") + 
  ylab("PC2 (27.24% Variance)") +
  labs(caption = "Principal Component Analysis with 20,825 SNPs \n of mosquitoes from 93 localities across 29 countries in Asia, the Americas, and Europe.") + 
guides(
    color = guide_legend(
      title = "Country",
      order = 2,
      ncol = 2
    ),
    shape = guide_legend(
      title = "Country",
      order = 2,
      ncol = 2
    ),
    fill = guide_legend(
      title = "Continent",
      order = 1,
      ncol = 1
    )
  ) +
  scale_fill_manual(values = continent_colors, labels = continent_labels) +
  scale_color_manual(values = colors) +
  scale_shape_manual(values = shapes) +
  guides(fill=guide_legend(ncol=2)) +
  my_theme() +
  theme(
    plot.caption = element_text(face = "italic"),
    legend.position = "right",
    legend.justification = "top",
    legend.box.just = "center",
    legend.box.background = element_blank(),
    legend.text=element_text(size=6),
    plot.margin = margin(5.5, 30, 5.5, 5.5, "points"),
    legend.margin = margin(5, 5, 5, 40) # move the legend a bit up
  )
```

```
#   save the pca plot                                                       ####
ggsave(
  here("scripts", "RMarkdowns",
    "output", "americas", "manuscript1", "figures", "PCA_manuscript1_r01_b_plink_PC1_2.pdf"
  ),
  width  = 10,
  height = 10,
  units  = "in"
)
```

Make plot 1 & 3

```
# source the plotting function
source(here("scripts", "RMarkdowns", "my_theme2.R"))

# Shapes
N = 100
M = 1000
good.shapes = c(1:25, 33:127)


# Compute the count for each country
country_count <- df4 |>
  group_by(country) |>
  summarize(count = n())

# Merge the count back to the main data
df4 <- df4 |>
  left_join(country_count, by = "country")

# Create a custom label for the legend
df4$country_label <-
  paste(df4$country, " (", df4$count, ")", sep = "")

# Define the color and shape manually
#palette23 <- c(palette1, palette2)
colors <- setNames(col_vector, unique(df4$country_label))
shapes <-
  setNames(good.shapes[c(1:25, 28:31, 55:57, 1:13)], unique(df4$country_label))


# Compute the center of ellipses for each continent
ellipse_centers <- df4 |>
  group_by(region) |>
  summarise(PC1_center = mean(PC1), PC3_center = mean(PC3))

# # Calculate the number of samples per region
continent_count <- df4 |>
  group_by(region) |>
  summarise(count = n())
continent_labels <-
  setNames(
    paste(continent_count$region, " (", continent_count$count, ")", sep = ""),
    continent_count$region
  )

continent_colors <-
  c("pink","#ffffaa","#bdb2ff","#9bf6ff","#baffc9","#ffffaa","#ffffaa","#bdb2ff","#bdb2ff")
  #Adjust these colors to your preference

#country_colors <-
#c("#bdb2ff","#baffc9","pink","#ffffaa","#baffc9","#ffffaa","#ffffaa","#baffc9","#bdb2ff","#bdb2ff","#bdb2ff","#ffffaa", "#ffffaa","#bdb2ff","pink","#ffffaa","#ffffaa","#ffffaa","#ffffaa","#bdb2ff","#bdb2ff","#bdb2ff","#ffffaa","#ffffaa","#ffffaa","pink","#ffffaa","#9bf6ff","#ffffaa")   #Adjust these colors to your preference

# Create the plot
ggplot(df4, aes(PC1, PC3)) +
  geom_point(aes(shape = country_label, color = country_label)) +
  stat_ellipse(
    aes(fill = region, group = region),
    geom = "polygon",
    alpha = 0.2,
    level = 0.8,
    segments = 40,
    color = "darkgray"
  ) +
  stat_ellipse(
    aes(group = region),
    geom = "path",
    level = 0.8,
    segments = 40,
    color = "darkgray"
  ) + # Line around the ellipse
  geom_text_repel(
    data = ellipse_centers,
    aes(x = PC1_center, y = PC3_center, label = region),
    color = "black"
  ) + # Add labels using ggrepel
  xlab("PC1 (74.97% Variance)") + 
  ylab("PC3 (16.55% Variance)") +
  labs(caption = "Principal Component Analysis with 20,825 SNPs \n of mosquitoes from 93 localities across 29 countries in Asia, the Americas, and Europe.") + 
guides(
    color = guide_legend(
      title = "Country",
      order = 2,
      ncol = 2
    ),
    shape = guide_legend(
      title = "Country",
      order = 2,
      ncol = 2
    ),
    fill = guide_legend(
      title = "Continent",
      order = 1,
      ncol = 1
    )
  ) +
  scale_fill_manual(values = continent_colors, labels = continent_labels) +
  scale_color_manual(values = colors) +
  scale_shape_manual(values = shapes) +
  guides(fill=guide_legend(ncol=2)) +
  my_theme() +
  theme(
    plot.caption = element_text(face = "italic"),
    legend.position = "right",
    legend.justification = "top",
    legend.box.just = "center",
    legend.box.background = element_blank(),
    legend.text=element_text(size=6),
    plot.margin = margin(5.5, 30, 5.5, 5.5, "points"),
    legend.margin = margin(5, 5, 5, 40) # move the legend a bit up
  )
```

```
#   save the pca plot                                                       ####
ggsave(
  here("scripts", "RMarkdowns",
    "output", "americas", "manuscript1", "figures", "PCA_manuscript1_r01_b_plink_PC1_3.pdf"
  ),
  width  = 10,
  height = 10,
  units  = "in"
)
```

Make plot 1 & 4

```
# source the plotting function
source(here("scripts", "RMarkdowns", "my_theme2.R"))

# Shapes
N = 100
M = 1000
good.shapes = c(1:25, 33:127)


# Compute the count for each country
country_count <- df4 |>
  group_by(country) |>
  summarize(count = n())

# Merge the count back to the main data
df4 <- df4 |>
  left_join(country_count, by = "country")

# Create a custom label for the legend
df4$country_label <-
  paste(df4$country, " (", df4$count, ")", sep = "")

# Define the color and shape manually
#palette23 <- c(palette1, palette2)
colors <- setNames(col_vector, unique(df4$country_label))
shapes <-
  setNames(good.shapes[c(1:25, 28:31, 55:57, 1:13)], unique(df4$country_label))


# Compute the center of ellipses for each continent
ellipse_centers <- df4 |>
  group_by(region) |>
  summarise(PC1_center = mean(PC1), PC4_center = mean(PC4))

# # Calculate the number of samples per region
continent_count <- df4 |>
  group_by(region) |>
  summarise(count = n())
continent_labels <-
  setNames(
    paste(continent_count$region, " (", continent_count$count, ")", sep = ""),
    continent_count$region
  )

continent_colors <-
  c("pink","#ffffaa","#bdb2ff","#9bf6ff","#baffc9","#ffffaa","#ffffaa","#bdb2ff","#bdb2ff")
  #Adjust these colors to your preference

#country_colors <-
#c("#bdb2ff","#baffc9","pink","#ffffaa","#baffc9","#ffffaa","#ffffaa","#baffc9","#bdb2ff","#bdb2ff","#bdb2ff","#ffffaa", "#ffffaa","#bdb2ff","pink","#ffffaa","#ffffaa","#ffffaa","#ffffaa","#bdb2ff","#bdb2ff","#bdb2ff","#ffffaa","#ffffaa","#ffffaa","pink","#ffffaa","#9bf6ff","#ffffaa")   #Adjust these colors to your preference

# Create the plot
ggplot(df4, aes(PC1, PC4)) +
  geom_point(aes(shape = country_label, color = country_label)) +
  stat_ellipse(
    aes(fill = region, group = region),
    geom = "polygon",
    alpha = 0.2,
    level = 0.8,
    segments = 40,
    color = "darkgray"
  ) +
  stat_ellipse(
    aes(group = region),
    geom = "path",
    level = 0.8,
    segments = 40,
    color = "darkgray"
  ) + # Line around the ellipse
  geom_text_repel(
    data = ellipse_centers,
    aes(x = PC1_center, y = PC4_center, label = region),
    color = "black"
  ) + # Add labels using ggrepel
  xlab("PC1 (74.97% Variance)") + 
  ylab("PC4 (11.82% Variance)") +
  labs(caption = "Principal Component Analysis with 20,825 SNPs \n of mosquitoes from 93 localities across 29 countries in Asia, the Americas, and Europe.") + 
guides(
    color = guide_legend(
      title = "Country",
      order = 2,
      ncol = 2
    ),
    shape = guide_legend(
      title = "Country",
      order = 2,
      ncol = 2
    ),
    fill = guide_legend(
      title = "Continent",
      order = 1,
      ncol = 1
    )
  ) +
  scale_fill_manual(values = continent_colors, labels = continent_labels) +
  scale_color_manual(values = colors) +
  scale_shape_manual(values = shapes) +
  guides(fill=guide_legend(ncol=2)) +
  my_theme() +
  theme(
    plot.caption = element_text(face = "italic"),
    legend.position = "right",
    legend.justification = "top",
    legend.box.just = "center",
    legend.box.background = element_blank(),
    legend.text=element_text(size=6),
    plot.margin = margin(5.5, 30, 5.5, 5.5, "points"),
    legend.margin = margin(5, 5, 5, 40) # move the legend a bit up
  )
```

```
#   save the pca plot                                                       ####
ggsave(
  here("scripts", "RMarkdowns",
    "output", "americas", "manuscript1", "figures", "PCA_manuscript1_r01_b_plink_PC1_4.pdf"
  ),
  width  = 10,
  height = 10,
  units  = "in"
)
```
