# Supplementary material for: Population Structure of the Invasive Asian Tiger Mosquito, Aedes albopictus, in the Americas
Source: Insects. 2026 Jul 23;17(8):755. doi: 10.3390/insects17080755 (PMC13512968; doi:10.3390/insects17080755)
Supplement: Supplementary file 1 [file insects-17-00755-s001.zip › RMarkdowns/S8 - American dataset structure analyses.html]

Americas - Structure Analyses


# Americas - Structure Analyses

#### Margaret Corley

#### 2024-04-05

# 1. LEA

Load LEA Libraries

```
library(LEA)
library(vcfR)
library(RColorBrewer)
library(adegenet)
library(ape)
library(tidyverse)
```

```
## Warning: Your system is mis-configured: '/etc/localtime' is not a symlink
```

```
library(here)
library(dplyr)
library(colorout)
library(extrafont)
library(scales)
library(stringr)
library(ggtext)
library(ggplot2)
```

## LEA analyses for r<0.01 LD and MAF 1%

Import the data for R2<0.01 (MAF 1% filtered)

```
genotype <- here(
   "americas/output/snps_sets/r2_0.01_b.vcf"
  )

d <- read.vcfR(
  genotype
)
```

```
## Scanning file to determine attributes.
## File attributes:
##   meta lines: 8
##   header_line: 9
##   variant count: 18964
##   column count: 554
## Meta line 8 read in.
## All meta lines processed.
## gt matrix initialized.
## Character matrix gt created.
##   Character matrix gt rows: 18964
##   Character matrix gt cols: 554
##   skip: 0
##   nrows: 18964
##   row_num: 0
## Processed variant 1000Processed variant 2000Processed variant 3000Processed variant 4000Processed variant 5000Processed variant 6000Processed variant 7000Processed variant 8000Processed variant 9000Processed variant 10000Processed variant 11000Processed variant 12000Processed variant 13000Processed variant 14000Processed variant 15000Processed variant 16000Processed variant 17000Processed variant 18000Processed variant: 18964
## All variants processed
```

Get population and individuals information

```
inds_full <- attr(d@gt,"dimnames")[[2]]
inds_full <- inds_full[-1]
a <- strsplit(inds_full, '_')
pops <- unname(sapply(a, FUN = function(x) return(as.character(x[1])))) 
table(pops)
```

```
## pops
## ARA BAF BCO BEA BEH BER BIT BPA BPV BRM CHI COL CUA DAL DEN FAY FIR FLO GRV HAL 
##  12   7  12  12   9  12  12  11  12  16   6  10   8  12  12  11  12   8  12  10 
## HAR HAT HOU IPO LIN LOS MAC MAN MAU MED MON NEB NEG NEH NEO NEW NOV NUE PAL PEO 
##  12  10  12   7  12  12  12  12  12   7  12  10   4   7  12  21   4  12  11  10 
## PLM POR REC RUS SAI SMI SPR STL TRA TUC WAV 
##   7  12  11  12  12  12   7  12  12  14   7
```

```
pops <- factor(pops)
inds <- unname(sapply(a, FUN = function(x) return(as.character(x[2]))))
```

Convert format

```
vcf2geno(genotype, gsub(".vcf", ".geno", genotype))
```

```
## 
##  - number of detected individuals:   545
##  - number of detected loci:      18964
## 
## For SNP info, please check /gpfs/gibbs/pi/caccone/mkc54/albo/americas/output/snps_sets/r2_0.01_b.vcfsnp.
## 
## 0 line(s) were removed because these are not SNPs.
## Please, check /gpfs/gibbs/pi/caccone/mkc54/albo/americas/output/snps_sets/r2_0.01_b.removed file, for more informations.
```

```
## [1] "/gpfs/gibbs/pi/caccone/mkc54/albo/americas/output/snps_sets/r2_0.01_b.geno"
```

```
vcf2lfmm(genotype, gsub(".vcf", ".lfmm", genotype))
```

```
## 
##  - number of detected individuals:   545
##  - number of detected loci:      18964
## 
## For SNP info, please check /gpfs/gibbs/pi/caccone/mkc54/albo/americas/output/snps_sets/r2_0.01_b.vcfsnp.
## 
## 0 line(s) were removed because these are not SNPs.
## Please, check /gpfs/gibbs/pi/caccone/mkc54/albo/americas/output/snps_sets/r2_0.01_b.removed file, for more informations.
## 
## 
##  - number of detected individuals:   545
##  - number of detected loci:      18964
```

```
## [1] "/gpfs/gibbs/pi/caccone/mkc54/albo/americas/output/snps_sets/r2_0.01_b.lfmm"
```

PCA for r2<0.01 snp set (1% MAF)

```
nPC <- length(inds)
pc <- pca(gsub(".vcf", ".lfmm", genotype), K = nPC)
```

```
## [1] "******************************"
## [1] " Principal Component Analysis "
## [1] "******************************"
## summary of the options:
## 
##         -n (number of individuals)          545
##         -L (number of loci)                 18964
##         -K (number of principal components) 545
##         -x (genotype file)                  /gpfs/gibbs/pi/caccone/mkc54/albo/americas/output/snps_sets/r2_0.01_b.lfmm
##         -a (eigenvalue file)                /gpfs/gibbs/pi/caccone/mkc54/albo/americas/output/snps_sets/r2_0.01_b.pca/r2_0.01_b.eigenvalues
##         -e (eigenvector file)               /gpfs/gibbs/pi/caccone/mkc54/albo/americas/output/snps_sets/r2_0.01_b.pca/r2_0.01_b.eigenvectors
##         -d (standard deviation file)        /gpfs/gibbs/pi/caccone/mkc54/albo/americas/output/snps_sets/r2_0.01_b.pca/r2_0.01_b.sdev
##         -p (projection file)                /gpfs/gibbs/pi/caccone/mkc54/albo/americas/output/snps_sets/r2_0.01_b.pca/r2_0.01_b.projections
##         -c data centered
```

```
show(pc)
```

```
## * pca class *
## 
## project directory:               /gpfs/gibbs/pi/caccone/mkc54/albo/americas/output/snps_sets/ 
## pca result directory:            r2_0.01_b.pca/ 
## input file:                      r2_0.01_b.lfmm 
## eigenvalue file:                 r2_0.01_b.eigenvalues 
## eigenvector file:                r2_0.01_b.eigenvectors 
## standard deviation file:         r2_0.01_b.sdev 
## projection file:                 r2_0.01_b.projections 
## pcaProject file:                   r2_0.01_b.pcaProject 
## number of individuals:           545 
## number of loci:                  18964 
## number of principal components:  545 
## centered:                        TRUE 
## scaled:                          FALSE
```

Test

```
# PC significant test: tracy-widom test
tw <- tracy.widom(pc)
```

```
## [1] "*******************"
## [1] " Tracy-Widom tests "
## [1] "*******************"
## summary of the options:
## 
##         -n (number of eigenvalues)          545
##         -i (input file)                     /gpfs/gibbs/pi/caccone/mkc54/albo/americas/output/snps_sets/r2_0.01_b.pca/r2_0.01_b.eigenvalues
##         -o (output file)                    /gpfs/gibbs/pi/caccone/mkc54/albo/americas/output/snps_sets/r2_0.01_b.pca/r2_0.01_b.tracywidom
```

```
# tw$pvalues
# plot the percentage of variance explained by each component
plot(tw$percentage, pch = 19, col = "blue", cex = .8)
```

Get values

```
# plot preparation
pc.coord <- as.data.frame(pc$projections)
colnames(pc.coord) <- paste0("PC", 1:nPC)
pc.coord$Individual <- inds
pc.coord$Population <- pops
# perc1 <- paste0(round(tw$percentage, digits = 3) * 100, "%")
perc <- paste0(round(pc$eigenvalues/sum(pc$eigenvalues), digits = 3) * 100, "%")
nb.cols <- 40
mycolors <- colorRampPalette(brewer.pal(8, "Set2"))(nb.cols)
```

Check R symbols for plot

```
#to see all shapes -> plot shapes - para escolher os simbolos
N = 100; M = 1000
good.shapes = c(1:25,33:127)
foo = data.frame( x = rnorm(M), y = rnorm(M), s = factor( sample(1:N, M, replace = TRUE) ) )
ggplot(aes(x,y,shape=s ), data=foo ) +
  scale_shape_manual(values=good.shapes[1:N]) +
  geom_point()
```

```
sampling_loc <- read.csv(
  here("scripts", "RMarkdowns", "output", "sampling_loc_americas_all.csv"
    ))
```

```
saveRDS(sampling_loc, here("scripts", "RMarkdowns", 
  "output", "sampling_loc_americas_all.rds"
))
```

Sample data

```
sampling_loc <- readRDS(here("scripts", "RMarkdowns", "output", "sampling_loc_americas_all.rds"))
head(sampling_loc)
```

```
##       Pop_City Country Latitude Longitude Continent Abbreviation Year
## 1   Berlin, NJ     USA 39.79081  -74.9291  Americas          BER 2018
## 2 Columbus, OH     USA 39.97170  -82.9071  Americas          COL 2015
## 3   Palm Beach     USA 26.70560  -80.0364  Americas          PAL 2018
## 4  Houston, TX     USA 29.75491  -95.3505  Americas          HOU 2018
## 5  Los Angeles     USA 34.05220 -118.2437  Americas          LOS 2018
## 6   Manaus, AM  Brazil -3.09161  -60.0325  Americas          MAU 2017
##          Region      Subregion order order2 orderold AM_order      state
## 1 North America Northeast, USA     1     NA       75        6    NJ, USA
## 2 North America   Midwest, USA     2     NA       76       18    OH, USA
## 3 North America Southeast, USA     3     NA       77       13    FL, USA
## 4 North America Southwest, USA     4     NA       78       23    TX, USA
## 5 North America      West, USA     5     NA       79       30    CA, USA
## 6 South America  South America     6     NA       80       38 AM, Brazil
```

More sample data

```
# import sample attributes
samples2 <- read.delim(
  here("scripts", "RMarkdowns",
    "population_metadata_all_1Apr2024.txt"
  ),
  head = TRUE
)

samples2<- samples2 |>
  dplyr::select(
    Subregion, country, pop
  )

# check head of the file
head(samples2)
```

```
##     Subregion             country pop
## 1   Caribbean             Bermuda BRM
## 2   Caribbean             Jamaica JAM
## 3   Caribbean Trinidad and Tobago SAI
## 4      Africa               Gabon GAB
## 5 East Africa          Madagascar DGV
## 6 East Africa          Madagascar ANT
```

Merge with sampling\_loc

```
merged_loc <- merge(samples2, sampling_loc, by.x = "pop", by.y = "Abbreviation")
head(merged_loc)
```

```
##   pop   Subregion.x    country           Pop_City    Country  Latitude
## 1 AIZ         Japan      Japan Aizuwakamatsu City      Japan  37.49240
## 2 ALD   East Europe    Albania             Durres    Albania  41.29704
## 3 ALU   East Europe    Ukraine            Alushta    Ukraine  44.68289
## 4 ALV   East Europe    Albania              Vlore    Albania  40.46600
## 5 ANT   East Africa Madagascar       Antananarivo Madagascar -18.87920
## 6 ARA South America     Brazil          Araguaina     Brazil  -7.19119
##   Longitude Continent    Year          Region   Subregion.y order order2
## 1 139.99360      Asia    2008       East Asia         Japan    NA     NA
## 2  19.50373    Europe    2018 Southern Europe   East Europe    33     25
## 3  34.40368    Europe    2021  Eastern Europe   East Europe    43     35
## 4  19.48970    Europe    2020 Southern Europe   East Europe    32     24
## 5  47.50790    Africa    2022     East Africa   East Africa    NA     76
## 6 -48.20810  Americas unknown   South America South America    NA     NA
##   orderold AM_order      state
## 1       NA       NA           
## 2       25       NA           
## 3       35       NA           
## 4       24       NA           
## 5       NA       NA           
## 6       NA       43 TO, Brazil
```

Check pops

```
head(pc.coord$Population)
```

```
## [1] MAU MAU MAU MAU MAU MAU
## 51 Levels: ARA BAF BCO BEA BEH BER BIT BPA BPV BRM CHI COL CUA DAL DEN ... WAV
```

```
#Check how many sampling localities
length(unique(pc.coord$Population))
```

```
## [1] 51
```

Check the regions

```
unique(merged_loc$Region)
```

```
##  [1] "East Asia"       "Southern Europe" "Eastern Europe"  "East Africa"    
##  [5] "South America"   "West Africa"     "North America"   "South Asia"     
##  [9] "Caribbean"       "Southeast Asia"  "Central Africa"  "Indian Ocean"   
## [13] "Western Europe"  "North Africa"
```

Merge

```
merged_data <- merge(pc.coord, merged_loc, by.x = "Population", by.y = "pop")
head(merged_data)
```

```
##   Population      PC1       PC2      PC3       PC4       PC5      PC6       PC7
## 1        ARA -37.9070  -9.61018  9.94773 -9.196120  3.464450 -2.36423  0.589113
## 2        ARA -36.5648 -11.34550  2.96039  0.640166 -2.826140 -1.84963 -4.199620
## 3        ARA -36.5969 -12.35130 13.42970 -3.476700 -9.852590  1.74641  5.662010
## 4        ARA -41.5976  -7.88095 -2.79000  0.858120  0.643241 -2.65064  5.130920
## 5        ARA -39.8731  -7.85610  9.74210 -6.314710  0.875901 -3.24501  3.724940
## 6        ARA -41.3347 -10.07700  1.88370  0.909860 -4.702400  3.23471 -1.834530
##         PC8       PC9      PC10      PC11     PC12      PC13      PC14
## 1  5.732670 -4.114930 -0.542681  -8.28962 -2.44458  -5.76569  2.330330
## 2  2.251590 -2.576180 -3.732350   5.05651 -5.89933 -17.12580 -1.759800
## 3 12.692200 -6.732400  2.948440 -15.84470 -9.78288  -2.28123  9.914320
## 4  1.479070 -2.427710 -2.226860  -3.06396 -4.18702   1.25832  7.529990
## 5  9.068670 -5.590320  3.781940 -11.76560 -5.03441  -4.24472  0.234659
## 6  0.299409 -0.963097 -4.257130  -0.28498 -3.30927  -4.94552  2.208080
##        PC15      PC16       PC17      PC18      PC19         PC20      PC21
## 1 -0.643058  0.987268  -2.649970  0.501899 -3.863630   4.06379000  4.334040
## 2 -2.728830 -4.567940  -6.997540 -1.186160 -6.016880 -11.36300000  8.083640
## 3  2.345180  0.138137  -7.629090 -1.374410  4.061870   8.51765000 -3.500900
## 4  1.591080 -6.676160  -0.112915 -0.733062 -2.071540  -0.00903307  0.438302
## 5  2.636800 -0.399105 -10.406800 -0.275686  0.225518   9.55475000  1.234540
## 6  0.318042 -2.113390  -4.488940 -1.207760 -2.716950  -1.22067000 -2.588480
##       PC22      PC23      PC24      PC25      PC26      PC27     PC28     PC29
## 1  2.01834 -0.897835 -1.956020  1.822500 -0.570732 -4.898150  2.72087  0.80630
## 2 -0.70718 -6.789230 -0.304502 -8.377160 -6.734230 -0.326494  7.59348 -7.18332
## 3  8.98791  5.174340  4.092580 -0.544960 -1.479450  6.296130 -1.44021 -1.36940
## 4 -2.15697  0.738792  6.764500  0.218494 -2.048830  2.093870 -2.96136 -3.86433
## 5  4.53145  1.725940 -2.056720  3.850930 -1.425800  1.693710  4.17852  4.28244
## 6  3.70234  3.059280  7.018610  4.014380 -3.705370 -4.538100  6.15959  2.20580
##        PC30      PC31     PC32     PC33       PC34      PC35      PC36     PC37
## 1 -4.733940  0.263034 1.787360  3.38063  0.2446050  0.191617  0.104094  2.64931
## 2  1.840970  2.726540 2.947410  2.45266 11.1271000 -7.402240 -1.527070  6.37666
## 3 -1.491300 -2.220170 5.213280  1.59842 -0.4262070  0.453386 -0.482883 -4.98909
## 4 -0.849582  4.012140 4.504560  2.41834 -4.8376600 -0.972463  1.355970  4.40864
## 5 -1.774260 -3.411930 0.805465 -2.10905 -0.0879769 -0.367516  0.674488  2.53181
## 6 -4.708710  2.692690 3.282350 -2.02362 -5.4665500  2.919310  2.006150  6.19209
##        PC38       PC39     PC40      PC41      PC42      PC43      PC44
## 1 -2.150060 -0.0331104 -2.63466 -2.119430 -0.824185  0.269032  0.651041
## 2  6.834190  5.5835000 -8.43281 -0.581894 -1.025690  2.791160  0.907117
## 3  0.711477 -4.8934500 -4.13755  2.307220 -0.607214 -1.165500 -6.300370
## 4 -5.712360  5.0677300 -4.01111 -2.199770 -3.634500 -1.892100 -0.260315
## 5 -7.001870 -0.2828400 -3.53528  1.253340 -0.621714  0.728653 -0.916191
## 6 -3.936760  6.4259900 -5.68249 -1.308170 -2.847190 -4.962860 -8.638020
##        PC45      PC46      PC47        PC48      PC49     PC50      PC51
## 1 -0.254626  4.003730  3.888250 -3.39219000 -1.507020  3.22283  6.108790
## 2 -4.389820  0.847831  0.115605 -0.88806200  2.728800 -2.07597 -0.968195
## 3  3.563110  2.764710  5.239370  0.00275125  5.958750  3.01967  8.462780
## 4 -1.307560  2.390500  1.037650 -4.33192000 -1.018360  3.67903  6.775290
## 5 -5.955920 -0.577598  0.519125 -5.51861000  4.349230  5.15577  1.311020
## 6 -0.074985  6.447980 -3.064570 -0.35745700  0.224836  2.14474 -1.991600
##        PC52       PC53      PC54      PC55       PC56      PC57       PC58
## 1 -0.211035   2.123420 -0.131438 -3.608070   1.627810  1.609460   1.424290
## 2  8.181740 -13.262100 -8.871410 -2.440050 -10.420500 16.687700 -14.142400
## 3 -3.283400   1.775390  4.413570 -2.656700  -0.685148 -0.840255  -0.167414
## 4  4.336170   5.842450 -3.201520  0.832952   0.977528 -0.296531  -4.319780
## 5  2.047100   2.069150  2.630670 -3.967640  -2.487730 -5.029050   1.447620
## 6 -8.414140  -0.430018  3.372410  1.355460   3.703930 10.159500   9.091780
##        PC59     PC60       PC61      PC62     PC63     PC64      PC65      PC66
## 1  2.073350 -2.56354 -3.2515100 -5.840920 -8.15472 -2.05068  0.269993 -1.239300
## 2 -0.490822 -3.11580 -0.0806427  1.200410  6.34282 -1.36162  3.169590 -1.970060
## 3  5.133510 -4.45706  1.0548700 -0.225541  3.56262  9.09849 -0.992340 -4.211420
## 4 -1.167760 -5.18144 -1.4034800  2.953400 -3.91285 -1.09490  0.744357 10.449600
## 5  5.306620  4.81811  2.5769000  2.203110  3.13866  2.76435 -0.986568 -2.099820
## 6  6.117740  3.75106 -0.4009790 -9.661840  3.14737 -4.35340  4.006650 -0.140657
##        PC67       PC68      PC69      PC70     PC71      PC72      PC73
## 1  -1.18652  -0.122224   4.49294  9.702590 -4.46715 -1.456730 -1.898670
## 2   3.32382 -16.350100 -11.92030  0.144891 12.11820  8.617320 11.074500
## 3  -1.80820   5.704690  -1.69796  0.213560 -6.49241 -3.107020 -4.199020
## 4  -3.15177   0.179072  -7.69161 -5.339410  3.04637 -0.918825 -2.729080
## 5   3.77198   1.698420  -2.90316  1.430460  1.07501 -3.017070 -0.579252
## 6 -14.68680  -3.618290  -4.38952 -4.369510 -6.91279 -9.126700 -0.545057
##        PC74       PC75      PC76      PC77      PC78     PC79      PC80
## 1  0.231327 -1.2754700  1.246270 -1.712270 -4.138900  3.55127 -2.107750
## 2 -0.301757  2.1860700 -0.338938 11.385100 -7.813710  1.72406  2.052750
## 3 -5.492980  0.4709130 -2.822900  4.922300  0.295016  3.81379  0.700492
## 4 -7.533550 -6.0956200  0.511370  9.824630  3.150140 -1.08349 -1.012090
## 5 -4.479130  4.8110400  5.132770 -0.487381  5.428150  4.94736  1.994890
## 6  3.151760  0.0613404 -3.584050  5.391250 -2.866810 -5.49361 -3.083570
##       PC81     PC82      PC83       PC84      PC85      PC86      PC87
## 1  5.28099 -5.14851  0.325022  4.1409400  5.970620 -2.040030  3.222030
## 2  6.11735 13.17850  4.125460 -0.0308223  4.900560 -5.411130  6.052500
## 3  1.12850 -2.08788 -3.665780 -4.3984300  2.186200 -1.826860 -5.381630
## 4  6.48777  4.83060  1.563840  2.3136900 -0.119046  4.730270  4.461860
## 5 -6.25650  7.00440  3.027570 -3.0624000  0.205956  0.945207 -7.438620
## 6 -3.87576 -3.11304  2.919190  2.1620900 -2.159540  2.203050 -0.987369
##        PC88     PC89       PC90      PC91     PC92      PC93      PC94
## 1 -0.530004 -3.26248   6.621100 -2.292360  5.96309  4.933470  2.019270
## 2  1.064260 17.52780 -11.714400 -1.504350 10.56350  0.844441 -8.834320
## 3 -3.578730  5.71201   1.794490 -2.503470 -2.87977 -6.947080 -5.751710
## 4  6.303570 -2.65151   0.835482 -0.285191 -3.76685  1.560660 -6.009790
## 5 -5.011200 -5.70159   2.422000 -3.103240  2.27191  5.264060  2.309380
## 6 -0.441108 -8.11054   5.522290  5.211170  8.73967 -2.563150 -0.950538
##        PC95      PC96      PC97      PC98      PC99     PC100     PC101
## 1  4.736320  7.088930 -0.668144  1.418180 -0.209002  5.573900  7.422300
## 2 -7.457650  9.249020  0.620138 -1.077260  5.164860  0.887652 -4.067360
## 3 -0.050995  0.210320 -4.473580 -0.530080  5.797650  0.933697  6.568110
## 4  1.812090  0.241315 -5.657580 -1.569250  2.898310  1.370890 -1.343930
## 5  6.419450 -7.960010  5.249710  0.907076  3.443990 -2.191660  0.126683
## 6  3.361170 -4.834090 -1.236420 -1.609650 -0.239708 -0.667317 -3.126350
##       PC102     PC103     PC104     PC105     PC106    PC107     PC108
## 1 -0.657106 -3.711560 -0.438403 -0.278933 -2.428000  8.74538 -1.669810
## 2 -5.054280  0.698882 -2.000590 18.384800  6.175650 -1.32465 -0.736191
## 3 -2.739290 -3.831850  1.486880 -1.110660 -1.484460  6.00566  0.566043
## 4  3.875400 -6.478600 -2.366960 -2.908140 -6.827090  4.02170 12.162400
## 5  1.152970  5.981510 -3.396170  2.919650  0.945428 -2.54601  6.273550
## 6  7.908580 -2.693450 -3.348920 -2.303650  4.349400 -6.73301 -0.227774
##       PC109     PC110     PC111     PC112     PC113    PC114    PC115     PC116
## 1  5.128620  2.623700   5.76585  2.153440 -5.403180 -9.23018  4.68654 -7.874060
## 2 -0.732338  2.724570 -11.45300 -5.535510  3.642740 -7.58595  5.31279 -0.903193
## 3 -1.546680  1.810810   4.37526 -4.876230 -0.263220 -2.31640 -8.14349 -9.092660
## 4 -5.044460 -0.147918   5.07623  2.870950 -1.447350 -8.36466  5.14888 12.214900
## 5 -4.160120 -5.535830  -3.66665  0.615182 -0.892667  1.72892  6.05721 -3.470050
## 6 -5.585610 -7.258660  -2.49314 -3.782220  7.399490 -4.22810 -2.68037 -3.162670
##       PC117    PC118    PC119     PC120    PC121     PC122     PC123     PC124
## 1 -3.397750 -0.93385  0.50628  0.347119  2.86372  5.934220 -2.482740  0.405653
## 2 -4.014740  4.46196  5.24106 -0.358426  3.39293 -2.139070  4.032960  3.234490
## 3  4.181420  6.67050 -1.96763 -5.326560  3.35979 -5.415750  6.361300 -6.086410
## 4  8.931160  0.45548 -5.44323 -2.771740 -4.67030  0.115008  3.087120  7.058140
## 5  2.407410  2.08450 -8.65159  0.378612  3.41776 -2.353890 -0.443451  2.647230
## 6  0.537601  4.45086 -2.76682  1.935190 -6.86496 -3.997410  9.988110  8.569230
##       PC125     PC126     PC127    PC128      PC129     PC130    PC131
## 1  -3.78267 -0.959596 -3.757260 -1.96536   7.021990  2.208040 -3.61928
## 2   3.29124 -3.311020  4.193640 -3.93472   1.893790 -3.445530  1.69709
## 3   6.43624 -4.762060  0.605246  1.93644   4.985220 -0.316501 -4.64517
## 4 -11.03310 -0.790885  4.800590 -5.71428  -0.300568 -4.922140  7.27934
## 5  -1.22976  3.204140  8.008470  3.36502   4.007540 11.590000  5.59730
## 6  -3.16003  5.024640  1.756030  2.16938 -10.337600  4.968010  7.46395
##       PC132     PC133      PC134      PC135    PC136     PC137     PC138
## 1  0.126642 -1.538510   1.925550  8.9379800  1.89565  2.209070  0.535142
## 2 -0.488046  6.694480 -10.678700 -1.2127300  7.45124  0.655187  1.424560
## 3  0.767543  3.975580  -6.630340  1.0714000  3.11975  0.561161 -8.591150
## 4  0.599019 -9.374260   4.156190  4.3622000 -2.98200  4.391440 -4.374870
## 5  5.294510  0.787674  -0.563205  2.8477500 -4.57320 -3.525510 -3.024150
## 6  1.844470  2.668930   1.713450  0.0209458  3.29515 -2.101650  7.270360
##       PC139     PC140     PC141     PC142     PC143     PC144      PC145
## 1 -7.270470  6.193760 -4.263530  3.571320 -8.128750  6.166230 -12.962400
## 2 -1.456680 -6.907710 -5.726080 -3.803910  1.947130  0.124438   2.707260
## 3  2.440360 -0.343067 -5.847430 -7.106190 -0.245547 -4.407020  -0.270334
## 4 -0.932771  2.898260  3.091390 -0.910775  5.657400 -4.268230   1.227760
## 5 -0.118163  4.718830  0.569474  4.309310 -5.917510  6.257050  -3.047350
## 6  4.306110  1.574640  2.963100  0.678810  8.397230  5.249990   3.141650
##       PC146    PC147     PC148    PC149       PC150    PC151     PC152
## 1  3.824830 -0.34796  0.897063  5.14368 -10.8492000 -3.20751 -4.395440
## 2  0.844867  2.15331 -0.662361 -4.03157   0.1604100 -5.17555 -4.002140
## 3  1.134390 -3.79978  2.666020  2.11743   0.2364290 -3.91545  0.171645
## 4 -8.375700  2.16469  1.924420  2.08842   2.2476900 -4.00978 -6.576010
## 5  2.353030 -4.06575  4.598330  2.40760   0.0587527 -4.10498  1.101240
## 6  0.985576  8.86362  0.962283 -6.63833   4.6051200 -2.12386  8.515810
##       PC153     PC154    PC155      PC156       PC157    PC158     PC159
## 1 -0.070366  5.578400 -1.40858 -12.908300  0.00153047  1.52865 -1.168470
## 2  3.234460  4.691950 -3.45407   0.317834  5.17370000 -4.19339 -0.549459
## 3 -0.361074  0.641574 -2.44387   4.954970 -1.10541000 -2.01641 -8.968140
## 4 -4.597700  0.455373 -5.61026  -0.855726  1.57089000  5.79094 -2.266990
## 5 -5.999400 -0.312694  3.55422  10.250700 -4.25919000 -4.61625  2.714850
## 6  1.732130 -2.364010 12.21300   4.663230  3.32824000  8.74713  1.962990
##      PC160     PC161     PC162       PC163    PC164     PC165     PC166
## 1 -3.83602  3.812720 -1.422160   2.9371900  1.68546  0.926122 -4.664940
## 2 -1.42145 -2.610990 -3.538420  -0.4157710 -2.66586 -4.274430 -2.970800
## 3 -5.26787  0.799908 -2.256170  -2.4472500  6.60211  0.755113  1.606370
## 4  2.15422  3.802200  4.054270  -0.0820288 -3.02239 -3.763660  0.693989
## 5 -4.72533 -6.349740  3.724550  -1.3795300 -6.52155 -5.940420 -0.618287
## 6 -2.55158 -0.229141 -0.941174 -10.3001000 -1.95706  0.464993  1.787080
##       PC167    PC168    PC169     PC170    PC171     PC172     PC173    PC174
## 1 -2.826100  4.94685  1.67453 -2.033910 -4.36814  4.216660 -3.032600 2.644150
## 2 -1.989890 -7.59111  3.74217 -0.989662 -2.25311  0.749917  3.063770 2.451950
## 3  0.874232 -1.14198  1.35277 -3.939150 -2.46070 -0.811147  3.125280 0.506294
## 4  5.394910  3.57772 -4.33867  3.613300  1.85866  8.128490 -2.106390 2.475020
## 5 -4.395380  8.17669 -5.73779 -0.148145  2.07008 -2.087590 -0.777014 2.327180
## 6  7.353930 -6.22182 -1.12385 -1.865050  1.63176  1.361190  0.579398 0.503605
##       PC175      PC176      PC177     PC178     PC179    PC180     PC181
## 1 -10.08210 -11.171400   0.346150  5.581240  -7.62956  1.19574 -2.684900
## 2  -3.49562   6.169750   2.367550  0.351617  -2.28339  2.06171  0.466939
## 3   3.26020  -0.858506  -0.116998  0.620531   3.77093 -1.71398 -4.879450
## 4  20.02420  -5.798240 -14.230000 -7.810930 -15.67740 -3.90270 -4.706230
## 5  -3.26434   4.875520  -2.114820  2.980280   0.33782  1.24366  4.174620
## 6  -0.94533   6.973000   2.370030  0.285371   0.89141 -1.06832  0.563296
##       PC182     PC183      PC184      PC185    PC186     PC187     PC188
## 1  0.188815  3.824320  3.7495500 -0.0762377 -2.44152 -6.533790 -0.558750
## 2  0.244412 -0.958576 -0.8419750  0.3213520  3.69853 -1.157560 -0.147196
## 3  5.322230  0.777626  0.0338642 -2.2389000  5.40728  0.147915  2.895620
## 4 -1.335540 -2.543830  5.9914800 -1.0922500  3.76932  3.623790  2.390010
## 5  4.330530  1.747200  0.2209830  6.7466600 -7.39639  6.701560  0.818724
## 6  3.736450 -2.783660  5.8324900 -6.3352500  2.11887  6.392940  0.776560
##       PC189      PC190     PC191     PC192      PC193     PC194     PC195
## 1 -0.934120  0.0183799 -5.069080  6.498130 -16.672800  1.259050  3.413680
## 2  2.519300 -2.4337600  0.657442  0.724264   1.480660 -0.320228 -0.837873
## 3 -1.550130  0.3627760 -3.347980  3.301950  -3.319740  4.796400  1.509340
## 4  1.854820  0.9784090  3.771850  0.229686  -0.348027  2.257410 -1.600570
## 5 -0.535695 -1.5930400  2.873770 -1.650060  -0.372643 -5.417190  0.794908
## 6  4.407340  3.9490400 -5.293300  7.669370  -5.523100 -3.675240 -2.319500
##       PC196     PC197     PC198    PC199     PC200     PC201      PC202
## 1  1.958970  5.078250  4.374910  4.88411 -9.560390 -3.662360 -2.3278900
## 2 -0.860606  0.166253 -5.586820 -2.25330 -2.627020  0.182664 -1.9602800
## 3  0.960446  3.743580  0.766434  3.44275 -3.748520 -1.428110  2.0858300
## 4  7.278420  3.874920 -5.661730  1.14982  0.311987 -0.466025 -0.0285923
## 5 -0.521352 -1.525440  3.766320 -3.70086  3.963850 -3.698540  0.2538910
## 6  1.794850 -7.356920  5.980530 -3.92504  1.147500  8.885740 -1.1791800
##       PC203     PC204    PC205      PC206   PC207     PC208    PC209     PC210
## 1  2.714630 -2.268830 -4.18325  0.0863424 4.48783  0.773759  3.47498 -2.395270
## 2 -0.714704 -0.906976  1.71859  1.5096200 1.77764  2.467790  1.91882  0.155145
## 3  4.598290  2.387320  4.56784  8.3002200 3.48119  2.454290 -1.77750 -1.488320
## 4  0.704324  1.551820 -3.23928 -8.5330000 2.26548 -6.543670  3.07688  1.470010
## 5 -1.211580 -4.694790 -2.51235 -8.1425000 2.02517  1.859470 -2.26240 -9.887920
## 6  4.217670 -0.409574  5.97681 -1.9443300 0.60955  2.662250  9.11333 -2.011450
##        PC211     PC212     PC213     PC214    PC215     PC216    PC217    PC218
## 1  -0.394640  1.964870 -2.891250  1.954000  3.00147  0.801920  7.64249  1.94504
## 2   0.397055 -2.459440 -0.496394  0.382127  0.38223 -0.590851 -4.30286 -2.07899
## 3 -12.582200  0.156506 -5.182850  1.236510  6.09963  3.004130  4.70278 -8.48045
## 4  -1.838200 -4.099660  2.169520 -0.679515 -4.15324 -1.522490  4.80866  1.03957
## 5   0.910792 -8.918050  2.090660 -5.846680  6.68996 -3.827740 -1.70307 -2.31545
## 6   6.291330  4.255100  8.727600 -3.081740  2.10520 -3.048320  4.77583  3.05839
##      PC219     PC220     PC221     PC222     PC223    PC224     PC225    PC226
## 1 -4.87437  2.756860 -2.383320 -7.712340 -3.780500 -1.63184 -5.328480 -3.62216
## 2  2.56293 -2.512570  0.361596 -8.900430  0.232982 -1.51885 -0.847336 -1.45739
## 3  3.87312  1.936920 -7.782400  5.655150 -2.440870  5.25928  1.260390 -3.85867
## 4  4.00814  5.281470  5.975540 -2.835100 -3.647670  4.21371 -1.660970  2.74487
## 5 -1.98218 -3.587100 -9.079720  2.570090  0.758585  2.32126 -0.212984  1.52988
## 6 -3.10807 -0.793185 -7.618740 -0.315435  3.136900  3.84785 -1.303400  5.88656
##       PC227     PC228     PC229    PC230     PC231    PC232    PC233     PC234
## 1 -5.088360 -1.402390  1.152390  4.29929  2.536530  1.97275 -0.70844  8.193560
## 2  0.932152 -0.832472  1.633190  1.14829  0.245581  1.16688  2.17409  4.517090
## 3  4.362440  0.620721 -3.194310  2.72995 -4.792450  4.31590  2.84225 -0.652315
## 4  8.973660  1.411660 -3.177760 -4.59293 -0.266155 -1.08403 -9.81122 -0.849969
## 5 -8.228860  2.544410  0.457872  7.21057 -2.597240 -2.92169 -1.75765  1.990410
## 6  1.810130  1.733080 -0.942504  4.17821  5.344730 -3.80194  4.82304  6.056470
##        PC235    PC236    PC237    PC238    PC239     PC240    PC241     PC242
## 1 -0.7464180  5.21632  8.55963 -6.96398  9.19198 -6.161310 -1.47464 -0.697168
## 2  3.9163500 -2.65302  4.30088 -4.14856  1.15105 -2.640680 -3.07768  0.264590
## 3  0.0131922  3.25342  0.43648 -2.03602  1.35833  0.581505  8.79982 -2.366340
## 4 13.1430000 -2.20540 -4.48812  1.36140  1.12208 -0.838196 -1.24277 -5.516580
## 5  5.7839900 -3.56584 10.04070  3.31063 -6.38369 -3.393640  8.07421  7.189270
## 6  6.1457500  0.76470 -3.20347  3.99327 -3.74169  4.550220 -5.77577 -1.694310
##       PC243     PC244     PC245     PC246      PC247     PC248     PC249
## 1 13.333700  5.576050 -1.239540 -0.357543  -6.111140  2.058980  3.196560
## 2  1.666060  1.036640  3.043390  2.141470  -2.915000 -0.792180 -3.192470
## 3 -7.132270  1.150110  7.370600 -0.553853   5.128080  3.367460  3.284580
## 4 10.569900 -0.964825  3.062040 -5.474730   2.086520  0.691722 -0.684603
## 5 -0.101616  6.692970 -0.700377 -2.968900  -0.382645  1.290580  4.061240
## 6 -1.375550  5.677340  0.626276  3.156770 -14.020900 -5.799920 -2.515760
##      PC250      PC251    PC252      PC253     PC254      PC255     PC256
## 1 -1.64400 -4.2447900  5.18989  1.6887400 -5.590000 -8.2287200  0.665394
## 2 -1.53014  0.1782530 -2.73905 -0.0352778 -1.614410  0.2304930 -0.179812
## 3  9.94392  1.1124700 -3.00201  4.6158900  6.086550  2.1221400  1.418120
## 4 -2.43596 -0.1692030 -2.69604 -0.8521460 -3.475850  5.4875000 -6.219280
## 5 -3.28026  2.7553400 -3.52933  0.8286970 -0.606345 -0.4613970  6.683000
## 6  3.55522  0.0144444 -1.66613  0.4372780 -4.197080 -0.0433868 -3.417270
##       PC257       PC258     PC259     PC260     PC261     PC262     PC263
## 1 -4.873870  0.93690500  0.683188 -1.709740  2.581100  1.445500 -5.543400
## 2  1.245080  0.68112600  1.314560 -0.994295  0.852577  0.661598  1.759250
## 3 -0.782761 -3.42413000 -7.292070  3.758990 -5.222550  3.710030 -2.525970
## 4  2.080470 -0.57011600  1.432980 12.559400 -7.763980 -3.665920 -0.765734
## 5  2.114870  1.23512000  3.013640  5.853750 -4.929640  1.999400 -5.659180
## 6  1.052780  0.00943249  3.016280  0.742379  1.285880 -8.763650  1.128630
##      PC264    PC265     PC266    PC267     PC268    PC269    PC270    PC271
## 1 -1.08590 -1.23627  0.512276 -2.28152  2.486330  1.30453 -4.28997 -7.83571
## 2  1.02876 -1.20561  0.670559  2.91095  2.565640  1.25886 -2.65574  1.58004
## 3 -4.10272  2.22547  1.586310  1.29866  3.481860  1.02314 -8.34621 -5.66141
## 4  2.30142  1.11644 -2.541310 -5.78525 -6.566840  6.96523  2.24790  2.82306
## 5  2.04473  1.15359  1.857860  7.94041  0.728914  1.82319  4.17096 -3.54045
## 6  3.52365 -4.67904  6.572090 -1.55929  6.331430 -2.86012 -6.13941  5.99306
##       PC272    PC273     PC274    PC275    PC276      PC277     PC278    PC279
## 1 -7.329760  2.00278 -3.184920 -4.31070 -6.37826   0.640856 -5.680590 -4.05434
## 2 -0.259489  1.56703 -0.310923  1.66031 -2.97948  -2.562480  0.320110 -3.08778
## 3  0.988663  7.21799  2.265160  1.16198  4.82665 -10.964000 -3.204590  8.09929
## 4  5.010890 -5.48531 -5.386810  7.51082 -3.08967  -0.971138 -3.689710  2.33970
## 5 -1.206710 -3.35213  3.916480  9.07744 -1.62662   8.115100 -7.093010 -1.82301
## 6 -4.511980 -0.32990  4.439410 -0.37246  4.06378   5.909520  0.167962  0.70806
##      PC280     PC281     PC282    PC283     PC284     PC285      PC286
## 1 -2.85328 -9.366700  1.117890  1.89382 -0.727465 -3.928260  -2.354420
## 2  1.56034 -1.511250  2.031110 -3.23271  1.628170 -0.935814  -0.407018
## 3 -6.45590  7.869240 -2.731720  5.28271  9.516220 -0.833463  -3.712410
## 4  4.96582  5.710100  0.956343 -1.17913 -4.919500  4.117690   0.703435
## 5 -1.69143 -3.096110  3.126550 -4.66241 -3.618860 -5.187550 -10.253400
## 6 -1.20028 -0.382945 -6.582990 -1.51687 -3.456520 -4.946500  -3.968110
##       PC287     PC288     PC289     PC290     PC291     PC292    PC293
## 1  1.039960  2.684830  2.746460 -2.933200 -7.507010 -4.071410  0.50190
## 2 -0.372507  1.035650 -2.614140 -0.655991  0.124817  0.994161 -1.57681
## 3  1.247410 -7.160670 -6.628460 -6.452660 -2.022710 -7.200850 -1.26003
## 4 -0.767015 -2.832750  0.699317 -0.279379  5.468550  2.813600  1.57068
## 5 -2.898720 -0.139782  1.847690 -3.395560  3.591530 -8.061070 -2.28912
## 6  3.673550 -1.772260  3.653460  0.202938 -5.472250  3.268560 -3.16638
##       PC294     PC295     PC296      PC297    PC298    PC299     PC300
## 1 -9.700060 -5.635800  -4.23754  2.3727600 -8.93242 -7.57506 -0.798471
## 2  0.676625 -0.328823  -2.89251 -0.0782211  1.60992  3.14753 -1.666310
## 3  4.825970  2.385540   3.71774  6.0621900  1.24246 -9.57963  3.857940
## 4  2.534950  3.719600 -10.66960  3.5386900  2.15046 -4.58325 -2.866330
## 5  4.713970  5.462290  -1.75159  0.0667062  2.84208  3.77450 -3.856600
## 6  1.907420  3.052700  -2.91875 -5.4598400 -2.08828  1.97889  2.714210
##       PC301     PC302     PC303     PC304     PC305     PC306    PC307    PC308
## 1  2.220230 -3.870880 -4.724740 -0.107905 -0.507053 -3.849490  3.92465  4.79518
## 2  1.157260 -0.120526  0.634384 -1.687660  0.810788  0.313134 -2.09255 -1.20537
## 3 -0.510808 -0.388956 10.090000 -1.578980 -0.139043 -2.274670  3.18762  2.22550
## 4  0.955660  1.381080  4.593670  8.942690 -0.914333  1.318540 -1.38461  6.48929
## 5  1.194590  1.094490  6.071650  5.519930 -1.102880 -2.233010  1.86325 -3.10091
## 6 -3.725400  3.556900  0.923282 -8.906120  1.889060 -4.111410  4.84727 -3.03325
##       PC309    PC310     PC311      PC312    PC313    PC314     PC315     PC316
## 1  3.746720 2.606690 -1.834290  0.0310485  5.55022 -1.76071  4.867500 -2.538650
## 2 -0.567429 0.268489  0.340253 -1.4928900 -1.61792  2.67783  0.992026  0.557487
## 3 -1.151400 3.956640  0.682425  3.3183500  4.49801  2.68907 -7.872540 -2.979830
## 4 -2.016240 5.905570 -2.568050  0.2623070 -1.77317 -1.32013 -4.956110 -2.095980
## 5  1.813430 1.440450  0.312618  2.4014200 -4.31461 -4.28057  3.168620  4.307760
## 6 -1.393420 7.340470 -1.580140  1.5007300 -5.46020 -6.51318 -1.696610 -2.725250
##         PC317     PC318     PC319     PC320    PC321     PC322     PC323
## 1  4.54382000  3.800180 -1.208250  3.079120  6.55892 -3.629130  5.653320
## 2  0.50242800  0.493770  2.040080 -1.011550 -1.26275 -0.610932  1.715450
## 3 -0.00158978 -0.772950 -1.994350 -0.737769  4.84859 -1.249720 -6.363120
## 4 -3.15956000  8.188940  0.803847  4.461260 -2.35992 -1.879360  1.494330
## 5 -1.87332000 -4.257530  1.785570  5.907030 -5.12051  0.392979 -0.892116
## 6  4.32389000  0.358546  2.474230 -0.424670  4.52301 -1.396210  2.271310
##         PC324    PC325      PC326     PC327     PC328    PC329     PC330
## 1 -0.00928339 -4.96102  2.2032800 -0.296827  3.675690 -4.07087 -0.118911
## 2  0.11172600  1.34768 -0.0843422  2.840040  1.373130  1.93845  0.149358
## 3  5.55116000 -5.60627 -7.6707900  0.899685 -1.002880 -5.41074  3.289640
## 4  3.96258000 -4.63609  2.2827700 -4.925030 -1.456080  4.53641  2.702060
## 5 -3.16660000 -4.14615  1.6121500  3.276770  0.360275  4.38704 -5.092310
## 6 -2.79535000 -2.10138  2.6838500 -5.748530  7.181800  2.64188  0.237702
##       PC331     PC332     PC333      PC334      PC335      PC336    PC337
## 1 -5.916770 -1.284790 -0.370394 -0.0854155  0.0583285  0.2255270 -2.21454
## 2 -0.496061 -3.003670  1.261230 -0.8773730  0.7563090 -0.9461730  1.59091
## 3  4.351740  5.636270  4.025760  1.3095400 -2.3302700  3.1714800  3.86130
## 4 -0.528953  1.053340  2.475160 -4.8448100 -1.5443000  0.1784730 -3.43205
## 5 11.614200  0.725883 10.518400 -1.0077500 -2.4056200 -7.1827700  3.32373
## 6  7.154210 -5.042230 -5.360170  4.1688800 -7.0550500 -0.0462976 -6.26455
##       PC338       PC339     PC340     PC341     PC342     PC343    PC344
## 1 -0.131367  3.42245000 -2.238460  3.634460 -2.209770  6.774310  6.76492
## 2  2.384670  0.00517625  0.779699 -0.645329 -0.411928  0.827153  1.74797
## 3  1.038590  0.32669100 -2.463330 -5.760680  2.815740 -3.536760 -2.77782
## 4 -3.716260  1.29159000 -1.454840  6.571660 -3.297520  3.027490 -2.77212
## 5 -7.184320 -3.31216000 -2.295250  0.103981 -1.783510  3.913100  1.65136
## 6 -0.369256  3.62249000  3.985880  5.579640  3.499480  2.766380  3.22730
##      PC345      PC346     PC347    PC348     PC349      PC350     PC351
## 1  5.02669  5.1567500  1.431210 -3.46819  2.876210 -1.4527900  1.236700
## 2  1.73634  0.0507539  1.411520 -1.81151 -0.101204  0.0881367 -1.731180
## 3  2.70743  3.8343000  0.647935 -2.64239  6.081570 -6.3936800 -0.874577
## 4  4.00267 -3.9824400  5.248790  2.62389  3.400820  1.9820200 -3.276660
## 5  6.71456  0.7873650  7.120620 -2.91862 -6.558170 -1.0085500  3.551040
## 6 -2.63697 -1.1218700 -8.225740 -3.21600 -2.183860  2.0877800  3.996860
##       PC352   PC353     PC354     PC355    PC356    PC357       PC358    PC359
## 1 -1.026970 1.59683 -1.266480  0.936827 -1.36178 -4.44377  0.53325300  2.58964
## 2  0.624564 2.03997  0.475887 -0.417468 -1.79375 -1.11459 -2.32379000  0.16934
## 3 -2.654370 5.49927  3.007570  3.829600 -2.20574  3.02302 -2.89492000  1.17256
## 4 -4.244400 6.55734  0.981958 -2.662100 -1.19389  1.42622  0.75886100  7.05569
## 5  0.121022 1.76212 -1.490250  2.973510  7.39017  4.55596 -1.27018000 -1.95396
## 6  3.936140 1.22538 -0.413484  3.687400  3.52455 -5.28928 -0.00928066 -5.27909
##      PC360     PC361      PC362    PC363    PC364      PC365     PC366
## 1  1.80545  2.074040 -7.1609600  3.35555  6.89543  0.0713204  0.914068
## 2  1.96724  0.861001 -0.0871115 -1.34003  1.88769  1.3980800  1.881620
## 3 -4.63348 -2.398610  4.7706600 -3.76113 -7.49972 -8.9488400  1.496590
## 4 -4.60031  0.482272 -3.9529500  1.81794  4.68378  6.9870400 -0.806710
## 5 -2.17590  1.393070  3.2540100  1.42312  1.52375  1.5608000 -6.720430
## 6 -3.35735  1.585900 -3.4970100 -5.20771 -1.81326  0.4215800  5.600050
##       PC367     PC368     PC369      PC370     PC371    PC372    PC373
## 1 -3.587460 -2.249690 -6.656450 -3.6056400 -7.342320 -7.84418  4.07623
## 2 -0.989233  2.684460 -1.821880  1.0809300  0.735125 -2.00916  2.40413
## 3 -2.624080  8.594780  0.809595  1.1641300  2.179140  2.43491 -3.40001
## 4  2.629040  3.062990  3.032370  0.0815035 -1.305000  4.72387  1.13859
## 5 -3.247780  0.612742 -3.151590  6.5409200 -2.661310  4.66729  3.74673
## 6 -0.183168  4.657970 -0.761995  0.1596030  3.933050  1.38905 -5.47376
##       PC374     PC375      PC376     PC377     PC378     PC379     PC380
## 1 -4.591550 -5.022200 -1.0135200  1.313610 -3.917120  5.124860 -3.993420
## 2 -0.632698 -0.272874  1.7949300  1.944780 -0.433715  1.593160 -1.427100
## 3 -4.157310  0.378142 -2.2721000  0.963764 -2.025670  3.119000 -4.854350
## 4 -7.211870  0.433675 -1.8826600 -1.872820 -6.335140 -0.783052  4.413950
## 5 -3.005350  0.727318 -6.6826900 -2.062950 -1.078970 -4.145030 -1.658480
## 6 -1.517090  1.865580 -0.0983782  6.003940 -3.023170  1.277820  0.710488
##       PC381    PC382     PC383      PC384    PC385     PC386    PC387     PC388
## 1 -4.450120 -1.31275  6.443110 -2.2341500  2.76875 -3.561390  2.99685  2.648290
## 2  0.835317  1.40917 -0.144225 -1.1657800  0.56764 -0.596765 -1.96106 -2.112930
## 3  0.150455  5.98921 -4.633640 -0.0923729 -7.08395 -1.860370  2.22308 -2.623300
## 4  1.462980  1.14635 -0.455234 -4.2428800 -2.51643  0.745813 -4.42161 -0.697312
## 5  5.690160 -4.80184 -2.023750  4.6224600 -3.08228 -2.107670 -6.52644 -2.167640
## 6 -5.291020  5.37094 -3.228220 -2.4320500 -4.80438 -1.704080 -1.53141 -2.485050
##        PC389    PC390     PC391     PC392   PC393    PC394     PC395     PC396
## 1  7.5006300  1.48616 -2.063460  2.412820 7.11575  2.47707  0.401125 -0.425991
## 2 -0.0395277  1.53476 -0.382647 -0.229714 1.62765  1.26007 -0.137781  2.693730
## 3 -3.3484500  5.77978 -0.803894 -0.286327 3.76709 -5.33421  4.208810  3.106550
## 4 -0.5856000 -0.16044  2.234450  5.153820 5.52632 -3.46772  2.839100  2.595530
## 5  1.3321000 -4.63974  2.294640 -2.482320 2.01988 -2.48352  5.488380 -6.900840
## 6 -1.6763400  4.83712  4.459910 -3.818600 6.16376 -4.14964  2.596470 -4.796800
##       PC397     PC398   PC399    PC400     PC401      PC402     PC403    PC404
## 1 -3.904130  2.762440 1.83816 -1.14342 -6.970280 -0.1990370 -0.576808  3.05738
## 2 -0.823795  0.848779 1.94796 -1.80482  1.804250 -0.2969750 -0.341593  1.94480
## 3  2.814470 -4.940510 1.16636 -0.20531 -3.314980  0.0154421 -0.323527  1.25116
## 4  1.323850 -0.227157 5.30331 -4.26518  0.046471  2.4223600 -0.327952  5.15449
## 5 -1.255800 -0.994436 3.99903  4.44287 -4.230280 -1.6497100 -7.900830  1.62633
## 6  3.330340  1.559890 1.83300 -2.80202 -3.454870 -5.2443900  0.590253 -5.85795
##       PC405     PC406    PC407      PC408     PC409     PC410     PC411
## 1  5.477130 -2.109060  2.74720 -0.0978993  1.452330  6.465730  3.967370
## 2 -0.281547  0.981071  1.14258 -0.3374640 -1.819810  2.840900 -2.252280
## 3  5.092410  1.622720  3.56474  3.2150000  2.972820 -1.789010 -1.083130
## 4 -1.966410  5.830110  2.49537 -4.2072200 -1.094300  2.606080  2.572190
## 5  0.525684 -4.052190 -4.36734 -1.3001800 -1.352620  0.589463  0.694678
## 6  2.372440  3.420280  2.97205  2.3440200 -0.771741  1.499190 -0.538753
##       PC412     PC413     PC414     PC415    PC416     PC417     PC418
## 1  1.513680 -1.484770  1.320590  2.431980  1.83314 -0.399934 -3.403670
## 2  0.385739  0.354422  2.567860 -1.170470 -1.37678 -1.561750  1.663020
## 3 -0.909813  0.649331  0.505687  0.514364  7.32527  2.168480  0.864703
## 4  2.123820  6.896970 -0.733283  0.276757 -1.25859 -0.109752  0.461330
## 5  6.590720 -2.425220 -1.185820 -3.826810 -3.44824 -2.486020 -5.963630
## 6  0.655289  2.878700  4.751480  3.815820 -3.79620  2.377710  6.448860
##       PC419     PC420        PC421    PC422    PC423     PC424    PC425
## 1  5.270210 -0.866381 -2.971030000 -4.62366  1.01594 -2.197620  1.88468
## 2  1.729510 -0.306836  0.000611313  1.15957 -1.60884  1.599530  1.32225
## 3  2.613200  2.027580  0.327797000 -2.03480 -2.34663 -1.211220 -1.31418
## 4 -0.552878  0.349375 -5.188730000  2.77966  2.88405 -0.475909 -2.36136
## 5 -0.172184  1.734890  1.673010000 -4.39768  7.65586  3.640860 -0.12778
## 6  3.044620 -2.050930 -0.718595000  3.90096 -3.38420  0.263874  7.09668
##       PC426      PC427      PC428     PC429     PC430     PC431    PC432
## 1  0.786094 -0.9682690  0.3035280  2.473340  1.462380 -0.294859 -3.97456
## 2 -0.156254  0.0885389  2.0728800  0.832777 -0.653042 -1.977130  1.19116
## 3  2.864680 -0.7400960 -9.1075900 -1.364460  2.159990  0.428196 -3.50034
## 4  4.559830 -0.0890836 -2.0206500 -2.571940  1.274010 -4.711740 -3.79749
## 5 -0.893310 -0.4068820 -0.0618767 -2.789820  2.280770  2.686980  3.02211
## 6  3.949970 -0.4510780 -4.0327600 -5.174590 -4.577900  2.668800 -4.76543
##       PC433     PC434     PC435    PC436      PC437     PC438      PC439
## 1  0.525039  5.683470  1.253790  4.16422  0.1514780  2.077590  2.7017200
## 2  1.064570 -0.219409  1.024550 -3.91454 -2.4946100  0.519972 -0.9443940
## 3  6.302120 -3.504660  1.363690  1.51890  4.0614900 -2.151770  0.2045140
## 4  4.375710  1.836880 -8.575780 -2.88672 -1.9595000 -1.185590  0.0171134
## 5 -3.778730  2.105780  5.074890  5.94410  0.0642657  1.053270  4.4514500
## 6  0.248621 -0.659361 -0.721335  3.44244 -5.9948200  2.036320  0.6988900
##       PC440     PC441      PC442     PC443     PC444     PC445     PC446
## 1  3.478130  2.650730  0.0265873  1.468740  0.551681  3.123980  0.274975
## 2  2.460990  2.047160  0.7655430  0.519738  0.185966  2.596920 -0.669185
## 3 -0.248653  0.737382  2.2010100  2.551490  2.540350 -7.065750 -4.656480
## 4 -4.013080  1.275380  2.4519300  4.656620 -1.938350  0.918655 -0.789969
## 5  0.590021 -0.958030  3.8177700  3.800150  4.233990  1.440120 -1.365290
## 6  1.934680  1.824700 -1.7210900 -2.831340 -2.676090  3.497210 -0.636471
##       PC447      PC448     PC449     PC450      PC451     PC452     PC453
## 1  0.943669  0.3222070 -0.610943 -3.352920 -0.7633900  2.578130 -5.221440
## 2  1.277840 -1.6045300  0.768711 -0.786382  1.3518200  0.732161 -0.119844
## 3 -5.637940 -0.0953424  4.231170  5.463110  3.2283700  1.847780 -1.295080
## 4  0.307288  3.5746300  1.157850  1.532080 -0.0135395 -2.670650 -1.964340
## 5 -1.246110 -0.3256300  1.704850 -0.492563 -3.4679700  4.029230  6.204570
## 6  0.730778 -0.1868030  1.459650 -1.856750  4.1085700 -0.945264 -1.932000
##        PC454      PC455     PC456     PC457     PC458    PC459     PC460
## 1  0.0171485  2.1844400  3.398670 -3.698940  4.762940  1.60250 -3.584420
## 2 -1.0791000 -0.8752630 -1.097660  0.373039 -0.627842  1.12619 -1.683720
## 3  4.1066600  0.0900784  1.721680 -2.295000 -1.189870  6.02753 -1.324260
## 4  0.0064710 -2.4846100  0.248448 -0.939491  1.972010 -1.06610 -0.769755
## 5 -3.3118100  3.7472000 -3.503320  2.940560 -2.927760  2.78826 -0.997233
## 6  1.2293700  3.1958900  1.535960 -2.113510  2.521750  2.79820 -0.851069
##        PC461     PC462      PC463     PC464     PC465     PC466      PC467
## 1  1.5306900  1.911410 -0.0364005  0.943737  2.919350  0.831800  1.3913100
## 2  0.3326610 -2.869140  0.7106310  0.480045 -0.295295  2.299330 -0.0499542
## 3  1.1660700  0.490193 -1.5101600 -1.164320 -2.443770 -3.735920 -0.2688100
## 4 -0.0429172  0.868060  3.4937400 -1.110990  0.419046  1.027710 -0.5818150
## 5 -3.6738900 -2.152260 -0.8994190 -0.796951 -0.176934  3.317660 -1.6170000
## 6  1.4718400 -0.269137  3.0548600  0.296205 -1.249220  0.684541  1.9096100
##        PC468     PC469    PC470     PC471      PC472     PC473     PC474
## 1 -0.5121040 -2.855870 -1.00368  0.644857  1.4843100  4.323210  1.398230
## 2  0.9673960 -0.417946 -1.50102 -0.642071  0.0320515 -1.165800 -2.161900
## 3 -2.3682800  2.628450 -1.27675 -2.530250 -0.0596221  1.692040  1.260510
## 4  1.0768700  0.442405  3.32010 -0.944915 -0.0597380  0.181597  0.165373
## 5 -0.0350924 -1.524740 -2.49437  5.923570 -1.0688300 -1.987910 -2.285380
## 6 -3.5762400  1.465610 -1.50356  1.895210 -0.8260880  3.486740 -1.486170
##       PC475     PC476       PC477     PC478     PC479     PC480      PC481
## 1  3.715460 -1.012370 -1.42339000 -0.744890  2.423410  0.342832 -1.4203500
## 2 -1.547620  0.212393  0.33058200 -0.788348  2.200980 -2.923410  0.5246630
## 3  3.923500  1.239600 -0.00690138 -1.602790  1.229390 -0.632059  0.8435760
## 4 -2.006380 -1.560820  1.03383000  0.932708 -0.884526  2.010790 -0.0572739
## 5  2.104310  5.155550 -2.63705000 -0.145459 -1.187000 -0.898976  0.3481790
## 6  0.413208 -0.565201 -2.12431000 -1.653950  1.821130  1.904920  0.7377760
##       PC482     PC483     PC484     PC485      PC486     PC487     PC488
## 1 -1.921070 -1.180040  1.491620 -3.037480  0.5616240 -0.367146 -1.230730
## 2 -1.259640 -1.835420  1.333970  1.255300  0.5401430  2.062000  2.505620
## 3  0.883542 -2.305480 -1.454620  0.444798  0.0131505 -0.257337 -0.770173
## 4  0.631029  1.066100 -1.398270 -0.902583  1.0746200 -0.552828  0.777716
## 5 -1.196620 -0.952978 -0.779617 -1.362290  1.5069300  0.924644  1.254930
## 6  2.439870 -1.561850  0.198646  1.374870 -1.9758300 -0.514576 -0.991689
##       PC489     PC490     PC491     PC492    PC493      PC494     PC495
## 1 -0.914278 -1.780120 -0.787901 -1.849540 1.112280  0.0604858  0.488617
## 2 -1.563050  1.541820 -1.500460 -0.404847 2.157330 -1.6461700  2.648630
## 3 -1.015670 -0.329919  0.864335 -0.409024 1.901290 -1.1644500  0.365874
## 4  3.154060 -1.436690 -0.843937 -1.527060 0.646669  0.3183540 -0.123275
## 5 -0.671605 -1.366240  0.508480  0.501858 1.245040  1.6365200 -0.560485
## 6 -1.142480  1.215340  0.293404  0.459897 0.302576  0.3611180 -1.397980
##       PC496     PC497     PC498     PC499     PC500      PC501     PC502
## 1  0.733093  1.271520  1.628350 -1.262700 -1.274350 -0.3207380 -1.554820
## 2  0.349932 -4.347060 -2.745970  0.259622 -5.797190  0.0225125 -4.413610
## 3  1.285120  1.421910  2.348570 -0.867816  1.185440  0.7684300 -0.421904
## 4 -0.773926  1.202400  2.657070 -1.500920  0.811657  0.5829690 -0.888610
## 5  0.114218 -0.510377  0.917460 -0.986374  1.874170  1.1962400  0.497156
## 6 -0.786868  2.696500 -0.217572  1.073060 -0.163080  0.0373848  0.330402
##        PC503     PC504     PC505      PC506      PC507     PC508     PC509
## 1 -1.5934900  0.133980  0.896410  0.5930310 -1.1191400  0.689315 -0.730230
## 2  5.8638500 -0.993055 -7.948870 -7.3764300 -8.4887600 -3.189690 14.247600
## 3  0.0365379  0.170388 -1.571270 -1.5294700 -0.2052220 -1.668360  0.544411
## 4  0.6280200  0.595855  0.343254 -0.0844626 -0.0403622  0.554432  1.947950
## 5  0.5657390 -0.879019 -0.214798  0.3038780 -0.8475740  1.053100  1.250290
## 6 -0.0831645 -0.896696  0.629570 -0.0382268  0.1587960 -0.888060 -0.551374
##        PC510      PC511     PC512     PC513     PC514     PC515     PC516
## 1 -0.0198567  0.0681992 -1.815880 -1.610840 -0.849271  0.152202  1.559870
## 2 -8.6044000 10.1392000 15.166300  0.487336 -6.557290 -7.005940  5.014560
## 3 -1.1960000  0.7957590  1.690710  0.924593 -0.456587 -0.148577 -0.126537
## 4  1.2824700 -0.2063060  0.524405  0.164443 -0.186641 -1.202890 -0.319647
## 5 -0.6514960  1.3201000  1.254440  0.267732 -0.207517  0.378442  0.856908
## 6  0.9272960 -0.9241340 -0.524830  0.327355 -0.619398 -0.492314 -0.621013
##       PC517       PC518     PC519      PC520      PC521       PC522      PC523
## 1 -0.309358  0.57516300 -0.143574  0.4618070 -0.0779483  0.20217800 -0.1599620
## 2  0.591402 -2.93672000 -0.368286  3.5286600 -2.3197400  1.44825000  2.1441700
## 3 -0.427306  0.29463500 -0.255634  0.8550410 -0.6665440  0.00428308 -0.0277785
## 4 -0.243684 -0.33429300 -0.615472 -0.0987814 -0.2142360  0.00112852 -0.4625900
## 5  1.031470  0.43049400  0.162072 -0.3905520  0.5406870 -0.32682600  0.3403160
## 6  1.148320 -0.00863989  0.145185 -0.3640900 -0.0883258 -0.46487900 -0.4387000
##        PC524      PC525      PC526       PC527     PC528       PC529     PC530
## 1 -0.3247470 -0.7280600 -0.4218040 -0.22605300 -0.194452 -0.10914900  0.212500
## 2  0.8782630 -0.0290323  0.2808440  0.22650500 -1.126300  1.64010000  0.827327
## 3 -0.0239706  0.6265620  0.2450890  0.78117300 -0.254694 -0.23633200 -0.393528
## 4 -0.1717960 -0.6326330  0.1976010  0.28149100 -0.815461  0.38163400 -0.723423
## 5  0.1675390  0.4443020  0.0251795  0.33256700 -0.209301 -0.00447175 -0.314321
## 6  0.0221470  0.1972900  0.8707020 -0.00838563  0.105209 -0.38784900 -0.576005
##         PC531     PC532     PC533     PC534      PC535      PC536       PC537
## 1  0.00792147  0.207631 -0.273052 -0.240891 -0.1789940 -0.1808580  0.24309700
## 2  0.37732300 -0.346809  0.687152  1.447790 -0.0652823  0.2152600  0.76768100
## 3  0.14538700  0.703666 -0.298500  0.177227  0.5091980 -0.3896330  0.00665415
## 4  0.36728800  0.292089 -0.174403  0.653600  0.7054080 -0.2683850  0.43984200
## 5  0.13104800 -0.335982  0.550267  0.549334 -1.0428500 -0.5650790  0.27482000
## 6 -0.41345500 -0.250526  0.485182  0.167528 -0.4751090  0.0795655 -0.37445000
##        PC538     PC539     PC540      PC541      PC542      PC543       PC544
## 1 -0.0904709 -0.438768  0.150273  0.0416606  0.2834150  0.0822470  0.00232307
## 2  0.2701830  0.719226 -1.018120 -0.4707690  0.1569560  0.1648990 -0.16635200
## 3 -0.1175110  0.888642 -0.873648  0.0314515 -0.0913483 -0.2918250  0.19215300
## 4 -0.0458135  0.558246 -0.907166  0.0162600  0.1593500 -0.1585100 -0.09235080
## 5 -0.0793495  0.677557 -0.615030  0.1186170  0.0534421 -0.1038610 -0.02058730
## 6  0.0609217 -0.583374  0.367820  0.3314690  0.1126820  0.0525785  0.12134800
##          PC545 Individual   Subregion.x country  Pop_City Country Latitude
## 1 -1.13213e-06       2571 South America  Brazil Araguaina  Brazil -7.19119
## 2 -1.13213e-06       2582 South America  Brazil Araguaina  Brazil -7.19119
## 3 -1.13213e-06       2574 South America  Brazil Araguaina  Brazil -7.19119
## 4 -1.13213e-06       2572 South America  Brazil Araguaina  Brazil -7.19119
## 5 -1.13213e-06       2573 South America  Brazil Araguaina  Brazil -7.19119
## 6 -1.13213e-06       2579 South America  Brazil Araguaina  Brazil -7.19119
##   Longitude Continent    Year        Region   Subregion.y order order2 orderold
## 1  -48.2081  Americas unknown South America South America    NA     NA       NA
## 2  -48.2081  Americas unknown South America South America    NA     NA       NA
## 3  -48.2081  Americas unknown South America South America    NA     NA       NA
## 4  -48.2081  Americas unknown South America South America    NA     NA       NA
## 5  -48.2081  Americas unknown South America South America    NA     NA       NA
## 6  -48.2081  Americas unknown South America South America    NA     NA       NA
##   AM_order      state
## 1       43 TO, Brazil
## 2       43 TO, Brazil
## 3       43 TO, Brazil
## 4       43 TO, Brazil
## 5       43 TO, Brazil
## 6       43 TO, Brazil
```

## 1.1 Create PCA plot

```
# #   save the pca plot                                                       ####
ggsave(
  here("scripts", "RMarkdowns", "output", "americas", "americas_all", "lea", "r_01_MAF_01", "PCA_lea_americas_all_pc1_pc2_r01.pdf"
  ),
  width  = 8,
  height = 8,
  units  = "in"
)
```

```
## Warning in MASS::cov.trob(data[, vars], wt = weight * nrow(data)): Probable
## convergence failure
```

```
# #   save the pca plot                                                       ####
ggsave(
  here("scripts", "RMarkdowns", "output", "americas", "americas_all", "lea", "r_01_MAF_01", "PCA_lea_americas_all_pc1_pc3_r01.pdf"
  ),
  width  = 8,
  height = 8,
  units  = "in"
)
```

Create PCA plot by country

```
# #   save the pca plot                                                       ####
ggsave(
  here("scripts", "RMarkdowns", "output", "americas", "americas_all", "lea", "r_01_MAF_01", "PCA_lea_americas_all_pc1_pc2_r01_country.pdf"
  ),
  width  = 8,
  height = 8,
  units  = "in"
)
```

## 1.2 Run LEA for r<0.01 dataset

We will do 5 repetitions

```
# set output dir
# main options
# K = number of ancestral populations
# entropy = TRUE computes the cross-entropy criterion, # CPU = 4 is the number of CPU used (hidden input) project = NULL
project = snmf(
  genotype,
  K = 1:25,
  project = "new",
  repetitions = 5,
  percentage = 0.25,
  iterations = 500,
  CPU = 10,
  entropy = TRUE
)
```

```
project = load.snmfProject("americas/output/snps_sets/r2_0.01_b.snmfProject")
```

Cross entropy

```
# Open a new pdf file
pdf(here("scripts", "RMarkdowns", "output", "americas", "americas_all", "lea", "r_01_MAF_01", "lea_cross_entropy_americas_all_r01.pdf"), width = 6, height = 4)

# Create your plot
plot(project, col = "blue", pch = 19, cex = 1.2)

# Close the pdf file
dev.off()
```

```
## png 
##   2
```

```
plot(project, col = "blue", pch = 19, cex = 1.2)
```

Summary of project

*check with run is best*

```
summary(project)
```

```
## $repetitions
##                       K = 1 K = 2 K = 3 K = 4 K = 5 K = 6 K = 7 K = 8 K = 9
## with cross-entropy        5     5     5     5     5     5     5     5     5
## without cross-entropy     0     0     0     0     0     0     0     0     0
## total                     5     5     5     5     5     5     5     5     5
##                       K = 10 K = 11 K = 12 K = 13 K = 14 K = 15 K = 16 K = 17
## with cross-entropy         5      5      5      5      5      5      5      5
## without cross-entropy      0      0      0      0      0      0      0      0
## total                      5      5      5      5      5      5      5      5
##                       K = 18 K = 19 K = 20 K = 21 K = 22 K = 23 K = 24 K = 25
## with cross-entropy         5      5      5      5      5      5      5      5
## without cross-entropy      0      0      0      0      0      0      0      0
## total                      5      5      5      5      5      5      5      5
## 
## $crossEntropy
##          K = 1     K = 2     K = 3     K = 4     K = 5     K = 6     K = 7
## min  0.9515870 0.9191440 0.9152363 0.9125754 0.9106960 0.9089523 0.9073094
## mean 0.9518369 0.9193693 0.9154532 0.9129443 0.9110569 0.9092439 0.9074733
## max  0.9519650 0.9195660 0.9156336 0.9131770 0.9112891 0.9096823 0.9076779
##          K = 8     K = 9    K = 10    K = 11    K = 12    K = 13    K = 14
## min  0.9060086 0.9044533 0.9040313 0.9030077 0.9023313 0.9012454 0.9010480
## mean 0.9062601 0.9051845 0.9042480 0.9033171 0.9027253 0.9017693 0.9013852
## max  0.9068592 0.9055170 0.9046460 0.9035275 0.9034082 0.9020793 0.9019317
##         K = 15    K = 16    K = 17    K = 18    K = 19    K = 20    K = 21
## min  0.9002859 0.9001952 0.8996875 0.8995482 0.8991271 0.8992491 0.8990559
## mean 0.9008959 0.9008361 0.9001319 0.9003851 0.9001518 0.8998171 0.9001056
## max  0.9012857 0.9011431 0.9003816 0.9009851 0.9017916 0.9004261 0.9010234
##         K = 22    K = 23    K = 24    K = 25
## min  0.8998592 0.8998618 0.8998106 0.9002683
## mean 0.9003114 0.9010423 0.9004165 0.9008393
## max  0.9013351 0.9030486 0.9006970 0.9013553
```

```
ce2 = cross.entropy(project, K = 2)
ce2
```

```
##           K = 2
## run 1 0.9191701
## run 2 0.9195660
## run 3 0.9191440
## run 4 0.9195346
## run 5 0.9194316
```

```
ce3 = cross.entropy(project, K = 3)
ce3
```

```
##           K = 3
## run 1 0.9153029
## run 2 0.9156336
## run 3 0.9152363
## run 4 0.9155924
## run 5 0.9155010
```

```
ce4 = cross.entropy(project, K = 4)
ce4
```

```
##           K = 4
## run 1 0.9127042
## run 2 0.9131130
## run 3 0.9125754
## run 4 0.9131517
## run 5 0.9131770
```

```
ce5 = cross.entropy(project, K = 5)
ce5
```

```
##           K = 5
## run 1 0.9106960
## run 2 0.9112855
## run 3 0.9108196
## run 4 0.9111941
## run 5 0.9112891
```

```
ce6 = cross.entropy(project, K = 6)
ce6
```

```
##           K = 6
## run 1 0.9089920
## run 2 0.9096823
## run 3 0.9089523
## run 4 0.9093118
## run 5 0.9092813
```

```
ce7 = cross.entropy(project, K = 7)
ce7
```

```
##           K = 7
## run 1 0.9073094
## run 2 0.9076779
## run 3 0.9073319
## run 4 0.9075216
## run 5 0.9075256
```

```
ce8 = cross.entropy(project, K = 8)
ce8
```

```
##           K = 8
## run 1 0.9060086
## run 2 0.9062414
## run 3 0.9061221
## run 4 0.9068592
## run 5 0.9060694
```

```
ce9 = cross.entropy(project, K = 9)
ce9
```

```
##           K = 9
## run 1 0.9044533
## run 2 0.9055170
## run 3 0.9049908
## run 4 0.9054561
## run 5 0.9055055
```

```
ce19 = cross.entropy(project, K = 19)
ce19
```

```
##          K = 19
## run 1 0.8991271
## run 2 0.9017916
## run 3 0.8995109
## run 4 0.8999248
## run 5 0.9004043
```

```
#run 1 is best for k=19

# get the cross-entropy of all runs for K = 20
ce20 = cross.entropy(project, K = 20)
ce20
```

```
##          K = 20
## run 1 0.8992491
## run 2 0.8998280
## run 3 0.8997749
## run 4 0.8998074
## run 5 0.9004261
```

```
#run 1 is best for k= 20

# get the cross-entropy of all runs for K = 21
ce21 = cross.entropy(project, K = 21)
ce21
```

```
##          K = 21
## run 1 0.8999492
## run 2 0.8998019
## run 3 0.8990559
## run 4 0.9010234
## run 5 0.9006978
```

```
#run 3 is best for k= 21
```

## 1.3 Plots for LEA r2<0.01 dataset

Because the cross-entropy plot does not have a clear inflection
point, we will plot a range of plausible/best supported Ks. In the
manuscript, K = 20 is shown in Figure S8.

### 1.3.1 K=20

Import the data for R2<0.01 (MAF 1% filtered)

```
genotype <- here(
   "americas/output/snps_sets/r2_0.01_b.vcf"
  )

d <- read.vcfR(
  genotype
)
```

```
## Scanning file to determine attributes.
## File attributes:
##   meta lines: 8
##   header_line: 9
##   variant count: 18964
##   column count: 554
## Meta line 8 read in.
## All meta lines processed.
## gt matrix initialized.
## Character matrix gt created.
##   Character matrix gt rows: 18964
##   Character matrix gt cols: 554
##   skip: 0
##   nrows: 18964
##   row_num: 0
## Processed variant 1000Processed variant 2000Processed variant 3000Processed variant 4000Processed variant 5000Processed variant 6000Processed variant 7000Processed variant 8000Processed variant 9000Processed variant 10000Processed variant 11000Processed variant 12000Processed variant 13000Processed variant 14000Processed variant 15000Processed variant 16000Processed variant 17000Processed variant 18000Processed variant: 18964
## All variants processed
```

Get population and individuals information

```
inds_full <- attr(d@gt,"dimnames")[[2]]
inds_full <- inds_full[-1]
a <- strsplit(inds_full, '_')
pops <- unname(sapply(a, FUN = function(x) return(as.character(x[1])))) 
table(pops)
```

```
## pops
## ARA BAF BCO BEA BEH BER BIT BPA BPV BRM CHI COL CUA DAL DEN FAY FIR FLO GRV HAL 
##  12   7  12  12   9  12  12  11  12  16   6  10   8  12  12  11  12   8  12  10 
## HAR HAT HOU IPO LIN LOS MAC MAN MAU MED MON NEB NEG NEH NEO NEW NOV NUE PAL PEO 
##  12  10  12   7  12  12  12  12  12   7  12  10   4   7  12  21   4  12  11  10 
## PLM POR REC RUS SAI SMI SPR STL TRA TUC WAV 
##   7  12  11  12  12  12   7  12  12  14   7
```

```
pops <- factor(pops)
inds <- unname(sapply(a, FUN = function(x) return(as.character(x[2]))))
```

Convert format

```
vcf2geno(genotype, gsub(".vcf", ".geno", genotype))
```

```
## 
##  - number of detected individuals:   545
##  - number of detected loci:      18964
## 
## For SNP info, please check /gpfs/gibbs/pi/caccone/mkc54/albo/americas/output/snps_sets/r2_0.01_b.vcfsnp.
## 
## 0 line(s) were removed because these are not SNPs.
## Please, check /gpfs/gibbs/pi/caccone/mkc54/albo/americas/output/snps_sets/r2_0.01_b.removed file, for more informations.
```

```
## [1] "/gpfs/gibbs/pi/caccone/mkc54/albo/americas/output/snps_sets/r2_0.01_b.geno"
```

```
vcf2lfmm(genotype, gsub(".vcf", ".lfmm", genotype))
```

```
## 
##  - number of detected individuals:   545
##  - number of detected loci:      18964
## 
## For SNP info, please check /gpfs/gibbs/pi/caccone/mkc54/albo/americas/output/snps_sets/r2_0.01_b.vcfsnp.
## 
## 0 line(s) were removed because these are not SNPs.
## Please, check /gpfs/gibbs/pi/caccone/mkc54/albo/americas/output/snps_sets/r2_0.01_b.removed file, for more informations.
## 
## 
##  - number of detected individuals:   545
##  - number of detected loci:      18964
```

```
## [1] "/gpfs/gibbs/pi/caccone/mkc54/albo/americas/output/snps_sets/r2_0.01_b.lfmm"
```

```
color_palette <-
  c(
    "#1E90FF",
    "#005C31", 
    "#2BCE48",
    "#5EF1F2",
    "blue",
    "#C20088",
    "#FF5005", 
    "#FFE100",
    "purple3",
    "#FF0010",
    "#FFA8BB",
    "#990000",
    "#003380",
    "#4C005C",
    "#808080",
    "#993F00",
    "magenta",
    "#9DCC00",
    "#F0A0FF",
    "#FFFC99"
)
```

Default plot

```
# select the best run for chosen K 
best = which.min(cross.entropy(project, K = 20))
# best is run 1

barchart(project, K = 20, run = best,
        border = NA, space = 0,
        col = color_palette,
        xlab = "Individuals",
        ylab = "Ancestry proportions",
        main = "Ancestry matrix") -> bp
axis(1, at = 1:length(bp$order),
     labels = bp$order, las=1,
     cex.axis = .4)
```

Mean admixture by country using ggplot

```
sampling_loc <- readRDS(here("scripts", "RMarkdowns", "output", "sampling_loc_americas_all.rds"))

library(reshape2)
```

```
## 
## Attaching package: 'reshape2'
```

```
## The following object is masked from 'package:tidyr':
## 
##     smiths
```

```
# Extract ancestry coefficients
Q_values <- as.data.frame(Q(project, K = 20, run = best))

# Create a named vector to map countries to regions

# Add individual IDs and pops ids
Q_values$ind <- inds
Q_values$pop <- pops

# Melt the data frame for plotting
Q_melted <- melt(Q_values, id.vars = c("ind", "pop"))

# Join with sampling_loc to get sampling localities
Q_joined <- Q_melted |>
  left_join(sampling_loc, by = c("pop" = "Abbreviation"))

# Create a combined variable for Region and Country
Q_joined <- Q_joined |>
  mutate(subregion_state = interaction(Subregion, state, sep = "_"))

# Order the combined variable by Region and Country
Q_ordered <- Q_joined |>
  arrange(Subregion, state) |>
  mutate(subregion_state = factor(subregion_state, levels = unique(subregion_state)))

# Group by Country and calculate mean ancestry proportions
Q_grouped <- Q_ordered |>
  group_by(subregion_state, variable) |>
  summarise(value = mean(value), .groups = "drop")

# Create a data frame for borders
borders <- data.frame(subregion_state = unique(Q_grouped$subregion_state))

# Add the order of each country to ensure correct placement of borders
borders$order <- 1:nrow(borders) + 0.5  # Shift borders to the right edge of the bars


# Function to filter and normalize data
normalize_data <- function(df, min_value) {
  df |>
    filter(value > min_value) |>
    group_by(subregion_state) |>
    mutate(value = value / sum(value))
}

# Use the function
Q_grouped_filtered <- normalize_data(Q_grouped, 0.1)

# source the plotting function
source(
  here("scripts", "RMarkdowns",
   "analyses", "my_theme2.R"
  )
)

color_palette <-
    c(
    "#FFFC99",
    "#F0A0FF",    
    "#9DCC00",    
    "#990000",
    "#4C005C",  
    "#FF5005", 
    "#FFA8BB",    
    "purple3",    
    "blue", 
    "#808080",
    "#003380",
    "#993F00",   
    "magenta",    
    "#FFE100",    
    "#2BCE48",    
    "#5EF1F2",    
    "#1E90FF",    
    "#C20088",
    "#005C31",     
    "#FF0010"    
    )
  

# Generate all potential variable names
all_variables <- paste0("V", 1:20)

# Create a data frame that pairs each potential variable with a color
color_mapping <- data.frame(variable = all_variables,
                           color = color_palette[1:length(all_variables)])

# Merge this data frame with Q_grouped_filtered to create the new color column
Q_grouped_filtered <- merge(Q_grouped_filtered, color_mapping, by = "variable")

# Create bar chart
ggplot(Q_grouped_filtered, aes(x = subregion_state, y = value, fill = color)) +
  geom_bar(stat = 'identity', width = 1) +
  geom_segment(data = borders, aes(x = order, xend = order, y = 0, yend = 1, fill = NULL), linetype = "solid", color = "#2C444A") +  # Add borders
  my_theme() +
  theme(axis.text.x = element_text(angle = 90, hjust = 1),
        legend.position = "none") +  # Hide legend
  xlab("") +  # Suppress x-axis label
  ylab("Ancestry proportions") +
  ggtitle("Ancestry matrix") +
  labs(caption = "Each bar represents the average ancestry proportions for individuals in a given location for k=20.") +
  # scale_fill_manual(values = color) +
  scale_x_discrete(labels = function(x) gsub(".*_", "", x)) + # Remove Region prefix from labels
  scale_fill_manual(values = color_palette)
```

```
ggsave(
  here("scripts", "RMarkdowns",
    "output", "americas", "americas_all", "lea", "r_01_MAF_01", "LEA_admixture_by_state_americas_k20_r01.pdf"
  ),
  width  = 10,
  height = 7,
  units  = "in"
)
```

Extract ancestry coefficients for k=20

*change to correct matrix*

```
leak20 <- read_delim(
  here("/gpfs/gibbs/pi/caccone/mkc54/albo/americas/output/snps_sets/r2_0.01_b.snmf/K20/run1/r2_0.01_b_r1.20.Q"),
  delim = " ", # Specify the delimiter if different from the default (comma)
  col_names = FALSE,
  show_col_types = FALSE
) 
head(leak20)
```

```
## # A tibble: 6 × 20
##      X1        X2      X3     X4      X5      X6      X7      X8      X9     X10
##   <dbl>     <dbl>   <dbl>  <dbl>   <dbl>   <dbl>   <dbl>   <dbl>   <dbl>   <dbl>
## 1 0.473 0.0239    1.49e-4 0.105  6.97e-2 9.99e-5 5.45e-2 1.64e-2 9.99e-5 9.14e-2
## 2 0.702 0.000419  9.99e-5 0.0711 9.99e-5 3.36e-2 9.99e-5 9.99e-5 9.99e-5 9.99e-5
## 3 0.537 0.0000999 3.02e-2 0.0895 5.66e-2 9.99e-5 9.99e-5 9.99e-5 5.35e-2 8.43e-2
## 4 0.476 0.0724    9.99e-5 0.180  9.99e-5 9.99e-5 9.99e-5 1.97e-3 9.99e-5 9.99e-5
## 5 0.564 0.0933    9.99e-5 0.0654 9.99e-5 9.99e-5 1.36e-2 3.16e-2 7.26e-3 3.29e-3
## 6 0.412 0.0227    9.99e-5 0.154  9.99e-5 2.04e-2 1.40e-1 8.82e-4 3.89e-2 9.99e-5
## # ℹ 10 more variables: X11 <dbl>, X12 <dbl>, X13 <dbl>, X14 <dbl>, X15 <dbl>,
## #   X16 <dbl>, X17 <dbl>, X18 <dbl>, X19 <dbl>, X20 <dbl>
```

The fam file

```
fam_file <- here("americas/output/snps_sets/r2_0.01_b.fam")

# Read the .fam file
fam_data <- read.table(fam_file, 
                       header = FALSE,
                       col.names = c("FamilyID", "IndividualID", "PaternalID", "MaternalID", "Sex", "Phenotype"))

# View the first few rows
head(fam_data)
```

```
##   FamilyID IndividualID PaternalID MaternalID Sex Phenotype
## 1      MAU          153          0          0   0        -9
## 2      MAU          154          0          0   0        -9
## 3      MAU          155          0          0   0        -9
## 4      MAU          156          0          0   0        -9
## 5      MAU          158          0          0   0        -9
## 6      MAU           16          0          0   0        -9
```

Create column ID

```
# Change column name
colnames(fam_data)[colnames(fam_data) == "IndividualID"] <- "ind"

# Change column name
colnames(fam_data)[colnames(fam_data) == "FamilyID"] <- "pop"

# Select ID
fam_data <- fam_data |>
  dplyr::select("ind", "pop")

# View the first few rows
head(fam_data)
```

```
##   ind pop
## 1 153 MAU
## 2 154 MAU
## 3 155 MAU
## 4 156 MAU
## 5 158 MAU
## 6  16 MAU
```

Add it to the matrix

```
leak20 <- fam_data |>
  dplyr::select(ind, pop) |>
  bind_cols(leak20)

head(leak20)
```

```
##   ind pop       X1          X2          X3        X4          X5          X6
## 1 153 MAU 0.473358 0.023895200 1.48781e-04 0.1051990 6.97350e-02 9.99370e-05
## 2 154 MAU 0.702265 0.000418555 9.98920e-05 0.0711284 9.98920e-05 3.36483e-02
## 3 155 MAU 0.536625 0.000099937 3.01508e-02 0.0895366 5.66425e-02 9.99370e-05
## 4 156 MAU 0.475596 0.072416600 9.99100e-05 0.1804070 9.99100e-05 9.99100e-05
## 5 158 MAU 0.564384 0.093271400 9.99101e-05 0.0653532 9.99101e-05 9.99101e-05
## 6  16 MAU 0.411931 0.022742600 9.99370e-05 0.1537390 9.99370e-05 2.03662e-02
##            X7          X8          X9         X10         X11         X12
## 1 0.054499700 0.016413100 0.000099937 0.091402000 4.56600e-02 9.99370e-05
## 2 0.000099892 0.000099892 0.000099892 0.000099892 9.98920e-05 9.98920e-05
## 3 0.000099937 0.000099937 0.053501900 0.084286900 1.48656e-04 9.99370e-05
## 4 0.000099910 0.001970130 0.000099910 0.000099910 9.24456e-03 9.99100e-05
## 5 0.013625000 0.031629800 0.007258370 0.003289720 9.99101e-05 9.99101e-05
## 6 0.139779000 0.000882050 0.038898900 0.000099937 9.59894e-04 9.99370e-05
##           X13         X14         X15         X16         X17         X18
## 1 0.003457710 9.99370e-05 4.73291e-02 9.99370e-05 3.48415e-02 9.99370e-05
## 2 0.000099892 8.51901e-02 9.98920e-05 2.90024e-02 9.98920e-05 9.98920e-05
## 3 0.023375300 2.56427e-02 9.99370e-05 9.99370e-05 1.92428e-02 6.91800e-03
## 4 0.026243900 9.99100e-05 9.99100e-05 5.40022e-03 3.70372e-02 1.01416e-01
## 5 0.045183700 9.99101e-05 9.99101e-05 9.99101e-05 9.99101e-05 9.99101e-05
## 6 0.010301700 7.44103e-02 9.99370e-05 9.99370e-05 3.11981e-02 9.99370e-05
##         X19         X20
## 1 0.0333609 0.000099937
## 2 0.0340467 0.043102300
## 3 0.0309365 0.042292700
## 4 0.0892689 0.000099910
## 5 0.1058170 0.069188700
## 6 0.0888973 0.005194760
```

Rename the columns

```
# Rename the columns starting from the third one
leak20 <- leak20 |>
  rename_with(~paste0("v", seq_along(.x)), .cols = -c(ind, pop))

# View the first few rows
head(leak20)
```

```
##   ind pop       v1          v2          v3        v4          v5          v6
## 1 153 MAU 0.473358 0.023895200 1.48781e-04 0.1051990 6.97350e-02 9.99370e-05
## 2 154 MAU 0.702265 0.000418555 9.98920e-05 0.0711284 9.98920e-05 3.36483e-02
## 3 155 MAU 0.536625 0.000099937 3.01508e-02 0.0895366 5.66425e-02 9.99370e-05
## 4 156 MAU 0.475596 0.072416600 9.99100e-05 0.1804070 9.99100e-05 9.99100e-05
## 5 158 MAU 0.564384 0.093271400 9.99101e-05 0.0653532 9.99101e-05 9.99101e-05
## 6  16 MAU 0.411931 0.022742600 9.99370e-05 0.1537390 9.99370e-05 2.03662e-02
##            v7          v8          v9         v10         v11         v12
## 1 0.054499700 0.016413100 0.000099937 0.091402000 4.56600e-02 9.99370e-05
## 2 0.000099892 0.000099892 0.000099892 0.000099892 9.98920e-05 9.98920e-05
## 3 0.000099937 0.000099937 0.053501900 0.084286900 1.48656e-04 9.99370e-05
## 4 0.000099910 0.001970130 0.000099910 0.000099910 9.24456e-03 9.99100e-05
## 5 0.013625000 0.031629800 0.007258370 0.003289720 9.99101e-05 9.99101e-05
## 6 0.139779000 0.000882050 0.038898900 0.000099937 9.59894e-04 9.99370e-05
##           v13         v14         v15         v16         v17         v18
## 1 0.003457710 9.99370e-05 4.73291e-02 9.99370e-05 3.48415e-02 9.99370e-05
## 2 0.000099892 8.51901e-02 9.98920e-05 2.90024e-02 9.98920e-05 9.98920e-05
## 3 0.023375300 2.56427e-02 9.99370e-05 9.99370e-05 1.92428e-02 6.91800e-03
## 4 0.026243900 9.99100e-05 9.99100e-05 5.40022e-03 3.70372e-02 1.01416e-01
## 5 0.045183700 9.99101e-05 9.99101e-05 9.99101e-05 9.99101e-05 9.99101e-05
## 6 0.010301700 7.44103e-02 9.99370e-05 9.99370e-05 3.11981e-02 9.99370e-05
##         v19         v20
## 1 0.0333609 0.000099937
## 2 0.0340467 0.043102300
## 3 0.0309365 0.042292700
## 4 0.0892689 0.000099910
## 5 0.1058170 0.069188700
## 6 0.0888973 0.005194760
```

Import Sample Locations

```
sampling_loc <- readRDS(here("scripts", "RMarkdowns", "output", "sampling_loc_americas_all.rds"))
head(sampling_loc)
```

```
##       Pop_City Country Latitude Longitude Continent Abbreviation Year
## 1   Berlin, NJ     USA 39.79081  -74.9291  Americas          BER 2018
## 2 Columbus, OH     USA 39.97170  -82.9071  Americas          COL 2015
## 3   Palm Beach     USA 26.70560  -80.0364  Americas          PAL 2018
## 4  Houston, TX     USA 29.75491  -95.3505  Americas          HOU 2018
## 5  Los Angeles     USA 34.05220 -118.2437  Americas          LOS 2018
## 6   Manaus, AM  Brazil -3.09161  -60.0325  Americas          MAU 2017
##          Region      Subregion order order2 orderold AM_order      state
## 1 North America Northeast, USA     1     NA       75        6    NJ, USA
## 2 North America   Midwest, USA     2     NA       76       18    OH, USA
## 3 North America Southeast, USA     3     NA       77       13    FL, USA
## 4 North America Southwest, USA     4     NA       78       23    TX, USA
## 5 North America      West, USA     5     NA       79       30    CA, USA
## 6 South America  South America     6     NA       80       38 AM, Brazil
```

```
source(
  here("scripts", "RMarkdowns",
    "my_theme3.R"
  )
)

# Melt the data frame for plotting
Q_melted <- leak20 |>
  pivot_longer(
    cols = -c(ind, pop),
    names_to = "variable",
    values_to = "value"
  )
# Join with sampling_loc to get sampling localities
Q_joined <- Q_melted |>
  left_join(sampling_loc, by = c("pop" = "Abbreviation"))

# Order the combined variable by Region and Country, then by individual
Q_ordered <- Q_joined |>
  arrange(AM_order, ind) |>
  mutate(ind = factor(ind, levels = unique(ind)))  # Convert ind to a factor with levels in the desired order

# Add labels: country names for the first individual in each country, NA for all other individuals
Q_ordered <- Q_ordered |>
  group_by(state) |>
  mutate(label = ifelse(row_number() == 1, as.character(state), NA))

# Group by individual and variable, calculate mean ancestry proportions
Q_grouped <- Q_ordered |>
  group_by(ind, variable) |>
  summarise(value = mean(value), .groups = "drop")

# Create a data frame for borders
borders <-
  data.frame(state = unique(Q_ordered$state))

# Add the order of the last individual of each country to ensure correct placement of borders
borders$order <-
  sapply(borders$state, function(rc)
    max(which(Q_ordered$state == rc))) + 0.5  # Shift borders to the right edge of the bars

# Select only the first occurrence of each country in the ordered data
label_df <- Q_ordered |>
  filter(!is.na(label)) |>
  distinct(label, .keep_all = TRUE)

# Create a custom label function
label_func <- function(x) {
  labels <- rep("", length(x))
  labels[x %in% label_df$ind] <- label_df$label
  labels
}

# Calculate the position of lines
border_positions <- Q_ordered |>
  group_by(state) |>
  summarise(pos = max(as.numeric(ind)) + 0)

# Calculate the position of population labels and bars
pop_labels <- Q_ordered |>
  mutate(Name = paste(pop, Pop_City, sep = " - ")) |>
  group_by(pop) |>
  slice_head(n = 1) |>
  ungroup() |>
  dplyr::select(ind, Pop_City, state, Name) |>
  mutate(pos = as.numeric(ind))  # calculate position of population labels

pop_labels_bars <- pop_labels |>
  mutate(pos = as.numeric(ind)  - .5)


# Calculate the position of lines
border_positions <- Q_ordered |>
  group_by(Country) |>
  summarise(pos = max(as.numeric(ind)) - 1)


pop_labels_bars <- pop_labels |>
  mutate(pos = as.numeric(ind)  - .5)

# Function to filter and normalize data
normalize_data <- function(df, min_value) {
  df |>
    filter(value > min_value) |>
    group_by(ind) |>
    mutate(value = value / sum(value))
}

# Use the function
Q_grouped_filtered <- normalize_data(Q_grouped, 0.1)
# 

color_palette <-
  c(
   "#CC0033",
   "cyan",
   "#AC7700", 
   "#1E90FF",  
   "magenta",
   "#FFFC99", 
   "#F0A0FF", 
   "#FF8005", 
   "#4C005C", 
   "#C20088",
   "purple3",  
   "#003380",
   "#808080", 
   "gray", 
   "green",
   "blue",
   "#2BCE48", 
   "#8c61cd",
   "#FF5005",
   "#FFE100"
  )

  
 
# Generate all potential variable names
all_variables <- paste0("v", 1:20)


# Create a data frame that pairs each potential variable with a color
color_mapping <- data.frame(variable = all_variables,
                           color = color_palette[1:length(all_variables)])

# Merge with Q_grouped_filtered
Q_grouped_filtered <- merge(Q_grouped_filtered, color_mapping, by = "variable")

# Create the plot
ggplot(Q_grouped_filtered, aes(x = as.factor(ind), y = value, fill = color)) +
  geom_bar(stat = 'identity', width = 1) +
  geom_vline(
    data = pop_labels_bars,
    aes(xintercept = pos),
    color = "#2C444A",
    linewidth = .2
  ) +
  geom_text(
    data = pop_labels,
    aes(x = as.numeric(ind), y = 1, label = Name),
    vjust = 1.5,
    hjust = 0,
    size = 2,
    angle = 90,
    inherit.aes = FALSE
  ) +
  my_theme() +
  theme(
    axis.text.x = element_text(
      angle = 90,
      hjust = 1,
      size = 12
    ),
    legend.position = "none",
    plot.margin = unit(c(3, 0.5, 0.5, 0.5), "cm")
  ) +
  xlab("Admixture matrix") +
  ylab("Ancestry proportions") +
  labs(caption = "Each bar represents the ancestry proportions for an individual for k=20.\n LEA inference with 18,964 SNPs.") +
  scale_x_discrete(labels = label_func) +
  scale_fill_manual(values = color_palette) +
  expand_limits(y = c(0, 1.5))
```

```
ggsave(
  here("scripts", "RMarkdowns", "output", "americas", "americas_all", "lea", "r_01_MAF_01", "lea_k=20_americas_all_r01_new.pdf"),
  width  = 12,
  height = 6,
  units  = "in",
  device = cairo_pdf
)
```

### 1.3.2 K=21

Default plot

```
# select the best run for chosen K 
best = which.min(cross.entropy(project, K = 21))
# best is run 1

barchart(project, K = 21, run = best,
        border = NA, space = 0,
        col = color_palette,
        xlab = "Individuals",
        ylab = "Ancestry proportions",
        main = "Ancestry matrix") -> bp
axis(1, at = 1:length(bp$order),
     labels = bp$order, las=1,
     cex.axis = .4)
```

Mean admixture by country using ggplot

```
sampling_loc <- readRDS(here("scripts", "RMarkdowns", "output", "sampling_loc_americas_all.rds"))

library(reshape2)

# Extract ancestry coefficients
Q_values <- as.data.frame(Q(project, K = 21, run = best))

# Create a named vector to map countries to regions

# Add individual IDs and pops ids
Q_values$ind <- inds
Q_values$pop <- pops

# Melt the data frame for plotting
Q_melted <- melt(Q_values, id.vars = c("ind", "pop"))

# Join with sampling_loc to get sampling localities
Q_joined <- Q_melted |>
  left_join(sampling_loc, by = c("pop" = "Abbreviation"))

# Create a combined variable for Region and Country
Q_joined <- Q_joined |>
  mutate(subregion_state = interaction(Subregion, state, sep = "_"))

# Order the combined variable by Region and Country
Q_ordered <- Q_joined |>
  arrange(Subregion, state) |>
  mutate(subregion_state = factor(subregion_state, levels = unique(subregion_state)))

# Group by Country and calculate mean ancestry proportions
Q_grouped <- Q_ordered |>
  group_by(subregion_state, variable) |>
  summarise(value = mean(value), .groups = "drop")

# Create a data frame for borders
borders <- data.frame(subregion_state = unique(Q_grouped$subregion_state))

# Add the order of each country to ensure correct placement of borders
borders$order <- 1:nrow(borders) + 0.5  # Shift borders to the right edge of the bars


# Function to filter and normalize data
normalize_data <- function(df, min_value) {
  df |>
    filter(value > min_value) |>
    group_by(subregion_state) |>
    mutate(value = value / sum(value))
}

# Use the function
Q_grouped_filtered <- normalize_data(Q_grouped, 0.1)

# source the plotting function
source(
  here("scripts", "RMarkdowns",
   "analyses", "my_theme2.R"
  )
)

color_palette <-
    c(
    "#1E90FF",  
    "gray",
    "#FFE100",  
    "#FFA8BB",  
    "#003380",
    "blue",
    "#005C31",   
    "#4C005C", 
    "magenta",
    "#FFFC99",
    "#F0A0FF",   
    "#2BCE48", 
    "#9DCC00",  
    "#990000",
    "#5EF1F2",   
    "purple3",  
    "#808080", 
    "#FF5005",     
    "#C20088",
    "#993F00",     
    "#FF0010"
    )
  
 
# Generate all potential variable names
all_variables <- paste0("V", 1:21)

# Create a data frame that pairs each potential variable with a color
color_mapping <- data.frame(variable = all_variables,
                           color = color_palette[1:length(all_variables)])

# Merge this data frame with Q_grouped_filtered to create the new color column
Q_grouped_filtered <- merge(Q_grouped_filtered, color_mapping, by = "variable")

# Create bar chart
ggplot(Q_grouped_filtered, aes(x = subregion_state, y = value, fill = color)) +
  geom_bar(stat = 'identity', width = 1) +
  geom_segment(data = borders, aes(x = order, xend = order, y = 0, yend = 1, fill = NULL), linetype = "solid", color = "#2C444A") +  # Add borders
  my_theme() +
  theme(axis.text.x = element_text(angle = 90, hjust = 1),
        legend.position = "none") +  # Hide legend
  xlab("") +  # Suppress x-axis label
  ylab("Ancestry proportions") +
  ggtitle("Ancestry matrix") +
  labs(caption = "Each bar represents the average ancestry proportions for individuals in a given location for k=21.") +
  # scale_fill_manual(values = color) +
  scale_x_discrete(labels = function(x) gsub(".*_", "", x)) + # Remove Region prefix from labels
  scale_fill_manual(values = color_palette)
```

```
ggsave(
  here("scripts", "RMarkdowns",
    "output", "americas", "americas_all", "lea", "r_01_MAF_01", "LEA_admixture_by_state_americas_k21_r01.pdf"
  ),
  width  = 10,
  height = 7,
  units  = "in"
)
```

Extract ancestry coefficients for k=21

*change to correct matrix*

```
leak21 <- read_delim(
  here("/gpfs/gibbs/pi/caccone/mkc54/albo/americas/output/snps_sets/r2_0.01_b.snmf/K21/run3/r2_0.01_b_r3.21.Q"),
  delim = " ", # Specify the delimiter if different from the default (comma)
  col_names = FALSE,
  show_col_types = FALSE
) 
head(leak21)
```

```
## # A tibble: 6 × 21
##        X1      X2      X3      X4      X5      X6      X7      X8     X9     X10
##     <dbl>   <dbl>   <dbl>   <dbl>   <dbl>   <dbl>   <dbl>   <dbl>  <dbl>   <dbl>
## 1 9.99e-5 9.99e-5 9.99e-5 8.88e-2 9.99e-5 9.99e-5 9.99e-5 9.99e-5 0.126  2.41e-2
## 2 3.17e-3 9.99e-5 2.67e-2 1.02e-1 1.05e-2 9.99e-5 9.99e-5 9.99e-5 0.0510 9.99e-5
## 3 5.78e-2 2.98e-2 2.39e-2 1.66e-2 9.99e-5 8.68e-4 9.99e-5 3.93e-2 0.102  2.97e-2
## 4 6.70e-2 9.99e-5 1.28e-3 2.67e-3 4.23e-2 9.99e-5 9.99e-5 1.87e-2 0.185  1.00e-2
## 5 9.99e-5 9.99e-5 2.33e-2 9.99e-5 9.99e-5 9.99e-5 3.49e-3 1.30e-2 0.0858 9.99e-5
## 6 1.82e-4 9.99e-5 3.18e-2 1.74e-2 2.99e-2 1.65e-2 1.39e-2 3.72e-2 0.170  5.58e-2
## # ℹ 11 more variables: X11 <dbl>, X12 <dbl>, X13 <dbl>, X14 <dbl>, X15 <dbl>,
## #   X16 <dbl>, X17 <dbl>, X18 <dbl>, X19 <dbl>, X20 <dbl>, X21 <dbl>
```

The fam file

```
fam_file <- here("americas/output/snps_sets/r2_0.01_b.fam")

# Read the .fam file
fam_data <- read.table(fam_file, 
                       header = FALSE,
                       col.names = c("FamilyID", "IndividualID", "PaternalID", "MaternalID", "Sex", "Phenotype"))

# View the first few rows
head(fam_data)
```

```
##   FamilyID IndividualID PaternalID MaternalID Sex Phenotype
## 1      MAU          153          0          0   0        -9
## 2      MAU          154          0          0   0        -9
## 3      MAU          155          0          0   0        -9
## 4      MAU          156          0          0   0        -9
## 5      MAU          158          0          0   0        -9
## 6      MAU           16          0          0   0        -9
```

Create column ID

```
# Change column name
colnames(fam_data)[colnames(fam_data) == "IndividualID"] <- "ind"

# Change column name
colnames(fam_data)[colnames(fam_data) == "FamilyID"] <- "pop"

# Select ID
fam_data <- fam_data |>
  dplyr::select("ind", "pop")

# View the first few rows
head(fam_data)
```

```
##   ind pop
## 1 153 MAU
## 2 154 MAU
## 3 155 MAU
## 4 156 MAU
## 5 158 MAU
## 6  16 MAU
```

Add it to the matrix

```
leak21 <- fam_data |>
  dplyr::select(ind, pop) |>
  bind_cols(leak21)

head(leak21)
```

```
##   ind pop          X1          X2          X3          X4          X5
## 1 153 MAU 9.98921e-05 9.98921e-05 9.98921e-05 0.088781700 9.98921e-05
## 2 154 MAU 3.16947e-03 9.99010e-05 2.66814e-02 0.102149000 1.04858e-02
## 3 155 MAU 5.77974e-02 2.98369e-02 2.39063e-02 0.016604500 9.99370e-05
## 4 156 MAU 6.70436e-02 9.99460e-05 1.27882e-03 0.002665500 4.23315e-02
## 5 158 MAU 9.99010e-05 9.99010e-05 2.33447e-02 0.000099901 9.99010e-05
## 6  16 MAU 1.82442e-04 9.99460e-05 3.18254e-02 0.017369900 2.99462e-02
##            X6          X7          X8        X9         X10         X11
## 1 9.98921e-05 9.98921e-05 9.98921e-05 0.1255750 0.024099600 9.98921e-05
## 2 9.99010e-05 9.99010e-05 9.99010e-05 0.0510237 0.000099901 9.99010e-05
## 3 8.68466e-04 9.99370e-05 3.93265e-02 0.1018090 0.029687800 2.83455e-02
## 4 9.99460e-05 9.99460e-05 1.87485e-02 0.1850020 0.010035100 1.90003e-02
## 5 9.99010e-05 3.49376e-03 1.29794e-02 0.0857662 0.000099901 9.41208e-02
## 6 1.65020e-02 1.39374e-02 3.71552e-02 0.1700760 0.055796100 9.99460e-05
##           X12         X13         X14         X15         X16         X17
## 1 0.039934000 0.036764600 9.98921e-05 9.98921e-05 9.98921e-05 0.037230100
## 2 0.000099901 0.019669700 9.99010e-05 9.99010e-05 2.79440e-02 0.000099901
## 3 0.093140500 0.001567970 9.99370e-05 9.99370e-05 9.99370e-05 0.000099937
## 4 0.000099946 0.000099946 4.92643e-02 2.37981e-02 6.28557e-04 0.000099946
## 5 0.000099901 0.000099901 9.99010e-05 2.99172e-03 9.99010e-05 0.040911900
## 6 0.000099946 0.000099946 9.99460e-05 3.46177e-02 9.99460e-05 0.082786800
##           X18        X19      X20         X21
## 1 9.98921e-05 0.07362870 0.520048 0.052739600
## 2 9.99010e-05 0.02914650 0.722852 0.005779850
## 3 9.99370e-05 0.04118820 0.510027 0.025193900
## 4 6.53391e-03 0.00497221 0.545736 0.022362500
## 5 1.00628e-01 0.02743970 0.607225 0.000099901
## 6 3.28627e-02 0.00543474 0.469898 0.001010160
```

Rename the columns

```
# Rename the columns starting from the third one
leak21 <- leak21 |>
  rename_with(~paste0("v", seq_along(.x)), .cols = -c(ind, pop))

# View the first few rows
head(leak21)
```

```
##   ind pop          v1          v2          v3          v4          v5
## 1 153 MAU 9.98921e-05 9.98921e-05 9.98921e-05 0.088781700 9.98921e-05
## 2 154 MAU 3.16947e-03 9.99010e-05 2.66814e-02 0.102149000 1.04858e-02
## 3 155 MAU 5.77974e-02 2.98369e-02 2.39063e-02 0.016604500 9.99370e-05
## 4 156 MAU 6.70436e-02 9.99460e-05 1.27882e-03 0.002665500 4.23315e-02
## 5 158 MAU 9.99010e-05 9.99010e-05 2.33447e-02 0.000099901 9.99010e-05
## 6  16 MAU 1.82442e-04 9.99460e-05 3.18254e-02 0.017369900 2.99462e-02
##            v6          v7          v8        v9         v10         v11
## 1 9.98921e-05 9.98921e-05 9.98921e-05 0.1255750 0.024099600 9.98921e-05
## 2 9.99010e-05 9.99010e-05 9.99010e-05 0.0510237 0.000099901 9.99010e-05
## 3 8.68466e-04 9.99370e-05 3.93265e-02 0.1018090 0.029687800 2.83455e-02
## 4 9.99460e-05 9.99460e-05 1.87485e-02 0.1850020 0.010035100 1.90003e-02
## 5 9.99010e-05 3.49376e-03 1.29794e-02 0.0857662 0.000099901 9.41208e-02
## 6 1.65020e-02 1.39374e-02 3.71552e-02 0.1700760 0.055796100 9.99460e-05
##           v12         v13         v14         v15         v16         v17
## 1 0.039934000 0.036764600 9.98921e-05 9.98921e-05 9.98921e-05 0.037230100
## 2 0.000099901 0.019669700 9.99010e-05 9.99010e-05 2.79440e-02 0.000099901
## 3 0.093140500 0.001567970 9.99370e-05 9.99370e-05 9.99370e-05 0.000099937
## 4 0.000099946 0.000099946 4.92643e-02 2.37981e-02 6.28557e-04 0.000099946
## 5 0.000099901 0.000099901 9.99010e-05 2.99172e-03 9.99010e-05 0.040911900
## 6 0.000099946 0.000099946 9.99460e-05 3.46177e-02 9.99460e-05 0.082786800
##           v18        v19      v20         v21
## 1 9.98921e-05 0.07362870 0.520048 0.052739600
## 2 9.99010e-05 0.02914650 0.722852 0.005779850
## 3 9.99370e-05 0.04118820 0.510027 0.025193900
## 4 6.53391e-03 0.00497221 0.545736 0.022362500
## 5 1.00628e-01 0.02743970 0.607225 0.000099901
## 6 3.28627e-02 0.00543474 0.469898 0.001010160
```

Import Sample Locations

```
sampling_loc <- readRDS(here("scripts", "RMarkdowns", "output", "sampling_loc_americas_all.rds"))
head(sampling_loc)
```

```
##       Pop_City Country Latitude Longitude Continent Abbreviation Year
## 1   Berlin, NJ     USA 39.79081  -74.9291  Americas          BER 2018
## 2 Columbus, OH     USA 39.97170  -82.9071  Americas          COL 2015
## 3   Palm Beach     USA 26.70560  -80.0364  Americas          PAL 2018
## 4  Houston, TX     USA 29.75491  -95.3505  Americas          HOU 2018
## 5  Los Angeles     USA 34.05220 -118.2437  Americas          LOS 2018
## 6   Manaus, AM  Brazil -3.09161  -60.0325  Americas          MAU 2017
##          Region      Subregion order order2 orderold AM_order      state
## 1 North America Northeast, USA     1     NA       75        6    NJ, USA
## 2 North America   Midwest, USA     2     NA       76       18    OH, USA
## 3 North America Southeast, USA     3     NA       77       13    FL, USA
## 4 North America Southwest, USA     4     NA       78       23    TX, USA
## 5 North America      West, USA     5     NA       79       30    CA, USA
## 6 South America  South America     6     NA       80       38 AM, Brazil
```

```
source(
  here("scripts", "RMarkdowns",
    "my_theme3.R"
  )
)

# Melt the data frame for plotting
Q_melted <- leak21 |>
  pivot_longer(
    cols = -c(ind, pop),
    names_to = "variable",
    values_to = "value"
  )
# Join with sampling_loc to get sampling localities
Q_joined <- Q_melted |>
  left_join(sampling_loc, by = c("pop" = "Abbreviation"))

# Order the combined variable by Region and Country, then by individual
Q_ordered <- Q_joined |>
  arrange(AM_order, ind) |>
  mutate(ind = factor(ind, levels = unique(ind)))  # Convert ind to a factor with levels in the desired order

# Add labels: country names for the first individual in each country, NA for all other individuals
Q_ordered <- Q_ordered |>
  group_by(state) |>
  mutate(label = ifelse(row_number() == 1, as.character(state), NA))

# Group by individual and variable, calculate mean ancestry proportions
Q_grouped <- Q_ordered |>
  group_by(ind, variable) |>
  summarise(value = mean(value), .groups = "drop")

# Create a data frame for borders
borders <-
  data.frame(state = unique(Q_ordered$state))

# Add the order of the last individual of each country to ensure correct placement of borders
borders$order <-
  sapply(borders$state, function(rc)
    max(which(Q_ordered$state == rc))) + 0.5  # Shift borders to the right edge of the bars

# Select only the first occurrence of each country in the ordered data
label_df <- Q_ordered |>
  filter(!is.na(label)) |>
  distinct(label, .keep_all = TRUE)

# Create a custom label function
label_func <- function(x) {
  labels <- rep("", length(x))
  labels[x %in% label_df$ind] <- label_df$label
  labels
}

# Calculate the position of lines
border_positions <- Q_ordered |>
  group_by(state) |>
  summarise(pos = max(as.numeric(ind)) + 0)

# Calculate the position of population labels and bars
pop_labels <- Q_ordered |>
  mutate(Name = paste(pop, Pop_City, sep = " - ")) |>
  group_by(pop) |>
  slice_head(n = 1) |>
  ungroup() |>
  dplyr::select(ind, Pop_City, state, Name) |>
  mutate(pos = as.numeric(ind))  # calculate position of population labels

pop_labels_bars <- pop_labels |>
  mutate(pos = as.numeric(ind)  - .5)


# Calculate the position of lines
border_positions <- Q_ordered |>
  group_by(Country) |>
  summarise(pos = max(as.numeric(ind)) - 1)


pop_labels_bars <- pop_labels |>
  mutate(pos = as.numeric(ind)  - .5)

# Function to filter and normalize data
normalize_data <- function(df, min_value) {
  df |>
    filter(value > min_value) |>
    group_by(ind) |>
    mutate(value = value / sum(value))
}

# Use the function
Q_grouped_filtered <- normalize_data(Q_grouped, 0.1)
# 

color_palette <-
    c(
    "#FFFC99",
    "#FF8005",  
    "#9DCC00",  
    "#2BCE48",
    "cyan",
    "blue",    
    "#FFA8BB",   
    "#FF0010",
    "#808080",    
    "orchid1",    
    "#4C005C",
    "#003380",
    "#990000", 
    "#8c61cd",
    "#FFE100",
    "purple3",  
    "#1E90FF",
    "#005C31", 
    "#993F00",
    "#C20088",   
    "magenta"   
    )

# Generate all potential variable names
all_variables <- paste0("v", 1:21)


# Create a data frame that pairs each potential variable with a color
color_mapping <- data.frame(variable = all_variables,
                          color = color_palette[1:length(all_variables)])

# Merge with Q_grouped_filtered
Q_grouped_filtered <- merge(Q_grouped_filtered, color_mapping, by = "variable")

# Create the plot
ggplot(Q_grouped_filtered, aes(x = as.factor(ind), y = value, fill = color)) +
  geom_bar(stat = 'identity', width = 1) +
  geom_vline(
    data = pop_labels_bars,
    aes(xintercept = pos),
    color = "#2C444A",
    linewidth = .2
  ) +
  geom_text(
    data = pop_labels,
    aes(x = as.numeric(ind), y = 1, label = Name),
    vjust = 1.5,
    hjust = 0,
    size = 2,
    angle = 90,
    inherit.aes = FALSE
  ) +
  my_theme() +
  theme(
    axis.text.x = element_text(
      angle = 90,
      hjust = 1,
      size = 12
    ),
    legend.position = "none",
    plot.margin = unit(c(3, 0.5, 0.5, 0.5), "cm")
  ) +
  xlab("Admixture matrix") +
  ylab("Ancestry proportions") +
  labs(caption = "Each bar represents the ancestry proportions for an individual for k=21.\n LEA inference with 18,964 SNPs.") +
  scale_x_discrete(labels = label_func) +
  scale_fill_manual(values = color_palette) +
  expand_limits(y = c(0, 1.5))
```

```
ggsave(
  here("scripts", "RMarkdowns", "output", "americas", "americas_all", "lea", "r_01_MAF_01", "lea_k=21_americas_all_r01.pdf"),
  width  = 12,
  height = 6,
  units  = "in",
  device = cairo_pdf
)
```

### 1.3.3 K=19

Extract ancestry coefficients for k=19

*change to correct matrix*

```
leak19 <- read_delim(
  here("/gpfs/gibbs/pi/caccone/mkc54/albo/americas/output/snps_sets/r2_0.01_b.snmf/K19/run1/r2_0.01_b_r1.19.Q"),
  delim = " ", # Specify the delimiter if different from the default (comma)
  col_names = FALSE,
  show_col_types = FALSE
) 
head(leak19)
```

```
## # A tibble: 6 × 19
##          X1        X2      X3        X4       X5      X6      X7      X8      X9
##       <dbl>     <dbl>   <dbl>     <dbl>    <dbl>   <dbl>   <dbl>   <dbl>   <dbl>
## 1 0.0169    0.0000999 0.0207  0.0670     7.12e-2 3.03e-3 9.99e-5 9.99e-5 3.74e-2
## 2 0.0000999 0.0000999 0.00299 0.0000999  9.99e-5 9.99e-5 6.40e-2 9.99e-5 9.99e-5
## 3 0.00853   0.0382    0.0122  0.0549     9.29e-2 2.22e-2 9.99e-5 9.99e-5 9.99e-5
## 4 0.0000999 0.00774   0.0275  0.0000999  9.99e-5 1.26e-2 9.99e-5 9.99e-5 1.35e-2
## 5 0.0000999 0.0197    0.0506  0.0000999  9.99e-5 9.99e-5 9.99e-5 9.99e-5 9.99e-5
## 6 0.0148    0.0239    0.0306  0.0000999  9.99e-5 9.99e-5 7.57e-2 9.99e-5 3.71e-2
## # ℹ 10 more variables: X10 <dbl>, X11 <dbl>, X12 <dbl>, X13 <dbl>, X14 <dbl>,
## #   X15 <dbl>, X16 <dbl>, X17 <dbl>, X18 <dbl>, X19 <dbl>
```

The fam file

```
fam_file <- here("americas/output/snps_sets/r2_0.01_b.fam")

# Read the .fam file
fam_data <- read.table(fam_file, 
                       header = FALSE,
                       col.names = c("FamilyID", "IndividualID", "PaternalID", "MaternalID", "Sex", "Phenotype"))

# View the first few rows
head(fam_data)
```

```
##   FamilyID IndividualID PaternalID MaternalID Sex Phenotype
## 1      MAU          153          0          0   0        -9
## 2      MAU          154          0          0   0        -9
## 3      MAU          155          0          0   0        -9
## 4      MAU          156          0          0   0        -9
## 5      MAU          158          0          0   0        -9
## 6      MAU           16          0          0   0        -9
```

Create column ID

```
# Change column name
colnames(fam_data)[colnames(fam_data) == "IndividualID"] <- "ind"

# Change column name
colnames(fam_data)[colnames(fam_data) == "FamilyID"] <- "pop"

# Select ID
fam_data <- fam_data |>
  dplyr::select("ind", "pop")

# View the first few rows
head(fam_data)
```

```
##   ind pop
## 1 153 MAU
## 2 154 MAU
## 3 155 MAU
## 4 156 MAU
## 5 158 MAU
## 6  16 MAU
```

Add it to the matrix

```
leak19 <- fam_data |>
  dplyr::select(ind, pop) |>
  bind_cols(leak19)

head(leak19)
```

```
##   ind pop          X1          X2         X3          X4          X5
## 1 153 MAU 0.016926100 0.000099946 0.02069720 0.067042300 0.071218000
## 2 154 MAU 0.000099901 0.000099901 0.00299027 0.000099901 0.000099901
## 3 155 MAU 0.008525010 0.038236700 0.01218000 0.054851500 0.092877700
## 4 156 MAU 0.000099928 0.007736350 0.02747860 0.000099928 0.000099928
## 5 158 MAU 0.000099910 0.019697800 0.05060670 0.000099910 0.000099910
## 6  16 MAU 0.014783500 0.023926900 0.03064890 0.000099928 0.000099928
##            X6          X7         X8          X9         X10       X11
## 1 0.003026840 0.000099946 9.9946e-05 0.037379600 0.057884400 0.0659411
## 2 0.000099901 0.063977400 9.9901e-05 0.000099901 0.027568700 0.0792092
## 3 0.022239200 0.000099928 9.9928e-05 0.000099928 0.000099928 0.1040440
## 4 0.012573900 0.000099928 9.9928e-05 0.013548000 0.089378900 0.1781310
## 5 0.000099910 0.000099910 9.9910e-05 0.000099910 0.069663300 0.0485606
## 6 0.000099928 0.075731200 9.9928e-05 0.037090400 0.057152400 0.1543820
##           X12         X13         X14      X15         X16         X17
## 1 0.000099946 0.000099946 0.000099946 0.536203 0.075514700 0.009111220
## 2 0.000099901 0.000099901 0.034912400 0.690580 0.000099901 0.035871400
## 3 0.057622300 0.004933040 0.076099600 0.527591 0.000099928 0.000099928
## 4 0.064844400 0.000099928 0.000099928 0.565093 0.001179680 0.020550600
## 5 0.000099910 0.029758000 0.066742200 0.656060 0.032688400 0.000099910
## 6 0.003156990 0.000099928 0.022724600 0.441793 0.137811000 0.000099928
##           X18         X19
## 1 0.022347200 0.016108200
## 2 0.000099901 0.063792200
## 3 0.000099928 0.000099928
## 4 0.000099928 0.018685800
## 5 0.025224000 0.000099910
## 6 0.000099928 0.000099928
```

Rename the columns

```
# Rename the columns starting from the third one
leak19 <- leak19 |>
  rename_with(~paste0("v", seq_along(.x)), .cols = -c(ind, pop))

# View the first few rows
head(leak19)
```

```
##   ind pop          v1          v2         v3          v4          v5
## 1 153 MAU 0.016926100 0.000099946 0.02069720 0.067042300 0.071218000
## 2 154 MAU 0.000099901 0.000099901 0.00299027 0.000099901 0.000099901
## 3 155 MAU 0.008525010 0.038236700 0.01218000 0.054851500 0.092877700
## 4 156 MAU 0.000099928 0.007736350 0.02747860 0.000099928 0.000099928
## 5 158 MAU 0.000099910 0.019697800 0.05060670 0.000099910 0.000099910
## 6  16 MAU 0.014783500 0.023926900 0.03064890 0.000099928 0.000099928
##            v6          v7         v8          v9         v10       v11
## 1 0.003026840 0.000099946 9.9946e-05 0.037379600 0.057884400 0.0659411
## 2 0.000099901 0.063977400 9.9901e-05 0.000099901 0.027568700 0.0792092
## 3 0.022239200 0.000099928 9.9928e-05 0.000099928 0.000099928 0.1040440
## 4 0.012573900 0.000099928 9.9928e-05 0.013548000 0.089378900 0.1781310
## 5 0.000099910 0.000099910 9.9910e-05 0.000099910 0.069663300 0.0485606
## 6 0.000099928 0.075731200 9.9928e-05 0.037090400 0.057152400 0.1543820
##           v12         v13         v14      v15         v16         v17
## 1 0.000099946 0.000099946 0.000099946 0.536203 0.075514700 0.009111220
## 2 0.000099901 0.000099901 0.034912400 0.690580 0.000099901 0.035871400
## 3 0.057622300 0.004933040 0.076099600 0.527591 0.000099928 0.000099928
## 4 0.064844400 0.000099928 0.000099928 0.565093 0.001179680 0.020550600
## 5 0.000099910 0.029758000 0.066742200 0.656060 0.032688400 0.000099910
## 6 0.003156990 0.000099928 0.022724600 0.441793 0.137811000 0.000099928
##           v18         v19
## 1 0.022347200 0.016108200
## 2 0.000099901 0.063792200
## 3 0.000099928 0.000099928
## 4 0.000099928 0.018685800
## 5 0.025224000 0.000099910
## 6 0.000099928 0.000099928
```

Import Sample Locations

```
sampling_loc <- readRDS(here("scripts", "RMarkdowns", "output", "sampling_loc_americas_all.rds"))
head(sampling_loc)
```

```
##       Pop_City Country Latitude Longitude Continent Abbreviation Year
## 1   Berlin, NJ     USA 39.79081  -74.9291  Americas          BER 2018
## 2 Columbus, OH     USA 39.97170  -82.9071  Americas          COL 2015
## 3   Palm Beach     USA 26.70560  -80.0364  Americas          PAL 2018
## 4  Houston, TX     USA 29.75491  -95.3505  Americas          HOU 2018
## 5  Los Angeles     USA 34.05220 -118.2437  Americas          LOS 2018
## 6   Manaus, AM  Brazil -3.09161  -60.0325  Americas          MAU 2017
##          Region      Subregion order order2 orderold AM_order      state
## 1 North America Northeast, USA     1     NA       75        6    NJ, USA
## 2 North America   Midwest, USA     2     NA       76       18    OH, USA
## 3 North America Southeast, USA     3     NA       77       13    FL, USA
## 4 North America Southwest, USA     4     NA       78       23    TX, USA
## 5 North America      West, USA     5     NA       79       30    CA, USA
## 6 South America  South America     6     NA       80       38 AM, Brazil
```

```
source(
  here("scripts", "RMarkdowns",
    "my_theme3.R"
  )
)

# Melt the data frame for plotting
Q_melted <- leak19 |>
  pivot_longer(
    cols = -c(ind, pop),
    names_to = "variable",
    values_to = "value"
  )
# Join with sampling_loc to get sampling localities
Q_joined <- Q_melted |>
  left_join(sampling_loc, by = c("pop" = "Abbreviation"))

# Order the combined variable by Region and Country, then by individual
Q_ordered <- Q_joined |>
  arrange(AM_order, ind) |>
  mutate(ind = factor(ind, levels = unique(ind)))  # Convert ind to a factor with levels in the desired order

# Add labels: country names for the first individual in each country, NA for all other individuals
Q_ordered <- Q_ordered |>
  group_by(state) |>
  mutate(label = ifelse(row_number() == 1, as.character(state), NA))

# Group by individual and variable, calculate mean ancestry proportions
Q_grouped <- Q_ordered |>
  group_by(ind, variable) |>
  summarise(value = mean(value), .groups = "drop")

# Create a data frame for borders
borders <-
  data.frame(state = unique(Q_ordered$state))

# Add the order of the last individual of each country to ensure correct placement of borders
borders$order <-
  sapply(borders$state, function(rc)
    max(which(Q_ordered$state == rc))) + 0.5  # Shift borders to the right edge of the bars

# Select only the first occurrence of each country in the ordered data
label_df <- Q_ordered |>
  filter(!is.na(label)) |>
  distinct(label, .keep_all = TRUE)

# Create a custom label function
label_func <- function(x) {
  labels <- rep("", length(x))
  labels[x %in% label_df$ind] <- label_df$label
  labels
}

# Calculate the position of lines
border_positions <- Q_ordered |>
  group_by(state) |>
  summarise(pos = max(as.numeric(ind)) + 0)

# Calculate the position of population labels and bars
pop_labels <- Q_ordered |>
  mutate(Name = paste(pop, Pop_City, sep = " - ")) |>
  group_by(pop) |>
  slice_head(n = 1) |>
  ungroup() |>
  dplyr::select(ind, Pop_City, state, Name) |>
  mutate(pos = as.numeric(ind))  # calculate position of population labels

pop_labels_bars <- pop_labels |>
  mutate(pos = as.numeric(ind)  - .5)


# Calculate the position of lines
border_positions <- Q_ordered |>
  group_by(Country) |>
  summarise(pos = max(as.numeric(ind)) - 1)


pop_labels_bars <- pop_labels |>
  mutate(pos = as.numeric(ind)  - .5)

# Function to filter and normalize data
normalize_data <- function(df, min_value) {
  df |>
    filter(value > min_value) |>
    group_by(ind) |>
    mutate(value = value / sum(value))
}

# Use the function
Q_grouped_filtered <- normalize_data(Q_grouped, 0.1)
# 

color_palette <-
  c(
    "#993F00",
    "#2BCE48",
    "#003380",
    "#C20088",
    "#F0A0FF",
    "#FF0010",
    "#4C005C",
    "#990000",  
    "#005C31", 
    "blue",
    "#FFFC99",
    "purple3",  
    "magenta",
    "#FFE100",
    "#808080",
    "#5EF1F2",
    "#FFA8BB",    
    "#9DCC00",  
    "#FF5005",
    "#1E90FF"    
  )
  
  
 
# Generate all potential variable names
all_variables <- paste0("v", 1:19)


# Create a data frame that pairs each potential variable with a color
color_mapping <- data.frame(variable = all_variables,
                           color = color_palette[1:length(all_variables)])

# Merge with Q_grouped_filtered
Q_grouped_filtered <- merge(Q_grouped_filtered, color_mapping, by = "variable")

# Create the plot
ggplot(Q_grouped_filtered, aes(x = as.factor(ind), y = value, fill = color)) +
  geom_bar(stat = 'identity', width = 1) +
  geom_vline(
    data = pop_labels_bars,
    aes(xintercept = pos),
    color = "#2C444A",
    linewidth = .2
  ) +
  geom_text(
    data = pop_labels,
    aes(x = as.numeric(ind), y = 1, label = Name),
    vjust = 1.5,
    hjust = 0,
    size = 2,
    angle = 90,
    inherit.aes = FALSE
  ) +
  my_theme() +
  theme(
    axis.text.x = element_text(
      angle = 90,
      hjust = 1,
      size = 12
    ),
    legend.position = "none",
    plot.margin = unit(c(3, 0.5, 0.5, 0.5), "cm")
  ) +
  xlab("Admixture matrix") +
  ylab("Ancestry proportions") +
  labs(caption = "Each bar represents the ancestry proportions for an individual for k=19.\n LEA inference with 18,964 SNPs.") +
  scale_x_discrete(labels = label_func) +
  scale_fill_manual(values = color_palette) +
  expand_limits(y = c(0, 1.5))
```

```
ggsave(
  here("scripts", "RMarkdowns", "output", "americas", "americas_all", "lea", "r_01_MAF_01", "lea_k=19_americas_all_r01.pdf"),
  width  = 12,
  height = 6,
  units  = "in",
  device = cairo_pdf
)
```

### 1.3.4 K=3

```
best = which.min(cross.entropy(project, K = 3))
best #3
```

```
## [1] 3
```

Extract ancestry coefficients for k=9

*change to correct matrix*

```
leak3 <- read_delim(
  here("/gpfs/gibbs/pi/caccone/mkc54/albo/americas/output/snps_sets/r2_0.01_b.snmf/K3/run3/r2_0.01_b_r3.3.Q"),
  delim = " ", # Specify the delimiter if different from the default (comma)
  col_names = FALSE,
  show_col_types = FALSE
) 
head(leak3)
```

```
## # A tibble: 6 × 3
##      X1    X2     X3
##   <dbl> <dbl>  <dbl>
## 1 0.296 0.611 0.0933
## 2 0.292 0.604 0.104 
## 3 0.282 0.566 0.152 
## 4 0.269 0.597 0.134 
## 5 0.265 0.618 0.116 
## 6 0.236 0.631 0.133
```

The fam file

```
fam_file <- here("americas/output/snps_sets/r2_0.01_b.fam")

# Read the .fam file
fam_data <- read.table(fam_file, 
                       header = FALSE,
                       col.names = c("FamilyID", "IndividualID", "PaternalID", "MaternalID", "Sex", "Phenotype"))

# View the first few rows
head(fam_data)
```

```
##   FamilyID IndividualID PaternalID MaternalID Sex Phenotype
## 1      MAU          153          0          0   0        -9
## 2      MAU          154          0          0   0        -9
## 3      MAU          155          0          0   0        -9
## 4      MAU          156          0          0   0        -9
## 5      MAU          158          0          0   0        -9
## 6      MAU           16          0          0   0        -9
```

Create column ID

```
# Change column name
colnames(fam_data)[colnames(fam_data) == "IndividualID"] <- "ind"

# Change column name
colnames(fam_data)[colnames(fam_data) == "FamilyID"] <- "pop"

# Select ID
fam_data <- fam_data |>
  dplyr::select("ind", "pop")

# View the first few rows
head(fam_data)
```

```
##   ind pop
## 1 153 MAU
## 2 154 MAU
## 3 155 MAU
## 4 156 MAU
## 5 158 MAU
## 6  16 MAU
```

Add it to the matrix

```
leak3 <- fam_data |>
  dplyr::select(ind, pop) |>
  bind_cols(leak3)

head(leak3)
```

```
##   ind pop       X1       X2        X3
## 1 153 MAU 0.296204 0.610515 0.0932805
## 2 154 MAU 0.291620 0.603928 0.1044520
## 3 155 MAU 0.281986 0.565868 0.1521470
## 4 156 MAU 0.268579 0.596936 0.1344850
## 5 158 MAU 0.265203 0.618297 0.1165000
## 6  16 MAU 0.235687 0.631356 0.1329570
```

Rename the columns

```
# Rename the columns starting from the third one
leak3 <- leak3 |>
  rename_with(~paste0("v", seq_along(.x)), .cols = -c(ind, pop))

# View the first few rows
head(leak3)
```

```
##   ind pop       v1       v2        v3
## 1 153 MAU 0.296204 0.610515 0.0932805
## 2 154 MAU 0.291620 0.603928 0.1044520
## 3 155 MAU 0.281986 0.565868 0.1521470
## 4 156 MAU 0.268579 0.596936 0.1344850
## 5 158 MAU 0.265203 0.618297 0.1165000
## 6  16 MAU 0.235687 0.631356 0.1329570
```

Import Sample Locations

```
sampling_loc <- readRDS(here("scripts", "RMarkdowns", "output", "sampling_loc_americas_all.rds"))
head(sampling_loc)
```

```
##       Pop_City Country Latitude Longitude Continent Abbreviation Year
## 1   Berlin, NJ     USA 39.79081  -74.9291  Americas          BER 2018
## 2 Columbus, OH     USA 39.97170  -82.9071  Americas          COL 2015
## 3   Palm Beach     USA 26.70560  -80.0364  Americas          PAL 2018
## 4  Houston, TX     USA 29.75491  -95.3505  Americas          HOU 2018
## 5  Los Angeles     USA 34.05220 -118.2437  Americas          LOS 2018
## 6   Manaus, AM  Brazil -3.09161  -60.0325  Americas          MAU 2017
##          Region      Subregion order order2 orderold AM_order      state
## 1 North America Northeast, USA     1     NA       75        6    NJ, USA
## 2 North America   Midwest, USA     2     NA       76       18    OH, USA
## 3 North America Southeast, USA     3     NA       77       13    FL, USA
## 4 North America Southwest, USA     4     NA       78       23    TX, USA
## 5 North America      West, USA     5     NA       79       30    CA, USA
## 6 South America  South America     6     NA       80       38 AM, Brazil
```

```
source(
  here("scripts", "RMarkdowns",
    "my_theme3.R"
  )
)

# Melt the data frame for plotting
Q_melted <- leak3 |>
  pivot_longer(
    cols = -c(ind, pop),
    names_to = "variable",
    values_to = "value"
  )
# Join with sampling_loc to get sampling localities
Q_joined <- Q_melted |>
  left_join(sampling_loc, by = c("pop" = "Abbreviation"))

# Order the combined variable by Region and Country, then by individual
Q_ordered <- Q_joined |>
  arrange(AM_order, ind) |>
  mutate(ind = factor(ind, levels = unique(ind)))  # Convert ind to a factor with levels in the desired order

# Add labels: country names for the first individual in each country, NA for all other individuals
Q_ordered <- Q_ordered |>
  group_by(state) |>
  mutate(label = ifelse(row_number() == 1, as.character(state), NA))

# Group by individual and variable, calculate mean ancestry proportions
Q_grouped <- Q_ordered |>
  group_by(ind, variable) |>
  summarise(value = mean(value), .groups = "drop")

# Create a data frame for borders
borders <-
  data.frame(state = unique(Q_ordered$state))

# Add the order of the last individual of each country to ensure correct placement of borders
borders$order <-
  sapply(borders$state, function(rc)
    max(which(Q_ordered$state == rc))) + 0.5  # Shift borders to the right edge of the bars

# Select only the first occurrence of each country in the ordered data
label_df <- Q_ordered |>
  filter(!is.na(label)) |>
  distinct(label, .keep_all = TRUE)

# Create a custom label function
label_func <- function(x) {
  labels <- rep("", length(x))
  labels[x %in% label_df$ind] <- label_df$label
  labels
}

# Calculate the position of lines
border_positions <- Q_ordered |>
  group_by(state) |>
  summarise(pos = max(as.numeric(ind)) + 0)

# Calculate the position of population labels and bars
pop_labels <- Q_ordered |>
  mutate(Name = paste(pop, Pop_City, sep = " - ")) |>
  group_by(pop) |>
  slice_head(n = 1) |>
  ungroup() |>
  dplyr::select(ind, Pop_City, state, Name) |>
  mutate(pos = as.numeric(ind))  # calculate position of population labels

pop_labels_bars <- pop_labels |>
  mutate(pos = as.numeric(ind)  - .5)


# Calculate the position of lines
border_positions <- Q_ordered |>
  group_by(Country) |>
  summarise(pos = max(as.numeric(ind)) - 1)


pop_labels_bars <- pop_labels |>
  mutate(pos = as.numeric(ind)  - .5)

# Function to filter and normalize data
normalize_data <- function(df, min_value) {
  df |>
    filter(value > min_value) |>
    group_by(ind) |>
    mutate(value = value / sum(value))
}

# Use the function
Q_grouped_filtered <- normalize_data(Q_grouped, 0.1)
# 

color_palette <-
  c(
    "#FF5005",
    "blue",
    "#FFE100"
    )
  
 
# Generate all potential variable names
all_variables <- paste0("v", 1:3)


# Create a data frame that pairs each potential variable with a color
color_mapping <- data.frame(variable = all_variables,
                           color = color_palette[1:length(all_variables)])

# Merge with Q_grouped_filtered
Q_grouped_filtered <- merge(Q_grouped_filtered, color_mapping, by = "variable")

# Create the plot
ggplot(Q_grouped_filtered, aes(x = as.factor(ind), y = value, fill = color)) +
  geom_bar(stat = 'identity', width = 1) +
  geom_vline(
    data = pop_labels_bars,
    aes(xintercept = pos),
    color = "#2C444A",
    linewidth = .2
  ) +
  geom_text(
    data = pop_labels,
    aes(x = as.numeric(ind), y = 1, label = Name),
    vjust = 1.5,
    hjust = 0,
    size = 2,
    angle = 90,
    inherit.aes = FALSE
  ) +
  my_theme() +
  theme(
    axis.text.x = element_text(
      angle = 90,
      hjust = 1,
      size = 12
    ),
    legend.position = "none",
    plot.margin = unit(c(3, 0.5, 0.5, 0.5), "cm")
  ) +
  xlab("Admixture matrix") +
  ylab("Ancestry proportions") +
  labs(caption = "Each bar represents the ancestry proportions for an individual for k=3.\n LEA inference with 18,964 SNPs.") +
  scale_x_discrete(labels = label_func) +
  scale_fill_manual(values = color_palette) +
  expand_limits(y = c(0, 1.5))
```

```
ggsave(
  here("scripts", "RMarkdowns", "output", "americas", "americas_all", "lea", "r_01_MAF_01", "lea_k=3_americas_all_r01.pdf"),
  width  = 12,
  height = 6,
  units  = "in",
  device = cairo_pdf
)
```

# 2. DAPC

Load DAPC Libraries

```
library(ade4)
library(adegenet)
library(here)
library(dartR) #to convert genind object
```

```
## Loading required package: dartR.data
```

```
## **** Welcome to dartR.data [Version 1.2.2 ] ****
```

```
## Registered S3 method overwritten by 'pegas':
##   method      from
##   print.amova ade4
```

```
## Registered S3 method overwritten by 'GGally':
##   method from   
##   +.gg   ggplot2
```

```
## Registered S3 method overwritten by 'genetics':
##   method      from 
##   [.haplotype pegas
```

```
## **** Welcome to the legacy dartR [Version 2.9.9.5 ] ****
```

```
## ##############################
##  Be aware this the legacy version of dartR, which is no longer developed. Please consider using the new version of dartR, called 'dartRverse'. As before you can find the latest version on CRAN. dartRverse has the same functionality as dartR, but is more up-to-date and maintained. To reduce dependencies, and improve the overall performance, dartRverse consists of several packacages, that are maintained seperately to reduce dependencies . To install dartRverse please download the package dartRverse and follow the instructions therein. For more information please visit: https://github.com/green-striped-gecko/dartRverse.
## ##############################
```

```
## Be aware that owing to CRAN requirements and compatibility reasons not all functions of the package may run after the basic installation, as some packages could still be missing. Hence for a most enjoyable experience we recommend to run the function
```

```
## gl.install.vanilla.dartR()
```

```
## This installs all missing and required packages for your version of dartR. In case something fails during installation please refer to this tutorial: https://github.com/green-striped-gecko/dartR/wiki/Installation-tutorial.
## 
## For information how to cite dartR, please use:
```

```
## citation('dartR')
```

```
## Global verbosity is set to: 2
```

```
## 
## **** Have fun using dartR! ****
```

```
## 
## Attaching package: 'dartR'
```

```
## The following objects are masked from 'package:dartR.data':
## 
##     bandicoot.gl, possums.gl, testset.gl, testset.gs
```

## 2.1. Create files & get sample locations

```
cd /gpfs/gibbs/pi/caccone/mkc54/albo/americas
plink \
--allow-extra-chr \
--keep-allele-order \
--bfile output/dapc/r2_0.1 \
--make-bed \
--out output/dapc/dapc_r1 \
--silent;
grep 'samples\|variants\|remaining' output/dapc/dapc_r1.log
```

43802 variants loaded from .bim file. 43802 variants and 544 people
pass filters and QC.

Convert to raw format

```
plink \
--allow-extra-chr \
--keep-allele-order \
--bfile output/dapc/dapc_r1 \
--recodeA \
--out output/dapc/dapc_r1 \
--silent;
grep 'samples\|variants\|remaining' output/dapc/dapc_r1.log
```

43802 variants loaded from .bim file. 43802 variants and 544 people
pass filters and QC.

Import the data and covert it to genind format

```
# import the data
snp <-
  read.PLINK(
    here("americas/output/dapc/dapc_r1.raw"),
    quiet = FALSE,
    chunkSize = 1000,
    parallel = require("parallel"),
    n.cores = 4
  )
```

```
## 
##  Reading PLINK raw format into a genlight object...
```

```
## Loading required package: parallel
```

```
## 
##  Reading loci information... 
## 
##  Reading and converting genotypes... 
## .
##  Building final object... 
## 
## ...done.
```

```
nInd(snp)
```

```
## [1] 544
```

```
nLoc(snp)
```

```
## [1] 43802
```

```
nPop(snp)
```

```
## [1] 51
```

```
indNames(snp)
```

```
##   [1] "153"  "154"  "155"  "156"  "158"  "16"   "174"  "175"  "176"  "177" 
##  [11] "178"  "17"   "180"  "181"  "182"  "183"  "184"  "185"  "186"  "187" 
##  [21] "188"  "189"  "18"   "190"  "191"  "2158" "2159" "2160" "2161" "2162"
##  [31] "2163" "2164" "2165" "2166" "2167" "2168" "2169" "2170" "2171" "2172"
##  [41] "2173" "2203" "2204" "2205" "2206" "2207" "2208" "2209" "2210" "2211"
##  [51] "2212" "2213" "2214" "2221" "2222" "2223" "2224" "2226" "2227" "2228"
##  [61] "2229" "2230" "2231" "2232" "2233" "2234" "2235" "2236" "2237" "2238"
##  [71] "2239" "2240" "2241" "2242" "2243" "2244" "2245" "2246" "2247" "2248"
##  [81] "2249" "2250" "2251" "2252" "2253" "2254" "2255" "2256" "2257" "2258"
##  [91] "2259" "2260" "2261" "2262" "2263" "2264" "2265" "2266" "2267" "2268"
## [101] "2269" "2270" "2271" "2272" "2273" "2275" "2288" "2289" "2290" "2291"
## [111] "2292" "2293" "2294" "2295" "2301" "2302" "2303" "2304" "2305" "2306"
## [121] "2307" "2308" "2309" "2310" "2311" "2312" "2313" "2314" "2315" "2316"
## [131] "2317" "2318" "2319" "2320" "2321" "2322" "2323" "2324" "2326" "2327"
## [141] "2329" "2330" "2331" "2332" "2333" "2334" "2335" "2336" "2337" "2338"
## [151] "2339" "2340" "2341" "2342" "2343" "2344" "2345" "2346" "2347" "2348"
## [161] "2349" "2350" "2351" "2352" "2353" "2354" "2355" "2357" "2358" "2360"
## [171] "2362" "2384" "2385" "2386" "2387" "2388" "2390" "2391" "2392" "2393"
## [181] "2394" "2401" "2402" "2403" "2404" "2405" "2406" "2407" "2408" "2409"
## [191] "2410" "2411" "2412" "2413" "2414" "2415" "2416" "2417" "2418" "2419"
## [201] "2420" "2421" "2422" "2423" "2424" "2425" "2426" "2427" "2428" "2429"
## [211] "2430" "2431" "2432" "2433" "2434" "2435" "2436" "2437" "2438" "2439"
## [221] "2440" "2441" "2442" "2443" "2444" "2445" "2446" "2447" "2448" "2449"
## [231] "2450" "2451" "2452" "2453" "2454" "2455" "2456" "2457" "2458" "2459"
## [241] "2461" "2462" "2463" "2464" "2465" "2466" "2467" "2468" "2469" "2470"
## [251] "2471" "2472" "2473" "2474" "2475" "2476" "2477" "2479" "2480" "2481"
## [261] "2482" "2483" "2484" "2485" "2486" "2487" "2488" "2489" "2490" "2491"
## [271] "2492" "2493" "2494" "2495" "2501" "2502" "2503" "2504" "2505" "2506"
## [281] "2507" "2508" "2509" "2510" "2511" "2512" "2513" "2514" "2515" "2516"
## [291] "2517" "2518" "2519" "2520" "2521" "2522" "2523" "2524" "2525" "2526"
## [301] "2527" "2528" "2529" "2530" "2531" "2532" "2533" "2534" "2535" "2536"
## [311] "2537" "2538" "2539" "2540" "2541" "2542" "2543" "2544" "2545" "2546"
## [321] "2547" "2548" "2550" "2551" "2552" "2553" "2554" "2561" "2562" "2564"
## [331] "2565" "2566" "2567" "2568" "2569" "2570" "2571" "2572" "2573" "2574"
## [341] "2575" "2576" "2577" "2578" "2579" "2580" "2581" "2582" "2583" "2584"
## [351] "2585" "2586" "2587" "2588" "2589" "2591" "2592" "2595" "294"  "295" 
## [361] "296"  "297"  "298"  "299"  "301"  "302"  "303"  "304"  "305"  "306" 
## [371] "307"  "308"  "309"  "310"  "311"  "312"  "313"  "314"  "315"  "316" 
## [381] "317"  "318"  "319"  "320"  "321"  "322"  "323"  "324"  "325"  "326" 
## [391] "327"  "328"  "329"  "330"  "331"  "332"  "333"  "334"  "335"  "336" 
## [401] "337"  "340"  "341"  "342"  "343"  "344"  "345"  "346"  "347"  "348" 
## [411] "349"  "350"  "351"  "352"  "353"  "354"  "356"  "357"  "358"  "359" 
## [421] "360"  "361"  "362"  "363"  "364"  "366"  "368"  "369"  "370"  "371" 
## [431] "372"  "373"  "374"  "375"  "376"  "377"  "378"  "379"  "380"  "381" 
## [441] "382"  "383"  "413"  "414"  "415"  "416"  "417"  "418"  "419"  "420" 
## [451] "421"  "422"  "424"  "425"  "426"  "427"  "428"  "429"  "430"  "431" 
## [461] "432"  "433"  "434"  "435"  "436"  "437"  "438"  "439"  "440"  "441" 
## [471] "442"  "443"  "444"  "445"  "446"  "447"  "448"  "449"  "450"  "451" 
## [481] "452"  "453"  "454"  "455"  "456"  "457"  "458"  "459"  "460"  "461" 
## [491] "463"  "464"  "466"  "467"  "468"  "469"  "470"  "472"  "473"  "474" 
## [501] "475"  "476"  "477"  "478"  "60"   "611"  "612"  "613"  "614"  "615" 
## [511] "616"  "617"  "618"  "619"  "620"  "621"  "622"  "634"  "635"  "636" 
## [521] "637"  "638"  "639"  "640"  "641"  "642"  "643"  "644"  "645"  "64"  
## [531] "684"  "685"  "686"  "687"  "688"  "689"  "690"  "691"  "692"  "693" 
## [541] "694"  "695"  "8"    "9"
```

```
# convert to genind
snp2 <- gl2gi(snp, probar = TRUE, verbose = NULL)
```

```
## Starting gl2gi 
##   Processing genlight object with SNP data
##   |                                                                              |                                                                      |   0%  |                                                                              |                                                                      |   1%  |                                                                              |=                                                                     |   1%  |                                                                              |=                                                                     |   2%  |                                                                              |==                                                                    |   2%  |                                                                              |==                                                                    |   3%  |                                                                              |===                                                                   |   4%  |                                                                              |===                                                                   |   5%  |                                                                              |====                                                                  |   5%  |                                                                              |====                                                                  |   6%  |                                                                              |=====                                                                 |   6%  |                                                                              |=====                                                                 |   7%  |                                                                              |=====                                                                 |   8%  |                                                                              |======                                                                |   8%  |                                                                              |======                                                                |   9%  |                                                                              |=======                                                               |   9%  |                                                                              |=======                                                               |  10%  |                                                                              |=======                                                               |  11%  |                                                                              |========                                                              |  11%  |                                                                              |========                                                              |  12%  |                                                                              |=========                                                             |  12%  |                                                                              |=========                                                             |  13%  |                                                                              |==========                                                            |  14%  |                                                                              |==========                                                            |  15%  |                                                                              |===========                                                           |  15%  |                                                                              |===========                                                           |  16%  |                                                                              |============                                                          |  17%  |                                                                              |============                                                          |  18%  |                                                                              |=============                                                         |  18%  |                                                                              |=============                                                         |  19%  |                                                                              |==============                                                        |  19%  |                                                                              |==============                                                        |  20%  |                                                                              |==============                                                        |  21%  |                                                                              |===============                                                       |  21%  |                                                                              |===============                                                       |  22%  |                                                                              |================                                                      |  22%  |                                                                              |================                                                      |  23%  |                                                                              |================                                                      |  24%  |                                                                              |=================                                                     |  24%  |                                                                              |=================                                                     |  25%  |                                                                              |==================                                                    |  25%  |                                                                              |==================                                                    |  26%  |                                                                              |===================                                                   |  26%  |                                                                              |===================                                                   |  27%  |                                                                              |===================                                                   |  28%  |                                                                              |====================                                                  |  28%  |                                                                              |====================                                                  |  29%  |                                                                              |=====================                                                 |  29%  |                                                                              |=====================                                                 |  30%  |                                                                              |=====================                                                 |  31%  |                                                                              |======================                                                |  31%  |                                                                              |======================                                                |  32%  |                                                                              |=======================                                               |  32%  |                                                                              |=======================                                               |  33%  |                                                                              |========================                                              |  34%  |                                                                              |========================                                              |  35%  |                                                                              |=========================                                             |  35%  |                                                                              |=========================                                             |  36%  |                                                                              |==========================                                            |  37%  |                                                                              |==========================                                            |  38%  |                                                                              |===========================                                           |  38%  |                                                                              |===========================                                           |  39%  |                                                                              |============================                                          |  39%  |                                                                              |============================                                          |  40%  |                                                                              |============================                                          |  41%  |                                                                              |=============================                                         |  41%  |                                                                              |=============================                                         |  42%  |                                                                              |==============================                                        |  42%  |                                                                              |==============================                                        |  43%  |                                                                              |==============================                                        |  44%  |                                                                              |===============================                                       |  44%  |                                                                              |===============================                                       |  45%  |                                                                              |================================                                      |  45%  |                                                                              |================================                                      |  46%  |                                                                              |=================================                                     |  47%  |                                                                              |=================================                                     |  48%  |                                                                              |==================================                                    |  48%  |                                                                              |==================================                                    |  49%  |                                                                              |===================================                                   |  49%  |                                                                              |===================================                                   |  50%  |                                                                              |===================================                                   |  51%  |                                                                              |====================================                                  |  51%  |                                                                              |====================================                                  |  52%  |                                                                              |=====================================                                 |  52%  |                                                                              |=====================================                                 |  53%  |                                                                              |======================================                                |  54%  |                                                                              |======================================                                |  55%  |                                                                              |=======================================                               |  55%  |                                                                              |=======================================                               |  56%  |                                                                              |========================================                              |  56%  |                                                                              |========================================                              |  57%  |                                                                              |========================================                              |  58%  |                                                                              |=========================================                             |  58%  |                                                                              |=========================================                             |  59%  |                                                                              |==========================================                            |  59%  |                                                                              |==========================================                            |  60%  |                                                                              |==========================================                            |  61%  |                                                                              |===========================================                           |  61%  |                                                                              |===========================================                           |  62%  |                                                                              |============================================                          |  62%  |                                                                              |============================================                          |  63%  |                                                                              |=============================================                         |  64%  |                                                                              |=============================================                         |  65%  |                                                                              |==============================================                        |  65%  |                                                                              |==============================================                        |  66%  |                                                                              |===============================================                       |  67%  |                                                                              |===============================================                       |  68%  |                                                                              |================================================                      |  68%  |                                                                              |================================================                      |  69%  |                                                                              |=================================================                     |  69%  |                                                                              |=================================================                     |  70%  |                                                                              |=================================================                     |  71%  |                                                                              |==================================================                    |  71%  |                                                                              |==================================================                    |  72%  |                                                                              |===================================================                   |  72%  |                                                                              |===================================================                   |  73%  |                                                                              |===================================================                   |  74%  |                                                                              |====================================================                  |  74%  |                                                                              |====================================================                  |  75%  |                                                                              |=====================================================                 |  75%  |                                                                              |=====================================================                 |  76%  |                                                                              |======================================================                |  76%  |                                                                              |======================================================                |  77%  |                                                                              |======================================================                |  78%  |                                                                              |=======================================================               |  78%  |                                                                              |=======================================================               |  79%  |                                                                              |========================================================              |  79%  |                                                                              |========================================================              |  80%  |                                                                              |========================================================              |  81%  |                                                                              |=========================================================             |  81%  |                                                                              |=========================================================             |  82%  |                                                                              |==========================================================            |  82%  |                                                                              |==========================================================            |  83%  |                                                                              |===========================================================           |  84%  |                                                                              |===========================================================           |  85%  |                                                                              |============================================================          |  85%  |                                                                              |============================================================          |  86%  |                                                                              |=============================================================         |  87%  |                                                                              |=============================================================         |  88%  |                                                                              |==============================================================        |  88%  |                                                                              |==============================================================        |  89%  |                                                                              |===============================================================       |  89%  |                                                                              |===============================================================       |  90%  |                                                                              |===============================================================       |  91%  |                                                                              |================================================================      |  91%  |                                                                              |================================================================      |  92%  |                                                                              |=================================================================     |  92%  |                                                                              |=================================================================     |  93%  |                                                                              |=================================================================     |  94%  |                                                                              |==================================================================    |  94%  |                                                                              |==================================================================    |  95%  |                                                                              |===================================================================   |  95%  |                                                                              |===================================================================   |  96%  |                                                                              |====================================================================  |  97%  |                                                                              |====================================================================  |  98%  |                                                                              |===================================================================== |  98%  |                                                                              |===================================================================== |  99%  |                                                                              |======================================================================|  99%  |                                                                              |======================================================================| 100%
## Matrix converted.. Prepare genind object...
## Completed: gl2gi
```

Save it

```
saveRDS(
  snp2, here(
    "americas/output/dapc/snp2.rds"
  )
)
```

To load it

```
snp2 <- readRDS(
  here(
    "americas/output/dapc/snp2.rds"
  )
)
```

Import sample data

```
sampling_loc <- readRDS(here("scripts", "RMarkdowns", "output", "sampling_loc_americas_all.rds"))
head(sampling_loc)
```

```
##       Pop_City Country Latitude Longitude Continent Abbreviation Year
## 1   Berlin, NJ     USA 39.79081  -74.9291  Americas          BER 2018
## 2 Columbus, OH     USA 39.97170  -82.9071  Americas          COL 2015
## 3   Palm Beach     USA 26.70560  -80.0364  Americas          PAL 2018
## 4  Houston, TX     USA 29.75491  -95.3505  Americas          HOU 2018
## 5  Los Angeles     USA 34.05220 -118.2437  Americas          LOS 2018
## 6   Manaus, AM  Brazil -3.09161  -60.0325  Americas          MAU 2017
##          Region      Subregion order order2 orderold AM_order      state
## 1 North America Northeast, USA     1     NA       75        6    NJ, USA
## 2 North America   Midwest, USA     2     NA       76       18    OH, USA
## 3 North America Southeast, USA     3     NA       77       13    FL, USA
## 4 North America Southwest, USA     4     NA       78       23    TX, USA
## 5 North America      West, USA     5     NA       79       30    CA, USA
## 6 South America  South America     6     NA       80       38 AM, Brazil
```

```
strata(snp2) <- data.frame(other(snp2))

# Currently set on just 
head(pop(snp2))
```

```
## [1] MAU MAU MAU MAU MAU MAU
## 51 Levels: ARA BAF BCO BEA BEH BER BIT BPA BPV BRM CHI COL CUA DAL DEN ... WAV
```

Load the csv

```
countr <- read.csv(here("scripts", "RMarkdowns", "output", "americas", "americas_all", "dapc", "DAPC_countries_americas_r1.csv"
))
df <- as.data.frame(countr)

head(df)
```

```
##   pop        region
## 1 MAU South America
## 2 MAU South America
## 3 MAU South America
## 4 MAU South America
## 5 MAU South America
## 6 MAU South America
```

```
snp2@pop <- as.factor(df$region)
```

```
snp2$pop
```

```
##   [1] South America South America South America South America South America
##   [6] South America South America South America South America South America
##  [11] South America South America South America South America South America
##  [16] South America South America South America South America South America
##  [21] South America South America South America South America South America
##  [26] Caribbean     Caribbean     Caribbean     Caribbean     Caribbean    
##  [31] Caribbean     Caribbean     Caribbean     Caribbean     Caribbean    
##  [36] Caribbean     Caribbean     Caribbean     Caribbean     Caribbean    
##  [41] Caribbean     South America South America South America South America
##  [46] South America South America South America South America South America
##  [51] South America South America South America North America North America
##  [56] North America North America North America North America North America
##  [61] North America North America North America North America North America
##  [66] North America North America North America North America North America
##  [71] North America North America North America North America North America
##  [76] North America North America North America North America North America
##  [81] North America North America North America North America North America
##  [86] North America North America North America North America North America
##  [91] North America North America North America North America North America
##  [96] North America North America North America North America North America
## [101] North America North America North America North America North America
## [106] North America North America North America North America North America
## [111] North America North America North America North America North America
## [116] North America North America North America North America North America
## [121] North America North America North America North America North America
## [126] North America North America North America North America North America
## [131] North America North America North America North America North America
## [136] North America North America North America North America North America
## [141] North America North America North America North America North America
## [146] North America North America North America North America North America
## [151] North America North America North America North America North America
## [156] North America North America North America North America North America
## [161] North America North America North America North America North America
## [166] North America North America North America North America North America
## [171] North America North America North America North America North America
## [176] North America North America North America North America North America
## [181] North America North America North America North America North America
## [186] North America North America North America North America North America
## [191] North America North America North America North America North America
## [196] North America North America North America North America North America
## [201] North America North America North America North America North America
## [206] South America South America South America South America South America
## [211] South America South America South America South America South America
## [216] South America South America South America South America South America
## [221] South America South America South America South America South America
## [226] South America South America South America South America South America
## [231] South America South America South America South America South America
## [236] South America South America South America South America South America
## [241] South America South America South America South America South America
## [246] South America South America South America South America South America
## [251] South America South America South America South America South America
## [256] South America South America South America South America South America
## [261] South America South America South America South America South America
## [266] South America South America South America South America South America
## [271] South America South America South America South America North America
## [276] North America North America North America North America North America
## [281] North America North America North America North America North America
## [286] North America North America North America North America North America
## [291] North America North America North America North America North America
## [296] North America North America North America North America North America
## [301] North America North America North America North America North America
## [306] North America North America North America North America North America
## [311] North America North America North America North America North America
## [316] North America North America North America North America North America
## [321] North America North America North America North America North America
## [326] North America North America South America South America South America
## [331] South America South America South America South America South America
## [336] South America South America South America South America South America
## [341] South America South America South America South America South America
## [346] South America South America South America South America South America
## [351] South America South America South America South America South America
## [356] South America South America South America North America North America
## [361] North America North America North America North America North America
## [366] North America North America North America North America North America
## [371] North America North America North America North America North America
## [376] North America North America North America North America North America
## [381] North America North America North America North America North America
## [386] North America North America North America North America North America
## [391] North America North America North America North America North America
## [396] North America North America North America North America North America
## [401] North America North America North America North America North America
## [406] North America North America North America North America North America
## [411] North America North America North America North America North America
## [416] North America North America North America North America North America
## [421] North America North America North America North America North America
## [426] North America Caribbean     Caribbean     Caribbean     Caribbean    
## [431] South America South America South America South America South America
## [436] South America South America South America South America South America
## [441] South America South America North America North America North America
## [446] North America North America North America North America North America
## [451] North America North America North America North America North America
## [456] North America North America North America North America North America
## [461] North America North America North America North America North America
## [466] North America North America North America North America North America
## [471] North America North America North America North America North America
## [476] North America North America North America North America North America
## [481] North America North America North America North America North America
## [486] North America North America North America North America North America
## [491] North America North America North America North America North America
## [496] North America North America North America North America North America
## [501] North America North America North America North America South America
## [506] Caribbean     Caribbean     Caribbean     Caribbean     Caribbean    
## [511] Caribbean     Caribbean     Caribbean     Caribbean     Caribbean    
## [516] Caribbean     Caribbean     South America South America South America
## [521] South America South America South America South America South America
## [526] South America South America South America South America South America
## [531] South America South America South America South America South America
## [536] South America South America South America South America South America
## [541] South America South America South America South America
## Levels: Caribbean North America South America
```

Save the genind object

```
saveRDS(snp2, here("scripts", "RMarkdowns",
  "output", "americas", "americas_all", "dapc", "snp_country.rds"
))
```

Load the genind object

```
snp_country <- readRDS(here("scripts", "RMarkdowns",
  "output", "americas", "americas_all", "dapc", "snp_country.rds"
))
```

### 2.1.1 Scale and find clusters

Scale

```
snp_country <- scaleGen(snp2, NA.method="mean")
class(snp_country)
```

```
## [1] "matrix" "array"
```

```
dim(snp_country)
```

```
## [1]   544 87604
```

```
snp_country[1:5,1:5]
```

```
##     AX-583033387_C.C AX-583033387_C.G AX-583035257_T.A AX-583035257_T.T
## 153       -0.3514624        0.3514624       -0.5220856        0.5220856
## 154       -0.3514624        0.3514624       -0.5220856        0.5220856
## 155        0.9856229       -0.9856229        0.9090120       -0.9090120
## 156        0.9856229       -0.9856229       -0.5220856        0.5220856
## 158        0.9856229       -0.9856229       -0.5220856        0.5220856
##     AX-583035268_A.A
## 153        0.6995577
## 154        0.6995577
## 155        0.6995577
## 156        0.6995577
## 158        0.6995577
```

```
# Get the populations from the genlight object
populations <- snp2$pop
```

### 2.1.2 Find clusters

```
grp <- find.clusters(snp_country, max.n.clust=25)
#retained 500
#Choose the number of clusters (>=2): 5
```

Save the genind object

```
saveRDS(grp, here("scripts", "RMarkdowns",
  "output", "americas", "americas_all", "dapc", "grp.rds"
))
```

Load the genind object

```
grp <- readRDS(here("scripts", "RMarkdowns",
  "output", "americas", "americas_all", "dapc", "grp.rds"
))
```

```
names(grp)
```

```
## [1] "Kstat" "stat"  "grp"   "size"
```

```
grp$size #group sizes for our clusters
```

```
## [1]  22  19 160 140 203
```

```
table(pop(snp2), grp$grp)
```

```
##                
##                   1   2   3   4   5
##   Caribbean       0   4  28   0   0
##   North America   0   0 132   0 203
##   South America  22  15   0 140   0
```

```
dapc1 <- dapc(snp_country, grp$grp)
#500 PCs retained
#4 discriminant functions retained
```

```
dapc1
```

Save the genind object

```
saveRDS(dapc1, here("scripts", "RMarkdowns",
  "output", "americas", "americas_all", "dapc", "dapc1.rds"
))
```

Load the genind object

```
dapc1 <- readRDS(here("scripts", "RMarkdowns",
  "output", "americas", "americas_all", "dapc", "dapc1.rds"
))
```

```
dapc1
```

```
##  #################################################
##  # Discriminant Analysis of Principal Components #
##  #################################################
## class: dapc
## $call: dapc.data.frame(x = as.data.frame(x), grp = ..1)
## 
## $n.pca: 500 first PCs of PCA used
## $n.da: 4 discriminant functions saved
## $var (proportion of conserved variance): 0.982
## 
## $eig (eigenvalues): 1180000 568500 83780 23480  vector    length content                   
## 1 $eig      4      eigenvalues               
## 2 $grp      544    prior group assignment    
## 3 $prior    5      prior group probabilities 
## 4 $assign   544    posterior group assignment
## 5 $pca.cent 87604  centring vector of PCA    
## 6 $pca.norm 87604  scaling vector of PCA     
## 7 $pca.eig  543    eigenvalues of PCA        
## 
##   data.frame    nrow  ncol content                                          
## 1 $tab          544   500  retained PCs of PCA                              
## 2 $means        5     500  group means                                      
## 3 $loadings     500   4    loadings of variables                            
## 4 $ind.coord    544   4    coordinates of individuals (principal components)
## 5 $grp.coord    5     4    coordinates of groups                            
## 6 $posterior    544   5    posterior membership probabilities               
## 7 $pca.loadings 87604 500  PCA loadings of original variables               
## 8 $var.contr    87604 4    contribution of original variables
```

```
scatter(dapc1)
```

### 2.1.3 Calculating the optimum PC number to rerun DAPC

```
optim.a.score(dapc1, n.pca=1:ncol(dapc1$tab), smart=TRUE, n=10, plot=TRUE, n.sim=10) #calculating optimal number of PCs 
#Optimal number = 34
```

### 2.1.4 Now do cross-validation

Cross-validation: The Discriminant Analysis of Principal Components
(DAPC) relies on dimension reduction of the data using PCA followed by a
linear discriminant analysis. How many PCA axes to retain is often a
non-trivial question. Cross validation provides an objective way to
decide how many axes to retain: different numbers are tried and the
quality of the corresponding DAPC is assessed by cross- validation: DAPC
is performed on a training set, typically made of 90% of the
observations (comprising 90% of the observations in each subpopulation)
, and then used to predict the groups of the 10% of remaining
observations. The current method uses the average prediction success per
group (result=“groupMean”), or the overall prediction success
(result=“overall”). The number of PCs associated with the lowest Mean
Squared Error is then retained in the DAPC.

```
xvalDapc(snp_country, populations, n.pca.max = 200, n.da = NULL,
              training.set = 0.9, result = c("groupMean", "overall"),
              center = TRUE, scale = FALSE,
              n.pca=NULL, n.rep = 10, xval.plot = TRUE)
```

180 pcas 2 discriminants functions proportion of conserved variance:
0.61

Run DAPC with object

```
dapc_snp1 <- dapc(snp_country, n.pca = 180, n.da = 2, grp = populations)
```

Save it

```
saveRDS(
  dapc_snp1, here("scripts", "RMarkdowns",
  "output", "americas", "americas_all", "dapc", "dapc_snp1.rds"
  )
)
```

To load it

```
dapc_snp1 <- readRDS(
  here("scripts", "RMarkdowns",
  "output", "americas", "americas_all", "dapc", "dapc_snp1.rds"
  )
)
```

Most contributing alleles

```
set.seed(4) 
contrib<-loadingplot(dapc_snp1$var.contr, axis=2, thres=.0002, lab.jitter=3)
```

```
scatter(dapc_snp1)
```

Run DAPC with optimised results

```
dapc_snp2 <- dapc(snp_country, n.pca = 34, n.da = 4, grp = populations)
```

Save it

```
saveRDS(
  dapc_snp2, here("scripts", "RMarkdowns",
  "output", "americas", "americas_all", "dapc", "dapc_snp2.rds"
  )
)
```

To load it

```
dapc_snp2 <- readRDS(
  here("scripts", "RMarkdowns",
  "output", "americas", "americas_all", "dapc", "dapc_snp2.rds"
  )
)
```

```
scatter(dapc_snp2)
```

Try again with only 4 clusters

```
grp4 <- find.clusters(snp_country, max.n.clust=15)
#retained 500
#Choose the number of clusters (>=2): 4
```

Save the genind object

```
saveRDS(grp4, here("scripts", "RMarkdowns",
  "output", "americas", "americas_all", "dapc", "grp4.rds"
))
```

Load the genind object

```
grp4 <- readRDS(here("scripts", "RMarkdowns",
  "output", "americas", "americas_all", "dapc", "grp4.rds"
))
```

```
names(grp4)
```

```
## [1] "Kstat" "stat"  "grp"   "size"
```

```
grp4$size #group sizes for our clusters
```

```
## [1] 130 204 162  48
```

```
table(pop(snp2), grp4$grp)
```

```
##                
##                   1   2   3   4
##   Caribbean      16   0   0  16
##   North America 114 204   0  17
##   South America   0   0 162  15
```

```
dapc2 <- dapc(snp_country, grp4$grp)
#500 PCs retained
#4 discriminant functions retained
```

```
dapc2

scatter(dapc2)
```

Cross-validation: The Discriminant Analysis of Principal Components
(DAPC) relies on dimension reduction of the data using PCA followed by a
linear discriminant analysis. How many PCA axes to retain is often a
non-trivial question. Cross validation provides an objective way to
decide how many axes to retain: different numbers are tried and the
quality of the corresponding DAPC is assessed by cross- validation: DAPC
is performed on a training set, typically made of 90% of the
observations (comprising 90% of the observations in each subpopulation)
, and then used to predict the groups of the 10% of remaining
observations. The current method uses the average prediction success per
group (result=“groupMean”), or the overall prediction success
(result=“overall”). The number of PCs associated with the lowest Mean
Squared Error is then retained in the DAPC.

```
xvalDapc(snp_country, populations, n.pca.max = 300, n.da = NULL,
              training.set = 0.9, result = c("groupMean", "overall"),
              center = TRUE, scale = FALSE,
              n.pca=NULL, n.rep = 10, xval.plot = TRUE)
```

200 pcas 2 discriminants functions proportion of conserved variance:
0.65

Most contributing alleles

```
set.seed(4) 
contrib<-loadingplot(dapc_snp2$var.contr, axis=2, thres=.0002, lab.jitter=3)
```

Run DAPC with object

```
dapc_snp3 <- dapc(snp_country, n.pca = 200, n.da = 4, grp = populations)
```

Save it

```
saveRDS(
  dapc_snp3, here("scripts", "RMarkdowns",
  "output", "americas", "americas_all", "dapc", "dapc_snp3.rds"
  )
)
```

To load it

```
dapc_snp3 <- readRDS(
  here("scripts", "RMarkdowns",
  "output", "americas", "americas_all", "dapc", "dapc_snp3.rds"
  )
)
```

```
scatter(dapc_snp3)
```

There truly do only seem to be 3 clusters, so re-do with 3

```
grp3 <- find.clusters(snp_country, max.n.clust=15)
#retained 500
#Choose the number of clusters (>=2): 3
```

Save the genind object

```
saveRDS(grp3, here("scripts", "RMarkdowns",
  "output", "americas", "americas_all", "dapc", "grp3.rds"
))
```

Load the genind object

```
grp3 <- readRDS(here("scripts", "RMarkdowns",
  "output", "americas", "americas_all", "dapc", "grp3.rds"
))
```

```
names(grp3)
```

```
## [1] "Kstat" "stat"  "grp"   "size"
```

```
grp3$size #group sizes for our clusters
```

```
## [1] 11 83
```

```
table(pop(snp2), grp3$grp)
```

```
dapc3 <- dapc(snp_country, grp3$grp)
#500 PCs retained
#3 discriminant functions retained
```

```
dapc3
```

Save the genind object

```
saveRDS(dapc3, here("scripts", "RMarkdowns",
  "output", "americas", "americas_all", "dapc", "dapc3.rds"
))
```

Load the genind object

```
dapc3 <- readRDS(here("scripts", "RMarkdowns",
  "output", "americas", "americas_all", "dapc", "dapc3.rds"
))
```

```
scatter(dapc3)
```

### 2.1.3 Calculating the optimum PC number to rerun DAPC

```
optim.a.score(dapc3, n.pca=1:ncol(dapc3$tab), smart=TRUE, n=10, plot=TRUE, n.sim=10) #calculating optimal number of PCs 
#Optimal number = 46
```

Run DAPC with object

```
dapc_snp3 <- dapc(snp_country, n.pca = 46, n.da = 2, grp = populations)
```

Save it

```
saveRDS(
  dapc_snp3, here("scripts", "RMarkdowns",
  "output", "americas", "americas_all", "dapc", "dapc_snp3.rds"
  )
)
```

To load it

```
dapc_snp3 <- readRDS(
  here("scripts", "RMarkdowns",
  "output", "americas", "americas_all", "dapc", "dapc_snp3.rds"
  )
)
```

Most contributing alleles

```
set.seed(4) 
contrib<-loadingplot(dapc_snp3$var.contr, axis=2, thres=.0002, lab.jitter=3)
```

```
scatter(dapc_snp3)
```

### 2.1.5 Make plots for DAPC

Check R symbols for plot

```
#to see all shapes -> plot shapes - para escolher os simbolos
N = 100; M = 1000
good.shapes = c(1:25,35:38,43,60,62:64)
foo = data.frame( x = rnorm(M), y = rnorm(M), s = factor( sample(1:N, M, replace = TRUE) ) )
ggplot(aes(x,y,shape=s ), data=foo ) +
  scale_shape_manual(values=good.shapes[1:N]) +
  geom_point()
```

```
## Warning: <ggplot> %+% x was deprecated in ggplot2 4.0.0.
## ℹ Please use <ggplot> + x instead.
## This warning is displayed once every 8 hours.
## Call `lifecycle::last_lifecycle_warnings()` to see where this warning was
## generated.
```

```
## Warning: Removed 672 rows containing missing values or values outside the scale range
## (`geom_point()`).
```

```
#1:25,28:31,36,55:57
```

Plot using different discriminant functions

1 & 2

```
pdf(file = "scripts/RMarkdowns/output/americas/americas_all/dapc/dapc_americas_all_r1_PC1_2.pdf",   # The directory you want to save the file in
    width = 7, # The width of the plot in inches
    height = 7) # The height of the plot in inches

myCol <- c( "#E41A1C", "orange", "#2524f9")

good.shapes = c(1:25,35:38,43,60,62:64)

op <- par(cex = 0.39)
scatter(dapc_snp3, pch = good.shapes, cstar = 0, col=myCol, label=NULL, mstree = FALSE, legend=TRUE, posi.da="topright", cex=1.0,  cex.lab=0.5, cex.main=0.5, cellipse=TRUE, posi.leg="topleft",  xax =1, yax=2)

dev.off()
```

```
myCol <- c( "#E41A1C", "orange", "#2524f9")
op <- par(cex = 0.39)
scatter(dapc_snp3, pch = good.shapes, cstar = 0, col=myCol, label=NULL, mstree = FALSE, legend=TRUE, posi.da="bottomleft", cex=1.0,  cex.lab=0.5, cex.main=0.5, cellipse=TRUE, posi.leg="topleft",  xax =1, yax=2)
```

### 2.1.6 Now plot by locality

```
cd /gpfs/gibbs/pi/caccone/mkc54/albo/americas
plink \
--allow-extra-chr \
--keep-allele-order \
--bfile output/dapc/50loc/modified/r2_0.01_b \
--make-bed \
--out output/dapc/50loc/dapc_r01 \
--silent;
grep 'samples\|variants\|remaining' output/dapc/50loc/dapc_r01.log
```

18964 variants loaded from .bim file. 18964 variants and 545 people
pass filters and QC.

Convert to raw format

```
plink \
--allow-extra-chr \
--keep-allele-order \
--bfile output/dapc/50loc/dapc_r01 \
--recodeA \
--out output/dapc/50loc/dapc_r01 \
--silent;
grep 'samples\|variants\|remaining' output/dapc/50loc/dapc_r01.log
```

18964 variants loaded from .bim file. 18964 variants and 545 people
pass filters and QC.

Import the data and covert it to genind format

```
# import the data
snp <-
  read.PLINK(
    here("americas/output/dapc/50loc/dapc_r01.raw"),
    quiet = FALSE,
    chunkSize = 1000,
    parallel = require("parallel"),
    n.cores = 4
  )
```

```
## 
##  Reading PLINK raw format into a genlight object...
```

```
## Loading required package: parallel
```

```
## 
##  Reading loci information... 
## 
##  Reading and converting genotypes... 
## .
##  Building final object... 
## 
## ...done.
```

```
nInd(snp)
```

```
## [1] 545
```

```
nLoc(snp)
```

```
## [1] 18964
```

```
nPop(snp)
```

```
## [1] 50
```

```
indNames(snp)
```

```
##   [1] "153"  "154"  "155"  "156"  "158"  "16"   "174"  "175"  "176"  "177" 
##  [11] "178"  "17"   "180"  "181"  "182"  "183"  "184"  "185"  "186"  "187" 
##  [21] "188"  "189"  "18"   "190"  "191"  "2158" "2159" "2160" "2161" "2162"
##  [31] "2163" "2164" "2165" "2166" "2167" "2168" "2169" "2170" "2171" "2172"
##  [41] "2173" "2203" "2204" "2205" "2206" "2207" "2208" "2209" "2210" "2211"
##  [51] "2212" "2213" "2214" "2221" "2222" "2223" "2224" "2226" "2227" "2228"
##  [61] "2229" "2230" "2231" "2232" "2233" "2234" "2235" "2236" "2237" "2238"
##  [71] "2239" "2240" "2241" "2242" "2243" "2244" "2245" "2246" "2247" "2248"
##  [81] "2249" "2250" "2251" "2252" "2253" "2254" "2255" "2256" "2257" "2258"
##  [91] "2259" "2260" "2261" "2262" "2263" "2264" "2265" "2266" "2267" "2268"
## [101] "2269" "2270" "2271" "2272" "2273" "2275" "2288" "2289" "2290" "2291"
## [111] "2292" "2293" "2294" "2295" "2301" "2302" "2303" "2304" "2305" "2306"
## [121] "2307" "2308" "2309" "2310" "2311" "2312" "2313" "2314" "2315" "2316"
## [131] "2317" "2318" "2319" "2320" "2321" "2322" "2323" "2324" "2326" "2327"
## [141] "2329" "2330" "2331" "2332" "2333" "2334" "2335" "2336" "2337" "2338"
## [151] "2339" "2340" "2341" "2342" "2343" "2344" "2345" "2346" "2347" "2348"
## [161] "2349" "2350" "2351" "2352" "2353" "2354" "2355" "2357" "2358" "2360"
## [171] "2362" "2384" "2385" "2386" "2387" "2388" "2390" "2391" "2392" "2393"
## [181] "2394" "2401" "2402" "2403" "2404" "2405" "2406" "2407" "2408" "2409"
## [191] "2410" "2411" "2412" "2413" "2414" "2415" "2416" "2417" "2418" "2419"
## [201] "2420" "2421" "2422" "2423" "2424" "2425" "2426" "2427" "2428" "2429"
## [211] "2430" "2431" "2432" "2433" "2434" "2435" "2436" "2437" "2438" "2439"
## [221] "2440" "2441" "2442" "2443" "2444" "2445" "2446" "2447" "2448" "2449"
## [231] "2450" "2451" "2452" "2453" "2454" "2455" "2456" "2457" "2458" "2459"
## [241] "2461" "2462" "2463" "2464" "2465" "2466" "2467" "2468" "2469" "2470"
## [251] "2471" "2472" "2473" "2474" "2475" "2476" "2477" "2479" "2480" "2481"
## [261] "2482" "2483" "2484" "2485" "2486" "2487" "2488" "2489" "2490" "2491"
## [271] "2492" "2493" "2494" "2495" "2501" "2502" "2503" "2504" "2505" "2506"
## [281] "2507" "2508" "2509" "2510" "2511" "2512" "2513" "2514" "2515" "2516"
## [291] "2517" "2518" "2519" "2520" "2521" "2522" "2523" "2524" "2525" "2526"
## [301] "2527" "2528" "2529" "2530" "2531" "2532" "2533" "2534" "2535" "2536"
## [311] "2537" "2538" "2539" "2540" "2541" "2542" "2543" "2544" "2545" "2546"
## [321] "2547" "2548" "2550" "2551" "2552" "2553" "2554" "2561" "2562" "2564"
## [331] "2565" "2566" "2567" "2568" "2569" "2570" "2571" "2572" "2573" "2574"
## [341] "2575" "2576" "2577" "2578" "2579" "2580" "2581" "2582" "2583" "2584"
## [351] "2585" "2586" "2587" "2588" "2589" "2591" "2592" "2595" "294"  "295" 
## [361] "296"  "297"  "298"  "299"  "301"  "302"  "303"  "304"  "305"  "306" 
## [371] "307"  "308"  "309"  "310"  "311"  "312"  "313"  "314"  "315"  "316" 
## [381] "317"  "318"  "319"  "320"  "321"  "322"  "323"  "324"  "325"  "326" 
## [391] "327"  "328"  "329"  "330"  "331"  "332"  "333"  "334"  "335"  "336" 
## [401] "337"  "340"  "341"  "342"  "343"  "344"  "345"  "346"  "347"  "348" 
## [411] "349"  "350"  "351"  "352"  "353"  "354"  "355"  "356"  "357"  "358" 
## [421] "359"  "360"  "361"  "362"  "363"  "364"  "366"  "368"  "369"  "370" 
## [431] "371"  "372"  "373"  "374"  "375"  "376"  "377"  "378"  "379"  "380" 
## [441] "381"  "382"  "383"  "413"  "414"  "415"  "416"  "417"  "418"  "419" 
## [451] "420"  "421"  "422"  "424"  "425"  "426"  "427"  "428"  "429"  "430" 
## [461] "431"  "432"  "433"  "434"  "435"  "436"  "437"  "438"  "439"  "440" 
## [471] "441"  "442"  "443"  "444"  "445"  "446"  "447"  "448"  "449"  "450" 
## [481] "451"  "452"  "453"  "454"  "455"  "456"  "457"  "458"  "459"  "460" 
## [491] "461"  "463"  "464"  "466"  "467"  "468"  "469"  "470"  "472"  "473" 
## [501] "474"  "475"  "476"  "477"  "478"  "60"   "611"  "612"  "613"  "614" 
## [511] "615"  "616"  "617"  "618"  "619"  "620"  "621"  "622"  "634"  "635" 
## [521] "636"  "637"  "638"  "639"  "640"  "641"  "642"  "643"  "644"  "645" 
## [531] "64"   "684"  "685"  "686"  "687"  "688"  "689"  "690"  "691"  "692" 
## [541] "693"  "694"  "695"  "8"    "9"
```

```
#pop(snp) <- factor(c())

# convert to genind
snp2 <- gl2gi(snp, probar = TRUE, verbose = NULL)
```

```
## Starting gl2gi 
##   Processing genlight object with SNP data
##   |                                                                              |                                                                      |   0%  |                                                                              |                                                                      |   1%  |                                                                              |=                                                                     |   1%  |                                                                              |=                                                                     |   2%  |                                                                              |==                                                                    |   2%  |                                                                              |==                                                                    |   3%  |                                                                              |===                                                                   |   4%  |                                                                              |===                                                                   |   5%  |                                                                              |====                                                                  |   5%  |                                                                              |====                                                                  |   6%  |                                                                              |=====                                                                 |   7%  |                                                                              |=====                                                                 |   8%  |                                                                              |======                                                                |   8%  |                                                                              |======                                                                |   9%  |                                                                              |=======                                                               |   9%  |                                                                              |=======                                                               |  10%  |                                                                              |=======                                                               |  11%  |                                                                              |========                                                              |  11%  |                                                                              |========                                                              |  12%  |                                                                              |=========                                                             |  12%  |                                                                              |=========                                                             |  13%  |                                                                              |==========                                                            |  14%  |                                                                              |==========                                                            |  15%  |                                                                              |===========                                                           |  15%  |                                                                              |===========                                                           |  16%  |                                                                              |============                                                          |  17%  |                                                                              |============                                                          |  18%  |                                                                              |=============                                                         |  18%  |                                                                              |=============                                                         |  19%  |                                                                              |==============                                                        |  19%  |                                                                              |==============                                                        |  20%  |                                                                              |==============                                                        |  21%  |                                                                              |===============                                                       |  21%  |                                                                              |===============                                                       |  22%  |                                                                              |================                                                      |  22%  |                                                                              |================                                                      |  23%  |                                                                              |=================                                                     |  24%  |                                                                              |=================                                                     |  25%  |                                                                              |==================                                                    |  25%  |                                                                              |==================                                                    |  26%  |                                                                              |===================                                                   |  27%  |                                                                              |===================                                                   |  28%  |                                                                              |====================                                                  |  28%  |                                                                              |====================                                                  |  29%  |                                                                              |=====================                                                 |  29%  |                                                                              |=====================                                                 |  30%  |                                                                              |=====================                                                 |  31%  |                                                                              |======================                                                |  31%  |                                                                              |======================                                                |  32%  |                                                                              |=======================                                               |  32%  |                                                                              |=======================                                               |  33%  |                                                                              |========================                                              |  34%  |                                                                              |========================                                              |  35%  |                                                                              |=========================                                             |  35%  |                                                                              |=========================                                             |  36%  |                                                                              |==========================                                            |  37%  |                                                                              |==========================                                            |  38%  |                                                                              |===========================                                           |  38%  |                                                                              |===========================                                           |  39%  |                                                                              |============================                                          |  39%  |                                                                              |============================                                          |  40%  |                                                                              |============================                                          |  41%  |                                                                              |=============================                                         |  41%  |                                                                              |=============================                                         |  42%  |                                                                              |==============================                                        |  42%  |                                                                              |==============================                                        |  43%  |                                                                              |===============================                                       |  44%  |                                                                              |===============================                                       |  45%  |                                                                              |================================                                      |  45%  |                                                                              |================================                                      |  46%  |                                                                              |=================================                                     |  47%  |                                                                              |=================================                                     |  48%  |                                                                              |==================================                                    |  48%  |                                                                              |==================================                                    |  49%  |                                                                              |===================================                                   |  49%  |                                                                              |===================================                                   |  50%  |                                                                              |===================================                                   |  51%  |                                                                              |====================================                                  |  51%  |                                                                              |====================================                                  |  52%  |                                                                              |=====================================                                 |  52%  |                                                                              |=====================================                                 |  53%  |                                                                              |======================================                                |  54%  |                                                                              |======================================                                |  55%  |                                                                              |=======================================                               |  55%  |                                                                              |=======================================                               |  56%  |                                                                              |========================================                              |  57%  |                                                                              |========================================                              |  58%  |                                                                              |=========================================                             |  58%  |                                                                              |=========================================                             |  59%  |                                                                              |==========================================                            |  59%  |                                                                              |==========================================                            |  60%  |                                                                              |==========================================                            |  61%  |                                                                              |===========================================                           |  61%  |                                                                              |===========================================                           |  62%  |                                                                              |============================================                          |  62%  |                                                                              |============================================                          |  63%  |                                                                              |=============================================                         |  64%  |                                                                              |=============================================                         |  65%  |                                                                              |==============================================                        |  65%  |                                                                              |==============================================                        |  66%  |                                                                              |===============================================                       |  67%  |                                                                              |===============================================                       |  68%  |                                                                              |================================================                      |  68%  |                                                                              |================================================                      |  69%  |                                                                              |=================================================                     |  69%  |                                                                              |=================================================                     |  70%  |                                                                              |=================================================                     |  71%  |                                                                              |==================================================                    |  71%  |                                                                              |==================================================                    |  72%  |                                                                              |===================================================                   |  72%  |                                                                              |===================================================                   |  73%  |                                                                              |====================================================                  |  74%  |                                                                              |====================================================                  |  75%  |                                                                              |=====================================================                 |  75%  |                                                                              |=====================================================                 |  76%  |                                                                              |======================================================                |  77%  |                                                                              |======================================================                |  78%  |                                                                              |=======================================================               |  78%  |                                                                              |=======================================================               |  79%  |                                                                              |========================================================              |  79%  |                                                                              |========================================================              |  80%  |                                                                              |========================================================              |  81%  |                                                                              |=========================================================             |  81%  |                                                                              |=========================================================             |  82%  |                                                                              |==========================================================            |  82%  |                                                                              |==========================================================            |  83%  |                                                                              |===========================================================           |  84%  |                                                                              |===========================================================           |  85%  |                                                                              |============================================================          |  85%  |                                                                              |============================================================          |  86%  |                                                                              |=============================================================         |  87%  |                                                                              |=============================================================         |  88%  |                                                                              |==============================================================        |  88%  |                                                                              |==============================================================        |  89%  |                                                                              |===============================================================       |  89%  |                                                                              |===============================================================       |  90%  |                                                                              |===============================================================       |  91%  |                                                                              |================================================================      |  91%  |                                                                              |================================================================      |  92%  |                                                                              |=================================================================     |  92%  |                                                                              |=================================================================     |  93%  |                                                                              |==================================================================    |  94%  |                                                                              |==================================================================    |  95%  |                                                                              |===================================================================   |  95%  |                                                                              |===================================================================   |  96%  |                                                                              |====================================================================  |  97%  |                                                                              |====================================================================  |  98%  |                                                                              |===================================================================== |  98%  |                                                                              |===================================================================== |  99%  |                                                                              |======================================================================|  99%  |                                                                              |======================================================================| 100%
## Matrix converted.. Prepare genind object...
## Completed: gl2gi
```

Save it

```
saveRDS(
  snp2, here(
    "americas/output/dapc/50loc/snp2_americas_2.rds"
  )
)
```

To load it

```
snp2 <- readRDS(
  here(
    "americas/output/dapc/50loc/snp2_americas_2.rds"
  )
)
```

### 2.2.1 Scale

```
snp3 <- scaleGen(snp2, NA.method="mean")
class(snp3)
```

Save it

```
saveRDS(
  snp3, here(
    "americas/output/dapc/50loc/snp3_americas_all.rds"
  )
)
```

To load it

```
snp3 <- readRDS(
  here(
    "americas/output/dapc/50loc/snp3_americas_all.rds"
  )
)
```

```
dim(snp3)
```

```
## [1]   545 37928
```

```
snp3[1:5,1:5]
```

```
##     AX-583034951_T.C AX-583034951_T.G AX-583036983_C.A AX-583036983_C.T
## 153        0.1710551       -0.1710551      -0.08792308       0.08792308
## 154        1.4658473       -1.4658473       1.23336536      -1.23336536
## 155        0.1710551       -0.1710551      -0.08792308       0.08792308
## 156        0.1710551       -0.1710551      -1.40921151       1.40921151
## 158        1.4658473       -1.4658473      -0.08792308       0.08792308
##     AX-583035671_G.A
## 153        0.0000000
## 154        0.0000000
## 155        0.9134782
## 156        0.9134782
## 158        0.9134782
```

```
# Get the populations from the genlight object
populations <- snp$pop
```

### 2.2.2 Find clusters

```
grp <- find.clusters(snp3, max.n.clust=15)
#retained 500
#Choose the number of clusters (>=2): 3
```

Save it

```
saveRDS(
  grp, here(
    "americas/output/dapc/50loc/grp_americas_all.rds"
  )
)
```

To load it

```
grp <- readRDS(
  here(
    "americas/output/dapc/50loc/grp_americas_all.rds"
  )
)
```

```
names(grp)
```

```
## [1] "Kstat" "stat"  "grp"   "size"
```

```
grp$size #group sizes for our clusters
```

```
## [1] 162 322  61
```

```
table(pop(snp2), grp$grp)
```

```
##      
##        1  2  3
##   ARA 12  0  0
##   BAF  7  0  0
##   BCO 12  0  0
##   BEA  0 12  0
##   BEH  9  0  0
##   BER  0 12  0
##   BIT 12  0  0
##   BPA 11  0  0
##   BPV 12  0  0
##   BRM  0  4 12
##   CHI  0  0  6
##   COL  0 10  0
##   CUA  0  0 15
##   DAL  0 12  0
##   DEN  0 12  0
##   FAY  0 11  0
##   FIR  0 12  0
##   FLO  8  0  0
##   GRV 12  0  0
##   HAL  0 10  0
##   HAR  0 12  0
##   HAT  0 10  0
##   HOU  0 12  0
##   IPO  7  0  0
##   LIN  0 12  0
##   LOS  0  0 12
##   MAC  0 12  0
##   MAN  0 12  0
##   MAU 12  0  0
##   MON  0 12  0
##   NEB  0 10  0
##   NEG  0  0  4
##   NEH  0  7  0
##   NEO  0 12  0
##   NEW  0 21  0
##   NOV  4  0  0
##   NUE  0 12  0
##   PAL  0 11  0
##   PEO  0 10  0
##   PLM  7  0  0
##   POR 12  0  0
##   REC 11  0  0
##   RUS  0 12  0
##   SAI  0  0 12
##   SMI  0 12  0
##   SPR  0  7  0
##   STL  0 12  0
##   TRA  0 12  0
##   TUC 14  0  0
##   WAV  0  7  0
```

```
xvalDapc(snp3, populations, n.pca.max = 200, n.da = NULL,
              training.set = 0.9, result = c("groupMean", "overall"),
              center = TRUE, scale = FALSE,
              n.pca=NULL, n.rep = 20, xval.plot = TRUE)
```

180 first PCs of PCA used 50 discriminant functions saved proportion
of conserved variance: 0.616

```
dapc1 <- dapc(snp3, snp$pop)
# PCs retained
# discriminant functions retained #5
```

Save the genind object

```
saveRDS(dapc1, here("americas", "output", "dapc", "50loc", "dapc1_americas_all_new.rds"
))
```

Load the genind object

```
dapc1 <- readRDS(here("americas", "output", "dapc", "50loc","dapc1_americas_all_new.rds"
))
```

```
scatter(dapc1)
```

Run DAPC with object

```
dapc_snp <- dapc(snp3, n.pca = 180, n.da = 50, grp = populations)
```

```
dapc_snp
```

Save the genind object

```
saveRDS(dapc_snp, here("americas", "output", "dapc", "50loc", "dapc_snp_americas_all_new.rds"
))
```

Load the genind object

```
dapc_snp <- readRDS(here("americas", "output", "dapc", "50loc", "dapc_snp_americas_all_new.rds"
))

dapc_snp
```

```
##  #################################################
##  # Discriminant Analysis of Principal Components #
##  #################################################
## class: dapc
## $call: dapc.data.frame(x = as.data.frame(x), grp = ..3, n.pca = 180, 
##     n.da = 50)
## 
## $n.pca: 180 first PCs of PCA used
## $n.da: 49 discriminant functions saved
## $var (proportion of conserved variance): 0.62
## 
## $eig (eigenvalues): 3016 1336 902.8 677.1 414.9 ...
## 
##   vector    length content                   
## 1 $eig      49     eigenvalues               
## 2 $grp      545    prior group assignment    
## 3 $prior    50     prior group probabilities 
## 4 $assign   545    posterior group assignment
## 5 $pca.cent 37928  centring vector of PCA    
## 6 $pca.norm 37928  scaling vector of PCA     
## 7 $pca.eig  544    eigenvalues of PCA        
## 
##   data.frame    nrow  ncol content                                          
## 1 $tab          545   180  retained PCs of PCA                              
## 2 $means        50    180  group means                                      
## 3 $loadings     180   49   loadings of variables                            
## 4 $ind.coord    545   49   coordinates of individuals (principal components)
## 5 $grp.coord    50    49   coordinates of groups                            
## 6 $posterior    545   50   posterior membership probabilities               
## 7 $pca.loadings 37928 180  PCA loadings of original variables               
## 8 $var.contr    37928 49   contribution of original variables
```

### 2.2.3 Plots for DAPC

```
myCol <- c("#146c45", "orange", "#2524f9", "#E41A1C", "purple", "#fd4e8b", "#51f310", "skyblue2", "#799d10", "#1F78B4", "#21a708", "magenta", "gold4",  "#a41415", "#fd5917", "#9d8d88", "#FB8072", "#6A3D9A", "#2c4a5e", "#a113b2", "gold2", "#66C2A5", "#332288", "#cf749b", "#8c61cd", "#52ef99", "#6a8fe0", "#871550", "pink2", "#784819", "#FF5005", "#1E90FF",  "#4C005C", "yellow",  "#993F00", "#F0A0FF")
```

```
pdf(file = "americas/output/dapc/50loc/dapc_americas_all_byloc_50pops_PC1_2.pdf",   # The directory you want to save the file in
    width = 7, # The width of the plot in inches
    height = 7) # The height of the plot in inches


good.shapes = c(1:25,35:38,43,60,62:64)

op <- par(cex = 0.39)
scatter(dapc_snp, pch = good.shapes, cstar = 0, col=myCol, label=NULL, mstree = FALSE, legend=TRUE, posi.da="topright", cex=1.0,  cex.lab=0.5, cex.main=0.5, cellipse=TRUE, posi.leg="topleft",  xax =1, yax=2)

dev.off()
```

Plot 1 & 2

```
good.shapes = c(1:25,35:38,43,60,62:64)
op <- par(cex = 0.39)
scatter(dapc_snp, pch = good.shapes, cstar = 0, col=myCol, label=NULL, mstree = FALSE, legend=TRUE, posi.da="topright", cex=1.0,  cex.lab=0.5, cex.main=0.5, cellipse=TRUE, posi.leg="topleft",  xax =1, yax=2)
```

```
pdf(file = "americas/output/dapc/50loc/dapc_americas_all_byloc_50pops_PC1_3.pdf",   # The directory you want to save the file in
    width = 7, # The width of the plot in inches
    height = 7) # The height of the plot in inches


good.shapes = c(1:25,35:38,43,60,62:64)

op <- par(cex = 0.39)
scatter(dapc_snp, pch = good.shapes, cstar = 0, col=myCol, label=NULL, mstree = FALSE, legend=TRUE, posi.da="topright", cex=1.0,  cex.lab=0.5, cex.main=0.5, cellipse=TRUE, posi.leg="topleft",  xax =1, yax=3)

dev.off()
```

Plot 1 & 3

```
op <- par(cex = 0.39)
scatter(dapc_snp, pch = good.shapes, cstar = 0, col=myCol, label=NULL, mstree = FALSE, legend=TRUE, posi.da="topright", cex=1.0,  cex.lab=0.5, cex.main=0.5, cellipse=TRUE, posi.leg="topleft",  xax =1, yax=3)
```

```
pdf(file = "americas/output/dapc/50loc/dapc_americas_all_byloc_50pops_PC1_4.pdf",   # The directory you want to save the file in
    width = 7, # The width of the plot in inches
    height = 7) # The height of the plot in inches


good.shapes = c(1:25,35:38,43,60,62:64)

op <- par(cex = 0.39)
scatter(dapc_snp, pch = good.shapes, cstar = 0, col=myCol, label=NULL, mstree = FALSE, legend=TRUE, posi.da="topright", cex=1.0,  cex.lab=0.5, cex.main=0.5, cellipse=TRUE, posi.leg="topleft",  xax =1, yax=4)

dev.off()
```

Plot 1 & 4

```
op <- par(cex = 0.39)
scatter(dapc_snp, pch = good.shapes, cstar = 0, col=myCol, label=NULL, mstree = FALSE, legend=TRUE, posi.da="topright", cex=1.0,  cex.lab=0.5, cex.main=0.5, cellipse=TRUE, posi.leg="topleft",  xax =1, yax=4)
```

```
pdf(file = "americas/output/dapc/50loc/dapc_americas_all_byloc_50pops_PC2_3.pdf",   # The directory you want to save the file in
    width = 7, # The width of the plot in inches
    height = 7) # The height of the plot in inches


good.shapes = c(1:25,35:38,43,60,62:64)

op <- par(cex = 0.39)
scatter(dapc_snp, pch = good.shapes, cstar = 0, col=myCol, label=NULL, mstree = FALSE, legend=TRUE, posi.da="topright", cex=1.0,  cex.lab=0.5, cex.main=0.5, cellipse=TRUE, posi.leg="topleft",  xax =2, yax=3)

dev.off()
```

Plot 2 & 3

```
op <- par(cex = 0.39)
scatter(dapc_snp, pch = good.shapes, cstar = 0, col=myCol, label=NULL, mstree = FALSE, legend=TRUE, posi.da="topright", cex=1.0,  cex.lab=0.5, cex.main=0.5, cellipse=TRUE, posi.leg="topleft",  xax =2, yax=3)
```

#### 2.2.3.1 Color by region

```
myCol2 <- c("blue",     "blue",     "blue",     "#66C2A5",  "blue",     "#66C2A5",  "blue",     "blue",     "blue",     "#a41415",  "#66C2A5",  "#66C2A5",  "blue",     "#66C2A5",  "#66C2A5",  "#66C2A5",  "#66C2A5",  "blue",     "blue",     "#66C2A5",  "#66C2A5",  "#66C2A5",  "#66C2A5",  "blue",     "#66C2A5",  "#66C2A5",  "#66C2A5",  "#66C2A5",  "blue",     "#66C2A5",  "#66C2A5",  "#a41415",  "#66C2A5",  "#66C2A5",  "#66C2A5",  "blue",     "#66C2A5",  "#66C2A5",  "#66C2A5",  "blue",     "blue",     "blue",     "#66C2A5",  "#a41415",  "#66C2A5",  "#66C2A5",  "#66C2A5",  "#66C2A5",  "blue",     "#66C2A5")
```

```
pdf(file = "americas/output/dapc/50loc/dapc_americas_all_byregion_PC1_2.pdf",   # The directory you want to save the file in
    width = 7, # The width of the plot in inches
    height = 7) # The height of the plot in inches


good.shapes = c(1:25,35:38,43,60,62:64)

op <- par(cex = 0.39)
scatter(dapc_snp, pch = good.shapes, cstar = 0, col=myCol2, label=NULL, mstree = FALSE, legend=TRUE, posi.da="topright", cex=1.0,  cex.lab=0.5, cex.main=0.5, cellipse=TRUE, posi.leg="topleft",  xax =1, yax=2)

dev.off()
```

Plot 1 & 2

```
good.shapes = c(1:25,35:38,43,60,62:64)
op <- par(cex = 0.39)
scatter(dapc_snp, pch = good.shapes, cstar = 0, col=myCol2, label=NULL, mstree = FALSE, legend=TRUE, posi.da="topright", cex=1.0,  cex.lab=0.5, cex.main=0.5, cellipse=TRUE, posi.leg="topleft",  xax =1, yax=2)
```

# 3. fastStructure

# Load libraries needed for fastStructure

```
library(tidyverse)          # many helpful things
library(here)               # to load data easily
library(dplyr)              # to manipulate data
library(ggplot2)            # plots
library(extrafont)          # probably won't work on openOnDemand on the clusters
library(hrbrthemes)         # not really needed, personal preference for plots
library(flextable)          # create tables
library(officer)            # export office format
library(here)
```

## 3.1. Run simple prior in fastStructure for file with LD pruning r2=0.01 & MAF 1%

### 3.1.1. Check populations in file

```
awk '{print $1}' americas/output/snps_sets/r2_0.01_b.fam | sort | uniq -c | awk '{print $2}'
```

```
## ARA
## BAF
## BCO
## BEA
## BEH
## BER
## BIT
## BPA
## BPV
## BRM
## CHI
## COL
## CUA
## DAL
## DEN
## FAY
## FIR
## FLO
## GRV
## HAL
## HAR
## HAT
## HOU
## IPO
## LIN
## LOS
## MAC
## MAN
## MAU
## MED
## MON
## NEB
## NEG
## NEH
## NEO
## NEW
## NOV
## NUE
## PAL
## PEO
## PLM
## POR
## REC
## RUS
## SAI
## SMI
## SPR
## STL
## TRA
## TUC
## WAV
```

Count the total number of population

```
awk '{print $1}' americas/output/snps_sets/r2_0.01_b.fam | sort | uniq -c | wc -l
#51
```

```
## 51
```

### 3.1.2 Create a directory for each run.

```
cd americas/output/fastStructure
for i in $(seq -w 1 100)
do
  mkdir run$i
done
```

Check

```
cd americas/output/fastStructure
ls 
#should have 100 folders now
```

### 3.1.3 Create the dsq files

We will run for K=1 to K=30

```
cd /gpfs/gibbs/pi/caccone/mkc54/albo/americas/output/fastStructure
module load dSQ/1.05 

for k in $(seq 1 30); do
    for run in $(seq -w 1 100); do
        echo "cd /gpfs/gibbs/pi/caccone/mkc54/albo/americas/output/fastStructure; source /vast/palmer/apps/software/miniconda/23.3.1/etc/profile.d/conda.sh; module load miniconda/4.12.0; conda activate fastStructure; export PYTHONPATH=/home/mkc54/fastStructure/; python -m structure -K $k --input=/gpfs/gibbs/pi/caccone/mkc54/albo/americas/output/snps_sets/r2_0.01_b --output=/gpfs/gibbs/pi/caccone/mkc54/albo/americas/output/fastStructure/run$run/simple --full --cv=10 --tol=10e-6"
    done
done > dsq_americas_all.txt


head dsq_americas_all.txt #check file

dsq --job-file dsq_americas_all.txt --output /dev/null --mail-type ALL -t 24:00:00 --partition=ycga --cpus-per-task=10 --job-name fS.simple_amall --batch-file simple_amall.sh

#Check the files to see if they have 3,000 jobs:
head simple_amall.sh
```

### 3.1.4 Run the jobs

Run a random job from our array to see if works

```
sbatch --array=$((1 + $RANDOM % 500)) simple_amall.sh

squeue -u mkc54

# check status
dsqa -j 35940482 #insert job number

#if it looks like its running ok, you can cancel it 
scancel 35940482
```

Submit all jobs.

```
sbatch simple_amall.sh
#Submitted batch job # 

#Check it
dsqa -j 35940507 #add job number here
```

### 3.1.5 Run autopsy once its done

```
cd /gpfs/gibbs/pi/caccone/mkc54/albo/americas/output/fastStructure
module load dSQ/1.05

dsqa -j 35940507 -f dsq_americas_all.txt -s TIMEOUT > re-run_jobs1.txt 2> 35940507_report1.txt; head 35940507_report1.txt
head re-run_jobs1.txt  #all completed
```

### 3.1.6. Find the optimal number of K.

you will see it varies from run to run. You can estimate mean, mode
and median values. Then you choose one that makes sense.

```
cd /gpfs/gibbs/pi/caccone/mkc54/albo/americas/output/fastStructure
srun -p ycga --pty -N 1 -n 1 -c 4 bash #start interactive session

module load miniconda
conda activate fastStructure 

#First check files
ls run*/* | wc -l # should be 15000

# chose k for manuscript data
export PYTHONPATH=/home/mkc54/fastStructure
python -m chooseK #see how we can use chooseK
#python /vast/palmer/home.mccleary/mkc54/fastStructure/chooseK.py
   #      --input=<file>

for i in cd /gpfs/gibbs/pi/caccone/mkc54/albo/americas/output/fastStructure/run*
do
    echo $i | sed 's@.*/@@'; python -m chooseK --input=$i/simple | grep 'likelihood\|structure'
done
```

Get outputs in a file

```
cd /gpfs/gibbs/pi/caccone/mkc54/albo/americas/output/fastStructure

cat <(echo 'run likelihood structure') \
<(for i in /gpfs/gibbs/pi/caccone/mkc54/albo/americas/output/fastStructure/run*
do
    echo $i | sed 's@.*/@@' | sed 's/run/run /' ; python -m chooseK --input=$i/simple | grep 'likelihood' | awk '{print $6, $8}'; python -m chooseK --input=$i/simple | grep 'structure' |  awk '{print $6, $10}'
done | xargs -n6 | awk '{print $2, $4, $6}') > simple_americas_all_r0.01_30k.txt

head simple_americas_all_r0.01_30k.txt #check the file
#ok

cd /gpfs/gibbs/pi/caccone/mkc54/albo/americas/output/fastStructure
cp -r simple_americas_all_r0.01_30k.txt /gpfs/gibbs/pi/caccone/mkc54/albo/scripts/RMarkdowns/output/americas/americas_all/fastStructure #copy file
```

### 3.1.7 Make a plot with the Ks.

Import the data in R.

```
# function to import our choosek.py data
import_fastStructure <- function(file) {
  # import as a tibble and set columns as integers
  dat <- read_delim(
    file,
    col_names      = TRUE,
    show_col_types = FALSE,
    col_types      = "iii"
  )

  # get columns we need and make it long for plotting
  dat <- dat |>
    gather(
      structure, likelihood, -run
    )

  # rename the columns by index
  dat <- dat |>
    rename(
      run   = 1,
      model = 2,
      k     = 3
    )
  return(dat)
}
```

Run (simple\_prior\_models file needs to be in the RMarkdown
folder)

```
choose_k_simple <- 
  import_fastStructure(
  here("scripts", "RMarkdowns", "output", "americas", "americas_all", "fastStructure", "simple_americas_all_r0.01_30k.txt"
  )
)
```

Function to plot fastStructure choosek.py results

```
# function to plot our choosek.py data
plot_fastStructure <- function(df) {
  df |>
    ggplot() +
    geom_line(
      aes(
        x              = run,
        y              = k,
        color          = model
      ),
      linewidth = 1
    ) +
    scale_colour_manual(
      "model",
      values = c(
        structure      = "#9d60ff",
        likelihood     = "#ffc08c"
      ),
      labels = c(
        "Maximizes \n Likelihood \n", "Explain \n Structure"
      )
    ) +
    labs(
      x                = "Run",
      y                = "K",
      title            = "fastStructure simple for all American populations (r2<0.01)",
      caption          = "algorithm runs for K ranging from 1 to 30"
    ) +
    theme(
      panel.grid.major = element_line(
        linetype       = "dashed",
        linewidth      = 0.2
      ),
      panel.grid.minor = element_line(
        linetype       = "dashed",
        linewidth      = 0.2
      ),
      legend.text = element_text(
        size = 12
      ),
      legend.title = element_text(
        size           = 14,
        face           = "bold"
      ),
      legend.position = "right"
    )
}
```

Use our function to plot k values

```
plot_fastStructure(
  choose_k_simple
) +
  labs(
    subtitle = "simple fastStructure for all American populations (r2<0.01, MAF>1%)"
  )
```

```
## Warning: <ggplot> %+% x was deprecated in ggplot2 4.0.0.
## ℹ Please use <ggplot> + x instead.
## This warning is displayed once every 8 hours.
## Call `lifecycle::last_lifecycle_warnings()` to see where this warning was
## generated.
```

```
# save plot
ggsave(
  here("scripts", "RMarkdowns",
    "output", "americas", "americas_all", "fastStructure", "fastStructure_simple_k30_americas_all_r0.01.pdf"
  ),
  width  = 8,
  height = 6,
  units  = "in"
)
```

### 3.1.8 Find mean K maximizing likelihood

```
choose_k_likelihood <- subset(choose_k_simple, model != "structure")
mean(choose_k_likelihood$k) #24.05
```

```
## [1] 24.05
```

```
median(choose_k_likelihood$k) #24
```

```
## [1] 24
```

Most common K

```
common <- table(choose_k_likelihood$k)
common #23, range 15-30
```

```
## 
## 15 17 18 19 20 21 22 23 24 25 26 27 28 29 30 
##  1  2  2  6 10  8  6 12  7 11  6  7  6  6 10
```

## 3.2 Plots for fastStructure simple r2<0.01, MAF 1%

We will plot a range of Ks with similar levels of support here. In
the manuscript, K = 23 and K=3 are shown in Figure 3B.

### 3.2.1 K=24

Extract ancestry coefficients for k=24

```
fast24 <- read_delim(
  here("/gpfs/gibbs/pi/caccone/mkc54/albo/americas/output/fastStructure/run095/simple.24.meanQ"),
  delim = "  ", # Specify the delimiter if different from the default (comma)
  col_names = FALSE,
  show_col_types = FALSE
) 
head(fast24)
```

```
## # A tibble: 6 × 24
##         X1      X2    X3    X4    X5      X6    X7    X8    X9   X10   X11   X12
##      <dbl>   <dbl> <dbl> <dbl> <dbl>   <dbl> <dbl> <dbl> <dbl> <dbl> <dbl> <dbl>
## 1 0.000001    1e-6  1e-6  1e-6  1e-6 1   e-6  1e-6  1e-6  1e-6 0.648  1e-6  1e-6
## 2 0.000001    1e-6  1e-6  1e-6  1e-6 1   e-6  1e-6  1e-6  1e-6 0.725  1e-6  1e-6
## 3 0.000001    1e-6  1e-6  1e-6  1e-6 1   e-6  1e-6  1e-6  1e-6 0.565  1e-6  1e-6
## 4 0.000001    1e-6  1e-6  1e-6  1e-6 1   e-6  1e-6  1e-6  1e-6 0.611  1e-6  1e-6
## 5 0.000001    1e-6  1e-6  1e-6  1e-6 1   e-6  1e-6  1e-6  1e-6 0.693  1e-6  1e-6
## 6 0.000001    1e-6  1e-6  1e-6  1e-6 3.44e-2  1e-6  1e-6  1e-6 0.461  1e-6  1e-6
## # ℹ 12 more variables: X13 <dbl>, X14 <dbl>, X15 <dbl>, X16 <dbl>, X17 <dbl>,
## #   X18 <dbl>, X19 <dbl>, X20 <dbl>, X21 <dbl>, X22 <dbl>, X23 <dbl>, X24 <dbl>
```

The fam file

```
fam_file <- here("americas/output/snps_sets/r2_0.01_b.fam")

# Read the .fam file
fam_data <- read.table(fam_file, 
                       header = FALSE,
                       col.names = c("FamilyID", "IndividualID", "PaternalID", "MaternalID", "Sex", "Phenotype"))

# View the first few rows
head(fam_data)
```

```
##   FamilyID IndividualID PaternalID MaternalID Sex Phenotype
## 1      MAU          153          0          0   0        -9
## 2      MAU          154          0          0   0        -9
## 3      MAU          155          0          0   0        -9
## 4      MAU          156          0          0   0        -9
## 5      MAU          158          0          0   0        -9
## 6      MAU           16          0          0   0        -9
```

Create column ID

```
# Change column name
colnames(fam_data)[colnames(fam_data) == "IndividualID"] <- "ind"

# Change column name
colnames(fam_data)[colnames(fam_data) == "FamilyID"] <- "pop"

# Select ID
fam_data <- fam_data |>
  dplyr::select("ind", "pop")

# View the first few rows
head(fam_data)
```

```
##   ind pop
## 1 153 MAU
## 2 154 MAU
## 3 155 MAU
## 4 156 MAU
## 5 158 MAU
## 6  16 MAU
```

Add it to the matrix

```
fast24 <- fam_data |>
  dplyr::select(ind, pop) |>
  bind_cols(fast24)

head(fast24)
```

```
##   ind pop    X1    X2    X3    X4    X5       X6    X7    X8    X9      X10
## 1 153 MAU 1e-06 1e-06 1e-06 1e-06 1e-06 0.000001 1e-06 1e-06 1e-06 0.647580
## 2 154 MAU 1e-06 1e-06 1e-06 1e-06 1e-06 0.000001 1e-06 1e-06 1e-06 0.725290
## 3 155 MAU 1e-06 1e-06 1e-06 1e-06 1e-06 0.000001 1e-06 1e-06 1e-06 0.564697
## 4 156 MAU 1e-06 1e-06 1e-06 1e-06 1e-06 0.000001 1e-06 1e-06 1e-06 0.611017
## 5 158 MAU 1e-06 1e-06 1e-06 1e-06 1e-06 0.000001 1e-06 1e-06 1e-06 0.692674
## 6  16 MAU 1e-06 1e-06 1e-06 1e-06 1e-06 0.034372 1e-06 1e-06 1e-06 0.461132
##     X11   X12      X13   X14   X15   X16   X17   X18      X19   X20   X21   X22
## 1 1e-06 1e-06 0.352393 1e-06 1e-06 1e-06 1e-06 1e-06 0.000001 1e-06 1e-06 1e-06
## 2 1e-06 1e-06 0.249297 1e-06 1e-06 1e-06 1e-06 1e-06 0.025389 1e-06 1e-06 1e-06
## 3 1e-06 1e-06 0.325010 1e-06 1e-06 1e-06 1e-06 1e-06 0.110269 1e-06 1e-06 1e-06
## 4 1e-06 1e-06 0.388958 1e-06 1e-06 1e-06 1e-06 1e-06 0.000001 1e-06 1e-06 1e-06
## 5 1e-06 1e-06 0.307301 1e-06 1e-06 1e-06 1e-06 1e-06 0.000001 1e-06 1e-06 1e-06
## 6 1e-06 1e-06 0.410675 1e-06 1e-06 1e-06 1e-06 1e-06 0.000001 1e-06 1e-06 1e-06
##        X23   X24
## 1 0.000001 1e-06
## 2 0.000001 1e-06
## 3 0.000001 1e-06
## 4 0.000001 1e-06
## 5 0.000001 1e-06
## 6 0.093797 1e-06
```

Rename the columns

```
# Rename the columns starting from the third one
fast24 <- fast24 |>
  rename_with(~paste0("v", seq_along(.x)), .cols = -c(ind, pop))

# View the first few rows
head(fast24)
```

```
##   ind pop    v1    v2    v3    v4    v5       v6    v7    v8    v9      v10
## 1 153 MAU 1e-06 1e-06 1e-06 1e-06 1e-06 0.000001 1e-06 1e-06 1e-06 0.647580
## 2 154 MAU 1e-06 1e-06 1e-06 1e-06 1e-06 0.000001 1e-06 1e-06 1e-06 0.725290
## 3 155 MAU 1e-06 1e-06 1e-06 1e-06 1e-06 0.000001 1e-06 1e-06 1e-06 0.564697
## 4 156 MAU 1e-06 1e-06 1e-06 1e-06 1e-06 0.000001 1e-06 1e-06 1e-06 0.611017
## 5 158 MAU 1e-06 1e-06 1e-06 1e-06 1e-06 0.000001 1e-06 1e-06 1e-06 0.692674
## 6  16 MAU 1e-06 1e-06 1e-06 1e-06 1e-06 0.034372 1e-06 1e-06 1e-06 0.461132
##     v11   v12      v13   v14   v15   v16   v17   v18      v19   v20   v21   v22
## 1 1e-06 1e-06 0.352393 1e-06 1e-06 1e-06 1e-06 1e-06 0.000001 1e-06 1e-06 1e-06
## 2 1e-06 1e-06 0.249297 1e-06 1e-06 1e-06 1e-06 1e-06 0.025389 1e-06 1e-06 1e-06
## 3 1e-06 1e-06 0.325010 1e-06 1e-06 1e-06 1e-06 1e-06 0.110269 1e-06 1e-06 1e-06
## 4 1e-06 1e-06 0.388958 1e-06 1e-06 1e-06 1e-06 1e-06 0.000001 1e-06 1e-06 1e-06
## 5 1e-06 1e-06 0.307301 1e-06 1e-06 1e-06 1e-06 1e-06 0.000001 1e-06 1e-06 1e-06
## 6 1e-06 1e-06 0.410675 1e-06 1e-06 1e-06 1e-06 1e-06 0.000001 1e-06 1e-06 1e-06
##        v23   v24
## 1 0.000001 1e-06
## 2 0.000001 1e-06
## 3 0.000001 1e-06
## 4 0.000001 1e-06
## 5 0.000001 1e-06
## 6 0.093797 1e-06
```

Import Sample Locations

```
sampling_loc <- readRDS(here("scripts", "RMarkdowns", "output", "sampling_loc_all_pops.rds"))
head(sampling_loc)
```

```
##      Pop_City Country Latitude Longitude Continent Abbreviation  Year    Region
## 1      Hainan   China  19.2000  109.6000      Asia          HAI  2014 East Asia
## 2      Yunnan   China  24.4753  101.3431      Asia          YUN  2014 East Asia
## 3       Hunan   China  27.6253  111.8569      Asia          HUN  2001 East Asia
## 4     Okinawa   Japan  26.5013  127.9454      Asia          OKI 2018? East Asia
## 5 Tanegashima   Japan  30.4564  130.9784      Asia          TAN  2008 East Asia
## 6   Kagoshima   Japan  31.5602  130.5581      Asia          KAG  2018 East Asia
##   Subregion order order2 orderold AM_order state FINAL_order
## 1     Japan    55     47       47       NA                 1
## 2     Japan    56     48       48       NA                 2
## 3     Japan    57     49       49       NA                 3
## 4     Japan    54     46       46       NA                 4
## 5     Japan    NA     NA       NA       NA                 5
## 6     Japan    53     45       45       NA                 6
```

```
source(
  here("scripts", "RMarkdowns",
    "my_theme3.R"
  )
)

# Melt the data frame for plotting
Q_melted <- fast24 |>
  pivot_longer(
    cols = -c(ind, pop),
    names_to = "variable",
    values_to = "value"
  )
# Join with sampling_loc to get sampling localities
Q_joined <- Q_melted |>
  left_join(sampling_loc, by = c("pop" = "Abbreviation"))


# Order the combined variable by Region and Country, then by individual
Q_ordered <- Q_joined |>
  arrange(FINAL_order, ind) |>
  mutate(ind = factor(ind, levels = unique(ind)))  # Convert ind to a factor with levels in the desired order

# Add labels: country names for the first individual in each country, NA for all other individuals
Q_ordered <- Q_ordered |>
  group_by(Country) |>
  mutate(label = ifelse(row_number() == 1, as.character(Country), NA))

# Group by individual and variable, calculate mean ancestry proportions
Q_grouped <- Q_ordered |>
  group_by(ind, variable) |>
  summarise(value = mean(value), .groups = "drop")

# Create a data frame for borders
borders <-
  data.frame(Country = unique(Q_ordered$Country))

# Add the order of the last individual of each country to ensure correct placement of borders
borders$order <-
  sapply(borders$Country, function(rc)
    max(which(Q_ordered$Country == rc))) + 0.5  # Shift borders to the right edge of the bars

# Select only the first occurrence of each country in the ordered data
label_df <- Q_ordered |>
  filter(!is.na(label)) |>
  distinct(label, .keep_all = TRUE)

# Create a custom label function
label_func <- function(x) {
  labels <- rep("", length(x))
  labels[x %in% label_df$ind] <- label_df$label
  labels
}

# Calculate the position of lines
border_positions <- Q_ordered |>
  group_by(Country) |>
  summarise(pos = max(as.numeric(ind)) + 0)

# Calculate the position of population labels and bars
pop_labels <- Q_ordered |>
  mutate(Name = paste(pop, Pop_City, sep = " - ")) |>
  group_by(pop) |>
  slice_head(n = 1) |>
  ungroup() |>
  dplyr::select(ind, Pop_City, Country, Name) |>
  mutate(pos = as.numeric(ind))  # calculate position of population labels

pop_labels_bars <- pop_labels |>
  mutate(pos = as.numeric(ind)  - .5)


# Calculate the position of lines
border_positions <- Q_ordered |>
  group_by(Country) |>
  summarise(pos = max(as.numeric(ind)) - 1)


pop_labels_bars <- pop_labels |>
  mutate(pos = as.numeric(ind)  - .5)

# Function to filter and normalize data
normalize_data <- function(df, min_value) {
  df |>
    filter(value > min_value) |>
    group_by(ind) |>
    mutate(value = value / sum(value))
}

# Use the function
Q_grouped_filtered <- normalize_data(Q_grouped, 0.1)
# 

color_palette <- c(
    "#993F00", 
    "#FFE100", 
    "#AC7700", 
    "orchid1", 
    "#808080",      
    "ivory",      
    "#005C31",       
    "blue",
    "#FFA8BB",   
    "#1E90FF",    
    "cadetblue",
    "#C20088",    
    "#8c61cd",
    "purple3",
    "#4C005C", 
    "#FFFC99", 
    "gray",
    "#2BCE48",
    "green",
    "#9DCC00",
    "#003380", 
    "magenta",
    "#FF8005",  
    "cyan",
    "orangered"
        ) 
    

# Generate all potential variable names
all_variables <- paste0("v", 1:24)


# Create a data frame that pairs each potential variable with a color
color_mapping <- data.frame(variable = all_variables,
                        color = color_palette[1:length(all_variables)])

# Merge with Q_grouped_filtered
Q_grouped_filtered <- merge(Q_grouped_filtered, color_mapping, by = "variable")

# Create the plot
ggplot(Q_grouped_filtered, aes(x = as.factor(ind), y = value, fill = color)) +
  geom_bar(stat = 'identity', width = 1) +
  geom_vline(
    data = pop_labels_bars,
    aes(xintercept = pos),
    color = "#2C444A",
    linewidth = .2
  ) +
  geom_text(
    data = pop_labels,
    aes(x = as.numeric(ind), y = 1, label = Name),
    vjust = 1.5,
    hjust = 0,
    size = 2,
    angle = 90,
    inherit.aes = FALSE
  ) +
  my_theme() +
  theme(
    axis.text.x = element_text(
      angle = 90,
      hjust = 1,
      size = 12
    ),
    legend.position = "none",
    plot.margin = unit(c(3, 0.5, 0.5, 0.5), "cm")
  ) +
  xlab("Admixture matrix") +
  ylab("Ancestry proportions") +
  labs(caption = "Each bar represents the ancestry proportions for an individual for k=24.\n fastStructure inference for 18,964 SNPs.") +
  scale_x_discrete(labels = label_func) +
  scale_fill_manual(values = color_palette) +
  expand_limits(y = c(0, 1.5))
```

```
ggsave(
  here("scripts", "RMarkdowns", "output", "americas", "americas_all", "fastStructure", "fastStructure_k=24_americas_all.pdf"),
  width  = 16,
  height = 6,
  units  = "in",
  device = cairo_pdf
)
```

### 3.2.2 K=23

Extract ancestry coefficients for k=23

```
fast23 <- read_delim(
  here("/gpfs/gibbs/pi/caccone/mkc54/albo/americas/output/fastStructure/run003/simple.23.meanQ"),
  delim = "  ", # Specify the delimiter if different from the default (comma)
  col_names = FALSE,
  show_col_types = FALSE
) 
head(fast23)
```

```
## # A tibble: 6 × 23
##         X1    X2      X3      X4    X5    X6    X7    X8    X9   X10   X11   X12
##      <dbl> <dbl>   <dbl>   <dbl> <dbl> <dbl> <dbl> <dbl> <dbl> <dbl> <dbl> <dbl>
## 1 0.000001 0.586    1e-6 1.81e-2 0.189  1e-6  1e-6  1e-6  1e-6  1e-6  1e-6  1e-6
## 2 0.000001 0.795    1e-6 1   e-6 0.205  1e-6  1e-6  1e-6  1e-6  1e-6  1e-6  1e-6
## 3 0.000001 0.532    1e-6 1   e-6 0.303  1e-6  1e-6  1e-6  1e-6  1e-6  1e-6  1e-6
## 4 0.000001 0.633    1e-6 1   e-6 0.306  1e-6  1e-6  1e-6  1e-6  1e-6  1e-6  1e-6
## 5 0.000001 0.656    1e-6 1   e-6 0.213  1e-6  1e-6  1e-6  1e-6  1e-6  1e-6  1e-6
## 6 0.000001 0.451    1e-6 1   e-6 0.364  1e-6  1e-6  1e-6  1e-6  1e-6  1e-6  1e-6
## # ℹ 11 more variables: X13 <dbl>, X14 <dbl>, X15 <dbl>, X16 <dbl>, X17 <dbl>,
## #   X18 <dbl>, X19 <dbl>, X20 <dbl>, X21 <dbl>, X22 <dbl>, X23 <dbl>
```

The fam file

```
fam_file <- here("americas/output/snps_sets/r2_0.01_b.fam")

# Read the .fam file
fam_data <- read.table(fam_file, 
                       header = FALSE,
                       col.names = c("FamilyID", "IndividualID", "PaternalID", "MaternalID", "Sex", "Phenotype"))

# View the first few rows
head(fam_data)
```

```
##   FamilyID IndividualID PaternalID MaternalID Sex Phenotype
## 1      MAU          153          0          0   0        -9
## 2      MAU          154          0          0   0        -9
## 3      MAU          155          0          0   0        -9
## 4      MAU          156          0          0   0        -9
## 5      MAU          158          0          0   0        -9
## 6      MAU           16          0          0   0        -9
```

Create column ID

```
# Change column name
colnames(fam_data)[colnames(fam_data) == "IndividualID"] <- "ind"

# Change column name
colnames(fam_data)[colnames(fam_data) == "FamilyID"] <- "pop"

# Select ID
fam_data <- fam_data |>
  dplyr::select("ind", "pop")

# View the first few rows
head(fam_data)
```

```
##   ind pop
## 1 153 MAU
## 2 154 MAU
## 3 155 MAU
## 4 156 MAU
## 5 158 MAU
## 6  16 MAU
```

Add it to the matrix

```
fast23 <- fam_data |>
  dplyr::select(ind, pop) |>
  bind_cols(fast23)

head(fast23)
```

```
##   ind pop    X1       X2    X3       X4       X5    X6    X7    X8    X9   X10
## 1 153 MAU 1e-06 0.586483 1e-06 0.018067 0.188686 1e-06 1e-06 1e-06 1e-06 1e-06
## 2 154 MAU 1e-06 0.794795 1e-06 0.000001 0.205180 1e-06 1e-06 1e-06 1e-06 1e-06
## 3 155 MAU 1e-06 0.531563 1e-06 0.000001 0.303131 1e-06 1e-06 1e-06 1e-06 1e-06
## 4 156 MAU 1e-06 0.632776 1e-06 0.000001 0.305619 1e-06 1e-06 1e-06 1e-06 1e-06
## 5 158 MAU 1e-06 0.655785 1e-06 0.000001 0.212982 1e-06 1e-06 1e-06 1e-06 1e-06
## 6  16 MAU 1e-06 0.450567 1e-06 0.000001 0.364450 1e-06 1e-06 1e-06 1e-06 1e-06
##     X11   X12   X13   X14      X15   X16      X17   X18   X19   X20      X21
## 1 1e-06 1e-06 1e-06 1e-06 0.000001 1e-06 0.000001 1e-06 1e-06 1e-06 0.206740
## 2 1e-06 1e-06 1e-06 1e-06 0.000001 1e-06 0.000001 1e-06 1e-06 1e-06 0.000001
## 3 1e-06 1e-06 1e-06 1e-06 0.000001 1e-06 0.041185 1e-06 1e-06 1e-06 0.000001
## 4 1e-06 1e-06 1e-06 1e-06 0.000001 1e-06 0.000001 1e-06 1e-06 1e-06 0.061581
## 5 1e-06 1e-06 1e-06 1e-06 0.000001 1e-06 0.000001 1e-06 1e-06 1e-06 0.131210
## 6 1e-06 1e-06 1e-06 1e-06 0.042753 1e-06 0.000001 1e-06 1e-06 1e-06 0.142207
##     X22      X23
## 1 1e-06 0.000001
## 2 1e-06 0.000001
## 3 1e-06 0.124098
## 4 1e-06 0.000001
## 5 1e-06 0.000001
## 6 1e-06 0.000001
```

Rename the columns

```
# Rename the columns starting from the third one
fast23 <- fast23 |>
  rename_with(~paste0("v", seq_along(.x)), .cols = -c(ind, pop))

# View the first few rows
head(fast23)
```

```
##   ind pop    v1       v2    v3       v4       v5    v6    v7    v8    v9   v10
## 1 153 MAU 1e-06 0.586483 1e-06 0.018067 0.188686 1e-06 1e-06 1e-06 1e-06 1e-06
## 2 154 MAU 1e-06 0.794795 1e-06 0.000001 0.205180 1e-06 1e-06 1e-06 1e-06 1e-06
## 3 155 MAU 1e-06 0.531563 1e-06 0.000001 0.303131 1e-06 1e-06 1e-06 1e-06 1e-06
## 4 156 MAU 1e-06 0.632776 1e-06 0.000001 0.305619 1e-06 1e-06 1e-06 1e-06 1e-06
## 5 158 MAU 1e-06 0.655785 1e-06 0.000001 0.212982 1e-06 1e-06 1e-06 1e-06 1e-06
## 6  16 MAU 1e-06 0.450567 1e-06 0.000001 0.364450 1e-06 1e-06 1e-06 1e-06 1e-06
##     v11   v12   v13   v14      v15   v16      v17   v18   v19   v20      v21
## 1 1e-06 1e-06 1e-06 1e-06 0.000001 1e-06 0.000001 1e-06 1e-06 1e-06 0.206740
## 2 1e-06 1e-06 1e-06 1e-06 0.000001 1e-06 0.000001 1e-06 1e-06 1e-06 0.000001
## 3 1e-06 1e-06 1e-06 1e-06 0.000001 1e-06 0.041185 1e-06 1e-06 1e-06 0.000001
## 4 1e-06 1e-06 1e-06 1e-06 0.000001 1e-06 0.000001 1e-06 1e-06 1e-06 0.061581
## 5 1e-06 1e-06 1e-06 1e-06 0.000001 1e-06 0.000001 1e-06 1e-06 1e-06 0.131210
## 6 1e-06 1e-06 1e-06 1e-06 0.042753 1e-06 0.000001 1e-06 1e-06 1e-06 0.142207
##     v22      v23
## 1 1e-06 0.000001
## 2 1e-06 0.000001
## 3 1e-06 0.124098
## 4 1e-06 0.000001
## 5 1e-06 0.000001
## 6 1e-06 0.000001
```

Import Sample Locations

```
sampling_loc <- readRDS(here("scripts", "RMarkdowns", "output", "sampling_loc_all_pops.rds"))
head(sampling_loc)
```

```
##      Pop_City Country Latitude Longitude Continent Abbreviation  Year    Region
## 1      Hainan   China  19.2000  109.6000      Asia          HAI  2014 East Asia
## 2      Yunnan   China  24.4753  101.3431      Asia          YUN  2014 East Asia
## 3       Hunan   China  27.6253  111.8569      Asia          HUN  2001 East Asia
## 4     Okinawa   Japan  26.5013  127.9454      Asia          OKI 2018? East Asia
## 5 Tanegashima   Japan  30.4564  130.9784      Asia          TAN  2008 East Asia
## 6   Kagoshima   Japan  31.5602  130.5581      Asia          KAG  2018 East Asia
##   Subregion order order2 orderold AM_order state FINAL_order
## 1     Japan    55     47       47       NA                 1
## 2     Japan    56     48       48       NA                 2
## 3     Japan    57     49       49       NA                 3
## 4     Japan    54     46       46       NA                 4
## 5     Japan    NA     NA       NA       NA                 5
## 6     Japan    53     45       45       NA                 6
```

```
source(
  here("scripts", "RMarkdowns",
    "my_theme3.R"
  )
)

# Melt the data frame for plotting
Q_melted <- fast23 |>
  pivot_longer(
    cols = -c(ind, pop),
    names_to = "variable",
    values_to = "value"
  )
# Join with sampling_loc to get sampling localities
Q_joined <- Q_melted |>
  left_join(sampling_loc, by = c("pop" = "Abbreviation"))


# Order the combined variable by Region and Country, then by individual
Q_ordered <- Q_joined |>
  arrange(FINAL_order, ind) |>
  mutate(ind = factor(ind, levels = unique(ind)))  # Convert ind to a factor with levels in the desired order

# Add labels: country names for the first individual in each country, NA for all other individuals
Q_ordered <- Q_ordered |>
  group_by(Country) |>
  mutate(label = ifelse(row_number() == 1, as.character(Country), NA))

# Group by individual and variable, calculate mean ancestry proportions
Q_grouped <- Q_ordered |>
  group_by(ind, variable) |>
  summarise(value = mean(value), .groups = "drop")

# Create a data frame for borders
borders <-
  data.frame(Country = unique(Q_ordered$Country))

# Add the order of the last individual of each country to ensure correct placement of borders
borders$order <-
  sapply(borders$Country, function(rc)
    max(which(Q_ordered$Country == rc))) + 0.5  # Shift borders to the right edge of the bars

# Select only the first occurrence of each country in the ordered data
label_df <- Q_ordered |>
  filter(!is.na(label)) |>
  distinct(label, .keep_all = TRUE)

# Create a custom label function
label_func <- function(x) {
  labels <- rep("", length(x))
  labels[x %in% label_df$ind] <- label_df$label
  labels
}

# Calculate the position of lines
border_positions <- Q_ordered |>
  group_by(Country) |>
  summarise(pos = max(as.numeric(ind)) + 0)

# Calculate the position of population labels and bars
pop_labels <- Q_ordered |>
  mutate(Name = paste(pop, Pop_City, sep = " - ")) |>
  group_by(pop) |>
  slice_head(n = 1) |>
  ungroup() |>
  dplyr::select(ind, Pop_City, Country, Name) |>
  mutate(pos = as.numeric(ind))  # calculate position of population labels

pop_labels_bars <- pop_labels |>
  mutate(pos = as.numeric(ind)  - .5)


# Calculate the position of lines
border_positions <- Q_ordered |>
  group_by(Country) |>
  summarise(pos = max(as.numeric(ind)) - 1)


pop_labels_bars <- pop_labels |>
  mutate(pos = as.numeric(ind)  - .5)

# Function to filter and normalize data
normalize_data <- function(df, min_value) {
  df |>
    filter(value > min_value) |>
    group_by(ind) |>
    mutate(value = value / sum(value))
}

# Use the function
Q_grouped_filtered <- normalize_data(Q_grouped, 0.1)
# 

color_palette <- c(
    "magenta",
    "#FFE100", 
    "#CC0033",
    "orangered",
    "#808080", 
    "purple3",
    "#2BCE48",  
    "#9DCC00", 
    "green",
    "gray", 
    "blue",
    "#AC7700", 
    "orchid1", 
    "#4C005C", 
    "#993F00", 
    "#FFFC99",
    "#8c61cd",
    "cyan",
    "ivory",   
    "#1E90FF",  
    "#C20088", 
    "#003380", 
    "#FF8005" 
        ) 
    

# Generate all potential variable names
all_variables <- paste0("v", 1:23)


# Create a data frame that pairs each potential variable with a color
color_mapping <- data.frame(variable = all_variables,
                        color = color_palette[1:length(all_variables)])

# Merge with Q_grouped_filtered
Q_grouped_filtered <- merge(Q_grouped_filtered, color_mapping, by = "variable")

# Create the plot
ggplot(Q_grouped_filtered, aes(x = as.factor(ind), y = value, fill = color)) +
  geom_bar(stat = 'identity', width = 1) +
  geom_vline(
    data = pop_labels_bars,
    aes(xintercept = pos),
    color = "#2C444A",
    linewidth = .2
  ) +
  geom_text(
    data = pop_labels,
    aes(x = as.numeric(ind), y = 1, label = Name),
    vjust = 1.5,
    hjust = 0,
    size = 2,
    angle = 90,
    inherit.aes = FALSE
  ) +
  my_theme() +
  theme(
    axis.text.x = element_text(
      angle = 90,
      hjust = 1,
      size = 12
    ),
    legend.position = "none",
    plot.margin = unit(c(3, 0.5, 0.5, 0.5), "cm")
  ) +
  xlab("Admixture matrix") +
  ylab("Ancestry proportions") +
  labs(caption = "Each bar represents the ancestry proportions for an individual for k=23.\n fastStructure inference for 18,964 SNPs.") +
  scale_x_discrete(labels = label_func) +
  scale_fill_manual(values = color_palette) +
  expand_limits(y = c(0, 1.5))
```

```
ggsave(
  here("scripts", "RMarkdowns", "output", "americas", "americas_all", "fastStructure", "fastStructure_k=23_americas_all.pdf"),
  width  = 16,
  height = 6,
  units  = "in",
  device = cairo_pdf
)
```

### 3.2.3 K=20

Extract ancestry coefficients for k=20

```
fast20 <- read_delim(
  here("/gpfs/gibbs/pi/caccone/mkc54/albo/americas/output/fastStructure/run081/simple.20.meanQ"),
  delim = "  ", # Specify the delimiter if different from the default (comma)
  col_names = FALSE,
  show_col_types = FALSE
) 
head(fast20)
```

```
## # A tibble: 6 × 20
##         X1    X2    X3    X4    X5    X6    X7      X8    X9   X10     X11   X12
##      <dbl> <dbl> <dbl> <dbl> <dbl> <dbl> <dbl>   <dbl> <dbl> <dbl>   <dbl> <dbl>
## 1 0.000001  1e-6  1e-6 0.598  1e-6  1e-6  1e-6 1   e-6  1e-6  1e-6 2.29e-1  1e-6
## 2 0.000001  1e-6  1e-6 0.756  1e-6  1e-6  1e-6 1   e-6  1e-6  1e-6 5.49e-2  1e-6
## 3 0.000001  1e-6  1e-6 0.595  1e-6  1e-6  1e-6 1   e-6  1e-6  1e-6 2.12e-1  1e-6
## 4 0.000001  1e-6  1e-6 0.642  1e-6  1e-6  1e-6 1   e-6  1e-6  1e-6 1   e-6  1e-6
## 5 0.000001  1e-6  1e-6 0.718  1e-6  1e-6  1e-6 1   e-6  1e-6  1e-6 1   e-6  1e-6
## 6 0.000001  1e-6  1e-6 0.456  1e-6  1e-6  1e-6 3.11e-2  1e-6  1e-6 1.62e-1  1e-6
## # ℹ 8 more variables: X13 <dbl>, X14 <dbl>, X15 <dbl>, X16 <dbl>, X17 <dbl>,
## #   X18 <dbl>, X19 <dbl>, X20 <dbl>
```

The fam file

```
fam_file <- here("americas/output/snps_sets/r2_0.01_b.fam")

# Read the .fam file
fam_data <- read.table(fam_file, 
                       header = FALSE,
                       col.names = c("FamilyID", "IndividualID", "PaternalID", "MaternalID", "Sex", "Phenotype"))

# View the first few rows
head(fam_data)
```

```
##   FamilyID IndividualID PaternalID MaternalID Sex Phenotype
## 1      MAU          153          0          0   0        -9
## 2      MAU          154          0          0   0        -9
## 3      MAU          155          0          0   0        -9
## 4      MAU          156          0          0   0        -9
## 5      MAU          158          0          0   0        -9
## 6      MAU           16          0          0   0        -9
```

Create column ID

```
# Change column name
colnames(fam_data)[colnames(fam_data) == "IndividualID"] <- "ind"

# Change column name
colnames(fam_data)[colnames(fam_data) == "FamilyID"] <- "pop"

# Select ID
fam_data <- fam_data |>
  dplyr::select("ind", "pop")

# View the first few rows
head(fam_data)
```

```
##   ind pop
## 1 153 MAU
## 2 154 MAU
## 3 155 MAU
## 4 156 MAU
## 5 158 MAU
## 6  16 MAU
```

Add it to the matrix

```
fast20 <- fam_data |>
  dplyr::select(ind, pop) |>
  bind_cols(fast20)

head(fast20)
```

```
##   ind pop    X1    X2    X3       X4    X5    X6    X7       X8    X9   X10
## 1 153 MAU 1e-06 1e-06 1e-06 0.598117 1e-06 1e-06 1e-06 0.000001 1e-06 1e-06
## 2 154 MAU 1e-06 1e-06 1e-06 0.756094 1e-06 1e-06 1e-06 0.000001 1e-06 1e-06
## 3 155 MAU 1e-06 1e-06 1e-06 0.595101 1e-06 1e-06 1e-06 0.000001 1e-06 1e-06
## 4 156 MAU 1e-06 1e-06 1e-06 0.641828 1e-06 1e-06 1e-06 0.000001 1e-06 1e-06
## 5 158 MAU 1e-06 1e-06 1e-06 0.718031 1e-06 1e-06 1e-06 0.000001 1e-06 1e-06
## 6  16 MAU 1e-06 1e-06 1e-06 0.455762 1e-06 1e-06 1e-06 0.031127 1e-06 1e-06
##        X11   X12      X13   X14   X15   X16   X17   X18   X19   X20
## 1 0.228814 1e-06 0.173045 1e-06 1e-06 1e-06 1e-06 1e-06 1e-06 1e-06
## 2 0.054918 1e-06 0.188965 1e-06 1e-06 1e-06 1e-06 1e-06 1e-06 1e-06
## 3 0.212020 1e-06 0.192856 1e-06 1e-06 1e-06 1e-06 1e-06 1e-06 1e-06
## 4 0.000001 1e-06 0.358148 1e-06 1e-06 1e-06 1e-06 1e-06 1e-06 1e-06
## 5 0.000001 1e-06 0.281945 1e-06 1e-06 1e-06 1e-06 1e-06 1e-06 1e-06
## 6 0.162294 1e-06 0.350795 1e-06 1e-06 1e-06 1e-06 1e-06 1e-06 1e-06
```

Rename the columns

```
# Rename the columns starting from the third one
fast20 <- fast20 |>
  rename_with(~paste0("v", seq_along(.x)), .cols = -c(ind, pop))

# View the first few rows
head(fast20)
```

```
##   ind pop    v1    v2    v3       v4    v5    v6    v7       v8    v9   v10
## 1 153 MAU 1e-06 1e-06 1e-06 0.598117 1e-06 1e-06 1e-06 0.000001 1e-06 1e-06
## 2 154 MAU 1e-06 1e-06 1e-06 0.756094 1e-06 1e-06 1e-06 0.000001 1e-06 1e-06
## 3 155 MAU 1e-06 1e-06 1e-06 0.595101 1e-06 1e-06 1e-06 0.000001 1e-06 1e-06
## 4 156 MAU 1e-06 1e-06 1e-06 0.641828 1e-06 1e-06 1e-06 0.000001 1e-06 1e-06
## 5 158 MAU 1e-06 1e-06 1e-06 0.718031 1e-06 1e-06 1e-06 0.000001 1e-06 1e-06
## 6  16 MAU 1e-06 1e-06 1e-06 0.455762 1e-06 1e-06 1e-06 0.031127 1e-06 1e-06
##        v11   v12      v13   v14   v15   v16   v17   v18   v19   v20
## 1 0.228814 1e-06 0.173045 1e-06 1e-06 1e-06 1e-06 1e-06 1e-06 1e-06
## 2 0.054918 1e-06 0.188965 1e-06 1e-06 1e-06 1e-06 1e-06 1e-06 1e-06
## 3 0.212020 1e-06 0.192856 1e-06 1e-06 1e-06 1e-06 1e-06 1e-06 1e-06
## 4 0.000001 1e-06 0.358148 1e-06 1e-06 1e-06 1e-06 1e-06 1e-06 1e-06
## 5 0.000001 1e-06 0.281945 1e-06 1e-06 1e-06 1e-06 1e-06 1e-06 1e-06
## 6 0.162294 1e-06 0.350795 1e-06 1e-06 1e-06 1e-06 1e-06 1e-06 1e-06
```

Import Sample Locations

```
sampling_loc <- readRDS(here("scripts", "RMarkdowns", "output", "sampling_loc_all_pops.rds"))
head(sampling_loc)
```

```
##      Pop_City Country Latitude Longitude Continent Abbreviation  Year    Region
## 1      Hainan   China  19.2000  109.6000      Asia          HAI  2014 East Asia
## 2      Yunnan   China  24.4753  101.3431      Asia          YUN  2014 East Asia
## 3       Hunan   China  27.6253  111.8569      Asia          HUN  2001 East Asia
## 4     Okinawa   Japan  26.5013  127.9454      Asia          OKI 2018? East Asia
## 5 Tanegashima   Japan  30.4564  130.9784      Asia          TAN  2008 East Asia
## 6   Kagoshima   Japan  31.5602  130.5581      Asia          KAG  2018 East Asia
##   Subregion order order2 orderold AM_order state FINAL_order
## 1     Japan    55     47       47       NA                 1
## 2     Japan    56     48       48       NA                 2
## 3     Japan    57     49       49       NA                 3
## 4     Japan    54     46       46       NA                 4
## 5     Japan    NA     NA       NA       NA                 5
## 6     Japan    53     45       45       NA                 6
```

```
source(
  here("scripts", "RMarkdowns",
    "my_theme3.R"
  )
)

# Melt the data frame for plotting
Q_melted <- fast20 |>
  pivot_longer(
    cols = -c(ind, pop),
    names_to = "variable",
    values_to = "value"
  )
# Join with sampling_loc to get sampling localities
Q_joined <- Q_melted |>
  left_join(sampling_loc, by = c("pop" = "Abbreviation"))


# Order the combined variable by Region and Country, then by individual
Q_ordered <- Q_joined |>
  arrange(FINAL_order, ind) |>
  mutate(ind = factor(ind, levels = unique(ind)))  # Convert ind to a factor with levels in the desired order

# Add labels: country names for the first individual in each country, NA for all other individuals
Q_ordered <- Q_ordered |>
  group_by(Country) |>
  mutate(label = ifelse(row_number() == 1, as.character(Country), NA))

# Group by individual and variable, calculate mean ancestry proportions
Q_grouped <- Q_ordered |>
  group_by(ind, variable) |>
  summarise(value = mean(value), .groups = "drop")

# Create a data frame for borders
borders <-
  data.frame(Country = unique(Q_ordered$Country))

# Add the order of the last individual of each country to ensure correct placement of borders
borders$order <-
  sapply(borders$Country, function(rc)
    max(which(Q_ordered$Country == rc))) + 0.5  # Shift borders to the right edge of the bars

# Select only the first occurrence of each country in the ordered data
label_df <- Q_ordered |>
  filter(!is.na(label)) |>
  distinct(label, .keep_all = TRUE)

# Create a custom label function
label_func <- function(x) {
  labels <- rep("", length(x))
  labels[x %in% label_df$ind] <- label_df$label
  labels
}

# Calculate the position of lines
border_positions <- Q_ordered |>
  group_by(Country) |>
  summarise(pos = max(as.numeric(ind)) + 0)

# Calculate the position of population labels and bars
pop_labels <- Q_ordered |>
  mutate(Name = paste(pop, Pop_City, sep = " - ")) |>
  group_by(pop) |>
  slice_head(n = 1) |>
  ungroup() |>
  dplyr::select(ind, Pop_City, Country, Name) |>
  mutate(pos = as.numeric(ind))  # calculate position of population labels

pop_labels_bars <- pop_labels |>
  mutate(pos = as.numeric(ind)  - .5)


# Calculate the position of lines
border_positions <- Q_ordered |>
  group_by(Country) |>
  summarise(pos = max(as.numeric(ind)) - 1)


pop_labels_bars <- pop_labels |>
  mutate(pos = as.numeric(ind)  - .5)

# Function to filter and normalize data
normalize_data <- function(df, min_value) {
  df |>
    filter(value > min_value) |>
    group_by(ind) |>
    mutate(value = value / sum(value))
}

# Use the function
Q_grouped_filtered <- normalize_data(Q_grouped, 0.1)
# 

color_palette <- c(
    "#003380",  
    "cadetblue", 
    "#AC7700",      
    "#FFA8BB",    
    "#8c61cd",     
    "#FF8005",  
    "green",
    "#1E90FF",     
    "#808080",  
    "cyan",
    "orchid1",
    "#4C005C", 
    "magenta",
    "#005C31",      
    "#FFE100",     
    "#FFFC99",
    "#2BCE48", 
    "purple3",
    "#9DCC00",     
    "#C20088"    
    )


# Generate all potential variable names
all_variables <- paste0("v", 1:20)


# Create a data frame that pairs each potential variable with a color
color_mapping <- data.frame(variable = all_variables,
                       color = color_palette[1:length(all_variables)])

# Merge with Q_grouped_filtered
Q_grouped_filtered <- merge(Q_grouped_filtered, color_mapping, by = "variable")

# Create the plot
ggplot(Q_grouped_filtered, aes(x = as.factor(ind), y = value, fill = color)) +
  geom_bar(stat = 'identity', width = 1) +
  geom_vline(
    data = pop_labels_bars,
    aes(xintercept = pos),
    color = "#2C444A",
    linewidth = .2
  ) +
  geom_text(
    data = pop_labels,
    aes(x = as.numeric(ind), y = 1, label = Name),
    vjust = 1.5,
    hjust = 0,
    size = 2,
    angle = 90,
    inherit.aes = FALSE
  ) +
  my_theme() +
  theme(
    axis.text.x = element_text(
      angle = 90,
      hjust = 1,
      size = 12
    ),
    legend.position = "none",
    plot.margin = unit(c(3, 0.5, 0.5, 0.5), "cm")
  ) +
  xlab("Admixture matrix") +
  ylab("Ancestry proportions") +
  labs(caption = "Each bar represents the ancestry proportions for an individual for k=20.\n fastStructure inference for 18,964 SNPs.") +
  scale_x_discrete(labels = label_func) +
  scale_fill_manual(values = color_palette) +
  expand_limits(y = c(0, 1.5))
```

```
ggsave(
  here("scripts", "RMarkdowns", "output", "americas", "americas_all", "fastStructure", "fastStructure_k=20_americas_all.pdf"),
  width  = 16,
  height = 6,
  units  = "in",
  device = cairo_pdf
)
```

### 3.2.4 K=7

Extract ancestry coefficients for k=7

```
fast7 <- read_delim(
  here("/gpfs/gibbs/pi/caccone/mkc54/albo/americas/output/fastStructure/run081/simple.7.meanQ"),
  delim = "  ", # Specify the delimiter if different from the default (comma)
  col_names = FALSE,
  show_col_types = FALSE
) 
head(fast7)
```

```
## # A tibble: 6 × 7
##      X1       X2       X3       X4    X5       X6       X7
##   <dbl>    <dbl>    <dbl>    <dbl> <dbl>    <dbl>    <dbl>
## 1 0.410 0.000004 0.000004 0.000004 0.367 0.000004 0.223   
## 2 0.300 0.000004 0.000004 0.000004 0.456 0.000004 0.244   
## 3 0.433 0.151    0.000004 0.0412   0.302 0.0733   0.000004
## 4 0.134 0.0114   0.0687   0.000004 0.601 0.000004 0.185   
## 5 0.373 0.106    0.131    0.000004 0.390 0.000004 0.000004
## 6 0.306 0.144    0.000004 0.000004 0.523 0.0274   0.000004
```

The fam file

```
fam_file <- here("americas/output/snps_sets/r2_0.01_b.fam")

# Read the .fam file
fam_data <- read.table(fam_file, 
                       header = FALSE,
                       col.names = c("FamilyID", "IndividualID", "PaternalID", "MaternalID", "Sex", "Phenotype"))

# View the first few rows
head(fam_data)
```

```
##   FamilyID IndividualID PaternalID MaternalID Sex Phenotype
## 1      MAU          153          0          0   0        -9
## 2      MAU          154          0          0   0        -9
## 3      MAU          155          0          0   0        -9
## 4      MAU          156          0          0   0        -9
## 5      MAU          158          0          0   0        -9
## 6      MAU           16          0          0   0        -9
```

Create column ID

```
# Change column name
colnames(fam_data)[colnames(fam_data) == "IndividualID"] <- "ind"

# Change column name
colnames(fam_data)[colnames(fam_data) == "FamilyID"] <- "pop"

# Select ID
fam_data <- fam_data |>
  dplyr::select("ind", "pop")

# View the first few rows
head(fam_data)
```

```
##   ind pop
## 1 153 MAU
## 2 154 MAU
## 3 155 MAU
## 4 156 MAU
## 5 158 MAU
## 6  16 MAU
```

Add it to the matrix

```
fast7 <- fam_data |>
  dplyr::select(ind, pop) |>
  bind_cols(fast7)

head(fast7)
```

```
##   ind pop       X1       X2       X3       X4       X5       X6       X7
## 1 153 MAU 0.409607 0.000004 0.000004 0.000004 0.367237 0.000004 0.223139
## 2 154 MAU 0.300098 0.000004 0.000004 0.000004 0.455591 0.000004 0.244295
## 3 155 MAU 0.433007 0.150578 0.000004 0.041160 0.301902 0.073345 0.000004
## 4 156 MAU 0.133939 0.011350 0.068717 0.000004 0.601306 0.000004 0.184681
## 5 158 MAU 0.372553 0.106369 0.130999 0.000004 0.390067 0.000004 0.000004
## 6  16 MAU 0.305620 0.143939 0.000004 0.000004 0.522988 0.027440 0.000004
```

Rename the columns

```
# Rename the columns starting from the third one
fast7 <- fast7 |>
  rename_with(~paste0("v", seq_along(.x)), .cols = -c(ind, pop))

# View the first few rows
head(fast7)
```

```
##   ind pop       v1       v2       v3       v4       v5       v6       v7
## 1 153 MAU 0.409607 0.000004 0.000004 0.000004 0.367237 0.000004 0.223139
## 2 154 MAU 0.300098 0.000004 0.000004 0.000004 0.455591 0.000004 0.244295
## 3 155 MAU 0.433007 0.150578 0.000004 0.041160 0.301902 0.073345 0.000004
## 4 156 MAU 0.133939 0.011350 0.068717 0.000004 0.601306 0.000004 0.184681
## 5 158 MAU 0.372553 0.106369 0.130999 0.000004 0.390067 0.000004 0.000004
## 6  16 MAU 0.305620 0.143939 0.000004 0.000004 0.522988 0.027440 0.000004
```

Import Sample Locations

```
sampling_loc <- readRDS(here("scripts", "RMarkdowns", "output", "sampling_loc_all_pops.rds"))
head(sampling_loc)
```

```
##      Pop_City Country Latitude Longitude Continent Abbreviation  Year    Region
## 1      Hainan   China  19.2000  109.6000      Asia          HAI  2014 East Asia
## 2      Yunnan   China  24.4753  101.3431      Asia          YUN  2014 East Asia
## 3       Hunan   China  27.6253  111.8569      Asia          HUN  2001 East Asia
## 4     Okinawa   Japan  26.5013  127.9454      Asia          OKI 2018? East Asia
## 5 Tanegashima   Japan  30.4564  130.9784      Asia          TAN  2008 East Asia
## 6   Kagoshima   Japan  31.5602  130.5581      Asia          KAG  2018 East Asia
##   Subregion order order2 orderold AM_order state FINAL_order
## 1     Japan    55     47       47       NA                 1
## 2     Japan    56     48       48       NA                 2
## 3     Japan    57     49       49       NA                 3
## 4     Japan    54     46       46       NA                 4
## 5     Japan    NA     NA       NA       NA                 5
## 6     Japan    53     45       45       NA                 6
```

```
source(
  here("scripts", "RMarkdowns",
    "my_theme3.R"
  )
)

# Melt the data frame for plotting
Q_melted <- fast7 |>
  pivot_longer(
    cols = -c(ind, pop),
    names_to = "variable",
    values_to = "value"
  )
# Join with sampling_loc to get sampling localities
Q_joined <- Q_melted |>
  left_join(sampling_loc, by = c("pop" = "Abbreviation"))


# Order the combined variable by Region and Country, then by individual
Q_ordered <- Q_joined |>
  arrange(FINAL_order, ind) |>
  mutate(ind = factor(ind, levels = unique(ind)))  # Convert ind to a factor with levels in the desired order

# Add labels: country names for the first individual in each country, NA for all other individuals
Q_ordered <- Q_ordered |>
  group_by(Country) |>
  mutate(label = ifelse(row_number() == 1, as.character(Country), NA))

# Group by individual and variable, calculate mean ancestry proportions
Q_grouped <- Q_ordered |>
  group_by(ind, variable) |>
  summarise(value = mean(value), .groups = "drop")

# Create a data frame for borders
borders <-
  data.frame(Country = unique(Q_ordered$Country))

# Add the order of the last individual of each country to ensure correct placement of borders
borders$order <-
  sapply(borders$Country, function(rc)
    max(which(Q_ordered$Country == rc))) + 0.5  # Shift borders to the right edge of the bars

# Select only the first occurrence of each country in the ordered data
label_df <- Q_ordered |>
  filter(!is.na(label)) |>
  distinct(label, .keep_all = TRUE)

# Create a custom label function
label_func <- function(x) {
  labels <- rep("", length(x))
  labels[x %in% label_df$ind] <- label_df$label
  labels
}

# Calculate the position of lines
border_positions <- Q_ordered |>
  group_by(Country) |>
  summarise(pos = max(as.numeric(ind)) + 0)

# Calculate the position of population labels and bars
pop_labels <- Q_ordered |>
  mutate(Name = paste(pop, Pop_City, sep = " - ")) |>
  group_by(pop) |>
  slice_head(n = 1) |>
  ungroup() |>
  dplyr::select(ind, Pop_City, Country, Name) |>
  mutate(pos = as.numeric(ind))  # calculate position of population labels

pop_labels_bars <- pop_labels |>
  mutate(pos = as.numeric(ind)  - .5)


# Calculate the position of lines
border_positions <- Q_ordered |>
  group_by(Country) |>
  summarise(pos = max(as.numeric(ind)) - 1)


pop_labels_bars <- pop_labels |>
  mutate(pos = as.numeric(ind)  - .5)

# Function to filter and normalize data
normalize_data <- function(df, min_value) {
  df |>
    filter(value > min_value) |>
    group_by(ind) |>
    mutate(value = value / sum(value))
}

# Use the function
Q_grouped_filtered <- normalize_data(Q_grouped, 0.1)
# 

color_palette <-
  c("green",
    "blue",
    "#FFE100",       
    "magenta",    
    "#FF8005",   
    "cyan",   
    "purple3"
    )


# Generate all potential variable names
all_variables <- paste0("v", 1:7)


# Create a data frame that pairs each potential variable with a color
color_mapping <- data.frame(variable = all_variables,
                       color = color_palette[1:length(all_variables)])

# Merge with Q_grouped_filtered
Q_grouped_filtered <- merge(Q_grouped_filtered, color_mapping, by = "variable")

# Create the plot
ggplot(Q_grouped_filtered, aes(x = as.factor(ind), y = value, fill = color)) +
  geom_bar(stat = 'identity', width = 1) +
  geom_vline(
    data = pop_labels_bars,
    aes(xintercept = pos),
    color = "#2C444A",
    linewidth = .2
  ) +
  geom_text(
    data = pop_labels,
    aes(x = as.numeric(ind), y = 1, label = Name),
    vjust = 1.5,
    hjust = 0,
    size = 2,
    angle = 90,
    inherit.aes = FALSE
  ) +
  my_theme() +
  theme(
    axis.text.x = element_text(
      angle = 90,
      hjust = 1,
      size = 12
    ),
    legend.position = "none",
    plot.margin = unit(c(3, 0.5, 0.5, 0.5), "cm")
  ) +
  xlab("Admixture matrix") +
  ylab("Ancestry proportions") +
  labs(caption = "Each bar represents the ancestry proportions for an individual for k=7.\n fastStructure inference for 18,964 SNPs.") +
  scale_x_discrete(labels = label_func) +
  scale_fill_manual(values = color_palette) +
  expand_limits(y = c(0, 1.5))
```

```
ggsave(
  here("scripts", "RMarkdowns", "output", "americas", "americas_all", "fastStructure", "fastStructure_k=7_americas_all.pdf"),
  width  = 16,
  height = 6,
  units  = "in",
  device = cairo_pdf
)
```

### 3.2.5 K=3

Extract ancestry coefficients for k=3

```
fast3 <- read_delim(
  here("/gpfs/gibbs/pi/caccone/mkc54/albo/americas/output/fastStructure/run081/simple.3.meanQ"),
  delim = "  ", # Specify the delimiter if different from the default (comma)
  col_names = FALSE,
  show_col_types = FALSE
) 
head(fast3)
```

```
## # A tibble: 6 × 3
##      X1      X2    X3
##   <dbl>   <dbl> <dbl>
## 1 0.745 0.00181 0.253
## 2 0.727 0.0117  0.262
## 3 0.660 0.0817  0.258
## 4 0.739 0.00543 0.256
## 5 0.734 0.0227  0.244
## 6 0.761 0.00482 0.235
```

The fam file

```
fam_file <- here("americas/output/snps_sets/r2_0.01_b.fam")

# Read the .fam file
fam_data <- read.table(fam_file, 
                       header = FALSE,
                       col.names = c("FamilyID", "IndividualID", "PaternalID", "MaternalID", "Sex", "Phenotype"))

# View the first few rows
head(fam_data)
```

```
##   FamilyID IndividualID PaternalID MaternalID Sex Phenotype
## 1      MAU          153          0          0   0        -9
## 2      MAU          154          0          0   0        -9
## 3      MAU          155          0          0   0        -9
## 4      MAU          156          0          0   0        -9
## 5      MAU          158          0          0   0        -9
## 6      MAU           16          0          0   0        -9
```

Create column ID

```
# Change column name
colnames(fam_data)[colnames(fam_data) == "IndividualID"] <- "ind"

# Change column name
colnames(fam_data)[colnames(fam_data) == "FamilyID"] <- "pop"

# Select ID
fam_data <- fam_data |>
  dplyr::select("ind", "pop")

# View the first few rows
head(fam_data)
```

```
##   ind pop
## 1 153 MAU
## 2 154 MAU
## 3 155 MAU
## 4 156 MAU
## 5 158 MAU
## 6  16 MAU
```

Add it to the matrix

```
fast3 <- fam_data |>
  dplyr::select(ind, pop) |>
  bind_cols(fast3)

head(fast3)
```

```
##   ind pop       X1       X2       X3
## 1 153 MAU 0.745416 0.001811 0.252773
## 2 154 MAU 0.726780 0.011695 0.261525
## 3 155 MAU 0.660403 0.081666 0.257931
## 4 156 MAU 0.738563 0.005434 0.256003
## 5 158 MAU 0.733775 0.022695 0.243531
## 6  16 MAU 0.760582 0.004816 0.234602
```

Rename the columns

```
# Rename the columns starting from the third one
fast3 <- fast3 |>
  rename_with(~paste0("v", seq_along(.x)), .cols = -c(ind, pop))

# View the first few rows
head(fast3)
```

```
##   ind pop       v1       v2       v3
## 1 153 MAU 0.745416 0.001811 0.252773
## 2 154 MAU 0.726780 0.011695 0.261525
## 3 155 MAU 0.660403 0.081666 0.257931
## 4 156 MAU 0.738563 0.005434 0.256003
## 5 158 MAU 0.733775 0.022695 0.243531
## 6  16 MAU 0.760582 0.004816 0.234602
```

Import Sample Locations

```
sampling_loc <- readRDS(here("scripts", "RMarkdowns", "output", "sampling_loc_all_pops.rds"))
head(sampling_loc)
```

```
##      Pop_City Country Latitude Longitude Continent Abbreviation  Year    Region
## 1      Hainan   China  19.2000  109.6000      Asia          HAI  2014 East Asia
## 2      Yunnan   China  24.4753  101.3431      Asia          YUN  2014 East Asia
## 3       Hunan   China  27.6253  111.8569      Asia          HUN  2001 East Asia
## 4     Okinawa   Japan  26.5013  127.9454      Asia          OKI 2018? East Asia
## 5 Tanegashima   Japan  30.4564  130.9784      Asia          TAN  2008 East Asia
## 6   Kagoshima   Japan  31.5602  130.5581      Asia          KAG  2018 East Asia
##   Subregion order order2 orderold AM_order state FINAL_order
## 1     Japan    55     47       47       NA                 1
## 2     Japan    56     48       48       NA                 2
## 3     Japan    57     49       49       NA                 3
## 4     Japan    54     46       46       NA                 4
## 5     Japan    NA     NA       NA       NA                 5
## 6     Japan    53     45       45       NA                 6
```

```
source(
  here("scripts", "RMarkdowns",
    "my_theme3.R"
  )
)

# Melt the data frame for plotting
Q_melted <- fast3 |>
  pivot_longer(
    cols = -c(ind, pop),
    names_to = "variable",
    values_to = "value"
  )
# Join with sampling_loc to get sampling localities
Q_joined <- Q_melted |>
  left_join(sampling_loc, by = c("pop" = "Abbreviation"))


# Order the combined variable by Region and Country, then by individual
Q_ordered <- Q_joined |>
  arrange(FINAL_order, ind) |>
  mutate(ind = factor(ind, levels = unique(ind)))  # Convert ind to a factor with levels in the desired order

# Add labels: country names for the first individual in each country, NA for all other individuals
Q_ordered <- Q_ordered |>
  group_by(Country) |>
  mutate(label = ifelse(row_number() == 1, as.character(Country), NA))

# Group by individual and variable, calculate mean ancestry proportions
Q_grouped <- Q_ordered |>
  group_by(ind, variable) |>
  summarise(value = mean(value), .groups = "drop")

# Create a data frame for borders
borders <-
  data.frame(Country = unique(Q_ordered$Country))

# Add the order of the last individual of each country to ensure correct placement of borders
borders$order <-
  sapply(borders$Country, function(rc)
    max(which(Q_ordered$Country == rc))) + 0.5  # Shift borders to the right edge of the bars

# Select only the first occurrence of each country in the ordered data
label_df <- Q_ordered |>
  filter(!is.na(label)) |>
  distinct(label, .keep_all = TRUE)

# Create a custom label function
label_func <- function(x) {
  labels <- rep("", length(x))
  labels[x %in% label_df$ind] <- label_df$label
  labels
}

# Calculate the position of lines
border_positions <- Q_ordered |>
  group_by(Country) |>
  summarise(pos = max(as.numeric(ind)) + 0)

# Calculate the position of population labels and bars
pop_labels <- Q_ordered |>
  mutate(Name = paste(pop, Pop_City, sep = " - ")) |>
  group_by(pop) |>
  slice_head(n = 1) |>
  ungroup() |>
  dplyr::select(ind, Pop_City, Country, Name) |>
  mutate(pos = as.numeric(ind))  # calculate position of population labels

pop_labels_bars <- pop_labels |>
  mutate(pos = as.numeric(ind)  - .5)


# Calculate the position of lines
border_positions <- Q_ordered |>
  group_by(Country) |>
  summarise(pos = max(as.numeric(ind)) - 1)


pop_labels_bars <- pop_labels |>
  mutate(pos = as.numeric(ind)  - .5)

# Function to filter and normalize data
normalize_data <- function(df, min_value) {
  df |>
    filter(value > min_value) |>
    group_by(ind) |>
    mutate(value = value / sum(value))
}

# Use the function
Q_grouped_filtered <- normalize_data(Q_grouped, 0.1)
# 

color_palette <-
  c("#FFE100",  
    "#FF8005",
    "blue"
    )


# Generate all potential variable names
all_variables <- paste0("v", 1:3)


# Create a data frame that pairs each potential variable with a color
color_mapping <- data.frame(variable = all_variables,
                       color = color_palette[1:length(all_variables)])

# Merge with Q_grouped_filtered
Q_grouped_filtered <- merge(Q_grouped_filtered, color_mapping, by = "variable")

# Create the plot
ggplot(Q_grouped_filtered, aes(x = as.factor(ind), y = value, fill = color)) +
  geom_bar(stat = 'identity', width = 1) +
  geom_vline(
    data = pop_labels_bars,
    aes(xintercept = pos),
    color = "#2C444A",
    linewidth = .2
  ) +
  geom_text(
    data = pop_labels,
    aes(x = as.numeric(ind), y = 1, label = Name),
    vjust = 1.5,
    hjust = 0,
    size = 2,
    angle = 90,
    inherit.aes = FALSE
  ) +
  my_theme() +
  theme(
    axis.text.x = element_text(
      angle = 90,
      hjust = 1,
      size = 12
    ),
    legend.position = "none",
    plot.margin = unit(c(3, 0.5, 0.5, 0.5), "cm")
  ) +
  xlab("Admixture matrix") +
  ylab("Ancestry proportions") +
  labs(caption = "Each bar represents the ancestry proportions for an individual for k=3.\n fastStructure inference for 18,964 SNPs.") +
  scale_x_discrete(labels = label_func) +
  scale_fill_manual(values = color_palette) +
  expand_limits(y = c(0, 1.5))
```

```
ggsave(
  here("scripts", "RMarkdowns", "output", "americas", "americas_all", "fastStructure", "fastStructure_k=3_americas_all_yellow.pdf"),
  width  = 16,
  height = 6,
  units  = "in",
  device = cairo_pdf
)
```

# 4. Fst

Load Libraries

```
library(StAMPP)
library(ggplot2)
library(tidyverse)
library(adegenet)
library(here)
library(flextable)
library(officer)
library(reshape2)
library(dplyr) 
library(tidyr)
library(geosphere)
library(flextable)
library(officer)
library(dartR) #do not load until Mantel test
#library(MASS) #do not load until Mantel test
library(Cairo)
```

# 4.1. Fst with Stampp

Estimate Fst for American populations with at least 4 individuals
using r<0.01 SNP set with 1% MAF pruning

Create list of populations

```
cd /gpfs/gibbs/pi/caccone/mkc54/albo/americas

awk '{print $1}' output/snps_sets/r2_0.01_b.fam | sort | uniq -c | awk '{print $2, $1}' | awk '$2 >= 4 {print}' | awk '{print $1}' > output/fst/pops_4fst.txt;
head  output/fst/pops_4fst.txt;
wc -l output/fst/pops_4fst.txt
```

```
## ARA
## BAF
## BCO
## BEA
## BEH
## BER
## BIT
## BPA
## BPV
## BRM
## 51 output/fst/pops_4fst.txt
```

We have 51 populations with 4 or more mosquitoes.

### 4.1.1 Convert to raw format and subset the bed file

First load plink

```
module load PLINK/1.9b_6.21-x86_64
```

```
cd /gpfs/gibbs/pi/caccone/mkc54/albo/americas
plink \
--allow-extra-chr \
--keep-allele-order \
--bfile output/snps_sets/r2_0.01_b \
--keep-fam output/fst/pops_4fst.txt \
--recodeA \
--out output/fst/r2_0.01_b \
--silent;
grep 'samples\|variants\|remaining' output/fst/r2_0.01_b.log
```

18964 variants loaded from .bim file. –keep-fam: 545 people
remaining. 18964 variants and 545 people pass filters and QC.

Look at https://rdrr.io/cran/StAMPP/man/stamppFst.html for
details of Fst estimations

```
LD2 <-
  read.PLINK(
    here(
      "americas/output/fst/r2_0.01_b.raw"
    ),
    quiet = FALSE,
    chunkSize = 1000,
    parallel = require("parallel"),
    n.cores = 4
  )

summary(LD2)
```

Save it

```
saveRDS(
  LD2, here(
    "americas/output/fst/LD2.rds"
  )
)
```

To load it

```
LD2 <- readRDS(
  here(
    "americas/output/fst/LD2.rds"
  )
)
```

### 4.1.2 Convert the genlight object to Stampp format, and estimate pairwide Fst values

The command below would also work, but you can simplify it and put
only the numbers: genome\_equal\_2 <- stamppFst(neutral, nboots=100,
percent=95 + nclusters==10)

This chunk will take a couple minutes to run. You may need to do it
in the terminal instead of RStudio. Install StAMPP for direct use in the
R module in the cluster

```
#start an interactive session and load R packages needed
#salloc -p scavenge -c 10 -t 2:00:00
salloc -p scavenge --cpus-per-task=5 -t 2:00:00 --mem-per-cpu=10000 #15640712   
module load R/4.2.0-foss-2020b
R
#install.packages("StAMPP", repos="http://cran.r-project.org")

library(StAMPP)
library(adegenet)
library(here)

LD2 <- readRDS(
  here(
    "/gpfs/gibbs/pi/caccone/mkc54/albo/americas/output/fst/LD2.rds"
  )
)

summary(LD2)

#The command below would also work, but you can simplify it and put only the numbers: genome_equal_2 <- stamppFst(neutral, nboots=100, percent=95 + nclusters==10)

#This chunk will take a couple minutes to run.

LD2_2 <- stamppConvert(LD2, type="genlight")

LD2_3 <- stamppFst(LD2_2, 1, 95, 1)

saveRDS(
  LD2_3, here(
    "/gpfs/gibbs/pi/caccone/mkc54/albo/americas/output/fst/LD2_3.rds"
  )
)
```

*The stamppFst function calculates pairwise Fst values along with
confidence intervals and p-values between populations according to the
method proposed by Wright(1949) and updated by Weir and Cockerham
(1984)*

To load it

```
LD2_3 <- readRDS(
  here(
    "/gpfs/gibbs/pi/caccone/mkc54/albo/americas/output/fst/LD2_3.rds"
  )
)
```

Now lets look at the object

```
summary(LD2_3)
```

```
##       MAU               TUC               REC               BRM         
##  Min.   :0.03501   Min.   :0.00705   Min.   :0.02637   Min.   :0.07753  
##  1st Qu.:0.08189   1st Qu.:0.07069   1st Qu.:0.08540   1st Qu.:0.09184  
##  Median :0.11341   Median :0.13238   Median :0.09929   Median :0.10815  
##  Mean   :0.10720   Mean   :0.11700   Mean   :0.10328   Mean   :0.12843  
##  3rd Qu.:0.13050   3rd Qu.:0.15631   3rd Qu.:0.12118   3rd Qu.:0.16445  
##  Max.   :0.19778   Max.   :0.19505   Max.   :0.21495   Max.   :0.25522  
##  NA's   :1         NA's   :2         NA's   :3         NA's   :4        
##       CUA                MED              FAY               DAL         
##  Min.   :0.000499   Min.   :0.1002   Min.   :0.01539   Min.   :0.01451  
##  1st Qu.:0.127032   1st Qu.:0.1216   1st Qu.:0.03249   1st Qu.:0.03414  
##  Median :0.136825   Median :0.1320   Median :0.04638   Median :0.05891  
##  Mean   :0.137352   Mean   :0.1359   Mean   :0.07592   Mean   :0.08244  
##  3rd Qu.:0.151250   3rd Qu.:0.1456   3rd Qu.:0.12453   3rd Qu.:0.13372  
##  Max.   :0.218593   Max.   :0.2129   Max.   :0.22579   Max.   :0.23537  
##  NA's   :5          NA's   :6        NA's   :7         NA's   :8        
##       NEO               RUS               CHI               NUE         
##  Min.   :0.03097   Min.   :0.06073   Min.   :0.08292   Min.   :0.01137  
##  1st Qu.:0.04811   1st Qu.:0.07649   1st Qu.:0.11445   1st Qu.:0.05051  
##  Median :0.06615   Median :0.09348   Median :0.13228   Median :0.06994  
##  Mean   :0.09188   Mean   :0.11847   Mean   :0.13177   Mean   :0.08466  
##  3rd Qu.:0.13922   3rd Qu.:0.16391   3rd Qu.:0.14361   3rd Qu.:0.11894  
##  Max.   :0.23948   Max.   :0.26079   Max.   :0.22780   Max.   :0.21124  
##  NA's   :9         NA's   :10        NA's   :11        NA's   :12       
##       TRA               HAL               STL               SMI         
##  Min.   :0.01157   Min.   :0.01660   Min.   :0.03602   Min.   :0.01591  
##  1st Qu.:0.03518   1st Qu.:0.03492   1st Qu.:0.05100   1st Qu.:0.05153  
##  Median :0.05299   Median :0.05006   Median :0.07427   Median :0.07748  
##  Mean   :0.07746   Mean   :0.08819   Mean   :0.10878   Mean   :0.09906  
##  3rd Qu.:0.11855   3rd Qu.:0.14014   3rd Qu.:0.16179   3rd Qu.:0.14185  
##  Max.   :0.21205   Max.   :0.23919   Max.   :0.26741   Max.   :0.24104  
##  NA's   :13        NA's   :14        NA's   :15        NA's   :16       
##       WAV               HAT               DEN               MON         
##  Min.   :0.02363   Min.   :0.02477   Min.   :0.02636   Min.   :0.01652  
##  1st Qu.:0.04449   1st Qu.:0.04464   1st Qu.:0.03427   1st Qu.:0.05082  
##  Median :0.07604   Median :0.08007   Median :0.07784   Median :0.08106  
##  Mean   :0.10690   Mean   :0.09978   Mean   :0.09714   Mean   :0.09211  
##  3rd Qu.:0.15898   3rd Qu.:0.14073   3rd Qu.:0.14373   3rd Qu.:0.12612  
##  Max.   :0.27248   Max.   :0.23848   Max.   :0.23915   Max.   :0.21189  
##  NA's   :17        NA's   :18        NA's   :19        NA's   :20       
##       BPV               BCO               BPA               BIT         
##  Min.   :0.04792   Min.   :0.02084   Min.   :0.06344   Min.   :0.04215  
##  1st Qu.:0.06845   1st Qu.:0.07834   1st Qu.:0.09243   1st Qu.:0.08121  
##  Median :0.12043   Median :0.12822   Median :0.10774   Median :0.13153  
##  Mean   :0.11567   Mean   :0.11730   Mean   :0.11009   Mean   :0.12017  
##  3rd Qu.:0.15327   3rd Qu.:0.15883   3rd Qu.:0.12270   3rd Qu.:0.15029  
##  Max.   :0.20678   Max.   :0.21037   Max.   :0.21619   Max.   :0.20893  
##  NA's   :21        NA's   :22        NA's   :23        NA's   :24       
##       FLO                BAF               PLM               NEH         
##  Min.   :0.009996   Min.   :0.00496   Min.   :0.01676   Min.   :0.02017  
##  1st Qu.:0.078937   1st Qu.:0.06388   1st Qu.:0.10535   1st Qu.:0.03504  
##  Median :0.140943   Median :0.13384   Median :0.14544   Median :0.06070  
##  Mean   :0.123673   Mean   :0.11688   Mean   :0.13034   Mean   :0.09251  
##  3rd Qu.:0.161640   3rd Qu.:0.15059   3rd Qu.:0.16140   3rd Qu.:0.14318  
##  Max.   :0.220637   Max.   :0.20892   Max.   :0.21471   Max.   :0.25331  
##  NA's   :25         NA's   :26        NA's   :27        NA's   :28       
##       LIN               HAR               FIR               NEB         
##  Min.   :0.05292   Min.   :0.03629   Min.   :0.06415   Min.   :0.02833  
##  1st Qu.:0.06240   1st Qu.:0.04956   1st Qu.:0.07539   1st Qu.:0.04588  
##  Median :0.08385   Median :0.07931   Median :0.10822   Median :0.07445  
##  Mean   :0.11761   Mean   :0.10751   Mean   :0.13937   Mean   :0.11086  
##  3rd Qu.:0.16521   3rd Qu.:0.15266   3rd Qu.:0.19103   3rd Qu.:0.16827  
##  Max.   :0.26368   Max.   :0.25029   Max.   :0.28852   Max.   :0.25481  
##  NA's   :29        NA's   :30        NA's   :31        NA's   :32       
##       BEH               ARA               IPO               PAL         
##  Min.   :0.03536   Min.   :0.05047   Min.   :0.03625   Min.   :0.03008  
##  1st Qu.:0.09285   1st Qu.:0.10787   1st Qu.:0.18427   1st Qu.:0.06070  
##  Median :0.15476   Median :0.12704   Median :0.20271   Median :0.07414  
##  Mean   :0.14022   Mean   :0.12167   Mean   :0.18453   Mean   :0.09449  
##  3rd Qu.:0.18451   3rd Qu.:0.14742   3rd Qu.:0.22891   3rd Qu.:0.12234  
##  Max.   :0.21721   Max.   :0.19793   Max.   :0.26530   Max.   :0.21919  
##  NA's   :33        NA's   :34        NA's   :35        NA's   :36       
##       MAN               BEA               PEO               HOU         
##  Min.   :0.02657   Min.   :0.01320   Min.   :0.02712   Min.   :0.01680  
##  1st Qu.:0.03027   1st Qu.:0.04250   1st Qu.:0.04310   1st Qu.:0.04186  
##  Median :0.04830   Median :0.05995   Median :0.07156   Median :0.05968  
##  Mean   :0.08738   Mean   :0.08060   Mean   :0.09761   Mean   :0.08673  
##  3rd Qu.:0.13320   3rd Qu.:0.11828   3rd Qu.:0.14585   3rd Qu.:0.12529  
##  Max.   :0.23630   Max.   :0.20292   Max.   :0.23403   Max.   :0.19797  
##  NA's   :37        NA's   :38        NA's   :39        NA's   :40       
##       LOS               NEG              NOV               NEW         
##  Min.   :0.09938   Min.   :0.1987   Min.   :0.03769   Min.   :0.05048  
##  1st Qu.:0.11937   1st Qu.:0.2110   1st Qu.:0.13545   1st Qu.:0.06032  
##  Median :0.13464   Median :0.2442   Median :0.19514   Median :0.07405  
##  Mean   :0.14303   Mean   :0.2356   Mean   :0.16726   Mean   :0.09768  
##  3rd Qu.:0.15981   3rd Qu.:0.2529   3rd Qu.:0.21659   3rd Qu.:0.12891  
##  Max.   :0.22181   Max.   :0.2894   Max.   :0.24970   Max.   :0.18080  
##  NA's   :41        NA's   :42       NA's   :43        NA's   :44       
##       BER               MAC               SPR              COL         
##  Min.   :0.04632   Min.   :0.05547   Min.   :0.0675   Min.   :0.09844  
##  1st Qu.:0.05668   1st Qu.:0.06481   1st Qu.:0.1090   1st Qu.:0.12639  
##  Median :0.08021   Median :0.07706   Median :0.1486   Median :0.15434  
##  Mean   :0.09824   Mean   :0.09456   Mean   :0.1422   Mean   :0.14517  
##  3rd Qu.:0.13674   3rd Qu.:0.12627   3rd Qu.:0.1818   3rd Qu.:0.16854  
##  Max.   :0.17825   Max.   :0.14919   Max.   :0.2041   Max.   :0.18273  
##  NA's   :45        NA's   :46        NA's   :47       NA's   :48       
##       SAI              POR               GRV     
##  Min.   :0.1292   Min.   :0.02975   Min.   : NA  
##  1st Qu.:0.1328   1st Qu.:0.02975   1st Qu.: NA  
##  Median :0.1363   Median :0.02975   Median : NA  
##  Mean   :0.1363   Mean   :0.02975   Mean   :NaN  
##  3rd Qu.:0.1399   3rd Qu.:0.02975   3rd Qu.: NA  
##  Max.   :0.1435   Max.   :0.02975   Max.   : NA  
##  NA's   :49       NA's   :50        NA's   :51
```

If you want you can save the fst values as csv.

```
# Convert to data frame
LD3_df <- data.frame(LD2_3)

# Save it
#write.csv(LD3_df, file = here("/gpfs/gibbs/pi/caccone/mkc54/albo/americas/output/fst/LD3_df.csv"))
```

### 4.1.3 Get matrix of Fst values

Check the Fst values

```
head(LD3_df)
```

```
##            MAU        TUC       REC       BRM          CUA MED FAY DAL NEO RUS
## MAU         NA         NA        NA        NA           NA  NA  NA  NA  NA  NA
## TUC 0.05874449         NA        NA        NA           NA  NA  NA  NA  NA  NA
## REC 0.07802916 0.07301274        NA        NA           NA  NA  NA  NA  NA  NA
## BRM 0.15008541 0.16898113 0.1350325        NA           NA  NA  NA  NA  NA  NA
## CUA 0.11305612 0.12229726 0.1324426 0.1637346           NA  NA  NA  NA  NA  NA
## MED 0.11376016 0.11971467 0.1250431 0.1613893 0.0004994715  NA  NA  NA  NA  NA
##     CHI NUE TRA HAL STL SMI WAV HAT DEN MON BPV BCO BPA BIT FLO BAF PLM NEH LIN
## MAU  NA  NA  NA  NA  NA  NA  NA  NA  NA  NA  NA  NA  NA  NA  NA  NA  NA  NA  NA
## TUC  NA  NA  NA  NA  NA  NA  NA  NA  NA  NA  NA  NA  NA  NA  NA  NA  NA  NA  NA
## REC  NA  NA  NA  NA  NA  NA  NA  NA  NA  NA  NA  NA  NA  NA  NA  NA  NA  NA  NA
## BRM  NA  NA  NA  NA  NA  NA  NA  NA  NA  NA  NA  NA  NA  NA  NA  NA  NA  NA  NA
## CUA  NA  NA  NA  NA  NA  NA  NA  NA  NA  NA  NA  NA  NA  NA  NA  NA  NA  NA  NA
## MED  NA  NA  NA  NA  NA  NA  NA  NA  NA  NA  NA  NA  NA  NA  NA  NA  NA  NA  NA
##     HAR FIR NEB BEH ARA IPO PAL MAN BEA PEO HOU LOS NEG NOV NEW BER MAC SPR COL
## MAU  NA  NA  NA  NA  NA  NA  NA  NA  NA  NA  NA  NA  NA  NA  NA  NA  NA  NA  NA
## TUC  NA  NA  NA  NA  NA  NA  NA  NA  NA  NA  NA  NA  NA  NA  NA  NA  NA  NA  NA
## REC  NA  NA  NA  NA  NA  NA  NA  NA  NA  NA  NA  NA  NA  NA  NA  NA  NA  NA  NA
## BRM  NA  NA  NA  NA  NA  NA  NA  NA  NA  NA  NA  NA  NA  NA  NA  NA  NA  NA  NA
## CUA  NA  NA  NA  NA  NA  NA  NA  NA  NA  NA  NA  NA  NA  NA  NA  NA  NA  NA  NA
## MED  NA  NA  NA  NA  NA  NA  NA  NA  NA  NA  NA  NA  NA  NA  NA  NA  NA  NA  NA
##     SAI POR GRV
## MAU  NA  NA  NA
## TUC  NA  NA  NA
## REC  NA  NA  NA
## BRM  NA  NA  NA
## CUA  NA  NA  NA
## MED  NA  NA  NA
```

Convert the data into a matrix.

```
aa <- as.matrix(LD3_df)
aa[upper.tri(aa)] <- t(aa)[upper.tri(t(aa))]
head(aa)
```

```
##            MAU        TUC        REC       BRM          CUA          MED
## MAU         NA 0.05874449 0.07802916 0.1500854 0.1130561233 0.1137601597
## TUC 0.05874449         NA 0.07301274 0.1689811 0.1222972604 0.1197146693
## REC 0.07802916 0.07301274         NA 0.1350325 0.1324426031 0.1250430640
## BRM 0.15008541 0.16898113 0.13503253        NA 0.1637346367 0.1613893394
## CUA 0.11305612 0.12229726 0.13244260 0.1637346           NA 0.0004994715
## MED 0.11376016 0.11971467 0.12504306 0.1613893 0.0004994715           NA
##            FAY        DAL        NEO       RUS       CHI        NUE        TRA
## MAU 0.10733517 0.11971393 0.11970018 0.1474338 0.1262039 0.10576034 0.10233905
## TUC 0.13238304 0.13540042 0.14382049 0.1694927 0.1411109 0.11871951 0.12238856
## REC 0.08581505 0.09284560 0.09564274 0.1251985 0.1267741 0.08679131 0.08037900
## BRM 0.08430082 0.08796953 0.09624635 0.1211850 0.1457402 0.08946586 0.08406597
## CUA 0.12600275 0.13684348 0.13869086 0.1677066 0.1276452 0.12092079 0.11811675
## MED 0.12163192 0.13201249 0.13459565 0.1620395 0.1209040 0.11459275 0.11407689
##            HAL       STL        SMI        WAV        HAT        DEN        MON
## MAU 0.11892614 0.1380262 0.12522128 0.13480481 0.11880244 0.11858553 0.10567083
## TUC 0.14467293 0.1682651 0.13595733 0.16324765 0.14449859 0.14696846 0.11755143
## REC 0.10024124 0.1203724 0.09522588 0.11199784 0.10218194 0.09779444 0.08359820
## BRM 0.08570106 0.1081485 0.10112489 0.09851417 0.09738253 0.09340006 0.08912405
## CUA 0.13840178 0.1630882 0.14274421 0.16157512 0.13876354 0.13852212 0.11931419
## MED 0.13280746 0.1595186 0.13905485 0.15480000 0.13549758 0.13558076 0.11267767
##            BPV        BCO        BPA        BIT        FLO         BAF
## MAU 0.03501251 0.06804723 0.08252511 0.04765931 0.06278763 0.052323488
## TUC 0.05660221 0.02368465 0.07068520 0.04333690 0.01980183 0.008855849
## REC 0.07208999 0.07872711 0.02637060 0.08554839 0.07613509 0.064383271
## BRM 0.16923303 0.17208156 0.14023970 0.16700487 0.17485369 0.165166063
## CUA 0.12829337 0.13326339 0.13736059 0.12945228 0.12939364 0.122525239
## MED 0.12599056 0.13128823 0.12929497 0.12736800 0.12834469 0.118472487
##            PLM        NEH       LIN       HAR       FIR        NEB        BEH
## MAU 0.06022929 0.12528167 0.1439245 0.1319357 0.1651315 0.13349600 0.06632462
## TUC 0.02107516 0.15157282 0.1684020 0.1573122 0.1843519 0.16118000 0.04881058
## REC 0.07911384 0.10520632 0.1236036 0.1123398 0.1435787 0.11358105 0.09770804
## BRM 0.17445638 0.08768557 0.1153785 0.1010850 0.1251620 0.09713417 0.19879330
## CUA 0.13085988 0.14394001 0.1633251 0.1539128 0.1867283 0.15102048 0.13303616
## MED 0.12730221 0.13808391 0.1583561 0.1486195 0.1827623 0.14592755 0.13118544
##            ARA        IPO        PAL        MAN        BEA        PEO
## MAU 0.06108938 0.09276131 0.11228001 0.12077987 0.09847090 0.11688628
## TUC 0.00705027 0.06586217 0.12996154 0.14587969 0.11841379 0.14157646
## REC 0.06824833 0.13921745 0.09532991 0.09588013 0.07574176 0.09408324
## BRM 0.16166053 0.23703017 0.10285204 0.09027734 0.07753377 0.08176778
## CUA 0.12142792 0.16945926 0.12748374 0.14080134 0.11088953 0.13516165
## MED 0.12018266 0.16723716 0.12107905 0.13511908 0.10504798 0.13005105
##            HOU       LOS       NEG        NOV       NEW        BER        MAC
## MAU 0.09155783 0.1342299 0.1977836 0.08167209 0.1345148 0.12512716 0.10201650
## TUC 0.11560369 0.1481454 0.1950481 0.06646059 0.1569134 0.15739000 0.12554776
## REC 0.07520335 0.1314667 0.2149498 0.13088323 0.1127245 0.10543635 0.08496253
## BRM 0.08136088 0.1384609 0.2552230 0.22448997 0.1039152 0.09341042 0.08853085
## CUA 0.10560457 0.1368059 0.2185932 0.14752285 0.1544329 0.15132650 0.11702443
## MED 0.10020120 0.1319973 0.2129320 0.14007178 0.1506226 0.14496958 0.11341919
##           SPR       COL        SAI        POR        GRV
## MAU 0.1499313 0.1327507 0.10787000 0.05713728 0.06628508
## TUC 0.1790321 0.1563057 0.12146607 0.04901419 0.06904212
## REC 0.1327278 0.1113696 0.09834113 0.11139192 0.10383445
## BRM 0.1239719 0.1037227 0.12153382 0.18792683 0.16685848
## CUA 0.1752530 0.1513846 0.11682959 0.12936694 0.12688132
## MED 0.1701583 0.1456257 0.11307860 0.12692984 0.12438445
```

Import sample locations

```
sampling_loc <- readRDS(here("scripts", "RMarkdowns", "output", "sampling_loc_americas_all.rds"))


# Arrange by region 
sampling_loc <- sampling_loc |>
  dplyr::arrange(
    order
  )

# Check it
head(sampling_loc)
```

```
##       Pop_City Country Latitude Longitude Continent Abbreviation Year
## 1   Berlin, NJ     USA 39.79081  -74.9291  Americas          BER 2018
## 2 Columbus, OH     USA 39.97170  -82.9071  Americas          COL 2015
## 3   Palm Beach     USA 26.70560  -80.0364  Americas          PAL 2018
## 4  Houston, TX     USA 29.75491  -95.3505  Americas          HOU 2018
## 5  Los Angeles     USA 34.05220 -118.2437  Americas          LOS 2018
## 6   Manaus, AM  Brazil -3.09161  -60.0325  Americas          MAU 2017
##          Region      Subregion order order2 orderold AM_order      state
## 1 North America Northeast, USA     1     NA       75        6    NJ, USA
## 2 North America   Midwest, USA     2     NA       76       18    OH, USA
## 3 North America Southeast, USA     3     NA       77       13    FL, USA
## 4 North America Southwest, USA     4     NA       78       23    TX, USA
## 5 North America      West, USA     5     NA       79       30    CA, USA
## 6 South America  South America     6     NA       80       38 AM, Brazil
```

Order

```
pops_4fst_order <- here(
  "/gpfs/gibbs/pi/caccone/mkc54/albo/americas/output/fst/pops_4fst_order.txt"
)

# Read the file
pops_4fst_order <- read.table(pops_4fst_order, 
                       header = FALSE,
                       col.names = c("FamilyID"))


order_pops <- as.vector(pops_4fst_order$FamilyID)
order_pops
```

```
##  [1] "BER" "COL" "PAL" "HOU" "LOS" "MAU" "REC" "GRV" "BRM" "JAM" "SAI" "BEA"
## [13] "CHI" "DAL" "FAY" "MAC" "MAN" "NEO" "NEW" "NUE" "PEO" "RUS" "SPR" "POR"
## [25] "NOV" "TUC" "MED" "NEG" "NEB" "NEH" "FIR" "LIN" "HAR" "WAV" "HAL" "HAT"
## [37] "STL" "TRA" "MON" "SMI" "DEN" "BPV" "BIT" "FLO" "ARA" "PLM" "BCO" "BAF"
## [49] "IPO" "BEH" "BPA" "CUA"
```

```
LD3_df_ordered <- read_csv(
  "/gpfs/gibbs/pi/caccone/mkc54/albo/americas/output/fst/LD3_df_ordered.csv"
)
```

```
## New names:
## Rows: 51 Columns: 52
## ── Column specification
## ──────────────────────────────────────────────────────── Delimiter: "," chr
## (1): ...1 dbl (51): NEB, NEH, FIR, LIN, NEW, BER, HAR, MAN, WAV, FAY, BEA, MAC,
## PAL, R...
## ℹ Use `spec()` to retrieve the full column specification for this data. ℹ
## Specify the column types or set `show_col_types = FALSE` to quiet this message.
## • `` -> `...1`
```

Create vector with order of populations

```
# Extract the populations that appear in LD2_df
populations_in_LD3 <- colnames(LD3_df_ordered)

# Reorder the populations based on order_pops
poporder <- populations_in_LD3[populations_in_LD3 %in% order_pops]

#LD2_df[match(poporder, LD2_df$Abbreviation),] #this also doesn't reorder it

# Print the reordered populations
print(poporder)
```

```
##  [1] "NEB" "NEH" "FIR" "LIN" "NEW" "BER" "HAR" "MAN" "WAV" "FAY" "BEA" "MAC"
## [13] "PAL" "RUS" "HAL" "HAT" "NEO" "COL" "PEO" "SPR" "STL" "NUE" "HOU" "TRA"
## [25] "MON" "DAL" "SMI" "DEN" "CHI" "LOS" "BRM" "NEG" "SAI" "CUA" "MED" "BPV"
## [37] "MAU" "BIT" "TUC" "FLO" "REC" "ARA" "PLM" "BCO" "BAF" "IPO" "NOV" "BEH"
## [49] "BPA" "GRV" "POR"
```

Lets check if the matrix is symmetric.

```
isSymmetric(aa)
```

```
## [1] TRUE
```

Order the matrix using poporder. We will also add NA on the upper
left side of the matrix.

```
aa <- aa[poporder, poporder]
aa[lower.tri(aa)] <- NA
```

## 4.1.4 Plot the matrix with ggplot

Now we have to convert the matrix to a data frame to plot it with
ggplot.

```
pairfst.long <- melt(aa)
summary(pairfst.long)
```

```
##       Var1           Var2          value       
##  NEB    :  51   NEB    :  51   Min.   :0.0005  
##  NEH    :  51   NEH    :  51   1st Qu.:0.0601  
##  FIR    :  51   FIR    :  51   Median :0.1136  
##  LIN    :  51   LIN    :  51   Mean   :0.1104  
##  NEW    :  51   NEW    :  51   3rd Qu.:0.1490  
##  BER    :  51   BER    :  51   Max.   :0.2895  
##  (Other):2295   (Other):2295   NA's   :1326
```

Now lets plot the data with ggplot. You can click in the little
square on the top left of the plot to open it on a new window. It will
have the right proportions.

```
pairfst.f <- ggplot(pairfst.long, aes(Var1, Var2)) +
  geom_tile(aes(fill = value), colour = "white") +
  scale_fill_gradient(
    low = "white",
    high = "#71b6ff",
    name = "Fst",
    na.value = "white",
    limits = c(0, 0.5)
  ) +
  scale_x_discrete(position = "top") +
  theme_bw() +
  geom_text(aes(label = ifelse(
    is.na(value), "", formatC(value, digits = 2, format = "f")
  )), size = 2.25) +
  theme(
    axis.text.x = element_text(angle = 90, hjust = 1),
    axis.title = element_blank(),
    panel.grid.major = element_blank(),
    panel.grid.minor = element_blank(),
    panel.border = element_blank(),
    panel.background = element_blank(),
    axis.text.y = element_text(hjust = 0)
  )
pairfst.f
```

Save it

```
ggsave(
  filename = here("scripts", "RMarkdowns", "output", "americas", "americas_all", "fst", "fst_matrix_americas_all_LD3_ordered.pdf"),
  pairfst.f,
  width = 14,
  height = 16,
  units = "in"
)
```

### 4.1.5 Fst by country for all Americas dataset

```
# Step 1: Map abbreviation to country
abbreviation_to_country <- sampling_loc %>% dplyr::select(Abbreviation, Country)
abbreviation_to_order <- sampling_loc %>% dplyr::select(Abbreviation, order)
# Step 2: Calculate mean Fst for each pair of countries

# Convert the matrix to a data frame and add row names as a new column
fst_df <- as.data.frame(as.matrix(LD3_df))
fst_df$Abbreviation1 <- rownames(fst_df)

# Gather columns into rows
fst_long <- fst_df %>% gather(key = "Abbreviation2", value = "Fst", -Abbreviation1)

# Merge with country mapping
fst_long <- merge(fst_long, abbreviation_to_country, by.x = "Abbreviation1", by.y = "Abbreviation")
fst_long <- merge(fst_long, abbreviation_to_country, by.x = "Abbreviation2", by.y = "Abbreviation", suffixes = c("_1", "_2"))

# Calculate mean Fst for each pair of countries
fst_summary <- fst_long %>% 
  group_by(Country_1, Country_2) %>% 
  summarize(Mean_Fst = mean(Fst, na.rm = TRUE), .groups = 'drop') %>% 
  filter(Country_1 != Country_2)


#save the fst values as csv
write.csv(fst_summary, file = here("/gpfs/gibbs/pi/caccone/mkc54/albo/americas/output/fst/fst_summary.csv"))

#remove spaces from Trinidad & Tobago and sort
fst_summary_ordered <- here(
  "/gpfs/gibbs/pi/caccone/mkc54/albo/americas/output/fst/fst_summary.txt")
#fst_summary_ordered <- data.frame(fst_summary_ordered)
fst_summary_ordered <- read.table(fst_summary_ordered,
                                  header=TRUE)


# Convert summary back to a matrix form, avoiding the use of tibbles for row names
fst_matrix_summary <- as.data.frame(spread(fst_summary_ordered, key = Country_2, value = Mean_Fst))
rownames(fst_matrix_summary) <- fst_matrix_summary$Country_1
fst_matrix_summary <- fst_matrix_summary[, -1]
fst_matrix_summary <- as.matrix(fst_matrix_summary)

# Make the matrix symmetric by averaging the off-diagonal elements
symmetric_fst_matrix <- matrix(nrow = nrow(fst_matrix_summary), ncol = ncol(fst_matrix_summary))
rownames(symmetric_fst_matrix) <- rownames(fst_matrix_summary)
colnames(symmetric_fst_matrix) <- colnames(fst_matrix_summary)

for(i in 1:nrow(fst_matrix_summary)) {
  for(j in i:nrow(fst_matrix_summary)) {
    if (i == j) {
      symmetric_fst_matrix[i, j] <- fst_matrix_summary[i, j]
    } else {
      avg_value <- mean(c(fst_matrix_summary[i, j], fst_matrix_summary[j, i]), na.rm = TRUE)
      symmetric_fst_matrix[i, j] <- avg_value
      symmetric_fst_matrix[j, i] <- avg_value
    }
  }
}

# Check if the matrix is symmetric
# print(isSymmetric(symmetric_fst_matrix))

# Your symmetric Fst matrix by country is now in symmetric_fst_matrix
print(symmetric_fst_matrix)
```

```
##                      Argentina   Bermuda     Brazil  Colombia   Jamaica
## Argentina                   NA 0.1879268 0.04472739 0.1281484 0.2080969
## Bermuda             0.18792683        NA 0.16534433 0.1625620 0.2552230
## Brazil              0.04472739 0.1653443         NA 0.1267886 0.2230572
## Colombia            0.12814839 0.1625620 0.12678863        NA 0.2157626
## Jamaica             0.20809690 0.2552230 0.22305720 0.2157626        NA
## Trinidad_and_Tobago 0.14347891 0.1215338 0.12809485 0.1149541 0.1987192
## USA                 0.17080272 0.0998206 0.15051870 0.1389210 0.2436315
##                     Trinidad_and_Tobago        USA
## Argentina                    0.14347891 0.17080272
## Bermuda                      0.12153382 0.09982060
## Brazil                       0.12809485 0.15051870
## Colombia                     0.11495409 0.13892102
## Jamaica                      0.19871920 0.24363152
## Trinidad_and_Tobago                  NA 0.09085887
## USA                          0.09085887         NA
```

#### 4.1.5.1 Reorder

```
#reorder 
#fst_matrix_summary <- fst_matrix_summary[match(order_countries, fst_matrix_summary$Country_1),]

# Read the file
country_order <- here(
  "/gpfs/gibbs/pi/caccone/mkc54/albo/americas/output/fst/country_order.txt")
country_order <- read.table(country_order, 
                       header = FALSE,
                       col.names = c("country"))

order_countries <- as.vector(country_order$country)
order_countries
```

```
## [1] "USA"                 "Bermuda"             "Jamaica"            
## [4] "Trinidad_and_Tobago" "Colombia"            "Brazil"             
## [7] "Argentina"
```

```
write.csv(symmetric_fst_matrix, file = here("/gpfs/gibbs/pi/caccone/mkc54/albo/americas/output/fst/symmetric_fst_matrix.csv"))

symmetric_fst_matrix_ordered <- read_csv(
 "/gpfs/gibbs/pi/caccone/mkc54/albo/americas/output/fst/symmetric_fst_matrix_ordered.csv"
)
```

```
## New names:
## Rows: 7 Columns: 8
## ── Column specification
## ──────────────────────────────────────────────────────── Delimiter: "," chr
## (1): ...1 dbl (7): USA, Bermuda, Jamaica, Trinidad_and_Tobago, Colombia,
## Brazil, Argen...
## ℹ Use `spec()` to retrieve the full column specification for this data. ℹ
## Specify the column types or set `show_col_types = FALSE` to quiet this message.
## • `` -> `...1`
```

```
symmetric_fst_matrix_ordered<- as.data.frame(symmetric_fst_matrix_ordered) #convert to dataframe
rownames(symmetric_fst_matrix_ordered) <- symmetric_fst_matrix_ordered$...1 #use first column as rownames
symmetric_fst_matrix_ordered <- symmetric_fst_matrix_ordered[ -c(1) ] #remove 1st column
symmetric_fst_matrix_ordered <-as.matrix(symmetric_fst_matrix_ordered) #convert to matrix
```

```
symmetric_fst_matrix_ordered[lower.tri(symmetric_fst_matrix_ordered)] <- NA
print(symmetric_fst_matrix_ordered)
```

```
##                     USA   Bermuda   Jamaica Trinidad_and_Tobago  Colombia
## USA                  NA 0.0998206 0.2436315          0.09085887 0.1389210
## Bermuda              NA        NA 0.2552230          0.12153382 0.1625620
## Jamaica              NA        NA        NA          0.19871920 0.2157626
## Trinidad_and_Tobago  NA        NA        NA                  NA 0.1149541
## Colombia             NA        NA        NA                  NA        NA
## Brazil               NA        NA        NA                  NA        NA
## Argentina            NA        NA        NA                  NA        NA
##                        Brazil  Argentina
## USA                 0.1505187 0.17080272
## Bermuda             0.1653443 0.18792683
## Jamaica             0.2230572 0.20809690
## Trinidad_and_Tobago 0.1280949 0.14347891
## Colombia            0.1267886 0.12814839
## Brazil                     NA 0.04472739
## Argentina                  NA         NA
```

Now we have to convert the matrix to a data frame to plot it with
ggplot.

```
pairfst.long2 <- melt(symmetric_fst_matrix_ordered)
summary(pairfst.long2)
```

```
##                   Var1                    Var2       value        
##  USA                :7   USA                :7   Min.   :0.04473  
##  Bermuda            :7   Bermuda            :7   1st Qu.:0.12679  
##  Jamaica            :7   Jamaica            :7   Median :0.15052  
##  Trinidad_and_Tobago:7   Trinidad_and_Tobago:7   Mean   :0.15805  
##  Colombia           :7   Colombia           :7   3rd Qu.:0.19872  
##  Brazil             :7   Brazil             :7   Max.   :0.25522  
##  Argentina          :7   Argentina          :7   NA's   :28
```

You can click in the little square on the top left of the plot to
open it on a new window. It will have the right proportions.

```
pairfst.f2 <- ggplot(pairfst.long2, aes(Var1, Var2)) +
  geom_tile(aes(fill = value), colour = "white") +
  scale_fill_gradient(
    low = "white",
    high = "#71b6ff",
    name = "Fst",
    na.value = "white",
    limits = c(0, 0.5)
  ) +
  scale_x_discrete(position = "top") +
  theme_bw() +
  geom_text(aes(label = ifelse(
    is.na(value), "", formatC(value, digits = 2, format = "f")
  )), size = 3) +
  theme(
    axis.text.x = element_text(angle = 90, hjust = 0),
    axis.title = element_blank(),
    panel.grid.major = element_blank(),
    panel.grid.minor = element_blank(),
    panel.border = element_blank(),
    panel.background = element_blank(),
    axis.text.y = element_text(hjust = 1)
  )
pairfst.f2
```

```
ggsave(
  filename = here("scripts", "RMarkdowns", "output", "americas", "americas_all", "fst", "fst_matrix_americas_all_LD3_by_country_ordered.pdf"),
  pairfst.f2, 
  width = 10,
  height = 9,
  units = "in"
)
```

Remove NAs and rename columns

```
# remove NAs
fst2 <-
  pairfst.long |>
  drop_na()

# rename columns
fst2 <-
  fst2 |>
  dplyr::rename(pop1 = 1,
         pop2 = 2,
         fst  = 3)


# Split the data into two data frames, one for pop1 and one for pop2
df_pop1 <- fst2 |>
  dplyr::select(pop = pop1, fst)
df_pop2 <- fst2 |>
  dplyr::select(pop = pop2, fst)

# Combine the two data frames
df_combined <- bind_rows(df_pop1, df_pop2)

# Calculate the mean fst for each population
mean_fst <- df_combined |>
  group_by(pop) |>
  summarise(mean_fst = mean(fst))

print(mean_fst)
```

```
## # A tibble: 51 × 2
##    pop   mean_fst
##    <fct>    <dbl>
##  1 NEB     0.0991
##  2 NEH     0.0910
##  3 FIR     0.130 
##  4 LIN     0.113 
##  5 NEW     0.101 
##  6 BER     0.0965
##  7 HAR     0.101 
##  8 MAN     0.0860
##  9 WAV     0.100 
## 10 FAY     0.0800
## # ℹ 41 more rows
```

Merge

```
fst3 <-
  sampling_loc |>
  left_join(
    mean_fst,
    by = c("Abbreviation" = "pop")
  ) |>
  drop_na(mean_fst) 
 #   dplyr::select(
#   -Region
#  )

# Remove " Asia" from the Region2 column
#fst3$Region2 <- gsub(" Asia", "", fst3$Region2)

# Rename the Region2 column to Region
#fst3 <- fst3 |>
#  dplyr::rename(Region = Region2)

# check output
head(fst3)
```

```
##       Pop_City Country Latitude Longitude Continent Abbreviation Year
## 1   Berlin, NJ     USA 39.79081  -74.9291  Americas          BER 2018
## 2 Columbus, OH     USA 39.97170  -82.9071  Americas          COL 2015
## 3   Palm Beach     USA 26.70560  -80.0364  Americas          PAL 2018
## 4  Houston, TX     USA 29.75491  -95.3505  Americas          HOU 2018
## 5  Los Angeles     USA 34.05220 -118.2437  Americas          LOS 2018
## 6   Manaus, AM  Brazil -3.09161  -60.0325  Americas          MAU 2017
##          Region      Subregion order order2 orderold AM_order      state
## 1 North America Northeast, USA     1     NA       75        6    NJ, USA
## 2 North America   Midwest, USA     2     NA       76       18    OH, USA
## 3 North America Southeast, USA     3     NA       77       13    FL, USA
## 4 North America Southwest, USA     4     NA       78       23    TX, USA
## 5 North America      West, USA     5     NA       79       30    CA, USA
## 6 South America  South America     6     NA       80       38 AM, Brazil
##     mean_fst
## 1 0.09645955
## 2 0.09914625
## 3 0.09729515
## 4 0.07100981
## 5 0.13062400
## 6 0.10720043
```

## 4.1.6. Means by category

Mean by region

```
# Group by Region and calculate the mean_fst by Region
region_means <- fst3 |>
  group_by(Region) |>
  summarize(mean_fst_by_region = round(mean(mean_fst, na.rm = TRUE), 2)) |>
  ungroup()  # Ungroup the data

# Add the mean_fst_by_region column to the fst3 tibble
fst3 <- fst3 |>
  left_join(region_means, by = "Region")

# Print the modified fst3 tibble
print(fst3)
```

```
##              Pop_City             Country   Latitude  Longitude Continent
## 1          Berlin, NJ                 USA  39.790810  -74.92910  Americas
## 2        Columbus, OH                 USA  39.971700  -82.90710  Americas
## 3          Palm Beach                 USA  26.705600  -80.03640  Americas
## 4         Houston, TX                 USA  29.754910  -95.35050  Americas
## 5         Los Angeles                 USA  34.052200 -118.24370  Americas
## 6          Manaus, AM              Brazil  -3.091610  -60.03250  Americas
## 7          Recife, PE              Brazil  -8.058820  -34.87810  Americas
## 8            Gravatai              Brazil -29.937600  -50.99070  Americas
## 9             Bermuda             Bermuda  32.299260  -64.78770  Americas
## 10    Saint Augustine Trinidad and Tobago  10.163600  -61.45940  Americas
## 11       Beaufort, SC                 USA  32.445620  -80.69110  Americas
## 12          Chino, CA                 USA  34.012880 -117.68900  Americas
## 13         Dallas, TX                 USA  32.774700  -96.80580  Americas
## 14   Fayetteville, NC                 USA  35.086260  -78.91100  Americas
## 15          Macon, GA                 USA  32.842300  -83.62890  Americas
## 16       Manassas, VA                 USA  38.771650  -77.39270  Americas
## 17    New Orleans, LA                 USA  29.994150  -90.01730  Americas
## 18         Newark, NJ                 USA  40.735840  -74.17280  Americas
## 19     Nueces Co., TX                 USA  27.723140  -97.59900  Americas
## 20         Peoria, IL                 USA  40.689000  -89.60890  Americas
## 21    Russelville, AR                 USA  35.277440  -93.13440  Americas
## 22    Springfield, MO                 USA  37.204340  -93.27860  Americas
## 23      Puerto Iguazu           Argentina -25.597200  -54.57860  Americas
## 24   Nova Venecia, ES              Brazil -18.708864  -40.39476  Americas
## 25        Tucurui, PA              Brazil  -3.689510  -49.67140  Americas
## 26           Medellin            Colombia   6.247878  -75.57190  Americas
## 27             Negirl             Jamaica  18.268100  -78.34670  Americas
## 28    New Bedford, MA                 USA  41.660000  -70.93000  Americas
## 29      New Haven, CT                 USA  41.320000  -72.93000  Americas
## 30    Fire Island, NY                 USA  40.710000  -72.94000  Americas
## 31        Lincoln, DE                 USA  38.869840  -75.42300  Americas
## 32     Harrisburg, PA                 USA  40.270000  -76.88000  Americas
## 33        Waverly, VA                 USA  37.036992  -77.09673  Americas
## 34     Huntsville, AL                 USA  34.762610  -86.52830  Americas
## 35    Hattiesburg, MS                 USA  31.348220  -89.23340  Americas
## 36      St. Louis, MO                 USA  38.624590  -90.20360  Americas
## 37     Travis Co., TX                 USA  30.324890  -97.77530  Americas
## 38 Montgomery Co., TX                 USA  30.395070  -95.70250  Americas
## 39      Smith Co., TX                 USA  32.369160  -95.21580  Americas
## 40     Denton Co., TX                 USA  33.215970  -97.13000  Americas
## 41        Porto Velho              Brazil  -8.714420  -63.88770  Americas
## 42           Itaituba              Brazil  -4.264200  -55.99220  Americas
## 43           Floriano              Brazil  -6.782860  -43.01590  Americas
## 44          Araguaina              Brazil  -7.191190  -48.20810  Americas
## 45             Palmas              Brazil -10.250900  -48.32840  Americas
## 46           Confresa              Brazil -10.653100  -51.54360  Americas
## 47      Alta Floresta              Brazil  -9.866830  -56.08740  Americas
## 48              Ipora              Brazil -16.444800  -51.12140  Americas
## 49     Belo Horizonte              Brazil -19.918700  -43.91750  Americas
## 50           Palmares              Brazil -22.457700  -43.39900  Americas
## 51           Medellin            Colombia   6.247878  -75.57190  Americas
##    Abbreviation    Year        Region      Subregion order order2 orderold
## 1           BER    2018 North America Northeast, USA     1     NA       75
## 2           COL    2015 North America   Midwest, USA     2     NA       76
## 3           PAL    2018 North America Southeast, USA     3     NA       77
## 4           HOU    2018 North America Southwest, USA     4     NA       78
## 5           LOS    2018 North America      West, USA     5     NA       79
## 6           MAU    2017 South America  South America     6     NA       80
## 7           REC    2017 South America  South America     7     NA       81
## 8           GRV    2018 South America  South America     8     NA       82
## 9           BRM    2015     Caribbean      Caribbean    NA     NA       NA
## 10          SAI    2021     Caribbean      Caribbean    NA     NA       NA
## 11          BEA    2015 North America Southeast, USA    NA     NA       NA
## 12          CHI    2002 North America      West, USA    NA     NA       NA
## 13          DAL    2015 North America Southwest, USA    NA     NA       NA
## 14          FAY    2018 North America Southeast, USA    NA     NA       NA
## 15          MAC    2015 North America Southeast, USA    NA     NA       NA
## 16          MAN    2018 North America Southeast, USA    NA     NA       NA
## 17          NEO    2015 North America Southeast, USA    NA     NA       NA
## 18          NEW    2018 North America Northeast, USA    NA     NA       NA
## 19          NUE    2015 North America Southwest, USA    NA     NA       NA
## 20          PEO    2015 North America   Midwest, USA    NA     NA       NA
## 21          RUS    2018 North America Southeast, USA    NA     NA       NA
## 22          SPR    2015 North America   Midwest, USA    NA     NA       NA
## 23          POR    2018 South America  South America    NA     NA       NA
## 24          NOV    2018 South America  South America    NA     NA       NA
## 25          TUC    2018 South America  South America    NA     NA       NA
## 26          MED    2021 South America  South America    NA     NA       NA
## 27          NEG    2020     Caribbean      Caribbean    NA     NA       NA
## 28          NEB    2015 North America Northeast, USA    NA     NA       NA
## 29          NEH unknown North America Northeast, USA    NA     NA       NA
## 30          FIR unknown North America Northeast, USA    NA     NA       NA
## 31          LIN unknown North America Northeast, USA    NA     NA       NA
## 32          HAR unknown North America Northeast, USA    NA     NA       NA
## 33          WAV unknown North America Southeast, USA    NA     NA       NA
## 34          HAL    2017 North America Southeast, USA    NA     NA       NA
## 35          HAT       0 North America Southeast, USA    NA     NA       NA
## 36          STL    2018 North America   Midwest, USA    NA     NA       NA
## 37          TRA       0 North America Southwest, USA    NA     NA       NA
## 38          MON    2009 North America Southwest, USA    NA     NA       NA
## 39          SMI    2017 North America Southwest, USA    NA     NA       NA
## 40          DEN unknown North America Southwest, USA    NA     NA       NA
## 41          BPV unknown South America  South America    NA     NA       NA
## 42          BIT unknown South America  South America    NA     NA       NA
## 43          FLO unknown South America  South America    NA     NA       NA
## 44          ARA unknown South America  South America    NA     NA       NA
## 45          PLM unknown South America  South America    NA     NA       NA
## 46          BCO unknown South America  South America    NA     NA       NA
## 47          BAF unknown South America  South America    NA     NA       NA
## 48          IPO unknown South America  South America    NA     NA       NA
## 49          BEH unknown South America  South America    NA     NA       NA
## 50          BPA unknown South America  South America    NA     NA       NA
## 51          CUA    2021 South America  South America    NA     NA       NA
##    AM_order               state   mean_fst mean_fst_by_region
## 1         6             NJ, USA 0.09645955               0.10
## 2        18             OH, USA 0.09914625               0.10
## 3        13             FL, USA 0.09729515               0.10
## 4        23             TX, USA 0.07100981               0.10
## 5        30             CA, USA 0.13062400               0.10
## 6        38          AM, Brazil 0.10720043               0.13
## 7        42          PE, Brazil 0.10217193               0.13
## 8        51          RS, Brazil 0.12346730               0.13
## 9        31             Bermuda 0.12980785               0.16
## 10       34 Trinidad and Tobago 0.10652197               0.16
## 11       11             SC, USA 0.07463889               0.10
## 12       29             CA, USA 0.13098716               0.10
## 13       26             TX, USA 0.08545401               0.10
## 14       10             NC, USA 0.07996046               0.10
## 15       12             GA, USA 0.08429231               0.10
## 16        8             VA, USA 0.08603304               0.10
## 17       17             LA, USA 0.09312426               0.10
## 18        5             NJ, USA 0.10140777               0.10
## 19       22             TX, USA 0.08436445               0.10
## 20       19             IL, USA 0.08577852               0.10
## 21       14             AR, USA 0.11917351               0.10
## 22       20             MO, USA 0.12054784               0.10
## 23       52           Argentina 0.13570942               0.13
## 24       48          ES, Brazil 0.16274369               0.13
## 25       40          PA, Brazil 0.11583224               0.13
## 26       36            Colombia 0.13272601               0.13
## 27       32             Jamaica 0.23038597               0.16
## 28        1             MA, USA 0.09910539               0.10
## 29        2             CT, USA 0.09098366               0.10
## 30        3             NY, USA 0.12990185               0.10
## 31        4             DE, USA 0.11311650               0.10
## 32        7             PA, USA 0.10053282               0.10
## 33        9             VA, USA 0.10045432               0.10
## 34       15             AL, USA 0.08725868               0.10
## 35       16             MS, USA 0.09191793               0.10
## 36       21             MO, USA 0.10615236               0.10
## 37       24             TX, USA 0.07638723               0.10
## 38       25             TX, USA 0.08013121               0.10
## 39       27             TX, USA 0.09355586               0.10
## 40       28             TX, USA 0.08649409               0.10
## 41       37          RO, Brazil 0.12025288               0.13
## 42       39          PA, Brazil 0.11848568               0.13
## 43       41          PI, Brazil 0.12242840               0.13
## 44       43          TO, Brazil 0.11147326               0.13
## 45       44          TO, Brazil 0.12044300               0.13
## 46       45          MT, Brazil 0.12273515               0.13
## 47       46          MT, Brazil 0.11144298               0.13
## 48       47          GO, Brazil 0.17684294               0.13
## 49       49          MG, Brazil 0.14209520               0.13
## 50       50          MG, Brazil 0.10624913               0.13
## 51       35            Colombia 0.13699453               0.13
```

Mean By country

```
# Group by Country and calculate the mean_fst by Country
country_means <- fst3 |>
  group_by(Country) |>
  summarize(mean_fst_by_country = round(mean(mean_fst, na.rm = TRUE), 2)) |>
  ungroup()  # Ungroup the data

# Add the mean_fst_by_country column to the fst3 tibble
fst3 <- fst3 |>
  left_join(country_means, by = "Country")

# Print the modified fst3 tibble
print(fst3)
```

```
##              Pop_City             Country   Latitude  Longitude Continent
## 1          Berlin, NJ                 USA  39.790810  -74.92910  Americas
## 2        Columbus, OH                 USA  39.971700  -82.90710  Americas
## 3          Palm Beach                 USA  26.705600  -80.03640  Americas
## 4         Houston, TX                 USA  29.754910  -95.35050  Americas
## 5         Los Angeles                 USA  34.052200 -118.24370  Americas
## 6          Manaus, AM              Brazil  -3.091610  -60.03250  Americas
## 7          Recife, PE              Brazil  -8.058820  -34.87810  Americas
## 8            Gravatai              Brazil -29.937600  -50.99070  Americas
## 9             Bermuda             Bermuda  32.299260  -64.78770  Americas
## 10    Saint Augustine Trinidad and Tobago  10.163600  -61.45940  Americas
## 11       Beaufort, SC                 USA  32.445620  -80.69110  Americas
## 12          Chino, CA                 USA  34.012880 -117.68900  Americas
## 13         Dallas, TX                 USA  32.774700  -96.80580  Americas
## 14   Fayetteville, NC                 USA  35.086260  -78.91100  Americas
## 15          Macon, GA                 USA  32.842300  -83.62890  Americas
## 16       Manassas, VA                 USA  38.771650  -77.39270  Americas
## 17    New Orleans, LA                 USA  29.994150  -90.01730  Americas
## 18         Newark, NJ                 USA  40.735840  -74.17280  Americas
## 19     Nueces Co., TX                 USA  27.723140  -97.59900  Americas
## 20         Peoria, IL                 USA  40.689000  -89.60890  Americas
## 21    Russelville, AR                 USA  35.277440  -93.13440  Americas
## 22    Springfield, MO                 USA  37.204340  -93.27860  Americas
## 23      Puerto Iguazu           Argentina -25.597200  -54.57860  Americas
## 24   Nova Venecia, ES              Brazil -18.708864  -40.39476  Americas
## 25        Tucurui, PA              Brazil  -3.689510  -49.67140  Americas
## 26           Medellin            Colombia   6.247878  -75.57190  Americas
## 27             Negirl             Jamaica  18.268100  -78.34670  Americas
## 28    New Bedford, MA                 USA  41.660000  -70.93000  Americas
## 29      New Haven, CT                 USA  41.320000  -72.93000  Americas
## 30    Fire Island, NY                 USA  40.710000  -72.94000  Americas
## 31        Lincoln, DE                 USA  38.869840  -75.42300  Americas
## 32     Harrisburg, PA                 USA  40.270000  -76.88000  Americas
## 33        Waverly, VA                 USA  37.036992  -77.09673  Americas
## 34     Huntsville, AL                 USA  34.762610  -86.52830  Americas
## 35    Hattiesburg, MS                 USA  31.348220  -89.23340  Americas
## 36      St. Louis, MO                 USA  38.624590  -90.20360  Americas
## 37     Travis Co., TX                 USA  30.324890  -97.77530  Americas
## 38 Montgomery Co., TX                 USA  30.395070  -95.70250  Americas
## 39      Smith Co., TX                 USA  32.369160  -95.21580  Americas
## 40     Denton Co., TX                 USA  33.215970  -97.13000  Americas
## 41        Porto Velho              Brazil  -8.714420  -63.88770  Americas
## 42           Itaituba              Brazil  -4.264200  -55.99220  Americas
## 43           Floriano              Brazil  -6.782860  -43.01590  Americas
## 44          Araguaina              Brazil  -7.191190  -48.20810  Americas
## 45             Palmas              Brazil -10.250900  -48.32840  Americas
## 46           Confresa              Brazil -10.653100  -51.54360  Americas
## 47      Alta Floresta              Brazil  -9.866830  -56.08740  Americas
## 48              Ipora              Brazil -16.444800  -51.12140  Americas
## 49     Belo Horizonte              Brazil -19.918700  -43.91750  Americas
## 50           Palmares              Brazil -22.457700  -43.39900  Americas
## 51           Medellin            Colombia   6.247878  -75.57190  Americas
##    Abbreviation    Year        Region      Subregion order order2 orderold
## 1           BER    2018 North America Northeast, USA     1     NA       75
## 2           COL    2015 North America   Midwest, USA     2     NA       76
## 3           PAL    2018 North America Southeast, USA     3     NA       77
## 4           HOU    2018 North America Southwest, USA     4     NA       78
## 5           LOS    2018 North America      West, USA     5     NA       79
## 6           MAU    2017 South America  South America     6     NA       80
## 7           REC    2017 South America  South America     7     NA       81
## 8           GRV    2018 South America  South America     8     NA       82
## 9           BRM    2015     Caribbean      Caribbean    NA     NA       NA
## 10          SAI    2021     Caribbean      Caribbean    NA     NA       NA
## 11          BEA    2015 North America Southeast, USA    NA     NA       NA
## 12          CHI    2002 North America      West, USA    NA     NA       NA
## 13          DAL    2015 North America Southwest, USA    NA     NA       NA
## 14          FAY    2018 North America Southeast, USA    NA     NA       NA
## 15          MAC    2015 North America Southeast, USA    NA     NA       NA
## 16          MAN    2018 North America Southeast, USA    NA     NA       NA
## 17          NEO    2015 North America Southeast, USA    NA     NA       NA
## 18          NEW    2018 North America Northeast, USA    NA     NA       NA
## 19          NUE    2015 North America Southwest, USA    NA     NA       NA
## 20          PEO    2015 North America   Midwest, USA    NA     NA       NA
## 21          RUS    2018 North America Southeast, USA    NA     NA       NA
## 22          SPR    2015 North America   Midwest, USA    NA     NA       NA
## 23          POR    2018 South America  South America    NA     NA       NA
## 24          NOV    2018 South America  South America    NA     NA       NA
## 25          TUC    2018 South America  South America    NA     NA       NA
## 26          MED    2021 South America  South America    NA     NA       NA
## 27          NEG    2020     Caribbean      Caribbean    NA     NA       NA
## 28          NEB    2015 North America Northeast, USA    NA     NA       NA
## 29          NEH unknown North America Northeast, USA    NA     NA       NA
## 30          FIR unknown North America Northeast, USA    NA     NA       NA
## 31          LIN unknown North America Northeast, USA    NA     NA       NA
## 32          HAR unknown North America Northeast, USA    NA     NA       NA
## 33          WAV unknown North America Southeast, USA    NA     NA       NA
## 34          HAL    2017 North America Southeast, USA    NA     NA       NA
## 35          HAT       0 North America Southeast, USA    NA     NA       NA
## 36          STL    2018 North America   Midwest, USA    NA     NA       NA
## 37          TRA       0 North America Southwest, USA    NA     NA       NA
## 38          MON    2009 North America Southwest, USA    NA     NA       NA
## 39          SMI    2017 North America Southwest, USA    NA     NA       NA
## 40          DEN unknown North America Southwest, USA    NA     NA       NA
## 41          BPV unknown South America  South America    NA     NA       NA
## 42          BIT unknown South America  South America    NA     NA       NA
## 43          FLO unknown South America  South America    NA     NA       NA
## 44          ARA unknown South America  South America    NA     NA       NA
## 45          PLM unknown South America  South America    NA     NA       NA
## 46          BCO unknown South America  South America    NA     NA       NA
## 47          BAF unknown South America  South America    NA     NA       NA
## 48          IPO unknown South America  South America    NA     NA       NA
## 49          BEH unknown South America  South America    NA     NA       NA
## 50          BPA unknown South America  South America    NA     NA       NA
## 51          CUA    2021 South America  South America    NA     NA       NA
##    AM_order               state   mean_fst mean_fst_by_region
## 1         6             NJ, USA 0.09645955               0.10
## 2        18             OH, USA 0.09914625               0.10
## 3        13             FL, USA 0.09729515               0.10
## 4        23             TX, USA 0.07100981               0.10
## 5        30             CA, USA 0.13062400               0.10
## 6        38          AM, Brazil 0.10720043               0.13
## 7        42          PE, Brazil 0.10217193               0.13
## 8        51          RS, Brazil 0.12346730               0.13
## 9        31             Bermuda 0.12980785               0.16
## 10       34 Trinidad and Tobago 0.10652197               0.16
## 11       11             SC, USA 0.07463889               0.10
## 12       29             CA, USA 0.13098716               0.10
## 13       26             TX, USA 0.08545401               0.10
## 14       10             NC, USA 0.07996046               0.10
## 15       12             GA, USA 0.08429231               0.10
## 16        8             VA, USA 0.08603304               0.10
## 17       17             LA, USA 0.09312426               0.10
## 18        5             NJ, USA 0.10140777               0.10
## 19       22             TX, USA 0.08436445               0.10
## 20       19             IL, USA 0.08577852               0.10
## 21       14             AR, USA 0.11917351               0.10
## 22       20             MO, USA 0.12054784               0.10
## 23       52           Argentina 0.13570942               0.13
## 24       48          ES, Brazil 0.16274369               0.13
## 25       40          PA, Brazil 0.11583224               0.13
## 26       36            Colombia 0.13272601               0.13
## 27       32             Jamaica 0.23038597               0.16
## 28        1             MA, USA 0.09910539               0.10
## 29        2             CT, USA 0.09098366               0.10
## 30        3             NY, USA 0.12990185               0.10
## 31        4             DE, USA 0.11311650               0.10
## 32        7             PA, USA 0.10053282               0.10
## 33        9             VA, USA 0.10045432               0.10
## 34       15             AL, USA 0.08725868               0.10
## 35       16             MS, USA 0.09191793               0.10
## 36       21             MO, USA 0.10615236               0.10
## 37       24             TX, USA 0.07638723               0.10
## 38       25             TX, USA 0.08013121               0.10
## 39       27             TX, USA 0.09355586               0.10
## 40       28             TX, USA 0.08649409               0.10
## 41       37          RO, Brazil 0.12025288               0.13
## 42       39          PA, Brazil 0.11848568               0.13
## 43       41          PI, Brazil 0.12242840               0.13
## 44       43          TO, Brazil 0.11147326               0.13
## 45       44          TO, Brazil 0.12044300               0.13
## 46       45          MT, Brazil 0.12273515               0.13
## 47       46          MT, Brazil 0.11144298               0.13
## 48       47          GO, Brazil 0.17684294               0.13
## 49       49          MG, Brazil 0.14209520               0.13
## 50       50          MG, Brazil 0.10624913               0.13
## 51       35            Colombia 0.13699453               0.13
##    mean_fst_by_country
## 1                 0.10
## 2                 0.10
## 3                 0.10
## 4                 0.10
## 5                 0.10
## 6                 0.12
## 7                 0.12
## 8                 0.12
## 9                 0.13
## 10                0.11
## 11                0.10
## 12                0.10
## 13                0.10
## 14                0.10
## 15                0.10
## 16                0.10
## 17                0.10
## 18                0.10
## 19                0.10
## 20                0.10
## 21                0.10
## 22                0.10
## 23                0.14
## 24                0.12
## 25                0.12
## 26                0.13
## 27                0.23
## 28                0.10
## 29                0.10
## 30                0.10
## 31                0.10
## 32                0.10
## 33                0.10
## 34                0.10
## 35                0.10
## 36                0.10
## 37                0.10
## 38                0.10
## 39                0.10
## 40                0.10
## 41                0.12
## 42                0.12
## 43                0.12
## 44                0.12
## 45                0.12
## 46                0.12
## 47                0.12
## 48                0.12
## 49                0.12
## 50                0.12
## 51                0.13
```

Mean By continent

```
# Group by Country and calculate the mean_fst by Country
country_means <- fst3 |>
  group_by(Continent) |>
  summarize(mean_fst_by_continent = round(mean(mean_fst, na.rm = TRUE), 2)) |>
  ungroup()  # Ungroup the data

# Add the mean_fst_by_country column to the fst3 tibble
fst3 <- fst3 |>
  left_join(country_means, by = "Continent")

# Print the modified fst3 tibble
print(fst3)
```

```
##              Pop_City             Country   Latitude  Longitude Continent
## 1          Berlin, NJ                 USA  39.790810  -74.92910  Americas
## 2        Columbus, OH                 USA  39.971700  -82.90710  Americas
## 3          Palm Beach                 USA  26.705600  -80.03640  Americas
## 4         Houston, TX                 USA  29.754910  -95.35050  Americas
## 5         Los Angeles                 USA  34.052200 -118.24370  Americas
## 6          Manaus, AM              Brazil  -3.091610  -60.03250  Americas
## 7          Recife, PE              Brazil  -8.058820  -34.87810  Americas
## 8            Gravatai              Brazil -29.937600  -50.99070  Americas
## 9             Bermuda             Bermuda  32.299260  -64.78770  Americas
## 10    Saint Augustine Trinidad and Tobago  10.163600  -61.45940  Americas
## 11       Beaufort, SC                 USA  32.445620  -80.69110  Americas
## 12          Chino, CA                 USA  34.012880 -117.68900  Americas
## 13         Dallas, TX                 USA  32.774700  -96.80580  Americas
## 14   Fayetteville, NC                 USA  35.086260  -78.91100  Americas
## 15          Macon, GA                 USA  32.842300  -83.62890  Americas
## 16       Manassas, VA                 USA  38.771650  -77.39270  Americas
## 17    New Orleans, LA                 USA  29.994150  -90.01730  Americas
## 18         Newark, NJ                 USA  40.735840  -74.17280  Americas
## 19     Nueces Co., TX                 USA  27.723140  -97.59900  Americas
## 20         Peoria, IL                 USA  40.689000  -89.60890  Americas
## 21    Russelville, AR                 USA  35.277440  -93.13440  Americas
## 22    Springfield, MO                 USA  37.204340  -93.27860  Americas
## 23      Puerto Iguazu           Argentina -25.597200  -54.57860  Americas
## 24   Nova Venecia, ES              Brazil -18.708864  -40.39476  Americas
## 25        Tucurui, PA              Brazil  -3.689510  -49.67140  Americas
## 26           Medellin            Colombia   6.247878  -75.57190  Americas
## 27             Negirl             Jamaica  18.268100  -78.34670  Americas
## 28    New Bedford, MA                 USA  41.660000  -70.93000  Americas
## 29      New Haven, CT                 USA  41.320000  -72.93000  Americas
## 30    Fire Island, NY                 USA  40.710000  -72.94000  Americas
## 31        Lincoln, DE                 USA  38.869840  -75.42300  Americas
## 32     Harrisburg, PA                 USA  40.270000  -76.88000  Americas
## 33        Waverly, VA                 USA  37.036992  -77.09673  Americas
## 34     Huntsville, AL                 USA  34.762610  -86.52830  Americas
## 35    Hattiesburg, MS                 USA  31.348220  -89.23340  Americas
## 36      St. Louis, MO                 USA  38.624590  -90.20360  Americas
## 37     Travis Co., TX                 USA  30.324890  -97.77530  Americas
## 38 Montgomery Co., TX                 USA  30.395070  -95.70250  Americas
## 39      Smith Co., TX                 USA  32.369160  -95.21580  Americas
## 40     Denton Co., TX                 USA  33.215970  -97.13000  Americas
## 41        Porto Velho              Brazil  -8.714420  -63.88770  Americas
## 42           Itaituba              Brazil  -4.264200  -55.99220  Americas
## 43           Floriano              Brazil  -6.782860  -43.01590  Americas
## 44          Araguaina              Brazil  -7.191190  -48.20810  Americas
## 45             Palmas              Brazil -10.250900  -48.32840  Americas
## 46           Confresa              Brazil -10.653100  -51.54360  Americas
## 47      Alta Floresta              Brazil  -9.866830  -56.08740  Americas
## 48              Ipora              Brazil -16.444800  -51.12140  Americas
## 49     Belo Horizonte              Brazil -19.918700  -43.91750  Americas
## 50           Palmares              Brazil -22.457700  -43.39900  Americas
## 51           Medellin            Colombia   6.247878  -75.57190  Americas
##    Abbreviation    Year        Region      Subregion order order2 orderold
## 1           BER    2018 North America Northeast, USA     1     NA       75
## 2           COL    2015 North America   Midwest, USA     2     NA       76
## 3           PAL    2018 North America Southeast, USA     3     NA       77
## 4           HOU    2018 North America Southwest, USA     4     NA       78
## 5           LOS    2018 North America      West, USA     5     NA       79
## 6           MAU    2017 South America  South America     6     NA       80
## 7           REC    2017 South America  South America     7     NA       81
## 8           GRV    2018 South America  South America     8     NA       82
## 9           BRM    2015     Caribbean      Caribbean    NA     NA       NA
## 10          SAI    2021     Caribbean      Caribbean    NA     NA       NA
## 11          BEA    2015 North America Southeast, USA    NA     NA       NA
## 12          CHI    2002 North America      West, USA    NA     NA       NA
## 13          DAL    2015 North America Southwest, USA    NA     NA       NA
## 14          FAY    2018 North America Southeast, USA    NA     NA       NA
## 15          MAC    2015 North America Southeast, USA    NA     NA       NA
## 16          MAN    2018 North America Southeast, USA    NA     NA       NA
## 17          NEO    2015 North America Southeast, USA    NA     NA       NA
## 18          NEW    2018 North America Northeast, USA    NA     NA       NA
## 19          NUE    2015 North America Southwest, USA    NA     NA       NA
## 20          PEO    2015 North America   Midwest, USA    NA     NA       NA
## 21          RUS    2018 North America Southeast, USA    NA     NA       NA
## 22          SPR    2015 North America   Midwest, USA    NA     NA       NA
## 23          POR    2018 South America  South America    NA     NA       NA
## 24          NOV    2018 South America  South America    NA     NA       NA
## 25          TUC    2018 South America  South America    NA     NA       NA
## 26          MED    2021 South America  South America    NA     NA       NA
## 27          NEG    2020     Caribbean      Caribbean    NA     NA       NA
## 28          NEB    2015 North America Northeast, USA    NA     NA       NA
## 29          NEH unknown North America Northeast, USA    NA     NA       NA
## 30          FIR unknown North America Northeast, USA    NA     NA       NA
## 31          LIN unknown North America Northeast, USA    NA     NA       NA
## 32          HAR unknown North America Northeast, USA    NA     NA       NA
## 33          WAV unknown North America Southeast, USA    NA     NA       NA
## 34          HAL    2017 North America Southeast, USA    NA     NA       NA
## 35          HAT       0 North America Southeast, USA    NA     NA       NA
## 36          STL    2018 North America   Midwest, USA    NA     NA       NA
## 37          TRA       0 North America Southwest, USA    NA     NA       NA
## 38          MON    2009 North America Southwest, USA    NA     NA       NA
## 39          SMI    2017 North America Southwest, USA    NA     NA       NA
## 40          DEN unknown North America Southwest, USA    NA     NA       NA
## 41          BPV unknown South America  South America    NA     NA       NA
## 42          BIT unknown South America  South America    NA     NA       NA
## 43          FLO unknown South America  South America    NA     NA       NA
## 44          ARA unknown South America  South America    NA     NA       NA
## 45          PLM unknown South America  South America    NA     NA       NA
## 46          BCO unknown South America  South America    NA     NA       NA
## 47          BAF unknown South America  South America    NA     NA       NA
## 48          IPO unknown South America  South America    NA     NA       NA
## 49          BEH unknown South America  South America    NA     NA       NA
## 50          BPA unknown South America  South America    NA     NA       NA
## 51          CUA    2021 South America  South America    NA     NA       NA
##    AM_order               state   mean_fst mean_fst_by_region
## 1         6             NJ, USA 0.09645955               0.10
## 2        18             OH, USA 0.09914625               0.10
## 3        13             FL, USA 0.09729515               0.10
## 4        23             TX, USA 0.07100981               0.10
## 5        30             CA, USA 0.13062400               0.10
## 6        38          AM, Brazil 0.10720043               0.13
## 7        42          PE, Brazil 0.10217193               0.13
## 8        51          RS, Brazil 0.12346730               0.13
## 9        31             Bermuda 0.12980785               0.16
## 10       34 Trinidad and Tobago 0.10652197               0.16
## 11       11             SC, USA 0.07463889               0.10
## 12       29             CA, USA 0.13098716               0.10
## 13       26             TX, USA 0.08545401               0.10
## 14       10             NC, USA 0.07996046               0.10
## 15       12             GA, USA 0.08429231               0.10
## 16        8             VA, USA 0.08603304               0.10
## 17       17             LA, USA 0.09312426               0.10
## 18        5             NJ, USA 0.10140777               0.10
## 19       22             TX, USA 0.08436445               0.10
## 20       19             IL, USA 0.08577852               0.10
## 21       14             AR, USA 0.11917351               0.10
## 22       20             MO, USA 0.12054784               0.10
## 23       52           Argentina 0.13570942               0.13
## 24       48          ES, Brazil 0.16274369               0.13
## 25       40          PA, Brazil 0.11583224               0.13
## 26       36            Colombia 0.13272601               0.13
## 27       32             Jamaica 0.23038597               0.16
## 28        1             MA, USA 0.09910539               0.10
## 29        2             CT, USA 0.09098366               0.10
## 30        3             NY, USA 0.12990185               0.10
## 31        4             DE, USA 0.11311650               0.10
## 32        7             PA, USA 0.10053282               0.10
## 33        9             VA, USA 0.10045432               0.10
## 34       15             AL, USA 0.08725868               0.10
## 35       16             MS, USA 0.09191793               0.10
## 36       21             MO, USA 0.10615236               0.10
## 37       24             TX, USA 0.07638723               0.10
## 38       25             TX, USA 0.08013121               0.10
## 39       27             TX, USA 0.09355586               0.10
## 40       28             TX, USA 0.08649409               0.10
## 41       37          RO, Brazil 0.12025288               0.13
## 42       39          PA, Brazil 0.11848568               0.13
## 43       41          PI, Brazil 0.12242840               0.13
## 44       43          TO, Brazil 0.11147326               0.13
## 45       44          TO, Brazil 0.12044300               0.13
## 46       45          MT, Brazil 0.12273515               0.13
## 47       46          MT, Brazil 0.11144298               0.13
## 48       47          GO, Brazil 0.17684294               0.13
## 49       49          MG, Brazil 0.14209520               0.13
## 50       50          MG, Brazil 0.10624913               0.13
## 51       35            Colombia 0.13699453               0.13
##    mean_fst_by_country mean_fst_by_continent
## 1                 0.10                  0.11
## 2                 0.10                  0.11
## 3                 0.10                  0.11
## 4                 0.10                  0.11
## 5                 0.10                  0.11
## 6                 0.12                  0.11
## 7                 0.12                  0.11
## 8                 0.12                  0.11
## 9                 0.13                  0.11
## 10                0.11                  0.11
## 11                0.10                  0.11
## 12                0.10                  0.11
## 13                0.10                  0.11
## 14                0.10                  0.11
## 15                0.10                  0.11
## 16                0.10                  0.11
## 17                0.10                  0.11
## 18                0.10                  0.11
## 19                0.10                  0.11
## 20                0.10                  0.11
## 21                0.10                  0.11
## 22                0.10                  0.11
## 23                0.14                  0.11
## 24                0.12                  0.11
## 25                0.12                  0.11
## 26                0.13                  0.11
## 27                0.23                  0.11
## 28                0.10                  0.11
## 29                0.10                  0.11
## 30                0.10                  0.11
## 31                0.10                  0.11
## 32                0.10                  0.11
## 33                0.10                  0.11
## 34                0.10                  0.11
## 35                0.10                  0.11
## 36                0.10                  0.11
## 37                0.10                  0.11
## 38                0.10                  0.11
## 39                0.10                  0.11
## 40                0.10                  0.11
## 41                0.12                  0.11
## 42                0.12                  0.11
## 43                0.12                  0.11
## 44                0.12                  0.11
## 45                0.12                  0.11
## 46                0.12                  0.11
## 47                0.12                  0.11
## 48                0.12                  0.11
## 49                0.12                  0.11
## 50                0.12                  0.11
## 51                0.13                  0.11
```

Mean by latitude

```
# Add a new column to indicate whether the latitude is above or below 30N
fst3 <- fst3 |>
  mutate(Latitude_group = ifelse(Latitude >= 30, "Above 30N", "Below 30N"))

# Summarize the data by Latitude_group and calculate the mean_fst
summary_by_latitude <- fst3 |>
  group_by(Latitude_group) |>
  summarize(mean_fst_by_latitude = mean(mean_fst, na.rm = TRUE)) |>
  ungroup()  # Ungroup the data

# Add the mean_fst_by_latitude column to the fst3 tibble
fst3 <- fst3 |>
  left_join(summary_by_latitude, by = "Latitude_group")


# Rename columns
fst3 <- fst3 |>
  dplyr::rename(
    City = Pop_City)

# Print the modified fst3 tibble
print(fst3)
```

```
##                  City             Country   Latitude  Longitude Continent
## 1          Berlin, NJ                 USA  39.790810  -74.92910  Americas
## 2        Columbus, OH                 USA  39.971700  -82.90710  Americas
## 3          Palm Beach                 USA  26.705600  -80.03640  Americas
## 4         Houston, TX                 USA  29.754910  -95.35050  Americas
## 5         Los Angeles                 USA  34.052200 -118.24370  Americas
## 6          Manaus, AM              Brazil  -3.091610  -60.03250  Americas
## 7          Recife, PE              Brazil  -8.058820  -34.87810  Americas
## 8            Gravatai              Brazil -29.937600  -50.99070  Americas
## 9             Bermuda             Bermuda  32.299260  -64.78770  Americas
## 10    Saint Augustine Trinidad and Tobago  10.163600  -61.45940  Americas
## 11       Beaufort, SC                 USA  32.445620  -80.69110  Americas
## 12          Chino, CA                 USA  34.012880 -117.68900  Americas
## 13         Dallas, TX                 USA  32.774700  -96.80580  Americas
## 14   Fayetteville, NC                 USA  35.086260  -78.91100  Americas
## 15          Macon, GA                 USA  32.842300  -83.62890  Americas
## 16       Manassas, VA                 USA  38.771650  -77.39270  Americas
## 17    New Orleans, LA                 USA  29.994150  -90.01730  Americas
## 18         Newark, NJ                 USA  40.735840  -74.17280  Americas
## 19     Nueces Co., TX                 USA  27.723140  -97.59900  Americas
## 20         Peoria, IL                 USA  40.689000  -89.60890  Americas
## 21    Russelville, AR                 USA  35.277440  -93.13440  Americas
## 22    Springfield, MO                 USA  37.204340  -93.27860  Americas
## 23      Puerto Iguazu           Argentina -25.597200  -54.57860  Americas
## 24   Nova Venecia, ES              Brazil -18.708864  -40.39476  Americas
## 25        Tucurui, PA              Brazil  -3.689510  -49.67140  Americas
## 26           Medellin            Colombia   6.247878  -75.57190  Americas
## 27             Negirl             Jamaica  18.268100  -78.34670  Americas
## 28    New Bedford, MA                 USA  41.660000  -70.93000  Americas
## 29      New Haven, CT                 USA  41.320000  -72.93000  Americas
## 30    Fire Island, NY                 USA  40.710000  -72.94000  Americas
## 31        Lincoln, DE                 USA  38.869840  -75.42300  Americas
## 32     Harrisburg, PA                 USA  40.270000  -76.88000  Americas
## 33        Waverly, VA                 USA  37.036992  -77.09673  Americas
## 34     Huntsville, AL                 USA  34.762610  -86.52830  Americas
## 35    Hattiesburg, MS                 USA  31.348220  -89.23340  Americas
## 36      St. Louis, MO                 USA  38.624590  -90.20360  Americas
## 37     Travis Co., TX                 USA  30.324890  -97.77530  Americas
## 38 Montgomery Co., TX                 USA  30.395070  -95.70250  Americas
## 39      Smith Co., TX                 USA  32.369160  -95.21580  Americas
## 40     Denton Co., TX                 USA  33.215970  -97.13000  Americas
## 41        Porto Velho              Brazil  -8.714420  -63.88770  Americas
## 42           Itaituba              Brazil  -4.264200  -55.99220  Americas
## 43           Floriano              Brazil  -6.782860  -43.01590  Americas
## 44          Araguaina              Brazil  -7.191190  -48.20810  Americas
## 45             Palmas              Brazil -10.250900  -48.32840  Americas
## 46           Confresa              Brazil -10.653100  -51.54360  Americas
## 47      Alta Floresta              Brazil  -9.866830  -56.08740  Americas
## 48              Ipora              Brazil -16.444800  -51.12140  Americas
## 49     Belo Horizonte              Brazil -19.918700  -43.91750  Americas
## 50           Palmares              Brazil -22.457700  -43.39900  Americas
## 51           Medellin            Colombia   6.247878  -75.57190  Americas
##    Abbreviation    Year        Region      Subregion order order2 orderold
## 1           BER    2018 North America Northeast, USA     1     NA       75
## 2           COL    2015 North America   Midwest, USA     2     NA       76
## 3           PAL    2018 North America Southeast, USA     3     NA       77
## 4           HOU    2018 North America Southwest, USA     4     NA       78
## 5           LOS    2018 North America      West, USA     5     NA       79
## 6           MAU    2017 South America  South America     6     NA       80
## 7           REC    2017 South America  South America     7     NA       81
## 8           GRV    2018 South America  South America     8     NA       82
## 9           BRM    2015     Caribbean      Caribbean    NA     NA       NA
## 10          SAI    2021     Caribbean      Caribbean    NA     NA       NA
## 11          BEA    2015 North America Southeast, USA    NA     NA       NA
## 12          CHI    2002 North America      West, USA    NA     NA       NA
## 13          DAL    2015 North America Southwest, USA    NA     NA       NA
## 14          FAY    2018 North America Southeast, USA    NA     NA       NA
## 15          MAC    2015 North America Southeast, USA    NA     NA       NA
## 16          MAN    2018 North America Southeast, USA    NA     NA       NA
## 17          NEO    2015 North America Southeast, USA    NA     NA       NA
## 18          NEW    2018 North America Northeast, USA    NA     NA       NA
## 19          NUE    2015 North America Southwest, USA    NA     NA       NA
## 20          PEO    2015 North America   Midwest, USA    NA     NA       NA
## 21          RUS    2018 North America Southeast, USA    NA     NA       NA
## 22          SPR    2015 North America   Midwest, USA    NA     NA       NA
## 23          POR    2018 South America  South America    NA     NA       NA
## 24          NOV    2018 South America  South America    NA     NA       NA
## 25          TUC    2018 South America  South America    NA     NA       NA
## 26          MED    2021 South America  South America    NA     NA       NA
## 27          NEG    2020     Caribbean      Caribbean    NA     NA       NA
## 28          NEB    2015 North America Northeast, USA    NA     NA       NA
## 29          NEH unknown North America Northeast, USA    NA     NA       NA
## 30          FIR unknown North America Northeast, USA    NA     NA       NA
## 31          LIN unknown North America Northeast, USA    NA     NA       NA
## 32          HAR unknown North America Northeast, USA    NA     NA       NA
## 33          WAV unknown North America Southeast, USA    NA     NA       NA
## 34          HAL    2017 North America Southeast, USA    NA     NA       NA
## 35          HAT       0 North America Southeast, USA    NA     NA       NA
## 36          STL    2018 North America   Midwest, USA    NA     NA       NA
## 37          TRA       0 North America Southwest, USA    NA     NA       NA
## 38          MON    2009 North America Southwest, USA    NA     NA       NA
## 39          SMI    2017 North America Southwest, USA    NA     NA       NA
## 40          DEN unknown North America Southwest, USA    NA     NA       NA
## 41          BPV unknown South America  South America    NA     NA       NA
## 42          BIT unknown South America  South America    NA     NA       NA
## 43          FLO unknown South America  South America    NA     NA       NA
## 44          ARA unknown South America  South America    NA     NA       NA
## 45          PLM unknown South America  South America    NA     NA       NA
## 46          BCO unknown South America  South America    NA     NA       NA
## 47          BAF unknown South America  South America    NA     NA       NA
## 48          IPO unknown South America  South America    NA     NA       NA
## 49          BEH unknown South America  South America    NA     NA       NA
## 50          BPA unknown South America  South America    NA     NA       NA
## 51          CUA    2021 South America  South America    NA     NA       NA
##    AM_order               state   mean_fst mean_fst_by_region
## 1         6             NJ, USA 0.09645955               0.10
## 2        18             OH, USA 0.09914625               0.10
## 3        13             FL, USA 0.09729515               0.10
## 4        23             TX, USA 0.07100981               0.10
## 5        30             CA, USA 0.13062400               0.10
## 6        38          AM, Brazil 0.10720043               0.13
## 7        42          PE, Brazil 0.10217193               0.13
## 8        51          RS, Brazil 0.12346730               0.13
## 9        31             Bermuda 0.12980785               0.16
## 10       34 Trinidad and Tobago 0.10652197               0.16
## 11       11             SC, USA 0.07463889               0.10
## 12       29             CA, USA 0.13098716               0.10
## 13       26             TX, USA 0.08545401               0.10
## 14       10             NC, USA 0.07996046               0.10
## 15       12             GA, USA 0.08429231               0.10
## 16        8             VA, USA 0.08603304               0.10
## 17       17             LA, USA 0.09312426               0.10
## 18        5             NJ, USA 0.10140777               0.10
## 19       22             TX, USA 0.08436445               0.10
## 20       19             IL, USA 0.08577852               0.10
## 21       14             AR, USA 0.11917351               0.10
## 22       20             MO, USA 0.12054784               0.10
## 23       52           Argentina 0.13570942               0.13
## 24       48          ES, Brazil 0.16274369               0.13
## 25       40          PA, Brazil 0.11583224               0.13
## 26       36            Colombia 0.13272601               0.13
## 27       32             Jamaica 0.23038597               0.16
## 28        1             MA, USA 0.09910539               0.10
## 29        2             CT, USA 0.09098366               0.10
## 30        3             NY, USA 0.12990185               0.10
## 31        4             DE, USA 0.11311650               0.10
## 32        7             PA, USA 0.10053282               0.10
## 33        9             VA, USA 0.10045432               0.10
## 34       15             AL, USA 0.08725868               0.10
## 35       16             MS, USA 0.09191793               0.10
## 36       21             MO, USA 0.10615236               0.10
## 37       24             TX, USA 0.07638723               0.10
## 38       25             TX, USA 0.08013121               0.10
## 39       27             TX, USA 0.09355586               0.10
## 40       28             TX, USA 0.08649409               0.10
## 41       37          RO, Brazil 0.12025288               0.13
## 42       39          PA, Brazil 0.11848568               0.13
## 43       41          PI, Brazil 0.12242840               0.13
## 44       43          TO, Brazil 0.11147326               0.13
## 45       44          TO, Brazil 0.12044300               0.13
## 46       45          MT, Brazil 0.12273515               0.13
## 47       46          MT, Brazil 0.11144298               0.13
## 48       47          GO, Brazil 0.17684294               0.13
## 49       49          MG, Brazil 0.14209520               0.13
## 50       50          MG, Brazil 0.10624913               0.13
## 51       35            Colombia 0.13699453               0.13
##    mean_fst_by_country mean_fst_by_continent Latitude_group
## 1                 0.10                  0.11      Above 30N
## 2                 0.10                  0.11      Above 30N
## 3                 0.10                  0.11      Below 30N
## 4                 0.10                  0.11      Below 30N
## 5                 0.10                  0.11      Above 30N
## 6                 0.12                  0.11      Below 30N
## 7                 0.12                  0.11      Below 30N
## 8                 0.12                  0.11      Below 30N
## 9                 0.13                  0.11      Above 30N
## 10                0.11                  0.11      Below 30N
## 11                0.10                  0.11      Above 30N
## 12                0.10                  0.11      Above 30N
## 13                0.10                  0.11      Above 30N
## 14                0.10                  0.11      Above 30N
## 15                0.10                  0.11      Above 30N
## 16                0.10                  0.11      Above 30N
## 17                0.10                  0.11      Below 30N
## 18                0.10                  0.11      Above 30N
## 19                0.10                  0.11      Below 30N
## 20                0.10                  0.11      Above 30N
## 21                0.10                  0.11      Above 30N
## 22                0.10                  0.11      Above 30N
## 23                0.14                  0.11      Below 30N
## 24                0.12                  0.11      Below 30N
## 25                0.12                  0.11      Below 30N
## 26                0.13                  0.11      Below 30N
## 27                0.23                  0.11      Below 30N
## 28                0.10                  0.11      Above 30N
## 29                0.10                  0.11      Above 30N
## 30                0.10                  0.11      Above 30N
## 31                0.10                  0.11      Above 30N
## 32                0.10                  0.11      Above 30N
## 33                0.10                  0.11      Above 30N
## 34                0.10                  0.11      Above 30N
## 35                0.10                  0.11      Above 30N
## 36                0.10                  0.11      Above 30N
## 37                0.10                  0.11      Above 30N
## 38                0.10                  0.11      Above 30N
## 39                0.10                  0.11      Above 30N
## 40                0.10                  0.11      Above 30N
## 41                0.12                  0.11      Below 30N
## 42                0.12                  0.11      Below 30N
## 43                0.12                  0.11      Below 30N
## 44                0.12                  0.11      Below 30N
## 45                0.12                  0.11      Below 30N
## 46                0.12                  0.11      Below 30N
## 47                0.12                  0.11      Below 30N
## 48                0.12                  0.11      Below 30N
## 49                0.12                  0.11      Below 30N
## 50                0.12                  0.11      Below 30N
## 51                0.13                  0.11      Below 30N
##    mean_fst_by_latitude
## 1            0.09927048
## 2            0.09927048
## 3            0.12299982
## 4            0.12299982
## 5            0.09927048
## 6            0.12299982
## 7            0.12299982
## 8            0.12299982
## 9            0.09927048
## 10           0.12299982
## 11           0.09927048
## 12           0.09927048
## 13           0.09927048
## 14           0.09927048
## 15           0.09927048
## 16           0.09927048
## 17           0.12299982
## 18           0.09927048
## 19           0.12299982
## 20           0.09927048
## 21           0.09927048
## 22           0.09927048
## 23           0.12299982
## 24           0.12299982
## 25           0.12299982
## 26           0.12299982
## 27           0.12299982
## 28           0.09927048
## 29           0.09927048
## 30           0.09927048
## 31           0.09927048
## 32           0.09927048
## 33           0.09927048
## 34           0.09927048
## 35           0.09927048
## 36           0.09927048
## 37           0.09927048
## 38           0.09927048
## 39           0.09927048
## 40           0.09927048
## 41           0.12299982
## 42           0.12299982
## 43           0.12299982
## 44           0.12299982
## 45           0.12299982
## 46           0.12299982
## 47           0.12299982
## 48           0.12299982
## 49           0.12299982
## 50           0.12299982
## 51           0.12299982
```

```
fst4 <- fst3 |>
  dplyr::select(
    Latitude_group, mean_fst_by_latitude, Region, mean_fst_by_region, Country, mean_fst_by_country, City, Abbreviation, mean_fst,
  )

fst4 <- fst4 |>
  arrange(
    Latitude_group, Region, Country, City
  )

# Round
fst4 <- fst4 |>
  mutate_if(is.numeric, ~ round(., 2))

head(fst4)
```

```
##   Latitude_group mean_fst_by_latitude        Region mean_fst_by_region Country
## 1      Above 30N                  0.1     Caribbean               0.16 Bermuda
## 2      Above 30N                  0.1 North America               0.10     USA
## 3      Above 30N                  0.1 North America               0.10     USA
## 4      Above 30N                  0.1 North America               0.10     USA
## 5      Above 30N                  0.1 North America               0.10     USA
## 6      Above 30N                  0.1 North America               0.10     USA
##   mean_fst_by_country         City Abbreviation mean_fst
## 1                0.13      Bermuda          BRM     0.13
## 2                0.10 Beaufort, SC          BEA     0.07
## 3                0.10   Berlin, NJ          BER     0.10
## 4                0.10    Chino, CA          CHI     0.13
## 5                0.10 Columbus, OH          COL     0.10
## 6                0.10   Dallas, TX          DAL     0.09
```

```
# Set theme if you want to use something different from the previous table
set_flextable_defaults(
  font.family = "Arial",
  font.size = 9,
  big.mark = ",",
  theme_fun = "theme_zebra" # try the themes: theme_alafoli(), theme_apa(), theme_booktabs(), theme_box(), theme_tron_legacy(), theme_tron(), theme_vader(), theme_vanilla(), theme_zebra()
)

# Then create the flextable object
flex_table <- flextable(fst4) |>
  set_caption(caption = as_paragraph(
    as_chunk(
      "Table 1. Fst values for Europe, Asia and Americas (using LD pruned r2<0.01 SNP set)",
      props = fp_text_default(color = "#000000", font.size = 14)
    )
  ),
  fp_p = fp_par(text.align = "center", padding = 5))

# Print the flextable
flex_table
```

Table 1. Fst values for Europe, Asia and Americas (using LD pruned r2<0.01 SNP set)

| Latitude\_group | mean\_fst\_by\_latitude | Region | mean\_fst\_by\_region | Country | mean\_fst\_by\_country | City | Abbreviation | mean\_fst |
| --- | --- | --- | --- | --- | --- | --- | --- | --- |
| Above 30N | 0.10 | Caribbean | 0.16 | Bermuda | 0.13 | Bermuda | BRM | 0.13 |
| Above 30N | 0.10 | North America | 0.10 | USA | 0.10 | Beaufort, SC | BEA | 0.07 |
| Above 30N | 0.10 | North America | 0.10 | USA | 0.10 | Berlin, NJ | BER | 0.10 |
| Above 30N | 0.10 | North America | 0.10 | USA | 0.10 | Chino, CA | CHI | 0.13 |
| Above 30N | 0.10 | North America | 0.10 | USA | 0.10 | Columbus, OH | COL | 0.10 |
| Above 30N | 0.10 | North America | 0.10 | USA | 0.10 | Dallas, TX | DAL | 0.09 |
| Above 30N | 0.10 | North America | 0.10 | USA | 0.10 | Denton Co., TX | DEN | 0.09 |
| Above 30N | 0.10 | North America | 0.10 | USA | 0.10 | Fayetteville, NC | FAY | 0.08 |
| Above 30N | 0.10 | North America | 0.10 | USA | 0.10 | Fire Island, NY | FIR | 0.13 |
| Above 30N | 0.10 | North America | 0.10 | USA | 0.10 | Harrisburg, PA | HAR | 0.10 |
| Above 30N | 0.10 | North America | 0.10 | USA | 0.10 | Hattiesburg, MS | HAT | 0.09 |
| Above 30N | 0.10 | North America | 0.10 | USA | 0.10 | Huntsville, AL | HAL | 0.09 |
| Above 30N | 0.10 | North America | 0.10 | USA | 0.10 | Lincoln, DE | LIN | 0.11 |
| Above 30N | 0.10 | North America | 0.10 | USA | 0.10 | Los Angeles | LOS | 0.13 |
| Above 30N | 0.10 | North America | 0.10 | USA | 0.10 | Macon, GA | MAC | 0.08 |
| Above 30N | 0.10 | North America | 0.10 | USA | 0.10 | Manassas, VA | MAN | 0.09 |
| Above 30N | 0.10 | North America | 0.10 | USA | 0.10 | Montgomery Co., TX | MON | 0.08 |
| Above 30N | 0.10 | North America | 0.10 | USA | 0.10 | New Bedford, MA | NEB | 0.10 |
| Above 30N | 0.10 | North America | 0.10 | USA | 0.10 | New Haven, CT | NEH | 0.09 |
| Above 30N | 0.10 | North America | 0.10 | USA | 0.10 | Newark, NJ | NEW | 0.10 |
| Above 30N | 0.10 | North America | 0.10 | USA | 0.10 | Peoria, IL | PEO | 0.09 |
| Above 30N | 0.10 | North America | 0.10 | USA | 0.10 | Russelville, AR | RUS | 0.12 |
| Above 30N | 0.10 | North America | 0.10 | USA | 0.10 | Smith Co., TX | SMI | 0.09 |
| Above 30N | 0.10 | North America | 0.10 | USA | 0.10 | Springfield, MO | SPR | 0.12 |
| Above 30N | 0.10 | North America | 0.10 | USA | 0.10 | St. Louis, MO | STL | 0.11 |
| Above 30N | 0.10 | North America | 0.10 | USA | 0.10 | Travis Co., TX | TRA | 0.08 |
| Above 30N | 0.10 | North America | 0.10 | USA | 0.10 | Waverly, VA | WAV | 0.10 |
| Below 30N | 0.12 | Caribbean | 0.16 | Jamaica | 0.23 | Negirl | NEG | 0.23 |
| Below 30N | 0.12 | Caribbean | 0.16 | Trinidad and Tobago | 0.11 | Saint Augustine | SAI | 0.11 |
| Below 30N | 0.12 | North America | 0.10 | USA | 0.10 | Houston, TX | HOU | 0.07 |
| Below 30N | 0.12 | North America | 0.10 | USA | 0.10 | New Orleans, LA | NEO | 0.09 |
| Below 30N | 0.12 | North America | 0.10 | USA | 0.10 | Nueces Co., TX | NUE | 0.08 |
| Below 30N | 0.12 | North America | 0.10 | USA | 0.10 | Palm Beach | PAL | 0.10 |
| Below 30N | 0.12 | South America | 0.13 | Argentina | 0.14 | Puerto Iguazu | POR | 0.14 |
| Below 30N | 0.12 | South America | 0.13 | Brazil | 0.12 | Alta Floresta | BAF | 0.11 |
| Below 30N | 0.12 | South America | 0.13 | Brazil | 0.12 | Araguaina | ARA | 0.11 |
| Below 30N | 0.12 | South America | 0.13 | Brazil | 0.12 | Belo Horizonte | BEH | 0.14 |
| Below 30N | 0.12 | South America | 0.13 | Brazil | 0.12 | Confresa | BCO | 0.12 |
| Below 30N | 0.12 | South America | 0.13 | Brazil | 0.12 | Floriano | FLO | 0.12 |
| Below 30N | 0.12 | South America | 0.13 | Brazil | 0.12 | Gravatai | GRV | 0.12 |
| Below 30N | 0.12 | South America | 0.13 | Brazil | 0.12 | Ipora | IPO | 0.18 |
| Below 30N | 0.12 | South America | 0.13 | Brazil | 0.12 | Itaituba | BIT | 0.12 |
| Below 30N | 0.12 | South America | 0.13 | Brazil | 0.12 | Manaus, AM | MAU | 0.11 |
| Below 30N | 0.12 | South America | 0.13 | Brazil | 0.12 | Nova Venecia, ES | NOV | 0.16 |
| Below 30N | 0.12 | South America | 0.13 | Brazil | 0.12 | Palmares | BPA | 0.11 |
| Below 30N | 0.12 | South America | 0.13 | Brazil | 0.12 | Palmas | PLM | 0.12 |
| Below 30N | 0.12 | South America | 0.13 | Brazil | 0.12 | Porto Velho | BPV | 0.12 |
| Below 30N | 0.12 | South America | 0.13 | Brazil | 0.12 | Recife, PE | REC | 0.10 |
| Below 30N | 0.12 | South America | 0.13 | Brazil | 0.12 | Tucurui, PA | TUC | 0.12 |
| Below 30N | 0.12 | South America | 0.13 | Colombia | 0.13 | Medellin | MED | 0.13 |
| Below 30N | 0.12 | South America | 0.13 | Colombia | 0.13 | Medellin | CUA | 0.14 |

```
# Initialize Word document
doc <- 
  read_docx() |>
  body_add_flextable(value = flex_table)

# Define the output path with 'here' library
output_path <- here("scripts", "RMarkdowns",
  "output",
  "americas",
  "americas_all",
  "fst", 
  "fst_americas_all_r01.docx"
  )

# Save the Word document
print(doc, target = output_path)
```

## 4.1.7. Scatter plots

To make scatter plot

```
# Group by Country and calculate the mean for mean_fst_by_country
aggregated_data <- fst4 |>
  dplyr::group_by(Country) |>
  dplyr::summarise(mean_fst = mean(mean_fst_by_country, na.rm = TRUE))

# save the data
saveRDS(aggregated_data, here("scripts", "RMarkdowns",
  "output", "americas", "americas_all", "fst", "LD2_country_america_all.rds"
))

# Order the aggregated data
aggregated_data <- aggregated_data[order(aggregated_data$mean_fst), ]

# Assign a numeric index for plotting
aggregated_data$index <- 1:nrow(aggregated_data)

# Fit a linear model
lm_fit <- lm(mean_fst ~ index, data = aggregated_data)

# Predicted values from the linear model
aggregated_data$fitted_values <- predict(lm_fit)

ggplot(aggregated_data, aes(x = index, y = mean_fst)) +
  geom_point(aes(color = Country), size = 3) +
  geom_line(aes(y = fitted_values), color = "blue") +  # Fitted line
  labs(
    title = "Mean Fst by Country",
    x = "Ordered Countries",
    y = "Mean Fst Value"
  ) +
  scale_x_continuous(breaks = aggregated_data$index, labels = aggregated_data$Country) +
  theme_classic() +
  theme(axis.text.x = element_text(angle = 45, hjust = 1)) +
  theme(legend.position = "none")
```

Estimate distances

```
# Grab the population names from the matrix aa
populations_with_fst <- colnames(aa)

# Subset the sampling_loc dataframe to only include populations with FST estimates
filtered_sampling_loc <- sampling_loc %>% filter(Abbreviation %in% populations_with_fst)

# Create an empty matrix to store the distances
n <- nrow(filtered_sampling_loc)
distance_matrix <- matrix(0, n, n)
rownames(distance_matrix) <- filtered_sampling_loc$Abbreviation
colnames(distance_matrix) <- filtered_sampling_loc$Abbreviation

# Calculate the distances
for (i in 1:n) {
  for (j in 1:n) {
    if (i != j) {
      coord1 <- c(filtered_sampling_loc$Longitude[i], filtered_sampling_loc$Latitude[i])
      coord2 <- c(filtered_sampling_loc$Longitude[j], filtered_sampling_loc$Latitude[j])
      distance_matrix[i, j] <- distHaversine(coord1, coord2) / 1000 # distance in km
    }
  }
}
# Print the distance matrix
head(distance_matrix)
```

```
##           BER       COL      PAL      HOU      LOS      MAU      REC       GRV
## BER    0.0000  681.5811 1531.526 2168.197 3869.221 5010.657 6733.168  8147.501
## COL  681.5811    0.0000 1500.468 1604.752 3187.646 5332.269 7272.529  8452.483
## PAL 1531.5257 1500.4678    0.000 1538.559 3736.715 3953.406 6222.233  7023.858
## HOU 2168.1972 1604.7524 1538.559    0.000 2210.698 5244.374 7710.420  8140.786
## LOS 3869.2209 3187.6459 3736.715 2210.698    0.000 7354.844 9914.923 10030.432
## MAU 5010.6569 5332.2689 3953.406 5244.374 7354.844    0.000 2840.159  3136.944
##          BRM      SAI       BEA        CHI       DAL       FAY       MAC
## BER 1234.854 3557.310  967.4761 3823.57400 2105.8658  630.8177 1097.6672
## COL 1834.421 3937.065  861.0315 3141.99576 1478.4203  648.0694  796.2625
## PAL 1601.195 2683.395  642.1071 3685.40896 1753.3347  939.0813  766.1127
## HOU 2919.355 4136.945 1427.7995 2159.35592  363.5529 1652.2966 1165.9449
## LOS 4926.755 6338.301 3480.9755   51.35935 1993.4465 3583.6693 3202.6468
## MAU 3971.576 1484.018 4516.8051 7305.75065 5561.5553 4684.9745 4709.5962
##           MAN       NEO       NEW       NUE      PEO       RUS       SPR
## BER  240.6821 1752.7895  123.2645 2480.0466 1249.957 1680.2747 1621.3667
## COL  492.8916 1285.0632  745.5006 1919.4940  574.158 1041.2023  952.8901
## PAL 1365.6197 1043.5958 1652.4417 1740.8520 1788.679 1570.0062 1708.4383
## HOU 1927.8001  515.4403 2271.2451  315.1368 1323.633  648.9484  851.2166
## LOS 3666.6095 2693.2540 3925.9493 2089.6509 2625.588 2296.9172 2279.2019
## MAU 4989.7745 4872.6090 5086.5174 5288.0717 5726.259 5491.1819 5650.8420
##          POR       NOV      TUC      MED       NEG      NEB       NEH       FIR
## BER 7579.277  7433.949 5486.324 3734.537 2418.3570  396.316  239.9150  197.5536
## COL 7868.169  7880.510 5927.677 3825.151 2455.4649 1025.512  855.4577  849.2138
## PAL 6431.363  6624.484 4702.789 2326.016  955.1584 1861.318 1752.4209 1690.2698
## HOU 7542.770  7988.458 6124.203 3338.628 2145.9325 2561.015 2393.4737 2364.3451
## LOS 9446.796 10110.775 8301.341 5365.924 4321.2878 4189.986 4026.1324 4029.9074
## MAU 2572.391  2756.252 1153.285 2016.015 3112.3904 5102.457 5114.0235 5049.7659
##           LIN       HAR       WAV       HAL       HAT       STL       TRA
## BER  110.9921  174.6320  360.1394 1168.6618 1596.9444 1322.2872 2323.9441
## COL  654.9661  514.0279  602.2321  662.3347 1116.7983  645.9952 1723.5200
## PAL 1420.7142 1537.8501 1182.9923 1090.3537 1033.0743 1631.0251 1779.2178
## HOU 2087.8104 2044.0692 1875.8593  999.5617  612.5473 1094.7102  242.1252
## LOS 3835.7943 3699.9409 3712.8163 2901.7616 2725.0655 2554.9081 1968.8560
## MAU 4931.2464 5123.5725 4803.9630 5042.6049 4925.4965 5582.7833 5469.1114
##           MON       SMI       DEN      BPV       BIT      FLO      ARA      PLM
## BER 2154.3152 1997.3713 2110.2195 5518.327 5273.7362 6134.118 5907.916 6210.779
## COL 1575.4412 1390.4694 1474.3014 5762.930 5641.3416 6619.995 6344.792 6630.544
## PAL 1584.4344 1597.5869 1797.8049 4311.594 4311.0212 5462.076 5101.924 5354.057
## HOU   78.9184  291.3002  420.6674 5448.579 5651.2030 6911.295 6502.972 6722.758
## LOS 2156.8227 2148.3759 1955.6448 7446.762 7785.3578 9099.400 8665.585 8862.229
## MAU 5315.0096 5417.5624 5615.8746  757.538  467.4255 1931.089 1387.862 1519.024
##          BCO       BAF      IPO      BEH       BPA      CUA
## BER 6113.023 5860.1227 6730.104 7386.220  7664.418 3734.537
## COL 6498.353 6198.9495 7092.144 7798.146  8068.479 3825.151
## PAL 5174.948 4824.2247 5731.015 6490.843  6746.764 2326.016
## HOU 6501.070 6094.4531 6997.734 7807.588  8042.548 3338.628
## LOS 8613.421 8173.4530 9055.763 9893.237 10106.270 5365.924
## MAU 1259.910  871.2168 1777.784 2563.793  2805.594 2016.015
```

Compare distance and FST

```
# Fill lower triangle of 'aa' matrix
aa[lower.tri(aa)] <- t(aa)[lower.tri(aa)]

# Fill diagonal with 0 (or another value that makes sense in your context)
diag(aa) <- 0


# Combine 'aa' and 'distance_matrix'
data <- data.frame(Distance = as.vector(distance_matrix), FST = as.vector(aa))

# Add row and column indices for easier tracking
data$row_index <- rep(rownames(distance_matrix), each = ncol(distance_matrix))
data$col_index <- rep(colnames(distance_matrix), nrow(distance_matrix))

data <- data |>
  dplyr::arrange(
    Distance
  )
head(data)
```

```
##   Distance FST row_index col_index
## 1        0   0       BER       BER
## 2        0   0       COL       COL
## 3        0   0       PAL       PAL
## 4        0   0       HOU       HOU
## 5        0   0       LOS       LOS
## 6        0   0       MAU       MAU
```

Fit linear regression

```
# Check for non-positive values in Distance
data <- data[data$Distance > 0, ]


# Fit linear model
lm_model <- lm(FST/(1-FST) ~ log(Distance), data = data)
equation_text <- sprintf("y = %.6fx + %.3f", coef(lm_model)[2], coef(lm_model)[1])
r2_text <- sprintf("R^2 = %.2f", summary(lm_model)$r.squared)


# source the plotting function
source(here("scripts", "RMarkdowns", "analyses", "my_theme2.R"))


# Plot
ggplot(data, aes(x = Distance, y = FST)) +
  geom_point() +
  geom_smooth(method = "lm", se = FALSE) +
  annotate("text", x = max(data$Distance) * 0.85, y = max(data$FST) * 0.95, label = paste(equation_text, r2_text, sep = "\n"), size = 4, color = "black") +
  labs(title = "FST vs Distance - All populations",
       x = "Distance (Km)",
       y = "FST/(1-FST)") +
  scale_x_continuous(labels = scales::comma) + 
  theme_classic()
```

```
## `geom_smooth()` using formula = 'y ~ x'
```

```
ggsave(
  filename = here("scripts", "RMarkdowns", "output", "americas", "americas_all", "fst", "fst_by_distance_americas_all.pdf"),
  width = 6,
  height = 4,
  units = "in"
)
```

## 4.1.8 Subset by countries with multiple pops

Select countries with at least 3 sampling localities

```
countries_with_3_pops <- filtered_sampling_loc %>%
  group_by(Country) %>%
  filter(n() >= 3) %>%
  pull(Country) %>%
  unique()
countries_with_3_pops
```

```
## [1] "USA"    "Brazil"
```

Do test for each country

```
results <- list()

for (country in countries_with_3_pops) {
  # Extract abbreviations for the country
  abbreviations <- filtered_sampling_loc %>%
    filter(Country == country) %>%
    pull(Abbreviation)
  
  # Subset the data
  subset_data <- data %>%
    filter(row_index %in% abbreviations & col_index %in% abbreviations)
  subset_data <- subset_data[subset_data$Distance > 0, ]
  # Perform linear regression
  lm_model <- lm(FST/(1-FST) ~ log(Distance), data = subset_data)
  results[[country]] <- list(
    equation = sprintf("y = %.5fx + %.3f", coef(lm_model)[2], coef(lm_model)[1]),
    r2 = sprintf("R^2 = %.2f", summary(lm_model)$r.squared)
  )
}

results
```

```
## $USA
## $USA$equation
## [1] "y = -0.00577x + 0.169"
## 
## $USA$r2
## [1] "R^2 = 0.00"
## 
## 
## $Brazil
## $Brazil$equation
## [1] "y = 0.01688x + -0.002"
## 
## $Brazil$r2
## [1] "R^2 = 0.02"
```

Merge the data

*need to update list*

```
data_merged <- data %>%
  left_join(filtered_sampling_loc[, c("Pop_City", "Country", "Abbreviation")], by = c("row_index" = "Abbreviation")) %>%
  rename(Country1 = Country) %>%
  left_join(filtered_sampling_loc[, c("Pop_City", "Country", "Abbreviation")], by = c("col_index" = "Abbreviation")) %>%
  dplyr::select(-Pop_City.x, -Pop_City.y) %>%
  filter(Country1 == Country) # Ensures the data is within the same country


# Filter to get the coutries with 3 or more sampling localities
countries_to_include <- c("USA", "Brazil")

# Filter
data_filtered <- data_merged %>%
  group_by(Country1) %>%
  filter(n() >= 3 & Country1 %in% countries_to_include) %>%
  ungroup()
```

Calculate linear regression for each country

```
regression_results <- data_filtered %>%
  group_by(Country1) %>%
  do(model = lm(FST/(1-FST) ~ log(Distance), data = .)) %>%
  rowwise() %>%
  mutate(equation = sprintf("italic(y) == %.3f * italic(x) + %.3f", coef(model)[2], coef(model)[1]),
         r2 = sprintf("italic(R)^2 == %.2f", summary(model)$r.squared))
```

Plot it

```
ggplot(data_filtered, aes(x = log(Distance), y = FST/(1-FST))) +
  geom_point() +
  geom_smooth(method = "lm", se = FALSE) +
  facet_wrap(~ Country1, scales = "free", ncol = 2) +
  geom_text(
    data = regression_results, 
    aes(label = paste(equation, r2, sep = "~~")), 
    x = Inf, y = Inf, 
    vjust = 2, hjust = 1.2, 
    size = 3.5, 
    parse = TRUE, 
    inherit.aes = FALSE
  ) +
  labs(title = "Fst vs Distance by Country",
       x = "Log(Distance)",
       y = "Fst/(1-Fst)") +
  scale_x_continuous(labels = scales::comma) + 
  theme_bw()
```

```
## `geom_smooth()` using formula = 'y ~ x'
```

```
ggsave(
  filename = here("scripts", "RMarkdowns", "output", "americas","americas_all", "fst", "fst_by_distance_USA_Brazil_LD3.pdf"),
  width = 8,
  height = 10,
  units = "in"
)
```

We can merge the FST and distance matrices

```
# Ensure the matrices have the same names in the same order
common_names <- intersect(rownames(distance_matrix), rownames(aa))
sorted_names <- sort(common_names)

# Reorder the matrices
distance_matrix <- distance_matrix[sorted_names, sorted_names]
aa <- aa[sorted_names, sorted_names]

# Initialize the final merged matrix
merged_matrix <- matrix(NA, nrow = nrow(aa), ncol = ncol(aa))
rownames(merged_matrix) <- sorted_names
colnames(merged_matrix) <- sorted_names

# Fill the upper triangular part from aa
merged_matrix[upper.tri(merged_matrix, diag = FALSE)] <- aa[upper.tri(aa, diag = FALSE)]

# Fill the lower triangular part from distance_matrix
merged_matrix[lower.tri(merged_matrix, diag = FALSE)] <- distance_matrix[lower.tri(distance_matrix, diag = FALSE)]

# Format the matrix (Fst two decimals and distance in Km with zero decimals)
# Format the elements based on their position in the matrix
for(i in 1:nrow(merged_matrix)) {
  for(j in 1:ncol(merged_matrix)) {
    if (i < j) {
      # Upper triangular - Fst values with two decimal places
      merged_matrix[i, j] <- sprintf("%.2f", as.numeric(merged_matrix[i, j]))
    } else if (i > j) {
      # Lower triangular - Distance values with zero decimal places
      merged_matrix[i, j] <- sprintf("%.0f", as.numeric(merged_matrix[i, j]))
    }
  }
}

# Now the merged_matrix should be formatted as you need
print(merged_matrix)
```

```
##     ARA    BAF    BCO    BEA    BEH    BER    BIT    BPA     BPV    BRM   
## ARA NA     "0.00" "0.02" "0.11" "0.05" "0.15" "0.04" "0.06"  "0.06" "0.16"
## BAF "917"  NA     "0.02" "0.11" "0.05" "0.15" "0.05" "0.06"  "0.05" "0.17"
## BCO "532"  "505"  NA     "0.12" "0.06" "0.16" "0.06" "0.08"  "0.07" "0.17"
## BEA "5596" "5387" "5711" NA     "0.15" "0.04" "0.12" "0.08"  "0.12" "0.08"
## BEH "1491" "1721" "1316" "7022" NA     "0.19" "0.07" "0.10"  "0.06" "0.20"
## BER "5908" "5860" "6113" "967"  "7386" NA     "0.15" "0.12"  "0.15" "0.09"
## BIT "922"  "624"  "864"  "4849" "2180" "5274" NA     "0.08"  "0.05" "0.17"
## BPA "1776" "1948" "1574" "7286" "288"  "7664" "2438" NA      "0.08" "0.14"
## BPV "1737" "866"  "1372" "4917" "2485" "5518" "1004" "2671"  NA     "0.17"
## BRM "4735" "4784" "4985" "1494" "6224" "1235" "4175" "6507"  "4567" NA    
## CHI "8615" "8125" "8564" "3431" "9845" "3824" "7736" "10059" "7400" "4878"
## COL "6345" "6199" "6498" "861"  "7798" "682"  "5641" "8068"  "5763" "1834"
## CUA "3387" "2808" "3261" "2964" "4527" "3735" "2471" "4743"  "2111" "3109"
## DAL "6797" "6418" "6814" "1510" "8125" "2106" "5959" "8365"  "5789" "2994"
## DEN "6849" "6473" "6868" "1538" "8179" "2110" "6013" "8419"  "5845" "3017"
## FAY "5704" "5551" "5851" "337"  "7152" "631"  "4994" "7422"  "5126" "1343"
## FIR "5903" "5891" "6127" "1151" "7387" "198"  "5296" "7668"  "5580" "1186"
## FLO "575"  "1480" "1032" "5915" "1466" "6134" "1465" "1745"  "2312" "4925"
## GRV "2549" "2296" "2148" "7619" "1323" "8148" "2906" "1126"  "2718" "7080"
## HAL "6165" "5914" "6258" "599"  "7573" "1169" "5394" "7831"  "5395" "2032"
## HAR "6047" "5978" "6241" "935"  "7521" "175"  "5397" "7798"  "5614" "1399"
## HAT "6119" "5791" "6166" "816"  "7482" "1597" "5305" "7730"  "5214" "2310"
## HOU "6503" "6094" "6501" "1428" "7808" "2168" "5651" "8043"  "5449" "2919"
## IPO "1078" "909"  "646"  "6288" "854"  "6730" "1457" "1051"  "1631" "5618"
## LIN "5847" "5784" "6044" "859"  "7322" "111"  "5201" "7599"  "5430" "1208"
## LOS "8666" "8173" "8613" "3481" "9893" "3869" "7785" "10106" "7447" "4927"
## MAC "5823" "5581" "5920" "279"  "7234" "1098" "5056" "7494"  "5078" "1766"
## MAN "5939" "5848" "6121" "765"  "7407" "241"  "5273" "7682"  "5468" "1348"
## MAU "1388" "871"  "1260" "4517" "2564" "5011" "467"  "2806"  "758"  "3972"
## MED "3387" "2808" "3261" "2964" "4527" "3735" "2471" "4743"  "2111" "3109"
## MON "6569" "6166" "6571" "1443" "7879" "2154" "5720" "8114"  "5524" "2937"
## NEB "5911" "5934" "6153" "1341" "7399" "396"  "5332" "7681"  "5654" "1176"
## NEG "4358" "3974" "4363" "1596" "5675" "2418" "3508" "5918"  "3398" "2070"
## NEH "5961" "5954" "6187" "1204" "7446" "240"  "5358" "7727"  "5646" "1238"
## NEO "6089" "5735" "6120" "928"  "7434" "1753" "5261" "7678"  "5137" "2412"
## NEW "5961" "5932" "6176" "1091" "7442" "123"  "5341" "7722"  "5605" "1258"
## NOV "1536" "1956" "1497" "7130" "394"  "7434" "2337" "522"   "2769" "6245"
## NUE "6580" "6124" "6548" "1709" "7844" "2480" "5709" "8070"  "5444" "3191"
## PAL "5102" "4824" "5175" "642"  "6491" "1532" "4311" "6747"  "4312" "1601"
## PEO "6806" "6597" "6926" "1214" "8236" "1250" "6063" "8500"  "6100" "2399"
## PLM "341"  "852"  "355"  "5867" "1176" "6211" "1077" "1457"  "1717" "5050"
## POR "2158" "1758" "1694" "7030" "1263" "7579" "2380" "1189"  "2122" "6535"
## REC "1474" "2340" "1853" "6626" "1641" "6733" "2374" "1844"  "3195" "5501"
## RUS "6671" "6358" "6729" "1192" "8045" "1680" "5866" "8295"  "5781" "2634"
## SAI "2427" "2308" "2564" "3171" "3861" "3557" "1717" "4132"  "2119" "2488"
## SMI "6647" "6276" "6669" "1364" "7981" "1997" "5813" "8222"  "5655" "2852"
## SPR "6808" "6520" "6881" "1265" "8197" "1621" "6018" "8451"  "5959" "2652"
## STL "6694" "6454" "6795" "1101" "8109" "1322" "5931" "8369"  "5933" "2401"
## TRA "6736" "6315" "6727" "1639" "8031" "2324" "5880" "8263"  "5657" "3133"
## TUC "422"  "988"  "802"  "5187" "1912" "5486" "705"  "2196"  "1669" "4313"
## WAV "5770" "5664" "5943" "608"  "7234" "360"  "5092" "7507"  "5276" "1243"
##     CHI     COL    CUA    DAL    DEN    FAY    FIR    FLO    GRV     HAL   
## ARA "0.14"  "0.15" "0.12" "0.13" "0.14" "0.12" "0.18" "0.02" "0.06"  "0.14"
## BAF "0.14"  "0.15" "0.12" "0.14" "0.14" "0.12" "0.18" "0.01" "0.06"  "0.14"
## BCO "0.15"  "0.16" "0.13" "0.15" "0.15" "0.13" "0.19" "0.03" "0.08"  "0.15"
## BEA "0.09"  "0.05" "0.11" "0.03" "0.03" "0.02" "0.08" "0.12" "0.12"  "0.03"
## BEH "0.16"  "0.19" "0.13" "0.18" "0.18" "0.17" "0.22" "0.06" "0.08"  "0.18"
## BER "0.13"  "0.05" "0.15" "0.04" "0.03" "0.04" "0.07" "0.16" "0.15"  "0.03"
## BIT "0.14"  "0.15" "0.13" "0.13" "0.14" "0.13" "0.18" "0.06" "0.07"  "0.14"
## BPA "0.13"  "0.12" "0.14" "0.10" "0.10" "0.09" "0.14" "0.07" "0.10"  "0.11"
## BPV "0.14"  "0.15" "0.13" "0.14" "0.14" "0.13" "0.19" "0.07" "0.08"  "0.14"
## BRM "0.15"  "0.10" "0.16" "0.09" "0.09" "0.08" "0.13" "0.17" "0.17"  "0.09"
## CHI NA      "0.13" "0.13" "0.12" "0.12" "0.10" "0.17" "0.15" "0.14"  "0.12"
## COL "3142"  NA     "0.15" "0.04" "0.04" "0.04" "0.08" "0.16" "0.15"  "0.02"
## CUA "5318"  "3825" NA     "0.14" "0.14" "0.13" "0.19" "0.13" "0.13"  "0.14"
## DAL "1942"  "1478" "3682" NA     "0.02" "0.02" "0.07" "0.15" "0.14"  "0.03"
## DEN "1905"  "1474" "3738" "58"   NA     "0.02" "0.07" "0.15" "0.14"  "0.03"
## FAY "3535"  "648"  "3228" "1670" "1689" NA     "0.07" "0.13" "0.13"  "0.03"
## FIR "3985"  "849"  "3845" "2296" "2298" "816"  NA     "0.19" "0.18"  "0.07"
## FLO "9049"  "6620" "3896" "7189" "7238" "5991" "6109" NA     "0.07"  "0.15"
## GRV "9987"  "8452" "4808" "8488" "8545" "7808" "8184" "2709" NA      "0.14"
## HAL "2852"  "662"  "3368" "976"  "993"  "696"  "1365" "6499" "8110"  NA    
## HAR "3655"  "514"  "3790" "1962" "1964" "604"  "337"  "6283" "8260"  "1049"
## HAT "2674"  "1117" "3136" "732"  "771"  "1047" "1794" "6491" "7930"  "456" 
## HOU "2159"  "1605" "3339" "364"  "421"  "1652" "2364" "6911" "8141"  "1000"
## IPO "9008"  "7092" "3692" "7323" "7379" "6444" "6750" "1391" "1502"  "6820"
## LIN "3789"  "655"  "3631" "2040" "2047" "523"  "295"  "6083" "8068"  "1089"
## LOS "51"    "3188" "5366" "1993" "1956" "3584" "4030" "9099" "10030" "2902"
## MAC "3152"  "796"  "3076" "1232" "1260" "502"  "1293" "6156" "7789"  "343" 
## MAN "3620"  "493"  "3625" "1872" "1878" "432"  "438"  "6189" "8125"  "928" 
## MAU "7306"  "5332" "2016" "5562" "5616" "4685" "5050" "1931" "3137"  "5043"
## MED "5318"  "3825" "0"    "3682" "3738" "3228" "3845" "3896" "4808"  "3368"
## MON "2105"  "1575" "3414" "285"  "342"  "1654" "2350" "6974" "8217"  "988" 
## NEB "4146"  "1026" "3969" "2486" "2487" "1009" "199"  "6095" "8229"  "1562"
## NEG "4271"  "2455" "1372" "2451" "2505" "1873" "2551" "4778" "6116"  "2007"
## NEH "3982"  "855"  "3913" "2315" "2316" "869"  "68"   "6162" "8247"  "1395"
## NEO "2642"  "1285" "3046" "715"  "764"  "1185" "1950" "6475" "7850"  "624" 
## NEW "3881"  "746"  "3842" "2196" "2198" "754"  "104"  "6175" "8222"  "1273"
## NOV "10062" "7881" "4756" "8295" "8348" "7240" "7422" "1358" "1647"  "7695"
## NUE "2039"  "1919" "3336" "567"  "613"  "1951" "2677" "7010" "8110"  "1312"
## PAL "3685"  "1500" "2326" "1753" "1798" "939"  "1690" "5462" "7024"  "1090"
## PEO "2581"  "574"  "4081" "1089" "1067" "1127" "1405" "7110" "8812"  "713" 
## PLM "8813"  "6631" "3535" "7026" "7079" "5986" "6212" "701"  "2209"  "6427"
## POR "9403"  "7868" "4210" "7890" "7947" "7225" "7622" "2428" "599"   "7517"
## REC "9864"  "7273" "4790" "7969" "8016" "6665" "6683" "909"  "2959"  "7221"
## RUS "2247"  "1041" "3698" "439"  "433"  "1293" "1867" "7030" "8497"  "605" 
## SAI "6287"  "3937" "1614" "4408" "4457" "3291" "3583" "2782" "4603"  "3739"
## SMI "2097"  "1390" "3551" "156"  "202"  "1538" "2190" "7036" "8359"  "848" 
## SPR "2231"  "953"  "3886" "589"  "565"  "1312" "1799" "7154" "8677"  "666" 
## STL "2508"  "646"  "3894" "883"  "867"  "1079" "1495" "7017" "8650"  "541" 
## TRA "1918"  "1724" "3546" "288"  "328"  "1841" "2518" "7149" "8340"  "1164"
## TUC "8251"  "5928" "3084" "6411" "6462" "5288" "5481" "814"  "2925"  "5759"
## WAV "3665"  "602"  "3431" "1857" "1869" "272"  "545"  "6030" "7937"  "887" 
##     HAR    HAT    HOU    IPO    LIN    LOS     MAC    MAN    MAU    MED   
## ARA "0.15" "0.14" "0.11" "0.07" "0.16" "0.14"  "0.12" "0.14" "0.06" "0.12"
## BAF "0.15" "0.14" "0.11" "0.06" "0.17" "0.15"  "0.12" "0.14" "0.05" "0.12"
## BCO "0.16" "0.15" "0.12" "0.09" "0.17" "0.16"  "0.13" "0.15" "0.07" "0.13"
## BEA "0.04" "0.03" "0.01" "0.19" "0.06" "0.09"  "0.02" "0.03" "0.10" "0.11"
## BEH "0.19" "0.18" "0.14" "0.06" "0.20" "0.17"  "0.15" "0.18" "0.07" "0.13"
## BER "0.05" "0.05" "0.04" "0.23" "0.07" "0.12"  "0.05" "0.03" "0.13" "0.14"
## BIT "0.15" "0.13" "0.11" "0.09" "0.16" "0.15"  "0.12" "0.14" "0.05" "0.13"
## BPA "0.12" "0.11" "0.08" "0.13" "0.13" "0.14"  "0.09" "0.10" "0.08" "0.13"
## BPV "0.16" "0.14" "0.11" "0.09" "0.16" "0.15"  "0.12" "0.14" "0.04" "0.13"
## BRM "0.10" "0.10" "0.08" "0.24" "0.12" "0.14"  "0.09" "0.09" "0.15" "0.16"
## CHI "0.13" "0.12" "0.08" "0.20" "0.14" "0.10"  "0.10" "0.11" "0.13" "0.12"
## COL "0.05" "0.05" "0.04" "0.24" "0.06" "0.12"  "0.06" "0.03" "0.13" "0.15"
## CUA "0.15" "0.14" "0.11" "0.17" "0.16" "0.14"  "0.12" "0.14" "0.11" "0.00"
## DAL "0.05" "0.03" "0.02" "0.22" "0.06" "0.11"  "0.05" "0.03" "0.12" "0.13"
## DEN "0.04" "0.02" "0.03" "0.22" "0.05" "0.11"  "0.05" "0.03" "0.12" "0.14"
## FAY "0.04" "0.03" "0.02" "0.21" "0.05" "0.10"  "0.03" "0.02" "0.11" "0.12"
## FIR "0.08" "0.08" "0.07" "0.26" "0.10" "0.15"  "0.09" "0.06" "0.17" "0.18"
## FLO "0.16" "0.15" "0.12" "0.08" "0.18" "0.16"  "0.13" "0.15" "0.06" "0.13"
## GRV "0.15" "0.14" "0.11" "0.06" "0.17" "0.15"  "0.13" "0.14" "0.07" "0.12"
## HAL "0.04" "0.04" "0.03" "0.22" "0.05" "0.11"  "0.05" "0.02" "0.12" "0.13"
## HAR NA     "0.04" "0.04" "0.23" "0.07" "0.12"  "0.06" "0.04" "0.13" "0.15"
## HAT "1491" NA     "0.03" "0.22" "0.07" "0.12"  "0.05" "0.04" "0.12" "0.14"
## HOU "2044" "613"  NA     "0.18" "0.06" "0.09"  "0.02" "0.03" "0.09" "0.10"
## IPO "6854" "6700" "6998" NA     "0.24" "0.21"  "0.20" "0.22" "0.09" "0.17"
## LIN "200"  "1508" "2088" "6658" NA     "0.13"  "0.07" "0.05" "0.14" "0.16"
## LOS "3700" "2725" "2211" "9056" "3836" NA      "0.10" "0.11" "0.13" "0.13"
## MAC "1023" "554"  "1166" "6486" "998"  "3203"  NA     "0.04" "0.10" "0.11"
## MAN "173"  "1357" "1928" "6729" "171"  "3667"  "867"  NA     "0.12" "0.14"
## MAU "5124" "4925" "5244" "1778" "4931" "7355"  "4710" "4990" NA     "0.11"
## MED "3790" "3136" "3339" "3692" "3631" "5366"  "3076" "3625" "2016" NA    
## MON "2026" "627"  "79"   "7070" "2077" "2157"  "1176" "1915" "5315" "3414"
## NEB "523"  "1993" "2561" "6782" "492"  "4190"  "1490" "636"  "5102" "3969"
## NEG "2453" "1822" "2146" "4882" "2311" "4321"  "1706" "2284" "3112" "1372"
## NEH "353"  "1830" "2393" "6812" "346"  "4026"  "1337" "474"  "5114" "3913"
## NEO "1651" "168"  "515"  "6643" "1661" "2693"  "685"  "1514" "4873" "3046"
## NEW "235"  "1707" "2271" "6797" "234"  "3926"  "1216" "352"  "5087" "3842"
## NOV "7576" "7632" "7988" "1166" "7376" "10111" "7354" "7471" "2756" "4756"
## NUE "2358" "905"  "315"  "7021" "2398" "2090"  "1457" "2239" "5288" "3336"
## PAL "1538" "1033" "1539" "5731" "1421" "3737"  "766"  "1366" "3953" "2326"
## PEO "1078" "1040" "1324" "7500" "1229" "2626"  "1023" "1066" "5726" "4081"
## PLM "6346" "6361" "6723" "753"  "6147" "8862"  "6086" "6234" "1519" "3535"
## POR "7688" "7333" "7543" "1080" "7498" "9447"  "7197" "7549" "2572" "4210"
## REC "6892" "7255" "7710" "1997" "6695" "9915"  "6880" "6817" "2840" "4790"
## RUS "1532" "568"  "649"  "7267" "1620" "2297"  "917"  "1450" "5491" "3698"
## SAI "3681" "3710" "4137" "3173" "3484" "6338"  "3396" "3558" "1484" "1614"
## SMI "1860" "577"  "291"  "7182" "1927" "2148"  "1087" "1760" "5418" "3551"
## SPR "1462" "750"  "851"  "7428" "1574" "2279"  "1004" "1403" "5651" "3886"
## STL "1159" "815"  "1095" "7360" "1282" "2555"  "875"  "1112" "5583" "3894"
## TRA "2189" "824"  "242"  "7217" "2250" "1969"  "1369" "2086" "5469" "3546"
## TUC "5626" "5724" "6124" "1429" "5426" "8301"  "5417" "5519" "1153" "3084"
## WAV "360"  "1283" "1876" "6548" "251"  "3713"  "757"  "195"  "4804" "3431"
##     MON    NEB    NEG    NEH    NEO    NEW    NOV    NUE    PAL    PEO   
## ARA "0.11" "0.15" "0.20" "0.14" "0.14" "0.15" "0.07" "0.12" "0.13" "0.13"
## BAF "0.12" "0.16" "0.21" "0.14" "0.14" "0.15" "0.06" "0.12" "0.13" "0.13"
## BCO "0.13" "0.16" "0.21" "0.15" "0.15" "0.16" "0.08" "0.13" "0.14" "0.14"
## BEA "0.02" "0.04" "0.20" "0.04" "0.04" "0.05" "0.17" "0.03" "0.04" "0.03"
## BEH "0.16" "0.19" "0.21" "0.19" "0.18" "0.19" "0.04" "0.15" "0.16" "0.17"
## BER "0.05" "0.05" "0.25" "0.03" "0.05" "0.05" "0.22" "0.06" "0.07" "0.04"
## BIT "0.11" "0.15" "0.21" "0.15" "0.14" "0.15" "0.09" "0.11" "0.13" "0.14"
## BPA "0.09" "0.12" "0.22" "0.11" "0.10" "0.12" "0.12" "0.09" "0.10" "0.10"
## BPV "0.12" "0.16" "0.21" "0.15" "0.14" "0.16" "0.08" "0.12" "0.13" "0.14"
## BRM "0.09" "0.10" "0.26" "0.09" "0.10" "0.10" "0.22" "0.09" "0.10" "0.08"
## CHI "0.10" "0.13" "0.23" "0.12" "0.12" "0.13" "0.17" "0.10" "0.11" "0.11"
## COL "0.05" "0.05" "0.25" "0.04" "0.06" "0.06" "0.22" "0.06" "0.08" "0.03"
## CUA "0.12" "0.15" "0.22" "0.14" "0.14" "0.15" "0.15" "0.12" "0.13" "0.14"
## DAL "0.01" "0.04" "0.24" "0.04" "0.04" "0.05" "0.20" "0.02" "0.06" "0.03"
## DEN "0.03" "0.03" "0.24" "0.03" "0.04" "0.03" "0.21" "0.04" "0.06" "0.03"
## FAY "0.03" "0.04" "0.23" "0.03" "0.03" "0.04" "0.19" "0.03" "0.05" "0.03"
## FIR "0.08" "0.07" "0.29" "0.07" "0.08" "0.08" "0.25" "0.09" "0.11" "0.07"
## FLO "0.13" "0.16" "0.22" "0.15" "0.15" "0.16" "0.08" "0.13" "0.14" "0.15"
## GRV "0.13" "0.15" "0.21" "0.14" "0.14" "0.16" "0.07" "0.12" "0.13" "0.14"
## HAL "0.04" "0.03" "0.24" "0.02" "0.04" "0.05" "0.20" "0.05" "0.06" "0.02"
## HAR "0.05" "0.05" "0.25" "0.03" "0.06" "0.05" "0.22" "0.06" "0.08" "0.04"
## HAT "0.04" "0.05" "0.24" "0.04" "0.05" "0.04" "0.20" "0.05" "0.07" "0.04"
## HOU "0.02" "0.04" "0.20" "0.03" "0.03" "0.04" "0.16" "0.02" "0.03" "0.03"
## IPO "0.20" "0.24" "0.26" "0.23" "0.22" "0.23" "0.04" "0.20" "0.20" "0.22"
## LIN "0.07" "0.06" "0.26" "0.06" "0.07" "0.07" "0.23" "0.08" "0.09" "0.05"
## LOS "0.10" "0.12" "0.22" "0.11" "0.12" "0.13" "0.18" "0.10" "0.11" "0.11"
## MAC "0.03" "0.06" "0.21" "0.05" "0.04" "0.06" "0.18" "0.03" "0.04" "0.05"
## MAN "0.04" "0.03" "0.24" "0.02" "0.04" "0.04" "0.20" "0.05" "0.06" "0.03"
## MAU "0.11" "0.13" "0.20" "0.13" "0.12" "0.13" "0.08" "0.11" "0.11" "0.12"
## MED "0.11" "0.15" "0.21" "0.14" "0.13" "0.15" "0.14" "0.11" "0.12" "0.13"
## MON NA     "0.05" "0.21" "0.04" "0.04" "0.06" "0.18" "0.01" "0.05" "0.04"
## NEB "2545" NA     "0.25" "0.02" "0.05" "0.05" "0.22" "0.06" "0.07" "0.04"
## NEG "2213" "2698" NA     "0.25" "0.24" "0.24" "0.25" "0.21" "0.22" "0.23"
## NEH "2377" "171"  "2618" NA     "0.05" "0.04" "0.21" "0.05" "0.06" "0.03"
## NEO "549"  "2149" "1761" "1988" NA     "0.05" "0.20" "0.05" "0.07" "0.04"
## NEW "2255" "290"  "2533" "123"  "1865" NA     "0.21" "0.07" "0.07" "0.05"
## NOV "8057" "7420" "5845" "7478" "7594" "7483" NA     "0.17" "0.18" "0.20"
## NUE "350"  "2874" "2232" "2707" "781"  "2584" "8042" NA     "0.05" "0.05"
## PAL "1584" "1861" "955"  "1752" "1044" "1652" "6624" "1741" NA     "0.06"
## PEO "1271" "1566" "2718" "1401" "1191" "1301" "8341" "1618" "1789" NA    
## PLM "6791" "6224" "4578" "6270" "6324" "6267" "1271" "6785" "5354" "7079"
## POR "7619" "7674" "5520" "7686" "7251" "7657" "1650" "7514" "6431" "8220"
## REC "7769" "6643" "5602" "6731" "7254" "6759" "1327" "7830" "6222" "7792"
## RUS "594"  "2054" "2391" "1883" "657"  "1766" "8189" "941"  "1570" "677" 
## SAI "4198" "3627" "2031" "3646" "3693" "3626" "3960" "4254" "2683" "4382"
## SMI "225"  "2383" "2306" "2212" "561"  "2092" "8148" "566"  "1598" "1053"
## SPR "790"  "1978" "2563" "1808" "858"  "1696" "8333" "1130" "1708" "501" 
## STL "1045" "1671" "2540" "1501" "961"  "1391" "8227" "1394" "1631" "235" 
## TRA "199"  "2711" "2379" "2542" "747"  "2421" "8217" "290"  "1779" "1369"
## TUC "6189" "5488" "3983" "5539" "5700" "5539" "1953" "6212" "4703" "6394"
## WAV "1871" "739"  "2093" "597"  "1431" "483"  "7303" "2182" "1183" "1157"
##     PLM    POR    REC    RUS    SAI    SMI    SPR    STL    TRA    TUC   
## ARA "0.02" "0.05" "0.07" "0.16" "0.12" "0.13" "0.17" "0.16" "0.12" "0.01"
## BAF "0.01" "0.04" "0.06" "0.17" "0.12" "0.14" "0.18" "0.16" "0.12" "0.01"
## BCO "0.02" "0.06" "0.08" "0.17" "0.13" "0.15" "0.18" "0.17" "0.13" "0.02"
## BEA "0.12" "0.14" "0.08" "0.06" "0.06" "0.04" "0.06" "0.05" "0.01" "0.12"
## BEH "0.05" "0.05" "0.10" "0.20" "0.14" "0.18" "0.22" "0.20" "0.15" "0.05"
## BER "0.16" "0.18" "0.11" "0.08" "0.10" "0.05" "0.06" "0.04" "0.04" "0.16"
## BIT "0.06" "0.07" "0.09" "0.16" "0.12" "0.13" "0.17" "0.16" "0.12" "0.04"
## BPA "0.07" "0.11" "0.03" "0.13" "0.11" "0.10" "0.14" "0.13" "0.09" "0.07"
## BPV "0.06" "0.06" "0.07" "0.17" "0.12" "0.14" "0.17" "0.16" "0.12" "0.06"
## BRM "0.17" "0.19" "0.14" "0.12" "0.12" "0.10" "0.12" "0.11" "0.08" "0.17"
## CHI "0.15" "0.15" "0.13" "0.15" "0.10" "0.12" "0.16" "0.14" "0.10" "0.14"
## COL "0.16" "0.18" "0.11" "0.08" "0.10" "0.06" "0.07" "0.04" "0.04" "0.16"
## CUA "0.13" "0.13" "0.13" "0.17" "0.12" "0.14" "0.18" "0.16" "0.12" "0.12"
## DAL "0.15" "0.17" "0.09" "0.07" "0.09" "0.02" "0.07" "0.05" "0.02" "0.14"
## DEN "0.15" "0.17" "0.10" "0.06" "0.09" "0.04" "0.05" "0.04" "0.02" "0.15"
## FAY "0.13" "0.16" "0.09" "0.07" "0.07" "0.04" "0.06" "0.05" "0.02" "0.13"
## FIR "0.19" "0.21" "0.14" "0.11" "0.13" "0.09" "0.11" "0.08" "0.08" "0.18"
## FLO "0.02" "0.06" "0.08" "0.18" "0.13" "0.15" "0.19" "0.17" "0.13" "0.02"
## GRV "0.07" "0.03" "0.10" "0.16" "0.13" "0.14" "0.17" "0.16" "0.12" "0.07"
## HAL "0.15" "0.17" "0.10" "0.07" "0.09" "0.05" "0.05" "0.04" "0.04" "0.14"
## HAR "0.16" "0.18" "0.11" "0.08" "0.10" "0.06" "0.07" "0.06" "0.04" "0.16"
## HAT "0.15" "0.17" "0.10" "0.07" "0.09" "0.04" "0.07" "0.06" "0.03" "0.14"
## HOU "0.12" "0.14" "0.08" "0.06" "0.06" "0.03" "0.06" "0.05" "0.01" "0.12"
## IPO "0.07" "0.04" "0.14" "0.25" "0.18" "0.22" "0.27" "0.25" "0.20" "0.07"
## LIN "0.18" "0.19" "0.12" "0.10" "0.11" "0.07" "0.08" "0.07" "0.06" "0.17"
## LOS "0.15" "0.16" "0.13" "0.14" "0.11" "0.12" "0.14" "0.13" "0.09" "0.15"
## MAC "0.12" "0.15" "0.08" "0.08" "0.06" "0.05" "0.08" "0.06" "0.03" "0.13"
## MAN "0.15" "0.17" "0.10" "0.06" "0.08" "0.05" "0.05" "0.04" "0.03" "0.15"
## MAU "0.06" "0.06" "0.08" "0.15" "0.11" "0.13" "0.15" "0.14" "0.10" "0.06"
## MED "0.13" "0.13" "0.13" "0.16" "0.11" "0.14" "0.17" "0.16" "0.11" "0.12"
## MON "0.13" "0.15" "0.08" "0.07" "0.07" "0.02" "0.07" "0.06" "0.02" "0.12"
## NEB "0.16" "0.18" "0.11" "0.08" "0.10" "0.06" "0.07" "0.05" "0.04" "0.16"
## NEG "0.21" "0.21" "0.21" "0.26" "0.20" "0.24" "0.29" "0.27" "0.21" "0.20"
## NEH "0.16" "0.18" "0.11" "0.07" "0.09" "0.05" "0.06" "0.04" "0.03" "0.15"
## NEO "0.15" "0.17" "0.10" "0.08" "0.09" "0.05" "0.07" "0.06" "0.03" "0.14"
## NEW "0.16" "0.18" "0.11" "0.08" "0.10" "0.05" "0.07" "0.06" "0.04" "0.16"
## NOV "0.06" "0.04" "0.13" "0.23" "0.16" "0.21" "0.25" "0.23" "0.18" "0.07"
## NUE "0.13" "0.15" "0.09" "0.08" "0.07" "0.03" "0.08" "0.07" "0.02" "0.12"
## PAL "0.14" "0.16" "0.10" "0.10" "0.07" "0.07" "0.10" "0.09" "0.05" "0.13"
## PEO "0.14" "0.17" "0.09" "0.06" "0.09" "0.05" "0.06" "0.04" "0.03" "0.14"
## PLM NA     "0.05" "0.08" "0.18" "0.13" "0.15" "0.18" "0.17" "0.13" "0.02"
## POR "1831" NA     "0.11" "0.19" "0.14" "0.17" "0.20" "0.19" "0.15" "0.05"
## REC "1498" "2858" NA     "0.13" "0.10" "0.10" "0.13" "0.12" "0.08" "0.07"
## RUS "6918" "7899" "7777" NA     "0.12" "0.08" "0.09" "0.08" "0.06" "0.17"
## SAI "2698" "4050" "3577" "4253" NA     "0.09" "0.12" "0.11" "0.07" "0.12"
## SMI "6878" "7760" "7814" "377"  "4254" NA     "0.08" "0.06" "0.03" "0.14"
## SPR "7062" "8080" "7884" "215"  "4385" "567"  NA     "0.06" "0.06" "0.18"
## STL "6960" "8055" "7721" "455"  "4268" "831"  "313"  NA     "0.05" "0.17"
## TRA "6953" "7742" "7951" "702"  "4376" "333"  "871"  "1155" NA     "0.12"
## TUC "745"  "2495" "1708" "6273" "2021" "6260" "6406" "6286" "6360" NA    
## WAV "6062" "7361" "6673" "1453" "3379" "1734" "1435" "1165" "2051" "5350"
##     WAV   
## ARA "0.15"
## BAF "0.16"
## BCO "0.16"
## BEA "0.05"
## BEH "0.20"
## BER "0.04"
## BIT "0.16"
## BPA "0.12"
## BPV "0.16"
## BRM "0.10"
## CHI "0.14"
## COL "0.04"
## CUA "0.16"
## DAL "0.03"
## DEN "0.03"
## FAY "0.03"
## FIR "0.07"
## FLO "0.16"
## GRV "0.16"
## HAL "0.03"
## HAR "0.05"
## HAT "0.04"
## HOU "0.04"
## IPO "0.25"
## LIN "0.07"
## LOS "0.13"
## MAC "0.05"
## MAN "0.02"
## MAU "0.13"
## MED "0.15"
## MON "0.05"
## NEB "0.04"
## NEG "0.27"
## NEH "0.02"
## NEO "0.05"
## NEW "0.05"
## NOV "0.23"
## NUE "0.06"
## PAL "0.08"
## PEO "0.03"
## PLM "0.17"
## POR "0.19"
## REC "0.11"
## RUS "0.08"
## SAI "0.10"
## SMI "0.06"
## SPR "0.07"
## STL "0.04"
## TRA "0.04"
## TUC "0.16"
## WAV NA
```

```
cities <- readRDS(here("scripts", "RMarkdowns", "output", "sampling_loc_americas_all.rds"))
cities <- as_tibble(cities)
head(cities)
```

```
## # A tibble: 6 × 14
##   Pop_City     Country Latitude Longitude Continent Abbreviation Year  Region   
##   <chr>        <chr>      <dbl>     <dbl> <chr>     <chr>        <chr> <chr>    
## 1 Berlin, NJ   USA        39.8      -74.9 Americas  BER          2018  North Am…
## 2 Columbus, OH USA        40.0      -82.9 Americas  COL          2015  North Am…
## 3 Palm Beach   USA        26.7      -80.0 Americas  PAL          2018  North Am…
## 4 Houston, TX  USA        29.8      -95.4 Americas  HOU          2018  North Am…
## 5 Los Angeles  USA        34.1     -118.  Americas  LOS          2018  North Am…
## 6 Manaus, AM   Brazil     -3.09     -60.0 Americas  MAU          2017  South Am…
## # ℹ 6 more variables: Subregion <chr>, order <int>, order2 <int>,
## #   orderold <int>, AM_order <int>, state <chr>
```

We can sort by distance

```
# Calculate row-wise mean distances (excluding diagonal)
row_means <- rowMeans(distance_matrix, na.rm=TRUE)

# Sort row names by mean distances
sorted_names_by_distance <- names(sort(row_means))

# Reorder distance_matrix and aa matrices based on these sorted names
distance_matrix <- distance_matrix[sorted_names_by_distance, sorted_names_by_distance]
aa <- aa[sorted_names_by_distance, sorted_names_by_distance]

# Your existing code to initialize and fill the merged_matrix
merged_matrix <- matrix(NA, nrow = nrow(aa), ncol = ncol(aa))
rownames(merged_matrix) <- sorted_names_by_distance
colnames(merged_matrix) <- sorted_names_by_distance

merged_matrix[upper.tri(merged_matrix, diag = FALSE)] <- aa[upper.tri(aa, diag = FALSE)]
merged_matrix[lower.tri(merged_matrix, diag = FALSE)] <- distance_matrix[lower.tri(distance_matrix, diag = FALSE)]

# Formatting code with absolute value for upper triangular part
for(i in 1:nrow(merged_matrix)) {
  for(j in 1:ncol(merged_matrix)) {
    if (i < j) {
      merged_matrix[i, j] <- sprintf("%.2f", abs(as.numeric(merged_matrix[i, j])))
    } else if (i > j) {
      merged_matrix[i, j] <- sprintf("%.0f", as.numeric(merged_matrix[i, j]))
    }
  }
}

# Print the merged matrix
print(merged_matrix)
```

```
##     BEA    PAL    MAC    FAY    WAV    HAL    HAT    NEO    NEG    MAN   
## BEA NA     "0.04" "0.02" "0.02" "0.05" "0.03" "0.03" "0.04" "0.20" "0.03"
## PAL "642"  NA     "0.04" "0.05" "0.08" "0.06" "0.07" "0.07" "0.22" "0.06"
## MAC "279"  "766"  NA     "0.03" "0.05" "0.05" "0.05" "0.04" "0.21" "0.04"
## FAY "337"  "939"  "502"  NA     "0.03" "0.03" "0.03" "0.03" "0.23" "0.02"
## WAV "608"  "1183" "757"  "272"  NA     "0.03" "0.04" "0.05" "0.27" "0.02"
## HAL "599"  "1090" "343"  "696"  "887"  NA     "0.04" "0.04" "0.24" "0.02"
## HAT "816"  "1033" "554"  "1047" "1283" "456"  NA     "0.05" "0.24" "0.04"
## NEO "928"  "1044" "685"  "1185" "1431" "624"  "168"  NA     "0.24" "0.04"
## NEG "1596" "955"  "1706" "1873" "2093" "2007" "1822" "1761" NA     "0.24"
## MAN "765"  "1366" "867"  "432"  "195"  "928"  "1357" "1514" "2284" NA    
## LIN "859"  "1421" "998"  "523"  "251"  "1089" "1508" "1661" "2311" "171" 
## BER "967"  "1532" "1098" "631"  "360"  "1169" "1597" "1753" "2418" "241" 
## HAR "935"  "1538" "1023" "604"  "360"  "1049" "1491" "1651" "2453" "173" 
## COL "861"  "1500" "796"  "648"  "602"  "662"  "1117" "1285" "2455" "493" 
## NEW "1091" "1652" "1216" "754"  "483"  "1273" "1707" "1865" "2533" "352" 
## FIR "1151" "1690" "1293" "816"  "545"  "1365" "1794" "1950" "2551" "438" 
## RUS "1192" "1570" "917"  "1293" "1453" "605"  "568"  "657"  "2391" "1450"
## BRM "1494" "1601" "1766" "1343" "1243" "2032" "2310" "2412" "2070" "1348"
## STL "1101" "1631" "875"  "1079" "1165" "541"  "815"  "961"  "2540" "1112"
## HOU "1428" "1539" "1166" "1652" "1876" "1000" "613"  "515"  "2146" "1928"
## SMI "1364" "1598" "1087" "1538" "1734" "848"  "577"  "561"  "2306" "1760"
## NEH "1204" "1752" "1337" "869"  "597"  "1395" "1830" "1988" "2618" "474" 
## MON "1443" "1584" "1176" "1654" "1871" "988"  "627"  "549"  "2213" "1915"
## SPR "1265" "1708" "1004" "1312" "1435" "666"  "750"  "858"  "2563" "1403"
## NEB "1341" "1861" "1490" "1009" "739"  "1562" "1993" "2149" "2698" "636" 
## PEO "1214" "1789" "1023" "1127" "1157" "713"  "1040" "1191" "2718" "1066"
## DAL "1510" "1753" "1232" "1670" "1857" "976"  "732"  "715"  "2451" "1872"
## DEN "1538" "1798" "1260" "1689" "1869" "993"  "771"  "764"  "2505" "1878"
## TRA "1639" "1779" "1369" "1841" "2051" "1164" "824"  "747"  "2379" "2086"
## NUE "1709" "1741" "1457" "1951" "2182" "1312" "905"  "781"  "2232" "2239"
## CUA "2964" "2326" "3076" "3228" "3431" "3368" "3136" "3046" "1372" "3625"
## MED "2964" "2326" "3076" "3228" "3431" "3368" "3136" "3046" "1372" "3625"
## SAI "3171" "2683" "3396" "3291" "3379" "3739" "3710" "3693" "2031" "3558"
## MAU "4517" "3953" "4710" "4685" "4804" "5043" "4925" "4873" "3112" "4990"
## BIT "4849" "4311" "5056" "4994" "5092" "5394" "5305" "5261" "3508" "5273"
## BPV "4917" "4312" "5078" "5126" "5276" "5395" "5214" "5137" "3398" "5468"
## TUC "5187" "4703" "5417" "5288" "5350" "5759" "5724" "5700" "3983" "5519"
## BAF "5387" "4824" "5581" "5551" "5664" "5914" "5791" "5735" "3974" "5848"
## ARA "5596" "5102" "5823" "5704" "5770" "6165" "6119" "6089" "4358" "5939"
## BCO "5711" "5175" "5920" "5851" "5943" "6258" "6166" "6120" "4363" "6121"
## PLM "5867" "5354" "6086" "5986" "6062" "6427" "6361" "6324" "4578" "6234"
## FLO "5915" "5462" "6156" "5991" "6030" "6499" "6491" "6475" "4778" "6189"
## IPO "6288" "5731" "6486" "6444" "6548" "6820" "6700" "6643" "4882" "6729"
## CHI "3431" "3685" "3152" "3535" "3665" "2852" "2674" "2642" "4271" "3620"
## LOS "3481" "3737" "3203" "3584" "3713" "2902" "2725" "2693" "4321" "3667"
## REC "6626" "6222" "6880" "6665" "6673" "7221" "7255" "7254" "5602" "6817"
## BEH "7022" "6491" "7234" "7152" "7234" "7573" "7482" "7434" "5675" "7407"
## POR "7030" "6431" "7197" "7225" "7361" "7517" "7333" "7251" "5520" "7549"
## NOV "7130" "6624" "7354" "7240" "7303" "7695" "7632" "7594" "5845" "7471"
## BPA "7286" "6747" "7494" "7422" "7507" "7831" "7730" "7678" "5918" "7682"
## GRV "7619" "7024" "7789" "7808" "7937" "8110" "7930" "7850" "6116" "8125"
##     LIN    BER    HAR    COL    NEW    FIR    RUS    BRM    STL    HOU   
## BEA "0.06" "0.04" "0.04" "0.05" "0.05" "0.08" "0.06" "0.08" "0.05" "0.01"
## PAL "0.09" "0.07" "0.08" "0.08" "0.07" "0.11" "0.10" "0.10" "0.09" "0.03"
## MAC "0.07" "0.05" "0.06" "0.06" "0.06" "0.09" "0.08" "0.09" "0.06" "0.02"
## FAY "0.05" "0.04" "0.04" "0.04" "0.04" "0.07" "0.07" "0.08" "0.05" "0.02"
## WAV "0.07" "0.04" "0.05" "0.04" "0.05" "0.07" "0.08" "0.10" "0.04" "0.04"
## HAL "0.05" "0.03" "0.04" "0.02" "0.05" "0.07" "0.07" "0.09" "0.04" "0.03"
## HAT "0.07" "0.05" "0.04" "0.05" "0.04" "0.08" "0.07" "0.10" "0.06" "0.03"
## NEO "0.07" "0.05" "0.06" "0.06" "0.05" "0.08" "0.08" "0.10" "0.06" "0.03"
## NEG "0.26" "0.25" "0.25" "0.25" "0.24" "0.29" "0.26" "0.26" "0.27" "0.20"
## MAN "0.05" "0.03" "0.04" "0.03" "0.04" "0.06" "0.06" "0.09" "0.04" "0.03"
## LIN NA     "0.07" "0.07" "0.06" "0.07" "0.10" "0.10" "0.12" "0.07" "0.06"
## BER "111"  NA     "0.05" "0.05" "0.05" "0.07" "0.08" "0.09" "0.04" "0.04"
## HAR "200"  "175"  NA     "0.05" "0.05" "0.08" "0.08" "0.10" "0.06" "0.04"
## COL "655"  "682"  "514"  NA     "0.06" "0.08" "0.08" "0.10" "0.04" "0.04"
## NEW "234"  "123"  "235"  "746"  NA     "0.08" "0.08" "0.10" "0.06" "0.04"
## FIR "295"  "198"  "337"  "849"  "104"  NA     "0.11" "0.13" "0.08" "0.07"
## RUS "1620" "1680" "1532" "1041" "1766" "1867" NA     "0.12" "0.08" "0.06"
## BRM "1208" "1235" "1399" "1834" "1258" "1186" "2634" NA     "0.11" "0.08"
## STL "1282" "1322" "1159" "646"  "1391" "1495" "455"  "2401" NA     "0.05"
## HOU "2088" "2168" "2044" "1605" "2271" "2364" "649"  "2919" "1095" NA    
## SMI "1927" "1997" "1860" "1390" "2092" "2190" "377"  "2852" "831"  "291" 
## NEH "346"  "240"  "353"  "855"  "123"  "68"   "1883" "1238" "1501" "2393"
## MON "2077" "2154" "2026" "1575" "2255" "2350" "594"  "2937" "1045" "79"  
## SPR "1574" "1621" "1462" "953"  "1696" "1799" "215"  "2652" "313"  "851" 
## NEB "492"  "396"  "523"  "1026" "290"  "199"  "2054" "1176" "1671" "2561"
## PEO "1229" "1250" "1078" "574"  "1301" "1405" "677"  "2399" "235"  "1324"
## DAL "2040" "2106" "1962" "1478" "2196" "2296" "439"  "2994" "883"  "364" 
## DEN "2047" "2110" "1964" "1474" "2198" "2298" "433"  "3017" "867"  "421" 
## TRA "2250" "2324" "2189" "1724" "2421" "2518" "702"  "3133" "1155" "242" 
## NUE "2398" "2480" "2358" "1919" "2584" "2677" "941"  "3191" "1394" "315" 
## CUA "3631" "3735" "3790" "3825" "3842" "3845" "3698" "3109" "3894" "3339"
## MED "3631" "3735" "3790" "3825" "3842" "3845" "3698" "3109" "3894" "3339"
## SAI "3484" "3557" "3681" "3937" "3626" "3583" "4253" "2488" "4268" "4137"
## MAU "4931" "5011" "5124" "5332" "5087" "5050" "5491" "3972" "5583" "5244"
## BIT "5201" "5274" "5397" "5641" "5341" "5296" "5866" "4175" "5931" "5651"
## BPV "5430" "5518" "5614" "5763" "5605" "5580" "5781" "4567" "5933" "5449"
## TUC "5426" "5486" "5626" "5928" "5539" "5481" "6273" "4313" "6286" "6124"
## BAF "5784" "5860" "5978" "6199" "5932" "5891" "6358" "4784" "6454" "6094"
## ARA "5847" "5908" "6047" "6345" "5961" "5903" "6671" "4735" "6694" "6503"
## BCO "6044" "6113" "6241" "6498" "6176" "6127" "6729" "4985" "6795" "6501"
## PLM "6147" "6211" "6346" "6631" "6267" "6212" "6918" "5050" "6960" "6723"
## FLO "6083" "6134" "6283" "6620" "6175" "6109" "7030" "4925" "7017" "6911"
## IPO "6658" "6730" "6854" "7092" "6797" "6750" "7267" "5618" "7360" "6998"
## CHI "3789" "3824" "3655" "3142" "3881" "3985" "2247" "4878" "2508" "2159"
## LOS "3836" "3869" "3700" "3188" "3926" "4030" "2297" "4927" "2555" "2211"
## REC "6695" "6733" "6892" "7273" "6759" "6683" "7777" "5501" "7721" "7710"
## BEH "7322" "7386" "7521" "7798" "7442" "7387" "8045" "6224" "8109" "7808"
## POR "7498" "7579" "7688" "7868" "7657" "7622" "7899" "6535" "8055" "7543"
## NOV "7376" "7434" "7576" "7881" "7483" "7422" "8189" "6245" "8227" "7988"
## BPA "7599" "7664" "7798" "8068" "7722" "7668" "8295" "6507" "8369" "8043"
## GRV "8068" "8148" "8260" "8452" "8222" "8184" "8497" "7080" "8650" "8141"
##     SMI    NEH    MON    SPR    NEB    PEO    DAL    DEN    TRA    NUE   
## BEA "0.04" "0.04" "0.02" "0.06" "0.04" "0.03" "0.03" "0.03" "0.01" "0.03"
## PAL "0.07" "0.06" "0.05" "0.10" "0.07" "0.06" "0.06" "0.06" "0.05" "0.05"
## MAC "0.05" "0.05" "0.03" "0.08" "0.06" "0.05" "0.05" "0.05" "0.03" "0.03"
## FAY "0.04" "0.03" "0.03" "0.06" "0.04" "0.03" "0.02" "0.02" "0.02" "0.03"
## WAV "0.06" "0.02" "0.05" "0.07" "0.04" "0.03" "0.03" "0.03" "0.04" "0.06"
## HAL "0.05" "0.02" "0.04" "0.05" "0.03" "0.02" "0.03" "0.03" "0.04" "0.05"
## HAT "0.04" "0.04" "0.04" "0.07" "0.05" "0.04" "0.03" "0.02" "0.03" "0.05"
## NEO "0.05" "0.05" "0.04" "0.07" "0.05" "0.04" "0.04" "0.04" "0.03" "0.05"
## NEG "0.24" "0.25" "0.21" "0.29" "0.25" "0.23" "0.24" "0.24" "0.21" "0.21"
## MAN "0.05" "0.02" "0.04" "0.05" "0.03" "0.03" "0.03" "0.03" "0.03" "0.05"
## LIN "0.07" "0.06" "0.07" "0.08" "0.06" "0.05" "0.06" "0.05" "0.06" "0.08"
## BER "0.05" "0.03" "0.05" "0.06" "0.05" "0.04" "0.04" "0.03" "0.04" "0.06"
## HAR "0.06" "0.03" "0.05" "0.07" "0.05" "0.04" "0.05" "0.04" "0.04" "0.06"
## COL "0.06" "0.04" "0.05" "0.07" "0.05" "0.03" "0.04" "0.04" "0.04" "0.06"
## NEW "0.05" "0.04" "0.06" "0.07" "0.05" "0.05" "0.05" "0.03" "0.04" "0.07"
## FIR "0.09" "0.07" "0.08" "0.11" "0.07" "0.07" "0.07" "0.07" "0.08" "0.09"
## RUS "0.08" "0.07" "0.07" "0.09" "0.08" "0.06" "0.07" "0.06" "0.06" "0.08"
## BRM "0.10" "0.09" "0.09" "0.12" "0.10" "0.08" "0.09" "0.09" "0.08" "0.09"
## STL "0.06" "0.04" "0.06" "0.06" "0.05" "0.04" "0.05" "0.04" "0.05" "0.07"
## HOU "0.03" "0.03" "0.02" "0.06" "0.04" "0.03" "0.02" "0.03" "0.01" "0.02"
## SMI NA     "0.05" "0.02" "0.08" "0.06" "0.05" "0.02" "0.04" "0.03" "0.03"
## NEH "2212" NA     "0.04" "0.06" "0.02" "0.03" "0.04" "0.03" "0.03" "0.05"
## MON "225"  "2377" NA     "0.07" "0.05" "0.04" "0.01" "0.03" "0.02" "0.01"
## SPR "567"  "1808" "790"  NA     "0.07" "0.06" "0.07" "0.05" "0.06" "0.08"
## NEB "2383" "171"  "2545" "1978" NA     "0.04" "0.04" "0.03" "0.04" "0.06"
## PEO "1053" "1401" "1271" "501"  "1566" NA     "0.03" "0.03" "0.03" "0.05"
## DAL "156"  "2315" "285"  "589"  "2486" "1089" NA     "0.02" "0.02" "0.02"
## DEN "202"  "2316" "342"  "565"  "2487" "1067" "58"   NA     "0.02" "0.04"
## TRA "333"  "2542" "199"  "871"  "2711" "1369" "288"  "328"  NA     "0.02"
## NUE "566"  "2707" "350"  "1130" "2874" "1618" "567"  "613"  "290"  NA    
## CUA "3551" "3913" "3414" "3886" "3969" "4081" "3682" "3738" "3546" "3336"
## MED "3551" "3913" "3414" "3886" "3969" "4081" "3682" "3738" "3546" "3336"
## SAI "4254" "3646" "4198" "4385" "3627" "4382" "4408" "4457" "4376" "4254"
## MAU "5418" "5114" "5315" "5651" "5102" "5726" "5562" "5616" "5469" "5288"
## BIT "5813" "5358" "5720" "6018" "5332" "6063" "5959" "6013" "5880" "5709"
## BPV "5655" "5646" "5524" "5959" "5654" "6100" "5789" "5845" "5657" "5444"
## TUC "6260" "5539" "6189" "6406" "5488" "6394" "6411" "6462" "6360" "6212"
## BAF "6276" "5954" "6166" "6520" "5934" "6597" "6418" "6473" "6315" "6124"
## ARA "6647" "5961" "6569" "6808" "5911" "6806" "6797" "6849" "6736" "6580"
## BCO "6669" "6187" "6571" "6881" "6153" "6926" "6814" "6868" "6727" "6548"
## PLM "6878" "6270" "6791" "7062" "6224" "7079" "7026" "7079" "6953" "6785"
## FLO "7036" "6162" "6974" "7154" "6095" "7110" "7189" "7238" "7149" "7010"
## IPO "7182" "6812" "7070" "7428" "6782" "7500" "7323" "7379" "7217" "7021"
## CHI "2097" "3982" "2105" "2231" "4146" "2581" "1942" "1905" "1918" "2039"
## LOS "2148" "4026" "2157" "2279" "4190" "2626" "1993" "1956" "1969" "2090"
## REC "7814" "6731" "7769" "7884" "6643" "7792" "7969" "8016" "7951" "7830"
## BEH "7981" "7446" "7879" "8197" "7399" "8236" "8125" "8179" "8031" "7844"
## POR "7760" "7686" "7619" "8080" "7674" "8220" "7890" "7947" "7742" "7514"
## NOV "8148" "7478" "8057" "8333" "7420" "8341" "8295" "8348" "8217" "8042"
## BPA "8222" "7727" "8114" "8451" "7681" "8500" "8365" "8419" "8263" "8070"
## GRV "8359" "8247" "8217" "8677" "8229" "8812" "8488" "8545" "8340" "8110"
##     CUA    MED    SAI    MAU    BIT    BPV    TUC    BAF    ARA    BCO   
## BEA "0.11" "0.11" "0.06" "0.10" "0.12" "0.12" "0.12" "0.11" "0.11" "0.12"
## PAL "0.13" "0.12" "0.07" "0.11" "0.13" "0.13" "0.13" "0.13" "0.13" "0.14"
## MAC "0.12" "0.11" "0.06" "0.10" "0.12" "0.12" "0.13" "0.12" "0.12" "0.13"
## FAY "0.13" "0.12" "0.07" "0.11" "0.13" "0.13" "0.13" "0.12" "0.12" "0.13"
## WAV "0.16" "0.15" "0.10" "0.13" "0.16" "0.16" "0.16" "0.16" "0.15" "0.16"
## HAL "0.14" "0.13" "0.09" "0.12" "0.14" "0.14" "0.14" "0.14" "0.14" "0.15"
## HAT "0.14" "0.14" "0.09" "0.12" "0.13" "0.14" "0.14" "0.14" "0.14" "0.15"
## NEO "0.14" "0.13" "0.09" "0.12" "0.14" "0.14" "0.14" "0.14" "0.14" "0.15"
## NEG "0.22" "0.21" "0.20" "0.20" "0.21" "0.21" "0.20" "0.21" "0.20" "0.21"
## MAN "0.14" "0.14" "0.08" "0.12" "0.14" "0.14" "0.15" "0.14" "0.14" "0.15"
## LIN "0.16" "0.16" "0.11" "0.14" "0.16" "0.16" "0.17" "0.17" "0.16" "0.17"
## BER "0.15" "0.14" "0.10" "0.13" "0.15" "0.15" "0.16" "0.15" "0.15" "0.16"
## HAR "0.15" "0.15" "0.10" "0.13" "0.15" "0.16" "0.16" "0.15" "0.15" "0.16"
## COL "0.15" "0.15" "0.10" "0.13" "0.15" "0.15" "0.16" "0.15" "0.15" "0.16"
## NEW "0.15" "0.15" "0.10" "0.13" "0.15" "0.16" "0.16" "0.15" "0.15" "0.16"
## FIR "0.19" "0.18" "0.13" "0.17" "0.18" "0.19" "0.18" "0.18" "0.18" "0.19"
## RUS "0.17" "0.16" "0.12" "0.15" "0.16" "0.17" "0.17" "0.17" "0.16" "0.17"
## BRM "0.16" "0.16" "0.12" "0.15" "0.17" "0.17" "0.17" "0.17" "0.16" "0.17"
## STL "0.16" "0.16" "0.11" "0.14" "0.16" "0.16" "0.17" "0.16" "0.16" "0.17"
## HOU "0.11" "0.10" "0.06" "0.09" "0.11" "0.11" "0.12" "0.11" "0.11" "0.12"
## SMI "0.14" "0.14" "0.09" "0.13" "0.13" "0.14" "0.14" "0.14" "0.13" "0.15"
## NEH "0.14" "0.14" "0.09" "0.13" "0.15" "0.15" "0.15" "0.14" "0.14" "0.15"
## MON "0.12" "0.11" "0.07" "0.11" "0.11" "0.12" "0.12" "0.12" "0.11" "0.13"
## SPR "0.18" "0.17" "0.12" "0.15" "0.17" "0.17" "0.18" "0.18" "0.17" "0.18"
## NEB "0.15" "0.15" "0.10" "0.13" "0.15" "0.16" "0.16" "0.16" "0.15" "0.16"
## PEO "0.14" "0.13" "0.09" "0.12" "0.14" "0.14" "0.14" "0.13" "0.13" "0.14"
## DAL "0.14" "0.13" "0.09" "0.12" "0.13" "0.14" "0.14" "0.14" "0.13" "0.15"
## DEN "0.14" "0.14" "0.09" "0.12" "0.14" "0.14" "0.15" "0.14" "0.14" "0.15"
## TRA "0.12" "0.11" "0.07" "0.10" "0.12" "0.12" "0.12" "0.12" "0.12" "0.13"
## NUE "0.12" "0.11" "0.07" "0.11" "0.11" "0.12" "0.12" "0.12" "0.12" "0.13"
## CUA NA     "0.00" "0.12" "0.11" "0.13" "0.13" "0.12" "0.12" "0.12" "0.13"
## MED "0"    NA     "0.11" "0.11" "0.13" "0.13" "0.12" "0.12" "0.12" "0.13"
## SAI "1614" "1614" NA     "0.11" "0.12" "0.12" "0.12" "0.12" "0.12" "0.13"
## MAU "2016" "2016" "1484" NA     "0.05" "0.04" "0.06" "0.05" "0.06" "0.07"
## BIT "2471" "2471" "1717" "467"  NA     "0.05" "0.04" "0.05" "0.04" "0.06"
## BPV "2111" "2111" "2119" "758"  "1004" NA     "0.06" "0.05" "0.06" "0.07"
## TUC "3084" "3084" "2021" "1153" "705"  "1669" NA     "0.01" "0.01" "0.02"
## BAF "2808" "2808" "2308" "871"  "624"  "866"  "988"  NA     "0.00" "0.02"
## ARA "3387" "3387" "2427" "1388" "922"  "1737" "422"  "917"  NA     "0.02"
## BCO "3261" "3261" "2564" "1260" "864"  "1372" "802"  "505"  "532"  NA    
## PLM "3535" "3535" "2698" "1519" "1077" "1717" "745"  "852"  "341"  "355" 
## FLO "3896" "3896" "2782" "1931" "1465" "2312" "814"  "1480" "575"  "1032"
## IPO "3692" "3692" "3173" "1778" "1457" "1631" "1429" "909"  "1078" "646" 
## CHI "5318" "5318" "6287" "7306" "7736" "7400" "8251" "8125" "8615" "8564"
## LOS "5366" "5366" "6338" "7355" "7785" "7447" "8301" "8173" "8666" "8613"
## REC "4790" "4790" "3577" "2840" "2374" "3195" "1708" "2340" "1474" "1853"
## BEH "4527" "4527" "3861" "2564" "2180" "2485" "1912" "1721" "1491" "1316"
## POR "4210" "4210" "4050" "2572" "2380" "2122" "2495" "1758" "2158" "1694"
## NOV "4756" "4756" "3960" "2756" "2337" "2769" "1953" "1956" "1536" "1497"
## BPA "4743" "4743" "4132" "2806" "2438" "2671" "2196" "1948" "1776" "1574"
## GRV "4808" "4808" "4603" "3137" "2906" "2718" "2925" "2296" "2549" "2148"
##     PLM    FLO    IPO    CHI     LOS     REC    BEH    POR    NOV    BPA   
## BEA "0.12" "0.12" "0.19" "0.09"  "0.09"  "0.08" "0.15" "0.14" "0.17" "0.08"
## PAL "0.14" "0.14" "0.20" "0.11"  "0.11"  "0.10" "0.16" "0.16" "0.18" "0.10"
## MAC "0.12" "0.13" "0.20" "0.10"  "0.10"  "0.08" "0.15" "0.15" "0.18" "0.09"
## FAY "0.13" "0.13" "0.21" "0.10"  "0.10"  "0.09" "0.17" "0.16" "0.19" "0.09"
## WAV "0.17" "0.16" "0.25" "0.14"  "0.13"  "0.11" "0.20" "0.19" "0.23" "0.12"
## HAL "0.15" "0.15" "0.22" "0.12"  "0.11"  "0.10" "0.18" "0.17" "0.20" "0.11"
## HAT "0.15" "0.15" "0.22" "0.12"  "0.12"  "0.10" "0.18" "0.17" "0.20" "0.11"
## NEO "0.15" "0.15" "0.22" "0.12"  "0.12"  "0.10" "0.18" "0.17" "0.20" "0.10"
## NEG "0.21" "0.22" "0.26" "0.23"  "0.22"  "0.21" "0.21" "0.21" "0.25" "0.22"
## MAN "0.15" "0.15" "0.22" "0.11"  "0.11"  "0.10" "0.18" "0.17" "0.20" "0.10"
## LIN "0.18" "0.18" "0.24" "0.14"  "0.13"  "0.12" "0.20" "0.19" "0.23" "0.13"
## BER "0.16" "0.16" "0.23" "0.13"  "0.12"  "0.11" "0.19" "0.18" "0.22" "0.12"
## HAR "0.16" "0.16" "0.23" "0.13"  "0.12"  "0.11" "0.19" "0.18" "0.22" "0.12"
## COL "0.16" "0.16" "0.24" "0.13"  "0.12"  "0.11" "0.19" "0.18" "0.22" "0.12"
## NEW "0.16" "0.16" "0.23" "0.13"  "0.13"  "0.11" "0.19" "0.18" "0.21" "0.12"
## FIR "0.19" "0.19" "0.26" "0.17"  "0.15"  "0.14" "0.22" "0.21" "0.25" "0.14"
## RUS "0.18" "0.18" "0.25" "0.15"  "0.14"  "0.13" "0.20" "0.19" "0.23" "0.13"
## BRM "0.17" "0.17" "0.24" "0.15"  "0.14"  "0.14" "0.20" "0.19" "0.22" "0.14"
## STL "0.17" "0.17" "0.25" "0.14"  "0.13"  "0.12" "0.20" "0.19" "0.23" "0.13"
## HOU "0.12" "0.12" "0.18" "0.08"  "0.09"  "0.08" "0.14" "0.14" "0.16" "0.08"
## SMI "0.15" "0.15" "0.22" "0.12"  "0.12"  "0.10" "0.18" "0.17" "0.21" "0.10"
## NEH "0.16" "0.15" "0.23" "0.12"  "0.11"  "0.11" "0.19" "0.18" "0.21" "0.11"
## MON "0.13" "0.13" "0.20" "0.10"  "0.10"  "0.08" "0.16" "0.15" "0.18" "0.09"
## SPR "0.18" "0.19" "0.27" "0.16"  "0.14"  "0.13" "0.22" "0.20" "0.25" "0.14"
## NEB "0.16" "0.16" "0.24" "0.13"  "0.12"  "0.11" "0.19" "0.18" "0.22" "0.12"
## PEO "0.14" "0.15" "0.22" "0.11"  "0.11"  "0.09" "0.17" "0.17" "0.20" "0.10"
## DAL "0.15" "0.15" "0.22" "0.12"  "0.11"  "0.09" "0.18" "0.17" "0.20" "0.10"
## DEN "0.15" "0.15" "0.22" "0.12"  "0.11"  "0.10" "0.18" "0.17" "0.21" "0.10"
## TRA "0.13" "0.13" "0.20" "0.10"  "0.09"  "0.08" "0.15" "0.15" "0.18" "0.09"
## NUE "0.13" "0.13" "0.20" "0.10"  "0.10"  "0.09" "0.15" "0.15" "0.17" "0.09"
## CUA "0.13" "0.13" "0.17" "0.13"  "0.14"  "0.13" "0.13" "0.13" "0.15" "0.14"
## MED "0.13" "0.13" "0.17" "0.12"  "0.13"  "0.13" "0.13" "0.13" "0.14" "0.13"
## SAI "0.13" "0.13" "0.18" "0.10"  "0.11"  "0.10" "0.14" "0.14" "0.16" "0.11"
## MAU "0.06" "0.06" "0.09" "0.13"  "0.13"  "0.08" "0.07" "0.06" "0.08" "0.08"
## BIT "0.06" "0.06" "0.09" "0.14"  "0.15"  "0.09" "0.07" "0.07" "0.09" "0.08"
## BPV "0.06" "0.07" "0.09" "0.14"  "0.15"  "0.07" "0.06" "0.06" "0.08" "0.08"
## TUC "0.02" "0.02" "0.07" "0.14"  "0.15"  "0.07" "0.05" "0.05" "0.07" "0.07"
## BAF "0.01" "0.01" "0.06" "0.14"  "0.15"  "0.06" "0.05" "0.04" "0.06" "0.06"
## ARA "0.02" "0.02" "0.07" "0.14"  "0.14"  "0.07" "0.05" "0.05" "0.07" "0.06"
## BCO "0.02" "0.03" "0.09" "0.15"  "0.16"  "0.08" "0.06" "0.06" "0.08" "0.08"
## PLM NA     "0.02" "0.07" "0.15"  "0.15"  "0.08" "0.05" "0.05" "0.06" "0.07"
## FLO "701"  NA     "0.08" "0.15"  "0.16"  "0.08" "0.06" "0.06" "0.08" "0.07"
## IPO "753"  "1391" NA     "0.20"  "0.21"  "0.14" "0.06" "0.04" "0.04" "0.13"
## CHI "8813" "9049" "9008" NA      "0.10"  "0.13" "0.16" "0.15" "0.17" "0.13"
## LOS "8862" "9099" "9056" "51"    NA      "0.13" "0.17" "0.16" "0.18" "0.14"
## REC "1498" "909"  "1997" "9864"  "9915"  NA     "0.10" "0.11" "0.13" "0.03"
## BEH "1176" "1466" "854"  "9845"  "9893"  "1641" NA     "0.05" "0.04" "0.10"
## POR "1831" "2428" "1080" "9403"  "9447"  "2858" "1263" NA     "0.04" "0.11"
## NOV "1271" "1358" "1166" "10062" "10111" "1327" "394"  "1650" NA     "0.12"
## BPA "1457" "1745" "1051" "10059" "10106" "1844" "288"  "1189" "522"  NA    
## GRV "2209" "2709" "1502" "9987"  "10030" "2959" "1323" "599"  "1647" "1126"
##     GRV   
## BEA "0.12"
## PAL "0.13"
## MAC "0.13"
## FAY "0.13"
## WAV "0.16"
## HAL "0.14"
## HAT "0.14"
## NEO "0.14"
## NEG "0.21"
## MAN "0.14"
## LIN "0.17"
## BER "0.15"
## HAR "0.15"
## COL "0.15"
## NEW "0.16"
## FIR "0.18"
## RUS "0.16"
## BRM "0.17"
## STL "0.16"
## HOU "0.11"
## SMI "0.14"
## NEH "0.14"
## MON "0.13"
## SPR "0.17"
## NEB "0.15"
## PEO "0.14"
## DAL "0.14"
## DEN "0.14"
## TRA "0.12"
## NUE "0.12"
## CUA "0.13"
## MED "0.12"
## SAI "0.13"
## MAU "0.07"
## BIT "0.07"
## BPV "0.08"
## TUC "0.07"
## BAF "0.06"
## ARA "0.06"
## BCO "0.08"
## PLM "0.07"
## FLO "0.07"
## IPO "0.06"
## CHI "0.14"
## LOS "0.15"
## REC "0.10"
## BEH "0.08"
## POR "0.03"
## NOV "0.07"
## BPA "0.10"
## GRV NA
```

Make a table and save it as a word document

```
# Convert the matrix to a data frame and add a column with row names
merged_df <- as.data.frame(merged_matrix)
merged_df$Population <- rownames(merged_matrix)

# Reorder columns to have RowNames as the first column
merged_df <- merged_df[, c("Population", colnames(merged_matrix))]


# Create a flextable object from the merged_matrix
ft <- qflextable(as.data.frame(merged_df))

ft
```

| Population | BEA | PAL | MAC | FAY | WAV | HAL | HAT | NEO | NEG | MAN | LIN | BER | HAR | COL | NEW | FIR | RUS | BRM | STL | HOU | SMI | NEH | MON | SPR | NEB | PEO | DAL | DEN | TRA | NUE | CUA | MED | SAI | MAU | BIT | BPV | TUC | BAF | ARA | BCO | PLM | FLO | IPO | CHI | LOS | REC | BEH | POR | NOV | BPA | GRV |
| --- | --- | --- | --- | --- | --- | --- | --- | --- | --- | --- | --- | --- | --- | --- | --- | --- | --- | --- | --- | --- | --- | --- | --- | --- | --- | --- | --- | --- | --- | --- | --- | --- | --- | --- | --- | --- | --- | --- | --- | --- | --- | --- | --- | --- | --- | --- | --- | --- | --- | --- | --- |
| BEA |  | 0.04 | 0.02 | 0.02 | 0.05 | 0.03 | 0.03 | 0.04 | 0.20 | 0.03 | 0.06 | 0.04 | 0.04 | 0.05 | 0.05 | 0.08 | 0.06 | 0.08 | 0.05 | 0.01 | 0.04 | 0.04 | 0.02 | 0.06 | 0.04 | 0.03 | 0.03 | 0.03 | 0.01 | 0.03 | 0.11 | 0.11 | 0.06 | 0.10 | 0.12 | 0.12 | 0.12 | 0.11 | 0.11 | 0.12 | 0.12 | 0.12 | 0.19 | 0.09 | 0.09 | 0.08 | 0.15 | 0.14 | 0.17 | 0.08 | 0.12 |
| PAL | 642 |  | 0.04 | 0.05 | 0.08 | 0.06 | 0.07 | 0.07 | 0.22 | 0.06 | 0.09 | 0.07 | 0.08 | 0.08 | 0.07 | 0.11 | 0.10 | 0.10 | 0.09 | 0.03 | 0.07 | 0.06 | 0.05 | 0.10 | 0.07 | 0.06 | 0.06 | 0.06 | 0.05 | 0.05 | 0.13 | 0.12 | 0.07 | 0.11 | 0.13 | 0.13 | 0.13 | 0.13 | 0.13 | 0.14 | 0.14 | 0.14 | 0.20 | 0.11 | 0.11 | 0.10 | 0.16 | 0.16 | 0.18 | 0.10 | 0.13 |
| MAC | 279 | 766 |  | 0.03 | 0.05 | 0.05 | 0.05 | 0.04 | 0.21 | 0.04 | 0.07 | 0.05 | 0.06 | 0.06 | 0.06 | 0.09 | 0.08 | 0.09 | 0.06 | 0.02 | 0.05 | 0.05 | 0.03 | 0.08 | 0.06 | 0.05 | 0.05 | 0.05 | 0.03 | 0.03 | 0.12 | 0.11 | 0.06 | 0.10 | 0.12 | 0.12 | 0.13 | 0.12 | 0.12 | 0.13 | 0.12 | 0.13 | 0.20 | 0.10 | 0.10 | 0.08 | 0.15 | 0.15 | 0.18 | 0.09 | 0.13 |
| FAY | 337 | 939 | 502 |  | 0.03 | 0.03 | 0.03 | 0.03 | 0.23 | 0.02 | 0.05 | 0.04 | 0.04 | 0.04 | 0.04 | 0.07 | 0.07 | 0.08 | 0.05 | 0.02 | 0.04 | 0.03 | 0.03 | 0.06 | 0.04 | 0.03 | 0.02 | 0.02 | 0.02 | 0.03 | 0.13 | 0.12 | 0.07 | 0.11 | 0.13 | 0.13 | 0.13 | 0.12 | 0.12 | 0.13 | 0.13 | 0.13 | 0.21 | 0.10 | 0.10 | 0.09 | 0.17 | 0.16 | 0.19 | 0.09 | 0.13 |
| WAV | 608 | 1183 | 757 | 272 |  | 0.03 | 0.04 | 0.05 | 0.27 | 0.02 | 0.07 | 0.04 | 0.05 | 0.04 | 0.05 | 0.07 | 0.08 | 0.10 | 0.04 | 0.04 | 0.06 | 0.02 | 0.05 | 0.07 | 0.04 | 0.03 | 0.03 | 0.03 | 0.04 | 0.06 | 0.16 | 0.15 | 0.10 | 0.13 | 0.16 | 0.16 | 0.16 | 0.16 | 0.15 | 0.16 | 0.17 | 0.16 | 0.25 | 0.14 | 0.13 | 0.11 | 0.20 | 0.19 | 0.23 | 0.12 | 0.16 |
| HAL | 599 | 1090 | 343 | 696 | 887 |  | 0.04 | 0.04 | 0.24 | 0.02 | 0.05 | 0.03 | 0.04 | 0.02 | 0.05 | 0.07 | 0.07 | 0.09 | 0.04 | 0.03 | 0.05 | 0.02 | 0.04 | 0.05 | 0.03 | 0.02 | 0.03 | 0.03 | 0.04 | 0.05 | 0.14 | 0.13 | 0.09 | 0.12 | 0.14 | 0.14 | 0.14 | 0.14 | 0.14 | 0.15 | 0.15 | 0.15 | 0.22 | 0.12 | 0.11 | 0.10 | 0.18 | 0.17 | 0.20 | 0.11 | 0.14 |
| HAT | 816 | 1033 | 554 | 1047 | 1283 | 456 |  | 0.05 | 0.24 | 0.04 | 0.07 | 0.05 | 0.04 | 0.05 | 0.04 | 0.08 | 0.07 | 0.10 | 0.06 | 0.03 | 0.04 | 0.04 | 0.04 | 0.07 | 0.05 | 0.04 | 0.03 | 0.02 | 0.03 | 0.05 | 0.14 | 0.14 | 0.09 | 0.12 | 0.13 | 0.14 | 0.14 | 0.14 | 0.14 | 0.15 | 0.15 | 0.15 | 0.22 | 0.12 | 0.12 | 0.10 | 0.18 | 0.17 | 0.20 | 0.11 | 0.14 |
| NEO | 928 | 1044 | 685 | 1185 | 1431 | 624 | 168 |  | 0.24 | 0.04 | 0.07 | 0.05 | 0.06 | 0.06 | 0.05 | 0.08 | 0.08 | 0.10 | 0.06 | 0.03 | 0.05 | 0.05 | 0.04 | 0.07 | 0.05 | 0.04 | 0.04 | 0.04 | 0.03 | 0.05 | 0.14 | 0.13 | 0.09 | 0.12 | 0.14 | 0.14 | 0.14 | 0.14 | 0.14 | 0.15 | 0.15 | 0.15 | 0.22 | 0.12 | 0.12 | 0.10 | 0.18 | 0.17 | 0.20 | 0.10 | 0.14 |
| NEG | 1596 | 955 | 1706 | 1873 | 2093 | 2007 | 1822 | 1761 |  | 0.24 | 0.26 | 0.25 | 0.25 | 0.25 | 0.24 | 0.29 | 0.26 | 0.26 | 0.27 | 0.20 | 0.24 | 0.25 | 0.21 | 0.29 | 0.25 | 0.23 | 0.24 | 0.24 | 0.21 | 0.21 | 0.22 | 0.21 | 0.20 | 0.20 | 0.21 | 0.21 | 0.20 | 0.21 | 0.20 | 0.21 | 0.21 | 0.22 | 0.26 | 0.23 | 0.22 | 0.21 | 0.21 | 0.21 | 0.25 | 0.22 | 0.21 |
| MAN | 765 | 1366 | 867 | 432 | 195 | 928 | 1357 | 1514 | 2284 |  | 0.05 | 0.03 | 0.04 | 0.03 | 0.04 | 0.06 | 0.06 | 0.09 | 0.04 | 0.03 | 0.05 | 0.02 | 0.04 | 0.05 | 0.03 | 0.03 | 0.03 | 0.03 | 0.03 | 0.05 | 0.14 | 0.14 | 0.08 | 0.12 | 0.14 | 0.14 | 0.15 | 0.14 | 0.14 | 0.15 | 0.15 | 0.15 | 0.22 | 0.11 | 0.11 | 0.10 | 0.18 | 0.17 | 0.20 | 0.10 | 0.14 |
| LIN | 859 | 1421 | 998 | 523 | 251 | 1089 | 1508 | 1661 | 2311 | 171 |  | 0.07 | 0.07 | 0.06 | 0.07 | 0.10 | 0.10 | 0.12 | 0.07 | 0.06 | 0.07 | 0.06 | 0.07 | 0.08 | 0.06 | 0.05 | 0.06 | 0.05 | 0.06 | 0.08 | 0.16 | 0.16 | 0.11 | 0.14 | 0.16 | 0.16 | 0.17 | 0.17 | 0.16 | 0.17 | 0.18 | 0.18 | 0.24 | 0.14 | 0.13 | 0.12 | 0.20 | 0.19 | 0.23 | 0.13 | 0.17 |
| BER | 967 | 1532 | 1098 | 631 | 360 | 1169 | 1597 | 1753 | 2418 | 241 | 111 |  | 0.05 | 0.05 | 0.05 | 0.07 | 0.08 | 0.09 | 0.04 | 0.04 | 0.05 | 0.03 | 0.05 | 0.06 | 0.05 | 0.04 | 0.04 | 0.03 | 0.04 | 0.06 | 0.15 | 0.14 | 0.10 | 0.13 | 0.15 | 0.15 | 0.16 | 0.15 | 0.15 | 0.16 | 0.16 | 0.16 | 0.23 | 0.13 | 0.12 | 0.11 | 0.19 | 0.18 | 0.22 | 0.12 | 0.15 |
| HAR | 935 | 1538 | 1023 | 604 | 360 | 1049 | 1491 | 1651 | 2453 | 173 | 200 | 175 |  | 0.05 | 0.05 | 0.08 | 0.08 | 0.10 | 0.06 | 0.04 | 0.06 | 0.03 | 0.05 | 0.07 | 0.05 | 0.04 | 0.05 | 0.04 | 0.04 | 0.06 | 0.15 | 0.15 | 0.10 | 0.13 | 0.15 | 0.16 | 0.16 | 0.15 | 0.15 | 0.16 | 0.16 | 0.16 | 0.23 | 0.13 | 0.12 | 0.11 | 0.19 | 0.18 | 0.22 | 0.12 | 0.15 |
| COL | 861 | 1500 | 796 | 648 | 602 | 662 | 1117 | 1285 | 2455 | 493 | 655 | 682 | 514 |  | 0.06 | 0.08 | 0.08 | 0.10 | 0.04 | 0.04 | 0.06 | 0.04 | 0.05 | 0.07 | 0.05 | 0.03 | 0.04 | 0.04 | 0.04 | 0.06 | 0.15 | 0.15 | 0.10 | 0.13 | 0.15 | 0.15 | 0.16 | 0.15 | 0.15 | 0.16 | 0.16 | 0.16 | 0.24 | 0.13 | 0.12 | 0.11 | 0.19 | 0.18 | 0.22 | 0.12 | 0.15 |
| NEW | 1091 | 1652 | 1216 | 754 | 483 | 1273 | 1707 | 1865 | 2533 | 352 | 234 | 123 | 235 | 746 |  | 0.08 | 0.08 | 0.10 | 0.06 | 0.04 | 0.05 | 0.04 | 0.06 | 0.07 | 0.05 | 0.05 | 0.05 | 0.03 | 0.04 | 0.07 | 0.15 | 0.15 | 0.10 | 0.13 | 0.15 | 0.16 | 0.16 | 0.15 | 0.15 | 0.16 | 0.16 | 0.16 | 0.23 | 0.13 | 0.13 | 0.11 | 0.19 | 0.18 | 0.21 | 0.12 | 0.16 |
| FIR | 1151 | 1690 | 1293 | 816 | 545 | 1365 | 1794 | 1950 | 2551 | 438 | 295 | 198 | 337 | 849 | 104 |  | 0.11 | 0.13 | 0.08 | 0.07 | 0.09 | 0.07 | 0.08 | 0.11 | 0.07 | 0.07 | 0.07 | 0.07 | 0.08 | 0.09 | 0.19 | 0.18 | 0.13 | 0.17 | 0.18 | 0.19 | 0.18 | 0.18 | 0.18 | 0.19 | 0.19 | 0.19 | 0.26 | 0.17 | 0.15 | 0.14 | 0.22 | 0.21 | 0.25 | 0.14 | 0.18 |
| RUS | 1192 | 1570 | 917 | 1293 | 1453 | 605 | 568 | 657 | 2391 | 1450 | 1620 | 1680 | 1532 | 1041 | 1766 | 1867 |  | 0.12 | 0.08 | 0.06 | 0.08 | 0.07 | 0.07 | 0.09 | 0.08 | 0.06 | 0.07 | 0.06 | 0.06 | 0.08 | 0.17 | 0.16 | 0.12 | 0.15 | 0.16 | 0.17 | 0.17 | 0.17 | 0.16 | 0.17 | 0.18 | 0.18 | 0.25 | 0.15 | 0.14 | 0.13 | 0.20 | 0.19 | 0.23 | 0.13 | 0.16 |
| BRM | 1494 | 1601 | 1766 | 1343 | 1243 | 2032 | 2310 | 2412 | 2070 | 1348 | 1208 | 1235 | 1399 | 1834 | 1258 | 1186 | 2634 |  | 0.11 | 0.08 | 0.10 | 0.09 | 0.09 | 0.12 | 0.10 | 0.08 | 0.09 | 0.09 | 0.08 | 0.09 | 0.16 | 0.16 | 0.12 | 0.15 | 0.17 | 0.17 | 0.17 | 0.17 | 0.16 | 0.17 | 0.17 | 0.17 | 0.24 | 0.15 | 0.14 | 0.14 | 0.20 | 0.19 | 0.22 | 0.14 | 0.17 |
| STL | 1101 | 1631 | 875 | 1079 | 1165 | 541 | 815 | 961 | 2540 | 1112 | 1282 | 1322 | 1159 | 646 | 1391 | 1495 | 455 | 2401 |  | 0.05 | 0.06 | 0.04 | 0.06 | 0.06 | 0.05 | 0.04 | 0.05 | 0.04 | 0.05 | 0.07 | 0.16 | 0.16 | 0.11 | 0.14 | 0.16 | 0.16 | 0.17 | 0.16 | 0.16 | 0.17 | 0.17 | 0.17 | 0.25 | 0.14 | 0.13 | 0.12 | 0.20 | 0.19 | 0.23 | 0.13 | 0.16 |
| HOU | 1428 | 1539 | 1166 | 1652 | 1876 | 1000 | 613 | 515 | 2146 | 1928 | 2088 | 2168 | 2044 | 1605 | 2271 | 2364 | 649 | 2919 | 1095 |  | 0.03 | 0.03 | 0.02 | 0.06 | 0.04 | 0.03 | 0.02 | 0.03 | 0.01 | 0.02 | 0.11 | 0.10 | 0.06 | 0.09 | 0.11 | 0.11 | 0.12 | 0.11 | 0.11 | 0.12 | 0.12 | 0.12 | 0.18 | 0.08 | 0.09 | 0.08 | 0.14 | 0.14 | 0.16 | 0.08 | 0.11 |
| SMI | 1364 | 1598 | 1087 | 1538 | 1734 | 848 | 577 | 561 | 2306 | 1760 | 1927 | 1997 | 1860 | 1390 | 2092 | 2190 | 377 | 2852 | 831 | 291 |  | 0.05 | 0.02 | 0.08 | 0.06 | 0.05 | 0.02 | 0.04 | 0.03 | 0.03 | 0.14 | 0.14 | 0.09 | 0.13 | 0.13 | 0.14 | 0.14 | 0.14 | 0.13 | 0.15 | 0.15 | 0.15 | 0.22 | 0.12 | 0.12 | 0.10 | 0.18 | 0.17 | 0.21 | 0.10 | 0.14 |
| NEH | 1204 | 1752 | 1337 | 869 | 597 | 1395 | 1830 | 1988 | 2618 | 474 | 346 | 240 | 353 | 855 | 123 | 68 | 1883 | 1238 | 1501 | 2393 | 2212 |  | 0.04 | 0.06 | 0.02 | 0.03 | 0.04 | 0.03 | 0.03 | 0.05 | 0.14 | 0.14 | 0.09 | 0.13 | 0.15 | 0.15 | 0.15 | 0.14 | 0.14 | 0.15 | 0.16 | 0.15 | 0.23 | 0.12 | 0.11 | 0.11 | 0.19 | 0.18 | 0.21 | 0.11 | 0.14 |
| MON | 1443 | 1584 | 1176 | 1654 | 1871 | 988 | 627 | 549 | 2213 | 1915 | 2077 | 2154 | 2026 | 1575 | 2255 | 2350 | 594 | 2937 | 1045 | 79 | 225 | 2377 |  | 0.07 | 0.05 | 0.04 | 0.01 | 0.03 | 0.02 | 0.01 | 0.12 | 0.11 | 0.07 | 0.11 | 0.11 | 0.12 | 0.12 | 0.12 | 0.11 | 0.13 | 0.13 | 0.13 | 0.20 | 0.10 | 0.10 | 0.08 | 0.16 | 0.15 | 0.18 | 0.09 | 0.13 |
| SPR | 1265 | 1708 | 1004 | 1312 | 1435 | 666 | 750 | 858 | 2563 | 1403 | 1574 | 1621 | 1462 | 953 | 1696 | 1799 | 215 | 2652 | 313 | 851 | 567 | 1808 | 790 |  | 0.07 | 0.06 | 0.07 | 0.05 | 0.06 | 0.08 | 0.18 | 0.17 | 0.12 | 0.15 | 0.17 | 0.17 | 0.18 | 0.18 | 0.17 | 0.18 | 0.18 | 0.19 | 0.27 | 0.16 | 0.14 | 0.13 | 0.22 | 0.20 | 0.25 | 0.14 | 0.17 |
| NEB | 1341 | 1861 | 1490 | 1009 | 739 | 1562 | 1993 | 2149 | 2698 | 636 | 492 | 396 | 523 | 1026 | 290 | 199 | 2054 | 1176 | 1671 | 2561 | 2383 | 171 | 2545 | 1978 |  | 0.04 | 0.04 | 0.03 | 0.04 | 0.06 | 0.15 | 0.15 | 0.10 | 0.13 | 0.15 | 0.16 | 0.16 | 0.16 | 0.15 | 0.16 | 0.16 | 0.16 | 0.24 | 0.13 | 0.12 | 0.11 | 0.19 | 0.18 | 0.22 | 0.12 | 0.15 |
| PEO | 1214 | 1789 | 1023 | 1127 | 1157 | 713 | 1040 | 1191 | 2718 | 1066 | 1229 | 1250 | 1078 | 574 | 1301 | 1405 | 677 | 2399 | 235 | 1324 | 1053 | 1401 | 1271 | 501 | 1566 |  | 0.03 | 0.03 | 0.03 | 0.05 | 0.14 | 0.13 | 0.09 | 0.12 | 0.14 | 0.14 | 0.14 | 0.13 | 0.13 | 0.14 | 0.14 | 0.15 | 0.22 | 0.11 | 0.11 | 0.09 | 0.17 | 0.17 | 0.20 | 0.10 | 0.14 |
| DAL | 1510 | 1753 | 1232 | 1670 | 1857 | 976 | 732 | 715 | 2451 | 1872 | 2040 | 2106 | 1962 | 1478 | 2196 | 2296 | 439 | 2994 | 883 | 364 | 156 | 2315 | 285 | 589 | 2486 | 1089 |  | 0.02 | 0.02 | 0.02 | 0.14 | 0.13 | 0.09 | 0.12 | 0.13 | 0.14 | 0.14 | 0.14 | 0.13 | 0.15 | 0.15 | 0.15 | 0.22 | 0.12 | 0.11 | 0.09 | 0.18 | 0.17 | 0.20 | 0.10 | 0.14 |
| DEN | 1538 | 1798 | 1260 | 1689 | 1869 | 993 | 771 | 764 | 2505 | 1878 | 2047 | 2110 | 1964 | 1474 | 2198 | 2298 | 433 | 3017 | 867 | 421 | 202 | 2316 | 342 | 565 | 2487 | 1067 | 58 |  | 0.02 | 0.04 | 0.14 | 0.14 | 0.09 | 0.12 | 0.14 | 0.14 | 0.15 | 0.14 | 0.14 | 0.15 | 0.15 | 0.15 | 0.22 | 0.12 | 0.11 | 0.10 | 0.18 | 0.17 | 0.21 | 0.10 | 0.14 |
| TRA | 1639 | 1779 | 1369 | 1841 | 2051 | 1164 | 824 | 747 | 2379 | 2086 | 2250 | 2324 | 2189 | 1724 | 2421 | 2518 | 702 | 3133 | 1155 | 242 | 333 | 2542 | 199 | 871 | 2711 | 1369 | 288 | 328 |  | 0.02 | 0.12 | 0.11 | 0.07 | 0.10 | 0.12 | 0.12 | 0.12 | 0.12 | 0.12 | 0.13 | 0.13 | 0.13 | 0.20 | 0.10 | 0.09 | 0.08 | 0.15 | 0.15 | 0.18 | 0.09 | 0.12 |
| NUE | 1709 | 1741 | 1457 | 1951 | 2182 | 1312 | 905 | 781 | 2232 | 2239 | 2398 | 2480 | 2358 | 1919 | 2584 | 2677 | 941 | 3191 | 1394 | 315 | 566 | 2707 | 350 | 1130 | 2874 | 1618 | 567 | 613 | 290 |  | 0.12 | 0.11 | 0.07 | 0.11 | 0.11 | 0.12 | 0.12 | 0.12 | 0.12 | 0.13 | 0.13 | 0.13 | 0.20 | 0.10 | 0.10 | 0.09 | 0.15 | 0.15 | 0.17 | 0.09 | 0.12 |
| CUA | 2964 | 2326 | 3076 | 3228 | 3431 | 3368 | 3136 | 3046 | 1372 | 3625 | 3631 | 3735 | 3790 | 3825 | 3842 | 3845 | 3698 | 3109 | 3894 | 3339 | 3551 | 3913 | 3414 | 3886 | 3969 | 4081 | 3682 | 3738 | 3546 | 3336 |  | 0.00 | 0.12 | 0.11 | 0.13 | 0.13 | 0.12 | 0.12 | 0.12 | 0.13 | 0.13 | 0.13 | 0.17 | 0.13 | 0.14 | 0.13 | 0.13 | 0.13 | 0.15 | 0.14 | 0.13 |
| MED | 2964 | 2326 | 3076 | 3228 | 3431 | 3368 | 3136 | 3046 | 1372 | 3625 | 3631 | 3735 | 3790 | 3825 | 3842 | 3845 | 3698 | 3109 | 3894 | 3339 | 3551 | 3913 | 3414 | 3886 | 3969 | 4081 | 3682 | 3738 | 3546 | 3336 | 0 |  | 0.11 | 0.11 | 0.13 | 0.13 | 0.12 | 0.12 | 0.12 | 0.13 | 0.13 | 0.13 | 0.17 | 0.12 | 0.13 | 0.13 | 0.13 | 0.13 | 0.14 | 0.13 | 0.12 |
| SAI | 3171 | 2683 | 3396 | 3291 | 3379 | 3739 | 3710 | 3693 | 2031 | 3558 | 3484 | 3557 | 3681 | 3937 | 3626 | 3583 | 4253 | 2488 | 4268 | 4137 | 4254 | 3646 | 4198 | 4385 | 3627 | 4382 | 4408 | 4457 | 4376 | 4254 | 1614 | 1614 |  | 0.11 | 0.12 | 0.12 | 0.12 | 0.12 | 0.12 | 0.13 | 0.13 | 0.13 | 0.18 | 0.10 | 0.11 | 0.10 | 0.14 | 0.14 | 0.16 | 0.11 | 0.13 |
| MAU | 4517 | 3953 | 4710 | 4685 | 4804 | 5043 | 4925 | 4873 | 3112 | 4990 | 4931 | 5011 | 5124 | 5332 | 5087 | 5050 | 5491 | 3972 | 5583 | 5244 | 5418 | 5114 | 5315 | 5651 | 5102 | 5726 | 5562 | 5616 | 5469 | 5288 | 2016 | 2016 | 1484 |  | 0.05 | 0.04 | 0.06 | 0.05 | 0.06 | 0.07 | 0.06 | 0.06 | 0.09 | 0.13 | 0.13 | 0.08 | 0.07 | 0.06 | 0.08 | 0.08 | 0.07 |
| BIT | 4849 | 4311 | 5056 | 4994 | 5092 | 5394 | 5305 | 5261 | 3508 | 5273 | 5201 | 5274 | 5397 | 5641 | 5341 | 5296 | 5866 | 4175 | 5931 | 5651 | 5813 | 5358 | 5720 | 6018 | 5332 | 6063 | 5959 | 6013 | 5880 | 5709 | 2471 | 2471 | 1717 | 467 |  | 0.05 | 0.04 | 0.05 | 0.04 | 0.06 | 0.06 | 0.06 | 0.09 | 0.14 | 0.15 | 0.09 | 0.07 | 0.07 | 0.09 | 0.08 | 0.07 |
| BPV | 4917 | 4312 | 5078 | 5126 | 5276 | 5395 | 5214 | 5137 | 3398 | 5468 | 5430 | 5518 | 5614 | 5763 | 5605 | 5580 | 5781 | 4567 | 5933 | 5449 | 5655 | 5646 | 5524 | 5959 | 5654 | 6100 | 5789 | 5845 | 5657 | 5444 | 2111 | 2111 | 2119 | 758 | 1004 |  | 0.06 | 0.05 | 0.06 | 0.07 | 0.06 | 0.07 | 0.09 | 0.14 | 0.15 | 0.07 | 0.06 | 0.06 | 0.08 | 0.08 | 0.08 |
| TUC | 5187 | 4703 | 5417 | 5288 | 5350 | 5759 | 5724 | 5700 | 3983 | 5519 | 5426 | 5486 | 5626 | 5928 | 5539 | 5481 | 6273 | 4313 | 6286 | 6124 | 6260 | 5539 | 6189 | 6406 | 5488 | 6394 | 6411 | 6462 | 6360 | 6212 | 3084 | 3084 | 2021 | 1153 | 705 | 1669 |  | 0.01 | 0.01 | 0.02 | 0.02 | 0.02 | 0.07 | 0.14 | 0.15 | 0.07 | 0.05 | 0.05 | 0.07 | 0.07 | 0.07 |
| BAF | 5387 | 4824 | 5581 | 5551 | 5664 | 5914 | 5791 | 5735 | 3974 | 5848 | 5784 | 5860 | 5978 | 6199 | 5932 | 5891 | 6358 | 4784 | 6454 | 6094 | 6276 | 5954 | 6166 | 6520 | 5934 | 6597 | 6418 | 6473 | 6315 | 6124 | 2808 | 2808 | 2308 | 871 | 624 | 866 | 988 |  | 0.00 | 0.02 | 0.01 | 0.01 | 0.06 | 0.14 | 0.15 | 0.06 | 0.05 | 0.04 | 0.06 | 0.06 | 0.06 |
| ARA | 5596 | 5102 | 5823 | 5704 | 5770 | 6165 | 6119 | 6089 | 4358 | 5939 | 5847 | 5908 | 6047 | 6345 | 5961 | 5903 | 6671 | 4735 | 6694 | 6503 | 6647 | 5961 | 6569 | 6808 | 5911 | 6806 | 6797 | 6849 | 6736 | 6580 | 3387 | 3387 | 2427 | 1388 | 922 | 1737 | 422 | 917 |  | 0.02 | 0.02 | 0.02 | 0.07 | 0.14 | 0.14 | 0.07 | 0.05 | 0.05 | 0.07 | 0.06 | 0.06 |
| BCO | 5711 | 5175 | 5920 | 5851 | 5943 | 6258 | 6166 | 6120 | 4363 | 6121 | 6044 | 6113 | 6241 | 6498 | 6176 | 6127 | 6729 | 4985 | 6795 | 6501 | 6669 | 6187 | 6571 | 6881 | 6153 | 6926 | 6814 | 6868 | 6727 | 6548 | 3261 | 3261 | 2564 | 1260 | 864 | 1372 | 802 | 505 | 532 |  | 0.02 | 0.03 | 0.09 | 0.15 | 0.16 | 0.08 | 0.06 | 0.06 | 0.08 | 0.08 | 0.08 |
| PLM | 5867 | 5354 | 6086 | 5986 | 6062 | 6427 | 6361 | 6324 | 4578 | 6234 | 6147 | 6211 | 6346 | 6631 | 6267 | 6212 | 6918 | 5050 | 6960 | 6723 | 6878 | 6270 | 6791 | 7062 | 6224 | 7079 | 7026 | 7079 | 6953 | 6785 | 3535 | 3535 | 2698 | 1519 | 1077 | 1717 | 745 | 852 | 341 | 355 |  | 0.02 | 0.07 | 0.15 | 0.15 | 0.08 | 0.05 | 0.05 | 0.06 | 0.07 | 0.07 |
| FLO | 5915 | 5462 | 6156 | 5991 | 6030 | 6499 | 6491 | 6475 | 4778 | 6189 | 6083 | 6134 | 6283 | 6620 | 6175 | 6109 | 7030 | 4925 | 7017 | 6911 | 7036 | 6162 | 6974 | 7154 | 6095 | 7110 | 7189 | 7238 | 7149 | 7010 | 3896 | 3896 | 2782 | 1931 | 1465 | 2312 | 814 | 1480 | 575 | 1032 | 701 |  | 0.08 | 0.15 | 0.16 | 0.08 | 0.06 | 0.06 | 0.08 | 0.07 | 0.07 |
| IPO | 6288 | 5731 | 6486 | 6444 | 6548 | 6820 | 6700 | 6643 | 4882 | 6729 | 6658 | 6730 | 6854 | 7092 | 6797 | 6750 | 7267 | 5618 | 7360 | 6998 | 7182 | 6812 | 7070 | 7428 | 6782 | 7500 | 7323 | 7379 | 7217 | 7021 | 3692 | 3692 | 3173 | 1778 | 1457 | 1631 | 1429 | 909 | 1078 | 646 | 753 | 1391 |  | 0.20 | 0.21 | 0.14 | 0.06 | 0.04 | 0.04 | 0.13 | 0.06 |
| CHI | 3431 | 3685 | 3152 | 3535 | 3665 | 2852 | 2674 | 2642 | 4271 | 3620 | 3789 | 3824 | 3655 | 3142 | 3881 | 3985 | 2247 | 4878 | 2508 | 2159 | 2097 | 3982 | 2105 | 2231 | 4146 | 2581 | 1942 | 1905 | 1918 | 2039 | 5318 | 5318 | 6287 | 7306 | 7736 | 7400 | 8251 | 8125 | 8615 | 8564 | 8813 | 9049 | 9008 |  | 0.10 | 0.13 | 0.16 | 0.15 | 0.17 | 0.13 | 0.14 |
| LOS | 3481 | 3737 | 3203 | 3584 | 3713 | 2902 | 2725 | 2693 | 4321 | 3667 | 3836 | 3869 | 3700 | 3188 | 3926 | 4030 | 2297 | 4927 | 2555 | 2211 | 2148 | 4026 | 2157 | 2279 | 4190 | 2626 | 1993 | 1956 | 1969 | 2090 | 5366 | 5366 | 6338 | 7355 | 7785 | 7447 | 8301 | 8173 | 8666 | 8613 | 8862 | 9099 | 9056 | 51 |  | 0.13 | 0.17 | 0.16 | 0.18 | 0.14 | 0.15 |
| REC | 6626 | 6222 | 6880 | 6665 | 6673 | 7221 | 7255 | 7254 | 5602 | 6817 | 6695 | 6733 | 6892 | 7273 | 6759 | 6683 | 7777 | 5501 | 7721 | 7710 | 7814 | 6731 | 7769 | 7884 | 6643 | 7792 | 7969 | 8016 | 7951 | 7830 | 4790 | 4790 | 3577 | 2840 | 2374 | 3195 | 1708 | 2340 | 1474 | 1853 | 1498 | 909 | 1997 | 9864 | 9915 |  | 0.10 | 0.11 | 0.13 | 0.03 | 0.10 |
| BEH | 7022 | 6491 | 7234 | 7152 | 7234 | 7573 | 7482 | 7434 | 5675 | 7407 | 7322 | 7386 | 7521 | 7798 | 7442 | 7387 | 8045 | 6224 | 8109 | 7808 | 7981 | 7446 | 7879 | 8197 | 7399 | 8236 | 8125 | 8179 | 8031 | 7844 | 4527 | 4527 | 3861 | 2564 | 2180 | 2485 | 1912 | 1721 | 1491 | 1316 | 1176 | 1466 | 854 | 9845 | 9893 | 1641 |  | 0.05 | 0.04 | 0.10 | 0.08 |
| POR | 7030 | 6431 | 7197 | 7225 | 7361 | 7517 | 7333 | 7251 | 5520 | 7549 | 7498 | 7579 | 7688 | 7868 | 7657 | 7622 | 7899 | 6535 | 8055 | 7543 | 7760 | 7686 | 7619 | 8080 | 7674 | 8220 | 7890 | 7947 | 7742 | 7514 | 4210 | 4210 | 4050 | 2572 | 2380 | 2122 | 2495 | 1758 | 2158 | 1694 | 1831 | 2428 | 1080 | 9403 | 9447 | 2858 | 1263 |  | 0.04 | 0.11 | 0.03 |
| NOV | 7130 | 6624 | 7354 | 7240 | 7303 | 7695 | 7632 | 7594 | 5845 | 7471 | 7376 | 7434 | 7576 | 7881 | 7483 | 7422 | 8189 | 6245 | 8227 | 7988 | 8148 | 7478 | 8057 | 8333 | 7420 | 8341 | 8295 | 8348 | 8217 | 8042 | 4756 | 4756 | 3960 | 2756 | 2337 | 2769 | 1953 | 1956 | 1536 | 1497 | 1271 | 1358 | 1166 | 10062 | 10111 | 1327 | 394 | 1650 |  | 0.12 | 0.07 |
| BPA | 7286 | 6747 | 7494 | 7422 | 7507 | 7831 | 7730 | 7678 | 5918 | 7682 | 7599 | 7664 | 7798 | 8068 | 7722 | 7668 | 8295 | 6507 | 8369 | 8043 | 8222 | 7727 | 8114 | 8451 | 7681 | 8500 | 8365 | 8419 | 8263 | 8070 | 4743 | 4743 | 4132 | 2806 | 2438 | 2671 | 2196 | 1948 | 1776 | 1574 | 1457 | 1745 | 1051 | 10059 | 10106 | 1844 | 288 | 1189 | 522 |  | 0.10 |
| GRV | 7619 | 7024 | 7789 | 7808 | 7937 | 8110 | 7930 | 7850 | 6116 | 8125 | 8068 | 8148 | 8260 | 8452 | 8222 | 8184 | 8497 | 7080 | 8650 | 8141 | 8359 | 8247 | 8217 | 8677 | 8229 | 8812 | 8488 | 8545 | 8340 | 8110 | 4808 | 4808 | 4603 | 3137 | 2906 | 2718 | 2925 | 2296 | 2549 | 2148 | 2209 | 2709 | 1502 | 9987 | 10030 | 2959 | 1323 | 599 | 1647 | 1126 |  |

```
dims <- flextable_dim(ft)
dims
```

```
## $widths
## [1] 32.00297
## 
## $heights
## [1] 19.77232
## 
## $aspect_ratio
## [1] 0.6178276
```

```
# Create a new Word document
doc <- read_docx()

# Add the flextable to the Word document
doc <- body_add_flextable(doc, ft)

# Save the Word document
print(doc, target =  here("scripts/RMarkdowns/output/americas/americas_all/fst/americas_all_LD3_flextable.docx"))
```

# 4.2. Mantel test

```
cd /gpfs/gibbs/pi/caccone/mkc54/albo/americas
plink \
--allow-extra-chr \
--keep-allele-order \
--bfile output/snps_sets/r2_0.01_b \
--keep-fam output/fst/pops_4fst.txt \
--make-bed \
--out output/fst/mantel;
grep 'samples\|variants\|remaining' output/fst/mantel.log
```

18964 variants loaded from .bim file. –keep-fam: 545 people
remaining. 18964 variants and 545 people pass filters and QC.

Then convert to raw format

```
plink \
--allow-extra-chr \
--keep-allele-order \
--bfile output/fst/mantel \
--recodeA \
--out output/fst/mantel \
--silent;
grep 'samples\|variants\|remaining' output/fst/mantel.log
```

18964 variants loaded from .bim file. 18964 variants and 545 people
pass filters and QC.

Import the data and covert it to genind format

```
# import the data
albo <-
  read.PLINK(
    here("/gpfs/gibbs/pi/caccone/mkc54/albo/americas/output/fst/mantel.raw"),
    quiet = FALSE,
    chunkSize = 1000,
    parallel = require("parallel"),
    n.cores = 4
  )

# convert to genind
albo2 <- gl2gi(albo, probar = TRUE, verbose = NULL)
```

Save

```
saveRDS(albo2, here("scripts", "RMarkdowns",
  "output", "americas", "americas_all", "fst", "americas_all_LD2.rds"
))
```

Load it

```
albo2 <- readRDS(here("scripts", "RMarkdowns",
  "output", "americas", "americas_all", "fst", "americas_all_LD2.rds"
))
```

Sample data

```
sampling_loc <- readRDS(here("scripts", "RMarkdowns", "output", "sampling_loc_americas_all.rds"))
head(sampling_loc)
```

```
##       Pop_City Country Latitude Longitude Continent Abbreviation Year
## 1   Berlin, NJ     USA 39.79081  -74.9291  Americas          BER 2018
## 2 Columbus, OH     USA 39.97170  -82.9071  Americas          COL 2015
## 3   Palm Beach     USA 26.70560  -80.0364  Americas          PAL 2018
## 4  Houston, TX     USA 29.75491  -95.3505  Americas          HOU 2018
## 5  Los Angeles     USA 34.05220 -118.2437  Americas          LOS 2018
## 6   Manaus, AM  Brazil -3.09161  -60.0325  Americas          MAU 2017
##          Region      Subregion order order2 orderold AM_order      state
## 1 North America Northeast, USA     1     NA       75        6    NJ, USA
## 2 North America   Midwest, USA     2     NA       76       18    OH, USA
## 3 North America Southeast, USA     3     NA       77       13    FL, USA
## 4 North America Southwest, USA     4     NA       78       23    TX, USA
## 5 North America      West, USA     5     NA       79       30    CA, USA
## 6 South America  South America     6     NA       80       38 AM, Brazil
```

The fam file

```
fam_file <- here(
  "/gpfs/gibbs/pi/caccone/mkc54/albo/americas/output/fst/mantel.fam"
)

# Read the .fam file
fam_data <- read.table(fam_file, 
                       header = FALSE,
                       col.names = c("FamilyID", "IndividualID", "PaternalID", "MaternalID", "Sex", "Phenotype"))

# # Replace the BEN a with BEN b (remember to never name samples with the same ID... I change it manually in the fam file.)
# fam_data <- fam_data %>% 
#   mutate(IndividualID = ifelse(FamilyID == "BEN" & IndividualID == "a", "b", IndividualID))

# View the first few rows
head(fam_data)
```

```
##   FamilyID IndividualID PaternalID MaternalID Sex Phenotype
## 1      MAU          153          0          0   0        -9
## 2      MAU          154          0          0   0        -9
## 3      MAU          155          0          0   0        -9
## 4      MAU          156          0          0   0        -9
## 5      MAU          158          0          0   0        -9
## 6      MAU           16          0          0   0        -9
```

Merge

```
# Join with sampling_loc to get sampling localities
loc_albo <- fam_data |>
  left_join(sampling_loc, by = c("FamilyID" = "Abbreviation"))

head(loc_albo)
```

```
##   FamilyID IndividualID PaternalID MaternalID Sex Phenotype   Pop_City Country
## 1      MAU          153          0          0   0        -9 Manaus, AM  Brazil
## 2      MAU          154          0          0   0        -9 Manaus, AM  Brazil
## 3      MAU          155          0          0   0        -9 Manaus, AM  Brazil
## 4      MAU          156          0          0   0        -9 Manaus, AM  Brazil
## 5      MAU          158          0          0   0        -9 Manaus, AM  Brazil
## 6      MAU           16          0          0   0        -9 Manaus, AM  Brazil
##   Latitude Longitude Continent Year        Region     Subregion order order2
## 1 -3.09161  -60.0325  Americas 2017 South America South America     6     NA
## 2 -3.09161  -60.0325  Americas 2017 South America South America     6     NA
## 3 -3.09161  -60.0325  Americas 2017 South America South America     6     NA
## 4 -3.09161  -60.0325  Americas 2017 South America South America     6     NA
## 5 -3.09161  -60.0325  Americas 2017 South America South America     6     NA
## 6 -3.09161  -60.0325  Americas 2017 South America South America     6     NA
##   orderold AM_order      state
## 1       80       38 AM, Brazil
## 2       80       38 AM, Brazil
## 3       80       38 AM, Brazil
## 4       80       38 AM, Brazil
## 5       80       38 AM, Brazil
## 6       80       38 AM, Brazil
```

Get the latitude and longitude

```
albo_dist2 <- cbind(loc_albo$Latitude, loc_albo$Longitude)
head(albo_dist2)
```

```
##          [,1]     [,2]
## [1,] -3.09161 -60.0325
## [2,] -3.09161 -60.0325
## [3,] -3.09161 -60.0325
## [4,] -3.09161 -60.0325
## [5,] -3.09161 -60.0325
## [6,] -3.09161 -60.0325
```

```
colnames(albo_dist2)<- c("x","y")
```

Add jitter

```
albo_dist2 <- jitter(albo_dist2, factor = 1, amount = NULL)
head(albo_dist2)
```

```
##              x         y
## [1,] -3.099679 -60.03806
## [2,] -3.085149 -60.04335
## [3,] -3.091166 -60.02168
## [4,] -3.103011 -60.02095
## [5,] -3.074193 -60.04446
## [6,] -3.106245 -60.03397
```

Add to object

```
albo2$other$xy <- albo_dist2
```

Save

```
saveRDS(
  albo2,
  here("scripts", "RMarkdowns",
    "output", "americas", "americas_all", "fst", "americas_all_LD2_MAF1.rds"
  )
)
```

### 4.2.1 Isolation By Distance for All American set

Convert the data

```
toto <- genind2genpop(albo2)
```

```
## 
##  Converting data from a genind to a genpop object... 
## 
## ...done.
```

Get 1 mosquito per population, it is just to get the geographical
coordinates

```
unique_populations <- unique(albo2@pop)
selected_individuals <- integer(length(unique_populations))
for (i in seq_along(unique_populations)) {
  inds_in_pop <- which(albo2@pop == unique_populations[i])
  selected_individuals[i] <- sample(inds_in_pop, 1)
}
albo2_subset <- albo2[selected_individuals, ]
```

Mantel test

```
Dgen <- dist.genpop(toto,method=2)
Dgeo <- dist(albo2_subset$other$xy)
ibd <- mantel.randtest(Dgen,Dgeo)
ibd
```

```
## Monte-Carlo test
## Call: mantel.randtest(m1 = Dgen, m2 = Dgeo)
## 
## Observation: 0.0695385 
## 
## Based on 999 replicates
## Simulated p-value: 0.122 
## Alternative hypothesis: greater 
## 
##      Std.Obs  Expectation     Variance 
## 1.2053498909 0.0006169272 0.0032695202
```

Plot

```
# Plot it
# Start the PDF device
CairoPDF(here("scripts", "RMarkdowns", 
     "output", "americas","americas_all", "fst", "sim_MAF01.pdf"))
plot(ibd)
dev.off()
```

```
plot(ibd)
```

```
#CairoPDF(here("scripts", "RMarkdowns", 
 #    "output", "americas","americas_all", "fst", "Genetic_vs_Geographic_Distance_MAF01.pdf"))

plot(Dgeo, Dgen)
# A linear regression model (lm stands for "linear model") is fitted, with the genetic distances (Dgen) as the response variable and the geographic distances (Dgeo) as the predictor. The distances are transformed into vectors using as.vector because the dist function produces a matrix-like structure, but the linear regression function lm requires vectors.
dist_lm <- lm(as.vector(Dgen) ~ as.vector(Dgeo))
abline(dist_lm, col="red", lty=2)
```

```
#dev.off()
```

Add the equation

```
CairoPDF(here("scripts", "RMarkdowns", 
     "output", "americas","americas_all", "fst", "Genetic_vs_Geographic_Distance_equ.pdf"))

# Plotting the data
plot(Dgeo, Dgen, main = "Genetic Distance vs Geographic Distance")
abline(dist_lm, col="red", lty=2)

# Extracting the coefficients from the linear model
intercept <- coef(dist_lm)[1]
slope <- coef(dist_lm)[2]
r2 <- summary(dist_lm)$r.squared

# Generating the equation string
equation <- sprintf("y = %.4fx + %.2f", slope, intercept)
r2_label <- sprintf("R^2 = %.2f", r2)

# Adding the equation and R^2 to the plot
# You can adjust the position (e.g., x and y values) as necessary
text(x = max(as.vector(Dgeo)) * 0.8, y = max(as.vector(Dgen)) * 0.9, labels = equation)
text(x = max(as.vector(Dgeo)) * 0.8, y = max(as.vector(Dgen)) * 0.85, labels = r2_label)

dev.off()
```

Use library MASS for plot

```
library(MASS)
dens <- kde2d(as.vector(Dgeo), as.vector(Dgen), n = 500)
myPal <-
  colorRampPalette(c("white", "blue", "gold", "orange", "red"))
# CairoPDF(here("output", "fst", "ibd.pdf"),
#     width = 5,
#     height = 4)
png(here("scripts", "RMarkdowns","output", "americas", "americas_all", "fst", "ibd2_MAF1.png"),
    width = 5,
    height = 4,
    units='in',
    res = 300)
myPal <-
  colorRampPalette(c("white", "purple", "gold", "orange", "red"))
plot(Dgeo, Dgen, pch = 20, cex = .3, bty = "n")
image(dens, col = transp(myPal(300), .7), add = TRUE)
abline(dist_lm)
# Extracting the coefficients and R^2 from the linear model
intercept <- coef(dist_lm)[1]
slope <- coef(dist_lm)[2]
r2 <- summary(dist_lm)$r.squared

# Constructing the equation and R^2 strings
equation <- sprintf("y = %.4fx + %.2f", slope, intercept)
r2_label <- sprintf("R^2 = %.2f", r2)

# Adding the equation and R^2 to the plot
text(x = max(as.vector(Dgeo)) * 0.8, y = max(as.vector(Dgen)) * 0.95, labels = equation)
text(x = max(as.vector(Dgeo)) * 0.8, y = max(as.vector(Dgen)) * 0.90, labels = r2_label)

title("Isolation by distance")
dev.off()
```

Use library MASS for plot Without equation

```
library(MASS)
dens <- kde2d(as.vector(Dgeo), as.vector(Dgen), n = 500)
myPal <-
  colorRampPalette(c("white", "blue", "gold", "orange", "red"))
# CairoPDF(here("output", "fst", "ibd.pdf"),
#     width = 5,
#     height = 4)
png(here("scripts", "RMarkdowns","output", "americas", "americas_all", "fst", "ibd2_MAF.png"),
    width = 5,
    height = 4,
    units='in',
    res = 300)
myPal <-
  colorRampPalette(c("white", "purple", "gold", "orange", "red"))
plot(Dgeo, Dgen, pch = 20, cex = .3, bty = "n")
image(dens, col = transp(myPal(300), .7), add = TRUE)
abline(dist_lm)
# Extracting the coefficients and R^2 from the linear model
intercept <- coef(dist_lm)[1]
slope <- coef(dist_lm)[2]
r2 <- summary(dist_lm)$r.squared

# Constructing the equation and R^2 strings
equation <- sprintf("y = %.4fx + %.2f", slope, intercept)
r2_label <- sprintf("R^2 = %.2f", r2)

# Adding the equation and R^2 to the plot
#text(x = max(as.vector(Dgeo)) * 0.8, y = max(as.vector(Dgen)) * 0.95, labels = equation)
#text(x = max(as.vector(Dgeo)) * 0.8, y = max(as.vector(Dgen)) * 0.90, labels = r2_label)

title("Isolation by distance")
dev.off()
```

```
library(MASS)
```

```
## 
## Attaching package: 'MASS'
```

```
## The following object is masked _by_ '.GlobalEnv':
## 
##     genotype
```

```
## The following object is masked from 'package:dplyr':
## 
##     select
```

```
dens <- kde2d(as.vector(Dgeo), as.vector(Dgen), n = 500)
myPal <-
  colorRampPalette(c("white", "blue", "gold", "orange", "red"))

myPal <-
  colorRampPalette(c("white", "purple", "gold", "orange", "red"))
plot(Dgeo, Dgen, pch = 20, cex = .3, bty = "n")
image(dens, col = transp(myPal(300), .7), add = TRUE)
abline(dist_lm)

# Extracting the coefficients and R^2 from the linear model
intercept <- coef(dist_lm)[1]
slope <- coef(dist_lm)[2]
r2 <- summary(dist_lm)$r.squared

# Constructing the equation and R^2 strings
equation <- sprintf("y = %.4fx + %.2f", slope, intercept)
r2_label <- sprintf("R^2 = %.2f", r2)

title("Isolation by distance")
```

# 4.3 Hudson Fst

Calcuate pairwise Hudson Fst on HPC

```
cd /gpfs/gibbs/pi/caccone/mkc54/albo/americas/output/fst

salloc -p scavenge --cpus-per-task=5 -t 2:00:00 --mem-per-cpu=10000    
module load R/4.2.0-foss-2020b
R

library(tidyverse)
library(adegenet)
library(here)
library(KRIS)
library(dartR)
```

Then in R (on the hpc)

```
pairwiseHudsonFst <- function(genind_object=gen, outout_file = NULL) {
  require("KRIS")
  populations <- unique(genind_object@pop)
  num_populations <- length(populations)
  fst_matrix <- matrix(NA, nrow = num_populations, ncol = num_populations, dimnames = list(populations, populations))
  calculate_fst <- function(subset_data, pop1, pop2) {
    idx_pop1 <- which(as.character(subset_data$pop) == pop1)
    idx_pop2 <- which(as.character(subset_data$pop) == pop2)
    tryCatch(
      KRIS::fst.hudson(subset_data$tab, idx_pop1, idx_pop2),
      error = function(e) {
        message(sprintf("Error calculating Fst for populations %s and %s: %s", pop1, pop2, e$message))
        return(NA)
      }
    )
  }
  for (i in 1:(num_populations - 1)) {
    for (j in (i + 1):num_populations) {
      pop1 <- populations[i]
      pop2 <- populations[j]
      subset_data <- genind_object[pop = c(pop1, pop2)]
      fst_value <- calculate_fst(subset_data, pop1, pop2)
      fst_matrix[i, j] <- fst_value
      fst_matrix[j, i] <- fst_value
    }
  }
  fst_dataframe <- as.data.frame(fst_matrix)
  if (!is.null(outout_file)) {
    write.csv(fst_dataframe, outout_file, row.names = TRUE)
  }
  return(fst_dataframe)
}
```

Convert genlight to genind object

```
LD2 <- readRDS(
  here(
    "/gpfs/gibbs/pi/caccone/mkc54/albo/americas/output/fst/LD2.rds"
  )
)

LD2_genind <- gl2gi(LD2, probar = TRUE, verbose = NULL)
```

```
pairwiseHudsonFst(genind_object=LD2_genind, outout_file = "/gpfs/gibbs/pi/caccone/mkc54/albo/americas/output/fst/hudson_fst_data_americas_all.csv")
```

# 5. IQ-tree for Americas

## 5.1 Get list of pops from Americas

```
samples1 <-  read.csv(here("scripts/RMarkdowns/output/sampling_loc_all_pops_9Apr2024.csv"), header = TRUE) |>
  dplyr::select(Continent, Subregion, Country, Abbreviation, Pop_City, Latitude, Longitude, Year)
head(samples1)
```

```
##   Continent Subregion Country Abbreviation    Pop_City Latitude Longitude  Year
## 1      Asia     China   China          HAI      Hainan  19.2000  109.6000  2014
## 2      Asia     China   China          YUN      Yunnan  24.4753  101.3431  2014
## 3      Asia     China   China          HUN       Hunan  27.6253  111.8569  2001
## 4      Asia  Southern   Japan          OKI     Okinawa  26.5013  127.9454 2018?
## 5      Asia  Southern   Japan          TAN Tanegashima  30.4564  130.9784  2008
## 6      Asia  Southern   Japan          KAG   Kagoshima  31.5602  130.5581  2018
```

Get all the populations from Americas

```
americas <- samples1$Abbreviation[samples1$Continent == "Americas"]

# Save the 'pop' data for all american locations to a text file
write.table(americas, here("americas/output/tree/americas_all.txt"), quote = FALSE, row.names = FALSE, col.names = FALSE)
```

## 5.2 PCA with Plink using the LD pruned data

I previously prepared the data with the families and individual ids,
set the reference alleles to match the AlbF3 genome assembly, and did QC
to produce file r2\_0.1\_b (With MAF filtering 1% and LD 10%)

```
cd /gpfs/gibbs/pi/caccone/mkc54/albo/americas
plink2 \
--allow-extra-chr \
--bfile output/snps_sets/r2_0.1_b \
--pca allele-wts \
--freq \
--out output/snps_sets/pca_americas \
--silent;
grep 'samples\|variants' output/snps_sets/pca_americas.log
```

545 samples (33 females, 31 males, 481 ambiguous; 545 founders)
loaded from 53638 variants loaded from
output/snps\_sets/r2\_0.1\_b.bim.

Check the files

```
cd /gpfs/gibbs/pi/caccone/mkc54/albo/americas
head -n 2 output/snps_sets/pca_americas.eigenvec
```

```
## #FID IID PC1 PC2 PC3 PC4 PC5 PC6 PC7 PC8 PC9 PC10
## MAU  153 -0.0512253  -0.0180611  -0.0228104  0.00820927  -0.0127452  -0.0216625  -0.00102254 -0.0362833  -0.00611653 0.00107263
```

```
cd /gpfs/gibbs/pi/caccone/mkc54/albo/americas
head -n 2 output/snps_sets/pca_americas.eigenval
```

```
## 36.0402
## 9.16268
```

Import PCA data

```
# import the data from Plink
pca <- read.delim(
  here(
    "americas/output/snps_sets/pca_americas.eigenvec"
  ),
  head = TRUE
)
 
# check head of the file
head(pca)
```

```
##   X.FID IID        PC1        PC2         PC3         PC4         PC5
## 1   MAU 153 -0.0512253 -0.0180611 -0.02281040  0.00820927 -0.01274520
## 2   MAU 154 -0.0491839 -0.0161037 -0.02461730 -0.00792746 -0.01938000
## 3   MAU 155 -0.0439404 -0.0146395  0.01146440  0.00155304  0.01433490
## 4   MAU 156 -0.0483692 -0.0109212 -0.03262880 -0.01615120 -0.03269590
## 5   MAU 158 -0.0504427 -0.0173971 -0.00134498 -0.02638470  0.00487328
## 6   MAU  16 -0.0532213 -0.0136383 -0.03436230 -0.00891607 -0.01382640
##           PC6         PC7        PC8          PC9        PC10
## 1 -0.02166250 -0.00102254 -0.0362833 -0.006116530  0.00107263
## 2  0.00154115  0.04522660 -0.0304253  0.014867700 -0.00878306
## 3  0.00140041  0.01961620 -0.0144866 -0.007020660 -0.00243671
## 4  0.02265900  0.05784050 -0.0191514 -0.001365880 -0.01527320
## 5 -0.00617669  0.03122540 -0.0465008  0.013875700 -0.01498130
## 6  0.00844360  0.03237400 -0.0176061  0.000400199 -0.01116950
```

```
# import the data from Plink
fam_pca <- read.delim(
  here(
    "americas/output/snps_sets/r2_0.1_b.fam"
  ),
  head = FALSE
)
 
# To see the changes
head(fam_pca)
```

```
##    V1  V2 V3 V4 V5 V6
## 1 MAU 153  0  0  0 -9
## 2 MAU 154  0  0  0 -9
## 3 MAU 155  0  0  0 -9
## 4 MAU 156  0  0  0 -9
## 5 MAU 158  0  0  0 -9
## 6 MAU  16  0  0  0 -9
```

Import sample attributes

```
# import sample attributes
samples2 <- read.delim(
  here("scripts", "RMarkdowns", "population_metadata_all_1Apr2024.txt"
  ),
  head = TRUE
)
#
# check head of the file
head(samples2)
```

```
##              geo    range continent         region             country pop
## 1      Caribbean invasive Caribbean      Caribbean             Bermuda BRM
## 2      Caribbean invasive Caribbean      Caribbean             Jamaica JAM
## 3      Caribbean invasive Caribbean      Caribbean Trinidad and Tobago SAI
## 4 Central Africa invasive    Africa Central Africa               Gabon GAB
## 5    East Africa invasive    Africa    East Africa          Madagascar DGV
## 6    East Africa invasive    Africa    East Africa          Madagascar ANT
##          pop_city               state   Subregion
## 1         Bermuda             Bermuda   Caribbean
## 2          Negirl             Jamaica   Caribbean
## 3 Saint Augustine Trinidad and Tobago   Caribbean
## 4     Franceville                <NA>      Africa
## 5     Diego ville                <NA> East Africa
## 6    Antananarivo                <NA> East Africa
```

Check number of samples per population

```
blocks_pca <- 
  pca |>
  group_by(X.FID) |>
  summarize(count_distinct = n_distinct(IID))

# check it
head(blocks_pca)
```

```
## # A tibble: 6 × 2
##   X.FID count_distinct
##   <chr>          <int>
## 1 ARA               12
## 2 BAF                7
## 3 BCO               12
## 4 BEA               12
## 5 BEH                9
## 6 BER               12
```

Merge the data

```
df4 <-
  merge(
    pca,
    samples2,
    by.x = "X.FID",
    by.y = "pop",
    all.x = T,
    all.y = F
  ) 
head(df4)
```

```
##   X.FID  IID        PC1        PC2        PC3         PC4         PC5
## 1   ARA 2571 -0.0614559 -0.0294697  0.0347773  0.02343130 -0.02826360
## 2   ARA 2582 -0.0599480 -0.0375594  0.0104330 -0.00302581  0.01183770
## 3   ARA 2574 -0.0597002 -0.0387225  0.0530016  0.00895457  0.02430710
## 4   ARA 2572 -0.0678255 -0.0235883 -0.0103110 -0.00634717  0.00134473
## 5   ARA 2573 -0.0635943 -0.0235650  0.0323129  0.01239430 -0.01581970
## 6   ARA 2579 -0.0682724 -0.0296955  0.0143650 -0.00197857  0.01461940
##            PC6         PC7        PC8         PC9        PC10           geo
## 1  0.009478990 -0.01001830  0.0122069  0.03118970  0.04164540 South America
## 2  0.003944660 -0.00299796 -0.0162203  0.01580860 -0.00289168 South America
## 3 -0.000659724 -0.04495030  0.0214710  0.04725060  0.05491530 South America
## 4  0.013393100  0.00253743  0.0209204 -0.00216233  0.00625948 South America
## 5  0.008279010 -0.01323220  0.0227525  0.03682550  0.04448760 South America
## 6 -0.008422170 -0.02044360 -0.0105984  0.01329110  0.00972138 South America
##      range     continent        region country  pop_city      state
## 1 invasive South America South America  Brazil Araguaína TO, Brazil
## 2 invasive South America South America  Brazil Araguaína TO, Brazil
## 3 invasive South America South America  Brazil Araguaína TO, Brazil
## 4 invasive South America South America  Brazil Araguaína TO, Brazil
## 5 invasive South America South America  Brazil Araguaína TO, Brazil
## 6 invasive South America South America  Brazil Araguaína TO, Brazil
##       Subregion
## 1 South America
## 2 South America
## 3 South America
## 4 South America
## 5 South America
## 6 South America
```

Get some data for the PCA plot

```
# How many populations
length(unique(df4$X.FID))
```

```
## [1] 51
```

```
# How many countries
length(unique(df4$country))
```

```
## [1] 7
```

```
# How many subregions
length(unique(df4$Subregion))
```

```
## [1] 7
```

```
# How many states
length(unique(df4$state))
```

```
## [1] 36
```

Make plot1 - by location (grouped by state)

```
library(ggrepel)
# source the plotting function
source(here("scripts", "RMarkdowns", "my_theme2.R"))

# Shapes
N = 100
M = 1000
good.shapes = c(1:25, 33:127)

# Colors
palette1 <- brewer.pal(12, "Paired")
palette2 <- brewer.pal(11, "Set3")
palette23 <- c(palette1, palette2)
colors2 <- 
  c("#52ef99", "#146c45", "#75d5e1", "#FB8072", "#2c4a5e", "#6a8fe0", "#8c61cd", "#f365e7", "#871550", "#a113b2", "#BF5B17", "#1F78B4", "#cf749b",  "#FF7F00","#2524f9", "#799d10", "#B3E2CD",  "#984EA3", "#754819", "#fda547", "#a41415", "#fd5917", "#fd4e8b", "#ead624", "#6A3D9A", "#21a708", "#332288", "#51f310", "#9d8d88", "#66C2A5", "#E41A1C", "#BC80BD", "#E7297A", "darkgray", "orange2", "magenta", "gold4", "purple", "#a7e831")


# Compute the count for each country
state_count <- df4 |>
  group_by(state) |>
  summarize(count = n())

# Merge the count back to the main data
df4 <- df4 |>
  left_join(state_count, by = "state")

# Create a custom label for the legend
df4$state_label <-
  paste(df4$state, " (", df4$count, ")", sep = "")

# Define the color and shape manually
#palette23 <- c(palette1, palette2)
colors <- setNames(colors2, unique(df4$state_label))
shapes <-
  setNames(good.shapes[c(1:25, 28:31, 55:57, 1:4)], unique(df4$state_label))


# Compute the center of ellipses for each continent
ellipse_centers <- df4 |>
  group_by(country) |>
  summarise(PC1_center = mean(PC1), PC2_center = mean(PC2))

# # Calculate the number of samples per region
country_count <- df4 |>
  group_by(country) |>
  summarise(count = n())
country_labels <-
  setNames(
    paste(country_count$country, " (", country_count$count, ")", sep = ""),
    country_count$country
  )

country_colors <-
  c("pink", "#fdc547", "#ffffaa", "#baffc9", "#9bf6ff", "#a0c4ff", "#bdb2ff")# Adjust these colors to your preference

  
# Create the plot
ggplot(df4, aes(PC1, PC2)) +
  geom_point(aes(shape = state_label, color = state_label)) +
  stat_ellipse(
    aes(fill = country, group = country),
    geom = "polygon",
    alpha = 0.25,
    level = 0.8,
    segments = 40,
    color = "darkgray"
  ) +
  stat_ellipse(
    aes(group = country),
    geom = "path",
    level = 0.8,
    segments = 40,
    color = "darkgray"
  ) + # Line around the ellipse
  geom_text_repel(
    data = ellipse_centers,
    aes(x = PC1_center, y = PC2_center, label = country),
    color = "black"
  ) + # Add labels using ggrepel
  xlab("PC1 (36.0% Variance)") + 
  ylab("PC2 (9.2% Variance)") +
  labs(caption = "Principal Component Analysis with 53,638 SNPs \n of 545 mosquitoes from 51 localities across the Americas.") + 
  guides(
    color = guide_legend(
      title = "State",
      order = 2,
      ncol = 2
    ),
    shape = guide_legend(
      title = "State",
      order = 2,
      ncol = 2
    ),
    fill = guide_legend(
      title = "Region",
      order = 1,
      ncol = 1
    )
  ) +
  scale_fill_manual(values = country_colors, labels = country_labels) +
  scale_color_manual(values = colors) +
  scale_shape_manual(values = shapes) +
  guides(fill=guide_legend(ncol=2)) +
  my_theme() +
  theme(
    plot.caption = element_text(face = "italic"),
    legend.position = "right",
    legend.justification = "top",
    legend.box.just = "center",
    legend.box.background = element_blank(),
    legend.text=element_text(size=6),
    plot.margin = margin(5.5, 30, 5.5, 5.5, "points"),
    legend.margin = margin(5, 5, 5, 40) # move the legend a bit up
  )
```

```
#   save the pca plot                                                       ####
ggsave(
  here("americas/output/tree/PCA_americas_all_r1_plink_bystate_PC1&2.pdf"
  ),
  width  = 10,
  height = 7,
  units  = "in"
)
```

Plot PCs 1 & 3

```
# source the plotting function
source(here("scripts", "RMarkdowns", "my_theme2.R"))

# Shapes
N = 100
M = 1000
good.shapes = c(1:25, 33:127)

# Colors
palette1 <- brewer.pal(12, "Paired")
palette2 <- brewer.pal(11, "Set3")
palette23 <- c(palette1, palette2)
colors2 <- 
  c("#52ef99", "#146c45", "#75d5e1", "#FB8072", "#2c4a5e", "#6a8fe0", "#8c61cd", "#f365e7", "#871550", "#a113b2", "#BF5B17", "#1F78B4", "#cf749b",  "#FF7F00","#2524f9", "#799d10", "#B3E2CD",  "#984EA3", "#754819", "#fda547", "#a41415", "#fd5917", "#fd4e8b", "#ead624", "#6A3D9A", "#21a708", "#332288", "#51f310", "#9d8d88", "#66C2A5", "#E41A1C", "#BC80BD", "#E7297A", "darkgray", "orange2", "magenta", "gold4", "purple", "#a7e831")


# Compute the count for each country
state_count <- df4 |>
  group_by(state) |>
  summarize(count = n())

# Merge the count back to the main data
df4 <- df4 |>
  left_join(state_count, by = "state")

# Create a custom label for the legend
df4$state_label <-
  paste(df4$state, " (", df4$count, ")", sep = "")

# Define the color and shape manually
#palette23 <- c(palette1, palette2)
colors <- setNames(colors2, unique(df4$state_label))
shapes <-
  setNames(good.shapes[c(1:25, 28:31, 55:57, 1:4)], unique(df4$state_label))


# Compute the center of ellipses for each continent
ellipse_centers <- df4 |>
  group_by(country) |>
  summarise(PC1_center = mean(PC1), PC3_center = mean(PC3))

# # Calculate the number of samples per region
country_count <- df4 |>
  group_by(country) |>
  summarise(count = n())
country_labels <-
  setNames(
    paste(country_count$country, " (", country_count$count, ")", sep = ""),
    country_count$country
  )


country_colors <-
  c("pink", "#fdc547", "#ffffaa", "#baffc9", "#9bf6ff", "#a0c4ff", "#bdb2ff")# Adjust these colors to your preference

  
# Create the plot
ggplot(df4, aes(PC1, PC3)) +
  geom_point(aes(shape = state_label, color = state_label)) +
  stat_ellipse(
    aes(fill = country, group = country),
    geom = "polygon",
    alpha = 0.25,
    level = 0.8,
    segments = 40,
    color = "darkgray"
  ) +
  stat_ellipse(
    aes(group = country),
    geom = "path",
    level = 0.8,
    segments = 40,
    color = "darkgray"
  ) + # Line around the ellipse
  geom_text_repel(
    data = ellipse_centers,
    aes(x = PC1_center, y = PC3_center, label = country),
    color = "black"
  ) + # Add labels using ggrepel
  xlab("PC1 (36.0% Variance)") + 
  ylab("PC3 (6.4% Variance)") +
  labs(caption = "Principal Component Analysis with 53,638 SNPs \n of 545 mosquitoes from 51 localities across the Americas.") + 
  guides(
    color = guide_legend(
      title = "State",
      order = 2,
      ncol = 2
    ),
    shape = guide_legend(
      title = "State",
      order = 2,
      ncol = 2
    ),
    fill = guide_legend(
      title = "Region",
      order = 1,
      ncol = 1
    )
  ) +
  scale_fill_manual(values = country_colors, labels = country_labels) +
  scale_color_manual(values = colors) +
  scale_shape_manual(values = shapes) +
  guides(fill=guide_legend(ncol=2)) +
  my_theme() +
  theme(
    plot.caption = element_text(face = "italic"),
    legend.position = "right",
    legend.justification = "top",
    legend.box.just = "center",
    legend.box.background = element_blank(),
    legend.text=element_text(size=6),
    plot.margin = margin(5.5, 30, 5.5, 5.5, "points"),
    legend.margin = margin(5, 5, 5, 40) # move the legend a bit up
  )
```

```
#   save the pca plot                                                       ####
ggsave(
  here("americas/output/tree/PCA_americas_all_r1_plink_bystate_PC1&3.pdf"
  ),
  width  = 10,
  height = 7,
  units  = "in"
)
```

## 5.3 Run iqtree

Create a vcf with the intergenic SNPs to run iqtree Using SNP set
with MAF pruning at 1% and LD pruning at 10%

```
cd /gpfs/gibbs/pi/caccone/mkc54/albo/americas

plink2 \
--allow-extra-chr \
--bfile output/snps_sets/r2_0.1_b \
--extract output/tree/intergenic.txt \
--recode vcf \
--out output/tree/file2 \
--silent;
grep 'samples\|variants\|remaining' output/tree/file2.log
```

545 samples (33 females, 31 males, 481 ambiguous; 545 founders)
loaded from 53638 variants loaded from output/snps\_sets/r2\_0.1\_b.bim.
–extract: 12772 variants remaining. 12772 variants remaining after main
filters.

Convert it to phylip format with python script

```
python /gpfs/gibbs/pi/caccone/mkc54/albo/americas/output/tree/vcf2phylip.py -i /gpfs/gibbs/pi/caccone/mkc54/albo/americas/output/tree/file2.vcf
```

Create run\_iqtree.sh file:

```
#!/bin/sh        
#SBATCH --ntasks=1
#SBATCH --cpus-per-task=10         
#SBATCH --mem-per-cpu=5gb
#SBATCH --time=100:00:00
#SBATCH --partition=week
#SBATCH --job-name=iqtree_albo_all
#SBATCH -o iqtree.%A_%a.albo.o.txt
#SBATCH -e iqtree.%A_%a.albo.ERROR.txt

# DO NOT EDIT LINE BELOW
cd /gpfs/gibbs/pi/caccone/mkc54/albo/americas/output/tree

# Load conda
module load miniconda
conda activate iqtree-env
export PATH=$PATH:/home/mkc54/.conda/envs/iqtree-env

# Run IQ-TREE
iqtree2 -s /gpfs/gibbs/pi/caccone/mkc54/albo/americas/output/tree/file2.min4.phy -m MFP -bb 1000 -bnni -st DNA -nt AUTO
```

Run it

```
cd /gpfs/gibbs/pi/caccone/mkc54/albo/americas/output/tree

sbatch run_iqtree.sh #53898071
 
squeue -u mkc54 #check it
scontrol show job 53898071
```

Make a populations metadata file to color the branches

```
library(ape)       # For reading/writing trees
library(dplyr)
```

Load the tree

```
tree <- read.tree("americas/output/tree/file2.min4.phy.treefile") 

#Load populations file
metadata <- read.delim("americas/output/tree/populations.txt", stringsAsFactors = FALSE)
```

```
# Assign numeric codes to populations
metadata <- metadata %>%
  mutate(pop_code = as.numeric(factor(population)))  # PopA = 1, PopB = 2, etc.

# Ensure order matches tree tip labels
pop_vector <- metadata$pop_code[match(tree$tip.label, metadata$taxon)]
```

Create and Write the NEXUS File

```
write.nexus_with_traits <- function(tree, tip_traits, trait_name = "population", file = "tree_with_traits.nex") {
  ntax <- length(tree$tip.label)
  
  # Start writing
  cat("#NEXUS\n\n", file = file)
  
  # TAXA block
  cat("Begin taxa;\n", file = file, append = TRUE)
  cat(paste0("    Dimensions ntax=", ntax, ";\n"), file = file, append = TRUE)
  cat("    Taxlabels\n", file = file, append = TRUE)
  write(paste("        ", tree$tip.label), file = file, append = TRUE)
  cat("    ;\nEnd;\n\n", file = file, append = TRUE)
  
  # TREES block
  cat("Begin trees;\n", file = file, append = TRUE)
  cat("    Tree TREE1 = [&R]", write.tree(tree), "\n", file = file, append = TRUE)
  cat("End;\n\n", file = file, append = TRUE)
  
  # CHARACTERS block for trait
  cat("Begin characters;\n", file = file, append = TRUE)
  cat(paste0("    Dimensions ntax=", ntax, " nchar=1;\n"), file = file, append = TRUE)
  cat("    Format datatype=Standard symbols=\"0123456789\" missing=? gap=-;\n", file = file, append = TRUE)
  cat(paste0("    Charstatelabels\n        1 ", trait_name, ";\n"), file = file, append = TRUE)
  cat("    Matrix\n", file = file, append = TRUE)
  for (i in seq_along(tree$tip.label)) {
    cat(paste0("        ", tree$tip.label[i], " ", tip_traits[i], "\n"), file = file, append = TRUE)
  }
  cat("    ;\nEnd;\n", file = file, append = TRUE)
}

# Write the file
write.nexus_with_traits(tree, pop_vector, file = "americas/output/tree/tree_with_population.nex")
```

Re-do by subregion

```
tree <- read.tree("americas/output/tree/file2.min4.phy.treefile") 

#Load populations file
metadata <- read.delim("americas/output/tree/populations_subregion.txt", stringsAsFactors = FALSE)
```

```
# Assign numeric codes to populations
metadata <- metadata %>%
  mutate(pop_code = as.numeric(factor(population)))  # PopA = 1, PopB = 2, etc.

# Ensure order matches tree tip labels
pop_vector <- metadata$pop_code[match(tree$tip.label, metadata$taxon)]
```

Create and Write the NEXUS File

```
write.nexus_with_traits <- function(tree, tip_traits, trait_name = "population", file = "tree_with_traits.nex") {
  ntax <- length(tree$tip.label)
  
  # Start writing
  cat("#NEXUS\n\n", file = file)
  
  # TAXA block
  cat("Begin taxa;\n", file = file, append = TRUE)
  cat(paste0("    Dimensions ntax=", ntax, ";\n"), file = file, append = TRUE)
  cat("    Taxlabels\n", file = file, append = TRUE)
  write(paste("        ", tree$tip.label), file = file, append = TRUE)
  cat("    ;\nEnd;\n\n", file = file, append = TRUE)
  
  # TREES block
  cat("Begin trees;\n", file = file, append = TRUE)
  cat("    Tree TREE1 = [&R]", write.tree(tree), "\n", file = file, append = TRUE)
  cat("End;\n\n", file = file, append = TRUE)
  
  # CHARACTERS block for trait
  cat("Begin characters;\n", file = file, append = TRUE)
  cat(paste0("    Dimensions ntax=", ntax, " nchar=1;\n"), file = file, append = TRUE)
  cat("    Format datatype=Standard symbols=\"0123456789\" missing=? gap=-;\n", file = file, append = TRUE)
  cat(paste0("    Charstatelabels\n        1 ", trait_name, ";\n"), file = file, append = TRUE)
  cat("    Matrix\n", file = file, append = TRUE)
  for (i in seq_along(tree$tip.label)) {
    cat(paste0("        ", tree$tip.label[i], " ", tip_traits[i], "\n"), file = file, append = TRUE)
  }
  cat("    ;\nEnd;\n", file = file, append = TRUE)
}

# Write the file
write.nexus_with_traits(tree, pop_vector, file = "americas/output/tree/tree_with_population_subregion.nex")
```

Then edit it in FigTree
