# Supplementary material for: Population Structure of the Invasive Asian Tiger Mosquito, Aedes albopictus, in the Americas
Source: Insects. 2026 Jul 23;17(8):755. doi: 10.3390/insects17080755 (PMC13512968; doi:10.3390/insects17080755)
Supplement: Supplementary file 1 [file insects-17-00755-s001.zip › RMarkdowns/S9 - Global dataset structure analyses.html]

Aedes albopictus all population structure analyses for Global dataset


# Aedes albopictus all population structure analyses for Global dataset

#### Margaret Corley

#### 2025-08-20

#For 1% LD dataset

# 1. LEA

Load LEA Libraries

```
library(LEA)
library(vcfR)
library(RColorBrewer)
library(adegenet)
library(ape)
library(tidyverse)
```

```
## Warning: Your system is mis-configured: '/etc/localtime' is not a symlink
```

```
library(here)
library(dplyr)
library(ggplot2)
library(colorout)
library(extrafont)
library(scales)
library(stringr)
library(ggtext)
```

LEA for all American populations + native range + selected European
populations (representing all 14 clusters identified in the European
analysis)

## 1.1 Check and import the data

Check data - we created 2 vcf files with LD pruning r2<0.01 (LD1)
and r2<0.1 (LD2) after QC for each MAF pruned dataset (MAF <1%
& <10%)

```
ls manuscript1/output/snps_sets/*.vcf
```

```
## manuscript1/output/snps_sets/r2_0.01.vcf
## manuscript1/output/snps_sets/r2_0.01_b.vcf
## manuscript1/output/snps_sets/r2_0.1.vcf
## manuscript1/output/snps_sets/r2_0.1_b.vcf
```

We will use the dataset filtered for r<0.01 LD and MAF 1% that
includes all samples (r2\_0.01\_b.vcf)

Import the data for R2<0.01 (MAF 10% filtered)

```
genotype <- here(
   "manuscript1/output/snps_sets/r2_0.01_b.vcf"
  )

d <- read.vcfR(
  genotype
)
```

```
## Scanning file to determine attributes.
## File attributes:
##   meta lines: 8
##   header_line: 9
##   variant count: 20825
##   column count: 942
## Meta line 8 read in.
## All meta lines processed.
## gt matrix initialized.
## Character matrix gt created.
##   Character matrix gt rows: 20825
##   Character matrix gt cols: 942
##   skip: 0
##   nrows: 20825
##   row_num: 0
## Processed variant 1000Processed variant 2000Processed variant 3000Processed variant 4000Processed variant 5000Processed variant 6000Processed variant 7000Processed variant 8000Processed variant 9000Processed variant 10000Processed variant 11000Processed variant 12000Processed variant 13000Processed variant 14000Processed variant 15000Processed variant 16000Processed variant 17000Processed variant 18000Processed variant 19000Processed variant 20000Processed variant: 20825
## All variants processed
```

Get population and individuals information

```
inds_full <- attr(d@gt,"dimnames")[[2]]
inds_full <- inds_full[-1]
a <- strsplit(inds_full, '_')
pops <- unname(sapply(a, FUN = function(x) return(as.character(x[1])))) 
table(pops)
```

```
## pops
## ALD ARA BAF BCO BEA BEH BEN BER BIT BPA BPV BRE BRM CAM CES CHA CHI COL CUA DAL 
##  10  12   7  12  12   9  12  12  12  11  12  13  16  12  15  12   6  10   8  12 
## DEN DES FAY FIR FLO FRS GEL GES GRA GRV HAI HAL HAN HAR HAT HOC HOU HUN INJ INW 
##  12  16  12  12   8  12   2  12  11  12  12  10   4  12  10   7  12  12  11   4 
## IPO JAF KAC KAG KAN KAT KLP KUN LAM LIN LOS MAC MAN MAT MAU MED MON NEB NEG NEH 
##   7   2   6  12  11   6   4   3   9  12  12  12  12  12  12   7  12  10   4   7 
## NEO NEW NOV NUE OKI PAL PEO PLM POR QNC RAR REC RUS SAI SER SMI SOC SON SPB SPR 
##  12  21   4  12  11  11  10   7  12  11  12  11  12  12   4  12  12   3   8   7 
## SPS SSK STL SUF SUU TAI TIK TRA TUC TUH UTS WAV YUN 
##   8  12  12   6   6   7  12  12  14  12  12   7   9
```

```
pops <- factor(pops)
inds <- unname(sapply(a, FUN = function(x) return(as.character(x[2]))))
```

Convert format

```
vcf2geno(genotype, gsub(".vcf", ".geno", genotype))
```

```
## 
##  - number of detected individuals:   933
##  - number of detected loci:      20825
## 
## For SNP info, please check /gpfs/gibbs/pi/caccone/mkc54/albo/manuscript1/output/snps_sets/r2_0.01_b.vcfsnp.
## 
## 0 line(s) were removed because these are not SNPs.
## Please, check /gpfs/gibbs/pi/caccone/mkc54/albo/manuscript1/output/snps_sets/r2_0.01_b.removed file, for more informations.
```

```
## [1] "/gpfs/gibbs/pi/caccone/mkc54/albo/manuscript1/output/snps_sets/r2_0.01_b.geno"
```

```
vcf2lfmm(genotype, gsub(".vcf", ".lfmm", genotype))
```

```
## 
##  - number of detected individuals:   933
##  - number of detected loci:      20825
## 
## For SNP info, please check /gpfs/gibbs/pi/caccone/mkc54/albo/manuscript1/output/snps_sets/r2_0.01_b.vcfsnp.
## 
## 0 line(s) were removed because these are not SNPs.
## Please, check /gpfs/gibbs/pi/caccone/mkc54/albo/manuscript1/output/snps_sets/r2_0.01_b.removed file, for more informations.
## 
## 
##  - number of detected individuals:   933
##  - number of detected loci:      20825
```

```
## [1] "/gpfs/gibbs/pi/caccone/mkc54/albo/manuscript1/output/snps_sets/r2_0.01_b.lfmm"
```

## 1.2 PCA

```
nPC <- length(inds)
pc <- pca(gsub(".vcf", ".lfmm", genotype), K = nPC)
```

```
## [1] "******************************"
## [1] " Principal Component Analysis "
## [1] "******************************"
## summary of the options:
## 
##         -n (number of individuals)          933
##         -L (number of loci)                 20825
##         -K (number of principal components) 933
##         -x (genotype file)                  /gpfs/gibbs/pi/caccone/mkc54/albo/manuscript1/output/snps_sets/r2_0.01_b.lfmm
##         -a (eigenvalue file)                /gpfs/gibbs/pi/caccone/mkc54/albo/manuscript1/output/snps_sets/r2_0.01_b.pca/r2_0.01_b.eigenvalues
##         -e (eigenvector file)               /gpfs/gibbs/pi/caccone/mkc54/albo/manuscript1/output/snps_sets/r2_0.01_b.pca/r2_0.01_b.eigenvectors
##         -d (standard deviation file)        /gpfs/gibbs/pi/caccone/mkc54/albo/manuscript1/output/snps_sets/r2_0.01_b.pca/r2_0.01_b.sdev
##         -p (projection file)                /gpfs/gibbs/pi/caccone/mkc54/albo/manuscript1/output/snps_sets/r2_0.01_b.pca/r2_0.01_b.projections
##         -c data centered
```

```
show(pc)
```

```
## * pca class *
## 
## project directory:               /gpfs/gibbs/pi/caccone/mkc54/albo/manuscript1/output/snps_sets/ 
## pca result directory:            r2_0.01_b.pca/ 
## input file:                      r2_0.01_b.lfmm 
## eigenvalue file:                 r2_0.01_b.eigenvalues 
## eigenvector file:                r2_0.01_b.eigenvectors 
## standard deviation file:         r2_0.01_b.sdev 
## projection file:                 r2_0.01_b.projections 
## pcaProject file:                   r2_0.01_b.pcaProject 
## number of individuals:           933 
## number of loci:                  20825 
## number of principal components:  933 
## centered:                        TRUE 
## scaled:                          FALSE
```

Test

```
# PC significant test: tracy-widom test
tw <- tracy.widom(pc)
```

```
## [1] "*******************"
## [1] " Tracy-Widom tests "
## [1] "*******************"
## summary of the options:
## 
##         -n (number of eigenvalues)          933
##         -i (input file)                     /gpfs/gibbs/pi/caccone/mkc54/albo/manuscript1/output/snps_sets/r2_0.01_b.pca/r2_0.01_b.eigenvalues
##         -o (output file)                    /gpfs/gibbs/pi/caccone/mkc54/albo/manuscript1/output/snps_sets/r2_0.01_b.pca/r2_0.01_b.tracywidom
```

```
# tw$pvalues
# plot the percentage of variance explained by each component
plot(tw$percentage, pch = 19, col = "blue", cex = .8)
```

Get values

```
# plot preparation
pc.coord <- as.data.frame(pc$projections)
colnames(pc.coord) <- paste0("PC", 1:nPC)
pc.coord$Individual <- inds
pc.coord$Population <- pops
# perc1 <- paste0(round(tw$percentage, digits = 3) * 100, "%")
perc <- paste0(round(pc$eigenvalues/sum(pc$eigenvalues), digits = 3) * 100, "%")
nb.cols <- 40
mycolors <- colorRampPalette(brewer.pal(8, "Set2"))(nb.cols)
```

Check R symbols for plot

```
#to see all shapes -> plot shapes - para escolher os simbolos
N = 100; M = 1000
good.shapes = c(1:20,22:25,35:38)
foo = data.frame( x = rnorm(M), y = rnorm(M), s = factor( sample(1:N, M, replace = TRUE) ) )
ggplot(aes(x,y,shape=s ), data=foo ) +
  scale_shape_manual(values=good.shapes[1:N]) +
  geom_point()
```

```
sampling_loc <- read.csv(
  here("scripts", "RMarkdowns", "output", "sampling_loc_all_pops_9Apr2024.csv"
    ))
```

```
saveRDS(sampling_loc, here("scripts", "RMarkdowns", 
  "output", "sampling_loc_all_pops.rds"
))
```

Sample data

```
sampling_loc <- readRDS(here("scripts", "RMarkdowns", "output", "sampling_loc_all_pops.rds"))
head(sampling_loc)
```

```
##      Pop_City Country Latitude Longitude Continent Abbreviation  Year    Region
## 1      Hainan   China  19.2000  109.6000      Asia          HAI  2014 East Asia
## 2      Yunnan   China  24.4753  101.3431      Asia          YUN  2014 East Asia
## 3       Hunan   China  27.6253  111.8569      Asia          HUN  2001 East Asia
## 4     Okinawa   Japan  26.5013  127.9454      Asia          OKI 2018? East Asia
## 5 Tanegashima   Japan  30.4564  130.9784      Asia          TAN  2008 East Asia
## 6   Kagoshima   Japan  31.5602  130.5581      Asia          KAG  2018 East Asia
##   Subregion order order2 orderold AM_order state FINAL_order
## 1     Japan    55     47       47       NA                 1
## 2     Japan    56     48       48       NA                 2
## 3     Japan    57     49       49       NA                 3
## 4     Japan    54     46       46       NA                 4
## 5     Japan    NA     NA       NA       NA                 5
## 6     Japan    53     45       45       NA                 6
```

More sample data

```
# import sample attributes
samples2 <- read.delim(
  here("scripts", "RMarkdowns",
    "population_metadata_all_1Apr2024.txt"
  ),
  head = TRUE
)

samples2<- samples2 |>
  dplyr::select(
    continent, region, pop
  )

# check head of the file
head(samples2)
```

```
##   continent         region pop
## 1 Caribbean      Caribbean BRM
## 2 Caribbean      Caribbean JAM
## 3 Caribbean      Caribbean SAI
## 4    Africa Central Africa GAB
## 5    Africa    East Africa DGV
## 6    Africa    East Africa ANT
```

Merge with sampling\_loc

```
merged_loc <- merge(samples2, sampling_loc, by.x = "pop", by.y = "Abbreviation")
head(merged_loc)
```

```
##   pop     continent          region           Pop_City    Country  Latitude
## 1 AIZ          Asia       East Asia Aizuwakamatsu City      Japan  37.49240
## 2 ALD        Europe Southern Europe             Durres    Albania  41.29704
## 3 ALU        Europe  Eastern Europe            Alushta    Ukraine  44.68289
## 4 ALV        Europe Southern Europe              Vlore    Albania  40.46600
## 5 ANT        Africa     East Africa       Antananarivo Madagascar -18.87920
## 6 ARA South America   South America          Araguaina     Brazil  -7.19119
##   Longitude Continent    Year          Region     Subregion order order2
## 1 139.99360      Asia    2008       East Asia         Japan    NA     NA
## 2  19.50373    Europe    2018 Southern Europe   East Europe    33     25
## 3  34.40368    Europe    2021  Eastern Europe   East Europe    43     35
## 4  19.48970    Europe    2020 Southern Europe   East Europe    32     24
## 5  47.50790    Africa    2022     East Africa   East Africa    NA     76
## 6 -48.20810  Americas unknown   South America South America    NA     NA
##   orderold AM_order      state FINAL_order
## 1       NA       NA                     16
## 2       25       NA                     80
## 3       35       NA                     90
## 4       24       NA                     79
## 5       NA       NA                     48
## 6       NA       43 TO, Brazil         140
```

Check pops

```
head(pc.coord$Population)
```

```
## [1] OKI OKI OKI OKI OKI OKI
## 93 Levels: ALD ARA BAF BCO BEA BEH BEN BER BIT BPA BPV BRE BRM CAM CES ... YUN
```

```
#Check how many sampling localities
length(unique(pc.coord$Population))
```

```
## [1] 93
```

Check the regions

```
unique(merged_loc$Region)
```

```
##  [1] "East Asia"       "Southern Europe" "Eastern Europe"  "East Africa"    
##  [5] "South America"   "West Africa"     "North America"   "South Asia"     
##  [9] "Caribbean"       "Southeast Asia"  "Central Africa"  "Indian Ocean"   
## [13] "Western Europe"  "North Africa"
```

Check the continents

```
unique(merged_loc$continent)
```

```
## [1] "Asia"          "Europe"        "Africa"        "South America"
## [5] "North America" "Caribbean"
```

Merge

```
merged_data <- merge(pc.coord, merged_loc, by.x = "Population", by.y = "pop")
head(merged_data)
```

```
##   Population      PC1      PC2      PC3      PC4      PC5      PC6      PC7
## 1        ALD 10.09330 -11.7630  9.12344 -5.86431 -13.0301 -18.8488 -5.12632
## 2        ALD  8.54143 -13.0372 10.32620 -6.40201 -14.5315 -22.4146 -3.12297
## 3        ALD  8.92085 -12.9362 11.52210 -6.46041 -10.1854 -17.1323 -5.44542
## 4        ALD  8.24525 -12.7354 10.10150 -8.06866 -12.2118 -19.7091 -4.04868
## 5        ALD  9.50405 -12.7986 10.00680 -8.98952 -13.0608 -21.6908 -4.70036
## 6        ALD  8.62124 -11.0422  7.19472 -6.58968 -10.7922 -15.8766 -4.02279
##        PC8      PC9     PC10    PC11      PC12     PC13     PC14     PC15
## 1 -6.31604 -1.03941 -4.24386 7.04841 -2.644350 -2.62419 -2.79673 2.242980
## 2 -2.94273 -1.52230 -6.30577 6.41723 -2.140240 -4.24134 -1.32372 2.408410
## 3 -4.46823 -4.17552 -5.28668 5.13351 -2.036730 -3.44339 -0.65362 4.571860
## 4 -1.10887 -4.33362 -4.62563 6.97150 -2.259420 -5.89913 -2.64679 0.456098
## 5 -1.49191 -3.80735 -3.35405 7.16143 -2.061190 -5.54987 -2.98937 1.435970
## 6 -3.90882 -1.26050 -4.06283 5.05843  0.142625 -3.68867 -2.71906 0.138041
##       PC16      PC17     PC18    PC19      PC20     PC21      PC22     PC23
## 1 -3.43622 -14.15530 11.85380 3.30346 -16.15630  2.55010 -4.072430 -4.78102
## 2 -5.20749 -14.29820  9.60743 4.54512 -16.05910  2.13572 -5.792870 -2.02435
## 3 -3.83459 -12.57310  3.37865 3.23015  -8.79551 -1.66476 -2.766390 -2.06919
## 4 -4.31231 -14.09340 12.96980 1.13248 -15.86030  4.03946 -2.305060 -5.79765
## 5 -2.78414 -14.74430 14.52460 3.93006 -17.26220  4.54176 -1.432990 -4.18861
## 6 -3.12785  -9.70071  7.49590 3.90202 -20.20350  5.00273 -0.665472 -3.76598
##       PC24     PC25      PC26     PC27     PC28     PC29     PC30      PC31
## 1 -8.62569 -4.01921 -3.515240 14.69240 10.09880 -3.94714 -6.63345  1.309920
## 2 -5.23623 -4.64081 -0.952301 14.01890 13.56990 -3.68035 -7.83646 -0.773477
## 3 -5.32721 -5.28797  0.272305  7.81639  7.39529 -1.62963 -3.75170  0.247673
## 4 -5.91187 -4.01517 -3.137300 16.48520 12.30940 -4.90670 -8.01304  0.936799
## 5 -5.17350 -4.43732 -2.725110 14.46390 14.85400 -4.64028 -7.80826  0.310018
## 6 -4.53911 -3.40582 -2.566750 16.01650 11.32430 -7.15740 -5.81391 -1.560200
##       PC32      PC33      PC34      PC35    PC36    PC37     PC38    PC39
## 1 -4.88248 -0.662480 -11.90520 -12.07740 17.6010 4.62771 -7.40209 8.01349
## 2 -1.76665  0.243663  -4.03353  -7.69360 12.1656 6.10563 -9.61658 8.58963
## 3 -2.49731 -0.973006  -9.96522  -5.59159 13.8082 3.06077 -3.66175 7.42779
## 4 -4.69555 -1.280600 -16.84870  -8.65705 20.4130 2.32176 -7.74898 7.40349
## 5 -5.34822 -0.853332 -15.71480  -6.43761 17.9431 2.03751 -6.32168 7.53385
## 6 -3.57475 -0.639002 -11.38560 -10.83120 15.7391 3.77538 -7.38436 5.26764
##       PC40     PC41     PC42      PC43    PC44    PC45      PC46    PC47
## 1 10.40320 -16.3536  2.88971  0.664126 3.49689 5.38696 -10.43230 7.35284
## 2  8.78577 -14.5574  5.07919 -1.208890 3.46336 5.22966 -10.84990 8.11188
## 3  6.06001 -13.2669 -2.02535  1.551140 1.56440 1.08894  -7.45707 6.69755
## 4 10.87300 -15.6962  4.43085  0.877073 4.95553 4.80767  -5.67341 7.59379
## 5 10.04470 -15.9452  7.27214  0.775980 5.84032 5.32759  -6.85311 8.46941
## 6  9.81581 -14.7652 -3.19968  1.195990 3.29015 4.20793  -5.84326 4.53722
##      PC48      PC49      PC50     PC51      PC52       PC53     PC54      PC55
## 1 4.27301  0.995720  4.336090 -5.05351 -0.451806  0.0601204 -2.53663 -1.955260
## 2 4.88125  2.196530  4.095070 -5.09077  1.149760 -0.5149370 -1.22175  0.531665
## 3 1.68132  1.401730  4.798590 -1.77313 -1.653250  2.0709100 -1.54323 -0.573185
## 4 5.17556  0.984952  1.622450 -4.95124 -1.154510  3.5080800 -3.38720 -0.922736
## 5 3.52888  1.142590  0.238119 -6.22600 -2.151940  1.1814200 -5.57678 -1.386600
## 6 4.42883 -2.112870 -1.023000 -8.42272  2.167520 -2.5087800 -2.48133 -0.886902
##        PC56      PC57      PC58     PC59     PC60     PC61     PC62       PC63
## 1  2.779480 -0.521845  1.328130 -1.30664 -1.26034  3.49284 -1.61945  1.5691300
## 2  2.891440 -2.791460  1.662880 -1.18467 -1.40521 -0.82100  1.04408 -2.0401800
## 3  4.437490 -5.321650  0.194928 -3.76281  0.28589  3.60743 -1.42117  0.3920520
## 4 -0.773035 -2.924080  3.233560  0.24864 -1.55491  2.93015  3.46361 -0.0561263
## 5 -0.536073 -3.941520  4.235960 -1.48453 -2.54475  3.09301  3.29698  0.1881920
## 6 -1.066190 -2.551790 -2.071610  2.35835  1.28365  3.20390  2.00791  4.1496100
##        PC64    PC65    PC66      PC67       PC68      PC69      PC70     PC71
## 1  1.480240 1.49981 5.91814 -0.475326  6.7161700  1.759570  0.699666 -5.28318
## 2 -0.308522 3.11432 6.56965  0.132029  5.9072100  3.106210 -2.140370 -5.20207
## 3 -2.404150 1.07071 6.48254 -1.856580  0.0146657  0.841348  1.141090 -3.67466
## 4  2.479560 1.94738 1.66543 -0.907799 -1.3501300 -2.474510 -6.211780 -4.65579
## 5  1.898830 2.10514 2.02940 -2.847680 -2.0645500 -2.312420 -7.239100 -6.68500
## 6  0.294738 3.27680 2.94510 -3.342140  5.4250200  3.172930 -2.339090 -4.29918
##       PC72      PC73       PC74      PC75      PC76      PC77     PC78
## 1 1.132390 -0.917510 -2.1556500 -2.125360  1.563610  1.382360  3.11634
## 2 0.273237  0.372440 -0.0719483 -0.827902 -0.376972  0.632275  1.32127
## 3 4.741490  2.644800 -2.9539100 -1.349670 -0.792902  0.213763  4.81271
## 4 3.239540  0.156160 -0.3565370 -3.274470  2.103870 -0.979774 -5.11838
## 5 1.667020 -0.161543  1.6766900 -1.816270  1.406000 -1.765400 -5.79117
## 6 0.998318 -3.122530 -0.5882820  1.059140 -0.371298  7.644050  2.15560
##        PC79    PC80      PC81       PC82      PC83     PC84      PC85      PC86
## 1 -3.412780 7.71662  4.369110 -0.6701740 -3.564000 -6.49000  0.756967 -3.340360
## 2  0.208293 9.17052  3.428610 -4.1270700 -4.305880 -2.60987  1.047490 -1.245870
## 3  0.352824 3.01740  6.243540  0.0158564  0.545566  3.04751  2.609550  0.560775
## 4  3.983880 6.73300 -0.342630  3.0611700 -0.233873 -4.76341 -0.723625  4.473030
## 5  1.243690 7.17807 -1.882390  2.8411200 -0.942992 -5.18604  0.987498  3.793950
## 6 -1.643320 5.29829  0.958735  4.0920000 -1.860970 -4.41087 -1.066590  1.309990
##        PC87     PC88      PC89      PC90      PC91    PC92      PC93     PC94
## 1 -1.322670  5.94645 -1.871890  0.429704   1.51261 8.05275  4.000420  1.32809
## 2 -0.174346  7.78799 -0.787578  0.444896   3.46242 5.29273  5.521330  4.57139
## 3  0.770578 -3.66322  1.013820 -1.009970   3.55854 0.11164  2.254360  3.69718
## 4  2.208900 -5.11703  0.756979  0.707765 -14.06640 3.39991 -1.824040 -4.72339
## 5  2.236620 -5.19498 -0.925681  0.637075 -14.28370 3.97848 -0.395965 -4.72165
## 6 -0.136030  3.74589  1.225850  0.579995   3.48448 6.12676  1.636820  2.59394
##        PC95      PC96      PC97       PC98       PC99      PC100     PC101
## 1  1.566930  1.818860 -0.715538   0.339149  0.0690415   0.343856  2.777620
## 2  3.187420  2.116630  0.635720   5.499100 -0.1348490   2.025760  1.478490
## 3  0.666683 -1.328010 -1.255830   2.238540  0.9522360   5.071020 -2.377790
## 4 -1.053940  4.054140  0.184419  -9.403800 -5.9190300 -12.787900  4.456070
## 5 -0.684541  4.618680 -0.501728 -10.272200 -4.9047100 -14.485000  4.683010
## 6  0.235971 -0.885724 -1.239480  -1.684890  0.3918780   1.488920  0.112608
##       PC102    PC103     PC104    PC105      PC106    PC107    PC108     PC109
## 1  2.538040 -5.12872  0.694282 -4.39654   5.068500 -0.24150 -3.72833 -0.923492
## 2  2.451830 -4.97632  3.976280 -4.93749   8.995070  4.94808 -2.48699 -1.740500
## 3 -2.224230 -3.22814  3.262900 -2.43652  -0.289143  2.53646  1.88570 -5.203050
## 4 -5.385240  3.46754 -4.696780  1.37274 -19.694700 -6.89268  5.63172  4.466930
## 5 -6.469010  2.84932 -4.177630  1.72027 -20.622300 -7.65578  6.93947  4.757590
## 6  0.142089 -1.21102  0.190264 -3.39762   4.461870 -2.57088 -2.17410 -0.439139
##       PC110     PC111     PC112    PC113    PC114     PC115      PC116    PC117
## 1  4.874550 -0.581097  1.148810 2.287680  4.08507 -3.577370  0.8564590  5.93341
## 2  6.084850  0.733213  1.959260 3.408620  6.45453  2.425030  1.5603100  8.28507
## 3  0.564625  4.988990  2.221290 2.797290  2.56496 -2.461960  2.8795800  6.09367
## 4 -3.565630 -3.186170 -6.858180 1.919010 -9.95828 -2.799240 -6.9385700 -9.76611
## 5 -5.663730 -3.334130 -5.556300 2.913330 -9.40137 -3.757040 -7.9505300 -9.99952
## 6  6.641430 -4.670310 -0.693891 0.253778  2.87706  0.916568 -0.0539251  2.75662
##       PC118     PC119     PC120      PC121     PC122    PC123     PC124
## 1 -1.926550 -4.108030   1.22630   3.695240 -10.85520 -5.23737  3.196130
## 2 -4.940950 -6.333000   1.83757   4.108390 -14.60400 -2.54095  0.241309
## 3 -2.507500  1.796370   1.20754   7.462600  -5.70045  3.73332 -5.811080
## 4  2.215510 -0.791354 -10.51650 -12.746700  20.01530  2.58512 -1.741820
## 5  4.113220 -1.228760 -10.45980 -10.043800  21.55720  3.41621 -3.060420
## 6 -0.570646 -3.501120  -4.37603  -0.451358  -5.12162 -4.18623 -0.770424
##      PC125    PC126     PC127     PC128     PC129      PC130     PC131
## 1 -5.04146 -2.59349  3.237120 -3.999420  1.397440  -5.493130   6.43064
## 2 -4.68080 -3.64402 -0.600661 -0.840055 -1.644940 -10.136700   5.34937
## 3 -5.61947 -6.26906  3.322780  0.404488  0.208432  -2.784410   8.45472
## 4 12.80860  9.21290 -4.885200 10.803200  1.687290  11.665400 -10.45130
## 5 14.80360  9.10190 -4.580810 11.112900  0.868551  11.957700 -11.71160
## 6  1.93143 -2.67672  7.218260 -0.891703  1.977850  -0.411091   2.75293
##        PC132     PC133     PC134     PC135     PC136     PC137    PC138
## 1 -0.5597020 -0.334286 -3.180930 -4.392260 -0.345361  0.602205 -3.89681
## 2  0.6319400  1.888510 -1.476620 -2.816160  3.191320  3.156410 -3.98407
## 3  0.1140110 -1.081730  0.751138  0.641509  1.252520  0.754353 -1.71212
## 4  0.0665431  2.590740 -2.322900  4.199000 -3.025570 -5.181920  9.74739
## 5  0.6427270  3.540590 -3.294340  3.982250 -3.501560 -6.975960 12.04460
## 6 -0.1298280  4.597020  0.223364 -1.748290 -1.710870  0.971168 -1.33489
##       PC139    PC140      PC141     PC142    PC143     PC144     PC145    PC146
## 1 -2.549150 3.265270 -0.6886430  1.180680  2.99787  1.299470 -0.116027  1.49773
## 2 -5.803300 4.563780  0.0280301 -0.604131  1.09687  0.337579  2.474420  7.24408
## 3  0.240692 2.353220 -1.8120100  1.555460  4.13035  0.236624 -1.713150 -1.40759
## 4 10.820300 1.791330  0.7920800 -1.884360 -3.86085 -6.002610  3.861230 -5.64295
## 5  9.661990 1.306470  0.4755300 -1.745370 -2.52793 -5.880440  2.232490 -4.42855
## 6 -3.637250 0.416339  0.9157790  1.609830  1.17370 -1.511240 -0.245127  2.40924
##       PC147      PC148     PC149     PC150      PC151    PC152     PC153
## 1  1.791560   3.609000 -3.911270  3.107870  1.5541300  0.22275 -0.730979
## 2  0.582894   4.280260 -2.566710  2.541150  6.5371000 -2.61586 -4.779410
## 3 -6.638920   5.095520 -4.249450  2.489030  0.0846622  3.83151  3.239790
## 4  4.189260  -7.833920  0.577805 -5.534770 -9.9985900  2.20603 -3.937350
## 5  4.657440 -10.589700  1.456160 -6.514900 -9.7589000  2.51704 -4.130060
## 6 -0.106420  -0.321046  5.948370 -0.248796  2.2838800 -1.88939  3.764900
##       PC154     PC155     PC156    PC157      PC158     PC159      PC160
## 1 -0.670278 -7.430020 -3.375740  7.63636 -3.4549000  1.637250 -5.2342400
## 2 -6.975830 -7.780590 -2.853440  4.00324  1.4152800 -1.870030 -2.0352900
## 3  4.514300 -0.752614 -2.275220  2.59495 -0.3306650  0.158886  0.0665491
## 4  3.511400  8.122330  0.671008 -8.85471  0.0133184 -1.607700 -0.9738770
## 5  3.312490  7.705030  1.003230 -6.91556  0.1063460 -1.588290 -0.8661500
## 6 -1.117320 -2.700510 -2.543710 -1.29663 -2.9668900  2.769880  0.5310170
##      PC161     PC162    PC163     PC164    PC165       PC166    PC167     PC168
## 1 -0.29099 -1.027980  2.28043  0.421740 -1.98826  -2.5455400 -1.73629  0.509014
## 2  4.38862 -0.306629 -5.50805 -0.492861  2.88236  -0.0485982  1.59834  7.812480
## 3 -7.24527 -1.090670  6.90358  9.360960 -3.93556 -10.3429000 -3.52079 -8.883370
## 4  1.42937  6.033110  0.80074 -0.689952  1.05529   0.1229300  4.88883  0.230709
## 5  1.88303  6.443010  1.96861 -1.435650  1.55201   1.2411200  3.93904 -2.268930
## 6  3.38277 -5.414230 -1.47620 -3.059460  1.76654   3.3227700 -1.79468 -1.610800
##       PC169    PC170     PC171     PC172     PC173     PC174     PC175
## 1 -1.063600 -0.26271 -0.188165 -5.189500 -1.483230  0.888230  1.335840
## 2 -0.454970 -3.35711 -3.606530 -2.690170  1.751560 -3.570020  6.253700
## 3  0.216617  5.43862  5.736930  6.609600 12.980000  7.922810 -1.328650
## 4  1.802430 -3.36994  4.753770  4.009390 -2.259360 -0.733258 -0.620989
## 5  2.689520 -2.80579  2.477100  5.112200  0.316553  1.794790 -1.582370
## 6 -1.069400 -2.63256  2.322370 -0.806284 -5.200430  3.604170 -0.555150
##       PC176    PC177     PC178     PC179     PC180     PC181     PC182
## 1  3.587540  5.36904  0.147675 -1.317130 -0.160468  2.956180 -3.684630
## 2  0.345610  6.64358 -0.111922 -0.790110 -3.168700  2.965230  3.118950
## 3 -1.729040  1.65099  0.111374  1.073420  3.308370 -3.284180  0.836803
## 4 -0.363967 -8.76532  1.429320 -2.814380  4.148880 -1.036500 -4.162950
## 5 -1.393850 -8.46119  2.046570 -0.414767  3.921880 -2.052880 -2.983040
## 6  3.997100  6.00017  1.451470 -1.265460 -3.324300  0.529274 -2.204260
##       PC183     PC184       PC185     PC186    PC187     PC188     PC189
## 1  2.702480 -0.695331 -5.71097000 -0.128649 -1.14198 -4.057660  4.182680
## 2 -0.940579 -3.253960  1.70992000 -1.351070 -2.61817  1.459400 -3.438740
## 3  1.063430 -5.368180 -2.03230000 -2.495050  4.21582  4.745840  7.761160
## 4  1.915930 -1.696390  1.87522000 -1.038990  2.33463 -1.418210  1.939820
## 5 -2.269000 -0.904741  0.00948089 -1.053290  2.13928 -1.696950  0.694116
## 6 -0.168362  3.678130  0.32683500 -0.477392  1.43925  0.455222 -1.896830
##       PC190    PC191      PC192     PC193       PC194     PC195    PC196
## 1 -0.373864  1.79696 -1.1428500 -0.160414 -2.46890000  3.888530  3.37523
## 2 -4.436510  3.24751 -1.9675800 -0.408644 -3.45502000 -0.677516  4.78764
## 3  5.791720  6.35655  0.4544560  1.230090  0.00563463  1.176860  4.68138
## 4  1.644270 -5.41008  0.0611654  2.582340  0.72686900 -2.964770 -5.63829
## 5  1.914380 -4.36393 -0.0952961  1.226050  1.00225000 -3.730520 -5.16672
## 6  3.058460 -2.29753  1.9708700  2.645180 -0.99098600  1.410680  2.48648
##      PC197      PC198     PC199     PC200     PC201     PC202     PC203
## 1 -1.24863  0.4058600 -2.501250  3.273910  4.204960 -0.819051 -2.918600
## 2 -4.48025  0.0409966 -0.053692 -0.717392  0.294762  1.797670  3.127130
## 3  1.85503 -0.8971950  8.072540  2.382580  2.764940 -7.586090 -1.023710
## 4  1.58026 -0.8838120  4.297590 -1.538230  1.210850  1.025020 -0.133480
## 5  1.96942  0.1751950  3.802260 -2.308350  2.877550  0.908530 -0.122748
## 6  4.65871  4.1719700 -2.640060  0.283069 -3.862460  3.509380 -1.996600
##       PC204      PC205     PC206      PC207     PC208    PC209     PC210
## 1 -2.914560 -0.8444530 -2.022560  3.3044200 -1.211920 -1.17036  1.857270
## 2 -1.231660 -1.2233100  4.573270 -2.1417100 -0.751690 -0.15116  1.165930
## 3  1.230530  3.4254300 -8.443980 -1.0209800 -0.383264  3.69025  7.669960
## 4  1.866210  0.0334422  1.203960  0.6514880  0.285750 -2.19467 -5.025700
## 5  2.544110  0.8511340 -0.771798  0.0568928  0.694968 -2.26999 -3.363280
## 6  0.196914 -1.5066300  0.384390  0.4569260 -0.161651 -1.92314  0.566084
##      PC211      PC212      PC213      PC214    PC215     PC216     PC217
## 1 -3.35688  1.2139400 -2.5415600  2.7934100 -3.90859  0.144195  0.916903
## 2  1.40589 -1.8805500  4.9745500 -0.6362910 -2.28449  1.899020 -1.659560
## 3 -2.88763 -9.5498200 -3.8737500 -0.0671873 -2.90871 -1.462400  6.466700
## 4  1.70643  1.6211600  0.3280230  4.5484200  1.57992 -1.655070 -1.125650
## 5  1.94073  0.0921334 -0.0805536  2.5061000  2.79579 -0.942561 -3.499490
## 6 -4.67270  4.4953400 -3.2102100  0.2953750  1.70969  1.230860 -0.662461
##       PC218      PC219     PC220    PC221     PC222    PC223     PC224
## 1  0.884269 -2.8894100 -0.445345 -1.96235  1.376290 -0.94927  1.501760
## 2  4.489590 -0.0244462  5.241670 -1.62306  0.670603  6.06242 -2.327030
## 3  4.977120 -0.6682370 -1.839740 -1.50101  2.922070 -1.10911 -3.065280
## 4 -2.683590  3.7073300 -2.139630  3.08671 -2.636650  3.23445 -1.307490
## 5 -4.789060  3.3315800 -2.346720  2.26077 -1.175760  3.65672 -0.465374
## 6  1.789090  1.4560800 -4.446490  7.11941 -0.678734 -5.57651  2.756280
##       PC225     PC226     PC227       PC228      PC229     PC230      PC231
## 1  2.424100 -0.428067  2.695600  0.00686375 -1.5230200  -3.82961  0.0185871
## 2 -4.384740 -2.077150  6.903430  1.99521000 -1.1161200 -10.04070  4.7565800
## 3  3.309920 -2.197240 -0.328339  3.63027000 -3.2210200   5.67300 -0.7589530
## 4  0.837389  2.367700 -1.151010  1.69398000 -1.5555200   3.35573 -7.6851600
## 5  2.453410  5.404550 -0.447502  1.44510000  0.0988835   2.09551 -6.5022400
## 6  0.134266 -0.361933 -3.434760 -1.58069000  3.3427700   4.82254 -0.4032720
##       PC232     PC233     PC234     PC235     PC236     PC237     PC238
## 1  0.879387  0.566458 -5.418730  2.610580 -3.830100  0.914808 -1.887380
## 2 -2.885080 -3.693610  5.959680 -0.346350  1.163080 -4.162700  0.826408
## 3 -1.786790  3.852860 -4.929630  1.714710 -0.812367  8.422090 -5.702620
## 4 -0.247226  2.036000  1.538890  0.894585  3.347880 -3.580750  2.088490
## 5 -0.229130  2.956630  0.317133  1.876850  3.867710 -0.511221  0.170749
## 6  8.964630 -1.284000 -0.458985 -0.233819 -3.185630  1.134010  5.608220
##       PC239     PC240     PC241     PC242     PC243     PC244     PC245
## 1 -2.317190 -0.940303  3.032290 -1.140570  1.024300  1.323050  0.176344
## 2 -1.333060  2.721710  3.403060  4.525830  1.022800  1.737620  1.164630
## 3  0.129378 -4.884690  4.528730  3.499000 -2.715730  6.621830 -6.577010
## 4  0.913815  0.901940 -1.868900  0.102578 -0.565465  1.056780 -4.723910
## 5  1.179410  0.480021 -0.309396  1.270810  0.169296 -0.466512 -1.899260
## 6  0.757847  3.067210 -8.429350 -5.440500  1.728030 -5.007100  3.805160
##       PC246     PC247      PC248    PC249     PC250     PC251        PC252
## 1  1.648410  0.774586  0.0701642  2.33555 -0.874590 -1.736700 -1.25046e+00
## 2  2.240940 -4.492390 -1.7047000  4.98987 -1.969620 -8.175520 -1.78991e-02
## 3  0.843471  7.909380  0.9914840 -2.92508 -3.733580  4.149460 -2.73392e+00
## 4 -0.423084  1.270900  3.3486600  4.37768  0.548588  0.955590 -6.79950e-07
## 5 -0.534941  1.015050  1.7409600  3.32864  0.540639  0.578096 -4.06904e-01
## 6 -4.058180 -0.656404  1.5661800 -3.67561 -0.488039  4.754280  3.81606e+00
##       PC253    PC254     PC255     PC256     PC257    PC258     PC259     PC260
## 1 -0.831706 -3.03624  4.160310  2.542780 -6.145210 -1.89153 -1.650710 -1.559800
## 2 -1.773010 -3.67767  2.770740  1.527670 -0.472337  2.94894 -0.288388 -2.395900
## 3  0.497660 -1.71662 -3.080570 -3.530370 -5.559940 -5.16890  5.760620  3.371550
## 4 -0.607777  2.80710 -1.821840  0.219795 -0.170391  1.72051 -2.486730 -1.611060
## 5 -0.661316  2.46786 -1.861340 -0.798455  1.039260  2.10654 -0.829630  0.143126
## 6  0.142759  2.11901  0.888233 -3.096790  8.197430 -1.31263 -4.938630 -1.351370
##       PC261     PC262     PC263     PC264     PC265     PC266     PC267
## 1  0.542685 -2.245450 -0.324445  1.326010 -1.568850 -4.985640  1.412310
## 2  1.146220 -1.019760  2.272700 -0.714534  6.105650 -1.358560  3.583270
## 3 -3.787550  3.139290 -6.832740 -1.090040  0.460056  2.741400 -1.441130
## 4 -0.142819  0.260692  1.054840 -0.951522 -1.112170  2.534910 -0.578523
## 5 -0.523008  2.867380  1.533000 -3.084370  0.101489  1.515200  0.092275
## 6  4.381680  2.058280  1.858660  1.836090 -3.880660  0.957482 -4.729340
##        PC268     PC269     PC270    PC271     PC272     PC273     PC274
## 1  1.7142600  6.357190  3.922370  1.71682 -0.458056  1.922980 -3.347790
## 2 -1.5173800 -0.621177 -2.188090 -5.18670 -0.255726  1.135560 -0.758583
## 3 -1.8174400  4.072070  8.163390 -4.33232  8.851890 -3.950580 -2.970470
## 4  0.0401501 -1.734820 -0.443746  1.02888 -1.388410 -0.964337 -1.610390
## 5 -1.2541500 -1.629830 -1.443690  1.72605  1.012980 -0.222194  0.110185
## 6  3.2080900 -4.369990 -1.277110  1.28105 -6.869630 -1.573580  7.084820
##        PC275     PC276      PC277    PC278      PC279      PC280     PC281
## 1  0.8531750 -4.242010  0.0956293 -1.61472 -2.1667100 -2.6018500 -4.199050
## 2 -0.9363060  0.214335 -1.9583600  4.86708 -4.6399900  0.0887393  0.485833
## 3  1.9876600  0.525506  0.3041670 -7.04688  3.1387200  5.8024400 -2.583070
## 4 -0.0821849  0.146828  1.6924400  1.21452 -0.3658660  0.5774830  1.725530
## 5 -0.9453490 -1.262990  1.3792500  1.33425  0.0980896  0.2025190  2.321420
## 6  0.5110010 -1.806260  2.5678100 -3.43257 -4.3541000 -3.0268200  4.022050
##       PC282    PC283      PC284     PC285     PC286     PC287      PC288
## 1  2.238510 -1.19179  0.5987880 -0.907409 -0.893618  4.325580   0.782097
## 2 -4.013830  4.79962 -5.2852600  2.793580  5.513840  0.247286   0.013555
## 3  4.042600 -8.85818  4.1992900  0.408026  4.457430 -7.716290 -10.232800
## 4  0.815628  1.44832  1.4745800  0.432124  0.990861  1.458530   0.588578
## 5  0.705189  1.49058  0.0215578  2.705750  1.627080  1.247540   0.159716
## 6 -0.713422 -1.96461 -0.0775187  0.934215 -4.200700  1.015600   5.917700
##       PC289     PC290     PC291     PC292     PC293     PC294     PC295
## 1 -0.309548  3.316900  3.174100 -2.689500 -0.920206  1.168960 -5.103880
## 2  1.538110 -3.191850  0.460200  2.423630  4.222590  2.483840  2.247620
## 3  4.727190  4.786020 -0.172211 -6.152900 -4.103160 -1.785810  6.362270
## 4 -0.190263 -0.554694 -3.391460 -0.254744  3.446880  1.376910  0.326889
## 5  0.324808 -1.191980 -2.470070 -0.250750 -0.570568  0.891394  0.614787
## 6 -7.928340  1.523370 -1.344660 -0.538572 -1.049220 -1.695150 -3.008910
##       PC296     PC297    PC298     PC299     PC300     PC301     PC302
## 1 -1.479510  3.925120 -3.43284  4.657100  0.768848 -0.653177  2.199090
## 2 -2.479630  0.703868  1.23798  3.724930 -1.415880  0.433164 -2.071370
## 3  3.296630  0.770974 -2.38424 -4.214310  1.537250 -6.486210  3.988650
## 4 -1.148700 -1.910470 -1.56398 -1.015920 -1.293740  0.553479  0.677321
## 5  0.168901 -1.409930 -1.16690 -1.426150 -1.806890 -0.355650  0.734791
## 6 -2.203570 -1.488190  2.58568  0.261778 -0.724129  5.311160 -1.842270
##       PC303     PC304     PC305     PC306     PC307     PC308     PC309
## 1 -3.182830 -2.163520  0.541463 -3.599900  2.722690  0.174623  2.349450
## 2  1.773620 -2.687790 -3.390940  1.080650  0.372144  1.958140 -0.119504
## 3 -4.494810  5.678850 -6.502590 -7.848180 -0.468627  5.874780  4.277540
## 4 -1.632370 -0.785093  1.000430  1.589690 -0.375141  0.658914  0.145633
## 5 -0.989485 -2.187180  0.955176  0.811074 -0.556287  0.256041  1.247650
## 6 -2.497000  2.551480  5.189890  9.008270 -5.232950 -6.518620 -2.657320
##       PC310     PC311     PC312     PC313     PC314    PC315    PC316     PC317
## 1  1.933220 -0.537458 -0.909060  0.691449  3.586470  0.36229  1.26737  2.300760
## 2 -0.662701 -0.930782  2.488890  5.559660 -1.663150 -1.61238  2.02602 -3.288640
## 3 -1.387170  1.834510  0.931623 -4.192510  0.322086 -2.44445  1.87035  1.036950
## 4 -1.163100 -0.773347  1.235410  0.216953 -0.212742 -1.53670  0.12868  2.085230
## 5 -0.999557  0.613967  1.072440 -1.690030 -1.002740 -1.51019 -0.41533  0.190448
## 6 -1.663400 -0.891624 -1.904780 -2.709560 -1.536360  3.16880 -1.19456  2.990650
##       PC318      PC319     PC320     PC321     PC322       PC323     PC324
## 1 -0.465216 -1.6704500  2.226130  1.532920  5.002530  5.43184000 1.7557200
## 2 -2.239130  0.0859792 -6.730450 -1.057210 -3.494650 -4.54932000 0.2582290
## 3  0.128684  4.2908500 -3.759110  7.470790 -0.292025  5.42512000 4.9684500
## 4  1.020050 -2.5966900 -0.816473 -0.931450 -3.434900  2.59069000 1.5002700
## 5  1.842610  1.4185400 -1.233670  0.615453 -1.503450 -0.00722114 0.6389790
## 6 -1.750460 -6.2246400  7.561550 -1.417740  3.239600  1.59185000 0.0398917
##      PC325     PC326     PC327     PC328     PC329     PC330     PC331
## 1 -1.55064  0.601156 -1.909670  1.377530  2.874840 -1.560850  0.714559
## 2 -1.63965 -3.844560 -3.831690  0.886180  1.274440 -1.823130  3.430730
## 3  8.48755  5.832200 -0.552764  5.424160 -7.009190  3.196230 -1.746720
## 4 -1.68258 -0.670774  2.937730 -0.681686 -1.660030 -0.169377  3.002860
## 5 -2.51904 -1.027460  1.779180 -1.326550 -0.887107 -0.118658 -0.200828
## 6  1.98460 -1.176770  1.981620 -3.149150  4.838520  3.126310  1.763570
##       PC332     PC333     PC334    PC335     PC336     PC337      PC338
## 1 -0.259504 -2.454650  0.996442 -1.61045 -1.770400  0.574471  3.2746400
## 2 -4.436250  3.774590 -0.496283 -1.20088 -4.198980  1.277540 -3.0265000
## 3 -0.982346 -7.147960  2.425710 -1.81307  4.170880 -7.529550 -0.0565951
## 4  2.266640  1.580660 -2.877860  3.71424 -1.160750  1.656160  0.4049800
## 5  0.675321  0.302406 -1.496930  2.17565  0.157950  1.467140 -1.9980900
## 6  4.191450  0.370335 -1.454490 -0.63827 -0.120136 -0.484560  4.0559600
##        PC339     PC340     PC341     PC342     PC343     PC344     PC345
## 1  0.0108402 -3.949140  0.383973  3.559280 -3.029220  0.325489  0.414494
## 2  1.5023400  0.525374 -0.711665 -2.640810 -0.879948  2.071340 -4.370450
## 3 -0.2427340  2.790690  0.685614  4.325370  1.056570 -2.918720  8.879920
## 4  2.2328200  1.952350  0.793959 -2.095690 -0.898855 -0.696723 -1.445510
## 5  1.0638800  3.687720  0.788011 -0.114715 -0.459635 -1.074850 -0.988687
## 6  0.1173760 -3.137210 -0.850899  0.782812  2.249040 -0.904149 -1.971420
##       PC346     PC347     PC348     PC349    PC350      PC351     PC352
## 1 -3.287120  6.353910 -4.509490  4.945450 -3.29983  1.1659200  0.364675
## 2 -1.044690 -3.552150 -3.039870 -7.476670  1.74514  3.1682700 -2.184290
## 3 -0.101469 -2.320380  8.212330  4.203970 -0.43648  1.6717200  1.720010
## 4  2.342220  1.193950 -1.432530  0.103589  1.95893  0.8692380 -2.581450
## 5  2.394830  0.398211 -0.768286 -1.249310  1.38168 -0.0483467 -1.246390
## 6 -0.695619 -0.519115  0.433737  5.627100 -6.34121  0.4838900  5.693480
##       PC353     PC354     PC355     PC356     PC357     PC358    PC359
## 1  0.275374 -0.404084  2.714260 -0.550385 -0.610103  3.121310 -1.16218
## 2  3.602700  2.294760  3.374030 -1.282590 -1.883330 -3.082210 -4.79642
## 3 -1.328650  1.737000 -2.097660  1.908420 -0.179202 -2.538170  1.85134
## 4 -2.818780 -0.188762 -1.650950  0.509258 -0.425722  0.480215 -1.32913
## 5 -1.274000 -1.448400  0.713621 -0.548037 -1.389260  0.422909 -2.66240
## 6 -4.150660 -1.172280 -2.794030  1.413820  7.132430  4.473120  6.05216
##       PC360    PC361     PC362     PC363    PC364     PC365     PC366     PC367
## 1  2.719680  2.21341  6.751830 -0.464810 -1.19729 -2.916290  0.354770 -1.263450
## 2  0.229673 -1.12705 -0.860891 -1.985260 -1.20924  4.039690 -0.652761 -0.404940
## 3 -4.030120 -2.25403 -1.522400  0.952070 -1.56565 -0.524858  0.985537  4.689960
## 4 -1.671090  1.40526 -1.328070 -1.282270  2.01538 -0.124195 -0.729827  0.798761
## 5 -1.980380  0.30505 -0.862815 -0.379753  1.44617 -1.197400  0.498571 -0.261867
## 6  2.213810 -1.46044 -7.760920  0.184307  1.64020  1.104900 -1.081290  4.758010
##        PC368    PC369      PC370     PC371     PC372     PC373     PC374
## 1 -0.7174670  2.21686 -4.3500600  2.409530 -3.126270  3.266080 -3.569210
## 2  2.7520700  1.13814 -3.3797500  3.283700 -0.376601  0.832266  0.968242
## 3 -2.7769600  4.27337  2.2761400 -3.444490  1.498600 -3.620930  1.653020
## 4  0.0571391  2.75438 -0.0578914  0.419103  0.566139 -0.121393  1.807940
## 5  0.5016140  1.65902  1.2229600  1.342980  0.926911 -1.309590  0.193695
## 6  0.1925790 -6.73919  7.5653800 -4.065080 -0.644576 -6.136870  2.523150
##       PC375     PC376     PC377     PC378     PC379     PC380     PC381
## 1  2.567300  6.221950 -0.989922 -1.713110 -2.569630 -0.160066  1.089540
## 2 -4.458170 -3.117900 -2.227290  2.478290 -0.215345  2.944230  3.762260
## 3  4.721290 -1.277200 -0.788629 -0.400317 -0.719255  5.636840 -0.308316
## 4  0.458039 -1.497360 -1.615810  1.904450 -0.111018 -0.998104  4.193420
## 5  0.172892 -0.607627 -2.450190 -0.175836 -2.263330  1.665030  3.806210
## 6  0.791259 -0.597713  4.497430  1.234390  1.650840 -1.924430 -4.766680
##      PC382     PC383      PC384     PC385     PC386    PC387     PC388
## 1  2.64288  3.663300 -0.7966610  0.415984  1.145320  0.44045 -0.142860
## 2 -1.68746  0.805294  3.4849200 -0.511606 -0.890222  4.72807 -3.276400
## 3  2.94976  0.226833  3.8195300  0.621174  2.415300 -5.47641  0.230073
## 4 -1.24469  0.539919  0.7954720  0.342927 -2.527320  2.00864 -1.343850
## 5 -2.45604  0.438824 -0.0361561  1.105480 -0.689992  2.33717  0.260416
## 6 -1.21576 -4.240900 -2.2945000  0.992153  4.802120 -2.94296 -0.281874
##       PC389     PC390     PC391    PC392      PC393      PC394      PC395
## 1  1.902120 -2.624350  0.299583 0.700538  4.1455000 -0.9785450 -0.5312250
## 2 -0.439681  0.653816  4.327010 3.552980  0.2435800  1.6476800 -0.1369510
## 3 -1.575890 -2.925620 -1.220680 1.519870 -5.5971600 -3.2340200 -0.0670594
## 4  1.424950 -0.117497 -2.067820 1.708240  0.5035180  2.8874200  0.1534910
## 5  1.410590  0.301313 -0.457852 0.350016 -0.0546749 -0.0505233 -1.1276800
## 6 -5.262170  3.352080 -7.005830 0.232504 -4.0519300  4.3181500  1.0485400
##        PC396      PC397      PC398       PC399     PC400     PC401     PC402
## 1  0.8659540 -1.2261200 -1.7142300  3.07456000  1.874730  6.472260  2.870440
## 2  0.4578910 -0.2554830  4.7598700 -0.22509200  2.820110 -0.217000  2.889730
## 3  4.7005900  0.1261140 -8.4274300 -7.28994000  1.646920 -2.452290 -3.178960
## 4  0.0777662  0.0551441 -0.0256262 -1.59314000 -1.822170  1.908610  0.896767
## 5 -0.3403050 -0.5251700 -1.9792300 -0.00650755 -0.223558 -1.015050  2.703620
## 6 -0.4236760  3.4684400 -1.3828400  4.60727000 -6.144420 -0.903542 -1.318640
##       PC403      PC404     PC405     PC406     PC407     PC408     PC409
## 1  0.727971  2.4900400  1.367500 -3.396330 -0.693761  3.832790  1.719710
## 2  0.801316 -4.8536100 -2.587210 -0.783654  0.215145 -1.365130  1.282210
## 3 -0.440331  4.6053500 -0.185855  0.635834 -1.742670  0.855052  1.661030
## 4  4.191010  0.0247995 -0.474136 -0.214636  0.249089  2.765060 -0.797186
## 5  2.079230  0.1013730  0.347095 -0.862246  1.009550  0.925715 -1.780530
## 6 -1.806210  1.5960800  5.912380  6.833510 -3.512680 -1.431930 -2.272750
##        PC410     PC411     PC412     PC413     PC414      PC415     PC416
## 1 -3.8529200  0.991206  0.804228  1.997960 -1.224300 -3.1296500  3.777630
## 2  1.9345900  1.462930  1.346350  5.766080 -0.139322  0.0501363 -4.247100
## 3  4.5380400  1.305950 -1.783200 -4.734530 -1.498840 -1.4989900 -2.013470
## 4 -0.0670273 -0.116476 -0.140863 -0.229715  0.576379 -0.6818830 -1.006560
## 5  1.5182000  0.490788 -2.122030 -0.860273  0.677164 -1.2176900 -2.385400
## 6  3.4889200 -0.484558  2.824440 -3.593910 -3.539810  1.7056300  0.415227
##       PC417     PC418     PC419     PC420      PC421     PC422     PC423
## 1  5.514010 -2.777100  4.741890 -3.646520  2.0528300 -1.859560  1.793530
## 2 -3.334990  1.627890  3.234690 -2.847870  1.2599000 -2.271810 -1.785870
## 3 -3.351090  1.146040  3.314440  3.594810  0.0832995 -0.881849  1.924760
## 4  1.180640 -3.626850 -0.352812 -0.976568 -0.4022390 -2.290430 -1.486760
## 5 -1.404580 -0.763074 -0.879987 -0.675664 -0.2265250 -1.471390 -0.713293
## 6 -0.463674  2.548030 -3.447960  6.872220 -3.5295900  5.942630 -2.776490
##      PC424     PC425    PC426     PC427     PC428     PC429      PC430
## 1  2.70672 -0.244327 -1.95145 -1.166260 -1.113350  4.499590  3.1872000
## 2  2.98253 -4.738990 -1.56793  4.447450 -2.530260  4.298800 -2.3087700
## 3 -1.37707 -0.549599 -3.51351  1.383930 -0.839479 -4.699030 -7.2388300
## 4  3.23094  0.418140 -4.40680  0.134194  0.691633  1.132580 -0.5365950
## 5  2.63757  1.518580 -2.96348  0.755831  0.214467  0.825183  0.0958914
## 6 -2.43367 -1.238610  4.14482 -2.136040  9.823910  2.179780  2.4382000
##       PC431    PC432     PC433     PC434     PC435     PC436      PC437
## 1  1.010380 -1.42228  0.265465  2.137230 -2.099080  4.305090 -1.7999500
## 2  3.010160  3.02598 -2.152070 -0.948899  0.560193 -3.746140  2.7094800
## 3 -6.717230 -3.72880  3.464120 -6.116600  2.557700 -0.785485 -0.4827310
## 4 -0.407869  1.00466 -2.077840 -1.710140  1.549940  0.802661  0.0122899
## 5 -0.444602 -1.59515 -1.557260 -1.782260  2.934630  1.680720  0.1495440
## 6  2.514300  4.80670 -0.223517  0.241169 -2.076640 -4.704640  3.9221200
##       PC438     PC439      PC440     PC441      PC442      PC443    PC444
## 1  3.009160 -2.282380 -0.6288940 -0.174383 -1.1496800 -4.2441200  1.04595
## 2  0.999137  2.615180 -0.4489710 -5.202510  0.2853910 -1.6672600 -4.83990
## 3 -2.027170  2.929950  0.3375440  4.578580 -0.0669933  0.0621375 -3.39830
## 4 -0.413276  0.672038  0.0183084 -1.253380  0.8709670  0.9752210 -2.09770
## 5 -1.058960  0.500133 -1.2273300 -0.651911  0.7593330 -0.6201030 -1.56370
## 6 -2.174460 -6.245420  6.5661200 -5.984090 -3.5877600 -0.6122270  5.17071
##       PC445      PC446     PC447      PC448     PC449     PC450     PC451
## 1  0.370447 -0.3157080 -8.769830 -1.0306400 -0.253000 -5.545050  1.957470
## 2 -3.690710  0.0591667 -2.031700 -0.0912076  4.360830  3.662260  1.572730
## 3 -1.299150 -7.5593000 11.418100  4.4692800 -7.941520  6.400560  0.133257
## 4  0.712873 -0.2941350  0.238441  0.5570790 -0.661187 -0.358439 -0.859377
## 5 -0.637456 -0.7989410  0.109801  1.9373100 -0.368006  1.521530  0.368244
## 6  9.078330  7.3899400  1.074220 -3.4584900 -1.930180 -4.328970  1.536510
##       PC452    PC453     PC454     PC455     PC456      PC457    PC458
## 1  0.106557  2.28870  5.185670  0.476058  0.860185 -0.0515014 -1.52748
## 2  2.919410  1.51619 -0.247866  0.950124  1.094090  2.3722000  3.53183
## 3  4.566070 -1.83711 -1.878430 -0.450207  0.974503 -2.7902700 -2.57766
## 4  3.354030 -1.99390 -0.874158  0.158202 -0.851141  0.1408380  3.47386
## 5  1.870210 -2.58921 -1.129780 -0.963834 -0.763508  0.1415130  1.72513
## 6 -3.322300  7.71662 -0.707759 -0.452496  0.273654 -1.3853900  2.73739
##       PC459      PC460     PC461    PC462     PC463     PC464     PC465
## 1  0.458375  0.4056330 -1.744530  3.02703 -1.640650 -3.451060 -0.412061
## 2  5.003710 -2.2902200 -2.131150 -4.84561 -1.766530  4.750890 -2.542240
## 3 -0.693656 -2.9773400 -3.061080  5.49228  2.403550 -6.613350  0.866179
## 4  2.490580  0.0242907  0.329785 -1.22987  3.304840  0.167176  1.898770
## 5  1.778180 -1.7410700  0.312236 -2.89206  1.829370 -0.438095  0.845840
## 6 -2.752500 10.5728000  4.523670  4.13351 -0.480155  3.518040  3.164060
##       PC466     PC467      PC468     PC469      PC470     PC471     PC472
## 1  0.608027  3.984070 -2.3597800  3.130410  2.2009200  1.667260 -4.676860
## 2 -0.575667 -0.591765 -2.6708900  0.290876 -3.5029300  1.803300 -0.913266
## 3  2.919130  2.720260  7.4525900  0.694249 -0.0217734 -0.888056  1.431880
## 4 -1.287940 -2.638740  0.0665417 -2.239320 -0.5231550  2.352570  0.880868
## 5 -2.243230 -1.963210  1.1994500 -2.065520 -0.5641920  1.149340  1.536310
## 6  6.184760 -1.334750 -6.8660500 -5.850280  2.7283900 -4.883400 -0.625743
##       PC473     PC474     PC475     PC476     PC477      PC478     PC479
## 1 -3.629930 -3.450970  1.770860  0.583894  0.671149  1.5936900 -2.859570
## 2  0.403816  2.775920 -1.143820  4.564630 -3.006510  1.5867300 -4.220650
## 3  3.567020  1.441650  0.342013 -1.504930 -3.421020 -3.7623300  0.640122
## 4 -0.543772 -2.571680  0.247481 -0.266556 -0.967774  0.0382954  0.388966
## 5 -0.209446 -0.940976  0.465440 -0.135196 -0.086343  0.3306810  1.157490
## 6 -2.336810 -2.231480 -1.984890  0.429775 -0.540701 -0.7354410  6.370170
##      PC480      PC481    PC482     PC483     PC484     PC485     PC486
## 1 -7.74602  1.2822900 -1.54064  2.065100  2.343380 -3.118510  0.182810
## 2  2.58700 -0.8257350  2.96328 -4.600970 -1.586330  0.541400  1.352710
## 3 -2.16541 -1.3845500 -3.88577  0.587622 -0.889029 -3.032680 -2.969990
## 4  0.75883 -0.4496780  2.41250  0.317241 -2.168980 -1.715470  0.753018
## 5 -1.27396  0.0886838  1.32481 -0.322677 -1.523310  0.542027 -0.558600
## 6  7.16256 -3.1926000 -1.70012  2.532670  5.612290 -2.219570  5.570040
##       PC487     PC488     PC489     PC490     PC491     PC492     PC493
## 1 -2.369160  0.347647  3.164070 -0.931841 -1.472410  2.973600  3.109130
## 2  2.589310 -3.123790  1.368960 -3.952490  2.932150 -0.231654  0.677852
## 3  2.710800 -0.835259 -1.794670  2.006570  1.747530  1.785650 -3.992280
## 4 -1.332630 -0.643923 -0.279731 -0.956002  1.675930 -0.245903  0.771552
## 5  2.174260  0.491265  1.176980 -0.730539 -0.214938 -1.046830 -0.569578
## 6 -0.310611  5.215820  1.497860  1.475120  4.479930  0.538192 -2.050610
##       PC494     PC495    PC496     PC497     PC498    PC499     PC500     PC501
## 1  0.848586  1.185560  3.95958 -4.294020  0.102847 -3.90031 -9.311350 -3.565650
## 2 -1.867780  4.721460  6.30913  0.978949 -0.676560  1.74134  2.397700 -1.148480
## 3  0.917599 -0.983508 -1.94543  0.896583 -5.110000 -1.00891 -0.221521  3.837800
## 4 -0.813731  0.984808  2.99429  1.377880 -0.523534 -1.93152 -0.747233  1.418650
## 5 -0.529761  1.568460  0.51823 -0.301758 -1.870340 -1.30480  0.143128  0.312042
## 6  3.763520 -1.627000 -1.31236 -1.734650  7.028540  6.61970 -1.065260  5.343140
##        PC502     PC503     PC504    PC505     PC506     PC507     PC508
## 1 -4.2900900  2.894250 -5.649900  3.62083 -1.232560  2.448910  2.300620
## 2  0.0305279  2.429620  0.205244 -2.36331 -0.865848 -2.132420  0.168307
## 3 -0.8209900 -2.131180 -2.369890 -4.39876  0.127694 -0.153604  3.678010
## 4 -0.3755950 -0.374189  1.255170  1.41406  1.514580 -0.974301 -2.398720
## 5  0.8667130  1.800370 -0.345318  1.05548  0.490970  0.826215 -0.960025
## 6 -0.9369000 -1.188950 -4.864690 -4.45128  2.045310 -2.562840 -0.471193
##       PC509    PC510     PC511      PC512    PC513      PC514    PC515
## 1  0.824394 -3.89775 -1.513420  5.6631600 -6.59384 -3.6965700  1.52801
## 2  0.226974 -1.21470  0.838374 -0.0723994 -1.56583  0.2068430 -2.14565
## 3  4.649790  2.94594 -3.571320  3.8065900  1.89948  1.3566800 -4.22433
## 4  1.084510  0.34494 -1.095130 -0.4689060  1.60281  0.0634357  1.46384
## 5  0.819577  1.28172 -0.723751  0.7199320  1.33524  0.2590050 -0.19600
## 6 -2.408920 -2.85718 -9.626010 -8.0796700 -1.45460 -1.8202300 -1.41543
##       PC516     PC517     PC518    PC519     PC520     PC521     PC522
## 1 -0.455283  3.435580  6.771620 -3.16149 -3.336160  0.231423  1.584160
## 2  2.658110 -0.540027  1.031750  2.61928  1.633430  1.584900 -2.992330
## 3 -4.297210  0.243740  4.266190 -7.41399  4.522940 -2.792840 -0.235453
## 4  2.030210 -1.404060 -0.224667  3.12633  0.208127  0.646241 -2.742080
## 5  0.890573 -0.238428 -0.303602  2.37130  0.440762 -0.376915 -1.033720
## 6 -1.132380  1.068760 -0.142322  1.48325 -2.503930  0.996432  2.039690
##       PC523     PC524     PC525     PC526     PC527      PC528    PC529
## 1  6.610710 -5.059790 -2.355830 -3.709400 -3.805340  1.2806400 -2.89815
## 2 -2.041910 -1.557920 -4.617790  0.687116  1.949970  0.1903990 -5.06388
## 3 -4.629030 -0.876052  0.402783  0.406645 -1.994210 -6.1622500 -2.69604
## 4  0.598167 -3.118580 -1.425070  1.551260  0.988679 -0.2563410 -1.31579
## 5 -1.090080 -2.340700 -0.394402  0.866166  1.737880  0.0486884 -1.19621
## 6  2.922430  4.620350  6.722230  4.604230  0.351315  0.9553100  3.12466
##        PC530    PC531     PC532      PC533     PC534     PC535     PC536
## 1  8.0820000  2.14284  0.356392 -3.9021400  0.136542  1.721590 -4.112960
## 2  0.0860068 -2.42212 -0.618165 -0.9240490  0.660113 -3.013780 -0.559195
## 3 -5.4218000 -0.97198  1.686130  6.4804400  3.966210  2.201550  1.699140
## 4  1.1433100  1.38176  0.206007  0.0551021  0.568925 -0.902673 -1.747150
## 5  0.4845570  1.52798 -1.983220  0.1863440 -0.333699 -1.967910 -3.627810
## 6 -0.4787490 -2.51709 -0.948417  1.2519400 10.030400  2.248940  6.066670
##       PC537    PC538     PC539     PC540     PC541    PC542      PC543
## 1  3.916810 -3.09193 -2.097390  5.216600 -0.402681  5.48729  2.8199200
## 2 -4.625950  6.27865  0.465266 -6.158420 -2.358730 -3.78803  0.8563390
## 3  0.795618  1.08013  1.897150  1.894710  1.774920  3.46375  1.9480900
## 4  1.528240  2.49938  2.086080 -0.346862 -0.714195 -1.84506  1.4864000
## 5 -0.436555  1.07275 -0.205475  0.379762 -1.400140 -1.14160 -0.0598783
## 6 -0.954780  1.77612  0.961356 -1.333400 -2.440400  3.54585  4.3945200
##       PC544    PC545     PC546      PC547     PC548    PC549    PC550     PC551
## 1  4.831030 3.294590  1.210160  5.0043200 -1.439150 -5.55341  8.19324  3.883270
## 2  0.603852 0.767939 -0.268910 -0.8933380  0.158255  2.65751  2.88644 -2.522190
## 3  5.991030 0.614752  3.468540 -1.0314900 -1.798880 -1.28793  1.23450  4.162840
## 4  3.387430 1.389200  1.105490  0.0904197 -0.843405 -1.00830  1.69082 -0.416450
## 5  1.753610 1.482520 -0.309973  0.8857630 -1.480100  0.43404  1.48365  0.352801
## 6 -1.747410 1.758180  1.399470  4.4295600  4.981910 -1.28135 -3.24572  0.845115
##       PC552     PC553     PC554     PC555    PC556      PC557      PC558
## 1 -6.468360  1.891250  7.100000  5.994140  7.47878 -0.8127630 -4.8502200
## 2 -0.871310 -1.901490  3.037800 -1.922560  1.17851  1.7784900  3.9293500
## 3  1.026880 -1.757370 -4.237240  5.387510 -2.39674 -1.7842000 -3.7994400
## 4 -1.378770  2.387870  0.215515 -0.864783  3.05762 -2.1675500  0.5600600
## 5 -0.996316 -0.516507  0.422518 -1.478760  0.83498 -0.0818735  0.0592081
## 6 11.956200  2.109910 -3.770220  2.732910  2.02038 -3.4009500  3.5130100
##       PC559      PC560    PC561     PC562     PC563     PC564    PC565
## 1  0.812316  3.6755900 -2.85072  6.914230 -5.738760  4.014530 2.680630
## 2 -0.128839 -1.0992300  8.64154 -6.799690  0.867847 -2.358670 3.358420
## 3 -2.265380 -0.4044050 -3.47597 -7.707960  0.450149  0.405758 3.863310
## 4 -1.640620 -0.0721689  1.90842 -0.402666 -0.250009  0.267418 0.285661
## 5 -1.085250  0.0640559  1.45869 -0.107262 -0.732080 -1.142690 1.477850
## 6  4.761000 -1.4499100  4.68335  4.502030  5.471380 -4.545670 0.167281
##       PC566      PC567     PC568       PC569     PC570    PC571      PC572
## 1 -1.081790 -1.8360400 -2.164930 -5.69401000 -4.491530  1.86444  2.5811200
## 2 -4.395080 -3.5719400  2.620270  3.82605000  0.361293  2.76242  0.0251406
## 3  2.645090  0.0597983  2.538120  0.39620600  1.371160  4.18541 -0.6681740
## 4 -1.110540  3.0251500 -0.172403  0.00741411 -1.969330  1.94691  2.9667900
## 5  0.375348  1.5656500 -1.154080 -1.04054000 -0.548161  2.19702  1.6111800
## 6  2.299250 -3.4006300  0.458353 -0.89594700 -5.465430 -1.53409  4.6510100
##       PC573     PC574     PC575      PC576      PC577      PC578     PC579
## 1 -0.982971 -0.571172 -1.030970 10.6615000  8.7089100 -2.9850100  3.352030
## 2  0.701841  0.149376  4.592840  3.4100800  4.8834400 -3.7489000 -0.155941
## 3 -0.508581 -5.125760 -1.907860 -1.9900900 -2.1992900 -5.7386900 -2.503440
## 4 -0.212586 -1.260670  0.253985 -0.0695024 -0.1184010  0.1096750  1.112960
## 5 -1.910860 -1.933930 -0.429592 -0.9446950  0.0428309 -0.0666836  0.357343
## 6 -4.350110  2.252710  2.744610  3.9031500 -0.2492460 -7.6527500 -5.934100
##      PC580     PC581     PC582    PC583      PC584      PC585     PC586
## 1 -7.41224  5.627760 -1.093960  9.02326 -0.1209290 -5.6890400  0.567859
## 2 -1.05451  0.837426 -1.389880 -1.56466 -2.4049200  0.6757770 -0.147722
## 3 -1.20398  3.623800  3.567330 -4.22099 -3.9927200 -1.1206900  3.155910
## 4 -2.11127  1.017360 -0.372258  1.26637  0.0138719  0.0726155 -0.468595
## 5 -1.22516  2.038060 -0.603193  1.07850  0.3755930 -0.3868940 -0.140550
## 6  2.57397 -4.680370  1.593660 -7.57047 -6.5256300  3.2996200  2.397050
##       PC587     PC588     PC589     PC590     PC591      PC592     PC593
## 1 10.357000  4.127150 -3.760940 -0.595460  2.387020 -5.3239800 -2.787660
## 2 -0.531240 -1.683240  5.993860  0.641731 -1.176510  2.2966800 -4.187150
## 3 -1.991790 -0.828086  0.142005  2.161370 -4.272690 -0.0769395 -1.165560
## 4 -0.293865 -0.726703  1.392090 -0.359644 -2.752560 -0.7642910  0.100628
## 5 -1.342820 -0.513588  0.330022  0.190186 -1.029880  0.5100500  1.748770
## 6  1.411660 -2.978310 -4.370550  6.701810 -0.379864  1.4904800 -1.125760
##       PC594     PC595     PC596      PC597     PC598     PC599     PC600
## 1 -1.816850  5.048700  3.271190 -3.0157700  2.979230  4.053340 -0.510472
## 2 -0.484474 -2.410620  2.030580  1.6115300 -1.648630  6.137480 -1.900410
## 3  0.123948 -1.911750 -1.047500  0.6914400 -0.529424  4.142120  2.394830
## 4  1.409380  0.233241  1.722070 -1.2605200 -0.814587  1.905090  2.301590
## 5  0.716077  1.541180  1.158500  0.0957914  0.358213  0.458067  0.781583
## 6 -2.167590 -3.149840  0.482401  4.9988300 -0.794234 -0.301116 -1.561370
##       PC601     PC602     PC603    PC604     PC605     PC606    PC607     PC608
## 1  2.620840 -0.674483 -2.821440 -3.71392 -0.879378  2.346130  5.02994  2.273300
## 2 -1.622450 -2.165240  0.875458 -1.61506  3.234420  0.186050 -4.15792  2.619940
## 3  1.053310  0.251868 -1.575670 -4.66055  2.403390  2.982140 -0.74683  2.586390
## 4  0.343212  0.394814  0.497673 -1.43009  0.760694  0.480594 -1.92535  0.394009
## 5 -1.054650 -1.558530 -0.314745 -0.29233  0.425098 -0.757425 -1.02666 -2.816100
## 6 -1.798740  3.153500  4.321160  3.37287 -3.603900  2.653830  7.76069  3.201330
##        PC609      PC610      PC611      PC612     PC613     PC614     PC615
## 1  8.3102000 -1.0048000 -0.0637395  4.3249500  2.177960  3.467130  2.511870
## 2  2.4146500  0.0773715 -1.2616800 -0.0189012  0.663779 -4.490300 -4.372440
## 3 -3.0900100 -4.7370300 -0.6411020 -0.8853690 -6.700280  1.172310 -2.598760
## 4  1.4875300 -1.2744100  0.0258766  1.0124700  0.612425  1.844000 -0.830306
## 5  0.0717385  1.1445600  1.4043500  0.2845190 -0.778562 -0.272696  0.223013
## 6 -1.7077900 -2.9161700  3.3200700  0.9429440 -1.857190 -2.475020  1.366890
##        PC616     PC617    PC618      PC619     PC620     PC621     PC622
## 1  5.2778900  8.442380  4.73148  2.5601000  1.817060  0.662594 -3.092570
## 2 -5.3449300 -0.617521 -1.10577 -2.3233300  5.484760  5.472010 -1.686820
## 3  1.6134200  1.285380  1.58438 -0.0507207 -4.427790 -2.362700 -0.159146
## 4 -1.0621500  2.420380 -2.06260 -1.5321400  1.944060  0.760535 -0.235201
## 5 -0.5499200  0.435517 -1.60272 -0.9116840  0.647403  0.155686 -0.677605
## 6 -0.0128066 -1.266930  1.86574  0.9237120 -6.183490 -0.695967  2.363100
##      PC623      PC624     PC625     PC626     PC627     PC628      PC629
## 1  4.24881 -0.4901100 -4.139310 -0.431547  1.699710 -1.602140 -6.1563900
## 2 -8.68322  0.0189036  3.868340 -0.701300  0.964387 -0.982793  0.0427711
## 3 -2.72887  3.1586700  3.276030  5.700990 -0.372446  3.466550  3.5372800
## 4 -1.74898  1.7660400 -0.172360  1.317780  1.123780 -0.424468 -0.1605700
## 5 -0.78971 -0.3074170 -0.572417 -0.127959 -0.795501 -0.770536 -0.1183250
## 6 -2.44683 -1.6888000  4.172820  1.518870 -0.920622  1.186540  4.3325000
##       PC630     PC631     PC632     PC633     PC634     PC635      PC636
## 1  3.492960  0.872802  1.235200  1.865430 -4.426350  6.303590  0.7636960
## 2 -3.901970  3.875950 -6.168280 -7.228900 -0.702705 -6.277760 -0.2826420
## 3 -1.899990  0.271997 -5.465230  1.048110  3.468760 -4.002380  1.8719300
## 4 -1.812260 -1.619840  0.177403  0.546287  1.448070 -0.847236 -1.0071200
## 5  0.038066 -2.422150 -0.158709 -0.212732  0.728823  0.746218 -0.0968246
## 6  1.378990 -2.224120  1.328560  0.768372 -1.038560 -0.579867 -2.6711000
##       PC637     PC638     PC639     PC640     PC641     PC642     PC643
## 1 -1.402100  0.600715 -1.735900 -0.230752 -1.986720  0.299349  0.474671
## 2  5.276400 -0.459857  4.209110  0.536550  6.176530  1.487850  3.315030
## 3 -0.208462 -0.362609  0.185939  2.640570  0.334790 -1.597060  0.360439
## 4  1.015050  1.605440  1.724400  0.943631  1.704850 -0.219151  0.920826
## 5  0.240487  1.835000  0.711994  0.245524  0.561314 -1.988760 -0.719945
## 6 -2.011970 -1.484410 -2.673090  0.769083  2.351170  0.271644  1.670870
##      PC644     PC645     PC646     PC647     PC648     PC649     PC650
## 1 -2.14021  1.573160  3.895980  2.851340  0.477696 -2.265080  5.963990
## 2  0.39460 -0.999247 -4.380650  0.591799 -2.650870 -1.651560 -3.574020
## 3  2.76086  2.233260 -2.996550  0.682888 -0.097747 -6.326860 -2.823750
## 4  1.41788  0.838238  0.332126 -0.908421 -0.971538  1.060200 -0.324290
## 5  0.62192 -0.938878  0.894614  0.411094  0.303673  0.954713 -0.423471
## 6  1.11174  0.556907 -1.517980 -4.363870  3.645730  0.650873  0.990181
##       PC651     PC652      PC653     PC654     PC655     PC656     PC657
## 1 -1.986040 -1.367430 -0.3591130 -3.315980 -4.617190  1.278690  3.670700
## 2 -0.222195 -3.024560  3.3706400  5.319200  0.776720  1.592660 -0.640050
## 3 -2.508530  2.664080 -1.4241400  2.333190 -0.381276 -2.635290 -4.465660
## 4 -1.651550 -1.524560 -0.0372698  0.781089 -1.736720 -0.247797 -1.935300
## 5 -0.965699 -0.675388  0.8279970 -0.241133  0.380474 -1.056740 -0.481147
## 6  0.809195  4.652910 -2.2675700  3.560520 -2.131460 -2.381940 -2.144700
##       PC658     PC659     PC660     PC661     PC662     PC663      PC664
## 1 -2.990700 -6.375400 -2.431580 -6.685370 -2.542260 -1.783140  5.0698200
## 2 -0.686228  0.857943 -0.759559  0.690270 -1.905050  1.234700 -3.1604000
## 3  1.838000 -0.537154 -4.273250 -1.589410  3.879870 -0.131847 -2.2650100
## 4 -1.756070 -2.158470 -1.237690 -0.680875 -0.510452  1.083530 -0.0897811
## 5 -1.261470 -0.449055  0.078126  0.814975 -0.714281 -0.899341 -0.1588930
## 6  0.894745 -0.485634 -2.752800  0.812416  2.270900  2.247150 -0.0543395
##       PC665     PC666      PC667     PC668     PC669     PC670     PC671
## 1  1.461580  5.637950  2.0119800  1.128520  4.469730  1.030870 -3.829000
## 2  4.240340  0.291592 -6.6032900  0.384615  3.270210  2.511960 -1.691460
## 3 -0.574336 -6.232870 -0.0905672 -0.739350 -0.620190  1.327870 -1.892980
## 4  1.311080  0.564276 -1.2475000  0.535032 -0.693397  0.380076 -1.882920
## 5 -0.175201 -1.208670  0.4599060 -1.487390 -0.857443  0.981732 -0.107214
## 6  1.561230 -2.228670 -0.2554010 -2.244410  0.495016 -0.269707  0.195192
##        PC672      PC673     PC674     PC675    PC676    PC677     PC678
## 1  6.1848700  1.9646200 -0.347458 -3.731380  0.56946 -2.01320  0.170475
## 2 -4.3631800  0.9314770  1.700190  7.584330 -5.75619 -2.12867 -3.214140
## 3 -2.9129600 -1.8802000  1.604010  1.701050  1.13935  3.06984  1.946700
## 4 -0.4258030 -0.9183410 -0.760727 -0.442025 -1.38517 -1.16635  0.035875
## 5  0.0104888  1.4939500  0.260795 -0.907028 -1.31072 -1.77747  0.177759
## 6 -1.9899200 -0.0800669  0.229716  4.420340  0.55052  4.20221  2.021550
##       PC679     PC680      PC681      PC682      PC683     PC684     PC685
## 1  4.199590 -1.427120  6.6895400 -1.1215400 -5.3892500 -1.282000  2.895820
## 2  3.246040 -2.928700 -4.1016000  2.7152700  1.3301000  3.040290  4.009080
## 3 -0.990312  0.640829 -1.3923900 -1.6245200  1.8577800 -2.590730 -0.784156
## 4 -0.434262 -0.359161 -0.8983400 -0.2265450  1.0972600 -0.738396  0.912234
## 5 -1.581640 -0.931023 -0.0306423 -0.7846950  0.0845458 -0.797718 -1.251240
## 6  4.125130  5.454370 -1.0194200 -0.0738183  0.5704130 -1.626680  4.261440
##       PC686     PC687     PC688     PC689     PC690     PC691     PC692
## 1  0.908344  4.078070 -1.124540  2.333490  0.990787  3.283620 -0.766332
## 2  3.929460 -2.774740  3.171160 -0.892275 -0.863196  0.593161 -4.903910
## 3 -2.966980  2.034090  2.317770 -0.341460 -0.849829 -1.356730  0.819097
## 4 -1.205190 -0.340567 -0.490077  0.395095  2.110870  0.425519  0.248788
## 5 -1.204020  0.415157  0.282212 -1.450860  0.265727  0.467793 -0.539335
## 6  3.043710  2.613250  0.901400  1.955820 -0.036801 -2.586370  1.013650
##       PC693     PC694    PC695     PC696      PC697     PC698     PC699
## 1 -3.033610 -1.988000 -2.15156 -5.231220 0.36213100 -2.745880  1.644580
## 2 -0.728987  4.265050  5.90974  1.391470 3.94776000 -0.772406 -3.479000
## 3  3.131670  2.566470  3.51399  0.208664 0.00989697  0.727316  3.029570
## 4  1.583050  1.002800  1.58529  0.336551 0.35960300  0.232825  0.786552
## 5  1.449110  0.632981  1.77627  0.236299 0.23181900 -0.777321 -0.815618
## 6  1.538250  1.680760  1.47288 -0.727824 1.02287000  1.718610  0.443659
##        PC700      PC701      PC702     PC703     PC704     PC705     PC706
## 1  1.7186700  3.4349500 -1.4333700 -2.872330  1.932240  4.776000  0.456057
## 2 -2.9616400 -5.1837100  4.5955200 -1.483760 -4.494830 -3.038030 -1.760650
## 3 -1.0090000  1.1423100  0.0176662  0.371206 -0.161055 -1.690450 -0.424346
## 4 -0.7017450 -1.0943900  0.6144490  0.220998 -0.496051 -0.826577  1.775960
## 5  0.0145609 -0.0276485 -0.8572430  0.217809  1.832830 -0.720910  0.854389
## 6  0.3414720 -2.9759900  1.6155500  0.352096  0.500868  1.545750 -0.743470
##        PC707     PC708     PC709      PC710    PC711     PC712      PC713
## 1 -2.2011100  2.319310  3.259430 -5.1853100  1.49182 -1.806100 -0.9329290
## 2 -1.0301000 -2.227740 -2.130440  5.6828100 -4.53717 -0.777382  2.9851700
## 3  2.0226400  0.630055 -1.894840  0.5070730 -1.44717  0.364113  0.8014700
## 4 -0.4658880 -0.387982  0.648217  0.0437275  0.19454 -1.147980  1.2543000
## 5  0.6300480  0.835386 -0.330107 -0.8302920 -2.19144 -0.118516  0.0597635
## 6 -0.0320584  0.753704 -1.494780 -0.4257440  2.74316  3.042760  1.4909600
##      PC714     PC715      PC716     PC717      PC718     PC719     PC720
## 1  3.87627 -1.574950 -0.0331527  2.213440 -2.4333100  3.242300 -1.319560
## 2 -6.33279  1.335180 -0.3396560  3.063910  5.2264800  0.962215  4.170100
## 3  1.84293 -0.234840 -1.1545600  2.384730  1.4964600 -2.112980  2.253910
## 4  1.85017  0.375517  0.0557549  0.399901  0.0124356 -0.931267 -1.184170
## 5 -2.14566 -0.466303 -1.2096500 -1.762030 -0.1958750 -1.527180  0.743151
## 6  2.82608  3.672360 -1.1207800  1.995900 -0.2134910  0.874753  2.624760
##       PC721     PC722     PC723     PC724     PC725     PC726     PC727
## 1  1.631440 -1.226410 -2.028420  0.964467 -1.213140  0.831599 -0.714503
## 2  0.844670  1.576340  5.883510 -1.559910 -6.085890 -0.930339 -1.299460
## 3  1.975880  2.750410  2.636320  0.752602 -2.385190 -0.615232 -0.949625
## 4  0.238852 -1.102590 -0.334264  0.934384 -0.250151  0.468645 -0.689875
## 5  1.483360 -0.188258  1.829030  0.262118  0.327645  0.220968 -1.480750
## 6 -0.815339  2.201860  3.161670  0.649065 -0.289726 -2.933730  3.319050
##        PC728     PC729     PC730     PC731     PC732      PC733      PC734
## 1  0.1589010 0.3151120 -0.364467  2.745420  0.665720  1.3090500 -1.2722000
## 2  1.1620200 0.4574050  5.518090 -2.140780  3.617600  2.0379300 -4.3610800
## 3 -3.7404300 2.4479300  0.299041  0.773940 -1.024260  0.0228225  0.4070380
## 4 -2.0616100 0.0407445 -0.829429  1.445240 -0.192752  0.5081210 -0.1604950
## 5  0.0803787 1.5984700 -0.252702  0.990827 -1.553450 -2.3438500  1.0618100
## 6 -2.5157400 0.5267630  0.949081 -1.484710  0.721351 -0.6979440 -0.0266725
##        PC735     PC736    PC737     PC738     PC739     PC740      PC741
## 1 -1.2184600  1.023040 1.487570 -3.084400 -0.904776 -1.256560  1.6669100
## 2  5.7793200 -0.441273 1.563680  4.044200 -3.306630  0.343537 -0.0973434
## 3  0.2669930  0.473931 2.539330 -0.448511  2.973410  2.774080  1.3423100
## 4 -0.3209460  0.952340 0.670179  0.715620  0.193107 -0.813580  1.4348700
## 5 -0.3549770  0.222206 0.103225 -0.369608 -0.419108  0.347774  0.3156630
## 6  0.0879263 -0.470775 2.161040 -1.016720  2.234300  2.624580  2.1708100
##       PC742     PC743      PC744     PC745     PC746     PC747     PC748
## 1 -3.335960  2.755190  1.4929200  0.507047  2.531600  2.698270 -0.762232
## 2 -0.441643 -5.107700  2.7271100 -1.829320 -2.291460 -2.895400  0.711699
## 3  1.807250 -2.248640  0.3602950 -2.810860  1.462200 -0.377290  3.018780
## 4 -0.292593  0.205874  0.5601290  0.475353  2.248270  0.665043  0.957200
## 5  0.699474  1.954510 -0.0895184  0.716150 -0.658713  0.185483  0.170516
## 6  0.463307  0.457399  0.8730990  1.057110  1.777700 -0.804537 -0.566584
##       PC749      PC750     PC751     PC752      PC753      PC754     PC755
## 1  3.262870  1.6077900  2.222870  2.050990 -2.0994600 -0.5668510 -2.311240
## 2 -0.111571 -2.2138500 -0.324312 -2.395000  2.2399200 -2.5337100 -1.923900
## 3  0.459146  1.2213800  2.652730  0.902386 -1.2403200 -0.0509261 -0.778213
## 4 -0.280354  0.9793260  1.958620  0.287545  0.0208127 -0.2522000 -0.495961
## 5 -1.209560 -0.0594633 -0.325593  0.417000 -0.8493790  0.7306970  0.441894
## 6  0.552564  1.0325700  1.809260 -1.908630  2.8310900 -0.5173440  0.818544
##        PC756     PC757     PC758     PC759     PC760     PC761     PC762
## 1 -0.2655360 -2.610330 -0.862674  0.318762 -0.160888 -1.741160  1.469510
## 2  1.7576500  0.625825 -2.062050 -4.570420  0.878063  3.363540 -0.428557
## 3 -0.0386657  0.350859  0.902827  0.458925  0.101214  0.872361  0.223561
## 4  0.3891480 -1.968590  1.610750  0.826272  0.118246 -1.220120 -0.487325
## 5 -0.5857230  1.090030 -1.296070 -1.257080 -0.209427  1.710550  0.508340
## 6  0.7470940  0.839219  0.134979 -1.314170  0.609773  0.558887 -0.834591
##       PC763     PC764     PC765      PC766     PC767     PC768     PC769
## 1 -0.669147 -0.869839  2.286950  0.9238720 -1.362640  0.573613 -2.847640
## 2 -1.766010  3.882340 -1.378810 -1.0042300 -2.151500  3.767060  5.846540
## 3  3.916770  0.374492  0.170029 -0.7498810 -1.674670  1.974050  0.502889
## 4 -1.930640 -0.139893 -0.900957 -0.2388270 -1.340310 -0.987495  1.094520
## 5  1.608020  0.519559 -0.073788  0.6739340 -0.153002 -0.484583 -1.646540
## 6 -0.339251  1.181640  0.399924 -0.0559249 -1.052390  0.413094 -0.228047
##       PC770     PC771     PC772     PC773       PC774     PC775     PC776
## 1 -0.146479  0.961767  0.654003 -0.591300 -0.10711500  1.173340  0.492012
## 2  4.394210 -1.375340  0.211226  2.803460  0.00864675 -1.415450 -2.264270
## 3 -0.963174 -1.764040  1.582530  0.282068 -1.47667000 -0.964485  0.986859
## 4 -0.657500  0.174889  1.722480 -0.652754 -0.38976000 -0.212473 -1.591400
## 5  0.207764  0.131707 -0.328130  0.562081  0.29686000  0.595939 -0.660228
## 6  0.339818  0.678013 -2.241570  0.916067 -0.37724000 -0.753339  0.573345
##       PC777     PC778      PC779     PC780     PC781      PC782      PC783
## 1 -1.077130  0.176791 -2.3825600  1.496420 -0.885966  2.0749900 -0.5787860
## 2 -1.666300 -2.210520  2.1585100 -0.872022  0.825802 -2.0828900 -0.4096460
## 3  1.038240  1.403030 -0.4714020  0.422030 -1.941470 -0.9000380 -0.8808400
## 4  0.488764 -0.320904 -1.8151100  0.558321  1.631540  0.3172100 -0.8915440
## 5 -0.143789 -0.388207  0.2214800 -0.139224 -1.280110 -0.3734990 -0.0977915
## 6 -1.320380  0.618521  0.0865774 -0.355430 -0.351996  0.0434186  0.7104320
##       PC784      PC785     PC786      PC787     PC788     PC789     PC790
## 1  0.245697 -1.7846800 -2.350450 -0.9874500 -1.392280 -0.319087 -0.857037
## 2 -2.138090 -0.0359143  0.098552 -0.8690950  4.918630 -0.407186  1.388720
## 3  0.627358 -0.4964810  0.970249 -0.6713680 -0.320788 -0.291777  0.966041
## 4  0.343208 -0.2074450  0.762541 -0.2112470 -0.524559 -0.529530 -0.768490
## 5 -1.621900 -1.2928300 -1.419980  0.0963472  0.656308  0.910648  0.445821
## 6  0.584934 -0.1396910 -0.861894  0.2182860  0.472015 -0.611699 -0.924108
##       PC791      PC792     PC793     PC794     PC795      PC796      PC797
## 1 -0.184434 -0.5361130  1.799650  0.338461  0.960771 -0.5745520 -0.5472940
## 2 -1.469080 -0.6933330 -1.776640 -0.455597 -1.481620 -1.5909100 -0.6776230
## 3  0.342840 -0.5600660  0.341989 -0.695241 -0.731789  0.4130200 -0.3557650
## 4 -1.042650 -0.3480740 -0.524386  0.900253 -0.100903  0.0381289  0.4951940
## 5  0.632266 -0.4431770  0.419004 -0.273413  0.173888  0.4074930 -0.4717630
## 6 -0.183955 -0.0584211  0.140570 -0.637520 -1.306320 -0.8085630  0.0926733
##        PC798     PC799     PC800     PC801     PC802     PC803      PC804
## 1  0.8815600 -1.214040 -0.275167 -0.759332 -0.566340 -1.329680 -0.1864820
## 2  0.0179318 -0.882084  1.218820  1.171860  1.492080 -0.703180  0.0799170
## 3  0.1736520  0.663205 -0.228483  0.327977 -1.681270 -1.096660 -0.9219640
## 4  0.8019760 -1.656420 -1.180090  0.299415  0.286678  0.992035  1.7291000
## 5 -1.0386200  1.783560  1.458340  0.833884 -0.624101 -0.869268 -1.2069000
## 6 -1.9743200  1.340260 -1.097350 -0.751737  0.409030 -0.811340  0.0549211
##        PC805     PC806     PC807     PC808     PC809     PC810     PC811
## 1 -0.1794890 -0.233709 -0.754882  0.159462 -0.374743 -1.804840 -0.495034
## 2 -1.2146400 -1.473950 -1.830110  2.180750 -0.414340  0.178959  0.945933
## 3  0.6189470  0.062435  1.545310  0.602263 -0.466002  0.290740 -1.778170
## 4 -0.2974400  1.046610  0.891659 -0.604585 -1.138030  0.584317 -0.857480
## 5  0.0987224 -0.551497  0.418141  1.468860 -0.958180  0.706654  1.348300
## 6  0.6726970  0.368318 -0.973819  0.314014  0.503378 -1.339990 -1.885480
##       PC812     PC813     PC814       PC815     PC816     PC817     PC818
## 1 -2.148390 -0.255627 -0.340198  0.13027000 -0.369321  0.589186  1.294670
## 2  1.117520  1.389970 -0.706944  1.38405000 -1.357650 -1.295260 -0.640422
## 3  1.845210 -0.476398  0.159611  0.00866575 -1.001250 -0.273187 -1.044890
## 4  0.541990 -0.154312  0.653820 -2.03637000  1.077600 -0.320027 -0.657330
## 5  0.151023  0.847159 -0.530045  0.94770900 -0.384360  0.101863  1.353950
## 6 -0.658134 -1.216310  0.349377  0.75927000 -0.268036 -1.350280 -1.087470
##       PC819     PC820     PC821      PC822      PC823      PC824     PC825
## 1  0.842219 -1.926590  2.049080 -0.0727288 -0.0485778 -0.1981350  0.616395
## 2  0.609827  0.239863  0.758208  1.0748100  1.0202900 -1.2505200 -0.284004
## 3 -1.588380  0.829787 -1.156820  1.1125300  1.2971200  0.0337264 -0.875991
## 4  0.666598 -0.716382  0.874565  0.0340905 -0.9347360  1.2249100  1.301250
## 5 -0.190915  1.220620 -0.177373 -0.4380870  0.9619230 -0.3615120 -1.547550
## 6  2.125620 -0.370864 -1.926270  1.9973600  1.5410900  0.9312680  0.074127
##       PC826     PC827     PC828      PC829      PC830      PC831     PC832
## 1  0.865272 -0.192247  0.279844  1.7832800  0.0200712 -0.9794230  2.061270
## 2 -2.125160  0.179309  0.243256  0.5585800  1.5760200 -0.5963800 -1.327540
## 3 -1.956220 -0.545897 -1.286450 -0.4751490  0.4416830 -0.6197920 -1.668660
## 4  0.539253  1.195750 -0.120745  0.0281689 -0.6070850 -0.1069320  0.130871
## 5 -1.204770 -1.500560  0.582675 -0.3064250  0.0767373  0.0252785 -0.790179
## 6 -0.251709 -1.409540 -0.921547 -0.6202460 -0.4262620  0.1901270  0.563899
##       PC833       PC834     PC835     PC836      PC837     PC838     PC839
## 1 -0.909492 -0.30225100  0.188160  0.676095 -1.0885900  0.123712 -0.406073
## 2  0.991478 -0.81682200 -0.226077  2.080310  0.7925940 -0.229000  0.361270
## 3  1.308150  0.00212058  1.219940 -0.344410  0.9659540  0.230251  1.488320
## 4  0.837077  1.47870000  1.388830 -0.162190  0.0245451 -1.097790 -0.538524
## 5 -0.677671 -2.49646000 -0.794235  0.159323 -1.0727600  0.530684 -0.286840
## 6 -0.812981  2.20944000 -1.261160  0.378836  0.8253750  1.620240 -0.627685
##       PC840      PC841      PC842       PC843       PC844     PC845     PC846
## 1  0.990540 -0.0841193 -0.9749180  0.29169600  0.00883489 -0.369751  0.912801
## 2 -2.051700 -0.2227680  0.4786750 -1.05714000  0.38805600 -0.228920 -1.845820
## 3  0.291579  0.3760600 -0.0210902  0.56234100 -0.42725100 -0.327148 -0.747908
## 4  0.439046  2.0539900  0.4644210  0.00642427  1.38986000 -0.195422  1.008580
## 5 -0.773073 -2.0417100 -0.0298319 -0.72037700 -1.42682000  0.642144 -1.416750
## 6  0.286393  0.1083070 -0.2603520  0.26317800 -0.58540700 -0.778223 -0.192863
##       PC847      PC848     PC849      PC850      PC851      PC852      PC853
## 1 -1.414200  0.1296720 -1.555180  1.0404700 -0.2944780 -0.0108651 -0.4654830
## 2  2.162370 -0.8545270  1.582770 -0.8584510  1.2113100  0.4985780  1.4864400
## 3 -0.682791 -0.6146020  0.486577 -1.7522200  0.5722230  0.5322310 -0.5487990
## 4  0.802539  2.5844100  2.474510 -0.0561106  1.6713000 -0.8121030 -1.2236700
## 5 -0.969429 -2.6742300 -2.595180 -0.7366720 -1.7044700  0.1281370  1.4771700
## 6  1.981040  0.0589784 -0.529355  0.0683757  0.0429685  0.5610440 -0.0379434
##       PC854     PC855      PC856      PC857     PC858      PC859     PC860
## 1  1.105170  0.273820  0.2917290  0.6105450  0.243642 -0.0720884  0.206551
## 2  0.518673  0.597173 -0.2146480  0.1219870  0.746575 -1.8088300 -0.244378
## 3 -0.412247 -0.270641  0.8259550  0.0823126  0.104485 -0.6186540 -0.308757
## 4 -3.719510 -1.711740  0.5700220  0.5995460  2.005670 -3.5151000  1.193890
## 5  3.542270  1.293810  0.0139799 -0.4195150 -2.033910  3.4807800 -0.387208
## 6  0.950468  0.343404  0.9904380 -0.1099770  0.238473 -0.1617820 -0.215466
##        PC861      PC862       PC863      PC864     PC865       PC866      PC867
## 1 -0.5870300  0.5530320  0.63739600 -0.2607980  0.692908 -0.08398400  0.0330389
## 2 -0.0439322  0.1771990 -0.00345984 -0.0392136 -1.021260 -0.23975000 -0.4762670
## 3  0.5972460  0.0616431  0.63016500  0.0834104  0.334656 -0.00489731 -0.4628170
## 4 -0.7604270 -1.4950100 -3.85840000  1.7653900  5.299650  4.44395000  1.5233000
## 5  0.2483800  1.3520500  3.98445000 -2.3236600 -5.131810 -4.03145000 -1.5889900
## 6  1.0910300  0.0618401  0.05699720 -0.1585990 -0.391887 -0.47113900  0.0188052
##         PC868     PC869      PC870     PC871      PC872      PC873      PC874
## 1  0.15223400 -0.319229  0.5924250 -0.611354  0.1598190 -0.5736370 -0.0244160
## 2  0.53184300  0.314985 -1.3799100  1.218800 -0.5877930 -1.0578200 -0.4535230
## 3 -0.19772200 -0.168979 -0.3897580  0.282107 -0.0946353 -0.0711884  0.0657764
## 4 -0.31440500 -2.344470  0.6791000  5.272740  5.9863200 -3.8069400  4.8321000
## 5  0.00560183  1.371210 -1.5862200 -5.008800 -6.2698300  3.8211600 -4.5737000
## 6 -0.00849512  1.201530 -0.0130047 -0.652534 -1.1019400 -0.1740170 -0.4453790
##         PC875       PC876      PC877     PC878     PC879      PC880     PC881
## 1  0.41543200  0.16221800  -0.895847  0.235586 -0.477548 -1.1529700 -0.249848
## 2 -0.34009500  0.52265200  -0.109974  0.830691  0.800841 -0.0661830 -0.367829
## 3 -0.00884115 -0.00873945   0.510265  0.670445 -0.222132  0.0386929 -0.123257
## 4 -3.46303000 -6.62802000  15.701400 -1.434800 -1.557470  9.2054300  2.308360
## 5  3.97414000  6.98487000 -15.505800  1.835430  1.855970 -9.9035100 -2.457090
## 6  0.97339100  0.60519300  -0.771924  0.421096 -0.181133  0.3224060 -0.111571
##        PC882      PC883     PC884      PC885      PC886      PC887     PC888
## 1 -0.0689516   0.495121 -0.369656   0.944117  0.3152060  0.0309335 -0.123319
## 2  0.0577736   0.245908  0.363758   0.171060 -0.8545390 -0.9933020  0.347411
## 3  0.2199360  -0.397829 -0.422959  -0.376337 -0.0393651  0.1172130  0.114682
## 4  0.4345150 -14.847500 -6.491130 -12.056900 -0.5928250 -0.6289720  1.836650
## 5 -0.2372150  15.178000  6.617840  12.853500  0.3411070  1.1277300 -1.779210
## 6  0.0993044   1.120850  0.352053   0.346316 -0.2215110 -0.1696950  0.614580
##        PC889     PC890      PC891      PC892     PC893      PC894      PC895
## 1 -0.6121290 -0.146702  0.0753581  0.6195410  0.821521 -0.3114300 -0.4736910
## 2 -0.1194540  0.336974 -0.2298310  0.0376849  0.117810  0.3600090 -0.7543990
## 3  0.0149206  0.455989 -0.3085640  0.1146130 -0.473137 -0.0333547  0.0605558
## 4  2.7195000 -1.149700  1.5972700  2.5586800 -3.804150  0.5398330  4.0250700
## 5 -2.5375000  0.837280 -1.2385500 -2.4431600  4.233620 -0.5789850 -3.9632000
## 6 -0.0480673 -0.240569 -0.7393560 -0.1066830 -0.252783 -0.8941990  0.0658015
##        PC896     PC897      PC898     PC899      PC900     PC901      PC902
## 1  0.0302765  0.700777 -0.1302060  0.275702  0.0152721  0.227990 -0.0414610
## 2 -0.3864010 -0.836917 -0.8027100  0.464461 -0.0103125  0.699584  0.0898253
## 3  0.0550200 -0.345351 -0.2729070 -0.220361 -0.2856780  0.256504 -0.1410660
## 4  0.7046230  1.482030  3.7307000 -2.268280  0.9484370  0.807987 -0.9106700
## 5 -0.8849550 -1.085340 -3.8564600  2.022900 -0.5561270 -0.850186  1.3939500
## 6 -0.5983010  0.020361 -0.0102515 -0.263858  0.0259823 -0.632275 -0.3801960
##        PC903     PC904      PC905       PC906     PC907       PC908      PC909
## 1  0.0976935 -0.548256  0.2254610 -0.24787400  0.472234 -0.00660823  0.0179293
## 2 -0.1094070 -0.341629 -0.2258720  0.00958437 -0.586128  0.11466200  1.0044400
## 3  0.3445910  0.136044  0.6033960  0.08799940  0.193445  0.44667900 -0.2598420
## 4 -0.4517310 -0.423242  1.8089200  0.29020900  0.944201 -1.11568000 -1.2105500
## 5  0.2697740  0.480520 -1.1733000 -0.46229700 -1.212610  1.04928000  1.0246900
## 6  0.3220040 -0.171739 -0.0354971 -0.26246400 -0.166229 -0.18172400 -0.0636656
##        PC910      PC911      PC912      PC913      PC914      PC915     PC916
## 1  0.2850600  0.1688830 -0.0231312 -0.0824134 -0.0117913  0.2226900  0.148575
## 2 -0.6303170 -0.2075820  0.1548870  0.1336210 -0.2414730  0.0285930  0.139923
## 3  0.1308360 -0.4002250 -0.0639613  0.1615440 -0.0883251 -0.0422935 -0.224916
## 4  0.0828278 -0.0418259  0.0894990 -0.2452240 -1.0399100 -0.8327160  0.319837
## 5 -0.1352140 -0.5657700 -0.2343600 -0.1974210  0.6927620  0.9344160 -0.361572
## 6 -0.1628120  0.1922250 -0.1174790  0.0760360  0.0658698  0.1351230  0.226722
##        PC917      PC918      PC919      PC920       PC921      PC922      PC923
## 1  0.1172470  0.1303090  0.1598570 -0.1274730 -0.00569113  0.0166240  0.0781717
## 2 -0.2252920 -0.1145590  0.0542411 -0.1765560  0.01912510  0.0443539 -0.3570420
## 3  0.0342032  0.0497810 -0.1416350  0.0505023  0.20158900  0.3113560 -0.1571120
## 4 -0.3034470  1.2604000  0.2816040 -1.3118700 -0.82847600  0.1529860  0.6187130
## 5  0.4661000 -1.5374300 -0.1578050  1.3215700  0.78442300 -0.2185550 -0.4696590
## 6  0.0536108 -0.0797995  0.3387910  0.4381890  0.17973200 -0.0325891  0.0290797
##        PC924     PC925      PC926      PC927      PC928      PC929      PC930
## 1 -0.2018670 -0.118119  0.0275344 -0.0748739 -0.2556450 -0.0659247 -0.1428410
## 2  0.1771850  0.148444  0.1504570 -0.1515850 -0.0430475 -0.0644432 -0.0984086
## 3 -0.0274635  0.490511  0.3939210  0.0952288 -0.2906840 -0.1142960 -0.2622020
## 4 -0.0370026  0.396221 -0.2022290  0.0725813  0.6199830  0.3341600  0.2898110
## 5 -0.0526970 -0.389421  0.4343140 -0.1976250 -0.5951060 -0.2457920 -0.1949460
## 6  0.3392440  0.178390 -0.1409580  0.3610940 -0.0398357  0.0445596 -0.1082030
##        PC931      PC932        PC933 Individual continent          region
## 1  0.2225370 -0.1224600 -5.37101e-07        802    Europe Southern Europe
## 2 -0.0717070  0.0273642 -5.37101e-07        803    Europe Southern Europe
## 3 -0.0166998 -0.0245194 -5.37101e-07        804    Europe Southern Europe
## 4 -0.4126520 -0.0853937 -5.37101e-07        806    Europe Southern Europe
## 5  0.2184870  0.0381609 -5.37101e-07        807    Europe Southern Europe
## 6  0.0178926 -0.0472435 -5.37101e-07        808    Europe Southern Europe
##   Pop_City Country Latitude Longitude Continent Year          Region
## 1   Durres Albania 41.29704  19.50373    Europe 2018 Southern Europe
## 2   Durres Albania 41.29704  19.50373    Europe 2018 Southern Europe
## 3   Durres Albania 41.29704  19.50373    Europe 2018 Southern Europe
## 4   Durres Albania 41.29704  19.50373    Europe 2018 Southern Europe
## 5   Durres Albania 41.29704  19.50373    Europe 2018 Southern Europe
## 6   Durres Albania 41.29704  19.50373    Europe 2018 Southern Europe
##     Subregion order order2 orderold AM_order state FINAL_order
## 1 East Europe    33     25       25       NA                80
## 2 East Europe    33     25       25       NA                80
## 3 East Europe    33     25       25       NA                80
## 4 East Europe    33     25       25       NA                80
## 5 East Europe    33     25       25       NA                80
## 6 East Europe    33     25       25       NA                80
```

### 1.2.1 Create PCA plots by country

PCs 1 and 2

```
# #   save the pca plot                                                       ####
ggsave(
  here("scripts", "RMarkdowns", "output", "americas", "manuscript1", "lea", "PCA_lea_man1_bycountry_pc1_pc2.pdf"
  ),
  width  = 8,
  height = 10,
  units  = "in"
)
```

USA clusters with Japan, Italy, Bermuda Jamaica clusters with
Southeast/South Asia. Brazil and Argentina form their own cluster;
Colombia is also separated, but is closest to Trinidad and Tobago

PCs 1 and 3

```
# #   save the pca plot                                                       ####
ggsave(
  here("scripts", "RMarkdowns", "output", "americas", "manuscript1", "lea", "PCA_lea_man1_bycountry_pc1_pc3.pdf"
  ),
  width  = 8,
  height = 10,
  units  = "in"
)
```

PCs 1 and 4

```
# #   save the pca plot                                                       ####
ggsave(
  here("scripts", "RMarkdowns", "output", "americas", "manuscript1", "lea", "PCA_lea_man1_bycountry_pc1_pc4.pdf"
  ),
  width  = 8,
  height = 10,
  units  = "in"
)
```

PCs 2 and 3

```
# #   save the pca plot                                                       ####
ggsave(
  here("scripts", "RMarkdowns", "output", "americas", "manuscript1", "lea", "PCA_lea_man1_bycountry_pc2_pc3.pdf"
  ),
  width  = 8,
  height = 10,
  units  = "in"
)
```

### 1.2.2. PCA plots by region

PCs 1 and 2

```
# #   save the pca plot                                                       ####
ggsave(
  here("scripts", "RMarkdowns", "output", "americas", "manuscript1", "lea", "PCA_lea_man1_byregion_pc1_pc2.pdf"
  ),
  width  = 8,
  height = 10,
  units  = "in"
)
```

PCs 1 and 3

```
# #   save the pca plot                                                       ####
ggsave(
  here("scripts", "RMarkdowns", "output", "americas", "manuscript1", "lea", "PCA_lea_man1_byregion_pc1_pc3.pdf"
  ),
  width  = 8,
  height = 10,
  units  = "in"
)
```

PCs 2 and 3

```
# #   save the pca plot                                                       ####
ggsave(
  here("scripts", "RMarkdowns", "output", "americas", "manuscript1", "lea", "PCA_lea_man1_byregion_pc2_pc3.pdf"
  ),
  width  = 8,
  height = 10,
  units  = "in"
)
```

PCs 1 and 4

```
# #   save the pca plot                                                       ####
ggsave(
  here("scripts", "RMarkdowns", "output", "americas", "manuscript1", "lea", "PCA_lea_man1_byregion_pc1_pc4.pdf"
  ),
  width  = 8,
  height = 10,
  units  = "in"
)
```

## 1.3. Run LEA structure analysis on Global dataset

Import the data again

```
genotype <- here(
   "manuscript1/output/snps_sets/r2_0.01_b.vcf"
  )

d <- read.vcfR(
  genotype
)
```

```
## Scanning file to determine attributes.
## File attributes:
##   meta lines: 8
##   header_line: 9
##   variant count: 20825
##   column count: 942
## Meta line 8 read in.
## All meta lines processed.
## gt matrix initialized.
## Character matrix gt created.
##   Character matrix gt rows: 20825
##   Character matrix gt cols: 942
##   skip: 0
##   nrows: 20825
##   row_num: 0
## Processed variant 1000Processed variant 2000Processed variant 3000Processed variant 4000Processed variant 5000Processed variant 6000Processed variant 7000Processed variant 8000Processed variant 9000Processed variant 10000Processed variant 11000Processed variant 12000Processed variant 13000Processed variant 14000Processed variant 15000Processed variant 16000Processed variant 17000Processed variant 18000Processed variant 19000Processed variant 20000Processed variant: 20825
## All variants processed
```

Get population and individuals information

```
inds_full <- attr(d@gt,"dimnames")[[2]]
inds_full <- inds_full[-1]
a <- strsplit(inds_full, '_')
pops <- unname(sapply(a, FUN = function(x) return(as.character(x[1])))) 
table(pops)
```

```
## pops
## ALD ARA BAF BCO BEA BEH BEN BER BIT BPA BPV BRE BRM CAM CES CHA CHI COL CUA DAL 
##  10  12   7  12  12   9  12  12  12  11  12  13  16  12  15  12   6  10   8  12 
## DEN DES FAY FIR FLO FRS GEL GES GRA GRV HAI HAL HAN HAR HAT HOC HOU HUN INJ INW 
##  12  16  12  12   8  12   2  12  11  12  12  10   4  12  10   7  12  12  11   4 
## IPO JAF KAC KAG KAN KAT KLP KUN LAM LIN LOS MAC MAN MAT MAU MED MON NEB NEG NEH 
##   7   2   6  12  11   6   4   3   9  12  12  12  12  12  12   7  12  10   4   7 
## NEO NEW NOV NUE OKI PAL PEO PLM POR QNC RAR REC RUS SAI SER SMI SOC SON SPB SPR 
##  12  21   4  12  11  11  10   7  12  11  12  11  12  12   4  12  12   3   8   7 
## SPS SSK STL SUF SUU TAI TIK TRA TUC TUH UTS WAV YUN 
##   8  12  12   6   6   7  12  12  14  12  12   7   9
```

```
pops <- factor(pops)
inds <- unname(sapply(a, FUN = function(x) return(as.character(x[2]))))
```

Convert format

```
vcf2geno(genotype, gsub(".vcf", ".geno", genotype))
vcf2lfmm(genotype, gsub(".vcf", ".lfmm", genotype))
```

We will do 5 repetitions

```
# main options
# K = number of ancestral populations
# CPU = 10 is the number of CPU used (hidden input) 
# entropy = TRUE computes the cross-entropy criterion
project = snmf(
  genotype,
  K = 1:30,
  project = "new",
  repetitions = 5,
  percentage = 0.25,
  iterations = 500,
  CPU = 10,
  entropy = TRUE
)
```

```
project = load.snmfProject("manuscript1/output/snps_sets/r2_0.01_b.snmfProject")
```

Cross entropy

```
plot(project, col = "blue", pch = 19, cex = 1.2)
```

Save

```
# Open a new pdf file
pdf(here("scripts", "RMarkdowns", "output", "americas", "manuscript1", "lea", "lea_cross_entropy_man1.pdf"), width = 6, height = 4)

# Create your plot
plot(project, col = "blue", pch = 19, cex = 1.2)

# Close the pdf file
dev.off()
```

Summary of project *check with run is best*

```
summary(project)
```

```
## $repetitions
##                       K = 1 K = 2 K = 3 K = 4 K = 5 K = 6 K = 7 K = 8 K = 9
## with cross-entropy        5     5     5     5     5     5     5     5     5
## without cross-entropy     0     0     0     0     0     0     0     0     0
## total                     5     5     5     5     5     5     5     5     5
##                       K = 10 K = 11 K = 12 K = 13 K = 14 K = 15 K = 16 K = 17
## with cross-entropy         5      5      5      5      5      5      5      5
## without cross-entropy      0      0      0      0      0      0      0      0
## total                      5      5      5      5      5      5      5      5
##                       K = 18 K = 19 K = 20 K = 21 K = 22 K = 23 K = 24 K = 25
## with cross-entropy         5      5      5      5      5      5      5      5
## without cross-entropy      0      0      0      0      0      0      0      0
## total                      5      5      5      5      5      5      5      5
##                       K = 26 K = 27 K = 28 K = 29 K = 30
## with cross-entropy         5      5      5      5      5
## without cross-entropy      0      0      0      0      0
## total                      5      5      5      5      5
## 
## $crossEntropy
##          K = 1     K = 2     K = 3     K = 4     K = 5     K = 6     K = 7
## min  0.9417444 0.9119608 0.9016317 0.8954289 0.8928168 0.8902474 0.8877078
## mean 0.9418537 0.9121585 0.9018055 0.8956336 0.8929859 0.8904319 0.8879179
## max  0.9420115 0.9123447 0.9020123 0.8958845 0.8931517 0.8907030 0.8881261
##          K = 8     K = 9    K = 10    K = 11    K = 12    K = 13    K = 14
## min  0.8862203 0.8849606 0.8836380 0.8825936 0.8818281 0.8807734 0.8800866
## mean 0.8867226 0.8855207 0.8840571 0.8828948 0.8819656 0.8812233 0.8803300
## max  0.8875317 0.8865999 0.8845850 0.8830997 0.8821758 0.8814918 0.8805922
##         K = 15    K = 16    K = 17    K = 18    K = 19    K = 20    K = 21
## min  0.8793331 0.8786371 0.8784613 0.8774766 0.8771674 0.8768258 0.8766588
## mean 0.8795028 0.8788363 0.8786550 0.8777594 0.8776834 0.8773234 0.8768022
## max  0.8797588 0.8791168 0.8788205 0.8782223 0.8787529 0.8776094 0.8769345
##         K = 22    K = 23    K = 24    K = 25    K = 26    K = 27    K = 28
## min  0.8758749 0.8753032 0.8757220 0.8752399 0.8749798 0.8746391 0.8742174
## mean 0.8762132 0.8758092 0.8759792 0.8755271 0.8754752 0.8752120 0.8747781
## max  0.8765692 0.8762365 0.8763969 0.8758072 0.8762137 0.8762397 0.8750602
##         K = 29    K = 30
## min  0.8740179 0.8741240
## mean 0.8750523 0.8746009
## max  0.8760351 0.8751445
```

```
# get the cross-entropy of all runs for:

best25 = which.min(cross.entropy(project, K = 25))
best25 #1
```

```
## [1] 1
```

```
best23 = which.min(cross.entropy(project, K = 23))
best23 #5
```

```
## [1] 5
```

```
best20 = which.min(cross.entropy(project, K = 20))
best20 #3
```

```
## [1] 3
```

```
best16 = which.min(cross.entropy(project, K = 16))
best16 #4
```

```
## [1] 4
```

```
best10 = which.min(cross.entropy(project, K = 10))
best10 #5
```

```
## [1] 5
```

```
best8 = which.min(cross.entropy(project, K = 8))
best8 #4
```

```
## [1] 4
```

```
best7 = which.min(cross.entropy(project, K = 7))
best7 #5
```

```
## [1] 5
```

```
best6 = which.min(cross.entropy(project, K = 6))
best6 #4
```

```
## [1] 4
```

```
best5 = which.min(cross.entropy(project, K = 5))
best5 #5
```

```
## [1] 5
```

# 2. FastStructure

Load libraries needed for fastStructure

```
library(tidyverse)          # many helpful things
library(here)               # to load data easily
library(dplyr)              # to manipulate data
library(ggplot2)            # plots
library(extrafont)          # probably won't work on openOnDemand on the clusters
library(hrbrthemes)         # not really needed, personal preference for plots
library(flextable)          # create tables
library(officer)            # export office format
```

## 2.1. Run simple prior in fastStructure for file with LD pruning r2=0.01 & MAF 1%

### 2.1.1. Check populations in file

```
awk '{print $1}' manuscript1/output/snps_sets/r2_0.01_b.fam | sort | uniq -c | awk '{print $2}'
```

```
## ALD
## ARA
## BAF
## BCO
## BEA
## BEH
## BEN
## BER
## BIT
## BPA
## BPV
## BRE
## BRM
## CAM
## CES
## CHA
## CHI
## COL
## CUA
## DAL
## DEN
## DES
## FAY
## FIR
## FLO
## FRS
## GEL
## GES
## GRA
## GRV
## HAI
## HAL
## HAN
## HAR
## HAT
## HOC
## HOU
## HUN
## INJ
## INW
## IPO
## JAF
## KAC
## KAG
## KAN
## KAT
## KLP
## KUN
## LAM
## LIN
## LOS
## MAC
## MAN
## MAT
## MAU
## MED
## MON
## NEB
## NEG
## NEH
## NEO
## NEW
## NOV
## NUE
## OKI
## PAL
## PEO
## PLM
## POR
## QNC
## RAR
## REC
## RUS
## SAI
## SER
## SMI
## SOC
## SON
## SPB
## SPR
## SPS
## SSK
## STL
## SUF
## SUU
## TAI
## TIK
## TRA
## TUC
## TUH
## UTS
## WAV
## YUN
```

Count the total number of population

```
awk '{print $1}' manuscript1/output/snps_sets/r2_0.01_b.fam | sort | uniq -c | wc -l
#93
```

```
## 93
```

### 2.1.2 Create a directory for each run.

```
cd manuscript1/output/fastStructure
for i in $(seq -w 1 100)
do
  mkdir run$i
done
```

### 2.1.3 Create the dsq files

We have 29 countries. We will run for K=2 to K=30 to be safe

```
cd /gpfs/gibbs/pi/caccone/mkc54/albo/manuscript1/output/fastStructure
module load dSQ/1.05 

for k in $(seq 2 30); do
    for run in $(seq -w 1 100); do
        echo "cd /gpfs/gibbs/pi/caccone/mkc54/albo/manuscript1/output/fastStructure; source /vast/palmer/apps/software/miniconda/23.3.1/etc/profile.d/conda.sh; module load miniconda/4.12.0; conda activate fastStructure; export PYTHONPATH=/home/mkc54/fastStructure/; python -m structure -K $k --input=/gpfs/gibbs/pi/caccone/mkc54/albo/manuscript1/output/snps_sets/r2_0.01_b --output=/gpfs/gibbs/pi/caccone/mkc54/albo/manuscript1/output/fastStructure/run$run/simple --full --cv=10 --tol=10e-6"
    done
done > dsq_manuscript1.txt


head dsq_manuscript1.txt #check file

dsq --job-file dsq_manuscript1.txt --output /dev/null --mail-type ALL -t 24:00:00 --partition=ycga --cpus-per-task=10 --job-name fS.simple_man1 --batch-file simple_man1.sh

#Check the files to see if they have 3,000 jobs:
head simple_man1.sh
```

### 2.1.4 Run the jobs

Run a random job from our array to see if works

```
sbatch --array=$((1 + $RANDOM % 100)) simple_man1.sh

squeue -u mkc54

# check status
dsqa -j 40238775 #insert job number

#if it looks like its running ok, you can cancel it 
scancel 40238775
```

Submit all jobs

```
sbatch simple_man1.sh
#Submitted batch job # 

#Check it
dsqa -j 40238869
#add job number here
```

### 2.1.5 Run autopsy once its done

```
cd /gpfs/gibbs/pi/caccone/mkc54/albo/manuscript1/output/fastStructure
module load dSQ/1.05

dsqa -j 40238869 -f dsq_manuscript1.txt -s TIMEOUT > re-run_jobs1.txt 2> 40238869_report1.txt; head 40238869_report1.txt
head re-run_jobs1.txt  #all completed
```

### 2.1.6. Find the optimal number of K.

you will see it varies from run to run. You can estimate mean, mode
and median values. Then you choose one that makes sense.

```
cd /gpfs/gibbs/pi/caccone/mkc54/albo/manuscript1/output/fastStructure
srun -p ycga --pty -N 1 -n 1 -c 4 bash #start interactive session

module load miniconda
conda activate fastStructure 

#First check files
ls run*/* | wc -l # should be 14500

# chose k for manuscript data
export PYTHONPATH=/home/mkc54/fastStructure
python -m chooseK #see how we can use chooseK
#python /vast/palmer/home.mccleary/mkc54/fastStructure/chooseK.py
   #      --input=<file>

for i in cd /gpfs/gibbs/pi/caccone/mkc54/albo/manuscript1/output/fastStructure/run*
do
    echo $i | sed 's@.*/@@'; python -m chooseK --input=$i/simple | grep 'likelihood\|structure'
done
```

Get outputs in a file

```
cd /gpfs/gibbs/pi/caccone/mkc54/albo/manuscript1/output/fastStructure

cat <(echo 'run likelihood structure') \
<(for i in /gpfs/gibbs/pi/caccone/mkc54/albo/manuscript1/output/fastStructure/run*
do
    echo $i | sed 's@.*/@@' | sed 's/run/run /' ; python -m chooseK --input=$i/simple | grep 'likelihood' | awk '{print $6, $8}'; python -m chooseK --input=$i/simple | grep 'structure' |  awk '{print $6, $10}'
done | xargs -n6 | awk '{print $2, $4, $6}') > simple_manuscript1_30k.txt

head simple_manuscript1_30k.txt #check the file
#ok

cd /gpfs/gibbs/pi/caccone/mkc54/albo/manuscript1/output/fastStructure
cp -r simple_manuscript1_30k.txt /gpfs/gibbs/pi/caccone/mkc54/albo/scripts/RMarkdowns/output/americas/manuscript1/fastStructure #copy file
```

### 2.1.7 Make a plot with the Ks.

Let’s import the data in R.

```
library(tidyverse)          # many helpful things
library(here)               # to load data easily
library(dplyr)              # to manipulate data
library(ggplot2)            # plots
library(extrafont)          # probably won't work on openOnDemand on the clusters
library(hrbrthemes)         # not really needed, personal preference for plots
library(flextable)          # create tables
library(officer)            # export office format
```

```
# function to import our choosek.py data
import_fastStructure <- function(file) {
  # import as a tibble and set columns as integers
  dat <- read_delim(
    file,
    col_names      = TRUE,
    show_col_types = FALSE,
    col_types      = "iii"
  )

  # get columns we need and make it long for plotting
  dat <- dat |>
    gather(
      structure, likelihood, -run
    )

  # rename the columns by index
  dat <- dat |>
    rename(
      run   = 1,
      model = 2,
      k     = 3
    )
  return(dat)
}
```

Run (simple\_prior\_models file needs to be in the RMarkdown
folder)

```
choose_k_simple <- 
  import_fastStructure(
  here("scripts/RMarkdowns/output/americas/manuscript1/fastStructure/simple_manuscript1_30k.txt"
  )
)
```

Function to plot fastStructure choosek.py results

```
# function to plot our choosek.py data
plot_fastStructure <- function(df) {
  df |>
    ggplot() +
    geom_line(
      aes(
        x              = run,
        y              = k,
        color          = model
      ),
      linewidth = 1
    ) +
    scale_colour_manual(
      "model",
      values = c(
        structure      = "#9d60ff",
        likelihood     = "#ffc08c"
      ),
      labels = c(
        "Maximizes \n Likelihood \n", "Explain \n Structure"
      )
    ) +
    labs(
      x                = "Run",
      y                = "K",
      title            = "fastStructure simple for the Americas, Europe & the native range",
      caption          = "algorithm runs for K ranging from 1 to 30"
    ) +
    theme(
      panel.grid.major = element_line(
        linetype       = "dashed",
        linewidth      = 0.2
      ),
      panel.grid.minor = element_line(
        linetype       = "dashed",
        linewidth      = 0.2
      ),
      legend.text = element_text(
        size = 12
      ),
      legend.title = element_text(
        size           = 14,
        face           = "bold"
      ),
      legend.position = "right"
    )
}
```

Use our function to plot k values

```
plot_fastStructure(
  choose_k_simple
) +
  labs(
    subtitle = "simple fastStructure for the Americas, Europe & the native range"
  )
```

```
# save plot
ggsave(
  here("scripts/RMarkdowns/output/americas/manuscript1/fastStructure/fastStructure_simple_manuscript1_30k.pdf"
  ),
  width  = 8,
  height = 6,
  units  = "in"
)
```

### 2.1.8 Find mean K maximizing likelihood

```
choose_k_likelihood <- subset(choose_k_simple, model != "structure")
mean(choose_k_likelihood$k) #26.79
```

```
## [1] 26.79
```

```
median(choose_k_likelihood$k) #27
```

```
## [1] 27
```

Most common K

```
common <- table(choose_k_likelihood$k)
common #27, range 20-30
```

```
## 
## 20 21 22 23 24 25 26 27 28 29 30 
##  1  1  5  7  4 12  8 19 13 14 16
```

Most common K among the 100 runs is 27 (range 20-30)

# 3. Figures for structure analyses

## 3.1. Figures from LEA

```
genotype <- here(
   "manuscript1/output/snps_sets/r2_0.01_b.vcf"
  )

d <- read.vcfR(
  genotype
)
```

```
## Scanning file to determine attributes.
## File attributes:
##   meta lines: 8
##   header_line: 9
##   variant count: 20825
##   column count: 942
## Meta line 8 read in.
## All meta lines processed.
## gt matrix initialized.
## Character matrix gt created.
##   Character matrix gt rows: 20825
##   Character matrix gt cols: 942
##   skip: 0
##   nrows: 20825
##   row_num: 0
## Processed variant 1000Processed variant 2000Processed variant 3000Processed variant 4000Processed variant 5000Processed variant 6000Processed variant 7000Processed variant 8000Processed variant 9000Processed variant 10000Processed variant 11000Processed variant 12000Processed variant 13000Processed variant 14000Processed variant 15000Processed variant 16000Processed variant 17000Processed variant 18000Processed variant 19000Processed variant 20000Processed variant: 20825
## All variants processed
```

Get population and individuals information

```
inds_full <- attr(d@gt,"dimnames")[[2]]
inds_full <- inds_full[-1]
a <- strsplit(inds_full, '_')
pops <- unname(sapply(a, FUN = function(x) return(as.character(x[1])))) 
table(pops)
```

```
## pops
## ALD ARA BAF BCO BEA BEH BEN BER BIT BPA BPV BRE BRM CAM CES CHA CHI COL CUA DAL 
##  10  12   7  12  12   9  12  12  12  11  12  13  16  12  15  12   6  10   8  12 
## DEN DES FAY FIR FLO FRS GEL GES GRA GRV HAI HAL HAN HAR HAT HOC HOU HUN INJ INW 
##  12  16  12  12   8  12   2  12  11  12  12  10   4  12  10   7  12  12  11   4 
## IPO JAF KAC KAG KAN KAT KLP KUN LAM LIN LOS MAC MAN MAT MAU MED MON NEB NEG NEH 
##   7   2   6  12  11   6   4   3   9  12  12  12  12  12  12   7  12  10   4   7 
## NEO NEW NOV NUE OKI PAL PEO PLM POR QNC RAR REC RUS SAI SER SMI SOC SON SPB SPR 
##  12  21   4  12  11  11  10   7  12  11  12  11  12  12   4  12  12   3   8   7 
## SPS SSK STL SUF SUU TAI TIK TRA TUC TUH UTS WAV YUN 
##   8  12  12   6   6   7  12  12  14  12  12   7   9
```

```
pops <- factor(pops)
inds <- unname(sapply(a, FUN = function(x) return(as.character(x[2]))))
```

Convert format

```
vcf2geno(genotype, gsub(".vcf", ".geno", genotype))
```

```
## 
##  - number of detected individuals:   933
##  - number of detected loci:      20825
## 
## For SNP info, please check /gpfs/gibbs/pi/caccone/mkc54/albo/manuscript1/output/snps_sets/r2_0.01_b.vcfsnp.
## 
## 0 line(s) were removed because these are not SNPs.
## Please, check /gpfs/gibbs/pi/caccone/mkc54/albo/manuscript1/output/snps_sets/r2_0.01_b.removed file, for more informations.
```

```
## [1] "/gpfs/gibbs/pi/caccone/mkc54/albo/manuscript1/output/snps_sets/r2_0.01_b.geno"
```

```
vcf2lfmm(genotype, gsub(".vcf", ".lfmm", genotype))
```

```
## 
##  - number of detected individuals:   933
##  - number of detected loci:      20825
## 
## For SNP info, please check /gpfs/gibbs/pi/caccone/mkc54/albo/manuscript1/output/snps_sets/r2_0.01_b.vcfsnp.
## 
## 0 line(s) were removed because these are not SNPs.
## Please, check /gpfs/gibbs/pi/caccone/mkc54/albo/manuscript1/output/snps_sets/r2_0.01_b.removed file, for more informations.
## 
## 
##  - number of detected individuals:   933
##  - number of detected loci:      20825
```

```
## [1] "/gpfs/gibbs/pi/caccone/mkc54/albo/manuscript1/output/snps_sets/r2_0.01_b.lfmm"
```

```
color_palette <-
c(  "blue",
    "#990000",    
    "magenta",
    "purple3",
    "#FF5005", 
    "#FFE100",
    "#5EF1F2",
    "#FF0010",
    "#005C31", 
    "#2BCE48",
    "#C20088",
    "#808080", 
    "#FFFC99",
    "#4C005C",
    "#993F00",
    "#FFA8BB",
    "#003380",
    "#1E90FF", 
    "orange",
    "#F0A0FF",    
    "#9DCC00",
    "#AC7700",
    "#52ef99",
    "orchid1",
    "#CB3D1C",
    "gray",
    "green",
    "cadetblue",
    "orchid4",
    "coral",
    "#fd4e8b",
    "orangered2",
    "ivory",
    "#B20CC9",
    "#AE9393",
    "#658B00", 
    "#8c61cd"
    )
```

```
project = load.snmfProject("manuscript1/output/snps_sets/r2_0.01_b.snmfProject")
```

### 3.1.1 LEA plots for k=6

Default plot

```
# select the best run for chosen K 
best6 = which.min(cross.entropy(project, K = 6))
# best is run 4

barchart(project, K = 6, run = best6,
        border = NA, space = 0,
        col = color_palette,
        xlab = "Individuals",
        ylab = "Ancestry proportions",
        main = "Ancestry matrix") -> bp
axis(1, at = 1:length(bp$order),
     labels = bp$order, las=1,
     cex.axis = .4)
```

#### 3.1.1.1 Mean admixture by country using ggplot

```
color_palette <-
c(  "blue",
    "purple3",  
    "#FF0010",   
    "magenta",
    "#FFE100",
    "#FF8005"
    ) 


sampling_loc <- readRDS(here("scripts", "RMarkdowns", "output", "sampling_loc_all_pops.rds"))

library(reshape2)
```

```
## 
## Attaching package: 'reshape2'
```

```
## The following object is masked from 'package:tidyr':
## 
##     smiths
```

```
# Extract ancestry coefficients
Q_values <- as.data.frame(Q(project, K = 6, run = best6))

# Add individual IDs and pops ids
Q_values$ind <- inds
Q_values$pop <- pops

# Melt the data frame for plotting
Q_melted <- melt(Q_values, id.vars = c("ind", "pop"))

# Join with sampling_loc to get sampling localities
Q_joined <- Q_melted |>
  left_join(sampling_loc, by = c("pop" = "Abbreviation"))

# Create a combined variable for Region and Country
Q_joined <- Q_joined |>
  mutate(Region_Country = interaction(Region, Country, sep = "_"))

# Order the combined variable by Region and Country
Q_ordered <- Q_joined |>
  arrange(Region, Country) |>
  mutate(Region_Country = factor(Region_Country, levels = unique(Region_Country)))

# Group by Country and calculate mean ancestry proportions
Q_grouped <- Q_ordered |>
  group_by(Region_Country, variable) |>
  summarise(value = mean(value), .groups = "drop")

# Create a data frame for borders
borders <- data.frame(Region_Country = unique(Q_grouped$Region_Country))

# Add the order of each country to ensure correct placement of borders
borders$order <- 1:nrow(borders) + 0.5  # Shift borders to the right edge of the bars


# Function to filter and normalize data
normalize_data <- function(df, min_value) {
  df |>
    filter(value > min_value) |>
    group_by(Region_Country) |>
    mutate(value = value / sum(value))
}

# Use the function
Q_grouped_filtered <- normalize_data(Q_grouped, 0.1)

# source the plotting function
source(
  here("scripts", "RMarkdowns",
   "analyses", "my_theme2.R"
  )
)

# Generate all potential variable names
all_variables <- paste0("V", 1:6)

# Create a data frame that pairs each potential variable with a color
color_mapping <- data.frame(variable = all_variables,
                           color = color_palette[1:length(all_variables)])

# Merge this data frame with Q_grouped_filtered to create the new color column
Q_grouped_filtered <- merge(Q_grouped_filtered, color_mapping, by = "variable")

# Create bar chart
ggplot(Q_grouped_filtered, aes(x = Region_Country, y = value, fill = color)) +
  geom_bar(stat = 'identity', width = 1) +
  geom_segment(data = borders, aes(x = order, xend = order, y = 0, yend = 1, fill = NULL), linetype = "solid", color = "#2C444A") +  # Add borders
  my_theme() +
  theme(axis.text.x = element_text(angle = 90, hjust = 1),
        legend.position = "none") +  # Hide legend
  xlab("") +  # Suppress x-axis label
  ylab("Ancestry proportions") +
  ggtitle("Ancestry matrix") +
  labs(caption = "Each bar represents the average ancestry proportions for individuals in a given country for k=6.") +
  # scale_fill_manual(values = color) +
  scale_x_discrete(labels = function(x) gsub(".*_", "", x)) + # Remove Region prefix from labels
  scale_fill_manual(values = color_palette)
```

```
ggsave(
  here("scripts", "RMarkdowns",
    "output", "americas", "manuscript1", "lea", "LEA_admix_by_country_man1_k6.pdf"
  ),
  width  = 10,
  height = 7,
  units  = "in"
)
```

Using ggplot2 for individual admixtures

#### 3.1.1.2 Extract ancestry coefficients for k=6

*change to correct matrix*

```
leak6 <- read_delim(
  here("/gpfs/gibbs/pi/caccone/mkc54/albo/manuscript1/output/snps_sets/r2_0.01_b.snmf/K6/run4/r2_0.01_b_r4.6.Q"),
  delim = " ", # Specify the delimiter if different from the default (comma)
  col_names = FALSE,
  show_col_types = FALSE
) 
head(leak6)
```

```
## # A tibble: 6 × 6
##       X1    X2    X3     X4       X5      X6
##    <dbl> <dbl> <dbl>  <dbl>    <dbl>   <dbl>
## 1 0.0905 0.160 0.604 0.0177 0.0257   0.102  
## 2 0.116  0.138 0.536 0.0264 0.0892   0.0946 
## 3 0.0631 0.152 0.632 0.0317 0.0395   0.0813 
## 4 0.0641 0.277 0.617 0.0380 0.000100 0.00400
## 5 0.0708 0.274 0.621 0.0166 0.000100 0.0179 
## 6 0.0837 0.211 0.590 0.0450 0.00150  0.0692
```

The fam file

```
fam_file <- here("manuscript1/output/snps_sets/r2_0.01_b.fam")

# Read the .fam file
fam_data <- read.table(fam_file, 
                       header = FALSE,
                       col.names = c("FamilyID", "IndividualID", "PaternalID", "MaternalID", "Sex", "Phenotype"))

# View the first few rows
head(fam_data)
```

```
##   FamilyID IndividualID PaternalID MaternalID Sex Phenotype
## 1      OKI         1001          0          0   2        -9
## 2      OKI         1002          0          0   2        -9
## 3      OKI         1003          0          0   2        -9
## 4      OKI         1004          0          0   2        -9
## 5      OKI         1005          0          0   2        -9
## 6      OKI         1006          0          0   1        -9
```

Create column ID

```
# Change column name
colnames(fam_data)[colnames(fam_data) == "IndividualID"] <- "ind"

# Change column name
colnames(fam_data)[colnames(fam_data) == "FamilyID"] <- "pop"

# Select ID
fam_data <- fam_data |>
  dplyr::select("ind", "pop")

# View the first few rows
head(fam_data)
```

```
##    ind pop
## 1 1001 OKI
## 2 1002 OKI
## 3 1003 OKI
## 4 1004 OKI
## 5 1005 OKI
## 6 1006 OKI
```

Add it to the matrix

```
leak6 <- fam_data |>
  dplyr::select(ind, pop) |>
  bind_cols(leak6)

head(leak6)
```

```
##    ind pop        X1       X2       X3        X4          X5         X6
## 1 1001 OKI 0.0905051 0.159599 0.604495 0.0177085 0.025702000 0.10199100
## 2 1002 OKI 0.1163120 0.137541 0.535974 0.0263565 0.089173100 0.09464330
## 3 1003 OKI 0.0631335 0.151922 0.632499 0.0317177 0.039450300 0.08127750
## 4 1004 OKI 0.0641155 0.277158 0.616665 0.0379619 0.000099991 0.00399984
## 5 1005 OKI 0.0708217 0.274003 0.620592 0.0166179 0.000099991 0.01786620
## 6 1006 OKI 0.0837076 0.211002 0.589626 0.0449908 0.001498250 0.06917520
```

Rename the columns

```
# Rename the columns starting from the third one
leak6 <- leak6 |>
  rename_with(~paste0("v", seq_along(.x)), .cols = -c(ind, pop))

# View the first few rows
head(leak6)
```

```
##    ind pop        v1       v2       v3        v4          v5         v6
## 1 1001 OKI 0.0905051 0.159599 0.604495 0.0177085 0.025702000 0.10199100
## 2 1002 OKI 0.1163120 0.137541 0.535974 0.0263565 0.089173100 0.09464330
## 3 1003 OKI 0.0631335 0.151922 0.632499 0.0317177 0.039450300 0.08127750
## 4 1004 OKI 0.0641155 0.277158 0.616665 0.0379619 0.000099991 0.00399984
## 5 1005 OKI 0.0708217 0.274003 0.620592 0.0166179 0.000099991 0.01786620
## 6 1006 OKI 0.0837076 0.211002 0.589626 0.0449908 0.001498250 0.06917520
```

Import Sample Locations

```
sampling_loc <- readRDS(here("scripts", "RMarkdowns", "output", "sampling_loc_all_pops.rds"))
head(sampling_loc)
```

```
##      Pop_City Country Latitude Longitude Continent Abbreviation  Year    Region
## 1      Hainan   China  19.2000  109.6000      Asia          HAI  2014 East Asia
## 2      Yunnan   China  24.4753  101.3431      Asia          YUN  2014 East Asia
## 3       Hunan   China  27.6253  111.8569      Asia          HUN  2001 East Asia
## 4     Okinawa   Japan  26.5013  127.9454      Asia          OKI 2018? East Asia
## 5 Tanegashima   Japan  30.4564  130.9784      Asia          TAN  2008 East Asia
## 6   Kagoshima   Japan  31.5602  130.5581      Asia          KAG  2018 East Asia
##   Subregion order order2 orderold AM_order state FINAL_order
## 1     Japan    55     47       47       NA                 1
## 2     Japan    56     48       48       NA                 2
## 3     Japan    57     49       49       NA                 3
## 4     Japan    54     46       46       NA                 4
## 5     Japan    NA     NA       NA       NA                 5
## 6     Japan    53     45       45       NA                 6
```

```
source(
  here("scripts", "RMarkdowns",
    "my_theme3.R"
  )
)

# Melt the data frame for plotting
Q_melted <- leak6 |>
  pivot_longer(
    cols = -c(ind, pop),
    names_to = "variable",
    values_to = "value"
  )
# Join with sampling_loc to get sampling localities
Q_joined <- Q_melted |>
  left_join(sampling_loc, by = c("pop" = "Abbreviation"))


# Order the combined variable by Region and Country, then by individual
Q_ordered <- Q_joined |>
  arrange(FINAL_order, ind) |>
  mutate(ind = factor(ind, levels = unique(ind)))  # Convert ind to a factor with levels in the desired order

# Add labels: country names for the first individual in each country, NA for all other individuals
Q_ordered <- Q_ordered |>
  group_by(Country) |>
  mutate(label = ifelse(row_number() == 1, as.character(Country), NA))

# Group by individual and variable, calculate mean ancestry proportions
Q_grouped <- Q_ordered |>
  group_by(ind, variable) |>
  summarise(value = mean(value), .groups = "drop")

# Create a data frame for borders
borders <-
  data.frame(Country = unique(Q_ordered$Country))

# Add the order of the last individual of each country to ensure correct placement of borders
borders$order <-
  sapply(borders$Country, function(rc)
    max(which(Q_ordered$Country == rc))) + 0.5  # Shift borders to the right edge of the bars

# Select only the first occurrence of each country in the ordered data
label_df <- Q_ordered |>
  filter(!is.na(label)) |>
  distinct(label, .keep_all = TRUE)

# Create a custom label function
label_func <- function(x) {
  labels <- rep("", length(x))
  labels[x %in% label_df$ind] <- label_df$label
  labels
}

# Calculate the position of lines
border_positions <- Q_ordered |>
  group_by(Country) |>
  summarise(pos = max(as.numeric(ind)) + 0)

# Calculate the position of population labels and bars
pop_labels <- Q_ordered |>
  mutate(Name = paste(pop, Pop_City, sep = " - ")) |>
  group_by(pop) |>
  slice_head(n = 1) |>
  ungroup() |>
  dplyr::select(ind, Pop_City, Country, Name) |>
  mutate(pos = as.numeric(ind))  # calculate position of population labels

pop_labels_bars <- pop_labels |>
  mutate(pos = as.numeric(ind)  - .5)


# Calculate the position of lines
border_positions <- Q_ordered |>
  group_by(Country) |>
  summarise(pos = max(as.numeric(ind)) - 1)


pop_labels_bars <- pop_labels |>
  mutate(pos = as.numeric(ind)  - .5)

# Function to filter and normalize data
normalize_data <- function(df, min_value) {
  df |>
    filter(value > min_value) |>
    group_by(ind) |>
    mutate(value = value / sum(value))
}

# Use the function
Q_grouped_filtered <- normalize_data(Q_grouped, 0.1)
# 

color_palette <-
c(  "blue",
    "purple3",  
    "#FF8005",    
    "#FF0010",    
    "magenta",
    "#FFE100"    
    )

# Generate all potential variable names
all_variables <- paste0("v", 1:6)


# Create a data frame that pairs each potential variable with a color
color_mapping <- data.frame(variable = all_variables,
                           color = color_palette[1:length(all_variables)])

# Merge with Q_grouped_filtered
Q_grouped_filtered <- merge(Q_grouped_filtered, color_mapping, by = "variable")

# Create the plot
ggplot(Q_grouped_filtered, aes(x = as.factor(ind), y = value, fill = color)) +
  geom_bar(stat = 'identity', width = 1) +
  geom_vline(
    data = pop_labels_bars,
    aes(xintercept = pos),
    color = "#2C444A",
    linewidth = .2
  ) +
  geom_text(
    data = pop_labels,
    aes(x = as.numeric(ind), y = 1, label = Name),
    vjust = 1.5,
    hjust = 0,
    size = 2,
    angle = 90,
    inherit.aes = FALSE
  ) +
  my_theme() +
  theme(
    axis.text.x = element_text(
      angle = 90,
      hjust = 1,
      size = 12
    ),
    legend.position = "none",
    plot.margin = unit(c(3, 0.5, 0.5, 0.5), "cm")
  ) +
  xlab("Admixture matrix") +
  ylab("Ancestry proportions") +
  labs(caption = "Each bar represents the ancestry proportions for an individual for k=6.\n LEA inference with 20,825 SNPs.") +
  scale_x_discrete(labels = label_func) +
  scale_fill_manual(values = color_palette) +
  expand_limits(y = c(0, 1.5))
```

```
ggsave(
  here("scripts", "RMarkdowns", "output", "americas", "manuscript1", "lea", "lea_k=6_manuscript1.pdf"),
  width  = 16,
  height = 6,
  units  = "in",
  device = cairo_pdf
)
```

### 3.1.2 LEA plots for k=7

Default plot

```
# select the best run for chosen K 
best7 = which.min(cross.entropy(project, K = 7))
# best is run 5

barchart(project, K = 7, run = best7,
        border = NA, space = 0,
        col = color_palette,
        xlab = "Individuals",
        ylab = "Ancestry proportions",
        main = "Ancestry matrix") -> bp
axis(1, at = 1:length(bp$order),
     labels = bp$order, las=1,
     cex.axis = .4)
```

#### 3.1.2.1 Mean admixture by country using ggplot

```
color_palette <-
c(  "#FFE100",   
    "purple3", 
    "magenta",    
    "blue",    
    "cyan",    
    "#FF8005",    
    "#FF0010")


sampling_loc <- readRDS(here("scripts", "RMarkdowns", "output", "sampling_loc_all_pops.rds"))

library(reshape2)

# Extract ancestry coefficients
Q_values <- as.data.frame(Q(project, K = 7, run = best7))

# Add individual IDs and pops ids
Q_values$ind <- inds
Q_values$pop <- pops

# Melt the data frame for plotting
Q_melted <- melt(Q_values, id.vars = c("ind", "pop"))

# Join with sampling_loc to get sampling localities
Q_joined <- Q_melted |>
  left_join(sampling_loc, by = c("pop" = "Abbreviation"))

# Create a combined variable for Region and Country
Q_joined <- Q_joined |>
  mutate(Region_Country = interaction(Region, Country, sep = "_"))

# Order the combined variable by Region and Country
Q_ordered <- Q_joined |>
  arrange(Region, Country) |>
  mutate(Region_Country = factor(Region_Country, levels = unique(Region_Country)))

# Group by Country and calculate mean ancestry proportions
Q_grouped <- Q_ordered |>
  group_by(Region_Country, variable) |>
  summarise(value = mean(value), .groups = "drop")

# Create a data frame for borders
borders <- data.frame(Region_Country = unique(Q_grouped$Region_Country))

# Add the order of each country to ensure correct placement of borders
borders$order <- 1:nrow(borders) + 0.5  # Shift borders to the right edge of the bars


# Function to filter and normalize data
normalize_data <- function(df, min_value) {
  df |>
    filter(value > min_value) |>
    group_by(Region_Country) |>
    mutate(value = value / sum(value))
}

# Use the function
Q_grouped_filtered <- normalize_data(Q_grouped, 0.1)

# source the plotting function
source(
  here("scripts", "RMarkdowns",
   "analyses", "my_theme2.R"
  )
)

# Generate all potential variable names
all_variables <- paste0("V", 1:7)

# Create a data frame that pairs each potential variable with a color
color_mapping <- data.frame(variable = all_variables,
                          color = color_palette[1:length(all_variables)])

# Merge this data frame with Q_grouped_filtered to create the new color column
Q_grouped_filtered <- merge(Q_grouped_filtered, color_mapping, by = "variable")

# Create bar chart
ggplot(Q_grouped_filtered, aes(x = Region_Country, y = value, fill = color)) +
  geom_bar(stat = 'identity', width = 1) +
  geom_segment(data = borders, aes(x = order, xend = order, y = 0, yend = 1, fill = NULL), linetype = "solid", color = "#2C444A") +  # Add borders
  my_theme() +
  theme(axis.text.x = element_text(angle = 90, hjust = 1),
        legend.position = "none") +  # Hide legend
  xlab("") +  # Suppress x-axis label
  ylab("Ancestry proportions") +
  ggtitle("Ancestry matrix") +
  labs(caption = "Each bar represents the average ancestry proportions for individuals in a given country for k=7.") +
  # scale_fill_manual(values = color) +
  scale_x_discrete(labels = function(x) gsub(".*_", "", x)) + # Remove Region prefix from labels
  scale_fill_manual(values = color_palette)
```

```
ggsave(
  here("scripts", "RMarkdowns",
    "output", "americas", "manuscript1", "lea", "LEA_admix_by_country_man1_k7.pdf"
  ),
  width  = 10,
  height = 7,
  units  = "in"
)
```

#### 3.1.2.2. Extract ancestry coefficients for k=7

*change to correct matrix*

```
leak7 <- read_delim(
  here("/gpfs/gibbs/pi/caccone/mkc54/albo/manuscript1/output/snps_sets/r2_0.01_b.snmf/K7/run5/r2_0.01_b_r5.7.Q"),
  delim = " ", # Specify the delimiter if different from the default (comma)
  col_names = FALSE,
  show_col_types = FALSE
) 
head(leak7)
```

```
## # A tibble: 6 × 7
##      X1       X2      X3       X4     X5    X6     X7
##   <dbl>    <dbl>   <dbl>    <dbl>  <dbl> <dbl>  <dbl>
## 1 0.184 0.000100 0.00117 0.0445   0.133  0.551 0.0855
## 2 0.175 0.0393   0.0242  0.0456   0.100  0.519 0.0967
## 3 0.182 0.0313   0.0256  0.0289   0.0520 0.619 0.0612
## 4 0.296 0.00608  0.0198  0.000100 0.0671 0.589 0.0212
## 5 0.287 0.000100 0.0138  0.000100 0.0701 0.612 0.0170
## 6 0.215 0.0234   0.0424  0.0124   0.0550 0.580 0.0714
```

The fam file

```
fam_file <- here("manuscript1/output/snps_sets/r2_0.01_b.fam")

# Read the .fam file
fam_data <- read.table(fam_file, 
                       header = FALSE,
                       col.names = c("FamilyID", "IndividualID", "PaternalID", "MaternalID", "Sex", "Phenotype"))

# View the first few rows
head(fam_data)
```

```
##   FamilyID IndividualID PaternalID MaternalID Sex Phenotype
## 1      OKI         1001          0          0   2        -9
## 2      OKI         1002          0          0   2        -9
## 3      OKI         1003          0          0   2        -9
## 4      OKI         1004          0          0   2        -9
## 5      OKI         1005          0          0   2        -9
## 6      OKI         1006          0          0   1        -9
```

Create column ID

```
# Change column name
colnames(fam_data)[colnames(fam_data) == "IndividualID"] <- "ind"

# Change column name
colnames(fam_data)[colnames(fam_data) == "FamilyID"] <- "pop"

# Select ID
fam_data <- fam_data |>
  dplyr::select("ind", "pop")

# View the first few rows
head(fam_data)
```

```
##    ind pop
## 1 1001 OKI
## 2 1002 OKI
## 3 1003 OKI
## 4 1004 OKI
## 5 1005 OKI
## 6 1006 OKI
```

Add it to the matrix

```
leak7 <- fam_data |>
  dplyr::select(ind, pop) |>
  bind_cols(leak7)

head(leak7)
```

```
##    ind pop       X1          X2         X3          X4        X5       X6
## 1 1001 OKI 0.184164 0.000099991 0.00117086 0.044463300 0.1332310 0.551359
## 2 1002 OKI 0.174551 0.039295900 0.02421110 0.045550700 0.1003170 0.519340
## 3 1003 OKI 0.182160 0.031312300 0.02560670 0.028937700 0.0519773 0.618842
## 4 1004 OKI 0.296351 0.006083610 0.01984520 0.000099991 0.0671124 0.589300
## 5 1005 OKI 0.286954 0.000099982 0.01380380 0.000099982 0.0700931 0.611977
## 6 1006 OKI 0.215049 0.023440100 0.04236610 0.012419700 0.0549962 0.580313
##          X7
## 1 0.0855124
## 2 0.0967351
## 3 0.0611637
## 4 0.0212082
## 5 0.0169716
## 6 0.0714154
```

Rename the columns

```
# Rename the columns starting from the third one
leak7 <- leak7 |>
  rename_with(~paste0("v", seq_along(.x)), .cols = -c(ind, pop))

# View the first few rows
head(leak7)
```

```
##    ind pop       v1          v2         v3          v4        v5       v6
## 1 1001 OKI 0.184164 0.000099991 0.00117086 0.044463300 0.1332310 0.551359
## 2 1002 OKI 0.174551 0.039295900 0.02421110 0.045550700 0.1003170 0.519340
## 3 1003 OKI 0.182160 0.031312300 0.02560670 0.028937700 0.0519773 0.618842
## 4 1004 OKI 0.296351 0.006083610 0.01984520 0.000099991 0.0671124 0.589300
## 5 1005 OKI 0.286954 0.000099982 0.01380380 0.000099982 0.0700931 0.611977
## 6 1006 OKI 0.215049 0.023440100 0.04236610 0.012419700 0.0549962 0.580313
##          v7
## 1 0.0855124
## 2 0.0967351
## 3 0.0611637
## 4 0.0212082
## 5 0.0169716
## 6 0.0714154
```

Import Sample Locations

```
sampling_loc <- readRDS(here("scripts", "RMarkdowns", "output", "sampling_loc_all_pops.rds"))
head(sampling_loc)
```

```
##      Pop_City Country Latitude Longitude Continent Abbreviation  Year    Region
## 1      Hainan   China  19.2000  109.6000      Asia          HAI  2014 East Asia
## 2      Yunnan   China  24.4753  101.3431      Asia          YUN  2014 East Asia
## 3       Hunan   China  27.6253  111.8569      Asia          HUN  2001 East Asia
## 4     Okinawa   Japan  26.5013  127.9454      Asia          OKI 2018? East Asia
## 5 Tanegashima   Japan  30.4564  130.9784      Asia          TAN  2008 East Asia
## 6   Kagoshima   Japan  31.5602  130.5581      Asia          KAG  2018 East Asia
##   Subregion order order2 orderold AM_order state FINAL_order
## 1     Japan    55     47       47       NA                 1
## 2     Japan    56     48       48       NA                 2
## 3     Japan    57     49       49       NA                 3
## 4     Japan    54     46       46       NA                 4
## 5     Japan    NA     NA       NA       NA                 5
## 6     Japan    53     45       45       NA                 6
```

```
source(
  here("scripts", "RMarkdowns",
    "my_theme3.R"
  )
)

# Melt the data frame for plotting
Q_melted <- leak7 |>
  pivot_longer(
    cols = -c(ind, pop),
    names_to = "variable",
    values_to = "value"
  )
# Join with sampling_loc to get sampling localities
Q_joined <- Q_melted |>
  left_join(sampling_loc, by = c("pop" = "Abbreviation"))


# Order the combined variable by Region and Country, then by individual
Q_ordered <- Q_joined |>
  arrange(FINAL_order, ind) |>
  mutate(ind = factor(ind, levels = unique(ind)))  # Convert ind to a factor with levels in the desired order

# Add labels: country names for the first individual in each country, NA for all other individuals
Q_ordered <- Q_ordered |>
  group_by(Country) |>
  mutate(label = ifelse(row_number() == 1, as.character(Country), NA))

# Group by individual and variable, calculate mean ancestry proportions
Q_grouped <- Q_ordered |>
  group_by(ind, variable) |>
  summarise(value = mean(value), .groups = "drop")

# Create a data frame for borders
borders <-
  data.frame(Country = unique(Q_ordered$Country))

# Add the order of the last individual of each country to ensure correct placement of borders
borders$order <-
  sapply(borders$Country, function(rc)
    max(which(Q_ordered$Country == rc))) + 0.5  # Shift borders to the right edge of the bars

# Select only the first occurrence of each country in the ordered data
label_df <- Q_ordered |>
  filter(!is.na(label)) |>
  distinct(label, .keep_all = TRUE)

# Create a custom label function
label_func <- function(x) {
  labels <- rep("", length(x))
  labels[x %in% label_df$ind] <- label_df$label
  labels
}

# Calculate the position of lines
border_positions <- Q_ordered |>
  group_by(Country) |>
  summarise(pos = max(as.numeric(ind)) + 0)

# Calculate the position of population labels and bars
pop_labels <- Q_ordered |>
  mutate(Name = paste(pop, Pop_City, sep = " - ")) |>
  group_by(pop) |>
  slice_head(n = 1) |>
  ungroup() |>
  dplyr::select(ind, Pop_City, Country, Name) |>
  mutate(pos = as.numeric(ind))  # calculate position of population labels

pop_labels_bars <- pop_labels |>
  mutate(pos = as.numeric(ind)  - .5)


# Calculate the position of lines
border_positions <- Q_ordered |>
  group_by(Country) |>
  summarise(pos = max(as.numeric(ind)) - 1)


pop_labels_bars <- pop_labels |>
  mutate(pos = as.numeric(ind)  - .5)

# Function to filter and normalize data
normalize_data <- function(df, min_value) {
  df |>
    filter(value > min_value) |>
    group_by(ind) |>
    mutate(value = value / sum(value))
}

# Use the function
Q_grouped_filtered <- normalize_data(Q_grouped, 0.1)
# 

color_palette <-
c(  
   "#FFE100",   
   "blue",
   "magenta", 
   "purple3",
   "cyan",   
   "#FF8005",
   "#FF0010"    
    )

# Generate all potential variable names
all_variables <- paste0("v", 1:7)


# Create a data frame that pairs each potential variable with a color
color_mapping <- data.frame(variable = all_variables,
                          color = color_palette[1:length(all_variables)])

# Merge with Q_grouped_filtered
Q_grouped_filtered <- merge(Q_grouped_filtered, color_mapping, by = "variable")

# Create the plot
ggplot(Q_grouped_filtered, aes(x = as.factor(ind), y = value, fill = color)) +
  geom_bar(stat = 'identity', width = 1) +
  geom_vline(
    data = pop_labels_bars,
    aes(xintercept = pos),
    color = "#2C444A",
    linewidth = .2
  ) +
  geom_text(
    data = pop_labels,
    aes(x = as.numeric(ind), y = 1, label = Name),
    vjust = 1.5,
    hjust = 0,
    size = 2,
    angle = 90,
    inherit.aes = FALSE
  ) +
  my_theme() +
  theme(
    axis.text.x = element_text(
      angle = 90,
      hjust = 1,
      size = 12
    ),
    legend.position = "none",
    plot.margin = unit(c(3, 0.5, 0.5, 0.5), "cm")
  ) +
  xlab("Admixture matrix") +
  ylab("Ancestry proportions") +
  labs(caption = "Each bar represents the ancestry proportions for an individual for k=7.\n LEA inference with 20,825 SNPs.") +
  scale_x_discrete(labels = label_func) +
  scale_fill_manual(values = color_palette) +
  expand_limits(y = c(0, 1.5))
```

```
ggsave(
  here("scripts", "RMarkdowns", "output", "americas", "manuscript1", "lea", "lea_k=7_manuscript1.pdf"),
  width  = 16,
  height = 6,
  units  = "in",
  device = cairo_pdf
)
```

### 3.1.3 LEA plots for k=8

Default plot

```
# select the best run for chosen K 
best8 = which.min(cross.entropy(project, K = 8))
# best is run 

color_palette <-
  c("blue",
    "magenta",
    "#FFE100",    
    "#FF0010",    
    "purple3", 
    "cyan",    
    "#FF8005",
    "green"     
    )


barchart(project, K = 8, run = best8,
        border = NA, space = 0,
        col = color_palette,
        xlab = "Individuals",
        ylab = "Ancestry proportions",
        main = "Ancestry matrix") -> bp
axis(1, at = 1:length(bp$order),
     labels = bp$order, las=1,
     cex.axis = .4)
```

#### 3.1.3.1 Mean admixture by country using ggplot

```
color_palette <-
      c(
      "#FF8005",
      "#FF0010",                  
      "green",  
      "blue",
      "magenta",
      "#FFE100",       
      "purple3", 
      "cyan"      
      )

sampling_loc <- readRDS(here("scripts", "RMarkdowns", "output", "sampling_loc_all_pops.rds"))

library(reshape2)

# Extract ancestry coefficients
Q_values <- as.data.frame(Q(project, K = 8, run = best8))

# Add individual IDs and pops ids
Q_values$ind <- inds
Q_values$pop <- pops

# Melt the data frame for plotting
Q_melted <- melt(Q_values, id.vars = c("ind", "pop"))

# Join with sampling_loc to get sampling localities
Q_joined <- Q_melted |>
  left_join(sampling_loc, by = c("pop" = "Abbreviation"))

# Create a combined variable for Region and Country
Q_joined <- Q_joined |>
  mutate(Region_Country = interaction(Region, Country, sep = "_"))

# Order the combined variable by Region and Country
Q_ordered <- Q_joined |>
  arrange(Region, Country) |>
  mutate(Region_Country = factor(Region_Country, levels = unique(Region_Country)))

# Group by Country and calculate mean ancestry proportions
Q_grouped <- Q_ordered |>
  group_by(Region_Country, variable) |>
  summarise(value = mean(value), .groups = "drop")

# Create a data frame for borders
borders <- data.frame(Region_Country = unique(Q_grouped$Region_Country))

# Add the order of each country to ensure correct placement of borders
borders$order <- 1:nrow(borders) + 0.5  # Shift borders to the right edge of the bars


# Function to filter and normalize data
normalize_data <- function(df, min_value) {
  df |>
    filter(value > min_value) |>
    group_by(Region_Country) |>
    mutate(value = value / sum(value))
}

# Use the function
Q_grouped_filtered <- normalize_data(Q_grouped, 0.1)

# source the plotting function
source(
  here("scripts", "RMarkdowns",
   "analyses", "my_theme2.R"
  )
)

# Generate all potential variable names
all_variables <- paste0("V", 1:8)

# Create a data frame that pairs each potential variable with a color
color_mapping <- data.frame(variable = all_variables,
                          color = color_palette[1:length(all_variables)])

# Merge this data frame with Q_grouped_filtered to create the new color column
Q_grouped_filtered <- merge(Q_grouped_filtered, color_mapping, by = "variable")

# Create bar chart
ggplot(Q_grouped_filtered, aes(x = Region_Country, y = value, fill = color)) +
  geom_bar(stat = 'identity', width = 1) +
  geom_segment(data = borders, aes(x = order, xend = order, y = 0, yend = 1, fill = NULL), linetype = "solid", color = "#2C444A") +  # Add borders
  my_theme() +
  theme(axis.text.x = element_text(angle = 90, hjust = 1),
        legend.position = "none") +  # Hide legend
  xlab("") +  # Suppress x-axis label
  ylab("Ancestry proportions") +
  ggtitle("Ancestry matrix") +
  labs(caption = "Each bar represents the average ancestry proportions for individuals in a given country for k=8.") +
  # scale_fill_manual(values = color) +
  scale_x_discrete(labels = function(x) gsub(".*_", "", x)) + # Remove Region prefix from labels
  scale_fill_manual(values = color_palette)
```

```
ggsave(
  here("scripts", "RMarkdowns",
    "output", "americas", "manuscript1", "lea", "LEA_admix_by_country_man1_k8.pdf"
  ),
  width  = 10,
  height = 7,
  units  = "in"
)
```

#### 3.1.3.2. Extract ancestry coefficients for k=8

*change to correct matrix*

```
leak8 <- read_delim(
  here("/gpfs/gibbs/pi/caccone/mkc54/albo/manuscript1/output/snps_sets/r2_0.01_b.snmf/K8/run4/r2_0.01_b_r4.8.Q"),
  delim = " ", # Specify the delimiter if different from the default (comma)
  col_names = FALSE,
  show_col_types = FALSE
) 
head(leak8)
```

```
## # A tibble: 6 × 8
##      X1    X2     X3     X4       X5       X6       X7       X8
##   <dbl> <dbl>  <dbl>  <dbl>    <dbl>    <dbl>    <dbl>    <dbl>
## 1 0.534 0.148 0.0919 0.0865 0.0234   0.000100 0.000410 0.116   
## 2 0.505 0.161 0.0923 0.0983 0.00429  0.0342   0.0277   0.0775  
## 3 0.615 0.135 0.0526 0.0391 0.0113   0.0377   0.0274   0.0824  
## 4 0.610 0.256 0.0599 0.0500 0.0195   0.000100 0.000100 0.00424 
## 5 0.618 0.251 0.0610 0.0698 0.000100 0.000100 0.000100 0.000319
## 6 0.579 0.175 0.0413 0.0548 0.0325   0.0240   0.0223   0.0713
```

The fam file

```
fam_file <- here("manuscript1/output/snps_sets/r2_0.01_b.fam")

# Read the .fam file
fam_data <- read.table(fam_file, 
                       header = FALSE,
                       col.names = c("FamilyID", "IndividualID", "PaternalID", "MaternalID", "Sex", "Phenotype"))

# View the first few rows
head(fam_data)
```

```
##   FamilyID IndividualID PaternalID MaternalID Sex Phenotype
## 1      OKI         1001          0          0   2        -9
## 2      OKI         1002          0          0   2        -9
## 3      OKI         1003          0          0   2        -9
## 4      OKI         1004          0          0   2        -9
## 5      OKI         1005          0          0   2        -9
## 6      OKI         1006          0          0   1        -9
```

Create column ID

```
# Change column name
colnames(fam_data)[colnames(fam_data) == "IndividualID"] <- "ind"

# Change column name
colnames(fam_data)[colnames(fam_data) == "FamilyID"] <- "pop"

# Select ID
fam_data <- fam_data |>
  dplyr::select("ind", "pop")

# View the first few rows
head(fam_data)
```

```
##    ind pop
## 1 1001 OKI
## 2 1002 OKI
## 3 1003 OKI
## 4 1004 OKI
## 5 1005 OKI
## 6 1006 OKI
```

Add it to the matrix

```
leak8 <- fam_data |>
  dplyr::select(ind, pop) |>
  bind_cols(leak8)

head(leak8)
```

```
##    ind pop       X1       X2        X3        X4          X5          X6
## 1 1001 OKI 0.534317 0.147752 0.0918703 0.0864944 0.023356400 0.000099991
## 2 1002 OKI 0.504875 0.160751 0.0923208 0.0983362 0.004293660 0.034192800
## 3 1003 OKI 0.614504 0.134991 0.0526376 0.0390554 0.011289000 0.037708600
## 4 1004 OKI 0.610151 0.256053 0.0598562 0.0500197 0.019482900 0.000099982
## 5 1005 OKI 0.617665 0.250944 0.0609641 0.0698077 0.000099973 0.000099973
## 6 1006 OKI 0.579058 0.174682 0.0413338 0.0547738 0.032549400 0.023994700
##            X7          X8
## 1 0.000410151 0.115700000
## 2 0.027710000 0.077520100
## 3 0.027379400 0.082434600
## 4 0.000099982 0.004237130
## 5 0.000099973 0.000318563
## 6 0.022344100 0.071264500
```

Rename the columns

```
# Rename the columns starting from the third one
leak8 <- leak8 |>
  rename_with(~paste0("v", seq_along(.x)), .cols = -c(ind, pop))

# View the first few rows
head(leak8)
```

```
##    ind pop       v1       v2        v3        v4          v5          v6
## 1 1001 OKI 0.534317 0.147752 0.0918703 0.0864944 0.023356400 0.000099991
## 2 1002 OKI 0.504875 0.160751 0.0923208 0.0983362 0.004293660 0.034192800
## 3 1003 OKI 0.614504 0.134991 0.0526376 0.0390554 0.011289000 0.037708600
## 4 1004 OKI 0.610151 0.256053 0.0598562 0.0500197 0.019482900 0.000099982
## 5 1005 OKI 0.617665 0.250944 0.0609641 0.0698077 0.000099973 0.000099973
## 6 1006 OKI 0.579058 0.174682 0.0413338 0.0547738 0.032549400 0.023994700
##            v7          v8
## 1 0.000410151 0.115700000
## 2 0.027710000 0.077520100
## 3 0.027379400 0.082434600
## 4 0.000099982 0.004237130
## 5 0.000099973 0.000318563
## 6 0.022344100 0.071264500
```

Import Sample Locations

```
sampling_loc <- readRDS(here("scripts", "RMarkdowns", "output", "sampling_loc_all_pops.rds"))
head(sampling_loc)
```

```
##      Pop_City Country Latitude Longitude Continent Abbreviation  Year    Region
## 1      Hainan   China  19.2000  109.6000      Asia          HAI  2014 East Asia
## 2      Yunnan   China  24.4753  101.3431      Asia          YUN  2014 East Asia
## 3       Hunan   China  27.6253  111.8569      Asia          HUN  2001 East Asia
## 4     Okinawa   Japan  26.5013  127.9454      Asia          OKI 2018? East Asia
## 5 Tanegashima   Japan  30.4564  130.9784      Asia          TAN  2008 East Asia
## 6   Kagoshima   Japan  31.5602  130.5581      Asia          KAG  2018 East Asia
##   Subregion order order2 orderold AM_order state FINAL_order
## 1     Japan    55     47       47       NA                 1
## 2     Japan    56     48       48       NA                 2
## 3     Japan    57     49       49       NA                 3
## 4     Japan    54     46       46       NA                 4
## 5     Japan    NA     NA       NA       NA                 5
## 6     Japan    53     45       45       NA                 6
```

```
source(
  here("scripts", "RMarkdowns",
    "my_theme3.R"
  )
)

# Melt the data frame for plotting
Q_melted <- leak8 |>
  pivot_longer(
    cols = -c(ind, pop),
    names_to = "variable",
    values_to = "value"
  )
# Join with sampling_loc to get sampling localities
Q_joined <- Q_melted |>
  left_join(sampling_loc, by = c("pop" = "Abbreviation"))


# Order the combined variable by Region and Country, then by individual
Q_ordered <- Q_joined |>
  arrange(FINAL_order, ind) |>
  mutate(ind = factor(ind, levels = unique(ind)))  # Convert ind to a factor with levels in the desired order

# Add labels: country names for the first individual in each country, NA for all other individuals
Q_ordered <- Q_ordered |>
  group_by(Country) |>
  mutate(label = ifelse(row_number() == 1, as.character(Country), NA))

# Group by individual and variable, calculate mean ancestry proportions
Q_grouped <- Q_ordered |>
  group_by(ind, variable) |>
  summarise(value = mean(value), .groups = "drop")

# Create a data frame for borders
borders <-
  data.frame(Country = unique(Q_ordered$Country))

# Add the order of the last individual of each country to ensure correct placement of borders
borders$order <-
  sapply(borders$Country, function(rc)
    max(which(Q_ordered$Country == rc))) + 0.5  # Shift borders to the right edge of the bars

# Select only the first occurrence of each country in the ordered data
label_df <- Q_ordered |>
  filter(!is.na(label)) |>
  distinct(label, .keep_all = TRUE)

# Create a custom label function
label_func <- function(x) {
  labels <- rep("", length(x))
  labels[x %in% label_df$ind] <- label_df$label
  labels
}

# Calculate the position of lines
border_positions <- Q_ordered |>
  group_by(Country) |>
  summarise(pos = max(as.numeric(ind)) + 0)

# Calculate the position of population labels and bars
pop_labels <- Q_ordered |>
  mutate(Name = paste(pop, Pop_City, sep = " - ")) |>
  group_by(pop) |>
  slice_head(n = 1) |>
  ungroup() |>
  dplyr::select(ind, Pop_City, Country, Name) |>
  mutate(pos = as.numeric(ind))  # calculate position of population labels

pop_labels_bars <- pop_labels |>
  mutate(pos = as.numeric(ind)  - .5)


# Calculate the position of lines
border_positions <- Q_ordered |>
  group_by(Country) |>
  summarise(pos = max(as.numeric(ind)) - 1)


pop_labels_bars <- pop_labels |>
  mutate(pos = as.numeric(ind)  - .5)

# Function to filter and normalize data
normalize_data <- function(df, min_value) {
  df |>
    filter(value > min_value) |>
    group_by(ind) |>
    mutate(value = value / sum(value))
}

# Use the function
Q_grouped_filtered <- normalize_data(Q_grouped, 0.1)
# 

color_palette <-
c(
  "magenta",  
  "blue",  
  "green",  
  "#FF8005",  
  "#FFE100", 
  "#FF0010",     
  "cyan", 
  "purple3"    
)

# Generate all potential variable names
all_variables <- paste0("v", 1:8)


# Create a data frame that pairs each potential variable with a color
color_mapping <- data.frame(variable = all_variables,
                           color = color_palette[1:length(all_variables)])

# Merge with Q_grouped_filtered
Q_grouped_filtered <- merge(Q_grouped_filtered, color_mapping, by = "variable")

# Create the plot
ggplot(Q_grouped_filtered, aes(x = as.factor(ind), y = value, fill = color)) +
  geom_bar(stat = 'identity', width = 1) +
  geom_vline(
    data = pop_labels_bars,
    aes(xintercept = pos),
    color = "#2C444A",
    linewidth = .2
  ) +
  geom_text(
    data = pop_labels,
    aes(x = as.numeric(ind), y = 1, label = Name),
    vjust = 1.5,
    hjust = 0,
    size = 2,
    angle = 90,
    inherit.aes = FALSE
  ) +
  my_theme() +
  theme(
    axis.text.x = element_text(
      angle = 90,
      hjust = 1,
      size = 12
    ),
    legend.position = "none",
    plot.margin = unit(c(3, 0.5, 0.5, 0.5), "cm")
  ) +
  xlab("Admixture matrix") +
  ylab("Ancestry proportions") +
  labs(caption = "Each bar represents the ancestry proportions for an individual for k=8.\n LEA inference with 20,825 SNPs.") +
  scale_x_discrete(labels = label_func) +
  scale_fill_manual(values = color_palette) +
  expand_limits(y = c(0, 1.5))
```

```
ggsave(
  here("scripts", "RMarkdowns", "output", "americas", "manuscript1", "lea", "lea_k=8_manuscript1.pdf"),
  width  = 16,
  height = 6,
  units  = "in",
  device = cairo_pdf
)
```

### 3.1.4 LEA plots for k=9

Default plot

```
# select the best run for chosen K 
best9 = which.min(cross.entropy(project, K = 9))
# best 5 is run 

color_palette <-
  c("blue",
    "magenta",
    "#FFE100",    
    "#FF0010",    
    "purple3", 
    "cyan",    
    "#FF8005",
    "green",
    "#990000"    
    )


barchart(project, K = 9, run = best9,
        border = NA, space = 0,
        col = color_palette,
        xlab = "Individuals",
        ylab = "Ancestry proportions",
        main = "Ancestry matrix") -> bp
axis(1, at = 1:length(bp$order),
     labels = bp$order, las=1,
     cex.axis = .4)
```

#### 3.1.4.1 Mean admixture by country using ggplot

```
color_palette <-
    c(
      "#FF0010", 
      "blue",  
      "green",        
      "magenta", 
      "#FF8005",
      "#990000",       
      "#FFE100",        
      "cyan",      
      "purple3"
      )

sampling_loc <- readRDS(here("scripts", "RMarkdowns", "output", "sampling_loc_all_pops.rds"))

library(reshape2)

# Extract ancestry coefficients
Q_values <- as.data.frame(Q(project, K = 9, run = best9))

# Add individual IDs and pops ids
Q_values$ind <- inds
Q_values$pop <- pops

# Melt the data frame for plotting
Q_melted <- melt(Q_values, id.vars = c("ind", "pop"))

# Join with sampling_loc to get sampling localities
Q_joined <- Q_melted |>
  left_join(sampling_loc, by = c("pop" = "Abbreviation"))

# Create a combined variable for Region and Country
Q_joined <- Q_joined |>
  mutate(Region_Country = interaction(Region, Country, sep = "_"))

# Order the combined variable by Region and Country
Q_ordered <- Q_joined |>
  arrange(Region, Country) |>
  mutate(Region_Country = factor(Region_Country, levels = unique(Region_Country)))

# Group by Country and calculate mean ancestry proportions
Q_grouped <- Q_ordered |>
  group_by(Region_Country, variable) |>
  summarise(value = mean(value), .groups = "drop")

# Create a data frame for borders
borders <- data.frame(Region_Country = unique(Q_grouped$Region_Country))

# Add the order of each country to ensure correct placement of borders
borders$order <- 1:nrow(borders) + 0.5  # Shift borders to the right edge of the bars


# Function to filter and normalize data
normalize_data <- function(df, min_value) {
  df |>
    filter(value > min_value) |>
    group_by(Region_Country) |>
    mutate(value = value / sum(value))
}

# Use the function
Q_grouped_filtered <- normalize_data(Q_grouped, 0.1)

# source the plotting function
source(
  here("scripts", "RMarkdowns",
   "analyses", "my_theme2.R"
  )
)

# Generate all potential variable names
all_variables <- paste0("V", 1:9)

# Create a data frame that pairs each potential variable with a color
color_mapping <- data.frame(variable = all_variables,
                          color = color_palette[1:length(all_variables)])

# Merge this data frame with Q_grouped_filtered to create the new color column
Q_grouped_filtered <- merge(Q_grouped_filtered, color_mapping, by = "variable")

# Create bar chart
ggplot(Q_grouped_filtered, aes(x = Region_Country, y = value, fill = color)) +
  geom_bar(stat = 'identity', width = 1) +
  geom_segment(data = borders, aes(x = order, xend = order, y = 0, yend = 1, fill = NULL), linetype = "solid", color = "#2C444A") +  # Add borders
  my_theme() +
  theme(axis.text.x = element_text(angle = 90, hjust = 1),
        legend.position = "none") +  # Hide legend
  xlab("") +  # Suppress x-axis label
  ylab("Ancestry proportions") +
  ggtitle("Ancestry matrix") +
  labs(caption = "Each bar represents the average ancestry proportions for individuals in a given country for k=9.") +
  # scale_fill_manual(values = color) +
  scale_x_discrete(labels = function(x) gsub(".*_", "", x)) + # Remove Region prefix from labels
  scale_fill_manual(values = color_palette)
```

```
ggsave(
  here("scripts", "RMarkdowns",
    "output", "americas", "manuscript1", "lea", "LEA_admix_by_country_man1_k9.pdf"
  ),
  width  = 10,
  height = 7,
  units  = "in"
)
```

#### 3.1.4.2. Extract ancestry coefficients for k=9

*change to correct matrix*

```
leak9 <- read_delim(
  here("/gpfs/gibbs/pi/caccone/mkc54/albo/manuscript1/output/snps_sets/r2_0.01_b.snmf/K9/run5/r2_0.01_b_r5.9.Q"),
  delim = " ", # Specify the delimiter if different from the default (comma)
  col_names = FALSE,
  show_col_types = FALSE
) 
head(leak9)
```

```
## # A tibble: 6 × 9
##      X1      X2     X3    X4       X5       X6     X7       X8     X9
##   <dbl>   <dbl>  <dbl> <dbl>    <dbl>    <dbl>  <dbl>    <dbl>  <dbl>
## 1 0.548 0.0411  0.0755 0.149 0.000100 0.00372  0.0934 0.000100 0.0884
## 2 0.526 0.0290  0.101  0.158 0.0246   0.0241   0.0756 0.00402  0.0574
## 3 0.599 0.0566  0.0704 0.154 0.0172   0.0116   0.0327 0.0150   0.0434
## 4 0.579 0.00181 0.0189 0.296 0.000100 0.000662 0.0394 0.0151   0.0488
## 5 0.586 0.00746 0.0237 0.272 0.000100 0.000100 0.0536 0.000100 0.0567
## 6 0.582 0.121   0.0596 0.132 0.000100 0.000100 0.0471 0.0140   0.0436
```

The fam file

```
fam_file <- here("manuscript1/output/snps_sets/r2_0.01_b.fam")

# Read the .fam file
fam_data <- read.table(fam_file, 
                       header = FALSE,
                       col.names = c("FamilyID", "IndividualID", "PaternalID", "MaternalID", "Sex", "Phenotype"))

# View the first few rows
head(fam_data)
```

```
##   FamilyID IndividualID PaternalID MaternalID Sex Phenotype
## 1      OKI         1001          0          0   2        -9
## 2      OKI         1002          0          0   2        -9
## 3      OKI         1003          0          0   2        -9
## 4      OKI         1004          0          0   2        -9
## 5      OKI         1005          0          0   2        -9
## 6      OKI         1006          0          0   1        -9
```

Create column ID

```
# Change column name
colnames(fam_data)[colnames(fam_data) == "IndividualID"] <- "ind"

# Change column name
colnames(fam_data)[colnames(fam_data) == "FamilyID"] <- "pop"

# Select ID
fam_data <- fam_data |>
  dplyr::select("ind", "pop")

# View the first few rows
head(fam_data)
```

```
##    ind pop
## 1 1001 OKI
## 2 1002 OKI
## 3 1003 OKI
## 4 1004 OKI
## 5 1005 OKI
## 6 1006 OKI
```

Add it to the matrix

```
leak9 <- fam_data |>
  dplyr::select(ind, pop) |>
  bind_cols(leak9)

head(leak9)
```

```
##    ind pop       X1         X2        X3       X4          X5          X6
## 1 1001 OKI 0.548495 0.04107270 0.0754920 0.149264 0.000099982 0.003722000
## 2 1002 OKI 0.525789 0.02897570 0.1012510 0.158269 0.024579600 0.024090000
## 3 1003 OKI 0.599466 0.05656070 0.0704482 0.153643 0.017193300 0.011555100
## 4 1004 OKI 0.579211 0.00181472 0.0189116 0.296029 0.000099991 0.000661754
## 5 1005 OKI 0.586429 0.00745800 0.0236510 0.271869 0.000099973 0.000099973
## 6 1006 OKI 0.582086 0.12145200 0.0596236 0.131923 0.000099982 0.000099982
##          X7          X8        X9
## 1 0.0933594 0.000099982 0.0883949
## 2 0.0755957 0.004020500 0.0574292
## 3 0.0327237 0.014982100 0.0434285
## 4 0.0393678 0.015119700 0.0487843
## 5 0.0535881 0.000099973 0.0567053
## 6 0.0470715 0.014026200 0.0436181
```

Rename the columns

```
# Rename the columns starting from the third one
leak9 <- leak9 |>
  rename_with(~paste0("v", seq_along(.x)), .cols = -c(ind, pop))

# View the first few rows
head(leak9)
```

```
##    ind pop       v1         v2        v3       v4          v5          v6
## 1 1001 OKI 0.548495 0.04107270 0.0754920 0.149264 0.000099982 0.003722000
## 2 1002 OKI 0.525789 0.02897570 0.1012510 0.158269 0.024579600 0.024090000
## 3 1003 OKI 0.599466 0.05656070 0.0704482 0.153643 0.017193300 0.011555100
## 4 1004 OKI 0.579211 0.00181472 0.0189116 0.296029 0.000099991 0.000661754
## 5 1005 OKI 0.586429 0.00745800 0.0236510 0.271869 0.000099973 0.000099973
## 6 1006 OKI 0.582086 0.12145200 0.0596236 0.131923 0.000099982 0.000099982
##          v7          v8        v9
## 1 0.0933594 0.000099982 0.0883949
## 2 0.0755957 0.004020500 0.0574292
## 3 0.0327237 0.014982100 0.0434285
## 4 0.0393678 0.015119700 0.0487843
## 5 0.0535881 0.000099973 0.0567053
## 6 0.0470715 0.014026200 0.0436181
```

Import Sample Locations

```
sampling_loc <- readRDS(here("scripts", "RMarkdowns", "output", "sampling_loc_all_pops.rds"))
head(sampling_loc)
```

```
##      Pop_City Country Latitude Longitude Continent Abbreviation  Year    Region
## 1      Hainan   China  19.2000  109.6000      Asia          HAI  2014 East Asia
## 2      Yunnan   China  24.4753  101.3431      Asia          YUN  2014 East Asia
## 3       Hunan   China  27.6253  111.8569      Asia          HUN  2001 East Asia
## 4     Okinawa   Japan  26.5013  127.9454      Asia          OKI 2018? East Asia
## 5 Tanegashima   Japan  30.4564  130.9784      Asia          TAN  2008 East Asia
## 6   Kagoshima   Japan  31.5602  130.5581      Asia          KAG  2018 East Asia
##   Subregion order order2 orderold AM_order state FINAL_order
## 1     Japan    55     47       47       NA                 1
## 2     Japan    56     48       48       NA                 2
## 3     Japan    57     49       49       NA                 3
## 4     Japan    54     46       46       NA                 4
## 5     Japan    NA     NA       NA       NA                 5
## 6     Japan    53     45       45       NA                 6
```

```
source(
  here("scripts", "RMarkdowns",
    "my_theme3.R"
  )
)

# Melt the data frame for plotting
Q_melted <- leak9 |>
  pivot_longer(
    cols = -c(ind, pop),
    names_to = "variable",
    values_to = "value"
  )
# Join with sampling_loc to get sampling localities
Q_joined <- Q_melted |>
  left_join(sampling_loc, by = c("pop" = "Abbreviation"))


# Order the combined variable by Region and Country, then by individual
Q_ordered <- Q_joined |>
  arrange(FINAL_order, ind) |>
  mutate(ind = factor(ind, levels = unique(ind)))  # Convert ind to a factor with levels in the desired order

# Add labels: country names for the first individual in each country, NA for all other individuals
Q_ordered <- Q_ordered |>
  group_by(Country) |>
  mutate(label = ifelse(row_number() == 1, as.character(Country), NA))

# Group by individual and variable, calculate mean ancestry proportions
Q_grouped <- Q_ordered |>
  group_by(ind, variable) |>
  summarise(value = mean(value), .groups = "drop")

# Create a data frame for borders
borders <-
  data.frame(Country = unique(Q_ordered$Country))

# Add the order of the last individual of each country to ensure correct placement of borders
borders$order <-
  sapply(borders$Country, function(rc)
    max(which(Q_ordered$Country == rc))) + 0.5  # Shift borders to the right edge of the bars

# Select only the first occurrence of each country in the ordered data
label_df <- Q_ordered |>
  filter(!is.na(label)) |>
  distinct(label, .keep_all = TRUE)

# Create a custom label function
label_func <- function(x) {
  labels <- rep("", length(x))
  labels[x %in% label_df$ind] <- label_df$label
  labels
}

# Calculate the position of lines
border_positions <- Q_ordered |>
  group_by(Country) |>
  summarise(pos = max(as.numeric(ind)) + 0)

# Calculate the position of population labels and bars
pop_labels <- Q_ordered |>
  mutate(Name = paste(pop, Pop_City, sep = " - ")) |>
  group_by(pop) |>
  slice_head(n = 1) |>
  ungroup() |>
  dplyr::select(ind, Pop_City, Country, Name) |>
  mutate(pos = as.numeric(ind))  # calculate position of population labels

pop_labels_bars <- pop_labels |>
  mutate(pos = as.numeric(ind)  - .5)


# Calculate the position of lines
border_positions <- Q_ordered |>
  group_by(Country) |>
  summarise(pos = max(as.numeric(ind)) - 1)


pop_labels_bars <- pop_labels |>
  mutate(pos = as.numeric(ind)  - .5)

# Function to filter and normalize data
normalize_data <- function(df, min_value) {
  df |>
    filter(value > min_value) |>
    group_by(ind) |>
    mutate(value = value / sum(value))
}

# Use the function
Q_grouped_filtered <- normalize_data(Q_grouped, 0.1)
# 

color_palette <-
  c(
    "#FF8005",  
    "magenta",   
    "purple3", 
    "#FFE100",
    "#990000",     
    "cyan",
    "blue",  
    "green",     
    "#FF0010"
    )

# Generate all potential variable names
all_variables <- paste0("v", 1:9)


# Create a data frame that pairs each potential variable with a color
color_mapping <- data.frame(variable = all_variables,
                           color = color_palette[1:length(all_variables)])

# Merge with Q_grouped_filtered
Q_grouped_filtered <- merge(Q_grouped_filtered, color_mapping, by = "variable")

# Create the plot
ggplot(Q_grouped_filtered, aes(x = as.factor(ind), y = value, fill = color)) +
  geom_bar(stat = 'identity', width = 1) +
  geom_vline(
    data = pop_labels_bars,
    aes(xintercept = pos),
    color = "#2C444A",
    linewidth = .2
  ) +
  geom_text(
    data = pop_labels,
    aes(x = as.numeric(ind), y = 1, label = Name),
    vjust = 1.5,
    hjust = 0,
    size = 2,
    angle = 90,
    inherit.aes = FALSE
  ) +
  my_theme() +
  theme(
    axis.text.x = element_text(
      angle = 90,
      hjust = 1,
      size = 12
    ),
    legend.position = "none",
    plot.margin = unit(c(3, 0.5, 0.5, 0.5), "cm")
  ) +
  xlab("Admixture matrix") +
  ylab("Ancestry proportions") +
  labs(caption = "Each bar represents the ancestry proportions for an individual for k=9.\n LEA inference with 20,825 SNPs.") +
  scale_x_discrete(labels = label_func) +
  scale_fill_manual(values = color_palette) +
  expand_limits(y = c(0, 1.5))
```

```
ggsave(
  here("scripts", "RMarkdowns", "output", "americas", "manuscript1", "lea", "lea_k=9_manuscript1.pdf"),
  width  = 16,
  height = 6,
  units  = "in",
  device = cairo_pdf
)
```

### 3.1.5 LEA plots for k=10

Default plot

```
# select the best run for chosen K 
best10 = which.min(cross.entropy(project, K = 10))
# best is run 2

color_palette <-
  c("blue",
    "magenta",
    "#FFE100",    
    "#FF0010",    
    "purple3", 
    "cyan",    
    "#FF8005",
    "green",
    "#990000",
    "#005C31"    
    )


barchart(project, K = 10, run = best10,
        border = NA, space = 0,
        col = color_palette,
        xlab = "Individuals",
        ylab = "Ancestry proportions",
        main = "Ancestry matrix") -> bp
axis(1, at = 1:length(bp$order),
     labels = bp$order, las=1,
     cex.axis = .4)
```

#### 3.1.5.1 Mean admixture by country using ggplot

```
sampling_loc <- readRDS(here("scripts", "RMarkdowns", "output", "sampling_loc_all_pops.rds"))

library(reshape2)

# Extract ancestry coefficients
Q_values <- as.data.frame(Q(project, K = 10, run = best10))

# Add individual IDs and pops ids
Q_values$ind <- inds
Q_values$pop <- pops

# Melt the data frame for plotting
Q_melted <- melt(Q_values, id.vars = c("ind", "pop"))

# Join with sampling_loc to get sampling localities
Q_joined <- Q_melted |>
  left_join(sampling_loc, by = c("pop" = "Abbreviation"))

# Create a combined variable for Region and Country
Q_joined <- Q_joined |>
  mutate(Region_Country = interaction(Region, Country, sep = "_"))

# Order the combined variable by Region and Country
Q_ordered <- Q_joined |>
  arrange(Region, Country) |>
  mutate(Region_Country = factor(Region_Country, levels = unique(Region_Country)))

# Group by Country and calculate mean ancestry proportions
Q_grouped <- Q_ordered |>
  group_by(Region_Country, variable) |>
  summarise(value = mean(value), .groups = "drop")

# Create a data frame for borders
borders <- data.frame(Region_Country = unique(Q_grouped$Region_Country))

# Add the order of each country to ensure correct placement of borders
borders$order <- 1:nrow(borders) + 0.5  # Shift borders to the right edge of the bars


# Function to filter and normalize data
normalize_data <- function(df, min_value) {
  df |>
    filter(value > min_value) |>
    group_by(Region_Country) |>
    mutate(value = value / sum(value))
}

# Use the function
Q_grouped_filtered <- normalize_data(Q_grouped, 0.1)

# source the plotting function
source(
  here("scripts", "RMarkdowns",
   "analyses", "my_theme2.R"
  )
)

# Generate all potential variable names
all_variables <- paste0("V", 1:10)

color_palette <-
      c(
      "cyan",
      "green",
      "blue",  
      "#005C31",
      "#FF0010",
      "purple3", 
      "#FFE100", 
      "#990000",  
      "magenta",             
      "#FF8005"
      )

# Create a data frame that pairs each potential variable with a color
color_mapping <- data.frame(variable = all_variables,
                        color = color_palette[1:length(all_variables)])

# Merge this data frame with Q_grouped_filtered to create the new color column
Q_grouped_filtered <- merge(Q_grouped_filtered, color_mapping, by = "variable")

# Create bar chart
ggplot(Q_grouped_filtered, aes(x = Region_Country, y = value, fill = color)) +
  geom_bar(stat = 'identity', width = 1) +
  geom_segment(data = borders, aes(x = order, xend = order, y = 0, yend = 1, fill = NULL), linetype = "solid", color = "#2C444A") +  # Add borders
  my_theme() +
  theme(axis.text.x = element_text(angle = 90, hjust = 1),
        legend.position = "none") +  # Hide legend
  xlab("") +  # Suppress x-axis label
  ylab("Ancestry proportions") +
  ggtitle("Ancestry matrix") +
  labs(caption = "Each bar represents the average ancestry proportions for individuals in a given country for k=10.") +
  # scale_fill_manual(values = color) +
  scale_x_discrete(labels = function(x) gsub(".*_", "", x)) + # Remove Region prefix from labels
  scale_fill_manual(values = color_palette)
```

```
ggsave(
  here("scripts", "RMarkdowns",
    "output", "americas", "manuscript1", "lea", "LEA_admix_by_country_man1_k10.pdf"
  ),
  width  = 10,
  height = 7,
  units  = "in"
)
```

#### 3.1.5.2. Extract ancestry coefficients for k=10

*change to correct matrix*

```
leak10 <- read_delim(
  here("/gpfs/gibbs/pi/caccone/mkc54/albo/manuscript1/output/snps_sets/r2_0.01_b.snmf/K10/run5/r2_0.01_b_r5.10.Q"),
  delim = " ", # Specify the delimiter if different from the default (comma)
  col_names = FALSE,
  show_col_types = FALSE
) 
head(leak10)
```

```
## # A tibble: 6 × 10
##         X1       X2    X3       X4     X5      X6      X7     X8       X9   X10
##      <dbl>    <dbl> <dbl>    <dbl>  <dbl>   <dbl>   <dbl>  <dbl>    <dbl> <dbl>
## 1 0.00674  0.0177   0.562 0.000100 0.0599 0.0502  0.0762  0.0850 0.000100 0.142
## 2 0.00127  0.00624  0.495 0.0242   0.0818 0.0571  0.0814  0.0881 0.0134   0.151
## 3 0.0121   0.0225   0.607 0.0167   0.0654 0.0300  0.0683  0.0246 0.0108   0.142
## 4 0.00975  0.000100 0.560 0.000100 0.149  0.0131  0.00849 0.0472 0.000100 0.212
## 5 0.000100 0.000100 0.587 0.000100 0.129  0.00418 0.0119  0.0319 0.000100 0.235
## 6 0.0149   0.0635   0.573 0.000725 0.0564 0.0403  0.0641  0.0380 0.000100 0.149
```

The fam file

```
fam_file <- here("manuscript1/output/snps_sets/r2_0.01_b.fam")

# Read the .fam file
fam_data <- read.table(fam_file, 
                       header = FALSE,
                       col.names = c("FamilyID", "IndividualID", "PaternalID", "MaternalID", "Sex", "Phenotype"))

# View the first few rows
head(fam_data)
```

```
##   FamilyID IndividualID PaternalID MaternalID Sex Phenotype
## 1      OKI         1001          0          0   2        -9
## 2      OKI         1002          0          0   2        -9
## 3      OKI         1003          0          0   2        -9
## 4      OKI         1004          0          0   2        -9
## 5      OKI         1005          0          0   2        -9
## 6      OKI         1006          0          0   1        -9
```

Create column ID

```
# Change column name
colnames(fam_data)[colnames(fam_data) == "IndividualID"] <- "ind"

# Change column name
colnames(fam_data)[colnames(fam_data) == "FamilyID"] <- "pop"

# Select ID
fam_data <- fam_data |>
  dplyr::select("ind", "pop")

# View the first few rows
head(fam_data)
```

```
##    ind pop
## 1 1001 OKI
## 2 1002 OKI
## 3 1003 OKI
## 4 1004 OKI
## 5 1005 OKI
## 6 1006 OKI
```

Add it to the matrix

```
leak10 <- fam_data |>
  dplyr::select(ind, pop) |>
  bind_cols(leak10)

head(leak10)
```

```
##    ind pop          X1          X2       X3          X4        X5         X6
## 1 1001 OKI 0.006739350 0.017667400 0.561711 0.000099982 0.0599229 0.05024760
## 2 1002 OKI 0.001272720 0.006240520 0.495318 0.024227800 0.0817529 0.05709620
## 3 1003 OKI 0.012113400 0.022513800 0.607259 0.016734500 0.0653686 0.02996800
## 4 1004 OKI 0.009748150 0.000099973 0.560145 0.000099973 0.1486840 0.01306270
## 5 1005 OKI 0.000099964 0.000099964 0.587338 0.000099964 0.1294560 0.00417851
## 6 1006 OKI 0.014944300 0.063508500 0.573303 0.000725139 0.0564384 0.04032890
##           X7        X8          X9      X10
## 1 0.07616310 0.0849749 0.000099982 0.142374
## 2 0.08136680 0.0881342 0.013408000 0.151183
## 3 0.06828460 0.0246311 0.010766200 0.142361
## 4 0.00849088 0.0471657 0.000099973 0.212404
## 5 0.01189990 0.0318517 0.000099964 0.234876
## 6 0.06410480 0.0380349 0.000099991 0.148512
```

Rename the columns

```
# Rename the columns starting from the third one
leak10 <- leak10 |>
  rename_with(~paste0("v", seq_along(.x)), .cols = -c(ind, pop))

# View the first few rows
head(leak10)
```

```
##    ind pop          v1          v2       v3          v4        v5         v6
## 1 1001 OKI 0.006739350 0.017667400 0.561711 0.000099982 0.0599229 0.05024760
## 2 1002 OKI 0.001272720 0.006240520 0.495318 0.024227800 0.0817529 0.05709620
## 3 1003 OKI 0.012113400 0.022513800 0.607259 0.016734500 0.0653686 0.02996800
## 4 1004 OKI 0.009748150 0.000099973 0.560145 0.000099973 0.1486840 0.01306270
## 5 1005 OKI 0.000099964 0.000099964 0.587338 0.000099964 0.1294560 0.00417851
## 6 1006 OKI 0.014944300 0.063508500 0.573303 0.000725139 0.0564384 0.04032890
##           v7        v8          v9      v10
## 1 0.07616310 0.0849749 0.000099982 0.142374
## 2 0.08136680 0.0881342 0.013408000 0.151183
## 3 0.06828460 0.0246311 0.010766200 0.142361
## 4 0.00849088 0.0471657 0.000099973 0.212404
## 5 0.01189990 0.0318517 0.000099964 0.234876
## 6 0.06410480 0.0380349 0.000099991 0.148512
```

Import Sample Locations

```
sampling_loc <- readRDS(here("scripts", "RMarkdowns", "output", "sampling_loc_all_pops.rds"))
head(sampling_loc)
```

```
##      Pop_City Country Latitude Longitude Continent Abbreviation  Year    Region
## 1      Hainan   China  19.2000  109.6000      Asia          HAI  2014 East Asia
## 2      Yunnan   China  24.4753  101.3431      Asia          YUN  2014 East Asia
## 3       Hunan   China  27.6253  111.8569      Asia          HUN  2001 East Asia
## 4     Okinawa   Japan  26.5013  127.9454      Asia          OKI 2018? East Asia
## 5 Tanegashima   Japan  30.4564  130.9784      Asia          TAN  2008 East Asia
## 6   Kagoshima   Japan  31.5602  130.5581      Asia          KAG  2018 East Asia
##   Subregion order order2 orderold AM_order state FINAL_order
## 1     Japan    55     47       47       NA                 1
## 2     Japan    56     48       48       NA                 2
## 3     Japan    57     49       49       NA                 3
## 4     Japan    54     46       46       NA                 4
## 5     Japan    NA     NA       NA       NA                 5
## 6     Japan    53     45       45       NA                 6
```

```
source(
  here("scripts", "RMarkdowns",
    "my_theme3.R"
  )
)

# Melt the data frame for plotting
Q_melted <- leak10 |>
  pivot_longer(
    cols = -c(ind, pop),
    names_to = "variable",
    values_to = "value"
  )
# Join with sampling_loc to get sampling localities
Q_joined <- Q_melted |>
  left_join(sampling_loc, by = c("pop" = "Abbreviation"))


# Order the combined variable by Region and Country, then by individual
Q_ordered <- Q_joined |>
  arrange(FINAL_order, ind) |>
  mutate(ind = factor(ind, levels = unique(ind)))  # Convert ind to a factor with levels in the desired order

# Add labels: country names for the first individual in each country, NA for all other individuals
Q_ordered <- Q_ordered |>
  group_by(Country) |>
  mutate(label = ifelse(row_number() == 1, as.character(Country), NA))

# Group by individual and variable, calculate mean ancestry proportions
Q_grouped <- Q_ordered |>
  group_by(ind, variable) |>
  summarise(value = mean(value), .groups = "drop")

# Create a data frame for borders
borders <-
  data.frame(Country = unique(Q_ordered$Country))

# Add the order of the last individual of each country to ensure correct placement of borders
borders$order <-
  sapply(borders$Country, function(rc)
    max(which(Q_ordered$Country == rc))) + 0.5  # Shift borders to the right edge of the bars

# Select only the first occurrence of each country in the ordered data
label_df <- Q_ordered |>
  filter(!is.na(label)) |>
  distinct(label, .keep_all = TRUE)

# Create a custom label function
label_func <- function(x) {
  labels <- rep("", length(x))
  labels[x %in% label_df$ind] <- label_df$label
  labels
}

# Calculate the position of lines
border_positions <- Q_ordered |>
  group_by(Country) |>
  summarise(pos = max(as.numeric(ind)) + 0)

# Calculate the position of population labels and bars
pop_labels <- Q_ordered |>
  mutate(Name = paste(pop, Pop_City, sep = " - ")) |>
  group_by(pop) |>
  slice_head(n = 1) |>
  ungroup() |>
  dplyr::select(ind, Pop_City, Country, Name) |>
  mutate(pos = as.numeric(ind))  # calculate position of population labels

pop_labels_bars <- pop_labels |>
  mutate(pos = as.numeric(ind)  - .5)


# Calculate the position of lines
border_positions <- Q_ordered |>
  group_by(Country) |>
  summarise(pos = max(as.numeric(ind)) - 1)


pop_labels_bars <- pop_labels |>
  mutate(pos = as.numeric(ind)  - .5)

# Function to filter and normalize data
normalize_data <- function(df, min_value) {
  df |>
    filter(value > min_value) |>
    group_by(ind) |>
    mutate(value = value / sum(value))
}

# Use the function
Q_grouped_filtered <- normalize_data(Q_grouped, 0.1)
# 

color_palette <-
  c(
      "magenta", 
      "blue", 
      "cyan", 
      "#005C31", 
      "#990000",
      "#FF0010",  
      "purple3",       
      "#FFE100", 
      "green",      
      "#FF8005"
      )
    

# Generate all potential variable names
all_variables <- paste0("v", 1:10)


# Create a data frame that pairs each potential variable with a color
color_mapping <- data.frame(variable = all_variables,
                           color = color_palette[1:length(all_variables)])

# Merge with Q_grouped_filtered
Q_grouped_filtered <- merge(Q_grouped_filtered, color_mapping, by = "variable")

# Create the plot
ggplot(Q_grouped_filtered, aes(x = as.factor(ind), y = value, fill = color)) +
  geom_bar(stat = 'identity', width = 1) +
  geom_vline(
    data = pop_labels_bars,
    aes(xintercept = pos),
    color = "#2C444A",
    linewidth = .2
  ) +
  geom_text(
    data = pop_labels,
    aes(x = as.numeric(ind), y = 1, label = Name),
    vjust = 1.5,
    hjust = 0,
    size = 2,
    angle = 90,
    inherit.aes = FALSE
  ) +
  my_theme() +
  theme(
    axis.text.x = element_text(
      angle = 90,
      hjust = 1,
      size = 12
    ),
    legend.position = "none",
    plot.margin = unit(c(3, 0.5, 0.5, 0.5), "cm")
  ) +
  xlab("Admixture matrix") +
  ylab("Ancestry proportions") +
  labs(caption = "Each bar represents the ancestry proportions for an individual for k=10.\n LEA inference with 20,825 SNPs.") +
  scale_x_discrete(labels = label_func) +
  scale_fill_manual(values = color_palette) +
  expand_limits(y = c(0, 1.5))
```

```
ggsave(
  here("scripts", "RMarkdowns", "output", "americas", "manuscript1", "lea", "lea_k=10_manuscript1.pdf"),
  width  = 16,
  height = 6,
  units  = "in",
  device = cairo_pdf
)
```

### 3.1.6 LEA plots for k=25

```
color_palette <-
  c(
    "green",
    "orangered",   
    "#FFFC99",  
    "#8c61cd",     
    "orchid1",
    "#003380",
    "#1E90FF",     
    "#FF8005", 
    "#9DCC00", 
    "#FFA8BB",   
    "#808080", 
    "purple3",   
    "#FFE100",
    "#990000",  
    "#AC7700",      
    "#4C005C",  
    "cyan",
    "#FF0010",
    "magenta",
    "blue",
    "cadetblue",   
    "gray",    
    "ivory", 
    "#993F00", 
    "#005C31"    
)
```

Default plot

```
# select the best run for chosen K 
best25 = which.min(cross.entropy(project, K = 25))
# best is run 1

barchart(project, K = 25, run = best25,
        border = NA, space = 0,
        col = color_palette,
        xlab = "Individuals",
        ylab = "Ancestry proportions",
        main = "Ancestry matrix") -> bp
axis(1, at = 1:length(bp$order),
     labels = bp$order, las=1,
     cex.axis = .4)
```

#### 3.1.6.1 Mean admixture by country using ggplot

```
sampling_loc <- readRDS(here("scripts", "RMarkdowns", "output", "sampling_loc_all_pops.rds"))

library(reshape2)

# Extract ancestry coefficients
Q_values <- as.data.frame(Q(project, K = 25, run = best25))

# Create a named vector to map countries to regions

# Add individual IDs and pops ids
Q_values$ind <- inds
Q_values$pop <- pops

# Melt the data frame for plotting
Q_melted <- melt(Q_values, id.vars = c("ind", "pop"))

# Join with sampling_loc to get sampling localities
Q_joined <- Q_melted |>
  left_join(sampling_loc, by = c("pop" = "Abbreviation"))

# Create a combined variable for Region and Country
Q_joined <- Q_joined |>
  mutate(Region_Country = interaction(Region, Country, sep = "_"))

# Order the combined variable by Region and Country
Q_ordered <- Q_joined |>
  arrange(Region, Country) |>
  mutate(Region_Country = factor(Region_Country, levels = unique(Region_Country)))

# Group by Country and calculate mean ancestry proportions
Q_grouped <- Q_ordered |>
  group_by(Region_Country, variable) |>
  summarise(value = mean(value), .groups = "drop")

# Create a data frame for borders
borders <- data.frame(Region_Country = unique(Q_grouped$Region_Country))

# Add the order of each country to ensure correct placement of borders
borders$order <- 1:nrow(borders) + 0.5  # Shift borders to the right edge of the bars


# Function to filter and normalize data
normalize_data <- function(df, min_value) {
  df |>
    filter(value > min_value) |>
    group_by(Region_Country) |>
    mutate(value = value / sum(value))
}

# Use the function
Q_grouped_filtered <- normalize_data(Q_grouped, 0.1)

# source the plotting function
source(
  here("scripts", "RMarkdowns",
   "analyses", "my_theme2.R"
  )
)

# Generate all potential variable names
all_variables <- paste0("V", 1:25)

color_palette <-
c(
"blue",
"#FF0010",
"#FFFC99", 
"#003380", 
"cyan",
"#FFA8BB", 
"orchid1",
"#005C31",
"#8c61cd",
"#9DCC00", 
"orangered",
"magenta",
"#1E90FF",
"#FFE100",
"green",
"#FF8005", 
"#990000",
"#808080", 
"#4C005C",
"#993F00", 
"purple3"
) 

# Create a data frame that pairs each potential variable with a color
color_mapping <- data.frame(variable = all_variables,
                          color = color_palette[1:length(all_variables)])

# Merge this data frame with Q_grouped_filtered to create the new color column
Q_grouped_filtered <- merge(Q_grouped_filtered, color_mapping, by = "variable")

# Create bar chart
ggplot(Q_grouped_filtered, aes(x = Region_Country, y = value, fill = color)) +
  geom_bar(stat = 'identity', width = 1) +
  geom_segment(data = borders, aes(x = order, xend = order, y = 0, yend = 1, fill = NULL), linetype = "solid", color = "#2C444A") +  # Add borders
  my_theme() +
  theme(axis.text.x = element_text(angle = 90, hjust = 1),
        legend.position = "none") +  # Hide legend
  xlab("") +  # Suppress x-axis label
  ylab("Ancestry proportions") +
  ggtitle("Ancestry matrix") +
  labs(caption = "Each bar represents the average ancestry proportions for individuals in a given country for k=25.") +
  # scale_fill_manual(values = color) +
  scale_x_discrete(labels = function(x) gsub(".*_", "", x)) + # Remove Region prefix from labels
  scale_fill_manual(values = color_palette)
```

```
ggsave(
  here("scripts", "RMarkdowns",
    "output", "americas", "manuscript1", "lea", "LEA_admix_by_country_man1_k25.pdf"
  ),
  width  = 10,
  height = 7,
  units  = "in"
)
```

#### 3.1.6.2. Extract ancestry coefficients for k=25

*change to correct matrix*

```
leak25 <- read_delim(
  here("/gpfs/gibbs/pi/caccone/mkc54/albo/manuscript1/output/snps_sets/r2_0.01_b.snmf/K25/run1/r2_0.01_b_r1.25.Q"),
  delim = " ", # Specify the delimiter if different from the default (comma)
  col_names = FALSE,
  show_col_types = FALSE
) 
head(leak25)
```

```
## # A tibble: 6 × 25
##          X1        X2        X3       X4      X5      X6      X7      X8      X9
##       <dbl>     <dbl>     <dbl>    <dbl>   <dbl>   <dbl>   <dbl>   <dbl>   <dbl>
## 1 0.0683    0.0000999 0.0000999  9.99e-5 9.99e-5 9.99e-5 3.83e-2 9.99e-5 7.89e-3
## 2 0.0289    0.0290    0.0000999  9.99e-5 9.99e-5 9.99e-5 3.11e-2 1.53e-2 4.39e-2
## 3 0.0361    0.0000998 0.0000998  9.98e-5 9.98e-5 9.98e-5 9.98e-5 9.98e-5 9.98e-5
## 4 0.0000998 0.0000998 0.0000998  9.98e-5 9.98e-5 9.98e-5 9.98e-5 9.98e-5 9.98e-5
## 5 0.0000998 0.00926   0.0000998  9.98e-5 9.98e-5 9.98e-5 9.98e-5 9.98e-5 9.98e-5
## 6 0.0000999 0.0000999 0.0165     9.99e-5 4.25e-2 9.99e-5 9.99e-5 9.99e-5 9.99e-5
## # ℹ 16 more variables: X10 <dbl>, X11 <dbl>, X12 <dbl>, X13 <dbl>, X14 <dbl>,
## #   X15 <dbl>, X16 <dbl>, X17 <dbl>, X18 <dbl>, X19 <dbl>, X20 <dbl>,
## #   X21 <dbl>, X22 <dbl>, X23 <dbl>, X24 <dbl>, X25 <dbl>
```

The fam file

```
fam_file <- here("manuscript1/output/snps_sets/r2_0.01_b.fam")

# Read the .fam file
fam_data <- read.table(fam_file, 
                       header = FALSE,
                       col.names = c("FamilyID", "IndividualID", "PaternalID", "MaternalID", "Sex", "Phenotype"))

# View the first few rows
head(fam_data)
```

```
##   FamilyID IndividualID PaternalID MaternalID Sex Phenotype
## 1      OKI         1001          0          0   2        -9
## 2      OKI         1002          0          0   2        -9
## 3      OKI         1003          0          0   2        -9
## 4      OKI         1004          0          0   2        -9
## 5      OKI         1005          0          0   2        -9
## 6      OKI         1006          0          0   1        -9
```

Create column ID

```
# Change column name
colnames(fam_data)[colnames(fam_data) == "IndividualID"] <- "ind"

# Change column name
colnames(fam_data)[colnames(fam_data) == "FamilyID"] <- "pop"

# Select ID
fam_data <- fam_data |>
  dplyr::select("ind", "pop")

# View the first few rows
head(fam_data)
```

```
##    ind pop
## 1 1001 OKI
## 2 1002 OKI
## 3 1003 OKI
## 4 1004 OKI
## 5 1005 OKI
## 6 1006 OKI
```

Add it to the matrix

```
leak25 <- fam_data |>
  dplyr::select(ind, pop) |>
  bind_cols(leak25)

head(leak25)
```

```
##    ind pop          X1          X2          X3          X4          X5
## 1 1001 OKI 6.83401e-02 9.98920e-05 9.98920e-05 9.98920e-05 9.98920e-05
## 2 1002 OKI 2.88506e-02 2.90059e-02 9.99011e-05 9.99011e-05 9.99011e-05
## 3 1003 OKI 3.60858e-02 9.98471e-05 9.98471e-05 9.98471e-05 9.98471e-05
## 4 1004 OKI 9.97930e-05 9.97930e-05 9.97930e-05 9.97930e-05 9.97930e-05
## 5 1005 OKI 9.98018e-05 9.26118e-03 9.98018e-05 9.98018e-05 9.98018e-05
## 6 1006 OKI 9.98560e-05 9.98560e-05 1.64973e-02 9.98560e-05 4.24552e-02
##            X6          X7          X8          X9         X10         X11
## 1 9.98920e-05 3.82734e-02 9.98920e-05 7.89184e-03 1.53355e-03 1.09065e-01
## 2 9.99011e-05 3.11126e-02 1.53449e-02 4.39140e-02 9.99011e-05 8.43381e-02
## 3 9.98471e-05 9.98471e-05 9.98471e-05 9.98471e-05 9.98471e-05 6.57253e-02
## 4 9.97930e-05 9.97930e-05 9.97930e-05 9.97930e-05 9.97930e-05 9.97930e-05
## 5 9.98018e-05 9.98018e-05 9.98018e-05 9.98018e-05 9.98018e-05 9.98018e-05
## 6 9.98560e-05 9.98560e-05 9.98560e-05 9.98560e-05 9.98560e-05 1.91078e-02
##           X12         X13         X14         X15         X16         X17
## 1 6.61881e-02 9.98920e-05 6.87678e-03 9.98920e-05 1.56187e-01 9.98920e-05
## 2 7.19765e-02 9.99011e-05 2.03292e-02 9.99011e-05 2.10676e-01 9.99011e-05
## 3 4.83053e-02 2.62326e-02 1.79040e-02 9.98471e-05 1.39357e-01 9.98471e-05
## 4 9.97930e-05 9.97930e-05 9.97930e-05 9.97930e-05 9.97930e-05 9.97930e-05
## 5 9.98018e-05 9.98018e-05 9.98018e-05 9.98018e-05 9.98018e-05 9.98018e-05
## 6 1.84922e-02 1.60868e-02 2.64452e-02 9.98560e-05 1.22493e-01 9.98560e-05
##        X18         X19         X20         X21         X22         X23
## 1 0.422075 7.16284e-02 9.98920e-05 9.98920e-05 1.09657e-02 3.16622e-03
## 2 0.299766 8.61968e-02 9.99011e-05 1.31821e-02 1.50884e-02 9.99011e-05
## 3 0.652993 9.98471e-05 9.98471e-05 1.16996e-02 9.98471e-05 9.98471e-05
## 4 0.940536 9.97930e-05 9.97930e-05 9.97930e-05 9.97930e-05 9.97930e-05
## 5 0.945292 9.98018e-05 9.98018e-05 9.98018e-05 9.98018e-05 9.98018e-05
## 6 0.709806 9.98560e-05 9.98560e-05 2.70194e-02 9.98560e-05 9.98560e-05
##           X24         X25
## 1 9.98920e-05 3.66105e-02
## 2 9.99011e-05 4.91198e-02
## 3 9.98471e-05 9.98471e-05
## 4 5.71688e-02 9.97930e-05
## 5 4.32509e-02 9.98018e-05
## 6 9.98560e-05 9.98560e-05
```

Rename the columns

```
# Rename the columns starting from the third one
leak25 <- leak25 |>
  rename_with(~paste0("v", seq_along(.x)), .cols = -c(ind, pop))

# View the first few rows
head(leak25)
```

```
##    ind pop          v1          v2          v3          v4          v5
## 1 1001 OKI 6.83401e-02 9.98920e-05 9.98920e-05 9.98920e-05 9.98920e-05
## 2 1002 OKI 2.88506e-02 2.90059e-02 9.99011e-05 9.99011e-05 9.99011e-05
## 3 1003 OKI 3.60858e-02 9.98471e-05 9.98471e-05 9.98471e-05 9.98471e-05
## 4 1004 OKI 9.97930e-05 9.97930e-05 9.97930e-05 9.97930e-05 9.97930e-05
## 5 1005 OKI 9.98018e-05 9.26118e-03 9.98018e-05 9.98018e-05 9.98018e-05
## 6 1006 OKI 9.98560e-05 9.98560e-05 1.64973e-02 9.98560e-05 4.24552e-02
##            v6          v7          v8          v9         v10         v11
## 1 9.98920e-05 3.82734e-02 9.98920e-05 7.89184e-03 1.53355e-03 1.09065e-01
## 2 9.99011e-05 3.11126e-02 1.53449e-02 4.39140e-02 9.99011e-05 8.43381e-02
## 3 9.98471e-05 9.98471e-05 9.98471e-05 9.98471e-05 9.98471e-05 6.57253e-02
## 4 9.97930e-05 9.97930e-05 9.97930e-05 9.97930e-05 9.97930e-05 9.97930e-05
## 5 9.98018e-05 9.98018e-05 9.98018e-05 9.98018e-05 9.98018e-05 9.98018e-05
## 6 9.98560e-05 9.98560e-05 9.98560e-05 9.98560e-05 9.98560e-05 1.91078e-02
##           v12         v13         v14         v15         v16         v17
## 1 6.61881e-02 9.98920e-05 6.87678e-03 9.98920e-05 1.56187e-01 9.98920e-05
## 2 7.19765e-02 9.99011e-05 2.03292e-02 9.99011e-05 2.10676e-01 9.99011e-05
## 3 4.83053e-02 2.62326e-02 1.79040e-02 9.98471e-05 1.39357e-01 9.98471e-05
## 4 9.97930e-05 9.97930e-05 9.97930e-05 9.97930e-05 9.97930e-05 9.97930e-05
## 5 9.98018e-05 9.98018e-05 9.98018e-05 9.98018e-05 9.98018e-05 9.98018e-05
## 6 1.84922e-02 1.60868e-02 2.64452e-02 9.98560e-05 1.22493e-01 9.98560e-05
##        v18         v19         v20         v21         v22         v23
## 1 0.422075 7.16284e-02 9.98920e-05 9.98920e-05 1.09657e-02 3.16622e-03
## 2 0.299766 8.61968e-02 9.99011e-05 1.31821e-02 1.50884e-02 9.99011e-05
## 3 0.652993 9.98471e-05 9.98471e-05 1.16996e-02 9.98471e-05 9.98471e-05
## 4 0.940536 9.97930e-05 9.97930e-05 9.97930e-05 9.97930e-05 9.97930e-05
## 5 0.945292 9.98018e-05 9.98018e-05 9.98018e-05 9.98018e-05 9.98018e-05
## 6 0.709806 9.98560e-05 9.98560e-05 2.70194e-02 9.98560e-05 9.98560e-05
##           v24         v25
## 1 9.98920e-05 3.66105e-02
## 2 9.99011e-05 4.91198e-02
## 3 9.98471e-05 9.98471e-05
## 4 5.71688e-02 9.97930e-05
## 5 4.32509e-02 9.98018e-05
## 6 9.98560e-05 9.98560e-05
```

Import Sample Locations

```
sampling_loc <- readRDS(here("scripts", "RMarkdowns", "output", "sampling_loc_all_pops.rds"))
head(sampling_loc)
```

```
##      Pop_City Country Latitude Longitude Continent Abbreviation  Year    Region
## 1      Hainan   China  19.2000  109.6000      Asia          HAI  2014 East Asia
## 2      Yunnan   China  24.4753  101.3431      Asia          YUN  2014 East Asia
## 3       Hunan   China  27.6253  111.8569      Asia          HUN  2001 East Asia
## 4     Okinawa   Japan  26.5013  127.9454      Asia          OKI 2018? East Asia
## 5 Tanegashima   Japan  30.4564  130.9784      Asia          TAN  2008 East Asia
## 6   Kagoshima   Japan  31.5602  130.5581      Asia          KAG  2018 East Asia
##   Subregion order order2 orderold AM_order state FINAL_order
## 1     Japan    55     47       47       NA                 1
## 2     Japan    56     48       48       NA                 2
## 3     Japan    57     49       49       NA                 3
## 4     Japan    54     46       46       NA                 4
## 5     Japan    NA     NA       NA       NA                 5
## 6     Japan    53     45       45       NA                 6
```

```
source(
  here("scripts", "RMarkdowns",
    "my_theme3.R"
  )
)

# Melt the data frame for plotting
Q_melted <- leak25 |>
  pivot_longer(
    cols = -c(ind, pop),
    names_to = "variable",
    values_to = "value"
  )
# Join with sampling_loc to get sampling localities
Q_joined <- Q_melted |>
  left_join(sampling_loc, by = c("pop" = "Abbreviation"))


# Order the combined variable by Region and Country, then by individual
Q_ordered <- Q_joined |>
  arrange(FINAL_order, ind) |>
  mutate(ind = factor(ind, levels = unique(ind)))  # Convert ind to a factor with levels in the desired order

# Add labels: country names for the first individual in each country, NA for all other individuals
Q_ordered <- Q_ordered |>
  group_by(Country) |>
  mutate(label = ifelse(row_number() == 1, as.character(Country), NA))

# Group by individual and variable, calculate mean ancestry proportions
Q_grouped <- Q_ordered |>
  group_by(ind, variable) |>
  summarise(value = mean(value), .groups = "drop")

# Create a data frame for borders
borders <-
  data.frame(Country = unique(Q_ordered$Country))

# Add the order of the last individual of each country to ensure correct placement of borders
borders$order <-
  sapply(borders$Country, function(rc)
    max(which(Q_ordered$Country == rc))) + 0.5  # Shift borders to the right edge of the bars

# Select only the first occurrence of each country in the ordered data
label_df <- Q_ordered |>
  filter(!is.na(label)) |>
  distinct(label, .keep_all = TRUE)

# Create a custom label function
label_func <- function(x) {
  labels <- rep("", length(x))
  labels[x %in% label_df$ind] <- label_df$label
  labels
}

# Calculate the position of lines
border_positions <- Q_ordered |>
  group_by(Country) |>
  summarise(pos = max(as.numeric(ind)) + 0)

# Calculate the position of population labels and bars
pop_labels <- Q_ordered |>
  mutate(Name = paste(pop, Pop_City, sep = " - ")) |>
  group_by(pop) |>
  slice_head(n = 1) |>
  ungroup() |>
  dplyr::select(ind, Pop_City, Country, Name) |>
  mutate(pos = as.numeric(ind))  # calculate position of population labels

pop_labels_bars <- pop_labels |>
  mutate(pos = as.numeric(ind)  - .5)


# Calculate the position of lines
border_positions <- Q_ordered |>
  group_by(Country) |>
  summarise(pos = max(as.numeric(ind)) - 1)


pop_labels_bars <- pop_labels |>
  mutate(pos = as.numeric(ind)  - .5)

# Function to filter and normalize data
normalize_data <- function(df, min_value) {
  df |>
    filter(value > min_value) |>
    group_by(ind) |>
    mutate(value = value / sum(value))
}

# Use the function
Q_grouped_filtered <- normalize_data(Q_grouped, 0.1)
# 


# Generate all potential variable names
all_variables <- paste0("v", 1:25)

color_palette <-
 c(
    "#FF8005", 
    "#808080", 
    "blue",    
    "#9DCC00", 
    "cyan",
    "#993F00", 
    "#AC7700",
    "#1E90FF",  
    "#8c61cd", 
    "orangered", 
    "purple3", 
    "cadetblue",    
    "magenta", 
    "#005C31", 
    "#FFFC99",   
    "ivory", 
    "#FF0010", 
    "green",     
    "#003380",  
    "#FFA8BB",      
    "#990000", 
    "gray",   
    "orchid1",
    "#4C005C", 
    "#FFE100"    
    )

# Create a data frame that pairs each potential variable with a color
color_mapping <- data.frame(variable = all_variables,
                           color = color_palette[1:length(all_variables)])

# Merge with Q_grouped_filtered
Q_grouped_filtered <- merge(Q_grouped_filtered, color_mapping, by = "variable")

# Create the plot
ggplot(Q_grouped_filtered, aes(x = as.factor(ind), y = value, fill = color)) +
  geom_bar(stat = 'identity', width = 1) +
  geom_vline(
    data = pop_labels_bars,
    aes(xintercept = pos),
    color = "#2C444A",
    linewidth = .2
  ) +
  geom_text(
    data = pop_labels,
    aes(x = as.numeric(ind), y = 1, label = Name),
    vjust = 1.5,
    hjust = 0,
    size = 2,
    angle = 90,
    inherit.aes = FALSE
  ) +
  my_theme() +
  theme(
    axis.text.x = element_text(
      angle = 90,
      hjust = 1,
      size = 12
    ),
    legend.position = "none",
    plot.margin = unit(c(3, 0.5, 0.5, 0.5), "cm")
  ) +
  xlab("Admixture matrix") +
  ylab("Ancestry proportions") +
  labs(caption = "Each bar represents the ancestry proportions for an individual for k=25.\n LEA inference with 20,825 SNPs.") +
  scale_x_discrete(labels = label_func) +
  scale_fill_manual(values = color_palette) +
  expand_limits(y = c(0, 1.5))
```

```
ggsave(
  here("scripts", "RMarkdowns", "output", "americas", "manuscript1", "lea", "lea_k=25_manuscript1.pdf"),
  width  = 16,
  height = 6,
  units  = "in",
  device = cairo_pdf
)
```

### 3.1.7 LEA plots for k=20

```
color_palette <-
  c(
    "green",
    "orangered",   
    "#FFFC99",  
    "#8c61cd",     
    "orchid1",
    "#003380",
    "#1E90FF",     
    "#FF8005", 
    "#9DCC00", 
    "#FFA8BB",   
    "#808080", 
    "purple3",   
    "#FFE100",
    "#990000",  
    "#AC7700",      
    "#4C005C",  
    "cyan",
    "#FF0010",
    "magenta",
    "blue",
    "cadetblue",   
    "gray",    
    "ivory", 
    "#993F00", 
    "#005C31"    
)
```

Default plot

```
# select the best run for chosen K 
best20 = which.min(cross.entropy(project, K = 20))
# best is run 1

barchart(project, K = 20, run = best20,
        border = NA, space = 0,
        col = color_palette,
        xlab = "Individuals",
        ylab = "Ancestry proportions",
        main = "Ancestry matrix") -> bp
axis(1, at = 1:length(bp$order),
     labels = bp$order, las=1,
     cex.axis = .4)
```

#### 3.1.7.1 Mean admixture by country using ggplot

```
sampling_loc <- readRDS(here("scripts", "RMarkdowns", "output", "sampling_loc_all_pops.rds"))

library(reshape2)

# Extract ancestry coefficients
Q_values <- as.data.frame(Q(project, K = 20, run = best20))

# Create a named vector to map countries to regions

# Add individual IDs and pops ids
Q_values$ind <- inds
Q_values$pop <- pops

# Melt the data frame for plotting
Q_melted <- melt(Q_values, id.vars = c("ind", "pop"))

# Join with sampling_loc to get sampling localities
Q_joined <- Q_melted |>
  left_join(sampling_loc, by = c("pop" = "Abbreviation"))

# Create a combined variable for Region and Country
Q_joined <- Q_joined |>
  mutate(Region_Country = interaction(Region, Country, sep = "_"))

# Order the combined variable by Region and Country
Q_ordered <- Q_joined |>
  arrange(Region, Country) |>
  mutate(Region_Country = factor(Region_Country, levels = unique(Region_Country)))

# Group by Country and calculate mean ancestry proportions
Q_grouped <- Q_ordered |>
  group_by(Region_Country, variable) |>
  summarise(value = mean(value), .groups = "drop")

# Create a data frame for borders
borders <- data.frame(Region_Country = unique(Q_grouped$Region_Country))

# Add the order of each country to ensure correct placement of borders
borders$order <- 1:nrow(borders) + 0.5  # Shift borders to the right edge of the bars


# Function to filter and normalize data
normalize_data <- function(df, min_value) {
  df |>
    filter(value > min_value) |>
    group_by(Region_Country) |>
    mutate(value = value / sum(value))
}

# Use the function
Q_grouped_filtered <- normalize_data(Q_grouped, 0.1)

# source the plotting function
source(
  here("scripts", "RMarkdowns",
   "analyses", "my_theme2.R"
  )
)

# Generate all potential variable names
all_variables <- paste0("V", 1:20)

color_palette <-
c(
"#FFE100",
"orchid1",
"#003380", 
"purple3",
"magenta",
"#9DCC00", 
"orangered",
"#FF0010",
"#808080", 
"#005C31",
"#FF8005", 
"#990000",
"#1E90FF",
"blue",
"cyan",
"cadetblue",
"#FFFC99"
)

# Create a data frame that pairs each potential variable with a color
color_mapping <- data.frame(variable = all_variables,
                          color = color_palette[1:length(all_variables)])

# Merge this data frame with Q_grouped_filtered to create the new color column
Q_grouped_filtered <- merge(Q_grouped_filtered, color_mapping, by = "variable")

# Create bar chart
ggplot(Q_grouped_filtered, aes(x = Region_Country, y = value, fill = color)) +
  geom_bar(stat = 'identity', width = 1) +
  geom_segment(data = borders, aes(x = order, xend = order, y = 0, yend = 1, fill = NULL), linetype = "solid", color = "#2C444A") +  # Add borders
  my_theme() +
  theme(axis.text.x = element_text(angle = 90, hjust = 1),
        legend.position = "none") +  # Hide legend
  xlab("") +  # Suppress x-axis label
  ylab("Ancestry proportions") +
  ggtitle("Ancestry matrix") +
  labs(caption = "Each bar represents the average ancestry proportions for individuals in a given country for k=20.") +
  # scale_fill_manual(values = color) +
  scale_x_discrete(labels = function(x) gsub(".*_", "", x)) + # Remove Region prefix from labels
  scale_fill_manual(values = color_palette)
```

```
ggsave(
  here("scripts", "RMarkdowns",
    "output", "americas", "manuscript1", "lea", "LEA_admix_by_country_man1_k20.pdf"
  ),
  width  = 10,
  height = 7,
  units  = "in"
)
```

#### 3.1.7.2. Extract ancestry coefficients for k=20

*change to correct matrix*

```
leak20 <- read_delim(
  here("/gpfs/gibbs/pi/caccone/mkc54/albo/manuscript1/output/snps_sets/r2_0.01_b.snmf/K20/run3/r2_0.01_b_r3.20.Q"),
  delim = " ", # Specify the delimiter if different from the default (comma)
  col_names = FALSE,
  show_col_types = FALSE
) 
head(leak20)
```

```
## # A tibble: 6 × 20
##         X1      X2      X3      X4      X5      X6    X7      X8      X9     X10
##      <dbl>   <dbl>   <dbl>   <dbl>   <dbl>   <dbl> <dbl>   <dbl>   <dbl>   <dbl>
## 1  3.06e-2 9.99e-5 9.99e-5 3.19e-2 9.99e-5 9.99e-5 0.427 9.99e-5 9.99e-5 9.99e-5
## 2  7.29e-2 2.39e-2 2.59e-2 1.00e-4 1.00e-4 1.71e-2 0.267 3.15e-2 1.00e-4 2.11e-2
## 3  9.99e-5 9.99e-5 1.67e-2 2.82e-2 1.72e-2 9.99e-5 0.672 1.83e-3 1.62e-2 9.99e-5
## 4  9.98e-5 9.98e-5 9.98e-5 9.98e-5 9.98e-5 9.98e-5 0.900 9.98e-5 9.98e-5 9.98e-5
## 5  9.98e-5 9.98e-5 9.98e-5 9.98e-5 9.98e-5 9.98e-5 0.936 9.98e-5 9.98e-5 9.98e-5
## 6  9.99e-5 9.99e-5 8.97e-3 1.95e-2 9.99e-5 9.99e-5 0.751 3.43e-3 1.02e-2 9.99e-5
## # ℹ 10 more variables: X11 <dbl>, X12 <dbl>, X13 <dbl>, X14 <dbl>, X15 <dbl>,
## #   X16 <dbl>, X17 <dbl>, X18 <dbl>, X19 <dbl>, X20 <dbl>
```

The fam file

```
fam_file <- here("manuscript1/output/snps_sets/r2_0.01_b.fam")

# Read the .fam file
fam_data <- read.table(fam_file, 
                       header = FALSE,
                       col.names = c("FamilyID", "IndividualID", "PaternalID", "MaternalID", "Sex", "Phenotype"))

# View the first few rows
head(fam_data)
```

```
##   FamilyID IndividualID PaternalID MaternalID Sex Phenotype
## 1      OKI         1001          0          0   2        -9
## 2      OKI         1002          0          0   2        -9
## 3      OKI         1003          0          0   2        -9
## 4      OKI         1004          0          0   2        -9
## 5      OKI         1005          0          0   2        -9
## 6      OKI         1006          0          0   1        -9
```

Create column ID

```
# Change column name
colnames(fam_data)[colnames(fam_data) == "IndividualID"] <- "ind"

# Change column name
colnames(fam_data)[colnames(fam_data) == "FamilyID"] <- "pop"

# Select ID
fam_data <- fam_data |>
  dplyr::select("ind", "pop")

# View the first few rows
head(fam_data)
```

```
##    ind pop
## 1 1001 OKI
## 2 1002 OKI
## 3 1003 OKI
## 4 1004 OKI
## 5 1005 OKI
## 6 1006 OKI
```

Add it to the matrix

```
leak20 <- fam_data |>
  dplyr::select(ind, pop) |>
  bind_cols(leak20)

head(leak20)
```

```
##    ind pop          X1          X2          X3          X4          X5
## 1 1001 OKI 3.06120e-02 9.99100e-05 9.99100e-05 3.19485e-02 9.99100e-05
## 2 1002 OKI 7.28606e-02 2.38574e-02 2.59435e-02 9.99550e-05 9.99550e-05
## 3 1003 OKI 9.99190e-05 9.99190e-05 1.66988e-02 2.81766e-02 1.72400e-02
## 4 1004 OKI 9.98378e-05 9.98378e-05 9.98378e-05 9.98378e-05 9.98378e-05
## 5 1005 OKI 9.98378e-05 9.98378e-05 9.98378e-05 9.98378e-05 9.98378e-05
## 6 1006 OKI 9.98920e-05 9.98920e-05 8.97185e-03 1.94965e-02 9.98920e-05
##            X6       X7          X8          X9         X10         X11
## 1 9.99100e-05 0.426981 9.99100e-05 9.99100e-05 9.99100e-05 2.70803e-02
## 2 1.71181e-02 0.267228 3.15209e-02 9.99550e-05 2.11178e-02 1.60308e-02
## 3 9.99190e-05 0.671843 1.83393e-03 1.62475e-02 9.99190e-05 9.99190e-05
## 4 9.98378e-05 0.899814 9.98378e-05 9.98378e-05 9.98378e-05 9.98378e-05
## 5 9.98378e-05 0.935938 9.98378e-05 9.98378e-05 9.98378e-05 9.98378e-05
## 6 9.98920e-05 0.751193 3.43474e-03 1.01842e-02 9.98920e-05 9.98920e-05
##           X12         X13         X14         X15         X16         X17
## 1 1.79172e-01 1.66674e-01 1.53596e-02 9.99100e-05 4.57297e-02 9.99100e-05
## 2 9.31839e-02 2.45387e-01 1.91602e-02 4.29681e-03 2.86808e-02 9.99550e-05
## 3 3.69289e-02 1.33751e-01 1.23076e-02 9.99190e-05 1.41762e-02 9.99190e-05
## 4 9.98378e-05 9.98378e-05 9.98378e-05 9.98378e-05 9.98378e-05 9.98378e-05
## 5 9.98378e-05 9.98378e-05 9.98378e-05 9.98378e-05 9.98378e-05 9.98378e-05
## 6 9.98920e-05 1.43390e-01 9.98920e-05 9.98920e-05 9.98920e-05 4.03119e-02
##           X18         X19         X20
## 1 5.21531e-02 0.023289500 9.99100e-05
## 2 5.88549e-02 0.000099955 7.42596e-02
## 3 4.98969e-02 0.000099919 9.99190e-05
## 4 9.98378e-05 0.098388700 9.98378e-05
## 5 9.98378e-05 0.062265200 9.98378e-05
## 6 2.18197e-02 0.000099892 9.98920e-05
```

Rename the columns

```
# Rename the columns starting from the third one
leak20 <- leak20 |>
  rename_with(~paste0("v", seq_along(.x)), .cols = -c(ind, pop))

# View the first few rows
head(leak20)
```

```
##    ind pop          v1          v2          v3          v4          v5
## 1 1001 OKI 3.06120e-02 9.99100e-05 9.99100e-05 3.19485e-02 9.99100e-05
## 2 1002 OKI 7.28606e-02 2.38574e-02 2.59435e-02 9.99550e-05 9.99550e-05
## 3 1003 OKI 9.99190e-05 9.99190e-05 1.66988e-02 2.81766e-02 1.72400e-02
## 4 1004 OKI 9.98378e-05 9.98378e-05 9.98378e-05 9.98378e-05 9.98378e-05
## 5 1005 OKI 9.98378e-05 9.98378e-05 9.98378e-05 9.98378e-05 9.98378e-05
## 6 1006 OKI 9.98920e-05 9.98920e-05 8.97185e-03 1.94965e-02 9.98920e-05
##            v6       v7          v8          v9         v10         v11
## 1 9.99100e-05 0.426981 9.99100e-05 9.99100e-05 9.99100e-05 2.70803e-02
## 2 1.71181e-02 0.267228 3.15209e-02 9.99550e-05 2.11178e-02 1.60308e-02
## 3 9.99190e-05 0.671843 1.83393e-03 1.62475e-02 9.99190e-05 9.99190e-05
## 4 9.98378e-05 0.899814 9.98378e-05 9.98378e-05 9.98378e-05 9.98378e-05
## 5 9.98378e-05 0.935938 9.98378e-05 9.98378e-05 9.98378e-05 9.98378e-05
## 6 9.98920e-05 0.751193 3.43474e-03 1.01842e-02 9.98920e-05 9.98920e-05
##           v12         v13         v14         v15         v16         v17
## 1 1.79172e-01 1.66674e-01 1.53596e-02 9.99100e-05 4.57297e-02 9.99100e-05
## 2 9.31839e-02 2.45387e-01 1.91602e-02 4.29681e-03 2.86808e-02 9.99550e-05
## 3 3.69289e-02 1.33751e-01 1.23076e-02 9.99190e-05 1.41762e-02 9.99190e-05
## 4 9.98378e-05 9.98378e-05 9.98378e-05 9.98378e-05 9.98378e-05 9.98378e-05
## 5 9.98378e-05 9.98378e-05 9.98378e-05 9.98378e-05 9.98378e-05 9.98378e-05
## 6 9.98920e-05 1.43390e-01 9.98920e-05 9.98920e-05 9.98920e-05 4.03119e-02
##           v18         v19         v20
## 1 5.21531e-02 0.023289500 9.99100e-05
## 2 5.88549e-02 0.000099955 7.42596e-02
## 3 4.98969e-02 0.000099919 9.99190e-05
## 4 9.98378e-05 0.098388700 9.98378e-05
## 5 9.98378e-05 0.062265200 9.98378e-05
## 6 2.18197e-02 0.000099892 9.98920e-05
```

Import Sample Locations

```
sampling_loc <- readRDS(here("scripts", "RMarkdowns", "output", "sampling_loc_all_pops.rds"))
head(sampling_loc)
```

```
##      Pop_City Country Latitude Longitude Continent Abbreviation  Year    Region
## 1      Hainan   China  19.2000  109.6000      Asia          HAI  2014 East Asia
## 2      Yunnan   China  24.4753  101.3431      Asia          YUN  2014 East Asia
## 3       Hunan   China  27.6253  111.8569      Asia          HUN  2001 East Asia
## 4     Okinawa   Japan  26.5013  127.9454      Asia          OKI 2018? East Asia
## 5 Tanegashima   Japan  30.4564  130.9784      Asia          TAN  2008 East Asia
## 6   Kagoshima   Japan  31.5602  130.5581      Asia          KAG  2018 East Asia
##   Subregion order order2 orderold AM_order state FINAL_order
## 1     Japan    55     47       47       NA                 1
## 2     Japan    56     48       48       NA                 2
## 3     Japan    57     49       49       NA                 3
## 4     Japan    54     46       46       NA                 4
## 5     Japan    NA     NA       NA       NA                 5
## 6     Japan    53     45       45       NA                 6
```

```
source(
  here("scripts", "RMarkdowns",
    "my_theme3.R"
  )
)

# Melt the data frame for plotting
Q_melted <- leak20 |>
  pivot_longer(
    cols = -c(ind, pop),
    names_to = "variable",
    values_to = "value"
  )
# Join with sampling_loc to get sampling localities
Q_joined <- Q_melted |>
  left_join(sampling_loc, by = c("pop" = "Abbreviation"))


# Order the combined variable by Region and Country, then by individual
Q_ordered <- Q_joined |>
  arrange(FINAL_order, ind) |>
  mutate(ind = factor(ind, levels = unique(ind)))  # Convert ind to a factor with levels in the desired order

# Add labels: country names for the first individual in each country, NA for all other individuals
Q_ordered <- Q_ordered |>
  group_by(Country) |>
  mutate(label = ifelse(row_number() == 1, as.character(Country), NA))

# Group by individual and variable, calculate mean ancestry proportions
Q_grouped <- Q_ordered |>
  group_by(ind, variable) |>
  summarise(value = mean(value), .groups = "drop")

# Create a data frame for borders
borders <-
  data.frame(Country = unique(Q_ordered$Country))

# Add the order of the last individual of each country to ensure correct placement of borders
borders$order <-
  sapply(borders$Country, function(rc)
    max(which(Q_ordered$Country == rc))) + 0.5  # Shift borders to the right edge of the bars

# Select only the first occurrence of each country in the ordered data
label_df <- Q_ordered |>
  filter(!is.na(label)) |>
  distinct(label, .keep_all = TRUE)

# Create a custom label function
label_func <- function(x) {
  labels <- rep("", length(x))
  labels[x %in% label_df$ind] <- label_df$label
  labels
}

# Calculate the position of lines
border_positions <- Q_ordered |>
  group_by(Country) |>
  summarise(pos = max(as.numeric(ind)) + 0)

# Calculate the position of population labels and bars
pop_labels <- Q_ordered |>
  mutate(Name = paste(pop, Pop_City, sep = " - ")) |>
  group_by(pop) |>
  slice_head(n = 1) |>
  ungroup() |>
  dplyr::select(ind, Pop_City, Country, Name) |>
  mutate(pos = as.numeric(ind))  # calculate position of population labels

pop_labels_bars <- pop_labels |>
  mutate(pos = as.numeric(ind)  - .5)


# Calculate the position of lines
border_positions <- Q_ordered |>
  group_by(Country) |>
  summarise(pos = max(as.numeric(ind)) - 1)


pop_labels_bars <- pop_labels |>
  mutate(pos = as.numeric(ind)  - .5)

# Function to filter and normalize data
normalize_data <- function(df, min_value) {
  df |>
    filter(value > min_value) |>
    group_by(ind) |>
    mutate(value = value / sum(value))
}

# Use the function
Q_grouped_filtered <- normalize_data(Q_grouped, 0.1)
# 


# Generate all potential variable names
all_variables <- paste0("v", 1:20)

color_palette <-
c(
"ivory", 
"#005C31",
"magenta",
"#990000", 
"orchid1",
"cadetblue",
"#FFE100",   
"#9DCC00", 
"#808080",
"#993F00", 
"#FFFC99",
"blue",
"#FF8005",
"cyan",
"#1E90FF",
"purple3",
"#FF0010", 
"#003380",  
"orangered",
"green")


# Create a data frame that pairs each potential variable with a color
color_mapping <- data.frame(variable = all_variables,
                           color = color_palette[1:length(all_variables)])

# Merge with Q_grouped_filtered
Q_grouped_filtered <- merge(Q_grouped_filtered, color_mapping, by = "variable")

# Create the plot
ggplot(Q_grouped_filtered, aes(x = as.factor(ind), y = value, fill = color)) +
  geom_bar(stat = 'identity', width = 1) +
  geom_vline(
    data = pop_labels_bars,
    aes(xintercept = pos),
    color = "#2C444A",
    linewidth = .2
  ) +
  geom_text(
    data = pop_labels,
    aes(x = as.numeric(ind), y = 1, label = Name),
    vjust = 1.5,
    hjust = 0,
    size = 2,
    angle = 90,
    inherit.aes = FALSE
  ) +
  my_theme() +
  theme(
    axis.text.x = element_text(
      angle = 90,
      hjust = 1,
      size = 12
    ),
    legend.position = "none",
    plot.margin = unit(c(3, 0.5, 0.5, 0.5), "cm")
  ) +
  xlab("Admixture matrix") +
  ylab("Ancestry proportions") +
  labs(caption = "Each bar represents the ancestry proportions for an individual for k=20.\n LEA inference with 20,825 SNPs.") +
  scale_x_discrete(labels = label_func) +
  scale_fill_manual(values = color_palette) +
  expand_limits(y = c(0, 1.5))
```

```
ggsave(
  here("scripts", "RMarkdowns", "output", "americas", "manuscript1", "lea", "lea_k=20_manuscript1.pdf"),
  width  = 16,
  height = 6,
  units  = "in",
  device = cairo_pdf
)
```

### 3.1.8 LEA plots for k=16

```
color_palette <-
  c(
    "green",
    "orangered",   
    "#FFFC99",  
    "#8c61cd",     
    "orchid1",
    "#003380",
    "#1E90FF",     
    "#FF8005", 
    "#9DCC00", 
    "#FFA8BB",   
    "#808080", 
    "purple3",   
    "#FFE100",
    "#990000",  
    "#AC7700",      
    "#4C005C",  
    "cyan",
    "#FF0010",
    "magenta",
    "blue",
    "cadetblue",   
    "gray",    
    "ivory", 
    "#993F00", 
    "#005C31"    
)
```

Default plot

```
# select the best run for chosen K 
best16 = which.min(cross.entropy(project, K = 16))
# best is run 1

barchart(project, K = 16, run = best16,
        border = NA, space = 0,
        col = color_palette,
        xlab = "Individuals",
        ylab = "Ancestry proportions",
        main = "Ancestry matrix") -> bp
axis(1, at = 1:length(bp$order),
     labels = bp$order, las=1,
     cex.axis = .4)
```

#### 3.1.8.1 Mean admixture by country using ggplot

```
sampling_loc <- readRDS(here("scripts", "RMarkdowns", "output", "sampling_loc_all_pops.rds"))

library(reshape2)

# Extract ancestry coefficients
Q_values <- as.data.frame(Q(project, K = 16, run = best16))

# Create a named vector to map countries to regions

# Add individual IDs and pops ids
Q_values$ind <- inds
Q_values$pop <- pops

# Melt the data frame for plotting
Q_melted <- melt(Q_values, id.vars = c("ind", "pop"))

# Join with sampling_loc to get sampling localities
Q_joined <- Q_melted |>
  left_join(sampling_loc, by = c("pop" = "Abbreviation"))

# Create a combined variable for Region and Country
Q_joined <- Q_joined |>
  mutate(Region_Country = interaction(Region, Country, sep = "_"))

# Order the combined variable by Region and Country
Q_ordered <- Q_joined |>
  arrange(Region, Country) |>
  mutate(Region_Country = factor(Region_Country, levels = unique(Region_Country)))

# Group by Country and calculate mean ancestry proportions
Q_grouped <- Q_ordered |>
  group_by(Region_Country, variable) |>
  summarise(value = mean(value), .groups = "drop")

# Create a data frame for borders
borders <- data.frame(Region_Country = unique(Q_grouped$Region_Country))

# Add the order of each country to ensure correct placement of borders
borders$order <- 1:nrow(borders) + 0.5  # Shift borders to the right edge of the bars


# Function to filter and normalize data
normalize_data <- function(df, min_value) {
  df |>
    filter(value > min_value) |>
    group_by(Region_Country) |>
    mutate(value = value / sum(value))
}

# Use the function
Q_grouped_filtered <- normalize_data(Q_grouped, 0.1)

# source the plotting function
source(
  here("scripts", "RMarkdowns",
   "analyses", "my_theme2.R"
  )
)

# Generate all potential variable names
all_variables <- paste0("V", 1:16)

color_palette <-
c(
"orchid1",
"blue",
"#005C31",
"cyan",
"#990000",
"#FF0010",
"#FFE100",
"#808080", 
"purple3",
"#FF8005", 
"orangered",
"#003380", 
"magenta",
"#1E90FF",
"#FFFC99",
"cadetblue"
)
  
# Create a data frame that pairs each potential variable with a color
color_mapping <- data.frame(variable = all_variables,
                          color = color_palette[1:length(all_variables)])

# Merge this data frame with Q_grouped_filtered to create the new color column
Q_grouped_filtered <- merge(Q_grouped_filtered, color_mapping, by = "variable")

# Create bar chart
ggplot(Q_grouped_filtered, aes(x = Region_Country, y = value, fill = color)) +
  geom_bar(stat = 'identity', width = 1) +
  geom_segment(data = borders, aes(x = order, xend = order, y = 0, yend = 1, fill = NULL), linetype = "solid", color = "#2C444A") +  # Add borders
  my_theme() +
  theme(axis.text.x = element_text(angle = 90, hjust = 1),
        legend.position = "none") +  # Hide legend
  xlab("") +  # Suppress x-axis label
  ylab("Ancestry proportions") +
  ggtitle("Ancestry matrix") +
  labs(caption = "Each bar represents the average ancestry proportions for individuals in a given country for k=16.") +
  # scale_fill_manual(values = color) +
  scale_x_discrete(labels = function(x) gsub(".*_", "", x)) + # Remove Region prefix from labels
  scale_fill_manual(values = color_palette)
```

```
ggsave(
  here("scripts", "RMarkdowns",
    "output", "americas", "manuscript1", "lea", "LEA_admix_by_country_man1_k16.pdf"
  ),
  width  = 10,
  height = 7,
  units  = "in"
)
```

#### 3.1.8.2. Extract ancestry coefficients for k=16

*change to correct matrix*

```
leak16 <- read_delim(
  here("/gpfs/gibbs/pi/caccone/mkc54/albo/manuscript1/output/snps_sets/r2_0.01_b.snmf/K16/run4/r2_0.01_b_r4.16.Q"),
  delim = " ", # Specify the delimiter if different from the default (comma)
  col_names = FALSE,
  show_col_types = FALSE
) 
head(leak16)
```

```
## # A tibble: 6 × 16
##          X1        X2        X3       X4      X5      X6      X7      X8      X9
##       <dbl>     <dbl>     <dbl>    <dbl>   <dbl>   <dbl>   <dbl>   <dbl>   <dbl>
## 1 0.0679    0.0513    0.0121     1.00e-4 1.49e-2 5.19e-2 1.00e-4 5.70e-2 1.67e-1
## 2 0.0643    0.000100  0.000100   1.00e-4 1.00e-4 5.85e-2 3.52e-2 8.90e-2 1.06e-1
## 3 0.0431    0.0000999 0.0000999  9.99e-5 1.13e-2 9.99e-5 9.99e-5 2.56e-2 5.91e-2
## 4 0.0000999 0.238     0.0000999  9.99e-5 9.99e-5 9.99e-5 9.99e-5 9.99e-5 9.99e-5
## 5 0.0000999 0.226     0.0000999  9.99e-5 9.99e-5 9.99e-5 9.99e-5 9.99e-5 9.99e-5
## 6 0.0356    0.0000999 0.00316    9.99e-5 9.99e-5 8.06e-3 9.99e-5 1.71e-2 3.12e-2
## # ℹ 7 more variables: X10 <dbl>, X11 <dbl>, X12 <dbl>, X13 <dbl>, X14 <dbl>,
## #   X15 <dbl>, X16 <dbl>
```

The fam file

```
fam_file <- here("manuscript1/output/snps_sets/r2_0.01_b.fam")

# Read the .fam file
fam_data <- read.table(fam_file, 
                       header = FALSE,
                       col.names = c("FamilyID", "IndividualID", "PaternalID", "MaternalID", "Sex", "Phenotype"))

# View the first few rows
head(fam_data)
```

```
##   FamilyID IndividualID PaternalID MaternalID Sex Phenotype
## 1      OKI         1001          0          0   2        -9
## 2      OKI         1002          0          0   2        -9
## 3      OKI         1003          0          0   2        -9
## 4      OKI         1004          0          0   2        -9
## 5      OKI         1005          0          0   2        -9
## 6      OKI         1006          0          0   1        -9
```

Create column ID

```
# Change column name
colnames(fam_data)[colnames(fam_data) == "IndividualID"] <- "ind"

# Change column name
colnames(fam_data)[colnames(fam_data) == "FamilyID"] <- "pop"

# Select ID
fam_data <- fam_data |>
  dplyr::select("ind", "pop")

# View the first few rows
head(fam_data)
```

```
##    ind pop
## 1 1001 OKI
## 2 1002 OKI
## 3 1003 OKI
## 4 1004 OKI
## 5 1005 OKI
## 6 1006 OKI
```

Add it to the matrix

```
leak16 <- fam_data |>
  dplyr::select(ind, pop) |>
  bind_cols(leak16)

head(leak16)
```

```
##    ind pop          X1          X2          X3         X4          X5
## 1 1001 OKI 0.067900300 0.051336000 0.012097400 9.9982e-05 0.014948000
## 2 1002 OKI 0.064283300 0.000099964 0.000099964 9.9964e-05 0.000099964
## 3 1003 OKI 0.043106800 0.000099937 0.000099937 9.9937e-05 0.011291800
## 4 1004 OKI 0.000099874 0.237556000 0.000099874 9.9874e-05 0.000099874
## 5 1005 OKI 0.000099874 0.226405000 0.000099874 9.9874e-05 0.000099874
## 6 1006 OKI 0.035563200 0.000099937 0.003157720 9.9937e-05 0.000099937
##            X6         X7          X8          X9         X10         X11
## 1 0.051905000 9.9982e-05 0.057041600 0.166829000 0.027252100 0.033793300
## 2 0.058514400 3.5184e-02 0.089031200 0.106170000 0.053903900 0.007800850
## 3 0.000099937 9.9937e-05 0.025605200 0.059132300 0.055061200 0.056263500
## 4 0.000099874 9.9874e-05 0.000099874 0.000099874 0.000099874 0.000099874
## 5 0.000099874 9.9874e-05 0.000099874 0.000099874 0.000099874 0.000099874
## 6 0.008057240 9.9937e-05 0.017102200 0.031152400 0.046486700 0.129289000
##           X12         X13         X14      X15         X16
## 1 0.004867160 0.026184600 0.009324250 0.474317 0.002004670
## 2 0.054129200 0.032287500 0.012620200 0.426339 0.059337000
## 3 0.000099937 0.014944000 0.000099937 0.686273 0.047622600
## 4 0.000099874 0.000099874 0.000099874 0.761045 0.000099874
## 5 0.000099874 0.000099874 0.000099874 0.772196 0.000099874
## 6 0.000099937 0.000099937 0.000099937 0.671949 0.056542500
```

Rename the columns

```
# Rename the columns starting from the third one
leak16 <- leak16 |>
  rename_with(~paste0("v", seq_along(.x)), .cols = -c(ind, pop))

# View the first few rows
head(leak16)
```

```
##    ind pop          v1          v2          v3         v4          v5
## 1 1001 OKI 0.067900300 0.051336000 0.012097400 9.9982e-05 0.014948000
## 2 1002 OKI 0.064283300 0.000099964 0.000099964 9.9964e-05 0.000099964
## 3 1003 OKI 0.043106800 0.000099937 0.000099937 9.9937e-05 0.011291800
## 4 1004 OKI 0.000099874 0.237556000 0.000099874 9.9874e-05 0.000099874
## 5 1005 OKI 0.000099874 0.226405000 0.000099874 9.9874e-05 0.000099874
## 6 1006 OKI 0.035563200 0.000099937 0.003157720 9.9937e-05 0.000099937
##            v6         v7          v8          v9         v10         v11
## 1 0.051905000 9.9982e-05 0.057041600 0.166829000 0.027252100 0.033793300
## 2 0.058514400 3.5184e-02 0.089031200 0.106170000 0.053903900 0.007800850
## 3 0.000099937 9.9937e-05 0.025605200 0.059132300 0.055061200 0.056263500
## 4 0.000099874 9.9874e-05 0.000099874 0.000099874 0.000099874 0.000099874
## 5 0.000099874 9.9874e-05 0.000099874 0.000099874 0.000099874 0.000099874
## 6 0.008057240 9.9937e-05 0.017102200 0.031152400 0.046486700 0.129289000
##           v12         v13         v14      v15         v16
## 1 0.004867160 0.026184600 0.009324250 0.474317 0.002004670
## 2 0.054129200 0.032287500 0.012620200 0.426339 0.059337000
## 3 0.000099937 0.014944000 0.000099937 0.686273 0.047622600
## 4 0.000099874 0.000099874 0.000099874 0.761045 0.000099874
## 5 0.000099874 0.000099874 0.000099874 0.772196 0.000099874
## 6 0.000099937 0.000099937 0.000099937 0.671949 0.056542500
```

Import Sample Locations

```
sampling_loc <- readRDS(here("scripts", "RMarkdowns", "output", "sampling_loc_all_pops.rds"))
head(sampling_loc)
```

```
##      Pop_City Country Latitude Longitude Continent Abbreviation  Year    Region
## 1      Hainan   China  19.2000  109.6000      Asia          HAI  2014 East Asia
## 2      Yunnan   China  24.4753  101.3431      Asia          YUN  2014 East Asia
## 3       Hunan   China  27.6253  111.8569      Asia          HUN  2001 East Asia
## 4     Okinawa   Japan  26.5013  127.9454      Asia          OKI 2018? East Asia
## 5 Tanegashima   Japan  30.4564  130.9784      Asia          TAN  2008 East Asia
## 6   Kagoshima   Japan  31.5602  130.5581      Asia          KAG  2018 East Asia
##   Subregion order order2 orderold AM_order state FINAL_order
## 1     Japan    55     47       47       NA                 1
## 2     Japan    56     48       48       NA                 2
## 3     Japan    57     49       49       NA                 3
## 4     Japan    54     46       46       NA                 4
## 5     Japan    NA     NA       NA       NA                 5
## 6     Japan    53     45       45       NA                 6
```

```
source(
  here("scripts", "RMarkdowns",
    "my_theme3.R"
  )
)

# Melt the data frame for plotting
Q_melted <- leak16 |>
  pivot_longer(
    cols = -c(ind, pop),
    names_to = "variable",
    values_to = "value"
  )
# Join with sampling_loc to get sampling localities
Q_joined <- Q_melted |>
  left_join(sampling_loc, by = c("pop" = "Abbreviation"))


# Order the combined variable by Region and Country, then by individual
Q_ordered <- Q_joined |>
  arrange(FINAL_order, ind) |>
  mutate(ind = factor(ind, levels = unique(ind)))  # Convert ind to a factor with levels in the desired order

# Add labels: country names for the first individual in each country, NA for all other individuals
Q_ordered <- Q_ordered |>
  group_by(Country) |>
  mutate(label = ifelse(row_number() == 1, as.character(Country), NA))

# Group by individual and variable, calculate mean ancestry proportions
Q_grouped <- Q_ordered |>
  group_by(ind, variable) |>
  summarise(value = mean(value), .groups = "drop")

# Create a data frame for borders
borders <-
  data.frame(Country = unique(Q_ordered$Country))

# Add the order of the last individual of each country to ensure correct placement of borders
borders$order <-
  sapply(borders$Country, function(rc)
    max(which(Q_ordered$Country == rc))) + 0.5  # Shift borders to the right edge of the bars

# Select only the first occurrence of each country in the ordered data
label_df <- Q_ordered |>
  filter(!is.na(label)) |>
  distinct(label, .keep_all = TRUE)

# Create a custom label function
label_func <- function(x) {
  labels <- rep("", length(x))
  labels[x %in% label_df$ind] <- label_df$label
  labels
}

# Calculate the position of lines
border_positions <- Q_ordered |>
  group_by(Country) |>
  summarise(pos = max(as.numeric(ind)) + 0)

# Calculate the position of population labels and bars
pop_labels <- Q_ordered |>
  mutate(Name = paste(pop, Pop_City, sep = " - ")) |>
  group_by(pop) |>
  slice_head(n = 1) |>
  ungroup() |>
  dplyr::select(ind, Pop_City, Country, Name) |>
  mutate(pos = as.numeric(ind))  # calculate position of population labels

pop_labels_bars <- pop_labels |>
  mutate(pos = as.numeric(ind)  - .5)


# Calculate the position of lines
border_positions <- Q_ordered |>
  group_by(Country) |>
  summarise(pos = max(as.numeric(ind)) - 1)


pop_labels_bars <- pop_labels |>
  mutate(pos = as.numeric(ind)  - .5)

# Function to filter and normalize data
normalize_data <- function(df, min_value) {
  df |>
    filter(value > min_value) |>
    group_by(ind) |>
    mutate(value = value / sum(value))
}

# Use the function
Q_grouped_filtered <- normalize_data(Q_grouped, 0.1)
# 

# Generate all potential variable names
all_variables <- paste0("v", 1:16)


color_palette <-
c(
"#990000",  
"magenta",  
"#FF8005",   
"#FFE100",
"orchid1",
"#FF0010",  
"blue",  
"cadetblue",  
"#003380",   
"#FFFC99",
"orangered",  
"#1E90FF",  
"#005C31",  
"#808080",   
"purple3",
"cyan"
)


# Create a data frame that pairs each potential variable with a color
color_mapping <- data.frame(variable = all_variables,
                           color = color_palette[1:length(all_variables)])

# Merge with Q_grouped_filtered
Q_grouped_filtered <- merge(Q_grouped_filtered, color_mapping, by = "variable")

# Create the plot
ggplot(Q_grouped_filtered, aes(x = as.factor(ind), y = value, fill = color)) +
  geom_bar(stat = 'identity', width = 1) +
  geom_vline(
    data = pop_labels_bars,
    aes(xintercept = pos),
    color = "#2C444A",
    linewidth = .2
  ) +
  geom_text(
    data = pop_labels,
    aes(x = as.numeric(ind), y = 1, label = Name),
    vjust = 1.5,
    hjust = 0,
    size = 2,
    angle = 90,
    inherit.aes = FALSE
  ) +
  my_theme() +
  theme(
    axis.text.x = element_text(
      angle = 90,
      hjust = 1,
      size = 12
    ),
    legend.position = "none",
    plot.margin = unit(c(3, 0.5, 0.5, 0.5), "cm")
  ) +
  xlab("Admixture matrix") +
  ylab("Ancestry proportions") +
  labs(caption = "Each bar represents the ancestry proportions for an individual for k=16.\n LEA inference with 20,825 SNPs.") +
  scale_x_discrete(labels = label_func) +
  scale_fill_manual(values = color_palette) +
  expand_limits(y = c(0, 1.5))
```

```
ggsave(
  here("scripts", "RMarkdowns", "output", "americas", "manuscript1", "lea", "lea_k=16_manuscript1.pdf"),
  width  = 16,
  height = 6,
  units  = "in",
  device = cairo_pdf
)
```

## 3.2. Figures from fastStructure

#### 3.2.1 K=25

Extract ancestry coefficients for k=25

```
fast25 <- read_delim(
  here("/gpfs/gibbs/pi/caccone/mkc54/albo/manuscript1/output/fastStructure/run045/simple.25.meanQ"),
  delim = "  ", # Specify the delimiter if different from the default (comma)
  col_names = FALSE,
  show_col_types = FALSE
) 
head(fast25)
```

```
## # A tibble: 6 × 25
##      X1       X2      X3    X4    X5    X6    X7    X8    X9     X10   X11   X12
##   <dbl>    <dbl>   <dbl> <dbl> <dbl> <dbl> <dbl> <dbl> <dbl>   <dbl> <dbl> <dbl>
## 1 0.844 0.000001    1e-6  1e-6  1e-6  1e-6  1e-6  1e-6  1e-6 6.95e-2  1e-6  1e-6
## 2 0.732 0.000001    1e-6  1e-6  1e-6  1e-6  1e-6  1e-6  1e-6 1.04e-1  1e-6  1e-6
## 3 0.911 0.000001    1e-6  1e-6  1e-6  1e-6  1e-6  1e-6  1e-6 1   e-6  1e-6  1e-6
## 4 0.927 0.000001    1e-6  1e-6  1e-6  1e-6  1e-6  1e-6  1e-6 1   e-6  1e-6  1e-6
## 5 0.932 0.000001    1e-6  1e-6  1e-6  1e-6  1e-6  1e-6  1e-6 1   e-6  1e-6  1e-6
## 6 0.862 0.000001    1e-6  1e-6  1e-6  1e-6  1e-6  1e-6  1e-6 4.74e-2  1e-6  1e-6
## # ℹ 13 more variables: X13 <dbl>, X14 <dbl>, X15 <dbl>, X16 <dbl>, X17 <dbl>,
## #   X18 <dbl>, X19 <dbl>, X20 <dbl>, X21 <dbl>, X22 <dbl>, X23 <dbl>,
## #   X24 <dbl>, X25 <dbl>
```

The fam file

```
fam_file <- here("manuscript1/output/snps_sets/r2_0.01_b.fam")

# Read the .fam file
fam_data <- read.table(fam_file, 
                       header = FALSE,
                       col.names = c("FamilyID", "IndividualID", "PaternalID", "MaternalID", "Sex", "Phenotype"))

# View the first few rows
head(fam_data)
```

```
##   FamilyID IndividualID PaternalID MaternalID Sex Phenotype
## 1      OKI         1001          0          0   2        -9
## 2      OKI         1002          0          0   2        -9
## 3      OKI         1003          0          0   2        -9
## 4      OKI         1004          0          0   2        -9
## 5      OKI         1005          0          0   2        -9
## 6      OKI         1006          0          0   1        -9
```

Create column ID

```
# Change column name
colnames(fam_data)[colnames(fam_data) == "IndividualID"] <- "ind"

# Change column name
colnames(fam_data)[colnames(fam_data) == "FamilyID"] <- "pop"

# Select ID
fam_data <- fam_data |>
  dplyr::select("ind", "pop")

# View the first few rows
head(fam_data)
```

```
##    ind pop
## 1 1001 OKI
## 2 1002 OKI
## 3 1003 OKI
## 4 1004 OKI
## 5 1005 OKI
## 6 1006 OKI
```

Add it to the matrix

```
fast25 <- fam_data |>
  dplyr::select(ind, pop) |>
  bind_cols(fast25)

head(fast25)
```

```
##    ind pop       X1    X2    X3    X4    X5    X6    X7    X8    X9      X10
## 1 1001 OKI 0.844082 1e-06 1e-06 1e-06 1e-06 1e-06 1e-06 1e-06 1e-06 0.069479
## 2 1002 OKI 0.732056 1e-06 1e-06 1e-06 1e-06 1e-06 1e-06 1e-06 1e-06 0.103982
## 3 1003 OKI 0.910566 1e-06 1e-06 1e-06 1e-06 1e-06 1e-06 1e-06 1e-06 0.000001
## 4 1004 OKI 0.926678 1e-06 1e-06 1e-06 1e-06 1e-06 1e-06 1e-06 1e-06 0.000001
## 5 1005 OKI 0.931821 1e-06 1e-06 1e-06 1e-06 1e-06 1e-06 1e-06 1e-06 0.000001
## 6 1006 OKI 0.862295 1e-06 1e-06 1e-06 1e-06 1e-06 1e-06 1e-06 1e-06 0.047355
##     X11   X12   X13   X14   X15   X16   X17   X18   X19      X20   X21   X22
## 1 1e-06 1e-06 1e-06 1e-06 1e-06 1e-06 1e-06 1e-06 1e-06 0.000001 1e-06 1e-06
## 2 1e-06 1e-06 1e-06 1e-06 1e-06 1e-06 1e-06 1e-06 1e-06 0.040550 1e-06 1e-06
## 3 1e-06 1e-06 1e-06 1e-06 1e-06 1e-06 1e-06 1e-06 1e-06 0.000001 1e-06 1e-06
## 4 1e-06 1e-06 1e-06 1e-06 1e-06 1e-06 1e-06 1e-06 1e-06 0.000001 1e-06 1e-06
## 5 1e-06 1e-06 1e-06 1e-06 1e-06 1e-06 1e-06 1e-06 1e-06 0.000001 1e-06 1e-06
## 6 1e-06 1e-06 1e-06 1e-06 1e-06 1e-06 1e-06 1e-06 1e-06 0.000001 1e-06 1e-06
##     X23   X24      X25
## 1 1e-06 1e-06 0.086417
## 2 1e-06 1e-06 0.123391
## 3 1e-06 1e-06 0.089411
## 4 1e-06 1e-06 0.073299
## 5 1e-06 1e-06 0.068156
## 6 1e-06 1e-06 0.090328
```

Rename the columns

```
# Rename the columns starting from the third one
fast25 <- fast25 |>
  rename_with(~paste0("v", seq_along(.x)), .cols = -c(ind, pop))

# View the first few rows
head(fast25)
```

```
##    ind pop       v1    v2    v3    v4    v5    v6    v7    v8    v9      v10
## 1 1001 OKI 0.844082 1e-06 1e-06 1e-06 1e-06 1e-06 1e-06 1e-06 1e-06 0.069479
## 2 1002 OKI 0.732056 1e-06 1e-06 1e-06 1e-06 1e-06 1e-06 1e-06 1e-06 0.103982
## 3 1003 OKI 0.910566 1e-06 1e-06 1e-06 1e-06 1e-06 1e-06 1e-06 1e-06 0.000001
## 4 1004 OKI 0.926678 1e-06 1e-06 1e-06 1e-06 1e-06 1e-06 1e-06 1e-06 0.000001
## 5 1005 OKI 0.931821 1e-06 1e-06 1e-06 1e-06 1e-06 1e-06 1e-06 1e-06 0.000001
## 6 1006 OKI 0.862295 1e-06 1e-06 1e-06 1e-06 1e-06 1e-06 1e-06 1e-06 0.047355
##     v11   v12   v13   v14   v15   v16   v17   v18   v19      v20   v21   v22
## 1 1e-06 1e-06 1e-06 1e-06 1e-06 1e-06 1e-06 1e-06 1e-06 0.000001 1e-06 1e-06
## 2 1e-06 1e-06 1e-06 1e-06 1e-06 1e-06 1e-06 1e-06 1e-06 0.040550 1e-06 1e-06
## 3 1e-06 1e-06 1e-06 1e-06 1e-06 1e-06 1e-06 1e-06 1e-06 0.000001 1e-06 1e-06
## 4 1e-06 1e-06 1e-06 1e-06 1e-06 1e-06 1e-06 1e-06 1e-06 0.000001 1e-06 1e-06
## 5 1e-06 1e-06 1e-06 1e-06 1e-06 1e-06 1e-06 1e-06 1e-06 0.000001 1e-06 1e-06
## 6 1e-06 1e-06 1e-06 1e-06 1e-06 1e-06 1e-06 1e-06 1e-06 0.000001 1e-06 1e-06
##     v23   v24      v25
## 1 1e-06 1e-06 0.086417
## 2 1e-06 1e-06 0.123391
## 3 1e-06 1e-06 0.089411
## 4 1e-06 1e-06 0.073299
## 5 1e-06 1e-06 0.068156
## 6 1e-06 1e-06 0.090328
```

Import Sample Locations

```
sampling_loc <- readRDS(here("scripts", "RMarkdowns", "output", "sampling_loc_all_pops.rds"))
head(sampling_loc)
```

```
##      Pop_City Country Latitude Longitude Continent Abbreviation  Year    Region
## 1      Hainan   China  19.2000  109.6000      Asia          HAI  2014 East Asia
## 2      Yunnan   China  24.4753  101.3431      Asia          YUN  2014 East Asia
## 3       Hunan   China  27.6253  111.8569      Asia          HUN  2001 East Asia
## 4     Okinawa   Japan  26.5013  127.9454      Asia          OKI 2018? East Asia
## 5 Tanegashima   Japan  30.4564  130.9784      Asia          TAN  2008 East Asia
## 6   Kagoshima   Japan  31.5602  130.5581      Asia          KAG  2018 East Asia
##   Subregion order order2 orderold AM_order state FINAL_order
## 1     Japan    55     47       47       NA                 1
## 2     Japan    56     48       48       NA                 2
## 3     Japan    57     49       49       NA                 3
## 4     Japan    54     46       46       NA                 4
## 5     Japan    NA     NA       NA       NA                 5
## 6     Japan    53     45       45       NA                 6
```

```
color_palette <-
  c(
    "orchid1",   
    "#FFE100",   
    "gray",    
    "#990000",     
    "magenta",   
    "#003380",   
    "orangered",    
    "cadetblue",   
    "blue",   
    "#9DCC00",    
    "cyan",    
    "#2BCE48",    
    "purple3",     
    "#005C31",    
    "#AC7700",   
    "#808080",    
    "#993F00",   
    "#FF8005",     
    "green",    
    "#8c61cd",      
    "#FFA8BB",      
    "#FFFC99",    
    "#4C005C",        
    "#FF0010",     
    "#1E90FF"    
  )
```

```
source(
  here("scripts", "RMarkdowns",
    "my_theme3.R"
  )
)

# Melt the data frame for plotting
Q_melted <- fast25 |>
  pivot_longer(
    cols = -c(ind, pop),
    names_to = "variable",
    values_to = "value"
  )
# Join with sampling_loc to get sampling localities
Q_joined <- Q_melted |>
  left_join(sampling_loc, by = c("pop" = "Abbreviation"))


# Order the combined variable by Region and Country, then by individual
Q_ordered <- Q_joined |>
  arrange(FINAL_order, ind) |>
  mutate(ind = factor(ind, levels = unique(ind)))  # Convert ind to a factor with levels in the desired order

# Add labels: country names for the first individual in each country, NA for all other individuals
Q_ordered <- Q_ordered |>
  group_by(Country) |>
  mutate(label = ifelse(row_number() == 1, as.character(Country), NA))

# Group by individual and variable, calculate mean ancestry proportions
Q_grouped <- Q_ordered |>
  group_by(ind, variable) |>
  summarise(value = mean(value), .groups = "drop")

# Create a data frame for borders
borders <-
  data.frame(Country = unique(Q_ordered$Country))

# Add the order of the last individual of each country to ensure correct placement of borders
borders$order <-
  sapply(borders$Country, function(rc)
    max(which(Q_ordered$Country == rc))) + 0.5  # Shift borders to the right edge of the bars

# Select only the first occurrence of each country in the ordered data
label_df <- Q_ordered |>
  filter(!is.na(label)) |>
  distinct(label, .keep_all = TRUE)

# Create a custom label function
label_func <- function(x) {
  labels <- rep("", length(x))
  labels[x %in% label_df$ind] <- label_df$label
  labels
}

# Calculate the position of lines
border_positions <- Q_ordered |>
  group_by(Country) |>
  summarise(pos = max(as.numeric(ind)) + 0)

# Calculate the position of population labels and bars
pop_labels <- Q_ordered |>
  mutate(Name = paste(pop, Pop_City, sep = " - ")) |>
  group_by(pop) |>
  slice_head(n = 1) |>
  ungroup() |>
  dplyr::select(ind, Pop_City, Country, Name) |>
  mutate(pos = as.numeric(ind))  # calculate position of population labels

pop_labels_bars <- pop_labels |>
  mutate(pos = as.numeric(ind)  - .5)


# Calculate the position of lines
border_positions <- Q_ordered |>
  group_by(Country) |>
  summarise(pos = max(as.numeric(ind)) - 1)


pop_labels_bars <- pop_labels |>
  mutate(pos = as.numeric(ind)  - .5)

# Function to filter and normalize data
normalize_data <- function(df, min_value) {
  df |>
    filter(value > min_value) |>
    group_by(ind) |>
    mutate(value = value / sum(value))
}

# Use the function
Q_grouped_filtered <- normalize_data(Q_grouped, 0.1)
# 


# Generate all potential variable names
all_variables <- paste0("v", 1:25)


# Create a data frame that pairs each potential variable with a color
color_mapping <- data.frame(variable = all_variables,
                          color = color_palette[1:length(all_variables)])

# Merge with Q_grouped_filtered
Q_grouped_filtered <- merge(Q_grouped_filtered, color_mapping, by = "variable")

# Create the plot
ggplot(Q_grouped_filtered, aes(x = as.factor(ind), y = value, fill = color)) +
  geom_bar(stat = 'identity', width = 1) +
  geom_vline(
    data = pop_labels_bars,
    aes(xintercept = pos),
    color = "#2C444A",
    linewidth = .2
  ) +
  geom_text(
    data = pop_labels,
    aes(x = as.numeric(ind), y = 1, label = Name),
    vjust = 1.5,
    hjust = 0,
    size = 2,
    angle = 90,
    inherit.aes = FALSE
  ) +
  my_theme() +
  theme(
    axis.text.x = element_text(
      angle = 90,
      hjust = 1,
      size = 12
    ),
    legend.position = "none",
    plot.margin = unit(c(3, 0.5, 0.5, 0.5), "cm")
  ) +
  xlab("Admixture matrix") +
  ylab("Ancestry proportions") +
  labs(caption = "Each bar represents the ancestry proportions for an individual for k=25.\n fastStructure inference for 20,825 SNPs.") +
  scale_x_discrete(labels = label_func) +
  scale_fill_manual(values = color_palette) +
  expand_limits(y = c(0, 1.5))
```

```
ggsave(
  here("scripts", "RMarkdowns", "output", "americas", "manuscript1", "fastStructure", "fastStructure_k=25_manuscript1.pdf"),
  width  = 16,
  height = 6,
  units  = "in",
  device = cairo_pdf
)
```

#### 3.2.2 K=27

Extract ancestry coefficients for k=27

```
fast27 <- read_delim(
  here("/gpfs/gibbs/pi/caccone/mkc54/albo/manuscript1/output/fastStructure/run095/simple.27.meanQ"),
  delim = "  ", # Specify the delimiter if different from the default (comma)
  col_names = FALSE,
  show_col_types = FALSE
) 
head(fast27)
```

```
## # A tibble: 6 × 27
##         X1    X2    X3    X4      X5    X6      X7      X8      X9     X10   X11
##      <dbl> <dbl> <dbl> <dbl>   <dbl> <dbl>   <dbl>   <dbl>   <dbl>   <dbl> <dbl>
## 1 0.000001  1e-6  1e-6  1e-6 3.79e-2  1e-6 5.20e-2 9.81e-3 1.44e-1 2.31e-1  1e-6
## 2 0.000001  1e-6  1e-6  1e-6 1   e-6  1e-6 1   e-6 1   e-6 1   e-6 3.38e-1  1e-6
## 3 0.000001  1e-6  1e-6  1e-6 1   e-6  1e-6 1   e-6 1   e-6 1   e-6 1   e-6  1e-6
## 4 0.000001  1e-6  1e-6  1e-6 1   e-6  1e-6 1   e-6 1   e-6 1   e-6 1   e-6  1e-6
## 5 0.000001  1e-6  1e-6  1e-6 1   e-6  1e-6 1   e-6 1   e-6 1   e-6 1   e-6  1e-6
## 6 0.000001  1e-6  1e-6  1e-6 1   e-6  1e-6 1   e-6 1   e-6 7.32e-2 1.52e-1  1e-6
## # ℹ 16 more variables: X12 <dbl>, X13 <dbl>, X14 <dbl>, X15 <dbl>, X16 <dbl>,
## #   X17 <dbl>, X18 <dbl>, X19 <dbl>, X20 <dbl>, X21 <dbl>, X22 <dbl>,
## #   X23 <dbl>, X24 <dbl>, X25 <dbl>, X26 <dbl>, X27 <dbl>
```

The fam file

```
fam_file <- here("manuscript1/output/snps_sets/r2_0.01_b.fam")

# Read the .fam file
fam_data <- read.table(fam_file, 
                       header = FALSE,
                       col.names = c("FamilyID", "IndividualID", "PaternalID", "MaternalID", "Sex", "Phenotype"))

# View the first few rows
head(fam_data)
```

```
##   FamilyID IndividualID PaternalID MaternalID Sex Phenotype
## 1      OKI         1001          0          0   2        -9
## 2      OKI         1002          0          0   2        -9
## 3      OKI         1003          0          0   2        -9
## 4      OKI         1004          0          0   2        -9
## 5      OKI         1005          0          0   2        -9
## 6      OKI         1006          0          0   1        -9
```

Create column ID

```
# Change column name
colnames(fam_data)[colnames(fam_data) == "IndividualID"] <- "ind"

# Change column name
colnames(fam_data)[colnames(fam_data) == "FamilyID"] <- "pop"

# Select ID
fam_data <- fam_data |>
  dplyr::select("ind", "pop")

# View the first few rows
head(fam_data)
```

```
##    ind pop
## 1 1001 OKI
## 2 1002 OKI
## 3 1003 OKI
## 4 1004 OKI
## 5 1005 OKI
## 6 1006 OKI
```

Add it to the matrix

```
fast27 <- fam_data |>
  dplyr::select(ind, pop) |>
  bind_cols(fast27)

head(fast27)
```

```
##    ind pop    X1    X2    X3    X4       X5    X6       X7       X8       X9
## 1 1001 OKI 1e-06 1e-06 1e-06 1e-06 0.037855 1e-06 0.051979 0.009812 0.143649
## 2 1002 OKI 1e-06 1e-06 1e-06 1e-06 0.000001 1e-06 0.000001 0.000001 0.000001
## 3 1003 OKI 1e-06 1e-06 1e-06 1e-06 0.000001 1e-06 0.000001 0.000001 0.000001
## 4 1004 OKI 1e-06 1e-06 1e-06 1e-06 0.000001 1e-06 0.000001 0.000001 0.000001
## 5 1005 OKI 1e-06 1e-06 1e-06 1e-06 0.000001 1e-06 0.000001 0.000001 0.000001
## 6 1006 OKI 1e-06 1e-06 1e-06 1e-06 0.000001 1e-06 0.000001 0.000001 0.073203
##        X10   X11   X12   X13   X14   X15      X16   X17      X18   X19      X20
## 1 0.230783 1e-06 1e-06 1e-06 1e-06 1e-06 0.000001 1e-06 0.000001 1e-06 0.525903
## 2 0.338142 1e-06 1e-06 1e-06 1e-06 1e-06 0.000001 1e-06 0.000001 1e-06 0.598897
## 3 0.000001 1e-06 1e-06 1e-06 1e-06 1e-06 0.040519 1e-06 0.274209 1e-06 0.685250
## 4 0.000001 1e-06 1e-06 1e-06 1e-06 1e-06 0.000001 1e-06 0.000001 1e-06 0.853847
## 5 0.000001 1e-06 1e-06 1e-06 1e-06 1e-06 0.000001 1e-06 0.000001 1e-06 0.999976
## 6 0.151671 1e-06 1e-06 1e-06 1e-06 1e-06 0.015622 1e-06 0.104201 1e-06 0.655282
##        X21   X22   X23   X24   X25      X26   X27
## 1 0.000001 1e-06 1e-06 1e-06 1e-06 0.000001 1e-06
## 2 0.000001 1e-06 1e-06 1e-06 1e-06 0.062939 1e-06
## 3 0.000001 1e-06 1e-06 1e-06 1e-06 0.000001 1e-06
## 4 0.146130 1e-06 1e-06 1e-06 1e-06 0.000001 1e-06
## 5 0.000001 1e-06 1e-06 1e-06 1e-06 0.000001 1e-06
## 6 0.000001 1e-06 1e-06 1e-06 1e-06 0.000001 1e-06
```

Rename the columns

```
# Rename the columns starting from the third one
fast27 <- fast27 |>
  rename_with(~paste0("v", seq_along(.x)), .cols = -c(ind, pop))

# View the first few rows
head(fast27)
```

```
##    ind pop    v1    v2    v3    v4       v5    v6       v7       v8       v9
## 1 1001 OKI 1e-06 1e-06 1e-06 1e-06 0.037855 1e-06 0.051979 0.009812 0.143649
## 2 1002 OKI 1e-06 1e-06 1e-06 1e-06 0.000001 1e-06 0.000001 0.000001 0.000001
## 3 1003 OKI 1e-06 1e-06 1e-06 1e-06 0.000001 1e-06 0.000001 0.000001 0.000001
## 4 1004 OKI 1e-06 1e-06 1e-06 1e-06 0.000001 1e-06 0.000001 0.000001 0.000001
## 5 1005 OKI 1e-06 1e-06 1e-06 1e-06 0.000001 1e-06 0.000001 0.000001 0.000001
## 6 1006 OKI 1e-06 1e-06 1e-06 1e-06 0.000001 1e-06 0.000001 0.000001 0.073203
##        v10   v11   v12   v13   v14   v15      v16   v17      v18   v19      v20
## 1 0.230783 1e-06 1e-06 1e-06 1e-06 1e-06 0.000001 1e-06 0.000001 1e-06 0.525903
## 2 0.338142 1e-06 1e-06 1e-06 1e-06 1e-06 0.000001 1e-06 0.000001 1e-06 0.598897
## 3 0.000001 1e-06 1e-06 1e-06 1e-06 1e-06 0.040519 1e-06 0.274209 1e-06 0.685250
## 4 0.000001 1e-06 1e-06 1e-06 1e-06 1e-06 0.000001 1e-06 0.000001 1e-06 0.853847
## 5 0.000001 1e-06 1e-06 1e-06 1e-06 1e-06 0.000001 1e-06 0.000001 1e-06 0.999976
## 6 0.151671 1e-06 1e-06 1e-06 1e-06 1e-06 0.015622 1e-06 0.104201 1e-06 0.655282
##        v21   v22   v23   v24   v25      v26   v27
## 1 0.000001 1e-06 1e-06 1e-06 1e-06 0.000001 1e-06
## 2 0.000001 1e-06 1e-06 1e-06 1e-06 0.062939 1e-06
## 3 0.000001 1e-06 1e-06 1e-06 1e-06 0.000001 1e-06
## 4 0.146130 1e-06 1e-06 1e-06 1e-06 0.000001 1e-06
## 5 0.000001 1e-06 1e-06 1e-06 1e-06 0.000001 1e-06
## 6 0.000001 1e-06 1e-06 1e-06 1e-06 0.000001 1e-06
```

Import Sample Locations

```
sampling_loc <- readRDS(here("scripts", "RMarkdowns", "output", "sampling_loc_all_pops.rds"))
head(sampling_loc)
```

```
##      Pop_City Country Latitude Longitude Continent Abbreviation  Year    Region
## 1      Hainan   China  19.2000  109.6000      Asia          HAI  2014 East Asia
## 2      Yunnan   China  24.4753  101.3431      Asia          YUN  2014 East Asia
## 3       Hunan   China  27.6253  111.8569      Asia          HUN  2001 East Asia
## 4     Okinawa   Japan  26.5013  127.9454      Asia          OKI 2018? East Asia
## 5 Tanegashima   Japan  30.4564  130.9784      Asia          TAN  2008 East Asia
## 6   Kagoshima   Japan  31.5602  130.5581      Asia          KAG  2018 East Asia
##   Subregion order order2 orderold AM_order state FINAL_order
## 1     Japan    55     47       47       NA                 1
## 2     Japan    56     48       48       NA                 2
## 3     Japan    57     49       49       NA                 3
## 4     Japan    54     46       46       NA                 4
## 5     Japan    NA     NA       NA       NA                 5
## 6     Japan    53     45       45       NA                 6
```

```
color_palette <-
  c(
    "#FF0010",    
    "#8c61cd", 
    "#4C005C",
    "#993F00",
    "purple3",
    "cyan",
    "blue",
    "orangered",
    "cadetblue",  
    "#9DCC00", 
    "magenta",
    "#FFFC99",    
    "#808080", 
    "#1E90FF", 
    "#005C31",
    "#003380",
    "green",
    "#AC7700", 
    "ivory",
    "#FFA8BB",  
    "#FF8005",  
    "#FFE100",
    "#990000",      
    "gray",
    "orchid1",
    "#2BCE48",
    "#B20CC9" )
```

```
source(
  here("scripts", "RMarkdowns",
    "my_theme3.R"
  )
)

# Melt the data frame for plotting
Q_melted <- fast27 |>
  pivot_longer(
    cols = -c(ind, pop),
    names_to = "variable",
    values_to = "value"
  )
# Join with sampling_loc to get sampling localities
Q_joined <- Q_melted |>
  left_join(sampling_loc, by = c("pop" = "Abbreviation"))


# Order the combined variable by Region and Country, then by individual
Q_ordered <- Q_joined |>
  arrange(FINAL_order, ind) |>
  mutate(ind = factor(ind, levels = unique(ind)))  # Convert ind to a factor with levels in the desired order

# Add labels: country names for the first individual in each country, NA for all other individuals
Q_ordered <- Q_ordered |>
  group_by(Country) |>
  mutate(label = ifelse(row_number() == 1, as.character(Country), NA))

# Group by individual and variable, calculate mean ancestry proportions
Q_grouped <- Q_ordered |>
  group_by(ind, variable) |>
  summarise(value = mean(value), .groups = "drop")

# Create a data frame for borders
borders <-
  data.frame(Country = unique(Q_ordered$Country))

# Add the order of the last individual of each country to ensure correct placement of borders
borders$order <-
  sapply(borders$Country, function(rc)
    max(which(Q_ordered$Country == rc))) + 0.5  # Shift borders to the right edge of the bars

# Select only the first occurrence of each country in the ordered data
label_df <- Q_ordered |>
  filter(!is.na(label)) |>
  distinct(label, .keep_all = TRUE)

# Create a custom label function
label_func <- function(x) {
  labels <- rep("", length(x))
  labels[x %in% label_df$ind] <- label_df$label
  labels
}

# Calculate the position of lines
border_positions <- Q_ordered |>
  group_by(Country) |>
  summarise(pos = max(as.numeric(ind)) + 0)

# Calculate the position of population labels and bars
pop_labels <- Q_ordered |>
  mutate(Name = paste(pop, Pop_City, sep = " - ")) |>
  group_by(pop) |>
  slice_head(n = 1) |>
  ungroup() |>
  dplyr::select(ind, Pop_City, Country, Name) |>
  mutate(pos = as.numeric(ind))  # calculate position of population labels

pop_labels_bars <- pop_labels |>
  mutate(pos = as.numeric(ind)  - .5)


# Calculate the position of lines
border_positions <- Q_ordered |>
  group_by(Country) |>
  summarise(pos = max(as.numeric(ind)) - 1)


pop_labels_bars <- pop_labels |>
  mutate(pos = as.numeric(ind)  - .5)

# Function to filter and normalize data
normalize_data <- function(df, min_value) {
  df |>
    filter(value > min_value) |>
    group_by(ind) |>
    mutate(value = value / sum(value))
}

# Use the function
Q_grouped_filtered <- normalize_data(Q_grouped, 0.1)
# 
color_palette <- c(
  "#B20CC9", 
  "#003380",
  "magenta",
  "#4C005C",  
  "#FFFC99",  
  "#1E90FF", 
  "green",
  "cadetblue",
  "#FFA8BB", 
  "#FFE100",  
  "cyan",
  "#AC7700",
  "orangered",
  "ivory",
  "#9DCC00", 
  "gray",
  "#993F00",
  "blue",
  "#FF0010", 
  "#8c61cd",
  "#005C31",
  "#2BCE48",   
  "#990000",     
  "#808080",  
  "orchid1",
  "#FF8005", 
  "purple3"
  )

# Generate all potential variable names
all_variables <- paste0("v", 1:27)


# Create a data frame that pairs each potential variable with a color
color_mapping <- data.frame(variable = all_variables,
                          color = color_palette[1:length(all_variables)])

# Merge with Q_grouped_filtered
Q_grouped_filtered <- merge(Q_grouped_filtered, color_mapping, by = "variable")

# Create the plot
ggplot(Q_grouped_filtered, aes(x = as.factor(ind), y = value, fill = color)) +
  geom_bar(stat = 'identity', width = 1) +
  geom_vline(
    data = pop_labels_bars,
    aes(xintercept = pos),
    color = "#2C444A",
    linewidth = .2
  ) +
  geom_text(
    data = pop_labels,
    aes(x = as.numeric(ind), y = 1, label = Name),
    vjust = 1.5,
    hjust = 0,
    size = 2,
    angle = 90,
    inherit.aes = FALSE
  ) +
  my_theme() +
  theme(
    axis.text.x = element_text(
      angle = 90,
      hjust = 1,
      size = 12
    ),
    legend.position = "none",
    plot.margin = unit(c(3, 0.5, 0.5, 0.5), "cm")
  ) +
  xlab("Admixture matrix") +
  ylab("Ancestry proportions") +
  labs(caption = "Each bar represents the ancestry proportions for an individual for k=27.\n fastStructure inference for 20,825 SNPs.") +
  scale_x_discrete(labels = label_func) +
  scale_fill_manual(values = color_palette) +
  expand_limits(y = c(0, 1.5))
```

```
ggsave(
  here("scripts", "RMarkdowns", "output", "americas", "manuscript1", "fastStructure", "fastStructure_k=27_manuscript1.pdf"),
  width  = 16,
  height = 6,
  units  = "in",
  device = cairo_pdf
)
```

#### 3.2.3 K=6

Extract ancestry coefficients for k=6

```
fast6 <- read_delim(
  here("/gpfs/gibbs/pi/caccone/mkc54/albo/manuscript1/output/fastStructure/run095/simple.6.meanQ"),
  delim = "  ", # Specify the delimiter if different from the default (comma)
  col_names = FALSE,
  show_col_types = FALSE
) 
head(fast6)
```

```
## # A tibble: 6 × 6
##         X1       X2       X3     X4       X5    X6
##      <dbl>    <dbl>    <dbl>  <dbl>    <dbl> <dbl>
## 1 0.0252   0.000004 0.000004 0.119  0.000004 0.856
## 2 0.0374   0.000004 0.000004 0.143  0.000004 0.820
## 3 0.0129   0.000004 0.00968  0.120  0.000004 0.857
## 4 0.000004 0.0458   0.000004 0.0666 0.000004 0.888
## 5 0.000004 0.000004 0.000004 0.0981 0.000004 0.902
## 6 0.0128   0.000004 0.0393   0.129  0.000004 0.819
```

The fam file

```
fam_file <- here("manuscript1/output/snps_sets/r2_0.01_b.fam")

# Read the .fam file
fam_data <- read.table(fam_file, 
                       header = FALSE,
                       col.names = c("FamilyID", "IndividualID", "PaternalID", "MaternalID", "Sex", "Phenotype"))

# View the first few rows
head(fam_data)
```

```
##   FamilyID IndividualID PaternalID MaternalID Sex Phenotype
## 1      OKI         1001          0          0   2        -9
## 2      OKI         1002          0          0   2        -9
## 3      OKI         1003          0          0   2        -9
## 4      OKI         1004          0          0   2        -9
## 5      OKI         1005          0          0   2        -9
## 6      OKI         1006          0          0   1        -9
```

Create column ID

```
# Change column name
colnames(fam_data)[colnames(fam_data) == "IndividualID"] <- "ind"

# Change column name
colnames(fam_data)[colnames(fam_data) == "FamilyID"] <- "pop"

# Select ID
fam_data <- fam_data |>
  dplyr::select("ind", "pop")

# View the first few rows
head(fam_data)
```

```
##    ind pop
## 1 1001 OKI
## 2 1002 OKI
## 3 1003 OKI
## 4 1004 OKI
## 5 1005 OKI
## 6 1006 OKI
```

Add it to the matrix

```
fast6 <- fam_data |>
  dplyr::select(ind, pop) |>
  bind_cols(fast6)

head(fast6)
```

```
##    ind pop       X1       X2       X3       X4    X5       X6
## 1 1001 OKI 0.025182 0.000004 0.000004 0.118735 4e-06 0.856070
## 2 1002 OKI 0.037366 0.000004 0.000004 0.143109 4e-06 0.819513
## 3 1003 OKI 0.012900 0.000004 0.009677 0.119955 4e-06 0.857460
## 4 1004 OKI 0.000004 0.045801 0.000004 0.066605 4e-06 0.887581
## 5 1005 OKI 0.000004 0.000004 0.000004 0.098072 4e-06 0.901911
## 6 1006 OKI 0.012825 0.000004 0.039329 0.129248 4e-06 0.818590
```

Rename the columns

```
# Rename the columns starting from the third one
fast6 <- fast6 |>
  rename_with(~paste0("v", seq_along(.x)), .cols = -c(ind, pop))

# View the first few rows
head(fast6)
```

```
##    ind pop       v1       v2       v3       v4    v5       v6
## 1 1001 OKI 0.025182 0.000004 0.000004 0.118735 4e-06 0.856070
## 2 1002 OKI 0.037366 0.000004 0.000004 0.143109 4e-06 0.819513
## 3 1003 OKI 0.012900 0.000004 0.009677 0.119955 4e-06 0.857460
## 4 1004 OKI 0.000004 0.045801 0.000004 0.066605 4e-06 0.887581
## 5 1005 OKI 0.000004 0.000004 0.000004 0.098072 4e-06 0.901911
## 6 1006 OKI 0.012825 0.000004 0.039329 0.129248 4e-06 0.818590
```

Import Sample Locations

```
sampling_loc <- readRDS(here("scripts", "RMarkdowns", "output", "sampling_loc_all_pops.rds"))
head(sampling_loc)
```

```
##      Pop_City Country Latitude Longitude Continent Abbreviation  Year    Region
## 1      Hainan   China  19.2000  109.6000      Asia          HAI  2014 East Asia
## 2      Yunnan   China  24.4753  101.3431      Asia          YUN  2014 East Asia
## 3       Hunan   China  27.6253  111.8569      Asia          HUN  2001 East Asia
## 4     Okinawa   Japan  26.5013  127.9454      Asia          OKI 2018? East Asia
## 5 Tanegashima   Japan  30.4564  130.9784      Asia          TAN  2008 East Asia
## 6   Kagoshima   Japan  31.5602  130.5581      Asia          KAG  2018 East Asia
##   Subregion order order2 orderold AM_order state FINAL_order
## 1     Japan    55     47       47       NA                 1
## 2     Japan    56     48       48       NA                 2
## 3     Japan    57     49       49       NA                 3
## 4     Japan    54     46       46       NA                 4
## 5     Japan    NA     NA       NA       NA                 5
## 6     Japan    53     45       45       NA                 6
```

```
source(
  here("scripts", "RMarkdowns",
    "my_theme3.R"
  )
)

# Melt the data frame for plotting
Q_melted <- fast6 |>
  pivot_longer(
    cols = -c(ind, pop),
    names_to = "variable",
    values_to = "value"
  )
# Join with sampling_loc to get sampling localities
Q_joined <- Q_melted |>
  left_join(sampling_loc, by = c("pop" = "Abbreviation"))


# Order the combined variable by Region and Country, then by individual
Q_ordered <- Q_joined |>
  arrange(FINAL_order, ind) |>
  mutate(ind = factor(ind, levels = unique(ind)))  # Convert ind to a factor with levels in the desired order

# Add labels: country names for the first individual in each country, NA for all other individuals
Q_ordered <- Q_ordered |>
  group_by(Country) |>
  mutate(label = ifelse(row_number() == 1, as.character(Country), NA))

# Group by individual and variable, calculate mean ancestry proportions
Q_grouped <- Q_ordered |>
  group_by(ind, variable) |>
  summarise(value = mean(value), .groups = "drop")

# Create a data frame for borders
borders <-
  data.frame(Country = unique(Q_ordered$Country))

# Add the order of the last individual of each country to ensure correct placement of borders
borders$order <-
  sapply(borders$Country, function(rc)
    max(which(Q_ordered$Country == rc))) + 0.5  # Shift borders to the right edge of the bars

# Select only the first occurrence of each country in the ordered data
label_df <- Q_ordered |>
  filter(!is.na(label)) |>
  distinct(label, .keep_all = TRUE)

# Create a custom label function
label_func <- function(x) {
  labels <- rep("", length(x))
  labels[x %in% label_df$ind] <- label_df$label
  labels
}

# Calculate the position of lines
border_positions <- Q_ordered |>
  group_by(Country) |>
  summarise(pos = max(as.numeric(ind)) + 0)

# Calculate the position of population labels and bars
pop_labels <- Q_ordered |>
  mutate(Name = paste(pop, Pop_City, sep = " - ")) |>
  group_by(pop) |>
  slice_head(n = 1) |>
  ungroup() |>
  dplyr::select(ind, Pop_City, Country, Name) |>
  mutate(pos = as.numeric(ind))  # calculate position of population labels

pop_labels_bars <- pop_labels |>
  mutate(pos = as.numeric(ind)  - .5)


# Calculate the position of lines
border_positions <- Q_ordered |>
  group_by(Country) |>
  summarise(pos = max(as.numeric(ind)) - 1)


pop_labels_bars <- pop_labels |>
  mutate(pos = as.numeric(ind)  - .5)

# Function to filter and normalize data
normalize_data <- function(df, min_value) {
  df |>
    filter(value > min_value) |>
    group_by(ind) |>
    mutate(value = value / sum(value))
}

# Use the function
Q_grouped_filtered <- normalize_data(Q_grouped, 0.1)
# 

color_palette <-
c( 
    "magenta",
    "green",
    "#FFE100",    
    "#FF8005",
    "purple3",
    "#FF0010"   ) 


# Generate all potential variable names
all_variables <- paste0("v", 1:6)


# Create a data frame that pairs each potential variable with a color
color_mapping <- data.frame(variable = all_variables,
                           color = color_palette[1:length(all_variables)])

# Merge with Q_grouped_filtered
Q_grouped_filtered <- merge(Q_grouped_filtered, color_mapping, by = "variable")

# Create the plot
ggplot(Q_grouped_filtered, aes(x = as.factor(ind), y = value, fill = color)) +
  geom_bar(stat = 'identity', width = 1) +
  geom_vline(
    data = pop_labels_bars,
    aes(xintercept = pos),
    color = "#2C444A",
    linewidth = .2
  ) +
  geom_text(
    data = pop_labels,
    aes(x = as.numeric(ind), y = 1, label = Name),
    vjust = 1.5,
    hjust = 0,
    size = 2,
    angle = 90,
    inherit.aes = FALSE
  ) +
  my_theme() +
  theme(
    axis.text.x = element_text(
      angle = 90,
      hjust = 1,
      size = 12
    ),
    legend.position = "none",
    plot.margin = unit(c(3, 0.5, 0.5, 0.5), "cm")
  ) +
  xlab("Admixture matrix") +
  ylab("Ancestry proportions") +
  labs(caption = "Each bar represents the ancestry proportions for an individual for k=6.\n fastStructure inference for 20,825 SNPs.") +
  scale_x_discrete(labels = label_func) +
  scale_fill_manual(values = color_palette) +
  expand_limits(y = c(0, 1.5))
```

```
ggsave(
  here("scripts", "RMarkdowns", "output", "americas", "manuscript1", "fastStructure", "fastStructure_k=6_manuscript1.pdf"),
  width  = 16,
  height = 6,
  units  = "in",
  device = cairo_pdf
)
```

#### 3.2.4 K=7

Extract ancestry coefficients for k=7

```
fast7 <- read_delim(
  here("/gpfs/gibbs/pi/caccone/mkc54/albo/manuscript1/output/fastStructure/run095/simple.7.meanQ"),
  delim = "  ", # Specify the delimiter if different from the default (comma)
  col_names = FALSE,
  show_col_types = FALSE
) 
head(fast7)
```

```
## # A tibble: 6 × 7
##      X1       X2       X3       X4       X5       X6       X7
##   <dbl>    <dbl>    <dbl>    <dbl>    <dbl>    <dbl>    <dbl>
## 1 0.891 0.000004 0.0600   0.0488   0.000004 0.000004 0.000004
## 2 0.782 0.0691   0.000004 0.0377   0.000004 0.111    0.000004
## 3 0.817 0.000004 0.000004 0.000004 0.000004 0.0733   0.109   
## 4 0.727 0.000004 0.000004 0.000004 0.000004 0.0506   0.223   
## 5 0.737 0.000004 0.000004 0.000004 0.000004 0.0496   0.214   
## 6 0.757 0.000004 0.000004 0.0146   0.000004 0.0816   0.147
```

The fam file

```
fam_file <- here("manuscript1/output/snps_sets/r2_0.01_b.fam")

# Read the .fam file
fam_data <- read.table(fam_file, 
                       header = FALSE,
                       col.names = c("FamilyID", "IndividualID", "PaternalID", "MaternalID", "Sex", "Phenotype"))

# View the first few rows
head(fam_data)
```

```
##   FamilyID IndividualID PaternalID MaternalID Sex Phenotype
## 1      OKI         1001          0          0   2        -9
## 2      OKI         1002          0          0   2        -9
## 3      OKI         1003          0          0   2        -9
## 4      OKI         1004          0          0   2        -9
## 5      OKI         1005          0          0   2        -9
## 6      OKI         1006          0          0   1        -9
```

Create column ID

```
# Change column name
colnames(fam_data)[colnames(fam_data) == "IndividualID"] <- "ind"

# Change column name
colnames(fam_data)[colnames(fam_data) == "FamilyID"] <- "pop"

# Select ID
fam_data <- fam_data |>
  dplyr::select("ind", "pop")

# View the first few rows
head(fam_data)
```

```
##    ind pop
## 1 1001 OKI
## 2 1002 OKI
## 3 1003 OKI
## 4 1004 OKI
## 5 1005 OKI
## 6 1006 OKI
```

Add it to the matrix

```
fast7 <- fam_data |>
  dplyr::select(ind, pop) |>
  bind_cols(fast7)

head(fast7)
```

```
##    ind pop       X1       X2       X3       X4    X5       X6       X7
## 1 1001 OKI 0.891224 0.000004 0.059978 0.048783 4e-06 0.000004 0.000004
## 2 1002 OKI 0.782013 0.069091 0.000004 0.037676 4e-06 0.111210 0.000004
## 3 1003 OKI 0.817254 0.000004 0.000004 0.000004 4e-06 0.073308 0.109425
## 4 1004 OKI 0.726599 0.000004 0.000004 0.000004 4e-06 0.050586 0.222801
## 5 1005 OKI 0.736735 0.000004 0.000004 0.000004 4e-06 0.049628 0.213623
## 6 1006 OKI 0.757141 0.000004 0.000004 0.014596 4e-06 0.081608 0.146644
```

Rename the columns

```
# Rename the columns starting from the third one
fast7 <- fast7 |>
  rename_with(~paste0("v", seq_along(.x)), .cols = -c(ind, pop))

# View the first few rows
head(fast7)
```

```
##    ind pop       v1       v2       v3       v4    v5       v6       v7
## 1 1001 OKI 0.891224 0.000004 0.059978 0.048783 4e-06 0.000004 0.000004
## 2 1002 OKI 0.782013 0.069091 0.000004 0.037676 4e-06 0.111210 0.000004
## 3 1003 OKI 0.817254 0.000004 0.000004 0.000004 4e-06 0.073308 0.109425
## 4 1004 OKI 0.726599 0.000004 0.000004 0.000004 4e-06 0.050586 0.222801
## 5 1005 OKI 0.736735 0.000004 0.000004 0.000004 4e-06 0.049628 0.213623
## 6 1006 OKI 0.757141 0.000004 0.000004 0.014596 4e-06 0.081608 0.146644
```

Import Sample Locations

```
sampling_loc <- readRDS(here("scripts", "RMarkdowns", "output", "sampling_loc_all_pops.rds"))
head(sampling_loc)
```

```
##      Pop_City Country Latitude Longitude Continent Abbreviation  Year    Region
## 1      Hainan   China  19.2000  109.6000      Asia          HAI  2014 East Asia
## 2      Yunnan   China  24.4753  101.3431      Asia          YUN  2014 East Asia
## 3       Hunan   China  27.6253  111.8569      Asia          HUN  2001 East Asia
## 4     Okinawa   Japan  26.5013  127.9454      Asia          OKI 2018? East Asia
## 5 Tanegashima   Japan  30.4564  130.9784      Asia          TAN  2008 East Asia
## 6   Kagoshima   Japan  31.5602  130.5581      Asia          KAG  2018 East Asia
##   Subregion order order2 orderold AM_order state FINAL_order
## 1     Japan    55     47       47       NA                 1
## 2     Japan    56     48       48       NA                 2
## 3     Japan    57     49       49       NA                 3
## 4     Japan    54     46       46       NA                 4
## 5     Japan    NA     NA       NA       NA                 5
## 6     Japan    53     45       45       NA                 6
```

```
source(
  here("scripts", "RMarkdowns",
    "my_theme3.R"
  )
)

# Melt the data frame for plotting
Q_melted <- fast7 |>
  pivot_longer(
    cols = -c(ind, pop),
    names_to = "variable",
    values_to = "value"
  )
# Join with sampling_loc to get sampling localities
Q_joined <- Q_melted |>
  left_join(sampling_loc, by = c("pop" = "Abbreviation"))


# Order the combined variable by Region and Country, then by individual
Q_ordered <- Q_joined |>
  arrange(FINAL_order, ind) |>
  mutate(ind = factor(ind, levels = unique(ind)))  # Convert ind to a factor with levels in the desired order

# Add labels: country names for the first individual in each country, NA for all other individuals
Q_ordered <- Q_ordered |>
  group_by(Country) |>
  mutate(label = ifelse(row_number() == 1, as.character(Country), NA))

# Group by individual and variable, calculate mean ancestry proportions
Q_grouped <- Q_ordered |>
  group_by(ind, variable) |>
  summarise(value = mean(value), .groups = "drop")

# Create a data frame for borders
borders <-
  data.frame(Country = unique(Q_ordered$Country))

# Add the order of the last individual of each country to ensure correct placement of borders
borders$order <-
  sapply(borders$Country, function(rc)
    max(which(Q_ordered$Country == rc))) + 0.5  # Shift borders to the right edge of the bars

# Select only the first occurrence of each country in the ordered data
label_df <- Q_ordered |>
  filter(!is.na(label)) |>
  distinct(label, .keep_all = TRUE)

# Create a custom label function
label_func <- function(x) {
  labels <- rep("", length(x))
  labels[x %in% label_df$ind] <- label_df$label
  labels
}

# Calculate the position of lines
border_positions <- Q_ordered |>
  group_by(Country) |>
  summarise(pos = max(as.numeric(ind)) + 0)

# Calculate the position of population labels and bars
pop_labels <- Q_ordered |>
  mutate(Name = paste(pop, Pop_City, sep = " - ")) |>
  group_by(pop) |>
  slice_head(n = 1) |>
  ungroup() |>
  dplyr::select(ind, Pop_City, Country, Name) |>
  mutate(pos = as.numeric(ind))  # calculate position of population labels

pop_labels_bars <- pop_labels |>
  mutate(pos = as.numeric(ind)  - .5)


# Calculate the position of lines
border_positions <- Q_ordered |>
  group_by(Country) |>
  summarise(pos = max(as.numeric(ind)) - 1)


pop_labels_bars <- pop_labels |>
  mutate(pos = as.numeric(ind)  - .5)

# Function to filter and normalize data
normalize_data <- function(df, min_value) {
  df |>
    filter(value > min_value) |>
    group_by(ind) |>
    mutate(value = value / sum(value))
}

# Use the function
Q_grouped_filtered <- normalize_data(Q_grouped, 0.1)
# 

color_palette <-
  c( 
    "purple3",
    "#FFE100",
    "#FF0010", 
    "green",
    "#FF8005",
    "magenta",
    "blue" 
    ) 

    
# Generate all potential variable names
all_variables <- paste0("v", 1:7)


# Create a data frame that pairs each potential variable with a color
color_mapping <- data.frame(variable = all_variables,
                           color = color_palette[1:length(all_variables)])

# Merge with Q_grouped_filtered
Q_grouped_filtered <- merge(Q_grouped_filtered, color_mapping, by = "variable")

# Create the plot
ggplot(Q_grouped_filtered, aes(x = as.factor(ind), y = value, fill = color)) +
  geom_bar(stat = 'identity', width = 1) +
  geom_vline(
    data = pop_labels_bars,
    aes(xintercept = pos),
    color = "#2C444A",
    linewidth = .2
  ) +
  geom_text(
    data = pop_labels,
    aes(x = as.numeric(ind), y = 1, label = Name),
    vjust = 1.5,
    hjust = 0,
    size = 2,
    angle = 90,
    inherit.aes = FALSE
  ) +
  my_theme() +
  theme(
    axis.text.x = element_text(
      angle = 90,
      hjust = 1,
      size = 12
    ),
    legend.position = "none",
    plot.margin = unit(c(3, 0.5, 0.5, 0.5), "cm")
  ) +
  xlab("Admixture matrix") +
  ylab("Ancestry proportions") +
  labs(caption = "Each bar represents the ancestry proportions for an individual for k=7.\n fastStructure inference for 20,825 SNPs.") +
  scale_x_discrete(labels = label_func) +
  scale_fill_manual(values = color_palette) +
  expand_limits(y = c(0, 1.5))
```

```
ggsave(
  here("scripts", "RMarkdowns", "output", "americas", "manuscript1", "fastStructure", "fastStructure_k=7_manuscript1.pdf"),
  width  = 16,
  height = 6,
  units  = "in",
  device = cairo_pdf
)
```

#### 3.2.5 K=8

Extract ancestry coefficients for k=8

```
fast8 <- read_delim(
  here("/gpfs/gibbs/pi/caccone/mkc54/albo/manuscript1/output/fastStructure/run095/simple.8.meanQ"),
  delim = "  ", # Specify the delimiter if different from the default (comma)
  col_names = FALSE,
  show_col_types = FALSE
) 
head(fast8)
```

```
## # A tibble: 6 × 8
##         X1    X2       X3       X4     X5       X6       X7       X8
##      <dbl> <dbl>    <dbl>    <dbl>  <dbl>    <dbl>    <dbl>    <dbl>
## 1 0.000003 0.764 0.0267   0.000003 0.0566 0.0613   0.0773   0.0140  
## 2 0.000003 0.723 0.0290   0.000003 0.0976 0.0878   0.0626   0.000003
## 3 0.000003 0.825 0.000003 0.0251   0.0743 0.0753   0.000003 0.000003
## 4 0.000003 0.725 0.00208  0.000003 0.0252 0.000003 0.235    0.0131  
## 5 0.000003 0.735 0.000427 0.000003 0.0251 0.000003 0.232    0.00803 
## 6 0.000003 0.774 0.00612  0.000003 0.0699 0.0438   0.106    0.000003
```

The fam file

```
fam_file <- here("manuscript1/output/snps_sets/r2_0.01_b.fam")

# Read the .fam file
fam_data <- read.table(fam_file, 
                       header = FALSE,
                       col.names = c("FamilyID", "IndividualID", "PaternalID", "MaternalID", "Sex", "Phenotype"))

# View the first few rows
head(fam_data)
```

```
##   FamilyID IndividualID PaternalID MaternalID Sex Phenotype
## 1      OKI         1001          0          0   2        -9
## 2      OKI         1002          0          0   2        -9
## 3      OKI         1003          0          0   2        -9
## 4      OKI         1004          0          0   2        -9
## 5      OKI         1005          0          0   2        -9
## 6      OKI         1006          0          0   1        -9
```

Create column ID

```
# Change column name
colnames(fam_data)[colnames(fam_data) == "IndividualID"] <- "ind"

# Change column name
colnames(fam_data)[colnames(fam_data) == "FamilyID"] <- "pop"

# Select ID
fam_data <- fam_data |>
  dplyr::select("ind", "pop")

# View the first few rows
head(fam_data)
```

```
##    ind pop
## 1 1001 OKI
## 2 1002 OKI
## 3 1003 OKI
## 4 1004 OKI
## 5 1005 OKI
## 6 1006 OKI
```

Add it to the matrix

```
fast8 <- fam_data |>
  dplyr::select(ind, pop) |>
  bind_cols(fast8)

head(fast8)
```

```
##    ind pop    X1       X2       X3       X4       X5       X6       X7       X8
## 1 1001 OKI 3e-06 0.764070 0.026748 0.000003 0.056618 0.061337 0.077266 0.013954
## 2 1002 OKI 3e-06 0.722951 0.029049 0.000003 0.097634 0.087777 0.062579 0.000003
## 3 1003 OKI 3e-06 0.825349 0.000003 0.025123 0.074255 0.075261 0.000003 0.000003
## 4 1004 OKI 3e-06 0.724841 0.002078 0.000003 0.025224 0.000003 0.234783 0.013064
## 5 1005 OKI 3e-06 0.734592 0.000427 0.000003 0.025096 0.000003 0.231849 0.008027
## 6 1006 OKI 3e-06 0.774395 0.006116 0.000003 0.069875 0.043758 0.105846 0.000003
```

Rename the columns

```
# Rename the columns starting from the third one
fast8 <- fast8 |>
  rename_with(~paste0("v", seq_along(.x)), .cols = -c(ind, pop))

# View the first few rows
head(fast8)
```

```
##    ind pop    v1       v2       v3       v4       v5       v6       v7       v8
## 1 1001 OKI 3e-06 0.764070 0.026748 0.000003 0.056618 0.061337 0.077266 0.013954
## 2 1002 OKI 3e-06 0.722951 0.029049 0.000003 0.097634 0.087777 0.062579 0.000003
## 3 1003 OKI 3e-06 0.825349 0.000003 0.025123 0.074255 0.075261 0.000003 0.000003
## 4 1004 OKI 3e-06 0.724841 0.002078 0.000003 0.025224 0.000003 0.234783 0.013064
## 5 1005 OKI 3e-06 0.734592 0.000427 0.000003 0.025096 0.000003 0.231849 0.008027
## 6 1006 OKI 3e-06 0.774395 0.006116 0.000003 0.069875 0.043758 0.105846 0.000003
```

Import Sample Locations

```
sampling_loc <- readRDS(here("scripts", "RMarkdowns", "output", "sampling_loc_all_pops.rds"))
head(sampling_loc)
```

```
##      Pop_City Country Latitude Longitude Continent Abbreviation  Year    Region
## 1      Hainan   China  19.2000  109.6000      Asia          HAI  2014 East Asia
## 2      Yunnan   China  24.4753  101.3431      Asia          YUN  2014 East Asia
## 3       Hunan   China  27.6253  111.8569      Asia          HUN  2001 East Asia
## 4     Okinawa   Japan  26.5013  127.9454      Asia          OKI 2018? East Asia
## 5 Tanegashima   Japan  30.4564  130.9784      Asia          TAN  2008 East Asia
## 6   Kagoshima   Japan  31.5602  130.5581      Asia          KAG  2018 East Asia
##   Subregion order order2 orderold AM_order state FINAL_order
## 1     Japan    55     47       47       NA                 1
## 2     Japan    56     48       48       NA                 2
## 3     Japan    57     49       49       NA                 3
## 4     Japan    54     46       46       NA                 4
## 5     Japan    NA     NA       NA       NA                 5
## 6     Japan    53     45       45       NA                 6
```

```
source(
  here("scripts", "RMarkdowns",
    "my_theme3.R"
  )
)

# Melt the data frame for plotting
Q_melted <- fast8 |>
  pivot_longer(
    cols = -c(ind, pop),
    names_to = "variable",
    values_to = "value"
  )
# Join with sampling_loc to get sampling localities
Q_joined <- Q_melted |>
  left_join(sampling_loc, by = c("pop" = "Abbreviation"))


# Order the combined variable by Region and Country, then by individual
Q_ordered <- Q_joined |>
  arrange(FINAL_order, ind) |>
  mutate(ind = factor(ind, levels = unique(ind)))  # Convert ind to a factor with levels in the desired order

# Add labels: country names for the first individual in each country, NA for all other individuals
Q_ordered <- Q_ordered |>
  group_by(Country) |>
  mutate(label = ifelse(row_number() == 1, as.character(Country), NA))

# Group by individual and variable, calculate mean ancestry proportions
Q_grouped <- Q_ordered |>
  group_by(ind, variable) |>
  summarise(value = mean(value), .groups = "drop")

# Create a data frame for borders
borders <-
  data.frame(Country = unique(Q_ordered$Country))

# Add the order of the last individual of each country to ensure correct placement of borders
borders$order <-
  sapply(borders$Country, function(rc)
    max(which(Q_ordered$Country == rc))) + 0.5  # Shift borders to the right edge of the bars

# Select only the first occurrence of each country in the ordered data
label_df <- Q_ordered |>
  filter(!is.na(label)) |>
  distinct(label, .keep_all = TRUE)

# Create a custom label function
label_func <- function(x) {
  labels <- rep("", length(x))
  labels[x %in% label_df$ind] <- label_df$label
  labels
}

# Calculate the position of lines
border_positions <- Q_ordered |>
  group_by(Country) |>
  summarise(pos = max(as.numeric(ind)) + 0)

# Calculate the position of population labels and bars
pop_labels <- Q_ordered |>
  mutate(Name = paste(pop, Pop_City, sep = " - ")) |>
  group_by(pop) |>
  slice_head(n = 1) |>
  ungroup() |>
  dplyr::select(ind, Pop_City, Country, Name) |>
  mutate(pos = as.numeric(ind))  # calculate position of population labels

pop_labels_bars <- pop_labels |>
  mutate(pos = as.numeric(ind)  - .5)


# Calculate the position of lines
border_positions <- Q_ordered |>
  group_by(Country) |>
  summarise(pos = max(as.numeric(ind)) - 1)


pop_labels_bars <- pop_labels |>
  mutate(pos = as.numeric(ind)  - .5)

# Function to filter and normalize data
normalize_data <- function(df, min_value) {
  df |>
    filter(value > min_value) |>
    group_by(ind) |>
    mutate(value = value / sum(value))
}

# Use the function
Q_grouped_filtered <- normalize_data(Q_grouped, 0.1)
# 

color_palette <-
  c( 
    "magenta",
    "cyan",
    "#FF0010", 
    "green",
    "#FF8005",      
    "#FFE100",
    "blue",    
    "purple3"
    ) 

    
# Generate all potential variable names
all_variables <- paste0("v", 1:8)


# Create a data frame that pairs each potential variable with a color
color_mapping <- data.frame(variable = all_variables,
                           color = color_palette[1:length(all_variables)])

# Merge with Q_grouped_filtered
Q_grouped_filtered <- merge(Q_grouped_filtered, color_mapping, by = "variable")

# Create the plot
ggplot(Q_grouped_filtered, aes(x = as.factor(ind), y = value, fill = color)) +
  geom_bar(stat = 'identity', width = 1) +
  geom_vline(
    data = pop_labels_bars,
    aes(xintercept = pos),
    color = "#2C444A",
    linewidth = .2
  ) +
  geom_text(
    data = pop_labels,
    aes(x = as.numeric(ind), y = 1, label = Name),
    vjust = 1.5,
    hjust = 0,
    size = 2,
    angle = 90,
    inherit.aes = FALSE
  ) +
  my_theme() +
  theme(
    axis.text.x = element_text(
      angle = 90,
      hjust = 1,
      size = 12
    ),
    legend.position = "none",
    plot.margin = unit(c(3, 0.5, 0.5, 0.5), "cm")
  ) +
  xlab("Admixture matrix") +
  ylab("Ancestry proportions") +
  labs(caption = "Each bar represents the ancestry proportions for an individual for k=8.\n fastStructure inference for 20,825 SNPs.") +
  scale_x_discrete(labels = label_func) +
  scale_fill_manual(values = color_palette) +
  expand_limits(y = c(0, 1.5))
```

```
ggsave(
  here("scripts", "RMarkdowns", "output", "americas", "manuscript1", "fastStructure", "fastStructure_k=8_manuscript1.pdf"),
  width  = 16,
  height = 6,
  units  = "in",
  device = cairo_pdf
)
```

#### 3.2.6 K=9

Extract ancestry coefficients for k=9

```
fast9 <- read_delim(
  here("/gpfs/gibbs/pi/caccone/mkc54/albo/manuscript1/output/fastStructure/run095/simple.9.meanQ"),
  delim = "  ", # Specify the delimiter if different from the default (comma)
  col_names = FALSE,
  show_col_types = FALSE
) 
head(fast9)
```

```
## # A tibble: 6 × 9
##         X1       X2       X3       X4    X5       X6       X7       X8       X9
##      <dbl>    <dbl>    <dbl>    <dbl> <dbl>    <dbl>    <dbl>    <dbl>    <dbl>
## 1 0.000003 0.000003 0.000003 0.000003 0.928 0.000003 0.000003 0.0292   0.0424  
## 2 0.000003 0.000003 0.000003 0.000003 0.841 0.0229   0.104    0.0328   0.000003
## 3 0.000003 0.000003 0.000003 0.000003 0.967 0.0203   0.000003 0.0127   0.000003
## 4 0.000003 0.000003 0.000003 0.000003 0.964 0.000003 0.000003 0.000205 0.0363  
## 5 0.000003 0.000003 0.000003 0.000003 1.00  0.000003 0.000003 0.000003 0.000003
## 6 0.000003 0.000003 0.000003 0.000003 0.957 0.0259   0.000003 0.0175   0.000003
```

The fam file

```
fam_file <- here("manuscript1/output/snps_sets/r2_0.01_b.fam")

# Read the .fam file
fam_data <- read.table(fam_file, 
                       header = FALSE,
                       col.names = c("FamilyID", "IndividualID", "PaternalID", "MaternalID", "Sex", "Phenotype"))

# View the first few rows
head(fam_data)
```

```
##   FamilyID IndividualID PaternalID MaternalID Sex Phenotype
## 1      OKI         1001          0          0   2        -9
## 2      OKI         1002          0          0   2        -9
## 3      OKI         1003          0          0   2        -9
## 4      OKI         1004          0          0   2        -9
## 5      OKI         1005          0          0   2        -9
## 6      OKI         1006          0          0   1        -9
```

Create column ID

```
# Change column name
colnames(fam_data)[colnames(fam_data) == "IndividualID"] <- "ind"

# Change column name
colnames(fam_data)[colnames(fam_data) == "FamilyID"] <- "pop"

# Select ID
fam_data <- fam_data |>
  dplyr::select("ind", "pop")

# View the first few rows
head(fam_data)
```

```
##    ind pop
## 1 1001 OKI
## 2 1002 OKI
## 3 1003 OKI
## 4 1004 OKI
## 5 1005 OKI
## 6 1006 OKI
```

Add it to the matrix

```
fast9 <- fam_data |>
  dplyr::select(ind, pop) |>
  bind_cols(fast9)

head(fast9)
```

```
##    ind pop    X1    X2    X3    X4       X5       X6       X7       X8       X9
## 1 1001 OKI 3e-06 3e-06 3e-06 3e-06 0.928370 0.000003 0.000003 0.029208 0.042405
## 2 1002 OKI 3e-06 3e-06 3e-06 3e-06 0.840580 0.022882 0.103694 0.032830 0.000003
## 3 1003 OKI 3e-06 3e-06 3e-06 3e-06 0.966979 0.020345 0.000003 0.012660 0.000003
## 4 1004 OKI 3e-06 3e-06 3e-06 3e-06 0.963509 0.000003 0.000003 0.000205 0.036270
## 5 1005 OKI 3e-06 3e-06 3e-06 3e-06 0.999978 0.000003 0.000003 0.000003 0.000003
## 6 1006 OKI 3e-06 3e-06 3e-06 3e-06 0.956582 0.025924 0.000003 0.017478 0.000003
```

Rename the columns

```
# Rename the columns starting from the third one
fast9 <- fast9 |>
  rename_with(~paste0("v", seq_along(.x)), .cols = -c(ind, pop))

# View the first few rows
head(fast9)
```

```
##    ind pop    v1    v2    v3    v4       v5       v6       v7       v8       v9
## 1 1001 OKI 3e-06 3e-06 3e-06 3e-06 0.928370 0.000003 0.000003 0.029208 0.042405
## 2 1002 OKI 3e-06 3e-06 3e-06 3e-06 0.840580 0.022882 0.103694 0.032830 0.000003
## 3 1003 OKI 3e-06 3e-06 3e-06 3e-06 0.966979 0.020345 0.000003 0.012660 0.000003
## 4 1004 OKI 3e-06 3e-06 3e-06 3e-06 0.963509 0.000003 0.000003 0.000205 0.036270
## 5 1005 OKI 3e-06 3e-06 3e-06 3e-06 0.999978 0.000003 0.000003 0.000003 0.000003
## 6 1006 OKI 3e-06 3e-06 3e-06 3e-06 0.956582 0.025924 0.000003 0.017478 0.000003
```

Import Sample Locations

```
sampling_loc <- readRDS(here("scripts", "RMarkdowns", "output", "sampling_loc_all_pops.rds"))
head(sampling_loc)
```

```
##      Pop_City Country Latitude Longitude Continent Abbreviation  Year    Region
## 1      Hainan   China  19.2000  109.6000      Asia          HAI  2014 East Asia
## 2      Yunnan   China  24.4753  101.3431      Asia          YUN  2014 East Asia
## 3       Hunan   China  27.6253  111.8569      Asia          HUN  2001 East Asia
## 4     Okinawa   Japan  26.5013  127.9454      Asia          OKI 2018? East Asia
## 5 Tanegashima   Japan  30.4564  130.9784      Asia          TAN  2008 East Asia
## 6   Kagoshima   Japan  31.5602  130.5581      Asia          KAG  2018 East Asia
##   Subregion order order2 orderold AM_order state FINAL_order
## 1     Japan    55     47       47       NA                 1
## 2     Japan    56     48       48       NA                 2
## 3     Japan    57     49       49       NA                 3
## 4     Japan    54     46       46       NA                 4
## 5     Japan    NA     NA       NA       NA                 5
## 6     Japan    53     45       45       NA                 6
```

```
source(
  here("scripts", "RMarkdowns",
    "my_theme3.R"
  )
)

# Melt the data frame for plotting
Q_melted <- fast9 |>
  pivot_longer(
    cols = -c(ind, pop),
    names_to = "variable",
    values_to = "value"
  )
# Join with sampling_loc to get sampling localities
Q_joined <- Q_melted |>
  left_join(sampling_loc, by = c("pop" = "Abbreviation"))


# Order the combined variable by Region and Country, then by individual
Q_ordered <- Q_joined |>
  arrange(FINAL_order, ind) |>
  mutate(ind = factor(ind, levels = unique(ind)))  # Convert ind to a factor with levels in the desired order

# Add labels: country names for the first individual in each country, NA for all other individuals
Q_ordered <- Q_ordered |>
  group_by(Country) |>
  mutate(label = ifelse(row_number() == 1, as.character(Country), NA))

# Group by individual and variable, calculate mean ancestry proportions
Q_grouped <- Q_ordered |>
  group_by(ind, variable) |>
  summarise(value = mean(value), .groups = "drop")

# Create a data frame for borders
borders <-
  data.frame(Country = unique(Q_ordered$Country))

# Add the order of the last individual of each country to ensure correct placement of borders
borders$order <-
  sapply(borders$Country, function(rc)
    max(which(Q_ordered$Country == rc))) + 0.5  # Shift borders to the right edge of the bars

# Select only the first occurrence of each country in the ordered data
label_df <- Q_ordered |>
  filter(!is.na(label)) |>
  distinct(label, .keep_all = TRUE)

# Create a custom label function
label_func <- function(x) {
  labels <- rep("", length(x))
  labels[x %in% label_df$ind] <- label_df$label
  labels
}

# Calculate the position of lines
border_positions <- Q_ordered |>
  group_by(Country) |>
  summarise(pos = max(as.numeric(ind)) + 0)

# Calculate the position of population labels and bars
pop_labels <- Q_ordered |>
  mutate(Name = paste(pop, Pop_City, sep = " - ")) |>
  group_by(pop) |>
  slice_head(n = 1) |>
  ungroup() |>
  dplyr::select(ind, Pop_City, Country, Name) |>
  mutate(pos = as.numeric(ind))  # calculate position of population labels

pop_labels_bars <- pop_labels |>
  mutate(pos = as.numeric(ind)  - .5)


# Calculate the position of lines
border_positions <- Q_ordered |>
  group_by(Country) |>
  summarise(pos = max(as.numeric(ind)) - 1)


pop_labels_bars <- pop_labels |>
  mutate(pos = as.numeric(ind)  - .5)

# Function to filter and normalize data
normalize_data <- function(df, min_value) {
  df |>
    filter(value > min_value) |>
    group_by(ind) |>
    mutate(value = value / sum(value))
}

# Use the function
Q_grouped_filtered <- normalize_data(Q_grouped, 0.1)
# 

color_palette <-
  c( 
    "#FFE100",  
    "#990000",
    "green", 
    "cyan",
    "purple3", 
    "#FF0010",    
    "blue",
    "#FF8005",     
    "magenta"  
    ) 

    
# Generate all potential variable names
all_variables <- paste0("v", 1:9)


# Create a data frame that pairs each potential variable with a color
color_mapping <- data.frame(variable = all_variables,
                          color = color_palette[1:length(all_variables)])

# Merge with Q_grouped_filtered
Q_grouped_filtered <- merge(Q_grouped_filtered, color_mapping, by = "variable")

# Create the plot
ggplot(Q_grouped_filtered, aes(x = as.factor(ind), y = value, fill = color)) +
  geom_bar(stat = 'identity', width = 1) +
  geom_vline(
    data = pop_labels_bars,
    aes(xintercept = pos),
    color = "#2C444A",
    linewidth = .2
  ) +
  geom_text(
    data = pop_labels,
    aes(x = as.numeric(ind), y = 1, label = Name),
    vjust = 1.5,
    hjust = 0,
    size = 2,
    angle = 90,
    inherit.aes = FALSE
  ) +
  my_theme() +
  theme(
    axis.text.x = element_text(
      angle = 90,
      hjust = 1,
      size = 12
    ),
    legend.position = "none",
    plot.margin = unit(c(3, 0.5, 0.5, 0.5), "cm")
  ) +
  xlab("Admixture matrix") +
  ylab("Ancestry proportions") +
  labs(caption = "Each bar represents the ancestry proportions for an individual for k=9.\n fastStructure inference for 20,825 SNPs.") +
  scale_x_discrete(labels = label_func) +
  scale_fill_manual(values = color_palette) +
  expand_limits(y = c(0, 1.5))
```

```
ggsave(
  here("scripts", "RMarkdowns", "output", "americas", "manuscript1", "fastStructure", "fastStructure_k=9_run95_manuscript1.pdf"),
  width  = 16,
  height = 6,
  units  = "in",
  device = cairo_pdf
)
```

#### 3.2.7 K=10

Extract ancestry coefficients for k=10

```
fast10 <- read_delim(
  here("/gpfs/gibbs/pi/caccone/mkc54/albo/manuscript1/output/fastStructure/run095/simple.10.meanQ"),
  delim = "  ", # Specify the delimiter if different from the default (comma)
  col_names = FALSE,
  show_col_types = FALSE
) 
head(fast10)
```

```
## # A tibble: 6 × 10
##         X1       X2       X3       X4     X5    X6      X7      X8    X9     X10
##      <dbl>    <dbl>    <dbl>    <dbl>  <dbl> <dbl>   <dbl>   <dbl> <dbl>   <dbl>
## 1 0.000002 0.000002 0.000002 0.000002   2e-6  2e-6 1.23e-2 2   e-6 0.946 4.18e-2
## 2 0.000002 0.0362   0.000002 0.000002   2e-6  2e-6 2   e-6 9.49e-2 0.869 2   e-6
## 3 0.000002 0.0156   0.000002 0.000002   2e-6  2e-6 2.49e-2 2   e-6 0.959 2   e-6
## 4 0.235    0.000002 0.000002 0.000002   2e-6  2e-6 2   e-6 2   e-6 0.765 2   e-6
## 5 0.217    0.000002 0.000002 0.000002   2e-6  2e-6 2   e-6 2   e-6 0.783 2   e-6
## 6 0.000002 0.00674  0.000002 0.000002   2e-6  2e-6 4.36e-2 2   e-6 0.907 4.28e-2
```

The fam file

```
fam_file <- here("manuscript1/output/snps_sets/r2_0.01_b.fam")

# Read the .fam file
fam_data <- read.table(fam_file, 
                       header = FALSE,
                       col.names = c("FamilyID", "IndividualID", "PaternalID", "MaternalID", "Sex", "Phenotype"))

# View the first few rows
head(fam_data)
```

```
##   FamilyID IndividualID PaternalID MaternalID Sex Phenotype
## 1      OKI         1001          0          0   2        -9
## 2      OKI         1002          0          0   2        -9
## 3      OKI         1003          0          0   2        -9
## 4      OKI         1004          0          0   2        -9
## 5      OKI         1005          0          0   2        -9
## 6      OKI         1006          0          0   1        -9
```

Create column ID

```
# Change column name
colnames(fam_data)[colnames(fam_data) == "IndividualID"] <- "ind"

# Change column name
colnames(fam_data)[colnames(fam_data) == "FamilyID"] <- "pop"

# Select ID
fam_data <- fam_data |>
  dplyr::select("ind", "pop")

# View the first few rows
head(fam_data)
```

```
##    ind pop
## 1 1001 OKI
## 2 1002 OKI
## 3 1003 OKI
## 4 1004 OKI
## 5 1005 OKI
## 6 1006 OKI
```

Add it to the matrix

```
fast10 <- fam_data |>
  dplyr::select(ind, pop) |>
  bind_cols(fast10)
```

Rename the columns

```
# Rename the columns starting from the third one
fast10 <- fast10 |>
  rename_with(~paste0("v", seq_along(.x)), .cols = -c(ind, pop))
```

Import Sample Locations

```
sampling_loc <- readRDS(here("scripts", "RMarkdowns", "output", "sampling_loc_all_pops.rds"))
head(sampling_loc)
```

```
##      Pop_City Country Latitude Longitude Continent Abbreviation  Year    Region
## 1      Hainan   China  19.2000  109.6000      Asia          HAI  2014 East Asia
## 2      Yunnan   China  24.4753  101.3431      Asia          YUN  2014 East Asia
## 3       Hunan   China  27.6253  111.8569      Asia          HUN  2001 East Asia
## 4     Okinawa   Japan  26.5013  127.9454      Asia          OKI 2018? East Asia
## 5 Tanegashima   Japan  30.4564  130.9784      Asia          TAN  2008 East Asia
## 6   Kagoshima   Japan  31.5602  130.5581      Asia          KAG  2018 East Asia
##   Subregion order order2 orderold AM_order state FINAL_order
## 1     Japan    55     47       47       NA                 1
## 2     Japan    56     48       48       NA                 2
## 3     Japan    57     49       49       NA                 3
## 4     Japan    54     46       46       NA                 4
## 5     Japan    NA     NA       NA       NA                 5
## 6     Japan    53     45       45       NA                 6
```

```
source(
  here("scripts", "RMarkdowns",
    "my_theme3.R"
  )
)

# Melt the data frame for plotting
Q_melted <- fast10 |>
  pivot_longer(
    cols = -c(ind, pop),
    names_to = "variable",
    values_to = "value"
  )
# Join with sampling_loc to get sampling localities
Q_joined <- Q_melted |>
  left_join(sampling_loc, by = c("pop" = "Abbreviation"))


# Order the combined variable by Region and Country, then by individual
Q_ordered <- Q_joined |>
  arrange(FINAL_order, ind) |>
  mutate(ind = factor(ind, levels = unique(ind)))  # Convert ind to a factor with levels in the desired order

# Add labels: country names for the first individual in each country, NA for all other individuals
Q_ordered <- Q_ordered |>
  group_by(Country) |>
  mutate(label = ifelse(row_number() == 1, as.character(Country), NA))

# Group by individual and variable, calculate mean ancestry proportions
Q_grouped <- Q_ordered |>
  group_by(ind, variable) |>
  summarise(value = mean(value), .groups = "drop")

# Create a data frame for borders
borders <-
  data.frame(Country = unique(Q_ordered$Country))

# Add the order of the last individual of each country to ensure correct placement of borders
borders$order <-
  sapply(borders$Country, function(rc)
    max(which(Q_ordered$Country == rc))) + 0.5  # Shift borders to the right edge of the bars

# Select only the first occurrence of each country in the ordered data
label_df <- Q_ordered |>
  filter(!is.na(label)) |>
  distinct(label, .keep_all = TRUE)

# Create a custom label function
label_func <- function(x) {
  labels <- rep("", length(x))
  labels[x %in% label_df$ind] <- label_df$label
  labels
}

# Calculate the position of lines
border_positions <- Q_ordered |>
  group_by(Country) |>
  summarise(pos = max(as.numeric(ind)) + 0)

# Calculate the position of population labels and bars
pop_labels <- Q_ordered |>
  mutate(Name = paste(pop, Pop_City, sep = " - ")) |>
  group_by(pop) |>
  slice_head(n = 1) |>
  ungroup() |>
  dplyr::select(ind, Pop_City, Country, Name) |>
  mutate(pos = as.numeric(ind))  # calculate position of population labels

pop_labels_bars <- pop_labels |>
  mutate(pos = as.numeric(ind)  - .5)


# Calculate the position of lines
border_positions <- Q_ordered |>
  group_by(Country) |>
  summarise(pos = max(as.numeric(ind)) - 1)


pop_labels_bars <- pop_labels |>
  mutate(pos = as.numeric(ind)  - .5)

# Function to filter and normalize data
normalize_data <- function(df, min_value) {
  df |>
    filter(value > min_value) |>
    group_by(ind) |>
    mutate(value = value / sum(value))
}

# Use the function
Q_grouped_filtered <- normalize_data(Q_grouped, 0.1)
# 

color_palette <-
  c(
    "purple3",
    "#FFE100",  
    "#FF0010",
    "magenta", 
    "#990000",     
    "#005C31", 
    "cyan", 
    "#FF8005",
    "green",
    "blue"    
      ) 

  
    
# Generate all potential variable names
all_variables <- paste0("v", 1:10)


# Create a data frame that pairs each potential variable with a color
color_mapping <- data.frame(variable = all_variables,
                         color = color_palette[1:length(all_variables)])

# Merge with Q_grouped_filtered
Q_grouped_filtered <- merge(Q_grouped_filtered, color_mapping, by = "variable")

# Create the plot
ggplot(Q_grouped_filtered, aes(x = as.factor(ind), y = value, fill = color)) +
  geom_bar(stat = 'identity', width = 1) +
  geom_vline(
    data = pop_labels_bars,
    aes(xintercept = pos),
    color = "#2C444A",
    linewidth = .2
  ) +
  geom_text(
    data = pop_labels,
    aes(x = as.numeric(ind), y = 1, label = Name),
    vjust = 1.5,
    hjust = 0,
    size = 2,
    angle = 90,
    inherit.aes = FALSE
  ) +
  my_theme() +
  theme(
    axis.text.x = element_text(
      angle = 90,
      hjust = 1,
      size = 12
    ),
    legend.position = "none",
    plot.margin = unit(c(3, 0.5, 0.5, 0.5), "cm")
  ) +
  xlab("Admixture matrix") +
  ylab("Ancestry proportions") +
  labs(caption = "Each bar represents the ancestry proportions for an individual for k=10.\n fastStructure inference for 20,825 SNPs.") +
  scale_x_discrete(labels = label_func) +
  scale_fill_manual(values = color_palette) +
  expand_limits(y = c(0, 1.5))
```

```
ggsave(
  here("scripts", "RMarkdowns", "output", "americas", "manuscript1", "fastStructure", "fastStructure_k=10_run95_manuscript1.pdf"),
  width  = 16,
  height = 6,
  units  = "in",
  device = cairo_pdf
)
```

# 4. DAPC

Load libraries

```
library(ade4)
library(adegenet)
library(MASS)        # load BEFORE tidyverse so tidyverse wins the conflicts
library(ape)         # same — load before tidyverse
library(reshape2)    # same

# Now load tidyverse — it will take priority over the above
library(tidyverse)

# Tidyverse-adjacent packages
library(here)
library(dartR)
library(dartR.base)
library(StAMPP)
library(geosphere)

# Table/output packages
library(flextable)   # only need to load once
library(officer)     # only need to load once

# Cairo last (it's a graphics device, not a data package)
library(Cairo)
```

## 4.1. Create files & get sample locations

```
module load PLINK/1.9b_6.21-x86_64

cd /gpfs/gibbs/pi/caccone/mkc54/albo/manuscript1

plink \
--allow-extra-chr \
--keep-allele-order \
--bfile output/snps_sets/r2_0.01_b \
--make-bed \
--out output/dapc/dapc_r01_b \
--silent;
grep 'samples\|variants\|remaining' output/dapc/dapc_r01_b.log
```

20825 variants loaded from .bim file. 20825 variants and 933 people
pass filters and QC.

Convert to raw format

```
plink \
--allow-extra-chr \
--keep-allele-order \
--bfile output/dapc/dapc_r01_b \
--recodeA \
--out output/dapc/dapc_r01_b \
--silent;
grep 'samples\|variants\|remaining' output/dapc/dapc_r01_b.log
```

20825 variants loaded from .bim file. 20825 variants and 933 people
pass filters and QC.

Import the data and covert it to genind format

```
# import the data
snp <-
  read.PLINK(
    here("manuscript1/output/dapc/dapc_r01_b.raw"),
    quiet = FALSE,
    chunkSize = 1000,
    parallel = require("parallel"),
    n.cores = 4
  )
```

```
## 
##  Reading PLINK raw format into a genlight object...
```

```
## Loading required package: parallel
```

```
## 
##  Reading loci information... 
## 
##  Reading and converting genotypes... 
## .
##  Building final object... 
## 
## ...done.
```

```
nInd(snp)
```

```
## [1] 933
```

```
nLoc(snp)
```

```
## [1] 20825
```

```
nPop(snp)
```

```
## [1] 93
```

```
indNames(snp)
```

```
##   [1] "1001" "1002" "1003" "1004" "1005" "1006" "1007" "1008" "1009" "1011"
##  [11] "1012" "1053" "1054" "1055" "1056" "1057" "1058" "1059" "1060" "1061"
##  [21] "1062" "1063" "1064" "1065" "1066" "1067" "1068" "1069" "1070" "1071"
##  [31] "1072" "1073" "1074" "1075" "1076" "1089" "1090" "1091" "1092" "1093"
##  [41] "1094" "1095" "1101" "1102" "1103" "1105" "1106" "1107" "1109" "1110"
##  [51] "1111" "1112" "1113" "1114" "1115" "1116" "1117" "1118" "1119" "1120"
##  [61] "1137" "1138" "1139" "1140" "1141" "1142" "1143" "1144" "1145" "1146"
##  [71] "1147" "1148" "1149" "1150" "1151" "1152" "1153" "1154" "1155" "1156"
##  [81] "1157" "1158" "1159" "1160" "1161" "1162" "1163" "1165" "1166" "1167"
##  [91] "1168" "1169" "1170" "1171" "1172" "1173" "1174" "1175" "1176" "1177"
## [101] "1178" "1179" "1180" "1181" "1182" "1183" "1184" "1214" "1215" "1216"
## [111] "1217" "1238" "1239" "1240" "1241" "1242" "1243" "1244" "1245" "1246"
## [121] "1247" "1249" "1250" "1251" "1252" "1253" "1254" "1255" "1256" "1257"
## [131] "1258" "1259" "1260" "1261" "1262" "1263" "1264" "1265" "1266" "1267"
## [141] "1268" "1270" "1271" "1272" "1273" "1274" "1276" "1282" "1283" "1285"
## [151] "1286" "1287" "1288" "1289" "1292" "1293" "1294" "1295" "1325" "1326"
## [161] "1328" "1329" "1330" "1331" "1332" "1333" "1334" "1335" "1336" "1373"
## [171] "1374" "1375" "1376" "1377" "1378" "1379" "1380" "1381" "1382" "1383"
## [181] "1384" "1430" "1431" "1432" "1433" "1434" "1435" "1436" "1437" "1438"
## [191] "1439" "1440" "1441" "1443" "1444" "1446" "1447" "1449" "1451" "1452"
## [201] "1453" "1454" "1456" "1458" "1460" "1461" "153"  "154"  "155"  "156" 
## [211] "158"  "16"   "174"  "175"  "176"  "177"  "178"  "17"   "180"  "181" 
## [221] "182"  "183"  "184"  "185"  "186"  "187"  "188"  "189"  "18"   "190" 
## [231] "191"  "2158" "2159" "2160" "2161" "2162" "2163" "2164" "2165" "2166"
## [241] "2167" "2168" "2169" "2170" "2171" "2172" "2173" "2174" "2175" "2176"
## [251] "2177" "2178" "2179" "217"  "2180" "2181" "2182" "2183" "2184" "2185"
## [261] "2187" "2188" "2189" "218"  "2191" "2192" "2193" "2194" "2195" "219" 
## [271] "2203" "2204" "2205" "2206" "2207" "2208" "2209" "220"  "2210" "2211"
## [281] "2212" "2213" "2214" "2215" "2216" "2217" "2218" "221"  "2221" "2222"
## [291] "2223" "2224" "2225" "2226" "2227" "2228" "2229" "222"  "2230" "2231"
## [301] "2232" "2233" "2234" "2235" "2236" "2237" "2238" "2239" "223"  "2240"
## [311] "2241" "2242" "2243" "2244" "2245" "2246" "2247" "2248" "2249" "224" 
## [321] "2250" "2251" "2252" "2253" "2254" "2255" "2256" "2257" "2258" "2259"
## [331] "225"  "2260" "2261" "2262" "2263" "2264" "2265" "2266" "2267" "2268"
## [341] "2269" "226"  "2270" "2271" "2272" "2273" "2275" "2288" "2289" "228" 
## [351] "2290" "2291" "2292" "2293" "2294" "2295" "2301" "2302" "2303" "2304"
## [361] "2305" "2306" "2307" "2308" "2309" "230"  "2310" "2311" "2312" "2313"
## [371] "2314" "2315" "2316" "2317" "2318" "2319" "2320" "2321" "2322" "2323"
## [381] "2324" "2326" "2327" "2329" "2330" "2331" "2332" "2333" "2334" "2335"
## [391] "2336" "2337" "2338" "2339" "2340" "2341" "2342" "2343" "2344" "2345"
## [401] "2346" "2347" "2348" "2349" "2350" "2351" "2352" "2353" "2354" "2355"
## [411] "2357" "2358" "2360" "2362" "2384" "2385" "2386" "2387" "2388" "2390"
## [421] "2391" "2392" "2393" "2394" "2401" "2402" "2403" "2404" "2405" "2406"
## [431] "2407" "2408" "2409" "2410" "2411" "2412" "2413" "2414" "2415" "2416"
## [441] "2417" "2418" "2419" "2420" "2421" "2422" "2423" "2424" "2425" "2426"
## [451] "2427" "2428" "2429" "2430" "2431" "2432" "2433" "2434" "2435" "2436"
## [461] "2437" "2438" "2439" "2440" "2441" "2442" "2443" "2444" "2445" "2446"
## [471] "2447" "2448" "2449" "2450" "2451" "2452" "2453" "2454" "2455" "2456"
## [481] "2457" "2458" "2459" "2461" "2462" "2463" "2464" "2465" "2466" "2467"
## [491] "2468" "2469" "2470" "2471" "2472" "2473" "2474" "2475" "2476" "2477"
## [501] "2479" "2480" "2481" "2482" "2483" "2484" "2485" "2486" "2487" "2488"
## [511] "2489" "2490" "2491" "2492" "2493" "2494" "2495" "2501" "2502" "2503"
## [521] "2504" "2505" "2506" "2507" "2508" "2509" "2510" "2511" "2512" "2513"
## [531] "2514" "2515" "2516" "2517" "2518" "2519" "2520" "2521" "2522" "2523"
## [541] "2524" "2525" "2526" "2527" "2528" "2529" "2530" "2531" "2532" "2533"
## [551] "2534" "2535" "2536" "2537" "2538" "2539" "2540" "2541" "2542" "2543"
## [561] "2544" "2545" "2546" "2547" "2548" "2550" "2551" "2552" "2553" "2554"
## [571] "255"  "2561" "2562" "2564" "2565" "2566" "2567" "2568" "2569" "256" 
## [581] "2570" "2571" "2572" "2573" "2574" "2575" "2576" "2577" "2578" "2579"
## [591] "257"  "2580" "2581" "2582" "2583" "2584" "2585" "2586" "2587" "2588"
## [601] "2589" "2591" "2592" "2595" "259"  "261"  "262"  "263"  "264"  "265" 
## [611] "266"  "267"  "268"  "269"  "270"  "271"  "272"  "273"  "275"  "276" 
## [621] "277"  "278"  "294"  "295"  "296"  "297"  "298"  "299"  "301"  "302" 
## [631] "303"  "304"  "305"  "306"  "307"  "308"  "309"  "310"  "311"  "312" 
## [641] "313"  "314"  "315"  "316"  "317"  "318"  "319"  "320"  "321"  "322" 
## [651] "323"  "324"  "325"  "326"  "327"  "328"  "329"  "330"  "331"  "332" 
## [661] "333"  "334"  "335"  "336"  "337"  "340"  "341"  "342"  "343"  "344" 
## [671] "345"  "346"  "347"  "348"  "349"  "350"  "351"  "352"  "353"  "354" 
## [681] "355"  "356"  "357"  "358"  "359"  "360"  "361"  "362"  "363"  "364" 
## [691] "366"  "368"  "369"  "370"  "371"  "372"  "373"  "374"  "375"  "376" 
## [701] "377"  "378"  "379"  "380"  "381"  "382"  "383"  "413"  "414"  "415" 
## [711] "416"  "417"  "418"  "419"  "420"  "421"  "422"  "424"  "425"  "426" 
## [721] "427"  "428"  "429"  "430"  "431"  "432"  "433"  "434"  "435"  "436" 
## [731] "437"  "438"  "439"  "440"  "441"  "442"  "443"  "444"  "445"  "446" 
## [741] "447"  "448"  "449"  "450"  "451"  "452"  "453"  "454"  "455"  "456" 
## [751] "457"  "458"  "459"  "460"  "461"  "463"  "464"  "466"  "467"  "468" 
## [761] "469"  "470"  "472"  "473"  "474"  "475"  "476"  "477"  "478"  "602" 
## [771] "603"  "604"  "607"  "609"  "60"   "610"  "611"  "612"  "613"  "614" 
## [781] "615"  "616"  "617"  "618"  "619"  "620"  "621"  "622"  "623"  "624" 
## [791] "625"  "626"  "627"  "628"  "629"  "630"  "631"  "632"  "633"  "634" 
## [801] "635"  "636"  "637"  "638"  "639"  "640"  "641"  "642"  "643"  "644" 
## [811] "645"  "64"   "666"  "669"  "670"  "671"  "672"  "673"  "674"  "675" 
## [821] "676"  "677"  "678"  "679"  "680"  "681"  "683"  "684"  "685"  "686" 
## [831] "687"  "688"  "689"  "690"  "691"  "692"  "693"  "694"  "695"  "723" 
## [841] "724"  "725"  "726"  "727"  "728"  "729"  "730"  "731"  "732"  "733" 
## [851] "784"  "785"  "786"  "787"  "788"  "789"  "790"  "791"  "792"  "793" 
## [861] "794"  "795"  "801"  "802"  "803"  "804"  "805"  "806"  "807"  "808" 
## [871] "809"  "810"  "811"  "812"  "813"  "814"  "815"  "816"  "817"  "818" 
## [881] "819"  "820"  "821"  "822"  "861"  "862"  "863"  "864"  "883"  "884" 
## [891] "885"  "886"  "887"  "888"  "889"  "890"  "891"  "892"  "893"  "894" 
## [901] "8"    "911"  "912"  "913"  "914"  "964"  "965"  "966"  "967"  "972" 
## [911] "973"  "975"  "976"  "977"  "978"  "979"  "980"  "981"  "982"  "983" 
## [921] "984"  "985"  "986"  "987"  "988"  "989"  "990"  "991"  "992"  "993" 
## [931] "994"  "995"  "9"
```

```
# convert to genind
snp2 <- gl2gi(snp, probar = TRUE, verbose = NULL)
```

```
## Starting gl2gi 
##   Processing genlight object with SNP data
##   |                                                                              |                                                                      |   0%  |                                                                              |                                                                      |   1%  |                                                                              |=                                                                     |   1%  |                                                                              |=                                                                     |   2%  |                                                                              |==                                                                    |   2%  |                                                                              |==                                                                    |   3%  |                                                                              |==                                                                    |   4%  |                                                                              |===                                                                   |   4%  |                                                                              |===                                                                   |   5%  |                                                                              |====                                                                  |   5%  |                                                                              |====                                                                  |   6%  |                                                                              |=====                                                                 |   6%  |                                                                              |=====                                                                 |   7%  |                                                                              |=====                                                                 |   8%  |                                                                              |======                                                                |   8%  |                                                                              |======                                                                |   9%  |                                                                              |=======                                                               |   9%  |                                                                              |=======                                                               |  10%  |                                                                              |=======                                                               |  11%  |                                                                              |========                                                              |  11%  |                                                                              |========                                                              |  12%  |                                                                              |=========                                                             |  12%  |                                                                              |=========                                                             |  13%  |                                                                              |=========                                                             |  14%  |                                                                              |==========                                                            |  14%  |                                                                              |==========                                                            |  15%  |                                                                              |===========                                                           |  15%  |                                                                              |===========                                                           |  16%  |                                                                              |============                                                          |  17%  |                                                                              |============                                                          |  18%  |                                                                              |=============                                                         |  18%  |                                                                              |=============                                                         |  19%  |                                                                              |==============                                                        |  19%  |                                                                              |==============                                                        |  20%  |                                                                              |==============                                                        |  21%  |                                                                              |===============                                                       |  21%  |                                                                              |===============                                                       |  22%  |                                                                              |================                                                      |  22%  |                                                                              |================                                                      |  23%  |                                                                              |=================                                                     |  24%  |                                                                              |=================                                                     |  25%  |                                                                              |==================                                                    |  25%  |                                                                              |==================                                                    |  26%  |                                                                              |===================                                                   |  26%  |                                                                              |===================                                                   |  27%  |                                                                              |===================                                                   |  28%  |                                                                              |====================                                                  |  28%  |                                                                              |====================                                                  |  29%  |                                                                              |=====================                                                 |  29%  |                                                                              |=====================                                                 |  30%  |                                                                              |=====================                                                 |  31%  |                                                                              |======================                                                |  31%  |                                                                              |======================                                                |  32%  |                                                                              |=======================                                               |  32%  |                                                                              |=======================                                               |  33%  |                                                                              |=======================                                               |  34%  |                                                                              |========================                                              |  34%  |                                                                              |========================                                              |  35%  |                                                                              |=========================                                             |  35%  |                                                                              |=========================                                             |  36%  |                                                                              |==========================                                            |  36%  |                                                                              |==========================                                            |  37%  |                                                                              |==========================                                            |  38%  |                                                                              |===========================                                           |  38%  |                                                                              |===========================                                           |  39%  |                                                                              |============================                                          |  39%  |                                                                              |============================                                          |  40%  |                                                                              |============================                                          |  41%  |                                                                              |=============================                                         |  41%  |                                                                              |=============================                                         |  42%  |                                                                              |==============================                                        |  42%  |                                                                              |==============================                                        |  43%  |                                                                              |==============================                                        |  44%  |                                                                              |===============================                                       |  44%  |                                                                              |===============================                                       |  45%  |                                                                              |================================                                      |  45%  |                                                                              |================================                                      |  46%  |                                                                              |=================================                                     |  47%  |                                                                              |=================================                                     |  48%  |                                                                              |==================================                                    |  48%  |                                                                              |==================================                                    |  49%  |                                                                              |===================================                                   |  49%  |                                                                              |===================================                                   |  50%  |                                                                              |===================================                                   |  51%  |                                                                              |====================================                                  |  51%  |                                                                              |====================================                                  |  52%  |                                                                              |=====================================                                 |  52%  |                                                                              |=====================================                                 |  53%  |                                                                              |======================================                                |  54%  |                                                                              |======================================                                |  55%  |                                                                              |=======================================                               |  55%  |                                                                              |=======================================                               |  56%  |                                                                              |========================================                              |  56%  |                                                                              |========================================                              |  57%  |                                                                              |========================================                              |  58%  |                                                                              |=========================================                             |  58%  |                                                                              |=========================================                             |  59%  |                                                                              |==========================================                            |  59%  |                                                                              |==========================================                            |  60%  |                                                                              |==========================================                            |  61%  |                                                                              |===========================================                           |  61%  |                                                                              |===========================================                           |  62%  |                                                                              |============================================                          |  62%  |                                                                              |============================================                          |  63%  |                                                                              |============================================                          |  64%  |                                                                              |=============================================                         |  64%  |                                                                              |=============================================                         |  65%  |                                                                              |==============================================                        |  65%  |                                                                              |==============================================                        |  66%  |                                                                              |===============================================                       |  66%  |                                                                              |===============================================                       |  67%  |                                                                              |===============================================                       |  68%  |                                                                              |================================================                      |  68%  |                                                                              |================================================                      |  69%  |                                                                              |=================================================                     |  69%  |                                                                              |=================================================                     |  70%  |                                                                              |=================================================                     |  71%  |                                                                              |==================================================                    |  71%  |                                                                              |==================================================                    |  72%  |                                                                              |===================================================                   |  72%  |                                                                              |===================================================                   |  73%  |                                                                              |===================================================                   |  74%  |                                                                              |====================================================                  |  74%  |                                                                              |====================================================                  |  75%  |                                                                              |=====================================================                 |  75%  |                                                                              |=====================================================                 |  76%  |                                                                              |======================================================                |  77%  |                                                                              |======================================================                |  78%  |                                                                              |=======================================================               |  78%  |                                                                              |=======================================================               |  79%  |                                                                              |========================================================              |  79%  |                                                                              |========================================================              |  80%  |                                                                              |========================================================              |  81%  |                                                                              |=========================================================             |  81%  |                                                                              |=========================================================             |  82%  |                                                                              |==========================================================            |  82%  |                                                                              |==========================================================            |  83%  |                                                                              |===========================================================           |  84%  |                                                                              |===========================================================           |  85%  |                                                                              |============================================================          |  85%  |                                                                              |============================================================          |  86%  |                                                                              |=============================================================         |  86%  |                                                                              |=============================================================         |  87%  |                                                                              |=============================================================         |  88%  |                                                                              |==============================================================        |  88%  |                                                                              |==============================================================        |  89%  |                                                                              |===============================================================       |  89%  |                                                                              |===============================================================       |  90%  |                                                                              |===============================================================       |  91%  |                                                                              |================================================================      |  91%  |                                                                              |================================================================      |  92%  |                                                                              |=================================================================     |  92%  |                                                                              |=================================================================     |  93%  |                                                                              |=================================================================     |  94%  |                                                                              |==================================================================    |  94%  |                                                                              |==================================================================    |  95%  |                                                                              |===================================================================   |  95%  |                                                                              |===================================================================   |  96%  |                                                                              |====================================================================  |  96%  |                                                                              |====================================================================  |  97%  |                                                                              |====================================================================  |  98%  |                                                                              |===================================================================== |  98%  |                                                                              |===================================================================== |  99%  |                                                                              |======================================================================|  99%  |                                                                              |======================================================================| 100%
## Matrix converted.. Prepare genind object...
## Completed: gl2gi
```

Save it

```
saveRDS(
  snp2, here(
    "manuscript1/output/dapc/snp2_manuscript1.rds"
  )
)
```

To load it

```
snp2 <- readRDS(
  here(
    "manuscript1/output/dapc/snp2_manuscript1.rds"
  )
)
```

### 4.1.1 Scale

```
snp3 <- scaleGen(snp2, NA.method="mean")
class(snp3)
```

Save it

```
saveRDS(
  snp3, here(
    "manuscript1/output/dapc/snp3_manuscriptl.rds"
  )
)
```

To load it

```
snp3 <- readRDS(
  here(
    "manuscript1/output/dapc/snp3_manuscriptl.rds"
  )
)
```

```
dim(snp3)
```

```
## [1]   933 41650
```

```
snp3[1:5,1:5]
```

```
##      AX-583033387_C.G AX-583033387_C.C AX-583034838_T.A AX-583034838_T.T
## 1001        1.6057210       -1.6057210        1.3041492       -1.3041492
## 1002       -0.9685527        0.9685527       -1.0945538        1.0945538
## 1003       -0.9685527        0.9685527       -1.0945538        1.0945538
## 1004        1.6057210       -1.6057210       -1.0945538        1.0945538
## 1005        1.6057210       -1.6057210        0.1047977       -0.1047977
##      AX-583036983_C.T
## 1001        1.4091172
## 1002        0.0845757
## 1003        1.4091172
## 1004        0.0845757
## 1005        1.4091172
```

```
# Get the populations from the genlight object
populations <- snp$pop
```

### 4.1.2 Find clusters

```
grp <- find.clusters(snp3, max.n.clust=15)
#retained 1000
#Choose the number of clusters (>=2): 7
```

Save it

```
saveRDS(
  grp, here(
    "manuscript1/output/dapc/grp_manuscript1.rds"
  )
)
```

To load it

```
grp <- readRDS(
  here(
    "manuscript1/output/dapc/grp_manuscript1.rds"
  )
)
```

```
names(grp)
```

```
## [1] "Kstat" "stat"  "grp"   "size"
```

```
grp$size #group sizes for our clusters
```

```
## [1]  29  48 162 154 253 117 170
```

```
table(pop(snp2), grp$grp)
```

```
##      
##        1  2  3  4  5  6  7
##   ALD  0  0  0 10  0  0  0
##   ARA  0  0 12  0  0  0  0
##   BAF  0  0  7  0  0  0  0
##   BCO  0  0 12  0  0  0  0
##   BEA  0  0  0  0  0  0 12
##   BEH  0  0  9  0  0  0  0
##   BEN  0  0  0  0  0 12  0
##   BER  0  0  0  0 12  0  0
##   BIT  0  0 12  0  0  0  0
##   BPA  0  0 11  0  0  0  0
##   BPV  0  0 12  0  0  0  0
##   BRE  0  0  0  0 13  0  0
##   BRM  0  0  0  0  0  0 16
##   CAM  0  0  0  0  0 12  0
##   CES  0  0  0  0 15  0  0
##   CHA  0  0  0  0  0 12  0
##   CHI  0  0  0  6  0  0  0
##   COL  0  0  0  0 10  0  0
##   CUA  0  0  0  8  0  0  0
##   DAL  0  0  0  0  3  0  9
##   DEN  0  0  0  0 10  0  2
##   DES  0  0  0  0 16  0  0
##   FAY  0  0  0  0  1  0 11
##   FIR  0  0  0  0 11  0  1
##   FLO  0  0  8  0  0  0  0
##   FRS  0  0  0 12  0  0  0
##   GEL  0  0  0  0  0  2  0
##   GES  0 12  0  0  0  0  0
##   GRA  0  0  0 11  0  0  0
##   GRV  0  0 12  0  0  0  0
##   HAI  0  0  0 12  0  0  0
##   HAL  0  0  0  0 10  0  0
##   HAN  0  0  0  4  0  0  0
##   HAR  0  0  0  0 12  0  0
##   HAT  0  0  0  0  4  0  6
##   HOC  0  0  0  7  0  0  0
##   HOU  0  0  0  0  0  0 12
##   HUN  0  0  0 12  0  0  0
##   INJ 11  0  0  0  0  0  0
##   INW  4  0  0  0  0  0  0
##   IPO  0  0  7  0  0  0  0
##   JAF  0  0  0  0  0  2  0
##   KAC  0  0  0  0  0  6  0
##   KAG  0  0  0  0 12  0  0
##   KAN  0  0  0  0 11  0  0
##   KAT  2  0  0  0  0  4  0
##   KLP  0  0  0  0  0  4  0
##   KUN  0  0  0  0  0  3  0
##   LAM  0  0  0  0  0  9  0
##   LIN  0  0  0  0 12  0  0
##   LOS  0  0  0 12  0  0  0
##   MAC  0  0  0  0  0  0 12
##   MAN  0  0  0  0 12  0  0
##   MAT  0  0  0  0  0 12  0
##   MAU  0  0 12  0  0  0  0
##   MED  0  0  0  7  0  0  0
##   MON  0  0  0  0  0  0 12
##   NEB  0  0  0  0 10  0  0
##   NEG  0  0  0  0  0  4  0
##   NEH  0  0  0  0  7  0  0
##   NEO  0  0  0  0  0  0 12
##   NEW  0  0  0  0 17  0  4
##   NOV  0  0  4  0  0  0  0
##   NUE  0  0  0  0  0  0 12
##   OKI  0  0  0 11  0  0  0
##   PAL  0  0  0  0  0  0 11
##   PEO  0  0  0  0  8  0  2
##   PLM  0  0  7  0  0  0  0
##   POR  0  0 12  0  0  0  0
##   QNC  0  0  0  0  0 11  0
##   RAR  0 12  0  0  0  0  0
##   REC  0  0 11  0  0  0  0
##   RUS  0  0  0  0  1  0 11
##   SAI  0  0  0 11  0  0  1
##   SER  0  0  0  4  0  0  0
##   SMI  0  0  0  0  0  0 12
##   SOC  0 12  0  0  0  0  0
##   SON  0  0  0  0  0  3  0
##   SPB  0  0  0  0  8  0  0
##   SPR  0  0  0  0  7  0  0
##   SPS  0  0  0  8  0  0  0
##   SSK  0  0  0  0  0 12  0
##   STL  0  0  0  0 12  0  0
##   SUF  6  0  0  0  0  0  0
##   SUU  6  0  0  0  0  0  0
##   TAI  0  0  0  7  0  0  0
##   TIK  0 12  0  0  0  0  0
##   TRA  0  0  0  0  0  0 12
##   TUC  0  0 14  0  0  0  0
##   TUH  0  0  0 12  0  0  0
##   UTS  0  0  0  0 12  0  0
##   WAV  0  0  0  0  7  0  0
##   YUN  0  0  0  0  0  9  0
```

```
xvalDapc(snp3, populations, n.pca.max = 800, n.da = NULL,
              training.set = 0.9, result = c("groupMean", "overall"),
              center = TRUE, scale = FALSE,
              n.pca=NULL, n.rep = 10, xval.plot = TRUE)
```

\(n.pca: 250 first PCs of PCA
used\)n.da: 92 discriminant functions saved $var (proportion of
conserved variance): 0.569

```
dapc1 <- dapc(snp3, snp$pop)
# PCs retained 250
# discriminant functions retained 92
```

Save the genind object

```
saveRDS(dapc1, here("scripts", "RMarkdowns",
  "output", "americas", "manuscript1", "dapc", "dapc1_manuscript1_loc.rds"
))
```

Load the genind object

```
dapc1 <- readRDS(here("scripts", "RMarkdowns",
  "output", "americas", "manuscript1", "dapc", "dapc1_manuscript1_loc.rds"
))
```

```
scatter(dapc1)
```

Run DAPC with object

```
dapc_snp <- dapc(snp3, n.pca = 250, n.da = 92, grp = populations)
```

```
dapc_snp
```

Save the genind object

```
saveRDS(dapc_snp, here("scripts", "RMarkdowns",
  "output", "americas", "manuscript1", "dapc", "dapc_snp_manuscript1_loc.rds"
))
```

Load the genind object

```
dapc_snp <- readRDS(here("scripts", "RMarkdowns",
  "output", "americas", "manuscript1", "dapc", "dapc_snp_manuscript1_loc.rds"
))
```

### 4.1.3 Plots for DAPC

```
myCol <- c("#146c45", "orange", "#2524f9", "#E41A1C", "purple", "#fd4e8b", "#51f310", "skyblue2", "#799d10", "#1F78B4", "#21a708", "magenta", "gold4",  "#a41415", "#fd5917", "#9d8d88", "#FB8072", "#6A3D9A", "#2c4a5e", "#a113b2", "gold2", "#66C2A5", "#332288", "#cf749b", "#8c61cd", "#52ef99", "#6a8fe0", "#871550", "pink2", "#784819", "#FF5005", "#1E90FF",  "#4C005C", "yellow",  "#993F00", "#F0A0FF")
```

Check R symbols for plot

```
#to see all shapes -> plot shapes - para escolher os simbolos
N = 100; M = 1000
good.shapes = c(1:25,35:38,43,60,62:64)
foo = data.frame( x = rnorm(M), y = rnorm(M), s = factor( sample(1:N, M, replace = TRUE) ) )
ggplot(aes(x,y,shape=s ), data=foo ) +
  scale_shape_manual(values=good.shapes[1:N]) +
  geom_point()
#1:25,28:31,36,55:57
```

Plot 1 & 2

```
op <- par(cex = 0.39)
scatter(dapc_snp, pch = good.shapes, cstar = 0, col=myCol, label=NULL, mstree = FALSE, legend=TRUE, posi.da="topright", cex=1.0,  cex.lab=0.5, cex.main=0.5, cellipse=TRUE, posi.leg="topleft",  xax =1, yax=2)
```

```
pdf(file = "scripts/RMarkdowns/output/americas/manuscript1/dapc/dapc_americas_byloc_PC1_2.pdf",   # The directory you want to save the file in
    width = 8, # The width of the plot in inches
    height = 8) # The height of the plot in inches


good.shapes = c(1:25,35:38,43,60,62:64)

op <- par(cex = 0.39)
scatter(dapc_snp, pch = good.shapes, cstar = 0, col=myCol, label=NULL, mstree = FALSE, legend=TRUE, posi.da="topright", cex=1.0,  cex.lab=0.5, cex.main=0.5, cellipse=TRUE, posi.leg="topleft",  xax =1, yax=2)

dev.off()
```

Plot 1 & 3

```
op <- par(cex = 0.39)
scatter(dapc_snp, pch = good.shapes, cstar = 0, col=myCol, label=NULL, mstree = FALSE, legend=TRUE, posi.da="topright", cex=1.0,  cex.lab=0.5, cex.main=0.5, cellipse=TRUE, posi.leg="topleft",  xax =1, yax=3)
```

```
pdf(file = "scripts/RMarkdowns/output/americas/manuscript1/dapc/dapc_americas_byloc_PC1_3.pdf",   # The directory you want to save the file in
    width = 8, # The width of the plot in inches
    height = 8) # The height of the plot in inches


good.shapes = c(1:25,35:38,43,60,62:64)

op <- par(cex = 0.39)
scatter(dapc_snp, pch = good.shapes, cstar = 0, col=myCol, label=NULL, mstree = FALSE, legend=TRUE, posi.da="bottomright", cex=1.0,  cex.lab=0.5, cex.main=0.5, cellipse=TRUE, posi.leg="topleft",  xax =1, yax=3)

dev.off()
```

Plot 1 & 4

```
op <- par(cex = 0.39)
scatter(dapc_snp, pch = good.shapes, cstar = 0, col=myCol, label=NULL, mstree = FALSE, legend=TRUE, posi.da="bottomright", cex=1.0,  cex.lab=0.5, cex.main=0.5, cellipse=TRUE, posi.leg="topleft",  xax =1, yax=4)
```

```
pdf(file = "scripts/RMarkdowns/output/americas/manuscript1/dapc/dapc_americas_byloc_PC1_4.pdf",   # The directory you want to save the file in
    width = 8, # The width of the plot in inches
    height = 8) # The height of the plot in inches


good.shapes = c(1:25,35:38,43,60,62:64)

op <- par(cex = 0.39)
scatter(dapc_snp, pch = good.shapes, cstar = 0, col=myCol, label=NULL, mstree = FALSE, legend=TRUE, posi.da="bottomright", cex=1.0,  cex.lab=0.5, cex.main=0.5, cellipse=TRUE, posi.leg="topleft",  xax =1, yax=4)

dev.off()
```

Plot 2 & 3

```
op <- par(cex = 0.39)
scatter(dapc_snp, pch = good.shapes, cstar = 0, col=myCol, label=NULL, mstree = FALSE, legend=TRUE, posi.da="bottomright", cex=1.0,  cex.lab=0.5, cex.main=0.5, cellipse=TRUE, posi.leg="topleft",  xax =2, yax=3)
```

```
pdf(file = "scripts/RMarkdowns/output/americas/manuscript1/dapc/dapc_americas_byloc_PC2_3.pdf",   # The directory you want to save the file in
    width = 8, # The width of the plot in inches
    height = 8) # The height of the plot in inches


good.shapes = c(1:25,35:38,43,60,62:64)

op <- par(cex = 0.39)
scatter(dapc_snp, pch = good.shapes, cstar = 0, col=myCol, label=NULL, mstree = FALSE, legend=TRUE, posi.da="bottomright", cex=1.0,  cex.lab=0.5, cex.main=0.5, cellipse=TRUE, posi.leg="topleft",  xax =2, yax=3)

dev.off()
```

### 4.1.4 Plots for DAPC colored be region

```
myCol <- c("purple","#005C31","#005C31","#005C31","#2524f9","#005C31","orange","#2524f9","#005C31","#005C31","#005C31","purple","#E41A1C","orange","purple","orange","#2524f9","#2524f9","#005C31",
"#2524f9","#2524f9","purple","#2524f9","#2524f9","#005C31","purple","orange","purple","purple","#005C31","orange","#2524f9","orange","#2524f9","#2524f9","orange","#2524f9","orange","orange","orange",
"#005C31","orange","orange","orange","orange","orange","orange","orange","orange","#2524f9","#2524f9","#2524f9","#2524f9","orange","#005C31","#005C31","#2524f9","#2524f9","#E41A1C","#2524f9","#2524f9",
"#2524f9","#005C31","#2524f9","orange","#2524f9","#2524f9","#005C31","#005C31","orange","purple","#005C31","#2524f9","#E41A1C","purple","#2524f9","purple","orange","purple","#2524f9","purple","orange",
"#2524f9","orange","orange","orange","purple","#2524f9","#005C31","purple","orange","#2524f9","orange")
```

Plot 1 & 2

```
op <- par(cex = 0.39)
scatter(dapc_snp, pch = good.shapes, cstar = 0, col=myCol, label=NULL, mstree = FALSE, legend=TRUE, posi.da="topright", cex=1.0,  cex.lab=0.5, cex.main=0.5, cellipse=TRUE, posi.leg="topleft",  xax =1, yax=2)
```

```
pdf(file = "scripts/RMarkdowns/output/americas/manuscript1/dapc/dapc_americas_byloc_colors_PC1_2.pdf",   # The directory you want to save the file in
    width = 8, # The width of the plot in inches
    height = 8) # The height of the plot in inches


good.shapes = c(1:25,35:38,43,60,62:64)

op <- par(cex = 0.39)
scatter(dapc_snp, pch = good.shapes, cstar = 0, col=myCol, label=NULL, mstree = FALSE, legend=TRUE, posi.da="topright", cex=1.0,  cex.lab=0.5, cex.main=0.5, cellipse=TRUE, posi.leg="topleft",  xax =1, yax=2)

dev.off()
```

Plot 1 & 3

```
op <- par(cex = 0.39)
scatter(dapc_snp, pch = good.shapes, cstar = 0, col=myCol, label=NULL, mstree = FALSE, legend=TRUE, posi.da="topright", cex=1.0,  cex.lab=0.5, cex.main=0.5, cellipse=TRUE, posi.leg="topleft",  xax =1, yax=3)
```

```
pdf(file = "scripts/RMarkdowns/output/americas/manuscript1/dapc/dapc_americas_byloc_colors_PC1_3.pdf",   # The directory you want to save the file in
    width = 8, # The width of the plot in inches
    height = 8) # The height of the plot in inches


good.shapes = c(1:25,35:38,43,60,62:64)

op <- par(cex = 0.39)
scatter(dapc_snp, pch = good.shapes, cstar = 0, col=myCol, label=NULL, mstree = FALSE, legend=TRUE, posi.da="topright", cex=1.0,  cex.lab=0.5, cex.main=0.5, cellipse=TRUE, posi.leg="topleft",  xax =1, yax=3)

dev.off()
```

Plot 1 & 4

```
op <- par(cex = 0.39)
scatter(dapc_snp, pch = good.shapes, cstar = 0, col=myCol, label=NULL, mstree = FALSE, legend=TRUE, posi.da="bottomright", cex=1.0,  cex.lab=0.5, cex.main=0.5, cellipse=TRUE, posi.leg="topleft",  xax =1, yax=4)
```

```
pdf(file = "scripts/RMarkdowns/output/americas/manuscript1/dapc/dapc_americas_byloc_colors_PC1_4.pdf",   # The directory you want to save the file in
    width = 8, # The width of the plot in inches
    height = 8) # The height of the plot in inches


good.shapes = c(1:25,35:38,43,60,62:64)

op <- par(cex = 0.39)
scatter(dapc_snp, pch = good.shapes, cstar = 0, col=myCol, label=NULL, mstree = FALSE, legend=TRUE, posi.da="bottomright", cex=1.0,  cex.lab=0.5, cex.main=0.5, cellipse=TRUE, posi.leg="topleft",  xax =1, yax=4)

dev.off()
```

Plot 2 & 3

```
op <- par(cex = 0.39)
scatter(dapc_snp, pch = good.shapes, cstar = 0, col=myCol, label=NULL, mstree = FALSE, legend=TRUE, posi.da="bottomright", cex=1.0,  cex.lab=0.5, cex.main=0.5, cellipse=TRUE, posi.leg="topleft",  xax =2, yax=3)
```

```
pdf(file = "scripts/RMarkdowns/output/americas/manuscript1/dapc/dapc_americas_byloc_colors_PC2_3.pdf",   # The directory you want to save the file in
    width = 8, # The width of the plot in inches
    height = 8) # The height of the plot in inches


good.shapes = c(1:25,35:38,43,60,62:64)

op <- par(cex = 0.39)
scatter(dapc_snp, pch = good.shapes, cstar = 0, col=myCol, label=NULL, mstree = FALSE, legend=TRUE, posi.da="bottomright", cex=1.0,  cex.lab=0.5, cex.main=0.5, cellipse=TRUE, posi.leg="topleft",  xax =2, yax=3)

dev.off()
```

## 4.2 DAPC by continent/major region

Import the data and covert it to genind format

```
# import the data
snp <-
  read.PLINK(
    here("manuscript1/output/dapc/dapc_r01_b.raw"),
    quiet = FALSE,
    chunkSize = 1000,
    parallel = require("parallel"),
    n.cores = 4
  )
```

```
## 
##  Reading PLINK raw format into a genlight object... 
## 
## 
##  Reading loci information... 
## 
##  Reading and converting genotypes... 
## .
##  Building final object... 
## 
## ...done.
```

```
nInd(snp)
```

```
## [1] 933
```

```
nLoc(snp)
```

```
## [1] 20825
```

```
nPop(snp)
```

```
## [1] 93
```

```
indNames(snp)
```

```
##   [1] "1001" "1002" "1003" "1004" "1005" "1006" "1007" "1008" "1009" "1011"
##  [11] "1012" "1053" "1054" "1055" "1056" "1057" "1058" "1059" "1060" "1061"
##  [21] "1062" "1063" "1064" "1065" "1066" "1067" "1068" "1069" "1070" "1071"
##  [31] "1072" "1073" "1074" "1075" "1076" "1089" "1090" "1091" "1092" "1093"
##  [41] "1094" "1095" "1101" "1102" "1103" "1105" "1106" "1107" "1109" "1110"
##  [51] "1111" "1112" "1113" "1114" "1115" "1116" "1117" "1118" "1119" "1120"
##  [61] "1137" "1138" "1139" "1140" "1141" "1142" "1143" "1144" "1145" "1146"
##  [71] "1147" "1148" "1149" "1150" "1151" "1152" "1153" "1154" "1155" "1156"
##  [81] "1157" "1158" "1159" "1160" "1161" "1162" "1163" "1165" "1166" "1167"
##  [91] "1168" "1169" "1170" "1171" "1172" "1173" "1174" "1175" "1176" "1177"
## [101] "1178" "1179" "1180" "1181" "1182" "1183" "1184" "1214" "1215" "1216"
## [111] "1217" "1238" "1239" "1240" "1241" "1242" "1243" "1244" "1245" "1246"
## [121] "1247" "1249" "1250" "1251" "1252" "1253" "1254" "1255" "1256" "1257"
## [131] "1258" "1259" "1260" "1261" "1262" "1263" "1264" "1265" "1266" "1267"
## [141] "1268" "1270" "1271" "1272" "1273" "1274" "1276" "1282" "1283" "1285"
## [151] "1286" "1287" "1288" "1289" "1292" "1293" "1294" "1295" "1325" "1326"
## [161] "1328" "1329" "1330" "1331" "1332" "1333" "1334" "1335" "1336" "1373"
## [171] "1374" "1375" "1376" "1377" "1378" "1379" "1380" "1381" "1382" "1383"
## [181] "1384" "1430" "1431" "1432" "1433" "1434" "1435" "1436" "1437" "1438"
## [191] "1439" "1440" "1441" "1443" "1444" "1446" "1447" "1449" "1451" "1452"
## [201] "1453" "1454" "1456" "1458" "1460" "1461" "153"  "154"  "155"  "156" 
## [211] "158"  "16"   "174"  "175"  "176"  "177"  "178"  "17"   "180"  "181" 
## [221] "182"  "183"  "184"  "185"  "186"  "187"  "188"  "189"  "18"   "190" 
## [231] "191"  "2158" "2159" "2160" "2161" "2162" "2163" "2164" "2165" "2166"
## [241] "2167" "2168" "2169" "2170" "2171" "2172" "2173" "2174" "2175" "2176"
## [251] "2177" "2178" "2179" "217"  "2180" "2181" "2182" "2183" "2184" "2185"
## [261] "2187" "2188" "2189" "218"  "2191" "2192" "2193" "2194" "2195" "219" 
## [271] "2203" "2204" "2205" "2206" "2207" "2208" "2209" "220"  "2210" "2211"
## [281] "2212" "2213" "2214" "2215" "2216" "2217" "2218" "221"  "2221" "2222"
## [291] "2223" "2224" "2225" "2226" "2227" "2228" "2229" "222"  "2230" "2231"
## [301] "2232" "2233" "2234" "2235" "2236" "2237" "2238" "2239" "223"  "2240"
## [311] "2241" "2242" "2243" "2244" "2245" "2246" "2247" "2248" "2249" "224" 
## [321] "2250" "2251" "2252" "2253" "2254" "2255" "2256" "2257" "2258" "2259"
## [331] "225"  "2260" "2261" "2262" "2263" "2264" "2265" "2266" "2267" "2268"
## [341] "2269" "226"  "2270" "2271" "2272" "2273" "2275" "2288" "2289" "228" 
## [351] "2290" "2291" "2292" "2293" "2294" "2295" "2301" "2302" "2303" "2304"
## [361] "2305" "2306" "2307" "2308" "2309" "230"  "2310" "2311" "2312" "2313"
## [371] "2314" "2315" "2316" "2317" "2318" "2319" "2320" "2321" "2322" "2323"
## [381] "2324" "2326" "2327" "2329" "2330" "2331" "2332" "2333" "2334" "2335"
## [391] "2336" "2337" "2338" "2339" "2340" "2341" "2342" "2343" "2344" "2345"
## [401] "2346" "2347" "2348" "2349" "2350" "2351" "2352" "2353" "2354" "2355"
## [411] "2357" "2358" "2360" "2362" "2384" "2385" "2386" "2387" "2388" "2390"
## [421] "2391" "2392" "2393" "2394" "2401" "2402" "2403" "2404" "2405" "2406"
## [431] "2407" "2408" "2409" "2410" "2411" "2412" "2413" "2414" "2415" "2416"
## [441] "2417" "2418" "2419" "2420" "2421" "2422" "2423" "2424" "2425" "2426"
## [451] "2427" "2428" "2429" "2430" "2431" "2432" "2433" "2434" "2435" "2436"
## [461] "2437" "2438" "2439" "2440" "2441" "2442" "2443" "2444" "2445" "2446"
## [471] "2447" "2448" "2449" "2450" "2451" "2452" "2453" "2454" "2455" "2456"
## [481] "2457" "2458" "2459" "2461" "2462" "2463" "2464" "2465" "2466" "2467"
## [491] "2468" "2469" "2470" "2471" "2472" "2473" "2474" "2475" "2476" "2477"
## [501] "2479" "2480" "2481" "2482" "2483" "2484" "2485" "2486" "2487" "2488"
## [511] "2489" "2490" "2491" "2492" "2493" "2494" "2495" "2501" "2502" "2503"
## [521] "2504" "2505" "2506" "2507" "2508" "2509" "2510" "2511" "2512" "2513"
## [531] "2514" "2515" "2516" "2517" "2518" "2519" "2520" "2521" "2522" "2523"
## [541] "2524" "2525" "2526" "2527" "2528" "2529" "2530" "2531" "2532" "2533"
## [551] "2534" "2535" "2536" "2537" "2538" "2539" "2540" "2541" "2542" "2543"
## [561] "2544" "2545" "2546" "2547" "2548" "2550" "2551" "2552" "2553" "2554"
## [571] "255"  "2561" "2562" "2564" "2565" "2566" "2567" "2568" "2569" "256" 
## [581] "2570" "2571" "2572" "2573" "2574" "2575" "2576" "2577" "2578" "2579"
## [591] "257"  "2580" "2581" "2582" "2583" "2584" "2585" "2586" "2587" "2588"
## [601] "2589" "2591" "2592" "2595" "259"  "261"  "262"  "263"  "264"  "265" 
## [611] "266"  "267"  "268"  "269"  "270"  "271"  "272"  "273"  "275"  "276" 
## [621] "277"  "278"  "294"  "295"  "296"  "297"  "298"  "299"  "301"  "302" 
## [631] "303"  "304"  "305"  "306"  "307"  "308"  "309"  "310"  "311"  "312" 
## [641] "313"  "314"  "315"  "316"  "317"  "318"  "319"  "320"  "321"  "322" 
## [651] "323"  "324"  "325"  "326"  "327"  "328"  "329"  "330"  "331"  "332" 
## [661] "333"  "334"  "335"  "336"  "337"  "340"  "341"  "342"  "343"  "344" 
## [671] "345"  "346"  "347"  "348"  "349"  "350"  "351"  "352"  "353"  "354" 
## [681] "355"  "356"  "357"  "358"  "359"  "360"  "361"  "362"  "363"  "364" 
## [691] "366"  "368"  "369"  "370"  "371"  "372"  "373"  "374"  "375"  "376" 
## [701] "377"  "378"  "379"  "380"  "381"  "382"  "383"  "413"  "414"  "415" 
## [711] "416"  "417"  "418"  "419"  "420"  "421"  "422"  "424"  "425"  "426" 
## [721] "427"  "428"  "429"  "430"  "431"  "432"  "433"  "434"  "435"  "436" 
## [731] "437"  "438"  "439"  "440"  "441"  "442"  "443"  "444"  "445"  "446" 
## [741] "447"  "448"  "449"  "450"  "451"  "452"  "453"  "454"  "455"  "456" 
## [751] "457"  "458"  "459"  "460"  "461"  "463"  "464"  "466"  "467"  "468" 
## [761] "469"  "470"  "472"  "473"  "474"  "475"  "476"  "477"  "478"  "602" 
## [771] "603"  "604"  "607"  "609"  "60"   "610"  "611"  "612"  "613"  "614" 
## [781] "615"  "616"  "617"  "618"  "619"  "620"  "621"  "622"  "623"  "624" 
## [791] "625"  "626"  "627"  "628"  "629"  "630"  "631"  "632"  "633"  "634" 
## [801] "635"  "636"  "637"  "638"  "639"  "640"  "641"  "642"  "643"  "644" 
## [811] "645"  "64"   "666"  "669"  "670"  "671"  "672"  "673"  "674"  "675" 
## [821] "676"  "677"  "678"  "679"  "680"  "681"  "683"  "684"  "685"  "686" 
## [831] "687"  "688"  "689"  "690"  "691"  "692"  "693"  "694"  "695"  "723" 
## [841] "724"  "725"  "726"  "727"  "728"  "729"  "730"  "731"  "732"  "733" 
## [851] "784"  "785"  "786"  "787"  "788"  "789"  "790"  "791"  "792"  "793" 
## [861] "794"  "795"  "801"  "802"  "803"  "804"  "805"  "806"  "807"  "808" 
## [871] "809"  "810"  "811"  "812"  "813"  "814"  "815"  "816"  "817"  "818" 
## [881] "819"  "820"  "821"  "822"  "861"  "862"  "863"  "864"  "883"  "884" 
## [891] "885"  "886"  "887"  "888"  "889"  "890"  "891"  "892"  "893"  "894" 
## [901] "8"    "911"  "912"  "913"  "914"  "964"  "965"  "966"  "967"  "972" 
## [911] "973"  "975"  "976"  "977"  "978"  "979"  "980"  "981"  "982"  "983" 
## [921] "984"  "985"  "986"  "987"  "988"  "989"  "990"  "991"  "992"  "993" 
## [931] "994"  "995"  "9"
```

```
# convert to genind
snp2 <- gl2gi(snp, probar = TRUE, verbose = NULL)
```

```
## Starting gl2gi 
##   Processing genlight object with SNP data
##   |                                                                              |                                                                      |   0%  |                                                                              |                                                                      |   1%  |                                                                              |=                                                                     |   1%  |                                                                              |=                                                                     |   2%  |                                                                              |==                                                                    |   2%  |                                                                              |==                                                                    |   3%  |                                                                              |==                                                                    |   4%  |                                                                              |===                                                                   |   4%  |                                                                              |===                                                                   |   5%  |                                                                              |====                                                                  |   5%  |                                                                              |====                                                                  |   6%  |                                                                              |=====                                                                 |   6%  |                                                                              |=====                                                                 |   7%  |                                                                              |=====                                                                 |   8%  |                                                                              |======                                                                |   8%  |                                                                              |======                                                                |   9%  |                                                                              |=======                                                               |   9%  |                                                                              |=======                                                               |  10%  |                                                                              |=======                                                               |  11%  |                                                                              |========                                                              |  11%  |                                                                              |========                                                              |  12%  |                                                                              |=========                                                             |  12%  |                                                                              |=========                                                             |  13%  |                                                                              |=========                                                             |  14%  |                                                                              |==========                                                            |  14%  |                                                                              |==========                                                            |  15%  |                                                                              |===========                                                           |  15%  |                                                                              |===========                                                           |  16%  |                                                                              |============                                                          |  17%  |                                                                              |============                                                          |  18%  |                                                                              |=============                                                         |  18%  |                                                                              |=============                                                         |  19%  |                                                                              |==============                                                        |  19%  |                                                                              |==============                                                        |  20%  |                                                                              |==============                                                        |  21%  |                                                                              |===============                                                       |  21%  |                                                                              |===============                                                       |  22%  |                                                                              |================                                                      |  22%  |                                                                              |================                                                      |  23%  |                                                                              |=================                                                     |  24%  |                                                                              |=================                                                     |  25%  |                                                                              |==================                                                    |  25%  |                                                                              |==================                                                    |  26%  |                                                                              |===================                                                   |  26%  |                                                                              |===================                                                   |  27%  |                                                                              |===================                                                   |  28%  |                                                                              |====================                                                  |  28%  |                                                                              |====================                                                  |  29%  |                                                                              |=====================                                                 |  29%  |                                                                              |=====================                                                 |  30%  |                                                                              |=====================                                                 |  31%  |                                                                              |======================                                                |  31%  |                                                                              |======================                                                |  32%  |                                                                              |=======================                                               |  32%  |                                                                              |=======================                                               |  33%  |                                                                              |=======================                                               |  34%  |                                                                              |========================                                              |  34%  |                                                                              |========================                                              |  35%  |                                                                              |=========================                                             |  35%  |                                                                              |=========================                                             |  36%  |                                                                              |==========================                                            |  36%  |                                                                              |==========================                                            |  37%  |                                                                              |==========================                                            |  38%  |                                                                              |===========================                                           |  38%  |                                                                              |===========================                                           |  39%  |                                                                              |============================                                          |  39%  |                                                                              |============================                                          |  40%  |                                                                              |============================                                          |  41%  |                                                                              |=============================                                         |  41%  |                                                                              |=============================                                         |  42%  |                                                                              |==============================                                        |  42%  |                                                                              |==============================                                        |  43%  |                                                                              |==============================                                        |  44%  |                                                                              |===============================                                       |  44%  |                                                                              |===============================                                       |  45%  |                                                                              |================================                                      |  45%  |                                                                              |================================                                      |  46%  |                                                                              |=================================                                     |  47%  |                                                                              |=================================                                     |  48%  |                                                                              |==================================                                    |  48%  |                                                                              |==================================                                    |  49%  |                                                                              |===================================                                   |  49%  |                                                                              |===================================                                   |  50%  |                                                                              |===================================                                   |  51%  |                                                                              |====================================                                  |  51%  |                                                                              |====================================                                  |  52%  |                                                                              |=====================================                                 |  52%  |                                                                              |=====================================                                 |  53%  |                                                                              |======================================                                |  54%  |                                                                              |======================================                                |  55%  |                                                                              |=======================================                               |  55%  |                                                                              |=======================================                               |  56%  |                                                                              |========================================                              |  56%  |                                                                              |========================================                              |  57%  |                                                                              |========================================                              |  58%  |                                                                              |=========================================                             |  58%  |                                                                              |=========================================                             |  59%  |                                                                              |==========================================                            |  59%  |                                                                              |==========================================                            |  60%  |                                                                              |==========================================                            |  61%  |                                                                              |===========================================                           |  61%  |                                                                              |===========================================                           |  62%  |                                                                              |============================================                          |  62%  |                                                                              |============================================                          |  63%  |                                                                              |============================================                          |  64%  |                                                                              |=============================================                         |  64%  |                                                                              |=============================================                         |  65%  |                                                                              |==============================================                        |  65%  |                                                                              |==============================================                        |  66%  |                                                                              |===============================================                       |  66%  |                                                                              |===============================================                       |  67%  |                                                                              |===============================================                       |  68%  |                                                                              |================================================                      |  68%  |                                                                              |================================================                      |  69%  |                                                                              |=================================================                     |  69%  |                                                                              |=================================================                     |  70%  |                                                                              |=================================================                     |  71%  |                                                                              |==================================================                    |  71%  |                                                                              |==================================================                    |  72%  |                                                                              |===================================================                   |  72%  |                                                                              |===================================================                   |  73%  |                                                                              |===================================================                   |  74%  |                                                                              |====================================================                  |  74%  |                                                                              |====================================================                  |  75%  |                                                                              |=====================================================                 |  75%  |                                                                              |=====================================================                 |  76%  |                                                                              |======================================================                |  77%  |                                                                              |======================================================                |  78%  |                                                                              |=======================================================               |  78%  |                                                                              |=======================================================               |  79%  |                                                                              |========================================================              |  79%  |                                                                              |========================================================              |  80%  |                                                                              |========================================================              |  81%  |                                                                              |=========================================================             |  81%  |                                                                              |=========================================================             |  82%  |                                                                              |==========================================================            |  82%  |                                                                              |==========================================================            |  83%  |                                                                              |===========================================================           |  84%  |                                                                              |===========================================================           |  85%  |                                                                              |============================================================          |  85%  |                                                                              |============================================================          |  86%  |                                                                              |=============================================================         |  86%  |                                                                              |=============================================================         |  87%  |                                                                              |=============================================================         |  88%  |                                                                              |==============================================================        |  88%  |                                                                              |==============================================================        |  89%  |                                                                              |===============================================================       |  89%  |                                                                              |===============================================================       |  90%  |                                                                              |===============================================================       |  91%  |                                                                              |================================================================      |  91%  |                                                                              |================================================================      |  92%  |                                                                              |=================================================================     |  92%  |                                                                              |=================================================================     |  93%  |                                                                              |=================================================================     |  94%  |                                                                              |==================================================================    |  94%  |                                                                              |==================================================================    |  95%  |                                                                              |===================================================================   |  95%  |                                                                              |===================================================================   |  96%  |                                                                              |====================================================================  |  96%  |                                                                              |====================================================================  |  97%  |                                                                              |====================================================================  |  98%  |                                                                              |===================================================================== |  98%  |                                                                              |===================================================================== |  99%  |                                                                              |======================================================================|  99%  |                                                                              |======================================================================| 100%
## Matrix converted.. Prepare genind object...
## Completed: gl2gi
```

Save it

```
saveRDS(
  snp2, here(
    "scripts/RMarkdowns/output/americas/manuscript1/dapc/continent/snp2.rds"
  )
)
```

To load it

```
snp2 <- readRDS(
  here(
    "scripts/RMarkdowns/output/americas/manuscript1/dapc/continent/snp2.rds"
  )
)
```

Import sample data

```
sampling_loc <- readRDS(here("scripts", "RMarkdowns", "output", "sampling_loc_all_pops.rds"))
head(sampling_loc)
```

```
##      Pop_City Country Latitude Longitude Continent Abbreviation  Year    Region
## 1      Hainan   China  19.2000  109.6000      Asia          HAI  2014 East Asia
## 2      Yunnan   China  24.4753  101.3431      Asia          YUN  2014 East Asia
## 3       Hunan   China  27.6253  111.8569      Asia          HUN  2001 East Asia
## 4     Okinawa   Japan  26.5013  127.9454      Asia          OKI 2018? East Asia
## 5 Tanegashima   Japan  30.4564  130.9784      Asia          TAN  2008 East Asia
## 6   Kagoshima   Japan  31.5602  130.5581      Asia          KAG  2018 East Asia
##   Subregion order order2 orderold AM_order state FINAL_order
## 1     Japan    55     47       47       NA                 1
## 2     Japan    56     48       48       NA                 2
## 3     Japan    57     49       49       NA                 3
## 4     Japan    54     46       46       NA                 4
## 5     Japan    NA     NA       NA       NA                 5
## 6     Japan    53     45       45       NA                 6
```

```
strata(snp2) <- data.frame(other(snp2))

# Currently set on just 
head(pop(snp2))
```

```
## [1] OKI OKI OKI OKI OKI OKI
## 93 Levels: ALD ARA BAF BCO BEA BEH BEN BER BIT BPA BPV BRE BRM CAM CES ... YUN
```

Load the csv

```
countr <- read.csv(here("scripts", "RMarkdowns", "output", "americas", "manuscript1", "dapc", "continent", "DAPC_countries_continents.csv"
))
df <- as.data.frame(countr)

head(df)
```

```
##   pop region
## 1 OKI   Asia
## 2 OKI   Asia
## 3 OKI   Asia
## 4 OKI   Asia
## 5 OKI   Asia
## 6 OKI   Asia
```

```
snp2@pop <- as.factor(df$region)
```

```
snp2$pop
```

```
##   [1] Asia          Asia          Asia          Asia          Asia         
##   [6] Asia          Asia          Asia          Asia          Asia         
##  [11] Asia          Asia          Asia          Asia          Asia         
##  [16] Asia          Asia          Asia          Asia          Asia         
##  [21] Asia          Asia          Asia          Europe        Europe       
##  [26] Europe        Europe        Europe        Europe        Europe       
##  [31] Europe        Europe        Europe        Europe        Europe       
##  [36] Asia          Asia          Asia          Asia          Asia         
##  [41] Asia          Asia          Asia          Asia          Asia         
##  [46] Asia          Asia          Asia          Europe        Europe       
##  [51] Europe        Europe        Europe        Europe        Europe       
##  [56] Europe        Europe        Europe        Europe        Europe       
##  [61] Europe        Europe        Europe        Europe        Europe       
##  [66] Europe        Europe        Europe        Europe        Europe       
##  [71] Europe        Europe        Europe        Europe        Europe       
##  [76] Europe        Europe        Europe        Europe        Europe       
##  [81] Europe        Europe        Europe        Europe        Asia         
##  [86] Asia          Asia          Asia          Asia          Asia         
##  [91] Asia          Asia          Asia          Asia          Asia         
##  [96] Asia          Asia          Asia          Asia          Asia         
## [101] Asia          Asia          Asia          Asia          Asia         
## [106] Asia          Asia          Asia          Asia          Asia         
## [111] Asia          Asia          Asia          Asia          Asia         
## [116] Asia          Asia          Asia          Asia          Asia         
## [121] Asia          Asia          Asia          Asia          Asia         
## [126] Asia          Asia          Asia          Asia          Asia         
## [131] Asia          Asia          Asia          Asia          Asia         
## [136] Asia          Asia          Asia          Asia          Asia         
## [141] Asia          Asia          Asia          Asia          Asia         
## [146] Asia          Asia          Asia          Asia          Asia         
## [151] Asia          Europe        Europe        Europe        Europe       
## [156] Europe        Europe        Europe        Asia          Asia         
## [161] Asia          Asia          Asia          Asia          Asia         
## [166] Asia          Asia          Asia          Asia          Asia         
## [171] Asia          Asia          Asia          Asia          Asia         
## [176] Asia          Asia          Asia          Asia          Asia         
## [181] Asia          Europe        Europe        Europe        Europe       
## [186] Europe        Europe        Europe        Europe        Europe       
## [191] Europe        Europe        Europe        Europe        Europe       
## [196] Europe        Europe        Europe        Europe        Europe       
## [201] Europe        Europe        Europe        Europe        Europe       
## [206] Europe        South America South America South America South America
## [211] South America South America South America South America South America
## [216] South America South America South America South America South America
## [221] South America South America South America South America South America
## [226] South America South America South America South America South America
## [231] South America Caribbean     Caribbean     Caribbean     Caribbean    
## [236] Caribbean     Caribbean     Caribbean     Caribbean     Caribbean    
## [241] Caribbean     Caribbean     Caribbean     Caribbean     Caribbean    
## [246] Caribbean     Caribbean     Asia          Asia          Asia         
## [251] Asia          Asia          Asia          Asia          Asia         
## [256] Asia          Asia          Asia          Asia          Asia         
## [261] Europe        Europe        Europe        Asia          Europe       
## [266] Europe        Europe        Europe        Europe        Asia         
## [271] South America South America South America South America South America
## [276] South America South America Asia          South America South America
## [281] South America South America South America Europe        Europe       
## [286] Europe        Europe        Asia          North America North America
## [291] North America North America North America North America North America
## [296] North America North America Asia          North America North America
## [301] North America North America North America North America North America
## [306] North America North America North America Asia          North America
## [311] North America North America North America North America North America
## [316] North America North America North America North America Asia         
## [321] North America North America North America North America North America
## [326] North America North America North America North America North America
## [331] Asia          North America North America North America North America
## [336] North America North America North America North America North America
## [341] North America Asia          North America North America North America
## [346] North America North America North America North America Asia         
## [351] North America North America North America North America North America
## [356] North America North America North America North America North America
## [361] North America North America North America North America North America
## [366] Asia          North America North America North America North America
## [371] North America North America North America North America North America
## [376] North America North America North America North America North America
## [381] North America North America North America North America North America
## [386] North America North America North America North America North America
## [391] North America North America North America North America North America
## [396] North America North America North America North America North America
## [401] North America North America North America North America North America
## [406] North America North America North America North America North America
## [411] North America North America North America North America North America
## [416] North America North America North America North America North America
## [421] North America North America North America North America North America
## [426] North America North America North America North America North America
## [431] North America North America North America North America North America
## [436] North America North America North America North America North America
## [441] North America North America North America North America North America
## [446] North America North America North America South America South America
## [451] South America South America South America South America South America
## [456] South America South America South America South America South America
## [461] South America South America South America South America South America
## [466] South America South America South America South America South America
## [471] South America South America South America South America South America
## [476] South America South America South America South America South America
## [481] South America South America South America South America South America
## [486] South America South America South America South America South America
## [491] South America South America South America South America South America
## [496] South America South America South America South America South America
## [501] South America South America South America South America South America
## [506] South America South America South America South America South America
## [511] South America South America South America South America South America
## [516] South America South America North America North America North America
## [521] North America North America North America North America North America
## [526] North America North America North America North America North America
## [531] North America North America North America North America North America
## [536] North America North America North America North America North America
## [541] North America North America North America North America North America
## [546] North America North America North America North America North America
## [551] North America North America North America North America North America
## [556] North America North America North America North America North America
## [561] North America North America North America North America North America
## [566] North America North America North America North America North America
## [571] Asia          South America South America South America South America
## [576] South America South America South America South America Asia         
## [581] South America South America South America South America South America
## [586] South America South America South America South America South America
## [591] Asia          South America South America South America South America
## [596] South America South America South America South America South America
## [601] South America South America South America South America Asia         
## [606] Asia          Asia          Asia          Asia          Asia         
## [611] Asia          Asia          Asia          Asia          Asia         
## [616] Asia          Asia          Asia          Asia          Asia         
## [621] Asia          Asia          North America North America North America
## [626] North America North America North America North America North America
## [631] North America North America North America North America North America
## [636] North America North America North America North America North America
## [641] North America North America North America North America North America
## [646] North America North America North America North America North America
## [651] North America North America North America North America North America
## [656] North America North America North America North America North America
## [661] North America North America North America North America North America
## [666] North America North America North America North America North America
## [671] North America North America North America North America North America
## [676] North America North America North America North America North America
## [681] North America North America North America North America North America
## [686] North America North America North America North America North America
## [691] North America Caribbean     Caribbean     Caribbean     Caribbean    
## [696] South America South America South America South America South America
## [701] South America South America South America South America South America
## [706] South America South America North America North America North America
## [711] North America North America North America North America North America
## [716] North America North America North America North America North America
## [721] North America North America North America North America North America
## [726] North America North America North America North America North America
## [731] North America North America North America North America North America
## [736] North America North America North America North America North America
## [741] North America North America North America North America North America
## [746] North America North America North America North America North America
## [751] North America North America North America North America North America
## [756] North America North America North America North America North America
## [761] North America North America North America North America North America
## [766] North America North America North America North America Asia         
## [771] Asia          Asia          Asia          Asia          South America
## [776] Asia          Caribbean     Caribbean     Caribbean     Caribbean    
## [781] Caribbean     Caribbean     Caribbean     Caribbean     Caribbean    
## [786] Caribbean     Caribbean     Caribbean     Asia          Asia         
## [791] Asia          Asia          Asia          Asia          Asia         
## [796] Asia          Asia          Asia          Asia          South America
## [801] South America South America South America South America South America
## [806] South America South America South America South America South America
## [811] South America South America Asia          Asia          Asia         
## [816] Asia          Asia          Asia          Asia          Asia         
## [821] Asia          Asia          Asia          Asia          Asia         
## [826] Asia          Asia          South America South America South America
## [831] South America South America South America South America South America
## [836] South America South America South America South America Europe       
## [841] Europe        Europe        Europe        Europe        Europe       
## [846] Europe        Europe        Europe        Europe        Europe       
## [851] Europe        Europe        Europe        Europe        Europe       
## [856] Europe        Europe        Europe        Europe        Europe       
## [861] Europe        Europe        Europe        Europe        Europe       
## [866] Europe        Europe        Europe        Europe        Europe       
## [871] Europe        Europe        Europe        Europe        Europe       
## [876] Europe        Europe        Europe        Europe        Europe       
## [881] Europe        Europe        Europe        Europe        Europe       
## [886] Europe        Europe        Europe        Europe        Europe       
## [891] Europe        Europe        Europe        Europe        Europe       
## [896] Europe        Europe        Europe        Europe        Europe       
## [901] South America Europe        Europe        Europe        Europe       
## [906] Asia          Asia          Asia          Asia          Asia         
## [911] Asia          Asia          Asia          Asia          Asia         
## [916] Asia          Asia          Asia          Asia          Asia         
## [921] Asia          Asia          Asia          Asia          Asia         
## [926] Asia          Asia          Asia          Asia          Asia         
## [931] Asia          Asia          South America
## Levels: Asia Caribbean Europe North America South America
```

Save the genind object

```
saveRDS(snp2, here("scripts", "RMarkdowns",
  "output", "americas", "manuscript1", "dapc", "continent", "snp_country.rds"
))
```

Load the genind object

```
snp_country <- readRDS(here("scripts", "RMarkdowns",
  "output", "americas", "manuscript1", "dapc", "continent", "snp_country.rds"
))
```

### 4.2.1 Scale and find clusters

Scale

```
snp_country <- scaleGen(snp2, NA.method="mean")
class(snp_country)
```

```
## [1] "matrix" "array"
```

```
dim(snp_country)
```

```
## [1]   933 41650
```

```
snp_country[1:5,1:5]
```

```
##      AX-583033387_C.G AX-583033387_C.C AX-583034838_T.A AX-583034838_T.T
## 1001        1.6057210       -1.6057210        1.3041492       -1.3041492
## 1002       -0.9685527        0.9685527       -1.0945538        1.0945538
## 1003       -0.9685527        0.9685527       -1.0945538        1.0945538
## 1004        1.6057210       -1.6057210       -1.0945538        1.0945538
## 1005        1.6057210       -1.6057210        0.1047977       -0.1047977
##      AX-583036983_C.T
## 1001        1.4091172
## 1002        0.0845757
## 1003        1.4091172
## 1004        0.0845757
## 1005        1.4091172
```

```
# Get the populations from the genlight object
populations <- snp2$pop
```

### 4.2.2 Find clusters

```
grp <- find.clusters(snp_country, max.n.clust=30)
#retained 1000
#Choose the number of clusters (>=2): 5
```

Save the genind object

```
saveRDS(grp, here("scripts", "RMarkdowns",
  "output", "americas", "manuscript1", "dapc", "continent", "grp.rds"
))
```

Load the genind object

```
grp <- readRDS(here("scripts", "RMarkdowns",
  "output", "americas", "manuscript1", "dapc", "continent", "grp.rds"
))
```

```
names(grp)
```

```
## [1] "Kstat" "stat"  "grp"   "size"
```

```
grp$size #group sizes for our clusters
```

```
## [1] 162 146 422 155  48
```

```
table(pop(snp2), grp$grp)
```

```
##                
##                   1   2   3   4   5
##   Asia            0 142  35  53   0
##   Caribbean       0   4  16  12   0
##   Europe          0   0  52  57  48
##   North America   0   0 319  18   0
##   South America 162   0   0  15   0
```

```
dapc1 <- dapc(snp_country, grp$grp)
#800 PCs retained
#4 discriminant functions retained
```

```
dapc1
```

```
##  #################################################
##  # Discriminant Analysis of Principal Components #
##  #################################################
## class: dapc
## $call: dapc.data.frame(x = as.data.frame(x), grp = ..1)
## 
## $n.pca: 250 first PCs of PCA used
## $n.da: 92 discriminant functions saved
## $var (proportion of conserved variance): 0.569
## 
## $eig (eigenvalues): 2961 1954 1510 715.8 612.7 ...
## 
##   vector    length content                   
## 1 $eig      92     eigenvalues               
## 2 $grp      933    prior group assignment    
## 3 $prior    93     prior group probabilities 
## 4 $assign   933    posterior group assignment
## 5 $pca.cent 41650  centring vector of PCA    
## 6 $pca.norm 41650  scaling vector of PCA     
## 7 $pca.eig  932    eigenvalues of PCA        
## 
##   data.frame    nrow  ncol content                                          
## 1 $tab          933   250  retained PCs of PCA                              
## 2 $means        93    250  group means                                      
## 3 $loadings     250   92   loadings of variables                            
## 4 $ind.coord    933   92   coordinates of individuals (principal components)
## 5 $grp.coord    93    92   coordinates of groups                            
## 6 $posterior    933   93   posterior membership probabilities               
## 7 $pca.loadings 41650 250  PCA loadings of original variables               
## 8 $var.contr    41650 92   contribution of original variables
```

Save the genind object

```
saveRDS(dapc1, here("scripts", "RMarkdowns",
  "output", "americas", "manuscript1", "dapc", "continent", "dapc1.rds"
))
```

Load the genind object

```
dapc1 <- readRDS(here("scripts", "RMarkdowns",
  "output", "americas", "manuscript1", "dapc", "continent", "dapc1.rds"
))
```

```
scatter(dapc1)
```

### 4.2.3 Calculating the optimum PC number to rerun DAPC

```
optim.a.score(dapc1, n.pca=1:ncol(dapc1$tab), smart=TRUE, n=10, plot=TRUE, n.sim=10) #calculating optimal number of PCs 
#Optimal number = 100
```

### 4.2.4 Now do cross-validation

Cross-validation: The Discriminant Analysis of Principal Components
(DAPC) relies on dimension reduction of the data using PCA followed by a
linear discriminant analysis. How many PCA axes to retain is often a
non-trivial question. Cross validation provides an objective way to
decide how many axes to retain: different numbers are tried and the
quality of the corresponding DAPC is assessed by cross- validation: DAPC
is performed on a training set, typically made of 90% of the
observations (comprising 90% of the observations in each subpopulation)
, and then used to predict the groups of the 10% of remaining
observations. The current method uses the average prediction success per
group (result=“groupMean”), or the overall prediction success
(result=“overall”). The number of PCs associated with the lowest Mean
Squared Error is then retained in the DAPC.

```
xvalDapc(snp_country, populations, n.pca.max = 800, n.da = NULL,
              training.set = 0.9, result = c("groupMean", "overall"),
              center = TRUE, scale = FALSE,
              n.pca=NULL, n.rep = 5, xval.plot = TRUE)
```

\(n.pca: 500 first PCs of PCA
used\)n.da: 4 discriminant functions saved $var (proportion of
conserved variance): 0.793

Run DAPC with object

```
dapc_snp1 <- dapc(snp_country, n.pca = 500, n.da = 4, grp = populations)
```

Save it

```
saveRDS(
  dapc_snp1, here("scripts", "RMarkdowns",
  "output", "americas", "manuscript1", "dapc", "continent", "dapc_snp1.rds"
  )
)
```

To load it

```
dapc_snp1 <- readRDS(
  here("scripts", "RMarkdowns",
  "output", "americas", "manuscript1", "dapc", "continent", "dapc_snp1.rds"
  )
)
```

Most contributing alleles

```
set.seed(4) 
contrib<-loadingplot(dapc_snp1$var.contr, axis=2, thres=.0002, lab.jitter=3)
```

```
scatter(dapc_snp1)
```

Run DAPC with optimised results

```
dapc_snp2 <- dapc(snp_country, n.pca = 100, n.da = 6, grp = populations)
```

Save it

```
saveRDS(
  dapc_snp2, here("scripts", "RMarkdowns",
  "output", "americas", "manuscript1", "dapc", "continent", "dapc_snp2.rds"
  )
)
```

To load it

```
dapc_snp2 <- readRDS(
  here("scripts", "RMarkdowns",
  "output", "americas", "manuscript1", "dapc", "continent", "dapc_snp2.rds"
  )
)
```

```
scatter(dapc_snp2)
```

### 4.2.5 Make plots for DAPC

Check R symbols for plot

```
#to see all shapes -> plot shapes - para escolher os simbolos
N = 100; M = 1000
good.shapes = c(1:25,35:38,43,60,62:64)
foo = data.frame( x = rnorm(M), y = rnorm(M), s = factor( sample(1:N, M, replace = TRUE) ) )
ggplot(aes(x,y,shape=s ), data=foo ) +
  scale_shape_manual(values=good.shapes[1:N]) +
  geom_point()
#1:25,28:31,36,55:57
```

Plot using different discriminant functions

1 & 2

```
pdf(file = "scripts/RMarkdowns/output/americas/manuscript1/dapc/continent/dapc_manuscript1_r01_PC1_2_continent.pdf",   # The directory you want to save the file in
    width = 7, # The width of the plot in inches
    height = 7) # The height of the plot in inches

myCol <- c("orange","#E41A1C","purple","#2524f9","#005C31")

good.shapes = c(1:25)

op <- par(cex = 0.39)
scatter(dapc_snp2, pch = good.shapes, cstar = 0, col=myCol, label=NULL, mstree = FALSE, legend=TRUE, posi.da="bottomright", cex=1.0,  cex.lab=0.5, cex.main=0.5, cellipse=TRUE, posi.leg="topleft",  xax =1, yax=2)

dev.off()
```

```
op <- par(cex = 0.39)
scatter(dapc_snp2, pch = good.shapes, cstar = 0, col=myCol, label=NULL, mstree = FALSE, legend=TRUE, posi.da="bottomright", cex=1.0,  cex.lab=0.5, cex.main=0.5, cellipse=TRUE, posi.leg="topleft",  xax =1, yax=2)
```

1 & 3

```
pdf(file = "scripts/RMarkdowns/output/americas/manuscript1/dapc/continent/dapc_manuscript1_r01_PC1_3_continent.pdf",   # The directory you want to save the file in
    width = 7, # The width of the plot in inches
    height = 7) # The height of the plot in inches

myCol <- c("orange","#E41A1C","purple","#2524f9","#005C31")

good.shapes = c(1:25)

op <- par(cex = 0.39)
scatter(dapc_snp2, pch = good.shapes, cstar = 0, col=myCol, label=NULL, mstree = FALSE, legend=TRUE, posi.da="topright", cex=1.0,  cex.lab=0.5, cex.main=0.5, cellipse=TRUE, posi.leg="topleft",  xax =1, yax=3)

dev.off()
```

```
op <- par(cex = 0.39)
scatter(dapc_snp2, pch = good.shapes, cstar = 0, col=myCol, label=NULL, mstree = FALSE, legend=TRUE, posi.da="topright", cex=1.0,  cex.lab=0.5, cex.main=0.5, cellipse=TRUE, posi.leg="topleft",  xax =1, yax=3)
```

1 & 4

```
pdf(file = "scripts/RMarkdowns/output/americas/manuscript1/dapc/continent/dapc_manuscript1_r01_PC1_4_continent.pdf",   # The directory you want to save the file in
    width = 7, # The width of the plot in inches
    height = 7) # The height of the plot in inches

myCol <- c("orange","#E41A1C","purple","#2524f9","#005C31")

good.shapes = c(1:25)

op <- par(cex = 0.39)
scatter(dapc_snp2, pch = good.shapes, cstar = 0, col=myCol, label=NULL, mstree = FALSE, legend=TRUE, posi.da="topright", cex=1.0,  cex.lab=0.5, cex.main=0.5, cellipse=TRUE, posi.leg="topleft",  xax =1, yax=4)

dev.off()
```

```
op <- par(cex = 0.39)
scatter(dapc_snp2, pch = good.shapes, cstar = 0, col=myCol, label=NULL, mstree = FALSE, legend=TRUE, posi.da="topright", cex=1.0,  cex.lab=0.5, cex.main=0.5, cellipse=TRUE, posi.leg="topleft",  xax =1, yax=4)
```

```
library(MASS)
library(ape)
library(reshape2)
library(tidyverse)
library(ade4)
library(adegenet)
library(here)
library(ggplot2)
library(dartR)
library(dartR.base)
library(StAMPP)
#library(geosphere)
library(flextable)
library(officer)
library(Cairo)
library(vcfR)
library(ggplot2)
```

# 5. Fst

## 5.1. Weir and Cockerham Fst

Estimate Fst for Global populations using stampp

We will estimate Fst for populations with at least 4 individuals
using r<0.01 SNP set with 1% MAF dataset

Create list of populations

```
cd /gpfs/gibbs/pi/caccone/mkc54/albo/manuscript1

awk '{print $1}' output/snps_sets/r2_0.01_b.fam | sort | uniq -c | awk '{print $2, $1}' | awk '$2 >= 4 {print}' | awk '{print $1}' > output/fst/pops_4fst.txt;
head  output/fst/pops_4fst.txt;
wc -l output/fst/pops_4fst.txt
```

```
## ALD
## ARA
## BAF
## BCO
## BEA
## BEH
## BEN
## BER
## BIT
## BPA
## 89 output/fst/pops_4fst.txt
```

We have 89 populations with 4 or more mosquitoes. We can convert to
raw format and subset the bed file

```
cd /gpfs/gibbs/pi/caccone/mkc54/albo
module load PLINK/1.9b_6.21-x86_64

plink \
--allow-extra-chr \
--keep-allele-order \
--bfile manuscript1/output/snps_sets/r2_0.01_b \
--keep-fam manuscript1/output/fst/pops_4fst.txt \
--recodeA \
--out manuscript1/output/fst/allpopsforfst \
--silent;
grep 'samples\|variants\|remaining' manuscript1/output/fst/allpopsforfst.log
```

20825 variants loaded from .bim file. –keep-fam: 923 people
remaining. Total genotyping rate in remaining samples is 0.967628. 20825
variants and 923 people pass filters and QC.

Look at https://rdrr.io/cran/StAMPP/man/stamppFst.html for
details of Fst estimations

```
allpopsforfst <-
  read.PLINK(
    here(
      "manuscript1", "output", "fst", "allpopsforfst.raw"
    ),
    quiet = FALSE,
    chunkSize = 1000,
    parallel = require("parallel"),
    n.cores = 4
  )

summary(allpopsforfst)
```

Save it

```
saveRDS(
  allpopsforfst, here(
    "manuscript1/output/fst/allpopsforfst.rds"
  )
)
```

Now lets convert the genlight object to Stampp format, and estimate
pairwide Fst values

```
#start an interactive session and load R packages needed
#salloc -p scavenge -c 10 -t 2:00:00
salloc -p scavenge --cpus-per-task=5 -t 2:00:00 --mem-per-cpu=10000    
module load R/4.2.0-foss-2020b
R

library(StAMPP)
library(adegenet)
library(here)

allpopsforfst <- readRDS(
  here(
    "/gpfs/gibbs/pi/caccone/mkc54/albo/manuscript1/output/fst/allpopsforfst.rds"
  )
)

summary(allpopsforfst)

#This chunk will take a couple minutes to run.

allpops_2 <- stamppConvert(allpopsforfst, type="genlight")

allpops_3 <- stamppFst(allpops_2, 1, 95, 1)

saveRDS(
  allpops_3, here(
    "/gpfs/gibbs/pi/caccone/mkc54/albo/manuscript1/output/fst/allpops_stamppFst.rds"
  )
)
```

To load it

```
allpops_3 <- readRDS(
  here(
    "manuscript1", "output", "fst", "allpops_stamppFst.rds"
  )
)
```

Now lets look at the object

```
summary(allpops_3)
```

```
##       OKI               HAI               SOC               YUN          
##  Min.   :0.07367   Min.   :0.03557   Min.   :0.01212   Min.   :0.001118  
##  1st Qu.:0.11289   1st Qu.:0.08171   1st Qu.:0.12024   1st Qu.:0.105623  
##  Median :0.14157   Median :0.10440   Median :0.14809   Median :0.129169  
##  Mean   :0.14194   Mean   :0.10432   Mean   :0.15048   Mean   :0.131233  
##  3rd Qu.:0.16040   3rd Qu.:0.11901   3rd Qu.:0.17614   3rd Qu.:0.169296  
##  Max.   :0.26068   Max.   :0.19298   Max.   :0.27609   Max.   :0.255105  
##  NA's   :1         NA's   :2         NA's   :3         NA's   :4         
##       KAT               GES               TIK               RAR         
##  Min.   :0.02791   Min.   :0.02787   Min.   :0.05166   Min.   :0.08671  
##  1st Qu.:0.11542   1st Qu.:0.12509   1st Qu.:0.13645   1st Qu.:0.12961  
##  Median :0.14134   Median :0.15178   Median :0.16309   Median :0.15488  
##  Mean   :0.14416   Mean   :0.15624   Mean   :0.16904   Mean   :0.16014  
##  3rd Qu.:0.18241   3rd Qu.:0.18132   3rd Qu.:0.19265   3rd Qu.:0.18191  
##  Max.   :0.28178   Max.   :0.28293   Max.   :0.29636   Max.   :0.28138  
##  NA's   :5         NA's   :6         NA's   :7         NA's   :8        
##       TAI              HUN               HAN               KLP         
##  Min.   :0.1289   Min.   :0.03941   Min.   :0.04755   Min.   :0.05108  
##  1st Qu.:0.1719   1st Qu.:0.06894   1st Qu.:0.09584   1st Qu.:0.14369  
##  Median :0.1977   Median :0.09341   Median :0.11723   Median :0.15450  
##  Mean   :0.2045   Mean   :0.09499   Mean   :0.12022   Mean   :0.16267  
##  3rd Qu.:0.2219   3rd Qu.:0.11519   3rd Qu.:0.13567   3rd Qu.:0.18959  
##  Max.   :0.3457   Max.   :0.20249   Max.   :0.24394   Max.   :0.31347  
##  NA's   :9        NA's   :10        NA's   :11        NA's   :12       
##       HOC               QNC               SSK               KAC         
##  Min.   :0.05221   Min.   :0.08175   Min.   :0.00359   Min.   :0.00112  
##  1st Qu.:0.09409   1st Qu.:0.18131   1st Qu.:0.11241   1st Qu.:0.11231  
##  Median :0.11478   Median :0.19859   Median :0.13385   Median :0.13360  
##  Mean   :0.11655   Mean   :0.20082   Mean   :0.13795   Mean   :0.13780  
##  3rd Qu.:0.12946   3rd Qu.:0.22971   3rd Qu.:0.17177   3rd Qu.:0.16854  
##  Max.   :0.20884   Max.   :0.32607   Max.   :0.25299   Max.   :0.26850  
##  NA's   :13        NA's   :14        NA's   :15        NA's   :16       
##       CHA                BRE               DES               CES        
##  Min.   :0.002963   Min.   :0.07084   Min.   :0.06195   Min.   :0.1169  
##  1st Qu.:0.113223   1st Qu.:0.09545   1st Qu.:0.08522   1st Qu.:0.1319  
##  Median :0.133173   Median :0.14358   Median :0.13646   Median :0.1792  
##  Mean   :0.139319   Mean   :0.15612   Mean   :0.14436   Mean   :0.1913  
##  3rd Qu.:0.170192   3rd Qu.:0.20132   3rd Qu.:0.19025   3rd Qu.:0.2354  
##  Max.   :0.252175   Max.   :0.31966   Max.   :0.30714   Max.   :0.3577  
##  NA's   :17         NA's   :18        NA's   :19        NA's   :20      
##       KAN               UTS               MAU               TUC          
##  Min.   :0.08847   Min.   :0.05283   Min.   :0.03648   Min.   :0.007459  
##  1st Qu.:0.11451   1st Qu.:0.07253   1st Qu.:0.09726   1st Qu.:0.107792  
##  Median :0.15616   Median :0.12563   Median :0.11786   Median :0.139953  
##  Mean   :0.17111   Mean   :0.13507   Mean   :0.11703   Mean   :0.125925  
##  3rd Qu.:0.21578   3rd Qu.:0.18281   3rd Qu.:0.13394   3rd Qu.:0.155229  
##  Max.   :0.33487   Max.   :0.29795   Max.   :0.23539   Max.   :0.249991  
##  NA's   :21        NA's   :22        NA's   :23        NA's   :24        
##       REC               BRM               CAM                KAG         
##  Min.   :0.02791   Min.   :0.07716   Min.   :0.008479   Min.   :0.06294  
##  1st Qu.:0.09275   1st Qu.:0.09634   1st Qu.:0.104477   1st Qu.:0.08323  
##  Median :0.11003   Median :0.12326   Median :0.120830   Median :0.11064  
##  Mean   :0.11479   Mean   :0.13864   Mean   :0.123932   Mean   :0.13097  
##  3rd Qu.:0.13079   3rd Qu.:0.17059   3rd Qu.:0.150325   3rd Qu.:0.17308  
##  Max.   :0.23495   Max.   :0.27467   Max.   :0.232982   Max.   :0.27461  
##  NA's   :25        NA's   :26        NA's   :27         NA's   :28       
##       CUA                MED              FAY               DAL         
##  Min.   :0.002043   Min.   :0.0937   Min.   :0.01769   Min.   :0.01464  
##  1st Qu.:0.127679   1st Qu.:0.1233   1st Qu.:0.03654   1st Qu.:0.04090  
##  Median :0.136685   Median :0.1330   Median :0.07269   Median :0.08197  
##  Mean   :0.142269   Mean   :0.1409   Mean   :0.09215   Mean   :0.09757  
##  3rd Qu.:0.151902   3rd Qu.:0.1495   3rd Qu.:0.13306   3rd Qu.:0.14326  
##  Max.   :0.255676   Max.   :0.2528   Max.   :0.24305   Max.   :0.25255  
##  NA's   :29         NA's   :30       NA's   :31        NA's   :32       
##       NEO               RUS               CHI               NUE         
##  Min.   :0.03057   Min.   :0.06128   Min.   :0.06956   Min.   :0.01106  
##  1st Qu.:0.05242   1st Qu.:0.07809   1st Qu.:0.11096   1st Qu.:0.05437  
##  Median :0.08387   Median :0.11641   Median :0.12855   Median :0.07972  
##  Mean   :0.10619   Mean   :0.13256   Mean   :0.13329   Mean   :0.09692  
##  3rd Qu.:0.14731   3rd Qu.:0.17609   3rd Qu.:0.14678   3rd Qu.:0.12959  
##  Max.   :0.25487   Max.   :0.27945   Max.   :0.24082   Max.   :0.23036  
##  NA's   :33        NA's   :34        NA's   :35        NA's   :36       
##       TRA               HAL               STL               SMI         
##  Min.   :0.01103   Min.   :0.01646   Min.   :0.03522   Min.   :0.01515  
##  1st Qu.:0.04050   1st Qu.:0.03999   1st Qu.:0.05862   1st Qu.:0.05496  
##  Median :0.07307   Median :0.08769   Median :0.11914   Median :0.10295  
##  Mean   :0.09164   Mean   :0.10222   Mean   :0.12500   Mean   :0.11341  
##  3rd Qu.:0.12987   3rd Qu.:0.15342   3rd Qu.:0.18292   3rd Qu.:0.16163  
##  Max.   :0.23340   Max.   :0.25416   Max.   :0.28324   Max.   :0.25668  
##  NA's   :37        NA's   :38        NA's   :39        NA's   :40       
##       WAV               HAT               DEN               MON         
##  Min.   :0.02447   Min.   :0.02523   Min.   :0.02710   Min.   :0.01685  
##  1st Qu.:0.05205   1st Qu.:0.05093   1st Qu.:0.04295   1st Qu.:0.05287  
##  Median :0.12119   Median :0.11360   Median :0.10995   Median :0.10668  
##  Mean   :0.12387   Mean   :0.11441   Mean   :0.11182   Mean   :0.10424  
##  3rd Qu.:0.18329   3rd Qu.:0.16040   3rd Qu.:0.16410   3rd Qu.:0.13592  
##  Max.   :0.28595   Max.   :0.25507   Max.   :0.25522   Max.   :0.22929  
##  NA's   :41        NA's   :42        NA's   :43        NA's   :44       
##       BPV               BCO               BPA               BIT         
##  Min.   :0.04873   Min.   :0.02047   Min.   :0.06348   Min.   :0.04198  
##  1st Qu.:0.08488   1st Qu.:0.10121   1st Qu.:0.10348   1st Qu.:0.11546  
##  Median :0.14021   Median :0.14359   Median :0.11942   Median :0.14212  
##  Mean   :0.13159   Mean   :0.13227   Mean   :0.12669   Mean   :0.13692  
##  3rd Qu.:0.15676   3rd Qu.:0.16033   3rd Qu.:0.13639   3rd Qu.:0.16116  
##  Max.   :0.25684   Max.   :0.25944   Max.   :0.23682   Max.   :0.25749  
##  NA's   :45        NA's   :46        NA's   :47        NA's   :48       
##       FLO               BAF               PLM               NEH         
##  Min.   :0.01003   Min.   :0.00612   Min.   :0.01682   Min.   :0.02120  
##  1st Qu.:0.11813   1st Qu.:0.10791   1st Qu.:0.11621   1st Qu.:0.04192  
##  Median :0.14739   Median :0.14010   Median :0.15077   Median :0.08899  
##  Mean   :0.13942   Mean   :0.13308   Mean   :0.14372   Mean   :0.11442  
##  3rd Qu.:0.16519   3rd Qu.:0.15901   3rd Qu.:0.17100   3rd Qu.:0.17198  
##  Max.   :0.27374   Max.   :0.26643   Max.   :0.27377   Max.   :0.26913  
##  NA's   :49        NA's   :50        NA's   :51        NA's   :52       
##       LIN               HAR               FIR               NEB         
##  Min.   :0.05155   Min.   :0.03659   Min.   :0.06303   Min.   :0.02835  
##  1st Qu.:0.07088   1st Qu.:0.05600   1st Qu.:0.08488   1st Qu.:0.05607  
##  Median :0.11197   Median :0.12040   Median :0.15125   Median :0.12031  
##  Mean   :0.13520   Mean   :0.12693   Mean   :0.15932   Mean   :0.13002  
##  3rd Qu.:0.19149   3rd Qu.:0.18256   3rd Qu.:0.21517   3rd Qu.:0.19063  
##  Max.   :0.27909   Max.   :0.26576   Max.   :0.30250   Max.   :0.27176  
##  NA's   :53        NA's   :54        NA's   :55        NA's   :56       
##       BEN               BEH               ARA               IPO         
##  Min.   :0.02771   Min.   :0.03704   Min.   :0.04841   Min.   :0.03632  
##  1st Qu.:0.11980   1st Qu.:0.13277   1st Qu.:0.11191   1st Qu.:0.18410  
##  Median :0.14357   Median :0.16653   Median :0.13823   Median :0.21016  
##  Mean   :0.14512   Mean   :0.15678   Mean   :0.13879   Mean   :0.20055  
##  3rd Qu.:0.17841   3rd Qu.:0.19185   3rd Qu.:0.16301   3rd Qu.:0.23686  
##  Max.   :0.27031   Max.   :0.28951   Max.   :0.24921   Max.   :0.34991  
##  NA's   :57        NA's   :58        NA's   :59        NA's   :60       
##       LAM               PAL               MAN               BEA         
##  Min.   :0.01865   Min.   :0.03001   Min.   :0.02570   Min.   :0.01324  
##  1st Qu.:0.12323   1st Qu.:0.06950   1st Qu.:0.03963   1st Qu.:0.04782  
##  Median :0.13815   Median :0.09797   Median :0.09531   Median :0.08800  
##  Mean   :0.14241   Mean   :0.11438   Mean   :0.11110   Mean   :0.10007  
##  3rd Qu.:0.17219   3rd Qu.:0.14624   3rd Qu.:0.16788   3rd Qu.:0.13975  
##  Max.   :0.26526   Max.   :0.23242   Max.   :0.25326   Max.   :0.21994  
##  NA's   :61        NA's   :62        NA's   :63        NA's   :64       
##       PEO               HOU               LOS               NEG        
##  Min.   :0.02749   Min.   :0.01650   Min.   :0.07693   Min.   :0.1457  
##  1st Qu.:0.05513   1st Qu.:0.05456   1st Qu.:0.11189   1st Qu.:0.2173  
##  Median :0.10621   Median :0.09677   Median :0.13047   Median :0.2484  
##  Mean   :0.11790   Mean   :0.10316   Mean   :0.14275   Mean   :0.2474  
##  3rd Qu.:0.16846   3rd Qu.:0.14872   3rd Qu.:0.17649   3rd Qu.:0.2628  
##  Max.   :0.25091   Max.   :0.21693   Max.   :0.23600   Max.   :0.4106  
##  NA's   :65        NA's   :66        NA's   :67        NA's   :68      
##       NOV               NEW               BER              MAC         
##  Min.   :0.03753   Min.   :0.05097   Min.   :0.0451   Min.   :0.05382  
##  1st Qu.:0.16865   1st Qu.:0.07712   1st Qu.:0.0715   1st Qu.:0.06960  
##  Median :0.20817   Median :0.12388   Median :0.1239   Median :0.11942  
##  Mean   :0.19113   Mean   :0.13348   Mean   :0.1326   Mean   :0.11932  
##  3rd Qu.:0.21804   3rd Qu.:0.17729   3rd Qu.:0.1747   3rd Qu.:0.14682  
##  Max.   :0.35018   Max.   :0.26293   Max.   :0.2652   Max.   :0.22627  
##  NA's   :69        NA's   :70        NA's   :71       NA's   :72       
##       SPR               COL               SAI               POR         
##  Min.   :0.06413   Min.   :0.04510   Min.   :0.06957   Min.   :0.02862  
##  1st Qu.:0.11455   1st Qu.:0.09591   1st Qu.:0.10240   1st Qu.:0.16178  
##  Median :0.16044   Median :0.15278   Median :0.12813   Median :0.17850  
##  Mean   :0.16739   Mean   :0.14963   Mean   :0.13483   Mean   :0.17243  
##  3rd Qu.:0.20727   3rd Qu.:0.18548   3rd Qu.:0.16729   3rd Qu.:0.20216  
##  Max.   :0.30425   Max.   :0.27035   Max.   :0.20992   Max.   :0.27560  
##  NA's   :73        NA's   :74        NA's   :75        NA's   :76       
##       SUF               SUU               GRV              GRA         
##  Min.   :0.03997   Min.   :0.09221   Min.   :0.1161   Min.   :0.06498  
##  1st Qu.:0.12475   1st Qu.:0.11713   1st Qu.:0.1425   1st Qu.:0.07877  
##  Median :0.20442   Median :0.14793   Median :0.1636   Median :0.09208  
##  Mean   :0.18230   Mean   :0.15306   Mean   :0.1741   Mean   :0.11325  
##  3rd Qu.:0.23647   3rd Qu.:0.17301   3rd Qu.:0.1998   3rd Qu.:0.15873  
##  Max.   :0.34977   Max.   :0.27772   Max.   :0.2562   Max.   :0.18911  
##  NA's   :77        NA's   :78        NA's   :79       NA's   :80       
##       TUH               ALD               FRS               SER        
##  Min.   :0.04608   Min.   :0.09138   Min.   :0.05779   Min.   :0.1922  
##  1st Qu.:0.08499   1st Qu.:0.12488   1st Qu.:0.10798   1st Qu.:0.2359  
##  Median :0.11746   Median :0.17099   Median :0.15529   Median :0.2579  
##  Mean   :0.13382   Mean   :0.17873   Mean   :0.15388   Mean   :0.2889  
##  3rd Qu.:0.18274   3rd Qu.:0.23047   3rd Qu.:0.20314   3rd Qu.:0.3403  
##  Max.   :0.24043   Max.   :0.27805   Max.   :0.24364   Max.   :0.4183  
##  NA's   :81        NA's   :82        NA's   :83        NA's   :84      
##       SPB              SPS              INW               INJ        
##  Min.   :0.1240   Min.   :0.1854   Min.   :0.06223   Min.   :0.1278  
##  1st Qu.:0.1634   1st Qu.:0.2223   1st Qu.:0.08703   1st Qu.:0.1278  
##  Median :0.2108   Median :0.2592   Median :0.11182   Median :0.1278  
##  Mean   :0.2047   Mean   :0.2476   Mean   :0.11182   Mean   :0.1278  
##  3rd Qu.:0.2521   3rd Qu.:0.2787   3rd Qu.:0.13662   3rd Qu.:0.1278  
##  Max.   :0.2734   Max.   :0.2982   Max.   :0.16142   Max.   :0.1278  
##  NA's   :85       NA's   :86       NA's   :87        NA's   :88      
##       MAT     
##  Min.   : NA  
##  1st Qu.: NA  
##  Median : NA  
##  Mean   :NaN  
##  3rd Qu.: NA  
##  Max.   : NA  
##  NA's   :89
```

```
# Convert to data frame
allpops_df <- data.frame(allpops_3)
```

If you want you can save the fst values as csv

```
# Save it
write.csv(allpops_df, file = here("manuscript1", "output", "fst", "allpops_3.csv"))
```

Check the Fst values

```
head(allpops_df)
```

```
##            OKI        HAI        SOC        YUN       KAT GES TIK RAR TAI HUN
## OKI         NA         NA         NA         NA        NA  NA  NA  NA  NA  NA
## HAI 0.09558814         NA         NA         NA        NA  NA  NA  NA  NA  NA
## SOC 0.13633551 0.11385993         NA         NA        NA  NA  NA  NA  NA  NA
## YUN 0.14594947 0.06147232 0.16663785         NA        NA  NA  NA  NA  NA  NA
## KAT 0.16182412 0.07881081 0.18188522 0.02757809        NA  NA  NA  NA  NA  NA
## GES 0.14091413 0.11836788 0.01211906 0.17292440 0.1893266  NA  NA  NA  NA  NA
##     HAN KLP HOC QNC SSK KAC CHA BRE DES CES KAN UTS MAU TUC REC BRM CAM KAG CUA
## OKI  NA  NA  NA  NA  NA  NA  NA  NA  NA  NA  NA  NA  NA  NA  NA  NA  NA  NA  NA
## HAI  NA  NA  NA  NA  NA  NA  NA  NA  NA  NA  NA  NA  NA  NA  NA  NA  NA  NA  NA
## SOC  NA  NA  NA  NA  NA  NA  NA  NA  NA  NA  NA  NA  NA  NA  NA  NA  NA  NA  NA
## YUN  NA  NA  NA  NA  NA  NA  NA  NA  NA  NA  NA  NA  NA  NA  NA  NA  NA  NA  NA
## KAT  NA  NA  NA  NA  NA  NA  NA  NA  NA  NA  NA  NA  NA  NA  NA  NA  NA  NA  NA
## GES  NA  NA  NA  NA  NA  NA  NA  NA  NA  NA  NA  NA  NA  NA  NA  NA  NA  NA  NA
##     MED FAY DAL NEO RUS CHI NUE TRA HAL STL SMI WAV HAT DEN MON BPV BCO BPA BIT
## OKI  NA  NA  NA  NA  NA  NA  NA  NA  NA  NA  NA  NA  NA  NA  NA  NA  NA  NA  NA
## HAI  NA  NA  NA  NA  NA  NA  NA  NA  NA  NA  NA  NA  NA  NA  NA  NA  NA  NA  NA
## SOC  NA  NA  NA  NA  NA  NA  NA  NA  NA  NA  NA  NA  NA  NA  NA  NA  NA  NA  NA
## YUN  NA  NA  NA  NA  NA  NA  NA  NA  NA  NA  NA  NA  NA  NA  NA  NA  NA  NA  NA
## KAT  NA  NA  NA  NA  NA  NA  NA  NA  NA  NA  NA  NA  NA  NA  NA  NA  NA  NA  NA
## GES  NA  NA  NA  NA  NA  NA  NA  NA  NA  NA  NA  NA  NA  NA  NA  NA  NA  NA  NA
##     FLO BAF PLM NEH LIN HAR FIR NEB BEN BEH ARA IPO LAM PAL MAN BEA PEO HOU LOS
## OKI  NA  NA  NA  NA  NA  NA  NA  NA  NA  NA  NA  NA  NA  NA  NA  NA  NA  NA  NA
## HAI  NA  NA  NA  NA  NA  NA  NA  NA  NA  NA  NA  NA  NA  NA  NA  NA  NA  NA  NA
## SOC  NA  NA  NA  NA  NA  NA  NA  NA  NA  NA  NA  NA  NA  NA  NA  NA  NA  NA  NA
## YUN  NA  NA  NA  NA  NA  NA  NA  NA  NA  NA  NA  NA  NA  NA  NA  NA  NA  NA  NA
## KAT  NA  NA  NA  NA  NA  NA  NA  NA  NA  NA  NA  NA  NA  NA  NA  NA  NA  NA  NA
## GES  NA  NA  NA  NA  NA  NA  NA  NA  NA  NA  NA  NA  NA  NA  NA  NA  NA  NA  NA
##     NEG NOV NEW BER MAC SPR COL SAI POR SUF SUU GRV GRA TUH ALD FRS SER SPB SPS
## OKI  NA  NA  NA  NA  NA  NA  NA  NA  NA  NA  NA  NA  NA  NA  NA  NA  NA  NA  NA
## HAI  NA  NA  NA  NA  NA  NA  NA  NA  NA  NA  NA  NA  NA  NA  NA  NA  NA  NA  NA
## SOC  NA  NA  NA  NA  NA  NA  NA  NA  NA  NA  NA  NA  NA  NA  NA  NA  NA  NA  NA
## YUN  NA  NA  NA  NA  NA  NA  NA  NA  NA  NA  NA  NA  NA  NA  NA  NA  NA  NA  NA
## KAT  NA  NA  NA  NA  NA  NA  NA  NA  NA  NA  NA  NA  NA  NA  NA  NA  NA  NA  NA
## GES  NA  NA  NA  NA  NA  NA  NA  NA  NA  NA  NA  NA  NA  NA  NA  NA  NA  NA  NA
##     INW INJ MAT
## OKI  NA  NA  NA
## HAI  NA  NA  NA
## SOC  NA  NA  NA
## YUN  NA  NA  NA
## KAT  NA  NA  NA
## GES  NA  NA  NA
```

We will convert the data into a matrix.

```
aa <- as.matrix(allpops_df)
aa[upper.tri(aa)] <- t(aa)[upper.tri(t(aa))]
head(aa)
```

```
##            OKI        HAI        SOC        YUN        KAT        GES
## OKI         NA 0.09558814 0.13633551 0.14594947 0.16182412 0.14091413
## HAI 0.09558814         NA 0.11385993 0.06147232 0.07881081 0.11836788
## SOC 0.13633551 0.11385993         NA 0.16663785 0.18188522 0.01211906
## YUN 0.14594947 0.06147232 0.16663785         NA 0.02757809 0.17292440
## KAT 0.16182412 0.07881081 0.18188522 0.02757809         NA 0.18932663
## GES 0.14091413 0.11836788 0.01211906 0.17292440 0.18932663         NA
##            TIK        RAR       TAI        HUN        HAN        KLP        HOC
## OKI 0.15298678 0.14221855 0.1624812 0.07367462 0.11262072 0.17208667 0.10209590
## HAI 0.12952901 0.11721834 0.1438048 0.04131371 0.03935239 0.08459820 0.03556564
## SOC 0.05912151 0.01989924 0.1961891 0.08231007 0.13396013 0.19140352 0.12172998
## YUN 0.18436091 0.16929635 0.2093213 0.09455389 0.05900262 0.05812901 0.07520378
## KAT 0.20259876 0.18597223 0.2316420 0.10792479 0.07860738 0.08135059 0.09354520
## GES 0.05432309 0.02787053 0.1986180 0.08639645 0.13951177 0.19762116 0.12629917
##            QNC         SSK         KAC         CHA       BRE       DES
## OKI 0.20816098 0.147614206 0.146291307 0.145417551 0.1732961 0.1605189
## HAI 0.13016038 0.064453269 0.058794970 0.061622890 0.1621626 0.1515315
## SOC 0.22754716 0.169222120 0.169132401 0.166503927 0.1735052 0.1632821
## YUN 0.08211356 0.004491156 0.001117613 0.001439952 0.2214530 0.2113039
## KAT 0.11566529 0.032655753 0.027910112 0.030487234 0.2356632 0.2244187
## GES 0.23486993 0.175728154 0.175086589 0.172317879 0.1753479 0.1662910
##           CES       KAN       UTS        MAU       TUC        REC       BRM
## OKI 0.2034129 0.1765158 0.1460260 0.14377638 0.1594006 0.13624179 0.1398983
## HAI 0.1929777 0.1662395 0.1358345 0.09502008 0.1028282 0.09866765 0.1235319
## SOC 0.2059232 0.1706158 0.1442653 0.16017389 0.1762622 0.15417512 0.1511297
## YUN 0.2544209 0.2287345 0.1998948 0.10690195 0.1033862 0.12321344 0.1716397
## KAT 0.2719183 0.2454021 0.2128193 0.11639467 0.1146626 0.13405590 0.1858164
## GES 0.2076713 0.1741899 0.1481145 0.16518769 0.1804067 0.15831344 0.1549302
##             CAM       KAG        CUA        MED        FAY       DAL       NEO
## OKI 0.131016865 0.1260474 0.14820809 0.14663578 0.10014439 0.1084890 0.1102706
## HAI 0.049755118 0.1166803 0.09610516 0.08921155 0.09067834 0.1009434 0.1023955
## SOC 0.151303233 0.1292758 0.16912438 0.16433917 0.11369321 0.1218228 0.1247596
## YUN 0.004125119 0.1725061 0.11277595 0.10530343 0.14642726 0.1552289 0.1565965
## KAT 0.028024545 0.1860823 0.12634461 0.11444477 0.15453315 0.1668447 0.1686488
## GES 0.157560709 0.1327545 0.17490132 0.17107125 0.11817163 0.1255112 0.1294903
##           RUS        CHI        NUE        TRA       HAL       STL       SMI
## OKI 0.1323524 0.11503695 0.10025146 0.09589567 0.1067001 0.1338220 0.1152633
## HAI 0.1276570 0.05891201 0.07839041 0.08221655 0.1013947 0.1231074 0.1020488
## SOC 0.1457495 0.13272942 0.11692530 0.10909637 0.1076025 0.1337008 0.1254611
## YUN 0.1784320 0.10810659 0.12858842 0.13308082 0.1560695 0.1863641 0.1573600
## KAT 0.1902152 0.12828818 0.14044260 0.14477684 0.1667415 0.1984763 0.1704798
## GES 0.1517791 0.13728710 0.12113085 0.11295424 0.1117998 0.1372284 0.1300365
##           WAV       HAT       DEN        MON       BPV       BCO       BPA
## OKI 0.1288128 0.1079221 0.1046447 0.09737039 0.1603635 0.1653289 0.1441479
## HAI 0.1199530 0.1036427 0.1030282 0.07885513 0.1067394 0.1114739 0.1061755
## SOC 0.1307224 0.1196601 0.1183906 0.11341963 0.1775241 0.1816329 0.1613069
## YUN 0.1790267 0.1539487 0.1603127 0.13005893 0.1130443 0.1145506 0.1258053
## KAT 0.1908540 0.1663502 0.1708964 0.14203610 0.1249593 0.1251151 0.1358078
## GES 0.1341381 0.1246739 0.1216414 0.11748504 0.1839108 0.1855865 0.1655459
##           BIT       FLO        BAF       PLM       NEH       LIN       HAR
## OKI 0.1590180 0.1683931 0.15870048 0.1682647 0.1143111 0.1280703 0.1212813
## HAI 0.1079176 0.1103403 0.09915842 0.1064906 0.1043950 0.1195671 0.1132978
## SOC 0.1774755 0.1853021 0.17551599 0.1862409 0.1180840 0.1320491 0.1264882
## YUN 0.1178931 0.1159422 0.10265725 0.1056234 0.1624636 0.1811374 0.1683406
## KAT 0.1298108 0.1258349 0.11346713 0.1163252 0.1736599 0.1927860 0.1804430
## GES 0.1819322 0.1896658 0.18117898 0.1901956 0.1208558 0.1362601 0.1311036
##           FIR       NEB        BEN        BEH       ARA       IPO         LAM
## OKI 0.1473220 0.1214149 0.16480162 0.18289716 0.1545442 0.2246868 0.150056765
## HAI 0.1422376 0.1119907 0.08119431 0.11299642 0.1003186 0.1475621 0.064812146
## SOC 0.1550713 0.1258977 0.18443443 0.19848522 0.1734538 0.2397570 0.172523618
## YUN 0.2001698 0.1720573 0.02233991 0.09834216 0.1065040 0.1264610 0.007137257
## KAT 0.2156081 0.1844052 0.03187799 0.11299195 0.1172988 0.1414378 0.033877867
## GES 0.1586044 0.1291062 0.19066744 0.20483388 0.1766602 0.2452972 0.179286918
##            PAL       MAN        BEA        PEO        HOU        LOS       NEG
## OKI 0.11298144 0.1085156 0.09161781 0.10694113 0.08864899 0.11450880 0.2424934
## HAI 0.08730472 0.0997583 0.07233305 0.09774803 0.06907390 0.07743439 0.1632479
## SOC 0.12670815 0.1112396 0.10475728 0.11102698 0.10053266 0.12657310 0.2576322
## YUN 0.12916878 0.1566688 0.12151888 0.15244264 0.11724201 0.12660762 0.1326778
## KAT 0.14087406 0.1684364 0.13239546 0.16294958 0.12884947 0.14123944 0.1632650
## GES 0.13113663 0.1150112 0.10780997 0.11563278 0.10595289 0.13117830 0.2658439
##            NOV       NEW       BER        MAC       SPR       COL        SAI
## OKI 0.20224951 0.1194139 0.1192349 0.10377704 0.1427641 0.1209269 0.11247446
## HAI 0.11846110 0.1182389 0.1098587 0.07851593 0.1313231 0.1091179 0.07498188
## SOC 0.22193780 0.1277548 0.1219931 0.11537023 0.1440148 0.1197429 0.13270703
## YUN 0.09269472 0.1712038 0.1668719 0.12601402 0.1913415 0.1698510 0.10492622
## KAT 0.10707880 0.1826798 0.1787213 0.13760812 0.2058800 0.1823181 0.11842615
## GES 0.22820527 0.1315438 0.1262965 0.11932998 0.1496688 0.1246398 0.13684611
##           POR        SUF        SUU       GRV        GRA        TUH       ALD
## OKI 0.1767596 0.21924545 0.15915034 0.1626244 0.09408549 0.10091373 0.1342470
## HAI 0.1158530 0.14180901 0.08734395 0.1079468 0.04660201 0.07795749 0.1105019
## SOC 0.1921536 0.23886778 0.17866612 0.1757704 0.10771104 0.10588536 0.1405699
## YUN 0.1075931 0.12838272 0.09996617 0.1183705 0.07751427 0.13460958 0.1666679
## KAT 0.1181223 0.06548768 0.06119742 0.1306540 0.09198048 0.14755170 0.1813161
## GES 0.1983565 0.24580096 0.18461460 0.1814535 0.11224050 0.11078263 0.1446401
##            FRS       SER       SPB       SPS        INW        INJ        MAT
## OKI 0.10385426 0.2190639 0.1191098 0.1469864 0.26067729 0.22685247 0.15235502
## HAI 0.08483983 0.1882815 0.1078702 0.1211492 0.18116251 0.14817013 0.06945987
## SOC 0.10224474 0.2246910 0.1163494 0.1504383 0.27608980 0.24167669 0.17192703
## YUN 0.14135486 0.2551050 0.1681615 0.1809089 0.15158505 0.12085119 0.01922669
## KAT 0.15470275 0.2817785 0.1794650 0.1962450 0.09782599 0.07814525 0.04558362
## GES 0.10564731 0.2295872 0.1209193 0.1554201 0.28292898 0.24851347 0.17814330
```

```
cities <- read_csv(here("manuscript1/output/fst/cities.csv"))
```

Save it

```
saveRDS(
  cities, here(
    "manuscript1/output/fst/citiesforfst.rds"
  )
)
```

Import sample locations

```
sampling_loc <- readRDS(here("manuscript1", "output", "fst", "citiesforfst.rds"))

# Arrange by region 
sampling_loc <- sampling_loc |>
  dplyr::arrange(
    continent, region, subregion
  ) 

# Check it
head(sampling_loc)
```

```
## # A tibble: 6 × 9
##   pop   city           state latitude longitude continent region subregion order
##   <chr> <chr>          <chr>    <dbl>     <dbl> <chr>     <chr>  <chr>     <dbl>
## 1 BRM   Bermuda        Berm…     32.3     -64.8 Americas  Carib… Caribbean   128
## 2 NEG   Negril         Jama…     18.3     -78.3 Americas  Carib… Caribbean   129
## 3 SAI   Saint Augusti… Trin…     10.2     -61.5 Americas  Carib… Caribbean   131
## 4 COL   Columbus, OH   OH, …     40.0     -82.9 Americas  North… Midwest,…   115
## 5 PEO   Peoria, IL     IL, …     40.7     -89.6 Americas  North… Midwest,…   116
## 6 SPR   Springfield, … MO, …     37.2     -93.3 Americas  North… Midwest,…   117
```

Order

```
order_pops <- as.vector(sampling_loc$pop)
order_pops
```

```
##  [1] "BRM" "NEG" "SAI" "COL" "PEO" "SPR" "STL" "NEB" "NEH" "FIR" "LIN" "NEW"
## [13] "BER" "HAR" "MAN" "WAV" "FAY" "BEA" "MAC" "PAL" "RUS" "HAL" "HAT" "NEO"
## [25] "NUE" "HOU" "TRA" "MON" "DAL" "SMI" "DEN" "CHI" "LOS" "CUA" "MED" "BPV"
## [37] "MAU" "BIT" "TUC" "FLO" "REC" "ARA" "PLM" "BCO" "BAF" "IPO" "NOV" "BEH"
## [49] "BPA" "GRV" "POR" "HAI" "YUN" "HUN" "TAI" "OKI" "UTS" "KAG" "KAN" "BEN"
## [61] "KAT" "CAM" "INW" "INJ" "SUF" "SUU" "KLP" "MAT" "CHA" "KAC" "SSK" "LAM"
## [73] "HOC" "QNC" "HAN" "GES" "SOC" "TIK" "RAR" "TUH" "SER" "GRA" "ALD" "CES"
## [85] "BRE" "DES" "SPB" "SPS" "FRS"
```

Lets check if the matrix is symmetric

```
isSymmetric(aa)
```

```
## [1] TRUE
```

Now lets order the matrix using poporder. We will also add NA on the
upper left side of the matrix.

```
aa <- aa[order_pops, order_pops]
aa[lower.tri(aa)] <- NA
```

Now we have to convert the matrix to a data frame to plot it with
ggplot.

```
pairfst.long <- melt(aa)
summary(pairfst.long)
```

```
##       Var1           Var2          value      
##  BRM    :  89   BRM    :  89   Min.   :0.001  
##  NEG    :  89   NEG    :  89   1st Qu.:0.096  
##  SAI    :  89   SAI    :  89   Median :0.133  
##  COL    :  89   COL    :  89   Mean   :0.137  
##  PEO    :  89   PEO    :  89   3rd Qu.:0.176  
##  SPR    :  89   SPR    :  89   Max.   :0.418  
##  (Other):7387   (Other):7387   NA's   :4005
```

Now lets plot the data with ggplot. You can click in the little
square on the top left of the plot to open it on a new window. It will
have the right proportions.

```
pairfst.f <- ggplot(pairfst.long, aes(Var1, Var2)) +
  geom_tile(aes(fill = value), colour = "white") +
  scale_fill_gradient(
    low = "white",
    high = "#71b6ff",
    name = "Fst",
    na.value = "white",
    limits = c(0, 0.5)
  ) +
  scale_x_discrete(position = "top") +
  theme_bw() +
  geom_text(aes(label = ifelse(
    is.na(value), "", formatC(value, digits = 2, format = "f")
  )), size = 1.5) +
  theme(
    axis.text.x = element_text(angle = 90, hjust = 1),
    axis.title = element_blank(),
    panel.grid.major = element_blank(),
    panel.grid.minor = element_blank(),
    panel.border = element_blank(),
    panel.background = element_blank(),
    axis.text.y = element_text(hjust = 0)
  )
pairfst.f
```

```
ggsave(
  filename = here("manuscript1", "output", "fst", "fst_matrix_allpops.pdf"),
  pairfst.f,
  width = 10,
  height = 10,
  units = "in"
)
```

Remove NAs and rename columns

```
# remove NAs
fst2 <-
  pairfst.long |>
  drop_na()

# rename columns
fst2 <-
  fst2 |>
  dplyr::rename(pop1 = 1,
         pop2 = 2,
         fst  = 3)


# Split the data into two data frames, one for pop1 and one for pop2
df_pop1 <- fst2 |>
  dplyr::select(pop = pop1, fst)
df_pop2 <- fst2 |>
  dplyr::select(pop = pop2, fst)

# Combine the two data frames
df_combined <- bind_rows(df_pop1, df_pop2)

# Calculate the mean fst for each population
mean_fst <- df_combined |>
  group_by(pop) |>
  summarise(mean_fst = mean(fst))

print(mean_fst)
```

```
## # A tibble: 89 × 2
##    pop   mean_fst
##    <fct>    <dbl>
##  1 BRM      0.144
##  2 NEG      0.232
##  3 SAI      0.114
##  4 COL      0.117
##  5 PEO      0.104
##  6 SPR      0.139
##  7 STL      0.127
##  8 NEB      0.118
##  9 NEH      0.111
## 10 FIR      0.149
## # ℹ 79 more rows
```

Merge

```
fst3 <-
  sampling_loc |>
  left_join(
    mean_fst,
    by = c("pop" = "pop")
  ) |>
  dplyr::select(
    -state
  )

# # Remove " Asia" from the Region2 column
# # fst3$Region2 <- gsub(" Asia", "", fst3$Region2)
# 
# # Rename the Region2 column to Region
# fst3 <- fst3 |>
#   # dplyr::rename(Region = Region2)
# 
# # check output
# head(fst3)
```

Mean by region

```
# Group by Region and calculate the mean_fst by Region
region_means <- fst3 |>
  group_by(region) |>
  summarize(mean_fst_by_region = round(mean(mean_fst, na.rm = TRUE), 2)) |>
  ungroup()  # Ungroup the data

# Add the mean_fst_by_region column to the fst3 tibble
fst3 <- fst3 |>
  left_join(region_means, by = "region")

# Print the modified fst3 tibble
print(fst3)
```

```
## # A tibble: 89 × 10
##    pop   city       latitude longitude continent region subregion order mean_fst
##    <chr> <chr>         <dbl>     <dbl> <chr>     <chr>  <chr>     <dbl>    <dbl>
##  1 BRM   Bermuda        32.3     -64.8 Americas  Carib… Caribbean   128    0.144
##  2 NEG   Negril         18.3     -78.3 Americas  Carib… Caribbean   129    0.232
##  3 SAI   Saint Aug…     10.2     -61.5 Americas  Carib… Caribbean   131    0.114
##  4 COL   Columbus,…     40.0     -82.9 Americas  North… Midwest,…   115    0.117
##  5 PEO   Peoria, IL     40.7     -89.6 Americas  North… Midwest,…   116    0.104
##  6 SPR   Springfie…     37.2     -93.3 Americas  North… Midwest,…   117    0.139
##  7 STL   St. Louis…     38.6     -90.2 Americas  North… Midwest,…   118    0.127
##  8 NEB   New Bedfo…     41.7     -70.9 Americas  North… Northeas…    98    0.118
##  9 NEH   New Haven…     41.3     -72.9 Americas  North… Northeas…    99    0.111
## 10 FIR   Fire Isla…     40.7     -72.9 Americas  North… Northeas…   100    0.149
## # ℹ 79 more rows
## # ℹ 1 more variable: mean_fst_by_region <dbl>
```

Mean by subregion

```
# Group by Country and calculate the mean_fst by Country
sub_means <- fst3 |>
  group_by(subregion) |>
  summarize(mean_fst_by_subregion = round(mean(mean_fst, na.rm = TRUE), 2)) |>
  ungroup()  # Ungroup the data

# Add the mean_fst_by_country column to the fst3 tibble
fst3 <- fst3 |>
  left_join(sub_means, by = "subregion")

# Print the modified fst3 tibble
print(fst3)
```

```
## # A tibble: 89 × 11
##    pop   city       latitude longitude continent region subregion order mean_fst
##    <chr> <chr>         <dbl>     <dbl> <chr>     <chr>  <chr>     <dbl>    <dbl>
##  1 BRM   Bermuda        32.3     -64.8 Americas  Carib… Caribbean   128    0.144
##  2 NEG   Negril         18.3     -78.3 Americas  Carib… Caribbean   129    0.232
##  3 SAI   Saint Aug…     10.2     -61.5 Americas  Carib… Caribbean   131    0.114
##  4 COL   Columbus,…     40.0     -82.9 Americas  North… Midwest,…   115    0.117
##  5 PEO   Peoria, IL     40.7     -89.6 Americas  North… Midwest,…   116    0.104
##  6 SPR   Springfie…     37.2     -93.3 Americas  North… Midwest,…   117    0.139
##  7 STL   St. Louis…     38.6     -90.2 Americas  North… Midwest,…   118    0.127
##  8 NEB   New Bedfo…     41.7     -70.9 Americas  North… Northeas…    98    0.118
##  9 NEH   New Haven…     41.3     -72.9 Americas  North… Northeas…    99    0.111
## 10 FIR   Fire Isla…     40.7     -72.9 Americas  North… Northeas…   100    0.149
## # ℹ 79 more rows
## # ℹ 2 more variables: mean_fst_by_region <dbl>, mean_fst_by_subregion <dbl>
```

Mean By continent

```
# Group by Country and calculate the mean_fst by Country
continent_means <- fst3 |>
  group_by(continent) |>
  summarize(mean_fst_by_continent = round(mean(mean_fst, na.rm = TRUE), 2)) |>
  ungroup()  # Ungroup the data

# Add the mean_fst_by_country column to the fst3 tibble
fst3 <- fst3 |>
  left_join(continent_means, by = "continent")

# Print the modified fst3 tibble
print(fst3)
```

```
## # A tibble: 89 × 12
##    pop   city       latitude longitude continent region subregion order mean_fst
##    <chr> <chr>         <dbl>     <dbl> <chr>     <chr>  <chr>     <dbl>    <dbl>
##  1 BRM   Bermuda        32.3     -64.8 Americas  Carib… Caribbean   128    0.144
##  2 NEG   Negril         18.3     -78.3 Americas  Carib… Caribbean   129    0.232
##  3 SAI   Saint Aug…     10.2     -61.5 Americas  Carib… Caribbean   131    0.114
##  4 COL   Columbus,…     40.0     -82.9 Americas  North… Midwest,…   115    0.117
##  5 PEO   Peoria, IL     40.7     -89.6 Americas  North… Midwest,…   116    0.104
##  6 SPR   Springfie…     37.2     -93.3 Americas  North… Midwest,…   117    0.139
##  7 STL   St. Louis…     38.6     -90.2 Americas  North… Midwest,…   118    0.127
##  8 NEB   New Bedfo…     41.7     -70.9 Americas  North… Northeas…    98    0.118
##  9 NEH   New Haven…     41.3     -72.9 Americas  North… Northeas…    99    0.111
## 10 FIR   Fire Isla…     40.7     -72.9 Americas  North… Northeas…   100    0.149
## # ℹ 79 more rows
## # ℹ 3 more variables: mean_fst_by_region <dbl>, mean_fst_by_subregion <dbl>,
## #   mean_fst_by_continent <dbl>
```

```
fst4 <- fst3 |>
  dplyr::select(
    continent, mean_fst_by_continent, region, mean_fst_by_region, subregion, mean_fst_by_subregion, city, pop, mean_fst,
  )

fst4 <- fst4 |>
  arrange(
    continent, region, subregion, city
  )

# Round
fst4 <- fst4 |>
  mutate_if(is.numeric, ~ round(., 2))

head(fst4)
```

```
## # A tibble: 6 × 9
##   continent mean_fst_by_continent region        mean_fst_by_region subregion   
##   <chr>                     <dbl> <chr>                      <dbl> <chr>       
## 1 Americas                   0.13 Caribbean                   0.16 Caribbean   
## 2 Americas                   0.13 Caribbean                   0.16 Caribbean   
## 3 Americas                   0.13 Caribbean                   0.16 Caribbean   
## 4 Americas                   0.13 North America               0.11 Midwest, USA
## 5 Americas                   0.13 North America               0.11 Midwest, USA
## 6 Americas                   0.13 North America               0.11 Midwest, USA
## # ℹ 4 more variables: mean_fst_by_subregion <dbl>, city <chr>, pop <chr>,
## #   mean_fst <dbl>
```

Fst by region

```
fst5 <- fst4 |>
  dplyr::select(region, mean_fst_by_region, subregion, mean_fst_by_subregion, city, pop, mean_fst ) |>
  dplyr::arrange(region, subregion)


# Set theme if you want to use something different from the previous table
set_flextable_defaults(
  font.family = "Arial",
  font.size = 9,
  big.mark = ",",
  theme_fun = "theme_zebra" # try the themes: theme_alafoli(), theme_apa(), theme_booktabs(), theme_box(), theme_tron_legacy(), theme_tron(), theme_vader(), theme_vanilla(), theme_zebra()
)

# Then create the flextable object
flex_table <- flextable(fst5) |>
  set_caption(caption = as_paragraph(
    as_chunk(
      "Table 1. Fst estimates for by regions.",
      props = fp_text_default(color = "#000000", font.size = 14)
    )
  ),
  fp_p = fp_par(text.align = "center", padding = 5))

# Print the flextable
flex_table
```

Table 1. Fst estimates for by regions.

| region | mean\_fst\_by\_region | subregion | mean\_fst\_by\_subregion | city | pop | mean\_fst |
| --- | --- | --- | --- | --- | --- | --- |
| Caribbean | 0.16 | Caribbean | 0.16 | Bermuda | BRM | 0.14 |
| Caribbean | 0.16 | Caribbean | 0.16 | Negril | NEG | 0.23 |
| Caribbean | 0.16 | Caribbean | 0.16 | Saint Augustine | SAI | 0.11 |
| East Asia | 0.14 | Japan | 0.14 | Hainan | HAI | 0.10 |
| East Asia | 0.14 | Japan | 0.14 | Hunan | HUN | 0.09 |
| East Asia | 0.14 | Japan | 0.14 | Kagoshima | KAG | 0.14 |
| East Asia | 0.14 | Japan | 0.14 | Kanazawa | KAN | 0.18 |
| East Asia | 0.14 | Japan | 0.14 | Okinawa | OKI | 0.14 |
| East Asia | 0.14 | Japan | 0.14 | Tainan | TAI | 0.20 |
| East Asia | 0.14 | Japan | 0.14 | Utsunomiya | UTS | 0.14 |
| East Asia | 0.14 | Japan | 0.14 | Yunnan | YUN | 0.13 |
| Eastern Europe | 0.16 | East Europe | 0.15 | Armavir | RAR | 0.16 |
| Eastern Europe | 0.16 | East Europe | 0.15 | Hopa | TUH | 0.12 |
| Eastern Europe | 0.16 | East Europe | 0.15 | Novi Sad | SER | 0.23 |
| Eastern Europe | 0.16 | East Europe | 0.15 | Sakhumi, Abkhazia | GES | 0.15 |
| Eastern Europe | 0.16 | East Europe | 0.15 | Sochi | SOC | 0.15 |
| Eastern Europe | 0.16 | East Europe | 0.15 | Tikhoretsk | TIK | 0.17 |
| North America | 0.11 | Midwest, USA | 0.12 | Columbus, OH | COL | 0.12 |
| North America | 0.11 | Midwest, USA | 0.12 | Peoria, IL | PEO | 0.10 |
| North America | 0.11 | Midwest, USA | 0.12 | Springfield, MO | SPR | 0.14 |
| North America | 0.11 | Midwest, USA | 0.12 | St. Louis, MO | STL | 0.13 |
| North America | 0.11 | Northeast, USA | 0.12 | Berlin, NJ | BER | 0.12 |
| North America | 0.11 | Northeast, USA | 0.12 | Fire Island, NY | FIR | 0.15 |
| North America | 0.11 | Northeast, USA | 0.12 | Harrisburg, PA | HAR | 0.12 |
| North America | 0.11 | Northeast, USA | 0.12 | Lincoln, DE | LIN | 0.13 |
| North America | 0.11 | Northeast, USA | 0.12 | New Bedford, MA | NEB | 0.12 |
| North America | 0.11 | Northeast, USA | 0.12 | New Haven, CT | NEH | 0.11 |
| North America | 0.11 | Northeast, USA | 0.12 | Newark, NJ | NEW | 0.12 |
| North America | 0.11 | Southeast, USA | 0.11 | Beaufort, SC | BEA | 0.09 |
| North America | 0.11 | Southeast, USA | 0.11 | Fayetteville, NC | FAY | 0.10 |
| North America | 0.11 | Southeast, USA | 0.11 | Hattiesburg, MS | HAT | 0.11 |
| North America | 0.11 | Southeast, USA | 0.11 | Huntsville, AL | HAL | 0.10 |
| North America | 0.11 | Southeast, USA | 0.11 | Macon, GA | MAC | 0.10 |
| North America | 0.11 | Southeast, USA | 0.11 | Manassas, VA | MAN | 0.10 |
| North America | 0.11 | Southeast, USA | 0.11 | New Orleans, LA | NEO | 0.11 |
| North America | 0.11 | Southeast, USA | 0.11 | Palm Beach | PAL | 0.11 |
| North America | 0.11 | Southeast, USA | 0.11 | Russelville, AR | RUS | 0.14 |
| North America | 0.11 | Southeast, USA | 0.11 | Waverly, VA | WAV | 0.12 |
| North America | 0.11 | Southwest, USA | 0.10 | Dallas, TX | DAL | 0.11 |
| North America | 0.11 | Southwest, USA | 0.10 | Denton Co., TX | DEN | 0.11 |
| North America | 0.11 | Southwest, USA | 0.10 | Houston, TX | HOU | 0.09 |
| North America | 0.11 | Southwest, USA | 0.10 | Montgomery Co., TX | MON | 0.10 |
| North America | 0.11 | Southwest, USA | 0.10 | Nueces Co., TX | NUE | 0.10 |
| North America | 0.11 | Southwest, USA | 0.10 | Smith Co., TX | SMI | 0.11 |
| North America | 0.11 | Southwest, USA | 0.10 | Travis Co., TX | TRA | 0.10 |
| North America | 0.11 | West, USA | 0.13 | Chino, CA | CHI | 0.13 |
| North America | 0.11 | West, USA | 0.13 | Los Angeles | LOS | 0.13 |
| South America | 0.14 | South America | 0.14 | Alta Floresta | BAF | 0.13 |
| South America | 0.14 | South America | 0.14 | Araguaina | ARA | 0.13 |
| South America | 0.14 | South America | 0.14 | Belo Horizonte | BEH | 0.15 |
| South America | 0.14 | South America | 0.14 | Confresa | BCO | 0.14 |
| South America | 0.14 | South America | 0.14 | Floriano | FLO | 0.14 |
| South America | 0.14 | South America | 0.14 | Gravatai | GRV | 0.14 |
| South America | 0.14 | South America | 0.14 | Ipora | IPO | 0.19 |
| South America | 0.14 | South America | 0.14 | Itaituba | BIT | 0.14 |
| South America | 0.14 | South America | 0.14 | Manaus, AM | MAU | 0.12 |
| South America | 0.14 | South America | 0.14 | Medellin | CUA | 0.14 |
| South America | 0.14 | South America | 0.14 | Medellin | MED | 0.14 |
| South America | 0.14 | South America | 0.14 | Nova Venecia, ES | NOV | 0.17 |
| South America | 0.14 | South America | 0.14 | Palmares | BPA | 0.13 |
| South America | 0.14 | South America | 0.14 | Palmas | PLM | 0.14 |
| South America | 0.14 | South America | 0.14 | Porto Velho | BPV | 0.14 |
| South America | 0.14 | South America | 0.14 | Puerto Iguazu | POR | 0.15 |
| South America | 0.14 | South America | 0.14 | Recife, PE | REC | 0.12 |
| South America | 0.14 | South America | 0.14 | Tucurui, PA | TUC | 0.13 |
| South Asia | 0.14 | South Asia | 0.14 | Bengaluru | BEN | 0.15 |
| South Asia | 0.14 | South Asia | 0.14 | Kathmandu | KAT | 0.14 |
| Southeast Asia | 0.16 | Southeast Asia | 0.16 | Chanthaburi | CHA | 0.13 |
| Southeast Asia | 0.16 | Southeast Asia | 0.16 | Hanoi | HAN | 0.12 |
| Southeast Asia | 0.16 | Southeast Asia | 0.16 | Ho Chi Minh City | HOC | 0.11 |
| Southeast Asia | 0.16 | Southeast Asia | 0.16 | Jakarta | INJ | 0.21 |
| Southeast Asia | 0.16 | Southeast Asia | 0.16 | Kanchanaburi | KAC | 0.13 |
| Southeast Asia | 0.16 | Southeast Asia | 0.16 | Kuala Lumpur | KLP | 0.16 |
| Southeast Asia | 0.16 | Southeast Asia | 0.16 | Lampang | LAM | 0.14 |
| Southeast Asia | 0.16 | Southeast Asia | 0.16 | Phnom Penh | CAM | 0.12 |
| Southeast Asia | 0.16 | Southeast Asia | 0.16 | Quy Nhon City | QNC | 0.20 |
| Southeast Asia | 0.16 | Southeast Asia | 0.16 | Sisaket | SSK | 0.13 |
| Southeast Asia | 0.16 | Southeast Asia | 0.16 | Sulawesi (Forest) | SUF | 0.21 |
| Southeast Asia | 0.16 | Southeast Asia | 0.16 | Sulawesi (Urban) | SUU | 0.15 |
| Southeast Asia | 0.16 | Southeast Asia | 0.16 | Tambun | MAT | 0.14 |
| Southeast Asia | 0.16 | Southeast Asia | 0.16 | Wainyapu | INW | 0.24 |
| Southern Europe | 0.15 | East Europe | 0.15 | Athens | GRA | 0.10 |
| Southern Europe | 0.15 | East Europe | 0.15 | Durres | ALD | 0.15 |
| Southern Europe | 0.15 | West Europe | 0.15 | Badajoz | SPB | 0.12 |
| Southern Europe | 0.15 | West Europe | 0.15 | Brescia | BRE | 0.16 |
| Southern Europe | 0.15 | West Europe | 0.15 | Cesena | CES | 0.20 |
| Southern Europe | 0.15 | West Europe | 0.15 | Desenzano | DES | 0.15 |
| Southern Europe | 0.15 | West Europe | 0.15 | San Roque | SPS | 0.16 |
| Western Europe | 0.11 | West Europe | 0.15 | Saint-Martin-d'Heres | FRS | 0.11 |

```
# Initialize Word document
doc <- 
  read_docx() |>
  body_add_flextable(value = flex_table)

# Define the output path with 'here' library
output_path <- here(
  "manuscript1",
  "output",
  "fst", 
  "fst_SNPS_region_subregion.docx"
  )

# Save the Word document
print(doc, target = output_path)
```

With continent

```
# Set theme if you want to use something different from the previous table
set_flextable_defaults(
  font.family = "Arial",
  font.size = 9,
  big.mark = ",",
  theme_fun = "theme_zebra" # try the themes: theme_alafoli(), theme_apa(), theme_booktabs(), theme_box(), theme_tron_legacy(), theme_tron(), theme_vader(), theme_vanilla(), theme_zebra()
)

# Then create the flextable object
flex_table <- flextable(fst4) |>
  set_caption(caption = as_paragraph(
    as_chunk(
      "Table 1. Fst values for Europe, Asia and Americas",
      props = fp_text_default(color = "#000000", font.size = 14)
    )
  ),
  fp_p = fp_par(text.align = "center", padding = 5))

# Print the flextable
flex_table
```

Table 1. Fst values for Europe, Asia and Americas

| continent | mean\_fst\_by\_continent | region | mean\_fst\_by\_region | subregion | mean\_fst\_by\_subregion | city | pop | mean\_fst |
| --- | --- | --- | --- | --- | --- | --- | --- | --- |
| Americas | 0.13 | Caribbean | 0.16 | Caribbean | 0.16 | Bermuda | BRM | 0.14 |
| Americas | 0.13 | Caribbean | 0.16 | Caribbean | 0.16 | Negril | NEG | 0.23 |
| Americas | 0.13 | Caribbean | 0.16 | Caribbean | 0.16 | Saint Augustine | SAI | 0.11 |
| Americas | 0.13 | North America | 0.11 | Midwest, USA | 0.12 | Columbus, OH | COL | 0.12 |
| Americas | 0.13 | North America | 0.11 | Midwest, USA | 0.12 | Peoria, IL | PEO | 0.10 |
| Americas | 0.13 | North America | 0.11 | Midwest, USA | 0.12 | Springfield, MO | SPR | 0.14 |
| Americas | 0.13 | North America | 0.11 | Midwest, USA | 0.12 | St. Louis, MO | STL | 0.13 |
| Americas | 0.13 | North America | 0.11 | Northeast, USA | 0.12 | Berlin, NJ | BER | 0.12 |
| Americas | 0.13 | North America | 0.11 | Northeast, USA | 0.12 | Fire Island, NY | FIR | 0.15 |
| Americas | 0.13 | North America | 0.11 | Northeast, USA | 0.12 | Harrisburg, PA | HAR | 0.12 |
| Americas | 0.13 | North America | 0.11 | Northeast, USA | 0.12 | Lincoln, DE | LIN | 0.13 |
| Americas | 0.13 | North America | 0.11 | Northeast, USA | 0.12 | New Bedford, MA | NEB | 0.12 |
| Americas | 0.13 | North America | 0.11 | Northeast, USA | 0.12 | New Haven, CT | NEH | 0.11 |
| Americas | 0.13 | North America | 0.11 | Northeast, USA | 0.12 | Newark, NJ | NEW | 0.12 |
| Americas | 0.13 | North America | 0.11 | Southeast, USA | 0.11 | Beaufort, SC | BEA | 0.09 |
| Americas | 0.13 | North America | 0.11 | Southeast, USA | 0.11 | Fayetteville, NC | FAY | 0.10 |
| Americas | 0.13 | North America | 0.11 | Southeast, USA | 0.11 | Hattiesburg, MS | HAT | 0.11 |
| Americas | 0.13 | North America | 0.11 | Southeast, USA | 0.11 | Huntsville, AL | HAL | 0.10 |
| Americas | 0.13 | North America | 0.11 | Southeast, USA | 0.11 | Macon, GA | MAC | 0.10 |
| Americas | 0.13 | North America | 0.11 | Southeast, USA | 0.11 | Manassas, VA | MAN | 0.10 |
| Americas | 0.13 | North America | 0.11 | Southeast, USA | 0.11 | New Orleans, LA | NEO | 0.11 |
| Americas | 0.13 | North America | 0.11 | Southeast, USA | 0.11 | Palm Beach | PAL | 0.11 |
| Americas | 0.13 | North America | 0.11 | Southeast, USA | 0.11 | Russelville, AR | RUS | 0.14 |
| Americas | 0.13 | North America | 0.11 | Southeast, USA | 0.11 | Waverly, VA | WAV | 0.12 |
| Americas | 0.13 | North America | 0.11 | Southwest, USA | 0.10 | Dallas, TX | DAL | 0.11 |
| Americas | 0.13 | North America | 0.11 | Southwest, USA | 0.10 | Denton Co., TX | DEN | 0.11 |
| Americas | 0.13 | North America | 0.11 | Southwest, USA | 0.10 | Houston, TX | HOU | 0.09 |
| Americas | 0.13 | North America | 0.11 | Southwest, USA | 0.10 | Montgomery Co., TX | MON | 0.10 |
| Americas | 0.13 | North America | 0.11 | Southwest, USA | 0.10 | Nueces Co., TX | NUE | 0.10 |
| Americas | 0.13 | North America | 0.11 | Southwest, USA | 0.10 | Smith Co., TX | SMI | 0.11 |
| Americas | 0.13 | North America | 0.11 | Southwest, USA | 0.10 | Travis Co., TX | TRA | 0.10 |
| Americas | 0.13 | North America | 0.11 | West, USA | 0.13 | Chino, CA | CHI | 0.13 |
| Americas | 0.13 | North America | 0.11 | West, USA | 0.13 | Los Angeles | LOS | 0.13 |
| Americas | 0.13 | South America | 0.14 | South America | 0.14 | Alta Floresta | BAF | 0.13 |
| Americas | 0.13 | South America | 0.14 | South America | 0.14 | Araguaina | ARA | 0.13 |
| Americas | 0.13 | South America | 0.14 | South America | 0.14 | Belo Horizonte | BEH | 0.15 |
| Americas | 0.13 | South America | 0.14 | South America | 0.14 | Confresa | BCO | 0.14 |
| Americas | 0.13 | South America | 0.14 | South America | 0.14 | Floriano | FLO | 0.14 |
| Americas | 0.13 | South America | 0.14 | South America | 0.14 | Gravatai | GRV | 0.14 |
| Americas | 0.13 | South America | 0.14 | South America | 0.14 | Ipora | IPO | 0.19 |
| Americas | 0.13 | South America | 0.14 | South America | 0.14 | Itaituba | BIT | 0.14 |
| Americas | 0.13 | South America | 0.14 | South America | 0.14 | Manaus, AM | MAU | 0.12 |
| Americas | 0.13 | South America | 0.14 | South America | 0.14 | Medellin | CUA | 0.14 |
| Americas | 0.13 | South America | 0.14 | South America | 0.14 | Medellin | MED | 0.14 |
| Americas | 0.13 | South America | 0.14 | South America | 0.14 | Nova Venecia, ES | NOV | 0.17 |
| Americas | 0.13 | South America | 0.14 | South America | 0.14 | Palmares | BPA | 0.13 |
| Americas | 0.13 | South America | 0.14 | South America | 0.14 | Palmas | PLM | 0.14 |
| Americas | 0.13 | South America | 0.14 | South America | 0.14 | Porto Velho | BPV | 0.14 |
| Americas | 0.13 | South America | 0.14 | South America | 0.14 | Puerto Iguazu | POR | 0.15 |
| Americas | 0.13 | South America | 0.14 | South America | 0.14 | Recife, PE | REC | 0.12 |
| Americas | 0.13 | South America | 0.14 | South America | 0.14 | Tucurui, PA | TUC | 0.13 |
| Asia | 0.15 | East Asia | 0.14 | Japan | 0.14 | Hainan | HAI | 0.10 |
| Asia | 0.15 | East Asia | 0.14 | Japan | 0.14 | Hunan | HUN | 0.09 |
| Asia | 0.15 | East Asia | 0.14 | Japan | 0.14 | Kagoshima | KAG | 0.14 |
| Asia | 0.15 | East Asia | 0.14 | Japan | 0.14 | Kanazawa | KAN | 0.18 |
| Asia | 0.15 | East Asia | 0.14 | Japan | 0.14 | Okinawa | OKI | 0.14 |
| Asia | 0.15 | East Asia | 0.14 | Japan | 0.14 | Tainan | TAI | 0.20 |
| Asia | 0.15 | East Asia | 0.14 | Japan | 0.14 | Utsunomiya | UTS | 0.14 |
| Asia | 0.15 | East Asia | 0.14 | Japan | 0.14 | Yunnan | YUN | 0.13 |
| Asia | 0.15 | South Asia | 0.14 | South Asia | 0.14 | Bengaluru | BEN | 0.15 |
| Asia | 0.15 | South Asia | 0.14 | South Asia | 0.14 | Kathmandu | KAT | 0.14 |
| Asia | 0.15 | Southeast Asia | 0.16 | Southeast Asia | 0.16 | Chanthaburi | CHA | 0.13 |
| Asia | 0.15 | Southeast Asia | 0.16 | Southeast Asia | 0.16 | Hanoi | HAN | 0.12 |
| Asia | 0.15 | Southeast Asia | 0.16 | Southeast Asia | 0.16 | Ho Chi Minh City | HOC | 0.11 |
| Asia | 0.15 | Southeast Asia | 0.16 | Southeast Asia | 0.16 | Jakarta | INJ | 0.21 |
| Asia | 0.15 | Southeast Asia | 0.16 | Southeast Asia | 0.16 | Kanchanaburi | KAC | 0.13 |
| Asia | 0.15 | Southeast Asia | 0.16 | Southeast Asia | 0.16 | Kuala Lumpur | KLP | 0.16 |
| Asia | 0.15 | Southeast Asia | 0.16 | Southeast Asia | 0.16 | Lampang | LAM | 0.14 |
| Asia | 0.15 | Southeast Asia | 0.16 | Southeast Asia | 0.16 | Phnom Penh | CAM | 0.12 |
| Asia | 0.15 | Southeast Asia | 0.16 | Southeast Asia | 0.16 | Quy Nhon City | QNC | 0.20 |
| Asia | 0.15 | Southeast Asia | 0.16 | Southeast Asia | 0.16 | Sisaket | SSK | 0.13 |
| Asia | 0.15 | Southeast Asia | 0.16 | Southeast Asia | 0.16 | Sulawesi (Forest) | SUF | 0.21 |
| Asia | 0.15 | Southeast Asia | 0.16 | Southeast Asia | 0.16 | Sulawesi (Urban) | SUU | 0.15 |
| Asia | 0.15 | Southeast Asia | 0.16 | Southeast Asia | 0.16 | Tambun | MAT | 0.14 |
| Asia | 0.15 | Southeast Asia | 0.16 | Southeast Asia | 0.16 | Wainyapu | INW | 0.24 |
| Europe | 0.15 | Eastern Europe | 0.16 | East Europe | 0.15 | Armavir | RAR | 0.16 |
| Europe | 0.15 | Eastern Europe | 0.16 | East Europe | 0.15 | Hopa | TUH | 0.12 |
| Europe | 0.15 | Eastern Europe | 0.16 | East Europe | 0.15 | Novi Sad | SER | 0.23 |
| Europe | 0.15 | Eastern Europe | 0.16 | East Europe | 0.15 | Sakhumi, Abkhazia | GES | 0.15 |
| Europe | 0.15 | Eastern Europe | 0.16 | East Europe | 0.15 | Sochi | SOC | 0.15 |
| Europe | 0.15 | Eastern Europe | 0.16 | East Europe | 0.15 | Tikhoretsk | TIK | 0.17 |
| Europe | 0.15 | Southern Europe | 0.15 | East Europe | 0.15 | Athens | GRA | 0.10 |
| Europe | 0.15 | Southern Europe | 0.15 | East Europe | 0.15 | Durres | ALD | 0.15 |
| Europe | 0.15 | Southern Europe | 0.15 | West Europe | 0.15 | Badajoz | SPB | 0.12 |
| Europe | 0.15 | Southern Europe | 0.15 | West Europe | 0.15 | Brescia | BRE | 0.16 |
| Europe | 0.15 | Southern Europe | 0.15 | West Europe | 0.15 | Cesena | CES | 0.20 |
| Europe | 0.15 | Southern Europe | 0.15 | West Europe | 0.15 | Desenzano | DES | 0.15 |
| Europe | 0.15 | Southern Europe | 0.15 | West Europe | 0.15 | San Roque | SPS | 0.16 |
| Europe | 0.15 | Western Europe | 0.11 | West Europe | 0.15 | Saint-Martin-d'Heres | FRS | 0.11 |

```
# Initialize Word document
doc <- 
  read_docx() |>
  body_add_flextable(value = flex_table)

# Define the output path with 'here' library
output_path <- here(
  "manuscript1",
  "output",
  "fst", 
  "fst_SNPS_continent_region_subregion.docx"
  )

# Save the Word document
print(doc, target = output_path)
```

## 5.2 Isolation by distance

```
plink \
--keep-allele-order \
--bfile manuscript1/output/snps_sets/r2_0.01_b \
--recodeA \
--make-bed \
--out manuscript1/output/fst/allpops_ibd \
--silent;
grep 'samples\|variants\|remaining' manuscript1/output/fst/allpops_ibd.log
```

20825 variants loaded from .bim file. 20825 variants and 933 people
pass filters and QC.

Import the data

```
allpopsidb <-
  read.PLINK(
    here(
     "manuscript1", "output", "fst", "allpops_ibd.raw"
    ),
    quiet = FALSE,
    chunkSize = 1000,
    parallel = require("parallel"),
    n.cores = 4
  )

summary(allpopsidb)
```

Now lets convert the genlight object to Stampp format, and estimate
pairwide Fst values

Save it

```
saveRDS(
  allpopsidb, here(
    "manuscript1/output/fst/allpopsidb.rds"
  )
)
```

Now lets convert the genlight object to Stampp format, and estimate
pairwide Fst values

```
#start an interactive session and load R packages needed
#salloc -p scavenge -c 10 -t 2:00:00
salloc -p scavenge --cpus-per-task=5 -t 2:00:00 --mem-per-cpu=10000    
module load R/4.2.0-foss-2020b
R

library(StAMPP)
library(adegenet)
library(here)

allpopsforidb <- readRDS(
  here(
    "/gpfs/gibbs/pi/caccone/mkc54/albo/manuscript1/output/fst/allpopsidb.rds"
  )
)

summary(allpopsforidb)

#This chunk will take a couple minutes to run.

allpopsidb_2 <- stamppConvert(allpopsforidb, type="genlight")

allpopsidb_3 <- stamppFst(allpopsidb_2, 1, 95, 1)

saveRDS(
  allpopsidb_3, here(
    "/gpfs/gibbs/pi/caccone/mkc54/albo/manuscript1/output/fst/allpopsidb_stamppFst2.rds"
  )
)
```

To load it

```
allpopsidb_3 <- readRDS(
  here(
    "manuscript1", "output", "fst", "allpopsidb_stamppFst2.rds"
  )
)
```

Now lets look at the object

```
summary(allpopsidb_3)
```

```
##       OKI               HAI               SOC               YUN          
##  Min.   :0.07367   Min.   :0.03557   Min.   :0.01212   Min.   :-0.00049  
##  1st Qu.:0.11398   1st Qu.:0.07883   1st Qu.:0.12175   1st Qu.: 0.10493  
##  Median :0.14327   Median :0.10364   Median :0.15122   Median : 0.12859  
##  Mean   :0.14355   Mean   :0.10340   Mean   :0.15267   Mean   : 0.12701  
##  3rd Qu.:0.16054   3rd Qu.:0.11901   3rd Qu.:0.17717   3rd Qu.: 0.16834  
##  Max.   :0.26068   Max.   :0.19298   Max.   :0.27609   Max.   : 0.25511  
##  NA's   :1         NA's   :2         NA's   :3         NA's   :4         
##       KAT               GES               TIK               RAR         
##  Min.   :0.01362   Min.   :0.02787   Min.   :0.05166   Min.   :0.08671  
##  1st Qu.:0.11420   1st Qu.:0.12590   1st Qu.:0.13672   1st Qu.:0.12977  
##  Median :0.14106   Median :0.15542   Median :0.16738   Median :0.15793  
##  Mean   :0.13992   Mean   :0.15858   Mean   :0.17168   Mean   :0.16225  
##  3rd Qu.:0.18157   3rd Qu.:0.18252   3rd Qu.:0.19548   3rd Qu.:0.18195  
##  Max.   :0.28178   Max.   :0.28293   Max.   :0.29676   Max.   :0.28138  
##  NA's   :5         NA's   :6         NA's   :7         NA's   :8        
##       TAI              HUN               HAN               KLP         
##  Min.   :0.1289   Min.   :0.03941   Min.   :0.04755   Min.   :0.05108  
##  1st Qu.:0.1738   1st Qu.:0.06968   1st Qu.:0.09226   1st Qu.:0.13832  
##  Median :0.2030   Median :0.09315   Median :0.11628   Median :0.15405  
##  Mean   :0.2078   Mean   :0.09589   Mean   :0.11897   Mean   :0.16015  
##  3rd Qu.:0.2254   3rd Qu.:0.11519   3rd Qu.:0.13567   3rd Qu.:0.18959  
##  Max.   :0.3487   Max.   :0.20249   Max.   :0.24394   Max.   :0.31347  
##  NA's   :9        NA's   :10        NA's   :11        NA's   :12       
##       HOC               QNC               SSK               KAC         
##  Min.   :0.05221   Min.   :0.08175   Min.   :0.00359   Min.   :0.00112  
##  1st Qu.:0.08960   1st Qu.:0.17927   1st Qu.:0.10992   1st Qu.:0.10694  
##  Median :0.11404   Median :0.19448   Median :0.13284   Median :0.13169  
##  Mean   :0.11581   Mean   :0.19799   Mean   :0.13296   Mean   :0.13283  
##  3rd Qu.:0.12946   3rd Qu.:0.22971   3rd Qu.:0.17122   3rd Qu.:0.16803  
##  Max.   :0.20884   Max.   :0.32607   Max.   :0.25299   Max.   :0.26850  
##  NA's   :13        NA's   :14        NA's   :15        NA's   :16       
##       SON                CHA                BRE               DES         
##  Min.   :0.002631   Min.   :0.000673   Min.   :0.07084   Min.   :0.06195  
##  1st Qu.:0.113504   1st Qu.:0.111205   1st Qu.:0.09662   1st Qu.:0.08815  
##  Median :0.139733   Median :0.131284   Median :0.14906   Median :0.13960  
##  Mean   :0.139830   Mean   :0.135700   Mean   :0.16057   Mean   :0.14866  
##  3rd Qu.:0.173584   3rd Qu.:0.169952   3rd Qu.:0.20531   3rd Qu.:0.19345  
##  Max.   :0.306673   Max.   :0.252175   Max.   :0.32326   Max.   :0.30714  
##  NA's   :17         NA's   :18         NA's   :19        NA's   :20       
##       CES              KAN               UTS               MAU         
##  Min.   :0.1169   Min.   :0.08847   Min.   :0.05283   Min.   :0.03648  
##  1st Qu.:0.1349   1st Qu.:0.11511   1st Qu.:0.07306   1st Qu.:0.09818  
##  Median :0.1807   Median :0.16296   Median :0.12867   Median :0.11733  
##  Mean   :0.1961   Mean   :0.17558   Mean   :0.13948   Mean   :0.11772  
##  3rd Qu.:0.2372   3rd Qu.:0.22144   3rd Qu.:0.18581   3rd Qu.:0.13398  
##  Max.   :0.3577   Max.   :0.33487   Max.   :0.29795   Max.   :0.23539  
##  NA's   :21       NA's   :22        NA's   :23        NA's   :24       
##       TUC                REC               BRM               CAM           
##  Min.   :0.007459   Min.   :0.02791   Min.   :0.07716   Min.   :-0.003462  
##  1st Qu.:0.106526   1st Qu.:0.09394   1st Qu.:0.09709   1st Qu.: 0.102135  
##  Median :0.137305   Median :0.11082   Median :0.12502   Median : 0.120785  
##  Mean   :0.126253   Mean   :0.11651   Mean   :0.14197   Mean   : 0.120292  
##  3rd Qu.:0.155990   3rd Qu.:0.13093   3rd Qu.:0.17525   3rd Qu.: 0.148330  
##  Max.   :0.249991   Max.   :0.23495   Max.   :0.27467   Max.   : 0.232982  
##  NA's   :25         NA's   :26        NA's   :27        NA's   :28         
##       KAG               CUA                MED              FAY         
##  Min.   :0.06294   Min.   :0.002043   Min.   :0.0937   Min.   :0.01769  
##  1st Qu.:0.08417   1st Qu.:0.126468   1st Qu.:0.1229   1st Qu.:0.03803  
##  Median :0.11702   Median :0.136637   Median :0.1329   Median :0.07478  
##  Mean   :0.13469   Mean   :0.142960   Mean   :0.1411   Mean   :0.09610  
##  3rd Qu.:0.17520   3rd Qu.:0.151997   3rd Qu.:0.1499   3rd Qu.:0.13585  
##  Max.   :0.27461   Max.   :0.255676   Max.   :0.2528   Max.   :0.24305  
##  NA's   :29        NA's   :30         NA's   :31       NA's   :32       
##       DAL               NEO               RUS               CHI         
##  Min.   :0.01464   Min.   :0.03057   Min.   :0.06128   Min.   :0.06956  
##  1st Qu.:0.04211   1st Qu.:0.05371   1st Qu.:0.07868   1st Qu.:0.11056  
##  Median :0.08732   Median :0.09026   Median :0.12329   Median :0.12796  
##  Mean   :0.10189   Mean   :0.11027   Mean   :0.13687   Mean   :0.13442  
##  3rd Qu.:0.14922   3rd Qu.:0.15436   3rd Qu.:0.18276   3rd Qu.:0.14722  
##  Max.   :0.25255   Max.   :0.25487   Max.   :0.27945   Max.   :0.24082  
##  NA's   :33        NA's   :34        NA's   :35        NA's   :36       
##       NUE               TRA               HAL               STL         
##  Min.   :0.01106   Min.   :0.01103   Min.   :0.01646   Min.   :0.03522  
##  1st Qu.:0.05711   1st Qu.:0.04184   1st Qu.:0.04080   1st Qu.:0.05985  
##  Median :0.09008   Median :0.08974   Median :0.10461   Median :0.12978  
##  Mean   :0.10017   Mean   :0.09534   Mean   :0.10674   Mean   :0.13031  
##  3rd Qu.:0.13014   3rd Qu.:0.13460   3rd Qu.:0.15909   3rd Qu.:0.18958  
##  Max.   :0.23036   Max.   :0.23340   Max.   :0.25416   Max.   :0.28324  
##  NA's   :37        NA's   :38        NA's   :39        NA's   :40       
##       SMI               WAV               HAT               DEN         
##  Min.   :0.01515   Min.   :0.02447   Min.   :0.02523   Min.   :0.02710  
##  1st Qu.:0.05620   1st Qu.:0.05285   1st Qu.:0.05234   1st Qu.:0.04612  
##  Median :0.11793   Median :0.13032   Median :0.12198   Median :0.11877  
##  Mean   :0.11779   Mean   :0.12912   Mean   :0.11858   Mean   :0.11646  
##  3rd Qu.:0.16165   3rd Qu.:0.18459   3rd Qu.:0.16096   3rd Qu.:0.16524  
##  Max.   :0.25668   Max.   :0.28595   Max.   :0.25507   Max.   :0.25522  
##  NA's   :41        NA's   :42        NA's   :43        NA's   :44       
##       MON               BPV               BCO               BPA         
##  Min.   :0.01685   Min.   :0.04873   Min.   :0.02047   Min.   :0.06348  
##  1st Qu.:0.05774   1st Qu.:0.09868   1st Qu.:0.11625   1st Qu.:0.10353  
##  Median :0.11303   Median :0.13757   Median :0.14182   Median :0.12159  
##  Mean   :0.10752   Mean   :0.13235   Mean   :0.13329   Mean   :0.12851  
##  3rd Qu.:0.13620   3rd Qu.:0.15678   3rd Qu.:0.16075   3rd Qu.:0.13656  
##  Max.   :0.22929   Max.   :0.25684   Max.   :0.25944   Max.   :0.23682  
##  NA's   :45        NA's   :46        NA's   :47        NA's   :48       
##       BIT               FLO               BAF               PLM         
##  Min.   :0.04198   Min.   :0.01003   Min.   :0.00612   Min.   :0.01682  
##  1st Qu.:0.11548   1st Qu.:0.11905   1st Qu.:0.10740   1st Qu.:0.11312  
##  Median :0.14151   Median :0.14731   Median :0.13984   Median :0.14811  
##  Mean   :0.13801   Mean   :0.14054   Mean   :0.13346   Mean   :0.14345  
##  3rd Qu.:0.16136   3rd Qu.:0.16758   3rd Qu.:0.16145   3rd Qu.:0.17392  
##  Max.   :0.25749   Max.   :0.27374   Max.   :0.26643   Max.   :0.27377  
##  NA's   :49        NA's   :50        NA's   :51        NA's   :52       
##       NEH               LIN               HAR               FIR         
##  Min.   :0.02120   Min.   :0.05155   Min.   :0.03659   Min.   :0.06303  
##  1st Qu.:0.04647   1st Qu.:0.07212   1st Qu.:0.06162   1st Qu.:0.08694  
##  Median :0.10817   Median :0.12402   Median :0.12813   Median :0.16539  
##  Mean   :0.12028   Mean   :0.14133   Mean   :0.13280   Mean   :0.16612  
##  3rd Qu.:0.17480   3rd Qu.:0.19377   3rd Qu.:0.18478   3rd Qu.:0.21891  
##  Max.   :0.26913   Max.   :0.27909   Max.   :0.26576   Max.   :0.30250  
##  NA's   :53        NA's   :54        NA's   :55        NA's   :56       
##       NEB               BEN               BEH               ARA         
##  Min.   :0.02835   Min.   :0.00319   Min.   :0.03704   Min.   :0.04841  
##  1st Qu.:0.06272   1st Qu.:0.11308   1st Qu.:0.11519   1st Qu.:0.10776  
##  Median :0.13864   Median :0.14105   Median :0.16590   Median :0.13794  
##  Mean   :0.13612   Mean   :0.13666   Mean   :0.15557   Mean   :0.13866  
##  3rd Qu.:0.19076   3rd Qu.:0.17405   3rd Qu.:0.19320   3rd Qu.:0.16754  
##  Max.   :0.27176   Max.   :0.27031   Max.   :0.28951   Max.   :0.24921  
##  NA's   :57        NA's   :58        NA's   :59        NA's   :60       
##       IPO               LAM               PAL               MAN         
##  Min.   :0.03632   Min.   :0.01102   Min.   :0.03001   Min.   :0.02570  
##  1st Qu.:0.17721   1st Qu.:0.11707   1st Qu.:0.07287   1st Qu.:0.04242  
##  Median :0.20887   Median :0.13428   Median :0.11496   Median :0.10696  
##  Mean   :0.19900   Mean   :0.13438   Mean   :0.11901   Mean   :0.11873  
##  3rd Qu.:0.23731   3rd Qu.:0.16759   3rd Qu.:0.14996   3rd Qu.:0.16898  
##  Max.   :0.34991   Max.   :0.26526   Max.   :0.23242   Max.   :0.25326  
##  NA's   :61        NA's   :62        NA's   :63        NA's   :64       
##       BEA               PEO               HOU               LOS         
##  Min.   :0.01324   Min.   :0.02749   Min.   :0.01650   Min.   :0.07693  
##  1st Qu.:0.05455   1st Qu.:0.05858   1st Qu.:0.05729   1st Qu.:0.11400  
##  Median :0.10177   Median :0.11635   Median :0.10306   Median :0.13200  
##  Mean   :0.10501   Mean   :0.12477   Mean   :0.10735   Mean   :0.14496  
##  3rd Qu.:0.14646   3rd Qu.:0.17311   3rd Qu.:0.15522   3rd Qu.:0.18084  
##  Max.   :0.21994   Max.   :0.25091   Max.   :0.21693   Max.   :0.23600  
##  NA's   :65        NA's   :66        NA's   :67        NA's   :68       
##       NEG              NOV               NEW               BER        
##  Min.   :0.1457   Min.   :0.03753   Min.   :0.05097   Min.   :0.0451  
##  1st Qu.:0.2167   1st Qu.:0.14564   1st Qu.:0.08199   1st Qu.:0.0820  
##  Median :0.2470   Median :0.20255   Median :0.14402   Median :0.1484  
##  Mean   :0.2481   Mean   :0.18657   Mean   :0.14288   Mean   :0.1418  
##  3rd Qu.:0.2661   3rd Qu.:0.21932   3rd Qu.:0.17732   3rd Qu.:0.1750  
##  Max.   :0.4106   Max.   :0.35018   Max.   :0.26293   Max.   :0.2652  
##  NA's   :69       NA's   :70        NA's   :71        NA's   :72      
##       MAC               SPR               COL              GEL        
##  Min.   :0.05382   Min.   :0.06413   Min.   :0.0451   Min.   :0.0010  
##  1st Qu.:0.07288   1st Qu.:0.11959   1st Qu.:0.1014   1st Qu.:0.1052  
##  Median :0.12178   Median :0.19775   Median :0.1697   Median :0.1326  
##  Mean   :0.12462   Mean   :0.17827   Mean   :0.1584   Mean   :0.1386  
##  3rd Qu.:0.15518   3rd Qu.:0.21910   3rd Qu.:0.1885   3rd Qu.:0.1741  
##  Max.   :0.22627   Max.   :0.30425   Max.   :0.2703   Max.   :0.3334  
##  NA's   :73        NA's   :74        NA's   :75       NA's   :76      
##       JAF               SAI               POR               SUF         
##  Min.   :0.03072   Min.   :0.06957   Min.   :0.02862   Min.   :0.03997  
##  1st Qu.:0.10880   1st Qu.:0.10537   1st Qu.:0.16181   1st Qu.:0.13672  
##  Median :0.14147   Median :0.12984   Median :0.18560   Median :0.20574  
##  Mean   :0.14988   Mean   :0.13970   Mean   :0.17464   Mean   :0.18911  
##  3rd Qu.:0.17444   3rd Qu.:0.17829   3rd Qu.:0.20302   3rd Qu.:0.23840  
##  Max.   :0.34128   Max.   :0.20992   Max.   :0.27560   Max.   :0.34977  
##  NA's   :77        NA's   :78        NA's   :79        NA's   :80       
##       SUU               KUN              GRV              GRA         
##  Min.   :0.09221   Min.   :0.1502   Min.   :0.1161   Min.   :0.06498  
##  1st Qu.:0.12182   1st Qu.:0.2235   1st Qu.:0.1425   1st Qu.:0.07877  
##  Median :0.15389   Median :0.2477   Median :0.1636   Median :0.09208  
##  Mean   :0.15924   Mean   :0.2611   Mean   :0.1741   Mean   :0.11325  
##  3rd Qu.:0.17899   3rd Qu.:0.2877   3rd Qu.:0.1998   3rd Qu.:0.15873  
##  Max.   :0.27772   Max.   :0.4336   Max.   :0.2562   Max.   :0.18911  
##  NA's   :81        NA's   :82       NA's   :83       NA's   :84       
##       TUH               ALD               FRS               SER        
##  Min.   :0.04608   Min.   :0.09138   Min.   :0.05779   Min.   :0.1922  
##  1st Qu.:0.08499   1st Qu.:0.12488   1st Qu.:0.10798   1st Qu.:0.2359  
##  Median :0.11746   Median :0.17099   Median :0.15529   Median :0.2579  
##  Mean   :0.13382   Mean   :0.17873   Mean   :0.15388   Mean   :0.2889  
##  3rd Qu.:0.18274   3rd Qu.:0.23047   3rd Qu.:0.20314   3rd Qu.:0.3403  
##  Max.   :0.24043   Max.   :0.27805   Max.   :0.24364   Max.   :0.4183  
##  NA's   :85        NA's   :86        NA's   :87        NA's   :88      
##       SPB              SPS              INW               INJ        
##  Min.   :0.1240   Min.   :0.1854   Min.   :0.06223   Min.   :0.1278  
##  1st Qu.:0.1634   1st Qu.:0.2223   1st Qu.:0.08703   1st Qu.:0.1278  
##  Median :0.2108   Median :0.2592   Median :0.11182   Median :0.1278  
##  Mean   :0.2047   Mean   :0.2476   Mean   :0.11182   Mean   :0.1278  
##  3rd Qu.:0.2521   3rd Qu.:0.2787   3rd Qu.:0.13662   3rd Qu.:0.1278  
##  Max.   :0.2734   Max.   :0.2982   Max.   :0.16142   Max.   :0.1278  
##  NA's   :89       NA's   :90       NA's   :91        NA's   :92      
##       MAT     
##  Min.   : NA  
##  1st Qu.: NA  
##  Median : NA  
##  Mean   :NaN  
##  3rd Qu.: NA  
##  Max.   : NA  
##  NA's   :93
```

```
# Convert to data frame
allpopsidb_df <- data.frame(allpopsidb_3)
```

If you want you can save the fst values as csv

```
# Save it
write.csv(allpopsidb_df, file = here("manuscript1", "output", "fst", "allpopsidb_3.csv"))
```

Check the Fst values

```
head(allpopsidb_df)
```

```
##            OKI        HAI        SOC        YUN       KAT GES TIK RAR TAI HUN
## OKI         NA         NA         NA         NA        NA  NA  NA  NA  NA  NA
## HAI 0.09558814         NA         NA         NA        NA  NA  NA  NA  NA  NA
## SOC 0.13633551 0.11385993         NA         NA        NA  NA  NA  NA  NA  NA
## YUN 0.14594947 0.06147232 0.16663785         NA        NA  NA  NA  NA  NA  NA
## KAT 0.16182412 0.07881081 0.18188522 0.02757809        NA  NA  NA  NA  NA  NA
## GES 0.14091413 0.11836788 0.01211906 0.17292440 0.1893266  NA  NA  NA  NA  NA
##     HAN KLP HOC QNC SSK KAC SON CHA BRE DES CES KAN UTS MAU TUC REC BRM CAM KAG
## OKI  NA  NA  NA  NA  NA  NA  NA  NA  NA  NA  NA  NA  NA  NA  NA  NA  NA  NA  NA
## HAI  NA  NA  NA  NA  NA  NA  NA  NA  NA  NA  NA  NA  NA  NA  NA  NA  NA  NA  NA
## SOC  NA  NA  NA  NA  NA  NA  NA  NA  NA  NA  NA  NA  NA  NA  NA  NA  NA  NA  NA
## YUN  NA  NA  NA  NA  NA  NA  NA  NA  NA  NA  NA  NA  NA  NA  NA  NA  NA  NA  NA
## KAT  NA  NA  NA  NA  NA  NA  NA  NA  NA  NA  NA  NA  NA  NA  NA  NA  NA  NA  NA
## GES  NA  NA  NA  NA  NA  NA  NA  NA  NA  NA  NA  NA  NA  NA  NA  NA  NA  NA  NA
##     CUA MED FAY DAL NEO RUS CHI NUE TRA HAL STL SMI WAV HAT DEN MON BPV BCO BPA
## OKI  NA  NA  NA  NA  NA  NA  NA  NA  NA  NA  NA  NA  NA  NA  NA  NA  NA  NA  NA
## HAI  NA  NA  NA  NA  NA  NA  NA  NA  NA  NA  NA  NA  NA  NA  NA  NA  NA  NA  NA
## SOC  NA  NA  NA  NA  NA  NA  NA  NA  NA  NA  NA  NA  NA  NA  NA  NA  NA  NA  NA
## YUN  NA  NA  NA  NA  NA  NA  NA  NA  NA  NA  NA  NA  NA  NA  NA  NA  NA  NA  NA
## KAT  NA  NA  NA  NA  NA  NA  NA  NA  NA  NA  NA  NA  NA  NA  NA  NA  NA  NA  NA
## GES  NA  NA  NA  NA  NA  NA  NA  NA  NA  NA  NA  NA  NA  NA  NA  NA  NA  NA  NA
##     BIT FLO BAF PLM NEH LIN HAR FIR NEB BEN BEH ARA IPO LAM PAL MAN BEA PEO HOU
## OKI  NA  NA  NA  NA  NA  NA  NA  NA  NA  NA  NA  NA  NA  NA  NA  NA  NA  NA  NA
## HAI  NA  NA  NA  NA  NA  NA  NA  NA  NA  NA  NA  NA  NA  NA  NA  NA  NA  NA  NA
## SOC  NA  NA  NA  NA  NA  NA  NA  NA  NA  NA  NA  NA  NA  NA  NA  NA  NA  NA  NA
## YUN  NA  NA  NA  NA  NA  NA  NA  NA  NA  NA  NA  NA  NA  NA  NA  NA  NA  NA  NA
## KAT  NA  NA  NA  NA  NA  NA  NA  NA  NA  NA  NA  NA  NA  NA  NA  NA  NA  NA  NA
## GES  NA  NA  NA  NA  NA  NA  NA  NA  NA  NA  NA  NA  NA  NA  NA  NA  NA  NA  NA
##     LOS NEG NOV NEW BER MAC SPR COL GEL JAF SAI POR SUF SUU KUN GRV GRA TUH ALD
## OKI  NA  NA  NA  NA  NA  NA  NA  NA  NA  NA  NA  NA  NA  NA  NA  NA  NA  NA  NA
## HAI  NA  NA  NA  NA  NA  NA  NA  NA  NA  NA  NA  NA  NA  NA  NA  NA  NA  NA  NA
## SOC  NA  NA  NA  NA  NA  NA  NA  NA  NA  NA  NA  NA  NA  NA  NA  NA  NA  NA  NA
## YUN  NA  NA  NA  NA  NA  NA  NA  NA  NA  NA  NA  NA  NA  NA  NA  NA  NA  NA  NA
## KAT  NA  NA  NA  NA  NA  NA  NA  NA  NA  NA  NA  NA  NA  NA  NA  NA  NA  NA  NA
## GES  NA  NA  NA  NA  NA  NA  NA  NA  NA  NA  NA  NA  NA  NA  NA  NA  NA  NA  NA
##     FRS SER SPB SPS INW INJ MAT
## OKI  NA  NA  NA  NA  NA  NA  NA
## HAI  NA  NA  NA  NA  NA  NA  NA
## SOC  NA  NA  NA  NA  NA  NA  NA
## YUN  NA  NA  NA  NA  NA  NA  NA
## KAT  NA  NA  NA  NA  NA  NA  NA
## GES  NA  NA  NA  NA  NA  NA  NA
```

We will convert the data into a matrix.

```
aa <- as.matrix(allpopsidb_df)
aa[upper.tri(aa)] <- t(aa)[upper.tri(t(aa))]
head(aa)
```

```
##            OKI        HAI        SOC        YUN        KAT        GES
## OKI         NA 0.09558814 0.13633551 0.14594947 0.16182412 0.14091413
## HAI 0.09558814         NA 0.11385993 0.06147232 0.07881081 0.11836788
## SOC 0.13633551 0.11385993         NA 0.16663785 0.18188522 0.01211906
## YUN 0.14594947 0.06147232 0.16663785         NA 0.02757809 0.17292440
## KAT 0.16182412 0.07881081 0.18188522 0.02757809         NA 0.18932663
## GES 0.14091413 0.11836788 0.01211906 0.17292440 0.18932663         NA
##            TIK        RAR       TAI        HUN        HAN        KLP        HOC
## OKI 0.15298678 0.14221855 0.1624812 0.07367462 0.11262072 0.17208667 0.10209590
## HAI 0.12952901 0.11721834 0.1438048 0.04131371 0.03935239 0.08459820 0.03556564
## SOC 0.05912151 0.01989924 0.1961891 0.08231007 0.13396013 0.19140352 0.12172998
## YUN 0.18436091 0.16929635 0.2093213 0.09455389 0.05900262 0.05812901 0.07520378
## KAT 0.20259876 0.18597223 0.2316420 0.10792479 0.07860738 0.08135059 0.09354520
## GES 0.05432309 0.02787053 0.1986180 0.08639645 0.13951177 0.19762116 0.12629917
##            QNC         SSK         KAC         SON         CHA       BRE
## OKI 0.20816098 0.147614206 0.146291307 0.151256704 0.145417551 0.1732961
## HAI 0.13016038 0.064453269 0.058794970 0.058418641 0.061622890 0.1621626
## SOC 0.22754716 0.169222120 0.169132401 0.175148129 0.166503927 0.1735052
## YUN 0.08211356 0.004491156 0.001117613 0.003388117 0.001439952 0.2214530
## KAT 0.11566529 0.032655753 0.027910112 0.027333949 0.030487234 0.2356632
## GES 0.23486993 0.175728154 0.175086589 0.183115762 0.172317879 0.1753479
##           DES       CES       KAN       UTS        MAU       TUC        REC
## OKI 0.1605189 0.2034129 0.1765158 0.1460260 0.14377638 0.1594006 0.13624179
## HAI 0.1515315 0.1929777 0.1662395 0.1358345 0.09502008 0.1028282 0.09866765
## SOC 0.1632821 0.2059232 0.1706158 0.1442653 0.16017389 0.1762622 0.15417512
## YUN 0.2113039 0.2544209 0.2287345 0.1998948 0.10690195 0.1033862 0.12321344
## KAT 0.2244187 0.2719183 0.2454021 0.2128193 0.11639467 0.1146626 0.13405590
## GES 0.1662910 0.2076713 0.1741899 0.1481145 0.16518769 0.1804067 0.15831344
##           BRM         CAM       KAG        CUA        MED        FAY       DAL
## OKI 0.1398983 0.131016865 0.1260474 0.14820809 0.14663578 0.10014439 0.1084890
## HAI 0.1235319 0.049755118 0.1166803 0.09610516 0.08921155 0.09067834 0.1009434
## SOC 0.1511297 0.151303233 0.1292758 0.16912438 0.16433917 0.11369321 0.1218228
## YUN 0.1716397 0.004125119 0.1725061 0.11277595 0.10530343 0.14642726 0.1552289
## KAT 0.1858164 0.028024545 0.1860823 0.12634461 0.11444477 0.15453315 0.1668447
## GES 0.1549302 0.157560709 0.1327545 0.17490132 0.17107125 0.11817163 0.1255112
##           NEO       RUS        CHI        NUE        TRA       HAL       STL
## OKI 0.1102706 0.1323524 0.11503695 0.10025146 0.09589567 0.1067001 0.1338220
## HAI 0.1023955 0.1276570 0.05891201 0.07839041 0.08221655 0.1013947 0.1231074
## SOC 0.1247596 0.1457495 0.13272942 0.11692530 0.10909637 0.1076025 0.1337008
## YUN 0.1565965 0.1784320 0.10810659 0.12858842 0.13308082 0.1560695 0.1863641
## KAT 0.1686488 0.1902152 0.12828818 0.14044260 0.14477684 0.1667415 0.1984763
## GES 0.1294903 0.1517791 0.13728710 0.12113085 0.11295424 0.1117998 0.1372284
##           SMI       WAV       HAT       DEN        MON       BPV       BCO
## OKI 0.1152633 0.1288128 0.1079221 0.1046447 0.09737039 0.1603635 0.1653289
## HAI 0.1020488 0.1199530 0.1036427 0.1030282 0.07885513 0.1067394 0.1114739
## SOC 0.1254611 0.1307224 0.1196601 0.1183906 0.11341963 0.1775241 0.1816329
## YUN 0.1573600 0.1790267 0.1539487 0.1603127 0.13005893 0.1130443 0.1145506
## KAT 0.1704798 0.1908540 0.1663502 0.1708964 0.14203610 0.1249593 0.1251151
## GES 0.1300365 0.1341381 0.1246739 0.1216414 0.11748504 0.1839108 0.1855865
##           BPA       BIT       FLO        BAF       PLM       NEH       LIN
## OKI 0.1441479 0.1590180 0.1683931 0.15870048 0.1682647 0.1143111 0.1280703
## HAI 0.1061755 0.1079176 0.1103403 0.09915842 0.1064906 0.1043950 0.1195671
## SOC 0.1613069 0.1774755 0.1853021 0.17551599 0.1862409 0.1180840 0.1320491
## YUN 0.1258053 0.1178931 0.1159422 0.10265725 0.1056234 0.1624636 0.1811374
## KAT 0.1358078 0.1298108 0.1258349 0.11346713 0.1163252 0.1736599 0.1927860
## GES 0.1655459 0.1819322 0.1896658 0.18117898 0.1901956 0.1208558 0.1362601
##           HAR       FIR       NEB        BEN        BEH       ARA       IPO
## OKI 0.1212813 0.1473220 0.1214149 0.16480162 0.18289716 0.1545442 0.2246868
## HAI 0.1132978 0.1422376 0.1119907 0.08119431 0.11299642 0.1003186 0.1475621
## SOC 0.1264882 0.1550713 0.1258977 0.18443443 0.19848522 0.1734538 0.2397570
## YUN 0.1683406 0.2001698 0.1720573 0.02233991 0.09834216 0.1065040 0.1264610
## KAT 0.1804430 0.2156081 0.1844052 0.03187799 0.11299195 0.1172988 0.1414378
## GES 0.1311036 0.1586044 0.1291062 0.19066744 0.20483388 0.1766602 0.2452972
##             LAM        PAL       MAN        BEA        PEO        HOU
## OKI 0.150056765 0.11298144 0.1085156 0.09161781 0.10694113 0.08864899
## HAI 0.064812146 0.08730472 0.0997583 0.07233305 0.09774803 0.06907390
## SOC 0.172523618 0.12670815 0.1112396 0.10475728 0.11102698 0.10053266
## YUN 0.007137257 0.12916878 0.1566688 0.12151888 0.15244264 0.11724201
## KAT 0.033877867 0.14087406 0.1684364 0.13239546 0.16294958 0.12884947
## GES 0.179286918 0.13113663 0.1150112 0.10780997 0.11563278 0.10595289
##            LOS       NEG        NOV       NEW       BER        MAC       SPR
## OKI 0.11450880 0.2424934 0.20224951 0.1194139 0.1192349 0.10377704 0.1427641
## HAI 0.07743439 0.1632479 0.11846110 0.1182389 0.1098587 0.07851593 0.1313231
## SOC 0.12657310 0.2576322 0.22193780 0.1277548 0.1219931 0.11537023 0.1440148
## YUN 0.12660762 0.1326778 0.09269472 0.1712038 0.1668719 0.12601402 0.1913415
## KAT 0.14123944 0.1632650 0.10707880 0.1826798 0.1787213 0.13760812 0.2058800
## GES 0.13117830 0.2658439 0.22820527 0.1315438 0.1262965 0.11932998 0.1496688
##           COL           GEL         JAF        SAI       POR        SUF
## OKI 0.1209269  0.1488435782 0.160616289 0.11247446 0.1767596 0.21924545
## HAI 0.1091179  0.0495294372 0.057898518 0.07498188 0.1158530 0.14180901
## SOC 0.1197429  0.1708151881 0.180660216 0.13270703 0.1921536 0.23886778
## YUN 0.1698510 -0.0004902834 0.008371674 0.10492622 0.1075931 0.12838272
## KAT 0.1823181  0.0178250690 0.013619668 0.11842615 0.1181223 0.06548768
## GES 0.1246398  0.1801347472 0.187305264 0.13684611 0.1983565 0.24580096
##            SUU       KUN       GRV        GRA        TUH       ALD        FRS
## OKI 0.15915034 0.2559533 0.1626244 0.09408549 0.10091373 0.1342470 0.10385426
## HAI 0.08734395 0.1679898 0.1079468 0.04660201 0.07795749 0.1105019 0.08483983
## SOC 0.17866612 0.2722068 0.1757704 0.10771104 0.10588536 0.1405699 0.10224474
## YUN 0.09996617 0.1377966 0.1183705 0.07751427 0.13460958 0.1666679 0.14135486
## KAT 0.06119742 0.1440158 0.1306540 0.09198048 0.14755170 0.1813161 0.15470275
## GES 0.18461460 0.2780361 0.1814535 0.11224050 0.11078263 0.1446401 0.10564731
##           SER       SPB       SPS        INW        INJ        MAT
## OKI 0.2190639 0.1191098 0.1469864 0.26067729 0.22685247 0.15235502
## HAI 0.1882815 0.1078702 0.1211492 0.18116251 0.14817013 0.06945987
## SOC 0.2246910 0.1163494 0.1504383 0.27608980 0.24167669 0.17192703
## YUN 0.2551050 0.1681615 0.1809089 0.15158505 0.12085119 0.01922669
## KAT 0.2817785 0.1794650 0.1962450 0.09782599 0.07814525 0.04558362
## GES 0.2295872 0.1209193 0.1554201 0.28292898 0.24851347 0.17814330
```

```
cities <- read_csv(here("manuscript1/output/fst/cities_man1.csv"))
```

Save it

```
saveRDS(
  cities, here(
    "manuscript1/output/fst/cities_man1.rds"
  )
)
```

Import sample locations

```
sampling_loc <- readRDS(here("manuscript1", "output", "fst", "cities_man1.rds"))

# Arrange by region 
sampling_loc <- sampling_loc |>
  dplyr::arrange(
    continent, region, subregion
  ) 

# Check it
head(sampling_loc)
```

```
## # A tibble: 6 × 10
##   pop   city   country state latitude longitude continent region subregion order
##   <chr> <chr>  <chr>   <chr>    <dbl>     <dbl> <chr>     <chr>  <chr>     <dbl>
## 1 BRM   Bermu… Bermuda Berm…     32.3     -64.8 Americas  Carib… Caribbean   128
## 2 NEG   Negril Jamaica Jama…     18.3     -78.3 Americas  Carib… Caribbean   129
## 3 SAI   Saint… Trinid… Trin…     10.2     -61.5 Americas  Carib… Caribbean   131
## 4 COL   Colum… USA     OH, …     40.0     -82.9 Americas  North… Midwest,…   115
## 5 PEO   Peori… USA     IL, …     40.7     -89.6 Americas  North… Midwest,…   116
## 6 SPR   Sprin… USA     MO, …     37.2     -93.3 Americas  North… Midwest,…   117
```

Order

```
order_pops <- as.vector(sampling_loc$pop)
order_pops
```

```
##  [1] "BRM" "NEG" "SAI" "COL" "PEO" "SPR" "STL" "NEB" "NEH" "FIR" "LIN" "NEW"
## [13] "BER" "HAR" "MAN" "WAV" "FAY" "BEA" "MAC" "PAL" "RUS" "HAL" "HAT" "NEO"
## [25] "NUE" "HOU" "TRA" "MON" "DAL" "SMI" "DEN" "CHI" "LOS" "CUA" "MED" "BPV"
## [37] "MAU" "BIT" "TUC" "FLO" "REC" "ARA" "PLM" "BCO" "BAF" "IPO" "NOV" "BEH"
## [49] "BPA" "GRV" "POR" "HAI" "YUN" "HUN" "TAI" "OKI" "UTS" "KAG" "KAN" "BEN"
## [61] "KAT" "GEL" "JAF" "KUN" "CAM" "INW" "INJ" "SUF" "SUU" "KLP" "MAT" "CHA"
## [73] "KAC" "SSK" "LAM" "HOC" "QNC" "HAN" "SON" "GES" "SOC" "TIK" "RAR" "TUH"
## [85] "SER" "GRA" "ALD" "CES" "BRE" "DES" "SPB" "SPS" "FRS"
```

Lets check if the matrix is symmetric.

```
isSymmetric(aa)
```

```
## [1] TRUE
```

Now lets order the matrix using poporder. We will also add NA on the
upper left side of the matrix.

```
aa <- aa[order_pops, order_pops]
aa[lower.tri(aa)] <- NA
```

Now we have to convert the matrix to a data frame to plot it with
ggplot.

```
pairfst.long <- melt(aa)
summary(pairfst.long)
```

```
##       Var1           Var2          value       
##  BRM    :  93   BRM    :  93   Min.   :-0.003  
##  NEG    :  93   NEG    :  93   1st Qu.: 0.099  
##  SAI    :  93   SAI    :  93   Median : 0.134  
##  COL    :  93   COL    :  93   Mean   : 0.139  
##  PEO    :  93   PEO    :  93   3rd Qu.: 0.179  
##  SPR    :  93   SPR    :  93   Max.   : 0.434  
##  (Other):8091   (Other):8091   NA's   :4371
```

Remove NAs and rename columns

```
# remove NAs
fst2 <-
  pairfst.long |>
  drop_na()

# rename columns
fst2 <-
  fst2 |>
  dplyr::rename(pop1 = 1,
         pop2 = 2,
         fst  = 3)


# Split the data into two data frames, one for pop1 and one for pop2
df_pop1 <- fst2 |>
  dplyr::select(pop = pop1, fst)
df_pop2 <- fst2 |>
  dplyr::select(pop = pop2, fst)

# Combine the two data frames
df_combined <- bind_rows(df_pop1, df_pop2)

# Calculate the mean fst for each population
mean_fst <- df_combined |>
  group_by(pop) |>
  summarise(mean_fst = mean(fst))

print(mean_fst)
```

```
## # A tibble: 93 × 2
##    pop   mean_fst
##    <fct>    <dbl>
##  1 BRM      0.147
##  2 NEG      0.232
##  3 SAI      0.115
##  4 COL      0.120
##  5 PEO      0.107
##  6 SPR      0.143
##  7 STL      0.130
##  8 NEB      0.121
##  9 NEH      0.114
## 10 FIR      0.153
## # ℹ 83 more rows
```

Merge

```
fst3 <-
  sampling_loc |>
  left_join(
    mean_fst,
    by = c("pop" = "pop")
  ) |>
  dplyr::select(
    -state
  )
```

Mean by region

```
# Group by Region and calculate the mean_fst by Region
region_means <- fst3 |>
  group_by(region) |>
  summarize(mean_fst_by_region = round(mean(mean_fst, na.rm = TRUE), 2)) |>
  ungroup()  # Ungroup the data

# Add the mean_fst_by_region column to the fst3 tibble
fst3 <- fst3 |>
  left_join(region_means, by = "region")

# Print the modified fst3 tibble
print(fst3)
```

```
## # A tibble: 93 × 11
##    pop   city        country latitude longitude continent region subregion order
##    <chr> <chr>       <chr>      <dbl>     <dbl> <chr>     <chr>  <chr>     <dbl>
##  1 BRM   Bermuda     Bermuda     32.3     -64.8 Americas  Carib… Caribbean   128
##  2 NEG   Negril      Jamaica     18.3     -78.3 Americas  Carib… Caribbean   129
##  3 SAI   Saint Augu… Trinid…     10.2     -61.5 Americas  Carib… Caribbean   131
##  4 COL   Columbus, … USA         40.0     -82.9 Americas  North… Midwest,…   115
##  5 PEO   Peoria, IL  USA         40.7     -89.6 Americas  North… Midwest,…   116
##  6 SPR   Springfiel… USA         37.2     -93.3 Americas  North… Midwest,…   117
##  7 STL   St. Louis,… USA         38.6     -90.2 Americas  North… Midwest,…   118
##  8 NEB   New Bedfor… USA         41.7     -70.9 Americas  North… Northeas…    98
##  9 NEH   New Haven,… USA         41.3     -72.9 Americas  North… Northeas…    99
## 10 FIR   Fire Islan… USA         40.7     -72.9 Americas  North… Northeas…   100
## # ℹ 83 more rows
## # ℹ 2 more variables: mean_fst <dbl>, mean_fst_by_region <dbl>
```

Mean by subregion

```
# Group by Country and calculate the mean_fst by Country
subregion_means <- fst3 |>
  group_by(subregion) |>
  summarize(mean_fst_by_subregion = round(mean(mean_fst, na.rm = TRUE), 2)) |>
  ungroup()  # Ungroup the data

# Add the mean_fst_by_country column to the fst3 tibble
fst3 <- fst3 |>
  left_join(subregion_means, by = "subregion")

# Print the modified fst3 tibble
print(fst3)
```

```
## # A tibble: 93 × 12
##    pop   city        country latitude longitude continent region subregion order
##    <chr> <chr>       <chr>      <dbl>     <dbl> <chr>     <chr>  <chr>     <dbl>
##  1 BRM   Bermuda     Bermuda     32.3     -64.8 Americas  Carib… Caribbean   128
##  2 NEG   Negril      Jamaica     18.3     -78.3 Americas  Carib… Caribbean   129
##  3 SAI   Saint Augu… Trinid…     10.2     -61.5 Americas  Carib… Caribbean   131
##  4 COL   Columbus, … USA         40.0     -82.9 Americas  North… Midwest,…   115
##  5 PEO   Peoria, IL  USA         40.7     -89.6 Americas  North… Midwest,…   116
##  6 SPR   Springfiel… USA         37.2     -93.3 Americas  North… Midwest,…   117
##  7 STL   St. Louis,… USA         38.6     -90.2 Americas  North… Midwest,…   118
##  8 NEB   New Bedfor… USA         41.7     -70.9 Americas  North… Northeas…    98
##  9 NEH   New Haven,… USA         41.3     -72.9 Americas  North… Northeas…    99
## 10 FIR   Fire Islan… USA         40.7     -72.9 Americas  North… Northeas…   100
## # ℹ 83 more rows
## # ℹ 3 more variables: mean_fst <dbl>, mean_fst_by_region <dbl>,
## #   mean_fst_by_subregion <dbl>
```

To make scatter plot

```
# Order the aggregated data
aggregated_data <- fst3[order(fst3$mean_fst), ]

# Assign a numeric index for plotting
aggregated_data$index <- 1:nrow(aggregated_data)

# Fit a linear model
lm_fit <- lm(mean_fst ~ index, data = aggregated_data)

# Predicted values from the linear model
aggregated_data$fitted_values <- predict(lm_fit)

ggplot(aggregated_data, aes(x = index, y = mean_fst)) +
  geom_point(aes(color = subregion), size = 3, shape = 16) + # shape = 16 for solid circle
  geom_line(aes(y = fitted_values), color = "black") +  # Fitted line
  labs(
    title = "Mean Fst",
    x = "Populations",
    y = "Mean Fst Value",
    color = "subregion"
  ) +
  scale_x_continuous(breaks = aggregated_data$index, labels = aggregated_data$pop) +
  scale_color_manual(values = c("#ff1e00", "#8c61cd", "#52ff02", "gold", "orange", "blue", "#9DCC00", "darkgreen", "#FF8005", "#ffb97f", "purple2", "#AC7700")) +
  theme_classic() +
  theme(axis.text.x = element_text(angle = 45, hjust = 1)) +
  theme(legend.position = "top")
```

```
ggsave(
  filename = here("manuscript1" ,"output", "fst", "figures", "fst_scatter_allpops_subregion_colored.pdf"),
  width = 7,
  height = 5,
  units = "in"
)
```

Mean By continent

```
# Group by Country and calculate the mean_fst by Country
continent_means <- fst3 |>
  group_by(continent) |>
  summarize(mean_fst_by_continent = round(mean(mean_fst, na.rm = TRUE), 2)) |>
  ungroup()  # Ungroup the data

# Add the mean_fst_by_country column to the fst3 tibble
fst3 <- fst3 |>
  left_join(continent_means, by = "continent")

# Print the modified fst3 tibble
print(fst3)
```

```
## # A tibble: 93 × 13
##    pop   city        country latitude longitude continent region subregion order
##    <chr> <chr>       <chr>      <dbl>     <dbl> <chr>     <chr>  <chr>     <dbl>
##  1 BRM   Bermuda     Bermuda     32.3     -64.8 Americas  Carib… Caribbean   128
##  2 NEG   Negril      Jamaica     18.3     -78.3 Americas  Carib… Caribbean   129
##  3 SAI   Saint Augu… Trinid…     10.2     -61.5 Americas  Carib… Caribbean   131
##  4 COL   Columbus, … USA         40.0     -82.9 Americas  North… Midwest,…   115
##  5 PEO   Peoria, IL  USA         40.7     -89.6 Americas  North… Midwest,…   116
##  6 SPR   Springfiel… USA         37.2     -93.3 Americas  North… Midwest,…   117
##  7 STL   St. Louis,… USA         38.6     -90.2 Americas  North… Midwest,…   118
##  8 NEB   New Bedfor… USA         41.7     -70.9 Americas  North… Northeas…    98
##  9 NEH   New Haven,… USA         41.3     -72.9 Americas  North… Northeas…    99
## 10 FIR   Fire Islan… USA         40.7     -72.9 Americas  North… Northeas…   100
## # ℹ 83 more rows
## # ℹ 4 more variables: mean_fst <dbl>, mean_fst_by_region <dbl>,
## #   mean_fst_by_subregion <dbl>, mean_fst_by_continent <dbl>
```

To make scatter plot by continent

```
# Order the aggregated data
aggregated_data <- fst3[order(fst3$mean_fst), ]

# Assign a numeric index for plotting
aggregated_data$index <- 1:nrow(aggregated_data)

# Fit a linear model
lm_fit <- lm(mean_fst ~ index, data = aggregated_data)

# Predicted values from the linear model
aggregated_data$fitted_values <- predict(lm_fit)

ggplot(aggregated_data, aes(x = index, y = mean_fst)) +
  geom_point(aes(color = continent), size = 3, shape = 16) + # shape = 16 for solid circle
  geom_line(aes(y = fitted_values), color = "black") +  # Fitted line
  labs(
    title = "Mean Fst",
    x = "Populations",
    y = "Mean Fst Value",
    color = "continent"
  ) +
  scale_x_continuous(breaks = aggregated_data$index, labels = aggregated_data$pop) +
  scale_color_manual(values = c("orange", "darkgreen", "purple2")) +
  theme_classic() +
  theme(axis.text.x = element_text(angle = 45, hjust = 1)) +
  theme(legend.position = "top")
```

```
ggsave(
  filename = here("manuscript1" ,"output", "fst", "figures", "fst_scatter_allpops_continent.pdf"),
  width = 7,
  height = 5,
  units = "in"
)
```

With continent

```
fst4 <- fst3 |>
  dplyr::select(
    continent, mean_fst_by_continent, region, mean_fst_by_region, subregion, mean_fst_by_subregion, city, pop, mean_fst,
  )

fst4 <- fst4 |>
  arrange(
    continent, region, subregion, city
  )

# Round
fst4 <- fst4 |>
  mutate_if(is.numeric, ~ round(., 2))

head(fst4)
```

```
# Set theme if you want to use something different from the previous table
set_flextable_defaults(
  font.family = "Arial",
  font.size = 9,
  big.mark = ",",
  theme_fun = "theme_zebra" # try the themes: theme_alafoli(), theme_apa(), theme_booktabs(), theme_box(), theme_tron_legacy(), theme_tron(), theme_vader(), theme_vanilla(), theme_zebra()
)

# Then create the flextable object
flex_table <- flextable(fst4) |>
  set_caption(caption = as_paragraph(
    as_chunk(
      "Table 1. Fst values for Europe, Asia and Americas (all pops for manuscript1",
      props = fp_text_default(color = "#000000", font.size = 14)
    )
  ),
  fp_p = fp_par(text.align = "center", padding = 5))

# Print the flextable
flex_table
```

```
# Initialize Word document
doc <- 
  read_docx() |>
  body_add_flextable(value = flex_table)

# Define the output path with 'here' library
output_path <- here(
  "manuscript1",
  "output",
  "fst", 
  "fst_SNPS_continent_region_subregion_allman1.docx"
  )

# Save the Word document
print(doc, target = output_path)
```

Estimate distances

```
# Grab the population names from the matrix aa
populations_with_fst <- colnames(aa)

# Create an empty matrix to store the distances
n <- nrow(sampling_loc)
distance_matrix <- matrix(0, n, n)
rownames(distance_matrix) <- sampling_loc$pop
colnames(distance_matrix) <- sampling_loc$pop

# Calculate the distances
for (i in 1:n) {
  for (j in 1:n) {
    if (i != j) {
      coord1 <- c(sampling_loc$longitude[i], sampling_loc$latitude[i])
      coord2 <- c(sampling_loc$longitude[j], sampling_loc$latitude[j])
      distance_matrix[i, j] <- distHaversine(coord1, coord2) / 1000 # distance in km
    }
  }
}

# Print the distance matrix
head(distance_matrix)
```

```
##          BRM      NEG      SAI       COL      PEO       SPR       STL      NEB
## BRM    0.000 2070.053 2487.778 1834.4207 2399.055 2651.8760 2400.6359 1175.708
## NEG 2070.053    0.000 2031.431 2455.4649 2718.388 2563.0260 2540.4791 2697.740
## SAI 2487.778 2031.431    0.000 3937.0649 4382.061 4384.6868 4267.5836 3626.677
## COL 1834.421 2455.465 3937.065    0.0000  574.158  952.8901  645.9952 1025.512
## PEO 2399.055 2718.388 4382.061  574.1580    0.000  501.3070  235.3912 1565.778
## SPR 2651.876 2563.026 4384.687  952.8901  501.307    0.0000  312.9047 1978.307
##           NEH       FIR       LIN       NEW       BER       HAR       MAN
## BRM 1237.6477 1185.7023 1207.7055 1258.2000 1234.8544 1399.3529 1348.2017
## NEG 2617.5152 2551.1955 2310.7563 2532.7579 2418.3570 2453.2786 2284.3127
## SAI 3646.2545 3583.3345 3484.4399 3626.0479 3557.3098 3680.7097 3557.5864
## COL  855.4577  849.2138  654.9661  745.5006  681.5811  514.0279  492.8916
## PEO 1400.7636 1404.6675 1228.9799 1300.8168 1249.9571 1077.8707 1066.4628
## SPR 1808.0043 1798.9308 1573.9007 1695.9361 1621.3667 1461.9026 1402.8245
##           WAV       FAY       BEA       MAC       PAL       RUS       HAL
## BRM 1242.9209 1343.1062 1493.9239 1766.2247 1601.1946 2634.4037 2031.9087
## NEG 2092.9162 1873.0196 1595.6076 1706.1567  955.1584 2390.5339 2006.8366
## SAI 3378.9144 3291.0740 3170.5178 3396.3147 2683.3948 4253.3103 3738.6261
## COL  602.2321  648.0694  861.0315  796.2625 1500.4678 1041.2023  662.3347
## PEO 1156.9277 1126.6718 1214.2582 1022.7848 1788.6790  677.0327  713.1727
## SPR 1434.7148 1311.5434 1264.9684 1004.0418 1708.4383  214.8918  665.8232
##           HAT      NEO      NUE       HOU       TRA       MON       DAL
## BRM 2309.6362 2411.764 3190.987 2919.3554 3132.7201 2936.6038 2993.7078
## NEG 1822.3601 1760.948 2231.740 2145.9325 2378.5564 2213.3079 2451.4847
## SAI 3709.6360 3693.272 4253.531 4136.9448 4376.2970 4197.7029 4407.5683
## COL 1116.7983 1285.063 1919.494 1604.7524 1723.5200 1575.4412 1478.4203
## PEO 1040.3572 1191.118 1618.198 1323.6330 1369.1747 1271.0627 1089.2702
## SPR  750.4193  857.540 1130.383  851.2166  871.2815  790.3832  588.6353
##           SMI       DEN      CHI      LOS      CUA      MED      BPV      MAU
## BRM 2852.2257 3017.3859 4877.947 4926.755 3108.604 3108.604 4566.624 3971.576
## NEG 2305.9090 2505.1609 4270.559 4321.288 1371.569 1371.569 3397.934 3112.390
## SAI 4254.3061 4456.7148 6287.222 6338.301 1614.468 1614.468 2118.647 1484.018
## COL 1390.4694 1474.3014 3141.996 3187.646 3825.151 3825.151 5762.930 5332.269
## PEO 1052.6527 1066.6571 2580.996 2625.588 4080.893 4080.893 6100.051 5726.259
## SPR  566.5981  565.4106 2231.253 2279.202 3886.188 3886.188 5959.367 5650.842
##          BIT      TUC      FLO      REC      ARA      PLM      BCO      BAF
## BRM 4175.196 4313.011 4925.257 5501.105 4734.976 5049.896 4984.800 4784.252
## NEG 3508.309 3983.353 4778.212 5602.224 4357.837 4577.611 4362.958 3973.695
## SAI 1716.680 2021.418 2781.995 3576.784 2426.968 2697.851 2564.149 2307.784
## COL 5641.342 5927.677 6619.995 7272.529 6344.792 6630.544 6498.353 6198.950
## PEO 6062.831 6393.802 7109.965 7791.737 6805.965 7079.408 6925.610 6597.286
## SPR 6017.579 6406.028 7154.091 7884.219 6808.124 7062.453 6881.078 6519.667
##          IPO      NOV      BEH      BPA      GRV      POR      HAI      YUN
## BRM 5618.381 6245.034 6223.685 6506.602 7079.580 6535.480 14273.51 13547.84
## NEG 4881.715 5844.981 5674.959 5917.680 6115.902 5519.648 15777.10 15279.22
## SAI 3173.326 3959.734 3861.362 4131.545 4602.851 4050.133 16624.74 15752.63
## COL 7092.144 7880.510 7798.146 8068.479 8452.483 7868.169 13323.72 12849.75
## PEO 7500.266 8340.976 8236.138 8499.913 8812.269 8220.035 13080.48 12695.39
## SPR 7428.023 8332.716 8197.314 8450.916 8676.875 8079.698 13315.37 13002.45
##          HUN      TAI      OKI      UTS      KAG      KAN      BEN      KAT
## BRM 13357.28 13858.90 13354.04 11953.86 12747.50 12053.57 13600.48 12645.06
## NEG 14811.52 15019.88 14300.61 12730.79 13689.57 12910.82 15670.48 14624.16
## SAI 15769.55 16341.49 15831.15 14376.97 15219.31 14500.97 14830.78 14505.97
## COL 12358.74 12628.17 11976.36 10460.14 11358.56 10605.60 13808.11 12404.40
## PEO 12115.94 12305.12 11610.98 10065.60 10995.26 10227.34 13919.91 12404.58
## SPR 12357.28 12471.89 11738.06 10168.47 11128.18 10348.02 14370.77 12809.00
##          GEL      JAF      KUN      CAM      INW      INJ      SUF      SUU
## BRM 12949.00 14054.36 13888.96 15034.24 17463.99 17006.01 16621.63 16501.68
## NEG 14870.33 16124.30 15914.98 16698.03 17812.70 18581.54 17333.30 17016.34
## SAI 14921.35 15234.65 14689.79 17188.94 19840.31 18676.06 19108.65 18932.33
## COL 12565.50 14238.75 14388.40 14244.52 15958.02 16154.49 15213.33 14995.29
## PEO 12517.05 14332.11 14579.70 14031.87 15501.27 15864.03 14796.87 14553.74
## SPR 12894.06 14773.78 15056.70 14285.20 15484.82 16028.37 14840.69 14568.04
##          KLP      MAT      CHA      KAC      SSK      LAM      HOC      QNC
## BRM 15841.91 15664.99 14846.95 14616.98 14632.81 14161.68 15158.25 14868.15
## NEG 17654.48 17486.89 16599.85 16435.38 16309.41 15960.77 16759.34 16374.34
## SAI 17653.49 17502.06 16918.44 16623.68 16815.64 16240.98 17362.43 17179.99
## COL 15215.55 15053.96 14161.24 14021.56 13859.26 13546.80 14303.99 13923.30
## PEO 15024.97 14873.24 13982.60 13874.30 13660.44 13404.49 14070.21 13670.05
## SPR 15286.89 15144.48 14263.17 14179.06 13927.71 13715.95 14305.71 13892.06
##          HAN      SON       GES       SOC       TIK       RAR       TUH
## BRM 14017.99 15375.05  8746.553  8637.369  8525.944  8646.400  8889.795
## NEG 15639.10 17200.16 10815.854 10706.652 10595.922 10716.448 10957.501
## SAI 16298.15 17263.89 10230.636 10126.025 10089.239 10191.982 10326.074
## COL 13186.29 14774.17  9203.877  9099.741  8929.074  9055.918  9377.642
## PEO 12983.00 14605.09  9472.022  9370.851  9185.711  9312.493  9652.034
## SPR 13251.34 14887.82  9970.018  9869.212  9682.431  9809.142 10150.636
##          SER      GRA      ALD      CES      BRE      DES      SPB      SPS
## BRM 7145.046 7784.124 7283.577 6617.836 6420.774 6448.794 5198.115 5399.642
## NEG 9201.952 9806.626 9319.404 8660.662 8469.453 8497.406 7163.460 7328.154
## SAI 8535.900 8924.900 8521.412 7933.883 7786.782 7812.741 6258.899 6323.681
## COL 7823.243 8605.886 8073.895 7407.144 7188.490 7215.452 6318.497 6585.458
## PEO 8171.793 8982.074 8448.348 7789.879 7568.771 7595.096 6790.645 7068.513
## SPR 8672.259 9480.074 8946.426 8286.580 8065.768 8092.196 7258.406 7530.201
##          FRS
## BRM 6093.691
## NEG 8136.147
## SAI 7437.665
## COL 6912.302
## PEO 7308.924
## SPR 7802.995
```

Compare distance and FST

```
# Fill lower triangle of 'aa' matrix
aa[lower.tri(aa)] <- t(aa)[lower.tri(aa)]

# Fill diagonal with 0 (or another value that makes sense in your context)
diag(aa) <- 0


# Combine 'aa' and 'distance_matrix'
data <- data.frame(Distance = as.vector(distance_matrix), FST = as.vector(aa))

# Add row and column indices for easier tracking
data$row_index <- rep(rownames(distance_matrix), each = ncol(distance_matrix))
data$col_index <- rep(colnames(distance_matrix), nrow(distance_matrix))

head(data)
```

```
##   Distance       FST row_index col_index
## 1    0.000 0.0000000       BRM       BRM
## 2 2070.053 0.2565221       BRM       NEG
## 3 2487.778 0.1194637       BRM       SAI
## 4 1834.421 0.1023216       BRM       COL
## 5 2399.055 0.0804618       BRM       PEO
## 6 2651.876 0.1242179       BRM       SPR
```

We can merge the FST and distance matrices

```
# Ensure the matrices have the same names in the same order
common_names <- intersect(rownames(distance_matrix), rownames(aa))
sorted_names <- sort(common_names)

# Reorder the matrices
distance_matrix <- distance_matrix[sorted_names, sorted_names]
aa <- aa[sorted_names, sorted_names]

# Initialize the final merged matrix
merged_matrix <- matrix(NA, nrow = nrow(aa), ncol = ncol(aa))
rownames(merged_matrix) <- sorted_names
colnames(merged_matrix) <- sorted_names

# Fill the upper triangular part from aa
merged_matrix[upper.tri(merged_matrix, diag = FALSE)] <- aa[upper.tri(aa, diag = FALSE)]

# Fill the lower triangular part from distance_matrix
merged_matrix[lower.tri(merged_matrix, diag = FALSE)] <- distance_matrix[lower.tri(distance_matrix, diag = FALSE)]

# Format the matrix (Fst two decimals and distance in Km with zero decimals)
# Format the elements based on their position in the matrix
for(i in 1:nrow(merged_matrix)) {
  for(j in 1:ncol(merged_matrix)) {
    if (i < j) {
      # Upper triangular - Fst values with two decimal places
      merged_matrix[i, j] <- sprintf("%.2f", as.numeric(merged_matrix[i, j]))
    } else if (i > j) {
      # Lower triangular - Distance values with zero decimal places
      merged_matrix[i, j] <- sprintf("%.0f", as.numeric(merged_matrix[i, j]))
    }
  }
}

# Now the merged_matrix should be formatted as you need
print(merged_matrix)
```

```
##     ALD     ARA     BAF     BCO     BEA     BEH     BEN     BER     BIT    
## ALD NA      "0.17"  "0.17"  "0.18"  "0.10"  "0.20"  "0.19"  "0.12"  "0.18" 
## ARA "8734"  NA      "0.01"  "0.02"  "0.11"  "0.05"  "0.11"  "0.14"  "0.04" 
## BAF "9565"  "917"   NA      "0.02"  "0.11"  "0.05"  "0.11"  "0.15"  "0.05" 
## BCO "9266"  "532"   "505"   NA      "0.12"  "0.06"  "0.12"  "0.15"  "0.06" 
## BEA "8461"  "5596"  "5387"  "5711"  NA      "0.14"  "0.14"  "0.04"  "0.12" 
## BEH "9436"  "1491"  "1721"  "1316"  "7022"  NA      "0.10"  "0.18"  "0.07" 
## BEN "6417"  "14073" "14978" "14498" "14478" "13774" NA      "0.19"  "0.12" 
## BER "7548"  "5908"  "5860"  "6113"  "967"   "7386"  "13512" NA      "0.15" 
## BIT "9132"  "922"   "624"   "864"   "4849"  "2180"  "14847" "5274"  NA     
## BPA "9609"  "1776"  "1948"  "1574"  "7286"  "288"   "13729" "7664"  "2438" 
## BPV "10111" "1737"  "866"   "1372"  "4917"  "2485"  "15804" "5518"  "1004" 
## BRE "887"   "8244"  "9025"  "8774"  "7576"  "9105"  "7236"  "6662"  "8546" 
## BRM "7284"  "4735"  "4784"  "4985"  "1494"  "6224"  "13600" "1235"  "4175" 
## CAM "8793"  "17049" "17953" "17466" "15103" "16579" "2976"  "14322" "17784"
## CES "673"   "8316"  "9112"  "8847"  "7788"  "9134"  "7056"  "6876"  "8646" 
## CHA "8483"  "16729" "17638" "17157" "15013" "16324" "2660"  "14196" "17455"
## CHI "10579" "8615"  "8125"  "8564"  "3431"  "9845"  "14562" "3824"  "7736" 
## COL "8074"  "6345"  "6199"  "6498"  "861"   "7798"  "13808" "682"   "5641" 
## CUA "9982"  "3387"  "2808"  "3261"  "2964"  "4527"  "16379" "3735"  "2471" 
## DAL "9525"  "6797"  "6418"  "6814"  "1510"  "8125"  "14910" "2106"  "5959" 
## DEN "9510"  "6849"  "6473"  "6868"  "1538"  "8179"  "14865" "2110"  "6013" 
## DES "859"   "8264"  "9046"  "8794"  "7604"  "9120"  "7208"  "6689"  "8568" 
## FAY "8146"  "5704"  "5551"  "5851"  "337"   "7152"  "14141" "631"   "4994" 
## FIR "7351"  "5903"  "5891"  "6127"  "1151"  "7387"  "13334" "198"   "5296" 
## FLO "8299"  "575"   "1480"  "1032"  "5915"  "1466"  "13501" "6134"  "1465" 
## FRS "1192"  "7924"  "8695"  "8453"  "7274"  "8817"  "7577"  "6370"  "8209" 
## GEL "6556"  "15159" "16051" "15671" "13369" "15340" "2050"  "12464" "15677"
## GES "1770"  "10492" "11332" "11022" "9740"  "11112" "4856"  "8782"  "10902"
## GRA "534"   "8976"  "9833"  "9505"  "8983"  "9572"  "5966"  "8076"  "9433" 
## GRV "10734" "2549"  "2296"  "2148"  "7619"  "1323"  "14438" "8148"  "2906" 
## HAI "8631"  "17291" "18180" "17800" "14185" "17261" "3487"  "13454" "17754"
## HAL "8696"  "6165"  "5914"  "6258"  "599"   "7573"  "14465" "1169"  "5394" 
## HAN "8199"  "16850" "17739" "17360" "14044" "16887" "3134"  "13267" "17329"
## HAR "7650"  "6047"  "5978"  "6241"  "935"   "7521"  "13551" "175"   "5397" 
## HAT "9141"  "6119"  "5791"  "6166"  "816"   "7482"  "14907" "1597"  "5305" 
## HOC "8995"  "17247" "18146" "17654" "15164" "16725" "3174"  "14408" "17992"
## HOU "9678"  "6503"  "6094"  "6501"  "1428"  "7808"  "15221" "2168"  "5651" 
## HUN "8217"  "16940" "17691" "17472" "13220" "17430" "3913"  "12500" "17135"
## INJ "10253" "16891" "17433" "16995" "17014" "15712" "3876"  "16295" "17802"
## INW "11624" "17768" "17827" "17601" "16709" "16317" "5347"  "16361" "18433"
## IPO "9686"  "1078"  "909"   "646"   "6288"  "854"   "14516" "6730"  "1457" 
## JAF "6860"  "14335" "15226" "14735" "14922" "13924" "454"   "13956" "15144"
## KAC "8168"  "16431" "17344" "16872" "14865" "16099" "2377"  "14020" "17140"
## KAG "9281"  "17322" "17527" "17700" "12190" "18629" "5773"  "11670" "16923"
## KAN "9266"  "16731" "16800" "17038" "11429" "18184" "6410"  "10944" "16216"
## KAT "6077"  "14643" "15539" "15154" "13179" "14831" "1828"  "12249" "15180"
## KLP "9135"  "16671" "17469" "16966" "16068" "15862" "2869"  "15246" "17555"
## KUN "6606"  "13536" "14407" "13909" "14963" "13047" "957"   "14002" "14380"
## LAM "7866"  "16327" "17243" "16807" "14389" "16192" "2419"  "13546" "16946"
## LIN "7643"  "5847"  "5784"  "6044"  "859"   "7322"  "13621" "111"   "5201" 
## LOS "10601" "8666"  "8173"  "8613"  "3481"  "9893"  "14541" "3869"  "7785" 
## MAC "8640"  "5823"  "5581"  "5920"  "279"   "7234"  "14561" "1098"  "5056" 
## MAN "7786"  "5939"  "5848"  "6121"  "765"   "7407"  "13717" "241"   "5273" 
## MAT "8981"  "16635" "17460" "16954" "15905" "15890" "2751"  "15075" "17502"
## MAU "9376"  "1388"  "871"   "1260"  "4517"  "2564"  "15247" "5011"  "467"  
## MED "9982"  "3387"  "2808"  "3261"  "2964"  "4527"  "16379" "3735"  "2471" 
## MON "9649"  "6569"  "6166"  "6571"  "1443"  "7879"  "15157" "2154"  "5720" 
## NEB "7152"  "5911"  "5934"  "6153"  "1341"  "7399"  "13152" "396"   "5332" 
## NEG "9319"  "4358"  "3974"  "4363"  "1596"  "5675"  "15670" "2418"  "3508" 
## NEH "7312"  "5961"  "5954"  "6187"  "1204"  "7446"  "13277" "240"   "5358" 
## NEO "9300"  "6089"  "5735"  "6120"  "928"   "7434"  "15073" "1753"  "5261" 
## NEW "7435"  "5961"  "5932"  "6176"  "1091"  "7442"  "13388" "123"   "5341" 
## NOV "9089"  "1536"  "1956"  "1497"  "7130"  "394"   "13396" "7434"  "2337" 
## NUE "9993"  "6580"  "6124"  "6548"  "1709"  "7844"  "15478" "2480"  "5709" 
## OKI "9496"  "17850" "18138" "18272" "12808" "18929" "5450"  "12286" "17527"
## PAL "8830"  "5102"  "4824"  "5175"  "642"   "6491"  "15001" "1532"  "4311" 
## PEO "8448"  "6806"  "6597"  "6926"  "1214"  "8236"  "13920" "1250"  "6063" 
## PLM "8984"  "341"   "852"   "355"   "5867"  "1176"  "14143" "6211"  "1077" 
## POR "10653" "2158"  "1758"  "1694"  "7030"  "1263"  "14849" "7579"  "2380" 
## QNC "8988"  "17462" "18379" "17916" "14784" "17099" "3424"  "14058" "18099"
## RAR "1798"  "10532" "11360" "11064" "9609"  "11195" "4954"  "8647"  "10915"
## REC "7801"  "1474"  "2340"  "1853"  "6626"  "1641"  "12645" "6733"  "2374" 
## RUS "9090"  "6671"  "6358"  "6729"  "1192"  "8045"  "14578" "1680"  "5866" 
## SAI "8521"  "2427"  "2308"  "2564"  "3171"  "3861"  "14831" "3557"  "1717" 
## SER "442"   "8914"  "9718"  "9445"  "8257"  "9694"  "6492"  "7326"  "9256" 
## SMI "9457"  "6647"  "6276"  "6669"  "1364"  "7981"  "14932" "1997"  "5813" 
## SOC "1678"  "10406" "11242" "10937" "9633"  "11042" "4965"  "8675"  "10808"
## SON "8747"  "16593" "17454" "16951" "15622" "15957" "2600"  "14786" "17424"
## SPB "2263"  "6662"  "7432"  "7190"  "6524"  "7593"  "8652"  "5703"  "6952" 
## SPR "8946"  "6808"  "6520"  "6881"  "1265"  "8197"  "14371" "1621"  "6018" 
## SPS "2226"  "6580"  "7377"  "7111"  "6758"  "7446"  "8546"  "5959"  "6921" 
## SSK "8488"  "16914" "17831" "17373" "14716" "16629" "2894"  "13925" "17566"
## STL "8643"  "6694"  "6454"  "6795"  "1101"  "8109"  "14155" "1322"  "5931" 
## SUF "11039" "18379" "18653" "18351" "16011" "17036" "4970"  "15562" "19215"
## SUU "11286" "18695" "18773" "18575" "15767" "17281" "5307"  "15387" "19394"
## TAI "9192"  "17885" "18524" "18408" "13477" "18359" "4631"  "12873" "17912"
## TIK "1733"  "10464" "11285" "10996" "9483"  "11150" "5075"  "8521"  "10833"
## TRA "9789"  "6736"  "6315"  "6727"  "1639"  "8031"  "15192" "2324"  "5880" 
## TUC "8577"  "422"   "988"   "802"   "5187"  "1912"  "14149" "5486"  "705"  
## TUH "1828"  "10518" "11369" "11047" "9904"  "11096" "4718"  "8948"  "10953"
## UTS "9458"  "16667" "16659" "16933" "11273" "18149" "6696"  "10822" "16093"
## WAV "7883"  "5770"  "5664"  "5943"  "608"   "7234"  "13872" "360"   "5092" 
## YUN "7598"  "16267" "17146" "16785" "13697" "16431" "2806"  "12873" "16728"
##     BPA     BPV     BRE     BRM     CAM     CES     CHA     CHI     COL    
## ALD "0.16"  "0.17"  "0.17"  "0.15"  "0.15"  "0.20"  "0.17"  "0.13"  "0.12" 
## ARA "0.06"  "0.06"  "0.19"  "0.16"  "0.10"  "0.23"  "0.11"  "0.14"  "0.15" 
## BAF "0.06"  "0.05"  "0.20"  "0.17"  "0.09"  "0.24"  "0.10"  "0.14"  "0.15" 
## BCO "0.08"  "0.07"  "0.20"  "0.17"  "0.11"  "0.23"  "0.11"  "0.15"  "0.15" 
## BEA "0.08"  "0.12"  "0.09"  "0.08"  "0.11"  "0.13"  "0.12"  "0.09"  "0.05" 
## BEH "0.10"  "0.06"  "0.24"  "0.20"  "0.09"  "0.27"  "0.10"  "0.16"  "0.19" 
## BEN "0.13"  "0.12"  "0.24"  "0.19"  "0.02"  "0.27"  "0.02"  "0.13"  "0.19" 
## BER "0.12"  "0.15"  "0.08"  "0.09"  "0.15"  "0.12"  "0.17"  "0.12"  "0.05" 
## BIT "0.08"  "0.05"  "0.20"  "0.17"  "0.11"  "0.23"  "0.12"  "0.15"  "0.15" 
## BPA NA      "0.08"  "0.16"  "0.14"  "0.12"  "0.20"  "0.13"  "0.13"  "0.12" 
## BPV "2671"  NA      "0.20"  "0.17"  "0.10"  "0.23"  "0.11"  "0.14"  "0.15" 
## BRE "9306"  "9497"  NA      "0.14"  "0.21"  "0.15"  "0.22"  "0.18"  "0.09" 
## BRM "6507"  "4567"  "6421"  NA      "0.16"  "0.18"  "0.17"  "0.14"  "0.10" 
## CAM "16464" "18772" "9467"  "15034" NA      "0.24"  "0.00"  "0.09"  "0.16" 
## CES "9328"  "9607"  "223"   "6618"  "9337"  NA      "0.25"  "0.21"  "0.13" 
## CHA "16226" "18445" "9167"  "14847" "329"   "9034"  NA      "0.11"  "0.17" 
## CHI "10059" "7400"  "9747"  "4878"  "13253" "9968"  "13354" NA      "0.12" 
## COL "8068"  "5763"  "7188"  "1834"  "14245" "7407"  "14161" "3142"  NA     
## CUA "4743"  "2111"  "9194"  "3109"  "18055" "9361"  "17923" "5318"  "3825" 
## DAL "8365"  "5789"  "8643"  "2994"  "14589" "8863"  "14600" "1942"  "1478" 
## DEN "8419"  "5845"  "8628"  "3017"  "14531" "8848"  "14542" "1905"  "1474" 
## DES "9321"  "9521"  "28"    "6449"  "9442"  "198"   "9142"  "9771"  "7215" 
## FAY "7422"  "5126"  "7261"  "1343"  "14830" "7473"  "14727" "3535"  "648"  
## FIR "7668"  "5580"  "6465"  "1186"  "14215" "6679"  "14080" "3985"  "849"  
## FLO "1745"  "2312"  "7859"  "4925"  "16477" "7916"  "16159" "9049"  "6620" 
## FRS "9025"  "9156"  "349"   "6094"  "9814"  "525"   "9515"  "9544"  "6912" 
## GEL "15372" "16666" "7198"  "12949" "2278"  "7076"  "1997"  "12636" "12565"
## GES "11262" "11879" "2444"  "8747"  "7041"  "2300"  "6736"  "11183" "9204" 
## GRA "9726"  "10425" "1417"  "7784"  "8445"  "1200"  "8128"  "11100" "8606" 
## GRV "1126"  "2718"  "10425" "7080"  "16815" "10450" "16661" "9987"  "8452" 
## HAI "17209" "18675" "9208"  "14274" "987"   "9108"  "1087"  "12279" "13324"
## HAL "7831"  "5395"  "7810"  "2032"  "14741" "8027"  "14686" "2852"  "662"  
## HAN "16860" "18279" "8788"  "14018" "1059"  "8684"  "1019"  "12368" "13186"
## HAR "7798"  "5614"  "6764"  "1399"  "14265" "6980"  "14150" "3655"  "514"  
## HAT "7730"  "5214"  "8254"  "2310"  "15028" "8470"  "14996" "2674"  "1117" 
## HOC "16598" "18976" "9662"  "15158" "209"   "9535"  "537"   "13196" "14304"
## HOU "8043"  "5449"  "8792"  "2919"  "14950" "9011"  "14963" "2159"  "1605" 
## HUN "17481" "17885" "8698"  "13357" "1929"  "8625"  "1956"  "11415" "12359"
## INJ "15489" "18084" "11029" "17006" "1989"  "10867" "2160"  "14514" "16154"
## INW "16043" "17941" "12344" "17464" "2908"  "12202" "3190"  "13562" "15958"
## IPO "1051"  "1631"  "9249"  "5618"  "17396" "9307"  "17123" "9008"  "7092" 
## JAF "13856" "16072" "7684"  "14054" "2732"  "7502"  "2433"  "14828" "14239"
## KAC "16019" "18135" "8859"  "14617" "647"   "8723"  "320"   "13394" "14022"
## KAG "18861" "17087" "9560"  "12747" "3446"  "9542"  "3595"  "9821"  "11359"
## KAN "18468" "16306" "9439"  "12054" "4223"  "9449"  "4357"  "9036"  "10606"
## KAT "14871" "16175" "6744"  "12645" "2724"  "6614"  "2424"  "12750" "12404"
## KLP "15701" "18325" "9885"  "15842" "1003"  "9731"  "1055"  "14196" "15216"
## KUN "12972" "15266" "7469"  "13889" "3572"  "7271"  "3288"  "15479" "14388"
## LAM "16147" "17949" "8524"  "14162" "951"   "8398"  "692"   "13006" "13547"
## LIN "7599"  "5430"  "6757"  "1208"  "14424" "6971"  "14301" "3789"  "655"  
## LOS "10106" "7447"  "9772"  "4927"  "13211" "9993"  "13314" "51"    "3188" 
## MAC "7494"  "5078"  "7754"  "1766"  "15012" "7968"  "14941" "3152"  "796"  
## MAN "7682"  "5468"  "6899"  "1348"  "14430" "7115"  "14318" "3620"  "493"  
## MAT "15739" "18324" "9725"  "15665" "875"   "9573"  "893"   "14109" "15054"
## MAU "2806"  "758"   "8747"  "3972"  "18130" "8861"  "17803" "7306"  "5332" 
## MED "4743"  "2111"  "9194"  "3109"  "18055" "9361"  "17923" "5318"  "3825" 
## MON "8114"  "5524"  "8764"  "2937"  "14871" "8983"  "14884" "2105"  "1575" 
## NEB "7681"  "5654"  "6266"  "1176"  "14098" "6481"  "13954" "4146"  "1026" 
## NEG "5918"  "3398"  "8469"  "2070"  "16698" "8661"  "16600" "4271"  "2455" 
## NEH "7727"  "5646"  "6425"  "1238"  "14147" "6640"  "14012" "3982"  "855"  
## NEO "7678"  "5137"  "8413"  "2412"  "15142" "8628"  "15120" "2642"  "1285" 
## NEW "7722"  "5605"  "6548"  "1258"  "14216" "6763"  "14087" "3881"  "746"  
## NOV "522"   "2769"  "8785"  "6245"  "16234" "8806"  "15969" "10062" "7881" 
## NUE "8070"  "5444"  "9107"  "3191"  "15035" "9326"  "15070" "2039"  "1919" 
## OKI "19052" "17697" "9849"  "13354" "2929"  "9812"  "3110"  "10379" "11976"
## PAL "6747"  "4312"  "7954"  "1601"  "15744" "8159"  "15655" "3685"  "1500" 
## PEO "8500"  "6100"  "7569"  "2399"  "14032" "7790"  "13983" "2581"  "574"  
## PLM "1457"  "1717"  "8520"  "5050"  "17112" "8585"  "16802" "8813"  "6631" 
## POR "1189"  "2122"  "10269" "6535"  "17376" "10314" "17198" "9403"  "7868" 
## QNC "16990" "19097" "9614"  "14868" "528"   "9499"  "782"   "12760" "13923"
## RAR "11353" "11884" "2410"  "8646"  "7058"  "2281"  "6757"  "10990" "9056" 
## REC "1844"  "3195"  "7466"  "5501"  "15615" "7493"  "15304" "9864"  "7273" 
## RUS "8295"  "5781"  "8207"  "2634"  "14487" "8427"  "14470" "2247"  "1041" 
## SAI "4132"  "2119"  "7787"  "2488"  "17189" "7934"  "16918" "6287"  "3937" 
## SER "9880"  "10219" "752"   "7145"  "8727"  "612"   "8425"  "10229" "7823" 
## SMI "8222"  "5655"  "8573"  "2852"  "14698" "8792"  "14702" "2097"  "1390" 
## SOC "11196" "11784" "2339"  "8637"  "7142"  "2197"  "6837"  "11099" "9100" 
## SON "15824" "18319" "9478"  "15375" "679"   "9330"  "625"   "13929" "14774"
## SPB "7811"  "7907"  "1595"  "5198"  "11036" "1702"  "10731" "9234"  "6318" 
## SPR "8451"  "5959"  "8066"  "2652"  "14285" "8287"  "14263" "2231"  "953"  
## SPS "7655"  "7892"  "1670"  "5400"  "11019" "1737"  "10707" "9533"  "6585" 
## SSK "16547" "18569" "9138"  "14633" "402"   "9016"  "368"   "12986" "13859"
## STL "8369"  "5933"  "7761"  "2401"  "14234" "7982"  "14192" "2508"  "646"  
## SUF "16777" "18775" "11705" "16622" "2245"  "11580" "2557"  "13080" "15213"
## SUU "17011" "18644" "11923" "16502" "2506"  "11808" "2827"  "12750" "14995"
## TAI "18355" "18388" "9647"  "13859" "2061"  "9582"  "2237"  "11218" "12628"
## TIK "11313" "11798" "2313"  "8526"  "7154"  "2191"  "6855"  "10871" "8929" 
## TRA "8263"  "5657"  "8905"  "3133"  "14779" "9125"  "14805" "1918"  "1724" 
## TUC "2196"  "1669"  "8043"  "4313"  "17104" "8127"  "16776" "8251"  "5928" 
## TUH "11239" "11937" "2549"  "8890"  "6966"  "2393"  "6657"  "11376" "9378" 
## UTS "18437" "16116" "9599"  "11954" "4470"  "9617"  "4613"  "8800"  "10460"
## WAV "7507"  "5276"  "6997"  "1243"  "14624" "7211"  "14510" "3665"  "602"  
## YUN "16440" "17684" "8189"  "13548" "1487"  "8083"  "1323"  "12329" "12850"
##     CUA     DAL     DEN     DES     FAY     FIR     FLO     FRS     GEL    
## ALD "0.17"  "0.12"  "0.11"  "0.15"  "0.11"  "0.16"  "0.18"  "0.09"  "0.17" 
## ARA "0.12"  "0.13"  "0.14"  "0.18"  "0.12"  "0.17"  "0.02"  "0.14"  "0.11" 
## BAF "0.13"  "0.13"  "0.14"  "0.19"  "0.12"  "0.18"  "0.01"  "0.14"  "0.10" 
## BCO "0.13"  "0.14"  "0.14"  "0.19"  "0.13"  "0.18"  "0.03"  "0.15"  "0.12" 
## BEA "0.11"  "0.03"  "0.03"  "0.08"  "0.02"  "0.07"  "0.12"  "0.06"  "0.11" 
## BEH "0.13"  "0.17"  "0.18"  "0.22"  "0.16"  "0.22"  "0.06"  "0.17"  "0.11" 
## BEN "0.13"  "0.17"  "0.18"  "0.23"  "0.17"  "0.22"  "0.12"  "0.16"  "0.02" 
## BER "0.15"  "0.04"  "0.04"  "0.08"  "0.04"  "0.07"  "0.16"  "0.07"  "0.16" 
## BIT "0.13"  "0.13"  "0.14"  "0.19"  "0.13"  "0.18"  "0.06"  "0.14"  "0.12" 
## BPA "0.14"  "0.10"  "0.10"  "0.15"  "0.09"  "0.14"  "0.07"  "0.12"  "0.12" 
## BPV "0.13"  "0.14"  "0.14"  "0.19"  "0.13"  "0.18"  "0.06"  "0.14"  "0.11" 
## BRE "0.20"  "0.09"  "0.08"  "0.09"  "0.08"  "0.13"  "0.21"  "0.12"  "0.23" 
## BRM "0.16"  "0.09"  "0.09"  "0.12"  "0.08"  "0.12"  "0.18"  "0.11"  "0.18" 
## CAM "0.10"  "0.14"  "0.15"  "0.20"  "0.13"  "0.18"  "0.11"  "0.13"  "-0.00"
## CES "0.24"  "0.13"  "0.12"  "0.13"  "0.13"  "0.16"  "0.24"  "0.15"  "0.28" 
## CHA "0.11"  "0.16"  "0.16"  "0.21"  "0.15"  "0.20"  "0.11"  "0.14"  "0.00" 
## CHI "0.13"  "0.11"  "0.12"  "0.16"  "0.10"  "0.16"  "0.15"  "0.10"  "0.11" 
## COL "0.15"  "0.04"  "0.04"  "0.08"  "0.04"  "0.08"  "0.16"  "0.06"  "0.17" 
## CUA NA      "0.13"  "0.14"  "0.19"  "0.13"  "0.18"  "0.13"  "0.14"  "0.12" 
## DAL "3682"  NA      "0.02"  "0.07"  "0.02"  "0.07"  "0.14"  "0.07"  "0.15" 
## DEN "3738"  "58"    NA      "0.07"  "0.02"  "0.07"  "0.15"  "0.06"  "0.15" 
## DES "9221"  "8670"  "8654"  NA      "0.07"  "0.11"  "0.19"  "0.11"  "0.22" 
## FAY "3228"  "1670"  "1689"  "7288"  NA      "0.07"  "0.13"  "0.06"  "0.14" 
## FIR "3845"  "2296"  "2298"  "6492"  "816"   NA      "0.19"  "0.10"  "0.21" 
## FLO "3896"  "7189"  "7238"  "7877"  "5991"  "6109"  NA      "0.15"  "0.12" 
## FRS "8847"  "8376"  "8363"  "375"   "6962"  "6173"  "7550"  NA      "0.13" 
## GEL "16057" "13353" "13301" "7174"  "13051" "12322" "14644" "7544"  NA     
## GES "11625" "10558" "10530" "2418"  "9408"  "8592"  "10042" "2794"  "4791" 
## GRA "10419" "10059" "10043" "1390"  "8671"  "7878"  "8513"  "1709"  "6244" 
## GRV "4808"  "8488"  "8545"  "10441" "7808"  "8184"  "2709"  "10140" "16268"
## HAI "17148" "13605" "13548" "9186"  "13928" "13363" "16769" "9546"  "2132" 
## HAL "3368"  "976"   "993"   "7837"  "696"   "1365"  "6499"  "7525"  "13170"
## HAN "16997" "13581" "13524" "8765"  "13771" "13164" "16330" "9128"  "1691" 
## HAR "3790"  "1962"  "1964"  "6791"  "604"   "337"   "6283"  "6477"  "12450"
## HAT "3136"  "732"   "771"   "8281"  "1047"  "1794"  "6491"  "7965"  "13557"
## HOC "18128" "14588" "14530" "9637"  "14900" "14307" "16674" "10009" "2468" 
## HOU "3339"  "364"   "421"   "8819"  "1652"  "2364"  "6911"  "8513"  "13704"
## HUN "16183" "12662" "12604" "8678"  "12965" "12414" "16515" "9021"  "2114" 
## INJ "19770" "16198" "16142" "11002" "16769" "16197" "16391" "11377" "4082" 
## INW "18251" "15449" "15400" "12319" "16602" "16344" "17432" "12693" "5184" 
## IPO "3692"  "7323"  "7379"  "9268"  "6444"  "6750"  "1391"  "8938"  "15918"
## JAF "16803" "15302" "15255" "7656"  "14585" "13780" "13760" "8024"  "2209" 
## KAC "17717" "14546" "14489" "8833"  "14568" "13895" "15864" "9207"  "1711" 
## KAG "14996" "11316" "11259" "9545"  "12004" "11637" "17199" "9838"  "3906" 
## KAN "14208" "10527" "10470" "9427"  "11253" "10920" "16722" "9689"  "4459" 
## KAT "15734" "13300" "13250" "6719"  "12852" "12096" "14125" "7091"  "520"  
## KLP "18949" "15590" "15532" "9858"  "15782" "15126" "16103" "10234" "2898" 
## KUN "16302" "15624" "15585" "7441"  "14630" "13814" "12961" "7795"  "3002" 
## LAM "17252" "14094" "14037" "8499"  "14093" "13423" "15778" "8870"  "1328" 
## LIN "3631"  "2040"  "2047"  "6784"  "523"   "295"   "6083"  "6463"  "12574"
## LOS "5366"  "1993"  "1956"  "9795"  "3584"  "4030"  "9099"  "9570"  "12609"
## MAC "3076"  "1232"  "1260"  "7781"  "502"   "1293"  "6156"  "7458"  "13361"
## MAN "3625"  "1872"  "1878"  "6927"  "432"   "438"   "6189"  "6609"  "12622"
## MAT "18772" "15461" "15403" "9699"  "15615" "14953" "16063" "10074" "2722" 
## MAU "2016"  "5562"  "5616"  "8771"  "4685"  "5050"  "1931"  "8405"  "15931"
## MED "0"     "3682"  "3738"  "9221"  "3228"  "3845"  "3896"  "8847"  "16057"
## MON "3414"  "285"   "342"   "8791"  "1654"  "2350"  "6974"  "8487"  "13629"
## NEB "3969"  "2486"  "2487"  "6293"  "1009"  "199"   "6095"  "5974"  "12173"
## NEG "1372"  "2451"  "2505"  "8497"  "1873"  "2551"  "4778"  "8136"  "14870"
## NEH "3913"  "2315"  "2316"  "6453"  "869"   "68"    "6162"  "6135"  "12257"
## NEO "3046"  "715"   "764"   "8440"  "1185"  "1950"  "6475"  "8123"  "13707"
## NEW "3842"  "2196"  "2198"  "6575"  "754"   "104"   "6175"  "6258"  "12347"
## NOV "4756"  "8295"  "8348"  "8800"  "7240"  "7422"  "1358"  "8506"  "14947"
## NUE "3336"  "567"   "613"   "9134"  "1951"  "2677"  "7010"  "8828"  "13899"
## OKI "15596" "11914" "11857" "9832"  "12621" "12250" "17643" "10143" "3712" 
## PAL "2326"  "1753"  "1798"  "7982"  "939"   "1690"  "5462"  "7638"  "13988"
## PEO "4081"  "1089"  "1067"  "7595"  "1127"  "1405"  "7110"  "7309"  "12517"
## PLM "3535"  "7026"  "7079"  "8539"  "5986"  "6212"  "701"   "8204"  "15343"
## POR "4210"  "7890"  "7947"  "10286" "7225"  "7622"  "2428"  "9967"  "16558"
## QNC "17746" "14162" "14104" "9590"  "14530" "13967" "16901" "9957"  "2432" 
## RAR "11559" "10397" "10368" "2385"  "9275"  "8459"  "10094" "2758"  "4795" 
## REC "4790"  "7969"  "8016"  "7481"  "6665"  "6683"  "909"   "7182"  "13923"
## RUS "3698"  "439"   "433"   "8234"  "1293"  "1867"  "7030"  "7938"  "13109"
## SAI "1614"  "4408"  "4457"  "7813"  "3291"  "3583"  "2782"  "7438"  "14921"
## SER "9945"  "9256"  "9237"  "725"   "7935"  "7130"  "8504"  "1100"  "6465" 
## SMI "3551"  "156"   "202"   "8600"  "1538"  "2190"  "7036"  "8300"  "13416"
## SOC "11517" "10457" "10430" "2313"  "9301"  "8485"  "9960"  "2689"  "4888" 
## SON "18481" "15221" "15164" "9452"  "15330" "14663" "16018" "9827"  "2436" 
## SPB "7733"  "7795"  "7793"  "1617"  "6241"  "5509"  "6296"  "1264"  "8778" 
## SPR "3886"  "589"   "565"   "8092"  "1312"  "1799"  "7154"  "7803"  "12894"
## SPS "7833"  "8059"  "8058"  "1689"  "6483"  "5766"  "6189"  "1371"  "8779" 
## SSK "17659" "14251" "14194" "9114"  "14438" "13816" "16354" "9484"  "1940" 
## STL "3894"  "883"   "867"   "7788"  "1079"  "1495"  "7017"  "7496"  "12747"
## SUF "18244" "14886" "14834" "11681" "15861" "15530" "17915" "12051" "4507" 
## SUU "17866" "14583" "14531" "11900" "15641" "15371" "18269" "12266" "4733" 
## TAI "16356" "12689" "12631" "9628"  "13264" "12816" "17491" "9964"  "3024" 
## TIK "11447" "10270" "10241" "2288"  "9150"  "8333"  "10033" "2660"  "4886" 
## TRA "3546"  "288"   "328"   "8932"  "1841"  "2518"  "7149"  "8633"  "13610"
## TUC "3084"  "6411"  "6462"  "8064"  "5288"  "5481"  "814"   "7714"  "15078"
## TUH "11741" "10739" "10712" "2522"  "9573"  "8757"  "10058" "2897"  "4730" 
## UTS "14009" "10328" "10272" "9588"  "11108" "10807" "16710" "9839"  "4747" 
## WAV "3431"  "1857"  "1869"  "7025"  "272"   "545"   "6030"  "6701"  "12806"
## YUN "16601" "13380" "13323" "8166"  "13407" "12757" "15760" "8529"  "1120" 
##     GES     GRA     GRV     HAI     HAL     HAN     HAR     HAT     HOC    
## ALD "0.14"  "0.08"  "0.18"  "0.11"  "0.11"  "0.13"  "0.12"  "0.12"  "0.12" 
## ARA "0.18"  "0.11"  "0.06"  "0.10"  "0.13"  "0.11"  "0.15"  "0.13"  "0.11" 
## BAF "0.18"  "0.11"  "0.06"  "0.10"  "0.14"  "0.11"  "0.15"  "0.14"  "0.11" 
## BCO "0.19"  "0.12"  "0.08"  "0.11"  "0.14"  "0.13"  "0.16"  "0.14"  "0.13" 
## BEA "0.11"  "0.06"  "0.12"  "0.07"  "0.04"  "0.08"  "0.04"  "0.03"  "0.08" 
## BEH "0.20"  "0.13"  "0.08"  "0.11"  "0.17"  "0.13"  "0.19"  "0.18"  "0.13" 
## BEN "0.19"  "0.10"  "0.13"  "0.08"  "0.18"  "0.08"  "0.19"  "0.17"  "0.10" 
## BER "0.13"  "0.10"  "0.15"  "0.11"  "0.03"  "0.13"  "0.05"  "0.05"  "0.12" 
## BIT "0.18"  "0.12"  "0.07"  "0.11"  "0.14"  "0.12"  "0.15"  "0.14"  "0.12" 
## BPA "0.17"  "0.11"  "0.10"  "0.11"  "0.11"  "0.12"  "0.12"  "0.11"  "0.12" 
## BPV "0.18"  "0.11"  "0.08"  "0.11"  "0.14"  "0.12"  "0.16"  "0.14"  "0.12" 
## BRE "0.18"  "0.15"  "0.20"  "0.16"  "0.09"  "0.19"  "0.10"  "0.10"  "0.17" 
## BRM "0.15"  "0.11"  "0.17"  "0.12"  "0.08"  "0.14"  "0.10"  "0.10"  "0.13" 
## CAM "0.16"  "0.07"  "0.11"  "0.05"  "0.14"  "0.05"  "0.16"  "0.14"  "0.06" 
## CES "0.21"  "0.18"  "0.23"  "0.19"  "0.12"  "0.22"  "0.13"  "0.14"  "0.20" 
## CHA "0.17"  "0.08"  "0.12"  "0.06"  "0.16"  "0.06"  "0.17"  "0.16"  "0.08" 
## CHI "0.14"  "0.07"  "0.14"  "0.06"  "0.11"  "0.08"  "0.13"  "0.12"  "0.07" 
## COL "0.12"  "0.10"  "0.15"  "0.11"  "0.02"  "0.13"  "0.05"  "0.05"  "0.12" 
## CUA "0.17"  "0.10"  "0.13"  "0.10"  "0.14"  "0.11"  "0.15"  "0.14"  "0.11" 
## DAL "0.13"  "0.09"  "0.14"  "0.10"  "0.03"  "0.11"  "0.05"  "0.03"  "0.11" 
## DEN "0.12"  "0.09"  "0.14"  "0.10"  "0.03"  "0.11"  "0.04"  "0.03"  "0.11" 
## DES "0.17"  "0.14"  "0.18"  "0.15"  "0.07"  "0.17"  "0.08"  "0.08"  "0.16" 
## FAY "0.12"  "0.08"  "0.13"  "0.09"  "0.03"  "0.10"  "0.04"  "0.03"  "0.09" 
## FIR "0.16"  "0.13"  "0.18"  "0.14"  "0.07"  "0.17"  "0.08"  "0.08"  "0.15" 
## FLO "0.19"  "0.12"  "0.07"  "0.11"  "0.14"  "0.13"  "0.16"  "0.15"  "0.13" 
## FRS "0.11"  "0.07"  "0.14"  "0.08"  "0.05"  "0.10"  "0.07"  "0.07"  "0.09" 
## GEL "0.18"  "0.07"  "0.12"  "0.05"  "0.15"  "0.06"  "0.17"  "0.15"  "0.06" 
## GES NA      "0.11"  "0.18"  "0.12"  "0.11"  "0.14"  "0.13"  "0.12"  "0.13" 
## GRA "1540"  NA      "0.12"  "0.05"  "0.08"  "0.06"  "0.10"  "0.09"  "0.05" 
## GRV "12379" "10845" NA      "0.11"  "0.14"  "0.12"  "0.15"  "0.14"  "0.12" 
## HAI "6861"  "8350"  "17743" NA      "0.10"  "0.04"  "0.11"  "0.10"  "0.04" 
## HAL "9860"  "9226"  "8110"  "13793" NA      "0.11"  "0.04"  "0.04"  "0.10" 
## HAN "6429"  "7913"  "17515" "441"   "13690" NA      "0.13"  "0.12"  "0.05" 
## HAR "8852"  "8180"  "8260"  "13383" "1049"  "13208" NA      "0.04"  "0.12" 
## HAT "10316" "9670"  "7930"  "14062" "456"   "13989" "1491"  NA      "0.11" 
## HOC "7240"  "8649"  "16888" "991"   "14782" "1146"  "14345" "15052" NA     
## HOU "10780" "10210" "8141"  "13966" "1000"  "13945" "2044"  "613"   "14947"
## HUN "6473"  "8005"  "18346" "966"   "12828" "954"   "12426" "13102" "1954" 
## INJ "8591"  "9825"  "15369" "2845"  "16575" "3034"  "16228" "16776" "1889" 
## INW "9903"  "11230" "15531" "3419"  "16135" "3762"  "16232" "16151" "2727" 
## IPO "11420" "9886"  "1502"  "17973" "6820"  "17560" "6854"  "6700"  "17554"
## JAF "5309"  "6403"  "14485" "3354"  "14893" "3041"  "13992" "15331" "2920" 
## KAC "6424"  "7810"  "16533" "1218"  "14569" "1028"  "13982" "14901" "856"  
## KAG "7663"  "9189"  "19804" "2510"  "11696" "2721"  "11556" "11874" "3377" 
## KAN "7753"  "9241"  "19013" "3270"  "10924" "3444"  "10823" "11093" "4161" 
## KAT "4319"  "5750"  "15795" "2648"  "13030" "2207"  "12248" "13437" "2921" 
## KLP "7441"  "8729"  "15892" "1984"  "15732" "2042"  "15203" "16028" "1011" 
## KUN "5223"  "6111"  "13592" "4241"  "15050" "3934"  "14067" "15505" "3749" 
## LAM "6106"  "7533"  "16783" "1070"  "14097" "733"   "13508" "14433" "1139" 
## LIN "8887"  "8170"  "8068"  "13552" "1089"  "13368" "200"   "1508"  "14508"
## LOS "11193" "11121" "10030" "12238" "2902"  "12330" "3700"  "2725"  "13154"
## MAC "9874"  "9166"  "7789"  "14076" "343"   "13956" "1023"  "554"   "15062"
## MAN "9007"  "8314"  "8125"  "13543" "928"   "13372" "173"   "1357"  "14507"
## MAT "7281"  "8580"  "15977" "1862"  "15578" "1894"  "15034" "15882" "912"  
## MAU "11141" "9703"  "3137"  "17919" "5043"  "17523" "5124"  "4925"  "18338"
## MED "11625" "10419" "4808"  "17148" "3368"  "16997" "3790"  "3136"  "18128"
## MON "10735" "10181" "8217"  "13887" "988"   "13866" "2026"  "627"   "14868"
## NEB "8399"  "7680"  "8229"  "13262" "1562"  "13051" "523"   "1993"  "14197"
## NEG "10816" "9807"  "6116"  "15777" "2007"  "15639" "2453"  "1822"  "16759"
## NEH "8542"  "7840"  "8247"  "13295" "1395"  "13096" "353"   "1830"  "14240"
## NEO "10483" "9828"  "7850"  "14171" "624"   "14109" "1651"  "168"   "15161"
## NEW "8661"  "7963"  "8222"  "13354" "1273"  "13162" "235"   "1707"  "14304"
## NOV "10749" "9211"  "1647"  "16882" "7695"  "16501" "7576"  "7632"  "16389"
## NUE "11085" "10525" "8110"  "14048" "1312"  "14052" "2358"  "905"   "15018"
## OKI "7817"  "9357"  "19641" "2047"  "12311" "2328"  "12173" "12483" "2839" 
## PAL "10200" "9341"  "7024"  "14822" "1090"  "14686" "1538"  "1033"  "15804"
## PEO "9472"  "8982"  "8812"  "13080" "713"   "12983" "1078"  "1040"  "14070"
## PLM "10733" "9207"  "2209"  "17464" "6427"  "17027" "6346"  "6361"  "17300"
## POR "12353" "10812" "599"   "18262" "7517"  "17976" "7688"  "7333"  "17462"
## QNC "7222"  "8672"  "17323" "604"   "14383" "882"   "13987" "14641" "436"  
## RAR "210"   "1629"  "12473" "6843"  "9714"  "6414"  "8714"  "10170" "7255" 
## REC "9495"  "7954"  "2959"  "16010" "7221"  "15582" "6892"  "7255"  "15806"
## RUS "10146" "9623"  "8497"  "13515" "605"   "13457" "1532"  "568"   "14505"
## SAI "10231" "8925"  "4603"  "16625" "3739"  "16298" "3681"  "3710"  "17362"
## SER "1695"  "884"   "11005" "8498"  "8458"  "8073"  "7419"  "8908"  "8924" 
## SMI "10522" "9990"  "8359"  "13717" "848"   "13684" "1860"  "577"   "14702"
## SOC "109"   "1470"  "12315" "6953"  "9756"  "6522"  "8747"  "10212" "7340" 
## SON "7034"  "8356"  "16136" "1653"  "15307" "1640"  "14747" "15621" "776"  
## SPB "3995"  "2687"  "8916"  "10803" "6871"  "10381" "5837"  "7282"  "11235"
## SPR "9970"  "9480"  "8677"  "13315" "666"   "13251" "1462"  "750"   "14306"
## SPS "3988"  "2601"  "8769"  "10836" "7124"  "10408" "6097"  "7527"  "11220"
## SSK "6727"  "8158"  "17022" "722"   "14367" "678"   "13870" "14666" "547"  
## STL "9692"  "9176"  "8650"  "13277" "541"   "13189" "1159"  "815"   "14267"
## SUF "9286"  "10683" "16361" "2614"  "15466" "2986"  "15444" "15550" "2046" 
## SUU "9524"  "10949" "16489" "2752"  "15206" "3151"  "15259" "15262" "2300" 
## TAI "7454"  "8988"  "18870" "1180"  "13014" "1497"  "12775" "13222" "1982" 
## TIK "315"   "1600"  "12435" "6922"  "9587"  "6496"  "8587"  "10044" "7349" 
## TRA "10841" "10322" "8340"  "13793" "1164"  "13786" "2189"  "824"   "14769"
## TUC "10345" "8849"  "2925"  "17199" "5759"  "16760" "5626"  "5724"  "17310"
## TUH "194"   "1544"  "12351" "6818"  "10033" "6383"  "9021"  "10489" "7167" 
## UTS "7974"  "9450"  "18787" "3527"  "10756" "3715"  "10696" "10909" "4399" 
## WAV "9137"  "8409"  "7937"  "13738" "887"   "13566" "360"   "1283"  "14702"
## YUN "5828"  "7315"  "17239" "1035"  "13391" "601"   "12828" "13722" "1627" 
##     HOU     HUN     INJ     INW     IPO     JAF     KAC     KAG     KAN    
## ALD "0.10"  "0.08"  "0.24"  "0.28"  "0.24"  "0.18"  "0.17"  "0.13"  "0.17" 
## ARA "0.11"  "0.11"  "0.19"  "0.22"  "0.07"  "0.10"  "0.11"  "0.16"  "0.21" 
## BAF "0.11"  "0.11"  "0.20"  "0.23"  "0.06"  "0.10"  "0.10"  "0.17"  "0.22" 
## BCO "0.12"  "0.12"  "0.20"  "0.23"  "0.09"  "0.12"  "0.11"  "0.17"  "0.22" 
## BEA "0.01"  "0.05"  "0.20"  "0.22"  "0.19"  "0.12"  "0.12"  "0.08"  "0.11" 
## BEH "0.14"  "0.13"  "0.19"  "0.23"  "0.06"  "0.11"  "0.10"  "0.20"  "0.25" 
## BEN "0.14"  "0.12"  "0.13"  "0.16"  "0.12"  "0.00"  "0.02"  "0.19"  "0.24" 
## BER "0.04"  "0.07"  "0.24"  "0.27"  "0.23"  "0.17"  "0.17"  "0.08"  "0.11" 
## BIT "0.11"  "0.12"  "0.21"  "0.24"  "0.09"  "0.12"  "0.12"  "0.17"  "0.21" 
## BPA "0.08"  "0.10"  "0.21"  "0.24"  "0.13"  "0.12"  "0.12"  "0.14"  "0.18" 
## BPV "0.11"  "0.12"  "0.20"  "0.23"  "0.09"  "0.11"  "0.11"  "0.17"  "0.22" 
## BRE "0.09"  "0.13"  "0.29"  "0.32"  "0.28"  "0.24"  "0.22"  "0.14"  "0.16" 
## BRM "0.08"  "0.10"  "0.24"  "0.27"  "0.24"  "0.18"  "0.17"  "0.13"  "0.16" 
## CAM "0.11"  "0.08"  "0.12"  "0.15"  "0.12"  "0.01"  "0.00"  "0.16"  "0.21" 
## CES "0.13"  "0.16"  "0.32"  "0.36"  "0.31"  "0.28"  "0.26"  "0.16"  "0.18" 
## CHA "0.12"  "0.10"  "0.12"  "0.15"  "0.12"  "0.01"  "0.00"  "0.17"  "0.23" 
## CHI "0.08"  "0.06"  "0.20"  "0.24"  "0.20"  "0.12"  "0.11"  "0.13"  "0.18" 
## COL "0.04"  "0.07"  "0.24"  "0.27"  "0.23"  "0.17"  "0.17"  "0.08"  "0.10" 
## CUA "0.11"  "0.10"  "0.19"  "0.23"  "0.17"  "0.12"  "0.12"  "0.16"  "0.21" 
## DAL "0.02"  "0.07"  "0.23"  "0.25"  "0.22"  "0.16"  "0.15"  "0.08"  "0.11" 
## DEN "0.03"  "0.07"  "0.23"  "0.26"  "0.22"  "0.16"  "0.16"  "0.08"  "0.11" 
## DES "0.08"  "0.12"  "0.28"  "0.31"  "0.27"  "0.22"  "0.21"  "0.12"  "0.14" 
## FAY "0.02"  "0.06"  "0.22"  "0.24"  "0.21"  "0.14"  "0.14"  "0.08"  "0.11" 
## FIR "0.07"  "0.11"  "0.27"  "0.30"  "0.26"  "0.22"  "0.20"  "0.12"  "0.15" 
## FLO "0.12"  "0.12"  "0.21"  "0.24"  "0.08"  "0.12"  "0.12"  "0.17"  "0.22" 
## FRS "0.05"  "0.05"  "0.22"  "0.24"  "0.21"  "0.14"  "0.14"  "0.08"  "0.11" 
## GEL "0.10"  "0.08"  "0.14"  "0.21"  "0.16"  "0.00"  "0.00"  "0.17"  "0.24" 
## GES "0.11"  "0.09"  "0.25"  "0.28"  "0.25"  "0.19"  "0.18"  "0.13"  "0.17" 
## GRA "0.06"  "0.04"  "0.16"  "0.19"  "0.16"  "0.08"  "0.08"  "0.10"  "0.15" 
## GRV "0.11"  "0.11"  "0.20"  "0.24"  "0.06"  "0.12"  "0.12"  "0.17"  "0.21" 
## HAI "0.07"  "0.04"  "0.15"  "0.18"  "0.15"  "0.06"  "0.06"  "0.12"  "0.17" 
## HAL "0.03"  "0.06"  "0.23"  "0.25"  "0.22"  "0.16"  "0.15"  "0.06"  "0.09" 
## HAN "0.08"  "0.05"  "0.17"  "0.21"  "0.17"  "0.06"  "0.06"  "0.13"  "0.19" 
## HAR "0.04"  "0.08"  "0.24"  "0.27"  "0.23"  "0.17"  "0.17"  "0.09"  "0.11" 
## HAT "0.03"  "0.07"  "0.23"  "0.26"  "0.22"  "0.15"  "0.15"  "0.08"  "0.12" 
## HOC "0.07"  "0.05"  "0.17"  "0.20"  "0.17"  "0.07"  "0.07"  "0.12"  "0.18" 
## HOU NA      "0.05"  "0.19"  "0.22"  "0.18"  "0.11"  "0.11"  "0.07"  "0.11" 
## HUN "13024" NA      "0.17"  "0.20"  "0.17"  "0.09"  "0.09"  "0.08"  "0.13" 
## INJ "16533" "3805"  NA      "0.06"  "0.22"  "0.14"  "0.13"  "0.25"  "0.30" 
## INW "15715" "4248"  "1528"  NA      "0.27"  "0.21"  "0.16"  "0.27"  "0.33" 
## IPO "6998"  "17889" "16538" "16981" NA      "0.15"  "0.13"  "0.24"  "0.30" 
## JAF "15622" "3888"  "3460"  "4950"  "14699" NA      "0.01"  "0.18"  "0.25" 
## KAC "14909" "1981"  "2393"  "3492"  "16877" "2179"  NA      "0.17"  "0.23" 
## KAG "11663" "1860"  "4901"  "4718"  "18346" "5739"  "3713"  NA      "0.13" 
## KAN "10874" "2532"  "5685"  "5424"  "17670" "6414"  "4456"  "791"   NA     
## KAT "13639" "2611"  "4429"  "5611"  "15399" "2085"  "2119"  "4367"  "4880" 
## KLP "15951" "2932"  "1188"  "2505"  "16714" "2502"  "1234"  "4376"  "5163" 
## KUN "15903" "4777"  "3986"  "5513"  "13835" "893"   "3052"  "6631"  "7300" 
## LAM "14457" "1637"  "2844"  "3859"  "16912" "2309"  "475"   "3450"  "4160" 
## LIN "2088"  "12597" "16395" "16430" "6658"  "14066" "14126" "11749" "11018"
## LOS "2211"  "11376" "14466" "13511" "9056"  "14802" "13356" "9778"  "8993" 
## MAC "1166"  "13110" "16880" "16478" "6486"  "14998" "14810" "12018" "11249"
## MAN "1928"  "12584" "16388" "16350" "6729"  "14160" "14152" "11690" "10952"
## MAT "15823" "2799"  "1363"  "2650"  "16738" "2400"  "1060"  "4289"  "5073" 
## MAU "5244"  "17174" "18249" "18618" "1778"  "15562" "17483" "16680" "15937"
## MED "3339"  "16183" "19770" "18251" "3692"  "16803" "17717" "14996" "14208"
## MON "79"    "12945" "16459" "15655" "7070"  "15555" "14830" "11586" "10797"
## NEB "2561"  "12319" "16085" "16310" "6782"  "13599" "13762" "11594" "10888"
## NEG "2146"  "14812" "18582" "17813" "4882"  "16124" "16435" "13690" "12911"
## NEH "2393"  "12347" "16129" "16282" "6812"  "13723" "13828" "11573" "10859"
## NEO "515"   "13214" "16855" "16164" "6643"  "15496" "15032" "11955" "11170"
## NEW "2271"  "12402" "16193" "16297" "6797"  "13833" "13907" "11597" "10875"
## NOV "7988"  "17038" "15497" "16232" "1166"  "13556" "15734" "18343" "18029"
## NUE "315"   "13119" "16516" "15594" "7021"  "15868" "15036" "11694" "10903"
## OKI "12259" "1599"  "4294"  "4110"  "18914" "5369"  "3265"  "618"   "1390" 
## PAL "1539"  "13857" "17642" "17181" "5731"  "15451" "15503" "12783" "12013"
## PEO "1324"  "12116" "15864" "15501" "7500"  "14332" "13874" "10995" "10227"
## PLM "6723"  "17201" "16731" "17486" "753"   "14381" "16517" "17663" "17066"
## POR "7543"  "18669" "15967" "16073" "1080"  "14936" "17044" "19205" "18416"
## QNC "14520" "1565"  "2241"  "2880"  "17905" "3213"  "1047"  "2942"  "3725" 
## RAR "10624" "6421"  "8650"  "9936"  "11478" "5408"  "6449"  "7561"  "7627" 
## REC "7710"  "15917" "15501" "16649" "1997"  "12886" "15020" "17017" "16751"
## RUS "649"   "12559" "16209" "15612" "7267"  "14983" "14389" "11308" "10525"
## SAI "4137"  "15770" "18676" "19840" "3173"  "15235" "16624" "15219" "14501"
## SER "9427"  "8027"  "10277" "11597" "9895"  "6941"  "8115"  "8998"  "8943" 
## SMI "291"   "12770" "16334" "15603" "7182"  "15331" "14639" "11445" "10657"
## SOC "10677" "6554"  "8698"  "10007" "11340" "5418"  "6526"  "7723"  "7801" 
## SON "15584" "2565"  "1646"  "2877"  "16793" "2281"  "768"   "4126"  "4903" 
## SPB "7870"  "10285" "12508" "13885" "7682"  "9088"  "10418" "11050" "10849"
## SPR "851"   "12357" "16028" "15485" "7428"  "14774" "14179" "11128" "10348"
## SPS "8121"  "10361" "12417" "13831" "7580"  "8975"  "10392" "11208" "11041"
## SSK "14614" "1595"  "2390"  "3271"  "17404" "2709"  "530"   "3232"  "3991" 
## STL "1095"  "12314" "16041" "15610" "7360"  "14567" "14090" "11157" "10384"
## SUF "15187" "3406"  "1535"  "859"   "17769" "4616"  "2874"  "3893"  "4621" 
## SUU "14874" "3468"  "1911"  "973"   "17953" "4964"  "3147"  "3756"  "4452" 
## TAI "13040" "986"   "3562"  "3635"  "18873" "4524"  "2397"  "1397"  "2185" 
## TIK "10497" "6485"  "8760"  "10037" "11418" "5529"  "6547"  "7594"  "7644" 
## TRA "242"   "12858" "16318" "15475" "7217"  "15580" "14764" "11464" "10674"
## TUC "6124"  "16729" "17204" "18179" "1429"  "14441" "16466" "16935" "16321"
## TUH "10957" "6461"  "8480"  "9812"  "11429" "5170"  "6343"  "7698"  "7813" 
## UTS "10671" "2811"  "5874"  "5539"  "17549" "6697"  "4722"  "1023"  "288"  
## WAV "1876"  "12779" "16582" "16525" "6548"  "14316" "14343" "11877" "11136"
## YUN "13743" "1108"  "3467"  "4320"  "17036" "2797"  "1179"  "2967"  "3619" 
##     KAT     KLP     KUN     LAM     LIN     LOS     MAC     MAN     MAT    
## ALD "0.18"  "0.19"  "0.28"  "0.17"  "0.11"  "0.12"  "0.11"  "0.11"  "0.17" 
## ARA "0.12"  "0.14"  "0.20"  "0.11"  "0.16"  "0.14"  "0.12"  "0.14"  "0.12" 
## BAF "0.11"  "0.14"  "0.21"  "0.11"  "0.16"  "0.15"  "0.12"  "0.14"  "0.11" 
## BCO "0.13"  "0.15"  "0.21"  "0.12"  "0.17"  "0.15"  "0.12"  "0.14"  "0.12" 
## BEA "0.13"  "0.14"  "0.21"  "0.13"  "0.06"  "0.09"  "0.02"  "0.03"  "0.13" 
## BEH "0.11"  "0.14"  "0.21"  "0.10"  "0.20"  "0.17"  "0.15"  "0.18"  "0.11" 
## BEN "0.03"  "0.08"  "0.11"  "0.03"  "0.20"  "0.15"  "0.15"  "0.18"  "0.04" 
## BER "0.18"  "0.18"  "0.26"  "0.17"  "0.06"  "0.12"  "0.05"  "0.03"  "0.17" 
## BIT "0.13"  "0.15"  "0.22"  "0.12"  "0.16"  "0.15"  "0.12"  "0.14"  "0.13" 
## BPA "0.14"  "0.15"  "0.22"  "0.13"  "0.13"  "0.14"  "0.09"  "0.10"  "0.14" 
## BPV "0.12"  "0.15"  "0.21"  "0.12"  "0.16"  "0.15"  "0.12"  "0.14"  "0.12" 
## BRE "0.24"  "0.24"  "0.32"  "0.23"  "0.11"  "0.17"  "0.11"  "0.07"  "0.23" 
## BRM "0.19"  "0.20"  "0.27"  "0.18"  "0.11"  "0.14"  "0.09"  "0.09"  "0.18" 
## CAM "0.03"  "0.05"  "0.13"  "0.01"  "0.17"  "0.11"  "0.11"  "0.14"  "0.02" 
## CES "0.27"  "0.28"  "0.36"  "0.26"  "0.14"  "0.20"  "0.14"  "0.12"  "0.26" 
## CHA "0.03"  "0.06"  "0.13"  "0.01"  "0.18"  "0.13"  "0.13"  "0.16"  "0.02" 
## CHI "0.13"  "0.14"  "0.24"  "0.12"  "0.13"  "0.10"  "0.09"  "0.11"  "0.12" 
## COL "0.18"  "0.19"  "0.27"  "0.18"  "0.06"  "0.11"  "0.05"  "0.03"  "0.18" 
## CUA "0.13"  "0.15"  "0.23"  "0.12"  "0.16"  "0.14"  "0.12"  "0.14"  "0.12" 
## DAL "0.17"  "0.17"  "0.25"  "0.16"  "0.06"  "0.11"  "0.04"  "0.03"  "0.16" 
## DEN "0.17"  "0.18"  "0.25"  "0.17"  "0.06"  "0.11"  "0.05"  "0.03"  "0.17" 
## DES "0.22"  "0.23"  "0.31"  "0.22"  "0.09"  "0.15"  "0.10"  "0.07"  "0.22" 
## FAY "0.15"  "0.16"  "0.24"  "0.15"  "0.06"  "0.10"  "0.03"  "0.03"  "0.15" 
## FIR "0.22"  "0.22"  "0.30"  "0.21"  "0.10"  "0.15"  "0.09"  "0.06"  "0.21" 
## FLO "0.13"  "0.15"  "0.22"  "0.12"  "0.17"  "0.15"  "0.13"  "0.15"  "0.13" 
## FRS "0.15"  "0.16"  "0.24"  "0.15"  "0.07"  "0.09"  "0.07"  "0.06"  "0.15" 
## GEL "0.02"  "0.08"  "0.20"  "0.01"  "0.18"  "0.12"  "0.12"  "0.15"  "0.02" 
## GES "0.19"  "0.20"  "0.28"  "0.18"  "0.14"  "0.13"  "0.12"  "0.12"  "0.18" 
## GRA "0.09"  "0.09"  "0.18"  "0.08"  "0.11"  "0.08"  "0.07"  "0.09"  "0.08" 
## GRV "0.13"  "0.15"  "0.22"  "0.12"  "0.17"  "0.15"  "0.12"  "0.14"  "0.13" 
## HAI "0.08"  "0.08"  "0.17"  "0.06"  "0.12"  "0.08"  "0.08"  "0.10"  "0.07" 
## HAL "0.17"  "0.17"  "0.25"  "0.16"  "0.05"  "0.10"  "0.05"  "0.02"  "0.16" 
## HAN "0.08"  "0.10"  "0.20"  "0.07"  "0.14"  "0.09"  "0.09"  "0.11"  "0.07" 
## HAR "0.18"  "0.19"  "0.26"  "0.17"  "0.07"  "0.12"  "0.06"  "0.04"  "0.18" 
## HAT "0.17"  "0.17"  "0.25"  "0.16"  "0.07"  "0.11"  "0.05"  "0.04"  "0.16" 
## HOC "0.09"  "0.10"  "0.19"  "0.08"  "0.12"  "0.09"  "0.08"  "0.10"  "0.08" 
## HOU "0.13"  "0.13"  "0.21"  "0.12"  "0.06"  "0.09"  "0.02"  "0.03"  "0.13" 
## HUN "0.11"  "0.11"  "0.19"  "0.10"  "0.08"  "0.06"  "0.06"  "0.06"  "0.10" 
## INJ "0.08"  "0.17"  "0.25"  "0.12"  "0.25"  "0.21"  "0.20"  "0.23"  "0.13" 
## INW "0.10"  "0.22"  "0.33"  "0.16"  "0.28"  "0.24"  "0.23"  "0.25"  "0.16" 
## IPO "0.14"  "0.18"  "0.25"  "0.13"  "0.24"  "0.21"  "0.20"  "0.22"  "0.14" 
## JAF "0.01"  "0.08"  "0.17"  "0.02"  "0.19"  "0.13"  "0.12"  "0.16"  "0.03" 
## KAC "0.03"  "0.06"  "0.15"  "0.01"  "0.18"  "0.13"  "0.12"  "0.16"  "0.02" 
## KAG "0.19"  "0.19"  "0.27"  "0.18"  "0.09"  "0.12"  "0.09"  "0.07"  "0.18" 
## KAN "0.25"  "0.26"  "0.33"  "0.23"  "0.10"  "0.17"  "0.12"  "0.10"  "0.23" 
## KAT NA      "0.08"  "0.14"  "0.03"  "0.19"  "0.14"  "0.14"  "0.17"  "0.05" 
## KLP "3242"  NA      "0.22"  "0.06"  "0.20"  "0.15"  "0.14"  "0.17"  "0.06" 
## KUN "2779"  "3196"  NA      "0.15"  "0.28"  "0.23"  "0.22"  "0.25"  "0.15" 
## LAM "1789"  "1704"  "3202"  NA      "0.19"  "0.13"  "0.13"  "0.16"  "0.02" 
## LIN "12359" "15352" "14108" "13652" NA      "0.12"  "0.07"  "0.05"  "0.19" 
## LOS "12726" "14153" "15460" "12970" "3836"  NA      "0.10"  "0.10"  "0.13" 
## MAC "13196" "15993" "15090" "14336" "998"   "3203"  NA      "0.04"  "0.13" 
## MAN "12421" "15371" "14225" "13677" "171"   "3667"  "867"   NA      "0.16" 
## MAT "3069"  "177"   "3124"  "1531"  "15181" "14066" "15834" "15203" NA     
## MAU "15450" "18006" "14818" "17237" "4931"  "7355"  "4710"  "4990"  "17942"
## MED "15734" "18949" "16302" "17252" "3631"  "5366"  "3076"  "3625"  "18772"
## MON "13568" "15872" "15846" "14378" "2077"  "2157"  "1176"  "1915"  "15744"
## NEB "11937" "14995" "13621" "13291" "492"   "4190"  "1490"  "636"   "14821"
## NEG "14624" "17654" "15915" "15961" "2311"  "4321"  "1706"  "2284"  "17487"
## NEH "12032" "15058" "13763" "13356" "346"   "4026"  "1337"  "474"   "14886"
## NEO "13594" "16144" "15673" "14566" "1661"  "2693"  "685"   "1514"  "16002"
## NEW "12128" "15135" "13881" "13434" "234"   "3926"  "1216"  "352"   "14964"
## NOV "14438" "15561" "12685" "15812" "7376"  "10111" "7354"  "7471"  "15578"
## NUE "13858" "16028" "16186" "14594" "2398"  "2090"  "1457"  "2239"  "15910"
## OKI "4204"  "3822"  "6262"  "3060"  "12365" "10335" "12634" "12307" "3747" 
## PAL "13780" "16710" "15408" "15028" "1421"  "3737"  "766"   "1366"  "16546"
## PEO "12405" "15025" "14580" "13404" "1229"  "2626"  "1023"  "1066"  "14873"
## PLM "14824" "16632" "13557" "16458" "6147"  "8862"  "6086"  "6234"  "16614"
## POR "16059" "16475" "14043" "17245" "7498"  "9447"  "7197"  "7549"  "16553"
## QNC "2922"  "1445"  "4070"  "1155"  "14156" "12718" "14670" "14147" "1348" 
## RAR "4335"  "7491"  "5355"  "6118"  "8753"  "10999" "9736"  "8871"  "7328" 
## REC "13404" "15197" "12072" "14977" "6695"  "9915"  "6880"  "6817"  "15161"
## RUS "13023" "15490" "15254" "13927" "1620"  "2297"  "917"   "1450"  "15349"
## SAI "14506" "17653" "14690" "16241" "3484"  "6338"  "3396"  "3558"  "17502"
## SER "6004"  "9134"  "6753"  "7788"  "7424"  "10249" "8422"  "7561"  "8974" 
## SMI "13348" "15701" "15623" "14184" "1927"  "2148"  "1087"  "1760"  "15568"
## SOC "4419"  "7547"  "5330"  "6206"  "8780"  "11109" "9768"  "8901"  "7386" 
## SON "2794"  "468"   "3057"  "1241"  "14893" "13888" "15558" "14916" "292"  
## SPB "8313"  "11397" "8755"  "10099" "5782"  "9269"  "6743"  "5943"  "11243"
## SPR "12809" "15287" "15057" "13716" "1574"  "2279"  "1004"  "1403"  "15144"
## SPS "8303"  "11330" "8603"  "10091" "6035"  "9569"  "6984"  "6198"  "11181"
## SSK "2412"  "1365"  "3582"  "624"   "14027" "12946" "14632" "14036" "1217" 
## STL "12638" "15230" "14811" "13621" "1282"  "2555"  "875"   "1112"  "15081"
## SUF "4967"  "2115"  "5293"  "3182"  "15638" "13030" "15804" "15573" "2220" 
## SUU "5209"  "2469"  "5658"  "3420"  "15457" "12699" "15547" "15379" "2565" 
## TAI "3537"  "2979"  "5414"  "2219"  "12960" "11174" "13325" "12920" "2894" 
## TIK "4431"  "7598"  "5481"  "6211"  "8627"  "10880" "9610"  "8744"  "7434" 
## TRA "13568" "15776" "15912" "14318" "2250"  "1969"  "1369"  "2086"  "15654"
## TUC "14570" "16854" "13675" "16307" "5426"  "8301"  "5417"  "5519"  "16798"
## TUH "4249"  "7339"  "5060"  "6038"  "9052"  "11386" "10042" "9175"  "7180" 
## UTS "5167"  "5396"  "7583"  "4435"  "10893" "8757"  "11085" "10821" "5311" 
## WAV "12598" "15563" "14359" "13868" "251"   "3713"  "757"   "195"   "15395"
## YUN "1640"  "2375"  "3681"  "714"   "12978" "12294" "13635" "12996" "2208" 
##     MAU     MED     MON     NEB     NEG     NEH     NEO     NEW     NOV    
## ALD "0.16"  "0.16"  "0.11"  "0.12"  "0.26"  "0.11"  "0.12"  "0.12"  "0.22" 
## ARA "0.06"  "0.12"  "0.11"  "0.15"  "0.20"  "0.14"  "0.13"  "0.15"  "0.07" 
## BAF "0.05"  "0.12"  "0.12"  "0.15"  "0.22"  "0.14"  "0.14"  "0.15"  "0.06" 
## BCO "0.07"  "0.13"  "0.13"  "0.16"  "0.21"  "0.15"  "0.15"  "0.16"  "0.08" 
## BEA "0.10"  "0.11"  "0.02"  "0.04"  "0.20"  "0.03"  "0.04"  "0.05"  "0.17" 
## BEH "0.07"  "0.13"  "0.15"  "0.19"  "0.22"  "0.18"  "0.18"  "0.19"  "0.04" 
## BEN "0.11"  "0.12"  "0.15"  "0.19"  "0.14"  "0.18"  "0.18"  "0.19"  "0.10" 
## BER "0.12"  "0.14"  "0.05"  "0.05"  "0.25"  "0.03"  "0.05"  "0.05"  "0.21" 
## BIT "0.05"  "0.13"  "0.11"  "0.15"  "0.22"  "0.15"  "0.14"  "0.15"  "0.09" 
## BPA "0.08"  "0.13"  "0.09"  "0.12"  "0.22"  "0.11"  "0.10"  "0.12"  "0.13" 
## BPV "0.04"  "0.13"  "0.12"  "0.16"  "0.21"  "0.15"  "0.14"  "0.16"  "0.08" 
## BRE "0.18"  "0.20"  "0.10"  "0.09"  "0.31"  "0.08"  "0.10"  "0.09"  "0.27" 
## BRM "0.15"  "0.16"  "0.09"  "0.10"  "0.26"  "0.09"  "0.10"  "0.10"  "0.23" 
## CAM "0.10"  "0.10"  "0.12"  "0.16"  "0.13"  "0.15"  "0.14"  "0.16"  "0.09" 
## CES "0.21"  "0.23"  "0.14"  "0.12"  "0.34"  "0.12"  "0.14"  "0.13"  "0.31" 
## CHA "0.11"  "0.11"  "0.13"  "0.17"  "0.13"  "0.16"  "0.16"  "0.17"  "0.09" 
## CHI "0.13"  "0.12"  "0.09"  "0.12"  "0.23"  "0.12"  "0.12"  "0.13"  "0.17" 
## COL "0.13"  "0.14"  "0.05"  "0.04"  "0.25"  "0.03"  "0.05"  "0.06"  "0.22" 
## CUA "0.11"  "0.00"  "0.12"  "0.15"  "0.22"  "0.14"  "0.14"  "0.15"  "0.15" 
## DAL "0.12"  "0.13"  "0.01"  "0.04"  "0.24"  "0.04"  "0.04"  "0.05"  "0.20" 
## DEN "0.12"  "0.14"  "0.03"  "0.03"  "0.24"  "0.03"  "0.04"  "0.03"  "0.20" 
## DES "0.17"  "0.19"  "0.09"  "0.07"  "0.29"  "0.07"  "0.09"  "0.09"  "0.26" 
## FAY "0.11"  "0.12"  "0.03"  "0.05"  "0.23"  "0.04"  "0.03"  "0.04"  "0.19" 
## FIR "0.16"  "0.18"  "0.08"  "0.07"  "0.29"  "0.07"  "0.08"  "0.08"  "0.25" 
## FLO "0.06"  "0.13"  "0.13"  "0.16"  "0.22"  "0.15"  "0.15"  "0.16"  "0.08" 
## FRS "0.12"  "0.13"  "0.06"  "0.06"  "0.23"  "0.06"  "0.07"  "0.08"  "0.19" 
## GEL "0.10"  "0.10"  "0.12"  "0.17"  "0.21"  "0.15"  "0.15"  "0.17"  "0.12" 
## GES "0.17"  "0.17"  "0.12"  "0.13"  "0.27"  "0.12"  "0.13"  "0.13"  "0.23" 
## GRA "0.10"  "0.09"  "0.07"  "0.10"  "0.18"  "0.09"  "0.09"  "0.10"  "0.13" 
## GRV "0.07"  "0.13"  "0.13"  "0.15"  "0.22"  "0.14"  "0.14"  "0.15"  "0.07" 
## HAI "0.10"  "0.09"  "0.08"  "0.11"  "0.16"  "0.10"  "0.10"  "0.12"  "0.12" 
## HAL "0.12"  "0.13"  "0.04"  "0.03"  "0.24"  "0.02"  "0.04"  "0.04"  "0.20" 
## HAN "0.10"  "0.10"  "0.09"  "0.13"  "0.20"  "0.12"  "0.12"  "0.13"  "0.14" 
## HAR "0.13"  "0.15"  "0.05"  "0.05"  "0.25"  "0.03"  "0.06"  "0.05"  "0.22" 
## HAT "0.12"  "0.14"  "0.04"  "0.05"  "0.24"  "0.04"  "0.05"  "0.05"  "0.20" 
## HOC "0.11"  "0.10"  "0.08"  "0.12"  "0.19"  "0.11"  "0.11"  "0.12"  "0.14" 
## HOU "0.09"  "0.10"  "0.02"  "0.04"  "0.20"  "0.03"  "0.03"  "0.04"  "0.16" 
## HUN "0.10"  "0.09"  "0.06"  "0.07"  "0.18"  "0.06"  "0.07"  "0.08"  "0.14" 
## INJ "0.19"  "0.18"  "0.20"  "0.25"  "0.25"  "0.24"  "0.23"  "0.24"  "0.20" 
## INW "0.22"  "0.22"  "0.23"  "0.27"  "0.33"  "0.27"  "0.25"  "0.26"  "0.26" 
## IPO "0.09"  "0.17"  "0.20"  "0.24"  "0.26"  "0.23"  "0.22"  "0.23"  "0.04" 
## JAF "0.10"  "0.11"  "0.13"  "0.17"  "0.22"  "0.16"  "0.16"  "0.18"  "0.11" 
## KAC "0.11"  "0.11"  "0.13"  "0.17"  "0.15"  "0.16"  "0.15"  "0.17"  "0.10" 
## KAG "0.15"  "0.16"  "0.08"  "0.08"  "0.26"  "0.07"  "0.09"  "0.09"  "0.22" 
## KAN "0.19"  "0.21"  "0.12"  "0.11"  "0.32"  "0.11"  "0.12"  "0.12"  "0.28" 
## KAT "0.12"  "0.11"  "0.14"  "0.18"  "0.16"  "0.17"  "0.17"  "0.18"  "0.11" 
## KLP "0.14"  "0.14"  "0.15"  "0.19"  "0.22"  "0.18"  "0.17"  "0.19"  "0.15" 
## KUN "0.20"  "0.22"  "0.22"  "0.27"  "0.32"  "0.26"  "0.25"  "0.26"  "0.24" 
## LAM "0.11"  "0.11"  "0.13"  "0.18"  "0.15"  "0.17"  "0.16"  "0.17"  "0.10" 
## LIN "0.14"  "0.16"  "0.07"  "0.06"  "0.26"  "0.06"  "0.07"  "0.07"  "0.23" 
## LOS "0.13"  "0.13"  "0.10"  "0.11"  "0.22"  "0.10"  "0.11"  "0.12"  "0.18" 
## MAC "0.10"  "0.11"  "0.03"  "0.06"  "0.21"  "0.05"  "0.04"  "0.06"  "0.17" 
## MAN "0.12"  "0.13"  "0.04"  "0.03"  "0.24"  "0.02"  "0.04"  "0.04"  "0.20" 
## MAT "0.12"  "0.11"  "0.14"  "0.18"  "0.15"  "0.17"  "0.16"  "0.18"  "0.10" 
## MAU NA      "0.12"  "0.11"  "0.13"  "0.20"  "0.13"  "0.12"  "0.13"  "0.08" 
## MED "2016"  NA      "0.11"  "0.15"  "0.22"  "0.14"  "0.13"  "0.15"  "0.14" 
## MON "5315"  "3414"  NA      "0.05"  "0.21"  "0.04"  "0.04"  "0.06"  "0.18" 
## NEB "5102"  "3969"  "2545"  NA      "0.26"  "0.02"  "0.05"  "0.05"  "0.22" 
## NEG "3112"  "1372"  "2213"  "2698"  NA      "0.25"  "0.24"  "0.25"  "0.25" 
## NEH "5114"  "3913"  "2377"  "171"   "2618"  NA      "0.05"  "0.04"  "0.21" 
## NEO "4873"  "3046"  "549"   "2149"  "1761"  "1988"  NA      "0.05"  "0.20" 
## NEW "5087"  "3842"  "2255"  "290"   "2533"  "123"   "1865"  NA      "0.21" 
## NOV "2756"  "4756"  "8057"  "7420"  "5845"  "7478"  "7594"  "7483"  NA     
## NUE "5288"  "3336"  "350"   "2874"  "2232"  "2707"  "781"   "2584"  "8042" 
## OKI "17296" "15596" "12183" "12205" "14301" "12187" "12560" "12211" "18560"
## PAL "3953"  "2326"  "1584"  "1861"  "955"   "1752"  "1044"  "1652"  "6624" 
## PEO "5726"  "4081"  "1271"  "1566"  "2718"  "1401"  "1191"  "1301"  "8341" 
## PLM "1519"  "3535"  "6791"  "6224"  "4578"  "6270"  "6324"  "6267"  "1271" 
## POR "2572"  "4210"  "7619"  "7674"  "5520"  "7686"  "7251"  "7657"  "1650" 
## QNC "18360" "17746" "14442" "13866" "16374" "13900" "14745" "13959" "16748"
## RAR "11140" "11559" "10579" "8268"  "10716" "8408"  "10338" "8526"  "10837"
## REC "2840"  "4790"  "7769"  "6643"  "5602"  "6731"  "7254"  "6759"  "1327" 
## RUS "5491"  "3698"  "594"   "2054"  "2391"  "1883"  "657"   "1766"  "8189" 
## SAI "1484"  "1614"  "4198"  "3627"  "2031"  "3646"  "3693"  "3626"  "3960" 
## SER "9472"  "9945"  "9394"  "6932"  "9202"  "7087"  "9070"  "7209"  "9358" 
## SMI "5418"  "3551"  "225"   "2383"  "2306"  "2212"  "561"   "2092"  "8148" 
## SOC "11044" "11517" "10633" "8292"  "10707" "8436"  "10379" "8555"  "10682"
## SON "17842" "18481" "15505" "14530" "17200" "14596" "15745" "14675" "15628"
## SPB "7160"  "7733"  "7858"  "5314"  "7163"  "5484"  "7426"  "5605"  "7295" 
## SPR "5651"  "3886"  "790"   "1978"  "2563"  "1808"  "858"   "1696"  "8333" 
## SPS "7152"  "7833"  "8112"  "5574"  "7328"  "5744"  "7668"  "5864"  "7135" 
## SSK "17861" "17659" "14535" "13698" "16309" "13748" "14785" "13818" "16264"
## STL "5583"  "3894"  "1045"  "1671"  "2540"  "1501"  "961"   "1391"  "8227" 
## SUF "19477" "18244" "15120" "15483" "17333" "15466" "15589" "15490" "16892"
## SUU "19401" "17866" "14808" "15340" "17016" "15310" "15291" "15323" "17174"
## TAI "17821" "16356" "12963" "12749" "15020" "12750" "13311" "12788" "17966"
## TIK "11052" "11447" "10452" "8142"  "10596" "8282"  "10211" "8400"  "10796"
## TRA "5469"  "3546"  "199"   "2711"  "2379"  "2542"  "747"   "2421"  "8217" 
## TUC "1153"  "3084"  "6189"  "5488"  "3983"  "5539"  "5700"  "5539"  "1953" 
## TUH "11203" "11741" "10914" "8564"  "10958" "8708"  "10655" "8827"  "10729"
## UTS "15789" "14009" "10595" "10783" "12731" "10746" "10982" "10758" "18051"
## WAV "4804"  "3431"  "1871"  "739"   "2093"  "597"   "1431"  "483"   "7303" 
## YUN "16929" "16601" "13664" "12632" "15279" "12689" "13854" "12763" "16038"
##     NUE     OKI     PAL     PEO     PLM     POR     QNC     RAR     REC    
## ALD "0.11"  "0.13"  "0.12"  "0.11"  "0.18"  "0.19"  "0.23"  "0.15"  "0.15" 
## ARA "0.11"  "0.15"  "0.13"  "0.13"  "0.02"  "0.05"  "0.17"  "0.18"  "0.07" 
## BAF "0.12"  "0.16"  "0.12"  "0.13"  "0.01"  "0.04"  "0.18"  "0.18"  "0.06" 
## BCO "0.13"  "0.17"  "0.13"  "0.14"  "0.02"  "0.06"  "0.18"  "0.18"  "0.08" 
## BEA "0.03"  "0.09"  "0.04"  "0.03"  "0.12"  "0.14"  "0.18"  "0.11"  "0.07" 
## BEH "0.15"  "0.18"  "0.16"  "0.17"  "0.05"  "0.04"  "0.17"  "0.20"  "0.10" 
## BEN "0.15"  "0.16"  "0.15"  "0.17"  "0.11"  "0.11"  "0.10"  "0.19"  "0.13" 
## BER "0.05"  "0.12"  "0.07"  "0.04"  "0.16"  "0.18"  "0.23"  "0.13"  "0.10" 
## BIT "0.11"  "0.16"  "0.13"  "0.14"  "0.06"  "0.06"  "0.19"  "0.18"  "0.09" 
## BPA "0.09"  "0.14"  "0.10"  "0.10"  "0.07"  "0.11"  "0.19"  "0.17"  "0.03" 
## BPV "0.12"  "0.16"  "0.13"  "0.14"  "0.06"  "0.06"  "0.18"  "0.18"  "0.07" 
## BRE "0.11"  "0.17"  "0.13"  "0.09"  "0.21"  "0.23"  "0.28"  "0.18"  "0.15" 
## BRM "0.09"  "0.14"  "0.10"  "0.08"  "0.18"  "0.19"  "0.23"  "0.16"  "0.14" 
## CAM "0.12"  "0.13"  "0.12"  "0.14"  "0.10"  "0.10"  "0.08"  "0.15"  "0.11" 
## CES "0.15"  "0.20"  "0.16"  "0.12"  "0.24"  "0.25"  "0.31"  "0.21"  "0.19" 
## CHA "0.13"  "0.15"  "0.13"  "0.15"  "0.11"  "0.11"  "0.08"  "0.17"  "0.13" 
## CHI "0.10"  "0.12"  "0.11"  "0.11"  "0.15"  "0.16"  "0.18"  "0.14"  "0.13" 
## COL "0.06"  "0.12"  "0.07"  "0.03"  "0.16"  "0.18"  "0.23"  "0.13"  "0.11" 
## CUA "0.12"  "0.15"  "0.13"  "0.13"  "0.13"  "0.13"  "0.19"  "0.17"  "0.13" 
## DAL "0.02"  "0.11"  "0.06"  "0.03"  "0.14"  "0.17"  "0.22"  "0.13"  "0.09" 
## DEN "0.04"  "0.10"  "0.06"  "0.03"  "0.15"  "0.17"  "0.22"  "0.13"  "0.10" 
## DES "0.10"  "0.16"  "0.11"  "0.07"  "0.20"  "0.21"  "0.27"  "0.17"  "0.15" 
## FAY "0.03"  "0.10"  "0.05"  "0.03"  "0.14"  "0.16"  "0.21"  "0.12"  "0.09" 
## FIR "0.09"  "0.15"  "0.11"  "0.07"  "0.19"  "0.21"  "0.26"  "0.16"  "0.14" 
## FLO "0.13"  "0.17"  "0.13"  "0.14"  "0.02"  "0.06"  "0.19"  "0.19"  "0.08" 
## FRS "0.07"  "0.10"  "0.08"  "0.06"  "0.15"  "0.16"  "0.20"  "0.11"  "0.11" 
## GEL "0.12"  "0.15"  "0.12"  "0.14"  "0.11"  "0.11"  "0.12"  "0.18"  "0.12" 
## GES "0.12"  "0.14"  "0.13"  "0.12"  "0.19"  "0.20"  "0.23"  "0.03"  "0.16" 
## GRA "0.07"  "0.09"  "0.08"  "0.08"  "0.11"  "0.13"  "0.15"  "0.11"  "0.10" 
## GRV "0.12"  "0.16"  "0.13"  "0.14"  "0.07"  "0.03"  "0.18"  "0.18"  "0.10" 
## HAI "0.08"  "0.10"  "0.09"  "0.10"  "0.11"  "0.12"  "0.13"  "0.12"  "0.10" 
## HAL "0.05"  "0.11"  "0.06"  "0.02"  "0.15"  "0.17"  "0.22"  "0.12"  "0.10" 
## HAN "0.09"  "0.11"  "0.10"  "0.11"  "0.12"  "0.13"  "0.15"  "0.14"  "0.11" 
## HAR "0.06"  "0.12"  "0.08"  "0.04"  "0.16"  "0.18"  "0.23"  "0.14"  "0.11" 
## HAT "0.05"  "0.11"  "0.07"  "0.04"  "0.15"  "0.17"  "0.22"  "0.13"  "0.10" 
## HOC "0.08"  "0.10"  "0.09"  "0.10"  "0.12"  "0.13"  "0.15"  "0.13"  "0.11" 
## HOU "0.02"  "0.09"  "0.03"  "0.03"  "0.12"  "0.14"  "0.18"  "0.11"  "0.07" 
## HUN "0.06"  "0.07"  "0.07"  "0.06"  "0.12"  "0.13"  "0.16"  "0.09"  "0.09" 
## INJ "0.20"  "0.23"  "0.20"  "0.23"  "0.20"  "0.20"  "0.19"  "0.25"  "0.21" 
## INW "0.23"  "0.26"  "0.23"  "0.25"  "0.24"  "0.23"  "0.24"  "0.28"  "0.23" 
## IPO "0.20"  "0.22"  "0.20"  "0.22"  "0.07"  "0.04"  "0.21"  "0.24"  "0.14" 
## JAF "0.13"  "0.16"  "0.13"  "0.15"  "0.10"  "0.11"  "0.12"  "0.19"  "0.12" 
## KAC "0.13"  "0.15"  "0.13"  "0.15"  "0.11"  "0.11"  "0.09"  "0.17"  "0.12" 
## KAG "0.09"  "0.13"  "0.10"  "0.07"  "0.18"  "0.19"  "0.23"  "0.14"  "0.13" 
## KAN "0.12"  "0.18"  "0.14"  "0.10"  "0.23"  "0.24"  "0.29"  "0.18"  "0.18" 
## KAT "0.14"  "0.16"  "0.14"  "0.16"  "0.12"  "0.12"  "0.12"  "0.19"  "0.13" 
## KLP "0.14"  "0.17"  "0.15"  "0.17"  "0.14"  "0.15"  "0.15"  "0.20"  "0.15" 
## KUN "0.22"  "0.26"  "0.23"  "0.25"  "0.21"  "0.20"  "0.23"  "0.28"  "0.22" 
## LAM "0.13"  "0.15"  "0.13"  "0.16"  "0.11"  "0.11"  "0.09"  "0.18"  "0.13" 
## LIN "0.07"  "0.13"  "0.09"  "0.05"  "0.17"  "0.19"  "0.24"  "0.14"  "0.12" 
## LOS "0.10"  "0.11"  "0.11"  "0.10"  "0.15"  "0.16"  "0.19"  "0.13"  "0.13" 
## MAC "0.03"  "0.10"  "0.04"  "0.04"  "0.12"  "0.15"  "0.19"  "0.12"  "0.08" 
## MAN "0.05"  "0.11"  "0.06"  "0.03"  "0.15"  "0.17"  "0.22"  "0.12"  "0.10" 
## MAT "0.13"  "0.15"  "0.14"  "0.16"  "0.12"  "0.12"  "0.10"  "0.17"  "0.13" 
## MAU "0.11"  "0.14"  "0.11"  "0.12"  "0.06"  "0.06"  "0.18"  "0.17"  "0.08" 
## MED "0.12"  "0.15"  "0.12"  "0.13"  "0.13"  "0.13"  "0.18"  "0.17"  "0.13" 
## MON "0.01"  "0.10"  "0.05"  "0.04"  "0.13"  "0.15"  "0.19"  "0.12"  "0.08" 
## NEB "0.06"  "0.12"  "0.07"  "0.04"  "0.16"  "0.18"  "0.23"  "0.13"  "0.11" 
## NEG "0.21"  "0.24"  "0.22"  "0.23"  "0.22"  "0.21"  "0.22"  "0.26"  "0.22" 
## NEH "0.05"  "0.11"  "0.06"  "0.03"  "0.15"  "0.17"  "0.23"  "0.13"  "0.11" 
## NEO "0.05"  "0.11"  "0.06"  "0.04"  "0.15"  "0.17"  "0.22"  "0.13"  "0.10" 
## NEW "0.07"  "0.12"  "0.07"  "0.05"  "0.16"  "0.18"  "0.22"  "0.13"  "0.11" 
## NOV "0.18"  "0.20"  "0.18"  "0.20"  "0.06"  "0.04"  "0.18"  "0.23"  "0.13" 
## NUE NA      "0.10"  "0.05"  "0.05"  "0.13"  "0.14"  "0.19"  "0.12"  "0.09" 
## OKI "12281" NA      "0.11"  "0.11"  "0.17"  "0.18"  "0.21"  "0.14"  "0.14" 
## PAL "1741"  "13399" NA      "0.06"  "0.13"  "0.15"  "0.19"  "0.13"  "0.09" 
## PEO "1618"  "11611" "1789"  NA      "0.14"  "0.16"  "0.22"  "0.12"  "0.09" 
## PLM "6785"  "18187" "5354"  "7079"  NA      "0.05"  "0.18"  "0.19"  "0.08" 
## POR "7514"  "19766" "6431"  "8220"  "1831"  NA      "0.18"  "0.20"  "0.11" 
## QNC "14586" "2410"  "15421" "13670" "17563" "17893" NA      "0.23"  "0.19" 
## RAR "10928" "7735"  "10080" "9312"  "10781" "12428" "7220"  NA      "0.16" 
## REC "7830"  "17297" "6222"  "7792"  "1498"  "2858"  "16066" "9570"  NA     
## RUS "941"   "11915" "1570"  "677"   "6918"  "7899"  "14089" "9988"  "7777" 
## SAI "4254"  "15831" "2683"  "4382"  "2698"  "4050"  "17180" "10192" "3577" 
## SER "9741"  "9245"  "8659"  "8172"  "9178"  "10890" "8888"  "1670"  "8054" 
## SMI "566"   "12046" "1598"  "1053"  "6878"  "7760"  "14278" "10364" "7814" 
## SOC "10983" "7887"  "10091" "9371"  "10650" "12280" "7318"  "187"   "9422" 
## SON "15685" "3604"  "16263" "14605" "16603" "16700" "1195"  "7076"  "15126"
## SPB "8179"  "11381" "6792"  "6791"  "6945"  "8719"  "11201" "3983"  "5967" 
## SPR "1130"  "11738" "1708"  "501"   "7062"  "8080"  "13892" "9809"  "7884" 
## SPS "8428"  "11514" "6999"  "7069"  "6852"  "8600"  "11210" "3995"  "5811" 
## SSK "14714" "2758"  "15358" "13660" "17021" "17551" "548"   "6735"  "15526"
## STL "1394"  "11771" "1631"  "235"   "6960"  "8055"  "13863" "9534"  "7721" 
## SUF "15116" "3280"  "16548" "14797" "18162" "16916" "2117"  "9299"  "17034"
## SUU "14788" "3154"  "16276" "14554" "18441" "17015" "2309"  "9527"  "17402"
## TAI "13082" "873"   "14091" "12305" "18167" "19435" "1548"  "7398"  "16900"
## TIK "10801" "7780"  "9956"  "9186"  "10717" "12378" "7308"  "127"   "9522" 
## TRA "290"   "12056" "1779"  "1369"  "6953"  "7742"  "14339" "10680" "7951" 
## TUC "6212"  "17485" "4703"  "6394"  "745"   "2495"  "17461" "10372" "1708" 
## TUH "11264" "7834"  "10356" "9652"  "10752" "12344" "7162"  "398"   "9488" 
## UTS "10691" "1590"  "11847" "10066" "16995" "18201" "3965"  "7844"  "16821"
## WAV "2182"  "12494" "1183"  "1157"  "6062"  "7361"  "14342" "9004"  "6673" 
## YUN "13882" "2678"  "14338" "12695" "16460" "17609" "1449"  "5812"  "15043"
##     RUS     SAI     SER     SMI     SOC     SON     SPB     SPR     SPS    
## ALD "0.14"  "0.13"  "0.22"  "0.12"  "0.14"  "0.18"  "0.11"  "0.13"  "0.14" 
## ARA "0.16"  "0.12"  "0.25"  "0.13"  "0.17"  "0.11"  "0.15"  "0.17"  "0.18" 
## BAF "0.17"  "0.12"  "0.27"  "0.14"  "0.18"  "0.11"  "0.15"  "0.18"  "0.19" 
## BCO "0.17"  "0.13"  "0.26"  "0.14"  "0.18"  "0.12"  "0.16"  "0.18"  "0.19" 
## BEA "0.06"  "0.06"  "0.17"  "0.04"  "0.10"  "0.12"  "0.05"  "0.06"  "0.11" 
## BEH "0.20"  "0.14"  "0.29"  "0.18"  "0.20"  "0.11"  "0.19"  "0.21"  "0.21" 
## BEN "0.19"  "0.12"  "0.27"  "0.18"  "0.18"  "0.03"  "0.19"  "0.21"  "0.20" 
## BER "0.08"  "0.09"  "0.19"  "0.05"  "0.12"  "0.17"  "0.05"  "0.07"  "0.13" 
## BIT "0.17"  "0.12"  "0.26"  "0.13"  "0.18"  "0.12"  "0.15"  "0.17"  "0.19" 
## BPA "0.13"  "0.11"  "0.24"  "0.10"  "0.16"  "0.13"  "0.12"  "0.14"  "0.17" 
## BPV "0.17"  "0.12"  "0.26"  "0.14"  "0.18"  "0.12"  "0.15"  "0.17"  "0.19" 
## BRE "0.13"  "0.14"  "0.24"  "0.10"  "0.17"  "0.23"  "0.09"  "0.12"  "0.18" 
## BRM "0.12"  "0.12"  "0.22"  "0.10"  "0.15"  "0.18"  "0.10"  "0.12"  "0.15" 
## CAM "0.16"  "0.09"  "0.23"  "0.14"  "0.15"  "0.00"  "0.15"  "0.18"  "0.16" 
## CES "0.17"  "0.18"  "0.28"  "0.14"  "0.21"  "0.28"  "0.13"  "0.14"  "0.21" 
## CHA "0.18"  "0.11"  "0.25"  "0.16"  "0.17"  "0.00"  "0.17"  "0.19"  "0.18" 
## CHI "0.14"  "0.10"  "0.23"  "0.12"  "0.13"  "0.11"  "0.12"  "0.15"  "0.14" 
## COL "0.08"  "0.10"  "0.19"  "0.06"  "0.12"  "0.17"  "0.05"  "0.07"  "0.13" 
## CUA "0.17"  "0.12"  "0.26"  "0.14"  "0.17"  "0.12"  "0.15"  "0.17"  "0.18" 
## DAL "0.07"  "0.09"  "0.18"  "0.02"  "0.12"  "0.15"  "0.05"  "0.07"  "0.12" 
## DEN "0.06"  "0.09"  "0.18"  "0.04"  "0.12"  "0.16"  "0.04"  "0.05"  "0.12" 
## DES "0.12"  "0.14"  "0.22"  "0.09"  "0.16"  "0.22"  "0.08"  "0.09"  "0.17" 
## FAY "0.07"  "0.07"  "0.18"  "0.04"  "0.11"  "0.14"  "0.05"  "0.07"  "0.12" 
## FIR "0.10"  "0.13"  "0.23"  "0.08"  "0.16"  "0.21"  "0.08"  "0.11"  "0.17" 
## FLO "0.18"  "0.13"  "0.27"  "0.15"  "0.19"  "0.12"  "0.16"  "0.18"  "0.19" 
## FRS "0.09"  "0.09"  "0.16"  "0.08"  "0.10"  "0.14"  "0.06"  "0.09"  "0.09" 
## GEL "0.18"  "0.10"  "0.33"  "0.15"  "0.17"  "0.00"  "0.16"  "0.20"  "0.19" 
## GES "0.15"  "0.14"  "0.23"  "0.13"  "0.01"  "0.18"  "0.12"  "0.15"  "0.16" 
## GRA "0.12"  "0.07"  "0.18"  "0.09"  "0.11"  "0.07"  "0.09"  "0.12"  "0.10" 
## GRV "0.16"  "0.13"  "0.26"  "0.14"  "0.18"  "0.12"  "0.15"  "0.17"  "0.19" 
## HAI "0.13"  "0.07"  "0.19"  "0.10"  "0.11"  "0.06"  "0.11"  "0.13"  "0.12" 
## HAL "0.07"  "0.09"  "0.18"  "0.05"  "0.11"  "0.15"  "0.02"  "0.05"  "0.11" 
## HAN "0.14"  "0.08"  "0.24"  "0.12"  "0.13"  "0.06"  "0.12"  "0.15"  "0.15" 
## HAR "0.08"  "0.10"  "0.20"  "0.05"  "0.13"  "0.17"  "0.05"  "0.07"  "0.13" 
## HAT "0.07"  "0.09"  "0.19"  "0.04"  "0.12"  "0.15"  "0.05"  "0.07"  "0.13" 
## HOC "0.13"  "0.08"  "0.21"  "0.11"  "0.12"  "0.07"  "0.11"  "0.14"  "0.13" 
## HOU "0.06"  "0.06"  "0.17"  "0.03"  "0.10"  "0.11"  "0.04"  "0.06"  "0.11" 
## HUN "0.09"  "0.07"  "0.16"  "0.07"  "0.08"  "0.09"  "0.07"  "0.10"  "0.09" 
## INJ "0.25"  "0.18"  "0.34"  "0.23"  "0.24"  "0.14"  "0.25"  "0.27"  "0.26" 
## INW "0.28"  "0.21"  "0.42"  "0.26"  "0.28"  "0.19"  "0.27"  "0.30"  "0.30" 
## IPO "0.25"  "0.18"  "0.35"  "0.22"  "0.24"  "0.15"  "0.24"  "0.26"  "0.26" 
## JAF "0.19"  "0.11"  "0.34"  "0.16"  "0.18"  "0.01"  "0.17"  "0.21"  "0.20" 
## KAC "0.18"  "0.10"  "0.27"  "0.16"  "0.17"  "0.00"  "0.17"  "0.19"  "0.18" 
## KAG "0.11"  "0.12"  "0.20"  "0.09"  "0.13"  "0.18"  "0.08"  "0.11"  "0.14" 
## KAN "0.14"  "0.16"  "0.25"  "0.12"  "0.17"  "0.24"  "0.10"  "0.13"  "0.18" 
## KAT "0.19"  "0.12"  "0.28"  "0.17"  "0.18"  "0.03"  "0.18"  "0.21"  "0.20" 
## KLP "0.20"  "0.12"  "0.31"  "0.17"  "0.19"  "0.06"  "0.19"  "0.22"  "0.21" 
## KUN "0.28"  "0.21"  "0.43"  "0.25"  "0.27"  "0.18"  "0.27"  "0.30"  "0.30" 
## LAM "0.18"  "0.11"  "0.27"  "0.16"  "0.17"  "0.01"  "0.17"  "0.20"  "0.19" 
## LIN "0.10"  "0.11"  "0.20"  "0.07"  "0.13"  "0.19"  "0.06"  "0.08"  "0.14" 
## LOS "0.14"  "0.11"  "0.21"  "0.11"  "0.13"  "0.13"  "0.11"  "0.14"  "0.13" 
## MAC "0.08"  "0.06"  "0.18"  "0.05"  "0.12"  "0.12"  "0.06"  "0.08"  "0.12" 
## MAN "0.06"  "0.08"  "0.18"  "0.04"  "0.11"  "0.16"  "0.03"  "0.06"  "0.11" 
## MAT "0.18"  "0.11"  "0.26"  "0.16"  "0.17"  "0.02"  "0.18"  "0.20"  "0.19" 
## MAU "0.15"  "0.11"  "0.24"  "0.13"  "0.16"  "0.11"  "0.13"  "0.15"  "0.17" 
## MED "0.16"  "0.11"  "0.25"  "0.14"  "0.16"  "0.11"  "0.15"  "0.17"  "0.18" 
## MON "0.07"  "0.07"  "0.18"  "0.02"  "0.11"  "0.13"  "0.05"  "0.07"  "0.11" 
## NEB "0.08"  "0.10"  "0.19"  "0.06"  "0.13"  "0.17"  "0.04"  "0.07"  "0.13" 
## NEG "0.26"  "0.20"  "0.41"  "0.24"  "0.26"  "0.19"  "0.26"  "0.29"  "0.28" 
## NEH "0.07"  "0.09"  "0.19"  "0.05"  "0.12"  "0.16"  "0.04"  "0.06"  "0.13" 
## NEO "0.08"  "0.08"  "0.19"  "0.05"  "0.12"  "0.16"  "0.06"  "0.07"  "0.13" 
## NEW "0.08"  "0.10"  "0.19"  "0.05"  "0.13"  "0.17"  "0.06"  "0.08"  "0.13" 
## NOV "0.23"  "0.16"  "0.35"  "0.21"  "0.22"  "0.11"  "0.22"  "0.25"  "0.24" 
## NUE "0.08"  "0.07"  "0.18"  "0.03"  "0.12"  "0.13"  "0.06"  "0.08"  "0.12" 
## OKI "0.13"  "0.11"  "0.22"  "0.12"  "0.14"  "0.15"  "0.12"  "0.14"  "0.15" 
## PAL "0.10"  "0.06"  "0.20"  "0.07"  "0.13"  "0.13"  "0.08"  "0.10"  "0.13" 
## PEO "0.06"  "0.08"  "0.18"  "0.05"  "0.11"  "0.15"  "0.04"  "0.06"  "0.12" 
## PLM "0.18"  "0.13"  "0.27"  "0.15"  "0.19"  "0.11"  "0.16"  "0.18"  "0.20" 
## POR "0.19"  "0.14"  "0.28"  "0.17"  "0.19"  "0.11"  "0.18"  "0.20"  "0.21" 
## QNC "0.24"  "0.17"  "0.33"  "0.22"  "0.23"  "0.11"  "0.23"  "0.26"  "0.25" 
## RAR "0.15"  "0.14"  "0.23"  "0.13"  "0.02"  "0.18"  "0.13"  "0.15"  "0.16" 
## REC "0.13"  "0.10"  "0.23"  "0.09"  "0.15"  "0.12"  "0.11"  "0.13"  "0.16" 
## RUS NA      "0.12"  "0.22"  "0.08"  "0.15"  "0.18"  "0.08"  "0.09"  "0.15" 
## SAI "4253"  NA      "0.20"  "0.09"  "0.13"  "0.10"  "0.10"  "0.12"  "0.14" 
## SER "8825"  "8536"  NA      "0.19"  "0.22"  "0.31"  "0.19"  "0.23"  "0.24" 
## SMI "377"   "4254"  "9196"  NA      "0.13"  "0.16"  "0.06"  "0.08"  "0.13" 
## SOC "10044" "10126" "1591"  "10421" NA      "0.18"  "0.12"  "0.14"  "0.15" 
## SON "15094" "17264" "8728"  "15324" "7139"  NA      "0.17"  "0.20"  "0.19" 
## SPB "7359"  "6259"  "2313"  "7696"  "3895"  "11008" NA      "0.06"  "0.12" 
## SPR "215"   "4385"  "8672"  "567"   "9869"  "14888" "7258"  NA      "0.15" 
## SPS "7625"  "6324"  "2341"  "7956"  "3892"  "10956" "332"   "7530"  NA     
## SSK "14133" "16816" "8405"  "14356" "6826"  "971"   "10717" "13928" "10713"
## STL "455"   "4268"  "8374"  "831"   "9590"  "14816" "6946"  "313"   "7217" 
## SUF "14990" "19109" "10969" "15035" "9386"  "2384"  "13280" "14841" "13264"
## SUU "14708" "18932" "11196" "14733" "9621"  "2710"  "13509" "14568" "13511"
## TAI "12658" "16341" "8990"  "12814" "7534"  "2739"  "11225" "12472" "11315"
## TIK "9861"  "10089" "1580"  "10237" "253"   "7180"  "3893"  "9682"  "3913" 
## TRA "702"   "4376"  "9526"  "333"   "10740" "15423" "8026"  "871"   "8283" 
## TUC "6273"  "2021"  "8732"  "6260"  "10255" "16720" "6451"  "6406"  "6391" 
## TUH "10325" "10326" "1797"  "10702" "283"   "6938"  "4076"  "10151" "4054" 
## UTS "10341" "14377" "9124"  "10461" "8019"  "5149"  "10979" "10168" "11182"
## WAV "1453"  "3379"  "7669"  "1734"  "9030"  "15107" "5999"  "1435"  "6246" 
## YUN "13213" "15753" "7472"  "13470" "5921"  "1925"  "9781"  "13002" "9807" 
##     SSK     STL     SUF     SUU     TAI     TIK     TRA     TUC     TUH   
## ALD "0.17"  "0.13"  "0.24"  "0.18"  "0.20"  "0.16"  "0.10"  "0.17"  "0.09"
## ARA "0.11"  "0.16"  "0.19"  "0.15"  "0.21"  "0.19"  "0.12"  "0.01"  "0.14"
## BAF "0.11"  "0.16"  "0.20"  "0.15"  "0.22"  "0.19"  "0.12"  "0.01"  "0.14"
## BCO "0.12"  "0.17"  "0.20"  "0.16"  "0.23"  "0.20"  "0.12"  "0.02"  "0.15"
## BEA "0.13"  "0.05"  "0.19"  "0.13"  "0.15"  "0.12"  "0.01"  "0.12"  "0.06"
## BEH "0.10"  "0.20"  "0.20"  "0.16"  "0.25"  "0.22"  "0.15"  "0.05"  "0.17"
## BEN "0.03"  "0.20"  "0.14"  "0.12"  "0.23"  "0.20"  "0.15"  "0.11"  "0.15"
## BER "0.17"  "0.04"  "0.23"  "0.17"  "0.18"  "0.14"  "0.04"  "0.15"  "0.08"
## BIT "0.12"  "0.16"  "0.20"  "0.16"  "0.22"  "0.19"  "0.12"  "0.04"  "0.14"
## BPA "0.13"  "0.13"  "0.20"  "0.16"  "0.20"  "0.18"  "0.09"  "0.07"  "0.12"
## BPV "0.12"  "0.16"  "0.20"  "0.16"  "0.22"  "0.19"  "0.12"  "0.06"  "0.14"
## BRE "0.22"  "0.09"  "0.28"  "0.23"  "0.23"  "0.19"  "0.09"  "0.20"  "0.13"
## BRM "0.17"  "0.11"  "0.24"  "0.18"  "0.20"  "0.16"  "0.08"  "0.17"  "0.11"
## CAM "0.01"  "0.17"  "0.12"  "0.09"  "0.19"  "0.17"  "0.12"  "0.10"  "0.12"
## CES "0.25"  "0.13"  "0.32"  "0.26"  "0.26"  "0.22"  "0.13"  "0.23"  "0.16"
## CHA "0.00"  "0.19"  "0.13"  "0.10"  "0.21"  "0.18"  "0.14"  "0.10"  "0.14"
## CHI "0.11"  "0.14"  "0.19"  "0.13"  "0.17"  "0.15"  "0.10"  "0.14"  "0.09"
## COL "0.17"  "0.04"  "0.23"  "0.17"  "0.18"  "0.14"  "0.04"  "0.15"  "0.08"
## CUA "0.12"  "0.16"  "0.19"  "0.14"  "0.22"  "0.19"  "0.12"  "0.12"  "0.13"
## DAL "0.16"  "0.05"  "0.22"  "0.16"  "0.17"  "0.14"  "0.02"  "0.13"  "0.08"
## DEN "0.16"  "0.04"  "0.22"  "0.16"  "0.17"  "0.13"  "0.02"  "0.14"  "0.07"
## DES "0.21"  "0.08"  "0.27"  "0.21"  "0.22"  "0.18"  "0.08"  "0.19"  "0.12"
## FAY "0.15"  "0.05"  "0.21"  "0.15"  "0.16"  "0.13"  "0.02"  "0.13"  "0.07"
## FIR "0.20"  "0.08"  "0.27"  "0.21"  "0.21"  "0.17"  "0.08"  "0.18"  "0.12"
## FLO "0.12"  "0.17"  "0.21"  "0.16"  "0.23"  "0.20"  "0.13"  "0.02"  "0.15"
## FRS "0.15"  "0.08"  "0.21"  "0.15"  "0.16"  "0.12"  "0.06"  "0.14"  "0.05"
## GEL "0.00"  "0.18"  "0.16"  "0.11"  "0.25"  "0.20"  "0.12"  "0.10"  "0.12"
## GES "0.18"  "0.14"  "0.25"  "0.18"  "0.20"  "0.05"  "0.11"  "0.18"  "0.11"
## GRA "0.08"  "0.11"  "0.15"  "0.10"  "0.15"  "0.12"  "0.07"  "0.11"  "0.06"
## GRV "0.12"  "0.16"  "0.21"  "0.16"  "0.22"  "0.19"  "0.12"  "0.07"  "0.14"
## HAI "0.06"  "0.12"  "0.14"  "0.09"  "0.14"  "0.13"  "0.08"  "0.10"  "0.08"
## HAL "0.16"  "0.04"  "0.22"  "0.16"  "0.17"  "0.13"  "0.04"  "0.14"  "0.07"
## HAN "0.06"  "0.14"  "0.16"  "0.10"  "0.18"  "0.15"  "0.09"  "0.12"  "0.09"
## HAR "0.17"  "0.06"  "0.23"  "0.17"  "0.18"  "0.14"  "0.04"  "0.16"  "0.08"
## HAT "0.16"  "0.06"  "0.22"  "0.16"  "0.18"  "0.14"  "0.03"  "0.14"  "0.08"
## HOC "0.08"  "0.13"  "0.16"  "0.10"  "0.16"  "0.14"  "0.09"  "0.12"  "0.08"
## HOU "0.12"  "0.05"  "0.18"  "0.12"  "0.14"  "0.11"  "0.01"  "0.11"  "0.06"
## HUN "0.10"  "0.09"  "0.16"  "0.10"  "0.13"  "0.10"  "0.06"  "0.12"  "0.04"
## INJ "0.12"  "0.25"  "0.08"  "0.09"  "0.29"  "0.26"  "0.21"  "0.19"  "0.21"
## INW "0.15"  "0.28"  "0.09"  "0.13"  "0.35"  "0.30"  "0.23"  "0.22"  "0.24"
## IPO "0.13"  "0.25"  "0.24"  "0.20"  "0.30"  "0.26"  "0.20"  "0.07"  "0.21"
## JAF "0.01"  "0.19"  "0.16"  "0.11"  "0.26"  "0.21"  "0.13"  "0.10"  "0.13"
## KAC "0.00"  "0.19"  "0.14"  "0.10"  "0.22"  "0.19"  "0.13"  "0.10"  "0.13"
## KAG "0.17"  "0.09"  "0.24"  "0.18"  "0.19"  "0.14"  "0.08"  "0.17"  "0.09"
## KAN "0.23"  "0.12"  "0.29"  "0.23"  "0.24"  "0.19"  "0.11"  "0.22"  "0.13"
## KAT "0.03"  "0.20"  "0.07"  "0.06"  "0.23"  "0.20"  "0.14"  "0.11"  "0.15"
## KLP "0.06"  "0.21"  "0.18"  "0.13"  "0.25"  "0.21"  "0.15"  "0.14"  "0.15"
## KUN "0.14"  "0.28"  "0.27"  "0.23"  "0.35"  "0.30"  "0.23"  "0.20"  "0.23"
## LAM "0.01"  "0.19"  "0.13"  "0.10"  "0.22"  "0.19"  "0.14"  "0.11"  "0.14"
## LIN "0.18"  "0.07"  "0.24"  "0.18"  "0.19"  "0.15"  "0.06"  "0.17"  "0.09"
## LOS "0.13"  "0.13"  "0.20"  "0.14"  "0.17"  "0.14"  "0.09"  "0.15"  "0.08"
## MAC "0.13"  "0.06"  "0.19"  "0.13"  "0.16"  "0.13"  "0.03"  "0.12"  "0.07"
## MAN "0.16"  "0.04"  "0.22"  "0.16"  "0.17"  "0.13"  "0.03"  "0.14"  "0.07"
## MAT "0.02"  "0.19"  "0.14"  "0.11"  "0.21"  "0.19"  "0.14"  "0.11"  "0.14"
## MAU "0.11"  "0.14"  "0.19"  "0.14"  "0.20"  "0.18"  "0.10"  "0.06"  "0.13"
## MED "0.11"  "0.16"  "0.19"  "0.13"  "0.21"  "0.18"  "0.11"  "0.12"  "0.13"
## MON "0.13"  "0.06"  "0.20"  "0.14"  "0.15"  "0.13"  "0.02"  "0.12"  "0.07"
## NEB "0.18"  "0.05"  "0.24"  "0.17"  "0.18"  "0.14"  "0.04"  "0.16"  "0.08"
## NEG "0.13"  "0.27"  "0.28"  "0.23"  "0.33"  "0.28"  "0.21"  "0.20"  "0.22"
## NEH "0.17"  "0.04"  "0.23"  "0.17"  "0.18"  "0.13"  "0.03"  "0.15"  "0.08"
## NEO "0.16"  "0.06"  "0.22"  "0.16"  "0.17"  "0.14"  "0.03"  "0.14"  "0.08"
## NEW "0.17"  "0.06"  "0.23"  "0.17"  "0.18"  "0.14"  "0.04"  "0.15"  "0.09"
## NOV "0.09"  "0.23"  "0.22"  "0.17"  "0.28"  "0.24"  "0.18"  "0.07"  "0.18"
## NUE "0.13"  "0.07"  "0.20"  "0.13"  "0.16"  "0.13"  "0.02"  "0.12"  "0.08"
## OKI "0.15"  "0.13"  "0.22"  "0.16"  "0.16"  "0.15"  "0.10"  "0.16"  "0.10"
## PAL "0.13"  "0.09"  "0.20"  "0.14"  "0.17"  "0.14"  "0.05"  "0.13"  "0.09"
## PEO "0.16"  "0.04"  "0.22"  "0.15"  "0.17"  "0.13"  "0.03"  "0.14"  "0.07"
## PLM "0.11"  "0.17"  "0.20"  "0.16"  "0.23"  "0.20"  "0.13"  "0.02"  "0.15"
## POR "0.11"  "0.19"  "0.20"  "0.16"  "0.24"  "0.21"  "0.15"  "0.05"  "0.16"
## QNC "0.08"  "0.25"  "0.21"  "0.18"  "0.28"  "0.24"  "0.19"  "0.17"  "0.20"
## RAR "0.17"  "0.14"  "0.24"  "0.18"  "0.20"  "0.05"  "0.12"  "0.18"  "0.11"
## REC "0.13"  "0.12"  "0.20"  "0.15"  "0.20"  "0.17"  "0.08"  "0.07"  "0.11"
## RUS "0.18"  "0.08"  "0.25"  "0.19"  "0.20"  "0.16"  "0.06"  "0.17"  "0.11"
## SAI "0.11"  "0.11"  "0.18"  "0.12"  "0.17"  "0.15"  "0.07"  "0.12"  "0.09"
## SER "0.25"  "0.20"  "0.35"  "0.28"  "0.31"  "0.25"  "0.17"  "0.25"  "0.17"
## SMI "0.16"  "0.06"  "0.22"  "0.16"  "0.18"  "0.14"  "0.03"  "0.13"  "0.09"
## SOC "0.17"  "0.13"  "0.24"  "0.18"  "0.20"  "0.06"  "0.11"  "0.18"  "0.11"
## SON "0.01"  "0.19"  "0.15"  "0.11"  "0.24"  "0.20"  "0.13"  "0.11"  "0.14"
## SPB "0.17"  "0.05"  "0.24"  "0.17"  "0.18"  "0.13"  "0.05"  "0.15"  "0.07"
## SPR "0.19"  "0.07"  "0.26"  "0.20"  "0.21"  "0.16"  "0.06"  "0.17"  "0.10"
## SPS "0.18"  "0.14"  "0.25"  "0.19"  "0.21"  "0.17"  "0.11"  "0.19"  "0.09"
## SSK NA      "0.19"  "0.13"  "0.10"  "0.21"  "0.19"  "0.14"  "0.11"  "0.14"
## STL "13866" NA      "0.25"  "0.19"  "0.19"  "0.15"  "0.05"  "0.17"  "0.09"
## SUF "2567"  "14929" NA      "0.04"  "0.29"  "0.26"  "0.20"  "0.19"  "0.20"
## SUU "2797"  "14673" "381"   NA      "0.22"  "0.20"  "0.14"  "0.15"  "0.14"
## TAI "1886"  "12479" "2777"  "2747"  NA      "0.22"  "0.16"  "0.22"  "0.16"
## TIK "6827"  "9407"  "9393"  "9616"  "7459"  NA      "0.12"  "0.19"  "0.12"
## TRA "14452" "1155"  "14956" "14639" "12846" "10554" NA      "0.12"  "0.07"
## TUC "16916" "6286"  "18728" "19074" "17628" "10298" "6360"  NA      "0.14"
## TUH "6660"  "9871"  "9211"  "9458"  "7445"  "508"   "11021" "10383" NA    
## UTS "4249"  "10216" "4755"  "4566"  "2418"  "7856"  "10467" "16250" "8040"
## WAV "14230" "1165"  "15756" "15556" "13112" "8878"  "2051"  "5350"  "9302"
## YUN "1087"  "12911" "3566"  "3744"  "1928"  "5894"  "13605" "16164" "5783"
##     UTS     WAV     YUN    
## ALD "0.13"  "0.13"  "0.17" 
## ARA "0.17"  "0.15"  "0.11" 
## BAF "0.18"  "0.15"  "0.10" 
## BCO "0.18"  "0.16"  "0.11" 
## BEA "0.06"  "0.05"  "0.12" 
## BEH "0.22"  "0.20"  "0.10" 
## BEN "0.22"  "0.20"  "0.02" 
## BER "0.07"  "0.04"  "0.17" 
## BIT "0.18"  "0.16"  "0.12" 
## BPA "0.15"  "0.12"  "0.13" 
## BPV "0.18"  "0.16"  "0.11" 
## BRE "0.11"  "0.07"  "0.22" 
## BRM "0.13"  "0.10"  "0.17" 
## CAM "0.18"  "0.16"  "0.00" 
## CES "0.13"  "0.14"  "0.25" 
## CHA "0.20"  "0.18"  "0.00" 
## CHI "0.15"  "0.13"  "0.11" 
## COL "0.07"  "0.04"  "0.17" 
## CUA "0.18"  "0.16"  "0.11" 
## DAL "0.07"  "0.03"  "0.16" 
## DEN "0.06"  "0.03"  "0.16" 
## DES "0.09"  "0.06"  "0.21" 
## FAY "0.07"  "0.04"  "0.15" 
## FIR "0.11"  "0.07"  "0.20" 
## FLO "0.19"  "0.16"  "0.12" 
## FRS "0.09"  "0.07"  "0.14" 
## GEL "0.20"  "0.17"  "-0.00"
## GES "0.15"  "0.13"  "0.17" 
## GRA "0.12"  "0.11"  "0.08" 
## GRV "0.18"  "0.16"  "0.12" 
## HAI "0.14"  "0.12"  "0.06" 
## HAL "0.05"  "0.03"  "0.16" 
## HAN "0.16"  "0.13"  "0.06" 
## HAR "0.07"  "0.05"  "0.17" 
## HAT "0.07"  "0.05"  "0.15" 
## HOC "0.14"  "0.12"  "0.08" 
## HOU "0.07"  "0.04"  "0.12" 
## HUN "0.10"  "0.08"  "0.09" 
## INJ "0.27"  "0.26"  "0.12" 
## INW "0.30"  "0.29"  "0.15" 
## IPO "0.26"  "0.25"  "0.13" 
## JAF "0.21"  "0.18"  "0.01" 
## KAC "0.20"  "0.18"  "0.00" 
## KAG "0.09"  "0.09"  "0.17" 
## KAN "0.11"  "0.12"  "0.23" 
## KAT "0.21"  "0.19"  "0.03" 
## KLP "0.22"  "0.20"  "0.06" 
## KUN "0.30"  "0.28"  "0.14" 
## LAM "0.21"  "0.19"  "0.01" 
## LIN "0.07"  "0.07"  "0.18" 
## LOS "0.14"  "0.13"  "0.13" 
## MAC "0.08"  "0.05"  "0.13" 
## MAN "0.06"  "0.03"  "0.16" 
## MAT "0.21"  "0.19"  "0.02" 
## MAU "0.16"  "0.13"  "0.11" 
## MED "0.17"  "0.15"  "0.11" 
## MON "0.08"  "0.05"  "0.13" 
## NEB "0.07"  "0.04"  "0.17" 
## NEG "0.28"  "0.27"  "0.13" 
## NEH "0.06"  "0.02"  "0.16" 
## NEO "0.08"  "0.05"  "0.16" 
## NEW "0.08"  "0.05"  "0.17" 
## NOV "0.25"  "0.23"  "0.09" 
## NUE "0.08"  "0.06"  "0.13" 
## OKI "0.15"  "0.13"  "0.15" 
## PAL "0.10"  "0.08"  "0.13" 
## PEO "0.06"  "0.03"  "0.15" 
## PLM "0.19"  "0.17"  "0.11" 
## POR "0.21"  "0.19"  "0.11" 
## QNC "0.26"  "0.25"  "0.08" 
## RAR "0.15"  "0.14"  "0.17" 
## REC "0.14"  "0.11"  "0.12" 
## RUS "0.11"  "0.08"  "0.18" 
## SAI "0.13"  "0.10"  "0.10" 
## SER "0.21"  "0.21"  "0.26" 
## SMI "0.08"  "0.05"  "0.16" 
## SOC "0.14"  "0.13"  "0.17" 
## SON "0.21"  "0.18"  "0.00" 
## SPB "0.06"  "0.04"  "0.17" 
## SPR "0.06"  "0.07"  "0.19" 
## SPS "0.14"  "0.14"  "0.18" 
## SSK "0.20"  "0.18"  "0.00" 
## STL "0.07"  "0.04"  "0.19" 
## SUF "0.26"  "0.25"  "0.13" 
## SUU "0.20"  "0.18"  "0.10" 
## TAI "0.20"  "0.19"  "0.21" 
## TIK "0.16"  "0.15"  "0.18" 
## TRA "0.07"  "0.04"  "0.13" 
## TUC "0.18"  "0.16"  "0.10" 
## TUH "0.10"  "0.09"  "0.13" 
## UTS NA      "0.07"  "0.20" 
## WAV "11002" NA      "0.18" 
## YUN "3902"  "13188" NA
```

```
cities <- readRDS(here("manuscript1", "output", "fst", "cities_man1.rds"))
head(cities)
```

```
## # A tibble: 6 × 10
##   pop   city   country state latitude longitude continent region subregion order
##   <chr> <chr>  <chr>   <chr>    <dbl>     <dbl> <chr>     <chr>  <chr>     <dbl>
## 1 HAI   Hainan China   <NA>      19.2     110.  Asia      East … Japan         1
## 2 YUN   Yunnan China   <NA>      24.5     101.  Asia      East … Japan         2
## 3 HUN   Hunan  China   <NA>      27.6     112.  Asia      East … Japan         3
## 4 TAI   Tainan Taiwan  <NA>      23.0     120.  Asia      East … Japan        20
## 5 BEN   Benga… India   <NA>      13.0      77.6 Asia      South… South As…    22
## 6 KAT   Kathm… Nepal   <NA>      27.7      85.3 Asia      South… South As…    23
```

We can sort by distance

```
# Calculate row-wise mean distances (excluding diagonal)
row_means <- rowMeans(distance_matrix, na.rm=TRUE)

# Sort row names by mean distances
sorted_names_by_distance <- names(sort(row_means))

# Reorder distance_matrix and aa matrices based on these sorted names
distance_matrix <- distance_matrix[sorted_names_by_distance, sorted_names_by_distance]
aa <- aa[sorted_names_by_distance, sorted_names_by_distance]

# Your existing code to initialize and fill the merged_matrix
merged_matrix <- matrix(NA, nrow = nrow(aa), ncol = ncol(aa))
rownames(merged_matrix) <- sorted_names_by_distance
colnames(merged_matrix) <- sorted_names_by_distance

merged_matrix[upper.tri(merged_matrix, diag = FALSE)] <- aa[upper.tri(aa, diag = FALSE)]
merged_matrix[lower.tri(merged_matrix, diag = FALSE)] <- distance_matrix[lower.tri(distance_matrix, diag = FALSE)]

# Formatting code with absolute value for upper triangular part
for(i in 1:nrow(merged_matrix)) {
  for(j in 1:ncol(merged_matrix)) {
    if (i < j) {
      merged_matrix[i, j] <- sprintf("%.2f", abs(as.numeric(merged_matrix[i, j])))
    } else if (i > j) {
      merged_matrix[i, j] <- sprintf("%.0f", as.numeric(merged_matrix[i, j]))
    }
  }
}

# Print the merged matrix
print(merged_matrix)
```

```
##     NEH     NEW     FIR     NEB     BER     HAR     LIN     MAN     WAV    
## NEH NA      "0.04"  "0.07"  "0.02"  "0.03"  "0.03"  "0.06"  "0.02"  "0.02" 
## NEW "123"   NA      "0.08"  "0.05"  "0.05"  "0.05"  "0.07"  "0.04"  "0.05" 
## FIR "68"    "104"   NA      "0.07"  "0.07"  "0.08"  "0.10"  "0.06"  "0.07" 
## NEB "171"   "290"   "199"   NA      "0.05"  "0.05"  "0.06"  "0.03"  "0.04" 
## BER "240"   "123"   "198"   "396"   NA      "0.05"  "0.06"  "0.03"  "0.04" 
## HAR "353"   "235"   "337"   "523"   "175"   NA      "0.07"  "0.04"  "0.05" 
## LIN "346"   "234"   "295"   "492"   "111"   "200"   NA      "0.05"  "0.07" 
## MAN "474"   "352"   "438"   "636"   "241"   "173"   "171"   NA      "0.03" 
## WAV "597"   "483"   "545"   "739"   "360"   "360"   "251"   "195"   NA     
## COL "855"   "746"   "849"   "1026"  "682"   "514"   "655"   "493"   "602"  
## FAY "869"   "754"   "816"   "1009"  "631"   "604"   "523"   "432"   "272"  
## PEO "1401"  "1301"  "1405"  "1566"  "1250"  "1078"  "1229"  "1066"  "1157" 
## STL "1501"  "1391"  "1495"  "1671"  "1322"  "1159"  "1282"  "1112"  "1165" 
## HAL "1395"  "1273"  "1365"  "1562"  "1169"  "1049"  "1089"  "928"   "887"  
## BEA "1204"  "1091"  "1151"  "1341"  "967"   "935"   "859"   "765"   "608"  
## MAC "1337"  "1216"  "1293"  "1490"  "1098"  "1023"  "998"   "867"   "757"  
## BRM "1238"  "1258"  "1186"  "1176"  "1235"  "1399"  "1208"  "1348"  "1243" 
## SPR "1808"  "1696"  "1799"  "1978"  "1621"  "1462"  "1574"  "1403"  "1435" 
## RUS "1883"  "1766"  "1867"  "2054"  "1680"  "1532"  "1620"  "1450"  "1453" 
## HAT "1830"  "1707"  "1794"  "1993"  "1597"  "1491"  "1508"  "1357"  "1283" 
## NEO "1988"  "1865"  "1950"  "2149"  "1753"  "1651"  "1661"  "1514"  "1431" 
## SMI "2212"  "2092"  "2190"  "2383"  "1997"  "1860"  "1927"  "1760"  "1734" 
## PAL "1752"  "1652"  "1690"  "1861"  "1532"  "1538"  "1421"  "1366"  "1183" 
## DEN "2316"  "2198"  "2298"  "2487"  "2110"  "1964"  "2047"  "1878"  "1869" 
## DAL "2315"  "2196"  "2296"  "2486"  "2106"  "1962"  "2040"  "1872"  "1857" 
## FRS "6135"  "6258"  "6173"  "5974"  "6370"  "6477"  "6463"  "6609"  "6701" 
## SPB "5484"  "5605"  "5509"  "5314"  "5703"  "5837"  "5782"  "5943"  "5999" 
## BRE "6425"  "6548"  "6465"  "6266"  "6662"  "6764"  "6757"  "6899"  "6997" 
## DES "6453"  "6575"  "6492"  "6293"  "6689"  "6791"  "6784"  "6927"  "7025" 
## MON "2377"  "2255"  "2350"  "2545"  "2154"  "2026"  "2077"  "1915"  "1871" 
## HOU "2393"  "2271"  "2364"  "2561"  "2168"  "2044"  "2088"  "1928"  "1876" 
## CES "6640"  "6763"  "6679"  "6481"  "6876"  "6980"  "6971"  "7115"  "7211" 
## SPS "5744"  "5864"  "5766"  "5574"  "5959"  "6097"  "6035"  "6198"  "6246" 
## TRA "2542"  "2421"  "2518"  "2711"  "2324"  "2189"  "2250"  "2086"  "2051" 
## SER "7087"  "7209"  "7130"  "6932"  "7326"  "7419"  "7424"  "7561"  "7669" 
## NUE "2707"  "2584"  "2677"  "2874"  "2480"  "2358"  "2398"  "2239"  "2182" 
## ALD "7312"  "7435"  "7351"  "7152"  "7548"  "7650"  "7643"  "7786"  "7883" 
## NEG "2618"  "2533"  "2551"  "2698"  "2418"  "2453"  "2311"  "2284"  "2093" 
## GRA "7840"  "7963"  "7878"  "7680"  "8076"  "8180"  "8170"  "8314"  "8409" 
## TIK "8282"  "8400"  "8333"  "8142"  "8521"  "8587"  "8627"  "8744"  "8878" 
## RAR "8408"  "8526"  "8459"  "8268"  "8647"  "8714"  "8753"  "8871"  "9004" 
## SOC "8436"  "8555"  "8485"  "8292"  "8675"  "8747"  "8780"  "8901"  "9030" 
## GES "8542"  "8661"  "8592"  "8399"  "8782"  "8852"  "8887"  "9007"  "9137" 
## TUH "8708"  "8827"  "8757"  "8564"  "8948"  "9021"  "9052"  "9175"  "9302" 
## CHI "3982"  "3881"  "3985"  "4146"  "3824"  "3655"  "3789"  "3620"  "3665" 
## LOS "4026"  "3926"  "4030"  "4190"  "3869"  "3700"  "3836"  "3667"  "3713" 
## SAI "3646"  "3626"  "3583"  "3627"  "3557"  "3681"  "3484"  "3558"  "3379" 
## CUA "3913"  "3842"  "3845"  "3969"  "3735"  "3790"  "3631"  "3625"  "3431" 
## MED "3913"  "3842"  "3845"  "3969"  "3735"  "3790"  "3631"  "3625"  "3431" 
## TUC "5539"  "5539"  "5481"  "5488"  "5486"  "5626"  "5426"  "5519"  "5350" 
## FLO "6162"  "6175"  "6109"  "6095"  "6134"  "6283"  "6083"  "6189"  "6030" 
## BIT "5358"  "5341"  "5296"  "5332"  "5274"  "5397"  "5201"  "5273"  "5092" 
## MAU "5114"  "5087"  "5050"  "5102"  "5011"  "5124"  "4931"  "4990"  "4804" 
## ARA "5961"  "5961"  "5903"  "5911"  "5908"  "6047"  "5847"  "5939"  "5770" 
## REC "6731"  "6759"  "6683"  "6643"  "6733"  "6892"  "6695"  "6817"  "6673" 
## PLM "6270"  "6267"  "6212"  "6224"  "6211"  "6346"  "6147"  "6234"  "6062" 
## BCO "6187"  "6176"  "6127"  "6153"  "6113"  "6241"  "6044"  "6121"  "5943" 
## BAF "5954"  "5932"  "5891"  "5934"  "5860"  "5978"  "5784"  "5848"  "5664" 
## BPV "5646"  "5605"  "5580"  "5654"  "5518"  "5614"  "5430"  "5468"  "5276" 
## IPO "6812"  "6797"  "6750"  "6782"  "6730"  "6854"  "6658"  "6729"  "6548" 
## KAT "12032" "12128" "12096" "11937" "12249" "12248" "12359" "12421" "12598"
## NOV "7478"  "7483"  "7422"  "7420"  "7434"  "7576"  "7376"  "7471"  "7303" 
## BEH "7446"  "7442"  "7387"  "7399"  "7386"  "7521"  "7322"  "7407"  "7234" 
## GEL "12257" "12347" "12322" "12173" "12464" "12450" "12574" "12622" "12806"
## BPA "7727"  "7722"  "7668"  "7681"  "7664"  "7798"  "7599"  "7682"  "7507" 
## KAN "10859" "10875" "10920" "10888" "10944" "10823" "11018" "10952" "11136"
## YUN "12689" "12763" "12757" "12632" "12873" "12828" "12978" "12996" "13188"
## UTS "10746" "10758" "10807" "10783" "10822" "10696" "10893" "10821" "11002"
## HUN "12347" "12402" "12414" "12319" "12500" "12426" "12597" "12584" "12779"
## POR "7686"  "7657"  "7622"  "7674"  "7579"  "7688"  "7498"  "7549"  "7361" 
## BEN "13277" "13388" "13334" "13152" "13512" "13551" "13621" "13717" "13872"
## KAG "11573" "11597" "11637" "11594" "11670" "11556" "11749" "11690" "11877"
## HAN "13096" "13162" "13164" "13051" "13267" "13208" "13368" "13372" "13566"
## LAM "13356" "13434" "13423" "13291" "13546" "13508" "13652" "13677" "13868"
## GRV "8247"  "8222"  "8184"  "8229"  "8148"  "8260"  "8068"  "8125"  "7937" 
## JAF "13723" "13833" "13780" "13599" "13956" "13992" "14066" "14160" "14316"
## HAI "13295" "13354" "13363" "13262" "13454" "13383" "13552" "13543" "13738"
## OKI "12187" "12211" "12250" "12205" "12286" "12173" "12365" "12307" "12494"
## KAC "13828" "13907" "13895" "13762" "14020" "13982" "14126" "14152" "14343"
## TAI "12750" "12788" "12816" "12749" "12873" "12775" "12960" "12920" "13112"
## KUN "13763" "13881" "13814" "13621" "14002" "14067" "14108" "14225" "14359"
## SSK "13748" "13818" "13816" "13698" "13925" "13870" "14027" "14036" "14230"
## CHA "14012" "14087" "14080" "13954" "14196" "14150" "14301" "14318" "14510"
## QNC "13900" "13959" "13967" "13866" "14058" "13987" "14156" "14147" "14342"
## CAM "14147" "14216" "14215" "14098" "14322" "14265" "14424" "14430" "14624"
## HOC "14240" "14304" "14307" "14197" "14408" "14345" "14508" "14507" "14702"
## SON "14596" "14675" "14663" "14530" "14786" "14747" "14893" "14916" "15107"
## MAT "14886" "14964" "14953" "14821" "15075" "15034" "15181" "15203" "15395"
## KLP "15058" "15135" "15126" "14995" "15246" "15203" "15352" "15371" "15563"
## SUF "15466" "15490" "15530" "15483" "15562" "15444" "15638" "15573" "15756"
## SUU "15310" "15323" "15371" "15340" "15387" "15259" "15457" "15379" "15556"
## INJ "16129" "16193" "16197" "16085" "16295" "16228" "16395" "16388" "16582"
## INW "16282" "16297" "16344" "16310" "16361" "16232" "16430" "16350" "16525"
##     COL     FAY     PEO     STL     HAL     BEA     MAC     BRM     SPR    
## NEH "0.03"  "0.04"  "0.03"  "0.04"  "0.02"  "0.03"  "0.05"  "0.09"  "0.06" 
## NEW "0.06"  "0.04"  "0.05"  "0.06"  "0.04"  "0.05"  "0.06"  "0.10"  "0.08" 
## FIR "0.08"  "0.07"  "0.07"  "0.08"  "0.07"  "0.07"  "0.09"  "0.12"  "0.11" 
## NEB "0.04"  "0.05"  "0.04"  "0.05"  "0.03"  "0.04"  "0.06"  "0.10"  "0.07" 
## BER "0.05"  "0.04"  "0.04"  "0.04"  "0.03"  "0.04"  "0.05"  "0.09"  "0.07" 
## HAR "0.05"  "0.04"  "0.04"  "0.06"  "0.04"  "0.04"  "0.06"  "0.10"  "0.07" 
## LIN "0.06"  "0.06"  "0.05"  "0.07"  "0.05"  "0.06"  "0.07"  "0.11"  "0.08" 
## MAN "0.03"  "0.03"  "0.03"  "0.04"  "0.02"  "0.03"  "0.04"  "0.09"  "0.06" 
## WAV "0.04"  "0.04"  "0.03"  "0.04"  "0.03"  "0.05"  "0.05"  "0.10"  "0.07" 
## COL NA      "0.04"  "0.03"  "0.04"  "0.02"  "0.05"  "0.05"  "0.10"  "0.07" 
## FAY "648"   NA      "0.03"  "0.05"  "0.03"  "0.02"  "0.03"  "0.08"  "0.07" 
## PEO "574"   "1127"  NA      "0.04"  "0.02"  "0.03"  "0.04"  "0.08"  "0.06" 
## STL "646"   "1079"  "235"   NA      "0.04"  "0.05"  "0.06"  "0.11"  "0.07" 
## HAL "662"   "696"   "713"   "541"   NA      "0.04"  "0.05"  "0.08"  "0.05" 
## BEA "861"   "337"   "1214"  "1101"  "599"   NA      "0.02"  "0.08"  "0.06" 
## MAC "796"   "502"   "1023"  "875"   "343"   "279"   NA      "0.09"  "0.08" 
## BRM "1834"  "1343"  "2399"  "2401"  "2032"  "1494"  "1766"  NA      "0.12" 
## SPR "953"   "1312"  "501"   "313"   "666"   "1265"  "1004"  "2652"  NA     
## RUS "1041"  "1293"  "677"   "455"   "605"   "1192"  "917"   "2634"  "215"  
## HAT "1117"  "1047"  "1040"  "815"   "456"   "816"   "554"   "2310"  "750"  
## NEO "1285"  "1185"  "1191"  "961"   "624"   "928"   "685"   "2412"  "858"  
## SMI "1390"  "1538"  "1053"  "831"   "848"   "1364"  "1087"  "2852"  "567"  
## PAL "1500"  "939"   "1789"  "1631"  "1090"  "642"   "766"   "1601"  "1708" 
## DEN "1474"  "1689"  "1067"  "867"   "993"   "1538"  "1260"  "3017"  "565"  
## DAL "1478"  "1670"  "1089"  "883"   "976"   "1510"  "1232"  "2994"  "589"  
## FRS "6912"  "6962"  "7309"  "7496"  "7525"  "7274"  "7458"  "6094"  "7803" 
## SPB "6318"  "6241"  "6791"  "6946"  "6871"  "6524"  "6743"  "5198"  "7258" 
## BRE "7188"  "7261"  "7569"  "7761"  "7810"  "7576"  "7754"  "6421"  "8066" 
## DES "7215"  "7288"  "7595"  "7788"  "7837"  "7604"  "7781"  "6449"  "8092" 
## MON "1575"  "1654"  "1271"  "1045"  "988"   "1443"  "1176"  "2937"  "790"  
## HOU "1605"  "1652"  "1324"  "1095"  "1000"  "1428"  "1166"  "2919"  "851"  
## CES "7407"  "7473"  "7790"  "7982"  "8027"  "7788"  "7968"  "6618"  "8287" 
## SPS "6585"  "6483"  "7069"  "7217"  "7124"  "6758"  "6984"  "5400"  "7530" 
## TRA "1724"  "1841"  "1369"  "1155"  "1164"  "1639"  "1369"  "3133"  "871"  
## SER "7823"  "7935"  "8172"  "8374"  "8458"  "8257"  "8422"  "7145"  "8672" 
## NUE "1919"  "1951"  "1618"  "1394"  "1312"  "1709"  "1457"  "3191"  "1130" 
## ALD "8074"  "8146"  "8448"  "8643"  "8696"  "8461"  "8640"  "7284"  "8946" 
## NEG "2455"  "1873"  "2718"  "2540"  "2007"  "1596"  "1706"  "2070"  "2563" 
## GRA "8606"  "8671"  "8982"  "9176"  "9226"  "8983"  "9166"  "7784"  "9480" 
## TIK "8929"  "9150"  "9186"  "9407"  "9587"  "9483"  "9610"  "8526"  "9682" 
## RAR "9056"  "9275"  "9312"  "9534"  "9714"  "9609"  "9736"  "8646"  "9809" 
## SOC "9100"  "9301"  "9371"  "9590"  "9756"  "9633"  "9768"  "8637"  "9869" 
## GES "9204"  "9408"  "9472"  "9692"  "9860"  "9740"  "9874"  "8747"  "9970" 
## TUH "9378"  "9573"  "9652"  "9871"  "10033" "9904"  "10042" "8890"  "10151"
## CHI "3142"  "3535"  "2581"  "2508"  "2852"  "3431"  "3152"  "4878"  "2231" 
## LOS "3188"  "3584"  "2626"  "2555"  "2902"  "3481"  "3203"  "4927"  "2279" 
## SAI "3937"  "3291"  "4382"  "4268"  "3739"  "3171"  "3396"  "2488"  "4385" 
## CUA "3825"  "3228"  "4081"  "3894"  "3368"  "2964"  "3076"  "3109"  "3886" 
## MED "3825"  "3228"  "4081"  "3894"  "3368"  "2964"  "3076"  "3109"  "3886" 
## TUC "5928"  "5288"  "6394"  "6286"  "5759"  "5187"  "5417"  "4313"  "6406" 
## FLO "6620"  "5991"  "7110"  "7017"  "6499"  "5915"  "6156"  "4925"  "7154" 
## BIT "5641"  "4994"  "6063"  "5931"  "5394"  "4849"  "5056"  "4175"  "6018" 
## MAU "5332"  "4685"  "5726"  "5583"  "5043"  "4517"  "4710"  "3972"  "5651" 
## ARA "6345"  "5704"  "6806"  "6694"  "6165"  "5596"  "5823"  "4735"  "6808" 
## REC "7273"  "6665"  "7792"  "7721"  "7221"  "6626"  "6880"  "5501"  "7884" 
## PLM "6631"  "5986"  "7079"  "6960"  "6427"  "5867"  "6086"  "5050"  "7062" 
## BCO "6498"  "5851"  "6926"  "6795"  "6258"  "5711"  "5920"  "4985"  "6881" 
## BAF "6199"  "5551"  "6597"  "6454"  "5914"  "5387"  "5581"  "4784"  "6520" 
## BPV "5763"  "5126"  "6100"  "5933"  "5395"  "4917"  "5078"  "4567"  "5959" 
## IPO "7092"  "6444"  "7500"  "7360"  "6820"  "6288"  "6486"  "5618"  "7428" 
## KAT "12404" "12852" "12405" "12638" "13030" "13179" "13196" "12645" "12809"
## NOV "7881"  "7240"  "8341"  "8227"  "7695"  "7130"  "7354"  "6245"  "8333" 
## BEH "7798"  "7152"  "8236"  "8109"  "7573"  "7022"  "7234"  "6224"  "8197" 
## GEL "12565" "13051" "12517" "12747" "13170" "13369" "13361" "12949" "12894"
## BPA "8068"  "7422"  "8500"  "8369"  "7831"  "7286"  "7494"  "6507"  "8451" 
## KAN "10606" "11253" "10227" "10384" "10924" "11429" "11249" "12054" "10348"
## YUN "12850" "13407" "12695" "12911" "13391" "13697" "13635" "13548" "13002"
## UTS "10460" "11108" "10066" "10216" "10756" "11273" "11085" "11954" "10168"
## HUN "12359" "12965" "12116" "12314" "12828" "13220" "13110" "13357" "12357"
## POR "7868"  "7225"  "8220"  "8055"  "7517"  "7030"  "7197"  "6535"  "8080" 
## BEN "13808" "14141" "13920" "14155" "14465" "14478" "14561" "13600" "14371"
## KAG "11359" "12004" "10995" "11157" "11696" "12190" "12018" "12747" "11128"
## HAN "13186" "13771" "12983" "13189" "13690" "14044" "13956" "14018" "13251"
## LAM "13547" "14093" "13404" "13621" "14097" "14389" "14336" "14162" "13716"
## GRV "8452"  "7808"  "8812"  "8650"  "8110"  "7619"  "7789"  "7080"  "8677" 
## JAF "14239" "14585" "14332" "14567" "14893" "14922" "14998" "14054" "14774"
## HAI "13324" "13928" "13080" "13277" "13793" "14185" "14076" "14274" "13315"
## OKI "11976" "12621" "11611" "11771" "12311" "12808" "12634" "13354" "11738"
## KAC "14022" "14568" "13874" "14090" "14569" "14865" "14810" "14617" "14179"
## TAI "12628" "13264" "12305" "12479" "13014" "13477" "13325" "13859" "12472"
## KUN "14388" "14630" "14580" "14811" "15050" "14963" "15090" "13889" "15057"
## SSK "13859" "14438" "13660" "13866" "14367" "14716" "14632" "14633" "13928"
## CHA "14161" "14727" "13983" "14192" "14686" "15013" "14941" "14847" "14263"
## QNC "13923" "14530" "13670" "13863" "14383" "14784" "14670" "14868" "13892"
## CAM "14245" "14830" "14032" "14234" "14741" "15103" "15012" "15034" "14285"
## HOC "14304" "14900" "14070" "14267" "14782" "15164" "15062" "15158" "14306"
## SON "14774" "15330" "14605" "14816" "15307" "15622" "15558" "15375" "14888"
## MAT "15054" "15615" "14873" "15081" "15578" "15905" "15834" "15665" "15144"
## KLP "15216" "15782" "15025" "15230" "15732" "16068" "15993" "15842" "15287"
## SUF "15213" "15861" "14797" "14929" "15466" "16011" "15804" "16622" "14841"
## SUU "14995" "15641" "14554" "14673" "15206" "15767" "15547" "16502" "14568"
## INJ "16154" "16769" "15864" "16041" "16575" "17014" "16880" "17006" "16028"
## INW "15958" "16602" "15501" "15610" "16135" "16709" "16478" "17464" "15485"
##     RUS     HAT     NEO     SMI     PAL     DEN     DAL     FRS     SPB    
## NEH "0.07"  "0.04"  "0.05"  "0.05"  "0.06"  "0.03"  "0.04"  "0.06"  "0.04" 
## NEW "0.08"  "0.05"  "0.05"  "0.05"  "0.07"  "0.03"  "0.05"  "0.08"  "0.06" 
## FIR "0.10"  "0.08"  "0.08"  "0.08"  "0.11"  "0.07"  "0.07"  "0.10"  "0.08" 
## NEB "0.08"  "0.05"  "0.05"  "0.06"  "0.07"  "0.03"  "0.04"  "0.06"  "0.04" 
## BER "0.08"  "0.05"  "0.05"  "0.05"  "0.07"  "0.04"  "0.04"  "0.07"  "0.05" 
## HAR "0.08"  "0.04"  "0.06"  "0.05"  "0.08"  "0.04"  "0.05"  "0.07"  "0.05" 
## LIN "0.10"  "0.07"  "0.07"  "0.07"  "0.09"  "0.06"  "0.06"  "0.07"  "0.06" 
## MAN "0.06"  "0.04"  "0.04"  "0.04"  "0.06"  "0.03"  "0.03"  "0.06"  "0.03" 
## WAV "0.08"  "0.05"  "0.05"  "0.05"  "0.08"  "0.03"  "0.03"  "0.07"  "0.04" 
## COL "0.08"  "0.05"  "0.05"  "0.06"  "0.07"  "0.04"  "0.04"  "0.06"  "0.05" 
## FAY "0.07"  "0.03"  "0.03"  "0.04"  "0.05"  "0.02"  "0.02"  "0.06"  "0.05" 
## PEO "0.06"  "0.04"  "0.04"  "0.05"  "0.06"  "0.03"  "0.03"  "0.06"  "0.04" 
## STL "0.08"  "0.06"  "0.06"  "0.06"  "0.09"  "0.04"  "0.05"  "0.08"  "0.05" 
## HAL "0.07"  "0.04"  "0.04"  "0.05"  "0.06"  "0.03"  "0.03"  "0.05"  "0.02" 
## BEA "0.06"  "0.03"  "0.04"  "0.04"  "0.04"  "0.03"  "0.03"  "0.06"  "0.05" 
## MAC "0.08"  "0.05"  "0.04"  "0.05"  "0.04"  "0.05"  "0.04"  "0.07"  "0.06" 
## BRM "0.12"  "0.10"  "0.10"  "0.10"  "0.10"  "0.09"  "0.09"  "0.11"  "0.10" 
## SPR "0.09"  "0.07"  "0.07"  "0.08"  "0.10"  "0.05"  "0.07"  "0.09"  "0.06" 
## RUS NA      "0.07"  "0.08"  "0.08"  "0.10"  "0.06"  "0.07"  "0.09"  "0.08" 
## HAT "568"   NA      "0.05"  "0.04"  "0.07"  "0.03"  "0.03"  "0.07"  "0.05" 
## NEO "657"   "168"   NA      "0.05"  "0.06"  "0.04"  "0.04"  "0.07"  "0.06" 
## SMI "377"   "577"   "561"   NA      "0.07"  "0.04"  "0.02"  "0.08"  "0.06" 
## PAL "1570"  "1033"  "1044"  "1598"  NA      "0.06"  "0.06"  "0.08"  "0.08" 
## DEN "433"   "771"   "764"   "202"   "1798"  NA      "0.02"  "0.06"  "0.04" 
## DAL "439"   "732"   "715"   "156"   "1753"  "58"    NA      "0.07"  "0.05" 
## FRS "7938"  "7965"  "8123"  "8300"  "7638"  "8363"  "8376"  NA      "0.06" 
## SPB "7359"  "7282"  "7426"  "7696"  "6792"  "7793"  "7795"  "1264"  NA     
## BRE "8207"  "8254"  "8413"  "8573"  "7954"  "8628"  "8643"  "349"   "1595" 
## DES "8234"  "8281"  "8440"  "8600"  "7982"  "8654"  "8670"  "375"   "1617" 
## MON "594"   "627"   "549"   "225"   "1584"  "342"   "285"   "8487"  "7858" 
## HOU "649"   "613"   "515"   "291"   "1539"  "421"   "364"   "8513"  "7870" 
## CES "8427"  "8470"  "8628"  "8792"  "8159"  "8848"  "8863"  "525"   "1702" 
## SPS "7625"  "7527"  "7668"  "7956"  "6999"  "8058"  "8059"  "1371"  "332"  
## TRA "702"   "824"   "747"   "333"   "1779"  "328"   "288"   "8633"  "8026" 
## SER "8825"  "8908"  "9070"  "9196"  "8659"  "9237"  "9256"  "1100"  "2313" 
## NUE "941"   "905"   "781"   "566"   "1741"  "613"   "567"   "8828"  "8179" 
## ALD "9090"  "9141"  "9300"  "9457"  "8830"  "9510"  "9525"  "1192"  "2263" 
## NEG "2391"  "1822"  "1761"  "2306"  "955"   "2505"  "2451"  "8136"  "7163" 
## GRA "9623"  "9670"  "9828"  "9990"  "9341"  "10043" "10059" "1709"  "2687" 
## TIK "9861"  "10044" "10211" "10237" "9956"  "10241" "10270" "2660"  "3893" 
## RAR "9988"  "10170" "10338" "10364" "10080" "10368" "10397" "2758"  "3983" 
## SOC "10044" "10212" "10379" "10421" "10091" "10430" "10457" "2689"  "3895" 
## GES "10146" "10316" "10483" "10522" "10200" "10530" "10558" "2794"  "3995" 
## TUH "10325" "10489" "10655" "10702" "10356" "10712" "10739" "2897"  "4076" 
## CHI "2247"  "2674"  "2642"  "2097"  "3685"  "1905"  "1942"  "9544"  "9234" 
## LOS "2297"  "2725"  "2693"  "2148"  "3737"  "1956"  "1993"  "9570"  "9269" 
## SAI "4253"  "3710"  "3693"  "4254"  "2683"  "4457"  "4408"  "7438"  "6259" 
## CUA "3698"  "3136"  "3046"  "3551"  "2326"  "3738"  "3682"  "8847"  "7733" 
## MED "3698"  "3136"  "3046"  "3551"  "2326"  "3738"  "3682"  "8847"  "7733" 
## TUC "6273"  "5724"  "5700"  "6260"  "4703"  "6462"  "6411"  "7714"  "6451" 
## FLO "7030"  "6491"  "6475"  "7036"  "5462"  "7238"  "7189"  "7550"  "6296" 
## BIT "5866"  "5305"  "5261"  "5813"  "4311"  "6013"  "5959"  "8209"  "6952" 
## MAU "5491"  "4925"  "4873"  "5418"  "3953"  "5616"  "5562"  "8405"  "7160" 
## ARA "6671"  "6119"  "6089"  "6647"  "5102"  "6849"  "6797"  "7924"  "6662" 
## REC "7777"  "7255"  "7254"  "7814"  "6222"  "8016"  "7969"  "7182"  "5967" 
## PLM "6918"  "6361"  "6324"  "6878"  "5354"  "7079"  "7026"  "8204"  "6945" 
## BCO "6729"  "6166"  "6120"  "6669"  "5175"  "6868"  "6814"  "8453"  "7190" 
## BAF "6358"  "5791"  "5735"  "6276"  "4824"  "6473"  "6418"  "8695"  "7432" 
## BPV "5781"  "5214"  "5137"  "5655"  "4312"  "5845"  "5789"  "9156"  "7907" 
## IPO "7267"  "6700"  "6643"  "7182"  "5731"  "7379"  "7323"  "8938"  "7682" 
## KAT "13023" "13437" "13594" "13348" "13780" "13250" "13300" "7091"  "8313" 
## NOV "8189"  "7632"  "7594"  "8148"  "6624"  "8348"  "8295"  "8506"  "7295" 
## BEH "8045"  "7482"  "7434"  "7981"  "6491"  "8179"  "8125"  "8817"  "7593" 
## GEL "13109" "13557" "13707" "13416" "13988" "13301" "13353" "7544"  "8778" 
## BPA "8295"  "7730"  "7678"  "8222"  "6747"  "8419"  "8365"  "9025"  "7811" 
## KAN "10525" "11093" "11170" "10657" "12013" "10470" "10527" "9689"  "10849"
## YUN "13213" "13722" "13854" "13470" "14338" "13323" "13380" "8529"  "9781" 
## UTS "10341" "10909" "10982" "10461" "11847" "10272" "10328" "9839"  "10979"
## HUN "12559" "13102" "13214" "12770" "13857" "12604" "12662" "9021"  "10285"
## POR "7899"  "7333"  "7251"  "7760"  "6431"  "7947"  "7890"  "9967"  "8719" 
## BEN "14578" "14907" "15073" "14932" "15001" "14865" "14910" "7577"  "8652" 
## KAG "11308" "11874" "11955" "11445" "12783" "11259" "11316" "9838"  "11050"
## HAN "13457" "13989" "14109" "13684" "14686" "13524" "13581" "9128"  "10381"
## LAM "13927" "14433" "14566" "14184" "15028" "14037" "14094" "8870"  "10099"
## GRV "8497"  "7930"  "7850"  "8359"  "7024"  "8545"  "8488"  "10140" "8916" 
## JAF "14983" "15331" "15496" "15331" "15451" "15255" "15302" "8024"  "9088" 
## HAI "13515" "14062" "14171" "13717" "14822" "13548" "13605" "9546"  "10803"
## OKI "11915" "12483" "12560" "12046" "13399" "11857" "11914" "10143" "11381"
## KAC "14389" "14901" "15032" "14639" "15503" "14489" "14546" "9207"  "10418"
## TAI "12658" "13222" "13311" "12814" "14091" "12631" "12689" "9964"  "11225"
## KUN "15254" "15505" "15673" "15623" "15408" "15585" "15624" "7795"  "8755" 
## SSK "14133" "14666" "14785" "14356" "15358" "14194" "14251" "9484"  "10717"
## CHA "14470" "14996" "15120" "14702" "15655" "14542" "14600" "9515"  "10731"
## QNC "14089" "14641" "14745" "14278" "15421" "14104" "14162" "9957"  "11201"
## CAM "14487" "15028" "15142" "14698" "15744" "14531" "14589" "9814"  "11036"
## HOC "14505" "15052" "15161" "14702" "15804" "14530" "14588" "10009" "11235"
## SON "15094" "15621" "15745" "15324" "16263" "15164" "15221" "9827"  "11008"
## MAT "15349" "15882" "16002" "15568" "16546" "15403" "15461" "10074" "11243"
## KLP "15490" "16028" "16144" "15701" "16710" "15532" "15590" "10234" "11397"
## SUF "14990" "15550" "15589" "15035" "16548" "14834" "14886" "12051" "13280"
## SUU "14708" "15262" "15291" "14733" "16276" "14531" "14583" "12266" "13509"
## INJ "16209" "16776" "16855" "16334" "17642" "16142" "16198" "11377" "12508"
## INW "15612" "16151" "16164" "15603" "17181" "15400" "15449" "12693" "13885"
##     BRE     DES     MON     HOU     CES     SPS     TRA     SER     NUE    
## NEH "0.08"  "0.07"  "0.04"  "0.03"  "0.12"  "0.13"  "0.03"  "0.19"  "0.05" 
## NEW "0.09"  "0.09"  "0.06"  "0.04"  "0.13"  "0.13"  "0.04"  "0.19"  "0.07" 
## FIR "0.13"  "0.11"  "0.08"  "0.07"  "0.16"  "0.17"  "0.08"  "0.23"  "0.09" 
## NEB "0.09"  "0.07"  "0.05"  "0.04"  "0.12"  "0.13"  "0.04"  "0.19"  "0.06" 
## BER "0.08"  "0.08"  "0.05"  "0.04"  "0.12"  "0.13"  "0.04"  "0.19"  "0.05" 
## HAR "0.10"  "0.08"  "0.05"  "0.04"  "0.13"  "0.13"  "0.04"  "0.20"  "0.06" 
## LIN "0.11"  "0.09"  "0.07"  "0.06"  "0.14"  "0.14"  "0.06"  "0.20"  "0.07" 
## MAN "0.07"  "0.07"  "0.04"  "0.03"  "0.12"  "0.11"  "0.03"  "0.18"  "0.05" 
## WAV "0.07"  "0.06"  "0.05"  "0.04"  "0.14"  "0.14"  "0.04"  "0.21"  "0.06" 
## COL "0.09"  "0.08"  "0.05"  "0.04"  "0.13"  "0.13"  "0.04"  "0.19"  "0.06" 
## FAY "0.08"  "0.07"  "0.03"  "0.02"  "0.13"  "0.12"  "0.02"  "0.18"  "0.03" 
## PEO "0.09"  "0.07"  "0.04"  "0.03"  "0.12"  "0.12"  "0.03"  "0.18"  "0.05" 
## STL "0.09"  "0.08"  "0.06"  "0.05"  "0.13"  "0.14"  "0.05"  "0.20"  "0.07" 
## HAL "0.09"  "0.07"  "0.04"  "0.03"  "0.12"  "0.11"  "0.04"  "0.18"  "0.05" 
## BEA "0.09"  "0.08"  "0.02"  "0.01"  "0.13"  "0.11"  "0.01"  "0.17"  "0.03" 
## MAC "0.11"  "0.10"  "0.03"  "0.02"  "0.14"  "0.12"  "0.03"  "0.18"  "0.03" 
## BRM "0.14"  "0.12"  "0.09"  "0.08"  "0.18"  "0.15"  "0.08"  "0.22"  "0.09" 
## SPR "0.12"  "0.09"  "0.07"  "0.06"  "0.14"  "0.15"  "0.06"  "0.23"  "0.08" 
## RUS "0.13"  "0.12"  "0.07"  "0.06"  "0.17"  "0.15"  "0.06"  "0.22"  "0.08" 
## HAT "0.10"  "0.08"  "0.04"  "0.03"  "0.14"  "0.13"  "0.03"  "0.19"  "0.05" 
## NEO "0.10"  "0.09"  "0.04"  "0.03"  "0.14"  "0.13"  "0.03"  "0.19"  "0.05" 
## SMI "0.10"  "0.09"  "0.02"  "0.03"  "0.14"  "0.13"  "0.03"  "0.19"  "0.03" 
## PAL "0.13"  "0.11"  "0.05"  "0.03"  "0.16"  "0.13"  "0.05"  "0.20"  "0.05" 
## DEN "0.08"  "0.07"  "0.03"  "0.03"  "0.12"  "0.12"  "0.02"  "0.18"  "0.04" 
## DAL "0.09"  "0.07"  "0.01"  "0.02"  "0.13"  "0.12"  "0.02"  "0.18"  "0.02" 
## FRS "0.12"  "0.11"  "0.06"  "0.05"  "0.15"  "0.09"  "0.06"  "0.16"  "0.07" 
## SPB "0.09"  "0.08"  "0.05"  "0.04"  "0.13"  "0.12"  "0.05"  "0.19"  "0.06" 
## BRE NA      "0.09"  "0.10"  "0.09"  "0.15"  "0.18"  "0.09"  "0.24"  "0.11" 
## DES "28"    NA      "0.09"  "0.08"  "0.13"  "0.17"  "0.08"  "0.22"  "0.10" 
## MON "8764"  "8791"  NA      "0.02"  "0.14"  "0.11"  "0.02"  "0.18"  "0.01" 
## HOU "8792"  "8819"  "79"    NA      "0.13"  "0.11"  "0.01"  "0.17"  "0.02" 
## CES "223"   "198"   "8983"  "9011"  NA      "0.21"  "0.13"  "0.28"  "0.15" 
## SPS "1670"  "1689"  "8112"  "8121"  "1737"  NA      "0.11"  "0.24"  "0.12" 
## TRA "8905"  "8932"  "199"   "242"   "9125"  "8283"  NA      "0.17"  "0.02" 
## SER "752"   "725"   "9394"  "9427"  "612"   "2341"  "9526"  NA      "0.18" 
## NUE "9107"  "9134"  "350"   "315"   "9326"  "8428"  "290"   "9741"  NA     
## ALD "887"   "859"   "9649"  "9678"  "673"   "2226"  "9789"  "442"   "9993" 
## NEG "8469"  "8497"  "2213"  "2146"  "8661"  "7328"  "2379"  "9202"  "2232" 
## GRA "1417"  "1390"  "10181" "10210" "1200"  "2601"  "10322" "884"   "10525"
## TIK "2313"  "2288"  "10452" "10497" "2191"  "3913"  "10554" "1580"  "10801"
## RAR "2410"  "2385"  "10579" "10624" "2281"  "3995"  "10680" "1670"  "10928"
## SOC "2339"  "2313"  "10633" "10677" "2197"  "3892"  "10740" "1591"  "10983"
## GES "2444"  "2418"  "10735" "10780" "2300"  "3988"  "10841" "1695"  "11085"
## TUH "2549"  "2522"  "10914" "10957" "2393"  "4054"  "11021" "1797"  "11264"
## CHI "9747"  "9771"  "2105"  "2159"  "9968"  "9533"  "1918"  "10229" "2039" 
## LOS "9772"  "9795"  "2157"  "2211"  "9993"  "9569"  "1969"  "10249" "2090" 
## SAI "7787"  "7813"  "4198"  "4137"  "7934"  "6324"  "4376"  "8536"  "4254" 
## CUA "9194"  "9221"  "3414"  "3339"  "9361"  "7833"  "3546"  "9945"  "3336" 
## MED "9194"  "9221"  "3414"  "3339"  "9361"  "7833"  "3546"  "9945"  "3336" 
## TUC "8043"  "8064"  "6189"  "6124"  "8127"  "6391"  "6360"  "8732"  "6212" 
## FLO "7859"  "7877"  "6974"  "6911"  "7916"  "6189"  "7149"  "8504"  "7010" 
## BIT "8546"  "8568"  "5720"  "5651"  "8646"  "6921"  "5880"  "9256"  "5709" 
## MAU "8747"  "8771"  "5315"  "5244"  "8861"  "7152"  "5469"  "9472"  "5288" 
## ARA "8244"  "8264"  "6569"  "6503"  "8316"  "6580"  "6736"  "8914"  "6580" 
## REC "7466"  "7481"  "7769"  "7710"  "7493"  "5811"  "7951"  "8054"  "7830" 
## PLM "8520"  "8539"  "6791"  "6723"  "8585"  "6852"  "6953"  "9178"  "6785" 
## BCO "8774"  "8794"  "6571"  "6501"  "8847"  "7111"  "6727"  "9445"  "6548" 
## BAF "9025"  "9046"  "6166"  "6094"  "9112"  "7377"  "6315"  "9718"  "6124" 
## BPV "9497"  "9521"  "5524"  "5449"  "9607"  "7892"  "5657"  "10219" "5444" 
## IPO "9249"  "9268"  "7070"  "6998"  "9307"  "7580"  "7217"  "9895"  "7021" 
## KAT "6744"  "6719"  "13568" "13639" "6614"  "8303"  "13568" "6004"  "13858"
## NOV "8785"  "8800"  "8057"  "7988"  "8806"  "7135"  "8217"  "9358"  "8042" 
## BEH "9105"  "9120"  "7879"  "7808"  "9134"  "7446"  "8031"  "9694"  "7844" 
## GEL "7198"  "7174"  "13629" "13704" "7076"  "8779"  "13610" "6465"  "13899"
## BPA "9306"  "9321"  "8114"  "8043"  "9328"  "7655"  "8263"  "9880"  "8070" 
## KAN "9439"  "9427"  "10797" "10874" "9449"  "11041" "10674" "8943"  "10903"
## YUN "8189"  "8166"  "13664" "13743" "8083"  "9807"  "13605" "7472"  "13882"
## UTS "9599"  "9588"  "10595" "10671" "9617"  "11182" "10467" "9124"  "10691"
## HUN "8698"  "8678"  "12945" "13024" "8625"  "10361" "12858" "8027"  "13119"
## POR "10269" "10286" "7619"  "7543"  "10314" "8600"  "7742"  "10890" "7514" 
## BEN "7236"  "7208"  "15157" "15221" "7056"  "8546"  "15192" "6492"  "15478"
## KAG "9560"  "9545"  "11586" "11663" "9542"  "11208" "11464" "8998"  "11694"
## HAN "8788"  "8765"  "13866" "13945" "8684"  "10408" "13786" "8073"  "14052"
## LAM "8524"  "8499"  "14378" "14457" "8398"  "10091" "14318" "7788"  "14594"
## GRV "10425" "10441" "8217"  "8141"  "10450" "8769"  "8340"  "11005" "8110" 
## JAF "7684"  "7656"  "15555" "15622" "7502"  "8975"  "15580" "6941"  "15868"
## HAI "9208"  "9186"  "13887" "13966" "9108"  "10836" "13793" "8498"  "14048"
## OKI "9849"  "9832"  "12183" "12259" "9812"  "11514" "12056" "9245"  "12281"
## KAC "8859"  "8833"  "14830" "14909" "8723"  "10392" "14764" "8115"  "15036"
## TAI "9647"  "9628"  "12963" "13040" "9582"  "11315" "12846" "8990"  "13082"
## KUN "7469"  "7441"  "15846" "15903" "7271"  "8603"  "15912" "6753"  "16186"
## SSK "9138"  "9114"  "14535" "14614" "9016"  "10713" "14452" "8405"  "14714"
## CHA "9167"  "9142"  "14884" "14963" "9034"  "10707" "14805" "8425"  "15070"
## QNC "9614"  "9590"  "14442" "14520" "9499"  "11210" "14339" "8888"  "14586"
## CAM "9467"  "9442"  "14871" "14950" "9337"  "11019" "14779" "8727"  "15035"
## HOC "9662"  "9637"  "14868" "14947" "9535"  "11220" "14769" "8924"  "15018"
## SON "9478"  "9452"  "15505" "15584" "9330"  "10956" "15423" "8728"  "15685"
## MAT "9725"  "9699"  "15744" "15823" "9573"  "11181" "15654" "8974"  "15910"
## KLP "9885"  "9858"  "15872" "15951" "9731"  "11330" "15776" "9134"  "16028"
## SUF "11705" "11681" "15120" "15187" "11580" "13264" "14956" "10969" "15116"
## SUU "11923" "11900" "14808" "14874" "11808" "13511" "14639" "11196" "14788"
## INJ "11029" "11002" "16459" "16533" "10867" "12417" "16318" "10277" "16516"
## INW "12344" "12319" "15655" "15715" "12202" "13831" "15475" "11597" "15594"
##     ALD     NEG     GRA     TIK     RAR     SOC     GES     TUH     CHI    
## NEH "0.11"  "0.25"  "0.09"  "0.13"  "0.13"  "0.12"  "0.12"  "0.08"  "0.12" 
## NEW "0.12"  "0.25"  "0.10"  "0.14"  "0.13"  "0.13"  "0.13"  "0.09"  "0.13" 
## FIR "0.16"  "0.29"  "0.13"  "0.17"  "0.16"  "0.16"  "0.16"  "0.12"  "0.16" 
## NEB "0.12"  "0.26"  "0.10"  "0.14"  "0.13"  "0.13"  "0.13"  "0.08"  "0.12" 
## BER "0.12"  "0.25"  "0.10"  "0.14"  "0.13"  "0.12"  "0.13"  "0.08"  "0.12" 
## HAR "0.12"  "0.25"  "0.10"  "0.14"  "0.14"  "0.13"  "0.13"  "0.08"  "0.13" 
## LIN "0.11"  "0.26"  "0.11"  "0.15"  "0.14"  "0.13"  "0.14"  "0.09"  "0.13" 
## MAN "0.11"  "0.24"  "0.09"  "0.13"  "0.12"  "0.11"  "0.12"  "0.07"  "0.11" 
## WAV "0.13"  "0.27"  "0.11"  "0.15"  "0.14"  "0.13"  "0.13"  "0.09"  "0.13" 
## COL "0.12"  "0.25"  "0.10"  "0.14"  "0.13"  "0.12"  "0.12"  "0.08"  "0.12" 
## FAY "0.11"  "0.23"  "0.08"  "0.13"  "0.12"  "0.11"  "0.12"  "0.07"  "0.10" 
## PEO "0.11"  "0.23"  "0.08"  "0.13"  "0.12"  "0.11"  "0.12"  "0.07"  "0.11" 
## STL "0.13"  "0.27"  "0.11"  "0.15"  "0.14"  "0.13"  "0.14"  "0.09"  "0.14" 
## HAL "0.11"  "0.24"  "0.08"  "0.13"  "0.12"  "0.11"  "0.11"  "0.07"  "0.11" 
## BEA "0.10"  "0.20"  "0.06"  "0.12"  "0.11"  "0.10"  "0.11"  "0.06"  "0.09" 
## MAC "0.11"  "0.21"  "0.07"  "0.13"  "0.12"  "0.12"  "0.12"  "0.07"  "0.09" 
## BRM "0.15"  "0.26"  "0.11"  "0.16"  "0.16"  "0.15"  "0.15"  "0.11"  "0.14" 
## SPR "0.13"  "0.29"  "0.12"  "0.16"  "0.15"  "0.14"  "0.15"  "0.10"  "0.15" 
## RUS "0.14"  "0.26"  "0.12"  "0.16"  "0.15"  "0.15"  "0.15"  "0.11"  "0.14" 
## HAT "0.12"  "0.24"  "0.09"  "0.14"  "0.13"  "0.12"  "0.12"  "0.08"  "0.12" 
## NEO "0.12"  "0.24"  "0.09"  "0.14"  "0.13"  "0.12"  "0.13"  "0.08"  "0.12" 
## SMI "0.12"  "0.24"  "0.09"  "0.14"  "0.13"  "0.13"  "0.13"  "0.09"  "0.12" 
## PAL "0.12"  "0.22"  "0.08"  "0.14"  "0.13"  "0.13"  "0.13"  "0.09"  "0.11" 
## DEN "0.11"  "0.24"  "0.09"  "0.13"  "0.13"  "0.12"  "0.12"  "0.07"  "0.12" 
## DAL "0.12"  "0.24"  "0.09"  "0.14"  "0.13"  "0.12"  "0.13"  "0.08"  "0.11" 
## FRS "0.09"  "0.23"  "0.07"  "0.12"  "0.11"  "0.10"  "0.11"  "0.05"  "0.10" 
## SPB "0.11"  "0.26"  "0.09"  "0.13"  "0.13"  "0.12"  "0.12"  "0.07"  "0.12" 
## BRE "0.17"  "0.31"  "0.15"  "0.19"  "0.18"  "0.17"  "0.18"  "0.13"  "0.18" 
## DES "0.15"  "0.29"  "0.14"  "0.18"  "0.17"  "0.16"  "0.17"  "0.12"  "0.16" 
## MON "0.11"  "0.21"  "0.07"  "0.13"  "0.12"  "0.11"  "0.12"  "0.07"  "0.09" 
## HOU "0.10"  "0.20"  "0.06"  "0.11"  "0.11"  "0.10"  "0.11"  "0.06"  "0.08" 
## CES "0.20"  "0.34"  "0.18"  "0.22"  "0.21"  "0.21"  "0.21"  "0.16"  "0.21" 
## SPS "0.14"  "0.28"  "0.10"  "0.17"  "0.16"  "0.15"  "0.16"  "0.09"  "0.14" 
## TRA "0.10"  "0.21"  "0.07"  "0.12"  "0.12"  "0.11"  "0.11"  "0.07"  "0.10" 
## SER "0.22"  "0.41"  "0.18"  "0.25"  "0.23"  "0.22"  "0.23"  "0.17"  "0.23" 
## NUE "0.11"  "0.21"  "0.07"  "0.13"  "0.12"  "0.12"  "0.12"  "0.08"  "0.10" 
## ALD NA      "0.26"  "0.08"  "0.16"  "0.15"  "0.14"  "0.14"  "0.09"  "0.13" 
## NEG "9319"  NA      "0.18"  "0.28"  "0.26"  "0.26"  "0.27"  "0.22"  "0.23" 
## GRA "534"   "9807"  NA      "0.12"  "0.11"  "0.11"  "0.11"  "0.06"  "0.07" 
## TIK "1733"  "10596" "1600"  NA      "0.05"  "0.06"  "0.05"  "0.12"  "0.15" 
## RAR "1798"  "10716" "1629"  "127"   NA      "0.02"  "0.03"  "0.11"  "0.14" 
## SOC "1678"  "10707" "1470"  "253"   "187"   NA      "0.01"  "0.11"  "0.13" 
## GES "1770"  "10816" "1540"  "315"   "210"   "109"   NA      "0.11"  "0.14" 
## TUH "1828"  "10958" "1544"  "508"   "398"   "283"   "194"   NA      "0.09" 
## CHI "10579" "4271"  "11100" "10871" "10990" "11099" "11183" "11376" NA     
## LOS "10601" "4321"  "11121" "10880" "10999" "11109" "11193" "11386" "51"   
## SAI "8521"  "2031"  "8925"  "10089" "10192" "10126" "10231" "10326" "6287" 
## CUA "9982"  "1372"  "10419" "11447" "11559" "11517" "11625" "11741" "5318" 
## MED "9982"  "1372"  "10419" "11447" "11559" "11517" "11625" "11741" "5318" 
## TUC "8577"  "3983"  "8849"  "10298" "10372" "10255" "10345" "10383" "8251" 
## FLO "8299"  "4778"  "8513"  "10033" "10094" "9960"  "10042" "10058" "9049" 
## BIT "9132"  "3508"  "9433"  "10833" "10915" "10808" "10902" "10953" "7736" 
## MAU "9376"  "3112"  "9703"  "11052" "11140" "11044" "11141" "11203" "7306" 
## ARA "8734"  "4358"  "8976"  "10464" "10532" "10406" "10492" "10518" "8615" 
## REC "7801"  "5602"  "7954"  "9522"  "9570"  "9422"  "9495"  "9488"  "9864" 
## PLM "8984"  "4578"  "9207"  "10717" "10781" "10650" "10733" "10752" "8813" 
## BCO "9266"  "4363"  "9505"  "10996" "11064" "10937" "11022" "11047" "8564" 
## BAF "9565"  "3974"  "9833"  "11285" "11360" "11242" "11332" "11369" "8125" 
## BPV "10111" "3398"  "10425" "11798" "11884" "11784" "11879" "11937" "7400" 
## IPO "9686"  "4882"  "9886"  "11418" "11478" "11340" "11420" "11429" "9008" 
## KAT "6077"  "14624" "5750"  "4431"  "4335"  "4419"  "4319"  "4249"  "12750"
## NOV "9089"  "5845"  "9211"  "10796" "10837" "10682" "10749" "10729" "10062"
## BEH "9436"  "5675"  "9572"  "11150" "11195" "11042" "11112" "11096" "9845" 
## GEL "6556"  "14870" "6244"  "4886"  "4795"  "4888"  "4791"  "4730"  "12636"
## BPA "9609"  "5918"  "9726"  "11313" "11353" "11196" "11262" "11239" "10059"
## KAN "9266"  "12911" "9241"  "7644"  "7627"  "7801"  "7753"  "7813"  "9036" 
## YUN "7598"  "15279" "7315"  "5894"  "5812"  "5921"  "5828"  "5783"  "12329"
## UTS "9458"  "12731" "9450"  "7856"  "7844"  "8019"  "7974"  "8040"  "8800" 
## HUN "8217"  "14812" "8005"  "6485"  "6421"  "6554"  "6473"  "6461"  "11415"
## POR "10653" "5520"  "10812" "12378" "12428" "12280" "12353" "12344" "9403" 
## BEN "6417"  "15670" "5966"  "5075"  "4954"  "4965"  "4856"  "4718"  "14562"
## KAG "9281"  "13690" "9189"  "7594"  "7561"  "7723"  "7663"  "7698"  "9821" 
## HAN "8199"  "15639" "7913"  "6496"  "6414"  "6522"  "6429"  "6383"  "12368"
## LAM "7866"  "15961" "7533"  "6211"  "6118"  "6206"  "6106"  "6038"  "13006"
## GRV "10734" "6116"  "10845" "12435" "12473" "12315" "12379" "12351" "9987" 
## JAF "6860"  "16124" "6403"  "5529"  "5408"  "5418"  "5309"  "5170"  "14828"
## HAI "8631"  "15777" "8350"  "6922"  "6843"  "6953"  "6861"  "6818"  "12279"
## OKI "9496"  "14301" "9357"  "7780"  "7735"  "7887"  "7817"  "7834"  "10379"
## KAC "8168"  "16435" "7810"  "6547"  "6449"  "6526"  "6424"  "6343"  "13394"
## TAI "9192"  "15020" "8988"  "7459"  "7398"  "7534"  "7454"  "7445"  "11218"
## KUN "6606"  "15915" "6111"  "5481"  "5355"  "5330"  "5223"  "5060"  "15479"
## SSK "8488"  "16309" "8158"  "6827"  "6735"  "6826"  "6727"  "6660"  "12986"
## CHA "8483"  "16600" "8128"  "6855"  "6757"  "6837"  "6736"  "6657"  "13354"
## QNC "8988"  "16374" "8672"  "7308"  "7220"  "7318"  "7222"  "7162"  "12760"
## CAM "8793"  "16698" "8445"  "7154"  "7058"  "7142"  "7041"  "6966"  "13253"
## HOC "8995"  "16759" "8649"  "7349"  "7255"  "7340"  "7240"  "7167"  "13196"
## SON "8747"  "17200" "8356"  "7180"  "7076"  "7139"  "7034"  "6938"  "13929"
## MAT "8981"  "17487" "8580"  "7434"  "7328"  "7386"  "7281"  "7180"  "14109"
## KLP "9135"  "17654" "8729"  "7598"  "7491"  "7547"  "7441"  "7339"  "14196"
## SUF "11039" "17333" "10683" "9393"  "9299"  "9386"  "9286"  "9211"  "13080"
## SUU "11286" "17016" "10949" "9616"  "9527"  "9621"  "9524"  "9458"  "12750"
## INJ "10253" "18582" "9825"  "8760"  "8650"  "8698"  "8591"  "8480"  "14514"
## INW "11624" "17813" "11230" "10037" "9936"  "10007" "9903"  "9812"  "13562"
##     LOS     SAI     CUA     MED     TUC     FLO     BIT     MAU     ARA    
## NEH "0.10"  "0.09"  "0.14"  "0.14"  "0.15"  "0.15"  "0.15"  "0.13"  "0.14" 
## NEW "0.12"  "0.10"  "0.15"  "0.15"  "0.15"  "0.16"  "0.15"  "0.13"  "0.15" 
## FIR "0.15"  "0.13"  "0.18"  "0.18"  "0.18"  "0.19"  "0.18"  "0.16"  "0.17" 
## NEB "0.11"  "0.10"  "0.15"  "0.15"  "0.16"  "0.16"  "0.15"  "0.13"  "0.15" 
## BER "0.12"  "0.09"  "0.15"  "0.14"  "0.15"  "0.16"  "0.15"  "0.12"  "0.14" 
## HAR "0.12"  "0.10"  "0.15"  "0.15"  "0.16"  "0.16"  "0.15"  "0.13"  "0.15" 
## LIN "0.12"  "0.11"  "0.16"  "0.16"  "0.17"  "0.17"  "0.16"  "0.14"  "0.16" 
## MAN "0.10"  "0.08"  "0.14"  "0.13"  "0.14"  "0.15"  "0.14"  "0.12"  "0.14" 
## WAV "0.13"  "0.10"  "0.16"  "0.15"  "0.16"  "0.16"  "0.16"  "0.13"  "0.15" 
## COL "0.11"  "0.10"  "0.15"  "0.14"  "0.15"  "0.16"  "0.15"  "0.13"  "0.15" 
## FAY "0.10"  "0.07"  "0.13"  "0.12"  "0.13"  "0.13"  "0.13"  "0.11"  "0.12" 
## PEO "0.10"  "0.08"  "0.13"  "0.13"  "0.14"  "0.14"  "0.14"  "0.12"  "0.13" 
## STL "0.13"  "0.11"  "0.16"  "0.16"  "0.17"  "0.17"  "0.16"  "0.14"  "0.16" 
## HAL "0.10"  "0.09"  "0.14"  "0.13"  "0.14"  "0.14"  "0.14"  "0.12"  "0.13" 
## BEA "0.09"  "0.06"  "0.11"  "0.11"  "0.12"  "0.12"  "0.12"  "0.10"  "0.11" 
## MAC "0.10"  "0.06"  "0.12"  "0.11"  "0.12"  "0.13"  "0.12"  "0.10"  "0.12" 
## BRM "0.14"  "0.12"  "0.16"  "0.16"  "0.17"  "0.18"  "0.17"  "0.15"  "0.16" 
## SPR "0.14"  "0.12"  "0.17"  "0.17"  "0.17"  "0.18"  "0.17"  "0.15"  "0.17" 
## RUS "0.14"  "0.12"  "0.17"  "0.16"  "0.17"  "0.18"  "0.17"  "0.15"  "0.16" 
## HAT "0.11"  "0.09"  "0.14"  "0.14"  "0.14"  "0.15"  "0.14"  "0.12"  "0.13" 
## NEO "0.11"  "0.08"  "0.14"  "0.13"  "0.14"  "0.15"  "0.14"  "0.12"  "0.13" 
## SMI "0.11"  "0.09"  "0.14"  "0.14"  "0.13"  "0.15"  "0.13"  "0.13"  "0.13" 
## PAL "0.11"  "0.06"  "0.13"  "0.12"  "0.13"  "0.13"  "0.13"  "0.11"  "0.13" 
## DEN "0.11"  "0.09"  "0.14"  "0.14"  "0.14"  "0.15"  "0.14"  "0.12"  "0.14" 
## DAL "0.11"  "0.09"  "0.13"  "0.13"  "0.13"  "0.14"  "0.13"  "0.12"  "0.13" 
## FRS "0.09"  "0.09"  "0.14"  "0.13"  "0.14"  "0.15"  "0.14"  "0.12"  "0.14" 
## SPB "0.11"  "0.10"  "0.15"  "0.15"  "0.15"  "0.16"  "0.15"  "0.13"  "0.15" 
## BRE "0.17"  "0.14"  "0.20"  "0.20"  "0.20"  "0.21"  "0.20"  "0.18"  "0.19" 
## DES "0.15"  "0.14"  "0.19"  "0.19"  "0.19"  "0.19"  "0.19"  "0.17"  "0.18" 
## MON "0.10"  "0.07"  "0.12"  "0.11"  "0.12"  "0.13"  "0.11"  "0.11"  "0.11" 
## HOU "0.09"  "0.06"  "0.11"  "0.10"  "0.11"  "0.12"  "0.11"  "0.09"  "0.11" 
## CES "0.20"  "0.18"  "0.24"  "0.23"  "0.23"  "0.24"  "0.23"  "0.21"  "0.23" 
## SPS "0.13"  "0.14"  "0.18"  "0.18"  "0.19"  "0.19"  "0.19"  "0.17"  "0.18" 
## TRA "0.09"  "0.07"  "0.12"  "0.11"  "0.12"  "0.13"  "0.12"  "0.10"  "0.12" 
## SER "0.21"  "0.20"  "0.26"  "0.25"  "0.25"  "0.27"  "0.26"  "0.24"  "0.25" 
## NUE "0.10"  "0.07"  "0.12"  "0.12"  "0.12"  "0.13"  "0.11"  "0.11"  "0.11" 
## ALD "0.12"  "0.13"  "0.17"  "0.16"  "0.17"  "0.18"  "0.18"  "0.16"  "0.17" 
## NEG "0.22"  "0.20"  "0.22"  "0.22"  "0.20"  "0.22"  "0.22"  "0.20"  "0.20" 
## GRA "0.08"  "0.07"  "0.10"  "0.09"  "0.11"  "0.12"  "0.12"  "0.10"  "0.11" 
## TIK "0.14"  "0.15"  "0.19"  "0.18"  "0.19"  "0.20"  "0.19"  "0.18"  "0.19" 
## RAR "0.13"  "0.14"  "0.17"  "0.17"  "0.18"  "0.19"  "0.18"  "0.17"  "0.18" 
## SOC "0.13"  "0.13"  "0.17"  "0.16"  "0.18"  "0.19"  "0.18"  "0.16"  "0.17" 
## GES "0.13"  "0.14"  "0.17"  "0.17"  "0.18"  "0.19"  "0.18"  "0.17"  "0.18" 
## TUH "0.08"  "0.09"  "0.13"  "0.13"  "0.14"  "0.15"  "0.14"  "0.13"  "0.14" 
## CHI "0.10"  "0.10"  "0.13"  "0.12"  "0.14"  "0.15"  "0.15"  "0.13"  "0.14" 
## LOS NA      "0.11"  "0.14"  "0.13"  "0.15"  "0.15"  "0.15"  "0.13"  "0.14" 
## SAI "6338"  NA      "0.12"  "0.11"  "0.12"  "0.13"  "0.12"  "0.11"  "0.12" 
## CUA "5366"  "1614"  NA      "0.00"  "0.12"  "0.13"  "0.13"  "0.11"  "0.12" 
## MED "5366"  "1614"  "0"     NA      "0.12"  "0.13"  "0.13"  "0.12"  "0.12" 
## TUC "8301"  "2021"  "3084"  "3084"  NA      "0.02"  "0.04"  "0.06"  "0.01" 
## FLO "9099"  "2782"  "3896"  "3896"  "814"   NA      "0.06"  "0.06"  "0.02" 
## BIT "7785"  "1717"  "2471"  "2471"  "705"   "1465"  NA      "0.05"  "0.04" 
## MAU "7355"  "1484"  "2016"  "2016"  "1153"  "1931"  "467"   NA      "0.06" 
## ARA "8666"  "2427"  "3387"  "3387"  "422"   "575"   "922"   "1388"  NA     
## REC "9915"  "3577"  "4790"  "4790"  "1708"  "909"   "2374"  "2840"  "1474" 
## PLM "8862"  "2698"  "3535"  "3535"  "745"   "701"   "1077"  "1519"  "341"  
## BCO "8613"  "2564"  "3261"  "3261"  "802"   "1032"  "864"   "1260"  "532"  
## BAF "8173"  "2308"  "2808"  "2808"  "988"   "1480"  "624"   "871"   "917"  
## BPV "7447"  "2119"  "2111"  "2111"  "1669"  "2312"  "1004"  "758"   "1737" 
## IPO "9056"  "3173"  "3692"  "3692"  "1429"  "1391"  "1457"  "1778"  "1078" 
## KAT "12726" "14506" "15734" "15734" "14570" "14125" "15180" "15450" "14643"
## NOV "10111" "3960"  "4756"  "4756"  "1953"  "1358"  "2337"  "2756"  "1536" 
## BEH "9893"  "3861"  "4527"  "4527"  "1912"  "1466"  "2180"  "2564"  "1491" 
## GEL "12609" "14921" "16057" "16057" "15078" "14644" "15677" "15931" "15159"
## BPA "10106" "4132"  "4743"  "4743"  "2196"  "1745"  "2438"  "2806"  "1776" 
## KAN "8993"  "14501" "14208" "14208" "16321" "16722" "16216" "15937" "16731"
## YUN "12294" "15753" "16601" "16601" "16164" "15760" "16728" "16929" "16267"
## UTS "8757"  "14377" "14009" "14009" "16250" "16710" "16093" "15789" "16667"
## HUN "11376" "15770" "16183" "16183" "16729" "16515" "17135" "17174" "16940"
## POR "9447"  "4050"  "4210"  "4210"  "2495"  "2428"  "2380"  "2572"  "2158" 
## BEN "14541" "14831" "16379" "16379" "14149" "13501" "14847" "15247" "14073"
## KAG "9778"  "15219" "14996" "14996" "16935" "17199" "16923" "16680" "17322"
## HAN "12330" "16298" "16997" "16997" "16760" "16330" "17329" "17523" "16850"
## LAM "12970" "16241" "17252" "17252" "16307" "15778" "16946" "17237" "16327"
## GRV "10030" "4603"  "4808"  "4808"  "2925"  "2709"  "2906"  "3137"  "2549" 
## JAF "14802" "15235" "16803" "16803" "14441" "13760" "15144" "15562" "14335"
## HAI "12238" "16625" "17148" "17148" "17199" "16769" "17754" "17919" "17291"
## OKI "10335" "15831" "15596" "15596" "17485" "17643" "17527" "17296" "17850"
## KAC "13356" "16624" "17717" "17717" "16466" "15864" "17140" "17483" "16431"
## TAI "11174" "16341" "16356" "16356" "17628" "17491" "17912" "17821" "17885"
## KUN "15460" "14690" "16302" "16302" "13675" "12961" "14380" "14818" "13536"
## SSK "12946" "16816" "17659" "17659" "16916" "16354" "17566" "17861" "16914"
## CHA "13314" "16918" "17923" "17923" "16776" "16159" "17455" "17803" "16729"
## QNC "12718" "17180" "17746" "17746" "17461" "16901" "18099" "18360" "17462"
## CAM "13211" "17189" "18055" "18055" "17104" "16477" "17784" "18130" "17049"
## HOC "13154" "17362" "18128" "18128" "17310" "16674" "17992" "18338" "17247"
## SON "13888" "17264" "18481" "18481" "16720" "16018" "17424" "17842" "16593"
## MAT "14066" "17502" "18772" "18772" "16798" "16063" "17502" "17942" "16635"
## KLP "14153" "17653" "18949" "18949" "16854" "16103" "17555" "18006" "16671"
## SUF "13030" "19109" "18244" "18244" "18728" "17915" "19215" "19477" "18379"
## SUU "12699" "18932" "17866" "17866" "19074" "18269" "19394" "19401" "18695"
## INJ "14466" "18676" "19770" "19770" "17204" "16391" "17802" "18249" "16891"
## INW "13511" "19840" "18251" "18251" "18179" "17432" "18433" "18618" "17768"
##     REC     PLM     BCO     BAF     BPV     IPO     KAT     NOV     BEH    
## NEH "0.11"  "0.15"  "0.15"  "0.14"  "0.15"  "0.23"  "0.17"  "0.21"  "0.18" 
## NEW "0.11"  "0.16"  "0.16"  "0.15"  "0.16"  "0.23"  "0.18"  "0.21"  "0.19" 
## FIR "0.14"  "0.19"  "0.18"  "0.18"  "0.18"  "0.26"  "0.22"  "0.25"  "0.22" 
## NEB "0.11"  "0.16"  "0.16"  "0.15"  "0.16"  "0.24"  "0.18"  "0.22"  "0.19" 
## BER "0.10"  "0.16"  "0.15"  "0.15"  "0.15"  "0.23"  "0.18"  "0.21"  "0.18" 
## HAR "0.11"  "0.16"  "0.16"  "0.15"  "0.16"  "0.23"  "0.18"  "0.22"  "0.19" 
## LIN "0.12"  "0.17"  "0.17"  "0.16"  "0.16"  "0.24"  "0.19"  "0.23"  "0.20" 
## MAN "0.10"  "0.15"  "0.14"  "0.14"  "0.14"  "0.22"  "0.17"  "0.20"  "0.18" 
## WAV "0.11"  "0.17"  "0.16"  "0.15"  "0.16"  "0.25"  "0.19"  "0.23"  "0.20" 
## COL "0.11"  "0.16"  "0.15"  "0.15"  "0.15"  "0.23"  "0.18"  "0.22"  "0.19" 
## FAY "0.09"  "0.14"  "0.13"  "0.12"  "0.13"  "0.21"  "0.15"  "0.19"  "0.16" 
## PEO "0.09"  "0.14"  "0.14"  "0.13"  "0.14"  "0.22"  "0.16"  "0.20"  "0.17" 
## STL "0.12"  "0.17"  "0.17"  "0.16"  "0.16"  "0.25"  "0.20"  "0.23"  "0.20" 
## HAL "0.10"  "0.15"  "0.14"  "0.14"  "0.14"  "0.22"  "0.17"  "0.20"  "0.17" 
## BEA "0.07"  "0.12"  "0.12"  "0.11"  "0.12"  "0.19"  "0.13"  "0.17"  "0.14" 
## MAC "0.08"  "0.12"  "0.12"  "0.12"  "0.12"  "0.20"  "0.14"  "0.17"  "0.15" 
## BRM "0.14"  "0.18"  "0.17"  "0.17"  "0.17"  "0.24"  "0.19"  "0.23"  "0.20" 
## SPR "0.13"  "0.18"  "0.18"  "0.18"  "0.17"  "0.26"  "0.21"  "0.25"  "0.21" 
## RUS "0.13"  "0.18"  "0.17"  "0.17"  "0.17"  "0.25"  "0.19"  "0.23"  "0.20" 
## HAT "0.10"  "0.15"  "0.14"  "0.14"  "0.14"  "0.22"  "0.17"  "0.20"  "0.18" 
## NEO "0.10"  "0.15"  "0.15"  "0.14"  "0.14"  "0.22"  "0.17"  "0.20"  "0.18" 
## SMI "0.09"  "0.15"  "0.14"  "0.14"  "0.14"  "0.22"  "0.17"  "0.21"  "0.18" 
## PAL "0.09"  "0.13"  "0.13"  "0.12"  "0.13"  "0.20"  "0.14"  "0.18"  "0.16" 
## DEN "0.10"  "0.15"  "0.14"  "0.14"  "0.14"  "0.22"  "0.17"  "0.20"  "0.18" 
## DAL "0.09"  "0.14"  "0.14"  "0.13"  "0.14"  "0.22"  "0.17"  "0.20"  "0.17" 
## FRS "0.11"  "0.15"  "0.15"  "0.14"  "0.14"  "0.21"  "0.15"  "0.19"  "0.17" 
## SPB "0.11"  "0.16"  "0.16"  "0.15"  "0.15"  "0.24"  "0.18"  "0.22"  "0.19" 
## BRE "0.15"  "0.21"  "0.20"  "0.20"  "0.20"  "0.28"  "0.24"  "0.27"  "0.24" 
## DES "0.15"  "0.20"  "0.19"  "0.19"  "0.19"  "0.27"  "0.22"  "0.26"  "0.22" 
## MON "0.08"  "0.13"  "0.13"  "0.12"  "0.12"  "0.20"  "0.14"  "0.18"  "0.15" 
## HOU "0.07"  "0.12"  "0.12"  "0.11"  "0.11"  "0.18"  "0.13"  "0.16"  "0.14" 
## CES "0.19"  "0.24"  "0.23"  "0.24"  "0.23"  "0.31"  "0.27"  "0.31"  "0.27" 
## SPS "0.16"  "0.20"  "0.19"  "0.19"  "0.19"  "0.26"  "0.20"  "0.24"  "0.21" 
## TRA "0.08"  "0.13"  "0.12"  "0.12"  "0.12"  "0.20"  "0.14"  "0.18"  "0.15" 
## SER "0.23"  "0.27"  "0.26"  "0.27"  "0.26"  "0.35"  "0.28"  "0.35"  "0.29" 
## NUE "0.09"  "0.13"  "0.13"  "0.12"  "0.12"  "0.20"  "0.14"  "0.18"  "0.15" 
## ALD "0.15"  "0.18"  "0.18"  "0.17"  "0.17"  "0.24"  "0.18"  "0.22"  "0.20" 
## NEG "0.22"  "0.22"  "0.21"  "0.22"  "0.21"  "0.26"  "0.16"  "0.25"  "0.22" 
## GRA "0.10"  "0.11"  "0.12"  "0.11"  "0.11"  "0.16"  "0.09"  "0.13"  "0.13" 
## TIK "0.17"  "0.20"  "0.20"  "0.19"  "0.19"  "0.26"  "0.20"  "0.24"  "0.22" 
## RAR "0.16"  "0.19"  "0.18"  "0.18"  "0.18"  "0.24"  "0.19"  "0.23"  "0.20" 
## SOC "0.15"  "0.19"  "0.18"  "0.18"  "0.18"  "0.24"  "0.18"  "0.22"  "0.20" 
## GES "0.16"  "0.19"  "0.19"  "0.18"  "0.18"  "0.25"  "0.19"  "0.23"  "0.20" 
## TUH "0.11"  "0.15"  "0.15"  "0.14"  "0.14"  "0.21"  "0.15"  "0.18"  "0.17" 
## CHI "0.13"  "0.15"  "0.15"  "0.14"  "0.14"  "0.20"  "0.13"  "0.17"  "0.16" 
## LOS "0.13"  "0.15"  "0.15"  "0.15"  "0.15"  "0.21"  "0.14"  "0.18"  "0.17" 
## SAI "0.10"  "0.13"  "0.13"  "0.12"  "0.12"  "0.18"  "0.12"  "0.16"  "0.14" 
## CUA "0.13"  "0.13"  "0.13"  "0.13"  "0.13"  "0.17"  "0.13"  "0.15"  "0.13" 
## MED "0.13"  "0.13"  "0.13"  "0.12"  "0.13"  "0.17"  "0.11"  "0.14"  "0.13" 
## TUC "0.07"  "0.02"  "0.02"  "0.01"  "0.06"  "0.07"  "0.11"  "0.07"  "0.05" 
## FLO "0.08"  "0.02"  "0.03"  "0.01"  "0.06"  "0.08"  "0.13"  "0.08"  "0.06" 
## BIT "0.09"  "0.06"  "0.06"  "0.05"  "0.05"  "0.09"  "0.13"  "0.09"  "0.07" 
## MAU "0.08"  "0.06"  "0.07"  "0.05"  "0.04"  "0.09"  "0.12"  "0.08"  "0.07" 
## ARA "0.07"  "0.02"  "0.02"  "0.01"  "0.06"  "0.07"  "0.12"  "0.07"  "0.05" 
## REC NA      "0.08"  "0.08"  "0.06"  "0.07"  "0.14"  "0.13"  "0.13"  "0.10" 
## PLM "1498"  NA      "0.02"  "0.01"  "0.06"  "0.07"  "0.12"  "0.06"  "0.05" 
## BCO "1853"  "355"   NA      "0.02"  "0.07"  "0.09"  "0.13"  "0.08"  "0.06" 
## BAF "2340"  "852"   "505"   NA      "0.05"  "0.06"  "0.11"  "0.06"  "0.05" 
## BPV "3195"  "1717"  "1372"  "866"   NA      "0.09"  "0.12"  "0.08"  "0.06" 
## IPO "1997"  "753"   "646"   "909"   "1631"  NA      "0.14"  "0.04"  "0.06" 
## KAT "13404" "14824" "15154" "15539" "16175" "15399" NA      "0.11"  "0.11" 
## NOV "1327"  "1271"  "1497"  "1956"  "2769"  "1166"  "14438" NA      "0.04" 
## BEH "1641"  "1176"  "1316"  "1721"  "2485"  "854"   "14831" "394"   NA     
## GEL "13923" "15343" "15671" "16051" "16666" "15918" "520"   "14947" "15340"
## BPA "1844"  "1457"  "1574"  "1948"  "2671"  "1051"  "14871" "522"   "288"  
## KAN "16751" "17066" "17038" "16800" "16306" "17670" "4880"  "18029" "18184"
## YUN "15043" "16460" "16785" "17146" "17684" "17036" "1640"  "16038" "16431"
## UTS "16821" "16995" "16933" "16659" "16116" "17549" "5167"  "18051" "18149"
## HUN "15917" "17201" "17472" "17691" "17885" "17889" "2611"  "17038" "17430"
## POR "2858"  "1831"  "1694"  "1758"  "2122"  "1080"  "16059" "1650"  "1263" 
## BEN "12645" "14143" "14498" "14978" "15804" "14516" "1828"  "13396" "13774"
## KAG "17017" "17663" "17700" "17527" "17087" "18346" "4367"  "18343" "18629"
## HAN "15582" "17027" "17360" "17739" "18279" "17560" "2207"  "16501" "16887"
## LAM "14977" "16458" "16807" "17243" "17949" "16912" "1789"  "15812" "16192"
## GRV "2959"  "2209"  "2148"  "2296"  "2718"  "1502"  "15795" "1647"  "1323" 
## JAF "12886" "14381" "14735" "15226" "16072" "14699" "2085"  "13556" "13924"
## HAI "16010" "17464" "17800" "18180" "18675" "17973" "2648"  "16882" "17261"
## OKI "17297" "18187" "18272" "18138" "17697" "18914" "4204"  "18560" "18929"
## KAC "15020" "16517" "16872" "17344" "18135" "16877" "2119"  "15734" "16099"
## TAI "16900" "18167" "18408" "18524" "18388" "18873" "3537"  "17966" "18359"
## KUN "12072" "13557" "13909" "14407" "15266" "13835" "2779"  "12685" "13047"
## SSK "15526" "17021" "17373" "17831" "18569" "17404" "2412"  "16264" "16629"
## CHA "15304" "16802" "17157" "17638" "18445" "17123" "2424"  "15969" "16324"
## QNC "16066" "17563" "17916" "18379" "19097" "17905" "2922"  "16748" "17099"
## CAM "15615" "17112" "17466" "17953" "18772" "17396" "2724"  "16234" "16579"
## HOC "15806" "17300" "17654" "18146" "18976" "17554" "2921"  "16389" "16725"
## SON "15126" "16603" "16951" "17454" "18319" "16793" "2794"  "15628" "15957"
## MAT "15161" "16614" "16954" "17460" "18324" "16738" "3069"  "15578" "15890"
## KLP "15197" "16632" "16966" "17469" "18325" "16714" "3242"  "15561" "15862"
## SUF "17034" "18162" "18351" "18653" "18775" "17769" "4967"  "16892" "17036"
## SUU "17402" "18441" "18575" "18773" "18644" "17953" "5209"  "17174" "17281"
## INJ "15501" "16731" "16995" "17433" "18084" "16538" "4429"  "15497" "15712"
## INW "16649" "17486" "17601" "17827" "17941" "16981" "5611"  "16232" "16317"
##     GEL     BPA     KAN     YUN     UTS     HUN     POR     BEN     KAG    
## NEH "0.15"  "0.11"  "0.11"  "0.16"  "0.06"  "0.06"  "0.17"  "0.18"  "0.07" 
## NEW "0.17"  "0.12"  "0.12"  "0.17"  "0.08"  "0.08"  "0.18"  "0.19"  "0.09" 
## FIR "0.21"  "0.14"  "0.15"  "0.20"  "0.11"  "0.11"  "0.21"  "0.22"  "0.12" 
## NEB "0.17"  "0.12"  "0.11"  "0.17"  "0.07"  "0.07"  "0.18"  "0.19"  "0.08" 
## BER "0.16"  "0.12"  "0.11"  "0.17"  "0.07"  "0.07"  "0.18"  "0.19"  "0.08" 
## HAR "0.17"  "0.12"  "0.11"  "0.17"  "0.07"  "0.08"  "0.18"  "0.19"  "0.09" 
## LIN "0.18"  "0.13"  "0.10"  "0.18"  "0.07"  "0.08"  "0.19"  "0.20"  "0.09" 
## MAN "0.15"  "0.10"  "0.10"  "0.16"  "0.06"  "0.06"  "0.17"  "0.18"  "0.07" 
## WAV "0.17"  "0.12"  "0.12"  "0.18"  "0.07"  "0.08"  "0.19"  "0.20"  "0.09" 
## COL "0.17"  "0.12"  "0.10"  "0.17"  "0.07"  "0.07"  "0.18"  "0.19"  "0.08" 
## FAY "0.14"  "0.09"  "0.11"  "0.15"  "0.07"  "0.06"  "0.16"  "0.17"  "0.08" 
## PEO "0.14"  "0.10"  "0.10"  "0.15"  "0.06"  "0.06"  "0.16"  "0.17"  "0.07" 
## STL "0.18"  "0.13"  "0.12"  "0.19"  "0.07"  "0.09"  "0.19"  "0.20"  "0.09" 
## HAL "0.15"  "0.11"  "0.09"  "0.16"  "0.05"  "0.06"  "0.17"  "0.18"  "0.06" 
## BEA "0.11"  "0.08"  "0.11"  "0.12"  "0.06"  "0.05"  "0.14"  "0.14"  "0.08" 
## MAC "0.12"  "0.09"  "0.12"  "0.13"  "0.08"  "0.06"  "0.15"  "0.15"  "0.09" 
## BRM "0.18"  "0.14"  "0.16"  "0.17"  "0.13"  "0.10"  "0.19"  "0.19"  "0.13" 
## SPR "0.20"  "0.14"  "0.13"  "0.19"  "0.06"  "0.10"  "0.20"  "0.21"  "0.11" 
## RUS "0.18"  "0.13"  "0.14"  "0.18"  "0.11"  "0.09"  "0.19"  "0.19"  "0.11" 
## HAT "0.15"  "0.11"  "0.12"  "0.15"  "0.07"  "0.07"  "0.17"  "0.17"  "0.08" 
## NEO "0.15"  "0.10"  "0.12"  "0.16"  "0.08"  "0.07"  "0.17"  "0.18"  "0.09" 
## SMI "0.15"  "0.10"  "0.12"  "0.16"  "0.08"  "0.07"  "0.17"  "0.18"  "0.09" 
## PAL "0.12"  "0.10"  "0.14"  "0.13"  "0.10"  "0.07"  "0.15"  "0.15"  "0.10" 
## DEN "0.15"  "0.10"  "0.11"  "0.16"  "0.06"  "0.07"  "0.17"  "0.18"  "0.08" 
## DAL "0.15"  "0.10"  "0.11"  "0.16"  "0.07"  "0.07"  "0.17"  "0.17"  "0.08" 
## FRS "0.13"  "0.12"  "0.11"  "0.14"  "0.09"  "0.05"  "0.16"  "0.16"  "0.08" 
## SPB "0.16"  "0.12"  "0.10"  "0.17"  "0.06"  "0.07"  "0.18"  "0.19"  "0.08" 
## BRE "0.23"  "0.16"  "0.16"  "0.22"  "0.11"  "0.13"  "0.23"  "0.24"  "0.14" 
## DES "0.22"  "0.15"  "0.14"  "0.21"  "0.09"  "0.12"  "0.21"  "0.23"  "0.12" 
## MON "0.12"  "0.09"  "0.12"  "0.13"  "0.08"  "0.06"  "0.15"  "0.15"  "0.08" 
## HOU "0.10"  "0.08"  "0.11"  "0.12"  "0.07"  "0.05"  "0.14"  "0.14"  "0.07" 
## CES "0.28"  "0.20"  "0.18"  "0.25"  "0.13"  "0.16"  "0.25"  "0.27"  "0.16" 
## SPS "0.19"  "0.17"  "0.18"  "0.18"  "0.14"  "0.09"  "0.21"  "0.20"  "0.14" 
## TRA "0.12"  "0.09"  "0.11"  "0.13"  "0.07"  "0.06"  "0.15"  "0.15"  "0.08" 
## SER "0.33"  "0.24"  "0.25"  "0.26"  "0.21"  "0.16"  "0.28"  "0.27"  "0.20" 
## NUE "0.12"  "0.09"  "0.12"  "0.13"  "0.08"  "0.06"  "0.14"  "0.15"  "0.09" 
## ALD "0.17"  "0.16"  "0.17"  "0.17"  "0.13"  "0.08"  "0.19"  "0.19"  "0.13" 
## NEG "0.21"  "0.22"  "0.32"  "0.13"  "0.28"  "0.18"  "0.21"  "0.14"  "0.26" 
## GRA "0.07"  "0.11"  "0.15"  "0.08"  "0.12"  "0.04"  "0.13"  "0.10"  "0.10" 
## TIK "0.20"  "0.18"  "0.19"  "0.18"  "0.16"  "0.10"  "0.21"  "0.20"  "0.14" 
## RAR "0.18"  "0.17"  "0.18"  "0.17"  "0.15"  "0.09"  "0.20"  "0.19"  "0.14" 
## SOC "0.17"  "0.16"  "0.17"  "0.17"  "0.14"  "0.08"  "0.19"  "0.18"  "0.13" 
## GES "0.18"  "0.17"  "0.17"  "0.17"  "0.15"  "0.09"  "0.20"  "0.19"  "0.13" 
## TUH "0.12"  "0.12"  "0.13"  "0.13"  "0.10"  "0.04"  "0.16"  "0.15"  "0.09" 
## CHI "0.11"  "0.13"  "0.18"  "0.11"  "0.15"  "0.06"  "0.16"  "0.13"  "0.13" 
## LOS "0.12"  "0.14"  "0.17"  "0.13"  "0.14"  "0.06"  "0.16"  "0.15"  "0.12" 
## SAI "0.10"  "0.11"  "0.16"  "0.10"  "0.13"  "0.07"  "0.14"  "0.12"  "0.12" 
## CUA "0.12"  "0.14"  "0.21"  "0.11"  "0.18"  "0.10"  "0.13"  "0.13"  "0.16" 
## MED "0.10"  "0.13"  "0.21"  "0.11"  "0.17"  "0.09"  "0.13"  "0.12"  "0.16" 
## TUC "0.10"  "0.07"  "0.22"  "0.10"  "0.18"  "0.12"  "0.05"  "0.11"  "0.17" 
## FLO "0.12"  "0.07"  "0.22"  "0.12"  "0.19"  "0.12"  "0.06"  "0.12"  "0.17" 
## BIT "0.12"  "0.08"  "0.21"  "0.12"  "0.18"  "0.12"  "0.06"  "0.12"  "0.17" 
## MAU "0.10"  "0.08"  "0.19"  "0.11"  "0.16"  "0.10"  "0.06"  "0.11"  "0.15" 
## ARA "0.11"  "0.06"  "0.21"  "0.11"  "0.17"  "0.11"  "0.05"  "0.11"  "0.16" 
## REC "0.12"  "0.03"  "0.18"  "0.12"  "0.14"  "0.09"  "0.11"  "0.13"  "0.13" 
## PLM "0.11"  "0.07"  "0.23"  "0.11"  "0.19"  "0.12"  "0.05"  "0.11"  "0.18" 
## BCO "0.12"  "0.08"  "0.22"  "0.11"  "0.18"  "0.12"  "0.06"  "0.12"  "0.17" 
## BAF "0.10"  "0.06"  "0.22"  "0.10"  "0.18"  "0.11"  "0.04"  "0.11"  "0.17" 
## BPV "0.11"  "0.08"  "0.22"  "0.11"  "0.18"  "0.12"  "0.06"  "0.12"  "0.17" 
## IPO "0.16"  "0.13"  "0.30"  "0.13"  "0.26"  "0.17"  "0.04"  "0.12"  "0.24" 
## KAT "0.02"  "0.14"  "0.25"  "0.03"  "0.21"  "0.11"  "0.12"  "0.03"  "0.19" 
## NOV "0.12"  "0.13"  "0.28"  "0.09"  "0.25"  "0.14"  "0.04"  "0.10"  "0.22" 
## BEH "0.11"  "0.10"  "0.25"  "0.10"  "0.22"  "0.13"  "0.04"  "0.10"  "0.20" 
## GEL NA      "0.12"  "0.24"  "0.00"  "0.20"  "0.08"  "0.11"  "0.02"  "0.17" 
## BPA "15372" NA      "0.18"  "0.13"  "0.15"  "0.10"  "0.11"  "0.13"  "0.14" 
## KAN "4459"  "18468" NA      "0.23"  "0.11"  "0.13"  "0.24"  "0.24"  "0.13" 
## YUN "1120"  "16440" "3619"  NA      "0.20"  "0.09"  "0.11"  "0.02"  "0.17" 
## UTS "4747"  "18437" "288"   "3902"  NA      "0.10"  "0.21"  "0.22"  "0.09" 
## HUN "2114"  "17481" "2532"  "1108"  "2811"  NA      "0.13"  "0.12"  "0.08" 
## POR "16558" "1189"  "18416" "17609" "18201" "18669" NA      "0.11"  "0.19" 
## BEN "2050"  "13729" "6410"  "2806"  "6696"  "3913"  "14849" NA      "0.19" 
## KAG "3906"  "18861" "791"   "2967"  "1023"  "1860"  "19205" "5773"  NA     
## HAN "1691"  "16860" "3444"  "601"   "3715"  "954"   "17976" "3134"  "2721" 
## LAM "1328"  "16147" "4160"  "714"   "4435"  "1637"  "17245" "2419"  "3450" 
## GRV "16268" "1126"  "19013" "17239" "18787" "18346" "599"   "14438" "19804"
## JAF "2209"  "13856" "6414"  "2797"  "6697"  "3888"  "14936" "454"   "5739" 
## HAI "2132"  "17209" "3270"  "1035"  "3527"  "966"   "18262" "3487"  "2510" 
## OKI "3712"  "19052" "1390"  "2678"  "1590"  "1599"  "19766" "5450"  "618"  
## KAC "1711"  "16019" "4456"  "1179"  "4722"  "1981"  "17044" "2377"  "3713" 
## TAI "3024"  "18355" "2185"  "1928"  "2418"  "986"   "19435" "4631"  "1397" 
## KUN "3002"  "12972" "7300"  "3681"  "7583"  "4777"  "14043" "957"   "6631" 
## SSK "1940"  "16547" "3991"  "1087"  "4249"  "1595"  "17551" "2894"  "3232" 
## CHA "1997"  "16226" "4357"  "1323"  "4613"  "1956"  "17198" "2660"  "3595" 
## QNC "2432"  "16990" "3725"  "1449"  "3965"  "1565"  "17893" "3424"  "2942" 
## CAM "2278"  "16464" "4223"  "1487"  "4470"  "1929"  "17376" "2976"  "3446" 
## HOC "2468"  "16598" "4161"  "1627"  "4399"  "1954"  "17462" "3174"  "3377" 
## SON "2436"  "15824" "4903"  "1925"  "5149"  "2565"  "16700" "2600"  "4126" 
## MAT "2722"  "15739" "5073"  "2208"  "5311"  "2799"  "16553" "2751"  "4289" 
## KLP "2898"  "15701" "5163"  "2375"  "5396"  "2932"  "16475" "2869"  "4376" 
## SUF "4507"  "16777" "4621"  "3566"  "4755"  "3406"  "16916" "4970"  "3893" 
## SUU "4733"  "17011" "4452"  "3744"  "4566"  "3468"  "17015" "5307"  "3756" 
## INJ "4082"  "15489" "5685"  "3467"  "5874"  "3805"  "15967" "3876"  "4901" 
## INW "5184"  "16043" "5424"  "4320"  "5539"  "4248"  "16073" "5347"  "4718" 
##     HAN     LAM     GRV     JAF    HAI    OKI    KAC    TAI    KUN    SSK   
## NEH "0.12"  "0.17"  "0.14"  "0.16" "0.10" "0.11" "0.16" "0.18" "0.26" "0.17"
## NEW "0.13"  "0.17"  "0.15"  "0.18" "0.12" "0.12" "0.17" "0.18" "0.26" "0.17"
## FIR "0.17"  "0.21"  "0.18"  "0.22" "0.14" "0.15" "0.20" "0.21" "0.30" "0.20"
## NEB "0.13"  "0.18"  "0.15"  "0.17" "0.11" "0.12" "0.17" "0.18" "0.27" "0.18"
## BER "0.13"  "0.17"  "0.15"  "0.17" "0.11" "0.12" "0.17" "0.18" "0.26" "0.17"
## HAR "0.13"  "0.17"  "0.15"  "0.17" "0.11" "0.12" "0.17" "0.18" "0.26" "0.17"
## LIN "0.14"  "0.19"  "0.17"  "0.19" "0.12" "0.13" "0.18" "0.19" "0.28" "0.18"
## MAN "0.11"  "0.16"  "0.14"  "0.16" "0.10" "0.11" "0.16" "0.17" "0.25" "0.16"
## WAV "0.13"  "0.19"  "0.16"  "0.18" "0.12" "0.13" "0.18" "0.19" "0.28" "0.18"
## COL "0.13"  "0.18"  "0.15"  "0.17" "0.11" "0.12" "0.17" "0.18" "0.27" "0.17"
## FAY "0.10"  "0.15"  "0.13"  "0.14" "0.09" "0.10" "0.14" "0.16" "0.24" "0.15"
## PEO "0.11"  "0.16"  "0.14"  "0.15" "0.10" "0.11" "0.15" "0.17" "0.25" "0.16"
## STL "0.14"  "0.19"  "0.16"  "0.19" "0.12" "0.13" "0.19" "0.19" "0.28" "0.19"
## HAL "0.11"  "0.16"  "0.14"  "0.16" "0.10" "0.11" "0.15" "0.17" "0.25" "0.16"
## BEA "0.08"  "0.13"  "0.12"  "0.12" "0.07" "0.09" "0.12" "0.15" "0.21" "0.13"
## MAC "0.09"  "0.13"  "0.12"  "0.12" "0.08" "0.10" "0.12" "0.16" "0.22" "0.13"
## BRM "0.14"  "0.18"  "0.17"  "0.18" "0.12" "0.14" "0.17" "0.20" "0.27" "0.17"
## SPR "0.15"  "0.20"  "0.17"  "0.21" "0.13" "0.14" "0.19" "0.21" "0.30" "0.19"
## RUS "0.14"  "0.18"  "0.16"  "0.19" "0.13" "0.13" "0.18" "0.20" "0.28" "0.18"
## HAT "0.12"  "0.16"  "0.14"  "0.15" "0.10" "0.11" "0.15" "0.18" "0.25" "0.16"
## NEO "0.12"  "0.16"  "0.14"  "0.16" "0.10" "0.11" "0.15" "0.17" "0.25" "0.16"
## SMI "0.12"  "0.16"  "0.14"  "0.16" "0.10" "0.12" "0.16" "0.18" "0.25" "0.16"
## PAL "0.10"  "0.13"  "0.13"  "0.13" "0.09" "0.11" "0.13" "0.17" "0.23" "0.13"
## DEN "0.11"  "0.17"  "0.14"  "0.16" "0.10" "0.10" "0.16" "0.17" "0.25" "0.16"
## DAL "0.11"  "0.16"  "0.14"  "0.16" "0.10" "0.11" "0.15" "0.17" "0.25" "0.16"
## FRS "0.10"  "0.15"  "0.14"  "0.14" "0.08" "0.10" "0.14" "0.16" "0.24" "0.15"
## SPB "0.12"  "0.17"  "0.15"  "0.17" "0.11" "0.12" "0.17" "0.18" "0.27" "0.17"
## BRE "0.19"  "0.23"  "0.20"  "0.24" "0.16" "0.17" "0.22" "0.23" "0.32" "0.22"
## DES "0.17"  "0.22"  "0.18"  "0.22" "0.15" "0.16" "0.21" "0.22" "0.31" "0.21"
## MON "0.09"  "0.13"  "0.13"  "0.13" "0.08" "0.10" "0.13" "0.15" "0.22" "0.13"
## HOU "0.08"  "0.12"  "0.11"  "0.11" "0.07" "0.09" "0.11" "0.14" "0.21" "0.12"
## CES "0.22"  "0.26"  "0.23"  "0.28" "0.19" "0.20" "0.26" "0.26" "0.36" "0.25"
## SPS "0.15"  "0.19"  "0.19"  "0.20" "0.12" "0.15" "0.18" "0.21" "0.30" "0.18"
## TRA "0.09"  "0.14"  "0.12"  "0.13" "0.08" "0.10" "0.13" "0.16" "0.23" "0.14"
## SER "0.24"  "0.27"  "0.26"  "0.34" "0.19" "0.22" "0.27" "0.31" "0.43" "0.25"
## NUE "0.09"  "0.13"  "0.12"  "0.13" "0.08" "0.10" "0.13" "0.16" "0.22" "0.13"
## ALD "0.13"  "0.17"  "0.18"  "0.18" "0.11" "0.13" "0.17" "0.20" "0.28" "0.17"
## NEG "0.20"  "0.15"  "0.22"  "0.22" "0.16" "0.24" "0.15" "0.33" "0.32" "0.13"
## GRA "0.06"  "0.08"  "0.12"  "0.08" "0.05" "0.09" "0.08" "0.15" "0.18" "0.08"
## TIK "0.15"  "0.19"  "0.19"  "0.21" "0.13" "0.15" "0.19" "0.22" "0.30" "0.19"
## RAR "0.14"  "0.18"  "0.18"  "0.19" "0.12" "0.14" "0.17" "0.20" "0.28" "0.17"
## SOC "0.13"  "0.17"  "0.18"  "0.18" "0.11" "0.14" "0.17" "0.20" "0.27" "0.17"
## GES "0.14"  "0.18"  "0.18"  "0.19" "0.12" "0.14" "0.18" "0.20" "0.28" "0.18"
## TUH "0.09"  "0.14"  "0.14"  "0.13" "0.08" "0.10" "0.13" "0.16" "0.23" "0.14"
## CHI "0.08"  "0.12"  "0.14"  "0.12" "0.06" "0.12" "0.11" "0.17" "0.24" "0.11"
## LOS "0.09"  "0.13"  "0.15"  "0.13" "0.08" "0.11" "0.13" "0.17" "0.23" "0.13"
## SAI "0.08"  "0.11"  "0.13"  "0.11" "0.07" "0.11" "0.10" "0.17" "0.21" "0.11"
## CUA "0.11"  "0.12"  "0.13"  "0.12" "0.10" "0.15" "0.12" "0.22" "0.23" "0.12"
## MED "0.10"  "0.11"  "0.13"  "0.11" "0.09" "0.15" "0.11" "0.21" "0.22" "0.11"
## TUC "0.12"  "0.11"  "0.07"  "0.10" "0.10" "0.16" "0.10" "0.22" "0.20" "0.11"
## FLO "0.13"  "0.12"  "0.07"  "0.12" "0.11" "0.17" "0.12" "0.23" "0.22" "0.12"
## BIT "0.12"  "0.12"  "0.07"  "0.12" "0.11" "0.16" "0.12" "0.22" "0.22" "0.12"
## MAU "0.10"  "0.11"  "0.07"  "0.10" "0.10" "0.14" "0.11" "0.20" "0.20" "0.11"
## ARA "0.11"  "0.11"  "0.06"  "0.10" "0.10" "0.15" "0.11" "0.21" "0.20" "0.11"
## REC "0.11"  "0.13"  "0.10"  "0.12" "0.10" "0.14" "0.12" "0.20" "0.22" "0.13"
## PLM "0.12"  "0.11"  "0.07"  "0.10" "0.11" "0.17" "0.11" "0.23" "0.21" "0.11"
## BCO "0.13"  "0.12"  "0.08"  "0.12" "0.11" "0.17" "0.11" "0.23" "0.21" "0.12"
## BAF "0.11"  "0.11"  "0.06"  "0.10" "0.10" "0.16" "0.10" "0.22" "0.21" "0.11"
## BPV "0.12"  "0.12"  "0.08"  "0.11" "0.11" "0.16" "0.11" "0.22" "0.21" "0.12"
## IPO "0.17"  "0.13"  "0.06"  "0.15" "0.15" "0.22" "0.13" "0.30" "0.25" "0.13"
## KAT "0.08"  "0.03"  "0.13"  "0.01" "0.08" "0.16" "0.03" "0.23" "0.14" "0.03"
## NOV "0.14"  "0.10"  "0.07"  "0.11" "0.12" "0.20" "0.10" "0.28" "0.24" "0.09"
## BEH "0.13"  "0.10"  "0.08"  "0.11" "0.11" "0.18" "0.10" "0.25" "0.21" "0.10"
## GEL "0.06"  "0.01"  "0.12"  "0.00" "0.05" "0.15" "0.00" "0.25" "0.20" "0.00"
## BPA "0.12"  "0.13"  "0.10"  "0.12" "0.11" "0.14" "0.12" "0.20" "0.22" "0.13"
## KAN "0.19"  "0.23"  "0.21"  "0.25" "0.17" "0.18" "0.23" "0.24" "0.33" "0.23"
## YUN "0.06"  "0.01"  "0.12"  "0.01" "0.06" "0.15" "0.00" "0.21" "0.14" "0.00"
## UTS "0.16"  "0.21"  "0.18"  "0.21" "0.14" "0.15" "0.20" "0.20" "0.30" "0.20"
## HUN "0.05"  "0.10"  "0.11"  "0.09" "0.04" "0.07" "0.09" "0.13" "0.19" "0.10"
## POR "0.13"  "0.11"  "0.03"  "0.11" "0.12" "0.18" "0.11" "0.24" "0.20" "0.11"
## BEN "0.08"  "0.03"  "0.13"  "0.00" "0.08" "0.16" "0.02" "0.23" "0.11" "0.03"
## KAG "0.13"  "0.18"  "0.17"  "0.18" "0.12" "0.13" "0.17" "0.19" "0.27" "0.17"
## HAN NA      "0.07"  "0.12"  "0.06" "0.04" "0.11" "0.06" "0.18" "0.20" "0.06"
## LAM "733"   NA      "0.12"  "0.02" "0.06" "0.15" "0.01" "0.22" "0.15" "0.01"
## GRV "17515" "16783" NA      "0.12" "0.11" "0.16" "0.12" "0.22" "0.22" "0.12"
## JAF "3041"  "2309"  "14485" NA     "0.06" "0.16" "0.01" "0.26" "0.17" "0.01"
## HAI "441"   "1070"  "17743" "3354" NA     "0.10" "0.06" "0.14" "0.17" "0.06"
## OKI "2328"  "3060"  "19641" "5369" "2047" NA     "0.15" "0.16" "0.26" "0.15"
## KAC "1028"  "475"   "16533" "2179" "1218" "3265" NA     "0.22" "0.15" "0.00"
## TAI "1497"  "2219"  "18870" "4524" "1180" "873"  "2397" NA     "0.35" "0.21"
## KUN "3934"  "3202"  "13592" "893"  "4241" "6262" "3052" "5414" NA     "0.14"
## SSK "678"   "624"   "17022" "2709" "722"  "2758" "530"  "1886" "3582" NA    
## CHA "1019"  "692"   "16661" "2433" "1087" "3110" "320"  "2237" "3288" "368" 
## QNC "882"   "1155"  "17323" "3213" "604"  "2410" "1047" "1548" "4070" "548" 
## CAM "1059"  "951"   "16815" "2732" "987"  "2929" "647"  "2061" "3572" "402" 
## HOC "1146"  "1139"  "16888" "2920" "991"  "2839" "856"  "1982" "3749" "547" 
## SON "1640"  "1241"  "16136" "2281" "1653" "3604" "768"  "2739" "3057" "971" 
## MAT "1894"  "1531"  "15977" "2400" "1862" "3747" "1060" "2894" "3124" "1217"
## KLP "2042"  "1704"  "15892" "2502" "1984" "3822" "1234" "2979" "3196" "1365"
## SUF "2986"  "3182"  "16361" "4616" "2614" "3280" "2874" "2777" "5293" "2567"
## SUU "3151"  "3420"  "16489" "4964" "2752" "3154" "3147" "2747" "5658" "2797"
## INJ "3034"  "2844"  "15369" "3460" "2845" "4294" "2393" "3562" "3986" "2390"
## INW "3762"  "3859"  "15531" "4950" "3419" "4110" "3492" "3635" "5513" "3271"
##     CHA    QNC    CAM    HOC    SON    MAT    KLP    SUF    SUU    INJ   
## NEH "0.16" "0.23" "0.15" "0.11" "0.16" "0.17" "0.18" "0.23" "0.17" "0.24"
## NEW "0.17" "0.22" "0.16" "0.12" "0.17" "0.18" "0.19" "0.23" "0.17" "0.24"
## FIR "0.20" "0.26" "0.18" "0.15" "0.21" "0.21" "0.22" "0.27" "0.21" "0.27"
## NEB "0.17" "0.23" "0.16" "0.12" "0.17" "0.18" "0.19" "0.24" "0.17" "0.25"
## BER "0.17" "0.23" "0.15" "0.12" "0.17" "0.17" "0.18" "0.23" "0.17" "0.24"
## HAR "0.17" "0.23" "0.16" "0.12" "0.17" "0.18" "0.19" "0.23" "0.17" "0.24"
## LIN "0.18" "0.24" "0.17" "0.12" "0.19" "0.19" "0.20" "0.24" "0.18" "0.25"
## MAN "0.16" "0.22" "0.14" "0.10" "0.16" "0.16" "0.17" "0.22" "0.16" "0.23"
## WAV "0.18" "0.25" "0.16" "0.12" "0.18" "0.19" "0.20" "0.25" "0.18" "0.26"
## COL "0.17" "0.23" "0.16" "0.12" "0.17" "0.18" "0.19" "0.23" "0.17" "0.24"
## FAY "0.15" "0.21" "0.13" "0.09" "0.14" "0.15" "0.16" "0.21" "0.15" "0.22"
## PEO "0.15" "0.22" "0.14" "0.10" "0.15" "0.16" "0.17" "0.22" "0.15" "0.23"
## STL "0.19" "0.25" "0.17" "0.13" "0.19" "0.19" "0.21" "0.25" "0.19" "0.25"
## HAL "0.16" "0.22" "0.14" "0.10" "0.15" "0.16" "0.17" "0.22" "0.16" "0.23"
## BEA "0.12" "0.18" "0.11" "0.08" "0.12" "0.13" "0.14" "0.19" "0.13" "0.20"
## MAC "0.13" "0.19" "0.11" "0.08" "0.12" "0.13" "0.14" "0.19" "0.13" "0.20"
## BRM "0.17" "0.23" "0.16" "0.13" "0.18" "0.18" "0.20" "0.24" "0.18" "0.24"
## SPR "0.19" "0.26" "0.18" "0.14" "0.20" "0.20" "0.22" "0.26" "0.20" "0.27"
## RUS "0.18" "0.24" "0.16" "0.13" "0.18" "0.18" "0.20" "0.25" "0.19" "0.25"
## HAT "0.16" "0.22" "0.14" "0.11" "0.15" "0.16" "0.17" "0.22" "0.16" "0.23"
## NEO "0.16" "0.22" "0.14" "0.11" "0.16" "0.16" "0.17" "0.22" "0.16" "0.23"
## SMI "0.16" "0.22" "0.14" "0.11" "0.16" "0.16" "0.17" "0.22" "0.16" "0.23"
## PAL "0.13" "0.19" "0.12" "0.09" "0.13" "0.14" "0.15" "0.20" "0.14" "0.20"
## DEN "0.16" "0.22" "0.15" "0.11" "0.16" "0.17" "0.18" "0.22" "0.16" "0.23"
## DAL "0.16" "0.22" "0.14" "0.11" "0.15" "0.16" "0.17" "0.22" "0.16" "0.23"
## FRS "0.14" "0.20" "0.13" "0.09" "0.14" "0.15" "0.16" "0.21" "0.15" "0.22"
## SPB "0.17" "0.23" "0.15" "0.11" "0.17" "0.18" "0.19" "0.24" "0.17" "0.25"
## BRE "0.22" "0.28" "0.21" "0.17" "0.23" "0.23" "0.24" "0.28" "0.23" "0.29"
## DES "0.21" "0.27" "0.20" "0.16" "0.22" "0.22" "0.23" "0.27" "0.21" "0.28"
## MON "0.13" "0.19" "0.12" "0.08" "0.13" "0.14" "0.15" "0.20" "0.14" "0.20"
## HOU "0.12" "0.18" "0.11" "0.07" "0.11" "0.13" "0.13" "0.18" "0.12" "0.19"
## CES "0.25" "0.31" "0.24" "0.20" "0.28" "0.26" "0.28" "0.32" "0.26" "0.32"
## SPS "0.18" "0.25" "0.16" "0.13" "0.19" "0.19" "0.21" "0.25" "0.19" "0.26"
## TRA "0.14" "0.19" "0.12" "0.09" "0.13" "0.14" "0.15" "0.20" "0.14" "0.21"
## SER "0.25" "0.33" "0.23" "0.21" "0.31" "0.26" "0.31" "0.35" "0.28" "0.34"
## NUE "0.13" "0.19" "0.12" "0.08" "0.13" "0.13" "0.14" "0.20" "0.13" "0.20"
## ALD "0.17" "0.23" "0.15" "0.12" "0.18" "0.17" "0.19" "0.24" "0.18" "0.24"
## NEG "0.13" "0.22" "0.13" "0.19" "0.19" "0.15" "0.22" "0.28" "0.23" "0.25"
## GRA "0.08" "0.15" "0.07" "0.05" "0.07" "0.08" "0.09" "0.15" "0.10" "0.16"
## TIK "0.18" "0.24" "0.17" "0.14" "0.20" "0.19" "0.21" "0.26" "0.20" "0.26"
## RAR "0.17" "0.23" "0.15" "0.13" "0.18" "0.17" "0.20" "0.24" "0.18" "0.25"
## SOC "0.17" "0.23" "0.15" "0.12" "0.18" "0.17" "0.19" "0.24" "0.18" "0.24"
## GES "0.17" "0.23" "0.16" "0.13" "0.18" "0.18" "0.20" "0.25" "0.18" "0.25"
## TUH "0.14" "0.20" "0.12" "0.08" "0.14" "0.14" "0.15" "0.20" "0.14" "0.21"
## CHI "0.11" "0.18" "0.09" "0.07" "0.11" "0.12" "0.14" "0.19" "0.13" "0.20"
## LOS "0.13" "0.19" "0.11" "0.09" "0.13" "0.13" "0.15" "0.20" "0.14" "0.21"
## SAI "0.11" "0.17" "0.09" "0.08" "0.10" "0.11" "0.12" "0.18" "0.12" "0.18"
## CUA "0.11" "0.19" "0.10" "0.11" "0.12" "0.12" "0.15" "0.19" "0.14" "0.19"
## MED "0.11" "0.18" "0.10" "0.10" "0.11" "0.11" "0.14" "0.19" "0.13" "0.18"
## TUC "0.10" "0.17" "0.10" "0.12" "0.11" "0.11" "0.14" "0.19" "0.15" "0.19"
## FLO "0.11" "0.19" "0.11" "0.13" "0.12" "0.13" "0.15" "0.21" "0.16" "0.21"
## BIT "0.12" "0.19" "0.11" "0.12" "0.12" "0.13" "0.15" "0.20" "0.16" "0.21"
## MAU "0.11" "0.18" "0.10" "0.11" "0.11" "0.12" "0.14" "0.19" "0.14" "0.19"
## ARA "0.11" "0.17" "0.10" "0.11" "0.11" "0.12" "0.14" "0.19" "0.15" "0.19"
## REC "0.13" "0.19" "0.11" "0.11" "0.12" "0.13" "0.15" "0.20" "0.15" "0.21"
## PLM "0.11" "0.18" "0.10" "0.12" "0.11" "0.12" "0.14" "0.20" "0.16" "0.20"
## BCO "0.11" "0.18" "0.11" "0.13" "0.12" "0.12" "0.15" "0.20" "0.16" "0.20"
## BAF "0.10" "0.18" "0.09" "0.11" "0.11" "0.11" "0.14" "0.20" "0.15" "0.20"
## BPV "0.11" "0.18" "0.10" "0.12" "0.12" "0.12" "0.15" "0.20" "0.16" "0.20"
## IPO "0.12" "0.21" "0.12" "0.17" "0.15" "0.14" "0.18" "0.24" "0.20" "0.22"
## KAT "0.03" "0.12" "0.03" "0.09" "0.03" "0.05" "0.08" "0.07" "0.06" "0.08"
## NOV "0.09" "0.18" "0.09" "0.14" "0.11" "0.10" "0.15" "0.22" "0.17" "0.20"
## BEH "0.10" "0.17" "0.09" "0.13" "0.11" "0.11" "0.14" "0.20" "0.16" "0.19"
## GEL "0.00" "0.12" "0.00" "0.06" "0.00" "0.02" "0.08" "0.16" "0.11" "0.14"
## BPA "0.13" "0.19" "0.12" "0.12" "0.13" "0.14" "0.15" "0.20" "0.16" "0.21"
## KAN "0.23" "0.29" "0.21" "0.18" "0.24" "0.23" "0.26" "0.29" "0.23" "0.30"
## YUN "0.00" "0.08" "0.00" "0.08" "0.00" "0.02" "0.06" "0.13" "0.10" "0.12"
## UTS "0.20" "0.26" "0.18" "0.14" "0.21" "0.21" "0.22" "0.26" "0.20" "0.27"
## HUN "0.10" "0.16" "0.08" "0.05" "0.09" "0.10" "0.11" "0.16" "0.10" "0.17"
## POR "0.11" "0.18" "0.10" "0.13" "0.11" "0.12" "0.15" "0.20" "0.16" "0.20"
## BEN "0.02" "0.10" "0.02" "0.10" "0.03" "0.04" "0.08" "0.14" "0.12" "0.13"
## KAG "0.17" "0.23" "0.16" "0.12" "0.18" "0.18" "0.19" "0.24" "0.18" "0.25"
## HAN "0.06" "0.15" "0.05" "0.05" "0.06" "0.07" "0.10" "0.16" "0.10" "0.17"
## LAM "0.01" "0.09" "0.01" "0.08" "0.01" "0.02" "0.06" "0.13" "0.10" "0.12"
## GRV "0.12" "0.18" "0.11" "0.12" "0.12" "0.13" "0.15" "0.21" "0.16" "0.20"
## JAF "0.01" "0.12" "0.01" "0.07" "0.01" "0.03" "0.08" "0.16" "0.11" "0.14"
## HAI "0.06" "0.13" "0.05" "0.04" "0.06" "0.07" "0.08" "0.14" "0.09" "0.15"
## OKI "0.15" "0.21" "0.13" "0.10" "0.15" "0.15" "0.17" "0.22" "0.16" "0.23"
## KAC "0.00" "0.09" "0.00" "0.07" "0.00" "0.02" "0.06" "0.14" "0.10" "0.13"
## TAI "0.21" "0.28" "0.19" "0.16" "0.24" "0.21" "0.25" "0.29" "0.22" "0.29"
## KUN "0.13" "0.23" "0.13" "0.19" "0.18" "0.15" "0.22" "0.27" "0.23" "0.25"
## SSK "0.00" "0.08" "0.01" "0.08" "0.01" "0.02" "0.06" "0.13" "0.10" "0.12"
## CHA NA     "0.08" "0.00" "0.08" "0.00" "0.02" "0.06" "0.13" "0.10" "0.12"
## QNC "782"  NA     "0.08" "0.15" "0.11" "0.10" "0.15" "0.21" "0.18" "0.19"
## CAM "329"  "528"  NA     "0.06" "0.00" "0.02" "0.05" "0.12" "0.09" "0.12"
## HOC "537"  "436"  "209"  NA     "0.07" "0.08" "0.10" "0.16" "0.10" "0.17"
## SON "625"  "1195" "679"  "776"  NA     "0.02" "0.06" "0.15" "0.11" "0.14"
## MAT "893"  "1348" "875"  "912"  "292"  NA     "0.06" "0.14" "0.11" "0.13"
## KLP "1055" "1445" "1003" "1011" "468"  "177"  NA     "0.18" "0.13" "0.17"
## SUF "2557" "2117" "2245" "2046" "2384" "2220" "2115" NA     "0.04" "0.08"
## SUU "2827" "2309" "2506" "2300" "2710" "2565" "2469" "381"  NA     "0.09"
## INJ "2160" "2241" "1989" "1889" "1646" "1363" "1188" "1535" "1911" NA    
## INW "3190" "2880" "2908" "2727" "2877" "2650" "2505" "859"  "973"  "1528"
##     INW   
## NEH "0.27"
## NEW "0.26"
## FIR "0.30"
## NEB "0.27"
## BER "0.27"
## HAR "0.27"
## LIN "0.28"
## MAN "0.25"
## WAV "0.29"
## COL "0.27"
## FAY "0.24"
## PEO "0.25"
## STL "0.28"
## HAL "0.25"
## BEA "0.22"
## MAC "0.23"
## BRM "0.27"
## SPR "0.30"
## RUS "0.28"
## HAT "0.26"
## NEO "0.25"
## SMI "0.26"
## PAL "0.23"
## DEN "0.26"
## DAL "0.25"
## FRS "0.24"
## SPB "0.27"
## BRE "0.32"
## DES "0.31"
## MON "0.23"
## HOU "0.22"
## CES "0.36"
## SPS "0.30"
## TRA "0.23"
## SER "0.42"
## NUE "0.23"
## ALD "0.28"
## NEG "0.33"
## GRA "0.19"
## TIK "0.30"
## RAR "0.28"
## SOC "0.28"
## GES "0.28"
## TUH "0.24"
## CHI "0.24"
## LOS "0.24"
## SAI "0.21"
## CUA "0.23"
## MED "0.22"
## TUC "0.22"
## FLO "0.24"
## BIT "0.24"
## MAU "0.22"
## ARA "0.22"
## REC "0.23"
## PLM "0.24"
## BCO "0.23"
## BAF "0.23"
## BPV "0.23"
## IPO "0.27"
## KAT "0.10"
## NOV "0.26"
## BEH "0.23"
## GEL "0.21"
## BPA "0.24"
## KAN "0.33"
## YUN "0.15"
## UTS "0.30"
## HUN "0.20"
## POR "0.23"
## BEN "0.16"
## KAG "0.27"
## HAN "0.21"
## LAM "0.16"
## GRV "0.24"
## JAF "0.21"
## HAI "0.18"
## OKI "0.26"
## KAC "0.16"
## TAI "0.35"
## KUN "0.33"
## SSK "0.15"
## CHA "0.15"
## QNC "0.24"
## CAM "0.15"
## HOC "0.20"
## SON "0.19"
## MAT "0.16"
## KLP "0.22"
## SUF "0.09"
## SUU "0.13"
## INJ "0.06"
## INW NA
```

Make a table and save as word document

```
# Convert the matrix to a data frame and add a column with row names
merged_df <- as.data.frame(merged_matrix)
merged_df$Population <- rownames(merged_matrix)

# Reorder columns to have RowNames as the first column
merged_df <- merged_df[, c("Population", colnames(merged_matrix))]


# Create a flextable object from the merged_matrix
ft <- qflextable(as.data.frame(merged_df))

ft
```

| Population | NEH | NEW | FIR | NEB | BER | HAR | LIN | MAN | WAV | COL | FAY | PEO | STL | HAL | BEA | MAC | BRM | SPR | RUS | HAT | NEO | SMI | PAL | DEN | DAL | FRS | SPB | BRE | DES | MON | HOU | CES | SPS | TRA | SER | NUE | ALD | NEG | GRA | TIK | RAR | SOC | GES | TUH | CHI | LOS | SAI | CUA | MED | TUC | FLO | BIT | MAU | ARA | REC | PLM | BCO | BAF | BPV | IPO | KAT | NOV | BEH | GEL | BPA | KAN | YUN | UTS | HUN | POR | BEN | KAG | HAN | LAM | GRV | JAF | HAI | OKI | KAC | TAI | KUN | SSK | CHA | QNC | CAM | HOC | SON | MAT | KLP | SUF | SUU | INJ | INW |
| --- | --- | --- | --- | --- | --- | --- | --- | --- | --- | --- | --- | --- | --- | --- | --- | --- | --- | --- | --- | --- | --- | --- | --- | --- | --- | --- | --- | --- | --- | --- | --- | --- | --- | --- | --- | --- | --- | --- | --- | --- | --- | --- | --- | --- | --- | --- | --- | --- | --- | --- | --- | --- | --- | --- | --- | --- | --- | --- | --- | --- | --- | --- | --- | --- | --- | --- | --- | --- | --- | --- | --- | --- | --- | --- | --- | --- | --- | --- | --- | --- | --- | --- | --- | --- | --- | --- | --- | --- | --- | --- | --- | --- | --- |
| NEH |  | 0.04 | 0.07 | 0.02 | 0.03 | 0.03 | 0.06 | 0.02 | 0.02 | 0.03 | 0.04 | 0.03 | 0.04 | 0.02 | 0.03 | 0.05 | 0.09 | 0.06 | 0.07 | 0.04 | 0.05 | 0.05 | 0.06 | 0.03 | 0.04 | 0.06 | 0.04 | 0.08 | 0.07 | 0.04 | 0.03 | 0.12 | 0.13 | 0.03 | 0.19 | 0.05 | 0.11 | 0.25 | 0.09 | 0.13 | 0.13 | 0.12 | 0.12 | 0.08 | 0.12 | 0.10 | 0.09 | 0.14 | 0.14 | 0.15 | 0.15 | 0.15 | 0.13 | 0.14 | 0.11 | 0.15 | 0.15 | 0.14 | 0.15 | 0.23 | 0.17 | 0.21 | 0.18 | 0.15 | 0.11 | 0.11 | 0.16 | 0.06 | 0.06 | 0.17 | 0.18 | 0.07 | 0.12 | 0.17 | 0.14 | 0.16 | 0.10 | 0.11 | 0.16 | 0.18 | 0.26 | 0.17 | 0.16 | 0.23 | 0.15 | 0.11 | 0.16 | 0.17 | 0.18 | 0.23 | 0.17 | 0.24 | 0.27 |
| NEW | 123 |  | 0.08 | 0.05 | 0.05 | 0.05 | 0.07 | 0.04 | 0.05 | 0.06 | 0.04 | 0.05 | 0.06 | 0.04 | 0.05 | 0.06 | 0.10 | 0.08 | 0.08 | 0.05 | 0.05 | 0.05 | 0.07 | 0.03 | 0.05 | 0.08 | 0.06 | 0.09 | 0.09 | 0.06 | 0.04 | 0.13 | 0.13 | 0.04 | 0.19 | 0.07 | 0.12 | 0.25 | 0.10 | 0.14 | 0.13 | 0.13 | 0.13 | 0.09 | 0.13 | 0.12 | 0.10 | 0.15 | 0.15 | 0.15 | 0.16 | 0.15 | 0.13 | 0.15 | 0.11 | 0.16 | 0.16 | 0.15 | 0.16 | 0.23 | 0.18 | 0.21 | 0.19 | 0.17 | 0.12 | 0.12 | 0.17 | 0.08 | 0.08 | 0.18 | 0.19 | 0.09 | 0.13 | 0.17 | 0.15 | 0.18 | 0.12 | 0.12 | 0.17 | 0.18 | 0.26 | 0.17 | 0.17 | 0.22 | 0.16 | 0.12 | 0.17 | 0.18 | 0.19 | 0.23 | 0.17 | 0.24 | 0.26 |
| FIR | 68 | 104 |  | 0.07 | 0.07 | 0.08 | 0.10 | 0.06 | 0.07 | 0.08 | 0.07 | 0.07 | 0.08 | 0.07 | 0.07 | 0.09 | 0.12 | 0.11 | 0.10 | 0.08 | 0.08 | 0.08 | 0.11 | 0.07 | 0.07 | 0.10 | 0.08 | 0.13 | 0.11 | 0.08 | 0.07 | 0.16 | 0.17 | 0.08 | 0.23 | 0.09 | 0.16 | 0.29 | 0.13 | 0.17 | 0.16 | 0.16 | 0.16 | 0.12 | 0.16 | 0.15 | 0.13 | 0.18 | 0.18 | 0.18 | 0.19 | 0.18 | 0.16 | 0.17 | 0.14 | 0.19 | 0.18 | 0.18 | 0.18 | 0.26 | 0.22 | 0.25 | 0.22 | 0.21 | 0.14 | 0.15 | 0.20 | 0.11 | 0.11 | 0.21 | 0.22 | 0.12 | 0.17 | 0.21 | 0.18 | 0.22 | 0.14 | 0.15 | 0.20 | 0.21 | 0.30 | 0.20 | 0.20 | 0.26 | 0.18 | 0.15 | 0.21 | 0.21 | 0.22 | 0.27 | 0.21 | 0.27 | 0.30 |
| NEB | 171 | 290 | 199 |  | 0.05 | 0.05 | 0.06 | 0.03 | 0.04 | 0.04 | 0.05 | 0.04 | 0.05 | 0.03 | 0.04 | 0.06 | 0.10 | 0.07 | 0.08 | 0.05 | 0.05 | 0.06 | 0.07 | 0.03 | 0.04 | 0.06 | 0.04 | 0.09 | 0.07 | 0.05 | 0.04 | 0.12 | 0.13 | 0.04 | 0.19 | 0.06 | 0.12 | 0.26 | 0.10 | 0.14 | 0.13 | 0.13 | 0.13 | 0.08 | 0.12 | 0.11 | 0.10 | 0.15 | 0.15 | 0.16 | 0.16 | 0.15 | 0.13 | 0.15 | 0.11 | 0.16 | 0.16 | 0.15 | 0.16 | 0.24 | 0.18 | 0.22 | 0.19 | 0.17 | 0.12 | 0.11 | 0.17 | 0.07 | 0.07 | 0.18 | 0.19 | 0.08 | 0.13 | 0.18 | 0.15 | 0.17 | 0.11 | 0.12 | 0.17 | 0.18 | 0.27 | 0.18 | 0.17 | 0.23 | 0.16 | 0.12 | 0.17 | 0.18 | 0.19 | 0.24 | 0.17 | 0.25 | 0.27 |
| BER | 240 | 123 | 198 | 396 |  | 0.05 | 0.06 | 0.03 | 0.04 | 0.05 | 0.04 | 0.04 | 0.04 | 0.03 | 0.04 | 0.05 | 0.09 | 0.07 | 0.08 | 0.05 | 0.05 | 0.05 | 0.07 | 0.04 | 0.04 | 0.07 | 0.05 | 0.08 | 0.08 | 0.05 | 0.04 | 0.12 | 0.13 | 0.04 | 0.19 | 0.05 | 0.12 | 0.25 | 0.10 | 0.14 | 0.13 | 0.12 | 0.13 | 0.08 | 0.12 | 0.12 | 0.09 | 0.15 | 0.14 | 0.15 | 0.16 | 0.15 | 0.12 | 0.14 | 0.10 | 0.16 | 0.15 | 0.15 | 0.15 | 0.23 | 0.18 | 0.21 | 0.18 | 0.16 | 0.12 | 0.11 | 0.17 | 0.07 | 0.07 | 0.18 | 0.19 | 0.08 | 0.13 | 0.17 | 0.15 | 0.17 | 0.11 | 0.12 | 0.17 | 0.18 | 0.26 | 0.17 | 0.17 | 0.23 | 0.15 | 0.12 | 0.17 | 0.17 | 0.18 | 0.23 | 0.17 | 0.24 | 0.27 |
| HAR | 353 | 235 | 337 | 523 | 175 |  | 0.07 | 0.04 | 0.05 | 0.05 | 0.04 | 0.04 | 0.06 | 0.04 | 0.04 | 0.06 | 0.10 | 0.07 | 0.08 | 0.04 | 0.06 | 0.05 | 0.08 | 0.04 | 0.05 | 0.07 | 0.05 | 0.10 | 0.08 | 0.05 | 0.04 | 0.13 | 0.13 | 0.04 | 0.20 | 0.06 | 0.12 | 0.25 | 0.10 | 0.14 | 0.14 | 0.13 | 0.13 | 0.08 | 0.13 | 0.12 | 0.10 | 0.15 | 0.15 | 0.16 | 0.16 | 0.15 | 0.13 | 0.15 | 0.11 | 0.16 | 0.16 | 0.15 | 0.16 | 0.23 | 0.18 | 0.22 | 0.19 | 0.17 | 0.12 | 0.11 | 0.17 | 0.07 | 0.08 | 0.18 | 0.19 | 0.09 | 0.13 | 0.17 | 0.15 | 0.17 | 0.11 | 0.12 | 0.17 | 0.18 | 0.26 | 0.17 | 0.17 | 0.23 | 0.16 | 0.12 | 0.17 | 0.18 | 0.19 | 0.23 | 0.17 | 0.24 | 0.27 |
| LIN | 346 | 234 | 295 | 492 | 111 | 200 |  | 0.05 | 0.07 | 0.06 | 0.06 | 0.05 | 0.07 | 0.05 | 0.06 | 0.07 | 0.11 | 0.08 | 0.10 | 0.07 | 0.07 | 0.07 | 0.09 | 0.06 | 0.06 | 0.07 | 0.06 | 0.11 | 0.09 | 0.07 | 0.06 | 0.14 | 0.14 | 0.06 | 0.20 | 0.07 | 0.11 | 0.26 | 0.11 | 0.15 | 0.14 | 0.13 | 0.14 | 0.09 | 0.13 | 0.12 | 0.11 | 0.16 | 0.16 | 0.17 | 0.17 | 0.16 | 0.14 | 0.16 | 0.12 | 0.17 | 0.17 | 0.16 | 0.16 | 0.24 | 0.19 | 0.23 | 0.20 | 0.18 | 0.13 | 0.10 | 0.18 | 0.07 | 0.08 | 0.19 | 0.20 | 0.09 | 0.14 | 0.19 | 0.17 | 0.19 | 0.12 | 0.13 | 0.18 | 0.19 | 0.28 | 0.18 | 0.18 | 0.24 | 0.17 | 0.12 | 0.19 | 0.19 | 0.20 | 0.24 | 0.18 | 0.25 | 0.28 |
| MAN | 474 | 352 | 438 | 636 | 241 | 173 | 171 |  | 0.03 | 0.03 | 0.03 | 0.03 | 0.04 | 0.02 | 0.03 | 0.04 | 0.09 | 0.06 | 0.06 | 0.04 | 0.04 | 0.04 | 0.06 | 0.03 | 0.03 | 0.06 | 0.03 | 0.07 | 0.07 | 0.04 | 0.03 | 0.12 | 0.11 | 0.03 | 0.18 | 0.05 | 0.11 | 0.24 | 0.09 | 0.13 | 0.12 | 0.11 | 0.12 | 0.07 | 0.11 | 0.10 | 0.08 | 0.14 | 0.13 | 0.14 | 0.15 | 0.14 | 0.12 | 0.14 | 0.10 | 0.15 | 0.14 | 0.14 | 0.14 | 0.22 | 0.17 | 0.20 | 0.18 | 0.15 | 0.10 | 0.10 | 0.16 | 0.06 | 0.06 | 0.17 | 0.18 | 0.07 | 0.11 | 0.16 | 0.14 | 0.16 | 0.10 | 0.11 | 0.16 | 0.17 | 0.25 | 0.16 | 0.16 | 0.22 | 0.14 | 0.10 | 0.16 | 0.16 | 0.17 | 0.22 | 0.16 | 0.23 | 0.25 |
| WAV | 597 | 483 | 545 | 739 | 360 | 360 | 251 | 195 |  | 0.04 | 0.04 | 0.03 | 0.04 | 0.03 | 0.05 | 0.05 | 0.10 | 0.07 | 0.08 | 0.05 | 0.05 | 0.05 | 0.08 | 0.03 | 0.03 | 0.07 | 0.04 | 0.07 | 0.06 | 0.05 | 0.04 | 0.14 | 0.14 | 0.04 | 0.21 | 0.06 | 0.13 | 0.27 | 0.11 | 0.15 | 0.14 | 0.13 | 0.13 | 0.09 | 0.13 | 0.13 | 0.10 | 0.16 | 0.15 | 0.16 | 0.16 | 0.16 | 0.13 | 0.15 | 0.11 | 0.17 | 0.16 | 0.15 | 0.16 | 0.25 | 0.19 | 0.23 | 0.20 | 0.17 | 0.12 | 0.12 | 0.18 | 0.07 | 0.08 | 0.19 | 0.20 | 0.09 | 0.13 | 0.19 | 0.16 | 0.18 | 0.12 | 0.13 | 0.18 | 0.19 | 0.28 | 0.18 | 0.18 | 0.25 | 0.16 | 0.12 | 0.18 | 0.19 | 0.20 | 0.25 | 0.18 | 0.26 | 0.29 |
| COL | 855 | 746 | 849 | 1026 | 682 | 514 | 655 | 493 | 602 |  | 0.04 | 0.03 | 0.04 | 0.02 | 0.05 | 0.05 | 0.10 | 0.07 | 0.08 | 0.05 | 0.05 | 0.06 | 0.07 | 0.04 | 0.04 | 0.06 | 0.05 | 0.09 | 0.08 | 0.05 | 0.04 | 0.13 | 0.13 | 0.04 | 0.19 | 0.06 | 0.12 | 0.25 | 0.10 | 0.14 | 0.13 | 0.12 | 0.12 | 0.08 | 0.12 | 0.11 | 0.10 | 0.15 | 0.14 | 0.15 | 0.16 | 0.15 | 0.13 | 0.15 | 0.11 | 0.16 | 0.15 | 0.15 | 0.15 | 0.23 | 0.18 | 0.22 | 0.19 | 0.17 | 0.12 | 0.10 | 0.17 | 0.07 | 0.07 | 0.18 | 0.19 | 0.08 | 0.13 | 0.18 | 0.15 | 0.17 | 0.11 | 0.12 | 0.17 | 0.18 | 0.27 | 0.17 | 0.17 | 0.23 | 0.16 | 0.12 | 0.17 | 0.18 | 0.19 | 0.23 | 0.17 | 0.24 | 0.27 |
| FAY | 869 | 754 | 816 | 1009 | 631 | 604 | 523 | 432 | 272 | 648 |  | 0.03 | 0.05 | 0.03 | 0.02 | 0.03 | 0.08 | 0.07 | 0.07 | 0.03 | 0.03 | 0.04 | 0.05 | 0.02 | 0.02 | 0.06 | 0.05 | 0.08 | 0.07 | 0.03 | 0.02 | 0.13 | 0.12 | 0.02 | 0.18 | 0.03 | 0.11 | 0.23 | 0.08 | 0.13 | 0.12 | 0.11 | 0.12 | 0.07 | 0.10 | 0.10 | 0.07 | 0.13 | 0.12 | 0.13 | 0.13 | 0.13 | 0.11 | 0.12 | 0.09 | 0.14 | 0.13 | 0.12 | 0.13 | 0.21 | 0.15 | 0.19 | 0.16 | 0.14 | 0.09 | 0.11 | 0.15 | 0.07 | 0.06 | 0.16 | 0.17 | 0.08 | 0.10 | 0.15 | 0.13 | 0.14 | 0.09 | 0.10 | 0.14 | 0.16 | 0.24 | 0.15 | 0.15 | 0.21 | 0.13 | 0.09 | 0.14 | 0.15 | 0.16 | 0.21 | 0.15 | 0.22 | 0.24 |
| PEO | 1401 | 1301 | 1405 | 1566 | 1250 | 1078 | 1229 | 1066 | 1157 | 574 | 1127 |  | 0.04 | 0.02 | 0.03 | 0.04 | 0.08 | 0.06 | 0.06 | 0.04 | 0.04 | 0.05 | 0.06 | 0.03 | 0.03 | 0.06 | 0.04 | 0.09 | 0.07 | 0.04 | 0.03 | 0.12 | 0.12 | 0.03 | 0.18 | 0.05 | 0.11 | 0.23 | 0.08 | 0.13 | 0.12 | 0.11 | 0.12 | 0.07 | 0.11 | 0.10 | 0.08 | 0.13 | 0.13 | 0.14 | 0.14 | 0.14 | 0.12 | 0.13 | 0.09 | 0.14 | 0.14 | 0.13 | 0.14 | 0.22 | 0.16 | 0.20 | 0.17 | 0.14 | 0.10 | 0.10 | 0.15 | 0.06 | 0.06 | 0.16 | 0.17 | 0.07 | 0.11 | 0.16 | 0.14 | 0.15 | 0.10 | 0.11 | 0.15 | 0.17 | 0.25 | 0.16 | 0.15 | 0.22 | 0.14 | 0.10 | 0.15 | 0.16 | 0.17 | 0.22 | 0.15 | 0.23 | 0.25 |
| STL | 1501 | 1391 | 1495 | 1671 | 1322 | 1159 | 1282 | 1112 | 1165 | 646 | 1079 | 235 |  | 0.04 | 0.05 | 0.06 | 0.11 | 0.07 | 0.08 | 0.06 | 0.06 | 0.06 | 0.09 | 0.04 | 0.05 | 0.08 | 0.05 | 0.09 | 0.08 | 0.06 | 0.05 | 0.13 | 0.14 | 0.05 | 0.20 | 0.07 | 0.13 | 0.27 | 0.11 | 0.15 | 0.14 | 0.13 | 0.14 | 0.09 | 0.14 | 0.13 | 0.11 | 0.16 | 0.16 | 0.17 | 0.17 | 0.16 | 0.14 | 0.16 | 0.12 | 0.17 | 0.17 | 0.16 | 0.16 | 0.25 | 0.20 | 0.23 | 0.20 | 0.18 | 0.13 | 0.12 | 0.19 | 0.07 | 0.09 | 0.19 | 0.20 | 0.09 | 0.14 | 0.19 | 0.16 | 0.19 | 0.12 | 0.13 | 0.19 | 0.19 | 0.28 | 0.19 | 0.19 | 0.25 | 0.17 | 0.13 | 0.19 | 0.19 | 0.21 | 0.25 | 0.19 | 0.25 | 0.28 |
| HAL | 1395 | 1273 | 1365 | 1562 | 1169 | 1049 | 1089 | 928 | 887 | 662 | 696 | 713 | 541 |  | 0.04 | 0.05 | 0.08 | 0.05 | 0.07 | 0.04 | 0.04 | 0.05 | 0.06 | 0.03 | 0.03 | 0.05 | 0.02 | 0.09 | 0.07 | 0.04 | 0.03 | 0.12 | 0.11 | 0.04 | 0.18 | 0.05 | 0.11 | 0.24 | 0.08 | 0.13 | 0.12 | 0.11 | 0.11 | 0.07 | 0.11 | 0.10 | 0.09 | 0.14 | 0.13 | 0.14 | 0.14 | 0.14 | 0.12 | 0.13 | 0.10 | 0.15 | 0.14 | 0.14 | 0.14 | 0.22 | 0.17 | 0.20 | 0.17 | 0.15 | 0.11 | 0.09 | 0.16 | 0.05 | 0.06 | 0.17 | 0.18 | 0.06 | 0.11 | 0.16 | 0.14 | 0.16 | 0.10 | 0.11 | 0.15 | 0.17 | 0.25 | 0.16 | 0.16 | 0.22 | 0.14 | 0.10 | 0.15 | 0.16 | 0.17 | 0.22 | 0.16 | 0.23 | 0.25 |
| BEA | 1204 | 1091 | 1151 | 1341 | 967 | 935 | 859 | 765 | 608 | 861 | 337 | 1214 | 1101 | 599 |  | 0.02 | 0.08 | 0.06 | 0.06 | 0.03 | 0.04 | 0.04 | 0.04 | 0.03 | 0.03 | 0.06 | 0.05 | 0.09 | 0.08 | 0.02 | 0.01 | 0.13 | 0.11 | 0.01 | 0.17 | 0.03 | 0.10 | 0.20 | 0.06 | 0.12 | 0.11 | 0.10 | 0.11 | 0.06 | 0.09 | 0.09 | 0.06 | 0.11 | 0.11 | 0.12 | 0.12 | 0.12 | 0.10 | 0.11 | 0.07 | 0.12 | 0.12 | 0.11 | 0.12 | 0.19 | 0.13 | 0.17 | 0.14 | 0.11 | 0.08 | 0.11 | 0.12 | 0.06 | 0.05 | 0.14 | 0.14 | 0.08 | 0.08 | 0.13 | 0.12 | 0.12 | 0.07 | 0.09 | 0.12 | 0.15 | 0.21 | 0.13 | 0.12 | 0.18 | 0.11 | 0.08 | 0.12 | 0.13 | 0.14 | 0.19 | 0.13 | 0.20 | 0.22 |
| MAC | 1337 | 1216 | 1293 | 1490 | 1098 | 1023 | 998 | 867 | 757 | 796 | 502 | 1023 | 875 | 343 | 279 |  | 0.09 | 0.08 | 0.08 | 0.05 | 0.04 | 0.05 | 0.04 | 0.05 | 0.04 | 0.07 | 0.06 | 0.11 | 0.10 | 0.03 | 0.02 | 0.14 | 0.12 | 0.03 | 0.18 | 0.03 | 0.11 | 0.21 | 0.07 | 0.13 | 0.12 | 0.12 | 0.12 | 0.07 | 0.09 | 0.10 | 0.06 | 0.12 | 0.11 | 0.12 | 0.13 | 0.12 | 0.10 | 0.12 | 0.08 | 0.12 | 0.12 | 0.12 | 0.12 | 0.20 | 0.14 | 0.17 | 0.15 | 0.12 | 0.09 | 0.12 | 0.13 | 0.08 | 0.06 | 0.15 | 0.15 | 0.09 | 0.09 | 0.13 | 0.12 | 0.12 | 0.08 | 0.10 | 0.12 | 0.16 | 0.22 | 0.13 | 0.13 | 0.19 | 0.11 | 0.08 | 0.12 | 0.13 | 0.14 | 0.19 | 0.13 | 0.20 | 0.23 |
| BRM | 1238 | 1258 | 1186 | 1176 | 1235 | 1399 | 1208 | 1348 | 1243 | 1834 | 1343 | 2399 | 2401 | 2032 | 1494 | 1766 |  | 0.12 | 0.12 | 0.10 | 0.10 | 0.10 | 0.10 | 0.09 | 0.09 | 0.11 | 0.10 | 0.14 | 0.12 | 0.09 | 0.08 | 0.18 | 0.15 | 0.08 | 0.22 | 0.09 | 0.15 | 0.26 | 0.11 | 0.16 | 0.16 | 0.15 | 0.15 | 0.11 | 0.14 | 0.14 | 0.12 | 0.16 | 0.16 | 0.17 | 0.18 | 0.17 | 0.15 | 0.16 | 0.14 | 0.18 | 0.17 | 0.17 | 0.17 | 0.24 | 0.19 | 0.23 | 0.20 | 0.18 | 0.14 | 0.16 | 0.17 | 0.13 | 0.10 | 0.19 | 0.19 | 0.13 | 0.14 | 0.18 | 0.17 | 0.18 | 0.12 | 0.14 | 0.17 | 0.20 | 0.27 | 0.17 | 0.17 | 0.23 | 0.16 | 0.13 | 0.18 | 0.18 | 0.20 | 0.24 | 0.18 | 0.24 | 0.27 |
| SPR | 1808 | 1696 | 1799 | 1978 | 1621 | 1462 | 1574 | 1403 | 1435 | 953 | 1312 | 501 | 313 | 666 | 1265 | 1004 | 2652 |  | 0.09 | 0.07 | 0.07 | 0.08 | 0.10 | 0.05 | 0.07 | 0.09 | 0.06 | 0.12 | 0.09 | 0.07 | 0.06 | 0.14 | 0.15 | 0.06 | 0.23 | 0.08 | 0.13 | 0.29 | 0.12 | 0.16 | 0.15 | 0.14 | 0.15 | 0.10 | 0.15 | 0.14 | 0.12 | 0.17 | 0.17 | 0.17 | 0.18 | 0.17 | 0.15 | 0.17 | 0.13 | 0.18 | 0.18 | 0.18 | 0.17 | 0.26 | 0.21 | 0.25 | 0.21 | 0.20 | 0.14 | 0.13 | 0.19 | 0.06 | 0.10 | 0.20 | 0.21 | 0.11 | 0.15 | 0.20 | 0.17 | 0.21 | 0.13 | 0.14 | 0.19 | 0.21 | 0.30 | 0.19 | 0.19 | 0.26 | 0.18 | 0.14 | 0.20 | 0.20 | 0.22 | 0.26 | 0.20 | 0.27 | 0.30 |
| RUS | 1883 | 1766 | 1867 | 2054 | 1680 | 1532 | 1620 | 1450 | 1453 | 1041 | 1293 | 677 | 455 | 605 | 1192 | 917 | 2634 | 215 |  | 0.07 | 0.08 | 0.08 | 0.10 | 0.06 | 0.07 | 0.09 | 0.08 | 0.13 | 0.12 | 0.07 | 0.06 | 0.17 | 0.15 | 0.06 | 0.22 | 0.08 | 0.14 | 0.26 | 0.12 | 0.16 | 0.15 | 0.15 | 0.15 | 0.11 | 0.14 | 0.14 | 0.12 | 0.17 | 0.16 | 0.17 | 0.18 | 0.17 | 0.15 | 0.16 | 0.13 | 0.18 | 0.17 | 0.17 | 0.17 | 0.25 | 0.19 | 0.23 | 0.20 | 0.18 | 0.13 | 0.14 | 0.18 | 0.11 | 0.09 | 0.19 | 0.19 | 0.11 | 0.14 | 0.18 | 0.16 | 0.19 | 0.13 | 0.13 | 0.18 | 0.20 | 0.28 | 0.18 | 0.18 | 0.24 | 0.16 | 0.13 | 0.18 | 0.18 | 0.20 | 0.25 | 0.19 | 0.25 | 0.28 |
| HAT | 1830 | 1707 | 1794 | 1993 | 1597 | 1491 | 1508 | 1357 | 1283 | 1117 | 1047 | 1040 | 815 | 456 | 816 | 554 | 2310 | 750 | 568 |  | 0.05 | 0.04 | 0.07 | 0.03 | 0.03 | 0.07 | 0.05 | 0.10 | 0.08 | 0.04 | 0.03 | 0.14 | 0.13 | 0.03 | 0.19 | 0.05 | 0.12 | 0.24 | 0.09 | 0.14 | 0.13 | 0.12 | 0.12 | 0.08 | 0.12 | 0.11 | 0.09 | 0.14 | 0.14 | 0.14 | 0.15 | 0.14 | 0.12 | 0.13 | 0.10 | 0.15 | 0.14 | 0.14 | 0.14 | 0.22 | 0.17 | 0.20 | 0.18 | 0.15 | 0.11 | 0.12 | 0.15 | 0.07 | 0.07 | 0.17 | 0.17 | 0.08 | 0.12 | 0.16 | 0.14 | 0.15 | 0.10 | 0.11 | 0.15 | 0.18 | 0.25 | 0.16 | 0.16 | 0.22 | 0.14 | 0.11 | 0.15 | 0.16 | 0.17 | 0.22 | 0.16 | 0.23 | 0.26 |
| NEO | 1988 | 1865 | 1950 | 2149 | 1753 | 1651 | 1661 | 1514 | 1431 | 1285 | 1185 | 1191 | 961 | 624 | 928 | 685 | 2412 | 858 | 657 | 168 |  | 0.05 | 0.06 | 0.04 | 0.04 | 0.07 | 0.06 | 0.10 | 0.09 | 0.04 | 0.03 | 0.14 | 0.13 | 0.03 | 0.19 | 0.05 | 0.12 | 0.24 | 0.09 | 0.14 | 0.13 | 0.12 | 0.13 | 0.08 | 0.12 | 0.11 | 0.08 | 0.14 | 0.13 | 0.14 | 0.15 | 0.14 | 0.12 | 0.13 | 0.10 | 0.15 | 0.15 | 0.14 | 0.14 | 0.22 | 0.17 | 0.20 | 0.18 | 0.15 | 0.10 | 0.12 | 0.16 | 0.08 | 0.07 | 0.17 | 0.18 | 0.09 | 0.12 | 0.16 | 0.14 | 0.16 | 0.10 | 0.11 | 0.15 | 0.17 | 0.25 | 0.16 | 0.16 | 0.22 | 0.14 | 0.11 | 0.16 | 0.16 | 0.17 | 0.22 | 0.16 | 0.23 | 0.25 |
| SMI | 2212 | 2092 | 2190 | 2383 | 1997 | 1860 | 1927 | 1760 | 1734 | 1390 | 1538 | 1053 | 831 | 848 | 1364 | 1087 | 2852 | 567 | 377 | 577 | 561 |  | 0.07 | 0.04 | 0.02 | 0.08 | 0.06 | 0.10 | 0.09 | 0.02 | 0.03 | 0.14 | 0.13 | 0.03 | 0.19 | 0.03 | 0.12 | 0.24 | 0.09 | 0.14 | 0.13 | 0.13 | 0.13 | 0.09 | 0.12 | 0.11 | 0.09 | 0.14 | 0.14 | 0.13 | 0.15 | 0.13 | 0.13 | 0.13 | 0.09 | 0.15 | 0.14 | 0.14 | 0.14 | 0.22 | 0.17 | 0.21 | 0.18 | 0.15 | 0.10 | 0.12 | 0.16 | 0.08 | 0.07 | 0.17 | 0.18 | 0.09 | 0.12 | 0.16 | 0.14 | 0.16 | 0.10 | 0.12 | 0.16 | 0.18 | 0.25 | 0.16 | 0.16 | 0.22 | 0.14 | 0.11 | 0.16 | 0.16 | 0.17 | 0.22 | 0.16 | 0.23 | 0.26 |
| PAL | 1752 | 1652 | 1690 | 1861 | 1532 | 1538 | 1421 | 1366 | 1183 | 1500 | 939 | 1789 | 1631 | 1090 | 642 | 766 | 1601 | 1708 | 1570 | 1033 | 1044 | 1598 |  | 0.06 | 0.06 | 0.08 | 0.08 | 0.13 | 0.11 | 0.05 | 0.03 | 0.16 | 0.13 | 0.05 | 0.20 | 0.05 | 0.12 | 0.22 | 0.08 | 0.14 | 0.13 | 0.13 | 0.13 | 0.09 | 0.11 | 0.11 | 0.06 | 0.13 | 0.12 | 0.13 | 0.13 | 0.13 | 0.11 | 0.13 | 0.09 | 0.13 | 0.13 | 0.12 | 0.13 | 0.20 | 0.14 | 0.18 | 0.16 | 0.12 | 0.10 | 0.14 | 0.13 | 0.10 | 0.07 | 0.15 | 0.15 | 0.10 | 0.10 | 0.13 | 0.13 | 0.13 | 0.09 | 0.11 | 0.13 | 0.17 | 0.23 | 0.13 | 0.13 | 0.19 | 0.12 | 0.09 | 0.13 | 0.14 | 0.15 | 0.20 | 0.14 | 0.20 | 0.23 |
| DEN | 2316 | 2198 | 2298 | 2487 | 2110 | 1964 | 2047 | 1878 | 1869 | 1474 | 1689 | 1067 | 867 | 993 | 1538 | 1260 | 3017 | 565 | 433 | 771 | 764 | 202 | 1798 |  | 0.02 | 0.06 | 0.04 | 0.08 | 0.07 | 0.03 | 0.03 | 0.12 | 0.12 | 0.02 | 0.18 | 0.04 | 0.11 | 0.24 | 0.09 | 0.13 | 0.13 | 0.12 | 0.12 | 0.07 | 0.12 | 0.11 | 0.09 | 0.14 | 0.14 | 0.14 | 0.15 | 0.14 | 0.12 | 0.14 | 0.10 | 0.15 | 0.14 | 0.14 | 0.14 | 0.22 | 0.17 | 0.20 | 0.18 | 0.15 | 0.10 | 0.11 | 0.16 | 0.06 | 0.07 | 0.17 | 0.18 | 0.08 | 0.11 | 0.17 | 0.14 | 0.16 | 0.10 | 0.10 | 0.16 | 0.17 | 0.25 | 0.16 | 0.16 | 0.22 | 0.15 | 0.11 | 0.16 | 0.17 | 0.18 | 0.22 | 0.16 | 0.23 | 0.26 |
| DAL | 2315 | 2196 | 2296 | 2486 | 2106 | 1962 | 2040 | 1872 | 1857 | 1478 | 1670 | 1089 | 883 | 976 | 1510 | 1232 | 2994 | 589 | 439 | 732 | 715 | 156 | 1753 | 58 |  | 0.07 | 0.05 | 0.09 | 0.07 | 0.01 | 0.02 | 0.13 | 0.12 | 0.02 | 0.18 | 0.02 | 0.12 | 0.24 | 0.09 | 0.14 | 0.13 | 0.12 | 0.13 | 0.08 | 0.11 | 0.11 | 0.09 | 0.13 | 0.13 | 0.13 | 0.14 | 0.13 | 0.12 | 0.13 | 0.09 | 0.14 | 0.14 | 0.13 | 0.14 | 0.22 | 0.17 | 0.20 | 0.17 | 0.15 | 0.10 | 0.11 | 0.16 | 0.07 | 0.07 | 0.17 | 0.17 | 0.08 | 0.11 | 0.16 | 0.14 | 0.16 | 0.10 | 0.11 | 0.15 | 0.17 | 0.25 | 0.16 | 0.16 | 0.22 | 0.14 | 0.11 | 0.15 | 0.16 | 0.17 | 0.22 | 0.16 | 0.23 | 0.25 |
| FRS | 6135 | 6258 | 6173 | 5974 | 6370 | 6477 | 6463 | 6609 | 6701 | 6912 | 6962 | 7309 | 7496 | 7525 | 7274 | 7458 | 6094 | 7803 | 7938 | 7965 | 8123 | 8300 | 7638 | 8363 | 8376 |  | 0.06 | 0.12 | 0.11 | 0.06 | 0.05 | 0.15 | 0.09 | 0.06 | 0.16 | 0.07 | 0.09 | 0.23 | 0.07 | 0.12 | 0.11 | 0.10 | 0.11 | 0.05 | 0.10 | 0.09 | 0.09 | 0.14 | 0.13 | 0.14 | 0.15 | 0.14 | 0.12 | 0.14 | 0.11 | 0.15 | 0.15 | 0.14 | 0.14 | 0.21 | 0.15 | 0.19 | 0.17 | 0.13 | 0.12 | 0.11 | 0.14 | 0.09 | 0.05 | 0.16 | 0.16 | 0.08 | 0.10 | 0.15 | 0.14 | 0.14 | 0.08 | 0.10 | 0.14 | 0.16 | 0.24 | 0.15 | 0.14 | 0.20 | 0.13 | 0.09 | 0.14 | 0.15 | 0.16 | 0.21 | 0.15 | 0.22 | 0.24 |
| SPB | 5484 | 5605 | 5509 | 5314 | 5703 | 5837 | 5782 | 5943 | 5999 | 6318 | 6241 | 6791 | 6946 | 6871 | 6524 | 6743 | 5198 | 7258 | 7359 | 7282 | 7426 | 7696 | 6792 | 7793 | 7795 | 1264 |  | 0.09 | 0.08 | 0.05 | 0.04 | 0.13 | 0.12 | 0.05 | 0.19 | 0.06 | 0.11 | 0.26 | 0.09 | 0.13 | 0.13 | 0.12 | 0.12 | 0.07 | 0.12 | 0.11 | 0.10 | 0.15 | 0.15 | 0.15 | 0.16 | 0.15 | 0.13 | 0.15 | 0.11 | 0.16 | 0.16 | 0.15 | 0.15 | 0.24 | 0.18 | 0.22 | 0.19 | 0.16 | 0.12 | 0.10 | 0.17 | 0.06 | 0.07 | 0.18 | 0.19 | 0.08 | 0.12 | 0.17 | 0.15 | 0.17 | 0.11 | 0.12 | 0.17 | 0.18 | 0.27 | 0.17 | 0.17 | 0.23 | 0.15 | 0.11 | 0.17 | 0.18 | 0.19 | 0.24 | 0.17 | 0.25 | 0.27 |
| BRE | 6425 | 6548 | 6465 | 6266 | 6662 | 6764 | 6757 | 6899 | 6997 | 7188 | 7261 | 7569 | 7761 | 7810 | 7576 | 7754 | 6421 | 8066 | 8207 | 8254 | 8413 | 8573 | 7954 | 8628 | 8643 | 349 | 1595 |  | 0.09 | 0.10 | 0.09 | 0.15 | 0.18 | 0.09 | 0.24 | 0.11 | 0.17 | 0.31 | 0.15 | 0.19 | 0.18 | 0.17 | 0.18 | 0.13 | 0.18 | 0.17 | 0.14 | 0.20 | 0.20 | 0.20 | 0.21 | 0.20 | 0.18 | 0.19 | 0.15 | 0.21 | 0.20 | 0.20 | 0.20 | 0.28 | 0.24 | 0.27 | 0.24 | 0.23 | 0.16 | 0.16 | 0.22 | 0.11 | 0.13 | 0.23 | 0.24 | 0.14 | 0.19 | 0.23 | 0.20 | 0.24 | 0.16 | 0.17 | 0.22 | 0.23 | 0.32 | 0.22 | 0.22 | 0.28 | 0.21 | 0.17 | 0.23 | 0.23 | 0.24 | 0.28 | 0.23 | 0.29 | 0.32 |
| DES | 6453 | 6575 | 6492 | 6293 | 6689 | 6791 | 6784 | 6927 | 7025 | 7215 | 7288 | 7595 | 7788 | 7837 | 7604 | 7781 | 6449 | 8092 | 8234 | 8281 | 8440 | 8600 | 7982 | 8654 | 8670 | 375 | 1617 | 28 |  | 0.09 | 0.08 | 0.13 | 0.17 | 0.08 | 0.22 | 0.10 | 0.15 | 0.29 | 0.14 | 0.18 | 0.17 | 0.16 | 0.17 | 0.12 | 0.16 | 0.15 | 0.14 | 0.19 | 0.19 | 0.19 | 0.19 | 0.19 | 0.17 | 0.18 | 0.15 | 0.20 | 0.19 | 0.19 | 0.19 | 0.27 | 0.22 | 0.26 | 0.22 | 0.22 | 0.15 | 0.14 | 0.21 | 0.09 | 0.12 | 0.21 | 0.23 | 0.12 | 0.17 | 0.22 | 0.18 | 0.22 | 0.15 | 0.16 | 0.21 | 0.22 | 0.31 | 0.21 | 0.21 | 0.27 | 0.20 | 0.16 | 0.22 | 0.22 | 0.23 | 0.27 | 0.21 | 0.28 | 0.31 |
| MON | 2377 | 2255 | 2350 | 2545 | 2154 | 2026 | 2077 | 1915 | 1871 | 1575 | 1654 | 1271 | 1045 | 988 | 1443 | 1176 | 2937 | 790 | 594 | 627 | 549 | 225 | 1584 | 342 | 285 | 8487 | 7858 | 8764 | 8791 |  | 0.02 | 0.14 | 0.11 | 0.02 | 0.18 | 0.01 | 0.11 | 0.21 | 0.07 | 0.13 | 0.12 | 0.11 | 0.12 | 0.07 | 0.09 | 0.10 | 0.07 | 0.12 | 0.11 | 0.12 | 0.13 | 0.11 | 0.11 | 0.11 | 0.08 | 0.13 | 0.13 | 0.12 | 0.12 | 0.20 | 0.14 | 0.18 | 0.15 | 0.12 | 0.09 | 0.12 | 0.13 | 0.08 | 0.06 | 0.15 | 0.15 | 0.08 | 0.09 | 0.13 | 0.13 | 0.13 | 0.08 | 0.10 | 0.13 | 0.15 | 0.22 | 0.13 | 0.13 | 0.19 | 0.12 | 0.08 | 0.13 | 0.14 | 0.15 | 0.20 | 0.14 | 0.20 | 0.23 |
| HOU | 2393 | 2271 | 2364 | 2561 | 2168 | 2044 | 2088 | 1928 | 1876 | 1605 | 1652 | 1324 | 1095 | 1000 | 1428 | 1166 | 2919 | 851 | 649 | 613 | 515 | 291 | 1539 | 421 | 364 | 8513 | 7870 | 8792 | 8819 | 79 |  | 0.13 | 0.11 | 0.01 | 0.17 | 0.02 | 0.10 | 0.20 | 0.06 | 0.11 | 0.11 | 0.10 | 0.11 | 0.06 | 0.08 | 0.09 | 0.06 | 0.11 | 0.10 | 0.11 | 0.12 | 0.11 | 0.09 | 0.11 | 0.07 | 0.12 | 0.12 | 0.11 | 0.11 | 0.18 | 0.13 | 0.16 | 0.14 | 0.10 | 0.08 | 0.11 | 0.12 | 0.07 | 0.05 | 0.14 | 0.14 | 0.07 | 0.08 | 0.12 | 0.11 | 0.11 | 0.07 | 0.09 | 0.11 | 0.14 | 0.21 | 0.12 | 0.12 | 0.18 | 0.11 | 0.07 | 0.11 | 0.13 | 0.13 | 0.18 | 0.12 | 0.19 | 0.22 |
| CES | 6640 | 6763 | 6679 | 6481 | 6876 | 6980 | 6971 | 7115 | 7211 | 7407 | 7473 | 7790 | 7982 | 8027 | 7788 | 7968 | 6618 | 8287 | 8427 | 8470 | 8628 | 8792 | 8159 | 8848 | 8863 | 525 | 1702 | 223 | 198 | 8983 | 9011 |  | 0.21 | 0.13 | 0.28 | 0.15 | 0.20 | 0.34 | 0.18 | 0.22 | 0.21 | 0.21 | 0.21 | 0.16 | 0.21 | 0.20 | 0.18 | 0.24 | 0.23 | 0.23 | 0.24 | 0.23 | 0.21 | 0.23 | 0.19 | 0.24 | 0.23 | 0.24 | 0.23 | 0.31 | 0.27 | 0.31 | 0.27 | 0.28 | 0.20 | 0.18 | 0.25 | 0.13 | 0.16 | 0.25 | 0.27 | 0.16 | 0.22 | 0.26 | 0.23 | 0.28 | 0.19 | 0.20 | 0.26 | 0.26 | 0.36 | 0.25 | 0.25 | 0.31 | 0.24 | 0.20 | 0.28 | 0.26 | 0.28 | 0.32 | 0.26 | 0.32 | 0.36 |
| SPS | 5744 | 5864 | 5766 | 5574 | 5959 | 6097 | 6035 | 6198 | 6246 | 6585 | 6483 | 7069 | 7217 | 7124 | 6758 | 6984 | 5400 | 7530 | 7625 | 7527 | 7668 | 7956 | 6999 | 8058 | 8059 | 1371 | 332 | 1670 | 1689 | 8112 | 8121 | 1737 |  | 0.11 | 0.24 | 0.12 | 0.14 | 0.28 | 0.10 | 0.17 | 0.16 | 0.15 | 0.16 | 0.09 | 0.14 | 0.13 | 0.14 | 0.18 | 0.18 | 0.19 | 0.19 | 0.19 | 0.17 | 0.18 | 0.16 | 0.20 | 0.19 | 0.19 | 0.19 | 0.26 | 0.20 | 0.24 | 0.21 | 0.19 | 0.17 | 0.18 | 0.18 | 0.14 | 0.09 | 0.21 | 0.20 | 0.14 | 0.15 | 0.19 | 0.19 | 0.20 | 0.12 | 0.15 | 0.18 | 0.21 | 0.30 | 0.18 | 0.18 | 0.25 | 0.16 | 0.13 | 0.19 | 0.19 | 0.21 | 0.25 | 0.19 | 0.26 | 0.30 |
| TRA | 2542 | 2421 | 2518 | 2711 | 2324 | 2189 | 2250 | 2086 | 2051 | 1724 | 1841 | 1369 | 1155 | 1164 | 1639 | 1369 | 3133 | 871 | 702 | 824 | 747 | 333 | 1779 | 328 | 288 | 8633 | 8026 | 8905 | 8932 | 199 | 242 | 9125 | 8283 |  | 0.17 | 0.02 | 0.10 | 0.21 | 0.07 | 0.12 | 0.12 | 0.11 | 0.11 | 0.07 | 0.10 | 0.09 | 0.07 | 0.12 | 0.11 | 0.12 | 0.13 | 0.12 | 0.10 | 0.12 | 0.08 | 0.13 | 0.12 | 0.12 | 0.12 | 0.20 | 0.14 | 0.18 | 0.15 | 0.12 | 0.09 | 0.11 | 0.13 | 0.07 | 0.06 | 0.15 | 0.15 | 0.08 | 0.09 | 0.14 | 0.12 | 0.13 | 0.08 | 0.10 | 0.13 | 0.16 | 0.23 | 0.14 | 0.14 | 0.19 | 0.12 | 0.09 | 0.13 | 0.14 | 0.15 | 0.20 | 0.14 | 0.21 | 0.23 |
| SER | 7087 | 7209 | 7130 | 6932 | 7326 | 7419 | 7424 | 7561 | 7669 | 7823 | 7935 | 8172 | 8374 | 8458 | 8257 | 8422 | 7145 | 8672 | 8825 | 8908 | 9070 | 9196 | 8659 | 9237 | 9256 | 1100 | 2313 | 752 | 725 | 9394 | 9427 | 612 | 2341 | 9526 |  | 0.18 | 0.22 | 0.41 | 0.18 | 0.25 | 0.23 | 0.22 | 0.23 | 0.17 | 0.23 | 0.21 | 0.20 | 0.26 | 0.25 | 0.25 | 0.27 | 0.26 | 0.24 | 0.25 | 0.23 | 0.27 | 0.26 | 0.27 | 0.26 | 0.35 | 0.28 | 0.35 | 0.29 | 0.33 | 0.24 | 0.25 | 0.26 | 0.21 | 0.16 | 0.28 | 0.27 | 0.20 | 0.24 | 0.27 | 0.26 | 0.34 | 0.19 | 0.22 | 0.27 | 0.31 | 0.43 | 0.25 | 0.25 | 0.33 | 0.23 | 0.21 | 0.31 | 0.26 | 0.31 | 0.35 | 0.28 | 0.34 | 0.42 |
| NUE | 2707 | 2584 | 2677 | 2874 | 2480 | 2358 | 2398 | 2239 | 2182 | 1919 | 1951 | 1618 | 1394 | 1312 | 1709 | 1457 | 3191 | 1130 | 941 | 905 | 781 | 566 | 1741 | 613 | 567 | 8828 | 8179 | 9107 | 9134 | 350 | 315 | 9326 | 8428 | 290 | 9741 |  | 0.11 | 0.21 | 0.07 | 0.13 | 0.12 | 0.12 | 0.12 | 0.08 | 0.10 | 0.10 | 0.07 | 0.12 | 0.12 | 0.12 | 0.13 | 0.11 | 0.11 | 0.11 | 0.09 | 0.13 | 0.13 | 0.12 | 0.12 | 0.20 | 0.14 | 0.18 | 0.15 | 0.12 | 0.09 | 0.12 | 0.13 | 0.08 | 0.06 | 0.14 | 0.15 | 0.09 | 0.09 | 0.13 | 0.12 | 0.13 | 0.08 | 0.10 | 0.13 | 0.16 | 0.22 | 0.13 | 0.13 | 0.19 | 0.12 | 0.08 | 0.13 | 0.13 | 0.14 | 0.20 | 0.13 | 0.20 | 0.23 |
| ALD | 7312 | 7435 | 7351 | 7152 | 7548 | 7650 | 7643 | 7786 | 7883 | 8074 | 8146 | 8448 | 8643 | 8696 | 8461 | 8640 | 7284 | 8946 | 9090 | 9141 | 9300 | 9457 | 8830 | 9510 | 9525 | 1192 | 2263 | 887 | 859 | 9649 | 9678 | 673 | 2226 | 9789 | 442 | 9993 |  | 0.26 | 0.08 | 0.16 | 0.15 | 0.14 | 0.14 | 0.09 | 0.13 | 0.12 | 0.13 | 0.17 | 0.16 | 0.17 | 0.18 | 0.18 | 0.16 | 0.17 | 0.15 | 0.18 | 0.18 | 0.17 | 0.17 | 0.24 | 0.18 | 0.22 | 0.20 | 0.17 | 0.16 | 0.17 | 0.17 | 0.13 | 0.08 | 0.19 | 0.19 | 0.13 | 0.13 | 0.17 | 0.18 | 0.18 | 0.11 | 0.13 | 0.17 | 0.20 | 0.28 | 0.17 | 0.17 | 0.23 | 0.15 | 0.12 | 0.18 | 0.17 | 0.19 | 0.24 | 0.18 | 0.24 | 0.28 |
| NEG | 2618 | 2533 | 2551 | 2698 | 2418 | 2453 | 2311 | 2284 | 2093 | 2455 | 1873 | 2718 | 2540 | 2007 | 1596 | 1706 | 2070 | 2563 | 2391 | 1822 | 1761 | 2306 | 955 | 2505 | 2451 | 8136 | 7163 | 8469 | 8497 | 2213 | 2146 | 8661 | 7328 | 2379 | 9202 | 2232 | 9319 |  | 0.18 | 0.28 | 0.26 | 0.26 | 0.27 | 0.22 | 0.23 | 0.22 | 0.20 | 0.22 | 0.22 | 0.20 | 0.22 | 0.22 | 0.20 | 0.20 | 0.22 | 0.22 | 0.21 | 0.22 | 0.21 | 0.26 | 0.16 | 0.25 | 0.22 | 0.21 | 0.22 | 0.32 | 0.13 | 0.28 | 0.18 | 0.21 | 0.14 | 0.26 | 0.20 | 0.15 | 0.22 | 0.22 | 0.16 | 0.24 | 0.15 | 0.33 | 0.32 | 0.13 | 0.13 | 0.22 | 0.13 | 0.19 | 0.19 | 0.15 | 0.22 | 0.28 | 0.23 | 0.25 | 0.33 |
| GRA | 7840 | 7963 | 7878 | 7680 | 8076 | 8180 | 8170 | 8314 | 8409 | 8606 | 8671 | 8982 | 9176 | 9226 | 8983 | 9166 | 7784 | 9480 | 9623 | 9670 | 9828 | 9990 | 9341 | 10043 | 10059 | 1709 | 2687 | 1417 | 1390 | 10181 | 10210 | 1200 | 2601 | 10322 | 884 | 10525 | 534 | 9807 |  | 0.12 | 0.11 | 0.11 | 0.11 | 0.06 | 0.07 | 0.08 | 0.07 | 0.10 | 0.09 | 0.11 | 0.12 | 0.12 | 0.10 | 0.11 | 0.10 | 0.11 | 0.12 | 0.11 | 0.11 | 0.16 | 0.09 | 0.13 | 0.13 | 0.07 | 0.11 | 0.15 | 0.08 | 0.12 | 0.04 | 0.13 | 0.10 | 0.10 | 0.06 | 0.08 | 0.12 | 0.08 | 0.05 | 0.09 | 0.08 | 0.15 | 0.18 | 0.08 | 0.08 | 0.15 | 0.07 | 0.05 | 0.07 | 0.08 | 0.09 | 0.15 | 0.10 | 0.16 | 0.19 |
| TIK | 8282 | 8400 | 8333 | 8142 | 8521 | 8587 | 8627 | 8744 | 8878 | 8929 | 9150 | 9186 | 9407 | 9587 | 9483 | 9610 | 8526 | 9682 | 9861 | 10044 | 10211 | 10237 | 9956 | 10241 | 10270 | 2660 | 3893 | 2313 | 2288 | 10452 | 10497 | 2191 | 3913 | 10554 | 1580 | 10801 | 1733 | 10596 | 1600 |  | 0.05 | 0.06 | 0.05 | 0.12 | 0.15 | 0.14 | 0.15 | 0.19 | 0.18 | 0.19 | 0.20 | 0.19 | 0.18 | 0.19 | 0.17 | 0.20 | 0.20 | 0.19 | 0.19 | 0.26 | 0.20 | 0.24 | 0.22 | 0.20 | 0.18 | 0.19 | 0.18 | 0.16 | 0.10 | 0.21 | 0.20 | 0.14 | 0.15 | 0.19 | 0.19 | 0.21 | 0.13 | 0.15 | 0.19 | 0.22 | 0.30 | 0.19 | 0.18 | 0.24 | 0.17 | 0.14 | 0.20 | 0.19 | 0.21 | 0.26 | 0.20 | 0.26 | 0.30 |
| RAR | 8408 | 8526 | 8459 | 8268 | 8647 | 8714 | 8753 | 8871 | 9004 | 9056 | 9275 | 9312 | 9534 | 9714 | 9609 | 9736 | 8646 | 9809 | 9988 | 10170 | 10338 | 10364 | 10080 | 10368 | 10397 | 2758 | 3983 | 2410 | 2385 | 10579 | 10624 | 2281 | 3995 | 10680 | 1670 | 10928 | 1798 | 10716 | 1629 | 127 |  | 0.02 | 0.03 | 0.11 | 0.14 | 0.13 | 0.14 | 0.17 | 0.17 | 0.18 | 0.19 | 0.18 | 0.17 | 0.18 | 0.16 | 0.19 | 0.18 | 0.18 | 0.18 | 0.24 | 0.19 | 0.23 | 0.20 | 0.18 | 0.17 | 0.18 | 0.17 | 0.15 | 0.09 | 0.20 | 0.19 | 0.14 | 0.14 | 0.18 | 0.18 | 0.19 | 0.12 | 0.14 | 0.17 | 0.20 | 0.28 | 0.17 | 0.17 | 0.23 | 0.15 | 0.13 | 0.18 | 0.17 | 0.20 | 0.24 | 0.18 | 0.25 | 0.28 |
| SOC | 8436 | 8555 | 8485 | 8292 | 8675 | 8747 | 8780 | 8901 | 9030 | 9100 | 9301 | 9371 | 9590 | 9756 | 9633 | 9768 | 8637 | 9869 | 10044 | 10212 | 10379 | 10421 | 10091 | 10430 | 10457 | 2689 | 3895 | 2339 | 2313 | 10633 | 10677 | 2197 | 3892 | 10740 | 1591 | 10983 | 1678 | 10707 | 1470 | 253 | 187 |  | 0.01 | 0.11 | 0.13 | 0.13 | 0.13 | 0.17 | 0.16 | 0.18 | 0.19 | 0.18 | 0.16 | 0.17 | 0.15 | 0.19 | 0.18 | 0.18 | 0.18 | 0.24 | 0.18 | 0.22 | 0.20 | 0.17 | 0.16 | 0.17 | 0.17 | 0.14 | 0.08 | 0.19 | 0.18 | 0.13 | 0.13 | 0.17 | 0.18 | 0.18 | 0.11 | 0.14 | 0.17 | 0.20 | 0.27 | 0.17 | 0.17 | 0.23 | 0.15 | 0.12 | 0.18 | 0.17 | 0.19 | 0.24 | 0.18 | 0.24 | 0.28 |
| GES | 8542 | 8661 | 8592 | 8399 | 8782 | 8852 | 8887 | 9007 | 9137 | 9204 | 9408 | 9472 | 9692 | 9860 | 9740 | 9874 | 8747 | 9970 | 10146 | 10316 | 10483 | 10522 | 10200 | 10530 | 10558 | 2794 | 3995 | 2444 | 2418 | 10735 | 10780 | 2300 | 3988 | 10841 | 1695 | 11085 | 1770 | 10816 | 1540 | 315 | 210 | 109 |  | 0.11 | 0.14 | 0.13 | 0.14 | 0.17 | 0.17 | 0.18 | 0.19 | 0.18 | 0.17 | 0.18 | 0.16 | 0.19 | 0.19 | 0.18 | 0.18 | 0.25 | 0.19 | 0.23 | 0.20 | 0.18 | 0.17 | 0.17 | 0.17 | 0.15 | 0.09 | 0.20 | 0.19 | 0.13 | 0.14 | 0.18 | 0.18 | 0.19 | 0.12 | 0.14 | 0.18 | 0.20 | 0.28 | 0.18 | 0.17 | 0.23 | 0.16 | 0.13 | 0.18 | 0.18 | 0.20 | 0.25 | 0.18 | 0.25 | 0.28 |
| TUH | 8708 | 8827 | 8757 | 8564 | 8948 | 9021 | 9052 | 9175 | 9302 | 9378 | 9573 | 9652 | 9871 | 10033 | 9904 | 10042 | 8890 | 10151 | 10325 | 10489 | 10655 | 10702 | 10356 | 10712 | 10739 | 2897 | 4076 | 2549 | 2522 | 10914 | 10957 | 2393 | 4054 | 11021 | 1797 | 11264 | 1828 | 10958 | 1544 | 508 | 398 | 283 | 194 |  | 0.09 | 0.08 | 0.09 | 0.13 | 0.13 | 0.14 | 0.15 | 0.14 | 0.13 | 0.14 | 0.11 | 0.15 | 0.15 | 0.14 | 0.14 | 0.21 | 0.15 | 0.18 | 0.17 | 0.12 | 0.12 | 0.13 | 0.13 | 0.10 | 0.04 | 0.16 | 0.15 | 0.09 | 0.09 | 0.14 | 0.14 | 0.13 | 0.08 | 0.10 | 0.13 | 0.16 | 0.23 | 0.14 | 0.14 | 0.20 | 0.12 | 0.08 | 0.14 | 0.14 | 0.15 | 0.20 | 0.14 | 0.21 | 0.24 |
| CHI | 3982 | 3881 | 3985 | 4146 | 3824 | 3655 | 3789 | 3620 | 3665 | 3142 | 3535 | 2581 | 2508 | 2852 | 3431 | 3152 | 4878 | 2231 | 2247 | 2674 | 2642 | 2097 | 3685 | 1905 | 1942 | 9544 | 9234 | 9747 | 9771 | 2105 | 2159 | 9968 | 9533 | 1918 | 10229 | 2039 | 10579 | 4271 | 11100 | 10871 | 10990 | 11099 | 11183 | 11376 |  | 0.10 | 0.10 | 0.13 | 0.12 | 0.14 | 0.15 | 0.15 | 0.13 | 0.14 | 0.13 | 0.15 | 0.15 | 0.14 | 0.14 | 0.20 | 0.13 | 0.17 | 0.16 | 0.11 | 0.13 | 0.18 | 0.11 | 0.15 | 0.06 | 0.16 | 0.13 | 0.13 | 0.08 | 0.12 | 0.14 | 0.12 | 0.06 | 0.12 | 0.11 | 0.17 | 0.24 | 0.11 | 0.11 | 0.18 | 0.09 | 0.07 | 0.11 | 0.12 | 0.14 | 0.19 | 0.13 | 0.20 | 0.24 |
| LOS | 4026 | 3926 | 4030 | 4190 | 3869 | 3700 | 3836 | 3667 | 3713 | 3188 | 3584 | 2626 | 2555 | 2902 | 3481 | 3203 | 4927 | 2279 | 2297 | 2725 | 2693 | 2148 | 3737 | 1956 | 1993 | 9570 | 9269 | 9772 | 9795 | 2157 | 2211 | 9993 | 9569 | 1969 | 10249 | 2090 | 10601 | 4321 | 11121 | 10880 | 10999 | 11109 | 11193 | 11386 | 51 |  | 0.11 | 0.14 | 0.13 | 0.15 | 0.15 | 0.15 | 0.13 | 0.14 | 0.13 | 0.15 | 0.15 | 0.15 | 0.15 | 0.21 | 0.14 | 0.18 | 0.17 | 0.12 | 0.14 | 0.17 | 0.13 | 0.14 | 0.06 | 0.16 | 0.15 | 0.12 | 0.09 | 0.13 | 0.15 | 0.13 | 0.08 | 0.11 | 0.13 | 0.17 | 0.23 | 0.13 | 0.13 | 0.19 | 0.11 | 0.09 | 0.13 | 0.13 | 0.15 | 0.20 | 0.14 | 0.21 | 0.24 |
| SAI | 3646 | 3626 | 3583 | 3627 | 3557 | 3681 | 3484 | 3558 | 3379 | 3937 | 3291 | 4382 | 4268 | 3739 | 3171 | 3396 | 2488 | 4385 | 4253 | 3710 | 3693 | 4254 | 2683 | 4457 | 4408 | 7438 | 6259 | 7787 | 7813 | 4198 | 4137 | 7934 | 6324 | 4376 | 8536 | 4254 | 8521 | 2031 | 8925 | 10089 | 10192 | 10126 | 10231 | 10326 | 6287 | 6338 |  | 0.12 | 0.11 | 0.12 | 0.13 | 0.12 | 0.11 | 0.12 | 0.10 | 0.13 | 0.13 | 0.12 | 0.12 | 0.18 | 0.12 | 0.16 | 0.14 | 0.10 | 0.11 | 0.16 | 0.10 | 0.13 | 0.07 | 0.14 | 0.12 | 0.12 | 0.08 | 0.11 | 0.13 | 0.11 | 0.07 | 0.11 | 0.10 | 0.17 | 0.21 | 0.11 | 0.11 | 0.17 | 0.09 | 0.08 | 0.10 | 0.11 | 0.12 | 0.18 | 0.12 | 0.18 | 0.21 |
| CUA | 3913 | 3842 | 3845 | 3969 | 3735 | 3790 | 3631 | 3625 | 3431 | 3825 | 3228 | 4081 | 3894 | 3368 | 2964 | 3076 | 3109 | 3886 | 3698 | 3136 | 3046 | 3551 | 2326 | 3738 | 3682 | 8847 | 7733 | 9194 | 9221 | 3414 | 3339 | 9361 | 7833 | 3546 | 9945 | 3336 | 9982 | 1372 | 10419 | 11447 | 11559 | 11517 | 11625 | 11741 | 5318 | 5366 | 1614 |  | 0.00 | 0.12 | 0.13 | 0.13 | 0.11 | 0.12 | 0.13 | 0.13 | 0.13 | 0.13 | 0.13 | 0.17 | 0.13 | 0.15 | 0.13 | 0.12 | 0.14 | 0.21 | 0.11 | 0.18 | 0.10 | 0.13 | 0.13 | 0.16 | 0.11 | 0.12 | 0.13 | 0.12 | 0.10 | 0.15 | 0.12 | 0.22 | 0.23 | 0.12 | 0.11 | 0.19 | 0.10 | 0.11 | 0.12 | 0.12 | 0.15 | 0.19 | 0.14 | 0.19 | 0.23 |
| MED | 3913 | 3842 | 3845 | 3969 | 3735 | 3790 | 3631 | 3625 | 3431 | 3825 | 3228 | 4081 | 3894 | 3368 | 2964 | 3076 | 3109 | 3886 | 3698 | 3136 | 3046 | 3551 | 2326 | 3738 | 3682 | 8847 | 7733 | 9194 | 9221 | 3414 | 3339 | 9361 | 7833 | 3546 | 9945 | 3336 | 9982 | 1372 | 10419 | 11447 | 11559 | 11517 | 11625 | 11741 | 5318 | 5366 | 1614 | 0 |  | 0.12 | 0.13 | 0.13 | 0.12 | 0.12 | 0.13 | 0.13 | 0.13 | 0.12 | 0.13 | 0.17 | 0.11 | 0.14 | 0.13 | 0.10 | 0.13 | 0.21 | 0.11 | 0.17 | 0.09 | 0.13 | 0.12 | 0.16 | 0.10 | 0.11 | 0.13 | 0.11 | 0.09 | 0.15 | 0.11 | 0.21 | 0.22 | 0.11 | 0.11 | 0.18 | 0.10 | 0.10 | 0.11 | 0.11 | 0.14 | 0.19 | 0.13 | 0.18 | 0.22 |
| TUC | 5539 | 5539 | 5481 | 5488 | 5486 | 5626 | 5426 | 5519 | 5350 | 5928 | 5288 | 6394 | 6286 | 5759 | 5187 | 5417 | 4313 | 6406 | 6273 | 5724 | 5700 | 6260 | 4703 | 6462 | 6411 | 7714 | 6451 | 8043 | 8064 | 6189 | 6124 | 8127 | 6391 | 6360 | 8732 | 6212 | 8577 | 3983 | 8849 | 10298 | 10372 | 10255 | 10345 | 10383 | 8251 | 8301 | 2021 | 3084 | 3084 |  | 0.02 | 0.04 | 0.06 | 0.01 | 0.07 | 0.02 | 0.02 | 0.01 | 0.06 | 0.07 | 0.11 | 0.07 | 0.05 | 0.10 | 0.07 | 0.22 | 0.10 | 0.18 | 0.12 | 0.05 | 0.11 | 0.17 | 0.12 | 0.11 | 0.07 | 0.10 | 0.10 | 0.16 | 0.10 | 0.22 | 0.20 | 0.11 | 0.10 | 0.17 | 0.10 | 0.12 | 0.11 | 0.11 | 0.14 | 0.19 | 0.15 | 0.19 | 0.22 |
| FLO | 6162 | 6175 | 6109 | 6095 | 6134 | 6283 | 6083 | 6189 | 6030 | 6620 | 5991 | 7110 | 7017 | 6499 | 5915 | 6156 | 4925 | 7154 | 7030 | 6491 | 6475 | 7036 | 5462 | 7238 | 7189 | 7550 | 6296 | 7859 | 7877 | 6974 | 6911 | 7916 | 6189 | 7149 | 8504 | 7010 | 8299 | 4778 | 8513 | 10033 | 10094 | 9960 | 10042 | 10058 | 9049 | 9099 | 2782 | 3896 | 3896 | 814 |  | 0.06 | 0.06 | 0.02 | 0.08 | 0.02 | 0.03 | 0.01 | 0.06 | 0.08 | 0.13 | 0.08 | 0.06 | 0.12 | 0.07 | 0.22 | 0.12 | 0.19 | 0.12 | 0.06 | 0.12 | 0.17 | 0.13 | 0.12 | 0.07 | 0.12 | 0.11 | 0.17 | 0.12 | 0.23 | 0.22 | 0.12 | 0.11 | 0.19 | 0.11 | 0.13 | 0.12 | 0.13 | 0.15 | 0.21 | 0.16 | 0.21 | 0.24 |
| BIT | 5358 | 5341 | 5296 | 5332 | 5274 | 5397 | 5201 | 5273 | 5092 | 5641 | 4994 | 6063 | 5931 | 5394 | 4849 | 5056 | 4175 | 6018 | 5866 | 5305 | 5261 | 5813 | 4311 | 6013 | 5959 | 8209 | 6952 | 8546 | 8568 | 5720 | 5651 | 8646 | 6921 | 5880 | 9256 | 5709 | 9132 | 3508 | 9433 | 10833 | 10915 | 10808 | 10902 | 10953 | 7736 | 7785 | 1717 | 2471 | 2471 | 705 | 1465 |  | 0.05 | 0.04 | 0.09 | 0.06 | 0.06 | 0.05 | 0.05 | 0.09 | 0.13 | 0.09 | 0.07 | 0.12 | 0.08 | 0.21 | 0.12 | 0.18 | 0.12 | 0.06 | 0.12 | 0.17 | 0.12 | 0.12 | 0.07 | 0.12 | 0.11 | 0.16 | 0.12 | 0.22 | 0.22 | 0.12 | 0.12 | 0.19 | 0.11 | 0.12 | 0.12 | 0.13 | 0.15 | 0.20 | 0.16 | 0.21 | 0.24 |
| MAU | 5114 | 5087 | 5050 | 5102 | 5011 | 5124 | 4931 | 4990 | 4804 | 5332 | 4685 | 5726 | 5583 | 5043 | 4517 | 4710 | 3972 | 5651 | 5491 | 4925 | 4873 | 5418 | 3953 | 5616 | 5562 | 8405 | 7160 | 8747 | 8771 | 5315 | 5244 | 8861 | 7152 | 5469 | 9472 | 5288 | 9376 | 3112 | 9703 | 11052 | 11140 | 11044 | 11141 | 11203 | 7306 | 7355 | 1484 | 2016 | 2016 | 1153 | 1931 | 467 |  | 0.06 | 0.08 | 0.06 | 0.07 | 0.05 | 0.04 | 0.09 | 0.12 | 0.08 | 0.07 | 0.10 | 0.08 | 0.19 | 0.11 | 0.16 | 0.10 | 0.06 | 0.11 | 0.15 | 0.10 | 0.11 | 0.07 | 0.10 | 0.10 | 0.14 | 0.11 | 0.20 | 0.20 | 0.11 | 0.11 | 0.18 | 0.10 | 0.11 | 0.11 | 0.12 | 0.14 | 0.19 | 0.14 | 0.19 | 0.22 |
| ARA | 5961 | 5961 | 5903 | 5911 | 5908 | 6047 | 5847 | 5939 | 5770 | 6345 | 5704 | 6806 | 6694 | 6165 | 5596 | 5823 | 4735 | 6808 | 6671 | 6119 | 6089 | 6647 | 5102 | 6849 | 6797 | 7924 | 6662 | 8244 | 8264 | 6569 | 6503 | 8316 | 6580 | 6736 | 8914 | 6580 | 8734 | 4358 | 8976 | 10464 | 10532 | 10406 | 10492 | 10518 | 8615 | 8666 | 2427 | 3387 | 3387 | 422 | 575 | 922 | 1388 |  | 0.07 | 0.02 | 0.02 | 0.01 | 0.06 | 0.07 | 0.12 | 0.07 | 0.05 | 0.11 | 0.06 | 0.21 | 0.11 | 0.17 | 0.11 | 0.05 | 0.11 | 0.16 | 0.11 | 0.11 | 0.06 | 0.10 | 0.10 | 0.15 | 0.11 | 0.21 | 0.20 | 0.11 | 0.11 | 0.17 | 0.10 | 0.11 | 0.11 | 0.12 | 0.14 | 0.19 | 0.15 | 0.19 | 0.22 |
| REC | 6731 | 6759 | 6683 | 6643 | 6733 | 6892 | 6695 | 6817 | 6673 | 7273 | 6665 | 7792 | 7721 | 7221 | 6626 | 6880 | 5501 | 7884 | 7777 | 7255 | 7254 | 7814 | 6222 | 8016 | 7969 | 7182 | 5967 | 7466 | 7481 | 7769 | 7710 | 7493 | 5811 | 7951 | 8054 | 7830 | 7801 | 5602 | 7954 | 9522 | 9570 | 9422 | 9495 | 9488 | 9864 | 9915 | 3577 | 4790 | 4790 | 1708 | 909 | 2374 | 2840 | 1474 |  | 0.08 | 0.08 | 0.06 | 0.07 | 0.14 | 0.13 | 0.13 | 0.10 | 0.12 | 0.03 | 0.18 | 0.12 | 0.14 | 0.09 | 0.11 | 0.13 | 0.13 | 0.11 | 0.13 | 0.10 | 0.12 | 0.10 | 0.14 | 0.12 | 0.20 | 0.22 | 0.13 | 0.13 | 0.19 | 0.11 | 0.11 | 0.12 | 0.13 | 0.15 | 0.20 | 0.15 | 0.21 | 0.23 |
| PLM | 6270 | 6267 | 6212 | 6224 | 6211 | 6346 | 6147 | 6234 | 6062 | 6631 | 5986 | 7079 | 6960 | 6427 | 5867 | 6086 | 5050 | 7062 | 6918 | 6361 | 6324 | 6878 | 5354 | 7079 | 7026 | 8204 | 6945 | 8520 | 8539 | 6791 | 6723 | 8585 | 6852 | 6953 | 9178 | 6785 | 8984 | 4578 | 9207 | 10717 | 10781 | 10650 | 10733 | 10752 | 8813 | 8862 | 2698 | 3535 | 3535 | 745 | 701 | 1077 | 1519 | 341 | 1498 |  | 0.02 | 0.01 | 0.06 | 0.07 | 0.12 | 0.06 | 0.05 | 0.11 | 0.07 | 0.23 | 0.11 | 0.19 | 0.12 | 0.05 | 0.11 | 0.18 | 0.12 | 0.11 | 0.07 | 0.10 | 0.11 | 0.17 | 0.11 | 0.23 | 0.21 | 0.11 | 0.11 | 0.18 | 0.10 | 0.12 | 0.11 | 0.12 | 0.14 | 0.20 | 0.16 | 0.20 | 0.24 |
| BCO | 6187 | 6176 | 6127 | 6153 | 6113 | 6241 | 6044 | 6121 | 5943 | 6498 | 5851 | 6926 | 6795 | 6258 | 5711 | 5920 | 4985 | 6881 | 6729 | 6166 | 6120 | 6669 | 5175 | 6868 | 6814 | 8453 | 7190 | 8774 | 8794 | 6571 | 6501 | 8847 | 7111 | 6727 | 9445 | 6548 | 9266 | 4363 | 9505 | 10996 | 11064 | 10937 | 11022 | 11047 | 8564 | 8613 | 2564 | 3261 | 3261 | 802 | 1032 | 864 | 1260 | 532 | 1853 | 355 |  | 0.02 | 0.07 | 0.09 | 0.13 | 0.08 | 0.06 | 0.12 | 0.08 | 0.22 | 0.11 | 0.18 | 0.12 | 0.06 | 0.12 | 0.17 | 0.13 | 0.12 | 0.08 | 0.12 | 0.11 | 0.17 | 0.11 | 0.23 | 0.21 | 0.12 | 0.11 | 0.18 | 0.11 | 0.13 | 0.12 | 0.12 | 0.15 | 0.20 | 0.16 | 0.20 | 0.23 |
| BAF | 5954 | 5932 | 5891 | 5934 | 5860 | 5978 | 5784 | 5848 | 5664 | 6199 | 5551 | 6597 | 6454 | 5914 | 5387 | 5581 | 4784 | 6520 | 6358 | 5791 | 5735 | 6276 | 4824 | 6473 | 6418 | 8695 | 7432 | 9025 | 9046 | 6166 | 6094 | 9112 | 7377 | 6315 | 9718 | 6124 | 9565 | 3974 | 9833 | 11285 | 11360 | 11242 | 11332 | 11369 | 8125 | 8173 | 2308 | 2808 | 2808 | 988 | 1480 | 624 | 871 | 917 | 2340 | 852 | 505 |  | 0.05 | 0.06 | 0.11 | 0.06 | 0.05 | 0.10 | 0.06 | 0.22 | 0.10 | 0.18 | 0.11 | 0.04 | 0.11 | 0.17 | 0.11 | 0.11 | 0.06 | 0.10 | 0.10 | 0.16 | 0.10 | 0.22 | 0.21 | 0.11 | 0.10 | 0.18 | 0.09 | 0.11 | 0.11 | 0.11 | 0.14 | 0.20 | 0.15 | 0.20 | 0.23 |
| BPV | 5646 | 5605 | 5580 | 5654 | 5518 | 5614 | 5430 | 5468 | 5276 | 5763 | 5126 | 6100 | 5933 | 5395 | 4917 | 5078 | 4567 | 5959 | 5781 | 5214 | 5137 | 5655 | 4312 | 5845 | 5789 | 9156 | 7907 | 9497 | 9521 | 5524 | 5449 | 9607 | 7892 | 5657 | 10219 | 5444 | 10111 | 3398 | 10425 | 11798 | 11884 | 11784 | 11879 | 11937 | 7400 | 7447 | 2119 | 2111 | 2111 | 1669 | 2312 | 1004 | 758 | 1737 | 3195 | 1717 | 1372 | 866 |  | 0.09 | 0.12 | 0.08 | 0.06 | 0.11 | 0.08 | 0.22 | 0.11 | 0.18 | 0.12 | 0.06 | 0.12 | 0.17 | 0.12 | 0.12 | 0.08 | 0.11 | 0.11 | 0.16 | 0.11 | 0.22 | 0.21 | 0.12 | 0.11 | 0.18 | 0.10 | 0.12 | 0.12 | 0.12 | 0.15 | 0.20 | 0.16 | 0.20 | 0.23 |
| IPO | 6812 | 6797 | 6750 | 6782 | 6730 | 6854 | 6658 | 6729 | 6548 | 7092 | 6444 | 7500 | 7360 | 6820 | 6288 | 6486 | 5618 | 7428 | 7267 | 6700 | 6643 | 7182 | 5731 | 7379 | 7323 | 8938 | 7682 | 9249 | 9268 | 7070 | 6998 | 9307 | 7580 | 7217 | 9895 | 7021 | 9686 | 4882 | 9886 | 11418 | 11478 | 11340 | 11420 | 11429 | 9008 | 9056 | 3173 | 3692 | 3692 | 1429 | 1391 | 1457 | 1778 | 1078 | 1997 | 753 | 646 | 909 | 1631 |  | 0.14 | 0.04 | 0.06 | 0.16 | 0.13 | 0.30 | 0.13 | 0.26 | 0.17 | 0.04 | 0.12 | 0.24 | 0.17 | 0.13 | 0.06 | 0.15 | 0.15 | 0.22 | 0.13 | 0.30 | 0.25 | 0.13 | 0.12 | 0.21 | 0.12 | 0.17 | 0.15 | 0.14 | 0.18 | 0.24 | 0.20 | 0.22 | 0.27 |
| KAT | 12032 | 12128 | 12096 | 11937 | 12249 | 12248 | 12359 | 12421 | 12598 | 12404 | 12852 | 12405 | 12638 | 13030 | 13179 | 13196 | 12645 | 12809 | 13023 | 13437 | 13594 | 13348 | 13780 | 13250 | 13300 | 7091 | 8313 | 6744 | 6719 | 13568 | 13639 | 6614 | 8303 | 13568 | 6004 | 13858 | 6077 | 14624 | 5750 | 4431 | 4335 | 4419 | 4319 | 4249 | 12750 | 12726 | 14506 | 15734 | 15734 | 14570 | 14125 | 15180 | 15450 | 14643 | 13404 | 14824 | 15154 | 15539 | 16175 | 15399 |  | 0.11 | 0.11 | 0.02 | 0.14 | 0.25 | 0.03 | 0.21 | 0.11 | 0.12 | 0.03 | 0.19 | 0.08 | 0.03 | 0.13 | 0.01 | 0.08 | 0.16 | 0.03 | 0.23 | 0.14 | 0.03 | 0.03 | 0.12 | 0.03 | 0.09 | 0.03 | 0.05 | 0.08 | 0.07 | 0.06 | 0.08 | 0.10 |
| NOV | 7478 | 7483 | 7422 | 7420 | 7434 | 7576 | 7376 | 7471 | 7303 | 7881 | 7240 | 8341 | 8227 | 7695 | 7130 | 7354 | 6245 | 8333 | 8189 | 7632 | 7594 | 8148 | 6624 | 8348 | 8295 | 8506 | 7295 | 8785 | 8800 | 8057 | 7988 | 8806 | 7135 | 8217 | 9358 | 8042 | 9089 | 5845 | 9211 | 10796 | 10837 | 10682 | 10749 | 10729 | 10062 | 10111 | 3960 | 4756 | 4756 | 1953 | 1358 | 2337 | 2756 | 1536 | 1327 | 1271 | 1497 | 1956 | 2769 | 1166 | 14438 |  | 0.04 | 0.12 | 0.13 | 0.28 | 0.09 | 0.25 | 0.14 | 0.04 | 0.10 | 0.22 | 0.14 | 0.10 | 0.07 | 0.11 | 0.12 | 0.20 | 0.10 | 0.28 | 0.24 | 0.09 | 0.09 | 0.18 | 0.09 | 0.14 | 0.11 | 0.10 | 0.15 | 0.22 | 0.17 | 0.20 | 0.26 |
| BEH | 7446 | 7442 | 7387 | 7399 | 7386 | 7521 | 7322 | 7407 | 7234 | 7798 | 7152 | 8236 | 8109 | 7573 | 7022 | 7234 | 6224 | 8197 | 8045 | 7482 | 7434 | 7981 | 6491 | 8179 | 8125 | 8817 | 7593 | 9105 | 9120 | 7879 | 7808 | 9134 | 7446 | 8031 | 9694 | 7844 | 9436 | 5675 | 9572 | 11150 | 11195 | 11042 | 11112 | 11096 | 9845 | 9893 | 3861 | 4527 | 4527 | 1912 | 1466 | 2180 | 2564 | 1491 | 1641 | 1176 | 1316 | 1721 | 2485 | 854 | 14831 | 394 |  | 0.11 | 0.10 | 0.25 | 0.10 | 0.22 | 0.13 | 0.04 | 0.10 | 0.20 | 0.13 | 0.10 | 0.08 | 0.11 | 0.11 | 0.18 | 0.10 | 0.25 | 0.21 | 0.10 | 0.10 | 0.17 | 0.09 | 0.13 | 0.11 | 0.11 | 0.14 | 0.20 | 0.16 | 0.19 | 0.23 |
| GEL | 12257 | 12347 | 12322 | 12173 | 12464 | 12450 | 12574 | 12622 | 12806 | 12565 | 13051 | 12517 | 12747 | 13170 | 13369 | 13361 | 12949 | 12894 | 13109 | 13557 | 13707 | 13416 | 13988 | 13301 | 13353 | 7544 | 8778 | 7198 | 7174 | 13629 | 13704 | 7076 | 8779 | 13610 | 6465 | 13899 | 6556 | 14870 | 6244 | 4886 | 4795 | 4888 | 4791 | 4730 | 12636 | 12609 | 14921 | 16057 | 16057 | 15078 | 14644 | 15677 | 15931 | 15159 | 13923 | 15343 | 15671 | 16051 | 16666 | 15918 | 520 | 14947 | 15340 |  | 0.12 | 0.24 | 0.00 | 0.20 | 0.08 | 0.11 | 0.02 | 0.17 | 0.06 | 0.01 | 0.12 | 0.00 | 0.05 | 0.15 | 0.00 | 0.25 | 0.20 | 0.00 | 0.00 | 0.12 | 0.00 | 0.06 | 0.00 | 0.02 | 0.08 | 0.16 | 0.11 | 0.14 | 0.21 |
| BPA | 7727 | 7722 | 7668 | 7681 | 7664 | 7798 | 7599 | 7682 | 7507 | 8068 | 7422 | 8500 | 8369 | 7831 | 7286 | 7494 | 6507 | 8451 | 8295 | 7730 | 7678 | 8222 | 6747 | 8419 | 8365 | 9025 | 7811 | 9306 | 9321 | 8114 | 8043 | 9328 | 7655 | 8263 | 9880 | 8070 | 9609 | 5918 | 9726 | 11313 | 11353 | 11196 | 11262 | 11239 | 10059 | 10106 | 4132 | 4743 | 4743 | 2196 | 1745 | 2438 | 2806 | 1776 | 1844 | 1457 | 1574 | 1948 | 2671 | 1051 | 14871 | 522 | 288 | 15372 |  | 0.18 | 0.13 | 0.15 | 0.10 | 0.11 | 0.13 | 0.14 | 0.12 | 0.13 | 0.10 | 0.12 | 0.11 | 0.14 | 0.12 | 0.20 | 0.22 | 0.13 | 0.13 | 0.19 | 0.12 | 0.12 | 0.13 | 0.14 | 0.15 | 0.20 | 0.16 | 0.21 | 0.24 |
| KAN | 10859 | 10875 | 10920 | 10888 | 10944 | 10823 | 11018 | 10952 | 11136 | 10606 | 11253 | 10227 | 10384 | 10924 | 11429 | 11249 | 12054 | 10348 | 10525 | 11093 | 11170 | 10657 | 12013 | 10470 | 10527 | 9689 | 10849 | 9439 | 9427 | 10797 | 10874 | 9449 | 11041 | 10674 | 8943 | 10903 | 9266 | 12911 | 9241 | 7644 | 7627 | 7801 | 7753 | 7813 | 9036 | 8993 | 14501 | 14208 | 14208 | 16321 | 16722 | 16216 | 15937 | 16731 | 16751 | 17066 | 17038 | 16800 | 16306 | 17670 | 4880 | 18029 | 18184 | 4459 | 18468 |  | 0.23 | 0.11 | 0.13 | 0.24 | 0.24 | 0.13 | 0.19 | 0.23 | 0.21 | 0.25 | 0.17 | 0.18 | 0.23 | 0.24 | 0.33 | 0.23 | 0.23 | 0.29 | 0.21 | 0.18 | 0.24 | 0.23 | 0.26 | 0.29 | 0.23 | 0.30 | 0.33 |
| YUN | 12689 | 12763 | 12757 | 12632 | 12873 | 12828 | 12978 | 12996 | 13188 | 12850 | 13407 | 12695 | 12911 | 13391 | 13697 | 13635 | 13548 | 13002 | 13213 | 13722 | 13854 | 13470 | 14338 | 13323 | 13380 | 8529 | 9781 | 8189 | 8166 | 13664 | 13743 | 8083 | 9807 | 13605 | 7472 | 13882 | 7598 | 15279 | 7315 | 5894 | 5812 | 5921 | 5828 | 5783 | 12329 | 12294 | 15753 | 16601 | 16601 | 16164 | 15760 | 16728 | 16929 | 16267 | 15043 | 16460 | 16785 | 17146 | 17684 | 17036 | 1640 | 16038 | 16431 | 1120 | 16440 | 3619 |  | 0.20 | 0.09 | 0.11 | 0.02 | 0.17 | 0.06 | 0.01 | 0.12 | 0.01 | 0.06 | 0.15 | 0.00 | 0.21 | 0.14 | 0.00 | 0.00 | 0.08 | 0.00 | 0.08 | 0.00 | 0.02 | 0.06 | 0.13 | 0.10 | 0.12 | 0.15 |
| UTS | 10746 | 10758 | 10807 | 10783 | 10822 | 10696 | 10893 | 10821 | 11002 | 10460 | 11108 | 10066 | 10216 | 10756 | 11273 | 11085 | 11954 | 10168 | 10341 | 10909 | 10982 | 10461 | 11847 | 10272 | 10328 | 9839 | 10979 | 9599 | 9588 | 10595 | 10671 | 9617 | 11182 | 10467 | 9124 | 10691 | 9458 | 12731 | 9450 | 7856 | 7844 | 8019 | 7974 | 8040 | 8800 | 8757 | 14377 | 14009 | 14009 | 16250 | 16710 | 16093 | 15789 | 16667 | 16821 | 16995 | 16933 | 16659 | 16116 | 17549 | 5167 | 18051 | 18149 | 4747 | 18437 | 288 | 3902 |  | 0.10 | 0.21 | 0.22 | 0.09 | 0.16 | 0.21 | 0.18 | 0.21 | 0.14 | 0.15 | 0.20 | 0.20 | 0.30 | 0.20 | 0.20 | 0.26 | 0.18 | 0.14 | 0.21 | 0.21 | 0.22 | 0.26 | 0.20 | 0.27 | 0.30 |
| HUN | 12347 | 12402 | 12414 | 12319 | 12500 | 12426 | 12597 | 12584 | 12779 | 12359 | 12965 | 12116 | 12314 | 12828 | 13220 | 13110 | 13357 | 12357 | 12559 | 13102 | 13214 | 12770 | 13857 | 12604 | 12662 | 9021 | 10285 | 8698 | 8678 | 12945 | 13024 | 8625 | 10361 | 12858 | 8027 | 13119 | 8217 | 14812 | 8005 | 6485 | 6421 | 6554 | 6473 | 6461 | 11415 | 11376 | 15770 | 16183 | 16183 | 16729 | 16515 | 17135 | 17174 | 16940 | 15917 | 17201 | 17472 | 17691 | 17885 | 17889 | 2611 | 17038 | 17430 | 2114 | 17481 | 2532 | 1108 | 2811 |  | 0.13 | 0.12 | 0.08 | 0.05 | 0.10 | 0.11 | 0.09 | 0.04 | 0.07 | 0.09 | 0.13 | 0.19 | 0.10 | 0.10 | 0.16 | 0.08 | 0.05 | 0.09 | 0.10 | 0.11 | 0.16 | 0.10 | 0.17 | 0.20 |
| POR | 7686 | 7657 | 7622 | 7674 | 7579 | 7688 | 7498 | 7549 | 7361 | 7868 | 7225 | 8220 | 8055 | 7517 | 7030 | 7197 | 6535 | 8080 | 7899 | 7333 | 7251 | 7760 | 6431 | 7947 | 7890 | 9967 | 8719 | 10269 | 10286 | 7619 | 7543 | 10314 | 8600 | 7742 | 10890 | 7514 | 10653 | 5520 | 10812 | 12378 | 12428 | 12280 | 12353 | 12344 | 9403 | 9447 | 4050 | 4210 | 4210 | 2495 | 2428 | 2380 | 2572 | 2158 | 2858 | 1831 | 1694 | 1758 | 2122 | 1080 | 16059 | 1650 | 1263 | 16558 | 1189 | 18416 | 17609 | 18201 | 18669 |  | 0.11 | 0.19 | 0.13 | 0.11 | 0.03 | 0.11 | 0.12 | 0.18 | 0.11 | 0.24 | 0.20 | 0.11 | 0.11 | 0.18 | 0.10 | 0.13 | 0.11 | 0.12 | 0.15 | 0.20 | 0.16 | 0.20 | 0.23 |
| BEN | 13277 | 13388 | 13334 | 13152 | 13512 | 13551 | 13621 | 13717 | 13872 | 13808 | 14141 | 13920 | 14155 | 14465 | 14478 | 14561 | 13600 | 14371 | 14578 | 14907 | 15073 | 14932 | 15001 | 14865 | 14910 | 7577 | 8652 | 7236 | 7208 | 15157 | 15221 | 7056 | 8546 | 15192 | 6492 | 15478 | 6417 | 15670 | 5966 | 5075 | 4954 | 4965 | 4856 | 4718 | 14562 | 14541 | 14831 | 16379 | 16379 | 14149 | 13501 | 14847 | 15247 | 14073 | 12645 | 14143 | 14498 | 14978 | 15804 | 14516 | 1828 | 13396 | 13774 | 2050 | 13729 | 6410 | 2806 | 6696 | 3913 | 14849 |  | 0.19 | 0.08 | 0.03 | 0.13 | 0.00 | 0.08 | 0.16 | 0.02 | 0.23 | 0.11 | 0.03 | 0.02 | 0.10 | 0.02 | 0.10 | 0.03 | 0.04 | 0.08 | 0.14 | 0.12 | 0.13 | 0.16 |
| KAG | 11573 | 11597 | 11637 | 11594 | 11670 | 11556 | 11749 | 11690 | 11877 | 11359 | 12004 | 10995 | 11157 | 11696 | 12190 | 12018 | 12747 | 11128 | 11308 | 11874 | 11955 | 11445 | 12783 | 11259 | 11316 | 9838 | 11050 | 9560 | 9545 | 11586 | 11663 | 9542 | 11208 | 11464 | 8998 | 11694 | 9281 | 13690 | 9189 | 7594 | 7561 | 7723 | 7663 | 7698 | 9821 | 9778 | 15219 | 14996 | 14996 | 16935 | 17199 | 16923 | 16680 | 17322 | 17017 | 17663 | 17700 | 17527 | 17087 | 18346 | 4367 | 18343 | 18629 | 3906 | 18861 | 791 | 2967 | 1023 | 1860 | 19205 | 5773 |  | 0.13 | 0.18 | 0.17 | 0.18 | 0.12 | 0.13 | 0.17 | 0.19 | 0.27 | 0.17 | 0.17 | 0.23 | 0.16 | 0.12 | 0.18 | 0.18 | 0.19 | 0.24 | 0.18 | 0.25 | 0.27 |
| HAN | 13096 | 13162 | 13164 | 13051 | 13267 | 13208 | 13368 | 13372 | 13566 | 13186 | 13771 | 12983 | 13189 | 13690 | 14044 | 13956 | 14018 | 13251 | 13457 | 13989 | 14109 | 13684 | 14686 | 13524 | 13581 | 9128 | 10381 | 8788 | 8765 | 13866 | 13945 | 8684 | 10408 | 13786 | 8073 | 14052 | 8199 | 15639 | 7913 | 6496 | 6414 | 6522 | 6429 | 6383 | 12368 | 12330 | 16298 | 16997 | 16997 | 16760 | 16330 | 17329 | 17523 | 16850 | 15582 | 17027 | 17360 | 17739 | 18279 | 17560 | 2207 | 16501 | 16887 | 1691 | 16860 | 3444 | 601 | 3715 | 954 | 17976 | 3134 | 2721 |  | 0.07 | 0.12 | 0.06 | 0.04 | 0.11 | 0.06 | 0.18 | 0.20 | 0.06 | 0.06 | 0.15 | 0.05 | 0.05 | 0.06 | 0.07 | 0.10 | 0.16 | 0.10 | 0.17 | 0.21 |
| LAM | 13356 | 13434 | 13423 | 13291 | 13546 | 13508 | 13652 | 13677 | 13868 | 13547 | 14093 | 13404 | 13621 | 14097 | 14389 | 14336 | 14162 | 13716 | 13927 | 14433 | 14566 | 14184 | 15028 | 14037 | 14094 | 8870 | 10099 | 8524 | 8499 | 14378 | 14457 | 8398 | 10091 | 14318 | 7788 | 14594 | 7866 | 15961 | 7533 | 6211 | 6118 | 6206 | 6106 | 6038 | 13006 | 12970 | 16241 | 17252 | 17252 | 16307 | 15778 | 16946 | 17237 | 16327 | 14977 | 16458 | 16807 | 17243 | 17949 | 16912 | 1789 | 15812 | 16192 | 1328 | 16147 | 4160 | 714 | 4435 | 1637 | 17245 | 2419 | 3450 | 733 |  | 0.12 | 0.02 | 0.06 | 0.15 | 0.01 | 0.22 | 0.15 | 0.01 | 0.01 | 0.09 | 0.01 | 0.08 | 0.01 | 0.02 | 0.06 | 0.13 | 0.10 | 0.12 | 0.16 |
| GRV | 8247 | 8222 | 8184 | 8229 | 8148 | 8260 | 8068 | 8125 | 7937 | 8452 | 7808 | 8812 | 8650 | 8110 | 7619 | 7789 | 7080 | 8677 | 8497 | 7930 | 7850 | 8359 | 7024 | 8545 | 8488 | 10140 | 8916 | 10425 | 10441 | 8217 | 8141 | 10450 | 8769 | 8340 | 11005 | 8110 | 10734 | 6116 | 10845 | 12435 | 12473 | 12315 | 12379 | 12351 | 9987 | 10030 | 4603 | 4808 | 4808 | 2925 | 2709 | 2906 | 3137 | 2549 | 2959 | 2209 | 2148 | 2296 | 2718 | 1502 | 15795 | 1647 | 1323 | 16268 | 1126 | 19013 | 17239 | 18787 | 18346 | 599 | 14438 | 19804 | 17515 | 16783 |  | 0.12 | 0.11 | 0.16 | 0.12 | 0.22 | 0.22 | 0.12 | 0.12 | 0.18 | 0.11 | 0.12 | 0.12 | 0.13 | 0.15 | 0.21 | 0.16 | 0.20 | 0.24 |
| JAF | 13723 | 13833 | 13780 | 13599 | 13956 | 13992 | 14066 | 14160 | 14316 | 14239 | 14585 | 14332 | 14567 | 14893 | 14922 | 14998 | 14054 | 14774 | 14983 | 15331 | 15496 | 15331 | 15451 | 15255 | 15302 | 8024 | 9088 | 7684 | 7656 | 15555 | 15622 | 7502 | 8975 | 15580 | 6941 | 15868 | 6860 | 16124 | 6403 | 5529 | 5408 | 5418 | 5309 | 5170 | 14828 | 14802 | 15235 | 16803 | 16803 | 14441 | 13760 | 15144 | 15562 | 14335 | 12886 | 14381 | 14735 | 15226 | 16072 | 14699 | 2085 | 13556 | 13924 | 2209 | 13856 | 6414 | 2797 | 6697 | 3888 | 14936 | 454 | 5739 | 3041 | 2309 | 14485 |  | 0.06 | 0.16 | 0.01 | 0.26 | 0.17 | 0.01 | 0.01 | 0.12 | 0.01 | 0.07 | 0.01 | 0.03 | 0.08 | 0.16 | 0.11 | 0.14 | 0.21 |
| HAI | 13295 | 13354 | 13363 | 13262 | 13454 | 13383 | 13552 | 13543 | 13738 | 13324 | 13928 | 13080 | 13277 | 13793 | 14185 | 14076 | 14274 | 13315 | 13515 | 14062 | 14171 | 13717 | 14822 | 13548 | 13605 | 9546 | 10803 | 9208 | 9186 | 13887 | 13966 | 9108 | 10836 | 13793 | 8498 | 14048 | 8631 | 15777 | 8350 | 6922 | 6843 | 6953 | 6861 | 6818 | 12279 | 12238 | 16625 | 17148 | 17148 | 17199 | 16769 | 17754 | 17919 | 17291 | 16010 | 17464 | 17800 | 18180 | 18675 | 17973 | 2648 | 16882 | 17261 | 2132 | 17209 | 3270 | 1035 | 3527 | 966 | 18262 | 3487 | 2510 | 441 | 1070 | 17743 | 3354 |  | 0.10 | 0.06 | 0.14 | 0.17 | 0.06 | 0.06 | 0.13 | 0.05 | 0.04 | 0.06 | 0.07 | 0.08 | 0.14 | 0.09 | 0.15 | 0.18 |
| OKI | 12187 | 12211 | 12250 | 12205 | 12286 | 12173 | 12365 | 12307 | 12494 | 11976 | 12621 | 11611 | 11771 | 12311 | 12808 | 12634 | 13354 | 11738 | 11915 | 12483 | 12560 | 12046 | 13399 | 11857 | 11914 | 10143 | 11381 | 9849 | 9832 | 12183 | 12259 | 9812 | 11514 | 12056 | 9245 | 12281 | 9496 | 14301 | 9357 | 7780 | 7735 | 7887 | 7817 | 7834 | 10379 | 10335 | 15831 | 15596 | 15596 | 17485 | 17643 | 17527 | 17296 | 17850 | 17297 | 18187 | 18272 | 18138 | 17697 | 18914 | 4204 | 18560 | 18929 | 3712 | 19052 | 1390 | 2678 | 1590 | 1599 | 19766 | 5450 | 618 | 2328 | 3060 | 19641 | 5369 | 2047 |  | 0.15 | 0.16 | 0.26 | 0.15 | 0.15 | 0.21 | 0.13 | 0.10 | 0.15 | 0.15 | 0.17 | 0.22 | 0.16 | 0.23 | 0.26 |
| KAC | 13828 | 13907 | 13895 | 13762 | 14020 | 13982 | 14126 | 14152 | 14343 | 14022 | 14568 | 13874 | 14090 | 14569 | 14865 | 14810 | 14617 | 14179 | 14389 | 14901 | 15032 | 14639 | 15503 | 14489 | 14546 | 9207 | 10418 | 8859 | 8833 | 14830 | 14909 | 8723 | 10392 | 14764 | 8115 | 15036 | 8168 | 16435 | 7810 | 6547 | 6449 | 6526 | 6424 | 6343 | 13394 | 13356 | 16624 | 17717 | 17717 | 16466 | 15864 | 17140 | 17483 | 16431 | 15020 | 16517 | 16872 | 17344 | 18135 | 16877 | 2119 | 15734 | 16099 | 1711 | 16019 | 4456 | 1179 | 4722 | 1981 | 17044 | 2377 | 3713 | 1028 | 475 | 16533 | 2179 | 1218 | 3265 |  | 0.22 | 0.15 | 0.00 | 0.00 | 0.09 | 0.00 | 0.07 | 0.00 | 0.02 | 0.06 | 0.14 | 0.10 | 0.13 | 0.16 |
| TAI | 12750 | 12788 | 12816 | 12749 | 12873 | 12775 | 12960 | 12920 | 13112 | 12628 | 13264 | 12305 | 12479 | 13014 | 13477 | 13325 | 13859 | 12472 | 12658 | 13222 | 13311 | 12814 | 14091 | 12631 | 12689 | 9964 | 11225 | 9647 | 9628 | 12963 | 13040 | 9582 | 11315 | 12846 | 8990 | 13082 | 9192 | 15020 | 8988 | 7459 | 7398 | 7534 | 7454 | 7445 | 11218 | 11174 | 16341 | 16356 | 16356 | 17628 | 17491 | 17912 | 17821 | 17885 | 16900 | 18167 | 18408 | 18524 | 18388 | 18873 | 3537 | 17966 | 18359 | 3024 | 18355 | 2185 | 1928 | 2418 | 986 | 19435 | 4631 | 1397 | 1497 | 2219 | 18870 | 4524 | 1180 | 873 | 2397 |  | 0.35 | 0.21 | 0.21 | 0.28 | 0.19 | 0.16 | 0.24 | 0.21 | 0.25 | 0.29 | 0.22 | 0.29 | 0.35 |
| KUN | 13763 | 13881 | 13814 | 13621 | 14002 | 14067 | 14108 | 14225 | 14359 | 14388 | 14630 | 14580 | 14811 | 15050 | 14963 | 15090 | 13889 | 15057 | 15254 | 15505 | 15673 | 15623 | 15408 | 15585 | 15624 | 7795 | 8755 | 7469 | 7441 | 15846 | 15903 | 7271 | 8603 | 15912 | 6753 | 16186 | 6606 | 15915 | 6111 | 5481 | 5355 | 5330 | 5223 | 5060 | 15479 | 15460 | 14690 | 16302 | 16302 | 13675 | 12961 | 14380 | 14818 | 13536 | 12072 | 13557 | 13909 | 14407 | 15266 | 13835 | 2779 | 12685 | 13047 | 3002 | 12972 | 7300 | 3681 | 7583 | 4777 | 14043 | 957 | 6631 | 3934 | 3202 | 13592 | 893 | 4241 | 6262 | 3052 | 5414 |  | 0.14 | 0.13 | 0.23 | 0.13 | 0.19 | 0.18 | 0.15 | 0.22 | 0.27 | 0.23 | 0.25 | 0.33 |
| SSK | 13748 | 13818 | 13816 | 13698 | 13925 | 13870 | 14027 | 14036 | 14230 | 13859 | 14438 | 13660 | 13866 | 14367 | 14716 | 14632 | 14633 | 13928 | 14133 | 14666 | 14785 | 14356 | 15358 | 14194 | 14251 | 9484 | 10717 | 9138 | 9114 | 14535 | 14614 | 9016 | 10713 | 14452 | 8405 | 14714 | 8488 | 16309 | 8158 | 6827 | 6735 | 6826 | 6727 | 6660 | 12986 | 12946 | 16816 | 17659 | 17659 | 16916 | 16354 | 17566 | 17861 | 16914 | 15526 | 17021 | 17373 | 17831 | 18569 | 17404 | 2412 | 16264 | 16629 | 1940 | 16547 | 3991 | 1087 | 4249 | 1595 | 17551 | 2894 | 3232 | 678 | 624 | 17022 | 2709 | 722 | 2758 | 530 | 1886 | 3582 |  | 0.00 | 0.08 | 0.01 | 0.08 | 0.01 | 0.02 | 0.06 | 0.13 | 0.10 | 0.12 | 0.15 |
| CHA | 14012 | 14087 | 14080 | 13954 | 14196 | 14150 | 14301 | 14318 | 14510 | 14161 | 14727 | 13983 | 14192 | 14686 | 15013 | 14941 | 14847 | 14263 | 14470 | 14996 | 15120 | 14702 | 15655 | 14542 | 14600 | 9515 | 10731 | 9167 | 9142 | 14884 | 14963 | 9034 | 10707 | 14805 | 8425 | 15070 | 8483 | 16600 | 8128 | 6855 | 6757 | 6837 | 6736 | 6657 | 13354 | 13314 | 16918 | 17923 | 17923 | 16776 | 16159 | 17455 | 17803 | 16729 | 15304 | 16802 | 17157 | 17638 | 18445 | 17123 | 2424 | 15969 | 16324 | 1997 | 16226 | 4357 | 1323 | 4613 | 1956 | 17198 | 2660 | 3595 | 1019 | 692 | 16661 | 2433 | 1087 | 3110 | 320 | 2237 | 3288 | 368 |  | 0.08 | 0.00 | 0.08 | 0.00 | 0.02 | 0.06 | 0.13 | 0.10 | 0.12 | 0.15 |
| QNC | 13900 | 13959 | 13967 | 13866 | 14058 | 13987 | 14156 | 14147 | 14342 | 13923 | 14530 | 13670 | 13863 | 14383 | 14784 | 14670 | 14868 | 13892 | 14089 | 14641 | 14745 | 14278 | 15421 | 14104 | 14162 | 9957 | 11201 | 9614 | 9590 | 14442 | 14520 | 9499 | 11210 | 14339 | 8888 | 14586 | 8988 | 16374 | 8672 | 7308 | 7220 | 7318 | 7222 | 7162 | 12760 | 12718 | 17180 | 17746 | 17746 | 17461 | 16901 | 18099 | 18360 | 17462 | 16066 | 17563 | 17916 | 18379 | 19097 | 17905 | 2922 | 16748 | 17099 | 2432 | 16990 | 3725 | 1449 | 3965 | 1565 | 17893 | 3424 | 2942 | 882 | 1155 | 17323 | 3213 | 604 | 2410 | 1047 | 1548 | 4070 | 548 | 782 |  | 0.08 | 0.15 | 0.11 | 0.10 | 0.15 | 0.21 | 0.18 | 0.19 | 0.24 |
| CAM | 14147 | 14216 | 14215 | 14098 | 14322 | 14265 | 14424 | 14430 | 14624 | 14245 | 14830 | 14032 | 14234 | 14741 | 15103 | 15012 | 15034 | 14285 | 14487 | 15028 | 15142 | 14698 | 15744 | 14531 | 14589 | 9814 | 11036 | 9467 | 9442 | 14871 | 14950 | 9337 | 11019 | 14779 | 8727 | 15035 | 8793 | 16698 | 8445 | 7154 | 7058 | 7142 | 7041 | 6966 | 13253 | 13211 | 17189 | 18055 | 18055 | 17104 | 16477 | 17784 | 18130 | 17049 | 15615 | 17112 | 17466 | 17953 | 18772 | 17396 | 2724 | 16234 | 16579 | 2278 | 16464 | 4223 | 1487 | 4470 | 1929 | 17376 | 2976 | 3446 | 1059 | 951 | 16815 | 2732 | 987 | 2929 | 647 | 2061 | 3572 | 402 | 329 | 528 |  | 0.06 | 0.00 | 0.02 | 0.05 | 0.12 | 0.09 | 0.12 | 0.15 |
| HOC | 14240 | 14304 | 14307 | 14197 | 14408 | 14345 | 14508 | 14507 | 14702 | 14304 | 14900 | 14070 | 14267 | 14782 | 15164 | 15062 | 15158 | 14306 | 14505 | 15052 | 15161 | 14702 | 15804 | 14530 | 14588 | 10009 | 11235 | 9662 | 9637 | 14868 | 14947 | 9535 | 11220 | 14769 | 8924 | 15018 | 8995 | 16759 | 8649 | 7349 | 7255 | 7340 | 7240 | 7167 | 13196 | 13154 | 17362 | 18128 | 18128 | 17310 | 16674 | 17992 | 18338 | 17247 | 15806 | 17300 | 17654 | 18146 | 18976 | 17554 | 2921 | 16389 | 16725 | 2468 | 16598 | 4161 | 1627 | 4399 | 1954 | 17462 | 3174 | 3377 | 1146 | 1139 | 16888 | 2920 | 991 | 2839 | 856 | 1982 | 3749 | 547 | 537 | 436 | 209 |  | 0.07 | 0.08 | 0.10 | 0.16 | 0.10 | 0.17 | 0.20 |
| SON | 14596 | 14675 | 14663 | 14530 | 14786 | 14747 | 14893 | 14916 | 15107 | 14774 | 15330 | 14605 | 14816 | 15307 | 15622 | 15558 | 15375 | 14888 | 15094 | 15621 | 15745 | 15324 | 16263 | 15164 | 15221 | 9827 | 11008 | 9478 | 9452 | 15505 | 15584 | 9330 | 10956 | 15423 | 8728 | 15685 | 8747 | 17200 | 8356 | 7180 | 7076 | 7139 | 7034 | 6938 | 13929 | 13888 | 17264 | 18481 | 18481 | 16720 | 16018 | 17424 | 17842 | 16593 | 15126 | 16603 | 16951 | 17454 | 18319 | 16793 | 2794 | 15628 | 15957 | 2436 | 15824 | 4903 | 1925 | 5149 | 2565 | 16700 | 2600 | 4126 | 1640 | 1241 | 16136 | 2281 | 1653 | 3604 | 768 | 2739 | 3057 | 971 | 625 | 1195 | 679 | 776 |  | 0.02 | 0.06 | 0.15 | 0.11 | 0.14 | 0.19 |
| MAT | 14886 | 14964 | 14953 | 14821 | 15075 | 15034 | 15181 | 15203 | 15395 | 15054 | 15615 | 14873 | 15081 | 15578 | 15905 | 15834 | 15665 | 15144 | 15349 | 15882 | 16002 | 15568 | 16546 | 15403 | 15461 | 10074 | 11243 | 9725 | 9699 | 15744 | 15823 | 9573 | 11181 | 15654 | 8974 | 15910 | 8981 | 17487 | 8580 | 7434 | 7328 | 7386 | 7281 | 7180 | 14109 | 14066 | 17502 | 18772 | 18772 | 16798 | 16063 | 17502 | 17942 | 16635 | 15161 | 16614 | 16954 | 17460 | 18324 | 16738 | 3069 | 15578 | 15890 | 2722 | 15739 | 5073 | 2208 | 5311 | 2799 | 16553 | 2751 | 4289 | 1894 | 1531 | 15977 | 2400 | 1862 | 3747 | 1060 | 2894 | 3124 | 1217 | 893 | 1348 | 875 | 912 | 292 |  | 0.06 | 0.14 | 0.11 | 0.13 | 0.16 |
| KLP | 15058 | 15135 | 15126 | 14995 | 15246 | 15203 | 15352 | 15371 | 15563 | 15216 | 15782 | 15025 | 15230 | 15732 | 16068 | 15993 | 15842 | 15287 | 15490 | 16028 | 16144 | 15701 | 16710 | 15532 | 15590 | 10234 | 11397 | 9885 | 9858 | 15872 | 15951 | 9731 | 11330 | 15776 | 9134 | 16028 | 9135 | 17654 | 8729 | 7598 | 7491 | 7547 | 7441 | 7339 | 14196 | 14153 | 17653 | 18949 | 18949 | 16854 | 16103 | 17555 | 18006 | 16671 | 15197 | 16632 | 16966 | 17469 | 18325 | 16714 | 3242 | 15561 | 15862 | 2898 | 15701 | 5163 | 2375 | 5396 | 2932 | 16475 | 2869 | 4376 | 2042 | 1704 | 15892 | 2502 | 1984 | 3822 | 1234 | 2979 | 3196 | 1365 | 1055 | 1445 | 1003 | 1011 | 468 | 177 |  | 0.18 | 0.13 | 0.17 | 0.22 |
| SUF | 15466 | 15490 | 15530 | 15483 | 15562 | 15444 | 15638 | 15573 | 15756 | 15213 | 15861 | 14797 | 14929 | 15466 | 16011 | 15804 | 16622 | 14841 | 14990 | 15550 | 15589 | 15035 | 16548 | 14834 | 14886 | 12051 | 13280 | 11705 | 11681 | 15120 | 15187 | 11580 | 13264 | 14956 | 10969 | 15116 | 11039 | 17333 | 10683 | 9393 | 9299 | 9386 | 9286 | 9211 | 13080 | 13030 | 19109 | 18244 | 18244 | 18728 | 17915 | 19215 | 19477 | 18379 | 17034 | 18162 | 18351 | 18653 | 18775 | 17769 | 4967 | 16892 | 17036 | 4507 | 16777 | 4621 | 3566 | 4755 | 3406 | 16916 | 4970 | 3893 | 2986 | 3182 | 16361 | 4616 | 2614 | 3280 | 2874 | 2777 | 5293 | 2567 | 2557 | 2117 | 2245 | 2046 | 2384 | 2220 | 2115 |  | 0.04 | 0.08 | 0.09 |
| SUU | 15310 | 15323 | 15371 | 15340 | 15387 | 15259 | 15457 | 15379 | 15556 | 14995 | 15641 | 14554 | 14673 | 15206 | 15767 | 15547 | 16502 | 14568 | 14708 | 15262 | 15291 | 14733 | 16276 | 14531 | 14583 | 12266 | 13509 | 11923 | 11900 | 14808 | 14874 | 11808 | 13511 | 14639 | 11196 | 14788 | 11286 | 17016 | 10949 | 9616 | 9527 | 9621 | 9524 | 9458 | 12750 | 12699 | 18932 | 17866 | 17866 | 19074 | 18269 | 19394 | 19401 | 18695 | 17402 | 18441 | 18575 | 18773 | 18644 | 17953 | 5209 | 17174 | 17281 | 4733 | 17011 | 4452 | 3744 | 4566 | 3468 | 17015 | 5307 | 3756 | 3151 | 3420 | 16489 | 4964 | 2752 | 3154 | 3147 | 2747 | 5658 | 2797 | 2827 | 2309 | 2506 | 2300 | 2710 | 2565 | 2469 | 381 |  | 0.09 | 0.13 |
| INJ | 16129 | 16193 | 16197 | 16085 | 16295 | 16228 | 16395 | 16388 | 16582 | 16154 | 16769 | 15864 | 16041 | 16575 | 17014 | 16880 | 17006 | 16028 | 16209 | 16776 | 16855 | 16334 | 17642 | 16142 | 16198 | 11377 | 12508 | 11029 | 11002 | 16459 | 16533 | 10867 | 12417 | 16318 | 10277 | 16516 | 10253 | 18582 | 9825 | 8760 | 8650 | 8698 | 8591 | 8480 | 14514 | 14466 | 18676 | 19770 | 19770 | 17204 | 16391 | 17802 | 18249 | 16891 | 15501 | 16731 | 16995 | 17433 | 18084 | 16538 | 4429 | 15497 | 15712 | 4082 | 15489 | 5685 | 3467 | 5874 | 3805 | 15967 | 3876 | 4901 | 3034 | 2844 | 15369 | 3460 | 2845 | 4294 | 2393 | 3562 | 3986 | 2390 | 2160 | 2241 | 1989 | 1889 | 1646 | 1363 | 1188 | 1535 | 1911 |  | 0.06 |
| INW | 16282 | 16297 | 16344 | 16310 | 16361 | 16232 | 16430 | 16350 | 16525 | 15958 | 16602 | 15501 | 15610 | 16135 | 16709 | 16478 | 17464 | 15485 | 15612 | 16151 | 16164 | 15603 | 17181 | 15400 | 15449 | 12693 | 13885 | 12344 | 12319 | 15655 | 15715 | 12202 | 13831 | 15475 | 11597 | 15594 | 11624 | 17813 | 11230 | 10037 | 9936 | 10007 | 9903 | 9812 | 13562 | 13511 | 19840 | 18251 | 18251 | 18179 | 17432 | 18433 | 18618 | 17768 | 16649 | 17486 | 17601 | 17827 | 17941 | 16981 | 5611 | 16232 | 16317 | 5184 | 16043 | 5424 | 4320 | 5539 | 4248 | 16073 | 5347 | 4718 | 3762 | 3859 | 15531 | 4950 | 3419 | 4110 | 3492 | 3635 | 5513 | 3271 | 3190 | 2880 | 2908 | 2727 | 2877 | 2650 | 2505 | 859 | 973 | 1528 |  |

```
# Create a new Word document
doc <- read_docx()

# Add the flextable to the Word document
doc <- body_add_flextable(doc, ft)

# Save the Word document
print(doc, target =  here("manuscript1", "output", "fst", "allpops_fst_distance.docx"))
```

Fit linear regression

```
# Check for non-positive values in Distance
data <- data[data$Distance > 0, ]

# Fit linear model
lm_model <- lm(FST/(1-FST) ~ log(Distance), data = data)
equation_text <- sprintf("y = %.6fx + %.3f", coef(lm_model)[2], coef(lm_model)[1])
r2_text <- sprintf("R^2 = %.2f", summary(lm_model)$r.squared)

# source the plotting function
source(here("scripts", "RMarkdowns", "my_theme2.R"))


# Plot
ggplot(data, aes(x = log(Distance), y = FST/(1-FST))) +
  geom_point() +
  geom_smooth(method = "lm", se = FALSE) +
  annotate("text", x = max(log((data$Distance))) * 0.85, y = max(data$FST/(1-data$FST)) * 0.95, label = paste(equation_text, r2_text, sep = "\n"), size = 4, color = "black") +
  labs(title = "FST vs Distance - All populations",
       x = "Log(Distance)",
       y = "FST(1-FST)") +
  scale_x_continuous(labels = scales::comma) + 
  theme_classic() +
  theme(axis.text.x = element_text(size = 14),
        axis.text.y = element_text(size = 14
                                   ))
```

```
## `geom_smooth()` using formula = 'y ~ x'
```

```
ggsave(
  filename = here("manuscript1", "output", "fst", "figures", "fst_by_distance.pdf"),
  width = 7,
  height = 5,
  units = "in"
)
```

## 5.3 Mantel test

Import sample locations

```
sampling_loc <- readRDS(here("manuscript1", "output", "fst", "cities_man1.rds"))

# Arrange by region 
sampling_loc <- sampling_loc |>
  dplyr::arrange(
    continent, region, subregion
  ) 

# Check it
head(sampling_loc)
```

```
## # A tibble: 6 × 10
##   pop   city   country state latitude longitude continent region subregion order
##   <chr> <chr>  <chr>   <chr>    <dbl>     <dbl> <chr>     <chr>  <chr>     <dbl>
## 1 BRM   Bermu… Bermuda Berm…     32.3     -64.8 Americas  Carib… Caribbean   128
## 2 NEG   Negril Jamaica Jama…     18.3     -78.3 Americas  Carib… Caribbean   129
## 3 SAI   Saint… Trinid… Trin…     10.2     -61.5 Americas  Carib… Caribbean   131
## 4 COL   Colum… USA     OH, …     40.0     -82.9 Americas  North… Midwest,…   115
## 5 PEO   Peori… USA     IL, …     40.7     -89.6 Americas  North… Midwest,…   116
## 6 SPR   Sprin… USA     MO, …     37.2     -93.3 Americas  North… Midwest,…   117
```

The fam file

```
fam_file <- here("manuscript1/output/snps_sets/r2_0.01_b.fam")

# Read the .fam file
fam_data <- read.table(fam_file, 
                       header = FALSE,
                       col.names = c("FamilyID", "IndividualID", "PaternalID", "MaternalID", "Sex", "Phenotype"))

# View the first few rows
head(fam_data)
```

```
##   FamilyID IndividualID PaternalID MaternalID Sex Phenotype
## 1      OKI         1001          0          0   2        -9
## 2      OKI         1002          0          0   2        -9
## 3      OKI         1003          0          0   2        -9
## 4      OKI         1004          0          0   2        -9
## 5      OKI         1005          0          0   2        -9
## 6      OKI         1006          0          0   1        -9
```

Merge

```
# Join with sampling_loc to get sampling localities
loc_2 <- fam_data |>
  left_join(sampling_loc, by = c("FamilyID" = "pop"))

head(loc_2)
```

```
##   FamilyID IndividualID PaternalID MaternalID Sex Phenotype    city country
## 1      OKI         1001          0          0   2        -9 Okinawa   Japan
## 2      OKI         1002          0          0   2        -9 Okinawa   Japan
## 3      OKI         1003          0          0   2        -9 Okinawa   Japan
## 4      OKI         1004          0          0   2        -9 Okinawa   Japan
## 5      OKI         1005          0          0   2        -9 Okinawa   Japan
## 6      OKI         1006          0          0   1        -9 Okinawa   Japan
##   state latitude longitude continent    region subregion order
## 1  <NA>  26.5013  127.9454      Asia East Asia     Japan     4
## 2  <NA>  26.5013  127.9454      Asia East Asia     Japan     4
## 3  <NA>  26.5013  127.9454      Asia East Asia     Japan     4
## 4  <NA>  26.5013  127.9454      Asia East Asia     Japan     4
## 5  <NA>  26.5013  127.9454      Asia East Asia     Japan     4
## 6  <NA>  26.5013  127.9454      Asia East Asia     Japan     4
```

Get the latitude and longitude

```
albo_dist2 <- cbind(loc_2$latitude, loc_2$longitude)
head(albo_dist2)
```

```
##         [,1]     [,2]
## [1,] 26.5013 127.9454
## [2,] 26.5013 127.9454
## [3,] 26.5013 127.9454
## [4,] 26.5013 127.9454
## [5,] 26.5013 127.9454
## [6,] 26.5013 127.9454
```

```
colnames(albo_dist2)<- c("x","y")
```

Add jitter

```
albo_dist2 <- jitter(albo_dist2, factor = 1, amount = NULL)
head(albo_dist2)
```

```
##             x        y
## [1,] 26.49767 127.9631
## [2,] 26.51855 127.9605
## [3,] 26.49698 127.9604
## [4,] 26.49043 127.9396
## [5,] 26.51985 127.9473
## [6,] 26.48197 127.9508
```

Add to object

```
allpopsidb$other$xy <- albo_dist2
```

Save

```
saveRDS(
  allpopsidb,
  here(
    "manuscript1", "output", "fst", "man1_allpops_mantel.rds"
  )
)
```

### 5.3.1 Isolation by distance

Convert to genid

```
mantel_allpops <- gl2gi(allpopsidb, probar = FALSE, verbose = NULL)
```

```
## Starting gl2gi 
##   Processing genlight object with SNP data
## Matrix converted.. Prepare genind object...
## Completed: gl2gi
```

Convert to genpop

```
toto <- genind2genpop(mantel_allpops)
```

```
## 
##  Converting data from a genind to a genpop object... 
## 
## ...done.
```

Get 1 mosquito per population, it is just to get the geographical
coordinates

```
unique_populations <- unique(mantel_allpops@pop)
selected_individuals <- integer(length(unique_populations))
for (i in seq_along(unique_populations)) {
  inds_in_pop <- which(mantel_allpops@pop == unique_populations[i])
  selected_individuals[i] <- sample(inds_in_pop, 1)
}
allpops2_subset <- mantel_allpops[selected_individuals, ]
```

Mantel test

```
Dgen <- dist.genpop(toto,method=2)
Dgeo <- dist(allpops2_subset$other$xy, method = "euclidean")
ibd <- mantel.randtest(Dgen,Dgeo,nrepet = 999)
ibd
```

```
## Monte-Carlo test
## Call: mantel.randtest(m1 = Dgen, m2 = Dgeo, nrepet = 999)
## 
## Observation: -0.05033947 
## 
## Based on 999 replicates
## Simulated p-value: 0.949 
## Alternative hypothesis: greater 
## 
##       Std.Obs   Expectation      Variance 
## -1.5266330388 -0.0006228983  0.0010605547
```

Plot

```
# Plot it
# Start the PDF device
CairoPDF(here("manuscript1", "output", "fst", "figures", "sim.pdf"))
plot(ibd)
dev.off()
plot(ibd)
```

```
plot(Dgeo, Dgen)
```

```
## Found more than one class "dist" in cache; using the first, from namespace 'spam'
```

```
## Also defined by 'BiocGenerics'
```

```
## Found more than one class "dist" in cache; using the first, from namespace 'spam'
```

```
## Also defined by 'BiocGenerics'
```

```
## Found more than one class "dist" in cache; using the first, from namespace 'spam'
```

```
## Also defined by 'BiocGenerics'
```

```
## Found more than one class "dist" in cache; using the first, from namespace 'spam'
```

```
## Also defined by 'BiocGenerics'
```

```
# A linear regression model (lm stands for "linear model") is fitted, with the genetic distances (Dgen) as the response variable and the geographic distances (Dgeo) as the predictor. The distances are transformed into vectors using as.vector because the dist function produces a matrix-like structure, but the linear regression function lm requires vectors.
dist_lm <- lm(as.vector(Dgen) ~ as.vector(Dgeo))
abline(dist_lm, col="red", lty=2)
```

```
CairoPDF(here("manuscript1", "output", "fst", "figures", "Genetic_vs_Geographic_Distance_noeqa.pdf"))

plot(Dgeo, Dgen)
# A linear regression model (lm stands for "linear model") is fitted, with the genetic distances (Dgen) as the response variable and the geographic distances (Dgeo) as the predictor. The distances are transformed into vectors using as.vector because the dist function produces a matrix-like structure, but the linear regression function lm requires vectors.
dist_lm <- lm(as.vector(Dgen) ~ as.vector(Dgeo))
abline(dist_lm, col="red", lty=2)

dev.off()
```

Add the equation

```
CairoPDF(here("manuscript1", "output", "fst", "figures", "Genetic_vs_Geographic_Distance.pdf"))

# Plotting the data
plot(Dgeo, Dgen, main = "Genetic Distance vs Geographic Distance")
abline(dist_lm, col="red", lty=2)

# Extracting the coefficients from the linear model
intercept <- coef(dist_lm)[1]
slope <- coef(dist_lm)[2]
r2 <- summary(dist_lm)$r.squared

# Generating the equation string
equation <- sprintf("y = %.4fx + %.2f", slope, intercept)
r2_label <- sprintf("R^2 = %.2f", r2)

# Adding the equation and R^2 to the plot
# You can adjust the position (e.g., x and y values) as necessary
 text(x = max(as.vector(Dgeo)) * 0.8, y = max(as.vector(Dgen)) * 0.9, labels = equation)
 text(x = max(as.vector(Dgeo)) * 0.8, y = max(as.vector(Dgen)) * 0.85, labels = r2_label)
dev.off()
```

Use library MASS for plot

```
dens <- kde2d(as.vector(Dgeo), as.vector(Dgen), n = 500)
myPal <-
  colorRampPalette(c("white", "blue", "gold", "orange", "red"))
# CairoPDF(here("output", "fst", "ibd.pdf"),
#     width = 5,
#     height = 4)
png(here("manuscript1", "output", "fst", "figures", "ibd2.png"),
    width = 5,
    height = 4,
    units='in',
    res = 300)
myPal <-
  colorRampPalette(c("white", "purple", "gold", "orange", "red"))
plot(Dgeo, Dgen, pch = 20, cex = .3, bty = "n")
image(dens, col = transp(myPal(300), .7), add = TRUE)
abline(dist_lm)
# Extracting the coefficients and R^2 from the linear model
intercept <- coef(dist_lm)[1]
slope <- coef(dist_lm)[2]
r2 <- summary(dist_lm)$r.squared

# Constructing the equation and R^2 strings
equation <- sprintf("y = %.4fx + %.2f", slope, intercept)
r2_label <- sprintf("R^2 = %.2f", r2)

# Adding the equation and R^2 to the plot
# text(x = max(as.vector(Dgeo)) * 0.8, y = max(as.vector(Dgen)) * 0.5, labels = equation)
# text(x = max(as.vector(Dgeo)) * 0.8, y = max(as.vector(Dgen)) * 0.45, labels = r2_label)

# title("Isolation by distance")
dev.off()
```

```
## png 
##   2
```

```
myPal <-
  colorRampPalette(c("white", "purple", "gold", "orange", "red"))
plot(Dgeo, Dgen, pch = 20, cex = .3, bty = "n")
image(dens, col = transp(myPal(300), .7), add = TRUE)
abline(dist_lm)
```

```
# Extracting the coefficients and R^2 from the linear model
intercept <- coef(dist_lm)[1]
slope <- coef(dist_lm)[2]
r2 <- summary(dist_lm)$r.squared

# Constructing the equation and R^2 strings
equation <- sprintf("y = %.4fx + %.2f", slope, intercept)
r2_label <- sprintf("R^2 = %.2f", r2)
```

## 5.4 Hudson Fst

Calcuate pairwise Hudson Fst on HPC

```
cd /gpfs/gibbs/pi/caccone/mkc54/albo/manuscript1/output/fst

salloc -p scavenge --cpus-per-task=5 -t 2:00:00 --mem-per-cpu=10000    
module load R/4.2.0-foss-2020b
R

library(tidyverse)
library(adegenet)
library(here)
library(KRIS)
library(dartR)
```

Then in R (on the hpc)

```
pairwiseHudsonFst <- function(genind_object=gen, outout_file = NULL) {
  require("KRIS")
  populations <- unique(genind_object@pop)
  num_populations <- length(populations)
  fst_matrix <- matrix(NA, nrow = num_populations, ncol = num_populations, dimnames = list(populations, populations))
  calculate_fst <- function(subset_data, pop1, pop2) {
    idx_pop1 <- which(as.character(subset_data$pop) == pop1)
    idx_pop2 <- which(as.character(subset_data$pop) == pop2)
    tryCatch(
      KRIS::fst.hudson(subset_data$tab, idx_pop1, idx_pop2),
      error = function(e) {
        message(sprintf("Error calculating Fst for populations %s and %s: %s", pop1, pop2, e$message))
        return(NA)
      }
    )
  }
  for (i in 1:(num_populations - 1)) {
    for (j in (i + 1):num_populations) {
      pop1 <- populations[i]
      pop2 <- populations[j]
      subset_data <- genind_object[pop = c(pop1, pop2)]
      fst_value <- calculate_fst(subset_data, pop1, pop2)
      fst_matrix[i, j] <- fst_value
      fst_matrix[j, i] <- fst_value
    }
  }
  fst_dataframe <- as.data.frame(fst_matrix)
  if (!is.null(outout_file)) {
    write.csv(fst_dataframe, outout_file, row.names = TRUE)
  }
  return(fst_dataframe)
}
```

Convert genlight to genind object

```
LD2 <- readRDS(
  here(
    "/gpfs/gibbs/pi/caccone/mkc54/albo/manuscript1/output/fst/allpopsforfst.rds"
  )
)

LD2_genind <- gl2gi(LD2, probar = TRUE, verbose = NULL)
```

```
pairwiseHudsonFst(genind_object=LD2_genind, outout_file = "/gpfs/gibbs/pi/caccone/mkc54/albo/manuscript1/output/fst/hudson_fst_data.csv")
```

Can continue in RStudio once calculated

```
# Load data from CSV into a matrix with row and column names
LD3 <- as.matrix(read.csv("/gpfs/gibbs/pi/caccone/mkc54/albo/manuscript1/output/fst/hudson_fst_data.csv", row.names = 1))
```

```
LD3[upper.tri(LD3)] <- NA
print(LD3)
```

```
saveRDS(
  LD3, here(
    "/gpfs/gibbs/pi/caccone/mkc54/albo/manuscript1/output/fst/hudson_fst_data_matrix.rds"
  )
)
```

To load it

```
LD3 <- readRDS(
  here(
    "/gpfs/gibbs/pi/caccone/mkc54/albo/manuscript1/output/fst/hudson_fst_data_matrix.rds"
  )
)
```

Now lets look at the object

```
summary(LD3)
```

```
##       OKI               HAI               SOC               YUN          
##  Min.   :0.06730   Min.   :0.03765   Min.   :0.01414   Min.   :0.009564  
##  1st Qu.:0.09524   1st Qu.:0.07261   1st Qu.:0.09998   1st Qu.:0.090484  
##  Median :0.11577   Median :0.08841   Median :0.12066   Median :0.107484  
##  Mean   :0.11689   Mean   :0.09053   Mean   :0.12272   Mean   :0.106589  
##  3rd Qu.:0.12955   3rd Qu.:0.09963   3rd Qu.:0.14141   3rd Qu.:0.131439  
##  Max.   :0.21908   Max.   :0.17482   Max.   :0.23077   Max.   :0.203287  
##  NA's   :1         NA's   :2         NA's   :3         NA's   :4         
##       KAT               GES               TIK               RAR         
##  Min.   :0.03573   Min.   :0.02794   Min.   :0.04589   Min.   :0.07786  
##  1st Qu.:0.09962   1st Qu.:0.10418   1st Qu.:0.11159   1st Qu.:0.10779  
##  Median :0.12228   Median :0.12588   Median :0.13052   Median :0.12607  
##  Mean   :0.11957   Mean   :0.12802   Mean   :0.13520   Mean   :0.12974  
##  3rd Qu.:0.14510   3rd Qu.:0.14622   3rd Qu.:0.15202   3rd Qu.:0.14437  
##  Max.   :0.21229   Max.   :0.23638   Max.   :0.23771   Max.   :0.23107  
##  NA's   :5         NA's   :6         NA's   :7         NA's   :8        
##       TAI              HUN               HAN               KLP         
##  Min.   :0.1179   Min.   :0.03964   Min.   :0.04880   Min.   :0.05625  
##  1st Qu.:0.1439   1st Qu.:0.06173   1st Qu.:0.08410   1st Qu.:0.11763  
##  Median :0.1609   Median :0.08250   Median :0.09756   Median :0.13176  
##  Mean   :0.1627   Mean   :0.08471   Mean   :0.09770   Mean   :0.13273  
##  3rd Qu.:0.1753   3rd Qu.:0.09872   3rd Qu.:0.10793   3rd Qu.:0.15421  
##  Max.   :0.2544   Max.   :0.19443   Max.   :0.17561   Max.   :0.21653  
##  NA's   :9        NA's   :10        NA's   :11        NA's   :12       
##       HOC               QNC               SSK               KAC         
##  Min.   :0.05089   Min.   :0.07075   Min.   :0.01056   Min.   :0.01210  
##  1st Qu.:0.08018   1st Qu.:0.14307   1st Qu.:0.09574   1st Qu.:0.09369  
##  Median :0.09433   Median :0.15908   Median :0.11072   Median :0.11000  
##  Mean   :0.09679   Mean   :0.15819   Mean   :0.11151   Mean   :0.11103  
##  3rd Qu.:0.10523   3rd Qu.:0.17870   3rd Qu.:0.13321   3rd Qu.:0.13228  
##  Max.   :0.17661   Max.   :0.24459   Max.   :0.20649   Max.   :0.20181  
##  NA's   :13        NA's   :14        NA's   :15        NA's   :16       
##       CHA               BRE               DES               CES         
##  Min.   :0.01040   Min.   :0.06057   Min.   :0.05505   Min.   :0.09637  
##  1st Qu.:0.09584   1st Qu.:0.08161   1st Qu.:0.07353   1st Qu.:0.10958  
##  Median :0.11022   Median :0.11942   Median :0.11199   Median :0.14216  
##  Mean   :0.11271   Mean   :0.12610   Mean   :0.11832   Mean   :0.15004  
##  3rd Qu.:0.13265   3rd Qu.:0.15819   3rd Qu.:0.15023   3rd Qu.:0.17771  
##  Max.   :0.20650   Max.   :0.25893   Max.   :0.25651   Max.   :0.27655  
##  NA's   :17        NA's   :18        NA's   :19        NA's   :20       
##       KAN              UTS               MAU               TUC         
##  Min.   :0.0769   Min.   :0.04796   Min.   :0.03681   Min.   :0.01157  
##  1st Qu.:0.0971   1st Qu.:0.06341   1st Qu.:0.08742   1st Qu.:0.09384  
##  Median :0.1273   Median :0.10383   Median :0.10031   Median :0.11356  
##  Mean   :0.1359   Mean   :0.10990   Mean   :0.10030   Mean   :0.10554  
##  3rd Qu.:0.1683   3rd Qu.:0.14420   3rd Qu.:0.11181   3rd Qu.:0.12685  
##  Max.   :0.2612   Max.   :0.24670   Max.   :0.20406   Max.   :0.21170  
##  NA's   :21       NA's   :22        NA's   :23        NA's   :24       
##       REC               BRM               CAM               KAG         
##  Min.   :0.02973   Min.   :0.06853   Min.   :0.01567   Min.   :0.05663  
##  1st Qu.:0.08004   1st Qu.:0.08132   1st Qu.:0.08970   1st Qu.:0.07272  
##  Median :0.09340   Median :0.10185   Median :0.10162   Median :0.09429  
##  Mean   :0.09829   Mean   :0.11503   Mean   :0.10250   Mean   :0.10854  
##  3rd Qu.:0.10963   3rd Qu.:0.13802   3rd Qu.:0.11968   3rd Qu.:0.13732  
##  Max.   :0.20902   Max.   :0.23702   Max.   :0.19625   Max.   :0.23244  
##  NA's   :25        NA's   :26        NA's   :27        NA's   :28       
##       CUA               MED               FAY               DAL         
##  Min.   :0.01128   Min.   :0.08536   Min.   :0.02041   Min.   :0.01777  
##  1st Qu.:0.10559   1st Qu.:0.10479   1st Qu.:0.03741   1st Qu.:0.03778  
##  Median :0.11311   Median :0.11227   Median :0.06523   Median :0.07220  
##  Mean   :0.11717   Mean   :0.11724   Mean   :0.08131   Mean   :0.08445  
##  3rd Qu.:0.12546   3rd Qu.:0.12375   3rd Qu.:0.11238   3rd Qu.:0.11971  
##  Max.   :0.20304   Max.   :0.20020   Max.   :0.22208   Max.   :0.22800  
##  NA's   :29        NA's   :30        NA's   :31        NA's   :32       
##       NEO               RUS               CHI               NUE         
##  Min.   :0.03194   Min.   :0.05719   Min.   :0.06647   Min.   :0.01439  
##  1st Qu.:0.04799   1st Qu.:0.06933   1st Qu.:0.09432   1st Qu.:0.04988  
##  Median :0.07353   Median :0.09900   Median :0.10467   Median :0.07159  
##  Mean   :0.09110   Mean   :0.11099   Mean   :0.11031   Mean   :0.08567  
##  3rd Qu.:0.12268   3rd Qu.:0.14139   3rd Qu.:0.11789   3rd Qu.:0.11012  
##  Max.   :0.22646   Max.   :0.23762   Max.   :0.19438   Max.   :0.21459  
##  NA's   :33        NA's   :34        NA's   :35        NA's   :36       
##       TRA               HAL               STL               SMI         
##  Min.   :0.01515   Min.   :0.01955   Min.   :0.03363   Min.   :0.01720  
##  1st Qu.:0.03915   1st Qu.:0.03811   1st Qu.:0.05268   1st Qu.:0.04986  
##  Median :0.06515   Median :0.07642   Median :0.09915   Median :0.08866  
##  Mean   :0.08216   Mean   :0.08719   Mean   :0.10326   Mean   :0.09572  
##  3rd Qu.:0.11244   3rd Qu.:0.12245   3rd Qu.:0.14319   3rd Qu.:0.12707  
##  Max.   :0.21897   Max.   :0.22243   Max.   :0.24013   Max.   :0.22763  
##  NA's   :37        NA's   :38        NA's   :39        NA's   :40       
##       WAV               HAT               DEN               MON         
##  Min.   :0.02823   Min.   :0.02696   Min.   :0.02855   Min.   :0.01979  
##  1st Qu.:0.04934   1st Qu.:0.04731   1st Qu.:0.04124   1st Qu.:0.04966  
##  Median :0.10108   Median :0.09603   Median :0.09412   Median :0.09121  
##  Mean   :0.10157   Mean   :0.09686   Mean   :0.09656   Mean   :0.09184  
##  3rd Qu.:0.14018   3rd Qu.:0.12594   3rd Qu.:0.13210   3rd Qu.:0.11263  
##  Max.   :0.23134   Max.   :0.22326   Max.   :0.22943   Max.   :0.21426  
##  NA's   :41        NA's   :42        NA's   :43        NA's   :44       
##       BPV               BCO               BPA               BIT         
##  Min.   :0.04640   Min.   :0.02358   Min.   :0.05745   Min.   :0.03975  
##  1st Qu.:0.08159   1st Qu.:0.09174   1st Qu.:0.08837   1st Qu.:0.09803  
##  Median :0.11611   Median :0.11752   Median :0.10194   Median :0.11770  
##  Mean   :0.11084   Mean   :0.11005   Mean   :0.10833   Mean   :0.11399  
##  3rd Qu.:0.12810   3rd Qu.:0.12892   3rd Qu.:0.11603   3rd Qu.:0.12937  
##  Max.   :0.21575   Max.   :0.21419   Max.   :0.20981   Max.   :0.21352  
##  NA's   :45        NA's   :46        NA's   :47        NA's   :48       
##       FLO               BAF               PLM               NEH         
##  Min.   :0.01539   Min.   :0.01302   Min.   :0.02323   Min.   :0.02502  
##  1st Qu.:0.10221   1st Qu.:0.09536   1st Qu.:0.10142   1st Qu.:0.04016  
##  Median :0.12187   Median :0.11787   Median :0.12441   Median :0.07707  
##  Mean   :0.11446   Mean   :0.11026   Mean   :0.11879   Mean   :0.09482  
##  3rd Qu.:0.13291   3rd Qu.:0.12894   3rd Qu.:0.13851   3rd Qu.:0.13577  
##  Max.   :0.21410   Max.   :0.20925   Max.   :0.21344   Max.   :0.22317  
##  NA's   :49        NA's   :50        NA's   :51        NA's   :52       
##       LIN               HAR               FIR               NEB         
##  Min.   :0.04756   Min.   :0.03600   Min.   :0.05651   Min.   :0.02945  
##  1st Qu.:0.06343   1st Qu.:0.05161   1st Qu.:0.07217   1st Qu.:0.05253  
##  Median :0.09413   Median :0.10207   Median :0.12181   Median :0.09971  
##  Mean   :0.11216   Mean   :0.10728   Mean   :0.12807   Mean   :0.10867  
##  3rd Qu.:0.15035   3rd Qu.:0.14449   3rd Qu.:0.16329   3rd Qu.:0.14649  
##  Max.   :0.23826   Max.   :0.23253   Max.   :0.24680   Max.   :0.23315  
##  NA's   :53        NA's   :54        NA's   :55        NA's   :56       
##       BEN               BEH               ARA               IPO         
##  Min.   :0.02734   Min.   :0.04362   Min.   :0.04620   Min.   :0.03418  
##  1st Qu.:0.10056   1st Qu.:0.11291   1st Qu.:0.09492   1st Qu.:0.15631  
##  Median :0.11886   Median :0.13649   Median :0.11455   Median :0.17298  
##  Mean   :0.11794   Mean   :0.12758   Mean   :0.11661   Mean   :0.16179  
##  3rd Qu.:0.14113   3rd Qu.:0.15049   3rd Qu.:0.12957   3rd Qu.:0.18683  
##  Max.   :0.21478   Max.   :0.22386   Max.   :0.20985   Max.   :0.25726  
##  NA's   :57        NA's   :58        NA's   :59        NA's   :60       
##       LAM               PAL               MAN               BEA         
##  Min.   :0.02251   Min.   :0.03229   Min.   :0.02808   Min.   :0.01752  
##  1st Qu.:0.10121   1st Qu.:0.06165   1st Qu.:0.03783   1st Qu.:0.04708  
##  Median :0.11227   Median :0.08483   Median :0.08235   Median :0.07911  
##  Mean   :0.11516   Mean   :0.10000   Mean   :0.09727   Mean   :0.09193  
##  3rd Qu.:0.13263   3rd Qu.:0.12240   3rd Qu.:0.13656   3rd Qu.:0.11872  
##  Max.   :0.20644   Max.   :0.21065   Max.   :0.22750   Max.   :0.21017  
##  NA's   :61        NA's   :62        NA's   :63        NA's   :64       
##       PEO               HOU               LOS               NEG        
##  Min.   :0.02782   Min.   :0.02026   Min.   :0.06978   Min.   :0.1394  
##  1st Qu.:0.05111   1st Qu.:0.05196   1st Qu.:0.09368   1st Qu.:0.1884  
##  Median :0.08967   Median :0.08693   Median :0.11051   Median :0.2029  
##  Mean   :0.10111   Mean   :0.09599   Mean   :0.12183   Mean   :0.2049  
##  3rd Qu.:0.14097   3rd Qu.:0.13528   3rd Qu.:0.15076   3rd Qu.:0.2215  
##  Max.   :0.22072   Max.   :0.21067   Max.   :0.20852   Max.   :0.2864  
##  NA's   :65        NA's   :66        NA's   :67        NA's   :68      
##       NOV               NEW               BER               MAC         
##  Min.   :0.04922   Min.   :0.04625   Min.   :0.04154   Min.   :0.04971  
##  1st Qu.:0.14240   1st Qu.:0.06907   1st Qu.:0.06343   1st Qu.:0.06289  
##  Median :0.16342   Median :0.10651   Median :0.10530   Median :0.10539  
##  Mean   :0.15621   Mean   :0.11510   Mean   :0.11206   Mean   :0.10545  
##  3rd Qu.:0.18221   3rd Qu.:0.14481   3rd Qu.:0.14110   3rd Qu.:0.12339  
##  Max.   :0.24556   Max.   :0.23806   Max.   :0.23316   Max.   :0.21151  
##  NA's   :69        NA's   :70        NA's   :71        NA's   :72       
##       SPR               COL               SAI               POR        
##  Min.   :0.05636   Min.   :0.04125   Min.   :0.06374   Min.   :0.0299  
##  1st Qu.:0.09742   1st Qu.:0.08148   1st Qu.:0.08803   1st Qu.:0.1280  
##  Median :0.12830   Median :0.12325   Median :0.10883   Median :0.1404  
##  Mean   :0.13297   Mean   :0.12268   Mean   :0.11795   Mean   :0.1398  
##  3rd Qu.:0.16182   3rd Qu.:0.15620   3rd Qu.:0.14208   3rd Qu.:0.1595  
##  Max.   :0.24164   Max.   :0.22938   Max.   :0.19634   Max.   :0.2232  
##  NA's   :73        NA's   :74        NA's   :75        NA's   :76      
##       SUF               SUU               GRV               GRA         
##  Min.   :0.04162   Min.   :0.07399   Min.   :0.09849   Min.   :0.06020  
##  1st Qu.:0.10405   1st Qu.:0.10166   1st Qu.:0.11845   1st Qu.:0.06963  
##  Median :0.16876   Median :0.12486   Median :0.13047   Median :0.07904  
##  Mean   :0.14600   Mean   :0.12422   Mean   :0.14325   Mean   :0.10200  
##  3rd Qu.:0.18395   3rd Qu.:0.13582   3rd Qu.:0.15867   3rd Qu.:0.13092  
##  Max.   :0.24740   Max.   :0.20884   Max.   :0.21228   Max.   :0.18060  
##  NA's   :77        NA's   :78        NA's   :79        NA's   :80       
##       TUH               ALD               FRS               SER        
##  Min.   :0.04437   Min.   :0.07909   Min.   :0.05312   Min.   :0.1670  
##  1st Qu.:0.07491   1st Qu.:0.10258   1st Qu.:0.09502   1st Qu.:0.1923  
##  Median :0.10012   Median :0.13367   Median :0.13823   Median :0.2094  
##  Mean   :0.11743   Mean   :0.14407   Mean   :0.13464   Mean   :0.2223  
##  3rd Qu.:0.16565   3rd Qu.:0.18201   3rd Qu.:0.16862   3rd Qu.:0.2510  
##  Max.   :0.21634   Max.   :0.22651   Max.   :0.21893   Max.   :0.2918  
##  NA's   :81        NA's   :82        NA's   :83        NA's   :84      
##       SPB              SPS              INW               INJ        
##  Min.   :0.1043   Min.   :0.1466   Min.   :0.06866   Min.   :0.1013  
##  1st Qu.:0.1272   1st Qu.:0.1701   1st Qu.:0.08825   1st Qu.:0.1013  
##  Median :0.1586   Median :0.1937   Median :0.10784   Median :0.1013  
##  Mean   :0.1623   Mean   :0.1929   Mean   :0.10784   Mean   :0.1013  
##  3rd Qu.:0.1937   3rd Qu.:0.2160   3rd Qu.:0.12743   3rd Qu.:0.1013  
##  Max.   :0.2278   Max.   :0.2384   Max.   :0.14702   Max.   :0.1013  
##  NA's   :85       NA's   :86       NA's   :87        NA's   :88      
##       MAT     
##  Min.   : NA  
##  1st Qu.: NA  
##  Median : NA  
##  Mean   :NaN  
##  3rd Qu.: NA  
##  Max.   : NA  
##  NA's   :89
```

Check the Fst values

```
head(LD3)
```

```
##            OKI        HAI        SOC        YUN       KAT GES TIK RAR TAI HUN
## OKI         NA         NA         NA         NA        NA  NA  NA  NA  NA  NA
## HAI 0.08268169         NA         NA         NA        NA  NA  NA  NA  NA  NA
## SOC 0.11228986 0.09582339         NA         NA        NA  NA  NA  NA  NA  NA
## YUN 0.11579214 0.05742478 0.13003132         NA        NA  NA  NA  NA  NA  NA
## KAT 0.12979869 0.07846689 0.14417007 0.03572613        NA  NA  NA  NA  NA  NA
## GES 0.11753346 0.10011811 0.01414008 0.13530983 0.1503583  NA  NA  NA  NA  NA
##     HAN KLP HOC QNC SSK KAC CHA BRE DES CES KAN UTS MAU TUC REC BRM CAM KAG CUA
## OKI  NA  NA  NA  NA  NA  NA  NA  NA  NA  NA  NA  NA  NA  NA  NA  NA  NA  NA  NA
## HAI  NA  NA  NA  NA  NA  NA  NA  NA  NA  NA  NA  NA  NA  NA  NA  NA  NA  NA  NA
## SOC  NA  NA  NA  NA  NA  NA  NA  NA  NA  NA  NA  NA  NA  NA  NA  NA  NA  NA  NA
## YUN  NA  NA  NA  NA  NA  NA  NA  NA  NA  NA  NA  NA  NA  NA  NA  NA  NA  NA  NA
## KAT  NA  NA  NA  NA  NA  NA  NA  NA  NA  NA  NA  NA  NA  NA  NA  NA  NA  NA  NA
## GES  NA  NA  NA  NA  NA  NA  NA  NA  NA  NA  NA  NA  NA  NA  NA  NA  NA  NA  NA
##     MED FAY DAL NEO RUS CHI NUE TRA HAL STL SMI WAV HAT DEN MON BPV BCO BPA BIT
## OKI  NA  NA  NA  NA  NA  NA  NA  NA  NA  NA  NA  NA  NA  NA  NA  NA  NA  NA  NA
## HAI  NA  NA  NA  NA  NA  NA  NA  NA  NA  NA  NA  NA  NA  NA  NA  NA  NA  NA  NA
## SOC  NA  NA  NA  NA  NA  NA  NA  NA  NA  NA  NA  NA  NA  NA  NA  NA  NA  NA  NA
## YUN  NA  NA  NA  NA  NA  NA  NA  NA  NA  NA  NA  NA  NA  NA  NA  NA  NA  NA  NA
## KAT  NA  NA  NA  NA  NA  NA  NA  NA  NA  NA  NA  NA  NA  NA  NA  NA  NA  NA  NA
## GES  NA  NA  NA  NA  NA  NA  NA  NA  NA  NA  NA  NA  NA  NA  NA  NA  NA  NA  NA
##     FLO BAF PLM NEH LIN HAR FIR NEB BEN BEH ARA IPO LAM PAL MAN BEA PEO HOU LOS
## OKI  NA  NA  NA  NA  NA  NA  NA  NA  NA  NA  NA  NA  NA  NA  NA  NA  NA  NA  NA
## HAI  NA  NA  NA  NA  NA  NA  NA  NA  NA  NA  NA  NA  NA  NA  NA  NA  NA  NA  NA
## SOC  NA  NA  NA  NA  NA  NA  NA  NA  NA  NA  NA  NA  NA  NA  NA  NA  NA  NA  NA
## YUN  NA  NA  NA  NA  NA  NA  NA  NA  NA  NA  NA  NA  NA  NA  NA  NA  NA  NA  NA
## KAT  NA  NA  NA  NA  NA  NA  NA  NA  NA  NA  NA  NA  NA  NA  NA  NA  NA  NA  NA
## GES  NA  NA  NA  NA  NA  NA  NA  NA  NA  NA  NA  NA  NA  NA  NA  NA  NA  NA  NA
##     NEG NOV NEW BER MAC SPR COL SAI POR SUF SUU GRV GRA TUH ALD FRS SER SPB SPS
## OKI  NA  NA  NA  NA  NA  NA  NA  NA  NA  NA  NA  NA  NA  NA  NA  NA  NA  NA  NA
## HAI  NA  NA  NA  NA  NA  NA  NA  NA  NA  NA  NA  NA  NA  NA  NA  NA  NA  NA  NA
## SOC  NA  NA  NA  NA  NA  NA  NA  NA  NA  NA  NA  NA  NA  NA  NA  NA  NA  NA  NA
## YUN  NA  NA  NA  NA  NA  NA  NA  NA  NA  NA  NA  NA  NA  NA  NA  NA  NA  NA  NA
## KAT  NA  NA  NA  NA  NA  NA  NA  NA  NA  NA  NA  NA  NA  NA  NA  NA  NA  NA  NA
## GES  NA  NA  NA  NA  NA  NA  NA  NA  NA  NA  NA  NA  NA  NA  NA  NA  NA  NA  NA
##     INW INJ MAT
## OKI  NA  NA  NA
## HAI  NA  NA  NA
## SOC  NA  NA  NA
## YUN  NA  NA  NA
## KAT  NA  NA  NA
## GES  NA  NA  NA
```

Convert the data into a matrix.

```
aa <- as.matrix(LD3)
aa[upper.tri(aa)] <- t(aa)[upper.tri(t(aa))]
head(aa)
```

```
##            OKI        HAI        SOC        YUN        KAT        GES
## OKI         NA 0.08268169 0.11228986 0.11579214 0.12979869 0.11753346
## HAI 0.08268169         NA 0.09582339 0.05742478 0.07846689 0.10011811
## SOC 0.11228986 0.09582339         NA 0.13003132 0.14417007 0.01414008
## YUN 0.11579214 0.05742478 0.13003132         NA 0.03572613 0.13530983
## KAT 0.12979869 0.07846689 0.14417007 0.03572613         NA 0.15035825
## GES 0.11753346 0.10011811 0.01414008 0.13530983 0.15035825         NA
##            TIK        RAR       TAI        HUN        HAN        KLP        HOC
## OKI 0.12371206 0.11705699 0.1335548 0.06729815 0.09141610 0.13943830 0.08570565
## HAI 0.10731189 0.09858151 0.1251949 0.04021476 0.04630969 0.08734436 0.03765262
## SOC 0.05207064 0.02063810 0.1573577 0.07396946 0.10500900 0.15195443 0.09857539
## YUN 0.14054421 0.13049713 0.1615414 0.08117588 0.05494547 0.05941637 0.06524127
## KAT 0.15460732 0.14480449 0.1721918 0.09948750 0.07176172 0.07434104 0.08440142
## GES 0.04925772 0.02794134 0.1600543 0.07813875 0.10957872 0.15677517 0.10265901
##            QNC        SSK        KAC         CHA       BRE       DES       CES
## OKI 0.16271362 0.11730198 0.11580491 0.115742453 0.1387992 0.1293697 0.1586276
## HAI 0.11294299 0.05865738 0.05858943 0.056507618 0.1307831 0.1218826 0.1509614
## SOC 0.17715022 0.13290421 0.13106145 0.130931794 0.1395691 0.1326901 0.1610333
## YUN 0.07126989 0.01177784 0.01207249 0.009563734 0.1675132 0.1609786 0.1863663
## KAT 0.09282384 0.03965856 0.03573132 0.038076402 0.1779542 0.1720546 0.1969942
## GES 0.18359105 0.13823935 0.13586223 0.135732658 0.1425648 0.1362589 0.1627626
##           KAN       UTS        MAU        TUC        REC       BRM        CAM
## OKI 0.1395602 0.1180465 0.11925414 0.12989700 0.11398793 0.1164645 0.10553710
## HAI 0.1313963 0.1078236 0.08351136 0.08837926 0.08604667 0.1028222 0.04667773
## SOC 0.1366748 0.1168696 0.13080801 0.14182174 0.12708620 0.1240746 0.12028843
## YUN 0.1678130 0.1493744 0.09349152 0.08911175 0.10368985 0.1354268 0.01244233
## KAT 0.1802954 0.1630360 0.10511907 0.10181151 0.11665125 0.1485751 0.03825775
## GES 0.1397995 0.1205597 0.13535629 0.14610856 0.13079183 0.1285723 0.12587912
##            KAG        CUA        MED        FAY        DAL        NEO       RUS
## OKI 0.10523386 0.12039803 0.12018690 0.08643722 0.09258665 0.09337691 0.1107434
## HAI 0.09573472 0.08571519 0.08216104 0.07725947 0.08413851 0.08517488 0.1061016
## SOC 0.10690651 0.13566811 0.13223746 0.09745339 0.10245126 0.10424628 0.1210305
## YUN 0.13231894 0.09429958 0.09001816 0.11821274 0.12335338 0.12403275 0.1390043
## KAT 0.14686073 0.10677257 0.09926118 0.13004971 0.13906261 0.13889583 0.1508654
## GES 0.11006928 0.14044833 0.13770274 0.10182070 0.10692432 0.10902333 0.1266632
##            CHI        NUE        TRA        HAL        STL        SMI
## OKI 0.09579221 0.08751335 0.08468852 0.09024111 0.11031763 0.09673045
## HAI 0.05853241 0.06906687 0.07155932 0.08340299 0.09907115 0.08489985
## SOC 0.10762513 0.09934153 0.09431803 0.09089918 0.11037404 0.10369924
## YUN 0.08991930 0.10730171 0.11018416 0.12023285 0.14211303 0.12435121
## KAT 0.10691381 0.12328740 0.12660200 0.13438085 0.15569136 0.14035432
## GES 0.11147736 0.10395966 0.09843576 0.09495313 0.11360289 0.10908845
##            WAV        HAT        DEN        MON        BPV        BCO
## OKI 0.10455193 0.09079702 0.08960815 0.08549006 0.13124716 0.13436578
## HAI 0.09959681 0.08639033 0.08571585 0.06896848 0.09270859 0.09587110
## SOC 0.10590488 0.09987098 0.10029013 0.09651836 0.14317088 0.14503130
## YUN 0.13616628 0.12096228 0.12723227 0.10810366 0.09615219 0.09664071
## KAT 0.14824250 0.13639734 0.14173410 0.12483943 0.10847733 0.10759240
## GES 0.10856053 0.10416131 0.10420531 0.10123504 0.14846771 0.14952026
##            BPA        BIT        FLO        BAF        PLM        NEH       LIN
## OKI 0.12048075 0.12947039 0.13528165 0.12825119 0.13581254 0.09357675 0.1061676
## HAI 0.09233415 0.09296947 0.09657258 0.08841429 0.09519624 0.08666284 0.0969583
## SOC 0.13317877 0.14236054 0.14686221 0.13984185 0.14720441 0.09667045 0.1098317
## YUN 0.10626986 0.09946437 0.09677759 0.08767629 0.09048376 0.12396725 0.1385981
## KAT 0.11772888 0.11125795 0.10495631 0.09837517 0.09966212 0.13715729 0.1513453
## GES 0.13649698 0.14678199 0.15095307 0.14450274 0.15132770 0.09964845 0.1135977
##            HAR       FIR        NEB        BEN        BEH        ARA       IPO
## OKI 0.10200936 0.1189740 0.10158298 0.12871724 0.14605030 0.12617930 0.1805418
## HAI 0.09417892 0.1146315 0.09239762 0.07083019 0.09856573 0.08683458 0.1313802
## SOC 0.10616213 0.1252538 0.10485624 0.14333738 0.15631315 0.13959597 0.1902597
## YUN 0.13143940 0.1509763 0.13351940 0.02412583 0.08384151 0.09133143 0.1074838
## KAT 0.14597234 0.1640393 0.14804091 0.03736564 0.09516177 0.10341814 0.1148694
## GES 0.11051990 0.1289002 0.10744015 0.14836310 0.16239220 0.14258493 0.1957003
##            LAM        PAL        MAN        BEA        PEO        HOU
## OKI 0.11837068 0.09648642 0.09310660 0.08160337 0.09062367 0.08013354
## HAI 0.06013534 0.07609765 0.08309913 0.06438147 0.08087216 0.06293295
## SOC 0.13409149 0.10598877 0.09523383 0.09152568 0.09379041 0.08911064
## YUN 0.01364806 0.10742494 0.12343238 0.10173982 0.11821552 0.10042463
## KAT 0.03825790 0.12099893 0.13943481 0.11798178 0.13240431 0.11760703
## GES 0.13967252 0.11001168 0.09917813 0.09508925 0.09805706 0.09428640
##            LOS       NEG        NOV       NEW        BER        MAC       SPR
## OKI 0.09785283 0.2073457 0.16627599 0.1013020 0.10026824 0.09065625 0.1156643
## HAI 0.06974945 0.1631205 0.11739015 0.0972630 0.09095991 0.06919882 0.1087890
## SOC 0.10651130 0.2181526 0.17893169 0.1093976 0.10255356 0.09895578 0.1151486
## YUN 0.10426232 0.1276615 0.08867712 0.1358189 0.13083385 0.10567617 0.1457691
## KAT 0.11999341 0.1393196 0.09631828 0.1498808 0.14473745 0.12126712 0.1580711
## GES 0.11067054 0.2244522 0.18452552 0.1133464 0.10668277 0.10256608 0.1206725
##            COL        SAI        POR        SUF        SUU        GRV
## OKI 0.09955760 0.09620034 0.14189354 0.17490633 0.12810973 0.13278755
## HAI 0.08907718 0.06834360 0.09965423 0.12735379 0.08150795 0.09326533
## SOC 0.09967069 0.11113482 0.15289496 0.18952076 0.14153628 0.14101661
## YUN 0.13001690 0.09186110 0.09134184 0.10505893 0.08492226 0.09952785
## KAT 0.14401723 0.10726345 0.10142757 0.06290249 0.06040939 0.11169221
## GES 0.10368791 0.11491625 0.15903332 0.19495552 0.14633784 0.14700686
##            GRA        TUH        ALD        FRS       SER        SPB       SPS
## OKI 0.08250387 0.08815829 0.10969689 0.08945351 0.1874987 0.09771466 0.1230115
## HAI 0.04524403 0.06885729 0.09377805 0.07365184 0.1744719 0.08873615 0.1057058
## SOC 0.09204692 0.09103124 0.11424222 0.08782914 0.1913205 0.09508764 0.1251840
## YUN 0.07055412 0.10894603 0.12899561 0.11345454 0.2032873 0.12888792 0.1421004
## KAT 0.08866006 0.12510165 0.14154458 0.12955439 0.2122911 0.14168292 0.1540249
## GES 0.09653626 0.09568680 0.11848594 0.09143679 0.1958411 0.09894882 0.1287373
##            INW        INJ        MAT
## OKI 0.21908356 0.17399947 0.12115236
## HAI 0.17481694 0.12336758 0.06308101
## SOC 0.23076837 0.18620299 0.13526651
## YUN 0.13773248 0.09466807 0.02325104
## KAT 0.09808361 0.06606488 0.04855740
## GES 0.23638319 0.19173036 0.14052271
```

Import sample locations

```
sampling_loc <- readRDS(here("manuscript1", "output", "fst", "citiesforfst.rds"))

# Arrange by region 
sampling_loc <- sampling_loc |>
  dplyr::arrange(
    continent, region, subregion
  ) 

# Check it
head(sampling_loc)
```

```
## # A tibble: 6 × 9
##   pop   city           state latitude longitude continent region subregion order
##   <chr> <chr>          <chr>    <dbl>     <dbl> <chr>     <chr>  <chr>     <dbl>
## 1 BRM   Bermuda        Berm…     32.3     -64.8 Americas  Carib… Caribbean   128
## 2 NEG   Negril         Jama…     18.3     -78.3 Americas  Carib… Caribbean   129
## 3 SAI   Saint Augusti… Trin…     10.2     -61.5 Americas  Carib… Caribbean   131
## 4 COL   Columbus, OH   OH, …     40.0     -82.9 Americas  North… Midwest,…   115
## 5 PEO   Peoria, IL     IL, …     40.7     -89.6 Americas  North… Midwest,…   116
## 6 SPR   Springfield, … MO, …     37.2     -93.3 Americas  North… Midwest,…   117
```

```
#sampling_loc <- readRDS(here("scripts", "RMarkdowns", "output", "sampling_loc_all_pops.rds"))
#head(sampling_loc)
```

Lets check if the matrix is symmetric

```
isSymmetric(aa)
```

```
## [1] TRUE
```

```
ordered_pops <- c("HAI", "YUN", "HUN", "OKI", "KAG", "UTS", "KAN", "TAI", "BEN", "KAT", "CAM", "INW", "INJ", "SUF", "SUU", "KLP", "MAT", "CHA", "KAC", "SSK", "LAM", "HOC", "QNC", "HAN", "FRS", "SPB", "SPS", "BRE", "DES", "CES", "ALD", "SER", "GRA", "TUH", "SOC", "TIK", "RAR", "GES", "NEB", "NEH", "FIR", "LIN", "NEW", "BER", "HAR", "MAN", "WAV", "FAY", "BEA", "MAC", "PAL", "RUS", "HAL", "HAT", "NEO", "COL", "PEO", "SPR", "STL", "NUE", "HOU", "TRA", "MON", "DAL", "SMI", "DEN", "CHI", "LOS", "BRM", "NEG", "SAI", "CUA", "MED", "BPV", "MAU", "BIT", "TUC", "FLO", "REC", "ARA", "PLM", "BCO", "BAF", "IPO", "NOV", "BEH", "BPA", "GRV", "POR")
```

```
aa <- aa[ordered_pops, ordered_pops]
```

```
# Load pheatmap
library(pheatmap)

# Plot the heatmap
pheatmap(aa,
         clustering_distance_rows = "euclidean",
         clustering_distance_cols = "euclidean",
         color = colorRampPalette(c("white", "red"))(100),
         display_numbers = TRUE,
         fontsize = 4,
         main = "Fst Heatmap")
```

```
pdf(file = "manuscript1/output/fst/hudson_fst_heatmap_man1.pdf",   # The directory you want to save the file in
    width = 7, # The width of the plot in inches
    height = 7)

pheatmap(aa,
         clustering_distance_rows = "euclidean",
         clustering_distance_cols = "euclidean",
         color = colorRampPalette(c("white", "red"))(100),
         display_numbers = TRUE,
         fontsize = 4,
         main = "Fst Heatmap")

dev.off()
```

### 5.4.1 Plot the matrix with ggplot

Now we have to convert the matrix to a data frame to plot it with
ggplot.

```
aa[lower.tri(aa)] <- NA
print(aa)
```

```
##     HAI        YUN        HUN        OKI        KAG        UTS        KAN
## HAI  NA 0.05742478 0.04021476 0.08268169 0.09573472 0.10782363 0.13139629
## YUN  NA         NA 0.08117588 0.11579214 0.13231894 0.14937436 0.16781304
## HUN  NA         NA         NA 0.06729815 0.07093168 0.08318253 0.10673106
## OKI  NA         NA         NA         NA 0.10523386 0.11804646 0.13956016
## KAG  NA         NA         NA         NA         NA 0.07806679 0.10617700
## UTS  NA         NA         NA         NA         NA         NA 0.08862305
## KAN  NA         NA         NA         NA         NA         NA         NA
## TAI  NA         NA         NA         NA         NA         NA         NA
## BEN  NA         NA         NA         NA         NA         NA         NA
## KAT  NA         NA         NA         NA         NA         NA         NA
## CAM  NA         NA         NA         NA         NA         NA         NA
## INW  NA         NA         NA         NA         NA         NA         NA
## INJ  NA         NA         NA         NA         NA         NA         NA
## SUF  NA         NA         NA         NA         NA         NA         NA
## SUU  NA         NA         NA         NA         NA         NA         NA
## KLP  NA         NA         NA         NA         NA         NA         NA
## MAT  NA         NA         NA         NA         NA         NA         NA
## CHA  NA         NA         NA         NA         NA         NA         NA
## KAC  NA         NA         NA         NA         NA         NA         NA
## SSK  NA         NA         NA         NA         NA         NA         NA
## LAM  NA         NA         NA         NA         NA         NA         NA
## HOC  NA         NA         NA         NA         NA         NA         NA
## QNC  NA         NA         NA         NA         NA         NA         NA
## HAN  NA         NA         NA         NA         NA         NA         NA
## FRS  NA         NA         NA         NA         NA         NA         NA
## SPB  NA         NA         NA         NA         NA         NA         NA
## SPS  NA         NA         NA         NA         NA         NA         NA
## BRE  NA         NA         NA         NA         NA         NA         NA
## DES  NA         NA         NA         NA         NA         NA         NA
## CES  NA         NA         NA         NA         NA         NA         NA
## ALD  NA         NA         NA         NA         NA         NA         NA
## SER  NA         NA         NA         NA         NA         NA         NA
## GRA  NA         NA         NA         NA         NA         NA         NA
## TUH  NA         NA         NA         NA         NA         NA         NA
## SOC  NA         NA         NA         NA         NA         NA         NA
## TIK  NA         NA         NA         NA         NA         NA         NA
## RAR  NA         NA         NA         NA         NA         NA         NA
## GES  NA         NA         NA         NA         NA         NA         NA
## NEB  NA         NA         NA         NA         NA         NA         NA
## NEH  NA         NA         NA         NA         NA         NA         NA
## FIR  NA         NA         NA         NA         NA         NA         NA
## LIN  NA         NA         NA         NA         NA         NA         NA
## NEW  NA         NA         NA         NA         NA         NA         NA
## BER  NA         NA         NA         NA         NA         NA         NA
## HAR  NA         NA         NA         NA         NA         NA         NA
## MAN  NA         NA         NA         NA         NA         NA         NA
## WAV  NA         NA         NA         NA         NA         NA         NA
## FAY  NA         NA         NA         NA         NA         NA         NA
## BEA  NA         NA         NA         NA         NA         NA         NA
## MAC  NA         NA         NA         NA         NA         NA         NA
## PAL  NA         NA         NA         NA         NA         NA         NA
## RUS  NA         NA         NA         NA         NA         NA         NA
## HAL  NA         NA         NA         NA         NA         NA         NA
## HAT  NA         NA         NA         NA         NA         NA         NA
## NEO  NA         NA         NA         NA         NA         NA         NA
## COL  NA         NA         NA         NA         NA         NA         NA
## PEO  NA         NA         NA         NA         NA         NA         NA
## SPR  NA         NA         NA         NA         NA         NA         NA
## STL  NA         NA         NA         NA         NA         NA         NA
## NUE  NA         NA         NA         NA         NA         NA         NA
## HOU  NA         NA         NA         NA         NA         NA         NA
## TRA  NA         NA         NA         NA         NA         NA         NA
## MON  NA         NA         NA         NA         NA         NA         NA
## DAL  NA         NA         NA         NA         NA         NA         NA
## SMI  NA         NA         NA         NA         NA         NA         NA
## DEN  NA         NA         NA         NA         NA         NA         NA
## CHI  NA         NA         NA         NA         NA         NA         NA
## LOS  NA         NA         NA         NA         NA         NA         NA
## BRM  NA         NA         NA         NA         NA         NA         NA
## NEG  NA         NA         NA         NA         NA         NA         NA
## SAI  NA         NA         NA         NA         NA         NA         NA
## CUA  NA         NA         NA         NA         NA         NA         NA
## MED  NA         NA         NA         NA         NA         NA         NA
## BPV  NA         NA         NA         NA         NA         NA         NA
## MAU  NA         NA         NA         NA         NA         NA         NA
## BIT  NA         NA         NA         NA         NA         NA         NA
## TUC  NA         NA         NA         NA         NA         NA         NA
## FLO  NA         NA         NA         NA         NA         NA         NA
## REC  NA         NA         NA         NA         NA         NA         NA
## ARA  NA         NA         NA         NA         NA         NA         NA
## PLM  NA         NA         NA         NA         NA         NA         NA
## BCO  NA         NA         NA         NA         NA         NA         NA
## BAF  NA         NA         NA         NA         NA         NA         NA
## IPO  NA         NA         NA         NA         NA         NA         NA
## NOV  NA         NA         NA         NA         NA         NA         NA
## BEH  NA         NA         NA         NA         NA         NA         NA
## BPA  NA         NA         NA         NA         NA         NA         NA
## GRV  NA         NA         NA         NA         NA         NA         NA
## POR  NA         NA         NA         NA         NA         NA         NA
##           TAI        BEN        KAT        CAM        INW        INJ        SUF
## HAI 0.1251949 0.07083019 0.07846689 0.04667773 0.17481694 0.12336758 0.12735379
## YUN 0.1615414 0.02412583 0.03572613 0.01244233 0.13773248 0.09466807 0.10505893
## HUN 0.1178945 0.09523127 0.09948750 0.06989559 0.19442642 0.14377505 0.14704760
## OKI 0.1335548 0.12871724 0.12979869 0.10553710 0.21908356 0.17399947 0.17490633
## KAG 0.1545910 0.14659286 0.14686073 0.12251727 0.23243852 0.18767116 0.18971067
## UTS 0.1630663 0.16213131 0.16303600 0.13889722 0.24670233 0.20268054 0.20135817
## KAN 0.1796272 0.18012886 0.18029545 0.15898385 0.26120806 0.21819803 0.21646518
## TAI        NA 0.17415512 0.17219184 0.15338029 0.25443040 0.20826277 0.20983924
## BEN        NA         NA 0.03736564 0.02601315 0.14307995 0.10187678 0.11186354
## KAT        NA         NA         NA 0.03825775 0.09808361 0.06606488 0.06290249
## CAM        NA         NA         NA         NA 0.14091902 0.09439381 0.10505843
## INW        NA         NA         NA         NA         NA 0.06866015 0.07804447
## INJ        NA         NA         NA         NA         NA         NA 0.06381918
## SUF        NA         NA         NA         NA         NA         NA         NA
## SUU        NA         NA         NA         NA         NA         NA         NA
## KLP        NA         NA         NA         NA         NA         NA         NA
## MAT        NA         NA         NA         NA         NA         NA         NA
## CHA        NA         NA         NA         NA         NA         NA         NA
## KAC        NA         NA         NA         NA         NA         NA         NA
## SSK        NA         NA         NA         NA         NA         NA         NA
## LAM        NA         NA         NA         NA         NA         NA         NA
## HOC        NA         NA         NA         NA         NA         NA         NA
## QNC        NA         NA         NA         NA         NA         NA         NA
## HAN        NA         NA         NA         NA         NA         NA         NA
## FRS        NA         NA         NA         NA         NA         NA         NA
## SPB        NA         NA         NA         NA         NA         NA         NA
## SPS        NA         NA         NA         NA         NA         NA         NA
## BRE        NA         NA         NA         NA         NA         NA         NA
## DES        NA         NA         NA         NA         NA         NA         NA
## CES        NA         NA         NA         NA         NA         NA         NA
## ALD        NA         NA         NA         NA         NA         NA         NA
## SER        NA         NA         NA         NA         NA         NA         NA
## GRA        NA         NA         NA         NA         NA         NA         NA
## TUH        NA         NA         NA         NA         NA         NA         NA
## SOC        NA         NA         NA         NA         NA         NA         NA
## TIK        NA         NA         NA         NA         NA         NA         NA
## RAR        NA         NA         NA         NA         NA         NA         NA
## GES        NA         NA         NA         NA         NA         NA         NA
## NEB        NA         NA         NA         NA         NA         NA         NA
## NEH        NA         NA         NA         NA         NA         NA         NA
## FIR        NA         NA         NA         NA         NA         NA         NA
## LIN        NA         NA         NA         NA         NA         NA         NA
## NEW        NA         NA         NA         NA         NA         NA         NA
## BER        NA         NA         NA         NA         NA         NA         NA
## HAR        NA         NA         NA         NA         NA         NA         NA
## MAN        NA         NA         NA         NA         NA         NA         NA
## WAV        NA         NA         NA         NA         NA         NA         NA
## FAY        NA         NA         NA         NA         NA         NA         NA
## BEA        NA         NA         NA         NA         NA         NA         NA
## MAC        NA         NA         NA         NA         NA         NA         NA
## PAL        NA         NA         NA         NA         NA         NA         NA
## RUS        NA         NA         NA         NA         NA         NA         NA
## HAL        NA         NA         NA         NA         NA         NA         NA
## HAT        NA         NA         NA         NA         NA         NA         NA
## NEO        NA         NA         NA         NA         NA         NA         NA
## COL        NA         NA         NA         NA         NA         NA         NA
## PEO        NA         NA         NA         NA         NA         NA         NA
## SPR        NA         NA         NA         NA         NA         NA         NA
## STL        NA         NA         NA         NA         NA         NA         NA
## NUE        NA         NA         NA         NA         NA         NA         NA
## HOU        NA         NA         NA         NA         NA         NA         NA
## TRA        NA         NA         NA         NA         NA         NA         NA
## MON        NA         NA         NA         NA         NA         NA         NA
## DAL        NA         NA         NA         NA         NA         NA         NA
## SMI        NA         NA         NA         NA         NA         NA         NA
## DEN        NA         NA         NA         NA         NA         NA         NA
## CHI        NA         NA         NA         NA         NA         NA         NA
## LOS        NA         NA         NA         NA         NA         NA         NA
## BRM        NA         NA         NA         NA         NA         NA         NA
## NEG        NA         NA         NA         NA         NA         NA         NA
## SAI        NA         NA         NA         NA         NA         NA         NA
## CUA        NA         NA         NA         NA         NA         NA         NA
## MED        NA         NA         NA         NA         NA         NA         NA
## BPV        NA         NA         NA         NA         NA         NA         NA
## MAU        NA         NA         NA         NA         NA         NA         NA
## BIT        NA         NA         NA         NA         NA         NA         NA
## TUC        NA         NA         NA         NA         NA         NA         NA
## FLO        NA         NA         NA         NA         NA         NA         NA
## REC        NA         NA         NA         NA         NA         NA         NA
## ARA        NA         NA         NA         NA         NA         NA         NA
## PLM        NA         NA         NA         NA         NA         NA         NA
## BCO        NA         NA         NA         NA         NA         NA         NA
## BAF        NA         NA         NA         NA         NA         NA         NA
## IPO        NA         NA         NA         NA         NA         NA         NA
## NOV        NA         NA         NA         NA         NA         NA         NA
## BEH        NA         NA         NA         NA         NA         NA         NA
## BPA        NA         NA         NA         NA         NA         NA         NA
## GRV        NA         NA         NA         NA         NA         NA         NA
## POR        NA         NA         NA         NA         NA         NA         NA
##            SUU        KLP        MAT         CHA        KAC        SSK
## HAI 0.08150795 0.08734436 0.06308101 0.056507618 0.05858943 0.05865738
## YUN 0.08492226 0.05941637 0.02325104 0.009563734 0.01207249 0.01177784
## HUN 0.09541383 0.10889629 0.08660325 0.080813861 0.08402483 0.08249639
## OKI 0.12810973 0.13943830 0.12115236 0.115742453 0.11580491 0.11730198
## KAG 0.14013580 0.15420821 0.13832483 0.132566780 0.13227653 0.13403757
## UTS 0.15664957 0.17011415 0.15505262 0.149498767 0.14907733 0.15049919
## KAN 0.17180514 0.18688856 0.17438099 0.169204190 0.16722918 0.17008670
## TAI 0.16499970 0.17746747 0.16564136 0.162565789 0.16042195 0.16458160
## BEN 0.09509288 0.07271140 0.03734625 0.025531073 0.02597544 0.02692757
## KAT 0.06040939 0.07434104 0.04855740 0.038076402 0.03573132 0.03965856
## CAM 0.07918113 0.05823680 0.02413289 0.010404092 0.01395882 0.01249426
## INW 0.11335855 0.16191344 0.14701732 0.140934934 0.13404122 0.14095875
## INJ 0.07399082 0.11984212 0.10126550 0.095899523 0.09272808 0.09798399
## SUF 0.04162011 0.12870223 0.11272363 0.108359593 0.10366349 0.10815863
## SUU         NA 0.10398942 0.08996548 0.085840388 0.08348579 0.08703515
## KLP         NA         NA 0.06382624 0.060523716 0.05788520 0.06236824
## MAT         NA         NA         NA 0.022934806 0.02483420 0.02458402
## CHA         NA         NA         NA          NA 0.01210413 0.01055937
## KAC         NA         NA         NA          NA         NA 0.01336643
## SSK         NA         NA         NA          NA         NA         NA
## LAM         NA         NA         NA          NA         NA         NA
## HOC         NA         NA         NA          NA         NA         NA
## QNC         NA         NA         NA          NA         NA         NA
## HAN         NA         NA         NA          NA         NA         NA
## FRS         NA         NA         NA          NA         NA         NA
## SPB         NA         NA         NA          NA         NA         NA
## SPS         NA         NA         NA          NA         NA         NA
## BRE         NA         NA         NA          NA         NA         NA
## DES         NA         NA         NA          NA         NA         NA
## CES         NA         NA         NA          NA         NA         NA
## ALD         NA         NA         NA          NA         NA         NA
## SER         NA         NA         NA          NA         NA         NA
## GRA         NA         NA         NA          NA         NA         NA
## TUH         NA         NA         NA          NA         NA         NA
## SOC         NA         NA         NA          NA         NA         NA
## TIK         NA         NA         NA          NA         NA         NA
## RAR         NA         NA         NA          NA         NA         NA
## GES         NA         NA         NA          NA         NA         NA
## NEB         NA         NA         NA          NA         NA         NA
## NEH         NA         NA         NA          NA         NA         NA
## FIR         NA         NA         NA          NA         NA         NA
## LIN         NA         NA         NA          NA         NA         NA
## NEW         NA         NA         NA          NA         NA         NA
## BER         NA         NA         NA          NA         NA         NA
## HAR         NA         NA         NA          NA         NA         NA
## MAN         NA         NA         NA          NA         NA         NA
## WAV         NA         NA         NA          NA         NA         NA
## FAY         NA         NA         NA          NA         NA         NA
## BEA         NA         NA         NA          NA         NA         NA
## MAC         NA         NA         NA          NA         NA         NA
## PAL         NA         NA         NA          NA         NA         NA
## RUS         NA         NA         NA          NA         NA         NA
## HAL         NA         NA         NA          NA         NA         NA
## HAT         NA         NA         NA          NA         NA         NA
## NEO         NA         NA         NA          NA         NA         NA
## COL         NA         NA         NA          NA         NA         NA
## PEO         NA         NA         NA          NA         NA         NA
## SPR         NA         NA         NA          NA         NA         NA
## STL         NA         NA         NA          NA         NA         NA
## NUE         NA         NA         NA          NA         NA         NA
## HOU         NA         NA         NA          NA         NA         NA
## TRA         NA         NA         NA          NA         NA         NA
## MON         NA         NA         NA          NA         NA         NA
## DAL         NA         NA         NA          NA         NA         NA
## SMI         NA         NA         NA          NA         NA         NA
## DEN         NA         NA         NA          NA         NA         NA
## CHI         NA         NA         NA          NA         NA         NA
## LOS         NA         NA         NA          NA         NA         NA
## BRM         NA         NA         NA          NA         NA         NA
## NEG         NA         NA         NA          NA         NA         NA
## SAI         NA         NA         NA          NA         NA         NA
## CUA         NA         NA         NA          NA         NA         NA
## MED         NA         NA         NA          NA         NA         NA
## BPV         NA         NA         NA          NA         NA         NA
## MAU         NA         NA         NA          NA         NA         NA
## BIT         NA         NA         NA          NA         NA         NA
## TUC         NA         NA         NA          NA         NA         NA
## FLO         NA         NA         NA          NA         NA         NA
## REC         NA         NA         NA          NA         NA         NA
## ARA         NA         NA         NA          NA         NA         NA
## PLM         NA         NA         NA          NA         NA         NA
## BCO         NA         NA         NA          NA         NA         NA
## BAF         NA         NA         NA          NA         NA         NA
## IPO         NA         NA         NA          NA         NA         NA
## NOV         NA         NA         NA          NA         NA         NA
## BEH         NA         NA         NA          NA         NA         NA
## BPA         NA         NA         NA          NA         NA         NA
## GRV         NA         NA         NA          NA         NA         NA
## POR         NA         NA         NA          NA         NA         NA
##            LAM        HOC        QNC        HAN        FRS        SPB
## HAI 0.06013534 0.03765262 0.11294299 0.04630969 0.07365184 0.08873615
## YUN 0.01364806 0.06524127 0.07126989 0.05494547 0.11345454 0.12888792
## HUN 0.08496449 0.04811887 0.13381467 0.05644654 0.04665981 0.06103595
## OKI 0.11837068 0.08570565 0.16271362 0.09141610 0.08945351 0.09771466
## KAG 0.13548837 0.09710343 0.17888844 0.10539377 0.07017478 0.06616876
## UTS 0.15354098 0.11023834 0.19603776 0.11893551 0.07425032 0.05504391
## KAN 0.17093180 0.13223974 0.21402001 0.13823164 0.09714231 0.07983521
## TAI 0.16435842 0.12587243 0.20584590 0.12756595 0.13699907 0.14441298
## BEN 0.02733954 0.07888185 0.08546797 0.06880643 0.12741750 0.14074399
## KAT 0.03825790 0.08440142 0.09282384 0.07176172 0.12955439 0.14168292
## CAM 0.01567233 0.05270395 0.07466036 0.04880240 0.10359753 0.11901499
## INW 0.14030753 0.17660582 0.18714906 0.16184539 0.21892551 0.22775749
## INJ 0.09615468 0.12785408 0.14442581 0.11745908 0.17242622 0.18239700
## SUF 0.10599068 0.13134362 0.15185107 0.12067588 0.17425423 0.18404272
## SUU 0.08575123 0.08385582 0.13245873 0.08203826 0.12486197 0.13441653
## KLP 0.05625029 0.09021072 0.10920905 0.07699495 0.13921833 0.15072183
## MAT 0.02251111 0.07158366 0.08522427 0.06227448 0.11926210 0.13486046
## CHA 0.01296504 0.06473432 0.07323084 0.05616349 0.11438376 0.12931967
## KAC 0.01431693 0.06602086 0.07075124 0.05374203 0.11418361 0.12877673
## SSK 0.01404728 0.06647350 0.07378103 0.05633151 0.11617244 0.13013722
## LAM         NA 0.06874471 0.07389917 0.05725079 0.11739369 0.13192849
## HOC         NA         NA 0.11738598 0.04885991 0.07829190 0.09245825
## QNC         NA         NA         NA 0.10502642 0.16354974 0.17536405
## HAN         NA         NA         NA         NA 0.08800520 0.10016927
## FRS         NA         NA         NA         NA         NA 0.05311811
## SPB         NA         NA         NA         NA         NA         NA
## SPS         NA         NA         NA         NA         NA         NA
## BRE         NA         NA         NA         NA         NA         NA
## DES         NA         NA         NA         NA         NA         NA
## CES         NA         NA         NA         NA         NA         NA
## ALD         NA         NA         NA         NA         NA         NA
## SER         NA         NA         NA         NA         NA         NA
## GRA         NA         NA         NA         NA         NA         NA
## TUH         NA         NA         NA         NA         NA         NA
## SOC         NA         NA         NA         NA         NA         NA
## TIK         NA         NA         NA         NA         NA         NA
## RAR         NA         NA         NA         NA         NA         NA
## GES         NA         NA         NA         NA         NA         NA
## NEB         NA         NA         NA         NA         NA         NA
## NEH         NA         NA         NA         NA         NA         NA
## FIR         NA         NA         NA         NA         NA         NA
## LIN         NA         NA         NA         NA         NA         NA
## NEW         NA         NA         NA         NA         NA         NA
## BER         NA         NA         NA         NA         NA         NA
## HAR         NA         NA         NA         NA         NA         NA
## MAN         NA         NA         NA         NA         NA         NA
## WAV         NA         NA         NA         NA         NA         NA
## FAY         NA         NA         NA         NA         NA         NA
## BEA         NA         NA         NA         NA         NA         NA
## MAC         NA         NA         NA         NA         NA         NA
## PAL         NA         NA         NA         NA         NA         NA
## RUS         NA         NA         NA         NA         NA         NA
## HAL         NA         NA         NA         NA         NA         NA
## HAT         NA         NA         NA         NA         NA         NA
## NEO         NA         NA         NA         NA         NA         NA
## COL         NA         NA         NA         NA         NA         NA
## PEO         NA         NA         NA         NA         NA         NA
## SPR         NA         NA         NA         NA         NA         NA
## STL         NA         NA         NA         NA         NA         NA
## NUE         NA         NA         NA         NA         NA         NA
## HOU         NA         NA         NA         NA         NA         NA
## TRA         NA         NA         NA         NA         NA         NA
## MON         NA         NA         NA         NA         NA         NA
## DAL         NA         NA         NA         NA         NA         NA
## SMI         NA         NA         NA         NA         NA         NA
## DEN         NA         NA         NA         NA         NA         NA
## CHI         NA         NA         NA         NA         NA         NA
## LOS         NA         NA         NA         NA         NA         NA
## BRM         NA         NA         NA         NA         NA         NA
## NEG         NA         NA         NA         NA         NA         NA
## SAI         NA         NA         NA         NA         NA         NA
## CUA         NA         NA         NA         NA         NA         NA
## MED         NA         NA         NA         NA         NA         NA
## BPV         NA         NA         NA         NA         NA         NA
## MAU         NA         NA         NA         NA         NA         NA
## BIT         NA         NA         NA         NA         NA         NA
## TUC         NA         NA         NA         NA         NA         NA
## FLO         NA         NA         NA         NA         NA         NA
## REC         NA         NA         NA         NA         NA         NA
## ARA         NA         NA         NA         NA         NA         NA
## PLM         NA         NA         NA         NA         NA         NA
## BCO         NA         NA         NA         NA         NA         NA
## BAF         NA         NA         NA         NA         NA         NA
## IPO         NA         NA         NA         NA         NA         NA
## NOV         NA         NA         NA         NA         NA         NA
## BEH         NA         NA         NA         NA         NA         NA
## BPA         NA         NA         NA         NA         NA         NA
## GRV         NA         NA         NA         NA         NA         NA
## POR         NA         NA         NA         NA         NA         NA
##            SPS        BRE        DES       CES        ALD       SER        GRA
## HAI 0.10570585 0.13078305 0.12188263 0.1509614 0.09377805 0.1744719 0.04524403
## YUN 0.14210044 0.16751321 0.16097858 0.1863663 0.12899561 0.2032873 0.07055412
## HUN 0.08439923 0.10773796 0.09795106 0.1273859 0.07002889 0.1570628 0.03964000
## OKI 0.12301151 0.13879922 0.12936966 0.1586276 0.10969689 0.1874987 0.08250387
## KAG 0.12011304 0.11175280 0.10176088 0.1288922 0.10595267 0.1775833 0.08793699
## UTS 0.11913089 0.09517856 0.07806213 0.1058680 0.10514670 0.1828231 0.10062395
## KAN 0.14471273 0.13074970 0.11742238 0.1421579 0.13280505 0.1982276 0.12320219
## TAI 0.16453821 0.18198789 0.17336236 0.1991115 0.15368859 0.2260168 0.12890126
## BEN 0.15307455 0.17880166 0.17108173 0.1986941 0.14228707 0.2147760 0.08606339
## KAT 0.15402491 0.17795423 0.17205460 0.1969942 0.14154458 0.2122911 0.08866006
## CAM 0.13220756 0.15831828 0.15035744 0.1781945 0.11934838 0.1962463 0.06044238
## INW 0.23836180 0.25893439 0.25650861 0.2765495 0.22651261 0.2917603 0.18060227
## INJ 0.19367617 0.21734231 0.21165942 0.2343703 0.18456819 0.2509775 0.13091795
## SUF 0.19331244 0.21745949 0.21292536 0.2339228 0.18391449 0.2474038 0.13532890
## SUU 0.14613460 0.17165363 0.16460142 0.1883579 0.13722493 0.2088382 0.08849481
## KLP 0.15968291 0.18437297 0.17971116 0.2026094 0.14872075 0.2165278 0.09414815
## MAT 0.14662648 0.17296682 0.16570129 0.1917333 0.13367497 0.2093496 0.07430198
## CHA 0.14191688 0.16778346 0.16093453 0.1877299 0.12870022 0.2065002 0.07089686
## KAC 0.14025540 0.16698032 0.16024554 0.1858350 0.12830497 0.2018100 0.07231981
## SSK 0.14432060 0.16857178 0.16178283 0.1877314 0.13025862 0.2064879 0.07185244
## LAM 0.14401040 0.16995074 0.16391071 0.1900563 0.13231919 0.2064382 0.07310944
## HOC 0.10759290 0.13153665 0.12243171 0.1501660 0.09390621 0.1743189 0.05089016
## QNC 0.18735285 0.21178907 0.20541915 0.2309495 0.17423047 0.2445931 0.12322596
## HAN 0.11461372 0.13902333 0.13285761 0.1577241 0.10110260 0.1756133 0.05816203
## FRS 0.08693678 0.10021005 0.09036630 0.1205001 0.07909065 0.1572011 0.06139607
## SPB 0.10431105 0.07820728 0.06876466 0.1004991 0.09364504 0.1670226 0.07903708
## SPS         NA 0.14255871 0.13684266 0.1637313 0.11151600 0.1923512 0.09197576
## BRE         NA         NA 0.07382486 0.1196141 0.13527402 0.1985094 0.12149866
## DES         NA         NA         NA 0.1098518 0.12559751 0.1927866 0.11412977
## CES         NA         NA         NA        NA 0.15022152 0.2172124 0.14209002
## ALD         NA         NA         NA        NA         NA 0.1794521 0.06963219
## SER         NA         NA         NA        NA         NA        NA 0.16989434
## GRA         NA         NA         NA        NA         NA        NA         NA
## TUH         NA         NA         NA        NA         NA        NA         NA
## SOC         NA         NA         NA        NA         NA        NA         NA
## TIK         NA         NA         NA        NA         NA        NA         NA
## RAR         NA         NA         NA        NA         NA        NA         NA
## GES         NA         NA         NA        NA         NA        NA         NA
## NEB         NA         NA         NA        NA         NA        NA         NA
## NEH         NA         NA         NA        NA         NA        NA         NA
## FIR         NA         NA         NA        NA         NA        NA         NA
## LIN         NA         NA         NA        NA         NA        NA         NA
## NEW         NA         NA         NA        NA         NA        NA         NA
## BER         NA         NA         NA        NA         NA        NA         NA
## HAR         NA         NA         NA        NA         NA        NA         NA
## MAN         NA         NA         NA        NA         NA        NA         NA
## WAV         NA         NA         NA        NA         NA        NA         NA
## FAY         NA         NA         NA        NA         NA        NA         NA
## BEA         NA         NA         NA        NA         NA        NA         NA
## MAC         NA         NA         NA        NA         NA        NA         NA
## PAL         NA         NA         NA        NA         NA        NA         NA
## RUS         NA         NA         NA        NA         NA        NA         NA
## HAL         NA         NA         NA        NA         NA        NA         NA
## HAT         NA         NA         NA        NA         NA        NA         NA
## NEO         NA         NA         NA        NA         NA        NA         NA
## COL         NA         NA         NA        NA         NA        NA         NA
## PEO         NA         NA         NA        NA         NA        NA         NA
## SPR         NA         NA         NA        NA         NA        NA         NA
## STL         NA         NA         NA        NA         NA        NA         NA
## NUE         NA         NA         NA        NA         NA        NA         NA
## HOU         NA         NA         NA        NA         NA        NA         NA
## TRA         NA         NA         NA        NA         NA        NA         NA
## MON         NA         NA         NA        NA         NA        NA         NA
## DAL         NA         NA         NA        NA         NA        NA         NA
## SMI         NA         NA         NA        NA         NA        NA         NA
## DEN         NA         NA         NA        NA         NA        NA         NA
## CHI         NA         NA         NA        NA         NA        NA         NA
## LOS         NA         NA         NA        NA         NA        NA         NA
## BRM         NA         NA         NA        NA         NA        NA         NA
## NEG         NA         NA         NA        NA         NA        NA         NA
## SAI         NA         NA         NA        NA         NA        NA         NA
## CUA         NA         NA         NA        NA         NA        NA         NA
## MED         NA         NA         NA        NA         NA        NA         NA
## BPV         NA         NA         NA        NA         NA        NA         NA
## MAU         NA         NA         NA        NA         NA        NA         NA
## BIT         NA         NA         NA        NA         NA        NA         NA
## TUC         NA         NA         NA        NA         NA        NA         NA
## FLO         NA         NA         NA        NA         NA        NA         NA
## REC         NA         NA         NA        NA         NA        NA         NA
## ARA         NA         NA         NA        NA         NA        NA         NA
## PLM         NA         NA         NA        NA         NA        NA         NA
## BCO         NA         NA         NA        NA         NA        NA         NA
## BAF         NA         NA         NA        NA         NA        NA         NA
## IPO         NA         NA         NA        NA         NA        NA         NA
## NOV         NA         NA         NA        NA         NA        NA         NA
## BEH         NA         NA         NA        NA         NA        NA         NA
## BPA         NA         NA         NA        NA         NA        NA         NA
## GRV         NA         NA         NA        NA         NA        NA         NA
## POR         NA         NA         NA        NA         NA        NA         NA
##            TUH        SOC        TIK        RAR        GES        NEB
## HAI 0.06885729 0.09582339 0.10731189 0.09858151 0.10011811 0.09239762
## YUN 0.10894603 0.13003132 0.14054421 0.13049713 0.13530983 0.13351940
## HUN 0.04410968 0.07396946 0.08725614 0.07786214 0.07813875 0.06543956
## OKI 0.08815829 0.11228986 0.12371206 0.11705699 0.11753346 0.10158298
## KAG 0.07770322 0.10690651 0.11766191 0.11110274 0.11006928 0.07119142
## UTS 0.08701728 0.11686961 0.12665053 0.12378191 0.12055967 0.05842827
## KAN 0.10830125 0.13667482 0.14697306 0.14387114 0.13979947 0.09108070
## TAI 0.13746640 0.15735766 0.16877633 0.16133233 0.16005426 0.14721586
## BEN 0.12230766 0.14333738 0.15408224 0.14373049 0.14836310 0.14648648
## KAT 0.12510165 0.14417007 0.15460732 0.14480449 0.15035825 0.14804091
## CAM 0.09920109 0.12028843 0.13148953 0.12152004 0.12587912 0.12222258
## INW 0.21634195 0.23076837 0.23770993 0.23107349 0.23638319 0.23314903
## INJ 0.16987593 0.18620299 0.19569249 0.18720601 0.19173036 0.18886910
## SUF 0.17129736 0.18952076 0.19752437 0.18927440 0.19495552 0.18867998
## SUU 0.12054396 0.14153628 0.15038440 0.14280370 0.14633784 0.13965192
## KLP 0.13368409 0.15195443 0.16072180 0.15274348 0.15677517 0.15548079
## MAT 0.11540239 0.13526651 0.14554394 0.13496733 0.14052271 0.13962277
## CHA 0.11024562 0.13093179 0.14040293 0.13115577 0.13573266 0.13369281
## KAC 0.11000257 0.13106145 0.14011069 0.13069409 0.13586223 0.13442578
## SSK 0.11229429 0.13290421 0.14243545 0.13257775 0.13823935 0.13497314
## LAM 0.11403101 0.13409149 0.14390404 0.13441806 0.13967252 0.13693827
## HOC 0.07427576 0.09857539 0.10886273 0.10089811 0.10265901 0.09555441
## QNC 0.15966197 0.17715022 0.18590118 0.17795247 0.18359105 0.18094060
## HAN 0.08309196 0.10500900 0.11502667 0.10779247 0.10957872 0.10467034
## FRS 0.04437323 0.08782914 0.10024179 0.09184065 0.09143679 0.05903475
## SPB 0.06671649 0.09508764 0.10738375 0.10199853 0.09894882 0.04265216
## SPS 0.08483326 0.12518398 0.13647788 0.12897574 0.12873726 0.10761797
## BRE 0.10821525 0.13956913 0.15017893 0.14436758 0.14256479 0.07836344
## DES 0.10120921 0.13269011 0.14325435 0.13964045 0.13625890 0.06546196
## CES 0.12954131 0.16103329 0.16982863 0.16505346 0.16276258 0.10129510
## ALD 0.07764388 0.11424222 0.12473636 0.11813694 0.11848594 0.09970913
## SER 0.16423503 0.19132045 0.20131266 0.19365149 0.19584108 0.17106138
## GRA 0.06019865 0.09204692 0.10402617 0.09541953 0.09653626 0.08425491
## TUH         NA 0.09103124 0.10240116 0.09564632 0.09568680 0.07158106
## SOC         NA         NA 0.05207064 0.02063810 0.01414008 0.10485624
## TIK         NA         NA         NA 0.04589549 0.04925772 0.11326409
## RAR         NA         NA         NA         NA 0.02794134 0.10985634
## GES         NA         NA         NA         NA         NA 0.10744015
## NEB         NA         NA         NA         NA         NA         NA
## NEH         NA         NA         NA         NA         NA         NA
## FIR         NA         NA         NA         NA         NA         NA
## LIN         NA         NA         NA         NA         NA         NA
## NEW         NA         NA         NA         NA         NA         NA
## BER         NA         NA         NA         NA         NA         NA
## HAR         NA         NA         NA         NA         NA         NA
## MAN         NA         NA         NA         NA         NA         NA
## WAV         NA         NA         NA         NA         NA         NA
## FAY         NA         NA         NA         NA         NA         NA
## BEA         NA         NA         NA         NA         NA         NA
## MAC         NA         NA         NA         NA         NA         NA
## PAL         NA         NA         NA         NA         NA         NA
## RUS         NA         NA         NA         NA         NA         NA
## HAL         NA         NA         NA         NA         NA         NA
## HAT         NA         NA         NA         NA         NA         NA
## NEO         NA         NA         NA         NA         NA         NA
## COL         NA         NA         NA         NA         NA         NA
## PEO         NA         NA         NA         NA         NA         NA
## SPR         NA         NA         NA         NA         NA         NA
## STL         NA         NA         NA         NA         NA         NA
## NUE         NA         NA         NA         NA         NA         NA
## HOU         NA         NA         NA         NA         NA         NA
## TRA         NA         NA         NA         NA         NA         NA
## MON         NA         NA         NA         NA         NA         NA
## DAL         NA         NA         NA         NA         NA         NA
## SMI         NA         NA         NA         NA         NA         NA
## DEN         NA         NA         NA         NA         NA         NA
## CHI         NA         NA         NA         NA         NA         NA
## LOS         NA         NA         NA         NA         NA         NA
## BRM         NA         NA         NA         NA         NA         NA
## NEG         NA         NA         NA         NA         NA         NA
## SAI         NA         NA         NA         NA         NA         NA
## CUA         NA         NA         NA         NA         NA         NA
## MED         NA         NA         NA         NA         NA         NA
## BPV         NA         NA         NA         NA         NA         NA
## MAU         NA         NA         NA         NA         NA         NA
## BIT         NA         NA         NA         NA         NA         NA
## TUC         NA         NA         NA         NA         NA         NA
## FLO         NA         NA         NA         NA         NA         NA
## REC         NA         NA         NA         NA         NA         NA
## ARA         NA         NA         NA         NA         NA         NA
## PLM         NA         NA         NA         NA         NA         NA
## BCO         NA         NA         NA         NA         NA         NA
## BAF         NA         NA         NA         NA         NA         NA
## IPO         NA         NA         NA         NA         NA         NA
## NOV         NA         NA         NA         NA         NA         NA
## BEH         NA         NA         NA         NA         NA         NA
## BPA         NA         NA         NA         NA         NA         NA
## GRV         NA         NA         NA         NA         NA         NA
## POR         NA         NA         NA         NA         NA         NA
##            NEH        FIR        LIN        NEW        BER        HAR
## HAI 0.08666284 0.11463155 0.09695830 0.09726300 0.09095991 0.09417892
## YUN 0.12396725 0.15097626 0.13859806 0.13581886 0.13083385 0.13143940
## HUN 0.05951251 0.09026610 0.07212713 0.07279694 0.06581941 0.06974761
## OKI 0.09357675 0.11897404 0.10616755 0.10130199 0.10026824 0.10200936
## KAG 0.06466883 0.09598736 0.07450472 0.08090600 0.07167084 0.07494259
## UTS 0.05602754 0.08774330 0.05900718 0.07068281 0.06063645 0.06366022
## KAN 0.09033379 0.11916027 0.08642891 0.10668041 0.09419677 0.09771312
## TAI 0.14285415 0.16952675 0.15384123 0.15666332 0.15060676 0.15288851
## BEN 0.13703696 0.16331326 0.15100151 0.14885700 0.14363054 0.14498985
## KAT 0.13715729 0.16403930 0.15134535 0.14988080 0.14473745 0.14597234
## CAM 0.11445743 0.14108577 0.12763902 0.12458446 0.12024210 0.12210128
## INW 0.22316908 0.24679589 0.23825855 0.23805832 0.23316107 0.23253382
## INJ 0.17857897 0.20469377 0.19170782 0.19029413 0.18552692 0.18720810
## SUF 0.18157417 0.20475717 0.19319579 0.19241933 0.18742885 0.18899372
## SUU 0.13279097 0.15791985 0.14409012 0.14346599 0.13833218 0.14081573
## KLP 0.14542713 0.17211725 0.15987918 0.15922756 0.15187095 0.15441763
## MAT 0.13062277 0.15758541 0.14468253 0.14100356 0.13581916 0.13872987
## CHA 0.12450749 0.15133674 0.13853562 0.13469331 0.13035962 0.13290483
## KAC 0.12525946 0.15058698 0.14013234 0.13709836 0.13216893 0.13242169
## SSK 0.12667966 0.15297299 0.13934585 0.13662153 0.13241201 0.13347826
## LAM 0.12795423 0.15390217 0.14121517 0.13837697 0.13389457 0.13589184
## HOC 0.08992395 0.11609842 0.09890926 0.10105048 0.09513500 0.09915859
## QNC 0.17182012 0.19735380 0.18538619 0.18331093 0.17850839 0.18067276
## HAN 0.09785534 0.12351536 0.10841739 0.10886079 0.10452605 0.10552105
## FRS 0.05413188 0.08767908 0.06631310 0.06937885 0.06053822 0.06347410
## SPB 0.03662397 0.07021486 0.05182213 0.05320781 0.04265011 0.04596192
## SPS 0.10541032 0.13394081 0.11644222 0.11906815 0.11054492 0.11371524
## BRE 0.06873487 0.10430885 0.09685470 0.08302041 0.07276202 0.08320298
## DES 0.05991533 0.08976551 0.08125442 0.07774881 0.06686052 0.06996969
## CES 0.09636846 0.13217441 0.11379215 0.11430991 0.10367701 0.10958420
## ALD 0.09205093 0.12433724 0.09520640 0.10650763 0.10004987 0.10376688
## SER 0.16285758 0.19401760 0.17688551 0.18250551 0.17435741 0.17913505
## GRA 0.07707271 0.10810891 0.09002041 0.08725094 0.08317784 0.08540687
## TUH 0.06796413 0.09789990 0.07666624 0.08001469 0.07210254 0.07444743
## SOC 0.09667045 0.12525379 0.10983168 0.10939765 0.10255356 0.10616213
## TIK 0.10509221 0.13318232 0.11917821 0.11910757 0.11376116 0.11300424
## RAR 0.10226089 0.12945775 0.11659555 0.11492873 0.10798521 0.11317255
## GES 0.09964845 0.12890017 0.11359766 0.11334636 0.10668277 0.11051990
## NEB 0.02640995 0.06388246 0.05566837 0.04864080 0.04288159 0.04619249
## NEH         NA 0.05507071 0.05383819 0.04015951 0.03194213 0.03417114
## FIR         NA         NA 0.08021402 0.07093811 0.06362977 0.07182716
## LIN         NA         NA         NA 0.06433358 0.05681389 0.06071131
## NEW         NA         NA         NA         NA 0.04625287 0.04957375
## BER         NA         NA         NA         NA         NA 0.04338151
## HAR         NA         NA         NA         NA         NA         NA
## MAN         NA         NA         NA         NA         NA         NA
## WAV         NA         NA         NA         NA         NA         NA
## FAY         NA         NA         NA         NA         NA         NA
## BEA         NA         NA         NA         NA         NA         NA
## MAC         NA         NA         NA         NA         NA         NA
## PAL         NA         NA         NA         NA         NA         NA
## RUS         NA         NA         NA         NA         NA         NA
## HAL         NA         NA         NA         NA         NA         NA
## HAT         NA         NA         NA         NA         NA         NA
## NEO         NA         NA         NA         NA         NA         NA
## COL         NA         NA         NA         NA         NA         NA
## PEO         NA         NA         NA         NA         NA         NA
## SPR         NA         NA         NA         NA         NA         NA
## STL         NA         NA         NA         NA         NA         NA
## NUE         NA         NA         NA         NA         NA         NA
## HOU         NA         NA         NA         NA         NA         NA
## TRA         NA         NA         NA         NA         NA         NA
## MON         NA         NA         NA         NA         NA         NA
## DAL         NA         NA         NA         NA         NA         NA
## SMI         NA         NA         NA         NA         NA         NA
## DEN         NA         NA         NA         NA         NA         NA
## CHI         NA         NA         NA         NA         NA         NA
## LOS         NA         NA         NA         NA         NA         NA
## BRM         NA         NA         NA         NA         NA         NA
## NEG         NA         NA         NA         NA         NA         NA
## SAI         NA         NA         NA         NA         NA         NA
## CUA         NA         NA         NA         NA         NA         NA
## MED         NA         NA         NA         NA         NA         NA
## BPV         NA         NA         NA         NA         NA         NA
## MAU         NA         NA         NA         NA         NA         NA
## BIT         NA         NA         NA         NA         NA         NA
## TUC         NA         NA         NA         NA         NA         NA
## FLO         NA         NA         NA         NA         NA         NA
## REC         NA         NA         NA         NA         NA         NA
## ARA         NA         NA         NA         NA         NA         NA
## PLM         NA         NA         NA         NA         NA         NA
## BCO         NA         NA         NA         NA         NA         NA
## BAF         NA         NA         NA         NA         NA         NA
## IPO         NA         NA         NA         NA         NA         NA
## NOV         NA         NA         NA         NA         NA         NA
## BEH         NA         NA         NA         NA         NA         NA
## BPA         NA         NA         NA         NA         NA         NA
## GRV         NA         NA         NA         NA         NA         NA
## POR         NA         NA         NA         NA         NA         NA
##            MAN        WAV        FAY        BEA        MAC        PAL
## HAI 0.08309913 0.09959681 0.07725947 0.06438147 0.06919882 0.07609765
## YUN 0.12343238 0.13616628 0.11821274 0.10173982 0.10567617 0.10742494
## HUN 0.05720447 0.07305447 0.05619857 0.04765379 0.05397341 0.06374790
## OKI 0.09310660 0.10455193 0.08643722 0.08160337 0.09065625 0.09648642
## KAG 0.06467420 0.07561717 0.07150346 0.06849597 0.07725548 0.08735208
## UTS 0.05537406 0.06385022 0.06323795 0.05918048 0.07345954 0.08890446
## KAN 0.08911324 0.09719452 0.09632757 0.09465081 0.10586754 0.11789871
## TAI 0.14297470 0.15070077 0.14045714 0.13631322 0.13819216 0.14398238
## BEN 0.13712730 0.14853944 0.13111926 0.11519229 0.11944739 0.12055284
## KAT 0.13943481 0.14824250 0.13004971 0.11798178 0.12126712 0.12099893
## CAM 0.11345835 0.12611400 0.10657575 0.09140331 0.09583195 0.09684322
## INW 0.22749837 0.23133581 0.22208078 0.21017186 0.21151266 0.21064701
## INJ 0.18026511 0.18748587 0.17277337 0.16070007 0.16156148 0.16301190
## SUF 0.18207064 0.18961078 0.17514917 0.16373843 0.16545802 0.16516442
## SUU 0.13281550 0.14150078 0.12502964 0.11490198 0.11589315 0.11803690
## KLP 0.14771071 0.15696731 0.13981163 0.12647828 0.13041765 0.12828817
## MAT 0.12946072 0.14209981 0.12273674 0.10673743 0.10991589 0.11278219
## CHA 0.12315605 0.13625616 0.11808220 0.10248924 0.10571253 0.10722552
## KAC 0.12546214 0.13664481 0.11911946 0.10323869 0.10672730 0.10817429
## SSK 0.12535356 0.13798988 0.11918985 0.10317233 0.10736491 0.10859228
## LAM 0.12807559 0.13973385 0.12024470 0.10508028 0.10846394 0.11051229
## HOC 0.08834302 0.10113928 0.08187341 0.07072372 0.07505420 0.08061234
## QNC 0.17241426 0.18256481 0.16645835 0.15338917 0.15543804 0.15730767
## HAN 0.09670646 0.10750869 0.09115918 0.07900095 0.08352262 0.08649535
## FRS 0.05358141 0.06627232 0.05822976 0.05341777 0.06087027 0.07225599
## SPB 0.03514768 0.03664514 0.04654682 0.04707986 0.05500300 0.06776279
## SPS 0.10102526 0.11438322 0.10617261 0.09795353 0.10673797 0.11469591
## BRE 0.06436941 0.06057041 0.07236521 0.08137917 0.09194614 0.10602467
## DES 0.05887218 0.05505537 0.06382017 0.07026102 0.08465420 0.09402044
## CES 0.09993965 0.10743870 0.10572993 0.11165637 0.11839160 0.12878408
## ALD 0.09194723 0.10464978 0.09295881 0.08693804 0.09569947 0.10251364
## SER 0.16914025 0.17379649 0.16967895 0.16757935 0.17260661 0.18001921
## GRA 0.07518513 0.09095898 0.07009208 0.05942881 0.06289223 0.06994973
## TUH 0.06430708 0.07760606 0.06668878 0.06028389 0.06721825 0.07845963
## SOC 0.09523383 0.10590488 0.09745339 0.09152568 0.09895578 0.10598877
## TIK 0.10574355 0.11760167 0.10987328 0.10125055 0.10778510 0.11770262
## RAR 0.10112712 0.11202011 0.10371489 0.09542263 0.10302045 0.11094729
## GES 0.09917813 0.10856053 0.10182070 0.09508925 0.10256608 0.11001168
## NEB 0.02945122 0.04385404 0.04402515 0.04226716 0.05252889 0.06470366
## NEH 0.02501622 0.02823010 0.03712910 0.03749075 0.04668568 0.05759399
## FIR 0.05650652 0.06240635 0.06376820 0.06709660 0.07584757 0.09115834
## LIN 0.04934516 0.06001046 0.05139462 0.05557262 0.06525134 0.07511391
## NEW 0.03678683 0.04978198 0.03875053 0.04388396 0.05571930 0.06422460
## BER 0.03335633 0.03803349 0.03826302 0.03991813 0.04858782 0.05908381
## HAR 0.03600320 0.04757507 0.04132528 0.03708578 0.05364548 0.06742308
## MAN         NA 0.03085513 0.02804762 0.03028193 0.04096542 0.05543616
## WAV         NA         NA 0.03839204 0.04801755 0.05281333 0.06971300
## FAY         NA         NA         NA 0.02434644 0.03350468 0.04603328
## BEA         NA         NA         NA         NA 0.02443356 0.04113139
## MAC         NA         NA         NA         NA         NA 0.04003899
## PAL         NA         NA         NA         NA         NA         NA
## RUS         NA         NA         NA         NA         NA         NA
## HAL         NA         NA         NA         NA         NA         NA
## HAT         NA         NA         NA         NA         NA         NA
## NEO         NA         NA         NA         NA         NA         NA
## COL         NA         NA         NA         NA         NA         NA
## PEO         NA         NA         NA         NA         NA         NA
## SPR         NA         NA         NA         NA         NA         NA
## STL         NA         NA         NA         NA         NA         NA
## NUE         NA         NA         NA         NA         NA         NA
## HOU         NA         NA         NA         NA         NA         NA
## TRA         NA         NA         NA         NA         NA         NA
## MON         NA         NA         NA         NA         NA         NA
## DAL         NA         NA         NA         NA         NA         NA
## SMI         NA         NA         NA         NA         NA         NA
## DEN         NA         NA         NA         NA         NA         NA
## CHI         NA         NA         NA         NA         NA         NA
## LOS         NA         NA         NA         NA         NA         NA
## BRM         NA         NA         NA         NA         NA         NA
## NEG         NA         NA         NA         NA         NA         NA
## SAI         NA         NA         NA         NA         NA         NA
## CUA         NA         NA         NA         NA         NA         NA
## MED         NA         NA         NA         NA         NA         NA
## BPV         NA         NA         NA         NA         NA         NA
## MAU         NA         NA         NA         NA         NA         NA
## BIT         NA         NA         NA         NA         NA         NA
## TUC         NA         NA         NA         NA         NA         NA
## FLO         NA         NA         NA         NA         NA         NA
## REC         NA         NA         NA         NA         NA         NA
## ARA         NA         NA         NA         NA         NA         NA
## PLM         NA         NA         NA         NA         NA         NA
## BCO         NA         NA         NA         NA         NA         NA
## BAF         NA         NA         NA         NA         NA         NA
## IPO         NA         NA         NA         NA         NA         NA
## NOV         NA         NA         NA         NA         NA         NA
## BEH         NA         NA         NA         NA         NA         NA
## BPA         NA         NA         NA         NA         NA         NA
## GRV         NA         NA         NA         NA         NA         NA
## POR         NA         NA         NA         NA         NA         NA
##            RUS        HAL        HAT        NEO        COL        PEO
## HAI 0.10610164 0.08340299 0.08639033 0.08517488 0.08907718 0.08087216
## YUN 0.13900426 0.12023285 0.12096228 0.12403275 0.13001690 0.11821552
## HUN 0.08229713 0.05532370 0.06336101 0.06510590 0.06378611 0.05677481
## OKI 0.11074337 0.09024111 0.09079702 0.09337691 0.09955760 0.09062367
## KAG 0.09429225 0.05663478 0.07272357 0.07598636 0.06489695 0.06092156
## UTS 0.09067769 0.04796216 0.06357344 0.06769179 0.05730593 0.05423544
## KAN 0.11835068 0.07690168 0.09744049 0.10290100 0.08676174 0.08669719
## TAI 0.16393251 0.14165072 0.14659651 0.14615680 0.14751800 0.14099805
## BEN 0.15011484 0.13287690 0.13456465 0.13762363 0.14246848 0.13076636
## KAT 0.15086539 0.13438085 0.13639734 0.13889583 0.14401723 0.13240431
## CAM 0.12878695 0.10968759 0.11144178 0.11291997 0.11978645 0.10798931
## INW 0.23762134 0.22242767 0.22326396 0.22645825 0.22937787 0.22071606
## INJ 0.19382152 0.17638028 0.17911082 0.17975919 0.18328685 0.17399537
## SUF 0.19583841 0.17948020 0.18141643 0.18074077 0.18441013 0.17469797
## SUU 0.15003833 0.13016437 0.13288368 0.13193194 0.13753697 0.12651614
## KLP 0.16155946 0.14506289 0.14431305 0.14534748 0.15267106 0.14261285
## MAT 0.14459809 0.12638389 0.12737052 0.12827638 0.13518344 0.12368871
## CHA 0.13929027 0.11975768 0.12097641 0.12371743 0.12967825 0.11782625
## KAC 0.13934757 0.12148362 0.12209106 0.12410909 0.12955198 0.11934791
## SSK 0.14049923 0.12235761 0.12321523 0.12489082 0.13162256 0.11945027
## LAM 0.14130508 0.12357170 0.12451171 0.12693213 0.13317598 0.12140262
## HOC 0.10880997 0.08737426 0.09119586 0.08920764 0.09387747 0.08478639
## QNC 0.18669835 0.16961334 0.16834006 0.17168216 0.17682288 0.16675714
## HAN 0.11450732 0.09510945 0.09768367 0.09744602 0.10203861 0.09364967
## FRS 0.08180171 0.04848533 0.06323905 0.06629307 0.05636710 0.05399448
## SPB 0.07188963 0.02581868 0.04765818 0.05436725 0.04125278 0.03618502
## SPS 0.12943858 0.09991117 0.10799693 0.11192628 0.10872685 0.10155510
## BRE 0.11277320 0.07549998 0.08183498 0.08537907 0.07661663 0.07593924
## DES 0.10117947 0.06531410 0.07215569 0.07785567 0.07149687 0.06450099
## CES 0.13441470 0.09722456 0.11342764 0.11347073 0.10745595 0.10231526
## ALD 0.11605761 0.08901394 0.09847767 0.10024456 0.09674550 0.09159779
## SER 0.18985704 0.16623072 0.17162449 0.17527011 0.17024318 0.16863657
## GRA 0.09899755 0.07195582 0.07858812 0.07737574 0.08107306 0.07317081
## TUH 0.09247191 0.06243556 0.07222040 0.07384047 0.06867450 0.06453884
## SOC 0.12103051 0.09089918 0.09987098 0.10424628 0.09967069 0.09379041
## TIK 0.12954378 0.10359104 0.11097551 0.11541975 0.11113045 0.10447956
## RAR 0.12606589 0.09840507 0.10583165 0.10945500 0.10648202 0.10016665
## GES 0.12666316 0.09495313 0.10416131 0.10902333 0.10368791 0.09805706
## NEB 0.06877635 0.03478789 0.04663423 0.04598922 0.04179558 0.03534443
## NEH 0.06549430 0.02666523 0.04214312 0.04474909 0.03387125 0.02909341
## FIR 0.08820228 0.05694859 0.06893157 0.06924559 0.06826640 0.05933661
## LIN 0.08441081 0.04252076 0.06260633 0.05951326 0.05170612 0.04755649
## NEW 0.07555348 0.04160091 0.04253117 0.04865503 0.05145387 0.04204470
## BER 0.06991678 0.03093049 0.04376030 0.04928720 0.04154226 0.03501200
## HAR 0.07054943 0.03877451 0.04092845 0.05150013 0.04426765 0.04076630
## MAN 0.05718953 0.02539043 0.03549813 0.03999877 0.02853875 0.02808086
## WAV 0.06678046 0.03082433 0.04389676 0.05090388 0.03894710 0.03564548
## FAY 0.06267253 0.03341324 0.02906104 0.03305540 0.04198697 0.03318266
## BEA 0.06007550 0.03608183 0.03408090 0.03657658 0.04466899 0.03157583
## MAC 0.07311064 0.04390558 0.04696302 0.04134456 0.04971357 0.04244574
## PAL 0.08492736 0.05697909 0.06110101 0.05855541 0.06421631 0.05426211
## RUS         NA 0.06122886 0.06537463 0.06964341 0.06988566 0.05773597
## HAL         NA         NA 0.03722794 0.03954068 0.02433199 0.01954986
## HAT         NA         NA         NA 0.04331353 0.04938027 0.04070654
## NEO         NA         NA         NA         NA 0.04893409 0.03854129
## COL         NA         NA         NA         NA         NA 0.02782054
## PEO         NA         NA         NA         NA         NA         NA
## SPR         NA         NA         NA         NA         NA         NA
## STL         NA         NA         NA         NA         NA         NA
## NUE         NA         NA         NA         NA         NA         NA
## HOU         NA         NA         NA         NA         NA         NA
## TRA         NA         NA         NA         NA         NA         NA
## MON         NA         NA         NA         NA         NA         NA
## DAL         NA         NA         NA         NA         NA         NA
## SMI         NA         NA         NA         NA         NA         NA
## DEN         NA         NA         NA         NA         NA         NA
## CHI         NA         NA         NA         NA         NA         NA
## LOS         NA         NA         NA         NA         NA         NA
## BRM         NA         NA         NA         NA         NA         NA
## NEG         NA         NA         NA         NA         NA         NA
## SAI         NA         NA         NA         NA         NA         NA
## CUA         NA         NA         NA         NA         NA         NA
## MED         NA         NA         NA         NA         NA         NA
## BPV         NA         NA         NA         NA         NA         NA
## MAU         NA         NA         NA         NA         NA         NA
## BIT         NA         NA         NA         NA         NA         NA
## TUC         NA         NA         NA         NA         NA         NA
## FLO         NA         NA         NA         NA         NA         NA
## REC         NA         NA         NA         NA         NA         NA
## ARA         NA         NA         NA         NA         NA         NA
## PLM         NA         NA         NA         NA         NA         NA
## BCO         NA         NA         NA         NA         NA         NA
## BAF         NA         NA         NA         NA         NA         NA
## IPO         NA         NA         NA         NA         NA         NA
## NOV         NA         NA         NA         NA         NA         NA
## BEH         NA         NA         NA         NA         NA         NA
## BPA         NA         NA         NA         NA         NA         NA
## GRV         NA         NA         NA         NA         NA         NA
## POR         NA         NA         NA         NA         NA         NA
##            SPR        STL        NUE        HOU        TRA        MON
## HAI 0.10878895 0.09907115 0.06906687 0.06293295 0.07155932 0.06896848
## YUN 0.14576915 0.14211303 0.10730171 0.10042463 0.11018416 0.10810366
## HUN 0.08541324 0.07465159 0.05397108 0.04556671 0.05166503 0.05193485
## OKI 0.11566434 0.11031763 0.08751335 0.08013354 0.08468852 0.08549006
## KAG 0.08743304 0.07437928 0.07695535 0.06606240 0.06842461 0.07294576
## UTS 0.05476146 0.06103356 0.07313386 0.06319187 0.06121621 0.06811266
## KAN 0.10274563 0.09677266 0.10559382 0.09675119 0.09515673 0.10096245
## TAI 0.16085012 0.15522801 0.13821663 0.13131595 0.13894192 0.13575748
## BEN 0.15845643 0.15442711 0.12046214 0.11444768 0.12407470 0.12153477
## KAT 0.15807107 0.15569136 0.12328740 0.11760703 0.12660200 0.12483943
## CAM 0.13584539 0.13099438 0.09679626 0.08954277 0.09964266 0.09750713
## INW 0.24164087 0.24012641 0.21459292 0.21067458 0.21897085 0.21425642
## INJ 0.19866216 0.19368918 0.16605592 0.15948772 0.16886803 0.16686183
## SUF 0.19763058 0.19557066 0.16947825 0.16352448 0.17325109 0.16915339
## SUU 0.15101057 0.14694406 0.11787330 0.11307293 0.12224038 0.11892467
## KLP 0.16548512 0.16320806 0.12925223 0.12521451 0.13455693 0.13086853
## MAT 0.15306905 0.14695642 0.11095488 0.10518952 0.11594144 0.11263434
## CHA 0.14661007 0.14152774 0.10760824 0.09992658 0.11019875 0.10797712
## KAC 0.14528067 0.14227094 0.10843028 0.10224514 0.11183941 0.10912357
## SSK 0.14832326 0.14334881 0.10881886 0.10149480 0.11177327 0.10966816
## LAM 0.15012595 0.14490148 0.10950097 0.10323255 0.11366401 0.11156401
## HOC 0.11074159 0.10311276 0.07450782 0.06787345 0.07661520 0.07436797
## QNC 0.19229755 0.18980821 0.15733403 0.15055216 0.16041738 0.15761157
## HAN 0.11711985 0.11188521 0.08438526 0.07719233 0.08531361 0.08290898
## FRS 0.07892435 0.06597360 0.06162491 0.05025887 0.05354467 0.05823884
## SPB 0.05636199 0.04693618 0.05809080 0.04415536 0.04724666 0.04966147
## SPS 0.12120153 0.11662168 0.10801595 0.09857549 0.10095129 0.10277433
## BRE 0.09450699 0.07519126 0.09455883 0.08108171 0.07998676 0.08620551
## DES 0.07655230 0.07277596 0.08273934 0.07252264 0.06937475 0.07819280
## CES 0.10883050 0.10761655 0.12154489 0.10942298 0.10770306 0.11490357
## ALD 0.10630430 0.10650187 0.09484847 0.08693389 0.08890111 0.09120518
## SER 0.18284995 0.17768535 0.17183560 0.16792044 0.16794270 0.16902509
## GRA 0.10025635 0.09147155 0.06336237 0.05585822 0.06361636 0.06311072
## TUH 0.08893069 0.07728821 0.06770880 0.05792125 0.06054785 0.06419636
## SOC 0.11514857 0.11037404 0.09934153 0.08911064 0.09431803 0.09651836
## TIK 0.12652496 0.12062059 0.10948532 0.10030080 0.10486303 0.10737400
## RAR 0.12319109 0.11487346 0.10578911 0.09566877 0.10023950 0.10188495
## GES 0.12067249 0.11360289 0.10395966 0.09428640 0.09843576 0.10123504
## NEB 0.06133364 0.04332474 0.05514948 0.04332334 0.04079917 0.04477338
## NEH 0.05497351 0.03708351 0.04988437 0.03809755 0.03388954 0.04280059
## FIR 0.08582838 0.06776713 0.07759054 0.06622426 0.06668462 0.07028184
## LIN 0.06943737 0.05839267 0.06663959 0.05357055 0.05312211 0.05964027
## NEW 0.06876421 0.05261958 0.05762768 0.04004588 0.04090672 0.05290687
## BER 0.05913472 0.03971603 0.04911121 0.03683237 0.03485692 0.04483070
## HAR 0.06307962 0.05443365 0.05280625 0.04298731 0.03633163 0.04759591
## MAN 0.05313687 0.03547294 0.04481838 0.02812508 0.03086322 0.03540733
## WAV 0.06222099 0.03797413 0.05566548 0.04295113 0.04579491 0.05162133
## FAY 0.06208117 0.04487586 0.03328757 0.02204416 0.02040738 0.03192769
## BEA 0.05834778 0.04791280 0.02805277 0.01752145 0.01514731 0.02416609
## MAC 0.07037622 0.05594409 0.03223665 0.02025767 0.02848225 0.03291010
## PAL 0.08482608 0.07503269 0.04670496 0.03229451 0.04365208 0.04782464
## RUS 0.07984891 0.07324647 0.06815275 0.05863950 0.05931465 0.06244032
## HAL 0.04886906 0.03744433 0.04750463 0.03264971 0.03565524 0.04027492
## HAT 0.06274300 0.05284310 0.04303971 0.03420074 0.03430407 0.03728119
## NEO 0.06335219 0.05139694 0.04461580 0.03193894 0.03288511 0.03947379
## COL 0.05864374 0.03882841 0.05562489 0.03971564 0.04009188 0.04809835
## PEO 0.05460295 0.03363120 0.04658681 0.03020884 0.03239711 0.04031155
## SPR         NA 0.05775883 0.07158790 0.05961184 0.05761344 0.06307257
## STL         NA         NA 0.06284945 0.04644813 0.04438570 0.05186684
## NUE         NA         NA         NA 0.02225161 0.02455459 0.01439337
## HOU         NA         NA         NA         NA 0.01702290 0.01978664
## TRA         NA         NA         NA         NA         NA 0.02085540
## MON         NA         NA         NA         NA         NA         NA
## DAL         NA         NA         NA         NA         NA         NA
## SMI         NA         NA         NA         NA         NA         NA
## DEN         NA         NA         NA         NA         NA         NA
## CHI         NA         NA         NA         NA         NA         NA
## LOS         NA         NA         NA         NA         NA         NA
## BRM         NA         NA         NA         NA         NA         NA
## NEG         NA         NA         NA         NA         NA         NA
## SAI         NA         NA         NA         NA         NA         NA
## CUA         NA         NA         NA         NA         NA         NA
## MED         NA         NA         NA         NA         NA         NA
## BPV         NA         NA         NA         NA         NA         NA
## MAU         NA         NA         NA         NA         NA         NA
## BIT         NA         NA         NA         NA         NA         NA
## TUC         NA         NA         NA         NA         NA         NA
## FLO         NA         NA         NA         NA         NA         NA
## REC         NA         NA         NA         NA         NA         NA
## ARA         NA         NA         NA         NA         NA         NA
## PLM         NA         NA         NA         NA         NA         NA
## BCO         NA         NA         NA         NA         NA         NA
## BAF         NA         NA         NA         NA         NA         NA
## IPO         NA         NA         NA         NA         NA         NA
## NOV         NA         NA         NA         NA         NA         NA
## BEH         NA         NA         NA         NA         NA         NA
## BPA         NA         NA         NA         NA         NA         NA
## GRV         NA         NA         NA         NA         NA         NA
## POR         NA         NA         NA         NA         NA         NA
##            DAL        SMI        DEN        CHI        LOS        BRM       NEG
## HAI 0.08413851 0.08489985 0.08571585 0.05853241 0.06974945 0.10282219 0.1631205
## YUN 0.12335338 0.12435121 0.12723227 0.08991930 0.10426232 0.13542682 0.1276615
## HUN 0.06202728 0.06469303 0.06143323 0.05884463 0.05858503 0.08544375 0.1812638
## OKI 0.09258665 0.09673045 0.08960815 0.09579221 0.09785283 0.11646448 0.2073457
## KAG 0.07172578 0.07624929 0.07005607 0.10750394 0.09992508 0.10508901 0.2223366
## UTS 0.06253356 0.06800468 0.05478074 0.11882782 0.11414219 0.10383464 0.2354324
## KAN 0.09696742 0.10235565 0.09229703 0.13602460 0.13620726 0.13297340 0.2503020
## TAI 0.14619857 0.14699357 0.14575371 0.13330791 0.14002497 0.16321432 0.2450688
## BEN 0.13644956 0.13760241 0.14010882 0.10433959 0.11828092 0.14821635 0.1343677
## KAT 0.13906261 0.14035432 0.14173410 0.10691381 0.11999341 0.14857512 0.1393196
## CAM 0.11323754 0.11456269 0.11625888 0.08101645 0.09488701 0.12521379 0.1299503
## INW 0.22800401 0.22763259 0.22942651 0.19438325 0.20851843 0.23701515 0.2299757
## INJ 0.18019258 0.18038552 0.18281642 0.14725312 0.16332409 0.18996301 0.1911622
## SUF 0.18202911 0.18274491 0.18419923 0.15006122 0.16546689 0.19262103 0.1973195
## SUU 0.13203087 0.13321228 0.13437845 0.10500049 0.11855653 0.14448819 0.1769015
## KLP 0.14577014 0.14463310 0.14955889 0.11184061 0.12729352 0.15707486 0.1561925
## MAT 0.12892167 0.12892954 0.13279090 0.09612198 0.10986554 0.14006771 0.1394073
## CHA 0.12346330 0.12452662 0.12714817 0.09129368 0.10480234 0.13518393 0.1312606
## KAC 0.12429725 0.12545147 0.12792201 0.09079984 0.10472626 0.13693684 0.1248980
## SSK 0.12523501 0.12603931 0.12910796 0.09240211 0.10662303 0.13630209 0.1322156
## LAM 0.12638038 0.12707452 0.13004025 0.09376890 0.10822534 0.13841051 0.1324493
## HOC 0.08881163 0.08922638 0.09041172 0.06191675 0.07659637 0.10543988 0.1651712
## QNC 0.17219858 0.17146114 0.17592687 0.13823975 0.15275254 0.18240673 0.1774166
## HAN 0.09780431 0.09771122 0.09862431 0.06796108 0.08065731 0.11238116 0.1509207
## FRS 0.06168772 0.06543134 0.05541279 0.08645166 0.07967171 0.09109560 0.2065255
## SPB 0.04461569 0.05421166 0.03781360 0.09885412 0.09329670 0.08360161 0.2165512
## SPS 0.10888982 0.11134249 0.10609523 0.11648276 0.11115542 0.12900293 0.2285402
## BRE 0.07743204 0.08100531 0.07187703 0.13870382 0.13485153 0.11619462 0.2514904
## DES 0.06355364 0.07577554 0.06135337 0.12951261 0.12522294 0.10459625 0.2441697
## CES 0.10847448 0.11627862 0.10055976 0.15771123 0.15455741 0.14454086 0.2658361
## ALD 0.09851137 0.10114120 0.09398633 0.10189580 0.09888393 0.12145854 0.2159331
## SER 0.17267365 0.17518684 0.16899447 0.18325479 0.18328068 0.19037767 0.2863980
## GRA 0.07628989 0.07868495 0.07864214 0.06646740 0.06978230 0.09573337 0.1715999
## TUH 0.07219580 0.07517320 0.06696953 0.08314756 0.07558854 0.09734188 0.2028902
## SOC 0.10245126 0.10369924 0.10029013 0.10762513 0.10651130 0.12407456 0.2181526
## TIK 0.11320408 0.11298818 0.10980313 0.12036853 0.11814489 0.13449596 0.2288710
## RAR 0.10890384 0.10998041 0.10584838 0.11089155 0.10909209 0.12979697 0.2204264
## GES 0.10692432 0.10908845 0.10420531 0.11147736 0.11067054 0.12857232 0.2244522
## NEB 0.04016259 0.04985863 0.03365538 0.10180286 0.09669156 0.08069989 0.2207520
## NEH 0.03548481 0.04287251 0.03557293 0.09658601 0.08750992 0.07325773 0.2105769
## FIR 0.05898866 0.07075348 0.06121971 0.12520615 0.11928745 0.10184975 0.2367339
## LIN 0.05257670 0.06242125 0.05176577 0.10766106 0.10310833 0.09621412 0.2284362
## NEW 0.04148019 0.04635866 0.03164402 0.11001498 0.10478379 0.08931772 0.2265972
## BER 0.03777735 0.04623294 0.03388664 0.10392620 0.09855385 0.07855387 0.2214968
## HAR 0.04490131 0.04852901 0.03614964 0.10746948 0.10207066 0.08507142 0.2213818
## MAN 0.02909044 0.04028504 0.02854577 0.09609599 0.08952182 0.07761879 0.2155600
## WAV 0.03642082 0.05005838 0.03576816 0.10741537 0.10421726 0.08255873 0.2218178
## FAY 0.02445453 0.03570371 0.02232307 0.09155670 0.08863422 0.07371230 0.2114527
## BEA 0.03082051 0.03559543 0.02904288 0.08394227 0.07911468 0.06853252 0.1974926
## MAC 0.04189748 0.04374821 0.04430356 0.08509463 0.08611893 0.07789620 0.2003488
## PAL 0.05443495 0.06160950 0.05607905 0.09213219 0.09335378 0.08626194 0.2002805
## RUS 0.05954608 0.06813912 0.05768779 0.11748739 0.11466660 0.10259187 0.2273289
## HAL 0.03294413 0.04297794 0.03037476 0.09597089 0.08850740 0.07306667 0.2123961
## HAT 0.03158054 0.03660347 0.02696071 0.09914768 0.09603454 0.08090814 0.2127373
## NEO 0.03503592 0.04236382 0.03939266 0.10002250 0.09652015 0.08173141 0.2154418
## COL 0.04056307 0.05172237 0.04022003 0.10230921 0.09483954 0.08560142 0.2184093
## PEO 0.03059637 0.04230223 0.03281171 0.09315593 0.08774627 0.06899067 0.2093423
## SPR 0.06119104 0.06512758 0.04983194 0.11791036 0.11378366 0.10140584 0.2299490
## STL 0.04200854 0.05464573 0.03821841 0.11033946 0.10600995 0.09024123 0.2294901
## NUE 0.02314034 0.02655853 0.04027558 0.08708409 0.08652197 0.07587552 0.2007050
## HOU 0.02597338 0.03122573 0.03155933 0.07880912 0.07775818 0.07235782 0.1973122
## TRA 0.01895256 0.02769179 0.02160174 0.08796328 0.08200134 0.07232074 0.2040480
## MON 0.01777369 0.01719825 0.03223206 0.08570514 0.08337358 0.07529130 0.2021933
## DAL         NA 0.01794244 0.02026327 0.09923920 0.09292481 0.07350994 0.2149322
## SMI         NA         NA 0.03686674 0.09877785 0.09483790 0.08234111 0.2162619
## DEN         NA         NA         NA 0.10000721 0.09426283 0.08016612 0.2189723
## CHI         NA         NA         NA         NA 0.08787056 0.11582856 0.1831915
## LOS         NA         NA         NA         NA         NA 0.11276254 0.1994158
## BRM         NA         NA         NA         NA         NA         NA 0.2241698
## NEG         NA         NA         NA         NA         NA         NA        NA
## SAI         NA         NA         NA         NA         NA         NA        NA
## CUA         NA         NA         NA         NA         NA         NA        NA
## MED         NA         NA         NA         NA         NA         NA        NA
## BPV         NA         NA         NA         NA         NA         NA        NA
## MAU         NA         NA         NA         NA         NA         NA        NA
## BIT         NA         NA         NA         NA         NA         NA        NA
## TUC         NA         NA         NA         NA         NA         NA        NA
## FLO         NA         NA         NA         NA         NA         NA        NA
## REC         NA         NA         NA         NA         NA         NA        NA
## ARA         NA         NA         NA         NA         NA         NA        NA
## PLM         NA         NA         NA         NA         NA         NA        NA
## BCO         NA         NA         NA         NA         NA         NA        NA
## BAF         NA         NA         NA         NA         NA         NA        NA
## IPO         NA         NA         NA         NA         NA         NA        NA
## NOV         NA         NA         NA         NA         NA         NA        NA
## BEH         NA         NA         NA         NA         NA         NA        NA
## BPA         NA         NA         NA         NA         NA         NA        NA
## GRV         NA         NA         NA         NA         NA         NA        NA
## POR         NA         NA         NA         NA         NA         NA        NA
##            SAI        CUA        MED        BPV        MAU        BIT
## HAI 0.06834360 0.08571519 0.08216104 0.09270859 0.08351136 0.09296947
## YUN 0.09186110 0.09429958 0.09001816 0.09615219 0.09349152 0.09946437
## HUN 0.06354950 0.09012034 0.08658990 0.09996038 0.08736955 0.09972531
## OKI 0.09620034 0.12039803 0.12018690 0.13124716 0.11925414 0.12947039
## KAG 0.10023771 0.13039157 0.12745781 0.13732413 0.12262277 0.13459979
## UTS 0.10471077 0.14009794 0.13810608 0.14406947 0.12810344 0.14059231
## KAN 0.13135861 0.16104148 0.15724428 0.16824726 0.15406869 0.16580639
## TAI 0.14376460 0.16579840 0.16430987 0.17665696 0.16507025 0.17500958
## BEN 0.10575970 0.10560345 0.10032681 0.10025141 0.09781351 0.10505625
## KAT 0.10726345 0.10677257 0.09926118 0.10847733 0.10511907 0.11125795
## CAM 0.08259694 0.08760331 0.08355001 0.08962724 0.08458462 0.09215941
## INW 0.19633874 0.19080617 0.18488357 0.19969786 0.19905859 0.20273120
## INJ 0.14947043 0.14523760 0.13964781 0.15711985 0.15426073 0.16119817
## SUF 0.15340702 0.15024077 0.14477550 0.16275118 0.15807314 0.16380354
## SUU 0.10814801 0.11173962 0.10812999 0.12717230 0.12028183 0.12936552
## KLP 0.11291076 0.11758527 0.11188545 0.12369581 0.11989939 0.12542047
## MAT 0.09537879 0.09963865 0.09531628 0.10305674 0.10049676 0.10536124
## CHA 0.09123235 0.09564679 0.09174600 0.09619065 0.09214600 0.09831966
## KAC 0.09304393 0.09530976 0.08918377 0.09550736 0.09368791 0.09832711
## SSK 0.09357507 0.09698788 0.09224911 0.09842381 0.09532877 0.10142913
## LAM 0.09438049 0.09530557 0.09061666 0.09869300 0.09582265 0.10126706
## HOC 0.07378702 0.09081199 0.08727751 0.10001898 0.09155301 0.10139894
## QNC 0.14247153 0.14367390 0.13867019 0.14827301 0.14560233 0.15083259
## HAN 0.07755303 0.08836663 0.08400386 0.09719880 0.08956555 0.09727659
## FRS 0.07916333 0.11398070 0.11226798 0.11875132 0.10441589 0.11930237
## SPB 0.08557794 0.12081244 0.11899420 0.12186351 0.10586934 0.12112688
## SPS 0.11838624 0.14226126 0.14122013 0.15207770 0.14009679 0.15250934
## BRE 0.11941901 0.15918894 0.15719478 0.15702234 0.14326814 0.15654027
## DES 0.11600517 0.14984222 0.14839928 0.15314170 0.13513554 0.14898195
## CES 0.14484074 0.17726979 0.17671321 0.17918846 0.16533519 0.17675150
## ALD 0.10648071 0.13212926 0.12891548 0.14008904 0.12811132 0.13997045
## SER 0.18338245 0.20304115 0.20019652 0.21575367 0.20405996 0.21351629
## GRA 0.06373532 0.08746585 0.08536480 0.09749162 0.08806894 0.09802694
## TUH 0.08238772 0.11208581 0.11064882 0.11971514 0.10639841 0.11770113
## SOC 0.11113482 0.13566811 0.13223746 0.14317088 0.13080801 0.14236054
## TIK 0.12210141 0.14631320 0.14298383 0.15476127 0.14216222 0.15248229
## RAR 0.11581691 0.13881202 0.13465222 0.14653481 0.13460883 0.14548466
## GES 0.11491625 0.14044833 0.13770274 0.14846771 0.13535629 0.14678199
## NEB 0.08395622 0.12148931 0.11967284 0.12744367 0.11022799 0.12483703
## NEH 0.07613602 0.11449699 0.11321233 0.12073792 0.10310958 0.11824601
## FIR 0.10836137 0.14453592 0.14235133 0.14760716 0.13286930 0.14310515
## LIN 0.09305910 0.12963909 0.12846508 0.13253794 0.11835817 0.13026953
## NEW 0.08742688 0.12707406 0.12692192 0.12958178 0.11234411 0.12545734
## BER 0.08056263 0.12297210 0.12022409 0.12316823 0.10311266 0.12030242
## HAR 0.08583425 0.12508247 0.12382091 0.12759963 0.10989286 0.12236330
## MAN 0.07246555 0.11627779 0.11507563 0.11890459 0.10205477 0.11615521
## WAV 0.08741609 0.12657253 0.12367018 0.12567302 0.10925621 0.12414736
## FAY 0.06717538 0.10795148 0.10770150 0.10818424 0.09363428 0.10655107
## BEA 0.05682825 0.09863567 0.09705807 0.10184910 0.08639256 0.09863748
## MAC 0.05842609 0.10084946 0.10048486 0.10285728 0.08823914 0.10263702
## PAL 0.05686658 0.10744135 0.10523896 0.10944519 0.09568542 0.11011350
## RUS 0.10010850 0.13544590 0.13349042 0.13719904 0.12320353 0.13480461
## HAL 0.07642139 0.11289476 0.11230339 0.11409349 0.09793282 0.11326409
## HAT 0.07846452 0.11332918 0.11439168 0.11607260 0.10079220 0.11143910
## NEO 0.07322078 0.11499135 0.11388631 0.11648469 0.10143454 0.11513986
## COL 0.08188500 0.11953641 0.11840604 0.12414803 0.10818827 0.12045687
## PEO 0.07400899 0.11084252 0.11002744 0.11347275 0.09789434 0.11235715
## SPR 0.10177934 0.13427085 0.13270784 0.13801223 0.12124649 0.13488507
## STL 0.09228175 0.13031205 0.12927151 0.13217426 0.11310290 0.12833888
## NUE 0.06155905 0.10407837 0.10164655 0.10295476 0.09133049 0.09475758
## HOU 0.05366231 0.09578751 0.09417752 0.09718300 0.08249297 0.09741963
## TRA 0.06055370 0.10290239 0.10211909 0.10327803 0.08952078 0.10196945
## MON 0.06239945 0.10353797 0.10145164 0.10363973 0.09105173 0.09619987
## DAL 0.07488561 0.11238214 0.11151553 0.11396728 0.10011282 0.10540462
## SMI 0.07554182 0.11656602 0.11650835 0.11588606 0.10444495 0.10561945
## DEN 0.07618820 0.11629273 0.11706659 0.11923669 0.10053695 0.11420117
## CHI 0.08724490 0.10553819 0.10237865 0.11657607 0.10737131 0.11783763
## LOS 0.09248468 0.11468480 0.11302958 0.12496631 0.11334555 0.12386270
## BRM 0.10061834 0.13370314 0.13271269 0.13937523 0.12491684 0.13697113
## NEG 0.18837082 0.18455258 0.17967090 0.18866389 0.18627698 0.19064523
## SAI         NA 0.10121718 0.10106795 0.10405592 0.09420979 0.10509951
## CUA         NA         NA 0.01128083 0.10712317 0.09702001 0.10834118
## MED         NA         NA         NA 0.10706741 0.09859930 0.10861739
## BPV         NA         NA         NA         NA 0.03681429 0.04833083
## MAU         NA         NA         NA         NA         NA 0.04426018
## BIT         NA         NA         NA         NA         NA         NA
## TUC         NA         NA         NA         NA         NA         NA
## FLO         NA         NA         NA         NA         NA         NA
## REC         NA         NA         NA         NA         NA         NA
## ARA         NA         NA         NA         NA         NA         NA
## PLM         NA         NA         NA         NA         NA         NA
## BCO         NA         NA         NA         NA         NA         NA
## BAF         NA         NA         NA         NA         NA         NA
## IPO         NA         NA         NA         NA         NA         NA
## NOV         NA         NA         NA         NA         NA         NA
## BEH         NA         NA         NA         NA         NA         NA
## BPA         NA         NA         NA         NA         NA         NA
## GRV         NA         NA         NA         NA         NA         NA
## POR         NA         NA         NA         NA         NA         NA
##            TUC        FLO        REC        ARA        PLM        BCO
## HAI 0.08837926 0.09657258 0.08604667 0.08683458 0.09519624 0.09587110
## YUN 0.08911175 0.09677759 0.10368985 0.09133143 0.09048376 0.09664071
## HUN 0.09838635 0.10442673 0.08303959 0.09438925 0.10418843 0.10249051
## OKI 0.12989700 0.13528165 0.11398793 0.12617930 0.13581254 0.13436578
## KAG 0.13646508 0.13930846 0.11051198 0.13154114 0.14280815 0.13837145
## UTS 0.14448179 0.14584543 0.11399698 0.13762243 0.14835644 0.14433845
## KAN 0.16848003 0.17031672 0.14134176 0.16331316 0.17486413 0.17006725
## TAI 0.17636798 0.17833534 0.16099527 0.17162176 0.17655826 0.17921863
## BEN 0.09383548 0.10160922 0.11136265 0.09660169 0.09304517 0.10116862
## KAT 0.10181151 0.10495631 0.11665125 0.10341814 0.09966212 0.10759240
## CAM 0.08262730 0.08993573 0.09565485 0.08390219 0.08628177 0.09042618
## INW 0.19472033 0.19731827 0.20902215 0.19667583 0.19041829 0.19962506
## INJ 0.15158493 0.15528424 0.16397766 0.15194756 0.14907776 0.15805246
## SUF 0.15722087 0.16015086 0.16683111 0.15800857 0.15345731 0.16301565
## SUU 0.12405423 0.12804983 0.12544106 0.12136355 0.12267965 0.12914732
## KLP 0.11762868 0.12197967 0.12918931 0.11844518 0.11587703 0.12548665
## MAT 0.09697649 0.10394243 0.11127168 0.09782971 0.09831280 0.10424138
## CHA 0.08872624 0.09630315 0.10472936 0.09045840 0.09116848 0.09603713
## KAC 0.08880380 0.09482038 0.10450847 0.09041754 0.08954004 0.09552601
## SSK 0.09095072 0.09890581 0.10541665 0.09263445 0.09408542 0.09882489
## LAM 0.09188743 0.09790933 0.10733127 0.09395627 0.09302404 0.09937593
## HOC 0.09654402 0.10376615 0.09208447 0.09474354 0.10171657 0.10345239
## QNC 0.14156228 0.14610901 0.15529027 0.14174706 0.14017772 0.14655613
## HAN 0.09419467 0.09806901 0.09461559 0.09391957 0.09687461 0.09998967
## FRS 0.11940649 0.12267207 0.09406564 0.11438762 0.12450495 0.12186962
## SPB 0.12320420 0.12600122 0.09428206 0.11733225 0.12761223 0.12454717
## SPS 0.15096932 0.15399676 0.13350288 0.14600210 0.15562450 0.15233802
## BRE 0.15966136 0.15941080 0.12247692 0.15278132 0.16242757 0.15436027
## DES 0.15268824 0.15194293 0.11952394 0.14396932 0.15331765 0.14959746
## CES 0.18069143 0.17801226 0.15168386 0.17376645 0.17723673 0.17771286
## ALD 0.13907317 0.14164519 0.11990414 0.13415927 0.14422881 0.14166351
## SER 0.21170135 0.21410243 0.19827473 0.20985178 0.21343763 0.21418944
## GRA 0.09520302 0.10240430 0.08766932 0.09210958 0.10006772 0.10029569
## TUH 0.11888119 0.12292083 0.09833982 0.11470341 0.12462605 0.12165956
## SOC 0.14182174 0.14686221 0.12708620 0.13959597 0.14720441 0.14503130
## TIK 0.15370069 0.15788162 0.13739856 0.14970966 0.15725900 0.15663374
## RAR 0.14498034 0.14994366 0.12966965 0.14171858 0.15071558 0.14807517
## GES 0.14610856 0.15095307 0.13079183 0.14258493 0.15132770 0.14952026
## NEB 0.12734373 0.12913611 0.09563037 0.12119361 0.13220376 0.12760554
## NEH 0.11971198 0.12040265 0.08942738 0.11287583 0.12430222 0.11895323
## FIR 0.14226489 0.14431682 0.11596394 0.13711218 0.14971013 0.14531932
## LIN 0.13295745 0.13780217 0.10229133 0.12878036 0.13997246 0.13478079
## NEW 0.12683537 0.13080513 0.09451656 0.12145349 0.13413890 0.12869413
## BER 0.12573036 0.12712962 0.08852651 0.11768471 0.13087518 0.12362915
## HAR 0.12684923 0.13128570 0.09482873 0.12005214 0.13190563 0.12728971
## MAN 0.11790001 0.12107230 0.08295051 0.11171059 0.12431706 0.11752281
## WAV 0.12787235 0.12703521 0.09417302 0.12125905 0.13231362 0.12624561
## FAY 0.10912929 0.11298744 0.07734062 0.10204811 0.11603483 0.10890861
## BEA 0.09837789 0.10351620 0.06814834 0.09214674 0.10576708 0.10126706
## MAC 0.10325921 0.10894702 0.07455312 0.09824175 0.10901738 0.10450119
## PAL 0.10708270 0.11216450 0.08214125 0.10495304 0.11458537 0.10927081
## RUS 0.13694468 0.14147247 0.10557630 0.13089993 0.14301724 0.13890138
## HAL 0.11419538 0.11863884 0.08433899 0.10939078 0.12132307 0.11630281
## HAT 0.11605138 0.12199951 0.08732557 0.10962484 0.12269312 0.11763962
## NEO 0.11657373 0.12217505 0.08252078 0.10985827 0.12418014 0.11943951
## COL 0.12277666 0.12652165 0.09250301 0.11819863 0.13024040 0.12290863
## PEO 0.11355660 0.11681290 0.08015477 0.10733909 0.11903041 0.11370925
## SPR 0.13851515 0.14124213 0.10907007 0.13227967 0.14044938 0.13953650
## STL 0.13263875 0.13372605 0.09993727 0.12556106 0.13806243 0.13264746
## NUE 0.09882002 0.11096062 0.07623864 0.09634748 0.11011844 0.10697759
## HOU 0.09759352 0.10391010 0.06810973 0.09268128 0.10547954 0.10057016
## TRA 0.10174825 0.10955946 0.07179817 0.09794937 0.11202990 0.10456046
## MON 0.09845267 0.11139511 0.07458112 0.09598720 0.10971630 0.10723825
## DAL 0.10849828 0.11970529 0.07970482 0.10582312 0.12092323 0.11598581
## SMI 0.10986802 0.12203179 0.08143479 0.10757851 0.12302715 0.11666544
## DEN 0.11876406 0.12170274 0.08493782 0.11175332 0.12362947 0.11844040
## CHI 0.11583456 0.12126699 0.10663045 0.11400493 0.11897887 0.12047623
## LOS 0.12243276 0.12735133 0.10957693 0.11917174 0.12828302 0.12567159
## BRM 0.13716736 0.14038143 0.11251617 0.13155166 0.14198584 0.13763707
## NEG 0.18224128 0.18432668 0.19759003 0.18414314 0.17892833 0.18695238
## SAI 0.10273586 0.11024862 0.08571140 0.10146172 0.10976247 0.10670362
## CUA 0.10240742 0.10547575 0.10979604 0.10204854 0.10809268 0.11001159
## MED 0.10231430 0.10631645 0.10812831 0.10292451 0.10773736 0.10923621
## BPV 0.05173018 0.05733959 0.06528422 0.05426692 0.05275556 0.05883313
## MAU 0.05182443 0.05565917 0.06922338 0.05411305 0.05703325 0.06001029
## BIT 0.04055607 0.05133756 0.07367062 0.03974566 0.05319098 0.05218582
## TUC         NA 0.02401856 0.06243614 0.01157325 0.02668868 0.02566308
## FLO         NA         NA 0.06609527 0.02173996 0.02099231 0.02831881
## REC         NA         NA         NA 0.05966375 0.07095640 0.06692402
## ARA         NA         NA         NA         NA 0.02322551 0.02494034
## PLM         NA         NA         NA         NA         NA 0.02738172
## BCO         NA         NA         NA         NA         NA         NA
## BAF         NA         NA         NA         NA         NA         NA
## IPO         NA         NA         NA         NA         NA         NA
## NOV         NA         NA         NA         NA         NA         NA
## BEH         NA         NA         NA         NA         NA         NA
## BPA         NA         NA         NA         NA         NA         NA
## GRV         NA         NA         NA         NA         NA         NA
## POR         NA         NA         NA         NA         NA         NA
##            BAF        IPO        NOV        BEH        BPA        GRV
## HAI 0.08841429 0.13138018 0.11739015 0.09856573 0.09233415 0.09326533
## YUN 0.08767629 0.10748383 0.08867712 0.08384151 0.10626986 0.09952785
## HUN 0.09788853 0.15108158 0.13748253 0.11326495 0.09021071 0.09904632
## OKI 0.12825119 0.18054177 0.16627599 0.14605030 0.12048075 0.13278755
## KAG 0.13364168 0.19227022 0.18134174 0.15627036 0.11746536 0.13305410
## UTS 0.14230472 0.20471215 0.19655834 0.16801344 0.12008545 0.14123158
## KAN 0.16472021 0.22177848 0.21167877 0.18928683 0.14694541 0.16527587
## TAI 0.17124149 0.21816447 0.20346518 0.18736986 0.16778043 0.17610241
## BEN 0.09151988 0.10548875 0.09062201 0.08934918 0.11126964 0.10571782
## KAT 0.09837517 0.11486944 0.09631828 0.09516177 0.11772888 0.11169221
## CAM 0.08196844 0.10601304 0.09053318 0.08134348 0.09822079 0.09265263
## INW 0.19027133 0.20605078 0.18695479 0.18725201 0.20980928 0.20370127
## INJ 0.14771547 0.16698605 0.14882993 0.14583693 0.16598296 0.16083195
## SUF 0.15358164 0.17332678 0.15404821 0.15168745 0.16806354 0.16622091
## SUU 0.12155679 0.15159113 0.13429524 0.12307417 0.12958637 0.12863974
## KLP 0.11535112 0.13398000 0.11327514 0.11329856 0.13175697 0.12690422
## MAT 0.09515026 0.11759823 0.09722642 0.09202249 0.11315148 0.10682943
## CHA 0.08745428 0.10802527 0.09010954 0.08550180 0.10634784 0.09962407
## KAC 0.08577721 0.10427830 0.08521056 0.08230602 0.10596975 0.09965826
## SSK 0.08946163 0.10939362 0.09212434 0.08681037 0.10828403 0.10205794
## LAM 0.08966695 0.10993623 0.09002448 0.08662790 0.10924813 0.10289396
## HOC 0.09574992 0.13670210 0.12072619 0.10515741 0.09849949 0.10105763
## QNC 0.13734751 0.15908343 0.14017640 0.13667005 0.15687596 0.14924478
## HAN 0.08981531 0.12560765 0.10807546 0.09733287 0.09865449 0.09826600
## FRS 0.11819792 0.17532955 0.16504515 0.13775104 0.10198612 0.11829694
## SPB 0.12135565 0.18598032 0.17608185 0.14890221 0.10217436 0.12145850
## SPS 0.14925350 0.20075087 0.19034309 0.16769767 0.14073893 0.15219571
## BRE 0.15480509 0.22086554 0.21102679 0.18232702 0.12969080 0.15806050
## DES 0.14912893 0.21098911 0.20513824 0.17660006 0.12384119 0.14636525
## CES 0.17627328 0.23555432 0.22744383 0.20084106 0.15969300 0.17734005
## ALD 0.13598775 0.18835458 0.17495942 0.15368670 0.12711893 0.13947303
## SER 0.20925009 0.25725653 0.24556473 0.22385625 0.20330728 0.21227892
## GRA 0.09556662 0.14190619 0.12835451 0.10710271 0.09263503 0.09849220
## TUH 0.11786682 0.17297504 0.16179177 0.13624846 0.10465576 0.11892354
## SOC 0.13984185 0.19025970 0.17893169 0.15631315 0.13317877 0.14101661
## TIK 0.15084536 0.20232461 0.18903458 0.16983566 0.14309416 0.15241779
## RAR 0.14334410 0.19286563 0.18055339 0.15929402 0.13567995 0.14560681
## GES 0.14450274 0.19570033 0.18452552 0.16239220 0.13649698 0.14700686
## NEB 0.12565797 0.19011046 0.18176819 0.15250610 0.10278381 0.12317065
## NEH 0.11568456 0.18133176 0.17287039 0.14428107 0.09633714 0.11464347
## FIR 0.14154467 0.20486147 0.19609241 0.17041508 0.11743550 0.14454178
## LIN 0.13221519 0.19367204 0.18643528 0.15751385 0.10820425 0.13366769
## NEW 0.12438277 0.19105956 0.18459991 0.15472001 0.10038983 0.12802102
## BER 0.12246823 0.18683018 0.18141458 0.14910636 0.09899688 0.12162707
## HAR 0.12319924 0.18984460 0.18236368 0.15278964 0.10189549 0.12501453
## MAN 0.11549585 0.18284566 0.17477572 0.14433771 0.08994100 0.11706172
## WAV 0.12322061 0.19161113 0.18243273 0.15302813 0.09793346 0.12454071
## FAY 0.10628387 0.17550583 0.16779028 0.13642410 0.08249772 0.11056678
## BEA 0.09634556 0.16325122 0.15562437 0.12307257 0.07730606 0.10129179
## MAC 0.10269208 0.16743481 0.15831093 0.12665337 0.08348250 0.10538696
## PAL 0.10553166 0.16758583 0.15977819 0.12931832 0.08996095 0.11225536
## RUS 0.13525209 0.19718638 0.18819249 0.16097225 0.10887250 0.13309667
## HAL 0.11334954 0.17953681 0.17012000 0.14015939 0.09109175 0.11519561
## HAT 0.11310997 0.17851811 0.17171744 0.14202140 0.09560626 0.11209919
## NEO 0.11600851 0.18130293 0.17271628 0.14407186 0.08937451 0.11735298
## COL 0.11979697 0.18571968 0.17736840 0.14928535 0.10018747 0.12325213
## PEO 0.11089530 0.17604120 0.16881159 0.13911949 0.08784424 0.11352623
## SPR 0.13709532 0.19814312 0.18986659 0.16238782 0.11604381 0.13538924
## STL 0.12938262 0.19530536 0.18872984 0.15848786 0.10833411 0.12917654
## NUE 0.10342440 0.16731475 0.15930558 0.12807271 0.08011411 0.10444699
## HOU 0.09646794 0.16229107 0.15447858 0.12225242 0.07382943 0.09834484
## TRA 0.10160839 0.16927825 0.16323380 0.13061077 0.08060494 0.10538069
## MON 0.10398787 0.16818822 0.16041326 0.12962536 0.08171467 0.10529702
## DAL 0.11252493 0.17887842 0.17333219 0.14189425 0.08422327 0.11432783
## SMI 0.11522381 0.18014426 0.17474298 0.14225096 0.08866037 0.11659406
## DEN 0.11697319 0.18461126 0.17734615 0.14581379 0.09157473 0.11838649
## CHI 0.11279023 0.15652148 0.14136298 0.12415450 0.11138212 0.11674611
## LOS 0.12069940 0.16927026 0.15642876 0.13649127 0.11565122 0.12279035
## BRM 0.13481540 0.19398156 0.18650046 0.15906441 0.11879891 0.13693122
## NEG 0.17718464 0.19772021 0.17904739 0.17794186 0.19703557 0.19135673
## SAI 0.10247996 0.15630951 0.14510771 0.11871955 0.09313536 0.10950768
## CUA 0.10187574 0.13673411 0.12415100 0.10631318 0.11448354 0.10574842
## MED 0.10148165 0.13461672 0.12068529 0.10657692 0.11125291 0.10433462
## BPV 0.04639937 0.08208887 0.08007546 0.05778027 0.06736470 0.06958986
## MAU 0.05031959 0.08739958 0.08748692 0.06117622 0.07256320 0.05893812
## BIT 0.04249875 0.08166907 0.08767338 0.06632044 0.07045894 0.06552508
## TUC 0.01583352 0.06838279 0.07463331 0.04677090 0.06260594 0.06047000
## FLO 0.01538883 0.07020249 0.07762703 0.05455045 0.06529767 0.06185629
## REC 0.05872394 0.11982781 0.12041806 0.08302292 0.02972773 0.08760378
## ARA 0.01302377 0.06909923 0.07720845 0.05091627 0.05744837 0.05594176
## PLM 0.02039582 0.06337996 0.06591053 0.04909436 0.06781302 0.06448888
## BCO 0.02358059 0.08024661 0.08410899 0.05206378 0.06701000 0.06863279
## BAF         NA 0.05943494 0.06406728 0.04295494 0.05853361 0.05369899
## IPO         NA         NA 0.03418368 0.05379457 0.11599574 0.06266484
## NOV         NA         NA         NA 0.04392378 0.11659673 0.07484300
## BEH         NA         NA         NA         NA 0.08745306 0.06756833
## BPA         NA         NA         NA         NA         NA 0.08768924
## GRV         NA         NA         NA         NA         NA         NA
## POR         NA         NA         NA         NA         NA         NA
##            POR
## HAI 0.09965423
## YUN 0.09134184
## HUN 0.11013410
## OKI 0.14189354
## KAG 0.15060535
## UTS 0.16043054
## KAN 0.18326122
## TAI 0.18598946
## BEN 0.09360701
## KAT 0.10142757
## CAM 0.08679927
## INW 0.19482956
## INJ 0.15321015
## SUF 0.15944791
## SUU 0.12799819
## KLP 0.11903087
## MAT 0.09870711
## CHA 0.09104684
## KAC 0.08965335
## SSK 0.09306120
## LAM 0.09424815
## HOC 0.10582854
## QNC 0.14208631
## HAN 0.09924034
## FRS 0.13351144
## SPB 0.14041478
## SPS 0.16453497
## BRE 0.17673512
## DES 0.16765546
## CES 0.19412978
## ALD 0.15155275
## SER 0.22318776
## GRA 0.10667818
## TUH 0.13284879
## SOC 0.15289496
## TIK 0.16419064
## RAR 0.15591017
## GES 0.15903332
## NEB 0.14423659
## NEH 0.13577029
## FIR 0.16320682
## LIN 0.15013016
## NEW 0.14615838
## BER 0.14202510
## HAR 0.14399686
## MAN 0.13780671
## WAV 0.14573853
## FAY 0.12833804
## BEA 0.11871603
## MAC 0.12338942
## PAL 0.12675855
## RUS 0.15449177
## HAL 0.13270655
## HAT 0.13335710
## NEO 0.13546787
## COL 0.14215852
## PEO 0.13175136
## SPR 0.15481384
## STL 0.15011716
## NUE 0.12106087
## HOU 0.11607324
## TRA 0.12362389
## MON 0.12401727
## DAL 0.13432317
## SMI 0.13705332
## DEN 0.13865854
## CHI 0.12432624
## LOS 0.13374364
## BRM 0.15151598
## NEG 0.18328975
## SAI 0.11992783
## CUA 0.10617063
## MED 0.10569761
## BPV 0.05532322
## MAU 0.05263241
## BIT 0.05785242
## TUC 0.04537586
## FLO 0.05105929
## REC 0.09272456
## ARA 0.04619664
## PLM 0.04887239
## BCO 0.05760008
## BAF 0.03972453
## IPO 0.04243890
## NOV 0.04921591
## BEH 0.04362418
## BPA 0.09228449
## GRV 0.02989984
## POR         NA
```

```
pairfst.long <- melt(aa)
summary(pairfst.long)
```

```
##       Var1           Var2          value      
##  HAI    :  89   HAI    :  89   Min.   :0.010  
##  YUN    :  89   YUN    :  89   1st Qu.:0.084  
##  HUN    :  89   HUN    :  89   Median :0.110  
##  OKI    :  89   OKI    :  89   Mean   :0.113  
##  KAG    :  89   KAG    :  89   3rd Qu.:0.141  
##  UTS    :  89   UTS    :  89   Max.   :0.292  
##  (Other):7387   (Other):7387   NA's   :4005
```

Now lets plot the data with ggplot. You can click in the little
square on the top left of the plot to open it on a new window. It will
have the right proportions.

```
pairfst.f <- ggplot(pairfst.long, aes(Var1, Var2)) +
  geom_tile(aes(fill = value), colour = "white") +
  scale_fill_gradient(
    low = "white",
    high = "#71b6ff",
    name = "Fst",
    na.value = "white",
    limits = c(0, 0.5)
  ) +
  scale_x_discrete(position = "top") +
  theme_bw() +
  geom_text(aes(label = ifelse(
    is.na(value), "", formatC(value, digits = 2, format = "f")
  )), size = 2.25) +
  theme(
    axis.text.x = element_text(angle = 90, hjust = 1),
    axis.title = element_blank(),
    panel.grid.major = element_blank(),
    panel.grid.minor = element_blank(),
    panel.border = element_blank(),
    panel.background = element_blank(),
    axis.text.y = element_text(hjust = 0)
  )
pairfst.f
```

Save it

```
ggsave(
  filename = here("manuscript1/output/fst/fst_hudson_matrix_man1_ordered.pdf"),
  pairfst.f,
  width = 14,
  height = 16,
  units = "in"
)
```

### 5.4.2 Fst by country for dataset

```
sampling_loc <- read.csv(
  here("scripts", "RMarkdowns", "output", "sampling_loc_all_pops_9Apr2024.csv"
    ))
```

## 5.5 Create NJ tree to use for local PCA analysis

Use Hudson Fst estimates

```
hudson_df <- readRDS(
  here(
    "manuscript1", "output", "fst", "hudson_fst_data_matrix.rds"
  )
)
```

We can estimate the Nei’s genetic distance using the formula:

D = Fst / (1 -Fst)

```
# Fill the upper triangle with the lower triangle values
hudson_df[upper.tri(hudson_df)] <- t(hudson_df)[upper.tri(hudson_df)]

# Apply Nei's genetic distance formula D = FST / (1 - FST)
nei_dist <- hudson_df / (1 - hudson_df)

# Replace any infinite values (in case FST is 1, which leads to division by zero)
nei_dist[is.infinite(nei_dist)] <- NA

# Ensure diagonal values are 0
diag(nei_dist) <- 0

# Build the NJ tree
nj_tree <- nj(nei_dist)

# Plot the NJ tree
plot(nj_tree, main="Neighbor-Joining Tree based on Hudson FST", type = "fan")
```

We can save it to use with FigTree

```
# Save the NJ tree in Newick format
write.tree(nj_tree, file =  here("manuscript1", "output", "fst", "figures", "nj_tree_hudson.newick"))

# Save the NJ tree in Nexus format
write.nexus(nj_tree, file = here("manuscript1", "output", "fst", "figures", "nj_tree_hudson.nexus"))
```

Now just using Hudson Fst (not Nei’s)

```
hudson_df <- readRDS(here("manuscript1", "output", "fst", "hudson_fst_data_matrix.rds"))

# Build the NJ tree
nj_tree <- nj(hudson_df)

# Plot the NJ tree
plot(nj_tree, main="Neighbor-Joining Tree based on Hudson FST", type = "fan")
```

Save

```
# Save the NJ tree in Newick format
write.tree(nj_tree, file =  here("manuscript1", "output", "fst", "figures", "nj_tree_hudson_Fst.newick"))

# Save the NJ tree in Nexus format
write.nexus(nj_tree, file = here("manuscript1", "output", "fst", "figures", "nj_tree_hudson_Fst.nexus"))
```

# 6. IQ tree

## 6.1 IQ-tree for Global dataset

For Global dataset (All Americas + 14 Europe + Asia)

Create a vcf with the intergenic SNPs to run iqtree Using SNP set
with MAF pruning at 1% and LD pruning at 10%

```
cd /gpfs/gibbs/pi/caccone/mkc54/albo/manuscript1

plink2 \
--allow-extra-chr \
--bfile output/snps_sets/r2_0.1_b \
--extract /gpfs/gibbs/pi/caccone/mkc54/albo/americas/output/tree/intergenic.txt \
--recode vcf \
--out output/tree/file2 \
--silent;
grep 'samples\|variants\|remaining' output/tree/file2.log
```

933 samples (61 females, 93 males, 779 ambiguous; 933 founders)
loaded from 61574 variants loaded from output/snps\_sets/r2\_0.1\_b.bim.
–extract: 14419 variants remaining. 14419 variants remaining after main
filters.

Convert it to phylip format with python script

```
python /gpfs/gibbs/pi/caccone/mkc54/albo/manuscript1/output/tree/vcf2phylip.py -i /gpfs/gibbs/pi/caccone/mkc54/albo/manuscript1/output/tree/file2.vcf
```

Check iqtree help

```
salloc -p ycga --cpus-per-task=5 -t 6:00:00 --mem-per-cpu=10000 #53734830
module load miniconda
conda activate iqtree-env  
iqtree --help
```

*could try increasing to >5 cpus/threads to speed up; or submit
as .sh*

Run iquetree

```
iqtree -s /gpfs/gibbs/pi/caccone/mkc54/albo/manuscript1/output/tree/file2.min4.phy -m MFP -bb 1000 -bnni -st DNA -nt AUTO
```

*Note: 113 sequences failed composition chi2 test (p-value<5%;
df=3)*

If it takes too long to run interactively, run on week partition

```
module load miniconda
conda env list #Path to iqtree = /home/mkc54/.conda/envs/iqtree-env
```

Create run\_iqtree.sh file:

```
#!/bin/sh        
#SBATCH --ntasks=1
#SBATCH --cpus-per-task=10         
#SBATCH --mem-per-cpu=5gb
#SBATCH --time=100:00:00
#SBATCH --partition=week
#SBATCH --job-name=iqtree_albo_man1
#SBATCH -o iqtree.%A_%a.albo.o.txt
#SBATCH -e iqtree.%A_%a.albo.ERROR.txt

# DO NOT EDIT LINE BELOW
cd /gpfs/gibbs/pi/caccone/mkc54/albo/manuscript1/output/tree

# Load conda
module load miniconda
conda activate iqtree-env
export PATH=$PATH:/home/mkc54/.conda/envs/iqtree-env

# Run IQ-TREE
iqtree2 -s /gpfs/gibbs/pi/caccone/mkc54/albo/manuscript1/output/tree/file2.min4.phy -m MFP -bb 1000 -bnni -st DNA -nt AUTO
```

CondaError: Run ‘conda init’ before ‘conda deactivate’

Run it

```
cd /gpfs/gibbs/pi/caccone/mkc54/albo/manuscript1/output/tree

sbatch run_iqtree.sh #55171235
 
squeue -u mkc54 #check it
scontrol show job 55171235
```

Then edit it in FigTree

Make a populations metadata file to color the branches

```
library(ape)       # For reading/writing trees
library(dplyr)
```

Load the tree

```
tree <- read.tree("all_pops/output/tree/file2.min4.phy.treefile") 

#Load populations file
metadata <- read.delim("all_pops/output/tree/populations.txt", stringsAsFactors = FALSE)
```

```
# Assign numeric codes to populations
metadata <- metadata %>%
  mutate(pop_code = as.numeric(factor(population)))  # PopA = 1, PopB = 2, etc.

# Ensure order matches tree tip labels
pop_vector <- metadata$pop_code[match(tree$tip.label, metadata$taxon)]
```

Create and Write the NEXUS File

```
write.nexus_with_traits <- function(tree, tip_traits, trait_name = "population", file = "tree_with_traits.nex") {
  ntax <- length(tree$tip.label)
  
  # Start writing
  cat("#NEXUS\n\n", file = file)
  
  # TAXA block
  cat("Begin taxa;\n", file = file, append = TRUE)
  cat(paste0("    Dimensions ntax=", ntax, ";\n"), file = file, append = TRUE)
  cat("    Taxlabels\n", file = file, append = TRUE)
  write(paste("        ", tree$tip.label), file = file, append = TRUE)
  cat("    ;\nEnd;\n\n", file = file, append = TRUE)
  
  # TREES block
  cat("Begin trees;\n", file = file, append = TRUE)
  cat("    Tree TREE1 = [&R]", write.tree(tree), "\n", file = file, append = TRUE)
  cat("End;\n\n", file = file, append = TRUE)
  
  # CHARACTERS block for trait
  cat("Begin characters;\n", file = file, append = TRUE)
  cat(paste0("    Dimensions ntax=", ntax, " nchar=1;\n"), file = file, append = TRUE)
  cat("    Format datatype=Standard symbols=\"0123456789\" missing=? gap=-;\n", file = file, append = TRUE)
  cat(paste0("    Charstatelabels\n        1 ", trait_name, ";\n"), file = file, append = TRUE)
  cat("    Matrix\n", file = file, append = TRUE)
  for (i in seq_along(tree$tip.label)) {
    cat(paste0("        ", tree$tip.label[i], " ", tip_traits[i], "\n"), file = file, append = TRUE)
  }
  cat("    ;\nEnd;\n", file = file, append = TRUE)
}

# Write the file
#write.nexus_with_traits(tree, pop_vector, file = "all_pops/output/tree/tree_with_population.nex")
```

Tree file appearance was further edited with FigTree

## 6.2 IQ-tree for subset of Global dataset

Create a vcf with the intergenic SNPs to run iqtree Using SNP set
with MAF pruning at 1% and LD pruning at 10%, for selected individuals
for each population in the Global dataset

```
cd /gpfs/gibbs/pi/caccone/mkc54/albo/manuscript1

plink2 \
--allow-extra-chr \
--keep output/tree/subset/subset.txt \
--bfile output/snps_sets/r2_0.1_b \
--extract /gpfs/gibbs/pi/caccone/mkc54/albo/americas/output/tree/intergenic.txt \
--recode vcf \
--out output/tree/subset/file2 \
--silent;
grep 'samples\|variants\|remaining' output/tree/subset/file2.log
```

933 samples (61 females, 93 males, 779 ambiguous; 933 founders)
loaded from 61574 variants loaded from output/snps\_sets/r2\_0.1\_b.bim.
–extract: 14419 variants remaining. –keep: 204 samples remaining. 204
samples (13 females, 14 males, 177 ambiguous; 204 founders) remaining
after 14419 variants remaining after main filters.

Convert it to phylip format with python script

```
python /gpfs/gibbs/pi/caccone/mkc54/albo/manuscript1/output/tree/vcf2phylip.py -i /gpfs/gibbs/pi/caccone/mkc54/albo/manuscript1/output/tree/subset/file2.vcf
```

Check iqtree help

```
salloc -p ycga --cpus-per-task=5 -t 6:00:00 --mem-per-cpu=10000 #53734830
module load miniconda
conda activate iqtree-env  
iqtree --help
```

*could try increasing to >5 cpus/threads to speed up; or submit
as .sh*

Run iquetree

```
iqtree -s /gpfs/gibbs/pi/caccone/mkc54/albo/manuscript1/output/tree/file2.min4.phy -m MFP -bb 1000 -bnni -st DNA -nt AUTO
```

*Note: 113 sequences failed composition chi2 test (p-value<5%;
df=3)*

If it takes too long to run interactively, run on week partition

```
module load miniconda
conda env list #Path to iqtree = /home/mkc54/.conda/envs/iqtree-env
```

Create run\_iqtree.sh file:

```
#!/bin/sh        
#SBATCH --ntasks=1
#SBATCH --cpus-per-task=10         
#SBATCH --mem-per-cpu=5gb
#SBATCH --time=100:00:00
#SBATCH --partition=week
#SBATCH --job-name=iqtree_albo_man1sub
#SBATCH -o iqtree.%A_%a.albo.o.txt
#SBATCH -e iqtree.%A_%a.albo.ERROR.txt

# DO NOT EDIT LINE BELOW
cd /gpfs/gibbs/pi/caccone/mkc54/albo/manuscript1/output/tree/subset

# Load conda
module load miniconda
conda activate iqtree-env
export PATH=$PATH:/home/mkc54/.conda/envs/iqtree-env

# Run IQ-TREE
iqtree2 -s /gpfs/gibbs/pi/caccone/mkc54/albo/manuscript1/output/tree/subset/file2.min4.phy -m MFP -bb 1000 -bnni -st DNA -nt AUTO
```

CondaError: Run ‘conda init’ before ‘conda deactivate’

Run it

```
cd /gpfs/gibbs/pi/caccone/mkc54/albo/manuscript1/output/tree/subset

sbatch run_iqtree.sh #55627393
 
squeue -u mkc54 #check it
scontrol show job 55627393
```

Then edit it in FigTree

Create and Write the NEXUS File

```
write.nexus_with_traits <- function(tree, tip_traits, trait_name = "population", file = "tree_with_traits.nex") {
  ntax <- length(tree$tip.label)
  
  # Start writing
  cat("#NEXUS\n\n", file = file)
  
  # TAXA block
  cat("Begin taxa;\n", file = file, append = TRUE)
  cat(paste0("    Dimensions ntax=", ntax, ";\n"), file = file, append = TRUE)
  cat("    Taxlabels\n", file = file, append = TRUE)
  write(paste("        ", tree$tip.label), file = file, append = TRUE)
  cat("    ;\nEnd;\n\n", file = file, append = TRUE)
  
  # TREES block
  cat("Begin trees;\n", file = file, append = TRUE)
  cat("    Tree TREE1 = [&R]", write.tree(tree), "\n", file = file, append = TRUE)
  cat("End;\n\n", file = file, append = TRUE)
  
  # CHARACTERS block for trait
  cat("Begin characters;\n", file = file, append = TRUE)
  cat(paste0("    Dimensions ntax=", ntax, " nchar=1;\n"), file = file, append = TRUE)
  cat("    Format datatype=Standard symbols=\"0123456789\" missing=? gap=-;\n", file = file, append = TRUE)
  cat(paste0("    Charstatelabels\n        1 ", trait_name, ";\n"), file = file, append = TRUE)
  cat("    Matrix\n", file = file, append = TRUE)
  for (i in seq_along(tree$tip.label)) {
    cat(paste0("        ", tree$tip.label[i], " ", tip_traits[i], "\n"), file = file, append = TRUE)
  }
  cat("    ;\nEnd;\n", file = file, append = TRUE)
}

# Write the file
write.nexus_with_traits(tree, pop_vector, file = "all_pops/output/tree/tree_with_population.nex")
```

# 7. TreeMix

```
library(tidyverse)
library(here)
library(dplyr)
library(ggplot2)
library(colorout)
library(extrafont)
library(reticulate)
#library(scales)
library(stringr)
library(grid)
library(flextable)
library(devtools)
library(readr)
library(purrr)
library(ggtext)
library(ggvenn)
library(data.table)
library(RColorBrewer)
library(ggrepel)
```

If given a set of allele frequencies from a number of populations, it
will return the maximum likelihood tree for the set of populations, and
optionally attempt to infer a number of admixture events. Note, that the
direction of introgression is often wrongly inferred and in general the
results of Treemix are more to be taken as a starting point for
investigating introgression in more detail than a final result. Treemix
assumes unlinked SNPs, so we must use input that has been pruned for
SNPs in high LD. Furthermore, treemix does not like missing data. We
will therefore remove sites with missing data.

Load treemix

```
module load TreeMix/1.13-GCC-10.2.0
module load VCFtools/0.1.16-GCCcore-10.2.0
module load PLINK/1.9b_6.21-x86_64
module load BCFtools/1.21-GCC-12.2.0
```

reeMix cannot use VCFs directly — it needs allele count summaries per
population (the .treemix.gz file), and requires a special input format.
We will use a script that generates this input file from a vcf and also
a clust file For more treemix details, see: https://speciationgenomics.github.io/Treemix/ https://ppp.readthedocs.io/en/jody/PPP\_pages/Input\_File\_Generators/vcf\_to\_treemix.html
https://www.gnxp.com/WordPress/2018/07/11/tutorial-to-run-pca-admixture-treemix-and-pairwise-fst-in-one-command/

General workflow is: -subset and prune dataset -convert plink files
to treemix format (treemix.gz) using plink2treemix.py -select root &
number of migrations (m) to test and run 5 repetitons for each m -Use
OptM to evaluate output and selection best m (number of migration
events) -Use python wrapper (treemix\_tree\_with\_bootreps\_NPS.py) to run
treemix with 100 bootstraps for best m -Use plotting\_funcs.R to plot
consensus tree, etc. -Can also visualize final max likelihood tree in
FigTree to edit appearance

We will run Treemix on two different subsets of data: 1) North
America (US & Caribbean) + native range, and 2) South America +
native range. To reduce the complexity/number of different populations,
we will group pops that are within the same cluster.

## 7.1 North American subset

### 7.1.1 Subset the populations from North America and Asia & prune

We want to group pops from same ancestry/cluster into just a few
different populations - select representative pops and group like-ones
together & remake the fam file. Then subset - we should have only 12
pop groups

```
cd /gpfs/gibbs/pi/caccone/mkc54/albo/manuscript1/output/treemix_subsets/north_america_simplified
module load PLINK/1.9b_6.21-x86_64

plink \
--keep-allele-order \
--keep-fam files/north_america_euro_native_simp.txt \
--bfile files/r2_0.1_b \
--make-bed \
--export vcf \
--out files/north_america_simp_subset_test_0.1b \
--silent
grep "samples\|variants" files/north_america_simp_subset_test_0.1b.log
```

61574 variants loaded from .bim file. Total genotyping rate in
remaining samples is 0.972158. 61574 variants and 318 people pass
filters and QC.

### 7.1.2 QC

Now need to do some QC again on the new subset before using it:

#### 7.1.2.1 Missingness

For treemix, a common threshold is max 5% missing per SNP (–geno
0.05)

```
cd /gpfs/gibbs/pi/caccone/mkc54/albo/manuscript1/output/treemix_subsets/north_america_simplified

module load PLINK/2_avx2_20221024

plink2 \
--allow-extra-chr \
--bfile files/north_america_simp_subset_test_0.1b \
--geno 0.05                `# we set genotype missiningness to 5% with this option` \
--make-bed \
--out files/file2 \
--silent \
--missing;                 # --missing produces sample-based and variant-based missing data reports. If run with --within/--family, the variant-based report is stratified by cluster.
grep "variants" files/file2.log
```

61574 variants loaded from
files/north\_america\_simp\_subset\_test\_0.1b.bim. –geno: 12674 variants
removed due to missing genotype data. 48900 variants remaining after
main filters.

Remove individuals missing more than 20% of SNPs. You can use the
threshold you want, change the flag –mind

```
cd /gpfs/gibbs/pi/caccone/mkc54/albo/manuscript1/output/treemix_subsets/north_america_simplified

plink2 \
--allow-extra-chr \
--bfile files/file2 \
--mind 0.2               `# here we set the individual missingness threshold of 20%`\
--make-bed \
--out files/file3 \
--silent;
grep "samples\|variants" files/file3.log
```

318 samples (15 females, 37 males, 266 ambiguous; 318 founders)
loaded from 48900 variants loaded from files/file2.bim. 0 samples
removed due to missing genotype data (–mind). 318 samples (15 females,
37 males, 266 ambiguous; 318 founders) remaining after

All individuals have <20% missingness

#### 7.1.2.2 Minor allele frequency

First, we estimate the allele frequency with Plink.

```
cd /gpfs/gibbs/pi/caccone/mkc54/albo/manuscript1/output/treemix_subsets/north_america_simplified/files
plink2 \
--allow-extra-chr \
--bfile file3 \
--freq \
--out MAF_check \
--silent
```

Now we apply the MAF filter for 0.01

```
# We will use MAF of 1%
plink2 \
--allow-extra-chr \
--bfile file3 \
--maf 0.01 \
--make-bed \
--out file4b \
--silent;
grep "variants" file4b.log
```

48900 variants loaded from file3.bim. 207 variants removed due to
allele frequency threshold(s) 48693 variants remaining after main
filters.

Preliminary LD pruning

```
plink2 \
--allow-extra-chr \
--bfile file4b \
--indep-pairwise 5 1 0.1 \
--out indepSNPb \
--silent;
grep 'pairwise\|variants\|samples' indepSNPb.log
```

–indep-pairwise 5 1 0.1 318 samples (15 females, 37 males, 266
ambiguous; 318 founders) loaded from 48693 variants loaded from
file4b.bim. –indep-pairwise (3 compute threads): 9060/48693 variants
removed.

#### 7.1.2.3 Heterozygosity

```
cd /gpfs/gibbs/pi/caccone/mkc54/albo/manuscript1/output/treemix_subsets/north_america_simplified/files
plink2 \
--allow-extra-chr \
--bfile file4b \
--extract indepSNPb.prune.in \
--het \
--out R_checkb \
--silent;
grep 'variants' R_checkb.log
```

48693 variants loaded from file4b.bim. –extract: 39633 variants
remaining. 39633 variants remaining after main filters.

```
#   find individuals with high heterozygosity                              ####
# import the data from Plink
het <- read.delim(
  here(
    "manuscript1/output/treemix_subsets/north_america_simplified/files/R_checkb.het"
  ),
  head = TRUE
)
#
# check head of the file
colnames(het)
```

```
## [1] "X.FID"  "IID"    "O.HOM." "E.HOM." "OBS_CT" "F"
```

Estimate heterozygosity

```
# create a column named HET_RATE and calculate the heterozygosity rate
het$HET_RATE <- (het$"OBS_CT" - het$"O.HOM") / het$"OBS_CT"
#
# use subset function to get values deviating from 4sd of the mean heterozygosity rate.
het_fail <-
  subset(
    het, (het$HET_RATE < mean(
      het$HET_RATE
    ) - 4 * sd(
      het$HET_RATE
    )) |
      (het$HET_RATE > mean(
        het$HET_RATE
      ) + 4 * sd(
        het$HET_RATE
      ))
  )
#
# get the list of individuals that failed our threshold of 4sd from the mean.
het_fail$HET_DST <-
  (het_fail$HET_RATE - mean(
    het$HET_RATE
  )) / sd(
    het$HET_RATE
  )
```

Save the files to use with Plink

```
#   save the data to use with Plink2                                        ####
#
write.table(
  het_fail,
  here(
    "manuscript1/output/treemix_subsets/north_america_simplified/files/fail-het-qc_b.txt"
  ),
  row.names = FALSE
)
```

Make plot

```
source(here("my_theme.R"))

#   make a heterozygosity plot                                              ####
#
ggplot(
  het,
  aes(
    HET_RATE
  )
) +
  geom_histogram(
    colour           = "black",
    fill             = "lightgray",
    bins             = 40
  ) +
  labs(
    x                = "Heterozygosity Rate",
    y                = "Number of Individuals"
  ) +
  geom_vline(
    aes(
      xintercept     = mean(
        HET_RATE
      )
    ),
    col              = "red",
  ) +
  geom_vline(
    aes(
      xintercept     = mean(
        HET_RATE
      ) + 4 * sd(
        HET_RATE
      )
    ),
    col              = "#BFB9B9",
  ) +
  geom_vline(
    aes(
      xintercept     = mean(
        HET_RATE
      ) - 4 * sd(
        HET_RATE
      )
    ),
    col              = "#BFB9B9",
  ) + 
  my_theme() +
  scale_y_continuous(
  )
```

The red line in the plot above indicates the mean, and the gray lines
indicate 4 standard deviation from the mean. We can see that some
mosquitoes do have excess heterozygous sites. We will remove them. We
can get their ID from the file “fail-het-qc.txt”. We can use the bash
script below to parse the file to use with Plink

```
cd /gpfs/gibbs/pi/caccone/mkc54/albo/manuscript1/output/treemix_subsets/north_america_simplified/files
sed 's/"// g' fail-het-qc_b.txt | awk '{print$1, $2}'> het_fail_ind_b.txt;
echo 'How many mosquitoes we need to remove from our data set:';
cat het_fail_ind_b.txt | tail -n +2 | wc -l;
echo 'Which mosquitoes we have to remove:';
tail -n +2 het_fail_ind_b.txt
```

```
## How many mosquitoes we need to remove from our data set:
## 0
## Which mosquitoes we have to remove:
```

```
cd /gpfs/gibbs/pi/caccone/mkc54/albo/manuscript1/output/treemix_subsets/north_america_simplified/files

plink2 \
--allow-extra-chr \
--bfile file4b \
--remove het_fail_ind_b.txt \
--make-bed \
--out file5b \
--silent;
grep 'variants\|samples' file5b.log
```

318 samples (15 females, 37 males, 266 ambiguous; 318 founders)
loaded from 48693 variants loaded from file4b.bim. –remove: 318 samples
remaining. 318 samples (15 females, 37 males, 266 ambiguous; 318
founders) remaining after

0 removed for heterozygosity

#### 7.1.2.4 LD Pruning r2 for datasets with MAF 1%

```
cd /gpfs/gibbs/pi/caccone/mkc54/albo/manuscript1/output/treemix_subsets/north_america_simplified/files

plink2 \
--allow-extra-chr \
--bfile file5b \
--indep-pairwise 5 1 0.1 \
--out indepSNP_chrb \
--silent;
grep 'pairwise\|variants\|samples' indepSNP_chrb.log
```

–indep-pairwise 5 1 0.1 318 samples (15 females, 37 males, 266
ambiguous; 318 founders) loaded from 48693 variants loaded from
file5b.bim. –indep-pairwise (3 compute threads): 9060/48693 variants
removed.

We can use the SNP set the LD pruning we just did and extract
them

```
cd /gpfs/gibbs/pi/caccone/mkc54/albo/manuscript1/output/treemix_subsets/north_america_simplified

plink2 \
--keep-allele-order \
--bfile files/file5b \
--make-bed \
--export vcf \
--out simplified_test/r2_0.1_b_north_america_subset_simplified \
--extract files/indepSNP_chrb.prune.in \
--silent
grep "variants\|samples" simplified_test/r2_0.1_b_north_america_subset_simplified.log
```

318 samples (15 females, 37 males, 266 ambiguous; 318 founders)
loaded from 48693 variants loaded from files/file5b.bim. –extract: 39633
variants remaining. 39633 variants remaining after main filters.

Ensure only biallelic SNPs

```
module load PLINK/1.9b_6.21-x86_64

cd /gpfs/gibbs/pi/caccone/mkc54/albo/manuscript1/output/treemix_subsets/north_america_simplified/simplified_test

plink \
--bfile r2_0.1_b_north_america_subset_simplified \
--biallelic-only strict \
--make-bed \
--out north_america_native_bial \
--silent;
grep 'pairwise\|variants\|samples' north_america_native_bial.log
```

39633 variants loaded from .bim file. 39633 variants and 318 people
pass filters and QC.

All are biallelic

### 7.1.3. Create files to use in treemix

We already have the .bed, .bim and .fam files, but we need to create
the cluster file

#### 7.1.3.1 create clust file

```
module load BCFtools/1.21-GCC-12.2.0
cd /gpfs/gibbs/pi/caccone/mkc54/albo/manuscript1/output/treemix_subsets/north_america_simplified/simplified_test

bcftools query -l r2_0.1_b_north_america_subset_simplified.vcf > samples.txt
#awk '{ split($1,a,"_"); print $1, a[1] }' samples.txt > man1.clust
awk '{ split($1,a,"_"); print a[2], a[1] }' samples.txt > man1_north.clust
```

Check it

```
cd /gpfs/gibbs/pi/caccone/mkc54/albo/manuscript1/output/treemix_subsets/north_america_simplified/simplified_test
head man1_north.clust
wc -l man1_north.clust
```

```
## 1001 OKI
## 1002 OKI
## 1003 OKI
## 1004 OKI
## 1005 OKI
## 1006 OKI
## 1007 OKI
## 1008 OKI
## 1009 OKI
## 1011 OKI
## 318 man1_north.clust
```

Need to add a 3rd column (with populations, not just family names).
Edit and upload, then check it

```
cd /gpfs/gibbs/pi/caccone/mkc54/albo/manuscript1/output/treemix_subsets/north_america_simplified/simplified_test
head man1_north_plink.txt
wc -l man1_north_plink.txt
```

```
## OKI  1001    Okinawa_Taiwan
## OKI  1002    Okinawa_Taiwan
## OKI  1003    Okinawa_Taiwan
## OKI  1004    Okinawa_Taiwan
## OKI  1005    Okinawa_Taiwan
## OKI  1006    Okinawa_Taiwan
## OKI  1007    Okinawa_Taiwan
## OKI  1008    Okinawa_Taiwan
## OKI  1009    Okinawa_Taiwan
## OKI  1011    Okinawa_Taiwan
## 317 man1_north_plink.txt
```

It must be in the same format as the fam file though Check fam file’s
current format

```
cd /gpfs/gibbs/pi/caccone/mkc54/albo/manuscript1/output/treemix_subsets/north_america_simplified/simplified_test
cut -d' ' -f1,2 r2_0.1_b_north_america_subset_simplified.fam | head
```

Need to re-arrange order of 1st 2 columns of clust file so its FID
IID POP Edit or upload the correctly formatted file

```
cd /gpfs/gibbs/pi/caccone/mkc54/albo/manuscript1/output/treemix_subsets/north_america_simplified/simplified_test
head man1_north_plink.txt
wc -l man1_north_plink.txt
```

```
## OKI  1001    Okinawa_Taiwan
## OKI  1002    Okinawa_Taiwan
## OKI  1003    Okinawa_Taiwan
## OKI  1004    Okinawa_Taiwan
## OKI  1005    Okinawa_Taiwan
## OKI  1006    Okinawa_Taiwan
## OKI  1007    Okinawa_Taiwan
## OKI  1008    Okinawa_Taiwan
## OKI  1009    Okinawa_Taiwan
## OKI  1011    Okinawa_Taiwan
## 317 man1_north_plink.txt
```

Ok now.

#### 7.1.3.2 convert to treemix format

Load conda environment with python, pandas and numpy

```
cd /gpfs/gibbs/pi/caccone/mkc54/albo/manuscript1/output/treemix_subsets/north_america_simplified/simplified_test
module load miniconda
conda activate treemix
module load TreeMix/1.13-GCC-10.2.0
```

```
#verify it is working
python --version #Python 3.10.18 
python -c "import pandas as pd; print(pd.__version__)" #2.3.3
```

Add the following .sh file to the directory

```
#!/bin/bash
# Script to convert PLINK files to TreeMix format
# Modified from Joana Meier's version by ChatGPT
# Requires: plink, plink2treemix.py (https://github.com/thomnelson/tools)
# Usage: ./vcf2treemix.sh <plink_prefix> <clust_file>

if [ $# -ne 2 ]; then
    echo "Usage: $0 <plink_prefix> <clust_file>"
    echo "Example: ./plink2treemix.sh r2_0.1_b_north_america_subset_simplified.clust"
    exit 1
fi

file=$1
clust=$2

# Check if required files exist
if [ ! -f "${file}.bed" ] || [ ! -f "${file}.bim" ] || [ ! -f "${file}.fam" ]; then
    echo "Error: Could not find PLINK files (.bed, .bim, .fam) with prefix '${file}'"
    exit 1
fi

if [ ! -f "$clust" ]; then
    echo "Error: Cluster file '$clust' not found"
    exit 1
fi

echo "=== Converting PLINK files to TreeMix format ==="
echo "Input PLINK prefix: $file"
echo "Cluster file: $clust"
echo "-----------------------------------------------"

# Step 1: Calculate population-stratified allele frequencies
plink --bfile $file \
      --freq --missing \
      --within $clust \
      --out $file \
      --allow-no-sex --allow-extra-chr 0

if [ ! -f "${file}.frq.strat" ]; then
    echo "Error: PLINK failed to produce ${file}.frq.strat"
    exit 1
fi

# Step 2: Compress the stratified frequency file
gzip -f ${file}.frq.strat

# Step 3: Convert to TreeMix input format using plink2treemix.py
echo "Running plink2treemix.py ..."
python plink2treemix.py ${file}.frq.strat.gz ${file}.treemix.frq.gz

if [ ! -s "${file}.treemix.frq.gz" ]; then
    echo "Error: TreeMix conversion failed or produced empty output."
    exit 1
fi

# Step 4: Done!
echo "Conversion complete!"
echo "Output file: ${file}.treemix.frq.gz"
echo "You can now run TreeMix using:"
echo "treemix -i ${file}.treemix.frq.gz -o ${file}_treemix_output"
```

Start interactive session and run plink2treemix.py

```
cd /gpfs/gibbs/pi/caccone/mkc54/albo/manuscript1/output/treemix_subsets/north_america_simplified/simplified_test

salloc -p scavenge --cpus-per-task=5 -t 2:00:00 --mem-per-cpu=10000
module load PLINK/1.9b_6.21-x86_64

#sed -i 's/\r$//' plink2treemix.sh #fix the .sh file if needed
chmod +x plink2treemix.sh
./plink2treemix.sh r2_0.1_b_north_america_subset_simplified man1_north_plink.txt
```

SNPs in file: 39633 Populations in input (n = 11): Athens\_Greece
California Eastern\_China Italy Japan Northeast\_US Okinawa\_Taiwan Russia
Southeast\_Asia Sulawesi\_Indonesia Texas

SNPs written: 39633

✅ Conversion complete! Output:
r2\_0.1\_b\_north\_america\_subset\_simplified.treemix.frq.gz You can now run
TreeMix using: treemix -i
r2\_0.1\_b\_north\_america\_subset\_simplified.treemix.frq.gz -o
r2\_0.1\_b\_north\_america\_subset\_simplified\_treemix\_output

Check it

```
cd /gpfs/gibbs/pi/caccone/mkc54/albo/manuscript1/output/treemix_subsets/north_america_simplified/simplified_test

zcat r2_0.1_b_north_america_subset_simplified.treemix.frq.gz | head
```

```
## Athens_Greece California Eastern_China Italy Japan Northeast_US Okinawa_Taiwan Russia Southeast_Asia Sulawesi_Indonesia Texas
## 20,2 29,7 44,4 84,4 58,12 108,4 33,3 46,2 38,18 17,7 96,0
## 16,6 26,10 45,3 46,38 66,4 77,35 36,0 46,2 54,2 24,0 75,21
## 19,3 25,11 31,17 80,6 25,43 78,34 26,10 24,18 42,10 14,10 63,27
## 6,16 26,10 32,16 43,39 25,43 58,50 23,13 33,15 38,18 17,5 45,45
## 11,11 13,19 10,38 60,28 55,15 90,22 19,17 18,30 9,47 2,22 80,16
## 22,0 36,0 47,1 88,0 70,0 112,0 36,0 48,0 51,5 14,6 94,2
## 9,11 8,28 34,14 41,43 50,20 71,41 27,9 15,33 24,32 6,18 46,50
## 15,7 36,0 43,5 85,3 67,3 109,3 36,0 48,0 35,21 9,13 91,5
## 13,9 27,9 20,28 69,13 43,21 90,20 29,7 36,12 44,10 24,0 61,29
```

### 7.1.4 Run tree mix

The treemix manual is here: https://software.cqls.oregonstate.edu/updates/docs/treemix/treemix\_manual\_10\_1\_2012.pdf

#### 7.1.4.1 Run repetitions to find best number of migrations

We will run tree mix with 5 reptitions (for evaluating best number of
edges/migration events later) We will also add a root - based on initial
run and previous structure analyses, we will use the most differentiated
pop as the outgroup (Indonesia)

Create the dsq files

```
cd /gpfs/gibbs/pi/caccone/mkc54/albo/manuscript1/output/treemix_subsets/north_america_simplified/simplified_test
module load dSQ/1.05 
module load TreeMix/1.13-GCC-10.2.0

# set variables
input="r2_0.1_b_north_america_subset_simplified.treemix.frq.gz"
stem="nasub_simp"   # base prefix for outputs
outdir="prelim"

for m in {0..5}; do
  for r in {1..5}; do
    echo "treemix -i $input -o ${outdir}/${stem}.m${m}.rep${r} -m $m -root Sulawesi_Indonesia -k 500 -seed $RANDOM"
  done
done > dsq_treemix_nasub.txt


head dsq_treemix_nasub.txt #check file

#create the .sh file
dsq --job-file dsq_treemix_nasub.txt --output /dev/null --mail-type ALL -t 24:00:00 --partition=ycga --mem-per-cpu=10G --cpus-per-task=10 --job-name fS.dsq_treemix_nasub --batch-file dsq_treemix_nasub.sh

#check the file
head dsq_treemix_nasub.sh
```

Run it

```
sbatch dsq_treemix_nasub.sh #63101810
dsqa -j 63101810
```

```
#autopsy
dsqa -j 63101810 -f dsq_treemix_nasub.txt -s FAILED > rerun_jobs.txt; wc -l rerun_jobs.txt
```

COMPLETED 30 0-29  
0 rerun\_jobs.txt

Plot treemix results in R

```
#install.packages("OptM", repos="https://cloud.r-project.org")
library(OptM)
library(RColorBrewer)
library(R.utils)
```

```
## Loading required package: R.oo
```

```
## Loading required package: R.methodsS3
```

```
## R.methodsS3 v1.8.2 (2022-06-13 22:00:14 UTC) successfully loaded. See ?R.methodsS3 for help.
```

```
## R.oo v1.26.0 (2024-01-24 05:12:50 UTC) successfully loaded. See ?R.oo for help.
```

```
## 
## Attaching package: 'R.oo'
```

```
## The following object is masked from 'package:R.methodsS3':
## 
##     throw
```

```
## The following objects are masked from 'package:devtools':
## 
##     check, unload
```

```
## The following objects are masked from 'package:methods':
## 
##     getClasses, getMethods
```

```
## The following objects are masked from 'package:base':
## 
##     attach, detach, load, save
```

```
## R.utils v2.12.3 (2023-11-18 01:00:02 UTC) successfully loaded. See ?R.utils for help.
```

```
## 
## Attaching package: 'R.utils'
```

```
## The following object is masked from 'package:tidyr':
## 
##     extract
```

```
## The following object is masked from 'package:utils':
## 
##     timestamp
```

```
## The following objects are masked from 'package:base':
## 
##     cat, commandArgs, getOption, isOpen, nullfile, parse, use, warnings
```

```
library(ggplot2)
```

#### 7.1.4.2 Plot using OptM

TreeMix produces the following output files per run:

.trees — inferred tree in Newick format .cov.gz —
covariance matrix .modelcov.gz — model-predicted covariance
.llik — log-likelihood value .vertices.gz, .edges.gz —
graph and migration edge data

Since we ran multiple m values and replicates, we want to: Evaluate
model fit (choose best m) Visualize trees and migration edges Check
residuals (how well the model fits population relationships)

Get plotting functions here - https://bitbucket.org/nygcresearch/treemix/src/master/src/plotting\_funcs.R
Alternately - https://github.com/carolindahms/TreeMix/blob/main/Step2%264\_TreeMix.R
Upload them to your directory on the cluster

Set source

```
# Load TreeMix plotting functions
source("/gpfs/gibbs/pi/caccone/mkc54/albo/manuscript1/output/treemix/north_america/plotting_funcs_fixed.R")  # Adjust this path as needed
```

Use OptM to choose best (need to have run multiple iterations for
each m)

```
#Analyzing the treemix results using the Evanno method
setwd("/gpfs/gibbs/pi/caccone/mkc54/albo/manuscript1/output/treemix_subsets/north_america_simplified/simplified_test/prelim")
optm_results <- optM(prefix = "nasub_simp", folder = ".", method = "Evanno")
#The maximum value for delta m was 1.4611 at m = 1 edges.
```

OptM throws and ‘error’: Error in optM(prefix = “nasub\_simp”, folder
= “.”, method = “Evanno”) : SD is zero for all runs! Check your
analysis. But this does not necessarily indicate an error in your
pipeline — it means your dataset is so strongly constrained that TreeMix
is deterministic (e.g. no variation among runs)

Run OptM using a method that does NOT require variance

```
setwd("/gpfs/gibbs/pi/caccone/mkc54/albo/manuscript1/output/treemix_subsets/north_america_simplified/simplified_test/prelim")
optM(prefix="nasub_simp", folder="/gpfs/gibbs/pi/caccone/mkc54/albo/manuscript1/output/treemix_subsets/north_america_simplified/simplified_test/prelim", method="linear")
```

```
## Finished reading .llik, .modelcov.goz, and .cov.gz files.
```

```
## No output file will be saved. To save an output file, run with 'tsv = "file.tsv"'
```

```
## All migration edges will be included.
```

```
## 'thresh' has been set to the default value of 0.05
```

```
## m ranges between 0 and 5.
```

```
## The average number of iterations per m (not including m = 0) was 5.
```

```
## Make sure these values are correct...
```

```
## Analyzing the treemix results using various linear models.
```

```
## Fitting piecewise linear model...
```

```
## Fitting bent cable model...
```

```
## Fitting a simple exponential model...
```

```
## Fitting a NLS model...
```

```
## 125555.4    (1.22e+01): par = (0 1)
## 17525.38    (4.88e+00): par = (-0.4051952 0.8796333)
## 2838.951    (3.38e+00): par = (0.1333185 0.56681)
## 446.2012    (5.05e+00): par = (1.878425 -0.0005279255)
## 47.17707    (9.33e-01): par = (1.673465 -0.1194177)
## 26.93544    (5.60e-02): par = (1.648545 -0.1954888)
## 26.85546    (4.59e-03): par = (1.64384 -0.2000602)
## 26.85499    (7.15e-04): par = (1.64351 -0.1994113)
## 26.85497    (1.11e-04): par = (1.643556 -0.1995124)
## 26.85497    (1.72e-05): par = (1.643548 -0.1994967)
## 26.85497    (2.65e-06): par = (1.64355 -0.1994991)
```

```
## Done fitting a NLS model...
```

```
## $out
##                       df       AIC delta.aic ChangePoints
## SimpleExponential      3  99.87764   0.00000     3.000000
## NonLinearLeastSquares  3 128.03901  28.16138     3.000000
## BentCable              6 215.69036 115.81272     3.282828
## PiecewiseLinear        5 224.74001 124.86237     2.752833
## 
## $PiecewiseLinear
## [1] "Threshold alpha: 2.75283259001759"
## [1] ""
## [1] "Model coefficients: Beta[0], Beta[1], Beta[2]"
## (Intercept)           x           w 
##   302.26647    42.62792   -25.42092 
##       Change.Point Initial.Slope Slope.Change Second.Slope
## 2.5%      2.561609      41.90168    -27.59003     16.43932
## 97.5%     2.860257      44.62001    -24.50353     17.96328
## 
## $BentCable
## $log.likelihood
##             [,1]      [,2]      [,3]      [,4]      [,5]      [,6]      [,7]
##   [1,] -179.9639 -179.9639 -179.9639 -179.9639 -179.9639 -179.9639 -179.9639
##   [2,] -179.9639 -179.9639 -179.9639 -179.9639 -179.9639 -179.9639 -179.9639
##   [3,] -179.9639 -179.9639 -179.9639 -179.9639 -179.9639 -179.9639 -179.9639
##   [4,] -179.9639 -179.9639 -179.9639 -179.9639 -179.9639 -179.9639 -179.9639
##   [5,] -179.9639 -179.9639 -179.9639 -179.9639 -179.9639 -179.9639 -179.9639
##   [6,] -179.9639 -179.9639 -179.9639 -179.9639 -179.9639 -179.9639 -179.9639
##   [7,] -179.9639 -179.9639 -179.9639 -179.9639 -179.9639 -179.9639 -179.9639
##   [8,] -179.9639 -179.9639 -179.9639 -179.9639 -179.9639 -179.9639 -179.9639
##   [9,] -179.9639 -179.9639 -179.9639 -179.9639 -179.9639 -179.9639 -179.9639
##  [10,] -179.9639 -179.9639 -179.9639 -179.9639 -179.9639 -179.9639 -179.9639
##  [11,] -179.9639 -179.9639 -179.9639 -179.9639 -179.9639 -179.9639 -179.9639
##  [12,] -179.9639 -179.9639 -179.9639 -179.9639 -179.9639 -179.9639 -179.9639
##  [13,] -179.9639 -179.9639 -179.9639 -179.9639 -179.9639 -179.9639 -179.9639
##  [14,] -179.9639 -179.9639 -179.9639 -179.9639 -179.9639 -179.9639 -179.9639
##  [15,] -179.9639 -179.9639 -179.9639 -179.9639 -179.9639 -179.9639 -179.9639
##  [16,] -179.9639 -179.9639 -179.9639 -179.9639 -179.9639 -179.9639 -179.9639
##  [17,] -179.9639 -179.9639 -179.9639 -179.9639 -179.9639 -179.9639 -179.9639
##  [18,] -179.9639 -179.9639 -179.9639 -179.9639 -179.9639 -179.9576 -179.8806
##  [19,] -179.9639 -179.9639 -179.9639 -179.9538 -179.8504 -179.6786 -179.4663
##  [20,] -179.9639 -179.9426 -179.7790 -179.5503 -179.2935 -179.0207 -178.7372
##  [21,] -179.4171 -179.1579 -178.8802 -178.5952 -178.3053 -178.0112 -177.7132
##  [22,] -177.2873 -177.2873 -177.2551 -177.1059 -176.8980 -176.6594 -176.4020
##  [23,] -175.0861 -175.0861 -175.0861 -175.0861 -175.0676 -174.9650 -174.8053
##  [24,] -172.9365 -172.9365 -172.9365 -172.9365 -172.9365 -172.9365 -172.9241
##  [25,] -170.8630 -170.8630 -170.8630 -170.8630 -170.8630 -170.8630 -170.8630
##  [26,] -168.8898 -168.8898 -168.8898 -168.8898 -168.8898 -168.8898 -168.8898
##  [27,] -167.0408 -167.0408 -167.0408 -167.0408 -167.0408 -167.0408 -167.0408
##  [28,] -165.3378 -165.3378 -165.3378 -165.3378 -165.3378 -165.3378 -165.3378
##  [29,] -163.8001 -163.8001 -163.8001 -163.8001 -163.8001 -163.8001 -163.8001
##  [30,] -162.4433 -162.4433 -162.4433 -162.4433 -162.4433 -162.4433 -162.4433
##  [31,] -161.2786 -161.2786 -161.2786 -161.2786 -161.2786 -161.2786 -161.2786
##  [32,] -160.3123 -160.3123 -160.3123 -160.3123 -160.3123 -160.3123 -160.3123
##  [33,] -159.5454 -159.5454 -159.5454 -159.5454 -159.5454 -159.5454 -159.5454
##  [34,] -158.9742 -158.9742 -158.9742 -158.9742 -158.9742 -158.9742 -158.9742
##  [35,] -158.5903 -158.5903 -158.5903 -158.5903 -158.5903 -158.5903 -158.5903
##  [36,] -158.3821 -158.3821 -158.3821 -158.3821 -158.3821 -158.3821 -158.3821
##  [37,] -158.3346 -158.3346 -158.3346 -158.3346 -158.3346 -158.3346 -158.3346
##  [38,] -158.4315 -158.4315 -158.4315 -158.4315 -158.4315 -158.3930 -158.2505
##  [39,] -158.6555 -158.6555 -158.6555 -158.5945 -158.3960 -158.1231 -157.8054
##  [40,] -158.9890 -158.8615 -158.5491 -158.1819 -157.7892 -157.3807 -156.9606
##  [41,] -158.0651 -157.7790 -157.3961 -156.9845 -156.5574 -156.1193 -155.6720
##  [42,] -155.0612 -155.0612 -155.0566 -154.9007 -154.6181 -154.2691 -153.8794
##  [43,] -151.8406 -151.8406 -151.8406 -151.8406 -151.8382 -151.7264 -151.4979
##  [44,] -148.4087 -148.4087 -148.4087 -148.4087 -148.4087 -148.4087 -148.4073
##  [45,] -144.7587 -144.7587 -144.7587 -144.7587 -144.7587 -144.7587 -144.7587
##  [46,] -140.8889 -140.8889 -140.8889 -140.8889 -140.8889 -140.8889 -140.8889
##  [47,] -136.8068 -136.8068 -136.8068 -136.8068 -136.8068 -136.8068 -136.8068
##  [48,] -132.5353 -132.5353 -132.5353 -132.5353 -132.5353 -132.5353 -132.5353
##  [49,] -128.1219 -128.1219 -128.1219 -128.1219 -128.1219 -128.1219 -128.1219
##  [50,] -123.6509 -123.6509 -123.6509 -123.6509 -123.6509 -123.6509 -123.6509
##  [51,] -119.2600 -119.2600 -119.2600 -119.2600 -119.2600 -119.2600 -119.2600
##  [52,] -115.1558 -115.1558 -115.1558 -115.1558 -115.1558 -115.1558 -115.1558
##  [53,] -111.6187 -111.6187 -111.6187 -111.6187 -111.6187 -111.6187 -111.6187
##  [54,] -108.9803 -108.9803 -108.9803 -108.9803 -108.9803 -108.9803 -108.9803
##  [55,] -107.5566 -107.5566 -107.5566 -107.5566 -107.5566 -107.5566 -107.5566
##  [56,] -107.5479 -107.5479 -107.5479 -107.5479 -107.5479 -107.5479 -107.5479
##  [57,] -108.9584 -108.9584 -108.9584 -108.9584 -108.9584 -108.9584 -108.9573
##  [58,] -111.5942 -111.5942 -111.5942 -111.5942 -111.5917 -111.4744 -111.2373
##  [59,] -115.1420 -115.1420 -115.1362 -114.9395 -114.5869 -114.1587 -113.6898
##  [60,] -119.2469 -118.8429 -118.3090 -117.7440 -117.1681 -116.5885 -116.0090
##  [61,] -120.8499 -120.6678 -120.2249 -119.7110 -119.1699 -118.6167 -118.0582
##  [62,] -120.9393 -120.9393 -120.9393 -120.8568 -120.5900 -120.2271 -119.8108
##  [63,] -121.5429 -121.5429 -121.5429 -121.5429 -121.5429 -121.4949 -121.3183
##  [64,] -122.7073 -122.7073 -122.7073 -122.7073 -122.7073 -122.7073 -122.7073
##  [65,] -124.4503 -124.4503 -124.4503 -124.4503 -124.4503 -124.4503 -124.4503
##  [66,] -126.7581 -126.7581 -126.7581 -126.7581 -126.7581 -126.7581 -126.7581
##  [67,] -129.5864 -129.5864 -129.5864 -129.5864 -129.5864 -129.5864 -129.5864
##  [68,] -132.8682 -132.8682 -132.8682 -132.8682 -132.8682 -132.8682 -132.8682
##  [69,] -136.5225 -136.5225 -136.5225 -136.5225 -136.5225 -136.5225 -136.5225
##  [70,] -140.4639 -140.4639 -140.4639 -140.4639 -140.4639 -140.4639 -140.4639
##  [71,] -144.6104 -144.6104 -144.6104 -144.6104 -144.6104 -144.6104 -144.6104
##  [72,] -148.8874 -148.8874 -148.8874 -148.8874 -148.8874 -148.8874 -148.8874
##  [73,] -153.2304 -153.2304 -153.2304 -153.2304 -153.2304 -153.2304 -153.2304
##  [74,] -157.5848 -157.5848 -157.5848 -157.5848 -157.5848 -157.5848 -157.5848
##  [75,] -161.9055 -161.9055 -161.9055 -161.9055 -161.9055 -161.9055 -161.9055
##  [76,] -166.1553 -166.1553 -166.1553 -166.1553 -166.1553 -166.1553 -166.1553
##  [77,] -170.3032 -170.3032 -170.3032 -170.3032 -170.3032 -170.3032 -170.2791
##  [78,] -174.3236 -174.3236 -174.3236 -174.3236 -174.2901 -174.1038 -173.8128
##  [79,] -178.1948 -178.1948 -178.1398 -177.8842 -177.5266 -177.1137 -176.6653
##  [80,] -181.7374 -181.3160 -180.8617 -180.3923 -179.9114 -179.4200 -178.9183
##  [81,] -182.6176 -182.5836 -182.3212 -181.9529 -181.5368 -181.0920 -180.6266
##  [82,] -182.6176 -182.6176 -182.6176 -182.6015 -182.4358 -182.1597 -181.8171
##  [83,] -182.6176 -182.6176 -182.6176 -182.6176 -182.6176 -182.6074 -182.4842
##  [84,] -182.6176 -182.6176 -182.6176 -182.6176 -182.6176 -182.6176 -182.6176
##  [85,] -182.6176 -182.6176 -182.6176 -182.6176 -182.6176 -182.6176 -182.6176
##  [86,] -182.6176 -182.6176 -182.6176 -182.6176 -182.6176 -182.6176 -182.6176
##  [87,] -182.6176 -182.6176 -182.6176 -182.6176 -182.6176 -182.6176 -182.6176
##  [88,] -182.6176 -182.6176 -182.6176 -182.6176 -182.6176 -182.6176 -182.6176
##  [89,] -182.6176 -182.6176 -182.6176 -182.6176 -182.6176 -182.6176 -182.6176
##  [90,] -182.6176 -182.6176 -182.6176 -182.6176 -182.6176 -182.6176 -182.6176
##  [91,] -182.6176 -182.6176 -182.6176 -182.6176 -182.6176 -182.6176 -182.6176
##  [92,] -182.6176 -182.6176 -182.6176 -182.6176 -182.6176 -182.6176 -182.6176
##  [93,] -182.6176 -182.6176 -182.6176 -182.6176 -182.6176 -182.6176 -182.6176
##  [94,] -182.6176 -182.6176 -182.6176 -182.6176 -182.6176 -182.6176 -182.6176
##  [95,] -182.6176 -182.6176 -182.6176 -182.6176 -182.6176 -182.6176 -182.6176
##  [96,] -182.6176 -182.6176 -182.6176 -182.6176 -182.6176 -182.6176 -182.6176
##  [97,] -182.6176 -182.6176 -182.6176 -182.6176 -182.6176 -182.6176 -182.6176
##  [98,] -182.6176 -182.6176 -182.6176 -182.6176 -182.6176 -182.6176 -182.6176
##  [99,] -182.6176 -182.6176 -182.6176 -182.6176 -182.6176 -182.6176 -182.6176
## [100,] -182.6176 -182.6176 -182.6176 -182.6176 -182.6176 -182.6176 -182.6176
##             [,8]      [,9]     [,10]     [,11]     [,12]     [,13]     [,14]
##   [1,] -179.9639 -179.9639 -179.9639 -179.9639 -179.9639 -179.9639 -179.9639
##   [2,] -179.9639 -179.9639 -179.9639 -179.9639 -179.9639 -179.9639 -179.9639
##   [3,] -179.9639 -179.9639 -179.9639 -179.9639 -179.9639 -179.9639 -179.9639
##   [4,] -179.9639 -179.9639 -179.9639 -179.9639 -179.9639 -179.9639 -179.9639
##   [5,] -179.9639 -179.9639 -179.9639 -179.9639 -179.9639 -179.9639 -179.9639
##   [6,] -179.9639 -179.9639 -179.9639 -179.9639 -179.9639 -179.9639 -179.9639
##   [7,] -179.9639 -179.9639 -179.9639 -179.9639 -179.9639 -179.9639 -179.9639
##   [8,] -179.9639 -179.9639 -179.9639 -179.9639 -179.9639 -179.9639 -179.9639
##   [9,] -179.9639 -179.9639 -179.9639 -179.9639 -179.9639 -179.9639 -179.9639
##  [10,] -179.9639 -179.9639 -179.9639 -179.9639 -179.9639 -179.9639 -179.9639
##  [11,] -179.9639 -179.9639 -179.9639 -179.9639 -179.9639 -179.9639 -179.9639
##  [12,] -179.9639 -179.9639 -179.9639 -179.9639 -179.9639 -179.9639 -179.9639
##  [13,] -179.9639 -179.9639 -179.9639 -179.9639 -179.9639 -179.9639 -179.9639
##  [14,] -179.9639 -179.9639 -179.9639 -179.9639 -179.9639 -179.9639 -179.9618
##  [15,] -179.9639 -179.9639 -179.9639 -179.9639 -179.9613 -179.9143 -179.8177
##  [16,] -179.9639 -179.9639 -179.9605 -179.9074 -179.8018 -179.6552 -179.4754
##  [17,] -179.9594 -179.8972 -179.7783 -179.6174 -179.4240 -179.2047 -178.9641
##  [18,] -179.7415 -179.5599 -179.3474 -179.1113 -178.8565 -178.5861 -178.3027
##  [19,] -179.2274 -178.9693 -178.6965 -178.4120 -178.1176 -177.8146 -177.5038
##  [20,] -178.4456 -178.1472 -177.8430 -177.5335 -177.2190 -176.8998 -176.5761
##  [21,] -177.4114 -177.1059 -176.7969 -176.4843 -176.1681 -175.8485 -175.5254
##  [22,] -176.1316 -175.8516 -175.5639 -175.2698 -174.9702 -174.6656 -174.3565
##  [23,] -174.6091 -174.3878 -174.1482 -173.8945 -173.6298 -173.3559 -173.0744
##  [24,] -172.8468 -172.7188 -172.5547 -172.3640 -172.1529 -171.9257 -171.6855
##  [25,] -170.8630 -170.8540 -170.7931 -170.6878 -170.5490 -170.3842 -170.1988
##  [26,] -168.8898 -168.8898 -168.8898 -168.8832 -168.8341 -168.7465 -168.6285
##  [27,] -167.0408 -167.0408 -167.0408 -167.0408 -167.0408 -167.0358 -166.9959
##  [28,] -165.3378 -165.3378 -165.3378 -165.3378 -165.3378 -165.3378 -165.3378
##  [29,] -163.8001 -163.8001 -163.8001 -163.8001 -163.8001 -163.8001 -163.8001
##  [30,] -162.4433 -162.4433 -162.4433 -162.4433 -162.4433 -162.4433 -162.4433
##  [31,] -161.2786 -161.2786 -161.2786 -161.2786 -161.2786 -161.2786 -161.2786
##  [32,] -160.3123 -160.3123 -160.3123 -160.3123 -160.3123 -160.3123 -160.3123
##  [33,] -159.5454 -159.5454 -159.5454 -159.5454 -159.5454 -159.5454 -159.5454
##  [34,] -158.9742 -158.9742 -158.9742 -158.9742 -158.9742 -158.9742 -158.9623
##  [35,] -158.5903 -158.5903 -158.5903 -158.5903 -158.5750 -158.5046 -158.3880
##  [36,] -158.3821 -158.3821 -158.3619 -158.2750 -158.1349 -157.9531 -157.7380
##  [37,] -158.3074 -158.1982 -158.0277 -157.8119 -157.5610 -157.2821 -156.9803
##  [38,] -158.0386 -157.7791 -157.4848 -157.1638 -156.8215 -156.4615 -156.0864
##  [39,] -157.4577 -157.0881 -156.7014 -156.3005 -155.8874 -155.4634 -155.0295
##  [40,] -156.5307 -156.0922 -155.6455 -155.1912 -154.7292 -154.2598 -153.7828
##  [41,] -155.2163 -154.7527 -154.2813 -153.8021 -153.3152 -152.8205 -152.3180
##  [42,] -153.4613 -153.0219 -152.5652 -152.0936 -151.6087 -151.1117 -150.6032
##  [43,] -151.1942 -150.8381 -150.4430 -150.0172 -149.5662 -149.0936 -148.6022
##  [44,] -148.3174 -148.1197 -147.8450 -147.5123 -147.1340 -146.7186 -146.2719
##  [45,] -144.7587 -144.7579 -144.6808 -144.5022 -144.2461 -143.9287 -143.5613
##  [46,] -140.8889 -140.8889 -140.8889 -140.8884 -140.8196 -140.6533 -140.4093
##  [47,] -136.8068 -136.8068 -136.8068 -136.8068 -136.8068 -136.8065 -136.7435
##  [48,] -132.5353 -132.5353 -132.5353 -132.5353 -132.5353 -132.5353 -132.5353
##  [49,] -128.1219 -128.1219 -128.1219 -128.1219 -128.1219 -128.1219 -128.1219
##  [50,] -123.6509 -123.6509 -123.6509 -123.6509 -123.6509 -123.6509 -123.6509
##  [51,] -119.2600 -119.2600 -119.2600 -119.2600 -119.2600 -119.2600 -119.2600
##  [52,] -115.1558 -115.1558 -115.1558 -115.1558 -115.1558 -115.1558 -115.1558
##  [53,] -111.6187 -111.6187 -111.6187 -111.6187 -111.6187 -111.6187 -111.6187
##  [54,] -108.9803 -108.9803 -108.9803 -108.9803 -108.9803 -108.9803 -108.9839
##  [55,] -107.5566 -107.5566 -107.5566 -107.5565 -107.5433 -107.5127 -107.4710
##  [56,] -107.5479 -107.5475 -107.5101 -107.4252 -107.3077 -107.1690 -107.0179
##  [57,] -108.8868 -108.7341 -108.5271 -108.2843 -108.0188 -107.7405 -107.4566
##  [58,] -110.9282 -110.5744 -110.1932 -109.7958 -109.3903 -108.9829 -108.5783
##  [59,] -113.1985 -112.6951 -112.1865 -111.6776 -111.1718 -110.6723 -110.1815
##  [60,] -115.4318 -114.8586 -114.2909 -113.7301 -113.1775 -112.6344 -112.1023
##  [61,] -117.4983 -116.9396 -116.3841 -115.8333 -115.2888 -114.7518 -114.2239
##  [62,] -119.3628 -118.8955 -118.4167 -117.9319 -117.4449 -116.9587 -116.4759
##  [63,] -121.0584 -120.7446 -120.3950 -120.0212 -119.6316 -119.2321 -118.8272
##  [64,] -122.6771 -122.5562 -122.3697 -122.1370 -121.8715 -121.5830 -121.2785
##  [65,] -124.4503 -124.4503 -124.4311 -124.3487 -124.2173 -124.0497 -123.8555
##  [66,] -126.7581 -126.7581 -126.7581 -126.7581 -126.7461 -126.6918 -126.6030
##  [67,] -129.5864 -129.5864 -129.5864 -129.5864 -129.5864 -129.5864 -129.5795
##  [68,] -132.8682 -132.8682 -132.8682 -132.8682 -132.8682 -132.8682 -132.8682
##  [69,] -136.5225 -136.5225 -136.5225 -136.5225 -136.5225 -136.5225 -136.5225
##  [70,] -140.4639 -140.4639 -140.4639 -140.4639 -140.4639 -140.4639 -140.4639
##  [71,] -144.6104 -144.6104 -144.6104 -144.6104 -144.6104 -144.6104 -144.6104
##  [72,] -148.8874 -148.8874 -148.8874 -148.8874 -148.8874 -148.8874 -148.8874
##  [73,] -153.2304 -153.2304 -153.2304 -153.2304 -153.2304 -153.2304 -153.2304
##  [74,] -157.5848 -157.5848 -157.5848 -157.5848 -157.5848 -157.5726 -157.4750
##  [75,] -161.9055 -161.9055 -161.9055 -161.8906 -161.7805 -161.5833 -161.3167
##  [76,] -166.1553 -166.1367 -166.0103 -165.7910 -165.5006 -165.1539 -164.7611
##  [77,] -170.1296 -169.8809 -169.5609 -169.1867 -168.7697 -168.3176 -167.8358
##  [78,] -173.4534 -173.0456 -172.6010 -172.1270 -171.6284 -171.1083 -170.5691
##  [79,] -176.1909 -175.6961 -175.1837 -174.6557 -174.1132 -173.5571 -172.9877
##  [80,] -178.4064 -177.8843 -177.3519 -176.8089 -176.2552 -175.6905 -175.1147
##  [81,] -180.1445 -179.6477 -179.1373 -178.6140 -178.0781 -177.5297 -176.9688
##  [82,] -181.4292 -181.0078 -180.5596 -180.0888 -179.5981 -179.0894 -178.5637
##  [83,] -182.2609 -181.9683 -181.6243 -181.2400 -180.8227 -180.3773 -179.9070
##  [84,] -182.6104 -182.5107 -182.3200 -182.0610 -181.7485 -181.3923 -180.9992
##  [85,] -182.6176 -182.6176 -182.6122 -182.5272 -182.3578 -182.1220 -181.8318
##  [86,] -182.6176 -182.6176 -182.6176 -182.6176 -182.6134 -182.5382 -182.3833
##  [87,] -182.6176 -182.6176 -182.6176 -182.6176 -182.6176 -182.6176 -182.6142
##  [88,] -182.6176 -182.6176 -182.6176 -182.6176 -182.6176 -182.6176 -182.6176
##  [89,] -182.6176 -182.6176 -182.6176 -182.6176 -182.6176 -182.6176 -182.6176
##  [90,] -182.6176 -182.6176 -182.6176 -182.6176 -182.6176 -182.6176 -182.6176
##  [91,] -182.6176 -182.6176 -182.6176 -182.6176 -182.6176 -182.6176 -182.6176
##  [92,] -182.6176 -182.6176 -182.6176 -182.6176 -182.6176 -182.6176 -182.6176
##  [93,] -182.6176 -182.6176 -182.6176 -182.6176 -182.6176 -182.6176 -182.6176
##  [94,] -182.6176 -182.6176 -182.6176 -182.6176 -182.6176 -182.6176 -182.6176
##  [95,] -182.6176 -182.6176 -182.6176 -182.6176 -182.6176 -182.6176 -182.6176
##  [96,] -182.6176 -182.6176 -182.6176 -182.6176 -182.6176 -182.6176 -182.6176
##  [97,] -182.6176 -182.6176 -182.6176 -182.6176 -182.6176 -182.6176 -182.6176
##  [98,] -182.6176 -182.6176 -182.6176 -182.6176 -182.6176 -182.6176 -182.6176
##  [99,] -182.6176 -182.6176 -182.6176 -182.6176 -182.6176 -182.6176 -182.6176
## [100,] -182.6176 -182.6176 -182.6176 -182.6176 -182.6176 -182.6176 -182.6176
##            [,15]     [,16]     [,17]     [,18]     [,19]     [,20]     [,21]
##   [1,] -179.9639 -179.9639 -179.9639 -179.9639 -179.9639 -179.9639 -179.9639
##   [2,] -179.9639 -179.9639 -179.9639 -179.9639 -179.9639 -179.9639 -179.9639
##   [3,] -179.9639 -179.9639 -179.9639 -179.9639 -179.9639 -179.9639 -179.9639
##   [4,] -179.9639 -179.9639 -179.9639 -179.9639 -179.9639 -179.9639 -179.9639
##   [5,] -179.9639 -179.9639 -179.9639 -179.9639 -179.9639 -179.9639 -179.9639
##   [6,] -179.9639 -179.9639 -179.9639 -179.9639 -179.9639 -179.9639 -179.9639
##   [7,] -179.9639 -179.9639 -179.9639 -179.9639 -179.9639 -179.9639 -179.9639
##   [8,] -179.9639 -179.9639 -179.9639 -179.9639 -179.9639 -179.9639 -179.9639
##   [9,] -179.9639 -179.9639 -179.9639 -179.9639 -179.9639 -179.9639 -179.9639
##  [10,] -179.9639 -179.9639 -179.9639 -179.9639 -179.9639 -179.9639 -179.9639
##  [11,] -179.9639 -179.9639 -179.9639 -179.9639 -179.9639 -179.9628 -179.9262
##  [12,] -179.9639 -179.9639 -179.9639 -179.9625 -179.9247 -179.8405 -179.7162
##  [13,] -179.9639 -179.9622 -179.9224 -179.8360 -179.7098 -179.5491 -179.3582
##  [14,] -179.9190 -179.8286 -179.6984 -179.5343 -179.3409 -179.1218 -178.8797
##  [15,] -179.6809 -179.5106 -179.3120 -179.0888 -178.8441 -178.5803 -178.2991
##  [16,] -179.2683 -179.0381 -178.7879 -178.5202 -178.2368 -177.9392 -177.6287
##  [17,] -178.7053 -178.4310 -178.1429 -177.8425 -177.5308 -177.2088 -176.8772
##  [18,] -178.0077 -177.7025 -177.3882 -177.0653 -176.7345 -176.3963 -176.0510
##  [19,] -177.1861 -176.8619 -176.5316 -176.1956 -175.8540 -175.5071 -175.1550
##  [20,] -176.2480 -175.9157 -175.5792 -175.2386 -174.8941 -174.5457 -174.1934
##  [21,] -175.1988 -174.8689 -174.5356 -174.1990 -173.8592 -173.5162 -173.1702
##  [22,] -174.0433 -173.7262 -173.4054 -173.0812 -172.7537 -172.4230 -172.0895
##  [23,] -172.7864 -172.4925 -172.1936 -171.8901 -171.5825 -171.2711 -170.9563
##  [24,] -171.4344 -171.1744 -170.9066 -170.6322 -170.3520 -170.0668 -169.7770
##  [25,] -169.9966 -169.7807 -169.5532 -169.3161 -169.0707 -168.8181 -168.5595
##  [26,] -168.4862 -168.3241 -168.1455 -167.9533 -167.7496 -167.5361 -167.3142
##  [27,] -166.9227 -166.8226 -166.7004 -166.5597 -166.4037 -166.2346 -166.0544
##  [28,] -165.3340 -165.3016 -165.2408 -165.1565 -165.0524 -164.9318 -164.7971
##  [29,] -163.8001 -163.8001 -163.7973 -163.7711 -163.7211 -163.6509 -163.5637
##  [30,] -162.4433 -162.4433 -162.4433 -162.4433 -162.4413 -162.4205 -162.3800
##  [31,] -161.2786 -161.2786 -161.2786 -161.2786 -161.2786 -161.2732 -161.2405
##  [32,] -160.3123 -160.3123 -160.3123 -160.3051 -160.2666 -160.1991 -160.1067
##  [33,] -159.5454 -159.5361 -159.4890 -159.4076 -159.2973 -159.1623 -159.0059
##  [34,] -158.9047 -158.8072 -158.6767 -158.5183 -158.3361 -158.1334 -157.9127
##  [35,] -158.2340 -158.0492 -157.8386 -157.6059 -157.3541 -157.0856 -156.8024
##  [36,] -157.4955 -157.2300 -156.9448 -156.6426 -156.3254 -155.9947 -155.6518
##  [37,] -156.6591 -156.3213 -155.9689 -155.6034 -155.2261 -154.8379 -154.4396
##  [38,] -155.6979 -155.2975 -154.8863 -154.4649 -154.0340 -153.5940 -153.1453
##  [39,] -154.5862 -154.1341 -153.6734 -153.2044 -152.7272 -152.2420 -151.7488
##  [40,] -153.2984 -152.8064 -152.3068 -151.7995 -151.2843 -150.7613 -150.2303
##  [41,] -151.8074 -151.2888 -150.7619 -150.2267 -149.6829 -149.1305 -148.5692
##  [42,] -150.0837 -149.5534 -149.0126 -148.4613 -147.8995 -147.3272 -146.7444
##  [43,] -148.0936 -147.5691 -147.0297 -146.4761 -145.9086 -145.3277 -144.7334
##  [44,] -145.7981 -145.3003 -144.7807 -144.2411 -143.6826 -143.1062 -142.5125
##  [45,] -143.1519 -142.7063 -142.2289 -141.7230 -141.1911 -140.6351 -140.0565
##  [46,] -140.1015 -139.7402 -139.3329 -138.8853 -138.4017 -137.8856 -137.3395
##  [47,] -136.5856 -136.3493 -136.0472 -135.6885 -135.2803 -134.8282 -134.3363
##  [48,] -132.5352 -132.4767 -132.3245 -132.0934 -131.7945 -131.4363 -131.0256
##  [49,] -128.1219 -128.1219 -128.1218 -128.0670 -127.9194 -127.6923 -127.3956
##  [50,] -123.6509 -123.6509 -123.6509 -123.6509 -123.6509 -123.5994 -123.4564
##  [51,] -119.2600 -119.2600 -119.2600 -119.2600 -119.2600 -119.2814 -119.3410
##  [52,] -115.1558 -115.1558 -115.1558 -115.1752 -115.2277 -115.3094 -115.4174
##  [53,] -111.6187 -111.6327 -111.6696 -111.7272 -111.8041 -111.8999 -112.0146
##  [54,] -108.9938 -109.0108 -109.0366 -109.0727 -109.1211 -109.1835 -109.2614
##  [55,] -107.4239 -107.3762 -107.3324 -107.2959 -107.2702 -107.2579 -107.2616
##  [56,] -106.8614 -106.7053 -106.5543 -106.4125 -106.2832 -106.1696 -106.0742
##  [57,] -107.1730 -106.8946 -106.6253 -106.3686 -106.1275 -105.9048 -105.7030
##  [58,] -108.1803 -107.7924 -107.4174 -107.0581 -106.7169 -106.3960 -106.0976
##  [59,] -109.7018 -109.2352 -108.7838 -108.3495 -107.9342 -107.5397 -107.1681
##  [60,] -111.5824 -111.0763 -110.5854 -110.1112 -109.6554 -109.2195 -108.8052
##  [61,] -113.7063 -113.2006 -112.7080 -112.2301 -111.7683 -111.3243 -110.8995
##  [62,] -115.9987 -115.5290 -115.0685 -114.6192 -114.1826 -113.7604 -113.3543
##  [63,] -118.4207 -118.0156 -117.6144 -117.2195 -116.8330 -116.4569 -116.0929
##  [64,] -120.9636 -120.6428 -120.3196 -119.9971 -119.6781 -119.3648 -119.0595
##  [65,] -123.6420 -123.4150 -123.1793 -122.9385 -122.6962 -122.4550 -122.2174
##  [66,] -126.4880 -126.3534 -126.2046 -126.0459 -125.8811 -125.7133 -125.5453
##  [67,] -129.5460 -129.4904 -129.4176 -129.3321 -129.2376 -129.1372 -129.0338
##  [68,] -132.8682 -132.8647 -132.8468 -132.8167 -132.7774 -132.7314 -132.6813
##  [69,] -136.5225 -136.5225 -136.5225 -136.5213 -136.5148 -136.5041 -136.4905
##  [70,] -140.4639 -140.4639 -140.4639 -140.4639 -140.4639 -140.4642 -140.4604
##  [71,] -144.6104 -144.6104 -144.6104 -144.6104 -144.6035 -144.5338 -144.3977
##  [72,] -148.8874 -148.8874 -148.8790 -148.8009 -148.6512 -148.4403 -148.1763
##  [73,] -153.2202 -153.1331 -152.9692 -152.7409 -152.4577 -152.1268 -151.7544
##  [74,] -157.2957 -157.0493 -156.7465 -156.3957 -156.0034 -155.5746 -155.1133
##  [75,] -160.9932 -160.6222 -160.2105 -159.7634 -159.2853 -158.7792 -158.2478
##  [76,] -164.3297 -163.8652 -163.3716 -162.8521 -162.3092 -161.7447 -161.1601
##  [77,] -167.3281 -166.7973 -166.2457 -165.6748 -165.0858 -164.4798 -163.8573
##  [78,] -170.0123 -169.4391 -168.8503 -168.2465 -167.6280 -166.9953 -166.3485
##  [79,] -172.4054 -171.8104 -171.2026 -170.5821 -169.9488 -169.3028 -168.6438
##  [80,] -174.5276 -173.9289 -173.3183 -172.6957 -172.0607 -171.4133 -170.7530
##  [81,] -176.3955 -175.8096 -175.2110 -174.5995 -173.9748 -173.3368 -172.6852
##  [82,] -178.0218 -177.4643 -176.8914 -176.3034 -175.7003 -175.0820 -174.4485
##  [83,] -179.4141 -178.9003 -178.3669 -177.8145 -177.2439 -176.6552 -176.0489
##  [84,] -180.5741 -180.1203 -179.6405 -179.1364 -178.6095 -178.0607 -177.4907
##  [85,] -181.4958 -181.1204 -180.7101 -180.2682 -179.7973 -179.2992 -178.7754
##  [86,] -182.1634 -181.8886 -181.5667 -181.2033 -180.8025 -180.3676 -179.9010
##  [87,] -182.5458 -182.4008 -182.1915 -181.9269 -181.6137 -181.2571 -180.8609
##  [88,] -182.6176 -182.6149 -182.5511 -182.4126 -182.2099 -181.9509 -181.6417
##  [89,] -182.6176 -182.6176 -182.6176 -182.6154 -182.5548 -182.4200 -182.2202
##  [90,] -182.6176 -182.6176 -182.6176 -182.6176 -182.6176 -182.6158 -182.5572
##  [91,] -182.6176 -182.6176 -182.6176 -182.6176 -182.6176 -182.6176 -182.6176
##  [92,] -182.6176 -182.6176 -182.6176 -182.6176 -182.6176 -182.6176 -182.6176
##  [93,] -182.6176 -182.6176 -182.6176 -182.6176 -182.6176 -182.6176 -182.6176
##  [94,] -182.6176 -182.6176 -182.6176 -182.6176 -182.6176 -182.6176 -182.6176
##  [95,] -182.6176 -182.6176 -182.6176 -182.6176 -182.6176 -182.6176 -182.6176
##  [96,] -182.6176 -182.6176 -182.6176 -182.6176 -182.6176 -182.6176 -182.6176
##  [97,] -182.6176 -182.6176 -182.6176 -182.6176 -182.6176 -182.6176 -182.6176
##  [98,] -182.6176 -182.6176 -182.6176 -182.6176 -182.6176 -182.6176 -182.6176
##  [99,] -182.6176 -182.6176 -182.6176 -182.6176 -182.6176 -182.6176 -182.6176
## [100,] -182.6176 -182.6176 -182.6176 -182.6176 -182.6176 -182.6176 -182.6176
##            [,22]     [,23]     [,24]     [,25]     [,26]     [,27]     [,28]
##   [1,] -179.9639 -179.9639 -179.9639 -179.9639 -179.9639 -179.9639 -179.9639
##   [2,] -179.9639 -179.9639 -179.9639 -179.9639 -179.9639 -179.9639 -179.9639
##   [3,] -179.9639 -179.9639 -179.9639 -179.9639 -179.9639 -179.9639 -179.9639
##   [4,] -179.9639 -179.9639 -179.9639 -179.9639 -179.9639 -179.9639 -179.9639
##   [5,] -179.9639 -179.9639 -179.9639 -179.9639 -179.9639 -179.9639 -179.9639
##   [6,] -179.9639 -179.9639 -179.9639 -179.9639 -179.9639 -179.9639 -179.9639
##   [7,] -179.9639 -179.9639 -179.9639 -179.9639 -179.9639 -179.9639 -179.9635
##   [8,] -179.9639 -179.9639 -179.9639 -179.9639 -179.9633 -179.9298 -179.8493
##   [9,] -179.9639 -179.9639 -179.9632 -179.9286 -179.8472 -179.7250 -179.5671
##  [10,] -179.9630 -179.9274 -179.8451 -179.7222 -179.5636 -179.3737 -179.1562
##  [11,] -179.8430 -179.7193 -179.5601 -179.3696 -179.1515 -178.9089 -178.6444
##  [12,] -179.5565 -179.3655 -179.1469 -178.9038 -178.6389 -178.3545 -178.0527
##  [13,] -179.1402 -178.8979 -178.6333 -178.3486 -178.0465 -177.7288 -177.3972
##  [14,] -178.6169 -178.3353 -178.0361 -177.7208 -177.3903 -177.0460 -176.6904
##  [15,] -178.0021 -177.6904 -177.3651 -177.0271 -176.6770 -176.3153 -175.9428
##  [16,] -177.3061 -176.9723 -176.6279 -176.2736 -175.9097 -175.5367 -175.1550
##  [17,] -176.5366 -176.1874 -175.8302 -175.4652 -175.0927 -174.7132 -174.3268
##  [18,] -175.6991 -175.3408 -174.9763 -174.6058 -174.2297 -173.8481 -173.4611
##  [19,] -174.7981 -174.4363 -174.0699 -173.6990 -173.3238 -172.9444 -172.5609
##  [20,] -173.8375 -173.4778 -173.1147 -172.7481 -172.3782 -172.0051 -171.6290
##  [21,] -172.8210 -172.4690 -172.1141 -171.7566 -171.3964 -171.0338 -170.6689
##  [22,] -171.7530 -171.4140 -171.0724 -170.7285 -170.3824 -170.0342 -169.6843
##  [23,] -170.6384 -170.3177 -169.9943 -169.6686 -169.3409 -169.0113 -168.6800
##  [24,] -169.4833 -169.1862 -168.8859 -168.5830 -168.2777 -167.9705 -167.6616
##  [25,] -168.2956 -168.0271 -167.7547 -167.4788 -167.2001 -166.9189 -166.6358
##  [26,] -167.0852 -166.8501 -166.6098 -166.3651 -166.1166 -165.8650 -165.6108
##  [27,] -165.8649 -165.6673 -165.4630 -165.2528 -165.0378 -164.8186 -164.5939
##  [28,] -164.6505 -164.4937 -164.3283 -164.1554 -163.9737 -163.7709 -163.5468
##  [29,] -163.4618 -163.3476 -163.2194 -163.0641 -162.8822 -162.6758 -162.4467
##  [30,] -162.3184 -162.2210 -162.0900 -161.9286 -161.7398 -161.5256 -161.2880
##  [31,] -161.1687 -161.0592 -160.9161 -160.7429 -160.5421 -160.3159 -160.0663
##  [32,] -159.9926 -159.8587 -159.6960 -159.5045 -159.2864 -159.0438 -158.7782
##  [33,] -158.8308 -158.6394 -158.4334 -158.2136 -157.9721 -157.7078 -157.4217
##  [34,] -157.6763 -157.4258 -157.1627 -156.8883 -156.6035 -156.3087 -155.9967
##  [35,] -156.5059 -156.1974 -155.8780 -155.5487 -155.2101 -154.8629 -154.5078
##  [36,] -155.2979 -154.9337 -154.5599 -154.1773 -153.7862 -153.3872 -152.9806
##  [37,] -154.0318 -153.6151 -153.1898 -152.7563 -152.3149 -151.8658 -151.4093
##  [38,] -152.6882 -152.2229 -151.7496 -151.2684 -150.7794 -150.2828 -149.7785
##  [39,] -151.2477 -150.7385 -150.2215 -149.6964 -149.1634 -148.6223 -148.0731
##  [40,] -149.6911 -149.1436 -148.5878 -148.0234 -147.4504 -146.8686 -146.2778
##  [41,] -147.9989 -147.4194 -146.8305 -146.2321 -145.6240 -145.0059 -144.3777
##  [42,] -146.1509 -145.5467 -144.9314 -144.3051 -143.6675 -143.0184 -142.3576
##  [43,] -144.1260 -143.5055 -142.8717 -142.2248 -141.5644 -140.8906 -140.2030
##  [44,] -141.9021 -141.2753 -140.6324 -139.9733 -139.2983 -138.6071 -137.8998
##  [45,] -139.4563 -138.8353 -138.1943 -137.5335 -136.8534 -136.1540 -135.4355
##  [46,] -136.7655 -136.1650 -135.5394 -134.8896 -134.2162 -133.5197 -132.8005
##  [47,] -133.8082 -133.2463 -132.6528 -132.0293 -131.3771 -130.6971 -130.0080
##  [48,] -130.5675 -130.0663 -129.5253 -128.9472 -128.3530 -127.7656 -127.1831
##  [49,] -127.0374 -126.6240 -126.1807 -125.7316 -125.2762 -124.8143 -124.3455
##  [50,] -123.2548 -123.0226 -122.7628 -122.4780 -122.1705 -121.8422 -121.4947
##  [51,] -119.3863 -119.3721 -119.3063 -119.1950 -119.0437 -118.8567 -118.6377
##  [52,] -115.5500 -115.7057 -115.8395 -115.9075 -115.9180 -115.8781 -115.7934
##  [53,] -112.1482 -112.3010 -112.4734 -112.6658 -112.8378 -112.9461 -112.9979
##  [54,] -109.3564 -109.4699 -109.6029 -109.7566 -109.9318 -110.1293 -110.3129
##  [55,] -107.2833 -107.3250 -107.3883 -107.4747 -107.5854 -107.7215 -107.8838
##  [56,] -105.9994 -105.9473 -105.9196 -105.9181 -105.9441 -105.9989 -106.0837
##  [57,] -105.5242 -105.3706 -105.2439 -105.1460 -105.0783 -105.0422 -105.0391
##  [58,] -105.8237 -105.5763 -105.3572 -105.1682 -105.0107 -104.8865 -104.7968
##  [59,] -106.8210 -106.5003 -106.2076 -105.9449 -105.7136 -105.5154 -105.3518
##  [60,] -108.4142 -108.0481 -107.7086 -107.3973 -107.1161 -106.8664 -106.6498
##  [61,] -110.4955 -110.1140 -109.7567 -109.4250 -109.1207 -108.8454 -108.6005
##  [62,] -112.9658 -112.5966 -112.2484 -111.9226 -111.6209 -111.3449 -111.0961
##  [63,] -115.7429 -115.4085 -115.0914 -114.7932 -114.5155 -114.2598 -114.0276
##  [64,] -118.7641 -118.4804 -118.2103 -117.9552 -117.7170 -117.4970 -117.2967
##  [65,] -121.9857 -121.7618 -121.5477 -121.3451 -121.1556 -120.9806 -120.8217
##  [66,] -125.3795 -125.2181 -125.0629 -124.9159 -124.7785 -124.6524 -124.5389
##  [67,] -128.9298 -128.8273 -128.7283 -128.6346 -128.5478 -128.4662 -128.3513
##  [68,] -132.6292 -132.5770 -132.5266 -132.4758 -132.3832 -132.2413 -132.0550
##  [69,] -136.4756 -136.4562 -136.3871 -136.2624 -136.0880 -135.8691 -135.6098
##  [70,] -140.4021 -140.2841 -140.1136 -139.8963 -139.6369 -139.3395 -139.0073
##  [71,] -144.2052 -143.9679 -143.6908 -143.3777 -143.0318 -142.6556 -142.2513
##  [72,] -147.8660 -147.5148 -147.1287 -146.7178 -146.2841 -145.8289 -145.3528
##  [73,] -151.3451 -150.9029 -150.4312 -149.9325 -149.4108 -148.8756 -148.3275
##  [74,] -154.6230 -154.1062 -153.5651 -153.0018 -152.4176 -151.8140 -151.1939
##  [75,] -157.6932 -157.1171 -156.5209 -155.9059 -155.2730 -154.6232 -153.9570
##  [76,] -160.5567 -159.9355 -159.2972 -158.6426 -157.9722 -157.2864 -156.5857
##  [77,] -163.2190 -162.5654 -161.8968 -161.2134 -160.5157 -159.8036 -159.0774
##  [78,] -165.6878 -165.0132 -164.3248 -163.6226 -162.9067 -162.1769 -161.4333
##  [79,] -167.9717 -167.2864 -166.5878 -165.8757 -165.1499 -164.4103 -163.6566
##  [80,] -170.0796 -169.3930 -168.6928 -167.9788 -167.2507 -166.5083 -165.7514
##  [81,] -172.0198 -171.3403 -170.6465 -169.9380 -169.2146 -168.4760 -167.7220
##  [82,] -173.7996 -173.1353 -172.4552 -171.7592 -171.0470 -170.3183 -169.5730
##  [83,] -175.4250 -174.7835 -174.1243 -173.4474 -172.7527 -172.0398 -171.3086
##  [84,] -176.9001 -176.2891 -175.6581 -175.0069 -174.3357 -173.6443 -172.9327
##  [85,] -178.2270 -177.6547 -177.0590 -176.4404 -175.7990 -175.1350 -174.4483
##  [86,] -179.4046 -178.8798 -178.3277 -177.7489 -177.1441 -176.5136 -175.8576
##  [87,] -180.4282 -179.9612 -179.4618 -178.9312 -178.3705 -177.7814 -177.1675
##  [88,] -181.2871 -180.8907 -180.4552 -179.9834 -179.4791 -178.9447 -178.3823
##  [89,] -181.9628 -181.6536 -181.2980 -180.9004 -180.4644 -179.9932 -179.4896
##  [90,] -182.4239 -182.2252 -181.9686 -181.6603 -181.3056 -180.9088 -180.4736
##  [91,] -182.6161 -182.5592 -182.4273 -182.2298 -181.9743 -181.6670 -181.3132
##  [92,] -182.6176 -182.6176 -182.6164 -182.5611 -182.4306 -182.2344 -181.9800
##  [93,] -182.6176 -182.6176 -182.6176 -182.6176 -182.6167 -182.5630 -182.4339
##  [94,] -182.6176 -182.6176 -182.6176 -182.6176 -182.6176 -182.6176 -182.6169
##  [95,] -182.6176 -182.6176 -182.6176 -182.6176 -182.6176 -182.6176 -182.6176
##  [96,] -182.6176 -182.6176 -182.6176 -182.6176 -182.6176 -182.6176 -182.6176
##  [97,] -182.6176 -182.6176 -182.6176 -182.6176 -182.6176 -182.6176 -182.6176
##  [98,] -182.6176 -182.6176 -182.6176 -182.6176 -182.6176 -182.6176 -182.6176
##  [99,] -182.6176 -182.6176 -182.6176 -182.6176 -182.6176 -182.6176 -182.6176
## [100,] -182.6176 -182.6176 -182.6176 -182.6176 -182.6176 -182.6176 -182.6176
##            [,29]     [,30]     [,31]     [,32]     [,33]     [,34]     [,35]
##   [1,] -179.9639 -179.9639 -179.9639 -179.9639 -179.9639 -179.9639 -179.9639
##   [2,] -179.9639 -179.9639 -179.9639 -179.9639 -179.9639 -179.9639 -179.9639
##   [3,] -179.9639 -179.9639 -179.9639 -179.9639 -179.9639 -179.9639 -179.9639
##   [4,] -179.9639 -179.9639 -179.9639 -179.9639 -179.9639 -179.9638 -179.9343
##   [5,] -179.9639 -179.9639 -179.9639 -179.9637 -179.9332 -179.8554 -179.7363
##   [6,] -179.9639 -179.9636 -179.9321 -179.8533 -179.7335 -179.5776 -179.3902
##   [7,] -179.9310 -179.8513 -179.7307 -179.5742 -179.3861 -179.1701 -178.9293
##   [8,] -179.7278 -179.5707 -179.3820 -179.1655 -178.9242 -178.6610 -178.3782
##   [9,] -179.3779 -179.1608 -178.9191 -178.6555 -178.3723 -178.0715 -177.7550
##  [10,] -178.9140 -178.6500 -178.3664 -178.0652 -177.7485 -177.4177 -177.0742
##  [11,] -178.3604 -178.0590 -177.7419 -177.4108 -177.0672 -176.7123 -176.3473
##  [12,] -177.7354 -177.4040 -177.0601 -176.7050 -176.3399 -175.9658 -175.5838
##  [13,] -177.0531 -176.6977 -176.3324 -175.9582 -175.5760 -175.1869 -174.7917
##  [14,] -176.3249 -175.9505 -175.5682 -175.1790 -174.7837 -174.3831 -173.9780
##  [15,] -175.5604 -175.1711 -174.7757 -174.3750 -173.9698 -173.5609 -173.1489
##  [16,] -174.7649 -174.3667 -173.9616 -173.5526 -173.1406 -172.7262 -172.3100
##  [17,] -173.9338 -173.5344 -173.1289 -172.7176 -172.3016 -171.8843 -171.4664
##  [18,] -173.0691 -172.6721 -172.2705 -171.8644 -171.4541 -171.0398 -170.6227
##  [19,] -172.1735 -171.7825 -171.3880 -170.9902 -170.5893 -170.1856 -169.7794
##  [20,] -171.2500 -170.8683 -170.4841 -170.0976 -169.7090 -169.3185 -168.9265
##  [21,] -170.3018 -169.9328 -169.5621 -169.1898 -168.8162 -168.4415 -168.0661
##  [22,] -169.3327 -168.9798 -168.6256 -168.2705 -167.9146 -167.5583 -167.2018
##  [23,] -168.3474 -168.0137 -167.6792 -167.3440 -167.0085 -166.6731 -166.3379
##  [24,] -167.3513 -167.0400 -166.7280 -166.4156 -166.1031 -165.7895 -165.4656
##  [25,] -166.3511 -166.0652 -165.7785 -165.4898 -165.1894 -164.8756 -164.5483
##  [26,] -165.3545 -165.0949 -164.8216 -164.5330 -164.2296 -163.9113 -163.5784
##  [27,] -164.3526 -164.0934 -163.8172 -163.5244 -163.2155 -162.8907 -162.5504
##  [28,] -163.3025 -163.0391 -162.7573 -162.4580 -162.1415 -161.8084 -161.4590
##  [29,] -162.1963 -161.9258 -161.6361 -161.3281 -161.0023 -160.6593 -160.2993
##  [30,] -161.0285 -160.7483 -160.4485 -160.1297 -159.7928 -159.4381 -159.0662
##  [31,] -159.7946 -159.5020 -159.1896 -158.8581 -158.5081 -158.1402 -157.7548
##  [32,] -158.4909 -158.1830 -157.8554 -157.5088 -157.1438 -156.7608 -156.3601
##  [33,] -157.1149 -156.7883 -156.4426 -156.0783 -155.6958 -155.2955 -154.8776
##  [34,] -155.6658 -155.3164 -154.9489 -154.5636 -154.1608 -153.7406 -153.3031
##  [35,] -154.1447 -153.7674 -153.3736 -152.9633 -152.5365 -152.0932 -151.6332
##  [36,] -152.5668 -152.1460 -151.7182 -151.2779 -150.8225 -150.3519 -149.8656
##  [37,] -150.9455 -150.4747 -149.9969 -149.5124 -149.0209 -148.5177 -148.0002
##  [38,] -149.2667 -148.7474 -148.2207 -147.6866 -147.1450 -146.5962 -146.0398
##  [39,] -147.5157 -146.9502 -146.3764 -145.7944 -145.2039 -144.6051 -143.9978
##  [40,] -145.6780 -145.0690 -144.4507 -143.8229 -143.1855 -142.5384 -141.8815
##  [41,] -143.7392 -143.0901 -142.4304 -141.7598 -141.0780 -140.3849 -139.6803
##  [42,] -141.6849 -141.0001 -140.3028 -139.5929 -138.8700 -138.1340 -137.3845
##  [43,] -139.5015 -138.7859 -138.0559 -137.3112 -136.5514 -135.7764 -134.9858
##  [44,] -137.1762 -136.4360 -135.6792 -134.9052 -134.1139 -133.3197 -132.5450
##  [45,] -134.6978 -133.9409 -133.1645 -132.3841 -131.6220 -130.8766 -130.1460
##  [46,] -132.0587 -131.3113 -130.5803 -129.8637 -129.1598 -128.4668 -127.7836
##  [47,] -129.3321 -128.6671 -128.0113 -127.3631 -126.7213 -126.0848 -125.4529
##  [48,] -126.6040 -126.0272 -125.4517 -124.8769 -124.3020 -123.7267 -123.1506
##  [49,] -123.8699 -123.3874 -122.8982 -122.4022 -121.8997 -121.3909 -120.8761
##  [50,] -121.1296 -120.7481 -120.3514 -119.9405 -119.5165 -119.0803 -118.6327
##  [51,] -118.3900 -118.1161 -117.8185 -117.4993 -117.1603 -116.8032 -116.4294
##  [52,] -115.6688 -115.5086 -115.3162 -115.0949 -114.8473 -114.5759 -114.2829
##  [53,] -112.9998 -112.9571 -112.8746 -112.7563 -112.6057 -112.4261 -112.2203
##  [54,] -110.4390 -110.5135 -110.5420 -110.5292 -110.4792 -110.3958 -110.2823
##  [55,] -108.0731 -108.2567 -108.3913 -108.4815 -108.5315 -108.5454 -108.5266
##  [56,] -106.1992 -106.3462 -106.5253 -106.7075 -106.8502 -106.9567 -107.0303
##  [57,] -105.0699 -105.1357 -105.2371 -105.3748 -105.5492 -105.7348 -105.8911
##  [58,] -104.7430 -104.7261 -104.7472 -104.8070 -104.9062 -105.0453 -105.2245
##  [59,] -105.2242 -105.1339 -105.0821 -105.0698 -105.0979 -105.1671 -105.2780
##  [60,] -106.4678 -106.3218 -106.2132 -106.1431 -106.1127 -106.1228 -106.1741
##  [61,] -108.3877 -108.2084 -108.0640 -107.9557 -107.8848 -107.8523 -107.8591
##  [62,] -110.8760 -110.6861 -110.5277 -110.4021 -110.3105 -110.2541 -110.2338
##  [63,] -113.8205 -113.6396 -113.4865 -113.3624 -113.2685 -113.2058 -113.1742
##  [64,] -117.1176 -116.9610 -116.8281 -116.7202 -116.6367 -116.5470 -116.4404
##  [65,] -120.6803 -120.5575 -120.4525 -120.3319 -120.1855 -120.0156 -119.8244
##  [66,] -124.4368 -124.3101 -124.1492 -123.9571 -123.7366 -123.4900 -123.2200
##  [67,] -128.1945 -127.9995 -127.7696 -127.5080 -127.2172 -126.8999 -126.5584
##  [68,] -131.8284 -131.5652 -131.2684 -130.9410 -130.5853 -130.2036 -129.7980
##  [69,] -135.3138 -134.9842 -134.6238 -134.2348 -133.8194 -133.3794 -132.9166
##  [70,] -138.6433 -138.2499 -137.8290 -137.3826 -136.9122 -136.4192 -135.9048
##  [71,] -141.8208 -141.3656 -140.8872 -140.3866 -139.8649 -139.3231 -138.7620
##  [72,] -144.8568 -144.3414 -143.8073 -143.2550 -142.6849 -142.0974 -141.4928
##  [73,] -147.7661 -147.1911 -146.6024 -145.9996 -145.3827 -144.7515 -144.1058
##  [74,] -150.5662 -149.9312 -149.2877 -148.6348 -147.9716 -147.2975 -146.6118
##  [75,] -153.2753 -152.5801 -151.8805 -151.1765 -150.4664 -149.7491 -149.0235
##  [76,] -155.8705 -155.1411 -154.3977 -153.6421 -152.8835 -152.1216 -151.3548
##  [77,] -158.3371 -157.5830 -156.8151 -156.0336 -155.2384 -154.4313 -153.6211
##  [78,] -160.6758 -159.9043 -159.1189 -158.3195 -157.5061 -156.6786 -155.8371
##  [79,] -162.8888 -162.1067 -161.3101 -160.4989 -159.6729 -158.8321 -157.9764
##  [80,] -164.9796 -164.1929 -163.3909 -162.5734 -161.7402 -160.8912 -160.0262
##  [81,] -166.9522 -166.1664 -165.3643 -164.5456 -163.7101 -162.8575 -161.9875
##  [82,] -168.8106 -168.0309 -167.2336 -166.4184 -165.5850 -164.7331 -163.8624
##  [83,] -170.5588 -169.7902 -169.0023 -168.1950 -167.3679 -166.5207 -165.6550
##  [84,] -172.2005 -171.4477 -170.6738 -169.8786 -169.0637 -168.2347 -167.3929
##  [85,] -173.7389 -173.0065 -172.2527 -171.4822 -170.6962 -169.8954 -169.0803
##  [86,] -175.1775 -174.4775 -173.7586 -173.0219 -172.2681 -171.4980 -170.7123
##  [87,] -176.5304 -175.8713 -175.1914 -174.4918 -173.7733 -173.0369 -172.2835
##  [88,] -177.7935 -177.1801 -176.5435 -175.8848 -175.2054 -174.5061 -173.7880
##  [89,] -178.9558 -178.3939 -177.8057 -177.1928 -176.5566 -175.8984 -175.2193
##  [90,] -180.0031 -179.5001 -178.9669 -178.4055 -177.8178 -177.2054 -176.5697
##  [91,] -180.9172 -180.4827 -180.0129 -179.5105 -178.9779 -178.4171 -177.8299
##  [92,] -181.6737 -181.3208 -180.9256 -180.4919 -180.0227 -179.5210 -178.9890
##  [93,] -182.2390 -181.9857 -181.6804 -181.3283 -180.9340 -180.5010 -180.0326
##  [94,] -182.5648 -182.4372 -182.2435 -181.9913 -181.6870 -181.3359 -180.9423
##  [95,] -182.6176 -182.6171 -182.5667 -182.4405 -182.2480 -181.9969 -181.6937
##  [96,] -182.6176 -182.6176 -182.6176 -182.6173 -182.5685 -182.4437 -182.2525
##  [97,] -182.6176 -182.6176 -182.6176 -182.6176 -182.6176 -182.6174 -182.5703
##  [98,] -182.6176 -182.6176 -182.6176 -182.6176 -182.6176 -182.6176 -182.6176
##  [99,] -182.6176 -182.6176 -182.6176 -182.6176 -182.6176 -182.6176 -182.6176
## [100,] -182.6176 -182.6176 -182.6176 -182.6176 -182.6176 -182.6176 -182.6176
##            [,36]     [,37]     [,38]     [,39]     [,40]     [,41]     [,42]
##   [1,] -179.9639 -179.9639 -179.9639 -179.9639 -179.9639 -179.9375 -179.8633
##   [2,] -179.9639 -179.9639 -179.9639 -179.9365 -179.8613 -179.7446 -179.5915
##   [3,] -179.9639 -179.9354 -179.8593 -179.7418 -179.5880 -179.4024 -179.1885
##   [4,] -179.8574 -179.7390 -179.5846 -179.3983 -179.1839 -178.9446 -178.6831
##   [5,] -179.5811 -179.3942 -179.1793 -178.9395 -178.6776 -178.3958 -178.0964
##   [6,] -179.1747 -178.9344 -178.6721 -178.3899 -178.0902 -177.7746 -177.4449
##   [7,] -178.6665 -178.3841 -178.0840 -177.7681 -177.4381 -177.0954 -176.7414
##   [8,] -178.0777 -177.7616 -177.4313 -177.0883 -176.7341 -176.3697 -175.9963
##   [9,] -177.4245 -177.0813 -176.7268 -176.3623 -175.9887 -175.6072 -175.2186
##  [10,] -176.7196 -176.3548 -175.9811 -175.5994 -175.2107 -174.8158 -174.4157
##  [11,] -175.9735 -175.5916 -175.2028 -174.8078 -174.4075 -174.0027 -173.5941
##  [12,] -175.1949 -174.7998 -174.3994 -173.9945 -173.5858 -173.1740 -172.7597
##  [13,] -174.3913 -173.9863 -173.5775 -173.1656 -172.7514 -172.3353 -171.9180
##  [14,] -173.5692 -173.1573 -172.7430 -172.3269 -171.9096 -171.4917 -171.0738
##  [15,] -172.7346 -172.3184 -171.9011 -171.4833 -171.0654 -170.6480 -170.2317
##  [16,] -171.8927 -171.4748 -171.0569 -170.6396 -170.2233 -169.8087 -169.3961
##  [17,] -171.0485 -170.6312 -170.2149 -169.8003 -169.3878 -168.9779 -168.5710
##  [18,] -170.2065 -169.7920 -169.3795 -168.9696 -168.5628 -168.1595 -167.7602
##  [19,] -169.3709 -168.9614 -168.5546 -168.1514 -167.7522 -167.3574 -166.9675
##  [20,] -168.5331 -168.1388 -167.7438 -167.3495 -166.9597 -166.5751 -166.1953
##  [21,] -167.6902 -167.3141 -166.9381 -166.5627 -166.1872 -165.8042 -165.4144
##  [22,] -166.8455 -166.4897 -166.1336 -165.7687 -165.3926 -165.0048 -164.6049
##  [23,] -166.0022 -165.6570 -165.3003 -164.9316 -164.5505 -164.1568 -163.7499
##  [24,] -165.1293 -164.7805 -164.4190 -164.0443 -163.6564 -163.2550 -162.8398
##  [25,] -164.2074 -163.8528 -163.4845 -163.1023 -162.7060 -162.2955 -161.8706
##  [26,] -163.2309 -162.8688 -162.4922 -162.1010 -161.6950 -161.2742 -160.8384
##  [27,] -162.1946 -161.8236 -161.4373 -161.0358 -160.6191 -160.1869 -159.7393
##  [28,] -161.0936 -160.7122 -160.3151 -159.9022 -159.4736 -159.0291 -158.5687
##  [29,] -159.9228 -159.5299 -159.1208 -158.6954 -158.2539 -157.7961 -157.3219
##  [30,] -158.6773 -158.2716 -157.8493 -157.4104 -156.9549 -156.4827 -155.9938
##  [31,] -157.3520 -156.9322 -156.4955 -156.0419 -155.5713 -155.0837 -154.5790
##  [32,] -155.9420 -155.5067 -155.0541 -154.5844 -154.0975 -153.5933 -153.0716
##  [33,] -154.4423 -153.9897 -153.5198 -153.0326 -152.5279 -152.0057 -151.4656
##  [34,] -152.8485 -152.3766 -151.8874 -151.3808 -150.8566 -150.3146 -149.7546
##  [35,] -151.1564 -150.6627 -150.1518 -149.6237 -149.0779 -148.5142 -147.9323
##  [36,] -149.3633 -148.8446 -148.3091 -147.7566 -147.1866 -146.5987 -145.9925
##  [37,] -147.4679 -146.9201 -146.3563 -145.7759 -145.1784 -144.5631 -143.9296
##  [38,] -145.4719 -144.8898 -144.2928 -143.6800 -143.0507 -142.4041 -141.7395
##  [39,] -143.3821 -142.7577 -142.1212 -141.4702 -140.8037 -140.1207 -139.4203
##  [40,] -141.2145 -140.5375 -139.8503 -139.1527 -138.4421 -137.7161 -136.9845
##  [41,] -138.9640 -138.2357 -137.4954 -136.7427 -135.9894 -135.2585 -134.5482
##  [42,] -136.6213 -135.8441 -135.0654 -134.3081 -133.5717 -132.8550 -132.1572
##  [43,] -134.1928 -133.4204 -132.6676 -131.9329 -131.2153 -130.5140 -129.8282
##  [44,] -131.7884 -131.0485 -130.3240 -129.6137 -128.9169 -128.2329 -127.5610
##  [45,] -129.4289 -128.7240 -128.0303 -127.3470 -126.6734 -126.0092 -125.3539
##  [46,] -127.1091 -126.4423 -125.7826 -125.1294 -124.4823 -123.8412 -123.2058
##  [47,] -124.8248 -124.2000 -123.5782 -122.9590 -122.3425 -121.7285 -121.1171
##  [48,] -122.5735 -121.9953 -121.4161 -120.8357 -120.2545 -119.6727 -119.0905
##  [49,] -120.3554 -119.8293 -119.2980 -118.7621 -118.2220 -117.6781 -117.1310
##  [50,] -118.1746 -117.7066 -117.2297 -116.7446 -116.2521 -115.7530 -115.2480
##  [51,] -116.0405 -115.6376 -115.2220 -114.7949 -114.3574 -113.9107 -113.4557
##  [52,] -113.9702 -113.6396 -113.2929 -112.9315 -112.5571 -112.1710 -111.7746
##  [53,] -111.9907 -111.7397 -111.4694 -111.1817 -110.8786 -110.5618 -110.2330
##  [54,] -110.1417 -109.9767 -109.7898 -109.5833 -109.3595 -109.1204 -108.8679
##  [55,] -108.4785 -108.4040 -108.3058 -108.1866 -108.0488 -107.8947 -107.7264
##  [56,] -107.0741 -107.0910 -107.0839 -107.0552 -107.0076 -106.9434 -106.8648
##  [57,] -106.0199 -106.1235 -106.2045 -106.2652 -106.3078 -106.3346 -106.3478
##  [58,] -105.4221 -105.6002 -105.7597 -105.9021 -106.0292 -106.1427 -106.2446
##  [59,] -105.4309 -105.6260 -105.8451 -106.0539 -106.2525 -106.4417 -106.6227
##  [60,] -106.2674 -106.4030 -106.5812 -106.8020 -107.0506 -107.2967 -107.5235
##  [61,] -107.9059 -107.9935 -108.1223 -108.2918 -108.4817 -108.6824 -108.8827
##  [62,] -110.2506 -110.3042 -110.3704 -110.4389 -110.5108 -110.5871 -110.6691
##  [63,] -113.1458 -113.1103 -113.0690 -113.0235 -112.9755 -112.9268 -112.8793
##  [64,] -116.3190 -116.1844 -116.0388 -115.8842 -115.7227 -115.5563 -115.3874
##  [65,] -119.6142 -119.3872 -119.1457 -118.8917 -118.6275 -118.3552 -118.0771
##  [66,] -122.9288 -122.6186 -122.2915 -121.9498 -121.5954 -121.2304 -120.8568
##  [67,] -126.1947 -125.8111 -125.4093 -124.9914 -124.5591 -124.1143 -123.6588
##  [68,] -129.3704 -128.9224 -128.4559 -127.9724 -127.4735 -126.9606 -126.4354
##  [69,] -132.4324 -131.9283 -131.4056 -130.8657 -130.3097 -129.7389 -129.1546
##  [70,] -135.3704 -134.8168 -134.2452 -133.6564 -133.0514 -132.4311 -131.7964
##  [71,] -138.1822 -137.5846 -136.9696 -136.3381 -135.6905 -135.0275 -134.3496
##  [72,] -140.8715 -140.2338 -139.5800 -138.9103 -138.2251 -137.5246 -136.8093
##  [73,] -143.4455 -142.7706 -142.0810 -141.3766 -140.6574 -139.9234 -139.1747
##  [74,] -145.9139 -145.2035 -144.4800 -143.7431 -142.9924 -142.2277 -141.4487
##  [75,] -148.2884 -147.5430 -146.7864 -146.0181 -145.2373 -144.4434 -143.6359
##  [76,] -150.5816 -149.8008 -149.0110 -148.2112 -147.4006 -146.5781 -145.7429
##  [77,] -152.8080 -151.9900 -151.1657 -150.3336 -149.4923 -148.6407 -147.7777
##  [78,] -154.9830 -154.1252 -153.2639 -152.3973 -151.5237 -150.6415 -149.7495
##  [79,] -157.1057 -156.2200 -155.3205 -154.4161 -153.5073 -152.5920 -151.6686
##  [80,] -159.1450 -158.2474 -157.3335 -156.4031 -155.4573 -154.5074 -153.5629
##  [81,] -161.1001 -160.1949 -159.2717 -158.3327 -157.3863 -156.4341 -155.4776
##  [82,] -162.9726 -162.0655 -161.1490 -160.2241 -159.2915 -158.3517 -157.4055
##  [83,] -164.7776 -163.8896 -162.9915 -162.0840 -161.1676 -160.2428 -159.3104
##  [84,] -166.5386 -165.6726 -164.7954 -163.9076 -163.0097 -162.1024 -161.1862
##  [85,] -168.2516 -167.4100 -166.5560 -165.6902 -164.8132 -163.9256 -163.0280
##  [86,] -169.9117 -169.0969 -168.2685 -167.4271 -166.5734 -165.7078 -164.8311
##  [87,] -171.5137 -170.7283 -169.9280 -169.1135 -168.2854 -167.4442 -166.5907
##  [88,] -173.0520 -172.2989 -171.5294 -170.7443 -169.9443 -169.1301 -168.3022
##  [89,] -174.5204 -173.8027 -173.0670 -172.3142 -171.5451 -170.7603 -169.9606
##  [90,] -175.9119 -175.2332 -174.5347 -173.8174 -173.0821 -172.3296 -171.5608
##  [91,] -177.2180 -176.5827 -175.9254 -175.2471 -174.5490 -173.8321 -173.0971
##  [92,] -178.4287 -177.8421 -177.2306 -176.5958 -175.9389 -175.2610 -174.5633
##  [93,] -179.5314 -179.0000 -178.4403 -177.8542 -177.2432 -176.6088 -175.9523
##  [94,] -180.5101 -180.0423 -179.5419 -179.0110 -178.4519 -177.8662 -177.2558
##  [95,] -181.3434 -180.9507 -180.5192 -180.0521 -179.5523 -179.0220 -178.4634
##  [96,] -182.0026 -181.7003 -181.3509 -180.9590 -180.5283 -180.0619 -179.5627
##  [97,] -182.4469 -182.2570 -182.0081 -181.7068 -181.3584 -180.9673 -180.5373
##  [98,] -182.6175 -182.5720 -182.4501 -182.2614 -182.0137 -181.7134 -181.3659
##  [99,] -182.6176 -182.6176 -182.6175 -182.5737 -182.4533 -182.2658 -182.0193
## [100,] -182.6176 -182.6176 -182.6176 -182.6176 -182.6176 -182.5754 -182.4564
##            [,43]     [,44]     [,45]     [,46]     [,47]     [,48]     [,49]
##   [1,] -179.7473 -179.5949 -179.4104 -179.1977 -178.9598 -178.6995 -178.4193
##   [2,] -179.4064 -179.1931 -178.9547 -178.6940 -178.4134 -178.1150 -177.8007
##   [3,] -178.9497 -178.6885 -178.4076 -178.1088 -177.7942 -177.4652 -177.1235
##   [4,] -178.4017 -178.1026 -177.7876 -177.4584 -177.1165 -176.7631 -176.3995
##   [5,] -177.7811 -177.4517 -177.1095 -176.7559 -176.3921 -176.0192 -175.6383
##   [6,] -177.1024 -176.7486 -176.3846 -176.0116 -175.6305 -175.2423 -174.8480
##   [7,] -176.3772 -176.0040 -175.6227 -175.2344 -174.8399 -174.4401 -174.0356
##   [8,] -175.6150 -175.2265 -174.8319 -174.4319 -174.0274 -173.6189 -173.2074
##   [9,] -174.8239 -174.4238 -174.0191 -173.6107 -173.1990 -172.7849 -172.3689
##  [10,] -174.0109 -173.6024 -173.1907 -172.7765 -172.3605 -171.9433 -171.5255
##  [11,] -173.1823 -172.7681 -172.3521 -171.9349 -171.5170 -171.0991 -170.6817
##  [12,] -172.3437 -171.9264 -171.5086 -171.0907 -170.6733 -170.2569 -169.8421
##  [13,] -171.5001 -171.0822 -170.6649 -170.2485 -169.8337 -169.4210 -169.0109
##  [14,] -170.6564 -170.2401 -169.8254 -169.4127 -169.0026 -168.5955 -168.1920
##  [15,] -169.8170 -169.4044 -168.9944 -168.5874 -168.1839 -167.7843 -167.3891
##  [16,] -168.9861 -168.5792 -168.1757 -167.7763 -167.3812 -166.9910 -166.6059
##  [17,] -168.1676 -167.7683 -167.3733 -166.9831 -166.5982 -166.2184 -165.8354
##  [18,] -167.3653 -166.9753 -166.5905 -166.2107 -165.8276 -165.4382 -165.0419
##  [19,] -166.5828 -166.2030 -165.8198 -165.4303 -165.0339 -164.6300 -164.2184
##  [20,] -165.8120 -165.4223 -165.0258 -164.6218 -164.2100 -163.7898 -163.3609
##  [21,] -165.0177 -164.6136 -164.2015 -163.7812 -163.3522 -162.9140 -162.4665
##  [22,] -164.1931 -163.7726 -163.3434 -162.9051 -162.4574 -161.9999 -161.5324
##  [23,] -163.3297 -162.8957 -162.4482 -161.9906 -161.5229 -161.0449 -160.5562
##  [24,] -162.4104 -161.9666 -161.5081 -161.0346 -160.5463 -160.0465 -159.5356
##  [25,] -161.4309 -160.9763 -160.5064 -160.0210 -159.5197 -159.0021 -158.4684
##  [26,] -160.3874 -159.9210 -159.4388 -158.9406 -158.4261 -157.8948 -157.3464
##  [27,] -159.2760 -158.7967 -158.3014 -157.7895 -157.2609 -156.7151 -156.1518
##  [28,] -158.0921 -157.5992 -157.0897 -156.5634 -156.0198 -155.4587 -154.8795
##  [29,] -156.8312 -156.3237 -155.7992 -155.2574 -154.6980 -154.1206 -153.5247
##  [30,] -155.4879 -154.9649 -154.4245 -153.8663 -153.2901 -152.6954 -152.0818
##  [31,] -154.0569 -153.5174 -152.9599 -152.3843 -151.7902 -151.1772 -150.5447
##  [32,] -152.5322 -151.9749 -151.3993 -150.8052 -150.1920 -149.5594 -148.9069
##  [33,] -150.9075 -150.3311 -149.7361 -149.1220 -148.4885 -147.8350 -147.1610
##  [34,] -149.1763 -148.5793 -147.9632 -147.3276 -146.6721 -145.9962 -145.2992
##  [35,] -147.3318 -146.7123 -146.0734 -145.4145 -144.7352 -144.0350 -143.3131
##  [36,] -145.3675 -144.7232 -144.0592 -143.3749 -142.6697 -141.9429 -141.1939
##  [37,] -143.2773 -142.6055 -141.9137 -141.2012 -140.4674 -139.7201 -138.9810
##  [38,] -141.0562 -140.3534 -139.6305 -138.8959 -138.1716 -137.4572 -136.7515
##  [39,] -138.7014 -137.9734 -137.2582 -136.5552 -135.8630 -135.1801 -134.5053
##  [40,] -136.2694 -135.5696 -134.8834 -134.2090 -133.5451 -132.8902 -132.2433
##  [41,] -133.8554 -133.1783 -132.5151 -131.8640 -131.2236 -130.5926 -129.9696
##  [42,] -131.4776 -130.8148 -130.1670 -129.5323 -128.9093 -128.2965 -127.6925
##  [43,] -129.1575 -128.5014 -127.8598 -127.2327 -126.6190 -126.0172 -125.4259
##  [44,] -126.9011 -126.2528 -125.6161 -124.9910 -124.3776 -123.7776 -123.1907
##  [45,] -124.7073 -124.0695 -123.4405 -122.8203 -122.2095 -121.6082 -121.0171
##  [46,] -122.5761 -121.9522 -121.3344 -120.7230 -120.1183 -119.5210 -118.9317
##  [47,] -120.5086 -119.9031 -119.3011 -118.7030 -118.1095 -117.5211 -116.9386
##  [48,] -118.5083 -117.9266 -117.3459 -116.7670 -116.1904 -115.6170 -115.0478
##  [49,] -116.5814 -116.0299 -115.4772 -114.9242 -114.3718 -113.8208 -113.2724
##  [50,] -114.7382 -114.2243 -113.7073 -113.1882 -112.6681 -112.1480 -111.6293
##  [51,] -112.9938 -112.5259 -112.0532 -111.5770 -111.0986 -110.6191 -110.1399
##  [52,] -111.3694 -110.9567 -110.5380 -110.1145 -109.6878 -109.2594 -108.8308
##  [53,] -109.8938 -109.5460 -109.1911 -108.8307 -108.4665 -108.1001 -107.7333
##  [54,] -108.6040 -108.3306 -108.0494 -107.7624 -107.4712 -107.1777 -106.8838
##  [55,] -107.5460 -107.3556 -107.1572 -106.9528 -106.7443 -106.5336 -106.3227
##  [56,] -106.7741 -106.6735 -106.5649 -106.4505 -106.3322 -106.2121 -106.0922
##  [57,] -106.3496 -106.3419 -106.3270 -106.3067 -106.2831 -106.2518 -106.2088
##  [58,] -106.3366 -106.4206 -106.4983 -106.5627 -106.6087 -106.6362 -106.6452
##  [59,] -106.7965 -106.9523 -107.0837 -107.1910 -107.2743 -107.3342 -107.3711
##  [60,] -107.7229 -107.8947 -108.0392 -108.1568 -108.2481 -108.3137 -108.3546
##  [61,] -109.0577 -109.2063 -109.3287 -109.4252 -109.4962 -109.5424 -109.5644
##  [62,] -110.7583 -110.8470 -110.9152 -110.9617 -110.9865 -110.9898 -110.9718
##  [63,] -112.8349 -112.7958 -112.7638 -112.7340 -112.6895 -112.6289 -112.5520
##  [64,] -115.2180 -115.0504 -114.8868 -114.7295 -114.5808 -114.4371 -114.2843
##  [65,] -117.7953 -117.5120 -117.2296 -116.9502 -116.6762 -116.4099 -116.1537
##  [66,] -120.4768 -120.0923 -119.7055 -119.3186 -118.9337 -118.5532 -118.1793
##  [67,] -123.1945 -122.7230 -122.2465 -121.7667 -121.2858 -120.8057 -120.3287
##  [68,] -125.8994 -125.3541 -124.8011 -124.2423 -123.6792 -123.1137 -122.5479
##  [69,] -128.5579 -127.9501 -127.3327 -126.7069 -126.0742 -125.4362 -124.7945
##  [70,] -131.1483 -130.4877 -129.8158 -129.1334 -128.4419 -127.7425 -127.0365
##  [71,] -133.6576 -132.9522 -132.2339 -131.5037 -130.7624 -130.0110 -129.2504
##  [72,] -136.0793 -135.3352 -134.5774 -133.8063 -133.0226 -132.2268 -131.4199
##  [73,] -138.4114 -137.6335 -136.8412 -136.0347 -135.2144 -134.3807 -133.5339
##  [74,] -140.6551 -139.8470 -139.0241 -138.1864 -137.3340 -136.4670 -135.5855
##  [75,] -142.8143 -141.9783 -141.1275 -140.2616 -139.3803 -138.4836 -137.5713
##  [76,] -144.8944 -144.0319 -143.1547 -142.2624 -141.3544 -140.4303 -139.4919
##  [77,] -146.9023 -146.0136 -145.1106 -144.1927 -143.2611 -142.3263 -141.3895
##  [78,] -148.8463 -147.9306 -147.0036 -146.0746 -145.1445 -144.2131 -143.2800
##  [79,] -150.7377 -149.8077 -148.8790 -147.9507 -147.0224 -146.0934 -145.1633
##  [80,] -152.6238 -151.6887 -150.7565 -149.8265 -148.8977 -147.9695 -147.0411
##  [81,] -154.5265 -153.5820 -152.6427 -151.7075 -150.7754 -149.8453 -148.9165
##  [82,] -156.4534 -155.4969 -154.5456 -153.6010 -152.6616 -151.7264 -150.7942
##  [83,] -158.3708 -157.4247 -156.4727 -155.5162 -154.5648 -153.6200 -152.6806
##  [84,] -160.2616 -159.3293 -158.3898 -157.4439 -156.4920 -155.5355 -154.5839
##  [85,] -162.1208 -161.2048 -160.2803 -159.3482 -158.4089 -157.4630 -156.5113
##  [86,] -163.9437 -163.0462 -162.1392 -161.2233 -160.2991 -159.3671 -158.4279
##  [87,] -165.7254 -164.8489 -163.9617 -163.0644 -162.1576 -161.2419 -160.3179
##  [88,] -167.4614 -166.6081 -165.7430 -164.8667 -163.9797 -163.0827 -162.1761
##  [89,] -169.1467 -168.3191 -167.4785 -166.6254 -165.7606 -164.8845 -163.9977
##  [90,] -170.7763 -169.9769 -169.1632 -168.3359 -167.4956 -166.6428 -165.7782
##  [91,] -172.3450 -171.5765 -170.7923 -169.9932 -169.1798 -168.3528 -167.5127
##  [92,] -173.8467 -173.1121 -172.3603 -171.5922 -170.8083 -170.0095 -169.1964
##  [93,] -175.2749 -174.5776 -173.8614 -173.1271 -172.3757 -171.6078 -170.8243
##  [94,] -176.6219 -175.9658 -175.2888 -174.5919 -173.8760 -173.1421 -172.3910
##  [95,] -177.8783 -177.2683 -176.6349 -175.9793 -175.3027 -174.6062 -173.8907
##  [96,] -179.0330 -178.4750 -177.8904 -177.2809 -176.6479 -175.9927 -175.3166
##  [97,] -180.0717 -179.5731 -179.0440 -178.4865 -177.9025 -177.2934 -176.6609
##  [98,] -180.9756 -180.5464 -180.0814 -179.5835 -179.0550 -178.4981 -177.9145
##  [99,] -181.7200 -181.3733 -180.9839 -180.5554 -180.0911 -179.5938 -179.0660
## [100,] -182.2702 -182.0248 -181.7265 -181.3808 -180.9922 -180.5644 -180.1008
##            [,50]     [,51]     [,52]     [,53]     [,54]     [,55]     [,56]
##   [1,] -178.1212 -177.8072 -177.4788 -177.1376 -176.7848 -176.4218 -176.0497
##   [2,] -177.4720 -177.1305 -176.7776 -176.4144 -176.0421 -175.6616 -175.2740
##   [3,] -176.7704 -176.4070 -176.0344 -175.6538 -175.2661 -174.8720 -174.4725
##   [4,] -176.0268 -175.6461 -175.2582 -174.8640 -174.4644 -174.0602 -173.6521
##   [5,] -175.2503 -174.8560 -174.4563 -174.0520 -173.6438 -173.2324 -172.8184
##   [6,] -174.4482 -174.0438 -173.6355 -173.2240 -172.8101 -172.3942 -171.9771
##   [7,] -173.6272 -173.2157 -172.8017 -172.3858 -171.9686 -171.5508 -171.1329
##   [8,] -172.7933 -172.3774 -171.9602 -171.5424 -171.1244 -170.7070 -170.2905
##   [9,] -171.9518 -171.5339 -171.1160 -170.6986 -170.2821 -169.8672 -169.4543
##  [10,] -171.1076 -170.6901 -170.2737 -169.8588 -169.4460 -169.0356 -168.6283
##  [11,] -170.2653 -169.8505 -169.4377 -169.0274 -168.6201 -168.2163 -167.8164
##  [12,] -169.4293 -169.0191 -168.6119 -168.2082 -167.8084 -167.4130 -167.0223
##  [13,] -168.6037 -168.2001 -167.8004 -167.4050 -167.0145 -166.6291 -166.2490
##  [14,] -167.7923 -167.3971 -167.0066 -166.6214 -166.2414 -165.8588 -165.4699
##  [15,] -166.9988 -166.6137 -166.2337 -165.8510 -165.4620 -165.0662 -164.6630
##  [16,] -166.2260 -165.8432 -165.4541 -165.0581 -164.6547 -164.2435 -163.8241
##  [17,] -165.4461 -165.0500 -164.6465 -164.2351 -163.8155 -163.3872 -162.9498
##  [18,] -164.6383 -164.2268 -163.8069 -163.3784 -162.9409 -162.4939 -162.0373
##  [19,] -163.7984 -163.3697 -162.9319 -162.4848 -162.0279 -161.5611 -161.0839
##  [20,] -162.9230 -162.4757 -162.0186 -161.5515 -161.0741 -160.5862 -160.0873
##  [21,] -162.0093 -161.5420 -161.0644 -160.5762 -160.0771 -159.5669 -159.0453
##  [22,] -161.0546 -160.5662 -160.0669 -159.5565 -159.0346 -158.5011 -157.9556
##  [23,] -160.0567 -159.5461 -159.0240 -158.4902 -157.9445 -157.3866 -156.8161
##  [24,] -159.0133 -158.4793 -157.9334 -157.3752 -156.8045 -156.2210 -155.6245
##  [25,] -157.9222 -157.3638 -156.7928 -156.2091 -155.6123 -155.0021 -154.3783
##  [26,] -156.7805 -156.1972 -155.6001 -154.9897 -154.3655 -153.7275 -153.0751
##  [27,] -155.5705 -154.9708 -154.3520 -153.7144 -153.0617 -152.3944 -151.7121
##  [28,] -154.2820 -153.6655 -153.0296 -152.3737 -151.6972 -151.0002 -150.2865
##  [29,] -152.9099 -152.2758 -151.6217 -150.9472 -150.2515 -149.5340 -148.7941
##  [30,] -151.4488 -150.7959 -150.1226 -149.4283 -148.7122 -147.9738 -147.2123
##  [31,] -149.8924 -149.2197 -148.5259 -147.8106 -147.0729 -146.3122 -145.5277
##  [32,] -148.2340 -147.5401 -146.8246 -146.0868 -145.3260 -144.5416 -143.7326
##  [33,] -146.4661 -145.7495 -145.0107 -144.2490 -143.4636 -142.6537 -141.8248
##  [34,] -144.5806 -143.8398 -143.0760 -142.2885 -141.4834 -140.6818 -139.8845
##  [35,] -142.5690 -141.8019 -141.0185 -140.2401 -139.4672 -138.6987 -137.9337
##  [36,] -140.4300 -139.6726 -138.9219 -138.1770 -137.4367 -136.7003 -135.9667
##  [37,] -138.2502 -137.5265 -136.8087 -136.0959 -135.3872 -134.6816 -133.9783
##  [38,] -136.0533 -135.3615 -134.6750 -133.9930 -133.3144 -132.6386 -131.9647
##  [39,] -133.8375 -133.1756 -132.5186 -131.8657 -131.2159 -130.5687 -129.9232
##  [40,] -131.6031 -130.9687 -130.3390 -129.7133 -129.0908 -128.4707 -127.8525
##  [41,] -129.3536 -128.7436 -128.1386 -127.5379 -126.9406 -126.3461 -125.7537
##  [42,] -127.0963 -126.5069 -125.9232 -125.3444 -124.7699 -124.1989 -123.6308
##  [43,] -124.8438 -124.2699 -123.7031 -123.1426 -122.5876 -122.0375 -121.4916
##  [44,] -122.6154 -122.0505 -121.4950 -120.9479 -120.4083 -119.8755 -119.3490
##  [45,] -120.4391 -119.8750 -119.3233 -118.7832 -118.2536 -117.7336 -117.2227
##  [46,] -118.3510 -117.7799 -117.2228 -116.6814 -116.1545 -115.6413 -115.1410
##  [47,] -116.3629 -115.7950 -115.2359 -114.6869 -114.1537 -113.6394 -113.1429
##  [48,] -114.4836 -113.9257 -113.3751 -112.8333 -112.3016 -111.7815 -111.2803
##  [49,] -112.7276 -112.1878 -111.6542 -111.1283 -110.6115 -110.1057 -109.6124
##  [50,] -111.1131 -110.6008 -110.0940 -109.5941 -109.1029 -108.6220 -108.1535
##  [51,] -109.6626 -109.1886 -108.7195 -108.2571 -107.8030 -107.3592 -106.9277
##  [52,] -108.4035 -107.9793 -107.5599 -107.1470 -106.7427 -106.3488 -105.9675
##  [53,] -107.3677 -107.0052 -106.6477 -106.2970 -105.9551 -105.6241 -105.3065
##  [54,] -106.5913 -106.3022 -106.0184 -105.7418 -105.4737 -105.2133 -104.9600
##  [55,] -106.1136 -105.9082 -105.7061 -105.5057 -105.3061 -105.1068 -104.9075
##  [56,] -105.9703 -105.8435 -105.7112 -105.5730 -105.4287 -105.2781 -105.1213
##  [57,] -106.1539 -106.0868 -106.0074 -105.9157 -105.8121 -105.6968 -105.5703
##  [58,] -106.6361 -106.6090 -106.5644 -106.5029 -106.4250 -106.3313 -106.2225
##  [59,] -107.3857 -107.3788 -107.3509 -107.3031 -107.2359 -107.1504 -107.0472
##  [60,] -108.3714 -108.3650 -108.3363 -108.2862 -108.2155 -108.1251 -108.0159
##  [61,] -109.5628 -109.5386 -109.4923 -109.4249 -109.3371 -109.2297 -109.1035
##  [62,] -110.9330 -110.8736 -110.7943 -110.6956 -110.5780 -110.4422 -110.2887
##  [63,] -112.4585 -112.3484 -112.2218 -112.0789 -111.9198 -111.7448 -111.5544
##  [64,] -114.1207 -113.9456 -113.7587 -113.5596 -113.3480 -113.1240 -112.8874
##  [65,] -115.9053 -115.6524 -115.3931 -115.1267 -114.8524 -114.5698 -114.2783
##  [66,] -117.8144 -117.4609 -117.1176 -116.7735 -116.4268 -116.0764 -115.7215
##  [67,] -119.8570 -119.3929 -118.9390 -118.4976 -118.0683 -117.6413 -117.2146
##  [68,] -121.9836 -121.4231 -120.8686 -120.3226 -119.7876 -119.2662 -118.7585
##  [69,] -124.1509 -123.5073 -122.8658 -122.2285 -121.5977 -120.9761 -120.3663
##  [70,] -126.3254 -125.6108 -124.8944 -124.1783 -123.4645 -122.7552 -122.0530
##  [71,] -128.4820 -127.7069 -126.9266 -126.1428 -125.3572 -124.5718 -123.7888
##  [72,] -130.6026 -129.7759 -128.9409 -128.0990 -127.2516 -126.4004 -125.5472
##  [73,] -132.6746 -131.8036 -130.9217 -130.0298 -129.1291 -128.2226 -127.3243
##  [74,] -134.6899 -133.7805 -132.8579 -131.9244 -130.9933 -130.0680 -129.1500
##  [75,] -136.6434 -135.7021 -134.7594 -133.8180 -132.8790 -131.9433 -131.0121
##  [76,] -138.5504 -137.6077 -136.6645 -135.7211 -134.7784 -133.8370 -132.8979
##  [77,] -140.4511 -139.5109 -138.5694 -137.6268 -136.6835 -135.7402 -134.7974
##  [78,] -142.3452 -141.4085 -140.4700 -139.5299 -138.5884 -137.6458 -136.7026
##  [79,] -144.2319 -143.2989 -142.3641 -141.4274 -140.4890 -139.5489 -138.6075
##  [80,] -146.1121 -145.1821 -144.2507 -143.3177 -142.3830 -141.4464 -140.5080
##  [81,] -147.9882 -147.0599 -146.1309 -145.2009 -144.2696 -143.3366 -142.4019
##  [82,] -149.8641 -148.9352 -148.0070 -147.0786 -146.1497 -145.2197 -144.2884
##  [83,] -151.7452 -150.8130 -149.8828 -148.9540 -148.0257 -147.0974 -146.1685
##  [84,] -153.6390 -152.6995 -151.7641 -150.8318 -149.9016 -148.9728 -148.0445
##  [85,] -155.5548 -154.6031 -153.6581 -152.7184 -151.7830 -150.8506 -149.9204
##  [86,] -157.4822 -156.5305 -155.5741 -154.6222 -153.6771 -152.7374 -151.8018
##  [87,] -159.3860 -158.4470 -157.5014 -156.5498 -155.5934 -154.6414 -153.6961
##  [88,] -161.2605 -160.3366 -159.4049 -158.4660 -157.5205 -156.5691 -155.6127
##  [89,] -163.1009 -162.1945 -161.2791 -160.3553 -159.4238 -158.4850 -157.5397
##  [90,] -164.9023 -164.0158 -163.1191 -162.2129 -161.2977 -160.3741 -159.4427
##  [91,] -166.6601 -165.7958 -164.9201 -164.0338 -163.1373 -162.2313 -161.3162
##  [92,] -168.3696 -167.5298 -166.6775 -165.8133 -164.9379 -164.0518 -163.1555
##  [93,] -170.0258 -169.2129 -168.3864 -167.5469 -166.6948 -165.8309 -164.9557
##  [94,] -171.6235 -170.8403 -170.0421 -169.2295 -168.4033 -167.5639 -166.7122
##  [95,] -173.1571 -172.4064 -171.6392 -170.8562 -170.0583 -169.2461 -168.4201
##  [96,] -174.6204 -173.9053 -173.1721 -172.4217 -171.6548 -170.8722 -170.0746
##  [97,] -176.0062 -175.3304 -174.6347 -173.9199 -173.1871 -172.4370 -171.6705
##  [98,] -177.3060 -176.6739 -176.0196 -175.3443 -174.6489 -173.9346 -173.2021
##  [99,] -178.5096 -177.9266 -177.3185 -176.6869 -176.0331 -175.3581 -174.6632
## [100,] -179.6042 -179.0769 -178.5211 -177.9386 -177.3310 -176.6999 -176.0465
##            [,57]     [,58]     [,59]     [,60]     [,61]     [,62]     [,63]
##   [1,] -175.6694 -175.2819 -174.8881 -174.4888 -174.0848 -173.6769 -173.2657
##   [2,] -174.8800 -174.4807 -174.0766 -173.6686 -173.2574 -172.8436 -172.4279
##   [3,] -174.0684 -173.6603 -173.2491 -172.8352 -172.4194 -172.0024 -171.5846
##   [4,] -173.2407 -172.8268 -172.4110 -171.9939 -171.5761 -171.1582 -170.7407
##   [5,] -172.4026 -171.9855 -171.5677 -171.1498 -170.7323 -170.3157 -169.9007
##   [6,] -171.5592 -171.1413 -170.7238 -170.3073 -169.8923 -169.4793 -169.0687
##   [7,] -170.7154 -170.2989 -169.8839 -169.4709 -169.0604 -168.6529 -168.2488
##   [8,] -169.8756 -169.4626 -169.0522 -168.6447 -168.2407 -167.8406 -167.4447
##   [9,] -169.0439 -168.6365 -168.2326 -167.8325 -167.4368 -167.0458 -166.6601
##  [10,] -168.2244 -167.8245 -167.4289 -167.0380 -166.6523 -166.2720 -165.8899
##  [11,] -167.4209 -167.0302 -166.6446 -166.2644 -165.8821 -165.4937 -165.0984
##  [12,] -166.6369 -166.2567 -165.8743 -165.4858 -165.0904 -164.6876 -164.2771
##  [13,] -165.8666 -165.4778 -165.0823 -164.6794 -164.2687 -163.8497 -163.4221
##  [14,] -165.0742 -164.6712 -164.2603 -163.8412 -163.4134 -162.9766 -162.5304
##  [15,] -164.2519 -163.8326 -163.4046 -162.9676 -162.5213 -162.0652 -161.5992
##  [16,] -163.3959 -162.9587 -162.5122 -162.0559 -161.5896 -161.1131 -160.6260
##  [17,] -162.5030 -162.0466 -161.5801 -161.1034 -160.6160 -160.1179 -159.6086
##  [18,] -161.5706 -161.0936 -160.6061 -160.1077 -159.5982 -159.0772 -158.5447
##  [19,] -160.5961 -160.0975 -159.5878 -159.0666 -158.5338 -157.9891 -157.4321
##  [20,] -159.5773 -159.0560 -158.5229 -157.9779 -157.4207 -156.8511 -156.2686
##  [21,] -158.5120 -157.9668 -157.4094 -156.8394 -156.2567 -155.6610 -155.0519
##  [22,] -157.3980 -156.8278 -156.2448 -155.6488 -155.0395 -154.4165 -153.7795
##  [23,] -156.2329 -155.6367 -155.0270 -154.4038 -153.7665 -153.1150 -152.4489
##  [24,] -155.0146 -154.3910 -153.7535 -153.1017 -152.4353 -151.7539 -151.0571
##  [25,] -153.7405 -153.0884 -152.4216 -151.7399 -151.0429 -150.3302 -149.6014
##  [26,] -152.4080 -151.7260 -151.0287 -150.3156 -149.5865 -148.8408 -148.0783
##  [27,] -151.0144 -150.3010 -149.5716 -148.8256 -148.0627 -147.2824 -146.4844
##  [28,] -149.5567 -148.8103 -148.0471 -147.2665 -146.4681 -145.6513 -144.8158
##  [29,] -148.0315 -147.2505 -146.4517 -145.6346 -144.7987 -143.9435 -143.0684
##  [30,] -146.4269 -145.6167 -144.7816 -143.9260 -143.0505 -142.1597 -141.2753
##  [31,] -144.7186 -143.8840 -143.0231 -142.1404 -141.2575 -140.3818 -139.5142
##  [32,] -142.8982 -142.0435 -141.1894 -140.3373 -139.4862 -138.6353 -137.7844
##  [33,] -140.9980 -140.1743 -139.3528 -138.5325 -137.7127 -136.8925 -136.0713
##  [34,] -139.0905 -138.2989 -137.5087 -136.7193 -135.9299 -135.1396 -134.3479
##  [35,] -137.1714 -136.4109 -135.6513 -134.8919 -134.1321 -133.3711 -132.6083
##  [36,] -135.2351 -134.5048 -133.7750 -133.0450 -132.3143 -131.5820 -130.8478
##  [37,] -133.2766 -132.5758 -131.8752 -131.1741 -130.4719 -129.7682 -129.0625
##  [38,] -131.2921 -130.6201 -129.9480 -129.2754 -128.6017 -127.9264 -127.2492
##  [39,] -129.2788 -128.6349 -127.9910 -127.3465 -126.7012 -126.0544 -125.4060
##  [40,] -127.2354 -126.6190 -126.0028 -125.3864 -124.7694 -124.1515 -123.5325
##  [41,] -125.1630 -124.5734 -123.9845 -123.3960 -122.8076 -122.2189 -121.6300
##  [42,] -123.0652 -122.5015 -121.9395 -121.3787 -120.8191 -120.2603 -119.7023
##  [43,] -120.9494 -120.4106 -119.8747 -119.3415 -118.8109 -118.2826 -117.7568
##  [44,] -118.8281 -118.3125 -117.8018 -117.2958 -116.7944 -116.2973 -115.8047
##  [45,] -116.7202 -116.2256 -115.7387 -115.2590 -114.7866 -114.3213 -113.8632
##  [46,] -114.6528 -114.1761 -113.7107 -113.2561 -112.8122 -112.3789 -111.9563
##  [47,] -112.6635 -112.2004 -111.7532 -111.3214 -110.9048 -110.5032 -110.1166
##  [48,] -110.8024 -110.3470 -109.9134 -109.5009 -109.1092 -108.7381 -108.3874
##  [49,] -109.1337 -108.6780 -108.2515 -107.8533 -107.4828 -107.1396 -106.8234
##  [50,] -107.6991 -107.2612 -106.8417 -106.4508 -106.0959 -105.7789 -105.5003
##  [51,] -106.5105 -106.1099 -105.7280 -105.3694 -105.0366 -104.7378 -104.4793
##  [52,] -105.6009 -105.2525 -104.9239 -104.6145 -104.3242 -104.0527 -103.8001
##  [53,] -105.0027 -104.7119 -104.4339 -104.1683 -103.9150 -103.6742 -103.4461
##  [54,] -104.7131 -104.4724 -104.2376 -104.0089 -103.7864 -103.5703 -103.3611
##  [55,] -104.7080 -104.5083 -104.3086 -104.1091 -103.9102 -103.7123 -103.5163
##  [56,] -104.9585 -104.7900 -104.6162 -104.4377 -104.2550 -104.0688 -103.8799
##  [57,] -105.4329 -105.2854 -105.1284 -104.9625 -104.7886 -104.6074 -104.4199
##  [58,] -106.0994 -105.9628 -105.8133 -105.6519 -105.4795 -105.2970 -105.1054
##  [59,] -106.9274 -106.7917 -106.6410 -106.4763 -106.2986 -106.1086 -105.9076
##  [60,] -107.8888 -107.7447 -107.5845 -107.4091 -107.2194 -107.0164 -106.8009
##  [61,] -108.9593 -108.7980 -108.6203 -108.4272 -108.2194 -107.9978 -107.7632
##  [62,] -110.1182 -109.9313 -109.7288 -109.5112 -109.2794 -109.0340 -108.7758
##  [63,] -111.3488 -111.1285 -110.8939 -110.6456 -110.3840 -110.1098 -109.8235
##  [64,] -112.6383 -112.3770 -112.1035 -111.8181 -111.5212 -111.2131 -110.8942
##  [65,] -113.9778 -113.6679 -113.3487 -113.0201 -112.6821 -112.3350 -111.9789
##  [66,] -115.3615 -114.9958 -114.6240 -114.2458 -113.8610 -113.4695 -113.0712
##  [67,] -116.7872 -116.3581 -115.9268 -115.4924 -115.0546 -114.6130 -114.1674
##  [68,] -118.2556 -117.7555 -117.2572 -116.7598 -116.2625 -115.7647 -115.2659
##  [69,] -119.7712 -119.1913 -118.6184 -118.0505 -117.4866 -116.9259 -116.3686
##  [70,] -121.3605 -120.6807 -120.0167 -119.3694 -118.7325 -118.1150 -117.5181
##  [71,] -123.0108 -122.2402 -121.4816 -120.7499 -120.0510 -119.3855 -118.7452
##  [72,] -124.6959 -123.8610 -123.0477 -122.2587 -121.4967 -120.7644 -120.0647
##  [73,] -126.4385 -125.5673 -124.7129 -123.8776 -123.0639 -122.2744 -121.5118
##  [74,] -128.2409 -127.3423 -126.4563 -125.5848 -124.7300 -123.8943 -123.0801
##  [75,] -130.0867 -129.1685 -128.2591 -127.3604 -126.4740 -125.6022 -124.7471
##  [76,] -131.9622 -131.0308 -130.1053 -129.1870 -128.2774 -127.3784 -126.4918
##  [77,] -133.8560 -132.9169 -131.9810 -131.0496 -130.1239 -129.2054 -128.2957
##  [78,] -135.7593 -134.8165 -133.8750 -132.9358 -131.9999 -131.0684 -130.1426
##  [79,] -137.6649 -136.7216 -135.7783 -134.8355 -133.8940 -132.9548 -132.0188
##  [80,] -139.5680 -138.6265 -137.6839 -136.7407 -135.7974 -134.8545 -133.9130
##  [81,] -141.4653 -140.5270 -139.5870 -138.6455 -137.7030 -136.7598 -135.8164
##  [82,] -143.3555 -142.4208 -141.4842 -140.5459 -139.6060 -138.6646 -137.7220
##  [83,] -145.2385 -144.3072 -143.3743 -142.4397 -141.5032 -140.5649 -139.6250
##  [84,] -147.1161 -146.1872 -145.2573 -144.3261 -143.3932 -142.4586 -141.5221
##  [85,] -148.9915 -148.0632 -147.1349 -146.2060 -145.2761 -144.3449 -143.4121
##  [86,] -150.8694 -149.9392 -149.0103 -148.0820 -147.1537 -146.2248 -145.2949
##  [87,] -152.7563 -151.8207 -150.8882 -149.9579 -149.0290 -148.1007 -147.1724
##  [88,] -154.6606 -153.7152 -152.7752 -151.8396 -150.9070 -149.9767 -149.0478
##  [89,] -156.5884 -155.6320 -154.6797 -153.7342 -152.7942 -151.8584 -150.9258
##  [90,] -158.5041 -157.5588 -156.6076 -155.6514 -154.6989 -153.7533 -152.8131
##  [91,] -160.3928 -159.4616 -158.5231 -157.5780 -156.6269 -155.6707 -154.7181
##  [92,] -162.2497 -161.3348 -160.4116 -159.4805 -158.5421 -157.5972 -156.6462
##  [93,] -164.0698 -163.1737 -162.2680 -161.3534 -160.4303 -159.4993 -158.5612
##  [94,] -165.8485 -164.9735 -164.0878 -163.1919 -162.2864 -161.3719 -160.4490
##  [95,] -167.5810 -166.7295 -165.8660 -164.9913 -164.1058 -163.2101 -162.3048
##  [96,] -169.2626 -168.4369 -167.5981 -166.7468 -165.8836 -165.0091 -164.1238
##  [97,] -170.8882 -170.0908 -169.2791 -168.4537 -167.6152 -166.7641 -165.9012
##  [98,] -172.4523 -171.6861 -170.9041 -170.1071 -169.2957 -168.4705 -167.6322
##  [99,] -173.9492 -173.2171 -172.4677 -171.7018 -170.9201 -170.1233 -169.3122
## [100,] -175.3719 -174.6774 -173.9638 -173.2320 -172.4830 -171.7174 -170.9360
##            [,64]     [,65]     [,66]     [,67]     [,68]     [,69]     [,70]
##   [1,] -172.8520 -172.4363 -172.0192 -171.6015 -171.1835 -170.7660 -170.3493
##   [2,] -172.0108 -171.5930 -171.1751 -170.7575 -170.3409 -169.9258 -169.5125
##   [3,] -171.1666 -170.7491 -170.3325 -169.9174 -169.5042 -169.0935 -168.6857
##   [4,] -170.3241 -169.9090 -169.4959 -169.0852 -168.6775 -168.2732 -167.8727
##   [5,] -169.4876 -169.0770 -168.6693 -168.2651 -167.8647 -167.4686 -167.0772
##   [6,] -168.6611 -168.2569 -167.8566 -167.4606 -167.0694 -166.6833 -166.3027
##   [7,] -167.8486 -167.4527 -167.0615 -166.6755 -166.2950 -165.9132 -165.5253
##   [8,] -167.0537 -166.6678 -166.2874 -165.9055 -165.5174 -165.1226 -164.7204
##   [9,] -166.2797 -165.8977 -165.5095 -165.1145 -164.7122 -164.3022 -163.8839
##  [10,] -165.5016 -165.1065 -164.7040 -164.2938 -163.8754 -163.4483 -163.0122
##  [11,] -164.6958 -164.2854 -163.8668 -163.4396 -163.0033 -162.5577 -162.1024
##  [12,] -163.8583 -163.4308 -162.9944 -162.5486 -162.0931 -161.6277 -161.1520
##  [13,] -162.9855 -162.5395 -162.0838 -161.6182 -161.1423 -160.6558 -160.1585
##  [14,] -162.0745 -161.6087 -161.1326 -160.6459 -160.1484 -159.6398 -159.1198
##  [15,] -161.1228 -160.6359 -160.1382 -159.6294 -159.1091 -158.5773 -158.0335
##  [16,] -160.1280 -159.6190 -159.0985 -158.5664 -158.0224 -157.4662 -156.8976
##  [17,] -159.0879 -158.5556 -158.0113 -157.4549 -156.8860 -156.3043 -155.7096
##  [18,] -158.0002 -157.4435 -156.8743 -156.2924 -155.6975 -155.0892 -154.4673
##  [19,] -156.8627 -156.2805 -155.6853 -155.0768 -154.4546 -153.8185 -153.1682
##  [20,] -155.6732 -155.0644 -154.4419 -153.8056 -153.1549 -152.4897 -151.8095
##  [21,] -154.4292 -153.7925 -153.1416 -152.4761 -151.7956 -151.0998 -150.3884
##  [22,] -153.1283 -152.4625 -151.7817 -151.0856 -150.3738 -149.6460 -148.9017
##  [23,] -151.7678 -151.0714 -150.3593 -149.6311 -148.8865 -148.1250 -147.3461
##  [24,] -150.3447 -149.6162 -148.8713 -148.1094 -147.3302 -146.5333 -145.7180
##  [25,] -148.8561 -148.0939 -147.3143 -146.5170 -145.7014 -144.8670 -144.0133
##  [26,] -147.2984 -146.5007 -145.6847 -144.8499 -143.9959 -143.1220 -142.2315
##  [27,] -145.6680 -144.8329 -143.9784 -143.1041 -142.2136 -141.3287 -140.4523
##  [28,] -143.9610 -143.0863 -142.1956 -141.3109 -140.4347 -139.5665 -138.7061
##  [29,] -142.1777 -141.2931 -140.4170 -139.5491 -138.6888 -137.8358 -136.9898
##  [30,] -140.3994 -139.5316 -138.6715 -137.8187 -136.9728 -136.1334 -135.3003
##  [31,] -138.6542 -137.8015 -136.9557 -136.1165 -135.2835 -134.4564 -133.6350
##  [32,] -136.9387 -136.0996 -135.2667 -134.4398 -133.6184 -132.8025 -131.9918
##  [33,] -135.2482 -134.4231 -133.6019 -132.7861 -131.9755 -131.1699 -130.3691
##  [34,] -133.5539 -132.7572 -131.9572 -131.1537 -130.3530 -129.5571 -128.7657
##  [35,] -131.8431 -131.0749 -130.3032 -129.5276 -128.7476 -127.9632 -127.1812
##  [36,] -130.1111 -129.3713 -128.6280 -127.8809 -127.1296 -126.3737 -125.6129
##  [37,] -128.3542 -127.6429 -126.9284 -126.2103 -125.4882 -124.7620 -124.0315
##  [38,] -126.5696 -125.8873 -125.2021 -124.5136 -123.8218 -123.1265 -122.4276
##  [39,] -124.7556 -124.1030 -123.4479 -122.7903 -122.1301 -121.4672 -120.8017
##  [40,] -122.9120 -122.2900 -121.6664 -121.0411 -120.4142 -119.7857 -119.1558
##  [41,] -121.0405 -120.4504 -119.8598 -119.2688 -118.6773 -118.0858 -117.4943
##  [42,] -119.1451 -118.5886 -118.0330 -117.4785 -116.9252 -116.3735 -115.8239
##  [43,] -117.2333 -116.7123 -116.1940 -115.6787 -115.1668 -114.6585 -114.1546
##  [44,] -115.3167 -114.8334 -114.3551 -113.8822 -113.4151 -112.9543 -112.5006
##  [45,] -113.4124 -112.9692 -112.5338 -112.1068 -111.6885 -111.2797 -110.8810
##  [46,] -111.5444 -111.1437 -110.7543 -110.3767 -110.0116 -109.6595 -109.3245
##  [47,] -109.7452 -109.3890 -109.0486 -108.7242 -108.4197 -108.1384 -107.8785
##  [48,] -108.0571 -107.7473 -107.4614 -107.2020 -106.9669 -106.7538 -106.5609
##  [49,] -106.5369 -106.2817 -106.0551 -105.8546 -105.6776 -105.5221 -105.3858
##  [50,] -105.2567 -105.0449 -104.8622 -104.7057 -104.5728 -104.4612 -104.3686
##  [51,] -104.2576 -104.0693 -103.9114 -103.7807 -103.6747 -103.5906 -103.5263
##  [52,] -103.5757 -103.3873 -103.2318 -103.1058 -103.0065 -102.9311 -102.8770
##  [53,] -103.2310 -103.0294 -102.8514 -102.7065 -102.5915 -102.5035 -102.4397
##  [54,] -103.1594 -102.9658 -102.7810 -102.6061 -102.4515 -102.3281 -102.2331
##  [55,] -103.3226 -103.1323 -102.9462 -102.7653 -102.5907 -102.4238 -102.2751
##  [56,] -103.6892 -103.4976 -103.3062 -103.1160 -102.9284 -102.7444 -102.5656
##  [57,] -104.2271 -104.0300 -103.8296 -103.6272 -103.4240 -103.2213 -103.0206
##  [58,] -104.9057 -104.6989 -104.4863 -104.2689 -104.0481 -103.8251 -103.6014
##  [59,] -105.6965 -105.4763 -105.2482 -105.0134 -104.7731 -104.5286 -104.2812
##  [60,] -106.5742 -106.3370 -106.0906 -105.8360 -105.5744 -105.3071 -105.0353
##  [61,] -107.5167 -107.2592 -106.9916 -106.7150 -106.4305 -106.1392 -105.8424
##  [62,] -108.5057 -108.2244 -107.9329 -107.6320 -107.3228 -107.0064 -106.6838
##  [63,] -109.5258 -109.2174 -108.8991 -108.5717 -108.2360 -107.8929 -107.5435
##  [64,] -110.5650 -110.2261 -109.8780 -109.5214 -109.1571 -108.7859 -108.4086
##  [65,] -111.6140 -111.2409 -110.8598 -110.4713 -110.0761 -109.6747 -109.2679
##  [66,] -112.6663 -112.2549 -111.8373 -111.4138 -110.9849 -110.5515 -110.1216
##  [67,] -113.7176 -113.2635 -112.8054 -112.3439 -111.8876 -111.4386 -110.9963
##  [68,] -114.7659 -114.2651 -113.7726 -113.2904 -112.8175 -112.3532 -111.8967
##  [69,] -115.8245 -115.2950 -114.7790 -114.2751 -113.7825 -113.3000 -112.8270
##  [70,] -116.9402 -116.3798 -115.8353 -115.3056 -114.7893 -114.2852 -113.7923
##  [71,] -118.1272 -117.5300 -116.9517 -116.3909 -115.8462 -115.3162 -114.7996
##  [72,] -119.3987 -118.7579 -118.1395 -117.5418 -116.9632 -116.4021 -115.8571
##  [73,] -120.7789 -120.0785 -119.4119 -118.7706 -118.1518 -117.5537 -116.9747
##  [74,] -122.2901 -121.5269 -120.7933 -120.0923 -119.4251 -118.7833 -118.1641
##  [75,] -123.9110 -123.0964 -122.3058 -121.5420 -120.8079 -120.1061 -119.4383
##  [76,] -125.6197 -124.7642 -123.9277 -123.1126 -122.3215 -121.5572 -120.8224
##  [77,] -127.3965 -126.5096 -125.6371 -124.7813 -123.9444 -123.1288 -122.3372
##  [78,] -129.2239 -128.3140 -127.4145 -126.5274 -125.6546 -124.7984 -123.9611
##  [79,] -131.0871 -130.1612 -129.2424 -128.3323 -127.4326 -126.5452 -125.6721
##  [80,] -132.9737 -132.0376 -131.1059 -130.1798 -129.2609 -128.3505 -127.4506
##  [81,] -134.8736 -133.9320 -132.9927 -132.0565 -131.1247 -130.1985 -129.2793
##  [82,] -136.7788 -135.8355 -134.8926 -133.9510 -133.0116 -132.0754 -131.1434
##  [83,] -138.6836 -137.7411 -136.7979 -135.8545 -134.9117 -133.9700 -133.0306
##  [84,] -140.5839 -139.6440 -138.7026 -137.7601 -136.8169 -135.8736 -134.9307
##  [85,] -142.4775 -141.5411 -140.6029 -139.6630 -138.7216 -137.7792 -136.8360
##  [86,] -144.3637 -143.4309 -142.4964 -141.5600 -140.6218 -139.6820 -138.7407
##  [87,] -146.2436 -145.3137 -144.3825 -143.4498 -142.5153 -141.5789 -140.6408
##  [88,] -148.1195 -147.1912 -146.2623 -145.3325 -144.4014 -143.4686 -142.5342
##  [89,] -149.9955 -149.0665 -148.1382 -147.2099 -146.2811 -145.3513 -144.4202
##  [90,] -151.8773 -150.9447 -150.0143 -149.0853 -148.1570 -147.2287 -146.2999
##  [91,] -153.7723 -152.8321 -151.8962 -150.9635 -150.0331 -149.1041 -148.1757
##  [92,] -155.6900 -154.7372 -153.7914 -152.8510 -151.9150 -150.9823 -150.0518
##  [93,] -157.6163 -156.6654 -155.7093 -154.7564 -153.8104 -152.8700 -151.9339
##  [94,] -159.5182 -158.5802 -157.6355 -156.6847 -155.7287 -154.7756 -153.8295
##  [95,] -161.3905 -160.4677 -159.5371 -158.5992 -157.6546 -156.7040 -155.7480
##  [96,] -163.2283 -162.3232 -161.4091 -160.4865 -159.5560 -158.6182 -157.6738
##  [97,] -165.0268 -164.1418 -163.2465 -162.3416 -161.4276 -160.5052 -159.5748
##  [98,] -166.7814 -165.9187 -165.0446 -164.1597 -163.2647 -162.3599 -161.4462
##  [99,] -168.4873 -167.6493 -166.7987 -165.9362 -165.0624 -164.1777 -163.2828
## [100,] -170.1396 -169.3287 -168.5041 -167.6664 -166.8160 -165.9538 -165.0801
##            [,71]     [,72]     [,73]     [,74]     [,75]     [,76]     [,77]
##   [1,] -169.9341 -169.5209 -169.1100 -168.7021 -168.2976 -167.8969 -167.5004
##   [2,] -169.1018 -168.6939 -168.2895 -167.8888 -167.4925 -167.1008 -166.7143
##   [3,] -168.2813 -167.8808 -167.4845 -167.0930 -166.7065 -166.3256 -165.9443
##   [4,] -167.4766 -167.0851 -166.6988 -166.3180 -165.9365 -165.5490 -165.1547
##   [5,] -166.6910 -166.3103 -165.9288 -165.5411 -165.1467 -164.7450 -164.3356
##   [6,] -165.9210 -165.5332 -165.1387 -164.7368 -164.3273 -163.9095 -163.4831
##   [7,] -165.1306 -164.7286 -164.3189 -163.9010 -163.4744 -163.0389 -162.5941
##   [8,] -164.3105 -163.8924 -163.4657 -163.0300 -162.5850 -162.1303 -161.6657
##   [9,] -163.4570 -163.0211 -162.5759 -162.1210 -161.6562 -161.1811 -160.6955
##  [10,] -162.5668 -162.1117 -161.6467 -161.1714 -160.6856 -160.1890 -159.6813
##  [11,] -161.6372 -161.1617 -160.6757 -160.1788 -159.6709 -159.1516 -158.6207
##  [12,] -160.6658 -160.1687 -159.6605 -159.1410 -158.6099 -158.0668 -157.5116
##  [13,] -159.6501 -159.1304 -158.5990 -158.0557 -157.5003 -156.9324 -156.3518
##  [14,] -158.5882 -158.0446 -157.4889 -156.9208 -156.3399 -155.7461 -155.1389
##  [15,] -157.4776 -156.9092 -156.3281 -155.7339 -155.1265 -154.5054 -153.8705
##  [16,] -156.3162 -155.7218 -155.1141 -154.4927 -153.8575 -153.2080 -152.5440
##  [17,] -155.1017 -154.4801 -153.8445 -153.1948 -152.5304 -151.8511 -151.1566
##  [18,] -153.8315 -153.1815 -152.5168 -151.8373 -151.1424 -150.4319 -149.7054
##  [19,] -152.5033 -151.8234 -151.1282 -150.4174 -149.6906 -148.9473 -148.1872
##  [20,] -151.1140 -150.4029 -149.6757 -148.9321 -148.1716 -147.3938 -146.5983
##  [21,] -149.6609 -148.9169 -148.1561 -147.3780 -146.5821 -145.7680 -144.9351
##  [22,] -148.1405 -147.3621 -146.5658 -145.7513 -144.9181 -144.0656 -143.1934
##  [23,] -146.5495 -145.7347 -144.9011 -144.0482 -143.1755 -142.2855 -141.3998
##  [24,] -144.8840 -144.0308 -143.1577 -142.2675 -141.3820 -140.5051 -139.6364
##  [25,] -143.1399 -142.2495 -141.3642 -140.4875 -139.6189 -138.7581 -137.9045
##  [26,] -141.3464 -140.4699 -139.6015 -138.7407 -137.8873 -137.0409 -136.2010
##  [27,] -139.5840 -138.7234 -137.8702 -137.0238 -136.1841 -135.3506 -134.5230
##  [28,] -137.8530 -137.0068 -136.1672 -135.3338 -134.5064 -133.6846 -132.8683
##  [29,] -136.1503 -135.3170 -134.4897 -133.6681 -132.8518 -132.0408 -131.2348
##  [30,] -134.4731 -133.6515 -132.8354 -132.0245 -131.2186 -130.4175 -129.6212
##  [31,] -132.8190 -132.0082 -131.2023 -130.4014 -129.6052 -128.8136 -128.0266
##  [32,] -131.1861 -130.3853 -129.5891 -128.7976 -128.0107 -127.2284 -126.4507
##  [33,] -129.5731 -128.7817 -127.9949 -127.2127 -126.4351 -125.6621 -124.8940
##  [34,] -127.9790 -127.1969 -126.4194 -125.6466 -124.8786 -124.1155 -123.3577
##  [35,] -126.4037 -125.6310 -124.8631 -124.1002 -123.3425 -122.5903 -121.8439
##  [36,] -124.8476 -124.0848 -123.3272 -122.5752 -121.8289 -121.0889 -120.3556
##  [37,] -123.2966 -122.5573 -121.8139 -121.0740 -120.3409 -119.6150 -118.8969
##  [38,] -121.7251 -121.0191 -120.3098 -119.5974 -118.8825 -118.1731 -117.4731
##  [39,] -120.1336 -119.4633 -118.7910 -118.1170 -117.4418 -116.7660 -116.0907
##  [40,] -118.5248 -117.8930 -117.2608 -116.6286 -115.9972 -115.3673 -114.7397
##  [41,] -116.9034 -116.3135 -115.7251 -115.1389 -114.5556 -113.9762 -113.4017
##  [42,] -115.2768 -114.7328 -114.1926 -113.6570 -113.1270 -112.6035 -112.0878
##  [43,] -113.6555 -113.1621 -112.6752 -112.1957 -111.7246 -111.2661 -110.8253
##  [44,] -112.0545 -111.6170 -111.1889 -110.7745 -110.3786 -110.0000 -109.6377
##  [45,] -110.4933 -110.1207 -109.7675 -109.4325 -109.1144 -108.8120 -108.5242
##  [46,] -109.0105 -108.7158 -108.4392 -108.1790 -107.9341 -107.7031 -107.4848
##  [47,] -107.6381 -107.4156 -107.2095 -107.0182 -106.8404 -106.6748 -106.5202
##  [48,] -106.3863 -106.2282 -106.0850 -105.9552 -105.8373 -105.7301 -105.6323
##  [49,] -105.2669 -105.1634 -105.0737 -104.9963 -104.9297 -104.8726 -104.8237
##  [50,] -104.2930 -104.2324 -104.1851 -104.1495 -104.1241 -104.1074 -104.0984
##  [51,] -103.4793 -103.4478 -103.4299 -103.4237 -103.4279 -103.4409 -103.4614
##  [52,] -102.8419 -102.8236 -102.8201 -102.8295 -102.8501 -102.8805 -102.9192
##  [53,] -102.3976 -102.3748 -102.3691 -102.3785 -102.4011 -102.4354 -102.4796
##  [54,] -102.1636 -102.1171 -102.0912 -102.0837 -102.0925 -102.1157 -102.1517
##  [55,] -102.1569 -102.0665 -102.0012 -101.9588 -101.9368 -101.9332 -101.9462
##  [56,] -102.3934 -102.2386 -102.1143 -102.0182 -101.9477 -101.9007 -101.8748
##  [57,] -102.8232 -102.6308 -102.4450 -102.2765 -102.1395 -102.0316 -101.9507
##  [58,] -103.3785 -103.1578 -102.9410 -102.7298 -102.5261 -102.3405 -102.1877
##  [59,] -104.0323 -103.7836 -103.5365 -103.2928 -103.0543 -102.8227 -102.6001
##  [60,] -104.7605 -104.4840 -104.2074 -103.9323 -103.6605 -103.3938 -103.1341
##  [61,] -105.5413 -105.2374 -104.9320 -104.6267 -104.3233 -104.0233 -103.7288
##  [62,] -106.3562 -106.0249 -105.6913 -105.3568 -105.0231 -104.6918 -104.3649
##  [63,] -107.1889 -106.8303 -106.4688 -106.1060 -105.7435 -105.3869 -105.0389
##  [64,] -108.0262 -107.6398 -107.2509 -106.8659 -106.4872 -106.1153 -105.7507
##  [65,] -108.8571 -108.4492 -108.0468 -107.6499 -107.2587 -106.8736 -106.4948
##  [66,] -109.6974 -109.2787 -108.8654 -108.4574 -108.0549 -107.6579 -107.2666
##  [67,] -110.5603 -110.1303 -109.7059 -109.2871 -108.8737 -108.4656 -108.0630
##  [68,] -111.4476 -111.0051 -110.5690 -110.1389 -109.7145 -109.2955 -108.8820
##  [69,] -112.3625 -111.9059 -111.4566 -111.0140 -110.5778 -110.1475 -109.7230
##  [70,] -113.3097 -112.8365 -112.3718 -111.9150 -111.4656 -111.0229 -110.5865
##  [71,] -114.2952 -113.8022 -113.3194 -112.8459 -112.3811 -111.9242 -111.4746
##  [72,] -115.3267 -114.8099 -114.3053 -113.8120 -113.3290 -112.8554 -112.3904
##  [73,] -116.4133 -115.8679 -115.3373 -114.8202 -114.3154 -113.8219 -113.3387
##  [74,] -117.5656 -116.9862 -116.4244 -115.8788 -115.3479 -114.8305 -114.3255
##  [75,] -118.7961 -118.1764 -117.5775 -116.9978 -116.4356 -115.8897 -115.3585
##  [76,] -120.1200 -119.4515 -118.8088 -118.1887 -117.5894 -117.0093 -116.4468
##  [77,] -121.5723 -120.8369 -120.1338 -119.4647 -118.8216 -118.2010 -117.6013
##  [78,] -123.1451 -122.3530 -121.5875 -120.8515 -120.1477 -119.4780 -118.8343
##  [79,] -124.8155 -123.9778 -123.1613 -122.3687 -121.6027 -120.8660 -120.1616
##  [80,] -126.5630 -125.6896 -124.8327 -123.9945 -123.1776 -122.3845 -121.6179
##  [81,] -128.3688 -127.4687 -126.5808 -125.7071 -124.8498 -124.0112 -123.1939
##  [82,] -130.2171 -129.2978 -128.3871 -127.4868 -126.5986 -125.7246 -124.8669
##  [83,] -132.0943 -131.1622 -130.2358 -129.3163 -128.4054 -127.5048 -126.6164
##  [84,] -133.9891 -133.0495 -132.1131 -131.1810 -130.2544 -129.3348 -128.4237
##  [85,] -135.8926 -134.9497 -134.0081 -133.0685 -132.1320 -131.1998 -130.2731
##  [86,] -137.7982 -136.8551 -135.9117 -134.9688 -134.0271 -133.0874 -132.1509
##  [87,] -139.7010 -138.7597 -137.8173 -136.8741 -135.9307 -134.9878 -134.0461
##  [88,] -141.5979 -140.6598 -139.7200 -138.7787 -137.8363 -136.8932 -135.9498
##  [89,] -143.4875 -142.5531 -141.6168 -140.6787 -139.7390 -138.7978 -137.8554
##  [90,] -145.3701 -144.4390 -143.5064 -142.5720 -141.6357 -140.6977 -139.7580
##  [91,] -147.2474 -146.3187 -145.3889 -144.4578 -143.5252 -142.5908 -141.6547
##  [92,] -149.1228 -148.1945 -147.2662 -146.3374 -145.4077 -144.4767 -143.5441
##  [93,] -151.0011 -150.0706 -149.1416 -148.2132 -147.2850 -146.3562 -145.4265
##  [94,] -152.8889 -151.9528 -151.0199 -150.0894 -149.1603 -148.2320 -147.3037
##  [95,] -154.7948 -153.8485 -152.9079 -151.9717 -151.0387 -150.1082 -149.1791
##  [96,] -156.7232 -155.7673 -154.8140 -153.8676 -152.9268 -151.9905 -151.0576
##  [97,] -158.6372 -157.6929 -156.7425 -155.7867 -154.8331 -153.8866 -152.9458
##  [98,] -160.5239 -159.5937 -158.6562 -157.7120 -156.7618 -155.8060 -154.8523
##  [99,] -162.3783 -161.4647 -160.5426 -159.6126 -158.6752 -157.7312 -156.7810
## [100,] -164.1957 -163.3010 -162.3967 -161.4833 -160.5613 -159.6314 -158.6942
##            [,78]     [,79]     [,80]     [,81]     [,82]     [,83]     [,84]
##   [1,] -167.1087 -166.7220 -166.3409 -165.9598 -165.5727 -165.1788 -164.7778
##   [2,] -166.3333 -165.9521 -165.5648 -165.1708 -164.7696 -164.3607 -163.9436
##   [3,] -165.5569 -165.1628 -164.7614 -164.3523 -163.9350 -163.5092 -163.0744
##   [4,] -164.7532 -164.3440 -163.9265 -163.5005 -163.0656 -162.6213 -162.1674
##   [5,] -163.9180 -163.4918 -163.0567 -162.6122 -162.1581 -161.6941 -161.2199
##   [6,] -163.0478 -162.6031 -162.1489 -161.6847 -161.2102 -160.7253 -160.2295
##   [7,] -162.1396 -161.6752 -161.2005 -160.7154 -160.2194 -159.7124 -159.1940
##   [8,] -161.1908 -160.7054 -160.2093 -159.7020 -159.1834 -158.6532 -158.1112
##   [9,] -160.1991 -159.6916 -159.1728 -158.6424 -158.1001 -157.5456 -156.9788
##  [10,] -159.1622 -158.6315 -158.0890 -157.5343 -156.9672 -156.3873 -155.7945
##  [11,] -158.0779 -157.5230 -156.9556 -156.3755 -155.7824 -155.1761 -154.5561
##  [12,] -156.9440 -156.3636 -155.7703 -155.1637 -154.5435 -153.9094 -153.2610
##  [13,] -155.7582 -155.1513 -154.5308 -153.8964 -153.2478 -152.5846 -151.9066
##  [14,] -154.5181 -153.8834 -153.2345 -152.5711 -151.8927 -151.1991 -150.4899
##  [15,] -153.2213 -152.5575 -151.8789 -151.1850 -150.4754 -149.7499 -149.0080
##  [16,] -151.8650 -151.1708 -150.4610 -149.7351 -148.9928 -148.2337 -147.4573
##  [17,] -150.4464 -149.7203 -148.9776 -148.2182 -147.4415 -146.6471 -145.8344
##  [18,] -148.9625 -148.2027 -147.4256 -146.6308 -145.8178 -144.9861 -144.1352
##  [19,] -147.4097 -146.6146 -145.8012 -144.9691 -144.1179 -143.2468 -142.3574
##  [20,] -145.7846 -144.9521 -144.1005 -143.2290 -142.3394 -141.4533 -140.5757
##  [21,] -144.0830 -143.2112 -142.3214 -141.4355 -140.5581 -139.6888 -138.8273
##  [22,] -142.3035 -141.4176 -140.5404 -139.6714 -138.8100 -137.9560 -137.1090
##  [23,] -140.5228 -139.6539 -138.7927 -137.9389 -137.0920 -136.2517 -135.4177
##  [24,] -138.7754 -137.9217 -137.0749 -136.2348 -135.4009 -134.5730 -133.7508
##  [25,] -137.0579 -136.2179 -135.3841 -134.5564 -133.7343 -132.9176 -132.1062
##  [26,] -135.3674 -134.5397 -133.7177 -132.9012 -132.0898 -131.2835 -130.4821
##  [27,] -133.7012 -132.8847 -132.0735 -131.2673 -130.4659 -129.6693 -128.8774
##  [28,] -132.0572 -131.2510 -130.4498 -129.6533 -128.8614 -128.0742 -127.2915
##  [29,] -130.4337 -129.6372 -128.8455 -128.0583 -127.2757 -126.4977 -125.7244
##  [30,] -128.8295 -128.0424 -127.2599 -126.4820 -125.7088 -124.9404 -124.1770
##  [31,] -127.2442 -126.4664 -125.6933 -124.9250 -124.1616 -123.4035 -122.6509
##  [32,] -125.6777 -124.9095 -124.1463 -123.3883 -122.6357 -121.8890 -121.1485
##  [33,] -124.1309 -123.3730 -122.6206 -121.8740 -121.1336 -120.3999 -119.6733
##  [34,] -122.6054 -121.8589 -121.1187 -120.3851 -119.6587 -118.9402 -118.2301
##  [35,] -121.1038 -120.3704 -119.6441 -118.9258 -118.2159 -117.5152 -116.8246
##  [36,] -119.6296 -118.9113 -118.2016 -117.5012 -116.8108 -116.1315 -115.4641
##  [37,] -118.1874 -117.4871 -116.7970 -116.1179 -115.4508 -114.7968 -114.1572
##  [38,] -116.7831 -116.1043 -115.4374 -114.7838 -114.1444 -113.5207 -112.9139
##  [39,] -115.4241 -114.7707 -114.1316 -113.5082 -112.9018 -112.3138 -111.7483
##  [40,] -114.1154 -113.4958 -112.8897 -112.3021 -111.7371 -111.2007 -110.6925
##  [41,] -112.8332 -112.2720 -111.7223 -111.1902 -110.6825 -110.2022 -109.7485
##  [42,] -111.5839 -111.0975 -110.6278 -110.1742 -109.7358 -109.3123 -108.9100
##  [43,] -110.4015 -109.9937 -109.6010 -109.2227 -108.8579 -108.5059 -108.1660
##  [44,] -109.2907 -108.9579 -108.6385 -108.3316 -108.0363 -107.7519 -107.4775
##  [45,] -108.2498 -107.9879 -107.7374 -107.4975 -107.2673 -107.0460 -106.8328
##  [46,] -107.2781 -107.0820 -106.8955 -106.7177 -106.5477 -106.3848 -106.2281
##  [47,] -106.3755 -106.2396 -106.1115 -105.9904 -105.8753 -105.7656 -105.6604
##  [48,] -105.5428 -105.4607 -105.3848 -105.3143 -105.2484 -105.1864 -105.1276
##  [49,] -104.7819 -104.7462 -104.7156 -104.6893 -104.6664 -104.6462 -104.6281
##  [50,] -104.0957 -104.0984 -104.1055 -104.1161 -104.1294 -104.1448 -104.1615
##  [51,] -103.4882 -103.5203 -103.5567 -103.5964 -103.6387 -103.6827 -103.7280
##  [52,] -102.9649 -103.0165 -103.0729 -103.1331 -103.1964 -103.2618 -103.3288
##  [53,] -102.5324 -102.5926 -102.6589 -102.7303 -102.8060 -102.8849 -102.9663
##  [54,] -102.1989 -102.2557 -102.3210 -102.3935 -102.4721 -102.5559 -102.6439
##  [55,] -101.9738 -102.0145 -102.0668 -102.1294 -102.2008 -102.2801 -102.3662
##  [56,] -101.8682 -101.8791 -101.9056 -101.9463 -101.9996 -102.0643 -102.1392
##  [57,] -101.8944 -101.8608 -101.8479 -101.8541 -101.8775 -101.9168 -101.9705
##  [58,] -102.0658 -101.9727 -101.9061 -101.8643 -101.8453 -101.8473 -101.8690
##  [59,] -102.3970 -102.2288 -102.0938 -101.9899 -101.9152 -101.8676 -101.8456
##  [60,] -102.8834 -102.6440 -102.4260 -102.2456 -102.1023 -101.9937 -101.9167
##  [61,] -103.4417 -103.1642 -102.9006 -102.6532 -102.4308 -102.2489 -102.1049
##  [62,] -104.0473 -103.7416 -103.4487 -103.1697 -102.9058 -102.6580 -102.4349
##  [63,] -104.7001 -104.3714 -104.0536 -103.7476 -103.4545 -103.1752 -102.9109
##  [64,] -105.3940 -105.0458 -104.7069 -104.3780 -104.0599 -103.7537 -103.4603
##  [65,] -106.1228 -105.7580 -105.4012 -105.0528 -104.7136 -104.3845 -104.0662
##  [66,] -106.8813 -106.5024 -106.1302 -105.7653 -105.4083 -105.0597 -104.7204
##  [67,] -107.6658 -107.2744 -106.8890 -106.5099 -106.1377 -105.7726 -105.4154
##  [68,] -108.4738 -108.0710 -107.6738 -107.2823 -106.8967 -106.5175 -106.1451
##  [69,] -109.3039 -108.8903 -108.4820 -108.0791 -107.6818 -107.2901 -106.9045
##  [70,] -110.1562 -109.7315 -109.3123 -108.8986 -108.4902 -108.0872 -107.6897
##  [71,] -111.0318 -110.5953 -110.1648 -109.7400 -109.3208 -108.9069 -108.4984
##  [72,] -111.9334 -111.4836 -111.0406 -110.6040 -110.1734 -109.7485 -109.3292
##  [73,] -112.8649 -112.3997 -111.9425 -111.4926 -111.0495 -110.6128 -110.1821
##  [74,] -113.8318 -113.3483 -112.8744 -112.4090 -111.9517 -111.5016 -111.0584
##  [75,] -114.8408 -114.3356 -113.8416 -113.3580 -112.8839 -112.4184 -111.9608
##  [76,] -115.9005 -115.3691 -114.8512 -114.3457 -113.8515 -113.3677 -112.8933
##  [77,] -117.0208 -116.4580 -115.9114 -115.3797 -114.8615 -114.3558 -113.8614
##  [78,] -118.2133 -117.6132 -117.0324 -116.4692 -115.9223 -115.3903 -114.8718
##  [79,] -119.4912 -118.8471 -118.2257 -117.6252 -117.0439 -116.4804 -115.9332
##  [80,] -120.8806 -120.1755 -119.5045 -118.8599 -118.2380 -117.6371 -117.0555
##  [81,] -122.4003 -121.6331 -120.8952 -120.1894 -119.5177 -118.8727 -118.2504
##  [82,] -124.0280 -123.2102 -122.4160 -121.6483 -120.9098 -120.2033 -119.5310
##  [83,] -125.7421 -124.8841 -124.0447 -123.2264 -122.4318 -121.6635 -120.9244
##  [84,] -127.5229 -126.6342 -125.7596 -124.9012 -124.0615 -123.2428 -122.4476
##  [85,] -129.3533 -128.4421 -127.5410 -126.6520 -125.7771 -124.9184 -124.0782
##  [86,] -131.2186 -130.2917 -129.3718 -128.4604 -127.5591 -126.6698 -125.7946
##  [87,] -133.1064 -132.1698 -131.2374 -130.3104 -129.3903 -128.4787 -127.5772
##  [88,] -135.0069 -134.0651 -133.1253 -132.1887 -131.2561 -130.3291 -129.4088
##  [89,] -136.9122 -135.9689 -135.0259 -134.0841 -133.1443 -132.2075 -131.2749
##  [90,] -138.8168 -137.8744 -136.9313 -135.9879 -135.0449 -134.1031 -133.1633
##  [91,] -140.7167 -139.7770 -138.8358 -137.8935 -136.9503 -136.0070 -135.0640
##  [92,] -142.6097 -141.6736 -140.7356 -139.7960 -138.8548 -137.9125 -136.9694
##  [93,] -144.4955 -143.5629 -142.6286 -141.6925 -140.7546 -139.8150 -138.8739
##  [94,] -146.3750 -145.4453 -144.5143 -143.5818 -142.6475 -141.7114 -140.7736
##  [95,] -148.2507 -147.3225 -146.3937 -145.4641 -144.5331 -143.6006 -142.6664
##  [96,] -150.1270 -149.1979 -148.2695 -147.3412 -146.4125 -145.4829 -144.5520
##  [97,] -152.0094 -151.0764 -150.1457 -149.2166 -148.2882 -147.3600 -146.4313
##  [98,] -153.9057 -152.9648 -152.0283 -151.0952 -150.1645 -149.2354 -148.3070
##  [99,] -155.8254 -154.8715 -153.9248 -152.9837 -152.0472 -151.1140 -150.1833
## [100,] -157.7503 -156.8003 -155.8447 -154.8907 -153.9438 -153.0027 -152.0661
##            [,85]     [,86]     [,87]     [,88]     [,89]     [,90]     [,91]
##   [1,] -164.3690 -163.9521 -163.5266 -163.0922 -162.6485 -162.1952 -161.7320
##   [2,] -163.5179 -163.0833 -162.6394 -162.1859 -161.7225 -161.2489 -160.7648
##   [3,] -162.6304 -162.1767 -161.7131 -161.2393 -160.7550 -160.2599 -159.7538
##   [4,] -161.7036 -161.2296 -160.7451 -160.2498 -159.7434 -159.2257 -158.6965
##   [5,] -160.7352 -160.2396 -159.7331 -159.2152 -158.6857 -158.1443 -157.5909
##   [6,] -159.7227 -159.2046 -158.6749 -158.1333 -157.5796 -157.0135 -156.4347
##   [7,] -158.6640 -158.1222 -157.5683 -157.0019 -156.4229 -155.8308 -155.2256
##   [8,] -157.5569 -156.9903 -156.4110 -155.8187 -155.2132 -154.5941 -153.9611
##   [9,] -156.3992 -155.8066 -155.2008 -154.5814 -153.9482 -153.3007 -152.6388
##  [10,] -155.1884 -154.5688 -153.9353 -153.2875 -152.6253 -151.9481 -151.2558
##  [11,] -153.9223 -153.2743 -152.6117 -151.9343 -151.2416 -150.5334 -149.8091
##  [12,] -152.5982 -151.9204 -151.2275 -150.5189 -149.7943 -149.0534 -148.2957
##  [13,] -151.2133 -150.5044 -149.7795 -149.0383 -148.2802 -147.5049 -146.7119
##  [14,] -149.7647 -149.0231 -148.2647 -147.4891 -146.6957 -145.8842 -145.0540
##  [15,] -148.2492 -147.4732 -146.6795 -145.8676 -145.0371 -144.1874 -143.3179
##  [16,] -146.6633 -145.8510 -145.0201 -144.1700 -143.3002 -142.4114 -141.5245
##  [17,] -145.0031 -144.1526 -143.2824 -142.3934 -141.5067 -140.6286 -139.7588
##  [18,] -143.2646 -142.3754 -141.4889 -140.6110 -139.7413 -138.8793 -138.0248
##  [19,] -141.4711 -140.5933 -139.7238 -138.8620 -138.0076 -137.1602 -136.3194
##  [20,] -139.7063 -138.8447 -137.9904 -137.1431 -136.3024 -135.4681 -134.6397
##  [21,] -137.9732 -137.1261 -136.2855 -135.4513 -134.6230 -133.8005 -132.9834
##  [22,] -136.2686 -135.4345 -134.6064 -133.7839 -132.9670 -132.1552 -131.3485
##  [23,] -134.5897 -133.7674 -132.9505 -132.1389 -131.3322 -130.5305 -129.7335
##  [24,] -132.9341 -132.1225 -131.3160 -130.5144 -129.7175 -128.9252 -128.1376
##  [25,] -131.2998 -130.4982 -129.7014 -128.9093 -128.1217 -127.3388 -126.5604
##  [26,] -129.6854 -128.8933 -128.1059 -127.3230 -126.5447 -125.7711 -125.0024
##  [27,] -128.0900 -127.3072 -126.5291 -125.7556 -124.9869 -124.2231 -123.4646
##  [28,] -126.5134 -125.7400 -124.9714 -124.2078 -123.4493 -122.6963 -121.9491
##  [29,] -124.9559 -124.1924 -123.4340 -122.6812 -121.9341 -121.1932 -120.4589
##  [30,] -123.4188 -122.6660 -121.9190 -121.1783 -120.4441 -119.7172 -118.9979
##  [31,] -121.9040 -121.1634 -120.4294 -119.7025 -118.9835 -118.2729 -117.5715
##  [32,] -120.4146 -119.6879 -118.9691 -118.2586 -117.5574 -116.8662 -116.1859
##  [33,] -118.9546 -118.2444 -117.5433 -116.8523 -116.1723 -115.5042 -114.8492
##  [34,] -117.5293 -116.8385 -116.1587 -115.4908 -114.8361 -114.1955 -113.5705
##  [35,] -116.1451 -115.4775 -114.8230 -114.1827 -113.5580 -112.9501 -112.3606
##  [36,] -114.8099 -114.1699 -113.5455 -112.9380 -112.3489 -111.7817 -111.2430
##  [37,] -113.5331 -112.9260 -112.3372 -111.7706 -111.2324 -110.7225 -110.2400
##  [38,] -112.3255 -111.7594 -111.2219 -110.7125 -110.2305 -109.7753 -109.3459
##  [39,] -111.2113 -110.7025 -110.2211 -109.7663 -109.3375 -108.9337 -108.5540
##  [40,] -110.2116 -109.7574 -109.3291 -108.9258 -108.5466 -108.1907 -107.8571
##  [41,] -109.3207 -108.9179 -108.5392 -108.1837 -107.8506 -107.5389 -107.2478
##  [42,] -108.5318 -108.1768 -107.8441 -107.5328 -107.2421 -106.9709 -106.7184
##  [43,] -107.8376 -107.5268 -107.2364 -106.9656 -106.7135 -106.4791 -106.2615
##  [44,] -107.2126 -106.9565 -106.7086 -106.4745 -106.2573 -106.0561 -105.8699
##  [45,] -106.6271 -106.4282 -106.2354 -106.0483 -105.8663 -105.6948 -105.5367
##  [46,] -106.0770 -105.9309 -105.7891 -105.6511 -105.5165 -105.3847 -105.2554
##  [47,] -105.5592 -105.4613 -105.3661 -105.2732 -105.1820 -105.0922 -105.0033
##  [48,] -105.0712 -105.0168 -104.9638 -104.9118 -104.8602 -104.8087 -104.7569
##  [49,] -104.6114 -104.5956 -104.5802 -104.5648 -104.5488 -104.5321 -104.5141
##  [50,] -104.1790 -104.1967 -104.2141 -104.2309 -104.2466 -104.2609 -104.2734
##  [51,] -103.7738 -103.8196 -103.8649 -103.9094 -103.9526 -103.9943 -104.0340
##  [52,] -103.3967 -103.4649 -103.5330 -103.6005 -103.6669 -103.7320 -103.7955
##  [53,] -103.0497 -103.1342 -103.2194 -103.3049 -103.3901 -103.4747 -103.5583
##  [54,] -102.7355 -102.8298 -102.9263 -103.0243 -103.1235 -103.2233 -103.3235
##  [55,] -102.4582 -102.5552 -102.6566 -102.7615 -102.8695 -102.9801 -103.0927
##  [56,] -102.2232 -102.3153 -102.4145 -102.5201 -102.6314 -102.7458 -102.8612
##  [57,] -102.0372 -102.1160 -102.2055 -102.3036 -102.4079 -102.5168 -102.6291
##  [58,] -101.9086 -101.9641 -102.0331 -102.1136 -102.2036 -102.3016 -102.4057
##  [59,] -101.8466 -101.8681 -101.9076 -101.9628 -102.0316 -102.1118 -102.2017
##  [60,] -101.8683 -101.8458 -101.8464 -101.8675 -101.9067 -101.9616 -102.0301
##  [61,] -101.9956 -101.9179 -101.8690 -101.8460 -101.8462 -101.8669 -101.9057
##  [62,] -102.2522 -102.1074 -101.9974 -101.9192 -101.8698 -101.8463 -101.8460
##  [63,] -102.6628 -102.4390 -102.2555 -102.1100 -101.9993 -101.9205 -101.8705
##  [64,] -103.1807 -102.9161 -102.6677 -102.4431 -102.2588 -102.1126 -102.0013
##  [65,] -103.7598 -103.4661 -103.1862 -102.9213 -102.6725 -102.4473 -102.2622
##  [66,] -104.3910 -104.0725 -103.7658 -103.4719 -103.1917 -102.9265 -102.6774
##  [67,] -105.0667 -104.7272 -104.3976 -104.0789 -103.7719 -103.4777 -103.1972
##  [68,] -105.7799 -105.4225 -105.0736 -104.7339 -104.4041 -104.0852 -103.7780
##  [69,] -106.5251 -106.1526 -105.7872 -105.4297 -105.0806 -104.7407 -104.4107
##  [70,] -107.2980 -106.9122 -106.5327 -106.1600 -105.7945 -105.4368 -105.0876
##  [71,] -108.0953 -107.6977 -107.3058 -106.9199 -106.5403 -106.1675 -105.8018
##  [72,] -108.9152 -108.5066 -108.1034 -107.7057 -107.3137 -106.9277 -106.5479
##  [73,] -109.7571 -109.3376 -108.9235 -108.5148 -108.1115 -107.7137 -107.3216
##  [74,] -110.6216 -110.1907 -109.7656 -109.3460 -108.9318 -108.5230 -108.1196
##  [75,] -111.5107 -111.0673 -110.6303 -110.1994 -109.7741 -109.3544 -108.9401
##  [76,] -112.4277 -111.9700 -111.5197 -111.0762 -110.6391 -110.2080 -109.7827
##  [77,] -113.3774 -112.9028 -112.4370 -111.9792 -111.5287 -111.0851 -110.6479
##  [78,] -114.3659 -113.8713 -113.3870 -112.9123 -112.4463 -111.9884 -111.5377
##  [79,] -115.4009 -114.8822 -114.3760 -113.8811 -113.3967 -112.9218 -112.4557
##  [80,] -116.4917 -115.9441 -115.4115 -114.8925 -114.3861 -113.8910 -113.4064
##  [81,] -117.6490 -117.0671 -116.5029 -115.9550 -115.4221 -114.9029 -114.3962
##  [82,] -118.8855 -118.2627 -117.6610 -117.0786 -116.5141 -115.9659 -115.4327
##  [83,] -120.2172 -119.5443 -118.8983 -118.2751 -117.6729 -117.0902 -116.5254
##  [84,] -121.6788 -120.9390 -120.2312 -119.5576 -118.9111 -118.2875 -117.6849
##  [85,] -123.2591 -122.4634 -121.6940 -120.9536 -120.2451 -119.5709 -118.9239
##  [86,] -124.9356 -124.0950 -123.2754 -122.4793 -121.7093 -120.9683 -120.2591
##  [87,] -126.6877 -125.8121 -124.9528 -124.1118 -123.2917 -122.4951 -121.7246
##  [88,] -128.4970 -127.5953 -126.7055 -125.8297 -124.9699 -124.1286 -123.3081
##  [89,] -130.3477 -129.4273 -128.5153 -127.6134 -126.7233 -125.8472 -124.9871
##  [90,] -132.2264 -131.2937 -130.3664 -129.4459 -128.5337 -127.6315 -126.7412
##  [91,] -134.1221 -133.1822 -132.2453 -131.3125 -130.3851 -129.4644 -128.5520
##  [92,] -136.0260 -135.0830 -134.1411 -133.2012 -132.2642 -131.3313 -130.4037
##  [93,] -137.9316 -136.9885 -136.0451 -135.1021 -134.1601 -133.2201 -132.2831
##  [94,] -139.8340 -138.8929 -137.9506 -137.0075 -136.0641 -135.1211 -134.1791
##  [95,] -141.7304 -140.7925 -139.8530 -138.9119 -137.9696 -137.0266 -136.0832
##  [96,] -143.6195 -142.6853 -141.7493 -140.8115 -139.8720 -138.9309 -137.9887
##  [97,] -145.5017 -144.5708 -143.6384 -142.7042 -141.7682 -140.8305 -139.8910
##  [98,] -147.3787 -146.4500 -145.5205 -144.5896 -143.6572 -142.7231 -141.7872
##  [99,] -149.2541 -148.3257 -147.3975 -146.4688 -145.5392 -144.6084 -143.6761
## [100,] -151.1329 -150.2021 -149.2729 -148.3445 -147.4163 -146.4876 -145.5580
##            [,92]     [,93]     [,94]     [,95]     [,96]     [,97]     [,98]
##   [1,] -161.2586 -160.7747 -160.2801 -159.7744 -159.2574 -158.7289 -158.1885
##   [2,] -160.2700 -159.7641 -159.2469 -158.7181 -158.1775 -157.6248 -157.0597
##   [3,] -159.2363 -158.7073 -158.1665 -157.6135 -157.0481 -156.4701 -155.8792
##   [4,] -158.1554 -157.6022 -157.0366 -156.4583 -155.8671 -155.2626 -154.6446
##   [5,] -157.0250 -156.4465 -155.8550 -155.2503 -154.6320 -153.9999 -153.3536
##   [6,] -155.8429 -155.2379 -154.6194 -153.9870 -153.3404 -152.6793 -152.0034
##   [7,] -154.6067 -153.9740 -153.3272 -152.6658 -151.9896 -151.2982 -150.5912
##   [8,] -153.3140 -152.6523 -151.9758 -151.2840 -150.5767 -149.8535 -149.1139
##   [9,] -151.9620 -151.2699 -150.5623 -149.8387 -149.0988 -148.3421 -147.5682
##  [10,] -150.5478 -149.8239 -149.0837 -148.3266 -147.5524 -146.7605 -145.9505
##  [11,] -149.0685 -148.3112 -147.5366 -146.7443 -145.9339 -145.1049 -144.2568
##  [12,] -147.5207 -146.7281 -145.9174 -145.0880 -144.2394 -143.3712 -142.4835
##  [13,] -145.9008 -145.0710 -144.2221 -143.3535 -142.4655 -141.5780 -140.6993
##  [14,] -144.2047 -143.3357 -142.4475 -141.5602 -140.6816 -139.8113 -138.9487
##  [15,] -142.4294 -141.5424 -140.6640 -139.7938 -138.9314 -138.0764 -137.2284
##  [16,] -140.6463 -139.7763 -138.9140 -138.0592 -137.2113 -136.3701 -135.5353
##  [17,] -138.8967 -138.0420 -137.1943 -136.3532 -135.5185 -134.6897 -133.8668
##  [18,] -137.1772 -136.3363 -135.5017 -134.6731 -133.8502 -133.0328 -132.2206
##  [19,] -135.4849 -134.6564 -133.8336 -133.0163 -132.2043 -131.3972 -130.5951
##  [20,] -133.8171 -132.9999 -132.1879 -131.3810 -130.5790 -129.7817 -128.9891
##  [21,] -132.1716 -131.3647 -130.5628 -129.7656 -128.9731 -128.1852 -127.4019
##  [22,] -130.5467 -129.7496 -128.9572 -128.1693 -127.3861 -126.6075 -125.8335
##  [23,] -128.9412 -128.1535 -127.3703 -126.5918 -125.8179 -125.0488 -124.2847
##  [24,] -127.3545 -126.5761 -125.8023 -125.0333 -124.2693 -123.5104 -122.7570
##  [25,] -125.7867 -125.0178 -124.2539 -123.4951 -122.7418 -121.9942 -121.2528
##  [26,] -124.2385 -123.4799 -122.7266 -121.9792 -121.2379 -120.5032 -119.7756
##  [27,] -122.7115 -121.9641 -121.2230 -120.4884 -119.7610 -119.0413 -118.3300
##  [28,] -121.2081 -120.4737 -119.7464 -119.0268 -118.3157 -117.6137 -116.9217
##  [29,] -119.7318 -119.0124 -118.3014 -117.5996 -116.9078 -116.2268 -115.5577
##  [30,] -118.2872 -117.5855 -116.8939 -116.2132 -115.5443 -114.8885 -114.2467
##  [31,] -116.8801 -116.1996 -115.5310 -114.8754 -114.2339 -113.6079 -112.9986
##  [32,] -115.5176 -114.8623 -114.2211 -113.5954 -112.9865 -112.3958 -111.8265
##  [33,] -114.2083 -113.5829 -112.9744 -112.3840 -111.8153 -111.2749 -110.7627
##  [34,] -112.9622 -112.3723 -111.8041 -111.2642 -110.7526 -110.2685 -109.8111
##  [35,] -111.7929 -111.2536 -110.7426 -110.2590 -109.8021 -109.3712 -108.9654
##  [36,] -110.7325 -110.2495 -109.7932 -109.3628 -108.9574 -108.5763 -108.2186
##  [37,] -109.7842 -109.3543 -108.9495 -108.5689 -108.2116 -107.8767 -107.5633
##  [38,] -108.9416 -108.5614 -108.2046 -107.8702 -107.5572 -107.2648 -106.9921
##  [39,] -108.1976 -107.8636 -107.5511 -107.2591 -106.9868 -106.7332 -106.4974
##  [40,] -107.5450 -107.2534 -106.9815 -106.7282 -106.4928 -106.2743 -106.0718
##  [41,] -106.9762 -106.7233 -106.4882 -106.2700 -106.0678 -105.8808 -105.7081
##  [42,] -106.4836 -106.2658 -106.0639 -105.8772 -105.7048 -105.5459 -105.3998
##  [43,] -106.0600 -105.8736 -105.7015 -105.5429 -105.3970 -105.2631 -105.1406
##  [44,] -105.6981 -105.5398 -105.3942 -105.2606 -105.1382 -105.0265 -104.9247
##  [45,] -105.3914 -105.2580 -105.1359 -105.0243 -104.9227 -104.8305 -104.7471
##  [46,] -105.1335 -105.0222 -104.9208 -104.8288 -104.7455 -104.6705 -104.6032
##  [47,] -104.9150 -104.8270 -104.7439 -104.6691 -104.6020 -104.5422 -104.4893
##  [48,] -104.7045 -104.6513 -104.5968 -104.5410 -104.4883 -104.4421 -104.4020
##  [49,] -104.4947 -104.4735 -104.4503 -104.4250 -104.3974 -104.3673 -104.3389
##  [50,] -104.2840 -104.2923 -104.2981 -104.3014 -104.3018 -104.2994 -104.2939
##  [51,] -104.0715 -104.1067 -104.1393 -104.1692 -104.1962 -104.2202 -104.2412
##  [52,] -103.8571 -103.9165 -103.9736 -104.0282 -104.0803 -104.1296 -104.1736
##  [53,] -103.6408 -103.7218 -103.8011 -103.8786 -103.9516 -104.0178 -104.0769
##  [54,] -103.4236 -103.5233 -103.6201 -103.7116 -103.7974 -103.8771 -103.9502
##  [55,] -103.2047 -103.3140 -103.4197 -103.5213 -103.6182 -103.7098 -103.7957
##  [56,] -102.9763 -103.0904 -103.2025 -103.3118 -103.4176 -103.5193 -103.6163
##  [57,] -102.7435 -102.8588 -102.9740 -103.0881 -103.2002 -103.3096 -103.4155
##  [58,] -102.5146 -102.6268 -102.7412 -102.8565 -102.9717 -103.0858 -103.1980
##  [59,] -102.2995 -102.4035 -102.5124 -102.6245 -102.7389 -102.8542 -102.9694
##  [60,] -102.1101 -102.1998 -102.2975 -102.4014 -102.5101 -102.6223 -102.7365
##  [61,] -101.9603 -102.0286 -102.1084 -102.1979 -102.2954 -102.3992 -102.5079
##  [62,] -101.8663 -101.9048 -101.9591 -102.0271 -102.1067 -102.1960 -102.2934
##  [63,] -101.8465 -101.8457 -101.8657 -101.9038 -101.9578 -102.0256 -102.1050
##  [64,] -101.9218 -101.8712 -101.8467 -101.8456 -101.8651 -101.9029 -101.9566
##  [65,] -102.1152 -102.0032 -101.9231 -101.8720 -101.8470 -101.8454 -101.8645
##  [66,] -102.4514 -102.2655 -102.1178 -102.0051 -101.9244 -101.8728 -101.8473
##  [67,] -102.9317 -102.6822 -102.4556 -102.2689 -102.1204 -102.0071 -101.9258
##  [68,] -103.4835 -103.2028 -102.9369 -102.6871 -102.4598 -102.2723 -102.1231
##  [69,] -104.0915 -103.7841 -103.4893 -103.2083 -102.9421 -102.6920 -102.4640
##  [70,] -104.7475 -104.4173 -104.0979 -103.7902 -103.4951 -103.2138 -102.9474
##  [71,] -105.4440 -105.0945 -104.7542 -104.4238 -104.1042 -103.7962 -103.5009
##  [72,] -106.1749 -105.8091 -105.4511 -105.1015 -104.7610 -104.4304 -104.1105
##  [73,] -106.9354 -106.5555 -106.1824 -105.8165 -105.4583 -105.1085 -104.7678
##  [74,] -107.7216 -107.3294 -106.9431 -106.5632 -106.1899 -105.8238 -105.4654
##  [75,] -108.5312 -108.1277 -107.7296 -107.3373 -106.9509 -106.5708 -106.1973
##  [76,] -109.3629 -108.9485 -108.5394 -108.1358 -107.7376 -107.3451 -106.9586
##  [77,] -110.2167 -109.7912 -109.3713 -108.9568 -108.5476 -108.1439 -107.7456
##  [78,] -111.0940 -110.6566 -110.2253 -109.7997 -109.3797 -108.9651 -108.5558
##  [79,] -111.9975 -111.5468 -111.1029 -110.6654 -110.2340 -109.8083 -109.3881
##  [80,] -112.9313 -112.4650 -112.0067 -111.5558 -111.1118 -110.6742 -110.2426
##  [81,] -113.9009 -113.4161 -112.9408 -112.4743 -112.0159 -111.5649 -111.1207
##  [82,] -114.9133 -114.4063 -113.9108 -113.4258 -112.9504 -112.4837 -112.0251
##  [83,] -115.9769 -115.4434 -114.9236 -114.4164 -113.9207 -113.4355 -112.9599
##  [84,] -117.1018 -116.5366 -115.9878 -115.4540 -114.9340 -114.4266 -113.9306
##  [85,] -118.2998 -117.6969 -117.1134 -116.5478 -115.9987 -115.4647 -114.9444
##  [86,] -119.5842 -118.9368 -118.3122 -117.7089 -117.1250 -116.5591 -116.0097
##  [87,] -120.9830 -120.2731 -119.5976 -118.9496 -118.3246 -117.7209 -117.1366
##  [88,] -122.5109 -121.7399 -120.9976 -120.2871 -119.6109 -118.9625 -118.3370
##  [89,] -124.1454 -123.3244 -122.5268 -121.7552 -121.0123 -120.3011 -119.6242
##  [90,] -125.8648 -125.0043 -124.1622 -123.3408 -122.5427 -121.7705 -121.0270
##  [91,] -127.6496 -126.7591 -125.8823 -125.0215 -124.1790 -123.3572 -122.5585
##  [92,] -129.4829 -128.5703 -127.6677 -126.7769 -125.8999 -125.0388 -124.1958
##  [93,] -131.3501 -130.4224 -129.5014 -128.5887 -127.6859 -126.7948 -125.9174
##  [94,] -133.2391 -132.3020 -131.3689 -130.4411 -129.5200 -128.6070 -127.7040
##  [95,] -135.1401 -134.1982 -133.2581 -132.3209 -131.3877 -130.4598 -129.5385
##  [96,] -137.0456 -136.1023 -135.1592 -134.2172 -133.2770 -132.3398 -131.4065
##  [97,] -138.9500 -138.0077 -137.0647 -136.1213 -135.1782 -134.2362 -133.2960
##  [98,] -140.8494 -139.9100 -138.9690 -138.0268 -137.0837 -136.1404 -135.1973
##  [99,] -142.7420 -141.8061 -140.8684 -139.9290 -138.9880 -138.0458 -137.1028
## [100,] -144.6272 -143.6949 -142.7609 -141.8250 -140.8873 -139.9480 -139.0070
##            [,99]    [,100]
##   [1,] -157.6361 -157.0712
##   [2,] -156.4819 -155.8912
##   [3,] -155.2750 -154.6573
##   [4,] -154.0128 -153.3668
##   [5,] -152.6928 -152.0172
##   [6,] -151.3123 -150.6056
##   [7,] -149.8683 -149.1290
##   [8,] -148.3576 -147.5840
##   [9,] -146.7767 -145.9670
##  [10,] -145.1218 -144.2741
##  [11,] -143.3889 -142.5015
##  [12,] -141.5959 -140.7170
##  [13,] -139.8288 -138.9661
##  [14,] -138.0936 -137.2455
##  [15,] -136.3871 -135.5521
##  [16,] -134.7064 -133.8833
##  [17,] -133.0492 -132.2370
##  [18,] -131.4135 -130.6113
##  [19,] -129.7978 -129.0051
##  [20,] -128.2011 -127.4176
##  [21,] -126.6231 -125.8491
##  [22,] -125.0643 -124.3001
##  [23,] -123.5257 -122.7721
##  [24,] -122.0093 -121.2677
##  [25,] -120.5180 -119.7902
##  [26,] -119.0558 -118.3443
##  [27,] -117.6278 -116.9355
##  [28,] -116.2405 -115.5711
##  [29,] -114.9016 -114.2595
##  [30,] -113.6204 -113.0107
##  [31,] -112.4075 -111.8377
##  [32,] -111.2855 -110.7727
##  [33,] -110.2780 -109.8201
##  [34,] -109.3797 -108.9733
##  [35,] -108.5838 -108.2256
##  [36,] -107.8833 -107.5695
##  [37,] -107.2706 -106.9974
##  [38,] -106.7381 -106.5020
##  [39,] -106.2785 -106.0757
##  [40,] -105.8844 -105.7115
##  [41,] -105.5490 -105.4027
##  [42,] -105.2657 -105.1430
##  [43,] -105.0286 -104.9267
##  [44,] -104.8323 -104.7487
##  [45,] -104.6719 -104.6045
##  [46,] -104.5433 -104.4903
##  [47,] -104.4429 -104.4028
##  [48,] -104.3679 -104.3394
##  [49,] -104.3159 -104.2982
##  [50,] -104.2854 -104.2779
##  [51,] -104.2591 -104.2713
##  [52,] -104.2099 -104.2383
##  [53,] -104.1286 -104.1728
##  [54,] -104.0166 -104.0758
##  [55,] -103.8755 -103.9488
##  [56,] -103.7080 -103.7941
##  [57,] -103.5173 -103.6144
##  [58,] -103.3074 -103.4134
##  [59,] -103.0835 -103.1957
##  [60,] -102.8518 -102.9670
##  [61,] -102.6200 -102.7342
##  [62,] -102.3971 -102.5056
##  [63,] -102.1942 -102.2914
##  [64,] -102.0241 -102.1033
##  [65,] -101.9019 -101.9554
##  [66,] -101.8452 -101.8639
##  [67,] -101.8735 -101.8475
##  [68,] -102.0090 -101.9271
##  [69,] -102.2757 -102.1257
##  [70,] -102.6969 -102.4682
##  [71,] -103.2194 -102.9526
##  [72,] -103.8023 -103.5068
##  [73,] -104.4370 -104.1169
##  [74,] -105.1155 -104.7746
##  [75,] -105.8311 -105.4726
##  [76,] -106.5784 -106.2048
##  [77,] -107.3530 -106.9664
##  [78,] -108.1520 -107.7536
##  [79,] -108.9734 -108.5640
##  [80,] -109.8168 -109.3966
##  [81,] -110.6830 -110.2513
##  [82,] -111.5739 -111.1296
##  [83,] -112.4930 -112.0343
##  [84,] -113.4452 -112.9694
##  [85,] -114.4367 -113.9405
##  [86,] -115.4753 -114.9548
##  [87,] -116.5704 -116.0206
##  [88,] -117.7329 -117.1483
##  [89,] -118.9753 -118.3495
##  [90,] -120.3152 -119.6376
##  [91,] -121.7858 -121.0418
##  [92,] -123.3735 -122.5744
##  [93,] -125.0560 -124.2127
##  [94,] -126.8127 -125.9350
##  [95,] -128.6254 -127.7221
##  [96,] -130.4785 -129.5570
##  [97,] -132.3587 -131.4253
##  [98,] -134.2552 -133.3150
##  [99,] -136.1594 -135.2163
## [100,] -138.0649 -137.1219
## 
## $SSE
##             [,1]      [,2]      [,3]      [,4]      [,5]      [,6]      [,7]
##   [1,] 1415.5368 1415.5368 1415.5368 1415.5368 1415.5368 1415.5368 1415.5368
##   [2,] 1415.5368 1415.5368 1415.5368 1415.5368 1415.5368 1415.5368 1415.5368
##   [3,] 1415.5368 1415.5368 1415.5368 1415.5368 1415.5368 1415.5368 1415.5368
##   [4,] 1415.5368 1415.5368 1415.5368 1415.5368 1415.5368 1415.5368 1415.5368
##   [5,] 1415.5368 1415.5368 1415.5368 1415.5368 1415.5368 1415.5368 1415.5368
##   [6,] 1415.5368 1415.5368 1415.5368 1415.5368 1415.5368 1415.5368 1415.5368
##   [7,] 1415.5368 1415.5368 1415.5368 1415.5368 1415.5368 1415.5368 1415.5368
##   [8,] 1415.5368 1415.5368 1415.5368 1415.5368 1415.5368 1415.5368 1415.5368
##   [9,] 1415.5368 1415.5368 1415.5368 1415.5368 1415.5368 1415.5368 1415.5368
##  [10,] 1415.5368 1415.5368 1415.5368 1415.5368 1415.5368 1415.5368 1415.5368
##  [11,] 1415.5368 1415.5368 1415.5368 1415.5368 1415.5368 1415.5368 1415.5368
##  [12,] 1415.5368 1415.5368 1415.5368 1415.5368 1415.5368 1415.5368 1415.5368
##  [13,] 1415.5368 1415.5368 1415.5368 1415.5368 1415.5368 1415.5368 1415.5368
##  [14,] 1415.5368 1415.5368 1415.5368 1415.5368 1415.5368 1415.5368 1415.5368
##  [15,] 1415.5368 1415.5368 1415.5368 1415.5368 1415.5368 1415.5368 1415.5368
##  [16,] 1415.5368 1415.5368 1415.5368 1415.5368 1415.5368 1415.5368 1415.5368
##  [17,] 1415.5368 1415.5368 1415.5368 1415.5368 1415.5368 1415.5368 1415.5368
##  [18,] 1415.5368 1415.5368 1415.5368 1415.5368 1415.5368 1415.2378 1411.6131
##  [19,] 1415.5368 1415.5368 1415.5368 1415.0619 1410.1906 1402.1388 1392.2535
##  [20,] 1415.5368 1414.5341 1406.8400 1396.1572 1384.2559 1371.7269 1358.8251
##  [21,] 1389.9723 1378.0135 1365.3148 1352.4080 1339.4018 1326.3340 1313.2227
##  [22,] 1294.7119 1294.7119 1293.3229 1286.9065 1278.0216 1267.8980 1257.0642
##  [23,] 1203.1160 1203.1160 1203.1160 1203.1160 1202.3742 1198.2701 1191.9081
##  [24,] 1119.9269 1119.9269 1119.9269 1119.9269 1119.9269 1119.9269 1119.4621
##  [25,] 1045.1337 1045.1337 1045.1337 1045.1337 1045.1337 1045.1337 1045.1337
##  [26,]  978.6054  978.6054  978.6054  978.6054  978.6054  978.6054  978.6054
##  [27,]  920.1101  920.1101  920.1101  920.1101  920.1101  920.1101  920.1101
##  [28,]  869.3335  869.3335  869.3335  869.3335  869.3335  869.3335  869.3335
##  [29,]  825.8974  825.8974  825.8974  825.8974  825.8974  825.8974  825.8974
##  [30,]  789.3776  789.3776  789.3776  789.3776  789.3776  789.3776  789.3776
##  [31,]  759.3189  759.3189  759.3189  759.3189  759.3189  759.3189  759.3189
##  [32,]  735.2496  735.2496  735.2496  735.2496  735.2496  735.2496  735.2496
##  [33,]  716.6927  716.6927  716.6927  716.6927  716.6927  716.6927  716.6927
##  [34,]  703.1752  703.1752  703.1752  703.1752  703.1752  703.1752  703.1752
##  [35,]  694.2363  694.2363  694.2363  694.2363  694.2363  694.2363  694.2363
##  [36,]  689.4328  689.4328  689.4328  689.4328  689.4328  689.4328  689.4328
##  [37,]  688.3433  688.3433  688.3433  688.3433  688.3433  688.3433  688.3433
##  [38,]  690.5709  690.5709  690.5709  690.5709  690.5709  689.6837  686.4153
##  [39,]  695.7453  695.7453  695.7453  694.3327  689.7540  683.5067  676.3074
##  [40,]  703.5230  700.5405  693.2833  684.8481  675.9418  666.8007  657.5271
##  [41,]  682.1869  675.7124  667.1438  658.0512  648.7496  639.3447  629.8829
##  [42,]  617.1884  617.1884  617.0941  613.8952  608.1391  601.1066  593.3472
##  [43,]  554.3627  554.3627  554.3627  554.3627  554.3190  552.2581  548.0666
##  [44,]  494.4400  494.4400  494.4400  494.4400  494.4400  494.4400  494.4168
##  [45,]  437.7987  437.7987  437.7987  437.7987  437.7987  437.7987  437.7987
##  [46,]  384.8155  384.8155  384.8155  384.8155  384.8155  384.8155  384.8155
##  [47,]  335.8597  335.8597  335.8597  335.8597  335.8597  335.8597  335.8597
##  [48,]  291.2879  291.2879  291.2879  291.2879  291.2879  291.2879  291.2879
##  [49,]  251.4382  251.4382  251.4382  251.4382  251.4382  251.4382  251.4382
##  [50,]  216.6242  216.6242  216.6242  216.6242  216.6242  216.6242  216.6242
##  [51,]  187.1296  187.1296  187.1296  187.1296  187.1296  187.1296  187.1296
##  [52,]  163.2027  163.2027  163.2027  163.2027  163.2027  163.2027  163.2027
##  [53,]  145.0516  145.0516  145.0516  145.0516  145.0516  145.0516  145.0516
##  [54,]  132.8399  132.8399  132.8399  132.8399  132.8399  132.8399  132.8399
##  [55,]  126.6830  126.6830  126.6830  126.6830  126.6830  126.6830  126.6830
##  [56,]  126.6462  126.6462  126.6462  126.6462  126.6462  126.6462  126.6462
##  [57,]  132.7427  132.7427  132.7427  132.7427  132.7427  132.7427  132.7378
##  [58,]  144.9334  144.9334  144.9334  144.9334  144.9214  144.3558  143.2192
##  [59,]  163.1278  163.1278  163.0962  162.0304  160.1372  157.8675  155.4196
##  [60,]  187.0475  184.5456  181.2907  177.9081  174.5252  171.1861  167.9110
##  [61,]  197.3145  196.1200  193.2459  189.9640  186.5685  183.1595  179.7812
##  [62,]  197.9029  197.9029  197.9029  197.3598  195.6123  193.2604  190.5969
##  [63,]  201.9254  201.9254  201.9254  201.9254  201.9254  201.6023  200.4190
##  [64,]  209.9165  209.9165  209.9165  209.9165  209.9165  209.9165  209.9165
##  [65,]  222.4742  222.4742  222.4742  222.4742  222.4742  222.4742  222.4742
##  [66,]  240.2634  240.2634  240.2634  240.2634  240.2634  240.2634  240.2634
##  [67,]  264.0174  264.0174  264.0174  264.0174  264.0174  264.0174  264.0174
##  [68,]  294.5380  294.5380  294.5380  294.5380  294.5380  294.5380  294.5380
##  [69,]  332.6918  332.6918  332.6918  332.6918  332.6918  332.6918  332.6918
##  [70,]  379.4028  379.4028  379.4028  379.4028  379.4028  379.4028  379.4028
##  [71,]  435.6390  435.6390  435.6390  435.6390  435.6390  435.6390  435.6390
##  [72,]  502.3920  502.3920  502.3920  502.3920  502.3920  502.3920  502.3920
##  [73,]  580.6487  580.6487  580.6487  580.6487  580.6487  580.6487  580.6487
##  [74,]  671.3523  671.3523  671.3523  671.3523  671.3523  671.3523  671.3523
##  [75,]  775.3531  775.3531  775.3531  775.3531  775.3531  775.3531  775.3531
##  [76,]  893.3486  893.3486  893.3486  893.3486  893.3486  893.3486  893.3486
##  [77,] 1025.8135 1025.8135 1025.8135 1025.8135 1025.8135 1025.8135 1024.9908
##  [78,] 1172.9234 1172.9234 1172.9234 1172.9234 1171.6129 1164.3622 1153.1207
##  [79,] 1334.4766 1334.4766 1332.0312 1320.7331 1305.0827 1287.2431 1268.1435
##  [80,] 1501.7410 1480.7968 1458.5383 1435.8965 1413.0620 1390.1022 1367.0494
##  [81,] 1546.4537 1544.7015 1531.2524 1512.5663 1491.7317 1469.7800 1447.1559
##  [82,] 1546.4537 1546.4537 1546.4537 1545.6238 1537.1102 1523.0309 1505.7343
##  [83,] 1546.4537 1546.4537 1546.4537 1546.4537 1546.4537 1545.9312 1539.5965
##  [84,] 1546.4537 1546.4537 1546.4537 1546.4537 1546.4537 1546.4537 1546.4537
##  [85,] 1546.4537 1546.4537 1546.4537 1546.4537 1546.4537 1546.4537 1546.4537
##  [86,] 1546.4537 1546.4537 1546.4537 1546.4537 1546.4537 1546.4537 1546.4537
##  [87,] 1546.4537 1546.4537 1546.4537 1546.4537 1546.4537 1546.4537 1546.4537
##  [88,] 1546.4537 1546.4537 1546.4537 1546.4537 1546.4537 1546.4537 1546.4537
##  [89,] 1546.4537 1546.4537 1546.4537 1546.4537 1546.4537 1546.4537 1546.4537
##  [90,] 1546.4537 1546.4537 1546.4537 1546.4537 1546.4537 1546.4537 1546.4537
##  [91,] 1546.4537 1546.4537 1546.4537 1546.4537 1546.4537 1546.4537 1546.4537
##  [92,] 1546.4537 1546.4537 1546.4537 1546.4537 1546.4537 1546.4537 1546.4537
##  [93,] 1546.4537 1546.4537 1546.4537 1546.4537 1546.4537 1546.4537 1546.4537
##  [94,] 1546.4537 1546.4537 1546.4537 1546.4537 1546.4537 1546.4537 1546.4537
##  [95,] 1546.4537 1546.4537 1546.4537 1546.4537 1546.4537 1546.4537 1546.4537
##  [96,] 1546.4537 1546.4537 1546.4537 1546.4537 1546.4537 1546.4537 1546.4537
##  [97,] 1546.4537 1546.4537 1546.4537 1546.4537 1546.4537 1546.4537 1546.4537
##  [98,] 1546.4537 1546.4537 1546.4537 1546.4537 1546.4537 1546.4537 1546.4537
##  [99,] 1546.4537 1546.4537 1546.4537 1546.4537 1546.4537 1546.4537 1546.4537
## [100,] 1546.4537 1546.4537 1546.4537 1546.4537 1546.4537 1546.4537 1546.4537
##             [,8]      [,9]     [,10]     [,11]     [,12]     [,13]     [,14]
##   [1,] 1415.5368 1415.5368 1415.5368 1415.5368 1415.5368 1415.5368 1415.5368
##   [2,] 1415.5368 1415.5368 1415.5368 1415.5368 1415.5368 1415.5368 1415.5368
##   [3,] 1415.5368 1415.5368 1415.5368 1415.5368 1415.5368 1415.5368 1415.5368
##   [4,] 1415.5368 1415.5368 1415.5368 1415.5368 1415.5368 1415.5368 1415.5368
##   [5,] 1415.5368 1415.5368 1415.5368 1415.5368 1415.5368 1415.5368 1415.5368
##   [6,] 1415.5368 1415.5368 1415.5368 1415.5368 1415.5368 1415.5368 1415.5368
##   [7,] 1415.5368 1415.5368 1415.5368 1415.5368 1415.5368 1415.5368 1415.5368
##   [8,] 1415.5368 1415.5368 1415.5368 1415.5368 1415.5368 1415.5368 1415.5368
##   [9,] 1415.5368 1415.5368 1415.5368 1415.5368 1415.5368 1415.5368 1415.5368
##  [10,] 1415.5368 1415.5368 1415.5368 1415.5368 1415.5368 1415.5368 1415.5368
##  [11,] 1415.5368 1415.5368 1415.5368 1415.5368 1415.5368 1415.5368 1415.5368
##  [12,] 1415.5368 1415.5368 1415.5368 1415.5368 1415.5368 1415.5368 1415.5368
##  [13,] 1415.5368 1415.5368 1415.5368 1415.5368 1415.5368 1415.5368 1415.5368
##  [14,] 1415.5368 1415.5368 1415.5368 1415.5368 1415.5368 1415.5368 1415.4388
##  [15,] 1415.5368 1415.5368 1415.5368 1415.5368 1415.4137 1413.1980 1408.6557
##  [16,] 1415.5368 1415.5368 1415.3785 1412.8750 1407.9101 1401.0441 1392.6753
##  [17,] 1415.3257 1412.3913 1406.8045 1399.2792 1390.2899 1380.1644 1369.1371
##  [18,] 1405.0819 1396.6013 1386.7441 1375.8746 1364.2362 1351.9981 1339.2824
##  [19,] 1381.2077 1369.3755 1356.9826 1344.1735 1331.0459 1317.6685 1304.0918
##  [20,] 1345.6791 1332.3621 1318.9192 1305.3806 1291.7677 1278.0965 1264.3798
##  [21,] 1300.0786 1286.9090 1273.7197 1260.5159 1247.3021 1234.0830 1220.8629
##  [22,] 1245.7856 1234.2114 1222.4322 1210.5072 1198.4769 1186.3707 1174.2108
##  [23,] 1184.1384 1175.4350 1166.0835 1156.2667 1146.1080 1135.6938 1125.0871
##  [24,] 1116.5830 1111.8265 1105.7629 1098.7566 1091.0522 1082.8201 1074.1834
##  [25,] 1045.1337 1044.8213 1042.7029 1039.0499 1034.2538 1028.5880 1022.2492
##  [26,]  978.6054  978.6054  978.6054  978.3881  976.7890  973.9405  970.1186
##  [27,]  920.1101  920.1101  920.1101  920.1101  920.1101  919.9564  918.7335
##  [28,]  869.3335  869.3335  869.3335  869.3335  869.3335  869.3335  869.3335
##  [29,]  825.8974  825.8974  825.8974  825.8974  825.8974  825.8974  825.8974
##  [30,]  789.3776  789.3776  789.3776  789.3776  789.3776  789.3776  789.3776
##  [31,]  759.3189  759.3189  759.3189  759.3189  759.3189  759.3189  759.3189
##  [32,]  735.2496  735.2496  735.2496  735.2496  735.2496  735.2496  735.2496
##  [33,]  716.6927  716.6927  716.6927  716.6927  716.6927  716.6927  716.6927
##  [34,]  703.1752  703.1752  703.1752  703.1752  703.1752  703.1752  702.8962
##  [35,]  694.2363  694.2363  694.2363  694.2363  693.8802  692.2540  689.5685
##  [36,]  689.4328  689.4328  688.9695  686.9780  683.7762  679.6461  674.7900
##  [37,]  687.7203  685.2203  681.3381  676.4532  670.8194  664.6128  657.9600
##  [38,]  681.5843  675.7136  669.1177  661.9973  654.4869  646.6797  638.6433
##  [39,]  668.5149  660.3293  651.8712  643.2178  634.4214  625.5185  616.5357
##  [40,]  648.1726  638.7667  629.3278  619.8680  610.3961  600.9186  591.4408
##  [41,]  620.3879  610.8737  601.3492  591.8208  582.2933  572.7704  563.2554
##  [42,]  585.1366  576.6284  567.9156  559.0579  550.0956  541.0573  531.9637
##  [43,]  542.5467  536.1447  529.1294  521.6722  513.8883  505.8576  497.6380
##  [44,]  492.9369  489.6997  485.2358  479.8840  473.8709  467.3541  460.4466
##  [45,]  437.7987  437.7860  436.6621  434.0702  430.3809  425.8519  420.6680
##  [46,]  384.8155  384.8155  384.8155  384.8087  383.9274  381.8057  378.7128
##  [47,]  335.8597  335.8597  335.8597  335.8597  335.8597  335.8563  335.1525
##  [48,]  291.2879  291.2879  291.2879  291.2879  291.2879  291.2879  291.2879
##  [49,]  251.4382  251.4382  251.4382  251.4382  251.4382  251.4382  251.4382
##  [50,]  216.6242  216.6242  216.6242  216.6242  216.6242  216.6242  216.6242
##  [51,]  187.1296  187.1296  187.1296  187.1296  187.1296  187.1296  187.1296
##  [52,]  163.2027  163.2027  163.2027  163.2027  163.2027  163.2027  163.2027
##  [53,]  145.0516  145.0516  145.0516  145.0516  145.0516  145.0516  145.0516
##  [54,]  132.8399  132.8399  132.8399  132.8399  132.8399  132.8399  132.8558
##  [55,]  126.6830  126.6830  126.6830  126.6826  126.6269  126.4976  126.3219
##  [56,]  126.6462  126.6444  126.4867  126.1293  125.6364  125.0568  124.4284
##  [57,]  132.4264  131.7543  130.8480  129.7932  128.6500  127.4618  126.2614
##  [58,]  141.7511  140.0896  138.3205  136.5003  134.6680  132.8514  131.0715
##  [59,]  152.8947  150.3507  147.8234  145.3366  142.9072  140.5474  138.2668
##  [60,]  164.7112  161.5941  158.5650  155.6284  152.7879  150.0470  147.4088
##  [61,]  176.4572  173.2015  170.0237  166.9306  163.9278  161.0200  158.2112
##  [62,]  187.7716  184.8694  181.9427  179.0260  176.1432  173.3117  170.5449
##  [63,]  198.6904  196.6231  194.3447  191.9386  189.4619  186.9555  184.4495
##  [64,]  209.7053  208.8623  207.5676  205.9637  204.1494  202.1953  200.1535
##  [65,]  222.4742  222.4742  222.3317  221.7221  220.7529  219.5230  218.1064
##  [66,]  240.2634  240.2634  240.2634  240.2634  240.1677  239.7333  239.0248
##  [67,]  264.0174  264.0174  264.0174  264.0174  264.0174  264.0174  263.9562
##  [68,]  294.5380  294.5380  294.5380  294.5380  294.5380  294.5380  294.5380
##  [69,]  332.6918  332.6918  332.6918  332.6918  332.6918  332.6918  332.6918
##  [70,]  379.4028  379.4028  379.4028  379.4028  379.4028  379.4028  379.4028
##  [71,]  435.6390  435.6390  435.6390  435.6390  435.6390  435.6390  435.6390
##  [72,]  502.3920  502.3920  502.3920  502.3920  502.3920  502.3920  502.3920
##  [73,]  580.6487  580.6487  580.6487  580.6487  580.6487  580.6487  580.6487
##  [74,]  671.3523  671.3523  671.3523  671.3523  671.3523  671.0784  668.8999
##  [75,]  775.3531  775.3531  775.3531  774.9672  772.1282  767.0706  760.2835
##  [76,]  893.3486  892.7950  889.0407  882.5660  874.0648  864.0208  852.7825
##  [77,] 1019.8947 1011.4753 1000.7410  988.3364  974.6941  960.1156  944.8182
##  [78,] 1139.3894 1124.0046 1107.4704 1090.1099 1072.1407 1053.7157 1034.9461
##  [79,] 1248.2514 1227.8291 1207.0368 1185.9789 1164.7275 1143.3347 1121.8403
##  [80,] 1343.9232 1320.7374 1297.5029 1274.2296 1250.9265 1227.6025 1204.2660
##  [81,] 1424.0850 1400.6955 1377.0673 1353.2537 1329.2931 1305.2146 1281.0415
##  [82,] 1486.3927 1465.6580 1443.9235 1421.4415 1398.3828 1374.8680 1350.9858
##  [83,] 1528.1781 1513.3434 1496.0891 1477.0491 1456.6468 1435.1771 1412.8523
##  [84,] 1546.0849 1540.9568 1531.1902 1518.0286 1502.2970 1484.5651 1465.2401
##  [85,] 1546.4537 1546.4537 1546.1771 1541.8021 1533.1234 1521.1161 1506.4725
##  [86,] 1546.4537 1546.4537 1546.4537 1546.4537 1546.2385 1542.3666 1534.4268
##  [87,] 1546.4537 1546.4537 1546.4537 1546.4537 1546.4537 1546.4537 1546.2824
##  [88,] 1546.4537 1546.4537 1546.4537 1546.4537 1546.4537 1546.4537 1546.4537
##  [89,] 1546.4537 1546.4537 1546.4537 1546.4537 1546.4537 1546.4537 1546.4537
##  [90,] 1546.4537 1546.4537 1546.4537 1546.4537 1546.4537 1546.4537 1546.4537
##  [91,] 1546.4537 1546.4537 1546.4537 1546.4537 1546.4537 1546.4537 1546.4537
##  [92,] 1546.4537 1546.4537 1546.4537 1546.4537 1546.4537 1546.4537 1546.4537
##  [93,] 1546.4537 1546.4537 1546.4537 1546.4537 1546.4537 1546.4537 1546.4537
##  [94,] 1546.4537 1546.4537 1546.4537 1546.4537 1546.4537 1546.4537 1546.4537
##  [95,] 1546.4537 1546.4537 1546.4537 1546.4537 1546.4537 1546.4537 1546.4537
##  [96,] 1546.4537 1546.4537 1546.4537 1546.4537 1546.4537 1546.4537 1546.4537
##  [97,] 1546.4537 1546.4537 1546.4537 1546.4537 1546.4537 1546.4537 1546.4537
##  [98,] 1546.4537 1546.4537 1546.4537 1546.4537 1546.4537 1546.4537 1546.4537
##  [99,] 1546.4537 1546.4537 1546.4537 1546.4537 1546.4537 1546.4537 1546.4537
## [100,] 1546.4537 1546.4537 1546.4537 1546.4537 1546.4537 1546.4537 1546.4537
##            [,15]     [,16]     [,17]     [,18]     [,19]     [,20]     [,21]
##   [1,] 1415.5368 1415.5368 1415.5368 1415.5368 1415.5368 1415.5368 1415.5368
##   [2,] 1415.5368 1415.5368 1415.5368 1415.5368 1415.5368 1415.5368 1415.5368
##   [3,] 1415.5368 1415.5368 1415.5368 1415.5368 1415.5368 1415.5368 1415.5368
##   [4,] 1415.5368 1415.5368 1415.5368 1415.5368 1415.5368 1415.5368 1415.5368
##   [5,] 1415.5368 1415.5368 1415.5368 1415.5368 1415.5368 1415.5368 1415.5368
##   [6,] 1415.5368 1415.5368 1415.5368 1415.5368 1415.5368 1415.5368 1415.5368
##   [7,] 1415.5368 1415.5368 1415.5368 1415.5368 1415.5368 1415.5368 1415.5368
##   [8,] 1415.5368 1415.5368 1415.5368 1415.5368 1415.5368 1415.5368 1415.5368
##   [9,] 1415.5368 1415.5368 1415.5368 1415.5368 1415.5368 1415.5368 1415.5368
##  [10,] 1415.5368 1415.5368 1415.5368 1415.5368 1415.5368 1415.5368 1415.5368
##  [11,] 1415.5368 1415.5368 1415.5368 1415.5368 1415.5368 1415.4840 1413.7579
##  [12,] 1415.5368 1415.5368 1415.5368 1415.4723 1413.6881 1409.7283 1403.8979
##  [13,] 1415.5368 1415.4577 1413.5797 1409.5125 1403.5960 1396.1001 1387.2422
##  [14,] 1413.4222 1409.1673 1403.0630 1395.4098 1386.4439 1376.3541 1365.2931
##  [15,] 1402.2453 1394.3088 1385.1070 1374.8424 1363.6751 1351.7336 1339.1230
##  [16,] 1383.0950 1372.5219 1361.1233 1349.0301 1336.3465 1323.1567 1309.5302
##  [17,] 1357.3804 1345.0245 1332.1697 1318.8953 1305.2648 1291.3304 1277.1354
##  [18,] 1326.1794 1312.7582 1299.0726 1285.1659 1271.0732 1256.8240 1242.4436
##  [19,] 1290.3538 1276.4844 1262.5076 1248.4434 1234.3086 1220.1181 1205.8851
##  [20,] 1250.6281 1236.8505 1223.0554 1209.2502 1195.4421 1181.6381 1167.8448
##  [21,] 1207.6463 1194.4377 1181.2419 1168.0634 1154.9072 1141.7784 1128.6821
##  [22,] 1162.0146 1149.7963 1137.5679 1125.3399 1113.1214 1100.9210 1088.7468
##  [23,] 1114.3350 1103.4741 1092.5334 1081.5364 1070.5029 1059.4495 1048.3908
##  [24,] 1065.2333 1056.0393 1046.6557 1037.1256 1027.4839 1017.7598 1007.9776
##  [25,] 1015.3832 1008.1005 1000.4865  992.6089  984.5219  976.2698  967.8895
##  [26,]  965.5277  960.3231  954.6251  948.5281  942.1081  935.4266  928.5345
##  [27,]  916.4969  913.4443  909.7297  905.4743  900.7755  895.7124  890.3501
##  [28,]  869.2242  868.2848  866.5273  864.0951  861.1042  857.6490  853.8075
##  [29,]  825.8974  825.8974  825.8199  825.1001  823.7255  821.8010  819.4138
##  [30,]  789.3776  789.3776  789.3776  789.3776  789.3232  788.7763  787.7122
##  [31,]  759.3189  759.3189  759.3189  759.3189  759.3189  759.1803  758.3546
##  [32,]  735.2496  735.2496  735.2496  735.0745  734.1312  732.4808  730.2273
##  [33,]  716.6927  716.4720  715.3458  713.4083  710.7907  707.5989  703.9195
##  [34,]  701.5483  699.2729  696.2366  692.5701  688.3772  683.7415  678.7314
##  [35,]  686.0385  681.8258  677.0552  671.8238  666.2093  660.2740  654.0690
##  [36,]  669.3569  663.4590  657.1828  650.5957  643.7518  636.6945  629.4594
##  [37,]  650.9540  643.6653  636.1481  628.4451  620.5905  612.6119  604.5322
##  [38,]  630.4274  622.0698  613.6001  605.0414  596.4127  587.7294  579.0046
##  [39,]  607.4932  598.4063  589.2875  580.1467  570.9923  561.8315  552.6703
##  [40,]  581.9669  572.5006  563.0453  553.6039  544.1793  534.7742  525.3911
##  [41,]  553.7512  544.2604  534.7855  525.3289  515.8928  506.4797  497.0917
##  [42,]  522.8307  513.6705  504.4931  495.3066  486.1178  476.9327  467.7565
##  [43,]  489.2724  480.7933  472.2261  463.5911  454.9047  446.1804  437.4296
##  [44,]  453.2320  445.7734  438.1196  430.3089  422.3721  414.3341  406.2158
##  [45,]  414.9657  408.8478  402.3937  395.6652  388.7121  381.5744  374.2848
##  [46,]  374.8474  370.3596  365.3650  359.9540  354.1987  348.1571  341.8769
##  [47,]  333.3923  330.7773  327.4628  323.5707  319.1982  314.4234  309.3105
##  [48,]  291.2865  290.7187  289.2483  287.0287  284.1829  280.8101  276.9915
##  [49,]  251.4382  251.4382  251.4377  250.9781  249.7470  247.8632  245.4243
##  [50,]  216.6242  216.6242  216.6242  216.6242  216.6241  216.2526  215.2241
##  [51,]  187.1296  187.1296  187.1296  187.1296  187.1296  187.2631  187.6354
##  [52,]  163.2027  163.2027  163.2028  163.3085  163.5946  164.0404  164.6324
##  [53,]  145.0518  145.1195  145.2982  145.5771  145.9509  146.4180  146.9786
##  [54,]  132.8995  132.9751  133.0891  133.2497  133.4648  133.7425  134.0904
##  [55,]  126.1237  125.9236  125.7395  125.5870  125.4792  125.4279  125.4432
##  [56,]  123.7810  123.1386  122.5204  121.9425  121.4184  120.9594  120.5754
##  [57,]  125.0736  123.9181  122.8106  121.7642  120.7898  119.8965  119.0925
##  [58,]  129.3443  127.6825  126.0966  124.5954  123.1861  121.8755  120.6693
##  [59,]  136.0733  133.9733  131.9724  130.0756  128.2873  126.6118  125.0529
##  [60,]  144.8764  142.4527  140.1406  137.9431  135.8631  133.9033  132.0669
##  [61,]  155.5052  152.9055  150.4154  148.0381  145.7770  143.6351  141.6156
##  [62,]  167.8533  165.2455  162.7289  160.3097  157.9936  155.7857  153.6909
##  [63,]  181.9670  179.5261  177.1414  174.8250  172.5872  170.4366  168.3812
##  [64,]  198.0637  195.9567  193.8570  191.7845  189.7557  187.7846  185.8831
##  [65,]  216.5598  214.9277  213.2452  211.5410  209.8389  208.1584  206.5164
##  [66,]  238.1103  237.0444  235.8712  234.6270  233.3416  232.0403  230.7446
##  [67,]  263.6623  263.1736  262.5362  261.7890  260.9654  260.0938  259.1987
##  [68,]  294.5380  294.5033  294.3280  294.0329  293.6473  293.1978  292.7083
##  [69,]  332.6918  332.6918  332.6918  332.6783  332.6070  332.4882  332.3380
##  [70,]  379.4028  379.4028  379.4028  379.4028  379.4028  379.4066  379.3581
##  [71,]  435.6390  435.6390  435.6390  435.6390  435.5386  434.5285  432.5620
##  [72,]  502.3920  502.3920  502.2515  500.9463  498.4529  494.9599  490.6236
##  [73,]  580.4527  578.7685  575.6165  571.2531  565.8843  559.6780  552.7725
##  [74,]  664.9148  659.4748  652.8526  645.2636  636.8798  627.8410  618.2620
##  [75,]  752.1295  742.8836  732.7583  721.9208  710.5051  698.6196  686.3537
##  [76,]  840.6075  827.6913  814.1851  800.2080  785.8557  771.2064  756.3247
##  [77,]  928.9634  912.6741  896.0460  879.1552  862.0635  844.8219  827.4732
##  [78,] 1015.9156  996.6888  977.3170  957.8420  938.2986  918.7164  899.1209
##  [79,] 1100.2752 1078.6652 1057.0317 1035.3935 1013.7674  992.1687  970.6116
##  [80,] 1180.9259 1157.5908 1134.2696 1110.9713 1087.7054 1064.4811 1041.3082
##  [81,] 1256.7931 1232.4865 1208.1371 1183.7589 1159.3655 1134.9699 1110.5848
##  [82,] 1326.8035 1302.3743 1277.7411 1252.9397 1228.0007 1202.9511 1177.8150
##  [83,] 1389.8305 1366.2324 1342.1527 1317.6675 1292.8391 1267.7201 1242.3553
##  [84,] 1444.6215 1422.9357 1400.3576 1377.0252 1353.0499 1328.5232 1303.5214
##  [85,] 1489.6986 1471.1733 1451.1873 1429.9677 1407.6954 1384.5173 1360.5543
##  [86,] 1523.2171 1509.3315 1493.2222 1475.2402 1455.6628 1434.7129 1412.5724
##  [87,] 1542.7584 1535.3213 1524.6474 1511.2583 1495.5634 1477.8892 1458.5002
##  [88,] 1546.4537 1546.3154 1543.0336 1535.9247 1525.5795 1512.4662 1496.9611
##  [89,] 1546.4537 1546.4537 1546.4537 1546.3410 1543.2232 1536.3020 1526.1075
##  [90,] 1546.4537 1546.4537 1546.4537 1546.4537 1546.4537 1546.3615 1543.3452
##  [91,] 1546.4537 1546.4537 1546.4537 1546.4537 1546.4537 1546.4537 1546.4537
##  [92,] 1546.4537 1546.4537 1546.4537 1546.4537 1546.4537 1546.4537 1546.4537
##  [93,] 1546.4537 1546.4537 1546.4537 1546.4537 1546.4537 1546.4537 1546.4537
##  [94,] 1546.4537 1546.4537 1546.4537 1546.4537 1546.4537 1546.4537 1546.4537
##  [95,] 1546.4537 1546.4537 1546.4537 1546.4537 1546.4537 1546.4537 1546.4537
##  [96,] 1546.4537 1546.4537 1546.4537 1546.4537 1546.4537 1546.4537 1546.4537
##  [97,] 1546.4537 1546.4537 1546.4537 1546.4537 1546.4537 1546.4537 1546.4537
##  [98,] 1546.4537 1546.4537 1546.4537 1546.4537 1546.4537 1546.4537 1546.4537
##  [99,] 1546.4537 1546.4537 1546.4537 1546.4537 1546.4537 1546.4537 1546.4537
## [100,] 1546.4537 1546.4537 1546.4537 1546.4537 1546.4537 1546.4537 1546.4537
##            [,22]     [,23]     [,24]     [,25]     [,26]     [,27]     [,28]
##   [1,] 1415.5368 1415.5368 1415.5368 1415.5368 1415.5368 1415.5368 1415.5368
##   [2,] 1415.5368 1415.5368 1415.5368 1415.5368 1415.5368 1415.5368 1415.5368
##   [3,] 1415.5368 1415.5368 1415.5368 1415.5368 1415.5368 1415.5368 1415.5368
##   [4,] 1415.5368 1415.5368 1415.5368 1415.5368 1415.5368 1415.5368 1415.5368
##   [5,] 1415.5368 1415.5368 1415.5368 1415.5368 1415.5368 1415.5368 1415.5368
##   [6,] 1415.5368 1415.5368 1415.5368 1415.5368 1415.5368 1415.5368 1415.5368
##   [7,] 1415.5368 1415.5368 1415.5368 1415.5368 1415.5368 1415.5368 1415.5177
##   [8,] 1415.5368 1415.5368 1415.5368 1415.5368 1415.5109 1413.9281 1410.1376
##   [9,] 1415.5368 1415.5368 1415.5030 1413.8723 1410.0405 1404.3096 1396.9393
##  [10,] 1415.4940 1413.8156 1409.9425 1404.1761 1396.7752 1387.9630 1377.9334
##  [11,] 1409.8438 1404.0420 1396.6104 1387.7718 1377.7195 1366.6220 1354.6271
##  [12,] 1396.4446 1387.5801 1377.5051 1366.3881 1354.3764 1341.5996 1328.1720
##  [13,] 1377.1998 1366.1195 1354.1239 1341.3344 1327.8948 1313.9079 1299.4637
##  [14,] 1353.3865 1340.7386 1327.4374 1313.5579 1299.1648 1284.3400 1269.2089
##  [15,] 1325.9299 1312.2272 1298.0766 1283.5316 1268.6384 1253.4383 1237.9679
##  [16,] 1295.5249 1281.1898 1266.5673 1251.6942 1236.6032 1221.3234 1205.8816
##  [17,] 1262.7167 1248.1058 1233.3306 1218.4157 1203.3834 1188.2542 1173.0469
##  [18,] 1227.9535 1213.3732 1198.7201 1184.0101 1169.2580 1154.4778 1139.6828
##  [19,] 1191.6216 1177.3386 1163.0469 1148.7566 1134.4774 1120.2191 1105.9911
##  [20,] 1154.0690 1140.3174 1126.5969 1112.9142 1099.2765 1085.6908 1072.1645
##  [21,] 1115.6237 1102.6087 1089.6430 1076.7323 1063.8828 1051.1008 1038.3929
##  [22,] 1076.6062 1064.5065 1052.4548 1040.4581 1028.5234 1016.6576 1004.8677
##  [23,] 1037.3397 1026.3077 1015.3056 1004.3432  993.4298  982.5743  971.7853
##  [24,]  998.1581  988.3195  978.4778  968.6475  958.8415  949.0717  939.3493
##  [25,]  959.4120  950.8637  942.2675  933.6434  925.0092  916.3809  907.7729
##  [26,]  921.4741  914.2815  906.9872  899.6180  892.1970  884.7448  877.2799
##  [27,]  884.7428  878.9362  872.9692  866.8754  860.6836  854.4195  848.0439
##  [28,]  849.6448  845.2156  840.5666  835.7378  830.6901  825.0952  818.9538
##  [29,]  816.6372  813.5331  810.0650  805.8814  801.0094  795.5181  789.4664
##  [30,]  786.0974  783.5507  780.1351  775.9505  771.0804  765.5957  759.5567
##  [31,]  756.5414  753.7851  750.1994  745.8789  740.9032  735.3395  729.2456
##  [32,]  727.4572  724.2162  720.3006  715.7164  710.5327  704.8089  698.5959
##  [33,]  699.8239  695.3717  690.6131  685.5725  680.0767  674.1095  667.7116
##  [34,]  673.4031  667.8037  661.9730  655.9445  649.7473  643.3934  636.7378
##  [35,]  647.6363  641.0113  634.2237  627.2990  620.2591  613.1231  605.9077
##  [36,]  622.0763  614.5698  606.9612  599.2686  591.5077  583.6924  575.8349
##  [37,]  596.3706  588.1433  579.8641  571.5452  563.1971  554.8294  546.4505
##  [38,]  570.2493  561.4729  552.6839  543.8896  535.0967  526.3113  517.5389
##  [39,]  543.5143  534.3684  525.2371  516.1246  507.0349  497.9717  488.9385
##  [40,]  516.0326  506.7012  497.3993  488.1296  478.8944  469.6964  460.5379
##  [41,]  487.7310  478.4001  469.1012  459.8367  450.6088  441.4200  432.2728
##  [42,]  458.5939  449.4491  440.3261  431.2286  422.1603  413.1243  404.1242
##  [43,]  428.6620  419.8861  411.1093  402.3383  393.5790  384.8370  376.1174
##  [44,]  398.0343  389.8044  381.5389  373.2487  364.9435  356.6321  348.3222
##  [45,]  366.8708  359.3552  351.7577  344.0950  336.3818  328.6309  320.8537
##  [46,]  335.3974  328.7514  321.9669  315.0676  308.0741  301.0044  293.8739
##  [47,]  303.9124  298.2732  292.4303  286.4154  280.2558  273.9751  267.7541
##  [48,]  272.7944  268.2749  263.4803  258.4509  253.3830  248.4697  243.6916
##  [49,]  242.5114  239.1922  235.6838  232.1819  228.6842  225.1897  221.6984
##  [50,]  213.7827  212.1343  210.3053  208.3186  206.1942  203.9499  201.6013
##  [51,]  187.9190  187.8303  187.4183  186.7247  185.7853  184.6308  183.2881
##  [52,]  165.3615  166.2221  166.9649  167.3437  167.4026  167.1797  166.7084
##  [53,]  147.6346  148.3887  149.2439  150.2038  151.0678  151.6138  151.8760
##  [54,]  134.5158  135.0254  135.6255  136.3220  137.1206  138.0263  138.8737
##  [55,]  125.5341  125.7087  125.9744  126.3377  126.8048  127.3813  128.0723
##  [56,]  120.2752  120.0663  119.9556  119.9495  120.0536  120.2732  120.6133
##  [57,]  118.3850  117.7802  117.2840  116.9016  116.6381  116.4981  116.4859
##  [58,]  119.5727  118.5907  117.7278  116.9882  116.3759  115.8950  115.5491
##  [59,]  123.6144  122.2999  121.1128  120.0566  119.1346  118.3501  117.7065
##  [60,]  130.3566  128.7754  127.3263  126.0122  124.8363  123.8015  122.9108
##  [61,]  139.7215  137.9561  136.3224  134.8237  133.4630  132.2436  131.1688
##  [62,]  151.7136  149.8581  148.1284  146.5286  145.0626  143.7341  142.5471
##  [63,]  166.4280  164.5831  162.8527  161.2420  159.7563  158.4006  157.1796
##  [64,]  184.0616  182.3294  180.6948  179.1653  177.7480  176.4494  175.2754
##  [65,]  204.9275  203.4042  201.9578  200.5984  199.3350  198.1760  197.1292
##  [66,]  229.4729  228.2413  227.0640  225.9535  224.9213  223.9777  223.1320
##  [67,]  258.3014  257.4205  256.5727  255.7726  255.0336  254.3409  253.3685
##  [68,]  292.2003  291.6928  291.2032  290.7104  289.8141  288.4468  286.6612
##  [69,]  332.1728  331.9574  331.1944  329.8203  327.9088  325.5241  322.7227
##  [70,]  378.6218  377.1360  374.9981  372.2911  369.0862  365.4452  361.4217
##  [71,]  429.7951  426.4078  422.4877  418.1020  413.3086  408.1582  402.6946
##  [72,]  485.5747  479.9231  473.7866  467.3411  460.6348  453.6970  446.5545
##  [73,]  545.2826  537.3049  528.9213  520.2018  511.2349  502.1943  493.1020
##  [74,]  608.2382  597.8498  587.1646  576.2409  565.1291  553.8731  542.5412
##  [75,]  673.7818  660.9668  647.9621  634.8138  621.5620  608.2419  594.8848
##  [76,]  741.2648  726.0728  710.7885  695.4466  680.0774  664.7082  649.3634
##  [77,]  810.0540  792.5959  775.1266  757.6709  740.2510  722.8872  705.5982
##  [78,]  879.5347  859.9782  840.4698  821.0266  801.6646  782.3987  763.2434
##  [79,]  949.1097  927.6758  906.3225  885.0620  863.9064  842.8675  821.9571
##  [80,] 1018.1967  995.1567  972.1986  949.3330  926.5709  903.9233  881.4015
##  [81,] 1086.2228 1061.8963 1037.6181 1013.4007  989.2569  965.1996  941.2420
##  [82,] 1152.6144 1127.3698 1102.1009 1076.8260 1051.5633 1026.3305 1001.1449
##  [83,] 1216.7839 1191.0407 1165.1572 1139.1625 1113.0836 1086.9458 1060.7736
##  [84,] 1278.1096 1252.3442 1226.2746 1199.9451 1173.3958 1146.6635 1119.7826
##  [85,] 1335.9078 1310.6641 1284.8976 1258.6735 1232.0500 1205.0792 1177.8088
##  [86,] 1389.3920 1365.2984 1340.3997 1314.7890 1288.5480 1261.7494 1234.4582
##  [87,] 1437.6135 1415.4098 1392.0416 1367.6390 1342.3150 1316.2129 1289.5535
##  [88,] 1479.3711 1459.9493 1438.9076 1416.4561 1392.8454 1368.2545 1342.8411
##  [89,] 1513.0691 1497.5528 1479.9061 1460.4204 1439.3507 1416.9218 1393.3326
##  [90,] 1536.5040 1526.3596 1513.3592 1497.8884 1480.2817 1460.8305 1439.7905
##  [91,] 1546.3789 1543.4459 1536.6765 1526.5942 1513.6475 1498.2232 1480.6565
##  [92,] 1546.4537 1546.4537 1546.3946 1543.5450 1536.8477 1526.8276 1513.9348
##  [93,] 1546.4537 1546.4537 1546.4537 1546.4537 1546.4084 1543.6426 1537.0175
##  [94,] 1546.4537 1546.4537 1546.4537 1546.4537 1546.4537 1546.4537 1546.4204
##  [95,] 1546.4537 1546.4537 1546.4537 1546.4537 1546.4537 1546.4537 1546.4537
##  [96,] 1546.4537 1546.4537 1546.4537 1546.4537 1546.4537 1546.4537 1546.4537
##  [97,] 1546.4537 1546.4537 1546.4537 1546.4537 1546.4537 1546.4537 1546.4537
##  [98,] 1546.4537 1546.4537 1546.4537 1546.4537 1546.4537 1546.4537 1546.4537
##  [99,] 1546.4537 1546.4537 1546.4537 1546.4537 1546.4537 1546.4537 1546.4537
## [100,] 1546.4537 1546.4537 1546.4537 1546.4537 1546.4537 1546.4537 1546.4537
##            [,29]     [,30]     [,31]     [,32]     [,33]     [,34]     [,35]
##   [1,] 1415.5368 1415.5368 1415.5368 1415.5368 1415.5368 1415.5368 1415.5368
##   [2,] 1415.5368 1415.5368 1415.5368 1415.5368 1415.5368 1415.5368 1415.5368
##   [3,] 1415.5368 1415.5368 1415.5368 1415.5368 1415.5368 1415.5368 1415.5368
##   [4,] 1415.5368 1415.5368 1415.5368 1415.5368 1415.5368 1415.5320 1414.1424
##   [5,] 1415.5368 1415.5368 1415.5368 1415.5283 1414.0902 1410.4244 1404.8368
##   [6,] 1415.5368 1415.5236 1414.0371 1410.3296 1404.7060 1397.4284 1388.7227
##   [7,] 1413.9830 1410.2340 1404.5746 1397.2660 1388.5335 1378.5727 1367.5540
##   [8,] 1404.4424 1397.1029 1388.3438 1378.3600 1367.3215 1355.3771 1342.6577
##   [9,] 1388.1536 1378.1470 1367.0887 1355.1274 1342.3936 1329.0024 1315.0562
##  [10,] 1366.8556 1354.8774 1342.1292 1328.7259 1314.7694 1300.3506 1285.5504
##  [11,] 1341.8645 1328.4491 1314.4824 1300.0551 1285.2480 1270.1333 1254.7761
##  [12,] 1314.1952 1299.7595 1284.9455 1269.8253 1254.4637 1238.9192 1223.2442
##  [13,] 1284.6428 1269.5171 1254.1513 1238.6037 1222.9266 1207.1675 1191.3694
##  [14,] 1253.8389 1238.2881 1222.6089 1206.8486 1191.0501 1175.2522 1159.4901
##  [15,] 1222.2911 1206.5297 1190.7308 1174.9333 1159.1723 1143.4796 1127.8843
##  [16,] 1190.3023 1174.6083 1158.8545 1143.1635 1127.5705 1112.1016 1096.7806
##  [17,] 1157.7795 1142.4689 1127.1314 1111.7829 1096.4728 1081.3251 1066.3671
##  [18,] 1124.8861 1110.1002 1095.3374 1080.6103 1065.9309 1051.3117 1036.7980
##  [19,] 1091.8030 1077.6645 1063.5851 1049.5746 1035.6430 1021.8003 1008.0569
##  [20,] 1058.7051 1045.3203 1032.0180 1018.8065 1005.6940  992.6892  979.8010
##  [21,] 1025.7657 1013.2261 1000.7814  988.4388  976.2059  964.0904  952.1005
##  [22,]  993.1607  981.5437  970.0238  958.6083  947.3048  936.1206  925.0635
##  [23,]  961.0710  950.4397  939.8991  929.4574  919.1223  908.9016  898.8032
##  [24,]  929.6847  920.0878  910.5681  901.1345  891.7960  882.5216  873.0440
##  [25,]  899.1985  890.6699  882.1985  873.7499  865.0453  856.0436  846.7542
##  [26,]  869.8187  862.3237  854.5026  846.3240  837.8058  828.9648  819.8163
##  [27,]  841.2476  834.0129  826.3693  818.3436  809.9594  801.2382  792.1995
##  [28,]  812.3121  805.2103  797.6837  789.7633  781.4765  772.8478  763.8990
##  [29,]  782.9049  775.8774  768.4221  760.5722  752.3571  743.8028  734.9323
##  [30,]  753.0148  746.0146  738.5947  730.7890  722.6270  714.1350  705.3362
##  [31,]  722.6705  715.6571  708.2425  700.4591  692.3355  683.8971  675.1664
##  [32,]  691.9378  684.8730  677.4352  669.6538  661.5552  653.1627  644.4973
##  [33,]  660.9188  653.7628  646.2717  638.4708  630.3825  622.0271  613.4230
##  [34,]  629.7526  622.4603  614.8816  607.0353  598.9389  590.6084  582.0589
##  [35,]  598.6187  591.1360  583.4271  575.5027  567.3737  559.0507  550.5439
##  [36,]  567.9461  560.0358  552.1068  544.0617  535.8669  527.5260  519.0435
##  [37,]  538.0681  529.6892  521.3204  512.9678  504.6328  496.2384  487.7525
##  [38,]  508.7847  500.0537  491.3505  482.6795  474.0449  465.4510  456.8990
##  [39,]  479.9389  470.9763  462.0540  453.1754  444.3436  435.5619  426.8337
##  [40,]  451.4217  442.3504  433.3265  424.3528  415.4320  406.5669  397.7604
##  [41,]  423.1697  414.1131  405.1057  396.1501  387.2489  378.4050  369.6210
##  [42,]  395.1630  386.2440  377.3703  368.5451  359.7713  351.0521  342.3906
##  [43,]  367.4248  358.7640  350.1390  341.5541  333.0133  324.5204  316.0792
##  [44,]  340.0209  331.7348  323.4699  315.2318  307.0260  299.0038  291.3815
##  [45,]  313.0603  305.2599  297.4609  289.8227  282.5537  275.6194  268.9886
##  [46,]  286.6968  279.6419  272.9104  266.4692  260.2892  254.3460  248.6191
##  [47,]  261.7886  256.0497  250.5131  245.1585  239.9693  234.9319  230.0347
##  [48,]  239.0329  234.4810  230.0260  225.6601  221.3769  217.1719  213.0413
##  [49,]  218.2114  214.7302  211.2565  207.7928  204.3414  200.9050  197.4864
##  [50,]  199.1624  196.6456  194.0624  191.4229  188.7366  186.0120  183.2573
##  [51,]  181.7805  180.1285  178.3506  176.4629  174.4801  172.4153  170.2808
##  [52,]  166.0177  165.1333  164.0780  162.8720  161.5334  160.0787  158.5227
##  [53,]  151.8854  151.6695  151.2528  150.6576  149.9035  149.0088  147.9899
##  [54,]  139.4585  139.8055  139.9383  139.8784  139.6456  139.2580  138.7321
##  [55,]  128.8829  129.6742  130.2574  130.6493  130.8674  130.9278  130.8459
##  [56,]  121.0786  121.6735  122.4022  123.1475  123.7350  124.1751  124.4800
##  [57,]  116.6057  116.8616  117.2574  117.7969  118.4837  119.2190  119.8416
##  [58,]  115.3419  115.2770  115.3579  115.5881  115.9709  116.5099  117.2082
##  [59,]  117.2070  116.8548  116.6531  116.6053  116.7146  116.9842  117.4174
##  [60,]  122.1675  121.5746  121.1352  120.8527  120.7300  120.7706  120.9776
##  [61,]  130.2417  129.4655  128.8438  128.3796  128.0764  127.9376  127.9666
##  [62,]  141.5051  140.6119  139.8713  139.2869  138.8625  138.6016  138.5081
##  [63,]  156.0978  155.1598  154.3700  153.7326  153.2520  152.9322  152.7709
##  [64,]  174.2320  173.3246  172.5586  171.9391  171.4613  170.9491  170.3433
##  [65,]  196.2016  195.4002  194.7176  193.9363  192.9922  191.9023  190.6833
##  [66,]  222.3738  221.4365  220.2525  218.8469  217.2437  215.4658  213.5350
##  [67,]  252.0475  250.4144  248.5031  246.3451  243.9692  241.4023  238.6695
##  [68,]  284.5043  282.0188  279.2430  276.2114  272.9558  269.5052  265.8859
##  [69,]  319.5543  316.0630  312.2882  308.2654  304.0264  299.6002  295.0134
##  [70,]  357.0628  352.4103  347.5010  342.3681  337.0414  331.5480  325.9125
##  [71,]  396.9569  390.9795  384.7933  378.4259  371.9026  365.2461  358.4777
##  [72,]  439.2317  431.7507  424.1322  416.3949  408.5564  400.6330  392.6402
##  [73,]  483.9605  474.7738  465.5473  456.2871  446.9998  437.6924  428.3724
##  [74,]  531.3083  520.1801  509.1410  498.1796  487.2877  476.4600  465.6931
##  [75,]  581.5184  568.1982  555.1017  542.2260  529.5436  517.0325  504.6757
##  [76,]  634.0651  618.8335  603.6874  588.6736  573.9735  559.5803  545.4589
##  [77,]  688.4012  671.3125  654.3475  637.5205  620.8456  604.3643  588.2617
##  [78,]  744.2124  725.3190  706.5762  687.9966  669.5925  651.3761  633.3595
##  [79,]  801.1871  780.5692  760.1151  739.8367  719.7459  699.8543  680.1741
##  [80,]  859.0172  836.7821  814.7081  792.8076  771.0928  749.5765  728.2712
##  [81,]  917.3974  893.6793  870.1015  846.6779  823.4228  800.3504  777.4755
##  [82,]  976.0238  950.9842  926.0434  901.2187  876.5272  851.9865  827.6140
##  [83,] 1034.5905 1008.4192  982.2823  956.2017  930.1993  904.2970  878.5745
##  [84,] 1092.7854 1065.7027 1038.5640 1011.3980  984.2926  957.4679  930.9721
##  [85,] 1150.2831 1122.5431 1094.6866 1066.9316 1039.3410 1011.9621  984.8377
##  [86,] 1206.7877 1178.9535 1151.0403 1123.1176 1095.2487 1067.4909 1039.8965
##  [87,] 1262.4559 1235.0232 1207.3487 1179.5169 1151.6046 1123.6814 1095.8108
##  [88,] 1316.7462 1290.0967 1263.0069 1235.5801 1207.9096 1180.0802 1152.1688
##  [89,] 1368.7599 1343.3617 1317.2793 1290.6398 1263.5579 1236.1369 1208.4704
##  [90,] 1417.3871 1393.8195 1369.2650 1343.8820 1317.8121 1291.1828 1264.1087
##  [91,] 1461.2400 1440.2298 1417.8518 1394.3059 1369.7698 1344.4020 1318.3447
##  [92,] 1498.5570 1481.0306 1461.6488 1440.6685 1418.3161 1394.7919 1370.2742
##  [93,] 1527.0599 1514.2210 1498.8900 1481.4040 1462.0570 1441.1066 1418.7799
##  [94,] 1543.7385 1537.1859 1527.2909 1514.5063 1499.2221 1481.7766 1462.4645
##  [95,] 1546.4537 1546.4305 1543.8329 1537.3530 1527.5209 1514.7905 1499.5533
##  [96,] 1546.4537 1546.4537 1546.4537 1546.4388 1543.9258 1537.5188 1527.7496
##  [97,] 1546.4537 1546.4537 1546.4537 1546.4537 1546.4537 1546.4453 1544.0170
##  [98,] 1546.4537 1546.4537 1546.4537 1546.4537 1546.4537 1546.4537 1546.4537
##  [99,] 1546.4537 1546.4537 1546.4537 1546.4537 1546.4537 1546.4537 1546.4537
## [100,] 1546.4537 1546.4537 1546.4537 1546.4537 1546.4537 1546.4537 1546.4537
##            [,36]     [,37]     [,38]     [,39]     [,40]     [,41]     [,42]
##   [1,] 1415.5368 1415.5368 1415.5368 1415.5368 1415.5368 1414.2935 1410.7958
##   [2,] 1415.5368 1415.5368 1415.5363 1414.2441 1410.7041 1405.2252 1398.0724
##   [3,] 1415.5347 1414.1937 1410.6116 1405.0964 1397.9123 1389.2872 1379.4191
##   [4,] 1410.5184 1404.9670 1397.7516 1389.0996 1379.2081 1368.2491 1356.3728
##   [5,] 1397.5903 1388.9114 1378.9967 1368.0177 1356.1243 1343.4484 1330.1064
##   [6,] 1378.7849 1367.7860 1355.8756 1343.1851 1329.8308 1315.9155 1301.5310
##   [7,] 1355.6265 1342.9215 1329.5549 1315.6293 1301.2361 1286.4569 1271.3645
##   [8,] 1329.2788 1315.3429 1300.9411 1286.1548 1271.0569 1255.7126 1240.1808
##   [9,] 1300.6459 1285.8527 1270.7491 1255.4005 1239.8655 1224.1971 1208.4430
##  [10,] 1270.4413 1255.0883 1239.5501 1223.8795 1208.1241 1192.3272 1176.5279
##  [11,] 1239.2347 1223.5619 1207.8053 1192.0080 1176.2090 1160.4438 1144.7445
##  [12,] 1207.4864 1191.6887 1175.8900 1160.1259 1144.4282 1128.8262 1113.3464
##  [13,] 1175.5711 1159.8080 1144.1120 1128.5122 1113.0351 1097.7046 1082.5424
##  [14,] 1143.7958 1128.1982 1112.7238 1097.3965 1082.2380 1067.2679 1052.5042
##  [15,] 1112.4127 1097.0885 1081.9336 1066.9676 1052.2082 1037.6715 1023.3721
##  [16,] 1081.6293 1066.6673 1051.9122 1037.3802 1023.0857 1009.0418  995.2601
##  [17,] 1051.6164 1037.0891 1022.7995 1008.7607  994.9844  981.4810  968.2596
##  [18,] 1022.5134 1008.4798  994.7089  981.2110  967.9955  955.0703  942.4427
##  [19,]  994.4234  980.9412  967.7315  954.8122  942.1907  929.8731  917.8647
##  [20,]  967.0384  954.4108  941.9279  929.6275  917.6254  905.9371  894.5413
##  [21,]  940.2442  928.5302  916.9669  905.5633  894.3007  882.9540  871.5562
##  [22,]  914.1414  903.3621  892.7028  881.9106  870.9237  859.7387  848.3536
##  [23,]  888.8002  878.6337  868.2478  857.6422  846.8174  835.7753  824.5180
##  [24,]  863.3138  853.3349  843.1116  832.6490  821.9523  811.0268  799.8781
##  [25,]  837.1869  827.3509  817.2554  806.9092  796.3207  785.4982  774.4497
##  [26,]  810.3748  800.6536  790.6651  780.4208  769.9316  759.2077  748.2588
##  [27,]  782.8607  773.2380  763.3460  753.1981  742.8069  732.1838  721.3398
##  [28,]  754.6498  745.1179  735.3193  725.2687  714.9797  704.4646  693.7350
##  [29,]  725.7663  716.3233  706.6202  696.6723  686.4936  676.0970  665.4944
##  [30,]  696.2515  686.8996  677.2975  667.4605  657.4027  647.1372  636.6760
##  [31,]  666.1636  656.9070  647.4130  637.6968  627.7720  617.6515  607.3471
##  [32,]  635.5777  626.4211  617.0430  607.4576  597.6781  587.7166  577.5847
##  [33,]  604.5869  595.5341  586.2786  576.8336  567.2109  557.4220  547.4774
##  [34,]  573.3039  564.3564  555.2284  545.9311  536.4751  526.8705  517.1268
##  [35,]  541.8635  533.0189  524.0195  514.8743  505.5919  496.1808  486.6492
##  [36,]  510.4247  501.6751  492.8006  483.8073  474.7015  465.4895  456.1778
##  [37,]  479.1743  470.5044  461.7445  452.8971  443.9654  434.9531  425.8645
##  [38,]  448.3301  439.7153  431.0512  422.3359  413.5689  404.7508  395.8830
##  [39,]  418.1622  409.5488  400.9513  392.3449  383.7247  375.0872  366.4307
##  [40,]  389.0153  380.3345  371.7212  363.1773  354.6753  346.1954  337.8557
##  [41,]  360.8999  352.2445  343.6578  335.1428  326.8320  318.9664  311.5021
##  [42,]  333.7900  325.2533  316.9190  309.0197  301.5264  294.4084  287.6393
##  [43,]  307.8342  300.0100  292.5746  285.4966  278.7489  272.3081  266.1542
##  [44,]  284.1254  277.2035  270.5888  264.2578  258.1906  252.3700  246.7813
##  [45,]  262.6348  256.5354  250.6714  245.0264  239.5865  234.3399  229.2768
##  [46,]  243.0912  237.7478  232.5767  227.5676  222.7119  218.0026  213.4337
##  [47,]  225.2685  220.6255  216.0996  211.6856  207.3795  203.1784  199.0798
##  [48,]  208.9825  204.9935  201.0733  197.2212  193.4371  189.7214  186.0749
##  [49,]  194.0885  190.7143  187.3669  184.0495  180.7654  177.5177  174.3100
##  [50,]  180.4799  177.6867  174.8844  172.0792  169.2772  166.4841  163.7055
##  [51,]  168.0873  165.8451  163.5635  161.2514  158.9169  156.5679  154.2115
##  [52,]  156.8789  155.1598  153.3767  151.5404  149.6607  147.7468  145.8077
##  [53,]  146.8616  145.6379  144.3315  142.9543  141.5171  140.0305  138.5041
##  [54,]  138.0833  137.3259  136.4730  135.5371  134.5296  133.4616  132.3432
##  [55,]  130.6362  130.3121  129.8866  129.3716  128.7787  128.1187  127.4019
##  [56,]  124.6618  124.7321  124.7023  124.5833  124.3857  124.1197  123.7951
##  [57,]  120.3571  120.7738  121.1003  121.3454  121.5179  121.6266  121.6801
##  [58,]  117.9827  118.6853  119.3178  119.8855  120.3944  120.8509  121.2619
##  [59,]  118.0173  118.7874  119.6578  120.4937  121.2938  122.0613  122.7999
##  [60,]  121.3544  121.9042  122.6303  123.5362  124.5640  125.5901  126.5434
##  [61,]  128.1668  128.5415  129.0945  129.8260  130.6504  131.5275  132.4083
##  [62,]  138.5856  138.8333  139.1399  139.4583  139.7929  140.1488  140.5324
##  [63,]  152.6266  152.4461  152.2365  152.0057  151.7626  151.5164  151.2766
##  [64,]  169.6550  168.8959  168.0781  167.2141  166.3161  165.3965  164.4676
##  [65,]  189.3520  187.9248  186.4176  184.8461  183.2253  181.5699  179.8944
##  [66,]  211.4721  209.2966  207.0275  204.6825  202.2787  199.8326  197.3598
##  [67,]  235.7940  232.7977  229.7010  226.5232  223.2827  219.9966  216.6816
##  [68,]  262.1227  258.2381  254.2534  250.1884  246.0618  241.8912  237.6932
##  [69,]  290.2902  285.4531  280.5229  275.5191  270.4601  265.3629  260.2441
##  [70,]  320.1576  314.3042  308.3718  302.3785  296.3416  290.2772  284.2007
##  [71,]  351.6166  344.6810  337.6879  330.6532  323.5920  316.5189  309.4475
##  [72,]  384.5925  376.5036  368.3868  360.2547  352.1196  343.9933  335.8875
##  [73,]  419.0474  409.7254  400.4142  391.1222  381.8575  372.6286  363.4442
##  [74,]  454.9856  444.3374  433.7498  423.2250  412.7662  402.3774  392.0632
##  [75,]  492.4598  480.3744  468.4122  456.5677  444.8376  433.2202  421.7151
##  [76,]  531.5808  517.9227  504.4660  491.1956  478.0999  465.1700  452.3992
##  [77,]  572.5307  557.1316  542.0315  527.2033  512.6249  498.2782  484.1489
##  [78,]  615.5809  598.2291  581.2985  564.7466  548.5380  532.6435  517.0387
##  [79,]  660.7171  641.4954  622.5455  604.0590  586.0331  568.4240  551.1951
##  [80,]  707.1900  686.3460  665.7523  645.4224  625.3919  605.8986  587.1218
##  [81,]  754.8129  732.3775  710.1846  688.2993  666.9256  646.0891  625.8147
##  [82,]  803.4277  779.4998  756.0439  733.0909  710.6520  688.7365  667.3521
##  [83,]  853.2508  828.3641  803.9342  779.9788  756.5128  733.5496  711.1002
##  [84,]  904.8369  879.0905  853.7581  828.8624  804.4231  780.4579  756.9820
##  [85,]  958.0066  931.5039  905.3611  879.6066  854.2656  829.3609  804.9122
##  [86,] 1012.5128  985.3829  958.5455  932.0359  905.8855  880.1229  854.7733
##  [87,] 1068.0502 1040.4520 1013.0637  985.9282  959.0846  932.5679  906.4100
##  [88,] 1124.2452 1096.3729 1068.6096 1041.0076 1013.6146  986.4736  959.6237
##  [89,] 1180.6434 1152.7330 1124.8090 1096.9351 1069.1690 1041.5633 1014.1656
##  [90,] 1236.6935 1209.0312 1181.2067 1153.2972 1125.3728 1097.4973 1069.7285
##  [91,] 1291.7255 1264.6593 1237.2501 1209.5919 1181.7699 1153.8614 1125.9367
##  [92,] 1344.9218 1318.8771 1292.2680 1265.2098 1237.8066 1210.1526 1182.3330
##  [93,] 1395.2776 1370.7783 1345.4413 1319.4093 1292.8104 1265.7602 1238.3630
##  [94,] 1441.5442 1419.2432 1395.7628 1371.2820 1345.9605 1319.9412 1293.3526
##  [95,] 1482.1484 1462.8714 1441.9812 1419.7060 1396.2476 1371.7854 1346.4794
##  [96,] 1515.0737 1499.8837 1482.5195 1463.2776 1442.4177 1420.1684 1396.7320
##  [97,] 1537.6831 1527.9771 1515.3559 1500.2131 1482.8898 1463.6832 1442.8536
##  [98,] 1546.4500 1544.1067 1537.8461 1528.2035 1515.6371 1500.5417 1483.2593
##  [99,] 1546.4537 1546.4537 1546.4528 1544.1947 1538.0077 1528.4287 1515.9172
## [100,] 1546.4537 1546.4537 1546.4537 1546.4537 1546.4537 1544.2812 1538.1680
##            [,43]     [,44]     [,45]     [,46]     [,47]     [,48]     [,49]
##   [1,] 1405.3532 1398.2319 1389.6611 1379.8396 1368.9408 1357.1161 1344.4989
##   [2,] 1389.4744 1379.6295 1368.7106 1356.8687 1344.2367 1330.9320 1317.0586
##   [3,] 1368.4800 1356.6209 1343.9742 1330.6571 1316.7731 1302.4146 1287.6637
##   [4,] 1343.7115 1330.3819 1316.4875 1302.1202 1287.3622 1272.2869 1256.9603
##   [5,] 1316.2016 1301.8257 1287.0605 1271.9796 1256.6485 1241.1265 1225.4670
##   [6,] 1286.7588 1271.6721 1256.3366 1240.8113 1225.1496 1209.3994 1193.6043
##   [7,] 1256.0246 1240.4961 1224.8321 1209.0806 1193.2851 1177.4849 1161.7157
##   [8,] 1224.5146 1208.7618 1192.9658 1177.1659 1161.3977 1145.6935 1130.0828
##   [9,] 1192.6465 1176.8469 1161.0797 1145.3771 1129.7686 1114.2806 1098.9374
##  [10,] 1160.7617 1145.0608 1129.4544 1113.9691 1098.6291 1083.4562 1068.4702
##  [11,] 1129.1403 1113.6577 1098.3208 1083.1515 1068.1695 1053.3928 1038.8375
##  [12,] 1098.0127 1082.8469 1067.8689 1053.0965 1038.5459 1024.2317 1010.1671
##  [13,] 1067.5684 1052.8003 1038.2543 1023.9451 1009.8856  996.0877  982.5618
##  [14,] 1037.9629 1023.6585 1009.6042  995.8117  982.2914  969.0527  956.1038
##  [15,] 1009.3229  995.5358  982.0211  968.7882  955.8452  943.1994  930.8571
##  [16,]  981.7510  968.5239  955.5868  942.9471  930.6109  918.5836  906.8699
##  [17,]  955.3285  942.6948  930.3648  918.3439  906.6365  895.2287  883.8732
##  [18,]  930.1189  918.1042  906.4032  894.9996  883.6434  872.2483  860.8028
##  [19,]  906.1701  894.7704  883.4136  872.0176  860.5710  849.0649  837.4928
##  [20,]  883.1838  871.7869  860.3391  848.8318  837.2583  825.6138  813.8951
##  [21,]  860.1073  848.5987  837.0238  825.3778  813.6576  801.8613  789.9882
##  [22,]  836.7892  825.1418  813.4200  801.6222  789.7476  777.7965  765.7701
##  [23,]  813.0486  801.3705  789.5069  777.5543  765.5264  753.4252  741.2531
##  [24,]  788.5120  776.9341  765.1504  753.1668  741.0065  728.7652  716.4596
##  [25,]  763.1828  751.7050  740.0236  728.1459  716.0788  703.8294  691.4202
##  [26,]  737.0939  725.7219  714.1512  702.3897  690.4454  678.3261  666.0391
##  [27,]  710.2850  699.0289  687.5808  675.9494  664.1431  652.1701  640.0384
##  [28,]  682.8018  671.6751  660.3646  648.8794  637.2283  625.4199  613.4624
##  [29,]  654.6970  643.7153  632.5592  621.2381  609.7610  598.1367  586.3737
##  [30,]  626.0304  615.2108  604.2272  593.0893  581.8060  570.3862  558.8383
##  [31,]  596.8699  586.2302  575.4381  564.5029  553.4336  542.2391  530.9277
##  [32,]  567.2928  556.8511  546.2693  535.5565  524.7215  513.7729  502.7191
##  [33,]  537.3872  527.1609  516.8077  506.3363  495.7554  485.0731  474.2976
##  [34,]  507.2532  497.2584  487.1510  476.9392  466.6311  456.2345  445.7571
##  [35,]  477.0050  467.2560  457.4098  447.4739  437.4557  427.3626  417.2019
##  [36,]  446.7727  437.2807  427.7085  418.0625  408.3493  398.5755  388.7479
##  [37,]  416.7042  407.4770  398.1880  388.8426  379.4464  370.1112  361.1050
##  [38,]  386.9678  378.0081  369.0073  360.0814  351.4917  343.2209  335.2416
##  [39,]  357.7546  349.1772  340.9517  333.0553  325.4580  318.1332  311.0573
##  [40,]  329.8976  322.2912  315.0021  308.0002  301.2587  294.7542  288.4660
##  [41,]  304.3916  297.5986  291.0913  284.8420  278.8261  273.0222  267.4112
##  [42,]  281.1962  275.0522  269.1764  263.5418  258.1251  252.9055  247.8651
##  [43,]  260.2696  254.6394  249.2509  244.0948  239.1523  234.4026  229.8276
##  [44,]  241.4119  236.2511  231.2897  226.5201  221.9360  217.5412  213.3262
##  [45,]  224.3885  219.6682  215.1101  210.7092  206.4619  202.3652  198.4170
##  [46,]  209.0005  204.6992  200.5269  196.4813  192.5609  188.7648  185.0926
##  [47,]  195.0821  191.1844  187.3861  183.6874  180.0888  176.5910  173.1954
##  [48,]  182.4987  178.9942  175.5631  172.2074  168.9293  165.7314  162.6163
##  [49,]  171.1456  168.0280  164.9609  161.9479  158.9928  156.0995  153.2718
##  [50,]  160.9468  158.2133  155.5101  152.8425  150.2154  147.6338  145.1029
##  [51,]  151.8550  149.5050  147.1681  144.8505  142.5585  140.2981  138.0753
##  [52,]  143.8515  141.8862  139.9193  137.9582  136.0100  134.0815  132.1793
##  [53,]  136.9472  135.3685  133.7764  132.1789  130.5839  128.9989  127.4311
##  [54,]  131.1842  129.9939  128.7813  127.5548  126.3227  125.0930  123.8735
##  [55,]  126.6382  125.8371  125.0077  124.1587  123.2987  122.4358  121.5782
##  [56,]  123.4215  123.0080  122.5636  122.0971  121.6168  121.1310  120.6477
##  [57,]  121.6872  121.6562  121.5955  121.5135  121.4178  121.2910  121.1177
##  [58,]  121.6343  121.9755  122.2920  122.5545  122.7426  122.8552  122.8923
##  [59,]  123.5134  124.1565  124.7018  125.1485  125.4966  125.7473  125.9021
##  [60,]  127.3871  128.1187  128.7372  129.2428  129.6367  129.9208  130.0978
##  [61,]  133.1828  133.8443  134.3916  134.8245  135.1442  135.3524  135.4515
##  [62,]  140.9506  141.3682  141.6898  141.9097  142.0272  142.0428  141.9577
##  [63,]  151.0532  150.8561  150.6956  150.5460  150.3227  150.0196  149.6353
##  [64,]  163.5416  162.6305  161.7461  160.9003  160.1044  159.3398  158.5301
##  [65,]  178.2125  176.5378  174.8835  173.2624  171.6871  170.1699  168.7227
##  [66,]  194.8753  192.3938  189.9294  187.4955  185.1055  182.7722  180.5083
##  [67,]  213.3533  210.0269  206.7169  203.4373  200.2019  197.0237  193.9157
##  [68,]  233.4838  229.2783  225.0912  220.9368  216.8286  212.7801  208.8041
##  [69,]  255.1191  250.0029  244.9099  239.8539  234.8484  229.9066  225.0412
##  [70,]  278.1269  272.0699  266.0435  260.0608  254.1348  248.2783  242.5036
##  [71,]  302.3913  295.3633  288.3759  281.4416  274.5724  267.7802  261.0769
##  [72,]  327.8135  319.7824  311.8054  303.8934  296.0572  288.3079  280.6563
##  [73,]  354.3128  345.2434  336.2449  327.3266  318.4976  309.7677  301.1463
##  [74,]  381.8288  371.6802  361.6236  351.6660  341.8146  332.0772  322.4619
##  [75,]  410.3231  399.0465  387.8882  376.8521  365.9430  355.1663  344.5284
##  [76,]  439.7830  427.3187  415.0052  402.8427  390.8328  378.9782  367.3071
##  [77,]  470.2255  456.4994  442.9642  429.6157  416.4801  403.7016  391.2914
##  [78,]  501.7037  486.6223  471.8151  457.4284  443.4647  429.9075  416.7421
##  [79,]  534.3542  518.0433  502.2509  486.9483  472.1102  457.7148  443.7428
##  [80,]  569.0261  551.5629  534.6893  518.3676  502.5650  487.2528  472.4056
##  [81,]  606.2853  587.4942  569.3853  551.9097  535.0246  518.6921  502.8793
##  [82,]  646.5047  626.2173  606.6724  587.8669  569.7447  552.2568  535.3601
##  [83,]  689.1740  667.7788  646.9205  626.6202  607.0597  588.2398  570.1044
##  [84,]  734.0084  711.5486  689.6118  668.2057  647.3366  627.0234  607.4473
##  [85,]  780.9373  757.4513  734.4675  711.9972  690.0497  668.6329  647.7528
##  [86,]  829.8595  805.4015  781.4168  757.9208  734.9268  712.4460  690.4879
##  [87,]  880.6393  855.2812  830.3584  805.8910  781.8966  758.3906  735.3863
##  [88,]  933.1002  906.9347  881.1559  855.7893  830.8575  806.3806  782.3765
##  [89,]  987.0192  960.1630  933.6326  907.4596  881.6727  856.2975  831.3567
##  [90,] 1042.1191 1014.7168  987.5648  960.7025  934.1651  907.9846  882.1897
##  [91,] 1098.0596 1070.2881 1042.6750 1015.2680  988.1106  961.2421  934.6978
##  [92,] 1154.4256 1126.5005 1098.6219 1070.8478 1043.2309 1015.8194  988.6565
##  [93,] 1210.7131 1182.8962 1154.9898 1127.0644 1099.1842 1071.4075 1043.7870
##  [94,] 1266.3105 1238.9193 1211.2736 1183.4593 1155.5539 1127.6283 1099.7466
##  [95,] 1320.4729 1293.8946 1266.8606 1239.4755 1211.8341 1184.0223 1156.1181
##  [96,] 1372.2884 1346.9980 1321.0043 1294.4364 1267.4105 1240.0315 1212.3945
##  [97,] 1420.6302 1397.2160 1372.7911 1347.5163 1321.5355 1294.9780 1267.9603
##  [98,] 1464.0881 1443.2889 1421.0916 1397.6996 1373.2935 1348.0344 1322.0665
##  [99,] 1500.8694 1483.6281 1464.4923 1443.7236 1421.5525 1398.1828 1373.7955
## [100,] 1528.6526 1516.1963 1501.1962 1483.9961 1464.8959 1444.1578 1422.0129
##            [,50]     [,51]     [,52]     [,53]     [,54]     [,55]     [,56]
##   [1,] 1331.2068 1317.3439 1303.0028 1288.2663 1273.2083 1257.8952 1242.3866
##   [2,] 1302.7088 1287.9651 1272.9013 1257.5836 1242.0716 1226.4190 1210.6743
##   [3,] 1272.5942 1257.2720 1241.7566 1226.1017 1210.3556 1194.5621 1178.7610
##   [4,] 1241.4416 1225.7844 1210.0369 1194.2429 1178.4419 1162.6699 1146.9595
##   [5,] 1209.7181 1193.9236 1178.1229 1162.3518 1146.6429 1131.0257 1115.5271
##   [6,] 1177.8039 1162.0337 1146.3264 1130.7114 1115.2154 1099.8628 1084.6756
##   [7,] 1146.0100 1130.3971 1114.9037 1099.5542 1084.3706 1069.3728 1054.5788
##   [8,] 1114.5921 1099.2458 1084.0657 1069.0718 1054.2822 1039.7131 1025.3793
##   [9,] 1083.7609 1068.7710 1053.9856 1039.4212 1025.0923 1011.0121  997.1927
##  [10,] 1053.6892 1039.1293 1024.8053 1010.7303  996.9163  983.3736  970.1118
##  [11,] 1024.5185 1010.4486  996.6399  983.1029  969.8469  956.8802  944.2101
##  [12,]  996.3637  982.8323  969.5820  956.6213  943.9573  931.5963  919.5439
##  [13,]  969.3173  956.3625  943.7045  931.3498  919.3037  907.5708  896.1450
##  [14,]  943.4519  931.1034  919.0635  907.3370  895.9159  884.5623  873.1708
##  [15,]  918.8235  907.1034  895.6869  884.3326  872.9402  861.4981  849.9971
##  [16,]  895.4578  884.1029  872.7096  861.2663  849.7641  838.1961  826.5575
##  [17,]  872.4790  861.0346  849.5311  837.9617  826.3216  814.6075  802.8174
##  [18,]  849.2980  837.7273  826.0857  814.3700  802.5784  790.7100  778.7651
##  [19,]  825.8498  814.1326  802.3394  790.4694  778.5230  766.5011  754.4057
##  [20,]  802.1003  790.2288  778.2808  766.2575  754.1606  741.9927  729.7568
##  [21,]  778.0387  766.0138  753.9155  741.7462  729.5089  717.2072  704.8448
##  [22,]  753.6704  741.4996  729.2610  716.9580  704.5945  692.1746  679.7029
##  [23,]  729.0131  716.7088  704.3441  691.9231  679.4505  666.9309  654.3694
##  [24,]  704.0937  691.6717  679.1980  666.6775  654.1152  641.5162  628.8860
##  [25,]  678.9455  666.4241  653.8610  641.2614  628.6306  615.9741  603.2975
##  [26,]  653.5921  641.0065  628.3751  615.7182  603.0413  590.3502  577.6507
##  [27,]  627.7559  615.3303  602.7695  590.0937  577.3941  564.6921  551.9938
##  [28,]  601.3641  589.1328  576.7766  564.3033  551.7210  539.0489  526.3764
##  [29,]  574.4801  562.4642  550.3340  538.0975  525.7629  513.3382  500.8316
##  [30,]  547.1708  535.3918  523.5095  511.5319  499.4672  487.3233  475.1087
##  [31,]  519.5078  507.9875  496.3751  484.6785  472.9059  461.0652  449.1649
##  [32,]  491.5683  480.3286  469.0079  457.6144  446.1559  434.6406  423.0766
##  [33,]  463.4369  452.4990  441.4916  430.4227  419.3001  408.1318  397.0095
##  [34,]  435.2068  424.5912  413.9178  403.1944  392.5176  382.1688  372.1455
##  [35,]  406.9808  396.7067  386.4815  376.5825  367.0040  357.7223  348.7163
##  [36,]  378.9735  369.5254  360.3941  351.5553  342.9869  334.6693  326.5847
##  [37,]  352.4145  344.0142  335.8812  327.9948  320.3368  312.8905  305.6411
##  [38,]  327.5295  320.0628  312.8222  305.7904  298.9517  292.2924  285.8000
##  [39,]  304.2095  297.5710  291.1253  284.8576  278.7547  272.8049  266.9976
##  [40,]  282.3755  276.4666  270.7246  265.1367  259.6916  254.3791  249.1902
##  [41,]  261.9765  256.7034  251.5786  246.5908  241.7297  236.9864  232.3530
##  [42,]  242.9880  238.2602  233.6693  229.2047  224.8569  220.6174  216.4792
##  [43,]  225.4114  221.1401  217.0015  212.9850  209.0811  205.2818  201.5801
##  [44,]  209.2743  205.3708  201.6029  197.9595  194.4309  191.0086  187.6854
##  [45,]  194.6309  191.0049  187.5250  184.1789  180.9558  177.8467  174.8434
##  [46,]  181.5446  178.1214  174.8439  171.7167  168.7275  165.8658  163.1223
##  [47,]  169.9036  166.7175  163.6393  160.6718  157.8413  155.1583  152.6118
##  [48,]  159.5869  156.6463  153.7978  151.0450  148.3914  145.8410  143.4248
##  [49,]  150.5138  147.8296  145.2235  142.6998  140.2630  137.9177  135.6686
##  [50,]  142.6276  140.2129  137.8639  135.5858  133.3837  131.2629  129.2286
##  [51,]  135.8958  133.7655  131.6902  129.6757  127.7278  125.8523  124.0550
##  [52,]  130.3102  128.4806  126.6968  124.9652  123.2922  121.6841  120.1472
##  [53,]  125.8878  124.3759  122.9023  121.4740  120.0976  118.7799  117.5290
##  [54,]  122.6718  121.4952  120.3511  119.2467  118.1857  117.1644  116.1790
##  [55,]  120.7336  119.9098  119.1049  118.3118  117.5272  116.7491  115.9761
##  [56,]  120.1582  119.6515  119.1251  118.5776  118.0084  117.4175  116.8055
##  [57,]  120.8962  120.6259  120.3069  119.9400  119.5264  119.0679  118.5667
##  [58,]  122.8548  122.7439  122.5617  122.3106  121.9934  121.6130  121.1729
##  [59,]  125.9635  125.9342  125.8175  125.6169  125.3361  124.9791  124.5502
##  [60,]  130.1707  130.1430  130.0186  129.8015  129.4960  129.1064  128.6374
##  [61,]  135.4446  135.3350  135.1265  134.8231  134.4290  133.9486  133.3864
##  [62,]  141.7739  141.4937  141.1202  140.6566  140.1064  139.4734  138.7616
##  [63,]  149.1697  148.6234  147.9976  147.2940  146.5149  145.6630  144.7411
##  [64,]  157.6678  156.7505  155.7768  154.7462  153.6588  152.5154  151.3174
##  [65,]  167.3316  165.9269  164.4992  163.0448  161.5609  160.0458  158.4984
##  [66,]  178.3259  176.2373  174.2319  172.2450  170.2656  168.2885  166.3095
##  [67,]  190.8905  187.9604  185.1376  182.4340  179.8419  177.3001  174.7962
##  [68,]  204.9134  201.1204  197.4374  193.8764  190.4494  187.1682  184.0273
##  [69,]  220.2648  215.5898  211.0285  206.5928  202.2947  198.1463  194.1593
##  [70,]  236.8230  231.2485  225.7923  220.4663  215.2823  210.2522  205.3879
##  [71,]  254.4742  247.9837  241.6170  235.3859  229.3019  223.3769  217.6226
##  [72,]  273.1132  265.6898  258.3972  251.2464  244.2489  237.4160  230.7594
##  [73,]  292.6435  284.2694  276.0342  267.9486  260.0233  252.2838  244.8414
##  [74,]  312.9773  303.6323  294.4363  285.4163  276.6939  268.2901  260.2046
##  [75,]  334.0360  323.7173  313.7030  304.0125  294.6440  285.5958  276.8669
##  [76,]  355.9580  344.9473  334.2703  323.9229  313.9021  304.2051  294.8301
##  [77,]  379.2401  367.5399  356.1839  345.1664  334.4827  324.1287  314.1013
##  [78,]  403.9560  391.5385  379.4801  367.7728  356.4100  345.3857  334.6953
##  [79,]  430.1774  417.0043  404.2107  391.7858  379.7202  368.0059  356.6361
##  [80,]  458.0014  444.0209  430.4475  417.2666  404.4654  392.0332  379.9604
##  [81,]  487.5574  472.7010  458.2881  444.2993  430.7178  417.5291  404.7204
##  [82,]  519.0168  503.1938  487.8623  472.9967  458.5750  444.5778  430.9883
##  [83,]  552.6041  535.6958  519.3417  503.5085  488.1673  473.2926  458.8621
##  [84,]  588.6131  570.4643  552.9517  536.0318  519.6668  503.8234  488.4725
##  [85,]  627.4269  607.8353  588.9866  570.8245  553.2994  536.3680  519.9922
##  [86,]  669.0602  648.1693  627.8308  608.2235  589.3604  571.1850  553.6475
##  [87,]  712.8950  690.9262  669.4878  648.5860  628.2350  608.6120  589.7345
##  [88,]  758.8605  735.8459  713.3442  691.3648  669.9156  649.0029  628.6396
##  [89,]  806.8705  782.8567  759.3307  736.3058  713.7936  691.8036  670.3437
##  [90,]  856.8059  831.8561  807.3606  783.3370  759.8010  736.7660  714.2433
##  [91,]  908.5098  882.7068  857.3145  832.3557  807.8508  783.8176  760.2716
##  [92,]  961.7818  935.2306  909.0351  883.2241  857.8232  832.8555  808.3412
##  [93,] 1016.3708  989.2025  962.3216  935.7636  909.5606  883.7415  858.3322
##  [94,] 1071.9672 1044.3431 1016.9224  989.7487  962.8616  936.2967  910.0863
##  [95,] 1128.1923 1100.3091 1072.5271 1044.8993 1017.4740  990.2949  963.4017
##  [96,] 1184.5853 1156.6823 1128.7562 1100.8716 1073.0870 1045.4556 1018.0258
##  [97,] 1240.5875 1212.9548 1185.1483 1157.2464 1129.3202 1101.4341 1073.6469
##  [98,] 1295.5195 1268.5100 1241.1434 1213.5150 1185.7113 1157.8106 1129.8841
##  [99,] 1348.5521 1322.5973 1296.0607 1269.0595 1241.6991 1214.0752 1186.2742
## [100,] 1398.6656 1374.2972 1349.0696 1323.1278 1296.6018 1269.6089 1242.2548
##            [,57]     [,58]     [,59]     [,60]     [,61]     [,62]     [,63]
##   [1,] 1226.7363 1210.9930 1195.2007 1179.3991 1163.6243 1147.9093 1132.2837
##   [2,] 1194.8814 1179.0800 1163.3062 1147.5926 1131.9691 1116.4626 1101.0975
##   [3,] 1162.9880 1147.2760 1131.6546 1116.1507 1100.7887 1085.5909 1070.5774
##   [4,] 1131.3402 1115.8389 1100.4800 1085.2857 1070.2761 1055.4693 1040.8819
##   [5,] 1100.1714 1084.9806 1069.9749 1055.1724 1040.5895 1026.2411 1012.1404
##   [6,] 1069.6738 1054.8756 1040.2973 1025.9537 1011.8582  998.0226  984.4577
##   [7,] 1040.0051 1025.6665 1011.5761  997.7458  984.1865  970.9074  957.9171
##   [8,] 1011.2940  997.4692  983.9154  970.6421  957.6577  944.9695  932.5838
##   [9,]  983.6445  970.3769  957.3984  944.7162  932.3368  920.2655  908.5072
##  [10,]  957.1392  944.4631  932.0898  920.0249  908.2729  896.8322  885.4810
##  [11,]  931.8430  919.7843  908.0387  896.6031  885.2514  873.8625  862.4248
##  [12,]  907.8047  896.3741  885.0217  873.6320  862.1931  850.6959  839.1335
##  [13,]  884.7920  873.4014  861.9615  850.4630  838.8992  827.2649  815.5569
##  [14,]  861.7298  850.2301  838.6649  827.0291  815.3196  803.5341  791.6719
##  [15,]  838.4305  826.7933  815.0822  803.2952  791.4315  779.4911  767.4753
##  [16,]  814.8449  803.0563  791.1910  779.2491  767.2318  755.1408  742.9786
##  [17,]  790.9505  779.0071  766.9883  754.8958  742.7321  730.5002  718.2036
##  [18,]  766.7447  754.6508  742.4857  730.2525  717.9545  705.5957  693.1802
##  [19,]  742.2392  730.0046  717.7054  705.3454  692.9288  680.4602  667.9442
##  [20,]  717.4563  705.0951  692.6774  680.2078  667.6909  655.1318  642.5356
##  [21,]  692.4260  679.9554  667.4376  654.8776  642.2808  629.6523  616.9976
##  [22,]  667.1842  654.6235  642.0259  629.3969  616.7418  604.0663  591.3761
##  [23,]  641.7711  629.1414  616.4859  603.8100  591.1196  578.4205  565.7185
##  [24,]  616.2300  603.5538  590.8632  578.1639  565.4619  552.7632  540.0737
##  [25,]  590.6067  577.9073  565.2053  552.5067  539.8175  527.1438  514.4917
##  [26,]  564.9487  552.2503  539.5613  526.8880  514.2364  501.6128  489.0232
##  [27,]  539.3051  526.6322  513.9811  501.3581  488.7693  476.2210  463.7194
##  [28,]  513.7258  501.1034  488.5154  475.9680  463.4674  451.0200  438.6320
##  [29,]  488.2615  475.7149  463.2154  450.7691  438.3824  426.0616  413.8129
##  [30,]  462.8316  450.5004  438.1329  425.8134  413.5662  401.4665  389.8040
##  [31,]  437.2130  425.2182  413.1890  401.2079  389.5731  378.3657  367.5797
##  [32,]  411.4721  399.9140  388.6901  377.8052  367.2376  356.9677  346.9844
##  [33,]  386.2178  375.7578  365.6073  355.7462  346.1562  336.8209  327.7253
##  [34,]  362.4249  352.9863  343.8111  334.8823  326.1847  317.7045  309.4293
##  [35,]  339.9667  331.4564  323.1696  315.0924  307.2119  299.5169  291.9971
##  [36,]  318.7171  311.0520  303.5766  296.2791  289.1491  282.1772  275.3550
##  [37,]  298.5754  291.6812  284.9479  278.3659  271.9266  265.6223  259.4463
##  [38,]  279.4634  273.2727  267.2190  261.2944  255.4919  249.8053  244.2292
##  [39,]  261.3237  255.7747  250.3433  245.0230  239.8082  234.6939  229.6757
##  [40,]  244.1171  239.1526  234.2906  229.5258  224.8535  220.2697  215.7708
##  [41,]  227.8224  223.3888  219.0466  214.7915  210.6195  206.5272  202.5120
##  [42,]  212.4357  208.4816  204.6122  200.8233  197.1116  193.4742  189.9090
##  [43,]  197.9698  194.4457  191.0033  187.6387  184.3489  181.1313  177.9839
##  [44,]  184.4549  181.3118  178.2515  175.2702  172.3647  169.5325  166.7716
##  [45,]  171.9390  169.1278  166.4047  163.7655  161.2068  158.7259  156.3205
##  [46,]  160.4891  157.9594  155.5276  153.1887  150.9387  148.7744  146.6932
##  [47,]  150.1924  147.8920  145.7036  143.6215  141.6408  139.7574  137.9680
##  [48,]  141.1583  139.0316  137.0363  135.1652  133.4120  131.7718  130.2403
##  [49,]  133.5206  131.5081  129.6515  127.9420  126.3718  124.9344  123.6243
##  [50,]  127.2863  125.4415  123.6999  122.0983  120.6623  119.3944  118.2906
##  [51,]  122.3418  120.7188  119.1919  117.7757  116.4764  115.3220  114.3326
##  [52,]  118.6878  117.3175  116.0394  114.8490  113.7428  112.7181  111.7731
##  [53,]  116.3446  115.2225  114.1595  113.1532  112.2020  111.3050  110.4618
##  [54,]  115.2269  114.3060  113.4152  112.5539  111.7221  110.9202  110.1495
##  [55,]  115.2075  114.4432  113.6838  112.9302  112.1839  111.4466  110.7205
##  [56,]  116.1734  115.5228  114.8555  114.1740  113.4807  112.7786  112.0708
##  [57,]  118.0253  117.4464  116.8332  116.1890  115.5173  114.8218  114.1065
##  [58,]  120.6767  120.1282  119.5312  118.8900  118.2087  117.4918  116.7437
##  [59,]  124.0536  123.4937  122.8751  122.2024  121.4804  120.7138  119.9076
##  [60,]  128.0937  127.4799  126.8009  126.0617  125.2670  124.4221  123.5319
##  [61,]  132.7470  132.0351  131.2555  130.4131  129.5128  128.5596  127.5586
##  [62,]  137.9752  137.1184  136.1957  135.2116  134.1708  133.0778  131.9375
##  [63,]  143.7526  142.7007  141.5893  140.4220  139.2030  137.9364  136.6264
##  [64,]  150.0665  148.7647  147.4147  146.0191  144.5810  143.1037  141.5906
##  [65,]  156.9184  155.3061  153.6623  151.9883  150.2857  148.5567  146.8036
##  [66,]  164.3257  162.3347  160.3353  158.3267  156.3088  154.2821  152.2475
##  [67,]  172.3235  169.8766  167.4513  165.0444  162.6534  160.2767  157.9135
##  [68,]  180.9681  177.9765  175.0448  172.1663  169.3358  166.5492  163.8032
##  [69,]  190.3457  186.7014  183.1697  179.7348  176.3881  173.1219  169.9359
##  [70,]  200.7014  196.2045  191.9093  187.8134  183.8677  180.1218  176.5735
##  [71,]  212.0509  206.6738  201.5128  196.6576  192.1288  187.9139  183.9456
##  [72,]  224.3026  218.1465  212.3123  206.8012  201.6143  196.7525  192.2170
##  [73,]  237.7180  230.9141  224.4303  218.2677  212.4270  206.9094  201.7158
##  [74,]  252.4374  244.9886  237.8588  231.0484  224.5581  218.3890  212.5418
##  [75,]  268.4567  260.3648  252.5911  245.1360  237.9996  231.1828  224.6861
##  [76,]  285.7754  277.0401  268.6234  260.5251  252.7450  245.2834  238.1406
##  [77,]  304.3978  295.0163  285.9552  277.2134  268.7903  260.6856  252.8991
##  [78,]  324.3347  314.3007  304.5907  295.2026  286.1351  277.3869  268.9573
##  [79,]  345.6051  334.9080  324.5407  314.5002  304.7836  295.3891  286.3151
##  [80,]  368.2391  356.8625  345.8246  335.1208  324.7469  314.6998  304.9767
##  [81,]  392.2807  380.2008  368.4725  357.0889  346.0443  335.3337  324.9533
##  [82,]  417.7917  404.9754  392.5285  380.4414  368.7060  357.3155  346.2641
##  [83,]  444.8565  431.2589  418.0545  405.2307  392.7763  380.6820  368.9396
##  [84,]  473.5886  459.1494  445.1354  431.5296  418.3175  405.4860  393.0243
##  [85,]  504.1385  488.7779  473.8848  459.4368  445.4144  431.8006  418.5806
##  [86,]  536.7044  520.3177  504.4538  489.0836  474.1812  459.7244  445.6936
##  [87,]  571.5457  553.9957  537.0411  520.6435  504.7694  489.3894  474.4778
##  [88,]  609.0009  590.1089  571.9066  554.3443  537.3780  520.9695  505.0851
##  [89,]  649.4200  629.0445  609.3900  590.4835  572.2679  554.6930  537.7151
##  [90,]  692.2426  670.7719  649.8374  629.4497  609.7795  590.8585  572.6294
##  [91,]  737.2263  714.6932  692.6819  671.2004  650.2550  629.8552  610.1692
##  [92,]  784.2983  760.7423  737.6868  715.1432  693.1213  671.6291  650.6728
##  [93,]  833.3555  808.8319  784.7792  761.2133  738.1475  715.5935  693.5610
##  [94,]  884.2592  858.8413  833.8556  809.3227  785.2604  761.6845  738.6085
##  [95,]  936.8300  910.6121  884.7769  859.3506  834.3560  809.8137  785.7417
##  [96,]  990.8413  963.9419  937.3634  911.1380  885.2949  859.8600  834.8565
##  [97,] 1046.0120 1018.5776  991.3878  964.4822  937.8969  911.6641  885.8130
##  [98,] 1101.9967 1074.2069 1046.5684 1019.1296  991.9344  965.0227  938.4306
##  [99,] 1158.3747 1130.4481 1102.5593 1074.7670 1047.1249 1019.6816  992.4811
## [100,] 1214.6352 1186.8370 1158.9388 1131.0121 1103.1219 1075.3272 1047.6816
##            [,64]     [,65]     [,66]     [,67]     [,68]     [,69]     [,70]
##   [1,] 1116.7745 1101.4064 1086.2015 1071.1802 1056.3606 1041.7594 1027.3915
##   [2,] 1085.8962 1070.8787 1056.0634 1041.4668 1027.1037 1012.9877  999.1307
##   [3,] 1055.7663 1041.1743 1026.8161 1012.7052  998.8535  985.2719  971.9699
##   [4,] 1026.5285 1012.4227  998.5764  985.0004  971.7041  958.6960  945.9836
##   [5,]  998.2995  984.7290  971.4384  958.4363  945.7299  933.3257  921.2294
##   [6,]  971.1729  958.1766  945.4763  933.0783  920.9882  909.2108  897.7483
##   [7,]  945.2228  932.8310  920.7472  908.9761  897.5193  886.1699  874.7845
##   [8,]  920.5063  908.7416  897.2902  885.9403  874.5541  863.1195  851.6273
##   [9,]  897.0612  885.7107  874.3236  862.8880  851.3945  839.8362  828.2078
##  [10,]  874.0931  862.6564  851.1617  839.6020  827.9721  816.2686  804.4893
##  [11,]  850.9288  839.3677  827.7364  816.0314  804.2505  792.3930  780.4588
##  [12,]  827.5007  815.7941  804.0118  792.1527  780.2170  768.2057  756.1206
##  [13,]  803.7729  791.9123  779.9751  767.9623  755.8757  743.7176  731.4911
##  [14,]  779.7331  767.7188  755.6308  743.4713  731.2435  718.9506  706.5965
##  [15,]  755.3858  743.2250  730.9957  718.7016  706.3464  693.9342  681.4696
##  [16,]  730.7480  718.4526  706.0962  693.6829  681.2172  668.7040  656.1481
##  [17,]  705.8459  693.4316  680.9649  668.4507  655.8940  643.2999  630.6738
##  [18,]  680.7125  668.1975  655.6400  643.0452  630.4184  617.7652  605.0912
##  [19,]  655.3859  642.7904  630.1631  617.5094  604.8350  592.1455  579.4469
##  [20,]  629.9077  617.2535  604.5787  591.8891  579.1903  566.4883  553.7891
##  [21,]  604.3225  591.6326  578.9337  566.2317  553.5326  540.8424  528.1672
##  [22,]  578.6771  565.9751  553.2761  540.5862  527.9114  515.2578  502.6317
##  [23,]  553.0197  540.3300  527.6555  515.0024  502.3769  489.7851  477.2334
##  [24,]  527.3997  514.7471  502.1222  489.5312  476.9802  464.4757  452.0237
##  [25,]  501.8675  489.2772  476.7271  464.2236  451.7728  439.3810  427.0546
##  [26,]  476.4741  463.9715  451.5218  439.1313  426.8063  414.5531  402.4292
##  [27,]  451.2709  438.8817  426.5580  414.3063  402.1882  390.4979  379.2554
##  [28,]  426.3098  414.0596  401.9475  390.2664  379.0327  368.2218  357.8110
##  [29,]  401.7069  390.0351  378.8102  368.0076  357.6047  347.5802  337.9147
##  [30,]  378.5879  367.7936  357.3985  347.3815  337.7230  328.4048  319.4102
##  [31,]  357.1924  347.1829  337.5314  328.2200  319.2317  310.5509  302.1631
##  [32,]  337.3400  328.0353  319.0533  310.3786  301.9966  293.8940  286.0582
##  [33,]  318.8560  310.2064  301.8302  293.7331  285.9025  278.3272  270.9963
##  [34,]  301.3480  293.4505  285.7278  278.1767  270.8506  263.7591  256.8929
##  [35,]  284.6433  277.4472  270.4017  263.5002  256.7371  250.1116  243.6759
##  [36,]  268.6751  262.1309  255.7164  249.4267  243.2573  237.2046  231.2653
##  [37,]  253.3926  247.4559  241.6317  235.9160  230.3056  224.7976  219.3900
##  [38,]  238.7588  233.3901  228.1196  222.9443  217.8618  212.8704  207.9685
##  [39,]  224.7499  219.9134  215.1637  210.4985  205.9164  201.4162  196.9971
##  [40,]  211.3541  207.0173  202.7584  198.5760  194.4693  190.4376  186.4809
##  [41,]  198.5716  194.7043  190.9087  187.1841  183.5301  179.9465  176.4337
##  [42,]  186.4141  182.9882  179.6305  176.3404  173.1181  169.9637  166.8781
##  [43,]  174.9052  171.8940  168.9499  166.0727  163.2625  160.5199  157.8460
##  [44,]  164.0806  161.4585  158.9048  156.4195  154.0028  151.6556  149.3789
##  [45,]  153.9892  151.7307  149.5447  147.4309  145.3897  143.4219  141.5286
##  [46,]  144.6932  142.7730  140.9318  139.1693  137.4857  135.8816  134.3727
##  [47,]  136.2702  134.6621  133.1425  131.7106  130.3806  129.1638  128.0494
##  [48,]  128.8141  127.4907  126.2817  125.1945  124.2170  123.3381  122.5475
##  [49,]  122.4493  121.4122  120.4986  119.6958  118.9919  118.3765  117.8400
##  [50,]  117.3338  116.5087  115.8011  115.1984  114.6893  114.2634  113.9113
##  [51,]  113.4907  112.7807  112.1884  111.7009  111.3067  110.9954  110.7574
##  [52,]  110.9400  110.2457  109.6755  109.2160  108.8551  108.5818  108.3863
##  [53,]  109.6727  108.9383  108.2938  107.7718  107.3595  107.0450  106.8177
##  [54,]  109.4112  108.7074  108.0401  107.4120  106.8598  106.4212  106.0846
##  [55,]  110.0082  109.3125  108.6364  107.9833  107.3569  106.7610  106.2333
##  [56,]  111.3606  110.6517  109.9479  109.2532  108.5719  107.9083  107.2671
##  [57,]  113.3754  112.6328  111.8831  111.1308  110.3806  109.6374  108.9062
##  [58,]  115.9690  115.1725  114.3590  113.5334  112.7008  111.8664  111.0354
##  [59,]  119.0666  118.1960  117.3008  116.3863  115.4577  114.5204  113.5799
##  [60,]  122.6015  121.6362  120.6411  119.6217  118.5833  117.5313  116.4714
##  [61,]  126.5147  125.4333  124.3195  123.1785  122.0159  120.8370  119.6473
##  [62,]  130.7548  129.5346  128.2819  127.0019  125.6997  124.3807  123.0502
##  [63,]  135.2774  133.8940  132.4809  131.0427  129.5844  128.1111  126.6278
##  [64,]  140.0455  138.4722  136.8749  135.2577  133.6251  131.9816  130.3321
##  [65,]  145.0292  143.2364  141.4285  139.6090  137.7816  135.9505  134.1197
##  [66,]  150.2064  148.1607  146.1125  144.0644  142.0193  139.9827  137.9910
##  [67,]  155.5634  153.2267  150.9044  148.6009  146.3578  144.1834  142.0733
##  [68,]  161.0953  158.4284  155.8491  153.3640  150.9655  148.6469  146.4024
##  [69,]  166.8816  163.9621  161.1656  158.4815  155.9003  153.4133  151.0131
##  [70,]  173.2048  169.9991  166.9419  164.0198  161.2210  158.5347  155.9514
##  [71,]  180.1955  176.6434  173.2711  170.0624  167.0023  164.0776  161.2764
##  [72,]  187.9965  184.0235  180.2692  176.7133  173.3376  170.1257  167.0627
##  [73,]  196.8475  192.3054  188.0792  184.1016  180.3430  176.7833  173.4041
##  [74,]  207.0176  201.8175  196.9426  192.3940  188.1619  184.1797  180.4169
##  [75,]  218.5104  212.6567  207.1260  201.9194  197.0379  192.4826  188.2448
##  [76,]  231.3173  224.8142  218.6320  212.7718  207.2346  202.0213  197.1333
##  [77,]  245.4310  238.2818  231.4520  224.9424  218.7537  212.8870  207.3432
##  [78,]  260.8461  253.0532  245.5787  238.4231  231.5868  225.0707  218.8755
##  [79,]  277.5604  269.1245  261.0069  253.2075  245.7266  238.5645  231.7217
##  [80,]  295.5757  286.4952  277.7341  269.2918  261.1677  253.3619  245.8745
##  [81,]  314.8995  305.1700  295.7625  286.6755  277.9080  269.4592  261.3287
##  [82,]  335.5468  325.1597  315.0994  305.3633  295.9493  286.8559  278.0819
##  [83,]  357.5423  346.4840  335.7601  325.3663  315.2994  305.5568  296.1363
##  [84,]  380.9229  369.1734  357.7691  346.7041  335.9734  325.5730  315.4996
##  [85,]  405.7416  393.2725  381.1638  369.4074  357.9961  346.9243  336.1869
##  [86,]  432.0716  418.8438  405.9972  393.5208  381.4049  369.6414  358.2233
##  [87,]  460.0122  445.9729  432.3429  419.1072  406.2531  393.7693  381.6462
##  [88,]  489.6953  474.7745  460.3001  446.2524  432.6143  419.3708  406.5091
##  [89,]  521.2957  505.4010  490.0015  475.0715  460.5883  446.5321  432.8858
##  [90,]  555.0420  538.0524  521.6221  505.7171  490.3079  475.3686  460.8766
##  [91,]  591.2337  572.9911  555.3913  538.3900  521.9487  506.0334  490.6145
##  [92,]  630.2611  610.5593  591.6092  573.3531  555.7408  538.7278  522.2755
##  [93,]  672.0580  651.0908  630.6673  610.9497  591.9850  573.7154  556.0905
##  [94,]  716.0440  694.0009  672.4871  651.5090  631.0738  611.3403  592.3610
##  [95,]  762.1558  739.0696  716.4948  694.4410  672.9164  651.9275  631.4807
##  [96,]  810.3049  786.2233  762.6274  739.5310  716.9457  694.8813  673.3460
##  [97,]  860.3697  835.3572  810.7963  786.7050  763.0992  739.9926  717.3968
##  [98,]  912.1904  886.3313  860.8795  835.8581  811.2879  787.1870  763.5712
##  [99,]  965.5634  938.9644  912.7168  886.8498  861.3895  836.3592  811.7797
## [100,] 1020.2338  993.0280  966.1041  939.4984  913.2434  887.3684  861.8996
##            [,71]    [,72]    [,73]    [,74]    [,75]    [,76]    [,77]    [,78]
##   [1,] 1013.2703 999.4080 985.8153 972.5018 959.4760 946.7455 934.3167 922.1953
##   [2,]  985.5435 972.2358 959.2159 946.4914 934.0688 921.9537 910.1508 898.6642
##   [3,]  958.9559 946.2375 933.8210 921.7121 909.9156 898.4352 887.0882 875.7062
##   [4,]  933.5733 921.4707 909.6806 898.2063 886.8586 875.4758 864.0456 852.5583
##   [5,]  909.4456 897.9773 886.6291 875.2454 863.8141 852.3256 840.7727 829.1501
##   [6,]  886.3995 875.0150 863.5826 852.0929 840.5386 828.9146 817.2171 805.4440
##   [7,]  863.3511 851.8601 840.3045 828.6790 816.9800 805.2054 793.3540 781.4260
##   [8,]  840.0704 828.4434 816.7429 804.9667 793.1138 781.1843 769.1791 757.0998
##   [9,]  816.5058 804.7280 792.8736 780.9425 768.9358 756.8551 744.7027 732.4816
##  [10,]  792.6333 780.7007 768.6924 756.6103 744.4565 732.2340 719.9463 707.5970
##  [11,]  768.4491 756.3654 744.2102 731.9864 719.6974 707.3469 694.9392 682.4786
##  [12,]  743.9640 731.7388 719.4485 707.0968 694.6880 682.2264 669.7168 657.1642
##  [13,]  719.1996 706.8467 694.4367 681.9741 669.4636 656.9102 644.3189 631.6952
##  [14,]  694.1855 681.7219 669.2104 656.6562 644.0642 631.4398 618.7886 606.1160
##  [15,]  668.9572 656.4021 643.8095 631.1845 618.5327 605.8598 593.1714 580.4732
##  [16,]  643.5547 630.9292 618.2769 605.6036 592.9149 580.2166 567.5147 554.8150
##  [17,]  618.0211 605.3474 592.6584 579.9601 567.2581 554.5585 541.8674 529.1908
##  [18,]  592.4020 579.7035 567.0015 554.3021 541.6112 528.9349 516.2794 503.6508
##  [19,]  566.7449 554.0456 541.3549 528.6790 516.0240 503.3960 490.8013 478.2460
##  [20,]  541.0987 528.4231 515.7686 503.1412 490.5472 477.9928 465.4843 453.0279
##  [21,]  515.5132 502.8864 490.2932 477.7396 465.2321 452.7768 440.3800 428.0481
##  [22,]  490.0392 477.4865 464.9799 452.5257 440.1302 427.7997 415.5404 403.3950
##  [23,]  464.7278 452.2747 439.8805 427.5513 415.2935 403.1532 391.4256 380.1478
##  [24,]  439.6307 427.3029 415.0467 402.9117 391.1934 379.9245 369.0803 358.6380
##  [25,]  414.7999 402.6703 390.9614 379.7013 368.8654 358.4310 348.3767 338.6828
##  [26,]  390.7295 379.4782 368.6507 358.2242 348.1774 338.4906 329.1456 320.1254
##  [27,]  368.4362 358.0175 347.9782 338.2985 328.9602 319.9464 311.2413 302.8304
##  [28,]  347.7791 338.1065 328.7749 319.7675 311.0685 302.6634 294.5386 286.6817
##  [29,]  328.5898 319.5888 310.8959 302.4965 294.3773 286.5257 278.9301 271.5798
##  [30,]  310.7233 302.3298 294.2161 286.3698 278.7792 271.4338 264.3236 257.4395
##  [31,]  294.0550 286.2139 278.6284 271.2879 264.1823 257.3027 250.6407 244.1884
##  [32,]  278.4778 271.1420 264.0412 257.1660 250.5083 244.0601 237.8146 231.7651
##  [33,]  263.9001 257.0294 250.3760 243.9320 237.6904 231.6448 225.7892 220.1182
##  [34,]  250.2437 243.8039 237.5664 231.5247 225.6728 220.0055 214.5177 209.2051
##  [35,]  237.4424 231.4046 225.5565 219.8929 214.4087 209.0996 203.9616 198.9912
##  [36,]  225.4403 219.7803 214.2997 208.9941 203.8596 198.8925 194.0899 189.4489
##  [37,]  214.0810 208.8695 203.7576 198.7939 193.9945 189.3568 184.8783 180.5570
##  [38,]  203.1553 198.4304 193.7937 189.2457 184.7894 180.4713 176.3087 172.3003
##  [39,]  192.6590 188.4020 184.2267 180.1339 176.1251 172.2021 168.3687 164.6690
##  [40,]  182.5995 178.7940 175.0654 171.4152 167.8452 164.3576 160.9549 157.6400
##  [41,]  172.9925 169.6239 166.3294 163.1109 159.9704 156.9105 153.9342 151.0446
##  [42,]  163.8623 160.9177 158.0461 155.2496 152.5308 149.8924 147.3376 144.8838
##  [43,]  155.2420 152.7097 150.2510 147.8684 145.5645 143.3567 141.2660 139.2844
##  [44,]  147.1742 145.0434 142.9886 141.0270 139.1778 137.4325 135.7828 134.2212
##  [45,]  139.7113 137.9866 136.3717 134.8573 133.4350 132.0968 130.8354 129.6443
##  [46,]  132.9734 131.6740 130.4652 129.3388 128.2871 127.3029 126.3800 125.5124
##  [47,]  127.0274 126.0889 125.2255 124.4297 123.6945 123.0137 122.3814 121.7924
##  [48,]  121.8363 121.1958 120.6187 120.0978 119.6268 119.2001 118.8122 118.4584
##  [49,]  117.3737 116.9696 116.6206 116.3201 116.0621 115.8413 115.6527 115.4917
##  [50,]  113.6245 113.3954 113.2168 113.0825 112.9867 112.9241 112.8899 112.8799
##  [51,]  110.5843 110.4682 110.4021 110.3796 110.3949 110.4427 110.5182 110.6172
##  [52,]  108.2595 108.1935 108.1807 108.2147 108.2892 108.3989 108.5388 108.7043
##  [53,]  106.6679 106.5869 106.5666 106.6000 106.6806 106.8023 106.9599 107.1484
##  [54,]  105.8391 105.6752 105.5840 105.5575 105.5885 105.6704 105.7973 105.9637
##  [55,]  105.8156 105.4971 105.2679 105.1190 105.0420 105.0296 105.0749 105.1718
##  [56,]  106.6531 106.1041 105.6655 105.3275 105.0804 104.9156 104.8253 104.8023
##  [57,]  108.1921 107.5004 106.8365 106.2383 105.7542 105.3747 105.0906 104.8937
##  [58,]  110.2132 109.4054 108.6177 107.8558 107.1258 106.4650 105.9243 105.4949
##  [59,]  112.6417 111.7116 110.7954 109.8990 109.0285 108.1902 107.3905 106.6659
##  [60,]  115.4092 114.3504 113.3010 112.2669 111.2543 110.2696 109.3191 108.4095
##  [61,]  118.4525 117.2584 116.0708 114.8958 113.7394 112.6079 111.5079 110.4458
##  [62,]  121.7138 120.3772 119.0460 117.7263 116.4240 115.1454 113.8973 112.6980
##  [63,]  125.1398 123.6527 122.1719 120.7031 119.2532 117.8443 116.4852 115.1772
##  [64,]  128.6815 127.0348 125.3986 123.7994 122.2466 120.7406 119.2822 117.8723
##  [65,]  132.2954 130.5090 128.7700 127.0775 125.4313 123.8313 122.2775 120.7706
##  [66,]  136.0535 134.1678 132.3320 130.5446 128.8046 127.1113 125.4641 123.8631
##  [67,]  140.0235 138.0307 136.0921 134.2054 132.3686 130.5803 128.8393 127.1450
##  [68,]  144.2267 142.1153 140.0643 138.0704 136.1308 134.2430 132.4052 130.6159
##  [69,]  148.6930 146.4470 144.2700 142.1573 140.1052 138.1102 136.1694 134.2806
##  [70,]  153.4627 151.0608 148.7391 146.4917 144.3133 142.1994 140.1461 138.1499
##  [71,]  158.5879 156.0027 153.5121 151.1085 148.7853 146.5364 144.3567 142.2415
##  [72,]  164.1354 161.3318 158.6412 156.0539 153.5615 151.1563 148.8315 146.5811
##  [73,]  170.1890 167.1232 164.1933 161.3873 158.6945 156.1052 153.6110 151.2040
##  [74,]  176.8534 173.4707 170.2525 167.1838 164.2512 161.4428 158.7478 156.1566
##  [75,]  184.2579 180.4909 176.9235 173.5374 170.3159 167.2444 164.3092 161.4984
##  [76,]  192.5714 188.3278 184.3362 180.5649 176.9937 173.6041 170.3795 167.3050
##  [77,]  202.1235 197.2288 192.6603 188.4108 184.4146 180.6391 177.0640 173.6709
##  [78,]  213.0023 207.4520 202.2257 197.3244 192.7494 188.4940 184.4930 180.7133
##  [79,]  225.1992 218.9975 213.1178 207.5609 202.3281 197.4202 192.8386 188.5772
##  [80,]  238.7060 231.8568 225.3277 219.1196 213.2334 207.6700 202.4306 197.5162
##  [81,]  253.5165 246.0226 238.8476 231.9920 225.4565 219.2419 213.3491 207.7792
##  [82,]  269.6267 261.4898 253.6711 246.1709 238.9894 232.1274 225.5853 219.3642
##  [83,]  287.0365 278.2560 269.7944 261.6510 253.8259 246.3192 239.1314 232.2628
##  [84,]  305.7504 296.3235 287.2171 278.4303 269.9621 261.8124 253.9809 246.4677
##  [85,]  325.7799 315.6999 305.9442 296.5107 287.3979 278.6046 270.1301 261.9739
##  [86,]  347.1447 336.4006 325.9869 315.9003 306.1381 296.6981 287.5789 278.7791
##  [87,]  369.8756 358.4506 347.3652 336.6143 326.1940 316.1009 306.3321 296.8857
##  [88,]  394.0178 381.8876 370.1100 358.6780 347.5858 336.8282 326.4013 316.3015
##  [89,]  419.6345 406.7652 394.2666 382.1291 370.3445 358.9056 347.8065 337.0423
##  [90,]  446.8120 433.1576 419.8984 407.0215 394.5155 382.3708 370.5791 359.1333
##  [91,]  475.6659 461.1650 447.0920 433.4295 420.1624 407.2779 394.7645 382.6126
##  [92,]  506.3499 490.9212 475.9634 461.4537 447.3722 433.7015 420.4266 407.5345
##  [93,]  539.0658 522.6026 506.6666 491.2282 476.2611 461.7425 447.6525 433.9737
##  [94,]  574.0780 556.4405 539.4041 522.9298 506.9835 491.5353 476.5589 462.0315
##  [95,]  611.7313 592.7374 574.4408 556.7907 539.7426 523.2573 507.3007 491.8426
##  [96,]  652.3462 631.8879 612.1226 593.1140 574.8038 557.1412 540.0813 523.5850
##  [97,]  695.3218 673.7757 652.7651 632.2954 612.5142 593.4909 575.1672 557.4919
##  [98,]  740.4544 717.8482 695.7626 674.2057 653.1842 632.7033 612.9061 593.8681
##  [99,]  787.6691 764.0434 740.9163 718.2998 696.2036 674.6360 653.6036 633.1115
## [100,]  836.8605 812.2716 788.1514 764.5158 741.3785 718.7515 696.6447 675.0664
##           [,79]    [,80]    [,81]    [,82]    [,83]    [,84]    [,85]    [,86]
##   [1,] 910.3862 898.8932 887.5472 876.1669 864.7398 853.2563 841.7088 830.0920
##   [2,] 887.3177 875.9366 864.5084 853.0237 841.4749 829.8566 818.1652 806.3982
##   [3,] 864.2770 852.7910 841.2408 829.6211 817.9282 806.1597 794.3146 782.3928
##   [4,] 841.0068 829.3857 817.6912 805.9212 794.0745 782.1511 770.1520 758.0786
##   [5,] 817.4542 805.6826 793.8344 781.9095 769.9088 757.8340 745.6873 733.4716
##   [6,] 793.5942 781.6678 769.6656 757.5893 745.4412 733.2242 720.9416 708.5971
##   [7,] 769.4223 757.3446 745.1951 732.9767 720.6928 708.3471 695.9439 683.4874
##   [8,] 744.9489 732.7292 720.4440 708.0971 695.6927 683.2352 670.7294 658.1800
##   [9,] 720.1951 707.8471 695.4416 682.9831 670.4762 657.9261 645.3377 632.7163
##  [10,] 695.1904 682.7309 670.2231 657.6721 645.0830 632.4611 619.8118 607.1407
##  [11,] 669.9700 657.4182 644.8283 632.2058 619.5560 606.8845 594.1971 581.4996
##  [12,] 644.5736 631.9505 619.3002 606.6284 593.9407 581.2430 568.5411 555.8410
##  [13,] 619.0444 606.3722 593.6842 580.9864 568.2845 555.5845 542.8925 530.2145
##  [14,] 593.4278 580.7298 568.0279 555.3280 542.6362 529.9586 517.3011 504.6701
##  [15,] 567.7713 555.0715 542.3799 529.7026 517.0457 504.4153 491.8176 479.2590
##  [16,] 542.1237 529.4467 516.7902 504.1604 491.5635 479.0057 466.4932 454.0323
##  [17,] 516.5348 503.9056 491.3094 478.7524 466.2409 453.7812 441.3795 429.0421
##  [18,] 491.0553 478.4992 465.9887 453.5301 441.1296 428.7935 416.5283 404.3639
##  [19,] 465.7365 453.2789 440.8797 428.5450 416.2813 404.1214 392.3562 381.0431
##  [20,] 440.6299 428.2966 416.0343 403.8791 392.1233 380.8190 369.9414 359.4674
##  [21,] 415.7873 403.6369 391.8905 380.5951 369.7259 359.2598 349.1755 339.4532
##  [22,] 391.6580 380.3714 369.5105 359.0524 348.9756 339.2604 329.8885 320.8426
##  [23,] 369.2953 358.8451 348.7758 339.0678 329.7025 320.6631 311.9336 303.4995
##  [24,] 348.5762 338.8752 329.5168 320.4837 311.7604 303.3320 295.1851 287.3070
##  [25,] 329.3311 320.3045 311.5872 303.1647 295.0233 287.1505 279.5346 272.1649
##  [26,] 311.4142 302.9975 294.8616 286.9941 279.3834 272.0185 264.8897 257.9876
##  [27,] 294.7001 286.8379 279.2322 271.8722 264.7480 257.8505 251.1711 244.7022
##  [28,] 279.0811 271.7260 264.6065 257.7134 251.0384 244.5736 238.3119 232.2468
##  [29,] 264.4650 257.5764 250.9057 244.4451 238.1875 232.1262 226.2555 220.5697
##  [30,] 250.7732 244.3167 238.0631 232.0058 226.1388 220.4567 214.9546 209.6280
##  [31,] 237.9388 231.8854 226.0222 220.3438 214.8453 209.5222 204.3705 199.3867
##  [32,] 225.9057 220.2310 214.7360 209.4164 204.2682 199.2878 194.4719 189.8180
##  [33,] 214.6268 209.3108 204.1659 199.1889 194.3763 189.7256 185.2343 180.9004
##  [34,] 204.0638 199.0900 194.2808 189.6333 185.1452 180.8144 176.6393 172.6185
##  [35,] 194.1853 189.5411 185.0562 180.7285 176.5565 172.5388 168.6743 164.9623
##  [36,] 184.9672 180.6427 176.4739 172.4592 168.5978 164.8889 161.3320 157.9269
##  [37,] 176.3912 172.3797 168.5214 164.8155 161.2617 157.8597 154.6097 151.5122
##  [38,] 168.4450 164.7422 161.1914 157.7925 154.5456 151.4512 148.5098 145.7337
##  [39,] 161.1213 157.7254 154.4816 151.3902 148.4519 145.6796 143.0953 140.6887
##  [40,] 154.4177 151.3294 148.3942 145.6256 143.0449 140.6419 138.4056 136.3259
##  [41,] 148.2454 145.5538 142.9947 140.5951 138.3620 136.2854 134.3557 132.5636
##  [42,] 142.5535 140.3391 138.2329 136.2278 134.3181 132.5288 130.8685 129.3290
##  [43,] 137.4038 135.6171 133.9176 132.2990 130.7557 129.2825 127.8751 126.5570
##  [44,] 132.7408 131.3351 129.9983 128.7250 127.5103 126.3496 125.2387 124.1739
##  [45,] 128.5173 127.4488 126.4337 125.4672 124.5450 123.6632 122.8181 122.0064
##  [46,] 124.6947 123.9220 123.1897 122.4936 121.8300 121.1954 120.5866 120.0006
##  [47,] 121.2419 120.7253 120.2388 119.7785 119.3412 118.9236 118.5230 118.1368
##  [48,] 118.1343 117.8359 117.5594 117.3016 117.0594 116.8299 116.6108 116.3995
##  [49,] 115.3543 115.2367 115.1356 115.0478 114.9704 114.9010 114.8372 114.7767
##  [50,] 112.8900 112.9166 112.9566 113.0068 113.0646 113.1276 113.1936 113.2604
##  [51,] 110.7356 110.8698 111.0167 111.1732 111.3367 111.5047 111.6750 111.8457
##  [52,] 108.8913 109.0962 109.3155 109.5462 109.7855 110.0309 110.2802 110.5313
##  [53,] 107.3635 107.6011 107.8577 108.1298 108.4147 108.7095 109.0118 109.3194
##  [54,] 106.1648 106.3960 106.6533 106.9332 107.2322 107.5474 107.8761 108.2158
##  [55,] 105.3146 105.4984 105.7184 105.9706 106.2511 106.5565 106.8837 107.2299
##  [56,] 104.8401 104.9328 105.0752 105.2622 105.4896 105.7533 106.0497 106.3756
##  [57,] 104.7763 104.7314 104.7528 104.8347 104.9720 105.1600 105.3943 105.6713
##  [58,] 105.1677 104.9348 104.7884 104.7220 104.7293 104.8048 104.9433 105.1377
##  [59,] 106.0696 105.5934 105.2283 104.9663 104.8001 104.7232 104.7268 104.8018
##  [60,] 107.5476 106.7690 106.1290 105.6232 105.2414 104.9716 104.8026 104.7239
##  [61,] 109.4289 108.4715 107.5805 106.7862 106.1406 105.6322 105.2480 104.9760
##  [62,] 111.5553 110.4715 109.4489 108.4902 107.5978 106.8007 106.1523 105.6412
##  [63,] 113.9221 112.7216 111.5778 110.4928 109.4690 108.5089 107.6152 106.8153
##  [64,] 116.5122 115.2032 113.9469 112.7453 111.6003 110.5141 109.4890 108.5276
##  [65,] 119.3112 117.9003 116.5391 115.2291 113.9718 112.7690 111.6229 110.5355
##  [66,] 122.3084 120.8006 119.3402 117.9283 116.5661 115.2550 113.9966 112.7928
##  [67,] 125.4970 123.8950 122.3394 120.8306 119.3692 117.9564 116.5931 115.2810
##  [68,] 128.8740 127.1788 125.5298 123.9269 122.3704 120.8606 119.3983 117.9844
##  [69,] 132.4418 130.6516 128.9088 127.2126 125.5627 123.9588 122.4013 120.8906
##  [70,] 136.2081 134.3183 132.4785 130.6873 128.9435 127.2464 125.5955 123.9908
##  [71,] 140.1870 138.1897 136.2468 134.3560 132.5152 130.7230 128.9783 127.2802
##  [72,] 144.4001 142.2836 140.2279 138.2295 136.2855 134.3936 132.5519 130.7587
##  [73,] 148.8777 146.6259 144.4435 142.3258 140.2689 138.2693 136.3243 134.4314
##  [74,] 153.6605 151.2518 148.9239 146.6707 144.4869 142.3679 140.3098 138.3092
##  [75,] 158.8012 156.2080 153.7100 151.2997 148.9702 146.7155 144.5304 142.4101
##  [76,] 164.3672 161.5540 158.8546 156.2594 153.7596 151.3476 149.0165 146.7604
##  [77,] 170.4431 167.3657 164.4253 161.6097 158.9081 156.3108 153.8092 151.3955
##  [78,] 177.1344 173.7377 170.5068 167.4265 164.4834 161.6654 158.9616 156.3623
##  [79,] 184.5716 180.7876 177.2048 173.8047 170.5705 167.4873 164.5416 161.7212
##  [80,] 192.9279 188.6606 184.6502 180.8620 177.2753 173.8717 170.6343 167.5482
##  [81,] 202.5332 197.6122 193.0173 188.7441 184.7290 180.9365 177.3459 173.9387
##  [82,] 213.4649 207.8885 202.6360 197.7084 193.1069 188.8276 184.8078 181.0110
##  [83,] 225.7143 219.4867 213.5809 207.9980 202.7389 197.8047 193.1967 188.9113
##  [84,] 239.2734 232.3984 225.8434 219.6093 213.6970 208.1075 202.8419 197.9012
##  [85,] 254.1359 246.6164 239.4156 232.5341 225.9727 219.7321 213.8133 208.2173
##  [86,] 270.2981 262.1355 254.2911 246.7651 239.5579 232.6700 226.1021 219.8550
##  [87,] 287.7599 278.9537 270.4663 262.2972 254.4464 246.9140 239.7003 232.8060
##  [88,] 306.5263 297.0733 287.9411 279.1285 270.6346 262.4591 254.6019 247.0630
##  [89,] 326.6087 316.5024 306.7205 297.2611 288.1224 279.3033 270.8030 262.6211
##  [90,] 348.0274 337.2565 326.8162 316.7033 306.9150 297.4490 288.3039 279.4783
##  [91,] 370.8139 359.3611 348.2485 337.4708 327.0239 316.9044 307.1095 297.6371
##  [92,] 395.0137 382.8546 371.0488 359.5891 348.4696 337.6852 327.2317 317.1056
##  [93,] 420.6910 407.7912 395.2630 383.0967 371.2838 359.8172 348.6909 337.8998
##  [94,] 447.9330 434.2461 420.9555 408.0481 395.5125 383.3390 371.5190 360.0455
##  [95,] 476.8570 462.3207 448.2137 434.5186 421.2202 408.3051 395.7622 383.5814
##  [96,] 507.6180 492.1502 477.1552 462.6100 448.4946 434.7913 421.4850 408.5623
##  [97,] 540.4202 523.9129 507.9355 492.4579 477.4536 462.8996 448.7756 435.0642
##  [98,] 575.5308 557.8428 540.7594 524.2410 508.2533 492.7658 477.7522 463.1893
##  [99,] 613.2984 594.2456 575.8946 558.1940 541.0988 524.5694 508.5712 493.0739
## [100,] 654.0231 633.5200 613.6909 594.6234 576.2588 558.5455 541.4384 524.8980
##           [,87]    [,88]    [,89]    [,90]    [,91]    [,92]    [,93]    [,94]
##   [1,] 818.4021 806.6367 794.7946 782.8759 770.8813 758.8124 746.6715 734.4613
##   [2,] 794.5546 782.6344 770.6382 758.5678 746.4255 734.2139 721.9364 709.5969
##   [3,] 770.3951 758.3233 746.1794 733.9665 721.6878 709.3470 696.9482 684.4959
##   [4,] 745.9334 733.7191 721.4391 709.0970 696.6971 684.2438 671.7416 659.1956
##   [5,] 721.1903 708.8471 696.4461 683.9917 671.4886 658.9417 646.3562 633.7373
##   [6,] 696.1950 683.7395 671.2355 658.6878 646.1016 633.4821 620.8348 608.1653
##   [7,] 670.9824 658.4339 645.8470 633.2269 620.5791 607.9092 595.2228 582.5259
##   [8,] 645.5923 632.9716 620.3233 607.6530 594.9664 582.2693 569.5675 556.8671
##   [9,] 620.0675 607.3969 594.7100 582.0127 569.3109 556.6106 543.9177 531.2383
##  [10,] 594.4536 581.7561 569.0543 556.3541 543.6614 530.9823 518.3231 505.6897
##  [11,] 568.7977 556.0976 543.4051 530.7264 518.0676 505.4348 492.8343 480.2722
##  [12,] 543.1488 530.4704 517.8121 505.1799 492.5801 480.0189 467.5025 455.0372
##  [13,] 517.5566 504.9250 492.3259 479.7655 467.2501 454.7860 442.3793 430.0365
##  [14,] 492.0718 479.5122 466.9978 454.5347 442.1293 429.7878 417.5166 405.3359
##  [15,] 466.7455 454.2835 441.8793 429.5392 417.2695 405.0926 393.2898 381.9411
##  [16,] 441.6294 429.2906 417.0224 404.8495 393.0561 381.7163 370.8051 360.2993
##  [17,] 416.7753 404.6066 392.8226 381.4917 370.5889 360.0911 349.9767 340.2259
##  [18,] 392.5893 381.2673 370.3729 359.8830 349.7762 340.0325 330.6335 321.5618
##  [19,] 370.1571 359.6751 349.5758 339.8393 330.4470 321.3818 312.6279 304.1704
##  [20,] 349.3756 339.6462 330.2607 321.2020 312.4541 304.0025 295.8333 287.9339
##  [21,] 330.0745 321.0222 312.2805 303.8347 295.6711 287.7770 280.1408 272.7516
##  [22,] 312.1070 303.6670 295.5090 287.6202 279.9891 272.6048 265.4572 258.5372
##  [23,] 295.3470 287.4635 279.8375 272.4581 265.3152 258.3996 251.7030 245.2173
##  [24,] 279.6860 272.3114 265.1732 258.2622 251.5699 245.0884 238.8105 232.7297
##  [25,] 265.0314 258.1249 251.4369 244.9595 238.6857 232.6089 226.7229 221.0224
##  [26,] 251.3040 244.8308 238.5611 232.4881 226.6060 220.9091 215.3927 210.0520
##  [27,] 238.4364 232.3674 226.4890 220.7959 215.2830 209.9459 204.7806 199.7833
##  [28,] 226.3722 220.6828 215.1735 209.8399 204.6780 199.6841 194.8551 190.1881
##  [29,] 215.0640 209.7339 204.5754 199.5849 194.7592 190.0955 185.5914 181.2448
##  [30,] 204.4729 199.4858 194.6634 190.0029 185.5020 181.1586 176.9709 172.9377
##  [31,] 194.5676 189.9104 185.4127 181.0724 176.8879 172.8578 168.9809 165.2566
##  [32,] 185.3235 180.9864 176.8050 172.7779 168.9042 165.1829 161.6137 158.1964
##  [33,] 176.7221 172.6982 168.8275 165.1093 161.5432 158.1290 154.8667 151.7568
##  [34,] 168.7509 165.0358 161.4727 158.0616 154.8024 151.6956 148.7418 145.9510
##  [35,] 161.4023 157.9942 154.7381 151.6344 148.6837 145.8966 143.2974 140.8768
##  [36,] 154.6739 151.5732 148.6256 145.8422 143.2467 140.8297 138.5803 136.4884
##  [37,] 148.5677 145.7879 143.1962 140.7826 138.5365 136.4477 134.5064 132.7035
##  [38,] 143.1457 140.7356 138.4928 136.4070 134.4686 132.6685 130.9980 129.4491
##  [39,] 138.4492 136.3664 134.4309 132.6335 130.9656 129.4190 127.9862 126.6598
##  [40,] 134.3933 132.5985 130.9331 129.3890 127.9583 126.6340 125.4093 124.2775
##  [41,] 130.9008 129.3590 127.9306 126.6083 125.3855 124.2556 123.2126 122.2508
##  [42,] 127.9028 126.5827 125.3618 124.2337 123.1924 122.2322 121.3477 120.5339
##  [43,] 125.3381 124.2118 123.1722 122.2136 121.3306 120.5181 119.7716 119.0864
##  [44,] 123.1521 122.1950 121.3135 120.5024 119.7571 119.0732 118.4464 117.8729
##  [45,] 121.2250 120.4712 119.7427 119.0600 118.4343 117.8618 117.3390 116.8623
##  [46,] 119.4348 118.8868 118.3545 117.8357 117.3289 116.8531 116.4203 116.0275
##  [47,] 117.7627 117.3985 117.0423 116.6924 116.3471 116.0051 115.6653 115.3454
##  [48,] 116.1941 115.9926 115.7933 115.5947 115.3954 115.1941 114.9897 114.7813
##  [49,] 114.7179 114.6587 114.5978 114.5337 114.4652 114.3911 114.3103 114.2222
##  [50,] 113.3263 113.3897 113.4490 113.5030 113.5505 113.5905 113.6219 113.6441
##  [51,] 112.0149 112.1811 112.3429 112.4988 112.6479 112.7890 112.9213 113.0441
##  [52,] 110.7824 111.0318 111.2780 111.5198 111.7559 111.9855 112.2075 112.4213
##  [53,] 109.6305 109.9431 110.2558 110.5671 110.8759 111.1810 111.4816 111.7768
##  [54,] 108.5643 108.9198 109.2805 109.6447 110.0113 110.3789 110.7466 111.1044
##  [55,] 107.5927 107.9698 108.3593 108.7593 109.1682 109.5766 109.9764 110.3648
##  [56,] 106.7281 107.1046 107.5026 107.9134 108.3290 108.7456 109.1599 109.5685
##  [57,] 105.9873 106.3343 106.7045 107.0927 107.4944 107.9050 108.3205 108.7372
##  [58,] 105.3798 105.6628 105.9805 106.3270 106.6968 107.0847 107.4862 107.8966
##  [59,] 104.9399 105.1332 105.3744 105.6567 105.9738 106.3198 106.6891 107.0768
##  [60,] 104.7260 104.7997 104.9366 105.1288 105.3691 105.6507 105.9671 106.3125
##  [61,] 104.8051 104.7247 104.7252 104.7975 104.9332 105.1244 105.3638 105.6446
##  [62,] 105.2546 104.9805 104.8076 104.7255 104.7245 104.7954 104.9299 105.1201
##  [63,] 106.1640 105.6502 105.2613 104.9850 104.8101 104.7263 104.7237 104.7933
##  [64,] 107.6326 106.8300 106.1757 105.6593 105.2680 104.9895 104.8127 104.7272
##  [65,] 109.5091 108.5464 107.6500 106.8447 106.1875 105.6685 105.2747 104.9941
##  [66,] 111.6455 110.5568 109.5292 108.5652 107.6674 106.8595 106.1994 105.6777
##  [67,] 114.0215 112.8166 111.6681 110.5782 109.5494 108.5840 107.6849 106.8744
##  [68,] 116.6202 115.3070 114.0464 112.8404 111.6907 110.5996 109.5695 108.6029
##  [69,] 119.4273 118.0125 116.6472 115.3330 114.0714 112.8642 111.7133 110.6211
##  [70,] 122.4323 120.9207 119.4564 118.0406 116.6743 115.3590 114.0963 112.8880
##  [71,] 125.6284 124.0227 122.4634 120.9507 119.4855 118.0687 116.7014 115.3851
##  [72,] 129.0131 127.3141 125.6613 124.0547 122.4944 120.9808 119.5146 118.0968
##  [73,] 132.5886 130.7945 129.0479 127.3479 125.6943 124.0867 122.5255 121.0109
##  [74,] 136.3631 134.4691 132.6254 130.8303 129.0827 127.3818 125.7272 124.1187
##  [75,] 140.3508 138.3490 136.4019 134.5069 132.6621 130.8660 129.1175 127.4157
##  [76,] 144.5738 142.4524 140.3919 138.3889 136.4407 134.5446 132.6989 130.9019
##  [77,] 149.0628 146.8052 144.6174 142.4946 140.4329 138.4288 136.4795 134.5824
##  [78,] 153.8588 151.4434 149.1092 146.8501 144.6609 142.5369 140.4740 138.4688
##  [79,] 159.0152 156.4139 153.9085 151.4914 149.1556 146.8951 144.7045 142.5792
##  [80,] 164.5998 161.7770 159.0688 156.4654 153.9582 151.5394 149.2020 146.9400
##  [81,] 170.6982 167.6091 164.6581 161.8328 159.1224 156.5170 154.0080 151.5874
##  [82,] 177.4166 174.0058 170.7621 167.6701 164.7164 161.8888 159.1761 156.5687
##  [83,] 184.8868 181.0857 177.4873 174.0730 170.8261 167.7312 164.7748 161.9447
##  [84,] 193.2865 188.9950 184.9658 181.1604 177.5581 174.1403 170.8901 167.7923
##  [85,] 202.9451 197.9978 193.3765 189.0789 185.0449 181.2352 177.6290 174.2076
##  [86,] 213.9297 208.3271 203.0484 198.0945 193.4666 189.1629 185.1241 181.3101
##  [87,] 226.2316 219.9780 214.0462 208.4371 203.1518 198.1913 193.5569 189.2469
##  [88,] 239.8429 232.9421 226.3612 220.1012 214.1628 208.5472 203.2553 198.2883
##  [89,] 254.7575 247.2122 239.9856 233.0783 226.4910 220.2245 214.2796 208.6574
##  [90,] 270.9716 262.7833 254.9132 247.3615 240.1285 233.2147 226.6209 220.3479
##  [91,] 288.4855 279.6535 271.1403 262.9455 255.0690 247.5109 240.2714 233.3512
##  [92,] 307.3042 297.8253 288.6672 279.8288 271.3092 263.1079 255.2250 247.6604
##  [93,] 327.4396 317.3069 307.4990 298.0136 288.8490 280.0041 271.4781 263.2705
##  [94,] 348.9124 338.1145 327.6477 317.5084 307.6939 298.2020 289.0310 280.1797
##  [95,] 371.7544 360.2739 349.1339 338.3294 327.8559 317.7100 307.8890 298.3906
##  [96,] 396.0119 383.8239 371.9898 360.5025 349.3557 338.5443 328.0642 317.9118
##  [97,] 421.7500 408.8197 396.2619 384.0666 372.2255 360.7311 349.5775 338.7595
##  [98,] 449.0568 435.3372 422.0151 409.0772 396.5119 384.3095 372.4612 360.9599
##  [99,] 478.0510 463.4791 449.3382 435.6104 422.2804 409.3348 396.7622 384.5524
## [100,] 508.8893 493.3822 478.3499 463.7692 449.6197 435.8837 422.5458 409.5926
##           [,95]    [,96]    [,97]    [,98]    [,99]   [,100]
##   [1,] 722.1851 709.8467 697.4502 685.0000 672.5006 659.9571
##   [2,] 697.1992 684.7479 672.2476 659.7033 647.1200 634.5030
##   [3,] 671.9946 659.4494 646.8654 634.2478 621.6020 608.9337
##   [4,] 646.6108 633.9926 621.3463 608.6775 595.9921 583.2956
##   [5,] 621.0905 608.4214 595.7356 583.0390 570.3374 557.6367
##   [6,] 595.4792 582.7824 570.0808 557.3801 544.6866 532.0063
##   [7,] 569.8241 557.1236 544.4303 531.7503 519.0896 506.4545
##   [8,] 544.1740 531.4943 518.8341 506.1996 493.5969 481.0323
##   [9,] 518.5786 505.9446 493.3427 480.7789 468.2597 455.7911
##  [10,] 493.0885 480.5256 468.0073 455.5398 443.1295 430.7826
##  [11,] 467.7549 455.2885 442.8794 430.5339 418.2582 406.0670
##  [12,] 442.6293 430.2852 418.0110 405.8231 393.9919 382.6164
##  [13,] 417.7638 405.5794 393.7577 382.3911 371.4546 360.9249
##  [14,] 393.5237 382.1660 371.2379 360.7162 350.5792 340.8068
##  [15,] 371.0214 360.5077 350.3782 340.6130 331.1936 322.1026
##  [16,] 350.1774 340.4194 331.0068 321.9222 313.1499 304.6748
##  [17,] 330.8200 321.7420 312.9757 304.5065 296.3206 288.4052
##  [18,] 312.8018 304.3384 296.1580 288.2480 280.5965 273.1926
##  [19,] 295.9956 288.0909 280.4445 273.0455 265.8838 258.9502
##  [20,] 280.2926 272.8985 265.7415 258.8125 252.1027 245.6045
##  [21,] 265.5993 258.6748 251.9694 245.4753 239.1853 233.0928
##  [22,] 251.8361 245.3463 239.0603 232.9717 227.0743 221.3627
##  [23,] 238.9354 232.8507 226.9571 221.2492 215.7219 210.3708
##  [24,] 226.8400 221.1358 215.6121 210.2645 205.0888 200.0815
##  [25,] 215.5023 210.1582 204.9860 199.9820 195.1431 190.4664
##  [26,] 204.8832 199.8826 195.0470 190.3736 185.8598 181.5038
##  [27,] 194.9510 190.2808 185.7703 181.4174 177.2203 173.1777
##  [28,] 185.6808 181.3310 177.1371 173.0977 169.2115 165.4780
##  [29,] 177.0540 173.0176 169.1346 165.4041 161.8257 158.3992
##  [30,] 169.0577 165.3303 161.7550 158.3316 155.0601 151.9409
##  [31,] 161.6843 158.2640 154.9956 151.8795 148.9164 146.1147
##  [32,] 154.9311 151.8181 148.8581 146.0601 143.4497 141.0187
##  [33,] 148.7999 146.0055 143.3988 140.9713 138.7121 136.6109
##  [34,] 143.3481 140.9240 138.6681 136.5700 134.6200 132.8090
##  [35,] 138.6242 136.5292 134.5821 132.7738 131.0957 129.5397
##  [36,] 134.5442 132.7387 131.0631 129.5094 128.0699 126.7373
##  [37,] 131.0305 129.4792 128.0419 126.7114 125.4808 124.3436
##  [38,] 128.0140 126.6856 125.4569 124.3215 123.2734 122.3069
##  [39,] 125.4331 124.2995 123.2531 122.2882 121.3992 120.5813
##  [40,] 123.2329 122.2695 121.3820 120.5655 119.8150 119.1263
##  [41,] 121.3648 120.5497 119.8005 119.1130 118.4828 117.9062
##  [42,] 119.7860 119.0997 118.4707 117.8951 117.3693 116.8899
##  [43,] 118.4585 117.8840 117.3592 116.8807 116.4453 116.0502
##  [44,] 117.3491 116.8715 116.4370 116.0426 115.6858 115.3638
##  [45,] 116.4286 116.0351 115.6789 115.3576 115.0690 114.8107
##  [46,] 115.6721 115.3515 115.0634 114.8058 114.5767 114.3744
##  [47,] 115.0579 114.8009 114.5724 114.3706 114.1940 114.0413
##  [48,] 114.5681 114.3668 114.1907 114.0384 113.9087 113.8006
##  [49,] 114.1259 114.0208 113.9063 113.7986 113.7116 113.6445
##  [50,] 113.6563 113.6580 113.6488 113.6282 113.5960 113.5677
##  [51,] 113.1567 113.2586 113.3493 113.4286 113.4962 113.5425
##  [52,] 112.6262 112.8218 113.0075 113.1734 113.3103 113.4177
##  [53,] 112.0660 112.3391 112.5872 112.8091 113.0038 113.1703
##  [54,] 111.4439 111.7631 112.0602 112.3338 112.5825 112.8049
##  [55,] 110.7392 111.0974 111.4372 111.7568 112.0544 112.3285
##  [56,] 109.9684 110.3570 110.7318 111.0903 111.4305 111.7506
##  [57,] 109.1515 109.5603 109.9605 110.3493 110.7244 111.0832
##  [58,] 108.3121 108.7288 109.1432 109.5521 109.9525 110.3416
##  [59,] 107.4779 107.8883 108.3037 108.7204 109.1349 109.5439
##  [60,] 106.6815 107.0688 107.4697 107.8799 108.2953 108.7120
##  [61,] 105.9604 106.3053 106.6738 107.0608 107.4615 107.8716
##  [62,] 105.3586 105.6386 105.9537 106.2981 106.6662 107.0528
##  [63,] 104.9266 105.1157 105.3533 105.6326 105.9470 106.2909
##  [64,] 104.7230 104.7913 104.9234 105.1114 105.3481 105.6265
##  [65,] 104.8154 104.7281 104.7224 104.7892 104.9201 105.1071
##  [66,] 105.2815 104.9987 104.8181 104.7290 104.7218 104.7872
##  [67,] 106.2113 105.6870 105.2884 105.0034 104.8208 104.7299
##  [68,] 107.7024 106.8893 106.2233 105.6963 105.2953 105.0081
##  [69,] 109.5897 108.6218 107.7199 106.9043 106.2353 105.7056
##  [70,] 111.7360 110.6426 109.6100 108.6407 107.7374 106.9193
##  [71,] 114.1213 112.9119 111.7587 110.6641 109.6302 108.6596
##  [72,] 116.7285 115.4112 114.1463 112.9357 111.7815 110.6856
##  [73,] 119.5438 118.1250 116.7557 115.4373 114.1713 112.9597
##  [74,] 122.5566 121.0411 119.5730 118.1532 116.7829 115.4634
##  [75,] 125.7602 124.1508 122.5877 121.0712 119.6021 118.1814
##  [76,] 129.1524 127.4496 125.7932 124.1828 122.6188 121.1014
##  [77,] 132.7357 130.9377 129.1873 127.4836 125.8262 124.2149
##  [78,] 136.5184 134.6203 132.7725 130.9735 129.2221 127.5175
##  [79,] 140.5151 138.5087 136.5572 134.6581 132.8094 131.0094
##  [80,] 144.7481 142.6215 140.5562 138.5487 136.5961 134.6960
##  [81,] 149.2485 146.9850 144.7917 142.6638 140.5973 138.5887
##  [82,] 154.0578 151.6355 149.2949 147.0300 144.8353 142.7062
##  [83,] 159.2298 156.6204 154.1076 151.6836 149.3415 147.0751
##  [84,] 164.8333 162.0007 159.2836 156.6721 154.1574 151.7317
##  [85,] 170.9542 167.8535 164.8918 162.0567 159.3374 156.7239
##  [86,] 177.7000 174.2750 171.0184 167.9147 164.9503 162.1128
##  [87,] 185.2034 181.3850 177.7710 174.3425 171.0826 167.9760
##  [88,] 193.6473 189.3311 185.2828 181.4601 177.8421 174.4101
##  [89,] 203.3590 198.3855 193.7378 189.4153 185.3622 181.5352
##  [90,] 214.3965 208.7678 203.4629 198.4827 193.8285 189.4997
##  [91,] 226.7509 220.4714 214.5136 208.8783 203.5668 198.5801
##  [92,] 240.4145 233.4879 226.8811 220.5951 214.6307 208.9890
##  [93,] 255.3811 247.8101 240.5577 233.6246 227.0114 220.7189
##  [94,] 271.6472 263.4331 255.5373 247.9599 240.7011 233.7615
##  [95,] 289.2131 280.3553 271.8164 263.5959 255.6937 248.1098
##  [96,] 308.0842 298.5793 289.3954 280.5311 271.9858 263.7589
##  [97,] 328.2727 318.1137 308.2796 298.7681 289.5777 280.7070
##  [98,] 349.7995 338.9747 328.4813 318.3157 308.4750 298.9571
##  [99,] 372.6971 361.1889 350.0216 339.1901 328.6900 318.5178
## [100,] 397.0125 384.7956 372.9332 361.4180 350.2439 339.4056
## 
## $alphas
##   [1] 0.00000000 0.05050505 0.10101010 0.15151515 0.20202020 0.25252525
##   [7] 0.30303030 0.35353535 0.40404040 0.45454545 0.50505051 0.55555556
##  [13] 0.60606061 0.65656566 0.70707071 0.75757576 0.80808081 0.85858586
##  [19] 0.90909091 0.95959596 1.01010101 1.06060606 1.11111111 1.16161616
##  [25] 1.21212121 1.26262626 1.31313131 1.36363636 1.41414141 1.46464646
##  [31] 1.51515152 1.56565657 1.61616162 1.66666667 1.71717172 1.76767677
##  [37] 1.81818182 1.86868687 1.91919192 1.96969697 2.02020202 2.07070707
##  [43] 2.12121212 2.17171717 2.22222222 2.27272727 2.32323232 2.37373737
##  [49] 2.42424242 2.47474747 2.52525253 2.57575758 2.62626263 2.67676768
##  [55] 2.72727273 2.77777778 2.82828283 2.87878788 2.92929293 2.97979798
##  [61] 3.03030303 3.08080808 3.13131313 3.18181818 3.23232323 3.28282828
##  [67] 3.33333333 3.38383838 3.43434343 3.48484848 3.53535354 3.58585859
##  [73] 3.63636364 3.68686869 3.73737374 3.78787879 3.83838384 3.88888889
##  [79] 3.93939394 3.98989899 4.04040404 4.09090909 4.14141414 4.19191919
##  [85] 4.24242424 4.29292929 4.34343434 4.39393939 4.44444444 4.49494949
##  [91] 4.54545455 4.59595960 4.64646465 4.69696970 4.74747475 4.79797980
##  [97] 4.84848485 4.89898990 4.94949495 5.00000000
## 
## $gammas
##   [1] 0.025 0.050 0.075 0.100 0.125 0.150 0.175 0.200 0.225 0.250 0.275 0.300
##  [13] 0.325 0.350 0.375 0.400 0.425 0.450 0.475 0.500 0.525 0.550 0.575 0.600
##  [25] 0.625 0.650 0.675 0.700 0.725 0.750 0.775 0.800 0.825 0.850 0.875 0.900
##  [37] 0.925 0.950 0.975 1.000 1.025 1.050 1.075 1.100 1.125 1.150 1.175 1.200
##  [49] 1.225 1.250 1.275 1.300 1.325 1.350 1.375 1.400 1.425 1.450 1.475 1.500
##  [61] 1.525 1.550 1.575 1.600 1.625 1.650 1.675 1.700 1.725 1.750 1.775 1.800
##  [73] 1.825 1.850 1.875 1.900 1.925 1.950 1.975 2.000 2.025 2.050 2.075 2.100
##  [85] 2.125 2.150 2.175 2.200 2.225 2.250 2.275 2.300 2.325 2.350 2.375 2.400
##  [97] 2.425 2.450 2.475 2.500
## 
## $alpha
## [1] 3.282828
## 
## $gamma
## [1] 2.475
## 
## $model
## 
## Call:
## stats::lm(formula = y ~ x + q)
## 
## Coefficients:
## (Intercept)            x            q  
##      301.96        46.33       -42.76  
## 
## 
## attr(,"class")
## [1] "bent_cable"
## 
## $SimpleExponential
## 
## Call:
## stats::lm(formula = y2 ~ x)
## 
## Coefficients:
## (Intercept)            x  
##      5.2020      -0.7791  
## 
## 
## $NonLinearLeastSquares
## Nonlinear regression model
##   model: y2 ~ I(exp(1)^(a + b * x))
##    data: data.frame(x = x, y2 = y2)
##       a       b 
##  1.6435 -0.1995 
##  residual sum-of-squares: 26.85
## 
## Number of iterations to convergence: 10 
## Achieved convergence tolerance: 2.652e-06
```

Get likelihoods for m 0-5

```
setwd("/gpfs/gibbs/pi/caccone/mkc54/albo/manuscript1/output/treemix_subsets/north_america_simplified/simplified_test/prelim")

# Get all llik files
files <- list.files(pattern = "nasub_simp\\.m[0-9]+\\.rep1\\.llik$")

# Extract m and likelihood
df <- data.frame(m = numeric(), lnL = numeric())

for(f in files){
  # extract m number from filename
  m <- as.numeric(sub("nasub_simp\\.m([0-9]+)\\.rep1\\.llik", "\\1", f))
  
  # read file
  lines <- readLines(f)
  
  # look for line containing "Exiting ln(likelihood)"
  lnL_line <- grep("Exiting ln\\(likelihood\\)", lines, value = TRUE)
  
  # extract numeric value after colon
  lnL <- as.numeric(sub(".*: *", "", lnL_line))
  
  df <- rbind(df, data.frame(m = m, lnL = lnL))
}

# Sort by m
df <- df[order(df$m), ]

# Compute delta lnL
df$delta_lnL <- c(NA, diff(df$lnL))

# Print table
print(df)
```

```
##   m     lnL delta_lnL
## 1 0 301.964        NA
## 2 1 349.129    47.165
## 3 2 385.405    36.276
## 4 3 423.272    37.867
## 5 4 442.264    18.992
## 6 5 457.686    15.422
```

Plot

```
setwd("/gpfs/gibbs/pi/caccone/mkc54/albo/manuscript1/output/treemix_subsets/north_america_simplified/simplified_test/prelim")

plot(df$m, df$lnL, type="b", pch=19, xlab="Migration edges (m)", ylab="ln(likelihood)")
abline(v = df$m[which.max(df$delta_lnL)], col="red", lty=2) # highlight largest gain
```

Check for m=0

```
setwd("/gpfs/gibbs/pi/caccone/mkc54/albo/manuscript1/output/treemix_subsets/north_america_simplified/simplified_test/prelim")
plot_tree("nasub_simp.m0.rep2")
```

```
## Warning in plot_tree("nasub_simp.m0.rep2"): NAs introduced by coercion
## Warning in plot_tree("nasub_simp.m0.rep2"): NAs introduced by coercion
## Warning in plot_tree("nasub_simp.m0.rep2"): NAs introduced by coercion
## Warning in plot_tree("nasub_simp.m0.rep2"): NAs introduced by coercion
```

```
##     V1                 V2       V3      V4      V5  V6  V7 V8  V9 V10
## 1    1      Athens_Greece NOT_ROOT NOT_MIG     TIP  76  NA NA  NA  NA
## 2    2               <NA> NOT_ROOT NOT_MIG NOT_TIP  76  31  1 212   7
## 3    3     Okinawa_Taiwan NOT_ROOT NOT_MIG     TIP  32  NA NA  NA  NA
## 4    4             Russia NOT_ROOT NOT_MIG     TIP  16  NA NA  NA  NA
## 5   15              Texas NOT_ROOT NOT_MIG     TIP  52  NA NA  NA  NA
## 6   16               <NA> NOT_ROOT NOT_MIG NOT_TIP  32   4  1 172   4
## 7   31      Eastern_China NOT_ROOT NOT_MIG     TIP   2  NA NA  NA  NA
## 8   32               <NA> NOT_ROOT NOT_MIG NOT_TIP 212   3  1  16   5
## 9   51              Italy NOT_ROOT NOT_MIG     TIP 136  NA NA  NA  NA
## 10  52               <NA> NOT_ROOT NOT_MIG NOT_TIP 172  15  1 136   2
## 11  75 Sulawesi_Indonesia NOT_ROOT NOT_MIG     TIP 213  NA NA  NA  NA
## 12  76               <NA> NOT_ROOT NOT_MIG NOT_TIP 104   1  1   2   8
## 13 103     Southeast_Asia NOT_ROOT NOT_MIG     TIP 104  NA NA  NA  NA
## 14 104               <NA> NOT_ROOT NOT_MIG NOT_TIP 213 103  1  76   9
## 15 135       Northeast_US NOT_ROOT NOT_MIG     TIP 136  NA NA  NA  NA
## 16 136               <NA> NOT_ROOT NOT_MIG NOT_TIP  52  51  1 135   1
## 17 171              Japan NOT_ROOT NOT_MIG     TIP 172  NA NA  NA  NA
## 18 172               <NA> NOT_ROOT NOT_MIG NOT_TIP  16  52  3 171   1
## 19 211         California NOT_ROOT NOT_MIG     TIP 212  NA NA  NA  NA
## 20 212               <NA> NOT_ROOT NOT_MIG NOT_TIP   2  32  6 211   1
## 21 213               <NA>     ROOT NOT_MIG NOT_TIP 213  75  1 104  10
##                                                                                                                                                                                                                                                                                                                                                                       V11
## 1                                                                                                                                                                                                                                                                                                                                                Athens_Greece:0.00581989
## 2                                                                                                            (Eastern_China:0.00109554,((Okinawa_Taiwan:0.0140044,(Russia:0.0199554,((Texas:0.0019019,(Italy:0.011679,Northeast_US:0.000981699):0.00263898):0.000210042,Japan:0.00727026):0.00779086):0.00193584):0.00229181,California:0.0081973):0.00161149):0.00224919
## 3                                                                                                                                                                                                                                                                                                                                                Okinawa_Taiwan:0.0140044
## 4                                                                                                                                                                                                                                                                                                                                                        Russia:0.0199554
## 5                                                                                                                                                                                                                                                                                                                                                         Texas:0.0019019
## 6                                                                                                                                                                                                                          (Russia:0.0199554,((Texas:0.0019019,(Italy:0.011679,Northeast_US:0.000981699):0.00263898):0.000210042,Japan:0.00727026):0.00779086):0.00193584
## 7                                                                                                                                                                                                                                                                                                                                                Eastern_China:0.00109554
## 8                                                                                                                                                                                    (Okinawa_Taiwan:0.0140044,(Russia:0.0199554,((Texas:0.0019019,(Italy:0.011679,Northeast_US:0.000981699):0.00263898):0.000210042,Japan:0.00727026):0.00779086):0.00193584):0.00229181
## 9                                                                                                                                                                                                                                                                                                                                                          Italy:0.011679
## 10                                                                                                                                                                                                                                                                                     (Texas:0.0019019,(Italy:0.011679,Northeast_US:0.000981699):0.00263898):0.000210042
## 11                                                                                                                                                                                                                                                                                                                                            Sulawesi_Indonesia:0.018019
## 12                                                                     (Athens_Greece:0.00581989,(Eastern_China:0.00109554,((Okinawa_Taiwan:0.0140044,(Russia:0.0199554,((Texas:0.0019019,(Italy:0.011679,Northeast_US:0.000981699):0.00263898):0.000210042,Japan:0.00727026):0.00779086):0.00193584):0.00229181,California:0.0081973):0.00161149):0.00224919):0.00826008
## 13                                                                                                                                                                                                                                                                                                                                              Southeast_Asia:0.00631183
## 14                                (Southeast_Asia:0.00631183,(Athens_Greece:0.00581989,(Eastern_China:0.00109554,((Okinawa_Taiwan:0.0140044,(Russia:0.0199554,((Texas:0.0019019,(Italy:0.011679,Northeast_US:0.000981699):0.00263898):0.000210042,Japan:0.00727026):0.00779086):0.00193584):0.00229181,California:0.0081973):0.00161149):0.00224919):0.00826008):0.018019
## 15                                                                                                                                                                                                                                                                                                                                               Northeast_US:0.000981699
## 16                                                                                                                                                                                                                                                                                                                   (Italy:0.011679,Northeast_US:0.000981699):0.00263898
## 17                                                                                                                                                                                                                                                                                                                                                       Japan:0.00727026
## 18                                                                                                                                                                                                                                                       ((Texas:0.0019019,(Italy:0.011679,Northeast_US:0.000981699):0.00263898):0.000210042,Japan:0.00727026):0.00779086
## 19                                                                                                                                                                                                                                                                                                                                                   California:0.0081973
## 20                                                                                                                                                 ((Okinawa_Taiwan:0.0140044,(Russia:0.0199554,((Texas:0.0019019,(Italy:0.011679,Northeast_US:0.000981699):0.00263898):0.000210042,Japan:0.00727026):0.00779086):0.00193584):0.00229181,California:0.0081973):0.00161149
## 21 (Sulawesi_Indonesia:0.018019,(Southeast_Asia:0.00631183,(Athens_Greece:0.00581989,(Eastern_China:0.00109554,((Okinawa_Taiwan:0.0140044,(Russia:0.0199554,((Texas:0.0019019,(Italy:0.011679,Northeast_US:0.000981699):0.00263898):0.000210042,Japan:0.00727026):0.00779086):0.00193584):0.00229181,California:0.0081973):0.00161149):0.00224919):0.00826008):0.018019);
##             x          y       ymin       ymax
## 1  0.03209897 0.77272727 0.72727273 0.81818182
## 2  0.02852827 0.63636364 0.00000000 0.72727273
## 3  0.04643597 0.59090909 0.54545455 0.63636364
## 4  0.05432281 0.50000000 0.45454545 0.54545455
## 5  0.04427021 0.40909091 0.36363636 0.45454545
## 6  0.03436741 0.45454545 0.09090909 0.54545455
## 7  0.02962381 0.68181818 0.63636364 0.72727273
## 8  0.03243157 0.54545455 0.09090909 0.63636364
## 9  0.05668629 0.31818182 0.27272727 0.36363636
## 10 0.04236831 0.36363636 0.18181818 0.45454545
## 11 0.01801900 0.95454545 0.90909091 1.00000000
## 12 0.02627908 0.72727273 0.00000000 0.81818182
## 13 0.02433083 0.86363636 0.81818182 0.90909091
## 14 0.01801900 0.81818182 0.00000000 0.90909091
## 15 0.04598899 0.22727273 0.18181818 0.27272727
## 16 0.04500729 0.27272727 0.18181818 0.36363636
## 17 0.04942853 0.13636364 0.09090909 0.18181818
## 18 0.04215827 0.18181818 0.09090909 0.45454545
## 19 0.03833706 0.04545455 0.00000000 0.09090909
## 20 0.03013976 0.09090909 0.00000000 0.63636364
## 21 0.00000000 0.90909091 0.00000000 1.00000000
```

```
## Warning in max(e[e[, 5] == "MIG", 4]): no non-missing arguments to max;
## returning -Inf
```

```
##  [1] 0.03209897 0.04643597 0.05432281 0.04427021 0.02962381 0.05668629
##  [7] 0.01801900 0.02433083 0.04598899 0.04942853 0.03833706
## [1] 0.003
## [1] "mse 0.000348772561983471"
```

```
## $d
##     V1                 V2       V3      V4      V5  V6  V7 V8  V9 V10
## 1    1      Athens_Greece NOT_ROOT NOT_MIG     TIP  76  NA NA  NA  NA
## 2    2               <NA> NOT_ROOT NOT_MIG NOT_TIP  76  31  1 212   7
## 3    3     Okinawa_Taiwan NOT_ROOT NOT_MIG     TIP  32  NA NA  NA  NA
## 4    4             Russia NOT_ROOT NOT_MIG     TIP  16  NA NA  NA  NA
## 5   15              Texas NOT_ROOT NOT_MIG     TIP  52  NA NA  NA  NA
## 6   16               <NA> NOT_ROOT NOT_MIG NOT_TIP  32   4  1 172   4
## 7   31      Eastern_China NOT_ROOT NOT_MIG     TIP   2  NA NA  NA  NA
## 8   32               <NA> NOT_ROOT NOT_MIG NOT_TIP 212   3  1  16   5
## 9   51              Italy NOT_ROOT NOT_MIG     TIP 136  NA NA  NA  NA
## 10  52               <NA> NOT_ROOT NOT_MIG NOT_TIP 172  15  1 136   2
## 11  75 Sulawesi_Indonesia NOT_ROOT NOT_MIG     TIP 213  NA NA  NA  NA
## 12  76               <NA> NOT_ROOT NOT_MIG NOT_TIP 104   1  1   2   8
## 13 103     Southeast_Asia NOT_ROOT NOT_MIG     TIP 104  NA NA  NA  NA
## 14 104               <NA> NOT_ROOT NOT_MIG NOT_TIP 213 103  1  76   9
## 15 135       Northeast_US NOT_ROOT NOT_MIG     TIP 136  NA NA  NA  NA
## 16 136               <NA> NOT_ROOT NOT_MIG NOT_TIP  52  51  1 135   1
## 17 171              Japan NOT_ROOT NOT_MIG     TIP 172  NA NA  NA  NA
## 18 172               <NA> NOT_ROOT NOT_MIG NOT_TIP  16  52  3 171   1
## 19 211         California NOT_ROOT NOT_MIG     TIP 212  NA NA  NA  NA
## 20 212               <NA> NOT_ROOT NOT_MIG NOT_TIP   2  32  6 211   1
## 21 213               <NA>     ROOT NOT_MIG NOT_TIP 213  75  1 104  10
##                                                                                                                                                                                                                                                                                                                                                                       V11
## 1                                                                                                                                                                                                                                                                                                                                                Athens_Greece:0.00581989
## 2                                                                                                            (Eastern_China:0.00109554,((Okinawa_Taiwan:0.0140044,(Russia:0.0199554,((Texas:0.0019019,(Italy:0.011679,Northeast_US:0.000981699):0.00263898):0.000210042,Japan:0.00727026):0.00779086):0.00193584):0.00229181,California:0.0081973):0.00161149):0.00224919
## 3                                                                                                                                                                                                                                                                                                                                                Okinawa_Taiwan:0.0140044
## 4                                                                                                                                                                                                                                                                                                                                                        Russia:0.0199554
## 5                                                                                                                                                                                                                                                                                                                                                         Texas:0.0019019
## 6                                                                                                                                                                                                                          (Russia:0.0199554,((Texas:0.0019019,(Italy:0.011679,Northeast_US:0.000981699):0.00263898):0.000210042,Japan:0.00727026):0.00779086):0.00193584
## 7                                                                                                                                                                                                                                                                                                                                                Eastern_China:0.00109554
## 8                                                                                                                                                                                    (Okinawa_Taiwan:0.0140044,(Russia:0.0199554,((Texas:0.0019019,(Italy:0.011679,Northeast_US:0.000981699):0.00263898):0.000210042,Japan:0.00727026):0.00779086):0.00193584):0.00229181
## 9                                                                                                                                                                                                                                                                                                                                                          Italy:0.011679
## 10                                                                                                                                                                                                                                                                                     (Texas:0.0019019,(Italy:0.011679,Northeast_US:0.000981699):0.00263898):0.000210042
## 11                                                                                                                                                                                                                                                                                                                                            Sulawesi_Indonesia:0.018019
## 12                                                                     (Athens_Greece:0.00581989,(Eastern_China:0.00109554,((Okinawa_Taiwan:0.0140044,(Russia:0.0199554,((Texas:0.0019019,(Italy:0.011679,Northeast_US:0.000981699):0.00263898):0.000210042,Japan:0.00727026):0.00779086):0.00193584):0.00229181,California:0.0081973):0.00161149):0.00224919):0.00826008
## 13                                                                                                                                                                                                                                                                                                                                              Southeast_Asia:0.00631183
## 14                                (Southeast_Asia:0.00631183,(Athens_Greece:0.00581989,(Eastern_China:0.00109554,((Okinawa_Taiwan:0.0140044,(Russia:0.0199554,((Texas:0.0019019,(Italy:0.011679,Northeast_US:0.000981699):0.00263898):0.000210042,Japan:0.00727026):0.00779086):0.00193584):0.00229181,California:0.0081973):0.00161149):0.00224919):0.00826008):0.018019
## 15                                                                                                                                                                                                                                                                                                                                               Northeast_US:0.000981699
## 16                                                                                                                                                                                                                                                                                                                   (Italy:0.011679,Northeast_US:0.000981699):0.00263898
## 17                                                                                                                                                                                                                                                                                                                                                       Japan:0.00727026
## 18                                                                                                                                                                                                                                                       ((Texas:0.0019019,(Italy:0.011679,Northeast_US:0.000981699):0.00263898):0.000210042,Japan:0.00727026):0.00779086
## 19                                                                                                                                                                                                                                                                                                                                                   California:0.0081973
## 20                                                                                                                                                 ((Okinawa_Taiwan:0.0140044,(Russia:0.0199554,((Texas:0.0019019,(Italy:0.011679,Northeast_US:0.000981699):0.00263898):0.000210042,Japan:0.00727026):0.00779086):0.00193584):0.00229181,California:0.0081973):0.00161149
## 21 (Sulawesi_Indonesia:0.018019,(Southeast_Asia:0.00631183,(Athens_Greece:0.00581989,(Eastern_China:0.00109554,((Okinawa_Taiwan:0.0140044,(Russia:0.0199554,((Texas:0.0019019,(Italy:0.011679,Northeast_US:0.000981699):0.00263898):0.000210042,Japan:0.00727026):0.00779086):0.00193584):0.00229181,California:0.0081973):0.00161149):0.00224919):0.00826008):0.018019);
##             x          y       ymin       ymax
## 1  0.03209897 0.77272727 0.72727273 0.81818182
## 2  0.02852827 0.63636364 0.00000000 0.72727273
## 3  0.04643597 0.59090909 0.54545455 0.63636364
## 4  0.05432281 0.50000000 0.45454545 0.54545455
## 5  0.04427021 0.40909091 0.36363636 0.45454545
## 6  0.03436741 0.45454545 0.09090909 0.54545455
## 7  0.02962381 0.68181818 0.63636364 0.72727273
## 8  0.03243157 0.54545455 0.09090909 0.63636364
## 9  0.05668629 0.31818182 0.27272727 0.36363636
## 10 0.04236831 0.36363636 0.18181818 0.45454545
## 11 0.01801900 0.95454545 0.90909091 1.00000000
## 12 0.02627908 0.72727273 0.00000000 0.81818182
## 13 0.02433083 0.86363636 0.81818182 0.90909091
## 14 0.01801900 0.81818182 0.00000000 0.90909091
## 15 0.04598899 0.22727273 0.18181818 0.27272727
## 16 0.04500729 0.27272727 0.18181818 0.36363636
## 17 0.04942853 0.13636364 0.09090909 0.18181818
## 18 0.04215827 0.18181818 0.09090909 0.45454545
## 19 0.03833706 0.04545455 0.00000000 0.09090909
## 20 0.03013976 0.09090909 0.00000000 0.63636364
## 21 0.00000000 0.90909091 0.00000000 1.00000000
## 
## $e
##     V1  V2          V3 V4      V5 V6 V7
## 1   52  15 0.001901900  1 NOT_MIG  0  1
## 2   16   4 0.019955400  1 NOT_MIG  0  1
## 3    2  31 0.001095540  1 NOT_MIG  0  1
## 4   32   3 0.014004400  1 NOT_MIG  0  1
## 5   76   1 0.005819890  1 NOT_MIG  0  1
## 6  104 103 0.006311830  1 NOT_MIG  0  1
## 7   52 136 0.002638980  1 NOT_MIG  0  1
## 8  136  51 0.011679000  1 NOT_MIG  0  1
## 9  136 135 0.000981699  1 NOT_MIG  0  1
## 10 172  52 0.000210042  1 NOT_MIG  0  1
## 11 172 171 0.007270260  1 NOT_MIG  0  1
## 12  16 172 0.007790860  1 NOT_MIG  0  1
## 13  32  16 0.001935840  1 NOT_MIG  0  1
## 14 212  32 0.002291810  1 NOT_MIG  0  1
## 15 212 211 0.008197300  1 NOT_MIG  0  1
## 16 104  76 0.008260080  1 NOT_MIG  0  1
## 17  76   2 0.002249190  1 NOT_MIG  0  1
## 18 213  75 0.018019000  1 NOT_MIG  0  1
## 19 213 104 0.018019000  1 NOT_MIG  0  1
## 20   2 212 0.001611490  1 NOT_MIG  0  1
```

```
setwd("/gpfs/gibbs/pi/caccone/mkc54/albo/manuscript1/output/treemix_subsets/north_america_simplified/simplified_test/prelim")

# Open PDF
pdf("treemix_prelim_m0.pdf", width = 12, height = 8)

plot_tree("nasub_simp.m0.rep2")

dev.off()
```

Check files for m=1

```
cd /gpfs/gibbs/pi/caccone/mkc54/albo/manuscript1/output/treemix_subsets/north_america_simplified/simplified_test/prelim
zcat nasub_simp.m1.rep1.treeout.gz
```

```
## (Sulawesi_Indonesia:0.018019,(Southeast_Asia:0.00631183,(Athens_Greece:0.0195533,(Eastern_China:0.000285662,(California:0.00774031,(Okinawa_Taiwan:0.0137794,(Russia:0.0198777,(Japan:0.00727026,(Texas:0.0019019,(Italy:0.011679,Northeast_US:0.000981699):0.00263898):0.000210042):0.00788444):0.00209508):0.0025636):0.00215994):0.00569165):0.00418375):0.018019);
## 0.354305 NA NA NA (Japan:0.00727026,(Texas:0.0019019,(Italy:0.011679,Northeast_US:0.000981699):0.00263898):0.000210042):0.00788444 Athens_Greece:0.0195533
```

```
setwd("/gpfs/gibbs/pi/caccone/mkc54/albo/manuscript1/output/treemix_subsets/north_america_simplified/simplified_test/prelim")
plot_tree("nasub_simp.m1.rep2")
```

```
## Warning in plot_tree("nasub_simp.m1.rep2"): NAs introduced by coercion
## Warning in plot_tree("nasub_simp.m1.rep2"): NAs introduced by coercion
## Warning in plot_tree("nasub_simp.m1.rep2"): NAs introduced by coercion
## Warning in plot_tree("nasub_simp.m1.rep2"): NAs introduced by coercion
```

```
##     V1                 V2       V3      V4      V5  V6  V7 V8  V9 V10
## 1    1              Italy NOT_ROOT NOT_MIG     TIP 104  NA NA  NA  NA
## 2    2               <NA> NOT_ROOT NOT_MIG NOT_TIP  76 104  2  75   1
## 3    3         California NOT_ROOT NOT_MIG     TIP  16  NA NA  NA  NA
## 4    4              Japan NOT_ROOT NOT_MIG     TIP  76  NA NA  NA  NA
## 5   15     Okinawa_Taiwan NOT_ROOT NOT_MIG     TIP  32  NA NA  NA  NA
## 6   16               <NA> NOT_ROOT NOT_MIG NOT_TIP 172   3  1  32   6
## 7   31      Athens_Greece NOT_ROOT NOT_MIG     TIP  52  NA NA  NA  NA
## 8   32               <NA> NOT_ROOT NOT_MIG NOT_TIP  16  15  1 136   5
## 9   51     Southeast_Asia NOT_ROOT NOT_MIG     TIP 212  NA NA  NA  NA
## 10  52               <NA> NOT_ROOT NOT_MIG NOT_TIP 212 172  8  31   1
## 11  75              Texas NOT_ROOT NOT_MIG     TIP   2  NA NA  NA  NA
## 12  76               <NA> NOT_ROOT NOT_MIG NOT_TIP 136   2  3   4   1
## 13 103       Northeast_US NOT_ROOT NOT_MIG     TIP 104  NA NA  NA  NA
## 14 104               <NA> NOT_ROOT NOT_MIG NOT_TIP   2   1  1 103   1
## 15 135             Russia NOT_ROOT NOT_MIG     TIP 136  NA NA  NA  NA
## 16 136               <NA> NOT_ROOT NOT_MIG NOT_TIP  32 135  1  76   4
## 17 171      Eastern_China NOT_ROOT NOT_MIG     TIP 172  NA NA  NA  NA
## 18 172               <NA> NOT_ROOT NOT_MIG NOT_TIP  52  16  7 171   1
## 19 211 Sulawesi_Indonesia NOT_ROOT NOT_MIG     TIP 213  NA NA  NA  NA
## 20 212               <NA> NOT_ROOT NOT_MIG NOT_TIP 213  51  1  52   9
## 21 213               <NA>     ROOT NOT_MIG NOT_TIP 213 211  1 212  10
## 22 217               <NA> NOT_ROOT     MIG NOT_TIP 136  76 NA  NA  NA
##                                                                                                                                                                                                                                                                                                                                                                       V11
## 1                                                                                                                                                                                                                                                                                                                                                          Italy:0.011679
## 2                                                                                                                                                                                                                                                                                      ((Italy:0.011679,Northeast_US:0.000981699):0.00263898,Texas:0.0019019):0.000210042
## 3                                                                                                                                                                                                                                                                                                                                                   California:0.00774031
## 4                                                                                                                                                                                                                                                                                                                                                        Japan:0.00727026
## 5                                                                                                                                                                                                                                                                                                                                                Okinawa_Taiwan:0.0137794
## 6                                                                                                                                                  (California:0.00774031,(Okinawa_Taiwan:0.0137794,(Russia:0.0198777,(((Italy:0.011679,Northeast_US:0.000981699):0.00263898,Texas:0.0019019):0.000210042,Japan:0.00727026):0.00788444):0.00209508):0.0025636):0.00215994
## 7                                                                                                                                                                                                                                                                                                                                                 Athens_Greece:0.0195533
## 8                                                                                                                                                                                     (Okinawa_Taiwan:0.0137794,(Russia:0.0198777,(((Italy:0.011679,Northeast_US:0.000981699):0.00263898,Texas:0.0019019):0.000210042,Japan:0.00727026):0.00788444):0.00209508):0.0025636
## 9                                                                                                                                                                                                                                                                                                                                               Southeast_Asia:0.00631183
## 10                                                                     (((California:0.00774031,(Okinawa_Taiwan:0.0137794,(Russia:0.0198777,(((Italy:0.011679,Northeast_US:0.000981699):0.00263898,Texas:0.0019019):0.000210042,Japan:0.00727026):0.00788444):0.00209508):0.0025636):0.00215994,Eastern_China:0.000285662):0.00569165,Athens_Greece:0.0195533):0.00418375
## 11                                                                                                                                                                                                                                                                                                                                                        Texas:0.0019019
## 12                                                                                                                                                                                                                                                       (((Italy:0.011679,Northeast_US:0.000981699):0.00263898,Texas:0.0019019):0.000210042,Japan:0.00727026):0.00788444
## 13                                                                                                                                                                                                                                                                                                                                               Northeast_US:0.000981699
## 14                                                                                                                                                                                                                                                                                                                   (Italy:0.011679,Northeast_US:0.000981699):0.00263898
## 15                                                                                                                                                                                                                                                                                                                                                       Russia:0.0198777
## 16                                                                                                                                                                                                                         (Russia:0.0198777,(((Italy:0.011679,Northeast_US:0.000981699):0.00263898,Texas:0.0019019):0.000210042,Japan:0.00727026):0.00788444):0.00209508
## 17                                                                                                                                                                                                                                                                                                                                              Eastern_China:0.000285662
## 18                                                                                                          ((California:0.00774031,(Okinawa_Taiwan:0.0137794,(Russia:0.0198777,(((Italy:0.011679,Northeast_US:0.000981699):0.00263898,Texas:0.0019019):0.000210042,Japan:0.00727026):0.00788444):0.00209508):0.0025636):0.00215994,Eastern_China:0.000285662):0.00569165
## 19                                                                                                                                                                                                                                                                                                                                            Sulawesi_Indonesia:0.018019
## 20                                (Southeast_Asia:0.00631183,(((California:0.00774031,(Okinawa_Taiwan:0.0137794,(Russia:0.0198777,(((Italy:0.011679,Northeast_US:0.000981699):0.00263898,Texas:0.0019019):0.000210042,Japan:0.00727026):0.00788444):0.00209508):0.0025636):0.00215994,Eastern_China:0.000285662):0.00569165,Athens_Greece:0.0195533):0.00418375):0.018019
## 21 (Sulawesi_Indonesia:0.018019,(Southeast_Asia:0.00631183,(((California:0.00774031,(Okinawa_Taiwan:0.0137794,(Russia:0.0198777,(((Italy:0.011679,Northeast_US:0.000981699):0.00263898,Texas:0.0019019):0.000210042,Japan:0.00727026):0.00788444):0.00209508):0.0025636):0.00215994,Eastern_China:0.000285662):0.00569165,Athens_Greece:0.0195533):0.00418375):0.018019);
## 22                                                                                                                                                                                                                                                                                                                                                                   <NA>
##             x          y       ymin       ymax
## 1  0.05712548 0.50000000 0.45454545 0.54545455
## 2  0.04280750 0.36363636 0.27272727 0.54545455
## 3  0.03779465 0.77272727 0.72727273 0.81818182
## 4  0.04986772 0.22727273 0.18181818 0.27272727
## 5  0.04639734 0.68181818 0.63636364 0.72727273
## 6  0.03005434 0.72727273 0.18181818 0.81818182
## 7  0.03035495 0.04545455 0.00000000 0.09090909
## 8  0.03261794 0.63636364 0.18181818 0.72727273
## 9  0.02433083 0.86363636 0.81818182 0.90909091
## 10 0.02220275 0.09090909 0.00000000 0.81818182
## 11 0.04470940 0.31818182 0.27272727 0.36363636
## 12 0.04259746 0.27272727 0.18181818 0.54545455
## 13 0.04642818 0.40909091 0.36363636 0.45454545
## 14 0.04544648 0.45454545 0.36363636 0.54545455
## 15 0.05459072 0.59090909 0.54545455 0.63636364
## 16 0.03471302 0.54545455 0.18181818 0.63636364
## 17 0.02818006 0.13636364 0.09090909 0.18181818
## 18 0.02789440 0.18181818 0.09090909 0.81818182
## 19 0.01801900 0.95454545 0.90909091 1.00000000
## 20 0.01801900 0.81818182 0.00000000 0.90909091
## 21 0.00000000 0.90909091 0.00000000 1.00000000
## 22 0.03609588         NA 0.18181818 0.54545455
## [1] "0.175391 0.03471302 0.04259746"
```

```
##  [1] 0.05712548 0.03779465 0.04986772 0.04639734 0.03035495 0.02433083
##  [7] 0.04470940 0.04642818 0.05459072 0.02818006 0.01801900
## [1] 0.003
## [1] "mse 0.000348772561983471"
```

```
## $d
##     V1                 V2       V3      V4      V5  V6  V7 V8  V9 V10
## 1    1              Italy NOT_ROOT NOT_MIG     TIP 104  NA NA  NA  NA
## 2    2               <NA> NOT_ROOT NOT_MIG NOT_TIP  76 104  2  75   1
## 3    3         California NOT_ROOT NOT_MIG     TIP  16  NA NA  NA  NA
## 4    4              Japan NOT_ROOT NOT_MIG     TIP  76  NA NA  NA  NA
## 5   15     Okinawa_Taiwan NOT_ROOT NOT_MIG     TIP  32  NA NA  NA  NA
## 6   16               <NA> NOT_ROOT NOT_MIG NOT_TIP 172   3  1  32   6
## 7   31      Athens_Greece NOT_ROOT NOT_MIG     TIP  52  NA NA  NA  NA
## 8   32               <NA> NOT_ROOT NOT_MIG NOT_TIP  16  15  1 136   5
## 9   51     Southeast_Asia NOT_ROOT NOT_MIG     TIP 212  NA NA  NA  NA
## 10  52               <NA> NOT_ROOT NOT_MIG NOT_TIP 212 172  8  31   1
## 11  75              Texas NOT_ROOT NOT_MIG     TIP   2  NA NA  NA  NA
## 12  76               <NA> NOT_ROOT NOT_MIG NOT_TIP 136   2  3   4   1
## 13 103       Northeast_US NOT_ROOT NOT_MIG     TIP 104  NA NA  NA  NA
## 14 104               <NA> NOT_ROOT NOT_MIG NOT_TIP   2   1  1 103   1
## 15 135             Russia NOT_ROOT NOT_MIG     TIP 136  NA NA  NA  NA
## 16 136               <NA> NOT_ROOT NOT_MIG NOT_TIP  32 135  1  76   4
## 17 171      Eastern_China NOT_ROOT NOT_MIG     TIP 172  NA NA  NA  NA
## 18 172               <NA> NOT_ROOT NOT_MIG NOT_TIP  52  16  7 171   1
## 19 211 Sulawesi_Indonesia NOT_ROOT NOT_MIG     TIP 213  NA NA  NA  NA
## 20 212               <NA> NOT_ROOT NOT_MIG NOT_TIP 213  51  1  52   9
## 21 213               <NA>     ROOT NOT_MIG NOT_TIP 213 211  1 212  10
## 22 217               <NA> NOT_ROOT     MIG NOT_TIP 136  76 NA  NA  NA
##                                                                                                                                                                                                                                                                                                                                                                       V11
## 1                                                                                                                                                                                                                                                                                                                                                          Italy:0.011679
## 2                                                                                                                                                                                                                                                                                      ((Italy:0.011679,Northeast_US:0.000981699):0.00263898,Texas:0.0019019):0.000210042
## 3                                                                                                                                                                                                                                                                                                                                                   California:0.00774031
## 4                                                                                                                                                                                                                                                                                                                                                        Japan:0.00727026
## 5                                                                                                                                                                                                                                                                                                                                                Okinawa_Taiwan:0.0137794
## 6                                                                                                                                                  (California:0.00774031,(Okinawa_Taiwan:0.0137794,(Russia:0.0198777,(((Italy:0.011679,Northeast_US:0.000981699):0.00263898,Texas:0.0019019):0.000210042,Japan:0.00727026):0.00788444):0.00209508):0.0025636):0.00215994
## 7                                                                                                                                                                                                                                                                                                                                                 Athens_Greece:0.0195533
## 8                                                                                                                                                                                     (Okinawa_Taiwan:0.0137794,(Russia:0.0198777,(((Italy:0.011679,Northeast_US:0.000981699):0.00263898,Texas:0.0019019):0.000210042,Japan:0.00727026):0.00788444):0.00209508):0.0025636
## 9                                                                                                                                                                                                                                                                                                                                               Southeast_Asia:0.00631183
## 10                                                                     (((California:0.00774031,(Okinawa_Taiwan:0.0137794,(Russia:0.0198777,(((Italy:0.011679,Northeast_US:0.000981699):0.00263898,Texas:0.0019019):0.000210042,Japan:0.00727026):0.00788444):0.00209508):0.0025636):0.00215994,Eastern_China:0.000285662):0.00569165,Athens_Greece:0.0195533):0.00418375
## 11                                                                                                                                                                                                                                                                                                                                                        Texas:0.0019019
## 12                                                                                                                                                                                                                                                       (((Italy:0.011679,Northeast_US:0.000981699):0.00263898,Texas:0.0019019):0.000210042,Japan:0.00727026):0.00788444
## 13                                                                                                                                                                                                                                                                                                                                               Northeast_US:0.000981699
## 14                                                                                                                                                                                                                                                                                                                   (Italy:0.011679,Northeast_US:0.000981699):0.00263898
## 15                                                                                                                                                                                                                                                                                                                                                       Russia:0.0198777
## 16                                                                                                                                                                                                                         (Russia:0.0198777,(((Italy:0.011679,Northeast_US:0.000981699):0.00263898,Texas:0.0019019):0.000210042,Japan:0.00727026):0.00788444):0.00209508
## 17                                                                                                                                                                                                                                                                                                                                              Eastern_China:0.000285662
## 18                                                                                                          ((California:0.00774031,(Okinawa_Taiwan:0.0137794,(Russia:0.0198777,(((Italy:0.011679,Northeast_US:0.000981699):0.00263898,Texas:0.0019019):0.000210042,Japan:0.00727026):0.00788444):0.00209508):0.0025636):0.00215994,Eastern_China:0.000285662):0.00569165
## 19                                                                                                                                                                                                                                                                                                                                            Sulawesi_Indonesia:0.018019
## 20                                (Southeast_Asia:0.00631183,(((California:0.00774031,(Okinawa_Taiwan:0.0137794,(Russia:0.0198777,(((Italy:0.011679,Northeast_US:0.000981699):0.00263898,Texas:0.0019019):0.000210042,Japan:0.00727026):0.00788444):0.00209508):0.0025636):0.00215994,Eastern_China:0.000285662):0.00569165,Athens_Greece:0.0195533):0.00418375):0.018019
## 21 (Sulawesi_Indonesia:0.018019,(Southeast_Asia:0.00631183,(((California:0.00774031,(Okinawa_Taiwan:0.0137794,(Russia:0.0198777,(((Italy:0.011679,Northeast_US:0.000981699):0.00263898,Texas:0.0019019):0.000210042,Japan:0.00727026):0.00788444):0.00209508):0.0025636):0.00215994,Eastern_China:0.000285662):0.00569165,Athens_Greece:0.0195533):0.00418375):0.018019);
## 22                                                                                                                                                                                                                                                                                                                                                                   <NA>
##             x          y       ymin       ymax
## 1  0.05712548 0.50000000 0.45454545 0.54545455
## 2  0.04280750 0.36363636 0.27272727 0.54545455
## 3  0.03779465 0.77272727 0.72727273 0.81818182
## 4  0.04986772 0.22727273 0.18181818 0.27272727
## 5  0.04639734 0.68181818 0.63636364 0.72727273
## 6  0.03005434 0.72727273 0.18181818 0.81818182
## 7  0.03035495 0.04545455 0.00000000 0.09090909
## 8  0.03261794 0.63636364 0.18181818 0.72727273
## 9  0.02433083 0.86363636 0.81818182 0.90909091
## 10 0.02220275 0.09090909 0.00000000 0.81818182
## 11 0.04470940 0.31818182 0.27272727 0.36363636
## 12 0.04259746 0.27272727 0.18181818 0.54545455
## 13 0.04642818 0.40909091 0.36363636 0.45454545
## 14 0.04544648 0.45454545 0.36363636 0.54545455
## 15 0.05459072 0.59090909 0.54545455 0.63636364
## 16 0.03471302 0.54545455 0.18181818 0.63636364
## 17 0.02818006 0.13636364 0.09090909 0.18181818
## 18 0.02789440 0.18181818 0.09090909 0.81818182
## 19 0.01801900 0.95454545 0.90909091 1.00000000
## 20 0.01801900 0.81818182 0.00000000 0.90909091
## 21 0.00000000 0.90909091 0.00000000 1.00000000
## 22 0.03609588 0.49762064 0.18181818 0.54545455
## 
## $e
##     V1  V2          V3       V4      V5       V6       V7
## 1   16   3 0.007740310 1.000000 NOT_MIG 0.000000 1.000000
## 2   32  15 0.013779400 1.000000 NOT_MIG 0.000000 1.000000
## 3   76   2 0.000210042 1.000000 NOT_MIG 0.000000 1.000000
## 4    2 104 0.002638980 1.000000 NOT_MIG 0.000000 1.000000
## 5  104   1 0.011679000 1.000000 NOT_MIG 0.000000 1.000000
## 6  104 103 0.000981699 1.000000 NOT_MIG 0.000000 1.000000
## 7   16  32 0.002563600 1.000000 NOT_MIG 0.000000 1.000000
## 8   32 136 0.002095080 1.000000 NOT_MIG 0.000000 1.000000
## 9  136 135 0.019877700 1.000000 NOT_MIG 0.000000 1.000000
## 10   2  75 0.001901900 1.000000 NOT_MIG 0.000000 1.000000
## 11  52 172 0.005691650 1.000000 NOT_MIG 0.000000 1.000000
## 12 172  16 0.002159940 1.000000 NOT_MIG 0.000000 1.000000
## 13 172 171 0.000285662 1.000000 NOT_MIG 0.000000 1.000000
## 14 212  51 0.006311830 1.000000 NOT_MIG 0.000000 1.000000
## 15 213 211 0.018019000 1.000000 NOT_MIG 0.000000 1.000000
## 16 213 212 0.018019000 1.000000 NOT_MIG 0.000000 1.000000
## 17  52  31 0.008152202 0.645695 NOT_MIG 0.000000 0.416922
## 18  76   4 0.007270260 1.000000 NOT_MIG 0.000000 1.000000
## 19 212  52 0.004183750 1.000000 NOT_MIG 0.000000 1.000000
## 20 136 217 0.001382860 1.000000 NOT_MIG 0.000000 1.000000
## 21 217  76 0.006501580 1.000000 NOT_MIG 0.175391 1.000000
## 22 217  31 0.000000000 0.354305     MIG 0.175391 0.000000
```

Save it

```
setwd("/gpfs/gibbs/pi/caccone/mkc54/albo/manuscript1/output/treemix_subsets/north_america_simplified/simplified_test/prelim")

# Open PDF
pdf("treemix_prelim_m1.pdf", width = 12, height = 8)

plot_tree("nasub_simp.m1.rep2")

dev.off()
```

Check for m=2

```
setwd("/gpfs/gibbs/pi/caccone/mkc54/albo/manuscript1/output/treemix_subsets/north_america_simplified/simplified_test/prelim")
plot_tree("nasub_simp.m2.rep2")
```

```
## Warning in plot_tree("nasub_simp.m2.rep2"): NAs introduced by coercion
## Warning in plot_tree("nasub_simp.m2.rep2"): NAs introduced by coercion
## Warning in plot_tree("nasub_simp.m2.rep2"): NAs introduced by coercion
## Warning in plot_tree("nasub_simp.m2.rep2"): NAs introduced by coercion
```

```
##     V1                 V2       V3      V4      V5  V6  V7 V8  V9 V10
## 1    1     Southeast_Asia NOT_ROOT NOT_MIG     TIP 104  NA NA  NA  NA
## 2    2               <NA> NOT_ROOT NOT_MIG NOT_TIP  52  15  1  16   2
## 3    3              Italy NOT_ROOT NOT_MIG     TIP  16  NA NA  NA  NA
## 4    4              Japan NOT_ROOT NOT_MIG     TIP  52  NA NA  NA  NA
## 5   15       Northeast_US NOT_ROOT NOT_MIG     TIP   2  NA NA  NA  NA
## 6   16               <NA> NOT_ROOT NOT_MIG NOT_TIP   2   3  1  51   1
## 7   31      Eastern_China NOT_ROOT NOT_MIG     TIP  32  NA NA  NA  NA
## 8   32               <NA> NOT_ROOT NOT_MIG NOT_TIP 136  31  1 212   7
## 9   51              Texas NOT_ROOT NOT_MIG     TIP  16  NA NA  NA  NA
## 10  52               <NA> NOT_ROOT NOT_MIG NOT_TIP  76   2  3   4   1
## 11  75             Russia NOT_ROOT NOT_MIG     TIP  76  NA NA  NA  NA
## 12  76               <NA> NOT_ROOT NOT_MIG NOT_TIP 172  75  1  52   4
## 13 103 Sulawesi_Indonesia NOT_ROOT NOT_MIG     TIP 213  NA NA  NA  NA
## 14 104               <NA> NOT_ROOT NOT_MIG NOT_TIP 213   1  1 136   9
## 15 135      Athens_Greece NOT_ROOT NOT_MIG     TIP 136  NA NA  NA  NA
## 16 136               <NA> NOT_ROOT NOT_MIG NOT_TIP 104 135  1  32   8
## 17 171     Okinawa_Taiwan NOT_ROOT NOT_MIG     TIP 172  NA NA  NA  NA
## 18 172               <NA> NOT_ROOT NOT_MIG NOT_TIP 212 171  1  76   5
## 19 211         California NOT_ROOT NOT_MIG     TIP 212  NA NA  NA  NA
## 20 212               <NA> NOT_ROOT NOT_MIG NOT_TIP  32 211  1 172   6
## 21 213               <NA>     ROOT NOT_MIG NOT_TIP 213 103  1 104  10
## 22 224               <NA> NOT_ROOT     MIG NOT_TIP  76  52 NA  NA  NA
## 23 269               <NA> NOT_ROOT     MIG NOT_TIP 212 172 NA  NA  NA
##                                                                                                                                                                                                                                                                                                                                                                         V11
## 1                                                                                                                                                                                                                                                                                                                                                 Southeast_Asia:0.00631183
## 2                                                                                                                                                                                                                                                                                      (Northeast_US:0.000554788,(Italy:0.00978441,Texas:0.00859993):0.00173882):0.00224092
## 3                                                                                                                                                                                                                                                                                                                                                          Italy:0.00978441
## 4                                                                                                                                                                                                                                                                                                                                                          Japan:0.00606759
## 5                                                                                                                                                                                                                                                                                                                                                  Northeast_US:0.000554788
## 6                                                                                                                                                                                                                                                                                                                            (Italy:0.00978441,Texas:0.00859993):0.00173882
## 7                                                                                                                                                                                                                                                                                                                                                 Eastern_China:0.000318175
## 8                                                                                                           (Eastern_China:0.000318175,(California:0.00774773,(Okinawa_Taiwan:0.0136561,(Russia:0.0195661,((Northeast_US:0.000554788,(Italy:0.00978441,Texas:0.00859993):0.00173882):0.00224092,Japan:0.00606759):0.00885809):0.00244078):0.00263578):0.0021316):0.00534473
## 9                                                                                                                                                                                                                                                                                                                                                          Texas:0.00859993
## 10                                                                                                                                                                                                                                                       ((Northeast_US:0.000554788,(Italy:0.00978441,Texas:0.00859993):0.00173882):0.00224092,Japan:0.00606759):0.00885809
## 11                                                                                                                                                                                                                                                                                                                                                         Russia:0.0195661
## 12                                                                                                                                                                                                                         (Russia:0.0195661,((Northeast_US:0.000554788,(Italy:0.00978441,Texas:0.00859993):0.00173882):0.00224092,Japan:0.00606759):0.00885809):0.00244078
## 13                                                                                                                                                                                                                                                                                                                                              Sulawesi_Indonesia:0.018019
## 14                                (Southeast_Asia:0.00631183,(Athens_Greece:0.0176195,(Eastern_China:0.000318175,(California:0.00774773,(Okinawa_Taiwan:0.0136561,(Russia:0.0195661,((Northeast_US:0.000554788,(Italy:0.00978441,Texas:0.00859993):0.00173882):0.00224092,Japan:0.00606759):0.00885809):0.00244078):0.00263578):0.0021316):0.00534473):0.00455171):0.018019
## 15                                                                                                                                                                                                                                                                                                                                                  Athens_Greece:0.0176195
## 16                                                                     (Athens_Greece:0.0176195,(Eastern_China:0.000318175,(California:0.00774773,(Okinawa_Taiwan:0.0136561,(Russia:0.0195661,((Northeast_US:0.000554788,(Italy:0.00978441,Texas:0.00859993):0.00173882):0.00224092,Japan:0.00606759):0.00885809):0.00244078):0.00263578):0.0021316):0.00534473):0.00455171
## 17                                                                                                                                                                                                                                                                                                                                                 Okinawa_Taiwan:0.0136561
## 18                                                                                                                                                                                   (Okinawa_Taiwan:0.0136561,(Russia:0.0195661,((Northeast_US:0.000554788,(Italy:0.00978441,Texas:0.00859993):0.00173882):0.00224092,Japan:0.00606759):0.00885809):0.00244078):0.00263578
## 19                                                                                                                                                                                                                                                                                                                                                    California:0.00774773
## 20                                                                                                                                                 (California:0.00774773,(Okinawa_Taiwan:0.0136561,(Russia:0.0195661,((Northeast_US:0.000554788,(Italy:0.00978441,Texas:0.00859993):0.00173882):0.00224092,Japan:0.00606759):0.00885809):0.00244078):0.00263578):0.0021316
## 21 (Sulawesi_Indonesia:0.018019,(Southeast_Asia:0.00631183,(Athens_Greece:0.0176195,(Eastern_China:0.000318175,(California:0.00774773,(Okinawa_Taiwan:0.0136561,(Russia:0.0195661,((Northeast_US:0.000554788,(Italy:0.00978441,Texas:0.00859993):0.00173882):0.00224092,Japan:0.00606759):0.00885809):0.00244078):0.00263578):0.0021316):0.00534473):0.00455171):0.018019);
## 22                                                                                                                                                                                                                                                                                                                                                                     <NA>
## 23                                                                                                                                                                                                                                                                                                                                                                     <NA>
##             x          y       ymin       ymax
## 1  0.02433083 0.86363636 0.81818182 0.90909091
## 2  0.04622261 0.27272727 0.09090909 0.36363636
## 3  0.05774584 0.22727273 0.18181818 0.27272727
## 4  0.05004928 0.04545455 0.00000000 0.09090909
## 5  0.04677740 0.31818182 0.27272727 0.36363636
## 6  0.04796143 0.18181818 0.09090909 0.27272727
## 7  0.02823362 0.68181818 0.63636364 0.72727273
## 8  0.02791544 0.63636364 0.00000000 0.72727273
## 9  0.05219696 0.13636364 0.09090909 0.18181818
## 10 0.04398169 0.09090909 0.00000000 0.36363636
## 11 0.05468970 0.40909091 0.36363636 0.45454545
## 12 0.03512360 0.36363636 0.00000000 0.45454545
## 13 0.01801900 0.95454545 0.90909091 1.00000000
## 14 0.01801900 0.81818182 0.00000000 0.90909091
## 15 0.03057518 0.77272727 0.72727273 0.81818182
## 16 0.02257071 0.72727273 0.00000000 0.81818182
## 17 0.04633892 0.50000000 0.45454545 0.54545455
## 18 0.03268282 0.45454545 0.00000000 0.54545455
## 19 0.03779477 0.59090909 0.54545455 0.63636364
## 20 0.03004704 0.54545455 0.00000000 0.63636364
## 21 0.00000000 0.90909091 0.00000000 1.00000000
## 22 0.03690991         NA 0.00000000 0.36363636
## 23 0.03075329         NA 0.00000000 0.54545455
## [1] "0.201659 0.0351236 0.04398169"
## [1] "0.267947 0.03004704 0.03268282"
```

```
##  [1] 0.02433083 0.05774584 0.05004928 0.04677740 0.02823362 0.05219696
##  [7] 0.05468970 0.01801900 0.03057518 0.04633892 0.03779477
## [1] 0.003
## [1] "mse 0.000348772561983471"
```

```
## $d
##     V1                 V2       V3      V4      V5  V6  V7 V8  V9 V10
## 1    1     Southeast_Asia NOT_ROOT NOT_MIG     TIP 104  NA NA  NA  NA
## 2    2               <NA> NOT_ROOT NOT_MIG NOT_TIP  52  15  1  16   2
## 3    3              Italy NOT_ROOT NOT_MIG     TIP  16  NA NA  NA  NA
## 4    4              Japan NOT_ROOT NOT_MIG     TIP  52  NA NA  NA  NA
## 5   15       Northeast_US NOT_ROOT NOT_MIG     TIP   2  NA NA  NA  NA
## 6   16               <NA> NOT_ROOT NOT_MIG NOT_TIP   2   3  1  51   1
## 7   31      Eastern_China NOT_ROOT NOT_MIG     TIP  32  NA NA  NA  NA
## 8   32               <NA> NOT_ROOT NOT_MIG NOT_TIP 136  31  1 212   7
## 9   51              Texas NOT_ROOT NOT_MIG     TIP  16  NA NA  NA  NA
## 10  52               <NA> NOT_ROOT NOT_MIG NOT_TIP  76   2  3   4   1
## 11  75             Russia NOT_ROOT NOT_MIG     TIP  76  NA NA  NA  NA
## 12  76               <NA> NOT_ROOT NOT_MIG NOT_TIP 172  75  1  52   4
## 13 103 Sulawesi_Indonesia NOT_ROOT NOT_MIG     TIP 213  NA NA  NA  NA
## 14 104               <NA> NOT_ROOT NOT_MIG NOT_TIP 213   1  1 136   9
## 15 135      Athens_Greece NOT_ROOT NOT_MIG     TIP 136  NA NA  NA  NA
## 16 136               <NA> NOT_ROOT NOT_MIG NOT_TIP 104 135  1  32   8
## 17 171     Okinawa_Taiwan NOT_ROOT NOT_MIG     TIP 172  NA NA  NA  NA
## 18 172               <NA> NOT_ROOT NOT_MIG NOT_TIP 212 171  1  76   5
## 19 211         California NOT_ROOT NOT_MIG     TIP 212  NA NA  NA  NA
## 20 212               <NA> NOT_ROOT NOT_MIG NOT_TIP  32 211  1 172   6
## 21 213               <NA>     ROOT NOT_MIG NOT_TIP 213 103  1 104  10
## 22 224               <NA> NOT_ROOT     MIG NOT_TIP  76  52 NA  NA  NA
## 23 269               <NA> NOT_ROOT     MIG NOT_TIP 212 172 NA  NA  NA
##                                                                                                                                                                                                                                                                                                                                                                         V11
## 1                                                                                                                                                                                                                                                                                                                                                 Southeast_Asia:0.00631183
## 2                                                                                                                                                                                                                                                                                      (Northeast_US:0.000554788,(Italy:0.00978441,Texas:0.00859993):0.00173882):0.00224092
## 3                                                                                                                                                                                                                                                                                                                                                          Italy:0.00978441
## 4                                                                                                                                                                                                                                                                                                                                                          Japan:0.00606759
## 5                                                                                                                                                                                                                                                                                                                                                  Northeast_US:0.000554788
## 6                                                                                                                                                                                                                                                                                                                            (Italy:0.00978441,Texas:0.00859993):0.00173882
## 7                                                                                                                                                                                                                                                                                                                                                 Eastern_China:0.000318175
## 8                                                                                                           (Eastern_China:0.000318175,(California:0.00774773,(Okinawa_Taiwan:0.0136561,(Russia:0.0195661,((Northeast_US:0.000554788,(Italy:0.00978441,Texas:0.00859993):0.00173882):0.00224092,Japan:0.00606759):0.00885809):0.00244078):0.00263578):0.0021316):0.00534473
## 9                                                                                                                                                                                                                                                                                                                                                          Texas:0.00859993
## 10                                                                                                                                                                                                                                                       ((Northeast_US:0.000554788,(Italy:0.00978441,Texas:0.00859993):0.00173882):0.00224092,Japan:0.00606759):0.00885809
## 11                                                                                                                                                                                                                                                                                                                                                         Russia:0.0195661
## 12                                                                                                                                                                                                                         (Russia:0.0195661,((Northeast_US:0.000554788,(Italy:0.00978441,Texas:0.00859993):0.00173882):0.00224092,Japan:0.00606759):0.00885809):0.00244078
## 13                                                                                                                                                                                                                                                                                                                                              Sulawesi_Indonesia:0.018019
## 14                                (Southeast_Asia:0.00631183,(Athens_Greece:0.0176195,(Eastern_China:0.000318175,(California:0.00774773,(Okinawa_Taiwan:0.0136561,(Russia:0.0195661,((Northeast_US:0.000554788,(Italy:0.00978441,Texas:0.00859993):0.00173882):0.00224092,Japan:0.00606759):0.00885809):0.00244078):0.00263578):0.0021316):0.00534473):0.00455171):0.018019
## 15                                                                                                                                                                                                                                                                                                                                                  Athens_Greece:0.0176195
## 16                                                                     (Athens_Greece:0.0176195,(Eastern_China:0.000318175,(California:0.00774773,(Okinawa_Taiwan:0.0136561,(Russia:0.0195661,((Northeast_US:0.000554788,(Italy:0.00978441,Texas:0.00859993):0.00173882):0.00224092,Japan:0.00606759):0.00885809):0.00244078):0.00263578):0.0021316):0.00534473):0.00455171
## 17                                                                                                                                                                                                                                                                                                                                                 Okinawa_Taiwan:0.0136561
## 18                                                                                                                                                                                   (Okinawa_Taiwan:0.0136561,(Russia:0.0195661,((Northeast_US:0.000554788,(Italy:0.00978441,Texas:0.00859993):0.00173882):0.00224092,Japan:0.00606759):0.00885809):0.00244078):0.00263578
## 19                                                                                                                                                                                                                                                                                                                                                    California:0.00774773
## 20                                                                                                                                                 (California:0.00774773,(Okinawa_Taiwan:0.0136561,(Russia:0.0195661,((Northeast_US:0.000554788,(Italy:0.00978441,Texas:0.00859993):0.00173882):0.00224092,Japan:0.00606759):0.00885809):0.00244078):0.00263578):0.0021316
## 21 (Sulawesi_Indonesia:0.018019,(Southeast_Asia:0.00631183,(Athens_Greece:0.0176195,(Eastern_China:0.000318175,(California:0.00774773,(Okinawa_Taiwan:0.0136561,(Russia:0.0195661,((Northeast_US:0.000554788,(Italy:0.00978441,Texas:0.00859993):0.00173882):0.00224092,Japan:0.00606759):0.00885809):0.00244078):0.00263578):0.0021316):0.00534473):0.00455171):0.018019);
## 22                                                                                                                                                                                                                                                                                                                                                                     <NA>
## 23                                                                                                                                                                                                                                                                                                                                                                     <NA>
##             x          y       ymin       ymax
## 1  0.02433083 0.86363636 0.81818182 0.90909091
## 2  0.04622261 0.27272727 0.09090909 0.36363636
## 3  0.05774584 0.22727273 0.18181818 0.27272727
## 4  0.05004928 0.04545455 0.00000000 0.09090909
## 5  0.04677740 0.31818182 0.27272727 0.36363636
## 6  0.04796143 0.18181818 0.09090909 0.27272727
## 7  0.02823362 0.68181818 0.63636364 0.72727273
## 8  0.02791544 0.63636364 0.00000000 0.72727273
## 9  0.05219696 0.13636364 0.09090909 0.18181818
## 10 0.04398169 0.09090909 0.00000000 0.36363636
## 11 0.05468970 0.40909091 0.36363636 0.45454545
## 12 0.03512360 0.36363636 0.00000000 0.45454545
## 13 0.01801900 0.95454545 0.90909091 1.00000000
## 14 0.01801900 0.81818182 0.00000000 0.90909091
## 15 0.03057518 0.77272727 0.72727273 0.81818182
## 16 0.02257071 0.72727273 0.00000000 0.81818182
## 17 0.04633892 0.50000000 0.45454545 0.54545455
## 18 0.03268282 0.45454545 0.00000000 0.54545455
## 19 0.03779477 0.59090909 0.54545455 0.63636364
## 20 0.03004704 0.54545455 0.00000000 0.63636364
## 21 0.00000000 0.90909091 0.00000000 1.00000000
## 22 0.03690991 0.30863845 0.00000000 0.36363636
## 23 0.03075329 0.52109573 0.00000000 0.54545455
## 
## $e
##     V1  V2          V3       V4      V5       V6       V7
## 1   76  75 0.019566100 1.000000 NOT_MIG 0.000000 1.000000
## 2   32  31 0.000318175 1.000000 NOT_MIG 0.000000 1.000000
## 3  172 171 0.013656100 1.000000 NOT_MIG 0.000000 1.000000
## 4  213 103 0.018019000 1.000000 NOT_MIG 0.000000 1.000000
## 5  213 104 0.018019000 1.000000 NOT_MIG 0.000000 1.000000
## 6  136 135 0.008004472 0.674015 NOT_MIG 0.000000 0.454296
## 7   52   2 0.002240920 1.000000 NOT_MIG 0.000000 1.000000
## 8   16   3 0.009784410 1.000000 NOT_MIG 0.000000 1.000000
## 9   52   4 0.006067590 1.000000 NOT_MIG 0.000000 1.000000
## 10 104   1 0.006311830 1.000000 NOT_MIG 0.000000 1.000000
## 11 104 136 0.004551710 1.000000 NOT_MIG 0.000000 1.000000
## 12 172  76 0.002440780 1.000000 NOT_MIG 0.000000 1.000000
## 13  76 224 0.001786310 1.000000 NOT_MIG 0.000000 1.000000
## 14 224  52 0.007071780 1.000000 NOT_MIG 0.201659 1.000000
## 15 224 135 0.000000000 0.325985     MIG 0.201659 0.000000
## 16 136  32 0.005344730 1.000000 NOT_MIG 0.000000 1.000000
## 17  32 212 0.002131600 1.000000 NOT_MIG 0.000000 1.000000
## 18   2  15 0.000554788 1.000000 NOT_MIG 0.000000 1.000000
## 19   2  16 0.001738820 1.000000 NOT_MIG 0.000000 1.000000
## 20  16  51 0.004235533 0.701789 NOT_MIG 0.000000 0.492508
## 21 212 211 0.007747730 1.000000 NOT_MIG 0.000000 1.000000
## 22 212 269 0.000706250 1.000000 NOT_MIG 0.000000 1.000000
## 23 269 172 0.001929530 1.000000 NOT_MIG 0.267947 1.000000
## 24 269  51 0.000000000 0.298211     MIG 0.267947 0.000000
```

```
setwd("/gpfs/gibbs/pi/caccone/mkc54/albo/manuscript1/output/treemix_subsets/north_america_simplified/simplified_test/prelim")

# Open PDF
pdf("treemix_prelim_m2.pdf", width = 12, height = 8)

plot_tree("nasub_simp.m2.rep2")

dev.off()
```

Check for m=3

```
setwd("/gpfs/gibbs/pi/caccone/mkc54/albo/manuscript1/output/treemix_subsets/north_america_simplified/simplified_test/prelim")
plot_tree("nasub_simp.m3.rep2")
```

```
## Warning in plot_tree("nasub_simp.m3.rep2"): NAs introduced by coercion
## Warning in plot_tree("nasub_simp.m3.rep2"): NAs introduced by coercion
## Warning in plot_tree("nasub_simp.m3.rep2"): NAs introduced by coercion
## Warning in plot_tree("nasub_simp.m3.rep2"): NAs introduced by coercion
```

```
##     V1                 V2       V3      V4      V5  V6  V7 V8  V9 V10
## 1    1       Northeast_US NOT_ROOT NOT_MIG     TIP  52  NA NA  NA  NA
## 2    2               <NA> NOT_ROOT NOT_MIG NOT_TIP 172  52  3   4   1
## 3    3      Athens_Greece NOT_ROOT NOT_MIG     TIP  16  NA NA  NA  NA
## 4    4              Japan NOT_ROOT NOT_MIG     TIP   2  NA NA  NA  NA
## 5   15 Sulawesi_Indonesia NOT_ROOT NOT_MIG     TIP 213  NA NA  NA  NA
## 6   16               <NA> NOT_ROOT NOT_MIG NOT_TIP 104   3  1  32   8
## 7   31      Eastern_China NOT_ROOT NOT_MIG     TIP  32  NA NA  NA  NA
## 8   32               <NA> NOT_ROOT NOT_MIG NOT_TIP  16 136  7  31   1
## 9   51              Texas NOT_ROOT NOT_MIG     TIP 212  NA NA  NA  NA
## 10  52               <NA> NOT_ROOT NOT_MIG NOT_TIP   2   1  1 212   2
## 11  75     Okinawa_Taiwan NOT_ROOT NOT_MIG     TIP  76  NA NA  NA  NA
## 12  76               <NA> NOT_ROOT NOT_MIG NOT_TIP 136  75  1 172   5
## 13 103     Southeast_Asia NOT_ROOT NOT_MIG     TIP 104  NA NA  NA  NA
## 14 104               <NA> NOT_ROOT NOT_MIG NOT_TIP 213 103  1  16   9
## 15 135         California NOT_ROOT NOT_MIG     TIP 136  NA NA  NA  NA
## 16 136               <NA> NOT_ROOT NOT_MIG NOT_TIP  32  76  6 135   1
## 17 171             Russia NOT_ROOT NOT_MIG     TIP 172  NA NA  NA  NA
## 18 172               <NA> NOT_ROOT NOT_MIG NOT_TIP  76 171  1   2   4
## 19 211              Italy NOT_ROOT NOT_MIG     TIP 212  NA NA  NA  NA
## 20 212               <NA> NOT_ROOT NOT_MIG NOT_TIP  52 211  1  51   1
## 21 213               <NA>     ROOT NOT_MIG NOT_TIP 213  15  1 104  10
## 22 269               <NA> NOT_ROOT     MIG NOT_TIP 136  76 NA  NA  NA
## 23 221               <NA> NOT_ROOT     MIG NOT_TIP  76 172 NA  NA  NA
## 24 359               <NA> NOT_ROOT     MIG NOT_TIP  32  31 NA  NA  NA
##                                                                                                                                                                                                                                                                                                                                                                         V11
## 1                                                                                                                                                                                                                                                                                                                                                  Northeast_US:0.000541981
## 2                                                                                                                                                                                                                                                         ((Northeast_US:0.000541981,(Italy:0.00971613,Texas:0.00883488):0.00179955):0.00224858,Japan:0.0060648):0.00564838
## 3                                                                                                                                                                                                                                                                                                                                                   Athens_Greece:0.0197113
## 4                                                                                                                                                                                                                                                                                                                                                           Japan:0.0060648
## 5                                                                                                                                                                                                                                                                                                                                               Sulawesi_Indonesia:0.018019
## 6                                                                      (Athens_Greece:0.0197113,(((Okinawa_Taiwan:0.0133686,(Russia:0.0501506,((Northeast_US:0.000541981,(Italy:0.00971613,Texas:0.00883488):0.00179955):0.00224858,Japan:0.0060648):0.00564838):0.00542495):0.00294073,California:0.00760474):0.00229985,Eastern_China:0.000280432):0.00575292):0.00410321
## 7                                                                                                                                                                                                                                                                                                                                                 Eastern_China:0.000280432
## 8                                                                                                           (((Okinawa_Taiwan:0.0133686,(Russia:0.0501506,((Northeast_US:0.000541981,(Italy:0.00971613,Texas:0.00883488):0.00179955):0.00224858,Japan:0.0060648):0.00564838):0.00542495):0.00294073,California:0.00760474):0.00229985,Eastern_China:0.000280432):0.00575292
## 9                                                                                                                                                                                                                                                                                                                                                          Texas:0.00883488
## 10                                                                                                                                                                                                                                                                                     (Northeast_US:0.000541981,(Italy:0.00971613,Texas:0.00883488):0.00179955):0.00224858
## 11                                                                                                                                                                                                                                                                                                                                                 Okinawa_Taiwan:0.0133686
## 12                                                                                                                                                                                    (Okinawa_Taiwan:0.0133686,(Russia:0.0501506,((Northeast_US:0.000541981,(Italy:0.00971613,Texas:0.00883488):0.00179955):0.00224858,Japan:0.0060648):0.00564838):0.00542495):0.00294073
## 13                                                                                                                                                                                                                                                                                                                                                Southeast_Asia:0.00631183
## 14                                (Southeast_Asia:0.00631183,(Athens_Greece:0.0197113,(((Okinawa_Taiwan:0.0133686,(Russia:0.0501506,((Northeast_US:0.000541981,(Italy:0.00971613,Texas:0.00883488):0.00179955):0.00224858,Japan:0.0060648):0.00564838):0.00542495):0.00294073,California:0.00760474):0.00229985,Eastern_China:0.000280432):0.00575292):0.00410321):0.018019
## 15                                                                                                                                                                                                                                                                                                                                                    California:0.00760474
## 16                                                                                                                                                 ((Okinawa_Taiwan:0.0133686,(Russia:0.0501506,((Northeast_US:0.000541981,(Italy:0.00971613,Texas:0.00883488):0.00179955):0.00224858,Japan:0.0060648):0.00564838):0.00542495):0.00294073,California:0.00760474):0.00229985
## 17                                                                                                                                                                                                                                                                                                                                                         Russia:0.0501506
## 18                                                                                                                                                                                                                          (Russia:0.0501506,((Northeast_US:0.000541981,(Italy:0.00971613,Texas:0.00883488):0.00179955):0.00224858,Japan:0.0060648):0.00564838):0.00542495
## 19                                                                                                                                                                                                                                                                                                                                                         Italy:0.00971613
## 20                                                                                                                                                                                                                                                                                                                           (Italy:0.00971613,Texas:0.00883488):0.00179955
## 21 (Sulawesi_Indonesia:0.018019,(Southeast_Asia:0.00631183,(Athens_Greece:0.0197113,(((Okinawa_Taiwan:0.0133686,(Russia:0.0501506,((Northeast_US:0.000541981,(Italy:0.00971613,Texas:0.00883488):0.00179955):0.00224858,Japan:0.0060648):0.00564838):0.00542495):0.00294073,California:0.00760474):0.00229985,Eastern_China:0.000280432):0.00575292):0.00410321):0.018019);
## 22                                                                                                                                                                                                                                                                                                                                                                     <NA>
## 23                                                                                                                                                                                                                                                                                                                                                                     <NA>
## 24                                                                                                                                                                                                                                                                                                                                                                     <NA>
##             x          y       ymin       ymax
## 1  0.04697959 0.50000000 0.45454545 0.54545455
## 2  0.04418903 0.27272727 0.18181818 0.54545455
## 3  0.03023127 0.77272727 0.72727273 0.81818182
## 4  0.05025383 0.22727273 0.18181818 0.27272727
## 5  0.01801900 0.95454545 0.90909091 1.00000000
## 6  0.02212221 0.72727273 0.00000000 0.81818182
## 7  0.02815556 0.04545455 0.00000000 0.09090909
## 8  0.02787513 0.09090909 0.00000000 0.72727273
## 9  0.05245883 0.31818182 0.27272727 0.36363636
## 10 0.04643761 0.45454545 0.27272727 0.54545455
## 11 0.04648431 0.68181818 0.63636364 0.72727273
## 12 0.03311571 0.63636364 0.18181818 0.72727273
## 13 0.02433083 0.86363636 0.81818182 0.90909091
## 14 0.01801900 0.81818182 0.00000000 0.90909091
## 15 0.03777972 0.13636364 0.09090909 0.18181818
## 16 0.03017498 0.18181818 0.09090909 0.72727273
## 17 0.05995283 0.59090909 0.54545455 0.63636364
## 18 0.03854065 0.54545455 0.18181818 0.63636364
## 19 0.05795329 0.40909091 0.36363636 0.45454545
## 20 0.04823716 0.36363636 0.27272727 0.45454545
## 21 0.00000000 0.90909091 0.00000000 1.00000000
## 22 0.03132090         NA 0.09090909 0.18181818
## 23 0.03664618         NA 0.18181818 0.63636364
## 24 0.02804465         NA 0.00000000 0.09090909
## [1] "0.389672 0.03017498 0.03311571"
## [1] "0.650785 0.03311571 0.03854065"
## [1] "0.604486 0.02787513 0.028155562"
```

```
##  [1] 0.04697959 0.03023127 0.05025383 0.01801900 0.02815556 0.05245883
##  [7] 0.04648431 0.02433083 0.03777972 0.05995283 0.05795329
## [1] 0.003
## [1] "mse 0.000348772561983471"
```

```
## $d
##     V1                 V2       V3      V4      V5  V6  V7 V8  V9 V10
## 1    1       Northeast_US NOT_ROOT NOT_MIG     TIP  52  NA NA  NA  NA
## 2    2               <NA> NOT_ROOT NOT_MIG NOT_TIP 172  52  3   4   1
## 3    3      Athens_Greece NOT_ROOT NOT_MIG     TIP  16  NA NA  NA  NA
## 4    4              Japan NOT_ROOT NOT_MIG     TIP   2  NA NA  NA  NA
## 5   15 Sulawesi_Indonesia NOT_ROOT NOT_MIG     TIP 213  NA NA  NA  NA
## 6   16               <NA> NOT_ROOT NOT_MIG NOT_TIP 104   3  1  32   8
## 7   31      Eastern_China NOT_ROOT NOT_MIG     TIP  32  NA NA  NA  NA
## 8   32               <NA> NOT_ROOT NOT_MIG NOT_TIP  16 136  7  31   1
## 9   51              Texas NOT_ROOT NOT_MIG     TIP 212  NA NA  NA  NA
## 10  52               <NA> NOT_ROOT NOT_MIG NOT_TIP   2   1  1 212   2
## 11  75     Okinawa_Taiwan NOT_ROOT NOT_MIG     TIP  76  NA NA  NA  NA
## 12  76               <NA> NOT_ROOT NOT_MIG NOT_TIP 136  75  1 172   5
## 13 103     Southeast_Asia NOT_ROOT NOT_MIG     TIP 104  NA NA  NA  NA
## 14 104               <NA> NOT_ROOT NOT_MIG NOT_TIP 213 103  1  16   9
## 15 135         California NOT_ROOT NOT_MIG     TIP 136  NA NA  NA  NA
## 16 136               <NA> NOT_ROOT NOT_MIG NOT_TIP  32  76  6 135   1
## 17 171             Russia NOT_ROOT NOT_MIG     TIP 172  NA NA  NA  NA
## 18 172               <NA> NOT_ROOT NOT_MIG NOT_TIP  76 171  1   2   4
## 19 211              Italy NOT_ROOT NOT_MIG     TIP 212  NA NA  NA  NA
## 20 212               <NA> NOT_ROOT NOT_MIG NOT_TIP  52 211  1  51   1
## 21 213               <NA>     ROOT NOT_MIG NOT_TIP 213  15  1 104  10
## 22 269               <NA> NOT_ROOT     MIG NOT_TIP 136  76 NA  NA  NA
## 23 221               <NA> NOT_ROOT     MIG NOT_TIP  76 172 NA  NA  NA
## 24 359               <NA> NOT_ROOT     MIG NOT_TIP  32  31 NA  NA  NA
##                                                                                                                                                                                                                                                                                                                                                                         V11
## 1                                                                                                                                                                                                                                                                                                                                                  Northeast_US:0.000541981
## 2                                                                                                                                                                                                                                                         ((Northeast_US:0.000541981,(Italy:0.00971613,Texas:0.00883488):0.00179955):0.00224858,Japan:0.0060648):0.00564838
## 3                                                                                                                                                                                                                                                                                                                                                   Athens_Greece:0.0197113
## 4                                                                                                                                                                                                                                                                                                                                                           Japan:0.0060648
## 5                                                                                                                                                                                                                                                                                                                                               Sulawesi_Indonesia:0.018019
## 6                                                                      (Athens_Greece:0.0197113,(((Okinawa_Taiwan:0.0133686,(Russia:0.0501506,((Northeast_US:0.000541981,(Italy:0.00971613,Texas:0.00883488):0.00179955):0.00224858,Japan:0.0060648):0.00564838):0.00542495):0.00294073,California:0.00760474):0.00229985,Eastern_China:0.000280432):0.00575292):0.00410321
## 7                                                                                                                                                                                                                                                                                                                                                 Eastern_China:0.000280432
## 8                                                                                                           (((Okinawa_Taiwan:0.0133686,(Russia:0.0501506,((Northeast_US:0.000541981,(Italy:0.00971613,Texas:0.00883488):0.00179955):0.00224858,Japan:0.0060648):0.00564838):0.00542495):0.00294073,California:0.00760474):0.00229985,Eastern_China:0.000280432):0.00575292
## 9                                                                                                                                                                                                                                                                                                                                                          Texas:0.00883488
## 10                                                                                                                                                                                                                                                                                     (Northeast_US:0.000541981,(Italy:0.00971613,Texas:0.00883488):0.00179955):0.00224858
## 11                                                                                                                                                                                                                                                                                                                                                 Okinawa_Taiwan:0.0133686
## 12                                                                                                                                                                                    (Okinawa_Taiwan:0.0133686,(Russia:0.0501506,((Northeast_US:0.000541981,(Italy:0.00971613,Texas:0.00883488):0.00179955):0.00224858,Japan:0.0060648):0.00564838):0.00542495):0.00294073
## 13                                                                                                                                                                                                                                                                                                                                                Southeast_Asia:0.00631183
## 14                                (Southeast_Asia:0.00631183,(Athens_Greece:0.0197113,(((Okinawa_Taiwan:0.0133686,(Russia:0.0501506,((Northeast_US:0.000541981,(Italy:0.00971613,Texas:0.00883488):0.00179955):0.00224858,Japan:0.0060648):0.00564838):0.00542495):0.00294073,California:0.00760474):0.00229985,Eastern_China:0.000280432):0.00575292):0.00410321):0.018019
## 15                                                                                                                                                                                                                                                                                                                                                    California:0.00760474
## 16                                                                                                                                                 ((Okinawa_Taiwan:0.0133686,(Russia:0.0501506,((Northeast_US:0.000541981,(Italy:0.00971613,Texas:0.00883488):0.00179955):0.00224858,Japan:0.0060648):0.00564838):0.00542495):0.00294073,California:0.00760474):0.00229985
## 17                                                                                                                                                                                                                                                                                                                                                         Russia:0.0501506
## 18                                                                                                                                                                                                                          (Russia:0.0501506,((Northeast_US:0.000541981,(Italy:0.00971613,Texas:0.00883488):0.00179955):0.00224858,Japan:0.0060648):0.00564838):0.00542495
## 19                                                                                                                                                                                                                                                                                                                                                         Italy:0.00971613
## 20                                                                                                                                                                                                                                                                                                                           (Italy:0.00971613,Texas:0.00883488):0.00179955
## 21 (Sulawesi_Indonesia:0.018019,(Southeast_Asia:0.00631183,(Athens_Greece:0.0197113,(((Okinawa_Taiwan:0.0133686,(Russia:0.0501506,((Northeast_US:0.000541981,(Italy:0.00971613,Texas:0.00883488):0.00179955):0.00224858,Japan:0.0060648):0.00564838):0.00542495):0.00294073,California:0.00760474):0.00229985,Eastern_China:0.000280432):0.00575292):0.00410321):0.018019);
## 22                                                                                                                                                                                                                                                                                                                                                                     <NA>
## 23                                                                                                                                                                                                                                                                                                                                                                     <NA>
## 24                                                                                                                                                                                                                                                                                                                                                                     <NA>
##             x          y       ymin       ymax
## 1  0.04697959 0.50000000 0.45454545 0.54545455
## 2  0.04418903 0.27272727 0.18181818 0.54545455
## 3  0.03023127 0.77272727 0.72727273 0.81818182
## 4  0.05025383 0.22727273 0.18181818 0.27272727
## 5  0.01801900 0.95454545 0.90909091 1.00000000
## 6  0.02212221 0.72727273 0.00000000 0.81818182
## 7  0.02815556 0.04545455 0.00000000 0.09090909
## 8  0.02787513 0.09090909 0.00000000 0.72727273
## 9  0.05245883 0.31818182 0.27272727 0.36363636
## 10 0.04643761 0.45454545 0.27272727 0.54545455
## 11 0.04648431 0.68181818 0.63636364 0.72727273
## 12 0.03311571 0.63636364 0.18181818 0.72727273
## 13 0.02433083 0.86363636 0.81818182 0.90909091
## 14 0.01801900 0.81818182 0.00000000 0.90909091
## 15 0.03777972 0.13636364 0.09090909 0.18181818
## 16 0.03017498 0.18181818 0.09090909 0.72727273
## 17 0.05995283 0.59090909 0.54545455 0.63636364
## 18 0.03854065 0.54545455 0.18181818 0.63636364
## 19 0.05795329 0.40909091 0.36363636 0.45454545
## 20 0.04823716 0.36363636 0.27272727 0.45454545
## 21 0.00000000 0.90909091 0.00000000 1.00000000
## 22 0.03132090 0.35894182 0.09090909 0.18181818
## 23 0.03664618 0.57720136 0.18181818 0.63636364
## 24 0.02804465 0.06343245 0.00000000 0.09090909
## 
## $e
##     V1  V2          V3       V4      V5       V6       V7
## 1   76  75 0.013368600 1.000000 NOT_MIG 0.000000 1.000000
## 2  172 171 0.021412185 0.653420 NOT_MIG 0.000000 0.426958
## 3  213  15 0.018019000 1.000000 NOT_MIG 0.000000 1.000000
## 4  213 104 0.018019000 1.000000 NOT_MIG 0.000000 1.000000
## 5  212 211 0.009716130 1.000000 NOT_MIG 0.000000 1.000000
## 6    2  52 0.002248580 1.000000 NOT_MIG 0.000000 1.000000
## 7  104 103 0.006311830 1.000000 NOT_MIG 0.000000 1.000000
## 8   52   1 0.000541981 1.000000 NOT_MIG 0.000000 1.000000
## 9   52 212 0.001799550 1.000000 NOT_MIG 0.000000 1.000000
## 10 212  51 0.004221675 0.691261 NOT_MIG 0.000000 0.477841
## 11 136 269 0.001145920 1.000000 NOT_MIG 0.000000 1.000000
## 12 269  76 0.001794810 1.000000 NOT_MIG 0.389672 1.000000
## 13 269  51 0.000000000 0.308739     MIG 0.389672 0.000000
## 14  16   3 0.008109059 0.641398 NOT_MIG 0.000000 0.411391
## 15 104  16 0.004103210 1.000000 NOT_MIG 0.000000 1.000000
## 16 136 135 0.007604740 1.000000 NOT_MIG 0.000000 1.000000
## 17   2   4 0.006064800 1.000000 NOT_MIG 0.000000 1.000000
## 18  16  32 0.005752920 1.000000 NOT_MIG 0.000000 1.000000
## 19 172   2 0.005648380 1.000000 NOT_MIG 0.000000 1.000000
## 20  76 221 0.003530470 1.000000 NOT_MIG 0.000000 1.000000
## 21 221 172 0.001894470 1.000000 NOT_MIG 0.650785 1.000000
## 22 221   3 0.000000000 0.358602     MIG 0.650785 0.000000
## 23  32 136 0.002299850 1.000000 NOT_MIG 0.000000 1.000000
## 24  32 359 0.000169517 1.000000 NOT_MIG 0.000000 1.000000
## 25 359  31 0.000110915 1.000000 NOT_MIG 0.604486 1.000000
## 26 359 171 0.000000000 0.346580     MIG 0.604486 0.000000
```

```
setwd("/gpfs/gibbs/pi/caccone/mkc54/albo/manuscript1/output/treemix_subsets/north_america_simplified/simplified_test/prelim")

# Open PDF
pdf("treemix_prelim_m3.pdf", width = 12, height = 8)

plot_tree("nasub_simp.m3.rep2")

dev.off()
```

Check for m=4

```
setwd("/gpfs/gibbs/pi/caccone/mkc54/albo/manuscript1/output/treemix_subsets/north_america_simplified/simplified_test/prelim")
plot_tree("nasub_simp.m4.rep4")
```

```
## Warning in plot_tree("nasub_simp.m4.rep4"): NAs introduced by coercion
## Warning in plot_tree("nasub_simp.m4.rep4"): NAs introduced by coercion
## Warning in plot_tree("nasub_simp.m4.rep4"): NAs introduced by coercion
## Warning in plot_tree("nasub_simp.m4.rep4"): NAs introduced by coercion
```

```
##     V1                 V2       V3      V4      V5  V6  V7 V8  V9 V10
## 1    1              Texas NOT_ROOT NOT_MIG     TIP 212  NA NA  NA  NA
## 2    2               <NA> NOT_ROOT NOT_MIG NOT_TIP 213   4  1  16   9
## 3    3 Sulawesi_Indonesia NOT_ROOT NOT_MIG     TIP 213  NA NA  NA  NA
## 4    4     Southeast_Asia NOT_ROOT NOT_MIG     TIP   2  NA NA  NA  NA
## 5   15      Athens_Greece NOT_ROOT NOT_MIG     TIP  16  NA NA  NA  NA
## 6   16               <NA> NOT_ROOT NOT_MIG NOT_TIP   2  15  1 443   8
## 7   31         California NOT_ROOT NOT_MIG     TIP 443  NA NA  NA  NA
## 8   32               <NA> NOT_ROOT NOT_MIG NOT_TIP  76 135  1 104   5
## 9   51       Northeast_US NOT_ROOT NOT_MIG     TIP  52  NA NA  NA  NA
## 10  52               <NA> NOT_ROOT NOT_MIG NOT_TIP 172  51  1 212   2
## 11  75      Eastern_China NOT_ROOT NOT_MIG     TIP  76  NA NA  NA  NA
## 12  76               <NA> NOT_ROOT NOT_MIG NOT_TIP 443  32  6  75   1
## 13 103             Russia NOT_ROOT NOT_MIG     TIP 104  NA NA  NA  NA
## 14 104               <NA> NOT_ROOT NOT_MIG NOT_TIP  32 103  1 172   4
## 15 135     Okinawa_Taiwan NOT_ROOT NOT_MIG     TIP  32  NA NA  NA  NA
## 16 136               <NA> NOT_ROOT     MIG NOT_TIP  32 104 NA  NA  NA
## 17 171              Japan NOT_ROOT NOT_MIG     TIP 172  NA NA  NA  NA
## 18 172               <NA> NOT_ROOT NOT_MIG NOT_TIP 104  52  3 171   1
## 19 211              Italy NOT_ROOT NOT_MIG     TIP 212  NA NA  NA  NA
## 20 212               <NA> NOT_ROOT NOT_MIG NOT_TIP  52 211  1   1   1
## 21 213               <NA>     ROOT NOT_MIG NOT_TIP 213   3  1   2  10
## 22 270               <NA> NOT_ROOT     MIG NOT_TIP  76  32 NA  NA  NA
## 23 443               <NA> NOT_ROOT NOT_MIG NOT_TIP  16  31  1  76   7
## 24 216               <NA> NOT_ROOT     MIG NOT_TIP  32 104 NA  NA  NA
## 25 359               <NA> NOT_ROOT     MIG NOT_TIP 443  76 NA  NA  NA
##                                                                                                                                                                                                                                                                                                                                                                V11
## 1                                                                                                                                                                                                                                                                                                                                                 Texas:0.00873751
## 2                                  (Southeast_Asia:0.00631201,(Athens_Greece:0.0221407,(California:0.0365741,((Okinawa_Taiwan:0.0130486,(Russia:0.0499766,((Northeast_US:0.000496528,(Italy:0.00961371,Texas:0.00873751):0.00186965):0.00227104,Japan:0.0060305):0.00551229):0.00585313):0.00512749,Eastern_China:0):0.00301495):0.00373597):0.00335599):0.0180191
## 3                                                                                                                                                                                                                                                                                                                                     Sulawesi_Indonesia:0.0180191
## 4                                                                                                                                                                                                                                                                                                                                        Southeast_Asia:0.00631201
## 5                                                                                                                                                                                                                                                                                                                                          Athens_Greece:0.0221407
## 6                                                                        (Athens_Greece:0.0221407,(California:0.0365741,((Okinawa_Taiwan:0.0130486,(Russia:0.0499766,((Northeast_US:0.000496528,(Italy:0.00961371,Texas:0.00873751):0.00186965):0.00227104,Japan:0.0060305):0.00551229):0.00585313):0.00512749,Eastern_China:0):0.00301495):0.00373597):0.00335599
## 7                                                                                                                                                                                                                                                                                                                                             California:0.0365741
## 8                                                                                                                                                                            (Okinawa_Taiwan:0.0130486,(Russia:0.0499766,((Northeast_US:0.000496528,(Italy:0.00961371,Texas:0.00873751):0.00186965):0.00227104,Japan:0.0060305):0.00551229):0.00585313):0.00512749
## 9                                                                                                                                                                                                                                                                                                                                         Northeast_US:0.000496528
## 10                                                                                                                                                                                                                                                                            (Northeast_US:0.000496528,(Italy:0.00961371,Texas:0.00873751):0.00186965):0.00227104
## 11                                                                                                                                                                                                                                                                                                                                                 Eastern_China:0
## 12                                                                                                                                              ((Okinawa_Taiwan:0.0130486,(Russia:0.0499766,((Northeast_US:0.000496528,(Italy:0.00961371,Texas:0.00873751):0.00186965):0.00227104,Japan:0.0060305):0.00551229):0.00585313):0.00512749,Eastern_China:0):0.00301495
## 13                                                                                                                                                                                                                                                                                                                                                Russia:0.0499766
## 14                                                                                                                                                                                                                 (Russia:0.0499766,((Northeast_US:0.000496528,(Italy:0.00961371,Texas:0.00873751):0.00186965):0.00227104,Japan:0.0060305):0.00551229):0.00585313
## 15                                                                                                                                                                                                                                                                                                                                        Okinawa_Taiwan:0.0130486
## 16                                                                                                                                                                                                                                                                                                                                                            <NA>
## 17                                                                                                                                                                                                                                                                                                                                                 Japan:0.0060305
## 18                                                                                                                                                                                                                                               ((Northeast_US:0.000496528,(Italy:0.00961371,Texas:0.00873751):0.00186965):0.00227104,Japan:0.0060305):0.00551229
## 19                                                                                                                                                                                                                                                                                                                                                Italy:0.00961371
## 20                                                                                                                                                                                                                                                                                                                  (Italy:0.00961371,Texas:0.00873751):0.00186965
## 21 (Sulawesi_Indonesia:0.0180191,(Southeast_Asia:0.00631201,(Athens_Greece:0.0221407,(California:0.0365741,((Okinawa_Taiwan:0.0130486,(Russia:0.0499766,((Northeast_US:0.000496528,(Italy:0.00961371,Texas:0.00873751):0.00186965):0.00227104,Japan:0.0060305):0.00551229):0.00585313):0.00512749,Eastern_China:0):0.00301495):0.00373597):0.00335599):0.0180191);
## 22                                                                                                                                                                                                                                                                                                                                                            <NA>
## 23                                                                                                            (California:0.0365741,((Okinawa_Taiwan:0.0130486,(Russia:0.0499766,((Northeast_US:0.000496528,(Italy:0.00961371,Texas:0.00873751):0.00186965):0.00227104,Japan:0.0060305):0.00551229):0.00585313):0.00512749,Eastern_China:0):0.00301495):0.00373597
## 24                                                                                                                                                                                                                                                                                                                                                            <NA>
## 25                                                                                                                                                                                                                                                                                                                                                            <NA>
##             x          y       ymin       ymax
## 1  0.05300360 0.22727273 0.18181818 0.27272727
## 2  0.01801910 0.81818182 0.00000000 0.90909091
## 3  0.01801910 0.95454545 0.90909091 1.00000000
## 4  0.02433111 0.86363636 0.81818182 0.90909091
## 5  0.02972411 0.77272727 0.72727273 0.81818182
## 6  0.02137509 0.72727273 0.00000000 0.81818182
## 7  0.03447416 0.68181818 0.63636364 0.72727273
## 8  0.03325350 0.54545455 0.09090909 0.63636364
## 9  0.04738648 0.40909091 0.36363636 0.45454545
## 10 0.04688995 0.36363636 0.18181818 0.45454545
## 11 0.02812601 0.04545455 0.00000000 0.09090909
## 12 0.02812601 0.09090909 0.00000000 0.63636364
## 13 0.06054842 0.50000000 0.45454545 0.54545455
## 14 0.03910662 0.45454545 0.09090909 0.54545455
## 15 0.04630210 0.59090909 0.54545455 0.63636364
## 16 0.03559389         NA 0.09090909 0.54545455
## 17 0.05064941 0.13636364 0.09090909 0.18181818
## 18 0.04461891 0.18181818 0.09090909 0.45454545
## 19 0.05837331 0.31818182 0.27272727 0.36363636
## 20 0.04875960 0.27272727 0.18181818 0.36363636
## 21 0.00000000 0.90909091 0.00000000 1.00000000
## 22 0.03032773         NA 0.00000000 0.09090909
## 23 0.02511106 0.63636364 0.00000000 0.72727273
## 24 0.03910662         NA 0.09090909 0.54545455
## 25 0.02717369         NA 0.00000000 0.63636364
## [1] "0.399853 0.033253499 0.039106619"
## [1] "0.429396 0.028126009 0.033253499"
## [1] "0.695147 0.033253499 0.039106619"
## [1] "0.684134 0.02511106 0.028126009"
```

```
##  [1] 0.05300360 0.01801910 0.02433111 0.02972411 0.03447416 0.04738648
##  [7] 0.02812601 0.06054842 0.04630210 0.05064941 0.05837331
## [1] 0.003
## [1] "mse 0.000348772561983471"
```

```
## $d
##     V1                 V2       V3      V4      V5  V6  V7 V8  V9 V10
## 1    1              Texas NOT_ROOT NOT_MIG     TIP 212  NA NA  NA  NA
## 2    2               <NA> NOT_ROOT NOT_MIG NOT_TIP 213   4  1  16   9
## 3    3 Sulawesi_Indonesia NOT_ROOT NOT_MIG     TIP 213  NA NA  NA  NA
## 4    4     Southeast_Asia NOT_ROOT NOT_MIG     TIP   2  NA NA  NA  NA
## 5   15      Athens_Greece NOT_ROOT NOT_MIG     TIP  16  NA NA  NA  NA
## 6   16               <NA> NOT_ROOT NOT_MIG NOT_TIP   2  15  1 443   8
## 7   31         California NOT_ROOT NOT_MIG     TIP 443  NA NA  NA  NA
## 8   32               <NA> NOT_ROOT NOT_MIG NOT_TIP  76 135  1 104   5
## 9   51       Northeast_US NOT_ROOT NOT_MIG     TIP  52  NA NA  NA  NA
## 10  52               <NA> NOT_ROOT NOT_MIG NOT_TIP 172  51  1 212   2
## 11  75      Eastern_China NOT_ROOT NOT_MIG     TIP  76  NA NA  NA  NA
## 12  76               <NA> NOT_ROOT NOT_MIG NOT_TIP 443  32  6  75   1
## 13 103             Russia NOT_ROOT NOT_MIG     TIP 104  NA NA  NA  NA
## 14 104               <NA> NOT_ROOT NOT_MIG NOT_TIP  32 103  1 172   4
## 15 135     Okinawa_Taiwan NOT_ROOT NOT_MIG     TIP  32  NA NA  NA  NA
## 16 136               <NA> NOT_ROOT     MIG NOT_TIP  32 104 NA  NA  NA
## 17 171              Japan NOT_ROOT NOT_MIG     TIP 172  NA NA  NA  NA
## 18 172               <NA> NOT_ROOT NOT_MIG NOT_TIP 104  52  3 171   1
## 19 211              Italy NOT_ROOT NOT_MIG     TIP 212  NA NA  NA  NA
## 20 212               <NA> NOT_ROOT NOT_MIG NOT_TIP  52 211  1   1   1
## 21 213               <NA>     ROOT NOT_MIG NOT_TIP 213   3  1   2  10
## 22 270               <NA> NOT_ROOT     MIG NOT_TIP  76  32 NA  NA  NA
## 23 443               <NA> NOT_ROOT NOT_MIG NOT_TIP  16  31  1  76   7
## 24 216               <NA> NOT_ROOT     MIG NOT_TIP  32 104 NA  NA  NA
## 25 359               <NA> NOT_ROOT     MIG NOT_TIP 443  76 NA  NA  NA
##                                                                                                                                                                                                                                                                                                                                                                V11
## 1                                                                                                                                                                                                                                                                                                                                                 Texas:0.00873751
## 2                                  (Southeast_Asia:0.00631201,(Athens_Greece:0.0221407,(California:0.0365741,((Okinawa_Taiwan:0.0130486,(Russia:0.0499766,((Northeast_US:0.000496528,(Italy:0.00961371,Texas:0.00873751):0.00186965):0.00227104,Japan:0.0060305):0.00551229):0.00585313):0.00512749,Eastern_China:0):0.00301495):0.00373597):0.00335599):0.0180191
## 3                                                                                                                                                                                                                                                                                                                                     Sulawesi_Indonesia:0.0180191
## 4                                                                                                                                                                                                                                                                                                                                        Southeast_Asia:0.00631201
## 5                                                                                                                                                                                                                                                                                                                                          Athens_Greece:0.0221407
## 6                                                                        (Athens_Greece:0.0221407,(California:0.0365741,((Okinawa_Taiwan:0.0130486,(Russia:0.0499766,((Northeast_US:0.000496528,(Italy:0.00961371,Texas:0.00873751):0.00186965):0.00227104,Japan:0.0060305):0.00551229):0.00585313):0.00512749,Eastern_China:0):0.00301495):0.00373597):0.00335599
## 7                                                                                                                                                                                                                                                                                                                                             California:0.0365741
## 8                                                                                                                                                                            (Okinawa_Taiwan:0.0130486,(Russia:0.0499766,((Northeast_US:0.000496528,(Italy:0.00961371,Texas:0.00873751):0.00186965):0.00227104,Japan:0.0060305):0.00551229):0.00585313):0.00512749
## 9                                                                                                                                                                                                                                                                                                                                         Northeast_US:0.000496528
## 10                                                                                                                                                                                                                                                                            (Northeast_US:0.000496528,(Italy:0.00961371,Texas:0.00873751):0.00186965):0.00227104
## 11                                                                                                                                                                                                                                                                                                                                                 Eastern_China:0
## 12                                                                                                                                              ((Okinawa_Taiwan:0.0130486,(Russia:0.0499766,((Northeast_US:0.000496528,(Italy:0.00961371,Texas:0.00873751):0.00186965):0.00227104,Japan:0.0060305):0.00551229):0.00585313):0.00512749,Eastern_China:0):0.00301495
## 13                                                                                                                                                                                                                                                                                                                                                Russia:0.0499766
## 14                                                                                                                                                                                                                 (Russia:0.0499766,((Northeast_US:0.000496528,(Italy:0.00961371,Texas:0.00873751):0.00186965):0.00227104,Japan:0.0060305):0.00551229):0.00585313
## 15                                                                                                                                                                                                                                                                                                                                        Okinawa_Taiwan:0.0130486
## 16                                                                                                                                                                                                                                                                                                                                                            <NA>
## 17                                                                                                                                                                                                                                                                                                                                                 Japan:0.0060305
## 18                                                                                                                                                                                                                                               ((Northeast_US:0.000496528,(Italy:0.00961371,Texas:0.00873751):0.00186965):0.00227104,Japan:0.0060305):0.00551229
## 19                                                                                                                                                                                                                                                                                                                                                Italy:0.00961371
## 20                                                                                                                                                                                                                                                                                                                  (Italy:0.00961371,Texas:0.00873751):0.00186965
## 21 (Sulawesi_Indonesia:0.0180191,(Southeast_Asia:0.00631201,(Athens_Greece:0.0221407,(California:0.0365741,((Okinawa_Taiwan:0.0130486,(Russia:0.0499766,((Northeast_US:0.000496528,(Italy:0.00961371,Texas:0.00873751):0.00186965):0.00227104,Japan:0.0060305):0.00551229):0.00585313):0.00512749,Eastern_China:0):0.00301495):0.00373597):0.00335599):0.0180191);
## 22                                                                                                                                                                                                                                                                                                                                                            <NA>
## 23                                                                                                            (California:0.0365741,((Okinawa_Taiwan:0.0130486,(Russia:0.0499766,((Northeast_US:0.000496528,(Italy:0.00961371,Texas:0.00873751):0.00186965):0.00227104,Japan:0.0060305):0.00551229):0.00585313):0.00512749,Eastern_China:0):0.00301495):0.00373597
## 24                                                                                                                                                                                                                                                                                                                                                            <NA>
## 25                                                                                                                                                                                                                                                                                                                                                            <NA>
##             x          y       ymin       ymax
## 1  0.05300360 0.22727273 0.18181818 0.27272727
## 2  0.01801910 0.81818182 0.00000000 0.90909091
## 3  0.01801910 0.95454545 0.90909091 1.00000000
## 4  0.02433111 0.86363636 0.81818182 0.90909091
## 5  0.02972411 0.77272727 0.72727273 0.81818182
## 6  0.02137509 0.72727273 0.00000000 0.81818182
## 7  0.03447416 0.68181818 0.63636364 0.72727273
## 8  0.03325350 0.54545455 0.09090909 0.63636364
## 9  0.04738648 0.40909091 0.36363636 0.45454545
## 10 0.04688995 0.36363636 0.18181818 0.45454545
## 11 0.02812601 0.04545455 0.00000000 0.09090909
## 12 0.02812601 0.09090909 0.00000000 0.63636364
## 13 0.06054842 0.50000000 0.45454545 0.54545455
## 14 0.03910662 0.45454545 0.09090909 0.54545455
## 15 0.04630210 0.59090909 0.54545455 0.63636364
## 16 0.03559389 0.50910427 0.09090909 0.54545455
## 17 0.05064941 0.13636364 0.09090909 0.18181818
## 18 0.04461891 0.18181818 0.09090909 0.45454545
## 19 0.05837331 0.31818182 0.27272727 0.36363636
## 20 0.04875960 0.27272727 0.18181818 0.36363636
## 21 0.00000000 0.90909091 0.00000000 1.00000000
## 22 0.03032773 0.28608909 0.00000000 0.09090909
## 23 0.02511106 0.63636364 0.00000000 0.72727273
## 24 0.03732228 0.48225936 0.09090909 0.54545455
## 25 0.02717369 0.26319964 0.00000000 0.63636364
## 
## $e
##     V1  V2          V3       V4      V5       V6       V7
## 1  104 103 0.021441800 0.655009 NOT_MIG 0.000000 0.429037
## 2  213   3 0.018019100 1.000000 NOT_MIG 0.000000 1.000000
## 3  213   2 0.018019100 1.000000 NOT_MIG 0.000000 1.000000
## 4  172  52 0.002271040 1.000000 NOT_MIG 0.000000 1.000000
## 5  212 211 0.009613710 1.000000 NOT_MIG 0.000000 1.000000
## 6    2   4 0.006312010 1.000000 NOT_MIG 0.000000 1.000000
## 7   52  51 0.000496528 1.000000 NOT_MIG 0.000000 1.000000
## 8   52 212 0.001869650 1.000000 NOT_MIG 0.000000 1.000000
## 9  212   1 0.004244006 0.696938 NOT_MIG 0.000000 0.485722
## 10 172 171 0.006030500 1.000000 NOT_MIG 0.000000 1.000000
## 11  76 270 0.002201720 1.000000 NOT_MIG 0.000000 1.000000
## 12 270  32 0.002925770 1.000000 NOT_MIG 0.429396 1.000000
## 13 270   1 0.000000000 0.303062     MIG 0.429396 0.000000
## 14   2  16 0.003355990 1.000000 NOT_MIG 0.000000 1.000000
## 15  32 135 0.013048600 1.000000 NOT_MIG 0.000000 1.000000
## 16  32 136 0.002340390 1.000000 NOT_MIG 0.000000 1.000000
## 17 136  31 0.000000000 0.494032     MIG 0.399853 0.000000
## 18  16  15 0.008349022 0.614076 NOT_MIG 0.000000 0.377089
## 19 443  31 0.009363102 0.505968 NOT_MIG 0.000000 0.256004
## 20  16 443 0.003735970 1.000000 NOT_MIG 0.000000 1.000000
## 21 104 172 0.005512290 1.000000 NOT_MIG 0.000000 1.000000
## 22 136 216 0.001728390 1.000000 NOT_MIG 0.399853 1.000000
## 23 216 104 0.001784340 1.000000 NOT_MIG 0.695147 1.000000
## 24 216  15 0.000000000 0.385924     MIG 0.695147 0.000000
## 25  76  75 0.000000000 1.000000 NOT_MIG 0.000000 1.000000
## 26 443 359 0.002062630 1.000000 NOT_MIG 0.000000 1.000000
## 27 359  76 0.000952319 1.000000 NOT_MIG 0.684134 1.000000
## 28 359 103 0.000000000 0.344991     MIG 0.684134 0.000000
```

```
setwd("/gpfs/gibbs/pi/caccone/mkc54/albo/manuscript1/output/treemix_subsets/north_america_simplified/simplified_test/prelim")

# Open PDF
pdf("treemix_prelim_m4.pdf", width = 12, height = 8)

plot_tree("nasub_simp.m4.rep4")

dev.off()
```

### 7.1.5 For migration events with 100 bootstraps

Use modified python wrapper used in Norah’s paper This uses the
pipeline ML tree → bootreps → combine → sumtrees

Uploaded to hpc as treemix\_tree\_with\_bootreps\_NPS.py It is the same
code used in the Saarman paper, but modified for python 3

```
module load dSQ/1.05 
module load TreeMix/1.13-GCC-10.2.0
```

It takes too long to run interactively, so save as .sh file as submit
via slurm treemix\_boot.sh

```
#!/bin/bash
#SBATCH --job-name=treemix_boot
#SBATCH --time=7-00:00:00           
#SBATCH --mem=40G                
#SBATCH --cpus-per-task=20         
#SBATCH --mail-type ALL
#SBATCH --partition=week        # need >24 hours

module load miniconda
conda activate dendro

# Load modules
module load TreeMix/1.13-GCC-10.2.0
module load Python/3.10.8-GCCcore-12.2.0

# Move to working directory
cd /gpfs/gibbs/pi/caccone/mkc54/albo/manuscript1/output/treemix_subsets/north_america_simplified/simplified_test


# Run Python script
python treemix_tree_with_bootreps_NPS_corrected.py \
  r2_0.1_b_north_america_subset_simplified.treemix.frq \
  4 \
  Sulawesi_Indonesia \
  results/ \
  100 \
  500

echo "Finished TreeMix bootstrap run at $(date)"
```

```
sbatch treemix_boot.sh #63102673
```

Set source

```
# Load TreeMix plotting functions
source("/gpfs/gibbs/pi/caccone/mkc54/albo/manuscript1/output/treemix/north_america/plotting_funcs_fixed.R")  # Adjust this path as needed
```

#### 7.1.5.1 Plot with R

Check cat\_trees.tre

```
salloc -p day --cpus-per-task=10 -t 24:00:00 --mem-per-cpu=10000

file /gpfs/gibbs/pi/caccone/mkc54/albo/manuscript1/output/treemix_subsets/north_america_simplified/simplified_test/results/cat_trees.tre
head -3 /gpfs/gibbs/pi/caccone/mkc54/albo/manuscript1/output/treemix_subsets/north_america_simplified/simplified_test/results/cat_trees.tre
#It has extra lines

#Remove metadata lines
#grep '(' /gpfs/gibbs/pi/caccone/mkc54/albo/manuscript1/output/treemix_subsets/north_america_simplified/simplified_test/results/cat_trees.tre > cat_trees_clean.tre
grep '^\s*(' /gpfs/gibbs/pi/caccone/mkc54/albo/manuscript1/output/treemix_subsets/north_america_simplified/simplified_test/results/cat_trees.tre > cat_trees_clean.tre
```

Clean cat\_trees

```
# Read the file line-by-line
lines <- readLines("/gpfs/gibbs/pi/caccone/mkc54/albo/manuscript1/output/treemix_subsets/north_america_simplified/simplified_test/results/cat_trees_clean.tre")

# Remove empty lines and whitespace
lines <- trimws(lines)
lines <- lines[nzchar(lines)]

# Try to parse each line as a tree
trees <- lapply(lines, function(x) {
  tryCatch(read.tree(text = x), error = function(e) NULL)
})

# Remove any failed parses
trees <- Filter(Negate(is.null), trees)

length(trees)  # number of successfully parsed trees
```

```
## [1] 100
```

Get Consensus tree

```
cons_tree <- consensus(trees, p = 0.5)
```

Plot consensus tree

```
plot(cons_tree, main = "TreeMix Bootstrap Consensus Tree")
```

Plot bootstrap support

```
# Load ape package
library(ape)

# Read all bootstrap trees
trees <- read.tree("/gpfs/gibbs/pi/caccone/mkc54/albo/manuscript1/output/treemix_subsets/north_america_simplified/simplified_test/results/cat_trees_clean.tre")

# Compute majority-rule consensus (branches present in >=50% of trees)
cons <- consensus(trees, p = 0.5)

# Compute bootstrap support values for each node
bs <- prop.clades(cons, trees)

# Plot consensus tree with bootstrap support
plot(cons, show.node.label = TRUE, cex = 0.8)
title("Consensus Tree with Bootstrap Support")
```

Convert support values to %

```
if(!is.null(cons$node.label)){
  cons$node.label <- as.numeric(cons$node.label) * 100
}
plot(cons, show.node.label = TRUE, cex = 0.7)
```

Save

```
pdf("/gpfs/gibbs/pi/caccone/mkc54/albo/manuscript1/output/treemix_subsets/north_america_simplified/simplified_test/results/consensus_tree.pdf", width=10, height=8)
plot(cons, show.node.label = TRUE, cex = 0.8)
dev.off()
```

#### 7.1.5.2 Plot with Treemix functions

Set source

```
# Load TreeMix plotting functions
source("/gpfs/gibbs/pi/caccone/mkc54/albo/manuscript1/output/treemix/plotting_funcs.R")  # Adjust this path as needed
```

Plot the maximum-likelihood tree structure, and the migration arrows
with their weights:

```
setwd("/gpfs/gibbs/pi/caccone/mkc54/albo/manuscript1/output/treemix_subsets/north_america_simplified/simplified_test/results")

plot_tree("_stem")
```

```
## Warning in plot_tree("_stem"): NAs introduced by coercion
## Warning in plot_tree("_stem"): NAs introduced by coercion
## Warning in plot_tree("_stem"): NAs introduced by coercion
## Warning in plot_tree("_stem"): NAs introduced by coercion
```

```
##     V1                 V2       V3      V4      V5  V6  V7 V8  V9 V10
## 1    0               <NA> NOT_ROOT NOT_MIG NOT_TIP  76  75  1 212   6
## 2    1      Athens_Greece NOT_ROOT NOT_MIG     TIP   2  NA NA  NA  NA
## 3    2               <NA> NOT_ROOT NOT_MIG NOT_TIP 104  76  8   1   1
## 4    3 Sulawesi_Indonesia NOT_ROOT NOT_MIG     TIP 213  NA NA  NA  NA
## 5    4         California NOT_ROOT NOT_MIG     TIP  76  NA NA  NA  NA
## 6   15              Texas NOT_ROOT NOT_MIG     TIP  32  NA NA  NA  NA
## 7   31              Italy NOT_ROOT NOT_MIG     TIP 172  NA NA  NA  NA
## 8   32               <NA> NOT_ROOT NOT_MIG NOT_TIP  52 136  3  15   1
## 9   51             Russia NOT_ROOT NOT_MIG     TIP  52  NA NA  NA  NA
## 10  52               <NA> NOT_ROOT NOT_MIG NOT_TIP 212  51  1  32   4
## 11  75      Eastern_China NOT_ROOT NOT_MIG     TIP   0  NA NA  NA  NA
## 12  76               <NA> NOT_ROOT NOT_MIG NOT_TIP   2   0  7   4   1
## 13 103     Southeast_Asia NOT_ROOT NOT_MIG     TIP 104  NA NA  NA  NA
## 14 104               <NA> NOT_ROOT NOT_MIG NOT_TIP 213 103  1   2   9
## 15 135              Japan NOT_ROOT NOT_MIG     TIP 172  NA NA  NA  NA
## 16 136               <NA> NOT_ROOT NOT_MIG NOT_TIP  32 172  2 171   1
## 17 171       Northeast_US NOT_ROOT NOT_MIG     TIP 136  NA NA  NA  NA
## 18 172               <NA> NOT_ROOT NOT_MIG NOT_TIP 136 135  1  31   1
## 19 211     Okinawa_Taiwan NOT_ROOT NOT_MIG     TIP 212  NA NA  NA  NA
## 20 212               <NA> NOT_ROOT NOT_MIG NOT_TIP   0 211  1  52   5
## 21 213               <NA>     ROOT NOT_MIG NOT_TIP 213 104 10   3   1
## 22 222               <NA> NOT_ROOT     MIG NOT_TIP 104 103 NA  NA  NA
## 23 295               <NA> NOT_ROOT     MIG NOT_TIP  52  51 NA  NA  NA
## 24 337               <NA> NOT_ROOT     MIG NOT_TIP  52  32 NA  NA  NA
## 25 422               <NA> NOT_ROOT     MIG NOT_TIP 104   2 NA  NA  NA
##                                                                                                                                                                                                                                                                                                                                                                        V11
## 1                                                                                                                                              (Eastern_China:0.00483568,(Okinawa_Taiwan:0.0136079,(Russia:0.0197603,(((Japan:0.013941,Italy:0.00990136):0.00168526,Northeast_US:0.000616116):0.00128105,Texas:0.00434392):0.00987843):0.00212482):0.000879899):0.00224622
## 2                                                                                                                                                                                                                                                                                                                                                  Athens_Greece:0.0188771
## 3                                                                      (((Eastern_China:0.00483568,(Okinawa_Taiwan:0.0136079,(Russia:0.0197603,(((Japan:0.013941,Italy:0.00990136):0.00168526,Northeast_US:0.000616116):0.00128105,Texas:0.00434392):0.00987843):0.00212482):0.000879899):0.00224622,California:0.00718492):0.00764789,Athens_Greece:0.0188771):0.00425953
## 4                                                                                                                                                                                                                                                                                                                                              Sulawesi_Indonesia:0.017958
## 5                                                                                                                                                                                                                                                                                                                                                    California:0.00718492
## 6                                                                                                                                                                                                                                                                                                                                                         Texas:0.00434392
## 7                                                                                                                                                                                                                                                                                                                                                         Italy:0.00990136
## 8                                                                                                                                                                                                                                                         (((Japan:0.013941,Italy:0.00990136):0.00168526,Northeast_US:0.000616116):0.00128105,Texas:0.00434392):0.00987843
## 9                                                                                                                                                                                                                                                                                                                                                         Russia:0.0197603
## 10                                                                                                                                                                                                                          (Russia:0.0197603,(((Japan:0.013941,Italy:0.00990136):0.00168526,Northeast_US:0.000616116):0.00128105,Texas:0.00434392):0.00987843):0.00212482
## 11                                                                                                                                                                                                                                                                                                                                                Eastern_China:0.00483568
## 12                                                                                                          ((Eastern_China:0.00483568,(Okinawa_Taiwan:0.0136079,(Russia:0.0197603,(((Japan:0.013941,Italy:0.00990136):0.00168526,Northeast_US:0.000616116):0.00128105,Texas:0.00434392):0.00987843):0.00212482):0.000879899):0.00224622,California:0.00718492):0.00764789
## 13                                                                                                                                                                                                                                                                                                                                               Southeast_Asia:0.00642381
## 14                                (Southeast_Asia:0.00642381,(((Eastern_China:0.00483568,(Okinawa_Taiwan:0.0136079,(Russia:0.0197603,(((Japan:0.013941,Italy:0.00990136):0.00168526,Northeast_US:0.000616116):0.00128105,Texas:0.00434392):0.00987843):0.00212482):0.000879899):0.00224622,California:0.00718492):0.00764789,Athens_Greece:0.0188771):0.00425953):0.017958
## 15                                                                                                                                                                                                                                                                                                                                                          Japan:0.013941
## 16                                                                                                                                                                                                                                                                                      ((Japan:0.013941,Italy:0.00990136):0.00168526,Northeast_US:0.000616116):0.00128105
## 17                                                                                                                                                                                                                                                                                                                                                Northeast_US:0.000616116
## 18                                                                                                                                                                                                                                                                                                                            (Japan:0.013941,Italy:0.00990136):0.00168526
## 19                                                                                                                                                                                                                                                                                                                                                Okinawa_Taiwan:0.0136079
## 20                                                                                                                                                                                   (Okinawa_Taiwan:0.0136079,(Russia:0.0197603,(((Japan:0.013941,Italy:0.00990136):0.00168526,Northeast_US:0.000616116):0.00128105,Texas:0.00434392):0.00987843):0.00212482):0.000879899
## 21 ((Southeast_Asia:0.00642381,(((Eastern_China:0.00483568,(Okinawa_Taiwan:0.0136079,(Russia:0.0197603,(((Japan:0.013941,Italy:0.00990136):0.00168526,Northeast_US:0.000616116):0.00128105,Texas:0.00434392):0.00987843):0.00212482):0.000879899):0.00224622,California:0.00718492):0.00764789,Athens_Greece:0.0188771):0.00425953):0.017958,Sulawesi_Indonesia:0.017958);
## 22                                                                                                                                                                                                                                                                                                                                                                    <NA>
## 23                                                                                                                                                                                                                                                                                                                                                                    <NA>
## 24                                                                                                                                                                                                                                                                                                                                                                    <NA>
## 25                                                                                                                                                                                                                                                                                                                                                                    <NA>
##             x          y       ymin       ymax
## 1  0.03211164 0.81818182 0.27272727 0.90909091
## 2  0.03015073 0.13636364 0.09090909 0.18181818
## 3  0.02221753 0.18181818 0.09090909 0.90909091
## 4  0.01795800 0.04545455 0.00000000 0.09090909
## 5  0.03705034 0.22727273 0.18181818 0.27272727
## 6  0.04830239 0.31818182 0.27272727 0.36363636
## 7  0.05786246 0.50000000 0.45454545 0.54545455
## 8  0.04499479 0.36363636 0.27272727 0.63636364
## 9  0.04499479 0.68181818 0.63636364 0.72727273
## 10 0.03511636 0.63636364 0.27272727 0.72727273
## 11 0.03469946 0.86363636 0.81818182 0.90909091
## 12 0.02986542 0.27272727 0.18181818 0.90909091
## 13 0.02221753 0.95454545 0.90909091 1.00000000
## 14 0.01795800 0.90909091 0.09090909 1.00000000
## 15 0.05577661 0.59090909 0.54545455 0.63636364
## 16 0.04627584 0.45454545 0.36363636 0.63636364
## 17 0.04689195 0.40909091 0.36363636 0.45454545
## 18 0.04796110 0.54545455 0.45454545 0.63636364
## 19 0.04659944 0.77272727 0.72727273 0.81818182
## 20 0.03299154 0.72727273 0.27272727 0.81818182
## 21 0.00000000 0.09090909 0.00000000 1.00000000
## 22 0.01996515         NA 0.09090909 0.90909091
## 23 0.03781688         NA 0.27272727 0.63636364
## 24 0.03642817         NA 0.27272727 0.63636364
## 25 0.02108497         NA 0.09090909 0.90909091
## [1] "0.312455 0.017958 0.02221753"
## [1] "0.136664 0.035116359 0.044994789"
## [1] "0.132795 0.035116359 0.044994789"
## [1] "0.734112 0.017958 0.02221753"
```

```
##  [1] 0.03015073 0.01795800 0.03705034 0.04830239 0.05786246 0.04499479
##  [7] 0.03469946 0.02221753 0.05577661 0.04689195 0.04659944
## [1] 0.003
## [1] "mse 0.000102502404132231"
```

```
## $d
##     V1                 V2       V3      V4      V5  V6  V7 V8  V9 V10
## 1    0               <NA> NOT_ROOT NOT_MIG NOT_TIP  76  75  1 212   6
## 2    1      Athens_Greece NOT_ROOT NOT_MIG     TIP   2  NA NA  NA  NA
## 3    2               <NA> NOT_ROOT NOT_MIG NOT_TIP 104  76  8   1   1
## 4    3 Sulawesi_Indonesia NOT_ROOT NOT_MIG     TIP 213  NA NA  NA  NA
## 5    4         California NOT_ROOT NOT_MIG     TIP  76  NA NA  NA  NA
## 6   15              Texas NOT_ROOT NOT_MIG     TIP  32  NA NA  NA  NA
## 7   31              Italy NOT_ROOT NOT_MIG     TIP 172  NA NA  NA  NA
## 8   32               <NA> NOT_ROOT NOT_MIG NOT_TIP  52 136  3  15   1
## 9   51             Russia NOT_ROOT NOT_MIG     TIP  52  NA NA  NA  NA
## 10  52               <NA> NOT_ROOT NOT_MIG NOT_TIP 212  51  1  32   4
## 11  75      Eastern_China NOT_ROOT NOT_MIG     TIP   0  NA NA  NA  NA
## 12  76               <NA> NOT_ROOT NOT_MIG NOT_TIP   2   0  7   4   1
## 13 103     Southeast_Asia NOT_ROOT NOT_MIG     TIP 104  NA NA  NA  NA
## 14 104               <NA> NOT_ROOT NOT_MIG NOT_TIP 213 103  1   2   9
## 15 135              Japan NOT_ROOT NOT_MIG     TIP 172  NA NA  NA  NA
## 16 136               <NA> NOT_ROOT NOT_MIG NOT_TIP  32 172  2 171   1
## 17 171       Northeast_US NOT_ROOT NOT_MIG     TIP 136  NA NA  NA  NA
## 18 172               <NA> NOT_ROOT NOT_MIG NOT_TIP 136 135  1  31   1
## 19 211     Okinawa_Taiwan NOT_ROOT NOT_MIG     TIP 212  NA NA  NA  NA
## 20 212               <NA> NOT_ROOT NOT_MIG NOT_TIP   0 211  1  52   5
## 21 213               <NA>     ROOT NOT_MIG NOT_TIP 213 104 10   3   1
## 22 222               <NA> NOT_ROOT     MIG NOT_TIP 104 103 NA  NA  NA
## 23 295               <NA> NOT_ROOT     MIG NOT_TIP  52  51 NA  NA  NA
## 24 337               <NA> NOT_ROOT     MIG NOT_TIP  52  32 NA  NA  NA
## 25 422               <NA> NOT_ROOT     MIG NOT_TIP 104   2 NA  NA  NA
##                                                                                                                                                                                                                                                                                                                                                                        V11
## 1                                                                                                                                              (Eastern_China:0.00483568,(Okinawa_Taiwan:0.0136079,(Russia:0.0197603,(((Japan:0.013941,Italy:0.00990136):0.00168526,Northeast_US:0.000616116):0.00128105,Texas:0.00434392):0.00987843):0.00212482):0.000879899):0.00224622
## 2                                                                                                                                                                                                                                                                                                                                                  Athens_Greece:0.0188771
## 3                                                                      (((Eastern_China:0.00483568,(Okinawa_Taiwan:0.0136079,(Russia:0.0197603,(((Japan:0.013941,Italy:0.00990136):0.00168526,Northeast_US:0.000616116):0.00128105,Texas:0.00434392):0.00987843):0.00212482):0.000879899):0.00224622,California:0.00718492):0.00764789,Athens_Greece:0.0188771):0.00425953
## 4                                                                                                                                                                                                                                                                                                                                              Sulawesi_Indonesia:0.017958
## 5                                                                                                                                                                                                                                                                                                                                                    California:0.00718492
## 6                                                                                                                                                                                                                                                                                                                                                         Texas:0.00434392
## 7                                                                                                                                                                                                                                                                                                                                                         Italy:0.00990136
## 8                                                                                                                                                                                                                                                         (((Japan:0.013941,Italy:0.00990136):0.00168526,Northeast_US:0.000616116):0.00128105,Texas:0.00434392):0.00987843
## 9                                                                                                                                                                                                                                                                                                                                                         Russia:0.0197603
## 10                                                                                                                                                                                                                          (Russia:0.0197603,(((Japan:0.013941,Italy:0.00990136):0.00168526,Northeast_US:0.000616116):0.00128105,Texas:0.00434392):0.00987843):0.00212482
## 11                                                                                                                                                                                                                                                                                                                                                Eastern_China:0.00483568
## 12                                                                                                          ((Eastern_China:0.00483568,(Okinawa_Taiwan:0.0136079,(Russia:0.0197603,(((Japan:0.013941,Italy:0.00990136):0.00168526,Northeast_US:0.000616116):0.00128105,Texas:0.00434392):0.00987843):0.00212482):0.000879899):0.00224622,California:0.00718492):0.00764789
## 13                                                                                                                                                                                                                                                                                                                                               Southeast_Asia:0.00642381
## 14                                (Southeast_Asia:0.00642381,(((Eastern_China:0.00483568,(Okinawa_Taiwan:0.0136079,(Russia:0.0197603,(((Japan:0.013941,Italy:0.00990136):0.00168526,Northeast_US:0.000616116):0.00128105,Texas:0.00434392):0.00987843):0.00212482):0.000879899):0.00224622,California:0.00718492):0.00764789,Athens_Greece:0.0188771):0.00425953):0.017958
## 15                                                                                                                                                                                                                                                                                                                                                          Japan:0.013941
## 16                                                                                                                                                                                                                                                                                      ((Japan:0.013941,Italy:0.00990136):0.00168526,Northeast_US:0.000616116):0.00128105
## 17                                                                                                                                                                                                                                                                                                                                                Northeast_US:0.000616116
## 18                                                                                                                                                                                                                                                                                                                            (Japan:0.013941,Italy:0.00990136):0.00168526
## 19                                                                                                                                                                                                                                                                                                                                                Okinawa_Taiwan:0.0136079
## 20                                                                                                                                                                                   (Okinawa_Taiwan:0.0136079,(Russia:0.0197603,(((Japan:0.013941,Italy:0.00990136):0.00168526,Northeast_US:0.000616116):0.00128105,Texas:0.00434392):0.00987843):0.00212482):0.000879899
## 21 ((Southeast_Asia:0.00642381,(((Eastern_China:0.00483568,(Okinawa_Taiwan:0.0136079,(Russia:0.0197603,(((Japan:0.013941,Italy:0.00990136):0.00168526,Northeast_US:0.000616116):0.00128105,Texas:0.00434392):0.00987843):0.00212482):0.000879899):0.00224622,California:0.00718492):0.00764789,Athens_Greece:0.0188771):0.00425953):0.017958,Sulawesi_Indonesia:0.017958);
## 22                                                                                                                                                                                                                                                                                                                                                                    <NA>
## 23                                                                                                                                                                                                                                                                                                                                                                    <NA>
## 24                                                                                                                                                                                                                                                                                                                                                                    <NA>
## 25                                                                                                                                                                                                                                                                                                                                                                    <NA>
##             x          y       ymin       ymax
## 1  0.03211164 0.81818182 0.27272727 0.90909091
## 2  0.03015073 0.13636364 0.09090909 0.18181818
## 3  0.02221753 0.18181818 0.09090909 0.90909091
## 4  0.01795800 0.04545455 0.00000000 0.09090909
## 5  0.03705034 0.22727273 0.18181818 0.27272727
## 6  0.04830239 0.31818182 0.27272727 0.36363636
## 7  0.05786246 0.50000000 0.45454545 0.54545455
## 8  0.04499479 0.36363636 0.27272727 0.63636364
## 9  0.04499479 0.68181818 0.63636364 0.72727273
## 10 0.03511636 0.63636364 0.27272727 0.72727273
## 11 0.03469946 0.86363636 0.81818182 0.90909091
## 12 0.02986542 0.27272727 0.18181818 0.90909091
## 13 0.02221753 0.95454545 0.90909091 1.00000000
## 14 0.01795800 0.90909091 0.09090909 1.00000000
## 15 0.05577661 0.59090909 0.54545455 0.63636364
## 16 0.04627584 0.45454545 0.36363636 0.63636364
## 17 0.04689195 0.40909091 0.36363636 0.45454545
## 18 0.04796110 0.54545455 0.45454545 0.63636364
## 19 0.04659944 0.77272727 0.72727273 0.81818182
## 20 0.03299154 0.72727273 0.27272727 0.81818182
## 21 0.00000000 0.09090909 0.00000000 1.00000000
## 22 0.01928891 0.92329341 0.09090909 0.90909091
## 23 0.03646638 0.64257564 0.27272727 0.63636364
## 24 0.03642817 0.60014682 0.27272727 0.63636364
## 25 0.02108497 0.37519127 0.09090909 0.90909091
## 
## $e
##     V1  V2          V3       V4      V5       V6       V7
## 1  104 222 0.002007150 1.000000 NOT_MIG 0.000000 1.000000
## 2  222  75 0.000000000 0.268460     MIG 0.312455 0.000000
## 3   76   0 0.002246220 1.000000 NOT_MIG 0.000000 1.000000
## 4    0  75 0.002587818 0.731540 NOT_MIG 0.000000 0.535151
## 5   76   4 0.007184920 1.000000 NOT_MIG 0.000000 1.000000
## 6    2  76 0.007647890 1.000000 NOT_MIG 0.000000 1.000000
## 7   52 295 0.002700520 1.000000 NOT_MIG 0.000000 1.000000
## 8  295 135 0.000000000 0.251259     MIG 0.136664 0.000000
## 9    2   1 0.007933201 0.648271 NOT_MIG 0.000000 0.420256
## 10  52 337 0.001311810 1.000000 NOT_MIG 0.000000 1.000000
## 11 337   1 0.000000000 0.351729     MIG 0.132795 0.000000
## 12 172 135 0.007815507 0.748741 NOT_MIG 0.000000 0.560613
## 13 212 211 0.013607900 1.000000 NOT_MIG 0.000000 1.000000
## 14   0 212 0.000879899 1.000000 NOT_MIG 0.000000 1.000000
## 15 172  31 0.009901360 1.000000 NOT_MIG 0.000000 1.000000
## 16 136 172 0.001685260 1.000000 NOT_MIG 0.000000 1.000000
## 17 136 171 0.000616116 1.000000 NOT_MIG 0.000000 1.000000
## 18  32 136 0.001281050 1.000000 NOT_MIG 0.000000 1.000000
## 19 295  51 0.017059800 1.000000 NOT_MIG 0.136664 1.000000
## 20 212  52 0.002124820 1.000000 NOT_MIG 0.000000 1.000000
## 21 222 103 0.004416660 1.000000 NOT_MIG 0.312455 1.000000
## 22  32  15 0.003307602 0.872601 NOT_MIG 0.000000 0.761433
## 23 213 104 0.017958000 1.000000 NOT_MIG 0.000000 1.000000
## 24 337  32 0.008566620 1.000000 NOT_MIG 0.132795 1.000000
## 25 213   3 0.017958000 1.000000 NOT_MIG 0.000000 1.000000
## 26 104 422 0.003126970 1.000000 NOT_MIG 0.000000 1.000000
## 27 422   2 0.001132560 1.000000 NOT_MIG 0.734112 1.000000
## 28 422  15 0.000000000 0.127399     MIG 0.734112 0.000000
```

Save the tree

```
setwd("/gpfs/gibbs/pi/caccone/mkc54/albo/manuscript1/output/treemix_subsets/north_america_simplified/simplified_test/results")

# Open PDF
pdf("treemix_consensustree_migration_northAmerica_simplified_test.pdf", width = 15, height = 8)

plot_tree("_stem")

dev.off()
```

Create pop\_order txt

```
cd /gpfs/gibbs/pi/caccone/mkc54/albo/manuscript1/output/treemix_subsets/north_america_simplified/simplified_test
zcat r2_0.1_b_north_america_subset_simplified.treemix.frq.gz | head -n 1 | tr ' ' '\n' > pop_order.txt
```

Plot residuals

```
setwd("/gpfs/gibbs/pi/caccone/mkc54/albo/manuscript1/output/treemix_subsets/north_america_simplified/simplified_test/results")

input_file <- "/gpfs/gibbs/pi/caccone/mkc54/albo/manuscript1/output/treemix_subsets/north_america_simplified/simplified_test/r2_0.1_b_north_america_subset_simplified.treemix.frq.gz"  # adjust if needed
outgroup_name <- "Sulawesi_Indonesia" 
pops_list <- "pop_order.txt"
run_prefix <- "_stem"
plot_resid(run_prefix, input_file, pop_order=pops_list)
```

```
## [1] 0.0001025024
## [1] "here"
```

```
## Warning in xy.coords(x, y, xlabel, ylabel, log): NAs introduced by coercion
```

```
## [1] "1 1 0"
## [1] "2 1 -3.7066e-05"
## [1] "2 2 0"
## [1] "3 1 0.00025568"
## [1] "3 2 -5.98000000000005e-06"
## [1] "3 3 0"
## [1] "4 1 9.91000000000013e-06"
## [1] "4 2 0.00022442"
## [1] "4 3 -5.80200000000002e-05"
## [1] "4 4 0"
## [1] "5 1 0.0001732"
## [1] "5 2 0.0003665"
## [1] "5 3 0.00016952"
## [1] "5 4 0"
## [1] "5 5 0"
## [1] "6 1 -9.14399999999999e-05"
## [1] "6 2 0.00017518"
## [1] "6 3 2.22e-05"
## [1] "6 4 -0.00022654"
## [1] "6 5 0.00030256"
## [1] "6 6 0"
## [1] "7 1 -5.8771e-05"
## [1] "7 2 -0.000470751"
## [1] "7 3 -0.000366238"
## [1] "7 4 2.01900000000002e-05"
## [1] "7 5 0.00020997"
## [1] "7 6 0.00025247"
## [1] "7 7 0"
## [1] "8 1 -0.00013473"
## [1] "8 2 -0.000206636"
## [1] "8 3 0.000210117"
## [1] "8 4 0.00019729"
## [1] "8 5 0"
## [1] "8 6 0.00019121"
## [1] "8 7 -0.001063238"
## [1] "8 8 0"
## [1] "9 1 6.13600000000003e-05"
## [1] "9 2 0.00030232"
## [1] "9 3 0"
## [1] "9 4 -0.00056653"
## [1] "9 5 -0.00058609"
## [1] "9 6 -0.00029521"
## [1] "9 7 0.00033561"
## [1] "9 8 0.00072155"
## [1] "9 9 0"
## [1] "10 1 -0.0001229706"
## [1] "10 2 -0.00026628"
## [1] "10 3 -0.000149915"
## [1] "10 4 -0.000115000000000001"
## [1] "10 5 -0.00034204"
## [1] "10 6 -3.59999999999996e-05"
## [1] "10 7 0.0004517"
## [1] "10 8 0.00050623"
## [1] "10 9 4.02500000000004e-05"
## [1] "10 10 0"
## [1] "11 1 -5.51900000000001e-05"
## [1] "11 2 -8.172e-05"
## [1] "11 3 -7.73600000000001e-05"
## [1] "11 4 0.000514280000000001"
## [1] "11 5 -0.00029362"
## [1] "11 6 -0.00029443"
## [1] "11 7 0.00068908"
## [1] "11 8 -0.00042181"
## [1] "11 9 -1.32700000000001e-05"
## [1] "11 10 3.403e-05"
## [1] "11 11 0"
##  [1] "#FF0000" "#FF0C00" "#FF1900" "#FF2500" "#FF3200" "#FF3E00" "#FF4B00"
##  [8] "#FF5700" "#FF6400" "#FF7000" "#FF7D00" "#FF8900" "#FF9600" "#FFA200"
## [15] "#FFAA00" "#FFB100" "#FFB800" "#FFBF00" "#FFC600" "#FFCC00" "#FFD300"
## [22] "#FFDA00" "#FFE100" "#FFE800" "#FFEF00" "#FFF500" "#FFFC00" "#FFFF0C"
## [29] "#FFFF20" "#FFFF33" "#FFFF47" "#FFFF5A" "#FFFF6D" "#FFFF81" "#FFFF94"
## [36] "#FFFFA7" "#FFFFBB" "#FFFFCE" "#FFFFE1" "#FFFFF5" "#F5FFF5" "#E1FFE1"
## [43] "#CEFFCE" "#BBFFBB" "#A7FFA7" "#94FF94" "#81FF81" "#6DFF6D" "#5AFF5A"
## [50] "#47FF47" "#33FF33" "#20FF20" "#0CFF0C" "#00F806" "#00E519" "#00D12D"
## [57] "#00BE40" "#00AB53" "#009767" "#00847A" "#00708E" "#005DA1" "#004AB4"
## [64] "#0036C8" "#0023DB" "#0010EE" "#0000FB" "#0000E8" "#0000D5" "#0000C1"
## [71] "#0000AE" "#00009A" "#000087" "#000074" "#000060" "#00004D" "#00003A"
## [78] "#000026" "#000013" "#000000"
##  [1] 0.500 0.505 0.510 0.515 0.520 0.525 0.530 0.535 0.540 0.545 0.550 0.555
## [13] 0.560 0.565 0.570 0.575 0.580 0.585 0.590 0.595 0.600 0.605 0.610 0.615
## [25] 0.620 0.625 0.630 0.635 0.640 0.645 0.650 0.655 0.660 0.665 0.670 0.675
## [37] 0.680 0.685 0.690 0.695 0.700 0.705 0.710 0.715 0.720 0.725 0.730 0.735
## [49] 0.740 0.745 0.750 0.755 0.760 0.765 0.770 0.775 0.780 0.785 0.790 0.795
## [61] 0.800 0.805 0.810 0.815 0.820 0.825 0.830 0.835 0.840 0.845 0.850 0.855
## [73] 0.860 0.865 0.870 0.875 0.880 0.885 0.890 0.895 0.900
```

```
##    Athens_Greece   California Eastern_China       Italy       Japan
## 1   0.0000000000 -0.000037066   0.000255680  0.00000991  0.00017320
## 2  -0.0000370660  0.000000000  -0.000005980  0.00022442  0.00036650
## 3   0.0002556800 -0.000005980   0.000000000 -0.00005802  0.00016952
## 4   0.0000099100  0.000224420  -0.000058020  0.00000000  0.00000000
## 5   0.0001732000  0.000366500   0.000169520  0.00000000  0.00000000
## 6  -0.0000914400  0.000175180   0.000022200 -0.00022654  0.00030256
## 7  -0.0000587710 -0.000470751  -0.000366238  0.00002019  0.00020997
## 8  -0.0001347300 -0.000206636   0.000210117  0.00019729  0.00000000
## 9   0.0000613600  0.000302320   0.000000000 -0.00056653 -0.00058609
## 10 -0.0001229706 -0.000266280  -0.000149915 -0.00011500 -0.00034204
## 11 -0.0000551900 -0.000081720  -0.000077360  0.00051428 -0.00029362
##    Northeast_US Okinawa_Taiwan       Russia Southeast_Asia Sulawesi_Indonesia
## 1   -0.00009144   -0.000058771 -0.000134730     0.00006136      -0.0001229706
## 2    0.00017518   -0.000470751 -0.000206636     0.00030232      -0.0002662800
## 3    0.00002220   -0.000366238  0.000210117     0.00000000      -0.0001499150
## 4   -0.00022654    0.000020190  0.000197290    -0.00056653      -0.0001150000
## 5    0.00030256    0.000209970  0.000000000    -0.00058609      -0.0003420400
## 6    0.00000000    0.000252470  0.000191210    -0.00029521      -0.0000360000
## 7    0.00025247    0.000000000 -0.001063238     0.00033561       0.0004517000
## 8    0.00019121   -0.001063238  0.000000000     0.00072155       0.0005062300
## 9   -0.00029521    0.000335610  0.000721550     0.00000000       0.0000402500
## 10  -0.00003600    0.000451700  0.000506230     0.00004025       0.0000000000
## 11  -0.00029443    0.000689080 -0.000421810    -0.00001327       0.0000340300
##          Texas
## 1  -0.00005519
## 2  -0.00008172
## 3  -0.00007736
## 4   0.00051428
## 5  -0.00029362
## 6  -0.00029443
## 7   0.00068908
## 8  -0.00042181
## 9  -0.00001327
## 10  0.00003403
## 11  0.00000000
```

```
setwd("/gpfs/gibbs/pi/caccone/mkc54/albo/manuscript1/output/treemix_subsets/north_america_simplified/simplified_test/results")

# Open PDF
pdf("treemix_residuals_na_simplified_test.pdf", width = 15, height = 8)

input_file <- "/gpfs/gibbs/pi/caccone/mkc54/albo/manuscript1/output/treemix_subsets/north_america_simplified/simplified_test/r2_0.1_b_north_america_subset_simplified.treemix.frq.gz"  # adjust if needed
outgroup_name <- "Sulawesi_Indonesia" 
pops_list <- "pop_order.txt"
run_prefix <- "_stem"
plot_resid(run_prefix, input_file, pop_order=pops_list)

dev.off()
```

## 7.2 South America subset

We will include 5 clusters from South America - which matches the
LEA/fastStructure & roughly corresponds to the structure of the
first tree from the original Treemix analysis (with each pop
independent)

I can also add a constraints file to set the tree structure more with
the argument -g constraints.dot But we will skip this for now

### 7.2.1 Subset the populations from South America and Asia & prune

First we need to copy the plink files from snps\_sets & upload a
new fam file to group our pops into 10 clusters

Then subset - we should have only 10 pop groups

```
cd /gpfs/gibbs/pi/caccone/mkc54/albo/manuscript1/output/treemix_subsets/south_america
module load PLINK/1.9b_6.21-x86_64

plink \
--keep-allele-order \
--keep-fam files/south_america_native.txt \
--bfile files/r2_0.1_b \
--make-bed \
--export vcf \
--out files/south_america_native_0.1b \
--silent
grep "samples\|variants" files/south_america_native_0.1b.log
```

61574 variants loaded from .bim file. Total genotyping rate in
remaining samples is 0.968132. 61574 variants and 270 people pass
filters and QC.

### 7.2.2 QC

Now need to do some QC again on the new subset before using it:

#### 7.2.2.1 Missingness

For treemix, a common threshold is max 5% missing per SNP (–geno
0.05)

```
cd /gpfs/gibbs/pi/caccone/mkc54/albo/manuscript1/output/treemix_subsets/south_america

module load PLINK/2_avx2_20221024

plink2 \
--allow-extra-chr \
--bfile files/south_america_native_0.1b \
--geno 0.05                `# we set genotype missiningness to 5% with this option` \
--make-bed \
--out files/file2 \
--silent \
--missing;                 # --missing produces sample-based and variant-based missing data reports. If run with --within/--family, the variant-based report is stratified by cluster.
grep "variants" files/file2.log
```

61574 variants loaded from files/south\_america\_native\_0.1b.bim.
–geno: 14718 variants removed due to missing genotype data. 46856
variants remaining after main filters.

Remove individuals missing more than 20% of SNPs. You can use the
threshold you want, change the flag –mind

```
cd /gpfs/gibbs/pi/caccone/mkc54/albo/manuscript1/output/treemix_subsets/south_america

plink2 \
--allow-extra-chr \
--bfile files/file2 \
--mind 0.2               `# here we set the individual missingness threshold of 20%`\
--make-bed \
--out files/file3 \
--silent;
grep "samples\|variants" files/file3.log
```

270 samples (18 females, 46 males, 206 ambiguous; 270 founders)
loaded from 46856 variants loaded from files/file2.bim. 0 samples
removed due to missing genotype data (–mind). 270 samples (18 females,
46 males, 206 ambiguous; 270 founders) remaining after

All individuals have <20% missingness

#### 7.2.2.2 Minor allele frequency

First, we estimate the allele frequency with Plink.

```
cd /gpfs/gibbs/pi/caccone/mkc54/albo/manuscript1/output/treemix_subsets/south_america/files
plink2 \
--allow-extra-chr \
--bfile file3 \
--freq \
--out MAF_check \
--silent
```

Now we apply the MAF filter for 0.01

```
# We will use MAF of 1%
plink2 \
--allow-extra-chr \
--bfile file3 \
--maf 0.01 \
--make-bed \
--out file4b \
--silent;
grep "variants" file4b.log
```

46856 variants loaded from file3.bim. 212 variants removed due to
allele frequency threshold(s) 46644 variants remaining after main
filters.

Preliminary LD pruning

```
plink2 \
--allow-extra-chr \
--bfile file4b \
--indep-pairwise 5 1 0.1 \
--out indepSNPb \
--silent;
grep 'pairwise\|variants\|samples' indepSNPb.log
```

–indep-pairwise 5 1 0.1 270 samples (18 females, 46 males, 206
ambiguous; 270 founders) loaded from 46644 variants loaded from
file4b.bim. –indep-pairwise (3 compute threads): 11274/46644 variants
removed.

#### 7.2.2.3 Heterozygosity

```
cd /gpfs/gibbs/pi/caccone/mkc54/albo/manuscript1/output/treemix_subsets/south_america/files
plink2 \
--allow-extra-chr \
--bfile file4b \
--extract indepSNPb.prune.in \
--het \
--out R_checkb \
--silent;
grep 'variants' R_checkb.log
```

46644 variants loaded from file4b.bim. –extract: 35370 variants
remaining. 35370 variants remaining after main filters.

```
#   find individuals with high heterozygosity                              ####
# import the data from Plink
het <- read.delim(
  here(
    "manuscript1/output/treemix_subsets/south_america/files/R_checkb.het"
  ),
  head = TRUE
)
#
# check head of the file
colnames(het)
```

```
## [1] "X.FID"  "IID"    "O.HOM." "E.HOM." "OBS_CT" "F"
```

Estimate heterozygosity

```
# create a column named HET_RATE and calculate the heterozygosity rate
het$HET_RATE <- (het$"OBS_CT" - het$"O.HOM") / het$"OBS_CT"
#
# use subset function to get values deviating from 4sd of the mean heterozygosity rate.
het_fail <-
  subset(
    het, (het$HET_RATE < mean(
      het$HET_RATE
    ) - 4 * sd(
      het$HET_RATE
    )) |
      (het$HET_RATE > mean(
        het$HET_RATE
      ) + 4 * sd(
        het$HET_RATE
      ))
  )
#
# get the list of individuals that failed our threshold of 4sd from the mean.
het_fail$HET_DST <-
  (het_fail$HET_RATE - mean(
    het$HET_RATE
  )) / sd(
    het$HET_RATE
  )
```

Save the files to use with Plink

```
#   save the data to use with Plink2                                        ####
#
write.table(
  het_fail,
  here(
    "manuscript1/output/treemix_subsets/south_america/files/fail-het-qc_b.txt"
  ),
  row.names = FALSE
)
```

Make plot

```
source(here("my_theme.R"))

#   make a heterozygosity plot                                              ####
#
ggplot(
  het,
  aes(
    HET_RATE
  )
) +
  geom_histogram(
    colour           = "black",
    fill             = "lightgray",
    bins             = 40
  ) +
  labs(
    x                = "Heterozygosity Rate",
    y                = "Number of Individuals"
  ) +
  geom_vline(
    aes(
      xintercept     = mean(
        HET_RATE
      )
    ),
    col              = "red",
  ) +
  geom_vline(
    aes(
      xintercept     = mean(
        HET_RATE
      ) + 4 * sd(
        HET_RATE
      )
    ),
    col              = "#BFB9B9",
  ) +
  geom_vline(
    aes(
      xintercept     = mean(
        HET_RATE
      ) - 4 * sd(
        HET_RATE
      )
    ),
    col              = "#BFB9B9",
  ) + 
  my_theme() +
  scale_y_continuous(
  )
```

The red line in the plot above indicates the mean, and the gray lines
indicate 4 standard deviation from the mean. We can see that some
mosquitoes do have excess heterozygous sites. We will remove them. We
can get their ID from the file “fail-het-qc.txt”. We can use the bash
script below to parse the file to use with Plink

```
cd /gpfs/gibbs/pi/caccone/mkc54/albo/manuscript1/output/treemix_subsets/south_america/files
sed 's/"// g' fail-het-qc_b.txt | awk '{print$1, $2}'> het_fail_ind_b.txt;
echo 'How many mosquitoes we need to remove from our data set:';
cat het_fail_ind_b.txt | tail -n +2 | wc -l;
echo 'Which mosquitoes we have to remove:';
tail -n +2 het_fail_ind_b.txt
```

```
## How many mosquitoes we need to remove from our data set:
## 1
## Which mosquitoes we have to remove:
## OKI 1161
```

1 mosquito needs to be removed

```
cd /gpfs/gibbs/pi/caccone/mkc54/albo/manuscript1/output/treemix_subsets/south_america/files

plink2 \
--allow-extra-chr \
--bfile file4b \
--remove het_fail_ind_b.txt \
--make-bed \
--out file5b \
--silent;
grep 'variants\|samples' file5b.log
```

270 samples (18 females, 46 males, 206 ambiguous; 270 founders)
loaded from 46644 variants loaded from file4b.bim. –remove: 269 samples
remaining. 269 samples (17 females, 46 males, 206 ambiguous; 269
founders) remaining after

1 removed for heterozygosity

#### 7.2.2.4 LD Pruning r2 for datasets with MAF 1%

```
cd /gpfs/gibbs/pi/caccone/mkc54/albo/manuscript1/output/treemix_subsets/south_america/files

plink2 \
--allow-extra-chr \
--bfile file5b \
--indep-pairwise 5 1 0.1 \
--out indepSNP_chrb \
--silent;
grep 'pairwise\|variants\|samples' indepSNP_chrb.log
```

–indep-pairwise 5 1 0.1 269 samples (17 females, 46 males, 206
ambiguous; 269 founders) loaded from 46644 variants loaded from
file5b.bim. –indep-pairwise (3 compute threads): 11299/46644 variants
removed.

We can use the SNP set the LD pruning we just did and extract
them

```
cd /gpfs/gibbs/pi/caccone/mkc54/albo/manuscript1/output/treemix_subsets/south_america

plink2 \
--keep-allele-order \
--bfile files/file5b \
--make-bed \
--export vcf \
--out r2_0.1_b_south_america_subset \
--extract files/indepSNP_chrb.prune.in \
--silent
grep "variants\|samples" r2_0.1_b_south_america_subset.log
```

269 samples (17 females, 46 males, 206 ambiguous; 269 founders)
loaded from 46644 variants loaded from files/file5b.bim. –extract: 35345
variants remaining. 35345 variants remaining after main filters.

Ensure only biallelic SNPs

```
module load PLINK/1.9b_6.21-x86_64

cd /gpfs/gibbs/pi/caccone/mkc54/albo/manuscript1/output/treemix_subsets/south_america

plink \
--bfile r2_0.1_b_south_america_subset \
--biallelic-only strict \
--make-bed \
--out south_america_native_bial \
--silent;
grep 'pairwise\|variants\|samples' south_america_native_bial.log
```

35345 variants loaded from .bim file. 35345 variants and 269 people
pass filters and QC.

All are biallelic

### 7.2.3 Create files to use in treemix

We already have the .bed, .bim and .fam files, but we need to create
the cluster file

#### 7.2.3.1 create clust file

```
module load BCFtools/1.21-GCC-12.2.0
cd /gpfs/gibbs/pi/caccone/mkc54/albo/manuscript1/output/treemix_subsets/south_america

bcftools query -l r2_0.1_b_south_america_subset.vcf > samples.txt
#awk '{ split($1,a,"_"); print $1, a[1] }' samples.txt > man1.clust
awk '{ split($1,a,"_"); print a[2], a[1] }' samples.txt > man1_south.clust
```

Check it

```
cd /gpfs/gibbs/pi/caccone/mkc54/albo/manuscript1/output/treemix_subsets/south_america
head man1_south.clust
wc -l man1_south.clust
```

```
## 1001 OKI
## 1002 OKI
## 1003 OKI
## 1004 OKI
## 1005 OKI
## 1006 OKI
## 1007 OKI
## 1008 OKI
## 1009 OKI
## 1011 OKI
## 269 man1_south.clust
```

Need to add a 3rd column (with populations, not just family names)
and put the columns in order (FID IID POP). Edit and upload, then check
it

```
cd /gpfs/gibbs/pi/caccone/mkc54/albo/manuscript1/output/treemix_subsets/south_america
head man1_south_plink.txt
wc -l man1_south_plink.txt
```

```
## OKI  1001    Okinawa_Taiwan
## OKI  1002    Okinawa_Taiwan
## OKI  1003    Okinawa_Taiwan
## OKI  1004    Okinawa_Taiwan
## OKI  1005    Okinawa_Taiwan
## OKI  1006    Okinawa_Taiwan
## OKI  1007    Okinawa_Taiwan
## OKI  1008    Okinawa_Taiwan
## OKI  1009    Okinawa_Taiwan
## OKI  1011    Okinawa_Taiwan
## 269 man1_south_plink.txt
```

It must be in the same format as the fam file though Check fam file’s
current format

```
cd /gpfs/gibbs/pi/caccone/mkc54/albo/manuscript1/output/treemix_subsets/south_america
cut -d' ' -f1,2 r2_0.1_b_south_america_subset.fam | head
```

It matches

#### 7.2.3.2 convert to treemix format

Load conda environment with python, pandas and numpy

```
cd /gpfs/gibbs/pi/caccone/mkc54/albo/manuscript1/output/treemix_subsets/south_america
module load miniconda
conda activate treemix
module load TreeMix/1.13-GCC-10.2.0
```

```
#verify it is working
python --version #Python 3.10.18 
python -c "import pandas as pd; print(pd.__version__)" #2.3.3
```

Add the following .sh file to the directory

```
#!/bin/bash
# Script to convert PLINK files to TreeMix format
# Modified from Joana Meier's version by ChatGPT
# Requires: plink, plink2treemix.py (https://github.com/thomnelson/tools)
# Usage: ./vcf2treemix.sh <plink_prefix> <clust_file>

if [ $# -ne 2 ]; then
    echo "Usage: $0 <plink_prefix> <clust_file>"
    echo "Example: ./plink2treemix.sh r2_0.1_b_south_america_subset.clust"
    exit 1
fi

file=$1
clust=$2

# Check if required files exist
if [ ! -f "${file}.bed" ] || [ ! -f "${file}.bim" ] || [ ! -f "${file}.fam" ]; then
    echo "Error: Could not find PLINK files (.bed, .bim, .fam) with prefix '${file}'"
    exit 1
fi

if [ ! -f "$clust" ]; then
    echo "Error: Cluster file '$clust' not found"
    exit 1
fi

echo "=== Converting PLINK files to TreeMix format ==="
echo "Input PLINK prefix: $file"
echo "Cluster file: $clust"
echo "-----------------------------------------------"

# Step 1: Calculate population-stratified allele frequencies
plink --bfile $file \
      --freq --missing \
      --within $clust \
      --out $file \
      --allow-no-sex --allow-extra-chr 0

if [ ! -f "${file}.frq.strat" ]; then
    echo "Error: PLINK failed to produce ${file}.frq.strat"
    exit 1
fi

# Step 2: Compress the stratified frequency file
gzip -f ${file}.frq.strat

# Step 3: Convert to TreeMix input format using plink2treemix.py
echo "Running plink2treemix.py ..."
python plink2treemix.py ${file}.frq.strat.gz ${file}.treemix.frq.gz

if [ ! -s "${file}.treemix.frq.gz" ]; then
    echo "Error: TreeMix conversion failed or produced empty output."
    exit 1
fi

# Step 4: Done!
echo "Conversion complete!"
echo "Output file: ${file}.treemix.frq.gz"
echo "You can now run TreeMix using:"
echo "treemix -i ${file}.treemix.frq.gz -o ${file}_treemix_output"
```

Start interactive session and run plink2treemix.py

```
cd /gpfs/gibbs/pi/caccone/mkc54/albo/manuscript1/output/treemix_subsets/south_america

salloc -p scavenge --cpus-per-task=5 -t 2:00:00 --mem-per-cpu=10000
module load PLINK/1.9b_6.21-x86_64

#sed -i 's/\r$//' plink2treemix.sh #fix the .sh file if needed
chmod +x plink2treemix.sh
./plink2treemix.sh r2_0.1_b_south_america_subset man1_south_plink.txt
```

SNPs in file: 35345 Populations in input (n = 10): Central\_Brazil
Eastern\_Brazil Eastern\_China Japan Medellin\_Colombia Okinawa\_Taiwan
Southeast\_Asia Southern\_SA Sulawesi\_Indonesia Western\_Brazil SNPs
written: 35345

✅ Conversion complete! Output:
r2\_0.1\_b\_south\_america\_subset.treemix.frq.gz You can now run TreeMix
using: treemix -i r2\_0.1\_b\_south\_america\_subset.treemix.frq.gz -o
r2\_0.1\_b\_south\_america\_subset\_treemix\_output

Check it

```
cd /gpfs/gibbs/pi/caccone/mkc54/albo/manuscript1/output/treemix_subsets/south_america

zcat r2_0.1_b_south_america_subset.treemix.frq.gz | head
```

### 7.2.4 Run tree mix

#### 7.2.4.1 Run repetitions to find best number of migrations

We will run tree mix with 5 reptitions (for evaluating best number of
edges/migration events later) We will also add a root - based on initial
run and previous structure analyses, we will use the most differentiated
pop as the outgroup (Indonesia)

Create the dsq files

```
cd /gpfs/gibbs/pi/caccone/mkc54/albo/manuscript1/output/treemix_subsets/south_america
module load dSQ/1.05 
module load TreeMix/1.13-GCC-10.2.0

# set variables
input="r2_0.1_b_south_america_subset.treemix.frq.gz"
stem="sasub_simp"   # base prefix for outputs
outdir="prelim"

for m in {0..5}; do
  for r in {1..5}; do
    echo "treemix -i $input -o ${outdir}/${stem}.m${m}.rep${r} -m $m -root Sulawesi_Indonesia -k 500 -seed $RANDOM"
  done
done > dsq_treemix_sasub.txt


head dsq_treemix_sasub.txt #check file

#create the .sh file
dsq --job-file dsq_treemix_sasub.txt --output /dev/null --mail-type ALL -t 24:00:00 --partition=ycga --mem-per-cpu=10G --cpus-per-task=10 --job-name fS.dsq_treemix_sasub --batch-file dsq_treemix_sasub.sh

#check the file
head dsq_treemix_sasub.sh
```

Run it

```
sbatch dsq_treemix_sasub.sh #63400893
dsqa -j 63400893
```

```
#autopsy
dsqa -j 63400893 -f dsq_treemix_sasub.sh -s FAILED > rerun_jobs.txt; wc -l rerun_jobs.txt
```

COMPLETED 30 0-29  
0 rerun\_jobs.txt

Plot treemix results in R

```
#install.packages("OptM", repos="https://cloud.r-project.org")
library(OptM)
library(RColorBrewer)
library(R.utils)
library(ggplot2)
```

#### 7.4.4.2 Plot using OptM

Get plotting functions here - https://bitbucket.org/nygcresearch/treemix/src/master/src/plotting\_funcs.R
Alternately - https://github.com/carolindahms/TreeMix/blob/main/Step2%264\_TreeMix.R
Upload them to your directory on the cluster

Set source

```
# Load TreeMix plotting functions
source("/gpfs/gibbs/pi/caccone/mkc54/albo/manuscript1/output/treemix/north_america/plotting_funcs_fixed.R")  # Adjust this path as needed
```

Use OptM to choose best (need to have run multiple iterations for
each m)

```
#Analyzing the treemix results using the Evanno method
setwd("/gpfs/gibbs/pi/caccone/mkc54/albo/manuscript1/output/treemix_subsets/south_america/prelim")
optm_results <- optM(prefix = "sasub_simp", folder = ".", method = "Evanno")
#The maximum value for delta m was 1.4611 at m = 1 edges.
```

OptM throws and ‘error’: Error in optM(prefix = “sasub\_simp”, folder
= “.”, method = “Evanno”) : SD is zero for all runs! Check your
analysis. But this does not necessarily indicate an error in your
pipeline — it means your dataset is so strongly constrained that TreeMix
is deterministic (e.g. no variation among runs)

Run OptM using a method that does NOT require variance

```
setwd("/gpfs/gibbs/pi/caccone/mkc54/albo/manuscript1/output/treemix_subsets/south_america/prelim")
optM(prefix="nasub_simp", folder="/gpfs/gibbs/pi/caccone/mkc54/albo/manuscript1/output/treemix_subsets/south_america/prelim", method="linear")
```

```
## Finished reading .llik, .modelcov.goz, and .cov.gz files.
```

```
## No output file will be saved. To save an output file, run with 'tsv = "file.tsv"'
```

```
## All migration edges will be included.
```

```
## 'thresh' has been set to the default value of 0.05
```

```
## m ranges between 0 and 5.
```

```
## The average number of iterations per m (not including m = 0) was 5.
```

```
## Make sure these values are correct...
```

```
## Analyzing the treemix results using various linear models.
```

```
## Fitting piecewise linear model...
```

```
## Fitting bent cable model...
```

```
## Fitting a simple exponential model...
```

```
## Fitting a NLS model...
```

```
## 125016.1    (1.08e+01): par = (0 1)
## 17598.69    (4.49e+00): par = (-0.2612376 0.8506854)
## 2829.024    (4.53e+00): par = (0.5777554 0.4769481)
## 518.6260    (3.50e+00): par = (2.077631 -0.03780368)
## 62.32494    (6.17e-01): par = (1.824247 -0.1237991)
## 46.02847    (2.65e-02): par = (1.782957 -0.1719427)
## 45.99617    (5.76e-04): par = (1.778818 -0.1725532)
## 45.99615    (8.26e-05): par = (1.778764 -0.1724684)
## 45.99615    (1.19e-05): par = (1.77877 -0.1724807)
## 45.99615    (1.73e-06): par = (1.778769 -0.1724789)
```

```
## Done fitting a NLS model...
```

```
## $out
##                       df      AIC delta.aic ChangePoints
## SimpleExponential      3 143.5054   0.00000     1.000000
## NonLinearLeastSquares  3 160.3254  16.81998     1.000000
## PiecewiseLinear        5 216.9291  73.42365     1.233254
## BentCable              6 218.9295  75.42403     1.060606
## 
## $PiecewiseLinear
## [1] "Threshold alpha: 1.23325431558243"
## [1] ""
## [1] "Model coefficients: Beta[0], Beta[1], Beta[2]"
## (Intercept)           x           w 
##     30.0773    120.0627    -73.2891 
##       Change.Point Initial.Slope Slope.Change Second.Slope
## 2.5%      1.186137      120.0621    -74.55838     45.52471
## 97.5%     1.262047      120.0633    -71.93414     47.95891
## 
## $BentCable
## $log.likelihood
##             [,1]      [,2]      [,3]      [,4]      [,5]      [,6]      [,7]
##   [1,] -158.6237 -158.6237 -158.6237 -158.6237 -158.6237 -158.6237 -158.6237
##   [2,] -158.6237 -158.6237 -158.6237 -158.6237 -158.6237 -158.6237 -158.6237
##   [3,] -158.6237 -158.6237 -158.6237 -158.6237 -158.6237 -158.6237 -158.6237
##   [4,] -158.6237 -158.6237 -158.6237 -158.6237 -158.6237 -158.6237 -158.6237
##   [5,] -158.6237 -158.6237 -158.6237 -158.6237 -158.6237 -158.6237 -158.6237
##   [6,] -158.6237 -158.6237 -158.6237 -158.6237 -158.6237 -158.6237 -158.6237
##   [7,] -158.6237 -158.6237 -158.6237 -158.6237 -158.6237 -158.6237 -158.6237
##   [8,] -158.6237 -158.6237 -158.6237 -158.6237 -158.6237 -158.6237 -158.6237
##   [9,] -158.6237 -158.6237 -158.6237 -158.6237 -158.6237 -158.6237 -158.6237
##  [10,] -158.6237 -158.6237 -158.6237 -158.6237 -158.6237 -158.6237 -158.6237
##  [11,] -158.6237 -158.6237 -158.6237 -158.6237 -158.6237 -158.6237 -158.6237
##  [12,] -158.6237 -158.6237 -158.6237 -158.6237 -158.6237 -158.6237 -158.6237
##  [13,] -158.6237 -158.6237 -158.6237 -158.6237 -158.6237 -158.6237 -158.6237
##  [14,] -158.6237 -158.6237 -158.6237 -158.6237 -158.6237 -158.6237 -158.6237
##  [15,] -158.6237 -158.6237 -158.6237 -158.6237 -158.6237 -158.6237 -158.6237
##  [16,] -158.6237 -158.6237 -158.6237 -158.6237 -158.6237 -158.6237 -158.6237
##  [17,] -158.6237 -158.6237 -158.6237 -158.6237 -158.6237 -158.6237 -158.6237
##  [18,] -158.6237 -158.6237 -158.6237 -158.6237 -158.6237 -158.5913 -158.1968
##  [19,] -158.6237 -158.6237 -158.6237 -158.5722 -158.0412 -157.1518 -156.0395
##  [20,] -158.6237 -158.5149 -157.6729 -156.4815 -155.1225 -153.6544 -152.1010
##  [21,] -155.7795 -154.3959 -152.8878 -151.3121 -149.6788 -147.9902 -146.2461
##  [22,] -143.6958 -143.6958 -143.5002 -142.5886 -141.3048 -139.8111 -138.1757
##  [23,] -129.4717 -129.4717 -129.4717 -129.4717 -129.3459 -128.6470 -127.5550
##  [24,] -114.8671 -114.8671 -114.8671 -114.8671 -114.8671 -114.8671 -114.7869
##  [25,] -104.6163 -104.6163 -104.6163 -104.6163 -104.6163 -104.6163 -104.6163
##  [26,] -105.5899 -105.5899 -105.5899 -105.5899 -105.5899 -105.5899 -105.5899
##  [27,] -116.1529 -116.1529 -116.1529 -116.1529 -116.1529 -116.1529 -116.1529
##  [28,] -129.2013 -129.2013 -129.2013 -129.2013 -129.2013 -129.2013 -129.2013
##  [29,] -141.2895 -141.2895 -141.2895 -141.2895 -141.2895 -141.2895 -141.2895
##  [30,] -151.7319 -151.7319 -151.7319 -151.7319 -151.7319 -151.7319 -151.7319
##  [31,] -160.6483 -160.6483 -160.6483 -160.6483 -160.6483 -160.6483 -160.6483
##  [32,] -168.2981 -168.2981 -168.2981 -168.2981 -168.2981 -168.2981 -168.2981
##  [33,] -174.9202 -174.9202 -174.9202 -174.9202 -174.9202 -174.9202 -174.9202
##  [34,] -180.7064 -180.7064 -180.7064 -180.7064 -180.7064 -180.7064 -180.7064
##  [35,] -185.8064 -185.8064 -185.8064 -185.8064 -185.8064 -185.8064 -185.8064
##  [36,] -190.3365 -190.3365 -190.3365 -190.3365 -190.3365 -190.3365 -190.3365
##  [37,] -194.3877 -194.3877 -194.3877 -194.3877 -194.3877 -194.3877 -194.3877
##  [38,] -198.0324 -198.0324 -198.0324 -198.0324 -198.0324 -198.0112 -197.9332
##  [39,] -201.3287 -201.3287 -201.3287 -201.2987 -201.2017 -201.0701 -200.9196
##  [40,] -204.3239 -204.2673 -204.1303 -203.9725 -203.8074 -203.6397 -203.4716
##  [41,] -206.5297 -206.4233 -206.2838 -206.1374 -205.9895 -205.8420 -205.6957
##  [42,] -207.9946 -207.9946 -207.9933 -207.9509 -207.8752 -207.7838 -207.6844
##  [43,] -209.5853 -209.5853 -209.5853 -209.5853 -209.5849 -209.5640 -209.5218
##  [44,] -211.2885 -211.2885 -211.2885 -211.2885 -211.2885 -211.2885 -211.2883
##  [45,] -213.0842 -213.0842 -213.0842 -213.0842 -213.0842 -213.0842 -213.0842
##  [46,] -214.9528 -214.9528 -214.9528 -214.9528 -214.9528 -214.9528 -214.9528
##  [47,] -216.8753 -216.8753 -216.8753 -216.8753 -216.8753 -216.8753 -216.8753
##  [48,] -218.8340 -218.8340 -218.8340 -218.8340 -218.8340 -218.8340 -218.8340
##  [49,] -220.8124 -220.8124 -220.8124 -220.8124 -220.8124 -220.8124 -220.8124
##  [50,] -222.7959 -222.7959 -222.7959 -222.7959 -222.7959 -222.7959 -222.7959
##  [51,] -224.7714 -224.7714 -224.7714 -224.7714 -224.7714 -224.7714 -224.7714
##  [52,] -226.7275 -226.7275 -226.7275 -226.7275 -226.7275 -226.7275 -226.7275
##  [53,] -228.6544 -228.6544 -228.6544 -228.6544 -228.6544 -228.6544 -228.6544
##  [54,] -230.5438 -230.5438 -230.5438 -230.5438 -230.5438 -230.5438 -230.5438
##  [55,] -232.3887 -232.3887 -232.3887 -232.3887 -232.3887 -232.3887 -232.3887
##  [56,] -234.1835 -234.1835 -234.1835 -234.1835 -234.1835 -234.1835 -234.1835
##  [57,] -235.9236 -235.9236 -235.9236 -235.9236 -235.9236 -235.9236 -235.9231
##  [58,] -237.6054 -237.6054 -237.6054 -237.6054 -237.6047 -237.5670 -237.4903
##  [59,] -239.2264 -239.2264 -239.2249 -239.1706 -239.0726 -238.9523 -238.8187
##  [60,] -240.7791 -240.6768 -240.5406 -240.3950 -240.2451 -240.0923 -239.9375
##  [61,] -241.5834 -241.5380 -241.4271 -241.2974 -241.1595 -241.0170 -240.8714
##  [62,] -241.9355 -241.9355 -241.9355 -241.9143 -241.8454 -241.7509 -241.6415
##  [63,] -242.3275 -242.3275 -242.3275 -242.3275 -242.3275 -242.3143 -242.2658
##  [64,] -242.7622 -242.7622 -242.7622 -242.7622 -242.7622 -242.7622 -242.7622
##  [65,] -243.2426 -243.2426 -243.2426 -243.2426 -243.2426 -243.2426 -243.2426
##  [66,] -243.7712 -243.7712 -243.7712 -243.7712 -243.7712 -243.7712 -243.7712
##  [67,] -244.3505 -244.3505 -244.3505 -244.3505 -244.3505 -244.3505 -244.3505
##  [68,] -244.9825 -244.9825 -244.9825 -244.9825 -244.9825 -244.9825 -244.9825
##  [69,] -245.6689 -245.6689 -245.6689 -245.6689 -245.6689 -245.6689 -245.6689
##  [70,] -246.4105 -246.4105 -246.4105 -246.4105 -246.4105 -246.4105 -246.4105
##  [71,] -247.2074 -247.2074 -247.2074 -247.2074 -247.2074 -247.2074 -247.2074
##  [72,] -248.0587 -248.0587 -248.0587 -248.0587 -248.0587 -248.0587 -248.0587
##  [73,] -248.9623 -248.9623 -248.9623 -248.9623 -248.9623 -248.9623 -248.9623
##  [74,] -249.9148 -249.9148 -249.9148 -249.9148 -249.9148 -249.9148 -249.9148
##  [75,] -250.9114 -250.9114 -250.9114 -250.9114 -250.9114 -250.9114 -250.9114
##  [76,] -251.9457 -251.9457 -251.9457 -251.9457 -251.9457 -251.9457 -251.9457
##  [77,] -253.0098 -253.0098 -253.0098 -253.0098 -253.0098 -253.0098 -253.0035
##  [78,] -254.0942 -254.0942 -254.0942 -254.0942 -254.0849 -254.0336 -253.9535
##  [79,] -255.1881 -255.1881 -255.1722 -255.0985 -254.9958 -254.8777 -254.7501
##  [80,] -256.2311 -256.1050 -255.9696 -255.8304 -255.6886 -255.5443 -255.3979
##  [81,] -256.4963 -256.4860 -256.4067 -256.2958 -256.1710 -256.0382 -255.8998
##  [82,] -256.4963 -256.4963 -256.4963 -256.4914 -256.4413 -256.3581 -256.2550
##  [83,] -256.4963 -256.4963 -256.4963 -256.4963 -256.4963 -256.4932 -256.4560
##  [84,] -256.4963 -256.4963 -256.4963 -256.4963 -256.4963 -256.4963 -256.4963
##  [85,] -256.4963 -256.4963 -256.4963 -256.4963 -256.4963 -256.4963 -256.4963
##  [86,] -256.4963 -256.4963 -256.4963 -256.4963 -256.4963 -256.4963 -256.4963
##  [87,] -256.4963 -256.4963 -256.4963 -256.4963 -256.4963 -256.4963 -256.4963
##  [88,] -256.4963 -256.4963 -256.4963 -256.4963 -256.4963 -256.4963 -256.4963
##  [89,] -256.4963 -256.4963 -256.4963 -256.4963 -256.4963 -256.4963 -256.4963
##  [90,] -256.4963 -256.4963 -256.4963 -256.4963 -256.4963 -256.4963 -256.4963
##  [91,] -256.4963 -256.4963 -256.4963 -256.4963 -256.4963 -256.4963 -256.4963
##  [92,] -256.4963 -256.4963 -256.4963 -256.4963 -256.4963 -256.4963 -256.4963
##  [93,] -256.4963 -256.4963 -256.4963 -256.4963 -256.4963 -256.4963 -256.4963
##  [94,] -256.4963 -256.4963 -256.4963 -256.4963 -256.4963 -256.4963 -256.4963
##  [95,] -256.4963 -256.4963 -256.4963 -256.4963 -256.4963 -256.4963 -256.4963
##  [96,] -256.4963 -256.4963 -256.4963 -256.4963 -256.4963 -256.4963 -256.4963
##  [97,] -256.4963 -256.4963 -256.4963 -256.4963 -256.4963 -256.4963 -256.4963
##  [98,] -256.4963 -256.4963 -256.4963 -256.4963 -256.4963 -256.4963 -256.4963
##  [99,] -256.4963 -256.4963 -256.4963 -256.4963 -256.4963 -256.4963 -256.4963
## [100,] -256.4963 -256.4963 -256.4963 -256.4963 -256.4963 -256.4963 -256.4963
##             [,8]      [,9]     [,10]     [,11]     [,12]     [,13]     [,14]
##   [1,] -158.6237 -158.6237 -158.6237 -158.6237 -158.6237 -158.6237 -158.6237
##   [2,] -158.6237 -158.6237 -158.6237 -158.6237 -158.6237 -158.6237 -158.6237
##   [3,] -158.6237 -158.6237 -158.6237 -158.6237 -158.6237 -158.6237 -158.6237
##   [4,] -158.6237 -158.6237 -158.6237 -158.6237 -158.6237 -158.6237 -158.6237
##   [5,] -158.6237 -158.6237 -158.6237 -158.6237 -158.6237 -158.6237 -158.6237
##   [6,] -158.6237 -158.6237 -158.6237 -158.6237 -158.6237 -158.6237 -158.6237
##   [7,] -158.6237 -158.6237 -158.6237 -158.6237 -158.6237 -158.6237 -158.6237
##   [8,] -158.6237 -158.6237 -158.6237 -158.6237 -158.6237 -158.6237 -158.6237
##   [9,] -158.6237 -158.6237 -158.6237 -158.6237 -158.6237 -158.6237 -158.6237
##  [10,] -158.6237 -158.6237 -158.6237 -158.6237 -158.6237 -158.6237 -158.6237
##  [11,] -158.6237 -158.6237 -158.6237 -158.6237 -158.6237 -158.6237 -158.6237
##  [12,] -158.6237 -158.6237 -158.6237 -158.6237 -158.6237 -158.6237 -158.6237
##  [13,] -158.6237 -158.6237 -158.6237 -158.6237 -158.6237 -158.6237 -158.6237
##  [14,] -158.6237 -158.6237 -158.6237 -158.6237 -158.6237 -158.6237 -158.6131
##  [15,] -158.6237 -158.6237 -158.6237 -158.6237 -158.6103 -158.3696 -157.8728
##  [16,] -158.6237 -158.6237 -158.6065 -158.3345 -157.7908 -157.0298 -156.0874
##  [17,] -158.6008 -158.2818 -157.6690 -156.8324 -155.8157 -154.6474 -153.3460
##  [18,] -157.4786 -156.5315 -155.4094 -154.1448 -152.7578 -151.2613 -149.6636
##  [19,] -154.7689 -153.3745 -151.8756 -150.2834 -148.6047 -146.8432 -145.0012
##  [20,] -150.4729 -148.7752 -147.0101 -145.1785 -143.2804 -141.3157 -139.2842
##  [21,] -144.4460 -142.5890 -140.6741 -138.7009 -136.6694 -134.5802 -132.4351
##  [22,] -136.4326 -134.6008 -132.6926 -130.7171 -128.6823 -126.5964 -124.4686
##  [23,] -126.2080 -124.6841 -123.0322 -121.2873 -119.4774 -117.6275 -115.7626
##  [24,] -114.2924 -113.4838 -112.4713 -111.3322 -110.1266 -108.9049 -107.7126
##  [25,] -104.6163 -104.5924 -104.4363 -104.1924 -103.9228 -103.6817 -103.5160
##  [26,] -105.5899 -105.5899 -105.5899 -105.6143 -105.7979 -106.1426 -106.6396
##  [27,] -116.1529 -116.1529 -116.1529 -116.1529 -116.1529 -116.1888 -116.4759
##  [28,] -129.2013 -129.2013 -129.2013 -129.2013 -129.2013 -129.2013 -129.2013
##  [29,] -141.2895 -141.2895 -141.2895 -141.2895 -141.2895 -141.2895 -141.2895
##  [30,] -151.7319 -151.7319 -151.7319 -151.7319 -151.7319 -151.7319 -151.7319
##  [31,] -160.6483 -160.6483 -160.6483 -160.6483 -160.6483 -160.6483 -160.6483
##  [32,] -168.2981 -168.2981 -168.2981 -168.2981 -168.2981 -168.2981 -168.2981
##  [33,] -174.9202 -174.9202 -174.9202 -174.9202 -174.9202 -174.9202 -174.9202
##  [34,] -180.7064 -180.7064 -180.7064 -180.7064 -180.7064 -180.7064 -180.6949
##  [35,] -185.8064 -185.8064 -185.8064 -185.8064 -185.7938 -185.7361 -185.6411
##  [36,] -190.3365 -190.3365 -190.3222 -190.2607 -190.1622 -190.0358 -189.8880
##  [37,] -194.3708 -194.3032 -194.1985 -194.0676 -193.9176 -193.7536 -193.5795
##  [38,] -197.8184 -197.6797 -197.5251 -197.3596 -197.1868 -197.0091 -196.8284
##  [39,] -200.7582 -200.5902 -200.4185 -200.2450 -200.0709 -199.8971 -199.7245
##  [40,] -203.3041 -203.1379 -202.9736 -202.8113 -202.6515 -202.4944 -202.3402
##  [41,] -205.5512 -205.4087 -205.2686 -205.1310 -204.9961 -204.8641 -204.7352
##  [42,] -207.5810 -207.4759 -207.3703 -207.2654 -207.1616 -207.0596 -206.9597
##  [43,] -209.4670 -209.4046 -209.3377 -209.2682 -209.1977 -209.1270 -209.0570
##  [44,] -211.2778 -211.2549 -211.2240 -211.1878 -211.1484 -211.1070 -211.0649
##  [45,] -213.0842 -213.0842 -213.0795 -213.0687 -213.0539 -213.0365 -213.0174
##  [46,] -214.9528 -214.9528 -214.9528 -214.9528 -214.9516 -214.9489 -214.9452
##  [47,] -216.8753 -216.8753 -216.8753 -216.8753 -216.8753 -216.8753 -216.8764
##  [48,] -218.8340 -218.8340 -218.8340 -218.8340 -218.8340 -218.8340 -218.8340
##  [49,] -220.8124 -220.8124 -220.8124 -220.8124 -220.8124 -220.8124 -220.8124
##  [50,] -222.7959 -222.7959 -222.7959 -222.7959 -222.7959 -222.7959 -222.7959
##  [51,] -224.7714 -224.7714 -224.7714 -224.7714 -224.7714 -224.7714 -224.7714
##  [52,] -226.7275 -226.7275 -226.7275 -226.7275 -226.7275 -226.7275 -226.7275
##  [53,] -228.6544 -228.6544 -228.6544 -228.6544 -228.6544 -228.6544 -228.6544
##  [54,] -230.5438 -230.5438 -230.5438 -230.5438 -230.5438 -230.5437 -230.5259
##  [55,] -232.3887 -232.3887 -232.3887 -232.3885 -232.3681 -232.3190 -232.2470
##  [56,] -234.1835 -234.1832 -234.1592 -234.1039 -234.0249 -233.9274 -233.8153
##  [57,] -235.8940 -235.8301 -235.7416 -235.6351 -235.5148 -235.3836 -235.2436
##  [58,] -237.3889 -237.2706 -237.1402 -237.0007 -236.8540 -236.7017 -236.5445
##  [59,] -238.6764 -238.5279 -238.3747 -238.2178 -238.0578 -237.8952 -237.7302
##  [60,] -239.7809 -239.6228 -239.4633 -239.3024 -239.1403 -238.9769 -238.8123
##  [61,] -240.7234 -240.5735 -240.4220 -240.2689 -240.1144 -239.9587 -239.8016
##  [62,] -241.5224 -241.3965 -241.2655 -241.1305 -240.9924 -240.8515 -240.7084
##  [63,] -242.1940 -242.1062 -242.0071 -241.8996 -241.7855 -241.6661 -241.5424
##  [64,] -242.7531 -242.7166 -242.6597 -242.5879 -242.5047 -242.4127 -242.3134
##  [65,] -243.2426 -243.2426 -243.2359 -243.2073 -243.1611 -243.1014 -243.0310
##  [66,] -243.7712 -243.7712 -243.7712 -243.7712 -243.7661 -243.7431 -243.7051
##  [67,] -244.3505 -244.3505 -244.3505 -244.3505 -244.3505 -244.3505 -244.3466
##  [68,] -244.9825 -244.9825 -244.9825 -244.9825 -244.9825 -244.9825 -244.9825
##  [69,] -245.6689 -245.6689 -245.6689 -245.6689 -245.6689 -245.6689 -245.6689
##  [70,] -246.4105 -246.4105 -246.4105 -246.4105 -246.4105 -246.4105 -246.4105
##  [71,] -247.2074 -247.2074 -247.2074 -247.2074 -247.2074 -247.2074 -247.2074
##  [72,] -248.0587 -248.0587 -248.0587 -248.0587 -248.0587 -248.0587 -248.0587
##  [73,] -248.9623 -248.9623 -248.9623 -248.9623 -248.9623 -248.9623 -248.9623
##  [74,] -249.9148 -249.9148 -249.9148 -249.9148 -249.9148 -249.9120 -249.8901
##  [75,] -250.9114 -250.9114 -250.9114 -250.9078 -250.8818 -250.8352 -250.7724
##  [76,] -251.9457 -251.9411 -251.9095 -251.8549 -251.7827 -251.6970 -251.6003
##  [77,] -252.9642 -252.8989 -252.8153 -252.7179 -252.6100 -252.4936 -252.3702
##  [78,] -253.8550 -253.7438 -253.6231 -253.4952 -253.3614 -253.2227 -253.0799
##  [79,] -254.6158 -254.4764 -254.3330 -254.1861 -254.0362 -253.8834 -253.7280
##  [80,] -255.2493 -255.0986 -254.9458 -254.7909 -254.6339 -254.4749 -254.3138
##  [81,] -255.7572 -255.6111 -255.4617 -255.3094 -255.1544 -254.9967 -254.8364
##  [82,] -256.1388 -256.0131 -255.8800 -255.7408 -255.5965 -255.4477 -255.2948
##  [83,] -256.3886 -256.3004 -256.1972 -256.0823 -255.9581 -255.8260 -255.6873
##  [84,] -256.4941 -256.4640 -256.4064 -256.3283 -256.2344 -256.1278 -256.0105
##  [85,] -256.4963 -256.4963 -256.4947 -256.4690 -256.4178 -256.3467 -256.2594
##  [86,] -256.4963 -256.4963 -256.4963 -256.4963 -256.4950 -256.4723 -256.4255
##  [87,] -256.4963 -256.4963 -256.4963 -256.4963 -256.4963 -256.4963 -256.4953
##  [88,] -256.4963 -256.4963 -256.4963 -256.4963 -256.4963 -256.4963 -256.4963
##  [89,] -256.4963 -256.4963 -256.4963 -256.4963 -256.4963 -256.4963 -256.4963
##  [90,] -256.4963 -256.4963 -256.4963 -256.4963 -256.4963 -256.4963 -256.4963
##  [91,] -256.4963 -256.4963 -256.4963 -256.4963 -256.4963 -256.4963 -256.4963
##  [92,] -256.4963 -256.4963 -256.4963 -256.4963 -256.4963 -256.4963 -256.4963
##  [93,] -256.4963 -256.4963 -256.4963 -256.4963 -256.4963 -256.4963 -256.4963
##  [94,] -256.4963 -256.4963 -256.4963 -256.4963 -256.4963 -256.4963 -256.4963
##  [95,] -256.4963 -256.4963 -256.4963 -256.4963 -256.4963 -256.4963 -256.4963
##  [96,] -256.4963 -256.4963 -256.4963 -256.4963 -256.4963 -256.4963 -256.4963
##  [97,] -256.4963 -256.4963 -256.4963 -256.4963 -256.4963 -256.4963 -256.4963
##  [98,] -256.4963 -256.4963 -256.4963 -256.4963 -256.4963 -256.4963 -256.4963
##  [99,] -256.4963 -256.4963 -256.4963 -256.4963 -256.4963 -256.4963 -256.4963
## [100,] -256.4963 -256.4963 -256.4963 -256.4963 -256.4963 -256.4963 -256.4963
##            [,15]     [,16]     [,17]     [,18]     [,19]     [,20]     [,21]
##   [1,] -158.6237 -158.6237 -158.6237 -158.6237 -158.6237 -158.6237 -158.6237
##   [2,] -158.6237 -158.6237 -158.6237 -158.6237 -158.6237 -158.6237 -158.6237
##   [3,] -158.6237 -158.6237 -158.6237 -158.6237 -158.6237 -158.6237 -158.6237
##   [4,] -158.6237 -158.6237 -158.6237 -158.6237 -158.6237 -158.6237 -158.6237
##   [5,] -158.6237 -158.6237 -158.6237 -158.6237 -158.6237 -158.6237 -158.6237
##   [6,] -158.6237 -158.6237 -158.6237 -158.6237 -158.6237 -158.6237 -158.6237
##   [7,] -158.6237 -158.6237 -158.6237 -158.6237 -158.6237 -158.6237 -158.6237
##   [8,] -158.6237 -158.6237 -158.6237 -158.6237 -158.6237 -158.6237 -158.6237
##   [9,] -158.6237 -158.6237 -158.6237 -158.6237 -158.6237 -158.6237 -158.6237
##  [10,] -158.6237 -158.6237 -158.6237 -158.6237 -158.6237 -158.6237 -158.6237
##  [11,] -158.6237 -158.6237 -158.6237 -158.6237 -158.6237 -158.6180 -158.4306
##  [12,] -158.6237 -158.6237 -158.6237 -158.6167 -158.4230 -157.9906 -157.3474
##  [13,] -158.6237 -158.6151 -158.4112 -157.9669 -157.3139 -156.4750 -155.4667
##  [14,] -158.3941 -157.9290 -157.2547 -156.3971 -155.3749 -154.2012 -152.8851
##  [15,] -157.1637 -156.2726 -155.2208 -154.0231 -152.6900 -151.2285 -149.6433
##  [16,] -154.9881 -153.7487 -152.3809 -150.8923 -149.2883 -147.5722 -145.7458
##  [17,] -151.9243 -150.3906 -148.7503 -147.0069 -145.1626 -143.2185 -141.1753
##  [18,] -147.9699 -146.1834 -144.3061 -142.3391 -140.2831 -138.1390 -135.9081
##  [19,] -143.0800 -141.0801 -139.0022 -136.8471 -134.6164 -132.3126 -129.9401
##  [20,] -137.1861 -135.0223 -132.7946 -130.5064 -128.1629 -125.7718 -123.3443
##  [21,] -130.2369 -127.9904 -125.7025 -123.3830 -121.0452 -118.7070 -116.3916
##  [22,] -122.3100 -120.1345 -117.9595 -115.8067 -113.7028 -111.6802 -109.7770
##  [23,] -113.9088 -112.0952 -110.3541 -108.7215 -107.2367 -105.9417 -104.8791
##  [24,] -106.5929 -105.5873 -104.7361 -104.0772 -103.6448 -103.4677 -103.5673
##  [25,] -103.4658 -103.5642 -103.8376 -104.3048 -104.9773 -105.8591 -106.9473
##  [26,] -107.2875 -108.0864 -109.0354 -110.1315 -111.3688 -112.7390 -114.2319
##  [27,] -117.0056 -117.7383 -118.6438 -119.6986 -120.8831 -122.1803 -123.5750
##  [28,] -129.2311 -129.4873 -129.9675 -130.6338 -131.4557 -132.4086 -133.4718
##  [29,] -141.2895 -141.2895 -141.3114 -141.5149 -141.9036 -142.4483 -143.1249
##  [30,] -151.7319 -151.7319 -151.7319 -151.7319 -151.7476 -151.9064 -152.2156
##  [31,] -160.6483 -160.6483 -160.6483 -160.6483 -160.6483 -160.6392 -160.5990
##  [32,] -168.2981 -168.2981 -168.2981 -168.2883 -168.2354 -168.1431 -168.0181
##  [33,] -174.9202 -174.9096 -174.8556 -174.7631 -174.6388 -174.4884 -174.3166
##  [34,] -180.6396 -180.5464 -180.4226 -180.2742 -180.1058 -179.9215 -179.7244
##  [35,] -185.5169 -185.3695 -185.2039 -185.0240 -184.8329 -184.6332 -184.4272
##  [36,] -189.7240 -189.5475 -189.3616 -189.1687 -188.9709 -188.7697 -188.5667
##  [37,] -193.3979 -193.2113 -193.0212 -192.8292 -192.6363 -192.4438 -192.2524
##  [38,] -196.6460 -196.4632 -196.2809 -196.0997 -195.9205 -195.7437 -195.5699
##  [39,] -199.5535 -199.3847 -199.2185 -199.0554 -198.8956 -198.7394 -198.5873
##  [40,] -202.1891 -202.0414 -201.8972 -201.7567 -201.6202 -201.4878 -201.3598
##  [41,] -204.6095 -204.4872 -204.3685 -204.2535 -204.1424 -204.0353 -203.9324
##  [42,] -206.8623 -206.7676 -206.6759 -206.5874 -206.5022 -206.4207 -206.3430
##  [43,] -208.9882 -208.9211 -208.8562 -208.7936 -208.7337 -208.6768 -208.6231
##  [44,] -211.0228 -210.9814 -210.9412 -210.9026 -210.8661 -210.8318 -210.8001
##  [45,] -212.9978 -212.9781 -212.9590 -212.9410 -212.9245 -212.9098 -212.8972
##  [46,] -214.9413 -214.9375 -214.9343 -214.9321 -214.9313 -214.9321 -214.9348
##  [47,] -216.8790 -216.8832 -216.8890 -216.8965 -216.9059 -216.9172 -216.9306
##  [48,] -218.8340 -218.8364 -218.8427 -218.8524 -218.8653 -218.8811 -218.8998
##  [49,] -220.8124 -220.8124 -220.8124 -220.8157 -220.8244 -220.8380 -220.8559
##  [50,] -222.7959 -222.7959 -222.7959 -222.7959 -222.7959 -222.7996 -222.8100
##  [51,] -224.7714 -224.7714 -224.7714 -224.7714 -224.7714 -224.7589 -224.7242
##  [52,] -226.7275 -226.7275 -226.7275 -226.7135 -226.6761 -226.6185 -226.5438
##  [53,] -228.6544 -228.6387 -228.5980 -228.5365 -228.4572 -228.3629 -228.2555
##  [54,] -230.4815 -230.4152 -230.3309 -230.2315 -230.1191 -229.9957 -229.8627
##  [55,] -232.1568 -232.0515 -231.9337 -231.8053 -231.6678 -231.5224 -231.3702
##  [56,] -233.6913 -233.5574 -233.4152 -233.2658 -233.1103 -232.9494 -232.7837
##  [57,] -235.0963 -234.9430 -234.7844 -234.6213 -234.4542 -234.2835 -234.1097
##  [58,] -236.3833 -236.2186 -236.0508 -235.8803 -235.7073 -235.5320 -235.3546
##  [59,] -237.5632 -237.3943 -237.2236 -237.0513 -236.8774 -236.7021 -236.5253
##  [60,] -238.6466 -238.4797 -238.3116 -238.1424 -237.9721 -237.8008 -237.6284
##  [61,] -239.6434 -239.4840 -239.3234 -239.1617 -238.9990 -238.8352 -238.6704
##  [62,] -240.5631 -240.4161 -240.2673 -240.1171 -239.9653 -239.8122 -239.6578
##  [63,] -241.4151 -241.2847 -241.1515 -241.0160 -240.8783 -240.7387 -240.5973
##  [64,] -242.2083 -242.0983 -241.9840 -241.8662 -241.7453 -241.6215 -241.4953
##  [65,] -242.9518 -242.8654 -242.7729 -242.6754 -242.5734 -242.4676 -242.3584
##  [66,] -243.6550 -243.5949 -243.5266 -243.4514 -243.3703 -243.2840 -243.1934
##  [67,] -244.3279 -244.2962 -244.2539 -244.2025 -244.1436 -244.0782 -244.0072
##  [68,] -244.9825 -244.9795 -244.9642 -244.9377 -244.9019 -244.8580 -244.8073
##  [69,] -245.6689 -245.6689 -245.6689 -245.6666 -245.6539 -245.6318 -245.6016
##  [70,] -246.4105 -246.4105 -246.4105 -246.4105 -246.4105 -246.4087 -246.3973
##  [71,] -247.2074 -247.2074 -247.2074 -247.2074 -247.2060 -247.1924 -247.1659
##  [72,] -248.0587 -248.0587 -248.0570 -248.0411 -248.0107 -247.9680 -247.9147
##  [73,] -248.9601 -248.9415 -248.9067 -248.8582 -248.7983 -248.7285 -248.6503
##  [74,] -249.8500 -249.7949 -249.7275 -249.6497 -249.5631 -249.4689 -249.3681
##  [75,] -250.6965 -250.6097 -250.5140 -250.4106 -250.3006 -250.1849 -250.0643
##  [76,] -251.4946 -251.3815 -251.2620 -251.1370 -251.0073 -250.8733 -250.7356
##  [77,] -252.2410 -252.1068 -251.9683 -251.8260 -251.6802 -251.5313 -251.3795
##  [78,] -252.9334 -252.7836 -252.6308 -252.4753 -252.3172 -252.1568 -251.9941
##  [79,] -253.5703 -253.4101 -253.2478 -253.0833 -252.9167 -252.7481 -252.5775
##  [80,] -254.1506 -253.9854 -253.8181 -253.6488 -253.4774 -253.3039 -253.1285
##  [81,] -254.6736 -254.5084 -254.3407 -254.1705 -253.9980 -253.8232 -253.6459
##  [82,] -255.1382 -254.9779 -254.8144 -254.6476 -254.4776 -254.3047 -254.1287
##  [83,] -255.5426 -255.3926 -255.2378 -255.0785 -254.9149 -254.7472 -254.5757
##  [84,] -255.8843 -255.7501 -255.6089 -255.4614 -255.3081 -255.1494 -254.9855
##  [85,] -256.1587 -256.0466 -255.9246 -255.7938 -255.6550 -255.5090 -255.3563
##  [86,] -256.3592 -256.2765 -256.1800 -256.0713 -255.9520 -255.8231 -255.6855
##  [87,] -256.4746 -256.4308 -256.3676 -256.2880 -256.1940 -256.0874 -255.9694
##  [88,] -256.4963 -256.4955 -256.4762 -256.4343 -256.3732 -256.2952 -256.2024
##  [89,] -256.4963 -256.4963 -256.4963 -256.4956 -256.4773 -256.4366 -256.3763
##  [90,] -256.4963 -256.4963 -256.4963 -256.4963 -256.4963 -256.4957 -256.4780
##  [91,] -256.4963 -256.4963 -256.4963 -256.4963 -256.4963 -256.4963 -256.4963
##  [92,] -256.4963 -256.4963 -256.4963 -256.4963 -256.4963 -256.4963 -256.4963
##  [93,] -256.4963 -256.4963 -256.4963 -256.4963 -256.4963 -256.4963 -256.4963
##  [94,] -256.4963 -256.4963 -256.4963 -256.4963 -256.4963 -256.4963 -256.4963
##  [95,] -256.4963 -256.4963 -256.4963 -256.4963 -256.4963 -256.4963 -256.4963
##  [96,] -256.4963 -256.4963 -256.4963 -256.4963 -256.4963 -256.4963 -256.4963
##  [97,] -256.4963 -256.4963 -256.4963 -256.4963 -256.4963 -256.4963 -256.4963
##  [98,] -256.4963 -256.4963 -256.4963 -256.4963 -256.4963 -256.4963 -256.4963
##  [99,] -256.4963 -256.4963 -256.4963 -256.4963 -256.4963 -256.4963 -256.4963
## [100,] -256.4963 -256.4963 -256.4963 -256.4963 -256.4963 -256.4963 -256.4963
##            [,22]     [,23]     [,24]     [,25]     [,26]     [,27]     [,28]
##   [1,] -158.6237 -158.6237 -158.6237 -158.6237 -158.6237 -158.6237 -158.6237
##   [2,] -158.6237 -158.6237 -158.6237 -158.6237 -158.6237 -158.6237 -158.6237
##   [3,] -158.6237 -158.6237 -158.6237 -158.6237 -158.6237 -158.6237 -158.6237
##   [4,] -158.6237 -158.6237 -158.6237 -158.6237 -158.6237 -158.6237 -158.6237
##   [5,] -158.6237 -158.6237 -158.6237 -158.6237 -158.6237 -158.6237 -158.6237
##   [6,] -158.6237 -158.6237 -158.6237 -158.6237 -158.6237 -158.6237 -158.6237
##   [7,] -158.6237 -158.6237 -158.6237 -158.6237 -158.6237 -158.6237 -158.6216
##   [8,] -158.6237 -158.6237 -158.6237 -158.6237 -158.6209 -158.4491 -158.0354
##   [9,] -158.6237 -158.6237 -158.6200 -158.4430 -158.0248 -157.3931 -156.5696
##  [10,] -158.6190 -158.4368 -158.0140 -157.3783 -156.5511 -155.5494 -154.3865
##  [11,] -158.0032 -157.3634 -156.5326 -155.5275 -154.3615 -153.0449 -151.5861
##  [12,] -156.5139 -155.5055 -154.3363 -153.0168 -151.5552 -149.9581 -148.2306
##  [13,] -154.3005 -152.9845 -151.5241 -149.9244 -148.1944 -146.3385 -144.3606
##  [14,] -151.4330 -149.8488 -148.1346 -146.2914 -144.3189 -142.2203 -140.0063
##  [15,] -147.9372 -146.1116 -144.1672 -142.1039 -139.9214 -137.6199 -135.2001
##  [16,] -143.8100 -141.7655 -139.6123 -137.3510 -134.9829 -132.5107 -129.9395
##  [17,] -139.0337 -136.7946 -134.4596 -132.0320 -129.5171 -126.9233 -124.2635
##  [18,] -133.5925 -131.1958 -128.7239 -126.1857 -123.5944 -120.9687 -118.3347
##  [19,] -127.5055 -125.0186 -122.4934 -119.9492 -117.4124 -114.9175 -112.5090
##  [20,] -120.8959 -118.4474 -116.0263 -113.6677 -111.4155 -109.3223 -107.4490
##  [21,] -114.1284 -111.9540 -109.9122 -108.0539 -106.4354 -105.1157 -104.1520
##  [22,] -108.0368 -106.5077 -105.2400 -104.2825 -103.6794 -103.4647 -103.6589
##  [23,] -104.0895 -103.6092 -103.4660 -103.6772 -104.2474 -105.1679 -106.4180
##  [24,] -103.9562 -104.6374 -105.6042 -106.8412 -108.3258 -110.0305 -111.9248
##  [25,] -108.2330 -109.7021 -111.3371 -113.1179 -115.0236 -117.0333 -119.1266
##  [26,] -115.8353 -117.5363 -119.3216 -121.1779 -123.0923 -125.0529 -127.0485
##  [27,] -125.0535 -126.6033 -128.2131 -129.8723 -131.5715 -133.3021 -135.0521
##  [28,] -134.6280 -135.8624 -137.1624 -138.5170 -139.9107 -141.3074 -142.6980
##  [29,] -143.9130 -144.7957 -145.7514 -146.7372 -147.7429 -148.7642 -149.7979
##  [30,] -152.6453 -153.1445 -153.6998 -154.3035 -154.9496 -155.6330 -156.3495
##  [31,] -160.6328 -160.7565 -160.9594 -161.2329 -161.5694 -161.9625 -162.4067
##  [32,] -167.8657 -167.6992 -167.6064 -167.6020 -167.6771 -167.8242 -168.0367
##  [33,] -174.1274 -173.9244 -173.7106 -173.4950 -173.3498 -173.2885 -173.3035
##  [34,] -179.5176 -179.3033 -179.0839 -178.8611 -178.6369 -178.4175 -178.2633
##  [35,] -184.2168 -184.0037 -183.7894 -183.5753 -183.3624 -183.1521 -182.9453
##  [36,] -188.3631 -188.1601 -187.9585 -187.7595 -187.5637 -187.3720 -187.1851
##  [37,] -192.0629 -191.8761 -191.6926 -191.5131 -191.3380 -191.1680 -191.0036
##  [38,] -195.3996 -195.2332 -195.0712 -194.9140 -194.7620 -194.6155 -194.4749
##  [39,] -198.4395 -198.2962 -198.1578 -198.0245 -197.8966 -197.7744 -197.6582
##  [40,] -201.2362 -201.1174 -201.0036 -200.8948 -200.7914 -200.6935 -200.6014
##  [41,] -203.8339 -203.7399 -203.6507 -203.5663 -203.4869 -203.4128 -203.3440
##  [42,] -206.2692 -206.1995 -206.1341 -206.0731 -206.0168 -205.9652 -205.9185
##  [43,] -208.5728 -208.5261 -208.4831 -208.4441 -208.4092 -208.3786 -208.3523
##  [44,] -210.7712 -210.7454 -210.7229 -210.7037 -210.6882 -210.6764 -210.6686
##  [45,] -212.8870 -212.8794 -212.8746 -212.8727 -212.8741 -212.8788 -212.8870
##  [46,] -214.9397 -214.9469 -214.9566 -214.9691 -214.9844 -215.0027 -215.0242
##  [47,] -216.9462 -216.9643 -216.9849 -217.0081 -217.0341 -217.0629 -217.0859
##  [48,] -218.9214 -218.9458 -218.9730 -219.0031 -219.0265 -219.0332 -219.0247
##  [49,] -220.8778 -220.9034 -220.9224 -220.9244 -220.9114 -220.8851 -220.8468
##  [50,] -222.8149 -222.8039 -222.7790 -222.7418 -222.6937 -222.6359 -222.5693
##  [51,] -224.6742 -224.6147 -224.5468 -224.4714 -224.3892 -224.3009 -224.2071
##  [52,] -226.4541 -226.3514 -226.2410 -226.1280 -226.0124 -225.8944 -225.7741
##  [53,] -228.1368 -228.0081 -227.8706 -227.7252 -227.5765 -227.4291 -227.2827
##  [54,] -229.7212 -229.5723 -229.4168 -229.2554 -229.0886 -228.9169 -228.7445
##  [55,] -231.2118 -231.0480 -230.8794 -230.7064 -230.5294 -230.3488 -230.1648
##  [56,] -232.6137 -232.4400 -232.2627 -232.0822 -231.8989 -231.7128 -231.5243
##  [57,] -233.9329 -233.7535 -233.5716 -233.3874 -233.2012 -233.0130 -232.8229
##  [58,] -235.1753 -234.9942 -234.8113 -234.6268 -234.4408 -234.2533 -234.0644
##  [59,] -236.3472 -236.1679 -235.9872 -235.8054 -235.6224 -235.4383 -235.2531
##  [60,] -237.4549 -237.2804 -237.1049 -236.9284 -236.7510 -236.5726 -236.3933
##  [61,] -238.5045 -238.3377 -238.1699 -238.0011 -237.8314 -237.6609 -237.4894
##  [62,] -239.5023 -239.3455 -239.1876 -239.0287 -238.8688 -238.7079 -238.5460
##  [63,] -240.4543 -240.3098 -240.1638 -240.0166 -239.8681 -239.7184 -239.5677
##  [64,] -241.3669 -241.2364 -241.1041 -240.9701 -240.8345 -240.6975 -240.5592
##  [65,] -242.2462 -242.1314 -242.0141 -241.8947 -241.7733 -241.6501 -241.5252
##  [66,] -243.0988 -243.0008 -242.8998 -242.7960 -242.6897 -242.5812 -242.4707
##  [67,] -243.9313 -243.8511 -243.7671 -243.6797 -243.5893 -243.4956 -243.3916
##  [68,] -244.7507 -244.6888 -244.6223 -244.5511 -244.4677 -244.3710 -244.2620
##  [69,] -245.5643 -245.5200 -245.4608 -245.3861 -245.2973 -245.1957 -245.0823
##  [70,] -246.3671 -246.3183 -246.2528 -246.1725 -246.0787 -245.9726 -245.8553
##  [71,] -247.1268 -247.0708 -246.9991 -246.9133 -246.8148 -246.7047 -246.5839
##  [72,] -247.8522 -247.7817 -247.7030 -247.6119 -247.5089 -247.3950 -247.2709
##  [73,] -248.5647 -248.4727 -248.3750 -248.2722 -248.1641 -248.0464 -247.9194
##  [74,] -249.2615 -249.1499 -249.0338 -248.9136 -248.7898 -248.6628 -248.5320
##  [75,] -249.9391 -249.8101 -249.6774 -249.5416 -249.4029 -249.2616 -249.1178
##  [76,] -250.5945 -250.4503 -250.3034 -250.1539 -250.0020 -249.8479 -249.6918
##  [77,] -251.2252 -251.0684 -250.9093 -250.7481 -250.5849 -250.4199 -250.2530
##  [78,] -251.8292 -251.6623 -251.4934 -251.3227 -251.1501 -250.9758 -250.7998
##  [79,] -252.4049 -252.2305 -252.0541 -251.8759 -251.6960 -251.5143 -251.3309
##  [80,] -252.9510 -252.7716 -252.5901 -252.4067 -252.2214 -252.0341 -251.8450
##  [81,] -253.4664 -253.2845 -253.1003 -252.9139 -252.7252 -252.5343 -252.3412
##  [82,] -253.9499 -253.7682 -253.5837 -253.3964 -253.2064 -253.0138 -252.8185
##  [83,] -254.4005 -254.2216 -254.0392 -253.8534 -253.6642 -253.4717 -253.2760
##  [84,] -254.8168 -254.6435 -254.4658 -254.2837 -254.0975 -253.9073 -253.7131
##  [85,] -255.1974 -255.0325 -254.8621 -254.6863 -254.5054 -254.3194 -254.1287
##  [86,] -255.5398 -255.3867 -255.2265 -255.0596 -254.8864 -254.7071 -254.5218
##  [87,] -255.8411 -255.7032 -255.5566 -255.4016 -255.2389 -255.0689 -254.8931
##  [88,] -256.0964 -255.9782 -255.8490 -255.7098 -255.5616 -255.4056 -255.2423
##  [89,] -256.2988 -256.2060 -256.0996 -255.9811 -255.8518 -255.7127 -255.5647
##  [90,] -256.4377 -256.3778 -256.3005 -256.2080 -256.1019 -255.9836 -255.8545
##  [91,] -256.4959 -256.4786 -256.4388 -256.3792 -256.3023 -256.2100 -256.1042
##  [92,] -256.4963 -256.4963 -256.4959 -256.4792 -256.4398 -256.3806 -256.3040
##  [93,] -256.4963 -256.4963 -256.4963 -256.4963 -256.4960 -256.4798 -256.4408
##  [94,] -256.4963 -256.4963 -256.4963 -256.4963 -256.4963 -256.4963 -256.4961
##  [95,] -256.4963 -256.4963 -256.4963 -256.4963 -256.4963 -256.4963 -256.4963
##  [96,] -256.4963 -256.4963 -256.4963 -256.4963 -256.4963 -256.4963 -256.4963
##  [97,] -256.4963 -256.4963 -256.4963 -256.4963 -256.4963 -256.4963 -256.4963
##  [98,] -256.4963 -256.4963 -256.4963 -256.4963 -256.4963 -256.4963 -256.4963
##  [99,] -256.4963 -256.4963 -256.4963 -256.4963 -256.4963 -256.4963 -256.4963
## [100,] -256.4963 -256.4963 -256.4963 -256.4963 -256.4963 -256.4963 -256.4963
##            [,29]     [,30]     [,31]     [,32]     [,33]     [,34]     [,35]
##   [1,] -158.6237 -158.6237 -158.6237 -158.6237 -158.6237 -158.6237 -158.6237
##   [2,] -158.6237 -158.6237 -158.6237 -158.6237 -158.6237 -158.6237 -158.6237
##   [3,] -158.6237 -158.6237 -158.6237 -158.6237 -158.6237 -158.6237 -158.6237
##   [4,] -158.6237 -158.6237 -158.6237 -158.6237 -158.6237 -158.6232 -158.4724
##   [5,] -158.6237 -158.6237 -158.6237 -158.6228 -158.4667 -158.0668 -157.4515
##   [6,] -158.6237 -158.6222 -158.4609 -158.0564 -157.4370 -156.6246 -155.6365
##   [7,] -158.4550 -158.0460 -157.4225 -156.6064 -155.6149 -154.4614 -153.1567
##   [8,] -157.4078 -156.5880 -155.5931 -154.4365 -153.1288 -151.6784 -150.0920
##   [9,] -155.5713 -154.4116 -153.1009 -151.6477 -150.0586 -148.3389 -146.4931
##  [10,] -153.0729 -151.6169 -150.0251 -148.3029 -146.4545 -144.4838 -142.3943
##  [11,] -149.9916 -148.2668 -146.4159 -144.4428 -142.3509 -140.1437 -137.8254
##  [12,] -146.3773 -144.4017 -142.3074 -140.0980 -137.7775 -135.3511 -132.8255
##  [13,] -142.2639 -140.0522 -137.7295 -135.3010 -132.7735 -130.1563 -127.4620
##  [14,] -137.6815 -135.2509 -132.7215 -130.1026 -127.4069 -124.6519 -121.8611
##  [15,] -132.6695 -130.0488 -127.3518 -124.5958 -121.8046 -119.0095 -116.2517
##  [16,] -127.2777 -124.5386 -121.7480 -118.9533 -116.1967 -113.5303 -111.0179
##  [17,] -121.5567 -118.8298 -116.1198 -113.4765 -110.9693 -108.6915 -106.7275
##  [18,] -115.7275 -113.1933 -110.7905 -108.5904 -106.6754 -105.1348 -104.0585
##  [19,] -110.2417 -108.1810 -106.4012 -104.9805 -103.9948 -103.5086 -103.5653
##  [20,] -105.8624 -104.6307 -103.8174 -103.4737 -103.6317 -104.2991 -105.4581
##  [21,] -103.5941 -103.4785 -103.8237 -104.6274 -105.8667 -107.5014 -109.4795
##  [22,] -104.2660 -105.2731 -106.6524 -108.3640 -110.3609 -112.5930 -115.0113
##  [23,] -107.9675 -109.7792 -111.8129 -114.0276 -116.3844 -118.8477 -121.3860
##  [24,] -113.9775 -116.1581 -118.4381 -120.7919 -123.1969 -125.6323 -128.0669
##  [25,] -121.2849 -123.4912 -125.7304 -127.9871 -130.2324 -132.4476 -134.6225
##  [26,] -129.0691 -131.1026 -133.1197 -135.1071 -137.0585 -138.9708 -140.8428
##  [27,] -136.7898 -138.5053 -140.1948 -141.8565 -143.4899 -145.0953 -146.6737
##  [28,] -144.0795 -145.4502 -146.8091 -148.1561 -149.4912 -150.8147 -152.1271
##  [29,] -150.8417 -151.8937 -152.9526 -154.0174 -155.0871 -156.1612 -157.2391
##  [30,] -157.0956 -157.8684 -158.6652 -159.4837 -160.3221 -161.1786 -162.0517
##  [31,] -162.8971 -163.4295 -164.0003 -164.6060 -165.2438 -165.9110 -166.6053
##  [32,] -168.3087 -168.6350 -169.0110 -169.4326 -169.8962 -170.3983 -170.9360
##  [33,] -173.3884 -173.5373 -173.7450 -174.0066 -174.3180 -174.6754 -175.0751
##  [34,] -178.1869 -178.1821 -178.2430 -178.3647 -178.5423 -178.7717 -179.0490
##  [35,] -182.7466 -182.6074 -182.5395 -182.5376 -182.5967 -182.7122 -182.8801
##  [36,] -187.0038 -186.8286 -186.6631 -186.5512 -186.5043 -186.5177 -186.5870
##  [37,] -190.8453 -190.6935 -190.5488 -190.4116 -190.2844 -190.2054 -190.1853
##  [38,] -194.3406 -194.2129 -194.0922 -193.9789 -193.8732 -193.7755 -193.6877
##  [39,] -197.5481 -197.4445 -197.3477 -197.2579 -197.1753 -197.1002 -197.0329
##  [40,] -200.5152 -200.4352 -200.3615 -200.2944 -200.2340 -200.1806 -200.1344
##  [41,] -203.2807 -203.2232 -203.1716 -203.1260 -203.0867 -203.0537 -203.0273
##  [42,] -205.8769 -205.8405 -205.8095 -205.7840 -205.7642 -205.7501 -205.7420
##  [43,] -208.3307 -208.3137 -208.3016 -208.2944 -208.2923 -208.2955 -208.3041
##  [44,] -210.6648 -210.6653 -210.6700 -210.6793 -210.6931 -210.7039 -210.6999
##  [45,] -212.8988 -212.9143 -212.9338 -212.9493 -212.9495 -212.9354 -212.9083
##  [46,] -215.0490 -215.0688 -215.0728 -215.0623 -215.0383 -215.0021 -214.9545
##  [47,] -217.0925 -217.0841 -217.0623 -217.0280 -216.9824 -216.9263 -216.8607
##  [48,] -219.0025 -218.9681 -218.9224 -218.8665 -218.8013 -218.7274 -218.6456
##  [49,] -220.7978 -220.7390 -220.6713 -220.5955 -220.5123 -220.4222 -220.3258
##  [50,] -222.4947 -222.4130 -222.3248 -222.2304 -222.1306 -222.0256 -221.9159
##  [51,] -224.1082 -224.0047 -223.8969 -223.7851 -223.6696 -223.5507 -223.4286
##  [52,] -225.6516 -225.5270 -225.4004 -225.2718 -225.1415 -225.0094 -224.8756
##  [53,] -227.1370 -226.9917 -226.8467 -226.7019 -226.5571 -226.4122 -226.2672
##  [54,] -228.5756 -228.4098 -228.2466 -228.0857 -227.9266 -227.7692 -227.6131
##  [55,] -229.9778 -229.7913 -229.6098 -229.4326 -229.2593 -229.0892 -228.9222
##  [56,] -231.3335 -231.1405 -230.9455 -230.7518 -230.5638 -230.3810 -230.2027
##  [57,] -232.6311 -232.4377 -232.2427 -232.0463 -231.8485 -231.6524 -231.4625
##  [58,] -233.8743 -233.6828 -233.4902 -233.2965 -233.1016 -232.9058 -232.7090
##  [59,] -235.0669 -234.8797 -234.6915 -234.5024 -234.3124 -234.1216 -233.9300
##  [60,] -236.2132 -236.0321 -235.8502 -235.6676 -235.4841 -235.3000 -235.1151
##  [61,] -237.3172 -237.1441 -236.9702 -236.7955 -236.6202 -236.4441 -236.2674
##  [62,] -238.3833 -238.2197 -238.0553 -237.8901 -237.7241 -237.5575 -237.3902
##  [63,] -239.4159 -239.2632 -239.1095 -238.9550 -238.7996 -238.6436 -238.4865
##  [64,] -240.4196 -240.2788 -240.1370 -239.9942 -239.8502 -239.6988 -239.5383
##  [65,] -241.3988 -241.2711 -241.1416 -241.0042 -240.8570 -240.7004 -240.5348
##  [66,] -242.3579 -242.2361 -242.1037 -241.9612 -241.8093 -241.6482 -241.4784
##  [67,] -243.2759 -243.1492 -243.0122 -242.8655 -242.7095 -242.5447 -242.3715
##  [68,] -244.1417 -244.0107 -243.8698 -243.7195 -243.5603 -243.3925 -243.2165
##  [69,] -244.9579 -244.8234 -244.6792 -244.5260 -244.3641 -244.1939 -244.0158
##  [70,] -245.7275 -245.5898 -245.4430 -245.2874 -245.1235 -244.9515 -244.7719
##  [71,] -246.4530 -246.3128 -246.1638 -246.0063 -245.8409 -245.6677 -245.4870
##  [72,] -247.1374 -246.9950 -246.8442 -246.6853 -246.5187 -246.3446 -246.1634
##  [73,] -247.7834 -247.6391 -247.4867 -247.3267 -247.1593 -246.9847 -246.8031
##  [74,] -248.3937 -248.2476 -248.0939 -247.9329 -247.7649 -247.5901 -247.4085
##  [75,] -248.9718 -248.8232 -248.6682 -248.5063 -248.3379 -248.1628 -247.9814
##  [76,] -249.5338 -249.3741 -249.2128 -249.0494 -248.8804 -248.7052 -248.5239
##  [77,] -250.0845 -249.9144 -249.7428 -249.5697 -249.3954 -249.2193 -249.0380
##  [78,] -250.6223 -250.4431 -250.2625 -250.0805 -249.8971 -249.7124 -249.5265
##  [79,] -251.1458 -250.9591 -250.7708 -250.5810 -250.3897 -250.1970 -250.0029
##  [80,] -251.6540 -251.4612 -251.2666 -251.0703 -250.8723 -250.6726 -250.4713
##  [81,] -252.1459 -251.9485 -251.7490 -251.5474 -251.3438 -251.1383 -250.9308
##  [82,] -252.6205 -252.4201 -252.2171 -252.0116 -251.8037 -251.5934 -251.3808
##  [83,] -253.0772 -252.8752 -252.6701 -252.4621 -252.2511 -252.0373 -251.8211
##  [84,] -253.5150 -253.3131 -253.1076 -252.8983 -252.6860 -252.4723 -252.2574
##  [85,] -253.9332 -253.7331 -253.5290 -253.3224 -253.1135 -252.9027 -252.6903
##  [86,] -254.3313 -254.1367 -253.9386 -253.7373 -253.5332 -253.3266 -253.1177
##  [87,] -254.7118 -254.5257 -254.3352 -254.1407 -253.9427 -253.7414 -253.5373
##  [88,] -255.0724 -254.8967 -254.7155 -254.5295 -254.3391 -254.1447 -253.9467
##  [89,] -255.4088 -255.2456 -255.0759 -254.9003 -254.7192 -254.5333 -254.3430
##  [90,] -255.7156 -255.5678 -255.4120 -255.2490 -255.0794 -254.9039 -254.7229
##  [91,] -255.9861 -255.8572 -255.7185 -255.5709 -255.4152 -255.2524 -255.0829
##  [92,] -256.2120 -256.1064 -255.9886 -255.8599 -255.7213 -255.5739 -255.4185
##  [93,] -256.3819 -256.3057 -256.2140 -256.1087 -255.9911 -255.8626 -255.7242
##  [94,] -256.4803 -256.4418 -256.3833 -256.3074 -256.2160 -256.1109 -255.9936
##  [95,] -256.4963 -256.4962 -256.4809 -256.4428 -256.3847 -256.3091 -256.2180
##  [96,] -256.4963 -256.4963 -256.4963 -256.4962 -256.4814 -256.4437 -256.3860
##  [97,] -256.4963 -256.4963 -256.4963 -256.4963 -256.4963 -256.4962 -256.4820
##  [98,] -256.4963 -256.4963 -256.4963 -256.4963 -256.4963 -256.4963 -256.4963
##  [99,] -256.4963 -256.4963 -256.4963 -256.4963 -256.4963 -256.4963 -256.4963
## [100,] -256.4963 -256.4963 -256.4963 -256.4963 -256.4963 -256.4963 -256.4963
##            [,36]     [,37]     [,38]     [,39]     [,40]     [,41]     [,42]
##   [1,] -158.6237 -158.6237 -158.6237 -158.6237 -158.6237 -158.4888 -158.1075
##   [2,] -158.6237 -158.6237 -158.6236 -158.4834 -158.0974 -157.4945 -156.6970
##   [3,] -158.6234 -158.4779 -158.0873 -157.4803 -156.6790 -155.7012 -154.5604
##   [4,] -158.0771 -157.4659 -156.6610 -155.6797 -154.5357 -153.2399 -151.8008
##   [5,] -156.6428 -155.6581 -154.5110 -153.2122 -151.7703 -150.1919 -148.4827
##   [6,] -154.4862 -153.1845 -151.7397 -150.1587 -148.4468 -146.6085 -144.6476
##   [7,] -151.7091 -150.1254 -148.4109 -146.5701 -144.6067 -142.5242 -140.3263
##   [8,] -148.3750 -146.5316 -144.5658 -142.4810 -140.2807 -137.9690 -135.5509
##   [9,] -144.5248 -142.4377 -140.2351 -137.9212 -135.5010 -132.9813 -130.3709
##  [10,] -140.1894 -137.8733 -135.4511 -132.9294 -130.3173 -127.6272 -124.8762
##  [11,] -135.4011 -132.8775 -130.2637 -127.5722 -124.8202 -122.0307 -119.2346
##  [12,] -130.2100 -127.5171 -124.7641 -121.9742 -119.1784 -116.4169 -113.7409
##  [13,] -124.7080 -121.9177 -119.1221 -116.3618 -113.6882 -111.1645 -108.8650
##  [14,] -119.0658 -116.3067 -113.6355 -111.1155 -108.8214 -106.8360 -105.2446
##  [15,] -113.5828 -111.0667 -108.7780 -106.7997 -105.2170 -104.1089 -103.5371
##  [16,] -108.7347 -106.7635 -105.1897 -104.0919 -103.5314 -103.5426 -104.1267
##  [17,] -105.1626 -104.0751 -103.5259 -103.5488 -104.1442 -105.2796 -106.8953
##  [18,] -103.5207 -103.5552 -104.1620 -105.3078 -106.9324 -108.9586 -111.3023
##  [19,] -104.1806 -105.3361 -106.9697 -109.0030 -111.3523 -113.9360 -116.6809
##  [20,] -107.0683 -109.0725 -111.4047 -113.9901 -116.7376 -119.5831 -122.4763
##  [21,] -111.7429 -114.2328 -116.8938 -119.6764 -122.5379 -125.4263 -128.2705
##  [22,] -117.5700 -120.2287 -122.9520 -125.7002 -128.4349 -131.1308 -133.7723
##  [23,] -123.9717 -126.5698 -129.1487 -131.6879 -134.1751 -136.6032 -138.9692
##  [24,] -130.4758 -132.8438 -135.1621 -137.4259 -139.6334 -141.7849 -143.8815
##  [25,] -136.7513 -138.8316 -140.8626 -142.8451 -144.7807 -146.6714 -148.5197
##  [26,] -142.6747 -144.4676 -146.2230 -147.9428 -149.6290 -151.2836 -152.9087
##  [27,] -148.2263 -149.7544 -151.2594 -152.7429 -154.2061 -155.6505 -157.0774
##  [28,] -153.4291 -154.7212 -156.0039 -157.2780 -158.5440 -159.8023 -161.0536
##  [29,] -158.3204 -159.4048 -160.4919 -161.5815 -162.6733 -163.7670 -164.8626
##  [30,] -162.9399 -163.8421 -164.7572 -165.6840 -166.6217 -167.5694 -168.5263
##  [31,] -167.3244 -168.0665 -168.8298 -169.6126 -170.4135 -171.2310 -172.0639
##  [32,] -171.5064 -172.1072 -172.7360 -173.3906 -174.0692 -174.7700 -175.4914
##  [33,] -175.5140 -175.9892 -176.4979 -177.0378 -177.6066 -178.2021 -178.8225
##  [34,] -179.3707 -179.7336 -180.1346 -180.5711 -181.0405 -181.5404 -182.0688
##  [35,] -183.0967 -183.3583 -183.6619 -184.0045 -184.3833 -184.7959 -185.2400
##  [36,] -186.7084 -186.8782 -187.0931 -187.3499 -187.6457 -187.9780 -188.3442
##  [37,] -190.2200 -190.3059 -190.4393 -190.6172 -190.8364 -191.0943 -191.3883
##  [38,] -193.6430 -193.6515 -193.7098 -193.8146 -193.9628 -194.1514 -194.3780
##  [39,] -196.9737 -196.9239 -196.9124 -196.9491 -197.0308 -197.1547 -197.3180
##  [40,] -200.0956 -200.0643 -200.0408 -200.0261 -200.0455 -200.1084 -200.2049
##  [41,] -203.0076 -202.9948 -202.9890 -202.9905 -202.9920 -202.9804 -202.9780
##  [42,] -205.7400 -205.7442 -205.7475 -205.7367 -205.7123 -205.6752 -205.6262
##  [43,] -208.3106 -208.3028 -208.2812 -208.2469 -208.2006 -208.1430 -208.0748
##  [44,] -210.6820 -210.6512 -210.6083 -210.5540 -210.4893 -210.4146 -210.3307
##  [45,] -212.8689 -212.8182 -212.7570 -212.6860 -212.6057 -212.5169 -212.4200
##  [46,] -214.8964 -214.8286 -214.7517 -214.6663 -214.5730 -214.4723 -214.3647
##  [47,] -216.7861 -216.7033 -216.6128 -216.5151 -216.4107 -216.3001 -216.1836
##  [48,] -218.5564 -218.4604 -218.3581 -218.2498 -218.1360 -218.0170 -217.8932
##  [49,] -220.2236 -220.1159 -220.0031 -219.8856 -219.7636 -219.6376 -219.5076
##  [50,] -221.8018 -221.6835 -221.5614 -221.4357 -221.3067 -221.1745 -221.0393
##  [51,] -223.3035 -223.1756 -223.0451 -222.9121 -222.7767 -222.6392 -222.4996
##  [52,] -224.7402 -224.6033 -224.4650 -224.3252 -224.1842 -224.0419 -223.8984
##  [53,] -226.1220 -225.9765 -225.8308 -225.6848 -225.5384 -225.3918 -225.2448
##  [54,] -227.4583 -227.3045 -227.1516 -226.9995 -226.8481 -226.6973 -226.5471
##  [55,] -228.7577 -228.5957 -228.4357 -228.2776 -228.1212 -227.9663 -227.8129
##  [56,] -230.0285 -229.8579 -229.6907 -229.5265 -229.3650 -229.2061 -229.0494
##  [57,] -231.2781 -231.0988 -230.9241 -230.7535 -230.5867 -230.4235 -230.2634
##  [58,] -232.5140 -232.3254 -232.1427 -231.9654 -231.7929 -231.6250 -231.4613
##  [59,] -233.7376 -233.5445 -233.3533 -233.1686 -232.9900 -232.8170 -232.6490
##  [60,] -234.9296 -234.7434 -234.5567 -234.3694 -234.1841 -234.0052 -233.8272
##  [61,] -236.0901 -235.9122 -235.7338 -235.5547 -235.3697 -235.1765 -234.9775
##  [62,] -237.2223 -237.0536 -236.8786 -236.6952 -236.5036 -236.3039 -236.0964
##  [63,] -238.3227 -238.1502 -237.9691 -237.7798 -237.5824 -237.3772 -237.1644
##  [64,] -239.3689 -239.1909 -239.0047 -238.8103 -238.6082 -238.3984 -238.1811
##  [65,] -240.3606 -240.1781 -239.9875 -239.7890 -239.5829 -239.3693 -239.1484
##  [66,] -241.3002 -241.1139 -240.9198 -240.7180 -240.5087 -240.2921 -240.0684
##  [67,] -242.1901 -242.0008 -241.8038 -241.5994 -241.3877 -241.1689 -240.9429
##  [68,] -243.0326 -242.8410 -242.6419 -242.4356 -242.2221 -242.0016 -241.7741
##  [69,] -243.8300 -243.6368 -243.4362 -243.2285 -243.0139 -242.7923 -242.5639
##  [70,] -244.5848 -244.3904 -244.1889 -243.9805 -243.7651 -243.5430 -243.3142
##  [71,] -245.2991 -245.1040 -244.9021 -244.6933 -244.4778 -244.2557 -244.0269
##  [72,] -245.9750 -245.7798 -245.5778 -245.3691 -245.1538 -244.9320 -244.7036
##  [73,] -246.6148 -246.4197 -246.2180 -246.0097 -245.7950 -245.5738 -245.3462
##  [74,] -247.2203 -247.0256 -246.8245 -246.6169 -246.4030 -246.1827 -245.9561
##  [75,] -247.7936 -247.5995 -247.3991 -247.1924 -246.9795 -246.7603 -246.5349
##  [76,] -248.3365 -248.1431 -247.9435 -247.7379 -247.5261 -247.3082 -247.0840
##  [77,] -248.8510 -248.6582 -248.4595 -248.2549 -248.0443 -247.8276 -247.6049
##  [78,] -249.3390 -249.1466 -248.9486 -248.7449 -248.5354 -248.3200 -248.0986
##  [79,] -249.8075 -249.6109 -249.4126 -249.2097 -249.0011 -248.7868 -248.5666
##  [80,] -250.2685 -250.0642 -249.8584 -249.6514 -249.4426 -249.2296 -249.0134
##  [81,] -250.7215 -250.5104 -250.2975 -250.0835 -249.8703 -249.6582 -249.4471
##  [82,] -251.1659 -250.9493 -250.7330 -250.5172 -250.3021 -250.0878 -249.8746
##  [83,] -251.6043 -251.3874 -251.1705 -250.9537 -250.7373 -250.5215 -250.3064
##  [84,] -252.0417 -251.8254 -251.6087 -251.3918 -251.1748 -250.9581 -250.7417
##  [85,] -252.4766 -252.2618 -252.0461 -251.8298 -251.6131 -251.3962 -251.1792
##  [86,] -252.9070 -252.6946 -252.4809 -252.2661 -252.0505 -251.8342 -251.6175
##  [87,] -253.3308 -253.1220 -252.9113 -252.6990 -252.4853 -252.2705 -252.0548
##  [88,] -253.7455 -253.5415 -253.3349 -253.1262 -252.9155 -252.7033 -252.4896
##  [89,] -254.1486 -253.9507 -253.7496 -253.5456 -253.3391 -253.1304 -252.9198
##  [90,] -254.5371 -254.3468 -254.1526 -253.9548 -253.7537 -253.5498 -253.3433
##  [91,] -254.9075 -254.7266 -254.5409 -254.3507 -254.1566 -253.9588 -253.7578
##  [92,] -255.2557 -255.0864 -254.9111 -254.7304 -254.5447 -254.3546 -254.1605
##  [93,] -255.5770 -255.4217 -255.2591 -255.0899 -254.9147 -254.7341 -254.5485
##  [94,] -255.8653 -255.7271 -255.5800 -255.4249 -255.2624 -255.0934 -254.9183
##  [95,] -256.1132 -255.9961 -255.8680 -255.7300 -255.5831 -255.4281 -255.2658
##  [96,] -256.3108 -256.2200 -256.1154 -255.9986 -255.8707 -255.7329 -255.5861
##  [97,] -256.4447 -256.3874 -256.3124 -256.2219 -256.1177 -256.0010 -255.8734
##  [98,] -256.4963 -256.4825 -256.4457 -256.3887 -256.3141 -256.2239 -256.1199
##  [99,] -256.4963 -256.4963 -256.4963 -256.4830 -256.4466 -256.3900 -256.3158
## [100,] -256.4963 -256.4963 -256.4963 -256.4963 -256.4963 -256.4835 -256.4476
##            [,43]     [,44]     [,45]     [,46]     [,47]     [,48]     [,49]
##   [1,] -157.5087 -156.7149 -155.7439 -154.6095 -153.3226 -151.8919 -150.3244
##   [2,] -155.7226 -154.5849 -153.2951 -151.8616 -150.2914 -148.5899 -146.7616
##   [3,] -153.2675 -151.8312 -150.2583 -148.5542 -146.7234 -144.7698 -142.6969
##   [4,] -150.2251 -148.5184 -146.6852 -144.7291 -142.6538 -140.4627 -138.1599
##   [5,] -146.6469 -144.6884 -142.6106 -140.4173 -138.1122 -135.7004 -133.1884
##   [6,] -142.5675 -140.3718 -138.0645 -135.6506 -133.1367 -130.5314 -127.8471
##   [7,] -138.0168 -135.6008 -133.0849 -130.4779 -127.7922 -125.0443 -122.2569
##   [8,] -133.0331 -130.4244 -127.7372 -124.9883 -122.2003 -119.4036 -116.6376
##   [9,] -127.6822 -124.9323 -122.1438 -119.3473 -116.5824 -113.8996 -111.3611
##  [10,] -122.0873 -119.2910 -116.5272 -113.8466 -111.3118 -108.9965 -106.9829
##  [11,] -116.4720 -113.7938 -111.2626 -108.9526 -106.9460 -105.3283 -104.1792
##  [12,] -111.2135 -108.9087 -106.9092 -105.3002 -104.1613 -103.5557 -103.5202
##  [13,] -106.8725 -105.2723 -104.1436 -103.5493 -103.5254 -104.0754 -105.1691
##  [14,] -104.1262 -103.5431 -103.5309 -104.0923 -105.1964 -106.7851 -108.7819
##  [15,] -103.5366 -104.1094 -105.2240 -106.8217 -108.8259 -111.1527 -113.7204
##  [16,] -105.2517 -106.8584 -108.8700 -111.2025 -113.7742 -116.5112 -119.3507
##  [17,] -108.9142 -111.2523 -113.8281 -116.5677 -119.4088 -122.3005 -125.1939
##  [18,] -113.8820 -116.6243 -119.4669 -122.3591 -125.2521 -128.1000 -130.8738
##  [19,] -119.5249 -122.4177 -125.3102 -128.1569 -130.9289 -133.6097 -136.1913
##  [20,] -125.3683 -128.2137 -130.9840 -133.6629 -136.2424 -138.7200 -141.0968
##  [21,] -131.0390 -133.7160 -136.2934 -138.7690 -141.1438 -143.4210 -145.6049
##  [22,] -136.3445 -138.8180 -141.1908 -143.4660 -145.6481 -147.7421 -149.7534
##  [23,] -141.2726 -143.5142 -145.6912 -147.7835 -149.7932 -151.7256 -153.5858
##  [24,] -145.9254 -147.9191 -149.8654 -151.7670 -153.6227 -155.4145 -157.1438
##  [25,] -150.3278 -152.0984 -153.8338 -155.5363 -157.2082 -158.8514 -160.4645
##  [26,] -154.5063 -156.0781 -157.6259 -159.1513 -160.6559 -162.1410 -163.6080
##  [27,] -158.4879 -159.8832 -161.2643 -162.6321 -163.9875 -165.3313 -166.6642
##  [28,] -162.2982 -163.5366 -164.7692 -165.9962 -167.2179 -168.4348 -169.6469
##  [29,] -165.9597 -167.0581 -168.1578 -169.2585 -170.3601 -171.4624 -172.5652
##  [30,] -169.4917 -170.4648 -171.4451 -172.4320 -173.4248 -174.4231 -175.4263
##  [31,] -172.9110 -173.7713 -174.6437 -175.5273 -176.4212 -177.3246 -178.2368
##  [32,] -176.2318 -176.9898 -177.7641 -178.5535 -179.3570 -180.1733 -181.0017
##  [33,] -179.4659 -180.1307 -180.8154 -181.5185 -182.2387 -182.9747 -183.7255
##  [34,] -182.6236 -183.2029 -183.8051 -184.4286 -185.0718 -185.7333 -186.4119
##  [35,] -185.7133 -186.2138 -186.7397 -187.2892 -187.8608 -188.4529 -189.0640
##  [36,] -188.7421 -189.1695 -189.6244 -190.1050 -190.6096 -191.1365 -191.6843
##  [37,] -191.7160 -192.0752 -192.4637 -192.8797 -193.3213 -193.7803 -194.2381
##  [38,] -194.6400 -194.9352 -195.2613 -195.6095 -195.9617 -196.3166 -196.6736
##  [39,] -197.5182 -197.7460 -197.9835 -198.2291 -198.4818 -198.7412 -199.0064
##  [40,] -200.3176 -200.4444 -200.5841 -200.7355 -200.8977 -201.0698 -201.2511
##  [41,] -202.9948 -203.0291 -203.0795 -203.1449 -203.2242 -203.3163 -203.4206
##  [42,] -205.5664 -205.5161 -205.4849 -205.4713 -205.4744 -205.4929 -205.5261
##  [43,] -207.9967 -207.9093 -207.8134 -207.7271 -207.6598 -207.6104 -207.5777
##  [44,] -210.2381 -210.1373 -210.0288 -209.9130 -209.7907 -209.6781 -209.5843
##  [45,] -212.3155 -212.2039 -212.0857 -211.9612 -211.8307 -211.6947 -211.5537
##  [46,] -214.2506 -214.1303 -214.0044 -213.8731 -213.7367 -213.5955 -213.4499
##  [47,] -216.0617 -215.9345 -215.8026 -215.6660 -215.5252 -215.3804 -215.2318
##  [48,] -217.7648 -217.6322 -217.4955 -217.3551 -217.2112 -217.0639 -216.9135
##  [49,] -219.3740 -219.2369 -219.0967 -218.9534 -218.8073 -218.6586 -218.5074
##  [50,] -220.9014 -220.7609 -220.6179 -220.4726 -220.3252 -220.1758 -220.0245
##  [51,] -222.3581 -222.2148 -222.0697 -221.9231 -221.7749 -221.6255 -221.4747
##  [52,] -223.7538 -223.6082 -223.4616 -223.3141 -223.1657 -223.0166 -222.8668
##  [53,] -225.0975 -224.9500 -224.8022 -224.6541 -224.5059 -224.3575 -224.2089
##  [54,] -226.3974 -226.2481 -226.0993 -225.9509 -225.8030 -225.6555 -225.5085
##  [55,] -227.6608 -227.5100 -227.3603 -227.2117 -227.0642 -226.9177 -226.7723
##  [56,] -228.8950 -228.7425 -228.5919 -228.4431 -228.2960 -228.1505 -228.0066
##  [57,] -230.1064 -229.9521 -229.8005 -229.6514 -229.5045 -229.3553 -229.2009
##  [58,] -231.3014 -231.1451 -230.9921 -230.8376 -230.6786 -230.5150 -230.3468
##  [59,] -232.4858 -232.3220 -232.1549 -231.9842 -231.8098 -231.6316 -231.4494
##  [60,] -233.6473 -233.4652 -233.2806 -233.0933 -232.9032 -232.7099 -232.5133
##  [61,] -234.7776 -234.5765 -234.3740 -234.1696 -233.9631 -233.7544 -233.5431
##  [62,] -235.8812 -235.6607 -235.4395 -235.2174 -234.9942 -234.7695 -234.5430
##  [63,] -236.9441 -236.7163 -236.4814 -236.2412 -236.0007 -235.7595 -235.5173
##  [64,] -237.9564 -237.7246 -237.4856 -237.2395 -236.9865 -236.7285 -236.4701
##  [65,] -238.9203 -238.6851 -238.4428 -238.1936 -237.9376 -237.6747 -237.4050
##  [66,] -239.8375 -239.5997 -239.3549 -239.1033 -238.8449 -238.5798 -238.3079
##  [67,] -240.7100 -240.4703 -240.2237 -239.9704 -239.7103 -239.4435 -239.1699
##  [68,] -241.5399 -241.2988 -241.0510 -240.7965 -240.5353 -240.2674 -239.9928
##  [69,] -242.3288 -242.0870 -241.8385 -241.5833 -241.3215 -241.0531 -240.7780
##  [70,] -243.0788 -242.8367 -242.5880 -242.3327 -242.0707 -241.8022 -241.5269
##  [71,] -243.7915 -243.5496 -243.3011 -243.0460 -242.7843 -242.5161 -242.2411
##  [72,] -244.4687 -244.2273 -243.9794 -243.7249 -243.4639 -243.1962 -242.9219
##  [73,] -245.1121 -244.8715 -244.6245 -244.3709 -244.1107 -243.8440 -243.5705
##  [74,] -245.7230 -245.4836 -245.2377 -244.9852 -244.7262 -244.4606 -244.1883
##  [75,] -246.3031 -246.0649 -245.8203 -245.5693 -245.3116 -245.0473 -244.7763
##  [76,] -246.8536 -246.6169 -246.3738 -246.1242 -245.8680 -245.6051 -245.3359
##  [77,] -247.3759 -247.1407 -246.8991 -246.6510 -246.3968 -246.1386 -245.8766
##  [78,] -247.8711 -247.6374 -247.3978 -247.1543 -246.9072 -246.6565 -246.4020
##  [79,] -248.3409 -248.1116 -247.8788 -247.6425 -247.4027 -247.1593 -246.9122
##  [80,] -248.7940 -248.5714 -248.3455 -248.1162 -247.8835 -247.6473 -247.4076
##  [81,] -249.2339 -249.0178 -248.7984 -248.5759 -248.3501 -248.1209 -247.8882
##  [82,] -249.6625 -249.4514 -249.2383 -249.0221 -248.8029 -248.5804 -248.3547
##  [83,] -250.0921 -249.8789 -249.6668 -249.4557 -249.2426 -249.0265 -248.8074
##  [84,] -250.5259 -250.3107 -250.0964 -249.8832 -249.6710 -249.4599 -249.2469
##  [85,] -250.9625 -250.7461 -250.5302 -250.3151 -250.1008 -249.8875 -249.6753
##  [86,] -251.4005 -251.1836 -250.9668 -250.7504 -250.5346 -250.3194 -250.1051
##  [87,] -251.8386 -251.6218 -251.4049 -251.1880 -250.9712 -250.7548 -250.5389
##  [88,] -252.2748 -252.0592 -251.8429 -251.6262 -251.4093 -251.1924 -250.9756
##  [89,] -252.7076 -252.4939 -252.2792 -252.0636 -251.8473 -251.6306 -251.4137
##  [90,] -253.1347 -252.9241 -252.7119 -252.4983 -252.2835 -252.0679 -251.8517
##  [91,] -253.5539 -253.3475 -253.1389 -252.9284 -252.7162 -252.5026 -252.2879
##  [92,] -253.9628 -253.7619 -253.5581 -253.3517 -253.1431 -252.9326 -252.7205
##  [93,] -254.3585 -254.1645 -253.9668 -253.7660 -253.5622 -253.3559 -253.1474
##  [94,] -254.7378 -254.5523 -254.3624 -254.1684 -253.9709 -253.7701 -253.5664
##  [95,] -255.0968 -254.9219 -254.7415 -254.5561 -254.3663 -254.1724 -253.9749
##  [96,] -255.4313 -255.2691 -255.1003 -254.9255 -254.7451 -254.5599 -254.3701
##  [97,] -255.7358 -255.5892 -255.4345 -255.2725 -255.1038 -254.9290 -254.7488
##  [98,] -256.0035 -255.8761 -255.7386 -255.5922 -255.4377 -255.2758 -255.1072
##  [99,] -256.2259 -256.1221 -256.0060 -255.8787 -255.7415 -255.5953 -255.4409
## [100,] -256.3914 -256.3174 -256.2278 -256.1243 -256.0084 -255.8814 -255.7444
##            [,50]     [,51]     [,52]     [,53]     [,54]     [,55]     [,56]
##   [1,] -148.6255 -146.7998 -144.8511 -142.7829 -140.5987 -138.3025 -135.8991
##   [2,] -144.8105 -142.7399 -140.5534 -138.2550 -135.8495 -133.3433 -130.7451
##   [3,] -140.5081 -138.2075 -135.7999 -133.2917 -130.6917 -128.0118 -125.2682
##   [4,] -135.7502 -133.2401 -130.6383 -127.9569 -125.2122 -122.4264 -119.6291
##   [5,] -130.5849 -127.9020 -125.1563 -122.3699 -119.5727 -116.8035 -114.1119
##   [6,] -125.1003 -122.3134 -119.5163 -116.7482 -114.0587 -111.5096 -109.1735
##   [7,] -119.4600 -116.6929 -114.0056 -111.4600 -109.1291 -107.0948 -105.4426
##   [8,] -113.9526 -111.4105 -109.0848 -107.0574 -105.4137 -104.2342 -103.5838
##   [9,] -109.0406 -107.0201 -105.3851 -104.2157 -103.5764 -103.5059 -104.0102
##  [10,] -105.3566 -104.1973 -103.5693 -103.5104 -104.0261 -105.0883 -106.6405
##  [11,] -103.5624 -103.5152 -104.0423 -105.1150 -106.6764 -108.6508 -110.9547
##  [12,] -104.0588 -105.1420 -106.7125 -108.6944 -111.0041 -113.5593 -116.2854
##  [13,] -106.7487 -108.7381 -111.0535 -113.6129 -116.3418 -119.1767 -122.0660
##  [14,] -111.1031 -113.6666 -116.3982 -119.2347 -122.1246 -125.0195 -127.8724
##  [15,] -116.4547 -119.2927 -122.1832 -125.0776 -127.9293 -130.7081 -133.3967
##  [16,] -122.2418 -125.1358 -127.9863 -130.7634 -133.4500 -136.0377 -138.5236
##  [17,] -128.0432 -130.8186 -133.5033 -136.0889 -138.5728 -140.9556 -143.2405
##  [18,] -133.5565 -136.1401 -138.6219 -141.0027 -143.2856 -145.4751 -147.5760
##  [19,] -138.6710 -141.0498 -143.3308 -145.5184 -147.6176 -149.6338 -151.5722
##  [20,] -143.3759 -145.5616 -147.6591 -149.6737 -151.6106 -153.4750 -155.2720
##  [21,] -147.7006 -149.7136 -151.6489 -153.5120 -155.3077 -157.0407 -158.7154
##  [22,] -151.6873 -153.5489 -155.3433 -157.0751 -158.7486 -160.3680 -161.9370
##  [23,] -155.3789 -157.1094 -158.7819 -160.4002 -161.9682 -163.4893 -164.9667
##  [24,] -158.8151 -160.4324 -161.9994 -163.5196 -164.9962 -166.4321 -167.8299
##  [25,] -162.0305 -163.5498 -165.0256 -166.4607 -167.8578 -169.2194 -170.5478
##  [26,] -165.0580 -166.4893 -167.8857 -169.2466 -170.5744 -171.8710 -173.1383
##  [27,] -167.9869 -169.2999 -170.6039 -171.8969 -173.1637 -174.4030 -175.6165
##  [28,] -170.8546 -172.0579 -173.2571 -174.4524 -175.6437 -176.8293 -177.9949
##  [29,] -173.6683 -174.7717 -175.8752 -176.9787 -178.0819 -179.1848 -180.2873
##  [30,] -176.4341 -177.4460 -178.4616 -179.4804 -180.5023 -181.5267 -182.5534
##  [31,] -179.1570 -180.0846 -181.0190 -181.9595 -182.9058 -183.8571 -184.8131
##  [32,] -181.8411 -182.6907 -183.5497 -184.4174 -185.2931 -186.1762 -187.0659
##  [33,] -184.4899 -185.2670 -186.0558 -186.8553 -187.6649 -188.4838 -189.3056
##  [34,] -187.1064 -187.8156 -188.5386 -189.2742 -190.0157 -190.7440 -191.4585
##  [35,] -189.6930 -190.3385 -190.9932 -191.6383 -192.2730 -192.8977 -193.5132
##  [36,] -192.2451 -192.8003 -193.3489 -193.8911 -194.4274 -194.9579 -195.4832
##  [37,] -194.6936 -195.1469 -195.5978 -196.0465 -196.4931 -196.9377 -197.3804
##  [38,] -197.0325 -197.3930 -197.7549 -198.1182 -198.4827 -198.8485 -199.2154
##  [39,] -199.2771 -199.5529 -199.8333 -200.1182 -200.4074 -200.7006 -200.9977
##  [40,] -201.4410 -201.6390 -201.8446 -202.0573 -202.2769 -202.5031 -202.7355
##  [41,] -203.5362 -203.6625 -203.7989 -203.9449 -204.1001 -204.2641 -204.4365
##  [42,] -205.5730 -205.6330 -205.7053 -205.7894 -205.8848 -205.9909 -206.1073
##  [43,] -207.5610 -207.5593 -207.5721 -207.5985 -207.6380 -207.6900 -207.7541
##  [44,] -209.5083 -209.4492 -209.4062 -209.3787 -209.3659 -209.3673 -209.3822
##  [45,] -211.4223 -211.3095 -211.2144 -211.1362 -211.0743 -211.0280 -210.9967
##  [46,] -213.3001 -213.1464 -213.0023 -212.8765 -212.7684 -212.6771 -212.6021
##  [47,] -215.0797 -214.9243 -214.7658 -214.6045 -214.4526 -214.3188 -214.2025
##  [48,] -216.7603 -216.6043 -216.4458 -216.2851 -216.1221 -215.9573 -215.8017
##  [49,] -218.3539 -218.1983 -218.0407 -217.8814 -217.7203 -217.5578 -217.3940
##  [50,] -219.8716 -219.7170 -219.5610 -219.4037 -219.2452 -219.0857 -218.9253
##  [51,] -221.3228 -221.1698 -221.0160 -220.8612 -220.7058 -220.5498 -220.3933
##  [52,] -222.7164 -222.5655 -222.4141 -222.2624 -222.1104 -221.9583 -221.8060
##  [53,] -224.0603 -223.9117 -223.7632 -223.6147 -223.4665 -223.3185 -223.1667
##  [54,] -225.3619 -225.2158 -225.0703 -224.9253 -224.7767 -224.6213 -224.4591
##  [55,] -226.6278 -226.4843 -226.3375 -226.1843 -226.0247 -225.8587 -225.6862
##  [56,] -227.8598 -227.7071 -227.5484 -227.3838 -227.2132 -227.0365 -226.8537
##  [57,] -229.0411 -228.8759 -228.7053 -228.5292 -228.3475 -228.1602 -227.9672
##  [58,] -230.1739 -229.9961 -229.8134 -229.6257 -229.4329 -229.2349 -229.0317
##  [59,] -231.2631 -231.0725 -230.8775 -230.6781 -230.4740 -230.2653 -230.0517
##  [60,] -232.3133 -232.1096 -231.9022 -231.6908 -231.4754 -231.2557 -231.0316
##  [61,] -233.3290 -233.1120 -232.8918 -232.6682 -232.4411 -232.2103 -231.9756
##  [62,] -234.3145 -234.0838 -233.8504 -233.6143 -233.3753 -233.1330 -232.8874
##  [63,] -235.2739 -235.0289 -234.7821 -234.5331 -234.2817 -234.0276 -233.7707
##  [64,] -236.2112 -235.9515 -235.6906 -235.4282 -235.1640 -234.8978 -234.6292
##  [65,] -237.1303 -236.8553 -236.5798 -236.3035 -236.0260 -235.7470 -235.4663
##  [66,] -238.0293 -237.7439 -237.4534 -237.1625 -236.8711 -236.5789 -236.2853
##  [67,] -238.8897 -238.6028 -238.3091 -238.0087 -237.7031 -237.3968 -237.0898
##  [68,] -239.7115 -239.4236 -239.1289 -238.8274 -238.5191 -238.2039 -237.8832
##  [69,] -240.4962 -240.2076 -239.9123 -239.6102 -239.3013 -238.9854 -238.6625
##  [70,] -241.2450 -240.9563 -240.6609 -240.3586 -240.0493 -239.7331 -239.4098
##  [71,] -241.9594 -241.6710 -241.3757 -241.0736 -240.7644 -240.4482 -240.1249
##  [72,] -242.6408 -242.3529 -242.0582 -241.7564 -241.4477 -241.1318 -240.8086
##  [73,] -243.2903 -243.0033 -242.7093 -242.4083 -242.1001 -241.7851 -241.4658
##  [74,] -243.9092 -243.6231 -243.3301 -243.0304 -242.7264 -242.4186 -242.1070
##  [75,] -244.4984 -244.2140 -243.9253 -243.6329 -243.3366 -243.0365 -242.7326
##  [76,] -245.0626 -244.7855 -244.5045 -244.2198 -243.9312 -243.6388 -243.3426
##  [77,] -245.6109 -245.3414 -245.0682 -244.7911 -244.5102 -244.2256 -243.9371
##  [78,] -246.1438 -245.8820 -245.6163 -245.3469 -245.0737 -244.7967 -244.5160
##  [79,] -246.6616 -246.4072 -246.1491 -245.8873 -245.6217 -245.3524 -245.0793
##  [80,] -247.1642 -246.9173 -246.6667 -246.4124 -246.1543 -245.8926 -245.6271
##  [81,] -247.6521 -247.4124 -247.1692 -246.9223 -246.6718 -246.4175 -246.1596
##  [82,] -248.1255 -247.8930 -247.6569 -247.4173 -247.1741 -246.9273 -246.6769
##  [83,] -248.5850 -248.3593 -248.1302 -247.8977 -247.6617 -247.4222 -247.1791
##  [84,] -249.0309 -248.8118 -248.5895 -248.3639 -248.1349 -247.9024 -247.6665
##  [85,] -249.4642 -249.2513 -249.0353 -248.8163 -248.5940 -248.3685 -248.1395
##  [86,] -249.8918 -249.6796 -249.4685 -249.2556 -249.0397 -248.8207 -248.5985
##  [87,] -250.3237 -250.1094 -249.8961 -249.6838 -249.4728 -249.2599 -249.0441
##  [88,] -250.7592 -250.5433 -250.3281 -250.1137 -249.9004 -249.6881 -249.4770
##  [89,] -251.1967 -250.9800 -250.7635 -250.5476 -250.3324 -250.1180 -249.9047
##  [90,] -251.6350 -251.4181 -251.2011 -250.9843 -250.7679 -250.5520 -250.3368
##  [91,] -252.0723 -251.8560 -251.6394 -251.4224 -251.2055 -250.9887 -250.7723
##  [92,] -252.5069 -252.2922 -252.0767 -251.8604 -251.6437 -251.4268 -251.2099
##  [93,] -252.9369 -252.7248 -252.5112 -252.2966 -252.0810 -251.8648 -251.6481
##  [94,] -253.3601 -253.1516 -252.9412 -252.7291 -252.5156 -252.3009 -252.0854
##  [95,] -253.7741 -253.5705 -253.3643 -253.1558 -252.9454 -252.7334 -252.5199
##  [96,] -254.1763 -253.9789 -253.7782 -253.5746 -253.3685 -253.1601 -252.9497
##  [97,] -254.5637 -254.3740 -254.1803 -253.9829 -253.7823 -253.5788 -253.3727
##  [98,] -254.9326 -254.7525 -254.5675 -254.3779 -254.1842 -253.9870 -253.7864
##  [99,] -255.2791 -255.1107 -254.9362 -254.7562 -254.5712 -254.3818 -254.1882
## [100,] -255.5983 -255.4441 -255.2825 -255.1142 -254.9398 -254.7599 -254.5750
##            [,57]     [,58]     [,59]     [,60]     [,61]     [,62]     [,63]
##   [1,] -133.3949 -130.7984 -128.1214 -125.3800 -122.5959 -119.7983 -117.0251
##   [2,] -128.0666 -125.3241 -122.5394 -119.7419 -116.9697 -114.2718 -111.7087
##   [3,] -122.4829 -119.6855 -116.9142 -114.2184 -111.6588 -109.3076 -107.2461
##   [4,] -116.8588 -114.1651 -111.6090 -109.2628 -107.2081 -105.5303 -104.3106
##   [5,] -111.5592 -109.2181 -107.1702 -105.5009 -104.2912 -103.6073 -103.4901
##   [6,] -107.1324 -105.4716 -104.2720 -103.5992 -103.4937 -103.9636 -104.9834
##   [7,] -104.2530 -103.5914 -103.4975 -103.9789 -105.0093 -106.5338 -108.4779
##   [8,] -103.5016 -103.9944 -105.0354 -106.5692 -108.5209 -110.8072 -113.3455
##   [9,] -105.0618 -106.6047 -108.5641 -110.8563 -113.3989 -116.1166 -118.9450
##  [10,] -108.6074 -110.9055 -113.4523 -116.1728 -119.0029 -121.8902 -124.7866
##  [11,] -113.5058 -116.2291 -119.0608 -121.9488 -124.8448 -127.7013 -130.4868
##  [12,] -119.1188 -122.0074 -124.9031 -127.7583 -130.5421 -133.2365 -135.8324
##  [13,] -124.9613 -127.8154 -130.5975 -133.2900 -135.8838 -138.3758 -140.7667
##  [14,] -130.6528 -133.3433 -135.9351 -138.4251 -140.8140 -143.1047 -145.3015
##  [15,] -135.9864 -138.4744 -140.8612 -143.1500 -145.3450 -147.4511 -149.4738
##  [16,] -140.9084 -143.1952 -145.3884 -147.4928 -149.5138 -151.4568 -153.3270
##  [17,] -145.4317 -147.5344 -149.5538 -151.4953 -153.3640 -155.1650 -156.9029
##  [18,] -149.5938 -151.5338 -153.4011 -155.2007 -156.9374 -158.6155 -160.2391
##  [19,] -153.4381 -155.2364 -156.9718 -158.6488 -160.2713 -161.8433 -163.3681
##  [20,] -157.0063 -158.6821 -160.3036 -161.8745 -163.3984 -164.8784 -166.3175
##  [21,] -160.3358 -161.9058 -163.4287 -164.9079 -166.3461 -167.7463 -169.1107
##  [22,] -163.4590 -164.9373 -166.3748 -167.7742 -169.1379 -170.4682 -171.7673
##  [23,] -166.4034 -167.8021 -169.1651 -170.4948 -171.7932 -173.0623 -174.3038
##  [24,] -169.1923 -170.5213 -171.8192 -173.0877 -174.3286 -175.5437 -176.7342
##  [25,] -171.8451 -173.1130 -174.3534 -175.5679 -176.7580 -177.9249 -179.0700
##  [26,] -174.3782 -175.5922 -176.7818 -177.9483 -179.0929 -180.2168 -181.3211
##  [27,] -176.8056 -177.9716 -179.1158 -180.2393 -181.3432 -182.4284 -183.4959
##  [28,] -179.1387 -180.2618 -181.3653 -182.4502 -183.5173 -184.5674 -185.6014
##  [29,] -181.3874 -182.4719 -183.5387 -184.5885 -185.6221 -186.6403 -187.6436
##  [30,] -183.5821 -184.6125 -185.6428 -186.6607 -187.6637 -188.6479 -189.5946
##  [31,] -185.7734 -186.7374 -187.7048 -188.6703 -189.6133 -190.5219 -191.3953
##  [32,] -187.9618 -188.8581 -189.7355 -190.5932 -191.4328 -192.2552 -193.0607
##  [33,] -190.1112 -190.8998 -191.6726 -192.4306 -193.1748 -193.9061 -194.6255
##  [34,] -192.1599 -192.8491 -193.5268 -194.1939 -194.8511 -195.4991 -196.1386
##  [35,] -194.1199 -194.7184 -195.3094 -195.8933 -196.4708 -197.0423 -197.6084
##  [36,] -196.0034 -196.5191 -197.0306 -197.5382 -198.0424 -198.5434 -199.0417
##  [37,] -197.8215 -198.2611 -198.6994 -199.1368 -199.5733 -200.0092 -200.4448
##  [38,] -199.5836 -199.9531 -200.3240 -200.6964 -201.0703 -201.4459 -201.8232
##  [39,] -201.2986 -201.6033 -201.9117 -202.2237 -202.5395 -202.8589 -203.1820
##  [40,] -202.9741 -203.2186 -203.4689 -203.7248 -203.9863 -204.2533 -204.5257
##  [41,] -204.6171 -204.8055 -205.0016 -205.2051 -205.4159 -205.6338 -205.8587
##  [42,] -206.2338 -206.3698 -206.5152 -206.6696 -206.8328 -207.0046 -207.1849
##  [43,] -207.8298 -207.9167 -208.0144 -208.1227 -208.2411 -208.3696 -208.5077
##  [44,] -209.4102 -209.4509 -209.5038 -209.5686 -209.6448 -209.7323 -209.8307
##  [45,] -210.9798 -210.9769 -210.9874 -211.0111 -211.0474 -211.0961 -211.1568
##  [46,] -212.5427 -212.4986 -212.4690 -212.4537 -212.4522 -212.4640 -212.4889
##  [47,] -214.1029 -214.0196 -213.9520 -213.8997 -213.8622 -213.8390 -213.8299
##  [48,] -215.6639 -215.5434 -215.4396 -215.3521 -215.2802 -215.2237 -215.1821
##  [49,] -217.2290 -217.0730 -216.9347 -216.8135 -216.7090 -216.6206 -216.5479
##  [50,] -218.7641 -218.6023 -218.4401 -218.2868 -218.1509 -218.0280 -217.9137
##  [51,] -220.2364 -220.0794 -219.9222 -219.7611 -219.5923 -219.4242 -219.2652
##  [52,] -221.6539 -221.4978 -221.3343 -221.1633 -220.9849 -220.7989 -220.6053
##  [53,] -223.0078 -222.8417 -222.6684 -222.4878 -222.3000 -222.1050 -221.9025
##  [54,] -224.2901 -224.1141 -223.9313 -223.7415 -223.5446 -223.3408 -223.1298
##  [55,] -225.5071 -225.3215 -225.1293 -224.9304 -224.7248 -224.5125 -224.2933
##  [56,] -226.6648 -226.4696 -226.2682 -226.0604 -225.8462 -225.6255 -225.3983
##  [57,] -227.7684 -227.5638 -227.3532 -227.1366 -226.9140 -226.6851 -226.4499
##  [58,] -228.8230 -228.6089 -228.3892 -228.1639 -227.9327 -227.6957 -227.4527
##  [59,] -229.8331 -229.6095 -229.3807 -229.1465 -228.9069 -228.6618 -228.4109
##  [60,] -230.8030 -230.5698 -230.3317 -230.0887 -229.8406 -229.5872 -229.3284
##  [61,] -231.7368 -231.4937 -231.2463 -230.9942 -230.7374 -230.4757 -230.2089
##  [62,] -232.6381 -232.3850 -232.1280 -231.8667 -231.6010 -231.3308 -231.0558
##  [63,] -233.5107 -233.2473 -232.9803 -232.7095 -232.4347 -232.1557 -231.8723
##  [64,] -234.3580 -234.0839 -233.8066 -233.5260 -233.2418 -232.9536 -232.6614
##  [65,] -235.1834 -234.8981 -234.6102 -234.3193 -234.0252 -233.7276 -233.4262
##  [66,] -235.9903 -235.6933 -235.3942 -235.0925 -234.7880 -234.4804 -234.1694
##  [67,] -236.7819 -236.4727 -236.1617 -235.8486 -235.5332 -235.2151 -234.8940
##  [68,] -237.5617 -237.2394 -236.9159 -236.5908 -236.2638 -235.9345 -235.6026
##  [69,] -238.3326 -237.9968 -237.6600 -237.3221 -236.9827 -236.6415 -236.2984
##  [70,] -239.0794 -238.7418 -238.3968 -238.0457 -237.6934 -237.3431 -236.9951
##  [71,] -239.7943 -239.4564 -239.1114 -238.7620 -238.4090 -238.0536 -237.7005
##  [72,] -240.4785 -240.1441 -239.8059 -239.4640 -239.1184 -238.7691 -238.4162
##  [73,] -241.1427 -240.8159 -240.4852 -240.1509 -239.8127 -239.4709 -239.1254
##  [74,] -241.7915 -241.4723 -241.1493 -240.8225 -240.4920 -240.1576 -239.8196
##  [75,] -242.4249 -242.1133 -241.7980 -241.4788 -241.1559 -240.8291 -240.4987
##  [76,] -243.0426 -242.7388 -242.4311 -242.1196 -241.8044 -241.4853 -241.1624
##  [77,] -243.6448 -243.3487 -243.0487 -242.7449 -242.4374 -242.1260 -241.8108
##  [78,] -244.2314 -243.9430 -243.6507 -243.3547 -243.0548 -242.7511 -242.4436
##  [79,] -244.8024 -244.5217 -244.2371 -243.9488 -243.6567 -243.3607 -243.0609
##  [80,] -245.3579 -245.0848 -244.8080 -244.5274 -244.2429 -243.9547 -243.6626
##  [81,] -245.8979 -245.6325 -245.3633 -245.0904 -244.8136 -244.5331 -244.2487
##  [82,] -246.4227 -246.1648 -245.9033 -245.6379 -245.3688 -245.0959 -244.8193
##  [83,] -246.9323 -246.6819 -246.4279 -246.1701 -245.9086 -245.6433 -245.3743
##  [84,] -247.4271 -247.1840 -246.9374 -246.6870 -246.4330 -246.1753 -245.9139
##  [85,] -247.9072 -247.6713 -247.4319 -247.1890 -246.9424 -246.6921 -246.4382
##  [86,] -248.3730 -248.1442 -247.9119 -247.6761 -247.4368 -247.1939 -246.9474
##  [87,] -248.8252 -248.6031 -248.3776 -248.1488 -247.9166 -247.6809 -247.4417
##  [88,] -249.2643 -249.0485 -248.8296 -248.6076 -248.3822 -248.1535 -247.9214
##  [89,] -249.6924 -249.4813 -249.2686 -249.0529 -248.8341 -248.6121 -248.3868
##  [90,] -250.1224 -249.9090 -249.6967 -249.4856 -249.2729 -249.0573 -248.8386
##  [91,] -250.5564 -250.3411 -250.1267 -249.9133 -249.7010 -249.4898 -249.2772
##  [92,] -250.9931 -250.7766 -250.5607 -250.3454 -250.1310 -249.9176 -249.7052
##  [93,] -251.4312 -251.2143 -250.9975 -250.7810 -250.5651 -250.3498 -250.1353
##  [94,] -251.8692 -251.6525 -251.4356 -251.2187 -251.0019 -250.7854 -250.5694
##  [95,] -252.3053 -252.0897 -251.8735 -251.6569 -251.4400 -251.2230 -251.0062
##  [96,] -252.7377 -252.5242 -252.3096 -252.0941 -251.8779 -251.6613 -251.4444
##  [97,] -253.1643 -252.9540 -252.7420 -252.5285 -252.3140 -252.0985 -251.8823
##  [98,] -253.5829 -253.3769 -253.1685 -252.9582 -252.7463 -252.5329 -252.3183
##  [99,] -253.9910 -253.7905 -253.5871 -253.3810 -253.1728 -252.9625 -252.7506
## [100,] -254.3856 -254.1921 -253.9950 -253.7946 -253.5912 -253.3852 -253.1770
##            [,64]     [,65]     [,66]     [,67]     [,68]     [,69]     [,70]
##   [1,] -114.3251 -111.7587 -109.3975 -107.3227 -105.6197 -104.3702 -103.6418
##   [2,] -109.3525 -107.2844 -105.5897 -104.3501 -103.6328 -103.4807 -103.9046
##   [3,] -105.5599 -104.3303 -103.6241 -103.4836 -103.9190 -104.9068 -106.3939
##   [4,] -103.6156 -103.4867 -103.9337 -104.9321 -106.4286 -108.3496 -110.6121
##   [5,] -103.9485 -104.9576 -106.4635 -108.3922 -110.6607 -113.1860 -115.8921
##   [6,] -106.4986 -108.4350 -110.7094 -113.2391 -115.9481 -118.7714 -121.6558
##   [7,] -110.7583 -113.2923 -116.0042 -118.8293 -121.7144 -124.6117 -127.4728
##   [8,] -116.0604 -118.8871 -121.7730 -124.6700 -127.5299 -130.3203 -133.0224
##   [9,] -121.8316 -124.7283 -127.5871 -130.3758 -133.0760 -135.6779 -138.1782
##  [10,] -127.6442 -130.4313 -133.1295 -135.7294 -138.2277 -140.6246 -142.9231
##  [11,] -133.1830 -135.7809 -138.2771 -140.6720 -142.9686 -145.1710 -147.2841
##  [12,] -138.3265 -140.7194 -143.0140 -145.2145 -147.3259 -149.3535 -151.3026
##  [13,] -143.0594 -145.2580 -147.3677 -149.3936 -151.3412 -153.2156 -155.0218
##  [14,] -147.4094 -149.4337 -151.3798 -153.2527 -155.0577 -156.7992 -158.4819
##  [15,] -151.4183 -153.2899 -155.0935 -156.8338 -158.5153 -160.1421 -161.7180
##  [16,] -155.1292 -156.8683 -158.5487 -160.1745 -161.7494 -163.2769 -164.7604
##  [17,] -158.5821 -160.2068 -161.7807 -163.3073 -164.7899 -166.2314 -167.6345
##  [18,] -161.8120 -163.3377 -164.8194 -166.2601 -167.6624 -169.0290 -170.3619
##  [19,] -164.8489 -166.2888 -167.6904 -169.0562 -170.3885 -171.6894 -172.9608
##  [20,] -167.7183 -169.0835 -170.4151 -171.7154 -172.9862 -174.2294 -175.4464
##  [21,] -170.4417 -171.7414 -173.0116 -174.2542 -175.4707 -176.6627 -177.8314
##  [22,] -173.0370 -174.2790 -175.4950 -176.6865 -177.8548 -179.0012 -180.1267
##  [23,] -175.5194 -176.7104 -177.8782 -179.0241 -180.1493 -181.2547 -182.3414
##  [24,] -177.9016 -179.0471 -180.1718 -181.2769 -182.3632 -183.4317 -184.4832
##  [25,] -180.1943 -181.2990 -182.3849 -183.4531 -184.5042 -185.5392 -186.5585
##  [26,] -182.4067 -183.4745 -184.5253 -185.5599 -186.5790 -187.5832 -188.5696
##  [27,] -184.5464 -185.5806 -186.5994 -187.6033 -188.5892 -189.5383 -190.4498
##  [28,] -186.6198 -187.6234 -188.6088 -189.5571 -190.4679 -191.3434 -192.1856
##  [29,] -188.6283 -189.5759 -190.4859 -191.3607 -192.2023 -193.0126 -193.7934
##  [30,] -190.5039 -191.3780 -192.2190 -193.0287 -193.8089 -194.5614 -195.2877
##  [31,] -192.2356 -193.0447 -193.8244 -194.5763 -195.3021 -196.0032 -196.6811
##  [32,] -193.8398 -194.5912 -195.3165 -196.0171 -196.6945 -197.3499 -197.9846
##  [33,] -195.3339 -196.0311 -196.7080 -197.3630 -197.9972 -198.6119 -199.2081
##  [34,] -196.7703 -197.3948 -198.0128 -198.6241 -199.2200 -199.7983 -200.3601
##  [35,] -198.1695 -198.7262 -199.2789 -199.8282 -200.3743 -200.9172 -201.4483
##  [36,] -199.5377 -200.0316 -200.5238 -201.0147 -201.5046 -201.9937 -202.4825
##  [37,] -200.8803 -201.3160 -201.7520 -202.1885 -202.6259 -203.0644 -203.5041
##  [38,] -202.2025 -202.5839 -202.9674 -203.3533 -203.7417 -204.1327 -204.5265
##  [39,] -203.5089 -203.8395 -204.1740 -204.5125 -204.8549 -205.2014 -205.5521
##  [40,] -204.8036 -205.0868 -205.3753 -205.6692 -205.9685 -206.2732 -206.5833
##  [41,] -206.0905 -206.3292 -206.5745 -206.8265 -207.0852 -207.3504 -207.6223
##  [42,] -207.3734 -207.5700 -207.7746 -207.9871 -208.2074 -208.4354 -208.6711
##  [43,] -208.6554 -208.8124 -208.9785 -209.1537 -209.3377 -209.5305 -209.7319
##  [44,] -209.9397 -210.0592 -210.1889 -210.3287 -210.4783 -210.6376 -210.8066
##  [45,] -211.2292 -211.3131 -211.4082 -211.5144 -211.6313 -211.7589 -211.8969
##  [46,] -212.5266 -212.5767 -212.6389 -212.7130 -212.7989 -212.8962 -213.0013
##  [47,] -213.8344 -213.8522 -213.8831 -213.9267 -213.9793 -214.0357 -214.0956
##  [48,] -215.1549 -215.1420 -215.1392 -215.1417 -215.1490 -215.1608 -215.1768
##  [49,] -216.4867 -216.4323 -216.3843 -216.3421 -216.3054 -216.2738 -216.2470
##  [50,] -217.8072 -217.7082 -217.6161 -217.5304 -217.4509 -217.3770 -217.3086
##  [51,] -219.1147 -218.9722 -218.8371 -218.7092 -218.5879 -218.4728 -218.3637
##  [52,] -220.4119 -220.2270 -220.0502 -219.8809 -219.7189 -219.5636 -219.4147
##  [53,] -221.6927 -221.4754 -221.2578 -221.0483 -220.8464 -220.6517 -220.4639
##  [54,] -222.9117 -222.6864 -222.4537 -222.2137 -221.9728 -221.7396 -221.5136
##  [55,] -224.0671 -223.8340 -223.5938 -223.3464 -223.0917 -222.8296 -222.5663
##  [56,] -225.1644 -224.9237 -224.6761 -224.4216 -224.1600 -223.8912 -223.6151
##  [57,] -226.2083 -225.9602 -225.7055 -225.4440 -225.1757 -224.9003 -224.6178
##  [58,] -227.2035 -226.9481 -226.6863 -226.4180 -226.1430 -225.8612 -225.5724
##  [59,] -228.1542 -227.8915 -227.6226 -227.3475 -227.0658 -226.7776 -226.4826
##  [60,] -229.0641 -228.7940 -228.5181 -228.2361 -227.9478 -227.6531 -227.3519
##  [61,] -229.9369 -229.6593 -229.3762 -229.0872 -228.7922 -228.4910 -228.1834
##  [62,] -230.7758 -230.4907 -230.2002 -229.9041 -229.6022 -229.2943 -228.9803
##  [63,] -231.5842 -231.2912 -230.9931 -230.6896 -230.3807 -230.0659 -229.7452
##  [64,] -232.3649 -232.0637 -231.7577 -231.4467 -231.1304 -230.8085 -230.4808
##  [65,] -233.1208 -232.8111 -232.4969 -232.1779 -231.8539 -231.5245 -231.1895
##  [66,] -233.8548 -233.5362 -233.2133 -232.8859 -232.5538 -232.2167 -231.8779
##  [67,] -234.5695 -234.2414 -233.9094 -233.5734 -233.2364 -232.8992 -232.5617
##  [68,] -235.2677 -234.9299 -234.5918 -234.2544 -233.9172 -233.5802 -233.2432
##  [69,] -235.9561 -235.6152 -235.2755 -234.9367 -234.5987 -234.2612 -233.9240
##  [70,] -236.6493 -236.3053 -235.9630 -235.6221 -235.2823 -234.9435 -234.6055
##  [71,] -237.3501 -237.0021 -236.6562 -236.3122 -235.9699 -235.6289 -235.2892
##  [72,] -238.0608 -237.7076 -237.3572 -237.0091 -236.6632 -236.3192 -235.9768
##  [73,] -238.7762 -238.4234 -238.0679 -237.7147 -237.3642 -237.0161 -236.6702
##  [74,] -239.4779 -239.1324 -238.7833 -238.4305 -238.0751 -237.7218 -237.3713
##  [75,] -240.1644 -239.8265 -239.4848 -239.1394 -238.7904 -238.4377 -238.0822
##  [76,] -240.8358 -240.5054 -240.1712 -239.8333 -239.4917 -239.1464 -238.7975
##  [77,] -241.4918 -241.1690 -240.8424 -240.5121 -240.1780 -239.8402 -239.4987
##  [78,] -242.1323 -241.8172 -241.4983 -241.1755 -240.8491 -240.5188 -240.1848
##  [79,] -242.7573 -242.4499 -242.1386 -241.8236 -241.5047 -241.1821 -240.8557
##  [80,] -243.3667 -243.0670 -242.7635 -242.4561 -242.1450 -241.8300 -241.5112
##  [81,] -243.9605 -243.6685 -243.3727 -243.0731 -242.7696 -242.4624 -242.1513
##  [82,] -244.5388 -244.2545 -243.9664 -243.6745 -243.3787 -243.0792 -242.7758
##  [83,] -245.1015 -244.8249 -244.5445 -244.2603 -243.9723 -243.6804 -243.3848
##  [84,] -245.6487 -245.3798 -245.1070 -244.8305 -244.5502 -244.2661 -243.9781
##  [85,] -246.1806 -245.9192 -245.6541 -245.3852 -245.1126 -244.8361 -244.5559
##  [86,] -246.6972 -246.4434 -246.1858 -245.9245 -245.6595 -245.3907 -245.1181
##  [87,] -247.1989 -246.9524 -246.7023 -246.4485 -246.1911 -245.9298 -245.6649
##  [88,] -247.6857 -247.4465 -247.2038 -246.9574 -246.7074 -246.4537 -246.1963
##  [89,] -248.1582 -247.9261 -247.6905 -247.4514 -247.2087 -246.9624 -246.7125
##  [90,] -248.6166 -248.3914 -248.1628 -247.9308 -247.6953 -247.4563 -247.2137
##  [91,] -249.0617 -248.8430 -248.6211 -248.3960 -248.1675 -247.9355 -247.7001
##  [92,] -249.4941 -249.2816 -249.0661 -248.8475 -248.6256 -248.4006 -248.1721
##  [93,] -249.9219 -249.7095 -249.4984 -249.2859 -249.0704 -248.8519 -248.6302
##  [94,] -250.3541 -250.1397 -249.9262 -249.7138 -249.5026 -249.2902 -249.0748
##  [95,] -250.7898 -250.5738 -250.3585 -250.1440 -249.9305 -249.7181 -249.5069
##  [96,] -251.2274 -251.0106 -250.7941 -250.5781 -250.3628 -250.1483 -249.9348
##  [97,] -251.6656 -251.4487 -251.2318 -251.0150 -250.7985 -250.5825 -250.3672
##  [98,] -252.1028 -251.8867 -251.6700 -251.4531 -251.2362 -251.0194 -250.8029
##  [99,] -252.5372 -252.3227 -252.1072 -251.8910 -251.6744 -251.4575 -251.2406
## [100,] -252.9668 -252.7549 -252.5415 -252.3270 -252.1115 -251.8954 -251.6788
##            [,71]     [,72]     [,73]     [,74]     [,75]     [,76]     [,77]
##   [1,] -103.4781 -103.8905 -104.8568 -106.3250 -108.2226 -110.4668 -112.9742
##   [2,] -104.8817 -106.3594 -108.2648 -110.5151 -113.0271 -115.7242 -118.5404
##   [3,] -108.3072 -110.5635 -113.0799 -115.7801 -118.5981 -121.4801 -124.3784
##   [4,] -113.1329 -115.8360 -118.6559 -121.5386 -124.4368 -127.3011 -130.0980
##   [5,] -118.7136 -121.5972 -124.4951 -127.3583 -130.1536 -132.8613 -135.4714
##   [6,] -124.5534 -127.4156 -130.2092 -132.9151 -135.5231 -138.0296 -140.4347
##   [7,] -130.2648 -132.9687 -135.5748 -138.0792 -140.4822 -142.7866 -144.9964
##   [8,] -135.6264 -138.1287 -140.5297 -142.8321 -145.0401 -147.1585 -149.1926
##   [9,] -140.5772 -142.8777 -145.0837 -147.2004 -149.2329 -151.1866 -153.0667
##  [10,] -145.1274 -147.2423 -149.2731 -151.2253 -153.1040 -154.9142 -156.6608
##  [11,] -149.3133 -151.2639 -153.1412 -154.9501 -156.6954 -158.3815 -160.0126
##  [12,] -153.1784 -154.9860 -156.7301 -158.4150 -160.0450 -161.6239 -163.1552
##  [13,] -156.7647 -158.4485 -160.0774 -161.6553 -163.1856 -164.6717 -166.1163
##  [14,] -160.1098 -161.6867 -163.2161 -164.7012 -166.1451 -167.5505 -168.9198
##  [15,] -163.2465 -164.7308 -166.1739 -167.5785 -168.9471 -170.2821 -171.5854
##  [16,] -166.2026 -167.6065 -168.9744 -170.3087 -171.6114 -172.8846 -174.1299
##  [17,] -169.0017 -170.3353 -171.6374 -172.9100 -174.1548 -175.3734 -176.5672
##  [18,] -171.6634 -172.9354 -174.1796 -175.3977 -176.5911 -177.7612 -178.9093
##  [19,] -174.2045 -175.4221 -176.6150 -177.7847 -178.9323 -180.0591 -181.1661
##  [20,] -176.6388 -177.8081 -178.9553 -180.0817 -181.1883 -182.2761 -183.3460
##  [21,] -178.9782 -180.1042 -181.2104 -182.2979 -183.3674 -184.4199 -185.4561
##  [22,] -181.2326 -182.3196 -183.3888 -184.4410 -185.4769 -186.4972 -187.5026
##  [23,] -183.4103 -184.4621 -185.4976 -186.5176 -187.5227 -188.5108 -189.4630
##  [24,] -185.5184 -186.5381 -187.5429 -188.5304 -189.4819 -190.3956 -191.2739
##  [25,] -187.5630 -188.5501 -189.5007 -190.4137 -191.2913 -192.1355 -192.9482
##  [26,] -189.5195 -190.4318 -191.3087 -192.1522 -192.9643 -193.7469 -194.5016
##  [27,] -191.3260 -192.1689 -192.9804 -193.7624 -194.5165 -195.2444 -195.9475
##  [28,] -192.9965 -193.7779 -194.5315 -195.2588 -195.9614 -196.6406 -197.2978
##  [29,] -194.5464 -195.2733 -195.9754 -196.6541 -197.3108 -197.9467 -198.5629
##  [30,] -195.9893 -196.6676 -197.3239 -197.9594 -198.5752 -199.1725 -199.7522
##  [31,] -197.3369 -197.9720 -198.5874 -199.1844 -199.7637 -200.3265 -200.8736
##  [32,] -198.5997 -199.1962 -199.7753 -200.3377 -200.8845 -201.4165 -201.9345
##  [33,] -199.7868 -200.3489 -200.8954 -201.4271 -201.9448 -202.4493 -202.9412
##  [34,] -200.9063 -201.4377 -201.9551 -202.4594 -202.9511 -203.4309 -203.8996
##  [35,] -201.9655 -202.4694 -202.9609 -203.4405 -203.9090 -204.3669 -204.8149
##  [36,] -202.9707 -203.4501 -203.9183 -204.3761 -204.8238 -205.2621 -205.6916
##  [37,] -203.9454 -204.3883 -204.8328 -205.2709 -205.7002 -206.1211 -206.5341
##  [38,] -204.9233 -205.3231 -205.7262 -206.1327 -206.5423 -206.9478 -207.3462
##  [39,] -205.9071 -206.2664 -206.6301 -206.9984 -207.3713 -207.7491 -208.1314
##  [40,] -206.8989 -207.2200 -207.5466 -207.8789 -208.2168 -208.5605 -208.9100
##  [41,] -207.9007 -208.1857 -208.4773 -208.7755 -209.0803 -209.3918 -209.7100
##  [42,] -208.9144 -209.1653 -209.4237 -209.6896 -209.9631 -210.2441 -210.5325
##  [43,] -209.9419 -210.1604 -210.3874 -210.6227 -210.8664 -211.1153 -211.3639
##  [44,] -210.9850 -211.1727 -211.3698 -211.5728 -211.7764 -211.9803 -212.1845
##  [45,] -212.0453 -212.2004 -212.3571 -212.5151 -212.6742 -212.8344 -212.9955
##  [46,] -213.1090 -213.2191 -213.3313 -213.4454 -213.5614 -213.6790 -213.7982
##  [47,] -214.1588 -214.2250 -214.2940 -214.3655 -214.4395 -214.5157 -214.5941
##  [48,] -215.1967 -215.2202 -215.2471 -215.2772 -215.3102 -215.3461 -215.3846
##  [49,] -216.2248 -216.2067 -216.1926 -216.1822 -216.1753 -216.1717 -216.1713
##  [50,] -217.2452 -217.1865 -217.1324 -217.0825 -217.0365 -216.9944 -216.9559
##  [51,] -218.2601 -218.1618 -218.0685 -217.9799 -217.8958 -217.8159 -217.7400
##  [52,] -219.2719 -219.1347 -219.0031 -218.8765 -218.7549 -218.6379 -218.5253
##  [53,] -220.2826 -220.1074 -219.9381 -219.7743 -219.6158 -219.4623 -219.3136
##  [54,] -221.2946 -221.0820 -220.8757 -220.6753 -220.4805 -220.2911 -220.1067
##  [55,] -222.3101 -222.0608 -221.8181 -221.5816 -221.3510 -221.1260 -220.9064
##  [56,] -223.3315 -223.0461 -222.7674 -222.4953 -222.2293 -221.9692 -221.7148
##  [57,] -224.3280 -224.0307 -223.7258 -223.4185 -223.1176 -222.8227 -222.5337
##  [58,] -225.2765 -224.9732 -224.6625 -224.3441 -224.0178 -223.6886 -223.3652
##  [59,] -226.1806 -225.8715 -225.5550 -225.2309 -224.8991 -224.5594 -224.2114
##  [60,] -227.0438 -226.7287 -226.4065 -226.0768 -225.7395 -225.3943 -225.0410
##  [61,] -227.8692 -227.5482 -227.2201 -226.8847 -226.5418 -226.1911 -225.8324
##  [62,] -228.6598 -228.3326 -227.9985 -227.6573 -227.3087 -226.9524 -226.5884
##  [63,] -229.4182 -229.0847 -228.7444 -228.3972 -228.0429 -227.6848 -227.3243
##  [64,] -230.1470 -229.8070 -229.4605 -229.1109 -228.7594 -228.4058 -228.0501
##  [65,] -230.8489 -230.5058 -230.1613 -229.8153 -229.4675 -229.1180 -228.7665
##  [66,] -231.5383 -231.1976 -230.8559 -230.5128 -230.1683 -229.8223 -229.4746
##  [67,] -232.2236 -231.8848 -231.5452 -231.2045 -230.8628 -230.5197 -230.1753
##  [68,] -232.9061 -232.5685 -232.2304 -231.8916 -231.5520 -231.2114 -230.8697
##  [69,] -233.5871 -233.2501 -232.9129 -232.5753 -232.2372 -231.8985 -231.5589
##  [70,] -234.2680 -233.9308 -233.5939 -233.2569 -232.9197 -232.5821 -232.2441
##  [71,] -234.9504 -234.6123 -234.2748 -233.9376 -233.6007 -233.2637 -232.9265
##  [72,] -235.6358 -235.2960 -234.9572 -234.6191 -234.2816 -233.9445 -233.6075
##  [73,] -236.3261 -235.9837 -235.6427 -235.3029 -234.9640 -234.6260 -234.2884
##  [74,] -237.0231 -236.6771 -236.3330 -235.9906 -235.6496 -235.3097 -234.9709
##  [75,] -237.7289 -237.3783 -237.0301 -236.6841 -236.3400 -235.9975 -235.6565
##  [76,] -238.4449 -238.0894 -237.7361 -237.3854 -237.0372 -236.6911 -236.3469
##  [77,] -239.1535 -238.8046 -238.4520 -238.0966 -237.7432 -237.3925 -237.0442
##  [78,] -239.8471 -239.5056 -239.1605 -238.8116 -238.4592 -238.1038 -237.7503
##  [79,] -240.5255 -240.1916 -239.8539 -239.5125 -239.1675 -238.8187 -238.4663
##  [80,] -241.1887 -240.8623 -240.5322 -240.1984 -239.8608 -239.5195 -239.1745
##  [81,] -241.8364 -241.5177 -241.1952 -240.8690 -240.5389 -240.2052 -239.8676
##  [82,] -242.4686 -242.1576 -241.8428 -241.5242 -241.2018 -240.8756 -240.5456
##  [83,] -243.0853 -242.7820 -242.4749 -242.1639 -241.8492 -241.5306 -241.2083
##  [84,] -243.6863 -243.3908 -243.0914 -242.7881 -242.4811 -242.1702 -241.8556
##  [85,] -244.2718 -243.9840 -243.6923 -243.3968 -243.0974 -242.7943 -242.4873
##  [86,] -244.8418 -244.5616 -244.2776 -243.9898 -243.6982 -243.4028 -243.1035
##  [87,] -245.3962 -245.1237 -244.8474 -244.5673 -244.2834 -243.9957 -243.7041
##  [88,] -245.9352 -245.6703 -245.4016 -245.1292 -244.8530 -244.5730 -244.2892
##  [89,] -246.4589 -246.2015 -245.9405 -245.6757 -245.4071 -245.1348 -244.8586
##  [90,] -246.9675 -246.7176 -246.4640 -246.2068 -245.9458 -245.6811 -245.4126
##  [91,] -247.4611 -247.2186 -246.9725 -246.7227 -246.4692 -246.2120 -245.9511
##  [92,] -247.9402 -247.7049 -247.4660 -247.2235 -246.9775 -246.7277 -246.4743
##  [93,] -248.4051 -248.1768 -247.9450 -247.7097 -247.4709 -247.2285 -246.9825
##  [94,] -248.8563 -248.6347 -248.4097 -248.1814 -247.9497 -247.7145 -247.4757
##  [95,] -249.2945 -249.0792 -248.8608 -248.6392 -248.4143 -248.1861 -247.9544
##  [96,] -249.7223 -249.5111 -249.2989 -249.0836 -248.8652 -248.6437 -248.4189
##  [97,] -250.1526 -249.9391 -249.7266 -249.5154 -249.3032 -249.0880 -248.8697
##  [98,] -250.5869 -250.3715 -250.1570 -249.9434 -249.7309 -249.5197 -249.3075
##  [99,] -251.0237 -250.8072 -250.5912 -250.3758 -250.1613 -249.9477 -249.7352
## [100,] -251.4619 -251.2449 -251.0281 -250.8116 -250.5956 -250.3802 -250.1656
##            [,78]     [,79]     [,80]     [,81]     [,82]     [,83]     [,84]
##   [1,] -115.6683 -118.4827 -121.3630 -124.2616 -127.1292 -129.9308 -132.6461
##   [2,] -121.4215 -124.3200 -127.1865 -129.9865 -132.6999 -135.3161 -137.8308
##   [3,] -127.2438 -130.0423 -132.7538 -135.3679 -137.8806 -140.2917 -142.6039
##   [4,] -132.8076 -135.4197 -137.9303 -140.3394 -142.6497 -144.8650 -146.9905
##   [5,] -137.9800 -140.3871 -142.6953 -144.9089 -147.0325 -149.0716 -151.0315
##   [6,] -142.7410 -144.9526 -147.0746 -149.1120 -151.0703 -152.9548 -154.7704
##   [7,] -147.1165 -149.1523 -151.1091 -152.9921 -154.8064 -156.5566 -158.2474
##   [8,] -151.1479 -153.0294 -154.8423 -156.5914 -158.2810 -159.9153 -161.4981
##   [9,] -154.8783 -156.6261 -158.3145 -159.9477 -161.5296 -163.0637 -164.5531
##  [10,] -158.3480 -159.9802 -161.5611 -163.0942 -164.5828 -166.0299 -167.4383
##  [11,] -161.5925 -163.1247 -164.6124 -166.0587 -167.4664 -168.8378 -170.1754
##  [12,] -164.6421 -166.0876 -167.4944 -168.8652 -170.2021 -171.5073 -172.7828
##  [13,] -167.5225 -168.8925 -170.2287 -171.5333 -172.8082 -174.0552 -175.2758
##  [14,] -170.2554 -171.5594 -172.8337 -174.0801 -175.3002 -176.4956 -177.6675
##  [15,] -172.8591 -174.1050 -175.3246 -176.5195 -177.6910 -178.8404 -179.9688
##  [16,] -175.3490 -176.5434 -177.7144 -178.8634 -179.9914 -181.0996 -182.1888
##  [17,] -177.7378 -178.8863 -180.0140 -181.1217 -182.2107 -183.2816 -184.3355
##  [18,] -180.0365 -181.1439 -182.2325 -183.3031 -184.3566 -185.3938 -186.4153
##  [19,] -182.2543 -183.3245 -184.3777 -185.4146 -186.4358 -187.4420 -188.4321
##  [20,] -184.3988 -185.4353 -186.4563 -187.4622 -188.4518 -189.4064 -190.3231
##  [21,] -186.4767 -187.4824 -188.4715 -189.4253 -190.3413 -191.2216 -192.0684
##  [22,] -188.4912 -189.4442 -190.3594 -191.2391 -192.0852 -192.8998 -193.6847
##  [23,] -190.3775 -191.2565 -192.1020 -192.9160 -193.7003 -194.4566 -195.1865
##  [24,] -192.1187 -192.9321 -193.7158 -194.4716 -195.2010 -195.9055 -196.5866
##  [25,] -193.7314 -194.4866 -195.2155 -195.9195 -196.6001 -197.2585 -197.8961
##  [26,] -195.2299 -195.9335 -196.6136 -197.2716 -197.9088 -198.5261 -199.1249
##  [27,] -196.6271 -197.2847 -197.9214 -198.5384 -199.1368 -199.7175 -200.2816
##  [28,] -197.9341 -198.5507 -199.1487 -199.7291 -200.2929 -200.8409 -201.3741
##  [29,] -199.1606 -199.7406 -200.3041 -200.8518 -201.3847 -201.9035 -202.4090
##  [30,] -200.3153 -200.8627 -201.3953 -201.9138 -202.4191 -202.9118 -203.3926
##  [31,] -201.4059 -201.9242 -202.4292 -202.9216 -203.4022 -203.8715 -204.3303
##  [32,] -202.4392 -202.9314 -203.4118 -203.8809 -204.3395 -204.7880 -205.2271
##  [33,] -203.4214 -203.8903 -204.3486 -204.7969 -205.2358 -205.6658 -206.0874
##  [34,] -204.3578 -204.8059 -205.2446 -205.6744 -206.0958 -206.5093 -206.9153
##  [35,] -205.2534 -205.6830 -206.1042 -206.5175 -206.9234 -207.3222 -207.7144
##  [36,] -206.1127 -206.5258 -206.9315 -207.3302 -207.7223 -208.1082 -208.4883
##  [37,] -206.9396 -207.3382 -207.7302 -208.1159 -208.4959 -208.8704 -209.2399
##  [38,] -207.7380 -208.1237 -208.5035 -208.8780 -209.2473 -209.6119 -209.9722
##  [39,] -208.5111 -208.8855 -209.2547 -209.6193 -209.9794 -210.3354 -210.6851
##  [40,] -209.2654 -209.6266 -209.9866 -210.3426 -210.6921 -211.0295 -211.3551
##  [41,] -210.0349 -210.3666 -210.7023 -211.0362 -211.3615 -211.6751 -211.9773
##  [42,] -210.8256 -211.1176 -211.4084 -211.6982 -211.9867 -212.2741 -212.5540
##  [43,] -211.6120 -211.8596 -212.1068 -212.3534 -212.5996 -212.8453 -213.0905
##  [44,] -212.3889 -212.5935 -212.7982 -213.0030 -213.2079 -213.4128 -213.6179
##  [45,] -213.1574 -213.3201 -213.4835 -213.6476 -213.8123 -213.9776 -214.1436
##  [46,] -213.9188 -214.0407 -214.1639 -214.2883 -214.4138 -214.5405 -214.6682
##  [47,] -214.6744 -214.7566 -214.8405 -214.9262 -215.0134 -215.1022 -215.1926
##  [48,] -215.4256 -215.4689 -215.5145 -215.5623 -215.6121 -215.6639 -215.7175
##  [49,] -216.1739 -216.1792 -216.1873 -216.1979 -216.2110 -216.2264 -216.2441
##  [50,] -216.9208 -216.8889 -216.8601 -216.8343 -216.8113 -216.7910 -216.7733
##  [51,] -217.6679 -217.5994 -217.5345 -217.4728 -217.4142 -217.3587 -217.3061
##  [52,] -218.4169 -218.3124 -218.2118 -218.1147 -218.0212 -217.9309 -217.8438
##  [53,] -219.1693 -219.0294 -218.8936 -218.7616 -218.6334 -218.5088 -218.3875
##  [54,] -219.9271 -219.7521 -219.5815 -219.4150 -219.2525 -219.0937 -218.9386
##  [55,] -220.6919 -220.4822 -220.2771 -220.0764 -219.8798 -219.6872 -219.4983
##  [56,] -221.4656 -221.2215 -220.9822 -220.7474 -220.5170 -220.2907 -220.0683
##  [57,] -222.2501 -221.9718 -221.6985 -221.4299 -221.1658 -220.9059 -220.6500
##  [58,] -223.0475 -222.7352 -222.4280 -222.1256 -221.8279 -221.5344 -221.2451
##  [59,] -223.8599 -223.5137 -223.1727 -222.8366 -222.5052 -222.1783 -221.8597
##  [60,] -224.6794 -224.3091 -223.9346 -223.5649 -223.2040 -222.8537 -222.5140
##  [61,] -225.4655 -225.0901 -224.7099 -224.3264 -223.9441 -223.5723 -223.2112
##  [62,] -226.2200 -225.8489 -225.4748 -225.0977 -224.7176 -224.3342 -223.9518
##  [63,] -226.9614 -226.5958 -226.2275 -225.8564 -225.4824 -225.1054 -224.7253
##  [64,] -227.6920 -227.3316 -226.9687 -226.6032 -226.2350 -225.8639 -225.4900
##  [65,] -228.4130 -228.0573 -227.6993 -227.3389 -226.9761 -226.6106 -226.2424
##  [66,] -229.1251 -228.7736 -228.4201 -228.0645 -227.7066 -227.3462 -226.9834
##  [67,] -229.8293 -229.4816 -229.1321 -228.7807 -228.4273 -228.0717 -227.7138
##  [68,] -230.5267 -230.1823 -229.8363 -229.4887 -229.1392 -228.7879 -228.4345
##  [69,] -231.2183 -230.8766 -230.5336 -230.1892 -229.8433 -229.4957 -229.1463
##  [70,] -231.9053 -231.5658 -231.2252 -230.8835 -230.5406 -230.1962 -229.8503
##  [71,] -232.5890 -232.2509 -231.9122 -231.5726 -231.2321 -230.8904 -230.5475
##  [72,] -233.2705 -232.9333 -232.5958 -232.2578 -231.9190 -231.5795 -231.2390
##  [73,] -233.9513 -233.6143 -233.2773 -232.9401 -232.6026 -232.2646 -231.9259
##  [74,] -234.6328 -234.2952 -233.9581 -233.6211 -233.2841 -232.9469 -232.6094
##  [75,] -235.3166 -234.9777 -234.6396 -234.3021 -233.9649 -233.6279 -233.2909
##  [76,] -236.0044 -235.6633 -235.3234 -234.9845 -234.6464 -234.3089 -233.9717
##  [77,] -236.6981 -236.3539 -236.0113 -235.6702 -235.3303 -234.9914 -234.6532
##  [78,] -237.3995 -237.0512 -236.7050 -236.3608 -236.0182 -235.6771 -235.3372
##  [79,] -238.1109 -237.7574 -237.4066 -237.0582 -236.7120 -236.3677 -236.0251
##  [80,] -238.8258 -238.4735 -238.1181 -237.7645 -237.4136 -237.0652 -236.7190
##  [81,] -239.5264 -239.1815 -238.8329 -238.4806 -238.1253 -237.7716 -237.4207
##  [82,] -240.2119 -239.8745 -239.5333 -239.1885 -238.8400 -238.4878 -238.1324
##  [83,] -240.8822 -240.5523 -240.2187 -239.8813 -239.5403 -239.1955 -238.8470
##  [84,] -241.5371 -241.2149 -240.8888 -240.5590 -240.2255 -239.8882 -239.5472
##  [85,] -242.1766 -241.8620 -241.5436 -241.2214 -240.8955 -240.5657 -240.2323
##  [86,] -242.8005 -242.4936 -242.1829 -241.8684 -241.5501 -241.2280 -240.9021
##  [87,] -243.4088 -243.1096 -242.8066 -242.4998 -242.1892 -241.8748 -241.5565
##  [88,] -244.0015 -243.7101 -243.4148 -243.1157 -242.8128 -242.5060 -242.1955
##  [89,] -244.5787 -244.2949 -244.0074 -243.7160 -243.4208 -243.1218 -242.8189
##  [90,] -245.1403 -244.8642 -244.5844 -244.3007 -244.0132 -243.7219 -243.4268
##  [91,] -245.6864 -245.4180 -245.1458 -244.8698 -244.5901 -244.3065 -244.0191
##  [92,] -246.2172 -245.9564 -245.6918 -245.4235 -245.1514 -244.8755 -244.5957
##  [93,] -246.7328 -246.4795 -246.2225 -245.9617 -245.6972 -245.4289 -245.1569
##  [94,] -247.2334 -246.9875 -246.7379 -246.4847 -246.2277 -245.9670 -245.7026
##  [95,] -247.7193 -247.4806 -247.2383 -246.9925 -246.7430 -246.4898 -246.2329
##  [96,] -248.1907 -247.9591 -247.7240 -247.4854 -247.2433 -246.9975 -246.7481
##  [97,] -248.6482 -248.4234 -248.1953 -247.9638 -247.7288 -247.4903 -247.2482
##  [98,] -249.0923 -248.8741 -248.6527 -248.4280 -248.2000 -247.9685 -247.7336
##  [99,] -249.5239 -249.3118 -249.0967 -248.8786 -248.6572 -248.4326 -248.2046
## [100,] -249.9520 -249.7395 -249.5282 -249.3161 -249.1011 -248.8830 -248.6617
##            [,85]     [,86]     [,87]     [,88]     [,89]     [,90]     [,91]
##   [1,] -135.2643 -137.7810 -140.1963 -142.5124 -144.7334 -146.8641 -148.9099
##   [2,] -140.2440 -142.5582 -144.7773 -146.9063 -148.9504 -150.9149 -152.8051
##   [3,] -144.8212 -146.9484 -148.9908 -150.9538 -152.8426 -154.6622 -156.4175
##   [4,] -149.0313 -150.9927 -152.8800 -154.6983 -156.4523 -158.1465 -159.7852
##   [5,] -152.9174 -154.7343 -156.4871 -158.1802 -159.8177 -161.4036 -162.9414
##   [6,] -156.5219 -158.2138 -159.8503 -161.4351 -162.9720 -164.4641 -165.9145
##   [7,] -159.8828 -161.4666 -163.0026 -164.4938 -165.9433 -167.3540 -168.7283
##   [8,] -163.0331 -164.5235 -165.9722 -167.3821 -168.7557 -170.0952 -171.4029
##   [9,] -166.0011 -167.4102 -168.7831 -170.1220 -171.4291 -172.7063 -173.9554
##  [10,] -168.8104 -170.1487 -171.4551 -172.7318 -173.9804 -175.2026 -176.3999
##  [11,] -171.4812 -172.7573 -174.0053 -175.2270 -176.4238 -177.5971 -178.7483
##  [12,] -174.0302 -175.2514 -176.4477 -177.6206 -178.7713 -179.9010 -181.0107
##  [13,] -176.4717 -177.6441 -178.7943 -179.9236 -181.0329 -182.1233 -183.1957
##  [14,] -178.8173 -179.9462 -181.0552 -182.1452 -183.2172 -184.2721 -185.3105
##  [15,] -181.0774 -182.1670 -183.2387 -184.2932 -185.3314 -186.3538 -187.3613
##  [16,] -183.2602 -184.3144 -185.3522 -186.3743 -187.3815 -188.3730 -189.3307
##  [17,] -185.3730 -186.3948 -187.4017 -188.3927 -189.3497 -190.2686 -191.1518
##  [18,] -187.4219 -188.4124 -189.3686 -190.2868 -191.1693 -192.0180 -192.8351
##  [19,] -189.3875 -190.3050 -191.1867 -192.0349 -192.8513 -193.6380 -194.3965
##  [20,] -191.2042 -192.0517 -192.8675 -193.6536 -194.4115 -195.1430 -195.8495
##  [21,] -192.8837 -193.6691 -194.4266 -195.1575 -195.8635 -196.5459 -197.2061
##  [22,] -194.4416 -195.1720 -195.8775 -196.5595 -197.2192 -197.8580 -198.4770
##  [23,] -195.8915 -196.5730 -197.2324 -197.8707 -198.4893 -199.0891 -199.6712
##  [24,] -197.2455 -197.8834 -198.5016 -199.1010 -199.6828 -200.2479 -200.7972
##  [25,] -198.5139 -199.1130 -199.6944 -200.2591 -200.8081 -201.3422 -201.8621
##  [26,] -199.7060 -200.2704 -200.8190 -201.3528 -201.8724 -202.3787 -202.8724
##  [27,] -200.8300 -201.3634 -201.8828 -202.3888 -202.8823 -203.3638 -203.8340
##  [28,] -201.8931 -202.3989 -202.8921 -203.3734 -203.8434 -204.3028 -204.7521
##  [29,] -202.9019 -203.3830 -203.8528 -204.3120 -204.7611 -205.2007 -205.6314
##  [30,] -203.8622 -204.3211 -204.7701 -205.2095 -205.6400 -206.0620 -206.4761
##  [31,] -204.7790 -205.2183 -205.6486 -206.0705 -206.4844 -206.8909 -207.2902
##  [32,] -205.6572 -206.0789 -206.4927 -206.8990 -207.2982 -207.6908 -208.0772
##  [33,] -206.5010 -206.9071 -207.3062 -207.6987 -208.0850 -208.4654 -208.8404
##  [34,] -207.3142 -207.7066 -208.0927 -208.4730 -208.8479 -209.2176 -209.5826
##  [35,] -208.1005 -208.4807 -208.8554 -209.2251 -209.5900 -209.9504 -210.3068
##  [36,] -208.8629 -209.2325 -209.5973 -209.9577 -210.3140 -210.6643 -211.0027
##  [37,] -209.6046 -209.9649 -210.3211 -210.6712 -211.0094 -211.3357 -211.6502
##  [38,] -210.3283 -210.6782 -211.0161 -211.3421 -211.6564 -211.9593 -212.2510
##  [39,] -211.0228 -211.3486 -211.6627 -211.9653 -212.2568 -212.5373 -212.8072
##  [40,] -211.6689 -211.9713 -212.2626 -212.5429 -212.8125 -213.0716 -213.3205
##  [41,] -212.2684 -212.5484 -212.8178 -213.0767 -213.3254 -213.5640 -213.7928
##  [42,] -212.8232 -213.0819 -213.3303 -213.5687 -213.7973 -214.0162 -214.2257
##  [43,] -213.3352 -213.5735 -213.8018 -214.0206 -214.2298 -214.4298 -214.6207
##  [44,] -213.8231 -214.0285 -214.2340 -214.4338 -214.6245 -214.8062 -214.9792
##  [45,] -214.3101 -214.4773 -214.6451 -214.8135 -214.9826 -215.1468 -215.3024
##  [46,] -214.7970 -214.9268 -215.0576 -215.1895 -215.3225 -215.4565 -215.5915
##  [47,] -215.2844 -215.3776 -215.4722 -215.5682 -215.6656 -215.7644 -215.8645
##  [48,] -215.7731 -215.8304 -215.8894 -215.9502 -216.0126 -216.0767 -216.1424
##  [49,] -216.2640 -216.2860 -216.3100 -216.3360 -216.3640 -216.3938 -216.4256
##  [50,] -216.7580 -216.7452 -216.7347 -216.7264 -216.7204 -216.7164 -216.7145
##  [51,] -217.2562 -217.2090 -217.1644 -217.1222 -217.0824 -217.0450 -217.0097
##  [52,] -217.7597 -217.6784 -217.6000 -217.5242 -217.4510 -217.3802 -217.3119
##  [53,] -218.2694 -218.1545 -218.0425 -217.9333 -217.8269 -217.7230 -217.6217
##  [54,] -218.7868 -218.6383 -218.4930 -218.3506 -218.2110 -218.0742 -217.9399
##  [55,] -219.3131 -219.1312 -218.9526 -218.7771 -218.6045 -218.4347 -218.2675
##  [56,] -219.8496 -219.6345 -219.4227 -219.2140 -219.0084 -218.8097 -218.6206
##  [57,] -220.3980 -220.1495 -219.9046 -219.6669 -219.4391 -219.2210 -219.0125
##  [58,] -220.9597 -220.6822 -220.4147 -220.1571 -219.9095 -219.6716 -219.4436
##  [59,] -221.5514 -221.2533 -220.9654 -220.6877 -220.4200 -220.1622 -219.9144
##  [60,] -222.1848 -221.8660 -221.5575 -221.2592 -220.9712 -220.6932 -220.4253
##  [61,] -222.8607 -222.5208 -222.1914 -221.8723 -221.5636 -221.2652 -220.9769
##  [62,] -223.5797 -223.2184 -222.8677 -222.5276 -222.1979 -221.8787 -221.5698
##  [63,] -224.3420 -223.9594 -223.5871 -223.2256 -222.8747 -222.5343 -222.2045
##  [64,] -225.1130 -224.7330 -224.3498 -223.9670 -223.5945 -223.2328 -222.8817
##  [65,] -225.8714 -225.4976 -225.1207 -224.7407 -224.3575 -223.9746 -223.6020
##  [66,] -226.6180 -226.2499 -225.8790 -225.5051 -225.1283 -224.7484 -224.3653
##  [67,] -227.3535 -226.9908 -226.6254 -226.2574 -225.8865 -225.5127 -225.1360
##  [68,] -228.0789 -227.7211 -227.3608 -226.9981 -226.6328 -226.2648 -225.8940
##  [69,] -228.7950 -228.4416 -228.0861 -227.7283 -227.3682 -227.0055 -226.6402
##  [70,] -229.5027 -229.1534 -228.8021 -228.4488 -228.0933 -227.7356 -227.3755
##  [71,] -230.2032 -229.8573 -229.5098 -229.1605 -228.8092 -228.4559 -228.1005
##  [72,] -230.8973 -230.5544 -230.2101 -229.8643 -229.5168 -229.1675 -228.8163
##  [73,] -231.5864 -231.2459 -230.9043 -230.5614 -230.2171 -229.8713 -229.5239
##  [74,] -232.2714 -231.9327 -231.5932 -231.2528 -230.9112 -230.5683 -230.2241
##  [75,] -232.9538 -232.6163 -232.2783 -231.9396 -231.6001 -231.2597 -230.9181
##  [76,] -233.6347 -233.2977 -232.9606 -232.6231 -232.2851 -231.9464 -231.6070
##  [77,] -234.3157 -233.9785 -233.6415 -233.3045 -232.9674 -232.6299 -232.2919
##  [78,] -234.9982 -234.6601 -234.3225 -233.9853 -233.6483 -233.3113 -232.9742
##  [79,] -235.6840 -235.3440 -235.0051 -234.6669 -234.3293 -233.9921 -233.6551
##  [80,] -236.3747 -236.0320 -235.6908 -235.3509 -235.0119 -234.6737 -234.3361
##  [81,] -237.0722 -236.7260 -236.3816 -236.0389 -235.6977 -235.3577 -235.0187
##  [82,] -237.7787 -237.4278 -237.0792 -236.7329 -236.3885 -236.0458 -235.7046
##  [83,] -238.4949 -238.1396 -237.7859 -237.4348 -237.0863 -236.7399 -236.3955
##  [84,] -239.2025 -238.8541 -238.5021 -238.1468 -237.7930 -237.4419 -237.0933
##  [85,] -239.8951 -239.5541 -239.2095 -238.8612 -238.5092 -238.1540 -237.8001
##  [86,] -240.5724 -240.2390 -239.9019 -239.5610 -239.2165 -238.8683 -238.5164
##  [87,] -241.2345 -240.9087 -240.5791 -240.2458 -239.9087 -239.5680 -239.2235
##  [88,] -241.8811 -241.5630 -241.2410 -240.9153 -240.5858 -240.2526 -239.9156
##  [89,] -242.5123 -242.2018 -241.8875 -241.5695 -241.2476 -240.9219 -240.5925
##  [90,] -243.1279 -242.8251 -242.5185 -242.2081 -241.8939 -241.5759 -241.2541
##  [91,] -243.7278 -243.4328 -243.1339 -242.8312 -242.5247 -242.2144 -241.9003
##  [92,] -244.3122 -244.0249 -243.7338 -243.4388 -243.1400 -242.8374 -242.5310
##  [93,] -244.8811 -244.6014 -244.3180 -244.0307 -243.7397 -243.4448 -243.1461
##  [94,] -245.4344 -245.1624 -244.8867 -244.6071 -244.3238 -244.0366 -243.7456
##  [95,] -245.9723 -245.7080 -245.4399 -245.1680 -244.8923 -244.6128 -244.3295
##  [96,] -246.4950 -246.2382 -245.9776 -245.7133 -245.4453 -245.1735 -244.8979
##  [97,] -247.0025 -246.7531 -246.5001 -246.2434 -245.9829 -245.7187 -245.4508
##  [98,] -247.4952 -247.2531 -247.0075 -246.7582 -246.5053 -246.2486 -245.9882
##  [99,] -247.9732 -247.7384 -247.5000 -247.2581 -247.0125 -246.7633 -246.5104
## [100,] -248.4372 -248.2093 -247.9780 -247.7432 -247.5049 -247.2630 -247.0175
##            [,92]     [,93]     [,94]     [,95]     [,96]     [,97]     [,98]
##   [1,] -150.8760 -152.7676 -154.5900 -156.3477 -158.0455 -159.6874 -161.2773
##   [2,] -154.6261 -156.3826 -158.0792 -159.7200 -161.3089 -162.8495 -164.3451
##   [3,] -158.1129 -159.7526 -161.3405 -162.8801 -164.3749 -165.8277 -167.2414
##   [4,] -161.3721 -162.9108 -164.4046 -165.8566 -167.2696 -168.6460 -169.9882
##   [5,] -164.4344 -165.8855 -167.2977 -168.6734 -170.0150 -171.3246 -172.6041
##   [6,] -167.3259 -168.7008 -170.0417 -171.3507 -172.6297 -173.8805 -175.1047
##   [7,] -170.0685 -171.3768 -172.6552 -173.9055 -175.1292 -176.3280 -177.5032
##   [8,] -172.6808 -173.9304 -175.1537 -176.3519 -177.5267 -178.6791 -179.8104
##   [9,] -175.1781 -176.3759 -177.5502 -178.7022 -179.8331 -180.9440 -182.0359
##  [10,] -177.5737 -178.7252 -179.8557 -180.9663 -182.0578 -183.1312 -184.1875
##  [11,] -179.8784 -180.9885 -182.0796 -183.1527 -184.2086 -185.2481 -186.2718
##  [12,] -182.1015 -183.1742 -184.2298 -185.2689 -186.2923 -187.3006 -188.2938
##  [13,] -184.2509 -185.2897 -186.3128 -187.3208 -188.3137 -189.2738 -190.1957
##  [14,] -186.3333 -187.3411 -188.3334 -189.2928 -190.2140 -191.0992 -191.9507
##  [15,] -188.3532 -189.3118 -190.2322 -191.1168 -191.9675 -192.7865 -193.5755
##  [16,] -190.2504 -191.1343 -191.9844 -192.8027 -193.5911 -194.3513 -195.0848
##  [17,] -192.0012 -192.8189 -193.6067 -194.3664 -195.0994 -195.8074 -196.4916
##  [18,] -193.6224 -194.3814 -195.1139 -195.8214 -196.5052 -197.1667 -197.8071
##  [19,] -195.1285 -195.8354 -196.5188 -197.1799 -197.8199 -198.4400 -199.0413
##  [20,] -196.5324 -197.1930 -197.8326 -198.4523 -199.0533 -199.6364 -200.2028
##  [21,] -197.8453 -198.4646 -199.0652 -199.6480 -200.2141 -200.7643 -201.2995
##  [22,] -199.0772 -199.6596 -200.2254 -200.7753 -201.3102 -201.8309 -202.3383
##  [23,] -200.2366 -200.7862 -201.3209 -201.8413 -202.3484 -202.8428 -203.3253
##  [24,] -201.3315 -201.8517 -202.3585 -202.8527 -203.3349 -203.8058 -204.2660
##  [25,] -202.3686 -202.8626 -203.3445 -203.8152 -204.2752 -204.7251 -205.1655
##  [26,] -203.3542 -203.8246 -204.2844 -204.7341 -205.1743 -205.6055 -206.0282
##  [27,] -204.2936 -204.7431 -205.1831 -205.6142 -206.0367 -206.4513 -206.8583
##  [28,] -205.1919 -205.6228 -206.0452 -206.4596 -206.8664 -207.2662 -207.6594
##  [29,] -206.0536 -206.4678 -206.8746 -207.2742 -207.6672 -208.0540 -208.4349
##  [30,] -206.8827 -207.2822 -207.6751 -208.0617 -208.4425 -208.8178 -209.1880
##  [31,] -207.6830 -208.0695 -208.4501 -208.8253 -209.1954 -209.5607 -209.9215
##  [32,] -208.4578 -208.8329 -209.2028 -209.5680 -209.9287 -210.2853 -210.6364
##  [33,] -209.2102 -209.5753 -209.9360 -210.2925 -210.6434 -210.9825 -211.3097
##  [34,] -209.9432 -210.2996 -210.6503 -210.9893 -211.3162 -211.6315 -211.9353
##  [35,] -210.6573 -210.9960 -211.3227 -211.6377 -211.9413 -212.2337 -212.5151
##  [36,] -211.3292 -211.6440 -211.9473 -212.2395 -212.5206 -212.7911 -213.0511
##  [37,] -211.9533 -212.2452 -212.5262 -212.7965 -213.0562 -213.3057 -213.5451
##  [38,] -212.5318 -212.8018 -213.0614 -213.3106 -213.5498 -213.7792 -213.9989
##  [39,] -213.0665 -213.3156 -213.5546 -213.7837 -214.0032 -214.2133 -214.4140
##  [40,] -213.5593 -213.7883 -214.0076 -214.2174 -214.4180 -214.6094 -214.7919
##  [41,] -214.0119 -214.2216 -214.4219 -214.6132 -214.7955 -214.9690 -215.1338
##  [42,] -214.4259 -214.6170 -214.7991 -214.9724 -215.1371 -215.2933 -215.4410
##  [43,] -214.8027 -214.9758 -215.1403 -215.2963 -215.4439 -215.5833 -215.7146
##  [44,] -215.1436 -215.2994 -215.4468 -215.5861 -215.7172 -215.8402 -215.9554
##  [45,] -215.4497 -215.5888 -215.7197 -215.8426 -215.9576 -216.0648 -216.1643
##  [46,] -215.7223 -215.8450 -215.9599 -216.0669 -216.1662 -216.2579 -216.3420
##  [47,] -215.9661 -216.0690 -216.1681 -216.2597 -216.3436 -216.4201 -216.4891
##  [48,] -216.2098 -216.2787 -216.3493 -216.4216 -216.4904 -216.5520 -216.6062
##  [49,] -216.4591 -216.4945 -216.5317 -216.5707 -216.6114 -216.6540 -216.6935
##  [50,] -216.7146 -216.7167 -216.7208 -216.7267 -216.7346 -216.7443 -216.7559
##  [51,] -216.9766 -216.9457 -216.9168 -216.8899 -216.8651 -216.8421 -216.8211
##  [52,] -217.2458 -217.1819 -217.1203 -217.0607 -217.0031 -216.9476 -216.8977
##  [53,] -217.5227 -217.4261 -217.3316 -217.2394 -217.1531 -217.0759 -217.0077
##  [54,] -217.8081 -217.6787 -217.5555 -217.4416 -217.3368 -217.2412 -217.1547
##  [55,] -218.1070 -217.9558 -217.8139 -217.6813 -217.5579 -217.4438 -217.3389
##  [56,] -218.4410 -218.2709 -218.1101 -217.9587 -217.8167 -217.6839 -217.5603
##  [57,] -218.8137 -218.6244 -218.4446 -218.2742 -218.1133 -217.9617 -217.8194
##  [58,] -219.2253 -219.0166 -218.8176 -218.6281 -218.4481 -218.2776 -218.1164
##  [59,] -219.6763 -219.4481 -219.2296 -219.0208 -218.8215 -218.6318 -218.4516
##  [60,] -220.1673 -219.9193 -219.6811 -219.4526 -219.2339 -219.0249 -218.8254
##  [61,] -220.6987 -220.4306 -220.1724 -219.9242 -219.6858 -219.4571 -219.2382
##  [62,] -221.2711 -220.9826 -220.7042 -220.4359 -220.1775 -219.9291 -219.6905
##  [63,] -221.8850 -221.5759 -221.2770 -220.9883 -220.7098 -220.4412 -220.1827
##  [64,] -222.5411 -222.2110 -221.8914 -221.5820 -221.2830 -220.9941 -220.7153
##  [65,] -223.2400 -222.8886 -222.5479 -222.2176 -221.8977 -221.5882 -221.2889
##  [66,] -223.9823 -223.6094 -223.2472 -222.8956 -222.5547 -222.2242 -221.9041
##  [67,] -224.7561 -224.3731 -223.9899 -223.6168 -223.2544 -222.9026 -222.5614
##  [68,] -225.5203 -225.1436 -224.7638 -224.3809 -223.9976 -223.6242 -223.2616
##  [69,] -226.2723 -225.9015 -225.5279 -225.1512 -224.7715 -224.3886 -224.0052
##  [70,] -227.0128 -226.6476 -226.2797 -225.9091 -225.5355 -225.1589 -224.7792
##  [71,] -227.7428 -227.3828 -227.0202 -226.6550 -226.2872 -225.9166 -225.5430
##  [72,] -228.4631 -228.1077 -227.7501 -227.3900 -227.0275 -226.6625 -226.2947
##  [73,] -229.1746 -228.8234 -228.4703 -228.1149 -227.7573 -227.3973 -227.0349
##  [74,] -229.8783 -229.5309 -229.1817 -228.8306 -228.4774 -228.1221 -227.7646
##  [75,] -230.5753 -230.2311 -229.8853 -229.5379 -229.1887 -228.8377 -228.4846
##  [76,] -231.2665 -230.9250 -230.5822 -230.2380 -229.8923 -229.5450 -229.1958
##  [77,] -231.9533 -231.6138 -231.2734 -230.9319 -230.5891 -230.2450 -229.8993
##  [78,] -232.6367 -232.2988 -231.9601 -231.6207 -231.2803 -230.9388 -230.5961
##  [79,] -233.3181 -232.9810 -232.6435 -232.3056 -231.9670 -231.6276 -231.2872
##  [80,] -233.9989 -233.6619 -233.3250 -232.9878 -232.6504 -232.3124 -231.9738
##  [81,] -234.6805 -234.3429 -234.0057 -233.6687 -233.3318 -232.9946 -232.6572
##  [82,] -235.3646 -235.0256 -234.6874 -234.3498 -234.0125 -233.6755 -233.3386
##  [83,] -236.0528 -235.7115 -235.3714 -235.0324 -234.6942 -234.3566 -234.0194
##  [84,] -236.7469 -236.4024 -236.0597 -235.7184 -235.3783 -235.0393 -234.7010
##  [85,] -237.4490 -237.1003 -236.7539 -236.4094 -236.0666 -235.7252 -235.3852
##  [86,] -238.1612 -237.8072 -237.4560 -237.1073 -236.7608 -236.4163 -236.0735
##  [87,] -238.8753 -238.5235 -238.1683 -237.8143 -237.4631 -237.1143 -236.7678
##  [88,] -239.5749 -239.2305 -238.8824 -238.5307 -238.1755 -237.8215 -237.4702
##  [89,] -240.2593 -239.9224 -239.5818 -239.2375 -238.8895 -238.5378 -238.1827
##  [90,] -240.9286 -240.5992 -240.2661 -239.9293 -239.5887 -239.2445 -238.8965
##  [91,] -241.5824 -241.2607 -240.9352 -240.6059 -240.2729 -239.9361 -239.5956
##  [92,] -242.2207 -241.9067 -241.5888 -241.2672 -240.9418 -240.6126 -240.2796
##  [93,] -242.8436 -242.5372 -242.2270 -241.9131 -241.5953 -241.2737 -240.9484
##  [94,] -243.4508 -243.1522 -242.8497 -242.5434 -242.2333 -241.9195 -241.6018
##  [95,] -244.0424 -243.7515 -243.4568 -243.1582 -242.8558 -242.5497 -242.2396
##  [96,] -244.6185 -244.3353 -244.0483 -243.7574 -243.4628 -243.1643 -242.8620
##  [97,] -245.1790 -244.9035 -244.6242 -244.3410 -244.0541 -243.7633 -243.4688
##  [98,] -245.7241 -245.4562 -245.1846 -244.9091 -244.6299 -244.3468 -244.0599
##  [99,] -246.2538 -245.9935 -245.7295 -245.4617 -245.1901 -244.9147 -244.6355
## [100,] -246.7684 -246.5155 -246.2590 -245.9988 -245.7348 -245.4671 -245.1956
##            [,99]    [,100]
##   [1,] -162.8188 -164.3153
##   [2,] -165.7987 -167.2132
##   [3,] -168.6185 -169.9614
##   [4,] -171.2984 -172.5786
##   [5,] -173.8555 -175.0803
##   [6,] -176.3040 -177.4796
##   [7,] -178.6560 -179.7878
##   [8,] -180.9218 -182.0140
##   [9,] -183.1097 -184.1663
##  [10,] -185.2272 -186.2512
##  [11,] -187.2804 -188.2740
##  [12,] -189.2548 -190.1775
##  [13,] -191.0817 -191.9338
##  [14,] -192.7702 -193.5598
##  [15,] -194.3362 -195.0703
##  [16,] -195.7933 -196.4780
##  [17,] -197.1536 -197.7944
##  [18,] -198.4276 -199.0293
##  [19,] -199.6248 -200.1916
##  [20,] -200.7534 -201.2889
##  [21,] -201.8206 -202.3282
##  [22,] -202.8330 -203.3157
##  [23,] -203.7964 -204.2568
##  [24,] -204.7162 -205.1567
##  [25,] -205.5969 -206.0198
##  [26,] -206.4430 -206.8501
##  [27,] -207.2582 -207.6515
##  [28,] -208.0462 -208.4272
##  [29,] -208.8103 -209.1805
##  [30,] -209.5533 -209.9142
##  [31,] -210.2781 -210.6294
##  [32,] -210.9758 -211.3032
##  [33,] -211.6252 -211.9292
##  [34,] -212.2279 -212.5095
##  [35,] -212.7857 -213.0459
##  [36,] -213.3008 -213.5404
##  [37,] -213.7747 -213.9946
##  [38,] -214.2091 -214.4100
##  [39,] -214.6056 -214.7883
##  [40,] -214.9656 -215.1306
##  [41,] -215.2902 -215.4381
##  [42,] -215.5806 -215.7120
##  [43,] -215.8378 -215.9531
##  [44,] -216.0627 -216.1623
##  [45,] -216.2561 -216.3404
##  [46,] -216.4186 -216.4878
##  [47,] -216.5508 -216.6052
##  [48,] -216.6531 -216.6928
##  [49,] -216.7259 -216.7511
##  [50,] -216.7694 -216.7802
##  [51,] -216.8020 -216.7884
##  [52,] -216.8569 -216.8251
##  [53,] -216.9487 -216.8987
##  [54,] -217.0773 -217.0090
##  [55,] -217.2431 -217.1564
##  [56,] -217.4460 -217.3409
##  [57,] -217.6865 -217.5628
##  [58,] -217.9647 -217.8222
##  [59,] -218.2809 -218.1196
##  [60,] -218.6356 -218.4552
##  [61,] -219.0290 -218.8294
##  [62,] -219.4617 -219.2426
##  [63,] -219.9340 -219.6952
##  [64,] -220.4466 -220.1878
##  [65,] -220.9998 -220.7208
##  [66,] -221.5943 -221.2948
##  [67,] -222.2307 -221.9104
##  [68,] -222.9096 -222.5682
##  [69,] -223.6317 -223.2688
##  [70,] -224.3964 -224.0129
##  [71,] -225.1665 -224.7869
##  [72,] -225.9241 -225.5506
##  [73,] -226.6699 -226.3021
##  [74,] -227.4046 -227.0422
##  [75,] -228.1293 -227.7718
##  [76,] -228.8448 -228.4917
##  [77,] -229.5520 -229.2029
##  [78,] -230.2520 -229.9063
##  [79,] -230.9457 -230.6030
##  [80,] -231.6344 -231.2941
##  [81,] -232.3193 -231.9807
##  [82,] -233.0014 -232.6640
##  [83,] -233.6824 -233.3454
##  [84,] -234.3634 -234.0262
##  [85,] -235.0461 -234.7079
##  [86,] -235.7321 -235.3920
##  [87,] -236.4232 -236.0804
##  [88,] -237.1214 -236.7748
##  [89,] -237.8286 -237.4772
##  [90,] -238.5450 -238.1899
##  [91,] -239.2515 -238.9036
##  [92,] -239.9430 -239.6026
##  [93,] -240.6193 -240.2864
##  [94,] -241.2803 -240.9550
##  [95,] -241.9258 -241.6082
##  [96,] -242.5559 -242.2459
##  [97,] -243.1704 -242.8681
##  [98,] -243.7693 -243.4748
##  [99,] -244.3526 -244.0658
## [100,] -244.9203 -244.6412
## 
## $SSE
##              [,1]       [,2]       [,3]       [,4]       [,5]       [,6]
##   [1,]   695.0079   695.0079   695.0079   695.0079   695.0079   695.0079
##   [2,]   695.0079   695.0079   695.0079   695.0079   695.0079   695.0079
##   [3,]   695.0079   695.0079   695.0079   695.0079   695.0079   695.0079
##   [4,]   695.0079   695.0079   695.0079   695.0079   695.0079   695.0079
##   [5,]   695.0079   695.0079   695.0079   695.0079   695.0079   695.0079
##   [6,]   695.0079   695.0079   695.0079   695.0079   695.0079   695.0079
##   [7,]   695.0079   695.0079   695.0079   695.0079   695.0079   695.0079
##   [8,]   695.0079   695.0079   695.0079   695.0079   695.0079   695.0079
##   [9,]   695.0079   695.0079   695.0079   695.0079   695.0079   695.0079
##  [10,]   695.0079   695.0079   695.0079   695.0079   695.0079   695.0079
##  [11,]   695.0079   695.0079   695.0079   695.0079   695.0079   695.0079
##  [12,]   695.0079   695.0079   695.0079   695.0079   695.0079   695.0079
##  [13,]   695.0079   695.0079   695.0079   695.0079   695.0079   695.0079
##  [14,]   695.0079   695.0079   695.0079   695.0079   695.0079   695.0079
##  [15,]   695.0079   695.0079   695.0079   695.0079   695.0079   695.0079
##  [16,]   695.0079   695.0079   695.0079   695.0079   695.0079   695.0079
##  [17,]   695.0079   695.0079   695.0079   695.0079   695.0079   695.0079
##  [18,]   695.0079   695.0079   695.0079   695.0079   695.0079   694.2575
##  [19,]   695.0079   695.0079   695.0079   693.8162   681.6445   661.7331
##  [20,]   695.0079   692.4929   673.3273   647.1104   618.4505   588.9148
##  [21,]   632.1436   603.6523   574.0554   544.6822   515.8214   487.5890
##  [22,]   422.5587   422.5587   419.8120   407.2476   390.1872   371.2354
##  [23,]   263.0100   263.0100   263.0100   263.0100   261.9091   255.8782
##  [24,]   161.6399   161.6399   161.6399   161.6399   161.6399   161.6399
##  [25,]   114.8560   114.8560   114.8560   114.8560   114.8560   114.8560
##  [26,]   118.6445   118.6445   118.6445   118.6445   118.6445   118.6445
##  [27,]   168.7182   168.7182   168.7182   168.7182   168.7182   168.7182
##  [28,]   260.6499   260.6499   260.6499   260.6499   260.6499   260.6499
##  [29,]   389.9888   389.9888   389.9888   389.9888   389.9888   389.9888
##  [30,]   552.3577   552.3577   552.3577   552.3577   552.3577   552.3577
##  [31,]   743.5309   743.5309   743.5309   743.5309   743.5309   743.5309
##  [32,]   959.4930   959.4930   959.4930   959.4930   959.4930   959.4930
##  [33,]  1196.4806  1196.4806  1196.4806  1196.4806  1196.4806  1196.4806
##  [34,]  1451.0098  1451.0098  1451.0098  1451.0098  1451.0098  1451.0098
##  [35,]  1719.8902  1719.8902  1719.8902  1719.8902  1719.8902  1719.8902
##  [36,]  2000.2292  2000.2292  2000.2292  2000.2292  2000.2292  2000.2292
##  [37,]  2289.4281  2289.4281  2289.4281  2289.4281  2289.4281  2289.4281
##  [38,]  2585.1718  2585.1718  2585.1718  2585.1718  2585.1718  2583.3437
##  [39,]  2885.4146  2885.4146  2885.4146  2882.5295  2873.2259  2860.6526
##  [40,]  3188.3625  3182.3556  3167.8613  3151.2347  3133.9412  3116.4770
##  [41,]  3431.6306  3419.4810  3403.6149  3387.0480  3370.3931  3353.8611
##  [42,]  3603.3545  3603.3545  3603.2037  3598.1103  3589.0390  3578.1218
##  [43,]  3799.5793  3799.5793  3799.5793  3799.5793  3799.5228  3796.8726
##  [44,]  4021.5299  4021.5299  4021.5299  4021.5299  4021.5299  4021.5299
##  [45,]  4269.5985  4269.5985  4269.5985  4269.5985  4269.5985  4269.5985
##  [46,]  4543.9936  4543.9936  4543.9936  4543.9936  4543.9936  4543.9936
##  [47,]  4844.7235  4844.7235  4844.7235  4844.7235  4844.7235  4844.7235
##  [48,]  5171.5815  5171.5815  5171.5815  5171.5815  5171.5815  5171.5815
##  [49,]  5524.1348  5524.1348  5524.1348  5524.1348  5524.1348  5524.1348
##  [50,]  5901.7174  5901.7174  5901.7174  5901.7174  5901.7174  5901.7174
##  [51,]  6303.4266  6303.4266  6303.4266  6303.4266  6303.4266  6303.4266
##  [52,]  6728.1254  6728.1254  6728.1254  6728.1254  6728.1254  6728.1254
##  [53,]  7174.4495  7174.4495  7174.4495  7174.4495  7174.4495  7174.4495
##  [54,]  7640.8188  7640.8188  7640.8188  7640.8188  7640.8188  7640.8188
##  [55,]  8125.4554  8125.4554  8125.4554  8125.4554  8125.4554  8125.4554
##  [56,]  8626.4046  8626.4046  8626.4046  8626.4046  8626.4046  8626.4046
##  [57,]  9141.5613  9141.5613  9141.5613  9141.5613  9141.5613  9141.5613
##  [58,]  9668.6995  9668.6995  9668.6995  9668.6995  9668.4425  9656.3276
##  [59,] 10205.5038 10205.5038 10204.9604 10186.5230 10153.3098 10112.6657
##  [60,] 10747.6034 10711.0034 10662.4791 10610.8763 10557.9671 10504.3458
##  [61,] 11039.6489 11022.9501 10982.2655 10934.8846 10884.7468 10833.1682
##  [62,] 11169.9695 11169.9695 11169.9695 11162.0730 11136.4557 11101.4521
##  [63,] 11316.8696 11316.8696 11316.8696 11316.8696 11316.8696 11311.9145
##  [64,] 11482.0632 11482.0632 11482.0632 11482.0632 11482.0632 11482.0632
##  [65,] 11667.3879 11667.3879 11667.3879 11667.3879 11667.3879 11667.3879
##  [66,] 11874.7941 11874.7941 11874.7941 11874.7941 11874.7941 11874.7941
##  [67,] 12106.3271 12106.3271 12106.3271 12106.3271 12106.3271 12106.3271
##  [68,] 12364.0972 12364.0972 12364.0972 12364.0972 12364.0972 12364.0972
##  [69,] 12650.2372 12650.2372 12650.2372 12650.2372 12650.2372 12650.2372
##  [70,] 12966.8433 12966.8433 12966.8433 12966.8433 12966.8433 12966.8433
##  [71,] 13315.8959 13315.8959 13315.8959 13315.8959 13315.8959 13315.8959
##  [72,] 13699.1586 13699.1586 13699.1586 13699.1586 13699.1586 13699.1586
##  [73,] 14118.0515 14118.0515 14118.0515 14118.0515 14118.0515 14118.0515
##  [74,] 14573.5015 14573.5015 14573.5015 14573.5015 14573.5015 14573.5015
##  [75,] 15065.7678 15065.7678 15065.7678 15065.7678 15065.7678 15065.7678
##  [76,] 15594.2519 15594.2519 15594.2519 15594.2519 15594.2519 15594.2519
##  [77,] 16157.3007 16157.3007 16157.3007 16157.3007 16157.3007 16157.3007
##  [78,] 16752.0193 16752.0193 16752.0193 16752.0193 16746.8472 16718.1923
##  [79,] 17374.1150 17374.1150 17364.9163 17322.3341 17263.1204 17195.2970
##  [80,] 17988.7962 17913.3371 17832.6832 17750.1470 17666.3964 17581.6592
##  [81,] 18148.5115 18142.2876 18094.4201 18027.6352 17952.7860 17873.4772
##  [82,] 18148.5115 18148.5115 18148.5115 18145.5639 18115.2893 18065.0761
##  [83,] 18148.5115 18148.5115 18148.5115 18148.5115 18148.5115 18146.6557
##  [84,] 18148.5115 18148.5115 18148.5115 18148.5115 18148.5115 18148.5115
##  [85,] 18148.5115 18148.5115 18148.5115 18148.5115 18148.5115 18148.5115
##  [86,] 18148.5115 18148.5115 18148.5115 18148.5115 18148.5115 18148.5115
##  [87,] 18148.5115 18148.5115 18148.5115 18148.5115 18148.5115 18148.5115
##  [88,] 18148.5115 18148.5115 18148.5115 18148.5115 18148.5115 18148.5115
##  [89,] 18148.5115 18148.5115 18148.5115 18148.5115 18148.5115 18148.5115
##  [90,] 18148.5115 18148.5115 18148.5115 18148.5115 18148.5115 18148.5115
##  [91,] 18148.5115 18148.5115 18148.5115 18148.5115 18148.5115 18148.5115
##  [92,] 18148.5115 18148.5115 18148.5115 18148.5115 18148.5115 18148.5115
##  [93,] 18148.5115 18148.5115 18148.5115 18148.5115 18148.5115 18148.5115
##  [94,] 18148.5115 18148.5115 18148.5115 18148.5115 18148.5115 18148.5115
##  [95,] 18148.5115 18148.5115 18148.5115 18148.5115 18148.5115 18148.5115
##  [96,] 18148.5115 18148.5115 18148.5115 18148.5115 18148.5115 18148.5115
##  [97,] 18148.5115 18148.5115 18148.5115 18148.5115 18148.5115 18148.5115
##  [98,] 18148.5115 18148.5115 18148.5115 18148.5115 18148.5115 18148.5115
##  [99,] 18148.5115 18148.5115 18148.5115 18148.5115 18148.5115 18148.5115
## [100,] 18148.5115 18148.5115 18148.5115 18148.5115 18148.5115 18148.5115
##              [,7]       [,8]       [,9]      [,10]      [,11]      [,12]
##   [1,]   695.0079   695.0079   695.0079   695.0079   695.0079   695.0079
##   [2,]   695.0079   695.0079   695.0079   695.0079   695.0079   695.0079
##   [3,]   695.0079   695.0079   695.0079   695.0079   695.0079   695.0079
##   [4,]   695.0079   695.0079   695.0079   695.0079   695.0079   695.0079
##   [5,]   695.0079   695.0079   695.0079   695.0079   695.0079   695.0079
##   [6,]   695.0079   695.0079   695.0079   695.0079   695.0079   695.0079
##   [7,]   695.0079   695.0079   695.0079   695.0079   695.0079   695.0079
##   [8,]   695.0079   695.0079   695.0079   695.0079   695.0079   695.0079
##   [9,]   695.0079   695.0079   695.0079   695.0079   695.0079   695.0079
##  [10,]   695.0079   695.0079   695.0079   695.0079   695.0079   695.0079
##  [11,]   695.0079   695.0079   695.0079   695.0079   695.0079   695.0079
##  [12,]   695.0079   695.0079   695.0079   695.0079   695.0079   695.0079
##  [13,]   695.0079   695.0079   695.0079   695.0079   695.0079   695.0079
##  [14,]   695.0079   695.0079   695.0079   695.0079   695.0079   695.0079
##  [15,]   695.0079   695.0079   695.0079   695.0079   695.0079   694.6989
##  [16,]   695.0079   695.0079   695.0079   694.6106   688.3403   675.9788
##  [17,]   695.0079   694.4781   687.1317   673.2393   654.7244   632.9082
##  [18,]   685.1890   668.9810   648.1910   624.3942   598.6195   571.5732
##  [19,]   637.6456   611.2041   583.4453   555.0102   526.3226   497.6794
##  [20,]   559.1958   529.6571   500.5161   471.9177   443.9674   416.7481
##  [21,]   460.0518   433.2590   407.2523   382.0699   357.7487   334.3250
##  [22,]   351.5405   331.6965   312.0491   292.8187   274.1579   256.1796
##  [23,]   246.7312   235.8987   224.2146   212.2025   200.2124   188.4906
##  [24,]   161.2085   158.5726   154.3560   149.2332   143.6733   138.0139
##  [25,]   114.8560   114.8560   114.7644   114.1687   113.2443   112.2310
##  [26,]   118.6445   118.6445   118.6445   118.6445   118.7411   119.4700
##  [27,]   168.7182   168.7182   168.7182   168.7182   168.7182   168.7182
##  [28,]   260.6499   260.6499   260.6499   260.6499   260.6499   260.6499
##  [29,]   389.9888   389.9888   389.9888   389.9888   389.9888   389.9888
##  [30,]   552.3577   552.3577   552.3577   552.3577   552.3577   552.3577
##  [31,]   743.5309   743.5309   743.5309   743.5309   743.5309   743.5309
##  [32,]   959.4930   959.4930   959.4930   959.4930   959.4930   959.4930
##  [33,]  1196.4806  1196.4806  1196.4806  1196.4806  1196.4806  1196.4806
##  [34,]  1451.0098  1451.0098  1451.0098  1451.0098  1451.0098  1451.0098
##  [35,]  1719.8902  1719.8902  1719.8902  1719.8902  1719.8902  1719.1648
##  [36,]  2000.2292  2000.2292  2000.2292  1999.2743  1995.1819  1988.6417
##  [37,]  2289.4281  2288.1401  2282.9875  2275.0390  2265.1299  2253.8309
##  [38,]  2576.6348  2566.7939  2554.9583  2541.8240  2527.8427  2513.3216
##  [39,]  2846.3379  2831.0584  2815.2527  2799.1896  2783.0445  2766.9385
##  [40,]  3099.0608  3081.8071  3064.7838  3048.0361  3031.5965  3015.4902
##  [41,]  3337.5478  3321.5060  3305.7695  3290.3627  3275.3047  3260.6120
##  [42,]  3566.2912  3554.0228  3541.5867  3529.1500  3516.8227  3504.6823
##  [43,]  3791.5362  3784.6189  3776.7560  3768.3401  3759.6281  3750.7966
##  [44,]  4021.5077  4020.0927  4017.0330  4012.8946  4008.0579  4002.7887
##  [45,]  4269.5985  4269.5985  4269.5909  4268.9188  4267.3949  4265.2887
##  [46,]  4543.9936  4543.9936  4543.9936  4543.9936  4543.9922  4543.8119
##  [47,]  4844.7235  4844.7235  4844.7235  4844.7235  4844.7235  4844.7235
##  [48,]  5171.5815  5171.5815  5171.5815  5171.5815  5171.5815  5171.5815
##  [49,]  5524.1348  5524.1348  5524.1348  5524.1348  5524.1348  5524.1348
##  [50,]  5901.7174  5901.7174  5901.7174  5901.7174  5901.7174  5901.7174
##  [51,]  6303.4266  6303.4266  6303.4266  6303.4266  6303.4266  6303.4266
##  [52,]  6728.1254  6728.1254  6728.1254  6728.1254  6728.1254  6728.1254
##  [53,]  7174.4495  7174.4495  7174.4495  7174.4495  7174.4495  7174.4495
##  [54,]  7640.8188  7640.8188  7640.8188  7640.8188  7640.8188  7640.8188
##  [55,]  8125.4554  8125.4554  8125.4554  8125.4554  8125.4132  8119.8916
##  [56,]  8626.4046  8626.4046  8626.3272  8619.4421  8603.5555  8580.9191
##  [57,]  9141.4225  9132.5458  9113.1158  9086.2931  9054.0886  9017.8436
##  [58,]  9631.6684  9599.1512  9561.3775  9519.9089  9475.7400  9429.5362
##  [59,] 10067.7364 10020.0961  9970.6214  9919.8404  9868.0919  9815.6037
##  [60,] 10450.2703 10395.8727 10341.2291 10286.3877 10231.3818 10176.2358
##  [61,] 10780.7156 10727.6755 10674.2097 10620.4178 10566.3657 10512.0996
##  [62,] 11061.0396 11017.2064 10971.0583 10923.2605 10874.2386 10824.2783
##  [63,] 11293.6482 11266.6160 11233.7138 11196.6773 11156.6116 11114.2544
##  [64,] 11482.0632 11478.5847 11464.6181 11442.9070 11415.5488 11383.9404
##  [65,] 11667.3879 11667.3879 11667.3879 11664.7996 11653.6711 11635.7615
##  [66,] 11874.7941 11874.7941 11874.7941 11874.7941 11874.7941 11872.8027
##  [67,] 12106.3271 12106.3271 12106.3271 12106.3271 12106.3271 12106.3271
##  [68,] 12364.0972 12364.0972 12364.0972 12364.0972 12364.0972 12364.0972
##  [69,] 12650.2372 12650.2372 12650.2372 12650.2372 12650.2372 12650.2372
##  [70,] 12966.8433 12966.8433 12966.8433 12966.8433 12966.8433 12966.8433
##  [71,] 13315.8959 13315.8959 13315.8959 13315.8959 13315.8959 13315.8959
##  [72,] 13699.1586 13699.1586 13699.1586 13699.1586 13699.1586 13699.1586
##  [73,] 14118.0515 14118.0515 14118.0515 14118.0515 14118.0515 14118.0515
##  [74,] 14573.5015 14573.5015 14573.5015 14573.5015 14573.5015 14573.5015
##  [75,] 15065.7678 15065.7678 15065.7678 15065.7678 15063.9911 15050.9087
##  [76,] 15594.2519 15594.2519 15591.8362 15575.4390 15547.1002 15509.7725
##  [77,] 16153.8898 16132.7386 16097.7088 16052.8913 16000.8794 15943.3986
##  [78,] 16673.6347 16618.9905 16557.4771 16491.0212 16420.8500 16347.7843
##  [79,] 17122.2915 17045.8182 16966.8325 16885.9122 16803.4295 16719.6375
##  [80,] 17496.0381 17409.5897 17322.3502 17234.3461 17145.5992 17056.1288
##  [81,] 17791.2537 17706.8905 17620.8221 17533.3130 17444.5355 17354.6092
##  [82,] 18003.1362 17933.5391 17858.5336 17779.4653 17697.1905 17612.2819
##  [83,] 18124.1377 18083.4550 18030.4191 17968.4739 17899.7906 17825.8075
##  [84,] 18148.5115 18147.2016 18128.9764 18094.1986 18047.1917 17990.7935
##  [85,] 18148.5115 18148.5115 18148.5115 18147.5291 18131.9823 18101.0894
##  [86,] 18148.5115 18148.5115 18148.5115 18148.5115 18148.5115 18147.7474
##  [87,] 18148.5115 18148.5115 18148.5115 18148.5115 18148.5115 18148.5115
##  [88,] 18148.5115 18148.5115 18148.5115 18148.5115 18148.5115 18148.5115
##  [89,] 18148.5115 18148.5115 18148.5115 18148.5115 18148.5115 18148.5115
##  [90,] 18148.5115 18148.5115 18148.5115 18148.5115 18148.5115 18148.5115
##  [91,] 18148.5115 18148.5115 18148.5115 18148.5115 18148.5115 18148.5115
##  [92,] 18148.5115 18148.5115 18148.5115 18148.5115 18148.5115 18148.5115
##  [93,] 18148.5115 18148.5115 18148.5115 18148.5115 18148.5115 18148.5115
##  [94,] 18148.5115 18148.5115 18148.5115 18148.5115 18148.5115 18148.5115
##  [95,] 18148.5115 18148.5115 18148.5115 18148.5115 18148.5115 18148.5115
##  [96,] 18148.5115 18148.5115 18148.5115 18148.5115 18148.5115 18148.5115
##  [97,] 18148.5115 18148.5115 18148.5115 18148.5115 18148.5115 18148.5115
##  [98,] 18148.5115 18148.5115 18148.5115 18148.5115 18148.5115 18148.5115
##  [99,] 18148.5115 18148.5115 18148.5115 18148.5115 18148.5115 18148.5115
## [100,] 18148.5115 18148.5115 18148.5115 18148.5115 18148.5115 18148.5115
##             [,13]      [,14]      [,15]      [,16]      [,17]      [,18]
##   [1,]   695.0079   695.0079   695.0079   695.0079   695.0079   695.0079
##   [2,]   695.0079   695.0079   695.0079   695.0079   695.0079   695.0079
##   [3,]   695.0079   695.0079   695.0079   695.0079   695.0079   695.0079
##   [4,]   695.0079   695.0079   695.0079   695.0079   695.0079   695.0079
##   [5,]   695.0079   695.0079   695.0079   695.0079   695.0079   695.0079
##   [6,]   695.0079   695.0079   695.0079   695.0079   695.0079   695.0079
##   [7,]   695.0079   695.0079   695.0079   695.0079   695.0079   695.0079
##   [8,]   695.0079   695.0079   695.0079   695.0079   695.0079   695.0079
##   [9,]   695.0079   695.0079   695.0079   695.0079   695.0079   695.0079
##  [10,]   695.0079   695.0079   695.0079   695.0079   695.0079   695.0079
##  [11,]   695.0079   695.0079   695.0079   695.0079   695.0079   695.0079
##  [12,]   695.0079   695.0079   695.0079   695.0079   695.0079   694.8460
##  [13,]   695.0079   695.0079   695.0079   694.8092   690.1027   679.9576
##  [14,]   695.0079   694.7619   689.7086   679.0996   664.0054   645.2933
##  [15,]   689.1478   677.8287   661.9947   642.6210   620.4807   596.1975
##  [16,]   659.0462   638.6652   615.6862   590.7692   564.4373   537.1146
##  [17,]   608.7328   582.8921   555.9128   528.2061   500.1010   471.8675
##  [18,]   543.7612   515.5601   487.2597   459.0909   431.2427   403.8742
##  [19,]   469.2994   441.3518   413.9727   387.2758   361.3595   336.3113
##  [20,]   390.3294   364.7724   340.1331   316.4642   293.8164   272.2393
##  [21,]   311.8352   290.3160   269.8045   250.3386   231.9568   214.6982
##  [22,]   238.9725   222.6098   207.1550   192.6645   179.1907   166.7824
##  [23,]   177.2189   166.5374   156.5582   147.3741   139.0644   131.6986
##  [24,]   132.5063   127.3435   122.6780   118.6342   115.3154   112.8102
##  [25,]   111.3327   110.7196   110.5344   110.8977   111.9128   113.6693
##  [26,]   120.8503   122.8692   125.5515   128.9399   133.0841   138.0363
##  [27,]   168.9205   170.5445   173.5830   177.8741   183.3251   189.8857
##  [28,]   260.6499   260.6499   260.9089   263.1465   267.3924   273.3972
##  [29,]   389.9888   389.9888   389.9888   389.9888   390.2735   392.9292
##  [30,]   552.3577   552.3577   552.3577   552.3577   552.3577   552.3577
##  [31,]   743.5309   743.5309   743.5309   743.5309   743.5309   743.5309
##  [32,]   959.4930   959.4930   959.4930   959.4930   959.4930   959.1782
##  [33,]  1196.4806  1196.4806  1196.4806  1196.0583  1193.9089  1190.2335
##  [34,]  1451.0098  1450.4546  1447.7794  1443.2893  1437.3491  1430.2551
##  [35,]  1715.8617  1710.4382  1703.3693  1695.0239  1685.6937  1675.6125
##  [36,]  1980.2783  1970.5513  1959.8068  1948.3117  1936.2753  1923.8652
##  [37,]  2241.5463  2228.5715  2215.1284  2201.3879  2187.4844  2173.5260
##  [38,]  2498.4782  2483.4723  2468.4244  2453.4286  2438.5599  2423.8796
##  [39,]  2750.9589  2735.1717  2719.6283  2704.3704  2689.4329  2674.8456
##  [40,]  2999.7382  2984.3587  2969.3680  2954.7815  2940.6136  2926.8784
##  [41,]  3246.2993  3232.3803  3218.8678  3205.7747  3193.1133  3180.8961
##  [42,]  3492.7854  3481.1758  3469.8887  3458.9531  3448.3941  3438.2337
##  [43,]  3741.9724  3733.2494  3724.6999  3716.3811  3708.3396  3700.6143
##  [44,]  3997.2789  3991.6713  3986.0753  3980.5769  3975.2448  3970.1358
##  [45,]  4262.8057  4260.1043  4257.3091  4254.5198  4251.8173  4249.2684
##  [46,]  4543.3982  4542.8469  4542.2452  4541.6702  4541.1890  4540.8600
##  [47,]  4844.7242  4844.8885  4845.3159  4845.9951  4846.9330  4848.1458
##  [48,]  5171.5815  5171.5815  5171.5825  5171.9960  5173.0811  5174.7582
##  [49,]  5524.1348  5524.1348  5524.1348  5524.1348  5524.1354  5524.7318
##  [50,]  5901.7174  5901.7174  5901.7174  5901.7174  5901.7174  5901.7174
##  [51,]  6303.4266  6303.4266  6303.4266  6303.4266  6303.4266  6303.4266
##  [52,]  6728.1254  6728.1254  6728.1254  6728.1254  6728.1226  6724.9889
##  [53,]  7174.4495  7174.4495  7174.4402  7170.6910  7160.9754  7146.2961
##  [54,]  7640.7975  7636.2768  7624.9664  7608.1464  7586.7991  7561.6910
##  [55,]  8106.5905  8087.1821  8062.8948  8034.6520  8003.1615  7968.9758
##  [56,]  8553.0928  8521.1920  8486.0365  8448.2424  8408.2829  8366.5274
##  [57,]  8978.4879  8936.6873  8892.9317  8847.5904  8800.9476  8753.2262
##  [58,]  9381.7597  9332.7429  9282.7314  9231.9113  9180.4272  9128.3931
##  [59,]  9762.5359  9709.0051  9655.0987  9600.8845  9546.4163  9491.7379
##  [60,] 10120.9690 10065.5974 10010.1345  9954.5930  9898.9842  9843.3189
##  [61,] 10457.6538 10403.0550 10348.3246 10293.4809 10238.5399 10183.5158
##  [62,] 10773.5796 10722.2873 10670.5098 10618.3303 10565.8143 10513.0147
##  [63,] 11070.1179 11024.5698 10977.8812 10930.2577 10881.8581 10832.8078
##  [64,] 11349.0488 11311.5655 11271.9989 11230.7316 11188.0579 11144.2080
##  [65,] 11612.6281 11585.3826 11554.8404 11521.6135 11486.1713 11448.8799
##  [66,] 11863.7063 11848.6706 11828.8807 11805.2255 11778.3851 11748.8894
##  [67,] 12106.3271 12104.7644 12097.2110 12084.4488 12067.3976 12046.7735
##  [68,] 12364.0972 12364.0972 12364.0972 12362.8575 12356.5293 12345.6331
##  [69,] 12650.2372 12650.2372 12650.2372 12650.2372 12650.2372 12649.2496
##  [70,] 12966.8433 12966.8433 12966.8433 12966.8433 12966.8433 12966.8433
##  [71,] 13315.8959 13315.8959 13315.8959 13315.8959 13315.8959 13315.8959
##  [72,] 13699.1586 13699.1586 13699.1586 13699.1586 13698.3801 13691.1417
##  [73,] 14118.0515 14118.0515 14117.0356 14108.2999 14091.9205 14069.1799
##  [74,] 14572.1664 14561.5389 14542.0620 14515.3965 14482.8142 14445.3058
##  [75,] 15027.5571 14996.1263 14958.2210 14915.0449 14867.5199 14816.3650
##  [76,] 15465.4949 15415.7223 15361.5219 15303.6962 15242.8613 15179.4994
##  [77,] 15881.6439 15816.4703 15748.5082 15678.2335 15606.0141 15532.1399
##  [78,] 16272.3960 16195.0977 16116.1964 16035.9268 15954.4728 15871.9820
##  [79,] 16634.7161 16548.7987 16461.9880 16374.3658 16285.9994 16196.9461
##  [80,] 16965.9531 16875.0897 16783.5565 16691.3717 16598.5541 16505.1229
##  [81,] 17263.6208 17171.6370 17078.7113 16984.8883 16890.2069 16794.7022
##  [82,] 17525.1388 17436.0498 17345.2304 17252.8462 17159.0281 17063.8825
##  [83,] 17747.5164 17665.6251 17580.6534 17492.9935 17402.9481 17310.7561
##  [84,] 17926.9445 17857.0174 17782.0118 17702.6741 17619.5744 17533.1566
##  [85,] 18058.2330 18005.7854 17945.4598 17878.5250 17805.9417 17728.4516
##  [86,] 18133.9891 18105.7335 18065.7417 18016.0412 17958.1544 17893.2474
##  [87,] 18148.5115 18147.9033 18135.3820 18108.9196 18070.8507 18022.9482
##  [88,] 18148.5115 18148.5115 18148.5115 18148.0203 18136.3603 18111.0687
##  [89,] 18148.5115 18148.5115 18148.5115 18148.5115 18148.5115 18148.1112
##  [90,] 18148.5115 18148.5115 18148.5115 18148.5115 18148.5115 18148.5115
##  [91,] 18148.5115 18148.5115 18148.5115 18148.5115 18148.5115 18148.5115
##  [92,] 18148.5115 18148.5115 18148.5115 18148.5115 18148.5115 18148.5115
##  [93,] 18148.5115 18148.5115 18148.5115 18148.5115 18148.5115 18148.5115
##  [94,] 18148.5115 18148.5115 18148.5115 18148.5115 18148.5115 18148.5115
##  [95,] 18148.5115 18148.5115 18148.5115 18148.5115 18148.5115 18148.5115
##  [96,] 18148.5115 18148.5115 18148.5115 18148.5115 18148.5115 18148.5115
##  [97,] 18148.5115 18148.5115 18148.5115 18148.5115 18148.5115 18148.5115
##  [98,] 18148.5115 18148.5115 18148.5115 18148.5115 18148.5115 18148.5115
##  [99,] 18148.5115 18148.5115 18148.5115 18148.5115 18148.5115 18148.5115
## [100,] 18148.5115 18148.5115 18148.5115 18148.5115 18148.5115 18148.5115
##             [,19]      [,20]      [,21]      [,22]      [,23]      [,24]
##   [1,]   695.0079   695.0079   695.0079   695.0079   695.0079   695.0079
##   [2,]   695.0079   695.0079   695.0079   695.0079   695.0079   695.0079
##   [3,]   695.0079   695.0079   695.0079   695.0079   695.0079   695.0079
##   [4,]   695.0079   695.0079   695.0079   695.0079   695.0079   695.0079
##   [5,]   695.0079   695.0079   695.0079   695.0079   695.0079   695.0079
##   [6,]   695.0079   695.0079   695.0079   695.0079   695.0079   695.0079
##   [7,]   695.0079   695.0079   695.0079   695.0079   695.0079   695.0079
##   [8,]   695.0079   695.0079   695.0079   695.0079   695.0079   695.0079
##   [9,]   695.0079   695.0079   695.0079   695.0079   695.0079   694.9230
##  [10,]   695.0079   695.0079   695.0079   694.9005   690.6931   681.0271
##  [11,]   695.0079   694.8754   690.5488   680.7816   666.4161   648.2131
##  [12,]   690.3741   680.4942   666.0610   647.8095   626.3969   602.4543
##  [13,]   665.3173   646.9713   625.5869   601.7354   575.9106   548.5455
##  [14,]   623.6757   599.7460   574.0055   546.8835   518.7532   489.9429
##  [15,]   570.2838   543.1673   515.2111   486.7285   457.9938   429.2511
##  [16,]   509.1513   480.8426   452.4419   424.1706   396.2253   368.7833
##  [17,]   443.7320   415.8887   388.5074   361.7397   335.7230   310.5841
##  [18,]   377.1230   351.1110   325.9479   301.7352   278.5674   256.5345
##  [19,]   312.2110   289.1333   267.1486   246.3251   226.7290   208.4252
##  [20,]   251.7818   232.4929   214.4214   197.6167   182.1287   168.0077
##  [21,]   198.6029   183.7116   170.0659   157.7083   146.6820   137.0311
##  [22,]   155.4868   145.3493   136.4147   128.7271   122.3304   117.2685
##  [23,]   125.3394   120.0440   115.8662   112.8567   111.0640   110.5352
##  [24,]   111.1959   110.5413   110.9089   112.3561   114.9367   118.7011
##  [25,]   116.2461   119.7138   124.1361   129.5717   136.0749   143.6966
##  [26,]   143.8484   150.5711   158.2532   166.9414   176.6806   187.5139
##  [27,]   197.5329   206.2612   216.0765   226.9924   239.0275   252.2037
##  [28,]   280.9914   290.0595   300.5235   312.3319   325.4522   339.8652
##  [29,]   398.0539   405.3479   414.5931   425.6291   438.3379   452.5270
##  [30,]   552.6481   555.5806   561.3362   569.4343   578.9901   589.8057
##  [31,]   743.5309   743.3054   742.3101   743.1475   746.2168   751.2829
##  [32,]   957.4874   954.5489   950.5777   945.7611   940.5264   937.6238
##  [33,]  1185.3131  1179.3845  1172.6485  1165.2767  1157.4170  1149.1975
##  [34,]  1422.2503  1413.5363  1404.2825  1394.6320  1384.7077  1374.6156
##  [35,]  1664.9707  1653.9254  1642.6078  1631.1292  1619.5848  1608.0570
##  [36,]  1911.2177  1898.4453  1885.6426  1872.8897  1860.2561  1847.8020
##  [37,]  2159.6012  2145.7839  2132.1366  2118.7133  2105.5613  2092.7225
##  [38,]  2409.4389  2395.2817  2381.4460  2367.9655  2354.8708  2342.1895
##  [39,]  2660.6348  2646.8242  2633.4356  2620.4892  2608.0042  2595.9988
##  [40,]  2913.5895  2900.7606  2888.4050  2876.5362  2865.1676  2854.3128
##  [41,]  3169.1353  3157.8434  3147.0327  3136.7159  3126.9055  3117.6144
##  [42,]  3428.4919  3419.1872  3410.3368  3401.9570  3394.0638  3386.6726
##  [43,]  3693.2383  3686.2406  3679.6471  3673.4810  3667.7641  3662.5162
##  [44,]  3965.2971  3960.7694  3956.5877  3952.7831  3949.3835  3946.4142
##  [45,]  4246.9293  4244.8481  4243.0661  4241.6199  4240.5419  4239.8613
##  [46,]  4540.7343  4540.8569  4541.2675  4542.0017  4543.0916  4544.5663
##  [47,]  4849.6543  4851.4814  4853.6507  4856.1856  4859.1088  4862.4423
##  [48,]  5176.9783  5179.7125  5182.9451  5186.6695  5190.8852  5195.5958
##  [49,]  5526.3420  5528.8414  5532.1422  5536.1818  5540.9161  5544.4134
##  [50,]  5901.7175  5902.4480  5904.4820  5905.4458  5903.2806  5898.3797
##  [51,]  6303.4263  6300.7963  6293.5100  6283.0296  6270.5826  6256.4052
##  [52,]  6716.5901  6703.7250  6687.0446  6667.0851  6644.2919  6619.8950
##  [53,]  7127.4489  7105.0731  7079.6891  7051.7247  7021.5355  6989.4198
##  [54,]  7533.4296  7502.5027  7469.3074  7434.1701  7397.3623  7359.1121
##  [55,]  7932.5327  7894.1844  7854.2176  7812.8685  7770.3341  7726.7802
##  [56,]  8323.2696  8278.7460  8233.1499  8186.6411  8139.3534  8091.4001
##  [57,]  8704.6040  8655.2251  8605.2077  8554.6507  8503.6373  8452.2387
##  [58,]  9075.9013  9023.0271  8969.8336  8916.3741  8862.6944  8808.8345
##  [59,]  9436.8857  9381.8908  9326.7802  9271.5779  9216.3054  9160.9824
##  [60,]  9787.6078  9731.8610  9676.0888  9620.3012  9564.5086  9508.7210
##  [61,] 10128.4220 10073.2708 10018.0742  9962.8437  9907.5908  9852.3265
##  [62,] 10459.9752 10406.7327 10353.3186 10299.7607 10246.0837 10192.3098
##  [63,] 10783.2076 10733.1400 10682.6735 10631.8661 10580.7673 10529.4202
##  [64,] 11099.3656 11053.6794 11007.2713 10960.2429 10912.6797 10864.6550
##  [65,] 11410.0299 11369.8553 11328.5470 11286.2629 11243.1349 11199.2748
##  [66,] 11717.1577 11683.5269 11648.2708 11611.6151 11573.7476 11534.8266
##  [67,] 12023.1427 11996.9594 11968.5922 11938.3442 11906.4670 11873.1718
##  [68,] 12330.8932 12312.8906 12292.0966 12268.8985 12243.6172 12216.5218
##  [69,] 12643.9287 12634.6110 12621.8725 12606.1864 12587.5847 12562.7858
##  [70,] 12966.8433 12966.0574 12961.1264 12948.1127 12927.0374 12898.8564
##  [71,] 13315.2973 13309.2664 13297.5026 13280.1724 13255.3908 13223.7498
##  [72,] 13677.2885 13657.8247 13633.5672 13605.1883 13573.2467 13537.6984
##  [73,] 14041.0936 14008.4772 13971.9936 13932.1880 13889.5135 13844.3501
##  [74,] 14403.6559 14358.4944 14310.3338 14259.5961 14206.6329 14151.7399
##  [75,] 14762.1504 14705.3351 14646.2935 14585.3352 14522.7191 14458.6641
##  [76,] 15113.9945 15046.6573 14977.7429 14907.4637 14835.9986 14763.5007
##  [77,] 15456.8440 15380.3169 15302.7170 15224.1778 15144.8139 15064.7250
##  [78,] 15788.5755 15704.3548 15619.4063 15533.8055 15447.6191 15360.9072
##  [79,] 16107.2559 16016.9733 15926.1396 15834.7929 15742.9698 15650.7054
##  [80,] 16411.0986 16316.5023 16221.3559 16125.6825 16029.5061 15932.8519
##  [81,] 16698.4067 16601.3515 16503.5667 16405.0821 16305.9275 16206.1329
##  [82,] 16967.4981 16869.9505 16771.3061 16671.6241 16570.9589 16469.3611
##  [83,] 17216.6102 17120.6686 17023.0640 16923.9100 16823.3052 16721.3375
##  [84,] 17443.7733 17351.7093 17257.1986 17160.4367 17061.5895 16960.8002
##  [85,] 17646.6378 17560.9663 17471.8151 17379.4956 17284.2683 17186.3536
##  [86,] 17822.2289 17745.8186 17664.5950 17579.0297 17489.5123 17396.3688
##  [87,] 17966.5821 17902.8286 17832.5449 17756.4237 17675.0315 17588.8375
##  [88,] 18074.1795 18027.2767 17971.6114 17908.1853 17837.8104 17761.1525
##  [89,] 18137.0341 18112.4118 18076.0646 18029.4365 17973.7400 17910.1187
##  [90,] 18148.5115 18148.1840 18137.4677 18113.1311 18076.9644 18030.4759
##  [91,] 18148.5115 18148.5115 18148.5115 18148.2460 18137.8255 18113.7454
##  [92,] 18148.5115 18148.5115 18148.5115 18148.5115 18148.5115 18148.3015
##  [93,] 18148.5115 18148.5115 18148.5115 18148.5115 18148.5115 18148.5115
##  [94,] 18148.5115 18148.5115 18148.5115 18148.5115 18148.5115 18148.5115
##  [95,] 18148.5115 18148.5115 18148.5115 18148.5115 18148.5115 18148.5115
##  [96,] 18148.5115 18148.5115 18148.5115 18148.5115 18148.5115 18148.5115
##  [97,] 18148.5115 18148.5115 18148.5115 18148.5115 18148.5115 18148.5115
##  [98,] 18148.5115 18148.5115 18148.5115 18148.5115 18148.5115 18148.5115
##  [99,] 18148.5115 18148.5115 18148.5115 18148.5115 18148.5115 18148.5115
## [100,] 18148.5115 18148.5115 18148.5115 18148.5115 18148.5115 18148.5115
##             [,25]      [,26]      [,27]      [,28]      [,29]      [,30]
##   [1,]   695.0079   695.0079   695.0079   695.0079   695.0079   695.0079
##   [2,]   695.0079   695.0079   695.0079   695.0079   695.0079   695.0079
##   [3,]   695.0079   695.0079   695.0079   695.0079   695.0079   695.0079
##   [4,]   695.0079   695.0079   695.0079   695.0079   695.0079   695.0079
##   [5,]   695.0079   695.0079   695.0079   695.0079   695.0079   695.0079
##   [6,]   695.0079   695.0079   695.0079   695.0079   695.0079   694.9746
##   [7,]   695.0079   695.0079   695.0079   694.9600   691.1124   681.7524
##   [8,]   695.0079   694.9428   690.9748   681.5125   667.4033   649.4126
##   [9,]   690.8351   681.2708   667.0758   649.0141   627.7725   603.9670
##  [10,]   666.7467   648.6142   627.3150   603.4636   577.6100   550.2460
##  [11,]   626.8565   602.9594   577.0706   549.6807   521.2285   492.1058
##  [12,]   576.5305   549.1151   520.6456   491.5130   462.0668   432.6190
##  [13,]   520.0624   490.9202   461.4709   432.0260   402.8634   374.2311
##  [14,]   460.7459   431.4273   402.2789   373.6598   345.7964   318.8853
##  [15,]   400.7206   372.6036   345.0868   318.3450   292.5931   268.1185
##  [16,]   342.0075   316.0487   291.0490   267.1434   244.4618   223.1299
##  [17,]   286.4416   263.4077   241.5902   221.0932   202.0181   184.4649
##  [18,]   235.7229   216.2164   198.0971   181.4458   166.3430   152.8685
##  [19,]   191.4784   175.9525   161.9119   149.4208   138.5442   129.3474
##  [20,]   155.3050   144.0724   134.3627   126.2294   119.7271   114.9111
##  [21,]   128.8005   122.0359   116.7839   113.0919   111.0080   110.5812
##  [22,]   113.5851   111.3244   110.5305   111.2482   113.5224   117.3983
##  [23,]   111.3162   113.4520   116.9870   121.9651   128.4299   136.4250
##  [24,]   123.6978   129.9733   137.5726   146.5395   156.9171   168.7475
##  [25,]   152.4848   162.4856   173.7429   186.2994   200.1964   215.4743
##  [26,]   199.4831   212.6282   226.9883   242.6012   259.5041   277.7032
##  [27,]   266.5453   282.0781   298.8289   316.7787   335.6702   355.4242
##  [28,]   355.5620   372.4706   390.2209   408.7347   427.9973   448.0058
##  [29,]   467.6437   483.5865   500.3332   517.8743   536.2096   555.3466
##  [30,]   601.7950   614.8956   629.0638   644.2696   660.4945   677.7287
##  [31,]   758.1632   766.7153   776.8280   788.4148   801.4087   815.7586
##  [32,]   937.4850   939.8356   944.4556   951.1680   959.8301   970.3268
##  [33,]  1140.9691  1135.4616  1133.1423  1133.7116  1136.9249  1142.5816
##  [34,]  1364.4484  1354.2876  1344.4181  1337.5265  1334.1266  1333.9106
##  [35,]  1596.6181  1585.3320  1574.2558  1563.4411  1553.1211  1545.9300
##  [36,]  1835.5810  1823.6409  1812.0249  1800.7723  1789.9197  1779.5009
##  [37,]  2080.2345  2068.1317  2056.4457  2045.2059  2034.4399  2024.1740
##  [38,]  2329.9473  2318.1683  2306.8754  2296.0902  2285.8339  2276.1268
##  [39,]  2584.4909  2573.4977  2563.0361  2553.1228  2543.7744  2535.0072
##  [40,]  2843.9853  2834.1989  2824.9674  2816.3048  2808.2252  2800.7431
##  [41,]  3108.8555  3100.6419  3092.9868  3085.9038  3079.4063  3073.5082
##  [42,]  3379.7982  3373.4555  3367.6593  3362.4239  3357.7641  3353.6941
##  [43,]  3657.7562  3653.5024  3649.7719  3646.5815  3643.9478  3641.8866
##  [44,]  3943.8985  3941.8583  3940.3139  3939.2847  3938.7895  3938.8460
##  [45,]  4239.6048  4239.7969  4240.4604  4241.6170  4243.2868  4245.4893
##  [46,]  4546.4528  4548.7757  4551.5582  4554.8221  4558.5879  4561.6014
##  [47,]  4866.2073  4870.4240  4875.1122  4878.8474  4879.9164  4878.5611
##  [48,]  5200.8079  5204.8820  5206.0319  5204.5564  5200.7189  5194.7492
##  [49,]  5544.7857  5542.3896  5537.5306  5530.4711  5521.4375  5510.6265
##  [50,]  5891.0723  5881.6367  5870.3095  5857.2934  5842.7633  5826.8711
##  [51,]  6240.6967  6223.6269  6205.3413  6185.9655  6165.6086  6144.3661
##  [52,]  6595.0006  6569.6427  6543.8533  6517.6629  6491.1006  6464.1937
##  [53,]  6955.6298  6921.2452  6887.3274  6853.7955  6820.5831  6787.6372
##  [54,]  7319.6127  7279.0290  7237.5034  7196.0151  7155.6239  7116.1880
##  [55,]  7682.3476  7637.1571  7591.3135  7544.9079  7498.0207  7451.5597
##  [56,]  8042.8778  7993.8701  7944.4498  7894.6811  7844.6207  7794.3197
##  [57,]  8400.5162  8348.5236  8296.3083  8243.9128  8191.3751  8138.7300
##  [58,]  8754.8298  8700.7118  8646.5092  8592.2482  8537.9532  8483.6469
##  [59,]  9105.6272  9050.2572  8994.8888  8939.5378  8884.2194  8828.9486
##  [60,]  9452.9490  9397.2031  9341.4939  9285.8324  9230.2296  9174.6970
##  [61,]  9797.0623  9741.8092  9686.5787  9631.3821  9576.2310  9521.1371
##  [62,] 10138.4596 10084.5522 10030.6056  9976.6370  9922.6629  9868.6994
##  [63,] 10477.8628 10426.1289 10374.2491 10322.2513 10270.1613 10218.0032
##  [64,] 10816.2316 10767.4649 10718.4034 10669.0905 10619.5655 10569.8639
##  [65,] 11154.7781 11109.7273 11064.1942 11018.2422 10971.9276 10925.3009
##  [66,] 11494.9863 11454.3422 11412.9942 11371.0294 11328.3445 11282.4516
##  [67,] 11838.6375 11803.0170 11766.2158 11725.5042 11680.3588 11631.1345
##  [68,] 12187.5551 12153.7221 12114.6013 12070.6733 12022.3510 11969.9916
##  [69,] 12531.5357 12494.5037 12452.2545 12405.2678 12353.9541 12298.6662
##  [70,] 12864.3603 12824.2083 12778.9544 12729.0675 12674.9469 12616.9350
##  [71,] 13186.0080 13142.7966 13094.6450 13042.0003 12985.2428 12924.6978
##  [72,] 13496.6621 13450.4095 13399.4301 13344.1377 13284.8841 13221.9706
##  [73,] 13797.0201 13747.3729 13693.5761 13635.6943 13574.0405 13508.8833
##  [74,] 14095.1682 14037.1335 13977.8220 13917.0376 13853.0153 13785.6836
##  [75,] 14393.3575 14326.9608 14259.6149 14191.4440 14122.5579 14052.7484
##  [76,] 14690.1021 14615.9182 14541.0509 14465.5913 14389.6214 14313.2159
##  [77,] 14983.9986 14902.7130 14820.9389 14738.7410 14656.1787 14573.3079
##  [78,] 15273.7246 15186.1223 15098.1480 15009.8471 14921.2630 14832.4376
##  [79,] 15558.0339 15464.9893 15371.6050 15277.9146 15183.9519 15089.7507
##  [80,] 15835.7458 15738.2153 15640.2885 15541.9949 15443.3651 15344.4307
##  [81,] 16105.7289 16004.7469 15903.2189 15801.1780 15698.6583 15595.6948
##  [82,] 16366.8787 16263.5578 16159.4438 16054.5810 15949.0139 15842.7869
##  [83,] 16618.0858 16513.6229 16408.0167 16301.3311 16193.6280 16084.9671
##  [84,] 16858.1942 16753.8833 16647.9684 16540.5423 16431.6913 16321.4969
##  [85,] 17085.9413 16983.1969 16878.2667 16771.2819 16662.3621 16551.6176
##  [86,] 17299.8756 17200.2702 17097.7591 16992.5245 16884.9394 16775.7910
##  [87,] 17498.2348 17403.5570 17305.2592 17204.1006 17100.4720 16994.7117
##  [88,] 17678.8781 17591.8116 17500.5275 17405.5307 17307.2749 17206.1698
##  [89,] 17839.5214 17762.7712 17680.5898 17593.6140 17502.4100 17407.4836
##  [90,] 17974.9470 17911.4760 17841.0111 17764.3778 17682.2995 17595.4148
##  [91,] 18077.8019 18031.5085 17976.1508 17912.8304 17842.4983 17765.9821
##  [92,] 18138.1778 18114.3548 18078.6351 18032.5374 17977.3514 17914.1820
##  [93,] 18148.5115 18148.3505 18138.5246 18114.9593 18079.4642 18033.5626
##  [94,] 18148.5115 18148.5115 18148.5115 18148.3931 18138.8657 18115.5590
##  [95,] 18148.5115 18148.5115 18148.5115 18148.5115 18148.5115 18148.4292
##  [96,] 18148.5115 18148.5115 18148.5115 18148.5115 18148.5115 18148.5115
##  [97,] 18148.5115 18148.5115 18148.5115 18148.5115 18148.5115 18148.5115
##  [98,] 18148.5115 18148.5115 18148.5115 18148.5115 18148.5115 18148.5115
##  [99,] 18148.5115 18148.5115 18148.5115 18148.5115 18148.5115 18148.5115
## [100,] 18148.5115 18148.5115 18148.5115 18148.5115 18148.5115 18148.5115
##             [,31]      [,32]      [,33]      [,34]      [,35]      [,36]
##   [1,]   695.0079   695.0079   695.0079   695.0079   695.0079   695.0079
##   [2,]   695.0079   695.0079   695.0079   695.0079   695.0079   695.0079
##   [3,]   695.0079   695.0079   695.0079   695.0079   695.0079   695.0026
##   [4,]   695.0079   695.0079   695.0079   694.9959   691.5115   682.4606
##   [5,]   695.0079   694.9866   691.3807   682.2264   668.3762   650.6004
##   [6,]   691.2477   681.9904   668.0535   650.2058   629.1387   605.4723
##   [7,]   667.7292   649.8098   628.6844   604.9714   579.2245   551.9391
##   [8,]   628.2290   604.4696   578.6870   551.3751   522.9757   493.8833
##   [9,]   578.1488   550.8108   522.3936   493.2909   463.8545   434.3985
##  [10,]   521.8112   492.6984   463.2586   433.8052   404.6181   375.9471
##  [11,]   462.6627   433.2120   404.0330   375.3748   347.4598   320.4858
##  [12,]   403.4481   374.8028   346.9049   319.9518   294.1191   269.5626
##  [13,]   346.3505   319.4183   293.6099   269.0807   245.9681   224.3936
##  [14,]   293.1012   268.5993   245.5167   223.9743   204.0785   185.9221
##  [15,]   245.0658   223.5558   203.6942   185.5738   169.2747   154.8663
##  [16,]   203.3106   185.2262   168.9647   154.5951   142.1759   131.7566
##  [17,]   168.5323   154.3181   141.9455   131.5670   123.2298   116.9664
##  [18,]   141.1022   131.1246   123.0162   116.8583   112.7400   110.7368
##  [19,]   121.8966   116.2585   112.5009   110.6921   110.9017   113.1998
##  [20,]   111.8376   110.5637   111.1474   113.6477   118.1242   124.6375
##  [21,]   111.8612   114.8984   119.7440   126.4500   135.0688   145.6536
##  [22,]   122.9217   130.1387   139.0959   149.8402   162.4188   176.8794
##  [23,]   145.9937   157.1794   170.0254   184.5750   200.8717   218.9530
##  [24,]   182.0726   196.9334   213.3708   231.4146   250.9777   271.9615
##  [25,]   232.1724   250.3114   269.7642   290.4372   312.2747   335.2396
##  [26,]   297.0175   317.3603   338.6901   360.9821   384.2249   408.4180
##  [27,]   376.0145   397.4292   419.6676   442.7381   466.6559   491.4422
##  [28,]   468.7666   490.2934   512.6054   535.7264   559.6838   584.5080
##  [29,]   575.2984   596.0828   617.7212   640.2381   663.6608   688.0184
##  [30,]   695.9697   715.2210   735.4907   756.7915   779.1394   802.5535
##  [31,]   831.4262   848.3841   866.6136   886.1035   906.8490   928.8508
##  [32,]   982.5659   996.4731  1011.9899  1029.0706  1047.6802  1067.7930
##  [33,]  1150.5169  1160.5953  1172.7056  1186.7569  1202.6748  1220.3998
##  [34,]  1336.6235  1342.0532  1350.0233  1360.3866  1373.0207  1387.8239
##  [35,]  1542.4368  1542.3373  1545.3765  1551.3403  1560.0482  1571.3485
##  [36,]  1769.7089  1763.1212  1760.3666  1761.1517  1765.2285  1772.3863
##  [37,]  2014.4331  2005.2413  1996.7601  1991.5069  1990.1727  1992.4789
##  [38,]  2266.9887  2258.4392  2250.4975  2243.1825  2236.6300  2233.2955
##  [39,]  2526.8377  2519.2823  2512.3574  2506.0793  2500.4648  2495.5303
##  [40,]  2793.8727  2787.6289  2782.0264  2777.0802  2772.8055  2769.2176
##  [41,]  3068.2233  3063.5659  3059.5501  3056.1906  3053.5018  3051.4987
##  [42,]  3350.2286  3347.3820  3345.1691  3343.6044  3342.7028  3342.4792
##  [43,]  3640.4139  3639.5453  3639.2962  3639.6821  3640.7182  3641.5135
##  [44,]  3939.4716  3940.6834  3942.4978  3943.9200  3943.3975  3941.0465
##  [45,]  4248.2430  4250.4340  4250.4608  4248.4744  4244.6288  4239.0645
##  [46,]  4562.2085  4560.6018  4556.9657  4551.4660  4544.2526  4535.4610
##  [47,]  4875.0027  4869.4373  4862.0394  4852.9650  4842.3540  4830.3325
##  [48,]  5186.8490  5177.1959  5165.9471  5153.2427  5139.2077  5123.9543
##  [49,]  5498.2099  5484.3386  5469.1461  5452.7507  5435.2580  5416.7629
##  [50,]  5809.7494  5791.5146  5772.2698  5752.1065  5731.1065  5709.3433
##  [51,]  6122.3220  6099.5507  6076.1186  6052.0849  6027.5034  6002.4227
##  [52,]  6436.9680  6409.4479  6381.6565  6353.6155  6325.3458  6296.8672
##  [53,]  6754.9150  6722.3826  6690.0126  6657.7834  6625.6783  6593.6840
##  [54,]  7077.5852  7039.7135  7002.4878  6965.8365  6929.6997  6894.0266
##  [55,]  7406.6078  7362.9938  7320.5658  7279.1952  7238.7725  7199.2043
##  [56,]  7743.8239  7693.9874  7645.9288  7599.4670  7554.4363  7510.6948
##  [57,]  8086.0094  8033.2428  7980.4577  7928.4651  7878.4255  7830.1603
##  [58,]  8429.3507  8375.0850  8320.8695  8266.7231  8212.6641  8159.4668
##  [59,]  8773.7401  8718.6085  8663.5682  8608.6336  8553.8189  8499.1388
##  [60,]  9119.2459  9063.8884  9008.6363  8953.5019  8898.4979  8843.6369
##  [61,]  9466.1124  9411.1689  9356.3191  9301.5754  9246.9507  9192.4580
##  [62,]  9814.7620  9760.8662  9707.0274  9653.2605  9599.5808  9546.0033
##  [63,] 10165.7996 10113.5721 10061.3413 10009.1272  9956.8592  9902.6517
##  [64,] 10520.0185 10470.0596 10419.9016 10367.4768 10312.1450 10254.0756
##  [65,] 10878.2642 10828.5495 10775.5367 10719.4366 10660.4392 10598.7164
##  [66,] 11232.7664 11179.5584 11123.0673 11063.5067 11001.0678 10935.9225
##  [67,] 11578.1416 11521.6526 11461.9082 11399.1215 11333.4822 11265.1598
##  [68,] 11913.9055 11854.3637 11791.6042 11725.8367 11657.2470 11586.0004
##  [69,] 12239.7092 12177.3487 12111.8166 12043.3167 11972.0287 11898.1120
##  [70,] 12555.3268 12490.3783 12422.3123 12351.3239 12277.5850 12201.2474
##  [71,] 12860.6450 12793.3267 12722.9535 12649.7094 12573.7562 12495.2364
##  [72,] 13155.6560 13086.1645 13013.6910 12938.4054 12860.4572 12779.9779
##  [73,] 13440.4544 13368.9547 13294.5590 13217.4202 13137.6727 13055.4349
##  [74,] 13715.2390 13641.8527 13565.6746 13486.8365 13405.4542 13321.6307
##  [75,] 13980.3212 13905.1128 13827.2416 13746.8130 13663.9207 13578.6481
##  [76,] 14236.4431 14159.1009 14079.5602 13997.5924 13913.2633 13826.6329
##  [77,] 14490.1808 14406.8470 14323.3542 14239.5153 14153.7570 14065.8018
##  [78,] 14743.4119 14654.2260 14564.9192 14475.5307 14386.0993 14296.4581
##  [79,] 14995.3454 14900.7708 14806.0622 14711.2554 14616.3870 14521.4939
##  [80,] 15245.2244 15145.7803 15046.1333 14946.3197 14846.3765 14746.3423
##  [81,] 15492.3239 15388.5830 15284.5109 15180.1474 15075.5339 14970.7127
##  [82,] 15735.9448 15628.5333 15520.5985 15412.1877 15303.3492 15194.1325
##  [83,] 15975.4070 15865.0058 15753.8213 15641.9116 15529.5908 15417.8014
##  [84,] 16210.0370 16097.3868 15983.8718 15870.3911 15757.1421 15644.2564
##  [85,] 16439.3889 16326.5194 16213.2431 16099.7371 15986.1658 15872.6818
##  [86,] 16665.3714 16553.9214 16441.6636 16328.8042 16215.5343 16102.0313
##  [87,] 16887.1295 16778.0095 16667.6131 16556.1816 16443.9380 16331.0889
##  [88,] 17102.5876 16996.8673 16889.3190 16780.2274 16669.8543 16558.4415
##  [89,] 17309.2894 17208.2381 17104.7025 16999.0222 16891.5079 16782.4448
##  [90,] 17504.2910 17409.4351 17311.3027 17210.3053 17106.8164 17001.1763
##  [91,] 17684.0072 17597.2138 17506.1704 17411.3852 17313.3148 17212.3715
##  [92,] 17843.9829 17767.5842 17685.7130 17599.0110 17508.0483 17413.3341
##  [93,] 17978.5487 17915.5307 17845.4651 17769.1840 17687.4168 17600.8066
##  [94,] 18080.2889 18034.5841 17979.7428 17916.8765 17846.9447 17770.7817
##  [95,] 18139.2012 18116.1539 18081.1095 18035.6019 17980.9336 17918.2195
##  [96,] 18148.5115 18148.4588 18139.5311 18116.7438 18081.9257 18036.6160
##  [97,] 18148.5115 18148.5115 18148.5115 18148.4818 18139.8553 18117.3289
##  [98,] 18148.5115 18148.5115 18148.5115 18148.5115 18148.5115 18148.4983
##  [99,] 18148.5115 18148.5115 18148.5115 18148.5115 18148.5115 18148.5115
## [100,] 18148.5115 18148.5115 18148.5115 18148.5115 18148.5115 18148.5115
##             [,37]      [,38]      [,39]      [,40]      [,41]      [,42]
##   [1,]   695.0079   695.0079   695.0079   695.0079   691.8901   683.1514
##   [2,]   695.0079   695.0066   691.7662   682.9231   669.3347   651.7764
##   [3,]   691.6400   682.6928   669.0168   651.3857   630.4954   606.9701
##   [4,]   668.6973   650.9937   630.0443   606.4717   580.8335   553.6281
##   [5,]   629.5920   605.9724   580.2978   553.0656   524.7204   495.6595
##   [6,]   579.7615   552.5025   524.1391   495.0676   465.6420   436.1789
##   [7,]   523.5576   494.4755   465.0462   435.5853   406.3747   377.6659
##   [8,]   464.4503   434.9918   405.7890   377.0927   349.1267   322.0905
##   [9,]   405.2034   376.5198   348.5707   321.5551   295.6499   271.0120
##  [10,]   348.0151   321.0202   295.1391   270.5283   247.3262   225.6553
##  [11,]   294.6289   270.0452   246.8729   225.2340   205.2357   186.9714
##  [12,]   246.4202   224.8135   204.8492   186.6209   170.2094   155.6846
##  [13,]   204.4635   186.2711   169.8971   155.4110   142.8721   132.3304
##  [14,]   169.5855   155.1382   142.6393   132.1383   123.6763   117.2865
##  [15,]   142.4072   131.9470   123.5266   117.1789   112.9296   110.7976
##  [16,]   123.3778   117.0722   112.8655   110.7765   110.8178   112.9965
##  [17,]   112.8023   110.7562   110.8406   113.0625   117.4237   123.9212
##  [18,]   110.8643   113.1295   117.5339   124.0744   132.7436   143.5300
##  [19,]   117.6449   124.2285   132.9406   143.7696   156.7003   171.7142
##  [20,]   133.2489   144.0206   156.9831   172.0388   189.1555   208.3066
##  [21,]   158.2582   172.9371   189.7449   208.7347   229.8312   252.6866
##  [22,]   193.2702   211.6359   231.9389   254.0755   277.9650   303.5489
##  [23,]   238.7608   260.1933   283.1750   307.6520   333.5883   360.9631
##  [24,]   294.2986   317.9423   342.8627   369.0438   396.4816   425.1820
##  [25,]   359.3104   384.4781   410.7442   438.1189   466.6200   496.2714
##  [26,]   433.5700   459.6964   486.8192   514.9653   544.1661   574.4569
##  [27,]   517.1227   543.7275   571.2895   599.8450   629.4327   660.0935
##  [28,]   610.2320   636.8910   664.5221   693.1641   722.8578   753.6450
##  [29,]   713.3425   739.6660   767.0236   795.4517   824.9879   855.6713
##  [30,]   827.0558   852.6706   879.4248   907.3468   936.4675   966.8194
##  [31,]   952.1143   976.6493  1002.4690  1029.5903  1058.0329  1087.8192
##  [32,]  1089.3913  1112.4642  1137.0068  1163.0197  1190.5078  1219.4806
##  [33,]  1239.8842  1261.0908  1283.9918  1308.5669  1334.8028  1362.6924
##  [34,]  1404.7120  1423.6158  1444.4792  1467.2570  1491.9138  1518.4227
##  [35,]  1585.1134  1601.2357  1619.6258  1640.2088  1662.9234  1687.7192
##  [36,]  1782.4464  1795.2571  1810.6895  1828.6347  1849.0006  1871.7100
##  [37,]  1998.1878  2007.0962  2019.0301  2033.8410  2051.4018  2071.6041
##  [38,]  2233.9324  2238.2785  2246.1083  2257.2277  2271.4698  2288.6908
##  [39,]  2491.3904  2490.4364  2493.4838  2500.2869  2510.6332  2524.3387
##  [40,]  2766.3322  2764.1648  2762.8118  2764.5988  2770.4022  2779.3304
##  [41,]  3050.1963  3049.6097  3049.7542  3049.9116  3048.7287  3048.4898
##  [42,]  3342.9484  3343.3108  3342.1042  3339.3923  3335.2693  3329.8247
##  [43,]  3640.5612  3637.9491  3633.7871  3628.1781  3621.2186  3612.9995
##  [44,]  3936.9965  3931.3674  3924.2700  3915.8074  3906.0755  3895.1635
##  [45,]  4231.9101  4223.2836  4213.2932  4202.0391  4189.6135  4176.1023
##  [46,]  4525.2145  4513.6254  4500.7964  4486.8217  4471.7881  4455.7759
##  [47,]  4817.0143  4802.5031  4786.8929  4770.2702  4752.7141  4734.2978
##  [48,]  5107.5839  5090.1882  5071.8505  5052.6473  5032.6484  5011.9185
##  [49,]  5397.3506  5377.0984  5356.0769  5334.3503  5311.9777  5289.0139
##  [50,]  5686.8835  5663.7872  5640.1097  5615.9016  5591.2095  5566.0770
##  [51,]  5976.8871  5950.9374  5924.6112  5897.9436  5870.9672  5843.7127
##  [52,]  6268.1989  6239.3593  6210.3661  6181.2367  6151.9881  6122.6369
##  [53,]  6561.7909  6529.9921  6498.2829  6466.6608  6435.1252  6403.6770
##  [54,]  6858.7749  6823.9089  6789.3986  6755.2195  6721.3509  6687.7762
##  [55,]  7160.4112  7122.3248  7084.8868  7048.0473  7011.7638  6975.9998
##  [56,]  7468.1205  7426.6083  7386.0676  7346.4202  7307.5985  7269.5441
##  [57,]  7783.4992  7738.2954  7694.4216  7651.7676  7610.2374  7569.7478
##  [58,]  8108.3376  8059.1069  8011.6058  7965.6877  7921.2245  7878.1049
##  [59,]  8444.6075  8390.9673  8339.4717  8289.9648  8242.2825  8196.2811
##  [60,]  8788.9321  8734.3969  8680.0448  8626.5886  8575.2890  8524.5659
##  [61,]  9138.1107  9083.9223  9029.8527  8974.3359  8916.7236  8857.7880
##  [62,]  9492.4731  9437.2711  9379.7461  9320.0163  9258.1924  9194.3782
##  [63,]  9845.8555  9786.6102  9725.0454  9661.2810  9595.4288  9527.5929
##  [64,] 10193.4236 10130.3308 10064.9278  9997.3345  9927.6622  9856.0138
##  [65,] 10534.4243 10467.7047 10398.6874 10327.4911 10254.2252 10178.9905
##  [66,] 10868.2257 10798.1176 10725.7257 10651.1663 10574.5457 10495.9620
##  [67,] 11194.3064 11121.0591 11045.5419 10967.8675 10888.1387 10806.4497
##  [68,] 11512.2448 11436.1126 11357.7235 11277.1857 11194.5977 11110.0496
##  [69,] 11821.7087 11742.9457 11661.9373 11578.7867 11493.5873 11406.4244
##  [70,] 12122.4460 12041.3013 11957.9212 11872.4030 11784.8346 11695.2964
##  [71,] 12414.2766 12330.9894 12245.4758 12157.8263 12068.1230 11976.4403
##  [72,] 12697.0843 12611.8799 12524.4577 12434.9008 12343.2844 12249.6769
##  [73,] 12970.8119 12883.8970 12794.7736 12703.5168 12610.1941 12514.8669
##  [74,] 13235.4569 13147.0143 13056.3756 12963.6061 12868.7649 12771.9056
##  [75,] 13491.0698 13401.2529 13309.2574 13215.1377 13118.9429 13020.7179
##  [76,] 13737.7555 13646.6809 13553.4541 13458.1162 13360.7049 13261.2549
##  [77,] 13975.6791 13883.4173 13789.0430 13692.5816 13594.0569 13493.4914
##  [78,] 14205.0775 14111.6401 14016.1499 13918.6126 13819.0352 13717.4253
##  [79,] 14426.6140 14331.6021 14234.9599 14136.3344 14035.7112 13933.0798
##  [80,] 14646.2564 14546.1593 14446.0925 14345.9336 14244.4259 14142.1154
##  [81,] 14865.7277 14760.6238 14655.6867 14551.8956 14449.3950 14348.0824
##  [82,] 15084.8388 14976.4377 14869.0853 14762.8506 14657.7964 14553.9798
##  [83,] 15306.7165 15196.4327 15087.0391 14978.6175 14871.2432 14764.9853
##  [84,] 15531.8558 15420.0531 15308.9530 15198.6522 15089.2397 14980.7977
##  [85,] 15759.4268 15646.5325 15534.1209 15422.3051 15311.1899 15200.8720
##  [86,] 15988.4598 15874.9726 15761.7117 15648.8089 15536.3863 15424.5575
##  [87,] 16217.8255 16104.3255 15990.7538 15877.2635 15763.9967 15651.0854
##  [88,] 16446.2123 16333.3734 16220.1166 16106.6197 15993.0479 15879.5544
##  [89,] 16672.0952 16560.7011 16448.4862 16335.6577 16222.4075 16108.9138
##  [90,] 16893.6962 16784.6617 16674.3356 16562.9603 16450.7599 16337.9419
##  [91,] 17108.9295 17003.3296 16895.8838 16786.8780 16676.5756 16565.2192
##  [92,] 17315.3258 17214.4367 17111.0417 17005.4823 16898.0708 16789.0938
##  [93,] 17509.9247 17415.2815 17317.3356 17216.5009 17113.1530 17007.6341
##  [94,] 17689.1186 17602.6004 17511.7995 17417.2277 17319.3442 17218.5640
##  [95,] 17848.4217 17772.3771 17690.8185 17604.3924 17513.6729 17419.1725
##  [96,] 17982.1211 17919.5595 17849.8962 17773.9702 17692.5163 17606.1827
##  [97,] 18082.7377 18037.6263 17983.3054 17920.8967 17851.3682 17775.5611
##  [98,] 18140.1739 18117.9090 18083.5454 18038.6328 17984.4863 17922.2310
##  [99,] 18148.5115 18148.5082 18140.4868 18118.4842 18084.3488 18039.6356
## [100,] 18148.5115 18148.5115 18148.5115 18148.5115 18140.7940 18119.0545
##             [,43]      [,44]      [,45]      [,46]      [,47]      [,48]
##   [1,]   669.6510   652.1657   631.3945   607.9644   582.4367   555.3131
##   [2,]   630.9455   607.4677   581.9029   554.7519   526.4624   497.4344
##   [3,]   581.3685   554.1903   525.8820   496.8429   467.4295   437.9603
##   [4,]   525.3014   496.2513   466.8337   437.3664   408.1332   379.3873
##   [5,]   466.2379   436.7726   407.5468   378.8132   350.7971   323.6992
##   [6,]   406.9606   378.2394   350.2399   323.1625   297.1853   272.4664
##   [7,]   349.6831   322.6263   296.6730   271.9810   248.6900   226.9231
##   [8,]   296.1612   271.4962   248.2348   226.4998   206.3992   188.0274
##   [9,]   247.7802   226.0772   206.0107   187.6747   171.1510   156.5100
##  [10,]   205.6228   187.3227   170.8363   156.2341   143.5757   132.9116
##  [11,]   170.5225   155.9589   143.3404   132.7170   124.1305   117.6142
##  [12,]   143.1058   132.5233   123.9782   117.5041   113.1270   110.8663
##  [13,]   123.8269   117.3949   113.0603   110.8425   110.7544   112.8035
##  [14,]   112.9945   110.8196   110.7747   112.8670   117.0985   123.4667
##  [15,]   110.7958   112.9313   117.2061   123.6173   132.1578   142.8163
##  [16,]   117.3145   123.7688   132.3522   143.0533   155.8572   170.7453
##  [17,]   132.5475   143.2912   156.1374   171.0674   188.0599   207.0891
##  [18,]   156.4184   171.3904   188.4243   207.4941   228.5001   251.2551
##  [19,]   188.7895   207.9000   228.9431   251.7316   276.1002   301.9087
##  [20,]   229.3868   252.2088   276.6077   302.4440   329.6000   357.9768
##  [21,]   277.1159   302.9799   330.1615   358.5620   388.0992   418.7047
##  [22,]   330.7235   359.1477   388.7071   419.3336   450.9711   483.5740
##  [23,]   389.7686   420.0080   451.6203   484.2424   517.7926   552.2424
##  [24,]   455.1595   486.4361   519.0402   553.0060   588.2924   624.4988
##  [25,]   527.1027   559.1482   592.4468   627.0408   662.9766   700.3036
##  [26,]   605.8765   638.4667   672.2720   707.3398   743.7198   781.4640
##  [27,]   691.8706   724.8091   758.9561   794.3603   831.0725   869.1450
##  [28,]   785.5694   818.6760   853.0112   888.6228   925.5601   963.8739
##  [29,]   887.5423   920.6429   955.0162   990.7068  1027.7606  1066.2249
##  [30,]   998.4367  1031.3552  1065.6128  1101.2484  1138.3030  1176.8188
##  [31,]  1118.9745  1151.5262  1185.5041  1220.9399  1257.8677  1296.3234
##  [32,]  1249.9510  1281.9358  1315.4545  1350.5298  1387.1870  1425.4542
##  [33,]  1392.2340  1423.4307  1456.2899  1490.8233  1527.0457  1564.9757
##  [34,]  1546.7641  1576.9251  1608.8989  1642.6837  1678.2825  1715.7026
##  [35,]  1714.5558  1743.4019  1774.2334  1807.0334  1841.7910  1878.5008
##  [36,]  1896.6984  1923.9127  1953.3096  1984.8545  2018.5206  2054.2881
##  [37,]  2094.3558  2119.5791  2147.2085  2177.1894  2209.4769  2243.5378
##  [38,]  2308.7669  2331.5922  2357.0758  2384.5961  2412.7568  2441.4635
##  [39,]  2541.2439  2560.6116  2580.9626  2602.1749  2624.1938  2646.9748
##  [40,]  2789.7894  2801.6078  2814.6816  2828.9226  2844.2566  2860.6209
##  [41,]  3050.1933  3053.6823  3058.8220  3065.4960  3073.6046  3083.0617
##  [42,]  3323.1945  3317.6277  3314.1723  3312.6777  3313.0129  3315.0637
##  [43,]  3603.6062  3593.1190  3581.6530  3571.3727  3563.3704  3557.4991
##  [44,]  3883.1550  3870.1281  3856.1563  3841.3087  3825.6796  3811.3465
##  [45,]  4161.5851  4146.1362  4129.8253  4112.7178  4094.8752  4076.3559
##  [46,]  4438.8593  4421.1077  4402.5857  4383.3538  4363.4693  4342.9860
##  [47,]  4715.0889  4695.1503  4674.5409  4653.3157  4631.5265  4609.2224
##  [48,]  4990.5173  4968.5003  4945.9197  4922.8240  4899.2594  4875.2693
##  [49,]  5265.5096  5241.5119  5217.0653  5192.2115  5166.9900  5141.4384
##  [50,]  5540.5444  5514.6498  5488.4290  5461.9161  5435.1435  5408.1423
##  [51,]  5816.2094  5788.4848  5760.5655  5732.4771  5704.2442  5675.8908
##  [52,]  6093.1996  6063.6924  6034.1316  6004.5334  5974.9140  5945.2899
##  [53,]  6372.3186  6341.0536  6309.8870  6278.8247  6247.8736  6217.0419
##  [54,]  6654.4823  6621.4587  6588.6978  6556.1942  6523.9449  6491.9485
##  [55,]  6940.7247  6905.9125  6871.5416  6837.5941  6804.0557  6770.9151
##  [56,]  7232.2067  7195.5427  7159.5152  7124.0923  7089.2471  7054.9572
##  [57,]  7530.2262  7491.6099  7453.8441  7416.8815  7380.6655  7344.0456
##  [58,]  7836.2315  7795.5191  7755.8713  7716.0119  7675.2226  7633.4926
##  [59,]  8151.8045  8107.4237  8062.3803  8016.6411  7970.1780  7922.9675
##  [60,]  8473.5996  8422.3182  8370.6599  8318.5711  8266.0051  8212.9213
##  [61,]  8798.9555  8740.1735  8681.3520  8622.4118  8563.2833  8503.9046
##  [62,]  9128.6715  9061.8036  8995.2274  8928.8935  8862.7042  8796.5723
##  [63,]  9457.8704  9386.3524  9313.1247  9238.8760  9165.0800  9091.6915
##  [64,]  9782.4851  9707.1654  9630.1383  9551.4824  9471.2717  9390.1530
##  [65,] 10101.8803 10022.9815  9942.3751  9860.1371  9776.3389  9691.0480
##  [66,] 10415.5053 10333.2595 10249.3025 10163.7073 10076.5421  9987.8718
##  [67,] 10722.8874 10637.5318 10550.4574 10461.7339 10371.4263 10279.5961
##  [68,] 11023.6241 10935.3973 10845.4399 10753.8179 10660.5929 10565.8231
##  [69,] 11317.3763 11226.5150 11133.9069 11039.6142 10943.6948 10846.2034
##  [70,] 11603.8614 11510.5972 11415.5658 11318.8254 11220.4298 11120.4302
##  [71,] 11882.8461 11787.4029 11690.1683 11591.1958 11490.5355 11388.2344
##  [72,] 12154.1403 12056.7317 11957.5039 11856.5057 11753.7827 11649.3782
##  [73,] 12417.5911 12318.4178 12217.3946 12114.5651 12009.9706 11903.6498
##  [74,] 12673.0771 12572.3243 12469.6885 12365.2084 12258.9200 12150.8574
##  [75,] 12920.5035 12818.3376 12714.2550 12608.2882 12500.4680 12390.8231
##  [76,] 13159.7978 13056.3632 12950.9786 12843.6699 12734.4614 12623.3767
##  [77,] 13390.9060 13286.3207 13179.7546 13071.2256 12960.9333 12849.8473
##  [78,] 13613.7911 13508.1411 13400.6791 13292.3597 13183.3228 13073.5864
##  [79,] 13828.6414 13723.3294 13617.2577 13510.4316 13402.8599 13294.5551
##  [80,] 14039.0786 13935.2975 13830.7610 13725.4644 13619.4081 13512.5971
##  [81,] 14246.4854 14144.1894 14041.1675 13937.4015 13832.8804 13727.5991
##  [82,] 14451.4526 14350.1279 14248.5447 14146.2631 14043.2561 13939.5053
##  [83,] 14659.9066 14556.0645 14453.5108 14352.1732 14250.6037 14148.3365
##  [84,] 14873.4016 14767.1204 14662.0174 14558.1497 14455.5695 14354.2181
##  [85,] 15091.4407 14982.9783 14875.5604 14769.2561 14664.1286 14560.2355
##  [86,] 15313.4271 15203.0921 15093.6421 14985.1594 14877.7196 14771.3922
##  [87,] 15538.6520 15426.8101 15315.6646 15205.3126 15095.8439 14987.3409
##  [88,] 15766.2818 15653.3621 15540.9179 15429.0629 15317.9024 15207.5335
##  [89,] 15995.3420 15881.8455 15768.5671 15655.6390 15543.1840 15431.3161
##  [90,] 16224.6984 16111.2080 15997.6361 15884.1367 15770.8525 15657.9160
##  [91,] 16453.0334 16340.2259 16226.9892 16113.5020 15999.9303 15886.4279
##  [92,] 16678.8151 16567.4778 16455.3066 16342.5097 16229.2799 16115.7961
##  [93,] 16900.2571 16791.3091 16681.0542 16569.7360 16457.5795 16344.7933
##  [94,] 17115.2634 17009.7852 16902.4428 16793.5239 16683.2929 16571.9939
##  [95,] 17321.3517 17220.6261 17117.3729 17011.9355 16904.6279 16795.7381
##  [96,] 17515.5446 17421.1159 17323.3579 17222.6872 17119.4816 17014.0851
##  [97,] 17694.2122 17607.9712 17517.4149 17423.0580 17325.3630 17224.7473
##  [98,] 17852.8376 17777.1497 17695.9061 17609.7580 17519.2835 17424.9987
##  [99,] 17985.6639 17923.5624 17854.3044 17778.7361 17697.5980 17611.5430
## [100,] 18085.1479 18040.6347 17986.8382 17924.8908 17855.7687 17780.3202
##             [,49]      [,50]      [,51]      [,52]      [,53]      [,54]
##   [1,]   527.0425   498.0257   468.6210   439.1483   409.8934   381.1113
##   [2,]   468.0253   438.5543   409.3065   380.5363   352.4708   325.3120
##   [3,]   408.7197   379.9616   351.9125   324.7739   298.7254   273.9261
##   [4,]   351.3546   324.2364   298.2115   273.4390   250.0594   228.1968
##   [5,]   297.6982   272.9524   249.6023   227.7716   207.5691   189.0901
##   [6,]   249.1458   227.3470   207.1784   188.7351   172.0995   157.3426
##   [7,]   206.7885   188.3809   171.7825   157.0643   144.2866   133.5003
##   [8,]   171.4664   156.7867   144.0488   133.3033   124.5922   117.9497
##   [9,]   143.8118   133.1070   124.4375   117.8370   113.3322   110.9428
##  [10,]   124.2836   117.7252   113.2630   110.9164   110.6990   112.6184
##  [11,]   113.1946   110.8909   110.7166   112.6793   116.7812   123.0200
##  [12,]   110.7351   112.7410   116.8861   123.1680   131.5798   142.1103
##  [13,]   116.9919   123.3169   131.7716   142.3447   155.0216   169.7841
##  [14,]   131.9643   142.5801   155.2993   170.1036   186.9718   205.8788
##  [15,]   155.5778   170.4241   187.3336   206.2814   227.1754   249.8294
##  [16,]   187.6963   206.6849   227.6162   250.3040   274.5810   300.3058
##  [17,]   228.0578   250.7792   275.0868   300.8396   327.9185   356.2238
##  [18,]   275.5932   301.3738   328.4785   356.8077   386.2780   416.8205
##  [19,]   329.0390   357.3920   386.8846   417.4482   449.0258   481.5713
##  [20,]   387.4917   418.0762   449.6738   482.2385   515.7334   550.1293
##  [21,]   450.3223   482.9061   516.4194   550.8333   586.1256   622.2801
##  [22,]   517.1058   551.5377   586.8475   623.0193   660.0419   697.9087
##  [23,]   587.5698   623.7589   660.7986   698.6824   737.4074   776.9741
##  [24,]   661.5556   699.4564   738.1983   777.7821   818.2109   859.4905
##  [25,]   738.9897   778.5904   819.0363   860.3332   902.4889   945.5131
##  [26,]   820.6267   861.2646   903.3495   946.3913   990.3129  1035.1268
##  [27,]   908.6318   949.5890   992.0739  1036.1462  1081.7794  1128.4378
##  [28,]  1003.6162  1044.8408  1087.6029  1131.9592  1177.9681  1225.6897
##  [29,]  1106.1484  1147.5815  1190.5756  1235.1841  1281.4614  1329.4640
##  [30,]  1216.8398  1258.4114  1301.5807  1346.3963  1392.9083  1441.1686
##  [31,]  1336.3445  1377.9708  1421.2436  1466.2061  1512.9032  1561.3815
##  [32,]  1465.3614  1506.9413  1550.2286  1595.2601  1642.0747  1690.7129
##  [33,]  1604.6347  1646.0470  1689.2397  1734.2422  1781.0865  1829.8067
##  [34,]  1754.9554  1796.0558  1839.0218  1883.8749  1930.6390  1978.9545
##  [35,]  1917.1625  1957.7802  2000.3620  2044.5013  2088.9400  2133.6016
##  [36,]  2092.1433  2131.6230  2171.4365  2211.5081  2251.8450  2292.4581
##  [37,]  2278.0368  2312.8935  2348.1030  2383.6657  2419.5862  2455.8728
##  [38,]  2470.6926  2500.4270  2530.6558  2561.3730  2592.5773  2624.2710
##  [39,]  2670.4824  2694.6884  2719.5714  2745.1156  2771.3104  2798.1492
##  [40,]  2877.9632  2896.2400  2915.4154  2935.4604  2956.3519  2978.0720
##  [41,]  3093.7936  3105.7373  3118.8389  3133.0530  3148.3411  3164.6714
##  [42,]  3318.7305  3323.9269  3330.5776  3338.6175  3347.9903  3358.6477
##  [43,]  3553.6292  3551.6470  3551.4526  3552.9583  3556.0870  3560.7715
##  [44,]  3799.4448  3789.8298  3782.3735  3776.9627  3773.4971  3771.8881
##  [45,]  4057.2354  4039.5045  4024.3418  4011.6055  4001.1694  3992.9211
##  [46,]  4321.9550  4300.4251  4278.4564  4257.9541  4240.1411  4224.8789
##  [47,]  4586.4499  4563.2533  4539.6752  4515.7563  4491.5441  4468.8605
##  [48,]  4850.8954  4826.1770  4801.1524  4775.8583  4750.3300  4724.6024
##  [49,]  5115.5927  5089.4873  5063.1556  5036.6299  5009.9416  4983.1215
##  [50,]  5380.9424  5353.5728  5326.0619  5298.4371  5270.7257  5242.9543
##  [51,]  5647.4405  5618.9163  5590.3411  5561.7375  5533.1280  5504.5350
##  [52,]  5915.6774  5886.0934  5856.5547  5827.0787  5797.6830  5768.3855
##  [53,]  6186.3383  6155.7728  6125.3558  6095.0991  6065.0148  6035.1161
##  [54,]  6460.2058  6428.7188  6397.4916  6366.5292  6335.8345  6304.5326
##  [55,]  6738.1637  6705.7955  6673.8000  6641.2228  6607.3980  6572.3398
##  [56,]  7021.1932  6986.9099  6951.4312  6914.7674  6876.9294  6837.9287
##  [57,]  7306.3290  7267.5185  7227.6182  7186.6335  7144.5702  7101.4350
##  [58,]  7590.8145  7547.1825  7502.5930  7457.0436  7410.5332  7363.0615
##  [59,]  7874.9895  7826.2272  7776.6662  7726.2942  7675.1006  7623.0764
##  [60,]  8159.2841  8105.0620  8050.2273  7994.7552  7938.6236  7881.8128
##  [61,]  8444.2209  8384.1829  8323.7466  8262.8723  8201.5243  8139.6699
##  [62,]  8730.4197  8664.1764  8597.7791  8531.1705  8464.2987  8397.1162
##  [63,]  9018.6054  8945.7271  8872.9710  8800.2595  8727.5220  8654.6938
##  [64,]  9309.6177  9229.6266  9150.0684  9070.8422  8991.8560  8913.0258
##  [65,]  9604.3283  9516.7862  9429.9289  9343.7255  9258.0597  9172.8255
##  [66,]  9897.7573  9806.2570  9713.4266  9619.8341  9527.0017  9434.9084
##  [67,] 10186.3015 10091.5979  9995.5382  9898.1732  9799.5522  9700.2077
##  [68,] 10469.5635 10371.8663 10272.7817 10172.3578 10070.6413  9967.6774
##  [69,] 10747.1915 10646.7085 10544.8013 10441.5154 10336.8947 10230.9820
##  [70,] 11018.8746 10915.8091 10811.2775 10705.3224 10597.9850 10489.3054
##  [71,] 11284.3371 11178.8861 11071.9222 10963.4848 10853.6123 10742.3422
##  [72,] 11543.3327 11435.6852 11326.4732 11215.7329 11103.4996 10989.8081
##  [73,] 11795.6393 11685.9744 11574.6888 11461.8156 11347.3869 11231.4346
##  [74,] 12041.0530 11929.5378 11816.3420 11701.4949 11585.1712 11468.3691
##  [75,] 12279.3815 12166.1697 12051.3713 11935.9775 11820.1840 11704.0231
##  [76,] 12510.6076 12397.1360 12283.1406 12168.6511 12053.6981 11938.3128
##  [77,] 12738.1284 12625.8019 12512.8943 12399.4336 12285.4485 12170.9687
##  [78,] 12963.1707 12852.0978 12740.3914 12628.0769 12515.1809 12401.7311
##  [79,] 13185.5326 13075.8101 12965.4079 12854.3481 12742.6542 12630.3517
##  [80,] 13405.0404 13296.7503 13187.7420 13078.0335 12967.6449 12856.5980
##  [81,] 13621.5581 13514.7623 13407.2205 13298.9451 13189.9512 13080.2567
##  [82,] 13834.9995 13729.7336 13623.7079 13516.9272 13409.4004 13301.1397
##  [83,] 14045.3443 13941.6087 13837.1182 13731.8677 13625.8573 13519.0918
##  [84,] 14252.6624 14150.4096 14047.4323 13943.7118 13839.2366 13734.0015
##  [85,] 14457.6288 14356.2628 14254.7208 14152.4824 14049.5199 13945.8146
##  [86,] 14666.2404 14562.3218 14459.6886 14358.3072 14256.7790 14154.5549
##  [87,] 14879.8794 14773.5288 14668.3526 14564.4086 14461.7490 14360.3514
##  [88,] 15098.0461 14989.5228 14882.0395 14775.6659 14670.4654 14566.4959
##  [89,] 15320.1405 15209.7548 15100.2487 14991.7051 14884.2001 14777.8034
##  [90,] 15545.4504 15433.5695 15322.3790 15211.9764 15102.4517 14993.8879
##  [91,] 15773.1380 15660.1933 15547.7170 15435.8233 15324.6178 15214.1983
##  [92,] 16002.2245 15888.7193 15775.4237 15662.4708 15549.9839 15438.0773
##  [93,] 16231.5704 16118.0901 16004.5187 15891.0107 15777.7096 15664.7485
##  [94,] 16459.8522 16347.0768 16233.8609 16120.3841 16006.8129 15893.3022
##  [95,] 16685.5312 16574.2514 16462.1247 16349.3600 16236.1512 16122.6780
##  [96,] 16906.8123 16797.9518 16687.7690 16576.5086 16464.3969 16351.6431
##  [97,] 17121.5893 17016.2339 16908.9961 16800.1649 16690.0063 16578.7654
##  [98,] 17327.3669 17226.8063 17123.6962 17018.3819 16911.1792 16802.3776
##  [99,] 17521.1507 17426.9380 17329.3695 17228.8643 17125.8021 17020.5292
## [100,] 17699.2879 17613.3263 17523.0162 17428.8760 17331.3710 17230.9212
##             [,55]      [,56]      [,57]      [,58]      [,59]      [,60]
##   [1,]   353.0294   325.8505   299.7546   274.9020   251.4343   229.4766
##   [2,]   299.2398   274.4138   250.9754   229.0493   208.7453   190.1594
##   [3,]   250.5170   228.6227   208.3526   189.8022   173.0549   158.1823
##   [4,]   207.9605   189.4458   172.7356   157.9016   145.0047   134.0965
##   [5,]   172.4172   157.6217   144.7645   133.8970   125.0616   118.2929
##   [6,]   144.5251   133.6983   124.9043   118.1776   113.5452   111.0271
##   [7,]   124.7479   118.0632   113.4733   110.9981   110.6514   112.4412
##   [8,]   113.4023   110.9700   110.6664   112.4994   116.4717   122.5812
##   [9,]   110.6823   112.5585   116.5740   122.7266   131.0095   141.4120
##  [10,]   116.6772   122.8729   131.1987   141.6439   154.1938   168.8304
##  [11,]   131.3888   141.8767   154.4689   169.1474   185.8911   204.6756
##  [12,]   154.7448   169.4653   186.2505   205.0759   225.8572   248.4096
##  [13,]   186.6107   205.4769   226.2958   248.8822   273.0672   298.7078
##  [14,]   226.7353   249.3555   273.5712   299.2399   326.2415   354.4750
##  [15,]   274.0758   299.7726   326.8000   355.0575   384.4608   414.9400
##  [16,]   327.3590   355.6404   385.0661   415.5664   447.0840   479.5720
##  [17,]   385.6718   416.1933   447.7309   480.2381   513.6775   548.0195
##  [18,]   448.3781   480.9045   514.3624   548.7224   583.9620   620.0646
##  [19,]   515.0477   549.4257   584.6829   620.8028   657.7741   695.5898
##  [20,]   585.4041   621.5413   658.5297   696.3624   735.0364   774.5521
##  [21,]   659.2856   697.1354   735.8264   775.3590   815.7365   856.9643
##  [22,]   736.6167   776.1664   816.5609   857.8060   899.9094   942.8806
##  [23,]   817.3857   858.6481   900.7689   943.7578   987.6257  1032.3852
##  [24,]   901.6287   944.6352   988.5211  1033.2987  1078.9814  1125.5836
##  [25,]   989.4168  1034.2125  1079.9137  1126.5346  1174.0904  1222.5965
##  [26,]  1080.8464  1127.4860  1175.0608  1223.5864  1273.0787  1323.5542
##  [27,]  1176.0317  1224.5766  1274.0886  1324.5842  1376.0799  1428.5924
##  [28,]  1275.0989  1325.6145  1377.1306  1429.6639  1483.2312  1537.8492
##  [29,]  1379.2495  1430.8776  1484.3241  1538.9635  1594.6707  1651.4621
##  [30,]  1491.2304  1543.1488  1596.9804  1652.7833  1710.5349  1769.5661
##  [31,]  1611.6892  1663.8763  1717.9946  1774.0972  1832.2391  1892.1663
##  [32,]  1741.2175  1793.6329  1848.0054  1904.0498  1960.5548  2017.4219
##  [33,]  1880.4391  1932.6640  1985.2614  2038.1422  2091.3277  2144.8424
##  [34,]  2027.5865  2076.4532  2125.5731  2174.9682  2224.6631  2274.6851
##  [35,]  2178.5000  2223.6531  2269.0814  2314.8083  2360.8594  2407.2626
##  [36,]  2333.3619  2374.5737  2416.1137  2458.0042  2500.2695  2542.9359
##  [37,]  2492.5371  2529.5935  2567.0589  2604.9525  2643.2953  2682.1105
##  [38,]  2656.4601  2689.1533  2722.3623  2756.1009  2790.3855  2825.2341
##  [39,]  2825.6297  2853.7528  2882.5229  2911.9467  2942.0339  2972.7963
##  [40,]  3000.6076  3023.9497  3048.0931  3073.0362  3098.7804  3125.3298
##  [41,]  3182.0178  3200.3593  3219.6796  3239.9667  3261.2123  3283.4116
##  [42,]  3370.5482  3383.6571  3397.9451  3413.3882  3429.9670  3447.6666
##  [43,]  3566.9533  3574.5813  3583.6118  3594.0071  3605.7355  3618.7705
##  [44,]  3772.0575  3773.9367  3777.4653  3782.5906  3789.2669  3797.4549
##  [45,]  3986.7609  3982.6000  3980.3600  3979.9715  3981.3732  3984.5117
##  [46,]  4212.0437  4201.5251  4193.2244  4187.0537  4182.9350  4180.7991
##  [47,]  4448.9739  4431.7498  4417.0675  4404.8191  4394.9080  4387.2478
##  [48,]  4698.7131  4674.4024  4652.9861  4634.3341  4618.3296  4604.8678
##  [49,]  4956.1997  4929.2062  4902.1719  4876.7571  4854.3266  4834.7554
##  [50,]  5215.1495  5187.3376  5159.5448  5131.7976  5104.1223  5078.1012
##  [51,]  5475.9812  5447.4892  5419.0819  5390.7825  5362.6146  5333.8971
##  [52,]  5739.2046  5710.1592  5681.2680  5651.7912  5621.0721  5589.1283
##  [53,]  6005.4151  5975.1079  5943.5371  5910.7200  5876.6740  5841.4167
##  [54,]  6271.9633  6238.1431  6203.0886  6166.8165  6129.3433  6090.6858
##  [55,]  6536.0631  6498.5827  6459.9136  6420.0705  6379.0681  6336.9209
##  [56,]  6797.7771  6756.4861  6714.0677  6670.5335  6625.8950  6580.1635
##  [57,]  7057.2346  7011.9762  6965.6670  6918.3142  6869.9251  6820.5069
##  [58,]  7314.6287  7265.2357  7214.8839  7163.5749  7111.3105  7058.0927
##  [59,]  7570.2136  7516.5055  7461.9459  7406.5297  7350.2521  7293.1089
##  [60,]  7824.3048  7766.0835  7707.1341  7647.4433  7586.9985  7525.7884
##  [61,]  8077.2795  8014.3259  7950.7842  7886.6314  7821.8464  7756.4094
##  [62,]  8329.5796  8261.6491  8193.2881  8124.4628  8055.1419  7985.2961
##  [63,]  8581.7159  8508.5340  8435.0982  8361.3623  8287.2834  8212.8220
##  [64,]  8834.2745  8755.5316  8676.7319  8597.8153  8518.7261  8439.4127
##  [65,]  9087.9256  9003.2710  8918.7793  8834.3749  8749.9877  8665.5526
##  [66,]  9343.4343  9252.4695  9161.9127  9071.6704  8981.6566  8891.7911
##  [67,]  9601.6753  9503.9465  9406.8987  9310.4188  9214.4025  9118.7532
##  [68,]  9863.5105  9758.6397  9654.6141  9551.4388  9448.9894  9347.1508
##  [69,] 10123.8193 10015.4478  9905.9084  9795.6696  9686.2928  9577.7989
##  [70,] 10379.3232 10268.0774 10155.6068 10041.9502  9927.1463  9811.6351
##  [71,] 10629.7115 10515.7569 10400.5150 10284.0225 10166.4277 10048.7308
##  [72,] 10874.6929 10758.1881 10640.4507 10522.4838 10404.5306 10286.6264
##  [73,] 11114.1243 10996.4585 10878.6648 10760.7781 10642.8338 10524.8670
##  [74,] 11351.3004 11233.9992 11116.4998 10998.8370 10881.0455 10763.1604
##  [75,] 11587.5279 11470.7315 11353.6679 11236.3710 11118.8753 11001.2154
##  [76,] 11822.5270 11706.3732 11589.8844 11473.0938 11356.0352 11238.7427
##  [77,] 12056.0248 11940.6479 11824.8699 11708.7232 11592.2408 11475.4560
##  [78,] 12287.7562 12173.2862 12058.3513 11942.9828 11827.2126 11711.0731
##  [79,] 12517.4672 12404.0283 12290.0638 12175.6034 12060.6776 11945.3176
##  [80,] 12744.9168 12632.6263 12519.7533 12406.3253 12292.3711 12177.9204
##  [81,] 12969.8815 12858.8478 12747.1791 12634.9007 12522.0391 12408.6220
##  [82,] 13192.1601 13082.4795 12972.1179 12861.0972 12749.4411 12637.1748
##  [83,] 13411.5800 13303.3339 13194.3687 13084.7021 12974.3541 12863.3465
##  [84,] 13628.0064 13521.2561 13413.7593 13305.5279 13196.5770 13086.9244
##  [85,] 13841.3548 13736.1350 13630.1552 13523.4201 13415.9382 13307.7215
##  [86,] 14051.6073 13947.9171 13843.4726 13738.2682 13632.3037 13525.5838
##  [87,] 14258.8368 14156.6271 14053.6943 13950.0193 13845.5901 13740.4010
##  [88,] 14463.8099 14362.3952 14260.8944 14158.6990 14055.7811 13952.1212
##  [89,] 14672.5787 14568.5838 14465.8713 14364.4388 14262.9517 14160.7706
##  [90,] 14886.3612 14779.9415 14674.6924 14570.6722 14467.9333 14366.4822
##  [91,] 15104.6551 14996.0711 14888.5227 14782.0800 14676.8067 14572.7611
##  [92,] 15326.8569 15216.4206 15106.8588 14998.2547 14890.6847 14784.2190
##  [93,] 15552.2510 15440.3316 15329.0963 15218.6433 15109.0630 15000.4387
##  [94,] 15779.9955 15667.0263 15554.5183 15442.5861 15331.3361 15220.8663
##  [95,] 16009.1072 15895.5938 15782.2817 15669.3044 15556.7859 15444.8410
##  [96,] 16238.4415 16124.9719 16011.4015 15897.8855 15784.5679 15671.5826
##  [97,] 16466.6688 16353.9261 16240.7316 16127.2657 16013.6958 15900.1772
##  [98,] 16692.2433 16581.0219 16468.9404 16356.2088 16243.0216 16129.5596
##  [99,] 16913.3617 16804.5896 16694.4798 16583.2781 16471.2118 16358.4913
## [100,] 17127.9072 17022.6757 16915.5435 16806.8011 16696.7158 16585.5339
##             [,61]      [,62]      [,63]      [,64]      [,65]      [,66]
##   [1,]   209.1388   190.5173   173.6956   158.7461   145.7302   134.7002
##   [2,]   173.3749   158.4638   145.4876   134.4982   125.5386   118.6437
##   [3,]   145.2458   134.2969   125.3787   118.5259   113.7659   111.1192
##   [4,]   125.2197   118.4090   113.6915   111.0877   110.6116   112.2718
##   [5,]   113.6179   111.0569   110.6240   112.3274   116.1701   122.1502
##   [6,]   110.6373   112.3839   116.2698   122.2930   130.4471   140.7215
##   [7,]   116.3703   122.4367   130.6337   140.9508   153.3736   167.8843
##   [8,]   130.8212   141.1810   153.6461   168.1988   184.8180   203.4798
##   [9,]   153.9195   168.5142   185.1749   203.8776   224.5454   246.9956
##  [10,]   185.5326   204.2762   224.9819   247.4663   271.5586   297.1147
##  [11,]   225.4192   247.9376   272.0609   297.6452   324.5691   352.7305
##  [12,]   272.5637   298.1762   325.1261   353.3115   382.6475   413.0633
##  [13,]   325.6836   353.8930   383.2515   413.6884   445.1458   477.5762
##  [14,]   383.8559   414.3140   445.7915   478.2411   511.6249   545.9129
##  [15,]   446.4376   478.9064   512.3087   546.6148   581.8016   617.8523
##  [16,]   512.9929   547.3170   582.5214   618.5894   655.5093   693.2740
##  [17,]   583.2415   619.3268   656.2639   694.0456   732.6686   772.1332
##  [18,]   657.0188   694.8175   733.4576   772.9391   813.2652   854.4413
##  [19,]   734.2468   773.7454   814.0886   855.2820   897.3331   940.2514
##  [20,]   814.9124   856.1230   898.1915   941.1275   984.9418  1029.6468
##  [21,]   899.0503   942.0039   985.8361  1030.5592  1076.1867  1122.7328
##  [22,]   986.7307  1031.4720  1077.1179  1123.6827  1171.1813  1219.6294
##  [23,]  1078.0495  1124.6329  1172.1506  1220.6181  1270.0513  1320.4668
##  [24,]  1173.1203  1221.6071  1271.0601  1321.4955  1372.9301  1425.3806
##  [25,]  1272.0692  1322.5247  1373.9796  1426.4508  1479.9549  1534.5088
##  [26,]  1375.0296  1427.5214  1481.0466  1535.6218  1591.2638  1647.9890
##  [27,]  1482.1387  1536.7353  1592.3990  1649.1463  1706.9934  1765.9563
##  [28,]  1593.5346  1650.3039  1708.1734  1767.1591  1827.2767  1888.2899
##  [29,]  1709.3540  1768.3623  1828.5029  1889.5205  1950.1525  2010.2175
##  [30,]  1829.7297  1890.7509  1951.3716  2011.4249  2070.8932  2129.7637
##  [31,]  1952.5905  2012.6321  2072.0885  2130.9468  2189.1985  2246.8391
##  [32,]  2074.6741  2132.3383  2190.3690  2247.9973  2305.0131  2361.4184
##  [33,]  2198.7139  2252.9725  2307.6508  2362.7839  2418.3387  2473.5267
##  [34,]  2325.0639  2375.8311  2427.0206  2478.6677  2530.8096  2583.4852
##  [35,]  2454.0475  2501.2456  2548.8899  2597.0150  2645.6569  2694.8533
##  [36,]  2586.0315  2629.5859  2673.6299  2718.1963  2763.3188  2809.0326
##  [37,]  2721.4228  2761.2585  2801.6454  2842.6129  2884.1916  2926.4135
##  [38,]  2860.6668  2896.7056  2933.3738  2970.6966  3008.7006  3047.4139
##  [39,]  3004.2482  3036.4056  3069.2867  3102.9115  3137.3016  3172.4805
##  [40,]  3152.6914  3180.8747  3209.8913  3239.7553  3270.4826  3302.0912
##  [41,]  3306.5634  3330.6690  3355.7330  3381.7624  3408.7668  3436.7580
##  [42,]  3466.4760  3486.3879  3507.3985  3529.5070  3552.7159  3577.0302
##  [43,]  3633.0904  3648.6779  3665.5199  3683.6071  3702.9339  3723.4979
##  [44,]  3807.1208  3818.2365  3830.7787  3844.7284  3860.0714  3876.7969
##  [45,]  3989.3401  3995.8180  4003.9110  4013.5899  4024.8303  4037.6129
##  [46,]  4180.5848  4182.2384  4185.7132  4190.9684  4197.9695  4206.6870
##  [47,]  4381.7617  4378.3816  4377.0471  4377.7053  4380.3099  4384.8210
##  [48,]  4593.8546  4585.2062  4578.8475  4574.7122  4572.7415  4572.3169
##  [49,]  4817.9310  4803.7522  4792.1282  4782.3700  4773.7102  4766.0682
##  [50,]  5055.1499  5034.4948  5015.3397  4997.5754  4981.1026  4965.8305
##  [51,]  5303.9722  5274.3376  5246.4534  5220.1966  5195.4547  5172.1244
##  [52,]  5555.9772  5521.6371  5486.1260  5450.8704  5417.3815  5385.5433
##  [53,]  5804.9657  5767.3386  5728.5529  5688.6261  5647.5753  5606.7574
##  [54,]  6050.8603  6009.8831  5967.7703  5924.5375  5880.2004  5834.7745
##  [55,]  6293.6433  6249.2490  6203.7520  6157.1657  6109.5033  6060.7779
##  [56,]  6533.3503  6485.4661  6436.5217  6386.5275  6335.4938  6283.4305
##  [57,]  6770.0667  6718.6114  6666.1479  6612.6829  6558.2231  6502.7750
##  [58,]  7003.9237  6948.8057  6892.7409  6835.7318  6777.7807  6718.8901
##  [59,]  7235.0965  7176.2114  7116.4507  7055.8116  6994.2919  6931.8893
##  [60,]  7463.8026  7401.0312  7337.4652  7273.0961  7207.9161  7141.9179
##  [61,]  7690.3021  7623.5076  7556.0099  7487.7941  7418.8462  7349.1532
##  [62,]  7914.8986  7843.9239  7772.3486  7700.1504  7627.3086  7553.8037
##  [63,]  8137.9409  8062.6058  7986.7845  7910.4467  7833.5641  7756.1103
##  [64,]  8359.8271  8279.9246  8199.6632  8119.0040  8037.9102  7956.3474
##  [65,]  8581.0093  8496.3015  8411.3768  8326.1862  8240.6838  8154.8265
##  [66,]  8802.0000  8712.2141  8622.3692  8532.4052  8442.2661  8351.8993
##  [67,]  9023.3814  8928.2038  8833.1432  8738.1271  8643.0882  8547.9633
##  [68,]  9245.8162  9144.8867  9044.2701  8943.8808  8843.7182  8744.6323
##  [69,]  9470.0622  9362.9660  9256.4915  9151.4778  9048.0758  8946.1906
##  [70,]  9697.0904  9584.5029  9473.9782  9365.3926  9258.6303  9153.5831
##  [71,]  9931.1899  9814.2152  9699.3870  9586.7566  9476.1915  9367.5678
##  [72,] 10168.8068 10051.1071  9933.5627  9816.5585  9701.6846  9589.0111
##  [73,] 10406.9131 10289.0076 10171.1859 10053.4834  9935.9356  9818.9029
##  [74,] 10645.2168 10527.2501 10409.2957 10291.3888 10173.5651 10055.8598
##  [75,] 10883.4261 10765.5426 10647.5999 10529.6333 10411.6782 10293.7701
##  [76,] 11121.2507 11003.5938 10885.8068 10767.9248 10649.9829 10532.0165
##  [77,] 11358.4024 11241.1143 11123.6260 11005.9721 10888.1874 10770.3069
##  [78,] 11594.5971 11477.8180 11360.7696 11243.4859 11126.0013 11008.3503
##  [79,] 11829.5551 11713.4227 11596.9532 11480.1800 11363.1367 11245.8574
##  [80,] 12063.0037 11947.6522 11831.8976 11715.7723 11599.3093 11482.5418
##  [81,] 12294.6782 12180.2373 12065.3297 11949.9866 11834.2398 11718.1217
##  [82,] 12524.3247 12410.9186 12296.9851 12182.5539 12067.6555 11952.3209
##  [83,] 12751.7029 12639.4487 12526.6101 12413.2150 12299.2918 12184.8704
##  [84,] 12976.5899 12865.5954 12753.9645 12641.7223 12528.8953 12415.5111
##  [85,] 13198.7851 13089.1464 12978.8255 12867.8441 12756.2258 12643.9957
##  [86,] 13418.1169 13309.9149 13200.9928 13091.3682 12981.0608 12870.0925
##  [87,] 13634.4519 13527.7472 13420.2953 13312.1080 13203.2003 13093.5897
##  [88,] 13847.7073 13742.5336 13636.5998 13529.9103 13422.4733 13314.3008
##  [89,] 14057.8675 13954.2228 13849.8241 13744.6658 13638.7474 13532.0731
##  [90,] 14265.0087 14162.8420 14059.9537 13956.3240 13851.9407 13746.7978
##  [91,] 14469.9959 14368.5253 14267.0654 14164.9130 14062.0395 13958.4250
##  [92,] 14678.9215 14574.8505 14472.0589 14370.5681 14269.1218 14166.9837
##  [93,] 14892.8471 14786.3584 14681.0367 14576.9405 14474.1225 14372.6106
##  [94,] 15111.2675 15002.6232 14895.0100 14788.4984 14683.1525 14579.0309
##  [95,] 15333.5762 15223.0897 15113.4725 15004.8081 14897.1734 14790.6388
##  [96,] 15559.0536 15447.0961 15335.8166 15225.3135 15115.6778 15006.9934
##  [97,] 15786.8543 15673.8610 15561.3217 15449.3515 15338.0573 15227.5376
##  [98,] 16015.9901 15902.4691 15789.1408 15676.1396 15563.5899 15451.6072
##  [99,] 16245.3116 16131.8533 16018.2845 15904.7610 15791.4275 15678.4184
## [100,] 16473.4829 16360.7737 16247.6014 16134.1471 16020.5789 15907.0530
##             [,67]      [,68]      [,69]      [,70]      [,71]      [,72]
##   [1,]   125.6992   118.7624   113.9174   111.1850   110.5797   112.1103
##   [2,]   113.8412   111.1517   110.5895   112.1633   115.8763   121.7270
##   [3,]   110.6001   112.2171   115.9734   121.8672   129.8924   140.0387
##   [4,]   116.0713   122.0083   130.0764   140.2654   152.5611   166.9458
##   [5,]   130.2613   140.4930   152.8310   167.2578   183.7524   202.2912
##   [6,]   153.1019   167.5706   184.1068   202.6865   223.2402   245.5875
##   [7,]   184.4620   203.0827   223.6745   246.0562   270.0555   295.5265
##   [8,]   224.1096   246.5256   270.5559   296.0554   322.9012   350.9901
##   [9,]   271.0570   296.5848   323.4566   351.5698   380.8382   411.1903
##  [10,]   324.0126   352.1499   381.4408   411.8142   443.2112   475.5838
##  [11,]   382.0440   412.4385   443.8557   476.2475   509.5756   543.8096
##  [12,]   444.5005   476.9117   510.2583   544.5103   579.6443   615.6430
##  [13,]   510.9414   545.2114   580.3631   616.3791   653.2476   690.9612
##  [14,]   581.0821   617.1155   654.0012   691.7318   730.3039   769.7174
##  [15,]   654.7551   692.5027   731.0918   770.5223   810.7971   851.9215
##  [16,]   731.8800   771.3276   811.6195   852.7611   894.7600   937.6254
##  [17,]   812.4422   853.6010   895.6173   938.5004   982.2612  1026.9119
##  [18,]   896.4750   939.3757   983.1544  1027.8231  1073.3954  1119.8854
##  [19,]   984.0479  1028.7348  1074.3255  1120.8341  1168.2757  1216.6658
##  [20,]  1075.2559  1121.7833  1169.2439  1217.6533  1267.0276  1317.3831
##  [21,]  1170.2124  1218.6412  1268.0351  1318.4106  1369.7841  1422.1726
##  [22,]  1269.0430  1319.4385  1370.8324  1423.2415  1476.6825  1531.1723
##  [23,]  1371.8811  1424.3108  1477.7729  1532.2840  1587.8609  1644.5200
##  [24,]  1478.8637  1533.3962  1588.9948  1645.6759  1703.4559  1762.3508
##  [25,]  1590.1291  1646.8322  1704.6346  1763.5522  1823.6007  1884.5969
##  [26,]  1705.8137  1764.7540  1824.8255  1885.8281  1946.4937  2006.5936
##  [27,]  1826.0509  1887.0591  1947.7136  2007.8018  2067.3060  2126.2130
##  [28,]  1948.9331  2009.0098  2068.5020  2127.3968  2185.6855  2243.3632
##  [29,]  2069.6977  2128.5804  2186.8567  2244.5221  2301.5750  2358.0172
##  [30,]  2188.0278  2245.6807  2302.7213  2359.1512  2414.9748  2470.1989
##  [31,]  2303.8674  2360.2849  2416.0963  2471.3084  2525.9301  2579.9722
##  [32,]  2417.2176  2472.4177  2527.0275  2581.0580  2634.5218  2687.4328
##  [33,]  2528.1247  2582.1437  2635.5961  2688.4961  2740.8591  2792.7015
##  [34,]  2636.6702  2689.5592  2741.9115  2793.7436  2845.0733  2895.9191
##  [35,]  2744.6428  2795.0658  2846.1052  2896.9414  2947.3136  2997.2418
##  [36,]  2855.3742  2902.3815  2950.0936  2998.5510  3047.7429  3096.8387
##  [37,]  2969.3121  3012.9222  3057.2797  3102.4222  3148.3886  3195.2192
##  [38,]  3086.8661  3127.0881  3168.1126  3209.9735  3252.7062  3296.3477
##  [39,]  3208.4733  3245.3067  3283.0091  3321.6106  3361.1427  3401.6387
##  [40,]  3334.6013  3368.0346  3402.4149  3437.7677  3474.1206  3511.5027
##  [41,]  3465.7505  3495.7606  3526.8070  3558.9103  3592.0934  3626.3811
##  [42,]  3602.4578  3629.0093  3656.6976  3685.5381  3715.5485  3746.7489
##  [43,]  3745.3000  3768.3442  3792.6371  3818.1885  3845.0106  3873.1183
##  [44,]  3894.8982  3914.3721  3935.2186  3957.4411  3981.0459  4006.0423
##  [45,]  4051.9225  4067.7479  4085.0819  4103.9212  4124.2656  4145.6514
##  [46,]  4217.0967  4229.1790  4242.9188  4257.8083  4273.1238  4288.8308
##  [47,]  4391.2043  4398.9011  4407.1787  4415.9920  4425.3034  4435.0790
##  [48,]  4572.6918  4573.8060  4575.6069  4578.0465  4581.0817  4584.6734
##  [49,]  4759.3710  4753.5525  4748.5524  4744.3157  4740.7925  4737.9372
##  [50,]  4951.6764  4938.5647  4926.4259  4915.1968  4904.8193  4895.2402
##  [51,]  5150.1113  5129.3286  5109.6965  5091.1419  5073.5974  5057.0008
##  [52,]  5355.2477  5326.3953  5298.8945  5272.6608  5247.6161  5223.6884
##  [53,]  5567.7304  5530.3847  5494.6151  5460.3245  5427.4231  5395.8276
##  [54,]  5788.2748  5741.9905  5697.5237  5654.7709  5613.6290  5574.0023
##  [55,]  6011.0021  5960.1886  5908.3498  5856.7100  5806.9131  5758.8620
##  [56,]  6230.3476  6176.2548  6121.1617  6065.0777  6008.0125  5951.1284
##  [57,]  6446.3450  6388.9395  6330.5650  6271.2277  6210.9340  6149.6906
##  [58,]  6659.0627  6598.3008  6536.6074  6473.9852  6410.4372  6345.9665
##  [59,]  6868.6019  6804.4281  6739.3667  6673.4164  6606.5764  6538.8463
##  [60,]  7075.0946  7007.4400  6938.9482  6869.6137  6799.4316  6728.3976
##  [61,]  7278.7027  7207.4833  7135.4841  7062.6950  6989.1066  6914.7102
##  [62,]  7479.6174  7404.7325  7329.1326  7252.8026  7175.7282  7097.8959
##  [63,]  7678.0601  7599.3901  7520.0781  7440.1032  7359.4459  7278.0876
##  [64,]  7874.2834  7791.6877  7708.5317  7624.7884  7540.4323  7455.4396
##  [65,]  8068.5740  7981.8882  7894.7333  7807.0759  7718.9373  7631.1636
##  [66,]  8261.2554  8170.2882  8079.0154  7988.2886  7898.3580  7809.1808
##  [67,]  8452.7635  8358.3421  8264.9205  8172.4428  8080.8568  7990.1135
##  [68,]  8646.8121  8550.1846  8454.6817  8360.2395  8266.7983  8174.3021
##  [69,]  8845.7335  8746.6213  8648.7762  8552.1253  8456.6003  8362.1373
##  [70,]  9050.1494  8948.2345  8847.7493  8748.6108  8650.7408  8554.0665
##  [71,]  9260.7698  9155.6889  9052.2236  8950.2789  8849.7657  8750.6008
##  [72,]  9478.4055  9369.7438  9262.9100  9157.7954  9054.2985  8952.3239
##  [73,]  9703.9830  9591.2664  9480.6203  9371.9205  9265.0509  9159.9026
##  [74,]  9938.3085  9821.2482  9706.2824  9593.5226  9482.8359  9374.0980
##  [75,] 10175.9443 10058.2363  9940.6815  9823.5944  9708.5826  9595.7796
##  [76,] 10414.0608 10296.1514 10178.3236 10060.6129  9943.0546  9825.9416
##  [77,] 10652.3659 10534.3996 10416.4434 10298.5327 10180.7029 10062.9895
##  [78,] 10890.5679 10772.6891 10654.7490 10536.7828 10418.8260 10300.9140
##  [79,] 11128.3765 11010.7285 10892.9484 10775.0712 10657.1320 10539.1660
##  [80,] 11365.5036 11248.2288 11130.7516 11013.1067 10895.3289 10777.4533
##  [81,] 11601.6652 11484.9035 11367.8705 11250.6001 11133.1266 11015.4848
##  [82,] 11836.5819 11720.4710 11604.0210 11487.2652 11370.2373 11252.9713
##  [83,] 12069.9811 11954.6550 11838.9239 11722.8201 11606.3766 11489.6267
##  [84,] 12301.5984 12187.1867 12072.3065 11956.9889 11841.2657 11725.1691
##  [85,] 12531.1802 12417.8070 12303.9047 12189.5028 12074.6317 11959.3227
##  [86,] 12758.4869 12646.2689 12533.4650 12420.1027 12306.2108 12191.8187
##  [87,] 12983.2959 12872.3407 12760.7477 12648.5418 12535.7494 12422.3982
##  [88,] 13205.4075 13095.8108 12985.5307 12874.5886 12763.0082 12650.8145
##  [89,] 13424.6511 13316.4933 13207.6144 13098.0318 12987.7652 12876.8363
##  [90,] 13640.8947 13534.2355 13426.8286 13318.6855 13209.8210 13100.2524
##  [91,] 13854.0570 13748.9294 13643.0416 13536.3977 13429.0057 13320.8774
##  [92,] 14064.1250 13960.5256 13856.1729 13751.0607 13645.1883 13538.5596
##  [93,] 14271.1780 14169.0542 14066.2103 13962.6260 13858.2886 13753.1917
##  [94,] 14476.1867 14374.6529 14273.2339 14171.1243 14068.2952 13964.7260
##  [95,] 14685.2688 14581.1219 14478.2514 14376.6949 14275.2894 14173.1942
##  [96,] 14899.3371 14792.7797 14687.3856 14583.2135 14480.3166 14378.7366
##  [97,] 15117.8835 15009.1791 14901.5014 14794.9211 14689.5028 14585.3055
##  [98,] 15340.2983 15229.7620 15120.0896 15011.3652 14903.6660 14797.0629
##  [99,] 15565.8584 15453.8632 15342.5396 15231.9868 15122.2961 15013.5518
## [100,] 15793.7143 15680.6974 15568.1272 15456.1194 15344.7813 15234.2120
##             [,73]      [,74]      [,75]      [,76]      [,77]      [,78]
##   [1,]   115.7801   121.5877   129.5269   139.5878   151.7562   166.0149
##   [2,]   129.7092   139.8128   152.0236   166.3243   182.6943   201.1099
##   [3,]   152.2919   166.6346   183.0461   201.5028   221.9416   244.1854
##   [4,]   183.3988   201.8966   222.3737   244.6521   268.5578   293.9433
##   [5,]   222.8066   245.1195   269.0564   294.4705   321.2378   349.2541
##   [6,]   269.5556   294.9982   321.7917   349.8323   379.0328   409.3211
##   [7,]   322.3462   350.4110   379.6342   409.9437   441.2801   473.5948
##   [8,]   380.2360   410.5668   441.9234   474.2574   507.5297   541.7095
##   [9,]   442.5671   474.9204   508.2113   542.4091   577.4902   613.4369
##  [10,]   508.8933   543.1092   578.2079   614.1719   650.9891   688.6516
##  [11,]   578.9259   614.9073   651.7416   689.4211   727.9423   767.3047
##  [12,]   652.4944   690.1910   728.7291   768.1086   808.3321   849.4048
##  [13,]   729.5163   768.9128   809.1534   850.2433   892.1900   935.0027
##  [14,]   809.9751   851.0822   893.0463   935.8766   979.5838  1024.1802
##  [15,]   893.9030   936.7508   980.4759  1025.0904  1070.6075  1117.0414
##  [16,]   981.3684  1026.0009  1071.5364  1117.9890  1165.3736  1213.7058
##  [17,]  1072.4657  1118.9370  1166.3406  1214.6921  1264.0074  1314.3030
##  [18,]  1167.3080  1215.6788  1265.0138  1315.3293  1366.6419  1418.9683
##  [19,]  1266.0205  1316.3560  1367.6889  1420.0360  1473.4140  1527.8397
##  [20,]  1368.7363  1421.1041  1474.5031  1528.9502  1584.4620  1641.0550
##  [21,]  1475.5926  1530.0610  1585.5945  1642.2095  1699.9225  1758.7493
##  [22,]  1586.7275  1643.3645  1701.0998  1759.9494  1819.9289  1880.9018
##  [23,]  1702.2776  1761.1498  1821.1523  1882.1337  1942.8329  2002.9677
##  [24,]  1822.3763  1883.3654  1944.0534  2004.1766  2063.7166  2122.6600
##  [25,]  1945.2737  2005.3852  2064.9133  2123.8446  2182.1702  2239.8850
##  [26,]  2066.1098  2125.0289  2183.3422  2241.0447  2298.1347  2354.6138
##  [27,]  2184.5140  2242.2041  2299.2817  2355.7485  2411.6087  2466.8689
##  [28,]  2300.4285  2356.8830  2412.7310  2467.9791  2522.6363  2576.7132
##  [29,]  2413.8530  2469.0891  2523.7344  2577.7997  2631.2974  2684.2415
##  [30,]  2524.8324  2578.8861  2632.3724  2685.3055  2737.7005  2789.5741
##  [31,]  2633.4472  2686.3692  2738.7536  2790.6168  2841.9764  2892.8509
##  [32,]  2739.8064  2791.6593  2843.0089  2893.8738  2944.2735  2994.2281
##  [33,]  2844.0412  2894.8966  2945.2871  2995.2328  3044.7548  3093.8742
##  [34,]  2946.3004  2996.2374  3045.7510  3094.8626  3143.5941  3191.9683
##  [35,]  3046.7470  3095.8507  3144.5749  3192.9420  3240.9750  3288.6974
##  [36,]  3145.5554  3193.9156  3241.9421  3289.6585  3337.0884  3384.2560
##  [37,]  3242.9091  3290.6194  3338.0438  3385.2064  3432.1316  3478.8442
##  [38,]  3340.9366  3386.5128  3433.0773  3479.7859  3526.3075  3572.6674
##  [39,]  3443.1335  3485.6637  3529.2677  3573.9856  3619.8240  3665.9358
##  [40,]  3549.9450  3589.4803  3630.1435  3671.9710  3715.0014  3759.2753
##  [41,]  3661.8001  3698.3793  3736.1495  3775.1438  3815.3969  3856.9460
##  [42,]  3779.1616  3812.8110  3847.7240  3883.9296  3921.4588  3959.9515
##  [43,]  3902.5291  3933.2627  3965.3417  3998.3750  4031.6436  4065.1280
##  [44,]  4032.4426  4059.8215  4087.4616  4115.3403  4143.4479  4171.7769
##  [45,]  4167.3598  4189.3633  4211.6450  4234.1904  4256.9878  4280.0276
##  [46,]  4304.9031  4321.3178  4338.0552  4355.0978  4372.4309  4390.0421
##  [47,]  4445.2889  4455.9061  4466.9071  4478.2706  4489.9782  4502.0136
##  [48,]  4588.7866  4593.3893  4598.4531  4603.9521  4609.8634  4616.1662
##  [49,]  4735.7085  4734.0686  4732.9830  4732.4207  4732.3532  4732.7546
##  [50,]  4886.4107  4878.2862  4870.8254  4863.9910  4857.7482  4852.0655
##  [51,]  5041.2950  5026.4272  5012.3489  4999.0152  4986.3846  4974.4190
##  [52,]  5200.8110  5178.9222  5157.9649  5137.8860  5118.6365  5100.1708
##  [53,]  5365.4607  5336.2507  5308.1310  5281.0397  5254.9192  5229.7158
##  [54,]  5535.8020  5498.9454  5463.3554  5428.9602  5395.6930  5363.4913
##  [55,]  5712.4553  5667.5980  5624.2018  5582.1843  5541.4683  5501.9818
##  [56,]  5896.1092  5842.8653  5791.2961  5741.3074  5692.8111  5645.7247
##  [57,]  6087.5038  6025.4771  5965.3333  5906.9909  5850.3500  5795.3169
##  [58,]  6280.5764  6214.2705  6147.0527  6079.9704  6014.7842  5951.4221
##  [59,]  6470.2259  6400.7153  6330.3150  6259.0260  6186.8497  6114.7804
##  [60,]  6656.5074  6583.7577  6510.1454  6435.6680  6360.3236  6284.1108
##  [61,]  6839.4975  6763.4611  6686.5942  6608.8907  6530.3450  6450.9524
##  [62,]  7019.2931  6939.9081  6859.7301  6778.7489  6696.9883  6615.2648
##  [63,]  7196.0110  7113.1998  7029.6778  6946.2729  6863.3096  6780.7714
##  [64,]  7369.8335  7284.4519  7199.5958  7115.2416  7031.3674  6947.9534
##  [65,]  7544.0312  7457.5080  7371.5640  7286.1715  7201.3050  7116.9409
##  [66,]  7720.7174  7632.9304  7545.7854  7459.2501  7373.2946  7287.8913
##  [67,]  7900.1672  7810.9752  7722.4977  7634.6974  7547.5397  7460.9924
##  [68,]  8082.6985  7991.9386  7901.9767  7812.7699  7724.2783  7636.4647
##  [69,]  8268.6764  8176.1617  8084.5406  7993.7641  7903.7865  7814.5648
##  [70,]  8458.5194  8364.0355  8270.5550  8178.0216  8086.3830  7995.5899
##  [71,]  8652.7059  8556.0081  8460.4389  8365.9342  8272.4339  8179.8820
##  [72,]  8851.7827  8752.5913  8654.6715  8557.9502  8462.3589  8367.8332
##  [73,]  9056.3740  8954.3696  8853.8002  8754.5823  8656.6376  8559.8928
##  [74,]  9267.1925  9162.0105  9058.4501  8956.4158  8855.8183  8756.5738
##  [75,]  9485.0523  9376.2762  9269.3348  9164.1190  9060.5268  8958.4626
##  [76,]  9710.8838  9598.0375  9487.2695  9378.4552  9271.4778  9166.2282
##  [77,]  9945.4278  9828.2898  9713.1858  9600.2962  9489.4875  9380.6349
##  [78,] 10183.0823 10065.3661  9947.8011  9830.6389  9715.4888  9602.5558
##  [79,] 10421.2087 10303.2954 10185.4617 10067.7429  9950.1744  9832.9890
##  [80,] 10659.5150 10541.5492 10423.5913 10305.6769 10187.8411 10070.1197
##  [81,] 10897.7094 10779.8354 10661.8979 10543.9324 10425.9740 10308.0583
##  [82,] 11135.5016 11017.8628 10900.0897 10782.2174 10664.2809 10546.3156
##  [83,] 11372.6039 11255.3424 11137.8765 11020.2408 10902.4701 10784.5994
##  [84,] 11608.7322 11491.9881 11374.9705 11257.7135 11140.2514 11022.6187
##  [85,] 11843.6073 11727.5180 11611.0876 11494.3494 11377.3370 11260.0845
##  [86,] 12076.9568 11961.6563 11845.9488 11729.8667 11613.4429 11496.7105
##  [87,] 12308.5167 12194.1344 12079.2817 11963.9897 11848.2901 11732.2153
##  [88,] 12538.0337 12424.6935 12310.8224 12196.4499 12081.6064 11966.3230
##  [89,] 12765.2685 12653.0869 12540.3177 12426.9885 12313.1278 12198.7652
##  [90,] 12989.9994 12879.0837 12767.5286 12655.3591 12542.6015 12429.2834
##  [91,] 13212.0273 13102.4727 12992.2334 12881.3308 12769.7884 12657.6311
##  [92,] 13431.1826 13323.0690 13214.2333 13104.6928 12994.4671 12883.5777
##  [93,] 13647.3346 13540.7211 13433.3592 13325.2603 13216.4390 13106.9126
##  [94,] 13860.4039 13755.3224 13649.4807 13542.8824 13435.5354 13327.4513
##  [95,] 14070.3798 13966.8257 13862.5189 13757.4528 13651.6264 13545.0433
##  [96,] 14277.3448 14175.2638 14072.4641 13968.9251 13864.6336 13759.5829
##  [97,] 14482.3824 14380.7781 14279.3998 14177.3330 14074.5482 13971.0243
##  [98,] 14691.6206 14587.3980 14484.4487 14382.8193 14281.4545 14179.4020
##  [99,] 14905.8312 14799.2052 14693.7389 14589.4911 14486.5156 14384.8603
## [100,] 15124.5030 15015.7387 14907.9967 14801.3480 14695.8576 14591.5847
##             [,79]      [,80]      [,81]      [,82]      [,83]      [,84]
##   [1,]   182.3432   200.7178   221.0795   243.2540   267.0654   292.3650
##   [2,]   221.5102   243.7194   267.5623   292.8906   319.5790   347.5222
##   [3,]   268.0597   293.4167   320.1314   348.0990   377.2315   407.4556
##   [4,]   320.6844   348.6763   377.8315   408.0770   439.3527   471.6092
##   [5,]   378.4319   408.6988   439.9947   472.2707   505.4871   539.6126
##   [6,]   440.6372   472.9325   506.1676   540.3112   575.3393   611.2339
##   [7,]   506.8484   541.0102   576.0559   611.9679   648.7336   686.3450
##   [8,]   576.7729   612.7022   649.4851   687.1135   725.5837   764.8951
##   [9,]   650.2369   687.8824   726.3695   765.6980   805.8702   846.8912
##  [10,]   727.1557   766.5012   806.6905   847.7287   889.6232   932.3831
##  [11,]   807.5111   848.5666   890.4785   933.2559   976.9097  1021.4518
##  [12,]   891.3341   934.1291   977.8007  1022.3609  1067.8229  1114.2009
##  [13,]   978.6921  1023.2704  1068.7507  1115.1473  1162.4750  1210.7493
##  [14,]  1069.6789  1116.0942  1163.4409  1211.7344  1260.9909  1311.2266
##  [15,]  1164.4071  1212.7199  1261.9960  1312.2516  1363.5033  1415.7678
##  [16,]  1263.0015  1313.2771  1364.5491  1416.8342  1470.1493  1524.5111
##  [17,]  1365.5953  1417.9011  1471.2371  1525.6202  1581.0670  1637.5940
##  [18,]  1472.3254  1526.7298  1582.1982  1638.7472  1696.3932  1755.1521
##  [19,]  1583.3299  1639.9009  1697.5692  1756.3507  1816.2613  1877.2048
##  [20,]  1698.7456  1757.5498  1817.4834  1878.4373  1939.1700  1999.3396
##  [21,]  1818.7059  1879.6697  1940.3912  2000.5492  2060.1250  2119.1049
##  [22,]  1941.6122  2001.7586  2061.3224  2120.2902  2178.6527  2236.4046
##  [23,]  2062.5196  2121.4752  2179.8254  2237.5650  2294.6921  2351.2082
##  [24,]  2180.9979  2238.7251  2295.8399  2352.3436  2408.2404  2463.5367
##  [25,]  2296.9874  2353.4788  2409.3634  2464.6477  2519.3404  2573.4521
##  [26,]  2410.4862  2465.7584  2520.4392  2574.5393  2628.0710  2681.0483
##  [27,]  2521.5379  2575.6264  2629.1467  2682.1129  2734.5401  2786.4447
##  [28,]  2630.2222  2683.1773  2735.5938  2787.4881  2838.8777  2889.7811
##  [29,]  2736.6473  2788.5312  2839.9108  2890.8046  2941.2319  2991.2128
##  [30,]  2840.9437  2891.8278  2942.2460  2992.2181  3041.7651  3090.9084
##  [31,]  2943.2598  2993.2232  3042.7618  3091.8971  3140.6511  3189.0462
##  [32,]  3043.7584  3092.8858  3141.6323  3190.0204  3238.0730  3285.8136
##  [33,]  3142.6133  3190.9944  3239.0405  3286.7750  3334.2216  3381.4044
##  [34,]  3240.0078  3287.7363  3335.1773  3382.3550  3429.2939  3476.0187
##  [35,]  3336.1329  3383.3056  3430.2399  3476.9606  3523.4928  3569.8618
##  [36,]  3431.1858  3477.9024  3524.4311  3570.7971  3617.0260  3663.1438
##  [37,]  3525.3693  3571.7323  3617.9587  3664.0745  3710.1059  3756.0792
##  [38,]  3618.8914  3665.0052  3711.0351  3757.0075  3802.9491  3848.8867
##  [39,]  3711.9643  3757.9358  3803.8770  3849.8149  3895.7767  3941.4527
##  [40,]  3804.8050  3850.7431  3896.7056  3942.3649  3986.9629  4030.4610
##  [41,]  3899.8302  3943.7170  3987.8526  4031.3284  4073.6926  4114.9353
##  [42,]  3998.6845  4037.6402  4076.8200  4116.2270  4155.8463  4194.7979
##  [43,]  4098.8245  4132.7312  4166.8484  4201.1782  4235.7243  4270.4923
##  [44,]  4200.3219  4229.0796  4258.0485  4287.2288  4316.6224  4346.2326
##  [45,]  4303.3020  4326.8056  4350.5345  4374.4866  4398.6611  4423.0589
##  [46,]  4407.9207  4426.0582  4444.4480  4463.0847  4481.9648  4501.0862
##  [47,]  4514.3628  4527.0137  4539.9558  4553.1806  4566.6810  4580.4514
##  [48,]  4622.8423  4629.8752  4637.2505  4644.9557  4652.9796  4661.3129
##  [49,]  4733.6017  4734.8733  4736.5503  4738.6155  4741.0536  4743.8507
##  [50,]  4846.9139  4842.2668  4838.0998  4834.3908  4831.1195  4828.2673
##  [51,]  4963.0829  4952.3438  4942.1716  4932.5385  4923.4188  4914.7889
##  [52,]  5082.4466  5065.4246  5049.0684  5033.3440  5018.2199  5003.6669
##  [53,]  5205.3796  5181.8639  5159.1254  5137.1234  5115.8201  5095.1802
##  [54,]  5332.2966  5302.0545  5272.7139  5244.2270  5216.5492  5189.6384
##  [55,]  5463.6572  5426.4314  5390.2452  5355.0429  5320.7724  5287.3846
##  [56,]  5599.9709  5555.4771  5512.1753  5470.0013  5428.8951  5388.8001
##  [57,]  5741.8036  5689.7274  5639.0108  5589.5807  5541.3683  5494.3087
##  [58,]  5889.7847  5829.7786  5771.3158  5714.3136  5658.6939  5604.3833
##  [59,]  6044.6163  5976.2955  5909.7193  5844.7944  5781.4506  5720.3731
##  [60,]  6207.0289  6130.0219  6054.9474  5982.5438  5913.1017  5846.5223
##  [61,]  6370.7362  6290.5005  6210.6153  6131.9731  6056.4413  5983.9769
##  [62,]  6533.9258  6452.9609  6372.3608  6292.1179  6212.2257  6133.5313
##  [63,]  6698.6433  6616.9119  6535.5653  6454.5929  6373.9856  6293.7355
##  [64,]  6864.9814  6782.4347  6700.2985  6618.5592  6537.2049  6456.2250
##  [65,]  7033.0572  6949.6341  6866.6533  6784.0982  6701.9538  6620.2067
##  [66,]  7203.0145  7118.6404  7034.7472  6951.3150  6868.3254  6785.7619
##  [67,]  7375.0256  7289.6114  7204.7241  7120.3401  7036.4374  6952.9961
##  [68,]  7549.2943  7462.7350  7376.7567  7291.3316  7206.4339  7122.0400
##  [69,]  7726.0592  7638.2322  7551.0492  7464.4778  7378.4881  7293.0521
##  [70,]  7905.5966  7816.3601  7727.8403  7640.0000  7552.8043  7466.2209
##  [71,]  8088.2257  7997.4161  7907.4070  7818.1556  7729.6217  7641.7681
##  [72,]  8274.3133  8181.7427  8090.0688  7999.2425  7909.2177  7819.9514
##  [73,]  8464.2792  8369.7327  8276.1930  8183.6037  8091.9123  8001.0693
##  [74,]  8658.6041  8561.8358  8466.2001  8371.6326  8278.0731  8185.4652
##  [75,]  8857.8369  8758.5659  8660.5712  8563.7793  8468.1213  8373.5328
##  [76,]  9062.6041  8960.5099  8859.8560  8760.5585  8662.5388  8565.7232
##  [77,]  9273.6215  9168.3380  9064.6821  8962.5579  8861.8757  8762.5516
##  [78,]  9491.7063  9382.8153  9275.7659  9170.4485  9066.7607  8964.6064
##  [79,]  9717.7926  9604.8162  9493.9259  9384.9965  9277.9110  9172.5597
##  [80,]  9952.5478  9835.3400  9720.0974  9607.0775  9496.1463  9387.1785
##  [81,] 10190.2207 10072.4965  9954.9213  9837.6920  9722.4030  9609.3396
##  [82,] 10428.3567 10310.4398 10192.6002 10074.8734  9957.2948  9840.0450
##  [83,] 10666.6639 10548.6988 10430.7394 10312.8213 10194.9798 10077.2504
##  [84,] 10904.8504 10786.9814 10669.0468 10551.0819 10433.1222 10315.2028
##  [85,] 11142.6261 11024.9966 10907.2307 10789.3634 10671.4297 10553.4651
##  [86,] 11379.7034 11262.4554 11145.0009 11027.3744 10909.6109 10791.7453
##  [87,] 11615.7981 11499.0716 11382.0696 11264.8262 11147.3755 11029.7522
##  [88,] 11850.6313 11734.5638 11618.1531 11501.4326 11384.4358 11267.1969
##  [89,] 12083.9309 11968.6561 11852.9724 11736.9121 11620.5080 11503.7934
##  [90,] 12315.4331 12201.0803 12086.2553 11970.9890 11855.3133 11739.2602
##  [91,] 12544.8851 12431.5780 12317.7382 12203.3953 12088.5795 11973.3218
##  [92,] 12772.0480 12659.9028 12547.1685 12433.8724 12320.0431 12205.7100
##  [93,] 12996.7005 12885.8243 12774.3073 12662.1743 12549.4516 12436.1666
##  [94,] 13218.6445 13109.1321 12998.9337 12888.0706 12776.5663 12664.4455
##  [95,] 13437.7114 13329.6420 13220.8496 13111.3513 13001.1665 12890.3167
##  [96,] 13653.7718 13547.2039 13439.8871 13331.8325 13223.0545 13113.5703
##  [97,] 13866.7480 13761.7127 13655.9169 13549.3643 13442.0624 13334.0226
##  [98,] 14076.6319 13973.1231 13868.8621 13763.8421 13658.0617 13551.5243
##  [99,] 14283.5090 14181.4707 14078.7153 13975.2216 13870.9759 13765.9713
## [100,] 14488.5830 14386.9010 14285.5632 14183.5391 14080.7984 13977.3197
##             [,85]      [,86]      [,87]      [,88]      [,89]      [,90]
##   [1,]   319.0271   346.9459   376.0328   406.2141   437.4288   469.6271
##   [2,]   376.6319   406.8346   438.0697   470.2874   503.4478   537.5190
##   [3,]   438.7110   470.9481   504.1272   538.2165   573.1916   609.0341
##   [4,]   504.8069   538.9144   573.9071   609.7670   646.4812   684.0416
##   [5,]   574.6230   610.5003   647.2317   684.8090   723.2282   762.4886
##   [6,]   647.9825   685.5769   724.0131   763.2904   803.4114   844.3808
##   [7,]   724.7982   764.0926   804.2306   845.2173   887.0596   929.7668
##   [8,]   805.0502   846.0541   887.9138   930.6385   974.2389  1018.7268
##   [9,]   888.7683   931.5106   975.1288  1019.6348  1065.0417  1111.3637
##  [10,]   976.0191  1020.5431  1065.9684  1112.3091  1159.5799  1207.7964
##  [11,]  1066.8955  1113.2548  1160.5446  1208.7803  1257.9780  1308.1538
##  [12,]  1161.5096  1209.7646  1258.9819  1309.1776  1360.3685  1412.5712
##  [13,]  1259.9862  1310.2019  1361.4130  1413.6363  1466.8885  1521.1863
##  [14,]  1362.4580  1414.7019  1467.9750  1522.2941  1577.6760  1634.1371
##  [15,]  1469.0619  1523.4024  1578.8059  1635.2890  1692.8680  1751.5590
##  [16,]  1579.9362  1636.4413  1694.0426  1752.7562  1812.5979  1873.5058
##  [17,]  1695.2176  1753.9539  1813.8186  1874.7390  1935.5051  1995.7094
##  [18,]  1815.0397  1875.9720  1936.7270  1996.9197  2056.5312  2115.5475
##  [19,]  1937.9486  1998.1298  2057.7293  2116.7335  2175.1329  2232.9219
##  [20,]  2058.9273  2117.9193  2176.3064  2234.0830  2291.2473  2347.8003
##  [21,]  2177.4797  2235.2439  2292.3958  2348.9365  2404.8699  2460.2024
##  [22,]  2293.5441  2350.0725  2405.9936  2461.3140  2516.0423  2570.1889
##  [23,]  2407.1171  2462.4255  2517.1419  2571.2768  2624.8426  2677.8530
##  [24,]  2518.2412  2572.3646  2625.9190  2678.9183  2731.3777  2783.3136
##  [25,]  2626.9951  2679.9834  2732.4321  2784.3575  2835.7772  2886.7095
##  [26,]  2733.4862  2785.4012  2836.8109  2887.7336  2938.1886  2988.1960
##  [27,]  2837.8444  2888.7574  2939.2032  2989.2018  3038.7740  3087.9411
##  [28,]  2940.2176  2990.2074  3039.7712  3088.9303  3137.7068  3186.1230
##  [29,]  3040.7682  3089.9194  3138.6884  3187.0976  3235.1699  3282.9287
##  [30,]  3139.6698  3188.0720  3236.1377  3283.8904  3331.3538  3378.5519
##  [31,]  3237.1054  3284.8520  3332.3098  3379.5028  3426.4555  3473.1925
##  [32,]  3333.2657  3380.4536  3427.4017  3474.1347  3520.6776  3567.0558
##  [33,]  3428.3479  3475.0767  3521.6161  3567.9912  3614.2277  3660.3515
##  [34,]  3522.5545  3568.9265  3615.1605  3661.2823  3707.3180  3753.2941
##  [35,]  3616.0933  3662.2131  3708.2473  3754.2225  3800.1651  3846.1022
##  [36,]  3709.1766  3755.1508  3801.0931  3847.0304  3892.9899  3938.7134
##  [37,]  3802.0211  3847.9586  3893.9188  3939.6269  3984.2912  4027.8563
##  [38,]  3894.8477  3940.5400  3985.1822  4028.7250  4071.1569  4112.4678
##  [39,]  3986.0728  4029.5933  4072.0026  4113.2907  4153.4490  4192.4698
##  [40,]  4072.8478  4114.1132  4154.2485  4193.2463  4231.1001  4267.8047
##  [41,]  4155.0477  4194.0223  4231.8530  4268.5343  4304.0618  4338.4318
##  [42,]  4232.6054  4269.2635  4304.7676  4339.1142  4372.3004  4404.3240
##  [43,]  4305.4729  4339.7961  4372.9589  4404.9589  4435.7946  4465.4649
##  [44,]  4376.0643  4406.1235  4436.4056  4466.0522  4494.5327  4521.8465
##  [45,]  4447.6823  4472.5346  4497.6207  4522.9464  4548.5101  4573.4677
##  [46,]  4520.4481  4540.0508  4559.8961  4579.9869  4600.3272  4620.9219
##  [47,]  4594.4876  4608.7867  4623.3471  4638.1681  4653.2505  4668.5959
##  [48,]  4669.9475  4678.8768  4688.0954  4697.5991  4707.3851  4717.4513
##  [49,]  4746.9947  4750.4748  4754.2817  4758.4072  4762.8446  4767.5881
##  [50,]  4825.8176  4823.7551  4822.0661  4820.7382  4819.7606  4819.1233
##  [51,]  4906.6271  4898.9132  4891.6289  4884.7574  4878.2831  4872.1921
##  [52,]  4989.6578  4976.1673  4963.1721  4950.6504  4938.5821  4926.9486
##  [53,]  5075.1707  5055.7606  5036.9213  5018.6257  5000.8487  4983.5669
##  [54,]  5163.4554  5137.9633  5113.1276  5088.9157  5065.2971  5042.2434
##  [55,]  5254.8336  5223.0760  5192.0710  5161.7802  5132.1676  5103.1990
##  [56,]  5349.6628  5311.4331  5274.0636  5237.5096  5201.7362  5167.4037
##  [57,]  5448.3410  5403.4074  5359.4638  5317.1820  5276.9532  5238.7253
##  [58,]  5551.3260  5500.2024  5451.3729  5404.7725  5360.3395  5318.0157
##  [59,]  5661.8889  5605.9176  5552.3829  5501.2123  5452.3371  5405.6923
##  [60,]  5782.7118  5721.5811  5663.0453  5607.0240  5553.4408  5502.2231
##  [61,]  5914.4759  5847.8396  5783.9741  5722.7901  5664.2027  5608.1314
##  [62,]  6057.9365  5985.4111  5915.8513  5849.1581  5785.2375  5724.0001
##  [63,]  6213.8362  6135.0908  6059.4329  5986.8466  5917.2278  5850.4777
##  [64,]  6375.6104  6295.3532  6215.4469  6136.6517  6060.9306  5988.2833
##  [65,]  6538.8447  6457.8574  6377.2354  6296.9711  6217.0577  6138.2138
##  [66,]  6703.6093  6621.8543  6540.4846  6459.4898  6378.8606  6298.5891
##  [67,]  6869.9977  6787.4257  6705.2650  6623.5020  6542.1247  6461.1224
##  [68,]  7038.1278  6954.6773  6871.6702  6789.0897  6706.9209  6625.1500
##  [69,]  7208.1440  7123.7400  7039.8183  6956.3587  6873.3428  6790.7539
##  [70,]  7380.2197  7294.7727  7209.8542  7125.4403  7041.5091  6958.0403
##  [71,]  7554.5597  7467.9642  7381.9515  7296.4936  7211.5647  7127.1408
##  [72,]  7731.4035  7643.5364  7556.3153  7469.7077  7383.6836  7298.2147
##  [73,]  7911.0288  7821.7475  7733.1854  7645.3050  7558.0712  7471.4515
##  [74,]  8093.7561  8002.8965  7912.8401  7823.5440  7734.9677  7647.0738
##  [75,]  8279.9536  8187.3270  8095.6002  8004.7239  7914.6518  7825.3407
##  [76,]  8470.0430  8375.4335  8281.8344  8189.1891  8097.4447  8006.5517
##  [77,]  8664.5068  8567.6676  8471.9651  8377.3346  8283.7157  8191.0517
##  [78,]  8863.8960  8764.5452  8666.4753  8569.6125  8473.8877  8379.2362
##  [79,]  9068.8399  8966.6556  8865.9168  8766.5394  8668.4443  8571.5578
##  [80,]  9280.0568  9174.6715  9070.9197  8968.7053  8867.9382  8768.5341
##  [81,]  9498.3675  9389.3612  9282.2034  9176.7840  9073.0002  8970.7556
##  [82,]  9724.7096  9611.6026  9500.5895  9391.5447  9284.3506  9178.8971
##  [83,]  9959.6684  9842.3990  9727.0171  9613.8664  9502.8123  9393.7289
##  [84,] 10197.3595 10079.6274  9962.0421  9844.7539  9729.3255  9616.1311
##  [85,] 10435.5049 10317.5844 10199.7391 10082.0045  9964.4159  9847.1098
##  [86,] 10673.8127 10555.8483 10437.8877 10319.9660 10202.1189 10084.3817
##  [87,] 10911.9911 10794.1272 10676.1956 10558.2315 10440.2704 10322.3477
##  [88,] 11149.7501 11032.1299 10914.3713 10796.5091 10678.5785 10560.6147
##  [89,] 11386.8019 11269.5675 11152.1246 11034.5076 10916.7514 10798.8909
##  [90,] 11622.8628 11506.1541 11389.1679 11271.9381 11154.4990 11036.8852
##  [91,] 11857.6540 11741.6083 11625.2174 11508.5147 11391.5338 11274.3085
##  [92,] 12090.9034 11975.6544 11859.9946 11743.9562 11627.5720 11510.8752
##  [93,] 12322.3477 12208.0246 12093.2273 11977.9868 11862.3350 11746.3039
##  [94,] 12551.7345 12438.4605 12324.6522 12210.3389 12095.5509 11980.3191
##  [95,] 12778.8251 12666.7165 12554.0171 12440.7543 12326.9565 12212.6531
##  [96,] 13003.3991 12892.5625 12781.0837 12668.9873 12556.2995 12443.0478
##  [97,] 13225.2591 13115.7890 13005.6315 12894.8081 12783.3419 12671.2578
##  [98,] 13444.2375 13336.2124 13227.4634 13118.0073 13007.8636 12897.0534
##  [99,] 13660.2062 13553.6840 13446.4122 13338.4020 13229.6674 13120.2255
## [100,] 13873.0894 13768.1001 13662.3504 13555.8434 13448.5867 13340.5912
##             [,91]      [,92]      [,93]      [,94]      [,95]      [,96]
##   [1,]   502.7688   536.8218   571.7615   607.5693   644.2320   681.7412
##   [2,]   572.4764   608.3015   644.9814   682.5076   720.8759   760.0852
##   [3,]   645.7311   683.2744   721.6596   760.8860   800.9557   841.8735
##   [4,]   722.4438   761.6871   801.7739   842.7089   884.4992   927.1537
##   [5,]   802.5925   843.5447   885.3523   928.0243   971.5713  1016.0050
##   [6,]   886.2058   928.8954   972.4601  1016.9119  1062.2639  1108.5300
##   [7,]   973.3493  1017.8192  1063.1895  1109.4742  1156.6882  1204.8470
##   [8,]  1064.1154  1110.4188  1157.6517  1205.8297  1254.9686  1305.0847
##   [9,]  1158.6156  1206.8128  1255.9713  1306.1073  1357.2374  1409.3783
##  [10,]  1256.9744  1307.1304  1358.2807  1410.4421  1463.6315  1517.8655
##  [11,]  1359.3244  1411.5064  1464.7167  1518.9720  1574.2890  1630.6842
##  [12,]  1465.8024  1520.0789  1575.4175  1631.8347  1689.3468  1747.9700
##  [13,]  1576.5466  1632.9857  1690.5201  1749.1658  1808.9388  1869.8048
##  [14,]  1691.6938  1750.3622  1810.1580  1871.0386  1931.8381  1992.0770
##  [15,]  1811.3777  1872.2723  1933.0606  1993.2880  2052.9352  2111.9879
##  [16,]  1934.2830  1994.4988  2054.1341  2113.1747  2171.6109  2229.4370
##  [17,]  2055.3327  2114.3612  2172.7851  2230.5989  2287.8002  2344.3902
##  [18,]  2173.9591  2231.7605  2288.9495  2345.5271  2401.4972  2456.8658
##  [19,]  2290.0985  2346.6638  2402.6216  2457.9782  2512.7421  2566.9236
##  [20,]  2403.7459  2459.0904  2513.8424  2568.0123  2621.6122  2674.6558
##  [21,]  2514.9425  2569.1007  2622.6892  2675.7218  2728.2134  2780.1805
##  [22,]  2623.7660  2676.7875  2729.2684  2781.2251  2832.6749  2883.6362
##  [23,]  2730.3232  2782.2694  2833.7092  2884.6609  2935.1437  2985.1776
##  [24,]  2834.7433  2885.6853  2936.1588  2986.1839  3035.7813  3084.9724
##  [25,]  2937.1738  2987.1901  3036.7790  3085.9621  3134.7611  3183.1985
##  [26,]  3037.7766  3086.9517  3135.7431  3184.1735  3232.2656  3280.0427
##  [27,]  3136.7250  3185.1483  3233.2338  3281.0048  3328.4850  3375.6985
##  [28,]  3234.2019  3281.9668  3329.4414  3376.6497  3423.6162  3470.3657
##  [29,]  3330.3976  3377.6008  3424.5627  3471.3080  3517.8618  3564.2492
##  [30,]  3425.5092  3472.2503  3518.8004  3565.1848  3611.4290  3657.5588
##  [31,]  3519.7390  3566.1203  3612.3619  3658.4898  3704.5299  3750.5089
##  [32,]  3613.2948  3659.4206  3705.4593  3751.4373  3797.3811  3843.3178
##  [33,]  3706.3887  3752.3657  3798.3091  3844.2460  3890.2034  3935.9701
##  [34,]  3799.2371  3845.1741  3891.1322  3936.8849  3981.6154  4025.2475
##  [35,]  3892.0610  3937.7994  3982.5078  4026.1176  4068.6171  4109.9962
##  [36,]  3983.3997  4026.9872  4069.4642  4110.8205  4151.0474  4190.1374
##  [37,]  4070.3108  4111.6444  4151.8484  4190.9153  4228.8386  4265.6129
##  [38,]  4152.6489  4191.6928  4229.5929  4266.3440  4301.9415  4336.3818
##  [39,]  4230.3467  4267.0746  4302.6487  4337.0656  4370.3222  4402.4163
##  [40,]  4303.3555  4337.7489  4370.9821  4403.0527  4433.9590  4463.6999
##  [41,]  4371.6415  4403.6886  4434.5713  4464.2887  4492.8398  4520.2244
##  [42,]  4435.1832  4464.8770  4493.4046  4520.7656  4546.9601  4571.9883
##  [43,]  4493.9688  4521.3063  4547.4772  4572.4819  4596.3209  4618.9950
##  [44,]  4547.9939  4572.9750  4596.7905  4619.4410  4640.9276  4661.2512
##  [45,]  4597.2596  4619.8866  4641.3497  4661.6498  4680.7881  4698.7658
##  [46,]  4641.7713  4662.0479  4681.1627  4699.1170  4715.9120  4731.5491
##  [47,]  4684.2071  4700.0879  4716.2394  4731.8530  4746.3100  4759.6115
##  [48,]  4727.7971  4738.4225  4749.3289  4760.5184  4771.9923  4782.9633
##  [49,]  4772.6331  4777.9761  4783.6147  4789.5473  4795.7735  4802.2938
##  [50,]  4818.8179  4818.8369  4819.1740  4819.8240  4820.7824  4822.0462
##  [51,]  4866.4716  4861.1101  4856.0972  4851.4235  4847.0811  4843.0627
##  [52,]  4915.7326  4904.9183  4894.4911  4884.4376  4874.7457  4865.4042
##  [53,]  4966.7583  4950.4026  4934.4808  4918.9751  4903.8707  4889.7831
##  [54,]  5019.7276  4997.7245  4976.2135  4955.8261  4937.0380  4919.8266
##  [55,]  5074.8472  5047.7590  5022.3825  4998.6865  4976.6420  4956.2220
##  [56,]  5134.9324  5104.2814  5075.4123  5048.2891  5022.8784  4999.1487
##  [57,]  5202.4492  5168.0787  5135.5703  5104.8829  5075.9781  5048.8200
##  [58,]  5277.7459  5239.4781  5203.1631  5168.7545  5136.2088  5105.4850
##  [59,]  5361.2161  5318.8502  5278.5395  5240.2317  5203.8777  5169.4311
##  [60,]  5453.3022  5406.6129  5362.0935  5319.6856  5279.3338  5240.9861
##  [61,]  5554.4996  5503.2348  5454.2682  5407.5345  5362.9718  5320.5218
##  [62,]  5665.3611  5609.2397  5555.5594  5504.2475  5455.2351  5408.4569
##  [63,]  5786.5020  5725.2113  5666.5205  5610.3491  5556.6202  5505.2611
##  [64,]  5918.6056  5851.7984  5787.7675  5726.4234  5667.6810  5611.4594
##  [65,]  6062.4296  5989.7212  5919.9845  5853.1202  5789.0342  5727.6367
##  [66,]  6218.6687  6139.7773  6063.9298  5991.1603  5921.3645  5854.4432
##  [67,]  6380.4859  6300.2073  6220.2798  6141.3420  6065.4312  5992.6006
##  [68,]  6543.7650  6462.7552  6382.1114  6301.8255  6221.8911  6142.9081
##  [69,]  6708.5768  6626.7980  6545.4054  6464.3881  6383.7370  6303.4440
##  [70,]  6875.0156  6792.4182  6710.2330  6628.4463  6547.0459  6466.0212
##  [71,]  7043.2000  6959.7221  6876.6886  6794.0828  6711.8893  6630.0947
##  [72,]  7213.2753  7128.8415  7044.8911  6961.4041  6878.3618  6795.7474
##  [73,]  7385.4158  7299.9360  7214.9862  7130.5424  7046.5825  6963.0862
##  [74,]  7559.8273  7473.1955  7387.1483  7301.6576  7216.6973  7132.2434
##  [75,]  7736.7503  7648.8429  7561.5836  7474.9398  7388.8811  7303.3793
##  [76,]  7916.4637  7827.1377  7738.5331  7650.6123  7563.3403  7476.6843
##  [77,]  8099.2895  8008.3798  7918.2760  7828.9350  7740.3162  7652.3819
##  [78,]  8285.5974  8192.9146  8101.1347  8010.2082  7920.0886  7830.7326
##  [79,]  8475.8107  8381.1381  8287.4794  8194.7778  8102.9803  8012.0370
##  [80,]  8670.4139  8573.5036  8477.7341  8383.0404  8289.3618  8196.6415
##  [81,]  8869.9601  8770.5293  8672.3839  8575.4499  8479.6580  8384.9432
##  [82,]  9075.0813  8972.8065  8871.9826  8772.5250  8674.3544  8577.3966
##  [83,]  9286.4986  9181.0110  9077.1630  8974.8580  8874.0057  8774.5212
##  [84,]  9505.0359  9395.9139  9288.6472  9183.1255  9079.2453  8976.9101
##  [85,]  9731.6348  9618.3966  9507.2603  9398.0996  9290.7966  9185.2406
##  [86,]  9966.7898  9849.4667  9733.9450  9620.6630  9509.4855  9400.2861
##  [87,] 10204.4987 10086.7589  9969.1637  9851.8245  9736.2562  9622.9303
##  [88,] 10442.6532 10324.7293 10206.8785 10089.1361  9971.5377  9854.1833
##  [89,] 10680.9614 10562.9980 10445.0360 10327.1110 10209.2584 10091.5135
##  [90,] 10919.1314 10801.2727 10683.3442 10565.3812 10447.4189 10329.4928
##  [91,] 11156.8733 11039.2627 10921.5114 10803.6545 10685.7271 10567.7644
##  [92,] 11393.8996 11276.6789 11159.2476 11041.6402 10923.8914 10806.0363
##  [93,] 11629.9264 11513.2356 11396.2652 11279.0492 11161.6218 11044.0176
##  [94,] 11864.6752 11748.6515 11632.2806 11515.5959 11398.6308 11281.4194
##  [95,] 12097.8743 11982.6512 11867.0153 11750.9990 11634.6348 11517.9560
##  [96,] 12329.2605 12214.9670 12100.1976 11984.9831 11869.3553 11753.3463
##  [97,] 12558.5817 12445.3411 12331.5644 12217.2808 12102.5207 11987.3149
##  [98,] 12785.6000 12673.5281 12560.8637 12447.6342 12333.8680 12219.5944
##  [99,] 13010.0954 12899.2984 12787.8578 12675.7981 12563.1454 12449.9271
## [100,] 13231.8711 13122.4433 13012.3269 12901.5432 12790.1153 12678.0679
##             [,97]      [,98]      [,99]     [,100]
##   [1,]   720.0924   759.2848   799.3203   840.2038
##   [2,]   800.1379   841.0385   882.7940   925.4134
##   [3,]   883.6465   926.2833   969.7947  1014.1924
##   [4,]   970.6828  1015.0985  1060.4138  1106.6428
##   [5,]  1061.3387  1107.5862  1154.7624  1202.8826
##   [6,]  1155.7251  1203.8646  1252.9644  1303.0406
##   [7,]  1253.9663  1304.0625  1355.1521  1407.2517
##   [8,]  1356.1945  1408.3148  1461.4623  1515.6537
##   [9,]  1462.5467  1516.7594  1572.0331  1628.3845
##  [10,]  1573.1608  1629.5341  1687.0017  1745.5796
##  [11,]  1688.1740  1746.7746  1806.5016  1867.3363
##  [12,]  1807.7200  1868.5707  1929.3922  1989.6542
##  [13,]  1930.6153  1990.8657  2050.5366  2109.6136
##  [14,]  2051.7360  2110.8008  2169.2616  2227.1124
##  [15,]  2170.4364  2228.2748  2285.5009  2342.1155
##  [16,]  2286.6507  2343.2530  2399.2475  2454.6403
##  [17,]  2400.3724  2455.7532  2510.5408  2564.7456
##  [18,]  2511.6416  2565.8347  2619.4574  2672.5232
##  [19,]  2620.5349  2673.5896  2726.1029  2778.0908
##  [20,]  2727.1583  2779.1358  2830.6057  2881.5864
##  [21,]  2831.6404  2882.6114  2933.1128  2983.1645
##  [22,]  2934.1283  2984.1712  3033.7854  3082.9925
##  [23,]  3034.7835  3083.9825  3132.7966  3181.2482
##  [24,]  3133.7789  3182.2234  3230.3287  3278.1181
##  [25,]  3231.2972  3279.0804  3326.5719  3373.7957
##  [26,]  3327.5285  3374.7472  3421.7230  3468.4807
##  [27,]  3422.6697  3469.4232  3515.9842  3562.3779
##  [28,]  3516.9230  3563.3136  3609.5629  3655.6969
##  [29,]  3610.4960  3656.6279  3702.6711  3748.6520
##  [30,]  3703.6005  3749.5804  3795.5251  3841.4616
##  [31,]  3796.4531  3842.3897  3888.3457  3934.1390
##  [32,]  3889.2745  3935.0548  3979.8293  4023.5060
##  [33,]  3980.7226  4024.3770  4066.9216  4108.3461
##  [34,]  4067.7696  4109.1714  4149.4441  4188.5801
##  [35,]  4150.2460  4189.3590  4227.3286  4264.1495
##  [36,]  4228.0838  4264.8814  4300.5256  4335.0127
##  [37,]  4301.2338  4335.6975  4369.0010  4401.1421
##  [38,]  4369.6618  4401.7794  4432.7328  4462.5208
##  [39,]  4433.3461  4463.1106  4491.7088  4519.1405
##  [40,]  4492.2745  4519.6827  4545.9243  4570.9997
##  [41,]  4546.4424  4571.4942  4595.3803  4618.1014
##  [42,]  4595.8509  4618.5484  4640.0820  4660.4525
##  [43,]  4640.5050  4660.8521  4680.0373  4698.0619
##  [44,]  4680.4129  4698.4141  4715.2559  4730.9397
##  [45,]  4715.5842  4731.2446  4745.7483  4759.0964
##  [46,]  4746.0294  4759.3542  4771.5248  4782.5424
##  [47,]  4771.7588  4782.7531  4792.5954  4801.2868
##  [48,]  4792.7824  4801.4506  4808.9687  4815.3377
##  [49,]  4809.1088  4815.4546  4820.6519  4824.7014
##  [50,]  4823.6128  4825.4810  4827.6503  4829.3821
##  [51,]  4839.3621  4835.9744  4832.8952  4830.7064
##  [52,]  4856.4036  4848.3427  4841.7525  4836.6251
##  [53,]  4877.2155  4866.1527  4856.5816  4848.4910
##  [54,]  4904.1711  4890.0526  4877.4544  4866.3614
##  [55,]  4937.4019  4920.1589  4904.4721  4890.3228
##  [56,]  4977.0711  4956.6187  4937.7665  4920.4917
##  [57,]  5023.3750  4999.6117  4977.5010  4957.0159
##  [58,]  5076.5446  5049.3516  5023.8723  5000.0753
##  [59,]  5136.8482  5106.0879  5077.1119  5049.8839
##  [60,]  5204.5931  5170.1084  5137.4882  5106.6916
##  [61,]  5280.1290  5241.7413  5205.3092  5170.7864
##  [62,]  5363.8510  5321.3588  5280.9250  5242.4973
##  [63,]  5456.2029  5409.3802  5364.7310  5322.1967
##  [64,]  5557.6819  5506.2757  5457.1717  5410.3044
##  [65,]  5668.8425  5612.5708  5558.7446  5507.2912
##  [66,]  5790.3020  5728.8510  5670.0050  5613.6831
##  [67,]  5922.7458  5855.7674  5791.5708  5730.0664
##  [68,]  6066.9339  5994.0421  5924.1282  5857.0926
##  [69,]  6223.5025  6144.4754  6068.4379  5995.4849
##  [70,]  6385.3627  6305.0626  6225.1140  6146.0441
##  [71,]  6548.6866  6467.6544  6386.9886  6306.6813
##  [72,]  6713.5458  6631.7432  6550.3275  6469.2877
##  [73,]  6880.0351  6797.4123  6715.2025  6633.3919
##  [74,]  7048.2740  6964.7685  6881.7086  6799.0773
##  [75,]  7218.4086  7133.9447  7049.9657  6966.4510
##  [76,]  7390.6140  7305.1013  7220.1200  7135.6462
##  [77,]  7565.0971  7478.4290  7392.3472  7306.8235
##  [78,]  7742.0996  7654.1519  7566.8543  7480.1740
##  [79,]  7921.9015  7832.5304  7743.8832  7655.9220
##  [80,]  8104.8261  8013.8661  7923.7147  7834.3286
##  [81,]  8291.2447  8198.5055  8106.6724  8015.6955
##  [82,]  8481.5823  8386.8464  8293.1279  8200.3699
##  [83,]  8676.3254  8579.3438  8483.5071  8388.7500
##  [84,]  8876.0293  8776.5180  8678.2969  8581.2915
##  [85,]  9081.3283  8978.9628  8878.0535  8778.5153
##  [86,]  9292.9467  9187.3565  9083.4119  8981.0161
##  [87,]  9511.7115  9402.4734  9295.0975  9189.4730
##  [88,]  9738.5682  9625.1984  9513.9383  9404.6614
##  [89,]  9973.9118  9856.5431  9740.8812  9627.4674
##  [90,] 10211.6383 10093.8909  9976.2859  9858.9038
##  [91,] 10449.8017 10331.8745 10214.0182 10096.2683
##  [92,] 10688.1099 10570.1476 10452.1846 10334.2563
##  [93,] 10926.2714 10808.4180 10690.4928 10572.5308
##  [94,] 11163.9960 11046.3950 10928.6512 10810.7997
##  [95,] 11400.9963 11283.7895 11166.3700 11048.7723
##  [96,] 11636.9888 11520.3161 11403.3617 11286.1596
##  [97,] 11871.6951 11755.6935 11639.3427 11522.6760
##  [98,] 12104.8436 11989.6465 11874.0347 11758.0405
##  [99,] 12336.1714 12221.9078 12107.1663 11991.9779
## [100,] 12565.4269 12452.2197 12338.4746 12224.2210
## 
## $alphas
##   [1] 0.00000000 0.05050505 0.10101010 0.15151515 0.20202020 0.25252525
##   [7] 0.30303030 0.35353535 0.40404040 0.45454545 0.50505051 0.55555556
##  [13] 0.60606061 0.65656566 0.70707071 0.75757576 0.80808081 0.85858586
##  [19] 0.90909091 0.95959596 1.01010101 1.06060606 1.11111111 1.16161616
##  [25] 1.21212121 1.26262626 1.31313131 1.36363636 1.41414141 1.46464646
##  [31] 1.51515152 1.56565657 1.61616162 1.66666667 1.71717172 1.76767677
##  [37] 1.81818182 1.86868687 1.91919192 1.96969697 2.02020202 2.07070707
##  [43] 2.12121212 2.17171717 2.22222222 2.27272727 2.32323232 2.37373737
##  [49] 2.42424242 2.47474747 2.52525253 2.57575758 2.62626263 2.67676768
##  [55] 2.72727273 2.77777778 2.82828283 2.87878788 2.92929293 2.97979798
##  [61] 3.03030303 3.08080808 3.13131313 3.18181818 3.23232323 3.28282828
##  [67] 3.33333333 3.38383838 3.43434343 3.48484848 3.53535354 3.58585859
##  [73] 3.63636364 3.68686869 3.73737374 3.78787879 3.83838384 3.88888889
##  [79] 3.93939394 3.98989899 4.04040404 4.09090909 4.14141414 4.19191919
##  [85] 4.24242424 4.29292929 4.34343434 4.39393939 4.44444444 4.49494949
##  [91] 4.54545455 4.59595960 4.64646465 4.69696970 4.74747475 4.79797980
##  [97] 4.84848485 4.89898990 4.94949495 5.00000000
## 
## $gammas
##   [1] 0.025 0.050 0.075 0.100 0.125 0.150 0.175 0.200 0.225 0.250 0.275 0.300
##  [13] 0.325 0.350 0.375 0.400 0.425 0.450 0.475 0.500 0.525 0.550 0.575 0.600
##  [25] 0.625 0.650 0.675 0.700 0.725 0.750 0.775 0.800 0.825 0.850 0.875 0.900
##  [37] 0.925 0.950 0.975 1.000 1.025 1.050 1.075 1.100 1.125 1.150 1.175 1.200
##  [49] 1.225 1.250 1.275 1.300 1.325 1.350 1.375 1.400 1.425 1.450 1.475 1.500
##  [61] 1.525 1.550 1.575 1.600 1.625 1.650 1.675 1.700 1.725 1.750 1.775 1.800
##  [73] 1.825 1.850 1.875 1.900 1.925 1.950 1.975 2.000 2.025 2.050 2.075 2.100
##  [85] 2.125 2.150 2.175 2.200 2.225 2.250 2.275 2.300 2.325 2.350 2.375 2.400
##  [97] 2.425 2.450 2.475 2.500
## 
## $alpha
## [1] 1.060606
## 
## $gamma
## [1] 0.675
## 
## $model
## 
## Call:
## stats::lm(formula = y ~ x + q)
## 
## Coefficients:
## (Intercept)            x            q  
##       30.08       131.98       -85.21  
## 
## 
## attr(,"class")
## [1] "bent_cable"
## 
## $SimpleExponential
## 
## Call:
## stats::lm(formula = y2 ~ x)
## 
## Coefficients:
## (Intercept)            x  
##      5.9617      -0.8095  
## 
## 
## $NonLinearLeastSquares
## Nonlinear regression model
##   model: y2 ~ I(exp(1)^(a + b * x))
##    data: data.frame(x = x, y2 = y2)
##       a       b 
##  1.7788 -0.1725 
##  residual sum-of-squares: 46
## 
## Number of iterations to convergence: 9 
## Achieved convergence tolerance: 1.727e-06
```

Get likelihoods for m 0-5

```
setwd("/gpfs/gibbs/pi/caccone/mkc54/albo/manuscript1/output/treemix_subsets/south_america/prelim")

# Get all llik files
files <- list.files(pattern = "sasub_simp\\.m[0-9]+\\.rep1\\.llik$")

# Extract m and likelihood
df <- data.frame(m = numeric(), lnL = numeric())

for(f in files){
  # extract m number from filename
  m <- as.numeric(sub("sasub_simp\\.m([0-9]+)\\.rep1\\.llik", "\\1", f))
  
  # read file
  lines <- readLines(f)
  
  # look for line containing "Exiting ln(likelihood)"
  lnL_line <- grep("Exiting ln\\(likelihood\\)", lines, value = TRUE)
  
  # extract numeric value after colon
  lnL <- as.numeric(sub(".*: *", "", lnL_line))
  
  df <- rbind(df, data.frame(m = m, lnL = lnL))
}

# Sort by m
df <- df[order(df$m), ]

# Compute delta lnL
df$delta_lnL <- c(NA, diff(df$lnL))

# Print table
print(df)
```

```
##   m      lnL delta_lnL
## 1 0  30.0773        NA
## 2 1 150.1400  120.0627
## 3 2 216.2100   66.0700
## 4 3 258.8430   42.6330
## 5 4 304.8300   45.9870
## 6 5 356.7930   51.9630
```

Plot

```
setwd("/gpfs/gibbs/pi/caccone/mkc54/albo/manuscript1/output/treemix_subsets/south_america/prelim")

plot(df$m, df$lnL, type="b", pch=19, xlab="Migration edges (m)", ylab="ln(likelihood)")
abline(v = df$m[which.max(df$delta_lnL)], col="red", lty=2) # highlight largest gain
```

It seems like m=1 is the best number of migration events

Check files for m=1

```
cd /gpfs/gibbs/pi/caccone/mkc54/albo/manuscript1/output/treemix_subsets/south_america/prelim
zcat sasub_simp.m1.rep1.treeout.gz
```

```
setwd("/gpfs/gibbs/pi/caccone/mkc54/albo/manuscript1/output/treemix_subsets/south_america/prelim")
plot_tree("sasub_simp.m1.rep2")
```

```
## Warning in plot_tree("sasub_simp.m1.rep2"): NAs introduced by coercion
## Warning in plot_tree("sasub_simp.m1.rep2"): NAs introduced by coercion
## Warning in plot_tree("sasub_simp.m1.rep2"): NAs introduced by coercion
## Warning in plot_tree("sasub_simp.m1.rep2"): NAs introduced by coercion
```

```
##     V1                 V2       V3      V4      V5  V6  V7 V8  V9 V10
## 1    1        Southern_SA NOT_ROOT NOT_MIG     TIP  16  NA NA  NA  NA
## 2    2               <NA> NOT_ROOT NOT_MIG NOT_TIP  16   3  1 103   1
## 3    3     Central_Brazil NOT_ROOT NOT_MIG     TIP   2  NA NA  NA  NA
## 4    4     Okinawa_Taiwan NOT_ROOT NOT_MIG     TIP  32  NA NA  NA  NA
## 5   15     Western_Brazil NOT_ROOT NOT_MIG     TIP 104  NA NA  NA  NA
## 6   16               <NA> NOT_ROOT NOT_MIG NOT_TIP 104   1  1   2   2
## 7   31              Japan NOT_ROOT NOT_MIG     TIP  32  NA NA  NA  NA
## 8   32               <NA> NOT_ROOT NOT_MIG NOT_TIP 172   4  1  31   1
## 9   51 Sulawesi_Indonesia NOT_ROOT NOT_MIG     TIP 173  NA NA  NA  NA
## 10  52               <NA> NOT_ROOT NOT_MIG NOT_TIP  76 104  4  75   1
## 11  75  Medellin_Colombia NOT_ROOT NOT_MIG     TIP  52  NA NA  NA  NA
## 12  76               <NA> NOT_ROOT NOT_MIG NOT_TIP 136  52  5 172   3
## 13 103     Eastern_Brazil NOT_ROOT NOT_MIG     TIP   2  NA NA  NA  NA
## 14 104               <NA> NOT_ROOT NOT_MIG NOT_TIP  52  15  1  16   3
## 15 135     Southeast_Asia NOT_ROOT NOT_MIG     TIP 136  NA NA  NA  NA
## 16 136               <NA> NOT_ROOT NOT_MIG NOT_TIP 173 135  1  76   8
## 17 171      Eastern_China NOT_ROOT NOT_MIG     TIP 172  NA NA  NA  NA
## 18 172               <NA> NOT_ROOT NOT_MIG NOT_TIP  76  32  2 171   1
## 19 173               <NA>     ROOT NOT_MIG NOT_TIP 173  51  1 136   9
## 20 175               <NA> NOT_ROOT     MIG NOT_TIP  32  31 NA  NA  NA
##                                                                                                                                                                                                                                                                                                                                                                V11
## 1                                                                                                                                                                                                                                                                                                                                           Southern_SA:0.00749437
## 2                                                                                                                                                                                                                                                                                                  (Central_Brazil:0.000957277,Eastern_Brazil:0.0210197):0.0028484
## 3                                                                                                                                                                                                                                                                                                                                       Central_Brazil:0.000957277
## 4                                                                                                                                                                                                                                                                                                                                         Okinawa_Taiwan:0.0140653
## 5                                                                                                                                                                                                                                                                                                                                        Western_Brazil:0.00383323
## 6                                                                                                                                                                                                                                                              (Southern_SA:0.00749437,(Central_Brazil:0.000957277,Eastern_Brazil:0.0210197):0.0028484):0.00178366
## 7                                                                                                                                                                                                                                                                                                                                                  Japan:0.0157704
## 8                                                                                                                                                                                                                                                                                                            (Okinawa_Taiwan:0.0140653,Japan:0.0157704):0.00313176
## 9                                                                                                                                                                                                                                                                                                                                     Sulawesi_Indonesia:0.0184639
## 10                                                                                                                                                                                ((Western_Brazil:0.00383323,(Southern_SA:0.00749437,(Central_Brazil:0.000957277,Eastern_Brazil:0.0210197):0.0028484):0.00178366):0.013085,Medellin_Colombia:0.019243):0.00110937
## 11                                                                                                                                                                                                                                                                                                                                      Medellin_Colombia:0.019243
## 12                                                                       (((Western_Brazil:0.00383323,(Southern_SA:0.00749437,(Central_Brazil:0.000957277,Eastern_Brazil:0.0210197):0.0028484):0.00178366):0.013085,Medellin_Colombia:0.019243):0.00110937,((Okinawa_Taiwan:0.0140653,Japan:0.0157704):0.00313176,Eastern_China:0.00219863):0.00605171):0.00417606
## 13                                                                                                                                                                                                                                                                                                                                        Eastern_Brazil:0.0210197
## 14                                                                                                                                                                                                                        (Western_Brazil:0.00383323,(Southern_SA:0.00749437,(Central_Brazil:0.000957277,Eastern_Brazil:0.0210197):0.0028484):0.00178366):0.013085
## 15                                                                                                                                                                                                                                                                                                                                       Southeast_Asia:0.00504721
## 16                                 (Southeast_Asia:0.00504721,(((Western_Brazil:0.00383323,(Southern_SA:0.00749437,(Central_Brazil:0.000957277,Eastern_Brazil:0.0210197):0.0028484):0.00178366):0.013085,Medellin_Colombia:0.019243):0.00110937,((Okinawa_Taiwan:0.0140653,Japan:0.0157704):0.00313176,Eastern_China:0.00219863):0.00605171):0.00417606):0.0184639
## 17                                                                                                                                                                                                                                                                                                                                        Eastern_China:0.00219863
## 18                                                                                                                                                                                                                                                                     ((Okinawa_Taiwan:0.0140653,Japan:0.0157704):0.00313176,Eastern_China:0.00219863):0.00605171
## 19 (Sulawesi_Indonesia:0.0184639,(Southeast_Asia:0.00504721,(((Western_Brazil:0.00383323,(Southern_SA:0.00749437,(Central_Brazil:0.000957277,Eastern_Brazil:0.0210197):0.0028484):0.00178366):0.013085,Medellin_Colombia:0.019243):0.00110937,((Okinawa_Taiwan:0.0140653,Japan:0.0157704):0.00313176,Eastern_China:0.00219863):0.00605171):0.00417606):0.0184639);
## 20                                                                                                                                                                                                                                                                                                                                                            <NA>
##             x    y ymin ymax
## 1  0.04611236 0.65  0.6  0.7
## 2  0.04146639 0.50  0.4  0.6
## 3  0.04242367 0.55  0.5  0.6
## 4  0.04588873 0.25  0.2  0.3
## 5  0.04066756 0.75  0.7  0.8
## 6  0.03861799 0.60  0.4  0.7
## 7  0.04759385 0.15  0.1  0.2
## 8  0.03182343 0.20  0.1  0.3
## 9  0.01846390 0.95  0.9  1.0
## 10 0.02374933 0.40  0.3  0.8
## 11 0.04299233 0.35  0.3  0.4
## 12 0.02263996 0.30  0.0  0.8
## 13 0.05204407 0.45  0.4  0.5
## 14 0.03683433 0.70  0.4  0.8
## 15 0.02351111 0.85  0.8  0.9
## 16 0.01846390 0.80  0.0  0.9
## 17 0.03089030 0.05  0.0  0.1
## 18 0.02869167 0.10  0.0  0.3
## 19 0.00000000 0.90  0.0  1.0
## 20 0.04624673   NA  0.1  0.2
## [1] "0.91458 0.03182343 0.04759385"
```

```
##  [1] 0.04611236 0.04242367 0.04588873 0.04066756 0.04759385 0.01846390
##  [7] 0.04299233 0.05204407 0.02351111 0.03089030
## [1] 0.003
## [1] "mse 0.0003551471"
```

```
## $d
##     V1                 V2       V3      V4      V5  V6  V7 V8  V9 V10
## 1    1        Southern_SA NOT_ROOT NOT_MIG     TIP  16  NA NA  NA  NA
## 2    2               <NA> NOT_ROOT NOT_MIG NOT_TIP  16   3  1 103   1
## 3    3     Central_Brazil NOT_ROOT NOT_MIG     TIP   2  NA NA  NA  NA
## 4    4     Okinawa_Taiwan NOT_ROOT NOT_MIG     TIP  32  NA NA  NA  NA
## 5   15     Western_Brazil NOT_ROOT NOT_MIG     TIP 104  NA NA  NA  NA
## 6   16               <NA> NOT_ROOT NOT_MIG NOT_TIP 104   1  1   2   2
## 7   31              Japan NOT_ROOT NOT_MIG     TIP  32  NA NA  NA  NA
## 8   32               <NA> NOT_ROOT NOT_MIG NOT_TIP 172   4  1  31   1
## 9   51 Sulawesi_Indonesia NOT_ROOT NOT_MIG     TIP 173  NA NA  NA  NA
## 10  52               <NA> NOT_ROOT NOT_MIG NOT_TIP  76 104  4  75   1
## 11  75  Medellin_Colombia NOT_ROOT NOT_MIG     TIP  52  NA NA  NA  NA
## 12  76               <NA> NOT_ROOT NOT_MIG NOT_TIP 136  52  5 172   3
## 13 103     Eastern_Brazil NOT_ROOT NOT_MIG     TIP   2  NA NA  NA  NA
## 14 104               <NA> NOT_ROOT NOT_MIG NOT_TIP  52  15  1  16   3
## 15 135     Southeast_Asia NOT_ROOT NOT_MIG     TIP 136  NA NA  NA  NA
## 16 136               <NA> NOT_ROOT NOT_MIG NOT_TIP 173 135  1  76   8
## 17 171      Eastern_China NOT_ROOT NOT_MIG     TIP 172  NA NA  NA  NA
## 18 172               <NA> NOT_ROOT NOT_MIG NOT_TIP  76  32  2 171   1
## 19 173               <NA>     ROOT NOT_MIG NOT_TIP 173  51  1 136   9
## 20 175               <NA> NOT_ROOT     MIG NOT_TIP  32  31 NA  NA  NA
##                                                                                                                                                                                                                                                                                                                                                                V11
## 1                                                                                                                                                                                                                                                                                                                                           Southern_SA:0.00749437
## 2                                                                                                                                                                                                                                                                                                  (Central_Brazil:0.000957277,Eastern_Brazil:0.0210197):0.0028484
## 3                                                                                                                                                                                                                                                                                                                                       Central_Brazil:0.000957277
## 4                                                                                                                                                                                                                                                                                                                                         Okinawa_Taiwan:0.0140653
## 5                                                                                                                                                                                                                                                                                                                                        Western_Brazil:0.00383323
## 6                                                                                                                                                                                                                                                              (Southern_SA:0.00749437,(Central_Brazil:0.000957277,Eastern_Brazil:0.0210197):0.0028484):0.00178366
## 7                                                                                                                                                                                                                                                                                                                                                  Japan:0.0157704
## 8                                                                                                                                                                                                                                                                                                            (Okinawa_Taiwan:0.0140653,Japan:0.0157704):0.00313176
## 9                                                                                                                                                                                                                                                                                                                                     Sulawesi_Indonesia:0.0184639
## 10                                                                                                                                                                                ((Western_Brazil:0.00383323,(Southern_SA:0.00749437,(Central_Brazil:0.000957277,Eastern_Brazil:0.0210197):0.0028484):0.00178366):0.013085,Medellin_Colombia:0.019243):0.00110937
## 11                                                                                                                                                                                                                                                                                                                                      Medellin_Colombia:0.019243
## 12                                                                       (((Western_Brazil:0.00383323,(Southern_SA:0.00749437,(Central_Brazil:0.000957277,Eastern_Brazil:0.0210197):0.0028484):0.00178366):0.013085,Medellin_Colombia:0.019243):0.00110937,((Okinawa_Taiwan:0.0140653,Japan:0.0157704):0.00313176,Eastern_China:0.00219863):0.00605171):0.00417606
## 13                                                                                                                                                                                                                                                                                                                                        Eastern_Brazil:0.0210197
## 14                                                                                                                                                                                                                        (Western_Brazil:0.00383323,(Southern_SA:0.00749437,(Central_Brazil:0.000957277,Eastern_Brazil:0.0210197):0.0028484):0.00178366):0.013085
## 15                                                                                                                                                                                                                                                                                                                                       Southeast_Asia:0.00504721
## 16                                 (Southeast_Asia:0.00504721,(((Western_Brazil:0.00383323,(Southern_SA:0.00749437,(Central_Brazil:0.000957277,Eastern_Brazil:0.0210197):0.0028484):0.00178366):0.013085,Medellin_Colombia:0.019243):0.00110937,((Okinawa_Taiwan:0.0140653,Japan:0.0157704):0.00313176,Eastern_China:0.00219863):0.00605171):0.00417606):0.0184639
## 17                                                                                                                                                                                                                                                                                                                                        Eastern_China:0.00219863
## 18                                                                                                                                                                                                                                                                     ((Okinawa_Taiwan:0.0140653,Japan:0.0157704):0.00313176,Eastern_China:0.00219863):0.00605171
## 19 (Sulawesi_Indonesia:0.0184639,(Southeast_Asia:0.00504721,(((Western_Brazil:0.00383323,(Southern_SA:0.00749437,(Central_Brazil:0.000957277,Eastern_Brazil:0.0210197):0.0028484):0.00178366):0.013085,Medellin_Colombia:0.019243):0.00110937,((Okinawa_Taiwan:0.0140653,Japan:0.0157704):0.00313176,Eastern_China:0.00219863):0.00605171):0.00417606):0.0184639);
## 20                                                                                                                                                                                                                                                                                                                                                            <NA>
##             x        y ymin ymax
## 1  0.04611236 0.650000  0.6  0.7
## 2  0.04146639 0.500000  0.4  0.6
## 3  0.04242367 0.550000  0.5  0.6
## 4  0.04588873 0.250000  0.2  0.3
## 5  0.04066756 0.750000  0.7  0.8
## 6  0.03861799 0.600000  0.4  0.7
## 7  0.04759385 0.150000  0.1  0.2
## 8  0.03182343 0.200000  0.1  0.3
## 9  0.01846390 0.950000  0.9  1.0
## 10 0.02374933 0.400000  0.3  0.8
## 11 0.04299233 0.350000  0.3  0.4
## 12 0.02263996 0.300000  0.0  0.8
## 13 0.05204407 0.450000  0.4  0.5
## 14 0.03683433 0.700000  0.4  0.8
## 15 0.02351111 0.850000  0.8  0.9
## 16 0.01846390 0.800000  0.0  0.9
## 17 0.03089030 0.050000  0.0  0.1
## 18 0.02869167 0.100000  0.0  0.3
## 19 0.00000000 0.900000  0.0  1.0
## 20 0.04624674 0.154271  0.1  0.2
## 
## $e
##     V1  V2          V3       V4      V5      V6       V7
## 1    2   3 0.000957277 1.000000 NOT_MIG 0.00000 1.000000
## 2   32   4 0.014065300 1.000000 NOT_MIG 0.00000 1.000000
## 3   52 104 0.013085000 1.000000 NOT_MIG 0.00000 1.000000
## 4  136 135 0.005047210 1.000000 NOT_MIG 0.00000 1.000000
## 5   76  52 0.001109370 1.000000 NOT_MIG 0.00000 1.000000
## 6   52  75 0.019243000 1.000000 NOT_MIG 0.00000 1.000000
## 7  172  32 0.003131760 1.000000 NOT_MIG 0.00000 1.000000
## 8  172 171 0.002198630 1.000000 NOT_MIG 0.00000 1.000000
## 9  136  76 0.004176060 1.000000 NOT_MIG 0.00000 1.000000
## 10 173  51 0.018463900 1.000000 NOT_MIG 0.00000 1.000000
## 11 173 136 0.018463900 1.000000 NOT_MIG 0.00000 1.000000
## 12  76 172 0.006051710 1.000000 NOT_MIG 0.00000 1.000000
## 13  32 175 0.014423300 1.000000 NOT_MIG 0.00000 1.000000
## 14 175  31 0.001347120 1.000000 NOT_MIG 0.91458 1.000000
## 15 175 103 0.000000000 0.290615     MIG 0.91458 0.000000
## 16 104  15 0.003833230 1.000000 NOT_MIG 0.00000 1.000000
## 17 104  16 0.001783660 1.000000 NOT_MIG 0.00000 1.000000
## 18  16   1 0.007494370 1.000000 NOT_MIG 0.00000 1.000000
## 19  16   2 0.002848400 1.000000 NOT_MIG 0.00000 1.000000
## 20   2 103 0.010577682 0.709385 NOT_MIG 0.00000 0.503227
```

Save it

```
setwd("/gpfs/gibbs/pi/caccone/mkc54/albo/manuscript1/output/treemix_subsets/south_america/prelim")

# Open PDF
pdf("treemix_prelim_m1.pdf", width = 12, height = 8)

plot_tree("sasub_simp.m1.rep2")

dev.off()
```

Check for m=0

```
setwd("/gpfs/gibbs/pi/caccone/mkc54/albo/manuscript1/output/treemix_subsets/south_america/prelim")
plot_tree("sasub_simp.m0.rep2")
```

```
## Warning in plot_tree("sasub_simp.m0.rep2"): NAs introduced by coercion
## Warning in plot_tree("sasub_simp.m0.rep2"): NAs introduced by coercion
## Warning in plot_tree("sasub_simp.m0.rep2"): NAs introduced by coercion
## Warning in plot_tree("sasub_simp.m0.rep2"): NAs introduced by coercion
```

```
##     V1                 V2       V3      V4      V5  V6  V7 V8  V9 V10
## 1    0               <NA> NOT_ROOT NOT_MIG NOT_TIP 104  76  3 172   5
## 2    1     Eastern_Brazil NOT_ROOT NOT_MIG     TIP  16  NA NA  NA  NA
## 3    2               <NA> NOT_ROOT NOT_MIG NOT_TIP  16  32  2   3   1
## 4    3     Western_Brazil NOT_ROOT NOT_MIG     TIP   2  NA NA  NA  NA
## 5    4        Southern_SA NOT_ROOT NOT_MIG     TIP  32  NA NA  NA  NA
## 6   15 Sulawesi_Indonesia NOT_ROOT NOT_MIG     TIP 173  NA NA  NA  NA
## 7   16               <NA> NOT_ROOT NOT_MIG NOT_TIP 172   1  1   2   3
## 8   31     Central_Brazil NOT_ROOT NOT_MIG     TIP  32  NA NA  NA  NA
## 9   32               <NA> NOT_ROOT NOT_MIG NOT_TIP   2  31  1   4   1
## 10  51      Eastern_China NOT_ROOT NOT_MIG     TIP  76  NA NA  NA  NA
## 11  75     Okinawa_Taiwan NOT_ROOT NOT_MIG     TIP 136  NA NA  NA  NA
## 12  76               <NA> NOT_ROOT NOT_MIG NOT_TIP   0  51  1 136   2
## 13 103     Southeast_Asia NOT_ROOT NOT_MIG     TIP 104  NA NA  NA  NA
## 14 104               <NA> NOT_ROOT NOT_MIG NOT_TIP 173 103  1   0   8
## 15 135              Japan NOT_ROOT NOT_MIG     TIP 136  NA NA  NA  NA
## 16 136               <NA> NOT_ROOT NOT_MIG NOT_TIP  76  75  1 135   1
## 17 171  Medellin_Colombia NOT_ROOT NOT_MIG     TIP 172  NA NA  NA  NA
## 18 172               <NA> NOT_ROOT NOT_MIG NOT_TIP   0 171  1  16   4
## 19 173               <NA>     ROOT NOT_MIG NOT_TIP 173  15  1 104   9
##                                                                                                                                                                                                                                                                                                                                                                     V11
## 1                                                                        ((Eastern_China:0.00263532,(Okinawa_Taiwan:0.0147928,Japan:0.0154501):0.00244058):0.00543424,(Medellin_Colombia:0.0198913,(Eastern_Brazil:0.00862157,((Central_Brazil:0.00281659,Southern_SA:0.00701044):0.00274911,Western_Brazil:0.00438972):0.00406141):0.00913182):0.000615636):0.00493291
## 2                                                                                                                                                                                                                                                                                                                                             Eastern_Brazil:0.00862157
## 3                                                                                                                                                                                                                                                                  ((Central_Brazil:0.00281659,Southern_SA:0.00701044):0.00274911,Western_Brazil:0.00438972):0.00406141
## 4                                                                                                                                                                                                                                                                                                                                             Western_Brazil:0.00438972
## 5                                                                                                                                                                                                                                                                                                                                                Southern_SA:0.00701044
## 6                                                                                                                                                                                                                                                                                                                                          Sulawesi_Indonesia:0.0184639
## 7                                                                                                                                                                                                                           (Eastern_Brazil:0.00862157,((Central_Brazil:0.00281659,Southern_SA:0.00701044):0.00274911,Western_Brazil:0.00438972):0.00406141):0.00913182
## 8                                                                                                                                                                                                                                                                                                                                             Central_Brazil:0.00281659
## 9                                                                                                                                                                                                                                                                                                         (Central_Brazil:0.00281659,Southern_SA:0.00701044):0.00274911
## 10                                                                                                                                                                                                                                                                                                                                             Eastern_China:0.00263532
## 11                                                                                                                                                                                                                                                                                                                                             Okinawa_Taiwan:0.0147928
## 12                                                                                                                                                                                                                                                                          (Eastern_China:0.00263532,(Okinawa_Taiwan:0.0147928,Japan:0.0154501):0.00244058):0.00543424
## 13                                                                                                                                                                                                                                                                                                                                            Southeast_Asia:0.00504721
## 14                                 (Southeast_Asia:0.00504721,((Eastern_China:0.00263532,(Okinawa_Taiwan:0.0147928,Japan:0.0154501):0.00244058):0.00543424,(Medellin_Colombia:0.0198913,(Eastern_Brazil:0.00862157,((Central_Brazil:0.00281659,Southern_SA:0.00701044):0.00274911,Western_Brazil:0.00438972):0.00406141):0.00913182):0.000615636):0.00493291):0.0184639
## 15                                                                                                                                                                                                                                                                                                                                                      Japan:0.0154501
## 16                                                                                                                                                                                                                                                                                                                (Okinawa_Taiwan:0.0147928,Japan:0.0154501):0.00244058
## 17                                                                                                                                                                                                                                                                                                                                          Medellin_Colombia:0.0198913
## 18                                                                                                                                                                                (Medellin_Colombia:0.0198913,(Eastern_Brazil:0.00862157,((Central_Brazil:0.00281659,Southern_SA:0.00701044):0.00274911,Western_Brazil:0.00438972):0.00406141):0.00913182):0.000615636
## 19 (Sulawesi_Indonesia:0.0184639,(Southeast_Asia:0.00504721,((Eastern_China:0.00263532,(Okinawa_Taiwan:0.0147928,Japan:0.0154501):0.00244058):0.00543424,(Medellin_Colombia:0.0198913,(Eastern_Brazil:0.00862157,((Central_Brazil:0.00281659,Southern_SA:0.00701044):0.00274911,Western_Brazil:0.00438972):0.00406141):0.00913182):0.000615636):0.00493291):0.0184639);
##             x    y ymin ymax
## 1  0.02339681 0.50  0.0  0.8
## 2  0.04176584 0.35  0.3  0.4
## 3  0.03720568 0.10  0.0  0.3
## 4  0.04159540 0.05  0.0  0.1
## 5  0.04696523 0.15  0.1  0.2
## 6  0.01846390 0.95  0.9  1.0
## 7  0.03314427 0.30  0.0  0.4
## 8  0.04277138 0.25  0.2  0.3
## 9  0.03995479 0.20  0.1  0.3
## 10 0.03146637 0.75  0.7  0.8
## 11 0.04606443 0.65  0.6  0.7
## 12 0.02883105 0.70  0.5  0.8
## 13 0.02351111 0.85  0.8  0.9
## 14 0.01846390 0.80  0.0  0.9
## 15 0.04672173 0.55  0.5  0.6
## 16 0.03127163 0.60  0.5  0.7
## 17 0.04390375 0.45  0.4  0.5
## 18 0.02401245 0.40  0.0  0.5
## 19 0.00000000 0.90  0.0  1.0
```

```
## Warning in max(e[e[, 5] == "MIG", 4]): no non-missing arguments to max;
## returning -Inf
```

```
##  [1] 0.04176584 0.04159540 0.04696523 0.01846390 0.04277138 0.03146637
##  [7] 0.04606443 0.02351111 0.04672173 0.04390375
## [1] 0.003
## [1] "mse 0.0003551471"
```

```
## $d
##     V1                 V2       V3      V4      V5  V6  V7 V8  V9 V10
## 1    0               <NA> NOT_ROOT NOT_MIG NOT_TIP 104  76  3 172   5
## 2    1     Eastern_Brazil NOT_ROOT NOT_MIG     TIP  16  NA NA  NA  NA
## 3    2               <NA> NOT_ROOT NOT_MIG NOT_TIP  16  32  2   3   1
## 4    3     Western_Brazil NOT_ROOT NOT_MIG     TIP   2  NA NA  NA  NA
## 5    4        Southern_SA NOT_ROOT NOT_MIG     TIP  32  NA NA  NA  NA
## 6   15 Sulawesi_Indonesia NOT_ROOT NOT_MIG     TIP 173  NA NA  NA  NA
## 7   16               <NA> NOT_ROOT NOT_MIG NOT_TIP 172   1  1   2   3
## 8   31     Central_Brazil NOT_ROOT NOT_MIG     TIP  32  NA NA  NA  NA
## 9   32               <NA> NOT_ROOT NOT_MIG NOT_TIP   2  31  1   4   1
## 10  51      Eastern_China NOT_ROOT NOT_MIG     TIP  76  NA NA  NA  NA
## 11  75     Okinawa_Taiwan NOT_ROOT NOT_MIG     TIP 136  NA NA  NA  NA
## 12  76               <NA> NOT_ROOT NOT_MIG NOT_TIP   0  51  1 136   2
## 13 103     Southeast_Asia NOT_ROOT NOT_MIG     TIP 104  NA NA  NA  NA
## 14 104               <NA> NOT_ROOT NOT_MIG NOT_TIP 173 103  1   0   8
## 15 135              Japan NOT_ROOT NOT_MIG     TIP 136  NA NA  NA  NA
## 16 136               <NA> NOT_ROOT NOT_MIG NOT_TIP  76  75  1 135   1
## 17 171  Medellin_Colombia NOT_ROOT NOT_MIG     TIP 172  NA NA  NA  NA
## 18 172               <NA> NOT_ROOT NOT_MIG NOT_TIP   0 171  1  16   4
## 19 173               <NA>     ROOT NOT_MIG NOT_TIP 173  15  1 104   9
##                                                                                                                                                                                                                                                                                                                                                                     V11
## 1                                                                        ((Eastern_China:0.00263532,(Okinawa_Taiwan:0.0147928,Japan:0.0154501):0.00244058):0.00543424,(Medellin_Colombia:0.0198913,(Eastern_Brazil:0.00862157,((Central_Brazil:0.00281659,Southern_SA:0.00701044):0.00274911,Western_Brazil:0.00438972):0.00406141):0.00913182):0.000615636):0.00493291
## 2                                                                                                                                                                                                                                                                                                                                             Eastern_Brazil:0.00862157
## 3                                                                                                                                                                                                                                                                  ((Central_Brazil:0.00281659,Southern_SA:0.00701044):0.00274911,Western_Brazil:0.00438972):0.00406141
## 4                                                                                                                                                                                                                                                                                                                                             Western_Brazil:0.00438972
## 5                                                                                                                                                                                                                                                                                                                                                Southern_SA:0.00701044
## 6                                                                                                                                                                                                                                                                                                                                          Sulawesi_Indonesia:0.0184639
## 7                                                                                                                                                                                                                           (Eastern_Brazil:0.00862157,((Central_Brazil:0.00281659,Southern_SA:0.00701044):0.00274911,Western_Brazil:0.00438972):0.00406141):0.00913182
## 8                                                                                                                                                                                                                                                                                                                                             Central_Brazil:0.00281659
## 9                                                                                                                                                                                                                                                                                                         (Central_Brazil:0.00281659,Southern_SA:0.00701044):0.00274911
## 10                                                                                                                                                                                                                                                                                                                                             Eastern_China:0.00263532
## 11                                                                                                                                                                                                                                                                                                                                             Okinawa_Taiwan:0.0147928
## 12                                                                                                                                                                                                                                                                          (Eastern_China:0.00263532,(Okinawa_Taiwan:0.0147928,Japan:0.0154501):0.00244058):0.00543424
## 13                                                                                                                                                                                                                                                                                                                                            Southeast_Asia:0.00504721
## 14                                 (Southeast_Asia:0.00504721,((Eastern_China:0.00263532,(Okinawa_Taiwan:0.0147928,Japan:0.0154501):0.00244058):0.00543424,(Medellin_Colombia:0.0198913,(Eastern_Brazil:0.00862157,((Central_Brazil:0.00281659,Southern_SA:0.00701044):0.00274911,Western_Brazil:0.00438972):0.00406141):0.00913182):0.000615636):0.00493291):0.0184639
## 15                                                                                                                                                                                                                                                                                                                                                      Japan:0.0154501
## 16                                                                                                                                                                                                                                                                                                                (Okinawa_Taiwan:0.0147928,Japan:0.0154501):0.00244058
## 17                                                                                                                                                                                                                                                                                                                                          Medellin_Colombia:0.0198913
## 18                                                                                                                                                                                (Medellin_Colombia:0.0198913,(Eastern_Brazil:0.00862157,((Central_Brazil:0.00281659,Southern_SA:0.00701044):0.00274911,Western_Brazil:0.00438972):0.00406141):0.00913182):0.000615636
## 19 (Sulawesi_Indonesia:0.0184639,(Southeast_Asia:0.00504721,((Eastern_China:0.00263532,(Okinawa_Taiwan:0.0147928,Japan:0.0154501):0.00244058):0.00543424,(Medellin_Colombia:0.0198913,(Eastern_Brazil:0.00862157,((Central_Brazil:0.00281659,Southern_SA:0.00701044):0.00274911,Western_Brazil:0.00438972):0.00406141):0.00913182):0.000615636):0.00493291):0.0184639);
##             x    y ymin ymax
## 1  0.02339681 0.50  0.0  0.8
## 2  0.04176584 0.35  0.3  0.4
## 3  0.03720568 0.10  0.0  0.3
## 4  0.04159540 0.05  0.0  0.1
## 5  0.04696523 0.15  0.1  0.2
## 6  0.01846390 0.95  0.9  1.0
## 7  0.03314427 0.30  0.0  0.4
## 8  0.04277138 0.25  0.2  0.3
## 9  0.03995479 0.20  0.1  0.3
## 10 0.03146637 0.75  0.7  0.8
## 11 0.04606443 0.65  0.6  0.7
## 12 0.02883105 0.70  0.5  0.8
## 13 0.02351111 0.85  0.8  0.9
## 14 0.01846390 0.80  0.0  0.9
## 15 0.04672173 0.55  0.5  0.6
## 16 0.03127163 0.60  0.5  0.7
## 17 0.04390375 0.45  0.4  0.5
## 18 0.02401245 0.40  0.0  0.5
## 19 0.00000000 0.90  0.0  1.0
## 
## $e
##     V1  V2          V3 V4      V5 V6 V7
## 1   16   1 0.008621570  1 NOT_MIG  0  1
## 2    2  32 0.002749110  1 NOT_MIG  0  1
## 3   32  31 0.002816590  1 NOT_MIG  0  1
## 4   32   4 0.007010440  1 NOT_MIG  0  1
## 5    2   3 0.004389720  1 NOT_MIG  0  1
## 6   76  51 0.002635320  1 NOT_MIG  0  1
## 7  104 103 0.005047210  1 NOT_MIG  0  1
## 8   76 136 0.002440580  1 NOT_MIG  0  1
## 9  136  75 0.014792800  1 NOT_MIG  0  1
## 10 136 135 0.015450100  1 NOT_MIG  0  1
## 11   0  76 0.005434240  1 NOT_MIG  0  1
## 12 172 171 0.019891300  1 NOT_MIG  0  1
## 13  16   2 0.004061410  1 NOT_MIG  0  1
## 14   0 172 0.000615636  1 NOT_MIG  0  1
## 15 104   0 0.004932910  1 NOT_MIG  0  1
## 16 173  15 0.018463900  1 NOT_MIG  0  1
## 17 173 104 0.018463900  1 NOT_MIG  0  1
## 18 172  16 0.009131820  1 NOT_MIG  0  1
```

```
setwd("/gpfs/gibbs/pi/caccone/mkc54/albo/manuscript1/output/treemix_subsets/south_america/prelim")

# Open PDF
pdf("treemix_prelim_m0.pdf", width = 12, height = 8)

plot_tree("sasub_simp.m0.rep2")

dev.off()
```

Check for m=2

```
setwd("/gpfs/gibbs/pi/caccone/mkc54/albo/manuscript1/output/treemix_subsets/south_america/prelim")
plot_tree("sasub_simp.m2.rep2")
```

```
## Warning in plot_tree("sasub_simp.m2.rep2"): NAs introduced by coercion
## Warning in plot_tree("sasub_simp.m2.rep2"): NAs introduced by coercion
## Warning in plot_tree("sasub_simp.m2.rep2"): NAs introduced by coercion
## Warning in plot_tree("sasub_simp.m2.rep2"): NAs introduced by coercion
```

```
##     V1                 V2       V3      V4      V5  V6  V7 V8  V9 V10
## 1    1 Sulawesi_Indonesia NOT_ROOT NOT_MIG     TIP 173  NA NA  NA  NA
## 2    2               <NA> NOT_ROOT NOT_MIG NOT_TIP 136  76  5 172   3
## 3    3     Okinawa_Taiwan NOT_ROOT NOT_MIG     TIP  16  NA NA  NA  NA
## 4    4     Western_Brazil NOT_ROOT NOT_MIG     TIP  32  NA NA  NA  NA
## 5   15              Japan NOT_ROOT NOT_MIG     TIP  16  NA NA  NA  NA
## 6   16               <NA> NOT_ROOT NOT_MIG NOT_TIP 172  15  1   3   1
## 7   31     Central_Brazil NOT_ROOT NOT_MIG     TIP  52  NA NA  NA  NA
## 8   32               <NA> NOT_ROOT NOT_MIG NOT_TIP 104  51  1   4   1
## 9   51        Southern_SA NOT_ROOT NOT_MIG     TIP  32  NA NA  NA  NA
## 10  52               <NA> NOT_ROOT NOT_MIG NOT_TIP 104 103  1  31   1
## 11  75  Medellin_Colombia NOT_ROOT NOT_MIG     TIP  76  NA NA  NA  NA
## 12  76               <NA> NOT_ROOT NOT_MIG NOT_TIP   2  75  1 104   4
## 13 103     Eastern_Brazil NOT_ROOT NOT_MIG     TIP  52  NA NA  NA  NA
## 14 104               <NA> NOT_ROOT NOT_MIG NOT_TIP  76  52  2  32   2
## 15 135     Southeast_Asia NOT_ROOT NOT_MIG     TIP 136  NA NA  NA  NA
## 16 136               <NA> NOT_ROOT NOT_MIG NOT_TIP 173   2  8 135   1
## 17 171      Eastern_China NOT_ROOT NOT_MIG     TIP 172  NA NA  NA  NA
## 18 172               <NA> NOT_ROOT NOT_MIG NOT_TIP   2 171  1  16   2
## 19 173               <NA>     ROOT NOT_MIG NOT_TIP 173 136  9   1   1
## 20 175               <NA> NOT_ROOT     MIG NOT_TIP  16  15 NA  NA  NA
## 21 233               <NA> NOT_ROOT     MIG NOT_TIP  16  15 NA  NA  NA
##                                                                                                                                                                                                                                                                                                                                                                V11
## 1                                                                                                                                                                                                                                                                                                                                      Sulawesi_Indonesia:0.018464
## 2                                                                      ((Medellin_Colombia:0.0188218,((Eastern_Brazil:0.0220818,Central_Brazil:0.000288198):0.00288911,(Southern_SA:0.0055347,Western_Brazil:0.00769172):0.00170155):0.0151086):0.00140696,(Eastern_China:0.00193434,(Japan:0.0161281,Okinawa_Taiwan:0.0136978):0.00347871):0.00642992):0.00371412
## 3                                                                                                                                                                                                                                                                                                                                         Okinawa_Taiwan:0.0136978
## 4                                                                                                                                                                                                                                                                                                                                        Western_Brazil:0.00769172
## 5                                                                                                                                                                                                                                                                                                                                                  Japan:0.0161281
## 6                                                                                                                                                                                                                                                                                                            (Japan:0.0161281,Okinawa_Taiwan:0.0136978):0.00347871
## 7                                                                                                                                                                                                                                                                                                                                       Central_Brazil:0.000288198
## 8                                                                                                                                                                                                                                                                                                     (Southern_SA:0.0055347,Western_Brazil:0.00769172):0.00170155
## 9                                                                                                                                                                                                                                                                                                                                            Southern_SA:0.0055347
## 10                                                                                                                                                                                                                                                                                                (Eastern_Brazil:0.0220818,Central_Brazil:0.000288198):0.00288911
## 11                                                                                                                                                                                                                                                                                                                                     Medellin_Colombia:0.0188218
## 12                                                                                                                                                                              (Medellin_Colombia:0.0188218,((Eastern_Brazil:0.0220818,Central_Brazil:0.000288198):0.00288911,(Southern_SA:0.0055347,Western_Brazil:0.00769172):0.00170155):0.0151086):0.00140696
## 13                                                                                                                                                                                                                                                                                                                                        Eastern_Brazil:0.0220818
## 14                                                                                                                                                                                                                       ((Eastern_Brazil:0.0220818,Central_Brazil:0.000288198):0.00288911,(Southern_SA:0.0055347,Western_Brazil:0.00769172):0.00170155):0.0151086
## 15                                                                                                                                                                                                                                                                                                                                       Southeast_Asia:0.00504738
## 16                                (((Medellin_Colombia:0.0188218,((Eastern_Brazil:0.0220818,Central_Brazil:0.000288198):0.00288911,(Southern_SA:0.0055347,Western_Brazil:0.00769172):0.00170155):0.0151086):0.00140696,(Eastern_China:0.00193434,(Japan:0.0161281,Okinawa_Taiwan:0.0136978):0.00347871):0.00642992):0.00371412,Southeast_Asia:0.00504738):0.018464
## 17                                                                                                                                                                                                                                                                                                                                        Eastern_China:0.00193434
## 18                                                                                                                                                                                                                                                                     (Eastern_China:0.00193434,(Japan:0.0161281,Okinawa_Taiwan:0.0136978):0.00347871):0.00642992
## 19 ((((Medellin_Colombia:0.0188218,((Eastern_Brazil:0.0220818,Central_Brazil:0.000288198):0.00288911,(Southern_SA:0.0055347,Western_Brazil:0.00769172):0.00170155):0.0151086):0.00140696,(Eastern_China:0.00193434,(Japan:0.0161281,Okinawa_Taiwan:0.0136978):0.00347871):0.00642992):0.00371412,Southeast_Asia:0.00504738):0.018464,Sulawesi_Indonesia:0.018464);
## 20                                                                                                                                                                                                                                                                                                                                                            <NA>
## 21                                                                                                                                                                                                                                                                                                                                                            <NA>
##             x    y ymin ymax
## 1  0.01846400 0.05  0.0  0.1
## 2  0.02217812 0.50  0.2  1.0
## 3  0.04578455 0.25  0.2  0.3
## 4  0.04612189 0.55  0.5  0.6
## 5  0.04821478 0.35  0.3  0.4
## 6  0.03208675 0.30  0.2  0.4
## 7  0.04187099 0.75  0.7  0.8
## 8  0.04039523 0.60  0.5  0.7
## 9  0.04592993 0.65  0.6  0.7
## 10 0.04158279 0.80  0.7  0.9
## 11 0.04240688 0.95  0.9  1.0
## 12 0.02358508 0.90  0.5  1.0
## 13 0.05224793 0.85  0.8  0.9
## 14 0.03869368 0.70  0.5  0.9
## 15 0.02351138 0.15  0.1  0.2
## 16 0.01846400 0.20  0.1  1.0
## 17 0.03054238 0.45  0.4  0.5
## 18 0.02860804 0.40  0.2  0.5
## 19 0.00000000 0.10  0.0  1.0
## 20 0.04754565   NA  0.2  0.3
## 21 0.04821478   NA  0.2  0.3
## [1] "0.958512 0.03208675 0.048214776"
## [1] "1 0.03208675 0.048214776"
```

```
##  [1] 0.01846400 0.04578455 0.04612189 0.04821478 0.04187099 0.04592993
##  [7] 0.04240688 0.05224793 0.02351138 0.03054238
## [1] 0.003
## [1] "mse 0.0003551471"
```

```
## $d
##     V1                 V2       V3      V4      V5  V6  V7 V8  V9 V10
## 1    1 Sulawesi_Indonesia NOT_ROOT NOT_MIG     TIP 173  NA NA  NA  NA
## 2    2               <NA> NOT_ROOT NOT_MIG NOT_TIP 136  76  5 172   3
## 3    3     Okinawa_Taiwan NOT_ROOT NOT_MIG     TIP  16  NA NA  NA  NA
## 4    4     Western_Brazil NOT_ROOT NOT_MIG     TIP  32  NA NA  NA  NA
## 5   15              Japan NOT_ROOT NOT_MIG     TIP  16  NA NA  NA  NA
## 6   16               <NA> NOT_ROOT NOT_MIG NOT_TIP 172  15  1   3   1
## 7   31     Central_Brazil NOT_ROOT NOT_MIG     TIP  52  NA NA  NA  NA
## 8   32               <NA> NOT_ROOT NOT_MIG NOT_TIP 104  51  1   4   1
## 9   51        Southern_SA NOT_ROOT NOT_MIG     TIP  32  NA NA  NA  NA
## 10  52               <NA> NOT_ROOT NOT_MIG NOT_TIP 104 103  1  31   1
## 11  75  Medellin_Colombia NOT_ROOT NOT_MIG     TIP  76  NA NA  NA  NA
## 12  76               <NA> NOT_ROOT NOT_MIG NOT_TIP   2  75  1 104   4
## 13 103     Eastern_Brazil NOT_ROOT NOT_MIG     TIP  52  NA NA  NA  NA
## 14 104               <NA> NOT_ROOT NOT_MIG NOT_TIP  76  52  2  32   2
## 15 135     Southeast_Asia NOT_ROOT NOT_MIG     TIP 136  NA NA  NA  NA
## 16 136               <NA> NOT_ROOT NOT_MIG NOT_TIP 173   2  8 135   1
## 17 171      Eastern_China NOT_ROOT NOT_MIG     TIP 172  NA NA  NA  NA
## 18 172               <NA> NOT_ROOT NOT_MIG NOT_TIP   2 171  1  16   2
## 19 173               <NA>     ROOT NOT_MIG NOT_TIP 173 136  9   1   1
## 20 175               <NA> NOT_ROOT     MIG NOT_TIP  16  15 NA  NA  NA
## 21 233               <NA> NOT_ROOT     MIG NOT_TIP  16  15 NA  NA  NA
##                                                                                                                                                                                                                                                                                                                                                                V11
## 1                                                                                                                                                                                                                                                                                                                                      Sulawesi_Indonesia:0.018464
## 2                                                                      ((Medellin_Colombia:0.0188218,((Eastern_Brazil:0.0220818,Central_Brazil:0.000288198):0.00288911,(Southern_SA:0.0055347,Western_Brazil:0.00769172):0.00170155):0.0151086):0.00140696,(Eastern_China:0.00193434,(Japan:0.0161281,Okinawa_Taiwan:0.0136978):0.00347871):0.00642992):0.00371412
## 3                                                                                                                                                                                                                                                                                                                                         Okinawa_Taiwan:0.0136978
## 4                                                                                                                                                                                                                                                                                                                                        Western_Brazil:0.00769172
## 5                                                                                                                                                                                                                                                                                                                                                  Japan:0.0161281
## 6                                                                                                                                                                                                                                                                                                            (Japan:0.0161281,Okinawa_Taiwan:0.0136978):0.00347871
## 7                                                                                                                                                                                                                                                                                                                                       Central_Brazil:0.000288198
## 8                                                                                                                                                                                                                                                                                                     (Southern_SA:0.0055347,Western_Brazil:0.00769172):0.00170155
## 9                                                                                                                                                                                                                                                                                                                                            Southern_SA:0.0055347
## 10                                                                                                                                                                                                                                                                                                (Eastern_Brazil:0.0220818,Central_Brazil:0.000288198):0.00288911
## 11                                                                                                                                                                                                                                                                                                                                     Medellin_Colombia:0.0188218
## 12                                                                                                                                                                              (Medellin_Colombia:0.0188218,((Eastern_Brazil:0.0220818,Central_Brazil:0.000288198):0.00288911,(Southern_SA:0.0055347,Western_Brazil:0.00769172):0.00170155):0.0151086):0.00140696
## 13                                                                                                                                                                                                                                                                                                                                        Eastern_Brazil:0.0220818
## 14                                                                                                                                                                                                                       ((Eastern_Brazil:0.0220818,Central_Brazil:0.000288198):0.00288911,(Southern_SA:0.0055347,Western_Brazil:0.00769172):0.00170155):0.0151086
## 15                                                                                                                                                                                                                                                                                                                                       Southeast_Asia:0.00504738
## 16                                (((Medellin_Colombia:0.0188218,((Eastern_Brazil:0.0220818,Central_Brazil:0.000288198):0.00288911,(Southern_SA:0.0055347,Western_Brazil:0.00769172):0.00170155):0.0151086):0.00140696,(Eastern_China:0.00193434,(Japan:0.0161281,Okinawa_Taiwan:0.0136978):0.00347871):0.00642992):0.00371412,Southeast_Asia:0.00504738):0.018464
## 17                                                                                                                                                                                                                                                                                                                                        Eastern_China:0.00193434
## 18                                                                                                                                                                                                                                                                     (Eastern_China:0.00193434,(Japan:0.0161281,Okinawa_Taiwan:0.0136978):0.00347871):0.00642992
## 19 ((((Medellin_Colombia:0.0188218,((Eastern_Brazil:0.0220818,Central_Brazil:0.000288198):0.00288911,(Southern_SA:0.0055347,Western_Brazil:0.00769172):0.00170155):0.0151086):0.00140696,(Eastern_China:0.00193434,(Japan:0.0161281,Okinawa_Taiwan:0.0136978):0.00347871):0.00642992):0.00371412,Southeast_Asia:0.00504738):0.018464,Sulawesi_Indonesia:0.018464);
## 20                                                                                                                                                                                                                                                                                                                                                            <NA>
## 21                                                                                                                                                                                                                                                                                                                                                            <NA>
##             x         y ymin ymax
## 1  0.01846400 0.0500000  0.0  0.1
## 2  0.02217812 0.5000000  0.2  1.0
## 3  0.04578455 0.2500000  0.2  0.3
## 4  0.04612189 0.5500000  0.5  0.6
## 5  0.04821478 0.3500000  0.3  0.4
## 6  0.03208675 0.3000000  0.2  0.4
## 7  0.04187099 0.7500000  0.7  0.8
## 8  0.04039523 0.6000000  0.5  0.7
## 9  0.04592993 0.6500000  0.6  0.7
## 10 0.04158279 0.8000000  0.7  0.9
## 11 0.04240688 0.9500000  0.9  1.0
## 12 0.02358508 0.9000000  0.5  1.0
## 13 0.05224793 0.8500000  0.8  0.9
## 14 0.03869368 0.7000000  0.5  0.9
## 15 0.02351138 0.1500000  0.1  0.2
## 16 0.01846400 0.2000000  0.1  1.0
## 17 0.03054238 0.4500000  0.4  0.5
## 18 0.02860804 0.4000000  0.2  0.5
## 19 0.00000000 0.1000000  0.0  1.0
## 20 0.04754566 0.3479256  0.2  0.3
## 21 0.04821478 0.3500000  0.2  0.3
## 
## $e
##     V1  V2          V3       V4      V5       V6       V7
## 1   76  75 0.018821800 1.000000 NOT_MIG 0.000000 1.000000
## 2  136   2 0.003714120 1.000000 NOT_MIG 0.000000 1.000000
## 3    2  76 0.001406960 1.000000 NOT_MIG 0.000000 1.000000
## 4   16 175 0.015458900 1.000000 NOT_MIG 0.000000 1.000000
## 5  175 103 0.000000000 0.305030     MIG 0.958512 0.000000
## 6   52 103 0.010665141 0.694970 NOT_MIG 0.000000 0.482983
## 7   52  31 0.000288198 1.000000 NOT_MIG 0.000000 1.000000
## 8   32  51 0.005534700 1.000000 NOT_MIG 0.000000 1.000000
## 9  136 135 0.005047380 1.000000 NOT_MIG 0.000000 1.000000
## 10 173 136 0.018464000 1.000000 NOT_MIG 0.000000 1.000000
## 11 172 171 0.001934340 1.000000 NOT_MIG 0.000000 1.000000
## 12 173   1 0.018464000 1.000000 NOT_MIG 0.000000 1.000000
## 13  16   3 0.013697800 1.000000 NOT_MIG 0.000000 1.000000
## 14 172  16 0.003478710 1.000000 NOT_MIG 0.000000 1.000000
## 15  76 104 0.015108600 1.000000 NOT_MIG 0.000000 1.000000
## 16   2 172 0.006429920 1.000000 NOT_MIG 0.000000 1.000000
## 17 175 233 0.000669126 1.000000 NOT_MIG 0.958512 1.000000
## 18 233  15 0.000000000 1.000000 NOT_MIG 1.000000 1.000000
## 19 233   4 0.000000000 0.137143     MIG 1.000000 0.000000
## 20 104  52 0.002889110 1.000000 NOT_MIG 0.000000 1.000000
## 21 104  32 0.001701550 1.000000 NOT_MIG 0.000000 1.000000
## 22  32   4 0.005726656 0.862857 NOT_MIG 0.000000 0.744522
```

```
setwd("/gpfs/gibbs/pi/caccone/mkc54/albo/manuscript1/output/treemix_subsets/south_america/prelim")

# Open PDF
pdf("treemix_prelim_m2.pdf", width = 12, height = 8)

plot_tree("sasub_simp.m2.rep2")

dev.off()
```

Check for m=3

```
setwd("/gpfs/gibbs/pi/caccone/mkc54/albo/manuscript1/output/treemix_subsets/south_america/prelim")
plot_tree("sasub_simp.m3.rep2")
```

```
## Warning in plot_tree("sasub_simp.m3.rep2"): NAs introduced by coercion
## Warning in plot_tree("sasub_simp.m3.rep2"): NAs introduced by coercion
## Warning in plot_tree("sasub_simp.m3.rep2"): NAs introduced by coercion
## Warning in plot_tree("sasub_simp.m3.rep2"): NAs introduced by coercion
```

```
##     V1                 V2       V3      V4      V5  V6  V7 V8  V9 V10
## 1    0               <NA> NOT_ROOT NOT_MIG NOT_TIP  76 136  5  16   3
## 2    1     Okinawa_Taiwan NOT_ROOT NOT_MIG     TIP   2  NA NA  NA  NA
## 3    2               <NA> NOT_ROOT NOT_MIG NOT_TIP  16   1  1  15   1
## 4    3              Japan NOT_ROOT NOT_MIG     TIP  16  NA NA  NA  NA
## 5    4     Southeast_Asia NOT_ROOT NOT_MIG     TIP  76  NA NA  NA  NA
## 6   15      Eastern_China NOT_ROOT NOT_MIG     TIP   2  NA NA  NA  NA
## 7   16               <NA> NOT_ROOT NOT_MIG NOT_TIP   0   3  1   2   2
## 8   31     Eastern_Brazil NOT_ROOT NOT_MIG     TIP 104  NA NA  NA  NA
## 9   51     Central_Brazil NOT_ROOT NOT_MIG     TIP 104  NA NA  NA  NA
## 10  52               <NA> NOT_ROOT NOT_MIG NOT_TIP 136 104  2 172   2
## 11  75 Sulawesi_Indonesia NOT_ROOT NOT_MIG     TIP 173  NA NA  NA  NA
## 12  76               <NA> NOT_ROOT NOT_MIG NOT_TIP 173   0  8   4   1
## 13 103        Southern_SA NOT_ROOT NOT_MIG     TIP 172  NA NA  NA  NA
## 14 104               <NA> NOT_ROOT NOT_MIG NOT_TIP  52  51  1  31   1
## 15 135  Medellin_Colombia NOT_ROOT NOT_MIG     TIP 136  NA NA  NA  NA
## 16 136               <NA> NOT_ROOT NOT_MIG NOT_TIP   0 135  1  52   4
## 17 171     Western_Brazil NOT_ROOT NOT_MIG     TIP 172  NA NA  NA  NA
## 18 172               <NA> NOT_ROOT NOT_MIG NOT_TIP  52 103  1 171   1
## 19 173               <NA>     ROOT NOT_MIG NOT_TIP 173  75  1  76   9
## 20 181               <NA> NOT_ROOT     MIG NOT_TIP  16   3 NA  NA  NA
## 21 221               <NA> NOT_ROOT     MIG NOT_TIP  16   3 NA  NA  NA
## 22 258               <NA> NOT_ROOT     MIG NOT_TIP 173  76 NA  NA  NA
##                                                                                                                                                                                                                                                                                                                                                                  V11
## 1                                                                       ((Medellin_Colombia:0.0189049,((Central_Brazil:0.000436048,Eastern_Brazil:0.0221593):0.00289092,(Southern_SA:0.0057985,Western_Brazil:0.00734554):0.00156216):0.0149873):0.000692209,(Japan:0.0157893,(Okinawa_Taiwan:0.0129093,Eastern_China:0.00922463):0.000736381):0.0103157):0.00455644
## 2                                                                                                                                                                                                                                                                                                                                           Okinawa_Taiwan:0.0129093
## 3                                                                                                                                                                                                                                                                                                    (Okinawa_Taiwan:0.0129093,Eastern_China:0.00922463):0.000736381
## 4                                                                                                                                                                                                                                                                                                                                                    Japan:0.0157893
## 5                                                                                                                                                                                                                                                                                                                                          Southeast_Asia:0.00480354
## 6                                                                                                                                                                                                                                                                                                                                           Eastern_China:0.00922463
## 7                                                                                                                                                                                                                                                                        (Japan:0.0157893,(Okinawa_Taiwan:0.0129093,Eastern_China:0.00922463):0.000736381):0.0103157
## 8                                                                                                                                                                                                                                                                                                                                           Eastern_Brazil:0.0221593
## 9                                                                                                                                                                                                                                                                                                                                         Central_Brazil:0.000436048
## 10                                                                                                                                                                                                                         ((Central_Brazil:0.000436048,Eastern_Brazil:0.0221593):0.00289092,(Southern_SA:0.0057985,Western_Brazil:0.00734554):0.00156216):0.0149873
## 11                                                                                                                                                                                                                                                                                                                                       Sulawesi_Indonesia:0.017055
## 12                                (((Medellin_Colombia:0.0189049,((Central_Brazil:0.000436048,Eastern_Brazil:0.0221593):0.00289092,(Southern_SA:0.0057985,Western_Brazil:0.00734554):0.00156216):0.0149873):0.000692209,(Japan:0.0157893,(Okinawa_Taiwan:0.0129093,Eastern_China:0.00922463):0.000736381):0.0103157):0.00455644,Southeast_Asia:0.00480354):0.0198668
## 13                                                                                                                                                                                                                                                                                                                                             Southern_SA:0.0057985
## 14                                                                                                                                                                                                                                                                                                  (Central_Brazil:0.000436048,Eastern_Brazil:0.0221593):0.00289092
## 15                                                                                                                                                                                                                                                                                                                                       Medellin_Colombia:0.0189049
## 16                                                                                                                                                                               (Medellin_Colombia:0.0189049,((Central_Brazil:0.000436048,Eastern_Brazil:0.0221593):0.00289092,(Southern_SA:0.0057985,Western_Brazil:0.00734554):0.00156216):0.0149873):0.000692209
## 17                                                                                                                                                                                                                                                                                                                                         Western_Brazil:0.00734554
## 18                                                                                                                                                                                                                                                                                                      (Southern_SA:0.0057985,Western_Brazil:0.00734554):0.00156216
## 19 (Sulawesi_Indonesia:0.017055,(((Medellin_Colombia:0.0189049,((Central_Brazil:0.000436048,Eastern_Brazil:0.0221593):0.00289092,(Southern_SA:0.0057985,Western_Brazil:0.00734554):0.00156216):0.0149873):0.000692209,(Japan:0.0157893,(Okinawa_Taiwan:0.0129093,Eastern_China:0.00922463):0.000736381):0.0103157):0.00455644,Southeast_Asia:0.00480354):0.0198668);
## 20                                                                                                                                                                                                                                                                                                                                                              <NA>
## 21                                                                                                                                                                                                                                                                                                                                                              <NA>
## 22                                                                                                                                                                                                                                                                                                                                                              <NA>
##             x    y ymin ymax
## 1  0.02442332 0.40  0.1  0.9
## 2  0.04838470 0.25  0.2  0.3
## 3  0.03547540 0.20  0.1  0.3
## 4  0.05052833 0.35  0.3  0.4
## 5  0.02467042 0.05  0.0  0.1
## 6  0.03951900 0.15  0.1  0.2
## 7  0.03473902 0.30  0.1  0.4
## 8  0.05362406 0.65  0.6  0.7
## 9  0.04342980 0.75  0.7  0.8
## 10 0.04010283 0.60  0.4  0.8
## 11 0.01705500 0.95  0.9  1.0
## 12 0.01986688 0.10  0.0  0.9
## 13 0.04746349 0.55  0.5  0.6
## 14 0.04299375 0.70  0.6  0.8
## 15 0.04402043 0.85  0.8  0.9
## 16 0.02511553 0.80  0.4  0.9
## 17 0.04724617 0.45  0.4  0.5
## 18 0.04166499 0.50  0.4  0.6
## 19 0.00000000 0.90  0.0  1.0
## 20 0.04874532   NA  0.1  0.3
## 21 0.05052833   NA  0.1  0.3
## 22 0.01705500   NA  0.0  0.9
## [1] "0.887075 0.03473902 0.05052833"
## [1] "1 0.03473902 0.05052833"
## [1] "0.858464 0 0.01986688"
```

```
##  [1] 0.04838470 0.05052833 0.02467042 0.03951900 0.05362406 0.04342980
##  [7] 0.01705500 0.04746349 0.04402043 0.04724617
## [1] 0.003
## [1] "mse 0.0003551471"
```

```
## $d
##     V1                 V2       V3      V4      V5  V6  V7 V8  V9 V10
## 1    0               <NA> NOT_ROOT NOT_MIG NOT_TIP  76 136  5  16   3
## 2    1     Okinawa_Taiwan NOT_ROOT NOT_MIG     TIP   2  NA NA  NA  NA
## 3    2               <NA> NOT_ROOT NOT_MIG NOT_TIP  16   1  1  15   1
## 4    3              Japan NOT_ROOT NOT_MIG     TIP  16  NA NA  NA  NA
## 5    4     Southeast_Asia NOT_ROOT NOT_MIG     TIP  76  NA NA  NA  NA
## 6   15      Eastern_China NOT_ROOT NOT_MIG     TIP   2  NA NA  NA  NA
## 7   16               <NA> NOT_ROOT NOT_MIG NOT_TIP   0   3  1   2   2
## 8   31     Eastern_Brazil NOT_ROOT NOT_MIG     TIP 104  NA NA  NA  NA
## 9   51     Central_Brazil NOT_ROOT NOT_MIG     TIP 104  NA NA  NA  NA
## 10  52               <NA> NOT_ROOT NOT_MIG NOT_TIP 136 104  2 172   2
## 11  75 Sulawesi_Indonesia NOT_ROOT NOT_MIG     TIP 173  NA NA  NA  NA
## 12  76               <NA> NOT_ROOT NOT_MIG NOT_TIP 173   0  8   4   1
## 13 103        Southern_SA NOT_ROOT NOT_MIG     TIP 172  NA NA  NA  NA
## 14 104               <NA> NOT_ROOT NOT_MIG NOT_TIP  52  51  1  31   1
## 15 135  Medellin_Colombia NOT_ROOT NOT_MIG     TIP 136  NA NA  NA  NA
## 16 136               <NA> NOT_ROOT NOT_MIG NOT_TIP   0 135  1  52   4
## 17 171     Western_Brazil NOT_ROOT NOT_MIG     TIP 172  NA NA  NA  NA
## 18 172               <NA> NOT_ROOT NOT_MIG NOT_TIP  52 103  1 171   1
## 19 173               <NA>     ROOT NOT_MIG NOT_TIP 173  75  1  76   9
## 20 181               <NA> NOT_ROOT     MIG NOT_TIP  16   3 NA  NA  NA
## 21 221               <NA> NOT_ROOT     MIG NOT_TIP  16   3 NA  NA  NA
## 22 258               <NA> NOT_ROOT     MIG NOT_TIP 173  76 NA  NA  NA
##                                                                                                                                                                                                                                                                                                                                                                  V11
## 1                                                                       ((Medellin_Colombia:0.0189049,((Central_Brazil:0.000436048,Eastern_Brazil:0.0221593):0.00289092,(Southern_SA:0.0057985,Western_Brazil:0.00734554):0.00156216):0.0149873):0.000692209,(Japan:0.0157893,(Okinawa_Taiwan:0.0129093,Eastern_China:0.00922463):0.000736381):0.0103157):0.00455644
## 2                                                                                                                                                                                                                                                                                                                                           Okinawa_Taiwan:0.0129093
## 3                                                                                                                                                                                                                                                                                                    (Okinawa_Taiwan:0.0129093,Eastern_China:0.00922463):0.000736381
## 4                                                                                                                                                                                                                                                                                                                                                    Japan:0.0157893
## 5                                                                                                                                                                                                                                                                                                                                          Southeast_Asia:0.00480354
## 6                                                                                                                                                                                                                                                                                                                                           Eastern_China:0.00922463
## 7                                                                                                                                                                                                                                                                        (Japan:0.0157893,(Okinawa_Taiwan:0.0129093,Eastern_China:0.00922463):0.000736381):0.0103157
## 8                                                                                                                                                                                                                                                                                                                                           Eastern_Brazil:0.0221593
## 9                                                                                                                                                                                                                                                                                                                                         Central_Brazil:0.000436048
## 10                                                                                                                                                                                                                         ((Central_Brazil:0.000436048,Eastern_Brazil:0.0221593):0.00289092,(Southern_SA:0.0057985,Western_Brazil:0.00734554):0.00156216):0.0149873
## 11                                                                                                                                                                                                                                                                                                                                       Sulawesi_Indonesia:0.017055
## 12                                (((Medellin_Colombia:0.0189049,((Central_Brazil:0.000436048,Eastern_Brazil:0.0221593):0.00289092,(Southern_SA:0.0057985,Western_Brazil:0.00734554):0.00156216):0.0149873):0.000692209,(Japan:0.0157893,(Okinawa_Taiwan:0.0129093,Eastern_China:0.00922463):0.000736381):0.0103157):0.00455644,Southeast_Asia:0.00480354):0.0198668
## 13                                                                                                                                                                                                                                                                                                                                             Southern_SA:0.0057985
## 14                                                                                                                                                                                                                                                                                                  (Central_Brazil:0.000436048,Eastern_Brazil:0.0221593):0.00289092
## 15                                                                                                                                                                                                                                                                                                                                       Medellin_Colombia:0.0189049
## 16                                                                                                                                                                               (Medellin_Colombia:0.0189049,((Central_Brazil:0.000436048,Eastern_Brazil:0.0221593):0.00289092,(Southern_SA:0.0057985,Western_Brazil:0.00734554):0.00156216):0.0149873):0.000692209
## 17                                                                                                                                                                                                                                                                                                                                         Western_Brazil:0.00734554
## 18                                                                                                                                                                                                                                                                                                      (Southern_SA:0.0057985,Western_Brazil:0.00734554):0.00156216
## 19 (Sulawesi_Indonesia:0.017055,(((Medellin_Colombia:0.0189049,((Central_Brazil:0.000436048,Eastern_Brazil:0.0221593):0.00289092,(Southern_SA:0.0057985,Western_Brazil:0.00734554):0.00156216):0.0149873):0.000692209,(Japan:0.0157893,(Okinawa_Taiwan:0.0129093,Eastern_China:0.00922463):0.000736381):0.0103157):0.00455644,Southeast_Asia:0.00480354):0.0198668);
## 20                                                                                                                                                                                                                                                                                                                                                              <NA>
## 21                                                                                                                                                                                                                                                                                                                                                              <NA>
## 22                                                                                                                                                                                                                                                                                                                                                              <NA>
##             x         y ymin ymax
## 1  0.02442332 0.4000000  0.1  0.9
## 2  0.04838470 0.2500000  0.2  0.3
## 3  0.03547540 0.2000000  0.1  0.3
## 4  0.05052833 0.3500000  0.3  0.4
## 5  0.02467042 0.0500000  0.0  0.1
## 6  0.03951900 0.1500000  0.1  0.2
## 7  0.03473902 0.3000000  0.1  0.4
## 8  0.05362406 0.6500000  0.6  0.7
## 9  0.04342980 0.7500000  0.7  0.8
## 10 0.04010283 0.6000000  0.4  0.8
## 11 0.01705500 0.9500000  0.9  1.0
## 12 0.01986688 0.1000000  0.0  0.9
## 13 0.04746349 0.5500000  0.5  0.6
## 14 0.04299375 0.7000000  0.6  0.8
## 15 0.04402043 0.8500000  0.8  0.9
## 16 0.02511553 0.8000000  0.4  0.9
## 17 0.04724617 0.4500000  0.4  0.5
## 18 0.04166499 0.5000000  0.4  0.6
## 19 0.00000000 0.9000000  0.0  1.0
## 20 0.04874532 0.3443538  0.1  0.3
## 21 0.05052833 0.3500000  0.1  0.3
## 22 0.01705500 0.2132288  0.0  0.9
## 
## $e
##     V1  V2          V3       V4      V5       V6       V7
## 1    2   1 0.012909300 1.000000 NOT_MIG 0.000000 1.000000
## 2  136 135 0.018904900 1.000000 NOT_MIG 0.000000 1.000000
## 3   76   0 0.004556440 1.000000 NOT_MIG 0.000000 1.000000
## 4    0 136 0.000692209 1.000000 NOT_MIG 0.000000 1.000000
## 5  136  52 0.014987300 1.000000 NOT_MIG 0.000000 1.000000
## 6  181  31 0.000000000 0.307380     MIG 0.887075 0.000000
## 7  172 103 0.005798500 1.000000 NOT_MIG 0.000000 1.000000
## 8  173  75 0.017055000 1.000000 NOT_MIG 0.000000 1.000000
## 9  104  51 0.000436048 1.000000 NOT_MIG 0.000000 1.000000
## 10 181 221 0.001783010 1.000000 NOT_MIG 0.887075 1.000000
## 11 221   3 0.000000000 1.000000 NOT_MIG 1.000000 1.000000
## 12 221 171 0.000000000 0.128332     MIG 1.000000 0.000000
## 13 104  31 0.010630314 0.692620 NOT_MIG 0.000000 0.479722
## 14 172 171 0.005581179 0.871668 NOT_MIG 0.000000 0.759804
## 15  52 104 0.002890920 1.000000 NOT_MIG 0.000000 1.000000
## 16  52 172 0.001562160 1.000000 NOT_MIG 0.000000 1.000000
## 17  76   4 0.004803540 1.000000 NOT_MIG 0.000000 1.000000
## 18   0  16 0.010315700 1.000000 NOT_MIG 0.000000 1.000000
## 19 173 258 0.017055000 1.000000 NOT_MIG 0.000000 1.000000
## 20 258  76 0.002811880 1.000000 NOT_MIG 0.858464 1.000000
## 21 258  15 0.000000000 0.337921     MIG 0.858464 0.000000
## 22  16 181 0.014006300 1.000000 NOT_MIG 0.000000 1.000000
## 23  16   2 0.000736381 1.000000 NOT_MIG 0.000000 1.000000
## 24   2  15 0.004043604 0.662079 NOT_MIG 0.000000 0.438348
```

```
setwd("/gpfs/gibbs/pi/caccone/mkc54/albo/manuscript1/output/treemix_subsets/south_america/prelim")

# Open PDF
pdf("treemix_prelim_m3.pdf", width = 12, height = 8)

plot_tree("sasub_simp.m3.rep2")

dev.off()
```

Check for m=4

```
setwd("/gpfs/gibbs/pi/caccone/mkc54/albo/manuscript1/output/treemix_subsets/south_america/prelim")
plot_tree("sasub_simp.m4.rep4")
```

```
## Warning in plot_tree("sasub_simp.m4.rep4"): NAs introduced by coercion
## Warning in plot_tree("sasub_simp.m4.rep4"): NAs introduced by coercion
## Warning in plot_tree("sasub_simp.m4.rep4"): NAs introduced by coercion
## Warning in plot_tree("sasub_simp.m4.rep4"): NAs introduced by coercion
```

```
##     V1                 V2       V3      V4      V5  V6  V7 V8  V9 V10
## 1    1     Eastern_Brazil NOT_ROOT NOT_MIG     TIP 104  NA NA  NA  NA
## 2    2               <NA> NOT_ROOT NOT_MIG NOT_TIP 136  16  5  32   3
## 3    3 Sulawesi_Indonesia NOT_ROOT NOT_MIG     TIP 173  NA NA  NA  NA
## 4    4  Medellin_Colombia NOT_ROOT NOT_MIG     TIP  16  NA NA  NA  NA
## 5   15     Okinawa_Taiwan NOT_ROOT NOT_MIG     TIP 172  NA NA  NA  NA
## 6   16               <NA> NOT_ROOT NOT_MIG NOT_TIP   2   4  1  52   4
## 7   31      Eastern_China NOT_ROOT NOT_MIG     TIP 172  NA NA  NA  NA
## 8   32               <NA> NOT_ROOT NOT_MIG NOT_TIP   2 171  1 172   2
## 9   51     Central_Brazil NOT_ROOT NOT_MIG     TIP 104  NA NA  NA  NA
## 10  52               <NA> NOT_ROOT NOT_MIG NOT_TIP  16 104  2  76   2
## 11  75     Western_Brazil NOT_ROOT NOT_MIG     TIP  76  NA NA  NA  NA
## 12  76               <NA> NOT_ROOT NOT_MIG NOT_TIP  52  75  1 103   1
## 13 103        Southern_SA NOT_ROOT NOT_MIG     TIP  76  NA NA  NA  NA
## 14 104               <NA> NOT_ROOT NOT_MIG NOT_TIP  52  51  1   1   1
## 15 135     Southeast_Asia NOT_ROOT NOT_MIG     TIP 136  NA NA  NA  NA
## 16 136               <NA> NOT_ROOT NOT_MIG NOT_TIP 173   2  8 135   1
## 17 171              Japan NOT_ROOT NOT_MIG     TIP  32  NA NA  NA  NA
## 18 172               <NA> NOT_ROOT NOT_MIG NOT_TIP  32  15  1  31   1
## 19 173               <NA>     ROOT NOT_MIG NOT_TIP 173   3  1 136   9
## 20 175               <NA> NOT_ROOT     MIG NOT_TIP  32 171 NA  NA  NA
## 21 233               <NA> NOT_ROOT     MIG NOT_TIP  32 171 NA  NA  NA
## 22 322               <NA> NOT_ROOT     MIG NOT_TIP  76 103 NA  NA  NA
## 23 271               <NA> NOT_ROOT     MIG NOT_TIP 136 135 NA  NA  NA
##                                                                                                                                                                                                                                                                                                                                                                    V11
## 1                                                                                                                                                                                                                                                                                                                                             Eastern_Brazil:0.0225477
## 2                                                                        ((Medellin_Colombia:0.0184242,((Central_Brazil:0.000241179,Eastern_Brazil:0.0225477):0.00271314,(Western_Brazil:0.00803396,Southern_SA:0.00552244):0.00200212):0.0157145):0.00129493,(Japan:0.0160291,(Okinawa_Taiwan:0.0135837,Eastern_China:0.00744591):0.000167547):0.00925979):0.00632743
## 3                                                                                                                                                                                                                                                                                                                                         Sulawesi_Indonesia:0.0173981
## 4                                                                                                                                                                                                                                                                                                                                          Medellin_Colombia:0.0184242
## 5                                                                                                                                                                                                                                                                                                                                             Okinawa_Taiwan:0.0135837
## 6                                                                                                                                                                                  (Medellin_Colombia:0.0184242,((Central_Brazil:0.000241179,Eastern_Brazil:0.0225477):0.00271314,(Western_Brazil:0.00803396,Southern_SA:0.00552244):0.00200212):0.0157145):0.00129493
## 7                                                                                                                                                                                                                                                                                                                                             Eastern_China:0.00744591
## 8                                                                                                                                                                                                                                                                         (Japan:0.0160291,(Okinawa_Taiwan:0.0135837,Eastern_China:0.00744591):0.000167547):0.00925979
## 9                                                                                                                                                                                                                                                                                                                                           Central_Brazil:0.000241179
## 10                                                                                                                                                                                                                          ((Central_Brazil:0.000241179,Eastern_Brazil:0.0225477):0.00271314,(Western_Brazil:0.00803396,Southern_SA:0.00552244):0.00200212):0.0157145
## 11                                                                                                                                                                                                                                                                                                                                           Western_Brazil:0.00803396
## 12                                                                                                                                                                                                                                                                                                       (Western_Brazil:0.00803396,Southern_SA:0.00552244):0.00200212
## 13                                                                                                                                                                                                                                                                                                                                              Southern_SA:0.00552244
## 14                                                                                                                                                                                                                                                                                                    (Central_Brazil:0.000241179,Eastern_Brazil:0.0225477):0.00271314
## 15                                                                                                                                                                                                                                                                                                                                           Southeast_Asia:0.00868521
## 16                                 (((Medellin_Colombia:0.0184242,((Central_Brazil:0.000241179,Eastern_Brazil:0.0225477):0.00271314,(Western_Brazil:0.00803396,Southern_SA:0.00552244):0.00200212):0.0157145):0.00129493,(Japan:0.0160291,(Okinawa_Taiwan:0.0135837,Eastern_China:0.00744591):0.000167547):0.00925979):0.00632743,Southeast_Asia:0.00868521):0.0173981
## 17                                                                                                                                                                                                                                                                                                                                                     Japan:0.0160291
## 18                                                                                                                                                                                                                                                                                                     (Okinawa_Taiwan:0.0135837,Eastern_China:0.00744591):0.000167547
## 19 (Sulawesi_Indonesia:0.0173981,(((Medellin_Colombia:0.0184242,((Central_Brazil:0.000241179,Eastern_Brazil:0.0225477):0.00271314,(Western_Brazil:0.00803396,Southern_SA:0.00552244):0.00200212):0.0157145):0.00129493,(Japan:0.0160291,(Okinawa_Taiwan:0.0135837,Eastern_China:0.00744591):0.000167547):0.00925979):0.00632743,Southeast_Asia:0.00868521):0.0173981);
## 20                                                                                                                                                                                                                                                                                                                                                                <NA>
## 21                                                                                                                                                                                                                                                                                                                                                                <NA>
## 22                                                                                                                                                                                                                                                                                                                                                                <NA>
## 23                                                                                                                                                                                                                                                                                                                                                                <NA>
##             x    y ymin ymax
## 1  0.05411329 0.65  0.6  0.7
## 2  0.02372553 0.40  0.1  0.9
## 3  0.01739810 0.95  0.9  1.0
## 4  0.04344466 0.85  0.8  0.9
## 5  0.04673657 0.25  0.2  0.3
## 6  0.02502046 0.80  0.4  0.9
## 7  0.03676968 0.15  0.1  0.2
## 8  0.03298532 0.30  0.1  0.4
## 9  0.04368928 0.75  0.7  0.8
## 10 0.04073496 0.60  0.4  0.8
## 11 0.04852795 0.55  0.5  0.6
## 12 0.04273708 0.50  0.4  0.6
## 13 0.04825952 0.45  0.4  0.5
## 14 0.04344810 0.70  0.6  0.8
## 15 0.02396962 0.05  0.0  0.1
## 16 0.01739810 0.10  0.0  0.9
## 17 0.04901441 0.35  0.3  0.4
## 18 0.03315287 0.20  0.1  0.3
## 19 0.00000000 0.90  0.0  1.0
## 20 0.04722282   NA  0.1  0.3
## 21 0.04901441   NA  0.1  0.3
## 22 0.04825952   NA  0.4  0.5
## 23 0.02077718   NA  0.0  0.1
## [1] "0.888228 0.03298532 0.0490144142"
## [1] "0.889607 0.03298532 0.0490144142"
## [1] "1 0.04273708 0.04825952"
## [1] "0.5142 0.0173981 0.0239696234757059"
```

```
##  [1] 0.05411329 0.01739810 0.04344466 0.04673657 0.03676968 0.04368928
##  [7] 0.04852795 0.04825952 0.02396962 0.04901441
## [1] 0.003
## [1] "mse 0.0003551471"
```

```
## $d
##     V1                 V2       V3      V4      V5  V6  V7 V8  V9 V10
## 1    1     Eastern_Brazil NOT_ROOT NOT_MIG     TIP 104  NA NA  NA  NA
## 2    2               <NA> NOT_ROOT NOT_MIG NOT_TIP 136  16  5  32   3
## 3    3 Sulawesi_Indonesia NOT_ROOT NOT_MIG     TIP 173  NA NA  NA  NA
## 4    4  Medellin_Colombia NOT_ROOT NOT_MIG     TIP  16  NA NA  NA  NA
## 5   15     Okinawa_Taiwan NOT_ROOT NOT_MIG     TIP 172  NA NA  NA  NA
## 6   16               <NA> NOT_ROOT NOT_MIG NOT_TIP   2   4  1  52   4
## 7   31      Eastern_China NOT_ROOT NOT_MIG     TIP 172  NA NA  NA  NA
## 8   32               <NA> NOT_ROOT NOT_MIG NOT_TIP   2 171  1 172   2
## 9   51     Central_Brazil NOT_ROOT NOT_MIG     TIP 104  NA NA  NA  NA
## 10  52               <NA> NOT_ROOT NOT_MIG NOT_TIP  16 104  2  76   2
## 11  75     Western_Brazil NOT_ROOT NOT_MIG     TIP  76  NA NA  NA  NA
## 12  76               <NA> NOT_ROOT NOT_MIG NOT_TIP  52  75  1 103   1
## 13 103        Southern_SA NOT_ROOT NOT_MIG     TIP  76  NA NA  NA  NA
## 14 104               <NA> NOT_ROOT NOT_MIG NOT_TIP  52  51  1   1   1
## 15 135     Southeast_Asia NOT_ROOT NOT_MIG     TIP 136  NA NA  NA  NA
## 16 136               <NA> NOT_ROOT NOT_MIG NOT_TIP 173   2  8 135   1
## 17 171              Japan NOT_ROOT NOT_MIG     TIP  32  NA NA  NA  NA
## 18 172               <NA> NOT_ROOT NOT_MIG NOT_TIP  32  15  1  31   1
## 19 173               <NA>     ROOT NOT_MIG NOT_TIP 173   3  1 136   9
## 20 175               <NA> NOT_ROOT     MIG NOT_TIP  32 171 NA  NA  NA
## 21 233               <NA> NOT_ROOT     MIG NOT_TIP  32 171 NA  NA  NA
## 22 322               <NA> NOT_ROOT     MIG NOT_TIP  76 103 NA  NA  NA
## 23 271               <NA> NOT_ROOT     MIG NOT_TIP 136 135 NA  NA  NA
##                                                                                                                                                                                                                                                                                                                                                                    V11
## 1                                                                                                                                                                                                                                                                                                                                             Eastern_Brazil:0.0225477
## 2                                                                        ((Medellin_Colombia:0.0184242,((Central_Brazil:0.000241179,Eastern_Brazil:0.0225477):0.00271314,(Western_Brazil:0.00803396,Southern_SA:0.00552244):0.00200212):0.0157145):0.00129493,(Japan:0.0160291,(Okinawa_Taiwan:0.0135837,Eastern_China:0.00744591):0.000167547):0.00925979):0.00632743
## 3                                                                                                                                                                                                                                                                                                                                         Sulawesi_Indonesia:0.0173981
## 4                                                                                                                                                                                                                                                                                                                                          Medellin_Colombia:0.0184242
## 5                                                                                                                                                                                                                                                                                                                                             Okinawa_Taiwan:0.0135837
## 6                                                                                                                                                                                  (Medellin_Colombia:0.0184242,((Central_Brazil:0.000241179,Eastern_Brazil:0.0225477):0.00271314,(Western_Brazil:0.00803396,Southern_SA:0.00552244):0.00200212):0.0157145):0.00129493
## 7                                                                                                                                                                                                                                                                                                                                             Eastern_China:0.00744591
## 8                                                                                                                                                                                                                                                                         (Japan:0.0160291,(Okinawa_Taiwan:0.0135837,Eastern_China:0.00744591):0.000167547):0.00925979
## 9                                                                                                                                                                                                                                                                                                                                           Central_Brazil:0.000241179
## 10                                                                                                                                                                                                                          ((Central_Brazil:0.000241179,Eastern_Brazil:0.0225477):0.00271314,(Western_Brazil:0.00803396,Southern_SA:0.00552244):0.00200212):0.0157145
## 11                                                                                                                                                                                                                                                                                                                                           Western_Brazil:0.00803396
## 12                                                                                                                                                                                                                                                                                                       (Western_Brazil:0.00803396,Southern_SA:0.00552244):0.00200212
## 13                                                                                                                                                                                                                                                                                                                                              Southern_SA:0.00552244
## 14                                                                                                                                                                                                                                                                                                    (Central_Brazil:0.000241179,Eastern_Brazil:0.0225477):0.00271314
## 15                                                                                                                                                                                                                                                                                                                                           Southeast_Asia:0.00868521
## 16                                 (((Medellin_Colombia:0.0184242,((Central_Brazil:0.000241179,Eastern_Brazil:0.0225477):0.00271314,(Western_Brazil:0.00803396,Southern_SA:0.00552244):0.00200212):0.0157145):0.00129493,(Japan:0.0160291,(Okinawa_Taiwan:0.0135837,Eastern_China:0.00744591):0.000167547):0.00925979):0.00632743,Southeast_Asia:0.00868521):0.0173981
## 17                                                                                                                                                                                                                                                                                                                                                     Japan:0.0160291
## 18                                                                                                                                                                                                                                                                                                     (Okinawa_Taiwan:0.0135837,Eastern_China:0.00744591):0.000167547
## 19 (Sulawesi_Indonesia:0.0173981,(((Medellin_Colombia:0.0184242,((Central_Brazil:0.000241179,Eastern_Brazil:0.0225477):0.00271314,(Western_Brazil:0.00803396,Southern_SA:0.00552244):0.00200212):0.0157145):0.00129493,(Japan:0.0160291,(Okinawa_Taiwan:0.0135837,Eastern_China:0.00744591):0.000167547):0.00925979):0.00632743,Southeast_Asia:0.00868521):0.0173981);
## 20                                                                                                                                                                                                                                                                                                                                                                <NA>
## 21                                                                                                                                                                                                                                                                                                                                                                <NA>
## 22                                                                                                                                                                                                                                                                                                                                                                <NA>
## 23                                                                                                                                                                                                                                                                                                                                                                <NA>
##             x         y ymin ymax
## 1  0.05411329 0.6500000  0.6  0.7
## 2  0.02372553 0.4000000  0.1  0.9
## 3  0.01739810 0.9500000  0.9  1.0
## 4  0.04344466 0.8500000  0.8  0.9
## 5  0.04673657 0.2500000  0.2  0.3
## 6  0.02502046 0.8000000  0.4  0.9
## 7  0.03676968 0.1500000  0.1  0.2
## 8  0.03298532 0.3000000  0.1  0.4
## 9  0.04368928 0.7500000  0.7  0.8
## 10 0.04073496 0.6000000  0.4  0.8
## 11 0.04852795 0.5500000  0.5  0.6
## 12 0.04273708 0.5000000  0.4  0.6
## 13 0.04825952 0.4500000  0.4  0.5
## 14 0.04344810 0.7000000  0.6  0.8
## 15 0.02396962 0.0500000  0.0  0.1
## 16 0.01739810 0.1000000  0.0  0.9
## 17 0.04901441 0.3500000  0.3  0.4
## 18 0.03315287 0.2000000  0.1  0.3
## 19 0.00000000 0.9000000  0.0  1.0
## 20 0.04722281 0.3444114  0.1  0.3
## 21 0.04724491 0.3444804  0.1  0.3
## 22 0.04825952 0.4500000  0.4  0.5
## 23 0.02077718 0.0742900  0.0  0.1
## 
## $e
##     V1  V2           V3       V4      V5       V6       V7
## 1   16   4 0.0184242000 1.000000 NOT_MIG 0.000000 1.000000
## 2    2  16 0.0012949300 1.000000 NOT_MIG 0.000000 1.000000
## 3  136   2 0.0063274300 1.000000 NOT_MIG 0.000000 1.000000
## 4  175   1 0.0000000000 0.312246     MIG 0.888228 0.000000
## 5  173   3 0.0173981000 1.000000 NOT_MIG 0.000000 1.000000
## 6  175 233 0.0000221042 1.000000 NOT_MIG 0.888228 1.000000
## 7  233  75 0.0000000000 0.151001     MIG 0.889607 0.000000
## 8  172  15 0.0135837000 1.000000 NOT_MIG 0.000000 1.000000
## 9   32 175 0.0142375000 1.000000 NOT_MIG 0.000000 1.000000
## 10  32 172 0.0001675470 1.000000 NOT_MIG 0.000000 1.000000
## 11 172  31 0.0036168126 0.696954 NOT_MIG 0.000000 0.485745
## 12 104  51 0.0002411790 1.000000 NOT_MIG 0.000000 1.000000
## 13 233 171 0.0017694900 1.000000 NOT_MIG 0.889607 1.000000
## 14   2  32 0.0092597900 1.000000 NOT_MIG 0.000000 1.000000
## 15 104   1 0.0106651876 0.687754 NOT_MIG 0.000000 0.473006
## 16  76  75 0.0057908728 0.848999 NOT_MIG 0.000000 0.720799
## 17  52 104 0.0027131400 1.000000 NOT_MIG 0.000000 1.000000
## 18  52  76 0.0020021200 1.000000 NOT_MIG 0.000000 1.000000
## 19  16  52 0.0157145000 1.000000 NOT_MIG 0.000000 1.000000
## 20  76 322 0.0055224400 1.000000 NOT_MIG 0.000000 1.000000
## 21 322 103 0.0000000000 1.000000 NOT_MIG 1.000000 1.000000
## 22 322 135 0.0000000000 0.130153     MIG 1.000000 0.000000
## 23 173 136 0.0173981000 1.000000 NOT_MIG 0.000000 1.000000
## 24 136 271 0.0033790812 0.869847 NOT_MIG 0.000000 0.756633
## 25 271 135 0.0031924423 0.869847 NOT_MIG 0.514200 0.756633
## 26 271  31 0.0000000000 0.303046     MIG 0.514200 0.000000
```

```
setwd("/gpfs/gibbs/pi/caccone/mkc54/albo/manuscript1/output/treemix_subsets/south_america/prelim")

# Open PDF
pdf("treemix_prelim_m4.pdf", width = 12, height = 8)

plot_tree("sasub_simp.m4.rep4")

dev.off()
```

### 7.2.5 For 1 migration event with 100 bootstraps

Use modified python wrapper used in Norah’s paper This uses the
pipeline ML tree → bootreps → combine → sumtrees

Uploaded to hpc as treemix\_tree\_with\_bootreps\_NPS.py It is the same
code used in the Saarman paper, but modified for python 3

```
module load dSQ/1.05 
module load TreeMix/1.13-GCC-10.2.0
```

It takes too long to run interactively, so save as .sh file as submit
via slurm treemix\_boot.sh

```
#!/bin/bash
#SBATCH --job-name=treemix_boot
#SBATCH --time=7-00:00:00           
#SBATCH --mem=40G                
#SBATCH --cpus-per-task=20         
#SBATCH --mail-type ALL
#SBATCH --partition=week        # need >24 hours

module load miniconda
conda activate dendro

# Load modules
module load TreeMix/1.13-GCC-10.2.0
module load Python/3.10.8-GCCcore-12.2.0

# Move to working directory
cd /gpfs/gibbs/pi/caccone/mkc54/albo/manuscript1/output/treemix_subsets/south_america


# Run Python script
python treemix_tree_with_bootreps_NPS_corrected.py \
  r2_0.1_b_south_america_subset.treemix.frq \
  1 \
  Sulawesi_Indonesia \
  results/ \
  100 \
  500

echo "Finished TreeMix bootstrap run at $(date)"
```

```
cd /gpfs/gibbs/pi/caccone/mkc54/albo/manuscript1/output/treemix_subsets/south_america

sbatch treemix_boot.sh #63401727
```

##### 7.2.5.1 Plots in R

Check cat\_trees.tre

```
salloc -p day --cpus-per-task=10 -t 24:00:00 --mem-per-cpu=10000

file /gpfs/gibbs/pi/caccone/mkc54/albo/manuscript1/output/treemix_subsets/south_america/results/cat_trees.tre
head -3 /gpfs/gibbs/pi/caccone/mkc54/albo/manuscript1/output/treemix_subsets/south_america/results/cat_trees.tre
#It has extra lines

#Remove metadata lines
#grep '(' /gpfs/gibbs/pi/caccone/mkc54/albo/manuscript1/output/treemix_subsets/north_america_simplified/simplified_test/results/cat_trees.tre > cat_trees_clean.tre
grep '^[[:space:]]*(' /gpfs/gibbs/pi/caccone/mkc54/albo/manuscript1/output/treemix_subsets/south_america/results/cat_trees.tre > bootstrap_trees_only.tre
```

Read all bootstrap trees

```
library(ape)
library(phytools)
```

```
## Loading required package: maps
```

```
## 
## Attaching package: 'maps'
```

```
## The following object is masked from 'package:purrr':
## 
##     map
```

```
## 
## Attaching package: 'phytools'
```

```
## The following object is masked from 'package:R.utils':
## 
##     getParent
```

```
## The following object is masked from 'package:scales':
## 
##     rescale
```

```
trees <- read.tree("/gpfs/gibbs/pi/caccone/mkc54/albo/manuscript1/output/treemix_subsets/south_america/results/bootstrap_trees_only.tre")
length(trees)  # number of successfully parsed trees
```

```
## [1] 100
```

Get Consensus tree

```
trees <- consensus(trees, p = 0.5)
```

Plot consensus tree

```
plot(trees, main = "TreeMix Bootstrap Consensus Tree")
```

Plot bootstrap support

```
# Load ape package
library(ape)

# Read all bootstrap trees
trees <- read.tree("/gpfs/gibbs/pi/caccone/mkc54/albo/manuscript1/output/treemix_subsets/south_america/results/bootstrap_trees_only.tre")

# Compute majority-rule consensus (branches present in >=50% of trees)
cons <- consensus(trees, p = 0.5)

# Compute bootstrap support values for each node
bs <- prop.clades(cons, trees)

# Plot consensus tree with bootstrap support
plot(cons, show.node.label = TRUE, cex = 0.8)
title("Consensus Tree with Bootstrap Support")
```

Convert support values to %

```
if(!is.null(cons$node.label)){
  cons$node.label <- as.numeric(cons$node.label) * 100
}
plot(cons, show.node.label = TRUE, cex = 0.7)
```

Save

```
pdf("/gpfs/gibbs/pi/caccone/mkc54/albo/manuscript1/output/treemix_subsets/south_america/results/consensus_tree.pdf", width=10, height=8)
plot(cons, show.node.label = TRUE, cex = 0.8)
dev.off()
```

##### 7.2.5.2 Plot with Treemix functions

Set source

```
# Load TreeMix plotting functions
source("/gpfs/gibbs/pi/caccone/mkc54/albo/manuscript1/output/treemix/plotting_funcs.R")  # Adjust this path as needed
```

### 7.2.6 Rerun with 1000 bootstraps to check (sensitivity analysis)

Use modified python wrapper used in Norah’s paper This uses the
pipeline ML tree → bootreps → combine → sumtrees

Uploaded to hpc as treemix\_tree\_with\_bootreps\_NPS.py It is the same
code used in the Saarman paper, but modified for python 3

```
module load dSQ/1.05 
module load TreeMix/1.13-GCC-10.2.0
```

It takes too long to run interactively, so save as .sh file as submit
via slurm treemix\_boot1000.sh

```
#!/bin/bash
#SBATCH --job-name=treemix_boot
#SBATCH --time=7-00:00:00           
#SBATCH --mem=40G                
#SBATCH --cpus-per-task=20         
#SBATCH --mail-type ALL
#SBATCH --partition=week        # need >24 hours

module load miniconda
conda activate dendro

# Load modules
module load TreeMix/1.13-GCC-10.2.0
module load Python/3.10.8-GCCcore-12.2.0

# Move to working directory
cd /gpfs/gibbs/pi/caccone/mkc54/albo/manuscript1/output/treemix_subsets/south_america


# Run Python script
python treemix_tree_with_bootreps_NPS_corrected.py \
  r2_0.1_b_south_america_subset.treemix.frq \
  1 \
  Sulawesi_Indonesia \
  results/ \
  1000 \
  500

echo "Finished TreeMix bootstrap run at $(date)"
```

```
cd /gpfs/gibbs/pi/caccone/mkc54/albo/manuscript1/output/treemix_subsets/south_america

sbatch treemix_boot1000.sh #63442500
```

#### 7.2.6.1 Plots in R

Check cat\_trees.tre

```
salloc -p day --cpus-per-task=10 -t 24:00:00 --mem-per-cpu=10000

file /gpfs/gibbs/pi/caccone/mkc54/albo/manuscript1/output/treemix_subsets/south_america/results1000/cat_trees.tre
head -3 /gpfs/gibbs/pi/caccone/mkc54/albo/manuscript1/output/treemix_subsets/south_america/results1000/cat_trees.tre
#It has extra lines

#Remove metadata lines
#grep '(' /gpfs/gibbs/pi/caccone/mkc54/albo/manuscript1/output/treemix_subsets/north_america_simplified/simplified_test/results/cat_trees.tre > cat_trees_clean.tre
cd /gpfs/gibbs/pi/caccone/mkc54/albo/manuscript1/output/treemix_subsets/south_america/results1000
grep '^(' cat_trees.tre > bootstrap_trees_only.tre

#grep '^\s*('/gpfs/gibbs/pi/caccone/mkc54/albo/manuscript1/output/treemix_subsets/south_america/results1000/cat_trees.tre > bootstrap_trees1000_only.tre
#grep '^[[:space:]]*(' /gpfs/gibbs/pi/caccone/mkc54/albo/manuscript1/output/treemix_subsets/south_america/results1000/cat_trees.tre > bootstrap_trees1000_only.tre
```

Read all bootstrap trees

```
library(ape)
library(phytools)

trees <- read.tree("/gpfs/gibbs/pi/caccone/mkc54/albo/manuscript1/output/treemix_subsets/south_america/results1000/bootstrap_trees_only.tre")
length(trees)  # number of successfully parsed trees
```

```
## [1] 1000
```

Get Consensus tree

```
trees <- consensus(trees, p = 0.5)
```

Plot consensus tree

```
plot(trees, main = "TreeMix Bootstrap Consensus Tree")
```

Plot bootstrap support

```
# Load ape package
library(ape)

# Read all bootstrap trees
trees <- read.tree("/gpfs/gibbs/pi/caccone/mkc54/albo/manuscript1/output/treemix_subsets/south_america/results1000/bootstrap_trees_only.tre")

# Compute majority-rule consensus (branches present in >=50% of trees)
cons <- consensus(trees, p = 0.5)

# Compute bootstrap support values for each node
bs <- prop.clades(cons, trees)

# Plot consensus tree with bootstrap support
plot(cons, show.node.label = TRUE, cex = 0.8)
title("Consensus Tree with Bootstrap Support")
```

Convert support values to %

```
if(!is.null(cons$node.label)){
  cons$node.label <- as.numeric(cons$node.label) * 100
}
plot(cons, show.node.label = TRUE, cex = 0.7)
```

Save

```
pdf("/gpfs/gibbs/pi/caccone/mkc54/albo/manuscript1/output/treemix_subsets/south_america/results1000/consensus_tree.pdf", width=10, height=8)
plot(cons, show.node.label = TRUE, cex = 0.8)
dev.off()
```

##### 7.2.6.2 Plot with Treemix functions

Set source

```
# Load TreeMix plotting functions
source("/gpfs/gibbs/pi/caccone/mkc54/albo/manuscript1/output/treemix/plotting_funcs.R")  # Adjust this path as needed
```

Plot the maximum-likelihood tree structure, and the migration arrows
with their weights:

```
setwd("/gpfs/gibbs/pi/caccone/mkc54/albo/manuscript1/output/treemix_subsets/south_america/results1000")

plot_tree("_stem")
```

```
## Warning in plot_tree("_stem"): NAs introduced by coercion
## Warning in plot_tree("_stem"): NAs introduced by coercion
## Warning in plot_tree("_stem"): NAs introduced by coercion
## Warning in plot_tree("_stem"): NAs introduced by coercion
```

```
##     V1                 V2       V3      V4      V5  V6  V7 V8  V9 V10
## 1    0               <NA> NOT_ROOT NOT_MIG NOT_TIP 172   2  8   4   1
## 2    1     Central_Brazil NOT_ROOT NOT_MIG     TIP 104  NA NA  NA  NA
## 3    2               <NA> NOT_ROOT NOT_MIG NOT_TIP   0  32  3 136   5
## 4    3      Eastern_China NOT_ROOT NOT_MIG     TIP  32  NA NA  NA  NA
## 5    4     Southeast_Asia NOT_ROOT NOT_MIG     TIP   0  NA NA  NA  NA
## 6   15     Eastern_Brazil NOT_ROOT NOT_MIG     TIP 104  NA NA  NA  NA
## 7   16               <NA> NOT_ROOT NOT_MIG NOT_TIP 136  75  1  76   3
## 8   31              Japan NOT_ROOT NOT_MIG     TIP  52  NA NA  NA  NA
## 9   32               <NA> NOT_ROOT NOT_MIG NOT_TIP   2   3  1  52   2
## 10  51     Okinawa_Taiwan NOT_ROOT NOT_MIG     TIP  52  NA NA  NA  NA
## 11  52               <NA> NOT_ROOT NOT_MIG NOT_TIP  32  51  1  31   1
## 12  75     Western_Brazil NOT_ROOT NOT_MIG     TIP  16  NA NA  NA  NA
## 13  76               <NA> NOT_ROOT NOT_MIG NOT_TIP  16 103  1 104   2
## 14 103        Southern_SA NOT_ROOT NOT_MIG     TIP  76  NA NA  NA  NA
## 15 104               <NA> NOT_ROOT NOT_MIG NOT_TIP  76   1  1  15   1
## 16 135  Medellin_Colombia NOT_ROOT NOT_MIG     TIP 136  NA NA  NA  NA
## 17 136               <NA> NOT_ROOT NOT_MIG NOT_TIP   2  16  4 135   1
## 18 172               <NA>     ROOT NOT_MIG NOT_TIP 172   0  9 171   1
## 19 171 Sulawesi_Indonesia NOT_ROOT NOT_MIG     TIP 172  NA NA  NA  NA
## 20 174               <NA> NOT_ROOT     MIG NOT_TIP  52  31 NA  NA  NA
##                                                                                                                                                                                                                                                                                                                                                                   V11
## 1                                  (((Eastern_China:0.00220752,(Okinawa_Taiwan:0.0141344,Japan:0.0156548):0.00310458):0.00601782,((Western_Brazil:0.00385428,(Southern_SA:0.00743487,(Central_Brazil:0.000946073,Eastern_Brazil:0.0210293):0.00286696):0.00175855):0.0130672,Medellin_Colombia:0.0192015):0.00108372):0.00414463,Southeast_Asia:0.00508702):0.0183341
## 2                                                                                                                                                                                                                                                                                                                                          Central_Brazil:0.000946073
## 3                                                                        ((Eastern_China:0.00220752,(Okinawa_Taiwan:0.0141344,Japan:0.0156548):0.00310458):0.00601782,((Western_Brazil:0.00385428,(Southern_SA:0.00743487,(Central_Brazil:0.000946073,Eastern_Brazil:0.0210293):0.00286696):0.00175855):0.0130672,Medellin_Colombia:0.0192015):0.00108372):0.00414463
## 4                                                                                                                                                                                                                                                                                                                                            Eastern_China:0.00220752
## 5                                                                                                                                                                                                                                                                                                                                           Southeast_Asia:0.00508702
## 6                                                                                                                                                                                                                                                                                                                                            Eastern_Brazil:0.0210293
## 7                                                                                                                                                                                                                          (Western_Brazil:0.00385428,(Southern_SA:0.00743487,(Central_Brazil:0.000946073,Eastern_Brazil:0.0210293):0.00286696):0.00175855):0.0130672
## 8                                                                                                                                                                                                                                                                                                                                                     Japan:0.0156548
## 9                                                                                                                                                                                                                                                                         (Eastern_China:0.00220752,(Okinawa_Taiwan:0.0141344,Japan:0.0156548):0.00310458):0.00601782
## 10                                                                                                                                                                                                                                                                                                                                           Okinawa_Taiwan:0.0141344
## 11                                                                                                                                                                                                                                                                                                              (Okinawa_Taiwan:0.0141344,Japan:0.0156548):0.00310458
## 12                                                                                                                                                                                                                                                                                                                                          Western_Brazil:0.00385428
## 13                                                                                                                                                                                                                                                               (Southern_SA:0.00743487,(Central_Brazil:0.000946073,Eastern_Brazil:0.0210293):0.00286696):0.00175855
## 14                                                                                                                                                                                                                                                                                                                                             Southern_SA:0.00743487
## 15                                                                                                                                                                                                                                                                                                   (Central_Brazil:0.000946073,Eastern_Brazil:0.0210293):0.00286696
## 16                                                                                                                                                                                                                                                                                                                                        Medellin_Colombia:0.0192015
## 17                                                                                                                                                                                ((Western_Brazil:0.00385428,(Southern_SA:0.00743487,(Central_Brazil:0.000946073,Eastern_Brazil:0.0210293):0.00286696):0.00175855):0.0130672,Medellin_Colombia:0.0192015):0.00108372
## 18 ((((Eastern_China:0.00220752,(Okinawa_Taiwan:0.0141344,Japan:0.0156548):0.00310458):0.00601782,((Western_Brazil:0.00385428,(Southern_SA:0.00743487,(Central_Brazil:0.000946073,Eastern_Brazil:0.0210293):0.00286696):0.00175855):0.0130672,Medellin_Colombia:0.0192015):0.00108372):0.00414463,Southeast_Asia:0.00508702):0.0183341,Sulawesi_Indonesia:0.0183341);
## 19                                                                                                                                                                                                                                                                                                                                       Sulawesi_Indonesia:0.0183341
## 20                                                                                                                                                                                                                                                                                                                                                               <NA>
##             x    y ymin ymax
## 1  0.01833410 0.20  0.1  1.0
## 2  0.04220123 0.45  0.4  0.5
## 3  0.02247873 0.70  0.2  1.0
## 4  0.03070407 0.95  0.9  1.0
## 5  0.02342112 0.15  0.1  0.2
## 6  0.05181307 0.35  0.3  0.4
## 7  0.03662965 0.60  0.3  0.7
## 8  0.04725590 0.75  0.7  0.8
## 9  0.02849655 0.90  0.7  1.0
## 10 0.04573553 0.85  0.8  0.9
## 11 0.03160113 0.80  0.7  0.9
## 12 0.04048393 0.65  0.6  0.7
## 13 0.03838820 0.50  0.3  0.6
## 14 0.04582307 0.55  0.5  0.6
## 15 0.04125516 0.40  0.3  0.5
## 16 0.04276395 0.25  0.2  0.3
## 17 0.02356245 0.30  0.2  0.7
## 18 0.00000000 0.10  0.0  1.0
## 19 0.01833410 0.05  0.0  0.1
## 20 0.04579203   NA  0.7  0.8
## [1] "0.90649 0.03160113 0.0472559"
```

```
##  [1] 0.04220123 0.03070407 0.02342112 0.05181307 0.04725590 0.04573553
##  [7] 0.04048393 0.04582307 0.04276395 0.01833410
## [1] 0.003
## [1] "mse 0.000120293369"
```

```
## $d
##     V1                 V2       V3      V4      V5  V6  V7 V8  V9 V10
## 1    0               <NA> NOT_ROOT NOT_MIG NOT_TIP 172   2  8   4   1
## 2    1     Central_Brazil NOT_ROOT NOT_MIG     TIP 104  NA NA  NA  NA
## 3    2               <NA> NOT_ROOT NOT_MIG NOT_TIP   0  32  3 136   5
## 4    3      Eastern_China NOT_ROOT NOT_MIG     TIP  32  NA NA  NA  NA
## 5    4     Southeast_Asia NOT_ROOT NOT_MIG     TIP   0  NA NA  NA  NA
## 6   15     Eastern_Brazil NOT_ROOT NOT_MIG     TIP 104  NA NA  NA  NA
## 7   16               <NA> NOT_ROOT NOT_MIG NOT_TIP 136  75  1  76   3
## 8   31              Japan NOT_ROOT NOT_MIG     TIP  52  NA NA  NA  NA
## 9   32               <NA> NOT_ROOT NOT_MIG NOT_TIP   2   3  1  52   2
## 10  51     Okinawa_Taiwan NOT_ROOT NOT_MIG     TIP  52  NA NA  NA  NA
## 11  52               <NA> NOT_ROOT NOT_MIG NOT_TIP  32  51  1  31   1
## 12  75     Western_Brazil NOT_ROOT NOT_MIG     TIP  16  NA NA  NA  NA
## 13  76               <NA> NOT_ROOT NOT_MIG NOT_TIP  16 103  1 104   2
## 14 103        Southern_SA NOT_ROOT NOT_MIG     TIP  76  NA NA  NA  NA
## 15 104               <NA> NOT_ROOT NOT_MIG NOT_TIP  76   1  1  15   1
## 16 135  Medellin_Colombia NOT_ROOT NOT_MIG     TIP 136  NA NA  NA  NA
## 17 136               <NA> NOT_ROOT NOT_MIG NOT_TIP   2  16  4 135   1
## 18 172               <NA>     ROOT NOT_MIG NOT_TIP 172   0  9 171   1
## 19 171 Sulawesi_Indonesia NOT_ROOT NOT_MIG     TIP 172  NA NA  NA  NA
## 20 174               <NA> NOT_ROOT     MIG NOT_TIP  52  31 NA  NA  NA
##                                                                                                                                                                                                                                                                                                                                                                   V11
## 1                                  (((Eastern_China:0.00220752,(Okinawa_Taiwan:0.0141344,Japan:0.0156548):0.00310458):0.00601782,((Western_Brazil:0.00385428,(Southern_SA:0.00743487,(Central_Brazil:0.000946073,Eastern_Brazil:0.0210293):0.00286696):0.00175855):0.0130672,Medellin_Colombia:0.0192015):0.00108372):0.00414463,Southeast_Asia:0.00508702):0.0183341
## 2                                                                                                                                                                                                                                                                                                                                          Central_Brazil:0.000946073
## 3                                                                        ((Eastern_China:0.00220752,(Okinawa_Taiwan:0.0141344,Japan:0.0156548):0.00310458):0.00601782,((Western_Brazil:0.00385428,(Southern_SA:0.00743487,(Central_Brazil:0.000946073,Eastern_Brazil:0.0210293):0.00286696):0.00175855):0.0130672,Medellin_Colombia:0.0192015):0.00108372):0.00414463
## 4                                                                                                                                                                                                                                                                                                                                            Eastern_China:0.00220752
## 5                                                                                                                                                                                                                                                                                                                                           Southeast_Asia:0.00508702
## 6                                                                                                                                                                                                                                                                                                                                            Eastern_Brazil:0.0210293
## 7                                                                                                                                                                                                                          (Western_Brazil:0.00385428,(Southern_SA:0.00743487,(Central_Brazil:0.000946073,Eastern_Brazil:0.0210293):0.00286696):0.00175855):0.0130672
## 8                                                                                                                                                                                                                                                                                                                                                     Japan:0.0156548
## 9                                                                                                                                                                                                                                                                         (Eastern_China:0.00220752,(Okinawa_Taiwan:0.0141344,Japan:0.0156548):0.00310458):0.00601782
## 10                                                                                                                                                                                                                                                                                                                                           Okinawa_Taiwan:0.0141344
## 11                                                                                                                                                                                                                                                                                                              (Okinawa_Taiwan:0.0141344,Japan:0.0156548):0.00310458
## 12                                                                                                                                                                                                                                                                                                                                          Western_Brazil:0.00385428
## 13                                                                                                                                                                                                                                                               (Southern_SA:0.00743487,(Central_Brazil:0.000946073,Eastern_Brazil:0.0210293):0.00286696):0.00175855
## 14                                                                                                                                                                                                                                                                                                                                             Southern_SA:0.00743487
## 15                                                                                                                                                                                                                                                                                                   (Central_Brazil:0.000946073,Eastern_Brazil:0.0210293):0.00286696
## 16                                                                                                                                                                                                                                                                                                                                        Medellin_Colombia:0.0192015
## 17                                                                                                                                                                                ((Western_Brazil:0.00385428,(Southern_SA:0.00743487,(Central_Brazil:0.000946073,Eastern_Brazil:0.0210293):0.00286696):0.00175855):0.0130672,Medellin_Colombia:0.0192015):0.00108372
## 18 ((((Eastern_China:0.00220752,(Okinawa_Taiwan:0.0141344,Japan:0.0156548):0.00310458):0.00601782,((Western_Brazil:0.00385428,(Southern_SA:0.00743487,(Central_Brazil:0.000946073,Eastern_Brazil:0.0210293):0.00286696):0.00175855):0.0130672,Medellin_Colombia:0.0192015):0.00108372):0.00414463,Southeast_Asia:0.00508702):0.0183341,Sulawesi_Indonesia:0.0183341);
## 19                                                                                                                                                                                                                                                                                                                                       Sulawesi_Indonesia:0.0183341
## 20                                                                                                                                                                                                                                                                                                                                                               <NA>
##             x         y ymin ymax
## 1  0.01833410 0.2000000  0.1  1.0
## 2  0.04220123 0.4500000  0.4  0.5
## 3  0.02247873 0.7000000  0.2  1.0
## 4  0.03070407 0.9500000  0.9  1.0
## 5  0.02342112 0.1500000  0.1  0.2
## 6  0.05181307 0.3500000  0.3  0.4
## 7  0.03662965 0.6000000  0.3  0.7
## 8  0.04725590 0.7500000  0.7  0.8
## 9  0.02849655 0.9000000  0.7  1.0
## 10 0.04573553 0.8500000  0.8  0.9
## 11 0.03160113 0.8000000  0.7  0.9
## 12 0.04048393 0.6500000  0.6  0.7
## 13 0.03838820 0.5000000  0.3  0.6
## 14 0.04582307 0.5500000  0.5  0.6
## 15 0.04125516 0.4000000  0.3  0.5
## 16 0.04276395 0.2500000  0.2  0.3
## 17 0.02356245 0.3000000  0.2  0.7
## 18 0.00000000 0.1000000  0.0  1.0
## 19 0.01833410 0.0500000  0.0  0.1
## 20 0.04579202 0.7546755  0.7  0.8
## 
## $e
##     V1  V2          V3      V4      V5      V6       V7
## 1    0   2 0.004144630 1.00000 NOT_MIG 0.00000 1.000000
## 2    2  32 0.006017820 1.00000 NOT_MIG 0.00000 1.000000
## 3   32   3 0.002207520 1.00000 NOT_MIG 0.00000 1.000000
## 4   32  52 0.003104580 1.00000 NOT_MIG 0.00000 1.000000
## 5   52  51 0.014134400 1.00000 NOT_MIG 0.00000 1.000000
## 6  104   1 0.000946073 1.00000 NOT_MIG 0.00000 1.000000
## 7    0   4 0.005087020 1.00000 NOT_MIG 0.00000 1.000000
## 8    2 136 0.001083720 1.00000 NOT_MIG 0.00000 1.000000
## 9  136  16 0.013067200 1.00000 NOT_MIG 0.00000 1.000000
## 10 136 135 0.019201500 1.00000 NOT_MIG 0.00000 1.000000
## 11 172   0 0.018334100 1.00000 NOT_MIG 0.00000 1.000000
## 12 172 171 0.018334100 1.00000 NOT_MIG 0.00000 1.000000
## 13  52 174 0.014190900 1.00000 NOT_MIG 0.00000 1.000000
## 14 174  31 0.001463870 1.00000 NOT_MIG 0.90649 1.000000
## 15 174  15 0.000000000 0.29144     MIG 0.90649 0.000000
## 16  16  75 0.003854280 1.00000 NOT_MIG 0.00000 1.000000
## 17  16  76 0.001758550 1.00000 NOT_MIG 0.00000 1.000000
## 18  76 103 0.007434870 1.00000 NOT_MIG 0.00000 1.000000
## 19  76 104 0.002866960 1.00000 NOT_MIG 0.00000 1.000000
## 20 104  15 0.010557913 0.70856 NOT_MIG 0.00000 0.502058
```

Save the tree

```
setwd("/gpfs/gibbs/pi/caccone/mkc54/albo/manuscript1/output/treemix_subsets/south_america/results1000")

# Open PDF
pdf("treemix_consensustree1000_migration_southAmerica_subset.pdf", width = 15, height = 8)

plot_tree("_stem")

dev.off()
```

This is virtually the same tree as when we did 100 bootstraps. Plot
the maximum-likelihood tree structure, and the migration arrows with
their weights:

```
setwd("/gpfs/gibbs/pi/caccone/mkc54/albo/manuscript1/output/treemix_subsets/south_america/results")

plot_tree("_stem")
```

```
## Warning in plot_tree("_stem"): NAs introduced by coercion
## Warning in plot_tree("_stem"): NAs introduced by coercion
## Warning in plot_tree("_stem"): NAs introduced by coercion
## Warning in plot_tree("_stem"): NAs introduced by coercion
```

```
##     V1                 V2       V3      V4      V5  V6  V7 V8  V9 V10
## 1    1     Central_Brazil NOT_ROOT NOT_MIG     TIP   2  NA NA  NA  NA
## 2    2               <NA> NOT_ROOT NOT_MIG NOT_TIP  76   1  1 171   1
## 3    3     Southeast_Asia NOT_ROOT NOT_MIG     TIP  52  NA NA  NA  NA
## 4    4        Southern_SA NOT_ROOT NOT_MIG     TIP  76  NA NA  NA  NA
## 5   15  Medellin_Colombia NOT_ROOT NOT_MIG     TIP  32  NA NA  NA  NA
## 6   16               <NA> NOT_ROOT NOT_MIG NOT_TIP  52  32  5 104   3
## 7   31      Eastern_China NOT_ROOT NOT_MIG     TIP 104  NA NA  NA  NA
## 8   32               <NA> NOT_ROOT NOT_MIG NOT_TIP  16  15  1 172   4
## 9   51 Sulawesi_Indonesia NOT_ROOT NOT_MIG     TIP 173  NA NA  NA  NA
## 10  52               <NA> NOT_ROOT NOT_MIG NOT_TIP 173   3  1  16   8
## 11  75     Western_Brazil NOT_ROOT NOT_MIG     TIP 172  NA NA  NA  NA
## 12  76               <NA> NOT_ROOT NOT_MIG NOT_TIP 172   4  1   2   2
## 13 103              Japan NOT_ROOT NOT_MIG     TIP 136  NA NA  NA  NA
## 14 104               <NA> NOT_ROOT NOT_MIG NOT_TIP  16  31  1 136   2
## 15 135     Okinawa_Taiwan NOT_ROOT NOT_MIG     TIP 136  NA NA  NA  NA
## 16 136               <NA> NOT_ROOT NOT_MIG NOT_TIP 104 135  1 103   1
## 17 171     Eastern_Brazil NOT_ROOT NOT_MIG     TIP   2  NA NA  NA  NA
## 18 172               <NA> NOT_ROOT NOT_MIG NOT_TIP  32  75  1  76   3
## 19 173               <NA>     ROOT NOT_MIG NOT_TIP 173  51  1  52   9
## 20 179               <NA> NOT_ROOT     MIG NOT_TIP 136 103 NA  NA  NA
##                                                                                                                                                                                                                                                                                                                                                                   V11
## 1                                                                                                                                                                                                                                                                                                                                          Central_Brazil:0.000946073
## 2                                                                                                                                                                                                                                                                                                    (Central_Brazil:0.000946073,Eastern_Brazil:0.0210293):0.00286696
## 3                                                                                                                                                                                                                                                                                                                                           Southeast_Asia:0.00508702
## 4                                                                                                                                                                                                                                                                                                                                              Southern_SA:0.00743487
## 5                                                                                                                                                                                                                                                                                                                                         Medellin_Colombia:0.0192015
## 6                                                                        ((Medellin_Colombia:0.0192015,(Western_Brazil:0.00385428,(Southern_SA:0.00743487,(Central_Brazil:0.000946073,Eastern_Brazil:0.0210293):0.00286696):0.00175855):0.0130672):0.00108372,(Eastern_China:0.00220752,(Okinawa_Taiwan:0.0141344,Japan:0.0156548):0.00310458):0.00601782):0.00414463
## 7                                                                                                                                                                                                                                                                                                                                            Eastern_China:0.00220752
## 8                                                                                                                                                                                 (Medellin_Colombia:0.0192015,(Western_Brazil:0.00385428,(Southern_SA:0.00743487,(Central_Brazil:0.000946073,Eastern_Brazil:0.0210293):0.00286696):0.00175855):0.0130672):0.00108372
## 9                                                                                                                                                                                                                                                                                                                                        Sulawesi_Indonesia:0.0183341
## 10                                 (Southeast_Asia:0.00508702,((Medellin_Colombia:0.0192015,(Western_Brazil:0.00385428,(Southern_SA:0.00743487,(Central_Brazil:0.000946073,Eastern_Brazil:0.0210293):0.00286696):0.00175855):0.0130672):0.00108372,(Eastern_China:0.00220752,(Okinawa_Taiwan:0.0141344,Japan:0.0156548):0.00310458):0.00601782):0.00414463):0.0183341
## 11                                                                                                                                                                                                                                                                                                                                          Western_Brazil:0.00385428
## 12                                                                                                                                                                                                                                                               (Southern_SA:0.00743487,(Central_Brazil:0.000946073,Eastern_Brazil:0.0210293):0.00286696):0.00175855
## 13                                                                                                                                                                                                                                                                                                                                                    Japan:0.0156548
## 14                                                                                                                                                                                                                                                                        (Eastern_China:0.00220752,(Okinawa_Taiwan:0.0141344,Japan:0.0156548):0.00310458):0.00601782
## 15                                                                                                                                                                                                                                                                                                                                           Okinawa_Taiwan:0.0141344
## 16                                                                                                                                                                                                                                                                                                              (Okinawa_Taiwan:0.0141344,Japan:0.0156548):0.00310458
## 17                                                                                                                                                                                                                                                                                                                                           Eastern_Brazil:0.0210293
## 18                                                                                                                                                                                                                         (Western_Brazil:0.00385428,(Southern_SA:0.00743487,(Central_Brazil:0.000946073,Eastern_Brazil:0.0210293):0.00286696):0.00175855):0.0130672
## 19 (Sulawesi_Indonesia:0.0183341,(Southeast_Asia:0.00508702,((Medellin_Colombia:0.0192015,(Western_Brazil:0.00385428,(Southern_SA:0.00743487,(Central_Brazil:0.000946073,Eastern_Brazil:0.0210293):0.00286696):0.00175855):0.0130672):0.00108372,(Eastern_China:0.00220752,(Okinawa_Taiwan:0.0141344,Japan:0.0156548):0.00310458):0.00601782):0.00414463):0.0183341);
## 20                                                                                                                                                                                                                                                                                                                                                               <NA>
##             x    y ymin ymax
## 1  0.04220123 0.45  0.4  0.5
## 2  0.04125516 0.40  0.3  0.5
## 3  0.02342112 0.85  0.8  0.9
## 4  0.04582307 0.55  0.5  0.6
## 5  0.04276395 0.75  0.7  0.8
## 6  0.02247873 0.30  0.0  0.8
## 7  0.03070407 0.25  0.2  0.3
## 8  0.02356245 0.70  0.3  0.8
## 9  0.01833410 0.95  0.9  1.0
## 10 0.01833410 0.80  0.0  0.9
## 11 0.04048393 0.65  0.6  0.7
## 12 0.03838820 0.50  0.3  0.6
## 13 0.04725590 0.05  0.0  0.1
## 14 0.02849655 0.20  0.0  0.3
## 15 0.04573553 0.15  0.1  0.2
## 16 0.03160113 0.10  0.0  0.2
## 17 0.05181307 0.35  0.3  0.4
## 18 0.03662965 0.60  0.3  0.7
## 19 0.00000000 0.90  0.0  1.0
## 20 0.04579203   NA  0.0  0.1
## [1] "0.90649 0.03160113 0.0472559"
```

```
##  [1] 0.04220123 0.02342112 0.04582307 0.04276395 0.03070407 0.01833410
##  [7] 0.04048393 0.04725590 0.04573553 0.05181307
## [1] 0.003
## [1] "mse 0.000120293369"
```

```
## $d
##     V1                 V2       V3      V4      V5  V6  V7 V8  V9 V10
## 1    1     Central_Brazil NOT_ROOT NOT_MIG     TIP   2  NA NA  NA  NA
## 2    2               <NA> NOT_ROOT NOT_MIG NOT_TIP  76   1  1 171   1
## 3    3     Southeast_Asia NOT_ROOT NOT_MIG     TIP  52  NA NA  NA  NA
## 4    4        Southern_SA NOT_ROOT NOT_MIG     TIP  76  NA NA  NA  NA
## 5   15  Medellin_Colombia NOT_ROOT NOT_MIG     TIP  32  NA NA  NA  NA
## 6   16               <NA> NOT_ROOT NOT_MIG NOT_TIP  52  32  5 104   3
## 7   31      Eastern_China NOT_ROOT NOT_MIG     TIP 104  NA NA  NA  NA
## 8   32               <NA> NOT_ROOT NOT_MIG NOT_TIP  16  15  1 172   4
## 9   51 Sulawesi_Indonesia NOT_ROOT NOT_MIG     TIP 173  NA NA  NA  NA
## 10  52               <NA> NOT_ROOT NOT_MIG NOT_TIP 173   3  1  16   8
## 11  75     Western_Brazil NOT_ROOT NOT_MIG     TIP 172  NA NA  NA  NA
## 12  76               <NA> NOT_ROOT NOT_MIG NOT_TIP 172   4  1   2   2
## 13 103              Japan NOT_ROOT NOT_MIG     TIP 136  NA NA  NA  NA
## 14 104               <NA> NOT_ROOT NOT_MIG NOT_TIP  16  31  1 136   2
## 15 135     Okinawa_Taiwan NOT_ROOT NOT_MIG     TIP 136  NA NA  NA  NA
## 16 136               <NA> NOT_ROOT NOT_MIG NOT_TIP 104 135  1 103   1
## 17 171     Eastern_Brazil NOT_ROOT NOT_MIG     TIP   2  NA NA  NA  NA
## 18 172               <NA> NOT_ROOT NOT_MIG NOT_TIP  32  75  1  76   3
## 19 173               <NA>     ROOT NOT_MIG NOT_TIP 173  51  1  52   9
## 20 179               <NA> NOT_ROOT     MIG NOT_TIP 136 103 NA  NA  NA
##                                                                                                                                                                                                                                                                                                                                                                   V11
## 1                                                                                                                                                                                                                                                                                                                                          Central_Brazil:0.000946073
## 2                                                                                                                                                                                                                                                                                                    (Central_Brazil:0.000946073,Eastern_Brazil:0.0210293):0.00286696
## 3                                                                                                                                                                                                                                                                                                                                           Southeast_Asia:0.00508702
## 4                                                                                                                                                                                                                                                                                                                                              Southern_SA:0.00743487
## 5                                                                                                                                                                                                                                                                                                                                         Medellin_Colombia:0.0192015
## 6                                                                        ((Medellin_Colombia:0.0192015,(Western_Brazil:0.00385428,(Southern_SA:0.00743487,(Central_Brazil:0.000946073,Eastern_Brazil:0.0210293):0.00286696):0.00175855):0.0130672):0.00108372,(Eastern_China:0.00220752,(Okinawa_Taiwan:0.0141344,Japan:0.0156548):0.00310458):0.00601782):0.00414463
## 7                                                                                                                                                                                                                                                                                                                                            Eastern_China:0.00220752
## 8                                                                                                                                                                                 (Medellin_Colombia:0.0192015,(Western_Brazil:0.00385428,(Southern_SA:0.00743487,(Central_Brazil:0.000946073,Eastern_Brazil:0.0210293):0.00286696):0.00175855):0.0130672):0.00108372
## 9                                                                                                                                                                                                                                                                                                                                        Sulawesi_Indonesia:0.0183341
## 10                                 (Southeast_Asia:0.00508702,((Medellin_Colombia:0.0192015,(Western_Brazil:0.00385428,(Southern_SA:0.00743487,(Central_Brazil:0.000946073,Eastern_Brazil:0.0210293):0.00286696):0.00175855):0.0130672):0.00108372,(Eastern_China:0.00220752,(Okinawa_Taiwan:0.0141344,Japan:0.0156548):0.00310458):0.00601782):0.00414463):0.0183341
## 11                                                                                                                                                                                                                                                                                                                                          Western_Brazil:0.00385428
## 12                                                                                                                                                                                                                                                               (Southern_SA:0.00743487,(Central_Brazil:0.000946073,Eastern_Brazil:0.0210293):0.00286696):0.00175855
## 13                                                                                                                                                                                                                                                                                                                                                    Japan:0.0156548
## 14                                                                                                                                                                                                                                                                        (Eastern_China:0.00220752,(Okinawa_Taiwan:0.0141344,Japan:0.0156548):0.00310458):0.00601782
## 15                                                                                                                                                                                                                                                                                                                                           Okinawa_Taiwan:0.0141344
## 16                                                                                                                                                                                                                                                                                                              (Okinawa_Taiwan:0.0141344,Japan:0.0156548):0.00310458
## 17                                                                                                                                                                                                                                                                                                                                           Eastern_Brazil:0.0210293
## 18                                                                                                                                                                                                                         (Western_Brazil:0.00385428,(Southern_SA:0.00743487,(Central_Brazil:0.000946073,Eastern_Brazil:0.0210293):0.00286696):0.00175855):0.0130672
## 19 (Sulawesi_Indonesia:0.0183341,(Southeast_Asia:0.00508702,((Medellin_Colombia:0.0192015,(Western_Brazil:0.00385428,(Southern_SA:0.00743487,(Central_Brazil:0.000946073,Eastern_Brazil:0.0210293):0.00286696):0.00175855):0.0130672):0.00108372,(Eastern_China:0.00220752,(Okinawa_Taiwan:0.0141344,Japan:0.0156548):0.00310458):0.00601782):0.00414463):0.0183341);
## 20                                                                                                                                                                                                                                                                                                                                                               <NA>
##             x         y ymin ymax
## 1  0.04220123 0.4500000  0.4  0.5
## 2  0.04125516 0.4000000  0.3  0.5
## 3  0.02342112 0.8500000  0.8  0.9
## 4  0.04582307 0.5500000  0.5  0.6
## 5  0.04276395 0.7500000  0.7  0.8
## 6  0.02247873 0.3000000  0.0  0.8
## 7  0.03070407 0.2500000  0.2  0.3
## 8  0.02356245 0.7000000  0.3  0.8
## 9  0.01833410 0.9500000  0.9  1.0
## 10 0.01833410 0.8000000  0.0  0.9
## 11 0.04048393 0.6500000  0.6  0.7
## 12 0.03838820 0.5000000  0.3  0.6
## 13 0.04725590 0.0500000  0.0  0.1
## 14 0.02849655 0.2000000  0.0  0.3
## 15 0.04573553 0.1500000  0.1  0.2
## 16 0.03160113 0.1000000  0.0  0.2
## 17 0.05181307 0.3500000  0.3  0.4
## 18 0.03662965 0.6000000  0.3  0.7
## 19 0.00000000 0.9000000  0.0  1.0
## 20 0.04579202 0.0546755  0.0  0.1
## 
## $e
##     V1  V2          V3      V4      V5      V6       V7
## 1    2   1 0.000946073 1.00000 NOT_MIG 0.00000 1.000000
## 2   52   3 0.005087020 1.00000 NOT_MIG 0.00000 1.000000
## 3   32  15 0.019201500 1.00000 NOT_MIG 0.00000 1.000000
## 4  104  31 0.002207520 1.00000 NOT_MIG 0.00000 1.000000
## 5  136 135 0.014134400 1.00000 NOT_MIG 0.00000 1.000000
## 6   16  32 0.001083720 1.00000 NOT_MIG 0.00000 1.000000
## 7   52  16 0.004144630 1.00000 NOT_MIG 0.00000 1.000000
## 8  173  51 0.018334100 1.00000 NOT_MIG 0.00000 1.000000
## 9  173  52 0.018334100 1.00000 NOT_MIG 0.00000 1.000000
## 10 104 136 0.003104580 1.00000 NOT_MIG 0.00000 1.000000
## 11  32 172 0.013067200 1.00000 NOT_MIG 0.00000 1.000000
## 12  16 104 0.006017820 1.00000 NOT_MIG 0.00000 1.000000
## 13 136 179 0.014190900 1.00000 NOT_MIG 0.00000 1.000000
## 14 179 103 0.001463870 1.00000 NOT_MIG 0.90649 1.000000
## 15 179 171 0.000000000 0.29144     MIG 0.90649 0.000000
## 16 172  75 0.003854280 1.00000 NOT_MIG 0.00000 1.000000
## 17 172  76 0.001758550 1.00000 NOT_MIG 0.00000 1.000000
## 18  76   4 0.007434870 1.00000 NOT_MIG 0.00000 1.000000
## 19  76   2 0.002866960 1.00000 NOT_MIG 0.00000 1.000000
## 20   2 171 0.010557913 0.70856 NOT_MIG 0.00000 0.502058
```

Save the tree

```
setwd("/gpfs/gibbs/pi/caccone/mkc54/albo/manuscript1/output/treemix_subsets/south_america/results")

# Open PDF
pdf("treemix_consensustree_migration_southAmerica_subset.pdf", width = 15, height = 8)

plot_tree("_stem")

dev.off()
```

Create pop\_order txt

```
cd /gpfs/gibbs/pi/caccone/mkc54/albo/manuscript1/output/treemix_subsets/south_america
zcat r2_0.1_b_south_america_subset.treemix.frq.gz | head -n 1 | tr ' ' '\n' > pop_order.txt
```

Plot residuals

```
setwd("/gpfs/gibbs/pi/caccone/mkc54/albo/manuscript1/output/treemix_subsets/south_america/results")

input_file <- "/gpfs/gibbs/pi/caccone/mkc54/albo/manuscript1/output/treemix_subsets/south_america/r2_0.1_b_south_america_subset.treemix.frq.gz"  # adjust if needed
outgroup_name <- "Sulawesi_Indonesia" 
pops_list <- "/gpfs/gibbs/pi/caccone/mkc54/albo/manuscript1/output/treemix_subsets/south_america/pop_order.txt"
run_prefix <- "_stem"
par(mar = c(10, 10, 4, 4))  # ← enlarge margins for long pop names

plot_resid(run_prefix, input_file, pop_order=pops_list)
```

```
## [1] 0.0001202934
## [1] "here"
```

```
## Warning in xy.coords(x, y, xlabel, ylabel, log): NAs introduced by coercion
```

```
## [1] "1 1 0"
## [1] "2 1 0"
## [1] "2 2 0"
## [1] "3 1 -0.00053164"
## [1] "3 2 -0.00016499"
## [1] "3 3 0"
## [1] "4 1 0.00168887"
## [1] "4 2 0"
## [1] "4 3 -0.00013567"
## [1] "4 4 0"
## [1] "5 1 -0.00026058"
## [1] "5 2 -0.00017151"
## [1] "5 3 -0.000376987"
## [1] "5 4 0.00054471"
## [1] "5 5 0"
## [1] "6 1 -5.345e-05"
## [1] "6 2 0.00066797"
## [1] "6 3 0.00018375"
## [1] "6 4 -0.00019467"
## [1] "6 5 -3.41999999999964e-06"
## [1] "6 6 0"
## [1] "7 1 0.0004589"
## [1] "7 2 -7.54900000000001e-05"
## [1] "7 3 0.001079951"
## [1] "7 4 -0.00214657"
## [1] "7 5 -0.000863396"
## [1] "7 6 -0.000352306"
## [1] "7 7 0"
## [1] "8 1 0.00072031"
## [1] "8 2 -0.00101658"
## [1] "8 3 -0.00104646"
## [1] "8 4 -0.00127844"
## [1] "8 5 0.00085813"
## [1] "8 6 -0.00146031"
## [1] "8 7 0.00237044"
## [1] "8 8 0"
## [1] "9 1 -0.00074273"
## [1] "9 2 -0.00034966"
## [1] "9 3 0.001546927"
## [1] "9 4 -0.00084541"
## [1] "9 5 0.00074908"
## [1] "9 6 0.00084131"
## [1] "9 7 0"
## [1] "9 8 0.0003599"
## [1] "9 9 0"
## [1] "10 1 -0.00127969"
## [1] "10 2 0.00111027"
## [1] "10 3 -0.00055488"
## [1] "10 4 0.00236718"
## [1] "10 5 -0.00047603"
## [1] "10 6 0.00037112"
## [1] "10 7 -0.00047153"
## [1] "10 8 0.000493"
## [1] "10 9 -0.00155944"
## [1] "10 10 0"
##  [1] "#FF0000" "#FF0C00" "#FF1900" "#FF2500" "#FF3200" "#FF3E00" "#FF4B00"
##  [8] "#FF5700" "#FF6400" "#FF7000" "#FF7D00" "#FF8900" "#FF9600" "#FFA200"
## [15] "#FFAA00" "#FFB100" "#FFB800" "#FFBF00" "#FFC600" "#FFCC00" "#FFD300"
## [22] "#FFDA00" "#FFE100" "#FFE800" "#FFEF00" "#FFF500" "#FFFC00" "#FFFF0C"
## [29] "#FFFF20" "#FFFF33" "#FFFF47" "#FFFF5A" "#FFFF6D" "#FFFF81" "#FFFF94"
## [36] "#FFFFA7" "#FFFFBB" "#FFFFCE" "#FFFFE1" "#FFFFF5" "#F5FFF5" "#E1FFE1"
## [43] "#CEFFCE" "#BBFFBB" "#A7FFA7" "#94FF94" "#81FF81" "#6DFF6D" "#5AFF5A"
## [50] "#47FF47" "#33FF33" "#20FF20" "#0CFF0C" "#00F806" "#00E519" "#00D12D"
## [57] "#00BE40" "#00AB53" "#009767" "#00847A" "#00708E" "#005DA1" "#004AB4"
## [64] "#0036C8" "#0023DB" "#0010EE" "#0000FB" "#0000E8" "#0000D5" "#0000C1"
## [71] "#0000AE" "#00009A" "#000087" "#000074" "#000060" "#00004D" "#00003A"
## [78] "#000026" "#000013" "#000000"
##  [1] 0.500 0.505 0.510 0.515 0.520 0.525 0.530 0.535 0.540 0.545 0.550 0.555
## [13] 0.560 0.565 0.570 0.575 0.580 0.585 0.590 0.595 0.600 0.605 0.610 0.615
## [25] 0.620 0.625 0.630 0.635 0.640 0.645 0.650 0.655 0.660 0.665 0.670 0.675
## [37] 0.680 0.685 0.690 0.695 0.700 0.705 0.710 0.715 0.720 0.725 0.730 0.735
## [49] 0.740 0.745 0.750 0.755 0.760 0.765 0.770 0.775 0.780 0.785 0.790 0.795
## [61] 0.800 0.805 0.810 0.815 0.820 0.825 0.830 0.835 0.840 0.845 0.850 0.855
## [73] 0.860 0.865 0.870 0.875 0.880 0.885 0.890 0.895 0.900
```

```
##    Central_Brazil Eastern_Brazil Eastern_China       Japan Medellin_Colombia
## 1      0.00000000     0.00000000  -0.000531640  0.00168887      -0.000260580
## 2      0.00000000     0.00000000  -0.000164990  0.00000000      -0.000171510
## 3     -0.00053164    -0.00016499   0.000000000 -0.00013567      -0.000376987
## 4      0.00168887     0.00000000  -0.000135670  0.00000000       0.000544710
## 5     -0.00026058    -0.00017151  -0.000376987  0.00054471       0.000000000
## 6     -0.00005345     0.00066797   0.000183750 -0.00019467      -0.000003420
## 7      0.00045890    -0.00007549   0.001079951 -0.00214657      -0.000863396
## 8      0.00072031    -0.00101658  -0.001046460 -0.00127844       0.000858130
## 9     -0.00074273    -0.00034966   0.001546927 -0.00084541       0.000749080
## 10    -0.00127969     0.00111027  -0.000554880  0.00236718      -0.000476030
##    Okinawa_Taiwan Southeast_Asia Southern_SA Sulawesi_Indonesia Western_Brazil
## 1    -0.000053450    0.000458900  0.00072031       -0.000742730    -0.00127969
## 2     0.000667970   -0.000075490 -0.00101658       -0.000349660     0.00111027
## 3     0.000183750    0.001079951 -0.00104646        0.001546927    -0.00055488
## 4    -0.000194670   -0.002146570 -0.00127844       -0.000845410     0.00236718
## 5    -0.000003420   -0.000863396  0.00085813        0.000749080    -0.00047603
## 6     0.000000000   -0.000352306 -0.00146031        0.000841310     0.00037112
## 7    -0.000352306    0.000000000  0.00237044        0.000000000    -0.00047153
## 8    -0.001460310    0.002370440  0.00000000        0.000359900     0.00049300
## 9     0.000841310    0.000000000  0.00035990        0.000000000    -0.00155944
## 10    0.000371120   -0.000471530  0.00049300       -0.001559440     0.00000000
```

```
setwd("/gpfs/gibbs/pi/caccone/mkc54/albo/manuscript1/output/treemix_subsets/south_america/results")

# Open PDF
pdf("treemix_residuals_sa_subset.pdf", width = 15, height = 8)

input_file <- "/gpfs/gibbs/pi/caccone/mkc54/albo/manuscript1/output/treemix_subsets/south_america/r2_0.1_b_south_america_subset.treemix.frq.gz"  # adjust if needed
outgroup_name <- "Sulawesi_Indonesia" 
pops_list <- "/gpfs/gibbs/pi/caccone/mkc54/albo/manuscript1/output/treemix_subsets/south_america/pop_order.txt"
run_prefix <- "_stem"
par(mar = c(10, 10, 4, 4))  # ← enlarge margins for long pop names

plot_resid(run_prefix, input_file, pop_order=pops_list)

dev.off()
```

# 8. Maps

Load libraries for pie-chart maps

```
# Load necessary libraries
library(raster)  # important to load before tidyverse, otherwise it masks select()
```

```
## Loading required package: sp
```

```
## 
## Attaching package: 'raster'
```

```
## The following object is masked from 'package:MASS':
## 
##     select
```

```
## The following object is masked from 'package:dplyr':
## 
##     select
```

```
library(tidyverse)
library(sf)
```

```
## Linking to GEOS 3.11.1, GDAL 3.6.2, PROJ 9.1.1; sf_use_s2() is TRUE
```

```
library(ggspatial)
library(dplyr)
library(colorout)
library(here)
library(extrafont)
library(ggplot2)
library(scatterpie)
```

```
## 
## Attaching package: 'scatterpie'
```

```
## The following object is masked from 'package:sp':
## 
##     recenter
```

```
library(rnaturalearth)
library(rnaturalearthdata)
```

```
## 
## Attaching package: 'rnaturalearthdata'
```

```
## The following object is masked from 'package:rnaturalearth':
## 
##     countries110
```

```
library(ggrepel)
library(Cairo)
```

## 8.1 Pie plots of LEA matricies with Global dataset

### 8.1.1 K=7

```
leak7 <- read_delim(
  here("manuscript1/output/snps_sets/r2_0.01_b.snmf/K7/run5/r2_0.01_b_r5.7.Q"),
  delim = " ", # Specify the delimiter if different from the default (comma)
  col_names = FALSE,
  show_col_types = FALSE
) 
head(leak7)
```

```
## # A tibble: 6 × 7
##      X1       X2      X3       X4     X5    X6     X7
##   <dbl>    <dbl>   <dbl>    <dbl>  <dbl> <dbl>  <dbl>
## 1 0.184 0.000100 0.00117 0.0445   0.133  0.551 0.0855
## 2 0.175 0.0393   0.0242  0.0456   0.100  0.519 0.0967
## 3 0.182 0.0313   0.0256  0.0289   0.0520 0.619 0.0612
## 4 0.296 0.00608  0.0198  0.000100 0.0671 0.589 0.0212
## 5 0.287 0.000100 0.0138  0.000100 0.0701 0.612 0.0170
## 6 0.215 0.0234   0.0424  0.0124   0.0550 0.580 0.0714
```

The fam file

```
fam_file <- here("manuscript1/output/snps_sets/r2_0.01_b.fam")

# Read the .fam file
fam_data <- read.table(fam_file, 
                       header = FALSE,
                       col.names = c("FamilyID", "IndividualID", "PaternalID", "MaternalID", "Sex", "Phenotype"))

# View the first few rows
head(fam_data)
```

```
##   FamilyID IndividualID PaternalID MaternalID Sex Phenotype
## 1      OKI         1001          0          0   2        -9
## 2      OKI         1002          0          0   2        -9
## 3      OKI         1003          0          0   2        -9
## 4      OKI         1004          0          0   2        -9
## 5      OKI         1005          0          0   2        -9
## 6      OKI         1006          0          0   1        -9
```

Create column ID

```
# Change column name
colnames(fam_data)[colnames(fam_data) == "IndividualID"] <- "ind"

# Change column name
colnames(fam_data)[colnames(fam_data) == "FamilyID"] <- "pop"

# Select ID
fam_data <- fam_data |>
  dplyr::select("ind", "pop")

# View the first few rows
head(fam_data)
```

```
##    ind pop
## 1 1001 OKI
## 2 1002 OKI
## 3 1003 OKI
## 4 1004 OKI
## 5 1005 OKI
## 6 1006 OKI
```

Add it to the matrix

```
leak7 <- fam_data |>
  dplyr::select(ind, pop) |>
  bind_cols(leak7)
```

Rename the columns

```
# Rename the columns starting from the third one
leak7 <- leak7 |>
  rename_with(~paste0("v", seq_along(.x)), .cols = -c(ind, pop))
```

Import Sample Locations

```
sampling_loc <- readRDS(here("scripts", "RMarkdowns", "output", "sampling_loc_all_pops.rds"))

pops <- sampling_loc |>
   dplyr::select(
    Abbreviation, Latitude, Longitude, Pop_City, Country, Region, Subregion, Year, FINAL_order
  )

pops <- pops |> filter(!is.na(order))
```

```
## Warning: There was 1 warning in `filter()`.
## ℹ In argument: `!is.na(order)`.
## Caused by warning in `is.na()`:
## ! is.na() applied to non-(list or vector) of type 'closure'
```

```
head(pops)
```

```
##   Abbreviation Latitude Longitude    Pop_City Country    Region Subregion  Year
## 1          HAI  19.2000  109.6000      Hainan   China East Asia     Japan  2014
## 2          YUN  24.4753  101.3431      Yunnan   China East Asia     Japan  2014
## 3          HUN  27.6253  111.8569       Hunan   China East Asia     Japan  2001
## 4          OKI  26.5013  127.9454     Okinawa   Japan East Asia     Japan 2018?
## 5          TAN  30.4564  130.9784 Tanegashima   Japan East Asia     Japan  2008
## 6          KAG  31.5602  130.5581   Kagoshima   Japan East Asia     Japan  2018
##   FINAL_order
## 1           1
## 2           2
## 3           3
## 4           4
## 5           5
## 6           6
```

Merge with pops

```
# Add an index column to Q_tibble
leak7$index <- seq_len(nrow(leak7))

# Perform the merge as before
df1 <-
  merge(
    leak7,
    pops,
    by.x = 2,
    by.y = 1,
    all.x = T,
    all.y = F
  ) |>
  na.omit()

# Order by the index column to ensure the order matches the original Q_tibble
df1 <- df1[order(df1$index),]

# Optionally, you can remove the index column if it's no longer needed
df1$index <- NULL

# Now the rows of df1 should be in the same order as the original Q_tibble
head(df1)
```

```
##     pop  ind       v1          v2         v3          v4        v5       v6
## 650 OKI 1001 0.184164 0.000099991 0.00117086 0.044463300 0.1332310 0.551359
## 651 OKI 1002 0.174551 0.039295900 0.02421110 0.045550700 0.1003170 0.519340
## 652 OKI 1003 0.182160 0.031312300 0.02560670 0.028937700 0.0519773 0.618842
## 653 OKI 1004 0.296351 0.006083610 0.01984520 0.000099991 0.0671124 0.589300
## 654 OKI 1005 0.286954 0.000099982 0.01380380 0.000099982 0.0700931 0.611977
## 655 OKI 1006 0.215049 0.023440100 0.04236610 0.012419700 0.0549962 0.580313
##            v7 Latitude Longitude Pop_City Country    Region Subregion  Year
## 650 0.0855124  26.5013  127.9454  Okinawa   Japan East Asia     Japan 2018?
## 651 0.0967351  26.5013  127.9454  Okinawa   Japan East Asia     Japan 2018?
## 652 0.0611637  26.5013  127.9454  Okinawa   Japan East Asia     Japan 2018?
## 653 0.0212082  26.5013  127.9454  Okinawa   Japan East Asia     Japan 2018?
## 654 0.0169716  26.5013  127.9454  Okinawa   Japan East Asia     Japan 2018?
## 655 0.0714154  26.5013  127.9454  Okinawa   Japan East Asia     Japan 2018?
##     FINAL_order
## 650           4
## 651           4
## 652           4
## 653           4
## 654           4
## 655           4
```

```
df1$Country[df1$Country == 'USA'] <- 'United States of America' #to get USA outline to show up
df1$Country[df1$Country == 'Serbia'] <- 'Republic of Serbia'
```

Make a new color palette

```
color_palette <-
  c(  
   "blue", 
   "#FF8005",
   "magenta", 
   "#FFE100", 
   "#FF0010",  
   "green",
   "purple3"
    )
```

Pie plots on world map

```
# Load necessary libraries
library(ggplot2)
library(scatterpie)
library(sf)
library(rnaturalearth)
library(rnaturalearthdata)
library(ggrepel)
library(Cairo)

world <- ne_countries(scale = "medium", returnclass = "sf")
countries_with_data <- unique(df1$Country)

# Calculate mean proportions for each population
df_mean <- df1 |>
  group_by(pop) |>
  summarise(across(starts_with("v"), \(x) mean(x, na.rm = TRUE)), 
            Longitude = mean(Longitude),
            Latitude = mean(Latitude))

source(
  here("scripts", "RMarkdowns",
    "analyses", "my_theme2.R"
  )
)

ggplot() +
  geom_sf(data = world, fill="white") +
  geom_scatterpie(data = df_mean, 
                  aes(x = Longitude, y = Latitude, r = 1.5), 
                  cols = c("v1", "v2", "v3", "v4", "v5", "v6", "v7"), color = NA) +
  geom_text_repel(data = df_mean,
                  aes(x = Longitude, y = Latitude, label = pop), 
                  size = 3, 
                  box.padding = unit(0.1, "lines"),
                  max.overlaps = 50) +
  scale_fill_manual(values = color_palette) +
  guides(fill = "none") +  # Hide legend
  coord_sf() +
  #coord_sf(xlim = c(-20, 150), ylim = c(-30, 60)) + #Europe & Asia only
  #coord_sf(xlim = c(-125, -60), ylim = c(25, 50)) + #just North America
  #coord_sf(xlim = c(-100, -20), ylim = c(-50, 30)) + #just South America
  my_theme()
```

```
## Warning: `aes_()` was deprecated in ggplot2 3.0.0.
## ℹ Please use tidy evaluation idioms with `aes()`
## ℹ The deprecated feature was likely used in the scatterpie package.
##   Please report the issue to the authors.
## This warning is displayed once every 8 hours.
## Call `lifecycle::last_lifecycle_warnings()` to see where this warning was
## generated.
```

```
ggsave(
  here("manuscript1/output/lea/man1_k7_lea_pie.pdf"),
  width  = 12,
  height = 6,
  units  = "in",
  device = cairo_pdf
)
```

### 8.1.2 K=20

```
leak20 <- read_delim(
  here("manuscript1/output/snps_sets/r2_0.01_b.snmf/K20/run3/r2_0.01_b_r3.20.Q"),
  delim = " ", # Specify the delimiter if different from the default (comma)
  col_names = FALSE,
  show_col_types = FALSE
) 
head(leak20)
```

```
## # A tibble: 6 × 20
##         X1      X2      X3      X4      X5      X6    X7      X8      X9     X10
##      <dbl>   <dbl>   <dbl>   <dbl>   <dbl>   <dbl> <dbl>   <dbl>   <dbl>   <dbl>
## 1  3.06e-2 9.99e-5 9.99e-5 3.19e-2 9.99e-5 9.99e-5 0.427 9.99e-5 9.99e-5 9.99e-5
## 2  7.29e-2 2.39e-2 2.59e-2 1.00e-4 1.00e-4 1.71e-2 0.267 3.15e-2 1.00e-4 2.11e-2
## 3  9.99e-5 9.99e-5 1.67e-2 2.82e-2 1.72e-2 9.99e-5 0.672 1.83e-3 1.62e-2 9.99e-5
## 4  9.98e-5 9.98e-5 9.98e-5 9.98e-5 9.98e-5 9.98e-5 0.900 9.98e-5 9.98e-5 9.98e-5
## 5  9.98e-5 9.98e-5 9.98e-5 9.98e-5 9.98e-5 9.98e-5 0.936 9.98e-5 9.98e-5 9.98e-5
## 6  9.99e-5 9.99e-5 8.97e-3 1.95e-2 9.99e-5 9.99e-5 0.751 3.43e-3 1.02e-2 9.99e-5
## # ℹ 10 more variables: X11 <dbl>, X12 <dbl>, X13 <dbl>, X14 <dbl>, X15 <dbl>,
## #   X16 <dbl>, X17 <dbl>, X18 <dbl>, X19 <dbl>, X20 <dbl>
```

The fam file

```
fam_file <- here("manuscript1/output/snps_sets/r2_0.01_b.fam")

# Read the .fam file
fam_data <- read.table(fam_file, 
                       header = FALSE,
                       col.names = c("FamilyID", "IndividualID", "PaternalID", "MaternalID", "Sex", "Phenotype"))

# View the first few rows
head(fam_data)
```

```
##   FamilyID IndividualID PaternalID MaternalID Sex Phenotype
## 1      OKI         1001          0          0   2        -9
## 2      OKI         1002          0          0   2        -9
## 3      OKI         1003          0          0   2        -9
## 4      OKI         1004          0          0   2        -9
## 5      OKI         1005          0          0   2        -9
## 6      OKI         1006          0          0   1        -9
```

Create column ID

```
# Change column name
colnames(fam_data)[colnames(fam_data) == "IndividualID"] <- "ind"

# Change column name
colnames(fam_data)[colnames(fam_data) == "FamilyID"] <- "pop"

# Select ID
fam_data <- fam_data |>
  dplyr::select("ind", "pop")

# View the first few rows
head(fam_data)
```

```
##    ind pop
## 1 1001 OKI
## 2 1002 OKI
## 3 1003 OKI
## 4 1004 OKI
## 5 1005 OKI
## 6 1006 OKI
```

Add it to the matrix

```
leak20 <- fam_data |>
  dplyr::select(ind, pop) |>
  bind_cols(leak20)
```

Rename the columns

```
# Rename the columns starting from the third one
leak20 <- leak20 |>
  rename_with(~paste0("v", seq_along(.x)), .cols = -c(ind, pop))
```

Import Sample Locations

```
sampling_loc <- readRDS(here("scripts", "RMarkdowns", "output", "sampling_loc_all_pops.rds"))

pops <- sampling_loc |>
   dplyr::select(
    Abbreviation, Latitude, Longitude, Pop_City, Country, Region, Subregion, Year, FINAL_order
  )

pops <- pops |> filter(!is.na(order))
```

```
## Warning: There was 1 warning in `filter()`.
## ℹ In argument: `!is.na(order)`.
## Caused by warning in `is.na()`:
## ! is.na() applied to non-(list or vector) of type 'closure'
```

```
head(pops)
```

```
##   Abbreviation Latitude Longitude    Pop_City Country    Region Subregion  Year
## 1          HAI  19.2000  109.6000      Hainan   China East Asia     Japan  2014
## 2          YUN  24.4753  101.3431      Yunnan   China East Asia     Japan  2014
## 3          HUN  27.6253  111.8569       Hunan   China East Asia     Japan  2001
## 4          OKI  26.5013  127.9454     Okinawa   Japan East Asia     Japan 2018?
## 5          TAN  30.4564  130.9784 Tanegashima   Japan East Asia     Japan  2008
## 6          KAG  31.5602  130.5581   Kagoshima   Japan East Asia     Japan  2018
##   FINAL_order
## 1           1
## 2           2
## 3           3
## 4           4
## 5           5
## 6           6
```

Merge with pops

```
# Add an index column to Q_tibble
leak20$index <- seq_len(nrow(leak20))

# Perform the merge as before
df1 <-
  merge(
    leak20,
    pops,
    by.x = 2,
    by.y = 1,
    all.x = T,
    all.y = F
  ) |>
  na.omit()

# Order by the index column to ensure the order matches the original Q_tibble
df1 <- df1[order(df1$index),]

# Optionally, you can remove the index column if it's no longer needed
df1$index <- NULL

# Now the rows of df1 should be in the same order as the original Q_tibble
head(df1)
```

```
##     pop  ind          v1          v2          v3          v4          v5
## 650 OKI 1001 3.06120e-02 9.99100e-05 9.99100e-05 3.19485e-02 9.99100e-05
## 651 OKI 1002 7.28606e-02 2.38574e-02 2.59435e-02 9.99550e-05 9.99550e-05
## 652 OKI 1003 9.99190e-05 9.99190e-05 1.66988e-02 2.81766e-02 1.72400e-02
## 653 OKI 1004 9.98378e-05 9.98378e-05 9.98378e-05 9.98378e-05 9.98378e-05
## 654 OKI 1005 9.98378e-05 9.98378e-05 9.98378e-05 9.98378e-05 9.98378e-05
## 655 OKI 1006 9.98920e-05 9.98920e-05 8.97185e-03 1.94965e-02 9.98920e-05
##              v6       v7          v8          v9         v10         v11
## 650 9.99100e-05 0.426981 9.99100e-05 9.99100e-05 9.99100e-05 2.70803e-02
## 651 1.71181e-02 0.267228 3.15209e-02 9.99550e-05 2.11178e-02 1.60308e-02
## 652 9.99190e-05 0.671843 1.83393e-03 1.62475e-02 9.99190e-05 9.99190e-05
## 653 9.98378e-05 0.899814 9.98378e-05 9.98378e-05 9.98378e-05 9.98378e-05
## 654 9.98378e-05 0.935938 9.98378e-05 9.98378e-05 9.98378e-05 9.98378e-05
## 655 9.98920e-05 0.751193 3.43474e-03 1.01842e-02 9.98920e-05 9.98920e-05
##             v12         v13         v14         v15         v16         v17
## 650 1.79172e-01 1.66674e-01 1.53596e-02 9.99100e-05 4.57297e-02 9.99100e-05
## 651 9.31839e-02 2.45387e-01 1.91602e-02 4.29681e-03 2.86808e-02 9.99550e-05
## 652 3.69289e-02 1.33751e-01 1.23076e-02 9.99190e-05 1.41762e-02 9.99190e-05
## 653 9.98378e-05 9.98378e-05 9.98378e-05 9.98378e-05 9.98378e-05 9.98378e-05
## 654 9.98378e-05 9.98378e-05 9.98378e-05 9.98378e-05 9.98378e-05 9.98378e-05
## 655 9.98920e-05 1.43390e-01 9.98920e-05 9.98920e-05 9.98920e-05 4.03119e-02
##             v18         v19         v20 Latitude Longitude Pop_City Country
## 650 5.21531e-02 0.023289500 9.99100e-05  26.5013  127.9454  Okinawa   Japan
## 651 5.88549e-02 0.000099955 7.42596e-02  26.5013  127.9454  Okinawa   Japan
## 652 4.98969e-02 0.000099919 9.99190e-05  26.5013  127.9454  Okinawa   Japan
## 653 9.98378e-05 0.098388700 9.98378e-05  26.5013  127.9454  Okinawa   Japan
## 654 9.98378e-05 0.062265200 9.98378e-05  26.5013  127.9454  Okinawa   Japan
## 655 2.18197e-02 0.000099892 9.98920e-05  26.5013  127.9454  Okinawa   Japan
##        Region Subregion  Year FINAL_order
## 650 East Asia     Japan 2018?           4
## 651 East Asia     Japan 2018?           4
## 652 East Asia     Japan 2018?           4
## 653 East Asia     Japan 2018?           4
## 654 East Asia     Japan 2018?           4
## 655 East Asia     Japan 2018?           4
```

```
df1$Country[df1$Country == 'USA'] <- 'United States of America' #to get USA outline to show up
df1$Country[df1$Country == 'Serbia'] <- 'Republic of Serbia'
```

Make a new color palette

```
color_palette <-
c(
"ivory", 
"#005C31",
"magenta",
"#990000", 
"orchid1",
"cadetblue",
"#FFE100",   
"#9DCC00", 
"#808080",
"#993F00", 
"#FFFC99",
"blue",
"#FF8005",
"cyan",     "#005C31",
"#1E90FF",
"purple3",
"#FF0010", 
"#003380",  
"orangered",
"green")
```

Pie plots on world map

```
# Load necessary libraries
library(ggplot2)
library(scatterpie)
library(sf)
library(rnaturalearth)
library(rnaturalearthdata)
library(ggrepel)
library(Cairo)

world <- ne_countries(scale = "medium", returnclass = "sf")
countries_with_data <- unique(df1$Country)

# Calculate mean proportions for each population
df_mean <- df1 |>
  group_by(pop) |>
  summarise(across(starts_with("v"), \(x) mean(x, na.rm = TRUE)), 
            Longitude = mean(Longitude),
            Latitude = mean(Latitude))

source(
  here("scripts", "RMarkdowns",
    "analyses", "my_theme2.R"
  )
)

ggplot() +
  geom_sf(data = world, fill="white") +
  geom_scatterpie(data = df_mean, 
                  aes(x = Longitude, y = Latitude, r = 1.5), 
                  cols = c("v1", "v2", "v3", "v4", "v5", "v6", "v7", "v8", "v9", "v10", "v11", "v12", "v13", "v14", "v15", "v16", "v17", 
                           "v18", "v19", "v20"), color = NA) +
  geom_text_repel(data = df_mean,
                  aes(x = Longitude, y = Latitude, label = pop), 
                 size = 3, 
                  box.padding = unit(0.1, "lines"),
                  max.overlaps = 50) +
  scale_fill_manual(values = color_palette) +
  guides(fill = "none") +  # Hide legend
  coord_sf() +
  #coord_sf(xlim = c(-20, 150), ylim = c(-30, 60)) + #Europe & Asia only
  #coord_sf(xlim = c(-125, -60), ylim = c(25, 50)) + #just North America
  #coord_sf(xlim = c(-100, -20), ylim = c(-50, 30)) + #just South America
  my_theme()
```

```
ggsave(
  here("manuscript1/output/lea/man1_k20_lea_pie.pdf"),
  width  = 12,
  height = 6,
  units  = "in",
  device = cairo_pdf
)
```

### 8.1.3 K=25

```
leak25 <- read_delim(
  here("manuscript1/output/snps_sets/r2_0.01_b.snmf/K25/run1/r2_0.01_b_r1.25.Q"),
  delim = " ", # Specify the delimiter if different from the default (comma)
  col_names = FALSE,
  show_col_types = FALSE
) 
head(leak25)
```

```
## # A tibble: 6 × 25
##          X1        X2        X3       X4      X5      X6      X7      X8      X9
##       <dbl>     <dbl>     <dbl>    <dbl>   <dbl>   <dbl>   <dbl>   <dbl>   <dbl>
## 1 0.0683    0.0000999 0.0000999  9.99e-5 9.99e-5 9.99e-5 3.83e-2 9.99e-5 7.89e-3
## 2 0.0289    0.0290    0.0000999  9.99e-5 9.99e-5 9.99e-5 3.11e-2 1.53e-2 4.39e-2
## 3 0.0361    0.0000998 0.0000998  9.98e-5 9.98e-5 9.98e-5 9.98e-5 9.98e-5 9.98e-5
## 4 0.0000998 0.0000998 0.0000998  9.98e-5 9.98e-5 9.98e-5 9.98e-5 9.98e-5 9.98e-5
## 5 0.0000998 0.00926   0.0000998  9.98e-5 9.98e-5 9.98e-5 9.98e-5 9.98e-5 9.98e-5
## 6 0.0000999 0.0000999 0.0165     9.99e-5 4.25e-2 9.99e-5 9.99e-5 9.99e-5 9.99e-5
## # ℹ 16 more variables: X10 <dbl>, X11 <dbl>, X12 <dbl>, X13 <dbl>, X14 <dbl>,
## #   X15 <dbl>, X16 <dbl>, X17 <dbl>, X18 <dbl>, X19 <dbl>, X20 <dbl>,
## #   X21 <dbl>, X22 <dbl>, X23 <dbl>, X24 <dbl>, X25 <dbl>
```

The fam file

```
fam_file <- here("manuscript1/output/snps_sets/r2_0.01_b.fam")

# Read the .fam file
fam_data <- read.table(fam_file, 
                       header = FALSE,
                       col.names = c("FamilyID", "IndividualID", "PaternalID", "MaternalID", "Sex", "Phenotype"))

# View the first few rows
head(fam_data)
```

```
##   FamilyID IndividualID PaternalID MaternalID Sex Phenotype
## 1      OKI         1001          0          0   2        -9
## 2      OKI         1002          0          0   2        -9
## 3      OKI         1003          0          0   2        -9
## 4      OKI         1004          0          0   2        -9
## 5      OKI         1005          0          0   2        -9
## 6      OKI         1006          0          0   1        -9
```

Create column ID

```
# Change column name
colnames(fam_data)[colnames(fam_data) == "IndividualID"] <- "ind"

# Change column name
colnames(fam_data)[colnames(fam_data) == "FamilyID"] <- "pop"

# Select ID
fam_data <- fam_data |>
  dplyr::select("ind", "pop")

# View the first few rows
head(fam_data)
```

```
##    ind pop
## 1 1001 OKI
## 2 1002 OKI
## 3 1003 OKI
## 4 1004 OKI
## 5 1005 OKI
## 6 1006 OKI
```

Add it to the matrix

```
leak25 <- fam_data |>
  dplyr::select(ind, pop) |>
  bind_cols(leak25)
```

Rename the columns

```
# Rename the columns starting from the third one
leak25 <- leak25 |>
  rename_with(~paste0("v", seq_along(.x)), .cols = -c(ind, pop))
```

Import Sample Locations

```
sampling_loc <- readRDS(here("scripts", "RMarkdowns", "output", "sampling_loc_all_pops.rds"))

pops <- sampling_loc |>
   dplyr::select(
    Abbreviation, Latitude, Longitude, Pop_City, Country, Region, Subregion, Year, FINAL_order
  )

pops <- pops |> filter(!is.na(order))
```

```
## Warning: There was 1 warning in `filter()`.
## ℹ In argument: `!is.na(order)`.
## Caused by warning in `is.na()`:
## ! is.na() applied to non-(list or vector) of type 'closure'
```

```
head(pops)
```

```
##   Abbreviation Latitude Longitude    Pop_City Country    Region Subregion  Year
## 1          HAI  19.2000  109.6000      Hainan   China East Asia     Japan  2014
## 2          YUN  24.4753  101.3431      Yunnan   China East Asia     Japan  2014
## 3          HUN  27.6253  111.8569       Hunan   China East Asia     Japan  2001
## 4          OKI  26.5013  127.9454     Okinawa   Japan East Asia     Japan 2018?
## 5          TAN  30.4564  130.9784 Tanegashima   Japan East Asia     Japan  2008
## 6          KAG  31.5602  130.5581   Kagoshima   Japan East Asia     Japan  2018
##   FINAL_order
## 1           1
## 2           2
## 3           3
## 4           4
## 5           5
## 6           6
```

Merge with pops

```
# Add an index column to Q_tibble
leak25$index <- seq_len(nrow(leak25))

# Perform the merge as before
df1 <-
  merge(
    leak25,
    pops,
    by.x = 2,
    by.y = 1,
    all.x = T,
    all.y = F
  ) |>
  na.omit()

# Order by the index column to ensure the order matches the original Q_tibble
df1 <- df1[order(df1$index),]

# Optionally, you can remove the index column if it's no longer needed
df1$index <- NULL

# Now the rows of df1 should be in the same order as the original Q_tibble
head(df1)
```

```
##     pop  ind          v1          v2          v3          v4          v5
## 650 OKI 1001 6.83401e-02 9.98920e-05 9.98920e-05 9.98920e-05 9.98920e-05
## 651 OKI 1002 2.88506e-02 2.90059e-02 9.99011e-05 9.99011e-05 9.99011e-05
## 652 OKI 1003 3.60858e-02 9.98471e-05 9.98471e-05 9.98471e-05 9.98471e-05
## 653 OKI 1004 9.97930e-05 9.97930e-05 9.97930e-05 9.97930e-05 9.97930e-05
## 654 OKI 1005 9.98018e-05 9.26118e-03 9.98018e-05 9.98018e-05 9.98018e-05
## 655 OKI 1006 9.98560e-05 9.98560e-05 1.64973e-02 9.98560e-05 4.24552e-02
##              v6          v7          v8          v9         v10         v11
## 650 9.98920e-05 3.82734e-02 9.98920e-05 7.89184e-03 1.53355e-03 1.09065e-01
## 651 9.99011e-05 3.11126e-02 1.53449e-02 4.39140e-02 9.99011e-05 8.43381e-02
## 652 9.98471e-05 9.98471e-05 9.98471e-05 9.98471e-05 9.98471e-05 6.57253e-02
## 653 9.97930e-05 9.97930e-05 9.97930e-05 9.97930e-05 9.97930e-05 9.97930e-05
## 654 9.98018e-05 9.98018e-05 9.98018e-05 9.98018e-05 9.98018e-05 9.98018e-05
## 655 9.98560e-05 9.98560e-05 9.98560e-05 9.98560e-05 9.98560e-05 1.91078e-02
##             v12         v13         v14         v15         v16         v17
## 650 6.61881e-02 9.98920e-05 6.87678e-03 9.98920e-05 1.56187e-01 9.98920e-05
## 651 7.19765e-02 9.99011e-05 2.03292e-02 9.99011e-05 2.10676e-01 9.99011e-05
## 652 4.83053e-02 2.62326e-02 1.79040e-02 9.98471e-05 1.39357e-01 9.98471e-05
## 653 9.97930e-05 9.97930e-05 9.97930e-05 9.97930e-05 9.97930e-05 9.97930e-05
## 654 9.98018e-05 9.98018e-05 9.98018e-05 9.98018e-05 9.98018e-05 9.98018e-05
## 655 1.84922e-02 1.60868e-02 2.64452e-02 9.98560e-05 1.22493e-01 9.98560e-05
##          v18         v19         v20         v21         v22         v23
## 650 0.422075 7.16284e-02 9.98920e-05 9.98920e-05 1.09657e-02 3.16622e-03
## 651 0.299766 8.61968e-02 9.99011e-05 1.31821e-02 1.50884e-02 9.99011e-05
## 652 0.652993 9.98471e-05 9.98471e-05 1.16996e-02 9.98471e-05 9.98471e-05
## 653 0.940536 9.97930e-05 9.97930e-05 9.97930e-05 9.97930e-05 9.97930e-05
## 654 0.945292 9.98018e-05 9.98018e-05 9.98018e-05 9.98018e-05 9.98018e-05
## 655 0.709806 9.98560e-05 9.98560e-05 2.70194e-02 9.98560e-05 9.98560e-05
##             v24         v25 Latitude Longitude Pop_City Country    Region
## 650 9.98920e-05 3.66105e-02  26.5013  127.9454  Okinawa   Japan East Asia
## 651 9.99011e-05 4.91198e-02  26.5013  127.9454  Okinawa   Japan East Asia
## 652 9.98471e-05 9.98471e-05  26.5013  127.9454  Okinawa   Japan East Asia
## 653 5.71688e-02 9.97930e-05  26.5013  127.9454  Okinawa   Japan East Asia
## 654 4.32509e-02 9.98018e-05  26.5013  127.9454  Okinawa   Japan East Asia
## 655 9.98560e-05 9.98560e-05  26.5013  127.9454  Okinawa   Japan East Asia
##     Subregion  Year FINAL_order
## 650     Japan 2018?           4
## 651     Japan 2018?           4
## 652     Japan 2018?           4
## 653     Japan 2018?           4
## 654     Japan 2018?           4
## 655     Japan 2018?           4
```

```
df1$Country[df1$Country == 'USA'] <- 'United States of America' #to get USA outline to show up
df1$Country[df1$Country == 'Serbia'] <- 'Republic of Serbia'
```

Make a new color palette

```
color_palette <-
  c(
    "#AC7700", 
    "gray",   
    "#FFE100",
    "#FFA8BB",  
    "#808080", 
    "blue",
    "#FFFC99",   
    "orangered",   
    "#005C31",
    "orchid1",
    "cyan",
    "purple3",  
    "#1E90FF", 
    "magenta",  
    "#8c61cd", 
    "#003380", 
    "#990000",
    "#9DCC00", 
    "#993F00", 
    "cadetblue", 
    "#FF8005", 
    "green",  
    "#4C005C",  
    "ivory", 
    "#FF0010"
)
```

Pie plots on world map

```
# Load necessary libraries
library(ggplot2)
library(scatterpie)
library(sf)
library(rnaturalearth)
library(rnaturalearthdata)
library(ggrepel)
library(Cairo)

world <- ne_countries(scale = "medium", returnclass = "sf")
countries_with_data <- unique(df1$Country)

# Calculate mean proportions for each population
df_mean <- df1 |>
  group_by(pop) |>
  summarise(across(starts_with("v"), \(x) mean(x, na.rm = TRUE)), 
            Longitude = mean(Longitude),
            Latitude = mean(Latitude))

source(
  here("scripts", "RMarkdowns",
    "analyses", "my_theme2.R"
  )
)

ggplot() +
  geom_sf(data = world, fill="white") +
  geom_scatterpie(data = df_mean, 
                  aes(x = Longitude, y = Latitude, r = 1.5), 
                  cols = c("v1", "v2", "v3", "v4", "v5", "v6", "v7", "v8", "v9", "v10", "v11", "v12", "v13", "v14", "v15", "v16", "v17", 
                           "v18", "v19", "v20", "v21", "v22", "v23", "v24", "v25"), color = NA) +
  geom_text_repel(data = df_mean,
                  aes(x = Longitude, y = Latitude, label = pop), 
                 size = 3, 
                  box.padding = unit(0.1, "lines"),
                  max.overlaps = 50) +
  scale_fill_manual(values = color_palette) +
  guides(fill = "none") +  # Hide legend
  coord_sf() +
  #coord_sf(xlim = c(-20, 150), ylim = c(-30, 60)) + #Europe & Asia only
  #coord_sf(xlim = c(-125, -60), ylim = c(25, 50)) + #just North America
  #coord_sf(xlim = c(-100, -20), ylim = c(-50, 30)) + #just South America
  my_theme()
```

```
ggsave(
  here("manuscript1/output/lea/man1_k25_lea_pie.pdf"),
  width  = 12,
  height = 6,
  units  = "in",
  device = cairo_pdf
)
```

## 8.2 Pie plots of fastStructure matricies with Global dataset

### 8.2.1 K=7

```
fast7 <- read_delim(
  here("/gpfs/gibbs/pi/caccone/mkc54/albo/manuscript1/output/fastStructure/run095/simple.7.meanQ"),
  delim = "  ", # Specify the delimiter if different from the default (comma)
  col_names = FALSE,
  show_col_types = FALSE
) 
head(fast7)
```

```
## # A tibble: 6 × 7
##      X1       X2       X3       X4       X5       X6       X7
##   <dbl>    <dbl>    <dbl>    <dbl>    <dbl>    <dbl>    <dbl>
## 1 0.891 0.000004 0.0600   0.0488   0.000004 0.000004 0.000004
## 2 0.782 0.0691   0.000004 0.0377   0.000004 0.111    0.000004
## 3 0.817 0.000004 0.000004 0.000004 0.000004 0.0733   0.109   
## 4 0.727 0.000004 0.000004 0.000004 0.000004 0.0506   0.223   
## 5 0.737 0.000004 0.000004 0.000004 0.000004 0.0496   0.214   
## 6 0.757 0.000004 0.000004 0.0146   0.000004 0.0816   0.147
```

The fam file

```
fam_file <- here("manuscript1/output/snps_sets/r2_0.01_b.fam")

# Read the .fam file
fam_data <- read.table(fam_file, 
                       header = FALSE,
                       col.names = c("FamilyID", "IndividualID", "PaternalID", "MaternalID", "Sex", "Phenotype"))

# View the first few rows
head(fam_data)
```

```
##   FamilyID IndividualID PaternalID MaternalID Sex Phenotype
## 1      OKI         1001          0          0   2        -9
## 2      OKI         1002          0          0   2        -9
## 3      OKI         1003          0          0   2        -9
## 4      OKI         1004          0          0   2        -9
## 5      OKI         1005          0          0   2        -9
## 6      OKI         1006          0          0   1        -9
```

Create column ID

```
# Change column name
colnames(fam_data)[colnames(fam_data) == "IndividualID"] <- "ind"

# Change column name
colnames(fam_data)[colnames(fam_data) == "FamilyID"] <- "pop"

# Select ID
fam_data <- fam_data |>
  dplyr::select("ind", "pop")

# View the first few rows
head(fam_data)
```

```
##    ind pop
## 1 1001 OKI
## 2 1002 OKI
## 3 1003 OKI
## 4 1004 OKI
## 5 1005 OKI
## 6 1006 OKI
```

Add it to the matrix

```
fast7 <- fam_data |>
  dplyr::select(ind, pop) |>
  bind_cols(fast7)

head(fast7)
```

```
##    ind pop       X1       X2       X3       X4    X5       X6       X7
## 1 1001 OKI 0.891224 0.000004 0.059978 0.048783 4e-06 0.000004 0.000004
## 2 1002 OKI 0.782013 0.069091 0.000004 0.037676 4e-06 0.111210 0.000004
## 3 1003 OKI 0.817254 0.000004 0.000004 0.000004 4e-06 0.073308 0.109425
## 4 1004 OKI 0.726599 0.000004 0.000004 0.000004 4e-06 0.050586 0.222801
## 5 1005 OKI 0.736735 0.000004 0.000004 0.000004 4e-06 0.049628 0.213623
## 6 1006 OKI 0.757141 0.000004 0.000004 0.014596 4e-06 0.081608 0.146644
```

Rename the columns

```
# Rename the columns starting from the third one
fast7 <- fast7 |>
  rename_with(~paste0("v", seq_along(.x)), .cols = -c(ind, pop))

# View the first few rows
head(fast7)
```

```
##    ind pop       v1       v2       v3       v4    v5       v6       v7
## 1 1001 OKI 0.891224 0.000004 0.059978 0.048783 4e-06 0.000004 0.000004
## 2 1002 OKI 0.782013 0.069091 0.000004 0.037676 4e-06 0.111210 0.000004
## 3 1003 OKI 0.817254 0.000004 0.000004 0.000004 4e-06 0.073308 0.109425
## 4 1004 OKI 0.726599 0.000004 0.000004 0.000004 4e-06 0.050586 0.222801
## 5 1005 OKI 0.736735 0.000004 0.000004 0.000004 4e-06 0.049628 0.213623
## 6 1006 OKI 0.757141 0.000004 0.000004 0.014596 4e-06 0.081608 0.146644
```

Import Sample Locations

```
sampling_loc <- readRDS(here("scripts", "RMarkdowns", "output", "sampling_loc_all_pops.rds"))

pops <- sampling_loc |>
   dplyr::select(
    Abbreviation, Latitude, Longitude, Pop_City, Country, Region, Subregion, Year, FINAL_order
  )

pops <- pops |> filter(!is.na(order))
```

```
## Warning: There was 1 warning in `filter()`.
## ℹ In argument: `!is.na(order)`.
## Caused by warning in `is.na()`:
## ! is.na() applied to non-(list or vector) of type 'closure'
```

```
head(pops)
```

```
##   Abbreviation Latitude Longitude    Pop_City Country    Region Subregion  Year
## 1          HAI  19.2000  109.6000      Hainan   China East Asia     Japan  2014
## 2          YUN  24.4753  101.3431      Yunnan   China East Asia     Japan  2014
## 3          HUN  27.6253  111.8569       Hunan   China East Asia     Japan  2001
## 4          OKI  26.5013  127.9454     Okinawa   Japan East Asia     Japan 2018?
## 5          TAN  30.4564  130.9784 Tanegashima   Japan East Asia     Japan  2008
## 6          KAG  31.5602  130.5581   Kagoshima   Japan East Asia     Japan  2018
##   FINAL_order
## 1           1
## 2           2
## 3           3
## 4           4
## 5           5
## 6           6
```

Merge with pops

```
# Add an index column to Q_tibble
fast7$index <- seq_len(nrow(fast7))

# Perform the merge as before
df1 <-
  merge(
    fast7,
    pops,
    by.x = 2,
    by.y = 1,
    all.x = T,
    all.y = F
  ) |>
  na.omit()

# Order by the index column to ensure the order matches the original Q_tibble
df1 <- df1[order(df1$index),]

# Optionally, you can remove the index column if it's no longer needed
df1$index <- NULL

# Now the rows of df1 should be in the same order as the original Q_tibble
head(df1)
```

```
##     pop  ind       v1       v2       v3       v4    v5       v6       v7
## 650 OKI 1001 0.891224 0.000004 0.059978 0.048783 4e-06 0.000004 0.000004
## 651 OKI 1002 0.782013 0.069091 0.000004 0.037676 4e-06 0.111210 0.000004
## 652 OKI 1003 0.817254 0.000004 0.000004 0.000004 4e-06 0.073308 0.109425
## 653 OKI 1004 0.726599 0.000004 0.000004 0.000004 4e-06 0.050586 0.222801
## 654 OKI 1005 0.736735 0.000004 0.000004 0.000004 4e-06 0.049628 0.213623
## 655 OKI 1006 0.757141 0.000004 0.000004 0.014596 4e-06 0.081608 0.146644
##     Latitude Longitude Pop_City Country    Region Subregion  Year FINAL_order
## 650  26.5013  127.9454  Okinawa   Japan East Asia     Japan 2018?           4
## 651  26.5013  127.9454  Okinawa   Japan East Asia     Japan 2018?           4
## 652  26.5013  127.9454  Okinawa   Japan East Asia     Japan 2018?           4
## 653  26.5013  127.9454  Okinawa   Japan East Asia     Japan 2018?           4
## 654  26.5013  127.9454  Okinawa   Japan East Asia     Japan 2018?           4
## 655  26.5013  127.9454  Okinawa   Japan East Asia     Japan 2018?           4
```

```
df1$Country[df1$Country == 'USA'] <- 'United States of America' #to get USA outline to show up
df1$Country[df1$Country == 'Serbia'] <- 'Republic of Serbia'
```

Make a new color palette

```
color_palette <-
  c(  
   "green",  
   "#FFE100", 
   "magenta", 
   "purple3", 
   "#FF8005",  
   "#FF0010", 
   "blue"
    )
```

Pie plots on world map

```
# Load necessary libraries
library(ggplot2)
library(scatterpie)
library(sf)
library(rnaturalearth)
library(rnaturalearthdata)
library(ggrepel)
library(Cairo)

world <- ne_countries(scale = "medium", returnclass = "sf")
countries_with_data <- unique(df1$Country)

# Calculate mean proportions for each population
df_mean <- df1 |>
  group_by(pop) |>
  summarise(across(starts_with("v"), \(x) mean(x, na.rm = TRUE)), 
            Longitude = mean(Longitude),
            Latitude = mean(Latitude))

source(
  here("scripts", "RMarkdowns",
    "analyses", "my_theme2.R"
  )
)

ggplot() +
  geom_sf(data = world, fill="white") +
  geom_scatterpie(data = df_mean, 
                  aes(x = Longitude, y = Latitude, r = 1.5), 
                  cols = c("v1", "v2", "v3", "v4", "v5", "v6", "v7"), color = NA) +
  geom_text_repel(data = df_mean,
                  aes(x = Longitude, y = Latitude, label = pop), 
                  size = 3, 
                  box.padding = unit(0.1, "lines"),
                  max.overlaps = 50) +
  scale_fill_manual(values = color_palette) +
  guides(fill = "none") +  # Hide legend
  coord_sf() +
  #coord_sf(xlim = c(-20, 150), ylim = c(-30, 60)) + #Europe & Asia only
  #coord_sf(xlim = c(-125, -60), ylim = c(25, 50)) + #just North America
  #coord_sf(xlim = c(-100, -20), ylim = c(-50, 30)) + #just South America
  my_theme()
```

```
ggsave(
  here("manuscript1/output/fastStructure/maps/man1_k7_fastStructure_pie.pdf"),
  width  = 12,
  height = 6,
  units  = "in",
  device = cairo_pdf
)
```

### 8.2.2 K=27

Extract ancestry coefficients for k=27

```
fast27 <- read_delim(
  here("/gpfs/gibbs/pi/caccone/mkc54/albo/manuscript1/output/fastStructure/run095/simple.27.meanQ"),
  delim = "  ", # Specify the delimiter if different from the default (comma)
  col_names = FALSE,
  show_col_types = FALSE
) 
head(fast27)
```

```
## # A tibble: 6 × 27
##         X1    X2    X3    X4      X5    X6      X7      X8      X9     X10   X11
##      <dbl> <dbl> <dbl> <dbl>   <dbl> <dbl>   <dbl>   <dbl>   <dbl>   <dbl> <dbl>
## 1 0.000001  1e-6  1e-6  1e-6 3.79e-2  1e-6 5.20e-2 9.81e-3 1.44e-1 2.31e-1  1e-6
## 2 0.000001  1e-6  1e-6  1e-6 1   e-6  1e-6 1   e-6 1   e-6 1   e-6 3.38e-1  1e-6
## 3 0.000001  1e-6  1e-6  1e-6 1   e-6  1e-6 1   e-6 1   e-6 1   e-6 1   e-6  1e-6
## 4 0.000001  1e-6  1e-6  1e-6 1   e-6  1e-6 1   e-6 1   e-6 1   e-6 1   e-6  1e-6
## 5 0.000001  1e-6  1e-6  1e-6 1   e-6  1e-6 1   e-6 1   e-6 1   e-6 1   e-6  1e-6
## 6 0.000001  1e-6  1e-6  1e-6 1   e-6  1e-6 1   e-6 1   e-6 7.32e-2 1.52e-1  1e-6
## # ℹ 16 more variables: X12 <dbl>, X13 <dbl>, X14 <dbl>, X15 <dbl>, X16 <dbl>,
## #   X17 <dbl>, X18 <dbl>, X19 <dbl>, X20 <dbl>, X21 <dbl>, X22 <dbl>,
## #   X23 <dbl>, X24 <dbl>, X25 <dbl>, X26 <dbl>, X27 <dbl>
```

The fam file

```
fam_file <- here("manuscript1/output/snps_sets/r2_0.01_b.fam")

# Read the .fam file
fam_data <- read.table(fam_file, 
                       header = FALSE,
                       col.names = c("FamilyID", "IndividualID", "PaternalID", "MaternalID", "Sex", "Phenotype"))

# View the first few rows
head(fam_data)
```

```
##   FamilyID IndividualID PaternalID MaternalID Sex Phenotype
## 1      OKI         1001          0          0   2        -9
## 2      OKI         1002          0          0   2        -9
## 3      OKI         1003          0          0   2        -9
## 4      OKI         1004          0          0   2        -9
## 5      OKI         1005          0          0   2        -9
## 6      OKI         1006          0          0   1        -9
```

Create column ID

```
# Change column name
colnames(fam_data)[colnames(fam_data) == "IndividualID"] <- "ind"

# Change column name
colnames(fam_data)[colnames(fam_data) == "FamilyID"] <- "pop"

# Select ID
fam_data <- fam_data |>
  dplyr::select("ind", "pop")

# View the first few rows
head(fam_data)
```

```
##    ind pop
## 1 1001 OKI
## 2 1002 OKI
## 3 1003 OKI
## 4 1004 OKI
## 5 1005 OKI
## 6 1006 OKI
```

Add it to the matrix

```
fast27 <- fam_data |>
  dplyr::select(ind, pop) |>
  bind_cols(fast27)

head(fast27)
```

```
##    ind pop    X1    X2    X3    X4       X5    X6       X7       X8       X9
## 1 1001 OKI 1e-06 1e-06 1e-06 1e-06 0.037855 1e-06 0.051979 0.009812 0.143649
## 2 1002 OKI 1e-06 1e-06 1e-06 1e-06 0.000001 1e-06 0.000001 0.000001 0.000001
## 3 1003 OKI 1e-06 1e-06 1e-06 1e-06 0.000001 1e-06 0.000001 0.000001 0.000001
## 4 1004 OKI 1e-06 1e-06 1e-06 1e-06 0.000001 1e-06 0.000001 0.000001 0.000001
## 5 1005 OKI 1e-06 1e-06 1e-06 1e-06 0.000001 1e-06 0.000001 0.000001 0.000001
## 6 1006 OKI 1e-06 1e-06 1e-06 1e-06 0.000001 1e-06 0.000001 0.000001 0.073203
##        X10   X11   X12   X13   X14   X15      X16   X17      X18   X19      X20
## 1 0.230783 1e-06 1e-06 1e-06 1e-06 1e-06 0.000001 1e-06 0.000001 1e-06 0.525903
## 2 0.338142 1e-06 1e-06 1e-06 1e-06 1e-06 0.000001 1e-06 0.000001 1e-06 0.598897
## 3 0.000001 1e-06 1e-06 1e-06 1e-06 1e-06 0.040519 1e-06 0.274209 1e-06 0.685250
## 4 0.000001 1e-06 1e-06 1e-06 1e-06 1e-06 0.000001 1e-06 0.000001 1e-06 0.853847
## 5 0.000001 1e-06 1e-06 1e-06 1e-06 1e-06 0.000001 1e-06 0.000001 1e-06 0.999976
## 6 0.151671 1e-06 1e-06 1e-06 1e-06 1e-06 0.015622 1e-06 0.104201 1e-06 0.655282
##        X21   X22   X23   X24   X25      X26   X27
## 1 0.000001 1e-06 1e-06 1e-06 1e-06 0.000001 1e-06
## 2 0.000001 1e-06 1e-06 1e-06 1e-06 0.062939 1e-06
## 3 0.000001 1e-06 1e-06 1e-06 1e-06 0.000001 1e-06
## 4 0.146130 1e-06 1e-06 1e-06 1e-06 0.000001 1e-06
## 5 0.000001 1e-06 1e-06 1e-06 1e-06 0.000001 1e-06
## 6 0.000001 1e-06 1e-06 1e-06 1e-06 0.000001 1e-06
```

Rename the columns

```
# Rename the columns starting from the third one
fast27 <- fast27 |>
  rename_with(~paste0("v", seq_along(.x)), .cols = -c(ind, pop))

# View the first few rows
head(fast27)
```

```
##    ind pop    v1    v2    v3    v4       v5    v6       v7       v8       v9
## 1 1001 OKI 1e-06 1e-06 1e-06 1e-06 0.037855 1e-06 0.051979 0.009812 0.143649
## 2 1002 OKI 1e-06 1e-06 1e-06 1e-06 0.000001 1e-06 0.000001 0.000001 0.000001
## 3 1003 OKI 1e-06 1e-06 1e-06 1e-06 0.000001 1e-06 0.000001 0.000001 0.000001
## 4 1004 OKI 1e-06 1e-06 1e-06 1e-06 0.000001 1e-06 0.000001 0.000001 0.000001
## 5 1005 OKI 1e-06 1e-06 1e-06 1e-06 0.000001 1e-06 0.000001 0.000001 0.000001
## 6 1006 OKI 1e-06 1e-06 1e-06 1e-06 0.000001 1e-06 0.000001 0.000001 0.073203
##        v10   v11   v12   v13   v14   v15      v16   v17      v18   v19      v20
## 1 0.230783 1e-06 1e-06 1e-06 1e-06 1e-06 0.000001 1e-06 0.000001 1e-06 0.525903
## 2 0.338142 1e-06 1e-06 1e-06 1e-06 1e-06 0.000001 1e-06 0.000001 1e-06 0.598897
## 3 0.000001 1e-06 1e-06 1e-06 1e-06 1e-06 0.040519 1e-06 0.274209 1e-06 0.685250
## 4 0.000001 1e-06 1e-06 1e-06 1e-06 1e-06 0.000001 1e-06 0.000001 1e-06 0.853847
## 5 0.000001 1e-06 1e-06 1e-06 1e-06 1e-06 0.000001 1e-06 0.000001 1e-06 0.999976
## 6 0.151671 1e-06 1e-06 1e-06 1e-06 1e-06 0.015622 1e-06 0.104201 1e-06 0.655282
##        v21   v22   v23   v24   v25      v26   v27
## 1 0.000001 1e-06 1e-06 1e-06 1e-06 0.000001 1e-06
## 2 0.000001 1e-06 1e-06 1e-06 1e-06 0.062939 1e-06
## 3 0.000001 1e-06 1e-06 1e-06 1e-06 0.000001 1e-06
## 4 0.146130 1e-06 1e-06 1e-06 1e-06 0.000001 1e-06
## 5 0.000001 1e-06 1e-06 1e-06 1e-06 0.000001 1e-06
## 6 0.000001 1e-06 1e-06 1e-06 1e-06 0.000001 1e-06
```

Import Sample Locations

```
sampling_loc <- readRDS(here("scripts", "RMarkdowns", "output", "sampling_loc_all_pops.rds"))

pops <- sampling_loc |>
   dplyr::select(
    Abbreviation, Latitude, Longitude, Pop_City, Country, Region, Subregion, Year, FINAL_order
  )

pops <- pops |> filter(!is.na(order))
```

```
## Warning: There was 1 warning in `filter()`.
## ℹ In argument: `!is.na(order)`.
## Caused by warning in `is.na()`:
## ! is.na() applied to non-(list or vector) of type 'closure'
```

```
head(pops)
```

```
##   Abbreviation Latitude Longitude    Pop_City Country    Region Subregion  Year
## 1          HAI  19.2000  109.6000      Hainan   China East Asia     Japan  2014
## 2          YUN  24.4753  101.3431      Yunnan   China East Asia     Japan  2014
## 3          HUN  27.6253  111.8569       Hunan   China East Asia     Japan  2001
## 4          OKI  26.5013  127.9454     Okinawa   Japan East Asia     Japan 2018?
## 5          TAN  30.4564  130.9784 Tanegashima   Japan East Asia     Japan  2008
## 6          KAG  31.5602  130.5581   Kagoshima   Japan East Asia     Japan  2018
##   FINAL_order
## 1           1
## 2           2
## 3           3
## 4           4
## 5           5
## 6           6
```

Merge with pops

```
# Add an index column to Q_tibble
fast27$index <- seq_len(nrow(fast27))

# Perform the merge as before
df1 <-
  merge(
    fast27,
    pops,
    by.x = 2,
    by.y = 1,
    all.x = T,
    all.y = F
  ) |>
  na.omit()

# Order by the index column to ensure the order matches the original Q_tibble
df1 <- df1[order(df1$index),]

# Optionally, you can remove the index column if it's no longer needed
df1$index <- NULL

# Now the rows of df1 should be in the same order as the original Q_tibble
head(df1)
```

```
##     pop  ind    v1    v2    v3    v4       v5    v6       v7       v8       v9
## 650 OKI 1001 1e-06 1e-06 1e-06 1e-06 0.037855 1e-06 0.051979 0.009812 0.143649
## 651 OKI 1002 1e-06 1e-06 1e-06 1e-06 0.000001 1e-06 0.000001 0.000001 0.000001
## 652 OKI 1003 1e-06 1e-06 1e-06 1e-06 0.000001 1e-06 0.000001 0.000001 0.000001
## 653 OKI 1004 1e-06 1e-06 1e-06 1e-06 0.000001 1e-06 0.000001 0.000001 0.000001
## 654 OKI 1005 1e-06 1e-06 1e-06 1e-06 0.000001 1e-06 0.000001 0.000001 0.000001
## 655 OKI 1006 1e-06 1e-06 1e-06 1e-06 0.000001 1e-06 0.000001 0.000001 0.073203
##          v10   v11   v12   v13   v14   v15      v16   v17      v18   v19
## 650 0.230783 1e-06 1e-06 1e-06 1e-06 1e-06 0.000001 1e-06 0.000001 1e-06
## 651 0.338142 1e-06 1e-06 1e-06 1e-06 1e-06 0.000001 1e-06 0.000001 1e-06
## 652 0.000001 1e-06 1e-06 1e-06 1e-06 1e-06 0.040519 1e-06 0.274209 1e-06
## 653 0.000001 1e-06 1e-06 1e-06 1e-06 1e-06 0.000001 1e-06 0.000001 1e-06
## 654 0.000001 1e-06 1e-06 1e-06 1e-06 1e-06 0.000001 1e-06 0.000001 1e-06
## 655 0.151671 1e-06 1e-06 1e-06 1e-06 1e-06 0.015622 1e-06 0.104201 1e-06
##          v20      v21   v22   v23   v24   v25      v26   v27 Latitude Longitude
## 650 0.525903 0.000001 1e-06 1e-06 1e-06 1e-06 0.000001 1e-06  26.5013  127.9454
## 651 0.598897 0.000001 1e-06 1e-06 1e-06 1e-06 0.062939 1e-06  26.5013  127.9454
## 652 0.685250 0.000001 1e-06 1e-06 1e-06 1e-06 0.000001 1e-06  26.5013  127.9454
## 653 0.853847 0.146130 1e-06 1e-06 1e-06 1e-06 0.000001 1e-06  26.5013  127.9454
## 654 0.999976 0.000001 1e-06 1e-06 1e-06 1e-06 0.000001 1e-06  26.5013  127.9454
## 655 0.655282 0.000001 1e-06 1e-06 1e-06 1e-06 0.000001 1e-06  26.5013  127.9454
##     Pop_City Country    Region Subregion  Year FINAL_order
## 650  Okinawa   Japan East Asia     Japan 2018?           4
## 651  Okinawa   Japan East Asia     Japan 2018?           4
## 652  Okinawa   Japan East Asia     Japan 2018?           4
## 653  Okinawa   Japan East Asia     Japan 2018?           4
## 654  Okinawa   Japan East Asia     Japan 2018?           4
## 655  Okinawa   Japan East Asia     Japan 2018?           4
```

```
df1$Country[df1$Country == 'USA'] <- 'United States of America' #to get USA outline to show up
df1$Country[df1$Country == 'Serbia'] <- 'Republic of Serbia'
```

```
color_palette <-
  c(
    "orangered",
    "green",  
    "#FFFC99",
    "#FFA8BB",  
    "#B20CC9",
    "#8c61cd", 
    "blue",
    "orchid1",
    "#FF0010", 
    "#FFE100",
    "#005C31",
    "#993F00",  
    "#1E90FF",
    "magenta",
    "#003380",
    "purple3",
    "#2BCE48", 
    "cyan", 
    "#990000", 
    "#9DCC00",  
    "#808080", 
    "gray",
    "cadetblue",     
    "#005C31",
    "#FF8005", 
    "#4C005C",
    "#AC7700")
```

Pie plots on world map

```
# Load necessary libraries
library(ggplot2)
library(scatterpie)
library(sf)
library(rnaturalearth)
library(rnaturalearthdata)
library(ggrepel)
library(Cairo)

world <- ne_countries(scale = "medium", returnclass = "sf")
countries_with_data <- unique(df1$Country)

# Calculate mean proportions for each population
df_mean <- df1 |>
  group_by(pop) |>
  summarise(across(starts_with("v"), \(x) mean(x, na.rm = TRUE)), 
            Longitude = mean(Longitude),
            Latitude = mean(Latitude))

source(
  here("scripts", "RMarkdowns",
    "analyses", "my_theme2.R"
  )
)

ggplot() +
  geom_sf(data = world, fill="white") +
  geom_scatterpie(data = df_mean, 
                  aes(x = Longitude, y = Latitude, r = 1.5), 
cols = c("v1", "v2", "v3", "v4", "v5", "v6", "v7", "v8", "v9", "v10", "v11", "v12", "v13", "v14", "v15", "v16", "v17", 
                           "v18", "v19", "v20", "v21", "v22", "v23", "v24", "v25", "v26", "v27"), color = NA) +
  geom_text_repel(data = df_mean,
                  aes(x = Longitude, y = Latitude, label = pop), 
                  size = 3, 
                  box.padding = unit(0.1, "lines"),
                  max.overlaps = 50) +
  scale_fill_manual(values = color_palette) +
  guides(fill = "none") +  # Hide legend
  coord_sf() +
  #coord_sf(xlim = c(-20, 150), ylim = c(-30, 60)) + #Europe & Asia only
  #coord_sf(xlim = c(-125, -60), ylim = c(25, 50)) + #just North America
  #coord_sf(xlim = c(-100, -20), ylim = c(-50, 30)) + #just South America
  my_theme()
```

```
ggsave(
  here("manuscript1/output/fastStructure/maps/man1_k27_fastStructure_pie.pdf"),
  width  = 12,
  height = 6,
  units  = "in",
  device = cairo_pdf
)
```

# Repeat for 10% LD dataset

# 1. LEA

Load LEA Libraries

```
library(LEA)
library(vcfR)
library(RColorBrewer)
library(adegenet)
library(ape)
library(tidyverse)
library(here)
library(dplyr)
library(ggplot2)
library(colorout)
library(extrafont)
library(scales)
library(stringr)
library(ggtext)
```

LEA for all American populations + native range + selected European
populations (representing all 14 clusters identified in the European
analysis)

## 1.1 Check and import the data

Check data - we created 2 vcf files with LD pruning r2<0.01 (LD1)
and r2<0.1 (LD2) after QC for each MAF pruned dataset (MAF <1%
& <10%)

```
ls manuscript1/output/snps_sets/*.vcf
```

```
## manuscript1/output/snps_sets/r2_0.01.vcf
## manuscript1/output/snps_sets/r2_0.01_b.vcf
## manuscript1/output/snps_sets/r2_0.1.vcf
## manuscript1/output/snps_sets/r2_0.1_b.vcf
```

We will use the dataset filtered for r<0.01 LD and MAF 1% that
includes all samples (r2\_0.01\_b.vcf)

Import the data for R2<0.01 (MAF 10% filtered)

```
genotype <- here(
   "manuscript1/output/snps_sets/r2_0.1_b.vcf"
  )

d <- read.vcfR(
  genotype
)
```

```
## Scanning file to determine attributes.
## File attributes:
##   meta lines: 8
##   header_line: 9
##   variant count: 61574
##   column count: 942
## Meta line 8 read in.
## All meta lines processed.
## gt matrix initialized.
## Character matrix gt created.
##   Character matrix gt rows: 61574
##   Character matrix gt cols: 942
##   skip: 0
##   nrows: 61574
##   row_num: 0
## Processed variant 1000Processed variant 2000Processed variant 3000Processed variant 4000Processed variant 5000Processed variant 6000Processed variant 7000Processed variant 8000Processed variant 9000Processed variant 10000Processed variant 11000Processed variant 12000Processed variant 13000Processed variant 14000Processed variant 15000Processed variant 16000Processed variant 17000Processed variant 18000Processed variant 19000Processed variant 20000Processed variant 21000Processed variant 22000Processed variant 23000Processed variant 24000Processed variant 25000Processed variant 26000Processed variant 27000Processed variant 28000Processed variant 29000Processed variant 30000Processed variant 31000Processed variant 32000Processed variant 33000Processed variant 34000Processed variant 35000Processed variant 36000Processed variant 37000Processed variant 38000Processed variant 39000Processed variant 40000Processed variant 41000Processed variant 42000Processed variant 43000Processed variant 44000Processed variant 45000Processed variant 46000Processed variant 47000Processed variant 48000Processed variant 49000Processed variant 50000Processed variant 51000Processed variant 52000Processed variant 53000Processed variant 54000Processed variant 55000Processed variant 56000Processed variant 57000Processed variant 58000Processed variant 59000Processed variant 60000Processed variant 61000Processed variant: 61574
## All variants processed
```

Get population and individuals information

```
inds_full <- attr(d@gt,"dimnames")[[2]]
inds_full <- inds_full[-1]
a <- strsplit(inds_full, '_')
pops <- unname(sapply(a, FUN = function(x) return(as.character(x[1])))) 
table(pops)
```

```
## pops
## ALD ARA BAF BCO BEA BEH BEN BER BIT BPA BPV BRE BRM CAM CES CHA CHI COL CUA DAL 
##  10  12   7  12  12   9  12  12  12  11  12  13  16  12  15  12   6  10   8  12 
## DEN DES FAY FIR FLO FRS GEL GES GRA GRV HAI HAL HAN HAR HAT HOC HOU HUN INJ INW 
##  12  16  12  12   8  12   2  12  11  12  12  10   4  12  10   7  12  12  11   4 
## IPO JAF KAC KAG KAN KAT KLP KUN LAM LIN LOS MAC MAN MAT MAU MED MON NEB NEG NEH 
##   7   2   6  12  11   6   4   3   9  12  12  12  12  12  12   7  12  10   4   7 
## NEO NEW NOV NUE OKI PAL PEO PLM POR QNC RAR REC RUS SAI SER SMI SOC SON SPB SPR 
##  12  21   4  12  11  11  10   7  12  11  12  11  12  12   4  12  12   3   8   7 
## SPS SSK STL SUF SUU TAI TIK TRA TUC TUH UTS WAV YUN 
##   8  12  12   6   6   7  12  12  14  12  12   7   9
```

```
pops <- factor(pops)
inds <- unname(sapply(a, FUN = function(x) return(as.character(x[2]))))
```

Convert format

```
vcf2geno(genotype, gsub(".vcf", ".geno", genotype))
```

```
## 
##  - number of detected individuals:   933
##  - number of detected loci:      61574
## 
## For SNP info, please check /gpfs/gibbs/pi/caccone/mkc54/albo/manuscript1/output/snps_sets/r2_0.1_b.vcfsnp.
## 
## 0 line(s) were removed because these are not SNPs.
## Please, check /gpfs/gibbs/pi/caccone/mkc54/albo/manuscript1/output/snps_sets/r2_0.1_b.removed file, for more informations.
```

```
## [1] "/gpfs/gibbs/pi/caccone/mkc54/albo/manuscript1/output/snps_sets/r2_0.1_b.geno"
```

```
vcf2lfmm(genotype, gsub(".vcf", ".lfmm", genotype))
```

```
## 
##  - number of detected individuals:   933
##  - number of detected loci:      61574
## 
## For SNP info, please check /gpfs/gibbs/pi/caccone/mkc54/albo/manuscript1/output/snps_sets/r2_0.1_b.vcfsnp.
## 
## 0 line(s) were removed because these are not SNPs.
## Please, check /gpfs/gibbs/pi/caccone/mkc54/albo/manuscript1/output/snps_sets/r2_0.1_b.removed file, for more informations.
## 
## 
##  - number of detected individuals:   933
##  - number of detected loci:      61574
```

```
## [1] "/gpfs/gibbs/pi/caccone/mkc54/albo/manuscript1/output/snps_sets/r2_0.1_b.lfmm"
```

## 1.2 PCA

```
nPC <- length(inds)
pc <- pca(gsub(".vcf", ".lfmm", genotype), K = nPC)
```

```
## [1] "******************************"
## [1] " Principal Component Analysis "
## [1] "******************************"
## summary of the options:
## 
##         -n (number of individuals)          933
##         -L (number of loci)                 61574
##         -K (number of principal components) 933
##         -x (genotype file)                  /gpfs/gibbs/pi/caccone/mkc54/albo/manuscript1/output/snps_sets/r2_0.1_b.lfmm
##         -a (eigenvalue file)                /gpfs/gibbs/pi/caccone/mkc54/albo/manuscript1/output/snps_sets/r2_0.1_b.pca/r2_0.1_b.eigenvalues
##         -e (eigenvector file)               /gpfs/gibbs/pi/caccone/mkc54/albo/manuscript1/output/snps_sets/r2_0.1_b.pca/r2_0.1_b.eigenvectors
##         -d (standard deviation file)        /gpfs/gibbs/pi/caccone/mkc54/albo/manuscript1/output/snps_sets/r2_0.1_b.pca/r2_0.1_b.sdev
##         -p (projection file)                /gpfs/gibbs/pi/caccone/mkc54/albo/manuscript1/output/snps_sets/r2_0.1_b.pca/r2_0.1_b.projections
##         -c data centered
```

```
show(pc)
```

```
## * pca class *
## 
## project directory:               /gpfs/gibbs/pi/caccone/mkc54/albo/manuscript1/output/snps_sets/ 
## pca result directory:            r2_0.1_b.pca/ 
## input file:                      r2_0.1_b.lfmm 
## eigenvalue file:                 r2_0.1_b.eigenvalues 
## eigenvector file:                r2_0.1_b.eigenvectors 
## standard deviation file:         r2_0.1_b.sdev 
## projection file:                 r2_0.1_b.projections 
## pcaProject file:                   r2_0.1_b.pcaProject 
## number of individuals:           933 
## number of loci:                  61574 
## number of principal components:  933 
## centered:                        TRUE 
## scaled:                          FALSE
```

Test

```
# PC significant test: tracy-widom test
tw <- tracy.widom(pc)
```

```
## [1] "*******************"
## [1] " Tracy-Widom tests "
## [1] "*******************"
## summary of the options:
## 
##         -n (number of eigenvalues)          933
##         -i (input file)                     /gpfs/gibbs/pi/caccone/mkc54/albo/manuscript1/output/snps_sets/r2_0.1_b.pca/r2_0.1_b.eigenvalues
##         -o (output file)                    /gpfs/gibbs/pi/caccone/mkc54/albo/manuscript1/output/snps_sets/r2_0.1_b.pca/r2_0.1_b.tracywidom
```

```
# tw$pvalues
# plot the percentage of variance explained by each component
plot(tw$percentage, pch = 19, col = "blue", cex = .8)
```

Get values

```
# plot preparation
pc.coord <- as.data.frame(pc$projections)
colnames(pc.coord) <- paste0("PC", 1:nPC)
pc.coord$Individual <- inds
pc.coord$Population <- pops
# perc1 <- paste0(round(tw$percentage, digits = 3) * 100, "%")
perc <- paste0(round(pc$eigenvalues/sum(pc$eigenvalues), digits = 3) * 100, "%")
nb.cols <- 40
mycolors <- colorRampPalette(brewer.pal(8, "Set2"))(nb.cols)
```

Check R symbols for plot

```
#to see all shapes -> plot shapes - para escolher os simbolos
N = 100; M = 1000
good.shapes = c(1:20,22:25,35:38)
foo = data.frame( x = rnorm(M), y = rnorm(M), s = factor( sample(1:N, M, replace = TRUE) ) )
ggplot(aes(x,y,shape=s ), data=foo ) +
  scale_shape_manual(values=good.shapes[1:N]) +
  geom_point()
```

```
sampling_loc <- read.csv(
  here("scripts", "RMarkdowns", "output", "sampling_loc_all_pops_9Apr2024.csv"
    ))
```

```
saveRDS(sampling_loc, here("scripts", "RMarkdowns", 
  "output", "sampling_loc_all_pops.rds"
))
```

Sample data

```
sampling_loc <- readRDS(here("scripts", "RMarkdowns", "output", "sampling_loc_all_pops.rds"))
head(sampling_loc)
```

```
##      Pop_City Country Latitude Longitude Continent Abbreviation  Year    Region
## 1      Hainan   China  19.2000  109.6000      Asia          HAI  2014 East Asia
## 2      Yunnan   China  24.4753  101.3431      Asia          YUN  2014 East Asia
## 3       Hunan   China  27.6253  111.8569      Asia          HUN  2001 East Asia
## 4     Okinawa   Japan  26.5013  127.9454      Asia          OKI 2018? East Asia
## 5 Tanegashima   Japan  30.4564  130.9784      Asia          TAN  2008 East Asia
## 6   Kagoshima   Japan  31.5602  130.5581      Asia          KAG  2018 East Asia
##   Subregion order order2 orderold AM_order state FINAL_order
## 1     Japan    55     47       47       NA                 1
## 2     Japan    56     48       48       NA                 2
## 3     Japan    57     49       49       NA                 3
## 4     Japan    54     46       46       NA                 4
## 5     Japan    NA     NA       NA       NA                 5
## 6     Japan    53     45       45       NA                 6
```

More sample data

```
# import sample attributes
samples2 <- read.delim(
  here("scripts", "RMarkdowns",
    "population_metadata_all_1Apr2024.txt"
  ),
  head = TRUE
)

samples2<- samples2 |>
  dplyr::select(
    continent, region, pop
  )

# check head of the file
head(samples2)
```

```
##   continent         region pop
## 1 Caribbean      Caribbean BRM
## 2 Caribbean      Caribbean JAM
## 3 Caribbean      Caribbean SAI
## 4    Africa Central Africa GAB
## 5    Africa    East Africa DGV
## 6    Africa    East Africa ANT
```

Merge with sampling\_loc

```
merged_loc <- merge(samples2, sampling_loc, by.x = "pop", by.y = "Abbreviation")
head(merged_loc)
```

```
##   pop     continent          region           Pop_City    Country  Latitude
## 1 AIZ          Asia       East Asia Aizuwakamatsu City      Japan  37.49240
## 2 ALD        Europe Southern Europe             Durres    Albania  41.29704
## 3 ALU        Europe  Eastern Europe            Alushta    Ukraine  44.68289
## 4 ALV        Europe Southern Europe              Vlore    Albania  40.46600
## 5 ANT        Africa     East Africa       Antananarivo Madagascar -18.87920
## 6 ARA South America   South America          Araguaina     Brazil  -7.19119
##   Longitude Continent    Year          Region     Subregion order order2
## 1 139.99360      Asia    2008       East Asia         Japan    NA     NA
## 2  19.50373    Europe    2018 Southern Europe   East Europe    33     25
## 3  34.40368    Europe    2021  Eastern Europe   East Europe    43     35
## 4  19.48970    Europe    2020 Southern Europe   East Europe    32     24
## 5  47.50790    Africa    2022     East Africa   East Africa    NA     76
## 6 -48.20810  Americas unknown   South America South America    NA     NA
##   orderold AM_order      state FINAL_order
## 1       NA       NA                     16
## 2       25       NA                     80
## 3       35       NA                     90
## 4       24       NA                     79
## 5       NA       NA                     48
## 6       NA       43 TO, Brazil         140
```

Check pops

```
head(pc.coord$Population)
```

```
## [1] OKI OKI OKI OKI OKI OKI
## 93 Levels: ALD ARA BAF BCO BEA BEH BEN BER BIT BPA BPV BRE BRM CAM CES ... YUN
```

```
#Check how many sampling localities
length(unique(pc.coord$Population))
```

```
## [1] 93
```

Check the regions

```
unique(merged_loc$Region)
```

```
##  [1] "East Asia"       "Southern Europe" "Eastern Europe"  "East Africa"    
##  [5] "South America"   "West Africa"     "North America"   "South Asia"     
##  [9] "Caribbean"       "Southeast Asia"  "Central Africa"  "Indian Ocean"   
## [13] "Western Europe"  "North Africa"
```

Check the continents

```
unique(merged_loc$continent)
```

```
## [1] "Asia"          "Europe"        "Africa"        "South America"
## [5] "North America" "Caribbean"
```

Merge

```
merged_data <- merge(pc.coord, merged_loc, by.x = "Population", by.y = "pop")
head(merged_data)
```

```
##   Population      PC1      PC2     PC3      PC4      PC5     PC6      PC7
## 1        ALD 12.47210 -17.3459 15.3715 -2.03914 -17.5181 29.4271  8.67642
## 2        ALD 11.70270 -18.9829 17.2753 -1.55201 -19.4285 35.1234  7.98931
## 3        ALD 11.87130 -17.8179 17.6462 -2.32098 -13.7110 28.2972  9.04092
## 4        ALD  9.95509 -17.7640 17.1607 -2.10500 -16.1996 32.1231  9.91590
## 5        ALD 12.43350 -17.3946 16.7447 -2.75256 -17.7517 35.3727 10.40750
## 6        ALD 11.48580 -16.1954 11.5519 -2.72355 -14.5214 25.6774  7.82205
##         PC8      PC9      PC10      PC11    PC12     PC13       PC14     PC15
## 1 -5.170070 -3.16559  -9.95041 -10.87760 4.28397 -2.45357 -4.0750500 -9.19432
## 2 -0.674442 -4.22693 -10.70950  -9.80780 4.19175 -4.19859 -0.6079330 -7.76688
## 3 -3.328340 -8.25317  -8.85598  -6.01130 3.22631 -3.11134 -0.0883012 -9.57125
## 4  0.675904 -5.74958  -9.52483 -12.15680 3.60446 -4.99037 -3.9176000 -4.87030
## 5  0.484387 -4.92715  -7.92123 -12.05770 3.91345 -5.42792 -4.0588500 -7.18650
## 6 -3.476920 -3.01476  -7.63438  -9.93542 2.14653 -2.01879 -2.5715900 -4.55891
##       PC16    PC17      PC18    PC19     PC20      PC21     PC22      PC23
## 1 -3.15854 16.0996 -12.00720 7.66511 -32.8951  4.531690 -1.59264  0.102413
## 2 -1.31815 17.5205  -9.89031 9.28258 -31.9964  6.019730  1.25888 -0.804603
## 3  2.45113 14.0448  -5.19429 8.64728 -17.3355 -0.286158  3.78432  0.619525
## 4 -5.10761 16.8699 -11.62190 5.73955 -34.0785  3.952390 -4.88544  4.730300
## 5 -3.23577 16.6454 -14.61410 7.48995 -35.7142  3.607730 -6.78271  4.081730
## 6 -5.35652 11.6194  -6.06302 9.91871 -33.0862  2.170390 -8.98188  0.927419
##       PC24     PC25      PC26      PC27    PC28     PC29     PC30    PC31
## 1 -8.57564 8.486470  0.820333  1.329430 30.6714 -2.63383 -7.66026 5.57388
## 2 -6.33156 1.463550 -1.777060  2.526950 30.1635 -9.27341 -6.05596 2.19556
## 3 -5.28300 0.591685  3.678450 -0.571494 17.2094 -3.99407 -4.50657 4.67916
## 4 -6.78118 7.991240  1.462960  5.007750 33.3670 -2.69857 -6.79597 5.83205
## 5 -6.18645 5.959110  1.141070  6.071760 31.5112 -8.46489 -4.60567 4.87215
## 6 -3.88095 4.609530  2.122890  4.878510 30.9498 -1.86787 -5.27658 0.65846
##       PC32     PC33    PC34      PC35     PC36    PC37      PC38      PC39
## 1 4.071330 14.88910 18.0315  -9.33594 -30.5081 12.8582 -11.57790 -13.70450
## 2 6.472600  9.50602 14.5320  -3.86030 -18.4456 12.3197 -11.76670 -13.60170
## 3 0.469717 10.31880 12.5926 -13.32040 -20.6154 10.6698  -5.17850 -11.02580
## 4 1.071100 16.77340 14.8018 -12.39760 -30.7984 12.1448  -9.08931 -18.00870
## 5 0.710753 17.09360 13.6596 -10.55280 -26.3430 11.3182  -7.21008 -16.04460
## 6 1.630530 12.81490 16.1492  -9.42359 -25.6295 13.0158 -10.10200  -9.85977
##        PC40     PC41      PC42       PC43     PC44      PC45    PC46      PC47
## 1 -0.587234 -28.8323  1.835170  0.0384991 -7.64847  5.293240 24.9085 -1.132090
## 2  1.908030 -25.3970  5.532530  4.2593000 -9.33399  5.752620 24.6796 -3.426270
## 3  0.521170 -23.6986  0.616582 -1.1900900 -6.01403 -0.825128 17.1390  0.224786
## 4 -0.416380 -33.0105  0.855150  4.4579500 -6.68400 -0.626926 18.9120  3.746940
## 5  0.232115 -32.2876  0.599561  8.3430900 -6.09456  0.733254 19.9615  3.470250
## 6 -0.915681 -27.5956 -2.551950 -4.0004500 -7.73024  2.556600 18.3099  1.039350
##        PC48      PC49      PC50       PC51      PC52     PC53      PC54
## 1 -4.360480  4.392310  0.681564  0.1880380  1.794900 3.237680 -0.928332
## 2 -3.139890  6.290850 -2.153170 -2.3910200  3.421670 1.490340  0.660591
## 3 -0.751560 -0.747995  2.175200 -0.0268773 -2.898900 1.410800  0.366135
## 4 -1.300180  5.779350 -0.329659 -1.6546000  1.637280 2.903390  0.907437
## 5  0.167128  6.530300 -3.340580 -1.6809600  0.973852 4.125180 -1.635360
## 6 -2.718520  8.802980  1.820840  2.2993600  6.504890 0.275305 -4.948840
##       PC55      PC56       PC57     PC58       PC59      PC60       PC61
## 1 -4.16921  3.273410  -8.342260  3.00285 -1.4486200  2.201970  0.3547510
## 2 -1.46150  2.009650  -9.196700  1.59162 -2.2417900 -2.091950 -3.6230700
## 3 -1.48645 -0.451974 -11.109700 -1.60505 -1.2909400 -2.291070  0.0246931
## 4 -1.22597  5.968140  -8.964380  6.16059  0.0920522  0.581733 -1.4602200
## 5 -2.83321  4.841150 -10.135700  4.38410  1.6161200  1.807520 -1.1118600
## 6 -2.77715 -2.913210   0.489801  5.77334 -3.6655900 -0.933340  0.1081650
##        PC62     PC63     PC64      PC65      PC66     PC67      PC68      PC69
## 1 -1.237010 -1.18475 -1.10734 -6.410970 -0.939701 4.658960 -1.776920 -3.265040
## 2 -0.230872  3.13794 -2.53963  0.110276 -3.181550 8.017560  1.601000 -2.767280
## 3  2.617740 -3.09886 -3.65672 -2.403640 -7.410530 2.958740 -0.529177 -1.541610
## 4 -0.986571 -2.99043  4.04356  4.054730 -1.682010 0.284102  0.116172  0.917741
## 5 -0.618095 -5.84041  2.72031  3.838820 -2.384890 1.034150 -1.279290 -0.688140
## 6 -2.349870 -2.49792 -1.86574  2.802150  5.216340 3.718490 -0.654553 -6.572550
##        PC70     PC71     PC72     PC73     PC74      PC75     PC76      PC77
## 1  1.904610 5.170640  5.48999  1.66407  1.46667  0.653395 -3.40975 -1.280700
## 2  3.756730 6.600860  2.03126  3.59956  5.38879 -1.652550 -5.84941 -3.781730
## 3 -0.411247 2.052990  6.65023 -1.56540 10.91580  1.833610  4.57139 -2.114940
## 4 -3.113170 0.496605 -3.92354  4.14673 -3.05857  4.105820 -3.34481  0.868898
## 5 -5.332120 3.809080 -4.23296  7.37401 -7.90029  3.642480 -3.38825 -0.433391
## 6  0.107085 4.826330  3.42373  6.58851  2.00521 -3.369450 -4.46056  4.865440
##         PC78     PC79     PC80    PC81       PC82        PC83     PC84     PC85
## 1  3.2130600  4.56050  8.81043 4.66404 -2.9191000 17.02930000  1.74178 -6.26756
## 2  7.3150000  6.73149  7.20350 6.13921  0.8557870 10.33450000 -1.15909 -4.88773
## 3  0.0455153  3.79497 -1.35781 2.81839 -5.2825500  0.00705801 -2.72962  6.06643
## 4  7.7155700 -2.88844  6.98450 1.61192 -7.1151000  4.12825000  6.95277 -5.12945
## 5  7.0177200 -4.60132  8.53960 2.21707 -6.3962100  5.53773000  6.01097 -6.25495
## 6 -0.8538970  3.28655  5.26362 3.56512 -0.0171874  9.21101000  2.17656 -5.25706
##        PC86      PC87      PC88       PC89      PC90      PC91      PC92
## 1  2.187420  0.591517  1.978320 -3.1480900  0.677793  6.338820 10.498900
## 2  4.015880 -0.754186  3.673220 -2.2769800 -0.509119  4.717090  9.257550
## 3 -3.751220 -0.692356  1.060030  0.8321190 -1.628860  1.108140  0.458722
## 4 -4.576720  7.131010 -0.577969 -0.0987706 -0.367819 -6.280500  0.505862
## 5 -5.901550  6.016590 -0.591979  1.7239300 -0.908827 -4.915650  0.188937
## 6  0.270983  2.754290  3.441800 -2.0190800 -1.209770  0.489177  8.917870
##       PC93     PC94      PC95      PC96       PC97      PC98       PC99
## 1  3.12486  5.88883  0.369937 -0.707514 -2.4154900 -0.754803   2.899340
## 2 -1.19889  6.75260 -1.892620  0.623602 -4.0416700 -1.622070   5.697230
## 3  1.28326 -4.39294  2.423370  0.932614  0.0183226 -6.362440   5.800040
## 4  1.45905 -5.65809 -8.701600 -2.458570 -3.0122700 13.103900 -22.066400
## 5  2.41877 -5.27139 -7.449840 -1.280090 -2.8643600 15.003500 -23.407000
## 6  5.07596  3.02677  1.230680  1.850730 -1.3031100 -1.508690   0.935088
##       PC100     PC101     PC102   PC103    PC104     PC105    PC106    PC107
## 1  2.763480   6.78773 -0.167617 7.16347 -1.39642  10.36860 -4.24550  1.89517
## 2  4.060740  11.18890 -1.302690 7.10377 -4.29362   9.81671 -4.82558  4.58001
## 3 -1.135060  11.61190  3.638440 2.29648 -3.71333   4.56109 -2.15596  0.60235
## 4 -6.186480 -21.90390  5.432840 2.15330  7.35406 -17.31530 -3.32797 -8.12568
## 5 -7.322470 -21.98400  5.957640 2.60806  6.19213 -18.75220 -2.87364 -9.44615
## 6 -0.804744   6.38337 -0.434590 7.77431  5.62614   5.97160 -2.41330  2.42791
##        PC108    PC109      PC110    PC111     PC112    PC113    PC114     PC115
## 1   3.790580  4.18476   3.806740 -6.54160   4.42059 3.145470 -2.01601 -4.298040
## 2   8.281100  6.22484   3.497200 -8.25477  14.34520 1.934300  1.16543 -4.094150
## 3  -0.439064  8.70047   5.864120 -4.42897   9.53725 1.702460 -3.75941 -1.540230
## 4 -18.613300 -9.58731 -20.258500 16.67950 -17.99800 0.904075  7.25515 -3.453410
## 5 -20.966900 -9.25753 -18.919600 17.93400 -20.70390 0.777590  7.11005 -4.880430
## 6   2.865310 -4.07016   0.330226 -2.54956   2.28428 0.105378  1.08168  0.559862
##       PC116     PC117    PC118     PC119     PC120     PC121    PC122    PC123
## 1  13.71890   6.44969  6.45516   6.26533 -11.41710   8.13677  6.75206 -3.78779
## 2  14.54490  11.17260  7.27779   3.94456 -14.46090   8.87256  4.49034 -6.17414
## 3   3.64129   6.44388 10.24920  -1.70589  -1.71659   3.61728  5.94133 -9.16811
## 4 -13.20620 -28.61340 -9.78750  -9.19302  22.40570 -19.00450 -5.28394 10.04950
## 5 -13.21390 -29.53310 -9.07402 -10.48350  27.25170 -19.67530 -6.36346  9.88740
## 6   6.18716   4.24633  1.07651   8.15438  -2.36496   3.53817  1.36879 -2.43487
##       PC124    PC125     PC126     PC127     PC128      PC129     PC130
## 1 -0.254138  4.98062  4.188610  2.344620 -2.052210   3.022460   4.60058
## 2 -6.565410  1.57070  1.925260  0.499353 -3.739810   5.036330   7.57632
## 3  3.169770  3.33599 11.147700  4.813400  1.839920   4.385330   7.86716
## 4 -2.636160 -8.92277 -5.351320  0.553464 -2.337420 -17.515700 -14.19110
## 5 -2.671220 -9.65653 -5.520680 -1.339190 -2.409860 -17.174900 -15.21800
## 6 -3.471640 -2.83466 -0.806266  5.659650  0.539852   0.619796   4.40731
##      PC131     PC132      PC133     PC134      PC135     PC136     PC137
## 1  3.73557  -7.05784  -3.404670 -4.486380   2.717930 -6.169370   6.44829
## 2  5.18431  -4.99908  -3.811010 -7.587890   7.744620 -1.954030  10.82500
## 3  7.67214 -14.82840 -11.120600 -1.624580   4.704320  2.414300  -6.43113
## 4 -2.71509   6.80187  12.415000  8.355680  -9.769480  1.900720 -13.63380
## 5 -3.90760   6.99191  14.438900 11.539000 -10.471900  0.494371 -16.04970
## 6  2.62929   4.61284  -0.577107  0.225313   0.293253 -3.462870   8.24996
##       PC138     PC139    PC140     PC141     PC142     PC143    PC144     PC145
## 1 -1.487430 -11.42420 -1.87373   5.17510  -7.45814   1.59361 -5.01190 -1.459520
## 2 -2.286890 -13.67290  4.07702   3.82733 -10.08060   2.79069 -5.52312 -1.593270
## 3  3.714430  -1.42509  1.72756   3.05629  -3.58368   2.23042 -5.08169  0.275348
## 4 -2.251370  19.93900 -7.02543 -13.60490  18.52260 -13.86530 19.52890  5.386820
## 5  0.744417  20.85290 -5.66362 -12.41730  18.34220 -11.58320 21.69100  5.174200
## 6 -5.137670  -9.72132 -3.26452  -1.07738  -2.24191   1.17387  2.49895  0.722375
##       PC146    PC147     PC148     PC149     PC150     PC151     PC152
## 1  0.255417  2.39231  0.511423 -0.841956  0.533351 -0.606113  1.756090
## 2  3.252920  6.90506 -1.755300 -1.317230  2.178410 -8.211490  2.430200
## 3  1.187120  1.40769  3.423100 -7.405340  3.760820  4.025550 -1.994780
## 4 -6.193440 -3.19125 -1.345150 -0.442917 -1.958000  0.864356 -3.943580
## 5 -7.968150 -3.34111 -3.363580  0.794268  0.481126  1.320530 -0.884502
## 6 -0.290400  2.71005 -4.667480  7.446490 -0.338663 -2.686220  2.857010
##        PC153    PC154    PC155    PC156    PC157    PC158      PC159      PC160
## 1  0.0373548 -4.44031 -4.28498 -3.83010 -5.71622 -4.68854   9.953090  0.0054397
## 2 -4.7974400 -1.35515 -8.20389 -2.67278 -7.60847 -5.67180   5.925970  7.4253200
## 3 -2.8435600  4.14554 -1.85707 -2.45021 -6.84394  7.03234   0.444077 -6.1882800
## 4  8.3373100 11.22310 13.76750  4.84734  8.96518  3.26969 -11.449500  1.9489100
## 5  9.4371700  9.34674 12.22500  5.38277 12.94650  5.91234  -8.968680  2.5138400
## 6 -1.6319700 -9.57413 -4.70452 -1.04007 -7.24512 -4.49017  -0.277788  3.0492000
##       PC161     PC162     PC163      PC164     PC165    PC166     PC167
## 1  1.252790 -0.794535  -2.39261  1.1257800  0.805601 -3.53083 -2.040810
## 2  4.953080  3.145190   7.71603 -0.0479613 -1.524750 -6.70223  0.215865
## 3 -3.347970 -6.849390 -10.69000 11.2927000  2.730140  7.08342  1.554640
## 4 -5.819500 -1.320000   4.88496 -4.8045700  0.966897  8.30516 -2.900980
## 5 -7.392380 -0.101183   5.83073 -5.7426400  1.632780  8.38883 -0.617469
## 6 -0.773739 -2.106360  -1.08542  1.9659400  2.031120 -4.72687  0.397220
##       PC168     PC169     PC170     PC171    PC172    PC173    PC174     PC175
## 1  -1.41530  3.956500 -0.287434 -5.020810 -2.03367 -3.40370  1.41045  4.720990
## 2 -10.57990  2.897010 -5.640700 -4.389750 -1.81438 -7.07297 -2.69015  8.892000
## 3   9.91340 -0.211275  4.408000 -3.943120  1.22319 10.92930 -1.47222 -4.857830
## 4   4.82110  3.522370  2.432610 -0.084975  7.25584  1.36230  2.63998 -8.552560
## 5   7.57137  0.356301  3.881810 -1.551690  5.67404  1.93774  4.03406 -8.010460
## 6  -5.93603 -0.660673  2.167150  2.785830 -4.62820  1.52273  1.05237  0.613194
##      PC176    PC177     PC178     PC179    PC180      PC181     PC182     PC183
## 1 -2.00159  6.19215  2.041390  1.834640 -2.65773  1.3549100   4.76350  2.173540
## 2 -6.92508  5.64175 -1.290270  0.165538  2.20229  1.7866100   2.61341 -1.296060
## 3 -1.99671 -1.79984  2.830790  4.743520 -3.25464 -7.2641500 -11.23700 -0.647134
## 4  2.03903 -2.26153  3.090870 -0.774695 -1.45875 -0.0984614   3.14168 -4.533180
## 5  1.34796 -4.71625  0.528938 -0.639184 -0.39441 -3.6538600   2.22941 -2.170840
## 6  5.75859 -2.65869 -2.047120  2.479840  4.81906  4.8641900   1.89585  4.509150
##       PC184      PC185     PC186      PC187     PC188     PC189       PC190
## 1  3.771880  1.0305300  2.699220   4.119280  0.966287 -2.453470  3.86609000
## 2 -0.467765 -4.7243900  0.262993   4.014280  0.999266 -3.900020 -6.82823000
## 3 -9.977800  0.9409580  5.366900  10.558800 -1.304360 -2.145500  6.18602000
## 4 -1.905220 -0.0725667 -1.583270  -4.188030  2.995520 -1.481300  4.53561000
## 5  2.189420  1.1023400 -0.675557  -0.397139  5.224960 -0.647945  0.00962616
## 6 -1.916890  4.8174100  2.659900 -11.226600 -3.082200  4.905210  0.74560400
##      PC191     PC192       PC193    PC194      PC195    PC196      PC197
## 1  5.54257  4.123410  2.65129000  7.09933  -2.208310  4.56766 -3.8015100
## 2  1.13095  2.021070 -2.06783000 -4.36921  -4.777900  6.64372  5.8557900
## 3 11.77170 -6.282700  9.21615000  8.04719 -15.505300  9.43444  0.1009980
## 4 -8.72379  0.852185 -4.36556000 -4.48808   0.162029 -1.53632 -0.3692860
## 5 -7.61701  2.298000 -0.00855056 -3.55160   1.395040 -5.95358  2.1863900
## 6 -3.67710  7.138320 -2.99596000  3.73958   8.146520 -4.81220  0.0988812
##       PC198     PC199     PC200     PC201     PC202     PC203    PC204    PC205
## 1  -6.64570  1.606720 -0.905456  2.759580 -0.723013 -0.807853  1.00199 -2.04222
## 2   2.56524  0.312666 -1.779890 -0.798943  2.923330  2.491800  5.38325 -4.37064
## 3 -15.64470  1.885900 -5.238360  5.626030  4.299770 13.866600 -9.06287 -7.84493
## 4  11.88690 -2.074090  4.546170  3.816480 -1.741190 -0.373681 -5.08396  6.30373
## 5   8.83142 -0.571147  4.215820  4.241820 -1.689610  1.744960 -7.66230  6.72477
## 6   2.45125 -2.904470 -3.425160  1.377710 -4.508120 -4.454350 -1.77934  2.47556
##       PC206     PC207     PC208     PC209      PC210     PC211    PC212
## 1 -0.423449 -1.781310 -2.718430  0.499711  0.0564189 -1.076330  2.37455
## 2 -2.212980  7.325380 -4.291420 -1.696700 -5.9851300  2.907710  8.25368
## 3 10.868800  4.946140 -0.319349 -6.548420  4.4783500  4.427760 -1.66921
## 4 -0.104402 -1.218560 -1.813880 -1.144920 -1.5435400 -3.261770 -6.08998
## 5 -1.770940  0.592556 -2.916220 -2.592820  0.6861830 -0.553201 -4.82399
## 6 -2.166760 -4.085220  3.899470  3.321140  3.3270400 -5.395810 -8.98527
##       PC213    PC214     PC215     PC216     PC217      PC218    PC219
## 1 -7.091970  1.17658  1.548480  0.576287  3.831140   1.464410  5.40389
## 2 -3.265010 -1.86041  0.462376  2.748250 -0.407777   5.242120  7.92652
## 3  0.677345  2.10459 -3.683740  1.328770 10.979700 -11.814500 -3.64973
## 4  5.108850  2.76619 -0.196268 -1.679860  2.990210   1.578070 -5.54948
## 5  4.991410  3.36367 -3.005580 -3.209370  2.899900   3.084450 -6.79024
## 6  1.760930  5.28127  2.130880  0.684292 -7.568870   0.260305  2.13556
##       PC220     PC221       PC222     PC223     PC224     PC225    PC226
## 1 -1.416100  -1.44189 -3.42400000  -5.21515  0.577804  1.860420  5.27210
## 2 -1.116800   3.39156  1.72128000   3.77216 -2.996600 -0.169884 -2.39942
## 3  0.898222 -11.92230  5.32621000 -18.52680 -2.993550 -1.189580 10.88790
## 4  9.755380   1.91583 -0.00828876   1.90407  0.600704 -2.656210 -5.55810
## 5  8.194460  -2.09433  0.18543500   2.27672 -0.996570 -0.784547 -4.58516
## 6 -6.560570   3.20275 -5.87010000   3.02144  6.843590  5.038690 -5.72116
##      PC227     PC228      PC229     PC230    PC231    PC232    PC233     PC234
## 1  2.21106  0.302302   0.519395  0.992303  6.83194  1.67831 -4.87230  0.222948
## 2  9.46577  4.035770  13.857800  3.009260  3.90221  7.62620 14.39700 -2.172050
## 3  1.42705 -4.504820 -21.221500 -3.348680 -6.94567 -5.85306 -7.30415 -0.637103
## 4 -3.35746 -1.728140  -3.562010  3.277080 -5.57469  5.48985  1.93675 -6.206020
## 5 -2.93576 -1.725550  -2.447100  1.460190 -4.44554  1.17798  2.02375 -5.291030
## 6 -6.61277 -2.969970  -0.872667 -0.289330  5.93416 -2.12949 -4.86614  7.648210
##        PC235      PC236     PC237     PC238      PC239     PC240     PC241
## 1   0.567484  -3.814030  5.759190  6.960130  0.7148090  1.364770  0.424481
## 2   0.653310   5.293270 -0.675218 -5.716690  3.1636400 -3.937780 -4.105680
## 3 -12.138700 -10.093800 -2.131500 -4.636060 -2.2967300  2.297430 -6.706670
## 4  -3.223050   1.134430 -2.857560 -0.497885  0.0133825  1.361480  1.493160
## 5  -1.126560   0.463989 -0.462921 -1.792890 -1.0236000  4.109020 -1.378550
## 6   2.502910   3.255050 -2.487290  8.427470 -2.8070700  0.890501  6.289090
##       PC242    PC243     PC244     PC245     PC246      PC247    PC248
## 1 -0.319599 -1.43470  6.116210 -5.042790  3.970260   0.440868  2.59491
## 2 -9.449650  6.89247  1.989630 -0.095161  1.315530   7.059030 -4.80703
## 3  5.603360 -2.63065 -2.488940  3.074890  5.669500 -10.007700  3.05268
## 4  0.873274 -1.85010 -0.562570 -0.156086  1.458760  -0.667995 -1.47361
## 5  0.713220 -1.00440  1.201830  0.212365  0.541066   1.418130 -1.20602
## 6  8.603510 -8.54961  0.947994  0.497500 -7.597980  -4.297820 -3.10996
##        PC249     PC250     PC251      PC252     PC253     PC254       PC255
## 1  0.0601088 -4.056190  3.281600   4.018910  1.829960  2.862270  -8.4268900
## 2  2.0487200 -0.757009  0.276534   1.928630 -0.187398  7.121140   0.8594930
## 3  1.3511600 -6.216420 12.358200   7.091980  2.619210 -4.551970 -10.3155000
## 4 -1.1668400 -0.453731  2.285090   2.805660 -1.498880  2.270280   0.0291084
## 5 -0.7557100  0.129251  2.086250  -0.011934 -0.503885  0.922913   2.4006300
## 6  0.0582419  4.474730 -9.520680 -11.136900 -0.210480 -0.547040   6.7057200
##      PC256     PC257     PC258     PC259    PC260     PC261     PC262
## 1  6.26587 -1.427400 -1.364020 -0.483539 -2.22377  2.851390 -0.653165
## 2 -4.03214  4.118090 -4.575950  1.702560 -4.85820  5.771620  6.266190
## 3  6.86902 -0.760599 10.801700  6.678700  9.41260 -7.680030 -8.461790
## 4 -2.69128  0.430376 -3.934650 -2.893590 -2.52620 -0.724484  0.726392
## 5 -2.34846  1.235770 -2.646580  1.052460 -2.25067 -0.237247 -1.177990
## 6 -4.53690 -3.610810  0.156929 -6.644540  2.57255 -3.032430  0.652874
##        PC263     PC264      PC265     PC266     PC267     PC268     PC269
## 1  4.5128400 -0.116734 -0.0262209 -2.032270 -0.517901  -1.68581  1.531850
## 2  0.2595160 -5.618050  1.7409800 -0.945241  2.549420 -10.16050 -4.721350
## 3 -6.1970800 15.143200 -3.9455900  1.453710 -7.792350  -4.86874  7.474580
## 4 -2.0394500  3.031660  2.6591800  0.649460  5.133390   4.38727  2.054180
## 5 -0.0382715 -0.213455  2.2766000  0.285463  2.196750   3.11127  0.529512
## 6 -0.7354090 -2.075360  2.1219200  0.794189  3.304320   5.35412  1.260370
##       PC270     PC271     PC272     PC273      PC274      PC275      PC276
## 1 -2.899600 -3.660400  0.416856  1.580550  -0.929610  4.9098500 -0.0660742
## 2  5.161390 -0.228787 -0.397251 -1.407380  -0.599395  5.7039900 -7.7682500
## 3 -8.003720  5.806960 -4.434920  4.728340 -13.442400 -4.5097500  3.8092100
## 4  1.327610  2.298300 -1.623020  0.480359   1.622090 -0.0911165 -1.2027900
## 5 -0.894352  0.535740 -3.983880  1.143020   1.800880  0.7457550 -0.0935000
## 6  0.390248 -3.114200  2.625140 -5.134770  14.006300 -2.1658000  0.0265242
##        PC277     PC278     PC279     PC280     PC281    PC282     PC283
## 1  0.6224920 -0.466127  2.142830  3.418780 -2.538900  4.37318 -3.746590
## 2  4.1139800 -1.192770  5.080340  2.082050  0.670263 -1.72296  0.713291
## 3  0.0234611  3.253900 -0.600073 -4.750480 -3.795320 14.12540 -4.734650
## 4 -1.0147400  3.370320 -1.450290 -2.015590 -0.503210 -3.57569  4.772720
## 5  2.4757300  0.748845  0.273054 -1.505900 -0.918943 -2.42148  3.468530
## 6 -1.3679600  2.048710 -9.122120  0.223173  3.187010 -6.23374  0.589888
##      PC284     PC285    PC286      PC287     PC288    PC289      PC290    PC291
## 1 -4.03237  -1.45206  2.86467  5.4434300  5.836350 -5.24135 -0.0765904 -2.81229
## 2 -6.59959   4.90951 -1.94733  4.9289500 -5.022320  4.40260 -5.7295700  3.76919
## 3  6.01638 -12.13400 10.27940 -5.4156300  9.853030 -4.88892  4.9905600  7.10318
## 4  3.99941   2.51943 -4.18728  0.3221460 -0.408947  2.27144  1.6252100 -1.43365
## 5  1.55163   2.09084 -3.03230 -0.0472207 -1.204880  2.57847 -1.1113100  3.12293
## 6  3.15984   1.14437 -1.05349 -5.1336300  0.296363 -1.14600  3.3956300 -5.18936
##        PC292      PC293     PC294     PC295      PC296       PC297    PC298
## 1 -1.2599200  -1.566710  -5.64641   1.63231  5.9712300   0.8273790 -0.84845
## 2 -1.2304300   5.696030  -2.68732   6.67740 -5.2146100   6.6377200 -3.49703
## 3  6.6666400 -15.845200 -13.68460 -10.75100  5.0241600   6.2026000  7.71184
## 4 -1.6765500   0.660885   2.88921  -2.41495 -0.9375180   0.8492660 -1.25335
## 5 -0.0469258  -1.278140   3.05224  -1.32164  0.0674804  -0.0368242 -2.39081
## 6 -5.3274700   5.979910   4.77636   2.48639  0.6789730 -11.8011000  3.12244
##       PC299    PC300      PC301     PC302     PC303    PC304     PC305
## 1 -0.380781 -0.11216  1.4409400 -1.228750 -0.487860  2.21696 -2.350780
## 2 -6.544540 -6.25533 -2.9521800 -0.709613  0.257047 -4.40067 -2.041460
## 3 -0.138067 11.25870  5.6179000  2.637340  4.832600  6.79548 10.991700
## 4 -4.914710 -1.76852  0.4908530 -0.299787 -2.578600 -3.09624 -1.075960
## 5 -2.567900 -1.30198 -0.0137013 -1.200780 -0.762078 -2.86012 -0.192828
## 6  1.259640  6.67217 -0.8722810  2.487470  0.521237  4.03967 -7.117020
##       PC306    PC307     PC308     PC309     PC310    PC311     PC312     PC313
## 1 -7.285300 -1.13842  6.421660  0.144481  6.506020 -2.42063 -1.573960  2.683240
## 2 -1.088030  5.26143  4.036070  1.010020  2.529180  4.06317 -0.228878 -2.201100
## 3  3.377060  2.10020 -4.267360 -4.060070 -1.115090  7.55665  9.838320  1.495360
## 4 -0.578170 -1.27182 -1.096920  2.144230 -0.976964 -2.72440 -1.783940 -1.809180
## 5  0.544196 -3.19914  0.178731 -0.430776 -0.392872 -1.39107  0.237933 -0.532791
## 6  1.590570 -7.48457 -3.027380 -0.814986 -4.036960 -1.59963 -6.382910  0.704372
##        PC314    PC315    PC316     PC317       PC318      PC319      PC320
## 1 -2.1715700 -2.26545  1.87903  0.048919  -0.6539000 -0.0858028  0.0634985
## 2 -0.0469358 -4.38619 -2.69552 -2.680300   6.1221700  4.0871400  2.4470500
## 3  3.4715900  1.70686  3.55337  6.454130 -16.3861000  2.4336700 11.2750000
## 4  1.9564400  2.65667  2.66972 -2.093240   1.5233100 -2.9434700 -4.0350200
## 5  0.7352040  1.64085  1.86205 -2.686830   0.0666389 -3.5849000  0.1822340
## 6 -1.9521700 -3.64090 -4.17515 -3.644160   2.5244000 -0.5484410 -8.6794600
##        PC321     PC322     PC323     PC324        PC325     PC326       PC327
## 1  3.1841600  0.313215  2.499090  8.257200  6.735780000  0.426050  -1.7594600
## 2  3.9440200  3.325020 -6.768400 -0.650677  0.387955000 -1.960630   0.0550073
## 3  2.6239300 -7.514350  1.673740 -3.427820 -1.086800000  0.906996 -10.7663000
## 4 -0.0412191 -2.304320 -1.163600 -0.967379  1.921790000 -0.847369   5.2116300
## 5 -0.3235800  0.598671 -2.043050 -0.633125  0.000866653 -1.222630   3.3655400
## 6 -2.0613000  0.704175  0.441571 -2.439170  8.203200000 -2.821800   5.5188100
##       PC328     PC329     PC330     PC331     PC332    PC333    PC334     PC335
## 1  4.100970  2.534050 -1.474600  1.307680 -7.233090  4.26246 -1.12176  0.372586
## 2 -1.227300 -1.313670  0.308595 -6.822580 -0.303221  2.89746 -5.02244  2.692370
## 3 -2.350080  2.767310 -3.189860  2.221610  2.708960  1.41139  7.86546  3.774360
## 4  0.971166 -0.658647 -1.851050 -3.398070 -0.357806 -3.31258 -3.53221 -0.251744
## 5  0.264885 -1.390660 -0.436948 -0.837843  1.553010 -1.48131 -2.42077  4.103830
## 6  0.197595 -3.160630  0.799300  3.962830  3.346890 -2.39221  3.83427 -4.780790
##       PC336    PC337     PC338     PC339     PC340     PC341      PC342
## 1  5.866980  1.30239  0.526834 -1.107230  3.019610 -1.111820  2.3632400
## 2 -4.256700 -3.43208  4.643950  1.323520 -1.986990  0.870715 -6.3637100
## 3  1.794150  6.72219 -1.483510 -2.899990  1.450600  5.135120  8.2203800
## 4  0.433595  1.19593 -2.971610  1.538850 -0.989238 -1.833990  1.5874700
## 5  1.483350  1.70277 -1.900300  1.292410 -2.639470 -2.761100 -0.0702725
## 6 -4.122610  3.04352 -6.178300 -0.227901 -2.213310  0.648995  1.7322900
##       PC343      PC344     PC345     PC346      PC347     PC348     PC349
## 1  1.250200  1.1618700  2.155410  2.134580 -4.7846900 -0.149676 -1.853740
## 2  0.374582 -2.9238100 -5.126650  3.931450 -0.0113821 -3.641620 -4.225300
## 3 -0.305223 -1.9347400  1.320990 -5.944650  2.4645600  4.167090  0.821082
## 4 -2.368420  0.3729620  0.520797  2.297740 -1.3927700 -2.910720  2.000350
## 5  1.093120  0.0427231  0.850082  0.806246  0.8369300  0.264753  0.317031
## 6 -0.133974  0.6719950  5.650300  0.337494 -0.4959550  1.217590 11.026000
##        PC350    PC351     PC352     PC353    PC354     PC355     PC356
## 1  1.9119100  3.33916 -2.928660 -3.167350 -0.66831  0.966432 -2.643700
## 2  0.7468550  5.87039 -0.263015 -1.179770 10.87660  0.223176  3.710530
## 3 -1.5108000 -6.86455  5.252980  0.346421 -2.71711  1.470390 -2.745890
## 4  2.2001000  0.15928  0.953818 -1.664980 -1.09961 -3.886120  0.752095
## 5  0.0564837 -1.89627  0.827445  1.294510 -1.75220 -0.986673  1.441250
## 6 -4.1069500  2.07181  2.780920  2.309450 -7.32127 -1.450000 -4.293230
##       PC357    PC358      PC359    PC360     PC361    PC362      PC363    PC364
## 1  0.216728 -7.52898 -1.9912900  6.54047 -6.905260 -1.25593  2.5075600 -3.61693
## 2 -0.234859  4.80045  2.8342200 -3.42859 -3.787650  4.59495 -2.4940900  3.95698
## 3 13.788400  1.00961  3.7116300 -4.09699  1.785230 -1.32592 -6.1763600 -2.17825
## 4 -0.818370  3.31784  0.0518406  1.57704  0.517772  5.33067 -0.0394817 -1.61180
## 5  2.400790  2.13047 -0.2397950  1.98999 -1.258120  3.50148 -0.7574140 -1.18145
## 6 -7.604190 -1.56056 -3.1431200  1.71085  2.929210 -8.56354  7.2353000  2.02088
##       PC365     PC366      PC367      PC368       PC369      PC370     PC371
## 1  5.743900 -0.196021  2.0388100 -0.4173740  -0.0131931 -1.3139100  0.844442
## 2 -5.819350  1.653600 -0.0922078  4.4171000   1.2819200  0.0827032 -5.414330
## 3 10.952400 -3.755260  3.2848000 -4.5053000   4.2444300  5.8895400 -2.702190
## 4 -2.642700  3.643790  1.0979300  0.0565652   0.4756870  0.4514890 -0.693002
## 5 -1.150510  2.426640  0.1244280  1.4058000   1.8904600 -0.3086300  0.278736
## 6 -0.728024 -2.781450 -5.1365000  1.9870400 -11.2914000 -4.5451100 -0.520310
##      PC372     PC373     PC374     PC375     PC376     PC377    PC378    PC379
## 1  1.01250 -2.165220  4.302880  0.250072  0.688157  0.547920  3.07148 -1.94854
## 2 -5.03846 -0.487427  6.073760 -3.999580 -3.332850 -1.487020 -6.62009  4.55177
## 3  6.45778  2.720560 -7.078930  5.991080 -2.593320  1.846770  3.89265 -1.97989
## 4  1.46960  1.175670  0.395864 -1.102400  1.272640  1.388030 -1.37821  4.53092
## 5  1.06038  0.427166 -0.842581 -1.620150  0.186343 -0.422873 -2.09686  4.09077
## 6  3.83800  1.062300  2.640610 -4.305640  8.330160  6.775910 -4.02844 -4.80348
##      PC380     PC381      PC382     PC383     PC384     PC385     PC386
## 1 -1.26041  1.876430   1.189230  3.421190 -9.267670 -1.760420  2.904220
## 2 -7.75964  1.505190 -10.306900 -8.170570  2.773600  2.782090  3.202560
## 3 -1.77873 -3.441260   3.490880  6.201090 -0.578744 -3.327610 -4.662420
## 4 -3.23523 -1.745430  -0.203669 -1.877130 -0.233976  0.656882  0.954479
## 5 -2.95613 -1.244160   0.733806 -0.719518  1.576940  2.433250 -1.193440
## 6  8.16373 -0.396948  12.073700 -1.104150 -2.295360 -4.728480 -4.300910
##       PC387     PC388     PC389     PC390     PC391     PC392     PC393
## 1  6.768260  0.364740 -4.103600  1.008540 -0.169599  0.610708 -2.326180
## 2 -4.560200 -2.260140  1.627940 -1.839640  8.393320  2.329390 -0.742307
## 3  1.596200 -0.343963  5.561930  0.379126  0.611365  3.518580 -0.900827
## 4  1.158940  2.120850  0.511827  1.657790  0.950310 -0.439784 -1.266560
## 5  0.209652  0.870856 -0.870021  0.289909  1.583230 -2.604700 -0.592347
## 6 -3.458170 -2.042760  1.381270  5.351220 -9.359500 -0.703677  6.831680
##       PC394      PC395     PC396     PC397    PC398     PC399     PC400
## 1  1.919960  0.0921915  1.423330 -1.427940 -3.61509  2.433320 -6.365900
## 2 -4.184660  0.4483690 -1.608260 -1.069310  2.08826 -6.899110 -7.549270
## 3  0.820582  4.1982600  6.141650  6.057690  7.35683  3.337330  4.157280
## 4 -3.055220  0.2803420  1.263620 -1.155620  1.46259  3.475550  0.967079
## 5 -1.512750 -0.4557770 -0.268563 -0.941020  1.19019  0.435961  0.364318
## 6  7.668510 -2.5265400 -0.582028  0.346225  1.35610 -1.005730  4.341290
##       PC401     PC402     PC403     PC404     PC405     PC406    PC407
## 1 -4.188510  8.895260 -1.267470  0.499873 -0.429464  3.943170  5.14353
## 2 -3.621970  0.123198 -1.852540  0.186583  0.517942 -5.447760 -1.25612
## 3 -6.995730 -8.215120 -0.657093 -4.143220 -2.023490  5.909050  5.46344
## 4 -0.921032 -1.072670 -2.331260 -5.099340  3.890400  1.563640  1.02128
## 5 -0.886964 -2.575290  0.101656 -1.188680  0.283069  0.480037  1.33045
## 6  2.657430  0.296446  2.815640 -0.766606  3.678240 -8.726200 -4.64404
##       PC408     PC409     PC410     PC411     PC412       PC413    PC414
## 1 -3.527520  0.796596 -5.558220   5.77749  3.000020  -0.0465376 -4.55751
## 2  1.586040  0.719133 -3.124030   6.94850  0.345273   0.4279660 -2.26064
## 3  1.629010  0.278307 -3.549420  -1.80311  0.635887 -12.8913000 -2.62527
## 4  0.392565  0.339440 -2.651540   2.14966  3.276770   3.1671500 -5.04053
## 5  2.578000 -2.110350 -0.866257   1.28584  2.668620  -0.1173300 -1.18262
## 6  2.925760  6.637650  9.627590 -15.70630 -3.585040   2.4036900  5.90805
##       PC415     PC416     PC417     PC418     PC419      PC420     PC421
## 1  3.050560  0.346242 -0.460687  2.598900 -6.962510 -9.0140200 -0.857158
## 2 -0.866159  1.896460 -6.230650  4.130440  0.511370  2.3656400 -1.209660
## 3 -9.903110  1.536100  1.672370 -7.526470  0.148073 -6.1058500  1.394200
## 4  1.890490 -0.606181  3.050990  0.713383  2.000930  0.0873058  0.161784
## 5 -1.400410 -0.197029  2.107700  0.288525  2.213180  0.5395620  0.597757
## 6  5.656030 -5.318940 -2.971930  5.628080 -3.415120  5.6915700  4.662840
##       PC422      PC423      PC424    PC425     PC426    PC427     PC428
## 1  2.890790 -3.9915200   3.480060 -3.43714  1.921160  2.78043  5.065330
## 2 -3.278600 -0.0733377   4.143300 -9.82253 -8.329210 -4.77920 -6.061280
## 3  4.115190  0.3705990 -11.005300  4.98669  2.004870  6.68411  4.495800
## 4  2.671030 -1.0175400  -0.480964 -1.28511  0.547166 -1.75151  0.984722
## 5  0.195058 -1.8988900  -1.442980 -1.60313 -0.733197  1.24669  1.942140
## 6 -1.057280  4.3726000   1.461940 11.88120 13.309400 -7.58519 -9.872750
##        PC429     PC430     PC431     PC432      PC433     PC434     PC435
## 1  2.2115800 -5.738500  4.953250  0.431328 -4.3620900  3.500060 -5.709400
## 2 -3.9937300  3.727500  0.191544  0.382687 -2.7080700 -3.832880  1.580620
## 3  6.7950000 -0.101663  1.175090 -3.738590  4.0216400  1.416360  6.763370
## 4  0.0267627  1.274010 -1.199690  1.900680  1.0069500  0.911206 -0.453357
## 5 -0.1085800  0.910201 -2.312260  0.919004  0.0511454 -0.792076  1.260900
## 6  5.6878900 11.273200  3.504040  0.606439  1.8837700  2.221310 -3.596040
##       PC436      PC437     PC438     PC439    PC440      PC441     PC442
## 1 -2.117060  1.3552100 -5.100340 -1.539730  8.32037   0.122489  5.861330
## 2  0.470262 -4.3963600  1.550150  4.324140 -4.80408   3.688780 -1.999580
## 3  1.615050 -7.0613500 -0.326242 -0.671576  1.60030  -1.346520 -5.453430
## 4 -1.324660 -0.0610928  2.803180  3.979070 -2.38851   1.345400  0.148125
## 5 -0.806985 -1.2721600  2.391170  1.606130 -2.00279   1.425360  2.034130
## 6  8.128620  9.7048800 -0.942066  3.106530  2.78461 -12.459800  7.163370
##       PC443    PC444     PC445     PC446     PC447     PC448       PC449
## 1  2.876720 -2.93052   1.71009 -2.365240  3.945100 -8.793920   0.0369488
## 2  0.360598  6.03197  -4.42558  1.167490 -1.928680 -2.455620  10.2919000
## 3  2.285740 -1.96073  10.27540  1.655950 -5.423250  6.898730   0.5140960
## 4  1.008360 -1.54334  -2.03943 -2.857460 -0.970752  3.060860   3.2078400
## 5  2.131280 -1.74694   1.12038 -0.550974 -2.002300  0.603121   2.1388700
## 6 -1.809600  5.46276 -10.37450 -2.035550  1.715150  0.973694 -13.7092000
##      PC450    PC451     PC452     PC453     PC454      PC455     PC456
## 1  3.89482  1.70770   1.36984  5.550500 -1.811490 -8.3163100  -2.42723
## 2  1.46273  1.08630  -1.42056  5.955280  3.086590  4.8522600   9.21781
## 3 -3.71684 -1.98311   1.61763 -2.542930  2.265030  0.0993377   7.48028
## 4 -3.68039  3.34363  -1.60247  0.577683  4.041530 -3.7683800   0.36257
## 5 -1.95677  1.02921   2.20556  0.738387  2.995340 -2.1615900   1.67325
## 6 -1.61001 -2.00091 -10.96310 -6.042930 -0.967949  5.1445500 -12.10730
##       PC457     PC458      PC459     PC460     PC461     PC462     PC463
## 1 -2.788030  1.339550  1.0835800 -2.131160  5.232790 -0.286953  8.422310
## 2 -0.421319  4.332130  6.5551200  2.312660  3.500680 -0.527712  0.209584
## 3 -1.485810 -0.568198 -9.3883700  0.596862 -9.653530 -1.624080  0.899350
## 4 -3.359780  5.067090  1.4706800  1.897570 -0.214532 -3.558510  1.026500
## 5 -1.234700  1.921300 -0.0465607  1.457740 -1.698820 -1.490630  0.959969
## 6  4.231430 -5.146420  2.3621000 -2.232170 11.361400  5.003230 -4.441690
##        PC464    PC465     PC466    PC467    PC468      PC469    PC470     PC471
## 1   2.490750  3.92988 -2.170000 -1.45296  1.34644  2.7442100 10.29210 -3.750380
## 2 -10.116200  1.83862 10.223200 -3.16550  3.97162  6.9190300  1.87046 -2.777330
## 3  -0.903255 -9.13568  1.089120 -1.13021  3.47280 -0.1879170 -1.03656 -0.208672
## 4  -4.021950  2.85460  0.497585 -2.24460  2.61183  2.8826000 -1.79659 -4.126210
## 5  -0.666641  2.66194 -0.681372 -0.71817  3.65854  0.0782989 -2.54452 -3.730320
## 6  -1.464310  8.37750 -1.636800 -6.98024 -9.02304 -3.9329800 -1.67418  9.703250
##       PC472     PC473    PC474    PC475     PC476     PC477     PC478     PC479
## 1  0.489817  -5.75057  5.77762 -1.78160  8.612230 -3.750520  1.817810  0.323695
## 2 -1.726990   1.24898 -2.15749  5.53092  5.146790  1.646400  3.799310  1.175360
## 3  4.885890  10.93900 -2.09301 -9.29722 -9.763470 -3.925390 -2.762930  7.969620
## 4  1.004990   1.79477  1.37325  4.38455  0.122302 -0.831890  0.911661 -1.666430
## 5  2.725930   1.94703  1.13234  2.93823  0.782463  1.181040  1.711210 -1.676520
## 6 -2.236720 -20.07420 -7.38515  5.79917 -5.969500 -0.077534 -1.688920 -9.480620
##       PC480     PC481     PC482     PC483     PC484     PC485     PC486
## 1  1.398580 -7.798920 -2.069070  0.593192 -3.064410 -0.683617 -8.174080
## 2 -4.855960 -6.210840 -2.982750 -3.529220 -2.336220 10.283600  0.398461
## 3 -0.914368  0.871876  3.169480  0.533333 -2.863500 -4.131380  4.746930
## 4 -3.885870 -2.821180 -0.416206 -1.109470  0.238069  5.677160 -0.340911
## 5 -6.588090 -0.711925  0.273927 -0.212729 -0.497965  5.321710 -2.466430
## 6 16.618600  7.839060 -1.919740 -7.631580 10.698700  1.713800  8.408030
##       PC487     PC488     PC489     PC490      PC491    PC492     PC493
## 1  5.275450   2.63577   2.63564 -0.658733 -9.8797000 -8.66753  1.585080
## 2 -1.917520   8.17993 -12.37540  7.901700  4.0822300  1.88824  3.037830
## 3 -7.264120  -1.99454   8.87511 -1.937640  0.6926640 -4.53122 -5.292970
## 4 -1.261650   4.17609  -2.12569  1.046030  0.0790462 -4.15026 -2.049780
## 5 -0.776933   3.84504  -1.11463  1.772100  0.8718370 -1.92905 -3.467220
## 6 11.451200 -13.37510  16.09320 -5.752300 -0.2571260  4.10084 -0.934313
##       PC494     PC495     PC496     PC497     PC498     PC499    PC500
## 1 -7.695260 -9.296680   8.33949 -3.411510 -4.760100 -1.565210 -1.91135
## 2  2.786010  7.153000   4.07705  2.732570 -8.288270 -1.104240  1.63679
## 3  4.429010 -0.820552 -11.31730 -3.325550 -5.002680  1.359710 -5.83626
## 4  0.958561  3.371570  -1.29290  0.542640 -3.068720  1.786170 -3.74472
## 5 -0.671600  1.865080  -1.32143 -0.626032 -2.257030  0.109776 -3.60837
## 6  2.409400  5.645510   6.86194 -3.194840 -0.351979 -2.154770  6.22448
##       PC501     PC502    PC503    PC504     PC505     PC506     PC507    PC508
## 1   4.12306  0.797225  1.16849 -8.52971 -7.855450 -0.953767  5.140790 -4.24345
## 2  -1.75553  2.788570  6.55212  7.95878  6.508520 -2.997830 -8.085950 -6.40956
## 3 -13.87010  0.412990 -6.43310 -5.65247 -0.304292 -3.686920 -0.757795  4.57484
## 4  -4.05833 -1.755510  3.73944  2.26075  0.338957 -0.436887 -2.720470 -1.99720
## 5  -4.27613 -2.435530  1.13713  2.85678  1.440370 -1.041010 -3.178770 -4.57325
## 6  17.21530 -9.247450 -8.46764  1.72544 -1.397460 -1.744410  4.182880 15.42140
##       PC509     PC510      PC511    PC512     PC513    PC514       PC515
## 1 -10.81130 -3.160920   5.684740  7.56945  2.401420  1.38137 -11.5427000
## 2   4.97663 -0.931841  -2.623520 -4.00188 -3.583060  7.02063   2.9680800
## 3   8.10907  2.466460 -11.594800  6.33174  3.248930 -6.04747   0.0529885
## 4   1.22438 -4.140830   1.977380 -1.37388  3.958010  3.30098   2.5968800
## 5   5.90894 -3.350140   0.608402  1.66597  3.648190  3.04438   0.7371930
## 6 -10.69660  6.460600  -2.973980 -6.99604  0.561209  3.13494  13.2513000
##       PC516     PC517     PC518    PC519      PC520     PC521      PC522
## 1  1.261720 -1.430320 -0.202636 -7.89507  -5.205330  2.324240   1.690690
## 2  3.767650  0.386057  1.249440  6.09587 -12.704600  3.948670  -1.171330
## 3 -5.386580 -4.630730 -6.227660  6.92659   4.510640  0.745487  10.303000
## 4 -0.543151  1.990040 -4.221460  3.33375  -0.490501  0.770362  -1.261050
## 5 -0.140016  0.107141 -2.182810  1.62048   0.180985  1.456920   0.743561
## 6  2.704500 -3.438750 10.309300  3.63902   5.883320 -8.685780 -11.425800
##       PC523    PC524     PC525      PC526      PC527     PC528     PC529
## 1  -3.98811  6.85142  0.136756 -8.7345500 -12.011400   9.97136  9.100590
## 2   5.49939  1.04053 -6.253370  6.0271900  -2.443780   1.86596 -1.401250
## 3 -10.19450 -8.19781 -1.435720 -3.0363800   0.737302 -10.16390 -4.208360
## 4   1.39042 -2.56012 -2.984650  0.0431859  -2.896450   3.47559  4.625250
## 5   1.29314 -2.56710  1.181600  2.2281300  -1.245420   3.23811 -0.115218
## 6   6.55285  2.02315  0.665386 -3.4662600   6.413740 -12.71170  9.446510
##        PC530    PC531     PC532    PC533     PC534     PC535      PC536
## 1 -15.656400  1.01756  7.105200  5.35051 -1.020000 -1.418510 -4.4623300
## 2  -1.643580  1.91591  0.121806  2.75398 -4.818360 -3.867780  1.4059700
## 3   3.199910 -7.27533  5.080250 -4.20534  6.813890  3.363240 -0.7734830
## 4   0.416985  3.56728 -1.006910 -2.60758 -0.681055  0.532116  2.0010400
## 5   1.605830  2.23123  1.790740 -2.38733  1.218370 -0.930840  0.0529179
## 6  -6.372720 -4.65376 -9.393190  4.15272 -2.665680 17.149200 10.9263000
##         PC537    PC538     PC539     PC540     PC541    PC542    PC543
## 1 -16.9401000  5.74674 -13.36670 -4.683140 -3.849220  3.02825  3.98110
## 2   6.1095400  2.08387   2.60035  2.197970  3.584710 -1.28570 -1.72725
## 3  -1.0908300  1.11981   1.54532 -1.128120 -5.427720  3.83918  0.84680
## 4  -3.3303800  1.75401   1.57644 -0.457477 -0.748972 -2.71678  4.40795
## 5  -3.7918300  1.59972   1.21434  0.957146 -0.258917 -2.82682  3.56973
## 6  -0.0150529 -3.98555   8.55511  4.268260  2.772100  6.77650  1.41799
##       PC544      PC545    PC546      PC547     PC548     PC549     PC550
## 1  0.955375  10.616600  5.85949 -2.3662200 -5.091870 -2.985820  7.750170
## 2  1.920650  -0.866365  3.87259  4.7452400 -0.508259  2.862490 -0.662142
## 3  7.716690 -10.186100 -4.42556 -3.2511200  0.816028 -2.358690 -0.161240
## 4 -1.781630  -1.626920  3.67095 -0.6619480 -1.684610  1.642340  2.930200
## 5 -1.304310  -1.233410  2.29688  0.0200007  1.107390  0.361807  1.104230
## 6 10.963100  -1.581070 -5.22263 -7.9951400 -5.284230 -4.572770  3.700340
##        PC551     PC552    PC553    PC554    PC555     PC556      PC557
## 1 -10.059800  1.396910 -2.42983  5.63017  3.10335  0.475876  4.2441500
## 2   7.011670 -2.071480  1.10114  2.73659 -4.59612 -0.878297 -1.2410500
## 3   2.287920 -3.103400  5.99100 -1.63698  2.59053  9.229780  4.4777300
## 4   0.933308 -0.501320  1.27979 -2.11544 -0.48322 -1.710310  2.5545500
## 5   1.415810  0.981691  2.27192 -2.20518 -1.36692  2.199170 -0.0571956
## 6  -3.274790 -4.031920  6.03491 -8.40832  7.28408  5.069280  2.0796100
##       PC558     PC559     PC560    PC561    PC562     PC563    PC564     PC565
## 1 -7.937780 -4.290980  7.430430 -1.39532 -6.63226  9.685640 -3.16250   6.71425
## 2  0.891109 -4.723890 -0.337483 -1.88267  1.87441 -4.265640 -3.80732  -6.25051
## 3 -1.581880 -5.794560  6.219830 -2.43592  3.05197 -4.849450 -3.37736 -10.50400
## 4  0.498649  0.171534  1.803750 -2.76788 -1.03714  1.876270 -1.97368   1.83713
## 5 -0.202879 -0.247173 -1.458600 -2.31684 -1.11552 -0.175665 -1.72786   2.36849
## 6 -4.839550 -6.492700 11.556400  4.24251 13.84380 -3.164670  8.74904 -17.74410
##       PC566     PC567      PC568      PC569      PC570     PC571      PC572
## 1 13.317200 -16.14020 -20.090200   4.157090 -6.4776000 -0.950869 -1.6304400
## 2 -2.157150   4.71525   3.014020   3.967560 -1.7742600 -2.029310 -0.0100381
## 3 -0.369705  -2.25847  -0.483762  -2.830480  5.2077600 -0.973007 -1.2491000
## 4  3.432480   1.47906  -2.461860   0.606850  1.4003200 -0.733995  0.5935490
## 5  0.349315   2.99420  -1.561790  -0.301647 -0.0985294  0.916523 -1.6766900
## 6  5.961150  -6.64463   0.952106 -13.507600  1.5452200 -6.986150 11.0420000
##       PC573       PC574      PC575    PC576     PC577     PC578      PC579
## 1 -0.592557 -17.7707000  17.630800 -2.22721  5.534500  7.278810 -10.929800
## 2  1.591070   0.0868532   2.556910 -2.53362 -4.567590 -5.914670   0.213551
## 3 -2.430920   5.8016000  -3.791740  2.08853  4.434230 -3.130830   0.847675
## 4  1.370660  -0.1345960   0.853780  2.32770  0.324467 -0.815664  -0.397939
## 5  1.559260   0.2664090  -0.598138  1.15370  1.060740 -1.719930   0.479418
## 6  4.860260   3.5468500 -11.469400 -2.37933 -2.914140 -2.415390   1.068860
##         PC580     PC581     PC582     PC583       PC584       PC585     PC586
## 1   0.0359472 -1.107710 -3.415380 -5.260570 -10.3529000 -8.10481000 -3.201920
## 2   0.4269450 -2.789610 -0.807337 -1.237900  -2.7487300 -4.44900000  2.405400
## 3  -9.4726900  9.202300 -7.084730  2.798030  -1.4405100 -2.22021000  0.982125
## 4   1.2513700  1.270310 -1.833950  0.341143  -0.0658079 -0.72088600 -0.437541
## 5   0.1184220  0.465456  0.853534  1.868550   0.5065780 -0.00821106 -0.989317
## 6 -10.0627000 -3.773440 -4.289100  0.397195  -2.6865000 -3.83930000  1.195240
##      PC587      PC588     PC589     PC590      PC591     PC592     PC593
## 1 14.36740 -1.7180100 -0.755609  9.392060  3.2737700  4.712940 11.241100
## 2  1.78962 -0.8697940  5.822260 -3.279600 -3.5977000  2.035540 -6.611250
## 3  2.97941  1.3390000  4.176340 -1.436920 -6.2969700 -4.420570  4.728760
## 4  2.11083 -1.4339000  0.397697 -0.247365 -1.4179500 -0.623943  0.435866
## 5  1.34196  0.0822553 -0.487529 -1.388640  0.1405980 -1.593960 -2.437120
## 6 -5.52573 -2.5427800  9.717560 -9.037110  0.0571758 -2.554230  7.602890
##        PC594      PC595     PC596     PC597     PC598     PC599      PC600
## 1 -11.560800   2.770630 -5.462680  3.833040  3.830670  3.313420 -10.622800
## 2   3.112210 -10.209200  4.458370  6.692390 -3.150310 -1.968770   3.739170
## 3  -3.254080  -2.327930  9.253910 -2.534510  5.425920 -0.572444   5.755990
## 4  -1.912380  -0.961684  1.291240 -0.308987 -0.701208  1.365840  -1.317810
## 5  -0.185481  -0.424246  0.579886 -2.480280 -0.517879 -0.785417  -0.785842
## 6  -3.155970   2.922670  5.629020 -4.738890  9.504470 -2.078180  -5.680580
##        PC601    PC602     PC603      PC604     PC605     PC606    PC607
## 1 -8.8322400 -8.59624 -9.497570  0.2956360 13.971700 -6.541410 -4.67057
## 2 -8.5202800 -9.17511  4.616980 -7.7889700 -2.002110  3.825750  1.23152
## 3  0.0494472  3.50554 -2.144850 -1.7273900  0.102602  1.940840 -1.10884
## 4 -1.4381900  1.93387 -0.430379  0.0194862 -0.767343 -0.200653 -2.57516
## 5  1.3544300  3.28147  1.150330  0.0937981 -0.832937 -0.466912 -2.27428
## 6  3.3902800  2.31591  4.974400  0.5849850  1.094800  4.196790  7.05405
##         PC608     PC609    PC610     PC611     PC612    PC613      PC614
## 1 10.94260000 -1.218220 -3.46139  6.904760 -2.021810 2.108850  1.4667100
## 2 -0.39940400 -5.890690 -3.59388  0.888628 -4.177040 2.232840 -1.1804100
## 3  7.14980000  0.260244 -1.81484 -2.190200  7.318090 3.564710 -5.9448700
## 4  4.25325000 -2.465990 -2.38950 -0.563611  0.460771 1.080420 -2.0850200
## 5 -0.00510144 -1.751790 -1.43053 -0.307399  0.763817 0.380909  0.0614424
## 6 -0.85318300  1.057590  6.14653  0.909803 -4.701390 2.252590  0.0470080
##       PC615     PC616     PC617     PC618     PC619      PC620     PC621
## 1 -1.869110 13.137200 -7.663560 -6.557330 -2.451080  0.0787073  1.806350
## 2 -4.976770 -1.299740  2.016960  1.544770  1.822990  5.4125600 -1.047270
## 3 -0.336235  3.146550  9.723650  1.710680  4.234520 -3.0789900 -0.982916
## 4  1.251400  0.315308 -0.381962 -1.574350 -3.502840  0.6575920  1.569150
## 5  0.553967 -1.501090  1.887620  0.105518 -0.592262 -1.6322300  1.533820
## 6  3.111690  0.309525  6.081890  1.470280  0.952028 -0.9379750 -1.988170
##        PC622     PC623     PC624    PC625      PC626     PC627    PC628
## 1  0.0830309 -5.984930  4.070250 -1.24352 -12.330600  2.200390  4.11488
## 2 -6.1785100  1.018450 -5.616030 -1.58828   5.388130 -8.321120 -5.71425
## 3  5.2019100  1.122500 -6.568640 -8.31032   4.963590 -1.736570  2.69567
## 4  0.6165250  0.526218 -2.699640 -3.16207  -0.171146 -2.890140  1.76633
## 5  0.6555010  0.137461 -0.759459 -3.05853   0.793462 -0.304722 -2.50649
## 6  2.7213100  0.453613  1.243160 -5.57160   2.091410 -4.033490  4.88815
##       PC629     PC630     PC631     PC632      PC633     PC634     PC635
## 1  7.842290  3.690640 -4.555820 -8.708840 -10.959100 -6.796220 -1.275590
## 2  5.781280  7.476760 -3.893620  4.676150  -1.076810  4.518170  8.590540
## 3  7.418910 -1.630520 -0.687097  6.668880  -5.992400 -3.399430 -6.146740
## 4  4.211020 -1.493410  1.424720 -1.759280  -3.869850 -0.397829 -0.179292
## 5  1.665210  0.245005  0.726547  0.727328  -0.879697 -1.060860  2.231140
## 6 -0.296072 -2.245820  0.648158  4.793650  -4.674840 -4.421890 -5.333700
##      PC636     PC637     PC638    PC639      PC640    PC641    PC642      PC643
## 1 -0.46957  2.563790  3.299190 -2.83702 -10.960000 -3.84175 -2.19971 -7.5826400
## 2 -5.77595 -6.807460  1.017240  4.50180   6.347470 -9.04221 -8.69728  5.3291600
## 3  6.73080 -5.516560 -0.106128  3.11926   2.296750 -2.81289 -7.64559 -0.8558800
## 4 -1.07245 -2.207150  0.772339  1.10464   0.256011 -2.65472 -4.01255  0.0281704
## 5 -0.45701 -0.350826  0.749323  1.34026  -1.327160 -1.98324 -1.55792 -0.8441300
## 6  4.01580 -1.615360  0.620013  3.17038   3.375620  5.17071 -2.15181 -0.9471420
##       PC644     PC645     PC646     PC647      PC648     PC649      PC650
## 1 -0.937945 -1.843720 -2.582970 -10.73890  3.7954200  2.855110 -0.2589810
## 2 -6.529410  6.006810  1.148680   5.06560  1.8991100  2.101600 -6.2700700
## 3  0.136030  2.429280  3.652820   8.23754 -4.0398200 -7.669620  4.5103500
## 4  1.369430  1.893500  0.217308   2.83880  0.7756960 -0.505091 -0.1935280
## 5 -0.790615  0.879196 -1.435110   1.31921 -0.3901950 -1.048880 -0.0646646
## 6  0.612677 -5.131560  5.635370  -1.17004  0.0239208 -2.801260  0.9213570
##       PC651     PC652     PC653     PC654      PC655     PC656     PC657
## 1 -2.277440  8.095210 -0.590145 -6.700960  8.1917700  5.218580 -2.377700
## 2 -0.723103 -2.044360 -8.799940  7.557590 -9.2962700 -5.298730  6.163190
## 3 -1.861290 -7.139070 -6.315390  2.288920  3.7429900 -2.960290  7.155420
## 4  0.733839 -1.659590 -2.787450 -0.184224 -1.0106900  0.633442 -0.104418
## 5  0.286248 -0.926919 -0.114351  0.813858 -0.7331730  0.528832  0.243730
## 6 -1.695660 -2.316580 -1.342170  0.687855 -0.0667946 -4.345210  3.930310
##      PC658     PC659    PC660     PC661    PC662    PC663     PC664     PC665
## 1 -6.72905 10.245500 -4.98574 -3.308890 5.752310  3.64647   7.94843 -9.795420
## 2 -9.73732 -6.805450  1.15989 -2.480330 3.510110  3.91034 -11.39320  2.639010
## 3 -3.61192 -1.178170 -2.19328 -0.115131 3.963130 -8.54738  -9.99102  6.340250
## 4 -2.78424  1.292840 -2.75815 -0.732759 2.434860 -1.06276  -2.44313  0.847658
## 5  1.26046  0.139417 -2.24198 -1.663460 2.254810 -2.60778  -1.97549 -1.013840
## 6 -1.43712  2.122950 -2.30078  0.384311 0.921404 -3.59456  -4.05171  4.754650
##       PC666     PC667      PC668     PC669     PC670     PC671     PC672
## 1 -0.978852 -7.685280  4.4173200  3.082660  5.156400 -1.854210 -2.332280
## 2 -2.153600  4.230980 -9.0736300 -4.866000 -1.320420  1.694480 11.925900
## 3  0.456456  1.805890 -1.7523700 -3.211490 -4.189360  4.687530  0.131379
## 4  0.142693  0.638929 -0.8172720 -0.625444  0.389608 -0.123802  0.514862
## 5  1.383310  1.173970 -0.0793292  1.211850  1.225400 -1.766580  0.285424
## 6  1.083460 -2.595800 -0.4180320  1.677620 -1.352900  2.576070  3.052710
##       PC673     PC674     PC675     PC676     PC677     PC678     PC679
## 1  7.714990  0.902983 -3.883970  3.517160 -2.888470  0.468737 -2.735040
## 2 -8.770240 -4.600020  4.612250 -9.491340  0.109318  0.464127 -5.452440
## 3 -2.053790  3.051010  0.782791  1.522370 -1.140540 -3.861170  1.060280
## 4 -0.960677 -0.996975 -0.876735 -1.650800 -0.212322  1.519270 -0.433806
## 5 -1.172580  0.632653  1.029420  0.237144 -0.384320  1.148320  1.298490
## 6  1.992090  4.382300 -0.486998 -1.571930  1.047320 -1.083300 -2.447950
##       PC680     PC681      PC682     PC683     PC684      PC685     PC686
## 1  3.559240  7.571770  0.0233162  4.201250 -2.857610 -3.0088600  0.142802
## 2 -2.254790 -7.770550 -6.4139700 -9.503610  0.734434  0.0214167  0.922537
## 3 -1.332280 -3.017710 -3.1139800 -5.776560 -1.799260 -2.1541100 -2.836470
## 4  0.354037  0.206872  1.0207900 -2.639840  0.501722 -1.0595800 -0.343254
## 5  0.890797  0.770138  1.9247100 -0.228412  0.391494 -0.3922560 -1.243100
## 6 -0.921721 -2.749610 -2.6931300 -1.203670 -1.722840  3.1239700 -3.015070
##        PC687     PC688     PC689     PC690      PC691      PC692     PC693
## 1  0.9631010 -1.850710 -8.103210 -2.753990   1.383770 -5.7830000 -2.392580
## 2 -4.0449500 -5.093930 -7.344620  5.664130 -13.139200 -1.3073300  7.367190
## 3  1.5678600  2.995680  3.689360 -0.727163  -0.377641  0.0422484  3.335250
## 4  0.1887190  0.223120  0.417790 -0.410303   1.428030  2.2496300  0.163116
## 5 -0.0112736 -0.260185  0.234566 -0.398761   0.185991 -0.2226260 -0.512066
## 6  0.5957400  3.837890  1.803740  0.853355  -1.590950  2.2629600 -0.283617
##       PC694      PC695     PC696     PC697      PC698      PC699     PC700
## 1  2.672030  3.1061700  0.616902  1.661150   4.304520  0.9408980  2.525140
## 2 -3.524050 -0.3402410  1.122280 -6.666780 -13.292400 -6.1220900 -7.996400
## 3 -4.423110 -3.1294800  1.456730 -2.289750  -2.670730  4.7457900 -0.654216
## 4 -1.058710 -0.0788688  1.257180  0.652511   0.522916 -0.0481975 -0.785245
## 5  0.543643 -1.7351600 -0.775722 -0.256420  -0.183184  2.4325000  0.759796
## 6 -4.015980 -0.8782250  2.879870 -3.955060  -3.867400  1.5343600 -2.560920
##       PC701     PC702     PC703     PC704     PC705     PC706     PC707
## 1  8.315710 -2.751580  1.091770 -5.456910  5.530970 -2.403810 -1.014950
## 2 -3.402650  7.727150  1.306800 15.873700 -0.339925  9.440910 -7.578310
## 3  2.577140 -1.242950 -1.410580  2.625250 -0.694065 -1.131340 -0.465066
## 4  2.030830  0.147544 -0.633884 -0.614220  0.211551  1.231170  0.465193
## 5  0.508467 -0.305402 -0.232083 -0.618519 -0.470989 -3.651930 -0.707635
## 6  0.477141 -1.078410  0.788460  4.157050 -1.144080  0.212263 -0.165601
##       PC708      PC709     PC710      PC711      PC712    PC713     PC714
## 1  3.092920  2.2228100  1.173030 -0.0781324 -2.7649000  1.04205  3.787530
## 2 -4.098670 -9.7812800  3.205430  7.1020200  6.7178300  6.45360 -6.418130
## 3  0.249806  1.8408400 -2.440510  2.8123600 -3.0757700  0.34986 -4.788850
## 4  1.156510  0.0337897 -0.151140 -0.8392580 -1.0255000 -1.58383 -0.899978
## 5 -0.547488  1.1084000 -0.633367 -0.1984590 -0.5816080  2.41489 -0.331643
## 6 -0.737585  2.2357700 -1.330920 -0.7080510  0.0323882  1.17648  0.262815
##       PC715      PC716     PC717      PC718     PC719     PC720     PC721
## 1  0.972488  0.6571170  1.144230  2.3198800  1.191150  1.090620  1.819680
## 2  4.935670  1.2694800 -9.558650 -5.4705800  2.286790  2.122820  5.128400
## 3 -1.015920 -2.7353600 -2.694190 -3.2520800 -0.986321 -0.645813  4.767740
## 4  0.178030 -0.0667111  1.434620  0.0984879  0.031049 -0.193997  1.423300
## 5  0.210236 -0.5518140 -2.145540 -0.8316130  0.650758 -1.225090  1.805580
## 6 -2.792650  0.1122550  0.118912  0.6374280  1.132260 -0.932538 -0.965396
##       PC722     PC723    PC724     PC725     PC726     PC727      PC728
## 1  1.442880  2.868730 2.082830 -1.045760  6.318320 -3.074210 -0.8773470
## 2 -7.410030 -1.593190 5.093140 -1.997920 -8.763980  3.591750  9.1125600
## 3 -6.972430 -0.595632 1.106040  2.257290 -2.013450  0.249988 -2.2318600
## 4  0.645839 -1.049580 0.262375 -1.546400  1.142240 -0.391041 -0.0377659
## 5 -1.822540  0.839740 0.497853  0.729818  0.210316 -1.268750 -0.0208362
## 6 -3.678980 -1.243660 1.560820  3.712500  1.178340  0.158514 -0.8389820
##        PC729     PC730     PC731     PC732     PC733      PC734     PC735
## 1  4.7446800 -2.507830  4.970770  1.653160 -1.565830 -0.0503822  2.964740
## 2 -1.4479000  8.603680 -1.859380  0.604591  3.172090 -1.8812300  0.163519
## 3 -1.0685200  4.017960 -4.637640 -1.033360  1.729150  2.8388000 -0.912366
## 4 -0.4191810 -0.116498 -0.128938 -2.035890  1.676760  1.6997400  0.664965
## 5  0.0920182 -0.685676 -0.352581 -1.181700  0.187980  1.5186900  0.814928
## 6  1.3785800  3.923050  0.916037  0.322230  0.879832  0.2346630  2.106700
##        PC736      PC737      PC738     PC739     PC740      PC741      PC742
## 1 -0.0545404  0.1080470 -0.1870690  1.949180 -2.124420  5.9693400 -0.3024650
## 2 -1.7641000  2.2447000  0.2883850  6.480370  2.993460  2.8673900  0.8210280
## 3  0.3356990 -1.0010300  3.9509300  2.943180  2.720330  0.0191857  0.0590165
## 4  2.5837700 -0.7925940  1.3339300  1.946990 -0.614367  0.6848490  1.1114700
## 5 -0.9146440 -0.0872072  0.3983760 -0.853748 -0.142914 -1.0270200 -1.0606000
## 6 -0.6634990  0.0404891  0.0355303  1.680390  4.190250 -0.2343540  1.5104500
##       PC743     PC744     PC745      PC746     PC747     PC748      PC749
## 1 -0.501076 -1.244670  0.418699 -1.7786300 -0.589285  1.530110  0.6540720
## 2 -4.721510 -6.210480 -1.630460 -0.2417410 -2.198180  4.258420  1.0140100
## 3  3.092970 -0.277524 -1.574570 -0.0303895 -1.865450  1.544670  0.5946640
## 4  0.681403  1.252200  1.445470 -2.4296000 -0.550373 -0.170530 -0.0902045
## 5 -0.276792  0.062173  0.310777 -0.2501430  0.222858 -0.622966 -1.3709200
## 6 -1.897840 -0.254566 -0.381911  2.2090800  1.642340  1.286210  1.5087700
##       PC750     PC751      PC752     PC753     PC754     PC755      PC756
## 1 -0.274253 -0.346528   0.640203 -0.844245  1.875370  3.024520 -1.7450800
## 2 -0.717583 -1.859920 -11.970900  5.161280 -0.465561 -2.368910 -0.9562030
## 3  0.391502  0.510731  -3.378530  1.152170 -1.154530 -1.222400  2.3879400
## 4  0.333235 -0.993200  -0.969760 -0.299044 -1.302790 -2.035420  1.2072800
## 5  0.481895  2.340660   0.458260 -2.456190  1.068670  0.963577 -1.4799400
## 6  1.913690 -0.606544  -4.470470  0.185839  0.207496 -1.899240 -0.0728127
##       PC757     PC758     PC759     PC760     PC761     PC762      PC763
## 1 -3.263270 -3.337780  0.565057 -0.315846  0.903968  1.516630 -2.6601900
## 2  1.979180  2.879390  3.379770 -0.188035 -0.408772 -0.244009  1.6930200
## 3  0.694249  0.445819 -0.762109  0.553738  1.170880  2.071300  1.6804600
## 4 -2.965020 -0.466331  1.883580  1.647440  1.613010 -1.519280 -1.1284600
## 5  0.702941  1.559340 -0.699376 -2.549360 -0.770142  0.460698 -0.0103156
## 6  0.765340  0.382530 -1.239420  0.289236 -0.168925  0.999421 -0.5162010
##       PC764      PC765     PC766     PC767     PC768     PC769     PC770
## 1  1.778650  0.5706570 -0.371561 -1.497570 -4.381020  1.243890  2.248130
## 2 -1.718710  1.2853400 -0.383236  2.841480  3.792060 -2.086870 -0.233220
## 3 -0.541596 -2.1706000  2.263310  0.906315  0.441441 -2.597210 -0.586944
## 4  0.697323  1.5837800 -0.108751 -1.220270 -0.813859 -0.523230  0.524438
## 5 -1.946630 -2.7691900  0.739136  0.668641  0.731291  1.031200 -0.369910
## 6 -0.514184 -0.0808515  0.789506  1.876790 -1.929900 -0.542094 -0.653419
##        PC771     PC772      PC773      PC774     PC775     PC776     PC777
## 1 -0.1732330 -2.160780 -3.6071600 -2.1047300  0.903033 -2.299320 -2.285610
## 2 -1.5126800 -2.117670  1.9892100  6.4225000 -2.904380 -0.420009  3.096540
## 3  0.4700860 -0.480577 -0.5465790  0.1378110 -0.224539  1.300750  1.181680
## 4  0.5710180 -0.339830  0.0795959  0.0475849  2.275360 -1.826620  0.549217
## 5 -0.0387854 -0.470205 -0.4903110 -0.5473870 -1.182890 -1.365740 -1.875030
## 6 -0.8940650 -0.687713 -0.6908740  0.1374900 -2.932100  1.367640  0.326895
##       PC778     PC779     PC780     PC781     PC782     PC783     PC784
## 1 -2.140190  1.993200  1.922120 -2.300720 -1.401900 -0.650031  1.468470
## 2  2.007960  4.308770 -0.029951  0.852218  0.991157  2.573250  1.778960
## 3 -0.196204 -0.101861  2.151260  1.762990 -0.138255  2.922610  0.478878
## 4 -0.439018 -0.790848 -2.889140  0.659955  1.096510 -0.727453 -1.608490
## 5  0.674263 -0.252367  2.405620  2.414220 -1.994280  2.262900  2.947360
## 6  3.224530  1.239790  1.490930 -0.383095  0.577615  1.551690  1.002660
##        PC785     PC786     PC787    PC788      PC789     PC790     PC791
## 1 -4.5830600  0.634860 -0.493373 0.848728  1.6121600 -0.179946  0.574275
## 2  1.1883100 -2.288390 -1.965380 3.544200 -2.0562100  2.378090  0.720809
## 3 -0.0889782 -0.841454  1.256180 0.632431 -1.0651800  1.143350  1.170450
## 4  0.3421180 -1.025950 -1.459980 1.674690 -0.1384270 -0.443186  1.455950
## 5 -2.0078200  0.902023 -0.451469 0.032456 -0.2001110  1.046260 -1.102550
## 6  0.5176040  1.659910  0.481274 0.306568  0.0611311  0.406007  1.752890
##        PC792     PC793     PC794     PC795     PC796     PC797     PC798
## 1 -0.9086910 -1.521030 -1.280350 -0.263426 -2.558830  0.410278  0.446194
## 2  1.5400600  3.303740 -0.445594 -0.466514  2.650090 -2.627330 -0.974133
## 3 -0.0897301 -0.409718 -1.409020  2.993300  0.805388 -0.644858 -0.824014
## 4  0.3920070 -1.340470  0.460055  0.691401  0.324568 -0.665665 -0.614820
## 5  0.0111024 -0.288279 -0.324706 -0.422375 -1.121810  1.547120 -0.421677
## 6 -0.7927890  1.188750 -0.221165 -0.823070 -1.580090  0.807039  0.403762
##       PC799       PC800     PC801      PC802     PC803      PC804      PC805
## 1  0.371371 -0.50014900 -3.679930  2.4586500  3.407620 -0.9333070  0.5079190
## 2  1.547310  0.33196300  2.262160 -3.1541100 -3.043970  0.9353850 -1.2108200
## 3  0.962603  0.95417300  2.133520 -2.2325800  0.949384 -0.7317120 -0.1281650
## 4 -2.744010 -0.00218451  0.942935 -2.6247000  0.579261  0.0468562  2.0866800
## 5  1.653000  0.19827500 -1.506750  1.9379300 -0.498526  0.3148670 -0.0545151
## 6  0.120140  0.96796900  0.192324  0.0515902  0.118370 -1.0597500 -1.3145800
##       PC806      PC807     PC808     PC809     PC810     PC811     PC812
## 1 -0.590068 -2.3145100  1.516270  1.377260 -1.677960 -1.131020  0.693753
## 2 -1.355310  1.1572100 -1.558810  1.085890  1.687050 -0.646803  1.135560
## 3 -1.049850  0.3250300  0.966564  0.520889 -1.008380  0.398579 -0.652057
## 4  0.120134  0.0834005  0.753011 -0.614749 -1.007760  0.151244 -2.109740
## 5 -0.365848 -1.3753300 -0.924156  1.169290  0.975947  0.548868  1.842450
## 6 -1.510720  0.2885410 -2.728760 -1.657470 -0.970105 -0.463773 -1.277150
##       PC813     PC814     PC815     PC816     PC817     PC818      PC819
## 1 -0.479574  0.809265 -0.104240 -0.143899  0.580870  0.647270 -0.1341280
## 2  0.661717  3.665220  2.058300  2.099630  0.538298 -0.481031 -0.7563290
## 3  2.077030  1.726050  0.577143  0.724074  0.404253 -0.700707  1.7796500
## 4  2.088330 -0.696383 -0.712691  0.768003  0.579239 -1.104520 -0.8320950
## 5 -1.179910  1.582040  1.282840 -1.126350 -0.215498  0.791934 -0.0167298
## 6 -0.589234  0.880270  1.563790  3.015480 -2.177780 -1.152880  1.8235300
##       PC820      PC821     PC822      PC823     PC824     PC825     PC826
## 1  0.480940 -0.0830959 -2.091510  0.4707690  0.633358  1.040920 -0.038308
## 2 -1.338720  1.7122200 -1.200040 -1.4610200 -0.207464  1.065610 -0.140433
## 3  0.787068  0.8729450 -0.303715 -0.3343050 -1.343680  0.412000  0.678000
## 4  0.780339  0.0316296 -0.032280 -0.1435880 -0.506939 -0.182709  0.731047
## 5 -0.344137 -0.3025920 -0.165304 -0.0219486  0.677284  0.290872 -0.562342
## 6  0.481720  0.1399090  0.494530 -1.1914800  0.349049 -0.253456  0.704457
##       PC827      PC828     PC829    PC830     PC831      PC832       PC833
## 1 -0.862525 -1.4404800 -0.428032 -1.43575 -1.238590 -0.9710950 -1.41158000
## 2 -0.844440  0.2181560 -4.032880 -1.16150  0.686576  1.0332300 -0.47404100
## 3 -1.498300  0.9043460 -0.653918  1.27200  1.072850  0.4510320 -1.00583000
## 4  0.261402 -1.7957500 -1.878530 -1.10461  0.196753  0.0595379  0.00494685
## 5 -1.199500  0.3234270  1.333570  1.06263 -0.394893  0.4562740 -0.38520000
## 6  0.811905  0.0819515 -1.046520  1.37089  1.274650 -0.1859690  0.57558100
##        PC834     PC835      PC836     PC837     PC838      PC839     PC840
## 1  0.8124630  0.278873  0.1561000 -0.145635  0.963983  0.3696650 -1.130520
## 2 -1.3607500  0.880028  0.2329190  0.016413  0.533442  1.2702500  0.580979
## 3  0.2798480 -0.739207 -0.0673526  1.135430 -1.484160 -0.3711200  0.547808
## 4  0.7301580 -1.921020  1.6838800  2.294690  1.456850 -0.4114090 -1.726830
## 5 -0.0658259  0.931774 -1.6381200 -2.357510 -0.924575  0.8267480  1.688780
## 6  0.3010610  3.627880  0.1406870  0.143266 -1.725910 -0.0974078 -0.636075
##        PC841      PC842     PC843    PC844     PC845     PC846     PC847
## 1  0.0517175 -0.1754660 -1.591790 -3.46131 -0.646382  0.881897  2.298750
## 2 -0.6889470  0.2921550  1.768790  1.17748  1.315010  0.113737 -0.740000
## 3  0.2773350 -0.1556390 -0.516030  2.15332  0.617726 -1.550710  0.021505
## 4 -1.2608300 -1.9557100 -0.421051  2.03634 -1.595060 -0.193341 -0.978483
## 5  1.0536300  2.5688500  1.057130 -1.72629  1.778970 -0.029983  0.316726
## 6 -1.0662400 -0.0315817  0.381516 -1.29748 -0.386187 -0.934134  1.104410
##         PC848     PC849     PC850     PC851      PC852     PC853     PC854
## 1 -0.86617600  0.833256 -0.947292  0.789946  0.9097950  0.727640 -0.134368
## 2 -0.00628969  0.185518  0.471186  0.834334  0.0232132  0.267786 -0.146894
## 3  0.98120500 -0.538732  0.836029 -0.137791 -0.1599210 -0.362267  0.171109
## 4 -0.67260800 -2.442290  0.383213 -1.895310  1.9753100  3.270190  0.281184
## 5  0.06974220  1.953620 -0.838027  1.598600 -1.9484600 -3.852550 -0.137394
## 6 -0.26036400  0.432592 -0.117560 -0.398783 -0.1335840 -0.630130 -0.043209
##       PC855     PC856     PC857      PC858      PC859      PC860     PC861
## 1  0.462540 -0.419273  0.345529 -0.1110000  0.9643680 -0.0221716  0.222090
## 2 -0.355702 -0.712416 -1.316310  0.3863270  0.3455210  1.4142700 -0.150463
## 3  0.315594  1.303490  0.377559  0.1151960 -0.0333118  0.4591570 -1.706500
## 4  1.440860  2.162990 -2.711350 -0.0606456  1.0731600 -2.8563300 -4.899760
## 5 -1.864780 -2.215440  1.848510  0.4619990 -0.2743920  2.6458300  4.618800
## 6  0.113808  0.676154 -0.370062 -0.2042910 -0.4950110  1.0522800 -0.329097
##       PC862      PC863     PC864      PC865      PC866     PC867     PC868
## 1  0.602023 -0.2723600 -0.091991  0.6272910  0.5053860 -0.481732  1.013840
## 2  0.626318  0.6389370  0.618471 -0.0596022 -0.2238020 -0.853176 -0.103744
## 3 -0.475023 -0.0388539 -0.387124 -0.1466920 -0.0675125 -0.564913  0.212671
## 4 -1.319810  1.2233900 -3.312020 -1.7664900 -7.3413700  6.278480  0.979295
## 5  1.395050 -1.4097400  3.663020  0.6009190  6.2869200 -5.206690 -0.173900
## 6 -0.082525 -0.4173050  0.289259  0.7486060  1.5795700 -1.537230 -0.699391
##       PC869      PC870     PC871       PC872      PC873     PC874     PC875
## 1 -0.597351  0.0348315  0.279573  -0.6004270  0.0126427  0.617458 -0.927539
## 2 -0.497966 -0.8729600 -1.219350  -0.8492770  0.0393202  0.770234  0.247693
## 3  0.355372  0.3199740 -0.251236  -0.0812435 -0.6572860 -0.534698  0.208263
## 4  2.911140 -0.1164440  2.069190  15.3537000 -1.3656600  6.036640 -5.488580
## 5 -2.726240 -0.6117230 -1.736570 -15.8492000  0.9582780 -5.156130  6.098650
## 6  0.100209  0.2397560  0.130314  -0.9128330  0.2525470 -0.403387  0.571104
##        PC876      PC877       PC878      PC879      PC880     PC881      PC882
## 1  -0.631859   1.513660   1.1961800  0.6836470   0.460662  0.632216   1.707890
## 2  -0.726893   0.469475   0.3915190 -1.2348200   0.845884  0.631475   0.438315
## 3   0.142138  -0.874589   0.0742214 -0.0794038  -0.567137 -0.422935  -0.282445
## 4  16.600800 -26.581000 -15.4344000 -0.3998270 -17.937000 -8.034170 -26.463200
## 5 -17.630500  26.581500  15.4714000 -0.2670720  18.405400  8.296410  27.332700
## 6  -0.689737   1.397320   0.2890440 -0.4018950   0.524233 -0.521840   0.858222
##       PC883      PC884       PC885     PC886       PC887     PC888     PC889
## 1  0.749692  0.6544530 -0.64663300  0.646122  0.56006700  0.103761  0.120574
## 2  0.114915 -0.1477560 -0.24229200  0.213023  0.80423300  0.261274  0.258073
## 3  0.192027 -0.0018532  0.00504095 -0.359653 -0.00595146  0.319100 -0.149307
## 4 -3.229590  1.9692400  4.05922000 -7.171380 -0.74636700 -4.380240  1.679370
## 5  3.448270 -1.7757700 -4.43171000  6.799210  0.45663100  4.319940 -1.693370
## 6  0.117226 -0.4708430  0.36195500  0.486131  0.13525300 -0.260488 -0.969128
##       PC890      PC891      PC892      PC893     PC894     PC895      PC896
## 1 -0.711144 -0.2090570 -0.0553978 -0.7743020 -0.316350 -0.516285  0.0926215
## 2 -0.118932 -0.4924700 -0.1433780 -0.1775270 -0.109243  0.131544  0.2617310
## 3 -0.285057  0.6285480  0.2030820 -0.0504705  0.065127 -0.276885 -0.3427320
## 4  4.948870  0.0464377 -0.8852940  1.4602900  0.767219 -2.898910 -1.5112800
## 5 -4.414240  0.0902607  0.5207640 -2.0055000 -0.506394  2.683650  1.5476100
## 6 -0.678645  0.6564850 -0.1400250 -0.0152137  0.356920  0.500773 -0.0655357
##        PC897     PC898      PC899      PC900      PC901      PC902      PC903
## 1  0.0547369 -0.340371  0.0894256  0.0627417  0.0127018 -0.1915890 -0.6847170
## 2 -1.6249100  0.975248  1.4359500  0.0579100 -0.5324990  0.0255975  0.4112400
## 3 -0.5111000  0.149732  0.0552835 -0.1675370 -0.0723522  0.3122280  0.3009030
## 4  3.3213600 -2.234540 -2.8166300  1.3891700  1.6883900  0.9685850 -1.1159000
## 5 -2.9725400  1.754680  2.8406800 -1.7609300 -1.2536700 -1.3268400  0.9217820
## 6  0.5611870 -0.359296 -0.2510140 -0.1819070 -0.4142500  0.3180630 -0.0125375
##        PC904      PC905       PC906      PC907      PC908      PC909
## 1  0.0180811  0.3439300 -0.41333600  0.0133251  0.4484590 -0.1666370
## 2  0.2269950  0.3999480  0.07723750  0.0704885 -0.4826130  0.0295396
## 3 -0.5396650  0.0780014 -0.00253384  0.2753340 -0.1481710  0.3274780
## 4  0.3199520  0.0864305  0.29278000  0.1390330 -0.0432160  0.0135659
## 5 -0.5212770  0.0568379 -0.64077400 -0.6181340 -0.2190930  0.5699180
## 6 -0.2544240 -0.1004000  0.34517400 -0.4610310  0.0905346 -0.2499610
##         PC910       PC911      PC912      PC913      PC914     PC915      PC916
## 1  0.00381806 -0.00287736 -0.0574551  0.0597161  0.0504773 -0.400598  0.0551224
## 2 -0.30589400  0.36426000  0.5040830  0.0872674 -0.4038840 -0.471562 -0.5794360
## 3  0.00307486  0.52434400  0.1508940  0.1849340  0.1450740 -0.125639  0.0988883
## 4  1.23971000  1.14183000  0.1078240  0.2911420 -0.3366520  0.425615 -0.9743780
## 5 -1.20905000 -0.70137800 -0.0185021 -0.4007010  0.3754090 -0.741609  0.9613330
## 6  0.05342590 -0.46471900  0.3326700 -0.1665290  0.1051710  0.141510 -0.0547851
##        PC917      PC918      PC919     PC920      PC921      PC922      PC923
## 1 -0.1875180 -0.0288136  0.0422655  0.225173 -0.2704020  0.0430636  0.2384520
## 2  0.2309330 -0.0911943  0.1847460  0.544520  0.2178110  0.2633010 -0.0708432
## 3  0.0337238 -0.0628511  0.2261640 -0.208037 -0.4779180  0.3304580 -0.0467790
## 4 -1.0503200 -1.0871300  0.9092910  1.135970  0.0182488  0.3556300  0.4910370
## 5  0.9730690  1.1598200 -1.3567900 -1.185600  0.3213970 -0.6840190 -0.5730510
## 6 -0.1743140  0.4769400 -0.3696610 -0.123960 -0.0727039 -0.1801580 -0.0373181
##        PC924      PC925      PC926      PC927      PC928       PC929      PC930
## 1  0.3927020  0.0341066 -0.0851779 -0.0938119 -0.0481371  0.21126200  0.0518456
## 2 -0.4271730  0.3957400 -0.2364750  0.2765870  0.1401900  0.13004400 -0.0816514
## 3 -0.0883277  0.4687910 -0.1713940 -0.2691790  0.1881190 -0.02020350 -0.2943930
## 4  0.2717850 -0.2212000  0.3098280 -0.1889900 -1.1889500 -0.34988300 -0.1490990
## 5 -0.4934060  0.2644150 -0.5650040  0.3460210  0.9129430  0.38676600  0.3286540
## 6 -0.2115970  0.0958226  0.1844200  0.1240490  0.1656380  0.00488192 -0.1637080
##        PC931      PC932        PC933 Individual continent          region
## 1 -0.2867540  0.0300271 -1.34671e-06        802    Europe Southern Europe
## 2  0.2920800 -0.0511823 -1.34671e-06        803    Europe Southern Europe
## 3 -0.1757190  0.1815670 -1.34671e-06        804    Europe Southern Europe
## 4  0.3227390  0.2202680 -1.34671e-06        806    Europe Southern Europe
## 5 -0.1358880 -0.1167550 -1.34671e-06        807    Europe Southern Europe
## 6 -0.0263995  0.0797279 -1.34671e-06        808    Europe Southern Europe
##   Pop_City Country Latitude Longitude Continent Year          Region
## 1   Durres Albania 41.29704  19.50373    Europe 2018 Southern Europe
## 2   Durres Albania 41.29704  19.50373    Europe 2018 Southern Europe
## 3   Durres Albania 41.29704  19.50373    Europe 2018 Southern Europe
## 4   Durres Albania 41.29704  19.50373    Europe 2018 Southern Europe
## 5   Durres Albania 41.29704  19.50373    Europe 2018 Southern Europe
## 6   Durres Albania 41.29704  19.50373    Europe 2018 Southern Europe
##     Subregion order order2 orderold AM_order state FINAL_order
## 1 East Europe    33     25       25       NA                80
## 2 East Europe    33     25       25       NA                80
## 3 East Europe    33     25       25       NA                80
## 4 East Europe    33     25       25       NA                80
## 5 East Europe    33     25       25       NA                80
## 6 East Europe    33     25       25       NA                80
```

### 1.2.1 Create PCA plots by country

PCs 1 and 2

```
# #   save the pca plot                                                       ####
ggsave(
  here("scripts", "RMarkdowns", "output", "americas", "manuscript1", "lea", "PCA_lea_man1_bycountry_LD10_pc1_pc2.pdf"
  ),
  width  = 8,
  height = 10,
  units  = "in"
)
```

USA clusters with Japan, Italy, Bermuda Jamaica clusters with
Southeast/South Asia. Brazil and Argentina form their own cluster;
Colombia is also separated, but is closest to Trinidad and Tobago

PCs 1 and 3

```
# #   save the pca plot                                                       ####
ggsave(
  here("scripts", "RMarkdowns", "output", "americas", "manuscript1", "lea", "PCA_lea_man1_bycountry_LD10_pc1_pc3.pdf"
  ),
  width  = 8,
  height = 10,
  units  = "in"
)
```

PCs 1 and 4

```
# #   save the pca plot                                                       ####
ggsave(
  here("scripts", "RMarkdowns", "output", "americas", "manuscript1", "lea", "PCA_lea_man1_bycountry_LD10_pc1_pc4.pdf"
  ),
  width  = 8,
  height = 10,
  units  = "in"
)
```

PCs 2 and 3

```
# #   save the pca plot                                                       ####
ggsave(
  here("scripts", "RMarkdowns", "output", "americas", "manuscript1", "lea", "PCA_lea_man1_bycountry_LD10_pc2_pc3.pdf"
  ),
  width  = 8,
  height = 10,
  units  = "in"
)
```

### 1.2.2. PCA plots by region

PCs 1 and 2

```
# #   save the pca plot                                                       ####
ggsave(
  here("scripts", "RMarkdowns", "output", "americas", "manuscript1", "lea", "PCA_lea_man1_byregion_LD10_pc1_pc2.pdf"
  ),
  width  = 8,
  height = 10,
  units  = "in"
)
```

PCs 1 and 3

```
# #   save the pca plot                                                       ####
ggsave(
  here("scripts", "RMarkdowns", "output", "americas", "manuscript1", "lea", "PCA_lea_man1_byregion_LD10_pc1_pc3.pdf"
  ),
  width  = 8,
  height = 10,
  units  = "in"
)
```

PCs 2 and 3

```
# #   save the pca plot                                                       ####
ggsave(
  here("scripts", "RMarkdowns", "output", "americas", "manuscript1", "lea", "PCA_lea_man1_byregion_LD10_pc2_pc3.pdf"
  ),
  width  = 8,
  height = 10,
  units  = "in"
)
```

PCs 1 and 4

```
# #   save the pca plot                                                       ####
ggsave(
  here("scripts", "RMarkdowns", "output", "americas", "manuscript1", "lea", "PCA_lea_man1_byregion_LD10_pc1_pc4.pdf"
  ),
  width  = 8,
  height = 10,
  units  = "in"
)
```

### 1.2.3 PCA colored by clades from Local PCA analyses

```
sampling_loc <- read.csv(
  here("scripts", "RMarkdowns", "output", "sampling_loc_all_pops_1Oct2025.csv"
    ))
```

```
saveRDS(sampling_loc, here("scripts", "RMarkdowns", 
  "output", "sampling_loc_all_pops_Oct2025.rds"
))
```

Sample data

```
sampling_loc <- readRDS(here("scripts", "RMarkdowns", "output", "sampling_loc_all_pops_Oct2025.rds"))
head(sampling_loc)
```

```
##      Pop_City Country Latitude Longitude Continent Abbreviation  Year    Region
## 1      Hainan   China  19.2000  109.6000      Asia          HAI  2014 East Asia
## 2      Yunnan   China  24.4753  101.3431      Asia          YUN  2014 East Asia
## 3       Hunan   China  27.6253  111.8569      Asia          HUN  2001 East Asia
## 4     Okinawa   Japan  26.5013  127.9454      Asia          OKI 2018? East Asia
## 5 Tanegashima   Japan  30.4564  130.9784      Asia          TAN  2008 East Asia
## 6   Kagoshima   Japan  31.5602  130.5581      Asia          KAG  2018 East Asia
##   Subregion order order2 orderold AM_order state FINAL_order Clade
## 1     China    55     47       47       NA                 1     3
## 2     China    56     48       48       NA                 2     4
## 3     China    57     49       49       NA                 3     3
## 4  Southern    54     46       46       NA                 4     3
## 5  Southern    NA     NA       NA       NA                 5    NA
## 6  Southern    53     45       45       NA                 6     1
```

More sample data

```
# import sample attributes
samples2 <- read.delim(
  here("scripts", "RMarkdowns",
    "population_metadata_all_1Apr2024.txt"
  ),
  head = TRUE
)

samples2<- samples2 |>
  dplyr::select(
    continent, region, pop
  )

# check head of the file
head(samples2)
```

```
##   continent         region pop
## 1 Caribbean      Caribbean BRM
## 2 Caribbean      Caribbean JAM
## 3 Caribbean      Caribbean SAI
## 4    Africa Central Africa GAB
## 5    Africa    East Africa DGV
## 6    Africa    East Africa ANT
```

Merge with sampling\_loc

```
merged_loc <- merge(samples2, sampling_loc, by.x = "pop", by.y = "Abbreviation")
head(merged_loc)
```

```
##   pop     continent          region           Pop_City    Country  Latitude
## 1 AIZ          Asia       East Asia Aizuwakamatsu City      Japan  37.49240
## 2 ALD        Europe Southern Europe             Durres    Albania  41.29704
## 3 ALU        Europe  Eastern Europe            Alushta    Ukraine  44.68289
## 4 ALV        Europe Southern Europe              Vlore    Albania  40.46600
## 5 ANT        Africa     East Africa       Antananarivo Madagascar -18.87920
## 6 ARA South America   South America          Araguaina     Brazil  -7.19119
##   Longitude Continent    Year          Region     Subregion order order2
## 1 139.99360      Asia    2008       East Asia      Northern    NA     NA
## 2  19.50373    Europe    2018 Southern Europe   East Europe    33     25
## 3  34.40368    Europe    2021  Eastern Europe   East Europe    43     35
## 4  19.48970    Europe    2020 Southern Europe   East Europe    32     24
## 5  47.50790    Africa    2022     East Africa   East Africa    NA     76
## 6 -48.20810  Americas unknown   South America South America    NA     NA
##   orderold AM_order      state FINAL_order Clade
## 1       NA       NA                     16    NA
## 2       25       NA                     80     3
## 3       35       NA                     90    NA
## 4       24       NA                     79    NA
## 5       NA       NA                     48    NA
## 6       NA       43 TO, Brazil         140     5
```

Check pops

```
head(pc.coord$Population)
```

```
## [1] OKI OKI OKI OKI OKI OKI
## 93 Levels: ALD ARA BAF BCO BEA BEH BEN BER BIT BPA BPV BRE BRM CAM CES ... YUN
```

```
#Check how many sampling localities
length(unique(pc.coord$Population))
```

```
## [1] 93
```

Check the regions

```
unique(merged_loc$Region)
```

```
##  [1] "East Asia"       "Southern Europe" "Eastern Europe"  "East Africa"    
##  [5] "South America"   "West Africa"     "North America"   "South Asia"     
##  [9] "Caribbean"       "Southeast Asia"  "Central Africa"  "Indian Ocean"   
## [13] "Western Europe"  "North Africa"
```

Check the continents

```
unique(merged_loc$continent)
```

```
## [1] "Asia"          "Europe"        "Africa"        "South America"
## [5] "North America" "Caribbean"
```

Check the clades

```
unique(merged_loc$Clade)
```

```
## [1] NA  3  5  1  4  2
```

Merge

```
merged_data <- merge(pc.coord, merged_loc, by.x = "Population", by.y = "pop")
head(merged_data)
```

```
##   Population      PC1      PC2     PC3      PC4      PC5     PC6      PC7
## 1        ALD 12.47210 -17.3459 15.3715 -2.03914 -17.5181 29.4271  8.67642
## 2        ALD 11.70270 -18.9829 17.2753 -1.55201 -19.4285 35.1234  7.98931
## 3        ALD 11.87130 -17.8179 17.6462 -2.32098 -13.7110 28.2972  9.04092
## 4        ALD  9.95509 -17.7640 17.1607 -2.10500 -16.1996 32.1231  9.91590
## 5        ALD 12.43350 -17.3946 16.7447 -2.75256 -17.7517 35.3727 10.40750
## 6        ALD 11.48580 -16.1954 11.5519 -2.72355 -14.5214 25.6774  7.82205
##         PC8      PC9      PC10      PC11    PC12     PC13       PC14     PC15
## 1 -5.170070 -3.16559  -9.95041 -10.87760 4.28397 -2.45357 -4.0750500 -9.19432
## 2 -0.674442 -4.22693 -10.70950  -9.80780 4.19175 -4.19859 -0.6079330 -7.76688
## 3 -3.328340 -8.25317  -8.85598  -6.01130 3.22631 -3.11134 -0.0883012 -9.57125
## 4  0.675904 -5.74958  -9.52483 -12.15680 3.60446 -4.99037 -3.9176000 -4.87030
## 5  0.484387 -4.92715  -7.92123 -12.05770 3.91345 -5.42792 -4.0588500 -7.18650
## 6 -3.476920 -3.01476  -7.63438  -9.93542 2.14653 -2.01879 -2.5715900 -4.55891
##       PC16    PC17      PC18    PC19     PC20      PC21     PC22      PC23
## 1 -3.15854 16.0996 -12.00720 7.66511 -32.8951  4.531690 -1.59264  0.102413
## 2 -1.31815 17.5205  -9.89031 9.28258 -31.9964  6.019730  1.25888 -0.804603
## 3  2.45113 14.0448  -5.19429 8.64728 -17.3355 -0.286158  3.78432  0.619525
## 4 -5.10761 16.8699 -11.62190 5.73955 -34.0785  3.952390 -4.88544  4.730300
## 5 -3.23577 16.6454 -14.61410 7.48995 -35.7142  3.607730 -6.78271  4.081730
## 6 -5.35652 11.6194  -6.06302 9.91871 -33.0862  2.170390 -8.98188  0.927419
##       PC24     PC25      PC26      PC27    PC28     PC29     PC30    PC31
## 1 -8.57564 8.486470  0.820333  1.329430 30.6714 -2.63383 -7.66026 5.57388
## 2 -6.33156 1.463550 -1.777060  2.526950 30.1635 -9.27341 -6.05596 2.19556
## 3 -5.28300 0.591685  3.678450 -0.571494 17.2094 -3.99407 -4.50657 4.67916
## 4 -6.78118 7.991240  1.462960  5.007750 33.3670 -2.69857 -6.79597 5.83205
## 5 -6.18645 5.959110  1.141070  6.071760 31.5112 -8.46489 -4.60567 4.87215
## 6 -3.88095 4.609530  2.122890  4.878510 30.9498 -1.86787 -5.27658 0.65846
##       PC32     PC33    PC34      PC35     PC36    PC37      PC38      PC39
## 1 4.071330 14.88910 18.0315  -9.33594 -30.5081 12.8582 -11.57790 -13.70450
## 2 6.472600  9.50602 14.5320  -3.86030 -18.4456 12.3197 -11.76670 -13.60170
## 3 0.469717 10.31880 12.5926 -13.32040 -20.6154 10.6698  -5.17850 -11.02580
## 4 1.071100 16.77340 14.8018 -12.39760 -30.7984 12.1448  -9.08931 -18.00870
## 5 0.710753 17.09360 13.6596 -10.55280 -26.3430 11.3182  -7.21008 -16.04460
## 6 1.630530 12.81490 16.1492  -9.42359 -25.6295 13.0158 -10.10200  -9.85977
##        PC40     PC41      PC42       PC43     PC44      PC45    PC46      PC47
## 1 -0.587234 -28.8323  1.835170  0.0384991 -7.64847  5.293240 24.9085 -1.132090
## 2  1.908030 -25.3970  5.532530  4.2593000 -9.33399  5.752620 24.6796 -3.426270
## 3  0.521170 -23.6986  0.616582 -1.1900900 -6.01403 -0.825128 17.1390  0.224786
## 4 -0.416380 -33.0105  0.855150  4.4579500 -6.68400 -0.626926 18.9120  3.746940
## 5  0.232115 -32.2876  0.599561  8.3430900 -6.09456  0.733254 19.9615  3.470250
## 6 -0.915681 -27.5956 -2.551950 -4.0004500 -7.73024  2.556600 18.3099  1.039350
##        PC48      PC49      PC50       PC51      PC52     PC53      PC54
## 1 -4.360480  4.392310  0.681564  0.1880380  1.794900 3.237680 -0.928332
## 2 -3.139890  6.290850 -2.153170 -2.3910200  3.421670 1.490340  0.660591
## 3 -0.751560 -0.747995  2.175200 -0.0268773 -2.898900 1.410800  0.366135
## 4 -1.300180  5.779350 -0.329659 -1.6546000  1.637280 2.903390  0.907437
## 5  0.167128  6.530300 -3.340580 -1.6809600  0.973852 4.125180 -1.635360
## 6 -2.718520  8.802980  1.820840  2.2993600  6.504890 0.275305 -4.948840
##       PC55      PC56       PC57     PC58       PC59      PC60       PC61
## 1 -4.16921  3.273410  -8.342260  3.00285 -1.4486200  2.201970  0.3547510
## 2 -1.46150  2.009650  -9.196700  1.59162 -2.2417900 -2.091950 -3.6230700
## 3 -1.48645 -0.451974 -11.109700 -1.60505 -1.2909400 -2.291070  0.0246931
## 4 -1.22597  5.968140  -8.964380  6.16059  0.0920522  0.581733 -1.4602200
## 5 -2.83321  4.841150 -10.135700  4.38410  1.6161200  1.807520 -1.1118600
## 6 -2.77715 -2.913210   0.489801  5.77334 -3.6655900 -0.933340  0.1081650
##        PC62     PC63     PC64      PC65      PC66     PC67      PC68      PC69
## 1 -1.237010 -1.18475 -1.10734 -6.410970 -0.939701 4.658960 -1.776920 -3.265040
## 2 -0.230872  3.13794 -2.53963  0.110276 -3.181550 8.017560  1.601000 -2.767280
## 3  2.617740 -3.09886 -3.65672 -2.403640 -7.410530 2.958740 -0.529177 -1.541610
## 4 -0.986571 -2.99043  4.04356  4.054730 -1.682010 0.284102  0.116172  0.917741
## 5 -0.618095 -5.84041  2.72031  3.838820 -2.384890 1.034150 -1.279290 -0.688140
## 6 -2.349870 -2.49792 -1.86574  2.802150  5.216340 3.718490 -0.654553 -6.572550
##        PC70     PC71     PC72     PC73     PC74      PC75     PC76      PC77
## 1  1.904610 5.170640  5.48999  1.66407  1.46667  0.653395 -3.40975 -1.280700
## 2  3.756730 6.600860  2.03126  3.59956  5.38879 -1.652550 -5.84941 -3.781730
## 3 -0.411247 2.052990  6.65023 -1.56540 10.91580  1.833610  4.57139 -2.114940
## 4 -3.113170 0.496605 -3.92354  4.14673 -3.05857  4.105820 -3.34481  0.868898
## 5 -5.332120 3.809080 -4.23296  7.37401 -7.90029  3.642480 -3.38825 -0.433391
## 6  0.107085 4.826330  3.42373  6.58851  2.00521 -3.369450 -4.46056  4.865440
##         PC78     PC79     PC80    PC81       PC82        PC83     PC84     PC85
## 1  3.2130600  4.56050  8.81043 4.66404 -2.9191000 17.02930000  1.74178 -6.26756
## 2  7.3150000  6.73149  7.20350 6.13921  0.8557870 10.33450000 -1.15909 -4.88773
## 3  0.0455153  3.79497 -1.35781 2.81839 -5.2825500  0.00705801 -2.72962  6.06643
## 4  7.7155700 -2.88844  6.98450 1.61192 -7.1151000  4.12825000  6.95277 -5.12945
## 5  7.0177200 -4.60132  8.53960 2.21707 -6.3962100  5.53773000  6.01097 -6.25495
## 6 -0.8538970  3.28655  5.26362 3.56512 -0.0171874  9.21101000  2.17656 -5.25706
##        PC86      PC87      PC88       PC89      PC90      PC91      PC92
## 1  2.187420  0.591517  1.978320 -3.1480900  0.677793  6.338820 10.498900
## 2  4.015880 -0.754186  3.673220 -2.2769800 -0.509119  4.717090  9.257550
## 3 -3.751220 -0.692356  1.060030  0.8321190 -1.628860  1.108140  0.458722
## 4 -4.576720  7.131010 -0.577969 -0.0987706 -0.367819 -6.280500  0.505862
## 5 -5.901550  6.016590 -0.591979  1.7239300 -0.908827 -4.915650  0.188937
## 6  0.270983  2.754290  3.441800 -2.0190800 -1.209770  0.489177  8.917870
##       PC93     PC94      PC95      PC96       PC97      PC98       PC99
## 1  3.12486  5.88883  0.369937 -0.707514 -2.4154900 -0.754803   2.899340
## 2 -1.19889  6.75260 -1.892620  0.623602 -4.0416700 -1.622070   5.697230
## 3  1.28326 -4.39294  2.423370  0.932614  0.0183226 -6.362440   5.800040
## 4  1.45905 -5.65809 -8.701600 -2.458570 -3.0122700 13.103900 -22.066400
## 5  2.41877 -5.27139 -7.449840 -1.280090 -2.8643600 15.003500 -23.407000
## 6  5.07596  3.02677  1.230680  1.850730 -1.3031100 -1.508690   0.935088
##       PC100     PC101     PC102   PC103    PC104     PC105    PC106    PC107
## 1  2.763480   6.78773 -0.167617 7.16347 -1.39642  10.36860 -4.24550  1.89517
## 2  4.060740  11.18890 -1.302690 7.10377 -4.29362   9.81671 -4.82558  4.58001
## 3 -1.135060  11.61190  3.638440 2.29648 -3.71333   4.56109 -2.15596  0.60235
## 4 -6.186480 -21.90390  5.432840 2.15330  7.35406 -17.31530 -3.32797 -8.12568
## 5 -7.322470 -21.98400  5.957640 2.60806  6.19213 -18.75220 -2.87364 -9.44615
## 6 -0.804744   6.38337 -0.434590 7.77431  5.62614   5.97160 -2.41330  2.42791
##        PC108    PC109      PC110    PC111     PC112    PC113    PC114     PC115
## 1   3.790580  4.18476   3.806740 -6.54160   4.42059 3.145470 -2.01601 -4.298040
## 2   8.281100  6.22484   3.497200 -8.25477  14.34520 1.934300  1.16543 -4.094150
## 3  -0.439064  8.70047   5.864120 -4.42897   9.53725 1.702460 -3.75941 -1.540230
## 4 -18.613300 -9.58731 -20.258500 16.67950 -17.99800 0.904075  7.25515 -3.453410
## 5 -20.966900 -9.25753 -18.919600 17.93400 -20.70390 0.777590  7.11005 -4.880430
## 6   2.865310 -4.07016   0.330226 -2.54956   2.28428 0.105378  1.08168  0.559862
##       PC116     PC117    PC118     PC119     PC120     PC121    PC122    PC123
## 1  13.71890   6.44969  6.45516   6.26533 -11.41710   8.13677  6.75206 -3.78779
## 2  14.54490  11.17260  7.27779   3.94456 -14.46090   8.87256  4.49034 -6.17414
## 3   3.64129   6.44388 10.24920  -1.70589  -1.71659   3.61728  5.94133 -9.16811
## 4 -13.20620 -28.61340 -9.78750  -9.19302  22.40570 -19.00450 -5.28394 10.04950
## 5 -13.21390 -29.53310 -9.07402 -10.48350  27.25170 -19.67530 -6.36346  9.88740
## 6   6.18716   4.24633  1.07651   8.15438  -2.36496   3.53817  1.36879 -2.43487
##       PC124    PC125     PC126     PC127     PC128      PC129     PC130
## 1 -0.254138  4.98062  4.188610  2.344620 -2.052210   3.022460   4.60058
## 2 -6.565410  1.57070  1.925260  0.499353 -3.739810   5.036330   7.57632
## 3  3.169770  3.33599 11.147700  4.813400  1.839920   4.385330   7.86716
## 4 -2.636160 -8.92277 -5.351320  0.553464 -2.337420 -17.515700 -14.19110
## 5 -2.671220 -9.65653 -5.520680 -1.339190 -2.409860 -17.174900 -15.21800
## 6 -3.471640 -2.83466 -0.806266  5.659650  0.539852   0.619796   4.40731
##      PC131     PC132      PC133     PC134      PC135     PC136     PC137
## 1  3.73557  -7.05784  -3.404670 -4.486380   2.717930 -6.169370   6.44829
## 2  5.18431  -4.99908  -3.811010 -7.587890   7.744620 -1.954030  10.82500
## 3  7.67214 -14.82840 -11.120600 -1.624580   4.704320  2.414300  -6.43113
## 4 -2.71509   6.80187  12.415000  8.355680  -9.769480  1.900720 -13.63380
## 5 -3.90760   6.99191  14.438900 11.539000 -10.471900  0.494371 -16.04970
## 6  2.62929   4.61284  -0.577107  0.225313   0.293253 -3.462870   8.24996
##       PC138     PC139    PC140     PC141     PC142     PC143    PC144     PC145
## 1 -1.487430 -11.42420 -1.87373   5.17510  -7.45814   1.59361 -5.01190 -1.459520
## 2 -2.286890 -13.67290  4.07702   3.82733 -10.08060   2.79069 -5.52312 -1.593270
## 3  3.714430  -1.42509  1.72756   3.05629  -3.58368   2.23042 -5.08169  0.275348
## 4 -2.251370  19.93900 -7.02543 -13.60490  18.52260 -13.86530 19.52890  5.386820
## 5  0.744417  20.85290 -5.66362 -12.41730  18.34220 -11.58320 21.69100  5.174200
## 6 -5.137670  -9.72132 -3.26452  -1.07738  -2.24191   1.17387  2.49895  0.722375
##       PC146    PC147     PC148     PC149     PC150     PC151     PC152
## 1  0.255417  2.39231  0.511423 -0.841956  0.533351 -0.606113  1.756090
## 2  3.252920  6.90506 -1.755300 -1.317230  2.178410 -8.211490  2.430200
## 3  1.187120  1.40769  3.423100 -7.405340  3.760820  4.025550 -1.994780
## 4 -6.193440 -3.19125 -1.345150 -0.442917 -1.958000  0.864356 -3.943580
## 5 -7.968150 -3.34111 -3.363580  0.794268  0.481126  1.320530 -0.884502
## 6 -0.290400  2.71005 -4.667480  7.446490 -0.338663 -2.686220  2.857010
##        PC153    PC154    PC155    PC156    PC157    PC158      PC159      PC160
## 1  0.0373548 -4.44031 -4.28498 -3.83010 -5.71622 -4.68854   9.953090  0.0054397
## 2 -4.7974400 -1.35515 -8.20389 -2.67278 -7.60847 -5.67180   5.925970  7.4253200
## 3 -2.8435600  4.14554 -1.85707 -2.45021 -6.84394  7.03234   0.444077 -6.1882800
## 4  8.3373100 11.22310 13.76750  4.84734  8.96518  3.26969 -11.449500  1.9489100
## 5  9.4371700  9.34674 12.22500  5.38277 12.94650  5.91234  -8.968680  2.5138400
## 6 -1.6319700 -9.57413 -4.70452 -1.04007 -7.24512 -4.49017  -0.277788  3.0492000
##       PC161     PC162     PC163      PC164     PC165    PC166     PC167
## 1  1.252790 -0.794535  -2.39261  1.1257800  0.805601 -3.53083 -2.040810
## 2  4.953080  3.145190   7.71603 -0.0479613 -1.524750 -6.70223  0.215865
## 3 -3.347970 -6.849390 -10.69000 11.2927000  2.730140  7.08342  1.554640
## 4 -5.819500 -1.320000   4.88496 -4.8045700  0.966897  8.30516 -2.900980
## 5 -7.392380 -0.101183   5.83073 -5.7426400  1.632780  8.38883 -0.617469
## 6 -0.773739 -2.106360  -1.08542  1.9659400  2.031120 -4.72687  0.397220
##       PC168     PC169     PC170     PC171    PC172    PC173    PC174     PC175
## 1  -1.41530  3.956500 -0.287434 -5.020810 -2.03367 -3.40370  1.41045  4.720990
## 2 -10.57990  2.897010 -5.640700 -4.389750 -1.81438 -7.07297 -2.69015  8.892000
## 3   9.91340 -0.211275  4.408000 -3.943120  1.22319 10.92930 -1.47222 -4.857830
## 4   4.82110  3.522370  2.432610 -0.084975  7.25584  1.36230  2.63998 -8.552560
## 5   7.57137  0.356301  3.881810 -1.551690  5.67404  1.93774  4.03406 -8.010460
## 6  -5.93603 -0.660673  2.167150  2.785830 -4.62820  1.52273  1.05237  0.613194
##      PC176    PC177     PC178     PC179    PC180      PC181     PC182     PC183
## 1 -2.00159  6.19215  2.041390  1.834640 -2.65773  1.3549100   4.76350  2.173540
## 2 -6.92508  5.64175 -1.290270  0.165538  2.20229  1.7866100   2.61341 -1.296060
## 3 -1.99671 -1.79984  2.830790  4.743520 -3.25464 -7.2641500 -11.23700 -0.647134
## 4  2.03903 -2.26153  3.090870 -0.774695 -1.45875 -0.0984614   3.14168 -4.533180
## 5  1.34796 -4.71625  0.528938 -0.639184 -0.39441 -3.6538600   2.22941 -2.170840
## 6  5.75859 -2.65869 -2.047120  2.479840  4.81906  4.8641900   1.89585  4.509150
##       PC184      PC185     PC186      PC187     PC188     PC189       PC190
## 1  3.771880  1.0305300  2.699220   4.119280  0.966287 -2.453470  3.86609000
## 2 -0.467765 -4.7243900  0.262993   4.014280  0.999266 -3.900020 -6.82823000
## 3 -9.977800  0.9409580  5.366900  10.558800 -1.304360 -2.145500  6.18602000
## 4 -1.905220 -0.0725667 -1.583270  -4.188030  2.995520 -1.481300  4.53561000
## 5  2.189420  1.1023400 -0.675557  -0.397139  5.224960 -0.647945  0.00962616
## 6 -1.916890  4.8174100  2.659900 -11.226600 -3.082200  4.905210  0.74560400
##      PC191     PC192       PC193    PC194      PC195    PC196      PC197
## 1  5.54257  4.123410  2.65129000  7.09933  -2.208310  4.56766 -3.8015100
## 2  1.13095  2.021070 -2.06783000 -4.36921  -4.777900  6.64372  5.8557900
## 3 11.77170 -6.282700  9.21615000  8.04719 -15.505300  9.43444  0.1009980
## 4 -8.72379  0.852185 -4.36556000 -4.48808   0.162029 -1.53632 -0.3692860
## 5 -7.61701  2.298000 -0.00855056 -3.55160   1.395040 -5.95358  2.1863900
## 6 -3.67710  7.138320 -2.99596000  3.73958   8.146520 -4.81220  0.0988812
##       PC198     PC199     PC200     PC201     PC202     PC203    PC204    PC205
## 1  -6.64570  1.606720 -0.905456  2.759580 -0.723013 -0.807853  1.00199 -2.04222
## 2   2.56524  0.312666 -1.779890 -0.798943  2.923330  2.491800  5.38325 -4.37064
## 3 -15.64470  1.885900 -5.238360  5.626030  4.299770 13.866600 -9.06287 -7.84493
## 4  11.88690 -2.074090  4.546170  3.816480 -1.741190 -0.373681 -5.08396  6.30373
## 5   8.83142 -0.571147  4.215820  4.241820 -1.689610  1.744960 -7.66230  6.72477
## 6   2.45125 -2.904470 -3.425160  1.377710 -4.508120 -4.454350 -1.77934  2.47556
##       PC206     PC207     PC208     PC209      PC210     PC211    PC212
## 1 -0.423449 -1.781310 -2.718430  0.499711  0.0564189 -1.076330  2.37455
## 2 -2.212980  7.325380 -4.291420 -1.696700 -5.9851300  2.907710  8.25368
## 3 10.868800  4.946140 -0.319349 -6.548420  4.4783500  4.427760 -1.66921
## 4 -0.104402 -1.218560 -1.813880 -1.144920 -1.5435400 -3.261770 -6.08998
## 5 -1.770940  0.592556 -2.916220 -2.592820  0.6861830 -0.553201 -4.82399
## 6 -2.166760 -4.085220  3.899470  3.321140  3.3270400 -5.395810 -8.98527
##       PC213    PC214     PC215     PC216     PC217      PC218    PC219
## 1 -7.091970  1.17658  1.548480  0.576287  3.831140   1.464410  5.40389
## 2 -3.265010 -1.86041  0.462376  2.748250 -0.407777   5.242120  7.92652
## 3  0.677345  2.10459 -3.683740  1.328770 10.979700 -11.814500 -3.64973
## 4  5.108850  2.76619 -0.196268 -1.679860  2.990210   1.578070 -5.54948
## 5  4.991410  3.36367 -3.005580 -3.209370  2.899900   3.084450 -6.79024
## 6  1.760930  5.28127  2.130880  0.684292 -7.568870   0.260305  2.13556
##       PC220     PC221       PC222     PC223     PC224     PC225    PC226
## 1 -1.416100  -1.44189 -3.42400000  -5.21515  0.577804  1.860420  5.27210
## 2 -1.116800   3.39156  1.72128000   3.77216 -2.996600 -0.169884 -2.39942
## 3  0.898222 -11.92230  5.32621000 -18.52680 -2.993550 -1.189580 10.88790
## 4  9.755380   1.91583 -0.00828876   1.90407  0.600704 -2.656210 -5.55810
## 5  8.194460  -2.09433  0.18543500   2.27672 -0.996570 -0.784547 -4.58516
## 6 -6.560570   3.20275 -5.87010000   3.02144  6.843590  5.038690 -5.72116
##      PC227     PC228      PC229     PC230    PC231    PC232    PC233     PC234
## 1  2.21106  0.302302   0.519395  0.992303  6.83194  1.67831 -4.87230  0.222948
## 2  9.46577  4.035770  13.857800  3.009260  3.90221  7.62620 14.39700 -2.172050
## 3  1.42705 -4.504820 -21.221500 -3.348680 -6.94567 -5.85306 -7.30415 -0.637103
## 4 -3.35746 -1.728140  -3.562010  3.277080 -5.57469  5.48985  1.93675 -6.206020
## 5 -2.93576 -1.725550  -2.447100  1.460190 -4.44554  1.17798  2.02375 -5.291030
## 6 -6.61277 -2.969970  -0.872667 -0.289330  5.93416 -2.12949 -4.86614  7.648210
##        PC235      PC236     PC237     PC238      PC239     PC240     PC241
## 1   0.567484  -3.814030  5.759190  6.960130  0.7148090  1.364770  0.424481
## 2   0.653310   5.293270 -0.675218 -5.716690  3.1636400 -3.937780 -4.105680
## 3 -12.138700 -10.093800 -2.131500 -4.636060 -2.2967300  2.297430 -6.706670
## 4  -3.223050   1.134430 -2.857560 -0.497885  0.0133825  1.361480  1.493160
## 5  -1.126560   0.463989 -0.462921 -1.792890 -1.0236000  4.109020 -1.378550
## 6   2.502910   3.255050 -2.487290  8.427470 -2.8070700  0.890501  6.289090
##       PC242    PC243     PC244     PC245     PC246      PC247    PC248
## 1 -0.319599 -1.43470  6.116210 -5.042790  3.970260   0.440868  2.59491
## 2 -9.449650  6.89247  1.989630 -0.095161  1.315530   7.059030 -4.80703
## 3  5.603360 -2.63065 -2.488940  3.074890  5.669500 -10.007700  3.05268
## 4  0.873274 -1.85010 -0.562570 -0.156086  1.458760  -0.667995 -1.47361
## 5  0.713220 -1.00440  1.201830  0.212365  0.541066   1.418130 -1.20602
## 6  8.603510 -8.54961  0.947994  0.497500 -7.597980  -4.297820 -3.10996
##        PC249     PC250     PC251      PC252     PC253     PC254       PC255
## 1  0.0601088 -4.056190  3.281600   4.018910  1.829960  2.862270  -8.4268900
## 2  2.0487200 -0.757009  0.276534   1.928630 -0.187398  7.121140   0.8594930
## 3  1.3511600 -6.216420 12.358200   7.091980  2.619210 -4.551970 -10.3155000
## 4 -1.1668400 -0.453731  2.285090   2.805660 -1.498880  2.270280   0.0291084
## 5 -0.7557100  0.129251  2.086250  -0.011934 -0.503885  0.922913   2.4006300
## 6  0.0582419  4.474730 -9.520680 -11.136900 -0.210480 -0.547040   6.7057200
##      PC256     PC257     PC258     PC259    PC260     PC261     PC262
## 1  6.26587 -1.427400 -1.364020 -0.483539 -2.22377  2.851390 -0.653165
## 2 -4.03214  4.118090 -4.575950  1.702560 -4.85820  5.771620  6.266190
## 3  6.86902 -0.760599 10.801700  6.678700  9.41260 -7.680030 -8.461790
## 4 -2.69128  0.430376 -3.934650 -2.893590 -2.52620 -0.724484  0.726392
## 5 -2.34846  1.235770 -2.646580  1.052460 -2.25067 -0.237247 -1.177990
## 6 -4.53690 -3.610810  0.156929 -6.644540  2.57255 -3.032430  0.652874
##        PC263     PC264      PC265     PC266     PC267     PC268     PC269
## 1  4.5128400 -0.116734 -0.0262209 -2.032270 -0.517901  -1.68581  1.531850
## 2  0.2595160 -5.618050  1.7409800 -0.945241  2.549420 -10.16050 -4.721350
## 3 -6.1970800 15.143200 -3.9455900  1.453710 -7.792350  -4.86874  7.474580
## 4 -2.0394500  3.031660  2.6591800  0.649460  5.133390   4.38727  2.054180
## 5 -0.0382715 -0.213455  2.2766000  0.285463  2.196750   3.11127  0.529512
## 6 -0.7354090 -2.075360  2.1219200  0.794189  3.304320   5.35412  1.260370
##       PC270     PC271     PC272     PC273      PC274      PC275      PC276
## 1 -2.899600 -3.660400  0.416856  1.580550  -0.929610  4.9098500 -0.0660742
## 2  5.161390 -0.228787 -0.397251 -1.407380  -0.599395  5.7039900 -7.7682500
## 3 -8.003720  5.806960 -4.434920  4.728340 -13.442400 -4.5097500  3.8092100
## 4  1.327610  2.298300 -1.623020  0.480359   1.622090 -0.0911165 -1.2027900
## 5 -0.894352  0.535740 -3.983880  1.143020   1.800880  0.7457550 -0.0935000
## 6  0.390248 -3.114200  2.625140 -5.134770  14.006300 -2.1658000  0.0265242
##        PC277     PC278     PC279     PC280     PC281    PC282     PC283
## 1  0.6224920 -0.466127  2.142830  3.418780 -2.538900  4.37318 -3.746590
## 2  4.1139800 -1.192770  5.080340  2.082050  0.670263 -1.72296  0.713291
## 3  0.0234611  3.253900 -0.600073 -4.750480 -3.795320 14.12540 -4.734650
## 4 -1.0147400  3.370320 -1.450290 -2.015590 -0.503210 -3.57569  4.772720
## 5  2.4757300  0.748845  0.273054 -1.505900 -0.918943 -2.42148  3.468530
## 6 -1.3679600  2.048710 -9.122120  0.223173  3.187010 -6.23374  0.589888
##      PC284     PC285    PC286      PC287     PC288    PC289      PC290    PC291
## 1 -4.03237  -1.45206  2.86467  5.4434300  5.836350 -5.24135 -0.0765904 -2.81229
## 2 -6.59959   4.90951 -1.94733  4.9289500 -5.022320  4.40260 -5.7295700  3.76919
## 3  6.01638 -12.13400 10.27940 -5.4156300  9.853030 -4.88892  4.9905600  7.10318
## 4  3.99941   2.51943 -4.18728  0.3221460 -0.408947  2.27144  1.6252100 -1.43365
## 5  1.55163   2.09084 -3.03230 -0.0472207 -1.204880  2.57847 -1.1113100  3.12293
## 6  3.15984   1.14437 -1.05349 -5.1336300  0.296363 -1.14600  3.3956300 -5.18936
##        PC292      PC293     PC294     PC295      PC296       PC297    PC298
## 1 -1.2599200  -1.566710  -5.64641   1.63231  5.9712300   0.8273790 -0.84845
## 2 -1.2304300   5.696030  -2.68732   6.67740 -5.2146100   6.6377200 -3.49703
## 3  6.6666400 -15.845200 -13.68460 -10.75100  5.0241600   6.2026000  7.71184
## 4 -1.6765500   0.660885   2.88921  -2.41495 -0.9375180   0.8492660 -1.25335
## 5 -0.0469258  -1.278140   3.05224  -1.32164  0.0674804  -0.0368242 -2.39081
## 6 -5.3274700   5.979910   4.77636   2.48639  0.6789730 -11.8011000  3.12244
##       PC299    PC300      PC301     PC302     PC303    PC304     PC305
## 1 -0.380781 -0.11216  1.4409400 -1.228750 -0.487860  2.21696 -2.350780
## 2 -6.544540 -6.25533 -2.9521800 -0.709613  0.257047 -4.40067 -2.041460
## 3 -0.138067 11.25870  5.6179000  2.637340  4.832600  6.79548 10.991700
## 4 -4.914710 -1.76852  0.4908530 -0.299787 -2.578600 -3.09624 -1.075960
## 5 -2.567900 -1.30198 -0.0137013 -1.200780 -0.762078 -2.86012 -0.192828
## 6  1.259640  6.67217 -0.8722810  2.487470  0.521237  4.03967 -7.117020
##       PC306    PC307     PC308     PC309     PC310    PC311     PC312     PC313
## 1 -7.285300 -1.13842  6.421660  0.144481  6.506020 -2.42063 -1.573960  2.683240
## 2 -1.088030  5.26143  4.036070  1.010020  2.529180  4.06317 -0.228878 -2.201100
## 3  3.377060  2.10020 -4.267360 -4.060070 -1.115090  7.55665  9.838320  1.495360
## 4 -0.578170 -1.27182 -1.096920  2.144230 -0.976964 -2.72440 -1.783940 -1.809180
## 5  0.544196 -3.19914  0.178731 -0.430776 -0.392872 -1.39107  0.237933 -0.532791
## 6  1.590570 -7.48457 -3.027380 -0.814986 -4.036960 -1.59963 -6.382910  0.704372
##        PC314    PC315    PC316     PC317       PC318      PC319      PC320
## 1 -2.1715700 -2.26545  1.87903  0.048919  -0.6539000 -0.0858028  0.0634985
## 2 -0.0469358 -4.38619 -2.69552 -2.680300   6.1221700  4.0871400  2.4470500
## 3  3.4715900  1.70686  3.55337  6.454130 -16.3861000  2.4336700 11.2750000
## 4  1.9564400  2.65667  2.66972 -2.093240   1.5233100 -2.9434700 -4.0350200
## 5  0.7352040  1.64085  1.86205 -2.686830   0.0666389 -3.5849000  0.1822340
## 6 -1.9521700 -3.64090 -4.17515 -3.644160   2.5244000 -0.5484410 -8.6794600
##        PC321     PC322     PC323     PC324        PC325     PC326       PC327
## 1  3.1841600  0.313215  2.499090  8.257200  6.735780000  0.426050  -1.7594600
## 2  3.9440200  3.325020 -6.768400 -0.650677  0.387955000 -1.960630   0.0550073
## 3  2.6239300 -7.514350  1.673740 -3.427820 -1.086800000  0.906996 -10.7663000
## 4 -0.0412191 -2.304320 -1.163600 -0.967379  1.921790000 -0.847369   5.2116300
## 5 -0.3235800  0.598671 -2.043050 -0.633125  0.000866653 -1.222630   3.3655400
## 6 -2.0613000  0.704175  0.441571 -2.439170  8.203200000 -2.821800   5.5188100
##       PC328     PC329     PC330     PC331     PC332    PC333    PC334     PC335
## 1  4.100970  2.534050 -1.474600  1.307680 -7.233090  4.26246 -1.12176  0.372586
## 2 -1.227300 -1.313670  0.308595 -6.822580 -0.303221  2.89746 -5.02244  2.692370
## 3 -2.350080  2.767310 -3.189860  2.221610  2.708960  1.41139  7.86546  3.774360
## 4  0.971166 -0.658647 -1.851050 -3.398070 -0.357806 -3.31258 -3.53221 -0.251744
## 5  0.264885 -1.390660 -0.436948 -0.837843  1.553010 -1.48131 -2.42077  4.103830
## 6  0.197595 -3.160630  0.799300  3.962830  3.346890 -2.39221  3.83427 -4.780790
##       PC336    PC337     PC338     PC339     PC340     PC341      PC342
## 1  5.866980  1.30239  0.526834 -1.107230  3.019610 -1.111820  2.3632400
## 2 -4.256700 -3.43208  4.643950  1.323520 -1.986990  0.870715 -6.3637100
## 3  1.794150  6.72219 -1.483510 -2.899990  1.450600  5.135120  8.2203800
## 4  0.433595  1.19593 -2.971610  1.538850 -0.989238 -1.833990  1.5874700
## 5  1.483350  1.70277 -1.900300  1.292410 -2.639470 -2.761100 -0.0702725
## 6 -4.122610  3.04352 -6.178300 -0.227901 -2.213310  0.648995  1.7322900
##       PC343      PC344     PC345     PC346      PC347     PC348     PC349
## 1  1.250200  1.1618700  2.155410  2.134580 -4.7846900 -0.149676 -1.853740
## 2  0.374582 -2.9238100 -5.126650  3.931450 -0.0113821 -3.641620 -4.225300
## 3 -0.305223 -1.9347400  1.320990 -5.944650  2.4645600  4.167090  0.821082
## 4 -2.368420  0.3729620  0.520797  2.297740 -1.3927700 -2.910720  2.000350
## 5  1.093120  0.0427231  0.850082  0.806246  0.8369300  0.264753  0.317031
## 6 -0.133974  0.6719950  5.650300  0.337494 -0.4959550  1.217590 11.026000
##        PC350    PC351     PC352     PC353    PC354     PC355     PC356
## 1  1.9119100  3.33916 -2.928660 -3.167350 -0.66831  0.966432 -2.643700
## 2  0.7468550  5.87039 -0.263015 -1.179770 10.87660  0.223176  3.710530
## 3 -1.5108000 -6.86455  5.252980  0.346421 -2.71711  1.470390 -2.745890
## 4  2.2001000  0.15928  0.953818 -1.664980 -1.09961 -3.886120  0.752095
## 5  0.0564837 -1.89627  0.827445  1.294510 -1.75220 -0.986673  1.441250
## 6 -4.1069500  2.07181  2.780920  2.309450 -7.32127 -1.450000 -4.293230
##       PC357    PC358      PC359    PC360     PC361    PC362      PC363    PC364
## 1  0.216728 -7.52898 -1.9912900  6.54047 -6.905260 -1.25593  2.5075600 -3.61693
## 2 -0.234859  4.80045  2.8342200 -3.42859 -3.787650  4.59495 -2.4940900  3.95698
## 3 13.788400  1.00961  3.7116300 -4.09699  1.785230 -1.32592 -6.1763600 -2.17825
## 4 -0.818370  3.31784  0.0518406  1.57704  0.517772  5.33067 -0.0394817 -1.61180
## 5  2.400790  2.13047 -0.2397950  1.98999 -1.258120  3.50148 -0.7574140 -1.18145
## 6 -7.604190 -1.56056 -3.1431200  1.71085  2.929210 -8.56354  7.2353000  2.02088
##       PC365     PC366      PC367      PC368       PC369      PC370     PC371
## 1  5.743900 -0.196021  2.0388100 -0.4173740  -0.0131931 -1.3139100  0.844442
## 2 -5.819350  1.653600 -0.0922078  4.4171000   1.2819200  0.0827032 -5.414330
## 3 10.952400 -3.755260  3.2848000 -4.5053000   4.2444300  5.8895400 -2.702190
## 4 -2.642700  3.643790  1.0979300  0.0565652   0.4756870  0.4514890 -0.693002
## 5 -1.150510  2.426640  0.1244280  1.4058000   1.8904600 -0.3086300  0.278736
## 6 -0.728024 -2.781450 -5.1365000  1.9870400 -11.2914000 -4.5451100 -0.520310
##      PC372     PC373     PC374     PC375     PC376     PC377    PC378    PC379
## 1  1.01250 -2.165220  4.302880  0.250072  0.688157  0.547920  3.07148 -1.94854
## 2 -5.03846 -0.487427  6.073760 -3.999580 -3.332850 -1.487020 -6.62009  4.55177
## 3  6.45778  2.720560 -7.078930  5.991080 -2.593320  1.846770  3.89265 -1.97989
## 4  1.46960  1.175670  0.395864 -1.102400  1.272640  1.388030 -1.37821  4.53092
## 5  1.06038  0.427166 -0.842581 -1.620150  0.186343 -0.422873 -2.09686  4.09077
## 6  3.83800  1.062300  2.640610 -4.305640  8.330160  6.775910 -4.02844 -4.80348
##      PC380     PC381      PC382     PC383     PC384     PC385     PC386
## 1 -1.26041  1.876430   1.189230  3.421190 -9.267670 -1.760420  2.904220
## 2 -7.75964  1.505190 -10.306900 -8.170570  2.773600  2.782090  3.202560
## 3 -1.77873 -3.441260   3.490880  6.201090 -0.578744 -3.327610 -4.662420
## 4 -3.23523 -1.745430  -0.203669 -1.877130 -0.233976  0.656882  0.954479
## 5 -2.95613 -1.244160   0.733806 -0.719518  1.576940  2.433250 -1.193440
## 6  8.16373 -0.396948  12.073700 -1.104150 -2.295360 -4.728480 -4.300910
##       PC387     PC388     PC389     PC390     PC391     PC392     PC393
## 1  6.768260  0.364740 -4.103600  1.008540 -0.169599  0.610708 -2.326180
## 2 -4.560200 -2.260140  1.627940 -1.839640  8.393320  2.329390 -0.742307
## 3  1.596200 -0.343963  5.561930  0.379126  0.611365  3.518580 -0.900827
## 4  1.158940  2.120850  0.511827  1.657790  0.950310 -0.439784 -1.266560
## 5  0.209652  0.870856 -0.870021  0.289909  1.583230 -2.604700 -0.592347
## 6 -3.458170 -2.042760  1.381270  5.351220 -9.359500 -0.703677  6.831680
##       PC394      PC395     PC396     PC397    PC398     PC399     PC400
## 1  1.919960  0.0921915  1.423330 -1.427940 -3.61509  2.433320 -6.365900
## 2 -4.184660  0.4483690 -1.608260 -1.069310  2.08826 -6.899110 -7.549270
## 3  0.820582  4.1982600  6.141650  6.057690  7.35683  3.337330  4.157280
## 4 -3.055220  0.2803420  1.263620 -1.155620  1.46259  3.475550  0.967079
## 5 -1.512750 -0.4557770 -0.268563 -0.941020  1.19019  0.435961  0.364318
## 6  7.668510 -2.5265400 -0.582028  0.346225  1.35610 -1.005730  4.341290
##       PC401     PC402     PC403     PC404     PC405     PC406    PC407
## 1 -4.188510  8.895260 -1.267470  0.499873 -0.429464  3.943170  5.14353
## 2 -3.621970  0.123198 -1.852540  0.186583  0.517942 -5.447760 -1.25612
## 3 -6.995730 -8.215120 -0.657093 -4.143220 -2.023490  5.909050  5.46344
## 4 -0.921032 -1.072670 -2.331260 -5.099340  3.890400  1.563640  1.02128
## 5 -0.886964 -2.575290  0.101656 -1.188680  0.283069  0.480037  1.33045
## 6  2.657430  0.296446  2.815640 -0.766606  3.678240 -8.726200 -4.64404
##       PC408     PC409     PC410     PC411     PC412       PC413    PC414
## 1 -3.527520  0.796596 -5.558220   5.77749  3.000020  -0.0465376 -4.55751
## 2  1.586040  0.719133 -3.124030   6.94850  0.345273   0.4279660 -2.26064
## 3  1.629010  0.278307 -3.549420  -1.80311  0.635887 -12.8913000 -2.62527
## 4  0.392565  0.339440 -2.651540   2.14966  3.276770   3.1671500 -5.04053
## 5  2.578000 -2.110350 -0.866257   1.28584  2.668620  -0.1173300 -1.18262
## 6  2.925760  6.637650  9.627590 -15.70630 -3.585040   2.4036900  5.90805
##       PC415     PC416     PC417     PC418     PC419      PC420     PC421
## 1  3.050560  0.346242 -0.460687  2.598900 -6.962510 -9.0140200 -0.857158
## 2 -0.866159  1.896460 -6.230650  4.130440  0.511370  2.3656400 -1.209660
## 3 -9.903110  1.536100  1.672370 -7.526470  0.148073 -6.1058500  1.394200
## 4  1.890490 -0.606181  3.050990  0.713383  2.000930  0.0873058  0.161784
## 5 -1.400410 -0.197029  2.107700  0.288525  2.213180  0.5395620  0.597757
## 6  5.656030 -5.318940 -2.971930  5.628080 -3.415120  5.6915700  4.662840
##       PC422      PC423      PC424    PC425     PC426    PC427     PC428
## 1  2.890790 -3.9915200   3.480060 -3.43714  1.921160  2.78043  5.065330
## 2 -3.278600 -0.0733377   4.143300 -9.82253 -8.329210 -4.77920 -6.061280
## 3  4.115190  0.3705990 -11.005300  4.98669  2.004870  6.68411  4.495800
## 4  2.671030 -1.0175400  -0.480964 -1.28511  0.547166 -1.75151  0.984722
## 5  0.195058 -1.8988900  -1.442980 -1.60313 -0.733197  1.24669  1.942140
## 6 -1.057280  4.3726000   1.461940 11.88120 13.309400 -7.58519 -9.872750
##        PC429     PC430     PC431     PC432      PC433     PC434     PC435
## 1  2.2115800 -5.738500  4.953250  0.431328 -4.3620900  3.500060 -5.709400
## 2 -3.9937300  3.727500  0.191544  0.382687 -2.7080700 -3.832880  1.580620
## 3  6.7950000 -0.101663  1.175090 -3.738590  4.0216400  1.416360  6.763370
## 4  0.0267627  1.274010 -1.199690  1.900680  1.0069500  0.911206 -0.453357
## 5 -0.1085800  0.910201 -2.312260  0.919004  0.0511454 -0.792076  1.260900
## 6  5.6878900 11.273200  3.504040  0.606439  1.8837700  2.221310 -3.596040
##       PC436      PC437     PC438     PC439    PC440      PC441     PC442
## 1 -2.117060  1.3552100 -5.100340 -1.539730  8.32037   0.122489  5.861330
## 2  0.470262 -4.3963600  1.550150  4.324140 -4.80408   3.688780 -1.999580
## 3  1.615050 -7.0613500 -0.326242 -0.671576  1.60030  -1.346520 -5.453430
## 4 -1.324660 -0.0610928  2.803180  3.979070 -2.38851   1.345400  0.148125
## 5 -0.806985 -1.2721600  2.391170  1.606130 -2.00279   1.425360  2.034130
## 6  8.128620  9.7048800 -0.942066  3.106530  2.78461 -12.459800  7.163370
##       PC443    PC444     PC445     PC446     PC447     PC448       PC449
## 1  2.876720 -2.93052   1.71009 -2.365240  3.945100 -8.793920   0.0369488
## 2  0.360598  6.03197  -4.42558  1.167490 -1.928680 -2.455620  10.2919000
## 3  2.285740 -1.96073  10.27540  1.655950 -5.423250  6.898730   0.5140960
## 4  1.008360 -1.54334  -2.03943 -2.857460 -0.970752  3.060860   3.2078400
## 5  2.131280 -1.74694   1.12038 -0.550974 -2.002300  0.603121   2.1388700
## 6 -1.809600  5.46276 -10.37450 -2.035550  1.715150  0.973694 -13.7092000
##      PC450    PC451     PC452     PC453     PC454      PC455     PC456
## 1  3.89482  1.70770   1.36984  5.550500 -1.811490 -8.3163100  -2.42723
## 2  1.46273  1.08630  -1.42056  5.955280  3.086590  4.8522600   9.21781
## 3 -3.71684 -1.98311   1.61763 -2.542930  2.265030  0.0993377   7.48028
## 4 -3.68039  3.34363  -1.60247  0.577683  4.041530 -3.7683800   0.36257
## 5 -1.95677  1.02921   2.20556  0.738387  2.995340 -2.1615900   1.67325
## 6 -1.61001 -2.00091 -10.96310 -6.042930 -0.967949  5.1445500 -12.10730
##       PC457     PC458      PC459     PC460     PC461     PC462     PC463
## 1 -2.788030  1.339550  1.0835800 -2.131160  5.232790 -0.286953  8.422310
## 2 -0.421319  4.332130  6.5551200  2.312660  3.500680 -0.527712  0.209584
## 3 -1.485810 -0.568198 -9.3883700  0.596862 -9.653530 -1.624080  0.899350
## 4 -3.359780  5.067090  1.4706800  1.897570 -0.214532 -3.558510  1.026500
## 5 -1.234700  1.921300 -0.0465607  1.457740 -1.698820 -1.490630  0.959969
## 6  4.231430 -5.146420  2.3621000 -2.232170 11.361400  5.003230 -4.441690
##        PC464    PC465     PC466    PC467    PC468      PC469    PC470     PC471
## 1   2.490750  3.92988 -2.170000 -1.45296  1.34644  2.7442100 10.29210 -3.750380
## 2 -10.116200  1.83862 10.223200 -3.16550  3.97162  6.9190300  1.87046 -2.777330
## 3  -0.903255 -9.13568  1.089120 -1.13021  3.47280 -0.1879170 -1.03656 -0.208672
## 4  -4.021950  2.85460  0.497585 -2.24460  2.61183  2.8826000 -1.79659 -4.126210
## 5  -0.666641  2.66194 -0.681372 -0.71817  3.65854  0.0782989 -2.54452 -3.730320
## 6  -1.464310  8.37750 -1.636800 -6.98024 -9.02304 -3.9329800 -1.67418  9.703250
##       PC472     PC473    PC474    PC475     PC476     PC477     PC478     PC479
## 1  0.489817  -5.75057  5.77762 -1.78160  8.612230 -3.750520  1.817810  0.323695
## 2 -1.726990   1.24898 -2.15749  5.53092  5.146790  1.646400  3.799310  1.175360
## 3  4.885890  10.93900 -2.09301 -9.29722 -9.763470 -3.925390 -2.762930  7.969620
## 4  1.004990   1.79477  1.37325  4.38455  0.122302 -0.831890  0.911661 -1.666430
## 5  2.725930   1.94703  1.13234  2.93823  0.782463  1.181040  1.711210 -1.676520
## 6 -2.236720 -20.07420 -7.38515  5.79917 -5.969500 -0.077534 -1.688920 -9.480620
##       PC480     PC481     PC482     PC483     PC484     PC485     PC486
## 1  1.398580 -7.798920 -2.069070  0.593192 -3.064410 -0.683617 -8.174080
## 2 -4.855960 -6.210840 -2.982750 -3.529220 -2.336220 10.283600  0.398461
## 3 -0.914368  0.871876  3.169480  0.533333 -2.863500 -4.131380  4.746930
## 4 -3.885870 -2.821180 -0.416206 -1.109470  0.238069  5.677160 -0.340911
## 5 -6.588090 -0.711925  0.273927 -0.212729 -0.497965  5.321710 -2.466430
## 6 16.618600  7.839060 -1.919740 -7.631580 10.698700  1.713800  8.408030
##       PC487     PC488     PC489     PC490      PC491    PC492     PC493
## 1  5.275450   2.63577   2.63564 -0.658733 -9.8797000 -8.66753  1.585080
## 2 -1.917520   8.17993 -12.37540  7.901700  4.0822300  1.88824  3.037830
## 3 -7.264120  -1.99454   8.87511 -1.937640  0.6926640 -4.53122 -5.292970
## 4 -1.261650   4.17609  -2.12569  1.046030  0.0790462 -4.15026 -2.049780
## 5 -0.776933   3.84504  -1.11463  1.772100  0.8718370 -1.92905 -3.467220
## 6 11.451200 -13.37510  16.09320 -5.752300 -0.2571260  4.10084 -0.934313
##       PC494     PC495     PC496     PC497     PC498     PC499    PC500
## 1 -7.695260 -9.296680   8.33949 -3.411510 -4.760100 -1.565210 -1.91135
## 2  2.786010  7.153000   4.07705  2.732570 -8.288270 -1.104240  1.63679
## 3  4.429010 -0.820552 -11.31730 -3.325550 -5.002680  1.359710 -5.83626
## 4  0.958561  3.371570  -1.29290  0.542640 -3.068720  1.786170 -3.74472
## 5 -0.671600  1.865080  -1.32143 -0.626032 -2.257030  0.109776 -3.60837
## 6  2.409400  5.645510   6.86194 -3.194840 -0.351979 -2.154770  6.22448
##       PC501     PC502    PC503    PC504     PC505     PC506     PC507    PC508
## 1   4.12306  0.797225  1.16849 -8.52971 -7.855450 -0.953767  5.140790 -4.24345
## 2  -1.75553  2.788570  6.55212  7.95878  6.508520 -2.997830 -8.085950 -6.40956
## 3 -13.87010  0.412990 -6.43310 -5.65247 -0.304292 -3.686920 -0.757795  4.57484
## 4  -4.05833 -1.755510  3.73944  2.26075  0.338957 -0.436887 -2.720470 -1.99720
## 5  -4.27613 -2.435530  1.13713  2.85678  1.440370 -1.041010 -3.178770 -4.57325
## 6  17.21530 -9.247450 -8.46764  1.72544 -1.397460 -1.744410  4.182880 15.42140
##       PC509     PC510      PC511    PC512     PC513    PC514       PC515
## 1 -10.81130 -3.160920   5.684740  7.56945  2.401420  1.38137 -11.5427000
## 2   4.97663 -0.931841  -2.623520 -4.00188 -3.583060  7.02063   2.9680800
## 3   8.10907  2.466460 -11.594800  6.33174  3.248930 -6.04747   0.0529885
## 4   1.22438 -4.140830   1.977380 -1.37388  3.958010  3.30098   2.5968800
## 5   5.90894 -3.350140   0.608402  1.66597  3.648190  3.04438   0.7371930
## 6 -10.69660  6.460600  -2.973980 -6.99604  0.561209  3.13494  13.2513000
##       PC516     PC517     PC518    PC519      PC520     PC521      PC522
## 1  1.261720 -1.430320 -0.202636 -7.89507  -5.205330  2.324240   1.690690
## 2  3.767650  0.386057  1.249440  6.09587 -12.704600  3.948670  -1.171330
## 3 -5.386580 -4.630730 -6.227660  6.92659   4.510640  0.745487  10.303000
## 4 -0.543151  1.990040 -4.221460  3.33375  -0.490501  0.770362  -1.261050
## 5 -0.140016  0.107141 -2.182810  1.62048   0.180985  1.456920   0.743561
## 6  2.704500 -3.438750 10.309300  3.63902   5.883320 -8.685780 -11.425800
##       PC523    PC524     PC525      PC526      PC527     PC528     PC529
## 1  -3.98811  6.85142  0.136756 -8.7345500 -12.011400   9.97136  9.100590
## 2   5.49939  1.04053 -6.253370  6.0271900  -2.443780   1.86596 -1.401250
## 3 -10.19450 -8.19781 -1.435720 -3.0363800   0.737302 -10.16390 -4.208360
## 4   1.39042 -2.56012 -2.984650  0.0431859  -2.896450   3.47559  4.625250
## 5   1.29314 -2.56710  1.181600  2.2281300  -1.245420   3.23811 -0.115218
## 6   6.55285  2.02315  0.665386 -3.4662600   6.413740 -12.71170  9.446510
##        PC530    PC531     PC532    PC533     PC534     PC535      PC536
## 1 -15.656400  1.01756  7.105200  5.35051 -1.020000 -1.418510 -4.4623300
## 2  -1.643580  1.91591  0.121806  2.75398 -4.818360 -3.867780  1.4059700
## 3   3.199910 -7.27533  5.080250 -4.20534  6.813890  3.363240 -0.7734830
## 4   0.416985  3.56728 -1.006910 -2.60758 -0.681055  0.532116  2.0010400
## 5   1.605830  2.23123  1.790740 -2.38733  1.218370 -0.930840  0.0529179
## 6  -6.372720 -4.65376 -9.393190  4.15272 -2.665680 17.149200 10.9263000
##         PC537    PC538     PC539     PC540     PC541    PC542    PC543
## 1 -16.9401000  5.74674 -13.36670 -4.683140 -3.849220  3.02825  3.98110
## 2   6.1095400  2.08387   2.60035  2.197970  3.584710 -1.28570 -1.72725
## 3  -1.0908300  1.11981   1.54532 -1.128120 -5.427720  3.83918  0.84680
## 4  -3.3303800  1.75401   1.57644 -0.457477 -0.748972 -2.71678  4.40795
## 5  -3.7918300  1.59972   1.21434  0.957146 -0.258917 -2.82682  3.56973
## 6  -0.0150529 -3.98555   8.55511  4.268260  2.772100  6.77650  1.41799
##       PC544      PC545    PC546      PC547     PC548     PC549     PC550
## 1  0.955375  10.616600  5.85949 -2.3662200 -5.091870 -2.985820  7.750170
## 2  1.920650  -0.866365  3.87259  4.7452400 -0.508259  2.862490 -0.662142
## 3  7.716690 -10.186100 -4.42556 -3.2511200  0.816028 -2.358690 -0.161240
## 4 -1.781630  -1.626920  3.67095 -0.6619480 -1.684610  1.642340  2.930200
## 5 -1.304310  -1.233410  2.29688  0.0200007  1.107390  0.361807  1.104230
## 6 10.963100  -1.581070 -5.22263 -7.9951400 -5.284230 -4.572770  3.700340
##        PC551     PC552    PC553    PC554    PC555     PC556      PC557
## 1 -10.059800  1.396910 -2.42983  5.63017  3.10335  0.475876  4.2441500
## 2   7.011670 -2.071480  1.10114  2.73659 -4.59612 -0.878297 -1.2410500
## 3   2.287920 -3.103400  5.99100 -1.63698  2.59053  9.229780  4.4777300
## 4   0.933308 -0.501320  1.27979 -2.11544 -0.48322 -1.710310  2.5545500
## 5   1.415810  0.981691  2.27192 -2.20518 -1.36692  2.199170 -0.0571956
## 6  -3.274790 -4.031920  6.03491 -8.40832  7.28408  5.069280  2.0796100
##       PC558     PC559     PC560    PC561    PC562     PC563    PC564     PC565
## 1 -7.937780 -4.290980  7.430430 -1.39532 -6.63226  9.685640 -3.16250   6.71425
## 2  0.891109 -4.723890 -0.337483 -1.88267  1.87441 -4.265640 -3.80732  -6.25051
## 3 -1.581880 -5.794560  6.219830 -2.43592  3.05197 -4.849450 -3.37736 -10.50400
## 4  0.498649  0.171534  1.803750 -2.76788 -1.03714  1.876270 -1.97368   1.83713
## 5 -0.202879 -0.247173 -1.458600 -2.31684 -1.11552 -0.175665 -1.72786   2.36849
## 6 -4.839550 -6.492700 11.556400  4.24251 13.84380 -3.164670  8.74904 -17.74410
##       PC566     PC567      PC568      PC569      PC570     PC571      PC572
## 1 13.317200 -16.14020 -20.090200   4.157090 -6.4776000 -0.950869 -1.6304400
## 2 -2.157150   4.71525   3.014020   3.967560 -1.7742600 -2.029310 -0.0100381
## 3 -0.369705  -2.25847  -0.483762  -2.830480  5.2077600 -0.973007 -1.2491000
## 4  3.432480   1.47906  -2.461860   0.606850  1.4003200 -0.733995  0.5935490
## 5  0.349315   2.99420  -1.561790  -0.301647 -0.0985294  0.916523 -1.6766900
## 6  5.961150  -6.64463   0.952106 -13.507600  1.5452200 -6.986150 11.0420000
##       PC573       PC574      PC575    PC576     PC577     PC578      PC579
## 1 -0.592557 -17.7707000  17.630800 -2.22721  5.534500  7.278810 -10.929800
## 2  1.591070   0.0868532   2.556910 -2.53362 -4.567590 -5.914670   0.213551
## 3 -2.430920   5.8016000  -3.791740  2.08853  4.434230 -3.130830   0.847675
## 4  1.370660  -0.1345960   0.853780  2.32770  0.324467 -0.815664  -0.397939
## 5  1.559260   0.2664090  -0.598138  1.15370  1.060740 -1.719930   0.479418
## 6  4.860260   3.5468500 -11.469400 -2.37933 -2.914140 -2.415390   1.068860
##         PC580     PC581     PC582     PC583       PC584       PC585     PC586
## 1   0.0359472 -1.107710 -3.415380 -5.260570 -10.3529000 -8.10481000 -3.201920
## 2   0.4269450 -2.789610 -0.807337 -1.237900  -2.7487300 -4.44900000  2.405400
## 3  -9.4726900  9.202300 -7.084730  2.798030  -1.4405100 -2.22021000  0.982125
## 4   1.2513700  1.270310 -1.833950  0.341143  -0.0658079 -0.72088600 -0.437541
## 5   0.1184220  0.465456  0.853534  1.868550   0.5065780 -0.00821106 -0.989317
## 6 -10.0627000 -3.773440 -4.289100  0.397195  -2.6865000 -3.83930000  1.195240
##      PC587      PC588     PC589     PC590      PC591     PC592     PC593
## 1 14.36740 -1.7180100 -0.755609  9.392060  3.2737700  4.712940 11.241100
## 2  1.78962 -0.8697940  5.822260 -3.279600 -3.5977000  2.035540 -6.611250
## 3  2.97941  1.3390000  4.176340 -1.436920 -6.2969700 -4.420570  4.728760
## 4  2.11083 -1.4339000  0.397697 -0.247365 -1.4179500 -0.623943  0.435866
## 5  1.34196  0.0822553 -0.487529 -1.388640  0.1405980 -1.593960 -2.437120
## 6 -5.52573 -2.5427800  9.717560 -9.037110  0.0571758 -2.554230  7.602890
##        PC594      PC595     PC596     PC597     PC598     PC599      PC600
## 1 -11.560800   2.770630 -5.462680  3.833040  3.830670  3.313420 -10.622800
## 2   3.112210 -10.209200  4.458370  6.692390 -3.150310 -1.968770   3.739170
## 3  -3.254080  -2.327930  9.253910 -2.534510  5.425920 -0.572444   5.755990
## 4  -1.912380  -0.961684  1.291240 -0.308987 -0.701208  1.365840  -1.317810
## 5  -0.185481  -0.424246  0.579886 -2.480280 -0.517879 -0.785417  -0.785842
## 6  -3.155970   2.922670  5.629020 -4.738890  9.504470 -2.078180  -5.680580
##        PC601    PC602     PC603      PC604     PC605     PC606    PC607
## 1 -8.8322400 -8.59624 -9.497570  0.2956360 13.971700 -6.541410 -4.67057
## 2 -8.5202800 -9.17511  4.616980 -7.7889700 -2.002110  3.825750  1.23152
## 3  0.0494472  3.50554 -2.144850 -1.7273900  0.102602  1.940840 -1.10884
## 4 -1.4381900  1.93387 -0.430379  0.0194862 -0.767343 -0.200653 -2.57516
## 5  1.3544300  3.28147  1.150330  0.0937981 -0.832937 -0.466912 -2.27428
## 6  3.3902800  2.31591  4.974400  0.5849850  1.094800  4.196790  7.05405
##         PC608     PC609    PC610     PC611     PC612    PC613      PC614
## 1 10.94260000 -1.218220 -3.46139  6.904760 -2.021810 2.108850  1.4667100
## 2 -0.39940400 -5.890690 -3.59388  0.888628 -4.177040 2.232840 -1.1804100
## 3  7.14980000  0.260244 -1.81484 -2.190200  7.318090 3.564710 -5.9448700
## 4  4.25325000 -2.465990 -2.38950 -0.563611  0.460771 1.080420 -2.0850200
## 5 -0.00510144 -1.751790 -1.43053 -0.307399  0.763817 0.380909  0.0614424
## 6 -0.85318300  1.057590  6.14653  0.909803 -4.701390 2.252590  0.0470080
##       PC615     PC616     PC617     PC618     PC619      PC620     PC621
## 1 -1.869110 13.137200 -7.663560 -6.557330 -2.451080  0.0787073  1.806350
## 2 -4.976770 -1.299740  2.016960  1.544770  1.822990  5.4125600 -1.047270
## 3 -0.336235  3.146550  9.723650  1.710680  4.234520 -3.0789900 -0.982916
## 4  1.251400  0.315308 -0.381962 -1.574350 -3.502840  0.6575920  1.569150
## 5  0.553967 -1.501090  1.887620  0.105518 -0.592262 -1.6322300  1.533820
## 6  3.111690  0.309525  6.081890  1.470280  0.952028 -0.9379750 -1.988170
##        PC622     PC623     PC624    PC625      PC626     PC627    PC628
## 1  0.0830309 -5.984930  4.070250 -1.24352 -12.330600  2.200390  4.11488
## 2 -6.1785100  1.018450 -5.616030 -1.58828   5.388130 -8.321120 -5.71425
## 3  5.2019100  1.122500 -6.568640 -8.31032   4.963590 -1.736570  2.69567
## 4  0.6165250  0.526218 -2.699640 -3.16207  -0.171146 -2.890140  1.76633
## 5  0.6555010  0.137461 -0.759459 -3.05853   0.793462 -0.304722 -2.50649
## 6  2.7213100  0.453613  1.243160 -5.57160   2.091410 -4.033490  4.88815
##       PC629     PC630     PC631     PC632      PC633     PC634     PC635
## 1  7.842290  3.690640 -4.555820 -8.708840 -10.959100 -6.796220 -1.275590
## 2  5.781280  7.476760 -3.893620  4.676150  -1.076810  4.518170  8.590540
## 3  7.418910 -1.630520 -0.687097  6.668880  -5.992400 -3.399430 -6.146740
## 4  4.211020 -1.493410  1.424720 -1.759280  -3.869850 -0.397829 -0.179292
## 5  1.665210  0.245005  0.726547  0.727328  -0.879697 -1.060860  2.231140
## 6 -0.296072 -2.245820  0.648158  4.793650  -4.674840 -4.421890 -5.333700
##      PC636     PC637     PC638    PC639      PC640    PC641    PC642      PC643
## 1 -0.46957  2.563790  3.299190 -2.83702 -10.960000 -3.84175 -2.19971 -7.5826400
## 2 -5.77595 -6.807460  1.017240  4.50180   6.347470 -9.04221 -8.69728  5.3291600
## 3  6.73080 -5.516560 -0.106128  3.11926   2.296750 -2.81289 -7.64559 -0.8558800
## 4 -1.07245 -2.207150  0.772339  1.10464   0.256011 -2.65472 -4.01255  0.0281704
## 5 -0.45701 -0.350826  0.749323  1.34026  -1.327160 -1.98324 -1.55792 -0.8441300
## 6  4.01580 -1.615360  0.620013  3.17038   3.375620  5.17071 -2.15181 -0.9471420
##       PC644     PC645     PC646     PC647      PC648     PC649      PC650
## 1 -0.937945 -1.843720 -2.582970 -10.73890  3.7954200  2.855110 -0.2589810
## 2 -6.529410  6.006810  1.148680   5.06560  1.8991100  2.101600 -6.2700700
## 3  0.136030  2.429280  3.652820   8.23754 -4.0398200 -7.669620  4.5103500
## 4  1.369430  1.893500  0.217308   2.83880  0.7756960 -0.505091 -0.1935280
## 5 -0.790615  0.879196 -1.435110   1.31921 -0.3901950 -1.048880 -0.0646646
## 6  0.612677 -5.131560  5.635370  -1.17004  0.0239208 -2.801260  0.9213570
##       PC651     PC652     PC653     PC654      PC655     PC656     PC657
## 1 -2.277440  8.095210 -0.590145 -6.700960  8.1917700  5.218580 -2.377700
## 2 -0.723103 -2.044360 -8.799940  7.557590 -9.2962700 -5.298730  6.163190
## 3 -1.861290 -7.139070 -6.315390  2.288920  3.7429900 -2.960290  7.155420
## 4  0.733839 -1.659590 -2.787450 -0.184224 -1.0106900  0.633442 -0.104418
## 5  0.286248 -0.926919 -0.114351  0.813858 -0.7331730  0.528832  0.243730
## 6 -1.695660 -2.316580 -1.342170  0.687855 -0.0667946 -4.345210  3.930310
##      PC658     PC659    PC660     PC661    PC662    PC663     PC664     PC665
## 1 -6.72905 10.245500 -4.98574 -3.308890 5.752310  3.64647   7.94843 -9.795420
## 2 -9.73732 -6.805450  1.15989 -2.480330 3.510110  3.91034 -11.39320  2.639010
## 3 -3.61192 -1.178170 -2.19328 -0.115131 3.963130 -8.54738  -9.99102  6.340250
## 4 -2.78424  1.292840 -2.75815 -0.732759 2.434860 -1.06276  -2.44313  0.847658
## 5  1.26046  0.139417 -2.24198 -1.663460 2.254810 -2.60778  -1.97549 -1.013840
## 6 -1.43712  2.122950 -2.30078  0.384311 0.921404 -3.59456  -4.05171  4.754650
##       PC666     PC667      PC668     PC669     PC670     PC671     PC672
## 1 -0.978852 -7.685280  4.4173200  3.082660  5.156400 -1.854210 -2.332280
## 2 -2.153600  4.230980 -9.0736300 -4.866000 -1.320420  1.694480 11.925900
## 3  0.456456  1.805890 -1.7523700 -3.211490 -4.189360  4.687530  0.131379
## 4  0.142693  0.638929 -0.8172720 -0.625444  0.389608 -0.123802  0.514862
## 5  1.383310  1.173970 -0.0793292  1.211850  1.225400 -1.766580  0.285424
## 6  1.083460 -2.595800 -0.4180320  1.677620 -1.352900  2.576070  3.052710
##       PC673     PC674     PC675     PC676     PC677     PC678     PC679
## 1  7.714990  0.902983 -3.883970  3.517160 -2.888470  0.468737 -2.735040
## 2 -8.770240 -4.600020  4.612250 -9.491340  0.109318  0.464127 -5.452440
## 3 -2.053790  3.051010  0.782791  1.522370 -1.140540 -3.861170  1.060280
## 4 -0.960677 -0.996975 -0.876735 -1.650800 -0.212322  1.519270 -0.433806
## 5 -1.172580  0.632653  1.029420  0.237144 -0.384320  1.148320  1.298490
## 6  1.992090  4.382300 -0.486998 -1.571930  1.047320 -1.083300 -2.447950
##       PC680     PC681      PC682     PC683     PC684      PC685     PC686
## 1  3.559240  7.571770  0.0233162  4.201250 -2.857610 -3.0088600  0.142802
## 2 -2.254790 -7.770550 -6.4139700 -9.503610  0.734434  0.0214167  0.922537
## 3 -1.332280 -3.017710 -3.1139800 -5.776560 -1.799260 -2.1541100 -2.836470
## 4  0.354037  0.206872  1.0207900 -2.639840  0.501722 -1.0595800 -0.343254
## 5  0.890797  0.770138  1.9247100 -0.228412  0.391494 -0.3922560 -1.243100
## 6 -0.921721 -2.749610 -2.6931300 -1.203670 -1.722840  3.1239700 -3.015070
##        PC687     PC688     PC689     PC690      PC691      PC692     PC693
## 1  0.9631010 -1.850710 -8.103210 -2.753990   1.383770 -5.7830000 -2.392580
## 2 -4.0449500 -5.093930 -7.344620  5.664130 -13.139200 -1.3073300  7.367190
## 3  1.5678600  2.995680  3.689360 -0.727163  -0.377641  0.0422484  3.335250
## 4  0.1887190  0.223120  0.417790 -0.410303   1.428030  2.2496300  0.163116
## 5 -0.0112736 -0.260185  0.234566 -0.398761   0.185991 -0.2226260 -0.512066
## 6  0.5957400  3.837890  1.803740  0.853355  -1.590950  2.2629600 -0.283617
##       PC694      PC695     PC696     PC697      PC698      PC699     PC700
## 1  2.672030  3.1061700  0.616902  1.661150   4.304520  0.9408980  2.525140
## 2 -3.524050 -0.3402410  1.122280 -6.666780 -13.292400 -6.1220900 -7.996400
## 3 -4.423110 -3.1294800  1.456730 -2.289750  -2.670730  4.7457900 -0.654216
## 4 -1.058710 -0.0788688  1.257180  0.652511   0.522916 -0.0481975 -0.785245
## 5  0.543643 -1.7351600 -0.775722 -0.256420  -0.183184  2.4325000  0.759796
## 6 -4.015980 -0.8782250  2.879870 -3.955060  -3.867400  1.5343600 -2.560920
##       PC701     PC702     PC703     PC704     PC705     PC706     PC707
## 1  8.315710 -2.751580  1.091770 -5.456910  5.530970 -2.403810 -1.014950
## 2 -3.402650  7.727150  1.306800 15.873700 -0.339925  9.440910 -7.578310
## 3  2.577140 -1.242950 -1.410580  2.625250 -0.694065 -1.131340 -0.465066
## 4  2.030830  0.147544 -0.633884 -0.614220  0.211551  1.231170  0.465193
## 5  0.508467 -0.305402 -0.232083 -0.618519 -0.470989 -3.651930 -0.707635
## 6  0.477141 -1.078410  0.788460  4.157050 -1.144080  0.212263 -0.165601
##       PC708      PC709     PC710      PC711      PC712    PC713     PC714
## 1  3.092920  2.2228100  1.173030 -0.0781324 -2.7649000  1.04205  3.787530
## 2 -4.098670 -9.7812800  3.205430  7.1020200  6.7178300  6.45360 -6.418130
## 3  0.249806  1.8408400 -2.440510  2.8123600 -3.0757700  0.34986 -4.788850
## 4  1.156510  0.0337897 -0.151140 -0.8392580 -1.0255000 -1.58383 -0.899978
## 5 -0.547488  1.1084000 -0.633367 -0.1984590 -0.5816080  2.41489 -0.331643
## 6 -0.737585  2.2357700 -1.330920 -0.7080510  0.0323882  1.17648  0.262815
##       PC715      PC716     PC717      PC718     PC719     PC720     PC721
## 1  0.972488  0.6571170  1.144230  2.3198800  1.191150  1.090620  1.819680
## 2  4.935670  1.2694800 -9.558650 -5.4705800  2.286790  2.122820  5.128400
## 3 -1.015920 -2.7353600 -2.694190 -3.2520800 -0.986321 -0.645813  4.767740
## 4  0.178030 -0.0667111  1.434620  0.0984879  0.031049 -0.193997  1.423300
## 5  0.210236 -0.5518140 -2.145540 -0.8316130  0.650758 -1.225090  1.805580
## 6 -2.792650  0.1122550  0.118912  0.6374280  1.132260 -0.932538 -0.965396
##       PC722     PC723    PC724     PC725     PC726     PC727      PC728
## 1  1.442880  2.868730 2.082830 -1.045760  6.318320 -3.074210 -0.8773470
## 2 -7.410030 -1.593190 5.093140 -1.997920 -8.763980  3.591750  9.1125600
## 3 -6.972430 -0.595632 1.106040  2.257290 -2.013450  0.249988 -2.2318600
## 4  0.645839 -1.049580 0.262375 -1.546400  1.142240 -0.391041 -0.0377659
## 5 -1.822540  0.839740 0.497853  0.729818  0.210316 -1.268750 -0.0208362
## 6 -3.678980 -1.243660 1.560820  3.712500  1.178340  0.158514 -0.8389820
##        PC729     PC730     PC731     PC732     PC733      PC734     PC735
## 1  4.7446800 -2.507830  4.970770  1.653160 -1.565830 -0.0503822  2.964740
## 2 -1.4479000  8.603680 -1.859380  0.604591  3.172090 -1.8812300  0.163519
## 3 -1.0685200  4.017960 -4.637640 -1.033360  1.729150  2.8388000 -0.912366
## 4 -0.4191810 -0.116498 -0.128938 -2.035890  1.676760  1.6997400  0.664965
## 5  0.0920182 -0.685676 -0.352581 -1.181700  0.187980  1.5186900  0.814928
## 6  1.3785800  3.923050  0.916037  0.322230  0.879832  0.2346630  2.106700
##        PC736      PC737      PC738     PC739     PC740      PC741      PC742
## 1 -0.0545404  0.1080470 -0.1870690  1.949180 -2.124420  5.9693400 -0.3024650
## 2 -1.7641000  2.2447000  0.2883850  6.480370  2.993460  2.8673900  0.8210280
## 3  0.3356990 -1.0010300  3.9509300  2.943180  2.720330  0.0191857  0.0590165
## 4  2.5837700 -0.7925940  1.3339300  1.946990 -0.614367  0.6848490  1.1114700
## 5 -0.9146440 -0.0872072  0.3983760 -0.853748 -0.142914 -1.0270200 -1.0606000
## 6 -0.6634990  0.0404891  0.0355303  1.680390  4.190250 -0.2343540  1.5104500
##       PC743     PC744     PC745      PC746     PC747     PC748      PC749
## 1 -0.501076 -1.244670  0.418699 -1.7786300 -0.589285  1.530110  0.6540720
## 2 -4.721510 -6.210480 -1.630460 -0.2417410 -2.198180  4.258420  1.0140100
## 3  3.092970 -0.277524 -1.574570 -0.0303895 -1.865450  1.544670  0.5946640
## 4  0.681403  1.252200  1.445470 -2.4296000 -0.550373 -0.170530 -0.0902045
## 5 -0.276792  0.062173  0.310777 -0.2501430  0.222858 -0.622966 -1.3709200
## 6 -1.897840 -0.254566 -0.381911  2.2090800  1.642340  1.286210  1.5087700
##       PC750     PC751      PC752     PC753     PC754     PC755      PC756
## 1 -0.274253 -0.346528   0.640203 -0.844245  1.875370  3.024520 -1.7450800
## 2 -0.717583 -1.859920 -11.970900  5.161280 -0.465561 -2.368910 -0.9562030
## 3  0.391502  0.510731  -3.378530  1.152170 -1.154530 -1.222400  2.3879400
## 4  0.333235 -0.993200  -0.969760 -0.299044 -1.302790 -2.035420  1.2072800
## 5  0.481895  2.340660   0.458260 -2.456190  1.068670  0.963577 -1.4799400
## 6  1.913690 -0.606544  -4.470470  0.185839  0.207496 -1.899240 -0.0728127
##       PC757     PC758     PC759     PC760     PC761     PC762      PC763
## 1 -3.263270 -3.337780  0.565057 -0.315846  0.903968  1.516630 -2.6601900
## 2  1.979180  2.879390  3.379770 -0.188035 -0.408772 -0.244009  1.6930200
## 3  0.694249  0.445819 -0.762109  0.553738  1.170880  2.071300  1.6804600
## 4 -2.965020 -0.466331  1.883580  1.647440  1.613010 -1.519280 -1.1284600
## 5  0.702941  1.559340 -0.699376 -2.549360 -0.770142  0.460698 -0.0103156
## 6  0.765340  0.382530 -1.239420  0.289236 -0.168925  0.999421 -0.5162010
##       PC764      PC765     PC766     PC767     PC768     PC769     PC770
## 1  1.778650  0.5706570 -0.371561 -1.497570 -4.381020  1.243890  2.248130
## 2 -1.718710  1.2853400 -0.383236  2.841480  3.792060 -2.086870 -0.233220
## 3 -0.541596 -2.1706000  2.263310  0.906315  0.441441 -2.597210 -0.586944
## 4  0.697323  1.5837800 -0.108751 -1.220270 -0.813859 -0.523230  0.524438
## 5 -1.946630 -2.7691900  0.739136  0.668641  0.731291  1.031200 -0.369910
## 6 -0.514184 -0.0808515  0.789506  1.876790 -1.929900 -0.542094 -0.653419
##        PC771     PC772      PC773      PC774     PC775     PC776     PC777
## 1 -0.1732330 -2.160780 -3.6071600 -2.1047300  0.903033 -2.299320 -2.285610
## 2 -1.5126800 -2.117670  1.9892100  6.4225000 -2.904380 -0.420009  3.096540
## 3  0.4700860 -0.480577 -0.5465790  0.1378110 -0.224539  1.300750  1.181680
## 4  0.5710180 -0.339830  0.0795959  0.0475849  2.275360 -1.826620  0.549217
## 5 -0.0387854 -0.470205 -0.4903110 -0.5473870 -1.182890 -1.365740 -1.875030
## 6 -0.8940650 -0.687713 -0.6908740  0.1374900 -2.932100  1.367640  0.326895
##       PC778     PC779     PC780     PC781     PC782     PC783     PC784
## 1 -2.140190  1.993200  1.922120 -2.300720 -1.401900 -0.650031  1.468470
## 2  2.007960  4.308770 -0.029951  0.852218  0.991157  2.573250  1.778960
## 3 -0.196204 -0.101861  2.151260  1.762990 -0.138255  2.922610  0.478878
## 4 -0.439018 -0.790848 -2.889140  0.659955  1.096510 -0.727453 -1.608490
## 5  0.674263 -0.252367  2.405620  2.414220 -1.994280  2.262900  2.947360
## 6  3.224530  1.239790  1.490930 -0.383095  0.577615  1.551690  1.002660
##        PC785     PC786     PC787    PC788      PC789     PC790     PC791
## 1 -4.5830600  0.634860 -0.493373 0.848728  1.6121600 -0.179946  0.574275
## 2  1.1883100 -2.288390 -1.965380 3.544200 -2.0562100  2.378090  0.720809
## 3 -0.0889782 -0.841454  1.256180 0.632431 -1.0651800  1.143350  1.170450
## 4  0.3421180 -1.025950 -1.459980 1.674690 -0.1384270 -0.443186  1.455950
## 5 -2.0078200  0.902023 -0.451469 0.032456 -0.2001110  1.046260 -1.102550
## 6  0.5176040  1.659910  0.481274 0.306568  0.0611311  0.406007  1.752890
##        PC792     PC793     PC794     PC795     PC796     PC797     PC798
## 1 -0.9086910 -1.521030 -1.280350 -0.263426 -2.558830  0.410278  0.446194
## 2  1.5400600  3.303740 -0.445594 -0.466514  2.650090 -2.627330 -0.974133
## 3 -0.0897301 -0.409718 -1.409020  2.993300  0.805388 -0.644858 -0.824014
## 4  0.3920070 -1.340470  0.460055  0.691401  0.324568 -0.665665 -0.614820
## 5  0.0111024 -0.288279 -0.324706 -0.422375 -1.121810  1.547120 -0.421677
## 6 -0.7927890  1.188750 -0.221165 -0.823070 -1.580090  0.807039  0.403762
##       PC799       PC800     PC801      PC802     PC803      PC804      PC805
## 1  0.371371 -0.50014900 -3.679930  2.4586500  3.407620 -0.9333070  0.5079190
## 2  1.547310  0.33196300  2.262160 -3.1541100 -3.043970  0.9353850 -1.2108200
## 3  0.962603  0.95417300  2.133520 -2.2325800  0.949384 -0.7317120 -0.1281650
## 4 -2.744010 -0.00218451  0.942935 -2.6247000  0.579261  0.0468562  2.0866800
## 5  1.653000  0.19827500 -1.506750  1.9379300 -0.498526  0.3148670 -0.0545151
## 6  0.120140  0.96796900  0.192324  0.0515902  0.118370 -1.0597500 -1.3145800
##       PC806      PC807     PC808     PC809     PC810     PC811     PC812
## 1 -0.590068 -2.3145100  1.516270  1.377260 -1.677960 -1.131020  0.693753
## 2 -1.355310  1.1572100 -1.558810  1.085890  1.687050 -0.646803  1.135560
## 3 -1.049850  0.3250300  0.966564  0.520889 -1.008380  0.398579 -0.652057
## 4  0.120134  0.0834005  0.753011 -0.614749 -1.007760  0.151244 -2.109740
## 5 -0.365848 -1.3753300 -0.924156  1.169290  0.975947  0.548868  1.842450
## 6 -1.510720  0.2885410 -2.728760 -1.657470 -0.970105 -0.463773 -1.277150
##       PC813     PC814     PC815     PC816     PC817     PC818      PC819
## 1 -0.479574  0.809265 -0.104240 -0.143899  0.580870  0.647270 -0.1341280
## 2  0.661717  3.665220  2.058300  2.099630  0.538298 -0.481031 -0.7563290
## 3  2.077030  1.726050  0.577143  0.724074  0.404253 -0.700707  1.7796500
## 4  2.088330 -0.696383 -0.712691  0.768003  0.579239 -1.104520 -0.8320950
## 5 -1.179910  1.582040  1.282840 -1.126350 -0.215498  0.791934 -0.0167298
## 6 -0.589234  0.880270  1.563790  3.015480 -2.177780 -1.152880  1.8235300
##       PC820      PC821     PC822      PC823     PC824     PC825     PC826
## 1  0.480940 -0.0830959 -2.091510  0.4707690  0.633358  1.040920 -0.038308
## 2 -1.338720  1.7122200 -1.200040 -1.4610200 -0.207464  1.065610 -0.140433
## 3  0.787068  0.8729450 -0.303715 -0.3343050 -1.343680  0.412000  0.678000
## 4  0.780339  0.0316296 -0.032280 -0.1435880 -0.506939 -0.182709  0.731047
## 5 -0.344137 -0.3025920 -0.165304 -0.0219486  0.677284  0.290872 -0.562342
## 6  0.481720  0.1399090  0.494530 -1.1914800  0.349049 -0.253456  0.704457
##       PC827      PC828     PC829    PC830     PC831      PC832       PC833
## 1 -0.862525 -1.4404800 -0.428032 -1.43575 -1.238590 -0.9710950 -1.41158000
## 2 -0.844440  0.2181560 -4.032880 -1.16150  0.686576  1.0332300 -0.47404100
## 3 -1.498300  0.9043460 -0.653918  1.27200  1.072850  0.4510320 -1.00583000
## 4  0.261402 -1.7957500 -1.878530 -1.10461  0.196753  0.0595379  0.00494685
## 5 -1.199500  0.3234270  1.333570  1.06263 -0.394893  0.4562740 -0.38520000
## 6  0.811905  0.0819515 -1.046520  1.37089  1.274650 -0.1859690  0.57558100
##        PC834     PC835      PC836     PC837     PC838      PC839     PC840
## 1  0.8124630  0.278873  0.1561000 -0.145635  0.963983  0.3696650 -1.130520
## 2 -1.3607500  0.880028  0.2329190  0.016413  0.533442  1.2702500  0.580979
## 3  0.2798480 -0.739207 -0.0673526  1.135430 -1.484160 -0.3711200  0.547808
## 4  0.7301580 -1.921020  1.6838800  2.294690  1.456850 -0.4114090 -1.726830
## 5 -0.0658259  0.931774 -1.6381200 -2.357510 -0.924575  0.8267480  1.688780
## 6  0.3010610  3.627880  0.1406870  0.143266 -1.725910 -0.0974078 -0.636075
##        PC841      PC842     PC843    PC844     PC845     PC846     PC847
## 1  0.0517175 -0.1754660 -1.591790 -3.46131 -0.646382  0.881897  2.298750
## 2 -0.6889470  0.2921550  1.768790  1.17748  1.315010  0.113737 -0.740000
## 3  0.2773350 -0.1556390 -0.516030  2.15332  0.617726 -1.550710  0.021505
## 4 -1.2608300 -1.9557100 -0.421051  2.03634 -1.595060 -0.193341 -0.978483
## 5  1.0536300  2.5688500  1.057130 -1.72629  1.778970 -0.029983  0.316726
## 6 -1.0662400 -0.0315817  0.381516 -1.29748 -0.386187 -0.934134  1.104410
##         PC848     PC849     PC850     PC851      PC852     PC853     PC854
## 1 -0.86617600  0.833256 -0.947292  0.789946  0.9097950  0.727640 -0.134368
## 2 -0.00628969  0.185518  0.471186  0.834334  0.0232132  0.267786 -0.146894
## 3  0.98120500 -0.538732  0.836029 -0.137791 -0.1599210 -0.362267  0.171109
## 4 -0.67260800 -2.442290  0.383213 -1.895310  1.9753100  3.270190  0.281184
## 5  0.06974220  1.953620 -0.838027  1.598600 -1.9484600 -3.852550 -0.137394
## 6 -0.26036400  0.432592 -0.117560 -0.398783 -0.1335840 -0.630130 -0.043209
##       PC855     PC856     PC857      PC858      PC859      PC860     PC861
## 1  0.462540 -0.419273  0.345529 -0.1110000  0.9643680 -0.0221716  0.222090
## 2 -0.355702 -0.712416 -1.316310  0.3863270  0.3455210  1.4142700 -0.150463
## 3  0.315594  1.303490  0.377559  0.1151960 -0.0333118  0.4591570 -1.706500
## 4  1.440860  2.162990 -2.711350 -0.0606456  1.0731600 -2.8563300 -4.899760
## 5 -1.864780 -2.215440  1.848510  0.4619990 -0.2743920  2.6458300  4.618800
## 6  0.113808  0.676154 -0.370062 -0.2042910 -0.4950110  1.0522800 -0.329097
##       PC862      PC863     PC864      PC865      PC866     PC867     PC868
## 1  0.602023 -0.2723600 -0.091991  0.6272910  0.5053860 -0.481732  1.013840
## 2  0.626318  0.6389370  0.618471 -0.0596022 -0.2238020 -0.853176 -0.103744
## 3 -0.475023 -0.0388539 -0.387124 -0.1466920 -0.0675125 -0.564913  0.212671
## 4 -1.319810  1.2233900 -3.312020 -1.7664900 -7.3413700  6.278480  0.979295
## 5  1.395050 -1.4097400  3.663020  0.6009190  6.2869200 -5.206690 -0.173900
## 6 -0.082525 -0.4173050  0.289259  0.7486060  1.5795700 -1.537230 -0.699391
##       PC869      PC870     PC871       PC872      PC873     PC874     PC875
## 1 -0.597351  0.0348315  0.279573  -0.6004270  0.0126427  0.617458 -0.927539
## 2 -0.497966 -0.8729600 -1.219350  -0.8492770  0.0393202  0.770234  0.247693
## 3  0.355372  0.3199740 -0.251236  -0.0812435 -0.6572860 -0.534698  0.208263
## 4  2.911140 -0.1164440  2.069190  15.3537000 -1.3656600  6.036640 -5.488580
## 5 -2.726240 -0.6117230 -1.736570 -15.8492000  0.9582780 -5.156130  6.098650
## 6  0.100209  0.2397560  0.130314  -0.9128330  0.2525470 -0.403387  0.571104
##        PC876      PC877       PC878      PC879      PC880     PC881      PC882
## 1  -0.631859   1.513660   1.1961800  0.6836470   0.460662  0.632216   1.707890
## 2  -0.726893   0.469475   0.3915190 -1.2348200   0.845884  0.631475   0.438315
## 3   0.142138  -0.874589   0.0742214 -0.0794038  -0.567137 -0.422935  -0.282445
## 4  16.600800 -26.581000 -15.4344000 -0.3998270 -17.937000 -8.034170 -26.463200
## 5 -17.630500  26.581500  15.4714000 -0.2670720  18.405400  8.296410  27.332700
## 6  -0.689737   1.397320   0.2890440 -0.4018950   0.524233 -0.521840   0.858222
##       PC883      PC884       PC885     PC886       PC887     PC888     PC889
## 1  0.749692  0.6544530 -0.64663300  0.646122  0.56006700  0.103761  0.120574
## 2  0.114915 -0.1477560 -0.24229200  0.213023  0.80423300  0.261274  0.258073
## 3  0.192027 -0.0018532  0.00504095 -0.359653 -0.00595146  0.319100 -0.149307
## 4 -3.229590  1.9692400  4.05922000 -7.171380 -0.74636700 -4.380240  1.679370
## 5  3.448270 -1.7757700 -4.43171000  6.799210  0.45663100  4.319940 -1.693370
## 6  0.117226 -0.4708430  0.36195500  0.486131  0.13525300 -0.260488 -0.969128
##       PC890      PC891      PC892      PC893     PC894     PC895      PC896
## 1 -0.711144 -0.2090570 -0.0553978 -0.7743020 -0.316350 -0.516285  0.0926215
## 2 -0.118932 -0.4924700 -0.1433780 -0.1775270 -0.109243  0.131544  0.2617310
## 3 -0.285057  0.6285480  0.2030820 -0.0504705  0.065127 -0.276885 -0.3427320
## 4  4.948870  0.0464377 -0.8852940  1.4602900  0.767219 -2.898910 -1.5112800
## 5 -4.414240  0.0902607  0.5207640 -2.0055000 -0.506394  2.683650  1.5476100
## 6 -0.678645  0.6564850 -0.1400250 -0.0152137  0.356920  0.500773 -0.0655357
##        PC897     PC898      PC899      PC900      PC901      PC902      PC903
## 1  0.0547369 -0.340371  0.0894256  0.0627417  0.0127018 -0.1915890 -0.6847170
## 2 -1.6249100  0.975248  1.4359500  0.0579100 -0.5324990  0.0255975  0.4112400
## 3 -0.5111000  0.149732  0.0552835 -0.1675370 -0.0723522  0.3122280  0.3009030
## 4  3.3213600 -2.234540 -2.8166300  1.3891700  1.6883900  0.9685850 -1.1159000
## 5 -2.9725400  1.754680  2.8406800 -1.7609300 -1.2536700 -1.3268400  0.9217820
## 6  0.5611870 -0.359296 -0.2510140 -0.1819070 -0.4142500  0.3180630 -0.0125375
##        PC904      PC905       PC906      PC907      PC908      PC909
## 1  0.0180811  0.3439300 -0.41333600  0.0133251  0.4484590 -0.1666370
## 2  0.2269950  0.3999480  0.07723750  0.0704885 -0.4826130  0.0295396
## 3 -0.5396650  0.0780014 -0.00253384  0.2753340 -0.1481710  0.3274780
## 4  0.3199520  0.0864305  0.29278000  0.1390330 -0.0432160  0.0135659
## 5 -0.5212770  0.0568379 -0.64077400 -0.6181340 -0.2190930  0.5699180
## 6 -0.2544240 -0.1004000  0.34517400 -0.4610310  0.0905346 -0.2499610
##         PC910       PC911      PC912      PC913      PC914     PC915      PC916
## 1  0.00381806 -0.00287736 -0.0574551  0.0597161  0.0504773 -0.400598  0.0551224
## 2 -0.30589400  0.36426000  0.5040830  0.0872674 -0.4038840 -0.471562 -0.5794360
## 3  0.00307486  0.52434400  0.1508940  0.1849340  0.1450740 -0.125639  0.0988883
## 4  1.23971000  1.14183000  0.1078240  0.2911420 -0.3366520  0.425615 -0.9743780
## 5 -1.20905000 -0.70137800 -0.0185021 -0.4007010  0.3754090 -0.741609  0.9613330
## 6  0.05342590 -0.46471900  0.3326700 -0.1665290  0.1051710  0.141510 -0.0547851
##        PC917      PC918      PC919     PC920      PC921      PC922      PC923
## 1 -0.1875180 -0.0288136  0.0422655  0.225173 -0.2704020  0.0430636  0.2384520
## 2  0.2309330 -0.0911943  0.1847460  0.544520  0.2178110  0.2633010 -0.0708432
## 3  0.0337238 -0.0628511  0.2261640 -0.208037 -0.4779180  0.3304580 -0.0467790
## 4 -1.0503200 -1.0871300  0.9092910  1.135970  0.0182488  0.3556300  0.4910370
## 5  0.9730690  1.1598200 -1.3567900 -1.185600  0.3213970 -0.6840190 -0.5730510
## 6 -0.1743140  0.4769400 -0.3696610 -0.123960 -0.0727039 -0.1801580 -0.0373181
##        PC924      PC925      PC926      PC927      PC928       PC929      PC930
## 1  0.3927020  0.0341066 -0.0851779 -0.0938119 -0.0481371  0.21126200  0.0518456
## 2 -0.4271730  0.3957400 -0.2364750  0.2765870  0.1401900  0.13004400 -0.0816514
## 3 -0.0883277  0.4687910 -0.1713940 -0.2691790  0.1881190 -0.02020350 -0.2943930
## 4  0.2717850 -0.2212000  0.3098280 -0.1889900 -1.1889500 -0.34988300 -0.1490990
## 5 -0.4934060  0.2644150 -0.5650040  0.3460210  0.9129430  0.38676600  0.3286540
## 6 -0.2115970  0.0958226  0.1844200  0.1240490  0.1656380  0.00488192 -0.1637080
##        PC931      PC932        PC933 Individual continent          region
## 1 -0.2867540  0.0300271 -1.34671e-06        802    Europe Southern Europe
## 2  0.2920800 -0.0511823 -1.34671e-06        803    Europe Southern Europe
## 3 -0.1757190  0.1815670 -1.34671e-06        804    Europe Southern Europe
## 4  0.3227390  0.2202680 -1.34671e-06        806    Europe Southern Europe
## 5 -0.1358880 -0.1167550 -1.34671e-06        807    Europe Southern Europe
## 6 -0.0263995  0.0797279 -1.34671e-06        808    Europe Southern Europe
##   Pop_City Country Latitude Longitude Continent Year          Region
## 1   Durres Albania 41.29704  19.50373    Europe 2018 Southern Europe
## 2   Durres Albania 41.29704  19.50373    Europe 2018 Southern Europe
## 3   Durres Albania 41.29704  19.50373    Europe 2018 Southern Europe
## 4   Durres Albania 41.29704  19.50373    Europe 2018 Southern Europe
## 5   Durres Albania 41.29704  19.50373    Europe 2018 Southern Europe
## 6   Durres Albania 41.29704  19.50373    Europe 2018 Southern Europe
##     Subregion order order2 orderold AM_order state FINAL_order Clade
## 1 East Europe    33     25       25       NA                80     3
## 2 East Europe    33     25       25       NA                80     3
## 3 East Europe    33     25       25       NA                80     3
## 4 East Europe    33     25       25       NA                80     3
## 5 East Europe    33     25       25       NA                80     3
## 6 East Europe    33     25       25       NA                80     3
```

PCs 1 and 2

```
# #   save the pca plot                                                       ####
ggsave(
  here("scripts", "RMarkdowns", "output", "americas", "manuscript1", "lea", "PCA_lea_man1_bypop_LD10_pc1_pc2.pdf"
  ),
  width  = 10,
  height = 16,
  units  = "in"
)
```

# 5. Fst

```
library(StAMPP)
library(ggplot2)
library(tidyverse)
library(adegenet)
library(here)
library(flextable)
library(officer)
library(reshape2)
library(dplyr)
library(tidyr)
library(geosphere)
library(flextable)
library(officer)
library(dartR.base)
library(MASS)
library(Cairo)
library(ape)
```

We will estimate Fst for populations with at least 4 individuals
using r<0.1 SNP set with 1% MAF dataset

Create list of populations

```
cd /gpfs/gibbs/pi/caccone/mkc54/albo/manuscript1

awk '{print $1}' output/snps_sets/r2_0.1_b.fam | sort | uniq -c | awk '{print $2, $1}' | awk '$2 >= 4 {print}' | awk '{print $1}' > output/fst/LD10/pops_4fst.txt;
head  output/fst/LD10/pops_4fst.txt;
wc -l output/fst/LD10/pops_4fst.txt
```

```
## ALD
## ARA
## BAF
## BCO
## BEA
## BEH
## BEN
## BER
## BIT
## BPA
## 89 output/fst/LD10/pops_4fst.txt
```

We have 89 populations with 4 or more mosquitoes. We can convert to
raw format and subset the bed file

```
cd /gpfs/gibbs/pi/caccone/mkc54/albo
module load PLINK/1.9b_6.21-x86_64

plink \
--allow-extra-chr \
--keep-allele-order \
--bfile manuscript1/output/snps_sets/r2_0.1_b \
--keep-fam manuscript1/output/fst/LD10/pops_4fst.txt \
--recodeA \
--out manuscript1/output/fst/LD10/allpopsforfst \
--silent;
grep 'samples\|variants\|remaining' manuscript1/output/fst/LD10/allpopsforfst.log
```

61574 variants loaded from .bim file. –keep-fam: 923 people
remaining. Total genotyping rate in remaining samples is 0.970995. 61574
variants and 923 people pass filters and QC.

```
allpopsforfst <-
  read.PLINK(
    here(
      "manuscript1", "output", "fst", "LD10", "allpopsforfst.raw"
    ),
    quiet = FALSE,
    chunkSize = 1000,
    parallel = require("parallel"),
    n.cores = 4
  )

summary(allpopsforfst)
```

Save it

```
saveRDS(
  allpopsforfst, here(
    "manuscript1/output/fst/LD10/allpopsforfst.rds"
  )
)
```

## 5.1 Hudson Fst

```
cd /gpfs/gibbs/pi/caccone/mkc54/albo/manuscript1/output/fst

salloc -p scavenge --cpus-per-task=5 -t 3:00:00 --mem-per-cpu=10000    
module load R/4.2.0-foss-2020b
R

library(tidyverse)
library(adegenet)
library(here)
library(KRIS)
library(dartR)
```

Then in R (on the hpc)

```
pairwiseHudsonFst <- function(genind_object=gen, outout_file = NULL) {
  require("KRIS")
  populations <- unique(genind_object@pop)
  num_populations <- length(populations)
  fst_matrix <- matrix(NA, nrow = num_populations, ncol = num_populations, dimnames = list(populations, populations))
  calculate_fst <- function(subset_data, pop1, pop2) {
    idx_pop1 <- which(as.character(subset_data$pop) == pop1)
    idx_pop2 <- which(as.character(subset_data$pop) == pop2)
    tryCatch(
      KRIS::fst.hudson(subset_data$tab, idx_pop1, idx_pop2),
      error = function(e) {
        message(sprintf("Error calculating Fst for populations %s and %s: %s", pop1, pop2, e$message))
        return(NA)
      }
    )
  }
  for (i in 1:(num_populations - 1)) {
    for (j in (i + 1):num_populations) {
      pop1 <- populations[i]
      pop2 <- populations[j]
      subset_data <- genind_object[pop = c(pop1, pop2)]
      fst_value <- calculate_fst(subset_data, pop1, pop2)
      fst_matrix[i, j] <- fst_value
      fst_matrix[j, i] <- fst_value
    }
  }
  fst_dataframe <- as.data.frame(fst_matrix)
  if (!is.null(outout_file)) {
    write.csv(fst_dataframe, outout_file, row.names = TRUE)
  }
  return(fst_dataframe)
}
```

Convert genlight to genind object

```
LD2 <- readRDS(
  here(
    "/gpfs/gibbs/pi/caccone/mkc54/albo/manuscript1/output/fst/LD10/allpopsforfst.rds"
  )
)

LD2_genind <- gl2gi(LD2, probar = TRUE, verbose = NULL)
```

```
pairwiseHudsonFst(genind_object=LD2_genind, outout_file = "/gpfs/gibbs/pi/caccone/mkc54/albo/manuscript1/output/fst/LD10/hudson_fst_data.csv")
```

Can continue in RStudio once calculated

```
# Load data from CSV into a matrix with row and column names
LD3 <- as.matrix(read.csv("/gpfs/gibbs/pi/caccone/mkc54/albo/manuscript1/output/fst/LD10/hudson_fst_data.csv", row.names = 1))
```

```
LD3[upper.tri(LD3)] <- NA
print(LD3)
```

```
saveRDS(
  LD3, here(
    "/gpfs/gibbs/pi/caccone/mkc54/albo/manuscript1/output/fst/LD10/hudson_fst_data_matrix.rds"
  )
)
```

To load it

```
LD3 <- readRDS(
  here(
    "/gpfs/gibbs/pi/caccone/mkc54/albo/manuscript1/output/fst/LD10/hudson_fst_data_matrix.rds"
  )
)
```

Now lets look at the object

```
summary(LD3)
```

```
##       OKI               HAI               SOC               YUN          
##  Min.   :0.06368   Min.   :0.03419   Min.   :0.01318   Min.   :0.009708  
##  1st Qu.:0.08660   1st Qu.:0.06266   1st Qu.:0.09095   1st Qu.:0.078568  
##  Median :0.10268   Median :0.07873   Median :0.10559   Median :0.087700  
##  Mean   :0.10627   Mean   :0.07928   Mean   :0.11023   Mean   :0.087678  
##  3rd Qu.:0.11803   3rd Qu.:0.08924   3rd Qu.:0.12609   3rd Qu.:0.104247  
##  Max.   :0.20818   Max.   :0.16964   Max.   :0.21688   Max.   :0.172933  
##  NA's   :1         NA's   :2         NA's   :3         NA's   :4         
##       KAT               GES               TIK               RAR         
##  Min.   :0.03863   Min.   :0.02701   Min.   :0.04415   Min.   :0.07189  
##  1st Qu.:0.09433   1st Qu.:0.09590   1st Qu.:0.10287   1st Qu.:0.09724  
##  Median :0.10385   Median :0.11021   Median :0.11640   Median :0.11013  
##  Mean   :0.10351   Mean   :0.11568   Mean   :0.12254   Mean   :0.11741  
##  3rd Qu.:0.12004   3rd Qu.:0.13145   3rd Qu.:0.13775   3rd Qu.:0.13190  
##  Max.   :0.18749   Max.   :0.22244   Max.   :0.22477   Max.   :0.21891  
##  NA's   :5         NA's   :6         NA's   :7         NA's   :8        
##       TAI              HUN               HAN               KLP        
##  Min.   :0.1083   Min.   :0.03748   Min.   :0.04279   Min.   :0.0527  
##  1st Qu.:0.1314   1st Qu.:0.05514   1st Qu.:0.07439   1st Qu.:0.1063  
##  Median :0.1415   Median :0.06525   Median :0.08517   Median :0.1135  
##  Mean   :0.1486   Mean   :0.07504   Mean   :0.08654   Mean   :0.1146  
##  3rd Qu.:0.1598   3rd Qu.:0.08913   3rd Qu.:0.09236   3rd Qu.:0.1278  
##  Max.   :0.2491   Max.   :0.17993   Max.   :0.16509   Max.   :0.1960  
##  NA's   :9        NA's   :10        NA's   :11        NA's   :12      
##       HOC               QNC               SSK                KAC         
##  Min.   :0.04395   Min.   :0.06771   Min.   :0.009783   Min.   :0.01170  
##  1st Qu.:0.06934   1st Qu.:0.12984   1st Qu.:0.083510   1st Qu.:0.08193  
##  Median :0.08412   Median :0.13599   Median :0.090023   Median :0.08916  
##  Mean   :0.08538   Mean   :0.13768   Mean   :0.091323   Mean   :0.09093  
##  3rd Qu.:0.09452   3rd Qu.:0.15076   3rd Qu.:0.104617   3rd Qu.:0.10388  
##  Max.   :0.17326   Max.   :0.22141   Max.   :0.174845   Max.   :0.17244  
##  NA's   :13        NA's   :14        NA's   :15         NA's   :16       
##       CHA                BRE               DES               CES         
##  Min.   :0.009783   Min.   :0.05713   Min.   :0.05113   Min.   :0.08777  
##  1st Qu.:0.082400   1st Qu.:0.07522   1st Qu.:0.06811   1st Qu.:0.10015  
##  Median :0.089027   Median :0.10748   Median :0.10093   Median :0.12731  
##  Mean   :0.092041   Mean   :0.11324   Mean   :0.10475   Mean   :0.13344  
##  3rd Qu.:0.103634   3rd Qu.:0.13757   3rd Qu.:0.12988   3rd Qu.:0.15497  
##  Max.   :0.173622   Max.   :0.23916   Max.   :0.23215   Max.   :0.25337  
##  NA's   :17         NA's   :18        NA's   :19        NA's   :20       
##       KAN               UTS               MAU               TUC         
##  Min.   :0.07085   Min.   :0.04478   Min.   :0.03353   Min.   :0.01108  
##  1st Qu.:0.08926   1st Qu.:0.05914   1st Qu.:0.07589   1st Qu.:0.08170  
##  Median :0.11425   Median :0.09318   Median :0.08706   Median :0.09593  
##  Mean   :0.12145   Mean   :0.09730   Mean   :0.08975   Mean   :0.09371  
##  3rd Qu.:0.14531   3rd Qu.:0.12344   3rd Qu.:0.09664   3rd Qu.:0.10804  
##  Max.   :0.24134   Max.   :0.22436   Max.   :0.19236   Max.   :0.19124  
##  NA's   :21        NA's   :22        NA's   :23        NA's   :24       
##       REC               BRM               CAM               KAG         
##  Min.   :0.02779   Min.   :0.06589   Min.   :0.01400   Min.   :0.05212  
##  1st Qu.:0.07188   1st Qu.:0.07712   1st Qu.:0.07652   1st Qu.:0.06733  
##  Median :0.08297   Median :0.09708   Median :0.08268   Median :0.08953  
##  Mean   :0.08945   Mean   :0.10584   Mean   :0.08496   Mean   :0.09778  
##  3rd Qu.:0.10007   3rd Qu.:0.12380   3rd Qu.:0.09538   3rd Qu.:0.11819  
##  Max.   :0.19851   Max.   :0.22110   Max.   :0.16722   Max.   :0.21435  
##  NA's   :25        NA's   :26        NA's   :27        NA's   :28       
##       CUA               MED               FAY               DAL         
##  Min.   :0.01147   Min.   :0.07879   Min.   :0.01905   Min.   :0.01664  
##  1st Qu.:0.09621   1st Qu.:0.09542   1st Qu.:0.03435   1st Qu.:0.03544  
##  Median :0.10172   Median :0.09977   Median :0.05928   Median :0.06646  
##  Mean   :0.10659   Mean   :0.10579   Mean   :0.07121   Mean   :0.07422  
##  3rd Qu.:0.11066   3rd Qu.:0.10813   3rd Qu.:0.09547   3rd Qu.:0.10010  
##  Max.   :0.18948   Max.   :0.18525   Max.   :0.19987   Max.   :0.20467  
##  NA's   :29        NA's   :30        NA's   :31        NA's   :32       
##       NEO               RUS               CHI               NUE         
##  Min.   :0.03032   Min.   :0.05446   Min.   :0.06384   Min.   :0.01344  
##  1st Qu.:0.04446   1st Qu.:0.06629   1st Qu.:0.08332   1st Qu.:0.04492  
##  Median :0.06688   Median :0.08807   Median :0.09432   Median :0.06622  
##  Mean   :0.08024   Mean   :0.10109   Mean   :0.10089   Mean   :0.07634  
##  3rd Qu.:0.10234   3rd Qu.:0.12233   3rd Qu.:0.10931   3rd Qu.:0.09533  
##  Max.   :0.20472   Max.   :0.22066   Max.   :0.19248   Max.   :0.19563  
##  NA's   :33        NA's   :34        NA's   :35        NA's   :36       
##       TRA               HAL               STL               SMI         
##  Min.   :0.01485   Min.   :0.01888   Min.   :0.03174   Min.   :0.01597  
##  1st Qu.:0.03677   1st Qu.:0.03567   1st Qu.:0.04856   1st Qu.:0.04620  
##  Median :0.05969   Median :0.06749   Median :0.08737   Median :0.08008  
##  Mean   :0.07281   Mean   :0.07592   Mean   :0.08992   Mean   :0.08340  
##  3rd Qu.:0.09376   3rd Qu.:0.10021   3rd Qu.:0.11469   3rd Qu.:0.10380  
##  Max.   :0.19761   Max.   :0.20050   Max.   :0.21573   Max.   :0.20466  
##  NA's   :37        NA's   :38        NA's   :39        NA's   :40       
##       WAV               HAT               DEN               MON         
##  Min.   :0.02665   Min.   :0.02577   Min.   :0.02611   Min.   :0.01837  
##  1st Qu.:0.04610   1st Qu.:0.04475   1st Qu.:0.03855   1st Qu.:0.04538  
##  Median :0.09052   Median :0.08703   Median :0.08458   Median :0.08226  
##  Mean   :0.08848   Mean   :0.08502   Mean   :0.08337   Mean   :0.08033  
##  3rd Qu.:0.11076   3rd Qu.:0.10415   3rd Qu.:0.10290   3rd Qu.:0.09372  
##  Max.   :0.21001   Max.   :0.20289   Max.   :0.20554   Max.   :0.19414  
##  NA's   :41        NA's   :42        NA's   :43        NA's   :44       
##       BPV               BCO               BPA               BIT        
##  Min.   :0.04367   Min.   :0.02247   Min.   :0.05130   Min.   :0.0368  
##  1st Qu.:0.07388   1st Qu.:0.08144   1st Qu.:0.08124   1st Qu.:0.0861  
##  Median :0.09999   Median :0.10080   Median :0.09233   Median :0.1007  
##  Mean   :0.09996   Mean   :0.09967   Mean   :0.09959   Mean   :0.1029  
##  3rd Qu.:0.11465   3rd Qu.:0.11683   3rd Qu.:0.10512   3rd Qu.:0.1146  
##  Max.   :0.19535   Max.   :0.19589   Max.   :0.20245   Max.   :0.1986  
##  NA's   :45        NA's   :46        NA's   :47        NA's   :48      
##       FLO               BAF               PLM               NEH         
##  Min.   :0.01505   Min.   :0.01261   Min.   :0.02273   Min.   :0.02352  
##  1st Qu.:0.08987   1st Qu.:0.08273   1st Qu.:0.09099   1st Qu.:0.03791  
##  Median :0.10578   Median :0.10007   Median :0.10844   Median :0.06781  
##  Mean   :0.10363   Mean   :0.09865   Mean   :0.10703   Mean   :0.08305  
##  3rd Qu.:0.12073   3rd Qu.:0.11544   3rd Qu.:0.12150   3rd Qu.:0.11459  
##  Max.   :0.19751   Max.   :0.19063   Max.   :0.19604   Max.   :0.20314  
##  NA's   :49        NA's   :50        NA's   :51        NA's   :52       
##       LIN               HAR               FIR               NEB         
##  Min.   :0.04489   Min.   :0.03407   Min.   :0.05359   Min.   :0.02875  
##  1st Qu.:0.05952   1st Qu.:0.04806   1st Qu.:0.06700   1st Qu.:0.04905  
##  Median :0.08625   Median :0.09182   Median :0.11042   Median :0.09158  
##  Mean   :0.09953   Mean   :0.09424   Mean   :0.11317   Mean   :0.09516  
##  3rd Qu.:0.12905   3rd Qu.:0.12457   3rd Qu.:0.14197   3rd Qu.:0.12573  
##  Max.   :0.21687   Max.   :0.21019   Max.   :0.22507   Max.   :0.21019  
##  NA's   :53        NA's   :54        NA's   :55        NA's   :56       
##       BEN               BEH               ARA               IPO         
##  Min.   :0.02515   Min.   :0.04024   Min.   :0.04399   Min.   :0.03406  
##  1st Qu.:0.08781   1st Qu.:0.10000   1st Qu.:0.08470   1st Qu.:0.13703  
##  Median :0.09825   Median :0.11880   Median :0.09949   Median :0.14938  
##  Mean   :0.09968   Mean   :0.11472   Mean   :0.10486   Mean   :0.14474  
##  3rd Qu.:0.11126   3rd Qu.:0.13289   3rd Qu.:0.11737   3rd Qu.:0.16519  
##  Max.   :0.18266   Max.   :0.20312   Max.   :0.19100   Max.   :0.23634  
##  NA's   :57        NA's   :58        NA's   :59        NA's   :60       
##       LAM               PAL               MAN               BEA         
##  Min.   :0.02159   Min.   :0.03148   Min.   :0.02660   Min.   :0.01654  
##  1st Qu.:0.08444   1st Qu.:0.05693   1st Qu.:0.03557   1st Qu.:0.04448  
##  Median :0.09142   Median :0.07818   Median :0.07253   Median :0.07225  
##  Mean   :0.09632   Mean   :0.09180   Mean   :0.08592   Mean   :0.08214  
##  3rd Qu.:0.10631   3rd Qu.:0.11289   3rd Qu.:0.11829   3rd Qu.:0.10571  
##  Max.   :0.17574   Max.   :0.19633   Max.   :0.20429   Max.   :0.19077  
##  NA's   :61        NA's   :62        NA's   :63        NA's   :64       
##       PEO               HOU               LOS               NEG        
##  Min.   :0.02609   Min.   :0.01974   Min.   :0.06545   Min.   :0.1302  
##  1st Qu.:0.04696   1st Qu.:0.04875   1st Qu.:0.08552   1st Qu.:0.1732  
##  Median :0.08079   Median :0.07994   Median :0.09856   Median :0.1834  
##  Mean   :0.08919   Mean   :0.08594   Mean   :0.11260   Mean   :0.1868  
##  3rd Qu.:0.11954   3rd Qu.:0.11601   3rd Qu.:0.13460   3rd Qu.:0.1940  
##  Max.   :0.19813   Max.   :0.19091   Max.   :0.20023   Max.   :0.2703  
##  NA's   :65        NA's   :66        NA's   :67        NA's   :68      
##       NOV               NEW               BER               MAC         
##  Min.   :0.04492   Min.   :0.04402   Min.   :0.03967   Min.   :0.04559  
##  1st Qu.:0.13069   1st Qu.:0.06446   1st Qu.:0.05886   1st Qu.:0.05794  
##  Median :0.14537   Median :0.09756   Median :0.09673   Median :0.08838  
##  Mean   :0.13944   Mean   :0.10281   Mean   :0.09970   Mean   :0.09623  
##  3rd Qu.:0.15670   3rd Qu.:0.12673   3rd Qu.:0.12292   3rd Qu.:0.10816  
##  Max.   :0.22310   Max.   :0.21330   Max.   :0.20886   Max.   :0.19360  
##  NA's   :69        NA's   :70        NA's   :71        NA's   :72       
##       SPR               COL               SAI               POR         
##  Min.   :0.05370   Min.   :0.04031   Min.   :0.06000   Min.   :0.02866  
##  1st Qu.:0.08645   1st Qu.:0.07149   1st Qu.:0.07785   1st Qu.:0.11603  
##  Median :0.11584   Median :0.10488   Median :0.10350   Median :0.12729  
##  Mean   :0.12044   Mean   :0.10892   Mean   :0.10973   Mean   :0.13051  
##  3rd Qu.:0.14629   3rd Qu.:0.13938   3rd Qu.:0.13517   3rd Qu.:0.15559  
##  Max.   :0.22340   Max.   :0.20648   Max.   :0.18752   Max.   :0.20257  
##  NA's   :73        NA's   :74        NA's   :75        NA's   :76       
##       SUF               SUU               GRV               GRA         
##  Min.   :0.04084   Min.   :0.07084   Min.   :0.09088   Min.   :0.05462  
##  1st Qu.:0.10981   1st Qu.:0.10029   1st Qu.:0.10505   1st Qu.:0.06191  
##  Median :0.15991   Median :0.11503   Median :0.11631   Median :0.06881  
##  Mean   :0.14035   Mean   :0.11843   Mean   :0.13211   Mean   :0.09366  
##  3rd Qu.:0.16859   3rd Qu.:0.12783   3rd Qu.:0.15418   3rd Qu.:0.12722  
##  Max.   :0.23762   Max.   :0.19748   Max.   :0.19942   Max.   :0.17356  
##  NA's   :77        NA's   :78        NA's   :79        NA's   :80       
##       TUH               ALD               FRS               SER        
##  Min.   :0.04194   Min.   :0.07304   Min.   :0.04952   Min.   :0.1547  
##  1st Qu.:0.07069   1st Qu.:0.09511   1st Qu.:0.08479   1st Qu.:0.1808  
##  Median :0.08792   Median :0.11170   Median :0.11989   Median :0.1864  
##  Mean   :0.10784   Mean   :0.13247   Mean   :0.12142   Mean   :0.2089  
##  3rd Qu.:0.15303   3rd Qu.:0.16993   3rd Qu.:0.15480   3rd Qu.:0.2372  
##  Max.   :0.20025   Max.   :0.21246   Max.   :0.19968   Max.   :0.2855  
##  NA's   :81        NA's   :82        NA's   :83        NA's   :84      
##       SPB               SPS              INW               INJ        
##  Min.   :0.09555   Min.   :0.1238   Min.   :0.06731   Min.   :0.1091  
##  1st Qu.:0.10353   1st Qu.:0.1536   1st Qu.:0.08867   1st Qu.:0.1091  
##  Median :0.13570   Median :0.1834   Median :0.11002   Median :0.1091  
##  Mean   :0.14354   Mean   :0.1785   Mean   :0.11002   Mean   :0.1091  
##  3rd Qu.:0.17571   3rd Qu.:0.2059   3rd Qu.:0.13138   3rd Qu.:0.1091  
##  Max.   :0.20722   Max.   :0.2284   Max.   :0.15274   Max.   :0.1091  
##  NA's   :85        NA's   :86       NA's   :87        NA's   :88      
##       MAT     
##  Min.   : NA  
##  1st Qu.: NA  
##  Median : NA  
##  Mean   :NaN  
##  3rd Qu.: NA  
##  Max.   : NA  
##  NA's   :89
```

Check the Fst values

```
head(LD3)
```

```
##            OKI        HAI        SOC        YUN       KAT GES TIK RAR TAI HUN
## OKI         NA         NA         NA         NA        NA  NA  NA  NA  NA  NA
## HAI 0.07376723         NA         NA         NA        NA  NA  NA  NA  NA  NA
## SOC 0.10512342 0.08422836         NA         NA        NA  NA  NA  NA  NA  NA
## YUN 0.09629156 0.04692943 0.10514513         NA        NA  NA  NA  NA  NA  NA
## KAT 0.11398214 0.07069831 0.12370034 0.03814367        NA  NA  NA  NA  NA  NA
## GES 0.11029577 0.08843467 0.01318313 0.11004072 0.1286342  NA  NA  NA  NA  NA
##     HAN KLP HOC QNC SSK KAC CHA BRE DES CES KAN UTS MAU TUC REC BRM CAM KAG CUA
## OKI  NA  NA  NA  NA  NA  NA  NA  NA  NA  NA  NA  NA  NA  NA  NA  NA  NA  NA  NA
## HAI  NA  NA  NA  NA  NA  NA  NA  NA  NA  NA  NA  NA  NA  NA  NA  NA  NA  NA  NA
## SOC  NA  NA  NA  NA  NA  NA  NA  NA  NA  NA  NA  NA  NA  NA  NA  NA  NA  NA  NA
## YUN  NA  NA  NA  NA  NA  NA  NA  NA  NA  NA  NA  NA  NA  NA  NA  NA  NA  NA  NA
## KAT  NA  NA  NA  NA  NA  NA  NA  NA  NA  NA  NA  NA  NA  NA  NA  NA  NA  NA  NA
## GES  NA  NA  NA  NA  NA  NA  NA  NA  NA  NA  NA  NA  NA  NA  NA  NA  NA  NA  NA
##     MED FAY DAL NEO RUS CHI NUE TRA HAL STL SMI WAV HAT DEN MON BPV BCO BPA BIT
## OKI  NA  NA  NA  NA  NA  NA  NA  NA  NA  NA  NA  NA  NA  NA  NA  NA  NA  NA  NA
## HAI  NA  NA  NA  NA  NA  NA  NA  NA  NA  NA  NA  NA  NA  NA  NA  NA  NA  NA  NA
## SOC  NA  NA  NA  NA  NA  NA  NA  NA  NA  NA  NA  NA  NA  NA  NA  NA  NA  NA  NA
## YUN  NA  NA  NA  NA  NA  NA  NA  NA  NA  NA  NA  NA  NA  NA  NA  NA  NA  NA  NA
## KAT  NA  NA  NA  NA  NA  NA  NA  NA  NA  NA  NA  NA  NA  NA  NA  NA  NA  NA  NA
## GES  NA  NA  NA  NA  NA  NA  NA  NA  NA  NA  NA  NA  NA  NA  NA  NA  NA  NA  NA
##     FLO BAF PLM NEH LIN HAR FIR NEB BEN BEH ARA IPO LAM PAL MAN BEA PEO HOU LOS
## OKI  NA  NA  NA  NA  NA  NA  NA  NA  NA  NA  NA  NA  NA  NA  NA  NA  NA  NA  NA
## HAI  NA  NA  NA  NA  NA  NA  NA  NA  NA  NA  NA  NA  NA  NA  NA  NA  NA  NA  NA
## SOC  NA  NA  NA  NA  NA  NA  NA  NA  NA  NA  NA  NA  NA  NA  NA  NA  NA  NA  NA
## YUN  NA  NA  NA  NA  NA  NA  NA  NA  NA  NA  NA  NA  NA  NA  NA  NA  NA  NA  NA
## KAT  NA  NA  NA  NA  NA  NA  NA  NA  NA  NA  NA  NA  NA  NA  NA  NA  NA  NA  NA
## GES  NA  NA  NA  NA  NA  NA  NA  NA  NA  NA  NA  NA  NA  NA  NA  NA  NA  NA  NA
##     NEG NOV NEW BER MAC SPR COL SAI POR SUF SUU GRV GRA TUH ALD FRS SER SPB SPS
## OKI  NA  NA  NA  NA  NA  NA  NA  NA  NA  NA  NA  NA  NA  NA  NA  NA  NA  NA  NA
## HAI  NA  NA  NA  NA  NA  NA  NA  NA  NA  NA  NA  NA  NA  NA  NA  NA  NA  NA  NA
## SOC  NA  NA  NA  NA  NA  NA  NA  NA  NA  NA  NA  NA  NA  NA  NA  NA  NA  NA  NA
## YUN  NA  NA  NA  NA  NA  NA  NA  NA  NA  NA  NA  NA  NA  NA  NA  NA  NA  NA  NA
## KAT  NA  NA  NA  NA  NA  NA  NA  NA  NA  NA  NA  NA  NA  NA  NA  NA  NA  NA  NA
## GES  NA  NA  NA  NA  NA  NA  NA  NA  NA  NA  NA  NA  NA  NA  NA  NA  NA  NA  NA
##     INW INJ MAT
## OKI  NA  NA  NA
## HAI  NA  NA  NA
## SOC  NA  NA  NA
## YUN  NA  NA  NA
## KAT  NA  NA  NA
## GES  NA  NA  NA
```

Convert the data into a matrix.

```
aa <- as.matrix(LD3)
aa[upper.tri(aa)] <- t(aa)[upper.tri(t(aa))]
head(aa)
```

```
##            OKI        HAI        SOC        YUN        KAT        GES
## OKI         NA 0.07376723 0.10512342 0.09629156 0.11398214 0.11029577
## HAI 0.07376723         NA 0.08422836 0.04692943 0.07069831 0.08843467
## SOC 0.10512342 0.08422836         NA 0.10514513 0.12370034 0.01318313
## YUN 0.09629156 0.04692943 0.10514513         NA 0.03814367 0.11004072
## KAT 0.11398214 0.07069831 0.12370034 0.03814367         NA 0.12863417
## GES 0.11029577 0.08843467 0.01318313 0.11004072 0.12863417         NA
##            TIK        RAR       TAI        HUN        HAN        KLP        HOC
## OKI 0.11643026 0.11006125 0.1269178 0.06367903 0.08322860 0.12053427 0.07727842
## HAI 0.09505142 0.08759342 0.1126920 0.03419236 0.04286215 0.07655417 0.03508590
## SOC 0.04846733 0.01969503 0.1461361 0.06802867 0.09265511 0.12934168 0.08722380
## YUN 0.11507030 0.10689212 0.1374139 0.06160361 0.04651939 0.05457312 0.05373712
## KAT 0.13330658 0.12547899 0.1538267 0.08329403 0.06680044 0.07303361 0.07583079
## GES 0.04649561 0.02700849 0.1503425 0.07249213 0.09693226 0.13435613 0.09128074
##            QNC        SSK        KAC         CHA       BRE       DES       CES
## OKI 0.14372810 0.09765271 0.09662465 0.096514927 0.1274990 0.1176353 0.1417788
## HAI 0.09966642 0.04773749 0.04839640 0.045681006 0.1110375 0.1019663 0.1267875
## SOC 0.15300163 0.10758634 0.10551656 0.105667501 0.1282711 0.1198481 0.1442225
## YUN 0.06844688 0.01103902 0.01177356 0.009708194 0.1334165 0.1257344 0.1489258
## KAT 0.09227759 0.04103359 0.03862814 0.039889410 0.1488866 0.1419480 0.1635790
## GES 0.15886631 0.11227977 0.10982828 0.110206010 0.1324414 0.1240128 0.1477453
##           KAN        UTS        MAU        TUC        REC       BRM        CAM
## OKI 0.1271190 0.10670042 0.10959879 0.11762064 0.10627181 0.1110292 0.08907568
## HAI 0.1102691 0.08961518 0.07704054 0.08154544 0.07873331 0.0917808 0.03916092
## SOC 0.1251452 0.10619203 0.11843449 0.12639833 0.11502974 0.1168058 0.09851215
## YUN 0.1342111 0.11579611 0.08025493 0.07856806 0.08721854 0.1099176 0.01176400
## KAT 0.1500692 0.13356854 0.09482363 0.09445662 0.10315110 0.1275230 0.04006233
## GES 0.1295614 0.11102462 0.12214372 0.13064175 0.11920667 0.1213941 0.10332952
##            KAG        CUA        MED        FAY        DAL        NEO
## OKI 0.09767664 0.11226621 0.11104901 0.07845046 0.08340571 0.08441784
## HAI 0.08167110 0.07988266 0.07614224 0.06388954 0.06966810 0.07107478
## SOC 0.10037113 0.12291256 0.11938639 0.08823947 0.09326005 0.09498720
## YUN 0.10424654 0.08355535 0.07861744 0.08912072 0.09378737 0.09420111
## KAT 0.12256822 0.09729450 0.09056707 0.10455734 0.11217979 0.11275857
## GES 0.10324781 0.12747963 0.12394816 0.09264022 0.09708824 0.09933866
##            RUS        CHI        NUE        TRA        HAL        STL
## OKI 0.10329209 0.08979964 0.08083509 0.07758484 0.08050965 0.09777750
## HAI 0.09154804 0.05403535 0.05968160 0.06009562 0.06744092 0.08080522
## SOC 0.11305514 0.09957006 0.09068379 0.08666643 0.08169408 0.09909253
## YUN 0.11143810 0.07688714 0.08289187 0.08359738 0.08976513 0.10773932
## KAT 0.12808224 0.09605349 0.10174713 0.10290147 0.10822461 0.12530336
## GES 0.11818016 0.10415203 0.09541820 0.09076599 0.08636054 0.10268115
##            SMI        WAV        HAT        DEN        MON        BPV
## OKI 0.08704837 0.09320919 0.08233125 0.08046139 0.07760929 0.11923147
## HAI 0.07000106 0.08126813 0.07191150 0.06970719 0.05765900 0.08545177
## SOC 0.09428830 0.09600350 0.09176426 0.08966963 0.08687259 0.12853493
## YUN 0.09419303 0.10302466 0.09265070 0.09499414 0.08159397 0.08418083
## KAT 0.11291579 0.11841260 0.11075111 0.11308143 0.10080508 0.09924213
## GES 0.09958252 0.09958866 0.09638160 0.09371092 0.09221033 0.13308804
##            BCO        BPA        BIT        FLO        BAF        PLM
## OKI 0.12245165 0.11324707 0.11938755 0.12361227 0.11659453 0.12381614
## HAI 0.08869450 0.08568039 0.08542868 0.08886188 0.08112439 0.08804585
## SOC 0.13026217 0.12196171 0.12879060 0.13162632 0.12437877 0.13108744
## YUN 0.08509623 0.09042176 0.08641829 0.08502248 0.07723015 0.08192344
## KAT 0.09958927 0.10592140 0.10166743 0.09709871 0.09130644 0.09474031
## GES 0.13422478 0.12535231 0.13225181 0.13583041 0.12857472 0.13524469
##            NEH        LIN        HAR        FIR        NEB        BEN
## OKI 0.08523650 0.09615606 0.09246523 0.10849900 0.09222318 0.10811518
## HAI 0.07059146 0.08056273 0.07785130 0.09656574 0.07586887 0.05874987
## SOC 0.08798395 0.10038534 0.09674167 0.11495400 0.09591646 0.11704763
## YUN 0.09349870 0.10782237 0.10077101 0.11815138 0.10181983 0.02210313
## KAT 0.11121502 0.12506080 0.11834217 0.13495781 0.11991794 0.04033671
## GES 0.09157870 0.10503314 0.10101987 0.11833238 0.09904113 0.12160552
##            BEH        ARA        IPO        LAM        PAL        MAN
## OKI 0.13105053 0.11402191 0.16091463 0.09831078 0.08983491 0.08396148
## HAI 0.09099089 0.07945282 0.11812127 0.04943733 0.06804376 0.06760088
## SOC 0.13851956 0.12364992 0.16717647 0.10829532 0.09782785 0.08728722
## YUN 0.07800263 0.07831550 0.09989017 0.01292215 0.08585570 0.09296262
## KAT 0.09206754 0.09395925 0.11189965 0.03952050 0.10230058 0.11203182
## GES 0.14338058 0.12661624 0.17263480 0.11327025 0.10194642 0.09106306
##            BEA        PEO        HOU        LOS       NEG        NOV        NEW
## OKI 0.07518427 0.08168734 0.07340192 0.09192136 0.1862097 0.14713641 0.09190158
## HAI 0.05396577 0.06596443 0.05277072 0.06328220 0.1453329 0.10511241 0.08058080
## SOC 0.08392032 0.08428878 0.08158761 0.09872076 0.1944836 0.15548416 0.09967017
## YUN 0.07668644 0.08815280 0.07593575 0.08621122 0.1187884 0.08354180 0.10345728
## KAT 0.09605462 0.10637076 0.09612151 0.10532313 0.1351688 0.09547091 0.12168804
## GES 0.08760082 0.08843206 0.08672919 0.10340641 0.2001288 0.16064527 0.10409245
##            BER        MAC        SPR        COL        SAI        POR
## OKI 0.09021827 0.08319013 0.10523654 0.08999373 0.09016156 0.12950254
## HAI 0.07415093 0.06029867 0.09132821 0.07263022 0.06165122 0.09143686
## SOC 0.09225229 0.09021276 0.10552215 0.09009897 0.10119384 0.13666876
## YUN 0.09925531 0.08195007 0.11366299 0.09756704 0.07565460 0.08286749
## KAT 0.11691061 0.10026956 0.13070337 0.11586459 0.09359962 0.09666469
## GES 0.09669515 0.09488879 0.11148834 0.09429929 0.10543802 0.14175663
##            SUF        SUU        GRV        GRA        TUH       ALD        FRS
## OKI 0.16793111 0.12357072 0.12202577 0.07622100 0.08254600 0.1020681 0.08270550
## HAI 0.12823890 0.08379587 0.08604480 0.04147636 0.05967171 0.0817037 0.06202915
## SOC 0.17744246 0.13219503 0.12760913 0.08351018 0.08560635 0.1057679 0.08145423
## YUN 0.11245368 0.08380156 0.08705798 0.05707034 0.08659450 0.1044009 0.08770007
## KAT 0.06393854 0.05316195 0.10299129 0.07706194 0.10580941 0.1204163 0.10745861
## GES 0.18288450 0.13720052 0.13249553 0.08794993 0.08993156 0.1102044 0.08517095
##           SER        SPB       SPS       INW        INJ        MAT
## OKI 0.1752938 0.08899001 0.1140170 0.2081844 0.16573046 0.10365965
## HAI 0.1551170 0.07299088 0.0925798 0.1696388 0.12376632 0.05445407
## SOC 0.1783002 0.08627710 0.1177965 0.2168754 0.17482427 0.11278953
## YUN 0.1729329 0.09801800 0.1163345 0.1424977 0.10166557 0.02288279
## KAT 0.1874853 0.11542150 0.1334770 0.1011008 0.06783486 0.04944736
## GES 0.1842830 0.09034415 0.1215557 0.2224370 0.17973711 0.11754803
```

Import sample locations

```
sampling_loc <- readRDS(here("manuscript1", "output", "fst", "citiesforfst.rds"))

# Arrange by region 
sampling_loc <- sampling_loc |>
  dplyr::arrange(
    continent, region, subregion
  ) 

# Check it
head(sampling_loc)
```

```
## # A tibble: 6 × 9
##   pop   city           state latitude longitude continent region subregion order
##   <chr> <chr>          <chr>    <dbl>     <dbl> <chr>     <chr>  <chr>     <dbl>
## 1 BRM   Bermuda        Berm…     32.3     -64.8 Americas  Carib… Caribbean   128
## 2 NEG   Negril         Jama…     18.3     -78.3 Americas  Carib… Caribbean   129
## 3 SAI   Saint Augusti… Trin…     10.2     -61.5 Americas  Carib… Caribbean   131
## 4 COL   Columbus, OH   OH, …     40.0     -82.9 Americas  North… Midwest,…   115
## 5 PEO   Peoria, IL     IL, …     40.7     -89.6 Americas  North… Midwest,…   116
## 6 SPR   Springfield, … MO, …     37.2     -93.3 Americas  North… Midwest,…   117
```

```
#sampling_loc <- readRDS(here("scripts", "RMarkdowns", "output", "sampling_loc_all_pops.rds"))
#head(sampling_loc)
```

Lets check if the matrix is symmetric

```
isSymmetric(aa)
```

```
## [1] TRUE
```

```
ordered_pops <- c("HAI", "YUN", "HUN", "OKI", "KAG", "UTS", "KAN", "TAI", "BEN", "KAT", "CAM", "INW", "INJ", "SUF", "SUU", "KLP", "MAT", "CHA", "KAC", "SSK", "LAM", "HOC", "QNC", "HAN", "FRS", "SPB", "SPS", "BRE", "DES", "CES", "ALD", "SER", "GRA", "TUH", "SOC", "TIK", "RAR", "GES", "NEB", "NEH", "FIR", "LIN", "NEW", "BER", "HAR", "MAN", "WAV", "FAY", "BEA", "MAC", "PAL", "RUS", "HAL", "HAT", "NEO", "COL", "PEO", "SPR", "STL", "NUE", "HOU", "TRA", "MON", "DAL", "SMI", "DEN", "CHI", "LOS", "BRM", "NEG", "SAI", "CUA", "MED", "BPV", "MAU", "BIT", "TUC", "FLO", "REC", "ARA", "PLM", "BCO", "BAF", "IPO", "NOV", "BEH", "BPA", "GRV", "POR")
```

```
aa <- aa[ordered_pops, ordered_pops]
```

```
# Load pheatmap
library(pheatmap)

# Plot the heatmap
pheatmap(aa,
         clustering_distance_rows = "euclidean",
         clustering_distance_cols = "euclidean",
         color = colorRampPalette(c("white", "red"))(100),
         display_numbers = TRUE,
         fontsize = 4,
         main = "Fst Heatmap")
```

```
pdf(file = "manuscript1/output/fst/LD10/hudson_fst_heatmap_man1.pdf",   # The directory you want to save the file in
    width = 7, # The width of the plot in inches
    height = 7)

pheatmap(aa,
         clustering_distance_rows = "euclidean",
         clustering_distance_cols = "euclidean",
         color = colorRampPalette(c("white", "red"))(100),
         display_numbers = TRUE,
         fontsize = 4,
         main = "Fst Heatmap")

dev.off()
```

### 5.1.1 Plot the matrix with ggplot

Now we have to convert the matrix to a data frame to plot it with
ggplot.

```
aa[lower.tri(aa)] <- NA
print(aa)
```

```
##     HAI        YUN        HUN        OKI        KAG        UTS        KAN
## HAI  NA 0.04692943 0.03419236 0.07376723 0.08167110 0.08961518 0.11026912
## YUN  NA         NA 0.06160361 0.09629156 0.10424654 0.11579611 0.13421107
## HUN  NA         NA         NA 0.06367903 0.06485788 0.07306270 0.09460797
## OKI  NA         NA         NA         NA 0.09767664 0.10670042 0.12711903
## KAG  NA         NA         NA         NA         NA 0.07242629 0.09825262
## UTS  NA         NA         NA         NA         NA         NA 0.08257492
## KAN  NA         NA         NA         NA         NA         NA         NA
## TAI  NA         NA         NA         NA         NA         NA         NA
## BEN  NA         NA         NA         NA         NA         NA         NA
## KAT  NA         NA         NA         NA         NA         NA         NA
## CAM  NA         NA         NA         NA         NA         NA         NA
## INW  NA         NA         NA         NA         NA         NA         NA
## INJ  NA         NA         NA         NA         NA         NA         NA
## SUF  NA         NA         NA         NA         NA         NA         NA
## SUU  NA         NA         NA         NA         NA         NA         NA
## KLP  NA         NA         NA         NA         NA         NA         NA
## MAT  NA         NA         NA         NA         NA         NA         NA
## CHA  NA         NA         NA         NA         NA         NA         NA
## KAC  NA         NA         NA         NA         NA         NA         NA
## SSK  NA         NA         NA         NA         NA         NA         NA
## LAM  NA         NA         NA         NA         NA         NA         NA
## HOC  NA         NA         NA         NA         NA         NA         NA
## QNC  NA         NA         NA         NA         NA         NA         NA
## HAN  NA         NA         NA         NA         NA         NA         NA
## FRS  NA         NA         NA         NA         NA         NA         NA
## SPB  NA         NA         NA         NA         NA         NA         NA
## SPS  NA         NA         NA         NA         NA         NA         NA
## BRE  NA         NA         NA         NA         NA         NA         NA
## DES  NA         NA         NA         NA         NA         NA         NA
## CES  NA         NA         NA         NA         NA         NA         NA
## ALD  NA         NA         NA         NA         NA         NA         NA
## SER  NA         NA         NA         NA         NA         NA         NA
## GRA  NA         NA         NA         NA         NA         NA         NA
## TUH  NA         NA         NA         NA         NA         NA         NA
## SOC  NA         NA         NA         NA         NA         NA         NA
## TIK  NA         NA         NA         NA         NA         NA         NA
## RAR  NA         NA         NA         NA         NA         NA         NA
## GES  NA         NA         NA         NA         NA         NA         NA
## NEB  NA         NA         NA         NA         NA         NA         NA
## NEH  NA         NA         NA         NA         NA         NA         NA
## FIR  NA         NA         NA         NA         NA         NA         NA
## LIN  NA         NA         NA         NA         NA         NA         NA
## NEW  NA         NA         NA         NA         NA         NA         NA
## BER  NA         NA         NA         NA         NA         NA         NA
## HAR  NA         NA         NA         NA         NA         NA         NA
## MAN  NA         NA         NA         NA         NA         NA         NA
## WAV  NA         NA         NA         NA         NA         NA         NA
## FAY  NA         NA         NA         NA         NA         NA         NA
## BEA  NA         NA         NA         NA         NA         NA         NA
## MAC  NA         NA         NA         NA         NA         NA         NA
## PAL  NA         NA         NA         NA         NA         NA         NA
## RUS  NA         NA         NA         NA         NA         NA         NA
## HAL  NA         NA         NA         NA         NA         NA         NA
## HAT  NA         NA         NA         NA         NA         NA         NA
## NEO  NA         NA         NA         NA         NA         NA         NA
## COL  NA         NA         NA         NA         NA         NA         NA
## PEO  NA         NA         NA         NA         NA         NA         NA
## SPR  NA         NA         NA         NA         NA         NA         NA
## STL  NA         NA         NA         NA         NA         NA         NA
## NUE  NA         NA         NA         NA         NA         NA         NA
## HOU  NA         NA         NA         NA         NA         NA         NA
## TRA  NA         NA         NA         NA         NA         NA         NA
## MON  NA         NA         NA         NA         NA         NA         NA
## DAL  NA         NA         NA         NA         NA         NA         NA
## SMI  NA         NA         NA         NA         NA         NA         NA
## DEN  NA         NA         NA         NA         NA         NA         NA
## CHI  NA         NA         NA         NA         NA         NA         NA
## LOS  NA         NA         NA         NA         NA         NA         NA
## BRM  NA         NA         NA         NA         NA         NA         NA
## NEG  NA         NA         NA         NA         NA         NA         NA
## SAI  NA         NA         NA         NA         NA         NA         NA
## CUA  NA         NA         NA         NA         NA         NA         NA
## MED  NA         NA         NA         NA         NA         NA         NA
## BPV  NA         NA         NA         NA         NA         NA         NA
## MAU  NA         NA         NA         NA         NA         NA         NA
## BIT  NA         NA         NA         NA         NA         NA         NA
## TUC  NA         NA         NA         NA         NA         NA         NA
## FLO  NA         NA         NA         NA         NA         NA         NA
## REC  NA         NA         NA         NA         NA         NA         NA
## ARA  NA         NA         NA         NA         NA         NA         NA
## PLM  NA         NA         NA         NA         NA         NA         NA
## BCO  NA         NA         NA         NA         NA         NA         NA
## BAF  NA         NA         NA         NA         NA         NA         NA
## IPO  NA         NA         NA         NA         NA         NA         NA
## NOV  NA         NA         NA         NA         NA         NA         NA
## BEH  NA         NA         NA         NA         NA         NA         NA
## BPA  NA         NA         NA         NA         NA         NA         NA
## GRV  NA         NA         NA         NA         NA         NA         NA
## POR  NA         NA         NA         NA         NA         NA         NA
##           TAI        BEN        KAT        CAM       INW        INJ        SUF
## HAI 0.1126920 0.05874987 0.07069831 0.03916092 0.1696388 0.12376632 0.12823890
## YUN 0.1374139 0.02210313 0.03814367 0.01176400 0.1424977 0.10166557 0.11245368
## HUN 0.1083448 0.07371343 0.08329403 0.05335141 0.1799285 0.13507239 0.13869483
## OKI 0.1269178 0.10811518 0.11398214 0.08907568 0.2081844 0.16573046 0.16793111
## KAG 0.1419169 0.11623093 0.12256822 0.09742419 0.2143544 0.17262638 0.17524516
## UTS 0.1478207 0.12713796 0.13356854 0.10871260 0.2243603 0.18304744 0.18282621
## KAN 0.1651866 0.14539815 0.15006925 0.12764046 0.2413425 0.19874298 0.19860409
## TAI        NA 0.14822197 0.15382672 0.13146723 0.2491277 0.20134332 0.20434179
## BEN        NA         NA 0.04033671 0.02366063 0.1471347 0.10771432 0.11748695
## KAT        NA         NA         NA 0.04006233 0.1011008 0.06783486 0.06393854
## CAM        NA         NA         NA         NA 0.1439715 0.10150385 0.11189991
## INW        NA         NA         NA         NA        NA 0.06730549 0.07805190
## INJ        NA         NA         NA         NA        NA         NA 0.06301294
## SUF        NA         NA         NA         NA        NA         NA         NA
## SUU        NA         NA         NA         NA        NA         NA         NA
## KLP        NA         NA         NA         NA        NA         NA         NA
## MAT        NA         NA         NA         NA        NA         NA         NA
## CHA        NA         NA         NA         NA        NA         NA         NA
## KAC        NA         NA         NA         NA        NA         NA         NA
## SSK        NA         NA         NA         NA        NA         NA         NA
## LAM        NA         NA         NA         NA        NA         NA         NA
## HOC        NA         NA         NA         NA        NA         NA         NA
## QNC        NA         NA         NA         NA        NA         NA         NA
## HAN        NA         NA         NA         NA        NA         NA         NA
## FRS        NA         NA         NA         NA        NA         NA         NA
## SPB        NA         NA         NA         NA        NA         NA         NA
## SPS        NA         NA         NA         NA        NA         NA         NA
## BRE        NA         NA         NA         NA        NA         NA         NA
## DES        NA         NA         NA         NA        NA         NA         NA
## CES        NA         NA         NA         NA        NA         NA         NA
## ALD        NA         NA         NA         NA        NA         NA         NA
## SER        NA         NA         NA         NA        NA         NA         NA
## GRA        NA         NA         NA         NA        NA         NA         NA
## TUH        NA         NA         NA         NA        NA         NA         NA
## SOC        NA         NA         NA         NA        NA         NA         NA
## TIK        NA         NA         NA         NA        NA         NA         NA
## RAR        NA         NA         NA         NA        NA         NA         NA
## GES        NA         NA         NA         NA        NA         NA         NA
## NEB        NA         NA         NA         NA        NA         NA         NA
## NEH        NA         NA         NA         NA        NA         NA         NA
## FIR        NA         NA         NA         NA        NA         NA         NA
## LIN        NA         NA         NA         NA        NA         NA         NA
## NEW        NA         NA         NA         NA        NA         NA         NA
## BER        NA         NA         NA         NA        NA         NA         NA
## HAR        NA         NA         NA         NA        NA         NA         NA
## MAN        NA         NA         NA         NA        NA         NA         NA
## WAV        NA         NA         NA         NA        NA         NA         NA
## FAY        NA         NA         NA         NA        NA         NA         NA
## BEA        NA         NA         NA         NA        NA         NA         NA
## MAC        NA         NA         NA         NA        NA         NA         NA
## PAL        NA         NA         NA         NA        NA         NA         NA
## RUS        NA         NA         NA         NA        NA         NA         NA
## HAL        NA         NA         NA         NA        NA         NA         NA
## HAT        NA         NA         NA         NA        NA         NA         NA
## NEO        NA         NA         NA         NA        NA         NA         NA
## COL        NA         NA         NA         NA        NA         NA         NA
## PEO        NA         NA         NA         NA        NA         NA         NA
## SPR        NA         NA         NA         NA        NA         NA         NA
## STL        NA         NA         NA         NA        NA         NA         NA
## NUE        NA         NA         NA         NA        NA         NA         NA
## HOU        NA         NA         NA         NA        NA         NA         NA
## TRA        NA         NA         NA         NA        NA         NA         NA
## MON        NA         NA         NA         NA        NA         NA         NA
## DAL        NA         NA         NA         NA        NA         NA         NA
## SMI        NA         NA         NA         NA        NA         NA         NA
## DEN        NA         NA         NA         NA        NA         NA         NA
## CHI        NA         NA         NA         NA        NA         NA         NA
## LOS        NA         NA         NA         NA        NA         NA         NA
## BRM        NA         NA         NA         NA        NA         NA         NA
## NEG        NA         NA         NA         NA        NA         NA         NA
## SAI        NA         NA         NA         NA        NA         NA         NA
## CUA        NA         NA         NA         NA        NA         NA         NA
## MED        NA         NA         NA         NA        NA         NA         NA
## BPV        NA         NA         NA         NA        NA         NA         NA
## MAU        NA         NA         NA         NA        NA         NA         NA
## BIT        NA         NA         NA         NA        NA         NA         NA
## TUC        NA         NA         NA         NA        NA         NA         NA
## FLO        NA         NA         NA         NA        NA         NA         NA
## REC        NA         NA         NA         NA        NA         NA         NA
## ARA        NA         NA         NA         NA        NA         NA         NA
## PLM        NA         NA         NA         NA        NA         NA         NA
## BCO        NA         NA         NA         NA        NA         NA         NA
## BAF        NA         NA         NA         NA        NA         NA         NA
## IPO        NA         NA         NA         NA        NA         NA         NA
## NOV        NA         NA         NA         NA        NA         NA         NA
## BEH        NA         NA         NA         NA        NA         NA         NA
## BPA        NA         NA         NA         NA        NA         NA         NA
## GRV        NA         NA         NA         NA        NA         NA         NA
## POR        NA         NA         NA         NA        NA         NA         NA
##            SUU        KLP        MAT         CHA        KAC         SSK
## HAI 0.08379587 0.07655417 0.05445407 0.045681006 0.04839640 0.047737487
## YUN 0.08380156 0.05457312 0.02288279 0.009708194 0.01177356 0.011039025
## HUN 0.09138594 0.08985394 0.06933609 0.061219498 0.06325614 0.062644317
## OKI 0.12357072 0.12053427 0.10365965 0.096514927 0.09662465 0.097652710
## KAG 0.12965474 0.12871408 0.11250201 0.104490592 0.10474999 0.105844126
## UTS 0.14030213 0.13875343 0.12387053 0.115878351 0.11564586 0.117061256
## KAN 0.15618077 0.15634128 0.14229614 0.134962676 0.13369768 0.136468650
## TAI 0.16032485 0.15890102 0.14391204 0.137488318 0.13685509 0.139824651
## BEN 0.09244728 0.06648869 0.03532170 0.023039701 0.02420628 0.023952964
## KAT 0.05316195 0.07303361 0.04944736 0.039889410 0.03862814 0.041033587
## CAM 0.07971266 0.05469883 0.02338573 0.009782863 0.01300467 0.011186249
## INW 0.11062449 0.17131671 0.15274399 0.144626754 0.14204652 0.144921450
## INJ 0.07084468 0.12844412 0.10906767 0.102440201 0.10139185 0.103554459
## SUF 0.04084024 0.13690218 0.12039852 0.114035638 0.11155893 0.114362200
## SUU         NA 0.10497003 0.08994619 0.084468267 0.08275533 0.085392493
## KLP         NA         NA 0.05946646 0.056349997 0.05300384 0.056586253
## MAT         NA         NA         NA 0.022264783 0.02413107 0.023277195
## CHA         NA         NA         NA          NA 0.01169909 0.009783403
## KAC         NA         NA         NA          NA         NA 0.012499266
## SSK         NA         NA         NA          NA         NA          NA
## LAM         NA         NA         NA          NA         NA          NA
## HOC         NA         NA         NA          NA         NA          NA
## QNC         NA         NA         NA          NA         NA          NA
## HAN         NA         NA         NA          NA         NA          NA
## FRS         NA         NA         NA          NA         NA          NA
## SPB         NA         NA         NA          NA         NA          NA
## SPS         NA         NA         NA          NA         NA          NA
## BRE         NA         NA         NA          NA         NA          NA
## DES         NA         NA         NA          NA         NA          NA
## CES         NA         NA         NA          NA         NA          NA
## ALD         NA         NA         NA          NA         NA          NA
## SER         NA         NA         NA          NA         NA          NA
## GRA         NA         NA         NA          NA         NA          NA
## TUH         NA         NA         NA          NA         NA          NA
## SOC         NA         NA         NA          NA         NA          NA
## TIK         NA         NA         NA          NA         NA          NA
## RAR         NA         NA         NA          NA         NA          NA
## GES         NA         NA         NA          NA         NA          NA
## NEB         NA         NA         NA          NA         NA          NA
## NEH         NA         NA         NA          NA         NA          NA
## FIR         NA         NA         NA          NA         NA          NA
## LIN         NA         NA         NA          NA         NA          NA
## NEW         NA         NA         NA          NA         NA          NA
## BER         NA         NA         NA          NA         NA          NA
## HAR         NA         NA         NA          NA         NA          NA
## MAN         NA         NA         NA          NA         NA          NA
## WAV         NA         NA         NA          NA         NA          NA
## FAY         NA         NA         NA          NA         NA          NA
## BEA         NA         NA         NA          NA         NA          NA
## MAC         NA         NA         NA          NA         NA          NA
## PAL         NA         NA         NA          NA         NA          NA
## RUS         NA         NA         NA          NA         NA          NA
## HAL         NA         NA         NA          NA         NA          NA
## HAT         NA         NA         NA          NA         NA          NA
## NEO         NA         NA         NA          NA         NA          NA
## COL         NA         NA         NA          NA         NA          NA
## PEO         NA         NA         NA          NA         NA          NA
## SPR         NA         NA         NA          NA         NA          NA
## STL         NA         NA         NA          NA         NA          NA
## NUE         NA         NA         NA          NA         NA          NA
## HOU         NA         NA         NA          NA         NA          NA
## TRA         NA         NA         NA          NA         NA          NA
## MON         NA         NA         NA          NA         NA          NA
## DAL         NA         NA         NA          NA         NA          NA
## SMI         NA         NA         NA          NA         NA          NA
[truncated: 81,443 more chars]
