# Supplementary material for: Population Structure of the Invasive Asian Tiger Mosquito, Aedes albopictus, in the Americas
Source: Insects. 2026 Jul 23;17(8):755. doi: 10.3390/insects17080755 (PMC13512968; doi:10.3390/insects17080755)
Supplement: Supplementary file 1 [file insects-17-00755-s001.zip › S1_Americas_supplementary_tables.pdf]

Table S1. List of supplementary files with additional methods and results. R Markdown files with the step-by-step details of analyses are available in the online repository (https://doi.org/10.5281/zenodo.20646598).

File	Description of file
File S1	Supplementary Tables (pdf)
File S2	Supplementary Figures (pdf)
File S3	Axiom Analysis Suites file for American dataset SNP genotype calls (pdf)
File S4	Axiom Analysis Suites file for Global dataset SNP genotype calls (pdf)
File S5	List of Collaborators Providing Samples (Excel)
File S6	Quality control for American dataset (R Markdown)
File S7	Quality control for Global dataset (R Markdown)
File S8	American dataset structure analyses (R Markdown)
File S9	Global dataset structure analyses (R Markdown)
File S10	Local PCA and KEGG ORA enrichment analyses (R Markdown)

Table S2. List of all sampling locations, unique codes assigned to each location ('Code'), including the year in which samples were collected, and the number of mosquitoes genotyped from each locality ('Samples Genotyped') and passing quality control filtering ('Samples passing all QC').

Continent	Region	Country	Locality	Code	Year	Samples Genotyped	Samples passing all QC
Americas	Caribbean	Bermuda	Bermuda	BRM	2015	16	16
Americas	Caribbean	Jamaica	Negril	NEG	2020	4	4
Americas	Caribbean	Trinidad and Tobago	Saint Augustine	SAI	2021	12	12
Americas	North America	USA	Beaufort, SC	BEA	2015	12	12
Americas	North America	USA	Berlin, NJ	BER	2018	12	12
Americas	North America	USA	Chino, CA	CHI	2002	7	6
Americas	North America	USA	Columbus, OH	COL	2015	11	10
Americas	North America	USA	Dallas, TX	DAL	2015	12	12
Americas	North America	USA	Denton Co., TX	DEN		12	12
Americas	North America	USA	Fayetteville, NC	FAY	2018	12	12
Americas	North America	USA	Fire Island, NY	FIR		12	12
Americas	North America	USA	Harrisburg, PA	HAR		12	12
Americas	North America	USA	Hattiesburg, MS	HAT		12	10
Americas	North America	USA	Houston, TX	HOU	2018	12	12
Americas	North America	USA	Huntsville, AL	HAL	2017	12	10
Americas	North America	USA	Lincoln, DE	LIN	2015	12	12
Americas	North America	USA	Los Angeles	LOS	2018	14	12
Americas	North America	USA	Macon, GA	MAC	2015	12	12
Americas	North America	USA	Manassas, VA	MAN	2018	12	12
Americas	North America	USA	Montgomery Co., TX	MON	2009	12	12
Americas	North America	USA	New Bedford, MA	NEB	2015	11	10
Americas	North America	USA	New Haven, CT	NEH		7	7
Americas	North America	USA	New Orleans, LA	NEO	2015	12	12
Americas	North America	USA	Newark, NJ	NEW	2018	22	21
Americas	North America	USA	Nueces Co., TX	NUE	2015	12	12
Americas	North America	USA	Palm Beach, CA	PAL	2018	12	11
Americas	North America	USA	Peoria, IL	PEO	2015	12	10
Americas	North America	USA	Russelville, AR	RUS	2018	12	12
Americas	North America	USA	Smith Co., TX	SMI	2017	12	12
Americas	North America	USA	Springfield, MO	SPR	2015	9	7
Americas	North America	USA	St. Louis, MO	STL	2018	12	12
Americas	North America	USA	Travis Co., TX	TRA		12	12
Americas	North America	USA	Waverly, VA	WAV		12	7
Americas	South America	Argentina	Puerto Iguazu	POR	2018	12	12
Americas	South America	Brazil	Alta Floresta	BAF	2017-18	7	7
Americas	South America	Brazil	Araguaina	ARA	2017-18	12	12
Americas	South America	Brazil	Belo Horizonte	BEH	2017-18	10	9
Americas	South America	Brazil	Confresa	BCO	2017-18	12	12
Americas	South America	Brazil	Floriano	FLO	2017-18	9	8
Americas	South America	Brazil	Gravatai	GRV	2018	12	12
Americas	South America	Brazil	Ipora	IPO	2017-18	8	7
Americas	South America	Brazil	Itaituba	BIT	2017-18	12	12
Americas	South America	Brazil	Manaus, AM	MAU	2017	12	12

Americas	South America	Brazil	Nova Venecia, ES	NOV	2018	4	4
Americas	South America	Brazil	Palmares	BPA	2017-18	12	11
Americas	South America	Brazil	Palmas	PLM	2017-18	7	7
Americas	South America	Brazil	Porto Velho	BPV	2017-18	12	12
Americas	South America	Brazil	Recife, PE	REC	2017	12	11
Americas	South America	Brazil	Tucurui, PA	TUC	2018	14	14
Americas	South America	Colombia	Medellin	CUA	2021	17	15
Asia	East Asia	China	Hainan	HAI	2014	12	12
Asia	East Asia	China	Hunan	HUN	2001	12	12
Asia	East Asia	China	Yunnan	YUN	2014	9	9
Asia	East Asia	Japan	Kagoshima	KAG	2018	14	12
Asia	East Asia	Japan	Kanazawa	KAN	2008	12	11
Asia	East Asia	Japan	Okinawa	OKI	2018	12	11
Asia	East Asia	Japan	Utsunomiya	UTS	2008	12	12
Asia	East Asia	Taiwan	Tainan	TAI	2018	8	7
Asia	South Asia	Bhutan	Gelephu	GEL	2009	2	2
Asia	South Asia	India	Bengaluru	BEN		14	12
Asia	South Asia	Maldives	Kunfunadhoo	KUN	2017	4	3
Asia	South Asia	Nepal	Kathmandu	KAT	2002	12	6
Asia	South Asia	Sri Lanka	Jaffna	JAF		2	2
Asia	Southeast Asia	Cambodia	Phnom Penh	CAM		12	12
Asia	Southeast Asia	Indonesia	Jakarta	INJ	2012	12	11
Asia	Southeast Asia	Indonesia	Sulawesi (Forest)	SUF	2008	6	6
Asia	Southeast Asia	Indonesia	Sulawesi (Urban)	SUU	2008	6	6
Asia	Southeast Asia	Indonesia	Wainyapu	INW	2013	8	4
Asia	Southeast Asia	Malaysia	Kuala Lumpur	KLP		4	4
Asia	Southeast Asia	Malaysia	Tambun	MAT	2013	12	12
Asia	Southeast Asia	Thailand	Chanthaburi	CHA	2016	12	12
Asia	Southeast Asia	Thailand	Kanchanaburi	KAC	2015	12	6
Asia	Southeast Asia	Thailand	Lampang	LAM	2011	10	9
Asia	Southeast Asia	Thailand	Sisaket	SSK	2018	12	12
Asia	Southeast Asia	Thailand	Songkhla	SON	2015	5	3
Asia	Southeast Asia	Vietnam	Hanoi	HAN		4	4
Asia	Southeast Asia	Vietnam	Ho Chi Minh City	HOC	2015	14	7
Asia	Southeast Asia	Vietnam	Quy Nhon City	QNC	2018	12	11
Europe	Eastern Europe	Georgia	Sakhumi, Abkhazia	GES	2021	12	12
Europe	Eastern Europe	Russia	Armavir	RAR	2021	12	12
Europe	Eastern Europe	Russia	Sochi	SOC	2021	12	12
Europe	Eastern Europe	Russia	Tikhoretsk	TIK	2021	12	12
Europe	Eastern Europe	Serbia	Novi Sad	SER	2019	4	4
Europe	Eastern Europe	Turkey	Hopa	TUH	2019	12	12
Europe	Southern Europe	Albania	Durres	ALD	2018	10	10
Europe	Southern Europe	Greece	Athens	GRA	2019	12	11
Europe	Southern Europe	Italy	Brescia	BRE	1995	13	13
Europe	Southern Europe	Italy	Cesena	CES	1995	20	15
Europe	Southern Europe	Italy	Desenzano	DES	1995	20	16
Europe	Southern Europe	Spain	Badajoz	SPB	2018	8	8
Europe	Southern Europe	Spain	San Roque	SPS	2017	10	8
Europe	Western Europe	France	Saint-Martin d'Heres	FRS	2019	12	12
**Total**						**1008**	**933**

Table S3. Number of individuals (N) and number of variants that passed quality control and were included in subsequent analyses for the American and Global datasets. Both datasets were filtered for linkage disequilibrium (LD) thresholds, $r^2 < 0.01$, and a minor allele frequency (MAF) of < 1%. Analyses were performed with these sets of SNPs unless otherwise noted in the text.

**Dataset**	**Set 1 N**	**Set 1 SNPs (MAF 1%)**
Global	933	20,825
American	545	18,964

Table S4. SNP density for each of the three *Aedes albopictus* chromosomes after filtering for missingness and minor allele frequency MAF<1%. The number of SNPs per chromosome (N) and per 1Mb window, as well as the number of windows per chromosome are shown for samples in the Global dataset (A) and the American dataset (B) after quality control, but before LD filtering.

Global

A)

**Chromosome**	**SNPs (N)**	**SNPs per 1Mb window**	**Number of windows**
1	21,337	57.98	368
2	40,408	69.67	580
3	34,523	70.60	489

American

B)

**Chromosome**	**SNPs (N)**	**SNPs per 1Mb window**	**Number of windows**
1	21,283	57.83	368
2	40,171	69.14	581
3	34,467	70.48	489

Table S5. Summary of algorithms and parameters used for analyses of population structure for the Global and America datasets. LEA: *"repetitions = 5"* sets the number of times to repeat the analysis, *"CPU=10"* allocates ten processing threads to computation, *"entropy=TRUE"* enables the calculation of entropy, providing an additional measure of genetic diversity or information, *"percentage=0.25"*= specifies 25% of the genotypes are masked when computing the cross-entropy criterion, "*iterations = 500*" sets the maximum number of iterations performed to 500. fastStructure: *"100 runs"* repeats the analysis 100 times for better reliability, *"--prior=simple"* sets the simple prior, affecting how the model handles ancestry estimates; *"--full"* computes a full model, rather than a simplified version; *"--cv=10"* enables 10-fold cross-validation for assessing the model's generalizability; *"--tol=10e-6"* sets the tolerance level for convergence at 10^-6, ensuring that the algorithm stops when the estimates are sufficiently accurate.

**Dataset**	**Algorithm**	**K tested**	**Parameters**
Global	LEA	1-30	repetitions = 5, CPU=10, entropy=TRUE, percentage = 0.25, iterations = 500
	fastStructure	1-30	100 runs, --prior=simple --full --cv=10 --tol=10e-6
American	LEA	1-25	repetitions = 5, CPU=10, entropy=TRUE, percentage = 0.25, iterations = 500
	fastStructure	1-30	100 runs, --prior=simple --full --cv=10 --tol=10e-6

Table S6. Clusters identified by DAPC for *Aedes albopictus* mosquitos in the A) Global dataset and B) American dataset.

A)

Code	1	2	3	4	5	6	7	Country	Region
BRM	0	0	0	16	0	0	0	Bermuda	Caribbean
NEG	4	0	0	0	0	0	0	Jamaica	Caribbean
SAI	0	0	0	1	11	0	0	Trinidad & Tobago	Caribbean
HAI	0	0	0	0	12	0	0	China	East Asia
HUN	0	0	0	0	12	0	0	China	East Asia
YUN	9	0	0	0	0	0	0	China	East Asia
KAG	0	0	12	0	0	0	0	Japan	East Asia
KAN	0	0	11	0	0	0	0	Japan	East Asia
OKI	0	0	0	0	11	0	0	Japan	East Asia
UTS	0	0	12	0	0	0	0	Japan	East Asia
TAI	0	0	0	0	7	0	0	Taiwan	East Asia
GES	0	0	0	0	0	12	0	Georgia	Eastern Europe
RAR	0	0	0	0	0	12	0	Russia	Eastern Europe
SOC	0	0	0	0	0	12	0	Russia	Eastern Europe
TIK	0	0	0	0	0	12	0	Russia	Eastern Europe
SER	0	0	0	0	4	0	0	Serbia	Eastern Europe
TUH	0	0	0	0	12	0	0	Turkey	Eastern Europe
BEA	0	0	0	12	0	0	0	USA	North America
BER	0	0	12	0	0	0	0	USA	North America
CHI	0	0	0	0	6	0	0	USA	North America
COL	0	0	10	0	0	0	0	USA	North America
DAL	0	0	3	9	0	0	0	USA	North America
DEN	0	0	10	2	0	0	0	USA	North America
FAY	0	0	1	11	0	0	0	USA	North America
FIR	0	0	11	1	0	0	0	USA	North America
HAL	0	0	10	0	0	0	0	USA	North America
HAR	0	0	12	0	0	0	0	USA	North America
HAT	0	0	4	6	0	0	0	USA	North America
HOU	0	0	0	12	0	0	0	USA	North America
LIN	0	0	12	0	0	0	0	USA	North America
LOS	0	0	0	0	12	0	0	USA	North America
MAC	0	0	0	12	0	0	0	USA	North America
MAN	0	0	12	0	0	0	0	USA	North America
MON	0	0	0	12	0	0	0	USA	North America
NEB	0	0	10	0	0	0	0	USA	North America
NEH	0	0	7	0	0	0	0	USA	North America
NEO	0	0	0	12	0	0	0	USA	North America
NEW	0	0	17	4	0	0	0	USA	North America
NUE	0	0	0	12	0	0	0	USA	North America
PAL	0	0	0	11	0	0	0	USA	North America
PEO	0	0	8	2	0	0	0	USA	North America
RUS	0	0	1	11	0	0	0	USA	North America
SMI	0	0	0	12	0	0	0	USA	North America

SPR	0	0	7	0	0	0	0	USA	North America
STL	0	0	12	0	0	0	0	USA	North America
TRA	0	0	0	12	0	0	0	USA	North America
WAV	0	0	7	0	0	0	0	USA	North America
POR	0	0	0	0	0	0	12	Argentina	South America
ARA	0	0	0	0	0	0	12	Brazil	South America
BAF	0	0	0	0	0	0	7	Brazil	South America
BCO	0	0	0	0	0	0	12	Brazil	South America
BEH	0	0	0	0	0	0	9	Brazil	South America
BIT	0	0	0	0	0	0	12	Brazil	South America
BPA	0	0	0	0	0	0	11	Brazil	South America
BPV	0	0	0	0	0	0	12	Brazil	South America
FLO	0	0	0	0	0	0	8	Brazil	South America
GRV	0	0	0	0	0	0	12	Brazil	South America
IPO	0	0	0	0	0	0	7	Brazil	South America
MAU	0	0	0	0	0	0	12	Brazil	South America
NOV	0	0	0	0	0	0	4	Brazil	South America
PLM	0	0	0	0	0	0	7	Brazil	South America
REC	0	0	0	0	0	0	11	Brazil	South America
TUC	0	0	0	0	0	0	14	Brazil	South America
CUA	0	0	0	0	8	0	0	Colombia	South America
MED	0	0	0	0	7	0	0	Colombia	South America
GEL	2	0	0	0	0	0	0	Bhutan	South Asia
BEN	12	0	0	0	0	0	0	India	South Asia
KUN	3	0	0	0	0	0	0	Maldives	South Asia
KAT	4	2	0	0	0	0	0	Nepal	South Asia
JAF	2	0	0	0	0	0	0	Sri Lanka	South Asia
CAM	12	0	0	0	0	0	0	Cambodia	Southeast Asia
INJ	0	11	0	0	0	0	0	Indonesia	Southeast Asia
INW	0	4	0	0	0	0	0	Indonesia	Southeast Asia
SUF	0	6	0	0	0	0	0	Indonesia	Southeast Asia
SUU	0	6	0	0	0	0	0	Indonesia	Southeast Asia
KLP	4	0	0	0	0	0	0	Malaysia	Southeast Asia
MAT	12	0	0	0	0	0	0	Malaysia	Southeast Asia
CHA	12	0	0	0	0	0	0	Thailand	Southeast Asia
KAC	6	0	0	0	0	0	0	Thailand	Southeast Asia
LAM	9	0	0	0	0	0	0	Thailand	Southeast Asia
SON	3	0	0	0	0	0	0	Thailand	Southeast Asia
SSK	12	0	0	0	0	0	0	Thailand	Southeast Asia
HAN	0	0	0	0	4	0	0	Vietnam	Southeast Asia
HOC	0	0	0	0	7	0	0	Vietnam	Southeast Asia
QNC	11	0	0	0	0	0	0	Vietnam	Southeast Asia
ALD	0	0	0	0	10	0	0	Albania	Southern Europe
GRA	0	0	0	0	11	0	0	Greece	Southern Europe
BRE	0	0	13	0	0	0	0	Italy	Southern Europe
CES	0	0	15	0	0	0	0	Italy	Southern Europe
DES	0	0	16	0	0	0	0	Italy	Southern Europe
SPB	0	0	8	0	0	0	0	Spain	Southern Europe

SPS	0	0	0	0	8	0	0	Spain	Southern Europe
FRS	0	0	0	0	12	0	0	France	Western Europe

B)

**Code**	**Locality**	**1**	**2**	**3**	**Country**	**Region**
BRM	Bermuda	0	0	16	Bermuda	Caribbean
NEG	Negril	0	4	0	Jamaica	Caribbean
SAI	Saint Augustine	0	12	0	Trinidad & Tobago	Caribbean
BEA	Beaufort, SC	0	0	12	USA	North America
BER	Berlin, NJ	0	0	12	USA	North America
CHI	Chino, CA	0	6	0	USA	North America
COL	Columbus, OH	0	0	10	USA	North America
DAL	Dallas, TX	0	0	12	USA	North America
DEN	Denton Co., TX	0	0	12	USA	North America
FAY	Fayetteville, NC	0	0	11	USA	North America
FIR	Fire Island, NY	0	0	12	USA	North America
HAR	Harrisburg, PA	0	0	12	USA	North America
HAT	Hattiesburg, MS	0	0	10	USA	North America
HOU	Houston, TX	0	0	12	USA	North America
HAL	Huntsville, AL	0	0	10	USA	North America
LIN	Lincoln, DE	0	0	12	USA	North America
LOS	Los Angeles	0	11	0	USA	North America
MAC	Macon, GA	0	0	12	USA	North America
MAN	Manassas, VA	0	0	12	USA	North America
MON	Montgomery Co., TX	0	0	12	USA	North America
NEB	New Bedford, MA	0	0	10	USA	North America
NEH	New Haven, CT	0	0	7	USA	North America
NEO	New Orleans, LA	0	0	12	USA	North America
NEW	Newark, NJ	0	0	21	USA	North America
NUE	Nueces Co., TX	0	0	12	USA	North America
PAL	Palm Beach	0	0	11	USA	North America
PEO	Peoria, IL	0	0	10	USA	North America
RUS	Russelville, AR	0	0	12	USA	North America
SMI	Smith Co., TX	0	0	12	USA	North America
SPR	Springfield, MO	0	0	7	USA	North America
STL	St. Louis, MO	0	0	12	USA	North America
TRA	Travis Co., TX	0	0	12	USA	North America
WAV	Waverly, VA	0	0	7	USA	North America
CUA	Medellin	0	15	0	Colombia	South America
POR	Puerto Iguazu	12	0	0	Argentina	South America
BAF	Alta Floresta	7	0	0	Brazil	South America
ARA	Araguaina	12	0	0	Brazil	South America
BEH	Belo Horizonte	9	0	0	Brazil	South America

BCO	Confresa	12	0	0	Brazil	South America
FLO	Floriano	8	0	0	Brazil	South America
GRV	Gravatai	12	0	0	Brazil	South America
IPO	Ipora	7	0	0	Brazil	South America
BIT	Itaituba	12	0	0	Brazil	South America
MAU	Manaus	12	0	0	Brazil	South America
NOV	Nova Venecia	4	0	0	Brazil	South America
BPA	Palmares	11	0	0	Brazil	South America
PLM	Palmas	7	0	0	Brazil	South America
BPV	Porto Velho	12	0	0	Brazil	South America
REC	Recife	11	0	0	Brazil	South America
TUC	Tucurui	14	0	0	Brazil	South America

Table S7. Mean FST values for Global locations in the SNP dataset with at least four individuals (N=89). Values were computed using the StAMPP package in R. Mean F_{ST} values for continents, regions, and subregions were determined by averaging F_{ST} across all populations within each region or country.

Continent	Mean Fst by continent	Region	Mean Fst by region	Subregion	Mean Fst by subregion	Locality	Code	Mean Fst
Americas	0.13	Caribbean	0.16			Bermuda	BRM	0.15
						Negril	NEG	0.23
						Saint Augustine	SAI	0.12
		North America	0.12	Midwest, USA	0.13	Columbus, OH	COL	0.12
						Peoria, IL	PEO	0.11
						Springfield, MO	SPR	0.14
						St. Louis, MO	STL	0.13
				Northeast, USA	0.13	Berlin, NJ	BER	0.12
						Fire Island, NY	FIR	0.15
						Harrisburg, PA	HAR	0.12
						Lincoln, DE	LIN	0.13
						New Bedford, MA	NEB	0.12
						New Haven, CT	NEH	0.11
						Newark, NJ	NEW	0.12
				Southeast, USA	0.11	Beaufort, SC	BEA	0.09
						Fayetteville, NC	FAY	0.10
						Huntsville, AL	HAL	0.11
						Hattiesburg, MS	HAT	0.11
						Macon, GA	MAC	0.10
						Manassas, VA	MAN	0.11
						New Orleans, LA	NEO	0.12
						Palm Beach	PAL	0.11
						Russelville, AR	RUS	0.14
						Waverly, VA	WAV	0.13
				Southwest, USA	0.10	Dallas, TX	DAL	0.11
						Denton Co., TX	DEN	0.11
						Houston, TX	HOU	0.09
						Montgomery Co. TX	MON	0.10
						Nueces Co., TX	NUE	0.10
						Smith Co., TX	SMI	0.12
						Travis Co., TX	TRA	0.10
				West, USA	0.13	Chino, CA	CHI	0.13
						Los Angeles	LOS	0.13
		South America	0.14			Araguaina	ARA	0.13
						Alta Floresta	BAF	0.13
						Confresa	BCO	0.14
						Belo Horizonte	BEH	0.15
						Itaituba	BIT	0.14
						Palmares	BPA	0.13
						Porto Velho	BPV	0.14

					Floriano	FLO	0.14
					Gravatai	GRV	0.14
					Ipora	IPO	0.19
					Manaus	MAU	0.12
					Nova Venecia	NOV	0.17
					Palmas	PLM	0.14
					Recife	REC	0.12
					Tucurui	TUC	0.13
					Puerto Iguazu	POR	0.15
					Medellin	CUA	0.14
Asia	0.15	East Asia	0.14		Hainan	HAI	0.10
					Hunan	HUN	0.10
					Kagoshima	KAG	0.14
					Kanazawa	KAN	0.18
					Okinawa	OKI	0.14
					Tainan	TAI	0.21
					Utsunomiya	UTS	0.15
					Yunnan	YUN	0.13
		South Asia	0.16		Bengaluru	BEN	0.14
					Gelephu	GEL	0.13
					Jaffna	JAF	0.14
					Kathmandu	KAT	0.14
					Kunfunadhoo	KUN	0.24
		Southeast Asia	0.15		Phnom Penh	CAM	0.12
					Chanthaburi	CHA	0.13
					Hanoi	HAN	0.12
					Ho Chi Minh City	HOC	0.11
					Jakarta	INJ	0.21
					Wainyapu	INW	0.24
					Kanchanaburi	KAC	0.13
					Kuala Lumpur	KLP	0.16
					Lampang	LAM	0.13
					Tambun	MAT	0.14
					Quy Nhon City	QNC	0.20
					Songkhla	SON	0.13
					Sisaket	SSK	0.13
					Sulawesi (Forest)	SUF	0.20
					Sulawesi (Urban)	SUU	0.15
Europe	0.16	Eastern Europe	0.17		Sakhumi, Abkhazia	GES	0.16
					Armavir	RAR	0.16
					Novi Sad	SER	0.24
					Sochi	SOC	0.15
					Tikhoretsk	TIK	0.17
					Hopa	TUH	0.12
		Southern Europe	0.15		Durres	ALD	0.15
					Athens	GRA	0.10
					Brescia	BRE	0.17
					Cesena	CES	0.20
					Desenzano	DES	0.16

			Badajoz	SPB	0.12
			San Roque	SPS	0.16
	Western Europe	0.11	Saint-Martin-d'Heres	FRS	0.11

Table S8. Results of KEGG Over-Representation Analysis (ORA).

ID	Description	Gene Ratio	Bg Ratio	p value	p.adjust	q value	Count	gene IDs	corner
aalb04214	Apoptosis - fly - Aedes albopictus (Asian tiger mosquito)	4/11	56/3976	0.0000	0.0002	0.0001	4	109408179/115257864/ 109428244/115271029	1
aalb04215	Apoptosis - multiple species - Aedes albopictus (Asian tiger mosquito)	2/11	28/3976	0.0025	0.0198	0.0131	2	109428244/115271029	1
aalb00600	Sphingolipid metabolism - Aedes albopictus (Asian tiger mosquito)	2/11	34/3976	0.0037	0.0198	0.0131	2	115266598/109430628	1
aalb04137	Mitophagy - animal - Aedes albopictus (Asian tiger mosquito)	2/11	59/3976	0.0109	0.0385	0.0253	2	109408179/109427928	1
aalb00564	Glycerophospholipid metabolism - Aedes albopictus (Asian tiger mosquito)	2/11	62/3976	0.0120	0.0385	0.0253	2	115266598/109430628	1
aalb04148	Efferocytosis - Aedes albopictus (Asian tiger mosquito)	2/11	74/3976	0.0169	0.0450	0.0296	2	109428244/115271029	1
aalb01230	Biosynthesis of amino acids - Aedes albopictus (Asian tiger mosquito)	3/18	57/3976	0.0020	0.0341	0.0293	3	109423532/109423534/ 109410517	2
aalb04512	ECM-receptor interaction - Aedes albopictus (Asian tiger mosquito)	2/18	17/3976	0.0025	0.0341	0.0293	2	109426653/115265272	2
