# Supplementary material for: Population Structure of the Invasive Asian Tiger Mosquito, Aedes albopictus, in the Americas
Source: Insects. 2026 Jul 23;17(8):755. doi: 10.3390/insects17080755 (PMC13512968; doi:10.3390/insects17080755)
Supplement: Supplementary file 1 [file insects-17-00755-s001.zip › S2_Americas_supplementary_figures.pdf]

Supplementary Figures

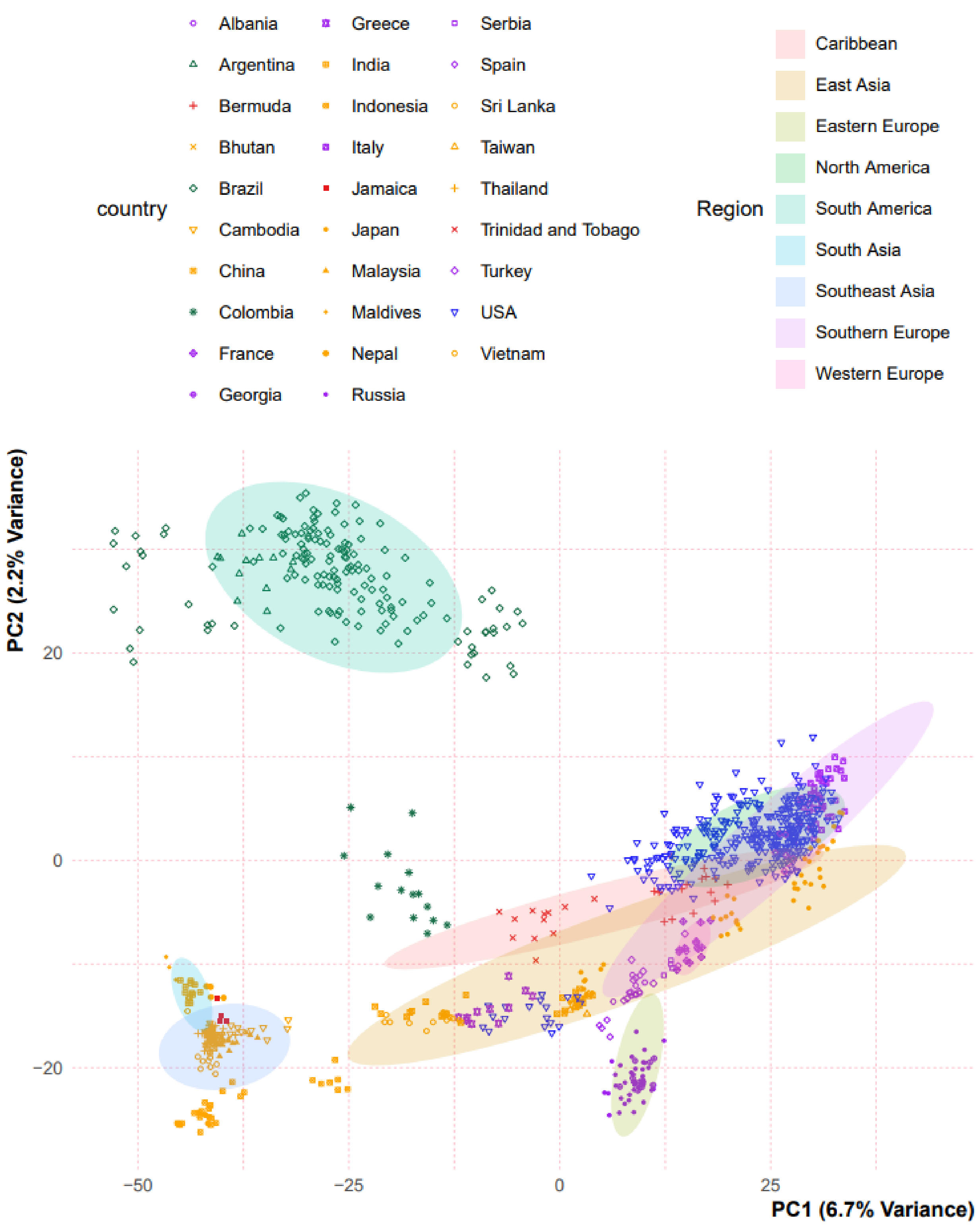

PCA with 20,825 SNPs for 933 mosquitoes from 93 locations in the Americas, Europe, and the native range

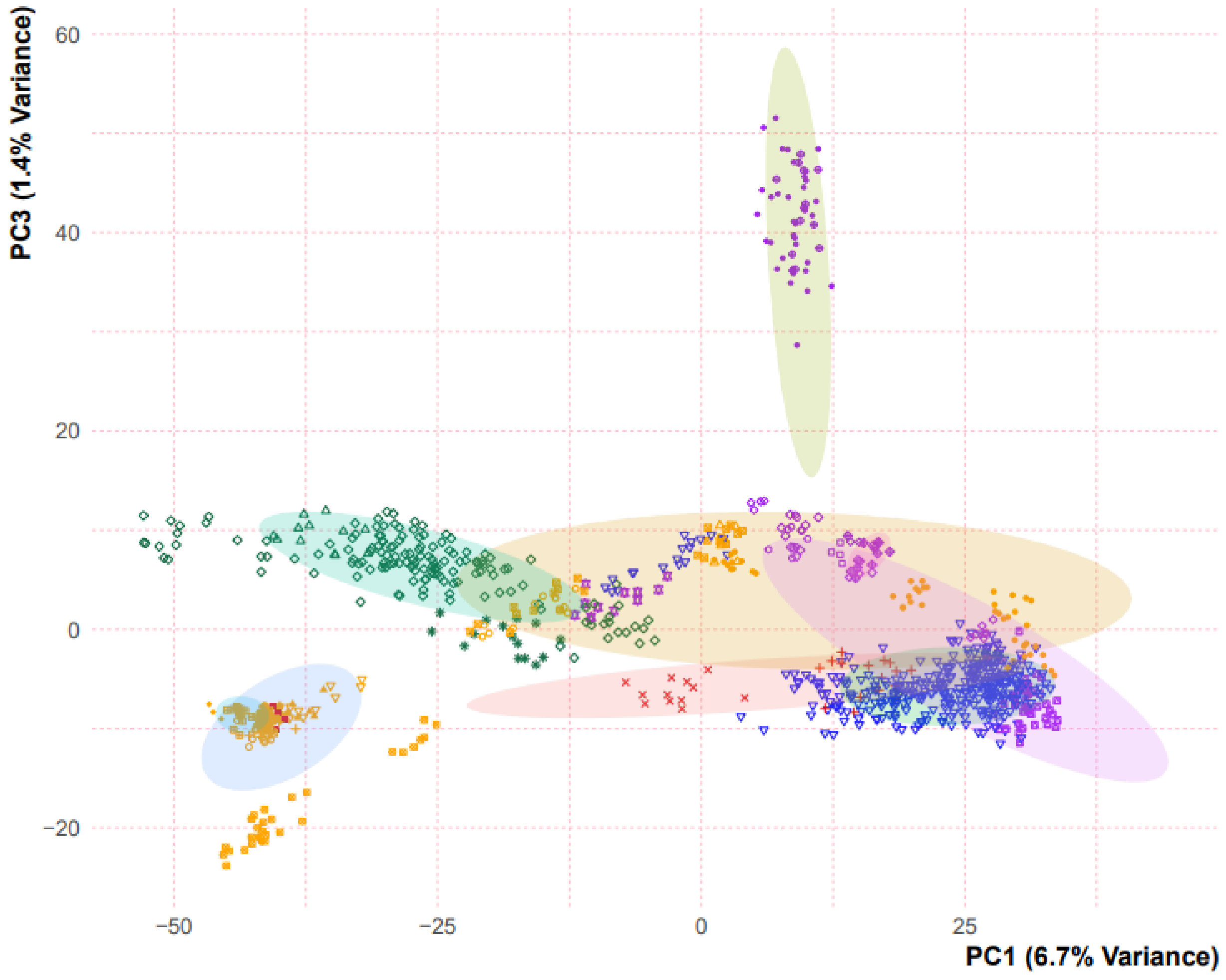

Figure S1. Scatterplots of principal component analysis (PCA) performed in the R package LEA showing the first three PCs for 933 *Ae. albopictus* mosquitoes in 92 localities in the Global dataset. In each plot the x-axis is principal component (PC) 1 and the y-axis is PC 2 in the top panel, and PC 3 in the bottom panel. The variance explained by each principal component is in parentheses on the y- and x-axes. Each symbol represents a mosquito, and its shape indicates the country from which it was sampled. Populations are colored by geographic region: orange=Asia, purple=Europe, red=Caribbean, blue=North America, green=South America. Ellipses mark the area covering 80% of samples in each region. Additional plots and details of the PCA analyses are shown in the Supplementary files.

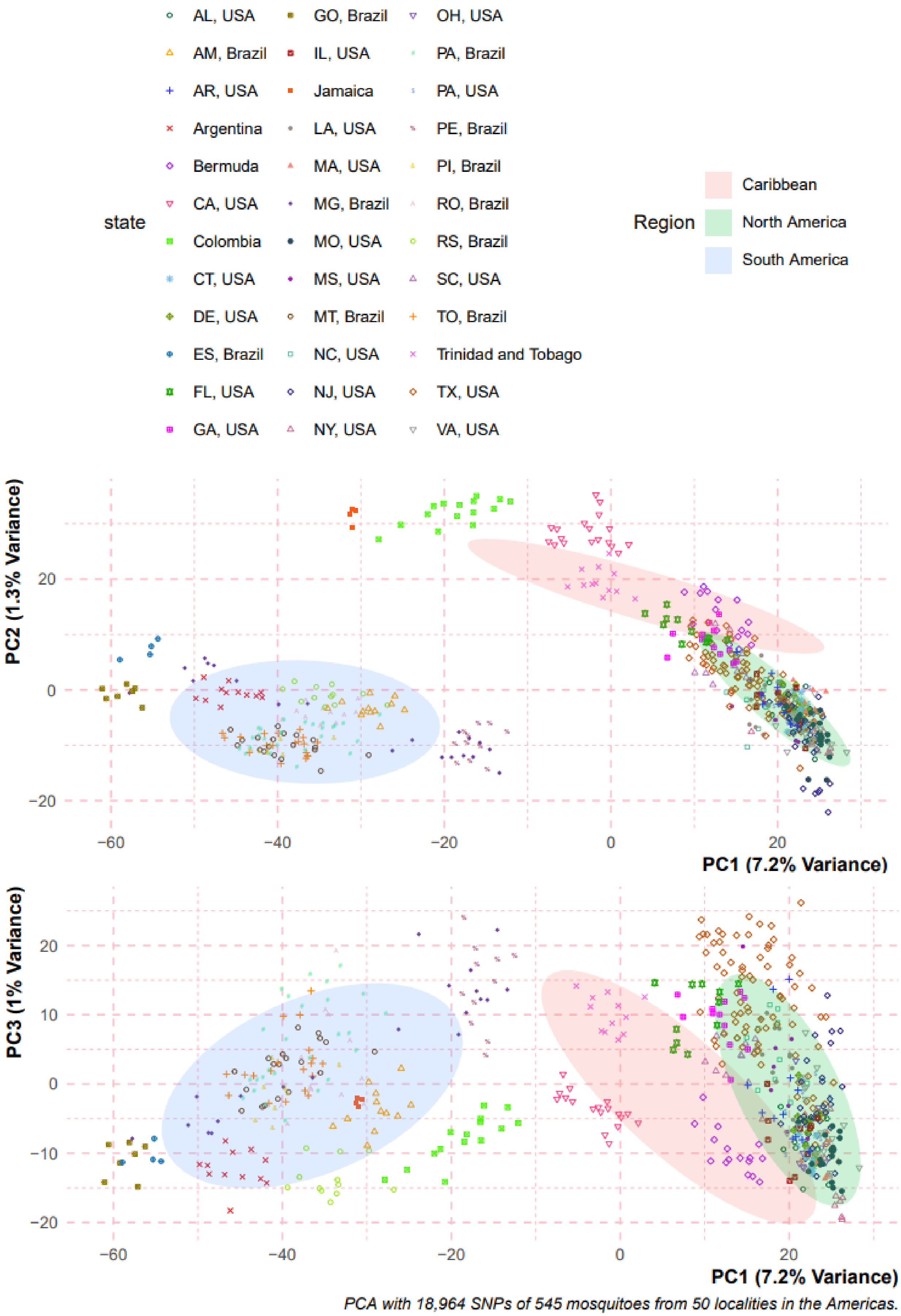

Figure S2. Scatterplots of principal component analysis (PCA) performed in the R package LEA showing the first three PCs for 545 *Ae. albopictus* mosquitoes in the American dataset. In each plot the x-axis is principal component (PC) 1 and the y-axis is PC 2 in the top panel and PC 3 in the bottom panel. The

variance explained by each principal component is in parentheses on the y- and x-axes. Each symbol represents a mosquito, and the color and shape indicate the location where it was sampled (state for Brazil and USA, country for all other locations). Ellipses mark 80% of the samples for each geographic region. Additional plots and details of the PCA analyses are shown in the Supplementary files.

DAPC analyses. DAPC uses discriminant functions to accentuate the genetic differences between groups. A DAPC of the Global dataset confirmed a clustering pattern similar to that identified by PCA (Figure 2). Specifically, the DAPC identified seven clusters consisting of 1) South and Southeast Asia with Jamaica, 2) Indonesia, 3) Japan, the Eastern US, and most of Southern and Western Europe, 4) Bermuda, parts of the Southern and Central US, 5) Southern parts of Eastern Asia (China, Taiwan, Okinawa, Vietnam), the Western US (California), some European populations (Greece, Albania, Turkey, some of Spain), Colombia, and Trinidad and Tobago, 6) Eastern Europe (Russia and Georgia), and 7) Brazil and Argentina (also see Table S6). Additional plots and details of DAPC analyses are shown in Supplementary File S9.

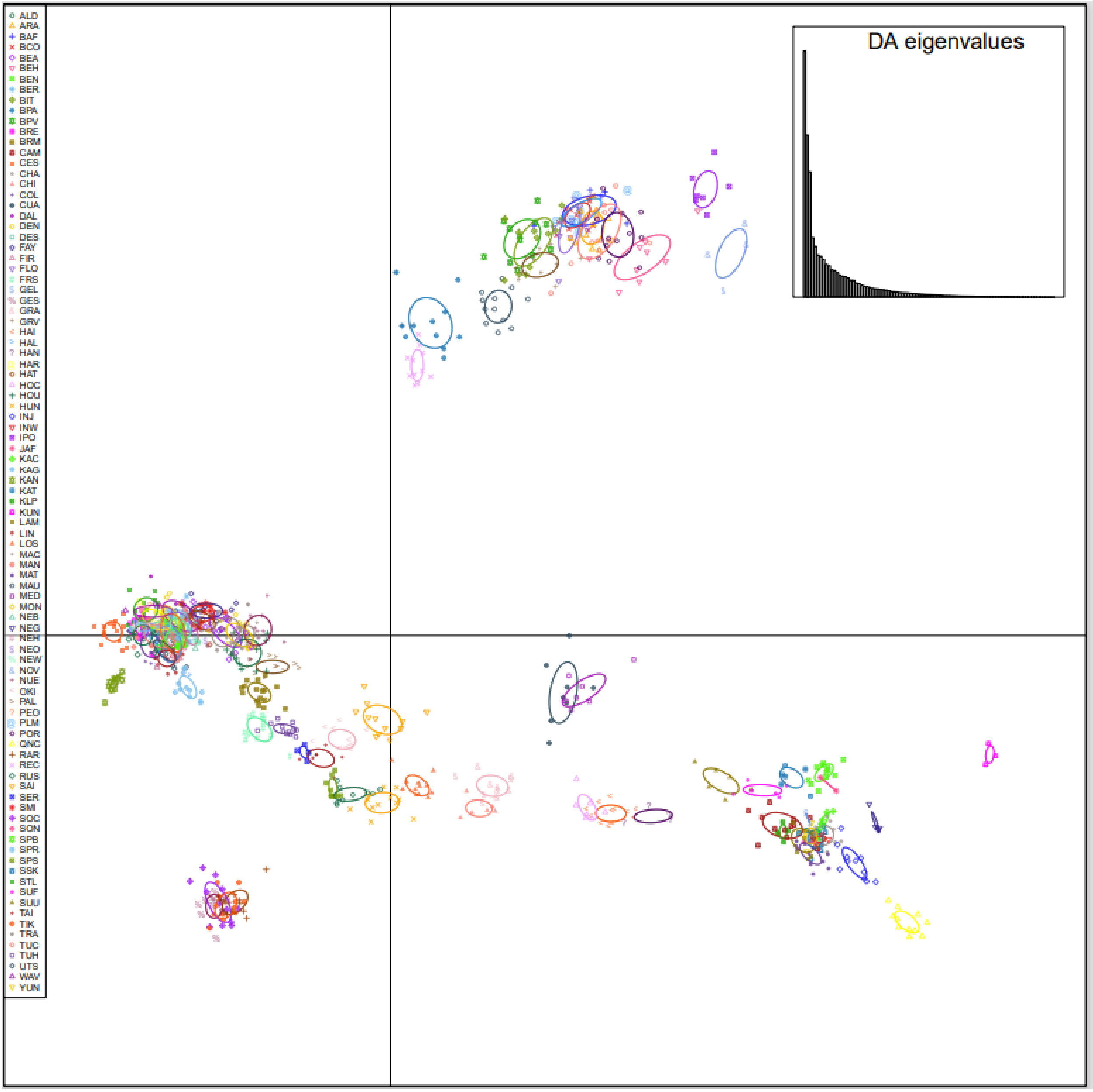

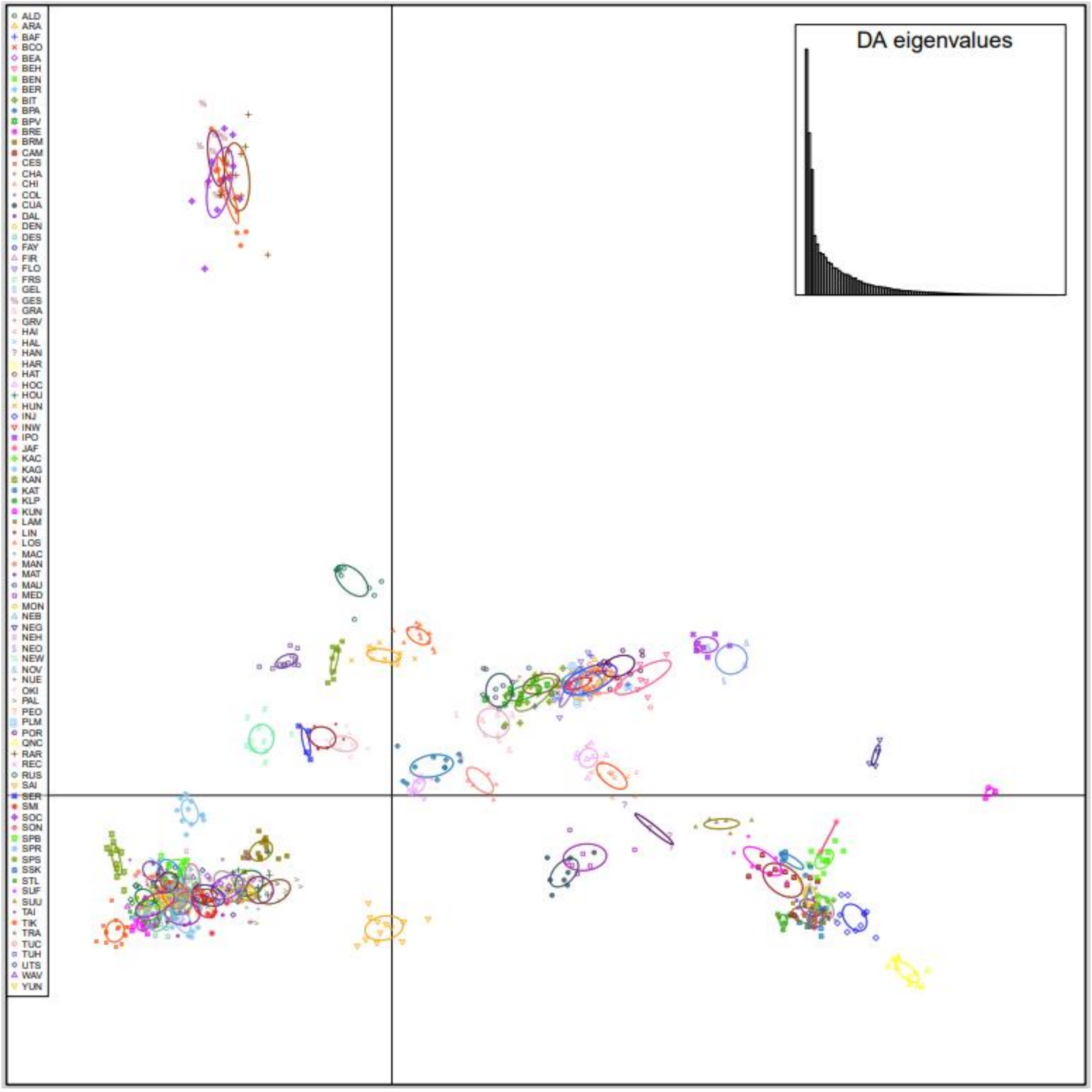

Figure S3. Scatterplots of the principal components (PCs) derived from DAPC for the Global dataset, showing each location in a different color. PCs 1 and 2 are shown in the top panel, and PCs 1 and 3 in the bottom panel. Each symbol represents a mosquito, and its color and shape indicate the location where it was sampled. Points are plotted according to their scores on the discriminant functions, and axes are scaled to the eigenvalues, which represent the relative contribution of each function (shown in the inset bar graph) to the total genetic variance observed.

A)

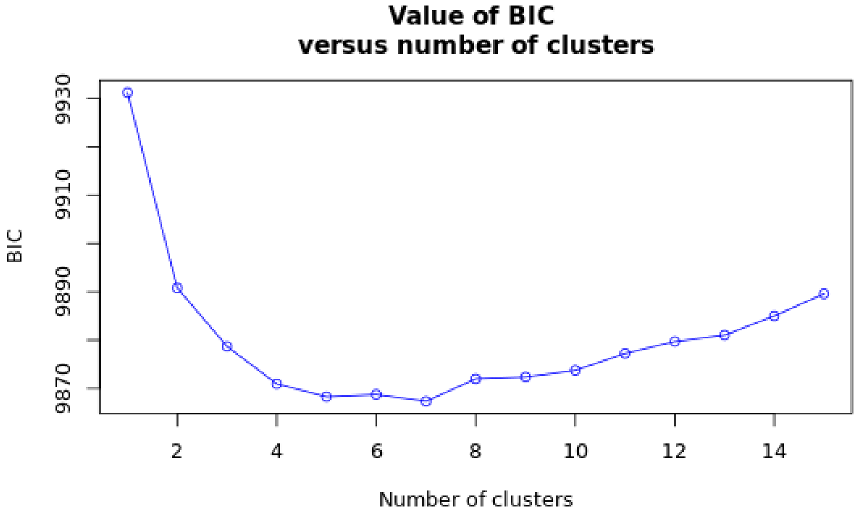
Value of BIC
versus number of clusters
BIC
9870
9890
9910
9930
2
4
6
8
10
12
14
Number of clusters

B)

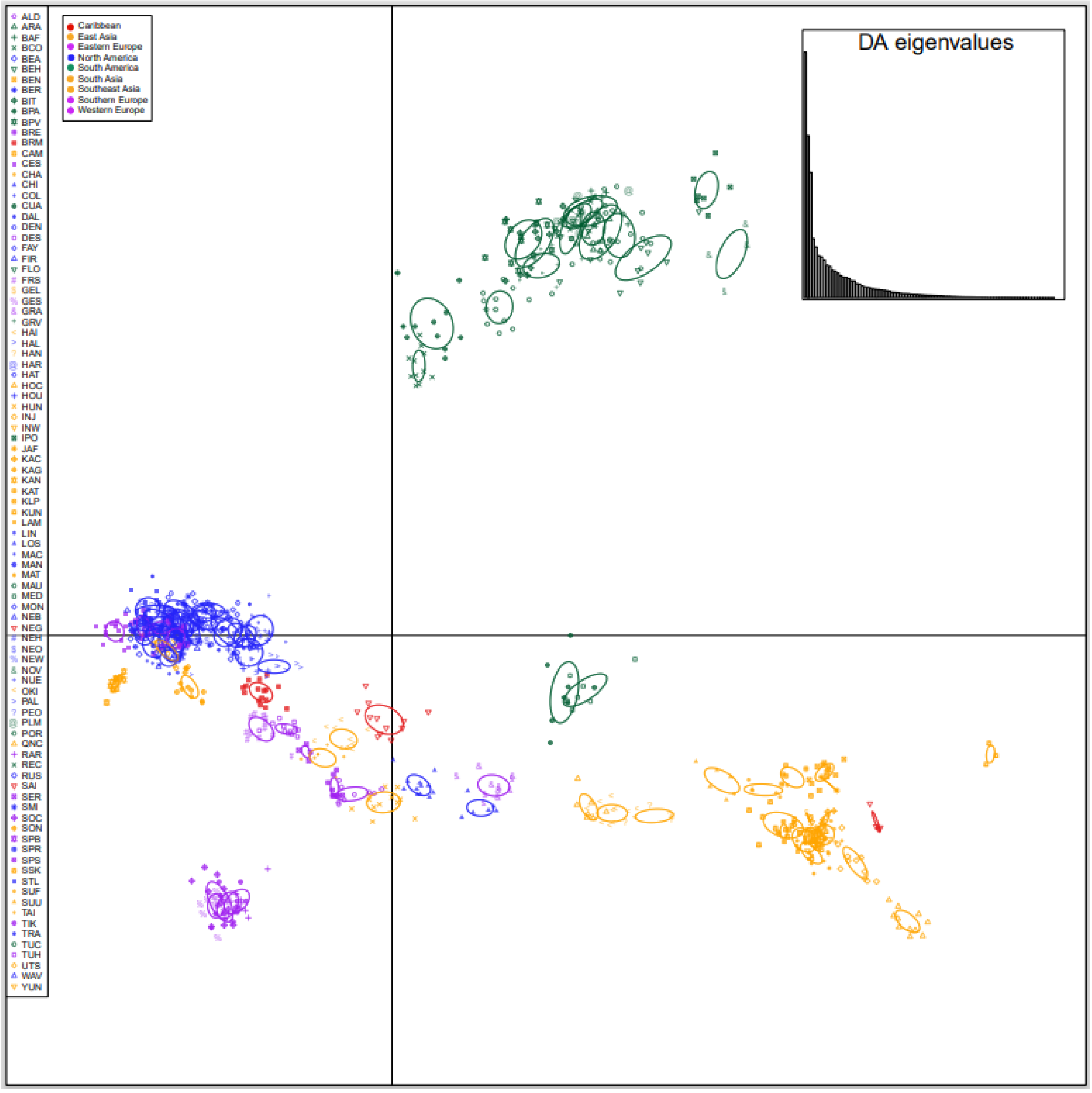
ALD
ARA
BAF
BCO
BEA
BEH
BEN
BER
BIT
BPA
BPV
BRE
BRM
CAM
CES
CHA
CHI
COL
CUA
DAL
DEN
DES
FAY
FIR
FLO
FRS
GEL
GES
GRA
GRV
HAI
HAL
HAN
HAR
HAT
HOC
HOU
HUN
INJ
INW
IPO
JAF
KAC
KAG
KAN
KAT
KLP
KUN
LAM
LIN
LOS
MAC
MAN
MAT
MAU
MED
MON
NEB
NEG
NEH
NEO
NEW
NOV
NUE
OKI
PAL
PEO
PLM
POR
QNC
RAR
REC
RUS
SAI
SER
SMI
SOC
SON
SPB
SPR
SPS
SSK
STL
SUF
SUU
TAI
TIK
TRA
TUC
TUH
UTS
WAV
YUN
Caribbean
East Asia
Eastern Europe
North America
South America
South Asia
Southeast Asia
Southern Europe
Western Europe
DA eigenvalues

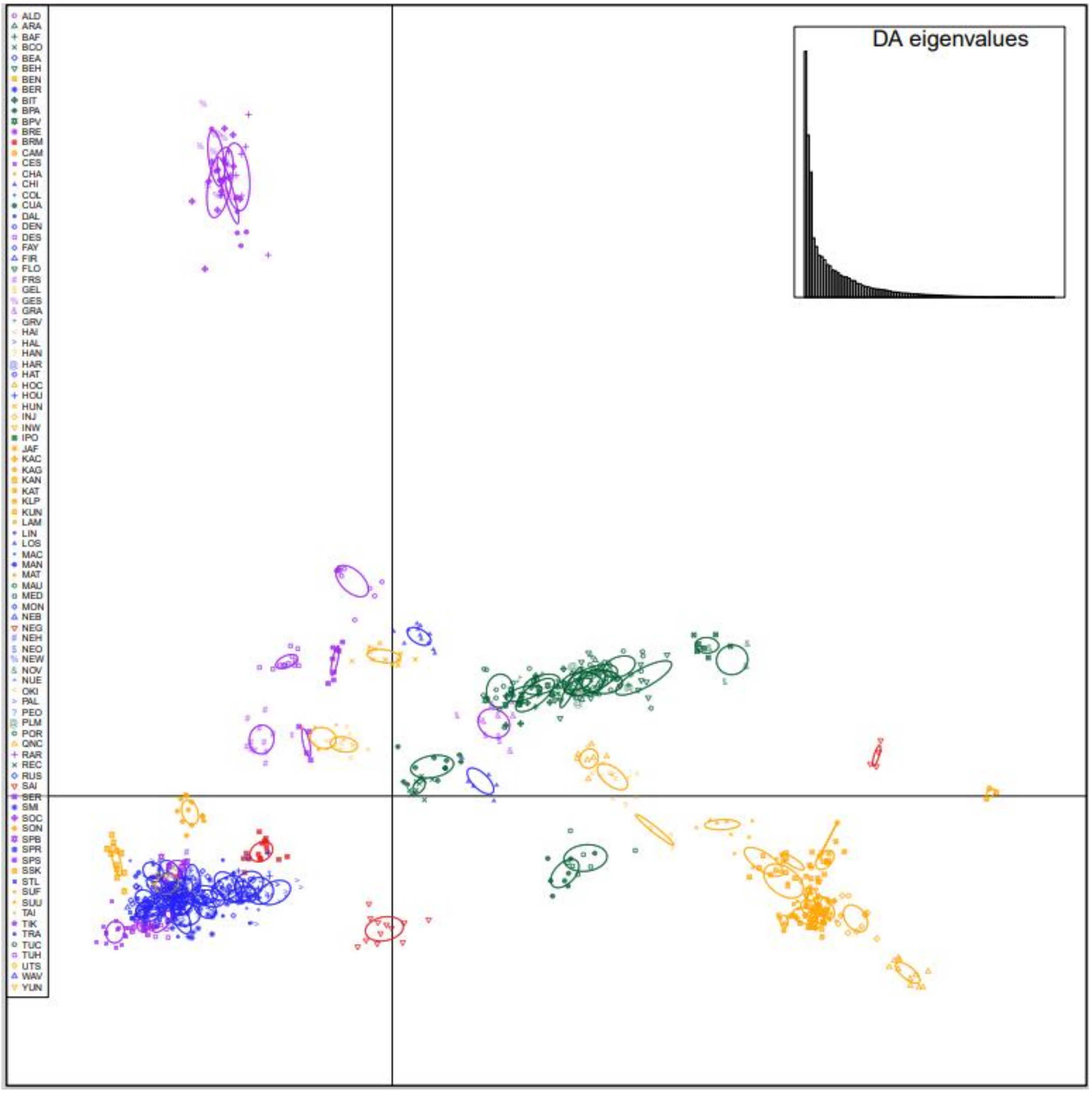
DA eigenvalues
ALD
ARA
BAF
BCO
BEA
BEH
BEN
BER
BIT
BPA
BPV
BRE
BRM
CAM
CES
CHA
CHI
COL
CUA
DAL
DEN
DES
FAY
FIR
FLO
FRS
GEL
GES
GRA
GRV
HAI
HAL
HAN
HAR
HAT
HOC
HOU
HUN
INJ
INW
IPO
JAF
KAC
KAG
KAN
KAT
KLP
KUN
LAM
LIN
LOS
MAC
MAN
MAT
MAU
MED
MON
NEB
NEG
NEH
NEO
NEW
NOV
NUE
OKI
PAL
PEO
PLM
POR
QNC
RAR
REC
RUS
SAI
SER
SMI
SOC
SON
SPB
SPR
SPS
SSK
STL
SUF
SUU
TAI
TIK
TRA
TUC
TUH
UTS
WAV
YUN

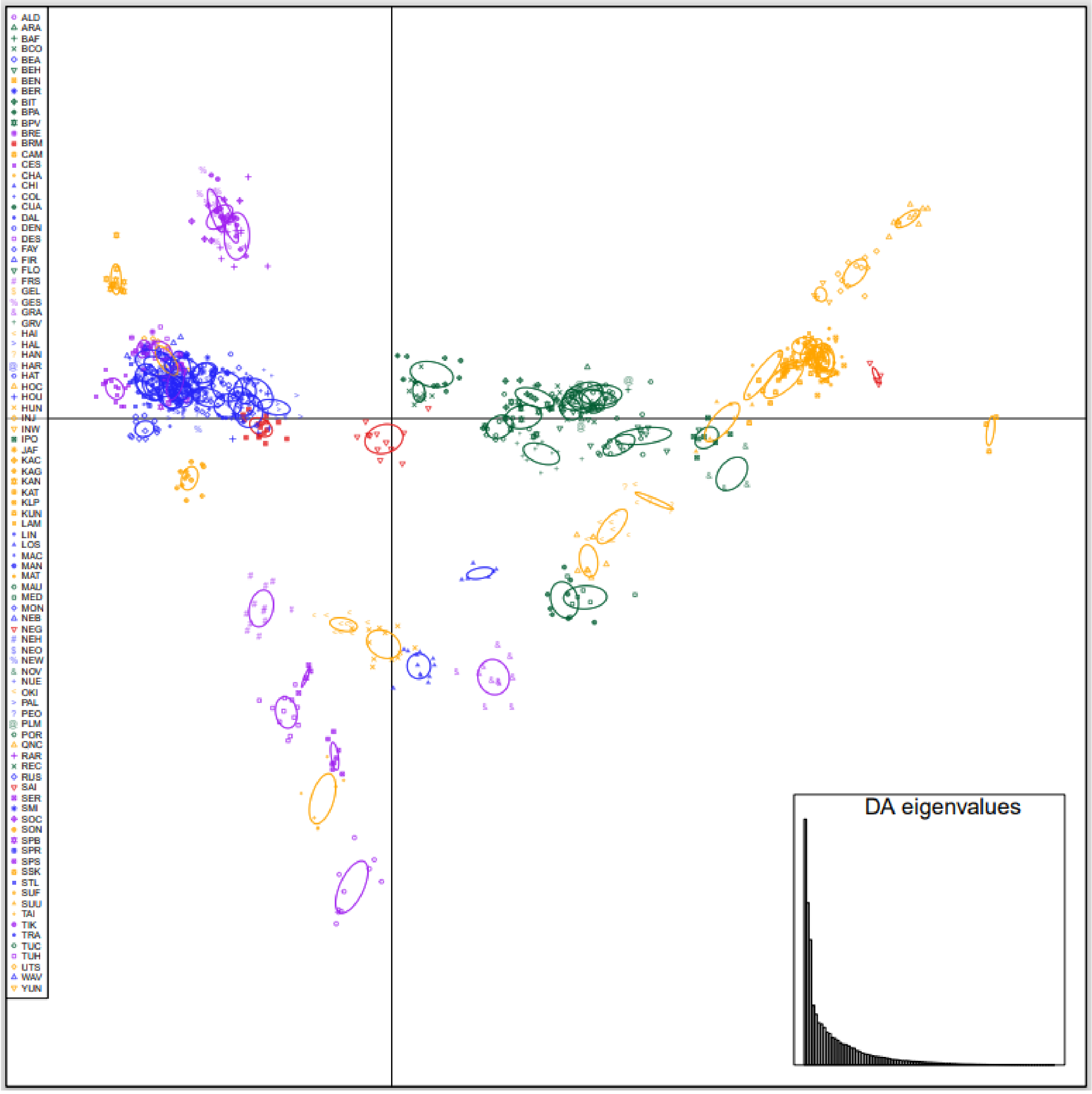

Figure S4. DAPC for the Global dataset. A) shows the plot indicating the best number of clusters (7). B) Scatterplots of the principal components derived from DAPC using the Global dataset showing each region in a different color. PCs 1 and 2 are shown in the top panel, PCs 1 and 3 in the center panel, and PCs 1 and 4 in the bottom panel. Each symbol represents a mosquito, and its color and shape indicate the location where it was sampled. Points are plotted according to scores on the discriminant functions, and axes are scaled to the eigenvalues, which represent the relative contribution of each function (shown in the inset bar graph) to the total genetic variance observed.

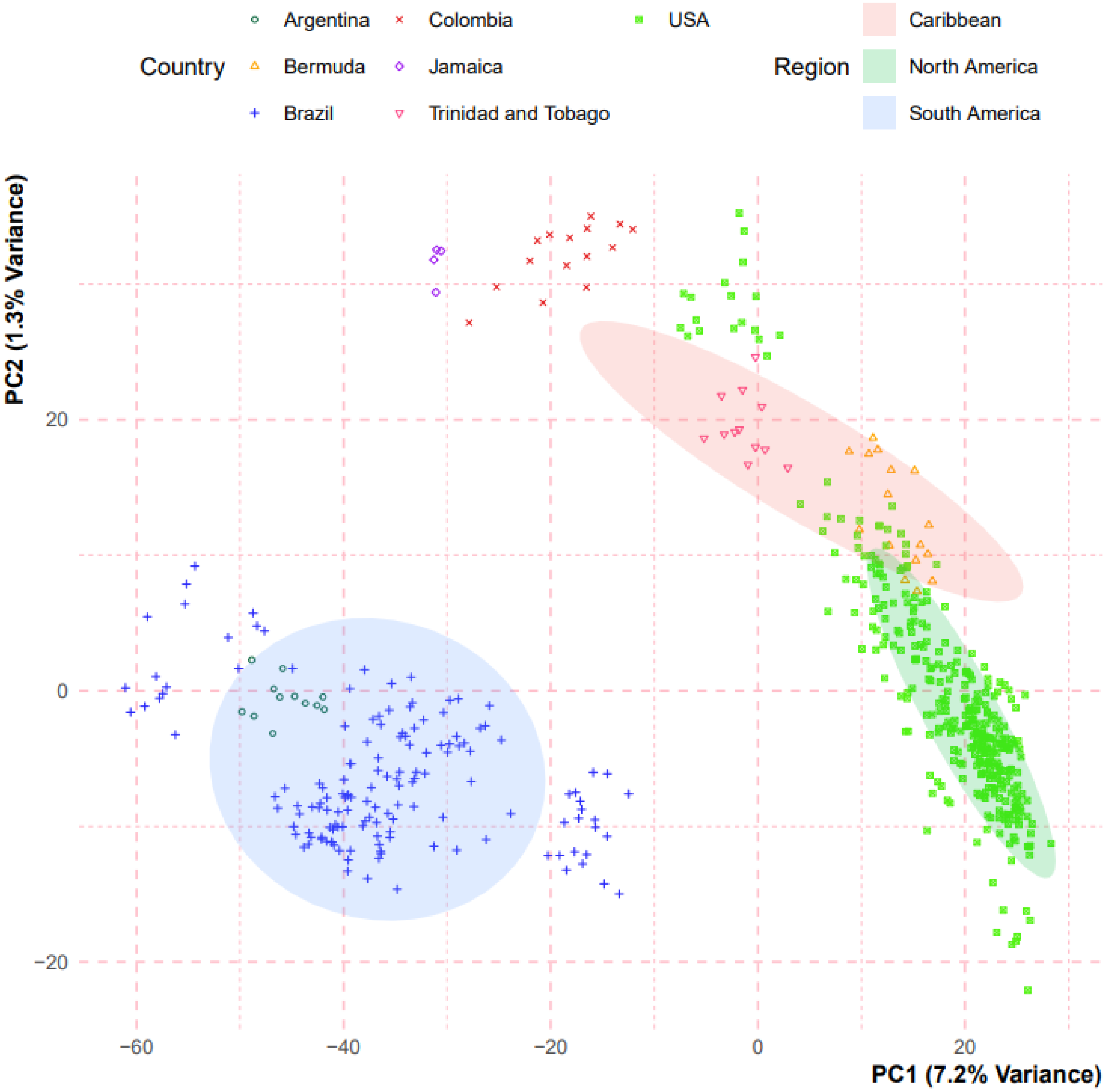

PCA with 18,964 SNPs of 545 mosquitoes from 50 localities in the Americas.

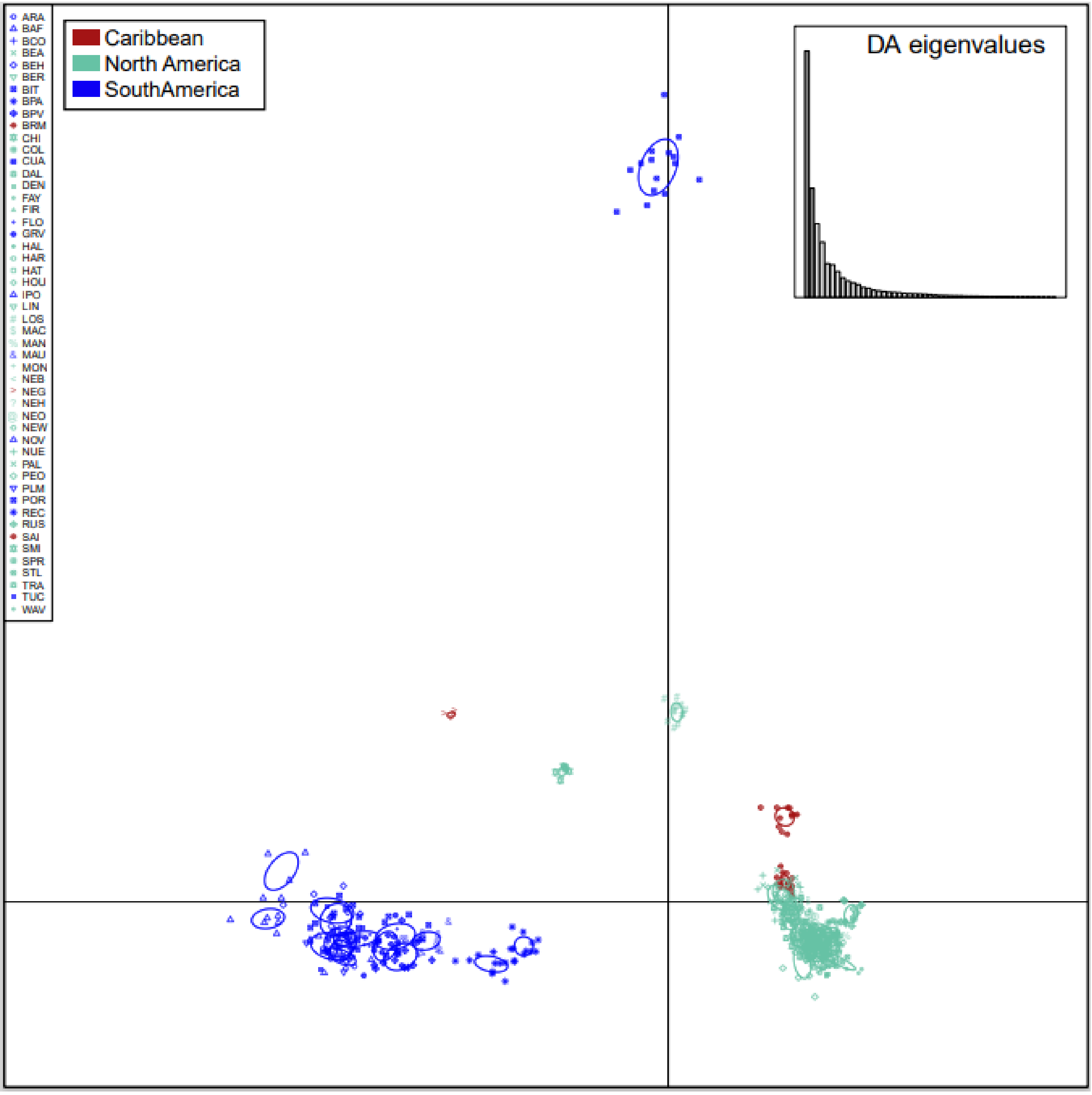

Figure S5. Scatterplots of principal component analysis (PCA, top) and Discriminant Analysis of Principal components (DAPC, bottom) for 545 *Ae. albopictus* mosquitos from 50 sampling sites in the American dataset. In both plots the x-axis is PC 1 and the y-axis is PC 2. Each symbol represents a mosquito, and its shape indicates the location where it was sampled. For the PCA (top), ellipses indicate the area covering 80% of the samples. For DAPC (bottom), the color of the symbols indicates the region where the mosquitoes were sampled. The points are plotted according to scores on the discriminant functions, and axes are scaled to the eigenvalues, which represent the relative contribution of each function (shown in the inset bar graph) to the total genetic variance observed. Additional plots and details of PCA and DAPC analyses are shown in Supplementary File S8.

A)

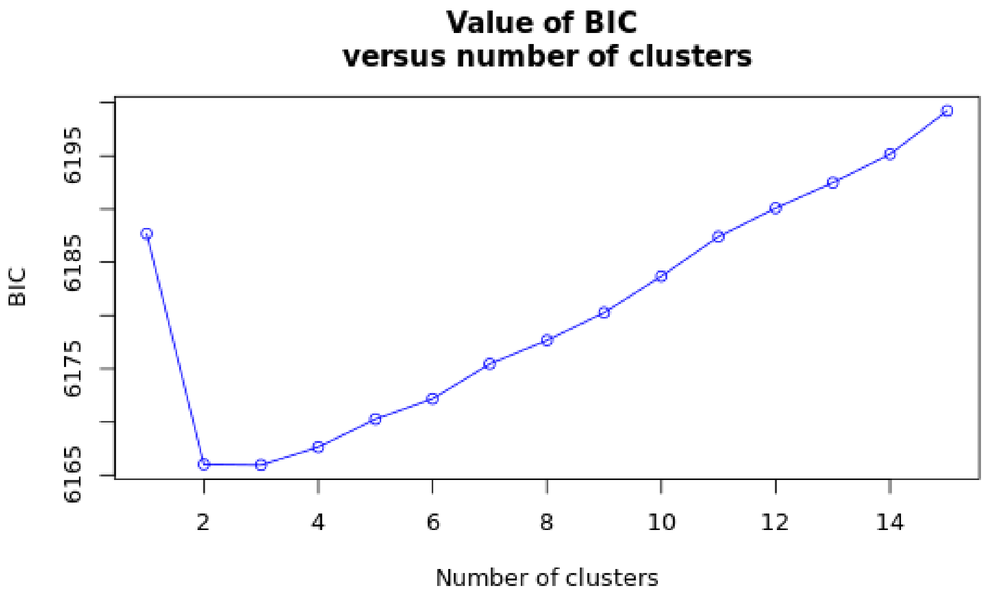
Value of BIC
versus number of clusters
BIC
6165
6175
6185
6195
2
4
6
8
10
12
14
Number of clusters

B)

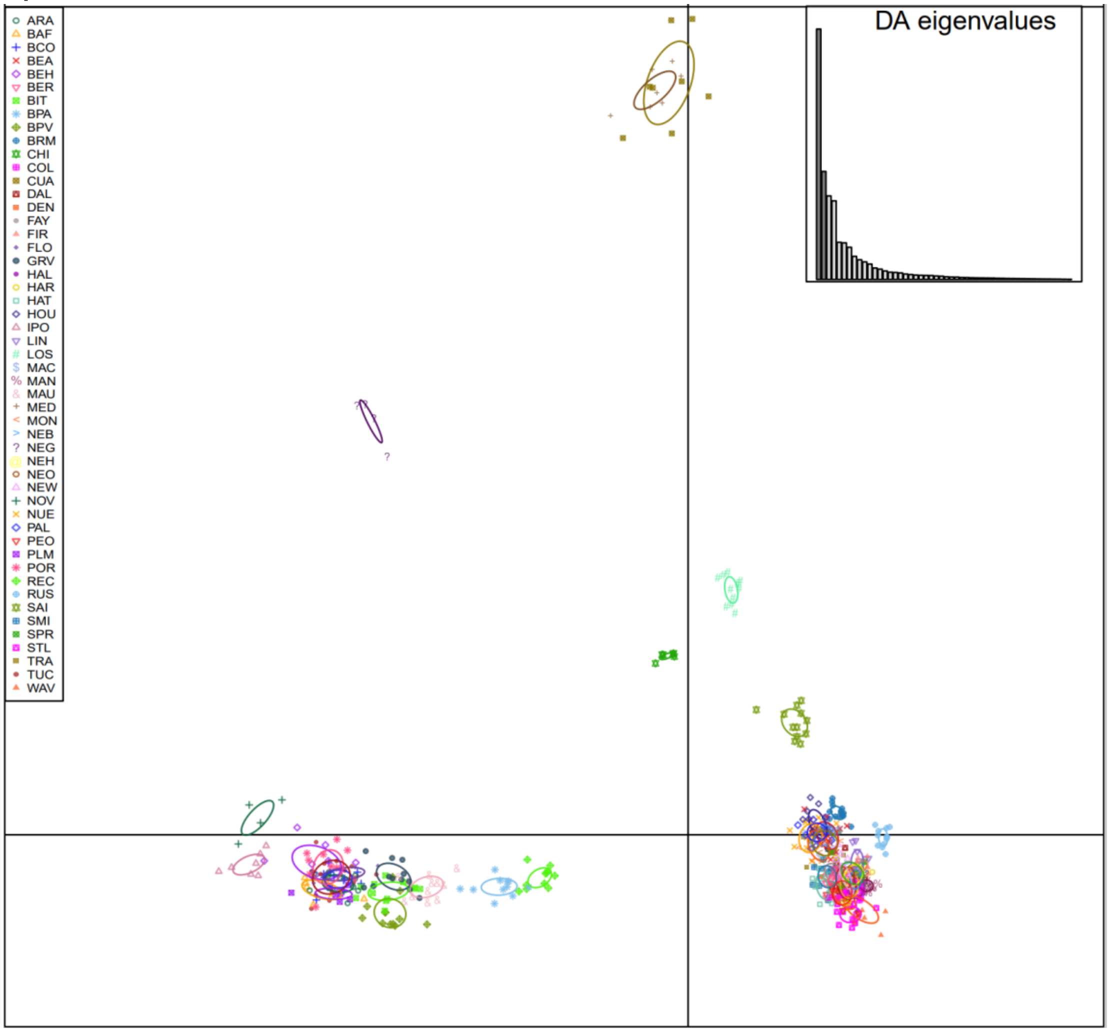
ARA
BAF
BCO
BEA
BEH
BER
BIT
BPA
BPV
BRM
CHI
COL
CUA
DAL
DEN
FAY
FIR
FLO
GRV
HAL
HAR
HAT
HOU
IPO
LIN
LOS
MAC
MAN
MAU
MED
MON
NEB
NEG
NEH
NEO
NEW
NOV
NUE
PAL
PEO
PLM
POR
REC
RUS
SAI
SMI
SPR
STL
TRA
TUC
WAV
DA eigenvalues

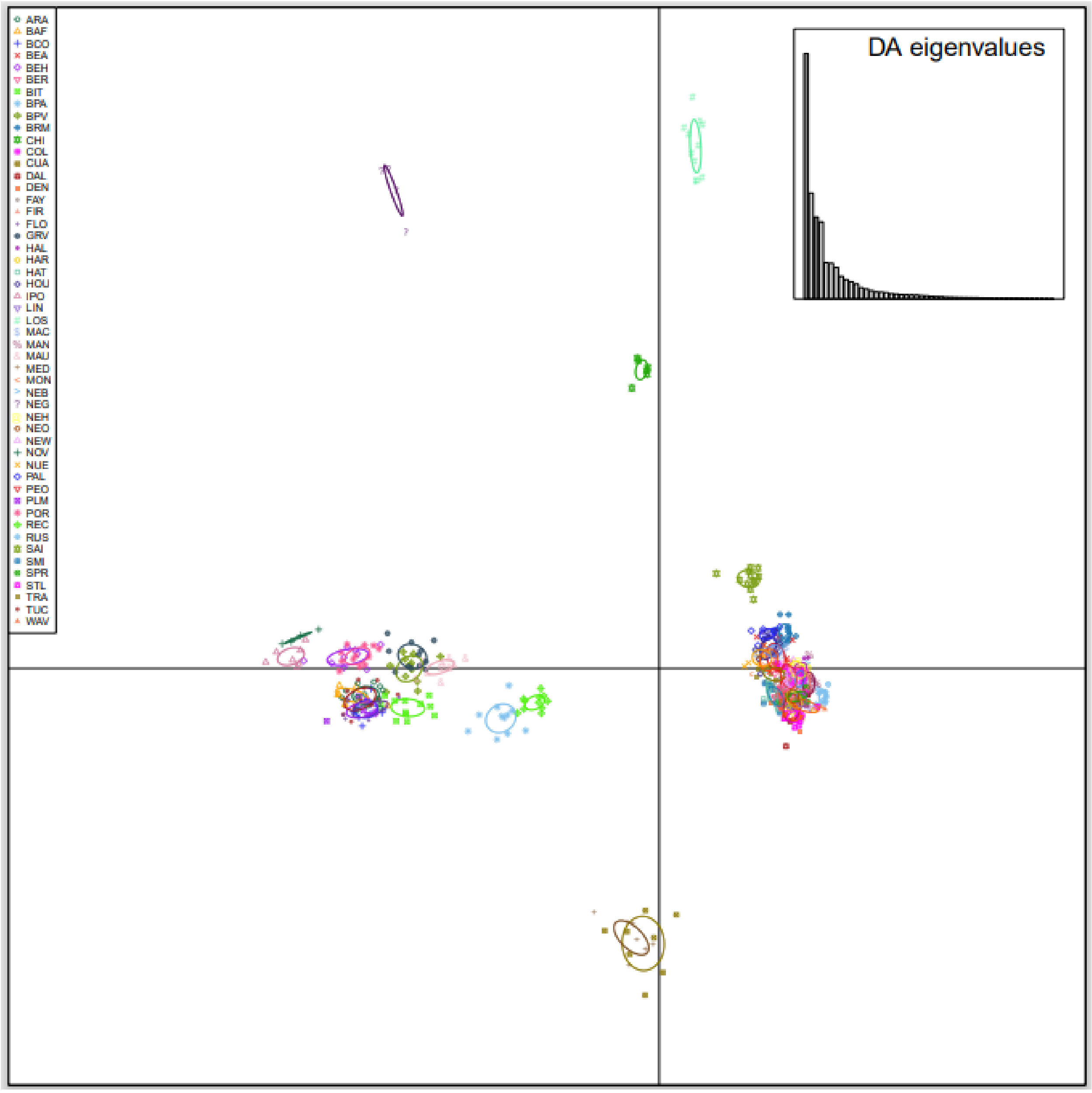
ARA
BAF
BOO
BEA
BEH
BER
BIT
BPA
BPV
BRM
CHI
COL
CUA
DAL
DEN
FAY
FIR
FLO
GRV
HAL
HAR
HAT
HOU
IPO
LIN
LOS
MAC
MAN
MAU
MED
MON
NEB
NEG
NEH
NEO
NEW
NOV
NUE
PAL
PEO
PLM
POR
REC
RUS
SAI
SMI
SPR
STL
TRA
TUC
WAV
DA eigenvalues

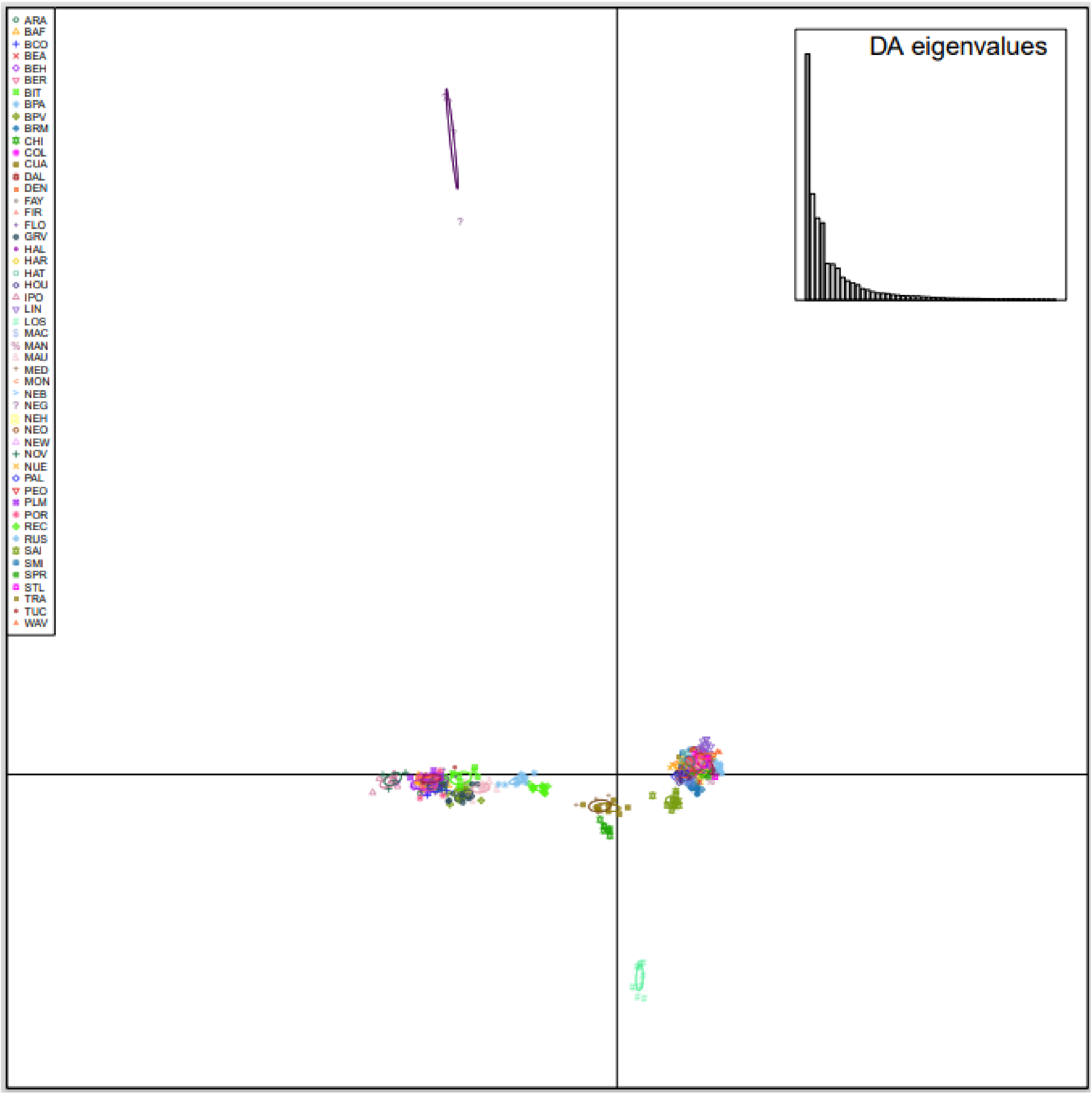

Figure S6. DAPC for the American dataset. A) shows the plot indicating the best number of clusters (3). B) shows scatterplots of the principal components derived from DAPC using the American dataset. PCs 1 and 2 are shown in the top panel, PCs 1 and 3 in the center panel, and PCs 1 and 4 in the bottom panel. Each symbol represents a mosquito, and its color and shape indicate the location where it was sampled. Points are plotted according to scores on the discriminant functions, and axes are scaled to the eigenvalues, which represent the relative contribution of each function (shown in the inset bar graph) to the total genetic variance observed.

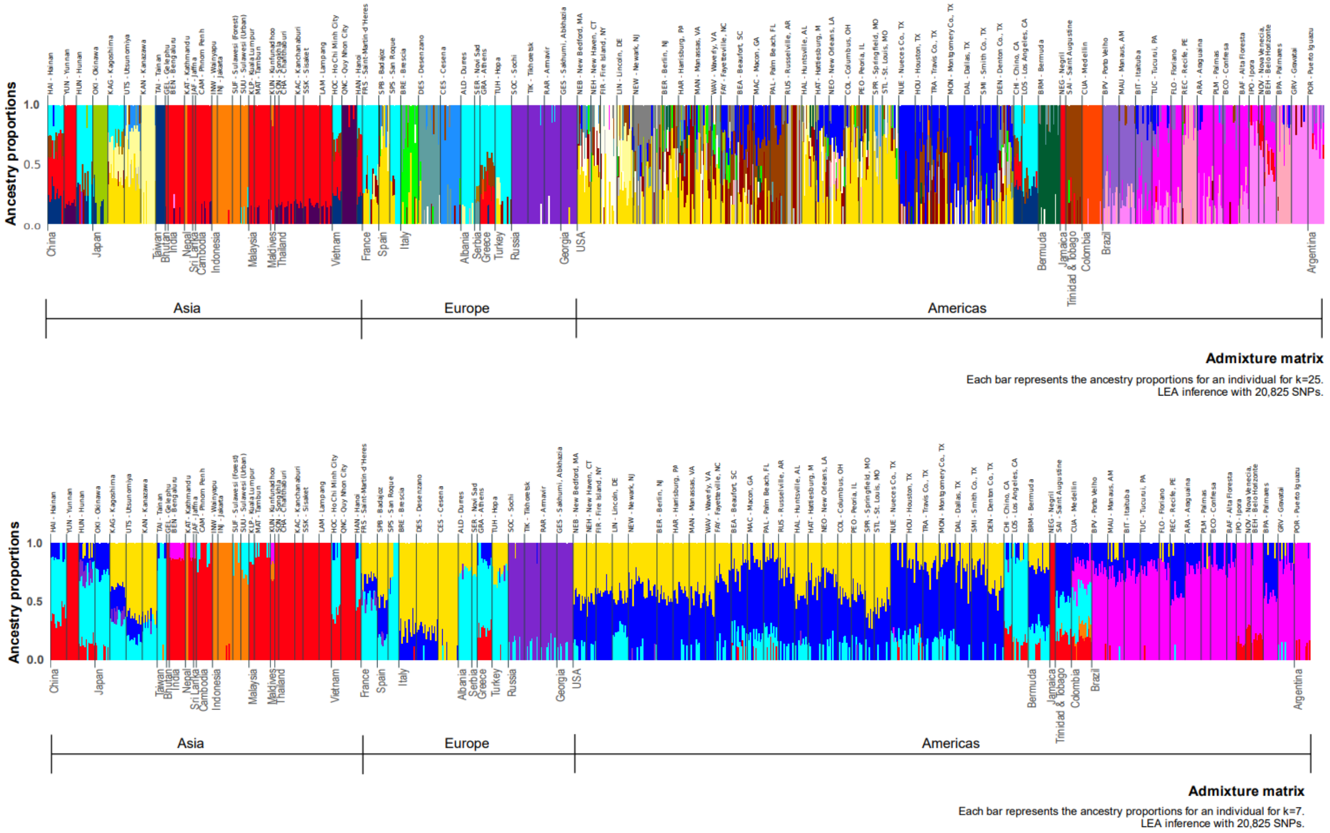

Figure S7. Plots showing the population structure of 933 *Ae. albopictus* mosquitos from 92 sampling sites in the Global dataset. Plots were created from Q matrices generated in LEA representing K=25 (top panel) and K=7 (bottom panel). In all plots, each vertical bar on the x-axis represents one mosquito and the y-axis shows the proportion of admixture for the ancestral genetic groups for each individual.

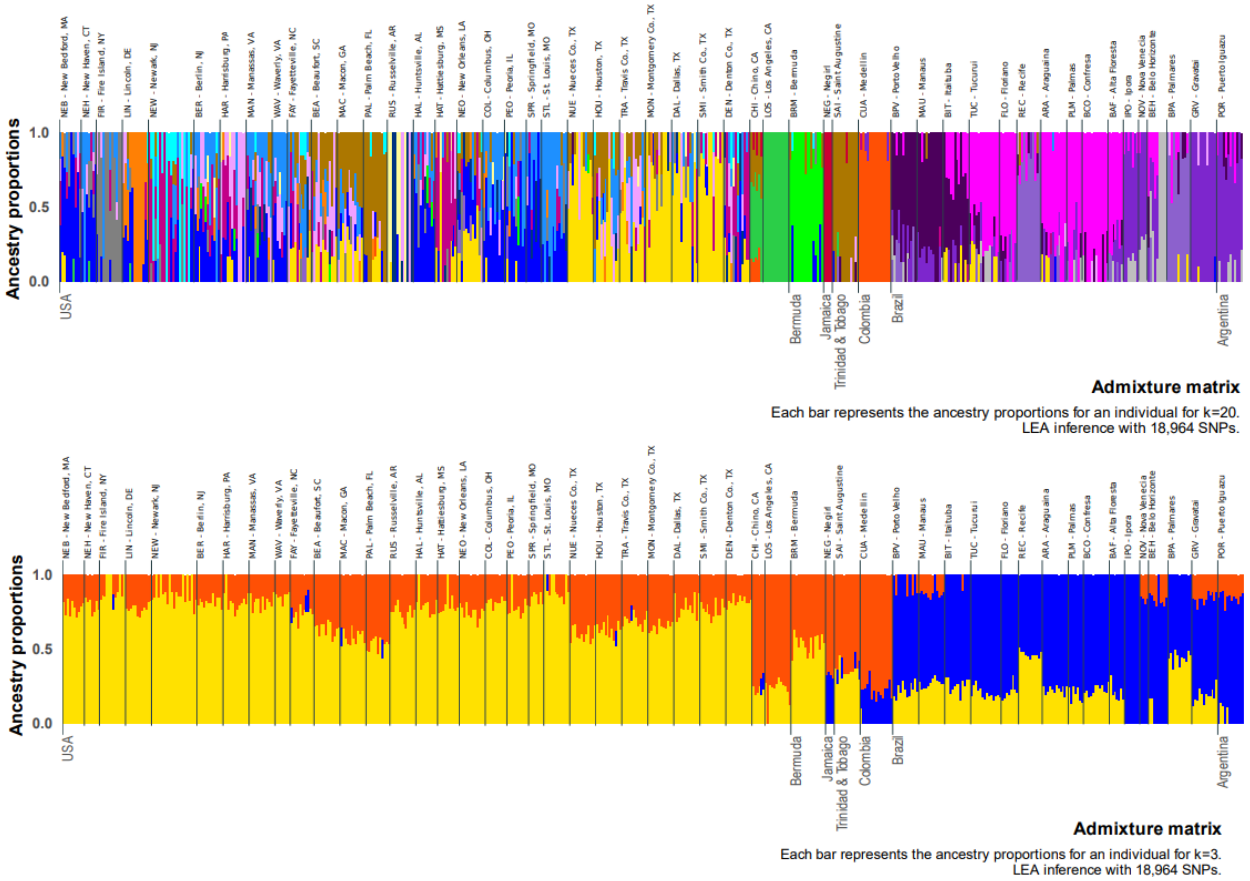

Figure S8. Populations structure of 545 Ae. albopictus mosquitos from 50 sampling sites in the American dataset. Plots were created from Q matrices generated in LEA representing K = 20 (top panel) and K = 3 (bottom panel). In all plots, each vertical bar on the x-axis represents one mosquito and the y-axis shows the proportion of admixture for the ancestral genetic groups for each individual.

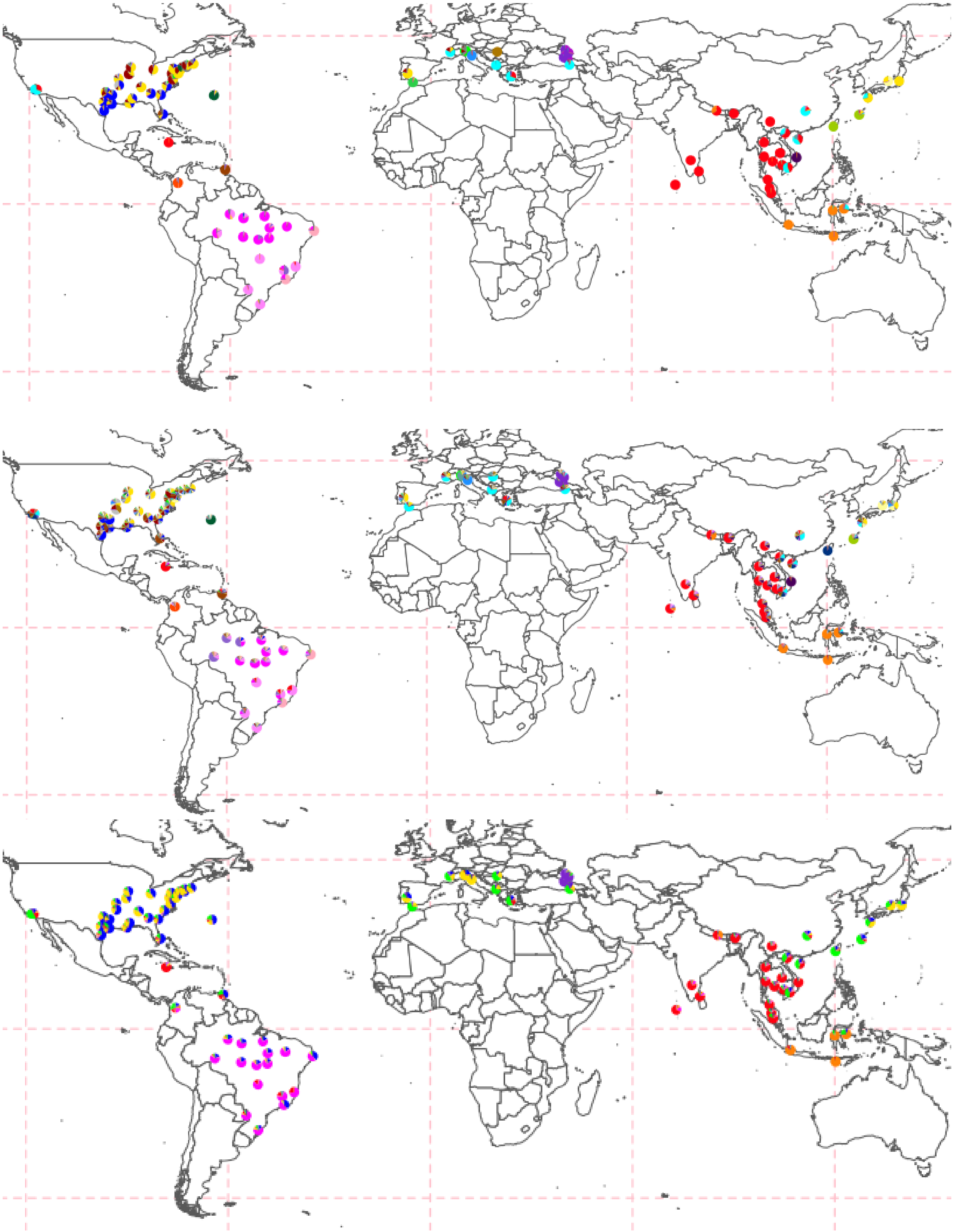

Figure S9. Map of the ancestry matrix for the Global dataset showing the best K obtained from fastStructure (K=27) in the top panel and from LEA (K=25) in the middle panel. The bottom panel shows the ancestry matrix from LEA for K=7 (the K=7 matrix from fastStructure is shown in Figure 3 of the main text). The colored pies at each sampling site reflect the proportions of clusters found at each location. See Figure 1 and Table S2 for full details on each sampling location.

A)

	China	Japan	Taiwan	India	Nepal	Cambodia	Indonesia	Malaysia	Thailand	Vietnam	France	Spain	Italy	Albania	Serbia	Greece	Turkey	Russia	Georgia	USA	Colombia	Bermuda	Jamaica	Trinidad and Tobago	Brazil	Argentina
Argentina	0.10	0.16	0.19	0.09	0.10	0.09	0.16	0.11	0.09	0.12	0.13	0.15	0.18	0.15	0.22	0.11	0.13	0.16	0.16	0.14	0.11	0.15	0.18	0.12	0.04	
Brazil	0.10	0.14	0.18	0.10	0.11	0.09	0.15	0.11	0.10	0.12	0.12	0.14	0.17	0.14	0.22	0.10	0.12	0.15	0.15	0.12	0.11	0.14	0.19	0.11		
Trinidad and Tobago	0.07	0.11	0.14	0.11	0.11	0.08	0.15	0.10	0.09	0.10	0.08	0.10	0.13	0.11	0.18	0.06	0.08	0.12	0.11	0.08	0.10	0.10	0.19			
Jamaica	0.16	0.23	0.25	0.13	0.14	0.13	0.20	0.15	0.13	0.16	0.21	0.22	0.25	0.22	0.29	0.17	0.20	0.22	0.22	0.22	0.18	0.22				
Bermuda	0.11	0.11	0.16	0.15	0.15	0.13	0.19	0.15	0.14	0.13	0.09	0.11	0.12	0.12	0.19	0.10	0.10	0.13	0.13	0.08	0.13					
Colombia	0.09	0.14	0.17	0.10	0.10	0.09	0.15	0.11	0.09	0.11	0.11	0.13	0.16	0.13	0.20	0.09	0.11	0.14	0.14	0.12						
USA	0.09	0.09	0.15	0.13	0.14	0.11	0.18	0.14	0.13	0.12	0.06	0.08	0.09	0.10	0.17	0.08	0.07	0.11	0.11							
Georgia	0.10	0.12	0.16	0.15	0.15	0.13	0.19	0.15	0.14	0.13	0.09	0.11	0.15	0.12	0.20	0.10	0.10	0.03								
Russia	0.10	0.12	0.16	0.15	0.15	0.12	0.19	0.15	0.14	0.13	0.09	0.12	0.15	0.12	0.20	0.10	0.10									
Turkey	0.07	0.09	0.14	0.12	0.13	0.10	0.17	0.12	0.11	0.11	0.04	0.08	0.11	0.08	0.16	0.06										
Greece	0.05	0.10	0.13	0.09	0.09	0.06	0.13	0.08	0.07	0.08	0.06	0.09	0.13	0.07	0.17											
Serbia	0.18	0.19	0.23	0.21	0.21	0.20	0.25	0.21	0.21	0.20	0.16	0.18	0.20	0.18												
Albania	0.10	0.11	0.15	0.14	0.14	0.12	0.18	0.14	0.13	0.12	0.08	0.10	0.14													
Italy	0.14	0.13	0.18	0.18	0.18	0.16	0.22	0.18	0.17	0.16	0.10	0.12														
Spain	0.10	0.10	0.15	0.15	0.15	0.13	0.19	0.15	0.14	0.13	0.07															
France	0.08	0.08	0.14	0.13	0.13	0.10	0.17	0.13	0.12	0.11																
Vietnam	0.07	0.13	0.15	0.08	0.08	0.06	0.13	0.09	0.07																	
Thailand	0.05	0.14	0.16	0.03	0.04	0.01	0.11	0.04																		
Malaysia	0.07	0.16	0.17	0.06	0.06	0.04	0.12																			
Indonesia	0.13	0.20	0.21	0.11	0.07	0.10																				
Cambodia	0.04	0.13	0.15	0.03	0.04																					
Nepal	0.08	0.15	0.17	0.04																						
India	0.06	0.15	0.17																							
Taiwan	0.13	0.15																								
Japan	0.10																									
China																										

Fst
0.5
0.4
0.3
0.2
0.1
0.0

B)

	NEB	NEH	FIR	LIN	NEW	BER	HAR	MAN	WAV	FAY	BEA	MAC	PAL	RUS	HAL	HAT	NEO	COL	PEO	SPR	STL	NUE	HOU	TRA	MON	DAL
POR	0.18	0.18	0.21	0.19	0.18	0.18	0.18	0.17	0.19	0.16	0.14	0.15	0.16	0.19	0.17	0.17	0.17	0.18	0.17	0.20	0.19	0.15	0.14	0.15	0.15	0.17
GRV	0.15	0.14	0.18	0.17	0.16	0.15	0.15	0.14	0.16	0.13	0.12	0.13	0.13	0.16	0.14	0.14	0.14	0.15	0.14	0.17	0.16	0.12	0.11	0.12	0.13	0.14
BPA	0.12	0.11	0.14	0.13	0.12	0.12	0.12	0.10	0.12	0.09	0.08	0.09	0.10	0.13	0.11	0.11	0.10	0.12	0.10	0.14	0.13	0.09	0.08	0.09	0.09	0.10
BEH	0.19	0.19	0.22	0.20	0.19	0.19	0.19	0.18	0.20	0.17	0.15	0.15	0.16	0.20	0.18	0.18	0.18	0.19	0.17	0.22	0.20	0.15	0.14	0.15	0.16	0.18
NOV	0.22	0.21	0.25	0.23	0.21	0.22	0.22	0.20	0.23	0.19	0.17	0.18	0.18	0.23	0.20	0.20	0.20	0.22	0.20	0.25	0.23	0.17	0.16	0.18	0.18	0.20
IPO	0.24	0.23	0.26	0.24	0.23	0.23	0.23	0.22	0.25	0.21	0.19	0.20	0.20	0.25	0.22	0.22	0.22	0.24	0.22	0.27	0.25	0.20	0.18	0.20	0.20	0.22
BAF	0.16	0.14	0.18	0.17	0.15	0.15	0.15	0.14	0.16	0.12	0.11	0.12	0.13	0.17	0.14	0.14	0.14	0.15	0.13	0.18	0.16	0.12	0.11	0.12	0.12	0.14
BCO	0.16	0.15	0.19	0.17	0.16	0.16	0.16	0.15	0.16	0.13	0.12	0.13	0.14	0.17	0.15	0.15	0.15	0.16	0.14	0.18	0.17	0.13	0.12	0.13	0.13	0.15
PLM	0.16	0.16	0.19	0.18	0.16	0.16	0.16	0.15	0.17	0.13	0.12	0.12	0.14	0.18	0.15	0.15	0.15	0.16	0.14	0.18	0.17	0.13	0.12	0.13	0.13	0.15
ARA	0.15	0.14	0.18	0.16	0.15	0.15	0.15	0.14	0.15	0.12	0.11	0.12	0.13	0.16	0.14	0.14	0.14	0.15	0.13	0.17	0.16	0.12	0.11	0.12	0.11	0.13
REC	0.11	0.11	0.14	0.12	0.11	0.11	0.11	0.10	0.11	0.09	0.08	0.08	0.10	0.13	0.10	0.10	0.10	0.11	0.09	0.13	0.12	0.09	0.08	0.08	0.08	0.09
FLO	0.16	0.15	0.19	0.18	0.16	0.16	0.16	0.15	0.16	0.13	0.12	0.13	0.14	0.18	0.15	0.15	0.15	0.16	0.15	0.19	0.17	0.13	0.12	0.13	0.13	0.15
TUC	0.16	0.15	0.18	0.17	0.16	0.16	0.16	0.15	0.16	0.13	0.12	0.13	0.13	0.17	0.14	0.14	0.14	0.16	0.14	0.18	0.17	0.12	0.12	0.12	0.12	0.14
BIT	0.15	0.15	0.18	0.16	0.15	0.15	0.15	0.14	0.16	0.13	0.12	0.12	0.13	0.16	0.14	0.13	0.14	0.15	0.14	0.17	0.16	0.11	0.11	0.12	0.11	0.13
MAU	0.13	0.13	0.17	0.14	0.13	0.13	0.13	0.12	0.13	0.11	0.10	0.10	0.11	0.15	0.12	0.12	0.12	0.13	0.12	0.15	0.14	0.11	0.09	0.10	0.11	0.12
BPV	0.16	0.15	0.19	0.16	0.16	0.15	0.16	0.14	0.16	0.13	0.12	0.12	0.13	0.17	0.14	0.14	0.14	0.15	0.14	0.17	0.16	0.12	0.11	0.12	0.12	0.14
MED	0.15	0.14	0.18	0.16	0.15	0.14	0.15	0.14	0.15	0.12	0.11	0.11	0.12	0.16	0.13	0.14	0.13	0.15	0.13	0.17	0.16	0.11	0.10	0.11	0.11	0.13
CUA	0.15	0.14	0.19	0.16	0.15	0.15	0.15	0.14	0.16	0.13	0.11	0.12	0.13	0.17	0.14	0.14	0.14	0.15	0.14	0.18	0.16	0.12	0.11	0.12	0.12	0.14
SAI	0.10	0.09	0.13	0.11	0.10	0.10	0.10	0.08	0.10	0.07	0.06	0.06	0.07	0.12	0.09	0.09	0.09	0.10	0.09	0.12	0.11	0.07	0.06	0.07	0.07	0.09
NEG	0.25	0.25	0.29	0.26	0.24	0.25	0.25	0.24	0.27	0.23	0.20	0.21	0.22	0.26	0.24	0.24	0.24	0.25	0.23	0.29	0.27	0.21	0.20	0.21	0.21	0.24
BRM	0.10	0.09	0.13	0.12	0.10	0.09	0.10	0.09	0.10	0.08	0.08	0.09	0.10	0.12	0.09	0.10	0.10	0.10	0.08	0.12	0.11	0.09	0.08	0.08	0.09	0.09
LOS	0.12	0.11	0.15	0.13	0.13	0.12	0.12	0.11	0.13	0.10	0.09	0.10	0.11	0.14	0.11	0.12	0.12	0.12	0.11	0.14	0.13	0.10	0.09	0.09	0.10	0.11
CHI	0.13	0.12	0.17	0.14	0.13	0.13	0.13	0.11	0.14	0.10	0.09	0.10	0.11	0.15	0.12	0.12	0.12	0.13	0.11	0.16	0.14	0.10	0.08	0.10	0.10	0.12
DEN	0.03	0.03	0.07	0.05	0.03	0.03	0.04	0.03	0.03	0.02	0.03	0.05	0.06	0.06	0.03	0.02	0.04	0.04	0.03	0.05	0.04	0.04	0.03	0.02	0.03	0.02
SMI	0.06	0.05	0.09	0.07	0.05	0.05	0.06	0.05	0.06	0.04	0.04	0.05	0.07	0.08	0.05	0.04	0.05	0.06	0.05	0.08	0.06	0.03	0.03	0.03	0.02	0.02
DAL	0.04	0.04	0.07	0.06	0.05	0.04	0.05	0.03	0.03	0.02	0.03	0.05	0.06	0.07	0.03	0.03	0.04	0.04	0.03	0.07	0.05	0.02	0.02	0.02	0.01	
MON	0.05	0.04	0.08	0.07	0.06	0.05	0.05	0.04	0.05	0.03	0.02	0.03	0.05	0.07	0.04	0.04	0.04	0.05	0.04	0.07	0.06	0.01	0.02	0.02		
TRA	0.04	0.03	0.08	0.06	0.04	0.04	0.04	0.03	0.04	0.02	0.01	0.03	0.05	0.06	0.04	0.03	0.03	0.04	0.03	0.06	0.05	0.02	0.01			
HOU	0.04	0.03	0.07	0.06	0.04	0.04	0.04	0.03	0.04	0.02	0.01	0.02	0.03	0.06	0.03	0.03	0.03	0.04	0.03	0.06	0.05	0.02				
NUE	0.06	0.05	0.09	0.08	0.07	0.06	0.06	0.05	0.06	0.03	0.03	0.03	0.05	0.08	0.05	0.05	0.05	0.06	0.05	0.08	0.07					
STL	0.05	0.04	0.08	0.07	0.06	0.04	0.06	0.04	0.04	0.05	0.05	0.06	0.09	0.08	0.04	0.06	0.06	0.04	0.04	0.06						
SPR	0.07	0.06	0.11	0.08	0.07	0.06	0.07	0.05	0.07	0.06	0.06	0.08	0.10	0.09	0.05	0.07	0.07	0.07	0.06							
PEO	0.04	0.03	0.07	0.05	0.05	0.04	0.04	0.03	0.03	0.03	0.03	0.05	0.06	0.06	0.02	0.04	0.04	0.03								
COL	0.05	0.04	0.08	0.06	0.06	0.05	0.05	0.03	0.04	0.04	0.05	0.06	0.08	0.08	0.02	0.05	0.06									
NEO	0.05	0.05	0.08	0.07	0.05	0.05	0.06	0.04	0.05	0.03	0.04	0.04	0.07	0.08	0.04	0.05										
HAT	0.05	0.04	0.08	0.07	0.04	0.05	0.04	0.04	0.04	0.03	0.03	0.05	0.07	0.07	0.04											
HAL	0.03	0.02	0.07	0.05	0.05	0.03	0.04	0.02	0.03	0.03	0.03	0.05	0.06	0.07												
RUS	0.08	0.07	0.11	0.10	0.08	0.08	0.08	0.06	0.08	0.07	0.06	0.08	0.10													
PAL	0.07	0.06	0.11	0.09	0.07	0.07	0.08	0.06	0.08	0.05	0.04	0.04														
MAC	0.06	0.05	0.09	0.07	0.06	0.05	0.06	0.04	0.05	0.03	0.02															
BEA	0.04	0.04	0.08	0.06	0.05	0.04	0.04	0.03	0.05	0.02																
FAY	0.04	0.03	0.07	0.05	0.04	0.04	0.04	0.02	0.03																	
WAV	0.04	0.02	0.07	0.07	0.05	0.04	0.05	0.02																		
MAN	0.03	0.02	0.06	0.05	0.04	0.03	0.04																			
HAR	0.05	0.03	0.08	0.07	0.05	0.05																				
BER	0.05	0.03	0.07	0.07	0.05																					
NEW	0.05	0.04	0.08	0.07																						
LIN	0.06	0.06	0.10																							
FIR	0.07	0.07																								
NEH	0.02																									
NEB																										

	SMI	DEN	CHI	LOS	BRM	NEG	SAI	CUA	MED	BPV	MAU	BIT	TUC	FLO	REC	ARA	PLM	BCO	BAF	IPO	NOV	BEH	BPA	GRV	POR
POR	0.17	0.17	0.15	0.16	0.19	0.21	0.14	0.13	0.13	0.06	0.06	0.07	0.05	0.06	0.11	0.05	0.05	0.06	0.04	0.04	0.04	0.05	0.11	0.03	
GRV	0.14	0.14	0.14	0.15	0.17	0.21	0.13	0.13	0.12	0.08	0.07	0.07	0.07	0.07	0.10	0.06	0.07	0.08	0.06	0.06	0.07	0.08	0.10		
BPA	0.10	0.10	0.13	0.14	0.14	0.22	0.11	0.14	0.13	0.08	0.08	0.08	0.07	0.07	0.03	0.06	0.07	0.08	0.06	0.13	0.12	0.10			
BEH	0.18	0.18	0.16	0.17	0.20	0.21	0.14	0.13	0.13	0.06	0.07	0.07	0.05	0.06	0.10	0.05	0.05	0.06	0.05	0.06	0.04				
NOV	0.21	0.21	0.17	0.18	0.22	0.25	0.16	0.15	0.14	0.08	0.08	0.09	0.07	0.08	0.13	0.07	0.06	0.08	0.06	0.04					
IPO	0.22	0.22	0.20	0.21	0.24	0.26	0.18	0.17	0.17	0.09	0.09	0.09	0.07	0.08	0.14	0.07	0.07	0.09	0.06						
BAF	0.14	0.14	0.14	0.15	0.17	0.21	0.12	0.12	0.12	0.05	0.05	0.05	0.01	0.01	0.06	0.00	0.01	0.02							
BCO	0.15	0.15	0.15	0.16	0.17	0.21	0.13	0.13	0.13	0.07	0.07	0.06	0.02	0.03	0.08	0.02	0.02								
PLM	0.15	0.15	0.15	0.15	0.17	0.21	0.13	0.13	0.13	0.06	0.06	0.06	0.02	0.02	0.08	0.02									
ARA	0.13	0.14	0.14	0.14	0.16	0.20	0.12	0.12	0.12	0.06	0.06	0.04	0.01	0.02	0.07										
REC	0.10	0.10	0.13	0.13	0.14	0.21	0.10	0.13	0.13	0.07	0.08	0.09	0.07	0.08											
FLO	0.15	0.15	0.15	0.16	0.17	0.22	0.13	0.13	0.13	0.07	0.06	0.06	0.02												
TUC	0.14	0.15	0.14	0.15	0.17	0.20	0.12	0.12	0.12	0.06	0.06	0.04													
BIT	0.13	0.14	0.14	0.15	0.17	0.21	0.12	0.13	0.13	0.05	0.05														
MAU	0.13	0.12	0.13	0.13	0.15	0.20	0.11	0.11	0.11	0.04															
BPV	0.14	0.14	0.14	0.15	0.17	0.21	0.12	0.13	0.13																
MED	0.14	0.14	0.12	0.13	0.16	0.21	0.11	0.00																	
CUA	0.14	0.14	0.13	0.14	0.16	0.22	0.12																		
SAI	0.09	0.09	0.10	0.11	0.12	0.20																			
NEG	0.24	0.24	0.23	0.22	0.26																				
BRM	0.10	0.09	0.15	0.14																					
LOS	0.12	0.11	0.10																						
CHI	0.12	0.12																							
DEN	0.04																								

C)

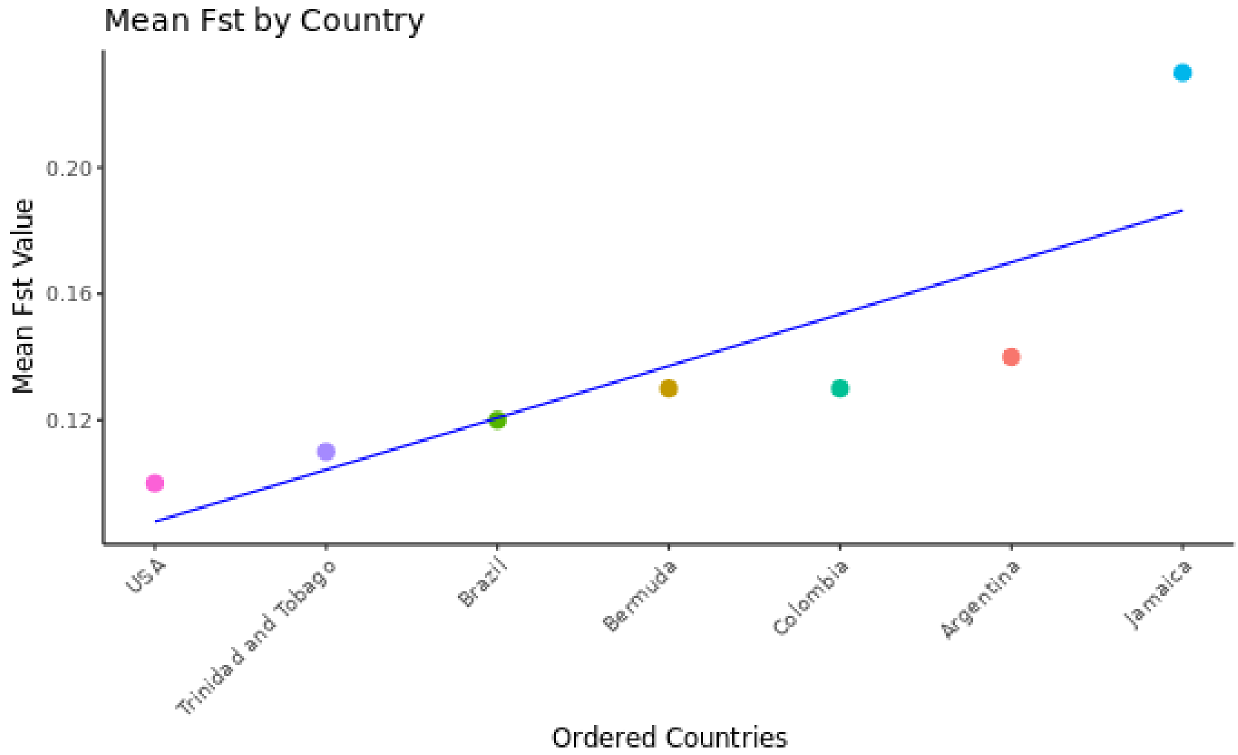

Figure S10. A) Pair-wise F_{ST} for each country in the Global dataset. B) Pair-wise F_{ST} for each locality in the American dataset. C) Mean pair-wise F_{ST} for countries in the American dataset. All F_{ST} estimates were calculated with the R package "StAMPP".

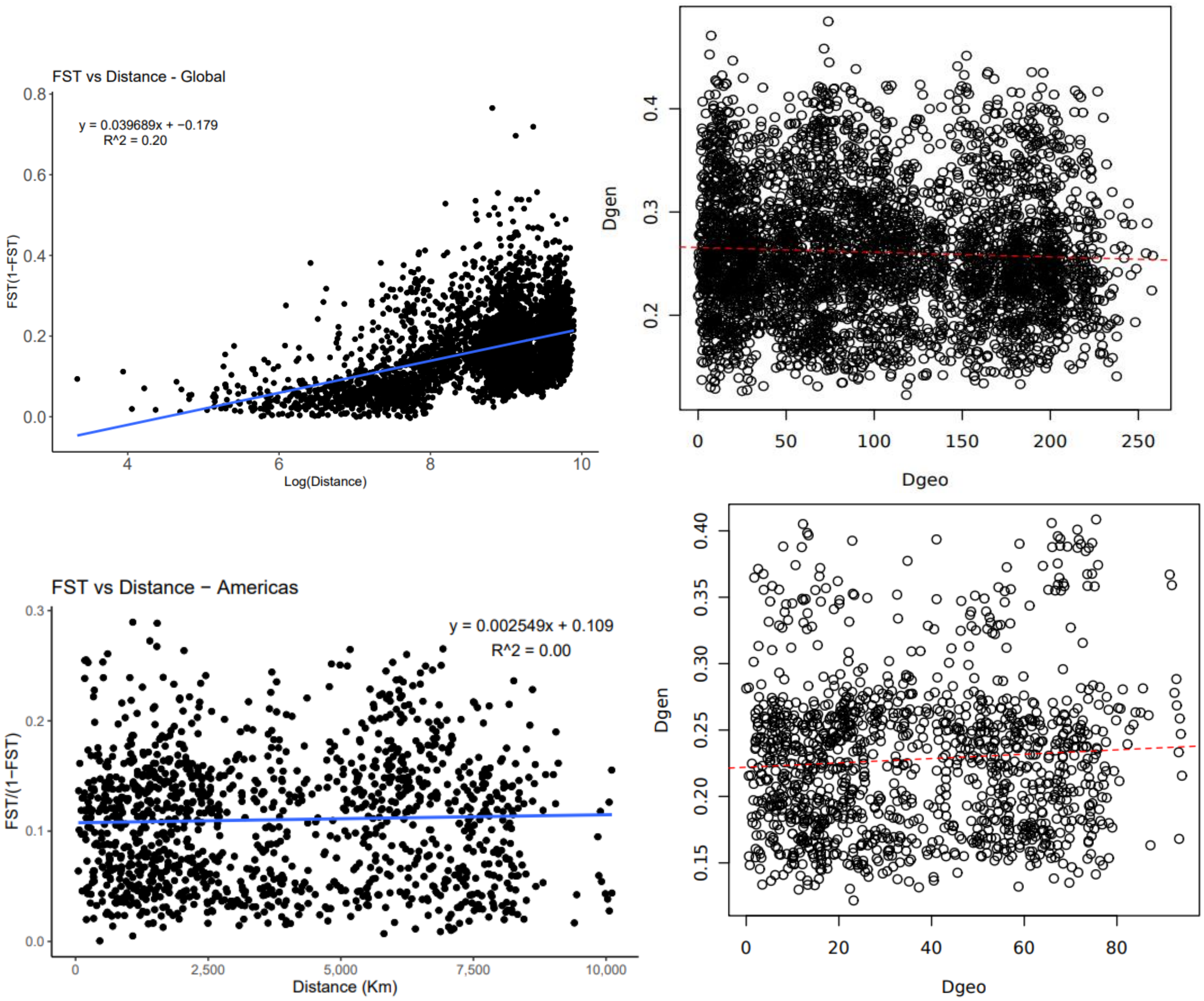

Figure S11. Relationships between genetic and geographic distance for the Global dataset (top) and the American dataset (bottom). In the panels on the left, Fst(1-Fst)) is plotted against log distance (Km). These F_{ST} estimates were calculated with the R package "StAMPP". For the Global dataset (top) the linear regression indicated R^2=0.20, and for the American dataset (bottom) R^2=0.00. In the panels on the right, genetic distance is plotted against geographical distance (Km) as calculated using the R package "geosphere". The linear regression here indicated R^2=0.00 for both the Global dataset (top) and the American dataset (bottom).

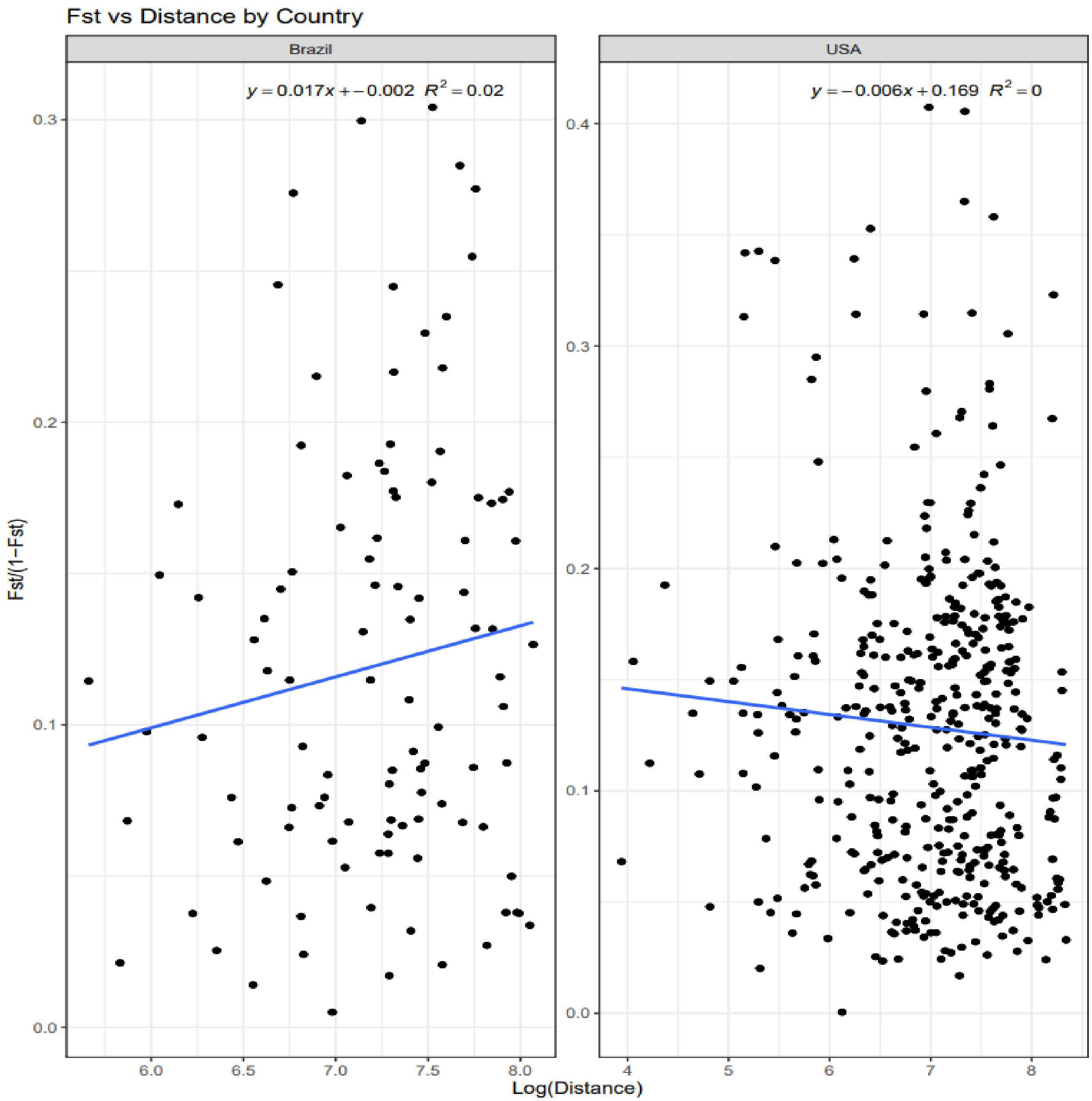

Figure S12. Relationships between genetic and geographic distance for Brazil (left) and the United States (right). In both panels, Fst(1-Fst)) is plotted against log distance (Km). F_{ST} estimates were calculated with the R package “StAMPP”.

A)

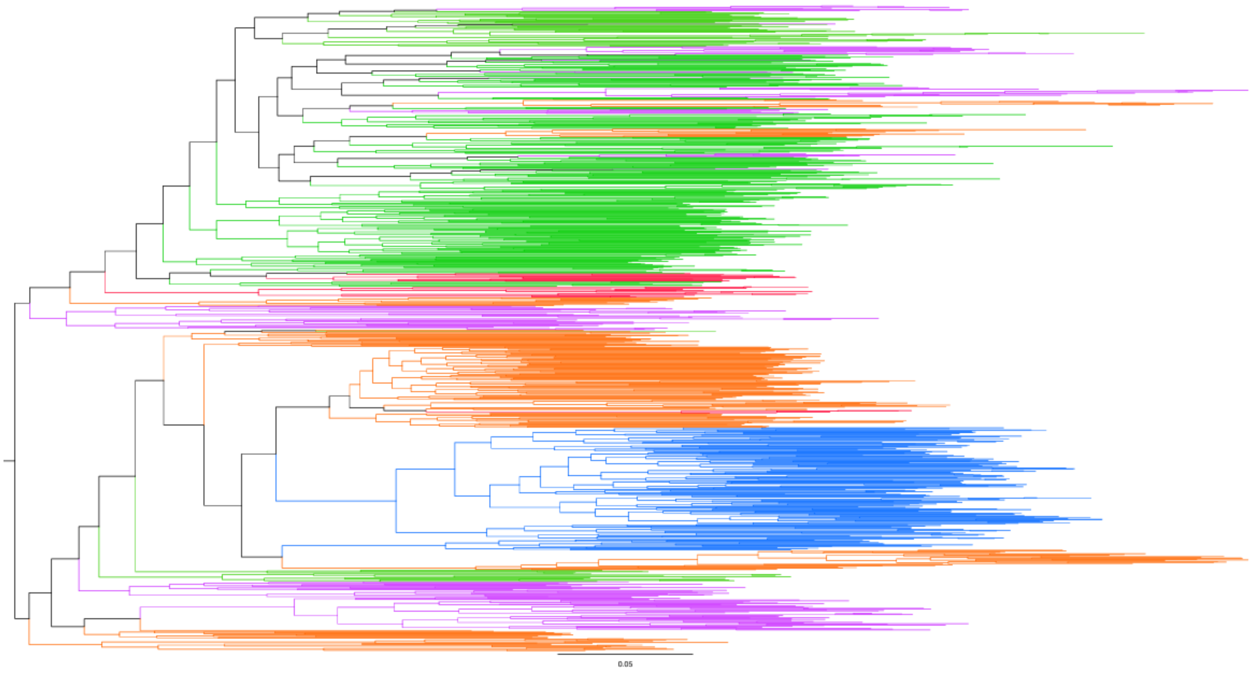

B)

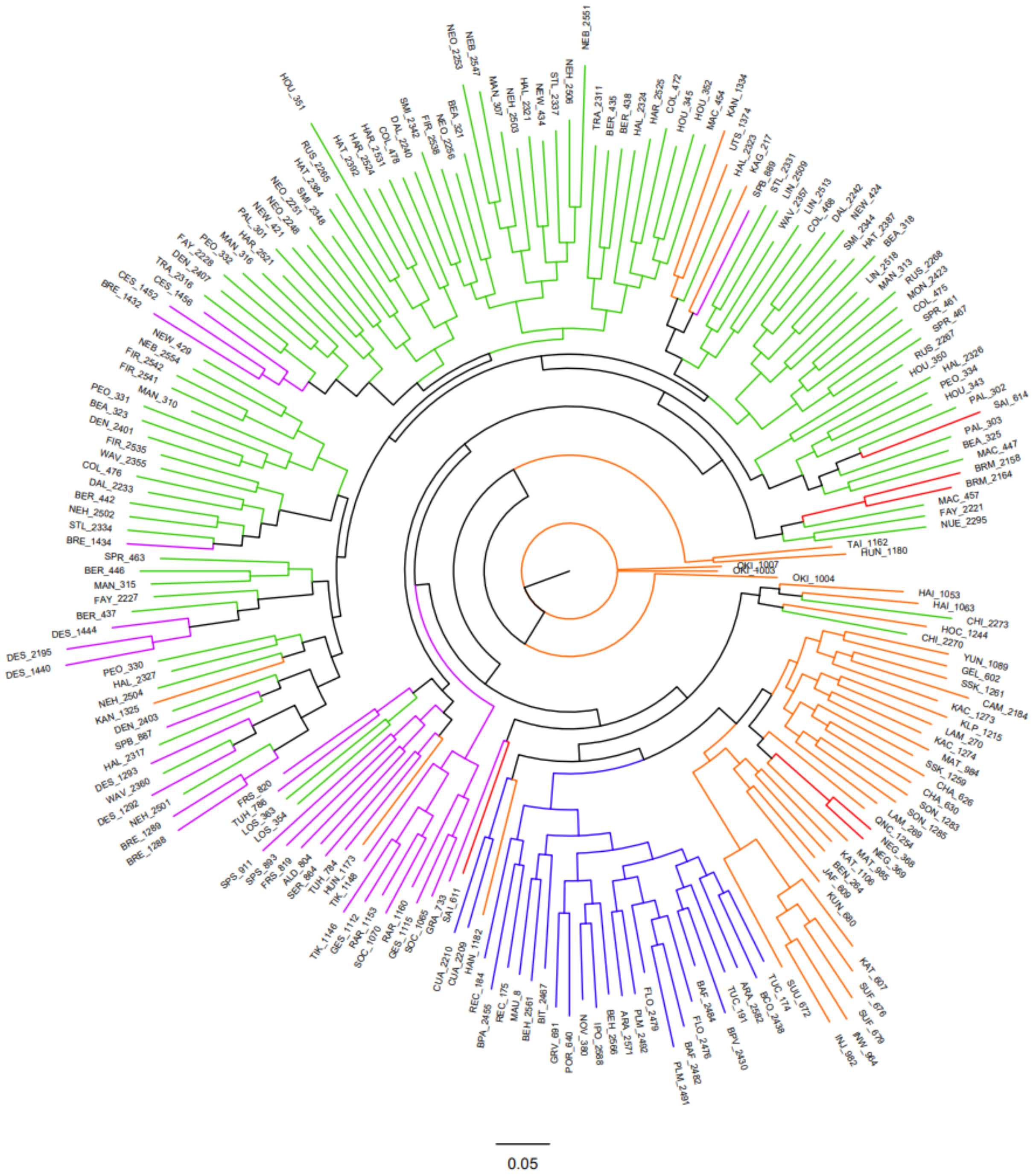

Figure S13. IQ-TREE for the full Global dataset (A) and a subset of 204 *Aedes albopictus* mosquitos in the Global dataset, for easier visualization (B). Labels are colored by region (orange = Asia, red = Atlantic Islands/Caribbean, purple = Europe, green = North America, blue = South America).

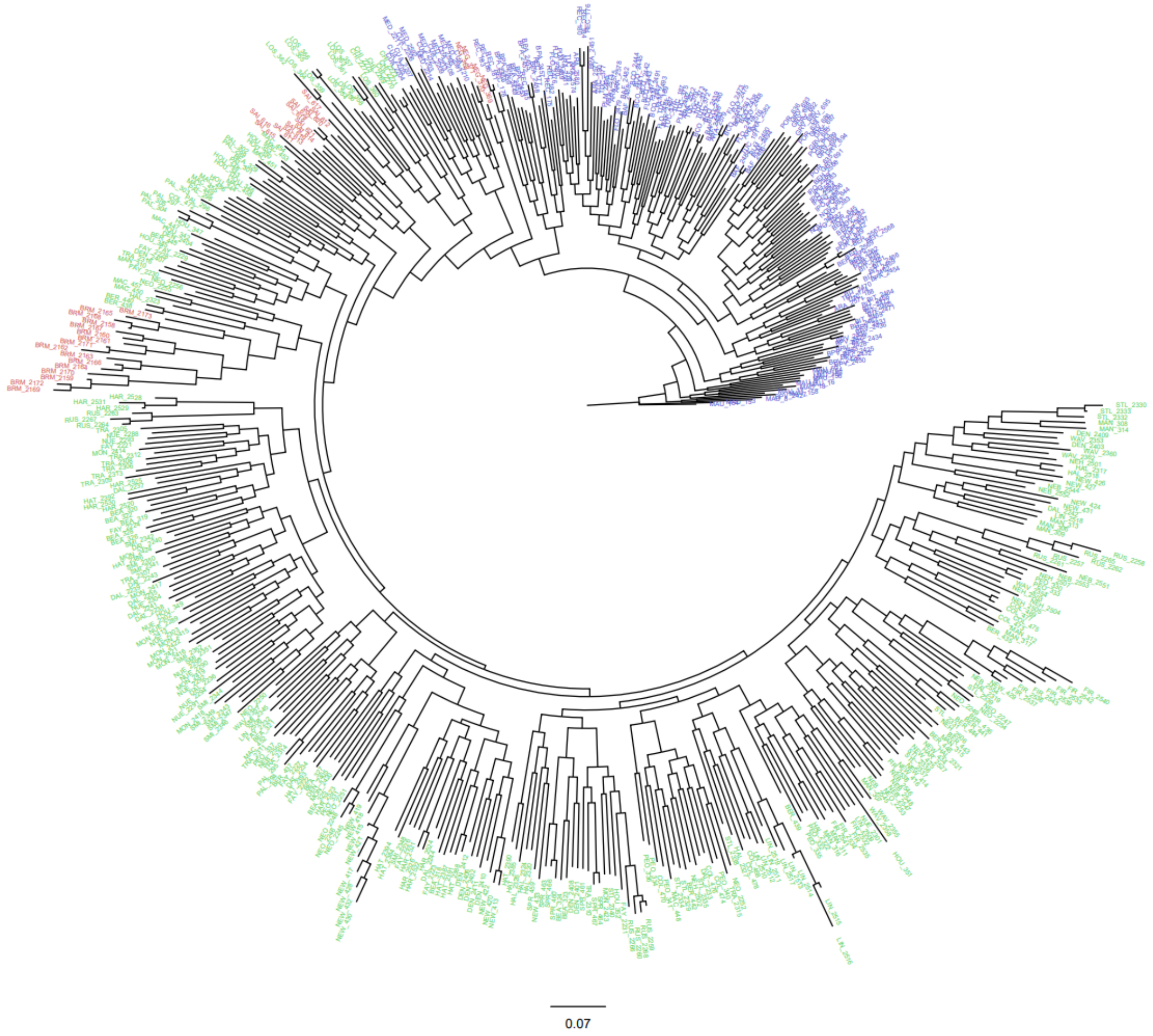

Figure S14. IQ-TREE for all individuals in the American dataset, with labels colored by region (red = Caribbean, green = North America, blue = South America).

A)

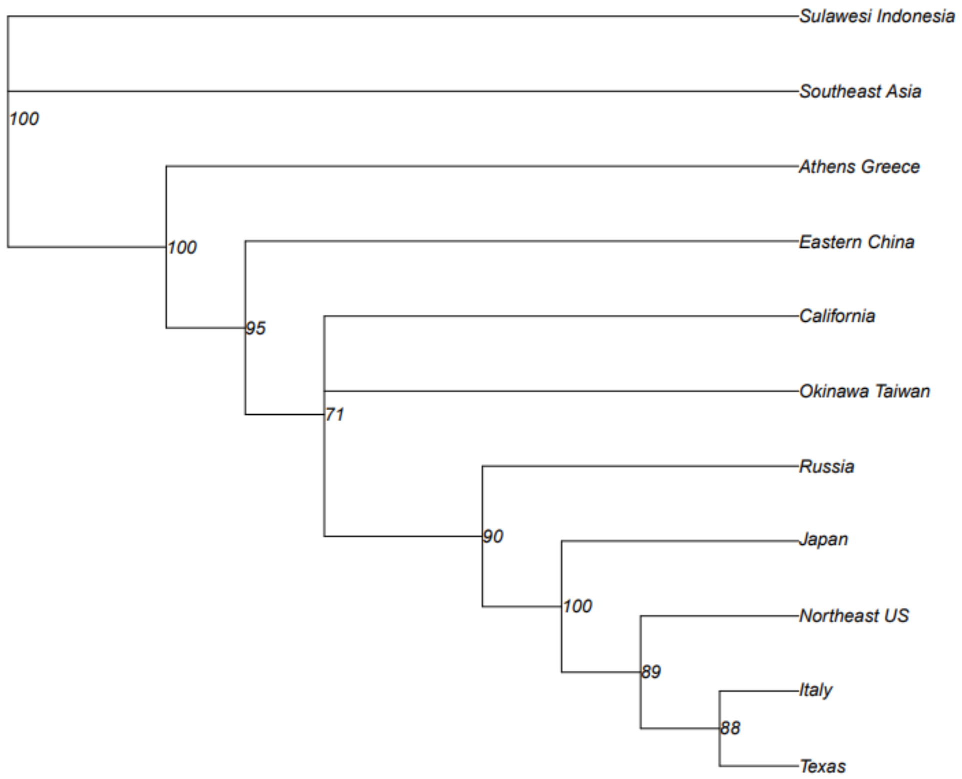

Sulawesi Indonesia
Southeast Asia
100
Athens Greece
100
Eastern China
95
California
Okinawa Taiwan
71
Russia
90
Japan
100
Northeast US
89
Italy
88
Texas

B)

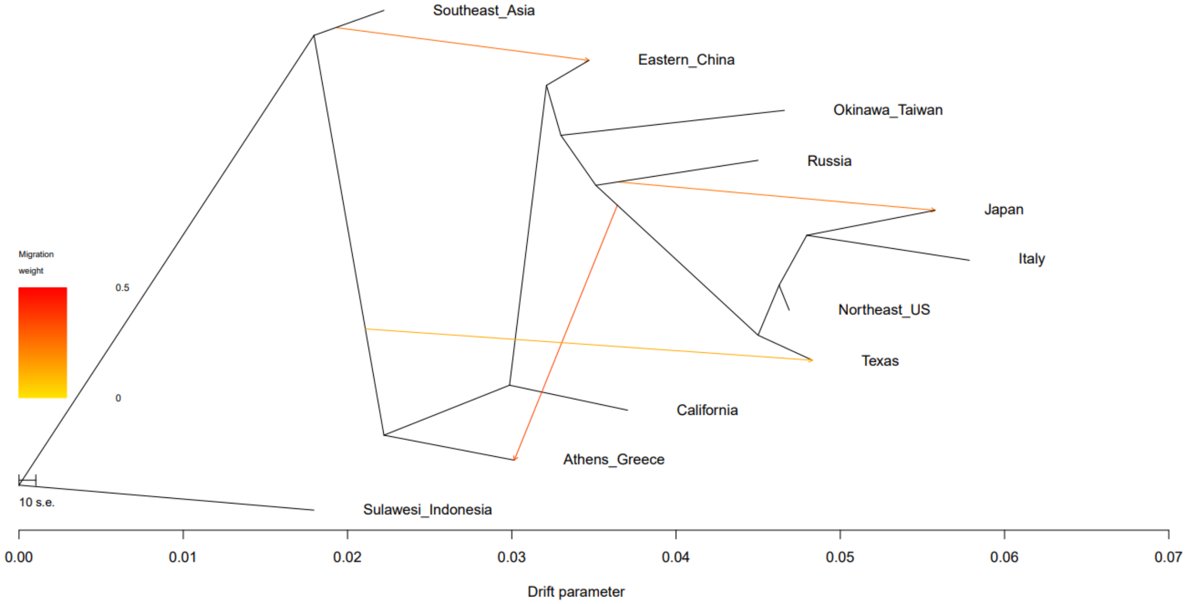

Southeast_Asia
Eastern_China
Okinawa_Taiwan
Russia
Japan
Italy
Northeast_US
Texas
California
Athens_Greece
Sulawesi_Indonesia
Migration weight
0.5
0
10 s.e.
0.00
0.01
0.02
0.03
0.04
0.05
0.06
0.07
Drift parameter

C)

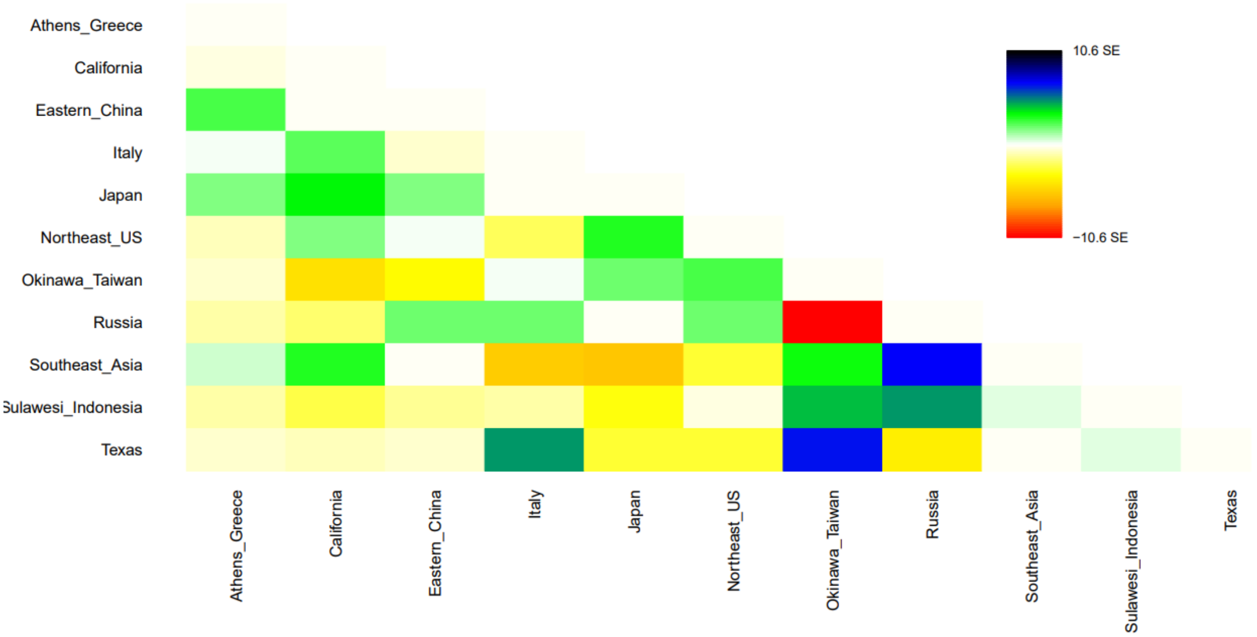

Figure S15. Results for admixture graphs created in TreeMix for grouped native range, North American, and European populations. Graphs are rooted with the Sulawesi, Indonesian population. A) A consensus tree showing bootstrap support values. B) Admixture graph with an arrow indicating the four inferred migration edges. The drift parameter is shown on the x-axis and the scale bar indicates ten times the average standard error of the entries in the sample covariance matrix, representing the amount of genetic drift within each population. C) Plot of residual covariance across population pairs (scaled by average standard error); values > 0 are candidates for additional migration/gene flow.

A)

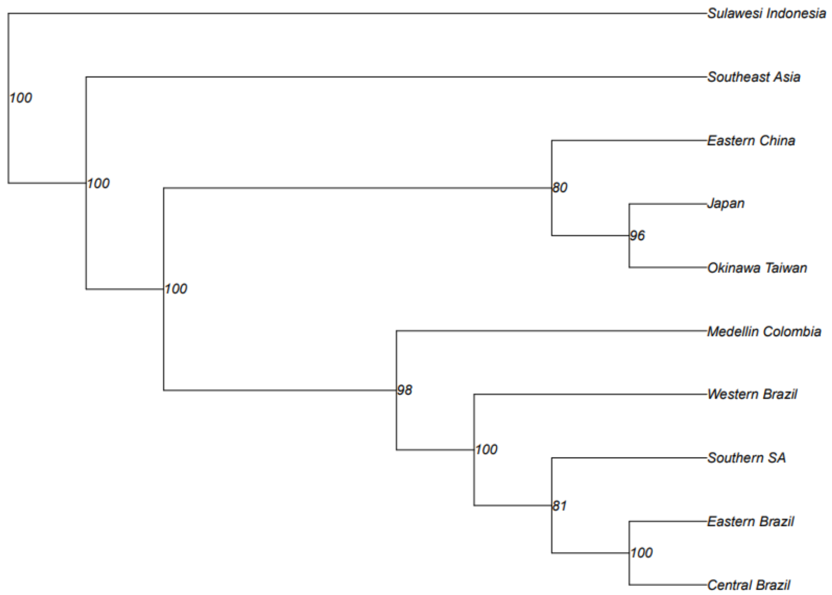
Sulawesi Indonesia
Southeast Asia
Eastern China
Japan
Okinawa Taiwan
Medellin Colombia
Western Brazil
Southern SA
Eastern Brazil
Central Brazil
100
100
80
96
100
98
100
81
100

B)

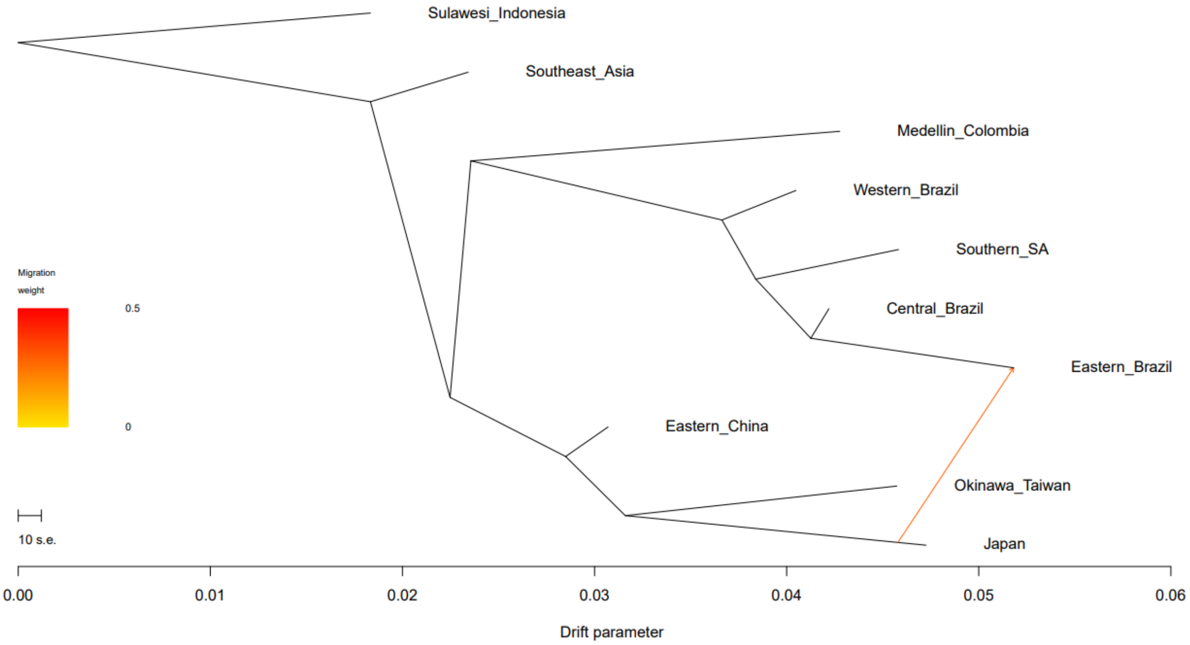
Sulawesi_Indonesia
Southeast_Asia
Medellin_Colombia
Western_Brazil
Southern_SA
Central_Brazil
Eastern_Brazil
Eastern_China
Okinawa_Taiwan
Japan
Migration weight
0.5
0
10 s.e.
0.00
0.01
0.02
0.03
0.04
0.05
0.06
Drift parameter

C)

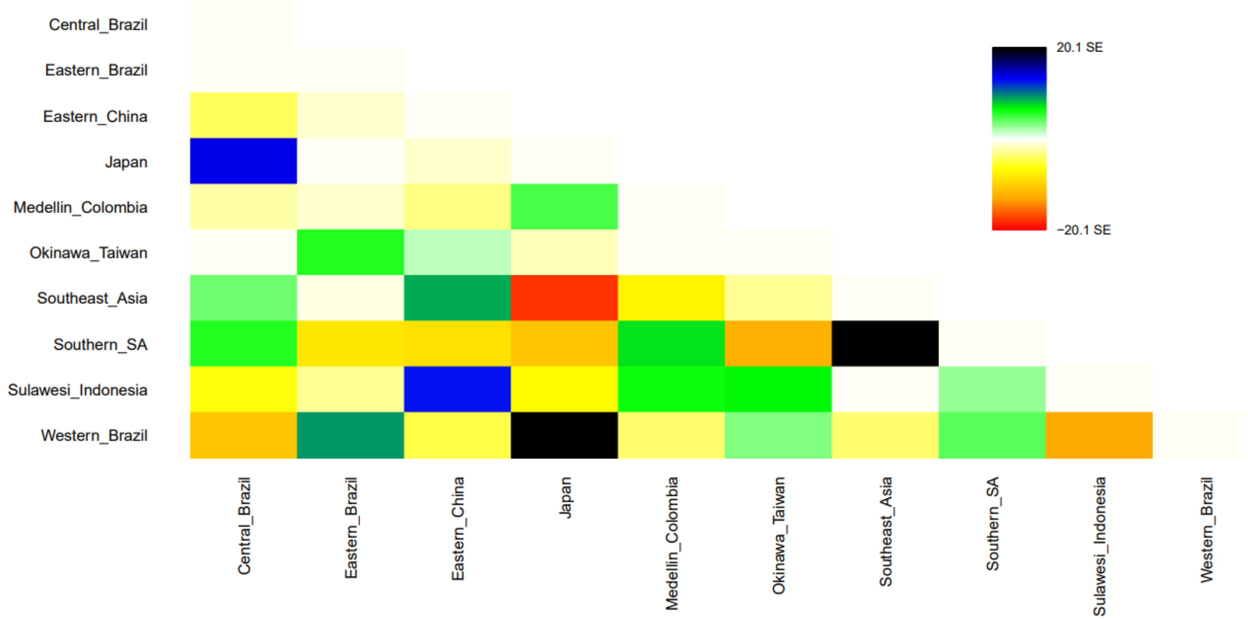

Figure S16. Results for admixture graphs created in TreeMix for grouped native range and South American populations. Admixture graphs are rooted with the Sulawesi, Indonesian population. A) A consensus tree showing bootstrap support values. B) Admixture graph with an arrow indicating the one inferred migration edge. The drift parameter is shown on the x-axis and the scale bar indicates ten times the average standard error of the entries in the sample covariance matrix, representing the amount of genetic drift within each population. C) Plot of residual covariance across population pairs (scaled by average standard error); values > 0 are candidates for additional migration/gene flow.

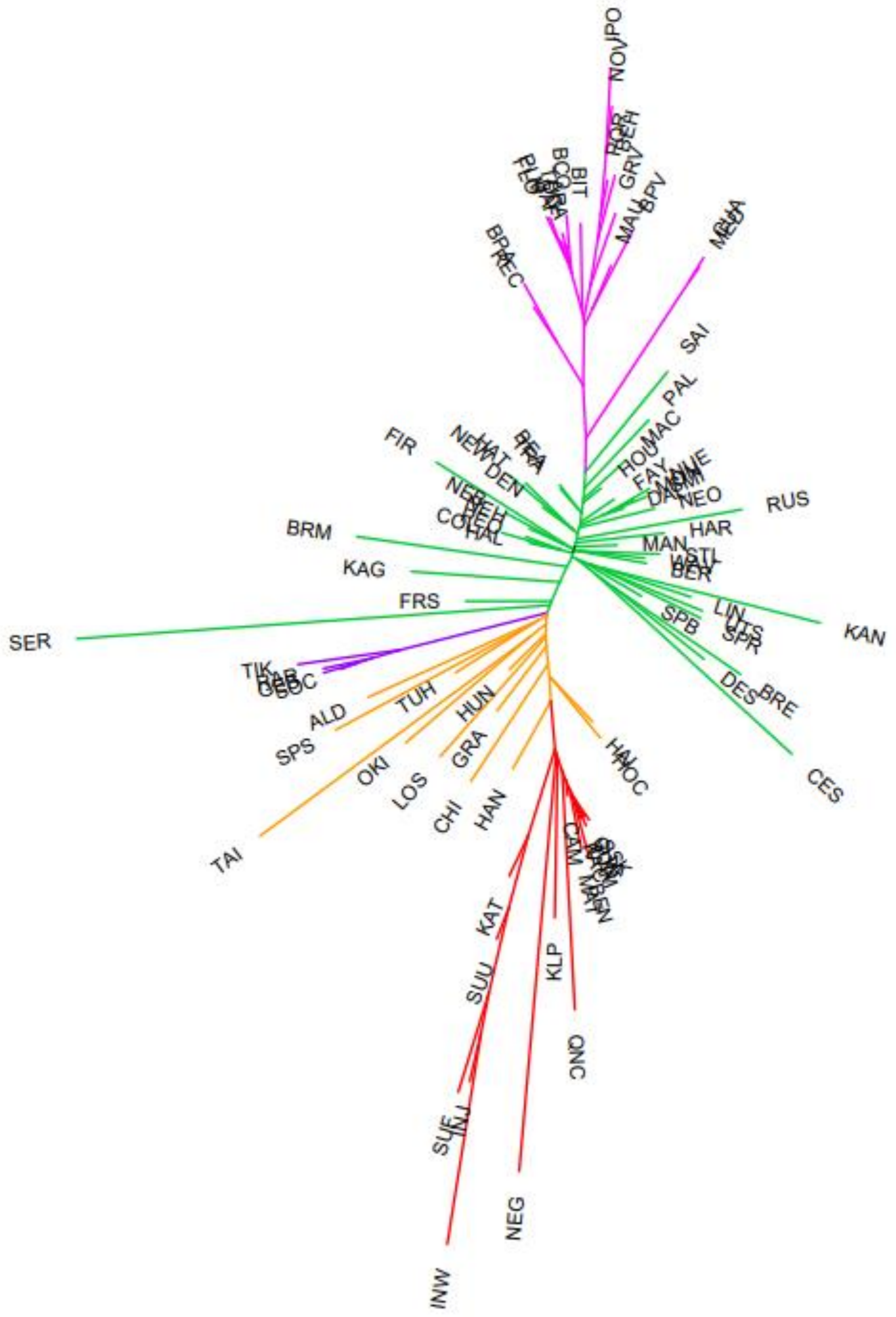

Figure S17. Neighbor-joining tree based on pair-wise Fst estimates. The tree was visualized in FigTree v1.4.4 (Rambaut, 2018), and the five clades identified were used for the Local PCA analysis.

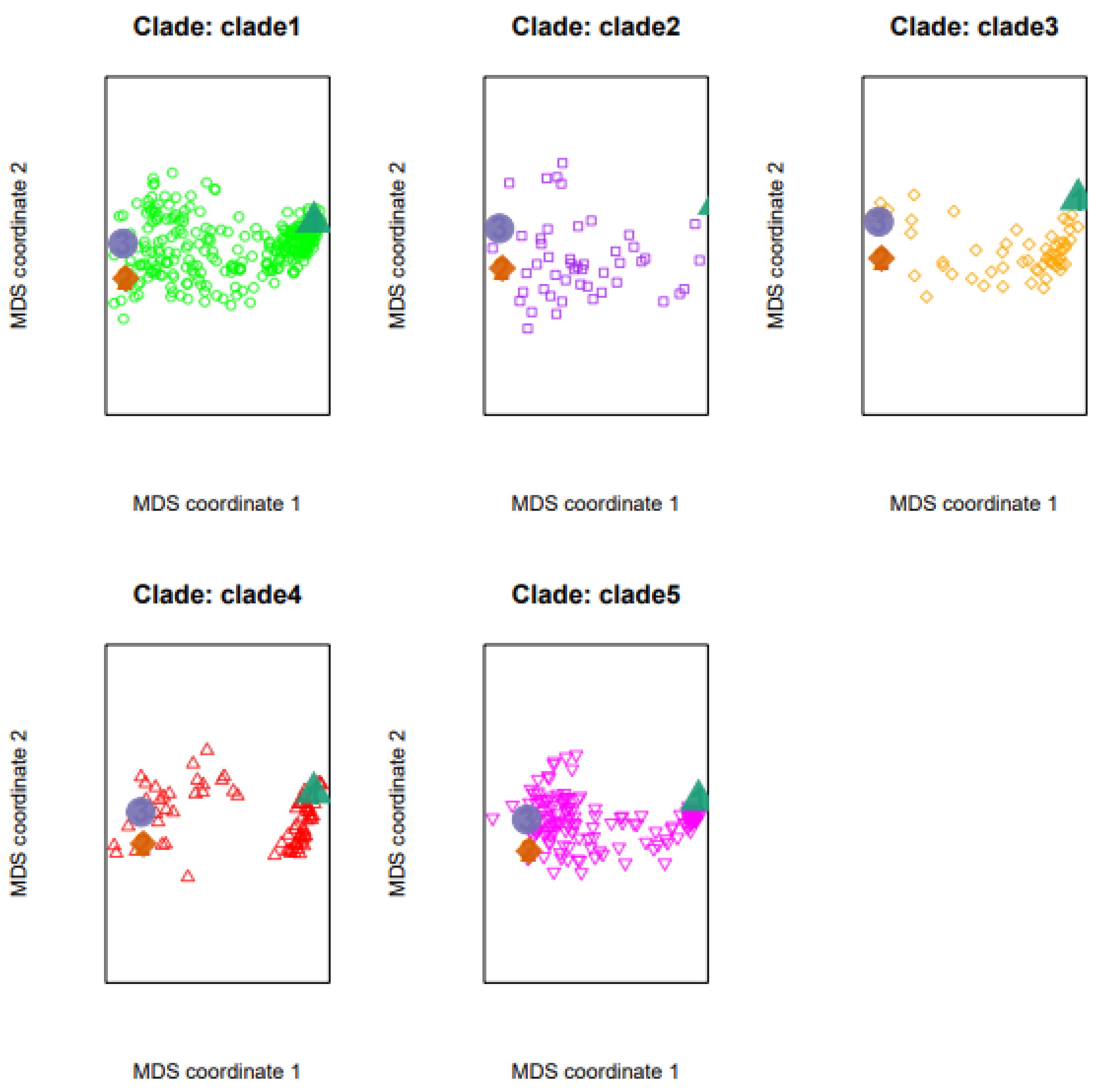

Figure S18. MDS coordinates for each of the five clades with the three most extreme genomic window corners identified (1=green triangle, 2=orange diamond, 3=purple circle).

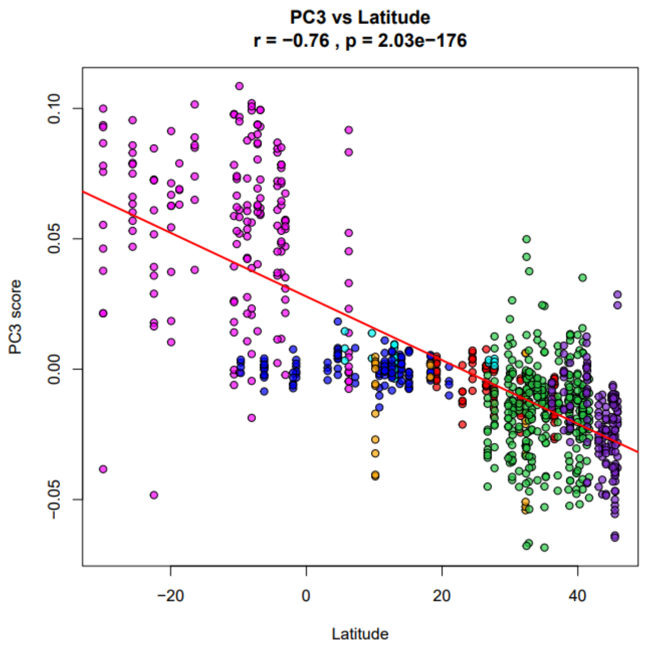
PC3 vs Latitude
r = −0.76 , p = 2.03e−176
PC3 score
0.10
0.05
0.00
−0.05
−20
0
20
40
Latitude

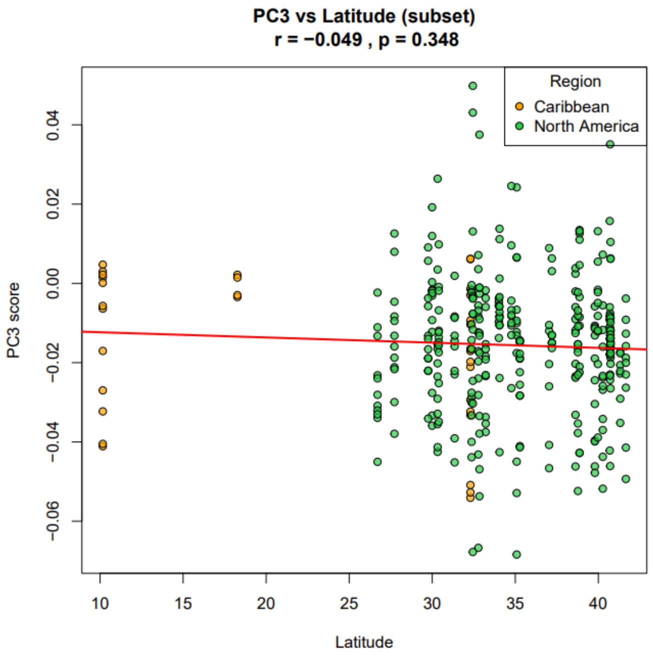
PC3 vs Latitude (subset)
r = −0.049 , p = 0.348
Region
Caribbean
North America
PC3 score
0.04
0.02
0.00
−0.02
−0.04
−0.06
10
15
20
25
30
35
40
Latitude

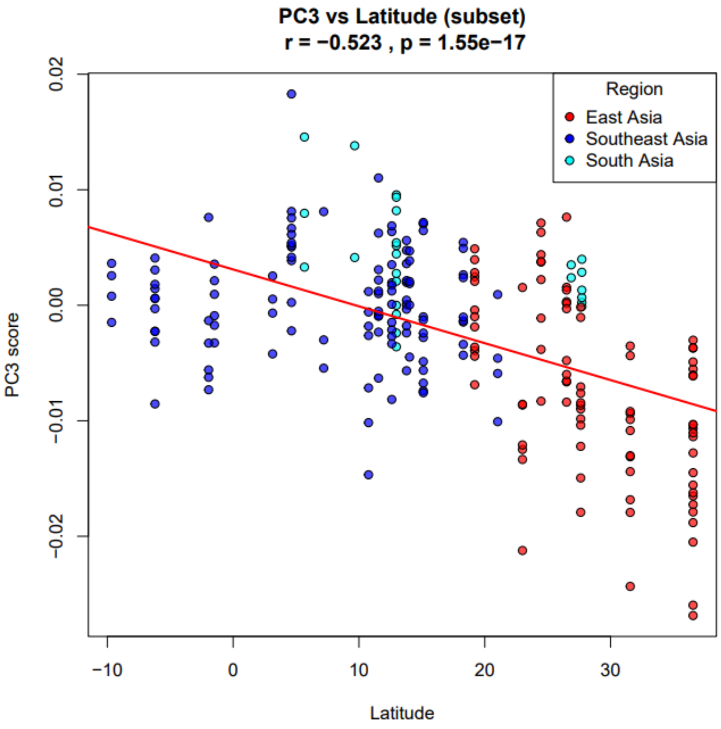

Figure S19. Principal component 3 (PC3) for corner 1 of our local PCA analysis plotted against the latitude of sampling locations of each mosquito, colored by region (magenta = South America, orange = Caribbean, blue = Southeast Asia, cyan = South Asia, red = East Asia, green = North America, purple = Europe). The top panel shows all regions, the middle panel the Caribbean and North America, and the bottom panel the regions in Asia.
