# Supplementary material for: Population Structure of the Invasive Asian Tiger Mosquito, Aedes albopictus, in the Americas
Source: Insects. 2026 Jul 23;17(8):755. doi: 10.3390/insects17080755 (PMC13512968; doi:10.3390/insects17080755)
Supplement: Supplementary file 1 [file insects-17-00755-s001.zip › S3_Axiom_Analysis_summary_americas_dataset.pdf]

Analysis Summary

- **Batch Name:** all_americas_1Apr2024_newprior
- **Array Package Name:** Axiom_Aealbo.r1
- **Array Display Name:** Axiom_Aealbo.r1
- **Workflow Type:** Best Practices Workflow
- **Date Created:** 4/2/2024 10:12:13 AM

Sample Summary

- Number of input samples: 569
- Samples passing DQC: 560 out of 569
- Samples passing DQC and QC CR: 555 out of 569
- Samples passing DQC, QC CR and Plate QC: 555 out of 569 (97.54%)
- Number of failing samples: 14
- Number of Samples Genotyped: 555
- Average QC CR for the passing samples: 98.95
- Inbred Penalty Applied: no
- Prior Model File: all_americas_1Apr2024_new_prior.models
- SNP List File: none

Plate QC Summary

Plate Barcode	Result	Number of files in a batch	Number of files failing dish QC	Number of files failing QC Call rate	Number of samples that passed	Percent of passing samples	Average call rate for passing samples	Filtered Call Rate
5513584436722042923428	PASSED	7	0	0	7	100	98.996	97.706
5513584436722042923430	PASSED	23	0	0	23	100	99.181	97.965
5513584436722042923432	PASSED	65	0	1	64	98.462	99.04	98.334
5513584436722042923435	PASSED	90	1	2	87	96.667	99.069	98.115
5513584452328010824448	PASSED	36	0	0	36	100	98.866	98.005
5513584478174020425866	PASSED	94	0	0	94	100	98.833	97.836
5513584478174020425867	PASSED	16	0	0	16	100	99.195	98.488
5513584478174020425868	PASSED	75	7	0	68	90.667	98.9	98.176
5513584478174020425873	PASSED	75	0	1	74	98.667	98.764	98
5513584478174020425874	PASSED	88	1	1	86	97.727	99.013	98.147

ProbeSet Metrics Summary

- Number of ProbeSets: 175396

ConversionType	Count	Percentage
PolyHighResolution	102629	58.513
Other	42294	24.113
OTV	19247	10.973
NoMinorHom	6678	3.807
MonoHighResolution	3548	2.023
CallRateBelowThreshold	1000	0.57

Marker Metrics Summary

- Number of Markers: 175396
- Number of BestandRecommended: 112855

- Percent BestandRecommended: 64.343

ConversionType	Count	Percentage
PolyHighResolution	102629	58.513
Other	42294	24.113
OTV	19247	10.973
NoMinorHom	6678	3.807
MonoHighResolution	3548	2.023
CallRateBelowThreshold	1000	0.57

Sample QC Thresholds

- DQC: ≥ 0.82
- QC call_rate: ≥ 90
- Average call rate for passing samples: ≥ 90
- Percent of passing samples: ≥ 90

SNP QC Thresholds

- species-type: Diploid
- cr-cutoff: ≥ 90
- fld-cutoff: ≥ 3.6
- het-so-cutoff: ≥ -0.1
- het-so-XChr-cutoff: ≥ -0.1
- het-so-ZChr-cutoff: ≥ -0.1
- het-so-otv-cutoff: ≥ -0.3
- hom-ro-1-cutoff: ≥ 0.6
- hom-ro-2-cutoff: ≥ 0.3
- hom-ro-3-cutoff: ≥ -0.9
- hom-ro: true
- num-minor-allele-cutoff: ≥ 2
- hom-ro-hap-1-XChr-cutoff: ≥ 0.1
- hom-ro-hap-1-MTChr-cutoff: ≥ 0.4
- hom-ro-hap-1-ZChr-cutoff: ≥ 0.1
- hom-ro-hap-2-XChr-cutoff: ≥ 0.05
- hom-ro-hap-2-MTChr-cutoff: ≥ 0.2
- hom-ro-hap-2-ZChr-cutoff: ≥ 0.05
- aaf-XChr-cut: < 0.36
- aaf-ZChr-cut: < 0.36
- fld-XChr-cut: ≥ 4
- fld-ZChr-cut: ≥ 4
- homfld-XChr-cut: ≥ 6.5
- homfld-ZChr-cut: ≥ 6.5
- homfld-YChr-cut: ≥ 6.5
- homfld-WChr-cut: ≥ 6.5
- min-YChr-samples-cut: ≥ 5
- min-WChr-samples-cut: ≥ 5
- priority-order: PolyHighResolution, NoMinorHom, MonoHighResolution, OTV, UnexpectedGenotypeFreq, CallRateBelowThreshold, Other, OtherMA

- recommended: PolyHighResolution, NoMinorHom, MonoHighResolution, Hemizygous
- y-restrict: ≤ 0.2
- min-genotype-freq-samples: ≥ 20
- genotype-p-value-cutoff: ≥ 1E-06

Multi-Allelic SNP QC Thresholds

- HomMMA-cutoff: > 10
- FLD-MA-cutoff: > 5.2
- FLD-MA-2-cutoff: > 5.2
- Min-FLD-MA-cutoff: > 0
- Min-FLD-MA-2-cutoff: > 0
- HetSO-MA-2-cutoff: > -0.1
- HomRO-MA-cutoff: > 0.5
- HomRO-MA-2-cutoff: > 0.5
- HomRO-MA-1-cutoff: > 1
- priority-order-MA: PolyHighResolution, NoMinorHom, MonoHighResolution, Hemizygous, UnexpectedGenotypeFreq, CallRateBelowThreshold, Other, OtherMA
- Best-CR-MA-cutoff: > 90
