# Supplementary material for: Population Structure of the Invasive Asian Tiger Mosquito, Aedes albopictus, in the Americas
Source: Insects. 2026 Jul 23;17(8):755. doi: 10.3390/insects17080755 (PMC13512968; doi:10.3390/insects17080755)
Supplement: Supplementary file 1 [file insects-17-00755-s001.zip › S4_Axiom_Analysis_summary_global_dataset.pdf]

Analysis Summary

- **Batch Name:** albo_manuscript1_19Aug2024_newprior
- **Array Package Name:** Axiom_Aealbo.r1
- **Array Display Name:** Axiom_Aealbo.r1
- **Workflow Type:** Best Practices Workflow
- **Date Created:** 8/19/2024 1:30:36 PM

Sample Summary

- Number of input samples: 983
- Samples passing DQC: 970 out of 983
- Samples passing DQC and QC CR: 959 out of 983
- Samples passing DQC, QC CR and Plate QC: 959 out of 983 (97.558%)
- Number of failing samples: 24
- Number of Samples Genotyped: 959
- Average QC CR for the passing samples: 98.738
- Inbred Penalty Applied: no
- Prior Model File: albo_manuscript1_19Aug2024_newprior.models
- SNP List File: none

Plate QC Summary

Plate Barcode	Result	Number of files in a batch	Number of files failing dish QC	Number of files failing QC Call rate	Number of samples that passed	Percent of passing samples	Average call rate for passing samples	Filtered Call Rate
5513584436722042923427	PASSED	38	0	1	37	97.368	98.805	97.653
5513584436722042923428	PASSED	7	0	0	7	100	98.944	97.501
5513584436722042923430	PASSED	23	0	0	23	100	99.154	97.817
5513584436722042923432	PASSED	65	0	0	65	100	98.916	98.184
5513584436722042923435	PASSED	90	1	2	87	96.667	99.121	98.131
5513584452328010824440	PASSED	52	0	0	52	100	98.528	97.725
5513584452328010824441	PASSED	24	0	0	24	100	99.107	98.387
5513584452328010824443	PASSED	38	0	0	38	100	99.005	98.275
5513584452328010824444	PASSED	43	0	0	43	100	98.87	98.114
5513584452328010824445	PASSED	68	1	2	65	95.588	98.572	97.829
5513584452328010824446	PASSED	24	0	0	24	100	98.443	97.748
5513584452328010824447	PASSED	32	0	0	32	100	98.324	97.471
5513584452328010824448	PASSED	73	0	0	73	100	98.5	97.596
5513584452328010824449	PASSED	32	3	3	26	81.25	97.382	96.692
5513584478174020425866	PASSED	94	0	0	94	100	98.717	97.61
5513584478174020425867	PASSED	38	0	2	36	94.737	98.425	97.653
5513584478174020425868	PASSED	75	7	0	68	90.667	98.873	98.087
5513584478174020425873	PASSED	79	0	0	79	100	98.669	97.898
5513584478174020425874	PASSED	88	1	1	86	97.727	98.983	98.047

ProbeSet Metrics Summary

- Number of ProbeSets: 175396

ConversionType	Count	Percentage
PolyHighResolution	103304	58.898
Other	42485	24.222
OTV	21550	12.286
NoMinorHom	5214	2.973
MonoHighResolution	1874	1.068
CallRateBelowThreshold	969	0.552

Marker Metrics Summary

- Number of Markers: 175396
- Number of BestandRecommended: 110392
- Percent BestandRecommended: 62.939

ConversionType	Count	Percentage
PolyHighResolution	103304	58.898
Other	42485	24.222
OTV	21550	12.286
NoMinorHom	5214	2.973
MonoHighResolution	1874	1.068
CallRateBelowThreshold	969	0.552

Sample QC Thresholds

- DQC: ≥ 0.82
- QC call_rate: ≥ 90
- Average call rate for passing samples: ≥ 90
- Percent of passing samples: ≥ 90

SNP QC Thresholds

- species-type: Diploid
- cr-cutoff: ≥ 90
- fld-cutoff: ≥ 3.6
- het-so-cutoff: ≥ -0.1
- het-so-XChr-cutoff: ≥ -0.1
- het-so-ZChr-cutoff: ≥ -0.1
- het-so-otv-cutoff: ≥ -0.3
- hom-ro-1-cutoff: ≥ 0.6
- hom-ro-2-cutoff: ≥ 0.3
- hom-ro-3-cutoff: ≥ -0.9
- hom-ro: true
- num-minor-allele-cutoff: ≥ 2
- hom-ro-hap-1-XChr-cutoff: ≥ 0.1
- hom-ro-hap-1-MTChr-cutoff: ≥ 0.4
- hom-ro-hap-1-ZChr-cutoff: ≥ 0.1
- hom-ro-hap-2-XChr-cutoff: ≥ 0.05
- hom-ro-hap-2-MTChr-cutoff: ≥ 0.2
- hom-ro-hap-2-ZChr-cutoff: ≥ 0.05
- aaf-XChr-cut: < 0.36
- aaf-ZChr-cut: < 0.36

- fld-XChr-cut: ≥ 4
- fld-ZChr-cut: ≥ 4
- homfld-XChr-cut: ≥ 6.5
- homfld-ZChr-cut: ≥ 6.5
- homfld-YChr-cut: ≥ 6.5
- homfld-WChr-cut: ≥ 6.5
- min-YChr-samples-cut: ≥ 5
- min-WChr-samples-cut: ≥ 5
- priority-order: PolyHighResolution, NoMinorHom, MonoHighResolution, OTV, UnexpectedGenotypeFreq, CallRateBelowThreshold, Other, OtherMA
- recommended: PolyHighResolution, NoMinorHom, MonoHighResolution, Hemizygous
- y-restrict: ≤ 0.2
- min-genotype-freq-samples: ≥ 20
- genotype-p-value-cutoff: ≥ 1E-06

Multi-Allelic SNP QC Thresholds

- HomMMA-cutoff: > 10
- FLD-MA-cutoff: > 5.2
- FLD-MA-2-cutoff: > 5.2
- Min-FLD-MA-cutoff: > 0
- Min-FLD-MA-2-cutoff: > 0
- HetSO-MA-2-cutoff: > -0.1
- HomRO-MA-cutoff: > 0.5
- HomRO-MA-2-cutoff: > 0.5
- HomRO-MA-1-cutoff: > 1
- priority-order-MA: PolyHighResolution, NoMinorHom, MonoHighResolution, Hemizygous, UnexpectedGenotypeFreq, CallRateBelowThreshold, Other, OtherMA
- Best-CR-MA-cutoff: > 90
